# Supplementary figures and images for: Investigating demic versus cultural diffusion and sex bias in the spread of Austronesian languages in Vietnam
Source: PLoS One. 2024 Jun 17;19(6):e0304964. doi: 10.1371/journal.pone.0304964 (PMC11182502; doi:10.1371/journal.pone.0304964)

**A**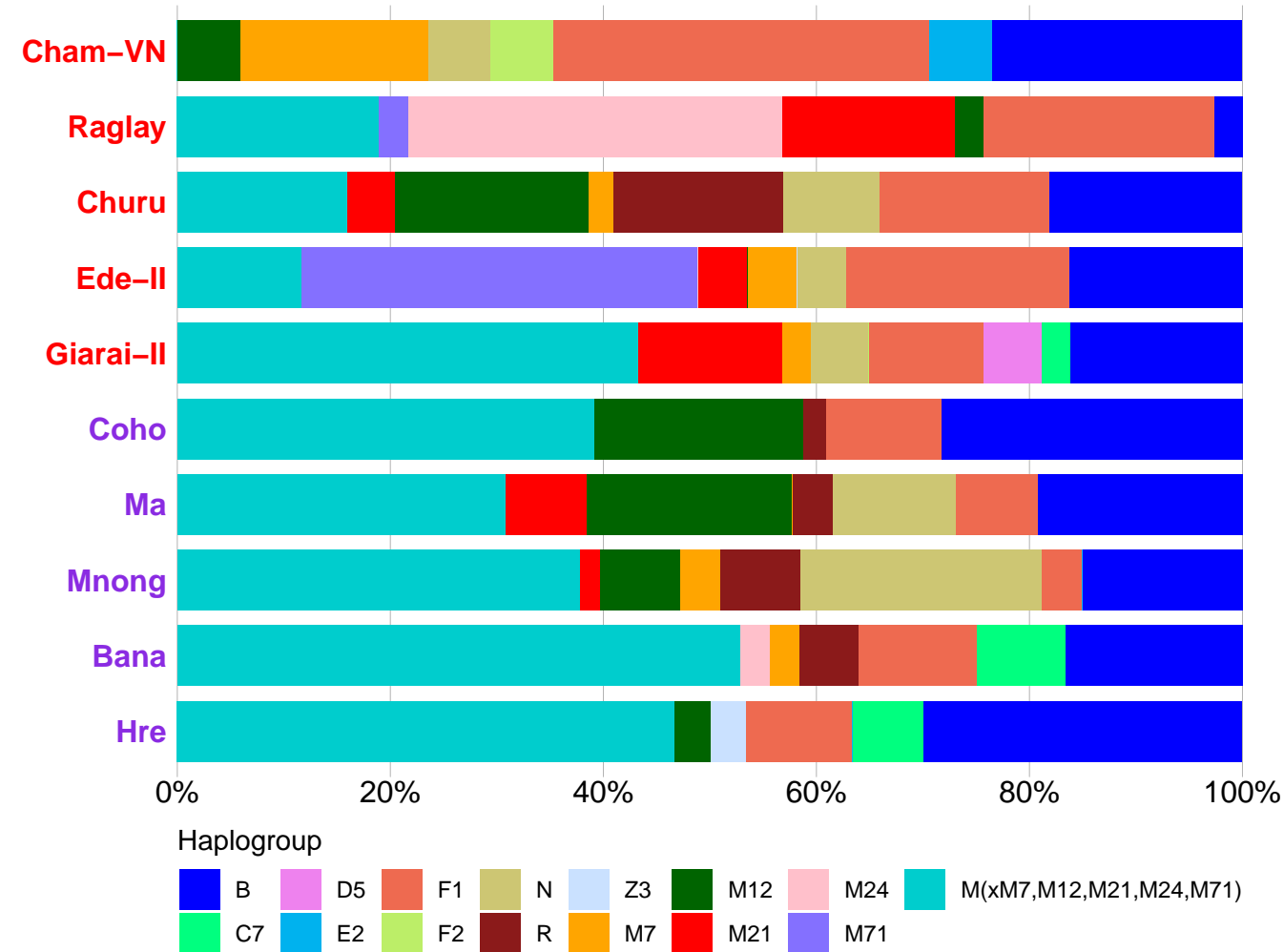**B**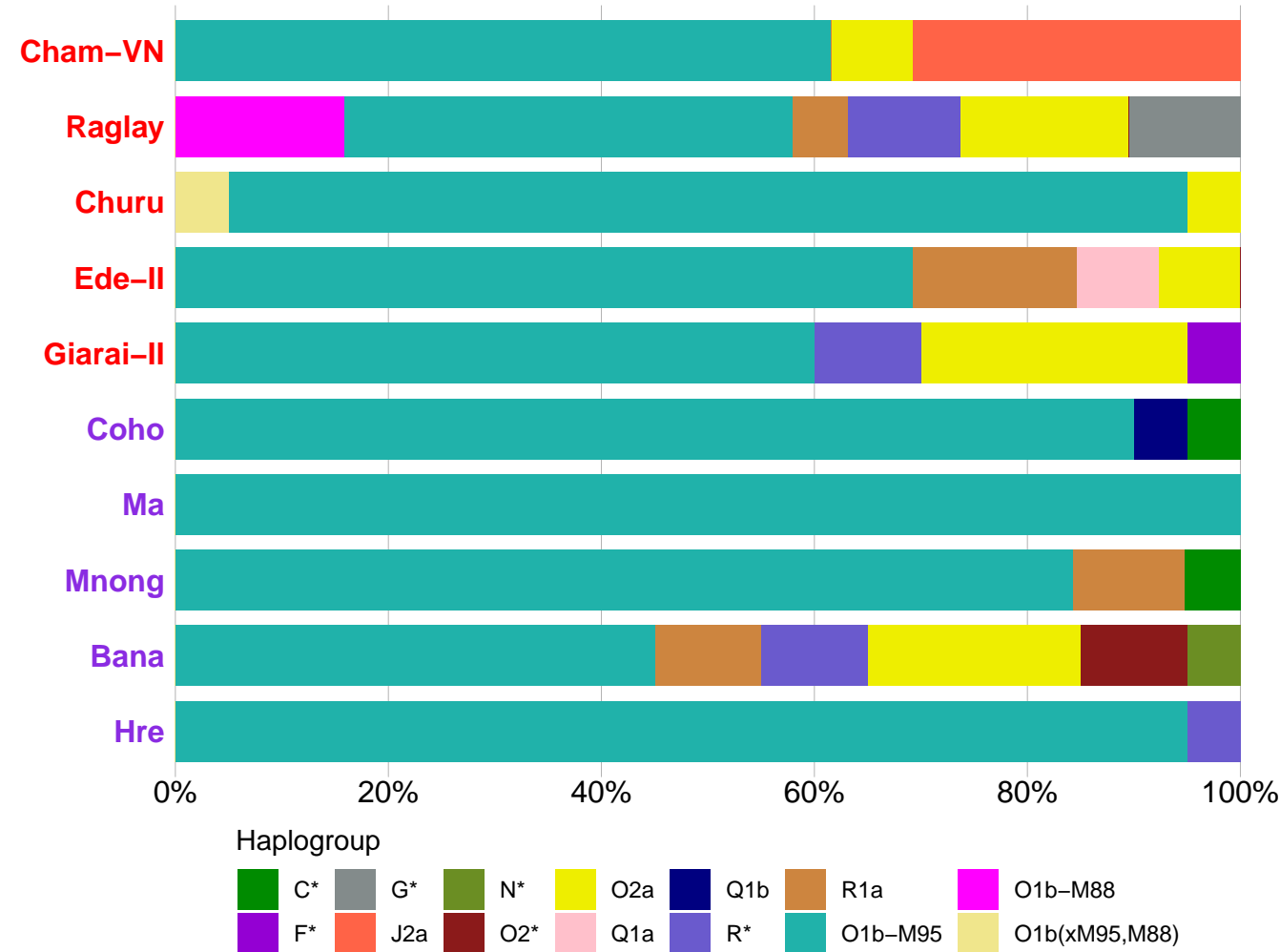

Supplement: S1 Fig — (A) mtDNA, (B) MSY. Austronesian-speaking groups are in red font and Austroasiatic-speaking groups are in purple font. (PDF) [file pone.0304964.s001.pdf]

Haplogroup

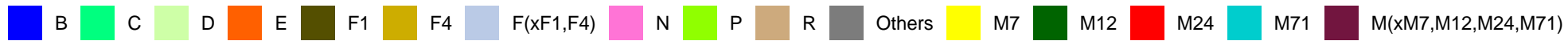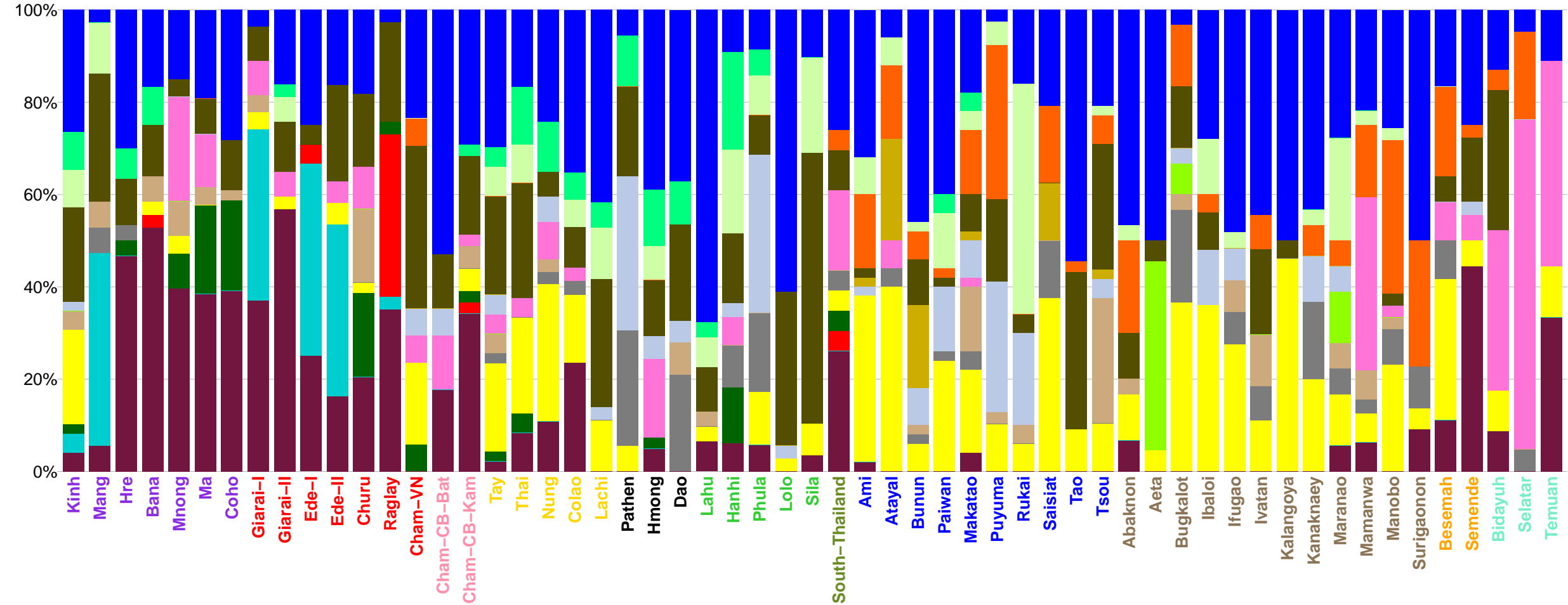

Supplement: S2 Fig — Population labels are color coded by language family with Vietnamese Austroasiatic in purple, Vietnamese Austronesian in red, Vietnamese Tai-Kadai in yellow, Vietnamese Hmong-Mien in black, Vietnamese Sino-Tibetan in lime, Cambodian Austronesian (Cham-CB-Bat and Cham-CB-Kam) in pink, Thai Austronesian (South-Thailand) in olive drab, Taiwanese Austronesian in blue, Philippine Austronesian in brown, Indonesian Austronesian in orange, and Malaysian Austronesian in turquoise. (PDF) [file pone.0304964.s002.pdf]

Haplogroup

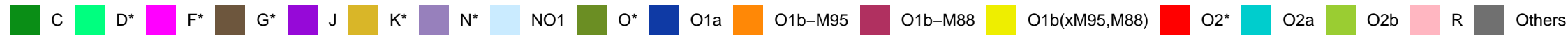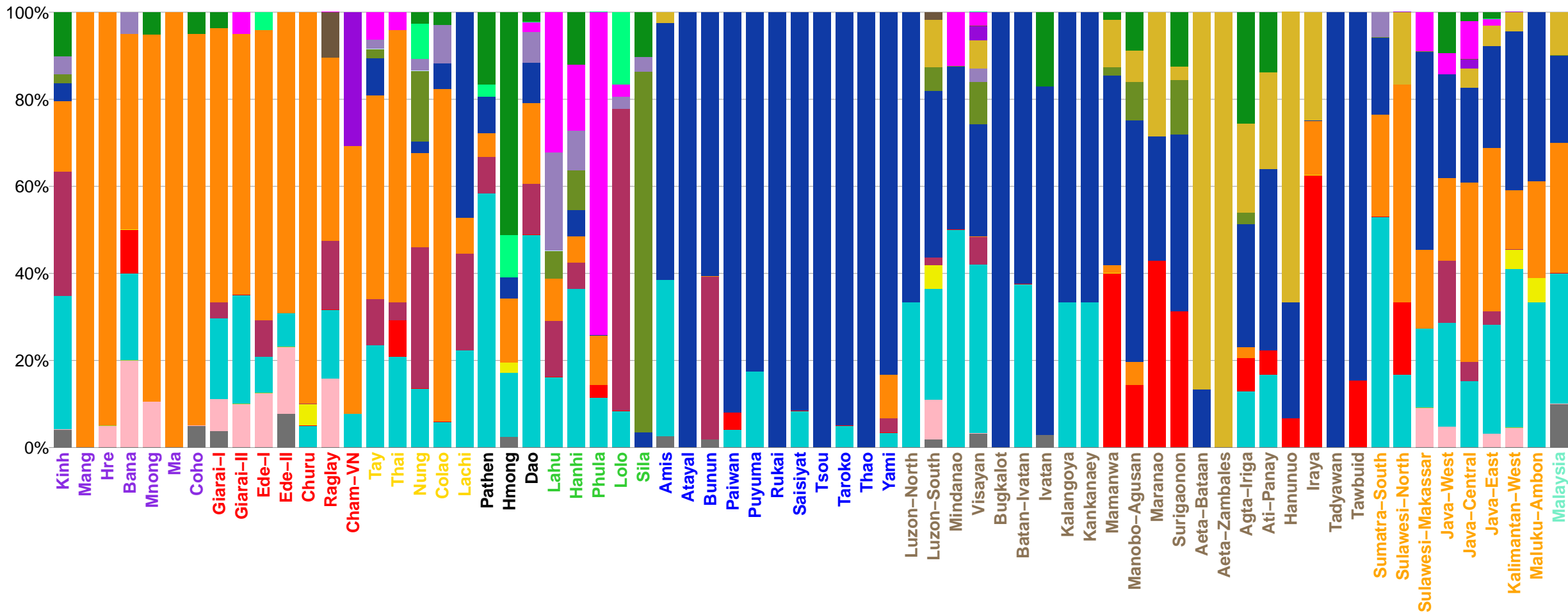

Supplement: S3 Fig — Population labels are color coded by language family with Vietnamese Austroasiatic in purple, Vietnamese Austronesian in red, Vietnamese Tai-Kadai in yellow, Vietnamese Hmong-Mien in black, Vietnamese Sino-Tibetan in lime, Taiwanese Austronesian in blue, Philippine Austronesian in brown, Indonesian Austronesian in orange, and Malaysian Austronesian in turquoise. (PDF) [file pone.0304964.s003.pdf]

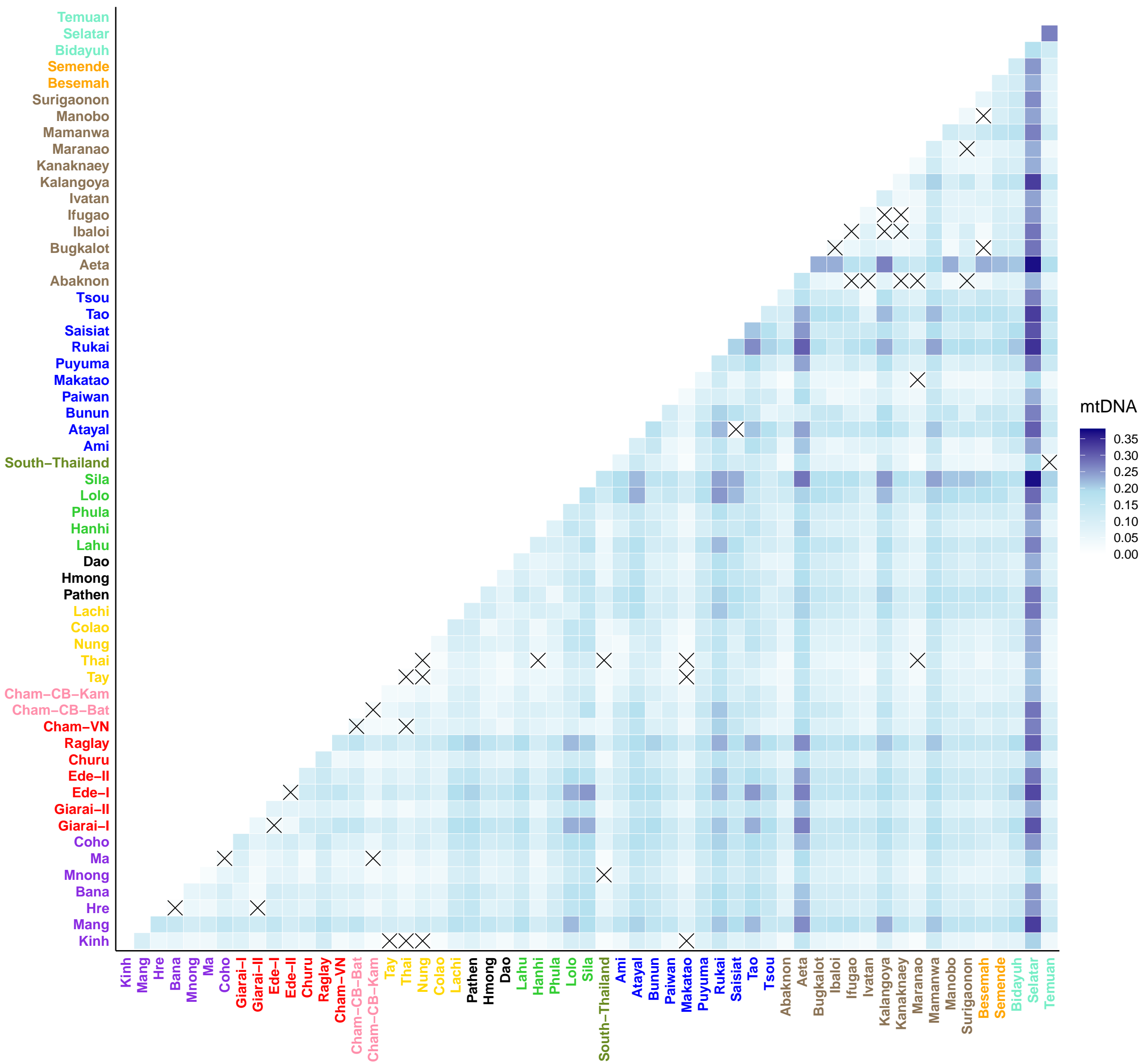

Supplement: S4 Fig — Distances that are not significantly different from zero are marked with a cross symbol (p> 0.05). Population labels are color coded by language family with Austroasiatic in purple, Vietnamese Austronesian in red, Tai-Kadai in yellow, Hmong-Mien in black, Sino-Tibetan in lime, Cambodian Austronesian in pink, Thai Austronesian in olive drab, Taiwanese Austronesian in blue, Philippine Austronesian in brown, Indonesian Austronesian in orange, and Malaysian Austronesian in turquoise. (PDF) [file pone.0304964.s004.pdf]

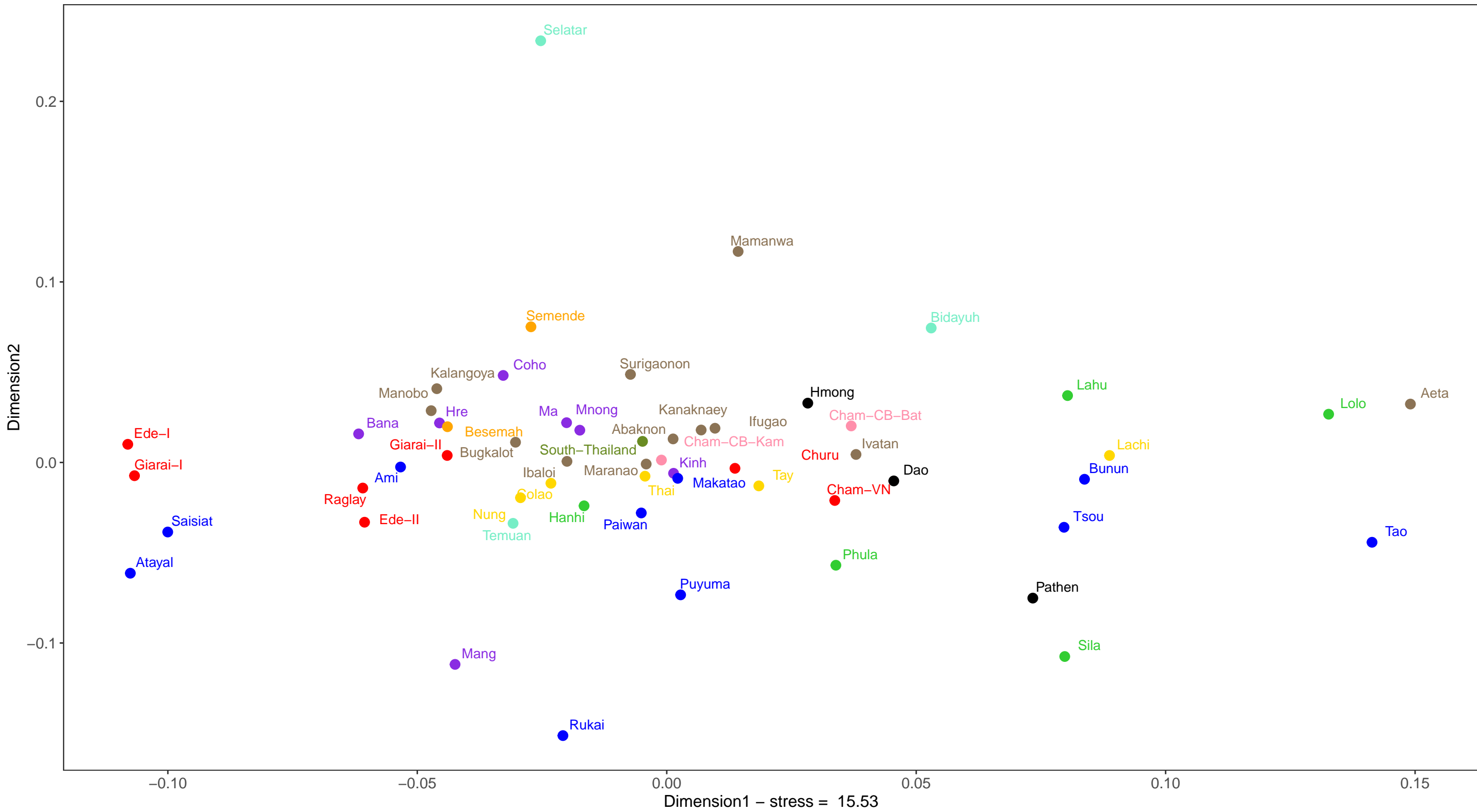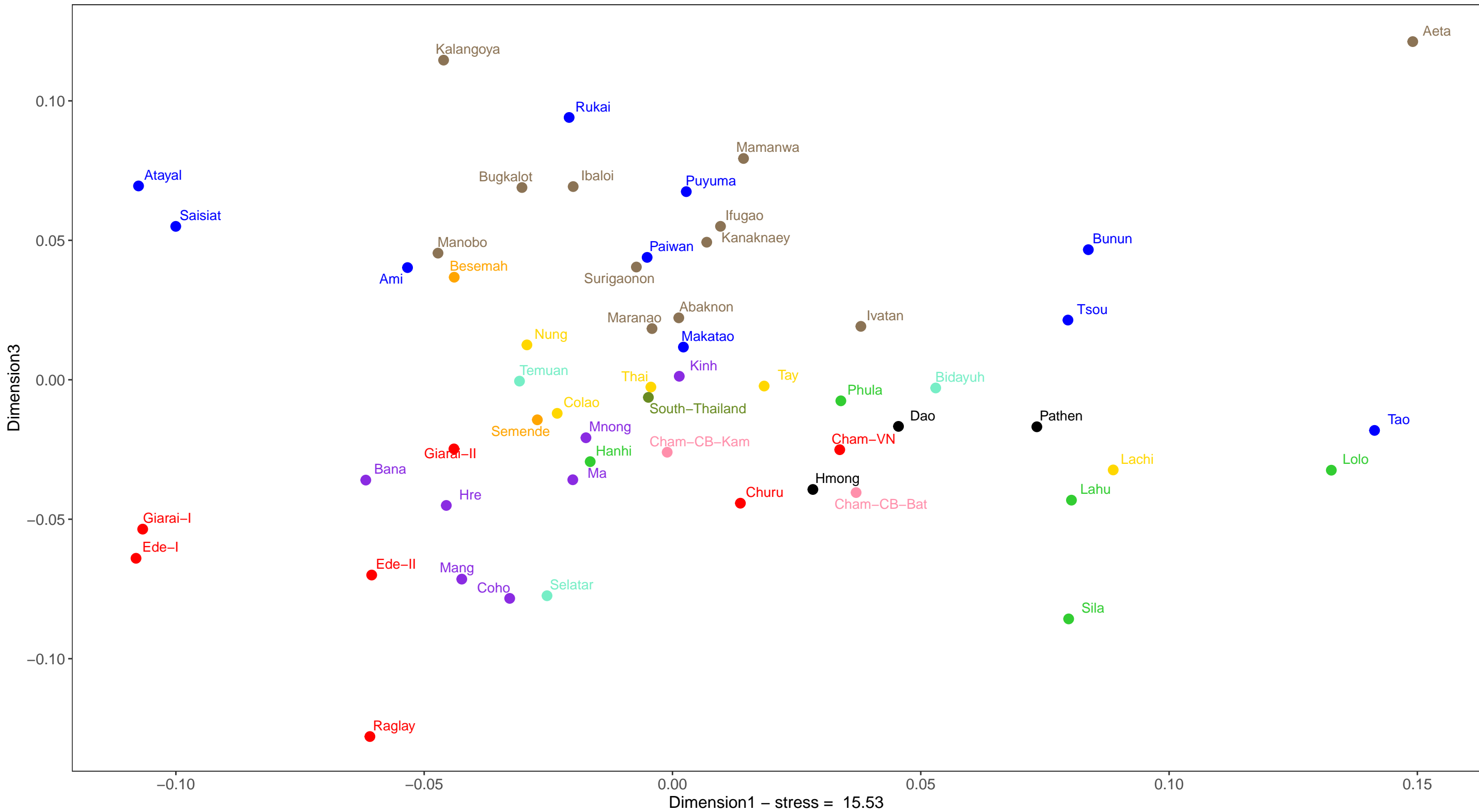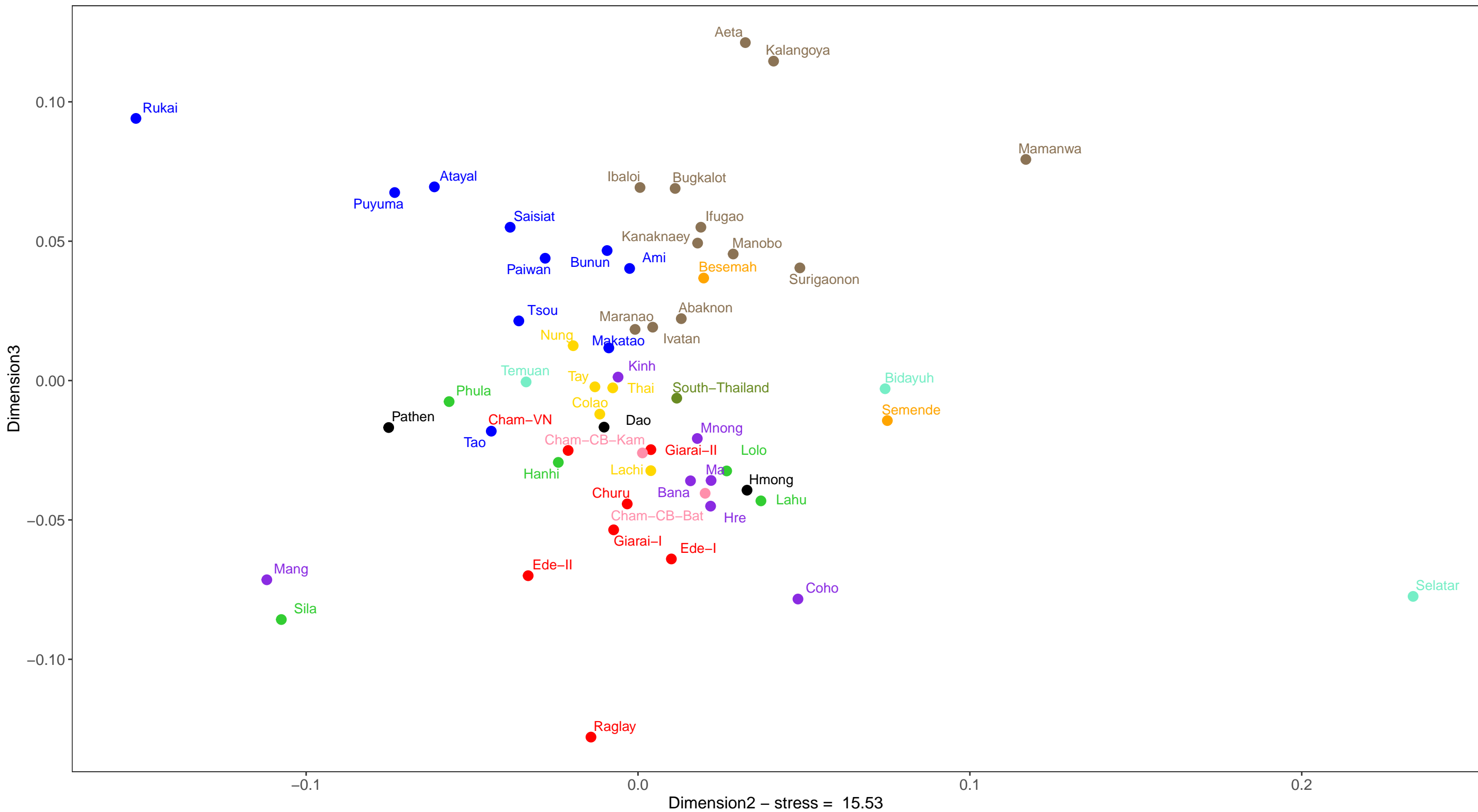

Supplement: S5 Fig — Results are shown as two-dimensional plots for each combination of the three dimensions, and the stress value is in percent. Population labels are color coded by language family with Vietnamese Austroasiatic in purple, Vietnamese Austronesian in red, Vietnamese Tai-Kadai in yellow, Vietnamese Hmong-Mien in black, Vietnamese Sino-Tibetan in lime, Cambodian Austronesian (Cham-CB-Bat and Cham-CB-Kam) in pink, Thai Austronesian in olive drab, Taiwanese Austronesian in blue, Philippine Austronesian in brown, Indonesian Austronesian in orange, and Malaysian Austronesian in turquoise. (PDF) [file pone.0304964.s005.pdf]
