# Supplementary material for: Investigating demic versus cultural diffusion and sex bias in the spread of Austronesian languages in Vietnam
Source: PLoS One. 2024 Jun 17;19(6):e0304964. doi: 10.1371/journal.pone.0304964 (PMC11182502; doi:10.1371/journal.pone.0304964)
Supplement: S1 Text — (DOCX) [file pone.0304964.s013.docx]

***Investigating demic versus cultural diffusion and sex bias in the spread of Austronesian languages in Vietnam***

***The spread of Austronesian languages in Vietnam***

Dinh Huong Thao^1^, Tran Huu Dinh^1^, Shigeki Mitsunaga^2^, La Duc Duy^1^, Nguyen Thanh Phuong^1,2^, Nguyen Phuong Anh^1^, Nguyen Tho Anh^1^, Bui Minh Duc^1^, Huynh Thi Thu Hue^1^, Nguyen Hai Ha^1^, Nguyen Dang Ton^1^, [Alexander Hübner](https://pubmed.ncbi.nlm.nih.gov/?term=H%C3%BCbner+A&cauthor_id=29483671)^3^, Brigitte Pakendorf^4^, Mark Stoneking^3,5*^, Ituro Inoue^2*^, Nguyen Thuy Duong^1*^ and Nong Van Hai^1*^

^1^Institute of Genome Research, Vietnam Academy of Science and Technology, 18 Hoang Quoc Viet, Cau Giay, Hanoi, Vietnam

^2^Division of Human Genetics, National Institute of Genetics, Shizuoka, 411-8540, Japan

^3^Department of Evolutionary Genetics, Max Planck Institute for Evolutionary Anthropology, Deutscher Platz 6, D04103 Leipzig, Germany

^4^Dynamique du Langage, UMR5596, CNRS & Université de Lyon, 69363 Lyon Cedex 07, France

^5^Laboratoire de Biométrie et Biologie Evolutive, Université Lyon 1, CNRS, UMR 5558, Villeurbanne 69622, France

**S1 Text**: Ethical approval and sampling procedure

In this note, we provide more details about the ethical approval and sampling procedure, as well as the criteria used to assess the ethnic affiliation of sample donors. After acquiring the ethical approval and authorization from the Institute of Genome Research, Vietnam Academy of Science and Technology, we sent the necessary legal documents and a copy of the study proposal, in which we included the objectives and relevant methods of the study, to the local authorities to obtain official permission to collect samples in their jurisdiction. The sampling criteria were also provided to the local/state authority, whose demographic database helped find individuals fulfilling the research criteria (all individuals born in Vietnam are required to register their ethnicity, and this information is kept in demographic databases). Once we received the authorization from the local/state authority, we travelled to the local villages and arranged with the local coordinators to recruit suitable study participants that met our criteria. At the sampling site, the involved communities were informed again about the study before giving their approval to take part in the study and donate their blood samples for genetic diversity and human history studies. We also interviewed the elders of the village, usually including the chief of the tribe, to confirm whether our gathered data were accurate and also to gain more insight into the history of the village/region/local people. Every participant was interviewed to fill in all the information in the sampling questionnaire and to re-assess the provided information. Upon completion of the research, we will prepare written reports summarizing the results using appropriate, non-technical language, and send these to the involved communities and the local/state authorities. To select appropriate participants for this study, we required three generations of the same ethnicity. This is a common requirement in such studies and reflects the fact that many (although certainly not all) people will reliably know the ethnicity, birthplace, and language(s) spoken by their parents and grandparents, as these are often people they have known. In our specific study, ethnicity (to three generations) was assessed not only by self-reporting during interviews, but also by official documents (as ethnicity is recorded on birth documents in Vietnam) and by talking with village elders (who, in our experience, are an excellent source of information about the ancestry of people residing in the village – they know who are the newcomers, vs. those whose parents and grandparents have always lived there). Furthermore, since minority languages and cultures are supported and valued, there are no particular incentives for individuals to claim a different ethnic identity than that of their parents and grandparents. We note that if, despite these precautions, there are nonetheless participants with misidentified ethnicity, then such individuals would be potential “outliers” with respect to the genetic ancestry of people who actually are of the specified ethnicity. We therefore utilized the genome-wide SNP data that we have for 170 individuals in this study and carried out a principle components analysis; practically no outliers were identified in the PCA plot. We also note that our results are consistent with historical information about the history of the populations analyzed, as discussed in the main text. While we cannot be sure that all individuals are of the specified ethnicity, we are confident that any such errors have little, if any, impact on our results and interpretations.
