# Supplementary material for: Investigating demic versus cultural diffusion and sex bias in the spread of Austronesian languages in Vietnam
Source: PLoS One. 2024 Jun 17;19(6):e0304964. doi: 10.1371/journal.pone.0304964 (PMC11182502; doi:10.1371/journal.pone.0304964)
Supplement: S2 Dataset — (PDF) [file pone.0304964.s015.pdf]

#Genotypes of 2088 SNPs for 170 newly genotyped individuals
>BANAN10
GTGTCCGGTAGCCCGCGCTAGTTAGACACCCCGGCAGGGGGGATTGCTTTCGAGACGGGAGATCCCTT
CGCCGACCCTGGAGGGCCGACGCCGAGGCATTCGGGCCCCTGCAACGTCAACAGCGGCAAGAAAACGG
GATGAATGGGCGTNATGGGGGGGGTCTGCTGGGGACCCGACGCGGTTGCCGTTTGCGGGGCCCCCGAC
CCATACCGACCCACCTAGGCGTCCAGTTACGGCGCACGTCGGGAGCGTGGTTGCCGTCAGAGCTGTGT
TTCTCGATCAGTCCCCCGCAGTGCCGCAGTATCTTGCCGTGGGCTGCTTTAATCTTGAAAGTGGTTTA
TACATTGGGCGACGAGGTGTCGACTCTCATTGGGGGTAACCGACGGGCACATGCAGTCCCCTCCCCGG
GCAGGCAGAGGCGGGGCCCCCGCGCGCCGGCCCCGGCCCACAATCTTACCAGGGTCCTCAAAGGAGCC
TTTGCATGGTACCCTTCGTGAATGGTTGCTTAAGAGGTCCACAACGTGGTCCGGGCACGGTCGACTAA
ACACTCAAAACAGCGACGGCAAATATAGGTACAAGGTCCAGGCCCTCACGGCACTAATTGCGATGACC
CAACTCACGGGGGCAGTCCCCGGCGCGGCGACCTTGATTACGTCCGGGGACAAGTATGTCGCTCCCCC
CGGGAGGGGTGCAGCCACATGGGAGATTCAAAGTTTCTCGTGACGTCGTTGTGATCACTGCAGCCTAG
CCGAGACTCCCGTACTACGCGAAGGTTGGTTATGTTAACCACTACAACGTGAGGCGCCGTAAGGGCCA
GTGTTGTGCCCGGCTCTTCAATGCGCCTTAGCGGCCTGATACACCCACCCAAGGAGATACTGCTAATC
ATATGGGTGGACAGAACCTCGCAACGCAGACGCCTCCCCGGCATGATATGGTTTTTTTCCGCTATTAT
CCAGCACGCAGCGCTATCATTCAAGAGAACCCAGTGACGCGTAAATCGTAAGATCTACCTGCCGCAGG
TGGACCTACTGCAAATACGGATTATGACTCGTAAAGGGGTCATGCGTATTTTCATCACTAGGCACGTT
CGAGAATAAATTAGTAGGTGTCCCGAGCCTTGTGGCGTTCCGCCTGACTCCTCATGAAGTCGACCTTC
TCACCGGCCCTATCTGCCGACGTAAGTCATAACCTAGATCTGTACCTCGGGGGGAGGGTCACTGTAAA
GGGATAATTGGAGGGTGATTTCCCACACTTTCCTAAGGGTACTTTTTGCCTGGCTTCGCAATTGGGTC
CAATGGATGTCGATCTCTGGTTTAGCAGTTGTGAAAGTGGCAAGGCGGGAGGTTAGACCTCCATTTAA
CATATACAAGCAAGTTAACTGCACTAGATGTGTAGACACTACAGTTACAGGAGTAGCCGAATAAGTCT
CCGACGTCAAGCGAATAAGCGTCATACGCGATTATCGCCTAAGAGCACGTATTGGCGGTAAAAGGCTA
GCTAGACGCTTATGGGTAGATTTCAAGGCGTTCGTAGTGGTATAATAGGATACTCTTTCACCAGCCTG
AGGGCCGAACGCTATACTAGTGGTCTGTGATGTAGGACCGAGTATCTCTCTAGGGACCATCTACTTGA
GCAATGGTGCGCAGGGGGAGACATAGACCAGCCTTGGGTGGCAAGCACTGCAATAAGTCCTGTTTAGC
CTTGGAGTTCACATGCCGGCACTGAAGCCGACCTACCTGAGCGTGTGCGATTACCGTTACAATGGCAT
CTGTCTAGTTCTGTTTACCTACGGCGCTCTTGGTTCCAGGTTAGGGGAAGTGTATGACCCATGTGTTT
TTATCGGCTTAACCACGAGTGATCCCCGGTCGTTTCCCCATTGAATCCCTGGTGCATCCTACTCCCAG
AATGATAGCTGACTGACTGGACTGGCTTTTCAAGTAATCGAGGGGGTATCGCGGTCACGGCCGTTAAC
AGATCCCGTCCTTAGTGTGGAATCCGCACCTGCTGACTAACGCTTCGCCGGCGTGTCTGCACAGCCGT
ATAGTGTTAATCATGACCCCAAGGAAGGATTAAACAAATATCTTGACG
>BANAN13
GTGTCCGGTAGCCCGCGCTAGTTAGACACCCCGGCAGGGGGGATTGCTTTCGAGACGGGAGATCCCTT
CGCCGACCCTGGAGGGCCGACGCCGAGGCATTCGGGCCCCTGCAACGTCAACAGCGGCAAGAAAACGG
GATGAATGGGCGTAATGGGGGGGGTCTGCTGGGGACCCGACGCGGTTGCCGTTTGCGGGGCCCCCGAC
CCATACCGACCCACCTAGGCGTCCAGTTACGGCGCACGGCGGGAGCGTGGTTGCCGTCAGAGCTGTGT
TTCTCGATCAGTCCCCCGCAGTGCCGCAGTATCTTGCCGTGGGCTGCTTTAATCTTGAAAGTGGTTTA
TACATTGGGCGACGAGGTGTCGACTCTCATTGGGGGTAACCGACGGGCACATGCAGTCCCCTCCCCGG
GCAGGCAGAGGCGGGGCCCCCGCGCGCCGGCCCCGGCCCACAATCTTACCAGGGTCCTCAAAGGAGCC
TTTGCATGGTACCCTTCGTGAATGGTTGCTTAAGAGGTCCACAACGTGGTCCGGGCACGGTCGACTAA
ACACTCAAAACAGCGACGGCAAATATAGGTACAAGGTCCAGGCCCTCACGGCACTAATTGCGATGACC
CAACTCACGGGGGCAGTCCCCGGCGCGGCGACCTTGATTACGTCCGGGGACAAGTATGTCGCTCCCCC
CGGGAGGGGTGCAGCCACATGGGAGATTCAAAGTTTCTCGTGACGTCGTTGTGATCACTGCAGCCTAG
CCGAGACTCCCGTACTACGCGAAGGTTGGTTATGTTAACCACTACAACGTGAGGCGCCGTAAGGGCCA
GTGTTGTGCCCGGCTCTTCAATGCGCCTTAGCGGCCTGATACACCCACCCAAGGAGATACTGCTAATC
ATATGGGTGGACAGAACCTCGCAACGCAGACGCCTCCCCGGCATGATATGGTTTTTTTCCGCTATTAT
CCAGCACGCAGCGCTATCATTCAAGAGAACCCAGTGACGCGTAAATCGTAAGATCTACCTGCCGCAGG
TGGACCTACTGCAAATACGGATTATGACTCGTAAAGGGGTCATGCGTATTTTCATCACTAGGCACGTT
CGAGAATAAATTAGTAGGTGTCCCGAGCCTTGTGGCGTTCCGCCTGACTCCTCATGAAGTCGACCTTC
TCACCGGCCCTATCTGCCGACGTAAGTCATAACCTAGATCTGTACCTCGGGGGGAGGGTCACTGTAAA
GGGATAATTGGAGGGTGATTTCCCACATTTTCCTAAGGGTACTTTTTGCCTGGCTTCGCAATTGGGTC
CAATGGATGTCGATCTCTGGTTTAGCAGTTGTGAAAGTGGCAAGGCGGGAGGTTAGACCTCCATTTAA

CATATACAAGCAAGTTAACTGCACTAGATGTGTAGACACTACAGTTACAGGAGTAGCCGAATAAGTCT
CCGACGTCAAGCGAATAAGCGTCATACGCGATTATCGCCTAAGAGCACGTATTGGCGGTAAAAGGCTA
GCTAGACGCTTGTGGGTAGATTTCAAGGCGTTCGTAGTGGTATAATAGGATACTCTTTCACCAGCCTG
AGGGCCGAACGCTATACTAGTGGTCTGTGATGTAGGACCGAGTATCTCTCTAGGGACCATCTACTTGA
GCAATGGTGCGCAGGGGGAGACATAGACCAGCCTTGGGTGGCAAGCACTGCAATAAGTCCTGTTTAGC
CTTGGAGTTCACACGCCGGCACTGAAGCCGACCTACCTGAGCGTGTGCGATTACCGTTACAATGGCAT
CTGTCTAGTTCTGTTTACCTACGGCGCTCTTGGTTCCAGGTTAGGGGAAGTGTATGACCCATGTGTTT
TTATCGGCTTAACCACGAGTGATCCCCGGTCGTTTCCCCATTGAATCCCTGGTGCATCCTACTCCCAG
AATGATAGCTGACTGACTGGACTGGCTTTTCAAGTAATCGAGGGGGTATCGCGGTCACGGCCGTTAAC
AGATCCCGTCCTTAGTGTGGAATCCGCACCTGCTGACTAACGCTTCGCCGGCGTGTCTGCACAGCCGT
ATAGTGTTAATCATGACCCCAAGGAAGGATTAAACAAATATCTTGACG
>BANAN14
GTGTCCGGTAGCCCGCGCTAGTTAGACACCCCGGCAGGGGGGATTGCTTTCGAGACGGGAGATCCCTT
CGCCGACCCTGGAGGGCCGACGCCGAGGCATTCGGGCCCCTGCAACGTCAACAGCGGCAAGAAAACGG
GATGAATGGGCGTAATGGGGGGGGTCTGCTGGGGACCCGACGCGGTTGCCGTTTGCGGGGCCCCCGAC
CCATACCGACCCACCTAGGCGTCCAGTTACGGCGCACGTCGGGAGCGTGGTTGCCGTCAGAGCTGTGT
TTCTCGATCAGTCCCCCGCAGTGCCGCAGTATCTTGCCGTGGGCTGCTTTAATCTTGAAAGTGGTTCA
TACATTGGGCGACGAGGTGTCGACTCTCATTGGGGGTAACCGACGGGCACATGCAGTCCCCTCCCCGG
GCAGGCAGAGGCGGGGCCCCCGCGCGCCGGCCCCGGCCCACAATCTTACCAGGGTCCTCAAAGGAGCC
TTTGCATGGTACCCTTCGTGAATGGTTGCTTAAGAGGTCCACCACGTGGTCCGGGCACGGTCGACTAA
ACACTCAAAACAGCGACGGCAAATATAGGTACAAGGTCCAGGCCCTCACGGCACTAATTGCGATGACC
CAACTCACGGGGGCAGTCCCCGGCGCGGCGACCTTGATTACGTCCGGGGACAAGTATGTCGTTCCCCC
CGGGAGGGGTGCAGCCACATGGGAGATTCAAAGTTTCTCGTGACGTCGTTGTGATCACTGCAGCCTAG
CCGAGACTCCCGTACTACGCGAAGGTTGGTTATGTTAACCACTACAACGTGAGGCGCCGTAAGGGCCA
GTGTTGTGCCCGGCTCTTCAATGCGCCTTAGCGGCCTGATACACCCACCCAAGGAGATACTGCTAATC
ATGTGGGTGGACAGAACCTCGCAACGCAGACGCCTCCCCGGCATGATATGGTTTTTTTCCGCTATTAT
CCAGCACGCAGCGCTATCATTCAAGAGAACCCAGTGACGCGTAAATCGTAAGATCTACCTGCCGCAGG
TGGACCTACTGCAAATACGGATTATGACTCGTAAAGAGGTTATNCGTATTTTCATCACTAGGCACGTT
CGAGAATAAATTAGTAGGTGTCCCGCGCCTTGTGGCGTTCCGCCTGACTCCTCATGAAGTCGACCTTC
TCACCGGCCCTATCTGCCGACGTAAGTCATAACCTAGATCTGTACCTCGGGGGGAGGGTCACTGTAAA
GGGATAATTGGAGGGTGATTTCCCACACTTTCCTAAGGGTACTTTTTGCCTGGCTTCGCAATTGGGTC
CAATGGATGTCGATCTCTGGTTTAGCAGTTGTGAAAGTGGCAAGGCGGGAGGTTAGACCTCCATTTAA
CATATACAAGCAAGTTAACTGCACTAGATGTGTAGACACTACAGTTACAGGAGTAGCCGAATAAGTCT
CCGACGTCAAGCGAATAAGCGTCATACGCGATTATCGCCTAAGAGCACGTATTGGCGGTAAAAGGCTA
GCTAGACGCTTGTGGGTAGATTTCAAGGCGTTCGTAGTGGTATAATAGGATACTCTTTCACCAGCCTG
AGGGCCGAACGCTATACTAGTGGTCTGTGATGTAGGACCGAGTATCTCTCTAGGGACCATCTACTTGA
GCAATGGTGCGCAGGGGGAGACATAGACCAGCCTTGGGTGGCAAGCACTGCAATAAGTCCTGTTTAGC
CTTGGAGTTCACACGCCGGCACTGAAGCCGACCTACCTGAGCGTGTGCGATTACCGTTACAATGGCAT
CTGTCTAGTTCTGTTTACCTACGGCGCTCTTGGTTCCAGGTTAGGGGAAGTGTATGACCCATGTGTTT
TTATCGGCTTAACCACGAGTGATCCCCGGTCGTTTCCCCATTGAATCCCTGGTGCATCCTACTCCCAG
AATGATAGCTGACTGACTGGACTGGCTTTTCAAGTAGTCGAGGGGGTATCGCGGTCACGGCCGTTAAC
AGATCCCGTCCTTAGTGTGGAATCCGCACCTGCTGACTAACGCTTCGCCGGCGTGTCTGCACAGCCGT
ATAGTGTTAATCATGACCCCAAGGAAGGATTAAACAAATATCTTGACG
>BANAN15
GTGTCCGGTAGCCCGCGCTAGTTAGACACCCCGGCAGGGGGGATTGCTTTCGGGACGGGAGATCCCTT
CGCCGACCCTGTAGGGCCGACGCCGAGGCATTCGGGCCCCTGCAACGTCAACAGCGGCAAGAAAACGG
GATGAATGGGCGTAATGGGGGGGGTCTGCTGGGGACCCGACGCGGTTGCCGTTTGCGGGGCCCCCGAC
CCATACCGACCCACCTAGGCGTCCAGTTACGGCGCACGTCGGGAGCGTGGTTGCCGTCAGAGCTGTGT
TTCTCGATCAGTCCCCCGCAGTGCCGCAGTATCTTGCCGTGGGCTGCTTTAATCTTGAAAGTGGTTCA
TACATTGGGCGACGAGGTGTCGGCTCTCATTGGGGGTAGCCGACGGACACATGCAGTCCCCTCCCCGG
GCAGGCAGAGGCGGGGCCCCCGCGCGCCGGCCCCGGCCCACAATCTTACCAGGGTCCTCAAAAGAGCC
TTTGCATGGTACCCTTCGTGAATGGTTGCTTAAGAGGTCCACCACGTAGTCCGGGCACGGTCGACTAA
ACACTCAAAACAGCGACGGCAAATAGAGGCACAAGGTCCAGGCCCTCACGGCACTAGTTGCGATGACC
CAACTCACGGGGGCAGTCCCCGGCGCGGCGACCTTGATTACGTCCGGGAACAAGTATGTCGTTTCCCC

CGGGAGGGGTGCAGCCACATGGGAGATTCAAAGTTTCTCGTGACGTCGTTGTGATCACTGCAGCCTAG
CCGAGACTCCCGTACTACGCGAAGGTTGGTTATGTTAACCACTACAACGTGAGGCGCCGTAAGGGCCA
GTGTTGTGCCCGGCTCTTCAATGCGCCTTAGCGGCCTGATACACCCACCCAAGGAGATACTGCTAATC
ATGTGGGTGGACAGAACCTCGCAACGCAGACGCATCCCCGGCATGATATGGTTTTTTTCCGCTATTAC
CCAGCACGCGGCGCTATCATTCAAGAGAACCCAGTGACGCGTAAATCGTAAGATCTACCTGCCGCAGG
TGGACCTACTGCAAATACGGATTATGACTCGTAAAGAGGTCATGCGTATTTTCATCACTAGGCACGTT
CGAGAGTAAATTAGTAGGTGTCCCGCGCCTTGTGGCGTTCCGCCTGACTCCTCATGAAGTCGACCTTC
TCATCGGCCCTATCTGCCGACGTAAGTCATAATCCAGATCTTCACCTCGGAGGGAGGGTCACTGTAAA
GGGATAATTGGAGGGCGATTTCCCACACTTTCCTAAGGGTACTTTTTGCTTGGCTTCGCAGTTGGGTC
CAATAGATGTTGATCTCTGGTTTAGCAGTTGTGAAAGTGGCAAGGCGGGAGGTTAGACCTCCATTTAA
CATATACAAGCAAGTTAACTGCACTAGATGTGTAGACACTACAGTTACAGGAGTAGCCGACTAAGTCT
CCGACGTCAAGCGAATAGGCGTCATACGCGATTATCGCCTAAGAGCACGTATTGGCGGTAAAAGGATA
ACTAGACGCTTGTGGGTAGATTTCAAGGCGCTCGTAGTGGTATAATAGGATACTCTTTCACCAGCCTG
AGAGCCGAACGCTATACTAGTGGTCTGTGATGTAGGACCAAGTAGCTCTCTAGGGACCATCTACTTGA
GCAATGGTGCGCAGGGGTAGACATAGACCAACCTTGGGTGGCAAGCACTGCAATAAGTCCTGTTTAGC
CTTGGAGTTCACACGCCGGCACTAAAGCCGACCTACCTGAGCTTGTGCGATTACCGTTACAATGGCAT
CTGTCTAGTTCTGTTTACCTACGGCGCTCTTGGTTCCATGTTAGGGGAAGTGTATGACCCATGTGTTT
TTATCGGCTTAACTACGAGTGATCCCCGGTCGTTTCCCCATTAAATCCCTGGTGCATCCTACTCCCAT
AATGATAGCTGACTGGCTGGACTGGCTTTTCAAGTAGTCGAGGGGGTATCGCGGTCACGGCCGTTAAC
AGATCCCGTCCTTAGTGTGGAATCCGCACCTGCTGACTAACGCTTCGCCGGCGTGTCTGCACATCCGT
ATAGTGTTAATCATGACCCCAAGGAAGGATTAAACAAATATCTTGACG
>BANAN18
GTGTCCGGTAGCCCGCGCTAGTCAGACACCCCGGCAGGGGGGATTGCTTTCGAGACGGGAGATCCCTT
CGCCGACCCTGGAGGGCCGACGCCGAGGCATTCGGGCCCCTGCAACGTCAACAGCGGCAAGAAAACGG
GATGAATGGGCGTAATGGGGGGGGTCTGCTGGGGACCCGACGCGGTTGCCGTTTGCGGGGCCCCCGAC
CCATACCGACCCACCTAGGCGTCCAGTTACGGCGCACGGCGGGAGCGTGGTTGCCGTCAGAGCTGTGT
TTCTCGATCAGTCCCCCGCAGTGCCGCAGTATCTTGCCGTGGGCTGCTTTAATCTTGAAAGTGGTTTA
TACATTGGGCGACGAGGTGTCGACTCTCATTGGGGGTAACCGACGGGCACATGCAGTCCCCTCCCCGG
GCAGGCAGAGGCGGGGCCCCCGCGCGCCGGCCCCGGCCCACAATCTTACCAGGGTCCTCAAAGGAGCC
TTTGCATGGTACCCTTCGTGAATGGTTGCTTAAGAGGTCCACAACGTGGTCCGGGCACGGTCGACTAA
ACACTCAAAACAGCGACGGCAAATATAGGTACAAGGTCCAGGCCCTCACGGCACTAATTGCGATGACC
CAACTCACGGGGGCAGTCCCCGGCGCGGCGACCTTGATTACGTCCGGGGACAAGTATGTCGCTCCCCC
CGGGAGGGGTGCAGCCACATGGGAGATTCAAAGTTTCTCGTGACGTCGTTGTGATCACTGCAGCCTAG
CCGAGACTCCCGTACTACGCGAAGGTTGGTTATGTTAACCACTACAACGTGAGGCGCCGTAAGGGCCA
GTGTTGTGCCCGGCTCTTCAATGCGCCTTAGCGGCCTGATACACCCACCCAAGGAGATACTGCTAATC
ATATGGGTGGACAGAACCTCGCAACGCAGACGCCTCCCCGGCATGATATGGTTTTTTTCCGCTATTAT
CCAGCACGCAGCGCTATCATTTAAGAGAACCCAGTGACGCGTAAATCGTAAGATCTACCTGCCGCAGG
TGGACCTACTGCAAATACGGATTATGACTCGTAAAGGGGTCATGCGTATTTTCATCACTAGGCACGTT
CGAGAATAAATTAGTAGGTGTCCCGAGCCTTGTGGCGTTCCGCCTGACTCCTCATGAAGTCGACCTTC
TCACCGGCCCTATCTGCCGACGTAAGTCATAACCTAGATCTGTACCTCGGGGGGAGGGTCACTGTAAA
GGGATAATTGGAGGGTGATTTCCCACATTTTCCTAAGGGTACTTTTTGCCTGGCTTCGCAATTGGGTC
CAATGGATGTCGATCTCTGGTTTAGCAGTTGTGAAAGTGGCAAGGCGGGAGGTTAGACCTCCATTTAA
CATATACAAGCAAGTTAACTGCACTAGATGTGTAGACACTACAGGTACAGGAGTAGCCGGATAAGTCT
CCGACGTCAAGCGAATAAGCGTCATACGCGATTATCGCCTAAGAGCACGTATTTGCGGTAAAAGGCTA
GCTAGACGCTTGTGGGTAGATTTCAAGGCGTTCGTAGTGGTATAATAGGATACTCTTTCACCAGCCTG
AGGGCCGAACGCTATACTAGTGGTCTGTGATGTAGGACCGAGTATCTCTCTAGGGACCATCTACTTGA
GCAATGGTGCGCAGGGGGAGACATAGACCAGCCTTGGGTGGCAAGCACTGCAATAAGTCCTGTTTAGC
CTTGGAGTTCACACGCCGGCACTGAAGCCGACCTACCTGAGCGTGTGCGATTACCGTTACAATGGCAT
CTGTCTAGTTCTGTTTACCTACGGCGCTCTTGGTTCCAGGTTAGGGGAAGTGTATGACCCATGTGTTT
TTATCGGCTTAACCACGAGTGATCCCCGGTCGTTTCCCCATTGAATCCCTGGTGCATCCTACTCCCAG
AATGATAGCTGACTGACTGGACTGGCTTTTCAAGTAATCGAGGGGGTATCGCGGTCACGGCCGTTAAC
AGATCCCGTCCTTAGTGTGGAATCCGCACCTGCTGACTAACGCTTCGCCGGCGTGTCTGCACAGCCGT
ATAGTGTTAGTCATGACCCCAAGGAAGGATTAAACAAATATCTTGACG
>BANAN19

GTGTCCGGTAGCCCGCGCTAGTCAGACACCCCGGCAGGGGGGATTGCTTTCGAGACGGGAGATCCCTT
CGCCGACCCTGGAGGGCCGACGCCGAGGCATTCGGGCCCCTGCAACGTCAACAGCGGCAAGAAAACGG
GATGAATGGGCGTAATGGGGGGGGTCTGCTGGGGACCCGACGCGGTTGCCGTTTGCGGGGCCCCCGAC
CCATACCGACCCACCTAGGCGTCCAGTTACGGCGCACGTCGGGAGCGTGGTTGCCGTCAGAGCTGTGT
TTCTCGATCAGTCCCCCGCAGTGCCGCAGTATCTTGCCGTGGGCTGCTTTAATCTTGAAAGTGGTTCA
TACATTGGGCGACGAGGTGTCGACTCTCATTGGGGGTAACCGACGGGCACATGCAGTCCCCTCCCCGG
GCAGGCAGAGGCGGGGCCCCCGCGCGCCGGCCCCGGCCCACAATCTTACCAGGGTCCTCAAAGGAGCC
TTTGCATGGTACCCTTCGTGAATGGTTGCTTAAGAGGTCCACAACGTGGTCCGGGCACGGTCGACTAA
ACACTCAAAACAGCGACGGCAAATATAGGTACAAGGTCCAGGCCCTCACGGCACTAATTGCGATGACC
CAACTCACGGGGGCAGTCCCCGGCGCGGCGACCTTGATTACGTCCGGGGACAAGTATGTCGCTCCCCC
CGGGAGGGGTGCAGCCACATGGGAGATTCAAAGTTTCTCGTGACGTCGTTGTGATCACTGCAGCCTAG
CCGAGACTCCCGTACTACGCGAAGGTTGGTTATGTTAACCACTACAACGTGAGGCGCCGTAAGGGCCA
GTGTTGTGCCCGGCTCTTCAATGCGCCTTAGCGGCCTGATACACCCACCCAAGGAGATACTGCTAATC
ATATGGGTGGACAGAACCTCGCAACGCAGACGCCTCCCCGGCATGATATGGTTTTTTTCCGCTATTAT
CCAGCACGCAGCGCTATCATTTAAGAGAACCCAGTGACGCGTAAATCGTAAGATCTACCTGCCGCAGG
TGGACCTACTGCAAATACGGATTATGACTCGTAAAGGGGTCATGCGTATTTTCATCACTAGGCACGTT
CGAGAATAAATTAGTAGGTGTCCCGAGCCTTGTGGCGTTCCGCCTGACTCCTCATGAAGTCGACCTTC
TCACCGGCCCTATCTGCCGACGTAAGTCATAACCTAGATCTGTACCTCGGGGGGAGGGTCACTGTAAA
GGGATAATTGGAGGGTGATTTCCCACATTTTCCTAAGGGTACTTTTTGCCTGGCTTCGCAATTGGGTC
CAATGGATGTCGATCTCTGGTTTAGCAGTTGTGAAAGTGGCAAGGCGGGAGGTTAGACCTCCATTTAA
CATATACAAGCAAGTTAACTGCACTAGATGTGTAGACACTACAGGTACAGGAGTAGCCGGATAAGTCT
CCGACGTCAAGCGAATAAGCGTCATACGCGATTATCGCCTAAGAGCACGTATTTGCGGTAAAAGGCTA
GCTAGACGCTTGTGGGTAGATTTCAAGGCGTTCGTAGTGGTATAATAGGATACTCTTTCACCAGCCTG
AGGGCCGAACGCTATACTAGTGGTCTGTGATGTAGGACCGAGTATCTCTCTAGGGACCATCTACTTGA
GCAATGGTGCGCAGGGGGAGACATAGACCAGCCTTGGGTGGCAAGCACTGCAATAAGTCCTGTTTAGC
CTTGGAGTTCACACGCCGGCACTGAAGCCGACCTACCTGAGCGTGTGCGATTACCGTTACAATGGCAT
CTGTCTAGTTCTGTTTACCTACGGCGCTCTTGGTTCCAGGTTAGGGGAAGTGTATGACCCATGTGTTT
TTATCGGCTTAACCACGAGTGATCCCCGGTCGTTTCCCCATTGAATCCCTGGTGCATCCTACTCCCAG
AATGATAGCTGACTGACTGGACTGGCTTTTCAAGTAATCGAGGGGGTATCGCGGTCACGGCCGTTAAC
AGATCCCGTCCTTAGTGTGGAATCCGCACCTGCTGACTAACGCTTCGCCGGCGTGTCTGCACAGCCGT
ATAGTGTTAGTCATGACCCCAAGGAAGGATTAAACAAATATCTTGACG
>BANAN20
GTGTCCGGTAGCCCGCGCTAGTTAGACACCCCGGCAGGGGGGATTGCTTTCGAGACGGGAGATCCCTT
CGCCGACCCTGGAGGGCCGACGCCGAGGCATTCGGGCCCCTGCAACGTCAACAGCGGCAAGAAAACGG
GATGAATGGGCGTAATGGGGGGGGTCTGCTGGGGACCCGACGCGGTTGCCGTTTGCGGGGCCCCCGAC
CCATACCGACCCACCTAGGCGTCCAGTTACGGCGCACGGCGGGAGCGTGGTTGCCGTCAGAGCTGTGT
TTCTCGATCAGTCCCCCGCAGTGCCGCAGTATCTTGCCGTGGGCTGCTTTAATCTTGAAAGTGGTTCA
TACATTGGGCGACGAGGTGTCGACTCTCATTGGGGGTAACCGACGGGCACATGCAGTCCCCTCCCCGG
GCAGGCAGAGGCGGGGCCCCCGCGCGCCGGCCCCGGCCCACAATCTTACCAGGGTCCTCAAAGGAGCC
TTTGCATGGTACCCTTCGTGAATGGTTGCTTAAGAGGTCCACAACGTGGTCCGGGCACGGTCGACTAA
ACACTCAAAACAGCGACGGCAAATATAGGTACAAGGTCCAGGCCCTCACGGCACTAATTGCGATGACC
CAACTCACGGGGGCAGTCCCCGGCGCGGCGACCTTGATTACGTCCGGGGACAAGTATGTCGCTCCCCC
CGGGAGGGGTGCAGCCACATGGGAGATTCAAAGTTTCTCGTGACGTCGTTGTGATCACTGCAGCCTAG
CCGAGACTCCCGTACTACGCGAAGGTTGGTTATGTTAACCACTACAACGTGAGGCGCCGTAAGGGCCA
GTGTTGTGCCCGGCTCTTCAATGCGCCTTAGCGGCCTGATACACCCACCCAAGGAGATACTGCTAATC
ATATGGGTGGACAGAACCTCGCAACGCAGACGCCTCCCCGGCATGATATGGTTTTTTTCCGCTATTAT
CCAGCACGCAGCGCTATCATTCAAGAGAACCCAGTGACGCGTAAATCGTAAGATCTACCTGCCGCAGG
TGGACCTACTGCAAATACGGATTATGACTCGTAAAGGGGTCATGCGTATTTTCATCACTAGGCACGTT
CGAGAATAAATTAGTAGGTGTCCCGAGCCTTGTGGCGTTCCGCCTGACTCCTCATGAAGTCGACCTTC
TCACCGGCCCTATCTGCCGACGTAAGTCATAACCTAGATCTGTACCTCGGGGGGAGGGTCACTGTAAA
GGGATAATTGGAGGGTGATTTCCCACACTTTCCTAAGGGTACTTTTTGCCTGGCTTCGCAATTGGGTC
CAATGGATGTCGATCTCTGGTTTAGCAGTTGTGAAAGTGGCAAGGCGGGAGGTTAGACCTCCATTTAA
CATATACAAGCAAGTTAACTGCACTAGATGTGTAGACACTACAGTTACAGGAGTAGCCGAATAAGTCT
CCGACGTCAAGCGAATAAGCGTCATACGCGATTATCGCCTAAGAGCACGTATTGGCGGTAAAAGGCTA

GCTAGACGCTTATGGGTAGATTTCAAGGCGTTCGTAGTGGTATAATAGGATACTCTTTCACCAGCCTG
AGGGCCGAACGCTATACTAGTGGTCTGTGATGTAGGACCGAGTATCTCTCTAGGGACCATCTACTTGA
GCAATGGTGCGCAGGGGGAGACATAGACCAGCCTTGGGTGGCAAGCACTGCAATAAGTCCTGTTTAGC
CTTGGAGTTCACATGCCGGCACTGAAGCCGACCTACCTGAGCGTGTGCGATTACCGTTACAATGGCAT
CTGTCTAGTTCTGTTTACCTACGGCGCTCTTGGTTCCAGGTTAGGGGAAGTGTATGACCCATGTGTTT
TTATCGGCTTAACCACGAGTGATCCCCGGTCGTTTCCCCATTGAATCCCTGGTGCATCCTACTCCCAG
AATGATAGCTGACTGACTGGACTGGCTTTTCAAGTAATCGAGGGGGTATCGCGGTCACGGCCGTTAAC
AGATCCCGTCCTTAGTGTGGAATCCGCACCTGCTGACTAACGCTTCGCCGGCGTGTCTGCACAGCCGT
ATAGTGTTAATCATGACCCCAAGGAAGGATTAAACAAATATCTTGACG
>BANAN21
GTGTCCGGTAGCCCGCGCTAGTTAGACACCCCGGCAGGGGGGATTGCTTTCGAGACGGGAGATCCCTT
CGCCGACCCTGGAGGGCCGACGCCGAGGCATTCGGGCCCCCGCAACGTCAACAGCGGCAAGAAAACGG
GATGAATGGGCGTAATGGGGGGGGTCTGCTGGGGACCCGACGCGGTTGCCGTTTGCGGGGCCCCCGAC
CCATACCGACCCACCTAGGCGTCCAGTTACGGCGCACGTCGGGAGCGTGGTTGCCGTCAGAGCTGTGT
TTCTCGATCAGTCCCCCGCAGTGCCGCAGTATCTTGCCGTGGGCTGCTTTAATCTTGAAAGTGGTTCA
TACATTGGGCGACGAGGTGTCGACTCTCATTGGGGGTAACCGACGGGCACATGCAGTCCCCTCCCCGG
GCAGGCAGAGGCGGGGCCCCCGCGCGCCGGCCCCGGCCCACAATCTTACCAGGGTCCTCAAAGGAGCC
TTTGCATGGTACCCTTCGTGAATGGTTGCTTAAGAGGTCCACAACGTGGTCCGGGCACGGTCGACTAA
ACACTCAAAACAGCGACGGCAAATATAGGTACAAGGTCCAGGCCCTCACGGCACTAATTGCGATGACC
CAACTCACGGGGGCAGTCCCCGGCGCGGCGACCTTGATTACGTCCGGGGACAAGTATGTCGCTCCCCC
CGGGAGGGGTGCAGCCACATGGGAGATTCAAAGTTTCTCGTGACGTCGTTGTGATCACTGCAGCCTAG
CCGAGACTCCCGTACTACGCGAAGGTTGGTTATGTTAACCACTACAACGTGAGGCGCCGTAAGGGCCA
GTGTTGTGCCCGGCTCTTCAATGCGCCTTAGCGGCCTGATACACCCACCCAAGGAGATACTGCTAATC
ATATGGGTGGACAGAACCTCGCAACGCAGACGCCTCCCCGGCATGATATGGTTTTTTTCCGCTATTAT
CCAGCACGCAGCGCTATCATTCAAGAGAACCCAGTGACGCGTAAATCGTAAGATCTACCTGCCGCAGG
TGGACCTACTGCAAATACGGATTATGACTCGTAAAGGGGTCATGCGTATTTTCATCACTAGGCACGTT
CGAGAATAAATTAGTAGGTGTCCCGAGCCTTGTGGCGTTCCGCCTGACTCCTCATGAAGTCGACCTTC
TCACCGGCCCTATCTGCCGACGTAAGTCATAACCTAGATCTGTACCTCGGGGGGAGGGTCACTGTAAA
GGGATAATTGGAGGGTGATTTCCCACATTTTCCTAAGGGTACTTTTTGCCTGGCTTCGCAATTGGGTC
CAATGGATGTCGATCTCTGGTTTAGCAGTTGTGAAAGTGGCAAGGCGGGAGGTTAGACCTCCATTTAA
CATATACAAGCAAGTTAACTGCACTAGATGTGTAGACACTACAGTTACAGGAGTAGCCGAATAAGTCT
CCGACGTCAAGCGAATAAGCGTCATACGCGATTATCGCCTAAGAGCACGTATTGGCGGTAAAAGGCTA
GCTAGACGCTTGTGGGTAGATTTCAAGGCGTTCGTAGTGGTATAATAGGATACTCTTTCACCAGCCTG
AGGGCCGAACGCTATACTAGTGGTCTGTGATGTAGGACCGAGTATCTCTCTAGGGACCATCTACTTGA
GCAATGGTGCGCAGGGGGAGACATAGACCAGCCTTGGGTGGCAAGCACTGCAATAAGTCCTGTTTAGC
CTTGGAGTTCACACGCCGGCACTGAAGCCGACCTACCTGAGCGTGTGCGATTACCGTTACAATGGCAT
CTGTCTAGTTCTGTTTACCTACGGCGCTCTTGGTTCCAGGTTAGGGGAAGTGTATGACCCATGTGTTT
TTATCGGCTTAACCACGAGTGATCCCCGGTCGTTTCCCCATTGAATCCCTGGTGCATCCTACTCCCAG
AATGATAGCTGACTGACTGGACTGGCTTTTCAAGTAATCGAGGGGGTATCGCGGTCACGGCCGTTAAC
AGATCCCGTCCTTAGTGTGGAATCCGCACCTGCTGACTAACGCTTCGCCGGCGTGTCTGCACAGCCGT
ATAGTGTTAATCATGACCCCAAGGAAGGATTAAACAAATATCTTGACG
>BANAN22
GTGTCCGGTAGCCCGCGCTAGTCAGACACCCCGGCAGGGGGGATTGCTTTCGAGACGGGAGATCCCTT
CGCCGACCCTGGAGGGCCGACGCCGAGGCATTCGGGCCCCCGCAACGTCAACAGCGGCAAGAAAACGG
GATGAATGGGCGTAATGGGGGGGGTCTGCTGGGGACCCGACGCGGTTGCCGTTTGCGGGGCCCCCGAC
CCATACCGACCCACCTAGGCGTCCAGTTACGGCGCACGGCGGGAGCGTGGTTGCCGTCAGAGCTGTGT
TTCTCGATCAGTCCCCCGCAGTGCCGCAGTATCTTGCCGTGGGCTGCTTTAATCTTGAAAGTGGTTCA
TACATTGGGCGACGAGGTGTCGACTCTCATTGGGGGTAACCGACGGGCACATGCAGTCCCCTCCCCGG
GCAGGCAGAGGCGGGGCCCCCGCGCGCCGGCCCCGGCCCACAATCTTACCAGGGTCCTCAAAGGAGCC
TTTGCATGGTACCCTTCGTGAATGGTTGCTTAAGAGGTCCACAACGTGGTCCGGGCACGGTCGACTAA
ACACTCAAAACAGCGACGGCAAATATAGGTACAAGGTCCAGGCCCTCACGGCACTAATTGCGATGACC
CAACTCACGGGGGCAGTCCCCGGCGCGGCGACCTTGATTACGTCCGGGGACAAGTATGTCGCTCCCCC
CGGGAGGGGTGCAGCCACATGGGAGATTCAAAGTTTCTCGTGACGTCGTTGTGATCACTGCAGCCTAG
CCGAGACTCCCGTACTACGCGAAGGTTGGTTATGTTAACCACTACAACGTGAGGCGCCGTAAGGGCCA

GTGTTGTGCCCGGCTCTTCAATGCGCCTTAGCGGCCTGATACACCCACCCAAGGAGATACTGCTAATC
ATATGGGTGGACAGAACCTCGCAACGCAGACGCCTCCCCGGCATGATATGGTTTTTTTCCGCTATTAT
CCAGCACGCAGCGCTATCATTTAAGAGAACCCAGTGACGCGTAAATCGTAAGATCTACCTGCCGCAGG
TGGACCTACTGCAAATACGGATTATGACTCGTAAAGGGGTCATGCGTATTTTCATCACTAGGCACGTT
CGAGAATAAATTAGTAGGTGTCCCGAGCCTTGTGGCGTTCCGCCTGACTCCTCATGAAGTCGACCTTC
TCACCGGCCCTATCTGCCGACGTAAGTCATAACCTAGATCTGTACCTCGGGGGGAGGGTCACTGTAAA
GGGATAATTGGAGGGTGATTTCCCACATTTTCCTAAGGGTACTTTTTGCCTGGCTTCGCAATTGGGTC
CAATGGATGTCGATCTCTGGTTTAGCAGTTGTGAAAGTGGCAAGGCGGGAGGTTAGACCTCCATTTAA
CATATACAAGCAAGTTAACTGCACTAGATGTGTAGACACTACAGGTACAGGAGTAGCCGGATAAGTCT
CCGACGTCAAGCGAATAAGCGTCATACGCGATTATCGCCTAAGAGCACGTATTTGCGGTAAAAGGCTA
GCTAGACGCTTGTGGGTAGATTTCAAGGCGTTCGTAGTGGTATAATAGGATACTCTTTCACCAGCCTG
AGGGCCGAACGCTATACTAGTGGTCTGTGATGTAGGACCGAGTATCTCTCTAGGGACCATCTACTTGA
GCAATGGTGCGCAGGGGGAGACATAGACCAGCCTTGGGTGGCAAGCACTGCAATAAGTCCTGTTTAGC
CTTGGAGTTCACACGCCGGCACTGAAGCCGACCTACCTGAGCGTGTGCGATTACCGTTACAATGGCAT
CTGTCTAGTTCTGTTTACCTACGGCGCTCTTGGTTCCAGGTTAGGGGAAGTGTATGACCCATGTGTTT
TTATCGGCTTAACCACGAGTGATCCCCGGTCGTTTCCCCATTGAATCCCTGGTGCATCCTACTCCCAG
AATGATAGCTGACTGACTGGACTGGCTTTTCAAGTAATCGAGGGGGTATCGCGGTCACGGCCGTTAAC
AGATCCCGTCCTTAGTGTGGAATCCGCACCTGCTGACTAACGCTTCGCCGGCGTGTCTGCACAGCCGT
ATAGTGTTAGTCATGACCCCAAGGAAGGATTAAACAAATATCTTGACG
>BANAN23
GTGTCCGGTAGCCCGCGCTAGTTAGACACCCCGGCAGGGGGGATTGCTTTCGAGACGGGAGATCCCTT
CGCCGACCCTGGAGGGCCGACGCCGAGGCATTCGGGCCCCTGCAACGTCAACAGCGGCAAGAAAACGG
GATGAATGGGCGTAATGGGGGGGGTCTGCTGGGGACCCGACGCGGTTGCCGTTTGCGGGGCCCCCGAC
CCATACCGACCCACCTAGGCGTCCAGTTACGGCGCACGTCGGGAGCGTGGTTGCCGTCAGAGCTGTGT
TTCTCGATCAGTCCCCCGCAGTGCCGCAGTATCTTGCCGTGGGCTGCTTTAATCTTGAAAGTGGTTTA
TACATTGGGCGACGAGGTGTCGACTCTCATTGGGGGTAACCGACGGGCACATGCAGTCCCCTCCCCGG
GCAGGCAGAGGCGGGGCCCCCGCGCGCCGGCCCCGGCCCACAATCTTACCAGGGTCCTCAAAGGAGCC
TTTGCATGGTACCCTTCGTGAATGGTTGCTTAAGAGGTCCACAACGTGGTCCGGGCACGGTCGACTAA
ACACTCAAAACAGCGACGGCAAATATAGGTACAAGGTCCAGGCCCTCACGGCACTAATTGCGATGACC
CAACTCACGGGGGCAGTCCCCGGCGCGGCGACCTTGATTACGTCCGGGGACAAGTATGTCGCTCCCCC
CGGGAGGGGTGCAGCCACATGGGAGATTCAAAGTTTCTCGTGACGTCGTTGTGATCACTGCAGCCTAG
CCGAGACTCCCGTACTACGCGAAGGTTGGTTATGTTAACCACTACAACGTGAGGCGCCGTAAGGGCCA
GTGTTGTGCCCGGCTCTTCAATGCGCCTTAGCGGCCTGATACACCCACCCAAGGAGATACTGCTAATC
ATATGGGTGGACAGAACCTCGCAACGCAGACGCCTCCCCGGCATGATATGGTTTTTTTCCGCTATTAT
CCAGCACGCAGCGCTATCATTCAAGAGAACCCAGTGACGCGTAAATCGTAAGATCTACCTGCCGCAGG
TGGACCTACTGCAAATACGGATTATGACTCGTAAAGGGGTCATGCGTATTTTCATCACTAGGCACGTT
CGAGAATAAATTAGTAGGTGTCCCGAGCCTTGTGGCGTTCCGCCTGACTCCTCATGAAGTCGACCTTC
TCACCGGCCCTATCTGCCGACGTAAGTCATAACCTAGATCTGTACCTCGGGGGGAGGGTCACTGTAAA
GGGATAATTGGAGGGTGATTTCCCACACTTTCCTAAGGGTACTTTTTGCCTGGCTTCGCAATTGGGTC
CAATGGATGTCGATCTCTGGTTTAGCAGTTGTGAAAGTGGCAAGGCGGGAGGTTAGACCTCCATTTAA
CATATACAAGCAAGTTAACTGCACTAGATGTGTAGACACTACAGTTACAGGAGTAGCCGAATAAGTCT
CCGACGTCAAGCGAATAAGCGTCATACGCGATTATCGCCTAAGAGCACGTATTGGCGGTAAAAGGCTA
GCTAGACGCTTATGGGTAGATTTCAAGGCGTTCGTAGTGGTATAATAGGATACTCTTTCACCAGCCTG
AGGGCCGAACGCTATACTAGTGGTCTGTGATGTAGGACCGAGTATCTCTCTAGGGACCATCTACTTGA
GCAATGGTGCGCAGGGGGAGACATAGACCAGCCTTGGGTGGCAAGCACTGCAATAAGTCCTGTTTAGC
CTTGGAGTTCACATGCCGGCACTGAAGCCGACCTACCTGAGCGTGTGCGATTACCGTTACAATGGCAT
CTGTCTAGTTCTGTTTACCTACGGCGCTCTTGGTTCCAGGTTAGGGGAAGTGTATGACCCATGTGTTT
TTATCGGCTTAACCACGAGTGATCCCCGGTCGTTTCCCCATTGAATCCCTGGTGCATCCTACTCCCAG
AATGATAGCTGACTGACTGGACTGGCTTTTCAAGTAATCGAGGGGGTATCGCGGTCACGGCCGTTAAC
AGATCCCGTCCTTAGTGTGGAATCCGCACCTGCTGACTAACGCTTCGCCGGCGTGTCTGCACAGCCGT
ATAGTGTTAATCATGACCCCAAGGAAGGATTAAACAAATATCTTGACG
>BANAN24
GTGTCCGGTAGCCCGCGCTAGTTAGACACCCCGGCAGGGGGGATTGCTTTCGGGACGGGAGATCCCTT
CGCCGACCCTGTAGGGCCGACGCCGAGGCATTCGGGCCCCCGCAACGTCAACAGCGGCAAGAAAACGG

GATGAATGGGCGTAATGGGGGGGGTCTGCTGGGGACCCGACGCGGTTGCCGTTTGCGGGGCCCCCGAC
CCATACCGACCCACCTAGGCGTCCAGTTACGGCGCACGTCGGGAGCGTGGTTGCCGTCAGAGCTGTGT
TTCTCGATCAGTCCCCCGCAGTGCCGCAGTATCTTGCCGTGGGCTGCTTTAATCTTGAAAGTGGTTCA
TACATTGGGCGACGAGGTGTCGGCTCTCATTGGGGGTAGCCGACGGACACATGCAGTCCCCTCCCCGG
GCAGGCAGAGGCGGGGCCCCCGCGCGCCGGCCCCGGCCCACAATCTTACCAGGGTCCTCAAAAGAGCC
TTTGCATGGTACCCTTCGTGAATGGTTGCTTAAGAGGTCCACCACGTAGTCCGGGCACGGTCGACTAA
ACACTCAAAACAGCGACGGCAAATAGAGGCACAAGGTCCAGGCCCTCACGGCACTAGTTGCGATGACC
CAACTCACGGGGGCAGTCCCCGGCGCGGCGACCTTGATTACGTCCGGGAACAAGTATGTCGTTTCCCC
CGGGAGGGGTGCAGCCACATGGGAGATTCAAAGTTTCTCGTGACGTCGTTGTGATCACTGCAGCCTAG
CCGAGACTCCCGTACTACGCGAAGGTTGGTTATGTTAACCACTACAACGTGAGGCGCCGTAAGGGCCA
GTGTTGTGCCCGGCTCTTCAATGCGCCTTAGCGGCCTGATACACCCACCCAAGGAGATACTGCTAATC
ATGTGGGTGGACAGAACCTCGCAACGCAGACGCATCCCCGGCATGATATGGTTTTTTTCCGCTATTAC
CCAGCACGCGGCGCTATCATTCAAGAGAACCCAGTGACGCGTAAATCGTAAGATCTACCTGCCGCAGG
TGGACCTACTGCAAATACGGATTATGACTCGTAAAGAGGTCATGCGTATTTTCATCACTAGGCACGTT
CGAGAGTAAATTAGTAGGTGTCCCGCGCCTTGTGGCGTTCCGCCTGACTCCTCATGAAGTCGACCTTC
TCATCGGCCCTATCTGCCGACGTAAGTCATAATCCAGATCTTCACCTCGGAGGGAGGGTCACTGTAAA
GGGATAATTGGAGGGCGATTTCCCACACTTTCCTAAGGGTACTTTTTGCTTGGCTTCGCAGTTGGGTC
CAATAGATGTTGATCTCTGGTTTAGCAGTTGTGAAAGTGGCAAGGCGGGAGGTTAGACCTCCATTTAA
CATATACAAGCAAGTTAACTGCACTAGATGTGTAGACACTACAGTTACAGGAGTAGCCGACTAAGTCT
CCGACGTCAAGCGAATAGGCGTCATACGCGATTATCGCCTAAGAGCACGTATTGGCGGTAAAAGGATA
ACTAGACGCTTGTGGGTAGATTTCAAGGCGCTCGTAGTGGTATAATAGGATACTCTTTCACCAGCCTG
AGAGCCGAACGCTATACTAGTGGTCTGTGATGTAGGACCAAGTAGCTCTCTAGGGACCATCTACTTGA
GCAATGGTGCGCAGGGGTAGACATAGACCAACCTTGGGTGGCAAGCACTGCAATAAGTCCTGTTTAGC
CTTGGAGTTCACACGCCGGCACTAAAGCCGACCTACCTGAGCTTGTGCGATTACCGTTACAATGGCAT
CTGTCTAGTTCTGTTTACCTACGGCGCTCTTGGTTCCATGTTAGGGGAAGTGTATGACCCATGTGTTT
TTATCGGCTTAACTACGAGTGATCCCCGGTCGTTTCCCCATTAAATCCCTGGTGCATCCTACTCCCAT
AATGATAGCTGACTGGCTGGACTGGCTTTTCAAGTAGTCGAGGGGGTATCGCGGTCACGGCCGTTAAC
AGATCCCGTCCTTAGTGTGGAATCCGCACCTGCTGACTAACGCTTCGCCGGCGTGTCTGCACATCCGT
ATAGTGTTAATCATGACCCCAAGGAAGGATTAAACAAATATCTTGACG
>BANAN25
GTGTCCGGTAGCCCGCGCTAGTTAGACACCCCGGCAGGGGGGATTGCTTTCGAGACGGGAGATCCCTT
CGCCGACCCTGGAGGGCCGACGCCGAGGCATTCGGGCCCCTGCAACGTCAACAGCGGCAAGAAAACGG
GATGAATGGGCGTAATGGGGGGGGTCTGCTGGGGACCCGACGCGGTTGCCGTTTGCGGGGCCCCCGAC
CCATACCGACCCACCTAGGCGTCCAGTTACGGCGCACGTCGGGAGCGTGGTTGCCGTCAGAGCTGTGT
TTCTCGATCAGTCCCCCGCAGTGCCGCAGTATCTTGCCGTGGGCTGCTTTAATCTTGAAAGTGGTTTA
TACATTGGGCGACGAGGTGTCGACTCTCATTGGGGGTAACCGACGGGCACATGCAGTCCCCTCCCCGG
GCAGGCAGAGGCGGGGCCCCCGCGCGCCGGCCCCGGCCCACAATCTTACCAGGGTCCTCAAAGGAGCC
TTTGCATGGTACCCTTCGTGAATGGTTGCTTAAGAGGTCCACAACGTGGTCCGGGCACGGTCGACTAA
ACACTCAAAACAGCGACGGCAAATATAGGTACAAGGTCCAGGCCCTCACGGCACTAATTGCGATGACC
CAACTCACGGGGGCAGTCCCCGGCGCGGCGACCTTGATTACGTCCGGGGACAAGTATGTCGCTCCCCC
CGGGAGGGGTGCAGCCACATGGGAGATTCAAAGTTTCTCGTGACGTCGTTGTGATCACTGCAGCCTAG
CCGAGACTCCCGTACTACGCGAAGGTTGGTTATGTTAACCACTACAACGTGAGGCGCCGTAAGGGCCA
GTGTTGTGCCCGGCTCTTCAATGCGCCTTAGCGGCCTGATACACCCACCCAAGGAGATACTGCTAATC
ATATGGGTGGACAGAACCTCGCAACGCAGACGCCTCCCCGGCATGATATGGTTTTTTTCCGCTATTAT
CCAGCACGCAGCGCTATCATTCAAGAGAACCCAGTGACGCGTAAATCGTAAGATCTACCTGCCGCAGG
TGGACCTACTGCAAATACGGATTATGACTCGTAAAGGGGTCATGCGTATTTTCATCACTAGGCACGTT
CGAGAATAAATTAGTAGGTGTCCCGAGCCTTGTGGCGTTCCGCCTGACTCCTCATGAAGTCGACCTTC
TCACCGGCCCTATCTGCCGACGTAAGTCATAACCTAGATCTGTACCTCGGGGGGAGGGTCACTGTAAA
GGGATAATTGGAGGGTGATTTCCCACACTTTCCTAAGGGTACTTTTTGCCTGGCTTCGCAATTGGGTC
CAATGGATGTCGATCTCTGGTTTAGCAGTTGTGAAAGTGGCAAGGCGGGAGGTTAGACCTCCATTTAA
CATATACAAGCAAGTTAACTGCACTAGATGTGTAGACACTACAGTTACAGGAGTAGCCGAATAAGTCT
CCGACGTCAAGCGAATAAGCGTCATACGCGATTATCGCCTAAGAGCACGTATTGGCGGTAAAAGGCTA
GCTAGACGCTTATGGGTAGATTTCAAGGCGTTCGTAGTGGTATAATAGGATACTCTTTCACCAGCCTG
AGGGCCGAACGCTATACTAGTGGTCTGTGATGTAGGACCGAGTATCTCTCTAGGGACCATCTACTTGA

GCAATGGTGCGCAGGGGGAGACATAGACCAGCCTTGGGTGGCAAGCACTGCAATAAGTCCTGTTTAGC
CTTGGAGTTCACATGCCGGCACTGAAGCCGACCTACCTGAGCGTGTGCGATTACCGTTACAATGGCAT
CTGTCTAGTTCTGTTTACCTACGGCGCTCTTGGTTCCAGGTTAGGGGAAGTGTATGACCCATGTGTTT
TTATCGGCTTAACCACGAGTGATCCCCGGTCGTTTCCCCATTGAATCCCTGGTGCATCCTACTCCCAG
AATGATAGCTGACTGACTGGACTGGCTTTTCAAGTAATCGAGGGGGTATCGCGGTCACGGCCGTTAAC
AGATCCCGTCCTTAGTGTGGAATCCGCACCTGCTGACTAACGCTTCGCCGGCGTGTCTGCACAGCCGT
ATAGTGTTAATCATGACCCCAAGGAAGGATTAAACAAATATCTTGACG
>BANAN26
GTGTCCGGTAGCCCGCGCTAGTTAGACACCCCGGCAGGGGGGATTGCTTTCGAGACGGGAGATCCCTT
CGCCGACCCTGGAGGGCCGACGCCGAGGCATTCGGGCCCCTGCAACGTCAACAGCGGCAAGAAAACGG
GATGAATGGGCGTAATGGGGGGGGTCTGCTGGGGACCCGACGCGGTTGCCGTTTGCGGGGCCCCCGAC
CCATACCGACCCACCTAGGCGTCCAGTTACGGCGCACGGCGGGAGCGTGGTTGCCGTCAGAGCTGTGT
TTCTCGATCAGTCCCCCGCAGTGCCGCAGTATCTTGCCGTGGGCTGCTTTAATCTTGAAAGTGGTTTA
TACATTGGGCGACGAGGTGTCGACTCTCATTGGGGGTAACCGACGGGCACATGCAGTCCCCTCCCCGG
GCAGGCAGAGGCGGGGCCCCCGCGCGCCGGCCCCGGCCCACAATCTTACCAGGGTCCTCANAGGAGCC
TTTGCATGGTACCCTTCGTGAATGGTTGCTTAAGAGGTCCACAACGTGGTCCGGGCACGGTCGACTAA
ACACTCAAAACAGCGACGGCAAATATAGGTACAAGGTCCAGGCCCTCACGGCACTAATTGCGATGACC
CAACTCACGGGGGCAGTCCCCGGCGCGGCGACCTTGATTACGTCCGGGGACAAGTATGTCGCTCCCCC
CGGGAGGGGTGCAGCCACATGGGAGATTCAAAGTTTCTCGTGACGTCGTTGTGATCACTGCAGCCTAG
CCGAGACTCCCGTACTACGCGAAGGTTGGTTATGTTAACCACTACAACGTGAGGCGCCGTAAGGGCCA
GTGTTGTGCCCGGCTCTTCAATGCGCCTTAGCGGCCTGATACACCCACCCAAGGAGATACTGCTAATC
ATATGGGTGGACAGAACCTCGCAACGCAGACGCCTCCCCGGCATGATATGGTTTTTTTCCGCTATTAT
CCAGCACGCAGCGCTATCATTCAAGAGAACCCAGTGACGCGTAAATCGTAAGATCTACCTGCCGCAGG
TGGACCTACTGCAAATACGGATTATGACTCGTAAAGGGGTCATGCGTATTTTCATCACTAGGCACGTT
CGAGAATAAATTAGTAGGTGTCCCGAGCCTTGTGGCGTTCCGCCTGACTCCTCATGAAGTCGACCTTC
TCACCGGCCCTATCTGCCGACGTAAGTCATAACCTAGATCTGTACCTCGGGGGGAGGGTCACTGTAAA
GGGATAATTGGAGGGTGATTTCCCACACTTTCCTAAGGGTACTTTTTGCCTGGCTTCGCAATTGGGTC
CAATGGATGTCGATCTCTGGTTTAGCAGTTGTGAAAGTGGCAAGGCGGGAGGTTAGACCTCCATTTAA
CATATACAAGCAAGTTAACTGCACTAGATGTGTAGACACTACAGTTACAGGAGTAGCCGAATAAGTCT
CCGACGTCAAGCGAATAAGCGTCATACGCGATTATCGCCTAAGAGCACGTATTGGCGGTAAAAGGCTA
GCTAGACGCTTATGGGTAGATTTCAAGGCGTTCGTAGTGGTATAATAGGATACTCTTTCACCAGCCTG
AGGGCCGAACGCTATACTAGTGGTCTGTGATGTAGGACCGAGTATCTCTCTAGGGACCATCTACTTGA
GCAATGGTGCGCAGGGGGAGACATAGACCAGCCTTGGGTGGCAAGCACTGCAATAAGTCCTGTTTAGC
CTTGGAGTTCACATGCCGGCACTGAAGCCGACCTACCTGAGCGTGTGCGATTACCGTTACAATGGCAT
CTGTCTAGTTCTGTTTACCTACGGCGCTCTTGGTTCCAGGTTAGGGGAAGTGTATGACCCATGTGTTT
TTATCGGCTTAACCACGAGTGATCCCCGGTCGTTTCCCCATTGAATCCCTGGTGCATCCTACTCCCAG
AATGATAGCTGACTGACTGGACTGGCTTTTCAAGTAATCGAGGGGGTATCGCGGTCACGGCCGTTAAC
AGATCCCGTCCTTAGTGTGGAATCCGCACCTGCTGACTAACGCTTCGCCGGCGTGTCTGCACAGCCGT
ATAGTGTTAATCATGACCCCAAGGAAGGATTAAACAAATATCTTGACG
>BANAN27
GTGTCCGGTAGCCCGCGCTAGTTAGACACCCCGGCAGGGGGGATTGCTTTCGAGACGGGAGATCCCTT
CGCCGACCCTGGAGGGCCGACGCCGAGGCATTCGGGCCCCTGCAACGTCAACAGCGGCAAGAAAACGG
GATGAATGGGCGTAATGGGGGGGGTCTGCTGGGGACCCGACGCGGTTGCCGTTTGCGGGGCCCCCGAC
CCATACCGACCCACCTAGGCGTCCAGTTACGGCGCACGTCGGGAGCGTGGTTGCCGTCAGAGCTGTGT
TTCTCGATCAGTCCCCCGCAGTGCCGCAGTATCTTGCCGTGGGCTGCTTTAATCTTGAAAGTGGTTTA
TACATTGGGCGACGAGGTGTCGACTCTCATTGGGGGTAACCGACGGGCACATGCAGTCCCCTCCCCGG
GCAGGCAGAGGCGGGGCCCCCGCGCGCCGGCCCCGGCCCACAATCTTACCAGGGTCCTCAAAGGAGCC
TTTGCATGGTACCCTTCGTGAATGGTTGCTTAAGAGGTCCACAACGTGGTCCGGGCACGGTCGACTAA
ACACTCAAAACAGCGACGGCAAATATAGGTACAAGGTCCAGGCCCTCACGGCACTAATTGCGATGACC
CAACTCACGGGGGCAGTCCCCGGCGCGGCGACCTTGATTACGTCCGGGGACAAGTATGTCGCTCCCCC
CGGGAGGGGTGCAGCCACATGGGAGATTCAAAGTTTCTCGTGACGTCGTTGTGATCACTGCAGCCTAG
CCGAGACTCCCGTACTACGCGAAGGTTGGTTATGTTAACCACTACAACGTGAGGCGCCGTAAGGGCCA
GTGTTGTGCCCGGCTCTTCAATGCGCCTTAGCGGCCTGATACACCCACCCAAGGAGATACTGCTAATC
ATATGGGTGGACAGAACCTCGCAACGCAGACGCCTCCCCGGCATGATATGGTTTTTTTCCGCTATTAT

CCAGCACGCAGCGCTATCATTCAAGAGAACCCAGTGACGCGTAAATCGTAAGATCTACCTGCCGCAGG
TGGACCTACTGCAAATACGGATTATGACTCGTAAAGGGGTCATGCGTATTTTCATCACTAGGCACGTT
CGAGAATAAATTAGTAGGTGTCCCGAGCCTTGTGGCGTTCCGCCTGACTCCTCATGAAGTCGACCTTC
TCACCGGCCCTATCTGCCGACGTAAGTCATAACCTAGATCTGTACCTCGGGGGGAGGGTCACTGTAAA
GGGATAATTGGAGGGTGATTTCCCACACTTTCCTAAGGGTACTTTTTGCCTGGCTTCGCAATTGGGTC
CAATGGATGTCGATCTCTGGTTTAGCAGTTGTGAAAGTGGCAAGGCGGGAGGTTAGACCTCCATTTAA
CATATACAAGCAAGTTAACTGCACTAGATGTGTAGACACTACAGTTACAGGAGTAGCCGAATAAGTCT
CCGACGTCAAGCGAATAAGCGTCATACGCGATTATCGCCTAAGAGCACGTATTGGCGGTAAAAGGCTA
GCTAGACGCTTATGGGTAGATTTCAAGGCGTTCGTAGTGGTATAATAGGATACTCTTTCACCAGCCTG
AGGGCCGAACGCTATACTAGTGGTCTGTGATGTAGGACCGAGTATCTCTCTAGGGACCATCTACTTGA
GCAATGGTGCGCAGGGGGAGACATAGACCAGCCTTGGGTGGCAAGCACTGCAATAAGTCCTGTTTAGC
CTTGGAGTTCACATGCCGGCACTGAAGCCGACCTACCTGAGCGTGTGCGATTACCGTTACAATGGCAT
CTGTCTAGTTCTGTTTACCTACGGCGCTCTTGGTTCCAGGTTAGGGGAAGTGTATGACCCATGTGTTT
TTATCGGCTTAACCACGAGTGATCCCCGGTCGTTTCCCCATTGAATCCCTGGTGCATCCTACTCCCAG
AATGATAGCTGACTGACTGGACTGGCTTTTCAAGTAATCGAGGGGGTATCGCGGTCACGGCCGTTAAC
AGATCCCGTCCTTAGTGTGGAATCCGCACCTGCTGACTAACGCTTCGCCGGCGTGTCTGCACAGCCGT
ATAGTGTTAATCATGACCCCAAGGAAGGATTAAACAAATATCTTGACG
>BANAN28
GTGTCCGGTAGCCCGCGCTAGTTAGACACCCCGGCAGGGGGGATTGCTTTCGAGACGGGAGATCCCTT
CGCCGACCCTGGAGGGCCGACGCCGAGGCATTCGGGCCCCTGCAACGTCAACAGCGGCAAGAAAACGG
GATGAATGGGCGTAATGGGGGGGGTCTGCTGGGGACCCGACGCGGTTGCCGTTTGCGGGGCCCCCGAC
CCATACCGACCCACCTAGGCGTCCAGTTACGGCGCACGTCGGGAGCGTGGTTGCCGTCAGAGCTGTGT
TTCTCGATCAGTCCCCCGCAGTGCCGCAGTATCTTGCCGTGGGCTGCTTTAATCTTGAAAGTGGTTTA
TACATTGGGCGACGAGGTGTCGACTCTCATTGGGGGTAACCGACGGGCACATGCAGTCCCCTCCCCGG
GCAGGCAGAGGCGGGGCCCCCGCGCGCCGGCCCCGGCCCACAATCTTACCAGGGTCCTCAAAGGAGCC
TTTGCATGGTACCCTTCGTGAATGGTTGCTTAAGAGGTCCACAACGTGGTCCGGGCACGGTCGACTAA
ACACTCAAAACAGCGACGGCAAATATAGGTACAAGGTCCAGGCCCTCACGGCACTAATTGCGATGACC
CAACTCACGGGGGCAGTCCCCGGCGCGGCGACCTTGATTACGTCCGGGGACAAGTATGTCGCTCCCCC
CGGGAGGGGTGCAGCCACATGGGAGATTCAAAGTTTCTCGTGACGTCGTTGTGATCACTGCAGCCTAG
CCGAGACTCCCGTACTACGCGAAGGTTGGTTATGTTAACCACTACAACGTGAGGCGCCGTAAGGGCCA
GTGTTGTGCCCGGCTCTTCAATGCGCCTTAGCGGCCTGATACACCCACCCAAGGAGATACTGCTAATC
ATATGGGTGGACAGAACCTCGCAACGCAGACGCCTCCCCGGCATGATATGGTTTTTTTCCGCTATTAT
CCAGCACGCAGCGCTATCATTCAAGAGAACCCAGTGACGCGTAAATCGTAAGATCTACCTGCCGCAGG
TGGACCTACTGCAAATACGGATTATGACTCGTAAAGGGGTCATGCGTATTTTCATCACTAGGCACGTT
CGAGAATAAATTAGTAGGTGTCCCGAGCCTTGTGGCGTTCCGCCTGACTCCTCATGAAGTCGACCTTC
TCACCGGCCCTATCTGCCGACGTAAGTCATAACCTAGATCTGTACCTCGGGGGGAGGGTCACTGTAAA
GGGATAATTGGAGGGTGATTTCCCACACTTTCCTAAGGGTACTTTTTGCCTGGCTTCGCAATTGGGTC
CAATGGATGTCGATCTCTGGTTTAGCAGTTGTGAAAGTGGCAAGGCGGGAGGTTAGACCTCCATTTAA
CATATACAAGCAAGTTAACTGCACTAGATGTGTAGACACTACAGTTACAGGAGTAGCCGAATAAGTCT
CCGACGTCAAGCGAATAAGCGTCATACGCGATTATCGCCTAAGAGCACGTATTGGCGGTAAAAGGCTA
GCTAGACGCTTATGGGTAGATTTCAAGGCGTTCGTAGTGGTATAATAGGATACTCTTTCACCAGCCTG
AGGGCCGAACGCTATACTAGTGGTCTGTGATGTAGGACCGAGTATCTCTCTAGGGACCATCTACTTGA
GCAATGGTGCGCAGGGGGAGACATAGACCAGCCTTGGGTGGCAAGCACTGCAATAAGTCCTGTTTAGC
CTTGGAGTTCACATGCCGGCACTGAAGCCGACCTACCTGAGCGTGTGCGATTACCGTTACAATGGCAT
CTGTCTAGTTCTGTTTACCTACGGCGCTCTTGGTTCCAGGTTAGGGGAAGTGTATGACCCATGTGTTT
TTATCGGCTTAACCACGAGTGATCCCCGGTCGTTTCCCCATTGAATCCCTGGTGCATCCTACTCCCAG
AATGATAGCTGACTGACTGGACTGGCTTTTCAAGTAATCGAGGGGGTATCGCGGTCACGGCCGTTAAC
AGATCCCGTCCTTAGTGTGGAATCCGCACCTGCTGACTAACGCTTCGCCGGCGTGTCTGCACAGCCGT
ATAGTGTTAATCATGACCCCAAGGAAGGATTAAACAAATATCTTGACG
>BANAN29
GTGTCCGGTAGCCCGCGCTAGTTAGACACCCCGGCAGGGGGGATTGCTTTCGAGACGGGAGATCCCTT
CGCCGACCCTGTAGGGCCGACGCCGAGGCATTCGGGCCCCCGCAACGTCAACAGCGGCAAGAAAACGG
GATGAATGGGCGTAATGGGGGGGGTCTGCTGGGGACCCGACGCGGTTGCCGTTTGCGGGGCCCCCGAC
CCATACCGACCCACCTAGGCGTCCAGTTACGGCGCACGTCGGGAGCGTGGTTGCCGTCAGAGCTGTGT

TTCTCGATCAGTCCCCCGCAGTGCCGCAGTATCTTGCCGTGGGCTGCTTTAATCTTGAAAGTGGTTTA
TACATTGGGCGACGAGGTGTCGGCTCTCATTGGGGGTAACCGACGGACACGTGCAGTCCCCTCCCCGG
GCAGGCAGAGGCGGGGCCCCCGCGCGCCGGCCCCGGCCCACAATCTTACCAGGGTCCTCAAAAGAGCC
TTTGCATGGTACCCTTCGTGAATGGTTGCTTAAGAGGTCCACCACGTAGTCCGGGCACGGTCAACTAA
ACACTCAAAACAGCGACGGCAAATAGAGGCACAAGGTCCAGGCCCTCACGGCACTAGTTGCGATGACC
CAACTCACGGGGGCAGTCCCCGGCGCAGCGACCTTGATTACGTCCGGGAACAAGTATGTCGTTTCCCC
CGGGAGGGGTGCAGCCACATGGGAGATTCAAAGTTTCTCGTGACGTCGTTGTGATCACTGCAGCCTAG
CCGAGACTCCCGTACTACGCGAAGGTTGGTTATGTTAACCACTACAACGTGAGGCGCCGTAAGGGCCA
GTGTTGTGCCCGGCTCTTCAATGCGCCTTAGCGGCCTGATACACCCACCCAAGGAGATACTGCTAATC
ATGTGGGTGGACAGAACCTCGCAACGCAGACGCATCCCCGGCATGATATGGTTTTTTTCCGCTATTAC
CCAGCACGCGGCGCTATCATTCAAGAGAACCCAGTGACGCGTAAATCGTCAGATCTACCTGCCGCAGG
TGGACCTACTGCAAATACGGATTATGACTCGTAAAGAGGTCATGCGTATTTTCATCACTAGGCACTTT
CGAGAGTAAATTAGTAGGTGTCCCGCGCCTTGTGGCGTTCCGCCTGGCTCCTCATGAAGTCGACCTTC
TCATCGGCCCTATTTGCCGACGTAAGTCATAATCCAGATCTTCACCTCGGAGGGAGGGTCACTGTAAA
GGGATAATTGGAGGGCGATTTCCCACACTTTCCTAAGGGTACTTTTTGCTTAGCTTCGCAGTTGGGTC
CAATAGATGTTGATCTCTGGTTTAGCAGTTGTGAAAGTGGCAAGGCGGGAGGTTAGGCCTCCATTTAA
CATATACAAGCAAGTTAACTGCACTAGATGTGTAGACACTACAGTTACAGGAGTAGCCGACTAAGTCT
CCGACGTCAAGCGAATAGGCGTCATACGCGATTATTGCCTAAGAGCACGTATTGGCGGTAAAAGGATA
ACTAGACGCTTGTGGGTAGATTTCAAGGCGCTCGTAGTGGTATAATAGGACACTCTTGCACCAGCCTG
AGAGCCGAACGCTATACTAGTGGTCTGTGATGTAGGACCAAGTAGCTCTCTAGGGACCATCTACTTGA
GCAATGGTGCGCAGGGGTAGACATAGACCAACCTTGGGTGGCAAGCACTGCAATAAGTCCTGTTTAGC
CTTGGAGTTCACACGCCGGCACTAAAGCCGACCTACCTGAGCTTGTGCGATTACCGTTACAATGGCAT
CTGTCTAGTTCTGTTTACCTACGGCGCTCTTGGTTCCATGTTAGGGGAAGTGTATGACCCATGTGTTT
TTATCGGCTTAACTACGAGTGATCCCCGGTCGTTTCCCCATTAAATCCCTGGTGCATCCTACTCCCAT
AATGATAGCTGACTGGCTGGACTGGCTTTTCAAGTAGTCGAGGGGGTATCGCGGTCACGGCCGTTAAC
AGATCCCGTCCTTAGTGTGGAATCCGCACCTGCTGACTAACGCTTCGCCGGCGTGTCTGCACATCCGT
ATAGTGTTAATCATGACCCCAAGGAAGGATTAAACAAATATCTTGACG
>BANAN30
GTGTCCGGTAGCCCGCGCTAGTTAGACACCCCGGCAGGGGGGATTGCTTTCGGGACGGGAGATCCCTT
CGCCGACCCTGGAGGGCCGACGCCGAGGCATTCGGGCCCCCGCAACGTCAACAGCGGCAAGAAAACGG
GATGAATGGGCGTAATGGGGGGGGTCTGCTGGGGACCCGACGCGGTTGCCGTTTGCGGGGCCCCCGAC
CCATACCGACCCACCTAGGCGTCCAGTTACGGCGCACGGCGGGAGCGTGGTTGCCGTCAGAGCTGTGT
TTCTCGATCAGTCCCCCGCAGTGCCGCAGTATCTTGCCGTGGGCTGCTTTAATCTTGAAAGTGGTTNA
TACATTGGGCGACGAGGTGTCGACTCTCATTGGGGGTAACCGACGGGCACATGCAGTCCCCTCCCCGG
GCAGGCAGAGGCGGGGCCCCCGCGCGCCGGCCCCGGCCCACAATCTTACCAGGGTCCTCAAAGGAGCC
TTTGCATGGTACCCTTCGTGAATGGTTGCTTAAGAGGTCCACAACGTGGTCCGGGCACGGTCGACTAA
ACACTCAAAACAGCGACGGCAAATATAGGTACAAGGTCCAGGCCCTCACGGCACTAATTGCGATGACC
CAACTCACGGGGGCAGTCCCCGGCGCGGCGACCTTGATTACGTCCGGGGACAAGTATGTCGCTCCCCC
CGGGAGGGGTGCAGCCACATGGGAGATTCAAAGTTTCTCGTGACGTCGTTGTGATCACTGCAGCCTAG
CCGAGACTCCCGTACTACGCGAAGGTTGGTTATGTTAACCACTACAACGTGAGGCGCCGTAAGGGCCA
GTGTTGTGCCCGGCTCTTCAATGCGCCTTAGCGGCCTGATACACCCACCCAAGGAGATACTGCTAATC
ATATGGGTGGACAGAACCTCGCAACGCAGACGCCTCCCCGGCATGATATGGTTTTTTTCCGCTATTAT
CCAGCACGCAGCGCTATCATTCAAGAGAACCCAGTGACGCGTAAATCGTAAGATCTACCTGCCGCAGG
TGGACCTACTGCAAATACGGATTATGACTCGTAAAGGGGTCATGCGTATTTTCATCACTAGGCACGTT
CGAGAATAAATTAGTAGGTGTCCCGAGCCTTGTGGCGTTCCGCCTGACTCCTCATGAAGTCGACCTTC
TCACCGGCCCTATCTGCCGACGTAAGTCATAACCTAGATCTGTACCTCGGGGGGAGGGTCACTGTAAA
GGGATAATTGGAGGGTGATTTCCCACACTTTCCTAAGGGTACTTTTTGCCTGGCTTCGCAATTGGGTC
CAATGGATGTCGATCTCTGGTTTAGCAGTTGTGAAAGTGGCAAGGCGGGAGGTTAGACCTCCATTTAA
CATATACAAGCAAGTTAACTGCACTAGATGTGTAGACACTACAGTTACAGGAGTAGCCGAATAAGTCT
CCGACGTCAAGCGAATAAGCGTCATACGCGATTATCGCCTAAGAGCACGTATTGGCGGTAAAAGGCTA
GCTAGACGCTTATGGGTAGATTTCAAGGCGTTCGTAGTGGTATAATAGGATACTCTTTCACCAGCCTG
AGGGCCGAACGCTATACTAGTGGTCTGTGATGTAGGACCGAGTATCTCTCTAGGGACCATCTACTTGA
GCAATGGTGCGCAGGGGGAGACATAGACCAGCCTTGGGTGGCAAGCACTGCAATAAGTCCTGTTTAGC
CTTGGAGTTCACATGCCGGCACTGAAGCCGACCTACCTGAGCGTGTGCGATTACCGTTACAATGGCAT

CTGTCTAGTTCTGTTTACCTACGGCGCTCTTGGTTCCAGGTTAGGGGAAGTGTATGACCCATGTGTTT
TTATCGGCTTAACCACGAGTGATCCCCGGTCGTTTCCCCATTGAATCCCTGGTGCATCCTACTCCCAG
AATGATAGCTGACTGACTGGACTGGCTTTTCAAGTAATCGAGGGGGTATCGCGGTCACGGCCGTTAAC
AGATCCCGTCCTTAGTGTGGAATCCGCACCTGCTGACTAACGCTTCGCCGGCGTGTCTGCACAGCCGT
ATAGTGTTAATCATGACCCCAAGGAAGGATTAAACAAATATCTTGACG
>BANAN31
GTGTCCGGTAGCCCGCGCTAGTTAGACACCCCGGCAGGGGGGATTGCTTTCGAGACGGGAGATCCCTT
CGCCGACCCTGGAGGGCCGACGCCGAGGCATTCGGGCCCCCGCAACGTCAACAGCGGCAAGAAAACGG
GATGAATGGGCGTAATGGGGGGGGTCTGCTGGGGACCCGACGCGGTTGCCGTTTGCGGGGCCCCCGAC
CCATACCGACCCACCTAGGCGTCCAGTTACGGCGCACGGCGGGAGCGTGGTTGCCGTCAGAGCTGTGT
TTCTCGATCAGTCCCCCGCAGTGCCGCAGTATCTTGCCGTGGGCTGCTTTAATCTTGAAAGTGGTTCA
TACATTGGGCGACGAGGTGTCGACTCTCATTGGGGGTAACCGACGGGCACATGCAGTCCCCTCCCCGG
GCAGGCAGAGGCGGGGCCCCCGCGCGCCGGCCCCGGCCCACAATCTTACCAGGGTCCTCAAAGGAGCC
TTTGCATGGTACCCTTCGTGAATGGTTGCTTAAGAGGTCCACAACGTGGTCCGGGCACGGTCGACTAA
ACACTCAAAACAGCGACGGCAAATATAGGTACAAGGTCCAGGCCCTCACGGCACTAATTGCGATGACC
CAACTCACGGGGGCAGTCCCCGGCGCGGCGACCTTGATTACGTCCGGGGACAAGTATGTCGCTCCCCC
CGGGAGGGGTGCAGCCACATGGGAGATTCAAAGTTTCTCGTGACGTCGTTGTGATCACTGCAGCCTAG
CCGAGACTCCCGTACTACGCGAAGGTTGGTTATGTTAACCACTACAACGTGAGGCGCCGTAAGGGCCA
GTGTTGTGCCCGGCTCTTCAATGCGCCTTAGCGGCCTGATACACCCACCCAAGGAGATACTGCTAATC
ATATGGGTGGACAGAACCTCGCAACGCAGACGCCTCCCCGGCATGATATGGTTTTTTTCCGCTATTAT
CCAGCACGCAGCGCTATCATTCAAGAGAACCCAGTGACGCGTAAATCGTAAGATCTACCTGCCGCAGG
TGGACCTACTGCAAATACGGATTATGACTCGTAAAGGGGTCATGCGTATTTTCATCACTAGGCACGTT
CGAGAATAAATTAGTAGGTGTCCCGAGCCTTGTGGCGTTCCGCCTGACTCCTCATGAAGTCGACCTTC
TCACCGGCCCTATCTGCCGACGTAAGTCATAACCTAGATCTGTACCTCGGGGGGAGGGTCACTGTAAA
GGGATAATTGGAGGGTGATTTCCCACACTTTCCTAAGGGTACTTTTTGCCTGGCTTCGCAATTGGGTC
CAATGGATGTCGATCTCTGGTTTAGCAGTTGTGAAAGTGGCAAGGCGGGAGGTTAGACCTCCATTTAA
CATATACAAGCAAGTTAACTGCACTAGATGTGTAGACACTACAGTTACAGGAGTAGCCGAATAAGTCT
CCGACGTCAAGCGAATAAGCGTCATACGCGATTATCGCCTAAGAGCACGTATTGGCGGTAAAAGGCTA
GCTAGACGCTTATGGGTAGATTTCAAGGCGTTCGTAGTGGTATAATAGGATACTCTTTCACCAGCCTG
AGGGCCGAACGCTATACTAGTGGTCTGTGATGTAGGACCGAGTATCTCTCTAGGGACCATCTACTTGA
GCAATGGTGCGCAGGGGGAGACATAGACCAGCCTTGGGTGGCAAGCACTGCAATAAGTCCTGTTTAGC
CTTGGAGTTCACATGCCGGCACTGAAGCCGACCTACCTGAGCGTGTGCGATTACCGTTACAATGGCAT
CTGTCTAGTTCTGTTTACCTACGGCGCTCTTGGTTCCAGGTTAGGGGAAGTGTATGACCCATGTGTTT
TTATCGGCTTAACCACGAGTGATCCCCGGTCGTTTCCCCATTGAATCCCTGGTGCATCCTACTCCCAG
AATGATAGCTGACTGACTGGACTGGCTTTTCAAGTAATCGAGGGGGTATCGCGGTCACGGCCGTTAAC
AGATCCCGTCCTTAGTGTGGAATCCGCACCTGCTGACTAACGCTTCGCCGGCGTGTCTGCACAGCCGT
ATAGTGTTAATCATGACCCCAAGGAAGGATTAAACAAATATCTTGACG
>BANAN34
GTGTCCGGTAGCCCGCGCTAGTTAGACACCCCGGCAGGGGGGATTGCTTTCGGGACGGGAGATCCCTT
CGCCGACCCTGTAGGGCCGACGCCGAGGCATTCGGGCCCCTGCAACGTCAACAGCGGCAAGAAAACGG
GATGAATGGGCGTAATGGGGGGGGNCTGCTGGNGACCCGACGCGGTNGCCGTTTGCGGGGCCCCCGAC
CCATACCGACCCACCTAGGCGTCCAGTTACGGCGCACGTCGGGAGCGTGGTTGCCGTCAGAGCTGTGT
TTCTCGATCAGTCCCCCGCAGTGCCGCAGTATCTTGCCGTGGGCTGCTTTAATCTTGAAAGTGGTTCA
TACATTGGGCGACGAGGTGTCGGCTCTCATTGGGGGTAACCGACGGACACGTGCAGTCCCCTCCCCGG
GCAGGCAGAGGCGGGGCCCCCGCGCGCCGGCCCCGGCCCACAATCTTACCAGGGTCCTCAAAAGAGCC
TTTGCATGGTACCCTTCGTGAATGGTTGCTTAAGAGGTCCACCACGTAGTCCGGGCACGGTCAACTAA
ACACTCAAAACAGCGACGGCAAATAGAGGCACAAGGTCCAGGCCCTCACGGCACTAGTTGCGATGACC
CAACTCACGGGGGCAGTCCCCGGCGCAGCGACCTTGATTACGTCCGGGAACAAGTATGTCGTTTCCCC
CGGGAGGGGTGCAGCCACATGGGAGATTCAAAGTTTCTCGTGACGTCGTTGTGATCACTGCAGCCTAG
CCGAGACTCCCGTACTACGCGAAGGTTGGTTATGTTAACCACTACAACGTGAGGCGCCGTAAGGGCCA
GTGTTGTGCCCGGCTCTTCAATGCGCCTTNGCGGCCTGATACACCCACCCAAGGAGATACTGCTAATC
ATGTGGGTGGACAGAACCTCGCAACGCAGACGCATCCCCGGCATGATATGGTTTTTTTCCGCTATTAC
CCAGCACGCGGCGCTATCATTCAAGAGAACCCAGTGACGCGTAAATCGTCAGATCTACCTGCCGCAGG
TGGACCTACTGCAAATACGGATTATGACTCGTAAAGAGGTCATGCGTATTTTCATCACTAGGCACTTT

CGAGAGTAAATTAGTAGGTGTCCCGCGCCTTGTGGCGTTCCGCCTGGCTCCTCATGAAGTCGACCTTC
TCATCGGCCCTATTTGCCGACGTAAGTCATAATCCAGATCTTCACCTCGGAGGGAGGGTCACTGTAAA
GGGATAATTGGAGGGCGATTTCCCACACTTTCCTAAGGGTACTTTTTGCTTAGCTTCGCAGTTGGGTC
CAATAGATGTTGATCTCTGGTTTAGCAGTTGTGAAAGTGGCAAGGCGGGAGGTTAGGCCTCCATTTAA
CATATACAAGCAAGTTAACTGCACTAGATGTGTAGACACTACAGTTACAGGAGTAGCCGACTAAGTCT
CCGACGTCAAGCGAATAGGCGTCATACGCGATTATTGCCTAAGAGCACGTATTGGCGGTAAAAGGATA
ACTAGACGCTTGTGGGTAGATTTCAAGGCGCTCGTAGTGGTATAATAGGACACTCTTGCACCAGCCTG
AGAGCCGAACGCTATACTAGTGGTCTGTGATGTAGGACCAAGTAGCTCTCTAGGGACCATCTACTTGA
GCAATGGTGCGCAGGGGTAGACATAGACCAACCTTGGGTGGCAAGCACTGCAATAAGTCCTGTTTAGC
CTTGGAGTTCACACGCCGGCACTAAAGCCGACCTACCTGAGCTTGTGCGATTACCGTTACAATGGCAT
CTGTCTAGTTCTGTTTACCTACGGCGCTCTTGGTTCCATGTTAGGGGAAGTGTATGACCCATGTGTTT
TTATCGGCTTAACTACGAGTGATCCCCGGTCGTTTCCCCATTAAATCCCTGGTGCATCCTACTCCCAT
AATGATAGCTGACTGGCTGGACTGGCTTTTCAAGTAGTCGAGGGGGTATCGCGGTCACGGCCGTTAAC
AGATCCCGTCCTTAGTGTGGAATCCGCACCTGCTGACTAACGCTTCGCCGGCGTGTCTGCACATCCGT
ATAGTGTTAATCATGACCCCAAGGAAGGATTAAACAAATATCTTGACG
>BANAN35
GTGTCCGGTAGCCCGCGCTAGTCAGACACCCCGGCAGGGGGGATTGCTTTCGAGACGGGAGATCCCTT
CGCCGACCCTGGAGGGCCGACGCCGAGGCATTCGGGCCCCCGCAACGTCAACAGCGGCAAGAAAACGG
GATGAATGGGCGTAATGGGGGGGGTCTGCTGGGGACCCGACGCGGTTGCCGTTTGCGGGGCCCCCGAC
CCATACCGACCCACCTAGGCGTCCAGTTACGGCGCACGTCGGGAGCGTGGTTGCCGTCAGAGCTGTGT
TTCTCGATCAGTCCCCCGCAGTGCCGCAGTATCTTGCCGTGGGCTGCTTTAATCTTGAAAGTGGTTTA
TACATTGGGCGACGAGGTGTCGACTCTCATTGGGGGTAACCGACGGGCACATGCAGTCCCCTCCCCGG
GCAGGCAGAGGCGGGGCCCCCGCGCGCCGGCCCCGGCCCACAATCTTACCAGGGTCCTCAAAGGAGCC
TTTGCATGGTACCCTTCGTGAATGGTTGCTTAAGAGGTCCACAACGTGGTCCGGGCACGGTCGACTAA
ACACTCAAAACAGCGACGGCAAATATAGGTACAAGGTCCAGGCCCTCACGGCACTAATTGCGATGACC
CAACTCACGGGGGCAGTCCCCGGCGCGGCGACCTTGATTACGTCCGGGGACAAGTATGTCGCTCCCCC
CGGGAGGGGTGCAGCCACATGGGAGATTCAAAGTTTCTCGTGACGTCGTTGTGATCACTGCAGCCTAG
CCGAGACTCCCGTACTACGCGAAGGTTGGTTATGTTAACCACTACAACGTGAGGCGCCGTAAGGGCCA
GTGTTGTGCCCGGCTCTTCAATGCGCCTTAGCGGCCTGATACACCCACCCAAGGAGATACTGCTAATC
ATATGGGTGGACAGAACCTCGCAACGCAGACGCCTCCCCGGCATGATATGGTTTTTTTCCGCTATTAT
CCAGCACGCAGCGCTATCATTCAAGAGAACCCAGTGACGCGTAAATCGTAAGATCTACCTGCCGCAGG
TGGACCTACTGCAAATACGGATTATGACTCGTAAAGGGGTCATGCGTATTTTCATCACTAGGCACGTT
CGAGAATAAATTAGTAGGTGTCCCGAGCCTTGTGGCGTTCCGCCTGACTCCTCATAAAGTCGACCTTC
TCACCGGCCCTATCTGCCGACGTAAGTCATAACCTAGATCTGTACCTCGGGGGGAGGGTCACTGTAAA
GGGATAATTGGAGGGTGATTTCCCACATTTTCCTAAGGGTACTTTTTGCCTGGCTTCGCAATTGGGTC
CAATGGATGTCGATCTCTGGTTTAGCAGTTGTGAAAGTGGCAAGGCGGGAGGTTAGACCTCCATTTAA
CATATACAAGCAAGTTAACTGCACTAGATGTGTAGACACTACAGGTACAGGAGTAGCCGGATAAGTCT
CCGACGTCAAGCGAATAAGCGTCATACGCGATTATCGCCTAAGAGCACGTATTTGCGGTAAAAGGCTA
GCTAGACGCTTGTGGGTAGATTTCAAGGCGTTCGTAGTGGTATAATAGGATACTCTTTCACCAGCCTG
AGGGCCGAACGCTATACTAGTGGTCTGTGATGTAGGACCGAGTATCTCTCTAGGGACCATCTACTTGA
GCAATGGTGCGCAGGGGGAGACATAGACCAGCCTTGGGTGGCAAGCACTGCAATAAGTCCTGTTTAGC
CTTGGAGTTCACACGCCGGCACTGAAGCCGACCTACCTGAGCGTGTGCGATTACCGTTACAATGGCAT
CTGTCTAGTTCTGTTTACCTACGGCGCTCTTGGTTCCAGGTTAGGGGAAGTGTATGACCCATGTGTTT
TTATCGGCTTAACCACGAGTGATCCCCGGTCGTTTCCCCATTGAATCCCTGGTGCATCCTACTCCCAG
AATGATAGCTGACTGACTGGACTGGCTTTTCAAGTAATCGAGGGGGTATCGCGGTCACGGCCGTTAAC
AGATCCCGTCCTTAGTGTGGAATCCGCACCTGCTGACTAACGCTTCGCCGGCGTGTCTGCACAGCCGT
ATAGTGTTAATCATGACCCCAAGGAAGGATTAAACAAATATCTTGACG
>GIRAN01
GTGTCCGGTAGCCCGCGCTAGTTAGACACCCCGGCAGGGGGGATTGCTTTCGAGACGGGAGATCCCTT
CGCCGACCCTGGAGGGCCGACGCCGAGGCATTCGGGCCCCTGCAACGTCAACAGCGGCAAGAAAACGG
GATGAATGGGCGTAATGGGGGGGGTCTGCTGGGGACCCGACGCGGTTGCCGTTTGCGGGGCCCCCGAC
CCATACCGACCCACCTAGGCGTCCAGTTACGGCGCACGTCGGGAGCGTGGTTGCCGTCAGAGCTGTGT
TTCTCGATCAGTCCCCCGCAGTGCCGCAGTATCTTGCCGTGGGCTGCTTTAATCTTGAAAGTGGTTCA
TACATTGGGCGACGAGGTGTCGACTCTCATTGGGGGTAACCGACGGGCACATGCAGTCCCCTCCCCGG

GCAGGCAGAGGCGGGGCCCCCGCGCGCCGGCCCCGGCCCACAATCTTACCAGGGTCCTCAAAGGAGCC
TTTGCATGGTACCCTTCGTGAATGGTTGCTTAAGAGGTCCACAACGTGGTCCGGGCACGGTCGACTAA
ACACTCAAAACAGCGACGGCAAATATAGGTACAAGGTCCAGGCCCTCACGGCACTAATTGCGATGACC
CAACTCACGGGGGCAGTCCCCGGCGCGGCGACCTTGATTACGTCCGGGGACAAGTATGTCGCTCCCCC
CGGGAGGGGTGCAGCCACATGGGAGATTCAAAGTTTCTCGTGACGTCGTTGTGATCACTGCAGCCTAG
CCGAGACTCCCGTACTACGCGAAGGTTGGTTATGTTAACCACTACAACGTGAGGCGCCGTAAGGGCCA
GTGTTGTGCCCGGCTCTTCAATGCGCCTTAGCGGCCTGATACACCCACCCAAGGAGATACTGCTAATC
ATATGGGTGGACAGAACCTCGCAACGCAGACGCCTCCCCGGCATGATATGGTTTTTTTCCGCTATTAT
CCAGCACGCAGCGCTATCATTCAAGAGAACCCAGTGACGCGTAAATCGTAAGATCTACCTGCCGCAGG
TGGACCTACTGCAAATACGGATTATGACTCGTAAAGGGGTCATGCGTATTTTCATCACTAGGCACGTT
CGAGAATAAATTAGTAGGTGTCCCGAGCCTTGTGGCGTTCCGCCTGACTCCTCATGAAGTCGACCTTC
TCACCGGCCCTATCTGCCGACGTAAGTCATAACCTAGATCTGTACCTCGGGGGGAGGGTCACTGTAAA
GGGATAATTGGAGGGTGATTTCCCACACTTTCCTAAGGGTACTTTTTGCCTGGCTTCGCAATTGGGTC
CAATGGATGTCGATCTCTGGTTTAGCAGTTGTGAAAGTGGCAAGGCGGGAGGTTAGACCTCCATTTAA
CATATACAAGCAAGTTAACTGCACTAGATGTGTAGACACTACAGTTACAGGAGTAGCCGAATAAGTCT
CCGACGTCAAGCGAATAAGCGTCATACGCGATTATCGCCTAAGAGCACGTATTGGCGGTAAAAGGCTA
GCTAGACGCTTATGGGTAGATTTCAAGGCGTTCGTAGTGGTATAATAGGATACTCTTTCACCAGCCTG
AGGGCCGAACGCTATACTAGTGGTCTGTGATGTAGGACCGAGTATCTCTCTAGGGACCATCTACTTGA
GCAATGGTGCGCAGGGGGAGACATAGACCAGCCTTGGGTGGCAAGCACTGCAATAAGTCCTGTTTAGC
CTTGGAGTTCACATGCCGGCACTGAAGCCGACCTACCTGAGCGTGTGCGATTACCGTTACAATGGCAT
CTGTCTAGTTCTGTTTACCTACGGCGCTCTTGGTTCCAGGTTAGGGGAAGTGTATGACCCATGTGTTT
TTATCGGCTTAACCACGAGTGATCCCCGGTCGTTTCCCCATTGAATCCCTGGTGCATCCTACTCCCAG
AATGATAGCTGACTGACTGGACTGGCTTTTCAAGTAATCGAGGGGGTATCGCGGTCACGGCCGTTAAC
AGATCCCGTCCTTAGTGTGGAATCCNCACCTGCTGACTAACGCTTCGCCGGCGTGTCTGCACAGCCGT
ATAGTGTTAATCATGACCCCAAGGAAGGATTAAACAAATATCTTGACG
>GIRAN02
GTGTCCGGTAGCCCGCGCTAGTTAGACACCCCGGCAGGGGGGATTGCTTTCGGGACGGGAGATCCCTT
CGCCGACCCTGGAGGGCCGACGCCGAGGCATTCGGGCCCCTGCAACGTCAACAGCGGCAAGAAAACGG
GATGAATGGGCGTAATGGGGGGGGTCTGCTGGGGACCCGACGCGGTTGCCGTTTGCGGGGCCCCCGAC
CCATACCGACCCACCTAGGCGTCCAGTTACGGCGCACGTCGGGAGCGTGGTTGCCGTCAGAGCTGTGT
TTCTCGATCAGTCCCCCGCAGTGCCGCAGTATCTTGCCGTGGGCTGCTTTAATCTTGAAAGTGGTTTA
TACATTGGGCGACGAGGTGTCGACTCTCATTGGGGGTAACCGACGGGCACATGCAGTCCCCTCCCCGG
GCAGGCAGAGGCGGGGCCCCCGCGCGCCGGCCCCGGCCCACAATCTTACCAGGGTCCTCAAAGGAGCC
TTTGCATGGTACCCTTCGTGAATGGTTGCTTAAGAGGTCCACAACGTGGTCCGGGCACGGTCGACTAA
ACACTCAAAACAGCGACGGCAAATATAGGTACAAGGTCCAGGCCCTCACGGCACTAATTGCGATGACC
CAACTCACGGGGGCAGTCCCCGGCGCGGCGACCTTGATTACGTCCGGGGACAAGTATGTCGCTCCCCC
CGGGAGGGGTGCAGCCACATGGGAGATTCAAAGTTTCTCGTGACGTCGTTGTGATCACTGCAGCCTAG
CCGAGACTCCCGTACTACGCGAAGGTTGGTTATGTTAACCACTACAACGTGAGGCGCCGTAAGGGCCA
GTGTTGTGCCCGGCTCTTCAATGCGCCTTAGCGGCCTGATACACCCACCCAAGGAGATACTGCTAATC
ATATGGGTGGACAGAACCTCGCAACGCAGACGCCTCCCCGGCATGATATGGTTTTTTTCCGCTATTAT
CCAGCACGCAGCGCTATCATTCAAGAGAACCCAGTGACGCGTAAATCGTAAGATCTACCTGCCGCAGG
TGGACCTACTGCAAATACGGATTATGACTCGTAAAGGGGTCATGCGTATTTTCATCACTAGGCACGTT
CGAGAATAAATTNGTAGGTGTCCCGAGCCTTGTGGCGTTCCGCCTGACTCCTCATGAAGTCGACCTTC
TCACCGGCCCTATCTGCCGACGTAAGTCATAACCTAGATCTGTACCTCGGGGGGAGGGTCACTGTAAA
GGGATAATTGGAGGGTGATTTCCCACACTTTCCTAAGGGTACTTTTTGCCTGGCTTCGCAATTGGGTC
CAATGGATGTCGATCTCTGGTTTAGCAGTTGTGAAAGTGGCAAGGCGGGAGGTTAGACCTCCATTTAA
CATATACAAGCAAGTTAACTGCACTAGATGTGNAGACACTACAGTTACAGGAGTAGCCGAATAAGTCT
CCGACGTCAAGCGAATAAGCGTCATACGCGATTATCGCCTAAGAGCACGTATTGGCGGTAAAAGGCTA
GCTAGACGCTTATGGGTAGATTTCAAGGCGTTCGTAGTGGTATAATAGGATACTCTTTCACCAGCCTG
AGGGCCGAACGCTATACTAGTGGTCTGTGATGTAGGACCGAGTATCTCTCTAGGGACCATCTACTTGA
GCAATGGTGCGCAGGGGGAGACATAGACCAGCCTTGGGTGGCAAGCACTGCAATAAGTCCTGTTTAGC
CTTGGAGTTCACATGCCGGCACTGAAGCCGACCTACCTGAGCGTGTGCGATTACCGTTACAATGGCAT
CTGTCTAGTTCTGTTTACCTACGGCGCTCTTGGTTCCAGGTTAGGGGAAGTGTATGACCCATGTGTTT
TTATCGGCTTAACCACGAGTGATCCCCGGTCGTTTCCCCATTGAATCCCTGGTGCATCCTACTCCCAG

AATGATAGCTGACTGACTGGACTGGCTTTTCAAGTAATCGAGGGGGTATCGCGGTCACGGCCGTTAAC
AGATCCCGTCCTTAGTGTGGAATCCGCACCTGCTGACTAACGCTTCGCCGGCGTGTCTGCACAGCCGT
ATAGTGTTAATCATGACCCCAAGGAAGGATTAAACAAATATCTTGACG
>GIRAN03
GTGTCCGGTAGCCCGCGCTAGTTAGACACCCCGGCAGGGGGGATTGCTTTCGAGACGGGAGATCCCTT
CGCCGACCCTGGAGGGCCGACGCCGAGGCATTCGGGCCCCCGCAACGTCAACAGCGGCAAGAAAACGG
GATGAATGGGCGTAATGGGGGGGGTCTGCTGGGGACCCGACGCGGTTGCCGTTTGCGGGGCCCCCGAC
CCATACCGACCCACCTAGGCGTCCAGTTACGGCGCACGTCGGGAGCGTGGTTGCCGTCAGAGCTGTGT
TTCTCGATCAGTCCCCCGCAGTGCCGCAGTATCTTGCCGTGGGCTGCTTTAATCTTGAAAGTGGTTTA
TACATTGGGCGACGAGGTGTCGACTCTCATTGGGGGTAACCGACGGGCACATGCAGTCCCCTCCCCGG
GCAGGCAGAGGCGGGGCCCCCGCGCGCCGGCCCCGGCCCACAATCTTACCAGGGTCCTCAAAGGAGCC
TTTGCATGGTACCCTTCGTGAATGGTTGCTTAAGAGGTCCACAACGTGGTCCGGGCACGGTCGACTAA
ACACTCAAAACAGCGACGGCAAATATAGGTACAAGGTCCAGGCCCTCACGGCACTAATTGCGATGACC
CAACTCACGGGGGCAGTCCCCGGCGCGGCGACCTTGATTACGTCCGGGGACAAGTATGTCGCTCCCCC
CGGGAGGGGTGCAGCCACATGGGAGATTCAAAGTTTCTCGTGACGTCGTTGTGATCACTGCAGCCTAG
CCGAGACTCCCGTACTACGCGAAGGTTGGTTATGTTAACCACTACAACGTGAGGCGCCGTAAGGGCCA
GTGTTGTGCCCGGCTCTTCAATGCGCCTTAGCGGCCTGATACACCCACCCAAGGAGATACTGCTAATC
ATATGGGTGGACAGAACCTCGCAACGCAGACGCCTCCCCGGCATGATATGGTTTTTTTCCGCTATTAT
CCAGCACGCAGCGCTATCATTCAAGAGAACCCAGTGACGCGTAAATCGTAAGATCTACCTGCCGCAGG
TGGACCTACTGCAAATACGGATTATGACTCGTAAAGGGGTCATGCGTATTTTCATCACTAGGCACGTT
CGAGAATAAATTAGTAGGTGTCCCGAGCCTTGTGGCGTTCCGCCTGACTCCTCATGAAGTCGACCTTC
TCACCGGCCCTATCTGCCGACGTAAGTCATAACCTAGATCTGTACCTCGGGGGGAGGGTCACTGTAAA
GGGATAATTGGAGGGTGATTTCCCACACTTTCCTAAGGGTACTTTTTGCCTGGCTTCGCAATTGGGTC
CAATGGATGTCGATCTCTGGTTTAGCAGTTGTGAAAGTGGCAAGGCGGGAGGTTAGACCTCCATTTAA
CATATACAAGCAAGTTAACTGCACTAGATGTGTAGACACTACAGTTACAGGAGTAGCCGAATAAGTCT
CCGACGTCAAGCGAATAAGCGTCATACGCGATTATCGCCTAAGAGCACGTATTGGCGGTAAAAGGCTA
GCTAGACGCTTATGGGTAGATTTCAAGGCGTTCGTAGTGGTATAATAGGATACTCTTTCACCAGCCTG
AGGGCCGAACGCTATACTAGTGGTCTGTGATGTAGGACCGAGTATCTCTCTAGGGACCATCTACTTGA
GCAATGGTGCGCAGGGGGAGACATAGACCAGCCTTGGGTGGCAAGCACTGCAATAAGTCCTGTTTAGC
CTTGGAGTTCACATGCCGGCACTGAAGCCGACCTACCTGAGCGTGTGCGATTACCGTTACAATGGCAT
CTGTCTAGTTCTGTTTACCTACGGCGCTCTTGGTTCCAGGTTAGGGGAAGTGTATGACCCATGTGTTT
TTATCGGCTTAACCACGAGTGATCCCCGGTCGTTTCCCCATTGAATCCCTGGTGCATCCTACTCCCAG
AATGATAGCTGACTGACTGGACTGGCTTTTCAAGTAATCGAGGGGGTATCGCGGTCACGGCCGTTAAC
AGATCCCGTCCTTAGTGTGGAATCCGCACCTGCTGACTAACGCTTCGCCGGCGTGTCTGCACAGCCGT
ATAGTGTTAATCATGACCCCAAGGAAGGATTAAACAAATATCTTGACG
>GIRAN04
GTGTCCGGTAGCCCGCGCTAGTTAGACACCCCGGCAGGGGGGATTGCTTTCGAGACGGGAGATCCCTT
CGCCGACCCTGGAGGGCCGACGCCGAGGCATTCGGGCCCCCGCAACGTCAACAGCGGCAAGAAAACGG
GATGAATGGGCGTAATGGGGGGGGTCTGCTGGGGACCCGACGCGGTTGCCGTTTGCGGGGCCCCCGAC
CCATACCGACCCACCTAGGCGTCCAGTTACGGCGCACGGCGGGAGCGTGGTTGCCGTCAGAGCTGTGT
TTCTCGATCAGTCCCCCGCAGTGCCGCAGTATCTTGCCGTGGGCTGCTTTAATCTTGAAAGTGGTTTA
TACATTGGGCGACGAGGTGTCGACTCTCATTGGGGGTAACCGACGGGCACATGCAGTCCCCTCCCCGG
GCAGGCAGAGGCGGGGCCCCCGCGCGCCGGCCCCGGCCCACAATCTTACCAGGGTCCTCAAAGGAGCC
TTTGCATGGTACCCTTCGTGAATGGTTGCTTAAGAGGTCCACAACGTGGTCCGGGCACGGTCGACTAA
ACACTCAAAACAGCGACGGCAAATATAGGTACAAGGTCCAGGCCCTCACGGCACTAATTGCGATGACC
CAACTCACGGGGGCAGTCCCCGGCGCGGCGACCTTGATTACGTCCGGGGACAAGTATGTCGCTCCCCC
CGGGAGGGGTGCAGCCACATGGGAGATTCAAAGTTTCTCGTGACGTCGTTGTGATCACTGCAGCCTAG
CCGAGACTCCCGTACTACGCGAAGGTTGGTTATGTTAACCACTACAACGTGAGGCGCCGTAAGGGCCA
GTGTTGTGCCCGGCTCTTCAATGCGCCTTAGCGGCCTGATACACCCACCCAAGGAGATACTGCTAATC
ATATGGGTGGACAGAACCTCGCAACGCAGACGCCTCCCCGGCATGATATGGTTTTTTTCCGCTATTAT
CCAGCACGCAGCGCTATCATTCAAGAGAACCCAGTGACGCGTAAATCGTAAGATCTACCTGCCGCAGG
TGGACCTACTGCAAATACGGATTATGACTCGTAAAGGGGTCATGCGTATTTTCATCACTAGGCACGTT
CGAGAATAAATTAGTAGGTGTCCCGAGCCTTGTGGCGTTCCGCCTGACTCCTCATGAAGTCGACCTTC
TCACCGGCCCTATCTGCCGACGTAAGTCATAACCTAGATCTGTACCTCGGGGGGAGGGTCACTGTAAA

GGGATAATTGGAGGGTGATTTCCCACACTTTCCTAAGGGTACTTTTTGCCTGGCTTCGCAATTGGGTC
CAATGGATGTCGATCTCTGGTTTAGCAGTTGTGAAAGTGGCAAGGCGGGAGGTTAGACCTCCATTTAA
CATATACAAGCAAGTTAACTGCACTAGATGTGTAGACACTACAGTTACAGGAGTAGCCGAATAAGTCT
CCGACGTCAAGCGAATAAGCGTCATACGCGATTATCGCCTAAGAGCACGTATTGGCGGTAAAAGGCTA
GCTAGACGCTTATGGGTAGATTTCAAGGCGTTCGTAGTGGTATAATAGGATACTCTTTCACCAGCCTG
AGGGCCGAACGCTATACTAGTGGTCTGTGATGTAGGACCGAGTATCTCTCTAGGGACCATCTACTTGA
GCAATGGTGCGCAGGGGGAGACATAGACCAGCCTTGGGTGGCAAGCACTGCAATAAGTCCTGTTTAGC
CTTGGAGTTCACATGCCGGCACTGAAGCCGACCTACCTGAGCGTGTGCGATTACCGTTACAATGGCAT
CTGTCTAGTTCTGTTTACCTACGGCGCTCTTGGTTCCAGGTTAGGGGAAGTGTATGACCCATGTGTTT
TTATCGGCTTAACCACGAGTGATCCCCGGTCGTTTCCCCATTGAATCCCTGGTGCATCCTACTCCCAG
AATGATAGCTGACTGACTGGACTGGCTTTTCAAGTAATCGAGGGGGTATCGCGGTCACGGCCGTTAAC
AGATCCCGTCCTTAGTGTGGAATCCGCACCTGCTGACTAACGCTTCGCCGGCGTGTCTGCACAGCCGT
ATAGTGTTAATCATGACCCCAAGGAAGGATTAAACAAATATCTTGACG
>GIRAN05
GTGTCCGGTAGCCCGCGCTAGTTAGACACCCCGGCAGGGGGGATTGCTTTCGGGACGGGAGATCCCTT
CGCCGACCCTGGAGGGCCGACGCCGAGGCATTCGGGCCCCTGCAACGTCAACAGCGGCAAGAAAACGG
GATGAATGGGCGTAATGGGGGGGGTCTGCTGGGGACCCGACGCGGTTGCCGTTTGCGGGGCCCCCGAC
CCATACCGACCCACCTAGGCGTCCAGTTACGGCGCACGTCGGGAGCGTGGTTGCCGTCAGAGCTGTGT
TTCTCGATCAGTCCCCCGCAGTGCCGCAGTATCTTGCCGTGGGCTGCTTTAATCTTGAAAGTGGTTCA
TACATTGGGCGACGAGGTGTCGACTCTCATTGGGGGTAACCGACGGGCACATGCAGTCCCCTCCCCGG
GCAGGCAGAGGCGGGGCCCCCGCGCGCCGGCCCCGGCCCACAATCTTACCAGGGTCCTCAAGGGAGCC
TTTGCATGGTACCCTTCGTGAATGGTTGCTTAAGAGGTCCACCACGTGGTCCGGGCACGGTCGACTAA
ACACTCAAAACAGCGACGGCAAATATAGGCACAAGGTCCAGGCCCTCACGGCACTAATTGCGATGACC
CAACTCACGGGGGCAGTCCCCGGCGCGGCGACCTTGATTACGGCCGGGGACAAGTATGTCGTTCCCCC
CGGGAGGGGTGCAGCCACATGGGAGATTCAAAGTTTCTCGTGACGTCGTTGTGATCACTGCAGCCTAG
CCGAGACTCCCGTACTACGCGAAGGTTGGTTATGTTAACCACTACAACGTGAGGCGCCGTAAGGGCCA
GTGTTGTGCCCGGCTCTTCAATGCGCCTTAGCGGCCTGATACACCCACCCAAGGAGATACTGCTAATC
ATGTGGGTGGACAGAACCTCGCAACGCAGACGCCTCCCCGGCATGATATGGTTTTTTTCCGCTATTAT
CCAGCACGCAGCGCTATCATTCAAGAGAACCCAGTGACGCGTAAATCGTAAGATCTACCTGCCGCAGG
TGGACCTACTGCAAATACGGATTATGACTCGTAAAGAGGTCATGCGTATTTTCATCACTAGGCACGTT
CGAGAATAAATTAGTAGGTGTCCCGCGCCTTGTGGCGTTCCGCCTGACTCCTCATGAAGTCGACCTTC
TCACCGGCCCTATCTGCCGACGTAAGTCATAACCTAGATCTGTACCTCGGGGGGAGGGTCACTGTAAA
GGGATAATTGGAGGGTGATTTCCCACACTTTCCTAAGGGTACTTTTTGCCTGGCTTCGCAATTGGGTC
CAATGGATGTCGATCTCTGGTTTAGCAGTTGTGAAAGTGGCAAGGCGGGAGGTTAGACCTCCATTTAA
CATATACAAGCAAGTTAACTGCACTAGATGTGTAGACACTACAGTTACAGGAGTAGCCGAATAAGTCT
CCGACGTCAAGCGAATAAGCGTCATACGCGATTATCGCCTAAGAGCACGTATTGGCGGTAAAAGGCTA
ACTAGACGCTTGTGGGTAGATTTCAAGGCGTTCGTAGTGGTATAACAGGATACTCTTTCACCAGCCTG
AGGGCCGAACGCTATACTAGTGGTCTGTGATGTAGGACCGAGTATCTCTCTAGGGACCATCTACTTGA
GCAATGGTGCGCAGGGGGAGACATAGACCAGCCTTGGGTGGCAAGCACTGCAATAAGTCCTGTTTAGC
CTTGGAGTTCACACGCCGGCACTGAAGCCGACCTACCTGAGCTTGTGCGATTACCGTTACAATGGCAT
CTGTCTAGTTCTGTTTACCTACGGCGCTCTTGGTTCCAGGTTAGGGGAAGTGTATGACCCATGTGTTT
TTATCGGCTTAACCACGAGTGATCCCCGGTCGTTTCCCCATTGAATCCCTGGTGCATCCTACTCCCAG
AATGATAGCTGACTGACTGGACTGGCTTTTCAAGTAGTCGAGGGGGTATCGCGGTCACGGCCGTTAAC
AGATCCCGTCCTTAGTGTGGAATCCGCACCTGCTGACTAACGCTTCGCCGGCGTGTCTGCACAGCCGT
ATAGTGTTAATCATGACCCCAAGGAAGGATTAAACAAATATCTTGACG
>GIRAN06
GTGTCCGGTAGCCCGCGCTAGTTAGACACCCCGGCAGGGGGGATTGCTTTCGAGACGGGAGATCCCTT
CGCCGACCCTGTAGGGCCGACGCCGAGGCATTCGGGCCCCTGCAACGTCAACAGCGGCAAGAAAACGG
GATGAATGGGCGTAATGGGGGGGGTCTGCTGGGGACCCGACGCGGTTGCCGTTTGCGGGGCCCCCGAC
CCATACCGACCCACCTAGGCGTCCAGTTACGGCGCACGGCGGGAGCGTGGTTGCCGTCAGAGCTGTGT
TTCTCGATCAGTCCCCCGCAGTGCCGCAGTATCTTGCCGTGGGCTGCTTTAATCTTGAAAGTGGTTTA
TACATTGGGCGACGAGGTGTCGGCTCTCATTGGGGGTAGCCGACGGACACATGCAGTCCCCTCCCCGG
GCAGGCAGAGGCGGGGCCCCCGCGCGCCGGCCCCGGCCCACAATCTTACCAGGGTCCTCAAAAGAGCC
TTTGCATGGTACCCTTCGTGAATGGTTGCTTAAGAGGTCCACCACGTAGTCCGGGCACGGTCGACTAA

ACACTCAAAACAGCGACGGCAAATAGAGGCACAAGGTCCAGGCCCTCACGGCACTAGTTGCGATGACC
CAACTCACGGGGGCAGTCCCCGGCGCGGCGACCTTGATTACGTCCGGGAACAAGTATGTCGTTTCCCC
CGGGAGGGGTGCAGCCACATGGGAGATTCAAAGTTTCTCGTGACGTCGTTGTGATCACTGCAGCCTAG
CCGAGACTCCCGTACTACGCGAAGGTTGGTTATGTTAACCACTACAACGTGAGGCGCCGTAAGGGCCA
GTGTTGTGCCCGGCTCTTCAATGCGCCTTAGCGGCCTGATACACCCACCCAAGGAGATACTGCTAATC
ATGTGGGTGGACAGAACCTCGCAACGCAGACGCATCCCCGGCNTGATATGGTTTTTTTCCGCTATTAC
CCAGCACGCGGCGCTATCATTCAAGAGAACCCAGTGACGCGTAAATCGTAAGATCTACCTGCCGCAGG
TGGACCTACTGCAAATACGGATTATGACTCGTAAAGAGGTCATGCGTATTTTCATCACTAGGCACGTT
CGAGAGTAAATTAGTAGGTGTCCCGCGCCTTGTGGCGTTCCGCCTGACTCCTCATGAAGTCGACCTTC
TCATCGGCCCTATCTGCCGACGTAAGTCATAATCCAGATCTTCACCTCGGAGGGAGGGTCACTGTAAA
GGGATAATTGGAGGGCGATTTCCCACACTTTCCTAAGNGTACTTTTTGCTTGGCTTCGCAGTTGGGTC
CAATAGATGTTGATCTCTGGTTTAGCAGTTGTGAAAGTGGCAAGGCGGGAGGTTAGACCTCCATTTAA
CATATACAAGCAAGTTAACTGCACTAGATGTGTAGACACTACAGTTACAGGAGTAGCCGACTAAGTCT
CCGACGTCAAGCGAATAGGCGTCATACGCGATTATCGCCTAAGAGCACGTATTGGCGGTAAAAGGATA
ACTAGACGCTTGTGGGTAGATTTCAAGGCGCTCGTAGTGGTATAATAGGATACTCTTTCACCAGCCTG
AGAGCCGAACGCTATACTAGTGGTCTGTGATGTAGGACCAAGTAGCTCTCTAGGGACCATCTACTTGA
GCAATGGTGCGCAGGGGTAGACATAGACCAACCTTGGGTGGCAAGCACTGCAATAAGTCCTGTTTAGC
CTTGGAGTTCACACGCCGGCACTAAAGCCGACCTACCTGAGCTTGTGCGATTACCGTTACAATGGCAT
CTGTCTAGTTCTGTTTACCTACGGCGCTCTTGGTTCCATGTTAGGGGAAGTGTATGACCCATGTGTTT
TTATCGGCTTAACTACGAGTGATCCCCGGTCGTTTCCCCATTAAATCCCTGGTGCATCCTACTCCCAT
AATGATAGCTGACTGGCTGGACTGGCTTTTCAAGTAGTCGAGGGGGTATCGCGGTCACGGCCGTTAAC
AGATCCCGTCCTTAGTGTGGAATCCGCACCTGCTGACTAACGCTTCGCCGGCGTGTCTGCACATCCGT
ATAGTGTTAATCATGACCCCAAGGAAGGATTAAACAAATATCTTGACG
>GIRAN08
GTGTCCGGTAGCCCGCGCTAGTTAGACACCCCGGCAGGGGGGATTGCTTTCGAGACGGGAGATCCCTT
CGCCGACCCTGTAGGGCCGACGCCGAGGCATTCGGGCCCCTGCAACGTCAACAGCGGCAAGAAAACGG
GATGAATGGGCGTAATGGGGGGGGTCTGCTGGGGACCCGACGCGGTTGCCGTTTGCGGGGCCCCCGAC
CCATACCGACCCACCTAGGCGTCCAGTTACGGCGCACGGCGGGAGCGTGGTTGCCGTCAGAGCTGTGT
TTCTCGATCAGTCCCCCGCAGTGCCGCAGTATCTTGCCGTGGGCTGCTTTAATCTTGAAAGTGGTTTA
TACATTGGGCGACGAGGTGTCGGCTCTCATTGGGGGTAGCCGACGGACACATGCAGTCCCCTCCCCGG
GCAGGCAGAGGCGGGGCCCCCGCGCGCCGGCCCCGGCCCACAATCTTACCAGGGTCCTCAAAAGAGCC
TTTGCATGGTACCCTTCGTGAATGGTTGCTTAAGAGGTCCACCACGTAGTCCGGGCACGGTCGACTAA
ACACTCAAAACAGCGACGGCAAATAGAGGCACAAGGTCCAGGCCCTCACGGCACTAGTTGCGATGACC
CAACTCACGGGGGCAGTCCCCGGCGCGGCGACCTTGATTACGTCCGGGAACAAGTATGTCGTTTCCCC
CGGGAGGGGTGCAGCCACATGGGAGATTCAAAGTTTCTCGTGACGTCGTTGTGATCACTGCAGCCTAG
CCGAGACTCCCGTACTACGCGAAGGTTGGTTATGTTAACCACTACAACGTGAGGCGCCGTAAGGGCCA
GTGTTGTGCCCGGCTCTTCAATGCGCCTTAGCGGCCTGATACACCCACCCAAGGAGATACTGCTAATC
ATGTGGGTGGACAGAACCTCGCAACGCAGACGCATCCCCGGCNTGATATGGTTTTTTTCCGCTATTAC
CCAGCACGCGGCGCTATCATTCAAGAGAACCCAGTGACGCGTAAATCGTAAGATCTACCTGCCGCAGG
TGGACCTACTGCAAATACGGATTATGACTCGTAAAGAGGTCATGCGTATTTTCATCACTAGGCACGTT
CGAGAGTAAATTAGTAGGTGTCCCGCGCCTTGTGGCGTTCCGCCTGACTCCTCATGAAGTCGACCTTC
TCATCGGCCCTATCTGCCGACGTAAGTCATAATCCAGATCTTCACCTCGGAGGGAGGGTCACTGTAAA
GGGATAATTGGAGGGCGATTTCCCACACTTTCCTAAGGGTACTTTTTGCTTGGCTTCGCAGTTGGGTC
CAATAGATGTTGATCTCTGGTTTAGCAGTTGTGAAAGTGGCAAGGCGGGAGGTTAGACCTCCATTTAA
CATATACAAGCAAGTTAACTGCACTAGATGTGTAGACACTACAGTTACAGGAGTAGCCGACTAAGTCT
CCGACGTCAAGCGAATAGGCGTCATACGCGATTATCGCCTAAGAGCACGTATTGGCGGTAAAAGGATA
ACTAGACGCTTGTGGGTAGATTTCAAGGCGCTCGTAGTGGTATAATAGGATACTCTTTCACCAGCCTG
AGAGCCGAACGCTATACTAGTGGTCTGTGATGTAGGACCAAGTAGCTCTCTAGGGACCATCTACTTGA
GCAATGGTGCGNAGGGGTAGACATAGACCAACCTTGGGTGGCAAGCACTGCAATAAGTCCTGTTTAGC
CTTGGAGTTCACACGCCGGCACTAAAGCCGACCTACCTGAGCTTGTGCGATTACCGTTACAATGGCAT
CTGTCTAGTTCTGTTTACCTACGGCGCTCTTGGTTCCATGTTAGGGGAAGTGTATGACCCATGTGTTT
TTATCGGCTTAACTACGAGTGATCCCCGGTCGTTTCCCCATTAAATCCCTGGTGCATCCTACTCCCAT
AATGATAGCTGACTGGCTGGACTGGCTTTTCAAGTAGTCGAGGGGGTATCGCGGTCACGGCCGTTAAC
AGATCCCGTCCTTAGTGTGGAATCCGCACCTGCTGACTAACGCTTCGCCGGCGTGTCTGCACATCCGT

ATAGTGTTAATCATGACCCCAAGGAAGGATTAAACAAATATCTTGACG
>GIRAN12
GTGTCCGGTAGCCCGCGCTAGTCAGACACCCCGGCAGGGGGGATTGCTTTCGAGACGGGAGATCCCTT
CGCCGACCCTGGAGGGCCGACGCCGAGGCATTCGGGCCCCTGCAACGTCAACAGCGGCAAAAAAACGG
GATGAATGGGCGTAATGGGGGGGGTCTGCTGGGGACCCGACGCGGTTGCCGTTTGCGGGGCCCCCGAC
CCATACCGACCCACCTAGGCGTCCAGTTACGGCGCACGGCGGGAGCGTGGTTGCCGTCAGAGCTGTGT
TTCTCGATCAGTCCCCCGCAGTGCCGCAGTATCTTGCCGTGGGCTGCTTTAATCTTGAAAGTGGTTTA
TACATTGGGCGACGAGGTGTCGACTCTCATTGGGGGTAACCGACGGGCACATGCAGTCCCCTCCCCGG
GCAGGCAGAGGCGGGGCCCCCGCGCGCCGGCCCCGGCCCACAATCTTACCAGGGTCCTCAAAGGAGCC
TTTGCATGGTACCCTTCGTGAATGGTTGCTTAAGAGGTCCACAACGTGGTCCGGGCACGGTCGACTAA
ACACTCAAAACAGCGACGGCAAATATAGGTACAAGGTCCAGGCCCTCACGGCACTAATTGCGATGACC
CAACTCACGGGGGCAGTCCCCGGCGCGGCGACCTTGATTACGTCCGGGGACAAGTATGTCGCTCCCCC
CGGGAGGGGTGCAGCCACATGGGAGATTCAAAGTTTCTCGTGACGTCGTTGTGATCACTGCAGCCTAG
CCGAGACTCCCGTACTACGCGAAGGTTGGTTATGTTAACCACTACAACGTGAGGCGCCGTAAGGGCCA
GTGTTGTGCCCGGCTCTTCAATGCGCCTTAGCGGCCTGATACACCCACCCAAGGAGATACTGCTAATC
ATATGGGTGGACAGAACCTCGCAACGCAGACGCCTCCCCGGCATGATATGGTTTTTTTCCGCTATTAT
CCAGCACGCAGCGCTATCATTTAAGAGAACCCAGTGACGCGTAAATCGTAAGATCTACCTGCCGCAGG
TGGACCTACTGCAAATACGGATTATGACTCGTAAAGGGGTCATGCGTATTTTCATCACTAGGCACGTT
CGAGAATAAATTAGTAGGTGTCCCGAGCCTTGTGGCGTTCCGCCTGACTCCTCATGAAGTCGACCTTC
TCACCGGCCCTATCTGCCGACGTAAGTCATAACCTAGATCTGTACCTCGGGGGGAGGGTCACTGTAAA
GGGATAATTGGAGGGTGATTTCCCACATTTTCCTAAGGGTACTTTTTGCCTGGCTTCGCAATTGGGTC
CAATGGATGTCGATCTCTGGTTTAGCAGTTGTGAAAGTGGCAAGGCGGGAGGTTAGACCTCCATTTAA
CATATACAAGCAAGTTNACTGCACTAGATGNGTAGACACTACAGGTACAGGAGTAGCCGGATAAGTCT
CCGACGTCAAGCGAATAAGCGTCATACGCGATTATCGCCTAAGAGCACGTATTTGCGGTAAAAGGCTA
GCTAGACGCTTGTGGGTAGATTTCAAGGCGTTCGTAGTGGTATAATAGGATACTCTTTCACCAGCCTG
AGGGCCGAACGCTATACTAGTGGTCTGTGATGTAGGACCGAGTATCTCTCTAGGGACCATCTACTTGA
GCAATGGTGCGCAGGGGGAGACATAGACCAGCCTTGGGTGGCAAGCACTGCAATAAGTCCTGTTTAGC
CTTGGAGTTCACACGCCGGCACTGAAGCCGACCTACCTGAGCGTGTGCGATTACCGTTACAATGGCAT
CTGTCTAGTTCTGTTTACCTACGGCGCTCTTGGTTCCAGGTTAGGGGAAGTGTATGACCCATGTGTTT
TTATCGGCTTAACCACGAGTGATCCCCGGTCGTTTCCCCATTGAATCCCTGGTGCATCCTACTCCCAG
AATGATAGCTGACTGACTGGACTGGCTTTTCAAGTAATCGAGGGGGTATCGCGGTCACGGCCGTTAAC
AGATCCCGTCCTTAGTGTGGAATCCGCACCTGCTGACTAACGCTTCGCCGGCGTGTCTGCACAGCCGT
ATAGTGTTAATCATGACCCCAAGGAAGGATTAAACAAATATCTTGACG
>GIRAN13
GTGTCCGGTAGCCCGCGCTAGTTAGACACCCCGGCAGGGGGGATTGCTTTCGAGACGGGAGATCCCTT
CGCCGACCCTGGAGGGCCGACGCCGAGGCATTCGGGCCCCCGCAACGTCAACAGCGGCAAGAAAACGG
GATGAATGGGCGTAATGGGGGGGGTCTGCTGGGGACCCGACGCGGTTGCCGTTTGCGGGGCCCCCGAC
CCATACCGACCCACCTAGGCGTCCAGTTACGGCGCACGGCGGGAGCGTGGTTGCCGTCAGAGCTGTGT
TTCTCGATCAGTCCCCCGCAGTGCCGCAGTATCTTGCCGTGGGCTGCTTTAATCTTGAAAGTGGTTTA
TACATTGGGCGACGAGGTGTCGACTCTCATTGGGGGTAACCGACGGGCACATGCAGTCCCCTCCCCGG
GCAGGCAGAGGCGGGGCCCCCGCGCGCCGGCCCCGGCCCACAATCTTACCAGGGTCCTCAAAGGAGCC
TTTGCATGGTACCCTTCGTGAATGGTTGCTTAAGAGGTCCACAACGTGGTCCGGGCACGGTCGACTAA
ACACTCAAAACAGCGACGGCAAATATAGGTACAAGGTCCAGGCCCTCACGGCACTAATTGCGATGACC
CAACTCACGGGGGCAGTCCCCGGCGCGGCGACCTTGATTACGTCCGGGGACAAGTATGTCGCTCCCCC
CGGGAGGGGTGCAGCCACATGGGAGATTCAAAGTTTCTCGTGACGTCGTTGTGATCACTGCAGCCTAG
CCGAGACTCCCGTACTACGCGAAGGTTGGTTATGTTAACCACTACAACGTGAGGCGCCGTAAGGGCCA
GTGTTGTGCCCGGCTCTTCAATGCGCCTTAGCGGCCTGATACACCCACCCAAGGAGATACTGCTAATC
ATATGGGTGGACAGAACCTCGCAACGCAGACGCCTCCCCGGCATGATATGGTTTTTTTCCGCTATTAT
CCAGCACGCAGCGCTATCATTCAAGAGAACCCAGTGACGCGTAAATCGTAAGATCTACCTGCCGCAGG
TGGACCTACTGCAAATACGGATTATGACTCGTAAAGGGGTCATGCGTATTTTCATCACTAGGCACGTT
CGAGAATAAATTAGTAGGTGTCCCGAGCCTTGTGGCGTTCCGCCTGACTCCTCATGAAGTCGACCTTC
TCACCGGCCCTATCTGCCGACGTAAGTCATAACCTAGATCTGTACCTCGGGGGGAGGGTCACTGTAAA
GGGATAATTGGAGGGTGATTTCCCACACTTTCCTAAGGGTACTTTTTGCCTGGCTTCGCAATTGGGTC
CAATGGATGTCGATCTCTGGTTTAGCAGTTGTGAAAGTGGCAAGGCGGGAGGTTAGACCTCCATTTAA

CATATACAAGCAAGTTAACTGCACTAGATGTGTAGACACTACAGTTACAGGAGTAGCCGAATAAGTCT
CCGACGTCAAGCGAATAAGCGTCATACGCGATTATCGCCTAAGAGCACGTATTGGCGGTAAAAGGCTA
GCTAGACGCTTATGGGTAGATTTCAAGGCGTTCGTAGTGGTATAATAGGATACTCTTTCACCAGCCTG
AGGGCCGAACGCTATACTAGTGGTCTGTGATGTAGGACCGAGTATCTCTCTAGGGACCATCTACTTGA
GCAATGGTGCGCAGGGGGAGACATAGACCAGCCTTGGGTGGCAAGCACTGCAATAAGTCCTGTTTAGC
CTTGGAGTTCACATGCCGGCACTGAAGCCGACCTACCTGAGCGTGTGCGATTACCGTTACAATGGCAT
CTGTCTAGTTCTGTTTACCTACGGCGCTCTTGGTTCCAGGTTAGGGGAAGTGTATGACCCATGTGTTT
TTATCGGCTTAACCACGAGTGATCCCCGGTCGTTTCCCCATTGAATCCCTGGTGCATCCTACTCCCAG
AATGATAGCTGACTGACTGGACTGGCTTTTCAAGTAATCGAGGGGGTATCGCGGTCACGGCCGTTAAC
AGATCCCGTCCTTAGTGTGGAATCCGCACCTGCTGACTAACGCTTCGCCGGCGTGTCTGCACAGCCGT
ATAGTGTTAATCATGACCCCAAGGAAGGATTAAACAAATATCTTGACG
>GIRAN14
GTGTCCGGTAGCCCGCGCTAGTCAGACACCCCGGCAGGGGGGATTGCTTTCGAGACGGGAGATCCCTT
CGCCGACCCTGGAGGGCCGACGCCGAGGCATTCGGGCCCCTGCAACGTCAACAGCGGCAAGAAAACGG
GATGAATGGGCGTAATGGGGGGGGTCTGCTGGGGACCCGACGCGGTTGCCGTTTGCGGGGCCCCCGAC
CCATACCGACCCACCTAGGCGTCCAGTTACGGCGCACGGCGGGAGCGTGGTTGCCGTCAGAGCTGTGT
TTCTCGATCAGTCCCCCGCAGTGCCGCAGTATCTTGCCGTGGGCTGCTTTAATCTTGAAAGTGGTTCA
TACATTGGGCGACGAGGTGTCGACTCTCATTGGGGGTAACCGACGGGCACATGCAGTCCCCTCCCCGG
GCAGGCAGAGGCGGGGCCCCCGCGCGCCGGCCCCGGCCCACAATCTTACCAGGGTCCTCAAAGGAGCC
TTTGCATGGTACCCTTCGTGAATGGTTGCTTAAGAGGTCCACAACGTGGTCCGGGCACGGTCGACTAA
ACACTCAAAACAGCGACGGCAAATATAGGTACAAGGTCCAGGCCCTCACGGCACTAATTGCGATGACC
CAACTCACGGGGGCAGTCCCCGGCGCGGCGACCTTGATTACGTCCGGGGACAAGTATGTCGCTCCCCC
CGGGAGGGGTGCAGCCACATGGGAGATTCAAAGTTTCTCGTGACGTCGTTGTGATCACTGCAGCCTAG
CCGAGACTCCCGTACTACGCGAAGGTTGGTTATGTTAACCACTACAACGTGAGGCGCCGTAAGGGCCA
GTGTTGTGCCCGGCTCTTCAATGCGCCTTAGCGGCCTGATACACCCACCCAAGGAGATACTGCTAATC
ATATGGGTGGACAGAACCTCGCAACGCAGACGCCTCCCCGGCATGATATGGTTTTTTTCCGCTATTAT
CCAGCACGCAGCGCTATCATTTAAGAGAACCCAGTGACGCGTAAATCGTAAGATCTACCTGCCGCAGG
TGGACCTACTGCAAATACGGATTATGACTCGTAAAGGGGTCATGCGTATTTTCATCACTAGGCACGTT
CGAGAATAAATTAGTAGGTGTCCCGAGCCTTGTGGCGTTCCGCCTGACTCCTCATGAAGTCGACCTTC
TCACCGGCCCTATCTGCCGACGTAAGTCATAACCTAGATCTGTACCTCGGGGGGAGGGTCACTGTAAA
GGGATAATTGGAGGGTGATTTCCCACATTTTCCTAAGGGTACTTTTTGCCTGGCTTCGCAATTGGGTC
CAATGGATGTCGATCTCTGGTTTAGCAGTTGTGAAAGTGGCAAGGCGGGAGGTTAGACCTCCATTTAA
CATATACAAGCAAGTTAACTGCACTAGATGTGTAGACACTACAGGTACAGGAGTAGCCGGATAAGTCT
CCGACGTCAAGCGAATAAGCGTCATACGCGATTATCGCCTAAGAGCACGTATTTGCGGTAAAAGGCTA
GCTAGACGCTTGTGGGTAGATTTCAAGGCGTTCGTAGTGGTATAATAGGATACTCTTTCACCAGCCTG
AGGGCCGAACGCTATACTAGTGGTCTGTGATGTAGGACCGAGTATCTCTCTAGGGACCATCTACTTGA
GCAATGGTGCGCAGGGGGAGACATAGACCAGCCTTGGGTGGCAAGCACTGCAATAAGTCCTGTTTAGC
CTTGGAGTTCACACGCCGGCACTGAAGCCGACCTACCTGAGCGTGTGCGATTACCGTTACAATGGCAT
CTGTCTAGTTCTGTTTACCTACGGCGCTCTTGGTTCCAGGTTAGGGGAAGTGTATGACCCATGTGTTT
TTATCGGCTTAACCACGAGTGATCCCCGGTCGTTTCCCCATTGAATCCCTGGTGCATCCTACTCCCAG
AATGATAGCTGACTGACTGGACTGGCTTTTCAAGTAATCGAGGGGGTATCGCGGTCACGGCCGTTAAC
AGATCCCGTCCTTAGTGTGGAATCCGCACCTGCTGACTAACGCTTCGCCGGCGTGTCTGCACAGCCGT
ATAGTGTTAGTCATGACCCCAAGGAAGGATTAAACAAATATCTTGACG
>GIRAN15
GTGTCCGGTAGCCCGCGCTAGTTAGACACCCCGGCAGGGGGGATTGCTTTCGGGACGGGAGATCCCTT
CGCCGACCCTGGAGGGCCGACGCCGAGGCATTCGGGCCCCNGCAACGTCAACAGCGGCAAGAAAACGG
GATGAATGGGCGTAATGGGGGGGGTCTGCTGGGGACCCGACGCGGTTGCCGTTTGCGGGGCCCCCGAC
CCATACCGACCCACCTAGGCGTCCAGTTACGGCGCACGTCGGGAGCGTGGTTGCCGTCAGAGCTGTGT
TTCTCGATCAGTCCCCCACAGTGCCGCAGTATCTTGCCGTGGGCTGCTTTAATCTTGAAAGTGGTTNA
TACATTGGGCGACGAGGTGTCGACTCTCATTGGGGGTAACCGACGGGCACATGCAGTCCCCTCCCCGG
GCAGGCAGAGGCGGGGCCCCCGCGCGCCGGCCCCGGCCCACAATCTTACCAGGGTCCTCAAAGGAGCC
TTTGCATGGTACCCTTCGTGAATGGTTGCTTAAGAAGTCCACAACGTGGTCCGGGCACGGTCGACTAA
ACACTCAAAACAGCGACGGCAAATATAGGTACAAGGTCCAGGCCCTCACGGCACTAATTGCGATGACC
CAACTCACGGGGGCAGTCCCCGGCGCGGCGACCTTGATTACGTCCGGGGACAAGTATGTCGCTCCCCC

CGGGAGGGGTGCAGCCACATGGGAGATTCAAAGTTTCTCGTGACGTCGTTGTGATCACTGCAGCCTAG
CCGAGACTCCCGTACTACGCGAAGGTTGGTTATGTTAACCACTACAACGTGAGGCGCCGTAAGGGCCA
GTGTTGTGCCCGGCTCTTCAATGCGCCTTAGCGGCCTGATACACCCACCCAAGGAGATACTGCTAATC
ATATGAGTGGACAGAACCTCGCAACGCAGACGCCTCCCCGGCATGATATGGTTTTTTTCCGCTATTAT
CCAGCACGCAGCGCTATCATTCAAGAGAACCCAGTGACGCGTAAATCGTAAGATCTACCTGCCGCAGG
TGGACCTACTGCAAATACGGATTATGACTCGTAAAGGGGTCATGCGTATTTTCATCACTAGGCACGTT
CGAGAATAAATTAGTAGGTGTCCCGAGCCTTGTGGCGTTCCGCCTGACTCCTCATGAAGTCGACCTTC
TCACCGGCCCTATCTGCCGACGTAAGTCATAACCTAGATCTGTACCTCGGGGGGAGGGTCACTGTAAA
GGGATAATTGGAGGGTGATTTCCCACATTTTCCTAAGGGTACTTTTTGCCTGGCTTCGCAATTGGGTC
CAATGGATGTCGATCTCTGGTTTAGCAGTTGTGAAAGTGGCAAGGCGGGAGGTTAGACCTCCATTTAA
CATATACAAGCAAGTTAACTGCACTAGATGTGTAGACACTACAGGTACAGGAGTAGCCGGATAAGTCT
CCGACGTCAAGCGAATAAGCTTCATACGCGATTATCGCCTAAGAGCACGTATTTGCGGTAAAAGGCTA
GCTAGACGCTTGTGGGTAGATTTCAAGGCGTTCGTAGTGGTATAATAGGATACTCTTTCACCAGCCTG
AGGGCCGAACGCTATACTAGTGGTCTGTGATGTAGGACCGAGTATCTCTCTAGGGACCATCTACTTGA
GCAATGGTGCGCAGGGGGAGACATAGACCAGCCTTGGGTGGCAAGCACTGCAATAAGTCCTGTTTAGC
CTTGGAGTTCACACGCCGGCACTGAAGCCGACCTACCTGAGCGTGTGCGATTACCGTTACAATGGCAT
CTGTCTAGTTCTGTTTACCTACGGCGCTCTTGGTTCCAGGTTAGGGGAAGTGTATGACCCATGTGTTT
TCATCGGCTTAACCACGAGTGATCCCCGGTCGTTTCCCCATTGAATCCCTGGTGCATCCTACTCCCAG
AATGATAGCTGACTGACTGGACTGGCTTTTCAAGTAATCGAGGGGGTATCGCGGTCACGGCCGTTAAC
AGATCCCGTCCTTAGTGTGGAATCCGCACCTGCTGACTAACGCTTCGCCGGCGTGTCTGCACAGCCGT
ATAGTGTTAATCATGACCCCAAGGAAGGATTAAACAAATATCTTGACG
>GIRAN16
GTGTCCGGTAGCCCGCGCTAGTTAGACACCCCGGCAGGGGGGATTGCTTTCGAGACGGGAGATCCCTT
CGCCGACCCTGGAGGGCCGACGCCGAGGCATTCGGGCCCCCGCAACGTCAACAGCGGCAAGAAAACGG
GATGAATGGGCGTAATGGGGGGGGTCTGCTGGGGACCCGACGCGGTTGCCGTTTGCGGGGCCCCCGAC
CCATACCGACCCACCTAGGCGTCCAGTTACGGCGCACGGCGGGAGCGTGGTTGCCGTCAGAGCTGTGT
TTCTCGATCAGTCCCCCGCAGTGCCGCAGTATCTTGCCGTGGGCTGCTTTAATCTTGAAAGTGGTTCA
TACATTGGGCGACGAGGTGTCGACTCTCATTGGGGGTAACCGACGGGCACATGCAGTCCCCTCCCCGG
GCAGGCAGAGGCGGGGCCCCCGCGCGCCGGCCCCGGCCCACAATCTTACCAGGGTCCTCAAAGGAGCC
TTTGCATGGTACCCTTCGTGAATGGTTGCTTAAGAGGTCCACAACGTGGTCCGGGCACGGTCGACTAA
ACACTCAAAACAGCGACGGCAAATATAGGTACAAGGTCCAGGCCCTCACGGCACTAATTGCGATGACC
CAACTCACGGGGGCAGTCCCCGGCGCGGCGACCTTGATTACGTCCGGGGACAAGTATGTCGCTCCCCC
CGGGAGGGGTGCAGCCACATGGGAGATTCAAAGTTTCTCGTGACGTCGTTGTGATCACTGCAGCCTAG
CCGAGACTCCCGTACTACGCGAAGGTTGGTTATGTTAACCACTACAACGTGAGGCGCCGTAAGGGCCA
GTGTTGTGCCCGGCTCTTCAATGCGCCTTAGCGGCCTGATACACCCACCCAAGGAGATACTGCTAATC
ATATGGGTGGACAGAACCTCGCAACGCAGACGCCTCCCCGGCATGATATGGTTTTTTTCCGCTATTAT
CCAGCACGCAGCGCTATCATTCAAGAGAACCCAGTGACGCGTAAATCGTAAGATCTACCTGCCGCAGG
TGGACCTACTGCAAATACGGATTATGACTCGTAAAGGGGTCATGCGTATTTTCATCACTAGGCACGTT
CGAGAATAAATTAGTAGGTGTCCCGAGCCTTGTGGCGTTCCGCCTGACTCCTCATGAAGTCGACCTTC
TCACCGGCCCTATCTGCCGACGTAAGTCATAACCTAGATCTGTACCTCGGGGGGAGGGTCACTGTAAA
GGGATAATTGGAGGGTGATTTCCCACACTTTCCTAAGGGTACTTTTTGCCTGGCTTCGCAATTGGGTC
CAATGGATGTCGATCTCTGGTTTAGCAGTTGTGAAAGTGGCAAGGCGGGAGGTTAGACCTCCATTTAA
CATATACAAGCAAGTTAACTGCACTAGATGTGTAGACACTACAGTTACAGGAGTAGCCGAATAAGTCT
CCGACGTCAAGCGAATAAGCGTCATACGCGATTATCGCCTAAGAGCACGTATTGGCGGTAAAAGGCTA
GCTAGACGCTTATGGGTAGATTTCAAGGCGTTCGTAGTGGTATAATAGGATACTCTTTCACCAGCCTG
AGGGCCGAACGCTATACTAGTGGTCTGTGATGTAGGACCGAGTATCTCTCTAGGGACCATCTACTTGA
GCAATGGTGCGCAGGGGGAGACATAGACCAGCCTTGGGTGGCAAGCACTGCAATAAGTCCTGTTTAGC
CTTGGAGTTCACATGCCGGCACTGAAGCCGACCTACCTGAGCGTGTGCGATTACCGTTACAATGGCAT
CTGTCTAGTTCTGTTTACCTACGGCGCTCTTGGTTCCAGGTTAGGGGAAGTGTATGACCCATGTGTTT
TTATCGGCTTAACCACGAGTGATCCCCGGTCGTTTCCCCATTGAATCCCTGGTGCATCCTACTCCCAG
AATGATAGCTGACTGACTGGACTGGCTTTTCAAGTAATCGAGGGGGTATCGCGGTCACGGCCGTTAAC
AGATCCCGTCCTTAGTGTGGAATCCGCACCTGCTGACTAACGCTTCGCCGGCGTGTCTGCACAGCCGT
ATAGTGTTAATCATGACCCCAAGGAAGGATTAAACAAATATCTTGACG
>GIRAN17

GTGTCCGGTAGCCCGCGCTAGTCAGACACCCCGGCAGGGGGGATTGCTTTCGAGACGGGAGATCCCTT
CGCCGACCCTGGAGGGCCGACGCCGAGGCATTCGGGCCCCTGCAACGTCAACAGCGGCAAAAAAACGG
GATGAATGGGCGTAATGGGGGGGGTCTGCTGGGGACCCGACGCGGTTGCCGTTTGCGGGGCCCCCGAC
CCATACCGACCCACCTAGGCGTCCAGTTACGGCGCACGGCGGGAGCGTGGTTGCCGTCAGAGCTGTGT
TTCTCGATCAGTCCCCCGCAGTGCCGCAGTATCTTGCCGTGGGCTGCTTTAATCTTGAAAGTGGTTTA
TACATTGGGCGACGAGGTGTCGACTCTCATTGGGGGTAACCGACGGGCACATGCAGTCCCCTCCCCGG
GCAGGCAGAGGCGGGGCCCCCGCGCGCCGGCCCCGGCCCACAATCTTACCAGGGTCCTCAAAGGAGCC
TTTGCATGGTACCCTTCGTGAATGGTTGCTTAAGAGGTCCACAACGTGGTCCGGGCACGGTCGACTAA
ACACTCAAAACAGCGACGGCAAATATAGGTACAAGGTCCAGGCCCTCACGGCACTAATTGCGATGACC
CAACTCACGGGGGCAGTCCCCGGCGCGGCGACCTTGATTACGTCCGGGGACAAGTATGTCGCTCCCCC
CGGGAGGGGTGCAGCCACATGGGAGATTCAAAGTTTCTCGTGACGTCGTTGTGATCACTGCAGCCTAG
CCGAGACTCCCGTACTACGCGAAGGTTGGTTATGTTAACCACTACAACGTGAGGCGCCGTAAGGGCCA
GTGTTGTGCCCGGCTCTTCAATGCGCCTTAGCGGCCTGATACACCCACCCAAGGAGATACTGCTAATC
ATATGGGTGGACAGAACCTCGCAACGCAGACGCCTCCCCGGCATGATATGGTTTTTTTCCGCTATTAT
CCAGCACGCAGCGCTATCATTTAAGAGAACCCAGTGACGCGTAAATCGTAAGATCTACCTGCCGCAGG
TGGACCTACTGCAAATACGGATTATGACTCGTAAAGGGGTCATGCGTATTTTCATCACTAGGCACGTT
CGAGAATAAATTAGTAGGTGTCCCGAGCCTTGTGGCGTTCCGCCTGACTCCTCATGAAGTCGACCTTC
TCACCGGCCCTATCTGCCGACGTAAGTCATAACCTAGATCTGTACCTCGGGGGGAGGGTCACTGTAAA
GGGATAATTGGAGGGTGATTTCCCACATTTTCCTAAGGGTACTTTTTGCCTGGCTTCGCAATTGGGTC
CAATGGATGTCGATCTCTGGTTTAGCAGTTGTGAAAGTGGCAAGGCGGGAGGTTAGACCTCCATTTAA
CATATACAAGCAAGTTAACTGCACTAGATGTGTAGACACTACAGGTACAGGAGTAGCCGGATAAGTCT
CCGACGTCAAGCGAATAAGCGTCATACGCGATTATCGCCTAAGAGCACGTATTTGCGGTAAAAGGCTA
GCTAGACGCTTGTGGGTAGATTTCAAGGCGTTCGTAGTGGTATAATAGGATACTCTTTCACCAGCCTG
AGGGCCGAACGCTATACTAGTGGTCTGTGATGTAGGACCGAGTATCTCTCTAGGGACCATCTACTTGA
GCAATGGTGCGCAGGGGGAGACATAGACCAGCCTTGGGTGGCAAGCACTGCAATAAGTCCTGTTTAGC
CTTGGAGTTCACACGCCGGCACTGAAGCCGACCTACCTGAGCGTGTGCGATTACCGTTACAATGGCAT
CTGTCTAGTTCTGTTTACCTACGGCGCTCTTGGTTCCAGGTTAGGGGAAGTGTATGACCCATGTGTTT
TTATCGGCTTAACCACGAGTGATCCCCGGTCGTTTCCCCATTGAATCCCTGGTGCATCCTACTCCCAG
AATGATAGCTGACTGACTGGACTGGCTTTTCAAGTAATCGAGGGGGTATCGCGGTCACGGCCGTTAAC
AGATCCCGTCCTTAGTGTGGAATCCGCACCTGCTGACTAACGCTTCGCCGGCGTGTCTGCACAGCCGT
ATAGTGTTAATCATGACCCCAAGGAAGGATTAAACAAATATCTTGACG
>GIRAN19
GTGTCCGGTAGCCCGCGCTAGTTAGACACCCCGGCAGGGGGGATTGCTTTCGAGACGGGAGATCCCTT
CGCCGACCCTGGAGGGCCGACGCCGAGGCATTCGGGCCCCCGCAACGTCAACAGCGGCAAGAAAACGG
GATGAATGGGCGTAATGGGGGGGGTCTGCTGGGGACCCGACGCGGTTGCCGTTTGCGGGGCCCCCGAC
CCATACCGACCCACCTAGGCGTCCAGTTACGGCGCACGGCGGGAGCGTGGTTGCCGTCAGAGCTGTGT
TTCTCGATCAGTCCCCCGCAGTGCCGCAGTATCTTGCCGTGGGCTGCTTTAATCTTGAAAGTGGTTTA
TACATTGGGCGACGAGGTGTCGACTCTCATTGGGGGTAACCGACGGGCACATGCAGTCCCCTCCCCGG
GCAGGCAGAGGCGGGGCCCCCGCGCGCCGGCCCCGGCCCACAATCTTACCAGGGTCCTCAAAGGAGCC
TTTGCATGGTACCCTTCGTGAATGGTTGCTTAAGAGGTCCACAACGTGGTCCGGGCACGGTCGACTAA
ACACTCAAAACAGCGACGGCAAATATAGGTACAAGGTCCAGGCCCTCACGGCACTAATTGCGATGACC
CAACTCACGGGGGCAGTCCCCGGCGCGGCGACCTTGATTACGTCCGGGGACAAGTATGTCGCTCCCCC
CGGGAGGGGTGCAGCCACATGGGAGATTCAAAGTTTCTCGTGACGTCGTTGTGATCACTGCAGCCTAG
CCGAGACTCCCGTACTACGCGAAGGTTGGTTATGTTAACCACTACAACGTGAGGCGCCGTAAGGGCCA
GTGTTGTGCCCGGCTCTTCAATGCGCCTTAGCGGCCTGATACACCCACCCAAGGAGATACTGCTAATC
ATATGGGTGGACAGAACCTCGCAACGCAGACGCCTCCCCGGCATGATATGGTTTTTTTCCGCTATTAT
CCAGCACGCAGCGCTATCATTCAAGAGAACCCAGTGACGCGTAAATCGTAAGATCTACCTGCCGCAGG
TGGACCTACTGCAAATACGGATTATGACTCGTAAAGGGGTCATGCGTATTTTCATCACTAGGCACGTT
CGAGAATAAATTAGTAGGTGTCCCGAGCCTTGTGGCGTTCCGCCTGACTCCTCATGAAGTCGACCTTC
TCACCGGCCCTATCTGCCGACGTAAGTCATAACCTAGATCTGTACCTCGGGGGGAGGGTCACTGTAAA
GGGATAATTGGAGGGTGATTTCCCACACTTTCCTAAGGGTACTTTTTGCCTGGCTTCGCAATTGGGTC
CAATGGATGTCGATCTCTGGTTTAGCAGTTGTGAAAGTGGCAAGGCGGGAGGTTAGACCTCCATTTAA
CATATACAAGCAAGTTAACTGCACTAGATGTGTAGACACTACAGTTACAGGAGTAGCCGAATAAGTCT
CCGACGTCAAGCGAATAAGCGTCATACGCGATTATCGCCTAAGAGCACGTATTGGCGGTAAAAGGCTA

GCTAGACGCTTATGGGTAGATTTCAAGGCGTTCGTAGTGGTATAATAGGATACTCTTTCACCAGCCTG
AGGGCCGAACGCTATACTAGTGGTCTGTGATGTAGGACCGAGTATCTCTCTAGGGACCATCTACTTGA
GCAATGGTGCGCAGGGGGAGACATAGACCAGCCTTGGGTGGCAAGCACTGCAATAAGTCCTGTTTAGC
CTTGGAGTTCACATGCCGGCACTGAAGCCGACCTACCTGAGCGTGTGCGATTACCGTTACAATGGCAT
CTGTCTAGTTCTGTTTACCTACGGCGCTCTTGGTTCCAGGTTAGGGGAAGTGTATGACCCATGTGTTT
TTATCGGCTTAACCACGAGTGATCCCCGGTCGTTTCCCCATTGAATCCCTGGTGCATCCTACTCCCAG
AATGATAGCTGACTGACTGGACTGGCTTTTCAAGTAATCGAGGGGGTATCGCGGTCACGGCCGTTAAC
AGATCCCGTCCTTAGTGTGGAATCCGCACCTGCTGACTAACGCTTCGCCGGCGTGTCTGCACAGCCGT
ATAGTGTTAATCATGACCCCAAGGAAGGATTAAACAAATATCTTGACG
>GIRAN23
GTGTCCGGTAGCCCGCGCTAGTCAGACACCCCGGCAGGGGGGATTGCTTTCGGGACGGGAGATCCCTT
CGCCGACCCTGGAGGGCCGACGCCGAGGCATTCGGGCCCCTGCAACGTCAACAGCGGCAAGAAAACGG
GATGAATGGGCGTAATGGGGGGGGTCTGCTGGGGACCCGACGCGGTTGCCGTTTGCGGGGCCCCCGAC
CCATACCGACCCACCTAGGCGTCCAGTTACGGCGCACGTCGGGAGCGTGGTTGCCGTCAGAGCTGTGT
TTCTCGATCAGTCCCCCGCAGTGCCGCAGTATCTTGCCGTGGGCTGCTTTAATCTTGAAAGTGGTTCA
TACATTGGGCGACGAGGTGTCGACTCTCATTGGGGGTAACCGACGGGCACATGCAGTCCCCTCCCCGG
GCAGGCAGAGGCGGGGCCCCCGCGCGCCGGCCCCGGCCCACAATCTTACCAGGGTCCTCAAAGGAGCC
TTTGCATGGTACCCTTCGTGAATGGTTGCTTAAGAGGTCCACAACGTGGTCCGGGCACGGTCGACTAA
ACACTCAAAACAGCGACGGCAAATATAGGTACAAGGTCCAGGCCCTCACGGCACTAATTGCGATGACC
CAACTCACGGGGGCAGTCCCCGGCGCGGCGACCTTGATTACGTCCGGGGACAAGTATGTCGCTCCCCC
CGGGAGGGGTGCAGCCACATGGGAGATTCAAAGTTTCTCGTGACGTCGTTGTGATCACTGCAGCCTAG
CCGAGACTCCCGTACTACGCGAAGGTTGGTTATGTTAACCACTACAACGTGAGGCGCCGTAAGGGCCA
GTGTTGTGCCCGGCTCTTCAATGCGCCTTAGCGGCCTGATACACCCACCCAAGGAGATACTGCTAATC
ATATGGGTGGACAGAACCTCGCAACGCAGACGCCTCCCCGGCATGATATGGTTTTTTTCCGCTATTAT
CCAGCACGCAGCGCTATCATTCAAGAGAACCCAGTGACGCGTAAATCGTAAGATCTACCTGCCGCAGG
TGGACCTACTGCAAATACGGATTATGACTCGTAAAGGGGTCATGCGTATTTTCATCACTAGGCACGTT
CGAGAATAAATTAGTAGGTGTCCCGAGCCTTGTGGCGTTCCGCCTGACTCCTCATAAAGTCGACCTTC
TCACCGGCCCTATCTGCCGACGTAAGTCATAACCTAGATCTGTACCTCGGGGGGAGGGTCACTGTAAA
GGGATAATTGGAGGGTGATTTCCCACATTTTCCTAAGGGTACTTTTTGCCTGGCTTCGCAATTGGGTC
CAATGGATGTCGATCTCTGGTTTAGCAGTTGTGAAAGTGGCAAGGCGGGAGGTTAGACCTCCATTTAA
CATATACAAGCAAGTTAACTGCACTAGATGTGTAGACACTACAGGTACAGGAGTAGCCGGATAAGTCT
CCGACGTCAAGCGAATAAGCGTCATACGCGATTATCGCCTAAGAGCACGTATTTGCGGTAAAAGGCTA
GCTAGACGCTTGTGGGTAGATTTCAAGGCGTTCGTAGTGGTATAATAGGATACTCTTTCACCAGCCTG
AGGGCCGAACGCTATACTAGTGGTCTGTGATGTAGGACCGAGTATCTCTCTAGGGACCATCTACTTGA
GCAATGGTGCGCAGGGGGAGACATAGACCAGCCTTGGGTGGCAAGCACTGCAATAAGTCCTGTTTAGC
CTTGGAGTTCACACGCCGGCACTGAAGCCGACCTACCTGAGCGTGTGCGATTACCGTTACAATGGCAT
CTGTCTAGTTCTGTTTACCTACGGCGCTCTTGGTTCCAGGTTAGGGGAAGTGTATGACCCATGTGTTT
TTATCGGCTTAACCACGAGTGATCCCCGGTCGTTTCCCCATTGAATCCCTGGTGCATCCTACTCCCAG
AATGATAGCTGACTGACTGGACTGGCTTTTCAAGTAATCGAGGGGGTATCGCGGTCACGGCCGTTAAC
AGATCCCGTCCTTAGTGTGGAATCCGCACCTGCTGACTAACGCTTCGCCGGCGTGTCTGCACAGCCGT
ATAGTGTTAATCATGACCCCAAGGAAGGATTAAACAAATATCTTGACG
>GIRAN26
GTGTCCGGTAGCCCGCGCTAGTTAGACACCCCGGCAGGGGGGATTGCTTTCGAGACGGGAGATCCCTT
CGCCGACCCTGGAGGGCCGACGCCGAGGCATTCGGGCCCCCGCAACGTCAACAGCGGCAAGAAAACGG
GATGAATGGGCGTAATGGGGGGGGTCTGCTGGGGACCCGACGCGGTTGCCGTTTGCGGGGCCCCCGAC
CCATACCGACCCACCTAGGCGTCCAGTTACGGCGCACGGCGGGAGCGTGGTTGCCGTCAGAGCTGTGT
TTCTCGATCAGTCCCCCGCAGTGCCGCAGTATCTTGCCGTGGGCTGCTTTAATCTTGAAAGTGGTTTA
TACATTGGGCGACGAGGTGTCGACTCTCATTGGGGGTAACCGACGGGCACATGCAGTCCCCTCCCCGG
GCAGGCAGAGGCGGGGCCCCCGCGCGCCGGCCCCGGCCCACAATCTTACCAGGGTCCTCAAAGGAGCC
TTTGCATGGTACCCTTCGTGAATGGTTGCTTAAGAGGTCCACAACGTGGTCCGGGCACGGTCGACTAA
ACACTCAAAACAGCGACGGCAAATATAGGTACAAGGTCCAGGCCCTCACGGCACTAATTGCGATGACC
CAACTCACGGGGGCAGTCCCCGGCGCGGCGACCTTGATTACGTCCGGGGACAAGTATGTCGCTCCCCC
CGGGAGGGGTGCAGCCACATGGGAGATTCAAAGTTTCTCGTGACGTCGTTGTGATCACTGCAGCCTAG
CCGAGACTCCCGTACTACGCGAAGGTTGGTTATGTTAACCACTACAACGTGAGGCGCCGTAAGGGCCA

GTGTTGTGCCCGGCTCTTCAATGCGCCTTAGCGGCCTGATACACCCACCCAAGGAGATACTGCTAATC
ATATGGGTGGACAGAACCTCGCAACGCAGACGCCTCCCCGGCATGATATGGTTTTTTTCCGCTATTAT
CCAGCACGCAGCGCTATCATTCAAGAGAACCCAGTGACGCGTAAATCGTAAGATCTACCTGCCGCAGG
TGGACCTACTGCAAATACGGATTATGACTCGTAAAGGGGTCATGCGTATTTTCATCACTAGGCACGTT
CGAGAATAAATTAGTAGGTGTCCCGAGCCTTGTGGCGTTCCGCCTGACTCCTCATGAAGTCGACCTTC
TCACCGGCCCTATCTGCCGACGTAAGTCATAACCTAGATCTGTACCTCGGGGGGAGGGTCACTGTAAA
GGGATAATTGGAGGGTGATTTCCCACACTTTCCTAAGGGTACTTTTTGCCTGGCTTCGCAATTGGGTC
CAATGGATGTCGATCTCTGGTTTAGCAGTTGTGAAAGTGGCAAGGCGGGAGGTTAGACCTCCATTTAA
CATATACAAGCAAGTTAACTGCACTAGATGTGTAGACACTACAGTTACAGGAGTAGCCGAATAAGTCT
CCGACGTCAAGCGAATAAGCGTCATACGCGATTATCGCCTAAGAGCACGTATTGGCGGTAAAAGGCTA
GCTAGACGCTTATGGGTAGATTTCAAGGCGTTCGTAGTGGTATAATAGGATACTCTTTCACCAGCCTG
AGGGCCGAACGCTATACTAGTGGTCTGTGATGTAGGACCGAGTATCTCTCTAGGGACCATCTACTTGA
GCAATGGTGCGCAGGGGGAGACATAGACCAGCCTTGGGTGGCAAGCACTGCAATAAGTCCTGTTTAGC
CTTGGAGTTCACATGCCGGCACTGAAGCCGACCTACCTGAGCGTGTGCGATTACCGTTACAATGGCAT
CTGTCTAGTTCTGTTTACCTACGGCGCTCTTGGTTCCAGGTTAGGGGAAGTGTATGACCCATGTGTTT
TTATCGGCTTAACCACGAGTGATCCCCGGTCGTTTCCCCATTGAATCCCTGGTGCATCCTACTCCCAG
AATGATAGCTGACTGACTGGACTGGCTTTTCAAGTAATCGAGGGGGTATCGCGGTCACGGCCGTTAAC
AGATCCCGTCCTTAGTGTGGAATCCGCACCTGCTGACTAACGCTTCGCCGGCGTGTCTGCACAGCCGT
ATAGTGTTAATCATGACCCCAAGGAAGGATTAAACAAATATCTTGACG
>GIRAN27
GTGTCCGGTAGCCCGCGCTAGTTAGACACCCCGGCAGGGGGGATTGCTTTCGAGACGGGAGATCCCTT
CGCCGACCCTGGAGGGCCGACGCCGAGGCATTCGGGCCCCTGCAACGTCAACAGCGGCAAGAAAACGG
GATGAATGGGCGTAATGGGGGGGGTCTGCTGGGGACCCGACGCGGTTGCCGTTTGCGGGGCCCCCGAC
CCATACCGACCCACCTAGGCGTCCAGTTACGGCGCACGTCGGGAGCGTGGTTGCCGTCAGAGCTGTGT
TTCTCGATCAGTCCCCCGCAGTGCCGCAGTATCTTGCCGTGGGCTGCTTTAATCTTGAAAGTGGTTTA
TACATTGGGCGACGAGGTGTCGACTCTCATTGGGGGTAACCGACGGGCACATGCAGTCCCCTCCCCGG
GCAGGCAGAGGCGGGGCCCCCGCGCGCCGGCCCCGGCCCACAATCTTACCAGGGTCCTCAAAGGAGCC
TTTGCATGGTACCCTTCGTGAATGGTTGCTTAAGAGGTCCACAACGTGGTCCGGGCACGGTCGACTAA
ACACTCAAAACAGCGACGGCAAATATAGGTACAAGGTCCAGGCCCTCACGGCACTAATTGCGATGACC
CAACTCACGGGGGCAGTCCCCGGCGCGGCGACCTTGATTACGTCCGGGGACAAGTATGTCGCTCCCCC
CGGGAGGGGTGCAGCCACATGGGAGATTCAAAGTTTCTCGTGACGTCGTTGTGATCACTGCAGCCTAG
CCGAGACTCCCGTACTACGCGAAGGTTGGTTATGTTAACCACTACAACGTGAGGCGCCGTAAGGGCCA
GTGTTGTGCCCGGCTCTTCAATGCGCCTTAGCGGCCTGATACACCCACCCAAGGAGATACTGCTAATC
ATATGGGTGGACAGAACCTCGCAACGCAGACGCCTCCCCGGCATGATATGGTTTTTTTCCGCTATTAT
CCAGCACGCAGCGCTATCATTCAAGAGAACCCAGTGACGCGTAAATCGTAAGATCTACCTGCCGCAGG
TGGACCTACTGCAAATACGGATTATGACTCGTAAAGGGGTCATGCGTATTTTCATCACTAGGCACGTT
CGAGAATAAATTAGTAGGTGTCCCGAGCCTTGTGGCGTTCCGCCTGACTCCTCATGAAGTCGACCTTC
TCACCGGCCCTATCTGCCGACGTAAGTCATAACCTAGATCTGTACCTCGGGGGGAGGGTCACTGTAAA
GGGATAATTGGAGGGTGATTTCCCACACTTTCCTAAGGGTACTTTTTGCCTGGCTTCGCAATTGGGTC
CAATGGATGTCGATCTCTGGTTTAGCAGTTGTGAAAGTGGCAAGGCGGGAGGTTAGACCTCCATTTAA
CATATACAAGCAAGTTAACTGCACTAGATGTGTAGACACTACAGTTACAGGAGTAGCCGAATAAGTCT
CCGACGTCAAGCGAATAAGCGTCATACGCGATTATCGCCTAAGAGCACGTATTGGCGGTAAAAGGCTA
GCTAGACGCTTATGGGTAGATTTCAAGGCGTTCGTAGTGGTATAATAGGATACTCTTTCACCAGCCTG
AGGGCCGAACGCTATACTAGTGGTCTGTGATGTAGGACCGAGTATCTCTCTAGGGACCATCTACTTGA
GCAATGGTGCGCAGGGGGAGACATAGACCAGCCTTGGGTGGCAAGCACTGCAATAAGTCCTGTTTAGC
CTTGGAGTTCACATGCCGGCACTGAAGCCGACCTACCTGAGCGTGTGCGATTACCGTTACAATGGCAT
CTGTCTAGTTCTGTTTACCTACGGCGCTCTTGGTTCCAGGTTAGGGGAAGTGTATGACCCATGTGTTT
TTATCGGCTTAACCACGAGTGATCCCCGGTCGTTTCCCCATTGAATCCCTGGTGCATCCTACTCCCAG
AATGATAGCTGACTGACTGGACTGGCTTTTCAAGTAATCGAGGGGGTATCGCGGTCACGGCCGTTAAC
AGATCCCGTCCTTAGTGTGGAATCCGCACCTGCTGACTAACGCTTCGCCGGCGTGTCTGCACAGCCGT
ATAGTGTTAATCATGACCCCAAGGAAGGATTAAACAAATATCTTGACG
>GIRAN29
GTGTCCGGTAGCCCGCGCTAGTTAGACACCCCGGCAGGGGGGATTGCTTTCGAGACGGGAGATCCCTT
CGCCGACCCTGGAGGGCCGACGCCGAGGCATTCGGGCCCCTGCAACGTCAACAGCGGCAAGAAAACGG

GATGAATGGGCGTAATGGGGGGGGTCTGCTGGGGACCCGACGCGGTTGCCGTTTGCGGGGCCCCCGAC
CCATACCGACCCACCTAGGCGTCCAGTTACGGCGCACGGCGGGAGCGTGGTTGCCGTCAGAGCTGTGT
TTCTCGATCAGTCCCCCGCAGTGCCGCAGTATCTTGCCGTGGGCTGCTTTAATCTTGAAAGTGGTTTA
TACATTGGGCGACGAGGTGTCGACTCTCATTGGGGGTAACCGACGGGCACATGCAGTCCCCTCCCCGG
GCAGGCAGAGGCGGGGCCCCCGCGCGCCGGCCCCGGCCCACAATCTTACCAGGGTCCTCAAAGGAGCC
TTTGCATGGTACCCTTCGTGAATGGTTGCTTAAGAGGTCCACAACGTGGTCCGGGCACGGTCGACTAA
ACACTCAAAACAGCGACGGCAAATATAGGTACAAGGTCCAGGCCCTCACGGCACTAATTGCGATGACC
CAACTCACGGGGGCAGTCCCCGGCGCGGCGACCTTGATTACGTCCGGGGACAAGTATGTCGCTCCCCC
CGGGAGGGGTGCAGCCACATGGGAGATTCAAAGTTTCTCGTGACGTCGTTGTGATCACTGCAGCCTAG
CCGAGACTCCCGTACTACGCGAAGGTTGGTTATGTTAACCACTACAACGTGAGGCGCCGTAAGGGCCA
GTGTTGTGCCCGGCTCTTCAATGCGCCTTAGCGGCCTGATACACCCACCCAAGGAGATACTGCTAATC
ATATGGGTGGACAGAACCTCGCAACGCAGACGCCTCCCCGGCATGATATGGTTTTTTTCCGCTATTAT
CCAGCACGCAGCGCTATCATTCAAGAGAACCCAGTGACGCGTAAATCGTAAGATCTACCTGCCGCAGG
TGGACCTACTGCAAATACGGATTATGACTCGTAAAGGGGTCATGCGTATTTTCATCACTAGGCACGTT
CGAGAATAAATTAGTAGGTGTCCCGAGCCTTGTGGCGTTCCGCCTGACTCCTCATGAAGTCGACCTTC
TCACCGGCCCTATCTGCCGACGTAAGTCATAACCTAGATCTGTACCTCGGGGGGAGGGTCACTGTAAA
GGGATAATTGGAGGGTGATTTCCCACACTTTCCTAAGGGTACTTTTTGCCTGGCTTCGCAATTGGGTC
CAATGGATGTCGATCTCTGGTTTAGCAGTTGTGAAAGTGGCAAGGCGGGAGGTTAGACCTCCATTTAA
CATATACAAGCAAGTTAACTGCACTAGATGTGTAGACACTACAGTTACAGGAGTAGCCGAATAAGTCT
CCGACGTCAAGCGAATAAGCGTCATACGCGATTATCGCCTAAGAGCACGTATTGGCGGTAAAAGGCTA
GCTAGACGCTTATGGGTAGATTTCAAGGCGTTCGTAGTGGTATAATAGGATACTCTTTCACCAGCCTG
AGGGCCGAACGCTATACTAGTGGTCTGTGATGTAGGACCGAGTATCTCTCTAGGGACCATCTACTTGA
GCAATGGTGCGCAGGGGGAGACATAGACCAGCCTTGGGTGGCAAGCACTGCAATAAGTCCTGTTTAGC
CTTGGAGTTCACATGCCGGCACTGAAGCCGACCTACCTGAGCGTGTGCGATTACCGTTACAATGGCAT
CTGTCTAGTTCTGTTTACCTACGGCGCTCTTGGTTCCAGGTTAGGGGAAGTGTATGACCCATGTGTTT
TTATCGGCTTAACCACGAGTGATCCCCGGTCGTTTCCCCATTGAATCCCTGGTGCATCCTACTCCCAG
AATGATAGCTGACTGACTGGACTGGCTTTTCAAGTAATCGAGGGGGTATCGCGGTCACGGCCGTTAAC
AGATCCCGTCCTTAGTGTGGAATCCGCACCTGCTGACTAACGCTTCGCCGGCGTGTCTGCACAGCCGT
ATAGTGTTAATCATGACCCCAAGGAAGGATTAAACAAATATCTTGACG
>GIRAN30
GTGTCCGGTAGCCCGCGCTAGTTAGACACCCCGGCAGGGGGGATTGCTTTCGAGACGGGAGATCCCTT
CGCCGACCCTGGAGGGCCGACGCCGAGGCATTCGGGCCCCCGCAACGTCAACAGCGGCAAGAAAACGG
GATGAATGGGCGTAATGGGGGGGGTCTGCTGGGGACCCGACGCGGTTGCCGTTTGCGGGGCCCCCGAC
CCATACCGACCCACCTAGGCGTCCAGTTACGGCGCACGGCGGGAGCGTGGTTGCCGTCAGAGCTGTGT
TTCTCGATCAGTCCCCCGCAGTGCCGCAGTATCTTGCCGTGGGCTGCTTTAATCTTGAAAGTGGTTTA
TACATTGGGCGACGAGGTGTCGACTCTCATTGGGGGTAACCGACGGGCACATGCAGTCCCCTCCCCGG
GCAGGCAGAGGCGGGGCCCCCGCGCGCCGGCCCCGGCCCACAATCTTACCAGGGTCCTCAAAGGAGCC
TTTGCATGGTACCCTTCGTGAATGGTTGCTTAAGAGGTCCACAACGTGGTCCGGGCACGGTCGACTAA
ACACTCAAAACAGCGACGGCAAATATAGGTACAAGGTCCAGGCCCTCACGGCACTAATTGCGATGACC
CAACTCACGGGGGCAGTCCCCGGCGCGGCGACCTTGATTACGTCCGGGGACAAGTATGTCGCTCCCCC
CGGGAGGGGTGCAGCCACATGGGAGATTCAAAGTTTCTCGTGACGTCGTTGTGATCACTGCAGCCTAG
CCGAGACTCCCGTACTACGCGAAGGTTGGTTATGTTAACCACTACAACGTGAGGCGCCGTAAGGGCCA
GTGTTGTGCCCGGCTCTTCAATGCGCCTTAGCGGCCTGATACACCCACCCAAGGAGATACTGCTAATC
ATATGGGTGGACAGAACCTCGCAACGCAGACGCCTCCCCGGCATGATATGGTTTTTTTCCGCTATTAT
CCAGCACGCAGCGCTATCATTCAAGAGAACCCAGTGACGCGTAAATCGTAAGATCTACCTGCCGCAGG
TGGACCTACTGCAAATACGGATTATGACTCGTAAAGGGGTCATGCGTATTTTCATCACTAGGCACGTT
CGAGAATAAATTAGTAGGTGTCCCGAGCCTTGTGGCGTTCCGCCTGACTCCTCATGAAGTCGACCTTC
TCACCGGCCCTATCTGCCGACGTAAGTCATAACCTAGATCTGTACCTCGGGGGGAGGGTCACTGTAAA
GGGATAATTGGAGGGTGATTTCCCACACTTTCCTAAGGGTACTTTTTGCCTGGCTTCGCAATTGGGTC
CAATGGATGTCGATCTCTGGTTTAGCAGTTGTGAAAGTGGCAAGGCGGGAGGTTAGACCTCCATTTAA
CATATACAAGCAAGTTAACTGCACTAGATGTGTAGACACTACAGTTACAGGAGTAGCCGAATAAGTCT
CCGACGTCAAGCGAATAAGCGTCATACGCGATTATCGCCTAAGAGCACGTATTGGCGGTAAAAGGCTA
GCTAGACGCTTATGGGTAGATTTCAAGGCGTTCGTAGTGGTATAATAGGATACTCTTTCACCAGCCTG
AGGGCCGAACGCTATACTAGTGGTCTGTGATGTAGGACCGAGTATCTCTCTAGGGACCATCTACTTGA

GCAATGGTGCGCAGGGGGAGACATAGACCAGCCTTGGGTGGCAAGCACTGCAATAAGTCCTGTTTAGC
CTTGGAGTTCACATGCCGGCACTGAAGCCGACCTACCTGAGCGTGTGCGATTACCGTTACAATGGCAT
CTGTCTAGTTCTGTTTACCTACGGCGCTCTTGGTTCCAGGTTAGGGGAAGTGTATGACCCATGTGTTT
TTATCGGCTTAACCACGAGTGATCCCCGGTCGTTTCCCCATTGAATCCCTGGTGCATCCTACTCCCAG
AATGATAGCTGACTGACTGGACTGGCTTTTCAAGTAATCGAGGGGGTATCGCGGTCACGGCCGTTAAC
AGATCCCGTCCTTAGTGTGGAATCCGCACCTGCTGACTAACGCTTCGCCGGCGTGTCTGCACAGCCGT
ATAGTGTTAATCATGACCCCAAGGAAGGATTAAACAAATATCTTGACG
>GIRAN31
GTGTCCGGTAGCCCGCGCTAGTTAGACACCCCGGCAGGGGGGATTGCTTTCGGGACGGGAGATCCCTT
CGCCGACCCTGGAGGGCCGACGCCGAGGCATTCGGGCCCCCGCAACGTCAACAGCGGCAAGAAAACGG
GATGAATGGGCGTAATGGGGGGGGTCTGCTGGGGACCCGACGCGGTTGCCGTTTGCGGGGCCCCCGAC
CCATACCGACCCACCTAGGCGTCCAGTTACGGCGCACGTCGGGAGCGTGGTTGCCGTCAGAGCTGTGT
TTCTCGATCAGTCCCCCGCAGTGCCGCAGTATCTTGCCGTGGGCTGCTTTAATCTTGAAAGTGGTTNA
TACATTGGGCGACGAGGTGTCGACTCTCATTGGGGGTAACCGACGGGCACATGCAGTCCCCTCCCCGG
GCAGGCAGAGGCGGGGCCCCCGCGCGCCGGCCCCGGCCCACAATCTTACCAGGGTCCTCAAAGGAGCC
TTTGCATGGTACCCTTCGTGAATGGTTGCTTAAGAGGTCCACAACGTGGTCCGGGCACGGTCGACTAA
ACACTCAAAACAGCGACGGCAAATATAGGTACAAGGTCCAGGCCCTCACGGCACTAATTGCGATGACC
CAACTCACGGGGGCAGTCCCCGGCGCGGCGACCTTGATTACGTCCGGGGACAAGTATGTCGCTCCCCC
CGGGAGGGGTGCAGCCACATGGGAGATTCAAAGTTTCTCGTGACGTCGTTGTGATCACTGCAGCCTAG
CCGAGACTCCCGTACTACGCGAAGGTTGGTTATGTTAACCACTACAACGTGAGGCGCCGTAAGGGCCA
GTGTTGTGCCCGGCTCTTCAATGCGCCTTAGCGGCCTGATACACCCACCCAAGGAGATACTGCTAATC
ATATGGGTGGACAGAACCTCGCAACGCAGACGCCTCCCCGGCATGATATGGTTTTTTTCCGCTATTAT
CCAGCACGCAGCGCTATCATTCAAGAGAACCCAGTGACGCGTAAATCGTAAGATCTACCTGCCGCAGG
TGGACCTACTGCAAATACGGATTATGACTCGTAAAGGGGTCATGCGTATTTTCATCACTAGGCACGTT
CGAGAATAAATTAGTAGGTGTCCCGAGCCTTGTGGCGTTCCGCCTGACTCCTCATGAAGTCGACCTTC
TCACCGGCCCTATCTGCCGACGTAAGTCATAACCTAGATCTGTACCTCGGGGGGAGGGTCACTGTAAA
GGGATAATTGGAGGGTGATTTCCCACACTTTCCTAAGGGTACTTTTTGCCTGGCTTCGCAATTGGGTC
CAATGGATGTCGATCTCTGGTTTAGCAGTTGTGAAAGTGGCAAGGCGGGAGGTTAGACCTCCATTTAA
CATATACAAGCAAGTTAACTGCACTAGATGTGTAGACACTACAGTTACAGGAGTAGCCGAATAAGTCT
CCGACGTCAAGCGAATAAGCGTCATACGCGATTATCGCCTAAGAGCACGTATTGGCGGTAAAAGGCTA
GCTAGACGCTTATGGGTAGATTTCAAGGCGTTCGTAGTGGTATAATAGGATACTCTTTCACCAGCCTG
AGGGCCGAACGCTATACTAGTGGTCTGTGATGTAGGACCGAGTATCTCTCTAGGGACCATCTACTTGA
GCAATGGTGCGCAGGGGGAGACATAGACCAGCCTTGGGTGGCAAGCACTGCAATAAGTCCTGTTTAGC
CTTGGAGTTCACATGCCGGCACTGAAGCCGACCTACCTGAGCGTGTGCGATTACCGTTACAATGGCAT
CTGTCTAGTTCTGTTTACCTACGGCGCTCTTGGTTCCAGGTTAGGGGAAGTGTATGACCCATGTGTTT
TTATCGGCTTAACCACGAGTGATCCCCGGTCGTTTCCCCATTGAATCCCTGGTGCATCCTACTCCCAG
AATGATAGCTGACTGACTGGACTGGCTTTTCAAGTAATCGAGGGGGTATCGCGGTCACGGCCGTTAAC
AGATCCCGTCCTTAGTGTGGAATCCGCACCTGCTGACTAACGCTTCGCCGGCGTGTCTGCACAGCCGT
ATAGTGTTAATCATGACCCCAAGGAAGGATTAAACAAATATCTTGACG
>HREN03
GTGTCCGGTAGCCCGCGCTAGTTAGACACCCCGGCAGGGGGGATTGCTTTCGAGACGGGAGATCCCTT
CGCCGACCCTGGAGGGCCGACGCCGAGGCATTCGGGCCCCCGCAACGTCAACAGCGGCAAGAAAACGG
GATGAATGGGCGTAATGGGGGGGGTCTGCTGGGGACCCGACGCGGTTGCCGTTTGCGGGGCCCCCGAC
CCATACCGACCCACCTAGGCGTCCAGTTACGGCGCACGTCGGGAGCGTGGTTGCCGTCAGAGCTGTGT
TTCTCGATCAGTCCCCCGCAGTGCCGCAGTATCTTGCCGTGGGCTGCTTTAATCTTGAAAGTGGTTTA
TACATTGGGCGACGAGGTGTCGACTCTCATTGGGGGTAACCGACGGGCACATGCAGTCCCCTCCCCGG
GCAGGCAGAGGCGGGGCCCCCGCGCGCCGGCCCCGGCCCACAATCTTACCAGGGTCCTCAAAGGAGCC
TTTGCATGGTACCCTTCGTGAATGGTTGCTTAAGAGGTCCACAACGTGGTCCGGGCACGGTCGACTAA
ACACTCAAAACAGCGACGGCAAATATAGGTACAAGGTCCAGGCCCTCACGGCACTAATTGCGATGACC
CAACTCACGGGGGCAGTCCCCGGCGCGGCGACCTTGATTACGTCCGGGGACAAGTATGTCGCTCCCCC
CGGGAGGGGTGCAGCCACATGGGAGATTCAAAGTTTCTCGTGACGTCGTTGTGATCACTGCAGCCTAG
CCGAGACTCCCGTACTACGCGAAGGTTGGTTATGTTAACCACTACAACGTGAGGCGCCGTAAGGGCCA
GTGTTGTGCCCGGCTCTTCAATGCGCCTTAGCGGCCTGATACACCCACCCAAGGAGATACTGCTAATC
ATATGGGTGGACAGAACCTCGCAACGCAGACGCCTCCCCGGCATGATATGGTTTTTTTCCGCTATTAT

CCAGCACGCAGCGCTATCATTCAAGAGAACCCAGTGACGCGTAAATCGTAAGATCTACCTGCCGCAGG
TGGACCTACTGCAAATACGGATTATGACTCGTAAAGGGGTCATGCGTATTTTCATCACTAGGCACGTT
CGAGAATAAATTAGTAGGTGTCCCGAGCCTTGTGGCGTTCCGCCTGACTCCTCATGAAGTCGACCTTC
TCACCGGCCCTATCTGCCGACGTAAGTCATAACCTAGATCTGTACCTCGGGGGGAGGGTCACTGTAAA
GGGATAATTGGAGGGTGATTTCCCACACTTTCCTAAGGGTACTTTTTGCCTGGCTTCGCAATTGGGTC
CAATGGATGTCGATCTCTGGTTTAGCAGTTGTGAAAGTGGCAAGGCGGGAGGTTAGACCTCCATTTAA
CATATACAAGCAAGTTAACTGCACTAGATGTGTAGACACTACAGTTACAGGAGTAGCCGAATAAGTCT
CCGACGTCAAGCGAATAAGCGTCATACGCGATTATCGCCTAAGAGCACGTATTGGCGGTAAAAGGCTA
GCTAGACGCTTATGGGTAGATTTCAAGGCGTTCGTAGTGGTATAATAGGATACTCTTTCACCAGCCTG
AGGGCCGAACGCTATACTAGTGGTCTGTGATGTAGGACCGAGTATCTCTCTAGGGACCATCTACTTGA
GCAATGGTGCGCAGGGGGAGACATAGACCAGCCTTGGGTGGCAAGCACTGCAATAAGTCCTGTTTAGC
CTTGGAGTTCACATGCCGGCACTGAAGCCGACCTACCTGAGCGTGTGCGATTACCGTTACAATGGCAT
CTGTCTAGTTCTGTTTACCTACGGCGCTCTTGGTTCCAGGTTAGGGGAAGTGTATGACCCATGTGTTT
TTATCGGCTTAACCACGAGTGATCCCCGGTCGTTTCCCCATTGAATCCCTGGTGCATCCTACTCCCAG
AATGATAGCTGACTGACTGGACTGGCTTTTCAAGTAATCGAGGGGGTATCGCGGTCACGGCCGTTAAC
AGATCCCGTCCTTAGTGTGGAATCCGCACCTGCTGACTAACGCTTCGCCGGCGTGTCTGCACAGCCGT
ATAGTGTTAATCATGACCCCAAGGAAGGATTAAACAAATATCTTGACG
>HREN05
GTGTCCGGTAGCCCGCGCTAGTTAGACACCCCGGCAGGGGGGATTGCTTTCGAGACGGGAGATCCCTT
CGCCGACCCTGGAGGGCCGACGCCGAGGCATTCGGGCCCCTGCAACGTCAACAGCGGCAAGAAAACGG
GATGAATGGGCGTAATGGGGGGGGTCTGCTGGGGACCCGACGCGGTTGCCGTTTGCGGGGCCCCCGAC
CCATACCGACCCACCTAGGCGTCCAGTTACGGCGCACGTCGGGAGCGTGGTTGCCGTCAGAGCTGTGT
TTCTCGATCAGTCCCCCGCAGTGCCGCAGTATCTTGCCGTGGGCTGCTTTAATCTTGAAAGTGGTTTA
TACATTGGGCGACGAGGTGTCGACTCTCATTGGGGGTAACCGACGGGCACATGCAGTCCCCTCCCCGG
GCAGGCAGAGGCGGGGCCCCCGCGCGCCGGCCCCGGCCCACAATCTTACCAGGGTCCTCAAAGGAGCC
TTTGCATGGTACCCTTCGTGAATGGTTGCTTAAGAGGTCCACAACGTGGTCCGGGCACGGTCGACTAA
ACACTCAAAACAGCGACGGCAAATATAGGTACAAGGTCCAGGCCCTCACGGCACTAATTGCGATGACC
CAACTCACGGGGGCAGTCCCCGGCGCGGCGACCTTGATTACGTCCGGGGACAAGTATGTCGCTCCCCC
CGGGAGGGGTGCAGCCACATGGGAGATTCAAAGTTTCTCGTGACGTCGTTGTGATCACTGCAGCCTAG
CCGAGACTCCCGTACTACGCGAAGGTTGGTTATGTTAACCACTACAACGTGAGGCGCCGTAAGGGCCA
GTGTTGTGCCCGGCTCTTCAATGCGCCTTAGCGGCCTGATACACCCACCCAAGGAGATACTGCTAATC
ATATGGGTGGACAGAACCTCGCAACGCAGACGCCTCCCCGGCATGATATGGTTTTTTTCCGCTATTAT
CCAGCACGCAGCGCTATCATTCAAGAGAACCCAGTGACGCGTAAATCGTAAGATCTACCTGCCGCAGG
TGGACCTACTGCAAATACGGATTATGACTCGTAAAGGGGTCATGCGTATTTTCATCACTAGGCACGTT
CGAGAATAAATTAGTAGGTGTCCCGAGCCTTGTGGCGTTCCGCCTGACTCCTCATGAAGTCGACCTTC
TCACCGGCCCTATCTGCCGACGTAAGTCATAACCTAGATCTGTACCTCGGGGGGAGGGTCACTGTAAA
GGGATAATTGGAGGGTGATTTCCCACACTTTCCTAAGGGTACTTTTTGCCTGGCTTCGCAATTGGGTC
CAATGGATGTCGATCTCTGGTTTAGCAGTTGTGAAAGTGGCAAGGCGGGAGGTTAGACCTCCATTTAA
CATATACAAGCAAGTTAACTGCACTAGATGTGTAGACACTACAGTTACAGGAGTAGCCGAATAAGTCT
CCGACGTCAAGCGAATAAGCGTCATACGCGATTATCGCCTAAGAGCACGTATTGGCGGTAAAAGGCTA
GCTAGACGCTTATGGGTAGATTTCAAGGCGTTCGTAGTGGTATAATAGGATACTCTTTCACCAGCCTG
AGGGCCGAACGCTATACTAGTGGTCTGTGATGTAGGACCGAGTATCTCTCTAGGGACCATCTACTTGA
GCAATGGTGCGCAGGGGGAGACATAGACCAGCCTTGGGTGGCAAGCACTGCAATAAGTCCTGTTTAGC
CTTGGAGTTCACATGCCGGCACTGAAGCCGACCTACCTGAGCGTGTGCGATTACCGTTACAATGGCAT
CTGTCTAGTTCTGTTTACCTACGGCGCTCTTGGTTCCAGGTTAGGGGAAGTGTATGACCCATGTGTTT
TTATCGGCTTAACCACGAGTGATCCCCGGTCGTTTCCCCATTGAATCCCTGGTGCATCCTACTCCCAG
AATGATAGCTGACTGACTGGACTGGCTTTTCAAGTAATCGAGGGGGTATCGCGGTCACGGCCGTTAAC
AGATCCCGTCCTTAGTGTGGAATCCGCACCTGCTGACTAACGCTTCGCCGGCGTGTCTGCACAGCCGT
ATAGTGTTAATCATGACCCCAAGGAAGGATTAAACAAATATCTTGACG
>HREN07
GTGTCCGGTAGCCCGCGCTAGTTAGACACCCCGGCAGGGGGGATTGCTTTCGAGACGGGAGATCCCTT
CGCCGACCCTGGAGGGCCGACGCCGAGGCATTCGGGCCCCTGCAACGTCAACAGCGGCAAGAAAACGG
GATGAATGGGCGTAATGGGGGGGGTCTGCTGGGGACCCGACGCGGTTGCCGTTTGCGGGGCCCCCGAC
CCATACCGACCCACCTAGGCGTCCAGTTACGGCGCACGTCGGGAGCGTGGTTGCCGTCAGAGCTGTGT

TTCTCGATCAGTCCCCCGCAGTGCCGCAGTATCTTGCCGTGGGCTGCTTTAATCTTGAAAGTGGTTCA
TACATTGGGCGACGAGGTGTCGACTCTCATTGGGGGTAACCGACGGGCACATGCAGTCCCCTCCCCGG
GCAGGCAGAGGCGGGGCCCCCGCGCGCCGGCCCCGGCCCACAATCTTACCAGGGTCCTCAAAGGAGCC
TTTGCATGGTACCCTTCGTGAATGGTTGCTTAAGAGGTCCACAACGTGGTCCGGGCACGGTCGACTAA
ACACTCAAAACAGCGACGGCAAATATAGGTACAAGGTCCAGGCCCTCACGGCACTAATTGCGATGACC
CAACTCACGGGGGCAGTCCCCGGCGCGGCGACCTTGATTACGTCCGGGGACAAGTATGTCGCTCCCCC
CGGGAGGGGTGCAGCCACATGGGAGATTCAAAGTTTCTCGTGACGTCGTTGTGATCACTGCAGCCTAG
CCGAGACTCCCGTACTACGCGAAGGTTGGTTATGTTAACCACTACAACGTGAGGCGCCGTAAGGGCCA
GTGTTGTGCCCGGCTCTTCAATGCGCCTTAGCGGCCTGATACACCCACCCAAGGAGATACTGCTAATC
ATATGGGTGGACAGAACCTCGCAACGCAGACGCCTCCCCGGCATGATATGGTTTTTTTCCGCTATTAT
CCAGCACGCAGCGCTATCATTCAAGAGAACCCAGTGACGCGTAAATCGTAAGATCTACCTGCCGCAGG
TGGACCTACTGCAAATACGGATTATGACTCGTAAAGGGGTCATGCGTATTTTCATCACTAGGCACGTT
CGAGAATAAATTAGTAGGTGTCCCGAGCCTTGTGGCGTTCCGCCTGACTCCTCATGAAGTCGACCTTC
TCACCGGCCCTATCTGCCGACGTAAGTCATAACCTAGATCTGTACCTCGGGGGGAGGGTCACTGTAAA
GGGATAATTGGAGGGTGATTTCCCACACTTTCCTAAGGGTACTTTTTGCCTGGCTTCGCAATTGGGTC
CAATGGATGTCGATCTCTGGTTTAGCAGTTGTGAAAGTGGCAAGGCGGGAGGTTAGACCTCCATTTAA
CATATACAAGCAAGTTAACTGCACTAGATGTGTAGACACTACAGTTACAGGAGTAGCCGAATAAGTCT
CCGACGTCAAGCGAATAAGCGTCATACGCGATTATCGCCTAAGAGCACGTATTGGCGGTAAAAGGCTA
GCTAGACGCTTATGGGTAGATTTCAAGGCGTTCGTAGTGGTATAATAGGATACTCTTTCACCAGCCTG
AGGGCCGAACGCTATACTAGTGGTCTGTGATGTAGGACCGAGTATCTCTCTAGGGACCATCTACTTGA
GCAATGGTGCGCAGGGGGAGACATAGACCAGCCTTGGGTGGCAAGCACTGCAATAAGTCCTGTTTAGC
CTTGGAGTTCACATGCCGGCACTGAAGCCGACCTACCTGAGCGTGTGCGATTACCGTTACAATGGCAT
CTGTCTAGTTCTGTTTACCTACGGCGCTCTTGGTTCCAGGTTAGGGGAAGTGTATGACCCATGTGTTT
TTATCGGCTTAACCACGAGTGATCCCCGGTCGTTTCCCCATTGAATCCCTGGTGCATCCTACTCCCAG
AATGATAGCTGACTGACTGGACTGGCTTTTCAAGTAATCGAGGGGGTATCGCGGTCACGGCCGTTAAC
AGATCCCGTCCTTAGTGTGGAATCCGCACCTGCTGACTAACGCTTCGCCGGCGTGTCTGCACAGCCGT
ATAGTGTTAATCATGACCCCAAGGAAGGATTAAACAAATATCTTGACG
>HREN08
GTGTCCGGTAGCCCGCGCTAGTTAGACACCCCGGCAGGGGGGATTGCTTTCGGGACGGGAGATCCCTT
CGCCGACCCTGGAGGGCCGACGCCGAGGCATTCGGGCCCCTGCAACGTCAACAGCGGCAAGAAAACGG
GATGAATGGGCGTAATGGGGGGGGTCTGCTGGGGACCCGACGCGGTTGCCGTTTGCGGGGCCCCCGAC
CCATACCGACCCACCTAGGCGTCCAGTTACGGCGCACGGCGGGAGCGTGGTTGCCGTCAGAGCTGTGT
TTCTCGATCAGTCCCCCGCAGTGCCGCAGTATCTTGCCGTGGGCTGCTTTAATCTTGAAAGTGGTTCA
TACATTGGGCGACGAGGTGTCGACTCTCATTGGGGGTAACCGACGGGCACATGCAGTCCCCTCCCCGG
GCAGGCAGAGGCGGGGCCCCCGCGCGCCGGCCCCGGCCCACAATCTTACCAGGGTCCTCAAAGGAGCC
TTTGCATGGTACCCTTCGTGAATGGTTGCTTAAGAGGTCCACAACGTGGTCCGGGCACGGTCGACTAA
ACACTCAAAACAGCGACGGCAAATATAGGTACAAGGTCCAGGCCCTCACGGCACTAATTGCGATGACC
CAACTCACGGGGGCAGTCCCCGGCGCGGCGACCTTGATTACGTCCGGGGACAAGTATGTCGCTCCCCC
CGGGAGGGGTGCAGCCACATGGGAGATTCAAAGTTTCTCGTGACGTCGTTGTGATCACTGCAGCCTAG
CCGAGACTCCCGTACTACGCGAAGGTTGGTTATGTTAACCACTACAACGTGAGGCGCCGTAAGGGCCA
GTGTTGTGCCCGGCTCTTCAATGCGCCTTAGCGGCCTGATACACCCACCCAAGGAGATACTGCTAATC
ATATGGGTGGACAGAACCTCGCAACGCAGACGCCTCCCCGGCATGATATGGTTTTTTTCCGCTATTAT
CCAGCACGCAGCGCTATCATTCAAGAGAACCCAGTGACGCGTAAATCGTAAGATCTACCTGCCGCAGG
TGGACCTACTGCAAATACGGATTATGACTCGTAAAGGGGTCATGCGTATTTTCATCACTAGGCACGTT
CGAGAATAAATTAGTAGGTGTCCCGAGCCTTGTGGCGTTCCGCCTGACTCCTCATGAAGTCGACCTTC
TCACCGGCCCTATCTGCCGACGTAAGTCATAACCTAGATCTGTACCTCGGGGGGAGGGTCACTGTAAA
GGGATAATTGGAGGGTGATTTCCCACACTTTCCTAAGGGTACTTTTTGCCTGGCTTCGCAATTGGGTC
CAATGGATGTCGATCTCTGGTTTAGCAGTTGTGAAAGTGGCAAGGCGGGAGGTTAGACCTCCATTTAA
CATATACAAGCAAGTTAACTGCACTAGATGTGTAGACACTACAGTTACAGGAGTAGCCGAATAAGTCT
CCGACGTCAAGCGAATAAGCGTCATACGCGATTATCGCCTAAGAGCACGTATTGGCGGTAAAAGGCTA
GCTAGACGCTTATGGGTAGATTTCAAGGCGTTCGTAGTGGTATAATAGGATACTCTTTCACCAGCCTG
AGGGCCGAACGCTATACTAGTGGTCTGTGATGTAGGACCGAGTATCTCTCTAGGGACCATCTACTTGA
GCAATGGTGCGCAGGGGGAGACATAGACCAGCCTTGGGTGGCAAGCACTGCAATAAGTCCTGTTTAGC
CTTGGAGTTCACATGCCGGCACTGAAGCCGACCTACCTGAGCGTGTGCGATTACCGTTACAATGGCAT

CTGTCTAGTTCTGTTTACCTACGGCGCTCTTGGTTCCAGGTTAGGGGAAGTGTATGACCCATGTGTTT
TTATCGGCTTAACCACGAGTGATCCCCGGTCGTTTCCCCATTGAATCCCTGGTGCATCCTACTCCCAG
AATGATAGCTGACTGACTGGACTGGCTTTTCAAGTAATCGAGGGGGTATCGCGGTCACGGCCGTTAAC
AGATCCCGTCCTTAGTGTGGAATCCGCACCTGCTGACTAACGCTTCGCCGGCGTGTCTGCACAGCCGT
ATAGTGTTAATCATGACCCCAAGGAAGGATTAAACAAATATCTTGACG
>HREN09
GTGTCCGGTAGCCCGCGCTAGTTAGACACCCCGGCAGGGGGGATTGCTTTCGGGACGGGAGATCCCTT
CGCCGACCCTGGAGGGCCGACGCCGAGGCATTCGGGCCCCTGCAACGTCAACAGCGGCAAGAAAACGG
GATGAATGGGCGTAATGGGGGGGGTCTGCTGGGGACCCGACGCGGTTGCCGTTTGCGGGGCCCCCGAC
CCATACCGACCCACCTAGGCGTCCAGTTACGGCGCACGGCGGGAGCGTGGTTGCCGTCAGAGCTGTGT
TTCTCGATCAGTCCCCCGCAGTGCCGCAGTATCTTGCCGTGGGCTGCTTTAATCTTGAAAGTGGTTCA
TACATTGGGCGACGAGGTGTCGACTCTCATTGGGGGTAACCGACGGGCACATGCAGTCCCCTCCCCGG
GCAGGCAGAGGCGGGGCCCCCGCGCGCCGGCCCCGGCCCACAATCTTACCAGGGTCCTCAAAGGAGCC
TTTGCATGGTACCCTTCGTGAATGGTTGCTTAAGAGGTCCACAACGTGGTCCGGGCACGGTCGACTAA
ACACTCAAAACAGCGACGGCAAATATAGGTACAAGGTCCAGGCCCTCACGGCACTAATTGCGATGACC
CAACTCACGGGGGCAGTCCCCGGCGCGGCGACCTTGATTACGTCCGGGGACAAGTATGTCGCTCCCCC
CGGGAGGGGTGCAGCCACATGGGAGATTCAAAGTTTCTCGTGACGTCGTTGTGATCACTGCAGCCTAG
CCGAGACTCCCGTACTACGCGAAGGTTGGTTATGTTAACCACTACAACGTGAGGCGCCGTAAGGGCCA
GTGTTGTGCCCGGCTCTTCAATGCGCCTTAGCGGCCTGATACACCCACCCAAGGAGATACTGCTAATC
ATATGGGTGGACAGAACCTCGCAACGCAGACGCCTCCCCGGCATGATATGGTTTTTTTCCGCTATTAT
CCAGCACGCAGCGCTATCATTCAAGAGAACCCAGTGACGCGTAAATCGTAAGATCTACCTGCCGCAGG
TGGACCTACTGCAAATACGGATTATGACTCGTAAAGGGGTCATGCGTATTTTCATCACTAGGCACGTT
CGAGAATAAATTAGTAGGTGTCCCGAGCCTTGTGGCGTTCCGCCTGACTCCTCATGAAGTCGACCTTC
TCACCGGCCCTATCTGCCGACGTAAGTCATAACCTAGATCTGTACCTCGGGGGGAGGGTCACTGTAAA
GGGATAATTGGAGGGTGATTTCCCACACTTTCCTAAGGGTACTTTTTGCCTGGCTTCGCAATTGGGTC
CAATGGATGTCGATCTCTGGTTTAGCAGTTGTGAAAGTGGCAAGGCGGGAGGTTAGACCTCCATTTAA
CATATACAAGCAAGTTAACTGCACTAGATGTGTAGACACTACAGTTACAGGAGTAGCNGAATAAGTCT
CCGACGTCAAGCGAATAAGCGTCATACGCGATTATCGCCTAAGAGCACGTATTGGCGGTAAAAGGCTA
GCTAGACGCTTATGGGTAGATTTCAAGGCGTTCGTAGTGGTATAATAGGATACTCTTTCACCAGCCTG
AGGGCCGAACGCTATACTAGTGGTCTGTGATGTAGGACCGAGTATCTCTCTAGGGACCATCTACTTGA
GCAATGGTGCGCAGGGGGAGACATAGACCAGCCTTGGGTGGCAAGCACTGCAATAAGTCCTGTTTAGC
CTTGGAGTTCACATGCCGGCACTGAAGCCGACCTACCTGAGCGTGTGCGATTACCGTTACAATGGCAT
CTGTCTAGTTCTGTTTACCTACGGCGCTCTTGGTTCCAGGTTAGGGGAAGTGTATGACCCATGTGTTT
TTATCGGCTTAACCACGAGTGATCCCCGGTCGTTTCCCCATTGAATCCCTGGTGCATCCTACTCCCAG
AATGATAGCTGACTGACTGGACTGGCTTTTCAAGTAATCGAGGGGGTATCGCGGTCACGGCCGTTAAC
AGATCCCGTCCTTAGTGTGGAATCCGCACCTGCTGACTAACGCTTCGCCGGCGTGTCTGCACAGCCGT
ATAGTGTTAATCATGACCCCAAGGAAGGATTAAACAAATATCTTGACG
>HREN11
GTGTCCGGTAGCCCGCGCTAGTTAGACACCCCGGCAGGGGGGATTGCTTTCGAGACGGGAGATCCCTT
CGCCGACCCTGGAGGGCCGACGCCGAGGCATTCGGGCCCCCGCAACGTCAACAGCGGCAAGAAAACGG
GATGAATGGGCGTAATGGGGGGGGTCTGCTGGGGACCCGACGCGGTTGCCGTTTGCGGGGCCCCCGAC
CCATACCGACCCACCTAGGCGTCCAGTTACGGCGCACGTCGGGAGCGTGGTTGCCGTCAGAGCTGTGT
TTCTCGATCAGTCCCCCGCAGTGCCGCAGTATCTTGCCGTGGGCTGCTTTAATCTTGAAAGTGGTTNA
TACATTGGGCGACGAGGTGTCGACTCTCATTGGGGGTAACCGACGGGCACATGCAGTCCCCTCCCCGG
GCAGGCAGAGGCGGGGCCCCCGCGCGCCGGCCCCGGCCCACAATCTTACCAGGGTCCTCAAAGGAGCC
TTTGCATGGTACCCTTCGTGAATGGTTGCTTAAGAGGTCCACAACGTGGTCCGGGCACGGTCGACTAA
ACACTCAAAACAGCGACGGCAAATATAGGTACAAGGTCCAGGCCCTCACGGCACTAATTGCGATGACC
CAACTCACGGGGGCAGTCCCCGGCGCGGCGACCTTGATTACGTCCGGGGACAAGTATGTCGCTCCCCC
CGGGAGGGGTGCAGCCACATGGGAGATTCAAAGTTTCTCGTGACGTCGTTGTGATCACTGCAGCCTAG
CCGAGACTCCCGTACTACGCGAAGGTTGGTTATGTTAACCACTACAACGTGAGGCGCCGTAAGGGCCA
GTGTTGTGCCCGGCTCTTCAATGCGCCTTAGCGGCCTGATACACCCACCCAAGGAGATACTGCTAATC
ATATGGGTGGACAGAACCTCGCAACGCAGACGCCTCCCCGGCATGATATGGTTTTTTTCCGCTATTAT
CCAGCACGCAGCGCTATCATTCAAGAGAACCCAGTGACGCGTAAATCGTAAGATCTACCTGCCGCAGG
TGGACCTACTGCAAATACGGATTATGACTCGTAAAGGGGTCATGCGTATTTTCATCACTAGGCACGTT

CGAGAATAAATTAGTAGGTGTCCCGAGCCTTGTGGCGTTCCGCCTGACTCCTCATGAAGTCGACCTTC
TCACCGGCCCTATCTGCCGACGTAAGTCATAACCTAGATCTGTACCTCGGGGGGAGGGTCACTGTAAA
GGGATAATTGGAGGGTGATTTCCCACACTTTCCTAAGGGTACTTTTTGCCTGGCTTCGCAATTGGGTC
CAATGGATGTCGATCTCTGGTTTAGCAGTTGTGAAAGTGGCAAGGCGGGAGGTTAGACCTCCATTTAA
CATATACAAGCAAGTTAACTGCACTAGATGTGTAGACACTACAGTTACAGGAGTAGCCGAATAAGTCT
CCGACGTCAAGCGAATAAGCGTCATACGCGATTATCGCCTAAGAGCACGTATTGGCGGTAAAAGGCTA
GCTAGACGCTTATGGGTAGATTTCAAGGCGTTCGTAGTGGTATAATAGGATACTCTTTCACCAGCCTG
AGGGCCGAACGCTATACTAGTGGTCTGTGATGTAGGACCGAGTATCTCTCTAGGGACCATCTACTTGA
GCAATGGTGCGCAGGGGGAGACATAGACCAGCCTTGGGTGGCAAGCACTGCAATAAGTCCTGTTTAGC
CTTGGAGTTCACATGCCGGCACTGAAGCCGACCTACCTGAGCGTGTGCGATTACCGTTACAATGGCAT
CTGTCTAGTTCTGTTTACCTACGGCGCTCTTGGTTCCAGGTTAGGGGAAGTGTATGACCCATGTGTTT
TTATCGGCTTAACCACGAGTGATCCCCGGTCGTTTCCCCATTGAATCCCTGGTGCATCCTACTCCCAG
AATGATAGCTGACTGACTGGACTGGCTTTTCAAGTAATCGAGGGGGTATCGCGGTCACGGCCGTTAAC
AGATCCCGTCCTTAGTGTGGAATCCGCACCTGCTGACTAACGCTTCGCCGGCGTGTCTGCACAGCCGT
ATAGTGTTAATCATGACCCCAAGGAAGGATTAAACAAATATCTTGACG
>HREN12
GTGTCCGGTAGCCCGCGCTAGTTAGACACCCCGGCAGGGGGGATTGCTTTCGAGACGGGAGATCCCTT
CGCCGACCCTGGAGGGCCGACGCCGAGGCATTCGGGCCCCTGCAACGTCAACAGCGGCAAGAAAACGG
GATGAATGGGCGTAATGGGGGGGGTCTGCTGGGGACCCGACGCGGTTGCCGTTTGCGGGGCCCCCGAC
CCATACCGACCCACCTAGGCGTCCAGTTACGGCGCACGTCGGGAGCGTGGTTGCCGTCAGAGCTGTGT
TTCTCGATCAGTCCCCCGCAGTGCCGCAGTATCTTGCCGTGGGCTGCTTTAATCTTGAAAGTGGTTTA
TACATTGGGCGACGAGGTGTCGACTCTCATTGGGGGTAACCGACGGGCACATGCAGTCCCCTCCCCGG
GCAGGCAGAGGCGGGGCCCCCGCGCGCCGGCCCCGGCCCACAATCTTACCAGGGTCCTCAAAGGAGCC
TTTGCATGGTACCCTTCGTGAATGGTTGCTTAAGAGGTCCACAACGTGGTCCGGGCACGGTCGACTAA
ACACTCAAAACAGCGACGGCAAATATAGGTACAAGGTCCAGGCCCTCACGGCACTAATTGCGATGACC
CAACTCACGGGGGCAGTCCCCGGCGCGGCGACCTTGATTACGTCCGGGGACAAGTATGTCGCTCCCCC
CGGGAGGGGTGCAGCCACATGGGAGATTCAAAGTTTCTCGTGACGTCGTTGTGATCACTGCAGCCTAG
CCGAGACTCCCGTACTACGCGAAGGTTGGTTATGTTAACCACTACAACGTGAGGCGCCGTAAGGGCCA
GTGTTGTGCCCGGCTCTTCAATGCGCCTTAGCGGCCTGATACACCCACCCAAGGAGATACTGCTAATC
ATATGGGTGGACAGAACCTCGCAACGCAGACGCCTCCCCGGCATGATATGGTTTTTTTCCGCTATTAT
CCAGCACGCAGCGCTATCATTCAAGAGAACCCAGTGACGCGTAAATCGTAAGATCTACCTGCCGCAGG
TGGACCTACTGCAAATACGGATTATGACTCGTAAAGGGGTCATGCGTATTTTCATCACTAGGCACGTT
CGAGAATAAATTAGTAGGTGTCCCGAGCCTTGTGGCGTTCCGCCTGACTCCTCATGAAGTCGACCTTC
TCACCGGCCCTATCTGCCGACGTAAGTCATAACCTAGATCTGTACCTCGGGGGGAGGGTCACTGTAAA
GGGATAATTGGAGGGTGATTTCCCACACTTTCCTAAGGGTACTTTTTGCCTGGCTTCGCAATTGGGTC
CAATGGATGTCGATCTCTGGTTTAGCAGTTGTGAAAGTGGCAAGGCGGGAGGTTAGACCTCCATTTAA
CATATACAAGCAAGTTAACTGCACTAGATGTGTAGACACTACAGTTACAGGAGTAGCCGAATAAGTCT
CCGACGTCAAGCGAATAAGCGTCATACGCGATTATCGCCTAAGAGCACGTATTGGCGGTAAAAGGCTA
GCTAGACGCTTATGGGTAGATTTCAAGGCGTTCGTAGTGGTATAATAGGATACTCTTTCACCAGCCTG
AGGGCCGAACGCTATACTAGTGGTCTGTGATGTAGGACCGAGTATCTCTCTAGGGACCATCTACTTGA
GCAATGGTGCGCAGGGGGAGACATAGACCAGCCTTGGGTGGCAAGCACTGCAATAAGTCCTGTTTAGC
CTTGGAGTTCACATGCCGGCACTGAAGCCGACCTACCTGAGCGTGTGCGATTACCGTTACAATGGCAT
CTGTCTAGTTCTGTTTACCTACGGCGCTCTTGGTTCCAGGTTAGGGGAAGTGTATGACCCATGTGTTT
TTATCGGCTTAACCACGAGTGATCCCCGGTCGTTTCCCCATTGAATCCCTGGTGCATCCTACTCCCAG
AATGATAGCTGACTGACTGGACTGGCTTTTCAAGTAATCGAGGGGGTATCGCGGTCACGGCCGTTAAC
AGATCCCGTCCTTAGTGTGGAATCCGCACCTGCTGACTAACGCTTCGCCGGCGTGTCTGCACAGCCGT
ATAGTGTTAATCATGACCCCAAGGAAGGATTAAACAAATATCTTGACG
>HREN13
GTGTCCGGTAGCCCGCGCTAGTTAGACACCCCGGCAGGGGGGATTGCTTTCGAGACGGGAGATCCCTT
CGCCGACCCTGGAGGGCCGACGCCGAGGCATTCGGGCCCCTGCAACGTCAACAGCGGCAAGAAAACGG
GATGAATGGGCGTAATGGGGGGGGTCTGCTGGGGACCCGACGCGGTTGCCGTTTGCGGGGCCCCCGAC
CCATACCGACCCACCTAGGCGTCCAGTTACGGCGCACGTCGGGAGCGTGGTTGCCGTCAGAGCTGTGT
TTCTCGATCAGTCCCCCGCAGTGCCGCAGTATCTTGCCGTGGGCTGCTTTAATCTTGAAAGTGGTTCA
TACATTGGGCGACGAGGTGTCGACTCTCATTGGGGGTAACCGACGGGCACATGCAGTCCCCTCCCCGG

GCAGGCAGAGGCGGGGCCCCCGCGCGCCGGCCCCGGCCCACAATCTTACCAGGGTCCTCAAAGGAGCC
TTTGCATGGTACCCTTCGTGAATGGTTGCTTAAGAGGTCCACAACGTGGTCCGGGCACGGTCGACTAA
ACACTCAAAACAGCGACGGCAAATATAGGTACAAGGTCCAGGCCCTCACGGCACTAATTGCGATGACC
CAACTCACGGGGGCAGTCCCCGGCGCGGCGACCTTGATTACGTCCGGGGACAAGTATGTCGCTCCCCC
CGGGAGGGGTGCAGCCACATGGGAGATTCAAAGTTTCTCGTGACGTCGTTGTGATCACTGCAGCCTAG
CCGAGACTCCCGTACTACGCGAAGGTTGGTTATGTTAACCACTACAACGTGAGGCGCCGTAAGGGCCA
GTGTTGTGCCCGGCTCTTCAATGCGCCTTAGCGGCCTGATACACCCACCCAAGGAGATACTGCTAATC
ATATGGGTGGACAGAACCTCGCAACGCAGACGCCTCCCCGGCATGATATGGTTTTTTTCCGCTATTAT
CCAGCACGCAGCGCTATCATTCAAGAGAACCCAGTGACGCGTAAATCGTAAGATCTACCTGCCGCAGG
TGGACCTACTGCAAATACGGATTATGACTCGTAAAGGGGTCATGCGTATTTTCATCACTAGGCACGTT
CGAGAATAAATTAGTAGGTGTCCCGAGCCTTGTGGCGTTCCGCCTGACTCCTCATGAAGTCGACCTTC
TCACCGGCCCTATCTGCCGACGTAAGTCATAACCTAGATCTGTACCTCGGGGGGAGGGTCACTGTAAA
GGGATAATTGGAGGGTGATTTCCCACACTTTCCTAAGGGTACTTTTTGCCTGGCTTCGCAATTGGGTC
CAATGGATGTCGATCTCTGGTTTAGCAGTTGTGAAAGTGGCAAGGCGGGAGGTTAGACCTCCATTTAA
CATATACAAGCAAGTTAACTGCACTAGATGTGTAGACACTACAGTTACAGGAGTAGCCGAATAAGTCT
CCGACGTCAAGCGAATAAGCGTCATACGCGATTATCGCCTAAGAGCACGTATTGGCGGTAAAAGGCTA
GCTAGACGCTTATGGGTAGATTTCAAGGCGTTCGTAGTGGTATAATAGGATACTCTTTCACCAGCCTG
AGGGCCGAACGCTATACTAGTGGTCTGTGATGTAGGACCGAGTATCTCTCTAGGGACCATCTACTTGA
GCAATGGTGCGCAGGGGGAGACATAGACCAGCCTTGGGTGGCAAGCACTGCAATAAGTCCTGTTTAGC
CTTGGAGTTCACATGCCGGCACTGAAGCCGACCTACCTGAGCGTGTGCGATTACCGTTACAATGGCAT
CTGTCTAGTTCTGTTTACCTACGGCGCTCTTGGTTCCAGGTTAGGGGAAGTGTATGACCCATGTGTTT
TTATCGGCTTAACCACGAGTGATCCCCGGTCGTTTCCCCATTGAATCCCTGGTGCATCCTACTCCCAG
AATGATAGCTGACTGACTGGACTGGCTTTTCAAGTAATCGAGGGGGTATCGCGGTCACGGCCGTTAAC
AGATCCCGTCCTTAGTGTGGAATCCGCACCTGCTGACTAACGCTTCGCCGGCGTGTCTGCACAGCCGT
ATAGTGTTAATCATGACCCCAAGGAAGGATTAAACAAATATCTTGACG
>HREN15
GTGTCCGGTAGCCCGCGCTAGTTAGACACCCCGGCAGGGGGGATTGCTTTCGAGACGGGAGATCCCTT
CGCCGACCCTGGAGGGCCGACGCCGAGGCATTCGGGCCCCCGCAACGTCAACAGCGGCAAGAAAACGG
GATGAATGGGCGTAATGGGGGGGGTCTGCTGGGGACCCGACGCGGTTGCCGTTTGCGGGGCCCCCGAC
CCATACCGACCCACCTAGGCGTCCAGTTACGGCGCACGTCGGGAGCGTGGTTGCCGTCAGAGCTGTGT
TTCTCGATCAGTCCCCCGCAGTGCCGCAGTATCTTGCCGTGGGCTGCTTTAATCTTGAAAGTGGTTCA
TACATTGGGCGACGAGGTGTCGACTCTCATTGGGGGTAACCGACGGGCACATGCAGTCCCCTCCCCGG
GCAGGCAGAGGCGGGGCCCCCGCGCGCCGGCCCCGGCCCACAATCTTACCAGGGTCCTCAAAGGAGCC
TTTGCATGGTACCCTTCGTGAATGGTTGCTTAAGAGGTCCACAACGTGGTCCGGGCACGGTCGACTAA
ACACTCAAAACAGCGACGGCAAATATAGGTACAAGGTCCAGGCCCTCACGGCACTAATTGCGATGACC
CAACTCACGGGGGCAGTCCCCGGCGCGGCGACCTTGNTTACGTCCGGGGACAAGTATGTCGCTCCCCC
CGGGAGGGGTGCAGCCACATGGGAGATTCAAAGTTTCTCGTGACGTCGTTGTGATCACTGCAGCCTAG
CCGAGACTCCCGTACTACGCGAAGGTTGGTTATGTTAACCACTACAACGTGAGGCGCCGTAAGGGCCA
GTGTTGTGCCCGGCTCTTCAATGCGCCTTAGCGGCCTGATACACCCACCCAAGGAGATACTGCTAATC
ATATGGGTNGACAGAACCTCGCAACGCAGACGCCTCCCCGGCATGATATGGTTTTTTTCCGCTATTAT
CCAGCACGCAGCGCTATCATTCAAGAGAACCCAGTGACGCGTAAATCGTAAGATCTACCTGCCGCAGG
TGGACCTACTGCAAATACGGATTATGACTCGTAAAGGGGTCATGCGTATTTTCATCACTAGGCACGTT
CGAGAATAAATTAGTAGGTGTCCCGAGCCTTGTGGCGTTCCGCCTGACTCCTCATGAAGTCGACCTTC
TCACCGGCCCTATCTGCCGACGTAAGTCATAACCTAGATCTGTACCTCGGGGGGAGGGTCACTGTNAA
GGGATAATTGGAGGGTGATTTCCCACACTTTCCTAAGGGTACTTTTTGCCTGGCTTCGCAATTGGGTC
CAATGGATGTCGATCTCTGGTTTAGCAGTTGTGAAAGTGGCAAGGCGGGAGGTTAGACCTCCATTTAA
CATATACAAGCAAGTTAACTGCACTAGATGTGTAGACACTACAGTTACAGGAGTAGCCGAATAAGTCT
CCGACGTCAAGCGAATAAGCGTCATACGCGATTATCGCCTAAGAGCACGTATTGGCGGTAAAAGGCTA
GCTAGACGCTTATGGGTAGATTTCAAGGCGTTCGTAGTGGTATAATAGGATACTCTTTCACCAGCCTG
AGGGCCGAACGCTATACTAGTGGTCTGTGATGTAGGACCGAGTATCTCTCTAGGGACCATCTACTTGA
GCAATGGTGCGCAGGGGGAGACATAGACCAGCCTTGGGTGGCAAGCACTGCAATAAGTCCTGTTTAGC
CTTGGAGTTCACATGCCGGCACTGAAGCCGACCTACCTGAGCGTGTGCGATTACCGTTACAATGGCAT
CTGTCTAGTTCTGTTTACCTACGGCGCTCTTGGTTCCAGGTTAGGGGAAGTGTATGACCCATGTGTTT
TTATCGGCTTAACCACGAGTGATCCCCGGTCGTTTCCCCATTGAATCCCTGGTGCATCCTACTCCCAG

AATGATAGCTGACTGACTGGACTGGCTTTTCAAGTAATCGAGGGGGTATCGCGGTCACGGCCGTTAAC
AGATCCCGTCCTTAGTGTGGAATCCGCACCTGCTGACTAACGCTTCGCCGGCGTGTCTGCACAGCCGT
ATAGTGTTAATCATGACCCCAAGGAAGGATTAAACAAATATCTTGACG
>HREN16
GTGTCCGGTAGCCCGCGCTAGTTAGACACCCCGGCAGGGGGGATTGCTTTCGAGACGGGAGATCCCTT
CGCCGACCCTGGAGGGCCGACGCCGAGGCATTCGGGCCCCTGCAACGTCAACAGCGGCAAGAAAACGG
GATGAATGGGCGTAATGGGGGGGGTCTGCTGGGGACCCGACGCGGTTGCCGTTTGCGGGGCCCCCGAC
CCATACCGACCCACCTAGGCGTCCAGTTACGGCGCACGGCGGGAGCGTGGTTGCCGTCAGAGCTGTGT
TTCTCGATCAGTCCCCCGCAGTGCCGCAGTATCTTGCCGTGGGCTGCTTTAATCTTGAAAGTGGTTCA
TACATTGGGCGACGAGGTGTCGACTCTCATTGGGGGTAACCGACGGGCACATGCAGTCCCCTCCCCGG
GCAGGCAGAGGCGGGGCCCCCGCGCGCCGGCCCCGGCCCACAATCTTACCAGGGTCCTCAAAGGAGCC
TTTGCATGGTACCCTTCGTGAATGGTTGCTTAAGAGGTCCACAACGTGGTCCGGGCACGGTCGACTAA
ACACTCAAAACAGCGACGGCAAATATAGGTACAAGGTCCAGGCCCTCACGGCACTAATTGCGATGACC
CAACTCACGGGGGCAGTCCCCGGCGCGGCGACCTTGATTACGTCCGGGGACAAGTATGTCGCTCCCCC
CGGGAGGGGTGCAGCCACATGGGAGATTCAAAGTTTCTCGTGACGTCGTTGTGATCACTGCAGCCTAG
CCGAGACTCCCGTACTACGCGAAGGTTGGTTATGTTAACCACTACAACGTGAGGCGCCGTAAGGGCCA
GTGTTGTGCCCGGCTCTTCAATGCGCCTTAGCGGCCTGATACACCCACCCAAGGAGATACTGCTAATC
ATATGGGTGGACAGAACCTCGCAACGCAGACGCCTCCCCGGCATGATATGGTTTTTTTCCGCTATTAT
CCAGCACGCAGCGCTATCATTCAAGAGAACCCAGTGACGCGTAAATCGTAAGATCTACCTGCCGCAGG
TGGACCTACTGCAAATACGGATTATGACTCGTAAAGGGGTCATGCGTATTTTCATCACTAGGCACGTT
CGAGAATAAATTAGTAGGTGTCCCGAGCCTTGTGGCGTTCCGCCTGACTCCTCATGAAGTCGACCTTC
TCACCGGCCCTATCTGCCGACGTAAGTCATAACCTAGATCTGTACCTCGGGGGGAGGGTCACTGTAAA
GGGATAATTGGAGGGTGATTTCCCACACTTTCCTAAGGGTACTTTTTGCCTGGCTTCGCAATTGGGTC
CAATGGATGTCGATCTCTGGTTTAGCAGTTGTGAAAGTGGCAAGGCGGGAGGTTAGACCTCCATTTAA
CATATACAAGCAAGTTAACTGCACTAGATGTGTAGACACTACAGTTACAGGAGTAGCCGAATAAGTCT
CCGACGTCAAGCGAATAAGCGTCATACGCGATTATCGCCTAAGAGCACGTATTGGCGGTAAAAGGCTA
GCTAGACGCTTATGGGTAGATTTCAAGGCGTTCGTAGTGGTATAATAGGATACTCTTTCACCAGCCTG
AGGGCCGAACGCTATACTAGTGGTCTGTGATGTAGGACCGAGTATCTCTCTAGGGACCATCTACTTGA
GCAATGGTGCGCAGGGGGAGACATAGACCAGCCTTGGGTGGCAAGCACTGCAATAAGTCCTGTTTAGC
CTTGGAGTTCACATGCCGGCACTGAAGCCGACCTACCTGAGCGTGTGCGATTACCGTTACAATGGCAT
CTGTCTAGTTCTGTTTACCTACGGCGCTCTTGGTTCCAGGTTAGGGGAAGTGTATGACCCATGTGTTT
TTATCGGCTTAACCACGAGTGATCCCCGGTCGTTTCCCCATTGAATCCCTGGTGCATCCTACTCCCAG
AATGATAGCTGACTGACTGGACTGGCTTTTCAAGTAATCGAGGGGGTATCGCGGTCACGGCCGTTAAC
AGATCCCGTCCTTAGTGTGGAATCCGCACCTGCTGACTAACGCTTCGCCGGCGTGTCTGCACAGCCGT
ATAGTGTTAATCATGACCCCAAGGAAGGATTAAACAAATATCTTGACG
>HREN17
GTGTCCGGTAGCCCGCGCTAGTTAGACACCCCGGCAGGGGGGATTGCTTTCGAGACGGGAGATCCCTT
CGCCGACCCTGGAGGGCCGACGCCGAGGCATTCGGGCCCCTGCAACGTCAACAGCGGCAAGAAAACGG
GATGAATGGGCGTAATGGGGGGGGTCTGCTGGGGACCCGACGCGGTTGCCGTTTGCGGGGCCCCCGAC
CCATACCGACCCACCTAGGCGTCCAGTTACGGCGCACGTCGGGAGCGTGGTTGCCGTCAGAGCTGTGT
TTCTCGATCAGTCCCCCGCAGTGCCGCAGTATCTTGCCGTGGGCTGCTTTAATCTTGAAAGTGGTTTA
TACATTGGGCGACGAGGTGTCGACTCTCATTGGGGGTAACCGACGGGCACATGCAGTCCCCTCCCCGG
GCAGGCAGAGGCGGGGCCCCCGCGCGCCGGCCCCGGCCCACAATCTTACCAGGGTCCTCAAAGGAGCC
TTTGCATGGTACCCTTCGTGAATGGTTGCTTAAGAGGTCCACAACGTGGTCCGGGCACGGTCGACTAA
ACACTCAAAACAGCGACGGCAAATATAGGTACAAGGTCCAGGCCCTCACGGCACTAATTGCGATGACC
CAACTCACGGGGGCAGTCCCCGGCGCGGCGACCTTGATTACGTCCGGGGACAAGTATGTCGCTCCCCC
CGGGAGGGGTGCAGCCACATGGGAGATTCAAAGTTTCTCGTGACGTCGTTGTGATCACTGCAGCCTAG
CCGAGACTCCCGTACTACGCGAAGGTTGGTTATGTTAACCACTACAACGTGAGGCGCCGTAAGGGCCA
GTGTTGTGCCCGGCTCTTCAATGCGCCTTAGCGGCCTGATACACCCACCCAAGGAGATACTGCTAATC
ATATGGGTGGACAGAACCTCGCAACGCAGACGCCTCCCCGGCATGATATGGTTTTTTTCCGCTATTAT
CCAGCACGCAGCGCTATCATTCAAGAGAACCCAGTGACGCGTAAATCGTAAGATCTACCTGCCGCAGG
TGGACCTACTGCAAATACGGATTATGACTCGTAAAGGGGTCATGCGTATTTTCATCACTAGGCACGTT
CGAGAATAAATTAGTAGGTGTCCCGAGCCTTGTGGCGTTCCGCCTGACTCCTCATGAAGTCGACCTTC
TCACCGGCCCTATCTGCCGACGTAAGTCATAACCTAGATCTGTACCTCGGGGGGAGGGTCACTGTAAA

GGGATAATTGGAGGGTGATTTCCCACACTTTCCTAAGGGTACTTTTTGCCTGGCTTCGCAATTGGGTC
CAATGGATGTCGATCTCTGGTTTAGCAGTTGTGAAAGTGGCAAGGCGGGAGGTTAGACCTCCATTTAA
CATATACAAGCAAGTTAACTGCACTAGATGTGTAGACACTACAGTTACAGGAGTAGCCGAATAAGTCT
CCGACGTCAAGCGAATAAGCGTCATACGCGATTATCGCCTAAGAGCACGTATTGGCGGTAAAAGGCTA
GCTAGACGCTTATGGGTAGATTTCAAGGCGTTCGTAGTGGTATAATAGGATACTCTTTCACCAGCCTG
AGGGCCGAACGCTATACTAGTGGTCTGTGATGTAGGACCGAGTATCTCTCTAGGGACCATCTACTTGA
GCAATGGTGCGCAGGGGGAGACATAGACCAGCCTTGGGTGGCAAGCACTGCAATAAGTCCTGTTTAGC
CTTGGAGTTCACATGCCGGCACTGAAGCCGACCTACCTGAGCGTGTGCGATTACCGTTACAATGGCAT
CTGTCTAGTTCTGTTTACCTACGGCGCTCTTGGTTCCAGGTTAGGGGAAGTGTATGACCCATGTGTTT
TTATCGGCTTAACCACGAGTGATCCCCGGTCGTTTCCCCATTGAATCCCTGGTGCATCCTACTCCCAG
AATGATAGCTGACTGACTGGACTGGCTTTTCAAGTAATCGAGGGGGTATCGCGGTCACGGCCGTTAAC
AGATCCCGTCCTTAGTGTGGAATCCGCACCTGCTGACTAACGCTTCGCCGGCGTGTCTGCACAGCCGT
ATAGTGTTAATCATGACCCCAAGGAAGGATTAAACAAATATCTTGACG
>HREN18
GTGTCCGGTAGCCCGCGCTAGTTAGACACCCCGGCAGGGGGGATTGCTTTCGAGACGGGAGATCCCTT
CGCCGACCCTGGAGGGCCGACGCCGAGGCATTCGGGCCCCCGCAACGTCAACAGCGGCAAGAAAACGG
GATGAATGGGCGTAATGGGGGGGGTCTGCTGGGGACCCGACGCGGTTGCCGTTTGCGGGGCCCCCGAC
CCATACCGACCCACCTAGGCGTCCAGTTACGGCGCACGGCGGGAGCGTGGTTGCCGTCAGAGCTGTGT
TTCTCGATCAGTCCCCCGCAGTGCCGCAGTATCTTGCCGTGGGCTGCTTTAATCTTGAAAGTGGTTCA
TACATTGGGCGACGAGGTGTCGACTCTCATTGGGGGTAACCGACGGGCACATGCAGTCCCCTCCCCGG
GCAGGCAGAGGCGGGGCCCCCGCGCGCCGGCCCCGGCCCACAATCTTACCAGGGTCCTCAAAGGAGCC
TTTGCATGGTACCCTTCGTGAATGGTTGCTTAAGAGGTCCACAACGTGGTCCGGGCACGGTCGACTAA
ACACTCAAAACAGCGACGGCAAATATAGGTACAAGGTCCAGGCCCTCACGGCACTAATTGCGATGACC
CAACTCACGGGGGCAGTCCCCGGCGCGGCGACCTTGATTACGTCCGGGGACAAGTATGTCGCTCCCCC
CGGGAGGGGTGCAGCCACATGGGAGATTCAAAGTTTCTCGTGACGTCGTTGTGATCACTGCAGCCTAG
CCGAGACTCCCGTACTACGCGAAGGTTGGTTATGTTAACCACTACAACGTGAGGCGCCGTAAGGGCCA
GTGTTGTGCCCGGCTCTTCAATGCGCCTTAGCGGCCTGATACACCCACCCAAGGAGATACTGCTAATC
ATATGGGTGGACAGAACCTCGCAACGCAGACGCCTCCCCGGCATGATATGGTTTTTTTCCGCTATTAT
CCAGCACGCAGCGCTATCATTCAAGAGAACCCAGTGACGCGTAAATCGTAAGATCTACCTGCCGCAGG
TGGACCTACTGCAAATACGGATTATGACTCGTAAAGGGGTCATGCGTATTTTCATCACTAGGCACGTT
CGAGAATAAATTAGTAGGTGTCCCGAGCCTTGTGGCGTTCCGCCTGACTCCTCATGAAGTCGACCTTC
TCACCGGCCCTATCTGCCGACGTAAGTCATAACCTAGATCTGTACCTCGGGGGGAGGGTCACTGTAAA
GGGATAATTGGAGGGTGATTTCCCACACTTTCCTAAGGGTACTTTTTGCCTGGCTTCGCAATTGGGTC
CAATGGATGTCGATCTCTGGTTTAGCAGTTGTGAAAGTGGCAAGGCGGGAGGTTAGACCTCCATTTAA
CATATACAAGCAAGTTAACTGCACTAGATGTGTAGACACTACAGTTACAGGAGTAGCCGAATAAGTCT
CCGACGTCAAGCGAATAAGCGTCATACGCGATTATCGCCTAAGAGCACGTATTGGCGGTAAAAGGCTA
GCTAGACGCTTATGGGTAGATTTCAAGGCGTTCGTAGTGGTATAATAGGATACTCTTTCACCAGCCTG
AGGGCCGAACGCTATACTAGTGGTCTGTGATGTAGGACCGAGTATCTCTCTAGGGACCATCTACTTGA
GCAATGGTGCGCAGGGGGAGACATAGACCAGCCTTGGGTGGCAAGCACTGCAATAAGTCCTGTTTAGC
CTTGGAGTTCACATGCCGGCACTGAAGCCGACCTACCTGAGCGTGTGCGATTACCGTTACAATGGCAT
CTGTCTAGTTCTGTTTACCTACGGCGCTCTTGGTTCCAGGTTAGGGGAAGTGTATGACCCATGTGTTT
TTATCGGCTTAACCACGAGTGATCCCCGGTCGTTTCCCCATTGAATCCCTGGTGCATCCTACTCCCAG
AATGATAGCTGACTGACTGGACTGGCTTTTCAAGTAATCGAGGGGGTATCGCGGTCACGGCCGTTAAC
AGATCCCGTCCTTAGTGTGGAATCCGCACCTGCTGACTAACGCTTCGCCGGCGTGTCTGCACAGCCGT
ATAGTGTTAATCATGACCCCAAGGAAGGATTAAACAAATATCTTGACG
>HREN19
GTGTCCGGTAGCCCGCGCTAGTTAGACACCCCGGCAGGGGGGATTGCTTTCGAGACGGGAGATCCCTT
CGCCGACCCTGTAGGGCCGACGCCGAGGCATTCGGGCCCCTGCAACGTCAACAGCGGCAAGAAAACGG
GATGAATGGGCGTAATGGGGGGGGTCTGCTGGGGACCCGACGCGGTTGCCGTTTGCGGGGCCCCCGAC
CCATACCGACCCACCTAGGCGTCCAGTTACGGCGCACGGCGGGAGCGTGGTTGCCGTCAGAGCTGTGT
TTCTCGATCAGTCCCCCGCAGTGCCGCAGTATCTTGCCGTGGGCTGCTTTAATCTTGAAAGTGGTTTA
TACATTGGGCGACGAGGTGTCGGCTCTCATTGGGGGTAGCCGACGGACACATGCAGTCCCCTCCCCGG
GCAGGCAGAGGCGGGGCCCCCGCGCGCCGGCCCCGGCCCACAATCTTACCAGGGTCCTCAAAAGAGCC
TTTGCATGGTACCCTTCGTGAATGGTTGCTTAAGAGGTCCACCACGTAGTCCGGGCACGGTCGACTAA

ACACTCAAAACAGCGACGGCAAATAGAGGCACAAGGTCCAGGCCCTCACGGCACTAGTTGCGATGACC
CAACTCACGGGGGCAGTCCCCGGCGCGGCGACCTTGATTACGTCCGGGAACAAGTATGTCGTTTCCCC
CGGGAGGGGTGCAGCCACATGGGAGATTCAAAGTTTCTCGTGACGTCGTTGTGATCACTGCAGCCTAG
CCGAGACTCCCGTACTACGCGAAGGTTGGTTATGTTAACCACTACAACGTGAGGCGCCGTAAGGGCCA
GTGTTGTGCCCGGCTCTTCAATGCGCCTTAGCGGCCTGATACACCCACCCAAGGAGATACTGCTAATC
ATGTGGGTGGACAGAACCTCGCAACGCAGACGCATCCCCGGCNTGATATGGTTTTTTTCCGCTATTAC
CCAGCACGCGGCGCTATCATTCAAGAGAACCCAGTGACGCGTAAATCGTAAGATCTACCTGCCGCAGG
TGGACCTACTGCAAATACGGATTATGACTCGTAAAGAGGTCATGCGTATTTTCATCACTAGGCACGTT
CGAGAGTAAATTAGTAGGTGTCCCGCGCCTTGTGGCGTTCCGCCTGACTCCTCATGAAGTCGACCTTC
TCATCGGCCCTATCTGCCGACGTAAGTCATAATCCAGATCTTCACCTCGGAGGGAGGGTCACTGTAAA
GGGATAATTGGAGGGCGATTTCCCACACTTTCCTAAGGGTACTTTTTGCTTGGCTTCGCAGTTGGGTC
CAATAGATGTTGATCTCTGGTTTAGCAGTTGTGAAAGTGGCAAGGCGGGAGGTTAGACCTCCATTTAA
CATATACAAGCAAGTTAACTGCACTAGATGTGTAGACACTACAGTTACAGGAGTAGCCGACTAAGTCT
CCGACGTCAAGCGAATAGGCGTCATACGCGATTATCGCCTAAGAGCACGTATTGGCGGTAAAAGGATA
ACTAGACGCTTGTGGGTAGATTTCAAGGCGCTCGTAGTGGTATAATAGGATACTCTTTCACCAGCCTG
AGAGCCGAACGCTATACTAGTGGTCTGTGATGTAGGACCAAGTAGCTCTCTAGGGACCATCTACTTGA
GCAATGGTGCGNAGGGGTAGACATAGACCAACCTTGGGTGGCAAGCACTGCAATAAGTCCTGTTTAGC
CTTGGAGTTCACACGCCGGCACTAAAGCCGACCTACCTGAGCTTGTGCGATTACCGTTACAATGGCAT
CTGTCTAGTTCTGTTTACCTACGGCGCTCTTGGTTCCATGTTAGGGGAAGTGTATGACCCATGTGTTT
TTATCGGCTTAACTACGAGTGATCCCCGGTCGTTTCCCCATTAAATCCCTGGTGCATCCTACTCCCAT
AATGATAGCTGACTGGCTGGACTGGCTTTTCAAGTAGTCGAGGGGGTATCGCGGTCACGGCCGTTAAC
AGATCCCGTCCTTAGTGTGGAATCCGCACCTGCTGACTAACGCTTCGCCGGCGTGTCTGCACATCCGT
ATAGTGTTAATCATGACCCCAAGGAAGGATTAAACAAATATCTTGACG
>HREN20
GTGTCCGGTAGCCCGCGCTAGTTAGACACCCCGGCAGGGGGGATTGCTTTCGAGACGGGAGATCCCTT
CGCCGACCCTGGAGGGCCGACGCCGAGGCATTCGGGCCCCTGCAACGTCAACAGCGGCAAGAAAACGG
GATGAATGGGCGTAATGGGGGGGGTCTGCTGGGGACCCGACGCGGTTGCCGTTTGCGGGGCCCCCGAC
CCATACCGACCCACCTAGGCGTCCAGTTACGGCGCACGTCGGGAGCGTGGTTGCCGTCAGAGCTGTGT
TTCTCGATCAGTCCCCCGCAGTGCCGCAGTATCTTGCCGTGGGCTGCTTTAATCTTGAAAGTGGTTTA
TACATTGGGCGACGAGGTGTCGACTCTCATTGGGGGTAACCGACGGGCACATGCAGTCCCCTCCCCGG
GCAGGCAGAGGCGGGGCCCCCGCGCGCCGGCCCCGGCCCACAATCTTACCAGGGTCCTCAAAGGAGCC
TTTGCATGGTACCCTTCGTGAATGGTTGCTTAAGAGGTCCACAACGTGGTCCGGGCACGGTCGACTAA
ACACTCAAAACAGCGACGGCAAATATAGGTACAAGGTCCAGGCCCTCACGGCACTAATTGCGATGACC
CAACTCACGGGGGCAGTCCCCGGCGCGGCGACCTTGATTACGTCCGGGGACAAGTATGTCGCTCCCCC
CGGGAGGGGTGCAGCCACATGGGAGATTCAAAGTTTCTCGTGACGTCGTTGTGATCACTGCAGCCTAG
CCGAGACTCCCGTACTACGCGAAGGTTGGTTATGTTAACCACTACAACGTGAGGCGCCGTAAGGGCCA
GTGTTGTGCCCGGCTCTTCAATGCGCCTTAGCGGCCTGATACACCCACCCAAGGAGATACTGCTAATC
ATATGGGTGGACAGAACCTCGCAACGCAGACGCCTCCCCGGCATGATATGGTTTTTTTCCGCTATTAT
CCAGCACGCAGCGCTATCATTCAAGAGAACCCAGTGACGCGTAAATCGTAAGATCTACCTGCCGCAGG
TGGACCTACTGCAAATACGGATTATGACTCGTAAAGGGGTCATGCGTATTTTCATCACTAGGCACGTT
CGAGAATAAATTAGTAGGTGTCCCGAGCCTTGTGGCGTTCCGCCTGACTCCTCATGAAGTCGACCTTC
TCACCGGCCCTATCTGCCGACGTAAGTCATAACCTAGATCTGTACCTCGGGGGGAGGGTCACTGTAAA
GGGATAATTGGAGGGTGATTTCCCACACTTTCCTAAGGGTACTTTTTGCCTGGCTTCGCAATTGGGTC
CAATGGATGTCGATCTCTGGTTTAGCAGTTGTGAAAGTGGCAAGGCGGGAGGTTAGACCTCCATTTAA
CATATACAAGCAAGTTAACTGCACTAGATGTGTAGACACTACAGTTACAGGAGTAGCCGAATAAGTCT
CCGACGTCAAGCGAATAAGCGTCATACGCGATTATCGCCTAAGAGCACGTATTGGCGGTAAAAGGCTA
GCTAGACGCTTATGGGTAGATTTCAAGGCGTTCGTAGTGGTATAATAGGATACTCTTTCACCAGCCTG
AGGGCCGAACGCTATACTAGTGGTCTGTGATGTAGGACCGAGTATCTCTCTAGGGACCATCTACTTGA
GCAATGGTGCGCAGGGGGAGACATAGACCAGCCTTGGGTGGCAAGCACTGCAATAAGTCCTGTTTAGC
CTTGGAGTTCACATGCCGGCACTGAAGCCGACCTACCTGAGCGTGTGCGATTACCGTTACAATGGCAT
CTGTCTAGTTCTGTTTACCTACGGCGCTCTTGGTTCCAGGTTAGGGGAAGTGTATGACCCATGTGTTT
TTATCGGCTTAACCACGAGTGATCCCCGGTCGTTTCCCCATTGAATCCCTGGTGCATCCTACTCCCAG
AATGATAGCTGACTGACTGGACTGGCTTTTCAAGTAATCGAGGGGGTATCGCGGTCACGGCCGTTAAC
AGATCCCGTCCTTAGTGTGGAATCCGCACCTGCTGACTAACGCTTCGCCGGCGTGTCTGCACAGCCGT

ATAGTGTTAATCATGACCCCAAGGAAGGATTAAACAAATATCTTGACG
>HREN22
GTGTCCGGTAGCCCGCGCTAGTTAGACACCCCGGCAGGGGGGATTGCTTTCGAGACGGGAGATCCCTT
CGCCGACCCTGGAGGGCCGACGCCGAGGCATTCGGGCCCCTGCAACGTCAACAGCGGCAAGAAAACGG
GATGAATGGGCGTAATGGGGGGGGTCTGCTGGGGACCCGACGCGGTTGCCGTTTGCGGGGCCCCCGAC
CCATACCGACCCACCTAGGCGTCCAGTTACGGCGCACGGCGGGAGCGTGGTTGCCGTCAGAGCTGTGT
TTCTCGATCAGTCCCCCGCAGTGCCGCAGTATCTTGCCGTGGGCTGCTTTAATCTTGAAAGTGGTTTA
TACATTGGGCGACGAGGTGTCGACTCTCATTGGGGGTAACCGACGGGCACATGCAGTCCCCTCCCCGG
GCAGGCAGAGGCGGGGCCCCCGCGCGCCGGCCCCGGCCCACAATCTTACCAGGGTCCTCAAAGGAGCC
TTTGCATGGTACCCTTCGTGAATGGTTGCTTAAGAGGTCCACAACGTGGTCCGGGCACGGTCGACTAA
ACACTCAAAACAGCGACGGCAAATATAGGTACAAGGTCCAGGCCCTCACGGCACTAATTGCGATGACC
CAACTCACGGGGGCAGTCCCCGGCGCGGCGACCTTGATTACGTCCGGGGACAAGTATGTCGCTCCCCC
CGGGAGGGGTGCAGCCACATGGGAGATTCAAAGTTTCTCGTGACGTCGTTGTGATCACTGCAGCCTAG
CCGAGACTCCCGTACTACGCGAAGGTTGGTTATGTTAACCACTACAACGTGAGGCGCCGTAAGGGCCA
GTGTTGTGCCCGGCTCTTCAATGCGCCTTAGCGGCCTGATACACCCACCCAAGGAGATACTGCTAATC
ATATGGGTGGACAGAACCTCGCAACGCAGACGCCTCCCCGGCATGATATGGTTTTTTTCCGCTATTAT
CCAGCACGCAGCGCTATCATTCAAGAGAACCCAGTGACGCGTAAATCGTAAGATCTACCTGCCGCAGG
TGGACCTACTGCAAATNCGGATTATGACTCGTAAAGGGGTCATGCGTATTTTCATCACTAGGCACGTT
CGAGAATAAATTAGTAGGTGTCCCGAGCCTTGTGGCGTTCCGCCTGACTCCTCATGAAGTCGACCTTC
TCACCGGCCCTATCTGCCGACGTAAGTCATAACCTAGATCTGTACCTCGGGGGGAGGGTCACTGTAAA
GGGATAATTGGAGGGTGATTTCCCACACTTTCCTAAGGGTACTTTTTGCCTGGCTTCGCAATTGGGTC
CAATGGATGTCGATCTCTGGTTTAGCAGTTGTGAAAGTGGCAAGGCGGGAGGTTAGACCTCCATTTAA
CATATACAAGCAAGTTAACTGCACTAGATGTGTAGACACTACAGTTACAGGAGTAGCCGAATAAGTCT
CCGACGTCAAGCGAATAAGCGTCATACGCGATTATCGCCTAAGAGCACGTATTGGCGGTAAAAGGCTA
GCTAGACGCTTATGGGTAGATTTCAAGGCGTTCGTAGTGGTATAATAGGATACTCTTTCACCAGCCTG
AGGGCCGAACGCTATACTAGTGGTCTGTGATGTAGGACCGAGTATCTCTCTAGGGACCATCTACTTGA
GCAATGGTGCGCAGGGGGAGACATAGACCAGCCTTGGGTGGCAAGCACTGCAATAAGTCCTGTTTAGC
CTTGGAGTTCACATGCCGGCACTGAAGCCGACCTACCTGAGCGTGTGCGATTACCGTTACAATGGCAT
CTGTCTAGTTCTGTTTACCTACGGCGCTCTTGGTTCCAGGTTAGGGGAAGTGTATGACCCATGTGTTT
TTATCGGCTTAACCACGAGTGATCCCCGGTCGTTTCCCCATTGAATCCCTGGTGCATCCTACTCCCAG
AATGATAGCTGACTGACTGGACTGGCTTTTCAAGTAATCGAGGGGGTATCGCGGTCACGGCCGTTAAC
AGATCCCGTCCTTAGTGTGGAATCCGCACCTGCTGACTAACGCTTCGCCGGCGTGTCTGCACAGCCGT
ATAGTGTTAATCATGACCCCAAGGAAGGATTAAACAAATATCTTGACG
>HREN23
GTGTCCGGTAGCCCGCGCTAGTTAGACACCCCGGCAGGGGGGATTGCTTTCGGGACGGGAGATCCCTT
CGCCGACCCTGGAGGGCCGACGCCGAGGCATTCGGGCCCCCGCAACGTCAACAGCGGCAAGAAAACGG
GATGAATGGGCGTAATGGGGGGGGTCTGCTGGGGACCCGACGCGGTTGCCGTTTGCGGGGCCCCCGAC
CCATACCGACCCACCTAGGCGTCCAGTTACGGCGCACGTCGGGAGCGTGGTTGCCGTCAGAGCTGTGT
TTCTCGATCAGTCCCCCGCAGTGCCGCAGTATCTTGCCGTGGGCTGCTTTAATCTTGAAAGTGGTTCA
TACATTGGGCGACGAGGTGTCGACTCTCATTGGGGGTAACCGACGGGCACATGCAGTCCCCTCCCCGG
GCAGGCAGAGGCGGGGCCCCCGCGCGCCGGCCCCGGCCCACAATCTTACCAGGGTCCTCAAAGGAGCC
TTTGCATGGTACCCTTCGTGAATGGTTGCTTAAGAGGTCCACAACGTGGTCCGGGCACGGTCGACTAA
ACACTCAAAACAGCGACGGCAAATATAGGTACAAGGTCCAGGCCCTCACGGCACTAATTGCGATGACC
CAACTCACGGGGGCAGTCCCCGGCGCGGCGACCTTGATTACGTCCGGGGACAAGTATGTCGCTCCCCC
CGGGAGGGGTGCAGCCACATGGGAGATTCAAAGTTTCTCGTGACGTCGTTGTGATCACTGCAGCCTAG
CCGAGACTCCCGTACTACGCGAAGGTTGGTTATGTTAACCACTACAACGTGAGGCGCCGTAAGGGCCA
GTGTTGTGCCCGGCTCTTCAATGCGCCTTAGCGGCCTGATACACCCACCCAAGGAGATACTGCTAATC
ATATGGGTGGACAGAACCTCGCAACGCAGACGCCTCCCCGGCATGATATGGTTTTTTTCCGCTATTAT
CCAGCACGCAGCGCTATCATTCAAGAGAACCCAGTGACGCGTAAATCGTAAGATCTACCTGCCGCAGG
TGGACCTACTGCAAATACGGATTATGACTCGTAAAGGGGTCATGCGTATTTTCATCACTAGGCACGTT
CGAGAATAAATTAGTAGGTGTCCCGAGCCTTGTGGCGTTCCGCCTGACTCCTCATGAAGTCGACCTTC
TCACCGGCCCTATCTGCCGACGTAAGTCATAACCTAGATCTGTACCTCGGGGGGAGGGTCACTGTAAA
GGGATAATTGGAGGGTGATTTCCCACACTTTCCTAAGGGTACTTTTTGCCTGGCTTCGCAATTGGGTC
CAATGGATGTCGATCTCTGGTTTAGCAGTTGTGAAAGTGGCAAGGCGGGAGGTTAGACCTCCATTTAA

CATATACAAGCAAGTTAACTGCACTAGATGTGTAGACACTACAGTTACAGGAGTAGCCGAATAAGTCT
CCGACGTCAAGCGAATAAGCGTCATACGCGATTATCGCCTAAGAGCACGTATTGGCGGTAAAAGGCTA
GCTAGACGCTTATGGGTAGATTTCAAGGCGTTCGTAGTGGTATAATAGGATACTCTTTCACCAGCCTG
AGGGCCGAACGCTATACTAGTGGTCTGTGATGTAGGACCGAGTATCTCTCTAGGGACCATCTACTTGA
GCAATGGTGCGCAGGGGGAGACATAGACCAGCCTTGGGTGGCAAGCACTGCAATAAGTCCTGTTTAGC
CTTGGAGTTCACATGCCGGCACTGAAGCCGACCTACCTGAGCGTGTGCGATTACCGTTACAATGGCAT
CTGTCTAGTTCTGTTTACCTACGGCGCTCTTGGTTCCAGGTTAGGGGAAGTGTATGACCCATGTGTTT
TTATCGGCTTAACCACGAGTGATCCCCGGTCGTTTCCCCATTGAATCCCTGGTGCATCCTACTCCCAG
AATGATAGCTGACTGACTGGACTGGCTTTTCAAGTAATCGAGGGGGTATCGCGGTCACGGCCGTTAAC
AGATCCCGTCCTTAGTGTGGAATCCGCACCTGCTGACTAACGCTTCGCCGGCGTGTCTGCACAGCCGT
ATAGTGTTAATCATGACCCCAAGGAAGGATTAAACAAATATCTTGACG
>HREN24
GTGTCCGGTAGCCCGCGCTAGTTAGACACCCCGGCAGGGGGGATTGCTTTCGAGACGGGAGATCCCTT
CGCCGACCCTGGAGGGCCGACGCCGAGGCATTCGGGCCCCTGCAACGTCAACAGCGGCAAGAAAACGG
GATGAATGGGCGTAATGGGGGGGGTCTGCTGGGGACCCGACGCGGTTGCCGTTTGCGGGGCCCCCGAC
CCATACCGACCCACCTAGGCGTCCAGTTACGGCGCACGTCGGGAGCGTGGTTGCCGTCAGAGCTGTGT
TTCTCGATCAGTCCCCCGCAGTGCCGCAGTATCTTGCCGTGGGCTGCTTTAATCTTGAAAGTGGTTCA
TACATTGGGCGACGAGGTGTCGACTCTCATTGGGGGTAACCGACGGGCACATGCAGTCCCCTCCCCGG
GCAGGCAGAGGCGGGGCCCCCGCGCGCCGGCCCCGGCCCACAATCTTACCAGGGTCCTCAAAGGAGCC
TTTGCATGGTACCCTTCGTGAATGGTTGCTTAAGAGGTCCACAACGTGGTCCGGGCACGGTCGACTAA
ACACTCAAAACAGCGACGGCAAATATAGGTACAAGGTCCAGGCCCTCACGGCACTAATTGCGATGACC
CAACTCACGGGGGCAGTCCCCGGCGCGGCGACCTTGATTACGTCCGGGGACAAGTATGTCGCTCCCCC
CGGGAGGGGTGCAGCCACATGGGAGATTCAAAGTTTCTCGTGACGTCGTTGTGATCACTGCAGCCTAG
CCGAGACTCCCGTACTACGCGAAGGTTGGTTATGTTAACCACTACAACGTGAGGCGCCGTAAGGGCCA
GTGTTGTGCCCGGCTCTTCAATGCGCCTTAGCGGCCTGATACACCCACCCAAGGAGATACTGCTAATC
ATATGGGTGGACAGAACCTCGCAACGCAGACGCCTCCCCGGCATGATATGGTTTTTTTCCGCTATTAT
CCAGCACGCAGCGCTATCATTCAAGAGAACCCAGTGACGCGTAAATCGTAAGATCTACCTGCCGCAGG
TGGACCTACTGCAAATACGGATTATGACTCGTAAAGGGGTCATGCGTATTTTCATCACTAGGCACGTT
CGAGAATAAATTAGTAGGTGTCCCGAGCCTTGTGGCGTTCCGCCTGACTCCTCATGAAGTCGACCTTC
TCACCGGCCCTATCTGCCGACGTAAGTCATAACCTAGATCTGTACCTCGGGGGGAGGGTCACTGTAAA
GGGATAATTGGAGGGTGATTTCCCACACTTTCCTAAGGGTACTTTTTGCCTGGCTTCGCAATTGGGTC
CAATGGATGTCGATCTCTGGTTTAGCAGTTGTGAAAGTGGCAAGGCGGGAGGTTAGACCTCCATTTAA
CATATACAAGCAAGTTAACTGCACTAGATGTGTAGACACTACAGTTACAGGAGTAGCCGAATAAGTCT
CCGACGTCAAGCGAATAAGCGTCATACGCGATTATCGCCTAAGAGCACGTATTGGCGGTAAAAGGCTA
GCTAGACGCTTATGGGTAGATTTCAAGGCGTTCGTAGTGGTATAATAGGATACTCTTTCACCAGCCTG
AGGGCCGAACGCTATACTAGTGGTCTGTGATGTAGGACCGAGTATCTCTCTAGGGACCATCTACTTGA
GCAATGGTGCGCAGGGGGAGACATAGACCAGCCTTGGGTGGCAAGCACTGCAATAAGTCCTGTTTAGC
CTTGGAGTTCACATGCCGGCACTGAAGCCGACCTACCTGAGCGTGTGCGATTACCGTTACAATGGCAT
CTGTCTAGTTCTGTTTACCTACGGCGCTCTTGGTTCCAGGTTAGGGGAAGTGTATGACCCATGTGTTT
TTATCGGCTTAACCACGAGTGATCCCCGGTCGTTTCCCCATTGAATCCCTGGTGCATCCTACTCCCAG
AATGATAGCTGACTGACTGGACTGGCTTTTCAAGTAATCGAGGGGGTATCGCGGTCACGGCCGTTAAC
AGATCCCGTCCTTAGTGTGGAATCCGCACCTGCTGACTAACGCTTCGCCGGCGTGTCTGCACAGCCGT
ATAGTGTTAATCATGACCCCAAGGAAGGATTAAACAAATATCTTGACG
>HREN25
GTGTCCGGTAGCCCGCGCTAGTTAGACACCCCGGCAGGGGGGATTGCTTTCGGGACGGGAGATCCCTT
CGCCGACCCTGGAGGGCCGACGCCGAGGCATTCGGGCCCCCGCAACGTCAACAGCGGCAAGAAAACGG
GATGAATGGGCGTAATGGGGGGGGTCTGCTGGGGACCCGACGCGGTTGCCGTTTGCGGGGCCCCCGAC
CCATACCGACCCACCTAGGCGTCCAGTTACGGCGCACGGCGGGAGCGTGGTTGCCGTCAGAGCTGTGT
TTCTCGATCAGTCCCCCGCAGTGCCGCAGTATCTTGCCGTGGGCTGCTTTAATCTTGAAAGTGGTTTA
TACATTGGGCGACGAGGTGTCGACTCTCATTGGGGGTAACCGACGGGCACATGCAGTCCCCTCCCCGG
GCAGGCAGAGGCGGGGCCCCCGCGCGCCGGCCCCGGCCCACAATCTTACCAGGGTCCTCAAAGGAGCC
TTTGCATGGTACCCTTCGTGAATGGTTGCTTAAGAGGTCCACAACGTGGTCCGGGCACGGTCGACTAA
ACACTCAAAACAGCGACGGCAAATATAGGTACAAGGTCCAGGCCCTCACGGCACTAATTGCGATGACC
CAACTCACGGGGGCAGTCCCCGGCGCGGCGACCTTGATTACGTCCGGGGACAAGTATGTCGCTCCCCC

CGGGAGGGGTGCAGCCACATGGGAGATTCAAAGTTTCTCGTGACGTCGTTGTGATCACTGCAGCCTAG
CCGAGACTCCCGTACTACGCGAAGGTTGGTTATGTTAACCACTACAACGTGAGGCGCCGTAAGGGCCA
GTGTTGTGCCCGGCTCTTCAATGCGCCTTAGCGGCCTGATACACCCACCCAAGGAGATACTGCTAATC
ATATGGGTGGACAGAACCTCGCAACGCAGACGCCTCCCCGGCATGATATGGTTTTTTTCCGCTATTAT
CCAGCACGCAGCGCTATCATTCAAGAGAACCCAGTGACGCGTAAATCGTAAGATCTACCTGCCGCAGG
TGGACCTACTGCAAATACGGATTATGACTCGTAAAGGGGTCATGCGTATTTTCATCACTAGGCACGTT
CGAGAATAAATTAGTAGGTGTCCCGAGCCTTGTGGCGTTCCGCCTGACTCCTCATGAAGTCGACCTTC
TCACCGGCCCTATCTGCCGACGTAAGTCATAACCTAGATCTGTACCTCGGGGGGAGGGTCACTGTAAA
GGGATAATTGGAGGGTGATTTCCCACACTTTCCTAAGGGTACTTTTTGCCTGGCTTCGCAATTGGGTC
CAATGGATGTCGATCTCTGGTTTAGCAGTTGTGAAAGTGGCAAGGCGGGAGGTTAGACCTCCATTTAA
CATATACAAGCAAGTTAACTGCACTAGATGTGTAGACACTACAGTTACAGGAGTAGCCGAATAAGTCT
CCGACGTCAAGCGAATAAGCGTCATACGCGATTATCGCCTAAGAGCACGTATTGGCGGTAAAAGGCTA
GCTAGACGCTTATGGGTAGATTTCAAGGCGTTCGTAGTGGTATAATAGGATACTCTTTCACCAGCCTG
AGGGCCGAACGCTATACTAGTGGTCTGTGATGTAGGACCGAGTATCTCTCTAGGGACCATCTACTTGA
GCAATGGTGCGCAGGGGGAGACATAGACCAGCCTTGGGTGGCAAGCACTGCAATAAGTCCTGTTTAGC
CTTGGAGTTCACATGCCGGCACTGAAGCCGACCTACCTGAGCGTGTGCGATTACCGTTACAATGGCAT
CTGTCTAGTTCTGTTTACCTACGGCGCTCTTGGTTCCAGGTTAGGGGAAGTGTATGACCCATGTGTTT
TTATCGGCTTAACCACGAGTGATCCCCGGTCGTTTCCCCATTGAATCCCTGGTGCATCCTACTCCCAG
AATGATAGCTGACTGACTGGACTGGCTTTTCAAGTAATCGAGGGGGTATCGCGGTCACGGCCGTTAAC
AGATCCCGTCCTTAGTGTGGAATCCGCACCTGCTGACTAACGCTTCGCCGGCGTGTCTGCACAGCCGT
ATAGTGTTAATCATGACCCCAAGGAAGGATTAAACAAATATCTTGACG
>HREN26
GTGTCCGGTAGCCCGCGCTAGTTAGACACCCCGGCAGGGGGGATTGCTTTCGAGACGGGAGATCCCTT
CGCCGACCCTGGAGGGCCGACGCCGAGGCATTCGGGCCCCCGCAACGTCAACAGCGGCAAGAAAACGG
GATGAATGGGCGTAATGGGGGGGGTCTGCTGGGGACCCGACGCGGTTGCCGTTTGCGGGGCCCCCGAC
CCATACCGACCCACCTAGGCGTCCAGTTACGGCGCACGTCGGGAGCGTGGTTGCCGTCAGAGCTGTGT
TTCTCGATCAGTCCCCCGCAGTGCCGCAGTATCTTGCCGTGGGCTGCTTTAATCTTGAAAGTGGTTTA
TACATTGGGCGACGAGGTGTCGACTCTCATTGGGGGTAACCGACGGGCACATGCAGTCCCCTCCCCGG
GCAGGCAGAGGCGGGGCCCCCGCGCGCCGGCCCCGGCCCACAATCTTACCAGGGTCCTCAAAGGAGCC
TTTGCATGGTACCCTTCGTGAATGGTTGCTTAAGAGGTCCACAACGTGGTCCGGGCACGGTCGACTAA
ACACTCAAAACAGCGACGGCAAATATAGGTACAAGGTCCAGGCCCTCACGGCACTAATTGCGATGACC
CAACTCACGGGGGCAGTCCCCGGCGCGGCGACCTTGATTACGTCCGGGGACAAGTATGTCGCTCCCCC
CGGGAGGGGTGCAGCCACATGGGAGATTCAAAGTTTCTCGTGACGTCGTTGTGATCACTGCAGCCTAG
CCGAGACTCCCGTACTACGCGAAGGTTGGTTATGTTAACCACTACAACGTGAGGCGCCGTAAGGGCCA
GTGTTGTGCCCGGCTCTTCAATGCGCCTTAGCGGCCTGATACACCCACCCAAGGAGATACTGCTAATC
ATATGGGTGGACAGAACCTCGCAACGCAGACGCCTCCCCGGCATGATATGGTTTTTTTCCGCTATTAT
CCAGCACGCAGCGCTATCATTCAAGAGAACCCAGTGACGCGTAAATCGTAAGATCTACCTGCCGCAGG
TGGACCTACTGCAAATACGGATTATGACTCGTAAAGGGGTCATGCGTATTTTCATCACTAGGCACGTT
CGAGAATAAATTAGTAGGTGTCCCGAGCCTTGTGGCGTTCCGCCTGACTCCTCATGAAGTCGACCTTC
TCACCGGCCCTATCTGCCGACGTAAGTCATAACCTAGATCTGTACCTCGGGGGGAGGGTCACTGTAAA
GGGATAATTGGAGGGTGATTTCCCACACTTTCCTAAGGGTACTTTTTGCCTGGCTTCGCAATTGGGTC
CAATGGATGTCGATCTCTGGTTTAGCAGTTGTGAAAGTGGCAAGGCGGGAGGTTAGACCTCCATTTAA
CATATACAAGCAAGTTAACTGCACTAGATGTGTAGACACTACAGTTACAGGAGTAGCCGAATAAGTCT
CCGACGTCAAGCGAATAAGCGTCATACGCGATTATCGCCTAAGAGCACGTATTGGCGGTAAAAGGCTA
GCTAGACGCTTATGGGTAGATTTCAAGGCGTTCGTAGTGGTATAATAGGATACTCTTTCACCAGCCTG
AGGGCCGAACGCTATACTAGTGGTCTGTGATGTAGGACCGAGTATCTCTCTAGGGACCATCTACTTGA
GCAATGGTGCGCAGGGGGAGACATAGACCAGCCTTGGGTGGCAAGCACTGCAATAAGTCCTGTTTAGC
CTTGGAGTTCACATGCCGGCACTGAAGCCGACCTACCTGAGCGTGTGCGATTACCGTTACAATGGCAT
CTGTCTAGTTCTGTTTACCTACGGCGCTCTTGGTTCCAGGTTAGGGGAAGTGTATGACCCATGTGTTT
TTATCGGCTTAACCACGAGTGATCCCCGGTCGTTTCCCCATTGAATCCCTGGTGCATCCTACTCCCAG
AATGATAGCTGACTGACTGGACTGGCTTTTCAAGTAATCGAGGGGGTATCGCGGTCACGGCCGTTAAC
AGATCCCGTCCTTAGTGTGGAATCCGCACCTGCTGACTAACGCTTCGCCGGCGTGTCTGCACAGCCGT
ATAGTGTTAATCATGACCCCAAGGAAGGATTAAACAAATATCTTGACG
>HREN27

GTGTCCGGTAGCCCGCGCTAGTTAGACACCCCGGCAGGGGGGATTGCTTTCGAGACGGGAGATCCCTT
CGCCGACCCTGGAGGGCCGACGCCGAGGCATTCGGGCCCCTGCAACGTCAACAGCGGCAAGAAAACGG
GATGAATGGGCGTAATGGGGGGGGTCTGCTGGGGACCCGACGCGGTTGCCGTTTGCGGGGCCCCCGAC
CCATACCGACCCACCTAGGCGTCCAGTTACGGCGCACGTCGGGAGCGTGGTTGCCGTCAGAGCTGTGT
TTCTCGATCAGTCCCCCGCAGTGCCGCAGTATCTTGCCGTGGGCTGCTTTAATCTTGAAAGTGGTTTA
TACATTGGGCGACGAGGTGTCGACTCTCATTGGGGGTAACCGACGGGCACATGCAGTCCCCTCCCCGG
GCAGGCAGAGGCGGGGCCCCCGCGCGCCGGCCCCGGCCCACAATCTTACCAGGGTCCTCAAAGGAGCC
TTTGCATGGTACCCTTCGTGAATGGTTGCTTAAGAGGTCCACAACGTGGTCCGGGCACGGTCGACTAA
ACACTCAAAACAGCGACGGCAAATATAGGTACAAGGTCCAGGCCCTCACGGCACTAATTGCGATGACC
CAACTCACGGGGGCAGTCCCCGGCGCGGCGACCTTGATTACGTCCGGGGACAAGTATGTCGCTCCCCC
CGGGAGGGGTGCAGCCACATGGGAGATTCAAAGTTTCTCGTGACGTCGTTGTGATCACTGCAGCCTAG
CCGAGACTCCCGTACTACGCGAAGGTTGGTTATGTTAACCACTACAACGTGAGGCGCCGTAAGGGCCA
GTGTTGTGCCCGGCTCTTCAATGCGCCTTAGCGGCCTGATACACCCACCCAAGGAGATACTGCTAATC
ATATGGGTGGACAGAACCTCGCAACGCAGACGCCTCCCCGGCATGATATGGTTTTTTTCCGCTATTAT
CCAGCACGCAGCGCTATCATTCAAGAGAACCCAGTGACGCGTAAATCGTAAGATCTACCTGCCGCAGG
TGGACCTACTGCAAATACGGATTATGACTCGTAAAGGGGTCATGCGTATTTTCATCACTAGGCACGTT
CGAGAATAAATTAGTAGGTGTCCCGAGCCTTGTGGCGTTCCGCCTGACTCCTCATGAAGTCGACCTTC
TCACCGGCCCTATCTGCCGACGTAAGTCATAACCTAGATCTGTACCTCGGGGGGAGGGTCACTGTAAA
GGGATAATTGGAGGGTGATTTCCCACACTTTCCTAAGGGTACTTTTTGCCTGGCTTCGCAATTGGGTC
CAATGGATGTCGATCTCTGGTTTAGCAGTTGTGAAAGTGGCAAGGCGGGAGGTTAGACCTCCATTTAA
CATATACAAGCAAGTTAACTGCACTAGATGTGTAGACACTACAGTTACAGGAGTAGCCGAATAAGTCT
CCGACGTCAAGCGAATAAGCGTCATACGCGATTATCGCCTAAGAGCACGTATTGGCGGTAAAAGGCTA
GCTAGACGCTTATGGGTAGATTTCAAGGCGTTCGTAGTGGTATAATAGGATACTCTTTCACCAGCCTG
AGGGCCGAACGCTATACTAGTGGTCTGTGATGTAGGACCGAGTATCTCTCTAGGGACCATCTACTTGA
GCAATGGTGCGCAGGGGGAGACATAGACCAGCCTTGGGTGGCAAGCACTGCAATAAGTCCTGTTTAGC
CTTGGAGTTCACATGCCGGCACTGAAGCCGACCTACCTGAGCGTGTGCGATTACCGTTACAATGGCAT
CTGTCTAGTTCTGTTTACCTACGGCGCTCTTGGTTCCAGGTTAGGGGAAGTGTATGACCCATGTGTTT
TTATCGGCTTAACCACGAGTGATCCCCGGTCGTTTCCCCATTGAATCCCTGGTGCATCCTACTCCCAG
AATGATAGCTGACTGACTGGACTGGCTTTTCAAGTAATCGAGGGGGTATCGCGGTCACGGCCGTTAAC
AGATCCCGTCCTTAGTGTGGAATCCGCACCTGCTGACTAACGCTTCGCCGGCGTGTCTGCACAGCCGT
ATAGTGTTAATCATGACCCCAAGGAAGGATTAAACAAATATCTTGACG
>MAN21
GTGTCCGGTAGCCCGCGCTAGTTAGACACCCCGGCAGGGGGGATTGCTTTCGAGACGGGAGATCCCTT
CGCCGACCCTGGAGGGCCGACGCCGAGGCATTCGGGCCCCCGCAACGTCAACAGCGGCAAGAAAACGG
GATGAATGGGCGTAATGGGGGGGGTCTGCTGGGGACCCGACGCGGTTGCCGTTTGCGGGGCCCCCGAC
CCATACCGACCCACCTAGGCGTCCAGTTACGGCGCACGTCGGGAGCGTGGTTGCCGTCAGAGCTGTGT
TTCTCGATCAGTCCCCCGCAGTGCCGCAGTATCTTGCCGTGGGCTGCTTTAATCTTGAAAGTGGTTCA
TACATTGGGCGACGAGGTGTCGACTCTCATTGGGGGTAACCGACGGGCACATGCAGTCCCCTCCCCGG
GCAGGCAGAGGCGGGGCCCCCGCGCGCCGGCCCCGGCCCACAATCTTACCAGGGTCCTCAAAGGAGCC
TTTGCATGGTACCCTTCGTGAATGGTTGCTTAAGAGGTCCACAACGTGGTCCGGGCACGGTCGACTAA
ACACTCAAAACAGCGACGGCAAATATAGGTACAAGGTCCAGGCCCTCACGGCACTAATTGCGATGACC
CAACTCACGGGGGCAGTCCCCGGCGCGGCGACCTTGATTACGTCCGGGGACAAGTATGTCGCTCCCCC
CGGGAGGGGTGCAGCCACATGGGAGATTCAAAGTTTCTCGTGACGTCGTTGTGATCACTGCAGCCTAG
CCGAGACTCCCGTACTACGCGAAGGTTGGTTATGTTAACCACTACAACGTGAGGCGCCGTAAGGGCCA
GTGTTGTGCCCGGCTCTTCAATGCGCCTTAGCGGCCTGATACACCCACCCAAGGAGATACTGCTAATC
ATATGGGTGGACAGAACCTCGCAACGCAGACGCCTCCCCGGCATGATATGGTTTTTTTCCGCTATTAT
CCAGCACGCAGCGCTATCATTCAAGAGAACCCAGTGACGCGTAAATCGTAAGATCTACCTGCCGCAGG
TGGACCTACTGCAAATACGGATTATGACTCGTAAAGGGGTCATGCGTATTTTCATCACTAGGCACGTT
CGAGAATAAATTAGTAGGTGTCCCGAGCCTTGTGGCGTTCCGCCTGACTCCTCATGAAGTCGACCTTC
TCACCGGCCCTATCTGCCGACGTAAGTCATAACCTAGATCTGTACCTCGGGGGGAGGGTCACTGTAAA
GGGATAATTGGAGGGTGATTTCCCACACTTTCCTAAGGGTACTTTTTGCCTGGCTTCGCAATTGGGTC
CAATGGATGTCGATCTCTGGTTTAGCAGTTGTGAAAGTGGCAAGGCGGGAGGTTAGACCTCCATTTAA
CATATACAAGCAAGTTAACTGCACTAGATGTGTAGACACTACAGTTACAGGAGTAGCCGAATAAGTCT
CCGACGTCAAGCGAATAAGCGTCATACGCGATTATCGCCTAAGAGCACGTATTGGCGGTAAAAGGCTA

GCTAGACGCTTATGGGTAGATTTCAAGGCGTTCGTAGTGGTATAATAGGATACTCTTTCACCAGCCTG
AGGGCCGAACGCTATACTAGTGGTCTGTGATGTAGGACCGAGTATCTCTCTAGGGACCATCTACTTGA
GCAATGGTGCGCAGGGGGAGACATAGACCAGCCTTGGGTGGCAAGCACTGCAATAAGTCCTGTTTAGC
CTTGGAGTTCACATGCCGGCACTGAAGCCGACCTACCTGAGCGTGTGCGATTACCGTTACAATGGCAT
CTGTCTAGTTCTGTTTACCTACGGCGCTCTTGGTTCCAGGTTAGGGGAAGTGTATGACCCATGTGTTT
TTATCGGCTTAACCACGAGTGATCCCCGGTCGTTTCCCCATTGAATCCCTGGTGCATCCTACTCCCAG
AATGATAGCTGACTGACTGGACTGGCTTTTCAAGTAATCGAGGGGGTATCGCGGTCACGGCCGTTAAC
AGATCCCGTCCTTAGTGTGGAATCCGCACCTGCTGACTAACGCTTCGCCGGCGTGTCTGCACAGCCGT
ATAGTGTTAATCATGACCCCAAGGAAGGATTAAACAAATATCTTGACG
>MAN23
GTGTCCGGTAGCCCGCGCTAGTTAGACACCCCGGCAGGGGGGATTGCTTTCGAGACGGGAGATCCCTT
CGCCGACCCTGGAGGGCCGACGCCGAGGCATTCGGGCCCCTGCAACGTCAACAGCGGCAAGAAAACGG
GATGAATGGGCGTAATGGGGGGGGTCTGCTGGGGACCCGACGCGGTTGCCGTTTGCGGGGCCCCCGAC
CCATACCGACCCACCTAGGCGTCCAGTTACGGCGCACGTCGGGAGCGTGGTTGCCGTCAGAGCTGTGT
TTCTCGATCAGTCCCCCGCAGTGCCGCAGTATCTTGCCGTGGGCTGCTTTAATCTTGAAAGTGGTTTA
TACATTGGGCGACGAGGTGTCGACTCTCATTGGGGGTAACCGACGGGCACATGCAGTCCCCTCCCCGG
GCAGGCAGAGGCGGGGCCCCCGCGCGCCGGCCCCGGCCCACAATCTTACCAGGGTCCTCAAAGGAGCC
TTTGCATGGTACCCTTCGTGAATGGTTGCTTAAGAGGTCCACAACGTGGTCCGGGCACGGTCGACTAA
ACACTCAAAACAGCGACGGCAAATATAGGTACAAGGTCCAGGCCCTCACGGCACTAATTGCGATGACC
CAACTCACGGGGGCAGTCCCCGGCGCGGCGACCTTGATTACGTCCGGGGACAAGTATGTCGCTCCCCC
CGGGAGGGGTGCAGCCACATGGGAGATTCAAAGTTTCTCGTGACGTCGTTGTGATCACTGCAGCCTAG
CCGAGACTCCCGTACTACGCGAAGGTTGGTTATGTTAACCACTACAACGTGAGGCGCCGTAAGGGCCA
GTGTTGTGCCCGGCTCTTCAATGCGCCTTAGCGGCCTGATACACCCACCCAAGGAGATACTGCTAATC
ATATGGGTGGACAGAACCTCGCAACGCAGACGCCTCCCCGGCATGATATGGTTTTTTTCCGCTATTAT
CCAGCACGCAGCGCTATCATTCAAGAGAACCCAGTGACGCGTAAATCGTAAGATCTACCTGCCGCAGG
TGGACCTACTGCAAATACGGATTATGACTCGTAAAGGGGTCATGCGTATTTTCATCACTAGGCACGTT
CGAGAATAAATTAGTAGGTGTCCCGAGCCTTGTGGCGTTCCGCCTGACTCCTCATGAAGTCGACCTTC
TCACCGGCCCTATCTGCCGACGTAAGTCATAACCTAGATCTGTACCTCGGGGGGAGGGTCACTGTAAA
GGGATAATTGGAGGGTGATTTCCCACACTTTCCTAAGGGTACTTTTTGCCTGGCTTCGCAATTGGGTC
CAATGGATGTCGATCTCTGGTTTAGCAGTTGTGAAAGTGGCAAGGCGGGAGGTTAGACCTCCATTTAA
CATATACAAGCAAGTTAACTGCACTAGATGTGTAGACACTACAGTTACAGGAGTAGCCGAATAAGTCT
CCGACGTCAAGCGAATAAGCGTCATACGCGATTATCGCCTAAGAGCACGTATTGGCGGTAAAAGGCTA
GCTAGACGCTTATGGGTAGATTTCAAGGCGTTCGTAGTGGTATAATAGGATACTCTTTCACCAGCCTG
AGGGCCGAACGCTATACTAGTGGTCTGTGATGTAGGACCGAGTATCTCTCTAGGGACCATCTACTTGA
GCAATGGTGCGCAGGGGGAGACATAGACCAGCCTTGGGTGGCAAGCACTGCAATAAGTCCTGTTTAGC
CTTGGAGTTCACATGCCGGCACTGAAGCCGACCTACCTGAGCGTGTGCGATTACCGTTACAATGGCAT
CTGTCTAGTTCTGTTTACCTACGGCGCTCTTGGTTCCAGGTTAGGGGAAGTGTATGACCCATGTGTTT
TTATCGGCTTAACCACGAGTGATCCCCGGTCGTTTCCCCATTGAATCCCTGGTGCATCCTACTCCCAG
AATGATAGCTGACTGACTGGACTGGCTTTTCAAGTAATCGAGGGGGTATCGCGGTCACGGCCGTTAAC
AGATCCCGTCCTTAGTGTGGAATCCGCACCTGCTGACTAACGCTTCGCCGGCGTGTCTGCACAGCCGT
ATAGTGTTAATCATGACCCCAAGGAAGGATTAAACAAATATCTTGACG
>MAN28
GTGTCCGGTAGCCCGCGCTAGTTAGACACCCCGGCAGGGGGGATTGCTTTCGAGACGGGAGATCCCTT
CGCCGACCCTGGAGGGCCGACGCCGAGGCATTCGGGCCCCTGCAACGTCAACAGCGGCAAGAAAACGG
GATGAATGGGCGTAATGGGGGGGGTCTGCTGGGGACCCGACGCGGTTGCCGTTTGCGGGGCCCCCGAC
CCATACCGACCCACCTAGGCGTCCAGTTACGGCGCACGGCGGGAGCGTGGTTGCCGTCAGAGCTGTGT
TTCTCGATCAGTCCCCCGCAGTGCCGCAGTATCTTGCCGTGGGCTGCTTTAATCTTGAAAGTGGTTTA
TACATTGGGCGACGAGGTGTCGACTCTCATTGGGGGTAACCGACGGGCACATGCAGTCCCCTCCCCGG
GCAGGCAGAGGCGGGGCCCCCGCGCGCCGGCCCCGGCCCACAATCTTACCAGGGTCCTCAAAGGAGCC
TTTGCATGGTACCCTTCGTGAATGGTTGCTTAAGAGGTCCACAACGTGGTCCGGGCACGGTCGACTAA
ACACTCAAAACAGCGACGGCAAATATAGGTACAAGGTCCAGGCCCTCACGGCACTAATTGCGATGACC
CAACTCACGGGGGCAGTCCCCGGCGCGGCGACCTTGATTACGTCCGGGGACAAGTATGTCGCTCCCCC
CGGGAGGGGTGCAGCCACATGGGAGATTCAAAGTTTCTCGTGACGTCGTTGTGATCACTGCAGCCTAG
CCGAGACTCCCGTACTACGCGAAGGTTGGTTATGTTAACCACTACAACGTGAGGCGCCGTAAGGGCCA

GTGTTGTGCCCGGCTCTTCAATGCGCCTTAGCGGCCTGATACACCCACCCAAGGAGATACTGCTAATC
ATATGGGTGGACAGAACCTCGCAACGCAGACGCCTCCCCGGCATGATATGGTTTTTTTCCGCTATTAT
CCAGCACGCAGCGCTATCATTCAAGAGAACCCAGTGACGCGTAAATCGTAAGATCTACCTGCCGCAGG
TGGACCTACTGCAAATACGGATTATGACTCGTAAAGGGGTCATGCGTATTTTCATCACTAGGCACGTT
CGAGAATAAATTAGTAGGTGTCCCGAGCCTTGTGGCGTTCCGCCTGACTCCTCATGAAGTCGACCTTC
TCACCGGCCCTATCTGCCGACGTAAGTCATAACCTAGATCTGTACCTCGGGGGGAGGGTCACTGTAAA
GGGATAATTGGAGGGTGATTTCCCACACTTTCCTAAGGGTACTTTTTGCCTGGCTTCGCAATTGGGTC
CAATGGATGTCGATCTCTGGTTTAGCAGTTGTGAAAGTGGCAAGGCGGGAGGTTAGACCTCCATTTAA
CATATACAAGCAAGTTAACTGCACTAGATGTGTAGACACTACAGTTACAGGAGTAGCCGAATAAGTCT
CCGACGTCAAGCGAATAAGCGTCATACGCGATTATCGCCTAAGAGCACGTATTGGCGGTAAAAGGCTA
GCTAGACGCTTATGGGTAGATTTCAAGGCGTTCGTAGTGGTATAATAGGATACTCTTTCACCAGCCTG
AGGGCCGAACGCTATACTAGTGGTCTGTGATGTAGGACCGAGTATCTCTCTAGGGACCATCTACTTGA
GCAATGGTGCGCAGGGGGAGACATAGACCAGCCTTGGGTGGCAAGCACTGCAATAAGTCCTGTTTAGC
CTTGGAGTTCACATGCCGGCACTGAAGCCGACCTACCTGAGCGTGTGCGATTACCGTTACAATGGCAT
CTGTCTAGTTCTGTTTACCTACGGCGCTCTTGGTTCCAGGTTAGGGGAAGTGTATGACCCATGTGTTT
TTATCGGCTTAACCANGAGTGATCCCCGGTCGTTTCCCCATTGAATCCCTGGTGCATCCTACTCCCAG
AATGATAGCTGACTGACTGGACTGGCTTTTCAAGTAATCGAGGGGGTATCGCGGTCACGGCCGTTAAC
AGATCCCGTCCTTAGTGTGGAATCCGCACCTGCTGACTAACGCTTCGCCGGCGTGTCTGCACAGCCGT
ATAGTGTTAATCATGACCCCAAGGAAGGATTAAACAAATATCTTGACG
>MAN30
GTGTCCGGTAGCCCGCGCTAGTTAGACACCCCGGCAGGGGGGATTGCTTTCGAGACGGGAGATCCCTT
CGCCGACCCTGGAGGGCCGACGCCGAGGCATTCGGGCCCCTGCAACGTCAACAGCGGCAAGAAAACGG
GATGAATGGGCGTAATGGGGGGGGTCTGCTGGGGACCCGACGCGGTTGCCGTTTGCGGGGCCCCCGAC
CCATACCGACCCACCTAGGCGTCCAGTTACGGCGCACGTCGGGAGCGTGGTTGCCGTCAGAGCTGTGT
TTCTCGATCAGTCCCCCGCAGTGCCGCAGTATCTTGCCGTGGGCTGCTTTAATCTTGAAAGTGGTTTA
TACATTGGGCGACGAGGTGTCGACTCTCATTGGGGGTAACCGACGGGCACATGCAGTCCCCTCCCCGG
GCAGGCAGAGGCGGGGCCCCCGCGCGCCGGCCCCGGCCCACAATCTTACCAGGGTCCTCAAAGGAGCC
TTTGCATGGTACCCTTCGTGAATGGTTGCTTAAGAGGTCCACAACGTGGTCCGGGCACGGTCGACTAA
ACACTCAAAACAGCGACGGCAAATATAGGTACAAGGTCCAGGCCCTCACGGCACTAATTGCGATGACC
CAACTCACGGGGGCAGTCCCCGGCGCGGCGACCTTGATTACGTCCGGGGACAAGTATGTCGCTCCCCC
CGGGAGGGGTGCAGCCACATGGGAGATTCAAAGTTTCTCGTGACGTCGTTGTGATCACTGCAGCCTAG
CCGAGACTCCCGTACTACGCGAAGGTTGGTTATGTTAACCACTACAACGTGAGGCGCCGTAAGGGCCA
GTGTTGTGCCCGGCTCTTCAATGCGCCTTAGCGGCCTGATACACCCACCCAAGGAGATACTGCTAATC
ATATGGGTGGACAGAACCTCGCAACGCAGACGCCTCCCCGGCATGATATGGTTTTTTTCCGCTATTAT
CCAGCACGCAGCGCTATCATTCAAGAGAACCCAGTGACGCGTAAATCGTAAGATCTACCTGCCGCAGG
TGGACCTACTGCAAATACGGATTATGACTCGTAAAGGGGTCATGCGTATTTTCATCACTAGGCACGTT
CGAGAATAAATTAGTAGGTGTCCCGAGCCTTGTGGCGTTCCGCCTGACTCCTCATGAAGTCGACCTTC
TCACCGGCCCTATCTGCCGACGTAAGTCATAACCTAGATCTGTACCTCGGGGGGAGGGTCACTGTAAA
GGGATAATTGGAGGGTGATTTCCCACACTTTCCTAAGGGTACTTTTTGCCTGGCTTCGCAATTGGGTC
CAATGGATGTCGATCTCTGGTTTAGCAGTTGTGAAAGTGGCAAGGCGGGAGGTTAGACCTCCATTTAA
CATATACAAGCAAGTTAACTGCACTAGATGTGTAGACACTACAGTTACAGGAGTAGCCGAATAAGTCT
CCGACGTCAAGCGAATAAGCGTCATACGCGATTATCGCCTAAGAGCACGTATTGGCGGTAAAAGGCTA
GCTAGACGCTTATGGGTAGATTTCAAGGCGTTCGTAGTGGTATAATAGGATACTCTTTCACCAGCCTG
AGGGCCGAACGCTATACTAGTGGTCTGTGATGTAGGACCGAGTATCTCTCTAGGGACCATCTACTTGA
GCAATGGTGCGCAGGGGGAGACATAGACCAGCCTTGGGTGGCAAGCACTGCAATAAGTCCTGTTTAGC
CTTGGAGTTCACATGCCGGCACTGAAGCCGACCTACCTGAGCGTGTGCGATTACCGTTACAATGGCAT
CTGTCTAGTTCTGTTTACCTACGGCGCTCTTGGTTCCAGGTTAGGGGAAGTGTATGACCCATGTGTTT
TTATCGGCTTAACCACGAGTGATCCCCGGTCGTTTCCCCATTGAATCCCTGGTGCATCCTACTCCCAG
AATGATAGCTGACTGACTGGACTGGCTTTTCAAGTAATCGAGGGGGTATCGCGGTCACGGCCGTTAAC
AGATCCCGTCCTTAGTGTGGAATCCGCACCTGCTGACTAACGCTTCGCCGGCGTGTCTGCACAGCCGT
ATAGTGTTAATCATGACCCCAAGGAAGGATTAAACAAATATCTTGACG
>MAN34
GTGTCCGGTAGCCCGCGCTAGTTAGACACCCCGGCAGGGGGGATTGCTTTCGAGACGGGAGATCCCTT
CGCCGACCCTGGAGGGCCGACGCCGAGGCATTCGGGCCCCTGCAACGTCAACAGCGGCAAGAAAACGG

GATGAATGGGCGTAATGGGGGGGGTCTGCTGGGGACCCGACGCGGTTGCCGTTTGCGGGGCCCCCGAC
CCATACCGACCCACCTAGGCGTCCAGTTACGGCGCACGTCGGGAGCGTGGTTGCCGTCAGAGCTGTGT
TTCTCGATCAGTCCCCCGCAGTGCCGCAGTATCTTGCCGTGGGCTGCTTTAATCTTGAAAGTGGTTCA
TACATTGGGCGACGAGGTGTCGACTCTCATTGGGGGTAACCGACGGGCACATGCAGTCCCCTCCCCGG
GCAGGCAGAGGCGGGGCCCCCGCGCGCCGGCCCCGGCCCACAATCTTACCAGGGTCCTCAAAGGAGCC
TTTGCATGGTACCCTTCGTGAATGGTTGCTTAAGAGGTCCACAACGTGGTCCGGGCACGGTCGACTAA
ACACTCAAAACAGCGACGGCAAATATAGGTACAAGGTCCAGGCCCTCACGGCACTAATTGCGATGACC
CAACTCACGGGGGCAGTCCCCGGCGCGGCGACCTTGATTACGTCCGGGGACAAGTATGTCGCTCCCCC
CGGGAGGGGTGCAGCCACATGGGAGATTCAAAGTTTCTCGTGACGTCGTTGTGATCACTGCAGCCTAG
CCGAGACTCCCGTACTACGCGAAGGTTGGTTATGTTAACCACTACAACGTGAGGCGCCGTAAGGGCCA
GTGTTGTGCCCGGCTCTTCAATGCGCCTTAGCGGCCTGATACACCCACCCAAGGAGATACTGCTAATC
ATATGGGTGGACAGAACCTCGCAACGCAGACGCCTCCCCGGCATGATATGGTTTTTTTCCGCTATTAT
CCAGCACGCAGCGCTATCATTCAAGAGAACCCAGTGACGCGTAAATCGTAAGATCTACCTGCCGCAGG
TGGACCTACTGCAAATACGGATTATGACTCGTAAAGGGGTCATGCGTATTTTCATCACTAGGCACGTT
CGAGAATAAATTAGTAGGTGTCCCGAGCCTTGTGGCGTTCCGCCTGACTCCTCATGAAGTCGACCTTC
TCACCGGCCCTATCTGCCGACGTAAGTCATAACCTAGATCTGTACCTCGGGGGGAGGGTCACTGTAAA
GGGATAATTGGAGGGTGATTTCCCACACTTTCCTAAGGGTACTTTTTGCCTGGCTTCGCAATTGGGTC
CAATGGATGTCGATCTCTGGTTTAGCAGTTGTGAAAGTGGCAAGGCGGGAGGTTAGACCTCCATTTAA
CATATACAAGCAAGTTAACTGCACTAGATGTGTAGACACTACAGTTACAGGAGTAGCCGAATAAGTCT
CCGACGTCAAGCGAATAAGCGTCATACGCGATTATCGCCTAAGAGCACGTATTGGCGGTAAAAGGCTA
GCTAGACGCTTATGGGTAGATTTCAAGGCGTTCGTAGTGGTATAATAGGATACTCTTTCACCAGCCTG
AGGGCCGAACGCTATACTAGTGGTCTGTGATGTAGGACCGAGTATCTCTCTAGGGACCATCTACTTGA
GCAATGGTGCGCAGGGGGAGACATAGACCAGCCTTGGGTGGCAAGCACTGCAATAAGTCCTGTTTAGC
CTTGGAGTTCACATGCCGGCACTGAAGCCGACCTACCTGAGCGTGTGCGATTACCGTTACAATGGCAT
CTGTCTAGTTCTGTTTACCTACGGCGCTCTTGGTTCCAGGTTAGGGGAAGTGTATGACCCATGTGTTT
TTATCGGCTTAACCACGAGTGATCCCCGGTCGTTTCCCCATTGAATCCCTGGTGCATCCTACTCCCAG
AATGATAGCTGACTGACTGGACTGGCTTTTCAAGTAATCGAGGGGGTATCGCGGTCACGGCCGTTAAC
AGATCCCGTCCTTAGTGTGGAATCCGCACCTGCTGACTAACGCTTCGCCGGCGTGTCTGCACAGCCGT
ATAGTGTTAATCATGACCCCAAGGAAGGATTAAACAAATATCTTGACG
>MAN31
GTGTCCGGTAGCCCGCGCTAGTTAGACACCCCGGCAGGGGGGATTGCTTTCGGGACGGGAGATCCCTT
CGCCGACCCTGGAGGGCCGACGCCGAGGCATTCGGGCCCCTGCAACGTCAACAGCGGCAAGAAAACGG
GATGAATGGGCGTAATGGGGGGGGTCTGCTGGGGACCCGACGCGGTTGCCGTTTGCGGGGCCCCCGAC
CCATACCGACCCACCTAGGCGTCCAGTTACGGCGCACGNCGGGAGCGTGGTTGCCGTCAGAGCTGTGT
TTCTCGATCAGTCCCCCGCAGTGCCGCAGTATCTTGCCGTGGGCTGCTTTAATCTTGAAAGTGGTTCA
TACATTGGGCGACGAGGTGTCGACTCTCATTGGGGGTAACCGACGGGCACATGCAGTCCCCTCCCCGG
GCAGGCAGAGGCGGGGCCCCCGCGCGCCGGCCCCGGCCCACAATCTTACCAGGGTCCTCAAAGGAGCC
TTTGCATGGTACCCTTCGTGAATGGTTGCTTAAGAGGTCCACAACGTGGTCCGGGCACGGTCGACTAA
ACACTCAAAACAGCGACGGCAAATATAGGTACAAGGTCCAGGCCCTCACGGCACTAATTGCGATGACC
CAACTCACGGGGGCAGTCCCCGGCGCGGCGACCTTGATTACGTCCGGGGACAAGTATGTCGCTCCCCC
CGGGAGGGGTGCAGCCACATGGGAGATTCAAAGTTTCTCGTGACGTCGTTGTGATCACTGCAGCCTAG
CCGAGACTCCCGTACTACGCGAAGGTTGGTTATGTTAACCACTACAACGTGAGGCGCCGTAAGGGCCA
GTGTTGTGCCCGGCTCTTCAATGCGCCTTAGCGGCCTGATACACCCACCCAAGGAGATACTGCTAATC
ATATGGGTGGACAGAACCTCGCAACGCAGACGCCTCCCCGGCATGATATGGTTTTTTTCCGCTATTAT
CCAGCACGCAGCGCTATCATTCAAGAGAACCCAGTGACGCGTAAATCGTAAGATCTACCTGCCGCAGG
TGGACCTACTGCAAATACGGATTATGACTCGTAAAGGGGTCATGCGTATTTTCATCACTAGGCACGTT
CGAGAATAAATTAGTAGGTGTCCCGAGCCTTGTGGCGTTCCGCCTGACTCCTCATGAAGTCGACCTTC
TCACCGGCCCTATCTGCCGACGTAAGTCATAACCTAGATCTGTACCTCGGGGGGAGGGTCACTGTAAA
GGGATAATTGGAGGGTGATTTCCCACACTTTCCTAAGGGTACTTTTTGCCTGGCTTCGCAATTGGGTC
CAATGGATGTCGATCTCTGGTTTAGCAGTTGTGAAAGTGGCAAGGCGGGAGGTTAGACCTCCATTTAA
CATATACAAGCAAGTTAACTGCACTAGATGTGTAGACACTACAGTTACAGGAGTAGCCGAATAAGTCT
CCGACGTCAAGCGAATAAGCGTCATACGCGATTATCGCCTAAGAGCACGTATTGGCGGTAAAAGGCTA
GCTAGACGCTTATGGGTAGATTTCAAGGCGTTCGTAGTGGTATAATAGGATACTCTTTCACCAGCCTG
AGGGCCGAACGCTATACTAGTGGTCTGTGATGTAGGACCGAGTATCTCTCTAGGGACCATCTACTTGA

GCAATGGTGCGCAGGGGGAGACATAGACCAGCCTTGGGTGGCAAGCACTGCAATAAGTCCTGTTTAGC
CTTGGAGTTCACATGCCGGCACTGAAGCCGACCTACCTGAGCGTGTGCGATTACCGTTACAATGGCAT
CTGTCTAGTTCTGTTTACCTACGGCGCTCTTGGTTCCAGGTTAGGGGAAGTGTATGACCCATGTGTTT
TTATCGGCTTAACCACGAGTGATCCCCGGTCGTTTCCCCATTGAATCCCTGGTGCATCCTACTCCCAG
AATGATAGCTGACTGACTGGACTGGCTTTTCAAGTAATCGAGGGGGTATCGCGGTCACGGCCGTTAAC
AGATCCCGTCCTTAGTGTGGAATCCGCACCTGCTGACTAACGCTTCGCCGGCGTGTCTGCACAGCCGT
ATAGTGTTAATCATGACCCCAAGGAAGGATTAAACAAATATCTTGACG
>EDEN27
GTGTCCGGTAGCCCGCGCTAGTTAGACACCCCGGCAGGGGGGATTGCTTTCGAGACGGGAGATCCCTT
CGCCGACCCTGGAGGGCCGACGCCGAGGCATTCGGGCCCCCGCAACGTCAACAGCGGCAAGAAAACGG
GATGAATGGGCGTAATGGGGGGGGTCTGCTGGGGACCCGACGCGGTTGCCGTTTGCGGGGCCCCCGAC
CCATACCGACCCACCTAGGCGTCCAGTTACGGCGCACGTCGGGAGCGTGGTTGCCGTCAGAGCTGTGT
TTCTCGATCAGTCCCCCGCAGTGCCGCAGTATCTTGCCGTGGGCTGCTTTAATCTTGAAAGTGGTTTA
TACATTGGGCGACGAGGTGTCGACTCTCATTGGGGGTAACCGACGGGCACATGCAGTCCCCTCCCCGG
GCAGGCAGAGGCGGGGCCCCCGCGCGCCGGCCCCGGCCCACAATCTTACCAGGGTCCTCAAAGGAGCC
TTTGCATGGTACCCTTCGTGAATGGTTGCNTAAGAGGTCCACAACGTGGTCCGGGCACGGTCGACTAA
ACACTCAAAACAGCGACGGCAAATATAGGTACAAGGTCCAGGCCCTCACGGCACTAATTGCGATGACC
CAACTCACGGGGGCAGTCCCCGGCGCGGCGACCTTGATTACGTCCGGGGACAAGTATGTCGCTCCCCC
CGGGAGGGGTGCAGCCACATGGGAGATTCAAAGTTTCTCGTGACGTCGTTGTGATCACTGCAGCCTAG
CCGAGACTCCCGTACTACGCGAAGGTTGGTTATGTTAACCACTACAACGTGAGGCGCCGTAAGGGCCA
GTGTTGTGCCCGGCTCTTCAATGCGCCTTAGCGGCCTGATACACCCACCCAAGGAGATACTGCTAATC
ATATGGGTGGACAGAACCTCGCAACGCAGACGCCTCCCCGGCATGATATGGTTTTTTTCCGCTATTAT
CCAGCACGCAGCGCTATCATTCAAGAGAACCCAGTGACGCGTAAATCGTAAGATCTACCTGCCGCAGG
TGGACCTACTGCAAATACGGATTATGACTCGTAAAGGGGTCATGCGTATTTTCATCACTAGGCACGTT
CGAGAATAAATTAGTAGGTGTCCCGAGCCTTGTGGCGTTCCGCCTGACTCCTCATGAAGTCGACCTTC
TCACCGGCCCTATCTGCCGACGTAAGTCATAACCTAGATCTGTACCTCGGGGGGAGGGTCACTGTAAA
GGGATAATTGGAGGGTGATTTCCCACACTTTCCTAAGGGTACTTTTTGCCTGGCTTCGCAATTGGGTC
CAATGGATGTCGATCTCTGGTTTAGCAGTTGTGAAAGTGGCAAGGCGGGAGGTTAGACCTCCATTTAA
CATATACAAGCAAGTTAACTGCACTAGATGTGTAGACACTACAGTTACAGGAGTAGCCGAATAAGTCT
CCGACGTCAAGCGAATAAGCGTCATACGCGATTATCGCCTAAGAGCACGTATTGGCGGTAAAAGGCTA
GCTAGACGCTTATGGGTAGATTTCAAGGCGTTCGTAGTGGTATAATAGGATACTCTTTCACCAGCCTG
AGGGCCGAACGCTATACTAGTGGTCTGTGATGTAGGACCGAGTATCTCTCTAGGGACCATCTACTTGA
GCAATGGTGCGCAGGGGGAGACATAGACCAGCCTTGGGTGGCAAGCACTGCAATAAGTCCTGTTTAGC
CTTGGAGTTCACATGCCGGCACTGAAGCCGACCTACCTGAGCGTGTGCGATTACCGTTACAATGGCAT
CTGTCTAGTTCTGTTTACCTACGGCGCTCTTGGTTCCAGGTTAGGGGAAGTGTATGACCCATGTGTTT
TTATCGGCTTAACCACGAGTGATCCCCGGTCGTTTCCCCATTGAATCCCTGGTGCATCCTACTCCCAG
AATGATAGCTGACTGACTGGACTGGCTTTTCAAGTAATCGAGGGGGTATCGCGGTCACGGCCGTTAAC
AGATCCCGTCCTTAGTGTGGAATCCGCACCTGCTGACTAACGCTTCGCCGGCGTGTCTGCACAGCCGT
ATAGTGTTAATCATGACCCCAAGGAAGGATTAAACAAATATCTTGACG
>EDEN29
GTGTCCGGTAGCCCGCGCTAGTTAGACACCCCGGCAGGGGGGATTGCTTTCGAGACGGGAGATCCCTT
CGCCGACCCTGGAGGGCCGACGCCGAGGCATTCGGGCCCCTGCAACGTCAACAGCGGCAAGAAAACGG
GATGAATGGGCGTAATGGGGGGGGTCTGCTGGGGACCCGACGCGGTTGCCGTTTGCGGGGCCCCCGAC
CCATACCGACCCACCTAGGCGTCCAGTTACGGCGCACGGCGGGAGCGTGGTTGCCGTCAGAGCTGTGT
TTCTCGATCAGTCCCCCGCAGTGCCGCAGTATCTTGCCGTGGGCTGCTTTAATCTTGAAAGTGGTTTA
TACATTGGGCGACGAGGTGTCGACTCTCATTGGGGGTAACCGACGGGCACATGCAGTCCCCTCCCCGG
GCAGGCAGAGGCGGGGCCCCCGCGCGCCGGCCCCGGCCCACAATCTTACCAGGGTCCTCAAAGGAGCC
TTTGCATGGTACCCTTCGTGAATGGTTGCTTAAGAGGTCCACAACGTGGTCCGGGCACGGTCGACTAA
ACACTCAAAACAGCGACGGCAAATATAGGTACAAGGTCCAGGCCCTCACGGCACTAATTGCGATGACC
CAACTCACGGGGGCAGTCCCCGGCGCGGCGACCTTGATTACGTCCGGGGACAAGTATGTCGCTCCCCC
CGGGAGGGGTGCAGCCACATGGGAGATTCAAAGTTTCTCGTGACGTCGTTGTGATCACTGCAGCCTAG
CCGAGACTCCCGTACTACGCGAAGGTTGGTTATGTTAACCACTACAACGTGAGGCGCCGTAAGGGCCA
GTGTTGTGCCCGGCTCTTCAATGCGCCTTAGCGGCCTGATACACCCACCCAAGGAGATACTGCTAATC
ATATGGGTGGACAGAACCTCGCAACGCAGACGCCTCCCCGGCATGATATGGTTTTTTTCCGCTATTAT

CCAGCACGCAGCGCTATCATTCAAGAGAACCCAGTGACGCGTAAATCGTAAGATCTACCTGCCGCAGG
TGGACCTACTGCAAATACGGATTATGACTCGTAAAGGGGTCATGCGTATTTTCATCACTAGGCACGTT
CGAGAATAAATTAGTAGGTGTCCCGAGCCTTGTGGCGTTCCGCCTGACTCCTCATGAAGTCGACCTTC
TCACCGGCCCTATCTGCCGACGTAAGTCATAACCTAGATCTGTACCTCGGGGGGAGGGTCACTGTAAA
GGGATAATTGGAGGGTGATTTCCCACACTTTCCTAAGGGTACTTTTTGCCTGGCTTCGCAATTGGGTC
CAATGGATGTCGATCTCTGGTTTAGCAGTTGTGAAAGTGGCAAGGCGGGAGGTTAGACCTCCATTTAA
CATATACAAGCAAGTTAACTGCACTAGATGTGTAGACACTACAGTTACAGGAGTAGCCGAATAAGTCT
CCGACGTCAAGCGAATAAGCGTCATACGCGATTATCGCCTAAGAGCACGTATTGGCGGTAAAAGGCTA
GCTAGACGCTTATGGGTAGATTTCAAGGCGTTCGTAGTGGTATAATAGGATACTCTTTCACCAGCCTG
AGGGCCGAACGCTATACTAGTGGTCTGTGATGTAGGACCGAGTATCTCTCTAGGGACCATCTACTTGA
GCAATGGTGCGCAGGGGGAGACATAGACCAGCCTTGGGTGGCAAGCACTGCAATAAGTCCTGTTTAGC
CTTGGAGTTCACATGCCGGCACTGAAGCCGACCTACCTGAGCGTGTGCGATTACCGTTACAATGGCAT
CTGTCTAGTTCTGTTTACCTACGGCGCTCTTGGTTCCAGGTTAGGGGAAGTGTATGACCCATGTGTTT
TTATCGGCTTAACCACGAGTGATCCCCGGTCGTTTCCCCATTGAATCCCTGGTGCATCCTACTCCCAG
AATGATAGCTGACTGACTGGACTGGCTTTTCAAGTAATCGAGGGGGTATCGCGGTCACGGCCGTTAAC
AGATCCCGTCCTTAGTGTGGAATCCGCACCTGCTGACTAACGCTTCGCCGGCGTGTCTGCACAGCCGT
ATAGTGTTAATCATGACCCCAAGGAAGGATTAAACAAATATCTTGACG
>EDEN30
GTGTCCGGTAGCCCGCGCTAGTTAGACACCCCGGCAGGGGGGATTGCTTTCGAGACGGGAGATCCCTT
CGCCGACCCTGGAGGGCCGACGCCGAGGCATTCGGGCCCCCGCAACGTCAACAGCGGCAAGAAAACGG
GATGAATGGGCGTAATGGGGGGGGTCTGCTGGGGACCCGACGCGGTTGCCGTTTGCGGGGCCCCCGAC
CCATACCGACCCACCTAGGCGTCCAGTTACGGCGCACGGCGGGAGCGTGGTTGCCGTCAGAGCTGTGT
TTCTCGATCAGTCCCCCGCAGTGCCGCAGTATCTTGCCGTGGGCTGCTTTAATCTTGAAAGTGGTTTA
TACATTGGGCGACGAGGTGTCGACTCTCATTGGGGGTAACCGACGGGCACATGCAGTCCCCTCCCCGG
GCANGCAGAGGCGGGGCCCCCGCGCGCCGGCCCCGGCCCACAATCTTACCAGGGTCCTCAAAGGAGCC
TTTGCATGGTACCCTTCGTGAATGGTTGCTTAAGAGGTCCACAACGTGGTCCGGGCACGGTCGACTAA
ACACTCAAAACAGCGACGGCAAATATAGGTACAAGGTCCAGGCCCTCACGGCACTAATTGCGATGACC
CAACTCACGGGGGCAGTCCCCGGCGCGGCGACCTTGATTACGTCCGGGGACAAGTATGTCGCTCCCCC
CGGGAGGGGTGCAGCCACATGGGAGATTCAAAGTTTCTCGTGACGTCGTTGTGATCACTGCAGCCTAG
CCGAGACTCCCGTACTACGCGAAGGTTGGTTATGTTAACCACTACAACGTGAGGCGCCGTAAGGGCCA
GTGTTGTGCCCGGCTCTTCAATGCGCCTTAGCGGCCTGATACACCCACCCAAGGAGATACTGCTAATC
ATATGGGTGGACAGAACCTCGCAACGCAGACGCCTCCCCGGCATGATATGGTTTTTTTCCGCTATTAT
CCAGCACGCAGCGCTATCATTCAAGAGAACCCAGTGACGCGTAAATCGTAAGATCTACCTGCCGCAGG
TGGACCTACTGCAAATACGGATTATGACTCGTAAAGGGGTCATGCGTATTTTCATCACTAGGCACGTT
CGAGAATAAATTAGTAGGTGTCCCGAGCCTTGTGGCGTTCCGCCTGACTCCTCATGAAGTCGACCTTC
TCACCGGCCCTATCTGCCGACGTAAGTCATAACCTAGATCTGTACCTCGGGGGGAGGGTCACTGTAAA
GGGATAATTGGAGGGTGATTTCCCACACTTTCCTAAGGGTACTTTTTGCCTGGCTTCGCAATTGGGTC
CAATGGATGTCGATCTCTGGTTTAGCAGTTGTGAAAGTGGCAAGGCGGGAGGTTAGACCTCCATTTAA
CATATACAAGCAAGTTAACTGCACTAGATGTGTAGACACTACAGTTACAGGAGTAGCCGAATAAGTCT
CCGACGTCAAGCGAATAAGCGTCATACGCGATTATCGCCTAAGAGCACGTATTGGCGGTAAAAGGCTA
GCTAGACGCTTATGGGTAGATTTCAAGGCGTTCGTAGTGGTATAATAGGATACTCTTTCACCAGCCTG
AGGGCCGAACGCTATACTAGTGGTCTGTGATGTAGGACCGAGTATCTCTCTAGGGACCATCTACTTGA
GCAATGGTGCGCAGGGGGAGACATAGACCAGCCTTGGGTGGCAAGCACTGCAATAAGTCCTGTTTAGC
CTTGGAGTTCACATGCCGGCACTGAAGCCGACCTACCTGAGCGTGTGCGATTACCGTTACAATGGCAT
CTGTCTAGTTCTGTTTACCTACGGCGCTCTTGGTTCCAGGTTAGGGGAAGTGTATGACCCATGTGTTT
TTATCGGCTTAACCACGAGTGATCCCCGGTCGTTTCCCCATTGAATCCCTGGTGCATCCTACTCCCAG
AATGATAGCTGACTGACTGGACTGGCTTTTCAAGTAATCGAGGGGGTATCGCGGTCACGGCCGTTAAC
AGATCCCGTCCTTAGTGTGGAATCCGCACCTGCTGACTAACGCTTCGCCGGCGTGTCTGCACAGCCGT
ATAGTGTTAATCATGACCCCAAGGAAGGATTAAACAAATATCTTGACG
>EDEN31
GTGTCCGGTAGCCCGCGCTAGTTAGACACCCCGGCAGGGGGGATTGCTTTCGAGACGGGAGATCCCTT
CGCCGACCCTGGAGGGCCGACGCCGAGGCATTCGGGCCCCTGCAACGTCAACAGCGGCAAGAAAACGG
GATGAATGGGCGTAATGGGGGGGGTCTGCTGGGGACCCGACGCGGTTGCCGTTTGCGGGGCCCCCGAC
CCATACCGACCCACCTAGGCGTCCAGTTACGGCGCACGTCGGGAGCGTGGTTGCCGTCAGAGCTGTGT

TTCTCGATCAGTCCCCCGCAGTGCCGCAGTATCTTGCCGTGGGCTGCTTTAATCTTGAAAGTGGTTTA
TACATTGGGCGACGAGGTGTCGACTCTCATTGGGGGTAACCGACGGGCACATGCAGTCCCCTCCCCGG
GCAGGCAGAGGCGGGGCCCCCGCGCGCCGGCCCCGGCCCACAATCTTACCAGGGTCCTCAAAGGAGCC
TTTGCATGGTACCCTTCGTGAATGGTTGCTTAAGAGGTCCACAACGTGGTCCGGGCACGGTCGACTAA
ACACTCAAAACAGCGACGGCAAATATAGGTACAAGGTCCAGGCCCTCACGGCACTAATTGCGATGACC
CAACTCACGGGGGCAGTCCCCGGCGCGGCGACCTTGATTACGTCCGGGGACAAGTATGTCGCTCCCCC
CGGGAGGGGTGCAGCCACATGGGAGATTCAAAGTTTCTCGTGACGTCGTTGTGATCACTGCAGCCTAG
CCGAGACTCCCGTACTACGCGAAGGTTGGTTATGTTAACCACTACAACGTGAGGCGCCGTAAGGGCCA
GTGTTGTGCCCGGCTCTTCAATGCGCCTTAGCGGCCTGATACACCCACCCAAGGAGATACTGCTAATC
ATATGGGTGGACAGAACCTCGCAACGCAGACGCCTCCCCGGCATGATATGGTTTTTTTCCGCTATTAT
CCAGCACGCAGCGCTATCATTCAAGAGAACCCAGTGACGCGTAAATCGTAAGATCTACCTGCCGCAGG
TGGACCTACTGCAAATACGGATTATGACTCGTAAAGGGGTCATGCGTATTTTCATCACTAGGCACGTT
CGAGAATAAATTAGTAGGTGTCCCGAGCCTTGTGGCGTTCCGCCTGACTCCTCATGAAGTCGACCTTC
TCACCGGCCCTATCTGCCGACGTAAGTCATAACCTAGATCTGTACCTCGGGGGGAGGGTCACTGTAAA
GGGATAATTGGAGGGTGATTTCCCACACTTTCCTAAGGGTACTTTTTGCCTGGCTTCGCAATTGGGTC
CAATGGATGTCGATCTCTGGTTTAGCAGTTGTGAAAGTGGCAAGGCGGGAGGTTAGACCTCCATTTAA
CATATACAAGCAAGTTAACTGCACTAGATGTGTAGACACTACAGTTACAGGAGTAGCCGAATAAGTCT
CCGACGTCAAGCGAATAAGCGTCATACGCGATTATCGCCTAAGAGCACGTATTGGCGGTAAAAGGCTA
GCTAGACGCTTATGGGTAGATTTCAAGGCGTTCGTAGTGGTATAATAGGATACTCTTTCACCAGCCTG
AGGGCCGAACGCTATACTAGTGGTCTGTGATGTAGGACCGAGTATCTCTCTAGGGACCATCTACTTGA
GCAATGGTGCGCAGGGGGAGACATAGACCAGCCTTGGGTGGCAAGCACTGCAATAAGTCCTGTTTAGC
CTTGGAGTTCACATGCCGGCACTGAAGCCGACCTACCTGAGCGTGTGCGATTACCGTTACAATGGCAT
CTGTCTAGTTCTGTTTACCTACGGCGCTCTTGGTTCCAGGTTAGGGGAAGTGTATGACCCATGTGTTT
TTATCGGCTTAACCACGAGTGATCCCCGGTCGTTTCCCCATTGAATCCCTGGTGCATCCTACTCCCAG
AATGATAGCTGACTGACTGGACTGGCTTTTCAAGTAATCGAGGGGGTATCGCGGTCACGGCCGTTAAC
AGATCCCGTCCTTAGTGTGGAATCCGCACCTGCTGACTAACGCTTCGCCGGCGTGTCTGCACAGCCGT
ATAGTGTTAATCATGACCCCAAGGAAGGATTAAACAAATATCTTGACG
>EDEN32
GTGTCCGGTAGCCCGCGCTAGTTAGACACCCCGGCAGGGGGGATTGCTTTCGAGACGGGAGATCCCTT
CGCCGACCCTGGAGGGCCGACGCCGAGGCATTCGGGCCCCTGCAACGTCAACAGCGGCAAGAAAACGG
GATGAATGGGCGTAATGGGGGGGGTCTGCTGGGGACCCGACGCGGTTGCCGTTTGCGGGGCCCCCGAC
CCATACCGACCCACCTAGGCGTCCAGTTACGGCGCACGTCGGGAGCGTGGTTGCCGTCAGAGCTGTGT
TTCTCGATCAGTCCCCCGCAGTGCCGCAGTATCTTGCCGTGGGCTGCTTTAATCTTGAAAGTGGTTTA
TACATTGGGCGACGAGGTGTCGACTCTCATTGGGGGTAACCGACGGGCACATGCAGTCCCCTCCCCGG
GCAGGCAGAGGCGGGGCCCCCGCGCGCCGGCCCCGGCCCACAATCTTACCAGGGTCCTCAAAGGAGCC
TTTGCATGGTACCCTTCGTGAATGGTTGCTTAAGAGGTCCACAACGTGGTCCGGGCACGGTCGACTAA
ACACTCAAAACAGCGACGGCAAATATAGGTACAAGGTCCAGGCCCTCACGGCACTAATTGCGATGACC
CAACTCACGGGGGCAGTCCCCGGCGCGGCGACCTTGATTACGTCCGGGGACAAGTATGTCGCTCCCCC
CGGGAGGGGTGCAGCCACATGGGAGATTCAAAGTTTCTCGTGACGTCGTTGTGATCACTGCAGCCTAG
CCGAGACTCCCGTACTACGCGAAGGTTGGTTATGTTAACCACTACAACGTGAGGCGCCGTAAGGGCCA
GTGTTGTGCCCGGCTCTTCAATGCGCCTTAGCGGCCTGATACACCCACCCAAGGAGATACTGCTAATC
ATATGGGTGGACAGAACCTCGCAACGCAGACGCCTCCCCGGCATGATATGGTTTTTTTCCGCTATTAT
CCAGCACGCAGCGCTATCATTCAAGAGAACCCAGTGACGCGTAAATCGTAAGATCTACCTGCCGCAGG
TGGACCTACTGCAAATACGGATTATGACTCGTAAAGGGGTCATGCGTATTTTCATCACTAGGCACGTT
CGAGAATAAATTAGTAGGTGTCCCGAGCCTTGTGGCGTTCCGCCTGACTCCTCATGAAGTCGACCTTC
TCACCGGCCCTATCTGCCGACGTAAGTCATAACCTAGATCTGTACCTCGGGGGGAGGGTCACTGTAAA
GGGATAATTGGAGGGTGATTTCCCACACTTTCCTAAGGGTACTTTTTGCCTGGCTTCGCAATTGGGTC
CAATGGATGTCGATCTCTGGTTTAGCAGTTGTGAAAGTGGCAAGGCGGGAGGTTAGACCTCCATTTAA
CATATACAAGCAAGTTAACTGCACTAGATGTGTAGACACTACAGTTACAGGAGTAGCCGAATAAGTCT
CCGACGTCAAGCGAATAAGCGTCATACGCGATTATCGCCTAAGAGCACGTATTGGCGGTAAAAGGCTA
GCTAGACGCTTATGGGTAGATTTCAAGGCGTTCGTAGTGGTATAATAGGATACTCTTTCACCAGCCTG
AGGGCCGAACGCTATACTAGTGGTCTGTGATGTAGGACCGAGTATCTCTCTAGGGACCATCTACTTGA
GCAATGGTGCGCAGGGGGAGACATAGACCAGCCTTGGGTGGCAAGCACTGCAATAAGTCCTGTTTAGC
CTTGGAGTTCACATGCCGGCACTGAAGCCGACCTACCTGAGCGTGTGCGATTACCGTTACAATGGCAT

CTGTCTAGTTCTGTTTACCTACGGCGCTCTTGGTTCCAGGTTAGGGGAAGTGTATGACCCATGTGTTT
TTATCGGCTTAACCACGAGTGATCCCCGGTCGTTTCCCCATTGAATCCCTGGTGCATCCTACTCCCAG
AATGATAGCTGACTGACTGGACTGGCTTTTCAAGTAATCGAGGGGGTATCGCGGTCACGGCCGTTAAC
AGATCCCGTCCTTAGTGTGGAATCCGCACCTGCTGACTAACGCTTCGCCGGCGTGTCTGCACAGCCGT
ATAGTGTTAATCATGACCCCAAGGAAGGATTAAACAAATATCTTGACG
>EDEN33
GTGTCCGGTAGCCCGCGCTAGTTAGACACCCCGGCAGGGGGGATTGCTTTCGAGACGGGAGATCCCTT
CGCCGACCCTGGAGGGCCGACGCCGAGGCATTCGGGCCCCTGCAACGTCAACAGCGGCAAGAAAACGG
GATGAATGGGCGTAATGGGGGGGGTCTGCTGGGGACCCGACGCGGTTGCCGTTTGCGGGGCCCCCGAC
CCATACCGACCCACCTAGGCGTCCAGTTACGGCGCACGGCGGGAGCGTGGTTGCCGTCAGAGCTGTGT
TTCTCGATCAGTCCCCCGCAGTGCCGCAGTATCTTGCCGTGGGCTGCTTTAATCTTGAAAGTGGTTTA
TACATTGGGCGACGAGGTGTCGACTCTCATTGGGGGTAACCGACGGGCACATGCAGTCCCCTCCCCGG
GCAGGCAGAGGCGGGGCCCCCGCGCGCCGGCCCCGGCCCACAATCTTACCAGGGTCCTCAAAGGAGCC
TTTGCATGGTACCCTTCGTGAATGGTTGCTTAAGAGGTCCACAACGTGGTCCGGGCACGGTCGACTAA
ACACTCAAAACAGCGACGGCAAATATAGGTACAAGGTCCAGGCCCTCACGGCACTAATTGCGATGACC
CAACTCACGGGGGCAGTCCCCGGCGCGGCGACCTNGATTACGTCCGGGGACAAGTATGTCGCTCCCCC
CGGGAGGGGTGCAGCCACATGGGAGATTCAAAGTTTCTCGTGACGTCGTTGTGATCACTGCAGCCTAG
CCGAGACTCCCGTACTACGCGAAGGTTGGTTATGTTAACCACTACAACGTGAGGCGCCGTAAGGGCCA
GTGTTGTGCCCGGCTCTTCAATGCGCCTTAGCGGCCTGATACACCCACCCAAGGAGATACTGCTAATC
ATATGGGTGGACAGAACCTCGCAACGCAGACGCCTCCCCGGCATGATATGGTTTTTTTCCGCTATTAT
CCAGCACGCAGCGCTATCATTCAAGAGAACCCAGTGACGCGTAAATCGTAAGATCTACCTGCCGCAGG
TGGACCTACTGCAAATACGGATTATGACTCGTAAAGGGGTCATGCGTATTTTCATCACTAGGCACGTT
CGAGAATAAATTAGTAGGTGTCCCGAGCCTTGTGGCGTTCCGCCTGACTCCTCATGAAGTCGACCTTC
TCACCGGCCCTATCTGCCGACGTAAGTCATAACCTAGATCTGTACCTCGGGGGGAGGGTCACTGTAAA
GGGATAATTGGAGGGTGATTTCCCACACTTTCCTAAGGGTACTTTTTGCCTGGCTTCGCAATTGGGTC
CAATGGATGTCGATCTCTGGTTTAGCAGTTGTGAAAGTGGCAAGGCGGGAGGTTAGACCTCCATTTAA
CATATACAAGCAAGTTAACTGCACTAGATGTGTAGACACTACAGTTACAGGAGTAGCCGAATAAGTCT
CCGACGTCAAGCGAATAAGCGTCATACGCGATTATCGCCTAAGAGCACGTATTGGCGGTAAAAGGCTA
GCTAGACGCTTATGGGTAGATTTCAAGGCGTTCGTAGTGGTATAATAGGATACTCTTTCACCAGCCTG
AGGGCCGAACGCTATACTAGTGGTCTGTGATGTAGGACCGAGTATCTCTCTAGGGACCATCTACTTGA
GCAATGGTGCGCAGGGGGAGACATAGACCAGCCTTGGGTGGCAAGCACTGCAATAAGTCCTGTTTAGC
CTTGGAGTTCACATGCCGGCACTGAAGCCGACCTACCTGAGCGTGTGCGATTACCGTTACAATGGCAT
CTGTCTAGTTCTGTTTACCTACGGCGCTCTTGGTTCCAGGTTAGGGGAAGTGTATGACCCATGTGTTT
TTATCGGCTTAACCACGAGTGATCCCCGGTCGTTTCCCCATTGAATCCCTGGTGCATCCTACTCCCAG
AATGATAGCTGACTGACTGGACTGGCTTTTCAAGTAATCGAGGGGGTATCGCGGTCACGGCCGTTAAC
AGATCCCGTCCTTAGTGTGGAATCCGCACCTGCTGACTAACGCTTCGCCGGCGTGTCTGCACAGCCGT
ATAGTGTTAATCATGACCCCAAGGAAGGATTAAACAAATATCTTGACG
>EDEN34
GTGTCCGGTAGCCCGCGCTAGTCAGACACCCCGGCAGGGGGGATTGCTTTCGGGACGGGAGATCCCTT
CGCCGACCCTGGAGGGCCGACGCCGAGGCATTCGGGCCCCTGCAACGTCAACAGCGGCAAGAAAACGG
GATGAATGGGCGTAATGGGGGGGGTCTGCTGGGGACCCGACGCGGTTGCCGTTTGCGGGGCCCCCGAC
CCATACCGACCCACCTAGGCGTCCAGTTACGGCGCACGGCGGGAGCGTGGTTGCCGTCAGAGCTGTGT
TTCTCGATCAGTCCCCCGCAGTGCCGCAGTATCTTGCCGTGGGCTGCTTTAATCTTGAAAGTGGTTCA
TACATTGGGCGACGAGGTGTCGACTCTCATTGGGGGTAACCGACGGGCACATGCAGTCCCCTCCCCGG
GCAGGCAGAGGCGGGGCCCCCGCGCGCCGGCCCCGGCCCACAATCTTACCAGGGTCCTCAAAGGAGCC
TTTGCATGGTACCCTTCGTGAATGGTTGCTTAAGAGGTCCACAACGTGGTCCGGGCACGGTCGACTAA
ACACTCAAAACAGCGACGGCAAATATAGGTACAAGGTCCAGGCCCTCACGGCACTAATTGCGATGACC
CAACTCACGGGGGCAGTCCCCGGCGCGGCGACCTTGATTACGTCCGGGGACAAGTATGTCGCTCCCCC
CGGGAGGGGTGCAGCCACATGGGAGATTCAAAGTTTCTCGTGACGTCGTTGTGATCACTGCAGCCTAG
CCGAGACTCCCGTACTACGCGAAGGTTGGTTATGTTAACCACTACAACGTGAGGCGCCGTAAGGGCCA
GTGTTGTGCCCGGCTCTTCAATGCGCCTTAGCGGCCTGATACACCCACCCAAGGAGATACTGCTAATC
ATATGGGTGGACAGAACCTCGCAACGCAGACGCCTCCCCGGCATGATATGGTTTTTTTCCGCTATTAT
CCAGCACGCAGCGCTATCATTCAAGAGAACCCAGTGACGCGTAAATCGTAAGATCTACCTGCCGCAGG
TGGACCTACTGCAAATACGGATTATGACTCGTAAAGGGGTCATGCGTATTTTCATCACTAGGCACGTT

CGAGAATAAATTAGTAGGTGTCCCGAGCCTTGTGGCGTTCCGCCTGACTCCTCATAAAGTCGACCTTC
TCACCGGCCCTATCTGCCGACGTAAGTCATAACCTAGATCTGTACCTCGGGGGGAGGGTCACTGTAAA
GGGATAATTGGAGGGTGATTTCCCACATTTTCCTAAGGGTACTTTTTGCCTGGCTTCGCAATTGGGTC
CAATGGATGTCGATCTCTGGTTTAGCAGTTGTGAAAGTGGCAAGGCGGGAGGTTAGACCTCCATTTAA
CATATACAAGCAAGTTAACTGCACTAGATGTGTAGACACTACAGGTACAGGAGTAGCCGGATAAGTCT
CCGACGTCAAGCGAATAAGCGTCATACGCGATTATCGCCTAAGAGCACGTATTTGCGGTAAAAGGCTA
GCTAGACGCTTGTGGGTAGATTTCAAGGCGTTCGTAGTGGTATAATAGGATACTCTTTCACCAGCCTG
AGGGCCGAACGCTATACTAGTGGTCTGTGATGTAGGACCGAGTATCTCTCTAGGGACCATCTACTTGA
GCAATGGTGCGCAGGGGGAGACATAGACCAGCCTTGGGTGGCAAGCACTGCAATAAGTCCTGTTTAGC
CTTGGAGTTCACACGCCGGCACTGAAGCCGACCTACCTGAGCGTGTGCGATTACCGTTACAATGGCAT
CTGTCTAGTTCTGTTTACCTACGGCGCTCTTGGTTCCAGGTTAGGGGAAGTGTATGACCCATGTGTTT
TTATCGGCTTAACCACGAGTGATCCCCGGTCGTTTCCCCATTGAATCCCTGGTGCATCCTACTCCCAG
AATGATAGCTGACTGACTGGACTGGCTTTTCAAGTAATCGAGGGGGTATCGCGGTCACGGCCGTTAAC
AGATCCCGTCCTTAGTGTGGAATCCGCACCTGCTGACTAACGCTTCGCCGGCGTGTCTGCACAGCCGT
ATAGTGTTAATCATGACCCCAAGGAAGGATTAAACAAATATCTTGACG
>EDEN37
GTGTCCGGTAGCCCGCGCTAGTTAGANACCCCGGCAGGGGGGATTGCTTTCGAGACGGGAGATCCCTT
CGCCGACCCTGTAGGGCCGACGCCGAGGCATTCGGGCCCCTGCAACGTCAACAGCGGCAAGAAAACGG
GATGAATGGGCGTAATGGGGGGGGTCTGCTGGGGACCCGACGCGGTTGCCGTTTGCGGGGCCCCCGAC
CCATACCGACCCACCTAGGCGTCCAGTTACGGCGCACGTCGGGAGCGTGGTTGCCGTCAGAGCTGTGT
TTCTCGATCAGTCCCCCGCAGTGCCGCAGTATCTTGCCGTGGGCTGCTTTAATCTTGAAAGTGGTTTA
TACATTGGGCGACGAGGTGTCGGCTCTCATTGGGGGTAACCGACGGACACGTGCAGTCCCCTCCCCGG
GCAGGCAGAGGCGGGGCCCCCGCGCGCCGGCCCCGGCCCACAATCTTACCAGGGTCCTCAAAAGAGCC
TTTGCATGGTACCCTTCGTGAATGGTTGCTTAAGAGGTCCACCACGTAGTCCGGGCACGGTCAACTAA
ACACTCAAAACAGCGACGGCAAATAGAGGCACAAGGTCCAGGCCCTCACGGCACTAGTTGCGATGACC
CAACTCACGGGGGCAGTCCCCGGCGCAGCGACCTTGATTACGTCCGGGAACAAGTATGTCGTTTCCCC
CGGGAGGGGTGCAGCCACATGGGAGATTCAAAGTTTCTCGTGACGTCGTTGTGATCACTGCAGCCTAG
CCGAGACTCCCGTACTACGCGAAGGTTGGTTATGTTAACCACTACAACGTGAGGCGCCGTAAGGGCCA
GTGTTGTGCCCGGCTCTTCAATGCGCCTTAGCGGCCTGATACACCCACCCAAGGAGATANTGCTAATC
ATGTGGGTGGACAGAACCTCGCAACGCAGACGCATCCCCGGCATGATATGGTTTTTTTCCGCTATTAC
CCAGCACGCGGCGCTATCATTCAAGAGAACCCAGTGACGCGTAAATCGTCAGATCTACCTGCCGCAGG
TGGACCTACTGCAAATACGGATTATGACTCGTAAAGAGGTCATGCGTATTTTCATCACTAGGCACTTT
CGAGAGTAAATTAGTAGGTGTCCCGCGCCTTGTGGCGTTCCGCCTGGCTCCTCATGAAGTCGACCTTC
TCATCGGCCCTATTTGCCGACGTAAGTCATAATCCAGATCTTCACCTCGGAGGGAGGGTCACTGTAAA
GGGATAATTGGAGGGCGATTTCCCACACTTTCCTAAGGGTACTTTTTGCTTAGCTTCGCAGTTGGGTC
CAATAGATGTTGATCTCTGGTTTAGCAGTTGTGAAAGTGGCAAGGCGGGAGGTTAGGCCTCCATTTAA
CATATACAAGCAAGTTAACTGCACTAGATGTGTAGACACTACAGTTACAGGAGTAGCCGACTAAGTCT
CCGACGTCAAGCGAATAGGCGTCATACGCGATTATTGCCTAAGAGCACGTATTGGCGGTAAAAGGATA
ACTAGACGCTTGTGGGTAGATTTCAAGGCGCTCGTAGTGGTATAATAGGACACTCTTGCACCAGCCTG
AGAGCCGAACGCTATACTAGTGGTCTGTGATGTAGGACCAAGTAGCTCTCTAGGGACCATCTACTTGA
GCAATGGTGCGCAGGGGTAGACATAGACCAACCTTGGGTGGCAAGCACTGCAATAAGTCCTGTTTAGC
CTTGGAGTTCACACGCCGGCACTAAAGCCGACCTACCTGAGCTTGTGCGATTACCGTTACAATGGCAT
CTGTCTAGTTCTGTTTACCTACGGCGCTCTTGGTTCCATGTTAGGGGAAGTGTATGACCCATGTGTTT
TTATCGGCTTAACTACGAGTGATCCCCGGTCGTTTCCCCATTAAATCCCTGGTGCATCCTACTCCCAT
AATGATAGCTGACTGGCTGGACTGGCTTTTCAAGTAGTCGAGGGGGTATCGCGGTCACGGCCGTTAAC
AGATCCCGTCCTTAGTGTGGAATCCGCACCTGCTGACTAACGCTTCGCCGGCGTGTCTGCACATCCGT
ATAGTGTTAATCATGACCCCAAGGAAGGATTAAACAAATATCTTGACG
>EDEN39
GTGTCCGGTAGCCCGCGCTAGTTAGACACCCCGGCAGGGGGGATTGCTTTCGGGACGGGAGATCCCTT
CGCCGACCCTGGAGGGCCGACGCCGAGGCATTCGGGCCCCTGCAACGTCAACAGCGGCAAGAAAACGG
GATGAATGGGCGTAATGGGGGGGGTCTGCTGGGGACCCGACGCGGTTGCCGTTTGCGGGGCCCCCGAC
CCATACCGACCCACCTAGGCGTCCAGTTACGGCGCACGGCGGGAGCGTGGTTGCCGTCAGAGCTGTGT
TTCTCGATCAGTCCCCCGCAGTGCCGCAGTATCTTGCCGTGGGCTGCTTTAATCTTGAAAGTGGTTTA
TACATTGGGCGACGAGGTGTCGACTCTCATTGGGGGTAACCGACGGGCACATGCAGTCCCCTCCCCGG

GCAGGCAGAGGCGGGGCCCCCGCGCGCCGGCCCCGGCCCACAATCTTACCAGGGTCCTCAAAGGAGCC
TTTGCATGGTACCCTTCGTGAATGGTTGCTTAAGAGGTCCACAACGTGGTCCGGGCACGGTCGACTAA
ACACTCAAAACAGCGACGGCAAATATAGGTACAAGGTCCAGGCCCTCACGGCACTAATTGCGATGACC
CAACTCACGGGGGCAGTCCCCGGCGCGGCGACCTTGATTACGTCCGGGGACAAGTATGTCGCTCCCCC
CGGGAGGGGTGCAGCCACATGGGAGATTCAAAGTTTCTCGTGACGTCGTTGTGATCACTGCAGCCTAG
CCGAGACTCCCGTACTACGCGAAGGTTGGTTATGTTAACCACTACAACGTGAGGCGCCGTAAGGGCCA
GTGTTGTGCCCGGCTCTTCAATGCGCCTTAGCGGCCTGATACACCCACCCAAGGAGATACTGCTAATC
ATATGGGTGGACAGAACCTCGCAACGCAGACGCCTCCCCGGCATGATATGGTTTTTTTCCGCTATTAT
CCAGCACGCANCGCTATCATTCAAGAGAACCCAGTGACGCGTAAATCGTAAGATCTACCTGCCGCAGG
TGGACCTACTGNGGNTNNNGATTATGACTCGTAAAGGGGTCATGCGTATTTTCATCACTAGGCACGTT
CGAGAATAAATTAGTAGGTGTCCCGAGCCTTGTGGCGTTCCGCCTGACTCCTCATGAAGTCGACCTTC
TCACCGGCCCTATCTGCCGACGTAAGTCATAACCTAGATCTGTACCTCGGGGGGAGGGTCACTGTAAA
GGGATAATTGGAGGGTGATTTCCCACACTTTCCTAAGGGTACTTTTTGCCTGGCTTCGCAATTGGGTC
CAATGGATGTCGATCTCTGGTTTAGCAGTTGTGAAAGTGGCAAGGCGGGAGGTTAGACCTCCATTTAA
CATATACAAGCAAGTTAACTGCACTAGATGTGTAGACACTACAGTTACAGGAGTAGCCGAATAAGTCT
CCGACGTCAAGCGAATAAGCGTCATACGCGATTATCGCCTAAGAGCACGTATTGGCGGTAAAAGGCTA
GCTAGACGCTTATGGGTAGATTTCAAGGCGTTCGTAGTGGTATAATAGGATACTCTTTCACCAGCCTG
AGGGCCGAACGCTATACTAGTGGTCTGTGATGTAGGACCGAGTATCTCTCTAGGGACCATCTACTTGA
GCAATGGTGCGCAGGGGGAGACATAGACCAGCCTTGGGTGGCAAGCACTGCAATAAGTCCTGTTTAGC
CTTGGAGTTCACATGCCGGCACTGAAGCCGACCTACCTGAGCGTGTGCGATTACCGTTACAATGGCAT
CTGTCTAGTTCTGTTTACCTACGGCGCTCTTGGTTCCAGGTTAGGGGAAGTGTATGACCCATGTGTTT
TTATCGGCTTAACCACGAGTGATCCCCGGTCGTTTCCCCATTGAATCCCTGGTGCATCCTACTCCCAG
AATGATAGCTGACTGACTGGACTGGCTTTTCAAGTAATCGAGGGGGTATCGCGGTCACGGCCGTTAAC
AGATCCCGTCCTTAGTGTGGAATCCGCACCTGCTGACTAACGCTTCGCCGGCGTGTCTGCACAGCCGT
ATAGTGTTAATCATGACCCCAAGGAAGGATTAAACAAATATCTTGACG
>EDEN40
GTGTCCGGTAGCCCGCGCTAGTTAGACACCCCGGCAGGGGGGATTGCTTTCGAGACGGGAGATCCCTT
CGCCGACCCTGTAGGGCCGACGCCGAGGCATTCGGGCCCCTGCAACGTCAACAGCGGCAAGAAAACGG
GATGAATGGGCGTAATGGGGGGGGTCTGCTGGGGACCCGACGCGGTTGCCGTTTGCGGGGCCCCCGAC
CCATACCGACCCACCTAGGCGTCCAGTTACGGCGCACGTCGGGAGCGTGGTTGCCGTCAGAGCTGTGT
TTCTCGATCAGTCCCCCGCAGTGCCGCAGTATCTTGCCGTGGGCTGCTTTAATCTTGAAAGTGGTTTA
TACATTGGGCGACGAGGTGTCGGCTCTCATTGGGGGTAACCGACGGACACGTGCAGTCCCCTCCCCGG
GCAGGCAGAGGCGGGGCCCCCGCGCGCCGGCCCCGGCCCACAATCTTACCAGGGTCCTCAAAAGAGCC
TTTGCATGGTACCCTTCGTGAATGGTTGCTTAAGAGGTCCACCACGTAGTCCGGGCACGGTCAACTAA
ACACTCAAAACAGCGACGGCAAATAGAGGCACAAGGTCCAGGCCCTCACGGCACTAGTTGCGATGACC
CAACTCACGGGGGCAGTCCCCGGCGCAGCGACCTTGATTACGTCCGGGAACAAGTATGTCGTTTCCCC
CGGGAGGGGTGCAGCCACATGGGAGATTCAAAGTTTCTCGTGACGTCGTTGTGATCACTGCAGCCTAG
CCGAGACTCCCGTACTACGCGAAGGTTGGTTATGTTAACCACTACAACGTGAGGCGCCGTAAGGGCCA
GTGTTGTGCCCGGCTCTTCAATGCGCCTTAGCGGCCTGATACACCCACCCAAGGAGATACTGCTAATC
ATGTGGGTGGACAGAACCTCGCAACGCAGACGCATCCCCGGCATGATATGGTTTTTTTCCGCTATTAC
CCAGCACGCGGCGCTATCATTCAAGAGAACCCAGTGACGCGTAAATCGTCAGATCTACCTGCCGCAGG
TGGACCTACTGCAAATACGGATTATGACTCGTAAAGAGGTCATGCGTATTTTCATCACTAGGCACTTT
CGAGAGTAAATTAGTAGGTGTCCCGCGCCTTGTGGCGTTCCGCCTGGCTCCTCATGAAGTCGACCTTC
TCATCGGCCCTATTTGCCGACGTAAGTCATAATCCAGATCTTCACCTCGGAGGGAGGGTCACTGTAAA
GGGATAATTGGAGGGCGATTTCCCACACTTTCCTAAGGGTACTTTTTGCTTAGCTTCGCAGTTGGGTC
CAATAGATGTTGATCTCTGGTTTAGCAGTTGTGAAAGTGGCAAGGCGGGAGGTTAGGCCTCCATTTAA
CATATACAAGCAAGTTAACTGCACTAGATGTGTAGACACTACAGTTACAGGAGTAGCCGACTAAGTCT
CCGACGTCAAGCGAATAGGCGTCATACGCGATTATTGCCTAAGAGCACGTATTGGCGGTAAAAGGATA
ACTAGACGCTTGTGGGTAGATTTCAAGGCGCTCGTAGTGGTATAATAGGACACTCTTGCACCAGCCTG
AGAGCCGAACGCTATACTAGTGGTCTGTGATGTAGGACCAAGTAGCTCTCTAGGGACCATCTACTTGA
GCAATGGTGCGCAGGGGTAGACATAGACCAACCTTGGGTGGCAAGCACTGCAATAAGTCCTGTTTAGC
CTTGGAGTTCACACGCCGGCACTAAAGCCGACCTACCTGAGCTTGTGCGATTACCGTTACAATGGCAT
CTGTCTAGTTCTGTTTACCTACGGCGCTCTTGGTTCCATGTTAGGGGAAGTGTATGACCCATGTGTTT
TTATCGGCTTAACTACGAGTGATCNCCGGTCGTTTCCCCATTAAATCCCTGGTGCATCCTACTCCCAT

AATGATAGCTGACTGGCTGGACTGGCTTTTCAAGTAGTCGAGGGGGTATCGCGGTCACGGCCGTTAAC
AGATCCCGTCCTTAGTGTGGAATCCGCACCTGCTGACTAACGCTTCGCCGGCGTGTCTGCACATCCGT
ATAGTGTTAATCATGACCCCAAGGAAGGATTAAACAAATATCTTGACG
>EDEN43
GTGTCCGGTAGCCCGCGCTAGTTAGACACCCCGGCAGGGGGGATTGCTTTCGAGACGGGAGATCCCTT
CGCCGACCCTGGAGGGCCGACGCCGAGGCATTCGGGCCCCCGCAACGTCAACAGCGGCAAGAAAACGG
GATGAATGGGCGTAATGGGGGGGGTCTGCTGGGGACCCGACGCGGTTGCCGTTTGCGGGGCCCCCGAC
CCATACCGACCCACCTAGGCGTCCAGTTACGGCGCACGTCGGGAGCGTGGTTGCCGTCAGAGCTGTGT
TTCTCGATCAGTCCCCCGCAGTGCCGCAGTATCTTGCCGTGGGCTGCTTTAATCTTGAAAGTGGTTTA
TACATTGGGCGACGAGGTGTCGACTCTCATTGGGGGTAACCGACGGGCACATGCAGTCCCCTCCCCGG
GCAGGCAGAGGCGGGGCCCCCGCGCGCCGGCCCCGGCCCACAATCTTACCAGGGTCCTCAAAGGAGCC
TTTGCATGGTACCCTTCGTGAATGGTTGCTTAAGAGGTCCACAACGTGGTCCGGGCACGGTCGACTAA
ACACTCAAAACAGCGACGGCAAATATAGGTACAAGGTCCAGGCCCTCACGGCACTAATTGCGATGACC
CAACTCACGGGGGCAGTCCCCGGCGCGGCGACCTTGATTACGTCCGGGGACAAGTATGTCGCTCCCCC
CGGGAGGGGTGCAGCCACATGGGAGATTCAAAGTTTCTCGTGACGTCGTTGTGATCACTGCAGCCTAG
CCGAGACTCCCGTACTACGCGAAGGTTGGTTATGTTAACCACTACAACGTGAGGCGCCGTAAGGGCCA
GTGTTGTGCCCGGCTCTTCAATGCGCCTTAGCGGCCTGATACACCCACCCAAGGAGATACTGCTAATC
ATATGGGTGGACAGAACCTCGCAACGCAGACGCCTCCCCGGCATGATATGGTTTTTTTCCGCTATTAT
CCAGCACGCAGCGCTATCATTCAAGAGAACCCAGTGACGCGTAAATCGTAAGATCTACCTGCCGCAGG
TGGACCTACTGCAAATACGGATTATGACTCGTAAAGGGGTCATGCGTATTTTCATCACTAGGCACGTT
CGAGAATAAATTAGTAGGTGTCCCGAGCCTTGTGGCGTTCCGCCTGACTCCTCATGAAGTCGACCTTC
TCACCGGCCCTATCTGCCGACGTAAGTCATAACCTAGATCTGTACCTCGGGGGGAGGGTCACTGTAAA
GGGATAATTGGAGGGTGATTTCCCACACTTTCCTAAGGGTACTTTTTGCCTGGCTTCGCAATTGGGTC
CAATGGATGTCGATCTCTGGTTTAGCAGTTGTGAAAGTGGCAAGGCGGGAGGTTAGACCTCCATTTAA
CATATACAAGCAAGTTAACTGCACTAGATGTGTAGACACTACAGTTACAGGAGTAGCCGAATAAGTCT
CCGACGTCAAGCGAATAAGCGTCATACGCGATTATCGCCTAAGAGCACGTATTGGCGGTAAAAGGCTA
GCTAGACGCTTATGGGTAGATTTCAAGGCGTTCGTAGTGGTATAATAGGATACTCTTTCACCAGCCTG
AGGGCCGAACGCTATACTAGTGGTCTGTGATGTAGGACCGAGTATCTCTCTAGGGACCATCTACTTGA
GCAATGGTGCGCAGGGGGAGACATAGACCAGCCTTGGGTGGCAAGCACTGCAATAAGTCCTGTTTAGC
CTTGGAGTTCACATGCCGGCACTGAAGCCGACCTACCTGANCGTGTGCGATTACCGTTACAATGGCAT
CTGTCTAGTTCTGTTTACCTACGGCGCTCTTGGTTCCAGGTTAGGGGAAGTGTATGACCCATGTGTTT
TTATCGGCTTAACCACGAGTGATCCCCGGTCGTTTCCCCATTGAATCCCTGGTGCATCCTACTCCCAG
AATGATAGCTGACTGACTGGACTGGCTTTTCAAGTAATCGAGGGGGTATCGCGGTCACGGCCGTTAAC
AGATCCCGTCCTTAGTGTGGAATCCGCACCTGCTGACTAACGCTTCGCCGGCGTGTCTGCACAGCCGT
ATAGTGTTAATCATGACCCCAAGGAAGGATTAAACAAATATCTTGACG
>EDEN52
GTGTCCGGTAGCCCGCGCTAGTTAGACACCCCGGCAGGGGGGATTGCTTTCGAGACGGGAGATCCCTT
CGCCGACCCTGGAGGGCCGACGCCGAGGCATTCGGGCCCCTGCAACGTCAACAGCGGCAAGAAAACGG
GATGAATGGGCGTAATGGGGGGGGTCTGCTGGGGACCCGACGCGGTTGCCGTTTGCGGGGCCCCCGAC
CCATACCGACCCACCTAGGCGTCCAGTTACGGCGCACGTCGGGAGCGTGGTTGCCGTCAGAGCTGTGT
TTCTCGATCAGTCCCCCGCAGTGCCGCAGTATCTTGCCGTGGGCTGCTTTAATCTTGAAAGTGGTTTA
TACATTGGGCGACGAGGTGTCGGCTCTCATTGGGGGTAACCGACGGGCACATGCAGTCCCCTCCCCGG
GCAGGCAGAGGCGGGGCCCCCGCGCGCCGGCCCCGGCCCACAATCTTACCAGGGTCCTCAAAGGAGCC
TTTGCATGGTACCCTTCGTGAATGGTTGCTTAAGAGGTCCACCACGTAGTCCGGGCACGGTCGACTAA
ACACTCAAAACAGCGACGGCAAATAGAGGCACAAAGTCCAGGCCCTCACGGCACTAGTTGCGATGACC
CAACTCACGGGGGCAGTCCCCGGCGCGGCGACCTTGATTACGTCCGGGAACAAGTATGTCGTTTCCCC
CGGGAGGGGTGCAGCCACATGGGAGATTCAAAGTTTCTCGTGACGTCGTTGTGATCACTGCAGCCTAG
CCGAGACTCCCGTACTACGCGAAGGTTGGTTATGTTAACCACTACAACGTGAGGCGCCGTAAGGGCCA
GTGTTGTGCCCGGCTCTTCAATGCGCCTTAGCGGCCTGATACACCCACCCAAGGAGATACTGCTAATC
ATGTGGGTGGACAGAACCTCGCAACGCAGACGCATCCCCGGCATGATATGGTTTTTTTCCGCTATTAC
CCAGCACGCGGCGCTATCATTCAAGAGAACCCAGTGACGTGTAAATCGTAAGATCTATCTGCCGCAGG
TGGACCTACTGCAAATACGGATTATGACTCGTAAAGAGGTCATGCGTATTTTCATCACTAGACACGTT
CGAGAATAAATTAGTAGGTGTCCCGCGCCTTGTGGCGTTCCGCCTGACTCCTCATGAAGTCGACCTTC
TCATCGGCCCTATCTGCCGACGTAAGTCATAACCCAGATCTGTACCTCGGAGGGAGGGTCACTGTAAA

GGGATAATTGGAGGGTGATTTCCCACACTTTCCTAAGGGTACTTTTTGCCTGGCTTCGCAGTTGGGTC
CAATAGATGTTGATCTCTGGTTTAGCAGTTGTGAAAGTGGCAAGGCGGGAGGTTAGACCTCCATTTAA
CATATACAAGCAAGTTAACTGCACTAGATGTGTAGACACTACAGTTACAGGAGTAGCCGACTAAGTCT
CCGACGTCAAGCGAATAGGCGTCATACGCGATTATCGCCTAAGAGCACGTATTGGCGGTAAAAGGCTA
ACTAGACGCTTGTGGGTAGATTTCAAGGCGCTCGTAGTGGTATAATAGGATACTCTTTCACCAGCCTG
AGGGCCGAACGCTATACTAGTGGTCTGTGATGTAGGACCAAGTAGCTCTCTAGGGACCATCTACTTGA
GCAATGGTGCGCAGGGGTAGACATAGACCAACCTTGGGTGGCAAGCACTGCAATAAGTCCTGTTTAGC
CTTGGAGTTCACACGCCGGCACTAAAGCCGACCTACCTGAGCTTGTGCGATTACCGTTACAATGGCAT
CTGTCTAGTTCTGTTTACCTACGGCGCTCTTGGTTCCATGTTAGGGGAAGTGTATGACCCATGTGTTT
TTATCGGCTTAACCACGAGTGATCCCCGGTCGTTTCCCCATTGAATCCCTGGTGCATCCTACTCCCAT
AATGATAGCTGACTGGCTGGACTGGCTTTTCAAGTAGTCGAGGGGGTATCGCGGTCACGGCCGTTAAC
AGATCCCGTCCTTAGTGTGGAATCCGCACCTGCTGACTAACGCTTCGCCGGCGTGTCTGCACATCCGT
ATAGTGTTAATCATGACCCCAAGGAAGGATTAAACAAATATCTTGACG
>EDEN55
GTGTCCGGTAGCCCGCGCTAGTTAGACACCCCGGCAGGGGGGATTGCTTTCGAGACGGGAGATCCCTT
CGCCGACCCTGGAGGGCCGACGCCGAGGCATTCGGGCCCCTGCAACGTCAACAGCGGCAAGAAAACGG
GATGAATGGGCGTAATGGGGGGGGTCTGCTGGGGACCCGACGCGGTTGCCGTTTGCGGGGCCCCCGAC
CCATACCGACCCACCTAGGCGTCCAGTTACGGCGCACGTCGGGAGCGTGGTTGCCGTCAGAGCTGTGT
TTCTCGATCAGTCCCCCGCAGTGCCGCAGTATCTTGCCGTGGGCTGCTTTAATCTTGAAAGTGGTTTA
TACATTGGGCGACGAGGTGTCGACTCTCATTGGGGGTAACCGACGGGCACATGCAGTCCCCTCCCCGG
GCAGGCAGAGGCGGGGCCCCCGCGCGCCGGCCCCGGCCCACAATCTTACCAGGGTCCTCAAAGGAGCC
TTTGCATGGTACCCTTCGTGAATGGTTGCTTAAGAGGTCCACAACGTGGTCCGGGCACGGTCGACTAA
ACACTCAAAACAGCGACGGCAAATATAGGTACAAGGTCCAGGCCCTCACGGCACTAATTGCGATGACC
CAANTCACGGGGGCAGTCCCCGGCGCGGCGACCTTGATTACGTCCGGGGACAAGTATGTCGCTCCCCC
CGGGAGGGGTGCAGCCACATGGGAGATTCAAAGTTTCTCGTGACGTCGTTGTGATCACTGCAGCCTAG
CCGAGACTCCCGTACTACGCGAAGGTTGGTTATGTTAACCACTACAACGTGAGGCGCCGTAAGGGCCA
GTGTTGTGCCCGGCTCTTCAATGCGCCTTAGCGGCCTGATACACCCACCCAAGGAGATACTGCTAATC
ATATGGGTGGACAGAACCTCGCAACGCAGACGCCTCCCCGGCATGATATGGTTTTTTTCCGCTATTAT
CCAGCACGCAGCGCTATCATTCAAGAGAACCCAGTGACGCGTAAATCGTAAGATCTACCTGCCGCAGG
TGGACCTACTGCAAATACGGATTATGACTCGTAAAGGGGTCATGCGTATTTTCATCACTAGGCACGTT
CGAGAATAAATTAGTAGGTGTCCCGAGCCTTGTGGCGTTCCGCCTGACTCCTCATGAAGTCGACCTTC
TCACCGGCCCTATCTGCCGACGTAAGTCATAACCTAGATCTGTACCTCGGGGGGAGGGTCACTGTAAA
NGGATAATTGGAGGGTGATTTCCCACACTTTCCTAAGGGTACTTTTTGCCTGGCTTCGCAATTGGGTC
CAATGGATGTCGATCTCTGGTTTAGCAGTTGTGAAAGTGGCAAGGCGGGAGGTTAGACCTCCATTTAA
CATATACAAGCAAGTTAACTGCACTAGATGTGTAGACACTACAGTTACAGGAGTAGCCGAATAAGTCT
CCGACGTCAAGCGAATAAGCGTCATACGCGATTATCGCCTAAGAGCACGTATTGGCGGTAAAAGGCTA
GCTAGACGCTTATGGGTAGATTTCAAGGCGTTCGTAGTGGTATAATAGGATACTCTTTCACCAGCCTG
AGGGCCGAACGCTATACTAGTGGTCTGTGATGTAGGACCGAGTATCTCTCTAGGGACCATCTACTTGA
GCAATGGTGCGCAGGGGGAGACATAGACCAGCCTTGGGTGGCAAGCACTGCAATAAGTCCTGTTTAGC
CTTGGAGTTCACATGCCGGCACTGAAGCCGACCTACCTGAGCGTGTGCGATTACCGTTACAATGGCAT
CTGTCTAGTTCTGTTTACCTACGGCGCTCTTGGTTCCAGGTTAGGGGAAGTGTATGACCCATGTGTTT
TTATCGGCTTAACCACGAGTGATCCCCGGTCGTTTCCCCATTGAATCCCTGGTGCATCCTACTCCCAG
AATGATAGCTGACTGACTGGACTGGCTTTTCAAGTAATCGAGGGGGTATCGCGGTCACGGCCGTTAAC
AGATCCCGTCCTTAGTGTGGAATCCGCACCTGCTGACTAACGCTTCGCCGGCGTGTCTGCACAGCCGT
ATAGTGTTAATCATGACCCCAAGGAAGGATTAAACAAATATCTTGACG
>CHAN01
GTGTCCGGTAGCCCGCGCTAGTTAGACACCCCGGCAGGGGGGATTGCTTTCGAGACGGGAGATCCCTT
CGCCGACCCTGGAGGGCCGACGCCGAGGCATTCGGGCCCCCGCAACGTCAACAGCGGCAAGAAAACGG
GATGAATGGGCGTAATGGGGGGGGTCTGCTGGGGACCCGACGCGGTTGCCGTTTGCGGGGCCCCCGAC
CCATACCGACCCACCTAGGCGTCCAGTTACGGCGCACGGCGGGAGCGTGGTTGCCGTCAGAGCTGTGT
TTCTCGATCAGTCCCCCGCAGTGCCGCAGTATCTTGCCGTGGGCTGCTTTAATCTTGAAAGTGGTTNA
TACATTGGGCGACGAGGTGTCGACTCTCATTGGGGGTAACCGACGGGCACATGCAGTCCCCTCCCCGG
GCAGGCAGAGGCGGGGCCCCCGCGCGCCGGCCCCGGCCCACAATCTTACCAGGGTCCTCAAAGGAGCC
TTTGCATGGTACCCTTCGTGAATGGTTGCTTAAGAGGTCCACAACGTGGTCCGGGCACGGTCGACTAA

ACACTCAAAACAGCGACGGCAAATATAGGTACAAGGTCCAGGCCCTCACGGCACTAATTGCGATGACC
CAACTCACGGGGGCAGTCCCCGGCGCGGCGACCTTGATTACGTCCGGGGACAAGTATGTCGCTCCCCC
CGGGAGGGGTGCAGCCACATGGGAGATTCAAAGTTTCTCGTGACGTCGTTGTGATCACTGCAGCCTAG
CCGAGACTCCCGTACTACGCGAAGGTTGGTTATGTTAACCACTACAACGTGAGGCGCCGTAAGGGCCA
GTGTTGTGCCCGGCTCTTCAATGCGCCTTAGCGGCCTGATACACCCACCCAAGGAGATACTGCTAATC
ATATGGGTGGACAGAACCTCGCAACGCAGACGCCTCCCCGGCATGATATGGTTTTTTTCCGCTATTAT
CCAGCACGCAGCGCTATCATTCAAGAGAACCCAGTGACGCGTAAATCGTAAGATCTACCTGCCGCAGG
TGGACCTACTGCAAATACGGATTATGACTCGTAAAGGGGTCATGCGTATTTTCATCACTAGGCACGTT
CGAGAATAAATTAGTAGGTGTCCCGAGCCTTGTGGCGTTCCGCCTGACTCCTCATGAAGTCGACCTTC
TCACCGGCCCTATCTGCCGACGTAAGTCATAACCTAGATCTGTACCTCGGGGGGAGGGTCACTGTAAA
GGGATAATTGGAGGGTGATTTCCCACACTTTCCTAAGGGTACTTTTTGCCTGGCTTCGCAATTGGGTC
CAATGGATGTCGATCTCTGGTTTAGCAGTTGTGAAAGTGGCAAGGCGGGAGGTTAGACCTCCATTTAA
CATATACAAGCAAGTTAACTGCACTAGATGTGTAGACACTACAGTTACAGGAGTAGCCGAATAAGTCT
CCGACGTCAAGCGAATAAGCGTCATACGCGATTATCGCCTAAGAGCACGTATTGGCGGTAAAAGGCTA
GCTAGACGCTTATGGGTAGATTTCAAGGCGTTCGTAGTGGTATAATAGGATACTCTTTCACCAGCCTG
AGGGCCGAACGCTATACTAGTGGTCTGTGATGTAGGACCGAGTATCTCTCTAGGGACCATCTACTTGA
GCAATGGTGCGCAGGGGGAGACATAGACCAGCCTTGGGTGGCAAGCACTGCAATAAGTCCTGTTTAGC
CTTGGAGTTCACATGCCGGCACTGAAGCCGACCTACCTGAGCGTGTGCGATTACCGTTACAATGGCAT
CTGTCTAGTTCTGTTTACCTACGGCGCTCTTGGTTCCAGGTTAGGGGAAGTGTATGACCCATGTGTTT
TTATCGGCTTAACCACGAGTGATCCCCGGTCGTTTCCCCATTGAATCCCTGGTGCATCCTACTCCCAG
AATGATAGCTGACTGACTGGACTGGCTTTTCAAGTAATCGAGGGGGTATCGCGGTCACGGCCGTTAAC
AGATCCCGTCCTTAGTGTGGAATCCGCACCTGCTGACTAACGCTTCGCCGGCGTGTCTGCACAGCCGT
ATAGTGTTAATCATGACCCCAAGGAAGGATTAAACAAATATCTTGACG
>CHAN03
GTGTCCGGTAGCCCGCGCTAGTTAGACACCCCGGCAGGGGGGATTGCNTTCGAGACGGGAGATCCCTT
CGCCGACCCTGGAGGGCCGACGCCGAGGCATTCGGGCCCCTGCAACGTCAACAGCGGCAAGAAAACGG
GATGAATGGGCGTAATGGGGGGGGTCTGCTGGGGACCCGACGCGGTTGCCGTTTGCGGGGCCCCCGAC
CCATACCGACCCACCTAGGCGTCCAGTTACGGCGCACGTCGGGAGCGTGGTTGCCGTCAGAGCTGTGT
TTCTCGATCAGTCCCCCGCAGTGCCGCAGTATCTTGCCGTGGGCTGCTTTAATCTTGAAAGTGGTTTA
TACATTGGGCGACGAGGTGTCGACTCTCATTGGGGGTAACCGACGGGCACATGCAGTCCCCTCCCCGG
GCAGGCAGAGGCGGGGCCCCCGCGCGCCGGCCCCGGCCCACAATCTTACCAGGGTCCTCAAAGGAGCC
TTTGCATGGTACCCTTCGTGAATGGTTGCTTAAGAGGTCCACAACGTGGTCCGGGCACGGTCGACTAA
ACACTCAAAACAGCGACGGCAAATATAGGTACAAGGTCCAGGCCCTCACGGCACTAATTGCGATGACC
CAACTCACGGGGGCAGTCCCCGGCGCGGCGACCTTGATTACGTCCGGGGACAAGTATGTCGCTCCCCC
CGGGAGGGGTGCAGCCACATGGGAGATTCAAAGTTTCTCGTGACGTCGTTGTGATCACTGCAGCCTAG
CCGAGACTCCCGTACTACGCGAAGGTTGGTTATGTTAACCACTACAACGTGAGGCGCCGTAAGGGCCA
GTGTTGTGCCCGGCTCTTCAATGCGCCTTAGCGGCCTGATACACCCACCCAAGGAGATACTGCTAATC
ATATGGGTGGACAGAACCTCGCAACGCAGACGCCTCCCCGGCATGATATGGTTTTTTTCCGCTATTAT
CCAGCACGCAGCGCTATCATTCAAGAGAACCCAGTGACGCGTAAATCGTAAGATCTACCTGCCGCAGG
TGGACCTACTGCAAATACGGATTATGACTCGTAAAGGGGTCATGCGTATTTTCATCACTAGGCACGTT
CGAGAATAAATTAGTAGGTGTCCCGAGCCTTGTGGCGTTCCGCCTGACTCCTCATGAAGTCGACCTTC
TCACCGGCCCTATCTGCCGACGTAAGTCATAACCTAGATCTGTACCTCGGGGGGAGGGTCACTGTAAA
GGGATAATTGGAGGGTGATTTCCCACACTTTCCTAAGGGTACTTTTTGCCTGGCTTCGCAATTGGGTC
CAATGGATGTCGATCTCTGGTTTAGCAGTTGTGAAAGTGGCAAGGCGGGAGGTTAGACCTCCATTTAA
CATATACAAGCAAGTTAACTGCACTAGATGTGTAGACACTACAGTTACAGGAGTAGCCGAATAAGTCT
CCGACGTCAAGCGAATAAGCGTCATACGCGATTATCGCCTAAGAGCACGTATTGGCGGTAAAAGGCTA
GCTAGACGCTTATGGGTAGATTTCAAGGCGTTCGTAGTGGTATAATAGGATACTCTTTCACCAGCCTG
AGGGCCGAACGCTATACTAGTGGTCTGTGATGTAGGACCGAGTATCTCTCTAGGGACCATCTACTTGA
GCAATGGTGCGCAGGGGGAGACATAGACCAGCCTTGGGTGGCAAGCACTGCAATAAGTCCTGTTTAGC
CTTGGAGTTCACATGCCGGCACTGAAGCCGACCTACCTGAGCGTGTGCGATTACCGTTACAATGGCAT
CTGTCTAGTTCTGTTTACCTACGGCGCTCTTGGTTCCAGGTTAGGGGAAGTGTATGACCCATGTGTTT
TTATCGGCTTAACCACGAGTGATCCCCGGTCGTTTCCCCATTGAATCCCTGGTGCATCCTACTCCCAG
AATGATAGCTGACTGACTGGACTGGCTTTTCAAGTAATCGAGGGGGTATCGCGGTCACGGCCGTTAAC
AGATCCCGTCCTTAGTGTGGAATCCGCACCTGCTGACTAACGCTTCGCCGGCGTGTCTGCACAGCCGT

ATAGTGTTAATCATGACCCCAAGGAAGGATTAAACAAATATCTTGACG
>CHAN05
GTGTCCGGTAGCCCGCGCTAGTTAGACACCCCGGCAGGGGGGATTGCTTTCGAGACGGGAGATCCCTT
CGCCGACCCTGGAGGGCCGACGCCGAGGCATTCGGGCCCCTGCAACGTCAACAGCGGCAAGAAAACGG
GATGAATGGGCGTAATGGGGGGGGTCTGCTGGGGACCCGACGCGGTTGCCGTTTGCGGGGCCCCCGAC
CCATACCGACCCACCTAGGCGTCCAGTTACGGCGCACGTCGGGAGCGTGGTTGCCGTCAGAGCTGTGT
TTCTCGATCAGTCCCCCGCAGTGCCGCAGTATCTTGCCGTGGGCTGCTTTAATCTTGAAAGTGGTTTA
TACATTGGGCGACGAGGTGTCGACTCTCATTGGGGGTAACCGACGGGCACATGCAGTCCCCTCCCCGG
GCAGGCAGAGGCGGGGCCCCCGCGCGCCGGCCCCGGCCCACAATCTTACCAGGGTCCTCAAAGGAGCC
TTTGCATGGTACCCTTCGTGAATGGTTGCTTAAGAGGTCCACAACGTGGTCCGGGCACGGTCGACTAA
ACACTCAAAACAGCGACGGCAAATATAGGTACAAGGTCCAGGCCCTCACGGCACTAATTGCGATGACC
CAACTCACGGGGGCAGTCCCCGGCGCGGCGACCTTGATTACGTCCGGGGACAAGTATGTCGCTCCCCC
CGGGAGGGGTGCAGCCACATGGGAGATTCAAAGTTTCTCGTGACGTCGTTGTGATCACTGCAGCCTAG
CCGAGACTCCCGTACTACGCGAAGGTTGGTTATGTTAACCACTACAACGTGAGGCGCCGTAAGGGCCA
GTGTTGTGCCCGGCTCTTCAATGCGCCTTAGCGGCCTGATACACCCACCCAAGGAGATACTGCTAATC
ATATGGGTGGACAGAACCTCGCAACGCAGACGCCTCCCCGGCATGATATGGTTTTTTTCCGCTATTAT
CCAGCACGCAGCGCTATCATTCAAGAGAACCCAGTGACGCGTAAATCGTAAGATCTACCTGCCGCAGG
TGGACCTACTGCAAATACGGATTATGACTCGTAAAGGGGTCATGCGTATTTTCATCACTAGGCACGTT
CGAGAATAAATTAGTAGGTGTCCCGAGCCTTGTGGCGTTCCGCCTGACTCCTCATGAAGTCGACCTTC
TCACCGGCCCTATCTGCCGACGTAAGTCATAACCTAGATCTGTACCTCGGGGGGAGGGTCACTGTAAA
GGGATAATTGGAGGGTGATTTCCCACACTTTCCTAAGGGTACTTTTTGCCTGGCTTCGCAATTGGGTC
CAATGGATGTCGATCTCTGGTTTAGCAGTTGTGAAAGTGGCAAGGCGGGAGGTTAGACCTCCATTTAA
CATATACAAGCAAGTTAACTGCACTAGATGTGTAGACACTACAGTTACAGGAGTAGCCGAATAAGTCT
CCGACGTCAAGCGAATAAGCGTCATACGCGATTATCGCCTAAGAGCACGTATTGGCGGTAAAAGGCTA
GCTAGACGCTTATGGGTAGATTTCAAGGCGTTCGTAGTGGTATAATAGGATACTCTTTCACCAGCCTG
AGGGCCGAACGCTATACTAGTGGTCTGTGATGTAGGACCGAGTATCTCTCTAGGGACCATCTACTTGA
GCAATGGTGCGCAGGGGGAGACATAGACCAGCCTTGGGTGGCAAGCACTGCAATAAGTCCTGTTTAGC
CTTGGAGTTCACATGCCGGCACTGAAGCCGACCTACCTGAGCGTGTGCGATTACCGTTACAATGGCAT
CTGTCTAGTTCTGTTTACCTACGGCGCTCTTGGTTCCAGGTTAGGGGAAGTGTATGACCCATGTGTTT
TTATCGGCTTAACCACGAGTGATCCCCGGTCGTTTCCCCATTGAATCCCTGGTGCATCCTACTCCCAG
AATGATAGCTGACTGACTGGACTGGCTTTTCAAGTAATCGAGGGGGTATCGCGGTCACGGCCGTTAAC
AGATCCCGTCCTTAGTGTGGAATCCGCACCTGCTGACTAACGCTTCGCCGGCGTGTCTGCACAGCCGT
ATAGTGTTAATCATGACCCCAAGGAAGGATTAAACAAATATCTTGACG
>CHAN06
GTGTCCGGTAGCCCGCGCTAGTTAGACACCCCGGCAGGGGGGATTGCTTTCGGGACGGGAGATCCCTT
CGCCGACCCTGGAGGGCCGACGCCGAGGCATTCGGGCCCCTGCAACGTCAACAGCGGCAAGAAAACGG
GATGAATGGGCGTAATGGGGGGGGTCTGCTGGGGACCCGACGCGGTTGCCGTTTGCGGGGCCCCCGAC
CCATACCGACCCACCTAGGCGTCCAGTTACGGCGCACGTCGGGAGCGTGGTTGCCGTCAGAGCTGTGT
TTCTCGATCAGTCCCCCGCAGTGCCGCAGTATCTTGCCGTGGGCTGCTTTAATCTTGAAAGTGGTTCA
TACATTGGGCGACGAGGTGTCGACTCTCATTGGGGGTAACCGACGGGCACATGCAGTCCCCTCCCCGG
GCAGGCAGAGGCGGGGCCCCCGCGCGCCGGCCCCGGCCCACAATCTTACCAGGGTCCTCAAAGGAGCC
TTTGCATGGTACCCTTCGTGAATGGTTGCTTAAGAGGTCCACCACGTGGTCCGGGCACGGTCGACTAA
ACACTCAAAACAGCGACGGCAAATATAGGCACAAGGTCCAGGCCCTCACGGCACTAATTGCGATGACC
CAACTCACGGGGGCAGTCCCCGGCGCGGCGATCTTGATTACGGCCGGGGACAAGTATGTCGTTCCCCC
CGGGAGGGGTGCAGCCACATGGGAGATTCAAAGTTTCTCGTGACGTCGTTGTGATCACTGCAGCCTAG
CCGAGACTCCCGTACTACGCGAAGGTTGGTTATGTTAACCACTACAACGTGAGGCGCCGTAAGGGCCA
GTGTTGTGCCCGGCTCTTCAATGCGCCTTAGCGGCCTGATACACCCACCCAAGGAGATGCTGCTAATC
ATGTGGGTGGACAGAACCTCGCAACGCAGACGCCTCCCCGGCATGATATGGTTTTTTTCCGCTATTAT
CCAGCACGCAGCGCTATCATTCAAGAGAACCCAGTGACGCGTAAATCGTAAGATCTACCTGCCGCAGG
TGGACCTACTGCAAATACGGATTATGACTCGTAAAGAGGTCATGCGTATTTTCATCACTAGGCACGTT
CGAGAATAAATTAGTAGGTGTCCCGCGCCTTGTGGCGTTCCGCCTGACTCCTCATGAAGTCGGCCTTC
TCACCGGCCCTATCTGCCGACGTAAGTCATAACCTAGATCTGTACCTCGGGGGGAGGGTCACTGTAAA
GGGATAATTGGAGGGTGATTTCCCACACTTTCCTAAGGGTACTTTTTGCCTGGCTTCGCAATTGGGTC
CAATGGATATCGATCTCTGGTTTAACAGTTGTGAAAGTGGCAAGGCGGGAGGTTAGACCTCCATTTAA

CATATACAAGCAAGTTAACTGCACTAGATGTGTAGACACTACAGTTACAGGAGTAGACGAATAAGTCT
CCGACGTCAAGCGAATAAGCGTCATACGCGATTATCGCCTAAGAGCACGTACCGGCGGTAAAAGGCTA
ACTAGACGCTTGTGGGTAGATTTCAAGGCGTTCGTAGTGGTATAACAGGATACTCTTTCACCAGCCTG
AGGGCCGAACGCTATACTAGTGGTCTATGATGTAGGACCGAGTATCTCTCTAGGGACCATCTACTTGA
GCAATGGTGCGCAGGGGGAGACATAGACCAGCCTTGGGTNGCAAGCACTGCAATAAGTCCTGTTTAGC
CTTGGAGTTCACACGCCGTCACTGAAGCCGACCTACCTGAGCTTGTGCGATTACCGTTACAATGGCAT
CTGTCTAGTTCTGTTTACCTACGGCGCTCTTGGTTCCAGGTTAGGGGAAGTGTATGACCCATGTGTTT
TTCTCGGCTTAACCACGAGTGATCCCCGGTCGTTTCCCCGTTGAATCCCTGGTGCATCCTACTCCCAG
AATGATAGCTGACTGACTGGACTGGCTTTTCAAGTAGTCGAGGGGGTATCGCGGTCACGGCCGTTAAC
AGATCCCGTCCTTAGTGTGGAATCCGCACCTGCTGACTAACGCTTCGCCGGCGTGTCTGCACAGCCGT
ATAGTGTTAATCATGACCCCAAGGAAGGATTAAACAAATATCTTGACG
>CHAN07
GTGTCCGGTAGCCCGCGCTAGTTAGACACCCCGGCAGGGGGGATTGCTTTCGGGACGGGAGATCCCTT
CGCCGACCCTGGAGGGCCGACGCCGAGGCATTCGGGCCCCTGCAACGTCAACAGCGGCAAGAAAACGG
GATGAATGGGCGTAATGGGGGGGGTCTGCTGGGGACCCGACGCGGTTGCCGTTTGCGGGGCCCCCGAC
CCATACCGACCCACCTAGGCGTCCAGTTACGGCGCACGTCGGGAGCGTGGTTGCCGTCAGAGCTGTGT
TTCTCGATCAGTCCCCCGCAGTGCCGCAGTATCTTGCCGTGGGCTGCTTTAATCTTGAAAGTGGTTCA
TACATTGGGCGACGAGGTGTCGACTCTCATTGGGGGTAACCGACGGGCACATGCAGTCCCCTCCCCGG
GCAGGCAGAGGCGGGGCCCCCGCGCGCCGGCCCCGGCCCACAATCTTACCAGGGTCCTCAAAGGAGCC
TTTGCATGGTACCCTTCGTGAATGGTTGCTTAAGAGGTCCACCACGTGGTCCGGGCACGGTCGACTAA
ACACTCAAAACAGCGACGGCAAATATAGGCACAAGGTCCAGGCCCTCACGGCACTAATTGCGATGACC
CAACTCACGGGGGCAGTCCCCGGCGCGGCGANCTTGATTACGGCCGGGGACAAGTATGTCGTTCCCCC
CGGGAGGGGTGCAGCCACATGGGAGATTCAAAGTTTCTCGTGACGTCGTTGTGATCACTGCAGCCTAG
CCGAGACTCCCGTACTACGCGAAGGTTGGTTATGTTAACCACTACAACGTGAGGCGCCGTAAGGGCCA
GTGTTGTGCCCGGCTCTTCAATGCGCCTTAGCGGCCTGATACACCCACCCAAGGAGATGCTGCTAATC
ATGTGGGTGGACAGAACCTCGCAACGCAGACGCCTCCCCGGCATGATATGGTTTTTTTCCGCTATTAT
CCAGCACGCAGCGCTATCATTCAAGAGAACCCAGTGACGCGTAAATCGTAAGATCTACCTGCCGCAGG
TGGACCTACTGCAAATACGGATTATGACTCGTAAAGAGGTCATGCGTATTTTCATCACTAGGCACGTT
CGAGAATAAATTAGTAGGTGTCCCGCGCCTTGTGGCGTTCCGCCTGACTCCTCATGAAGTCGGCCTTC
TCACCGGCCCTATCTGCCGACGTAAGTCATAACCTAGATCTGTACCTCGGGGGGAGGGTCACTGTAAA
GGGATAATTGGAGGGTGATTTCCCACACTTTCCTAAGGGTACTTTTTGCCTGGCTTCGCAATTGGGTC
CAATGGATATCGATCTCTGGTTTAACAGTTGTGAAAGTGGCAAGGCGGGAGGTTAGACCTCCATTTAA
CATATACAAGCAAGTTAACTGCACTAGATGTGTAGACACTACAGTTACAGGAGTAGACGAATAAGTCT
CCGACGTCAAGCGAATAAGCGTCATACGCGATTATCGCCTAAGAGCACGTACCGGCGGTAAAAGGCTA
ACTAGACGCTTGTGGGTAGATTTCAAGGCGTTCGTAGTGGTATAACAGGATACTCTTTCACCAGCCTG
AGGGCCGAACGCTATACTAGTGGTCTATGATGTAGGACCGAGTATCTCTCTAGGGACCATCTACTTGA
GCAATGGTGCGCAGGGGGAGACATAGACCAGCCTTGGGTNGCAAGCACTGCAATAAGTCCTGTTTAGC
CTTGGAGTTCACACGCCGTCACTGAAGCCGACCTACCTGAGCTTGTGCGATTACCGTTACAATGGCAT
CTGTCTAGTTCTGTTTACCTACGGCGCTCTTGGTTCCAGGTTAGGGGAAGTGTATGACCCATGTGTTT
TTCTCGGCTTAACCACGAGTGATCCCCGGTCGTTTCCCCGTTGAATCCCTGGTGCATCCTACTCCCAG
AATGATAGCTGACTGACTGGACTGGCTTTTCAAGTAGTCGAGGGGGTATCGCGGTCACGGCCGTTAAC
AGATCCCGTCCTTAGTGTGGAATCCGCACCTGCTGACTAACGCTTCGCCGGCGTGTCTGCACAGCCGT
ATAGTGTTAATCATGACCCCAAGGAAGGATTAAACAAATATCTTGACG
>CHAN08
GTGTCCGGTAGCCCGCGCTAGTTAGACACCCCGGCAGGGGGGATTGCTTTCGAGACGGGAGATCCCTT
CGCCGACCCTGGAGGGCCGACGCCGAGGCATTCGGGCCCCTGCAACGTCAACAGCGGCAAGAAAACGG
GATGAATGGGCGTAATGGGGGGGGTCTGCTGGGGACCCGACGCGGTTGCCGTTTGCGGGGCCCCCGAC
CCATACCGACCCACCTAGGCGTCCAGTTACGGCGCACGTCGGGAGCGTGGTTGCCGTCAGAGCTGTGT
TTCTCGATCAGTCCCCCGCAGTGCCGCAGTATCTTGCCGTGGGCTGCTTTAATCTTGAAAGTGGTTCA
TACATTGGGCGACGAGGTGTCGACTCTCATTGGGGGTAACCGACGGGCACATGCAGTCCCCTCCCCGG
GCAGGCAGAGGCGGGGCCCCCGCGCGCCGGCCCCGGCCCACAATCTTACCAGGGTCCTCAAAGGAGCC
TTTGCATGGTACCCTTCGTGAATGGTTGCTTAAGAGGTCCACCACGTGGTCCGGGCACGGTCGACTAA
ACACTCAAAACAGCGACGGCAAATATAGGCACAAGGTCCAGGCCCTCACGGCACTAATTGCGATGACC
CAACTCACGGGGGCAGTCCCCGGCGCGGCGATCTTGATTACGGCCGGGGACAAGTATGTCGTTCCCCC

CGGGAGGGGTGCAGCCACATGGGAGATTCAAAGTTTCTCGTGACGTCGTTGTGATCACTGCAGCCTAG
CCGAGACTCCCGTACTACGCGAAGGTTGGTTATGTTAACCACTACAACGTGAGGCGCCGTAAGGGCCA
GTGTTGTGCCCGGCTCTTCAATGCGCCTTAGCGGCCTGATACACCCACCCAAGGAGATGCTGCTAATC
ATGTGGGTGGACAGAACCTCGCAACGCAGACGCCTCCCCGGCATGATATGGTTTTTTTCCGCTATTAT
CCAGCACGCAGCGCTATCATTCAAGAGAACCCAGTGACGCGTAAATCGTAAGATCTACCTGCCGCAGG
TGGACCTACTGCAAATACGGATTATGACTCGTAAAGAGGTCATGCGTATTTTCATCACTAGGCACGTT
CGAGAATAAATTAGTAGGTGTCCCGCGCCTTGTGGCGTTCCGCCTGACTCCTCATGAAGTCGGCCTTC
TCACCGGCCCTATCTGCCGACGTAAGTCATAACCTAGATCTGTACCTCGGGGGGAGGGTCACTGTAAA
GGGATAATTGGAGGGTGATTTCCCACACTTTCCTAAGGGTACTTTTTGCCTGGCTTCGCAATTGGGTC
CAATGGATATCGATCTCTGGTTTAACAGTTGTGAAAGTGGCAAGGCGGGAGGTTAGACCTCCATTTAA
CATATACAAGCAAGTTAACTGCACTAGATGTGTAGACACTACAGTTACAGGAGTAGACGAATAAGTCT
CCGACGTCAAGCGAATAAGCGTCATACGCGATTATCGCCTAAGAGCACGTACCGGCGGTAAAAGGCTA
ACTAGACGCTTGTGGGTAGATTTCAAGGCGTTCGTAGTGGTATAACAGGATACTCTTTCACCAGCCTG
AGGGCCGAACGCTATACTAGTGGTCTATGATGTAGGACCGAGTATCTCTCTAGGGACCATCTACTTGA
GCAATGGTGCGCAGGGGGAGACATAGACCAGCCTTGGGTNGCAAGCACTGCAATAAGTCCTGTTTAGC
CTTGGAGTTCACACGCCGTCACTGAAGCCGACCTACCTGAGCTTGTGCGATTACCGTTACAATGGCAT
CTGTCTAGTTCTGTTTACCTACGGCGCTCTTGGTTCCAGGTTAGGGGAAGTGTATGACCCATGTGTTT
TTCTCGGCTTAACCACGAGTGATCCCCGGTCGTTTCCCCGTTGAATCCCTGGTGCATCCTACTCCCAG
AATGATAGCTGACTGACTGGACTGGCTTTTCAAGTAGTCGAGGGGGTATCGCGGTCACGGCCGTTAAC
AGATCCCGTCCTTAGTGTGGAATCCGCACCTGCTGACTAACGCTTCGCCGGCGTGTCTGCACAGCCGT
ATAGTGTTAATCATGACCCCAAGGAAGGATTAAACAAATATCTTGACG
>CHAN09
GTGTCCGGTAGCCCGCGCTAGTTAGACACCCCGGCAGGGGGGATTGCTTTCGAGACGGGAGATCCCTT
CGCCGACCCTGGAGGGCCGACGCCGAGGCATTCGGGCCCCCGCAACGTCAACAGCGGCAAGAAAACGG
GATGAATGGGCGTAATGGGGGGGGTCTGCTGGGGACCCGACGCGGTTGCCGTTTGCGGGGCCCCCGAC
CCATACCGACCCACCTAGGCGTCCAGTTACGGCGCACGGCGGGAGCGTGGTTGCCGTCAGAGCTGTGT
TTCTCGATCAGTCCCCCGCAGTGCCGCAGTATCTTGCCGTGGGCTGCTTTAATCTTGAAAGTGGTTNA
TACATTGGGCGACGAGGTGTCGACTCTCATTGGGGGTAACCGACGGGCACATGCAGTCCCCTCCCCGG
GCAGGCAGAGGCGGGGCCCCCGCGCGCCGGCCCCGGCCCACAATCTTACCAGGGTCCTCAAAGGAGCC
TTTGCATGGTACCCTTCGTGAATGGTTGCTTAAGAGGTCCACAACGTGGTCCGGGCACGGTCGACTAA
ACACTCAAAACAGCGACGGCAAATATAGGTACAAGGTCCAGGCCCTCACGGCACTAATTGCGATGACC
CAACTCACGGGGGCAGTCCCCGGCGCGGCGACCTTGATTACGTCCGGGGACAAGTATGTCGCTCCCCC
CGGGAGGGGTGCAGCCACATGGGAGATTCAAAGTTTCTCGTGACGTCGTTGTGATCACTGCAGCCTAG
CCGAGACTCCCGTACTACGCGAAGGTTGGTTATGTTAACCACTACAACGTGAGGCGCCGTAAGGGCCA
GTGTTGTGCCCGGCTCTTCAATGCGCCTTAGCGGCCTGATACACCCACCCAAGGAGATACTGCTAATC
ATATGGGTGGACAGAACCTCGCAACGCAGACGCCTCCCCGGCATGATATGGTTTTTTTCCGCTATTAT
CCAGCACGCAGCGCTATCATTCAAGAGAACCCAGTGACGCGTAAATCGTAAGATCTACCTGCCGCAGG
TGGACCTACTGCAAATACGGATTATGACTCGTAAAGGGGTCATGCGTATTTTCATCACTAGGCACGTT
CGAGAATAAATTAGTAGGTGTCCCGAGCCTTGTGGCGTTCCGCCTGACTCCTCATGAAGTCGACCTTC
TCACCGGCCCTATCTGCCGACGTAAGTCATAACCTAGATCTGTACCTCGGGGGGAGGGTCACTGTAAA
GGGATAATTGGAGGGTGATTTCCCACACTTTCCTAAGGGTACTTTTTGCCTGGCTTCGCAATTGGGTC
CAATGGATGTCGATCTCTGGTTTAGCAGTTGTGAAAGTGGCAAGGCGGGAGGTTAGACCTCCATTTAA
CATATACAAGCAAGTTAACTGCACTAGATGTGTAGACACTACAGTTACAGGAGTAGCCGAATAAGTCT
CCGACGTCAAGCGAATAAGCGTCATACGCGATTATCGCCTAAGAGCACGTATTGGCGGTAAAAGGCTA
GCTAGACGCTTATGGGTAGATTTCAAGGCGTTCGTAGTGGTATAATAGGATACTCTTTCACCAGCCTG
AGGGCCGAACGCTATACTAGTGGTCTGTGATGTAGGACCGAGTATCTCTCTAGGGACCATCTACTTGA
GCAATGGTGCGCAGGGGGAGACATAGACCAGCCTTGGGTGGCAAGCACTGCAATAAGTCCTGTTTAGC
CTTGGAGTTCACATGCCGGCACTGAAGCCGACCTACCTGAGCGTGTGCGATTACCGTTACAATGGCAT
CTGTCTAGTTCTGTTTACCTACGGCGCTCTTGGTTCCAGGTTAGGGGAAGTGTATGACCCATGTGTTT
TTATCGGCTTAACCACGAGTGATCCCCGGTCGTTTCCCCATTGAATCCCTGGTGCATCCTACTCCCAG
AATGATAGCTGACTGACTGGACTGGCTTTTCAAGTAATCGAGGGGGTATCGCGGTCACGGCCGTTAAC
AGATCCCGTCCTTAGTGTGGAATCCGCACCTGCTGACTAACGCTTCGCCGGCGTGTCTGCACAGCCGT
ATAGTGTTAATCATGACCCCAAGGAAGGATTAAACAAATATCTTGACG
>CHAN10

GTGTCCGGTAGCCCGCGCTAGTTAGACACCCCGGCAGGGGGGATTGCTTTCGAGACGGGAGATCCCTT
CGCCGACCCTGGAGGGCCGACGCCGAGGCATTCGGGCCCCTGCAACGTCAACAGCGGCAAGAAAACGG
GATGAATGGGCGTAATGGGGGGGGTCTGCTGGGGACCCGACGCGGTTGCCGTTTGCGGGGCCCCCGAC
CCATACCGACCCACCTAGGCGTCCAGTTACGGCGCACGTCGGGAGCGTGGTTGCCGTCAGAGCTGTGT
TTCTCGATCAGTCCCCCGCAGTGCCGCAGTATCTTGCCGTGGGCTGCTTTAATCTTGAAAGTGGTTTA
TACATTGGGCGACGAGGTGTCGACTCTCATTGGGGGTAACCGACGGGCACATGCAGTCCCCTCCCCGG
GCAGGCAGAGGCGGGGCCCCCGCGCGCCGGCCCCGGCCCACAATCTTACCAGGGTCCTCAAAGGAGCC
TTTGCATGGTACCCTTCGTGAATGGTTGCTTAAGAGGTCCACAACGTGGTCCGGGCACGGTCGACTAA
ACACTCAAAACAGCGACGGCAAATATAGGTACAAGGTCCAGGCCCTCACGGCACTAATTGCGATGACC
CAACTCACGGGGGCAGTCCCCGGCGCGGCGACCTTGATTACGTCCGGGGACAAGTATGTCGCTCCCCC
CGGGAGGGGTGCAGCCACATGGGAGATTCAAAGTTTCTCGTGACGTCGTTGTGATCACTGCAGCCTAG
CCGAGACTCCCGTACTACGCGAAGGTTGGTTATGTTAACCACTACAACGTGAGGCGCCGTAAGGGCCA
GTGTTGTGCCCGGCTCTTCAATGCGCCTTAGCGGCCTGATACACCCACCCAAGGAGATACTGCTAATC
ATATGGGTGGACAGAACCTCGCAACGCAGACGCCTCCCCGGCATGATATGGTTTTTTTCCGCTATTAT
CCAGCACGCAGCGCTATCATTCAAGAGAACCCAGTGACGCGTAAATCGTAAGATCTACCTGCCGCAGG
TGGACCTACTGCAAATACGGATTATGACTCGTAAAGGGGTCATGCGTATTTTCATCACTAGGCACGTT
CGAGAATAAATTAGTAGGTGTCCCGAGCCTTGTGGCGTTCCGCCTGACTCCTCATGAAGTCGACCTTC
TCACCGGCCCTATCTGCCGACGTAAGTCATAACCTAGATCTGTACCTCGGGGGGAGGGTCACTGTAAA
GGGATAATTGGAGGGTGATTTCCCACACTTTCCTAAGGGTACTTTTTGCCTGGCTTCGCAATTGGGTC
CAATGGATGTCGATCTCTGGTTTAGCAGTTGTGAAAGTGGCAAGGCGGGAGGTTAGACCTCCATTTAA
CATATACAAGCAAGTTAACTGCACTAGATGTGTAGACACTACAGTTACAGGAGTAGCCGAATAAGTCT
CCGACGTCAAGCGAATAAGCGTCATACGCGATTATCGCCTAAGAGCACGTATTGGCGGTAAAAGGCTA
GCTAGACGCTTATGGGTAGATTTCAAGGCGTTCGTAGTGGTATAATAGGATACTCTTTCACCAGCCTG
AGGGCCGAACGCTATACTAGTGGTCTGTGATGTAGGACCGAGTATCTCTCTAGGGACCATCTACTTGA
GCAATGGTGCGCAGGGGGAGACATAGACCAGCCTTGGGTGGCAAGCACTGCAATAAGTCCTGTTTAGC
CTTGGAGTTCACATGCCGGCACTGAAGCCGACCTACCTGAGCGTGTGCGATTACCGTTACAATGGCAT
CTGTCTAGTTCTGTTTACCTACGGCGCTCTTGGTTCCAGGTTAGGGGAAGTGTATGACCCATGTGTTT
TTATCGGCTTAACCACGAGTGATCCCCGGTCGTTTCCCCATTGAATCCCTGGTGCATCCTACTCCCAG
AATGATAGCTGACTGACTGGACTGGCTTTTCAAGTAATCGAGGGGGTATCGCGGTCACGGCCGTTAAC
AGATCCCGTCCTTAGTGTGGAATCCGCACCTGCTGACTAACGCTTCGCCGGCGTGTCTGCACAGCCGT
ATAGTGTTAATCATGACCCCAAGGAAGGATTAAACAAATATCTTGACG
>CHAN11
GTGTCCGGTAGCCCGCGCTAGTTAGACACCCCGGCAGGGGGGATTGCTTTCGGGACGGGAGATCCCTT
CGCCGACCCTGGAGGGCCGACGCCGAGGCATTCGGGCCCCTGCAACGTCAACAGCGGCAAGAAAACGG
GATGAATGGGCGTAATGGGGGGGGTCTGCTGGGGACCCGACGCGGTTGCCGTTTGCGGGGCCCCCGAC
CCATACCGACCCACCTAGGCGTCCAGTTACGGCGCACGTCGGGAGCGTGGTTGCCGTCAGAGCTGTGT
TTCTCGATCAGTCCCCCACAGTGCCGCAGTATCTTGCCGTGGGCTGCTTTAATCTTGAAAGTGGTTTA
TACATTGGGCGACGAGGTGTCGACTCTCATTGGGGGTAACCGACGGGCACATGCAGTCCCCTCCCCGG
GCAGGCAGAGGCGGGGCCCCCGCGCGCCGGCCCCGGCCCACAATCTTACCAGGGTCCTCAAAGGAGCC
TTTGCATGGTACCCTTCGTGAATGGTTGCTTAAGAAGTCCACAACGTGGTCCGGGCACGGTCGACTAA
ACACTCAAAACAGCGACGGCAAATATAGGTACAAGGTCCAGGCCCTCACGGCACTAATTGCGATGACC
CAACTCACGGGGGCAGTCCCCGGCGCGGCGACCTTGATTACGTCCGGGGACAAGTATGTCGCTCCCCC
CGGGAGGGGTGCAGCCACATGGGAGATTCAAAGTTTCTCGTGACGTCGTTGTGATCACTGCAGCCTAG
CCGAGACTCCCGTACTACGCGAAGGTTGGTTATGTTAACCACTACAACGTGAGGCGCCGTAAGGGCCA
GTGTTGNGCCCGGCTCTTCAATGCGCCTTAGCGGCCTGATACACCCACCCAAGGAGATACTGCTAATC
ATATGAGTGGACAGAACCTCGCAACGCAGACGCCTCCCCGGCATGATATGGTTTTTTTCCGCTATTAT
CCAGCACGCAGCGCTATCATTCAAGAGAACCCAGTGACGCGTAAATCGTAAGATCTACCTGCCGCAGG
TGGACCTACTGCAAATACGGATTATGACTCGTAAAGGGGTCATGCGTATTTTCATCACTAGGCACGTT
CGAGAATAAATTAGTAGGTGTCCCGAGCCTTGTGGCGTTCCGCCTGACTCCTCATGAAGTCGACCTTC
TCACCGGCCCTATCTGCCGACGTAAGTCATAACCTAGATCTGTACCTCGGGGGGAGGGTCACTGTAAA
GGGATAATTGGAGGGTGATTTCCCACATTTTCCTAAGGGTACTTTTTGCCTGGCTTCGCAATTGGGTC
CAATGGATGTCGATCTCTGGTTTAGCAGTTGTGAAAGTGGCAAGGCGGGAGGTTAGACCTCCATTTAA
CATATACAAGCAAGTTAACTGCACTAGATGTGTAGACACTACAGGTACAGGAGTAGCCGGATAAGTCT
CCGACGTCAAGCGAATAAGCTTCATACGCGATTATCGCCTAAGAGCACGTATTTGCGGTAAAAGGCTA

GCTAGACGCTTGTGGGTAGATTTCAAGGCGTTCGTAGTGGTATAATAGGATACTCTTTCACCAGCCTG
AGGGCCGAACGCTATACTAGTGGTCTGTGATGTAGGACCGAGTATCTCTCTAGGGACCATCTACTTGA
GCAATGGTGCGCAGGGGGAGACATAGACCAGCCTTGGGTGGCAAGCACTGCAATAAGTCCTGTTTAGC
CTTGGAGTTCACACGCCGGCACTGAAGCCGACCTACCTGAGCGTGTGCGATTACCGTTACAATGGCAT
CTGTCTAGTTCTGTTTACCTACGGCGCTCTTGGTTCCAGGTTAGGGGAAGTGTATGACCCATGTGTTT
TCATCGGCTTAACCACGAGTGATCCCCGGTCGTTTCCCCATTGAATCCCTGGTGCATCCTACTCCCAG
AATGATAGCTGACTGACTGGACTGGCTTTTCAAGTAATCGAGGGGGTATCGCGGTCACGGCCGTTAAC
AGATCCCGTCCTTAGTGTGGAATCCGCACCTGCTGACTAACGCTTCGCCGGCGTGTCTGCACAGCCGT
ATAGTGTTAATCATGACCCCAAGGAAGGATTAAACAAATATCTTGACG
>CHAN12
GTGTCCGGTAGCCCGCGCTAGTTAGACACCCCGGCAGGGGGGATTGCTTTCGAGACGGGAGATCCCTT
CGCCGACCCTGGAGGGCCGACGCCGAGGCATTCGGGCCCCTGCAACGTCAACAGCGGCAAGAAAACGG
GATGAATGGGCGTAATGGGGGGGGTCTGCTGGGGACCCGACGCGGTTGCCGTTTGCGGGGCCCCCGAC
CCATACCGACCCACCTAGGCGTCCAGTTACGGCGCACGGCGGGAGCGTGGTTGCCGTCAGAGCTGTGT
TTCTCGATCAGTCCCCCGCAGTGCCGCAGTATCTTGCCGTGGGCTGCTTTAATCTTGAAAGTGGTTTA
TACATTGGGCGACGAGGTGTCGACTCTCATTGGGGGTAACCGACGGGCACATGCAGTCCCCTCCCCGG
GCAGGCAGAGGCGGGGCCCCCGCGCGCCGGCCCCGGCCCACAATCTTACCAGGGTCCTCAAAGGAGCC
TTTGCATGGTACCCTTCGTGAATGGTTGCTTAAGAGGTCCACCACGTGGTCCGGGCACGGTCGACTAA
ACACTCAAAACAGCGACGGCAAATATAGGCACAAGGTCCAGGCCCTCACGGCACTAATTGCGATGACC
CAACTCACGGGGGCAGTCCCCGGCGCGGCGATCTTGATTACGGCCGGGGACAAGTATGTCGTTCCCCC
CGGGAGGGGTGCAGCCACATGGGAGATTCAAAGTTTCTCGTGACGTCGTTGTGATCACTGCAGCCTAG
CCGAGACTCCCGTACTACGCGAAGGTTGGTTATGTTAACCACTACAACGTGAGGCGCCGTAAGGGCCA
GTGTTGTGCCCGGCTCTTCAATGCGCCTTAGCGGCCTGATACACCCACCCAAGGAGATGCTGCTAATC
ATGTGGGTGGACAGAACCTCGCAACGCAGACGCCTCCCCGGCATGATATGGTTTTTTTCCGCTATTAT
CCAGCACGCAGCGCTATCATTCAAGAGAACCCAGTGACGCGTAAATCGTAAGATCTACCTGCCGCAGG
TGGACCTACTGCAAATACGGATTATGACTCGTAAAGAGGTCATGCGTATTTTCATCACTAGGCACGTT
CGAGAATAAATTAGTAGGTGTCCCGCGCCTTGTGGCGTTCCGCCTGACTCCTCATGAAGTCGGCCTTC
TCACCGGCCCTATCTGCCGACGTAAGTCATAACCTAGATCTGTACCTCGGGGGGAGGGTCACTGTAAA
GGGATAATTGGAGGGTGATTTCCCACACTTTCCTAAGGGTACTTTTTGCCTGGCTTCGCAATTGGGTC
CAATGGATATCGATCTCTGGTTTAACAGTTGTGAAAGTGGCAAGGCGGGAGGTTAGACCTCCATTTAA
CATATACAAGCAAGTTAACTGCACTAGATGTGTAGACACTACAGTTACAGGAGTAGACGAATAAGTCT
CCGACGTCAAGCGAATAAGCGTCATACGCGATTATCGCCTAAGAGCACGTACCGGCGGTAAAAGGCTA
ACTAGACGCTTGTGGGTAGATTTCAAGGCGTTCGTAGTGGTATAACAGGATACTCTTTCACCAGCCTG
AGGGCCGAACGCTATACTAGTGGTCTATGATGTAGGACCGAGTATCTCTCTAGGGACCATCTACTTGA
GCAATGGTGCGCAGGGGGAGACATAGACCAGCCTTGGGTNGCAAGCACTGCAATAAGTCCTGTTTAGC
CTTGGAGTTCACACGCCGTCACTGAAGCCGACCTACCTGAGCTTGTGCGATTACCGTTACAATGGCAT
CTGTCTAGTTCTGTTTACCTACGGCGCTCTTGGTTCCAGGTTAGGGGAAGTGTATGACCCATGTGTTT
TTCTCGGCTTAACCACGAGTGATCCCCGGTCGTTTCCCCGTTGAATCCCTGGTGCATCCTACTCCCAG
AATGATAGCTGACTGACTGGACTGGCTTTTCAAGTAGTCGAGGGGGTATCGCGGTCACGGCCGTTAAC
AGATCCCGTCCTTAGTGTGGAATCCGCACCTGCTGACTAACGCTTCGCCGGCGTGTCTGCACAGCCGT
ATAGTGTTAATCATGACCCCAAGGAAGGATTAAACAAATATCTTGACG
>CHAN15
GTGTCCGGTAGCCCGCGCTAGTTAGACACCCCGGCAGGGGGGATTGCTTTCGAGACGGGAGATCCCTT
CGCCGACCCTGGAGGGCCGACGCCGAGGCATTCGGGCCCCCGCAACGTCAACAGCGGCAAGAAAACGG
GATGAATGGGCGTAATGGGGGGGGTCTGCTGGGGACCCGACGCGGTTGCCGTTTGCGGGGCCCCCGAC
CCATACCGACCCACCTAGGCGTCCAGTTACGGCGCACGGCGGGAGCGTGGTTGCCGTCAGAGCTGTGT
TTCTCGATCAGTCCCCCGCAGTGCCGCAGTATCTTGCCGTGGGCTGCTTTAATCTTGAAAGTGGTTCA
TACATTGGGCGACGAGGTGTCGACTCTCATTGGGGGTAACCGACGGGCACATGCAGTCCCCTCCCCGG
GCAGGCAGAGGCGGGGCCCCCGCGCGCCGGCCCCGGCCCACAATCTTACCAGGGTCCTCAAAGGAGCC
TTTGCATGGTACCCTTCGTGAATGGTTGCTTAAGAGGTCCACAACGTGGTCCGGGCACGGTCGACTAA
ACACTCAAAACAGCGACGGCAAATATAGGTACAAGGTCCAGGCCCTCACGGCACTAATTGCGATGACC
CAACTCACGGGGGCAGTCCCCGGCGCGGCGACCTTGATTACGTCCGGGGACAAGTATGTCGCTCCCCC
CGGGAGGGGTGCAGCCACATGGGAGATTCAAAGTTTCTCGTGACGTCGTTGTGATCACTGCAGCCTAG
CCGAGACTCCCGTACTACGCGAAGGTTGGTTATGTTAACCACTACAACGTGAGGCGCCGTAAGGGCCA

GTGTTGTGCCCGGCTCTTCAATGCGCCTTAGCGGCCTGATACACCCACCCAAGGAGATACTGCTAATC
ATATGGGTGGACAGAACCTCGCAACGCAGACGCCTCCCCGGCATGATATGGTTTTTTTCCGCTATTAT
CCAGCACGCAGCGCTATCATTCAAGAGAACCCAGTGACGCGTAAATCGTAAGATCTACCTGCCGCAGG
TGGACCTACTGCAAATACGGATTATGACTCGTAAAGGGGTCATGCGTATTTTCATCACTAGGCACGTT
CGAGAATAAATTAGTAGGTGTCCCGAGCCTTGTGGCGTTCCGCCTGACTCCTCATGAAGTCGACCTTC
TCACCGGCCCTATCTGCCGACGTAAGTCATAACCTAGATCTGTACCTCGGGGGGAGGGTCACTGTAAA
GGGATAATTGGAGGGTGATTTCCCACACTTTCCTAAGGGTACTTTTTGCCTNGCTTCGCAATTGGGTC
CAATGGATGTCGATCTCTGGTTTAGCAGTTGTGAAAGTGGCAAGGCGGGAGGTTAGACCTCCATTTAA
CATATACAAGCAAGTTAACTGCACTAGATGTGTAGACACTACAGTTACAGGAGTAGCCGAATAAGTCT
CCGACGTCAAGCGAATAAGCGTCATACGCGATTATCGCCTAAGAGCACGTATTGGCGGTAAAAGGCTA
GCTAGACGCTTATGGGTAGATTTCAAGGCGTTCGTAGTGGTATAATAGGATACTCTTTCACCAGCCTG
AGGGCCGAACGCTATACTAGTGGTCTGTGATGTAGGACCGAGTATCTCTCTAGGGACCATCTACTTGA
GCAATGGTGCGCAGGGGGAGACATAGACCAGCCTTGGGTGGCAAGCACTGCAATAAGTCCTGTTTAGC
CTTGGAGTTCACATGCCGGCACTGAAGCCGACCTACCTGAGCGTGTGCGATTACCGTTACAATGGCAT
CTGTCTAGTTCTGTTTACCTACGGCGCTCTTGGTTCCAGGTTAGGGGAAGTGTATGACCCATGTGTTT
TTATCGGCTTAACCACGAGTGATCCCCGGTCGTTTCCCCATTGAATCCCTGGTGCATCCTACTCCCAG
AATGATAGCTGACTGACTGGACTGGCTTTTCAAGTAATCGAGGGGGTATCGCGGTCACGGCCGTTAAC
AGATCCCGTCCTTAGTGTGGAATCCGCACCTGCTGACTAACGCTTCGCCGGCGTGTCTGCACAGCCGT
ATAGTGTTAATCATGACCCCAAGGAAGGATTAAACAAATATCTTGACG
>CHAN17
GTGTCCGGTAGCCCGCGCTAGTTAGACACCCCGGCAGGGGGGATTGCTTTCGAGACGGGAGATCCCTT
CGCCGACCCTGGAGGGCCGACGCCGAGGCATTCGGGCCCCTGCAACGTCAACAGCGGCAAGAAAACGG
GATGAATGGGCGTAATGGGGGGGGTCTGCTGGGGACCCGACGCGGTTGCCGTTTGCGGGGCCCCCGAC
CCATACCGACCCACCTAGGCGTCCAGTTACGGCGCACGGCGGGAGCGTGGTTGCCGTCAGAGCTGTGT
TTCTCGATCAGTCCCCCGCAGTGCCGCAGTATCTTGCCGTGGGCTGCTTTAATCTTGAAAGTGGTTCA
TACATTGGGCGACGAGGTGTCGACTCTCATTGGGGGTAACCGACGGGCACATGCAGTCCCCTCCCCGG
GCAGGCAGAGGCGGGGCCCCCGCGCGCCGGCCCCGGCCCACAATCTTACCAGGGTCCTCAAAGGAGCC
TTTGCATGGTACCCTTCGTGAATGGTTGCTTAAGAGGTCCACAACGTGGTCCGGGCACGGTCGACTAA
ACACTCAAAACAGCGACGGCAAATATAGGTACAAGGTCCAGGCCCTCACGGCACTAATTGCGATGACC
CAACTCACGGGGGCAGTCCCCGGCGCGGCGACCTTGATTACGTCCGGGGACAAGTATGTCGCTCCCCC
CGGGAGGGGTGCAGCCACATGGGAGATTCAAAGTTTCTCGTGACGTCGTTGTGATCACTGCAGCCTAG
CCGAGACTCCCGTACTACGCGAAGGTTGGTTATGTTAACCACTACAACGTGAGGCGCCGTAAGGGCCA
GTGTTGTGCCCGGCTCTTCAATGCGCCTTAGCGGCCTGATACACCCACCCAAGGAGATACTGCTAATC
ATATGGGTGGACAGAACCTCGCAACGCAGACGCCTCCCCGGCATGATATGGTTTTTTTCCGCTATTAT
CCAGCACGCAGCGCTATCATTCAAGAGAACCCAGTGACGCGTAAATCGTAAGATCTACCTGCCGCAGG
TGGACCTACTGCAAATACGGATTATGACTCGTAAAGGGGTCATGCGTATTTTCATCACTAGGCACGTT
CGAGAATAAATTAGTAGGTGTCCCGAGCCTTGTGGCGTTCCGCCTGACTCCTCATGAAGTCGACCTTC
TCACCGGCCCTATCTGCCGACGTAAGTCATAACCTAGATCTGTACCTCGGGGGGAGGGTCACTGTAAA
GGGATAATTGGAGGGTGATTTCCCACACTTTCCTAAGGGTACTTTTTGCCTGGCTTCGCAATTGGGTC
CAATGGATGTCGATCTCTGGTTTAGCAGTTGTGAAAGTGGCAAGGCGGGAGGTTAGACCTCCATTTAA
CATATACAAGCAAGTTAACTGCACTAGATGTGTAGACACTACAGTTACAGGAGTAGCCGAATAAGTCT
CCGACGTCAAGCGAATAAGCGTCATACGCGATTATCGCCTAAGAGCACGTATTGGCGGTAAAAGGCTA
GCTAGACGCTTATGGGTAGATTTCAAGGCGTTCGTAGTGGTATAATAGGATACTCTTTCACCAGCCTG
AGGGCCGAACGCTATACTAGTGGTCTGTGATGTAGGACCGAGTATCTCTCTAGGGACCATCTACTTGA
GCAATGGTGCGCAGGGGGAGACATAGACCAGCCTTGGGTGGCAAGCACTGCAATAAGTCCTGTTTAGC
CTTGGAGTTCACATGCCGGCACTGAAGCCGACCTACCTGAGCGTGTGCGATTACCGTTACAATGGCAT
CTGTCTAGTTCTGTTTACCTACGGCGCTCTTGGTTCCAGGTTAGGGGAAGTGTATGACCCATGTGTTT
TTATCGGCTTAACCACGAGTGATCCCCGGTCGTTTCCCCATTGAATCCCTGGTGCATCCTACTCCCAG
AATGATAGCTGACTGACTGGACTGGCTTTTCAAGTAATCGAGGGGGTATCGCGGTCACGGCCGTTAAC
AGATCCCGTCCTTAGTGTGGAATCCGCACCTGCTGANTAACGCTTCGCCGGCGTGTCTGCACAGCCGT
ATAGTGTTAATCATGACCCCAAGGAAGGATTAAACAAATATCTTGACG
>CHAN18
GTGTCCGGTAGCCCGCGCTAGTTAGACACCCCGGCAGGGGGGATTGCTTTCGAGACGGGAGATCCCTT
CGCCGACCCTGGAGGGCCGACGCCGAGGCATTCGGGCCCCTGCAACGTCAACAGCGGCAAGAAAACGG

GATGAATGGGCGTAATGGGGGGGGTCTGCTGGGGACCCGACGCGGTTGCCGTTTGCGGGGCCCCCGAC
CCATACCGACCCACCTAGGCGTCCAGTTACGGCGCACGTCGGGAGCGTGGTTGCCGTCAGAGCTGTGT
TTCTCGATCAGTCCCCCGCAGTGCCGCAGTATCTTGCCGTGGGCTGCTTTAATCTTGAAAGTGGTTTA
TACATTGGGCGACGAGGTGTCGACTCTCATTGGGGGTAACCGACGGGCACATGCAGTCCCCTCCCCGG
GCAGGCAGAGGCGGGGCCCCCGCGCGCCGGCCCCGGCCCACAATCTTACCAGGGTCCTCAAAGGAGCC
TTTGCATGGTACCCTTCGTGAATGGTTGCTTAAGAGGTCCACAACGTGGTCCGGGCACGGTCGACTAA
ACACTCAAAACAGCGACGGCAAATATAGGTACAAGGTCCAGGCCCTCACGGCACTAATTGCGATGACC
CAACTCACGGGGGCAGTCCCCGGCGCGGCGACCTTGATTACGTCCGGGGACAAGTATGTCGCTCCCCC
CGGGAGGGGTGCAGCCACATGGGAGATTCAAAGTTTCTCGTGACGTCGTTGTGATCACTGCAGCCTAG
CCGAGACTCCCGTACTACGCGAAGGTTGGTTATGTTAACCACTACAACGTGAGGCGCCGTAAGGGCCA
GTGTTGTGCCCGGCTCTTCAATGCGCCTTAGCGGCCTGATACACCCACCCAAGGAGATACTGCTAATC
ATATGGGTGGACAGAACCTCGCAACGCAGACGCCTCCCCGGCATGATATGGTTTTTTTCCGCTATTAT
CCAGCACGCAGCGCTATCATTCAAGAGAACCCAGTGACGCGTAAATCGTAAGATCTACCTGCCGCAGG
TGGACCTACTGCAAATACGGATTATGACTCGTAAAGGGGTCATGCGTATTTTCATCACTAGGCACGTT
CGAGAATAAATTAGTAGGTGTCCCGAGCCTTGTGGCGTTCCGCCTGACTCCTCATGAAGTCGACCTTC
TCACCGGCCCTATCTGCCGACGTAAGTCATAACCTAGATCTGTACCTCGGGGGGAGGGTCACTGTAAA
GGGATAATTGGAGGGTGATTTCCCACACTTTCCTAAGGGTACTTTTTGCCTGGCTTCGCAATTGGGTC
CAATGGATGTCGATCTCTGGTTTAGCAGTTGTGAAAGTGGCAAGGCGGGAGGTTAGACCTCCATTTAA
CATATACAAGCAAGTTAACTGCACTAGATGTGTAGACACTACAGTTACAGGAGTAGCCGAATAAGTCT
CCGACGTCAAGCGAATAAGCGTCATACGCGATTATCGCCTAAGAGCACGTATTGGCGGTAAAAGGCTA
GCTAGACGCTTATGGGTAGATTTCAAGGCGTTCGTAGTGGTATAATAGGATACTCTTTCACCAGCCTG
AGGGCCGAACGCTATACTAGTGGTCTGTGATGTAGGACCGAGTATCTCTCTAGGGACCATCTACTTGA
GCAATGGTGCGCAGGGGGAGACATAGACCAGCCTTGGGTGGCAAGCACTGCAATAAGTCCTGTTTAGC
CTTGGAGTTCACATGCCGGCACTGAAGCCGACCTACCTGAGCGTGTGCGATTACCGTTACAATGGCAT
CTGTCTAGTTCTGTTTACCTACGGCGCTCTTGGTTCCAGGTTAGGGGAAGTGTATGACCCATGTGTTT
TTATCGGCTTAACCACGAGTGATCCCCGGTCGTTTCCCCATTGAATCCCTGGTGCATCCTACTCCCAG
AATGATAGCTGACTGACTGGACTGGCTTTTCAAGTAATCGAGGGGGTATCGCGGTCACGGCCGTTAAC
AGATCCCGTCCTTAGTGTGGAATCCGCACCTGCTGACTAACGCTTCGCCGGCGTGTCTGCACAGCCGT
ATAGTGTTAATCATGACCCCAAGGAAGGATTAAACAAATATCTTGACG
>MNON07
GTGTCCGGTAGCCCGCGCTAGTTAGACACCCCGGCAGGGGGGATTGCTTTCGAGACGGGAGATCCCTT
CGCCGACCCTGGAGGGCCGACGCCGAGGCATTCGGGCCCCTGCAACGTCAACAGCGGCAAGAAAACGG
GATGAATGGGCGTAATGGGGGGGGTCTGCTGGGGACCCGACGCGGTTGCCGTTTGCGGGGCCCCCGAC
CCATACCGACCCACCTAGGCGTCCAGTTACGGCGCACGTCGGGAGCGTGGTTGCCGTCAGAGCTGTGT
TTCTCGATCAGTCCCCCGCAGTGCCGCAGTATCTTGCCGTGGGCTGCTTTAATCTTGAAAGTGGTTTA
TACATTGGGCGACGAGGTGTCGACTCTCATTGGGGGTAACCGACGGGCACATGCAGTCCCCTCCCCGG
GCAGGCAGAGGCGGGGCCCCCGCGCGCCGGCCCCGGCCCACAATCTTACCAGGGTCCTCAAAGGAGCC
TTTGCATGGTACCCTTCGTGAATGGTTGCTTAAGAGGTCCACAACGTGGTCCGGGCACGGTCGACTAA
ACACTCAAAACAGCGACGGCAAATATAGGTACAAGGTCCAGGCCCTCACGGCACTAATTGCGATGACC
CAACTCACGGGGGCAGTCCCCGGCGCGGCGACCTTGATTACGTCCGGGGACAAGTATGTCGCTCCCCC
CGGGAGGGGTGCAGCCACATGGGAGATTCAAAGTTTCTCGTGACGTCGTTGTGATCACTGCAGCCTAG
CCGAGACTCCCGTACTACGCGAAGGTTGGTTATGTTAACCACTACAACGTGAGGCGCCGTAAGGGCCA
GTGTTGTGCCCGGCTCTTCAATGCGCCTTAGCGGCCTGATACACCCACCCAAGGAGATACTGCTAATC
ATATGGGTGGACAGAACCTCGCAACGCAGACGCCTCCCCGGCATGATATGGTTTTTTTCCGCTATTAT
CCAGCACGCAGCGCTATCATTCAAGAGAACCCAGTGACGCGTAAATCGTAAGATCTACCTGCCGCAGG
TGGACCTACTGCAAATACGGATTATGACTCGTAAAGGGGTCATGCGTATTTTCATCACTAGGCACGTT
CGAGAATAAATTAGTAGGTGTCCCGAGCCTTGTGGCGTTCCGCCTGACTCCTCATGAAGTCGACCTTC
TCACCGGCCCTATCTGCCGACGTAAGTCATAACCTAGATCTGTACCTCGGGGGGAGGGTCACTGTAAA
GGGATAATTGGAGGGTGATTTCCCACACTTTCCTAAGGGTACTTTTTGCCTGGCTTCGCAATTGGGTC
CAATGGATGTCGATCTCTGGTTTAGCAGTTGTGAAAGTGGCAAGGCGGGAGGTTAGACCTCCATTTAA
CATATACAAGCAAGTTAACTGCACTAGATGTGTAGACACTACAGTTACAGGAGTAGCCGAATAAGTCT
CCGACGTCAAGCGAATAAGCGTCATACGCGATTATCGCCTAAGAGCACGTATTGGCGGTAAAAGGCTA
GCTAGACGCTTATGGGTAGATTTCAAGGCGTTCGTAGTGGTATAATAGGATACTCTTTCACCAGCCTG
AGGGCCGAACGCTATACTAGTGGTCTGTGATGTAGGACCGAGTATCTCTCTAGGGACCATCTACTTGA

GCAATGGTGCGCAGGGGGAGACATAGACCAGCCTTGGGTGGCAAGCACTGCAATAAGTCCTGTTTAGC
CTTGGAGTTCACATGCCGGCACTGAAGCCGACCTACCTGAGCGTGTGCGATTACCGTTACAATGGCAT
CTGTCTAGTTCTGTTTACCTACGGCGCTCTTGGTTCCAGGTTAGGGGAAGTGTATGACCCATGTGTTT
TTATCGGCTTAACCACGAGTGATCCCCGGTCGTTTCCCCATTGAATCCCTGGTGCATCCTACTCCCAG
AATGATAGCTGACTGACTGGACTGGCTTTTCAAGTAATCGAGGGGGTATCGCGGTCACGGCCGTTAAC
AGATCCCGTCCTTAGTGTGGAATCCGCACCTGCTGACTAACGCTTCGCCGGCGTGTCTGCACAGCCGT
ATAGTGTTAATCATGACCCCAAGGAAGGATTAAACAAATATCTTGACG
>MNON09
GTGTCCGGTAGCCCGCGCTAGTTAGACACCCCGGCAGGGGGGATTGCTTTCGAGACGGGAGATCCCTT
CGCCGACCCTGGAGGGCCGACGCCGAGGCATTCGGGCCCCTGCAACGTCAACAGCGGCAAGAAAACGG
GATGAATGGGCGTAATGGGGGGGGTCTGCTGGGGACCCGACGCGGTTGCCGTTTGCGGGGCCCCCGAC
CCATACCGACCCACCTAGGCGTCCAGTTACGGCGCACGTCGGGAGCGTGGTTGCCGTCAGAGCTGTGT
TTCTCGATCAGTCCCCCGCAGTGCCGCAGTATCTTGCCGTGGGCTGCTTTAATCTTGAAAGTGGTTTA
TACATTGGGCGACGAGGTGTCGACTCTCATTGGGGGTAACCGACGGGCACATGCAGTCCCCTCCCCGG
GCAGGCAGAGGCGGGGCCCCCGCGCGCCGGCCCCGGCCCACAATCTTACCAGGGTCCTCAAAGGAGCC
TTTGCATGGTACCCTTCGTGAATGGTTGCTTAAGAGGTCCACAACGTGGTCCGGGCACGGTCGACTAA
ACACTCAAAACAGCGACGGCAAATATAGGTACAAGGTCCAGGCCCTCACGGCACTAATTGCGATGACC
CAACTCACGGGGGCAGTCCCCGGCGCGGCGACCTTGATTACGTCCGGGGACAAGTATGTCGCTCCCCC
CGGGAGGGGTGCAGCCACATGGGAGATTCAAAGTTTCTCGTGACGTCGTTGTGATCACTGCAGCCTAG
CCGAGACTCCCGTACTACGCGAAGGTTGGTTATGTTAACCACTACAACGTGAGGCGCCGTAAGGGCCA
GTGTTGTGCCCGGCTCTTCAATGCGCCTTAGCGGCCTGATACACCCACCCAAGGAGATACTGCTAATC
ATATGGGTGGACAGAACCTCGCAACGCAGACGCCTCCCCGGCATGATATGGTTTTTTTCCGCTATTAT
CCAGCACGCAGCGCTATCATTCAAGAGAACCCAGTGACGCGTAAATCGTAAGATCTACCTGCCGCAGG
TGGACCTACTGCAAATACGGATTATGACTCGTAAAGGGGTCATGCGTATTTTCATCACTAGGCACGTT
CGAGAATAAATTAGTAGGTGTCCCGAGCCTTGTGGCGTTCCGCCTGACTCCTCATGAAGTCGACCTTC
TCACCGGCCCTATCTGCCGACGTAAGTCATAACCTAGATCTGTACCTCGGGGGGAGGGTCACTGTAAA
GGGATAATTGGAGGGTGATTTCCCACACTTTCCTAAGGGTACTTTTTGCCTGGCTTCGCAATTGGGTC
CAATGGATGTCGATCTCTGGTTTAGCAGTTGTGAAAGTGGCAAGGCGGGAGGTTAGACCTCCATTTAA
CATATACAAGCAAGTTAACTGCACTAGATGTGTAGACACTACAGTTACAGGAGTAGCCGAATAAGTCT
CCGACGTCAAGCGAATAAGCGTCATACGCGATTATCGCCTAAGAGCACGTATTGGCGGTAAAAGGCTA
GCTAGACGCTTATGGGTAGATTTCAAGGCGTTCGTAGTGGTATAATAGGATACTCTTTCACCAGCCTG
AGGGCCGAACGCTATACTAGTGGTCTGTGATGTAGGACCGAGTATCTCTCTAGGGACCATCTACTTGA
GCAATGGTGCGCAGGGGGAGACATAGACCAGCCTTGGGTGGCAAGCACTGCAATAAGTCCTGTTTAGC
CTTGGAGTTCACATGCCGGCACTGAAGCCGACCTACCTGAGCGTGTGCGATTACCGTTACAATGGCAT
CTGTCTAGTTCTGTTTACCTACGGCGCTCTTGGTTCCAGGTTAGGGGAAGTGTATGACCCATGTGTTT
TTATCGGCTTAACCACGAGTGATCCCCGGTCGTTTCCCCATTGAATCCCTGGTGCATCCTACTCCCAG
AATGATAGCTGACTGACTGGACTGGCTTTTCAAGTAATCGAGGGGGTATCGCGGTCACGGCCGTTAAC
AGATCCCGTCCTTAGTGTGGAATCCGCACCTGCTGACTAACGCTTCGCCGGCGTGTCTGCACAGCCGT
ATAGTGTTAATCATGACCCCAAGGAAGGATTAAACAAATATCTTGACG
>MNON11
GTGTCCGGTAGCCCGCGCTAGTTAGACACCCCGGCAGGGGGGATTGCTTTCGAGACGGGAGATCCCTT
CGCCGACCCTGGAGGGCCGACGCCGAGGCATTCGGGCCCCCGCAACGTCAACAGCGGCAAGAAAACGG
GATGAATGGGCGTAATGGGGGGGGTCTGCTGGGGACCCGACGCGGTTGCCGTTTGCGGGGCCCCCGAC
CCATACCGACCCACCTAGGCGTCCAGTTACGGCGCACGGCGGGAGCGTGGTTGCCGTCAGAGCTGTGT
TTCTCGATCAGTCCCCCGCAGTGCCGCAGTATCTTGCCGTGGGCTGCTTTAATCTTGAAAGTGGTTTA
TACATTGGGCGACGAGGTGTCGACTCTCATTGGGGGTAACCGACGGGCACATGCAGTCCCCTCCCCGG
GCAGGCAGAGGCGGGGCCCCCGCGCGCCGGCCCCGGCCCACAATCTTACCAGGGTCCTCAAAGGAGCC
TTTGCATGGTACCCTTCGTGAATGGTTGCTTAAGAGGTCCACAACGTGGTCCGGGCACGGTCGACTAA
ACACTCAAAACAGCGACGGCAAATATAGGTACAAGGTCCAGGCCCTCACGGCACTAATTGCGATGACC
CAACTCACGGGGGCAGTCCCCGGCGCGGCGACCTTGATTACGTCCGGGGACAAGTATGTCGCTCCCCC
CGGGAGGGGTGCAGCCACATGGGAGATTCAAAGTTTCTCGTGACGTCGTTGTGATCACTGCAGCCTAG
CCGAGACTCCCGTACTACGCGAAGGTTGGTTATGTTANCCACTACAACGTGAGGCGCCGTAAGGGCCA
GTGTTGTGCCCGGCTCTTCAATGCGCCTTAGCGGCCTGATACACCCACCCAAGGAGATACTGCTAATC
ATATGGGTGGACAGAACCTCGCAACGCAGACGCCTCCCCGGCATGATATGGTTTTTTTCCGCTATTAT

CCAGCACGCAGCGCTATCATTCAAGAGAACCCAGTGACGCGTAAATCGTAAGATCTACCTGCCGCAGG
TGGACCTACTGCAAATACGGATTATGACTCGTAAAGGGGTCATGCGTATTTTCATCACTAGGCACGTT
CGAGAATAAATTAGTAGGTGTCCCGAGCCTTGTGGCGTTCCGCCTGACTCCTCATGAAGTCGACCTTC
TCACCGGCCCTATCTGCCGACGTAAGTCATAACCTAGATCTGTACCTCGGGGGGAGGGTCACTGTAAA
GGGATAATTGGAGGGTGATTTCCCACACTTTCCTAAGGGTACTTTTTGCCTGGCTTCGCAATTGGGTC
CAATGGATGTCGATCTCTGGTTTAGCAGTTGTGAAAGTGGCAAGGCGGGAGGTTAGACCTCCATTTAA
CATATACAAGCAAGTTAACTGCACTAGATGTGTAGACACTACAGTTACAGGAGTAGCCGAATAAGTCT
CCGACGTCAAGCGAATAAGCGTCATACGCGATTATCGCCTAAGAGCACGTATTGGCGGTAAAAGGCTA
GCTAGACGCTTATGGGTAGATTTCAAGGCGTTCGTAGTGGTATAATAGGATACTCTTTCACCAGCCTG
AGGGCCGAACGCTATACTAGTGGTCTGTGATGTAGGACCGAGTATCTCTCTAGGGACCATCTACTTGA
GCAATGGTGCGCAGGGGGAGACATAGACCAGCCTTGGGTGGCAAGCACTGCAATAAGTCCTGTTTAGC
CTTGGAGTTCACATGCCGGCACTGAAGCCGACCTACCTGAGCGTGTGCGATTACCGTTACAATGGCAT
CTGTCTAGTTCTGTTTACCTACGGCGCTCTTGGTTCCAGGTTAGGGGAAGTGTATGACCCATGTGTTT
TTATCGGCTTAACCACGAGTGATCCCCGGTCGTTTCCCCATTGAATCCCTGGTGCATCCTACTCCCAG
AATGATAGCTGACTGACTGGACTGGCTTTTCAAGTAATCGAGGGGGTATCGCGGTCACGGCCGTTAAC
AGATCCCGTCCTTAGTGTGGAATCCGCACCTGCTGACTAACGCTTCGCCGGCGTGTCTGCACAGCCGT
ATAGTGTTAATCATGACCCCAAGGAAGGATTAAACAAATATCTTGACG
>MNON12
GTGTCCGGTAGCCCGCGCTAGTTAGACACCCCGGCAGGGGGGATTGCTTTCGAGACGGGAGATCCCTT
CGCCGACCCTGGAGGGCCGACGCCGAGGCATTCGGGCCCCTGCAACGTCAACAGCGGCAAGAAAACGG
GATGAATGGGCGTAATGGGGGGGGTCTGCTGGGGACCCGACGCGGTTGCCGTTTGCGGGGCCCCCGAC
CCATACCGACCCACCTAGGCGTCCAGTTACGGCGCACGTCGGGAGCGTGGTTGCCGTCAGAGCTGTGT
TTCTCGATCAGTCCCCCGCAGTGCCGCAGTATCTTGCCGTGGGCTGCTTTAATCTTGAAAGTGGTTTA
TACATTGGGCGACGAGGTGTCGACTCTCATTGGGGGTAACCGACGGGCACATGCAGTCCCCTCCCCGG
GCAGGCAGAGGCGGGGCCCCCGCGCGCCGGCCCCGGCCCACAATCTTACCAGGGTCCTCAAAGGAGCC
TTTGCATGGTACCCTTCGTGAATGGTTGCTTAAGAGGTCCACAACGTGGTCCGGGCACGGTCGACTAA
ACACTCAAAACAGCGACGGCAAATATAGGTACAAGGTCCAGGCCCTCACGGCACTAATTGCGATGACC
CAACTCACGGGGGCAGTCCCCGGCGCGGCGACCTTGATTACGTCCGGGGACAAGTATGTCGCTCCCCC
CGGGAGGGGTGCAGCCACATGGGAGATTCAAAGTTTCTCGTGACGTCGTTGTGATCACTGCAGCCTAG
CCGAGACTCCCGTACTACGCGAAGGTTGGTTATGTTAACCACTACAACGTGAGGCGCCGTAAGGGCCA
GTGTTGTGCCCGGCTCTTCAATGCGCCTTAGCGGCCTGATACACCCACCCAAGGAGATACTGCTAATC
ATATGGGTGGACAGAACCTCGCAACGCAGACGCCTCCCCGGCATGATATGGTTTTTTTCCGCTATTAT
CCAGCACGCAGCGCTATCATTCAAGAGAACCCAGTGACGCGTAAATCGTAAGATCTACCTGCCGCAGG
TGGACCTACTGCAAATACGGATTATGACTCGTAAAGGGGTCATGCGTATTTTCATCACTAGGCACGTT
CGAGAATAAATTAGTAGGTGTCCCGAGCCTTGTGGCGTTCCGCCTGACTCCTCATGAAGTCGACCTTC
TCACCGGCCCTATCTGCCGACGTAAGTCATAACCTAGATCTGTACCTCGGGGGGAGGGTCACTGTAAA
GGGATAATTGGAGGGTGATTTCCCACACTTTCCTAAGGGTACTTTTTGCCTGGCTTCGCAATTGGGTC
CAATGGATGTCGATCTCTGGTTTAGCAGTTGTGAAAGTGGCAAGGCGGGAGGTTAGACCTCCATTTAA
CATATACAAGCAAGTTAACTGCACTAGATGTGTAGACACTACAGTTACAGGAGTAGCCGAATAAGTCT
CCGACGTCAAGCGAATAAGCGTCATACGCGATTATCGCCTAAGAGCACGTATTGGCGGTAAAAGGCTA
GCTAGACGCTTATGGGTAGATTTCAAGGCGTTCGTAGTGGTATAATAGGATACTCTTTCACCAGCCTG
AGGGCCGAACGCTATACTAGTGGTCTGTGATGTAGGACCGAGTATCTCTCTAGGGACCATCTACTTGA
GCAATGGTGCGCAGGGGGAGACATAGACCAGCCTTGGGTGGCAAGCACTGCAATAAGTCCTGTTTAGC
CTTGGAGTTCACATGCCGGCACTGAAGCCGACCTACCTGAGCGTGTGCGATTACCGTTACAATGGCAT
CTGTCTAGTTCTGTTTACCTACGGCGCTCTTGGTTCCAGGTTAGGGGAAGTGTATGACCCATGTGTTT
TTATCGGCTTAACCACGAGTGATCCCCGGTCGTTTCCCCATTGAATCCCTGGTGCATCCTACTCCCAG
AATGATAGCTGACTGACTGGACTGGCTTTTCAAGTAATCGAGGGGGTATCGCGGTCACGGCCGTTAAC
AGATCCCGTCCTTAGTGTGGAATCCGCACCTGCTGACTAACGCTTCGCCGGCGTGTCTGCACAGCCGT
ATAGTGTTAATCATGACCCCAAGGAAGGATTAAACAAATATCTTGACG
>MNON15
GTGTCCGGTAGCTCGCGCTAGTTAGACACCCCGGCAGGGGGGATTGCTTTCGAGACGGGAGATCCCTT
CGCCGACCCTGGAGGGCCGACGCCGAGGCATTCGGGCCCCTGCAACGTCAACAGCGGCAAGAAAACGG
GATGAATGGGCGTAATGGGGGGGGTCTGCTGGGGACCCGACGCGGTTGCCGTTTGCGGGGCCCCCGAC
CCATACCGACCCACCTAGGCGTCCAGTTACGGCGCACGTCGGGAGCGTGGTTGCCGTCAGAGCTGTGT

TTCTCGATCAGTCCCCCGCAGTGCCGCAGTATCTTGCCGTGGGCTGCTTTAATCTTGAAAGTGGTTCA
TACATTGGGCGACGAGGTGTCGACTCTCATTGGGGGTAACCGACGGGCACATGCAGTCCCCTCCCCGG
GCAGGCAGAGGCGGGGCCCCCGCGCGCCGGCCCCGGCCCACAATCTTACCAGGGTCCTCAAGGGAGCC
TTTGCATGGTACCCTTCGTGAATGGTTGCTTAAGAGGTCCACCACGTGGTCCGGGCACGGTCGACTAA
ACACTCAAAACAGCGACGGCAAATATAGGCACAAGGTCCAGACCCTCACGGCACTAATTGCGATGACC
CAACTCGCGGGGACAGTCACCGGCGCGGCGACCTTGATTACGGTCGGGGACAAGTATGTCGTTCCCCC
CGGGAGGGGTGCAGCCACATGGGAGATTCAAAGTTTCTCGTGACGTCGTTGTGATCACTGCAGCCTAG
CCGAGACTCCCGTACTACGCGAAGGTTGGTTATGTTAACCACTACAACGTGAGGCGCCGTAAGGGCCA
GTGTTGTGCCCGGCTCTTCAATGCGCCTTAGCGGCCTGATACACCCACCCAAGGAGATACCGTTAATC
ATGTGGGGGGACAGAACCTCGCAACGCAGACGCCTCCCCGGCATGATATGGTTTTTTTCCGCTATTAT
CCAGCACGCAGCGCTATCATTCAAGAGAACCCAGTGACGCGTAAATCGTAAGATCTACCTGCCGCAAG
TGGACCTACTGCAAATACGGATTATGACTCGTAAAGAGGTCATGCGTATTTTCATTACCAGGCACGTT
CGAGAATAAATTAGTAGGTGTCCCGCGCCTTGTGGCGTTCCGCCTGACTCCTCATGAAGTCGACCTTC
TCACCGGCCCTATCTGCCGACGTAAGCCATAACCTAGGTCTGTACCGTGGGGGGAGGGTCACTGTAAA
GGGATAATTGGAGGGTGATTTCCCACACTTTCCTAAGGGTACTTTTCGCCTGGCTTCGCAATTGGGCC
CANTGGATGTCGATCTCTGGTTTAGCAGTTGTGAAAGTGGTAAGGCGGGAGGTTAGACCTCCATTTAA
CATATNCAAGCAAGTTCACTGCACTAGATGTGTAGACACTACAGTTACGGGGGTAGCCGAATAAGTCT
CCGACGTCAAGCGAATAAGCGTCATACGCGATTATCGCCTAAGAGCACGTATTGGCGGTAAAAGGCTA
ACTAGACGCCTGTGGGTAGATTTCAAGGCGTTCGTAGTGGTATAACAAGATACTTTTTCACCAGCCTG
AGGGCCGAACGCTATACTAGTGGTCTGTGATGTAGGATCGAGTATCCCTCTAGGGACCATCTACTTGA
GCAATGGTGCGCAGGGGGAGACATAGACCAGCCTTGGGTGGCAAGCACTGCAATAAGTCCTGTTTAGC
CCTGGAGTTCACACGCCGGCACTGAAGCCGACCTACCTGAGCTTGTGCGATTACCGTTACAATGGCAT
CTGTCTAGTTCTGTTTACCTACGGCGCTCTTGGTTCCAGGTTAGGGGAAGTGTATGACCCATGTGTTT
TTATCGGCTTAACCACGAGTGATCCCCGGTCGTTTCCTCATTGAATCCCTGGTGCATCCTACTCCCAG
AATGATAGCTGACTGACTGGACTGGCTTTTCAAGTAGTCGAGGGGGTATCGCGGTCACGGCCGTTAAC
AGATCCCGTCCTTAGTGTGGAATCCGCACCTGCTGACTAACGCTTCGCCGGCGTGTTTGCACAGCCGT
ATAGTGTTAATCATGACCCCAAGGAAGGATTAAACAAATATCTTGACG
>MNON16
GTGTCCGGTAGCCCGCGCTAGTTAGACACCCCGGCAGGGGGGATTGCTTTCGAGACGGGAGATCCCTT
CGCCGACCCTGGAGGGCCGACGCCGAGGCATTCGGGCCCCTGCAACGTCAACAGCGGCAAGAAAACGG
GATGAATGGGCGTAATGGGGGGGGTCTGCTGGGGACCCGACGCGGTTGCCGTTTGCGGGGCCCCCGAC
CCATACCGACCCACCTAGGCGTCCAGTTACGGCGCACGTCGGGAGCGTGGTTGCCGTCAGAGCTGTGT
TTCTCGATCAGTCCCCCGCAGTGCCGCAGTATCTTGCCGTGGGCTGCTTTAATCTTGAAAGTGGTTTA
TACATTGGGCGACGAGGTGTCGACTCTCATTGGGGGTAACCGACGGGCACATGCAGTCCCCTCCCCGG
GCAGGCAGAGGCGGGGCCCCCGCGCGCCGGCCCCGGCCCACAATCTTACCAGGGTCCTCAAAGGAGCC
TTTGCATGGTACCCTTCGTGAATGGTTGCTTAAGAGGTCCACAACGTGGTCCGGGCACGGTCGACTAA
ACACTCAAAACAGCGACGGCAAATATAGGTACAAGGTCCAGGCCCTCACGGCACTAATTGCGATGACC
CAACTCACGGGGGCAGTCCCCGGCGCGGCGACCTTGATTACGTCCGGGGACAAGTATGTCGCTCCCCC
CGGGAGGGGTGCAGCCACATGGGAGATTCAAAGTTTCTCGTGACGTCGTTGTGATCACTGCAGCCTAG
CCGAGACTCCCGTACTACGCGAAGGTTGGTTATGTTAACCACTACAACGTGAGGCGCCGTAAGGGCCA
GTGTTGTGCCCGGCTCTTCAATGCGCCTTAGCGGCCTGATACACCCACCCAAGGAGATACTGCTAATC
ATATGGGTGGACAGAACCTCGCAACGCAGACGCCTCCCCGGCATGATATGGTTTTTTTCCGCTATTAT
CCAGCACGCAGCGCTATCATTCAAGAGAACCCAGTGACGCGTAAATCGTAAGATCTACCTGCCGCAGG
TGGACCTACTGCAAATACGGATTATGACTCGTAAAGGGGTCATGCGTATTTTCATCACTAGGCACGTT
CGAGAATAAATTAGTAGGTGTCCCGAGCCTTGTGGCGTTCCGCCTGACTCCTCATGAAGTCGACCTTC
TCACCGGCCCTATCTGCCGACGTAAGTCATAACCTAGATCTGTACCTCGGGGGGAGGGTCACTGTAAA
GGGATAATTGGAGGGTGATTTCCCACACTTTCCTAAGGGTACTTTTTGCCTGGCTTCGCAATTGGGTC
CAATGGATGTCGATCTCTGGTTTAGCAGTTGTGAAAGTGGCAAGGCGGGAGGTTAGACCTCCATTTAA
CATATACAAGCAAGTTAACTGCACTAGATGTGTAGACACTACAGTTACAGGAGTAGCCGAATAAGTCT
CCGACGTCAAGCGAATAAGCGTCATACGCGATTATCGCCTAAGAGCACGTATTGGCGGTAAAAGGCTA
GCTAGACGCTTATGGGTAGATTTCAAGGCGTTCGTAGTGGTATAATAGGATACTCTTTCACCAGCCTG
AGGGCCGAACGCTATACTAGTGGTCTGTGATGTAGGACCGAGTATCTCTCTAGGGACCATCTACTTGA
GCAATGGTGCGCAGGGGGAGACATAGACCAGCCTTGGGTGGCAAGCACTGCAATAAGTCCTGTTTAGC
CTTGGAGTTCACATGCCGGCACTGAAGCCGACCTACCTGAGCGTGTGCGATTACCGTTACAATGGCAT

CTGTCTAGTTCTGTTTACCTACGGCGCTCTTGGTTCCAGGTTAGGGGAAGTGTATGACCCATGTGTTT
TTATCGGCTTAACCACGAGTGATCCCCGGTCGTTTCCCCATTGAATCCCTGGTGCATCCTACTCCCAG
AATGATAGCTGACTGACTGGACTGGCTTTTCAAGTAATCGAGGGGGTATCGCGGTCACGGCCGTTAAC
AGATCCCGTCCTTAGTGTGGAATCCGCACCTGCTGACTAACGCTTCGCCGGCGTGTCTGCACAGCCGT
ATAGTGTTAATCATGACCCCAAGGAAGGATTAAACAAATATCTTGACG
>MNON21
GTGTCCGGTAGCCCGCGCTAGTTAGACACCCCGGCAGGGGGGATTGCTTTCGAGACGGGAGATCCCTT
CGCCGACCCTGGAGGGCCGACGCCGAGGCATTCGGGCCCCTGCAACGTCAACAGCGGCAAGAAAACGG
GATGAATGGGCGTAATGGGGGGGGTCTGCTGGGGACCCGACGCGGTTGCCGTTTGCGGGGCCCCCGAC
CCATACCGACCCACCTAGGCGTCCAGTTACGGCGCACGGCGGGAGCGTGGTTGCCGTCAGAGCTGTGT
TTCTCGATCAGTCCCCCGCAGTGCCGCAGTATCTTGCCGTGGGCTGCTTTAATCTTGAAAGTGGTTTA
TACATTGGGCGACGAGGTGTCGACTCTCATTGGGGGTAACCGACGGGCACATGCAGTCCCCTCCCCGG
GCAGGCAGAGGCGGGGCCCCCGCGCGCCGGCCCCGGCCCACAATCTTACCAGGGTCCTCAAAGGAGCC
TTTGCATGGTACCCTTCGTGAATGGTTGCTTAAGAGGTCCACAACGTGGTCCGGGCACGGTCGACTAA
ACACTCAAAACAGCGACGGCAAATATAGGTACAAGGTCCAGGCCCTCACGGCACTAATTGCGATGACC
CAACTCACGGGGGCAGTCCCCGGCGCGGCGACCTTGATTACGTCCGGGGACAAGTATGTCGCTCCCCC
CGGGAGGGGTGCAGCCACATGGGAGATTCAAAGTTTCTCGTGACGTCGTTGTGATCACTGCAGCCTAG
CCGAGACTCCCGTACTACGCGAAGGTTGGTTATGTTAACCACTACAACGTGAGGCGCCGTAAGGGCCA
GTGTTGTGCCCGGCTCTTCAATGCGCCTTAGCGGCCTGATACACCCACCCAAGGAGATACTGCTAATC
ATATGGGTGGACAGAACCTCGCAACGCAGACGCCTCCCCGGCATGATATGGTTTTTTTCCGCTATTAT
CCAGCACGCAGCGCTATCATTCAAGAGAACCCAGTGACGCGTAAATCGTAAGATCTACCTGCCGCAGG
TGGACCTACTGCAAATACGGATTATGACTCGTAAAGGGGTCATGCGTATTTTCATCACTAGGCACGTT
CGAGAATAAATTAGTAGGTGTCCCGAGCCTTGTGGCGTTCCGCCTGACTCCTCATGAAGTCGACCTTC
TCACCGGCCCTATCTGCCGACGTAAGTCATAACCTAGATCTGTACCTCGGGGGGAGGGTCACTGTAAA
GGGATAATTGGAGGGTGATTTCCCACACTTTCCTAAGGGTACTTTTTGCCTGGCTTCGCAATTGGGTC
CAATGGATGTCGATCTCTGGTTTAGCAGTTGTGAAAGTGGCAAGGCGGGAGGTTAGACCTCCATTTAA
CATATACAAGCAAGTTAACTGCACTAGATGTGTAGACACTACAGTTACAGGAGTAGCCGAATAAGTCT
CCGACGTCAAGCGAATAAGCGTCATACGCGATTATCGCCTAAGAGCACGTATTGGCGGTAAAAGGCTA
GCTAGACGCTTATGGGTAGATTTCAAGGCGTTCGTAGTGGTATAATAGGATACTCTTTCACCAGCCTG
AGGGCCGAACGCTATACTAGTGGTCTGTGATGTAGGACCGAGTATCTCTCTAGGGACCATCTACTTGA
GCAATGGTGCGCAGGGGGAGACATAGACCAGCCTTGGGTGGCAAGCACTGCAATAAGTCCTGTTTAGC
CTTGGAGTTCACATGCCGGCACTGAAGCCGACCTACCTGAGCGTGTGCGATTACCGTTACAATGGCAT
CTGTCTAGTTCTGTTTACCTACGGCGCTCTTGGTTCCAGGTTAGGGGAAGTGTATGACCCATGTGTTT
TTATCGGCTTAACCACGAGTGATCCCCGGTCGTTTCCCCATTGAATCCCTGGTGCATCCTACTCCCAG
AATGATAGCTGACTGACTGGACTGGCTTTTCAAGTAATCGAGGGGGTATCGCGGTCACGGCCGTTAAC
AGATCCCGTCCTTAGTGTGGAATCCGCACCTGCTGACTAACGCTTCGCCGGCGTGTCTGCACAGCCGT
ATAGTGTTAATCATGACCCCAAGGAAGGATTAAACAAATATCTTGACG
>MNON29
GTGTCCGGTAGCCCGCGCTAGTTAGACACCCCGGCAGGGGGGATTGCTTTCGAGACGGGAGATCCCTT
CGCCGACCCTGGAGGGCCGACGCCGAGGCATTCGGGCCCCNGCAACGTNAACAGCGGCAAGAAAACGG
GATGAATGGGCGTAATGGGGGGGGTCTGCTGGGGACCCGACGCGGTTGCCGTTTGCGGGGCCCCCGAC
CCATACCGACCCACCTAGGCGTCCAGTTACGGCGCACGTCGGGAGCGTGGTTGCCGTCAGAGCTGTGT
TTCTCGATCAGTCCCCCGCAGTGCCGCAGTATCTTGCCGTGGGCTGCTTTAATCTTGAAAGTGGTTTA
TACATTGGGCGACGAGGTGTCGACTCTCATTGGGGGTAACCGACGGGCACATGCAGTCCCCTCCCCGG
GCAGGCAGAGGCGGGGCCCCCGCGCGCCGGCCCCGGCCCACAATCTTACCAGGGTCCTCAAAGGAGCC
TTTGCATGGTACCCTTCGTGAATGGTTGCTTAAGAGGTCCACAACGTGGTCCGGGCACGGTCGACTAA
ACACTCAAAACAGCGACGGCAAATATAGGTACAAGGTCCAGGCCCTCACGGCACTAATTGCGATGACC
CAACTCACGGGGGCAGTCCCCGGCGCGGCGACCTTGATTACGTCCGGGGACAAGTATGTCGCTCCCCC
CGGGAGGGGTGCAGCCACATGGGAGATTCAAAGTTTCTCGTGACGTCGTTGTGATCACTGCAGCCTAG
CCGAGACTCCCGTACTACGCGAAGGTTGGTTATGTTAACCACTACAACGTGAGGCGCCGTAAGGGCCA
GTGTTGTGCCCGGCTCTTCAATGCGCCTTAGCGGCCTGATACACCCACCCAAGGAGATACTGCTAATC
ATATGGGTGGACAGAACCTCGCAACGCAGACGCCTCCCCGGCATGATATGGTTTTTTTCCGCTATTAT
CCAGCACGCAGCGCTATCATTCAAGAGAACCCAGTGACGCGTAAATCGTAAGATCTACCTGCCGCAGG
TGGACCTACTGCAAATACGGATTATGACTCGTAAAGGGGTCATGCGTATTTTCATCACTAGGCACGTT

CGAGAATAAATTAGTAGGTGTCCCGAGCCTTGTGGCGTTCCGCCTGACTCCTCATGAAGTCGACCTTC
TCACCGGCCCTATCTGCCGACGTAAGTCATAACCTAGATCTGTACCTCGGGGGGAGGGTCACTGTAAA
GGGATAATTGGAGGGTGATTTCCCACACTTTCCTAAGGGTACTTTTTGCCTGGCTTCGCAATTGGGTC
CAATGGATGTCGATCTCTGGTTTAGCAGTTGTGAAAGTGGCAAGGCGGGAGGTTAGACCTCCATTTAA
CATATACAAGCAAGTTAACTGCACTAGATGTGTAGACACTACAGTTACAGGAGTAGCCGAATAAGTCT
CCGACGTCAAGCGAATAAGCGTCATACGCGATTATCGCCTAAGAGCACGTATTGGCGGTAAAAGGCTA
GCTAGACGCTTATGGGTAGATTTCAAGGCGTTCGTAGTGGTATAATAGGATACTCTTTCACCAGCCTG
AGGGCCGAACGCTATACTAGTGGTCTGTGATGTAGGACCGAGTATCTCTCTAGGGACCATCTACTTGA
GCAATGGTGCGCAGGGGGAGACATAGACCAGCCTTGGGTGGCAAGCACTGCAATAAGTCCTGTTTAGC
CTTGGAGTTCACATGCCGGCACTGAAGCCGACCTACCTGAGCGTGTGCGATTACCGTTACAATGGCAT
CTGTCTAGTTCTGTTTACCTACGGCGCTCTTGGTTCCAGGTTAGGGGAAGTGTATGACCCATGTGTTT
TTATCGGCTTAACCACGAGTGATCCCCGGTCGTTTCCCCATTGAATCCCTGGTGCATCCTACTCCCAG
AATGATAGCTGACTGACTGGACTGGCTTTTCAAGTAATCGAGGGGGTATCGCGGTCACGGCCGTTAAC
AGATCCCGTCCTTAGTGTGGAATCCGCACCTGCTGACTAACGCTTCGCCGGCGTGTCTGCACAGCCGT
ATAGTGTTAATCATGACCCCAAGGAAGGATTAAACAAATATCTTGACG
>MNON32
GTGTCCGGTAGCCCGCGCTAGTTAGACACCCCGGCAGGGGGGATTGCTTTCGGGACGGGAGATCCCTT
CGCCGACCCTGGAGGGCCGACGCCGAGGCATTCGGGCCCCNGCAACGTCAACAGCGGCAAGAAAACGG
GATGAATGGGCGTAATGGGGGGGGTCTGCTGGGGACCCGACGCGGTTGCCGTTTGCGGGGCCCCCGAC
CCATACCGACCCACCTAGGCGTCCAGTTACGGCGCACGTCGGGAGCGTGGTTGCCGTCAGAGCTGTGT
TTCTCGATCAGTCCCCCGCAGTGCCGCAGTATCTTGCCGTGGGCTGCTTTAATCTTGAAAGTGGTTCA
TACATTGGGCGACGAGGTGTCGACTCTCATTGGGGGTAACCGACGGGCACATGCAGTCCCCTCCCCGG
GCAGGCAGAGGCGGGGCCCCCGCGCGCCGGCCCCGGCCCACAATCTTACCAGGGTCCTCAAAGGAGCC
TTTGCATGGTACCCTTCGTGAATGGTTGCTTAAGAGGTCCACAACGTGGTCCGGGCACGGTCGACTAA
ACACTCAAAACAGCGACGGCAAATATAGGTACAAGGTCCAGGCCCTCACGGCACTAATTGCGATGACC
CAACTCACGGGGGCAGTCCCCGGCGCGGCGACCTTGATTACGTCCGGGGACAAGTATGTCGCTCCCCC
CGGGAGGGGTGCAGCCACATGGGAGATTCAAAGTTTCTCGTGACGTCGTTGTGATCACTGCAGCCTAG
CCGAGACTCCCGTACTACGCGAAGGTTGGTTATGTTAACCACTACAACGTGAGGCGCCGTAAGGGCCA
GTGTTGTGCCCGGCTCTTCAATGCGCCTTAGCGGCCTGATACACCCACCCAAGGAGATACTGCTAATC
ATATGGGTGGACAGAACCTCGCAACGCAGACGCCTCCCCGGCATGATATGGTTTTTTTCCGCTATTAT
CCAGCACGCAGCGCTATCATTCAAGAGAACCCAGTGACGCGTAAATCGTAAGATCTACCTGCCGCAGG
TGGACCTACTGCAAATACGGATTATGACTCGTAAAGGGGTCATGCGTATTTTCATCACTAGGCACGTT
CGAGAATAAATTAGTAGGTGTCCCGAGCCTTGTGGCGTTCCGCCTGACTCCTCATGAAGTCGACCTTC
TCACCGGCCCTATCTGCCGACGTAAGTCATAACCTAGATCTGTACCTCGGGGGGAGGGTCACTGTAAA
GGGATAATTGGAGGGTGATTTCCCACACTTTCCTAAGGGTACTTTTTGCCTGGCTTCGCAATTGGGTC
CAATGGATGTCGATCTCTGGTTTAGCAGTTGTGAAAGTGGCAAGGCGGGAGGTTAGACCTCCATTTAA
CATATACAAGCAAGTTAACTGCACTAGATGTGTAGACACTACAGTTACAGGAGTAGCCGAATAAGTCT
CCGACGTCAAGCGAATAAGCGTCATACGCGATTATCGCCTAAGAGCACGTATTGGCGGTAAAAGGCTA
GCTAGACGCTTATGGGTAGATTTCAAGGCGTTCGTAGTGGTATAATAGGATACTCTTTCACCAGCCTG
AGGGCCGAACGCTATACTAGTGGTCTGTGATGTAGGACCGAGTATCTCTCTAGGGACCATCTACTTGA
GCAATGGTGCGCAGGGGGAGACATAGACCAGCCTTGGGTGGCAAGCACTGCAATAAGTCCTGTTTAGC
CTTGGAGTTCACATGCCGGCACTGAAGCCGACCTACCTGAGCGTGTGCGATTACCGTTACAATGGCAT
CTGTCTAGTTCTGTTTACCTACGGCGCTCTTGGTTCCAGGTTAGGGGAAGTGTATGACCCATGTGTTT
TTATCGGCTTAACCACGAGTGATCCCCGGTCGTTTCCCCATTGAATCCCTGGTGCATCCTACTCCCAG
AATGATAGCTGACTGACTGGACTGGCTTTTCAAGTAATCGAGGGGGTATCGCGGTCACGGCCGTTAAC
AGATCCCGTCCTTAGTGTGGAATCCGCACCTGCTGACTAACGCTTCGCCGGCGTGTCTGCACAGCCGT
ATAGTGTTAATCATGACCCCAAGGAAGGATTAAACAAATATCTTGACG
>MNON33
GTGTCCGGTAGCCCGCGCTAGTTAGACACCCCGGCAGGGGGGATTGCTTTCGAGACGGGAGATCCCTT
CGCCGACCCTGGAGGGCCGACGCCGAGGCATTCGGGCCCCTGCAACGTCAACAGCGGCAAGAAAACGG
GATGAATGGGCGTAATGGGGGGGGTCTGCTGGGGACCCGACGCGGTTGCCGTTTGCGGGGCCCCCGAC
CCATACCGACCCACCTAGGCGTCCAGTTACGGCGCACGTCGGGAGCGTGGTTGCCGTCAGAGCTGTGT
TTCTCGATCAGTCCCCCGCAGNGCCGCAGTATCTTGCCGTGGGCTGCTTTAATCTTGAAAGTGGTTCA
TACATTGGGCGACGAGGTGTCGACTCTCATTGGGGGTAACCGACGGGCACATGCAGTCCCCTCCCCGG

GCAGGCAGAGGCGGGGCCCCCGCGCGCCGGCCCCGGCCCACAATCTTACCAGGGTCCTCAAAGGAGCC
TTTGCATGGTACCCTTCGTGAATGGTTGCTTAAGAGGTCCACAACGTGGTCCGGGCACGGTCGACTAA
ACACTCAAAACAGCGACGGCAAATATAGGTACAAGGTCCAGGCCCTCACGGCACTAATTGCGATGACC
CAACTCACGGGGGCAGTCCCCGGCGCGGCGACCTTGATTACGTCCGGGGACAAGTATGTCGCTCCCCC
CGGGAGGGGTGCAGCCACATGGGAGATTCAAAGTTTCTCGTGACGTCGTTGTGATCACTGCAGCCTAG
CCGAGACTCCCGTACTACGCGAAGGTTGGTTATGTTAACCACTACAACGTGAGGCGCCGTAAGGGCCA
GTGTTGTGCCCGGCTCTTCAATGCGCCTTAGCGGCCTGATACACCCACCCAAGGAGATACTGCTAATC
ATATGGGTGGACAGAACCTCGCAACGCAGACGCCTCCCCGGCATGATATGGTTTTTTTCCGCTATTAT
CCAGCACGCAGCGCTATCATTCAAGAGAACCCAGTGACGCGTAAATCGTAAGATCTACCTGCCGCAGG
TGGACCTACTGCAAATACGGATTATGACTCGTAAAGGGGTCATGCGTATTTTCATCACTAGGCACGTT
CGAGAATAAATTAGTAGGTGTCCCGAGCCTTGTGGCGTTCCGCCTGACTCCTCATGAAGTCGACCTTC
TCACCGGCCCTATCTGCCGACGTAAGTCATAACCTAGATCTGTACCTCGGGGGGAGGGTCACTGTAAA
GGGATAATTGGAGGGTGATTTCCCACACTTTCCTAAGGGTACTTTTTGCCTGGCTTCGCAATTGGGTC
CAATGGATGTCGATCTCTGGTTTAGCAGTTGTGAAAGTGGCAAGGCGGGAGGTTAGACCTCCATTTAA
CATATACAAGCAAGTTAACTGCACTAGATGTGTAGACACTACAGTTACAGGAGTAGCCGAATAAGTCT
CCGACGTCAAGCGAATAAGCGTCATACGCGATTATCGCCTAAGAGCACGTATTGGCGGTAAAAGGCTA
GCTAGACGCTTATGGGTAGATTTCAAGGCGTTCGTAGTGGTATAATAGGATACTCTTTCACCAGCCTG
AGGGCCGAACGCTATACTAGTGGTCTGTGATGTAGGACCGAGTATCTCTCTAGGGACCATCTACTTGA
GCAATGGTGCGCAGGGGGAGACATAGACCAGCCTTGGGTGGCAAGCACTGCAATAAGTCCTGTTTAGC
CTTGGAGTTCACATGCCGGCACTGAAGCCGACCTACCTGAGCGTGTGCGATTACCGTTACAATGGCAT
CTGTCTAGTTCTGTTTACCTACGGCGCTCTTGGTTCCAGGTTAGGGGAAGTGTATGACCCATGTGTTT
TTATCGGCTTAACCACGAGTGATCCCCGGTCGTTTCCCCATTGAATCCCTGGTGCATCCTACTCCCAG
AATGATAGCTGACTGACTGGACTGGCTTTTCAAGTAATCGAGGGGGTATCGCGGTCACGGCCGTTAAC
AGATCCCGTCCTTAGTGTGGAATCCGCACCTGCTGACTAACGCTTCGCCGGCGTGTCTGCACAGCCGT
ATAGTGTTAATCATGACCCCAAGGAAGGATTAAACAAATATCTTGACG
>MNON36
GTGTCCGGTAGCCCGCGCTAGTTAGACACCCCGGCAGGGGGGATTGCTTTCGGGACGGGAGATCCCTT
CGCCGACCCTGGAGGGCCGACGCCGAGGCATTCGGGCCCCTGCAACGTCAACAGCGGCAAGAAAACGG
GATGAATGGGCGTAATGGGGGGGGTCTGCTGGGGACCCGACGCGGTTGCCGTTTGCGGGGCCCCCGAC
CCATACCGACCCACCTAGGCGTCCAGTTACGGCGCACGTCGGGAGCGTGGTTGCCGTCAGAGCTGTGT
TTCTCGATCAGTCCCCCGCAGTGCCGCAGTATCTTGCCGTGGGCTGCTTTAATCTTGAAAGTGGTTCA
TACATTGGGCGACGAGGTGTCGACTCTCATTGGGGGTAACCGACGGGCACATGCAGTCCCCTCCCCGG
GCAGGCAGAGGCGGGGCCCCCGCGCGCCGGCCCCGGCCCACAATCTTACCAGGGTCCTCAAAGGAGCC
TTTGCATGGTACCCTTCGTGAATGGTTGCTTAAGAGGTCCACAACGTGGTCCGGGCACGGTCGACTAA
ACACTCAAAACAGCGACGGCAAATATAGGTACAAGGTCCAGGCCCTCACGGCACTAATTGCGATGACC
CAACTCACGGGGGCAGTCCCCGGCGCGGCGACCTTGATTACGTCCGGGGACAAGTATGTCGCTCCCCC
CGGGAGGGGTGCAGCCACATGGGAGATTCAAAGTTTCTCGTGACGTCGTTGTGATCACTGCAGCCTAG
CCGAGACTCCCGTACTACGCGAAGGTTGGTTATGTTAACCACTACAACGTGAGGCGCCGTAAGGGCCA
GTGTTGTGCCCGGCTCTTCAATGCGCCTTAGCGGCCTGATACACCCACCCAAGGAGATACTGCTAATC
ATATGGGTGGACAGAACCTCGCAACGCAGACGCCTCCCCGGCATGATATGGTTTTTTTCCGCTATTAT
CCAGCACGCAGCGCTATCATTCAAGAGAACCCAGTGACGCGTAAATCGTAAGATCTACCTGCCGCAGG
TGGACCTACTGCAAATACGGATTATGACTCGTAAAGGGGTCATGCGTATTTTCATCACTAGGCACGTT
CGAGAATAAATTAGTAGGTGTCCCGAGCCTTGTGGCGTTCCGCCTGACTCCTCATGAAGTCGACCTTC
TCACCGGCCCTATCTGCCGACGTAAGTCATAACCTAGATCTGTACCTCGGGGGGAGGGTCACTGTAAA
GGGATAATTGGAGGGTGATTTCCCACACTTTCCTAAGGGTACTTTTTGCCTGGCTTCGCAATTGGGTC
CAATGGATGTCGATCTCTGGTTTAGCAGTTGTGAAAGTGGCAAGGCGGGAGGTTAGACCTCCATTTAA
CATATACAAGCAAGTTAACTGCACTAGATGTGTAGACACTACAGTTACAGGAGTAGCCGAATAAGTCT
CCGACGTCAAGCGAATAAGCGTCATACGCGATTATCGCCTAAGAGCACGTATTGGCGGTAAAAGGCTA
GCTAGACGCTTATGGGTAGATTTCAAGGCGTTCGTAGTGGTATAATAGGATACTCTTTCACCAGCCTG
AGGGCCGAACGCTATACTAGTGGTCTGTGATGTAGGACCGAGTATCTCTCTAGGGACCATCTACTTGA
GCAATGGTGCGCAGGGGGAGACATAGACCAGCCTTGGGTGGCAAGCACTGCAATAAGTCCTGTTTAGC
CTTGGAGTTCACATGCCGGCACTGAAGCCGACCTACCTGAGCGTGTGCGATTACCGTTACAATGGCAT
CTGTCTAGTTCTGTTTACCTACGGCGCTCTTGGTTCCAGGTTAGGGGAAGTGTATGACCCATGTGTTT
TTATCGGCTTAACCACGAGTGATCCCCGGTCGTTTCCCCATTGAATCCCTGGTGCATCCTACTCCCAG

AATGATAGCTGACTGACTGGACTGGCTTTTCAAGTAATCGAGGGGGTATCGCGGTCACGGCCGTTAAC
AGATCCCGTCCTTAGTGTGGAATCCGCACCTGCTGACTAACGCTTCGCCGGCGTGTCTGCACAGCCGT
ATAGTGTTAATCATGACCCCAAGGAAGGATTAAACAAATATCTTGACG
>MNON37
GTGTCCGGTAGCCCGCGCTAGTTAGACACCCCGGCAGGGGGGATTGCTTTCGAGACGGGAGATCCCTT
CGCCGACCCTGGAGGGCCGACGCCGAGGCATTCGGGCCCCTGCAACGTCAACAGCGGCAAGAAAACGG
GATGAATGGGCGTAATGGGGGGGGTCTGCTGGGGACCCGACGCGGTTGCCGTTTGCGGGGCCCCCGAC
CCATACCGACCCACCTAGGCGTCCAGTTACGGCGCACGGCGGGAGCGTGGTTGCCGTCAGAGCTGTGT
TTCTCGATCAGTCCCCCGCAGTGCCGCAGTATCTTGCCGTGGGCTGCTTTAATCTTGAAAGTGGTTCA
TACATTGGGCGACGAGGTGTCGACTCTCATTGGGGGTAACCGACGGGCACATGCAGTCCCCTCCCCGG
GCAGGCAGAGGCGGGGCCCCCGCGCGCCGGCCCCGGCCCACAATCTTACCAGGGTCCTCAAAGGAGCC
TTTGCATGGTACCCTTCGTGAATGGTTGCTTAAGAGGTCCACAACGTGGTCCGGGCACGGTCGACTAA
ACACTCAAAACAGCGACGGCAAATATAGGTACAAGGTCCAGGCCCTCACGGCACTAATTGCGATGACC
CAACTCACGGGGGCAGTCCCCGGCGCGGCGACCTTGATTACGTCCGGGGACAAGTATGTCGCTCCCCC
CGGGAGGGGTGCAGCCACATGGGAGATTCAAAGTTTCTCGTGACGTCGTTGTGATCACTGCAGCCTAG
CCGAGACTCCCGTACTACGCGAAGGTTGGTTATGTTAACCACTACAACGTGAGGCGCCGTAAGGGCCA
GTGTTGTGCCCGGCTCTTCAATGCGCCTTAGCGGCCTGATACACCCACCCAAGGAGATACTGCTAATC
ATATGGGTGGACAGAACCTCGCAACGCAGACGCCTCCCCGGCATGATATGGTTTTTTTCCGCTATTAT
CCAGCACGCAGCGCTATCATTCAAGAGAACCCAGTGACGCGTAAATCGTAAGATCTACCTGCCGCAGG
TGGACCTACTGCAAATACGGATTATGACTCGTAAAGGGGTCATGCGTATTTTCATCACTAGGCACGTT
CGAGAATAAATTAGTAGGTGTCCCGAGCCTTGTGGCGTTCCGCCTGACTCCTCATGAAGTCGACCTTC
TCACCGGCCCTATCTGCCGACGTAAGTCATAACCTAGATCTGTACCTCGGGGGGAGGGTCACTGTAAA
GGGATAATTGGAGGGTGATTTCCCACACTTTCCTAAGGGTACTTTTTGCCTGGCTTCGCAATTGGGTC
CAATGGATGTCGATCTCTGGTTTAGCAGTTGTGAAAGTGGCAAGGCGGGAGGTTAGACCTCCATTTAA
CATATACAAGCAAGTTAACTGCACTAGATGTGTAGACACTACAGTTACAGGAGTAGCCGAATAAGTCT
CCGACGTCAAGCGAATAAGCGTCATACGCGATTATCGCCTAAGAGCACGTATTGGCGGTAAAAGGCTA
GCTAGACGCTTATGGGTAGATTTCAAGGCGTTCGTAGTGGTATAATAGGATACTCTTTCACCAGCCTG
AGGGCCGAACGCTATACTAGTGGTCTGTGATGTAGGACCGAGTATCTCTCTAGGGACCATCTACTTGA
GCAATGGTGCGCAGGGGGAGACATAGACCAGCCTTGGGTGGCAAGCACTGCAATAAGTCCTGTTTAGC
CTTGGAGTTCACATGCCGGCACTGAAGCCGACCTACCTGAGCGTGTGCGATTACCGTTACAATGGCAT
CTGTCTAGTTCTGTTTACCTACGGCGCTCTTGGTTCCAGGTTAGGGGAAGTGTATGACCCATGTGTTT
TTATCGGCTTAACCACGAGTGATCCCCGGTCGTTTCCCCATTGAATCCCTGGTGCATCCTACTCCCAG
AATGATAGCTGACTGACTGGACTGGCTTTTCAAGTAATCGAGGGGGTATCGCGGTCACGGCCGTTAAC
AGATCCCGTCCTTAGTGTGGAATCCGCACCTGCTGACTAACGCTTCGCCGGCGTGTCTGCACAGCCGT
ATAGTGTTAATCATGACCCCAAGGAAGGATTAAACAAATATCTTGACG
>MNON39
GTGTCCGGTAGCCCGCGCTAGTTAGACACCCCGGCAGGGGGGATTGCTTTCGAGACGGGAGATCCCTT
CGCCGACCCTGTAGGGCCGACGCCGAGGCATTCGGGCCCCCGCAACGTCAACAGCGGCAAGAAAACGG
GATGAATGGGCGTAATGGGGGGGGTCTGCTGGGGACCCGACGCGGTTGCCGTTTGCGGGGCCCCCGAC
CCATACCNACCCACCTAGGCGTCCAGTTACGGCGCACGGCGGGAGCGTGGTTGCCGTCAGAGCTGTGT
TTCTCGATCAGTCCCCCGCAGTGCCGCAGTATCTTGCCGTGGGCTGCTTTAATCTTGAAAGTGGTTTA
TACATTGGGCGACGAGGTGTCGGCTCTCATTGGGGGTAACCGACGGACACGTGCAGTCCCCTCCCCGG
GCAGGCAGAGGCGGGGCCCCCGCGCGCCGGCCCCGGCCCACAATCTTACCAGGGTCCTCAAAAGAGCC
TTTGCATGGTACCCTTCGTGAATGGTTGCTTAAGAGGTCCACCACGTAGTCCGGGCACGGTCAACTAA
ACACTCAAAACAGCGACGGCAAATAGAGGCACAAGGTCCAGGCCCTCACGGCACTAGTTGCGATGACC
CAACTCACGGGGGCAGTCCCCGGCGCAGCGACCTTGATTACGTCCGGGAACAAGTATGTCGTTTCCCC
CGGGAGGGGTGCAGCCACATGGGAGATTCAAAGTTTCTCGTGACGTCGTTGTGATCACTGCAGCCTAG
CCGAGACTCCCGTACTACGCGAAGGTTGGTTATGTTAACCACTACAACGTGAGGCGCCGTAAGGGCCA
GTGTTGTGCCCGGCTCTTCAATGCGCCTTAGCGGCCTGATACACCCACCCAAGGAGATACTGCTAATC
ATGTGGGTGGACAGAACCTCGCAACGCAGACGCATCCCCGGCATGATATGGTTTTTTTCCGCTATTAC
CCAGCACGCGGCGCTATCATTCAAGAGAACCCAGTGACGCGTAAATCGTCAGATCTACCTGCCGCAGG
TGGACCTACTGCAAATACGGATTATGACTCGTAAAGAGGTCATGCGTATTTTCATCACTAGGCACTTT
CGAGAGTAAATTAGTAGGTGTCCCGCGCCTTGTGGCGTTCCGCCTGGCTCCTCATGAAGTCGACCTTC
TCATCGGCCCTATTTGCCGACGTAAGTCATAATCCAGATCTTCACCTCGGAGGGAGGGTCACTGTAAA

GGGATAATTGGAGGGCGATTTCCCACACTTTCCTAAGGGTACTTTTTGCTTAGCTTCGCAGTTGGGTC
CAATAGATGTTGATCTCTGGTTTAGCAGTTGTGAAAGTGGCAAGGCGGGAGGTTAGGCCTCCATTTAA
CATATACAAGCAAGTTAACTGCACTAGATGTGTAGACACTACAGTTACAGGAGTAGCCGACTAAGTCT
CCGACGTCAAGCGAATAGGCGTCATACGCGATTATTGCCTAAGAGCACGTATTGGCGGTAAAAGGATA
ACTAGACGCTTGTGGGTAGATTTCAAGGCGCTCGTAGTGGTATAATAGGACACTCTTGCACCAGCCTG
AGAGCCGAACGCTATACTAGTGGTCTGTGATGTAGGACCAAGTAGCTCTCTAGGGACCATCTACTTGA
GCAATGGTGCGCAGGGGTAGACATAGACCAACCTTGGGTGGCAAGCACTGCAATAAGTCCTGTTTAGC
CTTGGAGTTCACACGCCGGCACTAAAGCCGACCTACCTGAGCTTGTGCGATTACCGTTACAATGGCAT
CTGTCTAGTTCTGTTTACCTACGGCGCTCTTGGTTCCATGTTAGGGGAAGTGTATGACCCATGTGTTT
TTATCGGCTTAACTACGAGTGATCCCCGGTCGTTTCCCCATTAAATCCCTGGTGCATCCTACTCCCAT
AATGATAGCTGACTGGCTGGACTGGCTTTTCAAGTAGTCGAGGGGGTATCGCGGTCACGGCCGTTAAC
AGATCCCGTCCTTAGTGTGGAATCCGCACCTGCTGACTAACGCTTCGCCGGCGTGTCTGCACATCCGT
ATAGTGTTAATCATGACCCCAAGGAAGGATTAAACAAATATCTTGACG
>MNON40
GTGTCCGGTAGCCCGCGCTAGTTAGACACCCCGGCAGGGGGGATTGCTTTCGAGACGGGAGATCCCTT
CGCCGACCCTGGAGGGCCGACGCCGAGGCATTCGGGCCCCTGCAACGTCAACAGCGGCAAGAAAACGG
GATGAATGGGCGTAATGGGGGGGGTCTGCTGGGGACCCGACGCGGTTGCCGTTTGCGGGGCCCCCGAC
CCATACCGACCCACCTAGGCGTCCAGTTACGGCGCACGTCGGGAGCGTGGTTGCCGTCAGAGCTGTGT
TTCTCGATCAGTCCCCCGCAGTGCCGCAGTATCTTGCCGTGGGCTGCTTTAATCTTGAAAGTGGTTTA
TACATTGGGCGACGAGGTGTCGACTCTCATTGGGGGTAACCGACGGGCACATGCAGTCCCCTCCCCGG
GCAGGCAGAGGCGGGGCCCCCGCGCGCCGGCCCCGGCCCACAATCTTACCAGGGTCCTCAAAGGAGCC
TTTGCATGGTACCCTTCGTGAATGGTTGCTTAAGAGGTCCACAACGTGGTCCGGGCACGGTCGACTAA
ACACTCAAAACAGCGACGGCAAATATAGGTACAAGGTCCAGGCCCTCACGGCACTAATTGCGATGACC
CAACTCACGGGGGCAGTCCCCGGCGCGGCGACCTTGATTACGTCCGGGGACAAGTATGTCGCTCCCCC
CGGGAGGGGTGCAGCCACATGGGAGATTCAAAGTTTCTCGTGACGTCGTTGTGATCACTGCAGCCTAG
CCGAGACTCCCGTACTACGCGAAGGTTGGTTATGTTAACCACTACAACGTGAGGCGCCGTAAGGGCCA
GTGTTGTGCCCGGCTCTTCAATGCGCCTTAGCGGCCTGATACACCCACCCAAGGAGATACTGCTAATC
ATATGGGTGGACAGAACCTCGCAACGCAGACGCCTCCCCGGCATGATATGGTTTTTTTCCGCTATTAT
CCAGCACGCAGCGCTATCATTCAAGAGAACCCAGTGACGCGTAAATCGTAAGATCTACCTGCCGCAGG
TGGACCTACTGCAAATACGGATTATGACTCGTAAAGGGGTCATGCGTATTTTCATCACTAGGCACGTT
CGAGAATAAATTAGTAGGTGTCCCGAGCCTTGTGGCGTTCCGCCTGACTCCTCATGAAGTCGACCTTC
TCACCGGCCCTATCTGCCGACGTAAGTCATAACCTAGATCTGTACCTCGGGGGGAGGGTCACTGTAAA
GGGATAATTGGAGGGTGATTTCCCACACTTTCCTAAGGGTACTTTTTGCCTGGCTTCGCAATTGGGTC
CAATGGATGTCGATCTCTGGTTTAGCAGTTGTGAAAGTGGCAAGGCGGGAGGTTAGACCTCCATTTAA
CATATACAAGCAAGTTAACTGCACTAGATGTGTAGACACTACAGTTACAGGAGTAGCCGAATAAGTCT
CCGACGTCAAGCGAATAAGCGTCATACGCGATTATCGCCTAAGAGCACGTATTGGCGGTAAAAGGCTA
GCTAGACGCTTATGGGTAGATTTCAAGGCGTTCGTAGTGGTNTAATAGGATACTCTTTCACCAGCCTG
AGGGCCGAACGCTATACTAGTGGTCTGTGATGTAGGACCGAGTATCTCTCTAGGGACCATCTACTTGA
GCAATGGTGCGCAGGGGGAGACATAGACCAGCCTTGGGTGGCAAGCACTGCAATAAGTCCTGTTTAGC
CTTGGAGTTCACATGCCGGCACTGAAGCCGACCTACCTGAGCGTGTGCGATTACCGTTACAATGGCAT
CTGTCTAGTTCTGTTTACCTACGGCGCTCTTGGTTCCAGGTTAGGGGAAGTGTATGACCCATGTGTTT
TTATCGGCTTAACCACGAGTGATCCCCGGTCGTTTCCCCATTGAATCCCTGGTGCATCCTACTCCCAG
AATGATAGCTGACTGACTGGACTGGCTTTTCAAGTAATCGAGGGGGTATCGCGGTCACGGCCGTTAAC
AGATCCCGTCCTTAGTGTGGAATCCGCACCTGCTGACTAACGCTTCGCCGGCGTGTCTGCACAGCCGT
ATAGTGTTAATCATGACCCCAAGGAAGGATTAAACAAATATCTTGACG
>MNON48
GTGTCCGGTAGCCCGCGCTAGTTAGACACCCCGGCAGGGGGGATTGCTTTCGAGACGGGAGATCCCTT
CGCCGACCCTGGAGGGCCGACGCCGAGGCATTCGGGCCCCTGCAACGTCAACAGCGGCAAGAAAACGG
GATGAATGGGCGTAATGGGGGGGGTCTGCTGGGGACCCGACGCGGTTGCCGTTTGCGGGGCCCCCGAC
CCATACCGACCCACCTAGGCGTCCAGTTACGGCGCACGGCGGGAGCGTGGTTGCCGTCAGAGCTGTGT
TTCTCGATCAGTCCCCCGCAGTGCCGCAGTATCTTGCCGTGGGCTGCTTTAATCTTGAAAGTGGTTCA
TACATTGGGCGACGAGGTGTCGACTCTCATTGGGGGTAACCGACGGGCACATGCAGTCCCCTCCCCGG
GCAGGCAGAGGCGGGGCCCCCGCGCGCCGGCCCCGGCCCACAATCTTACCAGGGTCCTCAAAGGAGCC
TTTGCATGGTACCCTTCGTGAATGGTTGCTTAAGAGGTCCACAACGTGGTCCGGGCACGGTCGACTAA

ACACTCAAAACAGCGACGGCAAATATAGGTACAAGGTCCAGGCCCTCACGGCACTAATTGCGATGACC
CAACTCACGGGGGCAGTCCCCGGCGCGGCGACCTTGATTACGTCCGGGGACAAGTATGTCGCTCCCCC
CGGGAGGGGTGCAGCCACATGGGAGATTCAAAGTTTCTCGTGACGTCGTTGTGATCACTGCAGCCTAG
CCGAGACTCCCGTACTACGCGAAGGTTGGTTATGTTAACCACTACAACGTGAGGCGCCGTAAGGGCCA
GTGTTGTGCCCGGCTCTTCAATGCGCCTTAGCGGCCTGATACACCCACCCAAGGAGATACTGCTAATC
ATATGGGTGGACAGAACCTCGCAACGCAGACGCCTCCCCGGCATGATATGGTTTTTTTCCGCTATTAT
CCAGCACGCAGCGCTATCATTCAAGAGAACCCAGTGACGCGTAAATCGTAAGATCTACCTGCCGCAGG
TGGACCTACTGCAAATACGGATTATGACTCGTAAAGGGGTCATGCGTATTTTCATCACTAGGCACGTT
CGAGAATAAATTAGTAGGTGTCCCGAGCCTTGTGGCGTTCCGCCTGACTCCTCATGAAGTCGACCTTC
TCACCGGCCCTATCTGCCGACGTAAGTCATAACCTAGATCTGTACCTCGGGGGGAGGGTCACTGTAAA
GGGATAATTGGAGGGTGATTTCCCACACTTTCCTAAGGGTACTTTTTGCCTGGCTTCGCAATTGGGTC
CAATGGATGTCGATCTCTGGTTTAGCAGTTGTGAAAGTGGCAAGGCGGGAGGTTAGACCTCCATTTAA
CATATACAAGCAAGTTAACTGCACTAGATGTGTAGACACTACAGTTACAGGAGTAGCCGAATAAGTCT
CCGACGTCAAGCGAATAAGCGTCATACGCGATTATCGCCTAAGAGCACGTATTGGCGGTAAAAGGCTA
GCTAGACGCTTATGGGTAGATTTCAAGGCGTTCGTAGTGGTATAATAGGATACTCTTTCACCAGCCTG
AGGGCCGAACGCTATACTAGTGGTCTGTGATGTAGGACCGAGTATCTCTCTAGGGACCATCTACTTGA
GCAATGGTGCGCAGGGGGAGACATAGACCAGCCTTGGGTGGCAAGCACTGCAATAAGTCCTGTTTAGC
CTTGGAGTTCACATGCCGGCACTGAAGCCGACCTACCTGAGCGTGTGCGATTACCGTTACAATGGCAT
CTGTCTAGTTCTGTTTACCTACGGCGCTCTTGGTTCCAGGTTAGGGGAAGTGTATGACCCATGTGTTT
TTATCGGCTTAACCACGAGTGATCCCCGGTCGTTTCCCCATTGAATCCCTGGTGCATCCTACTCCCAG
AATGATAGCTGACTGACTGGACTGGCTTTTCAAGTAATCGAGGGGGTATCGCGGTCACGGCCGTTAAC
AGATCCCGTCCTTAGTGTGGAATCCGCACCTGCTGACTAACGCTTCGCCGGCGTGTCTGCACAGCCGT
ATAGTGTTAATCATGACCCCAAGGAAGGATTAAACAAATATCTTGACG
>MNON49
GTGTCCGGTAGCCCGCGCTAGTTAGACACCCCGGCAGGGGGGATTGCTTTCGAGACGGGAGATCCCTT
CGCCGACCCTGGAGGGCCGACGCCGAGGCATTCGGGCCCCCGCAACGTCAACAGCGGCAAGAAAACGG
GATGAATGGGCGTAATGGGGGGGGTCTGCTGGGGACCCGACGCGGTTGCCGTTTGCGGGGCCCCCGAC
CCATACCGACCCACCTAGGCGTCCAGTTACGGCGCACGGCGGGAGCGTGGTTGCCGTCAGAGCTGTGT
TTCTCGATCAGTCCCCCGCAGTGCCGCAGTATCTTGCCGTGGGCTGCTTTAATCTTGAAAGTGGTTCA
TACATTGGGCGACGAGGTGTCGACTCTCATTGGGGGTAACCGACGGGCACATGCAGTCCCCTCCCCGG
GCAGGCAGAGGCGGGGCCCCCGCGCGCCGGCCCCGGCCCACAATCTTACCAGGGTCCTCAAAGGAGCC
TTTGCATGGTACCCTTCGTGAATGGTTGCTTAAGAGGTCCACAACGTGGTCCGGGCACGGTCGACTAA
ACACTCAAAACAGCGACGGCAAATATAGGTACAAGGTCCAGGCCCTCACGGCACTAATTGCGATGACC
CAACTCACGGGGGCAGTCCCCGGCGCGGCGACCTTGATTACGTCCGGGGACAAGTATGTCGCTCCCCC
CGGGAGGGGTGCAGCCACATGGGAGATTCAAAGTTTCTCGTGACGTCGTTGTGATCACTGCAGCCTAG
CCGAGACTCCCGTACTACGCGAAGGTTGGTTATGTTAACCACTACAACGTGAGGCGCCGTAAGGGCCA
GTGTTGTGCCCGGCTCTTCAATGCGCCTTAGCGGCCTGATACACCCACCCAAGGAGATACTGCTAATC
ATATGGGTGGACAGAACCTCGCAACGCAGACGCCTCCCCGGCATGATATGGTTTTTTTCCGCTATTAT
CCAGCACGCAGCGCTATCATTCAAGAGAACCCAGTGACGCGTAAATCGTAAGATCTACCTGCCGCAGG
TGGACCTACTGCAAATACGGATTATGACTCGTAAAGGGGTCATGCGTATTTTCATCACTAGGCACGTT
CGAGAATAAATTAGTAGGTGTCCCGAGCCTTGTGGCGTTCCGCCTGACTCCTCATGAAGTCGACCTTC
TCACCGGCCCTATCTGCCGACGTAAGTCATAACCTAGATCTGTACCTCGGGGGGAGGGTCACTGTAAA
GGGATAATTGGAGGGTGATTTCCCACACTTTCCTAAGGGTACTTTTTGCCTGGCTTCGCAATTGGGTC
CAATGGATGTCGATCTCTGGTTTAGCAGTTGTGAAAGTGGCAAGGCGGGAGGTTAGACCTCCATTTAA
CATATACAAGCAAGTTAACTGCACTAGATGTGTAGACACTACAGTTACAGGAGTAGCCGAATAAGTCT
CCGACGTCAAGCGAATAAGCGTCATACGCGATTATCGCCTAAGAGCACGTATTGGCGGTAAAAGGCTA
GCTAGACGCTTATGGGTAGATTTCAAGGCGTTCGTAGTGGTATAATAGGATACTCTTTCACCAGCCTG
AGGGCCGAACGCTATACTAGTGGTCTGTGATGTAGGACCGAGTATCTCTCTAGGGACCATCTACTTGA
GCAATGGTGCGCAGGGGGAGACATAGACCAGCCTTGGGTGGCAAGCACTGCAATAAGTCCTGTTTAGC
CTTGGAGTTCACATGCCGGCACTGAAGCCGACCTACCTGAGCGTGTGCGATTACCGTTACAATGGCAT
CTGTCTAGTTCTGTTTACCTACGGCGCTCTTGGTTCCAGGTTAGGGGAAGTGTATGACCCATGTGTTT
TTATCGGCTTAACCACGAGTGATCCCCGGTCGTTTCCCCATTGAATCCCTGGTGCATCCTACTCCCAG
AATGATAGCTGACTGACTGGACTGGCTTTTCAAGTAATCGAGGGGGTATCGCGGTCACGGCCGTTAAC
AGATCCCGTCCTTAGTGTGGAATCCGCACCTGCTGACTAACGCTTCGCCGGCGTGTCTGCACAGCCGT

ATAGTGTTAATCATGACCCCAAGGAAGGATTAAACAAATATCTTGACG
>MNON50
GTGTCCGGTAGCCCGCGCTAGTTAGACACCCCGGCAGGGGGGATTGCTTTCGAGACGGGAGATCCCTT
CGCCGACCCTGTAGGGCCGACGCCGAGGCATTCGGGCCCCCGCAACGTCAACAGCGGCAAGAAAACGG
GATGAATGGGCGTAATGGGGGGGGTCTGCTGGGGACCCGACGCGGTTGCCGTTTGCGGGGCCCCCGAC
CCATACCGACCCACCTAGGCGTCCAGTTACGGCGCACGGCGGGAGCGTGGTTGCCGTCAGAGCTGTGT
TTCTCGATCAGTCCCCCGCAGTGCCGCAGTATCTTGCCGTGGGCTGCTTTAATCTTGAAAGTGGTTCA
TACATTGGGCGACGAGGTGTCGGCTCTCATTGGGGGTAACCGACGGACACGTGCAGTCCCCTCCCCGG
GCAGGCAGAGGCGGGGCCCCCGCGCGCCGGCCCCGGCCCACAATCTTACCAGGGTCCTCAAAAGAGCC
TTTGCATGGTACCCTTCGTGAATGGTTGCTTAAGAGGTCCACCACGTAGTCCGGGCACGGTCAACTAA
ACACTCAAAACAGCGACGGCAAATAGAGGCACAAGGTCCAGGCCCTCACGGCACTAGTTGCGATGACC
CAACTCACGGGGGCAGTCCCCGGCGCAGCGACCTTGATTACGTCCGGGAACAAGTATGTCGTTTCCCC
CGGGAGGGGTGCAGCCACATGGGAGATTCAAAGTTTCTCGTGACGTCGTTGTGATCACTGCAGCCTAG
CCGAGACTCCCGTACTACGCGAAGGTTGGTTATGTTAACCACTACAACGTGAGGCGCCGTAAGGGCCA
GTGTTGTGCCCGGCTCTTCAATGCGCCTTAGCGGCCTGATACACCCACCCAAGGAGATACTGCTAATC
ATGTGGGTGGACAGAACCTCGCAACGCAGACGCATCCCCGGCATGATATGGTTTTTTTCCGCTATTAC
CCAGCACGCGGCGCTATCATTCAAGAGAACCCAGTGACGCGTAAATCGTCAGATCTACCTGCCGCAGG
TGGACCTACTGCAAATACGGATTATGACTCGTAAAGAGGTCATGCGTATTTTCATCACTAGGCACTTT
CGAGAGTAAATTAGTAGGTGTCCCGCGCCTTGTGGCGTTCCGCCTGGCTCCTCATGAAGTCGACCTTC
TCATCGGCCCTATTTGCCGACGTAAGTCATAATCCAGATCTTCACCTCGGAGGGAGGGTCACTGTAAA
GGGATAATTGGAGGGCGATTTCCCACACTTTCCTAAGGGTACTTTTTGCTTAGCTTCGCAGTTGGGTC
CAATAGATGTTGATCTCTGGTTTAGCAGTTGTGAAAGTGGCAAGGCGGGAGGTTAGGCCTCCATTTAA
CATATACAAGCAAGTTAACTGCACTAGATGTGTAGACACTACAGTTACAGGAGTAGCCGACTAAGTCT
CCGACGTCAAGCGAATAGGCGTCATACGCGATTATTGCCTAAGAGCACGTATTGGCGGTAAAAGGATA
ACTAGACGCTTGTGGGTAGATTTCAAGGCGCTCGTAGTGGTATAATAGGACACTCTTGCACCAGCCTG
AGAGCCGAACGCTATACTAGTGGTCTGTGATGTAGGACCAAGTAGCTCTCTAGGGACCATCTACTTGA
GCAATGGTGCGCAGGGGTAGACATAGACCAACCTTGGGTGGCAAGCACTGCAATAAGTCCTGTTTAGC
CTTGGAGTTCACACGCCGGCACTAAAGCCGACCTACCTGAGCTTGTGCGATTACCGTTACAATGGCAT
CTGTCTAGTTCTGTTTACCTACGGCGCTCTTGGTTCCATGTTAGGGGAAGTGTATGACCCATGTGTTT
TTATCGGCTTAACTACGAGTGATCCCCGGTCGTTTCCCCATTAAATCCCTGGTGCATCCTACTCCCAT
AATGATAGCTGACTGGCTGGACTGGCTTTTCAAGTAGTCGAGGGGGTATCGCGGTCACGGCCGTTAAC
AGATCCCGTCCTTAGTGTGGAATCCGCACCTGCTGACTAACGCTTCGCCGGCGTGTCTGCACATCCGT
ATAGTGTTAATCATGACCCCAAGGAAGGATTAAACAAATATCTTGACG
>MNON51
GTGTCCGGTAGCCCGCGCTAGTTAGACACCCCGGCAGGGGGGATTGCTTTCGAGACGGGAGATCCCTT
CGCCGACCCTGGAGGGCCGACGCCGAGGCATTCGGGCCCCTGCAACGTCAACAGCGGCAAGAAAACGG
GATGAATGGGCGTAATGGGGGGGGTCTGCTGGGGACCCGACGCGGTTGCCGTTTGCGGGGCCCCCGAC
CCATACCGACCCACCTAGGCGTCCAGTTACGGCGCACGTCGGGAGCGTGGTTGCCGTCAGAGCTGTGT
TTCTCGATCAGTCCCCCGCAGTGCCGCAGTATCTTGCCGTGGGCTGCTTTAATCTTGAAAGTGGTTTA
TACATTGGGCGACGAGGTGTCGACTCTCATTGGGGGTAACCGACGGGCACATGCAGTCCCCTCCCCGG
GCAGGCAGAGGCGGGGCCCCCGCGCGCCGGCCCCGGCCCACAATCTTACCAGGGTCCTCAAAGGAGCC
TTTGCATGGTACCCTTCGTGAATGGTTGCTTAAGAGGTCCACAACGTGGTCCGGGCACGGTCGACTAA
ACACTCAAAACAGCGACGGCAAATATAGGTACAAGGTCCAGGCCCTCACGGCACTAATTGCGATGACC
CAACTCACGGGGGCAGTCCCCGGCGCGGCGACCTTGATTACGTCCGGGGACAAGTATGTCGCTCCCCC
CGGGAGGGGTGCAGCCACATGGGAGATTCAAAGTTTCTCGTGACGTCGTTGTGATCACTGCAGCCTAG
CCGAGACTCCCGTACTACGCGAAGGTTGGTTATGTTAACCACTACAACGTGAGGCGCCGTAAGGGCCA
GTGTTGTGCCCGGCTCTTCAATGCGCCTTAGCGGCCTGATACACCCACCCAAGGAGATACTGCTAATC
ATATGGGTGGACAGAACCTCGCAACGCAGACGCCTCCCCGGCATGATATGGTTTTTTTCCGCTATTAT
CCAGCACGCAGCGCTATCATTCAAGAGAACCCAGTGACGCGTAAATCGTAAGATCTACCTGCCGCAGG
TGGACCTACTGCAAATACGGATTATGACTCGTAAAGGGGTCATGCGTATTTTCATCACTAGGCACGTT
CGAGAATAAATTAGTAGGTGTCCCGAGCCTTGTGGCGTTCCGCCTGACTCCTCATGAAGTCGACCTTC
TCACCGGCCCTATCTGCCGACGTAAGTCATAACCTAGATCTGTACCTCGGGGGGAGGGTCACTGTAAA
GGGATAATTGGAGGGTGATTTCCCACACTTTCCTAAGGGTACTTTTTGCCTGGCTTCGCAATTGGGTC
CAATGGATGTCGATCTCTGGTTTAGCAGTTGTGAAAGTGGCAAGGCGGGAGGTTAGACCTCCATTTAA

CATATACAAGCAAGTTAACTGCACTAGATGTGTAGACACTACAGTTACAGGAGTAGCCGAATAAGTCT
CCGACGTCAAGCGAATAAGCGTCATACGCGATTATCGCCTAAGAGCACGTATTGGCGGTAAAAGGCTA
GCTAGACGCTTATGGGTAGATTTCAAGGCGTTCGTAGTGGTATAATAGGATACTCTTTCACCAGCCTG
AGGGCCGAACGCTATACTAGTGGTCTGTGATGTAGGACCGAGTATCTCTCTAGGGACCATCTACTTGA
GCAATGGTGCGCAGGGGGAGACATAGACCAGCCTTGGGTGGCAAGCACTGCAATAAGTCCTGTTTAGC
CTTGGAGTTCACATGCCGGCACTGAAGCCGACCTACCTGAGCGTGTGCGATTACCGTTACAATGGCAT
CTGTCTAGTTCTGTTTACCTACGGCGCTCTTGGTTCCAGGTTAGGGGAAGTGTATGACCCATGTGTTT
TTATCGGCTTAACCACGAGTGATCCCCGGTCGTTTCCCCATTGAATCCCTGGTGCATCCTACTCCCAG
AATGATAGCTGACTGACTGGACTGGCTTTTCAAGTAATCGAGGGGGTATCGCGGTCACGGCCGTTAAC
AGATCCCGTCCTTAGTGTGGAATCCGCACCTGCTGACTAACGCTTCGCCGGCGTGTCTGCACAGCCGT
ATAGTGTTAATCATGACCCCAAGGAAGGATTAAACAAATATCTTGACG
>MNON53
GTGTCCGGTAGCCCGCGCTAGTTAGACACCCCGGCAGGGGGGATTGCTTTCGAGACGGGAGATCCCTT
CGCCGACCCTGGAGGGCCGACGCCGAGGCATTCGGGCCCCTGCAACGTCAACAGCGGCAAGAAAACGG
GATGAATGGGCGTAATGGGGGGGGTCTGCTGGGGACCCGACGCGGTTGCCGTTTGCGGGGCCCCCGAC
CCATACCGACCCACCTAGGCGTCCAGTTACGGCGCACGTCGGGAGCGTGGTTGCCGTCAGAGCTGTGT
TTCTCGATCAGTCCCCCGCAGTGCCGCAGTATCTTGCCGTGGGCTGCTTTAATCTTGAAAGTGGTTTA
TACATTGGGCGACGAGGTGTCGACTCTCATTGGGGGTAACCGACGGGCACATGCAGTCCCCTCCCCGG
GCAGGCAGAGGCGGGGCCCCCGCGCGCCGGCCCCGGCCCACAATCTTACCAGGGTCCTCAAAGGAGCC
TTTGCATGGTACCCTTCGTGAATGGTTGCTTAAGAGGTCCACAACGTGGTCCGGGCACGGTCGACTAA
ACACTCAAAACAGCGACGGCAAATATAGGTACAAGGTCCAGGCCCTCACGGCACTAATTGCGATGACC
CAACTCACGGGGGCAGTCCCCGGCGCGGCGACCTTGATTACGTCCGGGGACAAGTATGTCGCTCCCCC
CGGGAGGGGTGCAGCCACATGGGAGATTCAAAGTTTCTCGTGACGTCGTTGTGATCACTGCAGCCTAG
CCGAGACTCCCGTACTACGCGAAGGTTGGTTATGTTAACCACTACAACGTGAGGCGCCGTAAGGGCCA
GTGTTGTGCCCGGCTCTTCAATGCGCCTTAGCGGCCTGATACACCCACCCAAGGAGATACTGCTAATC
ATATGGGTGGACAGAACCTCGCAACGCAGACGCCTCCCCGGCATGATATGGTTTTTTTCCGCTATTAT
CCAGCACGCAGCGCTATCATTCAAGAGAACCCAGTGACGCGTAAATCGTAAGATCTACCTGCCGCAGG
TGGACCTACTGCAAATACGGATTATGACTCGTAAAGGGGTCATGCGTATTTTCATCACTAGGCACGTT
CGAGAATAAATTAGTAGGTGTCCCGAGCCTTGTGGCGTTCCGCCTGACTCCTCATGAAGTCGACCTTC
TCACCGGCCCTATCTGCCGACGTAAGTCATAACCTAGATCTGTACCTCGGGGGGAGGGTCACTGTAAA
GGGATAATTGGAGGGTGATTTCCCACACTTTCCTAAGGGTACTTTTTGCCTGGCTTCGCAATTGGGTC
CAATGGATGTCGATCTCTGGTTTAGCAGTTGTGAAAGTGGCAAGGCGGGAGGTTAGACCTCCATTTAA
CATATACAAGCAAGTTAACTGCACTAGATGTGTAGACACTACAGTTACAGGAGTAGCCGAATAAGTCT
CCGACGTCAAGCGAATAAGCGTCATACGCGATTATCGCCTAAGAGCACGTATTGGCGGTAAAAGGCTA
GCTAGACGCTTATGGGTAGATTTCAAGGCGTTCGTAGTGGTATAATAGGATACTCTTTCACCAGCCTG
AGGGCCGAACGCTATACTAGTGGTCTGTGATGTAGGACCGAGTATCTCTCTAGGGACCATCTACTTGA
GCAATGGTGCGCAGGGGGAGACATAGACCAGCCTTGGGTGGCAAGCACTGCAATAAGTCCTGTTTAGC
CTTGGAGTTCACATGCCGGCACTGAAGCCGACCTACCTGAGCGTGTGCGATTACCGTTACAATGGCAT
CTGTCTAGTTCTGTTTACCTACGGCGCTCTTGGTTCCAGGTTAGGGGAAGTGTATGACCCATGTGTTT
TTATCGGCTTAACCACGAGTGATCCCCGGTCGTTTCCCCATTGAATCCCTGGTGCATCCTACTCCCAG
AATGATAGCTGACTGACTGGACTGGCTTTTCAAGTAATCGAGGGGGTATCGCGGTCACGGCCGTTAAC
AGATCCCGTCCTTAGTGTGGAATCCGCACCTGCTGACTAACGCTTCGCCGGCGTGTCTGCACAGCCGT
ATAGTGTTAATCATGACCCCAAGGAAGGATTAAACAAATATCTTGACG
>CHRUN1
GTGTCCGGTAGCCCGCGCTAGTTAGACACCCCGGCAGGGGGGATTGCTTTCGGGACGGGAGATCCCTT
CGCCGACCCTGGANGGCCGACGCCGAGGCATTCGGGCCCCTGCAACGTCAACAGCGGCAAGAAAACGG
GATGAATGGGCGTAATGGGGGGGGTCTGCTGGGGACCCGACGCGGTTGCCGTTTGCGGGGCCCCCGAC
CCATACCGACCCACCTAGGCGTCCAGTTACGGCGCACGTCGGGAGCGTGGTTGCCGTCAGAGCTGTGT
TTCTCGATCAGTCCCCCACAGTGCCGCAGTATCTTGCCGTGGGCTGCTTTAATCTTGAAAGTGGTTCA
TACATTGGGCGACGAGGTGTCGACTCTCATTGGGGGTAACCGACGGGCACATGCAGTCCCCTCCCCGG
GCAGGCAGAGGCGGGGCCCCCGCGCGCCGGCCCCGGCCCACAATCTTACCAGGGTCCTCAAAGGAGCC
TTTGCATGGTACCCTTCGTGAATGGTTGCTTAAGAAGTCCACAACGTGGTCCGGGCACGGTCGACTAA
ACACTCAAAACAGCGACGGCAAATATAGGTACAAGGTCCAGGCCCTCACGGCACTAATTGCGATGACC
CAACTCACGGGGGCAGTCCCCGGCGCGGCGACCTTGATTACGTCCGGGGACAAGTATGTCGCTCCCCC

CGGGAGGGGTGCAGCCACATGGGAGATTCAAAGTTTCTCGTGACGTCGTTGTGATCACTGCAGCCTAG
CCGAGACTCCCGTACTACGCGAAGGTTGGTTATGTTAACCACTACAACGTGAGGCGCCGTAAGGGCCA
GTGTTGTGCCCGGCTCTTCAATGCGCCTTAGCGGCCTGATACACCCACCCAAGGAGATACTGCTAATC
ATATGAGTGGACAGAACCTCGCAACGCAGACGCCTCCCCGGCATGATATGGTTTTTTTCCGCTATTAT
CCAGCACGCAGCGCTATCATTCAAGAGAACCCAGTGACGCGTAAATCGTAAGATCTACCTGCCGCAGG
TGGACCTACTGCAAATACGGATTATGACTCGTAAAGGGGTCATGCGTATTTTCATCACTAGGCACGTT
CGAGAATAAATTAGTAGGTGTCCCGAGCCTTGTGGCGTTCCGCCTGACTCCTCATGAAGTCGACCTTC
TCACCGGCCCTATCTGCCGACGTAAGTCATAACCTAGATCTGTACCTCGGGGGGAGGGTCACTGTAAA
GGGATAATTGGAGGGTGATTTCCCACATTTTCCTAAGGGTACTTTTTGCCTGGCTTCGCAATTGGGTC
CAATGGATGTCGATCTCTGGTTTAGCAGTTGTGAAAGTGGCAAGGCGGGAGGTTAGACCTCCATTTAA
CATATACAAGCAAGTTAACTGCACTAGATGTGTAGACACTACAGGTACAGGAGTAGCCGGATAAGTCT
CCGACGTCAAGCGAATAAGCTTCATACGCGATTATCGCCTAAGAGCACGTATTTGCGGTAAAAGGCTA
GCTAGACGCTTGTGGGTAGATTTCAAGGCGTTCGTAGTGGTATAATAGGATACTCTTTCACCAGCCTG
AGGGCCGAACGCTATACTAGTGGTCTGTGATGTAGGACCGAGTATCTCTCTAGGGACCATCTACTTGA
GCAATGGTGCGCAGGGGGAGACATAGACCAGCCTTGGGTGGCAAGCACTGCAATAAGTCCTGTTTAGC
CTTGGAGTTCACACGCCGGCACTGAAGCCGACCTACCTGAGCGTGTGCGATTACCGTTACAATGGCAT
CTGTCTAGTTCTGTTTACCTACGGCGCTCTTGGTTCCAGGTTAGGGGAAGTGTATGACCCATGTGTTT
TCATCGGCTTAACCACGAGTGATCCCCGGTNGTTTCCCCATTGAATCCCTGGTGCATCCTACTCCCAG
AATGATAGCTGACTGACTGGACTGGCTTTTCAAGTAATCGAGGGGGTATCGCGGTCACGGCCGTTAAC
AGATCCCGTCCTTAGTGTGGAATCCGCACCTGCTGACTAACGCTTCGCCGGCGTGTCTGCACAGCCGT
ATAGTGTTAANCATGACCCCAAGGAAGGATTAAACAAATATCTTGACG
>CHRUN2
GTGTCCGGTAGCCCGCGCTAGTTAGACACCCCGGCAGGGGGGATTGCTTTCGAGACGGGAGATCCCTT
CGCCGACCCTGGAGGGCCGACGCCGAGGCATTCGGGCCCCTGCAACGTCAACAGCGGCAAGAAAACGG
GATGAATGGGCGTAATGGGGGGGGTCTGCTGGGGACCCGACGCGGTTGCCGTTTGCGGGGCCCCCGAC
CCATACCGACCCACCTAGGCGTCCAGTTACGGCGCACGTCGGGAGCGTGGTTGCCGTCAGAGCTGTGT
TTCTCGATCAGTCCCCCGCAGTGCCGCAGTATCTTGCCGTGGGCTGCTTTAATCTTGAAAGTGGTTTA
TACATTGGGCGACGAGGTGTCGACTCTCATTGGGGGTAACCGACGGGCACATGCAGTCCCCTCCCCGG
GCAGGCAGAGGCGGGGCCCCCGCGCGCCGGCCCCGGCCCACAATCTTACCAGGGTCCTCAAAGGAGCC
TTTGCATGGTACCCTTCGTGAATGGTTGCTTAAGAGGTCCACAACGTGGTCCGGGCACGGTCGACTAA
ACACTCAAAACAGCGACGGCAAATATAGGTACAAGGTCCAGGCCCTCACGGCACTAATTGCGATGACC
CAACTCACGGGGGCAGTCCCCGGCGCGGCGACCTTGATTACGTCCGGGGACAAGTATGTCGCTCCCCC
CGGGAGGGGTGCAGCCACATGGGAGATTCAAAGTTTCTCGTGACGTCGTTGTGATCACTGCAGCCTAG
CCGAGACTCCCGTACTACGCGAAGGTTGGTTATGTTAACCACTACAACGTGAGGCGCCGTAAGGGCCA
GTGTTGTGCCCGGCTCTTCAATGCGCCTTAGCGGCCTGATACACCCACCCAAGGAGATACTGCTAATC
ATATGGGTGGACAGAACCTCGCAACGCAGACGCCTCCCCGGCATGATATGGTTTTTTTCCGCTATTAT
CCAGCACGCAGCGCTATCATTCAAGAGAACCCAGTGACGCGTAAATCGTAAGATCTACCTGCCGCAGG
TGGACCTACTGCAAATACGGATTATGACTCGTAAAGGGGTCATGCGTATTTTCATCACTAGGCACGTT
CGAGAATAAATTAGTAGGTGTCCCGAGCCTTGTGGCGTTCCGCCTGACTCCTCATGAAGTCGACCTTC
TCACCGGCCCTATCTGCCGACGTAAGTCATAACCTAGATCTGTACCTCGGGGGGAGGGTCACTGTAAA
GGGATAATTGGAGGGTGATTTCCCACACTTTCCTAAGGGTACTTTTTGCCTGGCTTCGCAATTGGGTC
CAATGGATGTCGATCTCTGGTTTAGCAGTTGTGAAAGTGGCAAGGCGGGAGGTTAGACCTCCATTTAA
CATATACAAGCAAGTTAACTGCACTAGATGTGTAGACACTACAGTTACAGGAGTAGCCGAATAAGTCT
CCGACGTCAAGCGAATAAGCGTCATACGCGATTATCGCCTAAGAGCACGTATTGGCGGTAAAAGGCTA
GCTAGACGCTTATGGGTAGATTTCAAGGCGTTCGTAGTGGTATAATAGGATACTCTTTCACCAGCCTG
AGGGCCGAACGCTATACTAGTGGTCTGTGATGTAGGACCGAGTATCTCTCTAGGGACCATCTACTTGA
GCAATGGTGCGCAGGGGGAGACATAGACCAGCCTTGGGTGGCAAGCACTGCAATAAGTCCTGTTTAGC
CTTGGAGTTCACATGCCGGCACTGAAGCCGACCTACCTGAGCGTGTGCGATTACCGTTACAATGGCAT
CTGTCTAGTTCTGTTTACCTACGGCGCTCTTGGTTCCAGGTTAGGGGAAGTGTATGACCCATGTGTTT
TTATCGGCTTAACCACGAGTGATCCCCGGTCGTTTCCCCATTGAATCCCTGGTGCATCCTACTCCCAG
AATGATAGCTGACTGACTGGACTGGCTTTTCAAGTAATCGAGGGGGTATCGCGGTCACGGCCGTTAAC
AGATCCCGTCCTTAGTGTGGAATCCGCACCTGCTGACTAACGCTTCGCCGGCGTGTCTGCACAGCCGT
ATAGTGTTAATCATGACCCCAAGGAAGGATTAAACAAATATCTTGACG
>CHRUN3

GTGTCCGGTAGCCCGCGCTAGTTAGACACCCCGGCAGGGGGGATTGCTTTCGGGACGGGAGATCCCTT
CGCCGACCCTGGAGGGCCGACGCCGAGGCATTCGGGCCCCTGCAACGTCAACAGCGGCAAGAAAACGG
GATGAATGGGCGTAATGGGGGGGGTCTGCTGGGGACCCGACGCGGTTGCCGTTTGCGGGGCCCCCGAC
CCATACCGACCCACCTAGGCGTCCAGTTACGGCGCACGTCGGGAGCGTGGTTGCCGTCAGAGCTGTGT
TTCTCGATCAGTCCCCCGCAGTGCCGCAGTATCTTGCCGTGGGCTGCTTTAATCTTGAAAGTGGTTCA
TACATTGGGCGACGAGGTGTCGACTCTCATTGGGGGTAACCGACGGGCACATGCAGTCCCCTCCCCGG
GCAGGCAGAGGCGGGGCCCCCGCGCGCCGGCCCCGGCCCACAATCTTACCAGGGTCCTCAAAGGAGCC
TTTGCATGGTACCCTTCGTGAATGGTTGCTTAAGAGGTCCACAACGTGGTCCGGGCACGGTCGACTAA
ACACTCAAAACAGCGACGGCAAATATAGGTACAAGGTCCAGGCCCTCACGGCACTAATTGCGATGACC
CAACTCACGGGGGCAGTCCCCGGCGCGGCGACCTTGATTACGTCCGGGGACAAGTATGTCGCTCCCCC
CGGGAGGGGTGCAGCCACATGGGAGATTCAAAGTTTCTCGTGACGTCGTTGTGATCACTGCAGCCTAG
CCGAGACTCCCGTACTACGCGAAGGTTGGTTATGTTAACCACTACAACGTGAGGCGCCGTAAGGGCCA
GTGTTGTGCCCGGCTCTTCAATGCGCCTTAGCGGCCTGATACACCCACCCAAGGAGATACTGCTAATC
ATATGGGTGGACAGAACCTCGCAACGCAGACGCCTCCCCGGCATGATATGGTTTTTTTCCGCTATTAT
CCAGCACGCAGCGCTATCATTCAAGAGAACCCAGTGACGCGTAAATCGTAAGATCTACCTGCCGCAGG
TGGACCTACTGCAAATACGGATTATGACTCGTAAAGGGGTCATGCGTATTTTCATCACTAGGCACGTT
CGAGAATAAATTAGTAGGTGTCCCGAGCCTTGTGGCGTTCCGCCTGACTCCTCATGAAGTCGACCTTC
TCACCGGCCCTATCTGCCGACGTAAGTCATAACCTAGATCTGTACCTCGGGGGGAGGGTCACTGTAAA
GGGATAATTGGAGGGTGATTTCCCACACTTTCCTAAGGGTACTTTTTGCCTGGCTTCGCAATTGGGTC
CAATGGATGTCGATCTCTGGTTTAGCAGTTGTGAAAGTGGCAAGGCGGGAGGTTAGACCTCCATTTAA
CATATACAAGCAAGTTAACTGCACTAGATGTGTAGACACTACAGTTACAGGAGTAGCCGAATAAGTCT
CCGACGTCAAGCGAATAAGCGTCATACGCGATTATCGCCTAAGAGCACGTATTGGCGGTAAAAGGCTA
GCTAGACGCTTATGGGTAGATTTCAAGGCGTTCGTAGTGGTATAATAGGATACTCTTTCACCAGCCTG
AGGGCCGAACGCTATACTAGTGGTCTGTGATGTAGGACCGAGTATCTCTCTAGGGACCATCTACTTGA
GCAATGGTGCGCAGGGGGAGACATAGACCAGCCTTGGGTGGCAAGCACTGCAATAAGTCCTGTTTAGC
CTTGGAGTTCACATGCCGGCACTGAAGCCGACCTACCTGAGCGTGTGCGATTACCGTTACAATGGCAT
CTGTCTAGTTCTGTTTACCTACGGCGCTCTTGGTTCCAGGTTAGGGGAAGTGTATGACCCATGTGTTT
TTATCGGCTTAACCACGAGTGATCCCCGGTCGTTTCCCCATTGAATCCCTGGTGCATCCTACTCCCAG
AATGATAGCTGACTGACTGGACTGGCTTTTCAAGTAATCGAGGGGGTATCGCGGTCACGGCCGTTAAC
AGATCCCGTCCTTAGTGTGGAATCCGCACCTGCTGACTAACGCTTCGCCGGCGTGTCTGCACAGCCGT
ATAGTGTTAATCATGACCCCAAGGAAGGATTAAACAAATATCTTGACG
>CHRUN4
GTGTCCGGTAGCCCGCGCTAGTTAGACACCCCGGCAGGGGGGATTGCTTTCGAGACGGGAGATCCCTT
CGCCGACCCTGGAGGGCCGACGCCGAGGCATTCGGGCCCCCGCAACGTCAACAGCGGCAAGAAAACGG
GATGAATGGGCGTAATGGGGGGGGTCTGCTGGGGACCCGACGCGGTTGCCGTTTGCGGGGCCCCCGAC
CCATACCGACCCACCTAGGCGTCCAGTTACGGCGCACGTCGGGAGCGTGGTTGCCGTCAGAGCTGTGT
TTCTCGATCAGTCCCCCGCAGTGCCGCAGTATCTTGCCGTGGGCTGCTTTAATCTTGAAAGTGGTTCA
TACATTGGGCGACGAGGTGTCGACTCTCATTGGGGGTAACCGACGGGCACATGCAGTCCCCTCCCCGG
GCAGGCAGAGGCGGGGCCCCCGCGCGCCGGCCCCGGCCCACAATCTTACCAGGGTCCTCAAAGGAGCC
TTTGCATGGTACCCTTCGTGAATGGTTGCTTAAGAGGTCCACAACGTGGTCCGGGCACGGTCGACTAA
ACACTCAAAACAGCGACGGCAAATATAGGTACAAGGTCCAGGCCCTCACGGCACTAATTGCGATGACC
CAACTCACGGGGGCAGTCCCCGGCGCGGCGACCTTGATTACGTCCGGGGACAAGTATGTCGCTCCCCC
CGGGAGGGGTGCAGCCACATGGGAGATTCAAAGTTTCTCGTGACGTCGTTGTGATCACTGCAGCCTAG
CCGAGACTCCCGTACTACGCGAAGGTTGGTTATGTTAACCACTACAACGTGAGGCGCCGTAAGGGCCA
GTGTTGTGCCCGGCTCTTCAATGCGCCTTAGCGGCCTGATACACCCACCCAAGGAGATACTGCTAATC
ATATGGGTGGACAGAACCTCGCAACGCAGACGCCTCCCCGGCATGATATGGTTTTTTTCCGCTATTAT
CCAGCACGCAGCGCTATCATTCAAGAGAACCCAGTGACGCGTAAATCGTAAGATCTACCTGCCGCAGG
TGGACCTACTGCAAATACGGATTATGACTCGTAAAGGGGTCATGCGTATTTTCATCACTAGGCACGTT
CGAGAATAAATTAGTAGGTGTCCCGAGCCTTGTGGCGTTCCGCCTGACTCCTCATGAAGTCGACCTTC
TCACCGGCCCTATCTGCCGACGTAAGTCATAACCTAGATCTGTACCTCGGGGGGAGGGTCACTGTAAA
GGGATAATTGGAGGGTGATTTCCCACACTTTCCTAAGGGTACTTTTTGCCTGGCTTCGCAATTGGGTC
CAATGGATGTCGATCTCTGGTTTAGCAGTTGTGAAAGTGGCAAGGCGGGAGGTTAGACCTCCATTTAA
CATATACAAGCAAGTTAACTGCACTAGATGTGTAGACACTACAGTTACAGGAGTAGCCGAATAAGTCT
CCGACGTCAAGCGAATAAGCGTCATACGCGATTATCGCCTAAGAGCACGTATTGGCGGTAAAAGGCTA

GCTAGACGCTTATGGGTAGATTTCAAGGCGTTCGTAGTGGTATAATAGGATACTCTTTCACCAGCCTG
AGGGCCGAACGCTATACTAGTGGTCTGTGATGTAGGACCGAGTATCTCTCTAGGGACCATCTACTTGA
GCAATGGTGCGCAGGGGGAGACATAGACCAGCCTTGGGTGGCAAGCACTGCAATAAGTCCTGTTTAGC
CTTGGAGTTCACATGCCGGCACTGAAGCCGACCTACCTGAGCGTGTGCGATTACCGTTACAATGGCAT
CTGTCTAGTTCTGTTTACCTACGGCGCTCTTGGTTCCAGGTTAGGGGAAGTGTATGACCCATGTGTTT
TTATCGGCTTAACCACGAGTGATCCCCGGTCGTTTCCCCATTGAATCCCTGGTGCATCCTACTCCCAG
AATGATAGCTGACTGACTGGACTGGCTTTTCAAGTAATCGAGGGGGTATCGCGGTCACGGCCGTTAAC
AGATCCCGTCCTTAGTGTGGAATCCGCACCTGCTGACTAACGCTTCGCCGGCGTGTCTGCACAGCCGT
ATAGTGTTAATCATGACCCCAAGGAAGGATTAAACAAATATCTTGACG
>CHRUN6
GTGTCCGGTAGCCCGCGCTAGTTAGACACCCCGGCAGGGGGGATTGCTTTCGAGACGGGAGATCCCTT
CGCCGACCCTGGAGGGCCGACGCCGAGGCATTCGGGCCCCTGCAACGTCAACAGCGGCAAGAAAACGG
GATGAATGGGCGTAATGGGGGGGGTCTGCTGGGGACCCGACGCGGTTGCCGTTTGCGGGGCCCCCGAC
CCATACCGACCCACCTAGGCGTCCAGTTACGGCGCACGGCGGGAGCGTGGTTGCCGTCAGAGCTGTGT
TTCTCGATCAGTCCCCCGCAGTGCCGCAGTATCTTGCCGTGGGCTGCTTTAATCTTGAAAGTGGTTCA
TACATTGGGCGACGAGGTGTCGACTCTCATTGGGGGTAACCGACGGGCACATGCAGTCCCCTCCCCGG
GCAGGCAGAGGCGGGGCCCCCGCGCGCCGGCCCCGGCCCACAATCTTACCAGGGTCCTCAAAGGAGCC
TTTGCATGGTACCCTTCGTGAATGGTTGCTTAAGAGGTCCACAACGTGGTCCGGGCACGGTCGACTAA
ACACTCAAAACAGCGACGGCAAATATAGGTACAAGGTCCAGGCCCTCACGGCACTAATTGCGATGACC
CAACTCACGGGGGCAGTCCCCGGCGCGGCGACCTTGATTACGTCCGGGGACAAGTATGTCGCTCCCCC
CGGGAGGGGTGCAGCCACATGGGAGATTCAAAGTTTCTCGTGACGTCGTTGTGATCACTGCAGCCTAG
CCGAGACTCCCGTACTACGCGAAGGTTGGTTATGTTAACCACTACAACGTGAGGCGCCGTAAGGGCCA
GTGTTGTGCCCGGCTCTTCAATGCGCCTTAGCGGCCTGATACACCCACCCAAGGAGATACTGCTAATC
ATATGGGTGGACAGAACCTCGCAACGCAGACGCCTCCCCGGCATGATATGGTTTTTTTCCGCTATTAT
CCAGCACGCAGCGCTATCATTCAAGAGAACCCAGTGACGCGTAAATCGTAAGATCTACCTGCCGCAGG
TGGACCTACTGCAAATACGGATTATGACTCGTAAAGGGGTCATGCGTATTTTCATCACTAGGCACGTT
CGAGAATAAATTAGTAGGTGTCCCGAGCCTTGTGGCGTTCCGCCTGACTCCTCATGAAGTCGACCTTC
TCACCGGCCCTATCTGCCGACGTAAGTCATAACCTAGATCTGTACCTCGGGGGGAGGGTCACTGTAAA
GGGATAATTGGAGGGTGATTTCCCACACTTTCCTAAGGGTACTTTTTGCCTGGCTTCGCAATTGGGTC
CAATGGATGTCGATCTCTGGTTTAGCAGTTGTGAAAGTGGCAAGGCGGGAGGTTAGACCTCCATTTAA
CATATACAAGCAAGTTAACTGCNCTAGATGTGTAGACACTACAGTTACAGGAGTAGCCGAATAAGTCT
CCGACGTCAAGCGAATAAGCGTCATACGCGATTATCGCCTAAGAGCACGTATTGGCGGTAAAAGGCTA
GCTAGACGCTTATGGGTAGATTTCAAGGCGTTCGTAGTGGTATAATAGGATACTCTTTCACCAGCCTG
AGGGCCGAACGCTATACTAGTGGTCTGTGATGTAGGACCGAGTATCTCTCTAGGGACCATCTACTTGA
GCAATGGTGCGCAGGGGGAGACATAGACCAGCCTTGGGTGGCAAGCACTGCAATAAGTCCTGTTTAGC
CTTGGAGTTCACATGCCGGCACTGAAGCCGACCTACCTGAGCGTGTGCGATTACCGTTACAATGGCAT
CTGTCTAGTTCTGTTTACCTACGGCGCTCTTGGTTCCAGGTTAGGGGAAGTGTATGACCCATGTGTTT
TTATCGGCTTAACCACGAGTGATCCCCGGTCGTTTCCCCATTGAATCCCTGGTGCATCCTACTCCCAG
AATGATAGCTGACTGACTGGACTGGCTTTTCAAGTAATCGAGGGGGTATCGCGGTCACGGCCGTTAAC
AGATCCCGTCCTTAGTGTGGAATCCGCACCTGCTGACTAACGCTTCGCCGGCGTGTCTGCACAGCCGT
ATAGTGTTAATCATGACCCCAAGGAAGGATTAAACAAATATCTTGACG
>CHRUN7
GTGTCCGGTAGCCCGCGCTAGTTAGACACCCCGGCAGGGGGGATTGCTTTCGAGACGGGAGATCCCTT
CGCCGACCCTGGAGGGCCGACGCCGAGGCATTCGGGCCCCCGCAACGTCAACAGCGGCAAGAAAACGG
GATGAATGGGCGTAATGGGGGGGGTCTGCTGGGGACCCGACGCGGTTGCCGTTTGCGGGGCCCCCGAC
CCATACCGACCCACCTAGGCGTCCAGTTACGGCGCACGGCGGGAGCGTGGTTGCCGTCAGAGCTGTGT
TTCTCGATCAGTCCCCCGCAGTGCCGCAGTATCTTGCCGTGGGCTGCTTTAATCTTGAAAGTGGTTCA
TACATTGGGCGACGAGGTGTCGACTCTCATTGGGGGTAACCGACGGGCACATGCAGTCCCCTCCCCGG
GCAGGCAGAGGCGGGGCCCCCGCGCGCCGGCCCCGGCCCACAATCTTACCAGGGTCCTCAAAGGAGCC
TTTGCATGGTACCCTTCGTGAATGGTTGCTTAAGAGGTCCACAACGTGGTCCGGGCACGGTCGACTAA
ACACTCAAAACAGCGACGGCAAATATAGGTACAAGGTCCAGGCCCTCACGGCACTAATTGCGATGACC
CAACTCACGGGGGCAGTCCCCGGCGCGGCGACCTTGATTACGTCCGGGGACAAGTATGTCGCTCCCCC
CGGGAGGGGTGCAGCCACATGGGAGATTCAAAGTTTCTCGTGACGTCGTTGTGATCACTGCAGCCTAG
CCGAGACTCCCGTACTACGCGAAGGTTGGTTATGTTAACCACTACAACGTGAGGCGCCGTAAGGGCCA

GTGTTGTGCCCGGCTCTTCAATGCGCCTTAGCGGCCTGATACACCCACCCAAGGAGATACTGCTAATC
ATATGGGTGGACAGAACCTCGCAACGCAGACGCCTCCCCGGCATGATATGGTTTTTTTCCGCTATTAT
CCAGCACGCAGCGCTATCATTCAAGAGAACCCAGTGACGCGTAAATCGTAAGATCTACCTGCCGCAGG
TGGACCTACTGCAAATACGGATTATGACTCGTAAAGGGGTCATGCGTATTTTCATCACTAGGCACGTT
CGAGAATAAATTAGTAGGTGTCCCGAGCCTTGTGGCGTTCCGCCTGACTCCTCATGAAGTCGACCTTC
TCACCGGCCCTATCTGCCGACGTAAGTCATAACCTAGATCTGTACCTCGGGGGGAGGGTCACTGTAAA
GGGATAATTGGAGGGTGATTTCCCACACTTTCCTAAGGGTACTTTTTGCCTGGCTTCGCAATTGGGTC
CAATGGATGTCGATCTCTGGTTTAGCAGTTGTGAAAGTGGCAAGGCGGGAGGTTAGACCTCCATTTAA
CATATACAAGCAAGTTAACTGCACTAGATGTGTAGACACTACAGTTACAGGAGTAGCCGAATAAGTCT
CCGACGTCAAGCGAATAAGCGTCATACGCGATTATCGCCTAAGAGCACGTATTGGCGGTAAAAGGCTA
GCTAGACGCTTATGGGTAGATTTCAAGGCGTTCGTAGTGGTATAATAGGATACTCTTTCACCAGCCTG
AGGGCCGAACGCTATACTAGTGGTCTGTGATGTAGGACCGAGTATCTCTCTAGGGACCATCTACTTGA
GCAATGGTGCGCAGGGGGAGACATAGACCAGCCTTGGGTGGCAAGCACTGCAATAAGTCCTGTTTAGC
CTTGGAGTTCACATGCCGGCACTGAAGCCGACCTACCTGAGCGTGTGCGATTACCGTTACAATGGCAT
CTGTCTAGTTCTGTTTACCTACGGCGCTCTTGGTTCCAGGTTAGGGGAAGTGTATGACCCATGTGTTT
TTATCGGCTTAACCACGAGTGATCCCCGGTCGTTTCCCCATTGAATCCCTGGTGCATCCTACTCCCAG
AATGATAGCTGACTGACTGGACTGGCTTTTCAAGTAATCGAGGGGGTATCGCGGTCACGGCCGTTAAC
AGATCCCGTCCTTAGTGTGGAATCCGCACCTGCTGACTAACGCTTCGCCGGCGTGTCTGCACAGCCGT
ATAGTGTTAATCATGACCCCAAGGAAGGATTAAACAAATATCTTGACG
>CHRUN8
GTGTCCGGTAGCCCGCGCTAGTTAGACACCCCGGCAGGGGGGATTGCTTTCGGGACGGGAGATCCCTT
CGCCGACCCTGGAGGGCCGACGCCGAGGCATTCGGGCCCCTGCAACGTCAACAGCGGCAAGAAAACGG
GATGAATGGGCGTAATGGGGGGGGTCTGCTGGGGACCCGACGCGGTTGCCGTTTGCGGGGCCCCCGAC
CCATACCGACCCACCTAGGCGTCCAGTTACGGCGCACGGCGGGAGCGTGGTTGCCGTCAGAGCTGTGT
TTCTCGATCAGTCCCCCGCAGTGCCGCAGTATCTTGCCGTGGGCTGCTTTAATCTTGAAAGTGGTTCA
TACATTGGGCGACGAGGTGTCGACTCTCATTGGGGGTAACCGACGGGCACATGCAGTCCCCTCCCCGG
GCAGGCAGAGGCGGGGCCCCCGCGCGCCGGCCCCGGCCCACAATCTTACCAGGGTCCTCAAAGGAGCC
TTTGCATGGTACCCTTCGTGAATGGTTGCTTAAGAGGTCCACAACGTGGTCCGGGCACGGTCGACTAA
ACACTCAAAACAGCGACGGCAAATATAGGTACAAGGTCCAGGCCCTCACGGCACTAATTGCGATGACC
CAACTCACGGGGGCAGTCCCCGGCGCGGCGACCTTGATTACGTCCGGGGACAAGTATGTCGCTCCCCC
CGGGAGGGGTGCAGCCACATGGGAGATTCAAAGTTTCTCGTGACGTCGTTGTGATCACTGCAGCCTAG
CCGAGACTCCCGTACTACGCGAAGGTTGGTTATGTTAACCACTACAACGTGAGGCGCCGTAAGGGCCA
GTGTTGTGCCCGGCTCTTCAATGCGCCTTAGCGGCCTGATACACCCACCCAAGGAGATACTGCTAATC
ATATGGGTGGACAGAACCTCGCAACGCAGACGCCTCCCCGGCATGATATGGTTTTTTTCCGCTATTAT
CCAGCACGCAGCGCTATCATTCAAGAGAACCCAGTGACGCGTAAATCGTAAGATCTACCTGCCGCAGG
TGGACCTACTGCAAATACGGATTATGACTCGTAAAGGGGTCATGCGTATTTTCATCACTAGGCACGTT
CGAGAATAAATTAGTAGGTGTCCCGAGCCTTGTGGCGTTCCGCCTGACTCCTCATGAAGTCGACCTTC
TCACCGGCCCTATCTGCCGACGTAAGTCATAACCTAGATCTGTACCTCGGGGGGAGGGTCACTGTAAA
GGGATAATTGGAGGGTGATTTCCCACACTTTCCTAAGGGTACTTTTTGCCTGGCTTCGCAATTGGGTC
CAATGGATGTCGATCTCTGGTTTAGCAGTTGTGAAAGTGGCAAGGCGGGAGGTTAGACCTCCATTTAA
CATATACAAGCAAGTTAACTGCACTAGATGTGTAGACACTACAGTTACAGGAGTAGCCGAATAAGTCT
CCGACGTCAAGCGAATAAGCGTCATACGCGATTATCGCCTAAGAGCACGTATTGGCGGTAAAAGGCTA
GCTAGACGCTTATGGGTAGATTTCAAGGCGTTCGTAGTGGTATAATAGGATACTCTTTCACCAGCCTG
AGGGCCGAACGCTATACTAGTGGTCTGTGATGTAGGACCGAGTATCTCTCTAGGGACCATCTACTTGA
GCAATGGTGCGCAGGGGGAGACATAGACCAGCCTTGGGTGGCAAGCACTGCAATAAGTCCTGTTTAGC
CTTGGAGTTCACATGCCGGCACTGAAGCCGACCTACCTGAGCGTGTGCGATTACCGTTACAATGGCAT
CTGTCTAGTTCTGTTTACCTACGGCGCTCTTGGTTCCAGGTTAGGGGAAGTGTATGACCCATGTGTTT
TTATCGGCTTAACCACGAGTGATCCCCGGTCGTTTCCCCATTGAATCCCTGGTGCATCCTACTCCCAG
AATGATAGCTGACTGACTGGACTGGCTTTTCAAGTAATCGAGGGGGTATCGCGGTCACGGCCGTTAAC
AGATCCCGTCCTTAGTGTGGAATCCGCACCTGCTGACTAACGCTTCGCCGGCGTGTCTGCACAGCCGT
ATAGTGTTAATCATGACCCCAAGGAAGGATTAAACAAATATCTTGACG
>CHRUN9
GTGTCCGGTAGCCCGCGCTAGTTAGACACCCCGGCAGGGGGGATTGCTTTCGAGACGGGAGATCCCTT
CGCCGACCCTGGAGGGCCGACGCCGAGGCATTCGGGCCCCTGCAACGTCAACAGCGGCAAGAAAACGG

GATGAATGGGCGTAATGGGGGGGGTCTGCTGGGGACCCGACGCGGTTGCCGTTTGCGGGGCCCCCGAC
CCATACCGACCCACCTAGGCGTCCAGTTACGGCGCACGTCGGGAGCGTGGTTGCCGTCAGAGCTGTGT
TTCTCGATCAGTCCCCCGCAGTGCCGCAGTATCTTGCCGTGGGCTGCTTTAATCTTGAAAGTGGTTTA
TACATTGGGCGACGAGGTGTCGACTCTCATTGGGGGTAACCGACGGGCACATGCAGTCCCCTCCCCGG
GCAGGCAGAGGCGGGGCCCCCGCGCGCCGGCCCCGGCCCACAATCTTACCAGGGTCCTCAAAGGAGCC
TTTGCATGGTACCCTTCGTGAATGGTTGCTTAAGAGGTCCACAACGTGGTCCGGGCACGGTCGACTAA
ACACTCAAAACAGCGACGGCAAATATAGGTACAAGGTCCAGGCCCTCACGGCACTAATTGCGATGACC
CAACTCACGGGGGCAGTCCCCGGCGCGGCGACCTTGATTACGTCCGGGGACAAGTATGTCGCTCCCCC
CGGGAGGGATGCAGCCACATGGGAGATTCAAAGTTTCTCGTGACGTCGTTGTGATCACTGCAGCCTAG
CCGAGACTCCCGTACTACGCGAAGGTTGGTTATGTTAACCACTACAACGTGAGGCGCCGTAAGGGCCA
GTGTTGTGCCCGGCTCTTNAATGCGCCTTAGCGGCCTGATACACCCACCCAAGGAGATACTGCTAATC
ATATGGGTGGACAGAACCTCGCAACGCAGACGCCTCCCCGGCATGATATGGTTTTTTTCCGCTATTAT
CCAGCACGCAGCGCTATCATTCAAGAGAACCCAGTGACGCGTAAATCGTAAGATCTACCTGCCGCAGG
TGGACCTACTGCAAATACGGATTATGACTCGTAAAGGGGTCATGCGTATTTTCATCACTAGGCACGTT
CGAGAATAAATTAGTAGGTGTCCCGAGCCTTGTGGCGTTCCGCCTGACTCCTCATGAAGTCGACCTTC
TCACCGGCCCTATCTGCCGACGTAAGTCATAACCTAGATCTGTACCTCGGGGGGAGGGTCACTGTAAA
GGGATAATTGGAGGGTGATTTCCCACACTTTCCTAAGGGTACTTTTTGCCTGGCTTCGCAATTGGGTC
CAATGGATGTCGATCTCTGGTTTAGCAGTTGTGAAAGTGGCAAGGCGGGAGGTTAGACCTCCATTTAA
CATATACAAGCAAGTTAACTGCACTAGATGTGTAGACACTACAGTTACAGGAGTAGCCGAATAAGTCT
CCGACGTCAAGCGAATAAGCGTCATACGCGATTATCGCCTAAGAGCACGTATTGGCGGTAAAAGGCTA
GCTAGACGCTTATGGGTAGATTTCAAGGCGTTCGTAGTGGTATAATAGGATACTCTTTCACCAGCCTG
AGGGCCGAACGCTATACTAGTGGTCTGTGATGTAGGACCGAGTATCTCTCTAGGGACCATCTACTTGA
GCAATGGTGCGCAGGGGGAGACATAGACCAGCCTTGGGTGGCAAGCACTGCAATAAGTCCTGTTTAGC
CTTGGAGTTCACATGCCGGCACTGAAGCCGACCTACCTGAGCGTGTGCGATTACCGTTACAATGGCAT
CTGTCTAGTTCTGTTTACCTACGGCGCTCTTGGTTCCAGGTTAGGGGAAGTGTATGACCCATGTGTTT
TTATCGGCTTAACCACGAGTGATCCCCGGTCGTTTCCCCATTGAATCCCTGGTGCATCCTACTCCCAG
AATGATAGCTGACTGACTGGACTGGCTTTTCAAGTAATCGAGGGGGTATCGCGGTCACGGCCGTTAAC
AGATCCCGTCCTTAGTGTGGAATCCGCACCTGCTGACTAACGCTTCGCCGGCGTGTCTGCACAGCCGT
ATAGTGTTAATCATGACCCCAAGGAAGGATTAAACAAATATCTTGACG
>CHRUN10
GTGTCCGGTAGCCCGCGCTAGTTAGACACCCCGGCAGGGGGGATTGCTTTCGAGACGGGAGATCCCTT
CGCCGACCCTGGAGGGCCGACGCCGAGGCATTCGGGCCCCTGCAACGTCAACAGCGGCAAGAAAACGG
GATGAATGGGCGTAATGGGGGGGGTCTGCTGGGGACCCGACGCGGTTGCCGTTTGCGGGGCCCCCGAC
CCATACCGACCCACCTAGGCGTCCAGTTACGGCGCACGTCGGGAGCGTGGTTGCCGTCAGAGCTGTGT
TTCTCGATCAGTCCCCCGCAGTGCCGCAGTATCTTGCCGTGGGCTGCTTTAATCTTGAAAGTGGTTTA
TACATTGGGCGACGAGGTGTCGACTCTCATTGGGGGTAACCGACGGGCACATGCAGTCCCCTCCCCGG
GCAGGCAGAGGCGGGGCCCCCGCGCGCCGGCCCCGGCCCACAATCTTACCAGGGTCCTCAAAGGAGCC
TTTGCATGGTACCCTTCGTGAATGGTTGCTTAAGAGGTCCACAACGTGGTCCGGGCACGGTCGACTAA
ACACTCAAAACAGCGACGGCAAATATAGGTACAAGGTCCAGGCCCTCACGGCACTAATTGCGATGACC
CAACTCACGGGGGCAGTCCCCGGCGCGGCGACCTTGATTACGTCCGGGGACAAGTATGTCGCTCCCCC
CGGGAGGGGTGCAGCCACATGGGAGATTCAAAGTTTCTCGTGACGTCGTTGTGATCACTGCAGCCTAG
CCGAGACTCCCGTACTACGCGAAGGTTGGTTATGTTAACCACTACAACGTGAGGCGCCGTAAGGGCCA
GTGTTGTGCCCGGCTCTTCAATGCGCCTTAGCGGCCTGATACACCCACCCAAGGAGATACTGCTAATC
ATATGGGTGGACAGAACCTCGCAACGCAGACGCCTCCCCGGCATGATATGGTTTTTTTCCGCTATTAT
CCAGCACGCAGCGCTATCATTCAAGAGAACCCAGTGACGCGTAAATCGTAAGATCTACCTGCCGCAGG
TGGACCTACTGCAAATACGGATTATGACTCGTAAAGGGGTCATGCGTATTTTCATCACTAGGCACGTT
CGAGAATAAATTAGTAGGTGTCCCGAGCCTTGTGGCGTTCCGCCTGACTCCTCATGAAGTCGACCTTC
TCACCGGCCCTATCTGCCGACGTAAGTCATAACCTAGATCTGTACCTCGGGGGGAGGGTCACTGTAAA
GGGATAATTGGAGGGTGATTTCCCACACTTTCCTAAGGGTACTTTTTGCCTGGCTTCGCAATTGGGTC
CAATGGATGTCGATCTCTGGTTTAGCAGTTGTGAAAGTGGCAAGGCGGGAGGTTAGACCTCCATTTAA
CATATACAAGCAAGTTAACTGCACTAGATGTGTAGACACTACAGTTACAGGAGTAGCCGAATAAGTCT
CCGACGTCAAGCGAATAAGCGTCATACGCGATTATCGCCTAAGAGCACGTATTGGCGGTAAAAGGCTA
GCTAGACGCTTATGGGTAGATTTCAAGGCGTTCGTAGTGGTATAATAGGATACTCTTTCACCAGCCTG
AGGGCCGAACGCTATACTAGTGGTCTGTGATGTAGGACCGAGTATCTCTCTAGGGACCATCTACTTGA

GCAATGGTGCGCAGGGGGAGACATAGACCAGCCTTGGGTGGCAAGCACTGCAATAAGTCCTGTTTAGC
CTTGGAGTTCACATGCCGGCACTGAAGCCGACCTACCTGAGCGTGTGCGATTACCGTTACAATGGCAT
CTGTCTAGTTCTGTTTACCTACGGCGCTCTTGGTTCCAGGTTAGGGGAAGTGTATGACCCATGTGTTT
TTATCGGCTTAACCACGAGTGATCCCCGGTCGTTTCCCCATTGAATCCCTGGTGCATCCTACTCCCAG
AATGATAGCTGACTGACTGGACTGGCTTTTCAAGTAATCGAGGGGGTATCGCGGTCACGGCCGTTAAC
AGATCCCGTCCTTAGTGTGGAATCCGCACCTGCTGACTAACGCTTCGCCGGCGTGTCTGCACAGCCGT
ATAGTGTTAATCATGACCCCAAGGAAGGATTAAACAAATATCTTGACG
>CHRUN11
GTGTCCGGTAGCCCGCGCTAGTTAGACACCCCGGCAGGGGGGATTGCTTTCGAGACGGGAGATCCCTT
CGCCGACCCTGGAGGGCCGACGCCGAGGCATTCGGGCCCCTGCAACGTCAACAGCGGCAAGAAAACGG
GATGAATGGGCGTAATGGGGGGGGTCTGCTGGGGACCCGACGCGGTTGCCGTTTGCGGGGCCCCCGAC
CCATACCGACCCANCTAGGCGTCCAGTTACGGCGCACGTCGGGAGCGTGGTTGCCGTCAGAGCTGTGT
TTCTCGATCAGTCCCCCGCAGTGCCGCAGTATCTTGCCGTGGGCTGCTTTAATCTTGAAAGTGGTTTA
TACATTGGGCGACGAGGTGTCGACTCTCATTGGGGGTAACCGACGGGCACATGCAGTCCCCTCCCCGG
GCAGGCAGAGGCGGGGCCCCCGCGCGCCGGCCCCGGCCCACAATCTTACCAGGGTCCTCAAAGGAGCC
TTTGCATGGTACCCTTCGTGAATGGTTGCTTAAGAGGTCCACAACGTGGTCCGGGCACGGTCGACTAA
ACACTCAAAACAGCGACGGCAAATATAGGTACAAGGTCCAGGCCCTCACGGCACTAATTGCGATGACC
CAACTCACGGGGGCAGTCCCCGGCGCGGCGACCTTGATTACGTCCGGGGACAAGTATGTCGCTCCCCC
CGGGAGGGGTGCAGCCACATGGGAGATTCAAAGTTTCTCGTGACGTCGTTGTGATCACTGCAGCCTAG
CCGAGACTCCCGTACTACGCGAAGGTTGGTTATGTTAACCACTACAACGTGAGGCGCCGTAAGGGCCA
GTGTTGTGCCCGGCTCTTCAATGCGCCTTAGCGGCCTGATACACCCACCCAAGGAGATACTGCTAATC
ATATGGGTGGACAGAACCTCGCAACGCAGACGCCTCCCCGGCATGATATGGTTTTTTTCCGCTATTAT
CCAGCACGCAGCGCTATCATTCAAGAGAACCCAGTGACGCGTAAATCGTAAGATCTACCTGCCGCAGG
TGGACCTACTGCAAATACGGATTATGACTCGTAAAGGGGTCATGCGTATTTTCATCACTAGGCACGTT
CGAGAATAAATTAGTAGGTGTCCCGAGCCTTGTGGCGTTCCGCCTGACTCCTCANGAAGTCGACCTTC
TCACCGGCCCTATCTGCCGACGTAAGTCATAACCTAGATCTGTACCTCGGGGGGAGGGTCACTGTAAA
GGGATAATTGGAGGGTGATTTCCCACACTTTCCTAAGGGTACTTTTTGCCTGGCTTCGCAATTGGGTC
CAATGGATGTCGATCTCTGGTTTAGCAGTTGTGAAAGTGGCAAGGCGGGAGGTTAGACCTCCATTTAA
CATATACAAGCAAGTTAACTGCACTAGATGTGTAGACACTACAGTTACAGGAGTAGCCGAATAAGTCT
CCGACGTCAAGCGAATAAGCGTCATACGCGATTATCGCCTAAGAGCACGTATTGGCGGTAAAAGGCTA
GCTAGACGCTTATGGGTAGATTTCAAGGCGTTCGTAGTGGTATAATAGGATACTCTTTCACCAGCCTG
AGGGCCGAACGCTATACTAGTGGTCTGTGATGTAGGACCGAGTATCTCTCTAGGGACCATCTACTTGA
GCAATGGTGCGCAGGGGGAGACATAGACCAGCCTTGGGTGGCAAGCACTGCAATAAGTCCTGTTTAGC
CTTGGAGTTCACATGCCGGCACTGAAGCCGACCTACCTGAGCGTGTGCGATTACCGTTACAATGGCAT
CTGTCTAGTTCTGTTTACCTACGGCGCTCTTGGTTCCAGGTTAGGGGAAGTGTATGACCCATGTGTTT
TTATCGGCTTAACCACGAGTGATCCCCGGTCGTTTCCCCATTGAATCCCTGGTGCATCCTACTCCCAG
AATGATAGCTGACTGACTGGACTGGCTTTTCAAGTAATCGAGGGGGTATCGCGGTCACGGCCGTTAAC
AGATCCCGTCCTTAGTGTGGAATCCGCACCTGCTGACTAACGCTTCGCCGGCGTGTCTGCACAGCCGT
ATAGTGTTAATCATGACCCCAAGGAAGGATTAAACAAATATCTTGACG
>CHRUN12
GTGTCCGGTAGCCCGCGCTAGTTAGACACCCCGGCAGGGGGGATTGCTTTCGGGACGGGAGATCCCTT
CGCCGACCCTGGAGGGCCGACGCCGAGGCATTCGGGCCCCTGCAACGTCAACAGCGGCAAGAAAACGG
GATGAATGGGCGTAATGGGGGGGGTCTGCTGGGGACCCGACGCGGTTGCCGTTTGCGGGGCCCCCGAC
CCATACCGACCCACCTAGGCGTCCAGTTACGGCGCACGTCGGGAGCGTGGTTGCCGTCAGAGCTGTGT
TTCTCGATCAGTCCCCCGCAGTGCCGCAGTATCTTGCCGTGGGCTGCTTTAATCTTGAAAGTGGTTCA
TACATTGGGCGACGAGGTGTCGACTCTCATTGGGGGTAACCGACGGGCACATGCAGTCCCCTCCCCGG
GCAGGCAGAGGCGGGGCCCCCGCGCGCCGGCCCCGGCCCACAATCTTACCAGGGTCCTCAAAGGAGCC
TTTGCATGGTACCCTTCGTGAATGGTTGCTTAAGAGGTCCACAACGTGGTCCGGGCACGGTCGACTAA
ACACTCAAAACAGCGACGGCAAATATAGGTACAAGGTCCAGGCCCTCACGGCACTAATTGCGATGACC
CAACTCACGGGGGCAGTCCCCGGCGCGGCGACCTTGATTACGTCCGGGGACAAGTATGTCGCTCCCCC
CGGGAGGGGTGCAGCCACATAGGAGATTCAAAGTTTCTCGTGACGTCGTTGTGATCACTGCAGCCTAG
CCGAGACTCCCGTACTACGCGAAGGTTGGTTATGTTAACCACTACAACGTGAGGCGCCGTAAGGGCCA
GTGTTGTGCCCGGCTCTTCAATGCGCCTTAGCGGCCTGATACACCCACCCAAGGAGATACTGCTAATC
ATATGGGTGGACAGAACCTCGCAACGCAGACGCCTCCCCGGCATGATATGGTTTTTTTCCGCTATTAT

CCAGCACGCAGCGCTATCATTCAAGAGAACCCAGTGACGCGTAAATCGTAAGATCTACCTGCCGCAGG
TGGACCTACTGCAAATACGGATTATGACTCGTAAAGGGGTCATGCGTATTTTCATCACTAGGCACGTT
CGAGAATAAATTAGTAGGTGTCCCGAGCCTTGTGGCGTTCCGCCTGACTCCTCATGAAGTCGACCTTC
TCACCGGCCCTATCTGCCGACGTAAGTCATAACCTAGATCTGTACCTCGGGGGGAGGGTCACTGTAAA
GGGATAATTGGAGGGTGATTTCCCACACTTTCCTAAGGGTACTTTTTGCCTGGCTTCGCAATTGGGTC
CAATGGATGTCGATCTCTGGTTTAGCAGTTGTGAAAGTGGCAAGGCGGGAGGTTAGACCTCCATTTAA
CATATACAAGCAAGTTAACTGCACTAGATGTGTAGACACTACAGTTACAGGAGTAGCCGAATAAGTCT
CCGACGTCAAGCGAATAAGCGTCATACGCGATTATCGCCTAAGAGCACGTATTGGCGGTAAAAGGCTA
GCTAGACGCTTATGGGTAGATTTCAAGGCGTTCGTAGTGGTATAATAGGATACTCTTTCACCAGCCTG
AGGGCCGAACGCTATACTAGTGGTCTGTGATGTAGGACCGAGTATCTCTCTAGGGACCATCTACTTGA
GCAATGGTGCGCAGGGGGAGACATAGACCAGCCTTGGGTGGCAAGCACTGCAATAAGTCCTGTTTAGC
CTTGGAGTTCACATGCCGGCACTGAAGCCGACCTACCTGAGCGTGTGCGATTACCGTTACAATGGCAT
CTGTCTAGTTCTGTTTACCTACGGCGCTCTTGGTTCCAGGTTAGGGGAAGTGTATGACCCATGTGTTT
TTATCGGCTTAACCACGAGTGATCCCCGGTCGTTTCCCCATTGAATCCCTGGTGCATCCTACTCCCAG
AATGATAGCTGACTGACTGGACTGGCTTTTCAAGTAATCGAGGGGGTATCGCGGTCACGGCCGTTAAC
AGATCCCGTCCTTAGTGTGGAATCCGCACCTGCTGACTAACGCTTCGCCGGCGTGTCTGCACAGCCGT
ATAGTGTTAATCATGACCCCAAGGAAGGATTAAACAAATATCTTGACG
>CHRUN13
GTGTCCGGTAGCCCGCGCTAGTTAGACACCCCGGCAGGGGGGATTGCTTTCGAGACGGGAGATCCCTT
CGCCGACCCTGGAGGGCCGACGCCGAGGCATTCGGGCCCCTGCAACGTCAACAGCGGCAAGAAAACGG
GATGAATGGGCGTAATGGGGGGGGTCTGCTGGGGACCCGACGCGGTTGCCGTTTGCGGGGCCCCCGAC
CCATACCGACCCACCTAGGCGTCCAGTTACGGCGCACGTCGGGAGCGTGGTTGCCGTCAGAGCTGTGT
TTCTCGATCAGTCCCCCGCAGTGCCGCAGTATCTTGCCGTGGGCTGCTTTAATCTTGAAAGTGGTTTA
TACATTGGGCGACGAGGTGTCGACTCTCATTGGGGGTAACCGACGGGCACATGCAGTCCCCTCCCCGG
GCAGGCAGAGGCGGGGCCCCCGCGCGCCGGCCCCGGCCCACAATCTTACCAGGGTCCTCAAAGGAGCC
TTTGCATGGTACCCTTCGTGAATGGTTGCTTAAGAGGTCCACAACGTGGTCCGGGCACGGTCGACTAA
ACACTCAAAACAGCGACGGCAAATATAGGTACAAGGTCCAGGCCCTCACGGCACTAATTGCGATGACC
CAACTCACGGGGGCAGTCCCCGGCGCGGCGACCTTGATTACGTCCGGGGACAAGTATGTCGCTCCCCC
CGGGAGGGGTGCAGCCACATGGGAGATTCAAAGTTTCTCGTGACGTCGTTGTGATCACTGCAGCCTAG
CCGAGACTCCCGTACTACGCGAAGGTTGGTTATGTTAACCACTACAACGTGAGGCGCCGTAAGGGCCA
GTGTTGTGCCCGGCTCTTCAATGCGCCTTAGCGGCCTGATACACCCACCCAAGGAGATACTGCTAATC
ATATGGGTGGACAGAACCTCGCAACGCAGACGCCTCCCCGGCATGATATGGTTTTTTTCCGCTATTAT
CCAGCACGCAGCGCTATCATTCAAGAGAACCCAGTGACGCGTAAATCGTAAGATCTACCTGCCGCAGG
TGGACCTACTGCAAATACGGATTATGACTCGTAAAGGGGTCATGCGTATTTTCATCACTAGGCACGTT
CGAGAATAAATTAGTAGGTGTCCCGAGCCTTGTGGCGTTCCGCCTGACTCCTCATGAAGTCGACCTTC
TCACCGGCCCTATCTGCCGACGTAAGTCATAACCTAGATCTGTACCTCGGGGGGAGGGTCACTGTAAA
GGGATAATTGGAGGGTGATTTCCCACACTTTCCTAAGGGTACTTTTTGCCTGGCTTCGCAATTGGGTC
CAATGGATGTCGATCTCTGGTTTAGCAGTTGTGAAAGTGGCAAGGCGGGAGGTTAGACCTCCATTTAA
CATATACAAGCAAGTTAACTGCACNAGATGTGTAGACACTACAGTTACAGGAGTAGCCGAATAAGTCT
CCGACGTCAAGCGAATAAGCGTCATACGCGATTATCGCCTAAGAGCACGTATTGGCGGTAAAAGGCTA
GCTAGACGCTTATGGGTAGATTTCAAGGCGTTCGTAGTGGTATAATAGGATACTCTTTCACCAGCCTG
AGGGCCGAACGCTATACTAGTGGTCTGTGATGTAGGACCGAGTATCTCTCTAGGGACCATCTACTTGA
GCAATGGTGCGCAGGGGGAGACATAGACCAGCCTTGGGTGGCAAGCACTGCAATAAGTCCTGTTTAGC
CTTGGAGTTCACATGCCGGCACTGAAGCCGACCTACCTGAGCGTGTGCGATTACCGTTACAATGGCAT
CTGTCTAGTTCTGTTTACCTACGGCGCTCTTGGTTCCAGGTTAGGGGAAGTGTATGACCCATGTGTTT
TTATCGGCTTAACCACGAGTGATCCCCGGTCGTTTCCCCATTGAATCCCTGGTGCATCCTACTCCCAG
AATGATAGCTGACTGACTGGACTGGCTTTTCAAGTAATCGAGGGGGTATCGCGGTCACGGCCGTTAAC
AGATCCCGTCCTTAGTGTGGAATCCGCACCTGCTGACTAACGCTTCGCCGGCGTGTCTGCACAGCCGT
ATAGTGTTAATCATGACCCCAAGGAAGGATTAAACAAATATCTTGACG
>CHRUN14
GTGTCCGGNAGCCCGCGCTAGTTAGACACCCCGGCAGGGGGGATTGCTTTCGAGACGGGAGATCCCTT
CGCCGACCCTGGAGGGCCGACGCCGAGGCATTCGGGCCCCTGCAACGTCAACAGCGGCAAGAAAACGG
GATGAATGGGCGTAATGGGGGGGGTCTGCTGGGGACCCGACGCGGTTGCCGTTTGCGGGGCCCCCGAC
CCATACCGACCCACCTAGGCGTCCAGTTACGGCGCACGTCGGGAGCGTGGTTGCCGTCAGAGCTGTGT

TTCTCGATCAGTCCCCCGCAGTGCCGCAGTATCTTGCCGTGGGCTGCTTTAATCTTGAAAGTGGTTCA
TACATTGGGCGACGAGGTGTCGACTCTCATTGGGGGTAACCGACGGGCACATGCAGTCCCCTCCCCGG
GCAGGCAGAGGCGGGGCCCCCGCGCGCCGGCCCCGGCCCACAATCTTACCAGGGTCCTCAAAGGAGCC
TTTGCATGGTACCCTTCGTGAATGGTTGCTTAAGAGGTCCACAACGTGGTCCGGGCACGGTCGACTAA
ACACTCAAAACAGCGACGGCAAATATAGGTACAAGGTCCAGGCCCTCACGGCACTAATTGCGATGACC
CAACTCACGGNGGCAGTCCCCGGCGCGGCGACCTTGATTACGTCCGGGGACAAGTATGTCGCTCCCCC
CGGGAGGGGTGCAGCCACATGGGAGATTCAAAGTTTCTCGTGACGTCGTTGTGATCACTGCAGCCTAG
CCGAGACTCCCGTACTACGCGAAGGTTGGTTATGTTAACCACTACAACGTGAGGCGCCGTAAGGGCCA
GTGTTGTGCCCGGCTCTTCAATGCGCCTTAGCGGCCTGATACACCCACCCAAGGAGATACTGCTAATC
ATATGGGTGGACAGAACCTCGCAACGCAGACGCCTCCCCGGCATGATATGGTTTTTTTCCGCTATTAT
CCAGCACGCAGCGCTATCATTCAAGAGAACCCAGTGACGCGTAAATCGTAAGATCTACCTGCCGCAGG
TGGACCTACTGCAAATACGGATTATGACTCGTAAAGGGGTCATGCGTATTTTCATCACTAGGCACGTT
CGAGAATAAATTAGTAGGTGTCCCGAGCCTTGTGGCGTTCCGCCTGACTCCTCATGAAGTCGACCTTC
TCACCGGCCCTATCTGCCGACGTAAGTCATAACCTAGATCTGTACCTCGGGGGGAGGGTCACTGTAAA
GGGATAATTGGAGGGTGATTTCCCACACTTTCCTAAGGGTACTTTTTGCCTGGCTTCGCAATTGGGTC
CAATGGATGTCGATCTCTGGTTTAGCAGTTGTGAAAGTGGCAAGGCGGGAGGTTAGACCTCCNTTTAA
CATATACAAGCAAGTTAACNGCACTAGATGTGTAGACACTACAGTTACAGGAGTAGCCGAATAAGTCT
CCGACGTCAAGCGAATAAGCGTCATACGCGATTATCGCCTAAGAGCACGTATTGGCGGTAAAAGGCTA
GCTAGACGCTTATGGGTAGATTTCAAGGCGTTCGTAGTGGTATAATAGGATACTCTTTCACCAGCCTG
AGGGCCGAACGCTATACTAGTGGTCTGTGATGTAGGACCGAGTATCTCTCTAGGGACCATCTACTTGA
GCAATGGTGCGCAGGGGGAGACATAGACCAGCCTTGGGTGGCAAGCACTGCAATAAGTCCTGTTTAGC
CTTGGAGTTCACATGCCGGCACTGAAGCCGACCTACCTGAGCGTGTGCGATTACCGTTACAATGGCAT
CTGTCTAGTTCTGTTTACCTACGGCGCTCTTGGTTCCAGGTTAGGGGAAGTGTATGACCCATGTGTTT
TTATCGGCTTAACCACGAGTGATCCCCGGTCGTTTCCCCATTGAATCCCTGGTGCATCCTACTCCCAG
AATGATAGCTGACTGACTGGACTGGCTTTTCAAGTAATCGAGGGGGTATCGCGGTCACGGCCGTTAAC
AGATCCCGTCCTTAGTGTGGAATCCGCACCTGCTGACTAACGCTTCGCCGGCGTGTCTGCACAGCCGT
ATAGTGTTAATCATGACCCCAAGGAAGGATTAAACAAATATCTTGACG
>CHRUN15
GTGTCCGGTAGCCCGCGCTAGTTAGACACCCCGGCAGGGGGGATTGCTTTCGAGACGGGAGATCCCTT
CGCCGACCCTGGAGGGCCGACGCCGAGGCATTCGGGCCCCTGCAACGTCAACAGCGGCAAGAAAACGG
GATGAATGGGCGTAATGGGGGGGGTCTGCTGGGGACCCGACGCGGTTGCCGTTTGCGGGGCCCCCGAC
CCATACCGACCCACCTAGGCGTCCAGTTACGGCGCACGTCGGGAGCGTGGTTGCCGTCAGAGCTGTGT
TTCTCGATCAGTCCCCCGCAGTGCCGCAGTATCTTGCCGTGGGCTGCTTTAATCTTGAAAGTGGTTTA
TACATTGGGCGACGAGGTGTCGACTCTCATTGGGGGTAACCGACGGGCACATGCAGTCCCCTCCCCGG
GCAGGCAGAGGCGGGGCCCCCGCGCGCCGGCCCCGGCCCACAATCTTACCAGGGTCCTCAAAGGAGCC
TTTGCATGGTACCCTTCGTGAATGGTTGCTTAAGAGGTCCACAACGTGGTCCGGGCACGGTCGACTAA
ACACTCAAAACAGCGACGGCAAATATAGGTACAAGGTCCAGGCCCTCACGGCACTAATTGCGATGACC
CAACTCACGGGGGCAGTCCCCGGCGCGGCGACCTTGATTACGTCCGGGGACAAGTATGTCGCTCCCCC
CGGGAGGGGTGCAGCCACATGGGAGATTCAAAGTTTCTCGTGACGTCGTTGTGATCACTGCAGCCTAG
CCGAGACTCCCGTACTACGCGAAGGTTGGTTATGTTAACCACTACAACGTGAGGCGCCGTAAGGGCCA
GTGTTGTGCCCGGCTCTTCAATGCGCCTTAGCGGCCTGATACACCCACCCAAGGAGATACTGCTAATC
ATATGGGTGGACAGAACCTCGCAACGCAGACGCCTCCCCGGCATGATATGGTTTTTTTCCGCTATTAT
CCAGCACGCAGCGCTATCATTCAAGAGAACCCAGTGACGCGTAAATCGTAAGATCTACCTGCCGCAGG
TGGACCTACTGCAAATACGGATTATGACTCGTAAAGGGGTCATGCGTATTTTCATCACTAGGCACGTT
CGAGAATAAATTAGTAGGTGTCCCGAGCCTTGTGGCGTTCCGCCTGACTCCTCATGAAGTCGACCTTC
TCACCGGCCCTATCTGCCGACGTAAGTCATAACCTAGATCTGTACCTCGGGGGGAGGGTCACTGTAAA
GGGATAATTGGAGGGTGATTTCCCACACTTTCCTAAGGGTACTTTTTGCCTGGCTTCGCAATTGGGTC
CAATGGATGTCGATCTCTGGTTTAGCAGTTGTGAAAGTGGCAAGGCGGGAGGTTAGACCTCCATTTAA
CATATACAAGCAAGTTAACTGCACTAGATGTGTAGACACTACAGTTACAGGAGTAGCCGAATAAGTCT
CCGACGTCAAGCGAATAAGCGTCATACGCGATTATCGCCTAAGAGCACGTATTGGCGGTAAAAGGCTA
GCTAGACGCTTATGGGTAGATTTCAAGGCGTTCGTAGTGGTATAATAGGATACTCTTTCACCAGCCTG
AGGGCCGAACGCTATACTAGTGGTCTGTGATGTAGGACCGAGTATCTCTCTAGGGACCATCTACTTGA
GCAATGGTGCGCAGGGGGAGACATAGACCAGCCTTGGGTGGCAAGCACTGCAATAAGTCCTGTTTAGC
CTTGGAGTTCACATGCCGGCACTGAAGCCGACCTACCTGAGCGTGTGCGATTACCGTTACAATGGCAT

CTGTCTAGTTCTGTTTACCTACGGCGCTCTTGGTTCCAGGTTAGGGGAAGTGTATGACCCATGTGTTT
TTATCGGCTTAACCACGAGTGATCCCCGGTCGTTTCCCCATTGAATCCCTGGTGCATCCTACTCCCAG
AATGATAGCTGACTGACTGGACTGGCTTTTCAAGTAATCGAGGGGGTATCGCGGTCACGGCCGTTAAC
AGATCCCGTCCTTAGTGTGGAATCCGCACCTGCTGACTAACGCTTCGCCGGCGTGTCTGCACAGCCGT
ATAGTGTTAATCATGACCCCAAGGAAGGATTAAACAAATATCTTGACG
>CHRUN16
GTGTCCGGTAGCCCGCGCTAGTTAGACACCCCGGCAGGGGGGATTGCTTTCGAGACGGGAGATCCCTT
CGCCGACCCTGGAGGGCCGACGCCGAGGCATTCGGGCCCCTGCAACGTCAACAGCGGCAAGAAAACGG
GATGAATGGGCGTAATGGGGGGGGTCTGCTGGGGACCCGACGCGGTTGCCGTTTGCGGGGCCCCCGAC
CCATACCGACCCACCTAGGCGTCCAGTTACGGCGCACGGCGGGAGCGTGGTTGCCGTCAGAGCTGTGT
TTCTCGATCAGTCCCCCGCAGTGCCGCAGTATCTTGCCGTGGGCTGCTTTAATCTTGAAAGTGGTTCA
TACATTGGGCGACGAGGTGTCGACTCTCATTGGGGGTAACCGACGGGCACATGCAGTCCCCTCCCCGG
GCAGGCAGAGGCGGGGCCCCCGCGCGCCGGCCCCGGCCCACAATCTTACCAGGGTCCTCAAAGGAGCC
TTTGCATGGTACCCTTCGTGAATGGTTGCTTAAGAGGTCCACAACGTGGTCCGGGCACGGTCGACTAA
ACACTCAAAACAGCGACGGCAAATATAGGTACAAGGTCCAGGCCCTCACGGCACTAATTGCGATGACC
CAACTCACGGGGGCAGTCCCCGGCGCGGCGACCTTGATTACGTCCGGGGACAAGTATGTCGCTCCCCC
CGGGAGGGGTGCAGCCACATGGGAGATTCAAAGTTTCTCGTGACGTCGTTGTGATCACTGCAGCCTAG
CCGAGACTCCCGTACTACGCGAAGGTTGGTTATGTTAACCACTACAACGTGAGGCGCCGTAAGGGCCA
GTGTTGTGCCCGGCTCTTCAATGCGCCTTAGCGGCCTGATACACCCACCCAAGGAGATACTGCTAATC
ATATGGGTGGACAGAACCTCGCAACGCAGACGCCTCCCCGGCATGATATGGTTTTTTTCCGCTATTAT
CCAGCACGCAGCGCTATCATTCAAGAGAACCCAGTGACGCGTAAATCGTAAGATCTACCTGCCGCAGG
TGGACCTACTGCAAATACGGATTATGACTCGTAAAGGGGTCATGCGTATTTTCATCACTAGGCACGTT
CGAGAATAAATTAGTAGGTGTCCCGAGCCTTGTGGCGTTCCGCCTGACTCCTCATGAAGTCGACCTTC
TCACCGGCCCTATCTGCCGACGTAAGTCATAACCTAGATCTGTACCTCGGGGGGAGGGTCACTGTAAA
GGGATAATTGGAGGGTGATTTCCCACACTTTCCTAAGGGTACTTTTTGCCTGGCTTCGCAATTGGGTC
CAATGGATGTCGATCTCTGGTTTAGCAGTTGTGAAAGTGGCAAGGCGGGAGGTTAGACCTCCATTTAA
CATATACAAGCAAGTTAACTGCACTAGATGTGTAGACACTACAGTTACAGGAGTAGCCGAATAAGTCT
CCGACGTCAAGCGAATAAGCGTCATACGCGATTATCGCCTAAGAGCACGTATTGGCGGTAAAAGGCTA
GCTAGACGCTTATGGGTAGATTTCAAGGCGTTCGTAGTGGTATAATAGGATACTCTTTCACCAGCCTG
AGGGCCGAACGCTATACTAGTGGTCTGTGATGTAGGACCGAGTATCTCTCTAGGGACCATCTACTTGA
GCAATGGTGCGCAGGGGGAGACATAGACCAGCCTTGGGTGGCAAGCACTGCAATAAGTCCTGTTTAGC
CTTGGAGTTCACACGCCGGCACTGAAGCCGACCTACCTGAGCGTGTGCGATTACCGTTACAATGGCAT
CTGTCTAGTTCTGTTTACCTACGGCGCTCTTGGTTCCAGGTTAGGGGAAGTGTATGACCCATGTGTTT
TTATCGGCTTAACCACGAGTGATCCCCGGTCGTTTCCCCATTGAATCCCTGGTGCATCCTACTCCCAG
AATGATAGCTGACTGACTGGACTGGCTTTTCAAGTAATCGAGGGGGTATCGCGGTCACGGCCGTTAAC
AGATCCCGTCCTTAGTGTGGAATCCGCACCTGCTGACTAACGCTTCGCCGGCGTGTCTGCACAGCCGT
ATAGTGTTAATCATGACCCCAAGGAAGGATTAAACAAATATCTTGACG
>CHRUN31
GTGTCCGGTAGCCCGCGCTAGTTAGACACCCCGGCAGGGGGGATTGCTTTCGAGACGGGAGATCCCTT
CGCCGACCCTGGAGGGCCGACGCCGAGGCATTCGGGCCCCTGCAACGTCAACAGCGGCAAGAAAACGG
GATGAATGGGCGTAATGGGGGGGGTCTGCTGGGGACCCGACGCGGTTGCCGTTTGCGGGGCCCCCGAC
CCATACCGACCCACCTAGGCGTCCAGTTACGGCGCACGTCGGGAGCGTGGTTGCCGTCAGAGCTGTGT
TTCTCGATCAGTCCCCCGCAGTGCCGCAGTATCTTGCCGTGGGCTGCTTTAATCTTGAAAGTGGTTTA
TACATTGGGCGACGAGGTGTCGACTCTCATTGGGGGTAACCGACGGGCACATGCAGTCCCCTCCCCGG
GCAGGCAGAGGCGGGGCCCCCGCGCGCCGGCCCCGGCCCACAATCTTACCAGGGTCCTCAAAGGAGCC
TTTGCATGGTACCCTTCGTGAATGGTTGCTTAAGAGGTCCACAACGTGGTCCGGGCACGGTCGACTAA
ACACTCAAAACAGCGACGGCAAATATAGGTACAAGGTCCAGGCCCTCACGGCACTAATTGCGATGACC
CAACTCACGGGGGCAGTCCCCGGCGCGGCGACCTTGATTACGTCCGGGGACAAGTATGTCGCTCCCCC
CGGGAGGGGTGCAGCCACATGGGAGATTCAAAGTTTCTCGTGACGTCGTTGTGATCACTGCAGCCTAG
CCGAGACTCCCGTACTACGCGAAGGTTGGTTATGTTAACCACTACAACGTGAGGCGCCGTAAGGGCCA
GTGTTGTGCCCGGCTCTTCAATGCGCCTTAGCGGCCTGATACACCCACCCAAGGAGATACTGCTAATC
ATATGGGTGGACAGAACCTCGCAACGCAGACGCCTCCCCGGCATGATATGGTTTTTTTCCGCTATTAT
CCAGCACGCAGCGCTATCATTCAAGAGAACCCAGTGACGCGTAAATCGTAAGATCTACCTGCCGCAGG
TGGACCTACTGCAAATACGGATTATGACTCGTAAAGGGGTCATGCGTATTTTCATCACTAGGCACGTT

CGAGAATAAATTAGTAGGTGTCCCGAGCCTTGTGGCGTTCCGCCTGACTCCTCATGAAGTCGACCTTC
TCACCGGCCCTATCTGCCGACGTAAGTCATAACCTAGATCTGTACCTCGGGGGGAGGGTCACTGTAAA
GGGATAATTGGAGGGTGATTTCCCACACTTTCCTAAGGGTACTTTTTGCCTGGCTTCGCAATTGGGTC
CAATGGATGTCGATCTCTGGTTTAGCAGTTGTGAAAGTGGCAAGGCGGGAGGTNNGACCTCCNTTTAA
CATATACAAGCAAGTTAACNGCACTAGATGTGTAGACACTACAGTTACAGGAGTAGCCGAATAAGTCT
CCGACGTCAAGCGAATAAGCGTCATACGCGATTATCGCCTAAGAGCACGTATTGGCGGTAAAAGGCTA
GCTAGACGCTTATGGGTAGATTTCAAGGCGTTCGTAGTGGTATAATAGGATACTCTTTCACCAGCCTG
AGGGCCGAACGCTATACTAGTGGTCTGTGATGTAGGACCGAGTATCTCTCTAGGGACCATCTACTTGA
GCAATGGTGCGCAGGGGGAGACATAGACCAGCCTTGGGTGGCAAGCACTGCAATAAGTCCTGTTTAGC
CTTGGAGTTCACATGCCGGCACTGAAGCCGACCTACCTGAGCGTGTGCGATTACCGTTACAATGGCAT
CTGTCTAGTTCTGTTTACCTACGGCGCTCTTGGTTCCAGGTTAGGGGAAGTGTATGACCCATGTGTTT
TTATCGGCTTAACCACGAGTGATCCCCGGTCGTTTCCCCATTGAATCCCTGGTGCATCCTACTCCCAG
AATGATAGCTGACTGACTGGACTGGCTTTTCAAGTAATCGAGGGGGTATCGCGGTCACGGCCGTTAAC
AGATCCCGTCCTTAGTGTGGAATCCGCACCTGCTGACTAACGCTTCGCCGGCGTGTCTGCACAGCCGT
ATAGTGTTAATCATGACCCCAAGGAAGGATTAAACAAATATCTTGACG
>CHRUN33
GTGTCCGGTAGCCCGCGCTAGTTAGACACCCCGGCAGGGGGGATTGCTTTCGGGACGGGAGATCCCTT
CGCCGACCCTGGAGGGCCGACGCCGAGGCATTCGGGCCCCTGCAACGTCAACAGCGGCAAGAAAACGG
GATGAATGGGCGTAATGGGGGGGGTCTGCTGGGGACCCGACGCGGTTGCCGTTTGCGGGGCCCCCGAC
CCATACCGACCCACCTAGGCGTCCAGTTACGGCGCACGTCGGGAGCGTGGTTGCCGTCAGAGCTGTGT
TTCTCGATCAGTCCCCCGCAGTGCCGCAGTATCTTGCCGTGGGCTGCTTTAATCTTGAAAGTGGTTNA
TACATTGGGCGACGAGGTGTCGACTCTCATTGGGGGTAACCGACGGGCACATGCAGTCCCCTCCCCGG
GCAGGCAGAGGCGGGGCCCCCGCGCGCCGGCCCCGGCCCACAATCTTACCAGGGTCCTCAAAGGAGCC
TTTGCATGGTACCCTTCGTGAATGGTTGCTTAAGAGGTCCACAACGTGGTCCGGGCACGGTCGACTAA
ACACTCAAAACAGCGACGGCAAATATAGGTACAAGGTCCAGGCCCTCACGGCACTAATTGCGATGACC
CAACTCACGGGGGCAGTCCCCGGCGCGGCGACCTTGATTACGTCCGGGGACAAGTATGTCGCTCCCCC
CGGGAGGGGTGCAGCCACATGGGAGATTCAAAGTTTCTCGTGACGTCGTTGTGATCACTGCAGCCTAG
CCGAGACTCCCGTACTACGCGAAGGTTGGTTATGTTAACCACTACAACGTGAGGCGCCGTAAGGGCCA
GTGTTGTGCCCGGCTCTTCAATGCGCCTTAGCGGCCTGATACACCCACCCAAGGAGATACTGCTAATC
ATATGGGTGGACAGAACCTCGCAACGCAGACGCCTCCCCGGCATGATATGGTTTTTTTCCGCTATTAT
CCAGCACGCAGCGCTATCATTCAAGAGAACCCAGTGACGCGTAAATCGTAAGATCTACCTGCCGCAGG
TGGACCTACTGCAAATACGGATTATGACTCGTAAAGGGGTCATGCGTATTTTCATCACTAGGCACGTT
CGAGAATAAATTAGTAGGTGTCCCGAGCCTTGTGGCGTTCCGCCTGACTCCTCATGAAGTCGACCTTC
TCACCGGCCCTATCTGCCGACGTAAGTCATAACCTAGATCTGTACCTCGGGGGGAGGGTCACTGTAAA
GGGATAATTGGAGGGTGATTTCCCACACTTTCCTAAGGGTACTTTTTGCCTGGCTTCGCAATTGGGTC
CAATGGATGTCGATCTCTGGTTTAGCAGTTGTGAAAGTGGCAAGGCGGGAGGTTAGACCTCCATTTAA
CATATACAAGCAAGTTAACTGCACTAGATGTGTAGACACTACAGTTACAGGAGTAGCCGAATAAGTCT
CCGACGTCAAGCGAATAAGCGTCATACGCGATTATCGCCTAAGAGCACGTATTGGCGGTAAAAGGCTA
GCTAGACGCTTATGGGTAGATTTCAAGGCGTTCGTAGTGGTATAATAGGATACTCTTTCACCAGCCTG
AGGGCCGAACGCTATACTAGTGGTCTGTGATGTAGGACCGAGTATCTCTCTAGGGACCATCTACTTGA
GCAATGGTGCGCAGGGGGAGACATAGACCAGCCTTGGGTGGCAAGCACTGCAATAAGTCCTGTTTAGC
CTTGGAGTTCACATGCCGGCACTGAAGCCGACCTACCTGAGCGTGTGCGATTACCGTTACAATGGCAT
CTGTCTAGTTCTGTTTACCTACGGCGCTCTTGGTTCCAGGTTAGGGGAAGTGTATGACCCATGTGTTT
TTATCGGCTTAACCACGAGTGATCCCCGGTCGTTTCCCCATTGAATCCCTGGTGCATCCTACTCCCAG
AATGATAGCTGACTGACTGGACTGGCTTTTCAAGTAATCGAGGGGGTATCGCGGTCACGGCCGTTAAC
AGATCCCGTCCTTAGTGTGGAATCCGCACCTGCTGACTAACGCTTCGCCGGCGTGTCTGCACAGCCGT
ATAGTGTTAATCATGACCCCAAGGAAGGATTAAACAAATATCTTGACG
>CHRUN37
GTGTCCGGTAGCCCGCGCTAGTTAGACACCCCGGCAGGGGGGATTGCTTTCGAGACGGGAGATCCCTT
CGCCGACCCTGGAGGGCCGACGCCGAGGCATTCGGGCCCCTGCAACGTCAACAGCGGCAAGAAAACGG
GATGAATGGGCGTAATGGGGGGGGTCTGCTGGGGACCCGACGCGGTTGCCGTTTGCGGGGCCCCCGAC
CCATACCGACCCACCTAGGCGTCCAGTTACGGCGCACGGCGGGAGCGTGGTTGCCGTCAGAGCTGTGT
TTCTCGATCAGTCCCCCGCAGTGCCGCAGTATCTTGCCGTGGGCTGCTTTAATCTTGAAAGTGGTTCA
TACATTGGGCGACGAGGTGTCGACTCTCATTGGGGGTAACCGACGGGCACATGCAGTCCCCTCCCCGG

GCAGGCAGAGGCGGGGCCCCCGCGCGCCGGCCCCGGCCCACAATCTTACCAGGGTCCTCAAAGGAGCC
TTTGCATGGTACCCTTCGTGAATGGTTGCTTAAGAGGTCCACAACGTGGTCCGGGCACGGTCGACTAA
ACACTCAAAACAGCGACGGCAAATATAGGTACAAGGTCCAGGCCCTCACGGCACTAATTGCGATGACC
CAACTCACGGGGGCAGTCCCCGGCGCGGCGACCTTGATTACGTCCGGGGACAAGTATGTCGCTCCCCC
CGGGAGGGGTGCAGCCACATGGGAGATTCAAAGTTTCTCGTGACGTCGTTGTGATCACTGCAGCCTAG
CCGAGACTCCCGTACTACGCGAAGGTTGGTTATGTTAACCACTACAACGTGAGGCGCCGTAAGGGCCA
GTGTTGTGCCCGGCTCTTCAATGCGCCTTAGCGGCCTGATACACCCACCCAAGGAGATACTGCTAATC
ATATGGGTGGACAGAACCTCGCAACGCAGACGCCTCCCCGGCATGATATGGTTTTTTTCCGCTATTAT
CCAGCACGCAGCGCTATCATTCAAGAGAACCCAGTGACGCGTAAATCGTAAGATCTACCTGCCGCAGG
TGGACCTACTGCAAATACGGATTATGACTCGTAAAGGGGTCATGCGTATTTTCATCACTAGGCACGTT
CGAGAATAAATTAGTAGGTGTCCCGAGCCTTGTGGCGTTCCGCCTGACTCCTCATGAAGTCGACCTTC
TCACCGGCCCTATCTGCCGACGTAAGTCATAACCTAGATCTGTACCTCGGGGGGAGGGTCACTGTAAA
GGGATAATTGGAGGGTGATTTCCCACACTTTCCTAAGGGTACTTTTTGCCTGGCTTCGCAATTGGGTC
CAATGGATGTCGATCTCTGGTTTAGCAGTTGTGAAAGTGGCAAGGCGGGAGGTTAGACCTCCATTTAA
CATATACAAGCAAGTTAACTGCACTAGATGTGTAGACACTACAGTTACAGGAGTAGCCGAATAAGTCT
CCGACGTCAAGCGAATAAGCGTCATACGCGATTATCGCCTAAGAGCACGTATTGGCGGTAAAAGGCTA
GCTAGACGCTTATGGGTAGATTTCAAGGCGTTCGTAGTGGTATAATAGGATACTCTTTCACCAGCCTG
AGGGCCGAACGCTATACTAGTGGTCTGTGATGTAGGACCGAGTATCTCTCTAGGGACCATCTACTTGA
GCAATGGTGCGCAGGGGGAGACATAGACCAGCCTTGGGTGGCAAGCACTGCAATAAGTCCTGTTTAGC
CTTGGAGTTCACATGCCGGCACTGAAGCCGACCTACCTGAGCGTGTGCGATTACCGTTACAATGGCAT
CTGTCTAGTTCTGTTTACCTACGGCGCTCTTGGTTCCAGGTTAGGGGAAGTGTATGACCCATGTGTTT
TTATCGGCTTAACCACGAGTGATCCCCGGTCGTTTCCCCGTTGAATCCCTGGTGCATCCTACTCCCAG
AATGATAGCTGACTGACTGGACTGGCTTTTCAAGTAATCGAGGGGGTATCGCGGTCACGGCCGTTAAC
AGATCCCGTCCTTAGTGTGGAATCCGCACCTGCTGACTAACGCTTCGCCGGCGTGTCTGCACAGCCGT
ATAGTGTTAATCATGACCCCAAGGAAGGATTAAACAAATATCTTGACG
>CHRUN41
GTGTCCGGTAGCCCGCGCTAGTTAGACACCCCGGCAGGGGGGATTGCTTTCGAGACGGGAGATCCCTT
CGCCGACCCTGGAGGGCCGACGCCGAGGCATTCGGGCCCCTGCAACGTCAACAGCGGCAAGAAAACGG
GATGAATGGGCGTAATGGGGGGGGTCTGCTGGGGACCCGACGCGGTTGCCGTTTGCGGGGCCCCCGAC
CCATACCGACCCACCTAGGCGTCCAGTTACGGCGCACGGCGGGAGCGTGGTTGCCGTCAGAGCTGTGT
TTCTCGATCAGTCCCCCGCAGTGCCGCAGTATCTTGCCGTGGGCTGCTTTAATCTTGAAAGTGGTTCA
TACATTGGGCGACGAGGTGTCGACTCTCATTGGGGGTAACCGACGGGCACATGCAGTCCCCTCCCCGG
GCAGGCAGAGGCGGGGCCCCCGCGCGCCGGCCCCGGCCCACAATCTTACCAGGGTCCTCAAAGGAGCC
TTTGCATGGTACCCTTCGTGAATGGTTGCTTAAGAGGTCCACAACGTGGTCCGGGCACGGTCGACTAA
ACACTCAAAACAGCGACGGCAAATATAGGTACAAGGTCCAGGCCCTCACGGCACTAATTGCGATGACC
CAACTCACGGGGGCAGTCCCCGGCGCGGCGACCTTGATTACGTCCGGGGACAAGTATGTCGCTCCCCC
CGGGAGGGATGCAGCCACATGGGAGATTCAAAGTTTCTCGTGACGTCGTTGTGATCACTGCAGCCTAG
CCGAGACTCCCGTACTACGCGAAGGTTGGTTATGTTAACCACTACAACGTGAGGCGCCGTAAGGGCCA
GTGTTGTGCCCGGCTCTTCAATGCGCCTTAGCGGCCTGATACACCCACCCAAGGAGATACTGCTAATC
ATATGGGTGGACAGAACCTCGCAACGCAGACGCCTCCCCGGCATGATANGGTTTTTTTCCGCTATTAT
CCAGCACGCAGCGCTATCATTCAAGAGAACCCAGTGACGCGTAAATCGTAAGATCTACCTGCCGCAGG
TGGACCTACTGCAAATACGGATTATGACTCGTAAAGGGGTCATGCGTATTTTCATCACTAGGCACGTT
CGAGAATAAATTAGTAGGTGTCCCGAGCCTTGTGGCGTTCCGCCTGACTCCTCATGAAGTCGACCTTC
TCACCGGCCCTATCTGCCGACGTAAGTCATAACCTAGATCTGTACCTCGGGGGGAGGGTCACTGTAAA
GGGATAATTGGAGGGTGATTTCCCACACTTTCCTAAGGGTACTTTTTGCCTGGCTTCGCAATTGGGTC
CAATGGATGTCGATCTCTGGTTTAGCAGTTGTGAAAGTGGCAAGGCGGGAGGTTAGACCTCCATTTAA
CATATACAAGCAAGTTAACTGCACTAGATGTGTAGACACTACAGTTACAGGAGTAGCCGAATAAGTCT
CCGACGTCAAGCGAATAAGCGTCATACGCGATTATCGCCTAAGAGCACGTATTGGCGGTAAAAGGCTA
GCTAGACGCTTATGGGTAGATTTCAAGGCGTTCGTAGTGGTATAATAGGATACTCTTTCACCAGCCTG
AGGGCCGAACGCTATACTAGTGGTCTGTGATGTAGGACCGAGTATCTCTCTAGGGACCATCTACTTGA
GCAATGGTGCGCAGGGGGAGACATAGACCAGCCTTGGGTGGCAAGCACTGCAATAAGTCCTGTTTAGC
CTTGGAGTTCACATGCCGGCACTGAAGCCGACCTACCTGAGCGTGTGCGATTACCGTTACAATGGCAT
CTGTCTAGTTCTGTTTACCTACGGCGCTCTTGGTTCCAGGTTAGGGGAAGTGTATGACCCATGTGTTT
TTATCGGCTTAACCACGAGTGATCCCCGGTCGTTTCCCCATTGAATCCCTGGTGCATCCTACTCCCAG

AATGATAGCTGACTGACTGGACTGGCTTTTCAAGTAATCGAGGGGGTATCGCGGTCACGGCCGTTAAC
AGATCCCGTCCTTAGTGTGGAATCCGCACCTGCTGACTAACGCTTCGCCGGCGTGTCTGCACAGCCGT
ATAGTGTTAATCATGACCCCAAGGAAGGATTAAACAAATATCTTGACG
>CHRUN42
GTGTCCGGTAGCCCGCGCTAGTTAGACACCCCGGCAGGGGGGATTGCTTTCGAGACGGGAGATCCCTT
CGCCGACCCTGGAGGGCCGACGCCGAGGCATTCGGGCCCCTGCAACGTCAACAGCGGCAAGAAAACGG
GATGAATGGGCGTAATGGGGGGGGTCTGCTGGGGACCCGACGCGGTTGCCGTTTGCGGGGCCCCCGAC
CCATACCGACCCACCTAGGCGTCCAGTTACGGCGCACGGCGGGAGCGTGGTTGCCGTCAGAGCTGTGT
TTCTCGATCAGTCCCCCGCAGTGCCGCAGTATCTTGCCGTGGGCTGCTTTAATCTTGAAAGTGGTTCA
TACATTGGGCGACGAGGTGTCGACTCTCATTGGGGGTAACCGACGGGCACATGCAGTCCCCTCCCCGG
GCAGGCAGAGGCGGGGCCCCCGCGCGCCGGCCCCGGCCCACAATCTTACCAGGGTCCTCAAAGGAGCC
TTTGCATGGTACCCTTCGTGAATGGTTGCTTAAGAGGTCCACAACGTGGTCCGGGCACGGTCGACTAA
ACACTCAAAACAGCGACGGCAAATATAGGTACAAGGTCCAGGCCCTCACGGCACTAATTGCGATGACC
CAACTCACGGGGGCAGTCCCCGGCGCGGCGACCTTGATTACGTCCGGGGACAAGTATGTCGCTCCCCC
CGGGAGGGGTGCAGCCACATGGGAGATTCAAAGTTTCTCGTGACGTCGTTGTGATCACTGCAGCCTAG
CCGAGACTCCCGTACTACGCGAAGGTTGGTTATGTTAACCACTACAACGTGAGGCGCCGTAAGGGCCA
GTGTTGTGCCCGGCTCTTCAATGCGCCTTAGCGGCCTGATACACCCACCCAAGGAGATACTGCTAATC
ATATGGGTGGACAGAACCTCGCAACGCAGACGCCTCCCCGGCATGATATGGTTTTTTTCCGCTATTAT
CCAGCACGCAGCGCTATCATTCAAGAGAACCCAGTGACGCGTAAATCGTAAGATCTACCTGCCGCAGG
TGGACCTACTGCAAATACGGATTATGACTCGTAAAGGGGTCATGCGTATTTTCATCACTAGGCACGTT
CGAGAATAAATTAGTAGGTGTCCCGAGCCTTGTGGCGTTCCGCCTGACTCCTCATGAAGTCGACCTTC
TCACCGGCCCTATCTGCCGACGTAAGTCATAACCTAGATCTGTACCTCGGGGGGAGGGTCACTGTAAA
GGGATAATTGGAGGGTGATTTCCCACACTTTCCTAAGGGTACTTTTTGCCTGGCTTCGCAATTGGGTC
CAATGGATGTCGATCTCTGGTTTAGCAGTTGTGAAAGTGGCAAGGCGGGAGGTTAGACCTCCATTTAA
CATATACAAGCAAGTTAACTGCACTAGATGTGTAGACACTACAGTTACAGGAGTAGCCGAATAAGTCT
CCGACGTCAAGCGAATAAGCGTCATACGCGATTATCGCCTAAGAGCACGTATTGGCGGTAAAAGGCTA
GCTAGACGCTTATGGGTAGATTTCAAGGCGTTCGTAGTGGTATAATAGGATACTCTTTCACCAGCCTG
AGGGCCGAACGCTATACTAGTGGTCTGTGATGTAGGACCGAGTATCTCTCTAGGGACCATCTACTTGA
GCAATGGTGCGCAGGGGGAGACATAGACCAGCCTTGGGTGGCAAGCACTGCAATAAGTCCTGTTTAGC
CTTGGAGTTCACATGCCGGCACTGAAGCCGACCTACCTGAGCGTGTGCGATTACCGTTACAATGGCAT
CTGTCTAGTTCTGTTTACCTACGGCGCTCTTGGTTCCAGGTTAGGGGAAGTGTATGACCCATGTGTTT
TTATCGGCTTAACCACGAGTGATCCCCGGTCGTTTCCCCATTGAATCCCTGGTGCATCCTACTCCCAG
AATGATAGCTGACTGACTGGACTGGCTTTTCAAGTAATCGAGGGGGTATCGCGGTCACGGCCGTTAAC
AGATCCCGTCCTTAGTGTGGAATCCGCACCTGCTGACTAACGCTTCGCCGGCGTGTCTGCACAGCCGT
ATAGTGTTAATCATGACCCCAAGGAAGGATTAAACAAATATCTTGACG
>COHON8
GTGTCCGGTAGCCCGCGCTAGTTAGACACCCCGGCAGGGGGGATTGCTTTCGAGACGGGAGATCCCTT
CGCCGACCCTGGAGGGCCGACGCCGAGGCATTCGGGCCCCTGCAACGTCAACAGCGGCAAGAAAACGG
GATGAATGGGCGTAATGGGGGGGGTCTGCTGGGGACCCGACGCGGTTGCCGTTTGCGGGGCCCCCGAC
CCATACCGACCCACCTAGGCGTCCAGTTACGGCGCACGGCGGGAGCGTGGTTGCCGTCAGAGCTGTGT
TTCTCGATCAGTCCCCCGCAGTGCCGCAGTATCTTGCCNTGGGCTGCTTTAATCTTGAAAGTGGTTTA
TACATTGGGCGACGAGGTGTCGACTCTCATTGGGGGTAACCGACGGGCACATGCAGTCCCCTCCCCGG
GCAGGCAGAGGCGGGGCCCCCGCGCGCCGGCCCCGGCCCACAATCTTACCAGGGTCCTCAAAGGAGCC
TTTGCATGGTACCCTTCGTGAATGGTTGCTTAAGAGGTCCACAACGTGGTCCGGGCACGGTCGACTAA
ACACTCAAAACAGCGACGGCAAATATAGGTACAAGGTCCAGGCCCTCACGGCACTAATTGCGATGACC
CAACTCACGGGGGCAGTCCCCGGCGCGGCGACCTTGATTACGTCCGGGGACAAGTATGTCGCTCCCCC
CGGGAGGGGTGCAGCCACATGGGAGATTCAAAGTTTCTCGTGACGTCGTTGTGATCACTGCAGCCTAG
CCGAGACTCCCGTACTACGCGAAGGTTGGTTATGTTAACCACTACAACGTGAGGCGCCGTAAGGGCCA
GTGTTGTGCCCGGCTCTTCAATGCGCCTTAGCGGCCTGATACACCCACCCAAGGAGATACTGCTAATC
ATATGGGTGGACAGAACCTCGCAACGCAGACGCCTCCCCGGCATGATATGGTTTTTTTCCGCTATTAT
CCAGCACGCAGCGCTATCATTCAAGAGAACCCAGTGACGCGTAAATCGTAAGATCTACCTGCCGCAGG
TGGACCTACTGCAAATACGGATTATGACTCGTAAAGGGGTCATGCGTATTTTCATCACTAGGCACGTT
CGAGAATAAATTAGTAGGTGTCCCGAGCCTTGTGGCGTTCCGCCTGACTCCTCATGAAGTCGACCTTC
TCACCGGCCCTATCTGCCGACGTAAGTCATAACCTAGATCTGTACCTCGGGGGGAGGGTCACTGTAAA

GGGATAATTGGAGGGTGATTTCCCACACTTTCCTAAGGGTACTTTTTGCCTGGCTTCGCAATTGGGTC
CAATGGATGTCGATCTCTGGTTTAGCAGTTGTGAAAGTGGCAAGGCGGGAGGTTAGACCTCCATTTAA
CATATACAAGCAAGTTAACTGCACTAGATGTGTAGACACTACAGTTACAGGAGTAGCCGAATAAGTCT
CCGACGTCAAGCGAATAAGCGTCATACGCGATTATCGCCTAAGAGCACGTATTGGCGGTAAAAGGCTA
GCTAGACGCTTATGGGTAGATTTCAAGGCGTTCGTAGTGGTATAATAGGATACTCTTTCACCAGCCTG
AGGGCCGAACGCTATACTAGTGGTCTGTGATGTAGGACCGAGTATCTCTCTAGGGACCATCTACTTGA
GCAATGGTGCGCAGGGGGAGACATAGACCAGCCTTGGGTGGCAAGCACTGCAATAAGTCCTGTTTAGC
CTTGGAGTTCACATGCCGGCACTGAAGCCGACCTACCTGAGCGTGTGCGATTACCGTTACAATGGCAT
CTGTCTAGTTCTGTTTACCTACGGCGCTCTTGGTTCCAGGTTAGGGGAAGTGTATGACCCATGTGTTT
TTATCGGCTTAACCACGAGTGATCCCCGGTCGTTTCCCCATTGAATCCCTGGTGCATCCTACTCCCAG
AATGATAGCTGACTGACTGGACTGGCTTTTCAAGTAATCGAGGGGGTATCGCGGTCACGGCCGTTAAC
AGATCCCGTCCTTAGTGTGGAATCCGCACCTGCTGACTAACGCTTCGCCGGCGTGTCTGCACAGCCGT
ATAGTGTTAATCATGACCCCAAGGAAGGATTAAACAAATATCTTGACG
>COHON9
GTGTCCGGTAGCCCGCGCTAGTTAGACACCCCGGCAGGGGGGATTGCTTTCGAGACGGGAGATCCCTT
CGCCGACCCTGGAGGGCCGACGCCGAGGCATTCGGGCCCCTGCAACGTCAACAGCGGCAAGAAAACGG
GATGAATGGGCGTAATGGGGGGGGTCTGCTGGGGACCCGACGCGGTTGCCGTTTGCGGGGCCCCCGAC
CCATACCGACCCACCTAGGCGTCCAGTTACGGCGCACGTCGGGAGCGTGGTTGCCGTCAGAGCTGTGT
TTCTCGATCAGTCCCCCGCAGTGCCGCAGTATCTTGCCGTGGGCTGCTTTAATCTTGAAAGTGGTTTA
TACATTGGGCGACGAGGTGTCGACTCTCATTGGGGGTAACCGACGGGCACATGCAGTCCCCTCCCCGG
GCAGGCAGAGGCGGGGCCCCCGCGCGCCGGCCCCGGCCCACAATCTTACCAGGGTCCTCAAAGGAGCC
TTTGCATGGTACCCTTCGTGAATGGTTGCTTAAGAGGTCCACAACGTGGTCCGGGCACGGTCGACTAA
ACACTCAAAACAGCGACGGCAAATATAGGTACAAGGTCCAGGCCCTCACGGCACTAATTGCGATGACC
CAACTCACGGGGGCAGTCCCCGGCGCGGCGACCTTGATTACGTCCGGGGACAAGTATGTCGCTCCCCC
CGGGAGGGGTGCAGCCACATGGGAGATTCAAAGTTTCTCGTGACGTCGTTGTGATCACTGCAGCCTAG
CCGAGACTCCCGTACTACGCGAAGGTTGGTTATGTTAACCACTACAACGTGAGGCGCCGTAAGGGCCA
GTGTTGTGCCCGGCTCTTCAATGCGCCTTAGCGGCCTGATACACCCACCCAAGGAGATACTGCTAATC
ATATGGGTGGACAGAACCTCGCAACGCAGACGCCTCCCCGGCATGATATGGTTTTTTTCCGCTATTAT
CCAGCACGCAGCGCTATCATTCAAGAGAACCCAGTGACGCGTAAATCGTAAGATCTACCTGCCGCAGG
TGGACCTACTGCAAATACGGATTATGACTCGTAAAGGGGTCATGCGTATTTTCATCACTAGGCACGTT
CGAGAATAAATTAGTAGGTGTCCCGAGCCTTGTGGCGTTCCGCCTGACTCCTCATGAAGTCGACCTTC
TCACCGGCCCTATCTGCCGACGTAAGTCATAACCTAGATCTGTACCTCGGGGGGAGGGTCACTGTAAA
GGGATAATTGGAGGGTGATTTCCCACACTTTCCTAAGGGTACTTTTTGCCTGGCTTCGCAATTGGGTC
CAATGGATGTCGATCTCTGGTTTAGCAGTTGTGAAAGTGGCAAGGCGGGAGGTTAGACCTCCATTTAA
CATATACAAGCAAGTTAACTGCACTAGATGTGTAGACACTACAGTTACAGGAGTAGCCGAATAAGTCT
CCGACGTCAAGCGAATAAGCGTCATACGCGATTATCGCCTAAGAGCACGTATTGGCGGTAAAAGGCTA
GCTAGACGCTTATGGGTAGATTTCAAGGCGTTCGTAGTGGTATAATAGGATACTCTTTCACCAGCCTG
AGGGCCGAACGCTATACTAGTGGTCTGTGATGTAGGACCGAGTATCTCTCTAGGGACCATCTACTTGA
GCAATGGTGCGCAGGGGGAGACATAGACCAGCCTTGGGTGGCAAGCACTGCAATAAGTCCTGTTTAGC
CTTGGAGTTCACATGCCGGCACTGAAGCCGACCTACCTGAGCGTGTGCGATTACCGTTACAATGGCAT
CTGTCTAGTTCTGTTTACCTACGGCGCTCTTGGTTCCAGGTTAGGGGAAGTGTATGACCCATGTGTTT
TTATCGGCTTAACCACGAGTGATCCCCGGTCGTTTCCCCATTGAATCCCTGGTGCATCCTACTCCCAG
AATGATAGCTGACTGACTGGACTGGCTTTTCAAGTAATCGAGGGGGTATCGCGGTCACGGCCGTTAAC
AGATCCCGTCCTTAGTGTGGAATCCGCACCTGCTGACTAACGCTTCGCCGGCGTGTCTGCACAGCCGT
ATAGTGTTAATCATGACCCCAAGGAAGGATTAAACAAATATCTTGACG
>COHON10
GTGTCCGGTAGCCCGCGCTAGTTAGACACCCCGGCAGGGGGGATTGCTTTCGAGACGGGAGATCCCTT
CGCCGACCCTGGAGGGCCGACGCCGAGGCATTCGGGCCCCTGCAACGTCAACAGCGGCAAGAAAACGG
GATGAATGGGCGTAATGGGGGGGGTCTGCTGGGGACCCGACGCGGTTGCCGTTTGCGGGGCCCCCGAC
CCATACCGACCCACCTAGGCGTCCAGTTACGGCGCACGTCGGGAGCGTGGTTGCCGTCAGAGCTGTGT
TTCTCGATCAGTCCCCCGCAGTGCCGCAGTATCTTGCCGTGGGCTGCTTTAATCTTGAAAGTGGTTTA
TACATTGGGCGACGAGGTGTCGACTCTCATTGGGGGTAACCGACGGGCACATGCAGTCCCCTCCCCGG
GCAGGCAGAGGCGGGGCCCCCGCGCGCCGGCCCCGGCCCACAATCTTACCAGGGTCCTCAAAGGAGCC
TTTGCATGGTACCCTTCGTGAATGGTTGCTTAAGAGGTCCACAACGTGGTCCGGGCACGGTCGACTAA

ACACTCAAAACAGCGACGGCAAATATAGGTACAAGGTCCAGGCCCTCACGGCACTAATTGCGATGACC
CAACTCACGGGGGCAGTCCCCGGCGCGGCGACCTTGATTACGTCCGGGGACAAGTATGTCGCTCCCCC
CGGGAGGGGTGCAGCCACATGGGAGATTCAAAGTTTCTCGTGACGTCGTTGTGATCACTGCAGCCTAG
CCGAGACTCCCGTACTACGCGAAGGTTGGTTATGTTAACCACTACAACGTGAGGCGCCGTAAGGGCCA
GTGTTGTGCCCGGCTCTTCAATGCGCCTTAGCGGCCTGATACACCCACCCAAGGAGATACTGCTAATC
ATATGGGTGGACAGAACCTCGCAACGCAGACGCCTCCCCGGCATGATATGGTTTTTTTCCGCTATTAT
CCAGCACGCAGCGCTATCATTCAAGAGAACCCAGTGACGCGTAAATCGTAAGATCTACCTGCCGCAGG
TGGACCTACTGCAAATACGGATTATGACTCGTAAAGGGGTCATGCGTATTTTCATCACTAGGCACGTT
CGAGAATAAATTAGTAGGTGTCCCGAGCCTTGTGGCGTTCCGCCTGACTCCTCATGAAGTCGACCTTC
TCACCGGCCCTATCTGCCGACGTAAGTCATAACCTAGATCTGTACCTCGGGGGGAGGGTCACTGTAAA
GGGATAATTGGAGGGTGATTTCCCACACTTTCCTAAGGGTACTTTTTGCCTGGCTTCGCAATTGGGTC
CAATGGATGTCGATCTCTGGTTTAGCAGTTGTGAAAGTGGCAAGGCGGGAGGTTAGACCTCCATTTAA
CATATACAAGCAAGTTAACTGCACTAGATGTGTAGACACTACAGTTACAGGAGTAGCCGAATAAGTCT
CCGACGTCAAGCGAATAAGCGTCATACGCGATTATCGCCTAAGAGCACGTATTGGCGGTAAAAGGCTA
GCTAGACGCTTATGGGTAGATTTCAAGGCGTTCGTAGTGGTATAATAGGATACTCTTTCACCAGCCTG
AGGGCCGAACGCTATACTAGTGGTCTGTGATGTAGGACCGAGTATCTCTCTAGGGACCATCTACTTGA
GCAATGGTGCGCAGGGGGAGACATAGACCAGCCTTGGGTGGCAAGCACTGCAATAAGTCCTGTTTAGC
CTTGGAGTTCACATGCCGGCACTGAAGCCGACCTACCTGAGCGTGTGCGATTACCGTTACAATGGCAT
CTGTCTAGTTCTGTTTACCTACGGCGCTCTTGGTTCCAGGTTAGGGGAAGTGTATGACCCATGTGTTT
TTATCGGCTTAACCACGAGTGATCCCCGGTCGTTTCCCCATTGAATCCCTGGTGCATCCTACTCCCAG
AATGATAGCTGACTGACTGGACTGGCTTTTCAAGTAATCGAGGGGGTATCGCGGTCACGGCCGTTAAC
AGATCCCGTCCTTAGTGTGGAATCCGCACCTGCTGACTAACGCTTCGCCGGCGTGTCTGCACAGCCGT
ATAGTGTTAATCATGACCCCAAGGAAGGATTAAACAAATATCTTGACG
>COHON11
GTGTCCGGTAGCCCGCGCTAGTTAGACACCCCGGCAGGGGGGATTGCTTTCGAGACGGGAGATCCCTT
CGCCGACCCTGGAGGGCCGACGCCGAGGCATTCGGGCCCCCGCAACGTCAACAGCGGCAAGAAAACGG
GATGAATGGGCGTAATGGGGGGGGTCTGCTGGGGACCCGACGCGGTTGCCGTTTGCGGGGCCCCCGAC
CCATACCGACCCACCTAGGCGTCCAGTTACGGCGCACGGCGGGAGCGTGGTTGCCGTCAGAGCTGTGT
TTCTCGATCAGTCCCCCGCAGTGCCGCAGTATCTTGCCGTGGGCTGCTTTAATCTTGAAAGTGGTTTA
TACATTGGGCGACGAGGTGTCGACTCTCATTGGGGGTAACCGACGGGCACATGCAGTCCCCTCCCCGG
GCAGGCAGAGGCGGGGCCCCCGCGCGCCGGCCCCGGCCCACAATCTTACCAGGGTCCTCAAAGGAGCC
TTTGCATGGTACCCTTCGTGAATGGTTGCTTAAGNGGTCCACAACGTGGTCCGGGCACGGTCGACTAA
ACACTCAAAACAGCGACGGCAAATATAGGTACAAGGTCCAGGCCCTCACGGCACTAATTGCGATGACC
CAACTCACGGGGGCAGTCCCCGGCGCGGCGACCTTGATTACGTCCGGGGACAAGTATGTCGCTCCCCC
CGGGAGGGGTGCAGCCACATGGGAGATTCAAAGTTTCTCGTGACGTCGTTGTGATCACTGCAGCCTAG
CCGAGACTCCCGTACTACGCGAAGGTTGGTTATGTTAACCACTACAACGTGAGGCGCCGTAAGGGCCA
GTGTTGTGCCCGGCTCTTCAATGCGCCTTAGCGGCCTGATACACCCACCCAAGGAGATACTGCTAATC
ATATGGGTGGACAGAACCTCGCAACGCAGACGCCTCCCCGGCATGATATGGTTTTTTTCCGCTATTAT
CCAGCACGCAGCGCTATCATTCAAGAGAACCCAGTGACGCGTAAATCGTAAGATCTACCTGCCGCAGG
TGGACCTACTGCAAATACGGATTATGACTCGTAAAGGGGTCATGCGTATTTTCATCACTAGGCACGTT
CGAGAATAAATTAGTAGGTGTCCCGAGCCTTGTGGCGTTCCGCCTGACTCCTCATGAAGTCGACCTTC
TCACCGGCCCTATCTGCCGACGTAAGTCATAACCTAGATCTGTACCTCGGGGGGAGGGTCACTGTAAA
GGGATAATTGGAGGGTGATTTCCCACACTTTCCTAAGGGTACTTTTTGCCTGGCTTCGCAATTGGGTC
CAATGGATGTCGATCTCTGGTTTAGCAGTTGTGAAAGTGGCAAGGCGGGAGGTTAGACCTCCATTTAA
CATATACAAGCAAGTTAACTGCACTAGATGTGTAGACACTACAGTTACAGGAGTAGCCGAATAAGTCT
CCGACGTCAAGCGAATAAGCGTCATACGCGATTATCGCCTAAGAGCACGTATTGGCGGTAAAAGGCTA
GCTAGACGCTTATGGGTAGATTTCAAGGCGTTCGTAGTGGTATAATAGGATACTCTTTCACCAGCCTG
AGGGCCGAACGCTATACTAGTGGTCTGTGATGTAGGACCGAGTATCTCTCTAGGGACCATCTACTTGA
GCAATGGTGCGCAGGGGGAGACATAGACCAGCCTTGGGTGGCAAGCACTGCAATAAGTCCTGTTTAGC
CTTGGAGTTCACATGCCGGCACTGAAGCCGACCTACCTGAGCGTGTGCGATTACCGTTACAATGGCAT
CTGTCTAGTTCTGTTTACCTACGGCGCTCTTGGTTCCAGGTTAGGGGAAGTGTATGACCCATGTGTTT
TTATCGGCTTAACCACGAGTGATCCCCGGTCGTTTCCCCATTGAATCCCTGGTGCATCCTACTCCCAG
AATGATAGCTGACTGACTGGACTGGCTTTTCAAGTAATCGAGGGGGTATCGCGGTCACGGCCGTTAAC
AGATCCCGTCCTTAGTGTGGAATCCGCACCTGCTGACTAACGCTTCGCCGGCGTGTCTGCACAGCCGT

ATAGTGTTAATCATGACCCCAAGGAAGGATTAAACAAATATCTTGACG
>COHON12
GTGTCCGGTAGCCCGCGCTAGTTAGACACCCCGGCAGGGGGGATTGCTTTCGAGACGGGAGATCCCTT
CGCCGACCCTGGAGGGCCGACGCCGAGGCATTCGGGCCCCTGCAACGTCAACAGCGGCAAGAAAACGG
GATGAATGGGCGTAATGGGGGGGGTCTGCTGGGGACCCGACGCGGTTGCCGTTTGCGGGGCCCCCGAC
CCATACCGACCCACCTAGGCGTCCAGTTACGGCGCACGTCGGGAGCGTGGTTGCCGTCAGAGCTGTGT
TTCTCGATCAGTCCCCCGCAGTGCCGCAGTATCTTGCCGTGGGCTGCTTTAATCTTGAAAGTGGTTCA
TACATTGGGCGACGAGGTGTCGACTCTCATTGGGGGTAACCGACGGGCACATGCAGTCCCCTCCCCGG
GCAGGCAGAGGCGGGGCCCCCGCGCGCCGGCCCCGGCCCACAATCTTACCAGGGTCCTCAAAGGAGCC
TTTGCATGGTACCCTTCGTGAATGGTTGCTTAAGAGGTCCACAACGTGGTCCGGGCACGGTCGACTAA
ACACTCAAAACAGCGACGGCAAATATAGGTACAAGGTCCAGGCCCTCACGGCACTAATTGCGATGACC
CAACTCACGGGGGCAGTCCCCGGCGCGGCGACCTTGATTACGTCCGGGGACAAGTATGTCGCTCCCCC
CGGGAGGGGTGCAGCCACATGGGAGATTCAAAGTTTCTCGTGACGTCGTTGTGATCACTGCAGCCTAG
CCGAGACTCCCGTACTACGCGAAGGTTGGTTATGTTAACCACTACAACGTGAGGCGCCGTAAGGGCCA
GTGTTGTGCCCGGCTCTTCAATGCGCCTTAGCGGCCTGATACACCCACCCAAGGAGATACTGCTAATC
ATATGGGTGGACAGAACCTCGCAACGCAGACGCCTCCCCGGCATGATATGGTTTTTTTCCGCTATTAT
CCAGCACGCAGCGCTATCATTCAAGAGAACCCAGTGACGCGTAAATCGTAAGATCTACCTGCCGCAGG
TGGACCTACTGCAAATACGGATTATGACTCGTAAAGGGGTCATGCGTATTTTCATCACTAGGCACGTT
CGAGAATAAATTAGTAGGTGTCCCGAGCCTTGTGGCGTTCCGCCTGACTCCTCATGAAGTCGACCTTC
TCACCGGCCCTATCTGCCGACGTAAGTCATAACCTAGATCTGTACCTCGGGGGGAGGGTCACTGTAAA
GGGATAATTGGAGGGTGATTTCCCACACTTTCCTAAGGGTACTTTTTGCCTGGCTTCGCAATTGGGTC
CAATGGATGTCGATCTCTGGTTTAGCAGTTGTGAAAGTGGCAAGGCGGGAGGTTAGACCTCCATTTAA
CATATACAAGCAAGTTAACTGCACTAGATGTGTAGACACTACAGTTACAGGAGTAGCCGAATAAGTCT
CCGACGTCAAGCGAATAAGCGTCATACGCGATTATCGCCTAAGAGCACGTATTGGCGGTAAAAGGCTA
GCTAGACGCTTATGGGTAGATTTCAAGGCGTTCGTAGTGGTATAATAGGATACTCTTTCACCAGCCTG
AGGGCCGAACGCTATACTAGTGGTCTGTGATGTAGGACCGAGTATCTCTCTAGGGACCATCTACTTGA
GCAATGGTGCGCAGGGGGAGACATAGACCAGCCTTGGGTGGCAAGCACTGCAATAAGTCCTGTTTAGC
CTTGGAGTTCACATGCCGGCACTGAAGCCGACCTACCTGAGCGTGTGCGATTACCGTTACAATGGCAT
CTGTCTAGTTCTGTTTACCTACGGCGCTCTTGGTTCCAGGTTAGGGGAAGTGTATGACCCATGTGTTT
TTATCGGCTTAACCACGAGTGATCCCCGGTCGTTTCCCCATTGAATCCCTGGTGCATCCTACTCCCAG
AATGATAGCTGACTGACTGGACTGGCTTTTCAAGTAATCGAGGGGGTATCGCGGTCACGGCCGTTAAC
AGATCCCGTCCTTAGTGTGGAATCCGCACCTGCTGACTAACGCTTCGCCGGCGTGTCTGCACAGCCGT
ATAGTGTTAATCATGACCCCAAGGAAGGATTAAACAAATATCTTGACG
>COHON19
GTGTCCGGTAGCCCGCGCTAGTTAGACACCCCGGCAGGGGGGATTGCTTTCGAGACGGGAGATCCCTT
CGCCGACCCTGGAGGGCCGACGCCGAGGCATTCGGGCCCCTGCAACGTCAACAGCGGCAAGAAAACGG
GATGAATGGGCGTAATGGGGGGGGTCTGCTGGGGACCCGACGCGGTTGCCGTTTGCGGGGCCCCCGAC
CCATACCGACCCACCTAGGCGTCCAGTTACGGCGCACGGCGGGAGCGTGGTTGCCGTCAGAGCTGTGT
TTCTCGATCAGTCCCCCGCAGTGCCGCAGTATCTTGCCGTGGGCTGCTTTAATCTTGAAAGTGGTTTA
TACATTGGGCGACGAGGTGTCGACTCTCATTGGGGGTAACCGACGGGCACATGCAGTCCCCTCCCCGG
GCAGGCAGAGGCGGGGCCCCCGCGCGCCGGCCCCGGCCCACAATCTTACCAGGGTCCTCAAAGGAGCC
TTTGCATGGTACCCTTCGTGAATGGTTGCTTAAGAGGTCCACAACGTGGTCCGGGCACGGTCGACTAA
ACACTCAAAACAGCGACGGCAAATATAGGTACAAGGTCCAGGCCCTCACGGCACTAATTGCGATGACC
CAACTCACGGGGGCAGTCCCCGGCGCGGCGACCTTGATTACGTCCGGGGACAAGTATGTCGCTCCCCC
CGGGAGGGGTGCAGCCACATGGGAGATTCAAAGTTTCTCGTGACGTCGTTGTGATCACTGCAGCCTAG
CCGAGACTCCCGTACTACGCGAAGGTTGGTTATGTTAACCACTACAACGTGAGGCGCCGTAAGGGCCA
GTGTTGTGCCCGGCTCTTCAATGCGCCTTAGCGGCCTGATACACCCACCCAAGGAGATACTGCTAATC
ATATGGGTGGACAGAACCTCGCAACGCAGACGCCTCCCCGGCATGATATGGTTTTTTTCCGCTATTAT
CCAGCACGCAGCGCTATCATTCAAGAGAACCCAGTGACGCGTAAATCGTAAGATCTACCTGCCGCAGG
TGGACCTACTGCAAATACGGATTATGACTCGTAAAGGGGTCATGCGTATTTTCATCACTAGGCACGTT
CGAGAATAAATTAGTAGGTGTCCCGAGCCTTGTGGCGTTCCGCCTGACTCCTCATGAAGTCGACCTTC
TCACCGGCCCTATCTGCCGACGTAAGTCATAACCTAGATCTGTACCTCGGGGGGAGGGTCACTGTAAA
GGGATAATTGGAGGGTGATTTCCCACACTTTCCTAAGGGTACTTTTTGCCTGGCTTCGCAATTGGGTC
CAATGGATGTCGATCTCTGGTTTAGCAGTTGTGAAAGTGGCAAGGCGGGAGGTTAGACCTCCATTTAA

CATATACAAGCAAGTTAACTGCACTAGATGTGTAGACACTACAGTTACAGGAGTAGCCGAATAAGTCT
CCGACGTCAAGCGAATAAGCGTCATACGCGATTATCGCCTAAGAGCACGTATTGGCGGTAAAAGGCTA
GCTAGACGCTTATGGGTAGATTTCAAGGCGTTCGTAGTGGTATAATAGGATACTCTTTCACCAGCCTG
AGGGCCGAACGCTATACTAGTGGTCTGTGATGTAGGACCGAGTATCTCTCTAGGGACCATCTACTTGA
GCAATGGTGCGCAGGGGGAGACATAGACCAGCCTTGGGTGGCAAGCACTGCAATAAGTCCTGTTTAGC
CTTGGAGTTCACATGCCGGCACTGAAGCCGACCTACCTGAGCGTGTGCGATTACCGTTACAATGGCAT
CTGTCTAGTTCTGTTTACCTACGGCGCTCTTGGTTCCAGGTTAGGGGAAGTGTATGACCCATGTGTTT
TTATCGGCTTAACCACGAGTGATCCCCGGTCGTTTCCCCATTGAATCCCTGGTGCATCCTACTCCCAG
AATGATAGCTGACTGACTGGACTGGCTTTTCAAGTAATCGAGGGGGTATCGCGGTCACGGCCGTTAAC
AGATCCCGTCCTTAGTGTGGAATCCGCACCTGCTGACTAACGCTTCGCCGGCGTGTCTGCACAGCCGT
ATAGTGTTAATCATGACCCCAAGGAAGGATTAAACAAATATCTTGACG
>COHON23
GTGTCCGGTAGCCCGCGCTAGTTAGACACCCCGGCAGGGGGGATTGCTTTCGAGACGGGAGATCCCTT
CGCCGACCCTGGAGGGCCGACGCCGAGGCATTCGGGCCCCTGCAACGTCAACAGCGGCAAGAAAACGG
GATGAATGGGCGTAATGGGGGGGGTCTGCTGGGGACCCGACGCGGTTGCCGTTTGCGGGGCCCCCGAC
CCATACCGACCCACCTAGGCGTCCAGTTACGGCGCACGGCGGGAGCGTGGTTGCCGTCAGAGCTGTGT
TTCTCGATCAGTCCCCCGCAGTGCCGCAGTATCTTGCCGTGGGCTGCTTTAATCTTGAAAGTGGTTCA
TACATTGGGCGACGAGGTGTCGACTCTCATTGGGGGTAACCGACGGGCACATGCAGTCCCCTCCCCGG
GCAGGCAGAGGCGGGGCCCCCGCGCGCCGGCCCCGGCCCACAATCTTACCAGGGTCCTCAAAGGAGCC
TTTGCATGGTACCCTTCGTGAATGGTTGCTTAAGAGGTCCACAACGTGGTCCGGGCACGGTCGACTAA
ACACTCAAAACAGCGACGGCAAATATAGGTACAAGGTCCAGGCCCTCACGGCACTAATTGCGATGACC
CAACTCACGGGGGCAGTCCCCGGCGCGGCGACCTTGATTACGTCCGGGGACAAGTATGTCGCTCCCCC
CGGGAGGGGTGCAGCCACATGGGAGATTCAAAGTTTCTCGTGACGTCGTTGTGATCACTGCAGCCTAG
CCGAGACTCCCGTACTACGCGAAGGTTGGTTATGTTAACCACTACAACGTGAGGCGCCGTAAGGGCCA
GTGTTGTGCCCGGCTCTTCAATGCGCCTTAGCGGCCTGATACACCCACCCAAGGAGATACTGCTAATC
ATATGGGTGGACAGAACCTCGCAACGCAGACGCCTCCCCGGCATGATATGGTTTTTTTCCGCTATTAT
CCAGCACGCAGCGCTATCATTCAAGAGAACCCAGTGACGCGTAAATCGTAAGATCTACCTGCCGCAGG
TGGACCTACTGCAAATACGGATTATGACTCGTAAAGGGGTCATGCGTATTTTCATCACTAGGCACGTT
CGAGAATAAATTAGTAGGTGTCCCGAGCCTTGTGGCGTTCCGCCTGACTCCTCATGAAGTCGACCTTC
TCACCGGCCCTATCTGCCGACGTAAGTCATAACCTAGATCTGTACCTCGGGGGGAGGGTCACTGTAAA
GGGATAATTGGAGGGTGATTTCCCACACTTTCCTAAGGGTACTTTTTGCCTGGCTTCGCAATTGGGTC
CAATGGATGTCGATCTCTGGTTTAGCAGTTGTGAAAGTGGCAAGGCGGGAGGTTAGACCTCCATTTAA
CATATACAAGCAAGTTAACTGCACTAGATGTGTAGACACTACAGTTACAGGAGTAGCCGAATAAGTCT
CCGACGTCAAGCGAATAAGCGTCATACGCGATTATCGCCTAAGAGCACGTATTGGCGGTAAAAGGCTA
GCTAGACGCTTATGGGTAGATTTCAAGGCGTTCGTAGTGGTATAATAGGATACTCTTTCACCAGCCTG
AGGGCCGAACGCTATACTAGTGGTCTGTGATGTAGGACCGAGTATCTCTCTAGGGACCATCTACTTGA
GCAATGGTGCGCAGGGGGAGACATAGACCAGCCTTGGGTGGCAAGCACTGCAATAAGTCCTGTTTAGC
CTTGGAGTTCACATGCCGGCACTGAAGCCGACCTACCTGAGCGTGTGCGATTACCGTTACAATGGCAT
CTGTCTAGTTCTGTTTACCTACGGCGCTCTTGGTTCCAGGTTAGGGGAAGTGTATGACCCATGTGTTT
TTATCGGCTTAACCACGAGTGATCCCCGGTCGTTTCCCCATTGAATCCCTGGTGCATCCTACTCCCAG
AATGATAGCTGACTGACTGGACTGGCTTTTCAAGTAATCGAGGGGGTATCGCGGTCACGGCCGTTAAC
AGATCCCGTCCTTAGTGTGGAATCCGCACCTGCTGACTAACGCTTCGCCGGCGTGTCTGCACAGCCGT
ATAGTGTTAATCATGACCCCAAGGAAGGATTAAACAAATATCTTGACG
>COHON27
GTGTCCGGTAGCCCGCGCTAGTTAGACACCCCGGCAGGGGGGATTGCTTTCGAGACGGGAGATCCCTT
CGCCGACCCTGGAGGGCCGACGCCGAGGCATTCGGGCCCCCGCAACGTCAACAGCGGCAAGAAAACGG
GATGAATGGGCGTAATGGGGGGGGTCTGCTGGGGACCCGACGCGGTTGCCGTTTGCGGGGCCCCCGAC
CCATACCGACCCACCTAGGCGTCCAGTTACGGCGCACGGCGGGAGCGTGGTTGCCGTCAGAGCTGTGT
TTCTCGATCAGTCCCCCGCAGTGCCGCAGTATCTTGCCGTGGGCTGCTTTAATCTTGAAAGTGGTTTA
TACATTGGGCGACGAGGTGTCGACTCTCATTGGGGGTAACCGACGGGCACATGCAGTCCCCTCCCCGG
GCAGGCAGAGGCGGGGCCCCCGCGCGCCGGCCCCGGCCCACAATCTTACCAGGGTCCTCAAAGGAGCC
TTTGCATGGTACCCTTCGTGAATGGTTGCTTAAGAGGTCCACAACGTGGTCCGGGCACGGTCGACTAA
ACACTCAAAACAGCGACGGCAAATATAGGTACAAGGTCCAGGCCCTCACGGCACTAATTGCGATGACC
CAACTCACGGGGGCAGTCCCCGGCGCGGCGACCTTGATTACGTCCGGGGACAAGTATGTCGCTCCCCC

CGGGAGGGGTGCAGCCACATGGGAGATTCAAAGTTTCTCGTGACGTCGTTGTGATCACTGCAGCCTAG
CCGAGACTCCCGTACTACGCGAAGGTTGGTTATGTTAACCACTACAACGTGAGGCGCCGTAAGGGCCA
GTGTTGTGCCCGGCTCTTCAATGCGCCTTAGCGGCCTGATACACCCACCCAAGGAGATACTGCTAATC
ATATGGGTGGACAGAACCTCGCAACGCAGACGCCTCCCCGGCATGATATGGTTTTTTTCCGCTATTAT
CCAGCACGCAGCGCTATCATTCAAGAGAACCCAGTGACGCGTAAATCGTAAGATCTACCTGCCGCAGG
TGGACCTACTGCAAATACGGATTATGACTCGTAAAGGGGTCATGCGTATTTTCATCACTAGGCACGTT
CGAGAATAAATTAGTAGGTGTCCCGAGCCTTGTGGCGTTCCGCCTGACTCCTCATGAAGTCGACCTTC
TCACCGGCCCTATCTGCCGACGTAAGTCATAACCTAGATCTGTACCTCGGGGGGAGGGTCACTGTAAA
GGGATAATTGGAGGGTGATTTCCCACACTTTCCTAAGGGTACTTTTTGCCTGGCTTCGCAATTGGGTC
CAATGGATGTCGATCTCTGGTTTAGCAGTTGTGAAAGTGGCAAGGCGGGAGGTTAGACCTCCATTTAA
CATATACAAGCAAGTTAACTGCACTAGATGTGTAGACACTACAGTTACAGGAGTAGCCGAATAAGTCT
CCGACGTCAAGCGAATAAGCGTCATACGCGATTATCGCCTAAGAGCACGTATTGGCGGTAAAAGGCTA
GCTAGACGCTTATGGGTAGATTTCAAGGCGTTCGTAGTGGTATAATAGGATACTCTTTCACCAGCCTG
AGGGCCGAACGCTATACTAGTGGTCTGTGATGTAGGACCGAGTATCTCTCTAGGGACCATCTACTTGA
GCAATGGTGCGCAGGGGGAGACATAGACCAGCCTTGGGTGGCAAGCACTGCAATAAGTCCTGTTTAGC
CTTGGAGTTCACATGCCGGCACTGAAGCCGACCTACCTGAGCGTGTGCGATTACCGTTACAATGGCAT
CTGTCTAGTTCTGTTTACCTACGGCGCTCTTGGTTCCAGGTTAGGGGAAGTGTATGACCCATGTGTTT
TTATCGGCTTAACCACGAGTGATCCCCGGTCGTTTCCCCATTGAATCCCTGGTGCATCCTACTCCCAG
AATGATAGCTGACTGACTGGACTGGCTTTTCAAGTAATCGAGGGGGTATCGCGGTCACGGCCGTTAAC
AGATCCCGTCCTTAGTGTGGAATCCGCACCTGCTGACTAACGCTTCGCCGGCGTGTCTGCACAGCCGT
ATAGTGTTAATCATGACCCCAAGGAAGGATTAAACAAATATCTTGACG
>COHON29
GTGTCCGGTAGCCCGCGCTAGTTAGACACCCCGGCAGGGGGGATTGCTTTCGAGACGGGAGATCCCTT
CGCCGACCCTGGAGGGCCGACGCCGAGGCATTCGGGCCCCTGCAACGTCAACAGCGGCAAGAAAACGG
GATGAATGGGCGTAATGGGGGGGGTCTGCTGGGGACCCGACGCGGTTGCCGTTTGCGGGGCCCCCGAC
CCATACCGACCCACCTAGGCGTCCAGTTACGGCGCACGGCGGGAGCGTGGTTGCCGTCAGAGCTGTGT
TTCTCGATCAGTCCCCCGCAGTGCCGCAGTATCTTGCCGTGGGCTGCTTTAATCTTGAAAGTGGTTTA
TACATTGGGCGACGAGGTGTCGACTCTCATTGGGGGTAACCGACGGGCACATGCAGTCCCCTCCCCGG
GCAGGCAGAGGCGGGGCCCCCGCGCGCCGGCCCCGGCCCACAATCTTACCAGGGTCCTCAAAGGAGCC
TTTGCATGGTACCCTTCGTGAATGGTTGCTTAAGAGGTCCACAACGTGGTCCGGGCACGGTCGACTAA
ACACTCAAAACAGCGACGGCAAATATAGGTACAAGGTCCAGGCCCTCACGGCACTAATTGCGATGACC
CAACTCACGGGGGCAGTCCCCGGCGCGGCGACCTTGATTACGTCCGGGGACAAGTATGTCGCTCCCCC
CGGGAGGGGTGCAGCCACATGGGAGATTCAAAGTTTCTCGTGACGTCGTTGTGATCACTGCAGCCTAG
CCGAGACTCCCGTACTACGCGAAGGTTGGTTATGTTAACCACTACAACGTGAGGCGCCGTAAGGGCCA
GTGTTGTGCCCGGCTCTTCAATGCGCCTTAGCGGCCTGATACACCCACCCAAGGAGATACTGCTAATC
ATATGGGTGGACAGAACCTCGCAACGCAGACGCCTCCCCGGCATGATATGGTTTTTTTCCGCTATTAT
CCAGCACGCAGCGCTATCATTCAAGAGAACCCAGTGACGCGTAAATCGTAAGATCTACCTGCCGCAGG
TGGACCTACTGCAAATACGGATTATGACTCGTAAAGGGGTCATGCGTATTTTCATCACTAGGCACGTT
CGAGAATAAATTAGTAGGTGTCCCGAGCCTTGTGGCGTTCCGCCTGACTCCTCATGAAGTCGACCTTC
TCACCGGCCCTATCTGCCGACGTAAGTCATAACCTAGATCTGTACCTCGGGGGGAGGGTCACTGTAAA
GGGATAATTGGAGGGTGATTTCCCACACTTTCCTAAGGGTACTTTTTGCCTGGCTTCGCAATTGGGTC
CAATGGATGTCGATCTCTGGTTTAGCAGTTGTGAAAGTGGCAAGGCGGGAGGTTAGACCTCCATTTAA
CATATACAAGCAAGTTAACTGCACTAGATGTGTAGACACTACAGTTACAGGAGTAGCCGAATAAGTCT
CCGACGTCAAGCGAATAAGCGTCATACGCGATTATCGCCTAAGAGCACGTATTGGCGGTAAAAGGCTA
GCTAGACGCTTATGGGTAGATTTCAAGGCGTTCGTAGTGGTATAATAGGATACTCTTTCACCAGCCTG
AGGGCCGAACGCTATACTAGTGGTCTGTGATGTAGGACCGAGTATCTCTCTAGGGACCATCTACTTGA
GCAATGGTGCGCAGGGGGAGACATAGACCAGCCTTGGGTGGCAAGCACTGCAATAAGTCCTGTTTAGC
CTTGGAGTTCACATGCCGGCACTGAAGCCGACCTACCTGAGCGTGTGCGATTACCGTTACAATGGCAT
CTGTCTAGTTCTGTTTACCTACGGCGCTCTTGGTTCCAGGTTAGGGGAAGTGTATGACCCATGTGTTT
TTATCGGCTTAACCACGAGTGATCCCCGGTCGTTTCCCCATTGAATCCCTGGTGCATCCTACTCCCAG
AATGATAGCTGACTGACTGGACTGGCTTTTCAAGTAATCGAGGGGGTATCGCGGTCACGGCCGTTAAC
AGATCCCGTCCTTAGTGTGGAATCCGCACCTGCTGACTAACGCTTCGCCGGCGTGTCTGCACAGCCGT
ATAGTGTTAATCATGACCCCAAGGAAGGATTAAACAAATATCTTGACG
>COHON32

GTGTCCGGTAGCCCGCGCTAGTTAGACACCCCGGCAGGGGGGATTGCTTTCGAGACGGGAGATCCCTT
CGCCGACCCTGGAGGGCCGACGCCGAGGCATTCGGGCCCCTGCAACGTCAACAGCGGCAAGAAAACGG
GATGAATGGGCGTAATGGGGGGGGTCTGCTGGGGACCCGACGCGGTTGCCGTTTGCGGGGCCCCCGAC
CCATACCGACCCACCTAGGCGTCCAGTTACGGCGCACGGCGGGAGCGTGGTTGCCGTCAGAGCTGTGT
TTCTCGATCAGTCCCCCGCAGTGCCGCAGTATCTTGCCGTGGGCTGCTTTAATCTTGAAAGTGGTTTA
TACATTGGGCGACGAGGTGTCGACTCTCATTGGGGGTAACCGACGGGCACATGCAGTCCCCTCCCCGG
GCAGGCAGAGGCGGGGCCCCCGCGCGCCGGCCCCGGCCCACAATCTTACCAGGGTCCTCAAAGGAGCC
TTTGCATGGTACCCTTCGTGAATGGTTGCTTAAGAGGTCCACAACGTGGTCCGGGCACGGTCGACTAA
ACACTCAAAACAGCGACGGCAAATATAGGTACAAGGTCCAGGCCCTCACGGCACTAATTGCGATGACC
CAACTCACGGGGGCAGTCCCCGGCGCGGCGACCTTGATTACGTCCGGGGACAAGTATGTCGCTCCCCC
CGGGAGGGGTGCAGCCACATGGGAGATTCAAAGTTTCTCGTGACGTCGTTGTGATCACTGCAGCCTAG
CCGAGACTCCCGTACTACGCGAAGGTTGGTTATGTTAACCACTACAACGTGAGGCGCCGTAAGGGCCA
GTGTTGTGCCCGGCTCTTCAATGCGCCTTAGCGGCCTGATACACCCACCCAAGGAGATACTGCTAATC
ATATGGGTGGACAGAACCTCGCAACGCAGACGCCTCCCCGGCATGATATGGTTTTTTTCCGCTATTAT
CCAGCACGCAGCGCTATCATTCAAGAGAACCCAGTGACGCGTAAATCGTAAGATCTACCTGCCGCAGG
TGGACCTACTGCAAATACGGATTATGACTCGTAAAGGGGTCATGCGTATTTTCATCACTAGGCACGTT
CGAGAATAAATTAGTAGGTGTCCCGAGCCTTGTGGCGTTCCGCCTGACTCCTCATGAAGTCGACCTTC
TCACCGGCCCTATCTGCCGACGTAAGTCATAACCTAGATCTGTACCTCGGGGGGAGGGTCACTGTAAA
GGGATAATTGGAGGGTGATTTCCCACACTTTCCTAAGGGTACTTTTTGCCTGGCTTCGCAATTGGGTC
CAATGGATGTCGATCTCTGGTTTAGCAGTTGTGAAAGTGGCAAGGCGGGAGGTTNGACCTCCNTTTAA
CATATACAAGCAAGTTAACNGCACTAGATGTGTAGACACTACAGTTACAGGAGTAGCCGAATAAGTCT
CCGACGTCAAGCGAATAAGCGTCATACGCGATTATCGCCTAAGAGCACGTATTGGCGGTAAAAGGCTA
GCTAGACGCTTATGGGTAGATTTCAAGGCGTTCGTAGTGGTATAATAGGATACTCTTTCACCAGCCTG
AGGGCCGAACGCTATACTAGTGGTCTGTGATGTAGGACCGAGTATCTCTCTAGGGACCATCTACTTGA
GCAATGGTGCGCAGGGGGAGACATAGACCAGCCTTGGGTGGCAAGCACTGCAATAAGTCCTGTTTAGC
CTTGGAGTTCACATGCCGGCACTGAAGCCGACCTACCTGAGCGTGTGCGATTACCGTTACAATGGCAT
CTGTCTAGTTCTGTTTACCTACGGCGCTCTTGGTTCCAGGTTAGGGGAAGTGTATGACCCATGTGTTT
TTATCGGCTTAACCACGAGTGATCCCCGGTCGTTTCCCCATTGAATCCCTGGTGCATCCTACTCCCAG
AATGATAGCTGACTGACTGGACTGGCTTTTCAAGTAATCGAGGGGGTATCGCGGTCACGGCCGTTAAC
AGATCCCGTCCTTAGTGTGGAATCCGCACCTGCTGACTAACGCTTCGCCGGCGTGTCTGCACAGCCGT
ATAGTGTTAATCATGACCCCAAGGAAGGATTAAACAAATATCTTGACG
>COHON33
GTGTCCGGTAGCCCGCGCTAGTTAGACACCCCGGCAGGGGGGATTGCTTTCGGGACGGGAGATCCCTT
CGCCGACCCTGGAGGGCCGACGCCGAGGCATTCGGGCCCCCGCAACGTCAACAGCGGCAAGAAAACGG
GATGAATGGGCGTAATGGGGGGGGTCTGCTGGGGACCCGACGCGGTTGCCGTTTGCGGGGCCCCCGAC
CCATACCGACCCACCTAGGCGTCCAGTTACGGCGCACGTCGGGAGCGTGGTTGCCGTCAGAGCTGTGT
TTCTCGATCAGTCCCCCGCAGTGCCGCAGTATCTTGCCGTGGGCTGCTTTAATCTTGAAAGTGGTTTA
TACATTGGGCGACGAGGTGTCGACTCTCATTGGGGGTAACCGACGGGCACATGCAGTCCCCTCCCCGG
GCAGGCAGAGGCGGGGCCCCCGCGCGCCGGCCCCGGCCCACAATCTTACCAGGGTCCTCAAAGGAGCC
TTTGCATGGTACCCTTCGTGAATGGTTGCTTAAGAGGTCCACAACGTGGTCCGGGCACGGTCGACTAA
ACACTCAAAACAGCGACGGCAAATATAGGTACAAGGTCCAGGCCCTCACGGCACTAATTGCGATGACC
CAACTCACGGGGGCAGTCCCCGGCGCGGCGACCTTGATTACGTCCGGGGACAAGTATGTCGCTCCCCC
CGGGAGGGGTGCAGCCACATGGGAGATTCAAAGTTTCTCGTGACGTCGTTGTGATCACTGCAGCCTAG
CCGAGACTCCCGTACTACGCGAAGGTTGGTTATGTTAACCACTACAACGTGAGGCGCCGTAAGGGCCA
GTGTTGTGCCCGGCTCTTCAATGCGCCTTAGCGGCCTGATACACCCACCCAAGGAGATACTGCTAATC
ATATGGGTGGACAGAACCTCGCAACGCAGACGCCTCCCCGGCATGATATGGTTTTTTTCCGCTATTAT
CCAGCACGCAGCGCTATCATTCAAGAGAACCCAGTGACGCGTAAATCGTAAGATCTACCTGCCGCAGG
TGGACCTACTGCAAATACGGATTATGACTCGTAAAGGGGTCATGCGTATTTTCATCACTAGGCACGTT
CGAGAATAAATTAGTAGGTGTCCCGAGCCTTGTGGCGTTCCGCCTGACTCCTCATGAAGTCGACCTTC
TCACCGGCCCTATCTGCCGACGTAAGTCATAACCTAGATCTGTACCTCGGGGGGAGGGTCACTGTAAA
GGGATAATTGGAGGGTGATTTCCCACACTTTCCTAAGGGTACTTTTTGCCTGGCTTCGCAATTGGGTC
CAATGGATGTCGATCTCTGGTTTAGCAGTTGTGAAAGTGGCAAGGCGGGAGGTTAGACCTCCATTTAA
CATATACAAGCAAGTTAACTGCACTAGATGTGTAGACACTACAGTTACAGGAGTAGCCGAATAAGTCT
CCGACGTCAAGCGAATAAGCGTCATACGCGATTATCGCCTAAGAGCACGTATTGGCGGTAAAAGGCTA

GCTAGACGCTTATGGGTAGATTTCAAGGCGTTCGTAGTGGTATAATAGGATACTCTTTCACCAGCCTG
AGGGCCGAACGCTATACTAGTGGTCTGTGATGTAGGACCGAGTATCTCTCTAGGGACCATCTACTTGA
GCAATGGTGCGCAGGGGGAGACATAGACCAGCCTTGGGTGGCAAGCACTGCAATAAGTCCTGTTTAGC
CTTGGAGTTCACATGCCGGCACTGAAGCCGACCTACCTGAGCGTGTGCGATTACCGTTACAATGGCAT
CTGTCTAGTTCTGTTTACCTACGGCGCTCTTGGTTCCAGGTTAGGGGAAGTGTATGACCCATGTGTTT
TTATCGGCTTAACCACGAGTGATCCCCGGTCGTTTCCCCATTGAATCCCTGGTGCATCCTACTCCCAG
AATGATAGCTGACTGACTGGACTGGCTTTTCAAGTAATCGAGGGGGTATCGCGGTCACGGCCGTTAAC
AGATCCCGTCCTTAGTGTGGAATCCGCACCTGCTGACTAACGCTTCGCCGGCGTGTCTGCACAGCCGT
ATAGTGTTAATCATGACCCCAAGGAAGGATTAAACAAATATCTTGACG
>COHON34
GTGTCCGGTAGCCCGCGCTAGTTAGACACCCCGGCAGGGGGGATTGCTTTCGAGACGGGAGATCCCTT
CGCCGACCCTGGAGGGCCGACGCCGAGGCATTCGGGCCCCTGCAACGTCAACAGCGGCAAGAAAACGG
GATGAATGGGCGTAATGGGGGGGGTCTGCTGGGGACCCGACGCGGTTGCCGTTTGCGGGGCCCCCGAC
CCATACCGACCCACCTAGGCGTCCAGTTACGGCGCACGTCGGGAGCGTGGTTGCCGTCAGAGCTGTGT
TTCTCGATCAGTCCCCCGCAGTGCCGCAGTATCTTGCCGTGGGCTGCTTTAATCTTGAAAGTGGTTTA
TACATTGGGCGACGAGGTGTCGACTCTCATTGGGGGTAACCGACGGGCACATGCAGTCCCCTCCCCGG
GCAGGCAGAGGCGGGGCCCCCGCGCGCCGGCCCCGGCCCACAATCTTACCAGGGTCCTCAAAGGAGCC
TTTGCATGGTACCCTTCGTGAATGGTTGCTTAAGAGGTCCACAACGTGGTCCGGGCACGGTCGACTAA
ACACTCAAAACAGCGACGGCAAATATAGGTACAAGGTCCAGGCCCTCACGGCACTAATTGCGATGACC
CAACTCACGGGGGCAGTCCCCGGCGCGGCGACCTTGATTACGTCCGGGGACAAGTATGTCGCTCCCCC
CGGGAGGGGTGCAGCCACATGGGAGATTCAAAGTTTCTCGTGACGTCGTTGTGATCACTGCAGCCTAG
CCGAGACTCCCGTACTACGCGAAGGTTGGTTATGTTAACCACTACAACGTGAGGCGCCGTAAGGGCCA
GTGTTGTGCCCGGCTCTTCAATGCGCCTTAGCGGCCTGATACACCCACCCAAGGAGATACTGCTAATC
ATATGGGTGGACAGAACCTCGCAACGCAGACGCCTCCCCGGCATGATATGGTTTTTTTCCGCTATTAT
CCAGCACGCAGCGCTATCATTCAAGAGAACCCAGTGACGCGTAAATCGTAAGATCTACCTGCCGCAGG
TGGACCTACTGCAAATACGGATTATGACTCGTAAAGGGGTCATGCGTATTTTCATCACTAGGCACGTT
CGAGAATAAATTAGTAGGTGTCCCGAGCCTTGTGGCGTTCCGCCTGACTCCTCATGAAGTCGACCTTC
TCACCGGCCCTATCTGCCGACGTAAGTCATAACCTAGATCTGTACCTCGGGGGGAGGGTCACTGTAAA
GGGATAATTGGAGGGTGATTTCCCACACTTTCCTAAGGGTACTTTTTGCCTGGCTTCGCAATTGGGTC
CAATGGATGTCGATCTCTGGTTTAGCAGTTGTGAAAGTGGCAAGGCGGGAGGTTAGACCTCCATTTAA
CATATACAAGCAAGTTAACTGCACTAGATGTGTAGACACTACAGTTACAGGAGTAGCCGAATAAGTCT
CCGACGTCAAGCGAATAAGCGTCATACGCGATTATCGCCTAAGAGCACGTATTGGCGGTAAAAGGCTA
GCTAGACGCTTATGGGTAGATTTCAAGGCGTTCGTAGTGGTATAATAGGATACTCTTTCACCAGCCTG
AGGGCCGAACGCTATACTAGTGGTCTGTGATGTAGGACCGAGTATCTCTCTAGGGACCATCTACTTGA
GCAATGGTGCGCAGGGGGAGACATAGACCAGCCTTGGGTGGCAAGCACTGCAATAAGTCCTGTTTAGC
CTTGGAGTTCACATGCCGGCACTGAAGCCGACCTACCTGAGCGTGTGCGATTACCGTTACAATGGCAT
CTGTCTAGTTCTGTTTACCTACGGCGCTCTTGGTTCCAGGTTAGGGGAAGTGTATGACCCATGTGTTT
TTATCGGCTTAACCACGAGTGATCCCCGGTCGTTTCCCCATTGAATCCCTGGTGCATCCTACTCCCAG
AATGATAGCTGACTGACTGGACTGGCTTTTCAAGTAATCGAGGGGGTATCGCGGTCACGGCCGTTAAC
AGATCCCGTCCTTAGTGTGGAATCCGCACCTGCTGACTAACGCTTCGCCGGCGTGTCTGCACAGCCGT
ATAGTGTTAATCATGACCCCAAGGAAGGATTAAACAAATATCTTGACG
>COHON36
GTGTCCGGTAGCCCGCGCTAGTTAGACACCCCGGCAGGGGGGATTGCTTTCGAGACGGGAGATCCCTT
CGCCGACCCTGGAGGGCCGACGCCGAGGCATTCGGGCCCCTGCAACGTCAACAGCGGCAAGAAAACGG
GATGAATGGGCGTAATGGGGGGGGTCTGCTGGGGACCCGACGCGGTTGCCGTTTGCGGGGCCCCCGAC
CCATACCGACCCACCTAGGCGTCCAGTTACGGCGCACGTCGGGAGCGTGGTTGCCGTCAGAGCTGTGT
TTCTCGATCAGTCCCCCGCAGTGCCGCAGTATCTTGCCGTGGGCTGCTTTAATCTTGAAAGTGGTTCA
TACATTGGGCGACGAGGTGTCGACTCTCATTGGGGGTAACCGACGGGCACATGCAGTCCCCTCCCCGG
GCAGGCAGAGGCGGGGCCCCCGCGCGCCGGCCCCGGCCCACAATCTTACCAGGGTCCTCAAAGGAGCC
TTTGCATGGTACCCTTCGTGAATGGTTGCTTAAGAGGTCCACAACGTGGTCCGGGCACGGTCGACTAA
ACACTCAAAACAGCGACGGCAAATATAGGTACAAGGTCCAGGCCCTCACGGCACTAATTGCGATGACC
CAACTCACGGGGGCAGTCCCCGGCGCGGCGACCTTGATTACGTCCGGGGACAAGTATGTCGCTCCCCC
CGGGAGGGGTGCAGCCACATGGGAGATTCAAAGTTTCTCGTGACGTCGTTGTGATCACTGCAGCCTAG
CCGAGACTCCCGTACTACGCGAAGGTTGGTTATGTTAACCACTACAACGTGAGGCGCCGTAAGGGCCA

GTGTTGTGCCCGGCTCTTCAATGCGCCTTAGCGGCCTGATACACCCACCCAAGGAGATACTGCTAATC
ATATGGGTGGACAGAACCTCGCAACGCAGACGCCTCCCCGGCATGATATGGTTTTTTTCCGCTATTAT
CCAGCACGCAGCGCTATCATTCAAGAGAACCCAGTGACGCGTAAATCGTAAGATCTACCTGCCGCAGG
TGGACCTACTGCAAATACGGATTATGACTCGTAAAGGGGTCATGCGTATTTTCATCACTAGGCACGTT
CGAGAATAAATTAGTAGGTGTCCCGAGCCTTGTGGCGTTCCGCCTGACTCCTCATGAAGTCGACCTTC
TCACCGGCCCTATCTGCCGACGTAAGTCATAACCTAGATCTGTACCTCGGGGGGAGGGTCACTGTAAA
GGGATAATTGGAGGGTGATTTCCCACACTTTCCTAAGGGTACTTTTTGCCTGGCTTCGCAATTGGGTC
CAATGGATGTCGATCTCTGGTTTAGCAGTTGTGAAAGTGGCAAGGCGGGAGGTTAGACCTCCATTTAA
CATATACAAGCAAGTTAACTGCACTAGATGTGTAGACACTACAGTTACAGGAGTAGCCGAATAAGTCT
CCGACGTCAAGCGAATAAGCGTCATACGCGATTATCGCCTAAGAGCACGTATTGGCGGTAAAAGGCTA
GCTAGACGCTTATGGGTAGATTTCAAGGCGTTCGTAGTGGTATAATAGGATACTCTTTCACCAGCCTG
AGGGCCGAACGCTATACTAGTGGTCTGTGATGTAGGACCGAGTATCTCTCTAGGGACCATCTACTTGA
GCAATGGTGCGCAGGGGGAGACATAGACCAGCCTTGGGTGGCAAGCACTGCAATAAGTCCTGTTTAGC
CTTGGAGTTCACATGCCGGCACTGAAGCCGACCTACCTGAGCGTGTGCGATTACCGTTACAATGGCAT
CTGTCTAGTTCTGTTTACCTACGGCGCTCTTGGTTCCAGGTTAGGGGAAGTGTATGACCCATGTGTTT
TTATCGGCTTAACCACGAGTGATCCCCGGTCGTTTCCCCATTGAATCCCTGGTGCATCCTACTCCCAG
AATGATAGCTGACTGACTGGACTGGCTTTTCAAGTAATCGAGGGGGTATCGCGGTCACGGCCGTTAAC
AGATCCCGTCCTTAGTGTGGAATCCGCACCTGCTGACTAACGCTTCGCCGGCGTGTCTGCACAGCCGT
ATAGTGTTAATCATGACCCCAAGGAAGGATTAAACAAATATCTTGACG
>COHON37
GTGTCCGGTAGCCCGCGCTAGTTAGACACCCCGGCAGGGGGGATTGCTTTCGAGACGGGAGATCCCTT
CGCCGACCCTGGAGGGCCGACGCCGAGGCATTCGGGCCCCTGCAACGTCAACAGCGGCAAGAAAACGG
GATGAATGGGCGTAATGGGGGGGGTCTGCTGGGGACCCGACGCGGTTGCCGTTTGCGGGGCCCCCGAC
CCATACCGACCCACCTAGGCGTCCAGTTACGGCGCACGTCGGGAGCGTGGTTGCCGTCAGAGCTGTGT
TTCTCGATCAGTCCCCCGCAGTGCCGCAGTATCTTGCCGTGGGCTGCTTTAATCTTGAAAGTGGTTCA
TACATTGGGCGACGAGGTGTCGACTCTCATTGGGGGTAACCGACGGGCACATGCAGTCCCCTCCCCGG
GCAGGCAGAGGCGGGGCCCCCGCGCGCCGGCCCCGGCCCACAATCTTACCAGGGTCCTCAAAGGAGCC
TTTGCATGGTACCCTTCGTGAATGGTTGCTTAAGAGGTCCACAACGTGGTCCGGGCACGGTCGACTAA
ACACTCAAAACAGCGACGGCAAATATAGGTACAAGGTCCAGGCCCTCACGGCACTAATTGCGATGACC
CAACTCACGGGGGCAGTCCCCGGCGCGGCGACCTTGATTACGTCCGGGGACAAGTATGTCGCTCCCCC
CGGGAGGGGTGCAGCCACATGGGAGATTCAAAGTTTCTCGTGACGTCGTTGTGATCACTGCAGCCTAG
CCGAGACTCCCGTACTACGCGAAGGTTGGTTATGTTAACCACTACAACGTGAGGCGCCGTAAGGGCCA
GTGTTGTGCCCGGCTCTTCAATGCGCCTTAGCGGCCTGATACACCCACCCAAGGAGATACTGCTAATC
ATATGGGTGGACAGAACCTCGCAACGCAGACGCCTCCCCGGCATGATATGGTTTTTTTCCGCTATTAT
CCAGCACGCAGCGCTATCATTCAAGAGAACCCAGTGACGCGTAAATCGTAAGATCTACCTGCCGCAGG
TGGACCTACTGCAAATACGGATTATGACTCGTAAAGGGGTCATGCGTATTTTCATCACTAGGCACGTT
CGAGAATAAATTAGTAGGTGTCCCGAGCCTTGTGGCGTTCCGCCTGACTCCTCATGAAGTCGACCTTC
TCACCGGCCCTATCTGCCGACGTAAGTCATAACCTAGATCTGTACCTCGGGGGGAGGGTCACTGTAAA
GGGATAATTGGAGGGTGATTTCCCACACTTTCCTAAGGGTACTTTTTGCCTGGCTTCGCAATTGGGTC
CAATGGATGTCGATCTCTGGTTTAGCAGTTGTGAAAGTGGCAAGGCGGGAGGTTAGACCTCCATTTAA
CATATACAAGCAAGTTAACTGCACTAGATGTGTAGACACTACAGTTACAGGAGTAGCCGAATAAGTCT
CCGACGTCAAGCGAATAAGCGTCATACGCGATTATCGCCTAAGAGCACGTATTGGCGGTAAAAGGCTA
GCTAGACGCTTATGGGTAGATTTCAAGGCGTTCGTAGTGGTATAATAGGATACTCTTTCACCAGCCTG
AGGGCCGAACGCTATACTAGTGGTCTGTGATGTAGGACCGAGTATCTCTCTAGGGACCATCTACTTGA
GCAATGGTGCGCAGGGGGAGACATAGACCAGCCTTGGGTGGCAAGCACTGCAATAAGTCCTGTTTAGC
CTTGGAGTTCACATGCCGGCACTGAAGCCGACCTACCTGAGCGTGTGCGATTACCGTTACAATGGCAT
CTGTCTAGTTCTGTTTACCTACGGCGCTCTTGGTTCCAGGTTAGGGGAAGTGTATGACCCATGTGTTT
TTATCGGCTTAACCACGAGTGATCCCCGGTCGTTTCCCCATTGAATCCCTGGTGCATCCTACTCCCAG
AATGATAGCTGACTGACTGGACTGGCTTTTCAAGTAATCGAGGGGGTATCGCGGTCACGGCCGTTAAC
AGATCCCGTCCTTAGTGTGGAATCCGCACCTGCTGACTAACGCTTCGCCGGCGTGTCTGCACAGCCGT
ATAGTGTTAATCATGACCCCAAGGAAGGATTAAACAAATATCTTGACG
>COHON38
GTGTCCGGTAGCCCGCGCTAGTTAGACACCCCGGCAGGGGGGATTGCTTTCGGGACGGGAGATCCCTT
CGCCGACCCTGGAGGGCCGACGCCGAGGCATTCGGGCCCCCGCAACGTCAACAGCGGCAAGAAAACGG

GATGAATGGGCGTAATGGGGGGGGTCTGCTGGGGACCCGACGCGGTTGCCGTTTGCGGGGCCCCCGAC
CCATACCGACCCACCTAGGCGTCCAGTTACGGCGCACGTCGGGAGCGTGGTTGCCGTCAGAGCTGTGT
TTCTCGATCAGTCCCCCGCAGTGCCGCAGTATCTTGCCGTGGGCTGCTTTAATCTTGAAAGTGGTTTA
TACATTGGGCGACGAGGTGTCGACTCTCATTGGGGGTAACCGACGGGCACATGCAGTCCCCTCCCCGG
GCAGGCAGAGGCGGGGCCCCCGCGCGCCGGCCCCGGCCCACAATCTTACCAGGGTCCTCAAAGGAGCC
TTTGCATGGTACCCTTCGTGAATGGTTGCTTAAGAGGTCCACAACGTGGTCCGGGCACGGTCGACTAA
ACACTCAAAACAGCGACGGCAAATATAGGTACAAGGTCCAGGCCCTCACGGCACTAATTGCGATGACC
CAACTCACGGGGGCAGTCCCCGGCGCGGCGACCTTGATTACGTCCGGGGACAAGTATGTCGCTCCCCC
CGGGAGGGGTGCAGCCACATGGGAGATTCAAAGTTTCTCGTGACGTCGTTGTGATCACTGCAGCCTAG
CCGAGACTCCCGTACTACGCGAAGGTTGGTTATGTTAACCACTACAACGTGAGGCGCCGTAAGGGCCA
GTGTTGTGCCCGGCTCTTCAATGCGCCTTAGCGGCCTGATACACCCACCCAAGGAGATACTGCTAATC
ATATGGGTGGACAGAACCTCGCAACGCAGACGCCTCCCCGGCATGATATGGTTTTTTTCCGCTATTAT
CCAGCACGCAGCGCTATCATTCAAGAGAACCCAGTGACGCGTAAATCGTAAGATCTACCTGCCGCAGG
TGGACCTACTGCAAATACGGATTATGACTCGTAAAGGGGTCATGCGTATTTTCATCACTAGGCACGTT
CGAGAATAAATTAGTAGGTGTCCCGAGCCTTGTGGCGTTCCGCCTGACTCCTCATGAAGTCGACCTTC
TCACCGGCCCTATCTGCCGACGTAAGTCATAACCTAGATCTGTACCTCGGGGGGAGGGTCACTGTAAA
GGGATAATTGGAGGGTGATTTCCCACACTTTCCTAAGGGTACTTTTTGCCTGGCTTCGCAATTGGGTC
CAATGGATGTCGATCTCTGGTTTAGCAGTTGTGAAAGTGGCAAGGCGGGAGGTTAGACCTCCATTTAA
CATATACAAGCAAGTTAACTGCACTAGATGTGTAGACACTACAGTTACAGGAGTAGCCGAATAAGTCT
CCGACGTCAAGCGAATAAGCGTCATACGCGATTATCGCCTAAGAGCACGTATTGGCGGTAAAAGGCTA
GCTAGACGCTTATGGGTAGATTTCAAGGCGTTCGTAGTGGTATAATAGGATACTCTTTCACCAGCCTG
AGGGCCGAACGCTATACTAGTGGTCTGTGATGTAGGACCGAGTATCTCTCTAGGGACCATCTACTTGA
GCAATGGTGCGCAGGGGGAGACATAGACCAGCCTTGGGTGGCAAGCACTGCAATAAGTCCTGTTTAGC
CTTGGAGTTCACATGCCGGCACTGAAGCCGACCTACCTGAGCGTGTGCGATTACCGTTACAATGGCAT
CTGTCTAGTTCTGTTTACCTACGGCGCTCTTGGTTCCAGGTTAGGGGAAGTGTATGACCCATGTGTTT
TTATCGGCTTAACCACGAGTGATCCCCGGTCGTTTCCCCATTGAATCCCTGGTGCATCCTACTCCCAG
AATGATAGCTGACTGACTGGACTGGCTTTTCAAGTAATCGAGGGGGTATCGCGGTCACGGCCGTTAAC
AGATCCCGTCCTTAGTGTGGAATCCGCACCTGCTGACTAACGCTTCGCCGGCGTGTCTGCACAGCCGT
ATAGTGTTAATCATGACCCCAAGGAAGGATTAAACAAATATCTTGACG
>COHON39
GTGTCCGGTAGCCCGCGCTAGTTAGACACCCCGGCAGGGGGGATTGCTTTCGAGACGGGAGATCCCTT
CGCCGACCCTGGAGGGCCGACGCCGAGGCATTCGGGCCCCTGCAACGTCAACAGCGGCAAGAAAACGG
GATGAATGGGCGTAATGGGGGGGGTCTGCTGGGGACCCGACGCGGTTGCCGTTTGCGGGGCCCCCGAC
CCATACCGACCCACCTAGGCGTCCAGTTACGGCGCACGGCGGGAGCGTGGTTGCCGTCAGAGCTGTGT
TTCTCGATCAGTCCCCCGCAGTGCCGCAGTATCTTGCCGTGGGCTGCTTTAATCTTGAAAGTGGTTTA
TACATTGGGCGACGAGGTGTCGACTCTCATTGGGGGTAACCGACGGGCACATGCAGTCCCCTCCCCGG
GCAGGCAGAGGCGGGGCCCCCGCGCGCCGGCCCCGGCCCACAATCTTACCAGGGTCCTCAAAGGAGCC
TTTGCATGGTACCCTTCGTGAATGGTTGCTTAAGAGGTCCACAACGTGGTCCGGGCACGGTCGACTAA
ACACTCAAAACAGCGACGGCAAATATAGGTACAAGGTCCAGGCCCTCACGGCACTAATTGCGATGACC
CAACTCACGGGGGCAGTCCCCGGCGCGGCGACCTTGATTACGTCCGGGGACAAGTATGTCGCTCCCCC
CGGGAGGGGTGCAGCCACATGGGAGATTCAAAGTTTCTCGTGACGTCGTTGTGATCACTGCAGCCTAG
CCGAGACTCCCGTACTACGCGAAGGTTGGTTATGTTAACCACTACAACGTGAGGCGCCGTAAGGGCCA
GTGTTGTGCCCGGCTCTTCAATGCGCCTTAGCGGCCTGATACACCCACCCAAGGAGATACTGCTAATC
ATATGGGTGGACAGAACCTCGCAACGCAGACGCCTCCCCGGCATGATATGGTTTTTTTCCGCTATTAT
CCAGCACGCAGCGCTATCATTCAAGAGAACCCAGTGACGCGTAAATCGTAAGATCTACCTGCCGCAGG
TGGACCTACTGCAAATACGGATTATGACTCGTAAAGGGGTCATGCGTATTTTCATCACTAGGCACGTT
CGAGAATAAATTAGTAGGTGTCCCGAGCCTTGTGGCGTTCCGCCTGACTCCTCATGAAGTCGACCTTC
TCACCGGCCCTATCTGCCGACGTAAGTCATAACCTAGATCTGTACCTCGGGGGGAGGGTCACTGTAAA
GGGATAATTGGAGGGTGATTTCCCACACTTTCCTAAGGGTACTTTTTGCCTGGCTTCGCAATTGGGTC
CAATGGATGTCGATCTCTGGTTTAGCAGTTGTGAAAGTGGCAAGGCGGGAGGTTAGACCTCCATTTAA
CATATACAAGCAAGTTAACTGCACTAGATGTGTAGACACTACAGTTACAGGAGTAGCCGAATAAGTCT
CCGACGTCAAGCGAATAAGCGTCATACGCGATTATCGCCTAAGAGCACGTATTGGCGGTAAAAGGCTA
GCTAGACGCTTATGGGTAGATTTCAAGGCGTTCGTAGTGGTATAATAGGATACTCTTTCACCAGCCTG
AGGGCCGAACGCTATACTAGTGGTCTGTGATGTAGGACCGAGTATCTCTCTAGGGACCATCTACTTGA

GCAATGGTGCGCAGGGGGAGACATAGACCAGCCTTGGGTGGCAAGCACTGCAATAAGTCCTGTTTAGC
CTTGGAGTTCACATGCCGGCACTGAAGCCGACCTACCTGAGCGTGTGCGATTACCGTTACAATGGCAT
CTGTCTAGTTCTGTTTACCTACGGCGCTCTTGGTTCCAGGTTAGGGGAAGTGTATGACCCATGTGTTT
TTATCGGCTTAACCACGAGTGATCCCCGGTCGTTTCCCCATTGAATCCCTGGTGCATCCTACTCCCAG
AATGATAGCTGACTGACTGGACTGGCTTTTCAAGTAATCGAGGGGGTATCGCGGTCACGGCCGTTAAC
AGATCCCGTCCTTAGTGTGGAATCCGCACCTGCTGACTAACGCTTCGCCGGCGTGTCTGCACAGCCGT
ATAGTGTTAATCATGACCCCAAGGAAGGATTAAACAAATATCTTGACG
>COHON40
GTGTCCGGTAGCTCGCGCTAGTTAGACACCCCGGCAGGGGGGATTGCTTTCGGGACGGGAGATCCCTT
CGCCGACCCTGGAGGGCCGACGCCGAGGCATTCGGGCCCCTGCAACGTCAACAGCGGCAAGAAAACGG
GATGAATGGGCGTAATGGGGGGGGTCTGCTGGGGACCCGACGCGGTTGCCGTTTGCGGGGCCCCCGAC
CCATACCGACCCACCTAGGCGTCCAGTTACGGCGCACGTCGGGAGCGTGGTTGCCGTCAGAGCTGTGT
TTCTCGATCAGTCCCCCGCAGTGCCGCAGTATCTTGCCGTGGGCTGCTTTAATCTTGAAAGTGGTTTA
TACATTGGGCGACGAGGTGTCGACTCTCATTGGGGGTAACCGACGGGCACATGCAGTCCCCTCCCCGG
GCAGGCAGAGGCGGGGCCCCCGCGCGCCGGCCCCGGCCCACAATCTTACCAGGGTCCTCAAGGGAGCC
TTTGCATGGTACCCTTCGTGAATGGTTGCTTAAGAGGTCCACCACGTGGTCCGGGCACGGTCGACTAA
ACACTCAAAACAGCGACGGCAAATATAGGCACAAGGTCCAGACCCTCACGGCACTAATTGCGATGACC
CAACTCGCGGGGACAGTCACCGGCGCGGCGACCTTGATTACGGTCGGGGACAAGTATGTCGTTCCCCC
CGGGAGGGGTGCAGCCACATGGGAGATTCAAAGTTTCTCGTGACGTCGTTGTGATCACTGCAGCCTAG
CCGAGACTCCCGTACTACGCGAAGGTTGGTTATGTTAACCACTACAACGTGAGGCGCCGTAAGGGCCA
GTGTTGTGCCCGGCTCTTCAATGCGCCTTAGCGGCCTGATACACCCACCCAAGGAGATACCGTTAATC
ATGTGGGGGGACAGAACCTCGCAACGCAGACGCCTCCCCGGCATGATATGGTTTTTTTCCGCTATTAT
CCAGCACGCAGCGCTATCATTCAAGAGAACCCAGTGACGCGTAAATCGTAAGATCTACCTGCCGCAAG
TGGACCTACTGCAAATACGGATTATGACTCGTAAAGAGGTCATGCGTATTTTCATTACCAGGCACGTT
CGAGAATAAATTAGTAGGTGTCCCGCGCCTTGTGGCGTTCCGCCTGACTCCTCATGAAGTCGACCTTC
TCACCGGCCCTATCTGCCGACGTAAGCCATAACCTAGGTCTGTACCGTGGGGGGAGGGTCACTGTAAA
GGGATAATTGGAGGGTGATTTCCCACACTTTCCTAAGGGTACTTTTCGCCTGGCTTCGCAATTGGGCC
CAATGGATGTCGATCTCTGGTTTAGCAGTTGTGAAAGTGGTAAGGCGGGAGGTTAGACCTCCATTTAA
CATATACAAGCAAGTTCACTGCACTAGATGTGTAGACACTACAGTTACGGGGGTAGCCGAATAAGTCT
CCGACGTCAAGCGAATAAGCGTCATACGCGATTATCGCCTAAGAGCACGTATTGGCGGTAAAAGGCTA
ACTAGACGCCTGTGGGTAGATTTCAAGGCGTTCGTAGTGGTATAACAAGATACTTTTTCACCAGCCTG
AGGGCCGAACGCTATACTAGTGGTCTGTGATGTAGGATCGAGTATCCCTCTAGGGACCATCTACTTGA
GCAATGGTGCGCAGGGGGAGACATAGACCAGCCTTGGGTGGCAAGCACTGCAATAAGTCCTGTTTAGC
CCTGGAGTTCACACGCCGGCACTGAAGCCGACCTACCTGAGCTTGTGCGATTACCGTTACAATGGCAT
CTGTCTAGTTCTGTTTACCTACGGCGCTCTTGGTTCCAGGTTAGGGGAAGTGTATGACCCATGTGTTT
TTATCGGCTTAACCACGAGTGATCCCCGGTCGTTTCCTCATTGAATCCCTGGTGCATCCTACTCCCAG
AATGATAGCTGACTGACTGGACTGGCTTTTCAAGTAGTCGAGGGGGTATCGCGGTCACGGCCGTTAAC
AGATCCCGTCCTTAGTGTGGAATCCGCACCTGCTGACTAACGCTTCGCCGGCGTGTTTGCACAGCCGT
ATAGTGTTAATCATGACCCCAAGGAAGGATTAAACAAATATCTTGACG
>COHON41
GTGTCCGGTAGCCCGCGCTAGTTAGACACCCCGGCAGGGGGGATTGCTTTCGAGACGGGAGATCCCTT
CGCCGACCCTGGAGGGCCGACGCCGAGGCATTCGGGCCCCTGCAACGTCAACAGCGGCAAGAAAACGG
GATGAATGGGCGTAATGGGGGGGGTCTGCTGGGGACCCGACGCGGTTGCCGTTTGCGGGGCCCCCGAC
CCATACCGACCCACCTAGGCGTCCAGTTACGGCGCACGTCGGGAGCGTGGTTGCCGTCAGAGCTGTGT
TTCTCGATCAGTCCCCCGCAGTGCCGCAGTATCTTGCCGTGGGCTGCTTTAATCTTGAAAGTGGTTNA
TACATTGGGCGACGAGGTGTCGGCTCTCATTGGGGGTAACCGACGGGCACATGCAGTCCCCTCCCCGG
GCAGGCAGAGGCGGGGCCCCCGCGCGCCGGCCCCGGCCCACAATCTTACCAGGGTCCTCAAAGGAGCC
TTTGCATGGTACCCTTCGTGAATGGTTGCTTAAGAGGTCCACCACGTAGTCCGGGCACGGTCGACTAA
ACACTCAAAACAGCGACGGCAAATAGAGGCACAAGGTCCAGGCCCTCACGGCACTAGTTGCGATGACC
CAACTCACGGGGGCAGTCCCCGGCGCGGCGACCTTGATTACGTCCGGGAACAAGTATGTCGTTTCCCC
CGGGAGGGGTGCAGCCACATGGGAGATTCAAAGTTTCTCGTGACGTCGTTGTGATCACTGCAGCCTAG
CCGAGACTCCCGTACTACGCGAAGGTTGGTTATGTTAACCACTACAACGTGAGGCGCCGTAAGGGCCA
GTGTTGTGCCCGGCTCTTCAATGCGCCTTAGCGGCCTGATACANCCACCCAAGGAGATACTGCTAATC
ATGTGGGTGGACAGAACCTCGCAACGCAGACGCATCCCCGGCATGATATGGTTTTTTTCCGCTATTAC

CCAGCATGCGGCGCTATCATTCAAGAGAACCCAGTGACGTGTAAATCGTAAGATCTACCTGCCGCAGG
TGGACCTACTGCAAATACGGATTATGACTCGTAAAGAGGTCATGCGTATTTTCATCACTAGGCACGTT
CGAGAATAAATTAGTAGGTGTCCCGCGCCTTGTGGCGTTCCGCCTGACTCCTCATGAAGTCGACCTTC
TCATCGGCCCTATCTGCCGACGTAAGTCATAACCCAGATCTGTACCTCGGAGGGAGGGTCACTGTAAA
GGGATAATTGGAGGGTGATTTCCCACACTTTCCTAAGGGTACTTTTTGCCTGGCTTCGCAGTTGGGTC
CAATAGATGTTGATCTCTGGTTTAGCAGTTGTGAAAGTGGCAAGGCGGGAGGTTAGACCTCCATTTAA
CATATACAAGCAAGTTAACTGCACTAGATGTGTAGACACTACAGTTACAGGAGTAGCCGACTAAGTCT
CCGACGTCAAGCGAATAGGCGTCATACGCGATTATCGCCTAAGAGCACGTATTGGCGGTAAAAGGCTA
ACTAGACGCTTGTGGGTAGATTTCAAGGCGCTCGTAGTGGTATAATAGGATACTCTTTCACCAGCCTG
AGGGCCGAACGCTATACTAGTGGTCTGTGATGTAGGACCAAGTAGCTCTCTAGGGACCATCTACTTGA
GCAATGGTGCGCAGGGGTAGACATAGACCAACCTTGGGTGGCAAGCACTGCAATAAGTCCTGTTCAGC
CTTGGAGTTCACACGCCGGCACTAAAGCCGACCTACCTGAGCTTGTGCGATTACCGTTACAATGGCAT
CTGTCTAGTTCTGTTTACCTACGGCGCTCTTGGTTCCATGTTAGGGGAAGTGTATGACCCATGTGTTT
TTATCGGCTTAACCACGAGTGATCCCCGGTCGTTTCCCCATTGAATCCCTGGTGCATCCTACTCCCAT
AATGATAGCTGACTGGCTGGACTGGCTTTTCAAGTAGTCGAGGGGGTATCGCGGTCACGGCCGTTAAC
AGATCCCGTCCTTAGTGTGGAATCCGCACCTGCTGACTAACGCTTCGCCNGCGTGTCTGCACATCCGT
ATAGTGTTAATCATGACCCCAAGGAAGGATTAAACAAATATCTTGACG
>COHON44
GTGTCCGGTAGCCCGCGCTAGTTAGACACCCCGGCAGGGGGGATTGCTTTCGAGACGGGAGATCCCTT
CGCCGACCCTGGAGGGCCGACGCCGAGGCATTCGGGCCCCTGCAACGTCAACAGCGGCAAGAAAACGG
GATGAATGGGCGTAATGGGGGGGGTCTGCTGGGGACCCGACGCGGTTGCCGTTTGCGGGGCCCCCGAC
CCATACCGACCCACCTAGGCGTCCAGTTACGGCGCACGTCGGGAGCGTGGTTGCCGTCAGAGCTGTGT
TTCTCGATCAGTCCCCCGCAGTGCCGCAGTATCTTGCCGTGGGCTGCTTTAATCTTGAAAGTGGTTTA
TACATTGGGCGACGAGGTGTCGACTCTCATTGGGGGTAACCGACGGGCACATGCAGTCCCCTCCCCGG
GCAGGCAGAGGCGGGGCCCCCGCGCGCCGGCCCCGGCCCACAATCTTACCAGGGTCCTCAAAGGAGCC
TTTGCATGGTACCCTTCGTGAATGGTTGCTTAAGAGGTCCACAACGTGGTCCGGGCACGGTCGACTAA
ACACTCAAAACAGCGACGGCAAATATAGGTACAAGGTCCAGGCCCTCACGGCACTAATTGCGATGACC
CAACTCACGGGGGCAGTCCCCGGCGCGGCGACCTTGATTACGTCCGGGGACAAGTATGTCGCTCCCCC
CGGGAGGGGTGCAGCCACATGGGAGATTCAAAGTTTCTCGTGACGTCGTTGTGATCACTGCAGCCTAG
CCGAGACTCCCGTACTACGCGAAGGTTGGTTATGTTAACCACTACAACGTGAGGCGCCGTAAGGGCCA
GTGTTGTGCCCGGCTCTTCAATGCGCCTTAGCGGCCTGATACACCCACCCAAGGAGATACTGCTAATC
ATATGGGTGGACAGAACCTCGCAACGCAGACGCCTCCCCGGCATGATATGGTTTTTTTCCGCTATTAT
CCAGCACGCAGCGCTATCATTCAAGAGAACCCAGTGACGCGTAAATCGTAAGATCTACCTGCCGCAGG
TGGACCTACTGCAAATACGGATTATGACTCGTAAAGGGGTCATGCGTATTTTCATCACTAGGCACGTT
CGAGAATAAATTAGTAGGTGTCCCGAGCCTTGTGGCGTTCCGCCTGACTCCTCATGAAGTCGACCTTC
TCACCGGCCCTATCTGCCGACGTAAGTCATAACCTAGATCTGTACCTCGGGGGGAGGGTCACTGTAAA
GGGATAATTGGAGGGTGATTTCCCACACTTTCCTAAGGGTACTTTTTGCCTGGCTTCGCAATTGGGTC
CAATGGATGTCGATCTCTGGTTTAGCAGTTGTGAAAGTGGCAAGGCGGGAGGTTAGACCTCCATTTAA
CATATACAAGCAAGTTAACTGCACTAGATGTGTAGACACTACAGTTACAGGAGTAGCCGAATAAGTCT
CCGACGTCAAGCGAATAAGCGTCATACGCGATTATCGCCTAAGAGCACGTATTGGCGGTAAAAGGCTA
GCTAGACGCTTATGGGTAGATTTCAAGGCGTTCGTAGTGGTATAATAGGATACTCTTTCACCAGCCTG
AGGGCCGAACGCTATACTAGTGGTCTGTGATGTAGGACCGAGTATCTCTCTAGGGACCATCTACTTGA
GCAATGGTGCGCAGGGGGAGACATAGACCAGCCTTGGGTGGCAAGCACTGCAATAAGTCCTGTTTAGC
CTTGGAGTTCACATGCCGGCACTGAAGCCGACCTACCTGAGCGTGTGCGATTACCGTTACAATGGCAT
CTGTCTAGTTCTGTTTACCTACGGCGCTCTTGGTTCCAGGTTAGGGGAAGTGTATGACCCATGTGTTT
TTATCGGCTTAACCACGAGTGATCCCCGGTCGTTTCCCCATTGAATCCCTGGTGCATCCTACTCCCAG
AATGATAGCTGACTGACTGGACTGGCTTTTCAAGTAATCGAGGGGGTATCGCGGTCACGGCCGTTAAC
AGATCCCGTCCTTAGTGTGGAATCCGCACCTGCTGACTAACGCTTCGCCGGCGTGTCTGCACAGCCGT
ATAGTGTTAATCATGACCCCAAGGAAGGATTAAACAAATATCTTGACG
>COHON45
GTGTCCGGTAGCCCGCGCTAGTTAGACACCCCGGCAGGGGGGATTGCTTTCGAGACGGGAGATCCCTT
CGCCGACCCTGGAGGGCCGACGCCGAGGCATTCGGGCCCCTGCAACGTCAACAGCGGCAAGAAAACGG
GATGAATGGGCGTAATGGGGGGGGTCTGCTGGGGACCCGACGCGGTTGCCGTTTGCGGGGCCCCCGAC
CCATACCGACCCACCTAGGCGTCCAGTTACGGCGCACGTCGGGAGCGTGGTTGCCGTCAGAGCTGTGT

TTCTCGATCAGTCCCCCGCAGTGCCGCAGTATCTTGCCGTGGGCTGCTTTAATCTTGAAAGTGGTTTA
TACATTGGGCGACGAGGTGTCGACTCTCATTGGGGGTAACCGACGGGCACATGCAGTCCCCTCCCCGG
GCAGGCAGAGGCGGGGCCCCCGCGCGCCGGCCCCGGCCCACAATCTTACCAGGGTCCTCAAAGGAGCC
TTTGCATGGTACCCTTCGTGAATGGTTGCTTAAGAGGTCCACAACGTGGTCCGGGCACGGTCGACTAA
ACACTCAAAACAGCGACGGCAAATATAGGTACAAGGTCCAGGCCCTCACGGCACTAATTGCGATGACC
CAACTCACGGGGGCAGTCCCCGGCGCGGCGACCTTGATTACGTCCGGGGACAAGTATGTCGCTCCCCC
CGGGAGGGGTGCAGCCACATGGGAGATTCAAAGTTTCTCGTGACGTCGTTGTGATCACTGCAGCCTAG
CCGAGACTCCCGTACTACGCGAAGGTTGGTTATGTTAACCACTACAACGTGAGGCGCCGTAAGGGCCA
GTGTTGTGCCCGGCTCTTCAATGCGCCTTAGCGGCCTGATACACCCACCCAAGGAGATACTGCTAATC
ATATGGGTGGACAGAACCTCGCAACGCAGACGCCTCCCCGGCATGATATGGTTTTTTTCCGCTATTAT
CCAGCACGCAGCGCTATCATTCAAGAGAACCCAGTGACGCGTAAATCGTAAGATCTACCTGCCGCAGG
TGGACCTACTGCAAATACGGATTATGACTCGTAAAGGGGTCATGCGTATTTTCATCACTAGGCACGTT
CGAGAATAAATTAGTAGGTGTCCCGAGCCTTGTGGCGTTCCGCCTGACTCCTCATGAAGTCGACCTTC
TCACCGGCCCTATCTGCCGACGTAAGTCATAACCTAGATCTGTACCTCGGGGGGAGGGTCACTGTAAA
GGGATAATTGGAGGGTGATTTCCCACACTTTCCTAAGGGTACTTTTTGCCTGGCTTCGCAATTGGGTC
CAATGGATGTCGATCTCTGGTTTAGCAGTTGTGAAAGTGGCAAGGCGGGAGGTTAGACCTCCATTTAA
CATATACAAGCAAGTTAACTGCACTAGATGTGTAGACACTACAGTTACAGGAGTAGCCGAATAAGTCT
CCGACGTCAAGCGAATAAGCGTCATACGCGATTATCGCCTAAGAGCACGTATTGGCGGTAAAAGGCTA
GCTAGACGCTTATGGGTAGATTTCAAGGCGTTCGTAGTGGTATAATAGGATACTCTTTCACCAGCCTG
AGGGCCGAACGCTATACTAGTGGTCTGTGATGTAGGACCGAGTATCTCTCTAGGGACCATCTACTTGA
GCAATGGTGCGCAGGGGGAGACATAGACCAGCCTTGGGTGGCAAGCACTGCAATAAGTCCTGTTTAGC
CTTGGAGTTCACATGCCGGCACTGAAGCCGACCTACCTGAGCGTGTGCGATTACCGTTACAATGGCAT
CTGTCTAGTTCTGTTTACCTACGGCGCTCTTGGTTCCAGGTTAGGGGAAGTGTATGACCCATGTGTTT
TTATCGGCTTAACCACGAGTGATCCCCGGTCGTTTCCCCATTGAATCCCTGGTGCATCCTACTCCCAG
AATGATAGCTGACTGACTGGACTGGCTTTTCAAGTAATCGAGGGGGTATCGCGGTCACGGCCGTTAAC
AGATCCCGTCCTTAGTGTGGAATCCGCACCTGCTGACTAACGCTTCGCCGGCGTGTCTGCACAGCCGT
ATAGTGTTAATCATGACCCCAAGGAAGGATTAAACAAATATCTTGACG
>RAGLN01
GTGTCCGGTAGCCCGCGCTAGTCAGACACCCCGGCAGGGGGGATTGCTTTCGAGACGGGAGATCCCTT
CGCCGACCCTGGAGGGCCGACGCCGAGGCATTCGGGCCCCTGCAACGTCAACAGCGGCAAGAAAACGG
GATGAATGGGCGTAATGGGGGGGGTCTGCTGGGGACCCGACGCGGTTGCCGTTTGCGGGGCCCCCGAC
CCATACCGACCCACCTAGGCGTCCAGTTACGGCGCACGGCGGGAGCGTGGTTGCCGTCAGAGCTGTGT
TTCTCGATCAGTCCCCCGCAGTGCCGCAGTATCTTGCCGTGGGCTGCTTTAATCTTGAAAGTGGTTTA
TACATTGGGCGACGAGGTGTCGACTCTCATTGGGGGTAACCGACGGGCACATGCAGTCCCCTCCCCGG
GCAGGCAGAGGCGGGGCCCCCGCGCGCCGGCCCCGGCCCACAATCTTACCAGGGTCCTCAAAGGAGCC
TTTGCATGGTACCCTTCGTGAATGGTTGCTTAAGAGGTCCACAACGTGGTCCGGGCACGGTCGACTAA
ACACTCAAAACAGCGACGGCAAATATAGGTACAAGGTCCAGGCCCTCACGGCACTAATTGCGATGACC
CAACTCACGGGGGCAGTCCCCGGCGCGGCGACCTTGATTACGTCCGGGGACAAGTATGTCGCTCCCCC
CGGGAGGGGTGCAGCCACATGGGAGATTCAAAGTTTCTCGTGACGTCGTTGTGATCACTGCAGCCTAG
CCGAGACTCCCGTACTACGCGAAGGTTGGTTATGTTAACCACTACAACGTGAGGCGCCGTAAGGGCCA
GTGTTGTGCCCGGCTCTTCAATGCGCCTTAGCGGCCTGATACACCCACCCAAGGAGATACTGCTAATC
ATATGGGTGGACAGAACCTCGCAACGCAGACGCCTCCCCGGCATGATATGGTTTTTTTCCGCTATNAT
CCAGCACGCAGCGCTATCATTTAAGAGAACCCAGTGACGCGTAAATCGTAAGATCTACCTGCCGCAGG
TGGACCTACTGCAAATACGGATTATGACTCGTAAAGGGGTCATGCGTATTTTCATCACTAGGCACGTT
CGAGAATAAATTAGTAGGTGTCCCGAGCCTTGTGGCGTTCCGCCTGACTCCTCATGAAGTCGACCTTC
TCACCGGCCCTATCTGCCGACGTAAGTCATAACCTAGATCTGTACCTCGGGGGGAGGGTCACTGTAAA
GGGATAATTGGAGGGTGATTTCCCACATTTTCCTAAGGGTACTTTTTGCCTGGCTTCGCAATTGGGTC
CAATGGATGTCGATCTCTGGTTTAGCAGTTGTGAAAGTGGCAAGGCGGGAGGTTAGACCTCCATTTAA
CATATACAAGCAAGTTAACTGCACTAGATGTGTAGACACTACAGGTACAGGAGTAGCCGGATAAGTCT
CCGACGTCAAGCGAATAAGCGTCATACGCGATTATCGCCTAAGAGCACGTATTTGCGGTAAAAGGCTA
GCTAGACGCTTGTGGGTAGATTTCAAGGCGTTCGTAGTGGTATAATAGGATACTCTTTCACCAGCCTG
AGGGCCGAACGCTATACTAGTGGTCTGTGATGTAGGACCGAGTATCTCTCTAGGGACCATCTACTTGA
GCAATGGTGCGCAGGGGGAGACATAGACCAGCCTTGGGTGGCAAGCACTGCAATAAGTCCTGTTTAGC
CTTGGAGTTCACACGCCGGCACTGAAGCCGACCTACCTGAGCGTGTGCGATTACCGTTACAATGGCAT

CTGTCTAGTTCTGTTTACCTACGGCGCTCTTGGTTCCAGGTTAGGGGAAGTGTATGACCCATGTGTTT
TTATCGGCTTAACCACGAGTGATCCCCGGTCGTTTCCCCATTGAATCCCTGGTGCATCCTACTCCCAG
AATGATAGCTGACTGACTGGACTGGCTTTTCAAGTAATCGAGGGGGTATCGCGGTCACGGCCGTTAAC
AGATCCCGTCCTTAGTGTGGAATCCGCACCTGCTGACTAACGCTTCGCCGGCGTGTCTGCACAGCCGT
ATAGTGTTAATCATGACCCCAAGGAAGGATTAAACAAATATCTTGACG
>RAGLN02
GTGTCCGGTAGCCCGCGCTAGTTAGACACCCCGGCAGGGGGGATTGCTTTCGAGACGGGAGATCCCTT
CGCCGACCCTGGAGGGCCGACGCCGAGGCATTCGGGCCCCTGCAACGTCAACAGCGGCAAGAAAACGG
GATGAATGGGCGTAATGGGGGGGGTCTGCTGGGGACCCGACGCGGTTGCCGTTTGCGGGGCCCCCGAC
CCATACCGACCCACCTAGGCGTCCAGTTACGGCGCACGTCGGGAGCGTGGTTGCCGTCAGAGCTGTGT
TTCTCGATCAGTCCCCCGCAGTGCCGCAGTATCTTGCCGTGGGCTGCTTTAATCTTGAAAGTGGTTCA
TACATTGGGCGACGAGGTGTCGACTCTCATTGGGGGTAACCGACGGGCACATGCAGTCCCCTCCCCGG
GCAGGCAGAGGCGGGGCCCCCGCGCGCCGGCCCCGGCCCACAATCTTACCAGGGTCCTCAAAGGAGCC
TTTGCATGGTACCCTTCGTGAATGGTTGCTTAAGAGGTCCACAACGTGGTCCGGGCACGGTCGACTAA
ACACTCAAAACAGCGACGGCAAATATAGGTACAAGGTCCAGGCCCTCACGGCACTAATTGCGATGACC
CAACTCACGGGGGCAGTCCCCGGCGCGGCGACCTTGATTACGTCCGGGGACAAGTATGTCGCTCCCCC
CGGGAGGGGTGCAGCCACATGGGAGATTCAAAGTTTCTCGTGACGTCGTTGTGATCACTGCAGCCTAG
CCGAGACTCCCGTACTACGCGAAGGTTGGTTATGTTAACCACTACAACGTGAGGCGCCGTAAGGGCCA
GTGTTGTGCCCGGCTCTTCAATGCGCCTTAGCGGCCTGATACACCCACCCAAGGAGATACTGCTAATC
ATATGGGTGGACAGAACCTCGCAACGCAGACGCCTCCCCGGCATGATATGGTTTTTTTCCGCTATTAT
CCAGCACGCAGCGCTATCATTCAAGAGAACCCAGTGACGCGTAAATCGTAAGATCTACCTGCCGCAGG
TGGACCTACTGCAAATACGGATTATGACTCGTAAAGGGGTCATGCGTATTTTCATCACTAGGCACGTT
CGAGAATAAATTAGTAGGTGTCCCGAGCCTTGTGGCGTTCCGCCTGACTCCTCATGAAGTCGACCTTC
TCACCGGCCCTATCTGCCGACGTAAGTCATAACCTAGATCTGTACCTCGGGGGGAGGGTCACTGTAAA
GGGATAATTGGAGGGTGATTTCCCACACTTTCCTAAGGGTACTTTTTGCCTGGCTTCGCAATTGGGTC
CAATGGATGTCGATCTCTGGTTTAGCAGTTGTGAAAGTGGCAAGGCGGGAGGTTAGACCTCCATTTAA
CATATACAAGCAAGTTAACTGCACTAGATGTGTAGACACTACAGTTACAGGAGTAGCCGAATAAGTCT
CCGACGTCAAGCGAATAAGCGTCATACGCGATTATCGCCTAAGAGCACGTATTGGCGGTAAAAGGCTA
GCTAGACGCTTATGGGTAGATTTCAAGGCGTTCGTAGTGGTATAATAGGATACTCTTTCACCAGCCTG
AGGGCCGAACGCTATACTAGTGGTCTGTGATGTAGGACCGAGTATCTCTCTAGGGACCATCTACTTGA
GCAATGGTGCGCAGGGGGAGACATAGACCAGCCTTGGGTGGCAAGCACTGCAATAAGTCCTGTTTAGC
CTTGGAGTTCACATGCCGGCACTGAAGCCGACCTACCTGAGCGTGTGCGATTACCGTTACAATGGCAT
CTGTCTAGTTCTGTTTACCTACGGCGCTCTTGGTTCCAGGTTAGGGGAAGTGTATGACCCATGTGTTT
TTATCGGCTTAACCACGAGTGATCCCCGGTCGTTTCCCCATTGAATCCCTGGTGCATCCTACTCCCAG
AATGATAGCTGACTGACTGGACTGGCTTTTCAAGTAATCGAGGGGGTATCGCGGTCACGGCCGTTAAC
AGATCCCGTCCTTAGTGTGGAATCCGCACCTGCTGACTAACGCTTCGCCGGCGTGTCTGCACAGCCGT
ATAGTGTTAATCATGACCCCAAGGAAGGATTAAACAAATATCTTGACG
>RAGLN03
GTGTCCGGTAGCCCGCGCTAGTTAGACACCCCGGCAGGGGGGATTGCTTTCGAGACGGGAGATCCCTT
CGCCGACCCTGGAGGGCCGACGCCGAGGCATTCGGGCCCCTGCAACGTCAACAGCGGCAAGAAAACGG
GATGAATGGGCGTAATGGGGGGGGTCTGCTGGGGACCCGACGCGGTTGCCGTTTGCGGGGCCCCCGAC
CCATACCGACCCACCTAGGCGTCCAGTTACGGCGCACGTCGGGAGCGTGGTTGCCGTCAGAGCTGTGT
TTCTCGATCAGTCCCCCGCAGTGCCGCAGTATCTTGCCGTGGGCTGCTTTAATCTTGAAAGTGGTTTA
TACATTGGGCGACGAGGTGTCGACTCTCATTGGGGGTAACCGACGGGCACATGCAGTCCCCTCCCCGG
GCAGGCAGAGGCGGGGCCCCCGCGCGCCGGCCCCGGCCCACAATCTTACCAGGGTCCTCAAAGGAGCC
TTTGCATGGTACCCTTCGTGAATGGTTGCTTAAGAGGTCCACAACGTGGTCCGGGCACGGTCGACTAA
ACACTCAAAACAGCGACGGCAAATATAGGTACAAGGTCCAGGCCCTCACGGCACTAATTGCGATGACC
CAACTCACGGGGGCAGTCCCCGGCGCGGCGACCTTGATTACGTCCGGGGACAAGTATGTCGCTCCCCC
CGGGAGGGGTGCAGCCACATGGGAGATTCAAAGTTTCTCGTGACGTCGTTGTGATCACTGCAGCCTAG
CCGAGACTCCCGTACTACGCGAAGGTTGGTTATGTTAACCACTACAACGTGAGGCGCCGTAAGGGCCA
GTGTTGTGCCCGGCTCTTCAATGCGCCTTAGCGGCCTGATACACCCACCCAAGGAGATACTGCTAATC
ATATGGGTGGACAGAACCTCGCAACGCAGACGCCTCCCCGGCATGATATGGTTTTTTTCCGCTATTAT
CCAGCACGCAGCGCTATCATTCAAGAGAACCCAGTGACGCGTAAATCGTAAGATCTACCTGCCGCAGG
TGGACCTACTGCAAATACGGATTATGACTCGTAAAGGGGTCATGCGTATTTTCATCACTAGGCACGTT

CGAGAATAAATTAGTAGGTGTCCCGAGCCTTGTGGCGTTCCGCCTGACTCCTCATGAAGTCGACCTTC
TCACCGGCCCTATCTGCCGACGTAAGTCATAACCTAGATCTGTACCTCGGGGGGAGGGTCACTGTAAA
GGGATAATTGGAGGGTGATTTCCCACACTTTCCTAAGGGTACTTTTTGCCTGGCTTCGCAATTGGGTC
CAATGGATGTCGATCTCTGGTTTAGCAGTTGTGAAAGTGGCAAGGCGGGAGGTTAGACCTCCATTTAA
CATATACAAGCAAGTTAACTGCACTAGATGTGTAGACACTACAGTTACAGGAGTAGCCGAATAAGTCT
CCGACGTCAAGCGAATAAGCGTCATACGCGATTATCGCCTAAGAGCACGTATTGGCGGTAAAAGGCTA
GCTAGACGCTTATGGGTAGATTTCAAGGCGTTCGTAGTGGTATAATAGGATACTCTTTCACCAGCCTG
AGGGCCGAACGCTATACTAGTGGTCTGTGATGTAGGACCGAGTATCTCTCTAGGGACCATCTACTTGA
GCAATGGTGCGCAGGGGGAGACATAGACCAGCCTTGGGTGGCAAGCACTGCAATAAGTCCTGTTTAGC
CTTGGAGTTCACATGCCGGCACTGAAGCCGACCTACCTGAGCGTGTGCGATTACCGTTACAATGGCAT
CTGTCTAGTTCTGTTTACCTACGGCGCTCTTGGTTCCAGGTTAGGGGAAGTGTATGANCCATGTGTTT
TTATCGGCTTAACCACGAGTGATCCCCGGTCGTTTCCCCATTGAATCCCTGGTGCATCCTACTCCCAG
AATGATAGCTGACTGACTGGACTGGCTTTTCAAGTAATCGAGGGGGTATCGCGGTCACGGCCGTTAAC
AGATCCCGTCCTTAGTGTGGAATCCGCACCTGCTGACTAACGCTTCGCCGGCGTGTCTGCACAGCCGT
ATAGTGTTAATCATGACCCCAAGGAAGGATTAAACAAATATCTTGACG
>RAGLN04
GTGTCCGGTAGCCCGCGCTAGTTAGACACCCCGGCAGGGGGGATTGCTTTCGAGACGGGAGATCCCTT
CGCCGACCCTGGAGGGCCGACGCCGAGGCATTCGGGCCCCTGCAACGTCAACAGCGGCAAGAAAACGG
GATGAATGGGCGTAATGGGGGGGGTCTGCTGGGGACCCGACGCGGTTGCCGTTTGCGGGGCCCCCGAC
CCATACCGACCCACCTAGGCGTCCAGTTACGGCGCACGTCGGGAGCGTGGTTGCCGTCAGAGCTGTGT
TTCTCGATCAGTCCCCCGCAGTGCCGCAGTATCTTGCCGTGGGCTGCTTTAATCTTGAAAGTGGTTTA
TACATTGGGCGACGAGGTGTCGACTCTCATTGGGGGTAACCGACGGGCACATGCAGTCCCCTCCCCGG
GCAGGCAGAGGCGGGGCCCCCGCGCGCCGGCCCCGGCCCACAATCTTACCAGGGTCCTCAAAGGAGCC
TTTGCATGGTACCCTTCGTGAATGGTTGCTTAAGAGGTCCACAACGTGGTCCGGGCACGGTCGACTAA
ACACTCAAAACAGCGACGGCAAATATAGGTACAAGGTCCAGGCCCTCACGGCACTAATTGCGATGACC
CAACTCACGGGGGCAGTCCCCGGCGCGGCGACCTTGATTACGTCCGGGGACAAGTATGTCGCTCCCCC
CGGGAGGGGTGCAGCCACATGGGAGATTCAAAGTTTCTCGTGACGTCGTTGTGATCACTGCAGCCTAG
CCGAGACTCCCGTACTACGCGAAGGTTGGTTATGTTAACCACTACAACGTGAGGCGCCGTAAGGGCCA
GTGTTGTGCCCGGCTCTTCAATGCGCCTTAGCGGCCTGATACACCCACCCAAGGAGATACTGCTAATC
ATATGGGTGGACAGAACCTCGCAACGCAGACGCCTCCCCGGCATGATATGGTTTTTTTCCGCTATTAT
CCAGCACGCAGCGCTATCNTTCAAGAGAACCCAGTGACGCGTAAATCGTAAGATCTACCTGCCGCAGG
TGGACCTACTGCAAATACGGATTATGACTCGTAAAGGGGTCATGCGTATTTTCATCACTAGGCACGTT
CGAGAATAAATTAGTAGGTGTCCCGAGCCTTGTGGCGTTCCGCCTGACTCCTCATGAAGTCGACCTTC
TCACCGGCCCTATCTGCCGACGTAAGTCATAACCTAGATCTGTACCTCGGGGGGAGGGTCACTGTAAA
GGGATAATTGGAGGGTGATTTCCCACACTTTCCTAAGGGTACTTTTTGCCTGGCTTCGCAATTGGGTC
CAATGGATGTCGATCTCTGGTTTAGCAGTTGTGAAAGTGGCAAGGCGGGAGGTTAGACCTCCATTTAA
CATATACAAGCAAGTTAACTGCACTAGATGTGTAGACACTACAGTTACAGGAGTAGCCGAATAAGTCT
CCGACGTCAAGCGAATAAGCGTCATACGCGATTATCGCCTAAGAGCACGTATTGGCGGTAAAAGGCTA
GCTAGACGCTTATGGGTAGATTTCAAGGCGTTCGTAGTGGTATAATAGGATACTCTTTCACCAGCCTG
AGGGCCGAACGCTATACTAGTGGTCTGTGATGTAGGACCGAGTATCTCTCNAGGGACCATCTACTTGA
GCAATGGTGCGCAGGGGGAGACATAGACCAGCCTTGGGTGGCAAGCACTGCAATAAGTCCTGTTTAGC
CTTGGAGTTCACATGCCGGCACTGAAGCCGACCTACCTGAGCGTGTGCGATTACCGTTACAATGGCAT
CTGTCTAGTTCTGTTTACCTACGGCGCTCTTGGTTCCAGGTTAGGGGAAGTGTATGACCCATGTGTTT
TTATCGGCTTAACCACGAGTGATCCCCGGTCGTTTCCCCATTGAATCCCTGGTGCATCCTACTCCCAG
AATGATAGCTGACTGACTGGACTGGCTTTTCAAGTAATCGAGGGGGTATCGCGGTCACGGCCGTTAAC
AGATCCCGTCCTTAGTGTGGAATCCGCACCTGCTGACTAACGCTTCGCCGGCGTGTCTGCACAGCCGT
ATAGTGTTAATCATGACCCCAAGGAAGGATTAAACAAATATCTTGACG
>RAGLN05
GTGTCCGGTAGCCCGCGCTAGTTAGACACCCCGGCAGGGGGGATTGCTTTCGGGACGGGAGATCCCTT
CGCCGACCCTGGAGGGCCGACGCCGAGGCATTCGGGCCCCTGCAACGTCAACAGCGGCAAGAAAACGG
GATGAATGGGCGTAATGGGGGGGGTCTGCTGGGGACCCGACGCGGTTGCCGTTTGCGGGGCCCCCGAC
CCATACCGACCCACCTAGGCGTCCAGTTACGGCGCACGGCGGGAGCGTGGTTGCCGTCAGAGCTGTGT
TTCTCGATCAGTCCCCCGCAGTGCCGCAGTATCTTGCCGTGGGCTGCTTTAATCTTGAAAGTGGTTCA
TACATTGGGCGACGAGGTGTCGACTCTCATTGGGGGTAACCGACGGGCACATGCAGTCCCCTCCCCGG

GCAGGCAGAGGCGGGGCCCCCGCGCGCCGGCCCCGGCCCACAATCTTACCAGGGTCCTCAAAGGAGCC
TTTGCATGGTACCCTTCGTGAATGGTTGCTTAAGAGGTCCACAACGTGGTCCGGGCACGGTCGACTAA
ACACTCAAAACAGCGACGGCAAATATAGGTACAAGGTCCAGGCCCTCACGGCACTAATTGCGATGACC
CAACTCACGGGGGCAGTCCCCGGCGCGGCGACCTTGATTACGTCCGGGGACAAGTATGTCGCTCCCCC
CGGGAGGGGTGCAGCCACATGGGAGATTCAAAGTTTCTCGTGACGTCGTTGTGATCACTGCAGCCTAG
CCGAGACTCCCGTACTACGCGAAGGTTGGTTATGTTAACCACTACAACGTGAGGCGCCGTAAGGGCCA
GTGTTGTGCCCGGCTCTTCAATGCGCCTTAGCGGCCTGATACACCCACCCAAGGAGATACTGCTAATC
ATATGGGTGGACAGAACCTCGCAACGCAGACGCCTCCCCGGCATGATATGGTTTTTTTCCGCTATTAT
CCAGCACGCAGCGCTATCATTCAAGAGAACCCAGTGACGCGTAAATCGTAAGATCTACCTGCCGCAGG
TGGACCTACTGCAAATACGGATTATGACTCGTAAAGGGGTCATGCGTATTTTCATCACTAGGCACGTT
CGAGAATAAATTAGTAGGTGTCCCGAGCCTTGTGGCGTTCCGCCTGACTCCTCATGAAGTCGACCTTC
TCACCGGCCCTATCTGCCGACGTAAGTCATAACCTAGATCTGTACCTCGGGGGGAGGGTCACTGTAAA
GGGATAATTGGAGGGTGATTTCCCACACTTTCCTAAGGGTACTTTTTGCCTGGCTTCGCAATTGGGTC
CAATGGATGTCGATCTCTGGTTTAGCAGTTGTGAAAGTGGCAAGGCGGGAGGTTAGACCTCCATTTAA
CATATACAAGCAAGTTAACTGCACTAGATGTGTAGACACTACAGTTACAGGAGTAGCCGAATAAGTCT
CCGACGTCAAGCGAATAAGCGTCATACGCGATTATCGCCTAAGAGCACGTATTGGCGGTAAAAGGCTA
GCTAGACGCTTATGGGTAGATTTCAAGGCGTTCGTAGTGGTATAATAGGATACTCTTTCACCAGCCTG
AGGGCCGAACGCTATACTAGTGGTCTGTGATGTAGGACCGAGTATCTCTCTAGGGACCATCTACTTGA
GCAATGGTGCGCAGGGGGAGACATAGACCAGCCTTGGGTGGCAAGCACTGCGATAAGTCCTGTTTAGC
CTTGGAGTTCACATGCCGGCACTGAAGCCGACCTACCTGAGCGTGTGCGATTACCGTTACAATGGCAT
CTGTCTAGTTCTGTTTACCTACGGCGCTCTTGGTTCCAGGTTAGGGGAAGTGTATGACCCATGTGTTT
TTATCGGCTTAACCACGAGTGATCCCCGGTCGTTTCCCCATTGAATCCCTGGTGCATCCTACTCCCAG
AATGATAGCTGACTGACTGGACTGGCTTTTCAAGTAATCGAGGGGGTATCGCGGTCACGGCCGTTAAC
AGATCCCGTCCTTAGTGTGGAATCCGCACCTGCTGACTAACGCTTCGCCGGCGTGTCTGCACAGCCGT
ATAGTGTTAATCATGACCCCAAGGAAGGATTAAACAAATATCTTGACG
>RAGLN06
GTGTCCGGTAGCCCGCGCTAGTTAGACACCCCGGCAGGGGGGATTGCTNTCGAGACGGGAGATCCCTT
CGCCGACCCTGTAGGGCCGACGCCGAGGCATTCGGGCCCCTGCAACGTCAACAGCGGCAAGAAAACGG
GATGAATGGGCGTAATGGGGGGGGTCTGCTGGGGACCCGACGCGGTTGCCGTTTGCGGGGCCCCCGAC
CCATACCGACCCACCTAGGCGTCCAGTTACGGCGCACGTCGGGAGCGTGGTTGCCGTCAGAGCTGTGT
TTCTCGATCAGTCCCCCGCAGTGCCGCAGTATCTTGCCGTGGGCTGCTTTAATCTTGAAAGTGGTTCA
TACATTGGGCGACGAGGTGTCGGCTCTCATTGGGGGTAGCCGACGGACACATGCAGTCCCCTCCCCGG
GCAGGCAGAGGCGGGGCCCCCGCGCGCCGGCCCCGGCCCACAATCTTACCAGGGTCCTCAAAAGAGCC
TTTGCATGGTACCCTTCGTGAATGGTTGCTTAAGAGGTCCACCACGTAGTCCGGGCACGGTCGACTAA
ACACTCAAAACAGCGACGGCAAATAGAGGCACAAGGTCCAGGCCCTCACGGCACTAGTTGCGATGACC
CAACTCACGGGGGCAGTCCCCGGCGCGGCGACCTTGATTACGTCCGGGAACAAGTATGTCGTTTCCCC
CGGGAGGGGTGCAGCCACATGGGAGATTCAAAGTTTCTCGTGACGTCGTTGTGATCACTGCAGCCTAG
CCGAGACTCCCGTACTACGCGAAGGTTGGTTATGTTAACCACTACAACGTGAGGCGCCGTAAGGGCCA
GTGTTGTGCCCGGCTCTTCAATGCGCCTTAGCGGCCTGATACACCCACCCAAGGAGATACTGCTAATC
ATGTGGGTGGACAGAACCTCGCAACGCAGACGCATCCCCGGCATGATATGGTTTTTTTCCGCTATTAC
CCAGCACGCGGCGCTATCATTCAAGAGAACCCAGTGACGCGTAAATCGTAAGATCTACCTGCCGCAGG
TGGACCTACTGCAAATACGGATTATGACTCGTAAAGAGGTCATGCGTATTTTCATCACTAGGCACGTT
CGAGAGTAAATTAGTAGGTGTCCCGCGCCNTGTGGCGTTCCGCCTGACTCCTCATGAAGTCGACCTTC
TCATCGGCCCTATCTGCCGACGTAAGTCATAATCCAGATCTTCACCTCGGAGGGAGGGTCACTGTAAA
GGGATAATTGGAGGGCGATTTCCCACACTTTCCTAAGGGTACTTTTTGCTTGGCTTCGCAGTTGGGTC
CAATAGATGTTGATCTCTGGTTTAGCAGTTGTGAAAGTGGCAAGGCGGGAGGTTAGACCTCCATTTAA
CATATACAAGCAAGTTAACTGCACTAGATGTGTAGACACTACAGTTACAGGAGTAGCCGACTAAGTCT
CCGACGTCAAGCGAATAGGCGTCATACGCGATTATCGCCTAAGAGCACGTATTGGCGGTAAAAGGATA
ACTAGACGCTTGTGGGTAGATTTCAAGGCGCTCGTAGTGGTATAATAGGATACTCTTTCACCAGCCTG
AGAGCCGAACGCTATACTAGTGGTCTGTGATGTAGGACCAAGTAGCTCTCTAGGGACCATCTACTTGA
GCAATGGTGCGNAGGGGTAGACATAGACCAACCTTGGGTGGCAAGCACTGCAATAAGTCCTGTTTAGC
CTTGGAGTTCACACGCCGGCACTAAAGCCGACCTACCTGAGCTTGTGCGATTACCGTTACAATGGCAT
CTGTCTAGTTCTGTTTACCTACGGCGCTCTTGGTTCCATGTTAGGGGAAGTGTATGACCCATGTGTTT
TTATCGGCTTAACTACGAGTGATCCCCGGTCGTTTCCCCATTAAATCCCTGGTGCATCCTACTCCCAT

AATGATAGCTGACTGGCTGGACTGGCTTTTCAAGTAGTCGAGGGGGTATCGCGGTCACGGCCGTTAAC
AGATCCCGTCCTTAGTGTGGAATCCGCACCTGCTGACTAACGCTTCGCCGGCGTGTCTGCACATCCGT
ATAGTGTTAATCATGACCCCAAGGAAGGATTAAACAAATATCTTGACG
>RAGLN07
GTGTCCGGTAGCCCGCGCTAGTCAGACACCCCGGCAGGGGGGATTGCTTTCGAGACGGGAGATCCCTT
CGCCGACCCTGGAGGGCCGACGCCGAGGCATTCGGGCCCCTGCAACGTCAACAGCGGCAAGAAAACGG
GATGAATGGGCGTAATGGGGGGGGTCTGCTGGGGACCCGACGCGGTTGCCGTTTGCGGGGCCCCCGAC
CCATACCGACCCACCTAGGCGTCCAGTTACGGCGCACGTCGGGAGCGTGGTTGCCGTCAGAGCTGTGT
TTCTCGATCAGTCCCCCGCAGTGCCGCAGTATCTTGCCGTGGGCTGCTTTAATCTTGAAAGTGGTTCA
TACATTGGGCGACGAGGTGTCGACTCTCATTGGGGGTAACCGACGGGCACATGCAGTCCCCTCCCCGG
GCAGGCAGAGGCGGGGCCCCCGCGCGCCGGCCCCGGCCCACAATCTTACCAGGGTCCTCAAAGGAGCC
TTTGCATGGTACCCTTCGTGAATGGTTGCTTAAGAGGTCCACAACGTGGTCCGGGCACGGTCGACTAA
ACACTCAAAACAGCGACGGCAAATATAGGTACAAGGTCCAGGCCCTCACGGCACTAATTGCGATGACC
CAACTCACGGGGGCAGTCCCCGGCGCGGCGACCTTGATTACGTCCGGGGACAAGTATGTCGCTCCCCC
CGGGAGGGGTGCAGCCACATGGGAGATTCAAAGTTTCTCGTGACGTCGTTGTGATCACTGCAGCCTAG
CCGAGACTCCCGTACTACGCGAAGGTTGGTTATGTTAACCACTACAACGTGAGGCGCCGTAAGGGCCA
GTGTTGTGCCCGGCTCTTCAATGCGCCTTAGCGGCCTGATACACCCACCCAAGGAGATACTGCTAATC
ATATGGGTGGACAGAACCTCGCAACGCAGACGCCTCCCCGGCATGATATGGTTTTTTTCCGCTATTAT
CCAGCACGCAGCGCTATCATTTAAGAGAACCCAGTGACGCGTAAATCGTAAGATCTACCTGCCGCAGG
TGGACCTACTGCAAATACGGATTATGACTCGTAAAGGGGTCATGCGTATTTTCATCACTAGGCACGTT
CGAGAATAAATTAGTAGGTGTCCCGAGCCNTGTGGCGTTCCGCCTGACTCCTCATGAAGTCGACCTTC
TCACCGGCCCTATCTGCCGACGTAAGTCATAACCTAGATCTGTACCTCGGGGGGAGGGTCACTGTAAA
GGGATAATTGGAGGGTGATTTCCCACATTTTCCTAAGGGTACTTTTTGCCTGGCTTCGCAATTGGGTC
CAATGGATGTCGATCTCTGGTTTAGCAGTTGTGAAAGTGGCAAGGCGGGAGGTTAGACCTCCATTTAA
CATATACAAGCAAGTTAACTGCACTAGATGTGTAGACACTACAGGTACAGGAGTAGCCGGATAAGTCT
CCGACGTCAAGCGAATAAGCGTCATACGCGATTATCGCCTAAGAGCACGTATTTGCGGTAAAAGGCTA
GCTAGACGCTTGTGGGTAGATTTCAAGGCGTTCGTAGTGGTATAATAGGATACTCTTTCACCAGCCTG
AGGGCCGAACGCTATACTAGTGGTCTGTGATGTAGGACCGAGTATCTCTCTAGGGACCATCTACTTGA
GCAATGGTGCGCAGGGGGAGACATAGACCAGCCTTGGGTGGCAAGCACTGCAATAAGTCCTGTTTAGC
CTTGGAGTTCACACGCCGGCACTGAAGCCGACCTACCTGAGCGTGTGCGATTACCGTTACAATGGCAT
CTGTCTAGTTCTGTTTACCTACGGCGCTCTTGGTTCCAGGTTAGGGGAAGTGTATGACCCATGTGTTT
TTATCGGCTTAACCACGAGTGATCCCCGGTCGTTTCCCCATTGAATCCCTGGTGCATCCTACTCCCAG
AATGATAGCTGACTGACTGGACTGGCTTTTCAAGTAATCGAGGGGGTATCGCGGTCACGGCCGTTAAC
AGATCCCGTCCTTAGTGTGGAATCCGCACCTGCTGACTAACGCTTCGCCGGCGTGTCTGCACAGCCGT
ATAGTGTTAATCATGACCCCAAGGAAGGATTAAACAAATATCTTGACG
>RAGLN08
GTGTCCGGTAGCCCGCGCTAGTTAGACACCCCGGCAGGGGGGATTGCTTTCGAGACGGGAGATCCCTT
CGCCGACCCTGTAGGGCCGACGCCGAGGCATTCGGGCCCCTGCAACGTCAACAGCGGCAAGAAAACGG
GATGAATGGGCGTAATGGGGGGGGTCTGCTGGGGACCCGACGCGGTTGCCGTTTGCGGGGCCCCCGAC
CCATACCGACCCACCTAGGCGTCCAGTTACGGCGCACGTCGGGAGCGTGGTTGCCGTCAGAGCTGTGT
TTCTCGATCAGTCCCCCGCAGTGCCGCAGTATCTTGCCGTGGGCTGCTTTAATCTTGAAAGTGGTTTA
TACATTGGGCGACGAGGTGTCGGCTCTCATTGGGGGTAACCGACGGACACGTGCAGTCCCCTCCCCGG
GCAGGCAGAGGCGGGGCCCCCGCGCGCCGGCCCCGGCCCACAATCTTACCAGGGTCCTCAAAAGAGCC
TTTGCATGGTACCCTTCGTGAATGGTTGCTTAAGAGGTCCACCACGTAGTCCGGGCACGGTCAACTAA
ACACTCAAAACAGCGACGGCAAATAGAGGCACAAGGTCCAGGCCCTCACGGCACTAGTTGCGATGACC
CAACTCACGGGGGCAGTCCCCGGCGCAGCGACCTTGATTACGTCCGGGAACAAGTATGTCGTTTCCCC
CGGGAGGGGTGCAGCCACATGGGAGATTCAAAGTTTCTCGTGACGTCGTTGTGATCACTGCAGCCTAG
CCGAGACTCCCGTACTACGCGAAGGTTGGTTATGTTAACCACTACAACGTGAGGCGCCGTAAGGGCCA
GTGTTGTGCCCGGCTCTTCAATGCGCCTTAGCGGCCTGATACACCCACCCAAGGAGATACTGCTAATC
ATGTGGGTGGACAGAACCTCGCAACGCAGACGCATCCCCGGCATGATATGGTTTTTTTCCGCTATTAC
CCAGCACGCGGCGCTATCATTCAAGAGAACCCAGTGACGCGTAAATCGTCAGATCTACCTGCCGCAGG
TGGACCTACTGCAAATACGGATTATGACTCGTAAAGAGGTCATGCGTATTTTCATCACTAGGCACTTT
CGAGAGTAAATTAGTAGGTGTCCCGCGCCTTGTGGCGTTCCGCCTGGCTCCTCATGAAGTCGACCTTC
TCATCGGCCCTATTTGCCGACGTAAGTCATAATCCAGATCTTCACCTCGGAGGGAGGGTCACTGTAAA

GGGATAATTGGAGGGCGATTTCCCACACTTTCCTAAGGGTACTTTTTGCTTAGCTTCGCAGTTGGGTC
CAATAGATGTTGATCTCTGGTTTAGCAGTTGTGAAAGTGGCAAGGCGGGAGGTTAGGCCTCCATTTAA
CATATACAAGCAAGTTAACTGCACTAGATGTGTAGACACTACAGTTACAGGAGTAGCCGACTAAGTCT
CCGACGTCAAGCGAATAGGCGTCATACGCGATTATTGCCTAAGAGCACGTATTGGCGGTAAAAGGATA
ACTAGACGCTTGTGGGTAGATTTCAAGGCGCTCGTAGTGGTATAATAGGACACTCTTGCACCAGCCTG
AGAGCCGAACGCTATACTAGTGGTCTGTGATGTAGGACCAAGTAGCTCTCTAGGGACCATCTACTTGA
GCAATGGTGCGCAGGGGTAGACATAGACCAACCTTGGGTGGCAAGCACTGCAATAAGTCCTGTTTAGC
CTTGGAGTTCACACGCCGGCACTAAAGCCGACCTACCTGAGCTTGTGCGATTACCGTTACAATGGCAT
CTGTCTAGTTCTGTTTACCTACGGCGCTCTTGGTTCCATGTTAGGGGAAGTGTATGACCCATGTGTTT
TTATCGGCTTAACTACGAGTGATCCCCGGTCGTTTCCCCATTAAATCCCTGGTGCATCCTACTCCCAT
AATGATAGCTGACTGGCTGGACTGGCTTTTCAAGTAGTCGAGGGGGTATCGCGGTCACGGCCGTTAAC
AGATCCCGTCCTTAGTGTGGAATCCGCACCTGCTGACTAACGCTTCGCCGGCGTGTCTGCACATCCGT
ATAGTGTTAATCATGACCCCAAGGAAGGATTAAACAAATATCTTGACG
>RAGLN09
GTGTCCGGTAGCCCGCGCTAGTTAGACACCCCGGCAGGGGGGATTGCTTTCGGGACGGGAGATCCCTT
CGCCGACCCTGGAGGGCCGACGCCGAGGCATTCGGGCCCCTGCAACGTCAACAGCGGCAAGAAAACGG
GATGAATGGGCGTAATGGGGGGGGTCTGCTGGGGACCCGACGCGGTTGCCGTTTGCGGGGCCCCCGAC
CCATACCGACCCACCTAGGCGTCCAGTTACGGCGCACGTCGGGAGCGTGGTTGCCGTCAGAGCTGTGT
TTCTCGATCAGTCCCCCGCAGTGCCGCAGTATCTTGCCGTGGGCTGCTTTAATCTTGAAAGTGGTTNA
TACATTGGGCGACGAGGTGTCGACTCTCATTGGGGGTAACCGACGGGCACATGCAGTCCCCTCCCCGG
GCAGGCAGAGGCGGGGCCCCCGCGCGCCGGCCCCGGCCCACAATCTTACCAGGGTCCTCAAAGGAGCC
TTTGCATGGTACCCTTCGTGAATGGTTGCTTAAGAGGTCCACAACGTGGTCCGGGCACGGTCGACTAA
ACACTCAAAACAGCGACGGCAAATATAGGTACAAGGTCCAGGCCCTCACGGCACTAATTGCGATGACC
CAACTCACGGGGGCAGTCCCCGGCGCGGCGACCTTGATTACGTCCGGGGACAAGTATGTCGCTCCCCC
CGGGAGGGGTGCAGCCACATGGGAGATTCAAAGTTTCTCGTGACGTCGTTGTGATCACTGCAGCCTAG
CCGAGACTCCCGTACTACGCGAAGNTTGGTTATGTTAACCACTACAACGTGAGGCGCCGTAAGGGCCA
GTGTTGTGCCCGGCTCTTCAATGCGCCTTAGCGGCCTGATACACCCACCCAAGGAGATACTGCTAATC
ATATGGGTGGACAGAACCTCGCAACGCAGACGCCTCCCCGGCATGATATGGTTTTTTTCCGCTATTAT
CCAGCACGCAGCGCTATCATTCAAGAGAACCCAGTGACGCGTAAATCGTAAGATCTACCTGCCGCAGG
TGGACCTACTGCAAATACGGATTATGACTCGTAAAGGGGTCATGCGTATTTTCATCACTAGGCACGTT
CGAGAATAAATTAGTAGGTGTCCCGAGCCTTGTGGCGTTCCGCCTGACTCCTCATGAAGTCGACCTTC
TCACCGGCCCTATCTGCCGACGTAAGTCATAACCTAGATCTGTACCTCGGGGGGAGGGTCACTGTAAA
GGGATAATTGGAGGGTGATTTCCCACACTTTCCTAAGGGTACTTTTTGCCTGGCTTCGCAATTGGGTC
CAATGGATGTCGATCTCTGGTTTAGCAGTTGTGAAAGTGGCAAGGCGGGAGGTTAGACCTCCATTTAA
CATATACAAGCAAGTTAACTGCACTAGATGTGTAGACACTACAGTTACAGGAGTAGCCGAATAAGTCT
CCGACGTCAAGCGAATAAGCGTCATACGCGATTATCGCCTAAGAGCACGTATTGGCGGTAAAAGGCTA
GCTAGACGCTTATGGGTAGATTTCAAGGCGTTCGTAGTGGTATAATAGGATACTCTTTCACCAGCCTG
AGGGCCGAACGCTATACTAGTGGTCTGTGATGTAGGACCGAGTATCTCTCTAGGGACCATCTACTTGA
GCAATGGTGCGCAGGGGGAGACATAGACCAGCCTTGGGTGGCAAGCACTGCGATAAGTCCTGTTTAGC
CTTGGAGTTCACATGCCGGCACTGAAGCCGACCTACCTGAGCGTGTGCGATTACCGTTACAATGGCAT
CTGTCTAGTTCTGTTTACCTACGGCGCTCTTGGTTCCAGGTTAGGGGAAGTGTATGACCCATGTGTTT
TTATCGGCTTAACCACGAGTGATCCCCGGTCGTTTCCCCATTGAATCCCTGGTGCATCCTACTCCCAG
AATGATAGCTGACTGACTGGACTGGCTTTTCAAGTAATCGAGGGGGTATCGCGGTCACGGCCGTTAAC
AGATCCCGTCCTTAGTGTGGAATCCGCACCTGCTGACTAACGCTTCGCCGGCGTGTCTGCACAGCCGT
ATAGTGTTAATCATGACCCCAAGGAAGGATTAAACAAATATCTTGACG
>RAGLN10
GTGTCCGGTAGCCCGCGCTAGTTAGATACCCCGGCAGGGGGGATTGCTTTCGAGACGGGAGATCCCTT
CGCCGACCCTGGAGGGCCGACGCCGAGGCATTCGGGCCCCTGCAACGTCAACAGCGGCAAGAAAACGG
GATGAATGGGCGTAATGGGGGGGGTCTGCTGGGGACCCGACGCGGTTGCCGTTTGCGGGGCCCCCGAC
CCATACCGACCCACCTAGGCGTCCAGTTACGGCGCACGTCGGGAGCGTGGTTGCCGTCAGAGCTGTGT
TTCTCGATCAGTCCCCCGCAGTGCCGCAGTATCTTGCCGTGGGCTGCTTTAANNTTGAAAGTGGTTCA
TACATTGGGCGACGAGGTGTCGACTCTCATTGGGGGTAACCGACGGGCACATGCAGTCCCCTCCCCGG
GCAGGCAGAGGCGGGGCCCCCGCGCGCCGGCCCCGGCCCACAATCTTACCAGGGTCCTCAAGGGAGCC
TTTGCATGGTACCCTTCGTGAATGGTTGCTTAAGAGGTCCACCACGTGGTCCGGGCACGGTCGACTAA

ACACTCAAAACAGCGACGGCAAATATAGGCACAAGGTCCAGGCTCTCACGGCACTAATTGCGATGACC
CAACTCACGGGGGCAGTCCCCGGCGCGGCGACCGTGATTACGGCCGGGGATAAGTATGTCGTTCCCCC
CGGGAGGGGTGCAGCCACATGGGAGATTCAAAGTTTCTCGTGACGTCGTTGTGATCACTGCAGCCTAG
CCGAGACTCCCGTGCTACGCGAAGGTTGGTTATGTTAACCACTACAACGTGAGGCGCCGTAAGGGCCA
GTGTTGTGCCCGGCTCTTCAATGCGCCTTAGCGGCCTGATACACCCACCAAAGGAGATACTGCTAATC
ATGTGGGTGGACAGAAACTCGCAACACAGACGCCTCCCCGGCATGATATGGTTTTTTTCCGCTATTAT
CCAGCACGCAGCGCTATCATTCAAGAGAACCCAGTGACGCGTAAATCGTAANATCTACCTGCCGCAGG
TGGACCTCCTGCAAATACGGATTATGACTCGTAAAGAGGTCATGCGTATTTTCATCACTAGGCACGTT
CGAGAATAAATTAGTAGGTGTCCCGCGCCTTGTGGCGTTCCGCCTGACTCCTCATGAAGTCGACCTTC
TCACCGGCCCTATCTGCCGACGTAAGTCATAACCTAGATCTGTACCTCGGGGGGAGGGTCACTGTAAA
GGGATAATTGGAGGGTGATTTCCCACACTTTCCTAAGGGTACTTTTTGCCTGGCTTCGCAATTGGGTC
CAATGGATGTCGCTCTCTGGTTTAGCAGTTGTGAAAGTGGCAAGGCGGGAGGTTAGACCTCCATTTAA
CATATACAAGCAAGTTAACTGCACTAGATGTGTAGACACTACAGTTACAGGAGTAGCCGAATNAGTCT
CCGACGNCAAGCGAATAAGCGTCATACGCGATTATCGCCTAAGAGCACGTATTGGCGGTAAAAGGCTA
ACTAGACGCTTGTGGGTAGATTTCAAGGCGTTCGTAGTGGTATAACAGGATACTCTTTCACCAGCCTG
AGGGCCGAACGCTATACTAGTGGTCTGTGATGTAGGACCGAGTATCTCTCTAGGGACCATCTACTTGA
GCAATGGTGCGCAGGGGGAGACATAGACCAGCCTTGGGTGGCAAGCACTGCAATAAGTCCTGTTTAGC
CTTGGAGTTCACACGCCGGCACTGAAGCCGACCTACCTGAGCTTGTGCGATTACCGTTACAATGGCAT
CTGTCTAGTTCTGTTTACCTACGGCGCTCTTGGTTCCAGGTTAGGGGAAGTGTATGACCCATGTGTTT
TTATCGGCTTAACCACGAGTGATCCCCGGTCGTTTCCCCATTGAATCCCTGGTGCATCCTACTCCCAG
AATGATAGCTGACTGACTGGACTGGCTTTTCAAGTAGTCGAGGGGGTATCGCGGTCACGGCCGTTAAC
AGATCCCGTCCTTAGTGTGGAATCCGCACCTGCTGACTAACGCTTCGCCGGCGTGTCTGCACAGCCGT
ATAGTGTTNATCATGACCCCAAGGAAGGATTAAACAAATATCTTGACG
>RAGLN11
GTGTCCGGTAGCCCGCGCTAGTTAGACACCCCGGCAGGGGGGATTGCTTTCGAGACGGGAGATCCCTT
CGCCGACCCTGGAGGGCCGACGCCGAGGCATTCGGGCCCCTGCAACGTCAACAGCGGCAAGAAAACGG
GATGAATGGGCGTAATGGGGGGGGTCTGCTGGGGACCCGACGCGGTTGCCGTTTGCGGGGCCCCCGAC
CCATACCGACCCACCTAGGCGTCCAGTTACGGCGCACGTCGGGAGCGTGGTTGCCGTCAGAGCTGTGT
TTCTCGATCAGTCCCCCGCAGTGCCGCAGTATCTTGCCGTGGGCTGCTTTAATCTTGAAAGTGGTTTA
TACATTGGGCGACGAGGTGTCGACTCTCATTGGGGGTAACCGACGGGCACATGCAGTCCCCTCCCCGG
GCAGGCAGAGGCGGGGCCCCCGCGCGCCGGCCCCGGCCCACAATCTTACCAGGGTCCTCAAAGGAGCC
TTTGCATGGTACCCTTCGTGAATGGTTGCTTAAGAGGTCCACAACGTGGTCCGGGCACGGTCGACTAA
ACACTCAAAACAGCGACGGCAAATATAGGTACAAGGTCCAGGCCCTCACGGCACTAATTGCGATGACC
CAACTCACGGGGGCAGTCCCCGGCGCGGCGACCTTGATTACGTCCGGGGACAAGTATGTCGCTCCCCC
CGGGAGGGGTGCAGCCACATGGGAGATTCAAAGTTTCTCGTGACGTCGTTGTGATCACTGCAGCCTAG
CCGAGACTCCCGTACTACGCGAAGGTTGGTTATGTTAACCACTACAACGTGAGGCGCCGTAAGGGCCA
GTGTTGTGCCCGGCTCTTCAATGCGCCTTAGCGGCCTGATACACCCACCCAAGGAGATACTGCTAATC
ATATGGGTGGACAGAACCTCGCAACGCAGACGCCTCCCCGGCATGATATGGTTTTTTTCCGCTATTAT
CCAGCACGCAGCGCTATCATTCAAGAGAACCCAGTGACGCGTAAATCGTAAGATCTACCTGCCGCAGG
TGGACCTACTGCAAATACGGATTATGACTCGTAAAGGGGTCATGCGTATTTTCATCACTAGGCACGTT
CGAGAATAAATTAGTAGGTGTCCCGAGCCTTGTGGCGTTCCGCCTGACTCCTCATGAAGTCGACCTTC
TCACCGGCCCTATCTGCCGACGTAAGTCATAACCTAGATCTGTACCTCGGGGGGAGGGTCACTGTAAA
GGGATAATTGGAGGGTGATTTCCCACACTTTCCTAAGGGTACTTTTTGCCTGGCTTCGCAATTGGGTC
CAATGGATGTCGATCTCTGGTTTAGCAGTTGTGAAAGTGGCAAGGCGGGAGGTTAGACCTCCATTTAA
CATATACAAGCAAGTTAACTGCACTAGATGTGTAGACACTACAGTTACAGGAGTAGCCGAATAAGTCT
CCGACGTCAAGCGAATAAGCGTCATACGCGATTATCGCCTAAGAGCACGTATTGGCGGTAAAAGGCTA
GCTAGACGCTTATGGGTAGATTTCAAGGCGTTCGTAGTGGTATAATAGGATACTCTTTCACCAGCCTG
AGGGCCGAACGCTATACTAGTGGTCTGTGATGTAGGACCGAGTATCTCTCTAGGGACCATCTACTTGA
GCAATGGTGCGCAGGGGGAGACATAGACCAGCCTTGGGTGGCAAGCACTGCAATAAGTCCTGTTTAGC
CTTGGAGTTCACATGCCGGCACTGAAGCCGACCTACCTGAGCGTGTGCGATTACCGTTACAATGGCAT
CTGTCTAGTTCTGTTTACCTACGGCGCTCTTGGTTCCAGGTTAGGGGAAGTGTATGACCCATGTGTTT
TTATCGGCTTAACCACGAGTGATCCCCGGTCGTTTCCCCATTGAATCCCTGGTGCATCCTACTCCCAG
AATGATAGCTGACTGACTGGACTGGCTTTTCAAGTAATCGAGGGGGTATCGCGGTCACGGCCGTTAAC
AGATCCCGTCCTTAGTGTGGAATCCGCACCTGCTGACTAACGCTTCGCCGGCGTGTCTGCACAGCCGT

ATAGTGTTAATCATGACCCCAAGGAAGGATTAAACAAATATCTTGACG
>RAGLN12
GTGTCCGGTAGCCCGCGCTAGTTAGACACCCCGGCAGGGGGGATTGCTTTCGAGACGGGAGATCCCTT
CGCCGACCCTGGAGGGCCGACGCCGAGGCATTCGGGCCCCTGCAACGTCAACAGCGGCAAGAAAACGG
GATGAATGGGCGTAATGGGGGGGGTCTGCTGGGGACCCGACGCGGTTGCCGTTTGCGGGGCCCCCGAC
CCATACCGACCCACCTAGGCGTCCAGTTACGGCGCACGTCGGGAGCGTGGTTGCCGTCAGAGCTGTGT
TTCTCGATCAGTCCCCCGCAGTGCCGCAGTATCTTGCCGTGGGCTGCTTTAATCTTGAAAGTGGTTCA
TACATTGGGCGACGAGGTGTCGACTCTCATTGGGGGTAACCGACGGGCACATGCAGTCCCCTCCCCGG
GCAGGCAGAGGCGGGGCCCCCGCGCNCCGGCCCCGGCCCACAATCTTACCAGGGTCCTCAAAGGAGCC
TTTGCATGGTACCCTTCGTGAATGGTTGCTTAAGAGGTCCACAACGTGGTCCGGGCACGGTCGACTAA
ACACTCAAAACAGCGACGGCAAATATAGGTACAAGGTCCAGGCCCTCACGGCACTAATTGCGATGACC
CAACTCACGGGGGCAGTCCCCGGCGCGGCGACCTTGATTACGTCCGGGGACAAGTATGTCGCTCCCCC
CGGGAGGGGTGCAGCCACATGGGAGATTCAAAGTTTCTCGTGACGTCGTTGTGATCACTGCAGCCTAG
CCGAGACTCCCGTACTACGCGAAGGTTGGTTATGTTAACCACTACAACGTGAGGCGCCGTAAGGGCCA
GTGTTGTGCCCGGCTCTTCAATGCGCCTTAGCGGCCTGATACACCCACCCAAGGAGATACTGCTAATC
ATATGGGTGGACAGAACCTCGCAACGCAGACGCCTCCCCGGCATGATATGGTTTTTTTCCGCTATTAT
CCAGCACGCAGCGCTATCATTCAAGAGAACCCAGTGACGCGTAAATCGTAAGATCTACCTGCCGCAGG
TGGACCTACTGCAAATACGGATTATGACTCGTAAAGGGGTCATGCGTATTTTCATCACTAGGCACGTT
CGAGAATAAATTAGTAGGTGTCCCGAGCCTTGTGGCGTTCCGCCTGACTCCTCATGAAGTCGACCTTC
TCACCGGCCCTATCTGCCGACGTAAGTCATAACCTAGATCTGTACCTCGGGGGGAGGGTCACTGTAAA
GGGATAATTGGAGGGTGATTTCCCACACTTTCCTAAGGGTACTTTTTGCCTGGCTTCGCAATTGGGTC
CAATGGATGTCGATCTCTGGTTTAGCAGTTGTGAAAGTGGCAAGGCGGGAGGTTAGACCTCCATTTAA
CATATACAAGCAAGTTAACTGCACTAGATGTGTAGACACTACAGTTACAGGAGTAGCCGAATAAGTCT
CCGACGTCAAGCGAATAAGCGTCATACGCGATTATCGCCTAAGAGCACGTATTGGCGGTAAAAGGCTA
GCTAGACGCTTATGGGTAGATTTCAAGGCGTTCGTAGTGGTATAATAGGATACTCTTTCACCAGCCTG
AGGGCCGAACGCTATACTAGTGGTCTGTGATGTAGGACCGAGTATCTCTCTAGGGACCATCTACTTGA
GCAATGGTGCGCAGGGGGAGACATAGACCAGCCTTGGGTGGCAAGCACTGCAATAAGTCCTGTTTAGC
CTTGGAGTTCACATGCCGGCACTGAAGCCGACCTACCTGAGCGTGTGCGATTACCGTTACAATGGCAT
CTGTCTAGTTCTGTTTACCTACGGCGCTCTTGGTTCCAGGTTAGGGGAAGTGTATGACCCATGTGTTT
TTATCGGCTTAACCACGAGTGATCCCCGGTCGTTTCCCCATTGAATCCCTGGTGCATCCTACTCCCAG
AATGATAGCTGACTGACTGGACTGGCTTTTCAAGTAATCGAGGGGGTATCGCGGTCACGGCCGTTAAC
AGATCCCGTCCTTAGTGTGGAATCCGCACCTGCTGACTAACGCTTCGCCGGCGTGTCTGCACAGCCGT
ATAGTGTTAATCATGACCCCAAGGAAGGATTAAACAAATATCTTGACG
>RAGLN14
GTGTCCGGTAGCCCGCGCTAGTTAGACACCCCGGCAGGGGGGATTGCTTTCGGGACGGGAGATCCCTT
CGCCGACCCTGTAGGGCCGACGCCGAGGCATTCGGGCCCCTGCAACGTCAACAGCGGCAAGAAAACGG
GATGAATGGGCGTAATGGGGGGGGTCTGCTGGGGACCCGACGCGGTTGCCGTTTGCGGGGCCCCCGAC
CCATACCGACCCACCTAGGCGTCCAGTTACGGCGCACGGCGGGAGCGTGGTTGCCGTCAGAGCTGTGT
TTCTCGATCAGTCCCCCGCAGTGCCGCAGTATCTTGCCGTGGGCTGCTTTAATCTTGAAAGTGGTTCA
TACATTGGGCGACGAGGTGTCGGCTCTCATTGGGGGTAGCCGACGGACACATGCAGTCCCCTCCCCGG
GCAGGCAGAGGCGGGGCCCCCGCGCGCCGGCCCCGGCCCACAATCTTACCAGGGTCCTCAAAAGAGCC
TTTGCATGGTACCCTTCGTGAATGGTTGCTTAAGAGGTCCACCACGTAGTCCGGGCACGGTCGACTAA
ACACTCAAAACAGCGACGGCAAATAGAGGCACAAGGTCCAGGCCCTCACGGCACTAGTTGCGATGACC
CAACTCACGGGGGCAGTCCCCGGCGCGGCGACCTTGATTACGTCCGGGAACAAGTATGTCGTTTCCCC
CGGGAGGGGTGCAGCCACATGGGAGATTCAAAGTTTCTCGTGACGTCGTTGTGATCACTGCAGCCTAG
CCGAGACTCCCGTACTACGCGAAGGTTGGTTATGTTAACCACTACAACGTGAGGCGCCGTAAGGGCCA
GTGTTGTGCCCGGCTCTTCAATGCGCCTTAGCGGCCTGATACACCCACCCAAGGAGATACTGCTAATC
ATGTGGGTGGACAGAACCTCGCAACGCAGACGCATCCCCGGCATGATATGGTTTTTTTCCGCTATTAC
CCAGCACGCGGCGCTATCATTCAAGAGAACCCAGTGACGCGTAAATCGTAAGATCTACCTGCCGCAGG
TGGACCTACTGCAAATACGGATTATGACTCGTAAAGAGGTCATGCGTATTTTCATCACTAGGCACGTT
CGAGAGTAAATTAGTAGGTGTCCCGCGCCTTGTGGCGTTCCGCCTGACTCCTCATGAAGTCGACCTTC
TCATCGGCCCTATCTGCCGACGTAAGTCATAATCCAGATCTTCACCTCGGAGGGAGGGTCACTGTAAA
GGGATAATTGGAGGGCGATTTCCCACACTTTCCTAAGGGTACTTTTTGCTTGGCTTCGCAGTTGGGTC
CAATAGATGTTGATCTCTGGTTTAGCAGTTGTGAAAGTGGCAAGGCGGGAGGTTAGACCTCCATTTAA

CATATACAAGCAAGTTAACTGCACTAGATGTGTAGACACTACAGTTACAGGAGTAGCCGACTAAGTCT
CCGACGTCAAGCGAATAGGCGTCATACGCGATTATCGCCTAAGAGCACGTATTGGCGGTAAAAGGATA
ACTAGACGCTTGTGGGTAGATTTCAAGGCGCTCGTAGTGGTATAATAGGATACTCTTTCACCAGCCTG
AGAGCCGAACGCTATACTAGTGGTCTGTGATGTAGGACCAAGTAGCTCTCTAGGGACCATCTACTTGA
GCAATGGTGCGCAGGGGTAGACATAGACCAACCTTGGGTGGCAAGCACTGCAATAAGTCCTGTTTAGC
CTTGGAGTTCACACGCCGGCACTAAAGCCGACCTACCTGAGCTTGTGCGATTACCGTTACAATGGCAT
CTGTCTAGTTCTGTTTACCTACGGCGCTCTTGGTTCCATGTTAGGGGAAGTGTATGACCCATGTGTTT
TTATCGGCTTAACTACGAGTGATCCCCGGTCGTTTCCCCATTAAATCCCTGGTGCATCCTACTCCCAT
AATGATAGCTGACTGGCTGGACTGGCTTTTCAAGTAGTCGAGGGGGTATCGCGGTCACGGCCGTTAAC
AGATCCCGTCCTTAGTGTGGAATCCGCACCTGCTGACTAACGCTTCGCCGGCGTGTCTGCACATCCGT
ATAGTGTTAATCATGACCCCAAGGAAGGATTAAACAAATATCTTGACG
>RAGLN16
GTGTCCGGTAGCCCGCGCTAGTTAGACACCCCGGCAGGGGGGATTGCTTTCGAGACGGGAGATCCCTT
CGCCGACCCTGGAGGGCCGACGCCGAGGCATTCGGGCCCCCGCAACGTCAACAGCGGCAAGAAAACGG
GATGAATGGGCGTAATGGGGGGGGTCTGCTGGGGACCCGACGCGGTTGCCGTTTGCGGGGCCCCCGAC
CCATACCGACCCACCTAGGCGTCCAGTTACGGCGCACGGCGGGAGCGTGGTTGCCGTCAGAGCTGTGT
TTCTCGATCAGTCCCCCGCAGTGCCGCAGTATCTTGCCGTGGGCTGCTTTAATCTTGAAAGTGGTTTA
TACATTGGGCGACGAGGTGTCGACTCTCATTGGGGGTAACCGACGGGCACATGCAGTCCCCTCCCCGG
GCAGGCAGAGGCGGGGCCCCCGCGCGCCGGCCCCGGCCCACAATCTTACCAGGGTCCTCAAAGGAGCC
TTTGCATGGTACCCTTCGTGAATGGTTGCTTAAGAGGTCCACAACGTGGTCCGGGCACGGTCGACTAA
ACACTCAAAACAGCGACGGCAAATATAGGTACAAGGTCCAGGCCCTCACGGCACTAATTGCGATGACC
CAACTCACGGGGGCAGTCCCCGGCGCGGCGACCTTGATTACGTCCGGGGACAAGTATGTCGCTCCCCC
CGGGAGGGGTGCAGCCACATGGGAGATTCAAAGTTTCTCGTGACGTCGTTGTGATCACTGCAGCCTAG
CCGAGACTCCCGTACTACGCGAAGGTTGGTTATGTTAACCACTACAACGTGAGGCGCCGTAAGGGCCA
GTGTTGTGCCCGGCTCTTCAATGCGCCTTAGCGGCCTGATACACCCACCCAAGGAGATACTGCTAATC
ATATGGGTGGACAGAACCTCGCAACGCAGACGCCTCCCCGGCATGATATGGTTTTTTTCCGCTATTAT
CCAGCACGCAGCGCTATCATTCAAGAGAACCCAGTGACGCGTAAATCGTAAGATCTACCTGCCGCAGG
TGGACCTACTGCAAATACGGATTATGACTCGTAAAGGGGTCATGCGTATTTTCATCACTAGGCACGTT
CGAGAATAAATTAGTAGGTGTCCCGAGCCTTGTGGCGTTCCGCCTGACTCCTCATGAAGTCGACCTTC
TCACCGGCCCTATCTGCCGACGTAAGTCATAACCTAGATCTGTACCTCGGGGGGAGGGTCACTGTAAA
GGGATAATTGGAGGGTGATTTCCCACACTTTCCTAAGGGTACTTTTTGCCTGGCTTCGCAATTGGGTC
CAATGGATGTCGATCTCTGGTTTAGCAGTTGTGAAAGTGGCAAGGCGGGAGGTTAGACCTCCATTTAA
CATATACAAGCAAGTTAACTGCACTAGATGTGTAGACACTACAGTTACAGGAGTAGCCGAATAAGTCT
CCGACGTCAAGCGAATAAGCGTCATACGCGATTATCGCCTAAGAGCACGTATTGGCGGTAAAAGGCTA
GCTAGACGCTTATGGGTAGATTTCAAGGCGTTCGTAGTGGTATAATAGGATACTCTTTCACCAGCCTG
AGGGCCGAACGCTATACTAGTGGTCTGTGATGTAGGACCGAGTATCTCTCTAGGGACCATCTACTTGA
GCAATGGTGCGCAGGGGGAGACATAGACCAGCCTTGGGTGGCAAGCACTGCGATAAGTCCTGTTTAGC
CTTGGAGTTCACATGCCGGCACTGAAGCCGACCTACCTGAGCGTGTGCGATTACCGTTACAATGGCAT
CTGTCTAGTTCTGTTTACCTACGGCGCTCTTGGTTCCAGGTTAGGGGAAGTGTATGACCCATGTGTTT
TTATCGGCTTAACCACGAGTGATCCCCGGTCGTTTCCCCATTGAATCCCTGGTGCATCCTACTCCCAG
AATGATAGCTGACTGACTGGACTGGCTTTTCAAGTAATCGAGGGGGTATCGCGGTCACGGCCGTTAAC
AGATCCCGTCCTTAGTGTGGAATCCGCACCTGCTGACTAACGCTTCGCCGGCGTGTCTGCACAGCCGT
ATAGTGTTAATCATGACCCCAAGGAAGGATTAAACAAATATCTTGACG
>RAGLN17
GTGTCCGGTAGCCCGCGCTAGTCAGACACCCCGGCAGGGGGGATTGCTTTCGGGACGGGAGATCCCTT
CGCCGACCCTGGAGGGCCGACGCCGAGGCATTCGGGCCCCTGCAACGTCAACAGCGGCAAGAAAACGG
GATGAATGGGCGTAATGGGGGGGGTCTGCTGGGGACCCGACGCGGTTGCCGTTTGCGGGGCCCCCGAC
CCATACCGACCCACCTAGGCGTCCAGTTACGGCGCACGGCGGGAGCGTGGTTGCCGTCAGAGCTGTGT
TTCTCGATCAGTCCCCCGCAGTGCCGCAGTATCTTGCCGTGGGCTGCTTTAATCTTGAAAGTGGTTCA
TACATTGGGCGACGAGGTGTCGACTCTCATTGGGGGTAACCGACGGGCACATGCAGTCCCCTCCCCGG
GCAGGCAGAGGCGGGGCCCCCGCGCGCCGGCCCCGGCCCACAATCTTACCAGGGTCCTCAAAGGAGCC
TTTGCATGGTACCCTTCGTGAATGGTTGCTTAAGAGGTCCACAACGTGGTCCGGGCACGGTCGACTAA
ACACTCAAAACAGCGACGGCAAATATAGGTACAAGGTCCAGGCCCTCACGGCACTAATTGCGATGACC
CAACTCACGGGGGCAGTCCCCGGCGCGGCGACCTTGATTACGTCCGGGGACAAGTATGTCGCTCCCCC

CGGGAGGGGTGCAGCCACATGGGAGATTCAAAGTTTCTCGTGACGTCGTTGTGATCACTGCAGCCTAG
CCGAGACTCCCGTACTACGCGAAGGTTGGTTATGTTAACCACTACAACGTGAGGCGCCGTAAGGGCCA
GTGTTGTGCCCGGCTCTTCAATGCGCCTTAGCGGCCTGATACACCCACCCAAGGAGATACTGCTAATC
ATATGGGTGGACAGAACCTCGCAACGCAGACGCCTCCCCGGCATGATATGGTTTTTTTCCGCTATTAT
CCAGCACGCAGCGCTATCATTTAAGAGAACCCAGTGACGCGTAAATCGTAAGATCTACCTGCCGCAGG
TGGACCTACTGCAAATACGGATTATGACTCGTAAAGGGGTCATGCGTATTTTCATCACTAGGCACGTT
CGAGAATAAATTAGTAGGTGTCCCGAGCCTTGTGGCGTTCCGCCTGACTCCTCATGAAGTCGACCTTC
TCACCGGCCCTATCTGCCGACGTAAGTCATAACCTAGATCTGTACCTCGGGGGGAGGGTCACTGTAAA
GGGATAATTGGAGGGTGATTTCCCACATTTTCCTAAGGGTACTTTTTGCCTGGCTTCGCAATTGGGTC
CAATGGATGTCGATCTCTGGTTTAGCAGTTGTGAAAGTGGCAAGGCGGGAGGTTAGACCTCCATTTAA
CATATACAAGCAAGTTAACTGCACTAGATGTGTAGACACTACAGGTACAGGAGTAGCCGGATAAGTCT
CCGACGTCAAGCGAATAAGCGTCATACGCGATTATCGCCTAAGAGCACGTATTTGCGGTAAAAGGCTA
GCTAGACGCTTGTGGGTAGATTTCAAGGCGTTCGTAGTGGTATAATAGGATACTCTTTCACCAGCCTG
AGGGCCGAACGCTATACTAGTGGTCTGTGATGTAGGACCGAGTATCTCTCTAGGGACCATCTACTTGA
GCAATGGTGCGCAGGGGGAGACATAGACCAGCCTTGGGTGGCAAGCACTGCAATAAGTCCTGTTTAGC
CTTGGAGTTCACACGCCGGCACTGAAGCCGACCTACCTGAGCGTGTGCGATTACCGTTACAATGGCAT
CTGTCTAGTTCTGTTTACCTACGGCGCTCTTGGTTCCAGGTTAGGGGAAGTGTATGACCCATGTGTTT
TTATCGGCTTAACCACGAGTGATCCCCGGTCGTTTCCCCATTGAATCCCTGGTGCATCCTACTCCCAG
AATGATAGCTGACTGACTGGACTGGCTTTTCAAGTAATCGAGGGGGTATCGCGGTCACGGCCGTTAAC
AGATCCCGTCCTTAGTGTGGAATCCGCACCTGCTGACTAACGCTTCGCCGGCGTGTCTGCACAGCCGT
ATAGTGTTAATCATGACCCCAAGGAAGGATTAAACAAATATCTTGACG
>RAGLN18
GTGTCCGGTAGCCCGCGCTAGTTAGACACCCCGGCAGGGGGGATTGCTTTCGAGACGGGAGATCCCTT
CGCCGACCCTGGAGGGCCGACGCCGAGGCATTCGGGCCCCNGCAACGTCAACAGCGGCAAGAAAACGG
GATGAATGGGCGTAATGGGGGGGGTCTGCTGGGGACCCGACGCGGTTGCCGTTTGCGGGGCCCCCGAC
CCATACCGACCCACCTAGGCGTCCAGTTACGGCGCACGGCGGGAGCGTGGTTGCCGTCAGAGCTGTGT
TTCTCGATCAGTCCCCCGCAGTGCCGCAGTATCTTGCCGTGGGCTGCTTTAATCTTGAAAGTGGTTTA
TACATTGGGCGACGAGGTGTCGACTCTCATTGGGGGTAACCGACGGGCACATGCAGTCCCCTCCCCGG
GCAGGCAGAGGCGGGGCCCCCGCGCGCCGGCCCCGGCCCACAATCTTACCAGGGTCCTCAAAGGAGCC
TTTGCATGGTACCCTTCGTGAATGGTTGCTTAAGAGGTCCACAACGTGGTCCGGGCACGGTCGACTAA
ACACTCAAAACAGCGACGGCAAATATAGGTACAAGGTCCAGGCCCTCACGGCACTAATTGCGATGACC
CAACTCACGGGGGCAGTCCCCGGCGCGGCGACCTTGATTACGTCCGGGGACAAGTATGTCGCTCCCCC
CGGGAGGGGTGCAGCCACATGGGAGATTCAAAGTTTCTCGTGACGTCGTTGTGATCACTGCAGCCTAG
CCGAGACTCCCGTACTACGCGAAGGTTGGTTATGTTAACCACTACAACGTGAGGCGCCGTAAGGGCCA
GTGTTGTGCCCGGCTCTTCAATGCGCCTTAGCGGCCTGATACACCCACCCAAGGAGATACTGCTAATC
ATATGGGTGGACAGAACCTCGCAACGCAGACGCCTCCCCGGCATGATATGGTTTTTTTCCGCTATTAT
CCAGCACGCAGCGCTATCATTCAAGAGAACCCAGTGACGCGTAAATCGTAAGATCTACCTGCCGCAGG
TGGACCTACTGCAAATACGGATTATGACTCGTAAAGGGGTCATGCGTATTTTCATCACTAGGCACGTT
CGAGAATAAATTAGTAGGTGTCCCGAGCCTTGTGGCGTTCCGCCTGACTCCTCATGAAGTCGACCTTC
TCACCGGCCCTATCTGCCGACGTAAGTCATAACCTAGATCTGTACCTCGGGGGGAGGGTCACTGTAAA
GGGATAATTGGAGGGTGATTTCCCACACTTTCCTAAGGGTACTTTTTGCCTGGCTTCGCAATTGGGTC
CAATGGATGTCGATCTCTGGTTTAGCAGTTGTGAAAGTGGCAAGGCGGGAGGTTAGACCTCCATTTAA
CATATACAAGCAAGTTAACTGCACTAGATGTGTAGACACTACAGTTACAGGAGTAGCCGAATAAGTCT
CCGACGTCAAGCGAATAAGCGTCATACGCGATTATCGCCTAAGAGCACGTATTGGCGGTAAAAGGCTA
GCTAGACGCTTATGGGTAGATTTCAAGGCGTTCGTAGTGGTATAATAGGATACTCTTTCACCAGCCTG
AGGGCCGAACGCTATACTAGTGGTCTGTGATGTAGGACCGAGTATCTCTCTAGGGACCATCTACTTGA
GCAATGGTGCGCAGGGGGAGACATAGACCAGCCTTGGGTGGCAAGCACTGCAATAAGTCCTGTTTAGC
CTTGGAGTTCACATGCCGGCACTGAAGCCGACCTACCTGAGCGTGTGCGATTACCGTTACAATGGCAT
CTGTCTAGTTCTGTTTACCTACGGCGCTCTTGGTTCCAGGTTAGGGGAAGNGTATGACCCATGTGTTT
TTATCGGCTTAACCACGAGTGATCCCCGGTCGTTTCCCCATTGAATCCCTGGTGCATCCTACTCCCAG
AATGATAGCTGACTGACTGGACTGGCTTTTCAAGTAATCGAGGGGGTATCGCGGTCACGGCCGTTAAC
AGATCCCGTCCTTAGTGTGGAATCCGCACCTGCTGACTAACGCTTCGCCGGCGTGTCTGCACAGCCGT
ATAGTGTTAATCATGACCCCAAGGAAGGATTAAACAAATATCTTGACG
>RAGLN19

GTGTCCGGTAGCCCGCGCTAGTTAGACACCCCGGCAGGGGGGATTGCTTTCGAGACGGGAGATCCCTT
CGCCGACCCTGGAGGGCCGACGCCGAGGCATTCGGGCCCCTGCAACGTCAACAGCGGCAAGAAAACGG
GATGAATGGGCGTAATGGGGGGGGTCTGCTGGGGACCCGACGCGGTTGCCGTTTGCGGGGCCCCCGAC
CCATACCGACCCACCTAGGCGTCCAGTTACGGCGCACGTCGGGAGCGTGGTTGCCGTCAGAGCTGTGT
TTCTCGATCAGTCCCCCGCAGTGCCGCAGTATCTTGCCGTGGGCTGCTTTAATCTTGAAAGTGGTTTA
TACATTGGGCGACGAGGTGTCGACTCTCATTGGGGGTAACCGACGGGCACATGCAGTCCCCTCCCCGG
GCAGGCAGAGGCGGGGCCCCCGCGCGCCGGCCCCGGCCCACAATCTTACCAGGGTCCTCAAAGGAGCC
TTTGCATGGTACCCTTCGTGAATGGTTGCTTAAGAGGTCCACAACGTGGTCCGGGCACGGTCGACTAA
ACACTCAAAACAGCGACGGCAAATATAGGTACAAGGTCCAGGCCCTCACGGCACTAATTGCGATGACC
CAACTCACGGGGGCAGTCCCCGGCGCGGCGACCTTGATTACGTCCGGGGACAAGTATGTCGCTCCCCC
CGGGAGGGGTGCAGCCACATGGGAGATTCAAAGTTTCTCGTGACGTCGTTGTGATCACTGCAGCCTAG
CCGAGACTCCCGTACTACGCGAAGGTTGGTTATGTTAACCACTACAACGTGAGGCGCCGTAAGGGCCA
GTGTTGTGCCCGGCTCTTCAATGCGCCTTAGCGGCCTGATACACCCACCCAAGGAGATACTGCTAATC
ATATGGGTGGACAGAACCTCGCAACGCAGACGCCTCCCCGGCATGATATGGTTTTTTTCCGCTATTAT
CCAGCACGCAGCGCTATCATTCAAGAGAACCCAGTGACGCGTAAATCGTAAGATCTACCTGCCGCAGG
TGGACCTACTGCAAATACGGATTATGACTCGTAAAGGGGTCATGCGTATTTTCATCACTAGGCACGTT
CGAGAATAAATTAGTAGGTGTCCCGAGCCTTGTGGCGTTCCGCCTGACTCCTCATGAAGTCGACCTTC
TCACCGGCCCTATCTGCCGACGTAAGTCATAACCTAGATCTGTACCTCGGGGGGAGGGTCACTGTAAA
GGGATAATTGGAGGGTGATTTCCCACACTTTCCTAAGGGTACTTTTTGCCTGGCTTCGCAATTGGGTC
CAATGGATGTCGATCTCTGGTTTAGCAGTTGTGAAAGTGGCAAGGCGGGAGGTTAGACCTCCATTTAA
CATATACAAGCAAGTTAACTGCACTAGATGTGTAGACACTACAGTTACAGGAGTAGCCGAATAAGTCT
CCGACGTCAAGCGAATAAGCGTCATACGCGATTATCGCCTAAGAGCACGTATTGGCGGTAAAAGGCTA
GCTAGACGCTTATGGGTAGATTTCAAGGCGTTCGTAGTGGTATAATAGGATACTCTTTCACCAGCCTG
AGGGCCGAACGCTATACTAGTGGTCTGTGATGTAGGACCGAGTATCTCTCTAGGGACCATCTACTTGA
GCAATGGTGCGCAGGGGGAGACATAGACCAGCCTTGGGTGGCAAGCACTGCAATAAGTCCTGTTTAGC
CTTGGAGTTCACATGCCGGCACTGAAGCCGACCTACCTGAGCGTGTGCGATTACCGTTACAATGGCAT
CTGTCTAGTTCTGTTTACCTACGGCGCTCTTGGTTCCAGGTTAGGGGAAGTGTATGACCCATGTGTTT
TTATCGGCTTAACCACGAGTGATCCCCGGTCGTTTCCCCATTGAATCCCTGGTGCATCCTACTCCCAG
AATGATAGCTGACTGACTGGACTGGCTTTTCAAGTAATCGAGGGGGTATCGCGGTCACGGCCGTTAAC
AGATCCCGTCCTTAGTGTGGAATCCGCACCTGCTGACTAACGCTTCGCCGGCGTGTCTGCACAGCCGT
ATAGTGTTAATCATGACCCCAAGGAAGGATTAAACAAATATCTTGACG
>RAGLN20
GTGTCCGGTAGCCCGCGCTAGTTAGATACCCCGGCAGGGGGGATTGCTTTCGGGACGGGAGATCCCTT
CGCCGACCCTGGAGGGCCGACGCCGAGGCATTCGGGCCCCTGCAACGTCAACAGCGGCAAGAAAACGG
GATGAATGGGCGTAATGGGGGGGGTCTGCTGGGGACCCGACGCGGTTGCCGTTTGCGGGGCCCCCGAC
CCATACCGACCCACCTAGGCGTCCAGTTACGGCGCACGGCGGGAGCGTGGTTGCCGTCAGAGCTGTGT
TTCTCGATCAGTCCCCCGCAGTGCCGCAGTATCTTGCCGTGGGCTGCTTTAANNTTGAAAGTGGTTCA
TACATTGGGCGACGAGGTGTCGACTCTCATTGGGGGTAACCGACGGGCACATGCAGTCCCCTCCCCGG
GCAGGCAGAGGCGGGGCCCCCGCGCGCCGGCCCCGGCCCACAATCTTACCAGGGTCCTCAAGGGAGCC
TTTGCATGGTACCCTTCGTGAATGGTTGCTTAAGAGGTCCACCACGTGGTCCGGGCACGGTCGACTAA
ACACTCAAAACAGCGACGGCAAATATAGGCACAAGGTCCAGGCTCTCACGGCACTAATTGCGATGACC
CAACTCACGGGGGCAGTCCCCGGCGCGGCGACCGTGATTACGGCCGGGGATAAGTATGTCGTTCCCCC
CGGGAGGGGTGCAGCCACATGGGAGATTCAAAGTTTCTCGTGACGTCGTTGTGATCACTGCAGCCTAG
CCGAGACTCCCGTGCTACGCGAAGGTTGGTTATGTTAACCACTACAACGTGAGGCGCCGTAAGGGCCA
GTGTTGTGCCCGGCTCTTCAATGCGCCTTAGCGGCCTGATACACCCACCAAAGGAGATACTGCTAATC
ATGTGGGTGGACAGAAACTCGCAACACAGACGCCTCCCCGGCATGATATGGTTTTTTTCCGCTATTAT
CCAGCACGCAGCGCTATCATTCAAGAGAACCCAGTGACGCGTAAATCGTAATATCTACCTGCCGCAGG
TGGACCTCCTGCAAATACGGATTATGACTCGTAAAGAGGTCATGCGTATTTTCATCACTAGGCACGTT
CGAGAATAAATTAGTAGGTGTCCCGCGCCTTGTGGCGTTCCGCCTGACTCCTCATGAAGTCGACCTTC
TCACCGGCCCTATCTGCCGACGTAAGTCATAACCTAGATCTGTACCTCGGGGGGAGGGTCACTGTAAA
GGGATAATTGGAGGGTGATTTCCCACACTTTCCTAAGGGTACTTTTTGCCTGGCTTCGCAATTGGGTC
CAATGGATGTCGCTCTCTGGTTTAGCAGTTGTGAAAGTGGCAAGGCGGGAGGTTAGACCTCCATTTAA
CATATACAAGCAAGTTAACTGCACTAGATGTGTAGACACTACAGTTACAGGAGTAGCCGAATAAGTCT
CCGACGNCAAGCGAATAAGCGTCATACGCGATTATCGCCTAAGAGCACGTATTGGCGGTAAAAGGCTA

ACTAGACGCTTGTGGGTAGATTTCAAGGCGTTCGTAGTGGTATAACAGGATACTCTTTCACCAGCCTG
AGGGCCGAACGCTATACTAGTGGTCTGTGATGTAGGACCGAGTATCTCTCTAGGGACCATCTACTTGA
GCAATGGTGCGCAGGGGGAGACATAGACCAGCCTTGGGTGGCAAGCACTGCAATAAGTCCTGTTTAGC
CTTGGAGTTCACACGCCGGCACTGAAGCCGACCTACCTGAGCTTGTGCGATTACCGTTACAATGGCAT
CTGTCTAGTTCTGTTTACCTACGGCGCTCTTGGTTCCAGGTTAGGGGAAGTGTATGACCCATGTGTTT
TTATCGGCTTAACCACGAGTGATCCCCGGTCGTTTCCCCATTGAATCCCTGGTGCATCCTACTCCCAG
AATGATAGCTGACTGACTGGACTGGCTTTTCAAGTAGTCGAGGGGGTATCGCGGTCACGGCCGTTAAC
AGATCCCGTCCTTAGTGTGGAATCCGCACCTGCTGACTAACGCTTCGCCGGCGTGTCTGCACAGCCGT
ATAGTGTTNATCATGACCCCAAGGAAGGATTAAACAAATATCTTGACG
>RAGLN21
GTGTCCGGTAGCCCGCGCTAGTTAGACACCCCGGCAGGGGGGATTGCTTTCGAGACGGGAGATCCCTT
CGCCGACCCTGGAGGGCCGACGCCGAGGCATTCGGGCCCCTGCAACGTCAACAGCGGCAAGAAAACGG
GATGAATGGGCGTAATGGGGGGGGTCTGCTGGGGACCCGACGCGGTTGCCGTTTGCGGGGCCCCCGAC
CCATACCGACCCACCTAGGCGTCCAGTTACGGCGCACGTCGGGAGCGTGGTTGCCGTCAGAGCTGTGT
TTCTCGATCAGTCCCCCGCAGTGCCGCAGTATCTTGCCGTGGGCTGCTTTAATCTTGAAAGTGGTTCA
TACATTGGGCGACGAGGTGTCGACTCTCATTGGGGGTAACCGACGGGCACATGCAGTCCCCTCCCCGG
GCAGGCAGAGGCGGGGCCCCCGCGCGCCGGCCCCGGCCCACAATCTTACCAGGGTCCTCAAAGGAGCC
TTTGCATGGTACCCTTCGTGAATGGTTGCTTAAGAGGTCCACAACGTGGTCCGGGCACGGTCGACTAA
ACACTCAAAACAGCGACGGCAAATATAGGTACAAGGTCCAGGCCCTCACGGCACTAATTGCGATGACC
CAACTCACGGGGGCAGTCCCCGGCGCGGCGACCTTGATTACGTCCGGGGACAAGTATGTCGCTCCCCC
CGGGAGGGGTGCAGCCACATGGGAGATTCAAAGTTTCTCGTGACGTCGTTGTGATCACTGCAGCCTAG
CCGAGACTCCCGTACTACGCGAAGGTTGGTTATGTTAACCACTACAACGTGAGGCGCCGTAAGGGCCA
GTGTTGTGCCCGGCTCTTCAATGCGCCTTAGCGGCCTGATACACCCACCCAAGGAGATACTGCTAATC
ATATGGGTGGACAGAACCTCGCAACGCAGACGCCTCCCCGGCATGATATGGTTTTTTTCCGCTATTAT
CCAGCACGCAGCGCTATCATTCAAGAGAACCCAGTGACGCGTAAATCGTAAGATCTACCTGCCGCAGG
TGGACCTACTGCAAATACGGATTATGACTCGTAAAGGGGTCATGCGTATTTTCATCACTAGGCACGTT
CGAGAATAAATTAGTAGGTGTCCCGAGCCTTGTGGCGTTCCGCCTGACTCCTCATGAAGTCGACCTTC
TCACCGGCCCTATCTGCCGACGTAAGTCATAACCTAGATCTGTACCTCGGGGGGAGGGTCACTGTAAA
GGGATAATTGGAGGGTGATTTCCCACACTTTCCTAAGGGTACTTTTTGCCTGGCTTCGCAATTGGGTC
CAATGGATGTCGATCTCTGGTTTAGCAGTTGTGAAAGTGGCAAGGCGGGAGGTTAGACCTCCATTTAA
CATATACAAGCAAGTTAACTGCACTAGATGTGTAGACACTACAGTTACAGGAGTAGCCGAATAAGTCT
CCGACGTCAAGCGAATAAGCGTCATACGCGATTATCGCCTAAGAGCACGTATTGGCGGTAAAAGGCTA
GCTAGACGCTTATGGGTAGATTTCAAGGCGTTCGTAGTGGTATAATAGGATACTCTTTCACCAGCCTG
AGGGCCGAACGCTATACTAGTGGTCTGTGATGTAGGACCGAGTATCTCTCTAGGGACCATCTACTTGA
GCAATGGTGCGCAGGGGGAGACATAGACCAGCCTTGGGTGGCAAGCACTGCAATAAGTCCTGTTTAGC
CTTGGAGTTCACATGCCGGCACTGAAGCCGACCTACCTGAGCGTGTGCGATTACCGTTACAATGGCAT
CTGTCTAGTTCTGTTTACCTACGGCGCTCTTGGTTCCAGGTTAGGGGAAGTGTATGACCCATGTGTTT
TTATCGGCTTAACCACGAGTGATCCCCGGTCGTTTCCCCATTGAATCCCTGGTGCATCCTACTCCCAG
AATGATAGCTGACTGACTGGACTGGCTTTTCAAGTAATCGAGGGGGTATCGCGGTCACGGCCGTTAAC
AGATCCCGTCCTTAGTGTGGAATCCGCACCTGCTGACTAACGCTTCGCCGGCGTGTCTGCACAGCCGT
ATAGTGTTAATCATGACCCCAAGGAAGGATTAAACAAATATCTTGACG
