# Supplementary material for: Investigating demic versus cultural diffusion and sex bias in the spread of Austronesian languages in Vietnam
Source: PLoS One. 2024 Jun 17;19(6):e0304964. doi: 10.1371/journal.pone.0304964 (PMC11182502; doi:10.1371/journal.pone.0304964)
Supplement: S3 Dataset — (PDF) [file pone.0304964.s016.pdf]

#Chromosomal positions on hg19 for 2079 SNPs
Y:2655180
Y:2661306
Y:2661694
Y:2667558
Y:2668456
Y:2668533
Y:2703038
Y:2705854
Y:2710309
Y:2712237
Y:2722252
Y:2731887
Y:2734854
Y:2739468
Y:2739796
Y:2743754
Y:2744747
Y:2794529
Y:2796314
Y:2815425
Y:2818883
Y:2825845
Y:2828196
Y:2828425
Y:2831112
Y:2843538
Y:2846401
Y:2847910
Y:2875993
Y:2878213
Y:2878752
Y:2887824
Y:2892150
Y:2911033
Y:2951972
Y:2972866
Y:2980217
Y:2980499
Y:2992912
Y:3000027
Y:3003092
Y:3051142
Y:3051164
Y:3104906
Y:3108652
Y:3117475
Y:3117489
Y:3118152
Y:3126754
Y:3146769
Y:3164319
Y:3176009
Y:3201208

Y:3215246
Y:3226500
Y:3235174
Y:3262326
Y:3262561
Y:3268355
Y:3277094
Y:3278407
Y:3278431
Y:3278444
Y:3281361
Y:3281371
Y:3286624
Y:3299112
Y:3305475
Y:3310270
Y:3332424
Y:3332449
Y:3347216
Y:3347234
Y:3347241
Y:3354421
Y:3354426
Y:3366693
Y:3389909
Y:3395936
Y:3397017
Y:3447851
Y:3453914
Y:3453980
Y:3455251
Y:3502823
Y:3502880
Y:3504766
Y:3504893
Y:3518221
Y:3560352
Y:3579383
Y:3581736
Y:3586567
Y:3593893
Y:3596903
Y:3597564
Y:3607291
Y:3609403
Y:3659251
Y:3667776
Y:3669272
Y:3678693
Y:3679598
Y:3706040
Y:3708061
Y:3713191
Y:3716332

Y:3718592
Y:3719373
Y:3720463
Y:3736252
Y:3736399
Y:3736407
Y:3765365
Y:3772901
Y:3773042
Y:3783509
Y:3794104
Y:3795597
Y:3796052
Y:3797733
Y:3803612
Y:3803619
Y:3834130
Y:3840123
Y:3844354
Y:3864743
Y:3865293
Y:3924998
Y:3933668
Y:3950276
Y:3966392
Y:3966398
Y:3987290
Y:4001897
Y:4002358
Y:4005991
Y:4008636
Y:4039748
Y:4054426
Y:4054430
Y:4110934
Y:4111277
Y:4111303
Y:4111794
Y:4112548
Y:4115966
Y:4115992
Y:4156680
Y:4172091
Y:4183257
Y:4184167
Y:4206268
Y:4210488
Y:4222921
Y:4232624
Y:4238929
Y:4245854
Y:4245874
Y:4247821
Y:4247842

Y:4250690
Y:4280570
Y:4312361
Y:4312373
Y:4312811
Y:4328695
Y:4338795
Y:4341612
Y:4352151
Y:4353235
Y:4369797
Y:4414465
Y:4417407
Y:4425581
Y:4429015
Y:4482533
Y:4487168
Y:4499679
Y:4501873
Y:4541197
Y:4541203
Y:4549897
Y:4559326
Y:4578080
Y:4578096
Y:4590834
Y:4606157
Y:4606167
Y:4611254
Y:4703379
Y:4703404
Y:4704109
Y:4705480
Y:4712154
Y:4712169
Y:4723982
Y:4731886
Y:4766990
Y:4768635
Y:4790899
Y:4804906
Y:4834238
Y:4839346
Y:4849444
Y:4865911
Y:4877002
Y:4896386
Y:4956432
Y:4959569
Y:4996777
Y:4996941
Y:5029024
Y:5032870
Y:5032888

Y:5051424
Y:5059913
Y:5092035
Y:5099869
Y:5104133
Y:5171370
Y:5171825
Y:5171850
Y:5203482
Y:5204145
Y:5204166
Y:5210434
Y:5212181
Y:5229038
Y:5231331
Y:5231362
Y:5231367
Y:5284540
Y:5298470
Y:5302075
Y:5302099
Y:5304720
Y:5305151
Y:5309859
Y:5309870
Y:5405246
Y:5419225
Y:5466621
Y:5472852
Y:5542290
Y:5557491
Y:5560349
Y:5560354
Y:5573895
Y:5589047
Y:5589156
Y:5589157
Y:5669085
Y:5677702
Y:5677713
Y:5677720
Y:5708197
Y:5727070
Y:5727073
Y:5727076
Y:5748428
Y:5795903
Y:5798365
Y:5798366
Y:5803952
Y:5807430
Y:5847376
Y:5852998
Y:5856662

Y:5858787
Y:5863244
Y:5864264
Y:5864299
Y:5869876
Y:5869884
Y:5877883
Y:5884878
Y:5887045
Y:5895574
Y:5895650
Y:5896908
Y:5898595
Y:5906439
Y:5908210
Y:5910810
Y:5916535
Y:5920778
Y:5920796
Y:5950471
Y:5950884
Y:5951972
Y:5965169
Y:5966941
Y:5967576
Y:5971228
Y:5973622
Y:5973640
Y:5974857
Y:5984066
Y:5993512
Y:5995188
Y:6000464
Y:6019917
Y:6019934
Y:6020192
Y:6023823
Y:6033570
Y:6033818
Y:6050955
Y:6055433
Y:6055441
Y:6074118
Y:6081803
Y:6081834
Y:6082075
Y:6082084
Y:6082930
Y:6095853
Y:6120437
Y:6120511
Y:6120566
Y:6121027
Y:6129369

Y:6145866
Y:6190103
Y:6190184
Y:6190215
Y:6351779
Y:6354971
Y:6356845
Y:6370460
Y:6372705
Y:6372857
Y:6385631
Y:6386320
Y:6396326
Y:6400042
Y:6401950
Y:6401964
Y:6415497
Y:6455440
Y:6466069
Y:6477392
Y:6481670
Y:6481684
Y:6481694
Y:6482140
Y:6485370
Y:6491292
Y:6491315
Y:6492311
Y:6493529
Y:6494174
Y:6537823
Y:6537833
Y:6541048
Y:6575897
Y:6588448
Y:6598473
Y:6598492
Y:6606696
Y:6615362
Y:6630931
Y:6631743
Y:6635162
Y:6636899
Y:6647632
Y:6659638
Y:6661837
Y:6662168
Y:6677619
Y:6697604
Y:6707890
Y:6728092
Y:6728094
Y:6736812
Y:6737845

Y:6740510
Y:6740704
Y:6781398
Y:6812031
Y:6835056
Y:6845657
Y:6854765
Y:6857616
Y:6861075
Y:6868118
Y:6883856
Y:6893093
Y:6894741
Y:6906074
Y:6932546
Y:6938119
Y:6938186
Y:6938665
Y:6941151
Y:6943622
Y:6951960
Y:6951995
Y:6952023
Y:6954470
Y:6961386
Y:6965215
Y:6976734
Y:6977849
Y:6980446
Y:6980501
Y:6980863
Y:6980931
Y:6986981
Y:6987038
Y:6989173
Y:6989176
Y:6992073
Y:6992130
Y:6992134
Y:6995523
Y:6995947
Y:6997136
Y:7009717
Y:7018543
Y:7028463
Y:7028917
Y:7028961
Y:7031761
Y:7032024
Y:7034770
Y:7035173
Y:7036112
Y:7036688
Y:7036962

Y:7045854
Y:7049541
Y:7054449
Y:7057319
Y:7057795
Y:7057887
Y:7064528
Y:7065032
Y:7067665
Y:7069017
Y:7069410
Y:7070002
Y:7070177
Y:7072035
Y:7072339
Y:7074320
Y:7075347
Y:7077142
Y:7083260
Y:7087303
Y:7087611
Y:7087623
Y:7091863
Y:7096458
Y:7106782
Y:7110209
Y:7117105
Y:7117232
Y:7128949
Y:7136012
Y:7143650
Y:7149924
Y:7151887
Y:7158945
Y:7167837
Y:7167858
Y:7167968
Y:7171069
Y:7173143
Y:7177189
Y:7188046
Y:7188055
Y:7188076
Y:7188470
Y:7188478
Y:7196878
Y:7196932
Y:7209616
Y:7218178
Y:7218552
Y:7220727
Y:7245239
Y:7279986
Y:7279988

Y:7280064
Y:7280070
Y:7280083
Y:7282885
Y:7304744
Y:7314381
Y:7314397
Y:7317911
Y:7331425
Y:7348186
Y:7358315
Y:7364282
Y:7377160
Y:7383553
Y:7401585
Y:7401836
Y:7420744
Y:7420756
Y:7420794
Y:7501465
Y:7508686
Y:7513676
Y:7518782
Y:7528917
Y:7533750
Y:7543038
Y:7546726
Y:7548915
Y:7555789
Y:7558795
Y:7559671
Y:7559753
Y:7565996
Y:7568568
Y:7571122
Y:7586919
Y:7602738
Y:7611416
Y:7628900
Y:7635648
Y:7638823
Y:7643967
Y:7654523
Y:7688366
Y:7690471
Y:7706834
Y:7707135
Y:7716262
Y:7730226
Y:7731874
Y:7736744
Y:7747954
Y:7756332
Y:7771131

Y:7794147
Y:7796708
Y:7812445
Y:7828081
Y:7844490
Y:7846935
Y:7847497
Y:7849278
Y:7855759
Y:7857743
Y:7872203
Y:7900431
Y:7900462
Y:7900560
Y:7900625
Y:7900883
Y:7907021
Y:7908950
Y:7911747
Y:7912411
Y:7912540
Y:7913195
Y:7913358
Y:7913691
Y:7914265
Y:7921525
Y:7926492
Y:7926775
Y:7934114
Y:7956783
Y:7963031
Y:7970612
Y:7989319
Y:8016785
Y:8021340
Y:8033198
Y:8039719
Y:8048543
Y:8086815
Y:8148869
Y:8149729
Y:8188581
Y:8194310
Y:8214827
Y:8217307
Y:8221741
Y:8227605
Y:8231698
Y:8240725
Y:8267746
Y:8281294
Y:8285596
Y:8286531
Y:8289210

Y:8294035
Y:8306262
Y:8311795
Y:8313414
Y:8315095
Y:8327732
Y:8331281
Y:8334875
Y:8348435
Y:8348932
Y:8352384
Y:8353707
Y:8360818
Y:8363884
Y:8364460
Y:8365549
Y:8380220
Y:8383516
Y:8383517
Y:8389216
Y:8392350
Y:8398483
Y:8405053
Y:8410428
Y:8422121
Y:8424089
Y:8424741
Y:8426277
Y:8428087
Y:8430685
Y:8432095
Y:8442341
Y:8442742
Y:8462028
Y:8467053
Y:8467095
Y:8473271
Y:8474189
Y:8483228
Y:8485542
Y:8490706
Y:8499467
Y:8501357
Y:8502236
Y:8532844
Y:8533735
Y:8538392
Y:8551166
Y:8568831
Y:8575495
Y:8590752
Y:8602518
Y:8614138
Y:8635928

Y:8637791
Y:8643763
Y:8647561
Y:8663204
Y:8667179
Y:8675410
Y:8675467
Y:8679843
Y:8680661
Y:8681209
Y:8684795
Y:8684890
Y:8694916
Y:8696594
Y:8696975
Y:8700380
Y:8714022
Y:8714163
Y:8727033
Y:8728974
Y:8758629
Y:8758632
Y:8759325
Y:8793919
Y:8796078
Y:8805525
Y:8809390
Y:8831694
Y:8845380
Y:8852858
Y:8871613
Y:8884016
Y:8890588
Y:8895656
Y:8898284
Y:8908270
Y:8965961
Y:8966028
Y:8966055
Y:8967006
Y:8967242
Y:8968890
Y:8978345
Y:8978986
Y:8980200
Y:8985065
Y:8989382
Y:8993466
Y:8998486
Y:8998738
Y:8998941
Y:9000384
Y:9002840
Y:9003725

Y:9004143
Y:9004925
Y:9005135
Y:9008216
Y:9008417
Y:9016999
Y:9017261
Y:9019637
Y:9027915
Y:9041864
Y:9046348
Y:9050522
Y:9051798
Y:9055509
Y:9060591
Y:9060781
Y:9065732
Y:9083228
Y:9083261
Y:9089886
Y:9098400
Y:9104370
Y:9105224
Y:9107406
Y:9115037
Y:9119543
Y:9142173
Y:9142204
Y:9144043
Y:9145499
Y:9147259
Y:9147265
Y:9148716
Y:9153466
Y:9153472
Y:9153639
Y:9161225
Y:9163445
Y:9171887
Y:9172309
Y:9172552
Y:9172643
Y:9172709
Y:9193040
Y:9198057
Y:9314387
Y:9373275
Y:9376415
Y:9378285
Y:9381487
Y:9381672
Y:9381846
Y:9382303
Y:9382917

Y:9383100
Y:9384825
Y:9385266
Y:9391033
Y:9438818
Y:9445220
Y:9448354
Y:9448625
Y:9464078
Y:9466878
Y:9469366
Y:9489987
Y:9493957
Y:9500700
Y:9507122
Y:9744845
Y:9745843
Y:9777755
Y:9786259
Y:9787327
Y:9791250
Y:9792145
Y:9792147
Y:9795077
Y:9819205
Y:9819227
Y:9819254
Y:9823383
Y:9839719
Y:9840350
Y:9840371
Y:9850515
Y:9868798
Y:9871009
Y:9939447
Y:9941770
Y:9952152
Y:9952426
Y:9954043
Y:9954250
Y:9954759
Y:9960721
Y:9960750
Y:9960766
Y:9961656
Y:9961659
Y:9961663
Y:9961666
Y:9963104
Y:9964094
Y:9964746
Y:9964928
Y:9967002
Y:9978914

Y:9981376
Y:9984212
Y:9984705
Y:9984713
Y:9987138
Y:9987152
Y:9987748
Y:9988278
Y:9988315
Y:9991927
Y:9991935
Y:9997296
Y:10004070
Y:10005480
Y:10007963
Y:10011250
Y:10015802
Y:10016648
Y:10043562
Y:10059692
Y:10059698
Y:10092834
Y:13131114
Y:13289640
Y:13291799
Y:13297363
Y:13408179
Y:13408186
Y:13410193
Y:13410203
Y:13417016
Y:13417216
Y:13443172
Y:13511147
Y:13539269
Y:13542620
Y:13565107
Y:13612329
Y:13617419
Y:13687186
Y:13687977
Y:13688325
Y:13700169
Y:13718424
Y:13896332
Y:13939613
Y:13939735
Y:13964620
Y:13964980
Y:13982822
Y:13982835
Y:13990448
Y:13996995
Y:14003516

Y:14016458
Y:14025213
Y:14028148
Y:14040500
Y:14060308
Y:14063609
Y:14092982
Y:14096577
Y:14099303
Y:14109237
Y:14140277
Y:14144593
Y:14159846
Y:14171665
Y:14193384
Y:14199284
Y:14199508
Y:14201316
Y:14219849
Y:14243581
Y:14243796
Y:14245444
Y:14247835
Y:14266799
Y:14280847
Y:14286528
Y:14288981
Y:14332407
Y:14334396
Y:14342918
Y:14342926
Y:14342945
Y:14404954
Y:14411232
Y:14416216
Y:14418592
Y:14461702
Y:14469411
Y:14484899
Y:14486667
Y:14496103
Y:14518533
Y:14548082
Y:14550606
Y:14550629
Y:14556878
Y:14577177
Y:14582555
Y:14596782
Y:14614341
Y:14640715
Y:14641193
Y:14643410
Y:14666569

Y:14679362
Y:14680213
Y:14681483
Y:14698928
Y:14699914
Y:14710265
Y:14734176
Y:14734195
Y:14753989
Y:14759054
Y:14763172
Y:14763240
Y:14777360
Y:14777842
Y:14790163
Y:14798272
Y:14804077
Y:14805581
Y:14813717
Y:14813991
Y:14814540
Y:14814550
Y:14814602
Y:14817309
Y:14827703
Y:14829423
Y:14838700
Y:14840113
Y:14842223
Y:14846030
Y:14847494
Y:14850341
Y:14851554
Y:14854941
Y:14864191
Y:14869076
Y:14871976
Y:14873551
Y:14874103
Y:14887218
Y:14888783
Y:14889974
Y:14890987
Y:14891532
Y:14898094
Y:14898570
Y:14901633
Y:14902414
Y:14904859
Y:14906886
Y:14917922
Y:14919172
Y:14920900
Y:14922396

Y:14922583
Y:14922817
Y:14924869
Y:14930316
Y:14945191
Y:14945705
Y:14967692
Y:14968331
Y:14968449
Y:14971330
Y:14976204
Y:14982984
Y:14984713
Y:14985806
Y:15008677
Y:15014550
Y:15015396
Y:15016536
Y:15017505
Y:15018459
Y:15018582
Y:15018696
Y:15019092
Y:15019124
Y:15021104
Y:15022005
Y:15022465
Y:15022707
Y:15025506
Y:15025605
Y:15026424
Y:15027510
Y:15027529
Y:15029433
Y:15030767
Y:15030878
Y:15030915
Y:15031385
Y:15033712
Y:15039955
Y:15042327
Y:15043822
Y:15053676
Y:15064733
Y:15067573
Y:15069836
Y:15082684
Y:15095345
Y:15095580
Y:15095760
Y:15126199
Y:15129415
Y:15156388
Y:15165471

Y:15192440
Y:15194083
Y:15204710
Y:15206662
Y:15222359
Y:15228609
Y:15242077
Y:15242249
Y:15255494
Y:15258913
Y:15262035
Y:15262779
Y:15265913
Y:15267960
Y:15273084
Y:15277795
Y:15302894
Y:15302901
Y:15309625
Y:15315047
Y:15317934
Y:15328735
Y:15350615
Y:15350616
Y:15360621
Y:15371669
Y:15371841
Y:15372130
Y:15393044
Y:15401647
Y:15404832
Y:15415115
Y:15420555
Y:15436316
Y:15436444
Y:15437152
Y:15437410
Y:15467364
Y:15469724
Y:15469740
Y:15470057
Y:15471258
Y:15483032
Y:15505259
Y:15506055
Y:15507686
Y:15510064
Y:15510490
Y:15510632
Y:15517851
Y:15526751
Y:15542883
Y:15544614
Y:15553123

Y:15562290
Y:15564625
Y:15574102
Y:15576203
Y:15576370
Y:15585558
Y:15590342
Y:15591201
Y:15591445
Y:15591447
Y:15592033
Y:15592610
Y:15592635
Y:15592640
Y:15594523
Y:15601246
Y:15601452
Y:15615429
Y:15625242
Y:15632294
Y:15633954
Y:15635497
Y:15635573
Y:15653114
Y:15668070
Y:15693300
Y:15704602
Y:15705458
Y:15724488
Y:15725184
Y:15726484
Y:15726577
Y:15744642
Y:15744876
Y:15754313
Y:15764177
Y:15768559
Y:15784412
Y:15784413
Y:15800326
Y:15809326
Y:15814487
Y:15815443
Y:15815616
Y:15816093
Y:15816094
Y:15816112
Y:15816113
Y:15816140
Y:15816142
Y:15816157
Y:15816206
Y:15850400
Y:15877654

Y:15877995
Y:15879017
Y:15929876
Y:15935524
Y:15939485
Y:15950750
Y:15951899
Y:15970265
Y:15977973
Y:15992237
Y:15999244
Y:16037481
Y:16045476
Y:16060785
Y:16074831
Y:16134080
Y:16172915
Y:16180103
Y:16182583
Y:16183412
Y:16190764
Y:16199051
Y:16200694
Y:16202267
Y:16213100
Y:16213163
Y:16222561
Y:16230262
Y:16251357
Y:16253694
Y:16263675
Y:16271151
Y:16278550
Y:16283856
Y:16291778
Y:16306764
Y:16315153
Y:16326080
Y:16338537
Y:16339916
Y:16340858
Y:16347425
Y:16348910
Y:16351523
Y:16354708
Y:16364417
Y:16371619
Y:16377198
Y:16388815
Y:16401339
Y:16404607
Y:16407250
Y:16417756
Y:16478520

Y:16490341
Y:16491135
Y:16494251
Y:16497020
Y:16519057
Y:16543462
Y:16567551
Y:16578072
Y:16596916
Y:16612163
Y:16615413
Y:16629782
Y:16638804
Y:16647978
Y:16660759
Y:16661107
Y:16690780
Y:16691822
Y:16692495
Y:16713287
Y:16713318
Y:16715315
Y:16735413
Y:16750812
Y:16751001
Y:16751825
Y:16758450
Y:16767517
Y:16772104
Y:16773870
Y:16774007
Y:16777011
Y:16779407
Y:16788383
Y:16788539
Y:16788664
Y:16788665
Y:16789997
Y:16792142
Y:16804852
Y:16809337
Y:16809478
Y:16817026
Y:16817030
Y:16817923
Y:16824270
Y:16834840
Y:16839499
Y:16839876
Y:16849860
Y:16849994
Y:16854801
Y:16856357
Y:16859582

Y:16903692
Y:16904772
Y:16922783
Y:16923335
Y:16925100
Y:16981954
Y:16984055
Y:16984545
Y:17004094
Y:17007767
Y:17053771
Y:17090108
Y:17117331
Y:17121032
Y:17132580
Y:17135154
Y:17135618
Y:17136797
Y:17141271
Y:17156422
Y:17191930
Y:17197796
Y:17209692
Y:17231905
Y:17236796
Y:17236990
Y:17242145
Y:17242819
Y:17252369
Y:17256018
Y:17274911
Y:17280486
Y:17285993
Y:17286288
Y:17301401
Y:17304901
Y:17310145
Y:17312195
Y:17317264
Y:17327936
Y:17333663
Y:17333664
Y:17340373
Y:17341676
Y:17358410
Y:17394830
Y:17396329
Y:17398083
Y:17400710
Y:17412198
Y:17420297
Y:17423320
Y:17446588
Y:17447638

Y:17461478
Y:17470112
Y:17471620
Y:17471820
Y:17493630
Y:17495914
Y:17500298
Y:17508337
Y:17510811
Y:17533325
Y:17536190
Y:17536230
Y:17550661
Y:17570599
Y:17571473
Y:17571692
Y:17583053
Y:17588338
Y:17589815
Y:17595842
Y:17609833
Y:17614366
Y:17650073
Y:17678693
Y:17678803
Y:17684717
Y:17685450
Y:17692832
Y:17696109
Y:17712208
Y:17717953
Y:17730752
Y:17730754
Y:17737313
Y:17742219
Y:17755905
Y:17758018
Y:17762668
Y:17765230
Y:17768441
Y:17789323
Y:17805633
Y:17807264
Y:17814087
Y:17821258
Y:17832361
Y:17853519
Y:17860729
Y:17871905
Y:17871912
Y:17872179
Y:17881712
Y:17882893
Y:17891241

Y:17894256
Y:17896264
Y:17897543
Y:17907131
Y:17916397
Y:17921495
Y:17922248
Y:17924825
Y:17937048
Y:17937365
Y:17938904
Y:17944430
Y:17951267
Y:17958770
Y:17958994
Y:17961512
Y:17974575
Y:17986687
Y:18019275
Y:18023975
Y:18026855
Y:18032753
Y:18039857
Y:18051212
Y:18061167
Y:18077016
Y:18087725
Y:18097251
Y:18099341
Y:18117193
Y:18137831
Y:18146921
Y:18167403
Y:18167479
Y:18170078
Y:18171845
Y:18179352
Y:18189773
Y:18224172
Y:18224184
Y:18224233
Y:18239854
Y:18240794
Y:18243999
Y:18248698
Y:18257568
Y:18266961
Y:18296453
Y:18385604
Y:18387494
Y:18397495
Y:18405504
Y:18412840
Y:18419910

Y:18537567
Y:18560005
Y:18561042
Y:18563482
Y:18573243
Y:18578476
Y:18585407
Y:18585419
Y:18596847
Y:18601274
Y:18608096
Y:18608276
Y:18615346
Y:18633084
Y:18637416
Y:18642688
Y:18647042
Y:18647146
Y:18667642
Y:18679818
Y:18684926
Y:18694500
Y:18706036
Y:18712055
Y:18719565
Y:18731810
Y:18736860
Y:18739828
Y:18742969
Y:18752773
Y:18755302
Y:18759669
Y:18771855
Y:18782266
Y:18785737
Y:18786174
Y:18803914
Y:18803916
Y:18820048
Y:18821912
Y:18823187
Y:18827301
Y:18831084
Y:18835911
Y:18839375
Y:18842841
Y:18871216
Y:18873866
Y:18881548
Y:18894151
Y:18907236
Y:18914441
Y:18927078
Y:18934621

Y:18958586
Y:19003145
Y:19022555
Y:19037067
Y:19044734
Y:19045124
Y:19045552
Y:19048602
Y:19053889
Y:19054889
Y:19061526
Y:19068036
Y:19088282
Y:19096363
Y:19105726
Y:19108908
Y:19113580
Y:19114679
Y:19114835
Y:19118027
Y:19120949
Y:19150014
Y:19166861
Y:19179335
Y:19179463
Y:19179540
Y:19183729
Y:19202489
Y:19206845
Y:19213252
Y:19218037
Y:19222231
Y:19230498
Y:19233673
Y:19236447
Y:19238511
Y:19267344
Y:19271372
Y:19272435
Y:19279765
Y:19285054
Y:19291359
Y:19292547
Y:19304761
Y:19305126
Y:19306942
Y:19323160
Y:19332760
Y:19349615
Y:19360642
Y:19372700
Y:19380619
Y:19413335
Y:19414474

Y:19422325
Y:19482416
Y:19496663
Y:19500107
Y:19501311
Y:19503812
Y:19503826
Y:19510566
Y:19515784
Y:19515794
Y:19550740
Y:19557145
Y:20066503
Y:20105854
Y:20804835
Y:20812228
Y:20812848
Y:20815225
Y:20828795
Y:20832034
Y:20832062
Y:20832084
Y:20832092
Y:20832125
Y:20832136
Y:20832137
Y:20836447
Y:20836471
Y:20836551
Y:20836567
Y:20837553
Y:20838224
Y:21080707
Y:21086211
Y:21088297
Y:21117888
Y:21132000
Y:21138077
Y:21138078
Y:21147336
Y:21150837
Y:21157531
Y:21166358
Y:21186263
Y:21215782
Y:21229621
Y:21229652
Y:21229653
Y:21229680
Y:21239980
Y:21242391
Y:21250252
Y:21252748
Y:21253443

Y:21255290
Y:21263029
Y:21264006
Y:21266479
Y:21282471
Y:21287315
Y:21293156
Y:21293261
Y:21294294
Y:21309376
Y:21312064
Y:21314704
Y:21327172
Y:21327234
Y:21327293
Y:21329363
Y:21331627
Y:21332123
Y:21358553
Y:21364755
Y:21365772
Y:21369873
Y:21402296
Y:21412205
Y:21438690
Y:21461202
Y:21462954
Y:21466597
Y:21480095
Y:21499188
Y:21499947
Y:21504449
Y:21506445
Y:21549921
Y:21554468
Y:21560122
Y:21567932
Y:21569908
Y:21570974
Y:21593470
Y:21595926
Y:21595927
Y:21595978
Y:21603164
Y:21610301
Y:21618856
Y:21622006
Y:21624208
Y:21624230
Y:21624316
Y:21641094
Y:21642296
Y:21643364
Y:21645133

Y:21646196
Y:21652284
Y:21658207
Y:21659451
Y:21672442
Y:21674068
Y:21674378
Y:21688300
Y:21717208
Y:21721419
Y:21722268
Y:21726388
Y:21728755
Y:21729056
Y:21729491
Y:21733133
Y:21735456
Y:21739006
Y:21740760
Y:21741920
Y:21747107
Y:21752037
Y:21754097
Y:21759161
Y:21764500
Y:21766113
Y:21777403
Y:21784286
Y:21798185
Y:21801722
Y:21811108
Y:21811903
Y:21841289
Y:21844896
Y:21847039
Y:21853357
Y:21854010
Y:21864291
Y:21865821
Y:21866424
Y:21866491
Y:21867718
Y:21867733
Y:21867787
Y:21868006
Y:21868068
Y:21868672
Y:21868726
Y:21868863
Y:21870638
Y:21872337
Y:21872738
Y:21878708
Y:21878762

Y:21878825
Y:21881573
Y:21881825
Y:21888486
Y:21892572
Y:21894407
Y:21894447
Y:21895077
Y:21896261
Y:21898279
Y:21900849
Y:21901035
Y:21902969
Y:21903202
Y:21903383
Y:21903853
Y:21904023
Y:21905043
Y:21905085
Y:21905206
Y:21905416
Y:21905917
Y:21906109
Y:21906607
Y:21907648
Y:21909842
Y:21916516
Y:21916548
Y:21917313
Y:21917939
Y:21919016
Y:21919037
Y:21925276
Y:21925314
Y:21930287
Y:21932935
Y:21934117
Y:21935753
Y:21936138
Y:21938158
Y:21938444
Y:21938955
Y:21939210
Y:21952388
Y:21969615
Y:21970721
Y:21978583
Y:21978600
Y:21983827
Y:22001252
Y:22003770
Y:22007989
Y:22026171
Y:22027847

Y:22042987
Y:22043291
Y:22047290
Y:22104501
Y:22104521
Y:22108050
Y:22115888
Y:22149593
Y:22157311
Y:22158010
Y:22163004
Y:22178569
Y:22181421
Y:22190712
Y:22203237
Y:22214221
Y:22218716
Y:22218735
Y:22218807
Y:22222328
Y:22230425
Y:22233360
Y:22239294
Y:22239506
Y:22239754
Y:22240008
Y:22240301
Y:22240354
Y:22240481
Y:22240673
Y:22241818
Y:22244026
Y:22247363
Y:22253036
Y:22256589
Y:22275260
Y:22287533
Y:22287602
Y:22287610
Y:22287640
Y:22287664
Y:22294541
Y:22299551
Y:22299833
Y:22301719
Y:22302537
Y:22303673
Y:22316088
Y:22316252
Y:22320314
Y:22325206
Y:22325248
Y:22345984
Y:22346014

Y:22362063
Y:22429567
Y:22430245
Y:22430269
Y:22439457
Y:22443470
Y:22444953
Y:22446064
Y:22446067
Y:22472931
Y:22473256
Y:22483349
Y:22483394
Y:22484137
Y:22509768
Y:22513726
Y:22514081
Y:22528821
Y:22528868
Y:22528886
Y:22574028
Y:22582201
Y:22596423
Y:22597106
Y:22602612
Y:22612832
Y:22634764
Y:22646728
Y:22648375
Y:22653287
Y:22677409
Y:22700848
Y:22702594
Y:22715814
Y:22718513
Y:22725379
Y:22729242
Y:22737801
Y:22738775
Y:22739367
Y:22741799
Y:22741818
Y:22741821
Y:22744939
Y:22744945
Y:22744993
Y:22745051
Y:22746786
Y:22749853
Y:22751581
Y:22751863
Y:22752620
Y:22754570
Y:22766265

Y:22771704
Y:22771797
Y:22774608
Y:22775209
Y:22792896
Y:22818334
Y:22822229
Y:22822230
Y:22822346
Y:22824532
Y:22833310
Y:22854091
Y:22889018
Y:22893888
Y:22914378
Y:22917995
Y:22918577
Y:22919819
Y:22919840
Y:22922129
Y:22923019
Y:22924348
Y:22928067
Y:22928699
Y:22938847
Y:22947915
Y:22968405
Y:22968980
Y:22973793
Y:22978713
Y:23005170
Y:23021978
Y:23030717
Y:23031377
Y:23035132
Y:23035483
Y:23035504
Y:23065366
Y:23113764
Y:23116369
Y:23121708
Y:23123790
Y:23124367
Y:23125435
Y:23144858
Y:23148103
Y:23148323
Y:23156865
Y:23165895
Y:23169767
Y:23185647
Y:23195236
Y:23202275
Y:23207709

Y:23208693
Y:23234163
Y:23235373
Y:23241568
Y:23248930
Y:23257819
Y:23265885
Y:23265887
Y:23265910
Y:23292782
Y:23300109
Y:23329763
Y:23348257
Y:23353025
Y:23353901
Y:23376463
Y:23379254
Y:23390480
Y:23394666
Y:23398687
Y:23403749
Y:23431541
Y:23446453
Y:23454394
Y:23470235
Y:23476936
Y:23482521
Y:23483755
Y:23490117
Y:23490670
Y:23531992
Y:23533521
Y:23543353
Y:23543442
Y:23543443
Y:23571026
Y:23571646
Y:23571841
Y:23577373
Y:23580343
Y:23584117
Y:23626712
Y:23634362
Y:23638549
Y:23654049
Y:23655582
Y:23735080
Y:23740892
Y:23742482
Y:23744218
Y:23748997
Y:23749157
Y:23749182
Y:23759180

Y:23781808
Y:23785257
Y:23785275
Y:23785284
Y:23785316
Y:23796497
Y:23799139
Y:23801231
Y:23807304
Y:23813365
Y:23813524
Y:23813660
Y:23814959
Y:23815059
Y:23815143
Y:23815163
Y:23815501
Y:23815530
Y:23816251
Y:23823069
Y:23839740
Y:23850353
Y:23851444
Y:23853320
Y:23865297
Y:23865327
Y:23865472
Y:23865873
Y:23869504
Y:23873042
Y:23873446
Y:23873465
Y:23873538
Y:23873680
Y:23873761
Y:23874186
Y:23874255
Y:23874448
Y:23880059
Y:23881350
Y:23881412
Y:23881528
Y:23882889
Y:23882907
Y:23883008
Y:23883016
Y:23883612
Y:23883634
Y:23891444
Y:23894015
Y:23894099
Y:23894215
Y:23894867
Y:23895012

Y:23897195
Y:23898108
Y:23898115
Y:23898319
Y:23898878
Y:23899208
Y:23899533
Y:23900003
Y:23955828
Y:23959349
Y:23971955
Y:23980011
Y:23984056
Y:23987612
Y:23992762
Y:24007524
Y:24046004
Y:24069959
Y:24070035
Y:24070054
Y:24072008
Y:24079994
Y:24359931
Y:24391629
Y:24394612
Y:24405862
Y:24429509
Y:24437979
Y:24439196
Y:24443836
Y:24444622
Y:24447989
Y:24452096
Y:24464547
Y:24478403
Y:24497922
Y:24502819
Y:24505075
Y:24522129
Y:24522333
Y:24880863
Y:24886437
Y:27390536
Y:27839135
Y:28472450
Y:28498442
Y:28498446
Y:28510138
Y:28526953
Y:28538592
Y:28539062
Y:28587511
Y:28661247
Y:28673390

Y:28738131
Y:28757590
Y:28760588
Y:28778687
Y:28793765
Y:28802547
Y:28807515
Y:28810702
Y:28810982
Y:28815656
Y:28815864
Y:28817000
Y:28817368
Y:28817442
Y:28817458
Y:28817636
Y:28817799
Y:58823397
Y:58844669
Y:58855394
Y:58859359
Y:58862608
Y:58866750
Y:58883690
Y:58969133
Y:58995123
Y:59028492
Y:59028650
