# Supplementary material for: Investigating demic versus cultural diffusion and sex bias in the spread of Austronesian languages in Vietnam
Source: PLoS One. 2024 Jun 17;19(6):e0304964. doi: 10.1371/journal.pone.0304964 (PMC11182502; doi:10.1371/journal.pone.0304964)
Supplement: S4 Dataset — (PDF) [file pone.0304964.s017.pdf]

#Genotypes of 2079 SNPs for 768 individuals (170 newly genotyped here individuals and 598 previously published)
>BANAN10
GTGTCCGGTAGCCCGCGCTAGTTAGACACCCCGGCAGGGGGGATTGCTTTCGAGACGGGAGATCCCTT
CGCCGACCCTGGAGGGCCGACGCCGAGGCATTCGGGCCCCTGCAACGTCAACAGCGGCAAGAAAACGG
GATGAATGGGCGTNATGGGGGGGGTCTGCTGGGGACCCGACGCGGTTGCCGTTTGCGGGGCCCCCGAC
CCATACCGACCCACCTAGGCGTCCAGTTACGGCGCACGTCGGGAGCGTGGTTGCCGTCAGAGCTGTGT
TTCTCGATCAGTCCCCCGCAGTGCCGCAGTATCTTGCCGTGGGCTGCTTTAATCTTGAAAGTGGTTTA
TACATTGGGCGACGAGGTGTCGACTCTCATTGGGGGTAACCGACGGGCACATGCAGTCCCCTCCCCGG
GCAGGCAGAGGCGGGGCCCCCGCGCGCCGGCCCCGGCCCACAATCTTACCAGGGTCCTCAAAGGAGCC
TTTGCATGGTACCCTTCGTGAATGGTTGCTTAAGAGGTCCACAACGTGGTCCGGGCACGGTCGACTAA
ACACTCAAAACAGCGACGGCAAATATAGGTACAAGGTCCAGGCCCTCACGGCACTAATTGCGATGACC
CAACTCACGGGGGCAGTCCCCGGCGCGGCGACCTTGATTACGTCCGGGGACAAGTATGTCGCTCCCCC
CGGGAGGGGTGCAGCCACATGGGAGATTCAAAGTTTCTCGTGACGTCGTTGTGATCACTGCAGCCTAG
CCGAGACTCCCGTACTACGCGAAGGTTGGTTATGTTAACCACTACAACGTGAGGCGCCGTAAGGGCCA
GTGTTGTGCCCGGCTCTTCAATGCGCCTTAGCGGCCTGATACACCCACCCAAGGAGATACTGCTAATC
ATATGGGTGGACAGAACCTCGCAACGCAGACGCCTCCCCGGCATGATATGGTTTTTTTCCGCTATTAT
CCAGCACGCAGCGCTATCATTCAAGAGAACCCAGTGACGCGTAAATCGTAAGATCTACCTGCCGCAGG
TGGACCTACTGCAAATACGGATTATGACTCGTAAAGGGGTCATGCGTATTTTCATCACTAGGCACGTT
CGAGAATAAATTAGTAGGTGTCCCGAGCCTTGTGGCGTTCCGCCTGACTCCTCATGAAGTCGACCTTC
TCACCGGCCCTATCTGCCGACGTAAGTCATAACCTAGATCTGTACCTCGGGGGGAGGGTCACTGTAAA
GGGATAATTGGAGGGTGATTTCCCACACTTTCCTAAGGGTACTTTTTGCCTGGCTTCGCAATTGGGTC
CAATGGATGTCGATCTCTGGTTTAGCAGTTGTGAAAGTGGCAAGGCGGGAGGTTAGACCTCCATTTAA
CATATACAAGCAAGTTAACTGCACTAGATGTGTAGACACTACAGTTACAGGAGTAGCCGAATAAGTCT
CCGACGTCAAGCGAATAAGCGTCATACGCGATTATCGCCTAAGAGCACGTATTGGCGGTAAAAGGCTA
GCTAGACGCTTATGGGTAGATTTCAAGGCGTTCGTAGTGGTATAATAGGATACTCTTTCACCAGCCTG
AGGGCCGAACGCTATACTAGTGGTCTGTGATGTAGGACCGAGTATCTCTCTAGGGACCATCTACTTGA
GCAATGGTGCGCAGGGGGAGACATAGACCAGCCTTGGGTGGCAAGCACTGCAATAAGTCCTGTTTAGC
CTTGGAGTTCACATGCCGGCACTGAAGCCGACCTACCTGAGCGTGTGATTACCGTTAAAGCATCTGTC
TAGTTCTGTTTCCGGCGCTCTTGGTTCCAGGTTAGGGGAAGTGTATGACCCATGTGTTTTTATCGGCT
TAACCACGAGTGATCCCCGGTCGTTTCCCCATTGAATCCCTGGTGCATCCTACTCCCAGAATGATAGC
TGACTGACTGGACTGGCTTTTCAAGTAATCGAGGGGGTATCGCGGTCACGGCCGTTAACAGATCCCGT
CCTTAGTGTGGAATCCGCACCTGCTGACTAACGCTTCGCCGGCGTGTCTGCACAGCCGTATAGTGTTA
ATCATGACCCCAAGGAAGGATTAAACAAATATCTTGACG
>BANAN13
GTGTCCGGTAGCCCGCGCTAGTTAGACACCCCGGCAGGGGGGATTGCTTTCGAGACGGGAGATCCCTT
CGCCGACCCTGGAGGGCCGACGCCGAGGCATTCGGGCCCCTGCAACGTCAACAGCGGCAAGAAAACGG
GATGAATGGGCGTAATGGGGGGGGTCTGCTGGGGACCCGACGCGGTTGCCGTTTGCGGGGCCCCCGAC
CCATACCGACCCACCTAGGCGTCCAGTTACGGCGCACGGCGGGAGCGTGGTTGCCGTCAGAGCTGTGT
TTCTCGATCAGTCCCCCGCAGTGCCGCAGTATCTTGCCGTGGGCTGCTTTAATCTTGAAAGTGGTTTA
TACATTGGGCGACGAGGTGTCGACTCTCATTGGGGGTAACCGACGGGCACATGCAGTCCCCTCCCCGG
GCAGGCAGAGGCGGGGCCCCCGCGCGCCGGCCCCGGCCCACAATCTTACCAGGGTCCTCAAAGGAGCC
TTTGCATGGTACCCTTCGTGAATGGTTGCTTAAGAGGTCCACAACGTGGTCCGGGCACGGTCGACTAA
ACACTCAAAACAGCGACGGCAAATATAGGTACAAGGTCCAGGCCCTCACGGCACTAATTGCGATGACC
CAACTCACGGGGGCAGTCCCCGGCGCGGCGACCTTGATTACGTCCGGGGACAAGTATGTCGCTCCCCC
CGGGAGGGGTGCAGCCACATGGGAGATTCAAAGTTTCTCGTGACGTCGTTGTGATCACTGCAGCCTAG
CCGAGACTCCCGTACTACGCGAAGGTTGGTTATGTTAACCACTACAACGTGAGGCGCCGTAAGGGCCA
GTGTTGTGCCCGGCTCTTCAATGCGCCTTAGCGGCCTGATACACCCACCCAAGGAGATACTGCTAATC
ATATGGGTGGACAGAACCTCGCAACGCAGACGCCTCCCCGGCATGATATGGTTTTTTTCCGCTATTAT
CCAGCACGCAGCGCTATCATTCAAGAGAACCCAGTGACGCGTAAATCGTAAGATCTACCTGCCGCAGG
TGGACCTACTGCAAATACGGATTATGACTCGTAAAGGGGTCATGCGTATTTTCATCACTAGGCACGTT
CGAGAATAAATTAGTAGGTGTCCCGAGCCTTGTGGCGTTCCGCCTGACTCCTCATGAAGTCGACCTTC
TCACCGGCCCTATCTGCCGACGTAAGTCATAACCTAGATCTGTACCTCGGGGGGAGGGTCACTGTAAA
GGGATAATTGGAGGGTGATTTCCCACATTTTCCTAAGGGTACTTTTTGCCTGGCTTCGCAATTGGGTC

CAATGGATGTCGATCTCTGGTTTAGCAGTTGTGAAAGTGGCAAGGCGGGAGGTTAGACCTCCATTTAA
CATATACAAGCAAGTTAACTGCACTAGATGTGTAGACACTACAGTTACAGGAGTAGCCGAATAAGTCT
CCGACGTCAAGCGAATAAGCGTCATACGCGATTATCGCCTAAGAGCACGTATTGGCGGTAAAAGGCTA
GCTAGACGCTTGTGGGTAGATTTCAAGGCGTTCGTAGTGGTATAATAGGATACTCTTTCACCAGCCTG
AGGGCCGAACGCTATACTAGTGGTCTGTGATGTAGGACCGAGTATCTCTCTAGGGACCATCTACTTGA
GCAATGGTGCGCAGGGGGAGACATAGACCAGCCTTGGGTGGCAAGCACTGCAATAAGTCCTGTTTAGC
CTTGGAGTTCACACGCCGGCACTGAAGCCGACCTACCTGAGCGTGTGATTACCGTTAAAGCATCTGTC
TAGTTCTGTTTCCGGCGCTCTTGGTTCCAGGTTAGGGGAAGTGTATGACCCATGTGTTTTTATCGGCT
TAACCACGAGTGATCCCCGGTCGTTTCCCCATTGAATCCCTGGTGCATCCTACTCCCAGAATGATAGC
TGACTGACTGGACTGGCTTTTCAAGTAATCGAGGGGGTATCGCGGTCACGGCCGTTAACAGATCCCGT
CCTTAGTGTGGAATCCGCACCTGCTGACTAACGCTTCGCCGGCGTGTCTGCACAGCCGTATAGTGTTA
ATCATGACCCCAAGGAAGGATTAAACAAATATCTTGACG
>BANAN14
GTGTCCGGTAGCCCGCGCTAGTTAGACACCCCGGCAGGGGGGATTGCTTTCGAGACGGGAGATCCCTT
CGCCGACCCTGGAGGGCCGACGCCGAGGCATTCGGGCCCCTGCAACGTCAACAGCGGCAAGAAAACGG
GATGAATGGGCGTAATGGGGGGGGTCTGCTGGGGACCCGACGCGGTTGCCGTTTGCGGGGCCCCCGAC
CCATACCGACCCACCTAGGCGTCCAGTTACGGCGCACGTCGGGAGCGTGGTTGCCGTCAGAGCTGTGT
TTCTCGATCAGTCCCCCGCAGTGCCGCAGTATCTTGCCGTGGGCTGCTTTAATCTTGAAAGTGGTTCA
TACATTGGGCGACGAGGTGTCGACTCTCATTGGGGGTAACCGACGGGCACATGCAGTCCCCTCCCCGG
GCAGGCAGAGGCGGGGCCCCCGCGCGCCGGCCCCGGCCCACAATCTTACCAGGGTCCTCAAAGGAGCC
TTTGCATGGTACCCTTCGTGAATGGTTGCTTAAGAGGTCCACCACGTGGTCCGGGCACGGTCGACTAA
ACACTCAAAACAGCGACGGCAAATATAGGTACAAGGTCCAGGCCCTCACGGCACTAATTGCGATGACC
CAACTCACGGGGGCAGTCCCCGGCGCGGCGACCTTGATTACGTCCGGGGACAAGTATGTCGTTCCCCC
CGGGAGGGGTGCAGCCACATGGGAGATTCAAAGTTTCTCGTGACGTCGTTGTGATCACTGCAGCCTAG
CCGAGACTCCCGTACTACGCGAAGGTTGGTTATGTTAACCACTACAACGTGAGGCGCCGTAAGGGCCA
GTGTTGTGCCCGGCTCTTCAATGCGCCTTAGCGGCCTGATACACCCACCCAAGGAGATACTGCTAATC
ATGTGGGTGGACAGAACCTCGCAACGCAGACGCCTCCCCGGCATGATATGGTTTTTTTCCGCTATTAT
CCAGCACGCAGCGCTATCATTCAAGAGAACCCAGTGACGCGTAAATCGTAAGATCTACCTGCCGCAGG
TGGACCTACTGCAAATACGGATTATGACTCGTAAAGAGGTTATNCGTATTTTCATCACTAGGCACGTT
CGAGAATAAATTAGTAGGTGTCCCGCGCCTTGTGGCGTTCCGCCTGACTCCTCATGAAGTCGACCTTC
TCACCGGCCCTATCTGCCGACGTAAGTCATAACCTAGATCTGTACCTCGGGGGGAGGGTCACTGTAAA
GGGATAATTGGAGGGTGATTTCCCACACTTTCCTAAGGGTACTTTTTGCCTGGCTTCGCAATTGGGTC
CAATGGATGTCGATCTCTGGTTTAGCAGTTGTGAAAGTGGCAAGGCGGGAGGTTAGACCTCCATTTAA
CATATACAAGCAAGTTAACTGCACTAGATGTGTAGACACTACAGTTACAGGAGTAGCCGAATAAGTCT
CCGACGTCAAGCGAATAAGCGTCATACGCGATTATCGCCTAAGAGCACGTATTGGCGGTAAAAGGCTA
GCTAGACGCTTGTGGGTAGATTTCAAGGCGTTCGTAGTGGTATAATAGGATACTCTTTCACCAGCCTG
AGGGCCGAACGCTATACTAGTGGTCTGTGATGTAGGACCGAGTATCTCTCTAGGGACCATCTACTTGA
GCAATGGTGCGCAGGGGGAGACATAGACCAGCCTTGGGTGGCAAGCACTGCAATAAGTCCTGTTTAGC
CTTGGAGTTCACACGCCGGCACTGAAGCCGACCTACCTGAGCGTGTGATTACCGTTAAAGCATCTGTC
TAGTTCTGTTTCCGGCGCTCTTGGTTCCAGGTTAGGGGAAGTGTATGACCCATGTGTTTTTATCGGCT
TAACCACGAGTGATCCCCGGTCGTTTCCCCATTGAATCCCTGGTGCATCCTACTCCCAGAATGATAGC
TGACTGACTGGACTGGCTTTTCAAGTAGTCGAGGGGGTATCGCGGTCACGGCCGTTAACAGATCCCGT
CCTTAGTGTGGAATCCGCACCTGCTGACTAACGCTTCGCCGGCGTGTCTGCACAGCCGTATAGTGTTA
ATCATGACCCCAAGGAAGGATTAAACAAATATCTTGACG
>BANAN15
GTGTCCGGTAGCCCGCGCTAGTTAGACACCCCGGCAGGGGGGATTGCTTTCGGGACGGGAGATCCCTT
CGCCGACCCTGTAGGGCCGACGCCGAGGCATTCGGGCCCCTGCAACGTCAACAGCGGCAAGAAAACGG
GATGAATGGGCGTAATGGGGGGGGTCTGCTGGGGACCCGACGCGGTTGCCGTTTGCGGGGCCCCCGAC
CCATACCGACCCACCTAGGCGTCCAGTTACGGCGCACGTCGGGAGCGTGGTTGCCGTCAGAGCTGTGT
TTCTCGATCAGTCCCCCGCAGTGCCGCAGTATCTTGCCGTGGGCTGCTTTAATCTTGAAAGTGGTTCA
TACATTGGGCGACGAGGTGTCGGCTCTCATTGGGGGTAGCCGACGGACACATGCAGTCCCCTCCCCGG
GCAGGCAGAGGCGGGGCCCCCGCGCGCCGGCCCCGGCCCACAATCTTACCAGGGTCCTCAAAAGAGCC
TTTGCATGGTACCCTTCGTGAATGGTTGCTTAAGAGGTCCACCACGTAGTCCGGGCACGGTCGACTAA
ACACTCAAAACAGCGACGGCAAATAGAGGCACAAGGTCCAGGCCCTCACGGCACTAGTTGCGATGACC

CAACTCACGGGGGCAGTCCCCGGCGCGGCGACCTTGATTACGTCCGGGAACAAGTATGTCGTTTCCCC
CGGGAGGGGTGCAGCCACATGGGAGATTCAAAGTTTCTCGTGACGTCGTTGTGATCACTGCAGCCTAG
CCGAGACTCCCGTACTACGCGAAGGTTGGTTATGTTAACCACTACAACGTGAGGCGCCGTAAGGGCCA
GTGTTGTGCCCGGCTCTTCAATGCGCCTTAGCGGCCTGATACACCCACCCAAGGAGATACTGCTAATC
ATGTGGGTGGACAGAACCTCGCAACGCAGACGCATCCCCGGCATGATATGGTTTTTTTCCGCTATTAC
CCAGCACGCGGCGCTATCATTCAAGAGAACCCAGTGACGCGTAAATCGTAAGATCTACCTGCCGCAGG
TGGACCTACTGCAAATACGGATTATGACTCGTAAAGAGGTCATGCGTATTTTCATCACTAGGCACGTT
CGAGAGTAAATTAGTAGGTGTCCCGCGCCTTGTGGCGTTCCGCCTGACTCCTCATGAAGTCGACCTTC
TCATCGGCCCTATCTGCCGACGTAAGTCATAATCCAGATCTTCACCTCGGAGGGAGGGTCACTGTAAA
GGGATAATTGGAGGGCGATTTCCCACACTTTCCTAAGGGTACTTTTTGCTTGGCTTCGCAGTTGGGTC
CAATAGATGTTGATCTCTGGTTTAGCAGTTGTGAAAGTGGCAAGGCGGGAGGTTAGACCTCCATTTAA
CATATACAAGCAAGTTAACTGCACTAGATGTGTAGACACTACAGTTACAGGAGTAGCCGACTAAGTCT
CCGACGTCAAGCGAATAGGCGTCATACGCGATTATCGCCTAAGAGCACGTATTGGCGGTAAAAGGATA
ACTAGACGCTTGTGGGTAGATTTCAAGGCGCTCGTAGTGGTATAATAGGATACTCTTTCACCAGCCTG
AGAGCCGAACGCTATACTAGTGGTCTGTGATGTAGGACCAAGTAGCTCTCTAGGGACCATCTACTTGA
GCAATGGTGCGCAGGGGTAGACATAGACCAACCTTGGGTGGCAAGCACTGCAATAAGTCCTGTTTAGC
CTTGGAGTTCACACGCCGGCACTAAAGCCGACCTACCTGAGCTTGTGATTACCGTTAAAGCATCTGTC
TAGTTCTGTTTCCGGCGCTCTTGGTTCCATGTTAGGGGAAGTGTATGACCCATGTGTTTTTATCGGCT
TAACTACGAGTGATCCCCGGTCGTTTCCCCATTAAATCCCTGGTGCATCCTACTCCCATAATGATAGC
TGACTGGCTGGACTGGCTTTTCAAGTAGTCGAGGGGGTATCGCGGTCACGGCCGTTAACAGATCCCGT
CCTTAGTGTGGAATCCGCACCTGCTGACTAACGCTTCGCCGGCGTGTCTGCACATCCGTATAGTGTTA
ATCATGACCCCAAGGAAGGATTAAACAAATATCTTGACG
>BANAN18
GTGTCCGGTAGCCCGCGCTAGTCAGACACCCCGGCAGGGGGGATTGCTTTCGAGACGGGAGATCCCTT
CGCCGACCCTGGAGGGCCGACGCCGAGGCATTCGGGCCCCTGCAACGTCAACAGCGGCAAGAAAACGG
GATGAATGGGCGTAATGGGGGGGGTCTGCTGGGGACCCGACGCGGTTGCCGTTTGCGGGGCCCCCGAC
CCATACCGACCCACCTAGGCGTCCAGTTACGGCGCACGGCGGGAGCGTGGTTGCCGTCAGAGCTGTGT
TTCTCGATCAGTCCCCCGCAGTGCCGCAGTATCTTGCCGTGGGCTGCTTTAATCTTGAAAGTGGTTTA
TACATTGGGCGACGAGGTGTCGACTCTCATTGGGGGTAACCGACGGGCACATGCAGTCCCCTCCCCGG
GCAGGCAGAGGCGGGGCCCCCGCGCGCCGGCCCCGGCCCACAATCTTACCAGGGTCCTCAAAGGAGCC
TTTGCATGGTACCCTTCGTGAATGGTTGCTTAAGAGGTCCACAACGTGGTCCGGGCACGGTCGACTAA
ACACTCAAAACAGCGACGGCAAATATAGGTACAAGGTCCAGGCCCTCACGGCACTAATTGCGATGACC
CAACTCACGGGGGCAGTCCCCGGCGCGGCGACCTTGATTACGTCCGGGGACAAGTATGTCGCTCCCCC
CGGGAGGGGTGCAGCCACATGGGAGATTCAAAGTTTCTCGTGACGTCGTTGTGATCACTGCAGCCTAG
CCGAGACTCCCGTACTACGCGAAGGTTGGTTATGTTAACCACTACAACGTGAGGCGCCGTAAGGGCCA
GTGTTGTGCCCGGCTCTTCAATGCGCCTTAGCGGCCTGATACACCCACCCAAGGAGATACTGCTAATC
ATATGGGTGGACAGAACCTCGCAACGCAGACGCCTCCCCGGCATGATATGGTTTTTTTCCGCTATTAT
CCAGCACGCAGCGCTATCATTTAAGAGAACCCAGTGACGCGTAAATCGTAAGATCTACCTGCCGCAGG
TGGACCTACTGCAAATACGGATTATGACTCGTAAAGGGGTCATGCGTATTTTCATCACTAGGCACGTT
CGAGAATAAATTAGTAGGTGTCCCGAGCCTTGTGGCGTTCCGCCTGACTCCTCATGAAGTCGACCTTC
TCACCGGCCCTATCTGCCGACGTAAGTCATAACCTAGATCTGTACCTCGGGGGGAGGGTCACTGTAAA
GGGATAATTGGAGGGTGATTTCCCACATTTTCCTAAGGGTACTTTTTGCCTGGCTTCGCAATTGGGTC
CAATGGATGTCGATCTCTGGTTTAGCAGTTGTGAAAGTGGCAAGGCGGGAGGTTAGACCTCCATTTAA
CATATACAAGCAAGTTAACTGCACTAGATGTGTAGACACTACAGGTACAGGAGTAGCCGGATAAGTCT
CCGACGTCAAGCGAATAAGCGTCATACGCGATTATCGCCTAAGAGCACGTATTTGCGGTAAAAGGCTA
GCTAGACGCTTGTGGGTAGATTTCAAGGCGTTCGTAGTGGTATAATAGGATACTCTTTCACCAGCCTG
AGGGCCGAACGCTATACTAGTGGTCTGTGATGTAGGACCGAGTATCTCTCTAGGGACCATCTACTTGA
GCAATGGTGCGCAGGGGGAGACATAGACCAGCCTTGGGTGGCAAGCACTGCAATAAGTCCTGTTTAGC
CTTGGAGTTCACACGCCGGCACTGAAGCCGACCTACCTGAGCGTGTGATTACCGTTAAAGCATCTGTC
TAGTTCTGTTTCCGGCGCTCTTGGTTCCAGGTTAGGGGAAGTGTATGACCCATGTGTTTTTATCGGCT
TAACCACGAGTGATCCCCGGTCGTTTCCCCATTGAATCCCTGGTGCATCCTACTCCCAGAATGATAGC
TGACTGACTGGACTGGCTTTTCAAGTAATCGAGGGGGTATCGCGGTCACGGCCGTTAACAGATCCCGT
CCTTAGTGTGGAATCCGCACCTGCTGACTAACGCTTCGCCGGCGTGTCTGCACAGCCGTATAGTGTTA
GTCATGACCCCAAGGAAGGATTAAACAAATATCTTGACG

>BANAN19
GTGTCCGGTAGCCCGCGCTAGTCAGACACCCCGGCAGGGGGGATTGCTTTCGAGACGGGAGATCCCTT
CGCCGACCCTGGAGGGCCGACGCCGAGGCATTCGGGCCCCTGCAACGTCAACAGCGGCAAGAAAACGG
GATGAATGGGCGTAATGGGGGGGGTCTGCTGGGGACCCGACGCGGTTGCCGTTTGCGGGGCCCCCGAC
CCATACCGACCCACCTAGGCGTCCAGTTACGGCGCACGTCGGGAGCGTGGTTGCCGTCAGAGCTGTGT
TTCTCGATCAGTCCCCCGCAGTGCCGCAGTATCTTGCCGTGGGCTGCTTTAATCTTGAAAGTGGTTCA
TACATTGGGCGACGAGGTGTCGACTCTCATTGGGGGTAACCGACGGGCACATGCAGTCCCCTCCCCGG
GCAGGCAGAGGCGGGGCCCCCGCGCGCCGGCCCCGGCCCACAATCTTACCAGGGTCCTCAAAGGAGCC
TTTGCATGGTACCCTTCGTGAATGGTTGCTTAAGAGGTCCACAACGTGGTCCGGGCACGGTCGACTAA
ACACTCAAAACAGCGACGGCAAATATAGGTACAAGGTCCAGGCCCTCACGGCACTAATTGCGATGACC
CAACTCACGGGGGCAGTCCCCGGCGCGGCGACCTTGATTACGTCCGGGGACAAGTATGTCGCTCCCCC
CGGGAGGGGTGCAGCCACATGGGAGATTCAAAGTTTCTCGTGACGTCGTTGTGATCACTGCAGCCTAG
CCGAGACTCCCGTACTACGCGAAGGTTGGTTATGTTAACCACTACAACGTGAGGCGCCGTAAGGGCCA
GTGTTGTGCCCGGCTCTTCAATGCGCCTTAGCGGCCTGATACACCCACCCAAGGAGATACTGCTAATC
ATATGGGTGGACAGAACCTCGCAACGCAGACGCCTCCCCGGCATGATATGGTTTTTTTCCGCTATTAT
CCAGCACGCAGCGCTATCATTTAAGAGAACCCAGTGACGCGTAAATCGTAAGATCTACCTGCCGCAGG
TGGACCTACTGCAAATACGGATTATGACTCGTAAAGGGGTCATGCGTATTTTCATCACTAGGCACGTT
CGAGAATAAATTAGTAGGTGTCCCGAGCCTTGTGGCGTTCCGCCTGACTCCTCATGAAGTCGACCTTC
TCACCGGCCCTATCTGCCGACGTAAGTCATAACCTAGATCTGTACCTCGGGGGGAGGGTCACTGTAAA
GGGATAATTGGAGGGTGATTTCCCACATTTTCCTAAGGGTACTTTTTGCCTGGCTTCGCAATTGGGTC
CAATGGATGTCGATCTCTGGTTTAGCAGTTGTGAAAGTGGCAAGGCGGGAGGTTAGACCTCCATTTAA
CATATACAAGCAAGTTAACTGCACTAGATGTGTAGACACTACAGGTACAGGAGTAGCCGGATAAGTCT
CCGACGTCAAGCGAATAAGCGTCATACGCGATTATCGCCTAAGAGCACGTATTTGCGGTAAAAGGCTA
GCTAGACGCTTGTGGGTAGATTTCAAGGCGTTCGTAGTGGTATAATAGGATACTCTTTCACCAGCCTG
AGGGCCGAACGCTATACTAGTGGTCTGTGATGTAGGACCGAGTATCTCTCTAGGGACCATCTACTTGA
GCAATGGTGCGCAGGGGGAGACATAGACCAGCCTTGGGTGGCAAGCACTGCAATAAGTCCTGTTTAGC
CTTGGAGTTCACACGCCGGCACTGAAGCCGACCTACCTGAGCGTGTGATTACCGTTAAAGCATCTGTC
TAGTTCTGTTTCCGGCGCTCTTGGTTCCAGGTTAGGGGAAGTGTATGACCCATGTGTTTTTATCGGCT
TAACCACGAGTGATCCCCGGTCGTTTCCCCATTGAATCCCTGGTGCATCCTACTCCCAGAATGATAGC
TGACTGACTGGACTGGCTTTTCAAGTAATCGAGGGGGTATCGCGGTCACGGCCGTTAACAGATCCCGT
CCTTAGTGTGGAATCCGCACCTGCTGACTAACGCTTCGCCGGCGTGTCTGCACAGCCGTATAGTGTTA
GTCATGACCCCAAGGAAGGATTAAACAAATATCTTGACG
>BANAN20
GTGTCCGGTAGCCCGCGCTAGTTAGACACCCCGGCAGGGGGGATTGCTTTCGAGACGGGAGATCCCTT
CGCCGACCCTGGAGGGCCGACGCCGAGGCATTCGGGCCCCTGCAACGTCAACAGCGGCAAGAAAACGG
GATGAATGGGCGTAATGGGGGGGGTCTGCTGGGGACCCGACGCGGTTGCCGTTTGCGGGGCCCCCGAC
CCATACCGACCCACCTAGGCGTCCAGTTACGGCGCACGGCGGGAGCGTGGTTGCCGTCAGAGCTGTGT
TTCTCGATCAGTCCCCCGCAGTGCCGCAGTATCTTGCCGTGGGCTGCTTTAATCTTGAAAGTGGTTCA
TACATTGGGCGACGAGGTGTCGACTCTCATTGGGGGTAACCGACGGGCACATGCAGTCCCCTCCCCGG
GCAGGCAGAGGCGGGGCCCCCGCGCGCCGGCCCCGGCCCACAATCTTACCAGGGTCCTCAAAGGAGCC
TTTGCATGGTACCCTTCGTGAATGGTTGCTTAAGAGGTCCACAACGTGGTCCGGGCACGGTCGACTAA
ACACTCAAAACAGCGACGGCAAATATAGGTACAAGGTCCAGGCCCTCACGGCACTAATTGCGATGACC
CAACTCACGGGGGCAGTCCCCGGCGCGGCGACCTTGATTACGTCCGGGGACAAGTATGTCGCTCCCCC
CGGGAGGGGTGCAGCCACATGGGAGATTCAAAGTTTCTCGTGACGTCGTTGTGATCACTGCAGCCTAG
CCGAGACTCCCGTACTACGCGAAGGTTGGTTATGTTAACCACTACAACGTGAGGCGCCGTAAGGGCCA
GTGTTGTGCCCGGCTCTTCAATGCGCCTTAGCGGCCTGATACACCCACCCAAGGAGATACTGCTAATC
ATATGGGTGGACAGAACCTCGCAACGCAGACGCCTCCCCGGCATGATATGGTTTTTTTCCGCTATTAT
CCAGCACGCAGCGCTATCATTCAAGAGAACCCAGTGACGCGTAAATCGTAAGATCTACCTGCCGCAGG
TGGACCTACTGCAAATACGGATTATGACTCGTAAAGGGGTCATGCGTATTTTCATCACTAGGCACGTT
CGAGAATAAATTAGTAGGTGTCCCGAGCCTTGTGGCGTTCCGCCTGACTCCTCATGAAGTCGACCTTC
TCACCGGCCCTATCTGCCGACGTAAGTCATAACCTAGATCTGTACCTCGGGGGGAGGGTCACTGTAAA
GGGATAATTGGAGGGTGATTTCCCACACTTTCCTAAGGGTACTTTTTGCCTGGCTTCGCAATTGGGTC
CAATGGATGTCGATCTCTGGTTTAGCAGTTGTGAAAGTGGCAAGGCGGGAGGTTAGACCTCCATTTAA
CATATACAAGCAAGTTAACTGCACTAGATGTGTAGACACTACAGTTACAGGAGTAGCCGAATAAGTCT

CCGACGTCAAGCGAATAAGCGTCATACGCGATTATCGCCTAAGAGCACGTATTGGCGGTAAAAGGCTA
GCTAGACGCTTATGGGTAGATTTCAAGGCGTTCGTAGTGGTATAATAGGATACTCTTTCACCAGCCTG
AGGGCCGAACGCTATACTAGTGGTCTGTGATGTAGGACCGAGTATCTCTCTAGGGACCATCTACTTGA
GCAATGGTGCGCAGGGGGAGACATAGACCAGCCTTGGGTGGCAAGCACTGCAATAAGTCCTGTTTAGC
CTTGGAGTTCACATGCCGGCACTGAAGCCGACCTACCTGAGCGTGTGATTACCGTTAAAGCATCTGTC
TAGTTCTGTTTCCGGCGCTCTTGGTTCCAGGTTAGGGGAAGTGTATGACCCATGTGTTTTTATCGGCT
TAACCACGAGTGATCCCCGGTCGTTTCCCCATTGAATCCCTGGTGCATCCTACTCCCAGAATGATAGC
TGACTGACTGGACTGGCTTTTCAAGTAATCGAGGGGGTATCGCGGTCACGGCCGTTAACAGATCCCGT
CCTTAGTGTGGAATCCGCACCTGCTGACTAACGCTTCGCCGGCGTGTCTGCACAGCCGTATAGTGTTA
ATCATGACCCCAAGGAAGGATTAAACAAATATCTTGACG
>BANAN21
GTGTCCGGTAGCCCGCGCTAGTTAGACACCCCGGCAGGGGGGATTGCTTTCGAGACGGGAGATCCCTT
CGCCGACCCTGGAGGGCCGACGCCGAGGCATTCGGGCCCCCGCAACGTCAACAGCGGCAAGAAAACGG
GATGAATGGGCGTAATGGGGGGGGTCTGCTGGGGACCCGACGCGGTTGCCGTTTGCGGGGCCCCCGAC
CCATACCGACCCACCTAGGCGTCCAGTTACGGCGCACGTCGGGAGCGTGGTTGCCGTCAGAGCTGTGT
TTCTCGATCAGTCCCCCGCAGTGCCGCAGTATCTTGCCGTGGGCTGCTTTAATCTTGAAAGTGGTTCA
TACATTGGGCGACGAGGTGTCGACTCTCATTGGGGGTAACCGACGGGCACATGCAGTCCCCTCCCCGG
GCAGGCAGAGGCGGGGCCCCCGCGCGCCGGCCCCGGCCCACAATCTTACCAGGGTCCTCAAAGGAGCC
TTTGCATGGTACCCTTCGTGAATGGTTGCTTAAGAGGTCCACAACGTGGTCCGGGCACGGTCGACTAA
ACACTCAAAACAGCGACGGCAAATATAGGTACAAGGTCCAGGCCCTCACGGCACTAATTGCGATGACC
CAACTCACGGGGGCAGTCCCCGGCGCGGCGACCTTGATTACGTCCGGGGACAAGTATGTCGCTCCCCC
CGGGAGGGGTGCAGCCACATGGGAGATTCAAAGTTTCTCGTGACGTCGTTGTGATCACTGCAGCCTAG
CCGAGACTCCCGTACTACGCGAAGGTTGGTTATGTTAACCACTACAACGTGAGGCGCCGTAAGGGCCA
GTGTTGTGCCCGGCTCTTCAATGCGCCTTAGCGGCCTGATACACCCACCCAAGGAGATACTGCTAATC
ATATGGGTGGACAGAACCTCGCAACGCAGACGCCTCCCCGGCATGATATGGTTTTTTTCCGCTATTAT
CCAGCACGCAGCGCTATCATTCAAGAGAACCCAGTGACGCGTAAATCGTAAGATCTACCTGCCGCAGG
TGGACCTACTGCAAATACGGATTATGACTCGTAAAGGGGTCATGCGTATTTTCATCACTAGGCACGTT
CGAGAATAAATTAGTAGGTGTCCCGAGCCTTGTGGCGTTCCGCCTGACTCCTCATGAAGTCGACCTTC
TCACCGGCCCTATCTGCCGACGTAAGTCATAACCTAGATCTGTACCTCGGGGGGAGGGTCACTGTAAA
GGGATAATTGGAGGGTGATTTCCCACATTTTCCTAAGGGTACTTTTTGCCTGGCTTCGCAATTGGGTC
CAATGGATGTCGATCTCTGGTTTAGCAGTTGTGAAAGTGGCAAGGCGGGAGGTTAGACCTCCATTTAA
CATATACAAGCAAGTTAACTGCACTAGATGTGTAGACACTACAGTTACAGGAGTAGCCGAATAAGTCT
CCGACGTCAAGCGAATAAGCGTCATACGCGATTATCGCCTAAGAGCACGTATTGGCGGTAAAAGGCTA
GCTAGACGCTTGTGGGTAGATTTCAAGGCGTTCGTAGTGGTATAATAGGATACTCTTTCACCAGCCTG
AGGGCCGAACGCTATACTAGTGGTCTGTGATGTAGGACCGAGTATCTCTCTAGGGACCATCTACTTGA
GCAATGGTGCGCAGGGGGAGACATAGACCAGCCTTGGGTGGCAAGCACTGCAATAAGTCCTGTTTAGC
CTTGGAGTTCACACGCCGGCACTGAAGCCGACCTACCTGAGCGTGTGATTACCGTTAAAGCATCTGTC
TAGTTCTGTTTCCGGCGCTCTTGGTTCCAGGTTAGGGGAAGTGTATGACCCATGTGTTTTTATCGGCT
TAACCACGAGTGATCCCCGGTCGTTTCCCCATTGAATCCCTGGTGCATCCTACTCCCAGAATGATAGC
TGACTGACTGGACTGGCTTTTCAAGTAATCGAGGGGGTATCGCGGTCACGGCCGTTAACAGATCCCGT
CCTTAGTGTGGAATCCGCACCTGCTGACTAACGCTTCGCCGGCGTGTCTGCACAGCCGTATAGTGTTA
ATCATGACCCCAAGGAAGGATTAAACAAATATCTTGACG
>BANAN22
GTGTCCGGTAGCCCGCGCTAGTCAGACACCCCGGCAGGGGGGATTGCTTTCGAGACGGGAGATCCCTT
CGCCGACCCTGGAGGGCCGACGCCGAGGCATTCGGGCCCCCGCAACGTCAACAGCGGCAAGAAAACGG
GATGAATGGGCGTAATGGGGGGGGTCTGCTGGGGACCCGACGCGGTTGCCGTTTGCGGGGCCCCCGAC
CCATACCGACCCACCTAGGCGTCCAGTTACGGCGCACGGCGGGAGCGTGGTTGCCGTCAGAGCTGTGT
TTCTCGATCAGTCCCCCGCAGTGCCGCAGTATCTTGCCGTGGGCTGCTTTAATCTTGAAAGTGGTTCA
TACATTGGGCGACGAGGTGTCGACTCTCATTGGGGGTAACCGACGGGCACATGCAGTCCCCTCCCCGG
GCAGGCAGAGGCGGGGCCCCCGCGCGCCGGCCCCGGCCCACAATCTTACCAGGGTCCTCAAAGGAGCC
TTTGCATGGTACCCTTCGTGAATGGTTGCTTAAGAGGTCCACAACGTGGTCCGGGCACGGTCGACTAA
ACACTCAAAACAGCGACGGCAAATATAGGTACAAGGTCCAGGCCCTCACGGCACTAATTGCGATGACC
CAACTCACGGGGGCAGTCCCCGGCGCGGCGACCTTGATTACGTCCGGGGACAAGTATGTCGCTCCCCC
CGGGAGGGGTGCAGCCACATGGGAGATTCAAAGTTTCTCGTGACGTCGTTGTGATCACTGCAGCCTAG

CCGAGACTCCCGTACTACGCGAAGGTTGGTTATGTTAACCACTACAACGTGAGGCGCCGTAAGGGCCA
GTGTTGTGCCCGGCTCTTCAATGCGCCTTAGCGGCCTGATACACCCACCCAAGGAGATACTGCTAATC
ATATGGGTGGACAGAACCTCGCAACGCAGACGCCTCCCCGGCATGATATGGTTTTTTTCCGCTATTAT
CCAGCACGCAGCGCTATCATTTAAGAGAACCCAGTGACGCGTAAATCGTAAGATCTACCTGCCGCAGG
TGGACCTACTGCAAATACGGATTATGACTCGTAAAGGGGTCATGCGTATTTTCATCACTAGGCACGTT
CGAGAATAAATTAGTAGGTGTCCCGAGCCTTGTGGCGTTCCGCCTGACTCCTCATGAAGTCGACCTTC
TCACCGGCCCTATCTGCCGACGTAAGTCATAACCTAGATCTGTACCTCGGGGGGAGGGTCACTGTAAA
GGGATAATTGGAGGGTGATTTCCCACATTTTCCTAAGGGTACTTTTTGCCTGGCTTCGCAATTGGGTC
CAATGGATGTCGATCTCTGGTTTAGCAGTTGTGAAAGTGGCAAGGCGGGAGGTTAGACCTCCATTTAA
CATATACAAGCAAGTTAACTGCACTAGATGTGTAGACACTACAGGTACAGGAGTAGCCGGATAAGTCT
CCGACGTCAAGCGAATAAGCGTCATACGCGATTATCGCCTAAGAGCACGTATTTGCGGTAAAAGGCTA
GCTAGACGCTTGTGGGTAGATTTCAAGGCGTTCGTAGTGGTATAATAGGATACTCTTTCACCAGCCTG
AGGGCCGAACGCTATACTAGTGGTCTGTGATGTAGGACCGAGTATCTCTCTAGGGACCATCTACTTGA
GCAATGGTGCGCAGGGGGAGACATAGACCAGCCTTGGGTGGCAAGCACTGCAATAAGTCCTGTTTAGC
CTTGGAGTTCACACGCCGGCACTGAAGCCGACCTACCTGAGCGTGTGATTACCGTTAAAGCATCTGTC
TAGTTCTGTTTCCGGCGCTCTTGGTTCCAGGTTAGGGGAAGTGTATGACCCATGTGTTTTTATCGGCT
TAACCACGAGTGATCCCCGGTCGTTTCCCCATTGAATCCCTGGTGCATCCTACTCCCAGAATGATAGC
TGACTGACTGGACTGGCTTTTCAAGTAATCGAGGGGGTATCGCGGTCACGGCCGTTAACAGATCCCGT
CCTTAGTGTGGAATCCGCACCTGCTGACTAACGCTTCGCCGGCGTGTCTGCACAGCCGTATAGTGTTA
GTCATGACCCCAAGGAAGGATTAAACAAATATCTTGACG
>BANAN23
GTGTCCGGTAGCCCGCGCTAGTTAGACACCCCGGCAGGGGGGATTGCTTTCGAGACGGGAGATCCCTT
CGCCGACCCTGGAGGGCCGACGCCGAGGCATTCGGGCCCCTGCAACGTCAACAGCGGCAAGAAAACGG
GATGAATGGGCGTAATGGGGGGGGTCTGCTGGGGACCCGACGCGGTTGCCGTTTGCGGGGCCCCCGAC
CCATACCGACCCACCTAGGCGTCCAGTTACGGCGCACGTCGGGAGCGTGGTTGCCGTCAGAGCTGTGT
TTCTCGATCAGTCCCCCGCAGTGCCGCAGTATCTTGCCGTGGGCTGCTTTAATCTTGAAAGTGGTTTA
TACATTGGGCGACGAGGTGTCGACTCTCATTGGGGGTAACCGACGGGCACATGCAGTCCCCTCCCCGG
GCAGGCAGAGGCGGGGCCCCCGCGCGCCGGCCCCGGCCCACAATCTTACCAGGGTCCTCAAAGGAGCC
TTTGCATGGTACCCTTCGTGAATGGTTGCTTAAGAGGTCCACAACGTGGTCCGGGCACGGTCGACTAA
ACACTCAAAACAGCGACGGCAAATATAGGTACAAGGTCCAGGCCCTCACGGCACTAATTGCGATGACC
CAACTCACGGGGGCAGTCCCCGGCGCGGCGACCTTGATTACGTCCGGGGACAAGTATGTCGCTCCCCC
CGGGAGGGGTGCAGCCACATGGGAGATTCAAAGTTTCTCGTGACGTCGTTGTGATCACTGCAGCCTAG
CCGAGACTCCCGTACTACGCGAAGGTTGGTTATGTTAACCACTACAACGTGAGGCGCCGTAAGGGCCA
GTGTTGTGCCCGGCTCTTCAATGCGCCTTAGCGGCCTGATACACCCACCCAAGGAGATACTGCTAATC
ATATGGGTGGACAGAACCTCGCAACGCAGACGCCTCCCCGGCATGATATGGTTTTTTTCCGCTATTAT
CCAGCACGCAGCGCTATCATTCAAGAGAACCCAGTGACGCGTAAATCGTAAGATCTACCTGCCGCAGG
TGGACCTACTGCAAATACGGATTATGACTCGTAAAGGGGTCATGCGTATTTTCATCACTAGGCACGTT
CGAGAATAAATTAGTAGGTGTCCCGAGCCTTGTGGCGTTCCGCCTGACTCCTCATGAAGTCGACCTTC
TCACCGGCCCTATCTGCCGACGTAAGTCATAACCTAGATCTGTACCTCGGGGGGAGGGTCACTGTAAA
GGGATAATTGGAGGGTGATTTCCCACACTTTCCTAAGGGTACTTTTTGCCTGGCTTCGCAATTGGGTC
CAATGGATGTCGATCTCTGGTTTAGCAGTTGTGAAAGTGGCAAGGCGGGAGGTTAGACCTCCATTTAA
CATATACAAGCAAGTTAACTGCACTAGATGTGTAGACACTACAGTTACAGGAGTAGCCGAATAAGTCT
CCGACGTCAAGCGAATAAGCGTCATACGCGATTATCGCCTAAGAGCACGTATTGGCGGTAAAAGGCTA
GCTAGACGCTTATGGGTAGATTTCAAGGCGTTCGTAGTGGTATAATAGGATACTCTTTCACCAGCCTG
AGGGCCGAACGCTATACTAGTGGTCTGTGATGTAGGACCGAGTATCTCTCTAGGGACCATCTACTTGA
GCAATGGTGCGCAGGGGGAGACATAGACCAGCCTTGGGTGGCAAGCACTGCAATAAGTCCTGTTTAGC
CTTGGAGTTCACATGCCGGCACTGAAGCCGACCTACCTGAGCGTGTGATTACCGTTAAAGCATCTGTC
TAGTTCTGTTTCCGGCGCTCTTGGTTCCAGGTTAGGGGAAGTGTATGACCCATGTGTTTTTATCGGCT
TAACCACGAGTGATCCCCGGTCGTTTCCCCATTGAATCCCTGGTGCATCCTACTCCCAGAATGATAGC
TGACTGACTGGACTGGCTTTTCAAGTAATCGAGGGGGTATCGCGGTCACGGCCGTTAACAGATCCCGT
CCTTAGTGTGGAATCCGCACCTGCTGACTAACGCTTCGCCGGCGTGTCTGCACAGCCGTATAGTGTTA
ATCATGACCCCAAGGAAGGATTAAACAAATATCTTGACG
>BANAN24
GTGTCCGGTAGCCCGCGCTAGTTAGACACCCCGGCAGGGGGGATTGCTTTCGGGACGGGAGATCCCTT

CGCCGACCCTGTAGGGCCGACGCCGAGGCATTCGGGCCCCCGCAACGTCAACAGCGGCAAGAAAACGG
GATGAATGGGCGTAATGGGGGGGGTCTGCTGGGGACCCGACGCGGTTGCCGTTTGCGGGGCCCCCGAC
CCATACCGACCCACCTAGGCGTCCAGTTACGGCGCACGTCGGGAGCGTGGTTGCCGTCAGAGCTGTGT
TTCTCGATCAGTCCCCCGCAGTGCCGCAGTATCTTGCCGTGGGCTGCTTTAATCTTGAAAGTGGTTCA
TACATTGGGCGACGAGGTGTCGGCTCTCATTGGGGGTAGCCGACGGACACATGCAGTCCCCTCCCCGG
GCAGGCAGAGGCGGGGCCCCCGCGCGCCGGCCCCGGCCCACAATCTTACCAGGGTCCTCAAAAGAGCC
TTTGCATGGTACCCTTCGTGAATGGTTGCTTAAGAGGTCCACCACGTAGTCCGGGCACGGTCGACTAA
ACACTCAAAACAGCGACGGCAAATAGAGGCACAAGGTCCAGGCCCTCACGGCACTAGTTGCGATGACC
CAACTCACGGGGGCAGTCCCCGGCGCGGCGACCTTGATTACGTCCGGGAACAAGTATGTCGTTTCCCC
CGGGAGGGGTGCAGCCACATGGGAGATTCAAAGTTTCTCGTGACGTCGTTGTGATCACTGCAGCCTAG
CCGAGACTCCCGTACTACGCGAAGGTTGGTTATGTTAACCACTACAACGTGAGGCGCCGTAAGGGCCA
GTGTTGTGCCCGGCTCTTCAATGCGCCTTAGCGGCCTGATACACCCACCCAAGGAGATACTGCTAATC
ATGTGGGTGGACAGAACCTCGCAACGCAGACGCATCCCCGGCATGATATGGTTTTTTTCCGCTATTAC
CCAGCACGCGGCGCTATCATTCAAGAGAACCCAGTGACGCGTAAATCGTAAGATCTACCTGCCGCAGG
TGGACCTACTGCAAATACGGATTATGACTCGTAAAGAGGTCATGCGTATTTTCATCACTAGGCACGTT
CGAGAGTAAATTAGTAGGTGTCCCGCGCCTTGTGGCGTTCCGCCTGACTCCTCATGAAGTCGACCTTC
TCATCGGCCCTATCTGCCGACGTAAGTCATAATCCAGATCTTCACCTCGGAGGGAGGGTCACTGTAAA
GGGATAATTGGAGGGCGATTTCCCACACTTTCCTAAGGGTACTTTTTGCTTGGCTTCGCAGTTGGGTC
CAATAGATGTTGATCTCTGGTTTAGCAGTTGTGAAAGTGGCAAGGCGGGAGGTTAGACCTCCATTTAA
CATATACAAGCAAGTTAACTGCACTAGATGTGTAGACACTACAGTTACAGGAGTAGCCGACTAAGTCT
CCGACGTCAAGCGAATAGGCGTCATACGCGATTATCGCCTAAGAGCACGTATTGGCGGTAAAAGGATA
ACTAGACGCTTGTGGGTAGATTTCAAGGCGCTCGTAGTGGTATAATAGGATACTCTTTCACCAGCCTG
AGAGCCGAACGCTATACTAGTGGTCTGTGATGTAGGACCAAGTAGCTCTCTAGGGACCATCTACTTGA
GCAATGGTGCGCAGGGGTAGACATAGACCAACCTTGGGTGGCAAGCACTGCAATAAGTCCTGTTTAGC
CTTGGAGTTCACACGCCGGCACTAAAGCCGACCTACCTGAGCTTGTGATTACCGTTAAAGCATCTGTC
TAGTTCTGTTTCCGGCGCTCTTGGTTCCATGTTAGGGGAAGTGTATGACCCATGTGTTTTTATCGGCT
TAACTACGAGTGATCCCCGGTCGTTTCCCCATTAAATCCCTGGTGCATCCTACTCCCATAATGATAGC
TGACTGGCTGGACTGGCTTTTCAAGTAGTCGAGGGGGTATCGCGGTCACGGCCGTTAACAGATCCCGT
CCTTAGTGTGGAATCCGCACCTGCTGACTAACGCTTCGCCGGCGTGTCTGCACATCCGTATAGTGTTA
ATCATGACCCCAAGGAAGGATTAAACAAATATCTTGACG
>BANAN25
GTGTCCGGTAGCCCGCGCTAGTTAGACACCCCGGCAGGGGGGATTGCTTTCGAGACGGGAGATCCCTT
CGCCGACCCTGGAGGGCCGACGCCGAGGCATTCGGGCCCCTGCAACGTCAACAGCGGCAAGAAAACGG
GATGAATGGGCGTAATGGGGGGGGTCTGCTGGGGACCCGACGCGGTTGCCGTTTGCGGGGCCCCCGAC
CCATACCGACCCACCTAGGCGTCCAGTTACGGCGCACGTCGGGAGCGTGGTTGCCGTCAGAGCTGTGT
TTCTCGATCAGTCCCCCGCAGTGCCGCAGTATCTTGCCGTGGGCTGCTTTAATCTTGAAAGTGGTTTA
TACATTGGGCGACGAGGTGTCGACTCTCATTGGGGGTAACCGACGGGCACATGCAGTCCCCTCCCCGG
GCAGGCAGAGGCGGGGCCCCCGCGCGCCGGCCCCGGCCCACAATCTTACCAGGGTCCTCAAAGGAGCC
TTTGCATGGTACCCTTCGTGAATGGTTGCTTAAGAGGTCCACAACGTGGTCCGGGCACGGTCGACTAA
ACACTCAAAACAGCGACGGCAAATATAGGTACAAGGTCCAGGCCCTCACGGCACTAATTGCGATGACC
CAACTCACGGGGGCAGTCCCCGGCGCGGCGACCTTGATTACGTCCGGGGACAAGTATGTCGCTCCCCC
CGGGAGGGGTGCAGCCACATGGGAGATTCAAAGTTTCTCGTGACGTCGTTGTGATCACTGCAGCCTAG
CCGAGACTCCCGTACTACGCGAAGGTTGGTTATGTTAACCACTACAACGTGAGGCGCCGTAAGGGCCA
GTGTTGTGCCCGGCTCTTCAATGCGCCTTAGCGGCCTGATACACCCACCCAAGGAGATACTGCTAATC
ATATGGGTGGACAGAACCTCGCAACGCAGACGCCTCCCCGGCATGATATGGTTTTTTTCCGCTATTAT
CCAGCACGCAGCGCTATCATTCAAGAGAACCCAGTGACGCGTAAATCGTAAGATCTACCTGCCGCAGG
TGGACCTACTGCAAATACGGATTATGACTCGTAAAGGGGTCATGCGTATTTTCATCACTAGGCACGTT
CGAGAATAAATTAGTAGGTGTCCCGAGCCTTGTGGCGTTCCGCCTGACTCCTCATGAAGTCGACCTTC
TCACCGGCCCTATCTGCCGACGTAAGTCATAACCTAGATCTGTACCTCGGGGGGAGGGTCACTGTAAA
GGGATAATTGGAGGGTGATTTCCCACACTTTCCTAAGGGTACTTTTTGCCTGGCTTCGCAATTGGGTC
CAATGGATGTCGATCTCTGGTTTAGCAGTTGTGAAAGTGGCAAGGCGGGAGGTTAGACCTCCATTTAA
CATATACAAGCAAGTTAACTGCACTAGATGTGTAGACACTACAGTTACAGGAGTAGCCGAATAAGTCT
CCGACGTCAAGCGAATAAGCGTCATACGCGATTATCGCCTAAGAGCACGTATTGGCGGTAAAAGGCTA
GCTAGACGCTTATGGGTAGATTTCAAGGCGTTCGTAGTGGTATAATAGGATACTCTTTCACCAGCCTG

AGGGCCGAACGCTATACTAGTGGTCTGTGATGTAGGACCGAGTATCTCTCTAGGGACCATCTACTTGA
GCAATGGTGCGCAGGGGGAGACATAGACCAGCCTTGGGTGGCAAGCACTGCAATAAGTCCTGTTTAGC
CTTGGAGTTCACATGCCGGCACTGAAGCCGACCTACCTGAGCGTGTGATTACCGTTAAAGCATCTGTC
TAGTTCTGTTTCCGGCGCTCTTGGTTCCAGGTTAGGGGAAGTGTATGACCCATGTGTTTTTATCGGCT
TAACCACGAGTGATCCCCGGTCGTTTCCCCATTGAATCCCTGGTGCATCCTACTCCCAGAATGATAGC
TGACTGACTGGACTGGCTTTTCAAGTAATCGAGGGGGTATCGCGGTCACGGCCGTTAACAGATCCCGT
CCTTAGTGTGGAATCCGCACCTGCTGACTAACGCTTCGCCGGCGTGTCTGCACAGCCGTATAGTGTTA
ATCATGACCCCAAGGAAGGATTAAACAAATATCTTGACG
>BANAN26
GTGTCCGGTAGCCCGCGCTAGTTAGACACCCCGGCAGGGGGGATTGCTTTCGAGACGGGAGATCCCTT
CGCCGACCCTGGAGGGCCGACGCCGAGGCATTCGGGCCCCTGCAACGTCAACAGCGGCAAGAAAACGG
GATGAATGGGCGTAATGGGGGGGGTCTGCTGGGGACCCGACGCGGTTGCCGTTTGCGGGGCCCCCGAC
CCATACCGACCCACCTAGGCGTCCAGTTACGGCGCACGGCGGGAGCGTGGTTGCCGTCAGAGCTGTGT
TTCTCGATCAGTCCCCCGCAGTGCCGCAGTATCTTGCCGTGGGCTGCTTTAATCTTGAAAGTGGTTTA
TACATTGGGCGACGAGGTGTCGACTCTCATTGGGGGTAACCGACGGGCACATGCAGTCCCCTCCCCGG
GCAGGCAGAGGCGGGGCCCCCGCGCGCCGGCCCCGGCCCACAATCTTACCAGGGTCCTCANAGGAGCC
TTTGCATGGTACCCTTCGTGAATGGTTGCTTAAGAGGTCCACAACGTGGTCCGGGCACGGTCGACTAA
ACACTCAAAACAGCGACGGCAAATATAGGTACAAGGTCCAGGCCCTCACGGCACTAATTGCGATGACC
CAACTCACGGGGGCAGTCCCCGGCGCGGCGACCTTGATTACGTCCGGGGACAAGTATGTCGCTCCCCC
CGGGAGGGGTGCAGCCACATGGGAGATTCAAAGTTTCTCGTGACGTCGTTGTGATCACTGCAGCCTAG
CCGAGACTCCCGTACTACGCGAAGGTTGGTTATGTTAACCACTACAACGTGAGGCGCCGTAAGGGCCA
GTGTTGTGCCCGGCTCTTCAATGCGCCTTAGCGGCCTGATACACCCACCCAAGGAGATACTGCTAATC
ATATGGGTGGACAGAACCTCGCAACGCAGACGCCTCCCCGGCATGATATGGTTTTTTTCCGCTATTAT
CCAGCACGCAGCGCTATCATTCAAGAGAACCCAGTGACGCGTAAATCGTAAGATCTACCTGCCGCAGG
TGGACCTACTGCAAATACGGATTATGACTCGTAAAGGGGTCATGCGTATTTTCATCACTAGGCACGTT
CGAGAATAAATTAGTAGGTGTCCCGAGCCTTGTGGCGTTCCGCCTGACTCCTCATGAAGTCGACCTTC
TCACCGGCCCTATCTGCCGACGTAAGTCATAACCTAGATCTGTACCTCGGGGGGAGGGTCACTGTAAA
GGGATAATTGGAGGGTGATTTCCCACACTTTCCTAAGGGTACTTTTTGCCTGGCTTCGCAATTGGGTC
CAATGGATGTCGATCTCTGGTTTAGCAGTTGTGAAAGTGGCAAGGCGGGAGGTTAGACCTCCATTTAA
CATATACAAGCAAGTTAACTGCACTAGATGTGTAGACACTACAGTTACAGGAGTAGCCGAATAAGTCT
CCGACGTCAAGCGAATAAGCGTCATACGCGATTATCGCCTAAGAGCACGTATTGGCGGTAAAAGGCTA
GCTAGACGCTTATGGGTAGATTTCAAGGCGTTCGTAGTGGTATAATAGGATACTCTTTCACCAGCCTG
AGGGCCGAACGCTATACTAGTGGTCTGTGATGTAGGACCGAGTATCTCTCTAGGGACCATCTACTTGA
GCAATGGTGCGCAGGGGGAGACATAGACCAGCCTTGGGTGGCAAGCACTGCAATAAGTCCTGTTTAGC
CTTGGAGTTCACATGCCGGCACTGAAGCCGACCTACCTGAGCGTGTGATTACCGTTAAAGCATCTGTC
TAGTTCTGTTTCCGGCGCTCTTGGTTCCAGGTTAGGGGAAGTGTATGACCCATGTGTTTTTATCGGCT
TAACCACGAGTGATCCCCGGTCGTTTCCCCATTGAATCCCTGGTGCATCCTACTCCCAGAATGATAGC
TGACTGACTGGACTGGCTTTTCAAGTAATCGAGGGGGTATCGCGGTCACGGCCGTTAACAGATCCCGT
CCTTAGTGTGGAATCCGCACCTGCTGACTAACGCTTCGCCGGCGTGTCTGCACAGCCGTATAGTGTTA
ATCATGACCCCAAGGAAGGATTAAACAAATATCTTGACG
>BANAN27
GTGTCCGGTAGCCCGCGCTAGTTAGACACCCCGGCAGGGGGGATTGCTTTCGAGACGGGAGATCCCTT
CGCCGACCCTGGAGGGCCGACGCCGAGGCATTCGGGCCCCTGCAACGTCAACAGCGGCAAGAAAACGG
GATGAATGGGCGTAATGGGGGGGGTCTGCTGGGGACCCGACGCGGTTGCCGTTTGCGGGGCCCCCGAC
CCATACCGACCCACCTAGGCGTCCAGTTACGGCGCACGTCGGGAGCGTGGTTGCCGTCAGAGCTGTGT
TTCTCGATCAGTCCCCCGCAGTGCCGCAGTATCTTGCCGTGGGCTGCTTTAATCTTGAAAGTGGTTTA
TACATTGGGCGACGAGGTGTCGACTCTCATTGGGGGTAACCGACGGGCACATGCAGTCCCCTCCCCGG
GCAGGCAGAGGCGGGGCCCCCGCGCGCCGGCCCCGGCCCACAATCTTACCAGGGTCCTCAAAGGAGCC
TTTGCATGGTACCCTTCGTGAATGGTTGCTTAAGAGGTCCACAACGTGGTCCGGGCACGGTCGACTAA
ACACTCAAAACAGCGACGGCAAATATAGGTACAAGGTCCAGGCCCTCACGGCACTAATTGCGATGACC
CAACTCACGGGGGCAGTCCCCGGCGCGGCGACCTTGATTACGTCCGGGGACAAGTATGTCGCTCCCCC
CGGGAGGGGTGCAGCCACATGGGAGATTCAAAGTTTCTCGTGACGTCGTTGTGATCACTGCAGCCTAG
CCGAGACTCCCGTACTACGCGAAGGTTGGTTATGTTAACCACTACAACGTGAGGCGCCGTAAGGGCCA
GTGTTGTGCCCGGCTCTTCAATGCGCCTTAGCGGCCTGATACACCCACCCAAGGAGATACTGCTAATC

ATATGGGTGGACAGAACCTCGCAACGCAGACGCCTCCCCGGCATGATATGGTTTTTTTCCGCTATTAT
CCAGCACGCAGCGCTATCATTCAAGAGAACCCAGTGACGCGTAAATCGTAAGATCTACCTGCCGCAGG
TGGACCTACTGCAAATACGGATTATGACTCGTAAAGGGGTCATGCGTATTTTCATCACTAGGCACGTT
CGAGAATAAATTAGTAGGTGTCCCGAGCCTTGTGGCGTTCCGCCTGACTCCTCATGAAGTCGACCTTC
TCACCGGCCCTATCTGCCGACGTAAGTCATAACCTAGATCTGTACCTCGGGGGGAGGGTCACTGTAAA
GGGATAATTGGAGGGTGATTTCCCACACTTTCCTAAGGGTACTTTTTGCCTGGCTTCGCAATTGGGTC
CAATGGATGTCGATCTCTGGTTTAGCAGTTGTGAAAGTGGCAAGGCGGGAGGTTAGACCTCCATTTAA
CATATACAAGCAAGTTAACTGCACTAGATGTGTAGACACTACAGTTACAGGAGTAGCCGAATAAGTCT
CCGACGTCAAGCGAATAAGCGTCATACGCGATTATCGCCTAAGAGCACGTATTGGCGGTAAAAGGCTA
GCTAGACGCTTATGGGTAGATTTCAAGGCGTTCGTAGTGGTATAATAGGATACTCTTTCACCAGCCTG
AGGGCCGAACGCTATACTAGTGGTCTGTGATGTAGGACCGAGTATCTCTCTAGGGACCATCTACTTGA
GCAATGGTGCGCAGGGGGAGACATAGACCAGCCTTGGGTGGCAAGCACTGCAATAAGTCCTGTTTAGC
CTTGGAGTTCACATGCCGGCACTGAAGCCGACCTACCTGAGCGTGTGATTACCGTTAAAGCATCTGTC
TAGTTCTGTTTCCGGCGCTCTTGGTTCCAGGTTAGGGGAAGTGTATGACCCATGTGTTTTTATCGGCT
TAACCACGAGTGATCCCCGGTCGTTTCCCCATTGAATCCCTGGTGCATCCTACTCCCAGAATGATAGC
TGACTGACTGGACTGGCTTTTCAAGTAATCGAGGGGGTATCGCGGTCACGGCCGTTAACAGATCCCGT
CCTTAGTGTGGAATCCGCACCTGCTGACTAACGCTTCGCCGGCGTGTCTGCACAGCCGTATAGTGTTA
ATCATGACCCCAAGGAAGGATTAAACAAATATCTTGACG
>BANAN28
GTGTCCGGTAGCCCGCGCTAGTTAGACACCCCGGCAGGGGGGATTGCTTTCGAGACGGGAGATCCCTT
CGCCGACCCTGGAGGGCCGACGCCGAGGCATTCGGGCCCCTGCAACGTCAACAGCGGCAAGAAAACGG
GATGAATGGGCGTAATGGGGGGGGTCTGCTGGGGACCCGACGCGGTTGCCGTTTGCGGGGCCCCCGAC
CCATACCGACCCACCTAGGCGTCCAGTTACGGCGCACGTCGGGAGCGTGGTTGCCGTCAGAGCTGTGT
TTCTCGATCAGTCCCCCGCAGTGCCGCAGTATCTTGCCGTGGGCTGCTTTAATCTTGAAAGTGGTTTA
TACATTGGGCGACGAGGTGTCGACTCTCATTGGGGGTAACCGACGGGCACATGCAGTCCCCTCCCCGG
GCAGGCAGAGGCGGGGCCCCCGCGCGCCGGCCCCGGCCCACAATCTTACCAGGGTCCTCAAAGGAGCC
TTTGCATGGTACCCTTCGTGAATGGTTGCTTAAGAGGTCCACAACGTGGTCCGGGCACGGTCGACTAA
ACACTCAAAACAGCGACGGCAAATATAGGTACAAGGTCCAGGCCCTCACGGCACTAATTGCGATGACC
CAACTCACGGGGGCAGTCCCCGGCGCGGCGACCTTGATTACGTCCGGGGACAAGTATGTCGCTCCCCC
CGGGAGGGGTGCAGCCACATGGGAGATTCAAAGTTTCTCGTGACGTCGTTGTGATCACTGCAGCCTAG
CCGAGACTCCCGTACTACGCGAAGGTTGGTTATGTTAACCACTACAACGTGAGGCGCCGTAAGGGCCA
GTGTTGTGCCCGGCTCTTCAATGCGCCTTAGCGGCCTGATACACCCACCCAAGGAGATACTGCTAATC
ATATGGGTGGACAGAACCTCGCAACGCAGACGCCTCCCCGGCATGATATGGTTTTTTTCCGCTATTAT
CCAGCACGCAGCGCTATCATTCAAGAGAACCCAGTGACGCGTAAATCGTAAGATCTACCTGCCGCAGG
TGGACCTACTGCAAATACGGATTATGACTCGTAAAGGGGTCATGCGTATTTTCATCACTAGGCACGTT
CGAGAATAAATTAGTAGGTGTCCCGAGCCTTGTGGCGTTCCGCCTGACTCCTCATGAAGTCGACCTTC
TCACCGGCCCTATCTGCCGACGTAAGTCATAACCTAGATCTGTACCTCGGGGGGAGGGTCACTGTAAA
GGGATAATTGGAGGGTGATTTCCCACACTTTCCTAAGGGTACTTTTTGCCTGGCTTCGCAATTGGGTC
CAATGGATGTCGATCTCTGGTTTAGCAGTTGTGAAAGTGGCAAGGCGGGAGGTTAGACCTCCATTTAA
CATATACAAGCAAGTTAACTGCACTAGATGTGTAGACACTACAGTTACAGGAGTAGCCGAATAAGTCT
CCGACGTCAAGCGAATAAGCGTCATACGCGATTATCGCCTAAGAGCACGTATTGGCGGTAAAAGGCTA
GCTAGACGCTTATGGGTAGATTTCAAGGCGTTCGTAGTGGTATAATAGGATACTCTTTCACCAGCCTG
AGGGCCGAACGCTATACTAGTGGTCTGTGATGTAGGACCGAGTATCTCTCTAGGGACCATCTACTTGA
GCAATGGTGCGCAGGGGGAGACATAGACCAGCCTTGGGTGGCAAGCACTGCAATAAGTCCTGTTTAGC
CTTGGAGTTCACATGCCGGCACTGAAGCCGACCTACCTGAGCGTGTGATTACCGTTAAAGCATCTGTC
TAGTTCTGTTTCCGGCGCTCTTGGTTCCAGGTTAGGGGAAGTGTATGACCCATGTGTTTTTATCGGCT
TAACCACGAGTGATCCCCGGTCGTTTCCCCATTGAATCCCTGGTGCATCCTACTCCCAGAATGATAGC
TGACTGACTGGACTGGCTTTTCAAGTAATCGAGGGGGTATCGCGGTCACGGCCGTTAACAGATCCCGT
CCTTAGTGTGGAATCCGCACCTGCTGACTAACGCTTCGCCGGCGTGTCTGCACAGCCGTATAGTGTTA
ATCATGACCCCAAGGAAGGATTAAACAAATATCTTGACG
>BANAN29
GTGTCCGGTAGCCCGCGCTAGTTAGACACCCCGGCAGGGGGGATTGCTTTCGAGACGGGAGATCCCTT
CGCCGACCCTGTAGGGCCGACGCCGAGGCATTCGGGCCCCCGCAACGTCAACAGCGGCAAGAAAACGG
GATGAATGGGCGTAATGGGGGGGGTCTGCTGGGGACCCGACGCGGTTGCCGTTTGCGGGGCCCCCGAC

CCATACCGACCCACCTAGGCGTCCAGTTACGGCGCACGTCGGGAGCGTGGTTGCCGTCAGAGCTGTGT
TTCTCGATCAGTCCCCCGCAGTGCCGCAGTATCTTGCCGTGGGCTGCTTTAATCTTGAAAGTGGTTTA
TACATTGGGCGACGAGGTGTCGGCTCTCATTGGGGGTAACCGACGGACACGTGCAGTCCCCTCCCCGG
GCAGGCAGAGGCGGGGCCCCCGCGCGCCGGCCCCGGCCCACAATCTTACCAGGGTCCTCAAAAGAGCC
TTTGCATGGTACCCTTCGTGAATGGTTGCTTAAGAGGTCCACCACGTAGTCCGGGCACGGTCAACTAA
ACACTCAAAACAGCGACGGCAAATAGAGGCACAAGGTCCAGGCCCTCACGGCACTAGTTGCGATGACC
CAACTCACGGGGGCAGTCCCCGGCGCAGCGACCTTGATTACGTCCGGGAACAAGTATGTCGTTTCCCC
CGGGAGGGGTGCAGCCACATGGGAGATTCAAAGTTTCTCGTGACGTCGTTGTGATCACTGCAGCCTAG
CCGAGACTCCCGTACTACGCGAAGGTTGGTTATGTTAACCACTACAACGTGAGGCGCCGTAAGGGCCA
GTGTTGTGCCCGGCTCTTCAATGCGCCTTAGCGGCCTGATACACCCACCCAAGGAGATACTGCTAATC
ATGTGGGTGGACAGAACCTCGCAACGCAGACGCATCCCCGGCATGATATGGTTTTTTTCCGCTATTAC
CCAGCACGCGGCGCTATCATTCAAGAGAACCCAGTGACGCGTAAATCGTCAGATCTACCTGCCGCAGG
TGGACCTACTGCAAATACGGATTATGACTCGTAAAGAGGTCATGCGTATTTTCATCACTAGGCACTTT
CGAGAGTAAATTAGTAGGTGTCCCGCGCCTTGTGGCGTTCCGCCTGGCTCCTCATGAAGTCGACCTTC
TCATCGGCCCTATTTGCCGACGTAAGTCATAATCCAGATCTTCACCTCGGAGGGAGGGTCACTGTAAA
GGGATAATTGGAGGGCGATTTCCCACACTTTCCTAAGGGTACTTTTTGCTTAGCTTCGCAGTTGGGTC
CAATAGATGTTGATCTCTGGTTTAGCAGTTGTGAAAGTGGCAAGGCGGGAGGTTAGGCCTCCATTTAA
CATATACAAGCAAGTTAACTGCACTAGATGTGTAGACACTACAGTTACAGGAGTAGCCGACTAAGTCT
CCGACGTCAAGCGAATAGGCGTCATACGCGATTATTGCCTAAGAGCACGTATTGGCGGTAAAAGGATA
ACTAGACGCTTGTGGGTAGATTTCAAGGCGCTCGTAGTGGTATAATAGGACACTCTTGCACCAGCCTG
AGAGCCGAACGCTATACTAGTGGTCTGTGATGTAGGACCAAGTAGCTCTCTAGGGACCATCTACTTGA
GCAATGGTGCGCAGGGGTAGACATAGACCAACCTTGGGTGGCAAGCACTGCAATAAGTCCTGTTTAGC
CTTGGAGTTCACACGCCGGCACTAAAGCCGACCTACCTGAGCTTGTGATTACCGTTAAAGCATCTGTC
TAGTTCTGTTTCCGGCGCTCTTGGTTCCATGTTAGGGGAAGTGTATGACCCATGTGTTTTTATCGGCT
TAACTACGAGTGATCCCCGGTCGTTTCCCCATTAAATCCCTGGTGCATCCTACTCCCATAATGATAGC
TGACTGGCTGGACTGGCTTTTCAAGTAGTCGAGGGGGTATCGCGGTCACGGCCGTTAACAGATCCCGT
CCTTAGTGTGGAATCCGCACCTGCTGACTAACGCTTCGCCGGCGTGTCTGCACATCCGTATAGTGTTA
ATCATGACCCCAAGGAAGGATTAAACAAATATCTTGACG
>BANAN30
GTGTCCGGTAGCCCGCGCTAGTTAGACACCCCGGCAGGGGGGATTGCTTTCGGGACGGGAGATCCCTT
CGCCGACCCTGGAGGGCCGACGCCGAGGCATTCGGGCCCCCGCAACGTCAACAGCGGCAAGAAAACGG
GATGAATGGGCGTAATGGGGGGGGTCTGCTGGGGACCCGACGCGGTTGCCGTTTGCGGGGCCCCCGAC
CCATACCGACCCACCTAGGCGTCCAGTTACGGCGCACGGCGGGAGCGTGGTTGCCGTCAGAGCTGTGT
TTCTCGATCAGTCCCCCGCAGTGCCGCAGTATCTTGCCGTGGGCTGCTTTAATCTTGAAAGTGGTTNA
TACATTGGGCGACGAGGTGTCGACTCTCATTGGGGGTAACCGACGGGCACATGCAGTCCCCTCCCCGG
GCAGGCAGAGGCGGGGCCCCCGCGCGCCGGCCCCGGCCCACAATCTTACCAGGGTCCTCAAAGGAGCC
TTTGCATGGTACCCTTCGTGAATGGTTGCTTAAGAGGTCCACAACGTGGTCCGGGCACGGTCGACTAA
ACACTCAAAACAGCGACGGCAAATATAGGTACAAGGTCCAGGCCCTCACGGCACTAATTGCGATGACC
CAACTCACGGGGGCAGTCCCCGGCGCGGCGACCTTGATTACGTCCGGGGACAAGTATGTCGCTCCCCC
CGGGAGGGGTGCAGCCACATGGGAGATTCAAAGTTTCTCGTGACGTCGTTGTGATCACTGCAGCCTAG
CCGAGACTCCCGTACTACGCGAAGGTTGGTTATGTTAACCACTACAACGTGAGGCGCCGTAAGGGCCA
GTGTTGTGCCCGGCTCTTCAATGCGCCTTAGCGGCCTGATACACCCACCCAAGGAGATACTGCTAATC
ATATGGGTGGACAGAACCTCGCAACGCAGACGCCTCCCCGGCATGATATGGTTTTTTTCCGCTATTAT
CCAGCACGCAGCGCTATCATTCAAGAGAACCCAGTGACGCGTAAATCGTAAGATCTACCTGCCGCAGG
TGGACCTACTGCAAATACGGATTATGACTCGTAAAGGGGTCATGCGTATTTTCATCACTAGGCACGTT
CGAGAATAAATTAGTAGGTGTCCCGAGCCTTGTGGCGTTCCGCCTGACTCCTCATGAAGTCGACCTTC
TCACCGGCCCTATCTGCCGACGTAAGTCATAACCTAGATCTGTACCTCGGGGGGAGGGTCACTGTAAA
GGGATAATTGGAGGGTGATTTCCCACACTTTCCTAAGGGTACTTTTTGCCTGGCTTCGCAATTGGGTC
CAATGGATGTCGATCTCTGGTTTAGCAGTTGTGAAAGTGGCAAGGCGGGAGGTTAGACCTCCATTTAA
CATATACAAGCAAGTTAACTGCACTAGATGTGTAGACACTACAGTTACAGGAGTAGCCGAATAAGTCT
CCGACGTCAAGCGAATAAGCGTCATACGCGATTATCGCCTAAGAGCACGTATTGGCGGTAAAAGGCTA
GCTAGACGCTTATGGGTAGATTTCAAGGCGTTCGTAGTGGTATAATAGGATACTCTTTCACCAGCCTG
AGGGCCGAACGCTATACTAGTGGTCTGTGATGTAGGACCGAGTATCTCTCTAGGGACCATCTACTTGA
GCAATGGTGCGCAGGGGGAGACATAGACCAGCCTTGGGTGGCAAGCACTGCAATAAGTCCTGTTTAGC

CTTGGAGTTCACATGCCGGCACTGAAGCCGACCTACCTGAGCGTGTGATTACCGTTAAAGCATCTGTC
TAGTTCTGTTTCCGGCGCTCTTGGTTCCAGGTTAGGGGAAGTGTATGACCCATGTGTTTTTATCGGCT
TAACCACGAGTGATCCCCGGTCGTTTCCCCATTGAATCCCTGGTGCATCCTACTCCCAGAATGATAGC
TGACTGACTGGACTGGCTTTTCAAGTAATCGAGGGGGTATCGCGGTCACGGCCGTTAACAGATCCCGT
CCTTAGTGTGGAATCCGCACCTGCTGACTAACGCTTCGCCGGCGTGTCTGCACAGCCGTATAGTGTTA
ATCATGACCCCAAGGAAGGATTAAACAAATATCTTGACG
>BANAN31
GTGTCCGGTAGCCCGCGCTAGTTAGACACCCCGGCAGGGGGGATTGCTTTCGAGACGGGAGATCCCTT
CGCCGACCCTGGAGGGCCGACGCCGAGGCATTCGGGCCCCCGCAACGTCAACAGCGGCAAGAAAACGG
GATGAATGGGCGTAATGGGGGGGGTCTGCTGGGGACCCGACGCGGTTGCCGTTTGCGGGGCCCCCGAC
CCATACCGACCCACCTAGGCGTCCAGTTACGGCGCACGGCGGGAGCGTGGTTGCCGTCAGAGCTGTGT
TTCTCGATCAGTCCCCCGCAGTGCCGCAGTATCTTGCCGTGGGCTGCTTTAATCTTGAAAGTGGTTCA
TACATTGGGCGACGAGGTGTCGACTCTCATTGGGGGTAACCGACGGGCACATGCAGTCCCCTCCCCGG
GCAGGCAGAGGCGGGGCCCCCGCGCGCCGGCCCCGGCCCACAATCTTACCAGGGTCCTCAAAGGAGCC
TTTGCATGGTACCCTTCGTGAATGGTTGCTTAAGAGGTCCACAACGTGGTCCGGGCACGGTCGACTAA
ACACTCAAAACAGCGACGGCAAATATAGGTACAAGGTCCAGGCCCTCACGGCACTAATTGCGATGACC
CAACTCACGGGGGCAGTCCCCGGCGCGGCGACCTTGATTACGTCCGGGGACAAGTATGTCGCTCCCCC
CGGGAGGGGTGCAGCCACATGGGAGATTCAAAGTTTCTCGTGACGTCGTTGTGATCACTGCAGCCTAG
CCGAGACTCCCGTACTACGCGAAGGTTGGTTATGTTAACCACTACAACGTGAGGCGCCGTAAGGGCCA
GTGTTGTGCCCGGCTCTTCAATGCGCCTTAGCGGCCTGATACACCCACCCAAGGAGATACTGCTAATC
ATATGGGTGGACAGAACCTCGCAACGCAGACGCCTCCCCGGCATGATATGGTTTTTTTCCGCTATTAT
CCAGCACGCAGCGCTATCATTCAAGAGAACCCAGTGACGCGTAAATCGTAAGATCTACCTGCCGCAGG
TGGACCTACTGCAAATACGGATTATGACTCGTAAAGGGGTCATGCGTATTTTCATCACTAGGCACGTT
CGAGAATAAATTAGTAGGTGTCCCGAGCCTTGTGGCGTTCCGCCTGACTCCTCATGAAGTCGACCTTC
TCACCGGCCCTATCTGCCGACGTAAGTCATAACCTAGATCTGTACCTCGGGGGGAGGGTCACTGTAAA
GGGATAATTGGAGGGTGATTTCCCACACTTTCCTAAGGGTACTTTTTGCCTGGCTTCGCAATTGGGTC
CAATGGATGTCGATCTCTGGTTTAGCAGTTGTGAAAGTGGCAAGGCGGGAGGTTAGACCTCCATTTAA
CATATACAAGCAAGTTAACTGCACTAGATGTGTAGACACTACAGTTACAGGAGTAGCCGAATAAGTCT
CCGACGTCAAGCGAATAAGCGTCATACGCGATTATCGCCTAAGAGCACGTATTGGCGGTAAAAGGCTA
GCTAGACGCTTATGGGTAGATTTCAAGGCGTTCGTAGTGGTATAATAGGATACTCTTTCACCAGCCTG
AGGGCCGAACGCTATACTAGTGGTCTGTGATGTAGGACCGAGTATCTCTCTAGGGACCATCTACTTGA
GCAATGGTGCGCAGGGGGAGACATAGACCAGCCTTGGGTGGCAAGCACTGCAATAAGTCCTGTTTAGC
CTTGGAGTTCACATGCCGGCACTGAAGCCGACCTACCTGAGCGTGTGATTACCGTTAAAGCATCTGTC
TAGTTCTGTTTCCGGCGCTCTTGGTTCCAGGTTAGGGGAAGTGTATGACCCATGTGTTTTTATCGGCT
TAACCACGAGTGATCCCCGGTCGTTTCCCCATTGAATCCCTGGTGCATCCTACTCCCAGAATGATAGC
TGACTGACTGGACTGGCTTTTCAAGTAATCGAGGGGGTATCGCGGTCACGGCCGTTAACAGATCCCGT
CCTTAGTGTGGAATCCGCACCTGCTGACTAACGCTTCGCCGGCGTGTCTGCACAGCCGTATAGTGTTA
ATCATGACCCCAAGGAAGGATTAAACAAATATCTTGACG
>BANAN34
GTGTCCGGTAGCCCGCGCTAGTTAGACACCCCGGCAGGGGGGATTGCTTTCGGGACGGGAGATCCCTT
CGCCGACCCTGTAGGGCCGACGCCGAGGCATTCGGGCCCCTGCAACGTCAACAGCGGCAAGAAAACGG
GATGAATGGGCGTAATGGGGGGGGNCTGCTGGNGACCCGACGCGGTNGCCGTTTGCGGGGCCCCCGAC
CCATACCGACCCACCTAGGCGTCCAGTTACGGCGCACGTCGGGAGCGTGGTTGCCGTCAGAGCTGTGT
TTCTCGATCAGTCCCCCGCAGTGCCGCAGTATCTTGCCGTGGGCTGCTTTAATCTTGAAAGTGGTTCA
TACATTGGGCGACGAGGTGTCGGCTCTCATTGGGGGTAACCGACGGACACGTGCAGTCCCCTCCCCGG
GCAGGCAGAGGCGGGGCCCCCGCGCGCCGGCCCCGGCCCACAATCTTACCAGGGTCCTCAAAAGAGCC
TTTGCATGGTACCCTTCGTGAATGGTTGCTTAAGAGGTCCACCACGTAGTCCGGGCACGGTCAACTAA
ACACTCAAAACAGCGACGGCAAATAGAGGCACAAGGTCCAGGCCCTCACGGCACTAGTTGCGATGACC
CAACTCACGGGGGCAGTCCCCGGCGCAGCGACCTTGATTACGTCCGGGAACAAGTATGTCGTTTCCCC
CGGGAGGGGTGCAGCCACATGGGAGATTCAAAGTTTCTCGTGACGTCGTTGTGATCACTGCAGCCTAG
CCGAGACTCCCGTACTACGCGAAGGTTGGTTATGTTAACCACTACAACGTGAGGCGCCGTAAGGGCCA
GTGTTGTGCCCGGCTCTTCAATGCGCCTTNGCGGCCTGATACACCCACCCAAGGAGATACTGCTAATC
ATGTGGGTGGACAGAACCTCGCAACGCAGACGCATCCCCGGCATGATATGGTTTTTTTCCGCTATTAC
CCAGCACGCGGCGCTATCATTCAAGAGAACCCAGTGACGCGTAAATCGTCAGATCTACCTGCCGCAGG

TGGACCTACTGCAAATACGGATTATGACTCGTAAAGAGGTCATGCGTATTTTCATCACTAGGCACTTT
CGAGAGTAAATTAGTAGGTGTCCCGCGCCTTGTGGCGTTCCGCCTGGCTCCTCATGAAGTCGACCTTC
TCATCGGCCCTATTTGCCGACGTAAGTCATAATCCAGATCTTCACCTCGGAGGGAGGGTCACTGTAAA
GGGATAATTGGAGGGCGATTTCCCACACTTTCCTAAGGGTACTTTTTGCTTAGCTTCGCAGTTGGGTC
CAATAGATGTTGATCTCTGGTTTAGCAGTTGTGAAAGTGGCAAGGCGGGAGGTTAGGCCTCCATTTAA
CATATACAAGCAAGTTAACTGCACTAGATGTGTAGACACTACAGTTACAGGAGTAGCCGACTAAGTCT
CCGACGTCAAGCGAATAGGCGTCATACGCGATTATTGCCTAAGAGCACGTATTGGCGGTAAAAGGATA
ACTAGACGCTTGTGGGTAGATTTCAAGGCGCTCGTAGTGGTATAATAGGACACTCTTGCACCAGCCTG
AGAGCCGAACGCTATACTAGTGGTCTGTGATGTAGGACCAAGTAGCTCTCTAGGGACCATCTACTTGA
GCAATGGTGCGCAGGGGTAGACATAGACCAACCTTGGGTGGCAAGCACTGCAATAAGTCCTGTTTAGC
CTTGGAGTTCACACGCCGGCACTAAAGCCGACCTACCTGAGCTTGTGATTACCGTTAAAGCATCTGTC
TAGTTCTGTTTCCGGCGCTCTTGGTTCCATGTTAGGGGAAGTGTATGACCCATGTGTTTTTATCGGCT
TAACTACGAGTGATCCCCGGTCGTTTCCCCATTAAATCCCTGGTGCATCCTACTCCCATAATGATAGC
TGACTGGCTGGACTGGCTTTTCAAGTAGTCGAGGGGGTATCGCGGTCACGGCCGTTAACAGATCCCGT
CCTTAGTGTGGAATCCGCACCTGCTGACTAACGCTTCGCCGGCGTGTCTGCACATCCGTATAGTGTTA
ATCATGACCCCAAGGAAGGATTAAACAAATATCTTGACG
>BANAN35
GTGTCCGGTAGCCCGCGCTAGTCAGACACCCCGGCAGGGGGGATTGCTTTCGAGACGGGAGATCCCTT
CGCCGACCCTGGAGGGCCGACGCCGAGGCATTCGGGCCCCCGCAACGTCAACAGCGGCAAGAAAACGG
GATGAATGGGCGTAATGGGGGGGGTCTGCTGGGGACCCGACGCGGTTGCCGTTTGCGGGGCCCCCGAC
CCATACCGACCCACCTAGGCGTCCAGTTACGGCGCACGTCGGGAGCGTGGTTGCCGTCAGAGCTGTGT
TTCTCGATCAGTCCCCCGCAGTGCCGCAGTATCTTGCCGTGGGCTGCTTTAATCTTGAAAGTGGTTTA
TACATTGGGCGACGAGGTGTCGACTCTCATTGGGGGTAACCGACGGGCACATGCAGTCCCCTCCCCGG
GCAGGCAGAGGCGGGGCCCCCGCGCGCCGGCCCCGGCCCACAATCTTACCAGGGTCCTCAAAGGAGCC
TTTGCATGGTACCCTTCGTGAATGGTTGCTTAAGAGGTCCACAACGTGGTCCGGGCACGGTCGACTAA
ACACTCAAAACAGCGACGGCAAATATAGGTACAAGGTCCAGGCCCTCACGGCACTAATTGCGATGACC
CAACTCACGGGGGCAGTCCCCGGCGCGGCGACCTTGATTACGTCCGGGGACAAGTATGTCGCTCCCCC
CGGGAGGGGTGCAGCCACATGGGAGATTCAAAGTTTCTCGTGACGTCGTTGTGATCACTGCAGCCTAG
CCGAGACTCCCGTACTACGCGAAGGTTGGTTATGTTAACCACTACAACGTGAGGCGCCGTAAGGGCCA
GTGTTGTGCCCGGCTCTTCAATGCGCCTTAGCGGCCTGATACACCCACCCAAGGAGATACTGCTAATC
ATATGGGTGGACAGAACCTCGCAACGCAGACGCCTCCCCGGCATGATATGGTTTTTTTCCGCTATTAT
CCAGCACGCAGCGCTATCATTCAAGAGAACCCAGTGACGCGTAAATCGTAAGATCTACCTGCCGCAGG
TGGACCTACTGCAAATACGGATTATGACTCGTAAAGGGGTCATGCGTATTTTCATCACTAGGCACGTT
CGAGAATAAATTAGTAGGTGTCCCGAGCCTTGTGGCGTTCCGCCTGACTCCTCATAAAGTCGACCTTC
TCACCGGCCCTATCTGCCGACGTAAGTCATAACCTAGATCTGTACCTCGGGGGGAGGGTCACTGTAAA
GGGATAATTGGAGGGTGATTTCCCACATTTTCCTAAGGGTACTTTTTGCCTGGCTTCGCAATTGGGTC
CAATGGATGTCGATCTCTGGTTTAGCAGTTGTGAAAGTGGCAAGGCGGGAGGTTAGACCTCCATTTAA
CATATACAAGCAAGTTAACTGCACTAGATGTGTAGACACTACAGGTACAGGAGTAGCCGGATAAGTCT
CCGACGTCAAGCGAATAAGCGTCATACGCGATTATCGCCTAAGAGCACGTATTTGCGGTAAAAGGCTA
GCTAGACGCTTGTGGGTAGATTTCAAGGCGTTCGTAGTGGTATAATAGGATACTCTTTCACCAGCCTG
AGGGCCGAACGCTATACTAGTGGTCTGTGATGTAGGACCGAGTATCTCTCTAGGGACCATCTACTTGA
GCAATGGTGCGCAGGGGGAGACATAGACCAGCCTTGGGTGGCAAGCACTGCAATAAGTCCTGTTTAGC
CTTGGAGTTCACACGCCGGCACTGAAGCCGACCTACCTGAGCGTGTGATTACCGTTAAAGCATCTGTC
TAGTTCTGTTTCCGGCGCTCTTGGTTCCAGGTTAGGGGAAGTGTATGACCCATGTGTTTTTATCGGCT
TAACCACGAGTGATCCCCGGTCGTTTCCCCATTGAATCCCTGGTGCATCCTACTCCCAGAATGATAGC
TGACTGACTGGACTGGCTTTTCAAGTAATCGAGGGGGTATCGCGGTCACGGCCGTTAACAGATCCCGT
CCTTAGTGTGGAATCCGCACCTGCTGACTAACGCTTCGCCGGCGTGTCTGCACAGCCGTATAGTGTTA
ATCATGACCCCAAGGAAGGATTAAACAAATATCTTGACG
>GIRAN01
GTGTCCGGTAGCCCGCGCTAGTTAGACACCCCGGCAGGGGGGATTGCTTTCGAGACGGGAGATCCCTT
CGCCGACCCTGGAGGGCCGACGCCGAGGCATTCGGGCCCCTGCAACGTCAACAGCGGCAAGAAAACGG
GATGAATGGGCGTAATGGGGGGGGTCTGCTGGGGACCCGACGCGGTTGCCGTTTGCGGGGCCCCCGAC
CCATACCGACCCACCTAGGCGTCCAGTTACGGCGCACGTCGGGAGCGTGGTTGCCGTCAGAGCTGTGT
TTCTCGATCAGTCCCCCGCAGTGCCGCAGTATCTTGCCGTGGGCTGCTTTAATCTTGAAAGTGGTTCA

TACATTGGGCGACGAGGTGTCGACTCTCATTGGGGGTAACCGACGGGCACATGCAGTCCCCTCCCCGG
GCAGGCAGAGGCGGGGCCCCCGCGCGCCGGCCCCGGCCCACAATCTTACCAGGGTCCTCAAAGGAGCC
TTTGCATGGTACCCTTCGTGAATGGTTGCTTAAGAGGTCCACAACGTGGTCCGGGCACGGTCGACTAA
ACACTCAAAACAGCGACGGCAAATATAGGTACAAGGTCCAGGCCCTCACGGCACTAATTGCGATGACC
CAACTCACGGGGGCAGTCCCCGGCGCGGCGACCTTGATTACGTCCGGGGACAAGTATGTCGCTCCCCC
CGGGAGGGGTGCAGCCACATGGGAGATTCAAAGTTTCTCGTGACGTCGTTGTGATCACTGCAGCCTAG
CCGAGACTCCCGTACTACGCGAAGGTTGGTTATGTTAACCACTACAACGTGAGGCGCCGTAAGGGCCA
GTGTTGTGCCCGGCTCTTCAATGCGCCTTAGCGGCCTGATACACCCACCCAAGGAGATACTGCTAATC
ATATGGGTGGACAGAACCTCGCAACGCAGACGCCTCCCCGGCATGATATGGTTTTTTTCCGCTATTAT
CCAGCACGCAGCGCTATCATTCAAGAGAACCCAGTGACGCGTAAATCGTAAGATCTACCTGCCGCAGG
TGGACCTACTGCAAATACGGATTATGACTCGTAAAGGGGTCATGCGTATTTTCATCACTAGGCACGTT
CGAGAATAAATTAGTAGGTGTCCCGAGCCTTGTGGCGTTCCGCCTGACTCCTCATGAAGTCGACCTTC
TCACCGGCCCTATCTGCCGACGTAAGTCATAACCTAGATCTGTACCTCGGGGGGAGGGTCACTGTAAA
GGGATAATTGGAGGGTGATTTCCCACACTTTCCTAAGGGTACTTTTTGCCTGGCTTCGCAATTGGGTC
CAATGGATGTCGATCTCTGGTTTAGCAGTTGTGAAAGTGGCAAGGCGGGAGGTTAGACCTCCATTTAA
CATATACAAGCAAGTTAACTGCACTAGATGTGTAGACACTACAGTTACAGGAGTAGCCGAATAAGTCT
CCGACGTCAAGCGAATAAGCGTCATACGCGATTATCGCCTAAGAGCACGTATTGGCGGTAAAAGGCTA
GCTAGACGCTTATGGGTAGATTTCAAGGCGTTCGTAGTGGTATAATAGGATACTCTTTCACCAGCCTG
AGGGCCGAACGCTATACTAGTGGTCTGTGATGTAGGACCGAGTATCTCTCTAGGGACCATCTACTTGA
GCAATGGTGCGCAGGGGGAGACATAGACCAGCCTTGGGTGGCAAGCACTGCAATAAGTCCTGTTTAGC
CTTGGAGTTCACATGCCGGCACTGAAGCCGACCTACCTGAGCGTGTGATTACCGTTAAAGCATCTGTC
TAGTTCTGTTTCCGGCGCTCTTGGTTCCAGGTTAGGGGAAGTGTATGACCCATGTGTTTTTATCGGCT
TAACCACGAGTGATCCCCGGTCGTTTCCCCATTGAATCCCTGGTGCATCCTACTCCCAGAATGATAGC
TGACTGACTGGACTGGCTTTTCAAGTAATCGAGGGGGTATCGCGGTCACGGCCGTTAACAGATCCCGT
CCTTAGTGTGGAATCCNCACCTGCTGACTAACGCTTCGCCGGCGTGTCTGCACAGCCGTATAGTGTTA
ATCATGACCCCAAGGAAGGATTAAACAAATATCTTGACG
>GIRAN02
GTGTCCGGTAGCCCGCGCTAGTTAGACACCCCGGCAGGGGGGATTGCTTTCGGGACGGGAGATCCCTT
CGCCGACCCTGGAGGGCCGACGCCGAGGCATTCGGGCCCCTGCAACGTCAACAGCGGCAAGAAAACGG
GATGAATGGGCGTAATGGGGGGGGTCTGCTGGGGACCCGACGCGGTTGCCGTTTGCGGGGCCCCCGAC
CCATACCGACCCACCTAGGCGTCCAGTTACGGCGCACGTCGGGAGCGTGGTTGCCGTCAGAGCTGTGT
TTCTCGATCAGTCCCCCGCAGTGCCGCAGTATCTTGCCGTGGGCTGCTTTAATCTTGAAAGTGGTTTA
TACATTGGGCGACGAGGTGTCGACTCTCATTGGGGGTAACCGACGGGCACATGCAGTCCCCTCCCCGG
GCAGGCAGAGGCGGGGCCCCCGCGCGCCGGCCCCGGCCCACAATCTTACCAGGGTCCTCAAAGGAGCC
TTTGCATGGTACCCTTCGTGAATGGTTGCTTAAGAGGTCCACAACGTGGTCCGGGCACGGTCGACTAA
ACACTCAAAACAGCGACGGCAAATATAGGTACAAGGTCCAGGCCCTCACGGCACTAATTGCGATGACC
CAACTCACGGGGGCAGTCCCCGGCGCGGCGACCTTGATTACGTCCGGGGACAAGTATGTCGCTCCCCC
CGGGAGGGGTGCAGCCACATGGGAGATTCAAAGTTTCTCGTGACGTCGTTGTGATCACTGCAGCCTAG
CCGAGACTCCCGTACTACGCGAAGGTTGGTTATGTTAACCACTACAACGTGAGGCGCCGTAAGGGCCA
GTGTTGTGCCCGGCTCTTCAATGCGCCTTAGCGGCCTGATACACCCACCCAAGGAGATACTGCTAATC
ATATGGGTGGACAGAACCTCGCAACGCAGACGCCTCCCCGGCATGATATGGTTTTTTTCCGCTATTAT
CCAGCACGCAGCGCTATCATTCAAGAGAACCCAGTGACGCGTAAATCGTAAGATCTACCTGCCGCAGG
TGGACCTACTGCAAATACGGATTATGACTCGTAAAGGGGTCATGCGTATTTTCATCACTAGGCACGTT
CGAGAATAAATTNGTAGGTGTCCCGAGCCTTGTGGCGTTCCGCCTGACTCCTCATGAAGTCGACCTTC
TCACCGGCCCTATCTGCCGACGTAAGTCATAACCTAGATCTGTACCTCGGGGGGAGGGTCACTGTAAA
GGGATAATTGGAGGGTGATTTCCCACACTTTCCTAAGGGTACTTTTTGCCTGGCTTCGCAATTGGGTC
CAATGGATGTCGATCTCTGGTTTAGCAGTTGTGAAAGTGGCAAGGCGGGAGGTTAGACCTCCATTTAA
CATATACAAGCAAGTTAACTGCACTAGATGTGNAGACACTACAGTTACAGGAGTAGCCGAATAAGTCT
CCGACGTCAAGCGAATAAGCGTCATACGCGATTATCGCCTAAGAGCACGTATTGGCGGTAAAAGGCTA
GCTAGACGCTTATGGGTAGATTTCAAGGCGTTCGTAGTGGTATAATAGGATACTCTTTCACCAGCCTG
AGGGCCGAACGCTATACTAGTGGTCTGTGATGTAGGACCGAGTATCTCTCTAGGGACCATCTACTTGA
GCAATGGTGCGCAGGGGGAGACATAGACCAGCCTTGGGTGGCAAGCACTGCAATAAGTCCTGTTTAGC
CTTGGAGTTCACATGCCGGCACTGAAGCCGACCTACCTGAGCGTGTGATTACCGTTAAAGCATCTGTC
TAGTTCTGTTTCCGGCGCTCTTGGTTCCAGGTTAGGGGAAGTGTATGACCCATGTGTTTTTATCGGCT

TAACCACGAGTGATCCCCGGTCGTTTCCCCATTGAATCCCTGGTGCATCCTACTCCCAGAATGATAGC
TGACTGACTGGACTGGCTTTTCAAGTAATCGAGGGGGTATCGCGGTCACGGCCGTTAACAGATCCCGT
CCTTAGTGTGGAATCCGCACCTGCTGACTAACGCTTCGCCGGCGTGTCTGCACAGCCGTATAGTGTTA
ATCATGACCCCAAGGAAGGATTAAACAAATATCTTGACG
>GIRAN03
GTGTCCGGTAGCCCGCGCTAGTTAGACACCCCGGCAGGGGGGATTGCTTTCGAGACGGGAGATCCCTT
CGCCGACCCTGGAGGGCCGACGCCGAGGCATTCGGGCCCCCGCAACGTCAACAGCGGCAAGAAAACGG
GATGAATGGGCGTAATGGGGGGGGTCTGCTGGGGACCCGACGCGGTTGCCGTTTGCGGGGCCCCCGAC
CCATACCGACCCACCTAGGCGTCCAGTTACGGCGCACGTCGGGAGCGTGGTTGCCGTCAGAGCTGTGT
TTCTCGATCAGTCCCCCGCAGTGCCGCAGTATCTTGCCGTGGGCTGCTTTAATCTTGAAAGTGGTTTA
TACATTGGGCGACGAGGTGTCGACTCTCATTGGGGGTAACCGACGGGCACATGCAGTCCCCTCCCCGG
GCAGGCAGAGGCGGGGCCCCCGCGCGCCGGCCCCGGCCCACAATCTTACCAGGGTCCTCAAAGGAGCC
TTTGCATGGTACCCTTCGTGAATGGTTGCTTAAGAGGTCCACAACGTGGTCCGGGCACGGTCGACTAA
ACACTCAAAACAGCGACGGCAAATATAGGTACAAGGTCCAGGCCCTCACGGCACTAATTGCGATGACC
CAACTCACGGGGGCAGTCCCCGGCGCGGCGACCTTGATTACGTCCGGGGACAAGTATGTCGCTCCCCC
CGGGAGGGGTGCAGCCACATGGGAGATTCAAAGTTTCTCGTGACGTCGTTGTGATCACTGCAGCCTAG
CCGAGACTCCCGTACTACGCGAAGGTTGGTTATGTTAACCACTACAACGTGAGGCGCCGTAAGGGCCA
GTGTTGTGCCCGGCTCTTCAATGCGCCTTAGCGGCCTGATACACCCACCCAAGGAGATACTGCTAATC
ATATGGGTGGACAGAACCTCGCAACGCAGACGCCTCCCCGGCATGATATGGTTTTTTTCCGCTATTAT
CCAGCACGCAGCGCTATCATTCAAGAGAACCCAGTGACGCGTAAATCGTAAGATCTACCTGCCGCAGG
TGGACCTACTGCAAATACGGATTATGACTCGTAAAGGGGTCATGCGTATTTTCATCACTAGGCACGTT
CGAGAATAAATTAGTAGGTGTCCCGAGCCTTGTGGCGTTCCGCCTGACTCCTCATGAAGTCGACCTTC
TCACCGGCCCTATCTGCCGACGTAAGTCATAACCTAGATCTGTACCTCGGGGGGAGGGTCACTGTAAA
GGGATAATTGGAGGGTGATTTCCCACACTTTCCTAAGGGTACTTTTTGCCTGGCTTCGCAATTGGGTC
CAATGGATGTCGATCTCTGGTTTAGCAGTTGTGAAAGTGGCAAGGCGGGAGGTTAGACCTCCATTTAA
CATATACAAGCAAGTTAACTGCACTAGATGTGTAGACACTACAGTTACAGGAGTAGCCGAATAAGTCT
CCGACGTCAAGCGAATAAGCGTCATACGCGATTATCGCCTAAGAGCACGTATTGGCGGTAAAAGGCTA
GCTAGACGCTTATGGGTAGATTTCAAGGCGTTCGTAGTGGTATAATAGGATACTCTTTCACCAGCCTG
AGGGCCGAACGCTATACTAGTGGTCTGTGATGTAGGACCGAGTATCTCTCTAGGGACCATCTACTTGA
GCAATGGTGCGCAGGGGGAGACATAGACCAGCCTTGGGTGGCAAGCACTGCAATAAGTCCTGTTTAGC
CTTGGAGTTCACATGCCGGCACTGAAGCCGACCTACCTGAGCGTGTGATTACCGTTAAAGCATCTGTC
TAGTTCTGTTTCCGGCGCTCTTGGTTCCAGGTTAGGGGAAGTGTATGACCCATGTGTTTTTATCGGCT
TAACCACGAGTGATCCCCGGTCGTTTCCCCATTGAATCCCTGGTGCATCCTACTCCCAGAATGATAGC
TGACTGACTGGACTGGCTTTTCAAGTAATCGAGGGGGTATCGCGGTCACGGCCGTTAACAGATCCCGT
CCTTAGTGTGGAATCCGCACCTGCTGACTAACGCTTCGCCGGCGTGTCTGCACAGCCGTATAGTGTTA
ATCATGACCCCAAGGAAGGATTAAACAAATATCTTGACG
>GIRAN04
GTGTCCGGTAGCCCGCGCTAGTTAGACACCCCGGCAGGGGGGATTGCTTTCGAGACGGGAGATCCCTT
CGCCGACCCTGGAGGGCCGACGCCGAGGCATTCGGGCCCCCGCAACGTCAACAGCGGCAAGAAAACGG
GATGAATGGGCGTAATGGGGGGGGTCTGCTGGGGACCCGACGCGGTTGCCGTTTGCGGGGCCCCCGAC
CCATACCGACCCACCTAGGCGTCCAGTTACGGCGCACGGCGGGAGCGTGGTTGCCGTCAGAGCTGTGT
TTCTCGATCAGTCCCCCGCAGTGCCGCAGTATCTTGCCGTGGGCTGCTTTAATCTTGAAAGTGGTTTA
TACATTGGGCGACGAGGTGTCGACTCTCATTGGGGGTAACCGACGGGCACATGCAGTCCCCTCCCCGG
GCAGGCAGAGGCGGGGCCCCCGCGCGCCGGCCCCGGCCCACAATCTTACCAGGGTCCTCAAAGGAGCC
TTTGCATGGTACCCTTCGTGAATGGTTGCTTAAGAGGTCCACAACGTGGTCCGGGCACGGTCGACTAA
ACACTCAAAACAGCGACGGCAAATATAGGTACAAGGTCCAGGCCCTCACGGCACTAATTGCGATGACC
CAACTCACGGGGGCAGTCCCCGGCGCGGCGACCTTGATTACGTCCGGGGACAAGTATGTCGCTCCCCC
CGGGAGGGGTGCAGCCACATGGGAGATTCAAAGTTTCTCGTGACGTCGTTGTGATCACTGCAGCCTAG
CCGAGACTCCCGTACTACGCGAAGGTTGGTTATGTTAACCACTACAACGTGAGGCGCCGTAAGGGCCA
GTGTTGTGCCCGGCTCTTCAATGCGCCTTAGCGGCCTGATACACCCACCCAAGGAGATACTGCTAATC
ATATGGGTGGACAGAACCTCGCAACGCAGACGCCTCCCCGGCATGATATGGTTTTTTTCCGCTATTAT
CCAGCACGCAGCGCTATCATTCAAGAGAACCCAGTGACGCGTAAATCGTAAGATCTACCTGCCGCAGG
TGGACCTACTGCAAATACGGATTATGACTCGTAAAGGGGTCATGCGTATTTTCATCACTAGGCACGTT
CGAGAATAAATTAGTAGGTGTCCCGAGCCTTGTGGCGTTCCGCCTGACTCCTCATGAAGTCGACCTTC

TCACCGGCCCTATCTGCCGACGTAAGTCATAACCTAGATCTGTACCTCGGGGGGAGGGTCACTGTAAA
GGGATAATTGGAGGGTGATTTCCCACACTTTCCTAAGGGTACTTTTTGCCTGGCTTCGCAATTGGGTC
CAATGGATGTCGATCTCTGGTTTAGCAGTTGTGAAAGTGGCAAGGCGGGAGGTTAGACCTCCATTTAA
CATATACAAGCAAGTTAACTGCACTAGATGTGTAGACACTACAGTTACAGGAGTAGCCGAATAAGTCT
CCGACGTCAAGCGAATAAGCGTCATACGCGATTATCGCCTAAGAGCACGTATTGGCGGTAAAAGGCTA
GCTAGACGCTTATGGGTAGATTTCAAGGCGTTCGTAGTGGTATAATAGGATACTCTTTCACCAGCCTG
AGGGCCGAACGCTATACTAGTGGTCTGTGATGTAGGACCGAGTATCTCTCTAGGGACCATCTACTTGA
GCAATGGTGCGCAGGGGGAGACATAGACCAGCCTTGGGTGGCAAGCACTGCAATAAGTCCTGTTTAGC
CTTGGAGTTCACATGCCGGCACTGAAGCCGACCTACCTGAGCGTGTGATTACCGTTAAAGCATCTGTC
TAGTTCTGTTTCCGGCGCTCTTGGTTCCAGGTTAGGGGAAGTGTATGACCCATGTGTTTTTATCGGCT
TAACCACGAGTGATCCCCGGTCGTTTCCCCATTGAATCCCTGGTGCATCCTACTCCCAGAATGATAGC
TGACTGACTGGACTGGCTTTTCAAGTAATCGAGGGGGTATCGCGGTCACGGCCGTTAACAGATCCCGT
CCTTAGTGTGGAATCCGCACCTGCTGACTAACGCTTCGCCGGCGTGTCTGCACAGCCGTATAGTGTTA
ATCATGACCCCAAGGAAGGATTAAACAAATATCTTGACG
>GIRAN05
GTGTCCGGTAGCCCGCGCTAGTTAGACACCCCGGCAGGGGGGATTGCTTTCGGGACGGGAGATCCCTT
CGCCGACCCTGGAGGGCCGACGCCGAGGCATTCGGGCCCCTGCAACGTCAACAGCGGCAAGAAAACGG
GATGAATGGGCGTAATGGGGGGGGTCTGCTGGGGACCCGACGCGGTTGCCGTTTGCGGGGCCCCCGAC
CCATACCGACCCACCTAGGCGTCCAGTTACGGCGCACGTCGGGAGCGTGGTTGCCGTCAGAGCTGTGT
TTCTCGATCAGTCCCCCGCAGTGCCGCAGTATCTTGCCGTGGGCTGCTTTAATCTTGAAAGTGGTTCA
TACATTGGGCGACGAGGTGTCGACTCTCATTGGGGGTAACCGACGGGCACATGCAGTCCCCTCCCCGG
GCAGGCAGAGGCGGGGCCCCCGCGCGCCGGCCCCGGCCCACAATCTTACCAGGGTCCTCAAGGGAGCC
TTTGCATGGTACCCTTCGTGAATGGTTGCTTAAGAGGTCCACCACGTGGTCCGGGCACGGTCGACTAA
ACACTCAAAACAGCGACGGCAAATATAGGCACAAGGTCCAGGCCCTCACGGCACTAATTGCGATGACC
CAACTCACGGGGGCAGTCCCCGGCGCGGCGACCTTGATTACGGCCGGGGACAAGTATGTCGTTCCCCC
CGGGAGGGGTGCAGCCACATGGGAGATTCAAAGTTTCTCGTGACGTCGTTGTGATCACTGCAGCCTAG
CCGAGACTCCCGTACTACGCGAAGGTTGGTTATGTTAACCACTACAACGTGAGGCGCCGTAAGGGCCA
GTGTTGTGCCCGGCTCTTCAATGCGCCTTAGCGGCCTGATACACCCACCCAAGGAGATACTGCTAATC
ATGTGGGTGGACAGAACCTCGCAACGCAGACGCCTCCCCGGCATGATATGGTTTTTTTCCGCTATTAT
CCAGCACGCAGCGCTATCATTCAAGAGAACCCAGTGACGCGTAAATCGTAAGATCTACCTGCCGCAGG
TGGACCTACTGCAAATACGGATTATGACTCGTAAAGAGGTCATGCGTATTTTCATCACTAGGCACGTT
CGAGAATAAATTAGTAGGTGTCCCGCGCCTTGTGGCGTTCCGCCTGACTCCTCATGAAGTCGACCTTC
TCACCGGCCCTATCTGCCGACGTAAGTCATAACCTAGATCTGTACCTCGGGGGGAGGGTCACTGTAAA
GGGATAATTGGAGGGTGATTTCCCACACTTTCCTAAGGGTACTTTTTGCCTGGCTTCGCAATTGGGTC
CAATGGATGTCGATCTCTGGTTTAGCAGTTGTGAAAGTGGCAAGGCGGGAGGTTAGACCTCCATTTAA
CATATACAAGCAAGTTAACTGCACTAGATGTGTAGACACTACAGTTACAGGAGTAGCCGAATAAGTCT
CCGACGTCAAGCGAATAAGCGTCATACGCGATTATCGCCTAAGAGCACGTATTGGCGGTAAAAGGCTA
ACTAGACGCTTGTGGGTAGATTTCAAGGCGTTCGTAGTGGTATAACAGGATACTCTTTCACCAGCCTG
AGGGCCGAACGCTATACTAGTGGTCTGTGATGTAGGACCGAGTATCTCTCTAGGGACCATCTACTTGA
GCAATGGTGCGCAGGGGGAGACATAGACCAGCCTTGGGTGGCAAGCACTGCAATAAGTCCTGTTTAGC
CTTGGAGTTCACACGCCGGCACTGAAGCCGACCTACCTGAGCTTGTGATTACCGTTAAAGCATCTGTC
TAGTTCTGTTTCCGGCGCTCTTGGTTCCAGGTTAGGGGAAGTGTATGACCCATGTGTTTTTATCGGCT
TAACCACGAGTGATCCCCGGTCGTTTCCCCATTGAATCCCTGGTGCATCCTACTCCCAGAATGATAGC
TGACTGACTGGACTGGCTTTTCAAGTAGTCGAGGGGGTATCGCGGTCACGGCCGTTAACAGATCCCGT
CCTTAGTGTGGAATCCGCACCTGCTGACTAACGCTTCGCCGGCGTGTCTGCACAGCCGTATAGTGTTA
ATCATGACCCCAAGGAAGGATTAAACAAATATCTTGACG
>GIRAN06
GTGTCCGGTAGCCCGCGCTAGTTAGACACCCCGGCAGGGGGGATTGCTTTCGAGACGGGAGATCCCTT
CGCCGACCCTGTAGGGCCGACGCCGAGGCATTCGGGCCCCTGCAACGTCAACAGCGGCAAGAAAACGG
GATGAATGGGCGTAATGGGGGGGGTCTGCTGGGGACCCGACGCGGTTGCCGTTTGCGGGGCCCCCGAC
CCATACCGACCCACCTAGGCGTCCAGTTACGGCGCACGGCGGGAGCGTGGTTGCCGTCAGAGCTGTGT
TTCTCGATCAGTCCCCCGCAGTGCCGCAGTATCTTGCCGTGGGCTGCTTTAATCTTGAAAGTGGTTTA
TACATTGGGCGACGAGGTGTCGGCTCTCATTGGGGGTAGCCGACGGACACATGCAGTCCCCTCCCCGG
GCAGGCAGAGGCGGGGCCCCCGCGCGCCGGCCCCGGCCCACAATCTTACCAGGGTCCTCAAAAGAGCC

TTTGCATGGTACCCTTCGTGAATGGTTGCTTAAGAGGTCCACCACGTAGTCCGGGCACGGTCGACTAA
ACACTCAAAACAGCGACGGCAAATAGAGGCACAAGGTCCAGGCCCTCACGGCACTAGTTGCGATGACC
CAACTCACGGGGGCAGTCCCCGGCGCGGCGACCTTGATTACGTCCGGGAACAAGTATGTCGTTTCCCC
CGGGAGGGGTGCAGCCACATGGGAGATTCAAAGTTTCTCGTGACGTCGTTGTGATCACTGCAGCCTAG
CCGAGACTCCCGTACTACGCGAAGGTTGGTTATGTTAACCACTACAACGTGAGGCGCCGTAAGGGCCA
GTGTTGTGCCCGGCTCTTCAATGCGCCTTAGCGGCCTGATACACCCACCCAAGGAGATACTGCTAATC
ATGTGGGTGGACAGAACCTCGCAACGCAGACGCATCCCCGGCNTGATATGGTTTTTTTCCGCTATTAC
CCAGCACGCGGCGCTATCATTCAAGAGAACCCAGTGACGCGTAAATCGTAAGATCTACCTGCCGCAGG
TGGACCTACTGCAAATACGGATTATGACTCGTAAAGAGGTCATGCGTATTTTCATCACTAGGCACGTT
CGAGAGTAAATTAGTAGGTGTCCCGCGCCTTGTGGCGTTCCGCCTGACTCCTCATGAAGTCGACCTTC
TCATCGGCCCTATCTGCCGACGTAAGTCATAATCCAGATCTTCACCTCGGAGGGAGGGTCACTGTAAA
GGGATAATTGGAGGGCGATTTCCCACACTTTCCTAAGNGTACTTTTTGCTTGGCTTCGCAGTTGGGTC
CAATAGATGTTGATCTCTGGTTTAGCAGTTGTGAAAGTGGCAAGGCGGGAGGTTAGACCTCCATTTAA
CATATACAAGCAAGTTAACTGCACTAGATGTGTAGACACTACAGTTACAGGAGTAGCCGACTAAGTCT
CCGACGTCAAGCGAATAGGCGTCATACGCGATTATCGCCTAAGAGCACGTATTGGCGGTAAAAGGATA
ACTAGACGCTTGTGGGTAGATTTCAAGGCGCTCGTAGTGGTATAATAGGATACTCTTTCACCAGCCTG
AGAGCCGAACGCTATACTAGTGGTCTGTGATGTAGGACCAAGTAGCTCTCTAGGGACCATCTACTTGA
GCAATGGTGCGCAGGGGTAGACATAGACCAACCTTGGGTGGCAAGCACTGCAATAAGTCCTGTTTAGC
CTTGGAGTTCACACGCCGGCACTAAAGCCGACCTACCTGAGCTTGTGATTACCGTTAAAGCATCTGTC
TAGTTCTGTTTCCGGCGCTCTTGGTTCCATGTTAGGGGAAGTGTATGACCCATGTGTTTTTATCGGCT
TAACTACGAGTGATCCCCGGTCGTTTCCCCATTAAATCCCTGGTGCATCCTACTCCCATAATGATAGC
TGACTGGCTGGACTGGCTTTTCAAGTAGTCGAGGGGGTATCGCGGTCACGGCCGTTAACAGATCCCGT
CCTTAGTGTGGAATCCGCACCTGCTGACTAACGCTTCGCCGGCGTGTCTGCACATCCGTATAGTGTTA
ATCATGACCCCAAGGAAGGATTAAACAAATATCTTGACG
>GIRAN08
GTGTCCGGTAGCCCGCGCTAGTTAGACACCCCGGCAGGGGGGATTGCTTTCGAGACGGGAGATCCCTT
CGCCGACCCTGTAGGGCCGACGCCGAGGCATTCGGGCCCCTGCAACGTCAACAGCGGCAAGAAAACGG
GATGAATGGGCGTAATGGGGGGGGTCTGCTGGGGACCCGACGCGGTTGCCGTTTGCGGGGCCCCCGAC
CCATACCGACCCACCTAGGCGTCCAGTTACGGCGCACGGCGGGAGCGTGGTTGCCGTCAGAGCTGTGT
TTCTCGATCAGTCCCCCGCAGTGCCGCAGTATCTTGCCGTGGGCTGCTTTAATCTTGAAAGTGGTTTA
TACATTGGGCGACGAGGTGTCGGCTCTCATTGGGGGTAGCCGACGGACACATGCAGTCCCCTCCCCGG
GCAGGCAGAGGCGGGGCCCCCGCGCGCCGGCCCCGGCCCACAATCTTACCAGGGTCCTCAAAAGAGCC
TTTGCATGGTACCCTTCGTGAATGGTTGCTTAAGAGGTCCACCACGTAGTCCGGGCACGGTCGACTAA
ACACTCAAAACAGCGACGGCAAATAGAGGCACAAGGTCCAGGCCCTCACGGCACTAGTTGCGATGACC
CAACTCACGGGGGCAGTCCCCGGCGCGGCGACCTTGATTACGTCCGGGAACAAGTATGTCGTTTCCCC
CGGGAGGGGTGCAGCCACATGGGAGATTCAAAGTTTCTCGTGACGTCGTTGTGATCACTGCAGCCTAG
CCGAGACTCCCGTACTACGCGAAGGTTGGTTATGTTAACCACTACAACGTGAGGCGCCGTAAGGGCCA
GTGTTGTGCCCGGCTCTTCAATGCGCCTTAGCGGCCTGATACACCCACCCAAGGAGATACTGCTAATC
ATGTGGGTGGACAGAACCTCGCAACGCAGACGCATCCCCGGCNTGATATGGTTTTTTTCCGCTATTAC
CCAGCACGCGGCGCTATCATTCAAGAGAACCCAGTGACGCGTAAATCGTAAGATCTACCTGCCGCAGG
TGGACCTACTGCAAATACGGATTATGACTCGTAAAGAGGTCATGCGTATTTTCATCACTAGGCACGTT
CGAGAGTAAATTAGTAGGTGTCCCGCGCCTTGTGGCGTTCCGCCTGACTCCTCATGAAGTCGACCTTC
TCATCGGCCCTATCTGCCGACGTAAGTCATAATCCAGATCTTCACCTCGGAGGGAGGGTCACTGTAAA
GGGATAATTGGAGGGCGATTTCCCACACTTTCCTAAGGGTACTTTTTGCTTGGCTTCGCAGTTGGGTC
CAATAGATGTTGATCTCTGGTTTAGCAGTTGTGAAAGTGGCAAGGCGGGAGGTTAGACCTCCATTTAA
CATATACAAGCAAGTTAACTGCACTAGATGTGTAGACACTACAGTTACAGGAGTAGCCGACTAAGTCT
CCGACGTCAAGCGAATAGGCGTCATACGCGATTATCGCCTAAGAGCACGTATTGGCGGTAAAAGGATA
ACTAGACGCTTGTGGGTAGATTTCAAGGCGCTCGTAGTGGTATAATAGGATACTCTTTCACCAGCCTG
AGAGCCGAACGCTATACTAGTGGTCTGTGATGTAGGACCAAGTAGCTCTCTAGGGACCATCTACTTGA
GCAATGGTGCGNAGGGGTAGACATAGACCAACCTTGGGTGGCAAGCACTGCAATAAGTCCTGTTTAGC
CTTGGAGTTCACACGCCGGCACTAAAGCCGACCTACCTGAGCTTGTGATTACCGTTAAAGCATCTGTC
TAGTTCTGTTTCCGGCGCTCTTGGTTCCATGTTAGGGGAAGTGTATGACCCATGTGTTTTTATCGGCT
TAACTACGAGTGATCCCCGGTCGTTTCCCCATTAAATCCCTGGTGCATCCTACTCCCATAATGATAGC
TGACTGGCTGGACTGGCTTTTCAAGTAGTCGAGGGGGTATCGCGGTCACGGCCGTTAACAGATCCCGT

CCTTAGTGTGGAATCCGCACCTGCTGACTAACGCTTCGCCGGCGTGTCTGCACATCCGTATAGTGTTA
ATCATGACCCCAAGGAAGGATTAAACAAATATCTTGACG
>GIRAN12
GTGTCCGGTAGCCCGCGCTAGTCAGACACCCCGGCAGGGGGGATTGCTTTCGAGACGGGAGATCCCTT
CGCCGACCCTGGAGGGCCGACGCCGAGGCATTCGGGCCCCTGCAACGTCAACAGCGGCAAAAAAACGG
GATGAATGGGCGTAATGGGGGGGGTCTGCTGGGGACCCGACGCGGTTGCCGTTTGCGGGGCCCCCGAC
CCATACCGACCCACCTAGGCGTCCAGTTACGGCGCACGGCGGGAGCGTGGTTGCCGTCAGAGCTGTGT
TTCTCGATCAGTCCCCCGCAGTGCCGCAGTATCTTGCCGTGGGCTGCTTTAATCTTGAAAGTGGTTTA
TACATTGGGCGACGAGGTGTCGACTCTCATTGGGGGTAACCGACGGGCACATGCAGTCCCCTCCCCGG
GCAGGCAGAGGCGGGGCCCCCGCGCGCCGGCCCCGGCCCACAATCTTACCAGGGTCCTCAAAGGAGCC
TTTGCATGGTACCCTTCGTGAATGGTTGCTTAAGAGGTCCACAACGTGGTCCGGGCACGGTCGACTAA
ACACTCAAAACAGCGACGGCAAATATAGGTACAAGGTCCAGGCCCTCACGGCACTAATTGCGATGACC
CAACTCACGGGGGCAGTCCCCGGCGCGGCGACCTTGATTACGTCCGGGGACAAGTATGTCGCTCCCCC
CGGGAGGGGTGCAGCCACATGGGAGATTCAAAGTTTCTCGTGACGTCGTTGTGATCACTGCAGCCTAG
CCGAGACTCCCGTACTACGCGAAGGTTGGTTATGTTAACCACTACAACGTGAGGCGCCGTAAGGGCCA
GTGTTGTGCCCGGCTCTTCAATGCGCCTTAGCGGCCTGATACACCCACCCAAGGAGATACTGCTAATC
ATATGGGTGGACAGAACCTCGCAACGCAGACGCCTCCCCGGCATGATATGGTTTTTTTCCGCTATTAT
CCAGCACGCAGCGCTATCATTTAAGAGAACCCAGTGACGCGTAAATCGTAAGATCTACCTGCCGCAGG
TGGACCTACTGCAAATACGGATTATGACTCGTAAAGGGGTCATGCGTATTTTCATCACTAGGCACGTT
CGAGAATAAATTAGTAGGTGTCCCGAGCCTTGTGGCGTTCCGCCTGACTCCTCATGAAGTCGACCTTC
TCACCGGCCCTATCTGCCGACGTAAGTCATAACCTAGATCTGTACCTCGGGGGGAGGGTCACTGTAAA
GGGATAATTGGAGGGTGATTTCCCACATTTTCCTAAGGGTACTTTTTGCCTGGCTTCGCAATTGGGTC
CAATGGATGTCGATCTCTGGTTTAGCAGTTGTGAAAGTGGCAAGGCGGGAGGTTAGACCTCCATTTAA
CATATACAAGCAAGTTNACTGCACTAGATGNGTAGACACTACAGGTACAGGAGTAGCCGGATAAGTCT
CCGACGTCAAGCGAATAAGCGTCATACGCGATTATCGCCTAAGAGCACGTATTTGCGGTAAAAGGCTA
GCTAGACGCTTGTGGGTAGATTTCAAGGCGTTCGTAGTGGTATAATAGGATACTCTTTCACCAGCCTG
AGGGCCGAACGCTATACTAGTGGTCTGTGATGTAGGACCGAGTATCTCTCTAGGGACCATCTACTTGA
GCAATGGTGCGCAGGGGGAGACATAGACCAGCCTTGGGTGGCAAGCACTGCAATAAGTCCTGTTTAGC
CTTGGAGTTCACACGCCGGCACTGAAGCCGACCTACCTGAGCGTGTGATTACCGTTAAAGCATCTGTC
TAGTTCTGTTTCCGGCGCTCTTGGTTCCAGGTTAGGGGAAGTGTATGACCCATGTGTTTTTATCGGCT
TAACCACGAGTGATCCCCGGTCGTTTCCCCATTGAATCCCTGGTGCATCCTACTCCCAGAATGATAGC
TGACTGACTGGACTGGCTTTTCAAGTAATCGAGGGGGTATCGCGGTCACGGCCGTTAACAGATCCCGT
CCTTAGTGTGGAATCCGCACCTGCTGACTAACGCTTCGCCGGCGTGTCTGCACAGCCGTATAGTGTTA
ATCATGACCCCAAGGAAGGATTAAACAAATATCTTGACG
>GIRAN13
GTGTCCGGTAGCCCGCGCTAGTTAGACACCCCGGCAGGGGGGATTGCTTTCGAGACGGGAGATCCCTT
CGCCGACCCTGGAGGGCCGACGCCGAGGCATTCGGGCCCCCGCAACGTCAACAGCGGCAAGAAAACGG
GATGAATGGGCGTAATGGGGGGGGTCTGCTGGGGACCCGACGCGGTTGCCGTTTGCGGGGCCCCCGAC
CCATACCGACCCACCTAGGCGTCCAGTTACGGCGCACGGCGGGAGCGTGGTTGCCGTCAGAGCTGTGT
TTCTCGATCAGTCCCCCGCAGTGCCGCAGTATCTTGCCGTGGGCTGCTTTAATCTTGAAAGTGGTTTA
TACATTGGGCGACGAGGTGTCGACTCTCATTGGGGGTAACCGACGGGCACATGCAGTCCCCTCCCCGG
GCAGGCAGAGGCGGGGCCCCCGCGCGCCGGCCCCGGCCCACAATCTTACCAGGGTCCTCAAAGGAGCC
TTTGCATGGTACCCTTCGTGAATGGTTGCTTAAGAGGTCCACAACGTGGTCCGGGCACGGTCGACTAA
ACACTCAAAACAGCGACGGCAAATATAGGTACAAGGTCCAGGCCCTCACGGCACTAATTGCGATGACC
CAACTCACGGGGGCAGTCCCCGGCGCGGCGACCTTGATTACGTCCGGGGACAAGTATGTCGCTCCCCC
CGGGAGGGGTGCAGCCACATGGGAGATTCAAAGTTTCTCGTGACGTCGTTGTGATCACTGCAGCCTAG
CCGAGACTCCCGTACTACGCGAAGGTTGGTTATGTTAACCACTACAACGTGAGGCGCCGTAAGGGCCA
GTGTTGTGCCCGGCTCTTCAATGCGCCTTAGCGGCCTGATACACCCACCCAAGGAGATACTGCTAATC
ATATGGGTGGACAGAACCTCGCAACGCAGACGCCTCCCCGGCATGATATGGTTTTTTTCCGCTATTAT
CCAGCACGCAGCGCTATCATTCAAGAGAACCCAGTGACGCGTAAATCGTAAGATCTACCTGCCGCAGG
TGGACCTACTGCAAATACGGATTATGACTCGTAAAGGGGTCATGCGTATTTTCATCACTAGGCACGTT
CGAGAATAAATTAGTAGGTGTCCCGAGCCTTGTGGCGTTCCGCCTGACTCCTCATGAAGTCGACCTTC
TCACCGGCCCTATCTGCCGACGTAAGTCATAACCTAGATCTGTACCTCGGGGGGAGGGTCACTGTAAA
GGGATAATTGGAGGGTGATTTCCCACACTTTCCTAAGGGTACTTTTTGCCTGGCTTCGCAATTGGGTC

CAATGGATGTCGATCTCTGGTTTAGCAGTTGTGAAAGTGGCAAGGCGGGAGGTTAGACCTCCATTTAA
CATATACAAGCAAGTTAACTGCACTAGATGTGTAGACACTACAGTTACAGGAGTAGCCGAATAAGTCT
CCGACGTCAAGCGAATAAGCGTCATACGCGATTATCGCCTAAGAGCACGTATTGGCGGTAAAAGGCTA
GCTAGACGCTTATGGGTAGATTTCAAGGCGTTCGTAGTGGTATAATAGGATACTCTTTCACCAGCCTG
AGGGCCGAACGCTATACTAGTGGTCTGTGATGTAGGACCGAGTATCTCTCTAGGGACCATCTACTTGA
GCAATGGTGCGCAGGGGGAGACATAGACCAGCCTTGGGTGGCAAGCACTGCAATAAGTCCTGTTTAGC
CTTGGAGTTCACATGCCGGCACTGAAGCCGACCTACCTGAGCGTGTGATTACCGTTAAAGCATCTGTC
TAGTTCTGTTTCCGGCGCTCTTGGTTCCAGGTTAGGGGAAGTGTATGACCCATGTGTTTTTATCGGCT
TAACCACGAGTGATCCCCGGTCGTTTCCCCATTGAATCCCTGGTGCATCCTACTCCCAGAATGATAGC
TGACTGACTGGACTGGCTTTTCAAGTAATCGAGGGGGTATCGCGGTCACGGCCGTTAACAGATCCCGT
CCTTAGTGTGGAATCCGCACCTGCTGACTAACGCTTCGCCGGCGTGTCTGCACAGCCGTATAGTGTTA
ATCATGACCCCAAGGAAGGATTAAACAAATATCTTGACG
>GIRAN14
GTGTCCGGTAGCCCGCGCTAGTCAGACACCCCGGCAGGGGGGATTGCTTTCGAGACGGGAGATCCCTT
CGCCGACCCTGGAGGGCCGACGCCGAGGCATTCGGGCCCCTGCAACGTCAACAGCGGCAAGAAAACGG
GATGAATGGGCGTAATGGGGGGGGTCTGCTGGGGACCCGACGCGGTTGCCGTTTGCGGGGCCCCCGAC
CCATACCGACCCACCTAGGCGTCCAGTTACGGCGCACGGCGGGAGCGTGGTTGCCGTCAGAGCTGTGT
TTCTCGATCAGTCCCCCGCAGTGCCGCAGTATCTTGCCGTGGGCTGCTTTAATCTTGAAAGTGGTTCA
TACATTGGGCGACGAGGTGTCGACTCTCATTGGGGGTAACCGACGGGCACATGCAGTCCCCTCCCCGG
GCAGGCAGAGGCGGGGCCCCCGCGCGCCGGCCCCGGCCCACAATCTTACCAGGGTCCTCAAAGGAGCC
TTTGCATGGTACCCTTCGTGAATGGTTGCTTAAGAGGTCCACAACGTGGTCCGGGCACGGTCGACTAA
ACACTCAAAACAGCGACGGCAAATATAGGTACAAGGTCCAGGCCCTCACGGCACTAATTGCGATGACC
CAACTCACGGGGGCAGTCCCCGGCGCGGCGACCTTGATTACGTCCGGGGACAAGTATGTCGCTCCCCC
CGGGAGGGGTGCAGCCACATGGGAGATTCAAAGTTTCTCGTGACGTCGTTGTGATCACTGCAGCCTAG
CCGAGACTCCCGTACTACGCGAAGGTTGGTTATGTTAACCACTACAACGTGAGGCGCCGTAAGGGCCA
GTGTTGTGCCCGGCTCTTCAATGCGCCTTAGCGGCCTGATACACCCACCCAAGGAGATACTGCTAATC
ATATGGGTGGACAGAACCTCGCAACGCAGACGCCTCCCCGGCATGATATGGTTTTTTTCCGCTATTAT
CCAGCACGCAGCGCTATCATTTAAGAGAACCCAGTGACGCGTAAATCGTAAGATCTACCTGCCGCAGG
TGGACCTACTGCAAATACGGATTATGACTCGTAAAGGGGTCATGCGTATTTTCATCACTAGGCACGTT
CGAGAATAAATTAGTAGGTGTCCCGAGCCTTGTGGCGTTCCGCCTGACTCCTCATGAAGTCGACCTTC
TCACCGGCCCTATCTGCCGACGTAAGTCATAACCTAGATCTGTACCTCGGGGGGAGGGTCACTGTAAA
GGGATAATTGGAGGGTGATTTCCCACATTTTCCTAAGGGTACTTTTTGCCTGGCTTCGCAATTGGGTC
CAATGGATGTCGATCTCTGGTTTAGCAGTTGTGAAAGTGGCAAGGCGGGAGGTTAGACCTCCATTTAA
CATATACAAGCAAGTTAACTGCACTAGATGTGTAGACACTACAGGTACAGGAGTAGCCGGATAAGTCT
CCGACGTCAAGCGAATAAGCGTCATACGCGATTATCGCCTAAGAGCACGTATTTGCGGTAAAAGGCTA
GCTAGACGCTTGTGGGTAGATTTCAAGGCGTTCGTAGTGGTATAATAGGATACTCTTTCACCAGCCTG
AGGGCCGAACGCTATACTAGTGGTCTGTGATGTAGGACCGAGTATCTCTCTAGGGACCATCTACTTGA
GCAATGGTGCGCAGGGGGAGACATAGACCAGCCTTGGGTGGCAAGCACTGCAATAAGTCCTGTTTAGC
CTTGGAGTTCACACGCCGGCACTGAAGCCGACCTACCTGAGCGTGTGATTACCGTTAAAGCATCTGTC
TAGTTCTGTTTCCGGCGCTCTTGGTTCCAGGTTAGGGGAAGTGTATGACCCATGTGTTTTTATCGGCT
TAACCACGAGTGATCCCCGGTCGTTTCCCCATTGAATCCCTGGTGCATCCTACTCCCAGAATGATAGC
TGACTGACTGGACTGGCTTTTCAAGTAATCGAGGGGGTATCGCGGTCACGGCCGTTAACAGATCCCGT
CCTTAGTGTGGAATCCGCACCTGCTGACTAACGCTTCGCCGGCGTGTCTGCACAGCCGTATAGTGTTA
GTCATGACCCCAAGGAAGGATTAAACAAATATCTTGACG
>GIRAN15
GTGTCCGGTAGCCCGCGCTAGTTAGACACCCCGGCAGGGGGGATTGCTTTCGGGACGGGAGATCCCTT
CGCCGACCCTGGAGGGCCGACGCCGAGGCATTCGGGCCCCNGCAACGTCAACAGCGGCAAGAAAACGG
GATGAATGGGCGTAATGGGGGGGGTCTGCTGGGGACCCGACGCGGTTGCCGTTTGCGGGGCCCCCGAC
CCATACCGACCCACCTAGGCGTCCAGTTACGGCGCACGTCGGGAGCGTGGTTGCCGTCAGAGCTGTGT
TTCTCGATCAGTCCCCCACAGTGCCGCAGTATCTTGCCGTGGGCTGCTTTAATCTTGAAAGTGGTTNA
TACATTGGGCGACGAGGTGTCGACTCTCATTGGGGGTAACCGACGGGCACATGCAGTCCCCTCCCCGG
GCAGGCAGAGGCGGGGCCCCCGCGCGCCGGCCCCGGCCCACAATCTTACCAGGGTCCTCAAAGGAGCC
TTTGCATGGTACCCTTCGTGAATGGTTGCTTAAGAAGTCCACAACGTGGTCCGGGCACGGTCGACTAA
ACACTCAAAACAGCGACGGCAAATATAGGTACAAGGTCCAGGCCCTCACGGCACTAATTGCGATGACC

CAACTCACGGGGGCAGTCCCCGGCGCGGCGACCTTGATTACGTCCGGGGACAAGTATGTCGCTCCCCC
CGGGAGGGGTGCAGCCACATGGGAGATTCAAAGTTTCTCGTGACGTCGTTGTGATCACTGCAGCCTAG
CCGAGACTCCCGTACTACGCGAAGGTTGGTTATGTTAACCACTACAACGTGAGGCGCCGTAAGGGCCA
GTGTTGTGCCCGGCTCTTCAATGCGCCTTAGCGGCCTGATACACCCACCCAAGGAGATACTGCTAATC
ATATGAGTGGACAGAACCTCGCAACGCAGACGCCTCCCCGGCATGATATGGTTTTTTTCCGCTATTAT
CCAGCACGCAGCGCTATCATTCAAGAGAACCCAGTGACGCGTAAATCGTAAGATCTACCTGCCGCAGG
TGGACCTACTGCAAATACGGATTATGACTCGTAAAGGGGTCATGCGTATTTTCATCACTAGGCACGTT
CGAGAATAAATTAGTAGGTGTCCCGAGCCTTGTGGCGTTCCGCCTGACTCCTCATGAAGTCGACCTTC
TCACCGGCCCTATCTGCCGACGTAAGTCATAACCTAGATCTGTACCTCGGGGGGAGGGTCACTGTAAA
GGGATAATTGGAGGGTGATTTCCCACATTTTCCTAAGGGTACTTTTTGCCTGGCTTCGCAATTGGGTC
CAATGGATGTCGATCTCTGGTTTAGCAGTTGTGAAAGTGGCAAGGCGGGAGGTTAGACCTCCATTTAA
CATATACAAGCAAGTTAACTGCACTAGATGTGTAGACACTACAGGTACAGGAGTAGCCGGATAAGTCT
CCGACGTCAAGCGAATAAGCTTCATACGCGATTATCGCCTAAGAGCACGTATTTGCGGTAAAAGGCTA
GCTAGACGCTTGTGGGTAGATTTCAAGGCGTTCGTAGTGGTATAATAGGATACTCTTTCACCAGCCTG
AGGGCCGAACGCTATACTAGTGGTCTGTGATGTAGGACCGAGTATCTCTCTAGGGACCATCTACTTGA
GCAATGGTGCGCAGGGGGAGACATAGACCAGCCTTGGGTGGCAAGCACTGCAATAAGTCCTGTTTAGC
CTTGGAGTTCACACGCCGGCACTGAAGCCGACCTACCTGAGCGTGTGATTACCGTTAAAGCATCTGTC
TAGTTCTGTTTCCGGCGCTCTTGGTTCCAGGTTAGGGGAAGTGTATGACCCATGTGTTTTCATCGGCT
TAACCACGAGTGATCCCCGGTCGTTTCCCCATTGAATCCCTGGTGCATCCTACTCCCAGAATGATAGC
TGACTGACTGGACTGGCTTTTCAAGTAATCGAGGGGGTATCGCGGTCACGGCCGTTAACAGATCCCGT
CCTTAGTGTGGAATCCGCACCTGCTGACTAACGCTTCGCCGGCGTGTCTGCACAGCCGTATAGTGTTA
ATCATGACCCCAAGGAAGGATTAAACAAATATCTTGACG
>GIRAN16
GTGTCCGGTAGCCCGCGCTAGTTAGACACCCCGGCAGGGGGGATTGCTTTCGAGACGGGAGATCCCTT
CGCCGACCCTGGAGGGCCGACGCCGAGGCATTCGGGCCCCCGCAACGTCAACAGCGGCAAGAAAACGG
GATGAATGGGCGTAATGGGGGGGGTCTGCTGGGGACCCGACGCGGTTGCCGTTTGCGGGGCCCCCGAC
CCATACCGACCCACCTAGGCGTCCAGTTACGGCGCACGGCGGGAGCGTGGTTGCCGTCAGAGCTGTGT
TTCTCGATCAGTCCCCCGCAGTGCCGCAGTATCTTGCCGTGGGCTGCTTTAATCTTGAAAGTGGTTCA
TACATTGGGCGACGAGGTGTCGACTCTCATTGGGGGTAACCGACGGGCACATGCAGTCCCCTCCCCGG
GCAGGCAGAGGCGGGGCCCCCGCGCGCCGGCCCCGGCCCACAATCTTACCAGGGTCCTCAAAGGAGCC
TTTGCATGGTACCCTTCGTGAATGGTTGCTTAAGAGGTCCACAACGTGGTCCGGGCACGGTCGACTAA
ACACTCAAAACAGCGACGGCAAATATAGGTACAAGGTCCAGGCCCTCACGGCACTAATTGCGATGACC
CAACTCACGGGGGCAGTCCCCGGCGCGGCGACCTTGATTACGTCCGGGGACAAGTATGTCGCTCCCCC
CGGGAGGGGTGCAGCCACATGGGAGATTCAAAGTTTCTCGTGACGTCGTTGTGATCACTGCAGCCTAG
CCGAGACTCCCGTACTACGCGAAGGTTGGTTATGTTAACCACTACAACGTGAGGCGCCGTAAGGGCCA
GTGTTGTGCCCGGCTCTTCAATGCGCCTTAGCGGCCTGATACACCCACCCAAGGAGATACTGCTAATC
ATATGGGTGGACAGAACCTCGCAACGCAGACGCCTCCCCGGCATGATATGGTTTTTTTCCGCTATTAT
CCAGCACGCAGCGCTATCATTCAAGAGAACCCAGTGACGCGTAAATCGTAAGATCTACCTGCCGCAGG
TGGACCTACTGCAAATACGGATTATGACTCGTAAAGGGGTCATGCGTATTTTCATCACTAGGCACGTT
CGAGAATAAATTAGTAGGTGTCCCGAGCCTTGTGGCGTTCCGCCTGACTCCTCATGAAGTCGACCTTC
TCACCGGCCCTATCTGCCGACGTAAGTCATAACCTAGATCTGTACCTCGGGGGGAGGGTCACTGTAAA
GGGATAATTGGAGGGTGATTTCCCACACTTTCCTAAGGGTACTTTTTGCCTGGCTTCGCAATTGGGTC
CAATGGATGTCGATCTCTGGTTTAGCAGTTGTGAAAGTGGCAAGGCGGGAGGTTAGACCTCCATTTAA
CATATACAAGCAAGTTAACTGCACTAGATGTGTAGACACTACAGTTACAGGAGTAGCCGAATAAGTCT
CCGACGTCAAGCGAATAAGCGTCATACGCGATTATCGCCTAAGAGCACGTATTGGCGGTAAAAGGCTA
GCTAGACGCTTATGGGTAGATTTCAAGGCGTTCGTAGTGGTATAATAGGATACTCTTTCACCAGCCTG
AGGGCCGAACGCTATACTAGTGGTCTGTGATGTAGGACCGAGTATCTCTCTAGGGACCATCTACTTGA
GCAATGGTGCGCAGGGGGAGACATAGACCAGCCTTGGGTGGCAAGCACTGCAATAAGTCCTGTTTAGC
CTTGGAGTTCACATGCCGGCACTGAAGCCGACCTACCTGAGCGTGTGATTACCGTTAAAGCATCTGTC
TAGTTCTGTTTCCGGCGCTCTTGGTTCCAGGTTAGGGGAAGTGTATGACCCATGTGTTTTTATCGGCT
TAACCACGAGTGATCCCCGGTCGTTTCCCCATTGAATCCCTGGTGCATCCTACTCCCAGAATGATAGC
TGACTGACTGGACTGGCTTTTCAAGTAATCGAGGGGGTATCGCGGTCACGGCCGTTAACAGATCCCGT
CCTTAGTGTGGAATCCGCACCTGCTGACTAACGCTTCGCCGGCGTGTCTGCACAGCCGTATAGTGTTA
ATCATGACCCCAAGGAAGGATTAAACAAATATCTTGACG

>GIRAN17
GTGTCCGGTAGCCCGCGCTAGTCAGACACCCCGGCAGGGGGGATTGCTTTCGAGACGGGAGATCCCTT
CGCCGACCCTGGAGGGCCGACGCCGAGGCATTCGGGCCCCTGCAACGTCAACAGCGGCAAAAAAACGG
GATGAATGGGCGTAATGGGGGGGGTCTGCTGGGGACCCGACGCGGTTGCCGTTTGCGGGGCCCCCGAC
CCATACCGACCCACCTAGGCGTCCAGTTACGGCGCACGGCGGGAGCGTGGTTGCCGTCAGAGCTGTGT
TTCTCGATCAGTCCCCCGCAGTGCCGCAGTATCTTGCCGTGGGCTGCTTTAATCTTGAAAGTGGTTTA
TACATTGGGCGACGAGGTGTCGACTCTCATTGGGGGTAACCGACGGGCACATGCAGTCCCCTCCCCGG
GCAGGCAGAGGCGGGGCCCCCGCGCGCCGGCCCCGGCCCACAATCTTACCAGGGTCCTCAAAGGAGCC
TTTGCATGGTACCCTTCGTGAATGGTTGCTTAAGAGGTCCACAACGTGGTCCGGGCACGGTCGACTAA
ACACTCAAAACAGCGACGGCAAATATAGGTACAAGGTCCAGGCCCTCACGGCACTAATTGCGATGACC
CAACTCACGGGGGCAGTCCCCGGCGCGGCGACCTTGATTACGTCCGGGGACAAGTATGTCGCTCCCCC
CGGGAGGGGTGCAGCCACATGGGAGATTCAAAGTTTCTCGTGACGTCGTTGTGATCACTGCAGCCTAG
CCGAGACTCCCGTACTACGCGAAGGTTGGTTATGTTAACCACTACAACGTGAGGCGCCGTAAGGGCCA
GTGTTGTGCCCGGCTCTTCAATGCGCCTTAGCGGCCTGATACACCCACCCAAGGAGATACTGCTAATC
ATATGGGTGGACAGAACCTCGCAACGCAGACGCCTCCCCGGCATGATATGGTTTTTTTCCGCTATTAT
CCAGCACGCAGCGCTATCATTTAAGAGAACCCAGTGACGCGTAAATCGTAAGATCTACCTGCCGCAGG
TGGACCTACTGCAAATACGGATTATGACTCGTAAAGGGGTCATGCGTATTTTCATCACTAGGCACGTT
CGAGAATAAATTAGTAGGTGTCCCGAGCCTTGTGGCGTTCCGCCTGACTCCTCATGAAGTCGACCTTC
TCACCGGCCCTATCTGCCGACGTAAGTCATAACCTAGATCTGTACCTCGGGGGGAGGGTCACTGTAAA
GGGATAATTGGAGGGTGATTTCCCACATTTTCCTAAGGGTACTTTTTGCCTGGCTTCGCAATTGGGTC
CAATGGATGTCGATCTCTGGTTTAGCAGTTGTGAAAGTGGCAAGGCGGGAGGTTAGACCTCCATTTAA
CATATACAAGCAAGTTAACTGCACTAGATGTGTAGACACTACAGGTACAGGAGTAGCCGGATAAGTCT
CCGACGTCAAGCGAATAAGCGTCATACGCGATTATCGCCTAAGAGCACGTATTTGCGGTAAAAGGCTA
GCTAGACGCTTGTGGGTAGATTTCAAGGCGTTCGTAGTGGTATAATAGGATACTCTTTCACCAGCCTG
AGGGCCGAACGCTATACTAGTGGTCTGTGATGTAGGACCGAGTATCTCTCTAGGGACCATCTACTTGA
GCAATGGTGCGCAGGGGGAGACATAGACCAGCCTTGGGTGGCAAGCACTGCAATAAGTCCTGTTTAGC
CTTGGAGTTCACACGCCGGCACTGAAGCCGACCTACCTGAGCGTGTGATTACCGTTAAAGCATCTGTC
TAGTTCTGTTTCCGGCGCTCTTGGTTCCAGGTTAGGGGAAGTGTATGACCCATGTGTTTTTATCGGCT
TAACCACGAGTGATCCCCGGTCGTTTCCCCATTGAATCCCTGGTGCATCCTACTCCCAGAATGATAGC
TGACTGACTGGACTGGCTTTTCAAGTAATCGAGGGGGTATCGCGGTCACGGCCGTTAACAGATCCCGT
CCTTAGTGTGGAATCCGCACCTGCTGACTAACGCTTCGCCGGCGTGTCTGCACAGCCGTATAGTGTTA
ATCATGACCCCAAGGAAGGATTAAACAAATATCTTGACG
>GIRAN19
GTGTCCGGTAGCCCGCGCTAGTTAGACACCCCGGCAGGGGGGATTGCTTTCGAGACGGGAGATCCCTT
CGCCGACCCTGGAGGGCCGACGCCGAGGCATTCGGGCCCCCGCAACGTCAACAGCGGCAAGAAAACGG
GATGAATGGGCGTAATGGGGGGGGTCTGCTGGGGACCCGACGCGGTTGCCGTTTGCGGGGCCCCCGAC
CCATACCGACCCACCTAGGCGTCCAGTTACGGCGCACGGCGGGAGCGTGGTTGCCGTCAGAGCTGTGT
TTCTCGATCAGTCCCCCGCAGTGCCGCAGTATCTTGCCGTGGGCTGCTTTAATCTTGAAAGTGGTTTA
TACATTGGGCGACGAGGTGTCGACTCTCATTGGGGGTAACCGACGGGCACATGCAGTCCCCTCCCCGG
GCAGGCAGAGGCGGGGCCCCCGCGCGCCGGCCCCGGCCCACAATCTTACCAGGGTCCTCAAAGGAGCC
TTTGCATGGTACCCTTCGTGAATGGTTGCTTAAGAGGTCCACAACGTGGTCCGGGCACGGTCGACTAA
ACACTCAAAACAGCGACGGCAAATATAGGTACAAGGTCCAGGCCCTCACGGCACTAATTGCGATGACC
CAACTCACGGGGGCAGTCCCCGGCGCGGCGACCTTGATTACGTCCGGGGACAAGTATGTCGCTCCCCC
CGGGAGGGGTGCAGCCACATGGGAGATTCAAAGTTTCTCGTGACGTCGTTGTGATCACTGCAGCCTAG
CCGAGACTCCCGTACTACGCGAAGGTTGGTTATGTTAACCACTACAACGTGAGGCGCCGTAAGGGCCA
GTGTTGTGCCCGGCTCTTCAATGCGCCTTAGCGGCCTGATACACCCACCCAAGGAGATACTGCTAATC
ATATGGGTGGACAGAACCTCGCAACGCAGACGCCTCCCCGGCATGATATGGTTTTTTTCCGCTATTAT
CCAGCACGCAGCGCTATCATTCAAGAGAACCCAGTGACGCGTAAATCGTAAGATCTACCTGCCGCAGG
TGGACCTACTGCAAATACGGATTATGACTCGTAAAGGGGTCATGCGTATTTTCATCACTAGGCACGTT
CGAGAATAAATTAGTAGGTGTCCCGAGCCTTGTGGCGTTCCGCCTGACTCCTCATGAAGTCGACCTTC
TCACCGGCCCTATCTGCCGACGTAAGTCATAACCTAGATCTGTACCTCGGGGGGAGGGTCACTGTAAA
GGGATAATTGGAGGGTGATTTCCCACACTTTCCTAAGGGTACTTTTTGCCTGGCTTCGCAATTGGGTC
CAATGGATGTCGATCTCTGGTTTAGCAGTTGTGAAAGTGGCAAGGCGGGAGGTTAGACCTCCATTTAA
CATATACAAGCAAGTTAACTGCACTAGATGTGTAGACACTACAGTTACAGGAGTAGCCGAATAAGTCT

CCGACGTCAAGCGAATAAGCGTCATACGCGATTATCGCCTAAGAGCACGTATTGGCGGTAAAAGGCTA
GCTAGACGCTTATGGGTAGATTTCAAGGCGTTCGTAGTGGTATAATAGGATACTCTTTCACCAGCCTG
AGGGCCGAACGCTATACTAGTGGTCTGTGATGTAGGACCGAGTATCTCTCTAGGGACCATCTACTTGA
GCAATGGTGCGCAGGGGGAGACATAGACCAGCCTTGGGTGGCAAGCACTGCAATAAGTCCTGTTTAGC
CTTGGAGTTCACATGCCGGCACTGAAGCCGACCTACCTGAGCGTGTGATTACCGTTAAAGCATCTGTC
TAGTTCTGTTTCCGGCGCTCTTGGTTCCAGGTTAGGGGAAGTGTATGACCCATGTGTTTTTATCGGCT
TAACCACGAGTGATCCCCGGTCGTTTCCCCATTGAATCCCTGGTGCATCCTACTCCCAGAATGATAGC
TGACTGACTGGACTGGCTTTTCAAGTAATCGAGGGGGTATCGCGGTCACGGCCGTTAACAGATCCCGT
CCTTAGTGTGGAATCCGCACCTGCTGACTAACGCTTCGCCGGCGTGTCTGCACAGCCGTATAGTGTTA
ATCATGACCCCAAGGAAGGATTAAACAAATATCTTGACG
>GIRAN23
GTGTCCGGTAGCCCGCGCTAGTCAGACACCCCGGCAGGGGGGATTGCTTTCGGGACGGGAGATCCCTT
CGCCGACCCTGGAGGGCCGACGCCGAGGCATTCGGGCCCCTGCAACGTCAACAGCGGCAAGAAAACGG
GATGAATGGGCGTAATGGGGGGGGTCTGCTGGGGACCCGACGCGGTTGCCGTTTGCGGGGCCCCCGAC
CCATACCGACCCACCTAGGCGTCCAGTTACGGCGCACGTCGGGAGCGTGGTTGCCGTCAGAGCTGTGT
TTCTCGATCAGTCCCCCGCAGTGCCGCAGTATCTTGCCGTGGGCTGCTTTAATCTTGAAAGTGGTTCA
TACATTGGGCGACGAGGTGTCGACTCTCATTGGGGGTAACCGACGGGCACATGCAGTCCCCTCCCCGG
GCAGGCAGAGGCGGGGCCCCCGCGCGCCGGCCCCGGCCCACAATCTTACCAGGGTCCTCAAAGGAGCC
TTTGCATGGTACCCTTCGTGAATGGTTGCTTAAGAGGTCCACAACGTGGTCCGGGCACGGTCGACTAA
ACACTCAAAACAGCGACGGCAAATATAGGTACAAGGTCCAGGCCCTCACGGCACTAATTGCGATGACC
CAACTCACGGGGGCAGTCCCCGGCGCGGCGACCTTGATTACGTCCGGGGACAAGTATGTCGCTCCCCC
CGGGAGGGGTGCAGCCACATGGGAGATTCAAAGTTTCTCGTGACGTCGTTGTGATCACTGCAGCCTAG
CCGAGACTCCCGTACTACGCGAAGGTTGGTTATGTTAACCACTACAACGTGAGGCGCCGTAAGGGCCA
GTGTTGTGCCCGGCTCTTCAATGCGCCTTAGCGGCCTGATACACCCACCCAAGGAGATACTGCTAATC
ATATGGGTGGACAGAACCTCGCAACGCAGACGCCTCCCCGGCATGATATGGTTTTTTTCCGCTATTAT
CCAGCACGCAGCGCTATCATTCAAGAGAACCCAGTGACGCGTAAATCGTAAGATCTACCTGCCGCAGG
TGGACCTACTGCAAATACGGATTATGACTCGTAAAGGGGTCATGCGTATTTTCATCACTAGGCACGTT
CGAGAATAAATTAGTAGGTGTCCCGAGCCTTGTGGCGTTCCGCCTGACTCCTCATAAAGTCGACCTTC
TCACCGGCCCTATCTGCCGACGTAAGTCATAACCTAGATCTGTACCTCGGGGGGAGGGTCACTGTAAA
GGGATAATTGGAGGGTGATTTCCCACATTTTCCTAAGGGTACTTTTTGCCTGGCTTCGCAATTGGGTC
CAATGGATGTCGATCTCTGGTTTAGCAGTTGTGAAAGTGGCAAGGCGGGAGGTTAGACCTCCATTTAA
CATATACAAGCAAGTTAACTGCACTAGATGTGTAGACACTACAGGTACAGGAGTAGCCGGATAAGTCT
CCGACGTCAAGCGAATAAGCGTCATACGCGATTATCGCCTAAGAGCACGTATTTGCGGTAAAAGGCTA
GCTAGACGCTTGTGGGTAGATTTCAAGGCGTTCGTAGTGGTATAATAGGATACTCTTTCACCAGCCTG
AGGGCCGAACGCTATACTAGTGGTCTGTGATGTAGGACCGAGTATCTCTCTAGGGACCATCTACTTGA
GCAATGGTGCGCAGGGGGAGACATAGACCAGCCTTGGGTGGCAAGCACTGCAATAAGTCCTGTTTAGC
CTTGGAGTTCACACGCCGGCACTGAAGCCGACCTACCTGAGCGTGTGATTACCGTTAAAGCATCTGTC
TAGTTCTGTTTCCGGCGCTCTTGGTTCCAGGTTAGGGGAAGTGTATGACCCATGTGTTTTTATCGGCT
TAACCACGAGTGATCCCCGGTCGTTTCCCCATTGAATCCCTGGTGCATCCTACTCCCAGAATGATAGC
TGACTGACTGGACTGGCTTTTCAAGTAATCGAGGGGGTATCGCGGTCACGGCCGTTAACAGATCCCGT
CCTTAGTGTGGAATCCGCACCTGCTGACTAACGCTTCGCCGGCGTGTCTGCACAGCCGTATAGTGTTA
ATCATGACCCCAAGGAAGGATTAAACAAATATCTTGACG
>GIRAN26
GTGTCCGGTAGCCCGCGCTAGTTAGACACCCCGGCAGGGGGGATTGCTTTCGAGACGGGAGATCCCTT
CGCCGACCCTGGAGGGCCGACGCCGAGGCATTCGGGCCCCCGCAACGTCAACAGCGGCAAGAAAACGG
GATGAATGGGCGTAATGGGGGGGGTCTGCTGGGGACCCGACGCGGTTGCCGTTTGCGGGGCCCCCGAC
CCATACCGACCCACCTAGGCGTCCAGTTACGGCGCACGGCGGGAGCGTGGTTGCCGTCAGAGCTGTGT
TTCTCGATCAGTCCCCCGCAGTGCCGCAGTATCTTGCCGTGGGCTGCTTTAATCTTGAAAGTGGTTTA
TACATTGGGCGACGAGGTGTCGACTCTCATTGGGGGTAACCGACGGGCACATGCAGTCCCCTCCCCGG
GCAGGCAGAGGCGGGGCCCCCGCGCGCCGGCCCCGGCCCACAATCTTACCAGGGTCCTCAAAGGAGCC
TTTGCATGGTACCCTTCGTGAATGGTTGCTTAAGAGGTCCACAACGTGGTCCGGGCACGGTCGACTAA
ACACTCAAAACAGCGACGGCAAATATAGGTACAAGGTCCAGGCCCTCACGGCACTAATTGCGATGACC
CAACTCACGGGGGCAGTCCCCGGCGCGGCGACCTTGATTACGTCCGGGGACAAGTATGTCGCTCCCCC
CGGGAGGGGTGCAGCCACATGGGAGATTCAAAGTTTCTCGTGACGTCGTTGTGATCACTGCAGCCTAG

CCGAGACTCCCGTACTACGCGAAGGTTGGTTATGTTAACCACTACAACGTGAGGCGCCGTAAGGGCCA
GTGTTGTGCCCGGCTCTTCAATGCGCCTTAGCGGCCTGATACACCCACCCAAGGAGATACTGCTAATC
ATATGGGTGGACAGAACCTCGCAACGCAGACGCCTCCCCGGCATGATATGGTTTTTTTCCGCTATTAT
CCAGCACGCAGCGCTATCATTCAAGAGAACCCAGTGACGCGTAAATCGTAAGATCTACCTGCCGCAGG
TGGACCTACTGCAAATACGGATTATGACTCGTAAAGGGGTCATGCGTATTTTCATCACTAGGCACGTT
CGAGAATAAATTAGTAGGTGTCCCGAGCCTTGTGGCGTTCCGCCTGACTCCTCATGAAGTCGACCTTC
TCACCGGCCCTATCTGCCGACGTAAGTCATAACCTAGATCTGTACCTCGGGGGGAGGGTCACTGTAAA
GGGATAATTGGAGGGTGATTTCCCACACTTTCCTAAGGGTACTTTTTGCCTGGCTTCGCAATTGGGTC
CAATGGATGTCGATCTCTGGTTTAGCAGTTGTGAAAGTGGCAAGGCGGGAGGTTAGACCTCCATTTAA
CATATACAAGCAAGTTAACTGCACTAGATGTGTAGACACTACAGTTACAGGAGTAGCCGAATAAGTCT
CCGACGTCAAGCGAATAAGCGTCATACGCGATTATCGCCTAAGAGCACGTATTGGCGGTAAAAGGCTA
GCTAGACGCTTATGGGTAGATTTCAAGGCGTTCGTAGTGGTATAATAGGATACTCTTTCACCAGCCTG
AGGGCCGAACGCTATACTAGTGGTCTGTGATGTAGGACCGAGTATCTCTCTAGGGACCATCTACTTGA
GCAATGGTGCGCAGGGGGAGACATAGACCAGCCTTGGGTGGCAAGCACTGCAATAAGTCCTGTTTAGC
CTTGGAGTTCACATGCCGGCACTGAAGCCGACCTACCTGAGCGTGTGATTACCGTTAAAGCATCTGTC
TAGTTCTGTTTCCGGCGCTCTTGGTTCCAGGTTAGGGGAAGTGTATGACCCATGTGTTTTTATCGGCT
TAACCACGAGTGATCCCCGGTCGTTTCCCCATTGAATCCCTGGTGCATCCTACTCCCAGAATGATAGC
TGACTGACTGGACTGGCTTTTCAAGTAATCGAGGGGGTATCGCGGTCACGGCCGTTAACAGATCCCGT
CCTTAGTGTGGAATCCGCACCTGCTGACTAACGCTTCGCCGGCGTGTCTGCACAGCCGTATAGTGTTA
ATCATGACCCCAAGGAAGGATTAAACAAATATCTTGACG
>GIRAN27
GTGTCCGGTAGCCCGCGCTAGTTAGACACCCCGGCAGGGGGGATTGCTTTCGAGACGGGAGATCCCTT
CGCCGACCCTGGAGGGCCGACGCCGAGGCATTCGGGCCCCTGCAACGTCAACAGCGGCAAGAAAACGG
GATGAATGGGCGTAATGGGGGGGGTCTGCTGGGGACCCGACGCGGTTGCCGTTTGCGGGGCCCCCGAC
CCATACCGACCCACCTAGGCGTCCAGTTACGGCGCACGTCGGGAGCGTGGTTGCCGTCAGAGCTGTGT
TTCTCGATCAGTCCCCCGCAGTGCCGCAGTATCTTGCCGTGGGCTGCTTTAATCTTGAAAGTGGTTTA
TACATTGGGCGACGAGGTGTCGACTCTCATTGGGGGTAACCGACGGGCACATGCAGTCCCCTCCCCGG
GCAGGCAGAGGCGGGGCCCCCGCGCGCCGGCCCCGGCCCACAATCTTACCAGGGTCCTCAAAGGAGCC
TTTGCATGGTACCCTTCGTGAATGGTTGCTTAAGAGGTCCACAACGTGGTCCGGGCACGGTCGACTAA
ACACTCAAAACAGCGACGGCAAATATAGGTACAAGGTCCAGGCCCTCACGGCACTAATTGCGATGACC
CAACTCACGGGGGCAGTCCCCGGCGCGGCGACCTTGATTACGTCCGGGGACAAGTATGTCGCTCCCCC
CGGGAGGGGTGCAGCCACATGGGAGATTCAAAGTTTCTCGTGACGTCGTTGTGATCACTGCAGCCTAG
CCGAGACTCCCGTACTACGCGAAGGTTGGTTATGTTAACCACTACAACGTGAGGCGCCGTAAGGGCCA
GTGTTGTGCCCGGCTCTTCAATGCGCCTTAGCGGCCTGATACACCCACCCAAGGAGATACTGCTAATC
ATATGGGTGGACAGAACCTCGCAACGCAGACGCCTCCCCGGCATGATATGGTTTTTTTCCGCTATTAT
CCAGCACGCAGCGCTATCATTCAAGAGAACCCAGTGACGCGTAAATCGTAAGATCTACCTGCCGCAGG
TGGACCTACTGCAAATACGGATTATGACTCGTAAAGGGGTCATGCGTATTTTCATCACTAGGCACGTT
CGAGAATAAATTAGTAGGTGTCCCGAGCCTTGTGGCGTTCCGCCTGACTCCTCATGAAGTCGACCTTC
TCACCGGCCCTATCTGCCGACGTAAGTCATAACCTAGATCTGTACCTCGGGGGGAGGGTCACTGTAAA
GGGATAATTGGAGGGTGATTTCCCACACTTTCCTAAGGGTACTTTTTGCCTGGCTTCGCAATTGGGTC
CAATGGATGTCGATCTCTGGTTTAGCAGTTGTGAAAGTGGCAAGGCGGGAGGTTAGACCTCCATTTAA
CATATACAAGCAAGTTAACTGCACTAGATGTGTAGACACTACAGTTACAGGAGTAGCCGAATAAGTCT
CCGACGTCAAGCGAATAAGCGTCATACGCGATTATCGCCTAAGAGCACGTATTGGCGGTAAAAGGCTA
GCTAGACGCTTATGGGTAGATTTCAAGGCGTTCGTAGTGGTATAATAGGATACTCTTTCACCAGCCTG
AGGGCCGAACGCTATACTAGTGGTCTGTGATGTAGGACCGAGTATCTCTCTAGGGACCATCTACTTGA
GCAATGGTGCGCAGGGGGAGACATAGACCAGCCTTGGGTGGCAAGCACTGCAATAAGTCCTGTTTAGC
CTTGGAGTTCACATGCCGGCACTGAAGCCGACCTACCTGAGCGTGTGATTACCGTTAAAGCATCTGTC
TAGTTCTGTTTCCGGCGCTCTTGGTTCCAGGTTAGGGGAAGTGTATGACCCATGTGTTTTTATCGGCT
TAACCACGAGTGATCCCCGGTCGTTTCCCCATTGAATCCCTGGTGCATCCTACTCCCAGAATGATAGC
TGACTGACTGGACTGGCTTTTCAAGTAATCGAGGGGGTATCGCGGTCACGGCCGTTAACAGATCCCGT
CCTTAGTGTGGAATCCGCACCTGCTGACTAACGCTTCGCCGGCGTGTCTGCACAGCCGTATAGTGTTA
ATCATGACCCCAAGGAAGGATTAAACAAATATCTTGACG
>GIRAN29
GTGTCCGGTAGCCCGCGCTAGTTAGACACCCCGGCAGGGGGGATTGCTTTCGAGACGGGAGATCCCTT

CGCCGACCCTGGAGGGCCGACGCCGAGGCATTCGGGCCCCTGCAACGTCAACAGCGGCAAGAAAACGG
GATGAATGGGCGTAATGGGGGGGGTCTGCTGGGGACCCGACGCGGTTGCCGTTTGCGGGGCCCCCGAC
CCATACCGACCCACCTAGGCGTCCAGTTACGGCGCACGGCGGGAGCGTGGTTGCCGTCAGAGCTGTGT
TTCTCGATCAGTCCCCCGCAGTGCCGCAGTATCTTGCCGTGGGCTGCTTTAATCTTGAAAGTGGTTTA
TACATTGGGCGACGAGGTGTCGACTCTCATTGGGGGTAACCGACGGGCACATGCAGTCCCCTCCCCGG
GCAGGCAGAGGCGGGGCCCCCGCGCGCCGGCCCCGGCCCACAATCTTACCAGGGTCCTCAAAGGAGCC
TTTGCATGGTACCCTTCGTGAATGGTTGCTTAAGAGGTCCACAACGTGGTCCGGGCACGGTCGACTAA
ACACTCAAAACAGCGACGGCAAATATAGGTACAAGGTCCAGGCCCTCACGGCACTAATTGCGATGACC
CAACTCACGGGGGCAGTCCCCGGCGCGGCGACCTTGATTACGTCCGGGGACAAGTATGTCGCTCCCCC
CGGGAGGGGTGCAGCCACATGGGAGATTCAAAGTTTCTCGTGACGTCGTTGTGATCACTGCAGCCTAG
CCGAGACTCCCGTACTACGCGAAGGTTGGTTATGTTAACCACTACAACGTGAGGCGCCGTAAGGGCCA
GTGTTGTGCCCGGCTCTTCAATGCGCCTTAGCGGCCTGATACACCCACCCAAGGAGATACTGCTAATC
ATATGGGTGGACAGAACCTCGCAACGCAGACGCCTCCCCGGCATGATATGGTTTTTTTCCGCTATTAT
CCAGCACGCAGCGCTATCATTCAAGAGAACCCAGTGACGCGTAAATCGTAAGATCTACCTGCCGCAGG
TGGACCTACTGCAAATACGGATTATGACTCGTAAAGGGGTCATGCGTATTTTCATCACTAGGCACGTT
CGAGAATAAATTAGTAGGTGTCCCGAGCCTTGTGGCGTTCCGCCTGACTCCTCATGAAGTCGACCTTC
TCACCGGCCCTATCTGCCGACGTAAGTCATAACCTAGATCTGTACCTCGGGGGGAGGGTCACTGTAAA
GGGATAATTGGAGGGTGATTTCCCACACTTTCCTAAGGGTACTTTTTGCCTGGCTTCGCAATTGGGTC
CAATGGATGTCGATCTCTGGTTTAGCAGTTGTGAAAGTGGCAAGGCGGGAGGTTAGACCTCCATTTAA
CATATACAAGCAAGTTAACTGCACTAGATGTGTAGACACTACAGTTACAGGAGTAGCCGAATAAGTCT
CCGACGTCAAGCGAATAAGCGTCATACGCGATTATCGCCTAAGAGCACGTATTGGCGGTAAAAGGCTA
GCTAGACGCTTATGGGTAGATTTCAAGGCGTTCGTAGTGGTATAATAGGATACTCTTTCACCAGCCTG
AGGGCCGAACGCTATACTAGTGGTCTGTGATGTAGGACCGAGTATCTCTCTAGGGACCATCTACTTGA
GCAATGGTGCGCAGGGGGAGACATAGACCAGCCTTGGGTGGCAAGCACTGCAATAAGTCCTGTTTAGC
CTTGGAGTTCACATGCCGGCACTGAAGCCGACCTACCTGAGCGTGTGATTACCGTTAAAGCATCTGTC
TAGTTCTGTTTCCGGCGCTCTTGGTTCCAGGTTAGGGGAAGTGTATGACCCATGTGTTTTTATCGGCT
TAACCACGAGTGATCCCCGGTCGTTTCCCCATTGAATCCCTGGTGCATCCTACTCCCAGAATGATAGC
TGACTGACTGGACTGGCTTTTCAAGTAATCGAGGGGGTATCGCGGTCACGGCCGTTAACAGATCCCGT
CCTTAGTGTGGAATCCGCACCTGCTGACTAACGCTTCGCCGGCGTGTCTGCACAGCCGTATAGTGTTA
ATCATGACCCCAAGGAAGGATTAAACAAATATCTTGACG
>GIRAN30
GTGTCCGGTAGCCCGCGCTAGTTAGACACCCCGGCAGGGGGGATTGCTTTCGAGACGGGAGATCCCTT
CGCCGACCCTGGAGGGCCGACGCCGAGGCATTCGGGCCCCCGCAACGTCAACAGCGGCAAGAAAACGG
GATGAATGGGCGTAATGGGGGGGGTCTGCTGGGGACCCGACGCGGTTGCCGTTTGCGGGGCCCCCGAC
CCATACCGACCCACCTAGGCGTCCAGTTACGGCGCACGGCGGGAGCGTGGTTGCCGTCAGAGCTGTGT
TTCTCGATCAGTCCCCCGCAGTGCCGCAGTATCTTGCCGTGGGCTGCTTTAATCTTGAAAGTGGTTTA
TACATTGGGCGACGAGGTGTCGACTCTCATTGGGGGTAACCGACGGGCACATGCAGTCCCCTCCCCGG
GCAGGCAGAGGCGGGGCCCCCGCGCGCCGGCCCCGGCCCACAATCTTACCAGGGTCCTCAAAGGAGCC
TTTGCATGGTACCCTTCGTGAATGGTTGCTTAAGAGGTCCACAACGTGGTCCGGGCACGGTCGACTAA
ACACTCAAAACAGCGACGGCAAATATAGGTACAAGGTCCAGGCCCTCACGGCACTAATTGCGATGACC
CAACTCACGGGGGCAGTCCCCGGCGCGGCGACCTTGATTACGTCCGGGGACAAGTATGTCGCTCCCCC
CGGGAGGGGTGCAGCCACATGGGAGATTCAAAGTTTCTCGTGACGTCGTTGTGATCACTGCAGCCTAG
CCGAGACTCCCGTACTACGCGAAGGTTGGTTATGTTAACCACTACAACGTGAGGCGCCGTAAGGGCCA
GTGTTGTGCCCGGCTCTTCAATGCGCCTTAGCGGCCTGATACACCCACCCAAGGAGATACTGCTAATC
ATATGGGTGGACAGAACCTCGCAACGCAGACGCCTCCCCGGCATGATATGGTTTTTTTCCGCTATTAT
CCAGCACGCAGCGCTATCATTCAAGAGAACCCAGTGACGCGTAAATCGTAAGATCTACCTGCCGCAGG
TGGACCTACTGCAAATACGGATTATGACTCGTAAAGGGGTCATGCGTATTTTCATCACTAGGCACGTT
CGAGAATAAATTAGTAGGTGTCCCGAGCCTTGTGGCGTTCCGCCTGACTCCTCATGAAGTCGACCTTC
TCACCGGCCCTATCTGCCGACGTAAGTCATAACCTAGATCTGTACCTCGGGGGGAGGGTCACTGTAAA
GGGATAATTGGAGGGTGATTTCCCACACTTTCCTAAGGGTACTTTTTGCCTGGCTTCGCAATTGGGTC
CAATGGATGTCGATCTCTGGTTTAGCAGTTGTGAAAGTGGCAAGGCGGGAGGTTAGACCTCCATTTAA
CATATACAAGCAAGTTAACTGCACTAGATGTGTAGACACTACAGTTACAGGAGTAGCCGAATAAGTCT
CCGACGTCAAGCGAATAAGCGTCATACGCGATTATCGCCTAAGAGCACGTATTGGCGGTAAAAGGCTA
GCTAGACGCTTATGGGTAGATTTCAAGGCGTTCGTAGTGGTATAATAGGATACTCTTTCACCAGCCTG

AGGGCCGAACGCTATACTAGTGGTCTGTGATGTAGGACCGAGTATCTCTCTAGGGACCATCTACTTGA
GCAATGGTGCGCAGGGGGAGACATAGACCAGCCTTGGGTGGCAAGCACTGCAATAAGTCCTGTTTAGC
CTTGGAGTTCACATGCCGGCACTGAAGCCGACCTACCTGAGCGTGTGATTACCGTTAAAGCATCTGTC
TAGTTCTGTTTCCGGCGCTCTTGGTTCCAGGTTAGGGGAAGTGTATGACCCATGTGTTTTTATCGGCT
TAACCACGAGTGATCCCCGGTCGTTTCCCCATTGAATCCCTGGTGCATCCTACTCCCAGAATGATAGC
TGACTGACTGGACTGGCTTTTCAAGTAATCGAGGGGGTATCGCGGTCACGGCCGTTAACAGATCCCGT
CCTTAGTGTGGAATCCGCACCTGCTGACTAACGCTTCGCCGGCGTGTCTGCACAGCCGTATAGTGTTA
ATCATGACCCCAAGGAAGGATTAAACAAATATCTTGACG
>GIRAN31
GTGTCCGGTAGCCCGCGCTAGTTAGACACCCCGGCAGGGGGGATTGCTTTCGGGACGGGAGATCCCTT
CGCCGACCCTGGAGGGCCGACGCCGAGGCATTCGGGCCCCCGCAACGTCAACAGCGGCAAGAAAACGG
GATGAATGGGCGTAATGGGGGGGGTCTGCTGGGGACCCGACGCGGTTGCCGTTTGCGGGGCCCCCGAC
CCATACCGACCCACCTAGGCGTCCAGTTACGGCGCACGTCGGGAGCGTGGTTGCCGTCAGAGCTGTGT
TTCTCGATCAGTCCCCCGCAGTGCCGCAGTATCTTGCCGTGGGCTGCTTTAATCTTGAAAGTGGTTNA
TACATTGGGCGACGAGGTGTCGACTCTCATTGGGGGTAACCGACGGGCACATGCAGTCCCCTCCCCGG
GCAGGCAGAGGCGGGGCCCCCGCGCGCCGGCCCCGGCCCACAATCTTACCAGGGTCCTCAAAGGAGCC
TTTGCATGGTACCCTTCGTGAATGGTTGCTTAAGAGGTCCACAACGTGGTCCGGGCACGGTCGACTAA
ACACTCAAAACAGCGACGGCAAATATAGGTACAAGGTCCAGGCCCTCACGGCACTAATTGCGATGACC
CAACTCACGGGGGCAGTCCCCGGCGCGGCGACCTTGATTACGTCCGGGGACAAGTATGTCGCTCCCCC
CGGGAGGGGTGCAGCCACATGGGAGATTCAAAGTTTCTCGTGACGTCGTTGTGATCACTGCAGCCTAG
CCGAGACTCCCGTACTACGCGAAGGTTGGTTATGTTAACCACTACAACGTGAGGCGCCGTAAGGGCCA
GTGTTGTGCCCGGCTCTTCAATGCGCCTTAGCGGCCTGATACACCCACCCAAGGAGATACTGCTAATC
ATATGGGTGGACAGAACCTCGCAACGCAGACGCCTCCCCGGCATGATATGGTTTTTTTCCGCTATTAT
CCAGCACGCAGCGCTATCATTCAAGAGAACCCAGTGACGCGTAAATCGTAAGATCTACCTGCCGCAGG
TGGACCTACTGCAAATACGGATTATGACTCGTAAAGGGGTCATGCGTATTTTCATCACTAGGCACGTT
CGAGAATAAATTAGTAGGTGTCCCGAGCCTTGTGGCGTTCCGCCTGACTCCTCATGAAGTCGACCTTC
TCACCGGCCCTATCTGCCGACGTAAGTCATAACCTAGATCTGTACCTCGGGGGGAGGGTCACTGTAAA
GGGATAATTGGAGGGTGATTTCCCACACTTTCCTAAGGGTACTTTTTGCCTGGCTTCGCAATTGGGTC
CAATGGATGTCGATCTCTGGTTTAGCAGTTGTGAAAGTGGCAAGGCGGGAGGTTAGACCTCCATTTAA
CATATACAAGCAAGTTAACTGCACTAGATGTGTAGACACTACAGTTACAGGAGTAGCCGAATAAGTCT
CCGACGTCAAGCGAATAAGCGTCATACGCGATTATCGCCTAAGAGCACGTATTGGCGGTAAAAGGCTA
GCTAGACGCTTATGGGTAGATTTCAAGGCGTTCGTAGTGGTATAATAGGATACTCTTTCACCAGCCTG
AGGGCCGAACGCTATACTAGTGGTCTGTGATGTAGGACCGAGTATCTCTCTAGGGACCATCTACTTGA
GCAATGGTGCGCAGGGGGAGACATAGACCAGCCTTGGGTGGCAAGCACTGCAATAAGTCCTGTTTAGC
CTTGGAGTTCACATGCCGGCACTGAAGCCGACCTACCTGAGCGTGTGATTACCGTTAAAGCATCTGTC
TAGTTCTGTTTCCGGCGCTCTTGGTTCCAGGTTAGGGGAAGTGTATGACCCATGTGTTTTTATCGGCT
TAACCACGAGTGATCCCCGGTCGTTTCCCCATTGAATCCCTGGTGCATCCTACTCCCAGAATGATAGC
TGACTGACTGGACTGGCTTTTCAAGTAATCGAGGGGGTATCGCGGTCACGGCCGTTAACAGATCCCGT
CCTTAGTGTGGAATCCGCACCTGCTGACTAACGCTTCGCCGGCGTGTCTGCACAGCCGTATAGTGTTA
ATCATGACCCCAAGGAAGGATTAAACAAATATCTTGACG
>HREN03
GTGTCCGGTAGCCCGCGCTAGTTAGACACCCCGGCAGGGGGGATTGCTTTCGAGACGGGAGATCCCTT
CGCCGACCCTGGAGGGCCGACGCCGAGGCATTCGGGCCCCCGCAACGTCAACAGCGGCAAGAAAACGG
GATGAATGGGCGTAATGGGGGGGGTCTGCTGGGGACCCGACGCGGTTGCCGTTTGCGGGGCCCCCGAC
CCATACCGACCCACCTAGGCGTCCAGTTACGGCGCACGTCGGGAGCGTGGTTGCCGTCAGAGCTGTGT
TTCTCGATCAGTCCCCCGCAGTGCCGCAGTATCTTGCCGTGGGCTGCTTTAATCTTGAAAGTGGTTTA
TACATTGGGCGACGAGGTGTCGACTCTCATTGGGGGTAACCGACGGGCACATGCAGTCCCCTCCCCGG
GCAGGCAGAGGCGGGGCCCCCGCGCGCCGGCCCCGGCCCACAATCTTACCAGGGTCCTCAAAGGAGCC
TTTGCATGGTACCCTTCGTGAATGGTTGCTTAAGAGGTCCACAACGTGGTCCGGGCACGGTCGACTAA
ACACTCAAAACAGCGACGGCAAATATAGGTACAAGGTCCAGGCCCTCACGGCACTAATTGCGATGACC
CAACTCACGGGGGCAGTCCCCGGCGCGGCGACCTTGATTACGTCCGGGGACAAGTATGTCGCTCCCCC
CGGGAGGGGTGCAGCCACATGGGAGATTCAAAGTTTCTCGTGACGTCGTTGTGATCACTGCAGCCTAG
CCGAGACTCCCGTACTACGCGAAGGTTGGTTATGTTAACCACTACAACGTGAGGCGCCGTAAGGGCCA
GTGTTGTGCCCGGCTCTTCAATGCGCCTTAGCGGCCTGATACACCCACCCAAGGAGATACTGCTAATC

ATATGGGTGGACAGAACCTCGCAACGCAGACGCCTCCCCGGCATGATATGGTTTTTTTCCGCTATTAT
CCAGCACGCAGCGCTATCATTCAAGAGAACCCAGTGACGCGTAAATCGTAAGATCTACCTGCCGCAGG
TGGACCTACTGCAAATACGGATTATGACTCGTAAAGGGGTCATGCGTATTTTCATCACTAGGCACGTT
CGAGAATAAATTAGTAGGTGTCCCGAGCCTTGTGGCGTTCCGCCTGACTCCTCATGAAGTCGACCTTC
TCACCGGCCCTATCTGCCGACGTAAGTCATAACCTAGATCTGTACCTCGGGGGGAGGGTCACTGTAAA
GGGATAATTGGAGGGTGATTTCCCACACTTTCCTAAGGGTACTTTTTGCCTGGCTTCGCAATTGGGTC
CAATGGATGTCGATCTCTGGTTTAGCAGTTGTGAAAGTGGCAAGGCGGGAGGTTAGACCTCCATTTAA
CATATACAAGCAAGTTAACTGCACTAGATGTGTAGACACTACAGTTACAGGAGTAGCCGAATAAGTCT
CCGACGTCAAGCGAATAAGCGTCATACGCGATTATCGCCTAAGAGCACGTATTGGCGGTAAAAGGCTA
GCTAGACGCTTATGGGTAGATTTCAAGGCGTTCGTAGTGGTATAATAGGATACTCTTTCACCAGCCTG
AGGGCCGAACGCTATACTAGTGGTCTGTGATGTAGGACCGAGTATCTCTCTAGGGACCATCTACTTGA
GCAATGGTGCGCAGGGGGAGACATAGACCAGCCTTGGGTGGCAAGCACTGCAATAAGTCCTGTTTAGC
CTTGGAGTTCACATGCCGGCACTGAAGCCGACCTACCTGAGCGTGTGATTACCGTTAAAGCATCTGTC
TAGTTCTGTTTCCGGCGCTCTTGGTTCCAGGTTAGGGGAAGTGTATGACCCATGTGTTTTTATCGGCT
TAACCACGAGTGATCCCCGGTCGTTTCCCCATTGAATCCCTGGTGCATCCTACTCCCAGAATGATAGC
TGACTGACTGGACTGGCTTTTCAAGTAATCGAGGGGGTATCGCGGTCACGGCCGTTAACAGATCCCGT
CCTTAGTGTGGAATCCGCACCTGCTGACTAACGCTTCGCCGGCGTGTCTGCACAGCCGTATAGTGTTA
ATCATGACCCCAAGGAAGGATTAAACAAATATCTTGACG
>HREN05
GTGTCCGGTAGCCCGCGCTAGTTAGACACCCCGGCAGGGGGGATTGCTTTCGAGACGGGAGATCCCTT
CGCCGACCCTGGAGGGCCGACGCCGAGGCATTCGGGCCCCTGCAACGTCAACAGCGGCAAGAAAACGG
GATGAATGGGCGTAATGGGGGGGGTCTGCTGGGGACCCGACGCGGTTGCCGTTTGCGGGGCCCCCGAC
CCATACCGACCCACCTAGGCGTCCAGTTACGGCGCACGTCGGGAGCGTGGTTGCCGTCAGAGCTGTGT
TTCTCGATCAGTCCCCCGCAGTGCCGCAGTATCTTGCCGTGGGCTGCTTTAATCTTGAAAGTGGTTTA
TACATTGGGCGACGAGGTGTCGACTCTCATTGGGGGTAACCGACGGGCACATGCAGTCCCCTCCCCGG
GCAGGCAGAGGCGGGGCCCCCGCGCGCCGGCCCCGGCCCACAATCTTACCAGGGTCCTCAAAGGAGCC
TTTGCATGGTACCCTTCGTGAATGGTTGCTTAAGAGGTCCACAACGTGGTCCGGGCACGGTCGACTAA
ACACTCAAAACAGCGACGGCAAATATAGGTACAAGGTCCAGGCCCTCACGGCACTAATTGCGATGACC
CAACTCACGGGGGCAGTCCCCGGCGCGGCGACCTTGATTACGTCCGGGGACAAGTATGTCGCTCCCCC
CGGGAGGGGTGCAGCCACATGGGAGATTCAAAGTTTCTCGTGACGTCGTTGTGATCACTGCAGCCTAG
CCGAGACTCCCGTACTACGCGAAGGTTGGTTATGTTAACCACTACAACGTGAGGCGCCGTAAGGGCCA
GTGTTGTGCCCGGCTCTTCAATGCGCCTTAGCGGCCTGATACACCCACCCAAGGAGATACTGCTAATC
ATATGGGTGGACAGAACCTCGCAACGCAGACGCCTCCCCGGCATGATATGGTTTTTTTCCGCTATTAT
CCAGCACGCAGCGCTATCATTCAAGAGAACCCAGTGACGCGTAAATCGTAAGATCTACCTGCCGCAGG
TGGACCTACTGCAAATACGGATTATGACTCGTAAAGGGGTCATGCGTATTTTCATCACTAGGCACGTT
CGAGAATAAATTAGTAGGTGTCCCGAGCCTTGTGGCGTTCCGCCTGACTCCTCATGAAGTCGACCTTC
TCACCGGCCCTATCTGCCGACGTAAGTCATAACCTAGATCTGTACCTCGGGGGGAGGGTCACTGTAAA
GGGATAATTGGAGGGTGATTTCCCACACTTTCCTAAGGGTACTTTTTGCCTGGCTTCGCAATTGGGTC
CAATGGATGTCGATCTCTGGTTTAGCAGTTGTGAAAGTGGCAAGGCGGGAGGTTAGACCTCCATTTAA
CATATACAAGCAAGTTAACTGCACTAGATGTGTAGACACTACAGTTACAGGAGTAGCCGAATAAGTCT
CCGACGTCAAGCGAATAAGCGTCATACGCGATTATCGCCTAAGAGCACGTATTGGCGGTAAAAGGCTA
GCTAGACGCTTATGGGTAGATTTCAAGGCGTTCGTAGTGGTATAATAGGATACTCTTTCACCAGCCTG
AGGGCCGAACGCTATACTAGTGGTCTGTGATGTAGGACCGAGTATCTCTCTAGGGACCATCTACTTGA
GCAATGGTGCGCAGGGGGAGACATAGACCAGCCTTGGGTGGCAAGCACTGCAATAAGTCCTGTTTAGC
CTTGGAGTTCACATGCCGGCACTGAAGCCGACCTACCTGAGCGTGTGATTACCGTTAAAGCATCTGTC
TAGTTCTGTTTCCGGCGCTCTTGGTTCCAGGTTAGGGGAAGTGTATGACCCATGTGTTTTTATCGGCT
TAACCACGAGTGATCCCCGGTCGTTTCCCCATTGAATCCCTGGTGCATCCTACTCCCAGAATGATAGC
TGACTGACTGGACTGGCTTTTCAAGTAATCGAGGGGGTATCGCGGTCACGGCCGTTAACAGATCCCGT
CCTTAGTGTGGAATCCGCACCTGCTGACTAACGCTTCGCCGGCGTGTCTGCACAGCCGTATAGTGTTA
ATCATGACCCCAAGGAAGGATTAAACAAATATCTTGACG
>HREN07
GTGTCCGGTAGCCCGCGCTAGTTAGACACCCCGGCAGGGGGGATTGCTTTCGAGACGGGAGATCCCTT
CGCCGACCCTGGAGGGCCGACGCCGAGGCATTCGGGCCCCTGCAACGTCAACAGCGGCAAGAAAACGG
GATGAATGGGCGTAATGGGGGGGGTCTGCTGGGGACCCGACGCGGTTGCCGTTTGCGGGGCCCCCGAC

CCATACCGACCCACCTAGGCGTCCAGTTACGGCGCACGTCGGGAGCGTGGTTGCCGTCAGAGCTGTGT
TTCTCGATCAGTCCCCCGCAGTGCCGCAGTATCTTGCCGTGGGCTGCTTTAATCTTGAAAGTGGTTCA
TACATTGGGCGACGAGGTGTCGACTCTCATTGGGGGTAACCGACGGGCACATGCAGTCCCCTCCCCGG
GCAGGCAGAGGCGGGGCCCCCGCGCGCCGGCCCCGGCCCACAATCTTACCAGGGTCCTCAAAGGAGCC
TTTGCATGGTACCCTTCGTGAATGGTTGCTTAAGAGGTCCACAACGTGGTCCGGGCACGGTCGACTAA
ACACTCAAAACAGCGACGGCAAATATAGGTACAAGGTCCAGGCCCTCACGGCACTAATTGCGATGACC
CAACTCACGGGGGCAGTCCCCGGCGCGGCGACCTTGATTACGTCCGGGGACAAGTATGTCGCTCCCCC
CGGGAGGGGTGCAGCCACATGGGAGATTCAAAGTTTCTCGTGACGTCGTTGTGATCACTGCAGCCTAG
CCGAGACTCCCGTACTACGCGAAGGTTGGTTATGTTAACCACTACAACGTGAGGCGCCGTAAGGGCCA
GTGTTGTGCCCGGCTCTTCAATGCGCCTTAGCGGCCTGATACACCCACCCAAGGAGATACTGCTAATC
ATATGGGTGGACAGAACCTCGCAACGCAGACGCCTCCCCGGCATGATATGGTTTTTTTCCGCTATTAT
CCAGCACGCAGCGCTATCATTCAAGAGAACCCAGTGACGCGTAAATCGTAAGATCTACCTGCCGCAGG
TGGACCTACTGCAAATACGGATTATGACTCGTAAAGGGGTCATGCGTATTTTCATCACTAGGCACGTT
CGAGAATAAATTAGTAGGTGTCCCGAGCCTTGTGGCGTTCCGCCTGACTCCTCATGAAGTCGACCTTC
TCACCGGCCCTATCTGCCGACGTAAGTCATAACCTAGATCTGTACCTCGGGGGGAGGGTCACTGTAAA
GGGATAATTGGAGGGTGATTTCCCACACTTTCCTAAGGGTACTTTTTGCCTGGCTTCGCAATTGGGTC
CAATGGATGTCGATCTCTGGTTTAGCAGTTGTGAAAGTGGCAAGGCGGGAGGTTAGACCTCCATTTAA
CATATACAAGCAAGTTAACTGCACTAGATGTGTAGACACTACAGTTACAGGAGTAGCCGAATAAGTCT
CCGACGTCAAGCGAATAAGCGTCATACGCGATTATCGCCTAAGAGCACGTATTGGCGGTAAAAGGCTA
GCTAGACGCTTATGGGTAGATTTCAAGGCGTTCGTAGTGGTATAATAGGATACTCTTTCACCAGCCTG
AGGGCCGAACGCTATACTAGTGGTCTGTGATGTAGGACCGAGTATCTCTCTAGGGACCATCTACTTGA
GCAATGGTGCGCAGGGGGAGACATAGACCAGCCTTGGGTGGCAAGCACTGCAATAAGTCCTGTTTAGC
CTTGGAGTTCACATGCCGGCACTGAAGCCGACCTACCTGAGCGTGTGATTACCGTTAAAGCATCTGTC
TAGTTCTGTTTCCGGCGCTCTTGGTTCCAGGTTAGGGGAAGTGTATGACCCATGTGTTTTTATCGGCT
TAACCACGAGTGATCCCCGGTCGTTTCCCCATTGAATCCCTGGTGCATCCTACTCCCAGAATGATAGC
TGACTGACTGGACTGGCTTTTCAAGTAATCGAGGGGGTATCGCGGTCACGGCCGTTAACAGATCCCGT
CCTTAGTGTGGAATCCGCACCTGCTGACTAACGCTTCGCCGGCGTGTCTGCACAGCCGTATAGTGTTA
ATCATGACCCCAAGGAAGGATTAAACAAATATCTTGACG
>HREN08
GTGTCCGGTAGCCCGCGCTAGTTAGACACCCCGGCAGGGGGGATTGCTTTCGGGACGGGAGATCCCTT
CGCCGACCCTGGAGGGCCGACGCCGAGGCATTCGGGCCCCTGCAACGTCAACAGCGGCAAGAAAACGG
GATGAATGGGCGTAATGGGGGGGGTCTGCTGGGGACCCGACGCGGTTGCCGTTTGCGGGGCCCCCGAC
CCATACCGACCCACCTAGGCGTCCAGTTACGGCGCACGGCGGGAGCGTGGTTGCCGTCAGAGCTGTGT
TTCTCGATCAGTCCCCCGCAGTGCCGCAGTATCTTGCCGTGGGCTGCTTTAATCTTGAAAGTGGTTCA
TACATTGGGCGACGAGGTGTCGACTCTCATTGGGGGTAACCGACGGGCACATGCAGTCCCCTCCCCGG
GCAGGCAGAGGCGGGGCCCCCGCGCGCCGGCCCCGGCCCACAATCTTACCAGGGTCCTCAAAGGAGCC
TTTGCATGGTACCCTTCGTGAATGGTTGCTTAAGAGGTCCACAACGTGGTCCGGGCACGGTCGACTAA
ACACTCAAAACAGCGACGGCAAATATAGGTACAAGGTCCAGGCCCTCACGGCACTAATTGCGATGACC
CAACTCACGGGGGCAGTCCCCGGCGCGGCGACCTTGATTACGTCCGGGGACAAGTATGTCGCTCCCCC
CGGGAGGGGTGCAGCCACATGGGAGATTCAAAGTTTCTCGTGACGTCGTTGTGATCACTGCAGCCTAG
CCGAGACTCCCGTACTACGCGAAGGTTGGTTATGTTAACCACTACAACGTGAGGCGCCGTAAGGGCCA
GTGTTGTGCCCGGCTCTTCAATGCGCCTTAGCGGCCTGATACACCCACCCAAGGAGATACTGCTAATC
ATATGGGTGGACAGAACCTCGCAACGCAGACGCCTCCCCGGCATGATATGGTTTTTTTCCGCTATTAT
CCAGCACGCAGCGCTATCATTCAAGAGAACCCAGTGACGCGTAAATCGTAAGATCTACCTGCCGCAGG
TGGACCTACTGCAAATACGGATTATGACTCGTAAAGGGGTCATGCGTATTTTCATCACTAGGCACGTT
CGAGAATAAATTAGTAGGTGTCCCGAGCCTTGTGGCGTTCCGCCTGACTCCTCATGAAGTCGACCTTC
TCACCGGCCCTATCTGCCGACGTAAGTCATAACCTAGATCTGTACCTCGGGGGGAGGGTCACTGTAAA
GGGATAATTGGAGGGTGATTTCCCACACTTTCCTAAGGGTACTTTTTGCCTGGCTTCGCAATTGGGTC
CAATGGATGTCGATCTCTGGTTTAGCAGTTGTGAAAGTGGCAAGGCGGGAGGTTAGACCTCCATTTAA
CATATACAAGCAAGTTAACTGCACTAGATGTGTAGACACTACAGTTACAGGAGTAGCCGAATAAGTCT
CCGACGTCAAGCGAATAAGCGTCATACGCGATTATCGCCTAAGAGCACGTATTGGCGGTAAAAGGCTA
GCTAGACGCTTATGGGTAGATTTCAAGGCGTTCGTAGTGGTATAATAGGATACTCTTTCACCAGCCTG
AGGGCCGAACGCTATACTAGTGGTCTGTGATGTAGGACCGAGTATCTCTCTAGGGACCATCTACTTGA
GCAATGGTGCGCAGGGGGAGACATAGACCAGCCTTGGGTGGCAAGCACTGCAATAAGTCCTGTTTAGC

CTTGGAGTTCACATGCCGGCACTGAAGCCGACCTACCTGAGCGTGTGATTACCGTTAAAGCATCTGTC
TAGTTCTGTTTCCGGCGCTCTTGGTTCCAGGTTAGGGGAAGTGTATGACCCATGTGTTTTTATCGGCT
TAACCACGAGTGATCCCCGGTCGTTTCCCCATTGAATCCCTGGTGCATCCTACTCCCAGAATGATAGC
TGACTGACTGGACTGGCTTTTCAAGTAATCGAGGGGGTATCGCGGTCACGGCCGTTAACAGATCCCGT
CCTTAGTGTGGAATCCGCACCTGCTGACTAACGCTTCGCCGGCGTGTCTGCACAGCCGTATAGTGTTA
ATCATGACCCCAAGGAAGGATTAAACAAATATCTTGACG
>HREN09
GTGTCCGGTAGCCCGCGCTAGTTAGACACCCCGGCAGGGGGGATTGCTTTCGGGACGGGAGATCCCTT
CGCCGACCCTGGAGGGCCGACGCCGAGGCATTCGGGCCCCTGCAACGTCAACAGCGGCAAGAAAACGG
GATGAATGGGCGTAATGGGGGGGGTCTGCTGGGGACCCGACGCGGTTGCCGTTTGCGGGGCCCCCGAC
CCATACCGACCCACCTAGGCGTCCAGTTACGGCGCACGGCGGGAGCGTGGTTGCCGTCAGAGCTGTGT
TTCTCGATCAGTCCCCCGCAGTGCCGCAGTATCTTGCCGTGGGCTGCTTTAATCTTGAAAGTGGTTCA
TACATTGGGCGACGAGGTGTCGACTCTCATTGGGGGTAACCGACGGGCACATGCAGTCCCCTCCCCGG
GCAGGCAGAGGCGGGGCCCCCGCGCGCCGGCCCCGGCCCACAATCTTACCAGGGTCCTCAAAGGAGCC
TTTGCATGGTACCCTTCGTGAATGGTTGCTTAAGAGGTCCACAACGTGGTCCGGGCACGGTCGACTAA
ACACTCAAAACAGCGACGGCAAATATAGGTACAAGGTCCAGGCCCTCACGGCACTAATTGCGATGACC
CAACTCACGGGGGCAGTCCCCGGCGCGGCGACCTTGATTACGTCCGGGGACAAGTATGTCGCTCCCCC
CGGGAGGGGTGCAGCCACATGGGAGATTCAAAGTTTCTCGTGACGTCGTTGTGATCACTGCAGCCTAG
CCGAGACTCCCGTACTACGCGAAGGTTGGTTATGTTAACCACTACAACGTGAGGCGCCGTAAGGGCCA
GTGTTGTGCCCGGCTCTTCAATGCGCCTTAGCGGCCTGATACACCCACCCAAGGAGATACTGCTAATC
ATATGGGTGGACAGAACCTCGCAACGCAGACGCCTCCCCGGCATGATATGGTTTTTTTCCGCTATTAT
CCAGCACGCAGCGCTATCATTCAAGAGAACCCAGTGACGCGTAAATCGTAAGATCTACCTGCCGCAGG
TGGACCTACTGCAAATACGGATTATGACTCGTAAAGGGGTCATGCGTATTTTCATCACTAGGCACGTT
CGAGAATAAATTAGTAGGTGTCCCGAGCCTTGTGGCGTTCCGCCTGACTCCTCATGAAGTCGACCTTC
TCACCGGCCCTATCTGCCGACGTAAGTCATAACCTAGATCTGTACCTCGGGGGGAGGGTCACTGTAAA
GGGATAATTGGAGGGTGATTTCCCACACTTTCCTAAGGGTACTTTTTGCCTGGCTTCGCAATTGGGTC
CAATGGATGTCGATCTCTGGTTTAGCAGTTGTGAAAGTGGCAAGGCGGGAGGTTAGACCTCCATTTAA
CATATACAAGCAAGTTAACTGCACTAGATGTGTAGACACTACAGTTACAGGAGTAGCNGAATAAGTCT
CCGACGTCAAGCGAATAAGCGTCATACGCGATTATCGCCTAAGAGCACGTATTGGCGGTAAAAGGCTA
GCTAGACGCTTATGGGTAGATTTCAAGGCGTTCGTAGTGGTATAATAGGATACTCTTTCACCAGCCTG
AGGGCCGAACGCTATACTAGTGGTCTGTGATGTAGGACCGAGTATCTCTCTAGGGACCATCTACTTGA
GCAATGGTGCGCAGGGGGAGACATAGACCAGCCTTGGGTGGCAAGCACTGCAATAAGTCCTGTTTAGC
CTTGGAGTTCACATGCCGGCACTGAAGCCGACCTACCTGAGCGTGTGATTACCGTTAAAGCATCTGTC
TAGTTCTGTTTCCGGCGCTCTTGGTTCCAGGTTAGGGGAAGTGTATGACCCATGTGTTTTTATCGGCT
TAACCACGAGTGATCCCCGGTCGTTTCCCCATTGAATCCCTGGTGCATCCTACTCCCAGAATGATAGC
TGACTGACTGGACTGGCTTTTCAAGTAATCGAGGGGGTATCGCGGTCACGGCCGTTAACAGATCCCGT
CCTTAGTGTGGAATCCGCACCTGCTGACTAACGCTTCGCCGGCGTGTCTGCACAGCCGTATAGTGTTA
ATCATGACCCCAAGGAAGGATTAAACAAATATCTTGACG
>HREN11
GTGTCCGGTAGCCCGCGCTAGTTAGACACCCCGGCAGGGGGGATTGCTTTCGAGACGGGAGATCCCTT
CGCCGACCCTGGAGGGCCGACGCCGAGGCATTCGGGCCCCCGCAACGTCAACAGCGGCAAGAAAACGG
GATGAATGGGCGTAATGGGGGGGGTCTGCTGGGGACCCGACGCGGTTGCCGTTTGCGGGGCCCCCGAC
CCATACCGACCCACCTAGGCGTCCAGTTACGGCGCACGTCGGGAGCGTGGTTGCCGTCAGAGCTGTGT
TTCTCGATCAGTCCCCCGCAGTGCCGCAGTATCTTGCCGTGGGCTGCTTTAATCTTGAAAGTGGTTNA
TACATTGGGCGACGAGGTGTCGACTCTCATTGGGGGTAACCGACGGGCACATGCAGTCCCCTCCCCGG
GCAGGCAGAGGCGGGGCCCCCGCGCGCCGGCCCCGGCCCACAATCTTACCAGGGTCCTCAAAGGAGCC
TTTGCATGGTACCCTTCGTGAATGGTTGCTTAAGAGGTCCACAACGTGGTCCGGGCACGGTCGACTAA
ACACTCAAAACAGCGACGGCAAATATAGGTACAAGGTCCAGGCCCTCACGGCACTAATTGCGATGACC
CAACTCACGGGGGCAGTCCCCGGCGCGGCGACCTTGATTACGTCCGGGGACAAGTATGTCGCTCCCCC
CGGGAGGGGTGCAGCCACATGGGAGATTCAAAGTTTCTCGTGACGTCGTTGTGATCACTGCAGCCTAG
CCGAGACTCCCGTACTACGCGAAGGTTGGTTATGTTAACCACTACAACGTGAGGCGCCGTAAGGGCCA
GTGTTGTGCCCGGCTCTTCAATGCGCCTTAGCGGCCTGATACACCCACCCAAGGAGATACTGCTAATC
ATATGGGTGGACAGAACCTCGCAACGCAGACGCCTCCCCGGCATGATATGGTTTTTTTCCGCTATTAT
CCAGCACGCAGCGCTATCATTCAAGAGAACCCAGTGACGCGTAAATCGTAAGATCTACCTGCCGCAGG

TGGACCTACTGCAAATACGGATTATGACTCGTAAAGGGGTCATGCGTATTTTCATCACTAGGCACGTT
CGAGAATAAATTAGTAGGTGTCCCGAGCCTTGTGGCGTTCCGCCTGACTCCTCATGAAGTCGACCTTC
TCACCGGCCCTATCTGCCGACGTAAGTCATAACCTAGATCTGTACCTCGGGGGGAGGGTCACTGTAAA
GGGATAATTGGAGGGTGATTTCCCACACTTTCCTAAGGGTACTTTTTGCCTGGCTTCGCAATTGGGTC
CAATGGATGTCGATCTCTGGTTTAGCAGTTGTGAAAGTGGCAAGGCGGGAGGTTAGACCTCCATTTAA
CATATACAAGCAAGTTAACTGCACTAGATGTGTAGACACTACAGTTACAGGAGTAGCCGAATAAGTCT
CCGACGTCAAGCGAATAAGCGTCATACGCGATTATCGCCTAAGAGCACGTATTGGCGGTAAAAGGCTA
GCTAGACGCTTATGGGTAGATTTCAAGGCGTTCGTAGTGGTATAATAGGATACTCTTTCACCAGCCTG
AGGGCCGAACGCTATACTAGTGGTCTGTGATGTAGGACCGAGTATCTCTCTAGGGACCATCTACTTGA
GCAATGGTGCGCAGGGGGAGACATAGACCAGCCTTGGGTGGCAAGCACTGCAATAAGTCCTGTTTAGC
CTTGGAGTTCACATGCCGGCACTGAAGCCGACCTACCTGAGCGTGTGATTACCGTTAAAGCATCTGTC
TAGTTCTGTTTCCGGCGCTCTTGGTTCCAGGTTAGGGGAAGTGTATGACCCATGTGTTTTTATCGGCT
TAACCACGAGTGATCCCCGGTCGTTTCCCCATTGAATCCCTGGTGCATCCTACTCCCAGAATGATAGC
TGACTGACTGGACTGGCTTTTCAAGTAATCGAGGGGGTATCGCGGTCACGGCCGTTAACAGATCCCGT
CCTTAGTGTGGAATCCGCACCTGCTGACTAACGCTTCGCCGGCGTGTCTGCACAGCCGTATAGTGTTA
ATCATGACCCCAAGGAAGGATTAAACAAATATCTTGACG
>HREN12
GTGTCCGGTAGCCCGCGCTAGTTAGACACCCCGGCAGGGGGGATTGCTTTCGAGACGGGAGATCCCTT
CGCCGACCCTGGAGGGCCGACGCCGAGGCATTCGGGCCCCTGCAACGTCAACAGCGGCAAGAAAACGG
GATGAATGGGCGTAATGGGGGGGGTCTGCTGGGGACCCGACGCGGTTGCCGTTTGCGGGGCCCCCGAC
CCATACCGACCCACCTAGGCGTCCAGTTACGGCGCACGTCGGGAGCGTGGTTGCCGTCAGAGCTGTGT
TTCTCGATCAGTCCCCCGCAGTGCCGCAGTATCTTGCCGTGGGCTGCTTTAATCTTGAAAGTGGTTTA
TACATTGGGCGACGAGGTGTCGACTCTCATTGGGGGTAACCGACGGGCACATGCAGTCCCCTCCCCGG
GCAGGCAGAGGCGGGGCCCCCGCGCGCCGGCCCCGGCCCACAATCTTACCAGGGTCCTCAAAGGAGCC
TTTGCATGGTACCCTTCGTGAATGGTTGCTTAAGAGGTCCACAACGTGGTCCGGGCACGGTCGACTAA
ACACTCAAAACAGCGACGGCAAATATAGGTACAAGGTCCAGGCCCTCACGGCACTAATTGCGATGACC
CAACTCACGGGGGCAGTCCCCGGCGCGGCGACCTTGATTACGTCCGGGGACAAGTATGTCGCTCCCCC
CGGGAGGGGTGCAGCCACATGGGAGATTCAAAGTTTCTCGTGACGTCGTTGTGATCACTGCAGCCTAG
CCGAGACTCCCGTACTACGCGAAGGTTGGTTATGTTAACCACTACAACGTGAGGCGCCGTAAGGGCCA
GTGTTGTGCCCGGCTCTTCAATGCGCCTTAGCGGCCTGATACACCCACCCAAGGAGATACTGCTAATC
ATATGGGTGGACAGAACCTCGCAACGCAGACGCCTCCCCGGCATGATATGGTTTTTTTCCGCTATTAT
CCAGCACGCAGCGCTATCATTCAAGAGAACCCAGTGACGCGTAAATCGTAAGATCTACCTGCCGCAGG
TGGACCTACTGCAAATACGGATTATGACTCGTAAAGGGGTCATGCGTATTTTCATCACTAGGCACGTT
CGAGAATAAATTAGTAGGTGTCCCGAGCCTTGTGGCGTTCCGCCTGACTCCTCATGAAGTCGACCTTC
TCACCGGCCCTATCTGCCGACGTAAGTCATAACCTAGATCTGTACCTCGGGGGGAGGGTCACTGTAAA
GGGATAATTGGAGGGTGATTTCCCACACTTTCCTAAGGGTACTTTTTGCCTGGCTTCGCAATTGGGTC
CAATGGATGTCGATCTCTGGTTTAGCAGTTGTGAAAGTGGCAAGGCGGGAGGTTAGACCTCCATTTAA
CATATACAAGCAAGTTAACTGCACTAGATGTGTAGACACTACAGTTACAGGAGTAGCCGAATAAGTCT
CCGACGTCAAGCGAATAAGCGTCATACGCGATTATCGCCTAAGAGCACGTATTGGCGGTAAAAGGCTA
GCTAGACGCTTATGGGTAGATTTCAAGGCGTTCGTAGTGGTATAATAGGATACTCTTTCACCAGCCTG
AGGGCCGAACGCTATACTAGTGGTCTGTGATGTAGGACCGAGTATCTCTCTAGGGACCATCTACTTGA
GCAATGGTGCGCAGGGGGAGACATAGACCAGCCTTGGGTGGCAAGCACTGCAATAAGTCCTGTTTAGC
CTTGGAGTTCACATGCCGGCACTGAAGCCGACCTACCTGAGCGTGTGATTACCGTTAAAGCATCTGTC
TAGTTCTGTTTCCGGCGCTCTTGGTTCCAGGTTAGGGGAAGTGTATGACCCATGTGTTTTTATCGGCT
TAACCACGAGTGATCCCCGGTCGTTTCCCCATTGAATCCCTGGTGCATCCTACTCCCAGAATGATAGC
TGACTGACTGGACTGGCTTTTCAAGTAATCGAGGGGGTATCGCGGTCACGGCCGTTAACAGATCCCGT
CCTTAGTGTGGAATCCGCACCTGCTGACTAACGCTTCGCCGGCGTGTCTGCACAGCCGTATAGTGTTA
ATCATGACCCCAAGGAAGGATTAAACAAATATCTTGACG
>HREN13
GTGTCCGGTAGCCCGCGCTAGTTAGACACCCCGGCAGGGGGGATTGCTTTCGAGACGGGAGATCCCTT
CGCCGACCCTGGAGGGCCGACGCCGAGGCATTCGGGCCCCTGCAACGTCAACAGCGGCAAGAAAACGG
GATGAATGGGCGTAATGGGGGGGGTCTGCTGGGGACCCGACGCGGTTGCCGTTTGCGGGGCCCCCGAC
CCATACCGACCCACCTAGGCGTCCAGTTACGGCGCACGTCGGGAGCGTGGTTGCCGTCAGAGCTGTGT
TTCTCGATCAGTCCCCCGCAGTGCCGCAGTATCTTGCCGTGGGCTGCTTTAATCTTGAAAGTGGTTCA

TACATTGGGCGACGAGGTGTCGACTCTCATTGGGGGTAACCGACGGGCACATGCAGTCCCCTCCCCGG
GCAGGCAGAGGCGGGGCCCCCGCGCGCCGGCCCCGGCCCACAATCTTACCAGGGTCCTCAAAGGAGCC
TTTGCATGGTACCCTTCGTGAATGGTTGCTTAAGAGGTCCACAACGTGGTCCGGGCACGGTCGACTAA
ACACTCAAAACAGCGACGGCAAATATAGGTACAAGGTCCAGGCCCTCACGGCACTAATTGCGATGACC
CAACTCACGGGGGCAGTCCCCGGCGCGGCGACCTTGATTACGTCCGGGGACAAGTATGTCGCTCCCCC
CGGGAGGGGTGCAGCCACATGGGAGATTCAAAGTTTCTCGTGACGTCGTTGTGATCACTGCAGCCTAG
CCGAGACTCCCGTACTACGCGAAGGTTGGTTATGTTAACCACTACAACGTGAGGCGCCGTAAGGGCCA
GTGTTGTGCCCGGCTCTTCAATGCGCCTTAGCGGCCTGATACACCCACCCAAGGAGATACTGCTAATC
ATATGGGTGGACAGAACCTCGCAACGCAGACGCCTCCCCGGCATGATATGGTTTTTTTCCGCTATTAT
CCAGCACGCAGCGCTATCATTCAAGAGAACCCAGTGACGCGTAAATCGTAAGATCTACCTGCCGCAGG
TGGACCTACTGCAAATACGGATTATGACTCGTAAAGGGGTCATGCGTATTTTCATCACTAGGCACGTT
CGAGAATAAATTAGTAGGTGTCCCGAGCCTTGTGGCGTTCCGCCTGACTCCTCATGAAGTCGACCTTC
TCACCGGCCCTATCTGCCGACGTAAGTCATAACCTAGATCTGTACCTCGGGGGGAGGGTCACTGTAAA
GGGATAATTGGAGGGTGATTTCCCACACTTTCCTAAGGGTACTTTTTGCCTGGCTTCGCAATTGGGTC
CAATGGATGTCGATCTCTGGTTTAGCAGTTGTGAAAGTGGCAAGGCGGGAGGTTAGACCTCCATTTAA
CATATACAAGCAAGTTAACTGCACTAGATGTGTAGACACTACAGTTACAGGAGTAGCCGAATAAGTCT
CCGACGTCAAGCGAATAAGCGTCATACGCGATTATCGCCTAAGAGCACGTATTGGCGGTAAAAGGCTA
GCTAGACGCTTATGGGTAGATTTCAAGGCGTTCGTAGTGGTATAATAGGATACTCTTTCACCAGCCTG
AGGGCCGAACGCTATACTAGTGGTCTGTGATGTAGGACCGAGTATCTCTCTAGGGACCATCTACTTGA
GCAATGGTGCGCAGGGGGAGACATAGACCAGCCTTGGGTGGCAAGCACTGCAATAAGTCCTGTTTAGC
CTTGGAGTTCACATGCCGGCACTGAAGCCGACCTACCTGAGCGTGTGATTACCGTTAAAGCATCTGTC
TAGTTCTGTTTCCGGCGCTCTTGGTTCCAGGTTAGGGGAAGTGTATGACCCATGTGTTTTTATCGGCT
TAACCACGAGTGATCCCCGGTCGTTTCCCCATTGAATCCCTGGTGCATCCTACTCCCAGAATGATAGC
TGACTGACTGGACTGGCTTTTCAAGTAATCGAGGGGGTATCGCGGTCACGGCCGTTAACAGATCCCGT
CCTTAGTGTGGAATCCGCACCTGCTGACTAACGCTTCGCCGGCGTGTCTGCACAGCCGTATAGTGTTA
ATCATGACCCCAAGGAAGGATTAAACAAATATCTTGACG
>HREN15
GTGTCCGGTAGCCCGCGCTAGTTAGACACCCCGGCAGGGGGGATTGCTTTCGAGACGGGAGATCCCTT
CGCCGACCCTGGAGGGCCGACGCCGAGGCATTCGGGCCCCCGCAACGTCAACAGCGGCAAGAAAACGG
GATGAATGGGCGTAATGGGGGGGGTCTGCTGGGGACCCGACGCGGTTGCCGTTTGCGGGGCCCCCGAC
CCATACCGACCCACCTAGGCGTCCAGTTACGGCGCACGTCGGGAGCGTGGTTGCCGTCAGAGCTGTGT
TTCTCGATCAGTCCCCCGCAGTGCCGCAGTATCTTGCCGTGGGCTGCTTTAATCTTGAAAGTGGTTCA
TACATTGGGCGACGAGGTGTCGACTCTCATTGGGGGTAACCGACGGGCACATGCAGTCCCCTCCCCGG
GCAGGCAGAGGCGGGGCCCCCGCGCGCCGGCCCCGGCCCACAATCTTACCAGGGTCCTCAAAGGAGCC
TTTGCATGGTACCCTTCGTGAATGGTTGCTTAAGAGGTCCACAACGTGGTCCGGGCACGGTCGACTAA
ACACTCAAAACAGCGACGGCAAATATAGGTACAAGGTCCAGGCCCTCACGGCACTAATTGCGATGACC
CAACTCACGGGGGCAGTCCCCGGCGCGGCGACCTTGNTTACGTCCGGGGACAAGTATGTCGCTCCCCC
CGGGAGGGGTGCAGCCACATGGGAGATTCAAAGTTTCTCGTGACGTCGTTGTGATCACTGCAGCCTAG
CCGAGACTCCCGTACTACGCGAAGGTTGGTTATGTTAACCACTACAACGTGAGGCGCCGTAAGGGCCA
GTGTTGTGCCCGGCTCTTCAATGCGCCTTAGCGGCCTGATACACCCACCCAAGGAGATACTGCTAATC
ATATGGGTNGACAGAACCTCGCAACGCAGACGCCTCCCCGGCATGATATGGTTTTTTTCCGCTATTAT
CCAGCACGCAGCGCTATCATTCAAGAGAACCCAGTGACGCGTAAATCGTAAGATCTACCTGCCGCAGG
TGGACCTACTGCAAATACGGATTATGACTCGTAAAGGGGTCATGCGTATTTTCATCACTAGGCACGTT
CGAGAATAAATTAGTAGGTGTCCCGAGCCTTGTGGCGTTCCGCCTGACTCCTCATGAAGTCGACCTTC
TCACCGGCCCTATCTGCCGACGTAAGTCATAACCTAGATCTGTACCTCGGGGGGAGGGTCACTGTNAA
GGGATAATTGGAGGGTGATTTCCCACACTTTCCTAAGGGTACTTTTTGCCTGGCTTCGCAATTGGGTC
CAATGGATGTCGATCTCTGGTTTAGCAGTTGTGAAAGTGGCAAGGCGGGAGGTTAGACCTCCATTTAA
CATATACAAGCAAGTTAACTGCACTAGATGTGTAGACACTACAGTTACAGGAGTAGCCGAATAAGTCT
CCGACGTCAAGCGAATAAGCGTCATACGCGATTATCGCCTAAGAGCACGTATTGGCGGTAAAAGGCTA
GCTAGACGCTTATGGGTAGATTTCAAGGCGTTCGTAGTGGTATAATAGGATACTCTTTCACCAGCCTG
AGGGCCGAACGCTATACTAGTGGTCTGTGATGTAGGACCGAGTATCTCTCTAGGGACCATCTACTTGA
GCAATGGTGCGCAGGGGGAGACATAGACCAGCCTTGGGTGGCAAGCACTGCAATAAGTCCTGTTTAGC
CTTGGAGTTCACATGCCGGCACTGAAGCCGACCTACCTGAGCGTGTGATTACCGTTAAAGCATCTGTC
TAGTTCTGTTTCCGGCGCTCTTGGTTCCAGGTTAGGGGAAGTGTATGACCCATGTGTTTTTATCGGCT

TAACCACGAGTGATCCCCGGTCGTTTCCCCATTGAATCCCTGGTGCATCCTACTCCCAGAATGATAGC
TGACTGACTGGACTGGCTTTTCAAGTAATCGAGGGGGTATCGCGGTCACGGCCGTTAACAGATCCCGT
CCTTAGTGTGGAATCCGCACCTGCTGACTAACGCTTCGCCGGCGTGTCTGCACAGCCGTATAGTGTTA
ATCATGACCCCAAGGAAGGATTAAACAAATATCTTGACG
>HREN16
GTGTCCGGTAGCCCGCGCTAGTTAGACACCCCGGCAGGGGGGATTGCTTTCGAGACGGGAGATCCCTT
CGCCGACCCTGGAGGGCCGACGCCGAGGCATTCGGGCCCCTGCAACGTCAACAGCGGCAAGAAAACGG
GATGAATGGGCGTAATGGGGGGGGTCTGCTGGGGACCCGACGCGGTTGCCGTTTGCGGGGCCCCCGAC
CCATACCGACCCACCTAGGCGTCCAGTTACGGCGCACGGCGGGAGCGTGGTTGCCGTCAGAGCTGTGT
TTCTCGATCAGTCCCCCGCAGTGCCGCAGTATCTTGCCGTGGGCTGCTTTAATCTTGAAAGTGGTTCA
TACATTGGGCGACGAGGTGTCGACTCTCATTGGGGGTAACCGACGGGCACATGCAGTCCCCTCCCCGG
GCAGGCAGAGGCGGGGCCCCCGCGCGCCGGCCCCGGCCCACAATCTTACCAGGGTCCTCAAAGGAGCC
TTTGCATGGTACCCTTCGTGAATGGTTGCTTAAGAGGTCCACAACGTGGTCCGGGCACGGTCGACTAA
ACACTCAAAACAGCGACGGCAAATATAGGTACAAGGTCCAGGCCCTCACGGCACTAATTGCGATGACC
CAACTCACGGGGGCAGTCCCCGGCGCGGCGACCTTGATTACGTCCGGGGACAAGTATGTCGCTCCCCC
CGGGAGGGGTGCAGCCACATGGGAGATTCAAAGTTTCTCGTGACGTCGTTGTGATCACTGCAGCCTAG
CCGAGACTCCCGTACTACGCGAAGGTTGGTTATGTTAACCACTACAACGTGAGGCGCCGTAAGGGCCA
GTGTTGTGCCCGGCTCTTCAATGCGCCTTAGCGGCCTGATACACCCACCCAAGGAGATACTGCTAATC
ATATGGGTGGACAGAACCTCGCAACGCAGACGCCTCCCCGGCATGATATGGTTTTTTTCCGCTATTAT
CCAGCACGCAGCGCTATCATTCAAGAGAACCCAGTGACGCGTAAATCGTAAGATCTACCTGCCGCAGG
TGGACCTACTGCAAATACGGATTATGACTCGTAAAGGGGTCATGCGTATTTTCATCACTAGGCACGTT
CGAGAATAAATTAGTAGGTGTCCCGAGCCTTGTGGCGTTCCGCCTGACTCCTCATGAAGTCGACCTTC
TCACCGGCCCTATCTGCCGACGTAAGTCATAACCTAGATCTGTACCTCGGGGGGAGGGTCACTGTAAA
GGGATAATTGGAGGGTGATTTCCCACACTTTCCTAAGGGTACTTTTTGCCTGGCTTCGCAATTGGGTC
CAATGGATGTCGATCTCTGGTTTAGCAGTTGTGAAAGTGGCAAGGCGGGAGGTTAGACCTCCATTTAA
CATATACAAGCAAGTTAACTGCACTAGATGTGTAGACACTACAGTTACAGGAGTAGCCGAATAAGTCT
CCGACGTCAAGCGAATAAGCGTCATACGCGATTATCGCCTAAGAGCACGTATTGGCGGTAAAAGGCTA
GCTAGACGCTTATGGGTAGATTTCAAGGCGTTCGTAGTGGTATAATAGGATACTCTTTCACCAGCCTG
AGGGCCGAACGCTATACTAGTGGTCTGTGATGTAGGACCGAGTATCTCTCTAGGGACCATCTACTTGA
GCAATGGTGCGCAGGGGGAGACATAGACCAGCCTTGGGTGGCAAGCACTGCAATAAGTCCTGTTTAGC
CTTGGAGTTCACATGCCGGCACTGAAGCCGACCTACCTGAGCGTGTGATTACCGTTAAAGCATCTGTC
TAGTTCTGTTTCCGGCGCTCTTGGTTCCAGGTTAGGGGAAGTGTATGACCCATGTGTTTTTATCGGCT
TAACCACGAGTGATCCCCGGTCGTTTCCCCATTGAATCCCTGGTGCATCCTACTCCCAGAATGATAGC
TGACTGACTGGACTGGCTTTTCAAGTAATCGAGGGGGTATCGCGGTCACGGCCGTTAACAGATCCCGT
CCTTAGTGTGGAATCCGCACCTGCTGACTAACGCTTCGCCGGCGTGTCTGCACAGCCGTATAGTGTTA
ATCATGACCCCAAGGAAGGATTAAACAAATATCTTGACG
>HREN17
GTGTCCGGTAGCCCGCGCTAGTTAGACACCCCGGCAGGGGGGATTGCTTTCGAGACGGGAGATCCCTT
CGCCGACCCTGGAGGGCCGACGCCGAGGCATTCGGGCCCCTGCAACGTCAACAGCGGCAAGAAAACGG
GATGAATGGGCGTAATGGGGGGGGTCTGCTGGGGACCCGACGCGGTTGCCGTTTGCGGGGCCCCCGAC
CCATACCGACCCACCTAGGCGTCCAGTTACGGCGCACGTCGGGAGCGTGGTTGCCGTCAGAGCTGTGT
TTCTCGATCAGTCCCCCGCAGTGCCGCAGTATCTTGCCGTGGGCTGCTTTAATCTTGAAAGTGGTTTA
TACATTGGGCGACGAGGTGTCGACTCTCATTGGGGGTAACCGACGGGCACATGCAGTCCCCTCCCCGG
GCAGGCAGAGGCGGGGCCCCCGCGCGCCGGCCCCGGCCCACAATCTTACCAGGGTCCTCAAAGGAGCC
TTTGCATGGTACCCTTCGTGAATGGTTGCTTAAGAGGTCCACAACGTGGTCCGGGCACGGTCGACTAA
ACACTCAAAACAGCGACGGCAAATATAGGTACAAGGTCCAGGCCCTCACGGCACTAATTGCGATGACC
CAACTCACGGGGGCAGTCCCCGGCGCGGCGACCTTGATTACGTCCGGGGACAAGTATGTCGCTCCCCC
CGGGAGGGGTGCAGCCACATGGGAGATTCAAAGTTTCTCGTGACGTCGTTGTGATCACTGCAGCCTAG
CCGAGACTCCCGTACTACGCGAAGGTTGGTTATGTTAACCACTACAACGTGAGGCGCCGTAAGGGCCA
GTGTTGTGCCCGGCTCTTCAATGCGCCTTAGCGGCCTGATACACCCACCCAAGGAGATACTGCTAATC
ATATGGGTGGACAGAACCTCGCAACGCAGACGCCTCCCCGGCATGATATGGTTTTTTTCCGCTATTAT
CCAGCACGCAGCGCTATCATTCAAGAGAACCCAGTGACGCGTAAATCGTAAGATCTACCTGCCGCAGG
TGGACCTACTGCAAATACGGATTATGACTCGTAAAGGGGTCATGCGTATTTTCATCACTAGGCACGTT
CGAGAATAAATTAGTAGGTGTCCCGAGCCTTGTGGCGTTCCGCCTGACTCCTCATGAAGTCGACCTTC

TCACCGGCCCTATCTGCCGACGTAAGTCATAACCTAGATCTGTACCTCGGGGGGAGGGTCACTGTAAA
GGGATAATTGGAGGGTGATTTCCCACACTTTCCTAAGGGTACTTTTTGCCTGGCTTCGCAATTGGGTC
CAATGGATGTCGATCTCTGGTTTAGCAGTTGTGAAAGTGGCAAGGCGGGAGGTTAGACCTCCATTTAA
CATATACAAGCAAGTTAACTGCACTAGATGTGTAGACACTACAGTTACAGGAGTAGCCGAATAAGTCT
CCGACGTCAAGCGAATAAGCGTCATACGCGATTATCGCCTAAGAGCACGTATTGGCGGTAAAAGGCTA
GCTAGACGCTTATGGGTAGATTTCAAGGCGTTCGTAGTGGTATAATAGGATACTCTTTCACCAGCCTG
AGGGCCGAACGCTATACTAGTGGTCTGTGATGTAGGACCGAGTATCTCTCTAGGGACCATCTACTTGA
GCAATGGTGCGCAGGGGGAGACATAGACCAGCCTTGGGTGGCAAGCACTGCAATAAGTCCTGTTTAGC
CTTGGAGTTCACATGCCGGCACTGAAGCCGACCTACCTGAGCGTGTGATTACCGTTAAAGCATCTGTC
TAGTTCTGTTTCCGGCGCTCTTGGTTCCAGGTTAGGGGAAGTGTATGACCCATGTGTTTTTATCGGCT
TAACCACGAGTGATCCCCGGTCGTTTCCCCATTGAATCCCTGGTGCATCCTACTCCCAGAATGATAGC
TGACTGACTGGACTGGCTTTTCAAGTAATCGAGGGGGTATCGCGGTCACGGCCGTTAACAGATCCCGT
CCTTAGTGTGGAATCCGCACCTGCTGACTAACGCTTCGCCGGCGTGTCTGCACAGCCGTATAGTGTTA
ATCATGACCCCAAGGAAGGATTAAACAAATATCTTGACG
>HREN18
GTGTCCGGTAGCCCGCGCTAGTTAGACACCCCGGCAGGGGGGATTGCTTTCGAGACGGGAGATCCCTT
CGCCGACCCTGGAGGGCCGACGCCGAGGCATTCGGGCCCCCGCAACGTCAACAGCGGCAAGAAAACGG
GATGAATGGGCGTAATGGGGGGGGTCTGCTGGGGACCCGACGCGGTTGCCGTTTGCGGGGCCCCCGAC
CCATACCGACCCACCTAGGCGTCCAGTTACGGCGCACGGCGGGAGCGTGGTTGCCGTCAGAGCTGTGT
TTCTCGATCAGTCCCCCGCAGTGCCGCAGTATCTTGCCGTGGGCTGCTTTAATCTTGAAAGTGGTTCA
TACATTGGGCGACGAGGTGTCGACTCTCATTGGGGGTAACCGACGGGCACATGCAGTCCCCTCCCCGG
GCAGGCAGAGGCGGGGCCCCCGCGCGCCGGCCCCGGCCCACAATCTTACCAGGGTCCTCAAAGGAGCC
TTTGCATGGTACCCTTCGTGAATGGTTGCTTAAGAGGTCCACAACGTGGTCCGGGCACGGTCGACTAA
ACACTCAAAACAGCGACGGCAAATATAGGTACAAGGTCCAGGCCCTCACGGCACTAATTGCGATGACC
CAACTCACGGGGGCAGTCCCCGGCGCGGCGACCTTGATTACGTCCGGGGACAAGTATGTCGCTCCCCC
CGGGAGGGGTGCAGCCACATGGGAGATTCAAAGTTTCTCGTGACGTCGTTGTGATCACTGCAGCCTAG
CCGAGACTCCCGTACTACGCGAAGGTTGGTTATGTTAACCACTACAACGTGAGGCGCCGTAAGGGCCA
GTGTTGTGCCCGGCTCTTCAATGCGCCTTAGCGGCCTGATACACCCACCCAAGGAGATACTGCTAATC
ATATGGGTGGACAGAACCTCGCAACGCAGACGCCTCCCCGGCATGATATGGTTTTTTTCCGCTATTAT
CCAGCACGCAGCGCTATCATTCAAGAGAACCCAGTGACGCGTAAATCGTAAGATCTACCTGCCGCAGG
TGGACCTACTGCAAATACGGATTATGACTCGTAAAGGGGTCATGCGTATTTTCATCACTAGGCACGTT
CGAGAATAAATTAGTAGGTGTCCCGAGCCTTGTGGCGTTCCGCCTGACTCCTCATGAAGTCGACCTTC
TCACCGGCCCTATCTGCCGACGTAAGTCATAACCTAGATCTGTACCTCGGGGGGAGGGTCACTGTAAA
GGGATAATTGGAGGGTGATTTCCCACACTTTCCTAAGGGTACTTTTTGCCTGGCTTCGCAATTGGGTC
CAATGGATGTCGATCTCTGGTTTAGCAGTTGTGAAAGTGGCAAGGCGGGAGGTTAGACCTCCATTTAA
CATATACAAGCAAGTTAACTGCACTAGATGTGTAGACACTACAGTTACAGGAGTAGCCGAATAAGTCT
CCGACGTCAAGCGAATAAGCGTCATACGCGATTATCGCCTAAGAGCACGTATTGGCGGTAAAAGGCTA
GCTAGACGCTTATGGGTAGATTTCAAGGCGTTCGTAGTGGTATAATAGGATACTCTTTCACCAGCCTG
AGGGCCGAACGCTATACTAGTGGTCTGTGATGTAGGACCGAGTATCTCTCTAGGGACCATCTACTTGA
GCAATGGTGCGCAGGGGGAGACATAGACCAGCCTTGGGTGGCAAGCACTGCAATAAGTCCTGTTTAGC
CTTGGAGTTCACATGCCGGCACTGAAGCCGACCTACCTGAGCGTGTGATTACCGTTAAAGCATCTGTC
TAGTTCTGTTTCCGGCGCTCTTGGTTCCAGGTTAGGGGAAGTGTATGACCCATGTGTTTTTATCGGCT
TAACCACGAGTGATCCCCGGTCGTTTCCCCATTGAATCCCTGGTGCATCCTACTCCCAGAATGATAGC
TGACTGACTGGACTGGCTTTTCAAGTAATCGAGGGGGTATCGCGGTCACGGCCGTTAACAGATCCCGT
CCTTAGTGTGGAATCCGCACCTGCTGACTAACGCTTCGCCGGCGTGTCTGCACAGCCGTATAGTGTTA
ATCATGACCCCAAGGAAGGATTAAACAAATATCTTGACG
>HREN19
GTGTCCGGTAGCCCGCGCTAGTTAGACACCCCGGCAGGGGGGATTGCTTTCGAGACGGGAGATCCCTT
CGCCGACCCTGTAGGGCCGACGCCGAGGCATTCGGGCCCCTGCAACGTCAACAGCGGCAAGAAAACGG
GATGAATGGGCGTAATGGGGGGGGTCTGCTGGGGACCCGACGCGGTTGCCGTTTGCGGGGCCCCCGAC
CCATACCGACCCACCTAGGCGTCCAGTTACGGCGCACGGCGGGAGCGTGGTTGCCGTCAGAGCTGTGT
TTCTCGATCAGTCCCCCGCAGTGCCGCAGTATCTTGCCGTGGGCTGCTTTAATCTTGAAAGTGGTTTA
TACATTGGGCGACGAGGTGTCGGCTCTCATTGGGGGTAGCCGACGGACACATGCAGTCCCCTCCCCGG
GCAGGCAGAGGCGGGGCCCCCGCGCGCCGGCCCCGGCCCACAATCTTACCAGGGTCCTCAAAAGAGCC

TTTGCATGGTACCCTTCGTGAATGGTTGCTTAAGAGGTCCACCACGTAGTCCGGGCACGGTCGACTAA
ACACTCAAAACAGCGACGGCAAATAGAGGCACAAGGTCCAGGCCCTCACGGCACTAGTTGCGATGACC
CAACTCACGGGGGCAGTCCCCGGCGCGGCGACCTTGATTACGTCCGGGAACAAGTATGTCGTTTCCCC
CGGGAGGGGTGCAGCCACATGGGAGATTCAAAGTTTCTCGTGACGTCGTTGTGATCACTGCAGCCTAG
CCGAGACTCCCGTACTACGCGAAGGTTGGTTATGTTAACCACTACAACGTGAGGCGCCGTAAGGGCCA
GTGTTGTGCCCGGCTCTTCAATGCGCCTTAGCGGCCTGATACACCCACCCAAGGAGATACTGCTAATC
ATGTGGGTGGACAGAACCTCGCAACGCAGACGCATCCCCGGCNTGATATGGTTTTTTTCCGCTATTAC
CCAGCACGCGGCGCTATCATTCAAGAGAACCCAGTGACGCGTAAATCGTAAGATCTACCTGCCGCAGG
TGGACCTACTGCAAATACGGATTATGACTCGTAAAGAGGTCATGCGTATTTTCATCACTAGGCACGTT
CGAGAGTAAATTAGTAGGTGTCCCGCGCCTTGTGGCGTTCCGCCTGACTCCTCATGAAGTCGACCTTC
TCATCGGCCCTATCTGCCGACGTAAGTCATAATCCAGATCTTCACCTCGGAGGGAGGGTCACTGTAAA
GGGATAATTGGAGGGCGATTTCCCACACTTTCCTAAGGGTACTTTTTGCTTGGCTTCGCAGTTGGGTC
CAATAGATGTTGATCTCTGGTTTAGCAGTTGTGAAAGTGGCAAGGCGGGAGGTTAGACCTCCATTTAA
CATATACAAGCAAGTTAACTGCACTAGATGTGTAGACACTACAGTTACAGGAGTAGCCGACTAAGTCT
CCGACGTCAAGCGAATAGGCGTCATACGCGATTATCGCCTAAGAGCACGTATTGGCGGTAAAAGGATA
ACTAGACGCTTGTGGGTAGATTTCAAGGCGCTCGTAGTGGTATAATAGGATACTCTTTCACCAGCCTG
AGAGCCGAACGCTATACTAGTGGTCTGTGATGTAGGACCAAGTAGCTCTCTAGGGACCATCTACTTGA
GCAATGGTGCGNAGGGGTAGACATAGACCAACCTTGGGTGGCAAGCACTGCAATAAGTCCTGTTTAGC
CTTGGAGTTCACACGCCGGCACTAAAGCCGACCTACCTGAGCTTGTGATTACCGTTAAAGCATCTGTC
TAGTTCTGTTTCCGGCGCTCTTGGTTCCATGTTAGGGGAAGTGTATGACCCATGTGTTTTTATCGGCT
TAACTACGAGTGATCCCCGGTCGTTTCCCCATTAAATCCCTGGTGCATCCTACTCCCATAATGATAGC
TGACTGGCTGGACTGGCTTTTCAAGTAGTCGAGGGGGTATCGCGGTCACGGCCGTTAACAGATCCCGT
CCTTAGTGTGGAATCCGCACCTGCTGACTAACGCTTCGCCGGCGTGTCTGCACATCCGTATAGTGTTA
ATCATGACCCCAAGGAAGGATTAAACAAATATCTTGACG
>HREN20
GTGTCCGGTAGCCCGCGCTAGTTAGACACCCCGGCAGGGGGGATTGCTTTCGAGACGGGAGATCCCTT
CGCCGACCCTGGAGGGCCGACGCCGAGGCATTCGGGCCCCTGCAACGTCAACAGCGGCAAGAAAACGG
GATGAATGGGCGTAATGGGGGGGGTCTGCTGGGGACCCGACGCGGTTGCCGTTTGCGGGGCCCCCGAC
CCATACCGACCCACCTAGGCGTCCAGTTACGGCGCACGTCGGGAGCGTGGTTGCCGTCAGAGCTGTGT
TTCTCGATCAGTCCCCCGCAGTGCCGCAGTATCTTGCCGTGGGCTGCTTTAATCTTGAAAGTGGTTTA
TACATTGGGCGACGAGGTGTCGACTCTCATTGGGGGTAACCGACGGGCACATGCAGTCCCCTCCCCGG
GCAGGCAGAGGCGGGGCCCCCGCGCGCCGGCCCCGGCCCACAATCTTACCAGGGTCCTCAAAGGAGCC
TTTGCATGGTACCCTTCGTGAATGGTTGCTTAAGAGGTCCACAACGTGGTCCGGGCACGGTCGACTAA
ACACTCAAAACAGCGACGGCAAATATAGGTACAAGGTCCAGGCCCTCACGGCACTAATTGCGATGACC
CAACTCACGGGGGCAGTCCCCGGCGCGGCGACCTTGATTACGTCCGGGGACAAGTATGTCGCTCCCCC
CGGGAGGGGTGCAGCCACATGGGAGATTCAAAGTTTCTCGTGACGTCGTTGTGATCACTGCAGCCTAG
CCGAGACTCCCGTACTACGCGAAGGTTGGTTATGTTAACCACTACAACGTGAGGCGCCGTAAGGGCCA
GTGTTGTGCCCGGCTCTTCAATGCGCCTTAGCGGCCTGATACACCCACCCAAGGAGATACTGCTAATC
ATATGGGTGGACAGAACCTCGCAACGCAGACGCCTCCCCGGCATGATATGGTTTTTTTCCGCTATTAT
CCAGCACGCAGCGCTATCATTCAAGAGAACCCAGTGACGCGTAAATCGTAAGATCTACCTGCCGCAGG
TGGACCTACTGCAAATACGGATTATGACTCGTAAAGGGGTCATGCGTATTTTCATCACTAGGCACGTT
CGAGAATAAATTAGTAGGTGTCCCGAGCCTTGTGGCGTTCCGCCTGACTCCTCATGAAGTCGACCTTC
TCACCGGCCCTATCTGCCGACGTAAGTCATAACCTAGATCTGTACCTCGGGGGGAGGGTCACTGTAAA
GGGATAATTGGAGGGTGATTTCCCACACTTTCCTAAGGGTACTTTTTGCCTGGCTTCGCAATTGGGTC
CAATGGATGTCGATCTCTGGTTTAGCAGTTGTGAAAGTGGCAAGGCGGGAGGTTAGACCTCCATTTAA
CATATACAAGCAAGTTAACTGCACTAGATGTGTAGACACTACAGTTACAGGAGTAGCCGAATAAGTCT
CCGACGTCAAGCGAATAAGCGTCATACGCGATTATCGCCTAAGAGCACGTATTGGCGGTAAAAGGCTA
GCTAGACGCTTATGGGTAGATTTCAAGGCGTTCGTAGTGGTATAATAGGATACTCTTTCACCAGCCTG
AGGGCCGAACGCTATACTAGTGGTCTGTGATGTAGGACCGAGTATCTCTCTAGGGACCATCTACTTGA
GCAATGGTGCGCAGGGGGAGACATAGACCAGCCTTGGGTGGCAAGCACTGCAATAAGTCCTGTTTAGC
CTTGGAGTTCACATGCCGGCACTGAAGCCGACCTACCTGAGCGTGTGATTACCGTTAAAGCATCTGTC
TAGTTCTGTTTCCGGCGCTCTTGGTTCCAGGTTAGGGGAAGTGTATGACCCATGTGTTTTTATCGGCT
TAACCACGAGTGATCCCCGGTCGTTTCCCCATTGAATCCCTGGTGCATCCTACTCCCAGAATGATAGC
TGACTGACTGGACTGGCTTTTCAAGTAATCGAGGGGGTATCGCGGTCACGGCCGTTAACAGATCCCGT

CCTTAGTGTGGAATCCGCACCTGCTGACTAACGCTTCGCCGGCGTGTCTGCACAGCCGTATAGTGTTA
ATCATGACCCCAAGGAAGGATTAAACAAATATCTTGACG
>HREN22
GTGTCCGGTAGCCCGCGCTAGTTAGACACCCCGGCAGGGGGGATTGCTTTCGAGACGGGAGATCCCTT
CGCCGACCCTGGAGGGCCGACGCCGAGGCATTCGGGCCCCTGCAACGTCAACAGCGGCAAGAAAACGG
GATGAATGGGCGTAATGGGGGGGGTCTGCTGGGGACCCGACGCGGTTGCCGTTTGCGGGGCCCCCGAC
CCATACCGACCCACCTAGGCGTCCAGTTACGGCGCACGGCGGGAGCGTGGTTGCCGTCAGAGCTGTGT
TTCTCGATCAGTCCCCCGCAGTGCCGCAGTATCTTGCCGTGGGCTGCTTTAATCTTGAAAGTGGTTTA
TACATTGGGCGACGAGGTGTCGACTCTCATTGGGGGTAACCGACGGGCACATGCAGTCCCCTCCCCGG
GCAGGCAGAGGCGGGGCCCCCGCGCGCCGGCCCCGGCCCACAATCTTACCAGGGTCCTCAAAGGAGCC
TTTGCATGGTACCCTTCGTGAATGGTTGCTTAAGAGGTCCACAACGTGGTCCGGGCACGGTCGACTAA
ACACTCAAAACAGCGACGGCAAATATAGGTACAAGGTCCAGGCCCTCACGGCACTAATTGCGATGACC
CAACTCACGGGGGCAGTCCCCGGCGCGGCGACCTTGATTACGTCCGGGGACAAGTATGTCGCTCCCCC
CGGGAGGGGTGCAGCCACATGGGAGATTCAAAGTTTCTCGTGACGTCGTTGTGATCACTGCAGCCTAG
CCGAGACTCCCGTACTACGCGAAGGTTGGTTATGTTAACCACTACAACGTGAGGCGCCGTAAGGGCCA
GTGTTGTGCCCGGCTCTTCAATGCGCCTTAGCGGCCTGATACACCCACCCAAGGAGATACTGCTAATC
ATATGGGTGGACAGAACCTCGCAACGCAGACGCCTCCCCGGCATGATATGGTTTTTTTCCGCTATTAT
CCAGCACGCAGCGCTATCATTCAAGAGAACCCAGTGACGCGTAAATCGTAAGATCTACCTGCCGCAGG
TGGACCTACTGCAAATNCGGATTATGACTCGTAAAGGGGTCATGCGTATTTTCATCACTAGGCACGTT
CGAGAATAAATTAGTAGGTGTCCCGAGCCTTGTGGCGTTCCGCCTGACTCCTCATGAAGTCGACCTTC
TCACCGGCCCTATCTGCCGACGTAAGTCATAACCTAGATCTGTACCTCGGGGGGAGGGTCACTGTAAA
GGGATAATTGGAGGGTGATTTCCCACACTTTCCTAAGGGTACTTTTTGCCTGGCTTCGCAATTGGGTC
CAATGGATGTCGATCTCTGGTTTAGCAGTTGTGAAAGTGGCAAGGCGGGAGGTTAGACCTCCATTTAA
CATATACAAGCAAGTTAACTGCACTAGATGTGTAGACACTACAGTTACAGGAGTAGCCGAATAAGTCT
CCGACGTCAAGCGAATAAGCGTCATACGCGATTATCGCCTAAGAGCACGTATTGGCGGTAAAAGGCTA
GCTAGACGCTTATGGGTAGATTTCAAGGCGTTCGTAGTGGTATAATAGGATACTCTTTCACCAGCCTG
AGGGCCGAACGCTATACTAGTGGTCTGTGATGTAGGACCGAGTATCTCTCTAGGGACCATCTACTTGA
GCAATGGTGCGCAGGGGGAGACATAGACCAGCCTTGGGTGGCAAGCACTGCAATAAGTCCTGTTTAGC
CTTGGAGTTCACATGCCGGCACTGAAGCCGACCTACCTGAGCGTGTGATTACCGTTAAAGCATCTGTC
TAGTTCTGTTTCCGGCGCTCTTGGTTCCAGGTTAGGGGAAGTGTATGACCCATGTGTTTTTATCGGCT
TAACCACGAGTGATCCCCGGTCGTTTCCCCATTGAATCCCTGGTGCATCCTACTCCCAGAATGATAGC
TGACTGACTGGACTGGCTTTTCAAGTAATCGAGGGGGTATCGCGGTCACGGCCGTTAACAGATCCCGT
CCTTAGTGTGGAATCCGCACCTGCTGACTAACGCTTCGCCGGCGTGTCTGCACAGCCGTATAGTGTTA
ATCATGACCCCAAGGAAGGATTAAACAAATATCTTGACG
>HREN23
GTGTCCGGTAGCCCGCGCTAGTTAGACACCCCGGCAGGGGGGATTGCTTTCGGGACGGGAGATCCCTT
CGCCGACCCTGGAGGGCCGACGCCGAGGCATTCGGGCCCCCGCAACGTCAACAGCGGCAAGAAAACGG
GATGAATGGGCGTAATGGGGGGGGTCTGCTGGGGACCCGACGCGGTTGCCGTTTGCGGGGCCCCCGAC
CCATACCGACCCACCTAGGCGTCCAGTTACGGCGCACGTCGGGAGCGTGGTTGCCGTCAGAGCTGTGT
TTCTCGATCAGTCCCCCGCAGTGCCGCAGTATCTTGCCGTGGGCTGCTTTAATCTTGAAAGTGGTTCA
TACATTGGGCGACGAGGTGTCGACTCTCATTGGGGGTAACCGACGGGCACATGCAGTCCCCTCCCCGG
GCAGGCAGAGGCGGGGCCCCCGCGCGCCGGCCCCGGCCCACAATCTTACCAGGGTCCTCAAAGGAGCC
TTTGCATGGTACCCTTCGTGAATGGTTGCTTAAGAGGTCCACAACGTGGTCCGGGCACGGTCGACTAA
ACACTCAAAACAGCGACGGCAAATATAGGTACAAGGTCCAGGCCCTCACGGCACTAATTGCGATGACC
CAACTCACGGGGGCAGTCCCCGGCGCGGCGACCTTGATTACGTCCGGGGACAAGTATGTCGCTCCCCC
CGGGAGGGGTGCAGCCACATGGGAGATTCAAAGTTTCTCGTGACGTCGTTGTGATCACTGCAGCCTAG
CCGAGACTCCCGTACTACGCGAAGGTTGGTTATGTTAACCACTACAACGTGAGGCGCCGTAAGGGCCA
GTGTTGTGCCCGGCTCTTCAATGCGCCTTAGCGGCCTGATACACCCACCCAAGGAGATACTGCTAATC
ATATGGGTGGACAGAACCTCGCAACGCAGACGCCTCCCCGGCATGATATGGTTTTTTTCCGCTATTAT
CCAGCACGCAGCGCTATCATTCAAGAGAACCCAGTGACGCGTAAATCGTAAGATCTACCTGCCGCAGG
TGGACCTACTGCAAATACGGATTATGACTCGTAAAGGGGTCATGCGTATTTTCATCACTAGGCACGTT
CGAGAATAAATTAGTAGGTGTCCCGAGCCTTGTGGCGTTCCGCCTGACTCCTCATGAAGTCGACCTTC
TCACCGGCCCTATCTGCCGACGTAAGTCATAACCTAGATCTGTACCTCGGGGGGAGGGTCACTGTAAA
GGGATAATTGGAGGGTGATTTCCCACACTTTCCTAAGGGTACTTTTTGCCTGGCTTCGCAATTGGGTC

CAATGGATGTCGATCTCTGGTTTAGCAGTTGTGAAAGTGGCAAGGCGGGAGGTTAGACCTCCATTTAA
CATATACAAGCAAGTTAACTGCACTAGATGTGTAGACACTACAGTTACAGGAGTAGCCGAATAAGTCT
CCGACGTCAAGCGAATAAGCGTCATACGCGATTATCGCCTAAGAGCACGTATTGGCGGTAAAAGGCTA
GCTAGACGCTTATGGGTAGATTTCAAGGCGTTCGTAGTGGTATAATAGGATACTCTTTCACCAGCCTG
AGGGCCGAACGCTATACTAGTGGTCTGTGATGTAGGACCGAGTATCTCTCTAGGGACCATCTACTTGA
GCAATGGTGCGCAGGGGGAGACATAGACCAGCCTTGGGTGGCAAGCACTGCAATAAGTCCTGTTTAGC
CTTGGAGTTCACATGCCGGCACTGAAGCCGACCTACCTGAGCGTGTGATTACCGTTAAAGCATCTGTC
TAGTTCTGTTTCCGGCGCTCTTGGTTCCAGGTTAGGGGAAGTGTATGACCCATGTGTTTTTATCGGCT
TAACCACGAGTGATCCCCGGTCGTTTCCCCATTGAATCCCTGGTGCATCCTACTCCCAGAATGATAGC
TGACTGACTGGACTGGCTTTTCAAGTAATCGAGGGGGTATCGCGGTCACGGCCGTTAACAGATCCCGT
CCTTAGTGTGGAATCCGCACCTGCTGACTAACGCTTCGCCGGCGTGTCTGCACAGCCGTATAGTGTTA
ATCATGACCCCAAGGAAGGATTAAACAAATATCTTGACG
>HREN24
GTGTCCGGTAGCCCGCGCTAGTTAGACACCCCGGCAGGGGGGATTGCTTTCGAGACGGGAGATCCCTT
CGCCGACCCTGGAGGGCCGACGCCGAGGCATTCGGGCCCCTGCAACGTCAACAGCGGCAAGAAAACGG
GATGAATGGGCGTAATGGGGGGGGTCTGCTGGGGACCCGACGCGGTTGCCGTTTGCGGGGCCCCCGAC
CCATACCGACCCACCTAGGCGTCCAGTTACGGCGCACGTCGGGAGCGTGGTTGCCGTCAGAGCTGTGT
TTCTCGATCAGTCCCCCGCAGTGCCGCAGTATCTTGCCGTGGGCTGCTTTAATCTTGAAAGTGGTTCA
TACATTGGGCGACGAGGTGTCGACTCTCATTGGGGGTAACCGACGGGCACATGCAGTCCCCTCCCCGG
GCAGGCAGAGGCGGGGCCCCCGCGCGCCGGCCCCGGCCCACAATCTTACCAGGGTCCTCAAAGGAGCC
TTTGCATGGTACCCTTCGTGAATGGTTGCTTAAGAGGTCCACAACGTGGTCCGGGCACGGTCGACTAA
ACACTCAAAACAGCGACGGCAAATATAGGTACAAGGTCCAGGCCCTCACGGCACTAATTGCGATGACC
CAACTCACGGGGGCAGTCCCCGGCGCGGCGACCTTGATTACGTCCGGGGACAAGTATGTCGCTCCCCC
CGGGAGGGGTGCAGCCACATGGGAGATTCAAAGTTTCTCGTGACGTCGTTGTGATCACTGCAGCCTAG
CCGAGACTCCCGTACTACGCGAAGGTTGGTTATGTTAACCACTACAACGTGAGGCGCCGTAAGGGCCA
GTGTTGTGCCCGGCTCTTCAATGCGCCTTAGCGGCCTGATACACCCACCCAAGGAGATACTGCTAATC
ATATGGGTGGACAGAACCTCGCAACGCAGACGCCTCCCCGGCATGATATGGTTTTTTTCCGCTATTAT
CCAGCACGCAGCGCTATCATTCAAGAGAACCCAGTGACGCGTAAATCGTAAGATCTACCTGCCGCAGG
TGGACCTACTGCAAATACGGATTATGACTCGTAAAGGGGTCATGCGTATTTTCATCACTAGGCACGTT
CGAGAATAAATTAGTAGGTGTCCCGAGCCTTGTGGCGTTCCGCCTGACTCCTCATGAAGTCGACCTTC
TCACCGGCCCTATCTGCCGACGTAAGTCATAACCTAGATCTGTACCTCGGGGGGAGGGTCACTGTAAA
GGGATAATTGGAGGGTGATTTCCCACACTTTCCTAAGGGTACTTTTTGCCTGGCTTCGCAATTGGGTC
CAATGGATGTCGATCTCTGGTTTAGCAGTTGTGAAAGTGGCAAGGCGGGAGGTTAGACCTCCATTTAA
CATATACAAGCAAGTTAACTGCACTAGATGTGTAGACACTACAGTTACAGGAGTAGCCGAATAAGTCT
CCGACGTCAAGCGAATAAGCGTCATACGCGATTATCGCCTAAGAGCACGTATTGGCGGTAAAAGGCTA
GCTAGACGCTTATGGGTAGATTTCAAGGCGTTCGTAGTGGTATAATAGGATACTCTTTCACCAGCCTG
AGGGCCGAACGCTATACTAGTGGTCTGTGATGTAGGACCGAGTATCTCTCTAGGGACCATCTACTTGA
GCAATGGTGCGCAGGGGGAGACATAGACCAGCCTTGGGTGGCAAGCACTGCAATAAGTCCTGTTTAGC
CTTGGAGTTCACATGCCGGCACTGAAGCCGACCTACCTGAGCGTGTGATTACCGTTAAAGCATCTGTC
TAGTTCTGTTTCCGGCGCTCTTGGTTCCAGGTTAGGGGAAGTGTATGACCCATGTGTTTTTATCGGCT
TAACCACGAGTGATCCCCGGTCGTTTCCCCATTGAATCCCTGGTGCATCCTACTCCCAGAATGATAGC
TGACTGACTGGACTGGCTTTTCAAGTAATCGAGGGGGTATCGCGGTCACGGCCGTTAACAGATCCCGT
CCTTAGTGTGGAATCCGCACCTGCTGACTAACGCTTCGCCGGCGTGTCTGCACAGCCGTATAGTGTTA
ATCATGACCCCAAGGAAGGATTAAACAAATATCTTGACG
>HREN25
GTGTCCGGTAGCCCGCGCTAGTTAGACACCCCGGCAGGGGGGATTGCTTTCGGGACGGGAGATCCCTT
CGCCGACCCTGGAGGGCCGACGCCGAGGCATTCGGGCCCCCGCAACGTCAACAGCGGCAAGAAAACGG
GATGAATGGGCGTAATGGGGGGGGTCTGCTGGGGACCCGACGCGGTTGCCGTTTGCGGGGCCCCCGAC
CCATACCGACCCACCTAGGCGTCCAGTTACGGCGCACGGCGGGAGCGTGGTTGCCGTCAGAGCTGTGT
TTCTCGATCAGTCCCCCGCAGTGCCGCAGTATCTTGCCGTGGGCTGCTTTAATCTTGAAAGTGGTTTA
TACATTGGGCGACGAGGTGTCGACTCTCATTGGGGGTAACCGACGGGCACATGCAGTCCCCTCCCCGG
GCAGGCAGAGGCGGGGCCCCCGCGCGCCGGCCCCGGCCCACAATCTTACCAGGGTCCTCAAAGGAGCC
TTTGCATGGTACCCTTCGTGAATGGTTGCTTAAGAGGTCCACAACGTGGTCCGGGCACGGTCGACTAA
ACACTCAAAACAGCGACGGCAAATATAGGTACAAGGTCCAGGCCCTCACGGCACTAATTGCGATGACC

CAACTCACGGGGGCAGTCCCCGGCGCGGCGACCTTGATTACGTCCGGGGACAAGTATGTCGCTCCCCC
CGGGAGGGGTGCAGCCACATGGGAGATTCAAAGTTTCTCGTGACGTCGTTGTGATCACTGCAGCCTAG
CCGAGACTCCCGTACTACGCGAAGGTTGGTTATGTTAACCACTACAACGTGAGGCGCCGTAAGGGCCA
GTGTTGTGCCCGGCTCTTCAATGCGCCTTAGCGGCCTGATACACCCACCCAAGGAGATACTGCTAATC
ATATGGGTGGACAGAACCTCGCAACGCAGACGCCTCCCCGGCATGATATGGTTTTTTTCCGCTATTAT
CCAGCACGCAGCGCTATCATTCAAGAGAACCCAGTGACGCGTAAATCGTAAGATCTACCTGCCGCAGG
TGGACCTACTGCAAATACGGATTATGACTCGTAAAGGGGTCATGCGTATTTTCATCACTAGGCACGTT
CGAGAATAAATTAGTAGGTGTCCCGAGCCTTGTGGCGTTCCGCCTGACTCCTCATGAAGTCGACCTTC
TCACCGGCCCTATCTGCCGACGTAAGTCATAACCTAGATCTGTACCTCGGGGGGAGGGTCACTGTAAA
GGGATAATTGGAGGGTGATTTCCCACACTTTCCTAAGGGTACTTTTTGCCTGGCTTCGCAATTGGGTC
CAATGGATGTCGATCTCTGGTTTAGCAGTTGTGAAAGTGGCAAGGCGGGAGGTTAGACCTCCATTTAA
CATATACAAGCAAGTTAACTGCACTAGATGTGTAGACACTACAGTTACAGGAGTAGCCGAATAAGTCT
CCGACGTCAAGCGAATAAGCGTCATACGCGATTATCGCCTAAGAGCACGTATTGGCGGTAAAAGGCTA
GCTAGACGCTTATGGGTAGATTTCAAGGCGTTCGTAGTGGTATAATAGGATACTCTTTCACCAGCCTG
AGGGCCGAACGCTATACTAGTGGTCTGTGATGTAGGACCGAGTATCTCTCTAGGGACCATCTACTTGA
GCAATGGTGCGCAGGGGGAGACATAGACCAGCCTTGGGTGGCAAGCACTGCAATAAGTCCTGTTTAGC
CTTGGAGTTCACATGCCGGCACTGAAGCCGACCTACCTGAGCGTGTGATTACCGTTAAAGCATCTGTC
TAGTTCTGTTTCCGGCGCTCTTGGTTCCAGGTTAGGGGAAGTGTATGACCCATGTGTTTTTATCGGCT
TAACCACGAGTGATCCCCGGTCGTTTCCCCATTGAATCCCTGGTGCATCCTACTCCCAGAATGATAGC
TGACTGACTGGACTGGCTTTTCAAGTAATCGAGGGGGTATCGCGGTCACGGCCGTTAACAGATCCCGT
CCTTAGTGTGGAATCCGCACCTGCTGACTAACGCTTCGCCGGCGTGTCTGCACAGCCGTATAGTGTTA
ATCATGACCCCAAGGAAGGATTAAACAAATATCTTGACG
>HREN26
GTGTCCGGTAGCCCGCGCTAGTTAGACACCCCGGCAGGGGGGATTGCTTTCGAGACGGGAGATCCCTT
CGCCGACCCTGGAGGGCCGACGCCGAGGCATTCGGGCCCCCGCAACGTCAACAGCGGCAAGAAAACGG
GATGAATGGGCGTAATGGGGGGGGTCTGCTGGGGACCCGACGCGGTTGCCGTTTGCGGGGCCCCCGAC
CCATACCGACCCACCTAGGCGTCCAGTTACGGCGCACGTCGGGAGCGTGGTTGCCGTCAGAGCTGTGT
TTCTCGATCAGTCCCCCGCAGTGCCGCAGTATCTTGCCGTGGGCTGCTTTAATCTTGAAAGTGGTTTA
TACATTGGGCGACGAGGTGTCGACTCTCATTGGGGGTAACCGACGGGCACATGCAGTCCCCTCCCCGG
GCAGGCAGAGGCGGGGCCCCCGCGCGCCGGCCCCGGCCCACAATCTTACCAGGGTCCTCAAAGGAGCC
TTTGCATGGTACCCTTCGTGAATGGTTGCTTAAGAGGTCCACAACGTGGTCCGGGCACGGTCGACTAA
ACACTCAAAACAGCGACGGCAAATATAGGTACAAGGTCCAGGCCCTCACGGCACTAATTGCGATGACC
CAACTCACGGGGGCAGTCCCCGGCGCGGCGACCTTGATTACGTCCGGGGACAAGTATGTCGCTCCCCC
CGGGAGGGGTGCAGCCACATGGGAGATTCAAAGTTTCTCGTGACGTCGTTGTGATCACTGCAGCCTAG
CCGAGACTCCCGTACTACGCGAAGGTTGGTTATGTTAACCACTACAACGTGAGGCGCCGTAAGGGCCA
GTGTTGTGCCCGGCTCTTCAATGCGCCTTAGCGGCCTGATACACCCACCCAAGGAGATACTGCTAATC
ATATGGGTGGACAGAACCTCGCAACGCAGACGCCTCCCCGGCATGATATGGTTTTTTTCCGCTATTAT
CCAGCACGCAGCGCTATCATTCAAGAGAACCCAGTGACGCGTAAATCGTAAGATCTACCTGCCGCAGG
TGGACCTACTGCAAATACGGATTATGACTCGTAAAGGGGTCATGCGTATTTTCATCACTAGGCACGTT
CGAGAATAAATTAGTAGGTGTCCCGAGCCTTGTGGCGTTCCGCCTGACTCCTCATGAAGTCGACCTTC
TCACCGGCCCTATCTGCCGACGTAAGTCATAACCTAGATCTGTACCTCGGGGGGAGGGTCACTGTAAA
GGGATAATTGGAGGGTGATTTCCCACACTTTCCTAAGGGTACTTTTTGCCTGGCTTCGCAATTGGGTC
CAATGGATGTCGATCTCTGGTTTAGCAGTTGTGAAAGTGGCAAGGCGGGAGGTTAGACCTCCATTTAA
CATATACAAGCAAGTTAACTGCACTAGATGTGTAGACACTACAGTTACAGGAGTAGCCGAATAAGTCT
CCGACGTCAAGCGAATAAGCGTCATACGCGATTATCGCCTAAGAGCACGTATTGGCGGTAAAAGGCTA
GCTAGACGCTTATGGGTAGATTTCAAGGCGTTCGTAGTGGTATAATAGGATACTCTTTCACCAGCCTG
AGGGCCGAACGCTATACTAGTGGTCTGTGATGTAGGACCGAGTATCTCTCTAGGGACCATCTACTTGA
GCAATGGTGCGCAGGGGGAGACATAGACCAGCCTTGGGTGGCAAGCACTGCAATAAGTCCTGTTTAGC
CTTGGAGTTCACATGCCGGCACTGAAGCCGACCTACCTGAGCGTGTGATTACCGTTAAAGCATCTGTC
TAGTTCTGTTTCCGGCGCTCTTGGTTCCAGGTTAGGGGAAGTGTATGACCCATGTGTTTTTATCGGCT
TAACCACGAGTGATCCCCGGTCGTTTCCCCATTGAATCCCTGGTGCATCCTACTCCCAGAATGATAGC
TGACTGACTGGACTGGCTTTTCAAGTAATCGAGGGGGTATCGCGGTCACGGCCGTTAACAGATCCCGT
CCTTAGTGTGGAATCCGCACCTGCTGACTAACGCTTCGCCGGCGTGTCTGCACAGCCGTATAGTGTTA
ATCATGACCCCAAGGAAGGATTAAACAAATATCTTGACG

>HREN27
GTGTCCGGTAGCCCGCGCTAGTTAGACACCCCGGCAGGGGGGATTGCTTTCGAGACGGGAGATCCCTT
CGCCGACCCTGGAGGGCCGACGCCGAGGCATTCGGGCCCCTGCAACGTCAACAGCGGCAAGAAAACGG
GATGAATGGGCGTAATGGGGGGGGTCTGCTGGGGACCCGACGCGGTTGCCGTTTGCGGGGCCCCCGAC
CCATACCGACCCACCTAGGCGTCCAGTTACGGCGCACGTCGGGAGCGTGGTTGCCGTCAGAGCTGTGT
TTCTCGATCAGTCCCCCGCAGTGCCGCAGTATCTTGCCGTGGGCTGCTTTAATCTTGAAAGTGGTTTA
TACATTGGGCGACGAGGTGTCGACTCTCATTGGGGGTAACCGACGGGCACATGCAGTCCCCTCCCCGG
GCAGGCAGAGGCGGGGCCCCCGCGCGCCGGCCCCGGCCCACAATCTTACCAGGGTCCTCAAAGGAGCC
TTTGCATGGTACCCTTCGTGAATGGTTGCTTAAGAGGTCCACAACGTGGTCCGGGCACGGTCGACTAA
ACACTCAAAACAGCGACGGCAAATATAGGTACAAGGTCCAGGCCCTCACGGCACTAATTGCGATGACC
CAACTCACGGGGGCAGTCCCCGGCGCGGCGACCTTGATTACGTCCGGGGACAAGTATGTCGCTCCCCC
CGGGAGGGGTGCAGCCACATGGGAGATTCAAAGTTTCTCGTGACGTCGTTGTGATCACTGCAGCCTAG
CCGAGACTCCCGTACTACGCGAAGGTTGGTTATGTTAACCACTACAACGTGAGGCGCCGTAAGGGCCA
GTGTTGTGCCCGGCTCTTCAATGCGCCTTAGCGGCCTGATACACCCACCCAAGGAGATACTGCTAATC
ATATGGGTGGACAGAACCTCGCAACGCAGACGCCTCCCCGGCATGATATGGTTTTTTTCCGCTATTAT
CCAGCACGCAGCGCTATCATTCAAGAGAACCCAGTGACGCGTAAATCGTAAGATCTACCTGCCGCAGG
TGGACCTACTGCAAATACGGATTATGACTCGTAAAGGGGTCATGCGTATTTTCATCACTAGGCACGTT
CGAGAATAAATTAGTAGGTGTCCCGAGCCTTGTGGCGTTCCGCCTGACTCCTCATGAAGTCGACCTTC
TCACCGGCCCTATCTGCCGACGTAAGTCATAACCTAGATCTGTACCTCGGGGGGAGGGTCACTGTAAA
GGGATAATTGGAGGGTGATTTCCCACACTTTCCTAAGGGTACTTTTTGCCTGGCTTCGCAATTGGGTC
CAATGGATGTCGATCTCTGGTTTAGCAGTTGTGAAAGTGGCAAGGCGGGAGGTTAGACCTCCATTTAA
CATATACAAGCAAGTTAACTGCACTAGATGTGTAGACACTACAGTTACAGGAGTAGCCGAATAAGTCT
CCGACGTCAAGCGAATAAGCGTCATACGCGATTATCGCCTAAGAGCACGTATTGGCGGTAAAAGGCTA
GCTAGACGCTTATGGGTAGATTTCAAGGCGTTCGTAGTGGTATAATAGGATACTCTTTCACCAGCCTG
AGGGCCGAACGCTATACTAGTGGTCTGTGATGTAGGACCGAGTATCTCTCTAGGGACCATCTACTTGA
GCAATGGTGCGCAGGGGGAGACATAGACCAGCCTTGGGTGGCAAGCACTGCAATAAGTCCTGTTTAGC
CTTGGAGTTCACATGCCGGCACTGAAGCCGACCTACCTGAGCGTGTGATTACCGTTAAAGCATCTGTC
TAGTTCTGTTTCCGGCGCTCTTGGTTCCAGGTTAGGGGAAGTGTATGACCCATGTGTTTTTATCGGCT
TAACCACGAGTGATCCCCGGTCGTTTCCCCATTGAATCCCTGGTGCATCCTACTCCCAGAATGATAGC
TGACTGACTGGACTGGCTTTTCAAGTAATCGAGGGGGTATCGCGGTCACGGCCGTTAACAGATCCCGT
CCTTAGTGTGGAATCCGCACCTGCTGACTAACGCTTCGCCGGCGTGTCTGCACAGCCGTATAGTGTTA
ATCATGACCCCAAGGAAGGATTAAACAAATATCTTGACG
>MAN21
GTGTCCGGTAGCCCGCGCTAGTTAGACACCCCGGCAGGGGGGATTGCTTTCGAGACGGGAGATCCCTT
CGCCGACCCTGGAGGGCCGACGCCGAGGCATTCGGGCCCCCGCAACGTCAACAGCGGCAAGAAAACGG
GATGAATGGGCGTAATGGGGGGGGTCTGCTGGGGACCCGACGCGGTTGCCGTTTGCGGGGCCCCCGAC
CCATACCGACCCACCTAGGCGTCCAGTTACGGCGCACGTCGGGAGCGTGGTTGCCGTCAGAGCTGTGT
TTCTCGATCAGTCCCCCGCAGTGCCGCAGTATCTTGCCGTGGGCTGCTTTAATCTTGAAAGTGGTTCA
TACATTGGGCGACGAGGTGTCGACTCTCATTGGGGGTAACCGACGGGCACATGCAGTCCCCTCCCCGG
GCAGGCAGAGGCGGGGCCCCCGCGCGCCGGCCCCGGCCCACAATCTTACCAGGGTCCTCAAAGGAGCC
TTTGCATGGTACCCTTCGTGAATGGTTGCTTAAGAGGTCCACAACGTGGTCCGGGCACGGTCGACTAA
ACACTCAAAACAGCGACGGCAAATATAGGTACAAGGTCCAGGCCCTCACGGCACTAATTGCGATGACC
CAACTCACGGGGGCAGTCCCCGGCGCGGCGACCTTGATTACGTCCGGGGACAAGTATGTCGCTCCCCC
CGGGAGGGGTGCAGCCACATGGGAGATTCAAAGTTTCTCGTGACGTCGTTGTGATCACTGCAGCCTAG
CCGAGACTCCCGTACTACGCGAAGGTTGGTTATGTTAACCACTACAACGTGAGGCGCCGTAAGGGCCA
GTGTTGTGCCCGGCTCTTCAATGCGCCTTAGCGGCCTGATACACCCACCCAAGGAGATACTGCTAATC
ATATGGGTGGACAGAACCTCGCAACGCAGACGCCTCCCCGGCATGATATGGTTTTTTTCCGCTATTAT
CCAGCACGCAGCGCTATCATTCAAGAGAACCCAGTGACGCGTAAATCGTAAGATCTACCTGCCGCAGG
TGGACCTACTGCAAATACGGATTATGACTCGTAAAGGGGTCATGCGTATTTTCATCACTAGGCACGTT
CGAGAATAAATTAGTAGGTGTCCCGAGCCTTGTGGCGTTCCGCCTGACTCCTCATGAAGTCGACCTTC
TCACCGGCCCTATCTGCCGACGTAAGTCATAACCTAGATCTGTACCTCGGGGGGAGGGTCACTGTAAA
GGGATAATTGGAGGGTGATTTCCCACACTTTCCTAAGGGTACTTTTTGCCTGGCTTCGCAATTGGGTC
CAATGGATGTCGATCTCTGGTTTAGCAGTTGTGAAAGTGGCAAGGCGGGAGGTTAGACCTCCATTTAA
CATATACAAGCAAGTTAACTGCACTAGATGTGTAGACACTACAGTTACAGGAGTAGCCGAATAAGTCT

CCGACGTCAAGCGAATAAGCGTCATACGCGATTATCGCCTAAGAGCACGTATTGGCGGTAAAAGGCTA
GCTAGACGCTTATGGGTAGATTTCAAGGCGTTCGTAGTGGTATAATAGGATACTCTTTCACCAGCCTG
AGGGCCGAACGCTATACTAGTGGTCTGTGATGTAGGACCGAGTATCTCTCTAGGGACCATCTACTTGA
GCAATGGTGCGCAGGGGGAGACATAGACCAGCCTTGGGTGGCAAGCACTGCAATAAGTCCTGTTTAGC
CTTGGAGTTCACATGCCGGCACTGAAGCCGACCTACCTGAGCGTGTGATTACCGTTAAAGCATCTGTC
TAGTTCTGTTTCCGGCGCTCTTGGTTCCAGGTTAGGGGAAGTGTATGACCCATGTGTTTTTATCGGCT
TAACCACGAGTGATCCCCGGTCGTTTCCCCATTGAATCCCTGGTGCATCCTACTCCCAGAATGATAGC
TGACTGACTGGACTGGCTTTTCAAGTAATCGAGGGGGTATCGCGGTCACGGCCGTTAACAGATCCCGT
CCTTAGTGTGGAATCCGCACCTGCTGACTAACGCTTCGCCGGCGTGTCTGCACAGCCGTATAGTGTTA
ATCATGACCCCAAGGAAGGATTAAACAAATATCTTGACG
>MAN23
GTGTCCGGTAGCCCGCGCTAGTTAGACACCCCGGCAGGGGGGATTGCTTTCGAGACGGGAGATCCCTT
CGCCGACCCTGGAGGGCCGACGCCGAGGCATTCGGGCCCCTGCAACGTCAACAGCGGCAAGAAAACGG
GATGAATGGGCGTAATGGGGGGGGTCTGCTGGGGACCCGACGCGGTTGCCGTTTGCGGGGCCCCCGAC
CCATACCGACCCACCTAGGCGTCCAGTTACGGCGCACGTCGGGAGCGTGGTTGCCGTCAGAGCTGTGT
TTCTCGATCAGTCCCCCGCAGTGCCGCAGTATCTTGCCGTGGGCTGCTTTAATCTTGAAAGTGGTTTA
TACATTGGGCGACGAGGTGTCGACTCTCATTGGGGGTAACCGACGGGCACATGCAGTCCCCTCCCCGG
GCAGGCAGAGGCGGGGCCCCCGCGCGCCGGCCCCGGCCCACAATCTTACCAGGGTCCTCAAAGGAGCC
TTTGCATGGTACCCTTCGTGAATGGTTGCTTAAGAGGTCCACAACGTGGTCCGGGCACGGTCGACTAA
ACACTCAAAACAGCGACGGCAAATATAGGTACAAGGTCCAGGCCCTCACGGCACTAATTGCGATGACC
CAACTCACGGGGGCAGTCCCCGGCGCGGCGACCTTGATTACGTCCGGGGACAAGTATGTCGCTCCCCC
CGGGAGGGGTGCAGCCACATGGGAGATTCAAAGTTTCTCGTGACGTCGTTGTGATCACTGCAGCCTAG
CCGAGACTCCCGTACTACGCGAAGGTTGGTTATGTTAACCACTACAACGTGAGGCGCCGTAAGGGCCA
GTGTTGTGCCCGGCTCTTCAATGCGCCTTAGCGGCCTGATACACCCACCCAAGGAGATACTGCTAATC
ATATGGGTGGACAGAACCTCGCAACGCAGACGCCTCCCCGGCATGATATGGTTTTTTTCCGCTATTAT
CCAGCACGCAGCGCTATCATTCAAGAGAACCCAGTGACGCGTAAATCGTAAGATCTACCTGCCGCAGG
TGGACCTACTGCAAATACGGATTATGACTCGTAAAGGGGTCATGCGTATTTTCATCACTAGGCACGTT
CGAGAATAAATTAGTAGGTGTCCCGAGCCTTGTGGCGTTCCGCCTGACTCCTCATGAAGTCGACCTTC
TCACCGGCCCTATCTGCCGACGTAAGTCATAACCTAGATCTGTACCTCGGGGGGAGGGTCACTGTAAA
GGGATAATTGGAGGGTGATTTCCCACACTTTCCTAAGGGTACTTTTTGCCTGGCTTCGCAATTGGGTC
CAATGGATGTCGATCTCTGGTTTAGCAGTTGTGAAAGTGGCAAGGCGGGAGGTTAGACCTCCATTTAA
CATATACAAGCAAGTTAACTGCACTAGATGTGTAGACACTACAGTTACAGGAGTAGCCGAATAAGTCT
CCGACGTCAAGCGAATAAGCGTCATACGCGATTATCGCCTAAGAGCACGTATTGGCGGTAAAAGGCTA
GCTAGACGCTTATGGGTAGATTTCAAGGCGTTCGTAGTGGTATAATAGGATACTCTTTCACCAGCCTG
AGGGCCGAACGCTATACTAGTGGTCTGTGATGTAGGACCGAGTATCTCTCTAGGGACCATCTACTTGA
GCAATGGTGCGCAGGGGGAGACATAGACCAGCCTTGGGTGGCAAGCACTGCAATAAGTCCTGTTTAGC
CTTGGAGTTCACATGCCGGCACTGAAGCCGACCTACCTGAGCGTGTGATTACCGTTAAAGCATCTGTC
TAGTTCTGTTTCCGGCGCTCTTGGTTCCAGGTTAGGGGAAGTGTATGACCCATGTGTTTTTATCGGCT
TAACCACGAGTGATCCCCGGTCGTTTCCCCATTGAATCCCTGGTGCATCCTACTCCCAGAATGATAGC
TGACTGACTGGACTGGCTTTTCAAGTAATCGAGGGGGTATCGCGGTCACGGCCGTTAACAGATCCCGT
CCTTAGTGTGGAATCCGCACCTGCTGACTAACGCTTCGCCGGCGTGTCTGCACAGCCGTATAGTGTTA
ATCATGACCCCAAGGAAGGATTAAACAAATATCTTGACG
>MAN28
GTGTCCGGTAGCCCGCGCTAGTTAGACACCCCGGCAGGGGGGATTGCTTTCGAGACGGGAGATCCCTT
CGCCGACCCTGGAGGGCCGACGCCGAGGCATTCGGGCCCCTGCAACGTCAACAGCGGCAAGAAAACGG
GATGAATGGGCGTAATGGGGGGGGTCTGCTGGGGACCCGACGCGGTTGCCGTTTGCGGGGCCCCCGAC
CCATACCGACCCACCTAGGCGTCCAGTTACGGCGCACGGCGGGAGCGTGGTTGCCGTCAGAGCTGTGT
TTCTCGATCAGTCCCCCGCAGTGCCGCAGTATCTTGCCGTGGGCTGCTTTAATCTTGAAAGTGGTTTA
TACATTGGGCGACGAGGTGTCGACTCTCATTGGGGGTAACCGACGGGCACATGCAGTCCCCTCCCCGG
GCAGGCAGAGGCGGGGCCCCCGCGCGCCGGCCCCGGCCCACAATCTTACCAGGGTCCTCAAAGGAGCC
TTTGCATGGTACCCTTCGTGAATGGTTGCTTAAGAGGTCCACAACGTGGTCCGGGCACGGTCGACTAA
ACACTCAAAACAGCGACGGCAAATATAGGTACAAGGTCCAGGCCCTCACGGCACTAATTGCGATGACC
CAACTCACGGGGGCAGTCCCCGGCGCGGCGACCTTGATTACGTCCGGGGACAAGTATGTCGCTCCCCC
CGGGAGGGGTGCAGCCACATGGGAGATTCAAAGTTTCTCGTGACGTCGTTGTGATCACTGCAGCCTAG

CCGAGACTCCCGTACTACGCGAAGGTTGGTTATGTTAACCACTACAACGTGAGGCGCCGTAAGGGCCA
GTGTTGTGCCCGGCTCTTCAATGCGCCTTAGCGGCCTGATACACCCACCCAAGGAGATACTGCTAATC
ATATGGGTGGACAGAACCTCGCAACGCAGACGCCTCCCCGGCATGATATGGTTTTTTTCCGCTATTAT
CCAGCACGCAGCGCTATCATTCAAGAGAACCCAGTGACGCGTAAATCGTAAGATCTACCTGCCGCAGG
TGGACCTACTGCAAATACGGATTATGACTCGTAAAGGGGTCATGCGTATTTTCATCACTAGGCACGTT
CGAGAATAAATTAGTAGGTGTCCCGAGCCTTGTGGCGTTCCGCCTGACTCCTCATGAAGTCGACCTTC
TCACCGGCCCTATCTGCCGACGTAAGTCATAACCTAGATCTGTACCTCGGGGGGAGGGTCACTGTAAA
GGGATAATTGGAGGGTGATTTCCCACACTTTCCTAAGGGTACTTTTTGCCTGGCTTCGCAATTGGGTC
CAATGGATGTCGATCTCTGGTTTAGCAGTTGTGAAAGTGGCAAGGCGGGAGGTTAGACCTCCATTTAA
CATATACAAGCAAGTTAACTGCACTAGATGTGTAGACACTACAGTTACAGGAGTAGCCGAATAAGTCT
CCGACGTCAAGCGAATAAGCGTCATACGCGATTATCGCCTAAGAGCACGTATTGGCGGTAAAAGGCTA
GCTAGACGCTTATGGGTAGATTTCAAGGCGTTCGTAGTGGTATAATAGGATACTCTTTCACCAGCCTG
AGGGCCGAACGCTATACTAGTGGTCTGTGATGTAGGACCGAGTATCTCTCTAGGGACCATCTACTTGA
GCAATGGTGCGCAGGGGGAGACATAGACCAGCCTTGGGTGGCAAGCACTGCAATAAGTCCTGTTTAGC
CTTGGAGTTCACATGCCGGCACTGAAGCCGACCTACCTGAGCGTGTGATTACCGTTAAAGCATCTGTC
TAGTTCTGTTTCCGGCGCTCTTGGTTCCAGGTTAGGGGAAGTGTATGACCCATGTGTTTTTATCGGCT
TAACCANGAGTGATCCCCGGTCGTTTCCCCATTGAATCCCTGGTGCATCCTACTCCCAGAATGATAGC
TGACTGACTGGACTGGCTTTTCAAGTAATCGAGGGGGTATCGCGGTCACGGCCGTTAACAGATCCCGT
CCTTAGTGTGGAATCCGCACCTGCTGACTAACGCTTCGCCGGCGTGTCTGCACAGCCGTATAGTGTTA
ATCATGACCCCAAGGAAGGATTAAACAAATATCTTGACG
>MAN30
GTGTCCGGTAGCCCGCGCTAGTTAGACACCCCGGCAGGGGGGATTGCTTTCGAGACGGGAGATCCCTT
CGCCGACCCTGGAGGGCCGACGCCGAGGCATTCGGGCCCCTGCAACGTCAACAGCGGCAAGAAAACGG
GATGAATGGGCGTAATGGGGGGGGTCTGCTGGGGACCCGACGCGGTTGCCGTTTGCGGGGCCCCCGAC
CCATACCGACCCACCTAGGCGTCCAGTTACGGCGCACGTCGGGAGCGTGGTTGCCGTCAGAGCTGTGT
TTCTCGATCAGTCCCCCGCAGTGCCGCAGTATCTTGCCGTGGGCTGCTTTAATCTTGAAAGTGGTTTA
TACATTGGGCGACGAGGTGTCGACTCTCATTGGGGGTAACCGACGGGCACATGCAGTCCCCTCCCCGG
GCAGGCAGAGGCGGGGCCCCCGCGCGCCGGCCCCGGCCCACAATCTTACCAGGGTCCTCAAAGGAGCC
TTTGCATGGTACCCTTCGTGAATGGTTGCTTAAGAGGTCCACAACGTGGTCCGGGCACGGTCGACTAA
ACACTCAAAACAGCGACGGCAAATATAGGTACAAGGTCCAGGCCCTCACGGCACTAATTGCGATGACC
CAACTCACGGGGGCAGTCCCCGGCGCGGCGACCTTGATTACGTCCGGGGACAAGTATGTCGCTCCCCC
CGGGAGGGGTGCAGCCACATGGGAGATTCAAAGTTTCTCGTGACGTCGTTGTGATCACTGCAGCCTAG
CCGAGACTCCCGTACTACGCGAAGGTTGGTTATGTTAACCACTACAACGTGAGGCGCCGTAAGGGCCA
GTGTTGTGCCCGGCTCTTCAATGCGCCTTAGCGGCCTGATACACCCACCCAAGGAGATACTGCTAATC
ATATGGGTGGACAGAACCTCGCAACGCAGACGCCTCCCCGGCATGATATGGTTTTTTTCCGCTATTAT
CCAGCACGCAGCGCTATCATTCAAGAGAACCCAGTGACGCGTAAATCGTAAGATCTACCTGCCGCAGG
TGGACCTACTGCAAATACGGATTATGACTCGTAAAGGGGTCATGCGTATTTTCATCACTAGGCACGTT
CGAGAATAAATTAGTAGGTGTCCCGAGCCTTGTGGCGTTCCGCCTGACTCCTCATGAAGTCGACCTTC
TCACCGGCCCTATCTGCCGACGTAAGTCATAACCTAGATCTGTACCTCGGGGGGAGGGTCACTGTAAA
GGGATAATTGGAGGGTGATTTCCCACACTTTCCTAAGGGTACTTTTTGCCTGGCTTCGCAATTGGGTC
CAATGGATGTCGATCTCTGGTTTAGCAGTTGTGAAAGTGGCAAGGCGGGAGGTTAGACCTCCATTTAA
CATATACAAGCAAGTTAACTGCACTAGATGTGTAGACACTACAGTTACAGGAGTAGCCGAATAAGTCT
CCGACGTCAAGCGAATAAGCGTCATACGCGATTATCGCCTAAGAGCACGTATTGGCGGTAAAAGGCTA
GCTAGACGCTTATGGGTAGATTTCAAGGCGTTCGTAGTGGTATAATAGGATACTCTTTCACCAGCCTG
AGGGCCGAACGCTATACTAGTGGTCTGTGATGTAGGACCGAGTATCTCTCTAGGGACCATCTACTTGA
GCAATGGTGCGCAGGGGGAGACATAGACCAGCCTTGGGTGGCAAGCACTGCAATAAGTCCTGTTTAGC
CTTGGAGTTCACATGCCGGCACTGAAGCCGACCTACCTGAGCGTGTGATTACCGTTAAAGCATCTGTC
TAGTTCTGTTTCCGGCGCTCTTGGTTCCAGGTTAGGGGAAGTGTATGACCCATGTGTTTTTATCGGCT
TAACCACGAGTGATCCCCGGTCGTTTCCCCATTGAATCCCTGGTGCATCCTACTCCCAGAATGATAGC
TGACTGACTGGACTGGCTTTTCAAGTAATCGAGGGGGTATCGCGGTCACGGCCGTTAACAGATCCCGT
CCTTAGTGTGGAATCCGCACCTGCTGACTAACGCTTCGCCGGCGTGTCTGCACAGCCGTATAGTGTTA
ATCATGACCCCAAGGAAGGATTAAACAAATATCTTGACG
>MAN34
GTGTCCGGTAGCCCGCGCTAGTTAGACACCCCGGCAGGGGGGATTGCTTTCGAGACGGGAGATCCCTT

CGCCGACCCTGGAGGGCCGACGCCGAGGCATTCGGGCCCCTGCAACGTCAACAGCGGCAAGAAAACGG
GATGAATGGGCGTAATGGGGGGGGTCTGCTGGGGACCCGACGCGGTTGCCGTTTGCGGGGCCCCCGAC
CCATACCGACCCACCTAGGCGTCCAGTTACGGCGCACGTCGGGAGCGTGGTTGCCGTCAGAGCTGTGT
TTCTCGATCAGTCCCCCGCAGTGCCGCAGTATCTTGCCGTGGGCTGCTTTAATCTTGAAAGTGGTTCA
TACATTGGGCGACGAGGTGTCGACTCTCATTGGGGGTAACCGACGGGCACATGCAGTCCCCTCCCCGG
GCAGGCAGAGGCGGGGCCCCCGCGCGCCGGCCCCGGCCCACAATCTTACCAGGGTCCTCAAAGGAGCC
TTTGCATGGTACCCTTCGTGAATGGTTGCTTAAGAGGTCCACAACGTGGTCCGGGCACGGTCGACTAA
ACACTCAAAACAGCGACGGCAAATATAGGTACAAGGTCCAGGCCCTCACGGCACTAATTGCGATGACC
CAACTCACGGGGGCAGTCCCCGGCGCGGCGACCTTGATTACGTCCGGGGACAAGTATGTCGCTCCCCC
CGGGAGGGGTGCAGCCACATGGGAGATTCAAAGTTTCTCGTGACGTCGTTGTGATCACTGCAGCCTAG
CCGAGACTCCCGTACTACGCGAAGGTTGGTTATGTTAACCACTACAACGTGAGGCGCCGTAAGGGCCA
GTGTTGTGCCCGGCTCTTCAATGCGCCTTAGCGGCCTGATACACCCACCCAAGGAGATACTGCTAATC
ATATGGGTGGACAGAACCTCGCAACGCAGACGCCTCCCCGGCATGATATGGTTTTTTTCCGCTATTAT
CCAGCACGCAGCGCTATCATTCAAGAGAACCCAGTGACGCGTAAATCGTAAGATCTACCTGCCGCAGG
TGGACCTACTGCAAATACGGATTATGACTCGTAAAGGGGTCATGCGTATTTTCATCACTAGGCACGTT
CGAGAATAAATTAGTAGGTGTCCCGAGCCTTGTGGCGTTCCGCCTGACTCCTCATGAAGTCGACCTTC
TCACCGGCCCTATCTGCCGACGTAAGTCATAACCTAGATCTGTACCTCGGGGGGAGGGTCACTGTAAA
GGGATAATTGGAGGGTGATTTCCCACACTTTCCTAAGGGTACTTTTTGCCTGGCTTCGCAATTGGGTC
CAATGGATGTCGATCTCTGGTTTAGCAGTTGTGAAAGTGGCAAGGCGGGAGGTTAGACCTCCATTTAA
CATATACAAGCAAGTTAACTGCACTAGATGTGTAGACACTACAGTTACAGGAGTAGCCGAATAAGTCT
CCGACGTCAAGCGAATAAGCGTCATACGCGATTATCGCCTAAGAGCACGTATTGGCGGTAAAAGGCTA
GCTAGACGCTTATGGGTAGATTTCAAGGCGTTCGTAGTGGTATAATAGGATACTCTTTCACCAGCCTG
AGGGCCGAACGCTATACTAGTGGTCTGTGATGTAGGACCGAGTATCTCTCTAGGGACCATCTACTTGA
GCAATGGTGCGCAGGGGGAGACATAGACCAGCCTTGGGTGGCAAGCACTGCAATAAGTCCTGTTTAGC
CTTGGAGTTCACATGCCGGCACTGAAGCCGACCTACCTGAGCGTGTGATTACCGTTAAAGCATCTGTC
TAGTTCTGTTTCCGGCGCTCTTGGTTCCAGGTTAGGGGAAGTGTATGACCCATGTGTTTTTATCGGCT
TAACCACGAGTGATCCCCGGTCGTTTCCCCATTGAATCCCTGGTGCATCCTACTCCCAGAATGATAGC
TGACTGACTGGACTGGCTTTTCAAGTAATCGAGGGGGTATCGCGGTCACGGCCGTTAACAGATCCCGT
CCTTAGTGTGGAATCCGCACCTGCTGACTAACGCTTCGCCGGCGTGTCTGCACAGCCGTATAGTGTTA
ATCATGACCCCAAGGAAGGATTAAACAAATATCTTGACG
>MAN31
GTGTCCGGTAGCCCGCGCTAGTTAGACACCCCGGCAGGGGGGATTGCTTTCGGGACGGGAGATCCCTT
CGCCGACCCTGGAGGGCCGACGCCGAGGCATTCGGGCCCCTGCAACGTCAACAGCGGCAAGAAAACGG
GATGAATGGGCGTAATGGGGGGGGTCTGCTGGGGACCCGACGCGGTTGCCGTTTGCGGGGCCCCCGAC
CCATACCGACCCACCTAGGCGTCCAGTTACGGCGCACGNCGGGAGCGTGGTTGCCGTCAGAGCTGTGT
TTCTCGATCAGTCCCCCGCAGTGCCGCAGTATCTTGCCGTGGGCTGCTTTAATCTTGAAAGTGGTTCA
TACATTGGGCGACGAGGTGTCGACTCTCATTGGGGGTAACCGACGGGCACATGCAGTCCCCTCCCCGG
GCAGGCAGAGGCGGGGCCCCCGCGCGCCGGCCCCGGCCCACAATCTTACCAGGGTCCTCAAAGGAGCC
TTTGCATGGTACCCTTCGTGAATGGTTGCTTAAGAGGTCCACAACGTGGTCCGGGCACGGTCGACTAA
ACACTCAAAACAGCGACGGCAAATATAGGTACAAGGTCCAGGCCCTCACGGCACTAATTGCGATGACC
CAACTCACGGGGGCAGTCCCCGGCGCGGCGACCTTGATTACGTCCGGGGACAAGTATGTCGCTCCCCC
CGGGAGGGGTGCAGCCACATGGGAGATTCAAAGTTTCTCGTGACGTCGTTGTGATCACTGCAGCCTAG
CCGAGACTCCCGTACTACGCGAAGGTTGGTTATGTTAACCACTACAACGTGAGGCGCCGTAAGGGCCA
GTGTTGTGCCCGGCTCTTCAATGCGCCTTAGCGGCCTGATACACCCACCCAAGGAGATACTGCTAATC
ATATGGGTGGACAGAACCTCGCAACGCAGACGCCTCCCCGGCATGATATGGTTTTTTTCCGCTATTAT
CCAGCACGCAGCGCTATCATTCAAGAGAACCCAGTGACGCGTAAATCGTAAGATCTACCTGCCGCAGG
TGGACCTACTGCAAATACGGATTATGACTCGTAAAGGGGTCATGCGTATTTTCATCACTAGGCACGTT
CGAGAATAAATTAGTAGGTGTCCCGAGCCTTGTGGCGTTCCGCCTGACTCCTCATGAAGTCGACCTTC
TCACCGGCCCTATCTGCCGACGTAAGTCATAACCTAGATCTGTACCTCGGGGGGAGGGTCACTGTAAA
GGGATAATTGGAGGGTGATTTCCCACACTTTCCTAAGGGTACTTTTTGCCTGGCTTCGCAATTGGGTC
CAATGGATGTCGATCTCTGGTTTAGCAGTTGTGAAAGTGGCAAGGCGGGAGGTTAGACCTCCATTTAA
CATATACAAGCAAGTTAACTGCACTAGATGTGTAGACACTACAGTTACAGGAGTAGCCGAATAAGTCT
CCGACGTCAAGCGAATAAGCGTCATACGCGATTATCGCCTAAGAGCACGTATTGGCGGTAAAAGGCTA
GCTAGACGCTTATGGGTAGATTTCAAGGCGTTCGTAGTGGTATAATAGGATACTCTTTCACCAGCCTG

AGGGCCGAACGCTATACTAGTGGTCTGTGATGTAGGACCGAGTATCTCTCTAGGGACCATCTACTTGA
GCAATGGTGCGCAGGGGGAGACATAGACCAGCCTTGGGTGGCAAGCACTGCAATAAGTCCTGTTTAGC
CTTGGAGTTCACATGCCGGCACTGAAGCCGACCTACCTGAGCGTGTGATTACCGTTAAAGCATCTGTC
TAGTTCTGTTTCCGGCGCTCTTGGTTCCAGGTTAGGGGAAGTGTATGACCCATGTGTTTTTATCGGCT
TAACCACGAGTGATCCCCGGTCGTTTCCCCATTGAATCCCTGGTGCATCCTACTCCCAGAATGATAGC
TGACTGACTGGACTGGCTTTTCAAGTAATCGAGGGGGTATCGCGGTCACGGCCGTTAACAGATCCCGT
CCTTAGTGTGGAATCCGCACCTGCTGACTAACGCTTCGCCGGCGTGTCTGCACAGCCGTATAGTGTTA
ATCATGACCCCAAGGAAGGATTAAACAAATATCTTGACG
>EDEN27
GTGTCCGGTAGCCCGCGCTAGTTAGACACCCCGGCAGGGGGGATTGCTTTCGAGACGGGAGATCCCTT
CGCCGACCCTGGAGGGCCGACGCCGAGGCATTCGGGCCCCCGCAACGTCAACAGCGGCAAGAAAACGG
GATGAATGGGCGTAATGGGGGGGGTCTGCTGGGGACCCGACGCGGTTGCCGTTTGCGGGGCCCCCGAC
CCATACCGACCCACCTAGGCGTCCAGTTACGGCGCACGTCGGGAGCGTGGTTGCCGTCAGAGCTGTGT
TTCTCGATCAGTCCCCCGCAGTGCCGCAGTATCTTGCCGTGGGCTGCTTTAATCTTGAAAGTGGTTTA
TACATTGGGCGACGAGGTGTCGACTCTCATTGGGGGTAACCGACGGGCACATGCAGTCCCCTCCCCGG
GCAGGCAGAGGCGGGGCCCCCGCGCGCCGGCCCCGGCCCACAATCTTACCAGGGTCCTCAAAGGAGCC
TTTGCATGGTACCCTTCGTGAATGGTTGCNTAAGAGGTCCACAACGTGGTCCGGGCACGGTCGACTAA
ACACTCAAAACAGCGACGGCAAATATAGGTACAAGGTCCAGGCCCTCACGGCACTAATTGCGATGACC
CAACTCACGGGGGCAGTCCCCGGCGCGGCGACCTTGATTACGTCCGGGGACAAGTATGTCGCTCCCCC
CGGGAGGGGTGCAGCCACATGGGAGATTCAAAGTTTCTCGTGACGTCGTTGTGATCACTGCAGCCTAG
CCGAGACTCCCGTACTACGCGAAGGTTGGTTATGTTAACCACTACAACGTGAGGCGCCGTAAGGGCCA
GTGTTGTGCCCGGCTCTTCAATGCGCCTTAGCGGCCTGATACACCCACCCAAGGAGATACTGCTAATC
ATATGGGTGGACAGAACCTCGCAACGCAGACGCCTCCCCGGCATGATATGGTTTTTTTCCGCTATTAT
CCAGCACGCAGCGCTATCATTCAAGAGAACCCAGTGACGCGTAAATCGTAAGATCTACCTGCCGCAGG
TGGACCTACTGCAAATACGGATTATGACTCGTAAAGGGGTCATGCGTATTTTCATCACTAGGCACGTT
CGAGAATAAATTAGTAGGTGTCCCGAGCCTTGTGGCGTTCCGCCTGACTCCTCATGAAGTCGACCTTC
TCACCGGCCCTATCTGCCGACGTAAGTCATAACCTAGATCTGTACCTCGGGGGGAGGGTCACTGTAAA
GGGATAATTGGAGGGTGATTTCCCACACTTTCCTAAGGGTACTTTTTGCCTGGCTTCGCAATTGGGTC
CAATGGATGTCGATCTCTGGTTTAGCAGTTGTGAAAGTGGCAAGGCGGGAGGTTAGACCTCCATTTAA
CATATACAAGCAAGTTAACTGCACTAGATGTGTAGACACTACAGTTACAGGAGTAGCCGAATAAGTCT
CCGACGTCAAGCGAATAAGCGTCATACGCGATTATCGCCTAAGAGCACGTATTGGCGGTAAAAGGCTA
GCTAGACGCTTATGGGTAGATTTCAAGGCGTTCGTAGTGGTATAATAGGATACTCTTTCACCAGCCTG
AGGGCCGAACGCTATACTAGTGGTCTGTGATGTAGGACCGAGTATCTCTCTAGGGACCATCTACTTGA
GCAATGGTGCGCAGGGGGAGACATAGACCAGCCTTGGGTGGCAAGCACTGCAATAAGTCCTGTTTAGC
CTTGGAGTTCACATGCCGGCACTGAAGCCGACCTACCTGAGCGTGTGATTACCGTTAAAGCATCTGTC
TAGTTCTGTTTCCGGCGCTCTTGGTTCCAGGTTAGGGGAAGTGTATGACCCATGTGTTTTTATCGGCT
TAACCACGAGTGATCCCCGGTCGTTTCCCCATTGAATCCCTGGTGCATCCTACTCCCAGAATGATAGC
TGACTGACTGGACTGGCTTTTCAAGTAATCGAGGGGGTATCGCGGTCACGGCCGTTAACAGATCCCGT
CCTTAGTGTGGAATCCGCACCTGCTGACTAACGCTTCGCCGGCGTGTCTGCACAGCCGTATAGTGTTA
ATCATGACCCCAAGGAAGGATTAAACAAATATCTTGACG
>EDEN29
GTGTCCGGTAGCCCGCGCTAGTTAGACACCCCGGCAGGGGGGATTGCTTTCGAGACGGGAGATCCCTT
CGCCGACCCTGGAGGGCCGACGCCGAGGCATTCGGGCCCCTGCAACGTCAACAGCGGCAAGAAAACGG
GATGAATGGGCGTAATGGGGGGGGTCTGCTGGGGACCCGACGCGGTTGCCGTTTGCGGGGCCCCCGAC
CCATACCGACCCACCTAGGCGTCCAGTTACGGCGCACGGCGGGAGCGTGGTTGCCGTCAGAGCTGTGT
TTCTCGATCAGTCCCCCGCAGTGCCGCAGTATCTTGCCGTGGGCTGCTTTAATCTTGAAAGTGGTTTA
TACATTGGGCGACGAGGTGTCGACTCTCATTGGGGGTAACCGACGGGCACATGCAGTCCCCTCCCCGG
GCAGGCAGAGGCGGGGCCCCCGCGCGCCGGCCCCGGCCCACAATCTTACCAGGGTCCTCAAAGGAGCC
TTTGCATGGTACCCTTCGTGAATGGTTGCTTAAGAGGTCCACAACGTGGTCCGGGCACGGTCGACTAA
ACACTCAAAACAGCGACGGCAAATATAGGTACAAGGTCCAGGCCCTCACGGCACTAATTGCGATGACC
CAACTCACGGGGGCAGTCCCCGGCGCGGCGACCTTGATTACGTCCGGGGACAAGTATGTCGCTCCCCC
CGGGAGGGGTGCAGCCACATGGGAGATTCAAAGTTTCTCGTGACGTCGTTGTGATCACTGCAGCCTAG
CCGAGACTCCCGTACTACGCGAAGGTTGGTTATGTTAACCACTACAACGTGAGGCGCCGTAAGGGCCA
GTGTTGTGCCCGGCTCTTCAATGCGCCTTAGCGGCCTGATACACCCACCCAAGGAGATACTGCTAATC

ATATGGGTGGACAGAACCTCGCAACGCAGACGCCTCCCCGGCATGATATGGTTTTTTTCCGCTATTAT
CCAGCACGCAGCGCTATCATTCAAGAGAACCCAGTGACGCGTAAATCGTAAGATCTACCTGCCGCAGG
TGGACCTACTGCAAATACGGATTATGACTCGTAAAGGGGTCATGCGTATTTTCATCACTAGGCACGTT
CGAGAATAAATTAGTAGGTGTCCCGAGCCTTGTGGCGTTCCGCCTGACTCCTCATGAAGTCGACCTTC
TCACCGGCCCTATCTGCCGACGTAAGTCATAACCTAGATCTGTACCTCGGGGGGAGGGTCACTGTAAA
GGGATAATTGGAGGGTGATTTCCCACACTTTCCTAAGGGTACTTTTTGCCTGGCTTCGCAATTGGGTC
CAATGGATGTCGATCTCTGGTTTAGCAGTTGTGAAAGTGGCAAGGCGGGAGGTTAGACCTCCATTTAA
CATATACAAGCAAGTTAACTGCACTAGATGTGTAGACACTACAGTTACAGGAGTAGCCGAATAAGTCT
CCGACGTCAAGCGAATAAGCGTCATACGCGATTATCGCCTAAGAGCACGTATTGGCGGTAAAAGGCTA
GCTAGACGCTTATGGGTAGATTTCAAGGCGTTCGTAGTGGTATAATAGGATACTCTTTCACCAGCCTG
AGGGCCGAACGCTATACTAGTGGTCTGTGATGTAGGACCGAGTATCTCTCTAGGGACCATCTACTTGA
GCAATGGTGCGCAGGGGGAGACATAGACCAGCCTTGGGTGGCAAGCACTGCAATAAGTCCTGTTTAGC
CTTGGAGTTCACATGCCGGCACTGAAGCCGACCTACCTGAGCGTGTGATTACCGTTAAAGCATCTGTC
TAGTTCTGTTTCCGGCGCTCTTGGTTCCAGGTTAGGGGAAGTGTATGACCCATGTGTTTTTATCGGCT
TAACCACGAGTGATCCCCGGTCGTTTCCCCATTGAATCCCTGGTGCATCCTACTCCCAGAATGATAGC
TGACTGACTGGACTGGCTTTTCAAGTAATCGAGGGGGTATCGCGGTCACGGCCGTTAACAGATCCCGT
CCTTAGTGTGGAATCCGCACCTGCTGACTAACGCTTCGCCGGCGTGTCTGCACAGCCGTATAGTGTTA
ATCATGACCCCAAGGAAGGATTAAACAAATATCTTGACG
>EDEN30
GTGTCCGGTAGCCCGCGCTAGTTAGACACCCCGGCAGGGGGGATTGCTTTCGAGACGGGAGATCCCTT
CGCCGACCCTGGAGGGCCGACGCCGAGGCATTCGGGCCCCCGCAACGTCAACAGCGGCAAGAAAACGG
GATGAATGGGCGTAATGGGGGGGGTCTGCTGGGGACCCGACGCGGTTGCCGTTTGCGGGGCCCCCGAC
CCATACCGACCCACCTAGGCGTCCAGTTACGGCGCACGGCGGGAGCGTGGTTGCCGTCAGAGCTGTGT
TTCTCGATCAGTCCCCCGCAGTGCCGCAGTATCTTGCCGTGGGCTGCTTTAATCTTGAAAGTGGTTTA
TACATTGGGCGACGAGGTGTCGACTCTCATTGGGGGTAACCGACGGGCACATGCAGTCCCCTCCCCGG
GCANGCAGAGGCGGGGCCCCCGCGCGCCGGCCCCGGCCCACAATCTTACCAGGGTCCTCAAAGGAGCC
TTTGCATGGTACCCTTCGTGAATGGTTGCTTAAGAGGTCCACAACGTGGTCCGGGCACGGTCGACTAA
ACACTCAAAACAGCGACGGCAAATATAGGTACAAGGTCCAGGCCCTCACGGCACTAATTGCGATGACC
CAACTCACGGGGGCAGTCCCCGGCGCGGCGACCTTGATTACGTCCGGGGACAAGTATGTCGCTCCCCC
CGGGAGGGGTGCAGCCACATGGGAGATTCAAAGTTTCTCGTGACGTCGTTGTGATCACTGCAGCCTAG
CCGAGACTCCCGTACTACGCGAAGGTTGGTTATGTTAACCACTACAACGTGAGGCGCCGTAAGGGCCA
GTGTTGTGCCCGGCTCTTCAATGCGCCTTAGCGGCCTGATACACCCACCCAAGGAGATACTGCTAATC
ATATGGGTGGACAGAACCTCGCAACGCAGACGCCTCCCCGGCATGATATGGTTTTTTTCCGCTATTAT
CCAGCACGCAGCGCTATCATTCAAGAGAACCCAGTGACGCGTAAATCGTAAGATCTACCTGCCGCAGG
TGGACCTACTGCAAATACGGATTATGACTCGTAAAGGGGTCATGCGTATTTTCATCACTAGGCACGTT
CGAGAATAAATTAGTAGGTGTCCCGAGCCTTGTGGCGTTCCGCCTGACTCCTCATGAAGTCGACCTTC
TCACCGGCCCTATCTGCCGACGTAAGTCATAACCTAGATCTGTACCTCGGGGGGAGGGTCACTGTAAA
GGGATAATTGGAGGGTGATTTCCCACACTTTCCTAAGGGTACTTTTTGCCTGGCTTCGCAATTGGGTC
CAATGGATGTCGATCTCTGGTTTAGCAGTTGTGAAAGTGGCAAGGCGGGAGGTTAGACCTCCATTTAA
CATATACAAGCAAGTTAACTGCACTAGATGTGTAGACACTACAGTTACAGGAGTAGCCGAATAAGTCT
CCGACGTCAAGCGAATAAGCGTCATACGCGATTATCGCCTAAGAGCACGTATTGGCGGTAAAAGGCTA
GCTAGACGCTTATGGGTAGATTTCAAGGCGTTCGTAGTGGTATAATAGGATACTCTTTCACCAGCCTG
AGGGCCGAACGCTATACTAGTGGTCTGTGATGTAGGACCGAGTATCTCTCTAGGGACCATCTACTTGA
GCAATGGTGCGCAGGGGGAGACATAGACCAGCCTTGGGTGGCAAGCACTGCAATAAGTCCTGTTTAGC
CTTGGAGTTCACATGCCGGCACTGAAGCCGACCTACCTGAGCGTGTGATTACCGTTAAAGCATCTGTC
TAGTTCTGTTTCCGGCGCTCTTGGTTCCAGGTTAGGGGAAGTGTATGACCCATGTGTTTTTATCGGCT
TAACCACGAGTGATCCCCGGTCGTTTCCCCATTGAATCCCTGGTGCATCCTACTCCCAGAATGATAGC
TGACTGACTGGACTGGCTTTTCAAGTAATCGAGGGGGTATCGCGGTCACGGCCGTTAACAGATCCCGT
CCTTAGTGTGGAATCCGCACCTGCTGACTAACGCTTCGCCGGCGTGTCTGCACAGCCGTATAGTGTTA
ATCATGACCCCAAGGAAGGATTAAACAAATATCTTGACG
>EDEN31
GTGTCCGGTAGCCCGCGCTAGTTAGACACCCCGGCAGGGGGGATTGCTTTCGAGACGGGAGATCCCTT
CGCCGACCCTGGAGGGCCGACGCCGAGGCATTCGGGCCCCTGCAACGTCAACAGCGGCAAGAAAACGG
GATGAATGGGCGTAATGGGGGGGGTCTGCTGGGGACCCGACGCGGTTGCCGTTTGCGGGGCCCCCGAC

CCATACCGACCCACCTAGGCGTCCAGTTACGGCGCACGTCGGGAGCGTGGTTGCCGTCAGAGCTGTGT
TTCTCGATCAGTCCCCCGCAGTGCCGCAGTATCTTGCCGTGGGCTGCTTTAATCTTGAAAGTGGTTTA
TACATTGGGCGACGAGGTGTCGACTCTCATTGGGGGTAACCGACGGGCACATGCAGTCCCCTCCCCGG
GCAGGCAGAGGCGGGGCCCCCGCGCGCCGGCCCCGGCCCACAATCTTACCAGGGTCCTCAAAGGAGCC
TTTGCATGGTACCCTTCGTGAATGGTTGCTTAAGAGGTCCACAACGTGGTCCGGGCACGGTCGACTAA
ACACTCAAAACAGCGACGGCAAATATAGGTACAAGGTCCAGGCCCTCACGGCACTAATTGCGATGACC
CAACTCACGGGGGCAGTCCCCGGCGCGGCGACCTTGATTACGTCCGGGGACAAGTATGTCGCTCCCCC
CGGGAGGGGTGCAGCCACATGGGAGATTCAAAGTTTCTCGTGACGTCGTTGTGATCACTGCAGCCTAG
CCGAGACTCCCGTACTACGCGAAGGTTGGTTATGTTAACCACTACAACGTGAGGCGCCGTAAGGGCCA
GTGTTGTGCCCGGCTCTTCAATGCGCCTTAGCGGCCTGATACACCCACCCAAGGAGATACTGCTAATC
ATATGGGTGGACAGAACCTCGCAACGCAGACGCCTCCCCGGCATGATATGGTTTTTTTCCGCTATTAT
CCAGCACGCAGCGCTATCATTCAAGAGAACCCAGTGACGCGTAAATCGTAAGATCTACCTGCCGCAGG
TGGACCTACTGCAAATACGGATTATGACTCGTAAAGGGGTCATGCGTATTTTCATCACTAGGCACGTT
CGAGAATAAATTAGTAGGTGTCCCGAGCCTTGTGGCGTTCCGCCTGACTCCTCATGAAGTCGACCTTC
TCACCGGCCCTATCTGCCGACGTAAGTCATAACCTAGATCTGTACCTCGGGGGGAGGGTCACTGTAAA
GGGATAATTGGAGGGTGATTTCCCACACTTTCCTAAGGGTACTTTTTGCCTGGCTTCGCAATTGGGTC
CAATGGATGTCGATCTCTGGTTTAGCAGTTGTGAAAGTGGCAAGGCGGGAGGTTAGACCTCCATTTAA
CATATACAAGCAAGTTAACTGCACTAGATGTGTAGACACTACAGTTACAGGAGTAGCCGAATAAGTCT
CCGACGTCAAGCGAATAAGCGTCATACGCGATTATCGCCTAAGAGCACGTATTGGCGGTAAAAGGCTA
GCTAGACGCTTATGGGTAGATTTCAAGGCGTTCGTAGTGGTATAATAGGATACTCTTTCACCAGCCTG
AGGGCCGAACGCTATACTAGTGGTCTGTGATGTAGGACCGAGTATCTCTCTAGGGACCATCTACTTGA
GCAATGGTGCGCAGGGGGAGACATAGACCAGCCTTGGGTGGCAAGCACTGCAATAAGTCCTGTTTAGC
CTTGGAGTTCACATGCCGGCACTGAAGCCGACCTACCTGAGCGTGTGATTACCGTTAAAGCATCTGTC
TAGTTCTGTTTCCGGCGCTCTTGGTTCCAGGTTAGGGGAAGTGTATGACCCATGTGTTTTTATCGGCT
TAACCACGAGTGATCCCCGGTCGTTTCCCCATTGAATCCCTGGTGCATCCTACTCCCAGAATGATAGC
TGACTGACTGGACTGGCTTTTCAAGTAATCGAGGGGGTATCGCGGTCACGGCCGTTAACAGATCCCGT
CCTTAGTGTGGAATCCGCACCTGCTGACTAACGCTTCGCCGGCGTGTCTGCACAGCCGTATAGTGTTA
ATCATGACCCCAAGGAAGGATTAAACAAATATCTTGACG
>EDEN32
GTGTCCGGTAGCCCGCGCTAGTTAGACACCCCGGCAGGGGGGATTGCTTTCGAGACGGGAGATCCCTT
CGCCGACCCTGGAGGGCCGACGCCGAGGCATTCGGGCCCCTGCAACGTCAACAGCGGCAAGAAAACGG
GATGAATGGGCGTAATGGGGGGGGTCTGCTGGGGACCCGACGCGGTTGCCGTTTGCGGGGCCCCCGAC
CCATACCGACCCACCTAGGCGTCCAGTTACGGCGCACGTCGGGAGCGTGGTTGCCGTCAGAGCTGTGT
TTCTCGATCAGTCCCCCGCAGTGCCGCAGTATCTTGCCGTGGGCTGCTTTAATCTTGAAAGTGGTTTA
TACATTGGGCGACGAGGTGTCGACTCTCATTGGGGGTAACCGACGGGCACATGCAGTCCCCTCCCCGG
GCAGGCAGAGGCGGGGCCCCCGCGCGCCGGCCCCGGCCCACAATCTTACCAGGGTCCTCAAAGGAGCC
TTTGCATGGTACCCTTCGTGAATGGTTGCTTAAGAGGTCCACAACGTGGTCCGGGCACGGTCGACTAA
ACACTCAAAACAGCGACGGCAAATATAGGTACAAGGTCCAGGCCCTCACGGCACTAATTGCGATGACC
CAACTCACGGGGGCAGTCCCCGGCGCGGCGACCTTGATTACGTCCGGGGACAAGTATGTCGCTCCCCC
CGGGAGGGGTGCAGCCACATGGGAGATTCAAAGTTTCTCGTGACGTCGTTGTGATCACTGCAGCCTAG
CCGAGACTCCCGTACTACGCGAAGGTTGGTTATGTTAACCACTACAACGTGAGGCGCCGTAAGGGCCA
GTGTTGTGCCCGGCTCTTCAATGCGCCTTAGCGGCCTGATACACCCACCCAAGGAGATACTGCTAATC
ATATGGGTGGACAGAACCTCGCAACGCAGACGCCTCCCCGGCATGATATGGTTTTTTTCCGCTATTAT
CCAGCACGCAGCGCTATCATTCAAGAGAACCCAGTGACGCGTAAATCGTAAGATCTACCTGCCGCAGG
TGGACCTACTGCAAATACGGATTATGACTCGTAAAGGGGTCATGCGTATTTTCATCACTAGGCACGTT
CGAGAATAAATTAGTAGGTGTCCCGAGCCTTGTGGCGTTCCGCCTGACTCCTCATGAAGTCGACCTTC
TCACCGGCCCTATCTGCCGACGTAAGTCATAACCTAGATCTGTACCTCGGGGGGAGGGTCACTGTAAA
GGGATAATTGGAGGGTGATTTCCCACACTTTCCTAAGGGTACTTTTTGCCTGGCTTCGCAATTGGGTC
CAATGGATGTCGATCTCTGGTTTAGCAGTTGTGAAAGTGGCAAGGCGGGAGGTTAGACCTCCATTTAA
CATATACAAGCAAGTTAACTGCACTAGATGTGTAGACACTACAGTTACAGGAGTAGCCGAATAAGTCT
CCGACGTCAAGCGAATAAGCGTCATACGCGATTATCGCCTAAGAGCACGTATTGGCGGTAAAAGGCTA
GCTAGACGCTTATGGGTAGATTTCAAGGCGTTCGTAGTGGTATAATAGGATACTCTTTCACCAGCCTG
AGGGCCGAACGCTATACTAGTGGTCTGTGATGTAGGACCGAGTATCTCTCTAGGGACCATCTACTTGA
GCAATGGTGCGCAGGGGGAGACATAGACCAGCCTTGGGTGGCAAGCACTGCAATAAGTCCTGTTTAGC

CTTGGAGTTCACATGCCGGCACTGAAGCCGACCTACCTGAGCGTGTGATTACCGTTAAAGCATCTGTC
TAGTTCTGTTTCCGGCGCTCTTGGTTCCAGGTTAGGGGAAGTGTATGACCCATGTGTTTTTATCGGCT
TAACCACGAGTGATCCCCGGTCGTTTCCCCATTGAATCCCTGGTGCATCCTACTCCCAGAATGATAGC
TGACTGACTGGACTGGCTTTTCAAGTAATCGAGGGGGTATCGCGGTCACGGCCGTTAACAGATCCCGT
CCTTAGTGTGGAATCCGCACCTGCTGACTAACGCTTCGCCGGCGTGTCTGCACAGCCGTATAGTGTTA
ATCATGACCCCAAGGAAGGATTAAACAAATATCTTGACG
>EDEN33
GTGTCCGGTAGCCCGCGCTAGTTAGACACCCCGGCAGGGGGGATTGCTTTCGAGACGGGAGATCCCTT
CGCCGACCCTGGAGGGCCGACGCCGAGGCATTCGGGCCCCTGCAACGTCAACAGCGGCAAGAAAACGG
GATGAATGGGCGTAATGGGGGGGGTCTGCTGGGGACCCGACGCGGTTGCCGTTTGCGGGGCCCCCGAC
CCATACCGACCCACCTAGGCGTCCAGTTACGGCGCACGGCGGGAGCGTGGTTGCCGTCAGAGCTGTGT
TTCTCGATCAGTCCCCCGCAGTGCCGCAGTATCTTGCCGTGGGCTGCTTTAATCTTGAAAGTGGTTTA
TACATTGGGCGACGAGGTGTCGACTCTCATTGGGGGTAACCGACGGGCACATGCAGTCCCCTCCCCGG
GCAGGCAGAGGCGGGGCCCCCGCGCGCCGGCCCCGGCCCACAATCTTACCAGGGTCCTCAAAGGAGCC
TTTGCATGGTACCCTTCGTGAATGGTTGCTTAAGAGGTCCACAACGTGGTCCGGGCACGGTCGACTAA
ACACTCAAAACAGCGACGGCAAATATAGGTACAAGGTCCAGGCCCTCACGGCACTAATTGCGATGACC
CAACTCACGGGGGCAGTCCCCGGCGCGGCGACCTNGATTACGTCCGGGGACAAGTATGTCGCTCCCCC
CGGGAGGGGTGCAGCCACATGGGAGATTCAAAGTTTCTCGTGACGTCGTTGTGATCACTGCAGCCTAG
CCGAGACTCCCGTACTACGCGAAGGTTGGTTATGTTAACCACTACAACGTGAGGCGCCGTAAGGGCCA
GTGTTGTGCCCGGCTCTTCAATGCGCCTTAGCGGCCTGATACACCCACCCAAGGAGATACTGCTAATC
ATATGGGTGGACAGAACCTCGCAACGCAGACGCCTCCCCGGCATGATATGGTTTTTTTCCGCTATTAT
CCAGCACGCAGCGCTATCATTCAAGAGAACCCAGTGACGCGTAAATCGTAAGATCTACCTGCCGCAGG
TGGACCTACTGCAAATACGGATTATGACTCGTAAAGGGGTCATGCGTATTTTCATCACTAGGCACGTT
CGAGAATAAATTAGTAGGTGTCCCGAGCCTTGTGGCGTTCCGCCTGACTCCTCATGAAGTCGACCTTC
TCACCGGCCCTATCTGCCGACGTAAGTCATAACCTAGATCTGTACCTCGGGGGGAGGGTCACTGTAAA
GGGATAATTGGAGGGTGATTTCCCACACTTTCCTAAGGGTACTTTTTGCCTGGCTTCGCAATTGGGTC
CAATGGATGTCGATCTCTGGTTTAGCAGTTGTGAAAGTGGCAAGGCGGGAGGTTAGACCTCCATTTAA
CATATACAAGCAAGTTAACTGCACTAGATGTGTAGACACTACAGTTACAGGAGTAGCCGAATAAGTCT
CCGACGTCAAGCGAATAAGCGTCATACGCGATTATCGCCTAAGAGCACGTATTGGCGGTAAAAGGCTA
GCTAGACGCTTATGGGTAGATTTCAAGGCGTTCGTAGTGGTATAATAGGATACTCTTTCACCAGCCTG
AGGGCCGAACGCTATACTAGTGGTCTGTGATGTAGGACCGAGTATCTCTCTAGGGACCATCTACTTGA
GCAATGGTGCGCAGGGGGAGACATAGACCAGCCTTGGGTGGCAAGCACTGCAATAAGTCCTGTTTAGC
CTTGGAGTTCACATGCCGGCACTGAAGCCGACCTACCTGAGCGTGTGATTACCGTTAAAGCATCTGTC
TAGTTCTGTTTCCGGCGCTCTTGGTTCCAGGTTAGGGGAAGTGTATGACCCATGTGTTTTTATCGGCT
TAACCACGAGTGATCCCCGGTCGTTTCCCCATTGAATCCCTGGTGCATCCTACTCCCAGAATGATAGC
TGACTGACTGGACTGGCTTTTCAAGTAATCGAGGGGGTATCGCGGTCACGGCCGTTAACAGATCCCGT
CCTTAGTGTGGAATCCGCACCTGCTGACTAACGCTTCGCCGGCGTGTCTGCACAGCCGTATAGTGTTA
ATCATGACCCCAAGGAAGGATTAAACAAATATCTTGACG
>EDEN34
GTGTCCGGTAGCCCGCGCTAGTCAGACACCCCGGCAGGGGGGATTGCTTTCGGGACGGGAGATCCCTT
CGCCGACCCTGGAGGGCCGACGCCGAGGCATTCGGGCCCCTGCAACGTCAACAGCGGCAAGAAAACGG
GATGAATGGGCGTAATGGGGGGGGTCTGCTGGGGACCCGACGCGGTTGCCGTTTGCGGGGCCCCCGAC
CCATACCGACCCACCTAGGCGTCCAGTTACGGCGCACGGCGGGAGCGTGGTTGCCGTCAGAGCTGTGT
TTCTCGATCAGTCCCCCGCAGTGCCGCAGTATCTTGCCGTGGGCTGCTTTAATCTTGAAAGTGGTTCA
TACATTGGGCGACGAGGTGTCGACTCTCATTGGGGGTAACCGACGGGCACATGCAGTCCCCTCCCCGG
GCAGGCAGAGGCGGGGCCCCCGCGCGCCGGCCCCGGCCCACAATCTTACCAGGGTCCTCAAAGGAGCC
TTTGCATGGTACCCTTCGTGAATGGTTGCTTAAGAGGTCCACAACGTGGTCCGGGCACGGTCGACTAA
ACACTCAAAACAGCGACGGCAAATATAGGTACAAGGTCCAGGCCCTCACGGCACTAATTGCGATGACC
CAACTCACGGGGGCAGTCCCCGGCGCGGCGACCTTGATTACGTCCGGGGACAAGTATGTCGCTCCCCC
CGGGAGGGGTGCAGCCACATGGGAGATTCAAAGTTTCTCGTGACGTCGTTGTGATCACTGCAGCCTAG
CCGAGACTCCCGTACTACGCGAAGGTTGGTTATGTTAACCACTACAACGTGAGGCGCCGTAAGGGCCA
GTGTTGTGCCCGGCTCTTCAATGCGCCTTAGCGGCCTGATACACCCACCCAAGGAGATACTGCTAATC
ATATGGGTGGACAGAACCTCGCAACGCAGACGCCTCCCCGGCATGATATGGTTTTTTTCCGCTATTAT
CCAGCACGCAGCGCTATCATTCAAGAGAACCCAGTGACGCGTAAATCGTAAGATCTACCTGCCGCAGG

TGGACCTACTGCAAATACGGATTATGACTCGTAAAGGGGTCATGCGTATTTTCATCACTAGGCACGTT
CGAGAATAAATTAGTAGGTGTCCCGAGCCTTGTGGCGTTCCGCCTGACTCCTCATAAAGTCGACCTTC
TCACCGGCCCTATCTGCCGACGTAAGTCATAACCTAGATCTGTACCTCGGGGGGAGGGTCACTGTAAA
GGGATAATTGGAGGGTGATTTCCCACATTTTCCTAAGGGTACTTTTTGCCTGGCTTCGCAATTGGGTC
CAATGGATGTCGATCTCTGGTTTAGCAGTTGTGAAAGTGGCAAGGCGGGAGGTTAGACCTCCATTTAA
CATATACAAGCAAGTTAACTGCACTAGATGTGTAGACACTACAGGTACAGGAGTAGCCGGATAAGTCT
CCGACGTCAAGCGAATAAGCGTCATACGCGATTATCGCCTAAGAGCACGTATTTGCGGTAAAAGGCTA
GCTAGACGCTTGTGGGTAGATTTCAAGGCGTTCGTAGTGGTATAATAGGATACTCTTTCACCAGCCTG
AGGGCCGAACGCTATACTAGTGGTCTGTGATGTAGGACCGAGTATCTCTCTAGGGACCATCTACTTGA
GCAATGGTGCGCAGGGGGAGACATAGACCAGCCTTGGGTGGCAAGCACTGCAATAAGTCCTGTTTAGC
CTTGGAGTTCACACGCCGGCACTGAAGCCGACCTACCTGAGCGTGTGATTACCGTTAAAGCATCTGTC
TAGTTCTGTTTCCGGCGCTCTTGGTTCCAGGTTAGGGGAAGTGTATGACCCATGTGTTTTTATCGGCT
TAACCACGAGTGATCCCCGGTCGTTTCCCCATTGAATCCCTGGTGCATCCTACTCCCAGAATGATAGC
TGACTGACTGGACTGGCTTTTCAAGTAATCGAGGGGGTATCGCGGTCACGGCCGTTAACAGATCCCGT
CCTTAGTGTGGAATCCGCACCTGCTGACTAACGCTTCGCCGGCGTGTCTGCACAGCCGTATAGTGTTA
ATCATGACCCCAAGGAAGGATTAAACAAATATCTTGACG
>EDEN37
GTGTCCGGTAGCCCGCGCTAGTTAGANACCCCGGCAGGGGGGATTGCTTTCGAGACGGGAGATCCCTT
CGCCGACCCTGTAGGGCCGACGCCGAGGCATTCGGGCCCCTGCAACGTCAACAGCGGCAAGAAAACGG
GATGAATGGGCGTAATGGGGGGGGTCTGCTGGGGACCCGACGCGGTTGCCGTTTGCGGGGCCCCCGAC
CCATACCGACCCACCTAGGCGTCCAGTTACGGCGCACGTCGGGAGCGTGGTTGCCGTCAGAGCTGTGT
TTCTCGATCAGTCCCCCGCAGTGCCGCAGTATCTTGCCGTGGGCTGCTTTAATCTTGAAAGTGGTTTA
TACATTGGGCGACGAGGTGTCGGCTCTCATTGGGGGTAACCGACGGACACGTGCAGTCCCCTCCCCGG
GCAGGCAGAGGCGGGGCCCCCGCGCGCCGGCCCCGGCCCACAATCTTACCAGGGTCCTCAAAAGAGCC
TTTGCATGGTACCCTTCGTGAATGGTTGCTTAAGAGGTCCACCACGTAGTCCGGGCACGGTCAACTAA
ACACTCAAAACAGCGACGGCAAATAGAGGCACAAGGTCCAGGCCCTCACGGCACTAGTTGCGATGACC
CAACTCACGGGGGCAGTCCCCGGCGCAGCGACCTTGATTACGTCCGGGAACAAGTATGTCGTTTCCCC
CGGGAGGGGTGCAGCCACATGGGAGATTCAAAGTTTCTCGTGACGTCGTTGTGATCACTGCAGCCTAG
CCGAGACTCCCGTACTACGCGAAGGTTGGTTATGTTAACCACTACAACGTGAGGCGCCGTAAGGGCCA
GTGTTGTGCCCGGCTCTTCAATGCGCCTTAGCGGCCTGATACACCCACCCAAGGAGATANTGCTAATC
ATGTGGGTGGACAGAACCTCGCAACGCAGACGCATCCCCGGCATGATATGGTTTTTTTCCGCTATTAC
CCAGCACGCGGCGCTATCATTCAAGAGAACCCAGTGACGCGTAAATCGTCAGATCTACCTGCCGCAGG
TGGACCTACTGCAAATACGGATTATGACTCGTAAAGAGGTCATGCGTATTTTCATCACTAGGCACTTT
CGAGAGTAAATTAGTAGGTGTCCCGCGCCTTGTGGCGTTCCGCCTGGCTCCTCATGAAGTCGACCTTC
TCATCGGCCCTATTTGCCGACGTAAGTCATAATCCAGATCTTCACCTCGGAGGGAGGGTCACTGTAAA
GGGATAATTGGAGGGCGATTTCCCACACTTTCCTAAGGGTACTTTTTGCTTAGCTTCGCAGTTGGGTC
CAATAGATGTTGATCTCTGGTTTAGCAGTTGTGAAAGTGGCAAGGCGGGAGGTTAGGCCTCCATTTAA
CATATACAAGCAAGTTAACTGCACTAGATGTGTAGACACTACAGTTACAGGAGTAGCCGACTAAGTCT
CCGACGTCAAGCGAATAGGCGTCATACGCGATTATTGCCTAAGAGCACGTATTGGCGGTAAAAGGATA
ACTAGACGCTTGTGGGTAGATTTCAAGGCGCTCGTAGTGGTATAATAGGACACTCTTGCACCAGCCTG
AGAGCCGAACGCTATACTAGTGGTCTGTGATGTAGGACCAAGTAGCTCTCTAGGGACCATCTACTTGA
GCAATGGTGCGCAGGGGTAGACATAGACCAACCTTGGGTGGCAAGCACTGCAATAAGTCCTGTTTAGC
CTTGGAGTTCACACGCCGGCACTAAAGCCGACCTACCTGAGCTTGTGATTACCGTTAAAGCATCTGTC
TAGTTCTGTTTCCGGCGCTCTTGGTTCCATGTTAGGGGAAGTGTATGACCCATGTGTTTTTATCGGCT
TAACTACGAGTGATCCCCGGTCGTTTCCCCATTAAATCCCTGGTGCATCCTACTCCCATAATGATAGC
TGACTGGCTGGACTGGCTTTTCAAGTAGTCGAGGGGGTATCGCGGTCACGGCCGTTAACAGATCCCGT
CCTTAGTGTGGAATCCGCACCTGCTGACTAACGCTTCGCCGGCGTGTCTGCACATCCGTATAGTGTTA
ATCATGACCCCAAGGAAGGATTAAACAAATATCTTGACG
>EDEN39
GTGTCCGGTAGCCCGCGCTAGTTAGACACCCCGGCAGGGGGGATTGCTTTCGGGACGGGAGATCCCTT
CGCCGACCCTGGAGGGCCGACGCCGAGGCATTCGGGCCCCTGCAACGTCAACAGCGGCAAGAAAACGG
GATGAATGGGCGTAATGGGGGGGGTCTGCTGGGGACCCGACGCGGTTGCCGTTTGCGGGGCCCCCGAC
CCATACCGACCCACCTAGGCGTCCAGTTACGGCGCACGGCGGGAGCGTGGTTGCCGTCAGAGCTGTGT
TTCTCGATCAGTCCCCCGCAGTGCCGCAGTATCTTGCCGTGGGCTGCTTTAATCTTGAAAGTGGTTTA

TACATTGGGCGACGAGGTGTCGACTCTCATTGGGGGTAACCGACGGGCACATGCAGTCCCCTCCCCGG
GCAGGCAGAGGCGGGGCCCCCGCGCGCCGGCCCCGGCCCACAATCTTACCAGGGTCCTCAAAGGAGCC
TTTGCATGGTACCCTTCGTGAATGGTTGCTTAAGAGGTCCACAACGTGGTCCGGGCACGGTCGACTAA
ACACTCAAAACAGCGACGGCAAATATAGGTACAAGGTCCAGGCCCTCACGGCACTAATTGCGATGACC
CAACTCACGGGGGCAGTCCCCGGCGCGGCGACCTTGATTACGTCCGGGGACAAGTATGTCGCTCCCCC
CGGGAGGGGTGCAGCCACATGGGAGATTCAAAGTTTCTCGTGACGTCGTTGTGATCACTGCAGCCTAG
CCGAGACTCCCGTACTACGCGAAGGTTGGTTATGTTAACCACTACAACGTGAGGCGCCGTAAGGGCCA
GTGTTGTGCCCGGCTCTTCAATGCGCCTTAGCGGCCTGATACACCCACCCAAGGAGATACTGCTAATC
ATATGGGTGGACAGAACCTCGCAACGCAGACGCCTCCCCGGCATGATATGGTTTTTTTCCGCTATTAT
CCAGCACGCANCGCTATCATTCAAGAGAACCCAGTGACGCGTAAATCGTAAGATCTACCTGCCGCAGG
TGGACCTACTGNGGNTNNNGATTATGACTCGTAAAGGGGTCATGCGTATTTTCATCACTAGGCACGTT
CGAGAATAAATTAGTAGGTGTCCCGAGCCTTGTGGCGTTCCGCCTGACTCCTCATGAAGTCGACCTTC
TCACCGGCCCTATCTGCCGACGTAAGTCATAACCTAGATCTGTACCTCGGGGGGAGGGTCACTGTAAA
GGGATAATTGGAGGGTGATTTCCCACACTTTCCTAAGGGTACTTTTTGCCTGGCTTCGCAATTGGGTC
CAATGGATGTCGATCTCTGGTTTAGCAGTTGTGAAAGTGGCAAGGCGGGAGGTTAGACCTCCATTTAA
CATATACAAGCAAGTTAACTGCACTAGATGTGTAGACACTACAGTTACAGGAGTAGCCGAATAAGTCT
CCGACGTCAAGCGAATAAGCGTCATACGCGATTATCGCCTAAGAGCACGTATTGGCGGTAAAAGGCTA
GCTAGACGCTTATGGGTAGATTTCAAGGCGTTCGTAGTGGTATAATAGGATACTCTTTCACCAGCCTG
AGGGCCGAACGCTATACTAGTGGTCTGTGATGTAGGACCGAGTATCTCTCTAGGGACCATCTACTTGA
GCAATGGTGCGCAGGGGGAGACATAGACCAGCCTTGGGTGGCAAGCACTGCAATAAGTCCTGTTTAGC
CTTGGAGTTCACATGCCGGCACTGAAGCCGACCTACCTGAGCGTGTGATTACCGTTAAAGCATCTGTC
TAGTTCTGTTTCCGGCGCTCTTGGTTCCAGGTTAGGGGAAGTGTATGACCCATGTGTTTTTATCGGCT
TAACCACGAGTGATCCCCGGTCGTTTCCCCATTGAATCCCTGGTGCATCCTACTCCCAGAATGATAGC
TGACTGACTGGACTGGCTTTTCAAGTAATCGAGGGGGTATCGCGGTCACGGCCGTTAACAGATCCCGT
CCTTAGTGTGGAATCCGCACCTGCTGACTAACGCTTCGCCGGCGTGTCTGCACAGCCGTATAGTGTTA
ATCATGACCCCAAGGAAGGATTAAACAAATATCTTGACG
>EDEN40
GTGTCCGGTAGCCCGCGCTAGTTAGACACCCCGGCAGGGGGGATTGCTTTCGAGACGGGAGATCCCTT
CGCCGACCCTGTAGGGCCGACGCCGAGGCATTCGGGCCCCTGCAACGTCAACAGCGGCAAGAAAACGG
GATGAATGGGCGTAATGGGGGGGGTCTGCTGGGGACCCGACGCGGTTGCCGTTTGCGGGGCCCCCGAC
CCATACCGACCCACCTAGGCGTCCAGTTACGGCGCACGTCGGGAGCGTGGTTGCCGTCAGAGCTGTGT
TTCTCGATCAGTCCCCCGCAGTGCCGCAGTATCTTGCCGTGGGCTGCTTTAATCTTGAAAGTGGTTTA
TACATTGGGCGACGAGGTGTCGGCTCTCATTGGGGGTAACCGACGGACACGTGCAGTCCCCTCCCCGG
GCAGGCAGAGGCGGGGCCCCCGCGCGCCGGCCCCGGCCCACAATCTTACCAGGGTCCTCAAAAGAGCC
TTTGCATGGTACCCTTCGTGAATGGTTGCTTAAGAGGTCCACCACGTAGTCCGGGCACGGTCAACTAA
ACACTCAAAACAGCGACGGCAAATAGAGGCACAAGGTCCAGGCCCTCACGGCACTAGTTGCGATGACC
CAACTCACGGGGGCAGTCCCCGGCGCAGCGACCTTGATTACGTCCGGGAACAAGTATGTCGTTTCCCC
CGGGAGGGGTGCAGCCACATGGGAGATTCAAAGTTTCTCGTGACGTCGTTGTGATCACTGCAGCCTAG
CCGAGACTCCCGTACTACGCGAAGGTTGGTTATGTTAACCACTACAACGTGAGGCGCCGTAAGGGCCA
GTGTTGTGCCCGGCTCTTCAATGCGCCTTAGCGGCCTGATACACCCACCCAAGGAGATACTGCTAATC
ATGTGGGTGGACAGAACCTCGCAACGCAGACGCATCCCCGGCATGATATGGTTTTTTTCCGCTATTAC
CCAGCACGCGGCGCTATCATTCAAGAGAACCCAGTGACGCGTAAATCGTCAGATCTACCTGCCGCAGG
TGGACCTACTGCAAATACGGATTATGACTCGTAAAGAGGTCATGCGTATTTTCATCACTAGGCACTTT
CGAGAGTAAATTAGTAGGTGTCCCGCGCCTTGTGGCGTTCCGCCTGGCTCCTCATGAAGTCGACCTTC
TCATCGGCCCTATTTGCCGACGTAAGTCATAATCCAGATCTTCACCTCGGAGGGAGGGTCACTGTAAA
GGGATAATTGGAGGGCGATTTCCCACACTTTCCTAAGGGTACTTTTTGCTTAGCTTCGCAGTTGGGTC
CAATAGATGTTGATCTCTGGTTTAGCAGTTGTGAAAGTGGCAAGGCGGGAGGTTAGGCCTCCATTTAA
CATATACAAGCAAGTTAACTGCACTAGATGTGTAGACACTACAGTTACAGGAGTAGCCGACTAAGTCT
CCGACGTCAAGCGAATAGGCGTCATACGCGATTATTGCCTAAGAGCACGTATTGGCGGTAAAAGGATA
ACTAGACGCTTGTGGGTAGATTTCAAGGCGCTCGTAGTGGTATAATAGGACACTCTTGCACCAGCCTG
AGAGCCGAACGCTATACTAGTGGTCTGTGATGTAGGACCAAGTAGCTCTCTAGGGACCATCTACTTGA
GCAATGGTGCGCAGGGGTAGACATAGACCAACCTTGGGTGGCAAGCACTGCAATAAGTCCTGTTTAGC
CTTGGAGTTCACACGCCGGCACTAAAGCCGACCTACCTGAGCTTGTGATTACCGTTAAAGCATCTGTC
TAGTTCTGTTTCCGGCGCTCTTGGTTCCATGTTAGGGGAAGTGTATGACCCATGTGTTTTTATCGGCT

TAACTACGAGTGATCNCCGGTCGTTTCCCCATTAAATCCCTGGTGCATCCTACTCCCATAATGATAGC
TGACTGGCTGGACTGGCTTTTCAAGTAGTCGAGGGGGTATCGCGGTCACGGCCGTTAACAGATCCCGT
CCTTAGTGTGGAATCCGCACCTGCTGACTAACGCTTCGCCGGCGTGTCTGCACATCCGTATAGTGTTA
ATCATGACCCCAAGGAAGGATTAAACAAATATCTTGACG
>EDEN43
GTGTCCGGTAGCCCGCGCTAGTTAGACACCCCGGCAGGGGGGATTGCTTTCGAGACGGGAGATCCCTT
CGCCGACCCTGGAGGGCCGACGCCGAGGCATTCGGGCCCCCGCAACGTCAACAGCGGCAAGAAAACGG
GATGAATGGGCGTAATGGGGGGGGTCTGCTGGGGACCCGACGCGGTTGCCGTTTGCGGGGCCCCCGAC
CCATACCGACCCACCTAGGCGTCCAGTTACGGCGCACGTCGGGAGCGTGGTTGCCGTCAGAGCTGTGT
TTCTCGATCAGTCCCCCGCAGTGCCGCAGTATCTTGCCGTGGGCTGCTTTAATCTTGAAAGTGGTTTA
TACATTGGGCGACGAGGTGTCGACTCTCATTGGGGGTAACCGACGGGCACATGCAGTCCCCTCCCCGG
GCAGGCAGAGGCGGGGCCCCCGCGCGCCGGCCCCGGCCCACAATCTTACCAGGGTCCTCAAAGGAGCC
TTTGCATGGTACCCTTCGTGAATGGTTGCTTAAGAGGTCCACAACGTGGTCCGGGCACGGTCGACTAA
ACACTCAAAACAGCGACGGCAAATATAGGTACAAGGTCCAGGCCCTCACGGCACTAATTGCGATGACC
CAACTCACGGGGGCAGTCCCCGGCGCGGCGACCTTGATTACGTCCGGGGACAAGTATGTCGCTCCCCC
CGGGAGGGGTGCAGCCACATGGGAGATTCAAAGTTTCTCGTGACGTCGTTGTGATCACTGCAGCCTAG
CCGAGACTCCCGTACTACGCGAAGGTTGGTTATGTTAACCACTACAACGTGAGGCGCCGTAAGGGCCA
GTGTTGTGCCCGGCTCTTCAATGCGCCTTAGCGGCCTGATACACCCACCCAAGGAGATACTGCTAATC
ATATGGGTGGACAGAACCTCGCAACGCAGACGCCTCCCCGGCATGATATGGTTTTTTTCCGCTATTAT
CCAGCACGCAGCGCTATCATTCAAGAGAACCCAGTGACGCGTAAATCGTAAGATCTACCTGCCGCAGG
TGGACCTACTGCAAATACGGATTATGACTCGTAAAGGGGTCATGCGTATTTTCATCACTAGGCACGTT
CGAGAATAAATTAGTAGGTGTCCCGAGCCTTGTGGCGTTCCGCCTGACTCCTCATGAAGTCGACCTTC
TCACCGGCCCTATCTGCCGACGTAAGTCATAACCTAGATCTGTACCTCGGGGGGAGGGTCACTGTAAA
GGGATAATTGGAGGGTGATTTCCCACACTTTCCTAAGGGTACTTTTTGCCTGGCTTCGCAATTGGGTC
CAATGGATGTCGATCTCTGGTTTAGCAGTTGTGAAAGTGGCAAGGCGGGAGGTTAGACCTCCATTTAA
CATATACAAGCAAGTTAACTGCACTAGATGTGTAGACACTACAGTTACAGGAGTAGCCGAATAAGTCT
CCGACGTCAAGCGAATAAGCGTCATACGCGATTATCGCCTAAGAGCACGTATTGGCGGTAAAAGGCTA
GCTAGACGCTTATGGGTAGATTTCAAGGCGTTCGTAGTGGTATAATAGGATACTCTTTCACCAGCCTG
AGGGCCGAACGCTATACTAGTGGTCTGTGATGTAGGACCGAGTATCTCTCTAGGGACCATCTACTTGA
GCAATGGTGCGCAGGGGGAGACATAGACCAGCCTTGGGTGGCAAGCACTGCAATAAGTCCTGTTTAGC
CTTGGAGTTCACATGCCGGCACTGAAGCCGACCTACCTGANCGTGTGATTACCGTTAAAGCATCTGTC
TAGTTCTGTTTCCGGCGCTCTTGGTTCCAGGTTAGGGGAAGTGTATGACCCATGTGTTTTTATCGGCT
TAACCACGAGTGATCCCCGGTCGTTTCCCCATTGAATCCCTGGTGCATCCTACTCCCAGAATGATAGC
TGACTGACTGGACTGGCTTTTCAAGTAATCGAGGGGGTATCGCGGTCACGGCCGTTAACAGATCCCGT
CCTTAGTGTGGAATCCGCACCTGCTGACTAACGCTTCGCCGGCGTGTCTGCACAGCCGTATAGTGTTA
ATCATGACCCCAAGGAAGGATTAAACAAATATCTTGACG
>EDEN52
GTGTCCGGTAGCCCGCGCTAGTTAGACACCCCGGCAGGGGGGATTGCTTTCGAGACGGGAGATCCCTT
CGCCGACCCTGGAGGGCCGACGCCGAGGCATTCGGGCCCCTGCAACGTCAACAGCGGCAAGAAAACGG
GATGAATGGGCGTAATGGGGGGGGTCTGCTGGGGACCCGACGCGGTTGCCGTTTGCGGGGCCCCCGAC
CCATACCGACCCACCTAGGCGTCCAGTTACGGCGCACGTCGGGAGCGTGGTTGCCGTCAGAGCTGTGT
TTCTCGATCAGTCCCCCGCAGTGCCGCAGTATCTTGCCGTGGGCTGCTTTAATCTTGAAAGTGGTTTA
TACATTGGGCGACGAGGTGTCGGCTCTCATTGGGGGTAACCGACGGGCACATGCAGTCCCCTCCCCGG
GCAGGCAGAGGCGGGGCCCCCGCGCGCCGGCCCCGGCCCACAATCTTACCAGGGTCCTCAAAGGAGCC
TTTGCATGGTACCCTTCGTGAATGGTTGCTTAAGAGGTCCACCACGTAGTCCGGGCACGGTCGACTAA
ACACTCAAAACAGCGACGGCAAATAGAGGCACAAAGTCCAGGCCCTCACGGCACTAGTTGCGATGACC
CAACTCACGGGGGCAGTCCCCGGCGCGGCGACCTTGATTACGTCCGGGAACAAGTATGTCGTTTCCCC
CGGGAGGGGTGCAGCCACATGGGAGATTCAAAGTTTCTCGTGACGTCGTTGTGATCACTGCAGCCTAG
CCGAGACTCCCGTACTACGCGAAGGTTGGTTATGTTAACCACTACAACGTGAGGCGCCGTAAGGGCCA
GTGTTGTGCCCGGCTCTTCAATGCGCCTTAGCGGCCTGATACACCCACCCAAGGAGATACTGCTAATC
ATGTGGGTGGACAGAACCTCGCAACGCAGACGCATCCCCGGCATGATATGGTTTTTTTCCGCTATTAC
CCAGCACGCGGCGCTATCATTCAAGAGAACCCAGTGACGTGTAAATCGTAAGATCTATCTGCCGCAGG
TGGACCTACTGCAAATACGGATTATGACTCGTAAAGAGGTCATGCGTATTTTCATCACTAGACACGTT
CGAGAATAAATTAGTAGGTGTCCCGCGCCTTGTGGCGTTCCGCCTGACTCCTCATGAAGTCGACCTTC

TCATCGGCCCTATCTGCCGACGTAAGTCATAACCCAGATCTGTACCTCGGAGGGAGGGTCACTGTAAA
GGGATAATTGGAGGGTGATTTCCCACACTTTCCTAAGGGTACTTTTTGCCTGGCTTCGCAGTTGGGTC
CAATAGATGTTGATCTCTGGTTTAGCAGTTGTGAAAGTGGCAAGGCGGGAGGTTAGACCTCCATTTAA
CATATACAAGCAAGTTAACTGCACTAGATGTGTAGACACTACAGTTACAGGAGTAGCCGACTAAGTCT
CCGACGTCAAGCGAATAGGCGTCATACGCGATTATCGCCTAAGAGCACGTATTGGCGGTAAAAGGCTA
ACTAGACGCTTGTGGGTAGATTTCAAGGCGCTCGTAGTGGTATAATAGGATACTCTTTCACCAGCCTG
AGGGCCGAACGCTATACTAGTGGTCTGTGATGTAGGACCAAGTAGCTCTCTAGGGACCATCTACTTGA
GCAATGGTGCGCAGGGGTAGACATAGACCAACCTTGGGTGGCAAGCACTGCAATAAGTCCTGTTTAGC
CTTGGAGTTCACACGCCGGCACTAAAGCCGACCTACCTGAGCTTGTGATTACCGTTAAAGCATCTGTC
TAGTTCTGTTTCCGGCGCTCTTGGTTCCATGTTAGGGGAAGTGTATGACCCATGTGTTTTTATCGGCT
TAACCACGAGTGATCCCCGGTCGTTTCCCCATTGAATCCCTGGTGCATCCTACTCCCATAATGATAGC
TGACTGGCTGGACTGGCTTTTCAAGTAGTCGAGGGGGTATCGCGGTCACGGCCGTTAACAGATCCCGT
CCTTAGTGTGGAATCCGCACCTGCTGACTAACGCTTCGCCGGCGTGTCTGCACATCCGTATAGTGTTA
ATCATGACCCCAAGGAAGGATTAAACAAATATCTTGACG
>EDEN55
GTGTCCGGTAGCCCGCGCTAGTTAGACACCCCGGCAGGGGGGATTGCTTTCGAGACGGGAGATCCCTT
CGCCGACCCTGGAGGGCCGACGCCGAGGCATTCGGGCCCCTGCAACGTCAACAGCGGCAAGAAAACGG
GATGAATGGGCGTAATGGGGGGGGTCTGCTGGGGACCCGACGCGGTTGCCGTTTGCGGGGCCCCCGAC
CCATACCGACCCACCTAGGCGTCCAGTTACGGCGCACGTCGGGAGCGTGGTTGCCGTCAGAGCTGTGT
TTCTCGATCAGTCCCCCGCAGTGCCGCAGTATCTTGCCGTGGGCTGCTTTAATCTTGAAAGTGGTTTA
TACATTGGGCGACGAGGTGTCGACTCTCATTGGGGGTAACCGACGGGCACATGCAGTCCCCTCCCCGG
GCAGGCAGAGGCGGGGCCCCCGCGCGCCGGCCCCGGCCCACAATCTTACCAGGGTCCTCAAAGGAGCC
TTTGCATGGTACCCTTCGTGAATGGTTGCTTAAGAGGTCCACAACGTGGTCCGGGCACGGTCGACTAA
ACACTCAAAACAGCGACGGCAAATATAGGTACAAGGTCCAGGCCCTCACGGCACTAATTGCGATGACC
CAANTCACGGGGGCAGTCCCCGGCGCGGCGACCTTGATTACGTCCGGGGACAAGTATGTCGCTCCCCC
CGGGAGGGGTGCAGCCACATGGGAGATTCAAAGTTTCTCGTGACGTCGTTGTGATCACTGCAGCCTAG
CCGAGACTCCCGTACTACGCGAAGGTTGGTTATGTTAACCACTACAACGTGAGGCGCCGTAAGGGCCA
GTGTTGTGCCCGGCTCTTCAATGCGCCTTAGCGGCCTGATACACCCACCCAAGGAGATACTGCTAATC
ATATGGGTGGACAGAACCTCGCAACGCAGACGCCTCCCCGGCATGATATGGTTTTTTTCCGCTATTAT
CCAGCACGCAGCGCTATCATTCAAGAGAACCCAGTGACGCGTAAATCGTAAGATCTACCTGCCGCAGG
TGGACCTACTGCAAATACGGATTATGACTCGTAAAGGGGTCATGCGTATTTTCATCACTAGGCACGTT
CGAGAATAAATTAGTAGGTGTCCCGAGCCTTGTGGCGTTCCGCCTGACTCCTCATGAAGTCGACCTTC
TCACCGGCCCTATCTGCCGACGTAAGTCATAACCTAGATCTGTACCTCGGGGGGAGGGTCACTGTAAA
NGGATAATTGGAGGGTGATTTCCCACACTTTCCTAAGGGTACTTTTTGCCTGGCTTCGCAATTGGGTC
CAATGGATGTCGATCTCTGGTTTAGCAGTTGTGAAAGTGGCAAGGCGGGAGGTTAGACCTCCATTTAA
CATATACAAGCAAGTTAACTGCACTAGATGTGTAGACACTACAGTTACAGGAGTAGCCGAATAAGTCT
CCGACGTCAAGCGAATAAGCGTCATACGCGATTATCGCCTAAGAGCACGTATTGGCGGTAAAAGGCTA
GCTAGACGCTTATGGGTAGATTTCAAGGCGTTCGTAGTGGTATAATAGGATACTCTTTCACCAGCCTG
AGGGCCGAACGCTATACTAGTGGTCTGTGATGTAGGACCGAGTATCTCTCTAGGGACCATCTACTTGA
GCAATGGTGCGCAGGGGGAGACATAGACCAGCCTTGGGTGGCAAGCACTGCAATAAGTCCTGTTTAGC
CTTGGAGTTCACATGCCGGCACTGAAGCCGACCTACCTGAGCGTGTGATTACCGTTAAAGCATCTGTC
TAGTTCTGTTTCCGGCGCTCTTGGTTCCAGGTTAGGGGAAGTGTATGACCCATGTGTTTTTATCGGCT
TAACCACGAGTGATCCCCGGTCGTTTCCCCATTGAATCCCTGGTGCATCCTACTCCCAGAATGATAGC
TGACTGACTGGACTGGCTTTTCAAGTAATCGAGGGGGTATCGCGGTCACGGCCGTTAACAGATCCCGT
CCTTAGTGTGGAATCCGCACCTGCTGACTAACGCTTCGCCGGCGTGTCTGCACAGCCGTATAGTGTTA
ATCATGACCCCAAGGAAGGATTAAACAAATATCTTGACG
>CHAN01
GTGTCCGGTAGCCCGCGCTAGTTAGACACCCCGGCAGGGGGGATTGCTTTCGAGACGGGAGATCCCTT
CGCCGACCCTGGAGGGCCGACGCCGAGGCATTCGGGCCCCCGCAACGTCAACAGCGGCAAGAAAACGG
GATGAATGGGCGTAATGGGGGGGGTCTGCTGGGGACCCGACGCGGTTGCCGTTTGCGGGGCCCCCGAC
CCATACCGACCCACCTAGGCGTCCAGTTACGGCGCACGGCGGGAGCGTGGTTGCCGTCAGAGCTGTGT
TTCTCGATCAGTCCCCCGCAGTGCCGCAGTATCTTGCCGTGGGCTGCTTTAATCTTGAAAGTGGTTNA
TACATTGGGCGACGAGGTGTCGACTCTCATTGGGGGTAACCGACGGGCACATGCAGTCCCCTCCCCGG
GCAGGCAGAGGCGGGGCCCCCGCGCGCCGGCCCCGGCCCACAATCTTACCAGGGTCCTCAAAGGAGCC

TTTGCATGGTACCCTTCGTGAATGGTTGCTTAAGAGGTCCACAACGTGGTCCGGGCACGGTCGACTAA
ACACTCAAAACAGCGACGGCAAATATAGGTACAAGGTCCAGGCCCTCACGGCACTAATTGCGATGACC
CAACTCACGGGGGCAGTCCCCGGCGCGGCGACCTTGATTACGTCCGGGGACAAGTATGTCGCTCCCCC
CGGGAGGGGTGCAGCCACATGGGAGATTCAAAGTTTCTCGTGACGTCGTTGTGATCACTGCAGCCTAG
CCGAGACTCCCGTACTACGCGAAGGTTGGTTATGTTAACCACTACAACGTGAGGCGCCGTAAGGGCCA
GTGTTGTGCCCGGCTCTTCAATGCGCCTTAGCGGCCTGATACACCCACCCAAGGAGATACTGCTAATC
ATATGGGTGGACAGAACCTCGCAACGCAGACGCCTCCCCGGCATGATATGGTTTTTTTCCGCTATTAT
CCAGCACGCAGCGCTATCATTCAAGAGAACCCAGTGACGCGTAAATCGTAAGATCTACCTGCCGCAGG
TGGACCTACTGCAAATACGGATTATGACTCGTAAAGGGGTCATGCGTATTTTCATCACTAGGCACGTT
CGAGAATAAATTAGTAGGTGTCCCGAGCCTTGTGGCGTTCCGCCTGACTCCTCATGAAGTCGACCTTC
TCACCGGCCCTATCTGCCGACGTAAGTCATAACCTAGATCTGTACCTCGGGGGGAGGGTCACTGTAAA
GGGATAATTGGAGGGTGATTTCCCACACTTTCCTAAGGGTACTTTTTGCCTGGCTTCGCAATTGGGTC
CAATGGATGTCGATCTCTGGTTTAGCAGTTGTGAAAGTGGCAAGGCGGGAGGTTAGACCTCCATTTAA
CATATACAAGCAAGTTAACTGCACTAGATGTGTAGACACTACAGTTACAGGAGTAGCCGAATAAGTCT
CCGACGTCAAGCGAATAAGCGTCATACGCGATTATCGCCTAAGAGCACGTATTGGCGGTAAAAGGCTA
GCTAGACGCTTATGGGTAGATTTCAAGGCGTTCGTAGTGGTATAATAGGATACTCTTTCACCAGCCTG
AGGGCCGAACGCTATACTAGTGGTCTGTGATGTAGGACCGAGTATCTCTCTAGGGACCATCTACTTGA
GCAATGGTGCGCAGGGGGAGACATAGACCAGCCTTGGGTGGCAAGCACTGCAATAAGTCCTGTTTAGC
CTTGGAGTTCACATGCCGGCACTGAAGCCGACCTACCTGAGCGTGTGATTACCGTTAAAGCATCTGTC
TAGTTCTGTTTCCGGCGCTCTTGGTTCCAGGTTAGGGGAAGTGTATGACCCATGTGTTTTTATCGGCT
TAACCACGAGTGATCCCCGGTCGTTTCCCCATTGAATCCCTGGTGCATCCTACTCCCAGAATGATAGC
TGACTGACTGGACTGGCTTTTCAAGTAATCGAGGGGGTATCGCGGTCACGGCCGTTAACAGATCCCGT
CCTTAGTGTGGAATCCGCACCTGCTGACTAACGCTTCGCCGGCGTGTCTGCACAGCCGTATAGTGTTA
ATCATGACCCCAAGGAAGGATTAAACAAATATCTTGACG
>CHAN03
GTGTCCGGTAGCCCGCGCTAGTTAGACACCCCGGCAGGGGGGATTGCNTTCGAGACGGGAGATCCCTT
CGCCGACCCTGGAGGGCCGACGCCGAGGCATTCGGGCCCCTGCAACGTCAACAGCGGCAAGAAAACGG
GATGAATGGGCGTAATGGGGGGGGTCTGCTGGGGACCCGACGCGGTTGCCGTTTGCGGGGCCCCCGAC
CCATACCGACCCACCTAGGCGTCCAGTTACGGCGCACGTCGGGAGCGTGGTTGCCGTCAGAGCTGTGT
TTCTCGATCAGTCCCCCGCAGTGCCGCAGTATCTTGCCGTGGGCTGCTTTAATCTTGAAAGTGGTTTA
TACATTGGGCGACGAGGTGTCGACTCTCATTGGGGGTAACCGACGGGCACATGCAGTCCCCTCCCCGG
GCAGGCAGAGGCGGGGCCCCCGCGCGCCGGCCCCGGCCCACAATCTTACCAGGGTCCTCAAAGGAGCC
TTTGCATGGTACCCTTCGTGAATGGTTGCTTAAGAGGTCCACAACGTGGTCCGGGCACGGTCGACTAA
ACACTCAAAACAGCGACGGCAAATATAGGTACAAGGTCCAGGCCCTCACGGCACTAATTGCGATGACC
CAACTCACGGGGGCAGTCCCCGGCGCGGCGACCTTGATTACGTCCGGGGACAAGTATGTCGCTCCCCC
CGGGAGGGGTGCAGCCACATGGGAGATTCAAAGTTTCTCGTGACGTCGTTGTGATCACTGCAGCCTAG
CCGAGACTCCCGTACTACGCGAAGGTTGGTTATGTTAACCACTACAACGTGAGGCGCCGTAAGGGCCA
GTGTTGTGCCCGGCTCTTCAATGCGCCTTAGCGGCCTGATACACCCACCCAAGGAGATACTGCTAATC
ATATGGGTGGACAGAACCTCGCAACGCAGACGCCTCCCCGGCATGATATGGTTTTTTTCCGCTATTAT
CCAGCACGCAGCGCTATCATTCAAGAGAACCCAGTGACGCGTAAATCGTAAGATCTACCTGCCGCAGG
TGGACCTACTGCAAATACGGATTATGACTCGTAAAGGGGTCATGCGTATTTTCATCACTAGGCACGTT
CGAGAATAAATTAGTAGGTGTCCCGAGCCTTGTGGCGTTCCGCCTGACTCCTCATGAAGTCGACCTTC
TCACCGGCCCTATCTGCCGACGTAAGTCATAACCTAGATCTGTACCTCGGGGGGAGGGTCACTGTAAA
GGGATAATTGGAGGGTGATTTCCCACACTTTCCTAAGGGTACTTTTTGCCTGGCTTCGCAATTGGGTC
CAATGGATGTCGATCTCTGGTTTAGCAGTTGTGAAAGTGGCAAGGCGGGAGGTTAGACCTCCATTTAA
CATATACAAGCAAGTTAACTGCACTAGATGTGTAGACACTACAGTTACAGGAGTAGCCGAATAAGTCT
CCGACGTCAAGCGAATAAGCGTCATACGCGATTATCGCCTAAGAGCACGTATTGGCGGTAAAAGGCTA
GCTAGACGCTTATGGGTAGATTTCAAGGCGTTCGTAGTGGTATAATAGGATACTCTTTCACCAGCCTG
AGGGCCGAACGCTATACTAGTGGTCTGTGATGTAGGACCGAGTATCTCTCTAGGGACCATCTACTTGA
GCAATGGTGCGCAGGGGGAGACATAGACCAGCCTTGGGTGGCAAGCACTGCAATAAGTCCTGTTTAGC
CTTGGAGTTCACATGCCGGCACTGAAGCCGACCTACCTGAGCGTGTGATTACCGTTAAAGCATCTGTC
TAGTTCTGTTTCCGGCGCTCTTGGTTCCAGGTTAGGGGAAGTGTATGACCCATGTGTTTTTATCGGCT
TAACCACGAGTGATCCCCGGTCGTTTCCCCATTGAATCCCTGGTGCATCCTACTCCCAGAATGATAGC
TGACTGACTGGACTGGCTTTTCAAGTAATCGAGGGGGTATCGCGGTCACGGCCGTTAACAGATCCCGT

CCTTAGTGTGGAATCCGCACCTGCTGACTAACGCTTCGCCGGCGTGTCTGCACAGCCGTATAGTGTTA
ATCATGACCCCAAGGAAGGATTAAACAAATATCTTGACG
>CHAN05
GTGTCCGGTAGCCCGCGCTAGTTAGACACCCCGGCAGGGGGGATTGCTTTCGAGACGGGAGATCCCTT
CGCCGACCCTGGAGGGCCGACGCCGAGGCATTCGGGCCCCTGCAACGTCAACAGCGGCAAGAAAACGG
GATGAATGGGCGTAATGGGGGGGGTCTGCTGGGGACCCGACGCGGTTGCCGTTTGCGGGGCCCCCGAC
CCATACCGACCCACCTAGGCGTCCAGTTACGGCGCACGTCGGGAGCGTGGTTGCCGTCAGAGCTGTGT
TTCTCGATCAGTCCCCCGCAGTGCCGCAGTATCTTGCCGTGGGCTGCTTTAATCTTGAAAGTGGTTTA
TACATTGGGCGACGAGGTGTCGACTCTCATTGGGGGTAACCGACGGGCACATGCAGTCCCCTCCCCGG
GCAGGCAGAGGCGGGGCCCCCGCGCGCCGGCCCCGGCCCACAATCTTACCAGGGTCCTCAAAGGAGCC
TTTGCATGGTACCCTTCGTGAATGGTTGCTTAAGAGGTCCACAACGTGGTCCGGGCACGGTCGACTAA
ACACTCAAAACAGCGACGGCAAATATAGGTACAAGGTCCAGGCCCTCACGGCACTAATTGCGATGACC
CAACTCACGGGGGCAGTCCCCGGCGCGGCGACCTTGATTACGTCCGGGGACAAGTATGTCGCTCCCCC
CGGGAGGGGTGCAGCCACATGGGAGATTCAAAGTTTCTCGTGACGTCGTTGTGATCACTGCAGCCTAG
CCGAGACTCCCGTACTACGCGAAGGTTGGTTATGTTAACCACTACAACGTGAGGCGCCGTAAGGGCCA
GTGTTGTGCCCGGCTCTTCAATGCGCCTTAGCGGCCTGATACACCCACCCAAGGAGATACTGCTAATC
ATATGGGTGGACAGAACCTCGCAACGCAGACGCCTCCCCGGCATGATATGGTTTTTTTCCGCTATTAT
CCAGCACGCAGCGCTATCATTCAAGAGAACCCAGTGACGCGTAAATCGTAAGATCTACCTGCCGCAGG
TGGACCTACTGCAAATACGGATTATGACTCGTAAAGGGGTCATGCGTATTTTCATCACTAGGCACGTT
CGAGAATAAATTAGTAGGTGTCCCGAGCCTTGTGGCGTTCCGCCTGACTCCTCATGAAGTCGACCTTC
TCACCGGCCCTATCTGCCGACGTAAGTCATAACCTAGATCTGTACCTCGGGGGGAGGGTCACTGTAAA
GGGATAATTGGAGGGTGATTTCCCACACTTTCCTAAGGGTACTTTTTGCCTGGCTTCGCAATTGGGTC
CAATGGATGTCGATCTCTGGTTTAGCAGTTGTGAAAGTGGCAAGGCGGGAGGTTAGACCTCCATTTAA
CATATACAAGCAAGTTAACTGCACTAGATGTGTAGACACTACAGTTACAGGAGTAGCCGAATAAGTCT
CCGACGTCAAGCGAATAAGCGTCATACGCGATTATCGCCTAAGAGCACGTATTGGCGGTAAAAGGCTA
GCTAGACGCTTATGGGTAGATTTCAAGGCGTTCGTAGTGGTATAATAGGATACTCTTTCACCAGCCTG
AGGGCCGAACGCTATACTAGTGGTCTGTGATGTAGGACCGAGTATCTCTCTAGGGACCATCTACTTGA
GCAATGGTGCGCAGGGGGAGACATAGACCAGCCTTGGGTGGCAAGCACTGCAATAAGTCCTGTTTAGC
CTTGGAGTTCACATGCCGGCACTGAAGCCGACCTACCTGAGCGTGTGATTACCGTTAAAGCATCTGTC
TAGTTCTGTTTCCGGCGCTCTTGGTTCCAGGTTAGGGGAAGTGTATGACCCATGTGTTTTTATCGGCT
TAACCACGAGTGATCCCCGGTCGTTTCCCCATTGAATCCCTGGTGCATCCTACTCCCAGAATGATAGC
TGACTGACTGGACTGGCTTTTCAAGTAATCGAGGGGGTATCGCGGTCACGGCCGTTAACAGATCCCGT
CCTTAGTGTGGAATCCGCACCTGCTGACTAACGCTTCGCCGGCGTGTCTGCACAGCCGTATAGTGTTA
ATCATGACCCCAAGGAAGGATTAAACAAATATCTTGACG
>CHAN06
GTGTCCGGTAGCCCGCGCTAGTTAGACACCCCGGCAGGGGGGATTGCTTTCGGGACGGGAGATCCCTT
CGCCGACCCTGGAGGGCCGACGCCGAGGCATTCGGGCCCCTGCAACGTCAACAGCGGCAAGAAAACGG
GATGAATGGGCGTAATGGGGGGGGTCTGCTGGGGACCCGACGCGGTTGCCGTTTGCGGGGCCCCCGAC
CCATACCGACCCACCTAGGCGTCCAGTTACGGCGCACGTCGGGAGCGTGGTTGCCGTCAGAGCTGTGT
TTCTCGATCAGTCCCCCGCAGTGCCGCAGTATCTTGCCGTGGGCTGCTTTAATCTTGAAAGTGGTTCA
TACATTGGGCGACGAGGTGTCGACTCTCATTGGGGGTAACCGACGGGCACATGCAGTCCCCTCCCCGG
GCAGGCAGAGGCGGGGCCCCCGCGCGCCGGCCCCGGCCCACAATCTTACCAGGGTCCTCAAAGGAGCC
TTTGCATGGTACCCTTCGTGAATGGTTGCTTAAGAGGTCCACCACGTGGTCCGGGCACGGTCGACTAA
ACACTCAAAACAGCGACGGCAAATATAGGCACAAGGTCCAGGCCCTCACGGCACTAATTGCGATGACC
CAACTCACGGGGGCAGTCCCCGGCGCGGCGATCTTGATTACGGCCGGGGACAAGTATGTCGTTCCCCC
CGGGAGGGGTGCAGCCACATGGGAGATTCAAAGTTTCTCGTGACGTCGTTGTGATCACTGCAGCCTAG
CCGAGACTCCCGTACTACGCGAAGGTTGGTTATGTTAACCACTACAACGTGAGGCGCCGTAAGGGCCA
GTGTTGTGCCCGGCTCTTCAATGCGCCTTAGCGGCCTGATACACCCACCCAAGGAGATGCTGCTAATC
ATGTGGGTGGACAGAACCTCGCAACGCAGACGCCTCCCCGGCATGATATGGTTTTTTTCCGCTATTAT
CCAGCACGCAGCGCTATCATTCAAGAGAACCCAGTGACGCGTAAATCGTAAGATCTACCTGCCGCAGG
TGGACCTACTGCAAATACGGATTATGACTCGTAAAGAGGTCATGCGTATTTTCATCACTAGGCACGTT
CGAGAATAAATTAGTAGGTGTCCCGCGCCTTGTGGCGTTCCGCCTGACTCCTCATGAAGTCGGCCTTC
TCACCGGCCCTATCTGCCGACGTAAGTCATAACCTAGATCTGTACCTCGGGGGGAGGGTCACTGTAAA
GGGATAATTGGAGGGTGATTTCCCACACTTTCCTAAGGGTACTTTTTGCCTGGCTTCGCAATTGGGTC

CAATGGATATCGATCTCTGGTTTAACAGTTGTGAAAGTGGCAAGGCGGGAGGTTAGACCTCCATTTAA
CATATACAAGCAAGTTAACTGCACTAGATGTGTAGACACTACAGTTACAGGAGTAGACGAATAAGTCT
CCGACGTCAAGCGAATAAGCGTCATACGCGATTATCGCCTAAGAGCACGTACCGGCGGTAAAAGGCTA
ACTAGACGCTTGTGGGTAGATTTCAAGGCGTTCGTAGTGGTATAACAGGATACTCTTTCACCAGCCTG
AGGGCCGAACGCTATACTAGTGGTCTATGATGTAGGACCGAGTATCTCTCTAGGGACCATCTACTTGA
GCAATGGTGCGCAGGGGGAGACATAGACCAGCCTTGGGTNGCAAGCACTGCAATAAGTCCTGTTTAGC
CTTGGAGTTCACACGCCGTCACTGAAGCCGACCTACCTGAGCTTGTGATTACCGTTAAAGCATCTGTC
TAGTTCTGTTTCCGGCGCTCTTGGTTCCAGGTTAGGGGAAGTGTATGACCCATGTGTTTTTCTCGGCT
TAACCACGAGTGATCCCCGGTCGTTTCCCCGTTGAATCCCTGGTGCATCCTACTCCCAGAATGATAGC
TGACTGACTGGACTGGCTTTTCAAGTAGTCGAGGGGGTATCGCGGTCACGGCCGTTAACAGATCCCGT
CCTTAGTGTGGAATCCGCACCTGCTGACTAACGCTTCGCCGGCGTGTCTGCACAGCCGTATAGTGTTA
ATCATGACCCCAAGGAAGGATTAAACAAATATCTTGACG
>CHAN07
GTGTCCGGTAGCCCGCGCTAGTTAGACACCCCGGCAGGGGGGATTGCTTTCGGGACGGGAGATCCCTT
CGCCGACCCTGGAGGGCCGACGCCGAGGCATTCGGGCCCCTGCAACGTCAACAGCGGCAAGAAAACGG
GATGAATGGGCGTAATGGGGGGGGTCTGCTGGGGACCCGACGCGGTTGCCGTTTGCGGGGCCCCCGAC
CCATACCGACCCACCTAGGCGTCCAGTTACGGCGCACGTCGGGAGCGTGGTTGCCGTCAGAGCTGTGT
TTCTCGATCAGTCCCCCGCAGTGCCGCAGTATCTTGCCGTGGGCTGCTTTAATCTTGAAAGTGGTTCA
TACATTGGGCGACGAGGTGTCGACTCTCATTGGGGGTAACCGACGGGCACATGCAGTCCCCTCCCCGG
GCAGGCAGAGGCGGGGCCCCCGCGCGCCGGCCCCGGCCCACAATCTTACCAGGGTCCTCAAAGGAGCC
TTTGCATGGTACCCTTCGTGAATGGTTGCTTAAGAGGTCCACCACGTGGTCCGGGCACGGTCGACTAA
ACACTCAAAACAGCGACGGCAAATATAGGCACAAGGTCCAGGCCCTCACGGCACTAATTGCGATGACC
CAACTCACGGGGGCAGTCCCCGGCGCGGCGANCTTGATTACGGCCGGGGACAAGTATGTCGTTCCCCC
CGGGAGGGGTGCAGCCACATGGGAGATTCAAAGTTTCTCGTGACGTCGTTGTGATCACTGCAGCCTAG
CCGAGACTCCCGTACTACGCGAAGGTTGGTTATGTTAACCACTACAACGTGAGGCGCCGTAAGGGCCA
GTGTTGTGCCCGGCTCTTCAATGCGCCTTAGCGGCCTGATACACCCACCCAAGGAGATGCTGCTAATC
ATGTGGGTGGACAGAACCTCGCAACGCAGACGCCTCCCCGGCATGATATGGTTTTTTTCCGCTATTAT
CCAGCACGCAGCGCTATCATTCAAGAGAACCCAGTGACGCGTAAATCGTAAGATCTACCTGCCGCAGG
TGGACCTACTGCAAATACGGATTATGACTCGTAAAGAGGTCATGCGTATTTTCATCACTAGGCACGTT
CGAGAATAAATTAGTAGGTGTCCCGCGCCTTGTGGCGTTCCGCCTGACTCCTCATGAAGTCGGCCTTC
TCACCGGCCCTATCTGCCGACGTAAGTCATAACCTAGATCTGTACCTCGGGGGGAGGGTCACTGTAAA
GGGATAATTGGAGGGTGATTTCCCACACTTTCCTAAGGGTACTTTTTGCCTGGCTTCGCAATTGGGTC
CAATGGATATCGATCTCTGGTTTAACAGTTGTGAAAGTGGCAAGGCGGGAGGTTAGACCTCCATTTAA
CATATACAAGCAAGTTAACTGCACTAGATGTGTAGACACTACAGTTACAGGAGTAGACGAATAAGTCT
CCGACGTCAAGCGAATAAGCGTCATACGCGATTATCGCCTAAGAGCACGTACCGGCGGTAAAAGGCTA
ACTAGACGCTTGTGGGTAGATTTCAAGGCGTTCGTAGTGGTATAACAGGATACTCTTTCACCAGCCTG
AGGGCCGAACGCTATACTAGTGGTCTATGATGTAGGACCGAGTATCTCTCTAGGGACCATCTACTTGA
GCAATGGTGCGCAGGGGGAGACATAGACCAGCCTTGGGTNGCAAGCACTGCAATAAGTCCTGTTTAGC
CTTGGAGTTCACACGCCGTCACTGAAGCCGACCTACCTGAGCTTGTGATTACCGTTAAAGCATCTGTC
TAGTTCTGTTTCCGGCGCTCTTGGTTCCAGGTTAGGGGAAGTGTATGACCCATGTGTTTTTCTCGGCT
TAACCACGAGTGATCCCCGGTCGTTTCCCCGTTGAATCCCTGGTGCATCCTACTCCCAGAATGATAGC
TGACTGACTGGACTGGCTTTTCAAGTAGTCGAGGGGGTATCGCGGTCACGGCCGTTAACAGATCCCGT
CCTTAGTGTGGAATCCGCACCTGCTGACTAACGCTTCGCCGGCGTGTCTGCACAGCCGTATAGTGTTA
ATCATGACCCCAAGGAAGGATTAAACAAATATCTTGACG
>CHAN08
GTGTCCGGTAGCCCGCGCTAGTTAGACACCCCGGCAGGGGGGATTGCTTTCGAGACGGGAGATCCCTT
CGCCGACCCTGGAGGGCCGACGCCGAGGCATTCGGGCCCCTGCAACGTCAACAGCGGCAAGAAAACGG
GATGAATGGGCGTAATGGGGGGGGTCTGCTGGGGACCCGACGCGGTTGCCGTTTGCGGGGCCCCCGAC
CCATACCGACCCACCTAGGCGTCCAGTTACGGCGCACGTCGGGAGCGTGGTTGCCGTCAGAGCTGTGT
TTCTCGATCAGTCCCCCGCAGTGCCGCAGTATCTTGCCGTGGGCTGCTTTAATCTTGAAAGTGGTTCA
TACATTGGGCGACGAGGTGTCGACTCTCATTGGGGGTAACCGACGGGCACATGCAGTCCCCTCCCCGG
GCAGGCAGAGGCGGGGCCCCCGCGCGCCGGCCCCGGCCCACAATCTTACCAGGGTCCTCAAAGGAGCC
TTTGCATGGTACCCTTCGTGAATGGTTGCTTAAGAGGTCCACCACGTGGTCCGGGCACGGTCGACTAA
ACACTCAAAACAGCGACGGCAAATATAGGCACAAGGTCCAGGCCCTCACGGCACTAATTGCGATGACC

CAACTCACGGGGGCAGTCCCCGGCGCGGCGATCTTGATTACGGCCGGGGACAAGTATGTCGTTCCCCC
CGGGAGGGGTGCAGCCACATGGGAGATTCAAAGTTTCTCGTGACGTCGTTGTGATCACTGCAGCCTAG
CCGAGACTCCCGTACTACGCGAAGGTTGGTTATGTTAACCACTACAACGTGAGGCGCCGTAAGGGCCA
GTGTTGTGCCCGGCTCTTCAATGCGCCTTAGCGGCCTGATACACCCACCCAAGGAGATGCTGCTAATC
ATGTGGGTGGACAGAACCTCGCAACGCAGACGCCTCCCCGGCATGATATGGTTTTTTTCCGCTATTAT
CCAGCACGCAGCGCTATCATTCAAGAGAACCCAGTGACGCGTAAATCGTAAGATCTACCTGCCGCAGG
TGGACCTACTGCAAATACGGATTATGACTCGTAAAGAGGTCATGCGTATTTTCATCACTAGGCACGTT
CGAGAATAAATTAGTAGGTGTCCCGCGCCTTGTGGCGTTCCGCCTGACTCCTCATGAAGTCGGCCTTC
TCACCGGCCCTATCTGCCGACGTAAGTCATAACCTAGATCTGTACCTCGGGGGGAGGGTCACTGTAAA
GGGATAATTGGAGGGTGATTTCCCACACTTTCCTAAGGGTACTTTTTGCCTGGCTTCGCAATTGGGTC
CAATGGATATCGATCTCTGGTTTAACAGTTGTGAAAGTGGCAAGGCGGGAGGTTAGACCTCCATTTAA
CATATACAAGCAAGTTAACTGCACTAGATGTGTAGACACTACAGTTACAGGAGTAGACGAATAAGTCT
CCGACGTCAAGCGAATAAGCGTCATACGCGATTATCGCCTAAGAGCACGTACCGGCGGTAAAAGGCTA
ACTAGACGCTTGTGGGTAGATTTCAAGGCGTTCGTAGTGGTATAACAGGATACTCTTTCACCAGCCTG
AGGGCCGAACGCTATACTAGTGGTCTATGATGTAGGACCGAGTATCTCTCTAGGGACCATCTACTTGA
GCAATGGTGCGCAGGGGGAGACATAGACCAGCCTTGGGTNGCAAGCACTGCAATAAGTCCTGTTTAGC
CTTGGAGTTCACACGCCGTCACTGAAGCCGACCTACCTGAGCTTGTGATTACCGTTAAAGCATCTGTC
TAGTTCTGTTTCCGGCGCTCTTGGTTCCAGGTTAGGGGAAGTGTATGACCCATGTGTTTTTCTCGGCT
TAACCACGAGTGATCCCCGGTCGTTTCCCCGTTGAATCCCTGGTGCATCCTACTCCCAGAATGATAGC
TGACTGACTGGACTGGCTTTTCAAGTAGTCGAGGGGGTATCGCGGTCACGGCCGTTAACAGATCCCGT
CCTTAGTGTGGAATCCGCACCTGCTGACTAACGCTTCGCCGGCGTGTCTGCACAGCCGTATAGTGTTA
ATCATGACCCCAAGGAAGGATTAAACAAATATCTTGACG
>CHAN09
GTGTCCGGTAGCCCGCGCTAGTTAGACACCCCGGCAGGGGGGATTGCTTTCGAGACGGGAGATCCCTT
CGCCGACCCTGGAGGGCCGACGCCGAGGCATTCGGGCCCCCGCAACGTCAACAGCGGCAAGAAAACGG
GATGAATGGGCGTAATGGGGGGGGTCTGCTGGGGACCCGACGCGGTTGCCGTTTGCGGGGCCCCCGAC
CCATACCGACCCACCTAGGCGTCCAGTTACGGCGCACGGCGGGAGCGTGGTTGCCGTCAGAGCTGTGT
TTCTCGATCAGTCCCCCGCAGTGCCGCAGTATCTTGCCGTGGGCTGCTTTAATCTTGAAAGTGGTTNA
TACATTGGGCGACGAGGTGTCGACTCTCATTGGGGGTAACCGACGGGCACATGCAGTCCCCTCCCCGG
GCAGGCAGAGGCGGGGCCCCCGCGCGCCGGCCCCGGCCCACAATCTTACCAGGGTCCTCAAAGGAGCC
TTTGCATGGTACCCTTCGTGAATGGTTGCTTAAGAGGTCCACAACGTGGTCCGGGCACGGTCGACTAA
ACACTCAAAACAGCGACGGCAAATATAGGTACAAGGTCCAGGCCCTCACGGCACTAATTGCGATGACC
CAACTCACGGGGGCAGTCCCCGGCGCGGCGACCTTGATTACGTCCGGGGACAAGTATGTCGCTCCCCC
CGGGAGGGGTGCAGCCACATGGGAGATTCAAAGTTTCTCGTGACGTCGTTGTGATCACTGCAGCCTAG
CCGAGACTCCCGTACTACGCGAAGGTTGGTTATGTTAACCACTACAACGTGAGGCGCCGTAAGGGCCA
GTGTTGTGCCCGGCTCTTCAATGCGCCTTAGCGGCCTGATACACCCACCCAAGGAGATACTGCTAATC
ATATGGGTGGACAGAACCTCGCAACGCAGACGCCTCCCCGGCATGATATGGTTTTTTTCCGCTATTAT
CCAGCACGCAGCGCTATCATTCAAGAGAACCCAGTGACGCGTAAATCGTAAGATCTACCTGCCGCAGG
TGGACCTACTGCAAATACGGATTATGACTCGTAAAGGGGTCATGCGTATTTTCATCACTAGGCACGTT
CGAGAATAAATTAGTAGGTGTCCCGAGCCTTGTGGCGTTCCGCCTGACTCCTCATGAAGTCGACCTTC
TCACCGGCCCTATCTGCCGACGTAAGTCATAACCTAGATCTGTACCTCGGGGGGAGGGTCACTGTAAA
GGGATAATTGGAGGGTGATTTCCCACACTTTCCTAAGGGTACTTTTTGCCTGGCTTCGCAATTGGGTC
CAATGGATGTCGATCTCTGGTTTAGCAGTTGTGAAAGTGGCAAGGCGGGAGGTTAGACCTCCATTTAA
CATATACAAGCAAGTTAACTGCACTAGATGTGTAGACACTACAGTTACAGGAGTAGCCGAATAAGTCT
CCGACGTCAAGCGAATAAGCGTCATACGCGATTATCGCCTAAGAGCACGTATTGGCGGTAAAAGGCTA
GCTAGACGCTTATGGGTAGATTTCAAGGCGTTCGTAGTGGTATAATAGGATACTCTTTCACCAGCCTG
AGGGCCGAACGCTATACTAGTGGTCTGTGATGTAGGACCGAGTATCTCTCTAGGGACCATCTACTTGA
GCAATGGTGCGCAGGGGGAGACATAGACCAGCCTTGGGTGGCAAGCACTGCAATAAGTCCTGTTTAGC
CTTGGAGTTCACATGCCGGCACTGAAGCCGACCTACCTGAGCGTGTGATTACCGTTAAAGCATCTGTC
TAGTTCTGTTTCCGGCGCTCTTGGTTCCAGGTTAGGGGAAGTGTATGACCCATGTGTTTTTATCGGCT
TAACCACGAGTGATCCCCGGTCGTTTCCCCATTGAATCCCTGGTGCATCCTACTCCCAGAATGATAGC
TGACTGACTGGACTGGCTTTTCAAGTAATCGAGGGGGTATCGCGGTCACGGCCGTTAACAGATCCCGT
CCTTAGTGTGGAATCCGCACCTGCTGACTAACGCTTCGCCGGCGTGTCTGCACAGCCGTATAGTGTTA
ATCATGACCCCAAGGAAGGATTAAACAAATATCTTGACG

>CHAN10
GTGTCCGGTAGCCCGCGCTAGTTAGACACCCCGGCAGGGGGGATTGCTTTCGAGACGGGAGATCCCTT
CGCCGACCCTGGAGGGCCGACGCCGAGGCATTCGGGCCCCTGCAACGTCAACAGCGGCAAGAAAACGG
GATGAATGGGCGTAATGGGGGGGGTCTGCTGGGGACCCGACGCGGTTGCCGTTTGCGGGGCCCCCGAC
CCATACCGACCCACCTAGGCGTCCAGTTACGGCGCACGTCGGGAGCGTGGTTGCCGTCAGAGCTGTGT
TTCTCGATCAGTCCCCCGCAGTGCCGCAGTATCTTGCCGTGGGCTGCTTTAATCTTGAAAGTGGTTTA
TACATTGGGCGACGAGGTGTCGACTCTCATTGGGGGTAACCGACGGGCACATGCAGTCCCCTCCCCGG
GCAGGCAGAGGCGGGGCCCCCGCGCGCCGGCCCCGGCCCACAATCTTACCAGGGTCCTCAAAGGAGCC
TTTGCATGGTACCCTTCGTGAATGGTTGCTTAAGAGGTCCACAACGTGGTCCGGGCACGGTCGACTAA
ACACTCAAAACAGCGACGGCAAATATAGGTACAAGGTCCAGGCCCTCACGGCACTAATTGCGATGACC
CAACTCACGGGGGCAGTCCCCGGCGCGGCGACCTTGATTACGTCCGGGGACAAGTATGTCGCTCCCCC
CGGGAGGGGTGCAGCCACATGGGAGATTCAAAGTTTCTCGTGACGTCGTTGTGATCACTGCAGCCTAG
CCGAGACTCCCGTACTACGCGAAGGTTGGTTATGTTAACCACTACAACGTGAGGCGCCGTAAGGGCCA
GTGTTGTGCCCGGCTCTTCAATGCGCCTTAGCGGCCTGATACACCCACCCAAGGAGATACTGCTAATC
ATATGGGTGGACAGAACCTCGCAACGCAGACGCCTCCCCGGCATGATATGGTTTTTTTCCGCTATTAT
CCAGCACGCAGCGCTATCATTCAAGAGAACCCAGTGACGCGTAAATCGTAAGATCTACCTGCCGCAGG
TGGACCTACTGCAAATACGGATTATGACTCGTAAAGGGGTCATGCGTATTTTCATCACTAGGCACGTT
CGAGAATAAATTAGTAGGTGTCCCGAGCCTTGTGGCGTTCCGCCTGACTCCTCATGAAGTCGACCTTC
TCACCGGCCCTATCTGCCGACGTAAGTCATAACCTAGATCTGTACCTCGGGGGGAGGGTCACTGTAAA
GGGATAATTGGAGGGTGATTTCCCACACTTTCCTAAGGGTACTTTTTGCCTGGCTTCGCAATTGGGTC
CAATGGATGTCGATCTCTGGTTTAGCAGTTGTGAAAGTGGCAAGGCGGGAGGTTAGACCTCCATTTAA
CATATACAAGCAAGTTAACTGCACTAGATGTGTAGACACTACAGTTACAGGAGTAGCCGAATAAGTCT
CCGACGTCAAGCGAATAAGCGTCATACGCGATTATCGCCTAAGAGCACGTATTGGCGGTAAAAGGCTA
GCTAGACGCTTATGGGTAGATTTCAAGGCGTTCGTAGTGGTATAATAGGATACTCTTTCACCAGCCTG
AGGGCCGAACGCTATACTAGTGGTCTGTGATGTAGGACCGAGTATCTCTCTAGGGACCATCTACTTGA
GCAATGGTGCGCAGGGGGAGACATAGACCAGCCTTGGGTGGCAAGCACTGCAATAAGTCCTGTTTAGC
CTTGGAGTTCACATGCCGGCACTGAAGCCGACCTACCTGAGCGTGTGATTACCGTTAAAGCATCTGTC
TAGTTCTGTTTCCGGCGCTCTTGGTTCCAGGTTAGGGGAAGTGTATGACCCATGTGTTTTTATCGGCT
TAACCACGAGTGATCCCCGGTCGTTTCCCCATTGAATCCCTGGTGCATCCTACTCCCAGAATGATAGC
TGACTGACTGGACTGGCTTTTCAAGTAATCGAGGGGGTATCGCGGTCACGGCCGTTAACAGATCCCGT
CCTTAGTGTGGAATCCGCACCTGCTGACTAACGCTTCGCCGGCGTGTCTGCACAGCCGTATAGTGTTA
ATCATGACCCCAAGGAAGGATTAAACAAATATCTTGACG
>CHAN11
GTGTCCGGTAGCCCGCGCTAGTTAGACACCCCGGCAGGGGGGATTGCTTTCGGGACGGGAGATCCCTT
CGCCGACCCTGGAGGGCCGACGCCGAGGCATTCGGGCCCCTGCAACGTCAACAGCGGCAAGAAAACGG
GATGAATGGGCGTAATGGGGGGGGTCTGCTGGGGACCCGACGCGGTTGCCGTTTGCGGGGCCCCCGAC
CCATACCGACCCACCTAGGCGTCCAGTTACGGCGCACGTCGGGAGCGTGGTTGCCGTCAGAGCTGTGT
TTCTCGATCAGTCCCCCACAGTGCCGCAGTATCTTGCCGTGGGCTGCTTTAATCTTGAAAGTGGTTTA
TACATTGGGCGACGAGGTGTCGACTCTCATTGGGGGTAACCGACGGGCACATGCAGTCCCCTCCCCGG
GCAGGCAGAGGCGGGGCCCCCGCGCGCCGGCCCCGGCCCACAATCTTACCAGGGTCCTCAAAGGAGCC
TTTGCATGGTACCCTTCGTGAATGGTTGCTTAAGAAGTCCACAACGTGGTCCGGGCACGGTCGACTAA
ACACTCAAAACAGCGACGGCAAATATAGGTACAAGGTCCAGGCCCTCACGGCACTAATTGCGATGACC
CAACTCACGGGGGCAGTCCCCGGCGCGGCGACCTTGATTACGTCCGGGGACAAGTATGTCGCTCCCCC
CGGGAGGGGTGCAGCCACATGGGAGATTCAAAGTTTCTCGTGACGTCGTTGTGATCACTGCAGCCTAG
CCGAGACTCCCGTACTACGCGAAGGTTGGTTATGTTAACCACTACAACGTGAGGCGCCGTAAGGGCCA
GTGTTGNGCCCGGCTCTTCAATGCGCCTTAGCGGCCTGATACACCCACCCAAGGAGATACTGCTAATC
ATATGAGTGGACAGAACCTCGCAACGCAGACGCCTCCCCGGCATGATATGGTTTTTTTCCGCTATTAT
CCAGCACGCAGCGCTATCATTCAAGAGAACCCAGTGACGCGTAAATCGTAAGATCTACCTGCCGCAGG
TGGACCTACTGCAAATACGGATTATGACTCGTAAAGGGGTCATGCGTATTTTCATCACTAGGCACGTT
CGAGAATAAATTAGTAGGTGTCCCGAGCCTTGTGGCGTTCCGCCTGACTCCTCATGAAGTCGACCTTC
TCACCGGCCCTATCTGCCGACGTAAGTCATAACCTAGATCTGTACCTCGGGGGGAGGGTCACTGTAAA
GGGATAATTGGAGGGTGATTTCCCACATTTTCCTAAGGGTACTTTTTGCCTGGCTTCGCAATTGGGTC
CAATGGATGTCGATCTCTGGTTTAGCAGTTGTGAAAGTGGCAAGGCGGGAGGTTAGACCTCCATTTAA
CATATACAAGCAAGTTAACTGCACTAGATGTGTAGACACTACAGGTACAGGAGTAGCCGGATAAGTCT

CCGACGTCAAGCGAATAAGCTTCATACGCGATTATCGCCTAAGAGCACGTATTTGCGGTAAAAGGCTA
GCTAGACGCTTGTGGGTAGATTTCAAGGCGTTCGTAGTGGTATAATAGGATACTCTTTCACCAGCCTG
AGGGCCGAACGCTATACTAGTGGTCTGTGATGTAGGACCGAGTATCTCTCTAGGGACCATCTACTTGA
GCAATGGTGCGCAGGGGGAGACATAGACCAGCCTTGGGTGGCAAGCACTGCAATAAGTCCTGTTTAGC
CTTGGAGTTCACACGCCGGCACTGAAGCCGACCTACCTGAGCGTGTGATTACCGTTAAAGCATCTGTC
TAGTTCTGTTTCCGGCGCTCTTGGTTCCAGGTTAGGGGAAGTGTATGACCCATGTGTTTTCATCGGCT
TAACCACGAGTGATCCCCGGTCGTTTCCCCATTGAATCCCTGGTGCATCCTACTCCCAGAATGATAGC
TGACTGACTGGACTGGCTTTTCAAGTAATCGAGGGGGTATCGCGGTCACGGCCGTTAACAGATCCCGT
CCTTAGTGTGGAATCCGCACCTGCTGACTAACGCTTCGCCGGCGTGTCTGCACAGCCGTATAGTGTTA
ATCATGACCCCAAGGAAGGATTAAACAAATATCTTGACG
>CHAN12
GTGTCCGGTAGCCCGCGCTAGTTAGACACCCCGGCAGGGGGGATTGCTTTCGAGACGGGAGATCCCTT
CGCCGACCCTGGAGGGCCGACGCCGAGGCATTCGGGCCCCTGCAACGTCAACAGCGGCAAGAAAACGG
GATGAATGGGCGTAATGGGGGGGGTCTGCTGGGGACCCGACGCGGTTGCCGTTTGCGGGGCCCCCGAC
CCATACCGACCCACCTAGGCGTCCAGTTACGGCGCACGGCGGGAGCGTGGTTGCCGTCAGAGCTGTGT
TTCTCGATCAGTCCCCCGCAGTGCCGCAGTATCTTGCCGTGGGCTGCTTTAATCTTGAAAGTGGTTTA
TACATTGGGCGACGAGGTGTCGACTCTCATTGGGGGTAACCGACGGGCACATGCAGTCCCCTCCCCGG
GCAGGCAGAGGCGGGGCCCCCGCGCGCCGGCCCCGGCCCACAATCTTACCAGGGTCCTCAAAGGAGCC
TTTGCATGGTACCCTTCGTGAATGGTTGCTTAAGAGGTCCACCACGTGGTCCGGGCACGGTCGACTAA
ACACTCAAAACAGCGACGGCAAATATAGGCACAAGGTCCAGGCCCTCACGGCACTAATTGCGATGACC
CAACTCACGGGGGCAGTCCCCGGCGCGGCGATCTTGATTACGGCCGGGGACAAGTATGTCGTTCCCCC
CGGGAGGGGTGCAGCCACATGGGAGATTCAAAGTTTCTCGTGACGTCGTTGTGATCACTGCAGCCTAG
CCGAGACTCCCGTACTACGCGAAGGTTGGTTATGTTAACCACTACAACGTGAGGCGCCGTAAGGGCCA
GTGTTGTGCCCGGCTCTTCAATGCGCCTTAGCGGCCTGATACACCCACCCAAGGAGATGCTGCTAATC
ATGTGGGTGGACAGAACCTCGCAACGCAGACGCCTCCCCGGCATGATATGGTTTTTTTCCGCTATTAT
CCAGCACGCAGCGCTATCATTCAAGAGAACCCAGTGACGCGTAAATCGTAAGATCTACCTGCCGCAGG
TGGACCTACTGCAAATACGGATTATGACTCGTAAAGAGGTCATGCGTATTTTCATCACTAGGCACGTT
CGAGAATAAATTAGTAGGTGTCCCGCGCCTTGTGGCGTTCCGCCTGACTCCTCATGAAGTCGGCCTTC
TCACCGGCCCTATCTGCCGACGTAAGTCATAACCTAGATCTGTACCTCGGGGGGAGGGTCACTGTAAA
GGGATAATTGGAGGGTGATTTCCCACACTTTCCTAAGGGTACTTTTTGCCTGGCTTCGCAATTGGGTC
CAATGGATATCGATCTCTGGTTTAACAGTTGTGAAAGTGGCAAGGCGGGAGGTTAGACCTCCATTTAA
CATATACAAGCAAGTTAACTGCACTAGATGTGTAGACACTACAGTTACAGGAGTAGACGAATAAGTCT
CCGACGTCAAGCGAATAAGCGTCATACGCGATTATCGCCTAAGAGCACGTACCGGCGGTAAAAGGCTA
ACTAGACGCTTGTGGGTAGATTTCAAGGCGTTCGTAGTGGTATAACAGGATACTCTTTCACCAGCCTG
AGGGCCGAACGCTATACTAGTGGTCTATGATGTAGGACCGAGTATCTCTCTAGGGACCATCTACTTGA
GCAATGGTGCGCAGGGGGAGACATAGACCAGCCTTGGGTNGCAAGCACTGCAATAAGTCCTGTTTAGC
CTTGGAGTTCACACGCCGTCACTGAAGCCGACCTACCTGAGCTTGTGATTACCGTTAAAGCATCTGTC
TAGTTCTGTTTCCGGCGCTCTTGGTTCCAGGTTAGGGGAAGTGTATGACCCATGTGTTTTTCTCGGCT
TAACCACGAGTGATCCCCGGTCGTTTCCCCGTTGAATCCCTGGTGCATCCTACTCCCAGAATGATAGC
TGACTGACTGGACTGGCTTTTCAAGTAGTCGAGGGGGTATCGCGGTCACGGCCGTTAACAGATCCCGT
CCTTAGTGTGGAATCCGCACCTGCTGACTAACGCTTCGCCGGCGTGTCTGCACAGCCGTATAGTGTTA
ATCATGACCCCAAGGAAGGATTAAACAAATATCTTGACG
>CHAN15
GTGTCCGGTAGCCCGCGCTAGTTAGACACCCCGGCAGGGGGGATTGCTTTCGAGACGGGAGATCCCTT
CGCCGACCCTGGAGGGCCGACGCCGAGGCATTCGGGCCCCCGCAACGTCAACAGCGGCAAGAAAACGG
GATGAATGGGCGTAATGGGGGGGGTCTGCTGGGGACCCGACGCGGTTGCCGTTTGCGGGGCCCCCGAC
CCATACCGACCCACCTAGGCGTCCAGTTACGGCGCACGGCGGGAGCGTGGTTGCCGTCAGAGCTGTGT
TTCTCGATCAGTCCCCCGCAGTGCCGCAGTATCTTGCCGTGGGCTGCTTTAATCTTGAAAGTGGTTCA
TACATTGGGCGACGAGGTGTCGACTCTCATTGGGGGTAACCGACGGGCACATGCAGTCCCCTCCCCGG
GCAGGCAGAGGCGGGGCCCCCGCGCGCCGGCCCCGGCCCACAATCTTACCAGGGTCCTCAAAGGAGCC
TTTGCATGGTACCCTTCGTGAATGGTTGCTTAAGAGGTCCACAACGTGGTCCGGGCACGGTCGACTAA
ACACTCAAAACAGCGACGGCAAATATAGGTACAAGGTCCAGGCCCTCACGGCACTAATTGCGATGACC
CAACTCACGGGGGCAGTCCCCGGCGCGGCGACCTTGATTACGTCCGGGGACAAGTATGTCGCTCCCCC
CGGGAGGGGTGCAGCCACATGGGAGATTCAAAGTTTCTCGTGACGTCGTTGTGATCACTGCAGCCTAG

CCGAGACTCCCGTACTACGCGAAGGTTGGTTATGTTAACCACTACAACGTGAGGCGCCGTAAGGGCCA
GTGTTGTGCCCGGCTCTTCAATGCGCCTTAGCGGCCTGATACACCCACCCAAGGAGATACTGCTAATC
ATATGGGTGGACAGAACCTCGCAACGCAGACGCCTCCCCGGCATGATATGGTTTTTTTCCGCTATTAT
CCAGCACGCAGCGCTATCATTCAAGAGAACCCAGTGACGCGTAAATCGTAAGATCTACCTGCCGCAGG
TGGACCTACTGCAAATACGGATTATGACTCGTAAAGGGGTCATGCGTATTTTCATCACTAGGCACGTT
CGAGAATAAATTAGTAGGTGTCCCGAGCCTTGTGGCGTTCCGCCTGACTCCTCATGAAGTCGACCTTC
TCACCGGCCCTATCTGCCGACGTAAGTCATAACCTAGATCTGTACCTCGGGGGGAGGGTCACTGTAAA
GGGATAATTGGAGGGTGATTTCCCACACTTTCCTAAGGGTACTTTTTGCCTNGCTTCGCAATTGGGTC
CAATGGATGTCGATCTCTGGTTTAGCAGTTGTGAAAGTGGCAAGGCGGGAGGTTAGACCTCCATTTAA
CATATACAAGCAAGTTAACTGCACTAGATGTGTAGACACTACAGTTACAGGAGTAGCCGAATAAGTCT
CCGACGTCAAGCGAATAAGCGTCATACGCGATTATCGCCTAAGAGCACGTATTGGCGGTAAAAGGCTA
GCTAGACGCTTATGGGTAGATTTCAAGGCGTTCGTAGTGGTATAATAGGATACTCTTTCACCAGCCTG
AGGGCCGAACGCTATACTAGTGGTCTGTGATGTAGGACCGAGTATCTCTCTAGGGACCATCTACTTGA
GCAATGGTGCGCAGGGGGAGACATAGACCAGCCTTGGGTGGCAAGCACTGCAATAAGTCCTGTTTAGC
CTTGGAGTTCACATGCCGGCACTGAAGCCGACCTACCTGAGCGTGTGATTACCGTTAAAGCATCTGTC
TAGTTCTGTTTCCGGCGCTCTTGGTTCCAGGTTAGGGGAAGTGTATGACCCATGTGTTTTTATCGGCT
TAACCACGAGTGATCCCCGGTCGTTTCCCCATTGAATCCCTGGTGCATCCTACTCCCAGAATGATAGC
TGACTGACTGGACTGGCTTTTCAAGTAATCGAGGGGGTATCGCGGTCACGGCCGTTAACAGATCCCGT
CCTTAGTGTGGAATCCGCACCTGCTGACTAACGCTTCGCCGGCGTGTCTGCACAGCCGTATAGTGTTA
ATCATGACCCCAAGGAAGGATTAAACAAATATCTTGACG
>CHAN17
GTGTCCGGTAGCCCGCGCTAGTTAGACACCCCGGCAGGGGGGATTGCTTTCGAGACGGGAGATCCCTT
CGCCGACCCTGGAGGGCCGACGCCGAGGCATTCGGGCCCCTGCAACGTCAACAGCGGCAAGAAAACGG
GATGAATGGGCGTAATGGGGGGGGTCTGCTGGGGACCCGACGCGGTTGCCGTTTGCGGGGCCCCCGAC
CCATACCGACCCACCTAGGCGTCCAGTTACGGCGCACGGCGGGAGCGTGGTTGCCGTCAGAGCTGTGT
TTCTCGATCAGTCCCCCGCAGTGCCGCAGTATCTTGCCGTGGGCTGCTTTAATCTTGAAAGTGGTTCA
TACATTGGGCGACGAGGTGTCGACTCTCATTGGGGGTAACCGACGGGCACATGCAGTCCCCTCCCCGG
GCAGGCAGAGGCGGGGCCCCCGCGCGCCGGCCCCGGCCCACAATCTTACCAGGGTCCTCAAAGGAGCC
TTTGCATGGTACCCTTCGTGAATGGTTGCTTAAGAGGTCCACAACGTGGTCCGGGCACGGTCGACTAA
ACACTCAAAACAGCGACGGCAAATATAGGTACAAGGTCCAGGCCCTCACGGCACTAATTGCGATGACC
CAACTCACGGGGGCAGTCCCCGGCGCGGCGACCTTGATTACGTCCGGGGACAAGTATGTCGCTCCCCC
CGGGAGGGGTGCAGCCACATGGGAGATTCAAAGTTTCTCGTGACGTCGTTGTGATCACTGCAGCCTAG
CCGAGACTCCCGTACTACGCGAAGGTTGGTTATGTTAACCACTACAACGTGAGGCGCCGTAAGGGCCA
GTGTTGTGCCCGGCTCTTCAATGCGCCTTAGCGGCCTGATACACCCACCCAAGGAGATACTGCTAATC
ATATGGGTGGACAGAACCTCGCAACGCAGACGCCTCCCCGGCATGATATGGTTTTTTTCCGCTATTAT
CCAGCACGCAGCGCTATCATTCAAGAGAACCCAGTGACGCGTAAATCGTAAGATCTACCTGCCGCAGG
TGGACCTACTGCAAATACGGATTATGACTCGTAAAGGGGTCATGCGTATTTTCATCACTAGGCACGTT
CGAGAATAAATTAGTAGGTGTCCCGAGCCTTGTGGCGTTCCGCCTGACTCCTCATGAAGTCGACCTTC
TCACCGGCCCTATCTGCCGACGTAAGTCATAACCTAGATCTGTACCTCGGGGGGAGGGTCACTGTAAA
GGGATAATTGGAGGGTGATTTCCCACACTTTCCTAAGGGTACTTTTTGCCTGGCTTCGCAATTGGGTC
CAATGGATGTCGATCTCTGGTTTAGCAGTTGTGAAAGTGGCAAGGCGGGAGGTTAGACCTCCATTTAA
CATATACAAGCAAGTTAACTGCACTAGATGTGTAGACACTACAGTTACAGGAGTAGCCGAATAAGTCT
CCGACGTCAAGCGAATAAGCGTCATACGCGATTATCGCCTAAGAGCACGTATTGGCGGTAAAAGGCTA
GCTAGACGCTTATGGGTAGATTTCAAGGCGTTCGTAGTGGTATAATAGGATACTCTTTCACCAGCCTG
AGGGCCGAACGCTATACTAGTGGTCTGTGATGTAGGACCGAGTATCTCTCTAGGGACCATCTACTTGA
GCAATGGTGCGCAGGGGGAGACATAGACCAGCCTTGGGTGGCAAGCACTGCAATAAGTCCTGTTTAGC
CTTGGAGTTCACATGCCGGCACTGAAGCCGACCTACCTGAGCGTGTGATTACCGTTAAAGCATCTGTC
TAGTTCTGTTTCCGGCGCTCTTGGTTCCAGGTTAGGGGAAGTGTATGACCCATGTGTTTTTATCGGCT
TAACCACGAGTGATCCCCGGTCGTTTCCCCATTGAATCCCTGGTGCATCCTACTCCCAGAATGATAGC
TGACTGACTGGACTGGCTTTTCAAGTAATCGAGGGGGTATCGCGGTCACGGCCGTTAACAGATCCCGT
CCTTAGTGTGGAATCCGCACCTGCTGANTAACGCTTCGCCGGCGTGTCTGCACAGCCGTATAGTGTTA
ATCATGACCCCAAGGAAGGATTAAACAAATATCTTGACG
>CHAN18
GTGTCCGGTAGCCCGCGCTAGTTAGACACCCCGGCAGGGGGGATTGCTTTCGAGACGGGAGATCCCTT

CGCCGACCCTGGAGGGCCGACGCCGAGGCATTCGGGCCCCTGCAACGTCAACAGCGGCAAGAAAACGG
GATGAATGGGCGTAATGGGGGGGGTCTGCTGGGGACCCGACGCGGTTGCCGTTTGCGGGGCCCCCGAC
CCATACCGACCCACCTAGGCGTCCAGTTACGGCGCACGTCGGGAGCGTGGTTGCCGTCAGAGCTGTGT
TTCTCGATCAGTCCCCCGCAGTGCCGCAGTATCTTGCCGTGGGCTGCTTTAATCTTGAAAGTGGTTTA
TACATTGGGCGACGAGGTGTCGACTCTCATTGGGGGTAACCGACGGGCACATGCAGTCCCCTCCCCGG
GCAGGCAGAGGCGGGGCCCCCGCGCGCCGGCCCCGGCCCACAATCTTACCAGGGTCCTCAAAGGAGCC
TTTGCATGGTACCCTTCGTGAATGGTTGCTTAAGAGGTCCACAACGTGGTCCGGGCACGGTCGACTAA
ACACTCAAAACAGCGACGGCAAATATAGGTACAAGGTCCAGGCCCTCACGGCACTAATTGCGATGACC
CAACTCACGGGGGCAGTCCCCGGCGCGGCGACCTTGATTACGTCCGGGGACAAGTATGTCGCTCCCCC
CGGGAGGGGTGCAGCCACATGGGAGATTCAAAGTTTCTCGTGACGTCGTTGTGATCACTGCAGCCTAG
CCGAGACTCCCGTACTACGCGAAGGTTGGTTATGTTAACCACTACAACGTGAGGCGCCGTAAGGGCCA
GTGTTGTGCCCGGCTCTTCAATGCGCCTTAGCGGCCTGATACACCCACCCAAGGAGATACTGCTAATC
ATATGGGTGGACAGAACCTCGCAACGCAGACGCCTCCCCGGCATGATATGGTTTTTTTCCGCTATTAT
CCAGCACGCAGCGCTATCATTCAAGAGAACCCAGTGACGCGTAAATCGTAAGATCTACCTGCCGCAGG
TGGACCTACTGCAAATACGGATTATGACTCGTAAAGGGGTCATGCGTATTTTCATCACTAGGCACGTT
CGAGAATAAATTAGTAGGTGTCCCGAGCCTTGTGGCGTTCCGCCTGACTCCTCATGAAGTCGACCTTC
TCACCGGCCCTATCTGCCGACGTAAGTCATAACCTAGATCTGTACCTCGGGGGGAGGGTCACTGTAAA
GGGATAATTGGAGGGTGATTTCCCACACTTTCCTAAGGGTACTTTTTGCCTGGCTTCGCAATTGGGTC
CAATGGATGTCGATCTCTGGTTTAGCAGTTGTGAAAGTGGCAAGGCGGGAGGTTAGACCTCCATTTAA
CATATACAAGCAAGTTAACTGCACTAGATGTGTAGACACTACAGTTACAGGAGTAGCCGAATAAGTCT
CCGACGTCAAGCGAATAAGCGTCATACGCGATTATCGCCTAAGAGCACGTATTGGCGGTAAAAGGCTA
GCTAGACGCTTATGGGTAGATTTCAAGGCGTTCGTAGTGGTATAATAGGATACTCTTTCACCAGCCTG
AGGGCCGAACGCTATACTAGTGGTCTGTGATGTAGGACCGAGTATCTCTCTAGGGACCATCTACTTGA
GCAATGGTGCGCAGGGGGAGACATAGACCAGCCTTGGGTGGCAAGCACTGCAATAAGTCCTGTTTAGC
CTTGGAGTTCACATGCCGGCACTGAAGCCGACCTACCTGAGCGTGTGATTACCGTTAAAGCATCTGTC
TAGTTCTGTTTCCGGCGCTCTTGGTTCCAGGTTAGGGGAAGTGTATGACCCATGTGTTTTTATCGGCT
TAACCACGAGTGATCCCCGGTCGTTTCCCCATTGAATCCCTGGTGCATCCTACTCCCAGAATGATAGC
TGACTGACTGGACTGGCTTTTCAAGTAATCGAGGGGGTATCGCGGTCACGGCCGTTAACAGATCCCGT
CCTTAGTGTGGAATCCGCACCTGCTGACTAACGCTTCGCCGGCGTGTCTGCACAGCCGTATAGTGTTA
ATCATGACCCCAAGGAAGGATTAAACAAATATCTTGACG
>MNON07
GTGTCCGGTAGCCCGCGCTAGTTAGACACCCCGGCAGGGGGGATTGCTTTCGAGACGGGAGATCCCTT
CGCCGACCCTGGAGGGCCGACGCCGAGGCATTCGGGCCCCTGCAACGTCAACAGCGGCAAGAAAACGG
GATGAATGGGCGTAATGGGGGGGGTCTGCTGGGGACCCGACGCGGTTGCCGTTTGCGGGGCCCCCGAC
CCATACCGACCCACCTAGGCGTCCAGTTACGGCGCACGTCGGGAGCGTGGTTGCCGTCAGAGCTGTGT
TTCTCGATCAGTCCCCCGCAGTGCCGCAGTATCTTGCCGTGGGCTGCTTTAATCTTGAAAGTGGTTTA
TACATTGGGCGACGAGGTGTCGACTCTCATTGGGGGTAACCGACGGGCACATGCAGTCCCCTCCCCGG
GCAGGCAGAGGCGGGGCCCCCGCGCGCCGGCCCCGGCCCACAATCTTACCAGGGTCCTCAAAGGAGCC
TTTGCATGGTACCCTTCGTGAATGGTTGCTTAAGAGGTCCACAACGTGGTCCGGGCACGGTCGACTAA
ACACTCAAAACAGCGACGGCAAATATAGGTACAAGGTCCAGGCCCTCACGGCACTAATTGCGATGACC
CAACTCACGGGGGCAGTCCCCGGCGCGGCGACCTTGATTACGTCCGGGGACAAGTATGTCGCTCCCCC
CGGGAGGGGTGCAGCCACATGGGAGATTCAAAGTTTCTCGTGACGTCGTTGTGATCACTGCAGCCTAG
CCGAGACTCCCGTACTACGCGAAGGTTGGTTATGTTAACCACTACAACGTGAGGCGCCGTAAGGGCCA
GTGTTGTGCCCGGCTCTTCAATGCGCCTTAGCGGCCTGATACACCCACCCAAGGAGATACTGCTAATC
ATATGGGTGGACAGAACCTCGCAACGCAGACGCCTCCCCGGCATGATATGGTTTTTTTCCGCTATTAT
CCAGCACGCAGCGCTATCATTCAAGAGAACCCAGTGACGCGTAAATCGTAAGATCTACCTGCCGCAGG
TGGACCTACTGCAAATACGGATTATGACTCGTAAAGGGGTCATGCGTATTTTCATCACTAGGCACGTT
CGAGAATAAATTAGTAGGTGTCCCGAGCCTTGTGGCGTTCCGCCTGACTCCTCATGAAGTCGACCTTC
TCACCGGCCCTATCTGCCGACGTAAGTCATAACCTAGATCTGTACCTCGGGGGGAGGGTCACTGTAAA
GGGATAATTGGAGGGTGATTTCCCACACTTTCCTAAGGGTACTTTTTGCCTGGCTTCGCAATTGGGTC
CAATGGATGTCGATCTCTGGTTTAGCAGTTGTGAAAGTGGCAAGGCGGGAGGTTAGACCTCCATTTAA
CATATACAAGCAAGTTAACTGCACTAGATGTGTAGACACTACAGTTACAGGAGTAGCCGAATAAGTCT
CCGACGTCAAGCGAATAAGCGTCATACGCGATTATCGCCTAAGAGCACGTATTGGCGGTAAAAGGCTA
GCTAGACGCTTATGGGTAGATTTCAAGGCGTTCGTAGTGGTATAATAGGATACTCTTTCACCAGCCTG

AGGGCCGAACGCTATACTAGTGGTCTGTGATGTAGGACCGAGTATCTCTCTAGGGACCATCTACTTGA
GCAATGGTGCGCAGGGGGAGACATAGACCAGCCTTGGGTGGCAAGCACTGCAATAAGTCCTGTTTAGC
CTTGGAGTTCACATGCCGGCACTGAAGCCGACCTACCTGAGCGTGTGATTACCGTTAAAGCATCTGTC
TAGTTCTGTTTCCGGCGCTCTTGGTTCCAGGTTAGGGGAAGTGTATGACCCATGTGTTTTTATCGGCT
TAACCACGAGTGATCCCCGGTCGTTTCCCCATTGAATCCCTGGTGCATCCTACTCCCAGAATGATAGC
TGACTGACTGGACTGGCTTTTCAAGTAATCGAGGGGGTATCGCGGTCACGGCCGTTAACAGATCCCGT
CCTTAGTGTGGAATCCGCACCTGCTGACTAACGCTTCGCCGGCGTGTCTGCACAGCCGTATAGTGTTA
ATCATGACCCCAAGGAAGGATTAAACAAATATCTTGACG
>MNON09
GTGTCCGGTAGCCCGCGCTAGTTAGACACCCCGGCAGGGGGGATTGCTTTCGAGACGGGAGATCCCTT
CGCCGACCCTGGAGGGCCGACGCCGAGGCATTCGGGCCCCTGCAACGTCAACAGCGGCAAGAAAACGG
GATGAATGGGCGTAATGGGGGGGGTCTGCTGGGGACCCGACGCGGTTGCCGTTTGCGGGGCCCCCGAC
CCATACCGACCCACCTAGGCGTCCAGTTACGGCGCACGTCGGGAGCGTGGTTGCCGTCAGAGCTGTGT
TTCTCGATCAGTCCCCCGCAGTGCCGCAGTATCTTGCCGTGGGCTGCTTTAATCTTGAAAGTGGTTTA
TACATTGGGCGACGAGGTGTCGACTCTCATTGGGGGTAACCGACGGGCACATGCAGTCCCCTCCCCGG
GCAGGCAGAGGCGGGGCCCCCGCGCGCCGGCCCCGGCCCACAATCTTACCAGGGTCCTCAAAGGAGCC
TTTGCATGGTACCCTTCGTGAATGGTTGCTTAAGAGGTCCACAACGTGGTCCGGGCACGGTCGACTAA
ACACTCAAAACAGCGACGGCAAATATAGGTACAAGGTCCAGGCCCTCACGGCACTAATTGCGATGACC
CAACTCACGGGGGCAGTCCCCGGCGCGGCGACCTTGATTACGTCCGGGGACAAGTATGTCGCTCCCCC
CGGGAGGGGTGCAGCCACATGGGAGATTCAAAGTTTCTCGTGACGTCGTTGTGATCACTGCAGCCTAG
CCGAGACTCCCGTACTACGCGAAGGTTGGTTATGTTAACCACTACAACGTGAGGCGCCGTAAGGGCCA
GTGTTGTGCCCGGCTCTTCAATGCGCCTTAGCGGCCTGATACACCCACCCAAGGAGATACTGCTAATC
ATATGGGTGGACAGAACCTCGCAACGCAGACGCCTCCCCGGCATGATATGGTTTTTTTCCGCTATTAT
CCAGCACGCAGCGCTATCATTCAAGAGAACCCAGTGACGCGTAAATCGTAAGATCTACCTGCCGCAGG
TGGACCTACTGCAAATACGGATTATGACTCGTAAAGGGGTCATGCGTATTTTCATCACTAGGCACGTT
CGAGAATAAATTAGTAGGTGTCCCGAGCCTTGTGGCGTTCCGCCTGACTCCTCATGAAGTCGACCTTC
TCACCGGCCCTATCTGCCGACGTAAGTCATAACCTAGATCTGTACCTCGGGGGGAGGGTCACTGTAAA
GGGATAATTGGAGGGTGATTTCCCACACTTTCCTAAGGGTACTTTTTGCCTGGCTTCGCAATTGGGTC
CAATGGATGTCGATCTCTGGTTTAGCAGTTGTGAAAGTGGCAAGGCGGGAGGTTAGACCTCCATTTAA
CATATACAAGCAAGTTAACTGCACTAGATGTGTAGACACTACAGTTACAGGAGTAGCCGAATAAGTCT
CCGACGTCAAGCGAATAAGCGTCATACGCGATTATCGCCTAAGAGCACGTATTGGCGGTAAAAGGCTA
GCTAGACGCTTATGGGTAGATTTCAAGGCGTTCGTAGTGGTATAATAGGATACTCTTTCACCAGCCTG
AGGGCCGAACGCTATACTAGTGGTCTGTGATGTAGGACCGAGTATCTCTCTAGGGACCATCTACTTGA
GCAATGGTGCGCAGGGGGAGACATAGACCAGCCTTGGGTGGCAAGCACTGCAATAAGTCCTGTTTAGC
CTTGGAGTTCACATGCCGGCACTGAAGCCGACCTACCTGAGCGTGTGATTACCGTTAAAGCATCTGTC
TAGTTCTGTTTCCGGCGCTCTTGGTTCCAGGTTAGGGGAAGTGTATGACCCATGTGTTTTTATCGGCT
TAACCACGAGTGATCCCCGGTCGTTTCCCCATTGAATCCCTGGTGCATCCTACTCCCAGAATGATAGC
TGACTGACTGGACTGGCTTTTCAAGTAATCGAGGGGGTATCGCGGTCACGGCCGTTAACAGATCCCGT
CCTTAGTGTGGAATCCGCACCTGCTGACTAACGCTTCGCCGGCGTGTCTGCACAGCCGTATAGTGTTA
ATCATGACCCCAAGGAAGGATTAAACAAATATCTTGACG
>MNON11
GTGTCCGGTAGCCCGCGCTAGTTAGACACCCCGGCAGGGGGGATTGCTTTCGAGACGGGAGATCCCTT
CGCCGACCCTGGAGGGCCGACGCCGAGGCATTCGGGCCCCCGCAACGTCAACAGCGGCAAGAAAACGG
GATGAATGGGCGTAATGGGGGGGGTCTGCTGGGGACCCGACGCGGTTGCCGTTTGCGGGGCCCCCGAC
CCATACCGACCCACCTAGGCGTCCAGTTACGGCGCACGGCGGGAGCGTGGTTGCCGTCAGAGCTGTGT
TTCTCGATCAGTCCCCCGCAGTGCCGCAGTATCTTGCCGTGGGCTGCTTTAATCTTGAAAGTGGTTTA
TACATTGGGCGACGAGGTGTCGACTCTCATTGGGGGTAACCGACGGGCACATGCAGTCCCCTCCCCGG
GCAGGCAGAGGCGGGGCCCCCGCGCGCCGGCCCCGGCCCACAATCTTACCAGGGTCCTCAAAGGAGCC
TTTGCATGGTACCCTTCGTGAATGGTTGCTTAAGAGGTCCACAACGTGGTCCGGGCACGGTCGACTAA
ACACTCAAAACAGCGACGGCAAATATAGGTACAAGGTCCAGGCCCTCACGGCACTAATTGCGATGACC
CAACTCACGGGGGCAGTCCCCGGCGCGGCGACCTTGATTACGTCCGGGGACAAGTATGTCGCTCCCCC
CGGGAGGGGTGCAGCCACATGGGAGATTCAAAGTTTCTCGTGACGTCGTTGTGATCACTGCAGCCTAG
CCGAGACTCCCGTACTACGCGAAGGTTGGTTATGTTANCCACTACAACGTGAGGCGCCGTAAGGGCCA
GTGTTGTGCCCGGCTCTTCAATGCGCCTTAGCGGCCTGATACACCCACCCAAGGAGATACTGCTAATC

ATATGGGTGGACAGAACCTCGCAACGCAGACGCCTCCCCGGCATGATATGGTTTTTTTCCGCTATTAT
CCAGCACGCAGCGCTATCATTCAAGAGAACCCAGTGACGCGTAAATCGTAAGATCTACCTGCCGCAGG
TGGACCTACTGCAAATACGGATTATGACTCGTAAAGGGGTCATGCGTATTTTCATCACTAGGCACGTT
CGAGAATAAATTAGTAGGTGTCCCGAGCCTTGTGGCGTTCCGCCTGACTCCTCATGAAGTCGACCTTC
TCACCGGCCCTATCTGCCGACGTAAGTCATAACCTAGATCTGTACCTCGGGGGGAGGGTCACTGTAAA
GGGATAATTGGAGGGTGATTTCCCACACTTTCCTAAGGGTACTTTTTGCCTGGCTTCGCAATTGGGTC
CAATGGATGTCGATCTCTGGTTTAGCAGTTGTGAAAGTGGCAAGGCGGGAGGTTAGACCTCCATTTAA
CATATACAAGCAAGTTAACTGCACTAGATGTGTAGACACTACAGTTACAGGAGTAGCCGAATAAGTCT
CCGACGTCAAGCGAATAAGCGTCATACGCGATTATCGCCTAAGAGCACGTATTGGCGGTAAAAGGCTA
GCTAGACGCTTATGGGTAGATTTCAAGGCGTTCGTAGTGGTATAATAGGATACTCTTTCACCAGCCTG
AGGGCCGAACGCTATACTAGTGGTCTGTGATGTAGGACCGAGTATCTCTCTAGGGACCATCTACTTGA
GCAATGGTGCGCAGGGGGAGACATAGACCAGCCTTGGGTGGCAAGCACTGCAATAAGTCCTGTTTAGC
CTTGGAGTTCACATGCCGGCACTGAAGCCGACCTACCTGAGCGTGTGATTACCGTTAAAGCATCTGTC
TAGTTCTGTTTCCGGCGCTCTTGGTTCCAGGTTAGGGGAAGTGTATGACCCATGTGTTTTTATCGGCT
TAACCACGAGTGATCCCCGGTCGTTTCCCCATTGAATCCCTGGTGCATCCTACTCCCAGAATGATAGC
TGACTGACTGGACTGGCTTTTCAAGTAATCGAGGGGGTATCGCGGTCACGGCCGTTAACAGATCCCGT
CCTTAGTGTGGAATCCGCACCTGCTGACTAACGCTTCGCCGGCGTGTCTGCACAGCCGTATAGTGTTA
ATCATGACCCCAAGGAAGGATTAAACAAATATCTTGACG
>MNON12
GTGTCCGGTAGCCCGCGCTAGTTAGACACCCCGGCAGGGGGGATTGCTTTCGAGACGGGAGATCCCTT
CGCCGACCCTGGAGGGCCGACGCCGAGGCATTCGGGCCCCTGCAACGTCAACAGCGGCAAGAAAACGG
GATGAATGGGCGTAATGGGGGGGGTCTGCTGGGGACCCGACGCGGTTGCCGTTTGCGGGGCCCCCGAC
CCATACCGACCCACCTAGGCGTCCAGTTACGGCGCACGTCGGGAGCGTGGTTGCCGTCAGAGCTGTGT
TTCTCGATCAGTCCCCCGCAGTGCCGCAGTATCTTGCCGTGGGCTGCTTTAATCTTGAAAGTGGTTTA
TACATTGGGCGACGAGGTGTCGACTCTCATTGGGGGTAACCGACGGGCACATGCAGTCCCCTCCCCGG
GCAGGCAGAGGCGGGGCCCCCGCGCGCCGGCCCCGGCCCACAATCTTACCAGGGTCCTCAAAGGAGCC
TTTGCATGGTACCCTTCGTGAATGGTTGCTTAAGAGGTCCACAACGTGGTCCGGGCACGGTCGACTAA
ACACTCAAAACAGCGACGGCAAATATAGGTACAAGGTCCAGGCCCTCACGGCACTAATTGCGATGACC
CAACTCACGGGGGCAGTCCCCGGCGCGGCGACCTTGATTACGTCCGGGGACAAGTATGTCGCTCCCCC
CGGGAGGGGTGCAGCCACATGGGAGATTCAAAGTTTCTCGTGACGTCGTTGTGATCACTGCAGCCTAG
CCGAGACTCCCGTACTACGCGAAGGTTGGTTATGTTAACCACTACAACGTGAGGCGCCGTAAGGGCCA
GTGTTGTGCCCGGCTCTTCAATGCGCCTTAGCGGCCTGATACACCCACCCAAGGAGATACTGCTAATC
ATATGGGTGGACAGAACCTCGCAACGCAGACGCCTCCCCGGCATGATATGGTTTTTTTCCGCTATTAT
CCAGCACGCAGCGCTATCATTCAAGAGAACCCAGTGACGCGTAAATCGTAAGATCTACCTGCCGCAGG
TGGACCTACTGCAAATACGGATTATGACTCGTAAAGGGGTCATGCGTATTTTCATCACTAGGCACGTT
CGAGAATAAATTAGTAGGTGTCCCGAGCCTTGTGGCGTTCCGCCTGACTCCTCATGAAGTCGACCTTC
TCACCGGCCCTATCTGCCGACGTAAGTCATAACCTAGATCTGTACCTCGGGGGGAGGGTCACTGTAAA
GGGATAATTGGAGGGTGATTTCCCACACTTTCCTAAGGGTACTTTTTGCCTGGCTTCGCAATTGGGTC
CAATGGATGTCGATCTCTGGTTTAGCAGTTGTGAAAGTGGCAAGGCGGGAGGTTAGACCTCCATTTAA
CATATACAAGCAAGTTAACTGCACTAGATGTGTAGACACTACAGTTACAGGAGTAGCCGAATAAGTCT
CCGACGTCAAGCGAATAAGCGTCATACGCGATTATCGCCTAAGAGCACGTATTGGCGGTAAAAGGCTA
GCTAGACGCTTATGGGTAGATTTCAAGGCGTTCGTAGTGGTATAATAGGATACTCTTTCACCAGCCTG
AGGGCCGAACGCTATACTAGTGGTCTGTGATGTAGGACCGAGTATCTCTCTAGGGACCATCTACTTGA
GCAATGGTGCGCAGGGGGAGACATAGACCAGCCTTGGGTGGCAAGCACTGCAATAAGTCCTGTTTAGC
CTTGGAGTTCACATGCCGGCACTGAAGCCGACCTACCTGAGCGTGTGATTACCGTTAAAGCATCTGTC
TAGTTCTGTTTCCGGCGCTCTTGGTTCCAGGTTAGGGGAAGTGTATGACCCATGTGTTTTTATCGGCT
TAACCACGAGTGATCCCCGGTCGTTTCCCCATTGAATCCCTGGTGCATCCTACTCCCAGAATGATAGC
TGACTGACTGGACTGGCTTTTCAAGTAATCGAGGGGGTATCGCGGTCACGGCCGTTAACAGATCCCGT
CCTTAGTGTGGAATCCGCACCTGCTGACTAACGCTTCGCCGGCGTGTCTGCACAGCCGTATAGTGTTA
ATCATGACCCCAAGGAAGGATTAAACAAATATCTTGACG
>MNON15
GTGTCCGGTAGCTCGCGCTAGTTAGACACCCCGGCAGGGGGGATTGCTTTCGAGACGGGAGATCCCTT
CGCCGACCCTGGAGGGCCGACGCCGAGGCATTCGGGCCCCTGCAACGTCAACAGCGGCAAGAAAACGG
GATGAATGGGCGTAATGGGGGGGGTCTGCTGGGGACCCGACGCGGTTGCCGTTTGCGGGGCCCCCGAC

CCATACCGACCCACCTAGGCGTCCAGTTACGGCGCACGTCGGGAGCGTGGTTGCCGTCAGAGCTGTGT
TTCTCGATCAGTCCCCCGCAGTGCCGCAGTATCTTGCCGTGGGCTGCTTTAATCTTGAAAGTGGTTCA
TACATTGGGCGACGAGGTGTCGACTCTCATTGGGGGTAACCGACGGGCACATGCAGTCCCCTCCCCGG
GCAGGCAGAGGCGGGGCCCCCGCGCGCCGGCCCCGGCCCACAATCTTACCAGGGTCCTCAAGGGAGCC
TTTGCATGGTACCCTTCGTGAATGGTTGCTTAAGAGGTCCACCACGTGGTCCGGGCACGGTCGACTAA
ACACTCAAAACAGCGACGGCAAATATAGGCACAAGGTCCAGACCCTCACGGCACTAATTGCGATGACC
CAACTCGCGGGGACAGTCACCGGCGCGGCGACCTTGATTACGGTCGGGGACAAGTATGTCGTTCCCCC
CGGGAGGGGTGCAGCCACATGGGAGATTCAAAGTTTCTCGTGACGTCGTTGTGATCACTGCAGCCTAG
CCGAGACTCCCGTACTACGCGAAGGTTGGTTATGTTAACCACTACAACGTGAGGCGCCGTAAGGGCCA
GTGTTGTGCCCGGCTCTTCAATGCGCCTTAGCGGCCTGATACACCCACCCAAGGAGATACCGTTAATC
ATGTGGGGGGACAGAACCTCGCAACGCAGACGCCTCCCCGGCATGATATGGTTTTTTTCCGCTATTAT
CCAGCACGCAGCGCTATCATTCAAGAGAACCCAGTGACGCGTAAATCGTAAGATCTACCTGCCGCAAG
TGGACCTACTGCAAATACGGATTATGACTCGTAAAGAGGTCATGCGTATTTTCATTACCAGGCACGTT
CGAGAATAAATTAGTAGGTGTCCCGCGCCTTGTGGCGTTCCGCCTGACTCCTCATGAAGTCGACCTTC
TCACCGGCCCTATCTGCCGACGTAAGCCATAACCTAGGTCTGTACCGTGGGGGGAGGGTCACTGTAAA
GGGATAATTGGAGGGTGATTTCCCACACTTTCCTAAGGGTACTTTTCGCCTGGCTTCGCAATTGGGCC
CANTGGATGTCGATCTCTGGTTTAGCAGTTGTGAAAGTGGTAAGGCGGGAGGTTAGACCTCCATTTAA
CATATNCAAGCAAGTTCACTGCACTAGATGTGTAGACACTACAGTTACGGGGGTAGCCGAATAAGTCT
CCGACGTCAAGCGAATAAGCGTCATACGCGATTATCGCCTAAGAGCACGTATTGGCGGTAAAAGGCTA
ACTAGACGCCTGTGGGTAGATTTCAAGGCGTTCGTAGTGGTATAACAAGATACTTTTTCACCAGCCTG
AGGGCCGAACGCTATACTAGTGGTCTGTGATGTAGGATCGAGTATCCCTCTAGGGACCATCTACTTGA
GCAATGGTGCGCAGGGGGAGACATAGACCAGCCTTGGGTGGCAAGCACTGCAATAAGTCCTGTTTAGC
CCTGGAGTTCACACGCCGGCACTGAAGCCGACCTACCTGAGCTTGTGATTACCGTTAAAGCATCTGTC
TAGTTCTGTTTCCGGCGCTCTTGGTTCCAGGTTAGGGGAAGTGTATGACCCATGTGTTTTTATCGGCT
TAACCACGAGTGATCCCCGGTCGTTTCCTCATTGAATCCCTGGTGCATCCTACTCCCAGAATGATAGC
TGACTGACTGGACTGGCTTTTCAAGTAGTCGAGGGGGTATCGCGGTCACGGCCGTTAACAGATCCCGT
CCTTAGTGTGGAATCCGCACCTGCTGACTAACGCTTCGCCGGCGTGTTTGCACAGCCGTATAGTGTTA
ATCATGACCCCAAGGAAGGATTAAACAAATATCTTGACG
>MNON16
GTGTCCGGTAGCCCGCGCTAGTTAGACACCCCGGCAGGGGGGATTGCTTTCGAGACGGGAGATCCCTT
CGCCGACCCTGGAGGGCCGACGCCGAGGCATTCGGGCCCCTGCAACGTCAACAGCGGCAAGAAAACGG
GATGAATGGGCGTAATGGGGGGGGTCTGCTGGGGACCCGACGCGGTTGCCGTTTGCGGGGCCCCCGAC
CCATACCGACCCACCTAGGCGTCCAGTTACGGCGCACGTCGGGAGCGTGGTTGCCGTCAGAGCTGTGT
TTCTCGATCAGTCCCCCGCAGTGCCGCAGTATCTTGCCGTGGGCTGCTTTAATCTTGAAAGTGGTTTA
TACATTGGGCGACGAGGTGTCGACTCTCATTGGGGGTAACCGACGGGCACATGCAGTCCCCTCCCCGG
GCAGGCAGAGGCGGGGCCCCCGCGCGCCGGCCCCGGCCCACAATCTTACCAGGGTCCTCAAAGGAGCC
TTTGCATGGTACCCTTCGTGAATGGTTGCTTAAGAGGTCCACAACGTGGTCCGGGCACGGTCGACTAA
ACACTCAAAACAGCGACGGCAAATATAGGTACAAGGTCCAGGCCCTCACGGCACTAATTGCGATGACC
CAACTCACGGGGGCAGTCCCCGGCGCGGCGACCTTGATTACGTCCGGGGACAAGTATGTCGCTCCCCC
CGGGAGGGGTGCAGCCACATGGGAGATTCAAAGTTTCTCGTGACGTCGTTGTGATCACTGCAGCCTAG
CCGAGACTCCCGTACTACGCGAAGGTTGGTTATGTTAACCACTACAACGTGAGGCGCCGTAAGGGCCA
GTGTTGTGCCCGGCTCTTCAATGCGCCTTAGCGGCCTGATACACCCACCCAAGGAGATACTGCTAATC
ATATGGGTGGACAGAACCTCGCAACGCAGACGCCTCCCCGGCATGATATGGTTTTTTTCCGCTATTAT
CCAGCACGCAGCGCTATCATTCAAGAGAACCCAGTGACGCGTAAATCGTAAGATCTACCTGCCGCAGG
TGGACCTACTGCAAATACGGATTATGACTCGTAAAGGGGTCATGCGTATTTTCATCACTAGGCACGTT
CGAGAATAAATTAGTAGGTGTCCCGAGCCTTGTGGCGTTCCGCCTGACTCCTCATGAAGTCGACCTTC
TCACCGGCCCTATCTGCCGACGTAAGTCATAACCTAGATCTGTACCTCGGGGGGAGGGTCACTGTAAA
GGGATAATTGGAGGGTGATTTCCCACACTTTCCTAAGGGTACTTTTTGCCTGGCTTCGCAATTGGGTC
CAATGGATGTCGATCTCTGGTTTAGCAGTTGTGAAAGTGGCAAGGCGGGAGGTTAGACCTCCATTTAA
CATATACAAGCAAGTTAACTGCACTAGATGTGTAGACACTACAGTTACAGGAGTAGCCGAATAAGTCT
CCGACGTCAAGCGAATAAGCGTCATACGCGATTATCGCCTAAGAGCACGTATTGGCGGTAAAAGGCTA
GCTAGACGCTTATGGGTAGATTTCAAGGCGTTCGTAGTGGTATAATAGGATACTCTTTCACCAGCCTG
AGGGCCGAACGCTATACTAGTGGTCTGTGATGTAGGACCGAGTATCTCTCTAGGGACCATCTACTTGA
GCAATGGTGCGCAGGGGGAGACATAGACCAGCCTTGGGTGGCAAGCACTGCAATAAGTCCTGTTTAGC

CTTGGAGTTCACATGCCGGCACTGAAGCCGACCTACCTGAGCGTGTGATTACCGTTAAAGCATCTGTC
TAGTTCTGTTTCCGGCGCTCTTGGTTCCAGGTTAGGGGAAGTGTATGACCCATGTGTTTTTATCGGCT
TAACCACGAGTGATCCCCGGTCGTTTCCCCATTGAATCCCTGGTGCATCCTACTCCCAGAATGATAGC
TGACTGACTGGACTGGCTTTTCAAGTAATCGAGGGGGTATCGCGGTCACGGCCGTTAACAGATCCCGT
CCTTAGTGTGGAATCCGCACCTGCTGACTAACGCTTCGCCGGCGTGTCTGCACAGCCGTATAGTGTTA
ATCATGACCCCAAGGAAGGATTAAACAAATATCTTGACG
>MNON21
GTGTCCGGTAGCCCGCGCTAGTTAGACACCCCGGCAGGGGGGATTGCTTTCGAGACGGGAGATCCCTT
CGCCGACCCTGGAGGGCCGACGCCGAGGCATTCGGGCCCCTGCAACGTCAACAGCGGCAAGAAAACGG
GATGAATGGGCGTAATGGGGGGGGTCTGCTGGGGACCCGACGCGGTTGCCGTTTGCGGGGCCCCCGAC
CCATACCGACCCACCTAGGCGTCCAGTTACGGCGCACGGCGGGAGCGTGGTTGCCGTCAGAGCTGTGT
TTCTCGATCAGTCCCCCGCAGTGCCGCAGTATCTTGCCGTGGGCTGCTTTAATCTTGAAAGTGGTTTA
TACATTGGGCGACGAGGTGTCGACTCTCATTGGGGGTAACCGACGGGCACATGCAGTCCCCTCCCCGG
GCAGGCAGAGGCGGGGCCCCCGCGCGCCGGCCCCGGCCCACAATCTTACCAGGGTCCTCAAAGGAGCC
TTTGCATGGTACCCTTCGTGAATGGTTGCTTAAGAGGTCCACAACGTGGTCCGGGCACGGTCGACTAA
ACACTCAAAACAGCGACGGCAAATATAGGTACAAGGTCCAGGCCCTCACGGCACTAATTGCGATGACC
CAACTCACGGGGGCAGTCCCCGGCGCGGCGACCTTGATTACGTCCGGGGACAAGTATGTCGCTCCCCC
CGGGAGGGGTGCAGCCACATGGGAGATTCAAAGTTTCTCGTGACGTCGTTGTGATCACTGCAGCCTAG
CCGAGACTCCCGTACTACGCGAAGGTTGGTTATGTTAACCACTACAACGTGAGGCGCCGTAAGGGCCA
GTGTTGTGCCCGGCTCTTCAATGCGCCTTAGCGGCCTGATACACCCACCCAAGGAGATACTGCTAATC
ATATGGGTGGACAGAACCTCGCAACGCAGACGCCTCCCCGGCATGATATGGTTTTTTTCCGCTATTAT
CCAGCACGCAGCGCTATCATTCAAGAGAACCCAGTGACGCGTAAATCGTAAGATCTACCTGCCGCAGG
TGGACCTACTGCAAATACGGATTATGACTCGTAAAGGGGTCATGCGTATTTTCATCACTAGGCACGTT
CGAGAATAAATTAGTAGGTGTCCCGAGCCTTGTGGCGTTCCGCCTGACTCCTCATGAAGTCGACCTTC
TCACCGGCCCTATCTGCCGACGTAAGTCATAACCTAGATCTGTACCTCGGGGGGAGGGTCACTGTAAA
GGGATAATTGGAGGGTGATTTCCCACACTTTCCTAAGGGTACTTTTTGCCTGGCTTCGCAATTGGGTC
CAATGGATGTCGATCTCTGGTTTAGCAGTTGTGAAAGTGGCAAGGCGGGAGGTTAGACCTCCATTTAA
CATATACAAGCAAGTTAACTGCACTAGATGTGTAGACACTACAGTTACAGGAGTAGCCGAATAAGTCT
CCGACGTCAAGCGAATAAGCGTCATACGCGATTATCGCCTAAGAGCACGTATTGGCGGTAAAAGGCTA
GCTAGACGCTTATGGGTAGATTTCAAGGCGTTCGTAGTGGTATAATAGGATACTCTTTCACCAGCCTG
AGGGCCGAACGCTATACTAGTGGTCTGTGATGTAGGACCGAGTATCTCTCTAGGGACCATCTACTTGA
GCAATGGTGCGCAGGGGGAGACATAGACCAGCCTTGGGTGGCAAGCACTGCAATAAGTCCTGTTTAGC
CTTGGAGTTCACATGCCGGCACTGAAGCCGACCTACCTGAGCGTGTGATTACCGTTAAAGCATCTGTC
TAGTTCTGTTTCCGGCGCTCTTGGTTCCAGGTTAGGGGAAGTGTATGACCCATGTGTTTTTATCGGCT
TAACCACGAGTGATCCCCGGTCGTTTCCCCATTGAATCCCTGGTGCATCCTACTCCCAGAATGATAGC
TGACTGACTGGACTGGCTTTTCAAGTAATCGAGGGGGTATCGCGGTCACGGCCGTTAACAGATCCCGT
CCTTAGTGTGGAATCCGCACCTGCTGACTAACGCTTCGCCGGCGTGTCTGCACAGCCGTATAGTGTTA
ATCATGACCCCAAGGAAGGATTAAACAAATATCTTGACG
>MNON29
GTGTCCGGTAGCCCGCGCTAGTTAGACACCCCGGCAGGGGGGATTGCTTTCGAGACGGGAGATCCCTT
CGCCGACCCTGGAGGGCCGACGCCGAGGCATTCGGGCCCCNGCAACGTNAACAGCGGCAAGAAAACGG
GATGAATGGGCGTAATGGGGGGGGTCTGCTGGGGACCCGACGCGGTTGCCGTTTGCGGGGCCCCCGAC
CCATACCGACCCACCTAGGCGTCCAGTTACGGCGCACGTCGGGAGCGTGGTTGCCGTCAGAGCTGTGT
TTCTCGATCAGTCCCCCGCAGTGCCGCAGTATCTTGCCGTGGGCTGCTTTAATCTTGAAAGTGGTTTA
TACATTGGGCGACGAGGTGTCGACTCTCATTGGGGGTAACCGACGGGCACATGCAGTCCCCTCCCCGG
GCAGGCAGAGGCGGGGCCCCCGCGCGCCGGCCCCGGCCCACAATCTTACCAGGGTCCTCAAAGGAGCC
TTTGCATGGTACCCTTCGTGAATGGTTGCTTAAGAGGTCCACAACGTGGTCCGGGCACGGTCGACTAA
ACACTCAAAACAGCGACGGCAAATATAGGTACAAGGTCCAGGCCCTCACGGCACTAATTGCGATGACC
CAACTCACGGGGGCAGTCCCCGGCGCGGCGACCTTGATTACGTCCGGGGACAAGTATGTCGCTCCCCC
CGGGAGGGGTGCAGCCACATGGGAGATTCAAAGTTTCTCGTGACGTCGTTGTGATCACTGCAGCCTAG
CCGAGACTCCCGTACTACGCGAAGGTTGGTTATGTTAACCACTACAACGTGAGGCGCCGTAAGGGCCA
GTGTTGTGCCCGGCTCTTCAATGCGCCTTAGCGGCCTGATACACCCACCCAAGGAGATACTGCTAATC
ATATGGGTGGACAGAACCTCGCAACGCAGACGCCTCCCCGGCATGATATGGTTTTTTTCCGCTATTAT
CCAGCACGCAGCGCTATCATTCAAGAGAACCCAGTGACGCGTAAATCGTAAGATCTACCTGCCGCAGG

TGGACCTACTGCAAATACGGATTATGACTCGTAAAGGGGTCATGCGTATTTTCATCACTAGGCACGTT
CGAGAATAAATTAGTAGGTGTCCCGAGCCTTGTGGCGTTCCGCCTGACTCCTCATGAAGTCGACCTTC
TCACCGGCCCTATCTGCCGACGTAAGTCATAACCTAGATCTGTACCTCGGGGGGAGGGTCACTGTAAA
GGGATAATTGGAGGGTGATTTCCCACACTTTCCTAAGGGTACTTTTTGCCTGGCTTCGCAATTGGGTC
CAATGGATGTCGATCTCTGGTTTAGCAGTTGTGAAAGTGGCAAGGCGGGAGGTTAGACCTCCATTTAA
CATATACAAGCAAGTTAACTGCACTAGATGTGTAGACACTACAGTTACAGGAGTAGCCGAATAAGTCT
CCGACGTCAAGCGAATAAGCGTCATACGCGATTATCGCCTAAGAGCACGTATTGGCGGTAAAAGGCTA
GCTAGACGCTTATGGGTAGATTTCAAGGCGTTCGTAGTGGTATAATAGGATACTCTTTCACCAGCCTG
AGGGCCGAACGCTATACTAGTGGTCTGTGATGTAGGACCGAGTATCTCTCTAGGGACCATCTACTTGA
GCAATGGTGCGCAGGGGGAGACATAGACCAGCCTTGGGTGGCAAGCACTGCAATAAGTCCTGTTTAGC
CTTGGAGTTCACATGCCGGCACTGAAGCCGACCTACCTGAGCGTGTGATTACCGTTAAAGCATCTGTC
TAGTTCTGTTTCCGGCGCTCTTGGTTCCAGGTTAGGGGAAGTGTATGACCCATGTGTTTTTATCGGCT
TAACCACGAGTGATCCCCGGTCGTTTCCCCATTGAATCCCTGGTGCATCCTACTCCCAGAATGATAGC
TGACTGACTGGACTGGCTTTTCAAGTAATCGAGGGGGTATCGCGGTCACGGCCGTTAACAGATCCCGT
CCTTAGTGTGGAATCCGCACCTGCTGACTAACGCTTCGCCGGCGTGTCTGCACAGCCGTATAGTGTTA
ATCATGACCCCAAGGAAGGATTAAACAAATATCTTGACG
>MNON32
GTGTCCGGTAGCCCGCGCTAGTTAGACACCCCGGCAGGGGGGATTGCTTTCGGGACGGGAGATCCCTT
CGCCGACCCTGGAGGGCCGACGCCGAGGCATTCGGGCCCCNGCAACGTCAACAGCGGCAAGAAAACGG
GATGAATGGGCGTAATGGGGGGGGTCTGCTGGGGACCCGACGCGGTTGCCGTTTGCGGGGCCCCCGAC
CCATACCGACCCACCTAGGCGTCCAGTTACGGCGCACGTCGGGAGCGTGGTTGCCGTCAGAGCTGTGT
TTCTCGATCAGTCCCCCGCAGTGCCGCAGTATCTTGCCGTGGGCTGCTTTAATCTTGAAAGTGGTTCA
TACATTGGGCGACGAGGTGTCGACTCTCATTGGGGGTAACCGACGGGCACATGCAGTCCCCTCCCCGG
GCAGGCAGAGGCGGGGCCCCCGCGCGCCGGCCCCGGCCCACAATCTTACCAGGGTCCTCAAAGGAGCC
TTTGCATGGTACCCTTCGTGAATGGTTGCTTAAGAGGTCCACAACGTGGTCCGGGCACGGTCGACTAA
ACACTCAAAACAGCGACGGCAAATATAGGTACAAGGTCCAGGCCCTCACGGCACTAATTGCGATGACC
CAACTCACGGGGGCAGTCCCCGGCGCGGCGACCTTGATTACGTCCGGGGACAAGTATGTCGCTCCCCC
CGGGAGGGGTGCAGCCACATGGGAGATTCAAAGTTTCTCGTGACGTCGTTGTGATCACTGCAGCCTAG
CCGAGACTCCCGTACTACGCGAAGGTTGGTTATGTTAACCACTACAACGTGAGGCGCCGTAAGGGCCA
GTGTTGTGCCCGGCTCTTCAATGCGCCTTAGCGGCCTGATACACCCACCCAAGGAGATACTGCTAATC
ATATGGGTGGACAGAACCTCGCAACGCAGACGCCTCCCCGGCATGATATGGTTTTTTTCCGCTATTAT
CCAGCACGCAGCGCTATCATTCAAGAGAACCCAGTGACGCGTAAATCGTAAGATCTACCTGCCGCAGG
TGGACCTACTGCAAATACGGATTATGACTCGTAAAGGGGTCATGCGTATTTTCATCACTAGGCACGTT
CGAGAATAAATTAGTAGGTGTCCCGAGCCTTGTGGCGTTCCGCCTGACTCCTCATGAAGTCGACCTTC
TCACCGGCCCTATCTGCCGACGTAAGTCATAACCTAGATCTGTACCTCGGGGGGAGGGTCACTGTAAA
GGGATAATTGGAGGGTGATTTCCCACACTTTCCTAAGGGTACTTTTTGCCTGGCTTCGCAATTGGGTC
CAATGGATGTCGATCTCTGGTTTAGCAGTTGTGAAAGTGGCAAGGCGGGAGGTTAGACCTCCATTTAA
CATATACAAGCAAGTTAACTGCACTAGATGTGTAGACACTACAGTTACAGGAGTAGCCGAATAAGTCT
CCGACGTCAAGCGAATAAGCGTCATACGCGATTATCGCCTAAGAGCACGTATTGGCGGTAAAAGGCTA
GCTAGACGCTTATGGGTAGATTTCAAGGCGTTCGTAGTGGTATAATAGGATACTCTTTCACCAGCCTG
AGGGCCGAACGCTATACTAGTGGTCTGTGATGTAGGACCGAGTATCTCTCTAGGGACCATCTACTTGA
GCAATGGTGCGCAGGGGGAGACATAGACCAGCCTTGGGTGGCAAGCACTGCAATAAGTCCTGTTTAGC
CTTGGAGTTCACATGCCGGCACTGAAGCCGACCTACCTGAGCGTGTGATTACCGTTAAAGCATCTGTC
TAGTTCTGTTTCCGGCGCTCTTGGTTCCAGGTTAGGGGAAGTGTATGACCCATGTGTTTTTATCGGCT
TAACCACGAGTGATCCCCGGTCGTTTCCCCATTGAATCCCTGGTGCATCCTACTCCCAGAATGATAGC
TGACTGACTGGACTGGCTTTTCAAGTAATCGAGGGGGTATCGCGGTCACGGCCGTTAACAGATCCCGT
CCTTAGTGTGGAATCCGCACCTGCTGACTAACGCTTCGCCGGCGTGTCTGCACAGCCGTATAGTGTTA
ATCATGACCCCAAGGAAGGATTAAACAAATATCTTGACG
>MNON33
GTGTCCGGTAGCCCGCGCTAGTTAGACACCCCGGCAGGGGGGATTGCTTTCGAGACGGGAGATCCCTT
CGCCGACCCTGGAGGGCCGACGCCGAGGCATTCGGGCCCCTGCAACGTCAACAGCGGCAAGAAAACGG
GATGAATGGGCGTAATGGGGGGGGTCTGCTGGGGACCCGACGCGGTTGCCGTTTGCGGGGCCCCCGAC
CCATACCGACCCACCTAGGCGTCCAGTTACGGCGCACGTCGGGAGCGTGGTTGCCGTCAGAGCTGTGT
TTCTCGATCAGTCCCCCGCAGNGCCGCAGTATCTTGCCGTGGGCTGCTTTAATCTTGAAAGTGGTTCA

TACATTGGGCGACGAGGTGTCGACTCTCATTGGGGGTAACCGACGGGCACATGCAGTCCCCTCCCCGG
GCAGGCAGAGGCGGGGCCCCCGCGCGCCGGCCCCGGCCCACAATCTTACCAGGGTCCTCAAAGGAGCC
TTTGCATGGTACCCTTCGTGAATGGTTGCTTAAGAGGTCCACAACGTGGTCCGGGCACGGTCGACTAA
ACACTCAAAACAGCGACGGCAAATATAGGTACAAGGTCCAGGCCCTCACGGCACTAATTGCGATGACC
CAACTCACGGGGGCAGTCCCCGGCGCGGCGACCTTGATTACGTCCGGGGACAAGTATGTCGCTCCCCC
CGGGAGGGGTGCAGCCACATGGGAGATTCAAAGTTTCTCGTGACGTCGTTGTGATCACTGCAGCCTAG
CCGAGACTCCCGTACTACGCGAAGGTTGGTTATGTTAACCACTACAACGTGAGGCGCCGTAAGGGCCA
GTGTTGTGCCCGGCTCTTCAATGCGCCTTAGCGGCCTGATACACCCACCCAAGGAGATACTGCTAATC
ATATGGGTGGACAGAACCTCGCAACGCAGACGCCTCCCCGGCATGATATGGTTTTTTTCCGCTATTAT
CCAGCACGCAGCGCTATCATTCAAGAGAACCCAGTGACGCGTAAATCGTAAGATCTACCTGCCGCAGG
TGGACCTACTGCAAATACGGATTATGACTCGTAAAGGGGTCATGCGTATTTTCATCACTAGGCACGTT
CGAGAATAAATTAGTAGGTGTCCCGAGCCTTGTGGCGTTCCGCCTGACTCCTCATGAAGTCGACCTTC
TCACCGGCCCTATCTGCCGACGTAAGTCATAACCTAGATCTGTACCTCGGGGGGAGGGTCACTGTAAA
GGGATAATTGGAGGGTGATTTCCCACACTTTCCTAAGGGTACTTTTTGCCTGGCTTCGCAATTGGGTC
CAATGGATGTCGATCTCTGGTTTAGCAGTTGTGAAAGTGGCAAGGCGGGAGGTTAGACCTCCATTTAA
CATATACAAGCAAGTTAACTGCACTAGATGTGTAGACACTACAGTTACAGGAGTAGCCGAATAAGTCT
CCGACGTCAAGCGAATAAGCGTCATACGCGATTATCGCCTAAGAGCACGTATTGGCGGTAAAAGGCTA
GCTAGACGCTTATGGGTAGATTTCAAGGCGTTCGTAGTGGTATAATAGGATACTCTTTCACCAGCCTG
AGGGCCGAACGCTATACTAGTGGTCTGTGATGTAGGACCGAGTATCTCTCTAGGGACCATCTACTTGA
GCAATGGTGCGCAGGGGGAGACATAGACCAGCCTTGGGTGGCAAGCACTGCAATAAGTCCTGTTTAGC
CTTGGAGTTCACATGCCGGCACTGAAGCCGACCTACCTGAGCGTGTGATTACCGTTAAAGCATCTGTC
TAGTTCTGTTTCCGGCGCTCTTGGTTCCAGGTTAGGGGAAGTGTATGACCCATGTGTTTTTATCGGCT
TAACCACGAGTGATCCCCGGTCGTTTCCCCATTGAATCCCTGGTGCATCCTACTCCCAGAATGATAGC
TGACTGACTGGACTGGCTTTTCAAGTAATCGAGGGGGTATCGCGGTCACGGCCGTTAACAGATCCCGT
CCTTAGTGTGGAATCCGCACCTGCTGACTAACGCTTCGCCGGCGTGTCTGCACAGCCGTATAGTGTTA
ATCATGACCCCAAGGAAGGATTAAACAAATATCTTGACG
>MNON36
GTGTCCGGTAGCCCGCGCTAGTTAGACACCCCGGCAGGGGGGATTGCTTTCGGGACGGGAGATCCCTT
CGCCGACCCTGGAGGGCCGACGCCGAGGCATTCGGGCCCCTGCAACGTCAACAGCGGCAAGAAAACGG
GATGAATGGGCGTAATGGGGGGGGTCTGCTGGGGACCCGACGCGGTTGCCGTTTGCGGGGCCCCCGAC
CCATACCGACCCACCTAGGCGTCCAGTTACGGCGCACGTCGGGAGCGTGGTTGCCGTCAGAGCTGTGT
TTCTCGATCAGTCCCCCGCAGTGCCGCAGTATCTTGCCGTGGGCTGCTTTAATCTTGAAAGTGGTTCA
TACATTGGGCGACGAGGTGTCGACTCTCATTGGGGGTAACCGACGGGCACATGCAGTCCCCTCCCCGG
GCAGGCAGAGGCGGGGCCCCCGCGCGCCGGCCCCGGCCCACAATCTTACCAGGGTCCTCAAAGGAGCC
TTTGCATGGTACCCTTCGTGAATGGTTGCTTAAGAGGTCCACAACGTGGTCCGGGCACGGTCGACTAA
ACACTCAAAACAGCGACGGCAAATATAGGTACAAGGTCCAGGCCCTCACGGCACTAATTGCGATGACC
CAACTCACGGGGGCAGTCCCCGGCGCGGCGACCTTGATTACGTCCGGGGACAAGTATGTCGCTCCCCC
CGGGAGGGGTGCAGCCACATGGGAGATTCAAAGTTTCTCGTGACGTCGTTGTGATCACTGCAGCCTAG
CCGAGACTCCCGTACTACGCGAAGGTTGGTTATGTTAACCACTACAACGTGAGGCGCCGTAAGGGCCA
GTGTTGTGCCCGGCTCTTCAATGCGCCTTAGCGGCCTGATACACCCACCCAAGGAGATACTGCTAATC
ATATGGGTGGACAGAACCTCGCAACGCAGACGCCTCCCCGGCATGATATGGTTTTTTTCCGCTATTAT
CCAGCACGCAGCGCTATCATTCAAGAGAACCCAGTGACGCGTAAATCGTAAGATCTACCTGCCGCAGG
TGGACCTACTGCAAATACGGATTATGACTCGTAAAGGGGTCATGCGTATTTTCATCACTAGGCACGTT
CGAGAATAAATTAGTAGGTGTCCCGAGCCTTGTGGCGTTCCGCCTGACTCCTCATGAAGTCGACCTTC
TCACCGGCCCTATCTGCCGACGTAAGTCATAACCTAGATCTGTACCTCGGGGGGAGGGTCACTGTAAA
GGGATAATTGGAGGGTGATTTCCCACACTTTCCTAAGGGTACTTTTTGCCTGGCTTCGCAATTGGGTC
CAATGGATGTCGATCTCTGGTTTAGCAGTTGTGAAAGTGGCAAGGCGGGAGGTTAGACCTCCATTTAA
CATATACAAGCAAGTTAACTGCACTAGATGTGTAGACACTACAGTTACAGGAGTAGCCGAATAAGTCT
CCGACGTCAAGCGAATAAGCGTCATACGCGATTATCGCCTAAGAGCACGTATTGGCGGTAAAAGGCTA
GCTAGACGCTTATGGGTAGATTTCAAGGCGTTCGTAGTGGTATAATAGGATACTCTTTCACCAGCCTG
AGGGCCGAACGCTATACTAGTGGTCTGTGATGTAGGACCGAGTATCTCTCTAGGGACCATCTACTTGA
GCAATGGTGCGCAGGGGGAGACATAGACCAGCCTTGGGTGGCAAGCACTGCAATAAGTCCTGTTTAGC
CTTGGAGTTCACATGCCGGCACTGAAGCCGACCTACCTGAGCGTGTGATTACCGTTAAAGCATCTGTC
TAGTTCTGTTTCCGGCGCTCTTGGTTCCAGGTTAGGGGAAGTGTATGACCCATGTGTTTTTATCGGCT

TAACCACGAGTGATCCCCGGTCGTTTCCCCATTGAATCCCTGGTGCATCCTACTCCCAGAATGATAGC
TGACTGACTGGACTGGCTTTTCAAGTAATCGAGGGGGTATCGCGGTCACGGCCGTTAACAGATCCCGT
CCTTAGTGTGGAATCCGCACCTGCTGACTAACGCTTCGCCGGCGTGTCTGCACAGCCGTATAGTGTTA
ATCATGACCCCAAGGAAGGATTAAACAAATATCTTGACG
>MNON37
GTGTCCGGTAGCCCGCGCTAGTTAGACACCCCGGCAGGGGGGATTGCTTTCGAGACGGGAGATCCCTT
CGCCGACCCTGGAGGGCCGACGCCGAGGCATTCGGGCCCCTGCAACGTCAACAGCGGCAAGAAAACGG
GATGAATGGGCGTAATGGGGGGGGTCTGCTGGGGACCCGACGCGGTTGCCGTTTGCGGGGCCCCCGAC
CCATACCGACCCACCTAGGCGTCCAGTTACGGCGCACGGCGGGAGCGTGGTTGCCGTCAGAGCTGTGT
TTCTCGATCAGTCCCCCGCAGTGCCGCAGTATCTTGCCGTGGGCTGCTTTAATCTTGAAAGTGGTTCA
TACATTGGGCGACGAGGTGTCGACTCTCATTGGGGGTAACCGACGGGCACATGCAGTCCCCTCCCCGG
GCAGGCAGAGGCGGGGCCCCCGCGCGCCGGCCCCGGCCCACAATCTTACCAGGGTCCTCAAAGGAGCC
TTTGCATGGTACCCTTCGTGAATGGTTGCTTAAGAGGTCCACAACGTGGTCCGGGCACGGTCGACTAA
ACACTCAAAACAGCGACGGCAAATATAGGTACAAGGTCCAGGCCCTCACGGCACTAATTGCGATGACC
CAACTCACGGGGGCAGTCCCCGGCGCGGCGACCTTGATTACGTCCGGGGACAAGTATGTCGCTCCCCC
CGGGAGGGGTGCAGCCACATGGGAGATTCAAAGTTTCTCGTGACGTCGTTGTGATCACTGCAGCCTAG
CCGAGACTCCCGTACTACGCGAAGGTTGGTTATGTTAACCACTACAACGTGAGGCGCCGTAAGGGCCA
GTGTTGTGCCCGGCTCTTCAATGCGCCTTAGCGGCCTGATACACCCACCCAAGGAGATACTGCTAATC
ATATGGGTGGACAGAACCTCGCAACGCAGACGCCTCCCCGGCATGATATGGTTTTTTTCCGCTATTAT
CCAGCACGCAGCGCTATCATTCAAGAGAACCCAGTGACGCGTAAATCGTAAGATCTACCTGCCGCAGG
TGGACCTACTGCAAATACGGATTATGACTCGTAAAGGGGTCATGCGTATTTTCATCACTAGGCACGTT
CGAGAATAAATTAGTAGGTGTCCCGAGCCTTGTGGCGTTCCGCCTGACTCCTCATGAAGTCGACCTTC
TCACCGGCCCTATCTGCCGACGTAAGTCATAACCTAGATCTGTACCTCGGGGGGAGGGTCACTGTAAA
GGGATAATTGGAGGGTGATTTCCCACACTTTCCTAAGGGTACTTTTTGCCTGGCTTCGCAATTGGGTC
CAATGGATGTCGATCTCTGGTTTAGCAGTTGTGAAAGTGGCAAGGCGGGAGGTTAGACCTCCATTTAA
CATATACAAGCAAGTTAACTGCACTAGATGTGTAGACACTACAGTTACAGGAGTAGCCGAATAAGTCT
CCGACGTCAAGCGAATAAGCGTCATACGCGATTATCGCCTAAGAGCACGTATTGGCGGTAAAAGGCTA
GCTAGACGCTTATGGGTAGATTTCAAGGCGTTCGTAGTGGTATAATAGGATACTCTTTCACCAGCCTG
AGGGCCGAACGCTATACTAGTGGTCTGTGATGTAGGACCGAGTATCTCTCTAGGGACCATCTACTTGA
GCAATGGTGCGCAGGGGGAGACATAGACCAGCCTTGGGTGGCAAGCACTGCAATAAGTCCTGTTTAGC
CTTGGAGTTCACATGCCGGCACTGAAGCCGACCTACCTGAGCGTGTGATTACCGTTAAAGCATCTGTC
TAGTTCTGTTTCCGGCGCTCTTGGTTCCAGGTTAGGGGAAGTGTATGACCCATGTGTTTTTATCGGCT
TAACCACGAGTGATCCCCGGTCGTTTCCCCATTGAATCCCTGGTGCATCCTACTCCCAGAATGATAGC
TGACTGACTGGACTGGCTTTTCAAGTAATCGAGGGGGTATCGCGGTCACGGCCGTTAACAGATCCCGT
CCTTAGTGTGGAATCCGCACCTGCTGACTAACGCTTCGCCGGCGTGTCTGCACAGCCGTATAGTGTTA
ATCATGACCCCAAGGAAGGATTAAACAAATATCTTGACG
>MNON39
GTGTCCGGTAGCCCGCGCTAGTTAGACACCCCGGCAGGGGGGATTGCTTTCGAGACGGGAGATCCCTT
CGCCGACCCTGTAGGGCCGACGCCGAGGCATTCGGGCCCCCGCAACGTCAACAGCGGCAAGAAAACGG
GATGAATGGGCGTAATGGGGGGGGTCTGCTGGGGACCCGACGCGGTTGCCGTTTGCGGGGCCCCCGAC
CCATACCNACCCACCTAGGCGTCCAGTTACGGCGCACGGCGGGAGCGTGGTTGCCGTCAGAGCTGTGT
TTCTCGATCAGTCCCCCGCAGTGCCGCAGTATCTTGCCGTGGGCTGCTTTAATCTTGAAAGTGGTTTA
TACATTGGGCGACGAGGTGTCGGCTCTCATTGGGGGTAACCGACGGACACGTGCAGTCCCCTCCCCGG
GCAGGCAGAGGCGGGGCCCCCGCGCGCCGGCCCCGGCCCACAATCTTACCAGGGTCCTCAAAAGAGCC
TTTGCATGGTACCCTTCGTGAATGGTTGCTTAAGAGGTCCACCACGTAGTCCGGGCACGGTCAACTAA
ACACTCAAAACAGCGACGGCAAATAGAGGCACAAGGTCCAGGCCCTCACGGCACTAGTTGCGATGACC
CAACTCACGGGGGCAGTCCCCGGCGCAGCGACCTTGATTACGTCCGGGAACAAGTATGTCGTTTCCCC
CGGGAGGGGTGCAGCCACATGGGAGATTCAAAGTTTCTCGTGACGTCGTTGTGATCACTGCAGCCTAG
CCGAGACTCCCGTACTACGCGAAGGTTGGTTATGTTAACCACTACAACGTGAGGCGCCGTAAGGGCCA
GTGTTGTGCCCGGCTCTTCAATGCGCCTTAGCGGCCTGATACACCCACCCAAGGAGATACTGCTAATC
ATGTGGGTGGACAGAACCTCGCAACGCAGACGCATCCCCGGCATGATATGGTTTTTTTCCGCTATTAC
CCAGCACGCGGCGCTATCATTCAAGAGAACCCAGTGACGCGTAAATCGTCAGATCTACCTGCCGCAGG
TGGACCTACTGCAAATACGGATTATGACTCGTAAAGAGGTCATGCGTATTTTCATCACTAGGCACTTT
CGAGAGTAAATTAGTAGGTGTCCCGCGCCTTGTGGCGTTCCGCCTGGCTCCTCATGAAGTCGACCTTC

TCATCGGCCCTATTTGCCGACGTAAGTCATAATCCAGATCTTCACCTCGGAGGGAGGGTCACTGTAAA
GGGATAATTGGAGGGCGATTTCCCACACTTTCCTAAGGGTACTTTTTGCTTAGCTTCGCAGTTGGGTC
CAATAGATGTTGATCTCTGGTTTAGCAGTTGTGAAAGTGGCAAGGCGGGAGGTTAGGCCTCCATTTAA
CATATACAAGCAAGTTAACTGCACTAGATGTGTAGACACTACAGTTACAGGAGTAGCCGACTAAGTCT
CCGACGTCAAGCGAATAGGCGTCATACGCGATTATTGCCTAAGAGCACGTATTGGCGGTAAAAGGATA
ACTAGACGCTTGTGGGTAGATTTCAAGGCGCTCGTAGTGGTATAATAGGACACTCTTGCACCAGCCTG
AGAGCCGAACGCTATACTAGTGGTCTGTGATGTAGGACCAAGTAGCTCTCTAGGGACCATCTACTTGA
GCAATGGTGCGCAGGGGTAGACATAGACCAACCTTGGGTGGCAAGCACTGCAATAAGTCCTGTTTAGC
CTTGGAGTTCACACGCCGGCACTAAAGCCGACCTACCTGAGCTTGTGATTACCGTTAAAGCATCTGTC
TAGTTCTGTTTCCGGCGCTCTTGGTTCCATGTTAGGGGAAGTGTATGACCCATGTGTTTTTATCGGCT
TAACTACGAGTGATCCCCGGTCGTTTCCCCATTAAATCCCTGGTGCATCCTACTCCCATAATGATAGC
TGACTGGCTGGACTGGCTTTTCAAGTAGTCGAGGGGGTATCGCGGTCACGGCCGTTAACAGATCCCGT
CCTTAGTGTGGAATCCGCACCTGCTGACTAACGCTTCGCCGGCGTGTCTGCACATCCGTATAGTGTTA
ATCATGACCCCAAGGAAGGATTAAACAAATATCTTGACG
>MNON40
GTGTCCGGTAGCCCGCGCTAGTTAGACACCCCGGCAGGGGGGATTGCTTTCGAGACGGGAGATCCCTT
CGCCGACCCTGGAGGGCCGACGCCGAGGCATTCGGGCCCCTGCAACGTCAACAGCGGCAAGAAAACGG
GATGAATGGGCGTAATGGGGGGGGTCTGCTGGGGACCCGACGCGGTTGCCGTTTGCGGGGCCCCCGAC
CCATACCGACCCACCTAGGCGTCCAGTTACGGCGCACGTCGGGAGCGTGGTTGCCGTCAGAGCTGTGT
TTCTCGATCAGTCCCCCGCAGTGCCGCAGTATCTTGCCGTGGGCTGCTTTAATCTTGAAAGTGGTTTA
TACATTGGGCGACGAGGTGTCGACTCTCATTGGGGGTAACCGACGGGCACATGCAGTCCCCTCCCCGG
GCAGGCAGAGGCGGGGCCCCCGCGCGCCGGCCCCGGCCCACAATCTTACCAGGGTCCTCAAAGGAGCC
TTTGCATGGTACCCTTCGTGAATGGTTGCTTAAGAGGTCCACAACGTGGTCCGGGCACGGTCGACTAA
ACACTCAAAACAGCGACGGCAAATATAGGTACAAGGTCCAGGCCCTCACGGCACTAATTGCGATGACC
CAACTCACGGGGGCAGTCCCCGGCGCGGCGACCTTGATTACGTCCGGGGACAAGTATGTCGCTCCCCC
CGGGAGGGGTGCAGCCACATGGGAGATTCAAAGTTTCTCGTGACGTCGTTGTGATCACTGCAGCCTAG
CCGAGACTCCCGTACTACGCGAAGGTTGGTTATGTTAACCACTACAACGTGAGGCGCCGTAAGGGCCA
GTGTTGTGCCCGGCTCTTCAATGCGCCTTAGCGGCCTGATACACCCACCCAAGGAGATACTGCTAATC
ATATGGGTGGACAGAACCTCGCAACGCAGACGCCTCCCCGGCATGATATGGTTTTTTTCCGCTATTAT
CCAGCACGCAGCGCTATCATTCAAGAGAACCCAGTGACGCGTAAATCGTAAGATCTACCTGCCGCAGG
TGGACCTACTGCAAATACGGATTATGACTCGTAAAGGGGTCATGCGTATTTTCATCACTAGGCACGTT
CGAGAATAAATTAGTAGGTGTCCCGAGCCTTGTGGCGTTCCGCCTGACTCCTCATGAAGTCGACCTTC
TCACCGGCCCTATCTGCCGACGTAAGTCATAACCTAGATCTGTACCTCGGGGGGAGGGTCACTGTAAA
GGGATAATTGGAGGGTGATTTCCCACACTTTCCTAAGGGTACTTTTTGCCTGGCTTCGCAATTGGGTC
CAATGGATGTCGATCTCTGGTTTAGCAGTTGTGAAAGTGGCAAGGCGGGAGGTTAGACCTCCATTTAA
CATATACAAGCAAGTTAACTGCACTAGATGTGTAGACACTACAGTTACAGGAGTAGCCGAATAAGTCT
CCGACGTCAAGCGAATAAGCGTCATACGCGATTATCGCCTAAGAGCACGTATTGGCGGTAAAAGGCTA
GCTAGACGCTTATGGGTAGATTTCAAGGCGTTCGTAGTGGTNTAATAGGATACTCTTTCACCAGCCTG
AGGGCCGAACGCTATACTAGTGGTCTGTGATGTAGGACCGAGTATCTCTCTAGGGACCATCTACTTGA
GCAATGGTGCGCAGGGGGAGACATAGACCAGCCTTGGGTGGCAAGCACTGCAATAAGTCCTGTTTAGC
CTTGGAGTTCACATGCCGGCACTGAAGCCGACCTACCTGAGCGTGTGATTACCGTTAAAGCATCTGTC
TAGTTCTGTTTCCGGCGCTCTTGGTTCCAGGTTAGGGGAAGTGTATGACCCATGTGTTTTTATCGGCT
TAACCACGAGTGATCCCCGGTCGTTTCCCCATTGAATCCCTGGTGCATCCTACTCCCAGAATGATAGC
TGACTGACTGGACTGGCTTTTCAAGTAATCGAGGGGGTATCGCGGTCACGGCCGTTAACAGATCCCGT
CCTTAGTGTGGAATCCGCACCTGCTGACTAACGCTTCGCCGGCGTGTCTGCACAGCCGTATAGTGTTA
ATCATGACCCCAAGGAAGGATTAAACAAATATCTTGACG
>MNON48
GTGTCCGGTAGCCCGCGCTAGTTAGACACCCCGGCAGGGGGGATTGCTTTCGAGACGGGAGATCCCTT
CGCCGACCCTGGAGGGCCGACGCCGAGGCATTCGGGCCCCTGCAACGTCAACAGCGGCAAGAAAACGG
GATGAATGGGCGTAATGGGGGGGGTCTGCTGGGGACCCGACGCGGTTGCCGTTTGCGGGGCCCCCGAC
CCATACCGACCCACCTAGGCGTCCAGTTACGGCGCACGGCGGGAGCGTGGTTGCCGTCAGAGCTGTGT
TTCTCGATCAGTCCCCCGCAGTGCCGCAGTATCTTGCCGTGGGCTGCTTTAATCTTGAAAGTGGTTCA
TACATTGGGCGACGAGGTGTCGACTCTCATTGGGGGTAACCGACGGGCACATGCAGTCCCCTCCCCGG
GCAGGCAGAGGCGGGGCCCCCGCGCGCCGGCCCCGGCCCACAATCTTACCAGGGTCCTCAAAGGAGCC

TTTGCATGGTACCCTTCGTGAATGGTTGCTTAAGAGGTCCACAACGTGGTCCGGGCACGGTCGACTAA
ACACTCAAAACAGCGACGGCAAATATAGGTACAAGGTCCAGGCCCTCACGGCACTAATTGCGATGACC
CAACTCACGGGGGCAGTCCCCGGCGCGGCGACCTTGATTACGTCCGGGGACAAGTATGTCGCTCCCCC
CGGGAGGGGTGCAGCCACATGGGAGATTCAAAGTTTCTCGTGACGTCGTTGTGATCACTGCAGCCTAG
CCGAGACTCCCGTACTACGCGAAGGTTGGTTATGTTAACCACTACAACGTGAGGCGCCGTAAGGGCCA
GTGTTGTGCCCGGCTCTTCAATGCGCCTTAGCGGCCTGATACACCCACCCAAGGAGATACTGCTAATC
ATATGGGTGGACAGAACCTCGCAACGCAGACGCCTCCCCGGCATGATATGGTTTTTTTCCGCTATTAT
CCAGCACGCAGCGCTATCATTCAAGAGAACCCAGTGACGCGTAAATCGTAAGATCTACCTGCCGCAGG
TGGACCTACTGCAAATACGGATTATGACTCGTAAAGGGGTCATGCGTATTTTCATCACTAGGCACGTT
CGAGAATAAATTAGTAGGTGTCCCGAGCCTTGTGGCGTTCCGCCTGACTCCTCATGAAGTCGACCTTC
TCACCGGCCCTATCTGCCGACGTAAGTCATAACCTAGATCTGTACCTCGGGGGGAGGGTCACTGTAAA
GGGATAATTGGAGGGTGATTTCCCACACTTTCCTAAGGGTACTTTTTGCCTGGCTTCGCAATTGGGTC
CAATGGATGTCGATCTCTGGTTTAGCAGTTGTGAAAGTGGCAAGGCGGGAGGTTAGACCTCCATTTAA
CATATACAAGCAAGTTAACTGCACTAGATGTGTAGACACTACAGTTACAGGAGTAGCCGAATAAGTCT
CCGACGTCAAGCGAATAAGCGTCATACGCGATTATCGCCTAAGAGCACGTATTGGCGGTAAAAGGCTA
GCTAGACGCTTATGGGTAGATTTCAAGGCGTTCGTAGTGGTATAATAGGATACTCTTTCACCAGCCTG
AGGGCCGAACGCTATACTAGTGGTCTGTGATGTAGGACCGAGTATCTCTCTAGGGACCATCTACTTGA
GCAATGGTGCGCAGGGGGAGACATAGACCAGCCTTGGGTGGCAAGCACTGCAATAAGTCCTGTTTAGC
CTTGGAGTTCACATGCCGGCACTGAAGCCGACCTACCTGAGCGTGTGATTACCGTTAAAGCATCTGTC
TAGTTCTGTTTCCGGCGCTCTTGGTTCCAGGTTAGGGGAAGTGTATGACCCATGTGTTTTTATCGGCT
TAACCACGAGTGATCCCCGGTCGTTTCCCCATTGAATCCCTGGTGCATCCTACTCCCAGAATGATAGC
TGACTGACTGGACTGGCTTTTCAAGTAATCGAGGGGGTATCGCGGTCACGGCCGTTAACAGATCCCGT
CCTTAGTGTGGAATCCGCACCTGCTGACTAACGCTTCGCCGGCGTGTCTGCACAGCCGTATAGTGTTA
ATCATGACCCCAAGGAAGGATTAAACAAATATCTTGACG
>MNON49
GTGTCCGGTAGCCCGCGCTAGTTAGACACCCCGGCAGGGGGGATTGCTTTCGAGACGGGAGATCCCTT
CGCCGACCCTGGAGGGCCGACGCCGAGGCATTCGGGCCCCCGCAACGTCAACAGCGGCAAGAAAACGG
GATGAATGGGCGTAATGGGGGGGGTCTGCTGGGGACCCGACGCGGTTGCCGTTTGCGGGGCCCCCGAC
CCATACCGACCCACCTAGGCGTCCAGTTACGGCGCACGGCGGGAGCGTGGTTGCCGTCAGAGCTGTGT
TTCTCGATCAGTCCCCCGCAGTGCCGCAGTATCTTGCCGTGGGCTGCTTTAATCTTGAAAGTGGTTCA
TACATTGGGCGACGAGGTGTCGACTCTCATTGGGGGTAACCGACGGGCACATGCAGTCCCCTCCCCGG
GCAGGCAGAGGCGGGGCCCCCGCGCGCCGGCCCCGGCCCACAATCTTACCAGGGTCCTCAAAGGAGCC
TTTGCATGGTACCCTTCGTGAATGGTTGCTTAAGAGGTCCACAACGTGGTCCGGGCACGGTCGACTAA
ACACTCAAAACAGCGACGGCAAATATAGGTACAAGGTCCAGGCCCTCACGGCACTAATTGCGATGACC
CAACTCACGGGGGCAGTCCCCGGCGCGGCGACCTTGATTACGTCCGGGGACAAGTATGTCGCTCCCCC
CGGGAGGGGTGCAGCCACATGGGAGATTCAAAGTTTCTCGTGACGTCGTTGTGATCACTGCAGCCTAG
CCGAGACTCCCGTACTACGCGAAGGTTGGTTATGTTAACCACTACAACGTGAGGCGCCGTAAGGGCCA
GTGTTGTGCCCGGCTCTTCAATGCGCCTTAGCGGCCTGATACACCCACCCAAGGAGATACTGCTAATC
ATATGGGTGGACAGAACCTCGCAACGCAGACGCCTCCCCGGCATGATATGGTTTTTTTCCGCTATTAT
CCAGCACGCAGCGCTATCATTCAAGAGAACCCAGTGACGCGTAAATCGTAAGATCTACCTGCCGCAGG
TGGACCTACTGCAAATACGGATTATGACTCGTAAAGGGGTCATGCGTATTTTCATCACTAGGCACGTT
CGAGAATAAATTAGTAGGTGTCCCGAGCCTTGTGGCGTTCCGCCTGACTCCTCATGAAGTCGACCTTC
TCACCGGCCCTATCTGCCGACGTAAGTCATAACCTAGATCTGTACCTCGGGGGGAGGGTCACTGTAAA
GGGATAATTGGAGGGTGATTTCCCACACTTTCCTAAGGGTACTTTTTGCCTGGCTTCGCAATTGGGTC
CAATGGATGTCGATCTCTGGTTTAGCAGTTGTGAAAGTGGCAAGGCGGGAGGTTAGACCTCCATTTAA
CATATACAAGCAAGTTAACTGCACTAGATGTGTAGACACTACAGTTACAGGAGTAGCCGAATAAGTCT
CCGACGTCAAGCGAATAAGCGTCATACGCGATTATCGCCTAAGAGCACGTATTGGCGGTAAAAGGCTA
GCTAGACGCTTATGGGTAGATTTCAAGGCGTTCGTAGTGGTATAATAGGATACTCTTTCACCAGCCTG
AGGGCCGAACGCTATACTAGTGGTCTGTGATGTAGGACCGAGTATCTCTCTAGGGACCATCTACTTGA
GCAATGGTGCGCAGGGGGAGACATAGACCAGCCTTGGGTGGCAAGCACTGCAATAAGTCCTGTTTAGC
CTTGGAGTTCACATGCCGGCACTGAAGCCGACCTACCTGAGCGTGTGATTACCGTTAAAGCATCTGTC
TAGTTCTGTTTCCGGCGCTCTTGGTTCCAGGTTAGGGGAAGTGTATGACCCATGTGTTTTTATCGGCT
TAACCACGAGTGATCCCCGGTCGTTTCCCCATTGAATCCCTGGTGCATCCTACTCCCAGAATGATAGC
TGACTGACTGGACTGGCTTTTCAAGTAATCGAGGGGGTATCGCGGTCACGGCCGTTAACAGATCCCGT

CCTTAGTGTGGAATCCGCACCTGCTGACTAACGCTTCGCCGGCGTGTCTGCACAGCCGTATAGTGTTA
ATCATGACCCCAAGGAAGGATTAAACAAATATCTTGACG
>MNON50
GTGTCCGGTAGCCCGCGCTAGTTAGACACCCCGGCAGGGGGGATTGCTTTCGAGACGGGAGATCCCTT
CGCCGACCCTGTAGGGCCGACGCCGAGGCATTCGGGCCCCCGCAACGTCAACAGCGGCAAGAAAACGG
GATGAATGGGCGTAATGGGGGGGGTCTGCTGGGGACCCGACGCGGTTGCCGTTTGCGGGGCCCCCGAC
CCATACCGACCCACCTAGGCGTCCAGTTACGGCGCACGGCGGGAGCGTGGTTGCCGTCAGAGCTGTGT
TTCTCGATCAGTCCCCCGCAGTGCCGCAGTATCTTGCCGTGGGCTGCTTTAATCTTGAAAGTGGTTCA
TACATTGGGCGACGAGGTGTCGGCTCTCATTGGGGGTAACCGACGGACACGTGCAGTCCCCTCCCCGG
GCAGGCAGAGGCGGGGCCCCCGCGCGCCGGCCCCGGCCCACAATCTTACCAGGGTCCTCAAAAGAGCC
TTTGCATGGTACCCTTCGTGAATGGTTGCTTAAGAGGTCCACCACGTAGTCCGGGCACGGTCAACTAA
ACACTCAAAACAGCGACGGCAAATAGAGGCACAAGGTCCAGGCCCTCACGGCACTAGTTGCGATGACC
CAACTCACGGGGGCAGTCCCCGGCGCAGCGACCTTGATTACGTCCGGGAACAAGTATGTCGTTTCCCC
CGGGAGGGGTGCAGCCACATGGGAGATTCAAAGTTTCTCGTGACGTCGTTGTGATCACTGCAGCCTAG
CCGAGACTCCCGTACTACGCGAAGGTTGGTTATGTTAACCACTACAACGTGAGGCGCCGTAAGGGCCA
GTGTTGTGCCCGGCTCTTCAATGCGCCTTAGCGGCCTGATACACCCACCCAAGGAGATACTGCTAATC
ATGTGGGTGGACAGAACCTCGCAACGCAGACGCATCCCCGGCATGATATGGTTTTTTTCCGCTATTAC
CCAGCACGCGGCGCTATCATTCAAGAGAACCCAGTGACGCGTAAATCGTCAGATCTACCTGCCGCAGG
TGGACCTACTGCAAATACGGATTATGACTCGTAAAGAGGTCATGCGTATTTTCATCACTAGGCACTTT
CGAGAGTAAATTAGTAGGTGTCCCGCGCCTTGTGGCGTTCCGCCTGGCTCCTCATGAAGTCGACCTTC
TCATCGGCCCTATTTGCCGACGTAAGTCATAATCCAGATCTTCACCTCGGAGGGAGGGTCACTGTAAA
GGGATAATTGGAGGGCGATTTCCCACACTTTCCTAAGGGTACTTTTTGCTTAGCTTCGCAGTTGGGTC
CAATAGATGTTGATCTCTGGTTTAGCAGTTGTGAAAGTGGCAAGGCGGGAGGTTAGGCCTCCATTTAA
CATATACAAGCAAGTTAACTGCACTAGATGTGTAGACACTACAGTTACAGGAGTAGCCGACTAAGTCT
CCGACGTCAAGCGAATAGGCGTCATACGCGATTATTGCCTAAGAGCACGTATTGGCGGTAAAAGGATA
ACTAGACGCTTGTGGGTAGATTTCAAGGCGCTCGTAGTGGTATAATAGGACACTCTTGCACCAGCCTG
AGAGCCGAACGCTATACTAGTGGTCTGTGATGTAGGACCAAGTAGCTCTCTAGGGACCATCTACTTGA
GCAATGGTGCGCAGGGGTAGACATAGACCAACCTTGGGTGGCAAGCACTGCAATAAGTCCTGTTTAGC
CTTGGAGTTCACACGCCGGCACTAAAGCCGACCTACCTGAGCTTGTGATTACCGTTAAAGCATCTGTC
TAGTTCTGTTTCCGGCGCTCTTGGTTCCATGTTAGGGGAAGTGTATGACCCATGTGTTTTTATCGGCT
TAACTACGAGTGATCCCCGGTCGTTTCCCCATTAAATCCCTGGTGCATCCTACTCCCATAATGATAGC
TGACTGGCTGGACTGGCTTTTCAAGTAGTCGAGGGGGTATCGCGGTCACGGCCGTTAACAGATCCCGT
CCTTAGTGTGGAATCCGCACCTGCTGACTAACGCTTCGCCGGCGTGTCTGCACATCCGTATAGTGTTA
ATCATGACCCCAAGGAAGGATTAAACAAATATCTTGACG
>MNON51
GTGTCCGGTAGCCCGCGCTAGTTAGACACCCCGGCAGGGGGGATTGCTTTCGAGACGGGAGATCCCTT
CGCCGACCCTGGAGGGCCGACGCCGAGGCATTCGGGCCCCTGCAACGTCAACAGCGGCAAGAAAACGG
GATGAATGGGCGTAATGGGGGGGGTCTGCTGGGGACCCGACGCGGTTGCCGTTTGCGGGGCCCCCGAC
CCATACCGACCCACCTAGGCGTCCAGTTACGGCGCACGTCGGGAGCGTGGTTGCCGTCAGAGCTGTGT
TTCTCGATCAGTCCCCCGCAGTGCCGCAGTATCTTGCCGTGGGCTGCTTTAATCTTGAAAGTGGTTTA
TACATTGGGCGACGAGGTGTCGACTCTCATTGGGGGTAACCGACGGGCACATGCAGTCCCCTCCCCGG
GCAGGCAGAGGCGGGGCCCCCGCGCGCCGGCCCCGGCCCACAATCTTACCAGGGTCCTCAAAGGAGCC
TTTGCATGGTACCCTTCGTGAATGGTTGCTTAAGAGGTCCACAACGTGGTCCGGGCACGGTCGACTAA
ACACTCAAAACAGCGACGGCAAATATAGGTACAAGGTCCAGGCCCTCACGGCACTAATTGCGATGACC
CAACTCACGGGGGCAGTCCCCGGCGCGGCGACCTTGATTACGTCCGGGGACAAGTATGTCGCTCCCCC
CGGGAGGGGTGCAGCCACATGGGAGATTCAAAGTTTCTCGTGACGTCGTTGTGATCACTGCAGCCTAG
CCGAGACTCCCGTACTACGCGAAGGTTGGTTATGTTAACCACTACAACGTGAGGCGCCGTAAGGGCCA
GTGTTGTGCCCGGCTCTTCAATGCGCCTTAGCGGCCTGATACACCCACCCAAGGAGATACTGCTAATC
ATATGGGTGGACAGAACCTCGCAACGCAGACGCCTCCCCGGCATGATATGGTTTTTTTCCGCTATTAT
CCAGCACGCAGCGCTATCATTCAAGAGAACCCAGTGACGCGTAAATCGTAAGATCTACCTGCCGCAGG
TGGACCTACTGCAAATACGGATTATGACTCGTAAAGGGGTCATGCGTATTTTCATCACTAGGCACGTT
CGAGAATAAATTAGTAGGTGTCCCGAGCCTTGTGGCGTTCCGCCTGACTCCTCATGAAGTCGACCTTC
TCACCGGCCCTATCTGCCGACGTAAGTCATAACCTAGATCTGTACCTCGGGGGGAGGGTCACTGTAAA
GGGATAATTGGAGGGTGATTTCCCACACTTTCCTAAGGGTACTTTTTGCCTGGCTTCGCAATTGGGTC

CAATGGATGTCGATCTCTGGTTTAGCAGTTGTGAAAGTGGCAAGGCGGGAGGTTAGACCTCCATTTAA
CATATACAAGCAAGTTAACTGCACTAGATGTGTAGACACTACAGTTACAGGAGTAGCCGAATAAGTCT
CCGACGTCAAGCGAATAAGCGTCATACGCGATTATCGCCTAAGAGCACGTATTGGCGGTAAAAGGCTA
GCTAGACGCTTATGGGTAGATTTCAAGGCGTTCGTAGTGGTATAATAGGATACTCTTTCACCAGCCTG
AGGGCCGAACGCTATACTAGTGGTCTGTGATGTAGGACCGAGTATCTCTCTAGGGACCATCTACTTGA
GCAATGGTGCGCAGGGGGAGACATAGACCAGCCTTGGGTGGCAAGCACTGCAATAAGTCCTGTTTAGC
CTTGGAGTTCACATGCCGGCACTGAAGCCGACCTACCTGAGCGTGTGATTACCGTTAAAGCATCTGTC
TAGTTCTGTTTCCGGCGCTCTTGGTTCCAGGTTAGGGGAAGTGTATGACCCATGTGTTTTTATCGGCT
TAACCACGAGTGATCCCCGGTCGTTTCCCCATTGAATCCCTGGTGCATCCTACTCCCAGAATGATAGC
TGACTGACTGGACTGGCTTTTCAAGTAATCGAGGGGGTATCGCGGTCACGGCCGTTAACAGATCCCGT
CCTTAGTGTGGAATCCGCACCTGCTGACTAACGCTTCGCCGGCGTGTCTGCACAGCCGTATAGTGTTA
ATCATGACCCCAAGGAAGGATTAAACAAATATCTTGACG
>MNON53
GTGTCCGGTAGCCCGCGCTAGTTAGACACCCCGGCAGGGGGGATTGCTTTCGAGACGGGAGATCCCTT
CGCCGACCCTGGAGGGCCGACGCCGAGGCATTCGGGCCCCTGCAACGTCAACAGCGGCAAGAAAACGG
GATGAATGGGCGTAATGGGGGGGGTCTGCTGGGGACCCGACGCGGTTGCCGTTTGCGGGGCCCCCGAC
CCATACCGACCCACCTAGGCGTCCAGTTACGGCGCACGTCGGGAGCGTGGTTGCCGTCAGAGCTGTGT
TTCTCGATCAGTCCCCCGCAGTGCCGCAGTATCTTGCCGTGGGCTGCTTTAATCTTGAAAGTGGTTTA
TACATTGGGCGACGAGGTGTCGACTCTCATTGGGGGTAACCGACGGGCACATGCAGTCCCCTCCCCGG
GCAGGCAGAGGCGGGGCCCCCGCGCGCCGGCCCCGGCCCACAATCTTACCAGGGTCCTCAAAGGAGCC
TTTGCATGGTACCCTTCGTGAATGGTTGCTTAAGAGGTCCACAACGTGGTCCGGGCACGGTCGACTAA
ACACTCAAAACAGCGACGGCAAATATAGGTACAAGGTCCAGGCCCTCACGGCACTAATTGCGATGACC
CAACTCACGGGGGCAGTCCCCGGCGCGGCGACCTTGATTACGTCCGGGGACAAGTATGTCGCTCCCCC
CGGGAGGGGTGCAGCCACATGGGAGATTCAAAGTTTCTCGTGACGTCGTTGTGATCACTGCAGCCTAG
CCGAGACTCCCGTACTACGCGAAGGTTGGTTATGTTAACCACTACAACGTGAGGCGCCGTAAGGGCCA
GTGTTGTGCCCGGCTCTTCAATGCGCCTTAGCGGCCTGATACACCCACCCAAGGAGATACTGCTAATC
ATATGGGTGGACAGAACCTCGCAACGCAGACGCCTCCCCGGCATGATATGGTTTTTTTCCGCTATTAT
CCAGCACGCAGCGCTATCATTCAAGAGAACCCAGTGACGCGTAAATCGTAAGATCTACCTGCCGCAGG
TGGACCTACTGCAAATACGGATTATGACTCGTAAAGGGGTCATGCGTATTTTCATCACTAGGCACGTT
CGAGAATAAATTAGTAGGTGTCCCGAGCCTTGTGGCGTTCCGCCTGACTCCTCATGAAGTCGACCTTC
TCACCGGCCCTATCTGCCGACGTAAGTCATAACCTAGATCTGTACCTCGGGGGGAGGGTCACTGTAAA
GGGATAATTGGAGGGTGATTTCCCACACTTTCCTAAGGGTACTTTTTGCCTGGCTTCGCAATTGGGTC
CAATGGATGTCGATCTCTGGTTTAGCAGTTGTGAAAGTGGCAAGGCGGGAGGTTAGACCTCCATTTAA
CATATACAAGCAAGTTAACTGCACTAGATGTGTAGACACTACAGTTACAGGAGTAGCCGAATAAGTCT
CCGACGTCAAGCGAATAAGCGTCATACGCGATTATCGCCTAAGAGCACGTATTGGCGGTAAAAGGCTA
GCTAGACGCTTATGGGTAGATTTCAAGGCGTTCGTAGTGGTATAATAGGATACTCTTTCACCAGCCTG
AGGGCCGAACGCTATACTAGTGGTCTGTGATGTAGGACCGAGTATCTCTCTAGGGACCATCTACTTGA
GCAATGGTGCGCAGGGGGAGACATAGACCAGCCTTGGGTGGCAAGCACTGCAATAAGTCCTGTTTAGC
CTTGGAGTTCACATGCCGGCACTGAAGCCGACCTACCTGAGCGTGTGATTACCGTTAAAGCATCTGTC
TAGTTCTGTTTCCGGCGCTCTTGGTTCCAGGTTAGGGGAAGTGTATGACCCATGTGTTTTTATCGGCT
TAACCACGAGTGATCCCCGGTCGTTTCCCCATTGAATCCCTGGTGCATCCTACTCCCAGAATGATAGC
TGACTGACTGGACTGGCTTTTCAAGTAATCGAGGGGGTATCGCGGTCACGGCCGTTAACAGATCCCGT
CCTTAGTGTGGAATCCGCACCTGCTGACTAACGCTTCGCCGGCGTGTCTGCACAGCCGTATAGTGTTA
ATCATGACCCCAAGGAAGGATTAAACAAATATCTTGACG
>CHRUN1
GTGTCCGGTAGCCCGCGCTAGTTAGACACCCCGGCAGGGGGGATTGCTTTCGGGACGGGAGATCCCTT
CGCCGACCCTGGANGGCCGACGCCGAGGCATTCGGGCCCCTGCAACGTCAACAGCGGCAAGAAAACGG
GATGAATGGGCGTAATGGGGGGGGTCTGCTGGGGACCCGACGCGGTTGCCGTTTGCGGGGCCCCCGAC
CCATACCGACCCACCTAGGCGTCCAGTTACGGCGCACGTCGGGAGCGTGGTTGCCGTCAGAGCTGTGT
TTCTCGATCAGTCCCCCACAGTGCCGCAGTATCTTGCCGTGGGCTGCTTTAATCTTGAAAGTGGTTCA
TACATTGGGCGACGAGGTGTCGACTCTCATTGGGGGTAACCGACGGGCACATGCAGTCCCCTCCCCGG
GCAGGCAGAGGCGGGGCCCCCGCGCGCCGGCCCCGGCCCACAATCTTACCAGGGTCCTCAAAGGAGCC
TTTGCATGGTACCCTTCGTGAATGGTTGCTTAAGAAGTCCACAACGTGGTCCGGGCACGGTCGACTAA
ACACTCAAAACAGCGACGGCAAATATAGGTACAAGGTCCAGGCCCTCACGGCACTAATTGCGATGACC

CAACTCACGGGGGCAGTCCCCGGCGCGGCGACCTTGATTACGTCCGGGGACAAGTATGTCGCTCCCCC
CGGGAGGGGTGCAGCCACATGGGAGATTCAAAGTTTCTCGTGACGTCGTTGTGATCACTGCAGCCTAG
CCGAGACTCCCGTACTACGCGAAGGTTGGTTATGTTAACCACTACAACGTGAGGCGCCGTAAGGGCCA
GTGTTGTGCCCGGCTCTTCAATGCGCCTTAGCGGCCTGATACACCCACCCAAGGAGATACTGCTAATC
ATATGAGTGGACAGAACCTCGCAACGCAGACGCCTCCCCGGCATGATATGGTTTTTTTCCGCTATTAT
CCAGCACGCAGCGCTATCATTCAAGAGAACCCAGTGACGCGTAAATCGTAAGATCTACCTGCCGCAGG
TGGACCTACTGCAAATACGGATTATGACTCGTAAAGGGGTCATGCGTATTTTCATCACTAGGCACGTT
CGAGAATAAATTAGTAGGTGTCCCGAGCCTTGTGGCGTTCCGCCTGACTCCTCATGAAGTCGACCTTC
TCACCGGCCCTATCTGCCGACGTAAGTCATAACCTAGATCTGTACCTCGGGGGGAGGGTCACTGTAAA
GGGATAATTGGAGGGTGATTTCCCACATTTTCCTAAGGGTACTTTTTGCCTGGCTTCGCAATTGGGTC
CAATGGATGTCGATCTCTGGTTTAGCAGTTGTGAAAGTGGCAAGGCGGGAGGTTAGACCTCCATTTAA
CATATACAAGCAAGTTAACTGCACTAGATGTGTAGACACTACAGGTACAGGAGTAGCCGGATAAGTCT
CCGACGTCAAGCGAATAAGCTTCATACGCGATTATCGCCTAAGAGCACGTATTTGCGGTAAAAGGCTA
GCTAGACGCTTGTGGGTAGATTTCAAGGCGTTCGTAGTGGTATAATAGGATACTCTTTCACCAGCCTG
AGGGCCGAACGCTATACTAGTGGTCTGTGATGTAGGACCGAGTATCTCTCTAGGGACCATCTACTTGA
GCAATGGTGCGCAGGGGGAGACATAGACCAGCCTTGGGTGGCAAGCACTGCAATAAGTCCTGTTTAGC
CTTGGAGTTCACACGCCGGCACTGAAGCCGACCTACCTGAGCGTGTGATTACCGTTAAAGCATCTGTC
TAGTTCTGTTTCCGGCGCTCTTGGTTCCAGGTTAGGGGAAGTGTATGACCCATGTGTTTTCATCGGCT
TAACCACGAGTGATCCCCGGTNGTTTCCCCATTGAATCCCTGGTGCATCCTACTCCCAGAATGATAGC
TGACTGACTGGACTGGCTTTTCAAGTAATCGAGGGGGTATCGCGGTCACGGCCGTTAACAGATCCCGT
CCTTAGTGTGGAATCCGCACCTGCTGACTAACGCTTCGCCGGCGTGTCTGCACAGCCGTATAGTGTTA
ANCATGACCCCAAGGAAGGATTAAACAAATATCTTGACG
>CHRUN2
GTGTCCGGTAGCCCGCGCTAGTTAGACACCCCGGCAGGGGGGATTGCTTTCGAGACGGGAGATCCCTT
CGCCGACCCTGGAGGGCCGACGCCGAGGCATTCGGGCCCCTGCAACGTCAACAGCGGCAAGAAAACGG
GATGAATGGGCGTAATGGGGGGGGTCTGCTGGGGACCCGACGCGGTTGCCGTTTGCGGGGCCCCCGAC
CCATACCGACCCACCTAGGCGTCCAGTTACGGCGCACGTCGGGAGCGTGGTTGCCGTCAGAGCTGTGT
TTCTCGATCAGTCCCCCGCAGTGCCGCAGTATCTTGCCGTGGGCTGCTTTAATCTTGAAAGTGGTTTA
TACATTGGGCGACGAGGTGTCGACTCTCATTGGGGGTAACCGACGGGCACATGCAGTCCCCTCCCCGG
GCAGGCAGAGGCGGGGCCCCCGCGCGCCGGCCCCGGCCCACAATCTTACCAGGGTCCTCAAAGGAGCC
TTTGCATGGTACCCTTCGTGAATGGTTGCTTAAGAGGTCCACAACGTGGTCCGGGCACGGTCGACTAA
ACACTCAAAACAGCGACGGCAAATATAGGTACAAGGTCCAGGCCCTCACGGCACTAATTGCGATGACC
CAACTCACGGGGGCAGTCCCCGGCGCGGCGACCTTGATTACGTCCGGGGACAAGTATGTCGCTCCCCC
CGGGAGGGGTGCAGCCACATGGGAGATTCAAAGTTTCTCGTGACGTCGTTGTGATCACTGCAGCCTAG
CCGAGACTCCCGTACTACGCGAAGGTTGGTTATGTTAACCACTACAACGTGAGGCGCCGTAAGGGCCA
GTGTTGTGCCCGGCTCTTCAATGCGCCTTAGCGGCCTGATACACCCACCCAAGGAGATACTGCTAATC
ATATGGGTGGACAGAACCTCGCAACGCAGACGCCTCCCCGGCATGATATGGTTTTTTTCCGCTATTAT
CCAGCACGCAGCGCTATCATTCAAGAGAACCCAGTGACGCGTAAATCGTAAGATCTACCTGCCGCAGG
TGGACCTACTGCAAATACGGATTATGACTCGTAAAGGGGTCATGCGTATTTTCATCACTAGGCACGTT
CGAGAATAAATTAGTAGGTGTCCCGAGCCTTGTGGCGTTCCGCCTGACTCCTCATGAAGTCGACCTTC
TCACCGGCCCTATCTGCCGACGTAAGTCATAACCTAGATCTGTACCTCGGGGGGAGGGTCACTGTAAA
GGGATAATTGGAGGGTGATTTCCCACACTTTCCTAAGGGTACTTTTTGCCTGGCTTCGCAATTGGGTC
CAATGGATGTCGATCTCTGGTTTAGCAGTTGTGAAAGTGGCAAGGCGGGAGGTTAGACCTCCATTTAA
CATATACAAGCAAGTTAACTGCACTAGATGTGTAGACACTACAGTTACAGGAGTAGCCGAATAAGTCT
CCGACGTCAAGCGAATAAGCGTCATACGCGATTATCGCCTAAGAGCACGTATTGGCGGTAAAAGGCTA
GCTAGACGCTTATGGGTAGATTTCAAGGCGTTCGTAGTGGTATAATAGGATACTCTTTCACCAGCCTG
AGGGCCGAACGCTATACTAGTGGTCTGTGATGTAGGACCGAGTATCTCTCTAGGGACCATCTACTTGA
GCAATGGTGCGCAGGGGGAGACATAGACCAGCCTTGGGTGGCAAGCACTGCAATAAGTCCTGTTTAGC
CTTGGAGTTCACATGCCGGCACTGAAGCCGACCTACCTGAGCGTGTGATTACCGTTAAAGCATCTGTC
TAGTTCTGTTTCCGGCGCTCTTGGTTCCAGGTTAGGGGAAGTGTATGACCCATGTGTTTTTATCGGCT
TAACCACGAGTGATCCCCGGTCGTTTCCCCATTGAATCCCTGGTGCATCCTACTCCCAGAATGATAGC
TGACTGACTGGACTGGCTTTTCAAGTAATCGAGGGGGTATCGCGGTCACGGCCGTTAACAGATCCCGT
CCTTAGTGTGGAATCCGCACCTGCTGACTAACGCTTCGCCGGCGTGTCTGCACAGCCGTATAGTGTTA
ATCATGACCCCAAGGAAGGATTAAACAAATATCTTGACG

>CHRUN3
GTGTCCGGTAGCCCGCGCTAGTTAGACACCCCGGCAGGGGGGATTGCTTTCGGGACGGGAGATCCCTT
CGCCGACCCTGGAGGGCCGACGCCGAGGCATTCGGGCCCCTGCAACGTCAACAGCGGCAAGAAAACGG
GATGAATGGGCGTAATGGGGGGGGTCTGCTGGGGACCCGACGCGGTTGCCGTTTGCGGGGCCCCCGAC
CCATACCGACCCACCTAGGCGTCCAGTTACGGCGCACGTCGGGAGCGTGGTTGCCGTCAGAGCTGTGT
TTCTCGATCAGTCCCCCGCAGTGCCGCAGTATCTTGCCGTGGGCTGCTTTAATCTTGAAAGTGGTTCA
TACATTGGGCGACGAGGTGTCGACTCTCATTGGGGGTAACCGACGGGCACATGCAGTCCCCTCCCCGG
GCAGGCAGAGGCGGGGCCCCCGCGCGCCGGCCCCGGCCCACAATCTTACCAGGGTCCTCAAAGGAGCC
TTTGCATGGTACCCTTCGTGAATGGTTGCTTAAGAGGTCCACAACGTGGTCCGGGCACGGTCGACTAA
ACACTCAAAACAGCGACGGCAAATATAGGTACAAGGTCCAGGCCCTCACGGCACTAATTGCGATGACC
CAACTCACGGGGGCAGTCCCCGGCGCGGCGACCTTGATTACGTCCGGGGACAAGTATGTCGCTCCCCC
CGGGAGGGGTGCAGCCACATGGGAGATTCAAAGTTTCTCGTGACGTCGTTGTGATCACTGCAGCCTAG
CCGAGACTCCCGTACTACGCGAAGGTTGGTTATGTTAACCACTACAACGTGAGGCGCCGTAAGGGCCA
GTGTTGTGCCCGGCTCTTCAATGCGCCTTAGCGGCCTGATACACCCACCCAAGGAGATACTGCTAATC
ATATGGGTGGACAGAACCTCGCAACGCAGACGCCTCCCCGGCATGATATGGTTTTTTTCCGCTATTAT
CCAGCACGCAGCGCTATCATTCAAGAGAACCCAGTGACGCGTAAATCGTAAGATCTACCTGCCGCAGG
TGGACCTACTGCAAATACGGATTATGACTCGTAAAGGGGTCATGCGTATTTTCATCACTAGGCACGTT
CGAGAATAAATTAGTAGGTGTCCCGAGCCTTGTGGCGTTCCGCCTGACTCCTCATGAAGTCGACCTTC
TCACCGGCCCTATCTGCCGACGTAAGTCATAACCTAGATCTGTACCTCGGGGGGAGGGTCACTGTAAA
GGGATAATTGGAGGGTGATTTCCCACACTTTCCTAAGGGTACTTTTTGCCTGGCTTCGCAATTGGGTC
CAATGGATGTCGATCTCTGGTTTAGCAGTTGTGAAAGTGGCAAGGCGGGAGGTTAGACCTCCATTTAA
CATATACAAGCAAGTTAACTGCACTAGATGTGTAGACACTACAGTTACAGGAGTAGCCGAATAAGTCT
CCGACGTCAAGCGAATAAGCGTCATACGCGATTATCGCCTAAGAGCACGTATTGGCGGTAAAAGGCTA
GCTAGACGCTTATGGGTAGATTTCAAGGCGTTCGTAGTGGTATAATAGGATACTCTTTCACCAGCCTG
AGGGCCGAACGCTATACTAGTGGTCTGTGATGTAGGACCGAGTATCTCTCTAGGGACCATCTACTTGA
GCAATGGTGCGCAGGGGGAGACATAGACCAGCCTTGGGTGGCAAGCACTGCAATAAGTCCTGTTTAGC
CTTGGAGTTCACATGCCGGCACTGAAGCCGACCTACCTGAGCGTGTGATTACCGTTAAAGCATCTGTC
TAGTTCTGTTTCCGGCGCTCTTGGTTCCAGGTTAGGGGAAGTGTATGACCCATGTGTTTTTATCGGCT
TAACCACGAGTGATCCCCGGTCGTTTCCCCATTGAATCCCTGGTGCATCCTACTCCCAGAATGATAGC
TGACTGACTGGACTGGCTTTTCAAGTAATCGAGGGGGTATCGCGGTCACGGCCGTTAACAGATCCCGT
CCTTAGTGTGGAATCCGCACCTGCTGACTAACGCTTCGCCGGCGTGTCTGCACAGCCGTATAGTGTTA
ATCATGACCCCAAGGAAGGATTAAACAAATATCTTGACG
>CHRUN4
GTGTCCGGTAGCCCGCGCTAGTTAGACACCCCGGCAGGGGGGATTGCTTTCGAGACGGGAGATCCCTT
CGCCGACCCTGGAGGGCCGACGCCGAGGCATTCGGGCCCCCGCAACGTCAACAGCGGCAAGAAAACGG
GATGAATGGGCGTAATGGGGGGGGTCTGCTGGGGACCCGACGCGGTTGCCGTTTGCGGGGCCCCCGAC
CCATACCGACCCACCTAGGCGTCCAGTTACGGCGCACGTCGGGAGCGTGGTTGCCGTCAGAGCTGTGT
TTCTCGATCAGTCCCCCGCAGTGCCGCAGTATCTTGCCGTGGGCTGCTTTAATCTTGAAAGTGGTTCA
TACATTGGGCGACGAGGTGTCGACTCTCATTGGGGGTAACCGACGGGCACATGCAGTCCCCTCCCCGG
GCAGGCAGAGGCGGGGCCCCCGCGCGCCGGCCCCGGCCCACAATCTTACCAGGGTCCTCAAAGGAGCC
TTTGCATGGTACCCTTCGTGAATGGTTGCTTAAGAGGTCCACAACGTGGTCCGGGCACGGTCGACTAA
ACACTCAAAACAGCGACGGCAAATATAGGTACAAGGTCCAGGCCCTCACGGCACTAATTGCGATGACC
CAACTCACGGGGGCAGTCCCCGGCGCGGCGACCTTGATTACGTCCGGGGACAAGTATGTCGCTCCCCC
CGGGAGGGGTGCAGCCACATGGGAGATTCAAAGTTTCTCGTGACGTCGTTGTGATCACTGCAGCCTAG
CCGAGACTCCCGTACTACGCGAAGGTTGGTTATGTTAACCACTACAACGTGAGGCGCCGTAAGGGCCA
GTGTTGTGCCCGGCTCTTCAATGCGCCTTAGCGGCCTGATACACCCACCCAAGGAGATACTGCTAATC
ATATGGGTGGACAGAACCTCGCAACGCAGACGCCTCCCCGGCATGATATGGTTTTTTTCCGCTATTAT
CCAGCACGCAGCGCTATCATTCAAGAGAACCCAGTGACGCGTAAATCGTAAGATCTACCTGCCGCAGG
TGGACCTACTGCAAATACGGATTATGACTCGTAAAGGGGTCATGCGTATTTTCATCACTAGGCACGTT
CGAGAATAAATTAGTAGGTGTCCCGAGCCTTGTGGCGTTCCGCCTGACTCCTCATGAAGTCGACCTTC
TCACCGGCCCTATCTGCCGACGTAAGTCATAACCTAGATCTGTACCTCGGGGGGAGGGTCACTGTAAA
GGGATAATTGGAGGGTGATTTCCCACACTTTCCTAAGGGTACTTTTTGCCTGGCTTCGCAATTGGGTC
CAATGGATGTCGATCTCTGGTTTAGCAGTTGTGAAAGTGGCAAGGCGGGAGGTTAGACCTCCATTTAA
CATATACAAGCAAGTTAACTGCACTAGATGTGTAGACACTACAGTTACAGGAGTAGCCGAATAAGTCT

CCGACGTCAAGCGAATAAGCGTCATACGCGATTATCGCCTAAGAGCACGTATTGGCGGTAAAAGGCTA
GCTAGACGCTTATGGGTAGATTTCAAGGCGTTCGTAGTGGTATAATAGGATACTCTTTCACCAGCCTG
AGGGCCGAACGCTATACTAGTGGTCTGTGATGTAGGACCGAGTATCTCTCTAGGGACCATCTACTTGA
GCAATGGTGCGCAGGGGGAGACATAGACCAGCCTTGGGTGGCAAGCACTGCAATAAGTCCTGTTTAGC
CTTGGAGTTCACATGCCGGCACTGAAGCCGACCTACCTGAGCGTGTGATTACCGTTAAAGCATCTGTC
TAGTTCTGTTTCCGGCGCTCTTGGTTCCAGGTTAGGGGAAGTGTATGACCCATGTGTTTTTATCGGCT
TAACCACGAGTGATCCCCGGTCGTTTCCCCATTGAATCCCTGGTGCATCCTACTCCCAGAATGATAGC
TGACTGACTGGACTGGCTTTTCAAGTAATCGAGGGGGTATCGCGGTCACGGCCGTTAACAGATCCCGT
CCTTAGTGTGGAATCCGCACCTGCTGACTAACGCTTCGCCGGCGTGTCTGCACAGCCGTATAGTGTTA
ATCATGACCCCAAGGAAGGATTAAACAAATATCTTGACG
>CHRUN6
GTGTCCGGTAGCCCGCGCTAGTTAGACACCCCGGCAGGGGGGATTGCTTTCGAGACGGGAGATCCCTT
CGCCGACCCTGGAGGGCCGACGCCGAGGCATTCGGGCCCCTGCAACGTCAACAGCGGCAAGAAAACGG
GATGAATGGGCGTAATGGGGGGGGTCTGCTGGGGACCCGACGCGGTTGCCGTTTGCGGGGCCCCCGAC
CCATACCGACCCACCTAGGCGTCCAGTTACGGCGCACGGCGGGAGCGTGGTTGCCGTCAGAGCTGTGT
TTCTCGATCAGTCCCCCGCAGTGCCGCAGTATCTTGCCGTGGGCTGCTTTAATCTTGAAAGTGGTTCA
TACATTGGGCGACGAGGTGTCGACTCTCATTGGGGGTAACCGACGGGCACATGCAGTCCCCTCCCCGG
GCAGGCAGAGGCGGGGCCCCCGCGCGCCGGCCCCGGCCCACAATCTTACCAGGGTCCTCAAAGGAGCC
TTTGCATGGTACCCTTCGTGAATGGTTGCTTAAGAGGTCCACAACGTGGTCCGGGCACGGTCGACTAA
ACACTCAAAACAGCGACGGCAAATATAGGTACAAGGTCCAGGCCCTCACGGCACTAATTGCGATGACC
CAACTCACGGGGGCAGTCCCCGGCGCGGCGACCTTGATTACGTCCGGGGACAAGTATGTCGCTCCCCC
CGGGAGGGGTGCAGCCACATGGGAGATTCAAAGTTTCTCGTGACGTCGTTGTGATCACTGCAGCCTAG
CCGAGACTCCCGTACTACGCGAAGGTTGGTTATGTTAACCACTACAACGTGAGGCGCCGTAAGGGCCA
GTGTTGTGCCCGGCTCTTCAATGCGCCTTAGCGGCCTGATACACCCACCCAAGGAGATACTGCTAATC
ATATGGGTGGACAGAACCTCGCAACGCAGACGCCTCCCCGGCATGATATGGTTTTTTTCCGCTATTAT
CCAGCACGCAGCGCTATCATTCAAGAGAACCCAGTGACGCGTAAATCGTAAGATCTACCTGCCGCAGG
TGGACCTACTGCAAATACGGATTATGACTCGTAAAGGGGTCATGCGTATTTTCATCACTAGGCACGTT
CGAGAATAAATTAGTAGGTGTCCCGAGCCTTGTGGCGTTCCGCCTGACTCCTCATGAAGTCGACCTTC
TCACCGGCCCTATCTGCCGACGTAAGTCATAACCTAGATCTGTACCTCGGGGGGAGGGTCACTGTAAA
GGGATAATTGGAGGGTGATTTCCCACACTTTCCTAAGGGTACTTTTTGCCTGGCTTCGCAATTGGGTC
CAATGGATGTCGATCTCTGGTTTAGCAGTTGTGAAAGTGGCAAGGCGGGAGGTTAGACCTCCATTTAA
CATATACAAGCAAGTTAACTGCNCTAGATGTGTAGACACTACAGTTACAGGAGTAGCCGAATAAGTCT
CCGACGTCAAGCGAATAAGCGTCATACGCGATTATCGCCTAAGAGCACGTATTGGCGGTAAAAGGCTA
GCTAGACGCTTATGGGTAGATTTCAAGGCGTTCGTAGTGGTATAATAGGATACTCTTTCACCAGCCTG
AGGGCCGAACGCTATACTAGTGGTCTGTGATGTAGGACCGAGTATCTCTCTAGGGACCATCTACTTGA
GCAATGGTGCGCAGGGGGAGACATAGACCAGCCTTGGGTGGCAAGCACTGCAATAAGTCCTGTTTAGC
CTTGGAGTTCACATGCCGGCACTGAAGCCGACCTACCTGAGCGTGTGATTACCGTTAAAGCATCTGTC
TAGTTCTGTTTCCGGCGCTCTTGGTTCCAGGTTAGGGGAAGTGTATGACCCATGTGTTTTTATCGGCT
TAACCACGAGTGATCCCCGGTCGTTTCCCCATTGAATCCCTGGTGCATCCTACTCCCAGAATGATAGC
TGACTGACTGGACTGGCTTTTCAAGTAATCGAGGGGGTATCGCGGTCACGGCCGTTAACAGATCCCGT
CCTTAGTGTGGAATCCGCACCTGCTGACTAACGCTTCGCCGGCGTGTCTGCACAGCCGTATAGTGTTA
ATCATGACCCCAAGGAAGGATTAAACAAATATCTTGACG
>CHRUN7
GTGTCCGGTAGCCCGCGCTAGTTAGACACCCCGGCAGGGGGGATTGCTTTCGAGACGGGAGATCCCTT
CGCCGACCCTGGAGGGCCGACGCCGAGGCATTCGGGCCCCCGCAACGTCAACAGCGGCAAGAAAACGG
GATGAATGGGCGTAATGGGGGGGGTCTGCTGGGGACCCGACGCGGTTGCCGTTTGCGGGGCCCCCGAC
CCATACCGACCCACCTAGGCGTCCAGTTACGGCGCACGGCGGGAGCGTGGTTGCCGTCAGAGCTGTGT
TTCTCGATCAGTCCCCCGCAGTGCCGCAGTATCTTGCCGTGGGCTGCTTTAATCTTGAAAGTGGTTCA
TACATTGGGCGACGAGGTGTCGACTCTCATTGGGGGTAACCGACGGGCACATGCAGTCCCCTCCCCGG
GCAGGCAGAGGCGGGGCCCCCGCGCGCCGGCCCCGGCCCACAATCTTACCAGGGTCCTCAAAGGAGCC
TTTGCATGGTACCCTTCGTGAATGGTTGCTTAAGAGGTCCACAACGTGGTCCGGGCACGGTCGACTAA
ACACTCAAAACAGCGACGGCAAATATAGGTACAAGGTCCAGGCCCTCACGGCACTAATTGCGATGACC
CAACTCACGGGGGCAGTCCCCGGCGCGGCGACCTTGATTACGTCCGGGGACAAGTATGTCGCTCCCCC
CGGGAGGGGTGCAGCCACATGGGAGATTCAAAGTTTCTCGTGACGTCGTTGTGATCACTGCAGCCTAG

CCGAGACTCCCGTACTACGCGAAGGTTGGTTATGTTAACCACTACAACGTGAGGCGCCGTAAGGGCCA
GTGTTGTGCCCGGCTCTTCAATGCGCCTTAGCGGCCTGATACACCCACCCAAGGAGATACTGCTAATC
ATATGGGTGGACAGAACCTCGCAACGCAGACGCCTCCCCGGCATGATATGGTTTTTTTCCGCTATTAT
CCAGCACGCAGCGCTATCATTCAAGAGAACCCAGTGACGCGTAAATCGTAAGATCTACCTGCCGCAGG
TGGACCTACTGCAAATACGGATTATGACTCGTAAAGGGGTCATGCGTATTTTCATCACTAGGCACGTT
CGAGAATAAATTAGTAGGTGTCCCGAGCCTTGTGGCGTTCCGCCTGACTCCTCATGAAGTCGACCTTC
TCACCGGCCCTATCTGCCGACGTAAGTCATAACCTAGATCTGTACCTCGGGGGGAGGGTCACTGTAAA
GGGATAATTGGAGGGTGATTTCCCACACTTTCCTAAGGGTACTTTTTGCCTGGCTTCGCAATTGGGTC
CAATGGATGTCGATCTCTGGTTTAGCAGTTGTGAAAGTGGCAAGGCGGGAGGTTAGACCTCCATTTAA
CATATACAAGCAAGTTAACTGCACTAGATGTGTAGACACTACAGTTACAGGAGTAGCCGAATAAGTCT
CCGACGTCAAGCGAATAAGCGTCATACGCGATTATCGCCTAAGAGCACGTATTGGCGGTAAAAGGCTA
GCTAGACGCTTATGGGTAGATTTCAAGGCGTTCGTAGTGGTATAATAGGATACTCTTTCACCAGCCTG
AGGGCCGAACGCTATACTAGTGGTCTGTGATGTAGGACCGAGTATCTCTCTAGGGACCATCTACTTGA
GCAATGGTGCGCAGGGGGAGACATAGACCAGCCTTGGGTGGCAAGCACTGCAATAAGTCCTGTTTAGC
CTTGGAGTTCACATGCCGGCACTGAAGCCGACCTACCTGAGCGTGTGATTACCGTTAAAGCATCTGTC
TAGTTCTGTTTCCGGCGCTCTTGGTTCCAGGTTAGGGGAAGTGTATGACCCATGTGTTTTTATCGGCT
TAACCACGAGTGATCCCCGGTCGTTTCCCCATTGAATCCCTGGTGCATCCTACTCCCAGAATGATAGC
TGACTGACTGGACTGGCTTTTCAAGTAATCGAGGGGGTATCGCGGTCACGGCCGTTAACAGATCCCGT
CCTTAGTGTGGAATCCGCACCTGCTGACTAACGCTTCGCCGGCGTGTCTGCACAGCCGTATAGTGTTA
ATCATGACCCCAAGGAAGGATTAAACAAATATCTTGACG
>CHRUN8
GTGTCCGGTAGCCCGCGCTAGTTAGACACCCCGGCAGGGGGGATTGCTTTCGGGACGGGAGATCCCTT
CGCCGACCCTGGAGGGCCGACGCCGAGGCATTCGGGCCCCTGCAACGTCAACAGCGGCAAGAAAACGG
GATGAATGGGCGTAATGGGGGGGGTCTGCTGGGGACCCGACGCGGTTGCCGTTTGCGGGGCCCCCGAC
CCATACCGACCCACCTAGGCGTCCAGTTACGGCGCACGGCGGGAGCGTGGTTGCCGTCAGAGCTGTGT
TTCTCGATCAGTCCCCCGCAGTGCCGCAGTATCTTGCCGTGGGCTGCTTTAATCTTGAAAGTGGTTCA
TACATTGGGCGACGAGGTGTCGACTCTCATTGGGGGTAACCGACGGGCACATGCAGTCCCCTCCCCGG
GCAGGCAGAGGCGGGGCCCCCGCGCGCCGGCCCCGGCCCACAATCTTACCAGGGTCCTCAAAGGAGCC
TTTGCATGGTACCCTTCGTGAATGGTTGCTTAAGAGGTCCACAACGTGGTCCGGGCACGGTCGACTAA
ACACTCAAAACAGCGACGGCAAATATAGGTACAAGGTCCAGGCCCTCACGGCACTAATTGCGATGACC
CAACTCACGGGGGCAGTCCCCGGCGCGGCGACCTTGATTACGTCCGGGGACAAGTATGTCGCTCCCCC
CGGGAGGGGTGCAGCCACATGGGAGATTCAAAGTTTCTCGTGACGTCGTTGTGATCACTGCAGCCTAG
CCGAGACTCCCGTACTACGCGAAGGTTGGTTATGTTAACCACTACAACGTGAGGCGCCGTAAGGGCCA
GTGTTGTGCCCGGCTCTTCAATGCGCCTTAGCGGCCTGATACACCCACCCAAGGAGATACTGCTAATC
ATATGGGTGGACAGAACCTCGCAACGCAGACGCCTCCCCGGCATGATATGGTTTTTTTCCGCTATTAT
CCAGCACGCAGCGCTATCATTCAAGAGAACCCAGTGACGCGTAAATCGTAAGATCTACCTGCCGCAGG
TGGACCTACTGCAAATACGGATTATGACTCGTAAAGGGGTCATGCGTATTTTCATCACTAGGCACGTT
CGAGAATAAATTAGTAGGTGTCCCGAGCCTTGTGGCGTTCCGCCTGACTCCTCATGAAGTCGACCTTC
TCACCGGCCCTATCTGCCGACGTAAGTCATAACCTAGATCTGTACCTCGGGGGGAGGGTCACTGTAAA
GGGATAATTGGAGGGTGATTTCCCACACTTTCCTAAGGGTACTTTTTGCCTGGCTTCGCAATTGGGTC
CAATGGATGTCGATCTCTGGTTTAGCAGTTGTGAAAGTGGCAAGGCGGGAGGTTAGACCTCCATTTAA
CATATACAAGCAAGTTAACTGCACTAGATGTGTAGACACTACAGTTACAGGAGTAGCCGAATAAGTCT
CCGACGTCAAGCGAATAAGCGTCATACGCGATTATCGCCTAAGAGCACGTATTGGCGGTAAAAGGCTA
GCTAGACGCTTATGGGTAGATTTCAAGGCGTTCGTAGTGGTATAATAGGATACTCTTTCACCAGCCTG
AGGGCCGAACGCTATACTAGTGGTCTGTGATGTAGGACCGAGTATCTCTCTAGGGACCATCTACTTGA
GCAATGGTGCGCAGGGGGAGACATAGACCAGCCTTGGGTGGCAAGCACTGCAATAAGTCCTGTTTAGC
CTTGGAGTTCACATGCCGGCACTGAAGCCGACCTACCTGAGCGTGTGATTACCGTTAAAGCATCTGTC
TAGTTCTGTTTCCGGCGCTCTTGGTTCCAGGTTAGGGGAAGTGTATGACCCATGTGTTTTTATCGGCT
TAACCACGAGTGATCCCCGGTCGTTTCCCCATTGAATCCCTGGTGCATCCTACTCCCAGAATGATAGC
TGACTGACTGGACTGGCTTTTCAAGTAATCGAGGGGGTATCGCGGTCACGGCCGTTAACAGATCCCGT
CCTTAGTGTGGAATCCGCACCTGCTGACTAACGCTTCGCCGGCGTGTCTGCACAGCCGTATAGTGTTA
ATCATGACCCCAAGGAAGGATTAAACAAATATCTTGACG
>CHRUN9
GTGTCCGGTAGCCCGCGCTAGTTAGACACCCCGGCAGGGGGGATTGCTTTCGAGACGGGAGATCCCTT

CGCCGACCCTGGAGGGCCGACGCCGAGGCATTCGGGCCCCTGCAACGTCAACAGCGGCAAGAAAACGG
GATGAATGGGCGTAATGGGGGGGGTCTGCTGGGGACCCGACGCGGTTGCCGTTTGCGGGGCCCCCGAC
CCATACCGACCCACCTAGGCGTCCAGTTACGGCGCACGTCGGGAGCGTGGTTGCCGTCAGAGCTGTGT
TTCTCGATCAGTCCCCCGCAGTGCCGCAGTATCTTGCCGTGGGCTGCTTTAATCTTGAAAGTGGTTTA
TACATTGGGCGACGAGGTGTCGACTCTCATTGGGGGTAACCGACGGGCACATGCAGTCCCCTCCCCGG
GCAGGCAGAGGCGGGGCCCCCGCGCGCCGGCCCCGGCCCACAATCTTACCAGGGTCCTCAAAGGAGCC
TTTGCATGGTACCCTTCGTGAATGGTTGCTTAAGAGGTCCACAACGTGGTCCGGGCACGGTCGACTAA
ACACTCAAAACAGCGACGGCAAATATAGGTACAAGGTCCAGGCCCTCACGGCACTAATTGCGATGACC
CAACTCACGGGGGCAGTCCCCGGCGCGGCGACCTTGATTACGTCCGGGGACAAGTATGTCGCTCCCCC
CGGGAGGGATGCAGCCACATGGGAGATTCAAAGTTTCTCGTGACGTCGTTGTGATCACTGCAGCCTAG
CCGAGACTCCCGTACTACGCGAAGGTTGGTTATGTTAACCACTACAACGTGAGGCGCCGTAAGGGCCA
GTGTTGTGCCCGGCTCTTNAATGCGCCTTAGCGGCCTGATACACCCACCCAAGGAGATACTGCTAATC
ATATGGGTGGACAGAACCTCGCAACGCAGACGCCTCCCCGGCATGATATGGTTTTTTTCCGCTATTAT
CCAGCACGCAGCGCTATCATTCAAGAGAACCCAGTGACGCGTAAATCGTAAGATCTACCTGCCGCAGG
TGGACCTACTGCAAATACGGATTATGACTCGTAAAGGGGTCATGCGTATTTTCATCACTAGGCACGTT
CGAGAATAAATTAGTAGGTGTCCCGAGCCTTGTGGCGTTCCGCCTGACTCCTCATGAAGTCGACCTTC
TCACCGGCCCTATCTGCCGACGTAAGTCATAACCTAGATCTGTACCTCGGGGGGAGGGTCACTGTAAA
GGGATAATTGGAGGGTGATTTCCCACACTTTCCTAAGGGTACTTTTTGCCTGGCTTCGCAATTGGGTC
CAATGGATGTCGATCTCTGGTTTAGCAGTTGTGAAAGTGGCAAGGCGGGAGGTTAGACCTCCATTTAA
CATATACAAGCAAGTTAACTGCACTAGATGTGTAGACACTACAGTTACAGGAGTAGCCGAATAAGTCT
CCGACGTCAAGCGAATAAGCGTCATACGCGATTATCGCCTAAGAGCACGTATTGGCGGTAAAAGGCTA
GCTAGACGCTTATGGGTAGATTTCAAGGCGTTCGTAGTGGTATAATAGGATACTCTTTCACCAGCCTG
AGGGCCGAACGCTATACTAGTGGTCTGTGATGTAGGACCGAGTATCTCTCTAGGGACCATCTACTTGA
GCAATGGTGCGCAGGGGGAGACATAGACCAGCCTTGGGTGGCAAGCACTGCAATAAGTCCTGTTTAGC
CTTGGAGTTCACATGCCGGCACTGAAGCCGACCTACCTGAGCGTGTGATTACCGTTAAAGCATCTGTC
TAGTTCTGTTTCCGGCGCTCTTGGTTCCAGGTTAGGGGAAGTGTATGACCCATGTGTTTTTATCGGCT
TAACCACGAGTGATCCCCGGTCGTTTCCCCATTGAATCCCTGGTGCATCCTACTCCCAGAATGATAGC
TGACTGACTGGACTGGCTTTTCAAGTAATCGAGGGGGTATCGCGGTCACGGCCGTTAACAGATCCCGT
CCTTAGTGTGGAATCCGCACCTGCTGACTAACGCTTCGCCGGCGTGTCTGCACAGCCGTATAGTGTTA
ATCATGACCCCAAGGAAGGATTAAACAAATATCTTGACG
>CHRUN10
GTGTCCGGTAGCCCGCGCTAGTTAGACACCCCGGCAGGGGGGATTGCTTTCGAGACGGGAGATCCCTT
CGCCGACCCTGGAGGGCCGACGCCGAGGCATTCGGGCCCCTGCAACGTCAACAGCGGCAAGAAAACGG
GATGAATGGGCGTAATGGGGGGGGTCTGCTGGGGACCCGACGCGGTTGCCGTTTGCGGGGCCCCCGAC
CCATACCGACCCACCTAGGCGTCCAGTTACGGCGCACGTCGGGAGCGTGGTTGCCGTCAGAGCTGTGT
TTCTCGATCAGTCCCCCGCAGTGCCGCAGTATCTTGCCGTGGGCTGCTTTAATCTTGAAAGTGGTTTA
TACATTGGGCGACGAGGTGTCGACTCTCATTGGGGGTAACCGACGGGCACATGCAGTCCCCTCCCCGG
GCAGGCAGAGGCGGGGCCCCCGCGCGCCGGCCCCGGCCCACAATCTTACCAGGGTCCTCAAAGGAGCC
TTTGCATGGTACCCTTCGTGAATGGTTGCTTAAGAGGTCCACAACGTGGTCCGGGCACGGTCGACTAA
ACACTCAAAACAGCGACGGCAAATATAGGTACAAGGTCCAGGCCCTCACGGCACTAATTGCGATGACC
CAACTCACGGGGGCAGTCCCCGGCGCGGCGACCTTGATTACGTCCGGGGACAAGTATGTCGCTCCCCC
CGGGAGGGGTGCAGCCACATGGGAGATTCAAAGTTTCTCGTGACGTCGTTGTGATCACTGCAGCCTAG
CCGAGACTCCCGTACTACGCGAAGGTTGGTTATGTTAACCACTACAACGTGAGGCGCCGTAAGGGCCA
GTGTTGTGCCCGGCTCTTCAATGCGCCTTAGCGGCCTGATACACCCACCCAAGGAGATACTGCTAATC
ATATGGGTGGACAGAACCTCGCAACGCAGACGCCTCCCCGGCATGATATGGTTTTTTTCCGCTATTAT
CCAGCACGCAGCGCTATCATTCAAGAGAACCCAGTGACGCGTAAATCGTAAGATCTACCTGCCGCAGG
TGGACCTACTGCAAATACGGATTATGACTCGTAAAGGGGTCATGCGTATTTTCATCACTAGGCACGTT
CGAGAATAAATTAGTAGGTGTCCCGAGCCTTGTGGCGTTCCGCCTGACTCCTCATGAAGTCGACCTTC
TCACCGGCCCTATCTGCCGACGTAAGTCATAACCTAGATCTGTACCTCGGGGGGAGGGTCACTGTAAA
GGGATAATTGGAGGGTGATTTCCCACACTTTCCTAAGGGTACTTTTTGCCTGGCTTCGCAATTGGGTC
CAATGGATGTCGATCTCTGGTTTAGCAGTTGTGAAAGTGGCAAGGCGGGAGGTTAGACCTCCATTTAA
CATATACAAGCAAGTTAACTGCACTAGATGTGTAGACACTACAGTTACAGGAGTAGCCGAATAAGTCT
CCGACGTCAAGCGAATAAGCGTCATACGCGATTATCGCCTAAGAGCACGTATTGGCGGTAAAAGGCTA
GCTAGACGCTTATGGGTAGATTTCAAGGCGTTCGTAGTGGTATAATAGGATACTCTTTCACCAGCCTG

AGGGCCGAACGCTATACTAGTGGTCTGTGATGTAGGACCGAGTATCTCTCTAGGGACCATCTACTTGA
GCAATGGTGCGCAGGGGGAGACATAGACCAGCCTTGGGTGGCAAGCACTGCAATAAGTCCTGTTTAGC
CTTGGAGTTCACATGCCGGCACTGAAGCCGACCTACCTGAGCGTGTGATTACCGTTAAAGCATCTGTC
TAGTTCTGTTTCCGGCGCTCTTGGTTCCAGGTTAGGGGAAGTGTATGACCCATGTGTTTTTATCGGCT
TAACCACGAGTGATCCCCGGTCGTTTCCCCATTGAATCCCTGGTGCATCCTACTCCCAGAATGATAGC
TGACTGACTGGACTGGCTTTTCAAGTAATCGAGGGGGTATCGCGGTCACGGCCGTTAACAGATCCCGT
CCTTAGTGTGGAATCCGCACCTGCTGACTAACGCTTCGCCGGCGTGTCTGCACAGCCGTATAGTGTTA
ATCATGACCCCAAGGAAGGATTAAACAAATATCTTGACG
>CHRUN11
GTGTCCGGTAGCCCGCGCTAGTTAGACACCCCGGCAGGGGGGATTGCTTTCGAGACGGGAGATCCCTT
CGCCGACCCTGGAGGGCCGACGCCGAGGCATTCGGGCCCCTGCAACGTCAACAGCGGCAAGAAAACGG
GATGAATGGGCGTAATGGGGGGGGTCTGCTGGGGACCCGACGCGGTTGCCGTTTGCGGGGCCCCCGAC
CCATACCGACCCANCTAGGCGTCCAGTTACGGCGCACGTCGGGAGCGTGGTTGCCGTCAGAGCTGTGT
TTCTCGATCAGTCCCCCGCAGTGCCGCAGTATCTTGCCGTGGGCTGCTTTAATCTTGAAAGTGGTTTA
TACATTGGGCGACGAGGTGTCGACTCTCATTGGGGGTAACCGACGGGCACATGCAGTCCCCTCCCCGG
GCAGGCAGAGGCGGGGCCCCCGCGCGCCGGCCCCGGCCCACAATCTTACCAGGGTCCTCAAAGGAGCC
TTTGCATGGTACCCTTCGTGAATGGTTGCTTAAGAGGTCCACAACGTGGTCCGGGCACGGTCGACTAA
ACACTCAAAACAGCGACGGCAAATATAGGTACAAGGTCCAGGCCCTCACGGCACTAATTGCGATGACC
CAACTCACGGGGGCAGTCCCCGGCGCGGCGACCTTGATTACGTCCGGGGACAAGTATGTCGCTCCCCC
CGGGAGGGGTGCAGCCACATGGGAGATTCAAAGTTTCTCGTGACGTCGTTGTGATCACTGCAGCCTAG
CCGAGACTCCCGTACTACGCGAAGGTTGGTTATGTTAACCACTACAACGTGAGGCGCCGTAAGGGCCA
GTGTTGTGCCCGGCTCTTCAATGCGCCTTAGCGGCCTGATACACCCACCCAAGGAGATACTGCTAATC
ATATGGGTGGACAGAACCTCGCAACGCAGACGCCTCCCCGGCATGATATGGTTTTTTTCCGCTATTAT
CCAGCACGCAGCGCTATCATTCAAGAGAACCCAGTGACGCGTAAATCGTAAGATCTACCTGCCGCAGG
TGGACCTACTGCAAATACGGATTATGACTCGTAAAGGGGTCATGCGTATTTTCATCACTAGGCACGTT
CGAGAATAAATTAGTAGGTGTCCCGAGCCTTGTGGCGTTCCGCCTGACTCCTCANGAAGTCGACCTTC
TCACCGGCCCTATCTGCCGACGTAAGTCATAACCTAGATCTGTACCTCGGGGGGAGGGTCACTGTAAA
GGGATAATTGGAGGGTGATTTCCCACACTTTCCTAAGGGTACTTTTTGCCTGGCTTCGCAATTGGGTC
CAATGGATGTCGATCTCTGGTTTAGCAGTTGTGAAAGTGGCAAGGCGGGAGGTTAGACCTCCATTTAA
CATATACAAGCAAGTTAACTGCACTAGATGTGTAGACACTACAGTTACAGGAGTAGCCGAATAAGTCT
CCGACGTCAAGCGAATAAGCGTCATACGCGATTATCGCCTAAGAGCACGTATTGGCGGTAAAAGGCTA
GCTAGACGCTTATGGGTAGATTTCAAGGCGTTCGTAGTGGTATAATAGGATACTCTTTCACCAGCCTG
AGGGCCGAACGCTATACTAGTGGTCTGTGATGTAGGACCGAGTATCTCTCTAGGGACCATCTACTTGA
GCAATGGTGCGCAGGGGGAGACATAGACCAGCCTTGGGTGGCAAGCACTGCAATAAGTCCTGTTTAGC
CTTGGAGTTCACATGCCGGCACTGAAGCCGACCTACCTGAGCGTGTGATTACCGTTAAAGCATCTGTC
TAGTTCTGTTTCCGGCGCTCTTGGTTCCAGGTTAGGGGAAGTGTATGACCCATGTGTTTTTATCGGCT
TAACCACGAGTGATCCCCGGTCGTTTCCCCATTGAATCCCTGGTGCATCCTACTCCCAGAATGATAGC
TGACTGACTGGACTGGCTTTTCAAGTAATCGAGGGGGTATCGCGGTCACGGCCGTTAACAGATCCCGT
CCTTAGTGTGGAATCCGCACCTGCTGACTAACGCTTCGCCGGCGTGTCTGCACAGCCGTATAGTGTTA
ATCATGACCCCAAGGAAGGATTAAACAAATATCTTGACG
>CHRUN12
GTGTCCGGTAGCCCGCGCTAGTTAGACACCCCGGCAGGGGGGATTGCTTTCGGGACGGGAGATCCCTT
CGCCGACCCTGGAGGGCCGACGCCGAGGCATTCGGGCCCCTGCAACGTCAACAGCGGCAAGAAAACGG
GATGAATGGGCGTAATGGGGGGGGTCTGCTGGGGACCCGACGCGGTTGCCGTTTGCGGGGCCCCCGAC
CCATACCGACCCACCTAGGCGTCCAGTTACGGCGCACGTCGGGAGCGTGGTTGCCGTCAGAGCTGTGT
TTCTCGATCAGTCCCCCGCAGTGCCGCAGTATCTTGCCGTGGGCTGCTTTAATCTTGAAAGTGGTTCA
TACATTGGGCGACGAGGTGTCGACTCTCATTGGGGGTAACCGACGGGCACATGCAGTCCCCTCCCCGG
GCAGGCAGAGGCGGGGCCCCCGCGCGCCGGCCCCGGCCCACAATCTTACCAGGGTCCTCAAAGGAGCC
TTTGCATGGTACCCTTCGTGAATGGTTGCTTAAGAGGTCCACAACGTGGTCCGGGCACGGTCGACTAA
ACACTCAAAACAGCGACGGCAAATATAGGTACAAGGTCCAGGCCCTCACGGCACTAATTGCGATGACC
CAACTCACGGGGGCAGTCCCCGGCGCGGCGACCTTGATTACGTCCGGGGACAAGTATGTCGCTCCCCC
CGGGAGGGGTGCAGCCACATAGGAGATTCAAAGTTTCTCGTGACGTCGTTGTGATCACTGCAGCCTAG
CCGAGACTCCCGTACTACGCGAAGGTTGGTTATGTTAACCACTACAACGTGAGGCGCCGTAAGGGCCA
GTGTTGTGCCCGGCTCTTCAATGCGCCTTAGCGGCCTGATACACCCACCCAAGGAGATACTGCTAATC

ATATGGGTGGACAGAACCTCGCAACGCAGACGCCTCCCCGGCATGATATGGTTTTTTTCCGCTATTAT
CCAGCACGCAGCGCTATCATTCAAGAGAACCCAGTGACGCGTAAATCGTAAGATCTACCTGCCGCAGG
TGGACCTACTGCAAATACGGATTATGACTCGTAAAGGGGTCATGCGTATTTTCATCACTAGGCACGTT
CGAGAATAAATTAGTAGGTGTCCCGAGCCTTGTGGCGTTCCGCCTGACTCCTCATGAAGTCGACCTTC
TCACCGGCCCTATCTGCCGACGTAAGTCATAACCTAGATCTGTACCTCGGGGGGAGGGTCACTGTAAA
GGGATAATTGGAGGGTGATTTCCCACACTTTCCTAAGGGTACTTTTTGCCTGGCTTCGCAATTGGGTC
CAATGGATGTCGATCTCTGGTTTAGCAGTTGTGAAAGTGGCAAGGCGGGAGGTTAGACCTCCATTTAA
CATATACAAGCAAGTTAACTGCACTAGATGTGTAGACACTACAGTTACAGGAGTAGCCGAATAAGTCT
CCGACGTCAAGCGAATAAGCGTCATACGCGATTATCGCCTAAGAGCACGTATTGGCGGTAAAAGGCTA
GCTAGACGCTTATGGGTAGATTTCAAGGCGTTCGTAGTGGTATAATAGGATACTCTTTCACCAGCCTG
AGGGCCGAACGCTATACTAGTGGTCTGTGATGTAGGACCGAGTATCTCTCTAGGGACCATCTACTTGA
GCAATGGTGCGCAGGGGGAGACATAGACCAGCCTTGGGTGGCAAGCACTGCAATAAGTCCTGTTTAGC
CTTGGAGTTCACATGCCGGCACTGAAGCCGACCTACCTGAGCGTGTGATTACCGTTAAAGCATCTGTC
TAGTTCTGTTTCCGGCGCTCTTGGTTCCAGGTTAGGGGAAGTGTATGACCCATGTGTTTTTATCGGCT
TAACCACGAGTGATCCCCGGTCGTTTCCCCATTGAATCCCTGGTGCATCCTACTCCCAGAATGATAGC
TGACTGACTGGACTGGCTTTTCAAGTAATCGAGGGGGTATCGCGGTCACGGCCGTTAACAGATCCCGT
CCTTAGTGTGGAATCCGCACCTGCTGACTAACGCTTCGCCGGCGTGTCTGCACAGCCGTATAGTGTTA
ATCATGACCCCAAGGAAGGATTAAACAAATATCTTGACG
>CHRUN13
GTGTCCGGTAGCCCGCGCTAGTTAGACACCCCGGCAGGGGGGATTGCTTTCGAGACGGGAGATCCCTT
CGCCGACCCTGGAGGGCCGACGCCGAGGCATTCGGGCCCCTGCAACGTCAACAGCGGCAAGAAAACGG
GATGAATGGGCGTAATGGGGGGGGTCTGCTGGGGACCCGACGCGGTTGCCGTTTGCGGGGCCCCCGAC
CCATACCGACCCACCTAGGCGTCCAGTTACGGCGCACGTCGGGAGCGTGGTTGCCGTCAGAGCTGTGT
TTCTCGATCAGTCCCCCGCAGTGCCGCAGTATCTTGCCGTGGGCTGCTTTAATCTTGAAAGTGGTTTA
TACATTGGGCGACGAGGTGTCGACTCTCATTGGGGGTAACCGACGGGCACATGCAGTCCCCTCCCCGG
GCAGGCAGAGGCGGGGCCCCCGCGCGCCGGCCCCGGCCCACAATCTTACCAGGGTCCTCAAAGGAGCC
TTTGCATGGTACCCTTCGTGAATGGTTGCTTAAGAGGTCCACAACGTGGTCCGGGCACGGTCGACTAA
ACACTCAAAACAGCGACGGCAAATATAGGTACAAGGTCCAGGCCCTCACGGCACTAATTGCGATGACC
CAACTCACGGGGGCAGTCCCCGGCGCGGCGACCTTGATTACGTCCGGGGACAAGTATGTCGCTCCCCC
CGGGAGGGGTGCAGCCACATGGGAGATTCAAAGTTTCTCGTGACGTCGTTGTGATCACTGCAGCCTAG
CCGAGACTCCCGTACTACGCGAAGGTTGGTTATGTTAACCACTACAACGTGAGGCGCCGTAAGGGCCA
GTGTTGTGCCCGGCTCTTCAATGCGCCTTAGCGGCCTGATACACCCACCCAAGGAGATACTGCTAATC
ATATGGGTGGACAGAACCTCGCAACGCAGACGCCTCCCCGGCATGATATGGTTTTTTTCCGCTATTAT
CCAGCACGCAGCGCTATCATTCAAGAGAACCCAGTGACGCGTAAATCGTAAGATCTACCTGCCGCAGG
TGGACCTACTGCAAATACGGATTATGACTCGTAAAGGGGTCATGCGTATTTTCATCACTAGGCACGTT
CGAGAATAAATTAGTAGGTGTCCCGAGCCTTGTGGCGTTCCGCCTGACTCCTCATGAAGTCGACCTTC
TCACCGGCCCTATCTGCCGACGTAAGTCATAACCTAGATCTGTACCTCGGGGGGAGGGTCACTGTAAA
GGGATAATTGGAGGGTGATTTCCCACACTTTCCTAAGGGTACTTTTTGCCTGGCTTCGCAATTGGGTC
CAATGGATGTCGATCTCTGGTTTAGCAGTTGTGAAAGTGGCAAGGCGGGAGGTTAGACCTCCATTTAA
CATATACAAGCAAGTTAACTGCACNAGATGTGTAGACACTACAGTTACAGGAGTAGCCGAATAAGTCT
CCGACGTCAAGCGAATAAGCGTCATACGCGATTATCGCCTAAGAGCACGTATTGGCGGTAAAAGGCTA
GCTAGACGCTTATGGGTAGATTTCAAGGCGTTCGTAGTGGTATAATAGGATACTCTTTCACCAGCCTG
AGGGCCGAACGCTATACTAGTGGTCTGTGATGTAGGACCGAGTATCTCTCTAGGGACCATCTACTTGA
GCAATGGTGCGCAGGGGGAGACATAGACCAGCCTTGGGTGGCAAGCACTGCAATAAGTCCTGTTTAGC
CTTGGAGTTCACATGCCGGCACTGAAGCCGACCTACCTGAGCGTGTGATTACCGTTAAAGCATCTGTC
TAGTTCTGTTTCCGGCGCTCTTGGTTCCAGGTTAGGGGAAGTGTATGACCCATGTGTTTTTATCGGCT
TAACCACGAGTGATCCCCGGTCGTTTCCCCATTGAATCCCTGGTGCATCCTACTCCCAGAATGATAGC
TGACTGACTGGACTGGCTTTTCAAGTAATCGAGGGGGTATCGCGGTCACGGCCGTTAACAGATCCCGT
CCTTAGTGTGGAATCCGCACCTGCTGACTAACGCTTCGCCGGCGTGTCTGCACAGCCGTATAGTGTTA
ATCATGACCCCAAGGAAGGATTAAACAAATATCTTGACG
>CHRUN14
GTGTCCGGNAGCCCGCGCTAGTTAGACACCCCGGCAGGGGGGATTGCTTTCGAGACGGGAGATCCCTT
CGCCGACCCTGGAGGGCCGACGCCGAGGCATTCGGGCCCCTGCAACGTCAACAGCGGCAAGAAAACGG
GATGAATGGGCGTAATGGGGGGGGTCTGCTGGGGACCCGACGCGGTTGCCGTTTGCGGGGCCCCCGAC

CCATACCGACCCACCTAGGCGTCCAGTTACGGCGCACGTCGGGAGCGTGGTTGCCGTCAGAGCTGTGT
TTCTCGATCAGTCCCCCGCAGTGCCGCAGTATCTTGCCGTGGGCTGCTTTAATCTTGAAAGTGGTTCA
TACATTGGGCGACGAGGTGTCGACTCTCATTGGGGGTAACCGACGGGCACATGCAGTCCCCTCCCCGG
GCAGGCAGAGGCGGGGCCCCCGCGCGCCGGCCCCGGCCCACAATCTTACCAGGGTCCTCAAAGGAGCC
TTTGCATGGTACCCTTCGTGAATGGTTGCTTAAGAGGTCCACAACGTGGTCCGGGCACGGTCGACTAA
ACACTCAAAACAGCGACGGCAAATATAGGTACAAGGTCCAGGCCCTCACGGCACTAATTGCGATGACC
CAACTCACGGNGGCAGTCCCCGGCGCGGCGACCTTGATTACGTCCGGGGACAAGTATGTCGCTCCCCC
CGGGAGGGGTGCAGCCACATGGGAGATTCAAAGTTTCTCGTGACGTCGTTGTGATCACTGCAGCCTAG
CCGAGACTCCCGTACTACGCGAAGGTTGGTTATGTTAACCACTACAACGTGAGGCGCCGTAAGGGCCA
GTGTTGTGCCCGGCTCTTCAATGCGCCTTAGCGGCCTGATACACCCACCCAAGGAGATACTGCTAATC
ATATGGGTGGACAGAACCTCGCAACGCAGACGCCTCCCCGGCATGATATGGTTTTTTTCCGCTATTAT
CCAGCACGCAGCGCTATCATTCAAGAGAACCCAGTGACGCGTAAATCGTAAGATCTACCTGCCGCAGG
TGGACCTACTGCAAATACGGATTATGACTCGTAAAGGGGTCATGCGTATTTTCATCACTAGGCACGTT
CGAGAATAAATTAGTAGGTGTCCCGAGCCTTGTGGCGTTCCGCCTGACTCCTCATGAAGTCGACCTTC
TCACCGGCCCTATCTGCCGACGTAAGTCATAACCTAGATCTGTACCTCGGGGGGAGGGTCACTGTAAA
GGGATAATTGGAGGGTGATTTCCCACACTTTCCTAAGGGTACTTTTTGCCTGGCTTCGCAATTGGGTC
CAATGGATGTCGATCTCTGGTTTAGCAGTTGTGAAAGTGGCAAGGCGGGAGGTTAGACCTCCNTTTAA
CATATACAAGCAAGTTAACNGCACTAGATGTGTAGACACTACAGTTACAGGAGTAGCCGAATAAGTCT
CCGACGTCAAGCGAATAAGCGTCATACGCGATTATCGCCTAAGAGCACGTATTGGCGGTAAAAGGCTA
GCTAGACGCTTATGGGTAGATTTCAAGGCGTTCGTAGTGGTATAATAGGATACTCTTTCACCAGCCTG
AGGGCCGAACGCTATACTAGTGGTCTGTGATGTAGGACCGAGTATCTCTCTAGGGACCATCTACTTGA
GCAATGGTGCGCAGGGGGAGACATAGACCAGCCTTGGGTGGCAAGCACTGCAATAAGTCCTGTTTAGC
CTTGGAGTTCACATGCCGGCACTGAAGCCGACCTACCTGAGCGTGTGATTACCGTTAAAGCATCTGTC
TAGTTCTGTTTCCGGCGCTCTTGGTTCCAGGTTAGGGGAAGTGTATGACCCATGTGTTTTTATCGGCT
TAACCACGAGTGATCCCCGGTCGTTTCCCCATTGAATCCCTGGTGCATCCTACTCCCAGAATGATAGC
TGACTGACTGGACTGGCTTTTCAAGTAATCGAGGGGGTATCGCGGTCACGGCCGTTAACAGATCCCGT
CCTTAGTGTGGAATCCGCACCTGCTGACTAACGCTTCGCCGGCGTGTCTGCACAGCCGTATAGTGTTA
ATCATGACCCCAAGGAAGGATTAAACAAATATCTTGACG
>CHRUN15
GTGTCCGGTAGCCCGCGCTAGTTAGACACCCCGGCAGGGGGGATTGCTTTCGAGACGGGAGATCCCTT
CGCCGACCCTGGAGGGCCGACGCCGAGGCATTCGGGCCCCTGCAACGTCAACAGCGGCAAGAAAACGG
GATGAATGGGCGTAATGGGGGGGGTCTGCTGGGGACCCGACGCGGTTGCCGTTTGCGGGGCCCCCGAC
CCATACCGACCCACCTAGGCGTCCAGTTACGGCGCACGTCGGGAGCGTGGTTGCCGTCAGAGCTGTGT
TTCTCGATCAGTCCCCCGCAGTGCCGCAGTATCTTGCCGTGGGCTGCTTTAATCTTGAAAGTGGTTTA
TACATTGGGCGACGAGGTGTCGACTCTCATTGGGGGTAACCGACGGGCACATGCAGTCCCCTCCCCGG
GCAGGCAGAGGCGGGGCCCCCGCGCGCCGGCCCCGGCCCACAATCTTACCAGGGTCCTCAAAGGAGCC
TTTGCATGGTACCCTTCGTGAATGGTTGCTTAAGAGGTCCACAACGTGGTCCGGGCACGGTCGACTAA
ACACTCAAAACAGCGACGGCAAATATAGGTACAAGGTCCAGGCCCTCACGGCACTAATTGCGATGACC
CAACTCACGGGGGCAGTCCCCGGCGCGGCGACCTTGATTACGTCCGGGGACAAGTATGTCGCTCCCCC
CGGGAGGGGTGCAGCCACATGGGAGATTCAAAGTTTCTCGTGACGTCGTTGTGATCACTGCAGCCTAG
CCGAGACTCCCGTACTACGCGAAGGTTGGTTATGTTAACCACTACAACGTGAGGCGCCGTAAGGGCCA
GTGTTGTGCCCGGCTCTTCAATGCGCCTTAGCGGCCTGATACACCCACCCAAGGAGATACTGCTAATC
ATATGGGTGGACAGAACCTCGCAACGCAGACGCCTCCCCGGCATGATATGGTTTTTTTCCGCTATTAT
CCAGCACGCAGCGCTATCATTCAAGAGAACCCAGTGACGCGTAAATCGTAAGATCTACCTGCCGCAGG
TGGACCTACTGCAAATACGGATTATGACTCGTAAAGGGGTCATGCGTATTTTCATCACTAGGCACGTT
CGAGAATAAATTAGTAGGTGTCCCGAGCCTTGTGGCGTTCCGCCTGACTCCTCATGAAGTCGACCTTC
TCACCGGCCCTATCTGCCGACGTAAGTCATAACCTAGATCTGTACCTCGGGGGGAGGGTCACTGTAAA
GGGATAATTGGAGGGTGATTTCCCACACTTTCCTAAGGGTACTTTTTGCCTGGCTTCGCAATTGGGTC
CAATGGATGTCGATCTCTGGTTTAGCAGTTGTGAAAGTGGCAAGGCGGGAGGTTAGACCTCCATTTAA
CATATACAAGCAAGTTAACTGCACTAGATGTGTAGACACTACAGTTACAGGAGTAGCCGAATAAGTCT
CCGACGTCAAGCGAATAAGCGTCATACGCGATTATCGCCTAAGAGCACGTATTGGCGGTAAAAGGCTA
GCTAGACGCTTATGGGTAGATTTCAAGGCGTTCGTAGTGGTATAATAGGATACTCTTTCACCAGCCTG
AGGGCCGAACGCTATACTAGTGGTCTGTGATGTAGGACCGAGTATCTCTCTAGGGACCATCTACTTGA
GCAATGGTGCGCAGGGGGAGACATAGACCAGCCTTGGGTGGCAAGCACTGCAATAAGTCCTGTTTAGC

CTTGGAGTTCACATGCCGGCACTGAAGCCGACCTACCTGAGCGTGTGATTACCGTTAAAGCATCTGTC
TAGTTCTGTTTCCGGCGCTCTTGGTTCCAGGTTAGGGGAAGTGTATGACCCATGTGTTTTTATCGGCT
TAACCACGAGTGATCCCCGGTCGTTTCCCCATTGAATCCCTGGTGCATCCTACTCCCAGAATGATAGC
TGACTGACTGGACTGGCTTTTCAAGTAATCGAGGGGGTATCGCGGTCACGGCCGTTAACAGATCCCGT
CCTTAGTGTGGAATCCGCACCTGCTGACTAACGCTTCGCCGGCGTGTCTGCACAGCCGTATAGTGTTA
ATCATGACCCCAAGGAAGGATTAAACAAATATCTTGACG
>CHRUN16
GTGTCCGGTAGCCCGCGCTAGTTAGACACCCCGGCAGGGGGGATTGCTTTCGAGACGGGAGATCCCTT
CGCCGACCCTGGAGGGCCGACGCCGAGGCATTCGGGCCCCTGCAACGTCAACAGCGGCAAGAAAACGG
GATGAATGGGCGTAATGGGGGGGGTCTGCTGGGGACCCGACGCGGTTGCCGTTTGCGGGGCCCCCGAC
CCATACCGACCCACCTAGGCGTCCAGTTACGGCGCACGGCGGGAGCGTGGTTGCCGTCAGAGCTGTGT
TTCTCGATCAGTCCCCCGCAGTGCCGCAGTATCTTGCCGTGGGCTGCTTTAATCTTGAAAGTGGTTCA
TACATTGGGCGACGAGGTGTCGACTCTCATTGGGGGTAACCGACGGGCACATGCAGTCCCCTCCCCGG
GCAGGCAGAGGCGGGGCCCCCGCGCGCCGGCCCCGGCCCACAATCTTACCAGGGTCCTCAAAGGAGCC
TTTGCATGGTACCCTTCGTGAATGGTTGCTTAAGAGGTCCACAACGTGGTCCGGGCACGGTCGACTAA
ACACTCAAAACAGCGACGGCAAATATAGGTACAAGGTCCAGGCCCTCACGGCACTAATTGCGATGACC
CAACTCACGGGGGCAGTCCCCGGCGCGGCGACCTTGATTACGTCCGGGGACAAGTATGTCGCTCCCCC
CGGGAGGGGTGCAGCCACATGGGAGATTCAAAGTTTCTCGTGACGTCGTTGTGATCACTGCAGCCTAG
CCGAGACTCCCGTACTACGCGAAGGTTGGTTATGTTAACCACTACAACGTGAGGCGCCGTAAGGGCCA
GTGTTGTGCCCGGCTCTTCAATGCGCCTTAGCGGCCTGATACACCCACCCAAGGAGATACTGCTAATC
ATATGGGTGGACAGAACCTCGCAACGCAGACGCCTCCCCGGCATGATATGGTTTTTTTCCGCTATTAT
CCAGCACGCAGCGCTATCATTCAAGAGAACCCAGTGACGCGTAAATCGTAAGATCTACCTGCCGCAGG
TGGACCTACTGCAAATACGGATTATGACTCGTAAAGGGGTCATGCGTATTTTCATCACTAGGCACGTT
CGAGAATAAATTAGTAGGTGTCCCGAGCCTTGTGGCGTTCCGCCTGACTCCTCATGAAGTCGACCTTC
TCACCGGCCCTATCTGCCGACGTAAGTCATAACCTAGATCTGTACCTCGGGGGGAGGGTCACTGTAAA
GGGATAATTGGAGGGTGATTTCCCACACTTTCCTAAGGGTACTTTTTGCCTGGCTTCGCAATTGGGTC
CAATGGATGTCGATCTCTGGTTTAGCAGTTGTGAAAGTGGCAAGGCGGGAGGTTAGACCTCCATTTAA
CATATACAAGCAAGTTAACTGCACTAGATGTGTAGACACTACAGTTACAGGAGTAGCCGAATAAGTCT
CCGACGTCAAGCGAATAAGCGTCATACGCGATTATCGCCTAAGAGCACGTATTGGCGGTAAAAGGCTA
GCTAGACGCTTATGGGTAGATTTCAAGGCGTTCGTAGTGGTATAATAGGATACTCTTTCACCAGCCTG
AGGGCCGAACGCTATACTAGTGGTCTGTGATGTAGGACCGAGTATCTCTCTAGGGACCATCTACTTGA
GCAATGGTGCGCAGGGGGAGACATAGACCAGCCTTGGGTGGCAAGCACTGCAATAAGTCCTGTTTAGC
CTTGGAGTTCACACGCCGGCACTGAAGCCGACCTACCTGAGCGTGTGATTACCGTTAAAGCATCTGTC
TAGTTCTGTTTCCGGCGCTCTTGGTTCCAGGTTAGGGGAAGTGTATGACCCATGTGTTTTTATCGGCT
TAACCACGAGTGATCCCCGGTCGTTTCCCCATTGAATCCCTGGTGCATCCTACTCCCAGAATGATAGC
TGACTGACTGGACTGGCTTTTCAAGTAATCGAGGGGGTATCGCGGTCACGGCCGTTAACAGATCCCGT
CCTTAGTGTGGAATCCGCACCTGCTGACTAACGCTTCGCCGGCGTGTCTGCACAGCCGTATAGTGTTA
ATCATGACCCCAAGGAAGGATTAAACAAATATCTTGACG
>CHRUN31
GTGTCCGGTAGCCCGCGCTAGTTAGACACCCCGGCAGGGGGGATTGCTTTCGAGACGGGAGATCCCTT
CGCCGACCCTGGAGGGCCGACGCCGAGGCATTCGGGCCCCTGCAACGTCAACAGCGGCAAGAAAACGG
GATGAATGGGCGTAATGGGGGGGGTCTGCTGGGGACCCGACGCGGTTGCCGTTTGCGGGGCCCCCGAC
CCATACCGACCCACCTAGGCGTCCAGTTACGGCGCACGTCGGGAGCGTGGTTGCCGTCAGAGCTGTGT
TTCTCGATCAGTCCCCCGCAGTGCCGCAGTATCTTGCCGTGGGCTGCTTTAATCTTGAAAGTGGTTTA
TACATTGGGCGACGAGGTGTCGACTCTCATTGGGGGTAACCGACGGGCACATGCAGTCCCCTCCCCGG
GCAGGCAGAGGCGGGGCCCCCGCGCGCCGGCCCCGGCCCACAATCTTACCAGGGTCCTCAAAGGAGCC
TTTGCATGGTACCCTTCGTGAATGGTTGCTTAAGAGGTCCACAACGTGGTCCGGGCACGGTCGACTAA
ACACTCAAAACAGCGACGGCAAATATAGGTACAAGGTCCAGGCCCTCACGGCACTAATTGCGATGACC
CAACTCACGGGGGCAGTCCCCGGCGCGGCGACCTTGATTACGTCCGGGGACAAGTATGTCGCTCCCCC
CGGGAGGGGTGCAGCCACATGGGAGATTCAAAGTTTCTCGTGACGTCGTTGTGATCACTGCAGCCTAG
CCGAGACTCCCGTACTACGCGAAGGTTGGTTATGTTAACCACTACAACGTGAGGCGCCGTAAGGGCCA
GTGTTGTGCCCGGCTCTTCAATGCGCCTTAGCGGCCTGATACACCCACCCAAGGAGATACTGCTAATC
ATATGGGTGGACAGAACCTCGCAACGCAGACGCCTCCCCGGCATGATATGGTTTTTTTCCGCTATTAT
CCAGCACGCAGCGCTATCATTCAAGAGAACCCAGTGACGCGTAAATCGTAAGATCTACCTGCCGCAGG

TGGACCTACTGCAAATACGGATTATGACTCGTAAAGGGGTCATGCGTATTTTCATCACTAGGCACGTT
CGAGAATAAATTAGTAGGTGTCCCGAGCCTTGTGGCGTTCCGCCTGACTCCTCATGAAGTCGACCTTC
TCACCGGCCCTATCTGCCGACGTAAGTCATAACCTAGATCTGTACCTCGGGGGGAGGGTCACTGTAAA
GGGATAATTGGAGGGTGATTTCCCACACTTTCCTAAGGGTACTTTTTGCCTGGCTTCGCAATTGGGTC
CAATGGATGTCGATCTCTGGTTTAGCAGTTGTGAAAGTGGCAAGGCGGGAGGTNNGACCTCCNTTTAA
CATATACAAGCAAGTTAACNGCACTAGATGTGTAGACACTACAGTTACAGGAGTAGCCGAATAAGTCT
CCGACGTCAAGCGAATAAGCGTCATACGCGATTATCGCCTAAGAGCACGTATTGGCGGTAAAAGGCTA
GCTAGACGCTTATGGGTAGATTTCAAGGCGTTCGTAGTGGTATAATAGGATACTCTTTCACCAGCCTG
AGGGCCGAACGCTATACTAGTGGTCTGTGATGTAGGACCGAGTATCTCTCTAGGGACCATCTACTTGA
GCAATGGTGCGCAGGGGGAGACATAGACCAGCCTTGGGTGGCAAGCACTGCAATAAGTCCTGTTTAGC
CTTGGAGTTCACATGCCGGCACTGAAGCCGACCTACCTGAGCGTGTGATTACCGTTAAAGCATCTGTC
TAGTTCTGTTTCCGGCGCTCTTGGTTCCAGGTTAGGGGAAGTGTATGACCCATGTGTTTTTATCGGCT
TAACCACGAGTGATCCCCGGTCGTTTCCCCATTGAATCCCTGGTGCATCCTACTCCCAGAATGATAGC
TGACTGACTGGACTGGCTTTTCAAGTAATCGAGGGGGTATCGCGGTCACGGCCGTTAACAGATCCCGT
CCTTAGTGTGGAATCCGCACCTGCTGACTAACGCTTCGCCGGCGTGTCTGCACAGCCGTATAGTGTTA
ATCATGACCCCAAGGAAGGATTAAACAAATATCTTGACG
>CHRUN33
GTGTCCGGTAGCCCGCGCTAGTTAGACACCCCGGCAGGGGGGATTGCTTTCGGGACGGGAGATCCCTT
CGCCGACCCTGGAGGGCCGACGCCGAGGCATTCGGGCCCCTGCAACGTCAACAGCGGCAAGAAAACGG
GATGAATGGGCGTAATGGGGGGGGTCTGCTGGGGACCCGACGCGGTTGCCGTTTGCGGGGCCCCCGAC
CCATACCGACCCACCTAGGCGTCCAGTTACGGCGCACGTCGGGAGCGTGGTTGCCGTCAGAGCTGTGT
TTCTCGATCAGTCCCCCGCAGTGCCGCAGTATCTTGCCGTGGGCTGCTTTAATCTTGAAAGTGGTTNA
TACATTGGGCGACGAGGTGTCGACTCTCATTGGGGGTAACCGACGGGCACATGCAGTCCCCTCCCCGG
GCAGGCAGAGGCGGGGCCCCCGCGCGCCGGCCCCGGCCCACAATCTTACCAGGGTCCTCAAAGGAGCC
TTTGCATGGTACCCTTCGTGAATGGTTGCTTAAGAGGTCCACAACGTGGTCCGGGCACGGTCGACTAA
ACACTCAAAACAGCGACGGCAAATATAGGTACAAGGTCCAGGCCCTCACGGCACTAATTGCGATGACC
CAACTCACGGGGGCAGTCCCCGGCGCGGCGACCTTGATTACGTCCGGGGACAAGTATGTCGCTCCCCC
CGGGAGGGGTGCAGCCACATGGGAGATTCAAAGTTTCTCGTGACGTCGTTGTGATCACTGCAGCCTAG
CCGAGACTCCCGTACTACGCGAAGGTTGGTTATGTTAACCACTACAACGTGAGGCGCCGTAAGGGCCA
GTGTTGTGCCCGGCTCTTCAATGCGCCTTAGCGGCCTGATACACCCACCCAAGGAGATACTGCTAATC
ATATGGGTGGACAGAACCTCGCAACGCAGACGCCTCCCCGGCATGATATGGTTTTTTTCCGCTATTAT
CCAGCACGCAGCGCTATCATTCAAGAGAACCCAGTGACGCGTAAATCGTAAGATCTACCTGCCGCAGG
TGGACCTACTGCAAATACGGATTATGACTCGTAAAGGGGTCATGCGTATTTTCATCACTAGGCACGTT
CGAGAATAAATTAGTAGGTGTCCCGAGCCTTGTGGCGTTCCGCCTGACTCCTCATGAAGTCGACCTTC
TCACCGGCCCTATCTGCCGACGTAAGTCATAACCTAGATCTGTACCTCGGGGGGAGGGTCACTGTAAA
GGGATAATTGGAGGGTGATTTCCCACACTTTCCTAAGGGTACTTTTTGCCTGGCTTCGCAATTGGGTC
CAATGGATGTCGATCTCTGGTTTAGCAGTTGTGAAAGTGGCAAGGCGGGAGGTTAGACCTCCATTTAA
CATATACAAGCAAGTTAACTGCACTAGATGTGTAGACACTACAGTTACAGGAGTAGCCGAATAAGTCT
CCGACGTCAAGCGAATAAGCGTCATACGCGATTATCGCCTAAGAGCACGTATTGGCGGTAAAAGGCTA
GCTAGACGCTTATGGGTAGATTTCAAGGCGTTCGTAGTGGTATAATAGGATACTCTTTCACCAGCCTG
AGGGCCGAACGCTATACTAGTGGTCTGTGATGTAGGACCGAGTATCTCTCTAGGGACCATCTACTTGA
GCAATGGTGCGCAGGGGGAGACATAGACCAGCCTTGGGTGGCAAGCACTGCAATAAGTCCTGTTTAGC
CTTGGAGTTCACATGCCGGCACTGAAGCCGACCTACCTGAGCGTGTGATTACCGTTAAAGCATCTGTC
TAGTTCTGTTTCCGGCGCTCTTGGTTCCAGGTTAGGGGAAGTGTATGACCCATGTGTTTTTATCGGCT
TAACCACGAGTGATCCCCGGTCGTTTCCCCATTGAATCCCTGGTGCATCCTACTCCCAGAATGATAGC
TGACTGACTGGACTGGCTTTTCAAGTAATCGAGGGGGTATCGCGGTCACGGCCGTTAACAGATCCCGT
CCTTAGTGTGGAATCCGCACCTGCTGACTAACGCTTCGCCGGCGTGTCTGCACAGCCGTATAGTGTTA
ATCATGACCCCAAGGAAGGATTAAACAAATATCTTGACG
>CHRUN37
GTGTCCGGTAGCCCGCGCTAGTTAGACACCCCGGCAGGGGGGATTGCTTTCGAGACGGGAGATCCCTT
CGCCGACCCTGGAGGGCCGACGCCGAGGCATTCGGGCCCCTGCAACGTCAACAGCGGCAAGAAAACGG
GATGAATGGGCGTAATGGGGGGGGTCTGCTGGGGACCCGACGCGGTTGCCGTTTGCGGGGCCCCCGAC
CCATACCGACCCACCTAGGCGTCCAGTTACGGCGCACGGCGGGAGCGTGGTTGCCGTCAGAGCTGTGT
TTCTCGATCAGTCCCCCGCAGTGCCGCAGTATCTTGCCGTGGGCTGCTTTAATCTTGAAAGTGGTTCA

TACATTGGGCGACGAGGTGTCGACTCTCATTGGGGGTAACCGACGGGCACATGCAGTCCCCTCCCCGG
GCAGGCAGAGGCGGGGCCCCCGCGCGCCGGCCCCGGCCCACAATCTTACCAGGGTCCTCAAAGGAGCC
TTTGCATGGTACCCTTCGTGAATGGTTGCTTAAGAGGTCCACAACGTGGTCCGGGCACGGTCGACTAA
ACACTCAAAACAGCGACGGCAAATATAGGTACAAGGTCCAGGCCCTCACGGCACTAATTGCGATGACC
CAACTCACGGGGGCAGTCCCCGGCGCGGCGACCTTGATTACGTCCGGGGACAAGTATGTCGCTCCCCC
CGGGAGGGGTGCAGCCACATGGGAGATTCAAAGTTTCTCGTGACGTCGTTGTGATCACTGCAGCCTAG
CCGAGACTCCCGTACTACGCGAAGGTTGGTTATGTTAACCACTACAACGTGAGGCGCCGTAAGGGCCA
GTGTTGTGCCCGGCTCTTCAATGCGCCTTAGCGGCCTGATACACCCACCCAAGGAGATACTGCTAATC
ATATGGGTGGACAGAACCTCGCAACGCAGACGCCTCCCCGGCATGATATGGTTTTTTTCCGCTATTAT
CCAGCACGCAGCGCTATCATTCAAGAGAACCCAGTGACGCGTAAATCGTAAGATCTACCTGCCGCAGG
TGGACCTACTGCAAATACGGATTATGACTCGTAAAGGGGTCATGCGTATTTTCATCACTAGGCACGTT
CGAGAATAAATTAGTAGGTGTCCCGAGCCTTGTGGCGTTCCGCCTGACTCCTCATGAAGTCGACCTTC
TCACCGGCCCTATCTGCCGACGTAAGTCATAACCTAGATCTGTACCTCGGGGGGAGGGTCACTGTAAA
GGGATAATTGGAGGGTGATTTCCCACACTTTCCTAAGGGTACTTTTTGCCTGGCTTCGCAATTGGGTC
CAATGGATGTCGATCTCTGGTTTAGCAGTTGTGAAAGTGGCAAGGCGGGAGGTTAGACCTCCATTTAA
CATATACAAGCAAGTTAACTGCACTAGATGTGTAGACACTACAGTTACAGGAGTAGCCGAATAAGTCT
CCGACGTCAAGCGAATAAGCGTCATACGCGATTATCGCCTAAGAGCACGTATTGGCGGTAAAAGGCTA
GCTAGACGCTTATGGGTAGATTTCAAGGCGTTCGTAGTGGTATAATAGGATACTCTTTCACCAGCCTG
AGGGCCGAACGCTATACTAGTGGTCTGTGATGTAGGACCGAGTATCTCTCTAGGGACCATCTACTTGA
GCAATGGTGCGCAGGGGGAGACATAGACCAGCCTTGGGTGGCAAGCACTGCAATAAGTCCTGTTTAGC
CTTGGAGTTCACATGCCGGCACTGAAGCCGACCTACCTGAGCGTGTGATTACCGTTAAAGCATCTGTC
TAGTTCTGTTTCCGGCGCTCTTGGTTCCAGGTTAGGGGAAGTGTATGACCCATGTGTTTTTATCGGCT
TAACCACGAGTGATCCCCGGTCGTTTCCCCGTTGAATCCCTGGTGCATCCTACTCCCAGAATGATAGC
TGACTGACTGGACTGGCTTTTCAAGTAATCGAGGGGGTATCGCGGTCACGGCCGTTAACAGATCCCGT
CCTTAGTGTGGAATCCGCACCTGCTGACTAACGCTTCGCCGGCGTGTCTGCACAGCCGTATAGTGTTA
ATCATGACCCCAAGGAAGGATTAAACAAATATCTTGACG
>CHRUN41
GTGTCCGGTAGCCCGCGCTAGTTAGACACCCCGGCAGGGGGGATTGCTTTCGAGACGGGAGATCCCTT
CGCCGACCCTGGAGGGCCGACGCCGAGGCATTCGGGCCCCTGCAACGTCAACAGCGGCAAGAAAACGG
GATGAATGGGCGTAATGGGGGGGGTCTGCTGGGGACCCGACGCGGTTGCCGTTTGCGGGGCCCCCGAC
CCATACCGACCCACCTAGGCGTCCAGTTACGGCGCACGGCGGGAGCGTGGTTGCCGTCAGAGCTGTGT
TTCTCGATCAGTCCCCCGCAGTGCCGCAGTATCTTGCCGTGGGCTGCTTTAATCTTGAAAGTGGTTCA
TACATTGGGCGACGAGGTGTCGACTCTCATTGGGGGTAACCGACGGGCACATGCAGTCCCCTCCCCGG
GCAGGCAGAGGCGGGGCCCCCGCGCGCCGGCCCCGGCCCACAATCTTACCAGGGTCCTCAAAGGAGCC
TTTGCATGGTACCCTTCGTGAATGGTTGCTTAAGAGGTCCACAACGTGGTCCGGGCACGGTCGACTAA
ACACTCAAAACAGCGACGGCAAATATAGGTACAAGGTCCAGGCCCTCACGGCACTAATTGCGATGACC
CAACTCACGGGGGCAGTCCCCGGCGCGGCGACCTTGATTACGTCCGGGGACAAGTATGTCGCTCCCCC
CGGGAGGGATGCAGCCACATGGGAGATTCAAAGTTTCTCGTGACGTCGTTGTGATCACTGCAGCCTAG
CCGAGACTCCCGTACTACGCGAAGGTTGGTTATGTTAACCACTACAACGTGAGGCGCCGTAAGGGCCA
GTGTTGTGCCCGGCTCTTCAATGCGCCTTAGCGGCCTGATACACCCACCCAAGGAGATACTGCTAATC
ATATGGGTGGACAGAACCTCGCAACGCAGACGCCTCCCCGGCATGATANGGTTTTTTTCCGCTATTAT
CCAGCACGCAGCGCTATCATTCAAGAGAACCCAGTGACGCGTAAATCGTAAGATCTACCTGCCGCAGG
TGGACCTACTGCAAATACGGATTATGACTCGTAAAGGGGTCATGCGTATTTTCATCACTAGGCACGTT
CGAGAATAAATTAGTAGGTGTCCCGAGCCTTGTGGCGTTCCGCCTGACTCCTCATGAAGTCGACCTTC
TCACCGGCCCTATCTGCCGACGTAAGTCATAACCTAGATCTGTACCTCGGGGGGAGGGTCACTGTAAA
GGGATAATTGGAGGGTGATTTCCCACACTTTCCTAAGGGTACTTTTTGCCTGGCTTCGCAATTGGGTC
CAATGGATGTCGATCTCTGGTTTAGCAGTTGTGAAAGTGGCAAGGCGGGAGGTTAGACCTCCATTTAA
CATATACAAGCAAGTTAACTGCACTAGATGTGTAGACACTACAGTTACAGGAGTAGCCGAATAAGTCT
CCGACGTCAAGCGAATAAGCGTCATACGCGATTATCGCCTAAGAGCACGTATTGGCGGTAAAAGGCTA
GCTAGACGCTTATGGGTAGATTTCAAGGCGTTCGTAGTGGTATAATAGGATACTCTTTCACCAGCCTG
AGGGCCGAACGCTATACTAGTGGTCTGTGATGTAGGACCGAGTATCTCTCTAGGGACCATCTACTTGA
GCAATGGTGCGCAGGGGGAGACATAGACCAGCCTTGGGTGGCAAGCACTGCAATAAGTCCTGTTTAGC
CTTGGAGTTCACATGCCGGCACTGAAGCCGACCTACCTGAGCGTGTGATTACCGTTAAAGCATCTGTC
TAGTTCTGTTTCCGGCGCTCTTGGTTCCAGGTTAGGGGAAGTGTATGACCCATGTGTTTTTATCGGCT

TAACCACGAGTGATCCCCGGTCGTTTCCCCATTGAATCCCTGGTGCATCCTACTCCCAGAATGATAGC
TGACTGACTGGACTGGCTTTTCAAGTAATCGAGGGGGTATCGCGGTCACGGCCGTTAACAGATCCCGT
CCTTAGTGTGGAATCCGCACCTGCTGACTAACGCTTCGCCGGCGTGTCTGCACAGCCGTATAGTGTTA
ATCATGACCCCAAGGAAGGATTAAACAAATATCTTGACG
>CHRUN42
GTGTCCGGTAGCCCGCGCTAGTTAGACACCCCGGCAGGGGGGATTGCTTTCGAGACGGGAGATCCCTT
CGCCGACCCTGGAGGGCCGACGCCGAGGCATTCGGGCCCCTGCAACGTCAACAGCGGCAAGAAAACGG
GATGAATGGGCGTAATGGGGGGGGTCTGCTGGGGACCCGACGCGGTTGCCGTTTGCGGGGCCCCCGAC
CCATACCGACCCACCTAGGCGTCCAGTTACGGCGCACGGCGGGAGCGTGGTTGCCGTCAGAGCTGTGT
TTCTCGATCAGTCCCCCGCAGTGCCGCAGTATCTTGCCGTGGGCTGCTTTAATCTTGAAAGTGGTTCA
TACATTGGGCGACGAGGTGTCGACTCTCATTGGGGGTAACCGACGGGCACATGCAGTCCCCTCCCCGG
GCAGGCAGAGGCGGGGCCCCCGCGCGCCGGCCCCGGCCCACAATCTTACCAGGGTCCTCAAAGGAGCC
TTTGCATGGTACCCTTCGTGAATGGTTGCTTAAGAGGTCCACAACGTGGTCCGGGCACGGTCGACTAA
ACACTCAAAACAGCGACGGCAAATATAGGTACAAGGTCCAGGCCCTCACGGCACTAATTGCGATGACC
CAACTCACGGGGGCAGTCCCCGGCGCGGCGACCTTGATTACGTCCGGGGACAAGTATGTCGCTCCCCC
CGGGAGGGGTGCAGCCACATGGGAGATTCAAAGTTTCTCGTGACGTCGTTGTGATCACTGCAGCCTAG
CCGAGACTCCCGTACTACGCGAAGGTTGGTTATGTTAACCACTACAACGTGAGGCGCCGTAAGGGCCA
GTGTTGTGCCCGGCTCTTCAATGCGCCTTAGCGGCCTGATACACCCACCCAAGGAGATACTGCTAATC
ATATGGGTGGACAGAACCTCGCAACGCAGACGCCTCCCCGGCATGATATGGTTTTTTTCCGCTATTAT
CCAGCACGCAGCGCTATCATTCAAGAGAACCCAGTGACGCGTAAATCGTAAGATCTACCTGCCGCAGG
TGGACCTACTGCAAATACGGATTATGACTCGTAAAGGGGTCATGCGTATTTTCATCACTAGGCACGTT
CGAGAATAAATTAGTAGGTGTCCCGAGCCTTGTGGCGTTCCGCCTGACTCCTCATGAAGTCGACCTTC
TCACCGGCCCTATCTGCCGACGTAAGTCATAACCTAGATCTGTACCTCGGGGGGAGGGTCACTGTAAA
GGGATAATTGGAGGGTGATTTCCCACACTTTCCTAAGGGTACTTTTTGCCTGGCTTCGCAATTGGGTC
CAATGGATGTCGATCTCTGGTTTAGCAGTTGTGAAAGTGGCAAGGCGGGAGGTTAGACCTCCATTTAA
CATATACAAGCAAGTTAACTGCACTAGATGTGTAGACACTACAGTTACAGGAGTAGCCGAATAAGTCT
CCGACGTCAAGCGAATAAGCGTCATACGCGATTATCGCCTAAGAGCACGTATTGGCGGTAAAAGGCTA
GCTAGACGCTTATGGGTAGATTTCAAGGCGTTCGTAGTGGTATAATAGGATACTCTTTCACCAGCCTG
AGGGCCGAACGCTATACTAGTGGTCTGTGATGTAGGACCGAGTATCTCTCTAGGGACCATCTACTTGA
GCAATGGTGCGCAGGGGGAGACATAGACCAGCCTTGGGTGGCAAGCACTGCAATAAGTCCTGTTTAGC
CTTGGAGTTCACATGCCGGCACTGAAGCCGACCTACCTGAGCGTGTGATTACCGTTAAAGCATCTGTC
TAGTTCTGTTTCCGGCGCTCTTGGTTCCAGGTTAGGGGAAGTGTATGACCCATGTGTTTTTATCGGCT
TAACCACGAGTGATCCCCGGTCGTTTCCCCATTGAATCCCTGGTGCATCCTACTCCCAGAATGATAGC
TGACTGACTGGACTGGCTTTTCAAGTAATCGAGGGGGTATCGCGGTCACGGCCGTTAACAGATCCCGT
CCTTAGTGTGGAATCCGCACCTGCTGACTAACGCTTCGCCGGCGTGTCTGCACAGCCGTATAGTGTTA
ATCATGACCCCAAGGAAGGATTAAACAAATATCTTGACG
>COHON8
GTGTCCGGTAGCCCGCGCTAGTTAGACACCCCGGCAGGGGGGATTGCTTTCGAGACGGGAGATCCCTT
CGCCGACCCTGGAGGGCCGACGCCGAGGCATTCGGGCCCCTGCAACGTCAACAGCGGCAAGAAAACGG
GATGAATGGGCGTAATGGGGGGGGTCTGCTGGGGACCCGACGCGGTTGCCGTTTGCGGGGCCCCCGAC
CCATACCGACCCACCTAGGCGTCCAGTTACGGCGCACGGCGGGAGCGTGGTTGCCGTCAGAGCTGTGT
TTCTCGATCAGTCCCCCGCAGTGCCGCAGTATCTTGCCNTGGGCTGCTTTAATCTTGAAAGTGGTTTA
TACATTGGGCGACGAGGTGTCGACTCTCATTGGGGGTAACCGACGGGCACATGCAGTCCCCTCCCCGG
GCAGGCAGAGGCGGGGCCCCCGCGCGCCGGCCCCGGCCCACAATCTTACCAGGGTCCTCAAAGGAGCC
TTTGCATGGTACCCTTCGTGAATGGTTGCTTAAGAGGTCCACAACGTGGTCCGGGCACGGTCGACTAA
ACACTCAAAACAGCGACGGCAAATATAGGTACAAGGTCCAGGCCCTCACGGCACTAATTGCGATGACC
CAACTCACGGGGGCAGTCCCCGGCGCGGCGACCTTGATTACGTCCGGGGACAAGTATGTCGCTCCCCC
CGGGAGGGGTGCAGCCACATGGGAGATTCAAAGTTTCTCGTGACGTCGTTGTGATCACTGCAGCCTAG
CCGAGACTCCCGTACTACGCGAAGGTTGGTTATGTTAACCACTACAACGTGAGGCGCCGTAAGGGCCA
GTGTTGTGCCCGGCTCTTCAATGCGCCTTAGCGGCCTGATACACCCACCCAAGGAGATACTGCTAATC
ATATGGGTGGACAGAACCTCGCAACGCAGACGCCTCCCCGGCATGATATGGTTTTTTTCCGCTATTAT
CCAGCACGCAGCGCTATCATTCAAGAGAACCCAGTGACGCGTAAATCGTAAGATCTACCTGCCGCAGG
TGGACCTACTGCAAATACGGATTATGACTCGTAAAGGGGTCATGCGTATTTTCATCACTAGGCACGTT
CGAGAATAAATTAGTAGGTGTCCCGAGCCTTGTGGCGTTCCGCCTGACTCCTCATGAAGTCGACCTTC

TCACCGGCCCTATCTGCCGACGTAAGTCATAACCTAGATCTGTACCTCGGGGGGAGGGTCACTGTAAA
GGGATAATTGGAGGGTGATTTCCCACACTTTCCTAAGGGTACTTTTTGCCTGGCTTCGCAATTGGGTC
CAATGGATGTCGATCTCTGGTTTAGCAGTTGTGAAAGTGGCAAGGCGGGAGGTTAGACCTCCATTTAA
CATATACAAGCAAGTTAACTGCACTAGATGTGTAGACACTACAGTTACAGGAGTAGCCGAATAAGTCT
CCGACGTCAAGCGAATAAGCGTCATACGCGATTATCGCCTAAGAGCACGTATTGGCGGTAAAAGGCTA
GCTAGACGCTTATGGGTAGATTTCAAGGCGTTCGTAGTGGTATAATAGGATACTCTTTCACCAGCCTG
AGGGCCGAACGCTATACTAGTGGTCTGTGATGTAGGACCGAGTATCTCTCTAGGGACCATCTACTTGA
GCAATGGTGCGCAGGGGGAGACATAGACCAGCCTTGGGTGGCAAGCACTGCAATAAGTCCTGTTTAGC
CTTGGAGTTCACATGCCGGCACTGAAGCCGACCTACCTGAGCGTGTGATTACCGTTAAAGCATCTGTC
TAGTTCTGTTTCCGGCGCTCTTGGTTCCAGGTTAGGGGAAGTGTATGACCCATGTGTTTTTATCGGCT
TAACCACGAGTGATCCCCGGTCGTTTCCCCATTGAATCCCTGGTGCATCCTACTCCCAGAATGATAGC
TGACTGACTGGACTGGCTTTTCAAGTAATCGAGGGGGTATCGCGGTCACGGCCGTTAACAGATCCCGT
CCTTAGTGTGGAATCCGCACCTGCTGACTAACGCTTCGCCGGCGTGTCTGCACAGCCGTATAGTGTTA
ATCATGACCCCAAGGAAGGATTAAACAAATATCTTGACG
>COHON9
GTGTCCGGTAGCCCGCGCTAGTTAGACACCCCGGCAGGGGGGATTGCTTTCGAGACGGGAGATCCCTT
CGCCGACCCTGGAGGGCCGACGCCGAGGCATTCGGGCCCCTGCAACGTCAACAGCGGCAAGAAAACGG
GATGAATGGGCGTAATGGGGGGGGTCTGCTGGGGACCCGACGCGGTTGCCGTTTGCGGGGCCCCCGAC
CCATACCGACCCACCTAGGCGTCCAGTTACGGCGCACGTCGGGAGCGTGGTTGCCGTCAGAGCTGTGT
TTCTCGATCAGTCCCCCGCAGTGCCGCAGTATCTTGCCGTGGGCTGCTTTAATCTTGAAAGTGGTTTA
TACATTGGGCGACGAGGTGTCGACTCTCATTGGGGGTAACCGACGGGCACATGCAGTCCCCTCCCCGG
GCAGGCAGAGGCGGGGCCCCCGCGCGCCGGCCCCGGCCCACAATCTTACCAGGGTCCTCAAAGGAGCC
TTTGCATGGTACCCTTCGTGAATGGTTGCTTAAGAGGTCCACAACGTGGTCCGGGCACGGTCGACTAA
ACACTCAAAACAGCGACGGCAAATATAGGTACAAGGTCCAGGCCCTCACGGCACTAATTGCGATGACC
CAACTCACGGGGGCAGTCCCCGGCGCGGCGACCTTGATTACGTCCGGGGACAAGTATGTCGCTCCCCC
CGGGAGGGGTGCAGCCACATGGGAGATTCAAAGTTTCTCGTGACGTCGTTGTGATCACTGCAGCCTAG
CCGAGACTCCCGTACTACGCGAAGGTTGGTTATGTTAACCACTACAACGTGAGGCGCCGTAAGGGCCA
GTGTTGTGCCCGGCTCTTCAATGCGCCTTAGCGGCCTGATACACCCACCCAAGGAGATACTGCTAATC
ATATGGGTGGACAGAACCTCGCAACGCAGACGCCTCCCCGGCATGATATGGTTTTTTTCCGCTATTAT
CCAGCACGCAGCGCTATCATTCAAGAGAACCCAGTGACGCGTAAATCGTAAGATCTACCTGCCGCAGG
TGGACCTACTGCAAATACGGATTATGACTCGTAAAGGGGTCATGCGTATTTTCATCACTAGGCACGTT
CGAGAATAAATTAGTAGGTGTCCCGAGCCTTGTGGCGTTCCGCCTGACTCCTCATGAAGTCGACCTTC
TCACCGGCCCTATCTGCCGACGTAAGTCATAACCTAGATCTGTACCTCGGGGGGAGGGTCACTGTAAA
GGGATAATTGGAGGGTGATTTCCCACACTTTCCTAAGGGTACTTTTTGCCTGGCTTCGCAATTGGGTC
CAATGGATGTCGATCTCTGGTTTAGCAGTTGTGAAAGTGGCAAGGCGGGAGGTTAGACCTCCATTTAA
CATATACAAGCAAGTTAACTGCACTAGATGTGTAGACACTACAGTTACAGGAGTAGCCGAATAAGTCT
CCGACGTCAAGCGAATAAGCGTCATACGCGATTATCGCCTAAGAGCACGTATTGGCGGTAAAAGGCTA
GCTAGACGCTTATGGGTAGATTTCAAGGCGTTCGTAGTGGTATAATAGGATACTCTTTCACCAGCCTG
AGGGCCGAACGCTATACTAGTGGTCTGTGATGTAGGACCGAGTATCTCTCTAGGGACCATCTACTTGA
GCAATGGTGCGCAGGGGGAGACATAGACCAGCCTTGGGTGGCAAGCACTGCAATAAGTCCTGTTTAGC
CTTGGAGTTCACATGCCGGCACTGAAGCCGACCTACCTGAGCGTGTGATTACCGTTAAAGCATCTGTC
TAGTTCTGTTTCCGGCGCTCTTGGTTCCAGGTTAGGGGAAGTGTATGACCCATGTGTTTTTATCGGCT
TAACCACGAGTGATCCCCGGTCGTTTCCCCATTGAATCCCTGGTGCATCCTACTCCCAGAATGATAGC
TGACTGACTGGACTGGCTTTTCAAGTAATCGAGGGGGTATCGCGGTCACGGCCGTTAACAGATCCCGT
CCTTAGTGTGGAATCCGCACCTGCTGACTAACGCTTCGCCGGCGTGTCTGCACAGCCGTATAGTGTTA
ATCATGACCCCAAGGAAGGATTAAACAAATATCTTGACG
>COHON10
GTGTCCGGTAGCCCGCGCTAGTTAGACACCCCGGCAGGGGGGATTGCTTTCGAGACGGGAGATCCCTT
CGCCGACCCTGGAGGGCCGACGCCGAGGCATTCGGGCCCCTGCAACGTCAACAGCGGCAAGAAAACGG
GATGAATGGGCGTAATGGGGGGGGTCTGCTGGGGACCCGACGCGGTTGCCGTTTGCGGGGCCCCCGAC
CCATACCGACCCACCTAGGCGTCCAGTTACGGCGCACGTCGGGAGCGTGGTTGCCGTCAGAGCTGTGT
TTCTCGATCAGTCCCCCGCAGTGCCGCAGTATCTTGCCGTGGGCTGCTTTAATCTTGAAAGTGGTTTA
TACATTGGGCGACGAGGTGTCGACTCTCATTGGGGGTAACCGACGGGCACATGCAGTCCCCTCCCCGG
GCAGGCAGAGGCGGGGCCCCCGCGCGCCGGCCCCGGCCCACAATCTTACCAGGGTCCTCAAAGGAGCC

TTTGCATGGTACCCTTCGTGAATGGTTGCTTAAGAGGTCCACAACGTGGTCCGGGCACGGTCGACTAA
ACACTCAAAACAGCGACGGCAAATATAGGTACAAGGTCCAGGCCCTCACGGCACTAATTGCGATGACC
CAACTCACGGGGGCAGTCCCCGGCGCGGCGACCTTGATTACGTCCGGGGACAAGTATGTCGCTCCCCC
CGGGAGGGGTGCAGCCACATGGGAGATTCAAAGTTTCTCGTGACGTCGTTGTGATCACTGCAGCCTAG
CCGAGACTCCCGTACTACGCGAAGGTTGGTTATGTTAACCACTACAACGTGAGGCGCCGTAAGGGCCA
GTGTTGTGCCCGGCTCTTCAATGCGCCTTAGCGGCCTGATACACCCACCCAAGGAGATACTGCTAATC
ATATGGGTGGACAGAACCTCGCAACGCAGACGCCTCCCCGGCATGATATGGTTTTTTTCCGCTATTAT
CCAGCACGCAGCGCTATCATTCAAGAGAACCCAGTGACGCGTAAATCGTAAGATCTACCTGCCGCAGG
TGGACCTACTGCAAATACGGATTATGACTCGTAAAGGGGTCATGCGTATTTTCATCACTAGGCACGTT
CGAGAATAAATTAGTAGGTGTCCCGAGCCTTGTGGCGTTCCGCCTGACTCCTCATGAAGTCGACCTTC
TCACCGGCCCTATCTGCCGACGTAAGTCATAACCTAGATCTGTACCTCGGGGGGAGGGTCACTGTAAA
GGGATAATTGGAGGGTGATTTCCCACACTTTCCTAAGGGTACTTTTTGCCTGGCTTCGCAATTGGGTC
CAATGGATGTCGATCTCTGGTTTAGCAGTTGTGAAAGTGGCAAGGCGGGAGGTTAGACCTCCATTTAA
CATATACAAGCAAGTTAACTGCACTAGATGTGTAGACACTACAGTTACAGGAGTAGCCGAATAAGTCT
CCGACGTCAAGCGAATAAGCGTCATACGCGATTATCGCCTAAGAGCACGTATTGGCGGTAAAAGGCTA
GCTAGACGCTTATGGGTAGATTTCAAGGCGTTCGTAGTGGTATAATAGGATACTCTTTCACCAGCCTG
AGGGCCGAACGCTATACTAGTGGTCTGTGATGTAGGACCGAGTATCTCTCTAGGGACCATCTACTTGA
GCAATGGTGCGCAGGGGGAGACATAGACCAGCCTTGGGTGGCAAGCACTGCAATAAGTCCTGTTTAGC
CTTGGAGTTCACATGCCGGCACTGAAGCCGACCTACCTGAGCGTGTGATTACCGTTAAAGCATCTGTC
TAGTTCTGTTTCCGGCGCTCTTGGTTCCAGGTTAGGGGAAGTGTATGACCCATGTGTTTTTATCGGCT
TAACCACGAGTGATCCCCGGTCGTTTCCCCATTGAATCCCTGGTGCATCCTACTCCCAGAATGATAGC
TGACTGACTGGACTGGCTTTTCAAGTAATCGAGGGGGTATCGCGGTCACGGCCGTTAACAGATCCCGT
CCTTAGTGTGGAATCCGCACCTGCTGACTAACGCTTCGCCGGCGTGTCTGCACAGCCGTATAGTGTTA
ATCATGACCCCAAGGAAGGATTAAACAAATATCTTGACG
>COHON11
GTGTCCGGTAGCCCGCGCTAGTTAGACACCCCGGCAGGGGGGATTGCTTTCGAGACGGGAGATCCCTT
CGCCGACCCTGGAGGGCCGACGCCGAGGCATTCGGGCCCCCGCAACGTCAACAGCGGCAAGAAAACGG
GATGAATGGGCGTAATGGGGGGGGTCTGCTGGGGACCCGACGCGGTTGCCGTTTGCGGGGCCCCCGAC
CCATACCGACCCACCTAGGCGTCCAGTTACGGCGCACGGCGGGAGCGTGGTTGCCGTCAGAGCTGTGT
TTCTCGATCAGTCCCCCGCAGTGCCGCAGTATCTTGCCGTGGGCTGCTTTAATCTTGAAAGTGGTTTA
TACATTGGGCGACGAGGTGTCGACTCTCATTGGGGGTAACCGACGGGCACATGCAGTCCCCTCCCCGG
GCAGGCAGAGGCGGGGCCCCCGCGCGCCGGCCCCGGCCCACAATCTTACCAGGGTCCTCAAAGGAGCC
TTTGCATGGTACCCTTCGTGAATGGTTGCTTAAGNGGTCCACAACGTGGTCCGGGCACGGTCGACTAA
ACACTCAAAACAGCGACGGCAAATATAGGTACAAGGTCCAGGCCCTCACGGCACTAATTGCGATGACC
CAACTCACGGGGGCAGTCCCCGGCGCGGCGACCTTGATTACGTCCGGGGACAAGTATGTCGCTCCCCC
CGGGAGGGGTGCAGCCACATGGGAGATTCAAAGTTTCTCGTGACGTCGTTGTGATCACTGCAGCCTAG
CCGAGACTCCCGTACTACGCGAAGGTTGGTTATGTTAACCACTACAACGTGAGGCGCCGTAAGGGCCA
GTGTTGTGCCCGGCTCTTCAATGCGCCTTAGCGGCCTGATACACCCACCCAAGGAGATACTGCTAATC
ATATGGGTGGACAGAACCTCGCAACGCAGACGCCTCCCCGGCATGATATGGTTTTTTTCCGCTATTAT
CCAGCACGCAGCGCTATCATTCAAGAGAACCCAGTGACGCGTAAATCGTAAGATCTACCTGCCGCAGG
TGGACCTACTGCAAATACGGATTATGACTCGTAAAGGGGTCATGCGTATTTTCATCACTAGGCACGTT
CGAGAATAAATTAGTAGGTGTCCCGAGCCTTGTGGCGTTCCGCCTGACTCCTCATGAAGTCGACCTTC
TCACCGGCCCTATCTGCCGACGTAAGTCATAACCTAGATCTGTACCTCGGGGGGAGGGTCACTGTAAA
GGGATAATTGGAGGGTGATTTCCCACACTTTCCTAAGGGTACTTTTTGCCTGGCTTCGCAATTGGGTC
CAATGGATGTCGATCTCTGGTTTAGCAGTTGTGAAAGTGGCAAGGCGGGAGGTTAGACCTCCATTTAA
CATATACAAGCAAGTTAACTGCACTAGATGTGTAGACACTACAGTTACAGGAGTAGCCGAATAAGTCT
CCGACGTCAAGCGAATAAGCGTCATACGCGATTATCGCCTAAGAGCACGTATTGGCGGTAAAAGGCTA
GCTAGACGCTTATGGGTAGATTTCAAGGCGTTCGTAGTGGTATAATAGGATACTCTTTCACCAGCCTG
AGGGCCGAACGCTATACTAGTGGTCTGTGATGTAGGACCGAGTATCTCTCTAGGGACCATCTACTTGA
GCAATGGTGCGCAGGGGGAGACATAGACCAGCCTTGGGTGGCAAGCACTGCAATAAGTCCTGTTTAGC
CTTGGAGTTCACATGCCGGCACTGAAGCCGACCTACCTGAGCGTGTGATTACCGTTAAAGCATCTGTC
TAGTTCTGTTTCCGGCGCTCTTGGTTCCAGGTTAGGGGAAGTGTATGACCCATGTGTTTTTATCGGCT
TAACCACGAGTGATCCCCGGTCGTTTCCCCATTGAATCCCTGGTGCATCCTACTCCCAGAATGATAGC
TGACTGACTGGACTGGCTTTTCAAGTAATCGAGGGGGTATCGCGGTCACGGCCGTTAACAGATCCCGT

CCTTAGTGTGGAATCCGCACCTGCTGACTAACGCTTCGCCGGCGTGTCTGCACAGCCGTATAGTGTTA
ATCATGACCCCAAGGAAGGATTAAACAAATATCTTGACG
>COHON12
GTGTCCGGTAGCCCGCGCTAGTTAGACACCCCGGCAGGGGGGATTGCTTTCGAGACGGGAGATCCCTT
CGCCGACCCTGGAGGGCCGACGCCGAGGCATTCGGGCCCCTGCAACGTCAACAGCGGCAAGAAAACGG
GATGAATGGGCGTAATGGGGGGGGTCTGCTGGGGACCCGACGCGGTTGCCGTTTGCGGGGCCCCCGAC
CCATACCGACCCACCTAGGCGTCCAGTTACGGCGCACGTCGGGAGCGTGGTTGCCGTCAGAGCTGTGT
TTCTCGATCAGTCCCCCGCAGTGCCGCAGTATCTTGCCGTGGGCTGCTTTAATCTTGAAAGTGGTTCA
TACATTGGGCGACGAGGTGTCGACTCTCATTGGGGGTAACCGACGGGCACATGCAGTCCCCTCCCCGG
GCAGGCAGAGGCGGGGCCCCCGCGCGCCGGCCCCGGCCCACAATCTTACCAGGGTCCTCAAAGGAGCC
TTTGCATGGTACCCTTCGTGAATGGTTGCTTAAGAGGTCCACAACGTGGTCCGGGCACGGTCGACTAA
ACACTCAAAACAGCGACGGCAAATATAGGTACAAGGTCCAGGCCCTCACGGCACTAATTGCGATGACC
CAACTCACGGGGGCAGTCCCCGGCGCGGCGACCTTGATTACGTCCGGGGACAAGTATGTCGCTCCCCC
CGGGAGGGGTGCAGCCACATGGGAGATTCAAAGTTTCTCGTGACGTCGTTGTGATCACTGCAGCCTAG
CCGAGACTCCCGTACTACGCGAAGGTTGGTTATGTTAACCACTACAACGTGAGGCGCCGTAAGGGCCA
GTGTTGTGCCCGGCTCTTCAATGCGCCTTAGCGGCCTGATACACCCACCCAAGGAGATACTGCTAATC
ATATGGGTGGACAGAACCTCGCAACGCAGACGCCTCCCCGGCATGATATGGTTTTTTTCCGCTATTAT
CCAGCACGCAGCGCTATCATTCAAGAGAACCCAGTGACGCGTAAATCGTAAGATCTACCTGCCGCAGG
TGGACCTACTGCAAATACGGATTATGACTCGTAAAGGGGTCATGCGTATTTTCATCACTAGGCACGTT
CGAGAATAAATTAGTAGGTGTCCCGAGCCTTGTGGCGTTCCGCCTGACTCCTCATGAAGTCGACCTTC
TCACCGGCCCTATCTGCCGACGTAAGTCATAACCTAGATCTGTACCTCGGGGGGAGGGTCACTGTAAA
GGGATAATTGGAGGGTGATTTCCCACACTTTCCTAAGGGTACTTTTTGCCTGGCTTCGCAATTGGGTC
CAATGGATGTCGATCTCTGGTTTAGCAGTTGTGAAAGTGGCAAGGCGGGAGGTTAGACCTCCATTTAA
CATATACAAGCAAGTTAACTGCACTAGATGTGTAGACACTACAGTTACAGGAGTAGCCGAATAAGTCT
CCGACGTCAAGCGAATAAGCGTCATACGCGATTATCGCCTAAGAGCACGTATTGGCGGTAAAAGGCTA
GCTAGACGCTTATGGGTAGATTTCAAGGCGTTCGTAGTGGTATAATAGGATACTCTTTCACCAGCCTG
AGGGCCGAACGCTATACTAGTGGTCTGTGATGTAGGACCGAGTATCTCTCTAGGGACCATCTACTTGA
GCAATGGTGCGCAGGGGGAGACATAGACCAGCCTTGGGTGGCAAGCACTGCAATAAGTCCTGTTTAGC
CTTGGAGTTCACATGCCGGCACTGAAGCCGACCTACCTGAGCGTGTGATTACCGTTAAAGCATCTGTC
TAGTTCTGTTTCCGGCGCTCTTGGTTCCAGGTTAGGGGAAGTGTATGACCCATGTGTTTTTATCGGCT
TAACCACGAGTGATCCCCGGTCGTTTCCCCATTGAATCCCTGGTGCATCCTACTCCCAGAATGATAGC
TGACTGACTGGACTGGCTTTTCAAGTAATCGAGGGGGTATCGCGGTCACGGCCGTTAACAGATCCCGT
CCTTAGTGTGGAATCCGCACCTGCTGACTAACGCTTCGCCGGCGTGTCTGCACAGCCGTATAGTGTTA
ATCATGACCCCAAGGAAGGATTAAACAAATATCTTGACG
>COHON19
GTGTCCGGTAGCCCGCGCTAGTTAGACACCCCGGCAGGGGGGATTGCTTTCGAGACGGGAGATCCCTT
CGCCGACCCTGGAGGGCCGACGCCGAGGCATTCGGGCCCCTGCAACGTCAACAGCGGCAAGAAAACGG
GATGAATGGGCGTAATGGGGGGGGTCTGCTGGGGACCCGACGCGGTTGCCGTTTGCGGGGCCCCCGAC
CCATACCGACCCACCTAGGCGTCCAGTTACGGCGCACGGCGGGAGCGTGGTTGCCGTCAGAGCTGTGT
TTCTCGATCAGTCCCCCGCAGTGCCGCAGTATCTTGCCGTGGGCTGCTTTAATCTTGAAAGTGGTTTA
TACATTGGGCGACGAGGTGTCGACTCTCATTGGGGGTAACCGACGGGCACATGCAGTCCCCTCCCCGG
GCAGGCAGAGGCGGGGCCCCCGCGCGCCGGCCCCGGCCCACAATCTTACCAGGGTCCTCAAAGGAGCC
TTTGCATGGTACCCTTCGTGAATGGTTGCTTAAGAGGTCCACAACGTGGTCCGGGCACGGTCGACTAA
ACACTCAAAACAGCGACGGCAAATATAGGTACAAGGTCCAGGCCCTCACGGCACTAATTGCGATGACC
CAACTCACGGGGGCAGTCCCCGGCGCGGCGACCTTGATTACGTCCGGGGACAAGTATGTCGCTCCCCC
CGGGAGGGGTGCAGCCACATGGGAGATTCAAAGTTTCTCGTGACGTCGTTGTGATCACTGCAGCCTAG
CCGAGACTCCCGTACTACGCGAAGGTTGGTTATGTTAACCACTACAACGTGAGGCGCCGTAAGGGCCA
GTGTTGTGCCCGGCTCTTCAATGCGCCTTAGCGGCCTGATACACCCACCCAAGGAGATACTGCTAATC
ATATGGGTGGACAGAACCTCGCAACGCAGACGCCTCCCCGGCATGATATGGTTTTTTTCCGCTATTAT
CCAGCACGCAGCGCTATCATTCAAGAGAACCCAGTGACGCGTAAATCGTAAGATCTACCTGCCGCAGG
TGGACCTACTGCAAATACGGATTATGACTCGTAAAGGGGTCATGCGTATTTTCATCACTAGGCACGTT
CGAGAATAAATTAGTAGGTGTCCCGAGCCTTGTGGCGTTCCGCCTGACTCCTCATGAAGTCGACCTTC
TCACCGGCCCTATCTGCCGACGTAAGTCATAACCTAGATCTGTACCTCGGGGGGAGGGTCACTGTAAA
GGGATAATTGGAGGGTGATTTCCCACACTTTCCTAAGGGTACTTTTTGCCTGGCTTCGCAATTGGGTC

CAATGGATGTCGATCTCTGGTTTAGCAGTTGTGAAAGTGGCAAGGCGGGAGGTTAGACCTCCATTTAA
CATATACAAGCAAGTTAACTGCACTAGATGTGTAGACACTACAGTTACAGGAGTAGCCGAATAAGTCT
CCGACGTCAAGCGAATAAGCGTCATACGCGATTATCGCCTAAGAGCACGTATTGGCGGTAAAAGGCTA
GCTAGACGCTTATGGGTAGATTTCAAGGCGTTCGTAGTGGTATAATAGGATACTCTTTCACCAGCCTG
AGGGCCGAACGCTATACTAGTGGTCTGTGATGTAGGACCGAGTATCTCTCTAGGGACCATCTACTTGA
GCAATGGTGCGCAGGGGGAGACATAGACCAGCCTTGGGTGGCAAGCACTGCAATAAGTCCTGTTTAGC
CTTGGAGTTCACATGCCGGCACTGAAGCCGACCTACCTGAGCGTGTGATTACCGTTAAAGCATCTGTC
TAGTTCTGTTTCCGGCGCTCTTGGTTCCAGGTTAGGGGAAGTGTATGACCCATGTGTTTTTATCGGCT
TAACCACGAGTGATCCCCGGTCGTTTCCCCATTGAATCCCTGGTGCATCCTACTCCCAGAATGATAGC
TGACTGACTGGACTGGCTTTTCAAGTAATCGAGGGGGTATCGCGGTCACGGCCGTTAACAGATCCCGT
CCTTAGTGTGGAATCCGCACCTGCTGACTAACGCTTCGCCGGCGTGTCTGCACAGCCGTATAGTGTTA
ATCATGACCCCAAGGAAGGATTAAACAAATATCTTGACG
>COHON23
GTGTCCGGTAGCCCGCGCTAGTTAGACACCCCGGCAGGGGGGATTGCTTTCGAGACGGGAGATCCCTT
CGCCGACCCTGGAGGGCCGACGCCGAGGCATTCGGGCCCCTGCAACGTCAACAGCGGCAAGAAAACGG
GATGAATGGGCGTAATGGGGGGGGTCTGCTGGGGACCCGACGCGGTTGCCGTTTGCGGGGCCCCCGAC
CCATACCGACCCACCTAGGCGTCCAGTTACGGCGCACGGCGGGAGCGTGGTTGCCGTCAGAGCTGTGT
TTCTCGATCAGTCCCCCGCAGTGCCGCAGTATCTTGCCGTGGGCTGCTTTAATCTTGAAAGTGGTTCA
TACATTGGGCGACGAGGTGTCGACTCTCATTGGGGGTAACCGACGGGCACATGCAGTCCCCTCCCCGG
GCAGGCAGAGGCGGGGCCCCCGCGCGCCGGCCCCGGCCCACAATCTTACCAGGGTCCTCAAAGGAGCC
TTTGCATGGTACCCTTCGTGAATGGTTGCTTAAGAGGTCCACAACGTGGTCCGGGCACGGTCGACTAA
ACACTCAAAACAGCGACGGCAAATATAGGTACAAGGTCCAGGCCCTCACGGCACTAATTGCGATGACC
CAACTCACGGGGGCAGTCCCCGGCGCGGCGACCTTGATTACGTCCGGGGACAAGTATGTCGCTCCCCC
CGGGAGGGGTGCAGCCACATGGGAGATTCAAAGTTTCTCGTGACGTCGTTGTGATCACTGCAGCCTAG
CCGAGACTCCCGTACTACGCGAAGGTTGGTTATGTTAACCACTACAACGTGAGGCGCCGTAAGGGCCA
GTGTTGTGCCCGGCTCTTCAATGCGCCTTAGCGGCCTGATACACCCACCCAAGGAGATACTGCTAATC
ATATGGGTGGACAGAACCTCGCAACGCAGACGCCTCCCCGGCATGATATGGTTTTTTTCCGCTATTAT
CCAGCACGCAGCGCTATCATTCAAGAGAACCCAGTGACGCGTAAATCGTAAGATCTACCTGCCGCAGG
TGGACCTACTGCAAATACGGATTATGACTCGTAAAGGGGTCATGCGTATTTTCATCACTAGGCACGTT
CGAGAATAAATTAGTAGGTGTCCCGAGCCTTGTGGCGTTCCGCCTGACTCCTCATGAAGTCGACCTTC
TCACCGGCCCTATCTGCCGACGTAAGTCATAACCTAGATCTGTACCTCGGGGGGAGGGTCACTGTAAA
GGGATAATTGGAGGGTGATTTCCCACACTTTCCTAAGGGTACTTTTTGCCTGGCTTCGCAATTGGGTC
CAATGGATGTCGATCTCTGGTTTAGCAGTTGTGAAAGTGGCAAGGCGGGAGGTTAGACCTCCATTTAA
CATATACAAGCAAGTTAACTGCACTAGATGTGTAGACACTACAGTTACAGGAGTAGCCGAATAAGTCT
CCGACGTCAAGCGAATAAGCGTCATACGCGATTATCGCCTAAGAGCACGTATTGGCGGTAAAAGGCTA
GCTAGACGCTTATGGGTAGATTTCAAGGCGTTCGTAGTGGTATAATAGGATACTCTTTCACCAGCCTG
AGGGCCGAACGCTATACTAGTGGTCTGTGATGTAGGACCGAGTATCTCTCTAGGGACCATCTACTTGA
GCAATGGTGCGCAGGGGGAGACATAGACCAGCCTTGGGTGGCAAGCACTGCAATAAGTCCTGTTTAGC
CTTGGAGTTCACATGCCGGCACTGAAGCCGACCTACCTGAGCGTGTGATTACCGTTAAAGCATCTGTC
TAGTTCTGTTTCCGGCGCTCTTGGTTCCAGGTTAGGGGAAGTGTATGACCCATGTGTTTTTATCGGCT
TAACCACGAGTGATCCCCGGTCGTTTCCCCATTGAATCCCTGGTGCATCCTACTCCCAGAATGATAGC
TGACTGACTGGACTGGCTTTTCAAGTAATCGAGGGGGTATCGCGGTCACGGCCGTTAACAGATCCCGT
CCTTAGTGTGGAATCCGCACCTGCTGACTAACGCTTCGCCGGCGTGTCTGCACAGCCGTATAGTGTTA
ATCATGACCCCAAGGAAGGATTAAACAAATATCTTGACG
>COHON27
GTGTCCGGTAGCCCGCGCTAGTTAGACACCCCGGCAGGGGGGATTGCTTTCGAGACGGGAGATCCCTT
CGCCGACCCTGGAGGGCCGACGCCGAGGCATTCGGGCCCCCGCAACGTCAACAGCGGCAAGAAAACGG
GATGAATGGGCGTAATGGGGGGGGTCTGCTGGGGACCCGACGCGGTTGCCGTTTGCGGGGCCCCCGAC
CCATACCGACCCACCTAGGCGTCCAGTTACGGCGCACGGCGGGAGCGTGGTTGCCGTCAGAGCTGTGT
TTCTCGATCAGTCCCCCGCAGTGCCGCAGTATCTTGCCGTGGGCTGCTTTAATCTTGAAAGTGGTTTA
TACATTGGGCGACGAGGTGTCGACTCTCATTGGGGGTAACCGACGGGCACATGCAGTCCCCTCCCCGG
GCAGGCAGAGGCGGGGCCCCCGCGCGCCGGCCCCGGCCCACAATCTTACCAGGGTCCTCAAAGGAGCC
TTTGCATGGTACCCTTCGTGAATGGTTGCTTAAGAGGTCCACAACGTGGTCCGGGCACGGTCGACTAA
ACACTCAAAACAGCGACGGCAAATATAGGTACAAGGTCCAGGCCCTCACGGCACTAATTGCGATGACC

CAACTCACGGGGGCAGTCCCCGGCGCGGCGACCTTGATTACGTCCGGGGACAAGTATGTCGCTCCCCC
CGGGAGGGGTGCAGCCACATGGGAGATTCAAAGTTTCTCGTGACGTCGTTGTGATCACTGCAGCCTAG
CCGAGACTCCCGTACTACGCGAAGGTTGGTTATGTTAACCACTACAACGTGAGGCGCCGTAAGGGCCA
GTGTTGTGCCCGGCTCTTCAATGCGCCTTAGCGGCCTGATACACCCACCCAAGGAGATACTGCTAATC
ATATGGGTGGACAGAACCTCGCAACGCAGACGCCTCCCCGGCATGATATGGTTTTTTTCCGCTATTAT
CCAGCACGCAGCGCTATCATTCAAGAGAACCCAGTGACGCGTAAATCGTAAGATCTACCTGCCGCAGG
TGGACCTACTGCAAATACGGATTATGACTCGTAAAGGGGTCATGCGTATTTTCATCACTAGGCACGTT
CGAGAATAAATTAGTAGGTGTCCCGAGCCTTGTGGCGTTCCGCCTGACTCCTCATGAAGTCGACCTTC
TCACCGGCCCTATCTGCCGACGTAAGTCATAACCTAGATCTGTACCTCGGGGGGAGGGTCACTGTAAA
GGGATAATTGGAGGGTGATTTCCCACACTTTCCTAAGGGTACTTTTTGCCTGGCTTCGCAATTGGGTC
CAATGGATGTCGATCTCTGGTTTAGCAGTTGTGAAAGTGGCAAGGCGGGAGGTTAGACCTCCATTTAA
CATATACAAGCAAGTTAACTGCACTAGATGTGTAGACACTACAGTTACAGGAGTAGCCGAATAAGTCT
CCGACGTCAAGCGAATAAGCGTCATACGCGATTATCGCCTAAGAGCACGTATTGGCGGTAAAAGGCTA
GCTAGACGCTTATGGGTAGATTTCAAGGCGTTCGTAGTGGTATAATAGGATACTCTTTCACCAGCCTG
AGGGCCGAACGCTATACTAGTGGTCTGTGATGTAGGACCGAGTATCTCTCTAGGGACCATCTACTTGA
GCAATGGTGCGCAGGGGGAGACATAGACCAGCCTTGGGTGGCAAGCACTGCAATAAGTCCTGTTTAGC
CTTGGAGTTCACATGCCGGCACTGAAGCCGACCTACCTGAGCGTGTGATTACCGTTAAAGCATCTGTC
TAGTTCTGTTTCCGGCGCTCTTGGTTCCAGGTTAGGGGAAGTGTATGACCCATGTGTTTTTATCGGCT
TAACCACGAGTGATCCCCGGTCGTTTCCCCATTGAATCCCTGGTGCATCCTACTCCCAGAATGATAGC
TGACTGACTGGACTGGCTTTTCAAGTAATCGAGGGGGTATCGCGGTCACGGCCGTTAACAGATCCCGT
CCTTAGTGTGGAATCCGCACCTGCTGACTAACGCTTCGCCGGCGTGTCTGCACAGCCGTATAGTGTTA
ATCATGACCCCAAGGAAGGATTAAACAAATATCTTGACG
>COHON29
GTGTCCGGTAGCCCGCGCTAGTTAGACACCCCGGCAGGGGGGATTGCTTTCGAGACGGGAGATCCCTT
CGCCGACCCTGGAGGGCCGACGCCGAGGCATTCGGGCCCCTGCAACGTCAACAGCGGCAAGAAAACGG
GATGAATGGGCGTAATGGGGGGGGTCTGCTGGGGACCCGACGCGGTTGCCGTTTGCGGGGCCCCCGAC
CCATACCGACCCACCTAGGCGTCCAGTTACGGCGCACGGCGGGAGCGTGGTTGCCGTCAGAGCTGTGT
TTCTCGATCAGTCCCCCGCAGTGCCGCAGTATCTTGCCGTGGGCTGCTTTAATCTTGAAAGTGGTTTA
TACATTGGGCGACGAGGTGTCGACTCTCATTGGGGGTAACCGACGGGCACATGCAGTCCCCTCCCCGG
GCAGGCAGAGGCGGGGCCCCCGCGCGCCGGCCCCGGCCCACAATCTTACCAGGGTCCTCAAAGGAGCC
TTTGCATGGTACCCTTCGTGAATGGTTGCTTAAGAGGTCCACAACGTGGTCCGGGCACGGTCGACTAA
ACACTCAAAACAGCGACGGCAAATATAGGTACAAGGTCCAGGCCCTCACGGCACTAATTGCGATGACC
CAACTCACGGGGGCAGTCCCCGGCGCGGCGACCTTGATTACGTCCGGGGACAAGTATGTCGCTCCCCC
CGGGAGGGGTGCAGCCACATGGGAGATTCAAAGTTTCTCGTGACGTCGTTGTGATCACTGCAGCCTAG
CCGAGACTCCCGTACTACGCGAAGGTTGGTTATGTTAACCACTACAACGTGAGGCGCCGTAAGGGCCA
GTGTTGTGCCCGGCTCTTCAATGCGCCTTAGCGGCCTGATACACCCACCCAAGGAGATACTGCTAATC
ATATGGGTGGACAGAACCTCGCAACGCAGACGCCTCCCCGGCATGATATGGTTTTTTTCCGCTATTAT
CCAGCACGCAGCGCTATCATTCAAGAGAACCCAGTGACGCGTAAATCGTAAGATCTACCTGCCGCAGG
TGGACCTACTGCAAATACGGATTATGACTCGTAAAGGGGTCATGCGTATTTTCATCACTAGGCACGTT
CGAGAATAAATTAGTAGGTGTCCCGAGCCTTGTGGCGTTCCGCCTGACTCCTCATGAAGTCGACCTTC
TCACCGGCCCTATCTGCCGACGTAAGTCATAACCTAGATCTGTACCTCGGGGGGAGGGTCACTGTAAA
GGGATAATTGGAGGGTGATTTCCCACACTTTCCTAAGGGTACTTTTTGCCTGGCTTCGCAATTGGGTC
CAATGGATGTCGATCTCTGGTTTAGCAGTTGTGAAAGTGGCAAGGCGGGAGGTTAGACCTCCATTTAA
CATATACAAGCAAGTTAACTGCACTAGATGTGTAGACACTACAGTTACAGGAGTAGCCGAATAAGTCT
CCGACGTCAAGCGAATAAGCGTCATACGCGATTATCGCCTAAGAGCACGTATTGGCGGTAAAAGGCTA
GCTAGACGCTTATGGGTAGATTTCAAGGCGTTCGTAGTGGTATAATAGGATACTCTTTCACCAGCCTG
AGGGCCGAACGCTATACTAGTGGTCTGTGATGTAGGACCGAGTATCTCTCTAGGGACCATCTACTTGA
GCAATGGTGCGCAGGGGGAGACATAGACCAGCCTTGGGTGGCAAGCACTGCAATAAGTCCTGTTTAGC
CTTGGAGTTCACATGCCGGCACTGAAGCCGACCTACCTGAGCGTGTGATTACCGTTAAAGCATCTGTC
TAGTTCTGTTTCCGGCGCTCTTGGTTCCAGGTTAGGGGAAGTGTATGACCCATGTGTTTTTATCGGCT
TAACCACGAGTGATCCCCGGTCGTTTCCCCATTGAATCCCTGGTGCATCCTACTCCCAGAATGATAGC
TGACTGACTGGACTGGCTTTTCAAGTAATCGAGGGGGTATCGCGGTCACGGCCGTTAACAGATCCCGT
CCTTAGTGTGGAATCCGCACCTGCTGACTAACGCTTCGCCGGCGTGTCTGCACAGCCGTATAGTGTTA
ATCATGACCCCAAGGAAGGATTAAACAAATATCTTGACG

>COHON32
GTGTCCGGTAGCCCGCGCTAGTTAGACACCCCGGCAGGGGGGATTGCTTTCGAGACGGGAGATCCCTT
CGCCGACCCTGGAGGGCCGACGCCGAGGCATTCGGGCCCCTGCAACGTCAACAGCGGCAAGAAAACGG
GATGAATGGGCGTAATGGGGGGGGTCTGCTGGGGACCCGACGCGGTTGCCGTTTGCGGGGCCCCCGAC
CCATACCGACCCACCTAGGCGTCCAGTTACGGCGCACGGCGGGAGCGTGGTTGCCGTCAGAGCTGTGT
TTCTCGATCAGTCCCCCGCAGTGCCGCAGTATCTTGCCGTGGGCTGCTTTAATCTTGAAAGTGGTTTA
TACATTGGGCGACGAGGTGTCGACTCTCATTGGGGGTAACCGACGGGCACATGCAGTCCCCTCCCCGG
GCAGGCAGAGGCGGGGCCCCCGCGCGCCGGCCCCGGCCCACAATCTTACCAGGGTCCTCAAAGGAGCC
TTTGCATGGTACCCTTCGTGAATGGTTGCTTAAGAGGTCCACAACGTGGTCCGGGCACGGTCGACTAA
ACACTCAAAACAGCGACGGCAAATATAGGTACAAGGTCCAGGCCCTCACGGCACTAATTGCGATGACC
CAACTCACGGGGGCAGTCCCCGGCGCGGCGACCTTGATTACGTCCGGGGACAAGTATGTCGCTCCCCC
CGGGAGGGGTGCAGCCACATGGGAGATTCAAAGTTTCTCGTGACGTCGTTGTGATCACTGCAGCCTAG
CCGAGACTCCCGTACTACGCGAAGGTTGGTTATGTTAACCACTACAACGTGAGGCGCCGTAAGGGCCA
GTGTTGTGCCCGGCTCTTCAATGCGCCTTAGCGGCCTGATACACCCACCCAAGGAGATACTGCTAATC
ATATGGGTGGACAGAACCTCGCAACGCAGACGCCTCCCCGGCATGATATGGTTTTTTTCCGCTATTAT
CCAGCACGCAGCGCTATCATTCAAGAGAACCCAGTGACGCGTAAATCGTAAGATCTACCTGCCGCAGG
TGGACCTACTGCAAATACGGATTATGACTCGTAAAGGGGTCATGCGTATTTTCATCACTAGGCACGTT
CGAGAATAAATTAGTAGGTGTCCCGAGCCTTGTGGCGTTCCGCCTGACTCCTCATGAAGTCGACCTTC
TCACCGGCCCTATCTGCCGACGTAAGTCATAACCTAGATCTGTACCTCGGGGGGAGGGTCACTGTAAA
GGGATAATTGGAGGGTGATTTCCCACACTTTCCTAAGGGTACTTTTTGCCTGGCTTCGCAATTGGGTC
CAATGGATGTCGATCTCTGGTTTAGCAGTTGTGAAAGTGGCAAGGCGGGAGGTTNGACCTCCNTTTAA
CATATACAAGCAAGTTAACNGCACTAGATGTGTAGACACTACAGTTACAGGAGTAGCCGAATAAGTCT
CCGACGTCAAGCGAATAAGCGTCATACGCGATTATCGCCTAAGAGCACGTATTGGCGGTAAAAGGCTA
GCTAGACGCTTATGGGTAGATTTCAAGGCGTTCGTAGTGGTATAATAGGATACTCTTTCACCAGCCTG
AGGGCCGAACGCTATACTAGTGGTCTGTGATGTAGGACCGAGTATCTCTCTAGGGACCATCTACTTGA
GCAATGGTGCGCAGGGGGAGACATAGACCAGCCTTGGGTGGCAAGCACTGCAATAAGTCCTGTTTAGC
CTTGGAGTTCACATGCCGGCACTGAAGCCGACCTACCTGAGCGTGTGATTACCGTTAAAGCATCTGTC
TAGTTCTGTTTCCGGCGCTCTTGGTTCCAGGTTAGGGGAAGTGTATGACCCATGTGTTTTTATCGGCT
TAACCACGAGTGATCCCCGGTCGTTTCCCCATTGAATCCCTGGTGCATCCTACTCCCAGAATGATAGC
TGACTGACTGGACTGGCTTTTCAAGTAATCGAGGGGGTATCGCGGTCACGGCCGTTAACAGATCCCGT
CCTTAGTGTGGAATCCGCACCTGCTGACTAACGCTTCGCCGGCGTGTCTGCACAGCCGTATAGTGTTA
ATCATGACCCCAAGGAAGGATTAAACAAATATCTTGACG
>COHON33
GTGTCCGGTAGCCCGCGCTAGTTAGACACCCCGGCAGGGGGGATTGCTTTCGGGACGGGAGATCCCTT
CGCCGACCCTGGAGGGCCGACGCCGAGGCATTCGGGCCCCCGCAACGTCAACAGCGGCAAGAAAACGG
GATGAATGGGCGTAATGGGGGGGGTCTGCTGGGGACCCGACGCGGTTGCCGTTTGCGGGGCCCCCGAC
CCATACCGACCCACCTAGGCGTCCAGTTACGGCGCACGTCGGGAGCGTGGTTGCCGTCAGAGCTGTGT
TTCTCGATCAGTCCCCCGCAGTGCCGCAGTATCTTGCCGTGGGCTGCTTTAATCTTGAAAGTGGTTTA
TACATTGGGCGACGAGGTGTCGACTCTCATTGGGGGTAACCGACGGGCACATGCAGTCCCCTCCCCGG
GCAGGCAGAGGCGGGGCCCCCGCGCGCCGGCCCCGGCCCACAATCTTACCAGGGTCCTCAAAGGAGCC
TTTGCATGGTACCCTTCGTGAATGGTTGCTTAAGAGGTCCACAACGTGGTCCGGGCACGGTCGACTAA
ACACTCAAAACAGCGACGGCAAATATAGGTACAAGGTCCAGGCCCTCACGGCACTAATTGCGATGACC
CAACTCACGGGGGCAGTCCCCGGCGCGGCGACCTTGATTACGTCCGGGGACAAGTATGTCGCTCCCCC
CGGGAGGGGTGCAGCCACATGGGAGATTCAAAGTTTCTCGTGACGTCGTTGTGATCACTGCAGCCTAG
CCGAGACTCCCGTACTACGCGAAGGTTGGTTATGTTAACCACTACAACGTGAGGCGCCGTAAGGGCCA
GTGTTGTGCCCGGCTCTTCAATGCGCCTTAGCGGCCTGATACACCCACCCAAGGAGATACTGCTAATC
ATATGGGTGGACAGAACCTCGCAACGCAGACGCCTCCCCGGCATGATATGGTTTTTTTCCGCTATTAT
CCAGCACGCAGCGCTATCATTCAAGAGAACCCAGTGACGCGTAAATCGTAAGATCTACCTGCCGCAGG
TGGACCTACTGCAAATACGGATTATGACTCGTAAAGGGGTCATGCGTATTTTCATCACTAGGCACGTT
CGAGAATAAATTAGTAGGTGTCCCGAGCCTTGTGGCGTTCCGCCTGACTCCTCATGAAGTCGACCTTC
TCACCGGCCCTATCTGCCGACGTAAGTCATAACCTAGATCTGTACCTCGGGGGGAGGGTCACTGTAAA
GGGATAATTGGAGGGTGATTTCCCACACTTTCCTAAGGGTACTTTTTGCCTGGCTTCGCAATTGGGTC
CAATGGATGTCGATCTCTGGTTTAGCAGTTGTGAAAGTGGCAAGGCGGGAGGTTAGACCTCCATTTAA
CATATACAAGCAAGTTAACTGCACTAGATGTGTAGACACTACAGTTACAGGAGTAGCCGAATAAGTCT

CCGACGTCAAGCGAATAAGCGTCATACGCGATTATCGCCTAAGAGCACGTATTGGCGGTAAAAGGCTA
GCTAGACGCTTATGGGTAGATTTCAAGGCGTTCGTAGTGGTATAATAGGATACTCTTTCACCAGCCTG
AGGGCCGAACGCTATACTAGTGGTCTGTGATGTAGGACCGAGTATCTCTCTAGGGACCATCTACTTGA
GCAATGGTGCGCAGGGGGAGACATAGACCAGCCTTGGGTGGCAAGCACTGCAATAAGTCCTGTTTAGC
CTTGGAGTTCACATGCCGGCACTGAAGCCGACCTACCTGAGCGTGTGATTACCGTTAAAGCATCTGTC
TAGTTCTGTTTCCGGCGCTCTTGGTTCCAGGTTAGGGGAAGTGTATGACCCATGTGTTTTTATCGGCT
TAACCACGAGTGATCCCCGGTCGTTTCCCCATTGAATCCCTGGTGCATCCTACTCCCAGAATGATAGC
TGACTGACTGGACTGGCTTTTCAAGTAATCGAGGGGGTATCGCGGTCACGGCCGTTAACAGATCCCGT
CCTTAGTGTGGAATCCGCACCTGCTGACTAACGCTTCGCCGGCGTGTCTGCACAGCCGTATAGTGTTA
ATCATGACCCCAAGGAAGGATTAAACAAATATCTTGACG
>COHON34
GTGTCCGGTAGCCCGCGCTAGTTAGACACCCCGGCAGGGGGGATTGCTTTCGAGACGGGAGATCCCTT
CGCCGACCCTGGAGGGCCGACGCCGAGGCATTCGGGCCCCTGCAACGTCAACAGCGGCAAGAAAACGG
GATGAATGGGCGTAATGGGGGGGGTCTGCTGGGGACCCGACGCGGTTGCCGTTTGCGGGGCCCCCGAC
CCATACCGACCCACCTAGGCGTCCAGTTACGGCGCACGTCGGGAGCGTGGTTGCCGTCAGAGCTGTGT
TTCTCGATCAGTCCCCCGCAGTGCCGCAGTATCTTGCCGTGGGCTGCTTTAATCTTGAAAGTGGTTTA
TACATTGGGCGACGAGGTGTCGACTCTCATTGGGGGTAACCGACGGGCACATGCAGTCCCCTCCCCGG
GCAGGCAGAGGCGGGGCCCCCGCGCGCCGGCCCCGGCCCACAATCTTACCAGGGTCCTCAAAGGAGCC
TTTGCATGGTACCCTTCGTGAATGGTTGCTTAAGAGGTCCACAACGTGGTCCGGGCACGGTCGACTAA
ACACTCAAAACAGCGACGGCAAATATAGGTACAAGGTCCAGGCCCTCACGGCACTAATTGCGATGACC
CAACTCACGGGGGCAGTCCCCGGCGCGGCGACCTTGATTACGTCCGGGGACAAGTATGTCGCTCCCCC
CGGGAGGGGTGCAGCCACATGGGAGATTCAAAGTTTCTCGTGACGTCGTTGTGATCACTGCAGCCTAG
CCGAGACTCCCGTACTACGCGAAGGTTGGTTATGTTAACCACTACAACGTGAGGCGCCGTAAGGGCCA
GTGTTGTGCCCGGCTCTTCAATGCGCCTTAGCGGCCTGATACACCCACCCAAGGAGATACTGCTAATC
ATATGGGTGGACAGAACCTCGCAACGCAGACGCCTCCCCGGCATGATATGGTTTTTTTCCGCTATTAT
CCAGCACGCAGCGCTATCATTCAAGAGAACCCAGTGACGCGTAAATCGTAAGATCTACCTGCCGCAGG
TGGACCTACTGCAAATACGGATTATGACTCGTAAAGGGGTCATGCGTATTTTCATCACTAGGCACGTT
CGAGAATAAATTAGTAGGTGTCCCGAGCCTTGTGGCGTTCCGCCTGACTCCTCATGAAGTCGACCTTC
TCACCGGCCCTATCTGCCGACGTAAGTCATAACCTAGATCTGTACCTCGGGGGGAGGGTCACTGTAAA
GGGATAATTGGAGGGTGATTTCCCACACTTTCCTAAGGGTACTTTTTGCCTGGCTTCGCAATTGGGTC
CAATGGATGTCGATCTCTGGTTTAGCAGTTGTGAAAGTGGCAAGGCGGGAGGTTAGACCTCCATTTAA
CATATACAAGCAAGTTAACTGCACTAGATGTGTAGACACTACAGTTACAGGAGTAGCCGAATAAGTCT
CCGACGTCAAGCGAATAAGCGTCATACGCGATTATCGCCTAAGAGCACGTATTGGCGGTAAAAGGCTA
GCTAGACGCTTATGGGTAGATTTCAAGGCGTTCGTAGTGGTATAATAGGATACTCTTTCACCAGCCTG
AGGGCCGAACGCTATACTAGTGGTCTGTGATGTAGGACCGAGTATCTCTCTAGGGACCATCTACTTGA
GCAATGGTGCGCAGGGGGAGACATAGACCAGCCTTGGGTGGCAAGCACTGCAATAAGTCCTGTTTAGC
CTTGGAGTTCACATGCCGGCACTGAAGCCGACCTACCTGAGCGTGTGATTACCGTTAAAGCATCTGTC
TAGTTCTGTTTCCGGCGCTCTTGGTTCCAGGTTAGGGGAAGTGTATGACCCATGTGTTTTTATCGGCT
TAACCACGAGTGATCCCCGGTCGTTTCCCCATTGAATCCCTGGTGCATCCTACTCCCAGAATGATAGC
TGACTGACTGGACTGGCTTTTCAAGTAATCGAGGGGGTATCGCGGTCACGGCCGTTAACAGATCCCGT
CCTTAGTGTGGAATCCGCACCTGCTGACTAACGCTTCGCCGGCGTGTCTGCACAGCCGTATAGTGTTA
ATCATGACCCCAAGGAAGGATTAAACAAATATCTTGACG
>COHON36
GTGTCCGGTAGCCCGCGCTAGTTAGACACCCCGGCAGGGGGGATTGCTTTCGAGACGGGAGATCCCTT
CGCCGACCCTGGAGGGCCGACGCCGAGGCATTCGGGCCCCTGCAACGTCAACAGCGGCAAGAAAACGG
GATGAATGGGCGTAATGGGGGGGGTCTGCTGGGGACCCGACGCGGTTGCCGTTTGCGGGGCCCCCGAC
CCATACCGACCCACCTAGGCGTCCAGTTACGGCGCACGTCGGGAGCGTGGTTGCCGTCAGAGCTGTGT
TTCTCGATCAGTCCCCCGCAGTGCCGCAGTATCTTGCCGTGGGCTGCTTTAATCTTGAAAGTGGTTCA
TACATTGGGCGACGAGGTGTCGACTCTCATTGGGGGTAACCGACGGGCACATGCAGTCCCCTCCCCGG
GCAGGCAGAGGCGGGGCCCCCGCGCGCCGGCCCCGGCCCACAATCTTACCAGGGTCCTCAAAGGAGCC
TTTGCATGGTACCCTTCGTGAATGGTTGCTTAAGAGGTCCACAACGTGGTCCGGGCACGGTCGACTAA
ACACTCAAAACAGCGACGGCAAATATAGGTACAAGGTCCAGGCCCTCACGGCACTAATTGCGATGACC
CAACTCACGGGGGCAGTCCCCGGCGCGGCGACCTTGATTACGTCCGGGGACAAGTATGTCGCTCCCCC
CGGGAGGGGTGCAGCCACATGGGAGATTCAAAGTTTCTCGTGACGTCGTTGTGATCACTGCAGCCTAG

CCGAGACTCCCGTACTACGCGAAGGTTGGTTATGTTAACCACTACAACGTGAGGCGCCGTAAGGGCCA
GTGTTGTGCCCGGCTCTTCAATGCGCCTTAGCGGCCTGATACACCCACCCAAGGAGATACTGCTAATC
ATATGGGTGGACAGAACCTCGCAACGCAGACGCCTCCCCGGCATGATATGGTTTTTTTCCGCTATTAT
CCAGCACGCAGCGCTATCATTCAAGAGAACCCAGTGACGCGTAAATCGTAAGATCTACCTGCCGCAGG
TGGACCTACTGCAAATACGGATTATGACTCGTAAAGGGGTCATGCGTATTTTCATCACTAGGCACGTT
CGAGAATAAATTAGTAGGTGTCCCGAGCCTTGTGGCGTTCCGCCTGACTCCTCATGAAGTCGACCTTC
TCACCGGCCCTATCTGCCGACGTAAGTCATAACCTAGATCTGTACCTCGGGGGGAGGGTCACTGTAAA
GGGATAATTGGAGGGTGATTTCCCACACTTTCCTAAGGGTACTTTTTGCCTGGCTTCGCAATTGGGTC
CAATGGATGTCGATCTCTGGTTTAGCAGTTGTGAAAGTGGCAAGGCGGGAGGTTAGACCTCCATTTAA
CATATACAAGCAAGTTAACTGCACTAGATGTGTAGACACTACAGTTACAGGAGTAGCCGAATAAGTCT
CCGACGTCAAGCGAATAAGCGTCATACGCGATTATCGCCTAAGAGCACGTATTGGCGGTAAAAGGCTA
GCTAGACGCTTATGGGTAGATTTCAAGGCGTTCGTAGTGGTATAATAGGATACTCTTTCACCAGCCTG
AGGGCCGAACGCTATACTAGTGGTCTGTGATGTAGGACCGAGTATCTCTCTAGGGACCATCTACTTGA
GCAATGGTGCGCAGGGGGAGACATAGACCAGCCTTGGGTGGCAAGCACTGCAATAAGTCCTGTTTAGC
CTTGGAGTTCACATGCCGGCACTGAAGCCGACCTACCTGAGCGTGTGATTACCGTTAAAGCATCTGTC
TAGTTCTGTTTCCGGCGCTCTTGGTTCCAGGTTAGGGGAAGTGTATGACCCATGTGTTTTTATCGGCT
TAACCACGAGTGATCCCCGGTCGTTTCCCCATTGAATCCCTGGTGCATCCTACTCCCAGAATGATAGC
TGACTGACTGGACTGGCTTTTCAAGTAATCGAGGGGGTATCGCGGTCACGGCCGTTAACAGATCCCGT
CCTTAGTGTGGAATCCGCACCTGCTGACTAACGCTTCGCCGGCGTGTCTGCACAGCCGTATAGTGTTA
ATCATGACCCCAAGGAAGGATTAAACAAATATCTTGACG
>COHON37
GTGTCCGGTAGCCCGCGCTAGTTAGACACCCCGGCAGGGGGGATTGCTTTCGAGACGGGAGATCCCTT
CGCCGACCCTGGAGGGCCGACGCCGAGGCATTCGGGCCCCTGCAACGTCAACAGCGGCAAGAAAACGG
GATGAATGGGCGTAATGGGGGGGGTCTGCTGGGGACCCGACGCGGTTGCCGTTTGCGGGGCCCCCGAC
CCATACCGACCCACCTAGGCGTCCAGTTACGGCGCACGTCGGGAGCGTGGTTGCCGTCAGAGCTGTGT
TTCTCGATCAGTCCCCCGCAGTGCCGCAGTATCTTGCCGTGGGCTGCTTTAATCTTGAAAGTGGTTCA
TACATTGGGCGACGAGGTGTCGACTCTCATTGGGGGTAACCGACGGGCACATGCAGTCCCCTCCCCGG
GCAGGCAGAGGCGGGGCCCCCGCGCGCCGGCCCCGGCCCACAATCTTACCAGGGTCCTCAAAGGAGCC
TTTGCATGGTACCCTTCGTGAATGGTTGCTTAAGAGGTCCACAACGTGGTCCGGGCACGGTCGACTAA
ACACTCAAAACAGCGACGGCAAATATAGGTACAAGGTCCAGGCCCTCACGGCACTAATTGCGATGACC
CAACTCACGGGGGCAGTCCCCGGCGCGGCGACCTTGATTACGTCCGGGGACAAGTATGTCGCTCCCCC
CGGGAGGGGTGCAGCCACATGGGAGATTCAAAGTTTCTCGTGACGTCGTTGTGATCACTGCAGCCTAG
CCGAGACTCCCGTACTACGCGAAGGTTGGTTATGTTAACCACTACAACGTGAGGCGCCGTAAGGGCCA
GTGTTGTGCCCGGCTCTTCAATGCGCCTTAGCGGCCTGATACACCCACCCAAGGAGATACTGCTAATC
ATATGGGTGGACAGAACCTCGCAACGCAGACGCCTCCCCGGCATGATATGGTTTTTTTCCGCTATTAT
CCAGCACGCAGCGCTATCATTCAAGAGAACCCAGTGACGCGTAAATCGTAAGATCTACCTGCCGCAGG
TGGACCTACTGCAAATACGGATTATGACTCGTAAAGGGGTCATGCGTATTTTCATCACTAGGCACGTT
CGAGAATAAATTAGTAGGTGTCCCGAGCCTTGTGGCGTTCCGCCTGACTCCTCATGAAGTCGACCTTC
TCACCGGCCCTATCTGCCGACGTAAGTCATAACCTAGATCTGTACCTCGGGGGGAGGGTCACTGTAAA
GGGATAATTGGAGGGTGATTTCCCACACTTTCCTAAGGGTACTTTTTGCCTGGCTTCGCAATTGGGTC
CAATGGATGTCGATCTCTGGTTTAGCAGTTGTGAAAGTGGCAAGGCGGGAGGTTAGACCTCCATTTAA
CATATACAAGCAAGTTAACTGCACTAGATGTGTAGACACTACAGTTACAGGAGTAGCCGAATAAGTCT
CCGACGTCAAGCGAATAAGCGTCATACGCGATTATCGCCTAAGAGCACGTATTGGCGGTAAAAGGCTA
GCTAGACGCTTATGGGTAGATTTCAAGGCGTTCGTAGTGGTATAATAGGATACTCTTTCACCAGCCTG
AGGGCCGAACGCTATACTAGTGGTCTGTGATGTAGGACCGAGTATCTCTCTAGGGACCATCTACTTGA
GCAATGGTGCGCAGGGGGAGACATAGACCAGCCTTGGGTGGCAAGCACTGCAATAAGTCCTGTTTAGC
CTTGGAGTTCACATGCCGGCACTGAAGCCGACCTACCTGAGCGTGTGATTACCGTTAAAGCATCTGTC
TAGTTCTGTTTCCGGCGCTCTTGGTTCCAGGTTAGGGGAAGTGTATGACCCATGTGTTTTTATCGGCT
TAACCACGAGTGATCCCCGGTCGTTTCCCCATTGAATCCCTGGTGCATCCTACTCCCAGAATGATAGC
TGACTGACTGGACTGGCTTTTCAAGTAATCGAGGGGGTATCGCGGTCACGGCCGTTAACAGATCCCGT
CCTTAGTGTGGAATCCGCACCTGCTGACTAACGCTTCGCCGGCGTGTCTGCACAGCCGTATAGTGTTA
ATCATGACCCCAAGGAAGGATTAAACAAATATCTTGACG
>COHON38
GTGTCCGGTAGCCCGCGCTAGTTAGACACCCCGGCAGGGGGGATTGCTTTCGGGACGGGAGATCCCTT

CGCCGACCCTGGAGGGCCGACGCCGAGGCATTCGGGCCCCCGCAACGTCAACAGCGGCAAGAAAACGG
GATGAATGGGCGTAATGGGGGGGGTCTGCTGGGGACCCGACGCGGTTGCCGTTTGCGGGGCCCCCGAC
CCATACCGACCCACCTAGGCGTCCAGTTACGGCGCACGTCGGGAGCGTGGTTGCCGTCAGAGCTGTGT
TTCTCGATCAGTCCCCCGCAGTGCCGCAGTATCTTGCCGTGGGCTGCTTTAATCTTGAAAGTGGTTTA
TACATTGGGCGACGAGGTGTCGACTCTCATTGGGGGTAACCGACGGGCACATGCAGTCCCCTCCCCGG
GCAGGCAGAGGCGGGGCCCCCGCGCGCCGGCCCCGGCCCACAATCTTACCAGGGTCCTCAAAGGAGCC
TTTGCATGGTACCCTTCGTGAATGGTTGCTTAAGAGGTCCACAACGTGGTCCGGGCACGGTCGACTAA
ACACTCAAAACAGCGACGGCAAATATAGGTACAAGGTCCAGGCCCTCACGGCACTAATTGCGATGACC
CAACTCACGGGGGCAGTCCCCGGCGCGGCGACCTTGATTACGTCCGGGGACAAGTATGTCGCTCCCCC
CGGGAGGGGTGCAGCCACATGGGAGATTCAAAGTTTCTCGTGACGTCGTTGTGATCACTGCAGCCTAG
CCGAGACTCCCGTACTACGCGAAGGTTGGTTATGTTAACCACTACAACGTGAGGCGCCGTAAGGGCCA
GTGTTGTGCCCGGCTCTTCAATGCGCCTTAGCGGCCTGATACACCCACCCAAGGAGATACTGCTAATC
ATATGGGTGGACAGAACCTCGCAACGCAGACGCCTCCCCGGCATGATATGGTTTTTTTCCGCTATTAT
CCAGCACGCAGCGCTATCATTCAAGAGAACCCAGTGACGCGTAAATCGTAAGATCTACCTGCCGCAGG
TGGACCTACTGCAAATACGGATTATGACTCGTAAAGGGGTCATGCGTATTTTCATCACTAGGCACGTT
CGAGAATAAATTAGTAGGTGTCCCGAGCCTTGTGGCGTTCCGCCTGACTCCTCATGAAGTCGACCTTC
TCACCGGCCCTATCTGCCGACGTAAGTCATAACCTAGATCTGTACCTCGGGGGGAGGGTCACTGTAAA
GGGATAATTGGAGGGTGATTTCCCACACTTTCCTAAGGGTACTTTTTGCCTGGCTTCGCAATTGGGTC
CAATGGATGTCGATCTCTGGTTTAGCAGTTGTGAAAGTGGCAAGGCGGGAGGTTAGACCTCCATTTAA
CATATACAAGCAAGTTAACTGCACTAGATGTGTAGACACTACAGTTACAGGAGTAGCCGAATAAGTCT
CCGACGTCAAGCGAATAAGCGTCATACGCGATTATCGCCTAAGAGCACGTATTGGCGGTAAAAGGCTA
GCTAGACGCTTATGGGTAGATTTCAAGGCGTTCGTAGTGGTATAATAGGATACTCTTTCACCAGCCTG
AGGGCCGAACGCTATACTAGTGGTCTGTGATGTAGGACCGAGTATCTCTCTAGGGACCATCTACTTGA
GCAATGGTGCGCAGGGGGAGACATAGACCAGCCTTGGGTGGCAAGCACTGCAATAAGTCCTGTTTAGC
CTTGGAGTTCACATGCCGGCACTGAAGCCGACCTACCTGAGCGTGTGATTACCGTTAAAGCATCTGTC
TAGTTCTGTTTCCGGCGCTCTTGGTTCCAGGTTAGGGGAAGTGTATGACCCATGTGTTTTTATCGGCT
TAACCACGAGTGATCCCCGGTCGTTTCCCCATTGAATCCCTGGTGCATCCTACTCCCAGAATGATAGC
TGACTGACTGGACTGGCTTTTCAAGTAATCGAGGGGGTATCGCGGTCACGGCCGTTAACAGATCCCGT
CCTTAGTGTGGAATCCGCACCTGCTGACTAACGCTTCGCCGGCGTGTCTGCACAGCCGTATAGTGTTA
ATCATGACCCCAAGGAAGGATTAAACAAATATCTTGACG
>COHON39
GTGTCCGGTAGCCCGCGCTAGTTAGACACCCCGGCAGGGGGGATTGCTTTCGAGACGGGAGATCCCTT
CGCCGACCCTGGAGGGCCGACGCCGAGGCATTCGGGCCCCTGCAACGTCAACAGCGGCAAGAAAACGG
GATGAATGGGCGTAATGGGGGGGGTCTGCTGGGGACCCGACGCGGTTGCCGTTTGCGGGGCCCCCGAC
CCATACCGACCCACCTAGGCGTCCAGTTACGGCGCACGGCGGGAGCGTGGTTGCCGTCAGAGCTGTGT
TTCTCGATCAGTCCCCCGCAGTGCCGCAGTATCTTGCCGTGGGCTGCTTTAATCTTGAAAGTGGTTTA
TACATTGGGCGACGAGGTGTCGACTCTCATTGGGGGTAACCGACGGGCACATGCAGTCCCCTCCCCGG
GCAGGCAGAGGCGGGGCCCCCGCGCGCCGGCCCCGGCCCACAATCTTACCAGGGTCCTCAAAGGAGCC
TTTGCATGGTACCCTTCGTGAATGGTTGCTTAAGAGGTCCACAACGTGGTCCGGGCACGGTCGACTAA
ACACTCAAAACAGCGACGGCAAATATAGGTACAAGGTCCAGGCCCTCACGGCACTAATTGCGATGACC
CAACTCACGGGGGCAGTCCCCGGCGCGGCGACCTTGATTACGTCCGGGGACAAGTATGTCGCTCCCCC
CGGGAGGGGTGCAGCCACATGGGAGATTCAAAGTTTCTCGTGACGTCGTTGTGATCACTGCAGCCTAG
CCGAGACTCCCGTACTACGCGAAGGTTGGTTATGTTAACCACTACAACGTGAGGCGCCGTAAGGGCCA
GTGTTGTGCCCGGCTCTTCAATGCGCCTTAGCGGCCTGATACACCCACCCAAGGAGATACTGCTAATC
ATATGGGTGGACAGAACCTCGCAACGCAGACGCCTCCCCGGCATGATATGGTTTTTTTCCGCTATTAT
CCAGCACGCAGCGCTATCATTCAAGAGAACCCAGTGACGCGTAAATCGTAAGATCTACCTGCCGCAGG
TGGACCTACTGCAAATACGGATTATGACTCGTAAAGGGGTCATGCGTATTTTCATCACTAGGCACGTT
CGAGAATAAATTAGTAGGTGTCCCGAGCCTTGTGGCGTTCCGCCTGACTCCTCATGAAGTCGACCTTC
TCACCGGCCCTATCTGCCGACGTAAGTCATAACCTAGATCTGTACCTCGGGGGGAGGGTCACTGTAAA
GGGATAATTGGAGGGTGATTTCCCACACTTTCCTAAGGGTACTTTTTGCCTGGCTTCGCAATTGGGTC
CAATGGATGTCGATCTCTGGTTTAGCAGTTGTGAAAGTGGCAAGGCGGGAGGTTAGACCTCCATTTAA
CATATACAAGCAAGTTAACTGCACTAGATGTGTAGACACTACAGTTACAGGAGTAGCCGAATAAGTCT
CCGACGTCAAGCGAATAAGCGTCATACGCGATTATCGCCTAAGAGCACGTATTGGCGGTAAAAGGCTA
GCTAGACGCTTATGGGTAGATTTCAAGGCGTTCGTAGTGGTATAATAGGATACTCTTTCACCAGCCTG

AGGGCCGAACGCTATACTAGTGGTCTGTGATGTAGGACCGAGTATCTCTCTAGGGACCATCTACTTGA
GCAATGGTGCGCAGGGGGAGACATAGACCAGCCTTGGGTGGCAAGCACTGCAATAAGTCCTGTTTAGC
CTTGGAGTTCACATGCCGGCACTGAAGCCGACCTACCTGAGCGTGTGATTACCGTTAAAGCATCTGTC
TAGTTCTGTTTCCGGCGCTCTTGGTTCCAGGTTAGGGGAAGTGTATGACCCATGTGTTTTTATCGGCT
TAACCACGAGTGATCCCCGGTCGTTTCCCCATTGAATCCCTGGTGCATCCTACTCCCAGAATGATAGC
TGACTGACTGGACTGGCTTTTCAAGTAATCGAGGGGGTATCGCGGTCACGGCCGTTAACAGATCCCGT
CCTTAGTGTGGAATCCGCACCTGCTGACTAACGCTTCGCCGGCGTGTCTGCACAGCCGTATAGTGTTA
ATCATGACCCCAAGGAAGGATTAAACAAATATCTTGACG
>COHON40
GTGTCCGGTAGCTCGCGCTAGTTAGACACCCCGGCAGGGGGGATTGCTTTCGGGACGGGAGATCCCTT
CGCCGACCCTGGAGGGCCGACGCCGAGGCATTCGGGCCCCTGCAACGTCAACAGCGGCAAGAAAACGG
GATGAATGGGCGTAATGGGGGGGGTCTGCTGGGGACCCGACGCGGTTGCCGTTTGCGGGGCCCCCGAC
CCATACCGACCCACCTAGGCGTCCAGTTACGGCGCACGTCGGGAGCGTGGTTGCCGTCAGAGCTGTGT
TTCTCGATCAGTCCCCCGCAGTGCCGCAGTATCTTGCCGTGGGCTGCTTTAATCTTGAAAGTGGTTTA
TACATTGGGCGACGAGGTGTCGACTCTCATTGGGGGTAACCGACGGGCACATGCAGTCCCCTCCCCGG
GCAGGCAGAGGCGGGGCCCCCGCGCGCCGGCCCCGGCCCACAATCTTACCAGGGTCCTCAAGGGAGCC
TTTGCATGGTACCCTTCGTGAATGGTTGCTTAAGAGGTCCACCACGTGGTCCGGGCACGGTCGACTAA
ACACTCAAAACAGCGACGGCAAATATAGGCACAAGGTCCAGACCCTCACGGCACTAATTGCGATGACC
CAACTCGCGGGGACAGTCACCGGCGCGGCGACCTTGATTACGGTCGGGGACAAGTATGTCGTTCCCCC
CGGGAGGGGTGCAGCCACATGGGAGATTCAAAGTTTCTCGTGACGTCGTTGTGATCACTGCAGCCTAG
CCGAGACTCCCGTACTACGCGAAGGTTGGTTATGTTAACCACTACAACGTGAGGCGCCGTAAGGGCCA
GTGTTGTGCCCGGCTCTTCAATGCGCCTTAGCGGCCTGATACACCCACCCAAGGAGATACCGTTAATC
ATGTGGGGGGACAGAACCTCGCAACGCAGACGCCTCCCCGGCATGATATGGTTTTTTTCCGCTATTAT
CCAGCACGCAGCGCTATCATTCAAGAGAACCCAGTGACGCGTAAATCGTAAGATCTACCTGCCGCAAG
TGGACCTACTGCAAATACGGATTATGACTCGTAAAGAGGTCATGCGTATTTTCATTACCAGGCACGTT
CGAGAATAAATTAGTAGGTGTCCCGCGCCTTGTGGCGTTCCGCCTGACTCCTCATGAAGTCGACCTTC
TCACCGGCCCTATCTGCCGACGTAAGCCATAACCTAGGTCTGTACCGTGGGGGGAGGGTCACTGTAAA
GGGATAATTGGAGGGTGATTTCCCACACTTTCCTAAGGGTACTTTTCGCCTGGCTTCGCAATTGGGCC
CAATGGATGTCGATCTCTGGTTTAGCAGTTGTGAAAGTGGTAAGGCGGGAGGTTAGACCTCCATTTAA
CATATACAAGCAAGTTCACTGCACTAGATGTGTAGACACTACAGTTACGGGGGTAGCCGAATAAGTCT
CCGACGTCAAGCGAATAAGCGTCATACGCGATTATCGCCTAAGAGCACGTATTGGCGGTAAAAGGCTA
ACTAGACGCCTGTGGGTAGATTTCAAGGCGTTCGTAGTGGTATAACAAGATACTTTTTCACCAGCCTG
AGGGCCGAACGCTATACTAGTGGTCTGTGATGTAGGATCGAGTATCCCTCTAGGGACCATCTACTTGA
GCAATGGTGCGCAGGGGGAGACATAGACCAGCCTTGGGTGGCAAGCACTGCAATAAGTCCTGTTTAGC
CCTGGAGTTCACACGCCGGCACTGAAGCCGACCTACCTGAGCTTGTGATTACCGTTAAAGCATCTGTC
TAGTTCTGTTTCCGGCGCTCTTGGTTCCAGGTTAGGGGAAGTGTATGACCCATGTGTTTTTATCGGCT
TAACCACGAGTGATCCCCGGTCGTTTCCTCATTGAATCCCTGGTGCATCCTACTCCCAGAATGATAGC
TGACTGACTGGACTGGCTTTTCAAGTAGTCGAGGGGGTATCGCGGTCACGGCCGTTAACAGATCCCGT
CCTTAGTGTGGAATCCGCACCTGCTGACTAACGCTTCGCCGGCGTGTTTGCACAGCCGTATAGTGTTA
ATCATGACCCCAAGGAAGGATTAAACAAATATCTTGACG
>COHON41
GTGTCCGGTAGCCCGCGCTAGTTAGACACCCCGGCAGGGGGGATTGCTTTCGAGACGGGAGATCCCTT
CGCCGACCCTGGAGGGCCGACGCCGAGGCATTCGGGCCCCTGCAACGTCAACAGCGGCAAGAAAACGG
GATGAATGGGCGTAATGGGGGGGGTCTGCTGGGGACCCGACGCGGTTGCCGTTTGCGGGGCCCCCGAC
CCATACCGACCCACCTAGGCGTCCAGTTACGGCGCACGTCGGGAGCGTGGTTGCCGTCAGAGCTGTGT
TTCTCGATCAGTCCCCCGCAGTGCCGCAGTATCTTGCCGTGGGCTGCTTTAATCTTGAAAGTGGTTNA
TACATTGGGCGACGAGGTGTCGGCTCTCATTGGGGGTAACCGACGGGCACATGCAGTCCCCTCCCCGG
GCAGGCAGAGGCGGGGCCCCCGCGCGCCGGCCCCGGCCCACAATCTTACCAGGGTCCTCAAAGGAGCC
TTTGCATGGTACCCTTCGTGAATGGTTGCTTAAGAGGTCCACCACGTAGTCCGGGCACGGTCGACTAA
ACACTCAAAACAGCGACGGCAAATAGAGGCACAAGGTCCAGGCCCTCACGGCACTAGTTGCGATGACC
CAACTCACGGGGGCAGTCCCCGGCGCGGCGACCTTGATTACGTCCGGGAACAAGTATGTCGTTTCCCC
CGGGAGGGGTGCAGCCACATGGGAGATTCAAAGTTTCTCGTGACGTCGTTGTGATCACTGCAGCCTAG
CCGAGACTCCCGTACTACGCGAAGGTTGGTTATGTTAACCACTACAACGTGAGGCGCCGTAAGGGCCA
GTGTTGTGCCCGGCTCTTCAATGCGCCTTAGCGGCCTGATACANCCACCCAAGGAGATACTGCTAATC

ATGTGGGTGGACAGAACCTCGCAACGCAGACGCATCCCCGGCATGATATGGTTTTTTTCCGCTATTAC
CCAGCATGCGGCGCTATCATTCAAGAGAACCCAGTGACGTGTAAATCGTAAGATCTACCTGCCGCAGG
TGGACCTACTGCAAATACGGATTATGACTCGTAAAGAGGTCATGCGTATTTTCATCACTAGGCACGTT
CGAGAATAAATTAGTAGGTGTCCCGCGCCTTGTGGCGTTCCGCCTGACTCCTCATGAAGTCGACCTTC
TCATCGGCCCTATCTGCCGACGTAAGTCATAACCCAGATCTGTACCTCGGAGGGAGGGTCACTGTAAA
GGGATAATTGGAGGGTGATTTCCCACACTTTCCTAAGGGTACTTTTTGCCTGGCTTCGCAGTTGGGTC
CAATAGATGTTGATCTCTGGTTTAGCAGTTGTGAAAGTGGCAAGGCGGGAGGTTAGACCTCCATTTAA
CATATACAAGCAAGTTAACTGCACTAGATGTGTAGACACTACAGTTACAGGAGTAGCCGACTAAGTCT
CCGACGTCAAGCGAATAGGCGTCATACGCGATTATCGCCTAAGAGCACGTATTGGCGGTAAAAGGCTA
ACTAGACGCTTGTGGGTAGATTTCAAGGCGCTCGTAGTGGTATAATAGGATACTCTTTCACCAGCCTG
AGGGCCGAACGCTATACTAGTGGTCTGTGATGTAGGACCAAGTAGCTCTCTAGGGACCATCTACTTGA
GCAATGGTGCGCAGGGGTAGACATAGACCAACCTTGGGTGGCAAGCACTGCAATAAGTCCTGTTCAGC
CTTGGAGTTCACACGCCGGCACTAAAGCCGACCTACCTGAGCTTGTGATTACCGTTAAAGCATCTGTC
TAGTTCTGTTTCCGGCGCTCTTGGTTCCATGTTAGGGGAAGTGTATGACCCATGTGTTTTTATCGGCT
TAACCACGAGTGATCCCCGGTCGTTTCCCCATTGAATCCCTGGTGCATCCTACTCCCATAATGATAGC
TGACTGGCTGGACTGGCTTTTCAAGTAGTCGAGGGGGTATCGCGGTCACGGCCGTTAACAGATCCCGT
CCTTAGTGTGGAATCCGCACCTGCTGACTAACGCTTCGCCNGCGTGTCTGCACATCCGTATAGTGTTA
ATCATGACCCCAAGGAAGGATTAAACAAATATCTTGACG
>COHON44
GTGTCCGGTAGCCCGCGCTAGTTAGACACCCCGGCAGGGGGGATTGCTTTCGAGACGGGAGATCCCTT
CGCCGACCCTGGAGGGCCGACGCCGAGGCATTCGGGCCCCTGCAACGTCAACAGCGGCAAGAAAACGG
GATGAATGGGCGTAATGGGGGGGGTCTGCTGGGGACCCGACGCGGTTGCCGTTTGCGGGGCCCCCGAC
CCATACCGACCCACCTAGGCGTCCAGTTACGGCGCACGTCGGGAGCGTGGTTGCCGTCAGAGCTGTGT
TTCTCGATCAGTCCCCCGCAGTGCCGCAGTATCTTGCCGTGGGCTGCTTTAATCTTGAAAGTGGTTTA
TACATTGGGCGACGAGGTGTCGACTCTCATTGGGGGTAACCGACGGGCACATGCAGTCCCCTCCCCGG
GCAGGCAGAGGCGGGGCCCCCGCGCGCCGGCCCCGGCCCACAATCTTACCAGGGTCCTCAAAGGAGCC
TTTGCATGGTACCCTTCGTGAATGGTTGCTTAAGAGGTCCACAACGTGGTCCGGGCACGGTCGACTAA
ACACTCAAAACAGCGACGGCAAATATAGGTACAAGGTCCAGGCCCTCACGGCACTAATTGCGATGACC
CAACTCACGGGGGCAGTCCCCGGCGCGGCGACCTTGATTACGTCCGGGGACAAGTATGTCGCTCCCCC
CGGGAGGGGTGCAGCCACATGGGAGATTCAAAGTTTCTCGTGACGTCGTTGTGATCACTGCAGCCTAG
CCGAGACTCCCGTACTACGCGAAGGTTGGTTATGTTAACCACTACAACGTGAGGCGCCGTAAGGGCCA
GTGTTGTGCCCGGCTCTTCAATGCGCCTTAGCGGCCTGATACACCCACCCAAGGAGATACTGCTAATC
ATATGGGTGGACAGAACCTCGCAACGCAGACGCCTCCCCGGCATGATATGGTTTTTTTCCGCTATTAT
CCAGCACGCAGCGCTATCATTCAAGAGAACCCAGTGACGCGTAAATCGTAAGATCTACCTGCCGCAGG
TGGACCTACTGCAAATACGGATTATGACTCGTAAAGGGGTCATGCGTATTTTCATCACTAGGCACGTT
CGAGAATAAATTAGTAGGTGTCCCGAGCCTTGTGGCGTTCCGCCTGACTCCTCATGAAGTCGACCTTC
TCACCGGCCCTATCTGCCGACGTAAGTCATAACCTAGATCTGTACCTCGGGGGGAGGGTCACTGTAAA
GGGATAATTGGAGGGTGATTTCCCACACTTTCCTAAGGGTACTTTTTGCCTGGCTTCGCAATTGGGTC
CAATGGATGTCGATCTCTGGTTTAGCAGTTGTGAAAGTGGCAAGGCGGGAGGTTAGACCTCCATTTAA
CATATACAAGCAAGTTAACTGCACTAGATGTGTAGACACTACAGTTACAGGAGTAGCCGAATAAGTCT
CCGACGTCAAGCGAATAAGCGTCATACGCGATTATCGCCTAAGAGCACGTATTGGCGGTAAAAGGCTA
GCTAGACGCTTATGGGTAGATTTCAAGGCGTTCGTAGTGGTATAATAGGATACTCTTTCACCAGCCTG
AGGGCCGAACGCTATACTAGTGGTCTGTGATGTAGGACCGAGTATCTCTCTAGGGACCATCTACTTGA
GCAATGGTGCGCAGGGGGAGACATAGACCAGCCTTGGGTGGCAAGCACTGCAATAAGTCCTGTTTAGC
CTTGGAGTTCACATGCCGGCACTGAAGCCGACCTACCTGAGCGTGTGATTACCGTTAAAGCATCTGTC
TAGTTCTGTTTCCGGCGCTCTTGGTTCCAGGTTAGGGGAAGTGTATGACCCATGTGTTTTTATCGGCT
TAACCACGAGTGATCCCCGGTCGTTTCCCCATTGAATCCCTGGTGCATCCTACTCCCAGAATGATAGC
TGACTGACTGGACTGGCTTTTCAAGTAATCGAGGGGGTATCGCGGTCACGGCCGTTAACAGATCCCGT
CCTTAGTGTGGAATCCGCACCTGCTGACTAACGCTTCGCCGGCGTGTCTGCACAGCCGTATAGTGTTA
ATCATGACCCCAAGGAAGGATTAAACAAATATCTTGACG
>COHON45
GTGTCCGGTAGCCCGCGCTAGTTAGACACCCCGGCAGGGGGGATTGCTTTCGAGACGGGAGATCCCTT
CGCCGACCCTGGAGGGCCGACGCCGAGGCATTCGGGCCCCTGCAACGTCAACAGCGGCAAGAAAACGG
GATGAATGGGCGTAATGGGGGGGGTCTGCTGGGGACCCGACGCGGTTGCCGTTTGCGGGGCCCCCGAC

CCATACCGACCCACCTAGGCGTCCAGTTACGGCGCACGTCGGGAGCGTGGTTGCCGTCAGAGCTGTGT
TTCTCGATCAGTCCCCCGCAGTGCCGCAGTATCTTGCCGTGGGCTGCTTTAATCTTGAAAGTGGTTTA
TACATTGGGCGACGAGGTGTCGACTCTCATTGGGGGTAACCGACGGGCACATGCAGTCCCCTCCCCGG
GCAGGCAGAGGCGGGGCCCCCGCGCGCCGGCCCCGGCCCACAATCTTACCAGGGTCCTCAAAGGAGCC
TTTGCATGGTACCCTTCGTGAATGGTTGCTTAAGAGGTCCACAACGTGGTCCGGGCACGGTCGACTAA
ACACTCAAAACAGCGACGGCAAATATAGGTACAAGGTCCAGGCCCTCACGGCACTAATTGCGATGACC
CAACTCACGGGGGCAGTCCCCGGCGCGGCGACCTTGATTACGTCCGGGGACAAGTATGTCGCTCCCCC
CGGGAGGGGTGCAGCCACATGGGAGATTCAAAGTTTCTCGTGACGTCGTTGTGATCACTGCAGCCTAG
CCGAGACTCCCGTACTACGCGAAGGTTGGTTATGTTAACCACTACAACGTGAGGCGCCGTAAGGGCCA
GTGTTGTGCCCGGCTCTTCAATGCGCCTTAGCGGCCTGATACACCCACCCAAGGAGATACTGCTAATC
ATATGGGTGGACAGAACCTCGCAACGCAGACGCCTCCCCGGCATGATATGGTTTTTTTCCGCTATTAT
CCAGCACGCAGCGCTATCATTCAAGAGAACCCAGTGACGCGTAAATCGTAAGATCTACCTGCCGCAGG
TGGACCTACTGCAAATACGGATTATGACTCGTAAAGGGGTCATGCGTATTTTCATCACTAGGCACGTT
CGAGAATAAATTAGTAGGTGTCCCGAGCCTTGTGGCGTTCCGCCTGACTCCTCATGAAGTCGACCTTC
TCACCGGCCCTATCTGCCGACGTAAGTCATAACCTAGATCTGTACCTCGGGGGGAGGGTCACTGTAAA
GGGATAATTGGAGGGTGATTTCCCACACTTTCCTAAGGGTACTTTTTGCCTGGCTTCGCAATTGGGTC
CAATGGATGTCGATCTCTGGTTTAGCAGTTGTGAAAGTGGCAAGGCGGGAGGTTAGACCTCCATTTAA
CATATACAAGCAAGTTAACTGCACTAGATGTGTAGACACTACAGTTACAGGAGTAGCCGAATAAGTCT
CCGACGTCAAGCGAATAAGCGTCATACGCGATTATCGCCTAAGAGCACGTATTGGCGGTAAAAGGCTA
GCTAGACGCTTATGGGTAGATTTCAAGGCGTTCGTAGTGGTATAATAGGATACTCTTTCACCAGCCTG
AGGGCCGAACGCTATACTAGTGGTCTGTGATGTAGGACCGAGTATCTCTCTAGGGACCATCTACTTGA
GCAATGGTGCGCAGGGGGAGACATAGACCAGCCTTGGGTGGCAAGCACTGCAATAAGTCCTGTTTAGC
CTTGGAGTTCACATGCCGGCACTGAAGCCGACCTACCTGAGCGTGTGATTACCGTTAAAGCATCTGTC
TAGTTCTGTTTCCGGCGCTCTTGGTTCCAGGTTAGGGGAAGTGTATGACCCATGTGTTTTTATCGGCT
TAACCACGAGTGATCCCCGGTCGTTTCCCCATTGAATCCCTGGTGCATCCTACTCCCAGAATGATAGC
TGACTGACTGGACTGGCTTTTCAAGTAATCGAGGGGGTATCGCGGTCACGGCCGTTAACAGATCCCGT
CCTTAGTGTGGAATCCGCACCTGCTGACTAACGCTTCGCCGGCGTGTCTGCACAGCCGTATAGTGTTA
ATCATGACCCCAAGGAAGGATTAAACAAATATCTTGACG
>RAGLN01
GTGTCCGGTAGCCCGCGCTAGTCAGACACCCCGGCAGGGGGGATTGCTTTCGAGACGGGAGATCCCTT
CGCCGACCCTGGAGGGCCGACGCCGAGGCATTCGGGCCCCTGCAACGTCAACAGCGGCAAGAAAACGG
GATGAATGGGCGTAATGGGGGGGGTCTGCTGGGGACCCGACGCGGTTGCCGTTTGCGGGGCCCCCGAC
CCATACCGACCCACCTAGGCGTCCAGTTACGGCGCACGGCGGGAGCGTGGTTGCCGTCAGAGCTGTGT
TTCTCGATCAGTCCCCCGCAGTGCCGCAGTATCTTGCCGTGGGCTGCTTTAATCTTGAAAGTGGTTTA
TACATTGGGCGACGAGGTGTCGACTCTCATTGGGGGTAACCGACGGGCACATGCAGTCCCCTCCCCGG
GCAGGCAGAGGCGGGGCCCCCGCGCGCCGGCCCCGGCCCACAATCTTACCAGGGTCCTCAAAGGAGCC
TTTGCATGGTACCCTTCGTGAATGGTTGCTTAAGAGGTCCACAACGTGGTCCGGGCACGGTCGACTAA
ACACTCAAAACAGCGACGGCAAATATAGGTACAAGGTCCAGGCCCTCACGGCACTAATTGCGATGACC
CAACTCACGGGGGCAGTCCCCGGCGCGGCGACCTTGATTACGTCCGGGGACAAGTATGTCGCTCCCCC
CGGGAGGGGTGCAGCCACATGGGAGATTCAAAGTTTCTCGTGACGTCGTTGTGATCACTGCAGCCTAG
CCGAGACTCCCGTACTACGCGAAGGTTGGTTATGTTAACCACTACAACGTGAGGCGCCGTAAGGGCCA
GTGTTGTGCCCGGCTCTTCAATGCGCCTTAGCGGCCTGATACACCCACCCAAGGAGATACTGCTAATC
ATATGGGTGGACAGAACCTCGCAACGCAGACGCCTCCCCGGCATGATATGGTTTTTTTCCGCTATNAT
CCAGCACGCAGCGCTATCATTTAAGAGAACCCAGTGACGCGTAAATCGTAAGATCTACCTGCCGCAGG
TGGACCTACTGCAAATACGGATTATGACTCGTAAAGGGGTCATGCGTATTTTCATCACTAGGCACGTT
CGAGAATAAATTAGTAGGTGTCCCGAGCCTTGTGGCGTTCCGCCTGACTCCTCATGAAGTCGACCTTC
TCACCGGCCCTATCTGCCGACGTAAGTCATAACCTAGATCTGTACCTCGGGGGGAGGGTCACTGTAAA
GGGATAATTGGAGGGTGATTTCCCACATTTTCCTAAGGGTACTTTTTGCCTGGCTTCGCAATTGGGTC
CAATGGATGTCGATCTCTGGTTTAGCAGTTGTGAAAGTGGCAAGGCGGGAGGTTAGACCTCCATTTAA
CATATACAAGCAAGTTAACTGCACTAGATGTGTAGACACTACAGGTACAGGAGTAGCCGGATAAGTCT
CCGACGTCAAGCGAATAAGCGTCATACGCGATTATCGCCTAAGAGCACGTATTTGCGGTAAAAGGCTA
GCTAGACGCTTGTGGGTAGATTTCAAGGCGTTCGTAGTGGTATAATAGGATACTCTTTCACCAGCCTG
AGGGCCGAACGCTATACTAGTGGTCTGTGATGTAGGACCGAGTATCTCTCTAGGGACCATCTACTTGA
GCAATGGTGCGCAGGGGGAGACATAGACCAGCCTTGGGTGGCAAGCACTGCAATAAGTCCTGTTTAGC

CTTGGAGTTCACACGCCGGCACTGAAGCCGACCTACCTGAGCGTGTGATTACCGTTAAAGCATCTGTC
TAGTTCTGTTTCCGGCGCTCTTGGTTCCAGGTTAGGGGAAGTGTATGACCCATGTGTTTTTATCGGCT
TAACCACGAGTGATCCCCGGTCGTTTCCCCATTGAATCCCTGGTGCATCCTACTCCCAGAATGATAGC
TGACTGACTGGACTGGCTTTTCAAGTAATCGAGGGGGTATCGCGGTCACGGCCGTTAACAGATCCCGT
CCTTAGTGTGGAATCCGCACCTGCTGACTAACGCTTCGCCGGCGTGTCTGCACAGCCGTATAGTGTTA
ATCATGACCCCAAGGAAGGATTAAACAAATATCTTGACG
>RAGLN02
GTGTCCGGTAGCCCGCGCTAGTTAGACACCCCGGCAGGGGGGATTGCTTTCGAGACGGGAGATCCCTT
CGCCGACCCTGGAGGGCCGACGCCGAGGCATTCGGGCCCCTGCAACGTCAACAGCGGCAAGAAAACGG
GATGAATGGGCGTAATGGGGGGGGTCTGCTGGGGACCCGACGCGGTTGCCGTTTGCGGGGCCCCCGAC
CCATACCGACCCACCTAGGCGTCCAGTTACGGCGCACGTCGGGAGCGTGGTTGCCGTCAGAGCTGTGT
TTCTCGATCAGTCCCCCGCAGTGCCGCAGTATCTTGCCGTGGGCTGCTTTAATCTTGAAAGTGGTTCA
TACATTGGGCGACGAGGTGTCGACTCTCATTGGGGGTAACCGACGGGCACATGCAGTCCCCTCCCCGG
GCAGGCAGAGGCGGGGCCCCCGCGCGCCGGCCCCGGCCCACAATCTTACCAGGGTCCTCAAAGGAGCC
TTTGCATGGTACCCTTCGTGAATGGTTGCTTAAGAGGTCCACAACGTGGTCCGGGCACGGTCGACTAA
ACACTCAAAACAGCGACGGCAAATATAGGTACAAGGTCCAGGCCCTCACGGCACTAATTGCGATGACC
CAACTCACGGGGGCAGTCCCCGGCGCGGCGACCTTGATTACGTCCGGGGACAAGTATGTCGCTCCCCC
CGGGAGGGGTGCAGCCACATGGGAGATTCAAAGTTTCTCGTGACGTCGTTGTGATCACTGCAGCCTAG
CCGAGACTCCCGTACTACGCGAAGGTTGGTTATGTTAACCACTACAACGTGAGGCGCCGTAAGGGCCA
GTGTTGTGCCCGGCTCTTCAATGCGCCTTAGCGGCCTGATACACCCACCCAAGGAGATACTGCTAATC
ATATGGGTGGACAGAACCTCGCAACGCAGACGCCTCCCCGGCATGATATGGTTTTTTTCCGCTATTAT
CCAGCACGCAGCGCTATCATTCAAGAGAACCCAGTGACGCGTAAATCGTAAGATCTACCTGCCGCAGG
TGGACCTACTGCAAATACGGATTATGACTCGTAAAGGGGTCATGCGTATTTTCATCACTAGGCACGTT
CGAGAATAAATTAGTAGGTGTCCCGAGCCTTGTGGCGTTCCGCCTGACTCCTCATGAAGTCGACCTTC
TCACCGGCCCTATCTGCCGACGTAAGTCATAACCTAGATCTGTACCTCGGGGGGAGGGTCACTGTAAA
GGGATAATTGGAGGGTGATTTCCCACACTTTCCTAAGGGTACTTTTTGCCTGGCTTCGCAATTGGGTC
CAATGGATGTCGATCTCTGGTTTAGCAGTTGTGAAAGTGGCAAGGCGGGAGGTTAGACCTCCATTTAA
CATATACAAGCAAGTTAACTGCACTAGATGTGTAGACACTACAGTTACAGGAGTAGCCGAATAAGTCT
CCGACGTCAAGCGAATAAGCGTCATACGCGATTATCGCCTAAGAGCACGTATTGGCGGTAAAAGGCTA
GCTAGACGCTTATGGGTAGATTTCAAGGCGTTCGTAGTGGTATAATAGGATACTCTTTCACCAGCCTG
AGGGCCGAACGCTATACTAGTGGTCTGTGATGTAGGACCGAGTATCTCTCTAGGGACCATCTACTTGA
GCAATGGTGCGCAGGGGGAGACATAGACCAGCCTTGGGTGGCAAGCACTGCAATAAGTCCTGTTTAGC
CTTGGAGTTCACATGCCGGCACTGAAGCCGACCTACCTGAGCGTGTGATTACCGTTAAAGCATCTGTC
TAGTTCTGTTTCCGGCGCTCTTGGTTCCAGGTTAGGGGAAGTGTATGACCCATGTGTTTTTATCGGCT
TAACCACGAGTGATCCCCGGTCGTTTCCCCATTGAATCCCTGGTGCATCCTACTCCCAGAATGATAGC
TGACTGACTGGACTGGCTTTTCAAGTAATCGAGGGGGTATCGCGGTCACGGCCGTTAACAGATCCCGT
CCTTAGTGTGGAATCCGCACCTGCTGACTAACGCTTCGCCGGCGTGTCTGCACAGCCGTATAGTGTTA
ATCATGACCCCAAGGAAGGATTAAACAAATATCTTGACG
>RAGLN03
GTGTCCGGTAGCCCGCGCTAGTTAGACACCCCGGCAGGGGGGATTGCTTTCGAGACGGGAGATCCCTT
CGCCGACCCTGGAGGGCCGACGCCGAGGCATTCGGGCCCCTGCAACGTCAACAGCGGCAAGAAAACGG
GATGAATGGGCGTAATGGGGGGGGTCTGCTGGGGACCCGACGCGGTTGCCGTTTGCGGGGCCCCCGAC
CCATACCGACCCACCTAGGCGTCCAGTTACGGCGCACGTCGGGAGCGTGGTTGCCGTCAGAGCTGTGT
TTCTCGATCAGTCCCCCGCAGTGCCGCAGTATCTTGCCGTGGGCTGCTTTAATCTTGAAAGTGGTTTA
TACATTGGGCGACGAGGTGTCGACTCTCATTGGGGGTAACCGACGGGCACATGCAGTCCCCTCCCCGG
GCAGGCAGAGGCGGGGCCCCCGCGCGCCGGCCCCGGCCCACAATCTTACCAGGGTCCTCAAAGGAGCC
TTTGCATGGTACCCTTCGTGAATGGTTGCTTAAGAGGTCCACAACGTGGTCCGGGCACGGTCGACTAA
ACACTCAAAACAGCGACGGCAAATATAGGTACAAGGTCCAGGCCCTCACGGCACTAATTGCGATGACC
CAACTCACGGGGGCAGTCCCCGGCGCGGCGACCTTGATTACGTCCGGGGACAAGTATGTCGCTCCCCC
CGGGAGGGGTGCAGCCACATGGGAGATTCAAAGTTTCTCGTGACGTCGTTGTGATCACTGCAGCCTAG
CCGAGACTCCCGTACTACGCGAAGGTTGGTTATGTTAACCACTACAACGTGAGGCGCCGTAAGGGCCA
GTGTTGTGCCCGGCTCTTCAATGCGCCTTAGCGGCCTGATACACCCACCCAAGGAGATACTGCTAATC
ATATGGGTGGACAGAACCTCGCAACGCAGACGCCTCCCCGGCATGATATGGTTTTTTTCCGCTATTAT
CCAGCACGCAGCGCTATCATTCAAGAGAACCCAGTGACGCGTAAATCGTAAGATCTACCTGCCGCAGG

TGGACCTACTGCAAATACGGATTATGACTCGTAAAGGGGTCATGCGTATTTTCATCACTAGGCACGTT
CGAGAATAAATTAGTAGGTGTCCCGAGCCTTGTGGCGTTCCGCCTGACTCCTCATGAAGTCGACCTTC
TCACCGGCCCTATCTGCCGACGTAAGTCATAACCTAGATCTGTACCTCGGGGGGAGGGTCACTGTAAA
GGGATAATTGGAGGGTGATTTCCCACACTTTCCTAAGGGTACTTTTTGCCTGGCTTCGCAATTGGGTC
CAATGGATGTCGATCTCTGGTTTAGCAGTTGTGAAAGTGGCAAGGCGGGAGGTTAGACCTCCATTTAA
CATATACAAGCAAGTTAACTGCACTAGATGTGTAGACACTACAGTTACAGGAGTAGCCGAATAAGTCT
CCGACGTCAAGCGAATAAGCGTCATACGCGATTATCGCCTAAGAGCACGTATTGGCGGTAAAAGGCTA
GCTAGACGCTTATGGGTAGATTTCAAGGCGTTCGTAGTGGTATAATAGGATACTCTTTCACCAGCCTG
AGGGCCGAACGCTATACTAGTGGTCTGTGATGTAGGACCGAGTATCTCTCTAGGGACCATCTACTTGA
GCAATGGTGCGCAGGGGGAGACATAGACCAGCCTTGGGTGGCAAGCACTGCAATAAGTCCTGTTTAGC
CTTGGAGTTCACATGCCGGCACTGAAGCCGACCTACCTGAGCGTGTGATTACCGTTAAAGCATCTGTC
TAGTTCTGTTTCCGGCGCTCTTGGTTCCAGGTTAGGGGAAGTGTATGANCCATGTGTTTTTATCGGCT
TAACCACGAGTGATCCCCGGTCGTTTCCCCATTGAATCCCTGGTGCATCCTACTCCCAGAATGATAGC
TGACTGACTGGACTGGCTTTTCAAGTAATCGAGGGGGTATCGCGGTCACGGCCGTTAACAGATCCCGT
CCTTAGTGTGGAATCCGCACCTGCTGACTAACGCTTCGCCGGCGTGTCTGCACAGCCGTATAGTGTTA
ATCATGACCCCAAGGAAGGATTAAACAAATATCTTGACG
>RAGLN04
GTGTCCGGTAGCCCGCGCTAGTTAGACACCCCGGCAGGGGGGATTGCTTTCGAGACGGGAGATCCCTT
CGCCGACCCTGGAGGGCCGACGCCGAGGCATTCGGGCCCCTGCAACGTCAACAGCGGCAAGAAAACGG
GATGAATGGGCGTAATGGGGGGGGTCTGCTGGGGACCCGACGCGGTTGCCGTTTGCGGGGCCCCCGAC
CCATACCGACCCACCTAGGCGTCCAGTTACGGCGCACGTCGGGAGCGTGGTTGCCGTCAGAGCTGTGT
TTCTCGATCAGTCCCCCGCAGTGCCGCAGTATCTTGCCGTGGGCTGCTTTAATCTTGAAAGTGGTTTA
TACATTGGGCGACGAGGTGTCGACTCTCATTGGGGGTAACCGACGGGCACATGCAGTCCCCTCCCCGG
GCAGGCAGAGGCGGGGCCCCCGCGCGCCGGCCCCGGCCCACAATCTTACCAGGGTCCTCAAAGGAGCC
TTTGCATGGTACCCTTCGTGAATGGTTGCTTAAGAGGTCCACAACGTGGTCCGGGCACGGTCGACTAA
ACACTCAAAACAGCGACGGCAAATATAGGTACAAGGTCCAGGCCCTCACGGCACTAATTGCGATGACC
CAACTCACGGGGGCAGTCCCCGGCGCGGCGACCTTGATTACGTCCGGGGACAAGTATGTCGCTCCCCC
CGGGAGGGGTGCAGCCACATGGGAGATTCAAAGTTTCTCGTGACGTCGTTGTGATCACTGCAGCCTAG
CCGAGACTCCCGTACTACGCGAAGGTTGGTTATGTTAACCACTACAACGTGAGGCGCCGTAAGGGCCA
GTGTTGTGCCCGGCTCTTCAATGCGCCTTAGCGGCCTGATACACCCACCCAAGGAGATACTGCTAATC
ATATGGGTGGACAGAACCTCGCAACGCAGACGCCTCCCCGGCATGATATGGTTTTTTTCCGCTATTAT
CCAGCACGCAGCGCTATCNTTCAAGAGAACCCAGTGACGCGTAAATCGTAAGATCTACCTGCCGCAGG
TGGACCTACTGCAAATACGGATTATGACTCGTAAAGGGGTCATGCGTATTTTCATCACTAGGCACGTT
CGAGAATAAATTAGTAGGTGTCCCGAGCCTTGTGGCGTTCCGCCTGACTCCTCATGAAGTCGACCTTC
TCACCGGCCCTATCTGCCGACGTAAGTCATAACCTAGATCTGTACCTCGGGGGGAGGGTCACTGTAAA
GGGATAATTGGAGGGTGATTTCCCACACTTTCCTAAGGGTACTTTTTGCCTGGCTTCGCAATTGGGTC
CAATGGATGTCGATCTCTGGTTTAGCAGTTGTGAAAGTGGCAAGGCGGGAGGTTAGACCTCCATTTAA
CATATACAAGCAAGTTAACTGCACTAGATGTGTAGACACTACAGTTACAGGAGTAGCCGAATAAGTCT
CCGACGTCAAGCGAATAAGCGTCATACGCGATTATCGCCTAAGAGCACGTATTGGCGGTAAAAGGCTA
GCTAGACGCTTATGGGTAGATTTCAAGGCGTTCGTAGTGGTATAATAGGATACTCTTTCACCAGCCTG
AGGGCCGAACGCTATACTAGTGGTCTGTGATGTAGGACCGAGTATCTCTCNAGGGACCATCTACTTGA
GCAATGGTGCGCAGGGGGAGACATAGACCAGCCTTGGGTGGCAAGCACTGCAATAAGTCCTGTTTAGC
CTTGGAGTTCACATGCCGGCACTGAAGCCGACCTACCTGAGCGTGTGATTACCGTTAAAGCATCTGTC
TAGTTCTGTTTCCGGCGCTCTTGGTTCCAGGTTAGGGGAAGTGTATGACCCATGTGTTTTTATCGGCT
TAACCACGAGTGATCCCCGGTCGTTTCCCCATTGAATCCCTGGTGCATCCTACTCCCAGAATGATAGC
TGACTGACTGGACTGGCTTTTCAAGTAATCGAGGGGGTATCGCGGTCACGGCCGTTAACAGATCCCGT
CCTTAGTGTGGAATCCGCACCTGCTGACTAACGCTTCGCCGGCGTGTCTGCACAGCCGTATAGTGTTA
ATCATGACCCCAAGGAAGGATTAAACAAATATCTTGACG
>RAGLN05
GTGTCCGGTAGCCCGCGCTAGTTAGACACCCCGGCAGGGGGGATTGCTTTCGGGACGGGAGATCCCTT
CGCCGACCCTGGAGGGCCGACGCCGAGGCATTCGGGCCCCTGCAACGTCAACAGCGGCAAGAAAACGG
GATGAATGGGCGTAATGGGGGGGGTCTGCTGGGGACCCGACGCGGTTGCCGTTTGCGGGGCCCCCGAC
CCATACCGACCCACCTAGGCGTCCAGTTACGGCGCACGGCGGGAGCGTGGTTGCCGTCAGAGCTGTGT
TTCTCGATCAGTCCCCCGCAGTGCCGCAGTATCTTGCCGTGGGCTGCTTTAATCTTGAAAGTGGTTCA

TACATTGGGCGACGAGGTGTCGACTCTCATTGGGGGTAACCGACGGGCACATGCAGTCCCCTCCCCGG
GCAGGCAGAGGCGGGGCCCCCGCGCGCCGGCCCCGGCCCACAATCTTACCAGGGTCCTCAAAGGAGCC
TTTGCATGGTACCCTTCGTGAATGGTTGCTTAAGAGGTCCACAACGTGGTCCGGGCACGGTCGACTAA
ACACTCAAAACAGCGACGGCAAATATAGGTACAAGGTCCAGGCCCTCACGGCACTAATTGCGATGACC
CAACTCACGGGGGCAGTCCCCGGCGCGGCGACCTTGATTACGTCCGGGGACAAGTATGTCGCTCCCCC
CGGGAGGGGTGCAGCCACATGGGAGATTCAAAGTTTCTCGTGACGTCGTTGTGATCACTGCAGCCTAG
CCGAGACTCCCGTACTACGCGAAGGTTGGTTATGTTAACCACTACAACGTGAGGCGCCGTAAGGGCCA
GTGTTGTGCCCGGCTCTTCAATGCGCCTTAGCGGCCTGATACACCCACCCAAGGAGATACTGCTAATC
ATATGGGTGGACAGAACCTCGCAACGCAGACGCCTCCCCGGCATGATATGGTTTTTTTCCGCTATTAT
CCAGCACGCAGCGCTATCATTCAAGAGAACCCAGTGACGCGTAAATCGTAAGATCTACCTGCCGCAGG
TGGACCTACTGCAAATACGGATTATGACTCGTAAAGGGGTCATGCGTATTTTCATCACTAGGCACGTT
CGAGAATAAATTAGTAGGTGTCCCGAGCCTTGTGGCGTTCCGCCTGACTCCTCATGAAGTCGACCTTC
TCACCGGCCCTATCTGCCGACGTAAGTCATAACCTAGATCTGTACCTCGGGGGGAGGGTCACTGTAAA
GGGATAATTGGAGGGTGATTTCCCACACTTTCCTAAGGGTACTTTTTGCCTGGCTTCGCAATTGGGTC
CAATGGATGTCGATCTCTGGTTTAGCAGTTGTGAAAGTGGCAAGGCGGGAGGTTAGACCTCCATTTAA
CATATACAAGCAAGTTAACTGCACTAGATGTGTAGACACTACAGTTACAGGAGTAGCCGAATAAGTCT
CCGACGTCAAGCGAATAAGCGTCATACGCGATTATCGCCTAAGAGCACGTATTGGCGGTAAAAGGCTA
GCTAGACGCTTATGGGTAGATTTCAAGGCGTTCGTAGTGGTATAATAGGATACTCTTTCACCAGCCTG
AGGGCCGAACGCTATACTAGTGGTCTGTGATGTAGGACCGAGTATCTCTCTAGGGACCATCTACTTGA
GCAATGGTGCGCAGGGGGAGACATAGACCAGCCTTGGGTGGCAAGCACTGCGATAAGTCCTGTTTAGC
CTTGGAGTTCACATGCCGGCACTGAAGCCGACCTACCTGAGCGTGTGATTACCGTTAAAGCATCTGTC
TAGTTCTGTTTCCGGCGCTCTTGGTTCCAGGTTAGGGGAAGTGTATGACCCATGTGTTTTTATCGGCT
TAACCACGAGTGATCCCCGGTCGTTTCCCCATTGAATCCCTGGTGCATCCTACTCCCAGAATGATAGC
TGACTGACTGGACTGGCTTTTCAAGTAATCGAGGGGGTATCGCGGTCACGGCCGTTAACAGATCCCGT
CCTTAGTGTGGAATCCGCACCTGCTGACTAACGCTTCGCCGGCGTGTCTGCACAGCCGTATAGTGTTA
ATCATGACCCCAAGGAAGGATTAAACAAATATCTTGACG
>RAGLN06
GTGTCCGGTAGCCCGCGCTAGTTAGACACCCCGGCAGGGGGGATTGCTNTCGAGACGGGAGATCCCTT
CGCCGACCCTGTAGGGCCGACGCCGAGGCATTCGGGCCCCTGCAACGTCAACAGCGGCAAGAAAACGG
GATGAATGGGCGTAATGGGGGGGGTCTGCTGGGGACCCGACGCGGTTGCCGTTTGCGGGGCCCCCGAC
CCATACCGACCCACCTAGGCGTCCAGTTACGGCGCACGTCGGGAGCGTGGTTGCCGTCAGAGCTGTGT
TTCTCGATCAGTCCCCCGCAGTGCCGCAGTATCTTGCCGTGGGCTGCTTTAATCTTGAAAGTGGTTCA
TACATTGGGCGACGAGGTGTCGGCTCTCATTGGGGGTAGCCGACGGACACATGCAGTCCCCTCCCCGG
GCAGGCAGAGGCGGGGCCCCCGCGCGCCGGCCCCGGCCCACAATCTTACCAGGGTCCTCAAAAGAGCC
TTTGCATGGTACCCTTCGTGAATGGTTGCTTAAGAGGTCCACCACGTAGTCCGGGCACGGTCGACTAA
ACACTCAAAACAGCGACGGCAAATAGAGGCACAAGGTCCAGGCCCTCACGGCACTAGTTGCGATGACC
CAACTCACGGGGGCAGTCCCCGGCGCGGCGACCTTGATTACGTCCGGGAACAAGTATGTCGTTTCCCC
CGGGAGGGGTGCAGCCACATGGGAGATTCAAAGTTTCTCGTGACGTCGTTGTGATCACTGCAGCCTAG
CCGAGACTCCCGTACTACGCGAAGGTTGGTTATGTTAACCACTACAACGTGAGGCGCCGTAAGGGCCA
GTGTTGTGCCCGGCTCTTCAATGCGCCTTAGCGGCCTGATACACCCACCCAAGGAGATACTGCTAATC
ATGTGGGTGGACAGAACCTCGCAACGCAGACGCATCCCCGGCATGATATGGTTTTTTTCCGCTATTAC
CCAGCACGCGGCGCTATCATTCAAGAGAACCCAGTGACGCGTAAATCGTAAGATCTACCTGCCGCAGG
TGGACCTACTGCAAATACGGATTATGACTCGTAAAGAGGTCATGCGTATTTTCATCACTAGGCACGTT
CGAGAGTAAATTAGTAGGTGTCCCGCGCCNTGTGGCGTTCCGCCTGACTCCTCATGAAGTCGACCTTC
TCATCGGCCCTATCTGCCGACGTAAGTCATAATCCAGATCTTCACCTCGGAGGGAGGGTCACTGTAAA
GGGATAATTGGAGGGCGATTTCCCACACTTTCCTAAGGGTACTTTTTGCTTGGCTTCGCAGTTGGGTC
CAATAGATGTTGATCTCTGGTTTAGCAGTTGTGAAAGTGGCAAGGCGGGAGGTTAGACCTCCATTTAA
CATATACAAGCAAGTTAACTGCACTAGATGTGTAGACACTACAGTTACAGGAGTAGCCGACTAAGTCT
CCGACGTCAAGCGAATAGGCGTCATACGCGATTATCGCCTAAGAGCACGTATTGGCGGTAAAAGGATA
ACTAGACGCTTGTGGGTAGATTTCAAGGCGCTCGTAGTGGTATAATAGGATACTCTTTCACCAGCCTG
AGAGCCGAACGCTATACTAGTGGTCTGTGATGTAGGACCAAGTAGCTCTCTAGGGACCATCTACTTGA
GCAATGGTGCGNAGGGGTAGACATAGACCAACCTTGGGTGGCAAGCACTGCAATAAGTCCTGTTTAGC
CTTGGAGTTCACACGCCGGCACTAAAGCCGACCTACCTGAGCTTGTGATTACCGTTAAAGCATCTGTC
TAGTTCTGTTTCCGGCGCTCTTGGTTCCATGTTAGGGGAAGTGTATGACCCATGTGTTTTTATCGGCT

TAACTACGAGTGATCCCCGGTCGTTTCCCCATTAAATCCCTGGTGCATCCTACTCCCATAATGATAGC
TGACTGGCTGGACTGGCTTTTCAAGTAGTCGAGGGGGTATCGCGGTCACGGCCGTTAACAGATCCCGT
CCTTAGTGTGGAATCCGCACCTGCTGACTAACGCTTCGCCGGCGTGTCTGCACATCCGTATAGTGTTA
ATCATGACCCCAAGGAAGGATTAAACAAATATCTTGACG
>RAGLN07
GTGTCCGGTAGCCCGCGCTAGTCAGACACCCCGGCAGGGGGGATTGCTTTCGAGACGGGAGATCCCTT
CGCCGACCCTGGAGGGCCGACGCCGAGGCATTCGGGCCCCTGCAACGTCAACAGCGGCAAGAAAACGG
GATGAATGGGCGTAATGGGGGGGGTCTGCTGGGGACCCGACGCGGTTGCCGTTTGCGGGGCCCCCGAC
CCATACCGACCCACCTAGGCGTCCAGTTACGGCGCACGTCGGGAGCGTGGTTGCCGTCAGAGCTGTGT
TTCTCGATCAGTCCCCCGCAGTGCCGCAGTATCTTGCCGTGGGCTGCTTTAATCTTGAAAGTGGTTCA
TACATTGGGCGACGAGGTGTCGACTCTCATTGGGGGTAACCGACGGGCACATGCAGTCCCCTCCCCGG
GCAGGCAGAGGCGGGGCCCCCGCGCGCCGGCCCCGGCCCACAATCTTACCAGGGTCCTCAAAGGAGCC
TTTGCATGGTACCCTTCGTGAATGGTTGCTTAAGAGGTCCACAACGTGGTCCGGGCACGGTCGACTAA
ACACTCAAAACAGCGACGGCAAATATAGGTACAAGGTCCAGGCCCTCACGGCACTAATTGCGATGACC
CAACTCACGGGGGCAGTCCCCGGCGCGGCGACCTTGATTACGTCCGGGGACAAGTATGTCGCTCCCCC
CGGGAGGGGTGCAGCCACATGGGAGATTCAAAGTTTCTCGTGACGTCGTTGTGATCACTGCAGCCTAG
CCGAGACTCCCGTACTACGCGAAGGTTGGTTATGTTAACCACTACAACGTGAGGCGCCGTAAGGGCCA
GTGTTGTGCCCGGCTCTTCAATGCGCCTTAGCGGCCTGATACACCCACCCAAGGAGATACTGCTAATC
ATATGGGTGGACAGAACCTCGCAACGCAGACGCCTCCCCGGCATGATATGGTTTTTTTCCGCTATTAT
CCAGCACGCAGCGCTATCATTTAAGAGAACCCAGTGACGCGTAAATCGTAAGATCTACCTGCCGCAGG
TGGACCTACTGCAAATACGGATTATGACTCGTAAAGGGGTCATGCGTATTTTCATCACTAGGCACGTT
CGAGAATAAATTAGTAGGTGTCCCGAGCCNTGTGGCGTTCCGCCTGACTCCTCATGAAGTCGACCTTC
TCACCGGCCCTATCTGCCGACGTAAGTCATAACCTAGATCTGTACCTCGGGGGGAGGGTCACTGTAAA
GGGATAATTGGAGGGTGATTTCCCACATTTTCCTAAGGGTACTTTTTGCCTGGCTTCGCAATTGGGTC
CAATGGATGTCGATCTCTGGTTTAGCAGTTGTGAAAGTGGCAAGGCGGGAGGTTAGACCTCCATTTAA
CATATACAAGCAAGTTAACTGCACTAGATGTGTAGACACTACAGGTACAGGAGTAGCCGGATAAGTCT
CCGACGTCAAGCGAATAAGCGTCATACGCGATTATCGCCTAAGAGCACGTATTTGCGGTAAAAGGCTA
GCTAGACGCTTGTGGGTAGATTTCAAGGCGTTCGTAGTGGTATAATAGGATACTCTTTCACCAGCCTG
AGGGCCGAACGCTATACTAGTGGTCTGTGATGTAGGACCGAGTATCTCTCTAGGGACCATCTACTTGA
GCAATGGTGCGCAGGGGGAGACATAGACCAGCCTTGGGTGGCAAGCACTGCAATAAGTCCTGTTTAGC
CTTGGAGTTCACACGCCGGCACTGAAGCCGACCTACCTGAGCGTGTGATTACCGTTAAAGCATCTGTC
TAGTTCTGTTTCCGGCGCTCTTGGTTCCAGGTTAGGGGAAGTGTATGACCCATGTGTTTTTATCGGCT
TAACCACGAGTGATCCCCGGTCGTTTCCCCATTGAATCCCTGGTGCATCCTACTCCCAGAATGATAGC
TGACTGACTGGACTGGCTTTTCAAGTAATCGAGGGGGTATCGCGGTCACGGCCGTTAACAGATCCCGT
CCTTAGTGTGGAATCCGCACCTGCTGACTAACGCTTCGCCGGCGTGTCTGCACAGCCGTATAGTGTTA
ATCATGACCCCAAGGAAGGATTAAACAAATATCTTGACG
>RAGLN08
GTGTCCGGTAGCCCGCGCTAGTTAGACACCCCGGCAGGGGGGATTGCTTTCGAGACGGGAGATCCCTT
CGCCGACCCTGTAGGGCCGACGCCGAGGCATTCGGGCCCCTGCAACGTCAACAGCGGCAAGAAAACGG
GATGAATGGGCGTAATGGGGGGGGTCTGCTGGGGACCCGACGCGGTTGCCGTTTGCGGGGCCCCCGAC
CCATACCGACCCACCTAGGCGTCCAGTTACGGCGCACGTCGGGAGCGTGGTTGCCGTCAGAGCTGTGT
TTCTCGATCAGTCCCCCGCAGTGCCGCAGTATCTTGCCGTGGGCTGCTTTAATCTTGAAAGTGGTTTA
TACATTGGGCGACGAGGTGTCGGCTCTCATTGGGGGTAACCGACGGACACGTGCAGTCCCCTCCCCGG
GCAGGCAGAGGCGGGGCCCCCGCGCGCCGGCCCCGGCCCACAATCTTACCAGGGTCCTCAAAAGAGCC
TTTGCATGGTACCCTTCGTGAATGGTTGCTTAAGAGGTCCACCACGTAGTCCGGGCACGGTCAACTAA
ACACTCAAAACAGCGACGGCAAATAGAGGCACAAGGTCCAGGCCCTCACGGCACTAGTTGCGATGACC
CAACTCACGGGGGCAGTCCCCGGCGCAGCGACCTTGATTACGTCCGGGAACAAGTATGTCGTTTCCCC
CGGGAGGGGTGCAGCCACATGGGAGATTCAAAGTTTCTCGTGACGTCGTTGTGATCACTGCAGCCTAG
CCGAGACTCCCGTACTACGCGAAGGTTGGTTATGTTAACCACTACAACGTGAGGCGCCGTAAGGGCCA
GTGTTGTGCCCGGCTCTTCAATGCGCCTTAGCGGCCTGATACACCCACCCAAGGAGATACTGCTAATC
ATGTGGGTGGACAGAACCTCGCAACGCAGACGCATCCCCGGCATGATATGGTTTTTTTCCGCTATTAC
CCAGCACGCGGCGCTATCATTCAAGAGAACCCAGTGACGCGTAAATCGTCAGATCTACCTGCCGCAGG
TGGACCTACTGCAAATACGGATTATGACTCGTAAAGAGGTCATGCGTATTTTCATCACTAGGCACTTT
CGAGAGTAAATTAGTAGGTGTCCCGCGCCTTGTGGCGTTCCGCCTGGCTCCTCATGAAGTCGACCTTC

TCATCGGCCCTATTTGCCGACGTAAGTCATAATCCAGATCTTCACCTCGGAGGGAGGGTCACTGTAAA
GGGATAATTGGAGGGCGATTTCCCACACTTTCCTAAGGGTACTTTTTGCTTAGCTTCGCAGTTGGGTC
CAATAGATGTTGATCTCTGGTTTAGCAGTTGTGAAAGTGGCAAGGCGGGAGGTTAGGCCTCCATTTAA
CATATACAAGCAAGTTAACTGCACTAGATGTGTAGACACTACAGTTACAGGAGTAGCCGACTAAGTCT
CCGACGTCAAGCGAATAGGCGTCATACGCGATTATTGCCTAAGAGCACGTATTGGCGGTAAAAGGATA
ACTAGACGCTTGTGGGTAGATTTCAAGGCGCTCGTAGTGGTATAATAGGACACTCTTGCACCAGCCTG
AGAGCCGAACGCTATACTAGTGGTCTGTGATGTAGGACCAAGTAGCTCTCTAGGGACCATCTACTTGA
GCAATGGTGCGCAGGGGTAGACATAGACCAACCTTGGGTGGCAAGCACTGCAATAAGTCCTGTTTAGC
CTTGGAGTTCACACGCCGGCACTAAAGCCGACCTACCTGAGCTTGTGATTACCGTTAAAGCATCTGTC
TAGTTCTGTTTCCGGCGCTCTTGGTTCCATGTTAGGGGAAGTGTATGACCCATGTGTTTTTATCGGCT
TAACTACGAGTGATCCCCGGTCGTTTCCCCATTAAATCCCTGGTGCATCCTACTCCCATAATGATAGC
TGACTGGCTGGACTGGCTTTTCAAGTAGTCGAGGGGGTATCGCGGTCACGGCCGTTAACAGATCCCGT
CCTTAGTGTGGAATCCGCACCTGCTGACTAACGCTTCGCCGGCGTGTCTGCACATCCGTATAGTGTTA
ATCATGACCCCAAGGAAGGATTAAACAAATATCTTGACG
>RAGLN09
GTGTCCGGTAGCCCGCGCTAGTTAGACACCCCGGCAGGGGGGATTGCTTTCGGGACGGGAGATCCCTT
CGCCGACCCTGGAGGGCCGACGCCGAGGCATTCGGGCCCCTGCAACGTCAACAGCGGCAAGAAAACGG
GATGAATGGGCGTAATGGGGGGGGTCTGCTGGGGACCCGACGCGGTTGCCGTTTGCGGGGCCCCCGAC
CCATACCGACCCACCTAGGCGTCCAGTTACGGCGCACGTCGGGAGCGTGGTTGCCGTCAGAGCTGTGT
TTCTCGATCAGTCCCCCGCAGTGCCGCAGTATCTTGCCGTGGGCTGCTTTAATCTTGAAAGTGGTTNA
TACATTGGGCGACGAGGTGTCGACTCTCATTGGGGGTAACCGACGGGCACATGCAGTCCCCTCCCCGG
GCAGGCAGAGGCGGGGCCCCCGCGCGCCGGCCCCGGCCCACAATCTTACCAGGGTCCTCAAAGGAGCC
TTTGCATGGTACCCTTCGTGAATGGTTGCTTAAGAGGTCCACAACGTGGTCCGGGCACGGTCGACTAA
ACACTCAAAACAGCGACGGCAAATATAGGTACAAGGTCCAGGCCCTCACGGCACTAATTGCGATGACC
CAACTCACGGGGGCAGTCCCCGGCGCGGCGACCTTGATTACGTCCGGGGACAAGTATGTCGCTCCCCC
CGGGAGGGGTGCAGCCACATGGGAGATTCAAAGTTTCTCGTGACGTCGTTGTGATCACTGCAGCCTAG
CCGAGACTCCCGTACTACGCGAAGNTTGGTTATGTTAACCACTACAACGTGAGGCGCCGTAAGGGCCA
GTGTTGTGCCCGGCTCTTCAATGCGCCTTAGCGGCCTGATACACCCACCCAAGGAGATACTGCTAATC
ATATGGGTGGACAGAACCTCGCAACGCAGACGCCTCCCCGGCATGATATGGTTTTTTTCCGCTATTAT
CCAGCACGCAGCGCTATCATTCAAGAGAACCCAGTGACGCGTAAATCGTAAGATCTACCTGCCGCAGG
TGGACCTACTGCAAATACGGATTATGACTCGTAAAGGGGTCATGCGTATTTTCATCACTAGGCACGTT
CGAGAATAAATTAGTAGGTGTCCCGAGCCTTGTGGCGTTCCGCCTGACTCCTCATGAAGTCGACCTTC
TCACCGGCCCTATCTGCCGACGTAAGTCATAACCTAGATCTGTACCTCGGGGGGAGGGTCACTGTAAA
GGGATAATTGGAGGGTGATTTCCCACACTTTCCTAAGGGTACTTTTTGCCTGGCTTCGCAATTGGGTC
CAATGGATGTCGATCTCTGGTTTAGCAGTTGTGAAAGTGGCAAGGCGGGAGGTTAGACCTCCATTTAA
CATATACAAGCAAGTTAACTGCACTAGATGTGTAGACACTACAGTTACAGGAGTAGCCGAATAAGTCT
CCGACGTCAAGCGAATAAGCGTCATACGCGATTATCGCCTAAGAGCACGTATTGGCGGTAAAAGGCTA
GCTAGACGCTTATGGGTAGATTTCAAGGCGTTCGTAGTGGTATAATAGGATACTCTTTCACCAGCCTG
AGGGCCGAACGCTATACTAGTGGTCTGTGATGTAGGACCGAGTATCTCTCTAGGGACCATCTACTTGA
GCAATGGTGCGCAGGGGGAGACATAGACCAGCCTTGGGTGGCAAGCACTGCGATAAGTCCTGTTTAGC
CTTGGAGTTCACATGCCGGCACTGAAGCCGACCTACCTGAGCGTGTGATTACCGTTAAAGCATCTGTC
TAGTTCTGTTTCCGGCGCTCTTGGTTCCAGGTTAGGGGAAGTGTATGACCCATGTGTTTTTATCGGCT
TAACCACGAGTGATCCCCGGTCGTTTCCCCATTGAATCCCTGGTGCATCCTACTCCCAGAATGATAGC
TGACTGACTGGACTGGCTTTTCAAGTAATCGAGGGGGTATCGCGGTCACGGCCGTTAACAGATCCCGT
CCTTAGTGTGGAATCCGCACCTGCTGACTAACGCTTCGCCGGCGTGTCTGCACAGCCGTATAGTGTTA
ATCATGACCCCAAGGAAGGATTAAACAAATATCTTGACG
>RAGLN10
GTGTCCGGTAGCCCGCGCTAGTTAGATACCCCGGCAGGGGGGATTGCTTTCGAGACGGGAGATCCCTT
CGCCGACCCTGGAGGGCCGACGCCGAGGCATTCGGGCCCCTGCAACGTCAACAGCGGCAAGAAAACGG
GATGAATGGGCGTAATGGGGGGGGTCTGCTGGGGACCCGACGCGGTTGCCGTTTGCGGGGCCCCCGAC
CCATACCGACCCACCTAGGCGTCCAGTTACGGCGCACGTCGGGAGCGTGGTTGCCGTCAGAGCTGTGT
TTCTCGATCAGTCCCCCGCAGTGCCGCAGTATCTTGCCGTGGGCTGCTTTAANNTTGAAAGTGGTTCA
TACATTGGGCGACGAGGTGTCGACTCTCATTGGGGGTAACCGACGGGCACATGCAGTCCCCTCCCCGG
GCAGGCAGAGGCGGGGCCCCCGCGCGCCGGCCCCGGCCCACAATCTTACCAGGGTCCTCAAGGGAGCC

TTTGCATGGTACCCTTCGTGAATGGTTGCTTAAGAGGTCCACCACGTGGTCCGGGCACGGTCGACTAA
ACACTCAAAACAGCGACGGCAAATATAGGCACAAGGTCCAGGCTCTCACGGCACTAATTGCGATGACC
CAACTCACGGGGGCAGTCCCCGGCGCGGCGACCGTGATTACGGCCGGGGATAAGTATGTCGTTCCCCC
CGGGAGGGGTGCAGCCACATGGGAGATTCAAAGTTTCTCGTGACGTCGTTGTGATCACTGCAGCCTAG
CCGAGACTCCCGTGCTACGCGAAGGTTGGTTATGTTAACCACTACAACGTGAGGCGCCGTAAGGGCCA
GTGTTGTGCCCGGCTCTTCAATGCGCCTTAGCGGCCTGATACACCCACCAAAGGAGATACTGCTAATC
ATGTGGGTGGACAGAAACTCGCAACACAGACGCCTCCCCGGCATGATATGGTTTTTTTCCGCTATTAT
CCAGCACGCAGCGCTATCATTCAAGAGAACCCAGTGACGCGTAAATCGTAANATCTACCTGCCGCAGG
TGGACCTCCTGCAAATACGGATTATGACTCGTAAAGAGGTCATGCGTATTTTCATCACTAGGCACGTT
CGAGAATAAATTAGTAGGTGTCCCGCGCCTTGTGGCGTTCCGCCTGACTCCTCATGAAGTCGACCTTC
TCACCGGCCCTATCTGCCGACGTAAGTCATAACCTAGATCTGTACCTCGGGGGGAGGGTCACTGTAAA
GGGATAATTGGAGGGTGATTTCCCACACTTTCCTAAGGGTACTTTTTGCCTGGCTTCGCAATTGGGTC
CAATGGATGTCGCTCTCTGGTTTAGCAGTTGTGAAAGTGGCAAGGCGGGAGGTTAGACCTCCATTTAA
CATATACAAGCAAGTTAACTGCACTAGATGTGTAGACACTACAGTTACAGGAGTAGCCGAATNAGTCT
CCGACGNCAAGCGAATAAGCGTCATACGCGATTATCGCCTAAGAGCACGTATTGGCGGTAAAAGGCTA
ACTAGACGCTTGTGGGTAGATTTCAAGGCGTTCGTAGTGGTATAACAGGATACTCTTTCACCAGCCTG
AGGGCCGAACGCTATACTAGTGGTCTGTGATGTAGGACCGAGTATCTCTCTAGGGACCATCTACTTGA
GCAATGGTGCGCAGGGGGAGACATAGACCAGCCTTGGGTGGCAAGCACTGCAATAAGTCCTGTTTAGC
CTTGGAGTTCACACGCCGGCACTGAAGCCGACCTACCTGAGCTTGTGATTACCGTTAAAGCATCTGTC
TAGTTCTGTTTCCGGCGCTCTTGGTTCCAGGTTAGGGGAAGTGTATGACCCATGTGTTTTTATCGGCT
TAACCACGAGTGATCCCCGGTCGTTTCCCCATTGAATCCCTGGTGCATCCTACTCCCAGAATGATAGC
TGACTGACTGGACTGGCTTTTCAAGTAGTCGAGGGGGTATCGCGGTCACGGCCGTTAACAGATCCCGT
CCTTAGTGTGGAATCCGCACCTGCTGACTAACGCTTCGCCGGCGTGTCTGCACAGCCGTATAGTGTTN
ATCATGACCCCAAGGAAGGATTAAACAAATATCTTGACG
>RAGLN11
GTGTCCGGTAGCCCGCGCTAGTTAGACACCCCGGCAGGGGGGATTGCTTTCGAGACGGGAGATCCCTT
CGCCGACCCTGGAGGGCCGACGCCGAGGCATTCGGGCCCCTGCAACGTCAACAGCGGCAAGAAAACGG
GATGAATGGGCGTAATGGGGGGGGTCTGCTGGGGACCCGACGCGGTTGCCGTTTGCGGGGCCCCCGAC
CCATACCGACCCACCTAGGCGTCCAGTTACGGCGCACGTCGGGAGCGTGGTTGCCGTCAGAGCTGTGT
TTCTCGATCAGTCCCCCGCAGTGCCGCAGTATCTTGCCGTGGGCTGCTTTAATCTTGAAAGTGGTTTA
TACATTGGGCGACGAGGTGTCGACTCTCATTGGGGGTAACCGACGGGCACATGCAGTCCCCTCCCCGG
GCAGGCAGAGGCGGGGCCCCCGCGCGCCGGCCCCGGCCCACAATCTTACCAGGGTCCTCAAAGGAGCC
TTTGCATGGTACCCTTCGTGAATGGTTGCTTAAGAGGTCCACAACGTGGTCCGGGCACGGTCGACTAA
ACACTCAAAACAGCGACGGCAAATATAGGTACAAGGTCCAGGCCCTCACGGCACTAATTGCGATGACC
CAACTCACGGGGGCAGTCCCCGGCGCGGCGACCTTGATTACGTCCGGGGACAAGTATGTCGCTCCCCC
CGGGAGGGGTGCAGCCACATGGGAGATTCAAAGTTTCTCGTGACGTCGTTGTGATCACTGCAGCCTAG
CCGAGACTCCCGTACTACGCGAAGGTTGGTTATGTTAACCACTACAACGTGAGGCGCCGTAAGGGCCA
GTGTTGTGCCCGGCTCTTCAATGCGCCTTAGCGGCCTGATACACCCACCCAAGGAGATACTGCTAATC
ATATGGGTGGACAGAACCTCGCAACGCAGACGCCTCCCCGGCATGATATGGTTTTTTTCCGCTATTAT
CCAGCACGCAGCGCTATCATTCAAGAGAACCCAGTGACGCGTAAATCGTAAGATCTACCTGCCGCAGG
TGGACCTACTGCAAATACGGATTATGACTCGTAAAGGGGTCATGCGTATTTTCATCACTAGGCACGTT
CGAGAATAAATTAGTAGGTGTCCCGAGCCTTGTGGCGTTCCGCCTGACTCCTCATGAAGTCGACCTTC
TCACCGGCCCTATCTGCCGACGTAAGTCATAACCTAGATCTGTACCTCGGGGGGAGGGTCACTGTAAA
GGGATAATTGGAGGGTGATTTCCCACACTTTCCTAAGGGTACTTTTTGCCTGGCTTCGCAATTGGGTC
CAATGGATGTCGATCTCTGGTTTAGCAGTTGTGAAAGTGGCAAGGCGGGAGGTTAGACCTCCATTTAA
CATATACAAGCAAGTTAACTGCACTAGATGTGTAGACACTACAGTTACAGGAGTAGCCGAATAAGTCT
CCGACGTCAAGCGAATAAGCGTCATACGCGATTATCGCCTAAGAGCACGTATTGGCGGTAAAAGGCTA
GCTAGACGCTTATGGGTAGATTTCAAGGCGTTCGTAGTGGTATAATAGGATACTCTTTCACCAGCCTG
AGGGCCGAACGCTATACTAGTGGTCTGTGATGTAGGACCGAGTATCTCTCTAGGGACCATCTACTTGA
GCAATGGTGCGCAGGGGGAGACATAGACCAGCCTTGGGTGGCAAGCACTGCAATAAGTCCTGTTTAGC
CTTGGAGTTCACATGCCGGCACTGAAGCCGACCTACCTGAGCGTGTGATTACCGTTAAAGCATCTGTC
TAGTTCTGTTTCCGGCGCTCTTGGTTCCAGGTTAGGGGAAGTGTATGACCCATGTGTTTTTATCGGCT
TAACCACGAGTGATCCCCGGTCGTTTCCCCATTGAATCCCTGGTGCATCCTACTCCCAGAATGATAGC
TGACTGACTGGACTGGCTTTTCAAGTAATCGAGGGGGTATCGCGGTCACGGCCGTTAACAGATCCCGT

CCTTAGTGTGGAATCCGCACCTGCTGACTAACGCTTCGCCGGCGTGTCTGCACAGCCGTATAGTGTTA
ATCATGACCCCAAGGAAGGATTAAACAAATATCTTGACG
>RAGLN12
GTGTCCGGTAGCCCGCGCTAGTTAGACACCCCGGCAGGGGGGATTGCTTTCGAGACGGGAGATCCCTT
CGCCGACCCTGGAGGGCCGACGCCGAGGCATTCGGGCCCCTGCAACGTCAACAGCGGCAAGAAAACGG
GATGAATGGGCGTAATGGGGGGGGTCTGCTGGGGACCCGACGCGGTTGCCGTTTGCGGGGCCCCCGAC
CCATACCGACCCACCTAGGCGTCCAGTTACGGCGCACGTCGGGAGCGTGGTTGCCGTCAGAGCTGTGT
TTCTCGATCAGTCCCCCGCAGTGCCGCAGTATCTTGCCGTGGGCTGCTTTAATCTTGAAAGTGGTTCA
TACATTGGGCGACGAGGTGTCGACTCTCATTGGGGGTAACCGACGGGCACATGCAGTCCCCTCCCCGG
GCAGGCAGAGGCGGGGCCCCCGCGCNCCGGCCCCGGCCCACAATCTTACCAGGGTCCTCAAAGGAGCC
TTTGCATGGTACCCTTCGTGAATGGTTGCTTAAGAGGTCCACAACGTGGTCCGGGCACGGTCGACTAA
ACACTCAAAACAGCGACGGCAAATATAGGTACAAGGTCCAGGCCCTCACGGCACTAATTGCGATGACC
CAACTCACGGGGGCAGTCCCCGGCGCGGCGACCTTGATTACGTCCGGGGACAAGTATGTCGCTCCCCC
CGGGAGGGGTGCAGCCACATGGGAGATTCAAAGTTTCTCGTGACGTCGTTGTGATCACTGCAGCCTAG
CCGAGACTCCCGTACTACGCGAAGGTTGGTTATGTTAACCACTACAACGTGAGGCGCCGTAAGGGCCA
GTGTTGTGCCCGGCTCTTCAATGCGCCTTAGCGGCCTGATACACCCACCCAAGGAGATACTGCTAATC
ATATGGGTGGACAGAACCTCGCAACGCAGACGCCTCCCCGGCATGATATGGTTTTTTTCCGCTATTAT
CCAGCACGCAGCGCTATCATTCAAGAGAACCCAGTGACGCGTAAATCGTAAGATCTACCTGCCGCAGG
TGGACCTACTGCAAATACGGATTATGACTCGTAAAGGGGTCATGCGTATTTTCATCACTAGGCACGTT
CGAGAATAAATTAGTAGGTGTCCCGAGCCTTGTGGCGTTCCGCCTGACTCCTCATGAAGTCGACCTTC
TCACCGGCCCTATCTGCCGACGTAAGTCATAACCTAGATCTGTACCTCGGGGGGAGGGTCACTGTAAA
GGGATAATTGGAGGGTGATTTCCCACACTTTCCTAAGGGTACTTTTTGCCTGGCTTCGCAATTGGGTC
CAATGGATGTCGATCTCTGGTTTAGCAGTTGTGAAAGTGGCAAGGCGGGAGGTTAGACCTCCATTTAA
CATATACAAGCAAGTTAACTGCACTAGATGTGTAGACACTACAGTTACAGGAGTAGCCGAATAAGTCT
CCGACGTCAAGCGAATAAGCGTCATACGCGATTATCGCCTAAGAGCACGTATTGGCGGTAAAAGGCTA
GCTAGACGCTTATGGGTAGATTTCAAGGCGTTCGTAGTGGTATAATAGGATACTCTTTCACCAGCCTG
AGGGCCGAACGCTATACTAGTGGTCTGTGATGTAGGACCGAGTATCTCTCTAGGGACCATCTACTTGA
GCAATGGTGCGCAGGGGGAGACATAGACCAGCCTTGGGTGGCAAGCACTGCAATAAGTCCTGTTTAGC
CTTGGAGTTCACATGCCGGCACTGAAGCCGACCTACCTGAGCGTGTGATTACCGTTAAAGCATCTGTC
TAGTTCTGTTTCCGGCGCTCTTGGTTCCAGGTTAGGGGAAGTGTATGACCCATGTGTTTTTATCGGCT
TAACCACGAGTGATCCCCGGTCGTTTCCCCATTGAATCCCTGGTGCATCCTACTCCCAGAATGATAGC
TGACTGACTGGACTGGCTTTTCAAGTAATCGAGGGGGTATCGCGGTCACGGCCGTTAACAGATCCCGT
CCTTAGTGTGGAATCCGCACCTGCTGACTAACGCTTCGCCGGCGTGTCTGCACAGCCGTATAGTGTTA
ATCATGACCCCAAGGAAGGATTAAACAAATATCTTGACG
>RAGLN14
GTGTCCGGTAGCCCGCGCTAGTTAGACACCCCGGCAGGGGGGATTGCTTTCGGGACGGGAGATCCCTT
CGCCGACCCTGTAGGGCCGACGCCGAGGCATTCGGGCCCCTGCAACGTCAACAGCGGCAAGAAAACGG
GATGAATGGGCGTAATGGGGGGGGTCTGCTGGGGACCCGACGCGGTTGCCGTTTGCGGGGCCCCCGAC
CCATACCGACCCACCTAGGCGTCCAGTTACGGCGCACGGCGGGAGCGTGGTTGCCGTCAGAGCTGTGT
TTCTCGATCAGTCCCCCGCAGTGCCGCAGTATCTTGCCGTGGGCTGCTTTAATCTTGAAAGTGGTTCA
TACATTGGGCGACGAGGTGTCGGCTCTCATTGGGGGTAGCCGACGGACACATGCAGTCCCCTCCCCGG
GCAGGCAGAGGCGGGGCCCCCGCGCGCCGGCCCCGGCCCACAATCTTACCAGGGTCCTCAAAAGAGCC
TTTGCATGGTACCCTTCGTGAATGGTTGCTTAAGAGGTCCACCACGTAGTCCGGGCACGGTCGACTAA
ACACTCAAAACAGCGACGGCAAATAGAGGCACAAGGTCCAGGCCCTCACGGCACTAGTTGCGATGACC
CAACTCACGGGGGCAGTCCCCGGCGCGGCGACCTTGATTACGTCCGGGAACAAGTATGTCGTTTCCCC
CGGGAGGGGTGCAGCCACATGGGAGATTCAAAGTTTCTCGTGACGTCGTTGTGATCACTGCAGCCTAG
CCGAGACTCCCGTACTACGCGAAGGTTGGTTATGTTAACCACTACAACGTGAGGCGCCGTAAGGGCCA
GTGTTGTGCCCGGCTCTTCAATGCGCCTTAGCGGCCTGATACACCCACCCAAGGAGATACTGCTAATC
ATGTGGGTGGACAGAACCTCGCAACGCAGACGCATCCCCGGCATGATATGGTTTTTTTCCGCTATTAC
CCAGCACGCGGCGCTATCATTCAAGAGAACCCAGTGACGCGTAAATCGTAAGATCTACCTGCCGCAGG
TGGACCTACTGCAAATACGGATTATGACTCGTAAAGAGGTCATGCGTATTTTCATCACTAGGCACGTT
CGAGAGTAAATTAGTAGGTGTCCCGCGCCTTGTGGCGTTCCGCCTGACTCCTCATGAAGTCGACCTTC
TCATCGGCCCTATCTGCCGACGTAAGTCATAATCCAGATCTTCACCTCGGAGGGAGGGTCACTGTAAA
GGGATAATTGGAGGGCGATTTCCCACACTTTCCTAAGGGTACTTTTTGCTTGGCTTCGCAGTTGGGTC

CAATAGATGTTGATCTCTGGTTTAGCAGTTGTGAAAGTGGCAAGGCGGGAGGTTAGACCTCCATTTAA
CATATACAAGCAAGTTAACTGCACTAGATGTGTAGACACTACAGTTACAGGAGTAGCCGACTAAGTCT
CCGACGTCAAGCGAATAGGCGTCATACGCGATTATCGCCTAAGAGCACGTATTGGCGGTAAAAGGATA
ACTAGACGCTTGTGGGTAGATTTCAAGGCGCTCGTAGTGGTATAATAGGATACTCTTTCACCAGCCTG
AGAGCCGAACGCTATACTAGTGGTCTGTGATGTAGGACCAAGTAGCTCTCTAGGGACCATCTACTTGA
GCAATGGTGCGCAGGGGTAGACATAGACCAACCTTGGGTGGCAAGCACTGCAATAAGTCCTGTTTAGC
CTTGGAGTTCACACGCCGGCACTAAAGCCGACCTACCTGAGCTTGTGATTACCGTTAAAGCATCTGTC
TAGTTCTGTTTCCGGCGCTCTTGGTTCCATGTTAGGGGAAGTGTATGACCCATGTGTTTTTATCGGCT
TAACTACGAGTGATCCCCGGTCGTTTCCCCATTAAATCCCTGGTGCATCCTACTCCCATAATGATAGC
TGACTGGCTGGACTGGCTTTTCAAGTAGTCGAGGGGGTATCGCGGTCACGGCCGTTAACAGATCCCGT
CCTTAGTGTGGAATCCGCACCTGCTGACTAACGCTTCGCCGGCGTGTCTGCACATCCGTATAGTGTTA
ATCATGACCCCAAGGAAGGATTAAACAAATATCTTGACG
>RAGLN16
GTGTCCGGTAGCCCGCGCTAGTTAGACACCCCGGCAGGGGGGATTGCTTTCGAGACGGGAGATCCCTT
CGCCGACCCTGGAGGGCCGACGCCGAGGCATTCGGGCCCCCGCAACGTCAACAGCGGCAAGAAAACGG
GATGAATGGGCGTAATGGGGGGGGTCTGCTGGGGACCCGACGCGGTTGCCGTTTGCGGGGCCCCCGAC
CCATACCGACCCACCTAGGCGTCCAGTTACGGCGCACGGCGGGAGCGTGGTTGCCGTCAGAGCTGTGT
TTCTCGATCAGTCCCCCGCAGTGCCGCAGTATCTTGCCGTGGGCTGCTTTAATCTTGAAAGTGGTTTA
TACATTGGGCGACGAGGTGTCGACTCTCATTGGGGGTAACCGACGGGCACATGCAGTCCCCTCCCCGG
GCAGGCAGAGGCGGGGCCCCCGCGCGCCGGCCCCGGCCCACAATCTTACCAGGGTCCTCAAAGGAGCC
TTTGCATGGTACCCTTCGTGAATGGTTGCTTAAGAGGTCCACAACGTGGTCCGGGCACGGTCGACTAA
ACACTCAAAACAGCGACGGCAAATATAGGTACAAGGTCCAGGCCCTCACGGCACTAATTGCGATGACC
CAACTCACGGGGGCAGTCCCCGGCGCGGCGACCTTGATTACGTCCGGGGACAAGTATGTCGCTCCCCC
CGGGAGGGGTGCAGCCACATGGGAGATTCAAAGTTTCTCGTGACGTCGTTGTGATCACTGCAGCCTAG
CCGAGACTCCCGTACTACGCGAAGGTTGGTTATGTTAACCACTACAACGTGAGGCGCCGTAAGGGCCA
GTGTTGTGCCCGGCTCTTCAATGCGCCTTAGCGGCCTGATACACCCACCCAAGGAGATACTGCTAATC
ATATGGGTGGACAGAACCTCGCAACGCAGACGCCTCCCCGGCATGATATGGTTTTTTTCCGCTATTAT
CCAGCACGCAGCGCTATCATTCAAGAGAACCCAGTGACGCGTAAATCGTAAGATCTACCTGCCGCAGG
TGGACCTACTGCAAATACGGATTATGACTCGTAAAGGGGTCATGCGTATTTTCATCACTAGGCACGTT
CGAGAATAAATTAGTAGGTGTCCCGAGCCTTGTGGCGTTCCGCCTGACTCCTCATGAAGTCGACCTTC
TCACCGGCCCTATCTGCCGACGTAAGTCATAACCTAGATCTGTACCTCGGGGGGAGGGTCACTGTAAA
GGGATAATTGGAGGGTGATTTCCCACACTTTCCTAAGGGTACTTTTTGCCTGGCTTCGCAATTGGGTC
CAATGGATGTCGATCTCTGGTTTAGCAGTTGTGAAAGTGGCAAGGCGGGAGGTTAGACCTCCATTTAA
CATATACAAGCAAGTTAACTGCACTAGATGTGTAGACACTACAGTTACAGGAGTAGCCGAATAAGTCT
CCGACGTCAAGCGAATAAGCGTCATACGCGATTATCGCCTAAGAGCACGTATTGGCGGTAAAAGGCTA
GCTAGACGCTTATGGGTAGATTTCAAGGCGTTCGTAGTGGTATAATAGGATACTCTTTCACCAGCCTG
AGGGCCGAACGCTATACTAGTGGTCTGTGATGTAGGACCGAGTATCTCTCTAGGGACCATCTACTTGA
GCAATGGTGCGCAGGGGGAGACATAGACCAGCCTTGGGTGGCAAGCACTGCGATAAGTCCTGTTTAGC
CTTGGAGTTCACATGCCGGCACTGAAGCCGACCTACCTGAGCGTGTGATTACCGTTAAAGCATCTGTC
TAGTTCTGTTTCCGGCGCTCTTGGTTCCAGGTTAGGGGAAGTGTATGACCCATGTGTTTTTATCGGCT
TAACCACGAGTGATCCCCGGTCGTTTCCCCATTGAATCCCTGGTGCATCCTACTCCCAGAATGATAGC
TGACTGACTGGACTGGCTTTTCAAGTAATCGAGGGGGTATCGCGGTCACGGCCGTTAACAGATCCCGT
CCTTAGTGTGGAATCCGCACCTGCTGACTAACGCTTCGCCGGCGTGTCTGCACAGCCGTATAGTGTTA
ATCATGACCCCAAGGAAGGATTAAACAAATATCTTGACG
>RAGLN17
GTGTCCGGTAGCCCGCGCTAGTCAGACACCCCGGCAGGGGGGATTGCTTTCGGGACGGGAGATCCCTT
CGCCGACCCTGGAGGGCCGACGCCGAGGCATTCGGGCCCCTGCAACGTCAACAGCGGCAAGAAAACGG
GATGAATGGGCGTAATGGGGGGGGTCTGCTGGGGACCCGACGCGGTTGCCGTTTGCGGGGCCCCCGAC
CCATACCGACCCACCTAGGCGTCCAGTTACGGCGCACGGCGGGAGCGTGGTTGCCGTCAGAGCTGTGT
TTCTCGATCAGTCCCCCGCAGTGCCGCAGTATCTTGCCGTGGGCTGCTTTAATCTTGAAAGTGGTTCA
TACATTGGGCGACGAGGTGTCGACTCTCATTGGGGGTAACCGACGGGCACATGCAGTCCCCTCCCCGG
GCAGGCAGAGGCGGGGCCCCCGCGCGCCGGCCCCGGCCCACAATCTTACCAGGGTCCTCAAAGGAGCC
TTTGCATGGTACCCTTCGTGAATGGTTGCTTAAGAGGTCCACAACGTGGTCCGGGCACGGTCGACTAA
ACACTCAAAACAGCGACGGCAAATATAGGTACAAGGTCCAGGCCCTCACGGCACTAATTGCGATGACC

CAACTCACGGGGGCAGTCCCCGGCGCGGCGACCTTGATTACGTCCGGGGACAAGTATGTCGCTCCCCC
CGGGAGGGGTGCAGCCACATGGGAGATTCAAAGTTTCTCGTGACGTCGTTGTGATCACTGCAGCCTAG
CCGAGACTCCCGTACTACGCGAAGGTTGGTTATGTTAACCACTACAACGTGAGGCGCCGTAAGGGCCA
GTGTTGTGCCCGGCTCTTCAATGCGCCTTAGCGGCCTGATACACCCACCCAAGGAGATACTGCTAATC
ATATGGGTGGACAGAACCTCGCAACGCAGACGCCTCCCCGGCATGATATGGTTTTTTTCCGCTATTAT
CCAGCACGCAGCGCTATCATTTAAGAGAACCCAGTGACGCGTAAATCGTAAGATCTACCTGCCGCAGG
TGGACCTACTGCAAATACGGATTATGACTCGTAAAGGGGTCATGCGTATTTTCATCACTAGGCACGTT
CGAGAATAAATTAGTAGGTGTCCCGAGCCTTGTGGCGTTCCGCCTGACTCCTCATGAAGTCGACCTTC
TCACCGGCCCTATCTGCCGACGTAAGTCATAACCTAGATCTGTACCTCGGGGGGAGGGTCACTGTAAA
GGGATAATTGGAGGGTGATTTCCCACATTTTCCTAAGGGTACTTTTTGCCTGGCTTCGCAATTGGGTC
CAATGGATGTCGATCTCTGGTTTAGCAGTTGTGAAAGTGGCAAGGCGGGAGGTTAGACCTCCATTTAA
CATATACAAGCAAGTTAACTGCACTAGATGTGTAGACACTACAGGTACAGGAGTAGCCGGATAAGTCT
CCGACGTCAAGCGAATAAGCGTCATACGCGATTATCGCCTAAGAGCACGTATTTGCGGTAAAAGGCTA
GCTAGACGCTTGTGGGTAGATTTCAAGGCGTTCGTAGTGGTATAATAGGATACTCTTTCACCAGCCTG
AGGGCCGAACGCTATACTAGTGGTCTGTGATGTAGGACCGAGTATCTCTCTAGGGACCATCTACTTGA
GCAATGGTGCGCAGGGGGAGACATAGACCAGCCTTGGGTGGCAAGCACTGCAATAAGTCCTGTTTAGC
CTTGGAGTTCACACGCCGGCACTGAAGCCGACCTACCTGAGCGTGTGATTACCGTTAAAGCATCTGTC
TAGTTCTGTTTCCGGCGCTCTTGGTTCCAGGTTAGGGGAAGTGTATGACCCATGTGTTTTTATCGGCT
TAACCACGAGTGATCCCCGGTCGTTTCCCCATTGAATCCCTGGTGCATCCTACTCCCAGAATGATAGC
TGACTGACTGGACTGGCTTTTCAAGTAATCGAGGGGGTATCGCGGTCACGGCCGTTAACAGATCCCGT
CCTTAGTGTGGAATCCGCACCTGCTGACTAACGCTTCGCCGGCGTGTCTGCACAGCCGTATAGTGTTA
ATCATGACCCCAAGGAAGGATTAAACAAATATCTTGACG
>RAGLN18
GTGTCCGGTAGCCCGCGCTAGTTAGACACCCCGGCAGGGGGGATTGCTTTCGAGACGGGAGATCCCTT
CGCCGACCCTGGAGGGCCGACGCCGAGGCATTCGGGCCCCNGCAACGTCAACAGCGGCAAGAAAACGG
GATGAATGGGCGTAATGGGGGGGGTCTGCTGGGGACCCGACGCGGTTGCCGTTTGCGGGGCCCCCGAC
CCATACCGACCCACCTAGGCGTCCAGTTACGGCGCACGGCGGGAGCGTGGTTGCCGTCAGAGCTGTGT
TTCTCGATCAGTCCCCCGCAGTGCCGCAGTATCTTGCCGTGGGCTGCTTTAATCTTGAAAGTGGTTTA
TACATTGGGCGACGAGGTGTCGACTCTCATTGGGGGTAACCGACGGGCACATGCAGTCCCCTCCCCGG
GCAGGCAGAGGCGGGGCCCCCGCGCGCCGGCCCCGGCCCACAATCTTACCAGGGTCCTCAAAGGAGCC
TTTGCATGGTACCCTTCGTGAATGGTTGCTTAAGAGGTCCACAACGTGGTCCGGGCACGGTCGACTAA
ACACTCAAAACAGCGACGGCAAATATAGGTACAAGGTCCAGGCCCTCACGGCACTAATTGCGATGACC
CAACTCACGGGGGCAGTCCCCGGCGCGGCGACCTTGATTACGTCCGGGGACAAGTATGTCGCTCCCCC
CGGGAGGGGTGCAGCCACATGGGAGATTCAAAGTTTCTCGTGACGTCGTTGTGATCACTGCAGCCTAG
CCGAGACTCCCGTACTACGCGAAGGTTGGTTATGTTAACCACTACAACGTGAGGCGCCGTAAGGGCCA
GTGTTGTGCCCGGCTCTTCAATGCGCCTTAGCGGCCTGATACACCCACCCAAGGAGATACTGCTAATC
ATATGGGTGGACAGAACCTCGCAACGCAGACGCCTCCCCGGCATGATATGGTTTTTTTCCGCTATTAT
CCAGCACGCAGCGCTATCATTCAAGAGAACCCAGTGACGCGTAAATCGTAAGATCTACCTGCCGCAGG
TGGACCTACTGCAAATACGGATTATGACTCGTAAAGGGGTCATGCGTATTTTCATCACTAGGCACGTT
CGAGAATAAATTAGTAGGTGTCCCGAGCCTTGTGGCGTTCCGCCTGACTCCTCATGAAGTCGACCTTC
TCACCGGCCCTATCTGCCGACGTAAGTCATAACCTAGATCTGTACCTCGGGGGGAGGGTCACTGTAAA
GGGATAATTGGAGGGTGATTTCCCACACTTTCCTAAGGGTACTTTTTGCCTGGCTTCGCAATTGGGTC
CAATGGATGTCGATCTCTGGTTTAGCAGTTGTGAAAGTGGCAAGGCGGGAGGTTAGACCTCCATTTAA
CATATACAAGCAAGTTAACTGCACTAGATGTGTAGACACTACAGTTACAGGAGTAGCCGAATAAGTCT
CCGACGTCAAGCGAATAAGCGTCATACGCGATTATCGCCTAAGAGCACGTATTGGCGGTAAAAGGCTA
GCTAGACGCTTATGGGTAGATTTCAAGGCGTTCGTAGTGGTATAATAGGATACTCTTTCACCAGCCTG
AGGGCCGAACGCTATACTAGTGGTCTGTGATGTAGGACCGAGTATCTCTCTAGGGACCATCTACTTGA
GCAATGGTGCGCAGGGGGAGACATAGACCAGCCTTGGGTGGCAAGCACTGCAATAAGTCCTGTTTAGC
CTTGGAGTTCACATGCCGGCACTGAAGCCGACCTACCTGAGCGTGTGATTACCGTTAAAGCATCTGTC
TAGTTCTGTTTCCGGCGCTCTTGGTTCCAGGTTAGGGGAAGNGTATGACCCATGTGTTTTTATCGGCT
TAACCACGAGTGATCCCCGGTCGTTTCCCCATTGAATCCCTGGTGCATCCTACTCCCAGAATGATAGC
TGACTGACTGGACTGGCTTTTCAAGTAATCGAGGGGGTATCGCGGTCACGGCCGTTAACAGATCCCGT
CCTTAGTGTGGAATCCGCACCTGCTGACTAACGCTTCGCCGGCGTGTCTGCACAGCCGTATAGTGTTA
ATCATGACCCCAAGGAAGGATTAAACAAATATCTTGACG

>RAGLN19
GTGTCCGGTAGCCCGCGCTAGTTAGACACCCCGGCAGGGGGGATTGCTTTCGAGACGGGAGATCCCTT
CGCCGACCCTGGAGGGCCGACGCCGAGGCATTCGGGCCCCTGCAACGTCAACAGCGGCAAGAAAACGG
GATGAATGGGCGTAATGGGGGGGGTCTGCTGGGGACCCGACGCGGTTGCCGTTTGCGGGGCCCCCGAC
CCATACCGACCCACCTAGGCGTCCAGTTACGGCGCACGTCGGGAGCGTGGTTGCCGTCAGAGCTGTGT
TTCTCGATCAGTCCCCCGCAGTGCCGCAGTATCTTGCCGTGGGCTGCTTTAATCTTGAAAGTGGTTTA
TACATTGGGCGACGAGGTGTCGACTCTCATTGGGGGTAACCGACGGGCACATGCAGTCCCCTCCCCGG
GCAGGCAGAGGCGGGGCCCCCGCGCGCCGGCCCCGGCCCACAATCTTACCAGGGTCCTCAAAGGAGCC
TTTGCATGGTACCCTTCGTGAATGGTTGCTTAAGAGGTCCACAACGTGGTCCGGGCACGGTCGACTAA
ACACTCAAAACAGCGACGGCAAATATAGGTACAAGGTCCAGGCCCTCACGGCACTAATTGCGATGACC
CAACTCACGGGGGCAGTCCCCGGCGCGGCGACCTTGATTACGTCCGGGGACAAGTATGTCGCTCCCCC
CGGGAGGGGTGCAGCCACATGGGAGATTCAAAGTTTCTCGTGACGTCGTTGTGATCACTGCAGCCTAG
CCGAGACTCCCGTACTACGCGAAGGTTGGTTATGTTAACCACTACAACGTGAGGCGCCGTAAGGGCCA
GTGTTGTGCCCGGCTCTTCAATGCGCCTTAGCGGCCTGATACACCCACCCAAGGAGATACTGCTAATC
ATATGGGTGGACAGAACCTCGCAACGCAGACGCCTCCCCGGCATGATATGGTTTTTTTCCGCTATTAT
CCAGCACGCAGCGCTATCATTCAAGAGAACCCAGTGACGCGTAAATCGTAAGATCTACCTGCCGCAGG
TGGACCTACTGCAAATACGGATTATGACTCGTAAAGGGGTCATGCGTATTTTCATCACTAGGCACGTT
CGAGAATAAATTAGTAGGTGTCCCGAGCCTTGTGGCGTTCCGCCTGACTCCTCATGAAGTCGACCTTC
TCACCGGCCCTATCTGCCGACGTAAGTCATAACCTAGATCTGTACCTCGGGGGGAGGGTCACTGTAAA
GGGATAATTGGAGGGTGATTTCCCACACTTTCCTAAGGGTACTTTTTGCCTGGCTTCGCAATTGGGTC
CAATGGATGTCGATCTCTGGTTTAGCAGTTGTGAAAGTGGCAAGGCGGGAGGTTAGACCTCCATTTAA
CATATACAAGCAAGTTAACTGCACTAGATGTGTAGACACTACAGTTACAGGAGTAGCCGAATAAGTCT
CCGACGTCAAGCGAATAAGCGTCATACGCGATTATCGCCTAAGAGCACGTATTGGCGGTAAAAGGCTA
GCTAGACGCTTATGGGTAGATTTCAAGGCGTTCGTAGTGGTATAATAGGATACTCTTTCACCAGCCTG
AGGGCCGAACGCTATACTAGTGGTCTGTGATGTAGGACCGAGTATCTCTCTAGGGACCATCTACTTGA
GCAATGGTGCGCAGGGGGAGACATAGACCAGCCTTGGGTGGCAAGCACTGCAATAAGTCCTGTTTAGC
CTTGGAGTTCACATGCCGGCACTGAAGCCGACCTACCTGAGCGTGTGATTACCGTTAAAGCATCTGTC
TAGTTCTGTTTCCGGCGCTCTTGGTTCCAGGTTAGGGGAAGTGTATGACCCATGTGTTTTTATCGGCT
TAACCACGAGTGATCCCCGGTCGTTTCCCCATTGAATCCCTGGTGCATCCTACTCCCAGAATGATAGC
TGACTGACTGGACTGGCTTTTCAAGTAATCGAGGGGGTATCGCGGTCACGGCCGTTAACAGATCCCGT
CCTTAGTGTGGAATCCGCACCTGCTGACTAACGCTTCGCCGGCGTGTCTGCACAGCCGTATAGTGTTA
ATCATGACCCCAAGGAAGGATTAAACAAATATCTTGACG
>RAGLN20
GTGTCCGGTAGCCCGCGCTAGTTAGATACCCCGGCAGGGGGGATTGCTTTCGGGACGGGAGATCCCTT
CGCCGACCCTGGAGGGCCGACGCCGAGGCATTCGGGCCCCTGCAACGTCAACAGCGGCAAGAAAACGG
GATGAATGGGCGTAATGGGGGGGGTCTGCTGGGGACCCGACGCGGTTGCCGTTTGCGGGGCCCCCGAC
CCATACCGACCCACCTAGGCGTCCAGTTACGGCGCACGGCGGGAGCGTGGTTGCCGTCAGAGCTGTGT
TTCTCGATCAGTCCCCCGCAGTGCCGCAGTATCTTGCCGTGGGCTGCTTTAANNTTGAAAGTGGTTCA
TACATTGGGCGACGAGGTGTCGACTCTCATTGGGGGTAACCGACGGGCACATGCAGTCCCCTCCCCGG
GCAGGCAGAGGCGGGGCCCCCGCGCGCCGGCCCCGGCCCACAATCTTACCAGGGTCCTCAAGGGAGCC
TTTGCATGGTACCCTTCGTGAATGGTTGCTTAAGAGGTCCACCACGTGGTCCGGGCACGGTCGACTAA
ACACTCAAAACAGCGACGGCAAATATAGGCACAAGGTCCAGGCTCTCACGGCACTAATTGCGATGACC
CAACTCACGGGGGCAGTCCCCGGCGCGGCGACCGTGATTACGGCCGGGGATAAGTATGTCGTTCCCCC
CGGGAGGGGTGCAGCCACATGGGAGATTCAAAGTTTCTCGTGACGTCGTTGTGATCACTGCAGCCTAG
CCGAGACTCCCGTGCTACGCGAAGGTTGGTTATGTTAACCACTACAACGTGAGGCGCCGTAAGGGCCA
GTGTTGTGCCCGGCTCTTCAATGCGCCTTAGCGGCCTGATACACCCACCAAAGGAGATACTGCTAATC
ATGTGGGTGGACAGAAACTCGCAACACAGACGCCTCCCCGGCATGATATGGTTTTTTTCCGCTATTAT
CCAGCACGCAGCGCTATCATTCAAGAGAACCCAGTGACGCGTAAATCGTAATATCTACCTGCCGCAGG
TGGACCTCCTGCAAATACGGATTATGACTCGTAAAGAGGTCATGCGTATTTTCATCACTAGGCACGTT
CGAGAATAAATTAGTAGGTGTCCCGCGCCTTGTGGCGTTCCGCCTGACTCCTCATGAAGTCGACCTTC
TCACCGGCCCTATCTGCCGACGTAAGTCATAACCTAGATCTGTACCTCGGGGGGAGGGTCACTGTAAA
GGGATAATTGGAGGGTGATTTCCCACACTTTCCTAAGGGTACTTTTTGCCTGGCTTCGCAATTGGGTC
CAATGGATGTCGCTCTCTGGTTTAGCAGTTGTGAAAGTGGCAAGGCGGGAGGTTAGACCTCCATTTAA
CATATACAAGCAAGTTAACTGCACTAGATGTGTAGACACTACAGTTACAGGAGTAGCCGAATAAGTCT

CCGACGNCAAGCGAATAAGCGTCATACGCGATTATCGCCTAAGAGCACGTATTGGCGGTAAAAGGCTA
ACTAGACGCTTGTGGGTAGATTTCAAGGCGTTCGTAGTGGTATAACAGGATACTCTTTCACCAGCCTG
AGGGCCGAACGCTATACTAGTGGTCTGTGATGTAGGACCGAGTATCTCTCTAGGGACCATCTACTTGA
GCAATGGTGCGCAGGGGGAGACATAGACCAGCCTTGGGTGGCAAGCACTGCAATAAGTCCTGTTTAGC
CTTGGAGTTCACACGCCGGCACTGAAGCCGACCTACCTGAGCTTGTGATTACCGTTAAAGCATCTGTC
TAGTTCTGTTTCCGGCGCTCTTGGTTCCAGGTTAGGGGAAGTGTATGACCCATGTGTTTTTATCGGCT
TAACCACGAGTGATCCCCGGTCGTTTCCCCATTGAATCCCTGGTGCATCCTACTCCCAGAATGATAGC
TGACTGACTGGACTGGCTTTTCAAGTAGTCGAGGGGGTATCGCGGTCACGGCCGTTAACAGATCCCGT
CCTTAGTGTGGAATCCGCACCTGCTGACTAACGCTTCGCCGGCGTGTCTGCACAGCCGTATAGTGTTN
ATCATGACCCCAAGGAAGGATTAAACAAATATCTTGACG
>RAGLN21
GTGTCCGGTAGCCCGCGCTAGTTAGACACCCCGGCAGGGGGGATTGCTTTCGAGACGGGAGATCCCTT
CGCCGACCCTGGAGGGCCGACGCCGAGGCATTCGGGCCCCTGCAACGTCAACAGCGGCAAGAAAACGG
GATGAATGGGCGTAATGGGGGGGGTCTGCTGGGGACCCGACGCGGTTGCCGTTTGCGGGGCCCCCGAC
CCATACCGACCCACCTAGGCGTCCAGTTACGGCGCACGTCGGGAGCGTGGTTGCCGTCAGAGCTGTGT
TTCTCGATCAGTCCCCCGCAGTGCCGCAGTATCTTGCCGTGGGCTGCTTTAATCTTGAAAGTGGTTCA
TACATTGGGCGACGAGGTGTCGACTCTCATTGGGGGTAACCGACGGGCACATGCAGTCCCCTCCCCGG
GCAGGCAGAGGCGGGGCCCCCGCGCGCCGGCCCCGGCCCACAATCTTACCAGGGTCCTCAAAGGAGCC
TTTGCATGGTACCCTTCGTGAATGGTTGCTTAAGAGGTCCACAACGTGGTCCGGGCACGGTCGACTAA
ACACTCAAAACAGCGACGGCAAATATAGGTACAAGGTCCAGGCCCTCACGGCACTAATTGCGATGACC
CAACTCACGGGGGCAGTCCCCGGCGCGGCGACCTTGATTACGTCCGGGGACAAGTATGTCGCTCCCCC
CGGGAGGGGTGCAGCCACATGGGAGATTCAAAGTTTCTCGTGACGTCGTTGTGATCACTGCAGCCTAG
CCGAGACTCCCGTACTACGCGAAGGTTGGTTATGTTAACCACTACAACGTGAGGCGCCGTAAGGGCCA
GTGTTGTGCCCGGCTCTTCAATGCGCCTTAGCGGCCTGATACACCCACCCAAGGAGATACTGCTAATC
ATATGGGTGGACAGAACCTCGCAACGCAGACGCCTCCCCGGCATGATATGGTTTTTTTCCGCTATTAT
CCAGCACGCAGCGCTATCATTCAAGAGAACCCAGTGACGCGTAAATCGTAAGATCTACCTGCCGCAGG
TGGACCTACTGCAAATACGGATTATGACTCGTAAAGGGGTCATGCGTATTTTCATCACTAGGCACGTT
CGAGAATAAATTAGTAGGTGTCCCGAGCCTTGTGGCGTTCCGCCTGACTCCTCATGAAGTCGACCTTC
TCACCGGCCCTATCTGCCGACGTAAGTCATAACCTAGATCTGTACCTCGGGGGGAGGGTCACTGTAAA
GGGATAATTGGAGGGTGATTTCCCACACTTTCCTAAGGGTACTTTTTGCCTGGCTTCGCAATTGGGTC
CAATGGATGTCGATCTCTGGTTTAGCAGTTGTGAAAGTGGCAAGGCGGGAGGTTAGACCTCCATTTAA
CATATACAAGCAAGTTAACTGCACTAGATGTGTAGACACTACAGTTACAGGAGTAGCCGAATAAGTCT
CCGACGTCAAGCGAATAAGCGTCATACGCGATTATCGCCTAAGAGCACGTATTGGCGGTAAAAGGCTA
GCTAGACGCTTATGGGTAGATTTCAAGGCGTTCGTAGTGGTATAATAGGATACTCTTTCACCAGCCTG
AGGGCCGAACGCTATACTAGTGGTCTGTGATGTAGGACCGAGTATCTCTCTAGGGACCATCTACTTGA
GCAATGGTGCGCAGGGGGAGACATAGACCAGCCTTGGGTGGCAAGCACTGCAATAAGTCCTGTTTAGC
CTTGGAGTTCACATGCCGGCACTGAAGCCGACCTACCTGAGCGTGTGATTACCGTTAAAGCATCTGTC
TAGTTCTGTTTCCGGCGCTCTTGGTTCCAGGTTAGGGGAAGTGTATGACCCATGTGTTTTTATCGGCT
TAACCACGAGTGATCCCCGGTCGTTTCCCCATTGAATCCCTGGTGCATCCTACTCCCAGAATGATAGC
TGACTGACTGGACTGGCTTTTCAAGTAATCGAGGGGGTATCGCGGTCACGGCCGTTAACAGATCCCGT
CCTTAGTGTGGAATCCGCACCTGCTGACTAACGCTTCGCCGGCGTGTCTGCACAGCCGTATAGTGTTA
ATCATGACCCCAAGGAAGGATTAAACAAATATCTTGACG
>CoLao566
GTNNNNNNTAGCCCNNNNNAGTTAGACANNNCNNNNNNNNNNNNTNNNNNNNNNNNNNNNNNNNNNNN
NNNNNNNNNNNNNNNNNNNNNNNNNNNNNNNNNNNNNNNNNNNNNNNNNNNNNNNNNNNNNNNNNNNN
NNNNNNNNNNNNNNNNNNNNNNNNNNNNNNNNNNNNNNNNNNNNNNNNNNNNNNNNNNNNNNNNNNNN
NNNNNNNNNNNNNNNNNNNNNNNNNNNNNNNNNNNNNNNNNNNNNNNNNNNNNNNNNNNNNNNNNNNN
NNNNNNNNNNNNNNNNNNNNNNNNNNNNNNNNNNNNNNNNNNNNNNNNNNNNCTNNNAAANNNTNNN
NNNNNNNNNNNNNNNNNNNNNNNNNCNCNNTGNNNGTAANCNANNNGNACATGCAGTCCCCTCNNNNN
NNNNNCAGAGNNNNNNNCCNNNNNNNCCGGCCCCNNCCNNNNNNNNNNNNNNNGNNNNNNNANNNNNN
NNNNNNTGGTACNNNNCGNNNNNGGTTGNNTAAGNGGTCCACAACGNGNTCNNGNNACNNNNGNNNAN
NNNCNNNNAACNNNNNNNNNNNANATNNNTANNAGGNCNNNNCCNTNNNGNNNNNNANTGCNATGANN
CANNNNNCGGNGGNNGTNCCCGGNGCGGCGACCTTGANNACGTCNGGGGNCAAGTATGGCNNNCNCCC
CGNGAAGGANNCNGCNGTANGTGCNNNNNANNNNNTCNTGNGANGTCGCCGNNATTNNNNTGGTCNNN

CNNGANTGNCCNNACCANNCGNANNNTGGTNATGNNAGTANNNNNNNNNNNNNNNNNNNN
NNNNNNNNNNNNNNNNNNNNNNNNGNNNNGNNNNNNCNANNNAAGGNNNTANNNCTNNTN
NNNNNNNNGGACNNNACNNNNCNNNNNNNACNCNNNCNCNNNNNNNNNNGGTTNNNNNNCNNTATTNT
CCAGNACGCNGNGCNNNCNTTCANGNGAACNNNGNNACGCGNNAATCGTANNATCTACCTGCCGCNNG
NNNNNNTNCNGNNNNNNCGGANNNTNNNNCGTANNNNNNNCANGCGTNNTNTNNTCNCNAGNCNNGTT
CGNNNANANANTAGNANGTNTNNNNNNNNNNTGTNNCGTTCCGCNTGANNCNNCNNNNNGNNGNNNTTN
NCANCGGCNNTANCTGCNGANNTAAGNCATANNNTNNNTCNNTANCTNNNNNGGNGGGNNACTGTAAA
NGGNNAATTNGNNNNTNANTNCNNNNACNTNNCNNNGNNTNCTNNNTNCCTGNNTNCNNAATTGGNNC
CANTGNNTGNCNATCTCTGGNTTNGNNNNNNTNNNNNTNNCANNNNGGNNNNNNANANNNCNATNTAA
NNTANANNNNCNNNNNNANNNCACNNNNNNNNNAGNNNCNANNNTNANANNNGTAGNNNANTNAGTCT
CCGANNTNNNNNNNATAAGCGNNANACNNNNNNNNNNNNNANGANNNCGNNTTGGNNNTNNNNNNCNA
GNNANACNNTNATGGNTNNNNNTCAANNNNTTNGTAGTGGTATAATNGNNNNCTNTTTCNCCAGNNTN
NNNGNNNNNNNCNATANNANNNNNNNGNGANNNNNNNNNNNANNNNCTCNNNAGGNANCANCTACTNNA
GCANNGGNGCGNAGNNNGAGANNNNGACNNNCCTTGGGTGGCAANCACNGNAATAAGTNNNGTTNANC
CTNNNAGTNNACATNNNNNCANNGNNNCNNNNNNANCNGANNGNNNNNNNNNNNNNNNNNNNNNN
NNNNNNNNNNNNNNNNNNNNNNNNNNNNANNNNANGGNNNGTNTNTGNCNCNTGTGTTTTTANNNGNN
NNNNNNNGNNNGANCCCCGGTCGNNTNCNNATTGAANNCCTGNNGCATNNNNNTNCNAGAATGANNGC
NNNNNGNNTNGNCTNNCNNNTCAAGNAATCANNNAGNNNNNNNNNTCNNNNNNNNNNNNCNNTCCCNN
ACTGAGGGTNNNNNNNNCATNNGCTNNNNNNNNCTNCGCNGGNGTNNCNNNNNNGCNGTATNNNGTNA
NNCANNNNNNNNNNNNNNNNNTNAAACAANNNNNNNTNNNN
>CoLao567
GNNNNNNNNAGCCCNNNNNAGNNNGNCANNNCNNNNNNNNNNNNNNNNNNNNNNNNNNNNNNNNNN
NNNNNNNNNNNNNNNNNNNNNNNNNNNNNNNNNNNNNNNNNNNNNNNNNNNNNNNNNNNNNNNNNN
NNNNNNNNNNNNNNNNNNNNNNNNNNNNNNNNNNNNNNNNNNNNNNNNNNNNNNNNNNNNNNNNNN
NNNNNNNNNNNNNNNNNNNNNNNNNNNNNNNNNNNNNNNNNNNNNNNNNNNNNNNNNNNNNNNNNN
NNNNNNNNNNNNNNNNNNNNNNNNNNNNNNNNNNNNNNNNNNNNNNNNNNNNNNNNNAAANNNTNNN
NNNNNNNNNNNNNNNNNNNNNNNNNNNNCNNNNNNNGTAANCNANNNGNNCATGCAGTNNNCTCNNNNN
NNNNNCAGAGNNNNNNNNNNNNNNNNNNCCGNCNCCNNCCNNNNNNNNNNNNNNNNGNNNNNNNANNNNNN
NNNNNNTGGTACNNNNCGNNNNNGGNNGNNNNNGAGGTCCACAACGNGNTCNNNNNACNNNNGNNNAN
NNNNNNNNAANNNNNNNNNNNNNNNNNTNNNTNNNAGGNNNNNNCCNNNNNNNNNNNNNANNGCNATNANN
CANNNNNCNGNNNCNGTNCCCGGNNNNGNNACCNTNNNNANNNCNGGNGNCAAGTATNGCNNNCNCNC
NGNGAAGGANNCNNCNGTANGTGCNNNNNANNNNNNCNTGNGANNTNGCNGNNNNNNNNNTNNNCNNN
CNNNNNTGNNCNNACCANNCGNNNNNTGGTNATGNNAGTNNNNNNNNNNNNNNNNNNNNNNNNNNNNN
NNNNNNNNNNNNNNNNNNNNNNNNNNNNNNNNNNNGNNNNGNNNNNNCNANNNANGGNNNTANNNCTNNNN
NNNNNNNNNNNCNNNNCNNNNNNNNNNNNNNCNNNNNCNNNNNNNNNNNNNGGTTNNNNNNCNNTNNNNN
CNANNANGCNGNGNNNNCNNTNANNNGAACNNNGNNANGNGNNNNNNNTNNNNNCTNNCTGCNNCNNG
NNNNNNTNCNGNNNNNNCGGNNNNNNNNNCGTANNNNNNNCNNGCNNNNNNNNNNNNNCNAGNCNNGTT
CGNNNANNNANTAGNNNGTNNNNNNNNNNNNNGTNNCGTTNNNNNTGANNCNNCNNNNNGNNGNNNTTN
NCANCGGNNNTANCTNCNGANNTAAGNCANANNNTNNNTCNNTNNCTNNNNNNGNNGGNNACTGNAAA
NGNNNAATTNGNNNNTNANTNCNNNCATNTNNCNNNGNNNNCTNNNTNCNNGNNNNCNNNNTTGGNNN
NANTGNNTGNNNATCTCTNGNTTNGNNNNNNTNNNNNTNNCANNNNGGNNNNNNNNANNNNNATNNAA
NNTANNNNNNCNNNNNNANNNCACNNNNNNNNNAGNNNNNNNNNNNANANNNNTAGNNNNNTNAGTCN
CCGNNNTNNNNNNNNTANGCGNNANACNNNNNNNNNNNNNANNANNNCGNNTTTGNNNNNNNNNNNN
GNNNNACNNTNGTGGNTNNNNNTNNANNNNTTNNNAGTGGTATAATNGNNNNNNNTTNCNCCAGNNTN
NNNNNNNNNNNCNNNNNNANNNNNNNGNNANNNNNNNNNNNNNNNNNTCNNNAGGNANCANCNANNNNA
NCANNGGNNCNNNNNNNNNGANNNNGACNNNCCTTGGGTGGNANNNACNGNANNAAGNNNNNTTNNNN
NTNNNNNTNNNNACNNNNNNANNGNNNCNNNNNNANCNGNNNGNNNNNNNNNNNNNNNNNNNNNNNNN
NNNNNNNNNNNNNNNNNNNNNNNNNNNNNNNNNNNANNGNNNGTNTNNGNCNNNNNNNNNNNTNNNNGNN
NNNNNNNGNNNNNNCCCCGGTCNNNNNCNNATTGAANNCCNGNNGCNNNNNNNNNNNANNNNNANNNN
NNNNNGNNTNNNNTNNNNNNNNNANGNAATNANNNANNNNNNNNNNNCNNNNNNNNNNNNCNNTNNNNN
ACTGAGNNNNNNNNNNNCANNNGCTNNNNNNNNNTNCGCNNGNNTNNNNNNNNNGCNGTATNNNGNNN
NNCANNNNNNNNNNNNNNNNNNNNNNNNNNNNNNNTNNNN
>CoLao568
GTGNNNNNTAGCCCNNNNNAGTTAGACANNNCNNNNNNNNNNNNTNNNNNNNNNNNNNNNNNNNNNN

NNNNNNNNNNNNNNNNNNNNNNNNNNNNNTNNNNNNNNNNNNNNNNNNNNNNNNNNNNNNNN
NNNNNNNNNNNNNNNNNNNNNNNNNNNNNNNNNNNNNNNNNNNNNNNNNNNNNNNNNNNN
NNNNNNNNNNNNNNNNNNNNNNNNNNNNNNNNNNNNNNNNNNNNNNNNNNNNNNNNNNNN
NNNNNNNNNNNNNNNNNNNNNNNNNNNNNNNNNNNNNNNNNNNNNNCNTCTNNNAAANNNTNNN
NNNNNNNNNNNNNNNNNNNNNNNNNNNCNCNTTGNNNGTAANCNANNNGNACATGCAGTCCCCTCNNNNN
NNNGGCAGAGNNNNNNCCCNNNNNNNCCGNCCCCNNCCNNNNNNNNNNNNNNNNNNNNNNNNNANNNNNN
NNNNNNTGGTACNNNNCGNNNNNGGTTGTTTAAGAGGTCCACAACGNGNTCNNGNCACNNNNGNNNAN
NNNCNNNNAACNNNNNNNNNNNNANNTNNNTANNAGGNCNNNGCCNTCNNNNNNNNNANTGCNATGANN
CANNNCNCGGNGGNNGTCCCCGGCGCGGNGACCTTGNNNANNTCCGGGGNCAAGTATGGCNNNCNCCC
CGGGAAGGANNCNGCNGTANGTGCNNNNNANNNNNTCNTGNGANGTCGCCGNNATTNNNNTGGTCNNN
CNAGAATGNCCNNACCANNCGNANNNTGGTNATGNNAGTNNNNNNNNNNNNNNNNNNNNNNNNNNNN
NNNNNNNNNNNNNNNNNNNNNNNNNNNNNNNNNGNNNNGNNNNNNCNANNNAAGGNNNTANNNCTNNTN
NTNNNNNNNGGACNNNACNNNNCNNNNNNNACNCNNNCNCNNNNNNANNNGGTTTTTNNNCNNTANTNT
CCAGNACGCNGNGCNNNCNTTCANGNGAACNNNGNNACGCGNNAATCGTANNATCTACCTGCCGCNNG
TNNNNNTNCNGNNNNNNCGGANNNTNNNNNGTANNNNNNNCANGCGTNNTNTNNTNNCNAGNCNNGTT
CGNNNANANANTAGNANGTNTNNNNNNNNNNTGTNNCGTTCCGCNTGANNCNNCANNNNGNNGNNCTTN
NCANCGGCNNTANCTGCNGANNTAAGNCATANNNTNNNTCNNTANCTNNNNNGGNGGGNNACTGTAAA
NGGNNAATTNGNNGNTNANTNCCNNNACNTNNCTNNGNNTNCTNNNTNCCTGNNTNCNNAATTGGNNC
CANTGNNTGNCNATCTCTGGNTTNGNNNNNNNTNNNNNTNNCANNNNGGNNNNNNNNANNNCNATNTAA
NNTATANNNNCNNNNNNANNNCACNNNNNNNGNAGNNNCNANNNTTANANNNGTAGNNNANTNAGTCT
CCGANNTNNNNNNNNATAAGCGNNANACNNNNNNNNNNNNNNANGANNNCGNNTTGGNNNTNNNNNNNNNA
GNNANACNNTNATGGNTNNNNNTCAANNNNTTNGTAGTGGTATAATNGNNNNNNNNTTTCNCCAGNNTN
NNNGNNNNNNNNCNATANNANNNNNNNGNGANNNANNNNNNANNNNCTCNNNAGGGANCANCTACTTNA
GCANNGGNGCGNAGNNNGAGANNNNGACNNNCCTTGGGTGGCAANNACNGCAATAAGTNNNGTTNNNN
CTNNNAGTNNACATNNNNNCANNGNNNCNNNNNNANCNGANNGNNNNNNNNNNNNNNNNNNNNNNNNN
NNNNNNNNNNNNNNNNNNNNNNNNNNNNNNNANNNNANGGNNNGTNTNTGNCNCATNNNTTTTTANNNGNN
NNNNNNNGNNNNANCCCCGGTCGNNTNCNNATTGAANNCCTGNNGCATNNNNNTNCNAGAATGANNNC
NNNNNGNNTNGNCTNNCNNNTNAAGNAATCAANNAGNNNNNNNNNTCNNNGNNNNNNNNCNNNNNCNN
ACTGAGGGNNNNNNNNNCATCNGCTNNNNNNNNNCTNCGCNGGNGTNNCNNNNNNGCNGTATNGNGNNA
NNCANNNNNNNNNNNNNNNNNNTNAAACNANNNNNCTNNNN
>CoLao569
GTGNNNNNNNAGCCCNNNNNAGTTAGACANNNCNNNNNNNNNNNNNTNNNNNNNNNNNNNNNNNNNNNNN
NNNNNNNNNNNNNNNNNNNNNNNNNNNNNNNNNNNNNNNNNNNNNNNNNNNNNNNNNNNN
NNNNNNNNNNNNNNNNNNNNNNNNNNNNNNNNNNNNNNNNNNNNNNNNNNNNNNNNNNNN
NNNNNNNNNNNNNNNNNNNNNNNNNNNNNNNNNNNNNNNNNNNNNNNNNNNNNNNNNNNN
NNNNNNNNNNNNNNNNNNNNNNNNNNNNNNNNNNNNNNNNNNNNNNNNNTCTNNNAAANNNTNNN
NNNNNNNNNNNNNNNNNNNNNNNNNNNCNCNNTGNNNGTAANCNANNNGNACATGCAGTNCCCTCNNNNN
NNNGGCAGAGNNNNNNNNNNNNNNNCNCCGNCCCCNNCCNNNNNNNNNNNNNNNNNGNNNNNNNANNNNNN
NNNNNNTGGTACNNNNCGNNNNNGGTTGTNTAAGNGGTCCACAACGNGNTCNGNNNACNNNNGNNNAN
NNNCNNNNANNNNNNNNNNNNNNNANNTNNNTANNAGGNCNNNNCCNTNNNNNNNNNNNANTGCNATGANN
CANNNNNCNGNNGNNGTNCCCGGCGNGGCGACCTTGANNANGTCCGGGNNCAAGTATGGCGNNCNCCC
CGGGAAGGANNCNGCNGTANGTGCNNNNNANNNNNTCNTGNGANGTNGCCGNNANNCNNGTGNNCNNN
NNNGAATGCNCNNACCANNCGNANNNTGGTNATGNNAGTNNNNNNNNNNNNNNNNNNNNNNNNNNNN
NNNNNNNNNNNNNNNNNNNNANNNNNNNNNNNNNGNNNNGNNNNNNCNANNNAAGGNNNTANNNCTNNTN
NNNNNNNNNGGACNNNACNNNNCNNNNNNNACNCNNNCNCNNNNNNNNNNNGNTTTTNNNNNCNNTANTNT
CCAGNACGCNGNGCNNNCNTTCANGNGAACNNNGNNACGCGNNAATCGTANNATCTACCTGCCGCNNG
NNNNNNTACNGNNNNNNCGGANNNTNNNNNCGTANNNNNNNTCATGCGTNNTNTNNTNNCNAGNCNNGTT
CGNNNANANANTAGNANGTNTNNNNNNNNNNTGTNNCGTTCCGNNTGANNCNNCANNNNGNNGNNNTTN
TCANCGGCNNTANCTGCNGANNTAAGNCATANNNTNNNTCNNTANCTNNNNNGGNGGGNNACTGTAAA
NGGNNAATTNGNNNNTNANTNCNNNCACNTNNCNNNGNNNNCTNNNTNCCTGNNTNCNNAATTGGNNN
CANTGNNTGNCNATCTCTNGNTTNGNNNNNNNTNNNNNTNNCANNNNGGNNNNNNNNANNNCNATNTAA
NNTANANNNNCNNNNNNANNNCACNANNNNNGNAGNNNCNANNNTNANANNNGTAGNNNNNTAAGTCN
CCGANNTNNNNNNNNATAAGCGNNANACNNNNNNNNNNNNNNANGANNNCGNNTTGGNNNTNNNNNNNNNA
GNNAGACNNTNATGGNTNNNNNTCAANNNNTTNGTAGTGGTATAATNGNNNNNNNNTTTCNCCAGNNTN

NNNGNNNNNNNNCNATANNANNNNNNNGNGANNNNNNNNNNNANNNNCTCNNNAGGNANCANCTACTNNA
GCANNGGNGCGNAGNNNGAGANNNNGACNNNCCTTGGGTGGCAANNACNGNAATAAGTNNNGTTNNNN
CTNNNAGTNNACATNNNNNCACNGNNNCNNNNNNANCNGANNGNNNNNNNNNNNNNNNNNNNNNNNN
NNNNNNNNNNNNNNNNNNNNNNNNNNNNNNNNNANNGNNNGTNTNTGNCNCNTGTGTTTTTANNNGNN
NNNNNNNGNNNNANCCCCGGTCGNNNNCNNATTGAANNCCTGNNGCATNNNNNTNCNAGAATGANNNC
NNNNNGNNTNGNCTNNCNNNTCAAGNAATCANNNAGGTNNNNNNNTCNNNNNNNNNNNNNCNNTCNCNN
ACTGAGGGNNNNNNNNNCATNNGCTNNNNNNNNNTNCGCNGGNNNNNCNNNNNNGCNGTATNGNGNNA
NNCANNNNNNNNNNNNNNNNNTNAAACGANNNNNNNTNNNN
>CoLao570
GTGNNNNNNAGCCCNNNNNAGTTAGACANNNCNNNNNNNNNNNNNTNNNNNNNNNNNNNNNNNNNNNNNN
NNNNNNNNNNNNNNNNNNNNNNNNNNNNNNNTNNNNNNNNNNNNNNNNNNNNNNNNNNNNNNNNNNNNNNNN
NNNNNNNNNNNNNNNNNNNNNNNNNNNNNNNNNNNNNNNNNNNNNNNNNNNNNNNNNNNNNNNNNNNNNNN
NNNNNNNNNNNNNNNNNNNNNNNNNNNNNNNNNNNNNNNNNNNNNNNNNNNNNNNNNNNNNNNNNNNNNNN
NNNNNNNNNNNNNNNNNNNNNNNNNNNNNNNNNNNNNNNNNNNNNNNNNNNNNCNTCTNNNAAANNNTNNN
NNNNNNNNNNNNNNNNNNNNNNNNNNNNCNCNTTGNNNGTAANCNANNNGNACATGCAGTNCCCTCNNNNN
NNNNNCAGAGNNNNNNNCNNNNNNCNCCGGCCCCNNCCNNNNNNNNNNNNNNNNNGNNNNNNNANNNNNN
NNNNNNTGGTACNNNNCGNNNNNGGTTGTNNAAGAGGTCCACAACGNGGTCNNGNCACNNNNGNNNAN
NNNCNNAAAANNNNNNNNNNNNNNNNNNTNNNTANNAGGNNNNNNCCNTCNNNNNNNNNNANTGCNATGACC
CAANNNNCGGGGGCNGTNCCCGGNGNGGCGACCTTGNNNACGTCCGGGGNCAAGTATGGCNNNCNCCC
CGNGAAGGANNCNGCCGTANGTGCNNNNNANNNNNTCNTGNGANGTCGCCGNNATTNNNNTGGTCNNN
CNAGAATGNCCNNACCANNCGNANNNTGGTNATGNNAGTNNNNNNNNNNNNNNNNNNNNNNNNNNNNN
NNNNNNNNNNNNNNNNNNNNNNNNNNNNNNNNNNNGNNNNGNNNNNNCNANNNAAGGANNTANNNCTNNTN
NTNNNNNNNGGACNNNACNNNNCNNNNNNNACNCNNNCNCNNNNNNNNNNNGNTTTTNNNNNCNNTATTNT
CCAGNACGCNGNGCNNNCATTCANGNGAACNNNGNNACGCGNNAATCGTANNATCTACCTGCCGCNNG
TNNNNNTNCNGNNNNNNNCGGANNNTNNNNCGTANNNNNNTCATGCGTNNTNTNNTCNCNAGNCANGTT
CGNNNANANANTAGNANGTNTNNNNNNNNNNNGTNNCGTTCCGCNTGANNCNNCANNNNGNNGNNNTTN
NCANCGGCNNTANCTGCNGANNTAAGNCATANNNTNNNTCNNTANCTNNNNNGGNGGGNNACTGTAAA
NGGNNAATTGGNNGNTNANTNCNNNCACNTNNCTNNGNNTNCTNNNTNCCTGNNTNCNNAATTGGNNC
CANTGNNTGNCNATCTCTGGNTTNGCNNNNNTNNNNNTNNCAANNNGGNNNNNNANANNNCNATNTAA
NNTANANNNNCNNNNNNANNNCACNNNNNNNGNAGNNNNNANNNTNANANNNGTAGNNNANTAAGTCT
CCGANNTNNNNNNNATAAGCGNNANACNNNNNNNNNNNNNANGANNACGNNTTGGNNNTNNNNNNNNA
GNNAGACNNTNATGGNTNNNNNTCAANNNNTTCGTAGTGGTATAATNGNNNNNNNTTTCNCCAGNNTN
NNNGNNNNNNNNCNATANNANNNNNNNGNGANNNNNNNNNNNANNNNNTCNNNAGGNANCANCTACTNNA
GCANNGGNGCGNAGNNNGAGANNNNGACNNNCCTTGGGTGGCAANNACNGCAATAAGTNNNGTTNANC
CTNNNAGTNNACATNNNNNCANNGNNNCNNNNNNANCNGANNGNNNNNNNNNNNNNNNNNNNNNNNN
NNNNNNNNNNNNNNNNNNNNNNNNNNNNNANNNNANGGNNNGTNTNTGACNCNTGTGTTTTTANNNGNN
NNNNNNNGNNNNANCCCCGGTCGTNNNCNNATTGAANNCCTGNNGCATNNNNNTNCNAGAATGANNNC
NNNNNGNNTNGNCTNNCNNNTCAAGNAATCANNNAGNNANNNNNNTCNNNGNNNNNNNNCNNTCNCNN
ACTGAGGGTNNNNNNNNCATNTGCTNNNNNNNNCTNCGCNGGNNNNNCNNNNNAGCNGTATNNNGTNA
NNCANNNNNNNNNNNNNNNNNTNNAACNANNNNNNNTNNNN
>CoLao571
GTGNNNNNTAGCCCNNNNNAGTTAGACANNNCNNNNNNNNNNNNNNNNNNNNNNNNNNNNNNNNNNNNNN
NNNNNNNNNNNNNNNNNNNNNNNNNNNNNNNTNNNNNNNNNNNNNNNNNNNNNNNNNNNNNNNNNNNNNNNN
NNNNNNNNNNNNNNNNNNNNNNNNNNNNNNNNNNNNNNNNNNNNNNNNNNNNNNNNNNNNNNNNNNNNNNN
NNNNNNNNNNNNNNNNNNNNNNNNNNNNNNNNNNNNNNNNNNNNNNNNNNNNNNNNNNNNNNNNNNNNNNN
NNNNNNNNNNNNNNNNNNNNNNNNNNNNNNNNNNNNNNNNNNNNNNNNNNNNNCNTCTNNNAAANNNTNNN
NNNNNNNNNNNNNNNNNNNNNNNNNNNNNNNCNNTGNNNGTAANCNANNNGNACATGCAGTCCCCTCNNNNN
NNNNNCAGAGNNNNNNNCCNNNNNNNCCGGCCCCGNCCNNNNNNNNNNNNCNNNGNNNNNNNNANNNNNN
NNNNNNTGGTACNNNNCGNNNNNGGTTGTTTNAGAGGTCCACAACGNGGTCNNGNNACNNNNGNNNAN
NNNCNNNNAACNNNNNNNNNNNNNANATNNNTANNAGGNNNNNNCCNTCNNNNNNNNNNATTGCNATGANN
CANNNCACGGGGGCNGTNCCCGGCGCGGCGACCTTGANNACGTCCGGGGNCAAGTATGGCNNNCNCCC
CGNGAAGGANNCNGCNGTANGTGCNNNNNANNNNNTCNTGNGANGTCGCCGNNATTNNCGTGNTCNNN
NNAGAATGNCCNNACCANNCGNANNNTGGTNATGNNAGTNNNNNNNNNNNNNNNNNNNNNNNNNNNNN
NNNNNNNNNNNNNNNNNNNNNNNNNNNNNNNNNNNGNNNNGNNNNNNCNANNNAAGGNNNTANNNCTNNTN

NTNNNNNNNGGACNNNACNNNNCNNNNNNNNACNCNNNCNCNNNNNNANNNGNTTTTNNNNCNNTANTNT
CCAGNANGCNGNGCNNNCNTTCANGNGAACNNNGNNACGCGNNAATCGTANNATCTACCTGCCGCNNG
TNNNNNTACNGNNNNNNCGGANNNTNNNNCGTANNNNNNNCATGCGTNNTNTNNTCNCNAGNCNNGTT
CGNNNANANANTAGNANGTNTNNNNNNNNNNTGTNNCGTTCCGCNTGANNCNNCNNNNNGNNGNNCTTN
NCANCGGCNNTANCTGCNGANNTAAGNCATANNNTNNNTCNNTANCTNNNNNGGNGGGNNACTGTAAA
NGGNNAATTNGNNNNTNATTNCCNNCACNTNNCNNNGNNTNCTNNNTNCCTGNNTNCNNAATTGGNNC
CANTGNNTGNCNATCTCTGGNTTNGNNNNNNTNNNNNTNNCANNNNGGNNNGTNNNANNNCNATNTAA
NNTANANNNNCNNNNNNANNNCACNNNNNNNGNAGNNNCNANNNTTANANNNGTAGNNNANTAAGTCT
CCGANGTNNNNNNNATAAGCGNNANACNNNNNNNNNNNNNNANGANNNCGNNTTGGNNNTNNNNNNCNA
GNNAGACNNTNATGGNTNNNNNTCAANNNNTTNGTAGTGGTATAATNGNNNNNNNTTTCNCCAGNNNN
NNNGNNNNNNNCNATANNANNNNNNNGNGANNNNNNNNNNNANNNNCTCNNNAGGNANCANCTACTNNA
GCAATGGNGCGCAGNNNGAGANNNNGACNNGCCTTGGGTGGCAANNACNGCAATAAGTNNNGTTNNNN
CTNNNAGTNNACATNNNNNCANNGNNNCNNNNNNANCNGANNGNNNNNNNNNNNNNNNNNNNNNNNNN
NNNNNNNNNNNNNNNNNNNNNNNNNNNNNNANNNNANGGNNNGTNTNTGACNCNTNNNNTTTNTANNNGNN
NNNNNNNGNNNNANCCCCGGTCGNNTNCNNATTGAANNCCTGNNGCATNNNNNTNCNAGAATGANNGC
NNNNNGNNTNGNCTNNCNNNTCAAGNAATCANNTANGTNNNNNNNTCNNNNNNNNNNNNNCNNTCCCNN
ACTGAGGGTNNNNNNNNCATCNGCTNNNNNNNNNNTNCGCNGGNGTNNCNNNNNNGCNGTATNNNGTNN
NNCANNNNNNNNNNNNNNNNNNTNAAACGANNNNNNNTNNNN
>CoLao572
GTGNNNNNTAGCCCNNNNNAGTTAGACANNNCNNNNNNNNNNNNNTNNNNNNNNNNNNNNNNNNNNNNNN
NNNNNNNNNNNNNNNNNNNNNNNNNNNNNNNNTNNNNNNNNNNNNNNNNNNNNNNNNNNNNNNNNNNNNNN
NNNNNNNNNNNNNNNNNNNNNNNNNNNNNNNNNNNNNNNNNNNNNNNNNNNNNNNNNNNNNNNNNNNNNN
NNNNNNNNNNNNNNNNNNNNNNNNNNNNNNNNNNNNNNNNNNNNNNNNNNNNNNNNNNNNNNNNNNNNNN
NNNNNNNNNNNNNNNNNNNNNNNNNNNNNNNNNNNNNNNNNNNNNNNNNNNNNNTCNNNNAAANNNTNNN
NNNNNNNNNNNNNNNNNNNNNNNNNNCNCNNTNNNNGTAANCNANNNGNACATGCAGTCCCCTCNNNNN
NNNGGCAGAGNNNNNNNCCNNNNNNNCCGGCCCCNNCCNNNNNNNNNNNNNNNNNGNNNNNNNANNNNNN
NNNNNNTGGTACNNNNCGNNNNNGGTTGNNTAAGAGGTCCACAACGNGGTCNNGNNACNNNNGNNNAN
NNNCNNNNAACNNNNNNNNNNNNANNTNNNTANNAGGNNNNNNCCNTCNNGNNNNNNANNGCNATGACC
CNNNTNNCNGGGGCNGTNCCCGGNGNGGCNACCTTGNNNANNTCCGGGGACAAGTATGGCGNNCNCCC
CGGGAAGGANNCNGCNGTANGTGCNNNNNANNNNNTCNTGNGANGTNGCCGNNATTNNNNTGGTCNNN
NNAGAATGNCCNNACCANNCGNANNNTGGTNATGNNAGTNNNNNNNNNNNNNNNNNNNNNNNNNNNNNN
NNNNNNNNNNNNNNNNNNNNNNNNNNNNNNNNNNNGNNNNGNNNNNNCNANNNAAGGNNNTANNNCTNNTN
NTNNNNNNNGGACNNNACNNNNNNNNNNNNNACNCNNNCNCNNNNNNNNNNGNTTTTTNNNCNNTANTNT
CCAGNACGCNGNGCNNNCATTCANGNGAACNNNGNNACGCGNNAATCGTANNATCTACCTGCCGCNNG
NNNNNNTNCNGNNNNNNCGGANNNTNNNNCGTANNNNNNNCATGCGTNNTNTNNTCNCNAGNCNNGTT
CGANNANANANTAGNANGTNTNNNNNNNNNNTGTNNCGTTCCGCNTGANNCNNCANNNNGNNGNNCTTN
NCANCGGCNNTANCTNCNGANNTAAGNCATANNNTNNNTCNNTANCTNNNNNGGNGGGNNACTGTAAA
NGGNNAATTNGNNNNTNATTNCNNNCACNTNNCTNNGNNTNCTNNNTNCCTGNNTNCNNAATTGGGNN
CANTGNNTGNCNATCTCTNGNTTNGCNNNNNTGNNNGTNNCANNNNGGNNNNNNNNANNNCNATNTAA
NNTANANNNNCNNNNNNANNNCACNNNNNNNGNAGNNNCNANNNTTANANNNGTAGNNNANTAAGTCT
CCGANNTNNNNNNNATAAGCGNNANACNNNNNNNNNNNNNNANGANNNCGNNTTGGNNNTNNNNNNCNA
GNNAGACNNTNATGGNTNNNNNTCAANNNNTTNGTAGTGGTATAATNGNNNNNNNTTTCNCCAGNNTN
NNNGNNNNNNNGCNATANNANNNNNNNGNGANNNNNNNNNNNANNNNCTCNNNAGGGANCANCTACTTNA
GCANTGGNNCGNAGNNNGAGANNNNGACNNNCCTTGGGTGGCAANNACNGCAATAAGTNNNGTTNANN
NTNNNAGTNNACATNNNNNCANNGNNNCNNNNNNANCNGANNGNNNNNNNNNNNNNNNNNNNNNNNNN
NNNNNNNNNNNNNNNNNNNNNNNNNNNNNNNNNNNNANNGNNNGTNTNTGACNCATGTGTTTTTANNNGNN
NNNNNNNGNNNNANCCCNGGTCGNNNNCNNATTGAANNCCTGNNGCATNNNNNTNCNAGAATGANNNC
NNNNNGNNTNGNCTNNCNNNTCAAGNAATCANNNANGTNNNNNGNTCNNNNNNNNNNNNNCNNNCNCNN
ACTGAGGGTNNNNNNNNCATNTGCTNNNNNNNNNNTNCGCNGGNGTNNCNNNNNAGCNGTATNNNGTNA
NNCANNNNNNNNNNNNNNNNNNTNAAACNANNNNNNNTNNNN
>CoLao573
GTGNNNNNNAGCCCNNNNNAGTTAGACANNNCNNNNNNNNNNNNNTNNNNNNNNNNNNNNNNNNNNNNNN
NNNNNNNNNNNNNNNNNNNNNNNNNNNNNNNNNNNNNNNNNNNNNNNNNNNNNNNNNNNNNNNNNNNNNN
NNNNNNNNNNNNNNNNNNNNNNNNNNNNNNNNNNNNNNNNNNNNNNNNNNNNNNNNNNNNNNNNNNNNNN

NNNNNNNNNNNNNNNNNNNNNNNNNNNNNNNNNNNNNNNNNNNNNNNNNNNNNNNNNNNNNNNNNNNN
NNNNNNNNNNNNNNNNNNNNNNNNNNNNNNNNNNNNNNNNNNNNNNNNNNNNTCTNNNAAANNNTNNN
NNNNNNNNNNNNNNNNNNNNNNNNNCNCNTTNNNNGTAANCNANNNGNACATGCAGTCCCCTCNNNNN
NNNGGCAGAGNNNNNNNCCNNNNNNNCCGGCCCCGNCCNNNNNNNNNNNNNNNGNNNNNNNANNNNNN
NNNNNNTGGTACNNNNCGNNNNNGGTTGTTTNAGAGGTCCACAACGNGGTCNNGNNACNNNNGNNNAN
NNNCNNNNANCNNNNNNNNNNNNNNTNNNTANNAGGNCNNNNCCNTCNNGNNNNTAATNGCNATGANN
CANNNCNCGGGGGCNGTNCCCGGCGCGGNGACCTTGANNANNTCCGGGGNCAAGTATGGCGNNCNCCC
CGGGAAGGANNCNGCCGTANGTGCNNNNNANNNNNTCNTGNGANGTCGCCGNNATTNNCNTGGTCNNN
NNAGAATGNNCNNACCANNCGNANNNTGGTTATGNNAGTNNNNNNNNNNNNNNNNNNNNNNNNNNNNN
NNNNNNNNNNNNNNNNNNNNNNNNNNNNNNNNGNNNNGNNNNNNCNANNNAAGGNNNTANNNCTNNTN
NTNNNNNNGGACNNNACNNNNCNNNNNNNACNCNNNCNCNNNNNNNNNNNGNTTTTNNNNCNNTATTNT
CCAGNACGCNGNGCNNNCATTCANGNGAACNNNGNNACGCGNNAATCGTANNATCTACCTGCCGCNNG
NNNNNNTACNGNNNNNNCGGANNNTNNNNCGTANNNNNNNCATGCGTNNTNTNNTCNCNAGNCNNGTT
CGNNNANANANTAGNANGTNTNNNNNNNNNTGTNNCGTTCCGCNTGANNCNNCANNNNGNNGNNCTTN
NCANCGGCNNTANCTGCNGANNTAAGNCATANNNTNNNTCNNTANCTNNNNNGGNGGGNNACTGTAAA
NGGNNAATTNGNNGNTNANTNCNNNCACNTNNCNNNGNNTNCTNNNTNCCTGNNTNCNNAATTGGNNC
CANTGNNTGNCATCTCTGGNTTNGNNNNNNTNNNNNNTNNCANNNNGGNNNGTNANANNNCNATNTAA
NNTANANNNNCNNNNNNANNNCACNNNNNNNNNAGNNACNANNNTTANANNNGTAGNNNANTAAGTCT
CCGANGTNNNNNNATAAGCGNNANACNNNNNNNNNNNNNNANGANNNCGNNTTGGNNNTNNNNNNCNA
GNNAGACNNTNATGGNTNNNNNTCAANNNNTTNGTAGTGGTATAATNGNNNNNNNTTTCNCCAGNNTN
NNNGNNNNNNNCNATANNANNNNNNNGNGANNNNNNNNNNANNNNCTCNNNAGGGANCANCTACTTNA
GCANNGGNGCGCAGNNNGAGANNNNGACNNNCCTTGGGTGGCAANNACNGNAATAAGTNNNGTTNNNC
CTNNNAGTNNACATNNNNNCANNGNNNCNNNNNNANCNGANNGNNNNNNNNNNNNNNNNNNNNNNNNN
NNNNNNNNNNNNNNNNNNNNNNNNNNNNNNNNNANNGNNNGTNTNTGACNCATGTGTTTTTANNNGNN
NNNNNACGNNNNANCCCCGGTCGNNNNCNNATTGAANNCCTGNNGCATNNNNNNTNCNAGAATGANNNC
NNNNNGNNTNNNCTNNNNNNNTCAAGNAATCAANNAGGTNNNNNNNTCNNNGNNNNNNNCNNTCNCNN
ACTGAGGGTNNNNNNNNCATCNNCTNNNNNNNNCTNCGCNGGNGTNNCNNNNNNGCNGTATNGNGTNA
NNCANNNNNNNNNNGNNNNNNTNAAACANNNNNNCTNNNN
>CoLao574
GTGNNNNNNAGCCCNNNNNAGTTAGACANNNCNNNNNNNNNNNNTNNNNNNNNNNNNNNNNNNNNNNN
NNNNNNNNNNNNNNNNNNNNNNNNNNNNNNNNNNNNNNNNNNNNNNNNNNNNNNNNNNNNNNNNNNNN
NNNNNNNNNNNNNNNNNNNNNNNNNNNNNNNNNNNNNNNNNNNNNNNNNNNNNNNNNNNNNNNNNNNN
NNNNNNNNNNNNNNNNNNNNNNNNNNNNNNNNNNNNNNNNNNNNNNNNNNNNNNNNNNNNNNNNNNNN
NNNNNNNNNNNNNNNNNNNNNNNNNNNNNNNNNNNNNNNNNNNNNNNNNNCNTCTNNNAAANNNTNNN
NNNNNNNNNNNNNNNNNNNNNNNNNCNCNNTGNNNGTAANCNANNNGNACATGCAGTCCCCTCNNNNN
NNNNNCAGAGNNNNNNCCCNNNNNNNCCGNCCCCNNCCNNNNNNNNNNNNNNNGNNNNNNNANNNNNN
NNNNNNTGGTACNNNNCGNNNNNGGTTGTNTNAGAGGTCCACAACGNGNTCNGNNNACNNNNGNNNAN
NNNCNNNNAANNNNNNNNNNNNANNTNNNTANNAGGNCNNNNCCNTCNNNNNNNNAANTGCNATGANN
CAANNCNCNGGGGNNGTNCCCGGNNCGGCGACCTTGANNANGTCCGGGGNCAAGTATGGCNNNCNCCC
CGGGAAGGANNCNGCNGTANGTGCNNNNNANNNNNTCNTGNGANGTNGCCGNNANNCNCGTGGTCNNN
CNNGANTGNNCNNACCANNCGNANNNTGGTNATGNNAGTNNNNNNNNNNNNNNNNNNNNNNNNNNNNN
NNNNNNNNNNNNNNNNNNNNNNNNNNNNNNNNNNNNNGNNNNNNCNANNNAAGGNNNTANNNCTNNTN
NNNNNNNNGGACNNNACNNNNCNNNNNNNACNCNNNCNCNNNNNNNNNNGNTTNNNNNNCNNTATTNT
CCAGNACGCNGNGCNNNCNTTCANGNGAACNNNGNNACGCGNNAATCGTANNATCTACCTGCCGCNNG
NNNNNNTACNGNNNNNNCGGANNNTNNNNCGTANNNNNNTCANGCGTNNTNTNNTCNCNAGNCNNGTT
CGNNNANANANTANNANGTNTNNNNNNNNNTGTNNCGTTCCGCNTGANNCNNCNNNNNGNNGNNNTTN
NCANCGGCNNTANCTGCNGANNTAAGNCATANNNTNNNTCNNTANCTNNNNNGGNGGGNNACTGTAAA
NGGNNAATTNGNNNNTNANTNCNNNNACNTNNCTNNGNNNNCTNNNTNCCTGNNTNCNNAATTGGNNN
CANTGNNTGNCATCTCTNGNTTNGCNNNNNTNNNNNNTNNCANNNNGGNNNGTNNNANNNCNATNTAA
NNTANANNNNCNNNNNNANNNCACNNNNNNNNNAGNNNCNANNNTNANANNNGTAGNNNANTAAGTCT
CCGANNTNNNNNNATAAGCGNNANACNNNNNNNNNNNNNNANGANNNCGNNTTGGNNNTNNNNNNCNA
GNNAGACNNTNATGGNTNNNNNTCAANNNNTTNGTAGTGGTATAATNGNNNNNNNTTTCNCCAGNNTN
NNNGNNNNNNNCNATANNANNNNNNNGNGANNNNNNNNNNANNNNCTCNNNAGGNANCANCTACTNNA
GCANTGGNGCGCAGNNNGAGANNNNGACNNNCCTTGGGTGGCAANNACNGCAATAAGTNNNGTTNNNC

CTNNNAGTNNACATNNNNNCANNGNNNCNNNNNNANCNGANNGNNNNNNNNNNNNNNNNNNNNNNNNNNN
NNNNNNNNNNNNNNNNNNNNNNNNNNNNNNNANNNNANNGNNNGTNTNTGNCNCATNNNTTTTANNNGNN
NNNNNNNGNNNNNNCCCCGGTCGNNTNCNNATTGAANNCCTGNNGCATNNNNNTNCNAGAATGANNNC
NNNNNGNNTNNNCTNNCNNNTNAAGNAATCANNNAGGNNNNNNNNTCNNNNNNNNNNNNCNNTCNCNN
ACTGAGGGTNNNNNNNNCANCNGCTNNNNNNNNNNTNCGCNGGNGTNNCNNNNNNGCNGTATNNNGTNN
NNCANNNNNNNNNNNNNNNNNNTNAAACNANNNNNNNTNNNN
>CoLao575
GTGNNNNNNAGCTCNNNNNAGTTAGACANNNCNNNNNNNNNNNNNTNNNNNNNNNNNNNNNNNNNNNNNN
NNNNNNNNNNNNNNNNNNNNNNNNNNNNNNNTNNNNNNNNNNNNNNNNNNNNNNNNNNNNNNNNNNNNNN
NNNNNNNNNNNNNNNNNNNNNNNNNNNNNNNNNNNNNNNNNNNNNNNNNNNNNNNNNNNNNNNNNNNNNN
NNNNNNNNNNNNNNNNNNNNNNNNNNNNNNNNNNNNNNNNNNNNNNNNNNNNNNNNNNNNNNNNNNNNNN
NNNNNNNNNNNNNNNNNNNNNNNNNNNNNNNNNNNNNNNNNNNNNNNNNNNCNTCTNNNAAANNNTNNN
NNNNNNNNNNNNNNNNNNNNNNNNNNNCNCNNTNNNNGTAANCNANNNGNACATGCAGTCCCCTCNNNNN
NNNNNCAGAGNNNNNNNCCNNNNNCNCCGGCCCCNNCCNNNNNNNNNNNNNNNNNNNNNNNNNGNNNNNN
NNNNNNTGGTACNNNNCGNNNNNGGTTGNNTNAGAGGTCCACCACGNGNTCNGNNNACNNNNGNNNAN
NNNCNNNAANCNGNNNNNNNNNNANNTNNNCANNAGGNNNNNNCCNTCNNNNNNNNNNANNGCNATGACN
CANNNNNCNGGGACNGTNACCGGCGNGGCGACCTTGATNANGGTCGGGNACAAGTATGTCGNNCNCCC
CGGGAAGGANNCNGCNGTANGTGCNNNNNANNNNNTCNTGNGANGTNGCCGNNATTCNCNTGGTCNNN
CNAGAATGNCCNNACCANNCGNANNNTGGTTATGNNAGTNNNNNNNNNNNNNNNNNNNNNNNNNNNNNNN
NNNNNNNNNNNNNNNNNNNNNNNNNNNNNNNNNNNNNCNGNNNNNNCNANNNAAGGNNNTANNNTTNNTN
NTNNNNNNGGACNNNACNNNNCNNNNNNNACNCNNNCNCNNNNNNNNNNGNTTNNNNNNCNNTANTNT
CCAGNANGCNGNGCNNNCNTTCANGNGAACNNNGNNACGCGNNAATCGTANNNTCTACCTGCCGCNNG
NNNNNNTNCNGNNNNNNCGGANNNTNNCTCGTANNNNNNNCANGCGTNNNNTNNTNNCNAGNCNNGTT
CGNNNANANANTAGNANGTNTNNNNNNNNNNTGTNNCGTTCCGCNTGANNCNNCNNNNNGNNGNNNTTN
NCANCGGCNNTANCTGCNGANNTAAGNCATANNNTNNNTCNNTANCGNNNNNGGNGGGNNACTGTAAA
NGGNNAATTNGNNNNTNATTNCCNNCACNTNNCTNNGNNNNCTNNNCNCCTGNNTNCNNAATTGGNNN
CANTGNNTGNCNATCTCTNGNTTNGNNNNNNTNNNNNTNNTANNNNGGNNNNNNANANNNCNATNTAA
CNTANANNNNCNNNNNNANNNCACNNNNNNNGNAGNNNCNANNNTNANGNNNGTAGNNNANTAAGTCT
CCGANNTNNNNNNNATAAGCGNNANACNNNNNNNNNNNNNANGANNNCGNNTTGGNNNTNNNNNNNNA
ANNAGACNNCNGTGGNTNNNNNTCAANNNNTTNGTAGTGGTATAACNANNNNNNNTTTCNCCAGNNTN
NNNGNNNNNNNCNATANNANNNNNNNGNGANNNNNNNNNNNANNNNCCCNNNAGGGANCANCTACTTNA
NCANNGGNGCGNAGNNNGAGANNNNGACNNNCCTTGGGTGGCANNNACNGAATAAGTNNGTTNNNN
NCNNNAGTNNACACNNNNNCANNGNNNCNNNNNNANCNGANNTNNNNNNNNNNNNNNNNNNNNNNNNNNN
NNNNNNNNNNNNNNNNNNNNNNNNNNNNNNANNNNANGGNNNGTNTNTGNCNCATGNNTTTNTANNNGNN
NNNNNNNGNNNNANCCCCGGTCGNNNNCNNATTGAANNCCTGNNGCATNNNNNTNCNAGAATGANNNC
NNNNNGNNTNGNCTNNCNNNTNAAGNAGTCNANNAGNNNNNNNGNTCNNNGNNNNNNNNCNNTCNCNN
ACTGAGGGTNNNNNNNNCATCTGCTNNNNNNNNNNTNCGCNGNNNNNNTNNNNNAGCNGTATNGNGNNN
NNCANNNNNNNNNNNNNNNNNNTNNAACGANNNNNNNTNNNN
>CoLao576
GTGNNNNNNAGCCCNNNNNAGTTAGACANNNCNNNNNNNNNNNNNTNNNNNNNNNNNNNNNNNNNNNNNN
NNNNNNNNNNNNNNNNNNNNNNNNNNNNNNNTNNNNNNNNNNNNNNNNNNNNNNNNNNNNNNNNNNNNNN
NNNNNNNNNNNNNNNNNNNNNNNNNNNNNNNNNNNNNNNNNNNNNNNNNNNNNNNNNNNNNNNNNNNNNN
NNNNNNNNNNNNNNNNNNNNNNNNNNNNNNNNNNNNNNNNNNNNNNNNNNNNNNNNNNNNNNNNNNNNNN
NNNNNNNNNNNNNNNNNNNNNNNNNNNNNNNNNNNNNNNNNNNNNNNNNNNNNNNNTNNNAAANNNTNNN
NNNNNNNNNNNNNNNNNNNNNNNNNNNCNCNNTNNNNGTAANCNANNNGNACATGCAGTNCCCTCNNNNN
NNNGGCAGAGNNNNNNNCNNNNNNNNCCGNCCCCNNCCNNNNNNNNNNNNNNNNNGNNNNNNNANNNNNN
NNNNNNTGGTACNNNNCGNNNNNGGTTGTNTNAGAGGTCCACAACGNGNTCNGNNNACNNNNGNNNAN
NNNCNNNAAANNNNNNNNNNNNNNNNNTNNNTANNAGGNNNNNNCCNTNNNNNNNNNNNATNGCNATGANN
CANNNNNCGGGGGNNGTNCCCGGCGNGGCGACCTTGNNNANGTCNGGGNNCAAGTATGGCGNNCNCCC
CGNGAAGGANNCNGCNGTANGTGCNNNNNANNNNNTCNTGNGANGTNGCCGNNATTCNCGTGGTCNNN
CNAGAATGNCCNNACCANNCGNANNNTGGTNATGNNAGTNNNNNNNNNNNNNNNNNNNNNNNNNNNNNNN
NNNNNNNNNNNNNNNNNNNNNNNNNNNNNNNNNNGNNNNGNNNNNNCNANNNAAGGNNNTANNNCTNNTN
NTNNNNNNGNNCNNNACNNNNCNNNNNNNACNCNNNCNCNNNNNNANNNGGTTNNNNNNCNNTANTNT
CCAGNACGCNGNGCNNNCATTCANGNGAACNNNGNNACGCGNNAATCGTANNATCTACCTGCCGCNNG

NNNNNNTNCNGNNNNNNCGGANNNTNNNNCGTANNNNNNNCANGCGTNNTNTNNTNNCNAGNCNNGTT
CGNNNANANANTAGNANGTNTNNNNNNNNNNTGTNNCGTTCCGCNTGANNCNNCNNNNNGNNGNNNTTN
NCANCGGCNNTANCTNCNGANNTAAGNCATANNNTNNNTCNNTANCTNNNNNGGNGGGNNACTGTAAA
NGGNNAATTNGNNNNTNANTNCNNNCACNTNNCNNNGNNTNCTNNNTNCCTGNNTNCNNAATTGGNNC
CANTGNNTGNCNATCTCTNGNTTNGNNNNNNTNNNNNTNNCANNNNGGNNNGTNNNANNNCNATNTAA
NNTANANNNNCNNNNNNANNNCACNNNNNNNNNAGNNNCNANNNTNANANNNGTAGNNNANTAAGTCT
CCGANNTNNNNNNNATAAGCGNNANACNNNNNNNNNNNNNNANGANNACGNNTTGGNNNTNNNNNNNNA
GNNAGACNNTNATGGNTNNNNNTCAANNNNTTNGTAGTGGTATAATNGNNNNNNNTTTCNCCAGNNTN
NNNGNNNNNNNCNATANNANNNNNNNGNGANNNNNNNNNNANNNNCTCNNNAGGGANCANCTACTNNA
GCANNGGNGCGNAGNNNGAGANNNNGACNNNCCTTGGGTGGCAANNACNGNAATAAGTNNGTTNNNN
CTNNNAGTNNACATNNNNNCANNGNNNCNNNNNNANCNGANNGNNNNNNNNNNNNNNNNNNNNNNNNN
NNNNNNNNNNNNNNNNNNNNNNNNNNNNNNNNNNNANNGNNNGTNTNTGACNCATGTGTTTTTANNNGNN
NNNNNNNGNNNNANCCCCGGTCGNNNNCNNATTGAANNCCTGNNGCATNNNNNTNCNAGAATGANNNC
NNNNNGNNTNNNCTGNCNNNTCAAGNAATCNNNNAGNNNNNNNNNTCNNNNNNNNNNNNCNNNNNCNN
ACTGAGGGTNNNNNNNNCATNNGCTNNNNNNNNNNTNCGCNGGNGTNNCNNNNNAGCNGTATNGNGTNN
NNCANNNNNNNNNNNNNNNNNTNAAACAANNNNNNNTNNNN
>CoLao577
GTGNNNNNTAGCCCNNNNNAGTTAGACANNNCNNNNNNNNNNNNNTNNNNNNNNNNNNNNNNNNNNNNN
NNNNNNNNNNNNNNNNNNNNNNNNNNNNNNNNNNNNNNNNNNNNNNNNNNNNNNNNNNNNNNNNNNNN
NNNNNNNNNNNNNNNNNNNNNNNNNNNNNNNNNNNNNNNNNNNNNNNNNNNNNNNNNNNNNNNNNNNN
NNNNNNNNNNNNNNNNNNNNNNNNNNNNNNNNNNNNNNNNNNNNNNNNNNNNNNNNNNNNNNNNNNNN
NNNNNNNNNNNNNNNNNNNNNNNNNNNNNNNNNNNNNNNNNNNNNNNNNNNNNCNTCTNNNAAANNNTNNN
NNNNNNNNNNNNNNNNNNNNNNNNNNNNNCNCNNTGNNNGTAANCNANNNGNACATGCAGTCCCCTCNNNNN
NNNNNCAGAGNNNNNNNCCNNNNNNNCCGNCCCCNNCCNNNNNNNNNNNNCNNNGNNNNNNNANNNNNN
NNNNNNTGGTACNNNNCGNNNNNGGTTGTNTAAGAGGTCCACAACGNGNTCNNGNNACNNNNGNNNAN
NNNCNNNNNANNNNNNNNNNNNNANNTNNNTANNAGGNCNNNNCCNTCNNNNNNNNNNANNGCNATGACC
CANNNNNCGGNGGCNGTNCCCGGCGCGGCGACCTTGNNNANGTCCGGGGNCAAGTATGGCGNNCNCCC
CGNGAAGGANNCNGCNGTANGTGCNNNNNANNNNNTCNTGNGANGTCGCCGNNATTCNCNTGGTCNNN
CNAGAATGNCCNNACCANNCGNANNNTGGTNATGNNAGTNNNNNNNNNNNNNNNNNNNNNNNNNNNNN
NNNNNNNNNNNNNNNNNNNNNNNNNNNNNNNNNNNNNNNGNNNNNNCNANNNAAGGNNNTANNNCTNNTN
NTNNNNNNNGGACNNNACNNNNCNNNNNNNACNCNNNCNCNNNNNNANNNGGTTTTNNNNCNNTANTNT
CCAGNACGCNGNGCNNNCNTTCANGNGAACNNNGNNACGCGNNAATCGTANNATCTACCTGCCGCNNG
NNNNNNTNCNGNNNNNNCGGANNNTNNNNCGTANNNNNNTCANGCGTNNTNTNNTCNCNAGNCNNGTT
CGNNNANANANTAGNANGTNTNNNNNNNNNNTGTNNCGTTCCGCNTGANNCNNCANNNNGNNGNNNTTN
NCANCGGCNNTANCTGCNGANNTAAGNCATANNNTNNNTCNNTANCTNNNNNGGNGGGNNACTGTAAA
NGNNNAATTNGNNNNTNANTNCNNNCACNTNNCNNNGNNTNCTNNNTNCCTGNNTNCNNAATTGGNNN
CANTGNNTGNCNATCTCTGGNTTNGNNNNNNTGNNNNTNNCANNNNGGNNNNNNNNANNNCNATNTAA
NNTANANNNNCNNNNNNANNNCACNNNNNNNNNAGNNNNNANNNTTANANNNGTAGNNNANTAAGTCT
CCGANNTNNNNNNNATAAGCGNNANACNNNNNNNNNNNNNNANGANNNCGNNTTGGNNNTNNNNNNNNA
GNNAGACNNTNATGGNTNNNNNTCAANNNNTTNGTAGTGGTATAATNGNNNNNNNTTTCNCCAGNNTN
NNNGNNNNNNNCNATANNANNNNNNNGNGANNNNNNNNNNANNNNCTCNNNAGGNANCANCTACTNNA
GCANNGGNGCGNAGNNNGAGANNNNGACNNGCCTTGGGTGGCAANNACNGCAANAAGTNNNGTTNNNN
CTNNNAGTNNACATNNNNNCANNGNNNCNNNNNNANCNGANNGNNNNNNNNNNNNNNNNNNNNNNNNN
NNNNNNNNNNNNNNNNNNNNNNNNNNNNNNNNNNNANNGNNNGTNTNTGNCNCATGTGTTTTTANNNGNN
NNNNNACGNNNNANCCCCGGTCGNNNNCNNATTGAANNCCTGNNGCATNNNNNTNCNAGAATGANNNC
NNNNNGNNTNGNCTNNCNNNTCAAGNAATCANNTAGGNNNNNNNNTCNNNNNNNNNNNNNCNNTNNCNN
ACTGAGGGTNNNNNNNNCATCTGCTNNNNNNNNNNTNCGCNGGNGTNNCNNNNNNGCNGTATNGNGTNA
NNCANNNNNNNNNNNNNNNNNTNAAACNANNNNNCTNNNN
>CoLao578
GTGNNNNNTAGCCCNNNNNAGTTAGACANNNCNNNNNNNNNNNNNTNNNNNNNNNNNNNNNNNNNNNNN
NNNNNNNNNNNNNNNNNNNNNNNNNNNNNNNNNNNNNNNNNNNNNNNNNNNNNNNNNNNNNNNNNNNN
NNNNNNNNNNNNNNNNNNNNNNNNNNNNNNNNNNNNNNNNNNNNNNNNNNNNNNNNNNNNNNNNNNNN
NNNNNNNNNNNNNNNNNNNNNNNNNNNNNNNNNNNNNNNNNNNNNNNNNNNNNNNNNNNNNNNNNNNN
NNNNNNNNNNNNNNNNNNNNNNNNNNNNNNNNNNNNNNNNNNNNNNNNNNNNNCNTCTNNNAAANNNTNNN

NNNNNNNNNNNNNNNNNNNNNNNNNNCNCNTTGNNNGTAANCNANNNGNACATGCAGTCCCCTCNNNNN
NNNGGCAGAGNNNNNNNCNNNNNNNNCCGGCCCCGNCCNNNNNNNNNNNCNNNNNNNNNNNANNNNNN
NNNNNNTGGTACNNNNCGNNNNNGGTTGNNTAAGAGGTCCACAACGNGGTCNNGNNACNNNNGNNNAN
NNNCNNNNNNCNNNNNNNNNNNNANNTNNNTANNAGGNNNNNNCCNTCNNNNNNNNNAATNGCNATGACC
CANNNCACGGGGGNNGTCCCCGGCGNGGCGACCTTGANNACGTCCGGGGNCAAGTATGGCNNNCNCCC
CGGGAAGGANNCNGCCGTANGTGCNNNNNANNNNNTCNTGNGANGTCGCCGNNATTNNCGTGGTCNNN
CNAGAATGNCCNNACCANNCGNANNNTGGTNATGNNAGTNNNNNNNNNNNNNNNNNNNNNNNNNNNNNNN
NNNNNNNNNNNNNNNNNNNNNNNNNNNNNNNNNNGNNNNGNNNNNNCNANNNAAGGNNNTANNNCTNNTN
NNNNNNNNGGACNNNACNNNNCNNNNNNNACNCNNNCNCNNNNNNANNNGGTTTTNNNNCNNTANTNT
CCAGNACGCNGNGCNNNCATTCANGNGAACNNNGNNACGCGNNAATCGTANNATCTACCTGCCGCNNG
NNNNNNTACNGNNNNNNCGGANNNTNNCTCGTANNNNNNNCATGCGTNNTNTNNTCNCNAGNCANGTT
CGNNNANANANTAGNANGTNTNNNNNNNNNNTGTNNCGTTCCGCNTGANNCNNCANNNNGNNGNNCTTN
NCANCGGCNNTANCTGCNGANNTAAGNCATANNNTNNNTCNNTANCTNNNNNGGNGGGNNACTGTAAA
NGGNNAATTGGNNNNTNANTNCNNNCACNTNNCTNNGNNNNCTNNNTNCCTGNNTNCNNAATTGGNNN
CANTGNNTGNCNATCTCTNGNTTNGNNNNNNTGNNNNTNNCANNNNGGNNNNNNANANNNCNATNTAA
NNTANANNNNCNNNNNNANNNCACNNNNNNNGNAGNNNCNANNNTTANANNNGTAGNNNAATAAGTCT
CCGANNTNNNNNNNATAAGCGNNANACNNNNNNNNNNNNNNANGANNACGNNTTGGNNNTNNNNNNNNA
GNNAGACNNTNATGGNTNNNNNTCAANNNNTTNGTAGTGGTATAATNGNNNNNNNTTTCNCCAGNNTN
NNNGNNNNNNNCNATANNANNNNNNNGNGANNNNNNNNNNNANNNNCTCNNNAGGGANCANCTACTNNA
GCANNGGNGCGNAGNNNGAGANNNNGACNNGCCTTGGGTGGCAANNACNGCAATAAGTNNNGTTNANC
CTNNNAGTNNACATNNNNNCANNGNNNCNNNNNNANCNGANNGNNNNNNNNNNNNNNNNNNNNNNNNNN
NNNNNNNNNNNNNNNNNNNNNNNNNNNNANNNNANGGNNNGTNTNTGNCNCATGTGTTTTTANNNGNN
NNNNNACGNNNGANCCCCGGTCGNNNNCNNATTGAANNCCTGNNGCATNNNNNTNCNAGAATGANNGC
NNNNNGNNTNGNCTNNCNNNTCAAGNAATCANNNAGNNNNNNNGNTCNNNNNNNNNNNNNCNNTCCCNN
ACTGAGGGTNNNNNNNNCATCTGCTNNNNNNNNNNTNCGCNGGNGTNNCNNNNNNGCNGTATNGTGTNA
NNCANNNNNNNNNNNNNNNNNTNAAACNANNNNNNTNNNN
>CoLao580
GTGNNNNNTAGCCCNNNNNAGTTAGACANNNCNNNNNNNNNNNNTNNNNNNNNNNNNNNNNNNNNNNNNN
NNNNNNNNNNNNNNNNNNNNNNNNNNNNNNNNNNNNNNNNNNNNNNNNNNNNNNNNNNNNNNNNNNNNNN
NNNNNNNNNNNNNNNNNNNNNNNNNNNNNNNNNNNNNNNNNNNNNNNNNNNNNNNNNNNNNNNCNNNNNN
NNNNNNNNNNNNNNNNNNNNNNNNNNNNNNNNNNNNNNNNNNNNNNNNNNNNNNNNNNNNNNNNNNNNNN
NNNNNNNNNNNNNNNNNNNNNNNNNNNNNNNNNNNNNNNNNNNNNNNNNNNNNNTCTNNNAAANNNTNNN
NNNNNNNNNNNNNNNNNNNNNNNNNNCNCNTTNNNNGTAANCNANNNGNACATGCAGTCCCCTCNNNNN
NNNGGCAGAGNNNNNNNNCNNNNNNNCCGNCCCCNNCCNNNNNNNNNNNNNNNNNGNNNNNNNANNNNNN
NNNNNNTGGTACNNNNCGNNNNNGGTTGTTTNAGAGGTCCACAACGNGNTCNNGNNACNNNNGNNNAN
NNNCNNNNAANNNNNNNNNNNNNANNTNNNTANNAGGNCNNNNCCNTCNNGNNNNNNATNGCNATGACC
CANNNCNCNGNGGNNGTNCCCGGCGCGGCGACCTTGNNNACGTCCGGGGNCAAGTATGGCNNNCNCCC
CGGGAAGGANNCNGCNGTANGTGCNNNNNANNNNNTCNTGNGANGTCGCCGNNATTCNNGTGGTCNNN
CNAGAATGNNCNNACCANNCGNANNNTGGTNATGTNAGTANNNNNNNNNNNNNNNNNNNNNNNNNNNNNN
NNNNNNNNNNNNNNNNNNNNNNNNNNNNNNNNNNNNNNNGNNNNNNCNANNNAAGGNNNTANNNCTNNTN
NNNNNNNNGNNCNNNACNNNNCNNNNNNNACNCNNNCNCNNNNNNNNNNNGGTTTTTNNNCNNTANTNT
CCAGNACGCNGNGCNNNCATTCANGNGAACNNNGNNACGCGNNAATCGTANNATCTACCTGCCGCNNG
NNNNNNTNCNGNNNNNNCGGANNNTNNCTCGTANNNNNNNCATGCGTNNTNTNNTCNCNAGNCNNGTT
CGNNNANANANTAGNANGTNTNNNNNNNNNNTGTNNCGTTCCGCNTGANNCNNCNNNNNGNNGNNNTTN
TCANCGGCNNTANCTGCNGANNTAAGNCATANNNTNNNTCNNTANCTNNNNNGGNGGGNNACTGTAAA
NGGNNAATTNGNNGNTNATTNCNNNCACNTNNCTNNGNNNNCTNNNTNCCTGNNTNCNNAATTGGNNC
CANTGNNTGNCNATCTCTGGNTTNGNNNNNNTNNNNNTNNCANNNNGGNNNNNNNNANNNCNATNTAA
NNTANANNNNCNNNNNNANNNCACNNNNNNNNNAGNNACNANNNTNANANNNGTAGNNNANTAAGTCT
CCGANNTNNNNNNNATAAGCGNNANACNNNNNNNNNNNNNNANGANNACGNNTTGGNNNTNNNNNNCNA
GNNAGACNNTNATGGNTNNNNNTCAANNNNTTNGTAGTGGTATAATNGNNNNNNNTTTCNCCAGNNTN
NNNGNNNNNNNCNATANNANNNNNNNGNGANNNNNNNNNNNANNNNCTCNNNAGGNANCANCTACTNNA
GCANTGGNGCGNAGNNNGAGANNNNGACNNGCCTTGGGTGGCAANNACNGCAATAAGTNNNGTTNNNC
CTNNNAGTNNACATNNNNNCANNGNNNCNNNNNNANCNGANNGNNNNNNNNNNNNNNNNNNNNNNNNNN
NNNNNNNNNNNNNNNNNNNNNNNNNNNNANNNNANGGNNNGTNTNTGACNCATGTGTTTTTANNNGNN

NNNNNACGNNNNANCCCCGGTCGNNNNCNNATTGAANNCCTGNNGCATNNNNNTNCNAGAATGANNGC
NNNNNGNNTNGNCTNNCNNNTCAAGNAATCANNNAGGTNNNNNNNTCNNNNNNNNNNNNCNNTCNCNN
ACTGAGGGTNNNNNNNNCATNNGCTNNNNNNNNCTNCGCNGGCGTNNCNNNNNAGCNGTATNGNGTNN
NNCANNNNNNNNNNNNNNNNNNTNAAACGANNNNNNNTNNNN
>CoLao581
GTNNNNNNTAGCCCNNNNNAGTTNGACANNNCNNNNNNNNNNNNNNNNNNNNNNNNNNNNNNNNNNNNNN
NNNNNNNNNNNNNNNNNNNNNNNNNNNNNNNNNNNNNNNNNNNNNNNNNNNNNNNNNNNNNNNNNNNNNN
NNNNNNNNNNNNNNNNNNNNNNNNNNNNNNNNNNNNNNNNNNNNNNNNNNNNNNNNNNNNNNNNNNNNNN
NNNNNNNNNNNNNNNNNNNNNNNNNNNNNNNNNNNNNNNNNNNNNNNNNNNNNNNNNNNNNNNNNNNNNN
NNNNNNNNNNNNNNNNNNNNNNNNNNNNNNNNNNNNNNNNNNNNNNNNNNNNNCNTCTNNNAAANNNTNNN
NNNNNNNNNNNNNNNNNNNNNNNNNNNCNCNTTNNNNGTAANCNANNNGNACATGCAGTCCCCTCNNNNN
NNNGGCAGAGNNNNNNNNCNNNNNNNNCCGNCCCCNNCCNNNNNNNNNNNNCNNNNNNNNNNNNANNNNNN
NNNNNNTGGTACNNNNCGNNNNNGGTTGTNTAAGAGGTCCACAACGNGGTCNGNNNACNGNNGNNNAN
NNNCNNNAAANNGNNNNNNNNNNANNTNNNTANNAGGNCNNNNCCNTCNNNNNNNNNNATNGCNATGANN
CANNNCNCNGNGGNNGTNCCCGGCGNGGCGACCTTGNNNACGTCCGGGNNCAAGTATGGCNNNCNCCC
CGGGAAGGANNCNGCNGTANGTGCNNNNNANNNNNTCNTGNGANGTNGCCGNNATTNNCGTGGNCNNN
CNAGANTGNNCNNACCANNCGNANNNTGGTNATGNNAGTNNNNNNNNNNNNNNNNNNNNNNNNNNNNN
NNNNNNNNNNNNNNNNNNNNNNNNNNNNNNNNNNNGNNNNGNNNNNNCNANNNAAGGNNNTANNNCTNNNN
NNNNNNNNNNNNCNNNACNNNNCNNNNNNNNACNCNNNCNCNNNNNNANNNGGTTNNNNNNNCNNTANTNN
CCAGNANGCNGNGCNNNCNTTCANGNGAACNNNGNNACGCGNNAATCGTANNATCTACCTGCCGCNNG
NNNNNNTNCNGNNNNNNNCGGANNNTNNCNNGTANNNNNNNNCATGCGTNNNNNTNNTCNCNAGNCANGTT
CGNNNANANANTAGNANGTNTNNNNNNNNNNNTGTNNCGTTCCGCNTGANNCNNCANNNNGNNGNNNTTN
NCANCGGCNNTANCTGCNGANNTAAGNCATANNNTNNNTCTNTANCTNNNNNGGNGGGNNACTGTAAA
NGGNNAATTNGNNNNTNANTNCNNNCACNTNNCNNNGNNNNCTNNNTNCCTGNNTNCNNAATTGGNNN
CANTGNNTGNCNATCTCTNGNTTNGNNNNNNNTNNNNNNTNNCANNNNGGNNNGTNNNANNNCNATNTAA
NNTANANNNNCNNNNNNANNNCACNNNNNNNNNNAGNNNCNANNNTNANANNNGTAGNNNANTAAGTCN
CCGANNTNNNNNNNNATAAGCGNNANACNNNNNNNNNNNNNNNANGANNACGNNTTGGNNNTNNNNNNNNNA
GNNAGACNNTNATGGNTNNNNNNTNNANNNNTTNGTAGTGGTATAATNGNNNNNNNNTTNCNCCAGNNTN
NNNGNNNNNNNNCNATANNANNNNNNNNGNGANNNNNNNNNNNNANNNNNNTCNNNAGGNANCANCTACTNNA
GCANNGGNNCGNAGNNNGAGANNNNGACNNNCCTTGGGTGGCANNNACNGNANTAAGTNNNGTTNNNN
CTNNNAGTNNACATNNNNNCANNGNNNCNNNNNNANCNGANNGNNNNNNNNNNNNNNNNNNNNNNNNNN
NNNNNNNNNNNNNNNNNNNNNNNNNNNNNNNNNNNNNNNANNGNNNGTNTNTGACNCNTNNNTTTTTANNNGNN
NNNNNNNGNNNGANCCCCGGTCGNNNNCNNATTGAANNCCTGNNGCATNNNNNTNCNAGAATGANNNC
NNNNNGNNTNGNCTNNNNNNNTCAAGNAATCNNNNANNNNNNNNNNNNTCNNNGNNNNNNNNNCNNNCNCNN
ACTGAGGGNNNNNNNNNCATCTGCTNNNNNNNNNNTNCNCNGNNNNNNNCNNNNNNGCNGTATNNNGNNN
NNCANNNNNNNNNNNNNNNNNNTNAAACAANNNNNNNTNNNN
>CoLao582
GTGNNNNNTAGCCCNNNNNAGTTAGACANNNCNNNNNNNNNNNNNTNNNNNNNNNNNNNNNNNNNNNNN
NNNNNNNNNNNNNNNNNNNNNNNNNNNNNNNNNNNNNNNNNNNNNNNNNNNNNNNNNNNNNNNNNNNNNN
NNNNNNNNNNNNNNNNNNNNNNNNNNNNNNNNNNNNNNNNNNNNNNNNNNNNNNNNNNNNNNNNNNNNNN
NNNNNNNNNNNNNNNNNNNNNNNNNNNNNNNNNNNNNNNNNNNNNNNNNNNNNNNNNNNNNNNNNNNNNN
NNNNNNNNNNNNNNNNNNNNNNNNNNNNNNNNNNNNNNNNNNNNNNNNNNNNNNTCTNNNAAANNNTNNN
NNNNNNNNNNNNNNNNNNNNNNNNNNNCNCNTTGNNNGTAANCNANNNGNACATGCAGTCCCCTCNNNNN
NNNNNCAGAGNNNNNNNNCCNNNNNCNCCGNCCCCNNCCNNNNNNNNTNNNCNNNGNNNNNNNNANNNNNN
NNNNNNTGGTACNNNNCGNNNNNGGTTGTNTAAGAGGTCCACAACGNGGTCNNGNCACGNNNGNNNAN
NNNCNNNNAANNNNNNNNNNNNNAANNTNNNTANNAGGNNNNNNCCNTCNNGNNNNNNANNGCNATGACC
CANNNCACGGNGGCNGTCCCCGGNGCGGCGACCTTGANNANGTCCGGGGNCAAGTATGGCNNNCNCCC
CGGGAAGGANNCNGCCGTANGTGCNNNNNANNNNNTCNTGNGANGTCGCCGNNATTNNCGTGGTCNNN
CNAGAATGNCCNNACCANNCGNANNNTGGTNATGNNAGTNNNNNNNNNNNNNNNNNNNNNNNNNNNNN
NNNNNNNNNNNNNNNNNNNNNNNNNNNNNNNNNNNGNNNNGNNNNNNCNANNNAAGGNNNTANNNCTNNTN
NTNNNNNNNGGACNNNACNNNNCNNNNNNNNACNCNNNCNCNNNNNNANNNGNTTTTNNNNNCNNTANTNT
CCAGNACGCNGNGCNNNCNTTCANGNGAACNNNGNNACGCGNNAATCGTANNATCTACCTGCCGCNNG
NNNNNNTACNGNNNNNNNCGGANNNTNNNNNCGTANNNNNNNNCATGCGTNNTNTNNTNNCNAGNCANGTT
CGNNNANANANTAGNANGTNTNNNNNNNNNNNTGTNNCGTTCCGCNTGACNCNNCANNNNGNNGNNCTTN

TCANCGGCNNTANCTGCNGANNTAAGNCATANNNTNNNNTCNNTANCTNNNNNNGGNGGGNNACTGTAAA
NGGNNAATTNGNNNNNTNATTNCNNNCACNTNNCNNNGNNTNCTNNNNTNCCTGNNTNCNNAATTGGNNC
CANTGNNTGNCNATCTCTNGNTTNGNNNNNNTNNNNNNTNNCANNNNGGNNNGTNNNANNNCNATNTAA
NNTANANNNNCNNNNNNANNNCACNNNNNNNNNAGNNNCNANNNNTTANANNNGTAGNNNNAATAAGTCT
CCGANNTNNNNNNNATAAGCGNNANACNNNNNNNNNNNNNNANGANNACGNNTTGGNNNTNNNNNNCNA
GNNAGACNNTNATGGNTNNNNNTCAANNNNTTNGTAGTGGTATAATNGNNNNNNNTTTCNCCAGNNTN
NNNGNNNNNNNCNATANNANNNNNNNGNGANNNNNNNNNNNANNNNCTCNNNAGGGANCANCTACTTNA
NCANNGGNGCGNAGNNNGAGANNNNGACNNNCCTTGGGTGGCAANNACNGNAATAAGTNNNGTTNNNC
CTNNNAGTNNACATNNNNNCANNGNNNCNNNNNNANCNGANNGNNNNNNNNNNNNNNNNNNNNNNNNNNN
NNNNNNNNNNNNNNNNNNNNNNNNNNNNNNAGNNNANNGNNNGTNTNTGACNCNTGTGTTTTTANNNGNN
NNNNNACGNNNNANCCCCGGTCGNNNNCNNATTGAANNCCTGNNGCATNNNNNTNCNAGAATGANNNC
NNNNNGNNTNGNCTNNNNNNTCAAGNAATCANNNAGGTANNNNNNTCNNNGNNNNNNNNCNNTCCCNN
ACTGAGGGTNNNNNNNNCATCTGCTNNNNNNNNCTNCGCNGGCGTNNCNNNNNNGCNGTATNGNGTNA
NNCANNNNNNNNNNNNNNNNNTNNAACGANNNNNNNTNNNN
>CoLao584
GTGNNNNNTAGCCCNNNNNAGTTAGACANNNCNNNNNNNNNNNNNTNNNNNNNNNNNNNNNNNNNNNNNN
NNNNNNNNNNNNNNNNNNNNNNNNNNNNNNNNNNNNNNNNNNNNNNNNNNNNNNNNNNNNNNNNNNNNNN
NNNNNNNNNNNNNNNNNNNNNNNNNNNNNNNNNNNNNNNNNNNNNNNNNNNNNNNNNNNNNNNNNNNNNN
NNNNNNNNNNNNNNNNNNNNNNNNNNNNNNNNNNNNNNNNNNNNNNNNNNNNNNNNNNNNNNNNNNNNNN
NNNNNNNNNNNNNNNNNNNNNNNNNNNNNNNNNNNNNNNNNNNNNNNNNNNCNNCTNNNAAANNNTNNN
NNNNNNNNNNNNNNNNNNNNNNNNNNNCNCNTTNNNNGTAANCNANNNGNACATGCAGTCCCCTCNNNNN
NNNNNCAGAGNNNNNNNCCNNNNNNNCCGGCCCCNNCCNNNNNNNNNNNNCNNNGNNNNNNNANNNNNN
NNNNCNTGGTACNNNNCGNNNNNGGTTGTTTNAGAGGTCCACAACGNGNTCNNNNNACNNNNGNNNAN
NNNCNNNNANNNNNNNNNNNNNNNNNTNNNTANNAGGNCNNNNCCNTNNNGNNNNNNANNGCNATGACC
CANNTCNCNGGGGCNGTNCCCGGNGCGGNGACCTTGNNNANGTCCGGGNNCAAGTATGGCNNNCNCCC
CGGGAAGGANNCNGCNGTANGTGCNNNNNANNNNNTCNTGNGANGTCGCCGNNATTCNCGTGGTCNNN
CNAGAATGNNCNNACCANNCGNANNNTGGTTATGNNAGTNNNNNNNNNNNNNNNNNNNNNNNNNNNNNNN
NNNNNNNNNNNNNNNNNNNNNNNNNNNNNNNNNNGNNNNGNNNNNNCNANNNAAGGNNNTANNNCTNNTN
NTNNNNNNNGGACNNNACNNNNCNNNNNNNNACNCNNNCNCNNNNNNNNNNNGGTTTTNNNNCNNTANTNT
CCAGNACGCNGNGCNNNCNTTCANGNGAACNNNGNNACGCGNNAATCGTANNATCTACCTGCCGCNNG
NNNNNNTACNGNNNNNNCGGANNNTNNNNNGTANNNNNNNNCANGCGTNNTNTNNTCNCNAGNCNNGTT
CGNNNANANANTAGNANGTNTNNNNNNNNNNTGTNNCGTTCCGCNTGANNCNNCANNNNGNNGNNNTTN
TCANCGGCNNTANCTNNNGANNTAAGNCATANNNTNNNTCNNTANCTNNNNNGGNGGGNNACTGTAAA
NGGNNAATTNGNNNNTNATTNCNNNCACNTNNCTNNGNNNNCTNNNTNCCTGNNTNCNNAATTGGNNN
CANTGNNTGNCNATCTCTNGNTTNGNNNNNNTNNNNNTNNCANNNNGGNNNGTNANANNNCNATNTAA
NNTANANNNNCNNNNNNANNNCACNNNNNNNNNNAGNNNCNANNNTNANANNNGTAGNNNANTAAGTCT
CCGANNTNNNNNNNATAAGCGNNANACNNNNNNNNNNNNNNANGANNACGNNTTGGNNNTNNNNNNNNA
GNNANACNNTNATGGNTNNNNNTCAANNNNTTCGTAGTGGTATAATNGNNNNNNNTTTCNCCAGNNTN
NNNGNNNNNNNCNATANNANNNNNNNGNGANNNNNNNNNNNANNNNCTCNNNAGGGANCANCTACTNNA
NCANTGGNGCGNAGNNNGAGANNNNGACNNNCCTTGGGTGGCAANNACNGNAATAAGTNNNGTTNNNC
CTNNNAGTNNACATNNNNNCANNGNNNCNNNNNNANCNGANNGNNNNNNNNNNNNNNNNNNNNNNNNNNN
NNNNNNNNNNNNNNNNNNNNNNNNNNNNNNNNNNNANGGNNNGTNTNTGNCNCATGTGTTTTTANNNGNN
NNNNNACGNNNGANCCCCGGTCGNNNNCNNATTGAANNCCTGNNGCATNNNNNTNCNAGAATGANNGC
NNNNNGNNTNGNCTNNCNNNTCAAGNAATCANNNAGGTNNNNNGNTCNNNNNNNNNNNNNCNNNCNCNN
ACTGAGGGTNNNNNNNNCATNNGCTNNNNNNNNCTNCGCNGNNGTNNCNNNNNAGCNGTATNGNGTNA
NNCANNNNNNNNNNNNNNNNNTNAAACAANNNNNNNTNNNN
>CoLao585
GTGNNNNNTAGCCCNNNNNAGTTAGACANNNCNNNNNNNNNNNNNTNNNNNNNNNNNNNNNNNNNNNNNN
NNNNNNNNNNNNNNNNNNNNNNNNNNNNNNNNNNNNNNNNNNNNNNNNNNNNNNNNNNNNNNNNNNNNNN
NNNNNNNNNNNNNNNNNNNNNNNNNNNNNNNNNNNNNNNNNNNNNNNNNNNNNNNNNNNNNNNNNNNNNN
NNNNNNNNNNNNNNNNNNNNNNNNNNNNNNNNNNNNNNNNNNNNNNNNNNNNNNNNNNNNNNNNNNNNNN
NNNNNNNNNNNNNNNNNNNNNNNNNNNNNNNNNNNNNNNNNNNNNNNNNNNNNNTCTNNNAAANNNTNNN
NNNNNNNNNNNNNNNNNNNNNNNNNNNCNCNTTNNNNGTAANCNANNNGNACATGCAGTCCCCTCNNNNN
NNNNNCAGAGNNNNNNCCCNNNNNNNNCCGNCCCCNNCCNNNNNNNNNNNNNNNNNNNNNNNNANNNNNN

NNNNNNTGGTACNNNNCGNNNNNGGTTGTNTAAGAGGTCCACAACGNGGTCNNNNNACNNNNGNNNAN
NNNCNNNNNNCNNNNNNNNNNNANNTNNNTANNAGGNCNNNNCCNTCNNGNNANTNANNGCNATGACC
CANNNCNCGGGGGCNGTNCCCGGCGNGGCGACCTTGANNACGTCNGGGGNCAAGTATGGCGNNCNCCC
CGGGAAGGANNCNGCNGTANGTGCNNNNNANNNNNTCNTGNGANGTCGCCGNNATTNNNNTGGTCNNN
CNAGAATGNNCNNACCANNCGNANNNTGGTNATGNNAGTNNNNNNNNNNNNNNNNNNNNNNNNNNNNN
NNNNNNNNNNNNNNNNNNNNNNNNNNNNNNNNNNNNNNGNNNNNNCNANNNAAGGNNNTANNNCTNNTN
NTNNNNNNNNNCNNNACNNNNNNNNNNNNNACNCNNNCNCNNNNNNANNNGNTTTTNNNNCNNTANTNT
CCAGNANGCNGNGCNNNCNTTCANGNGAACNNNGNNACGCGNNAATCGTANNATCTACCTGCCGCNNG
NNNNNNTACNGNNNNNNCGGANNNTNNNNCGTANNNNNNNCANGCGTNNTNTNNTCNCNAGNCNNGTT
CGNNNANANANTAGNANGTNTNNNNNNNNNNTGTNNCGTTCCGCNTGANNCNNCANNNNGNNGNNCTTN
TCANCGGCNNTANCTNCNGANNTAAGNCATANNNTNNNTCNNTANCTNNNNNGGNGGGNNACTGTAAA
NGGNNAATTNGNNNNTNANTNCNNNCACNTNNCNNNGNNNNCTNNNTNCCTGNNTNCNNAATTGGNNC
CANTGNNTGNCNATCTCTNGNTTNGNNNNNNTNNNNNTNNCANNNNGGNNNNNNNNANNNCNANNTAA
NNTANANNNNCNNNNNNANNNCACNNNNNNNGNAGNNNCNANNNTTANANNNGTAGNNNANTAAGTCT
CCGANNTNNNNNNNATAAGCGNNANACNNNNNNNNNNNNNANGANNNCGNNTTGGNNNTNNNNNNNNA
GNNAGACNNTNATGGNTNNNNNTCAANNNNTTNGTAGTGGTATAATNGNNNNNNNTTTCNCCAGNNTN
NNNGNNNNNNNCNATANNANNNNNNNGNGANNNNNNNNNNANNNNCTCNNNAGGGANCANCTACTNNA
GCANTGGNGCGNAGNNNGAGANNNNGACNNNCCTTGGGTGGCAANNACNGCAATAAGTNNGTTNNNN
CTNNNAGTNNACATNNNNNCANNGNNNCNNNNNNANCNGANNGNNNNNNNNNNNNNNNNNNNNNNNNN
NNNNNNNNNNNNNNNNNNNNNNNNNNNNNNNNNNNNNANGGNNNGTNTNTGNCNCATGTGTTTTTANNNGNN
NNNNNNNGNNNNANCCCCGGTCGNNNNCNNATTGAANNCCTGNNGCATNNNNNTNCNAGAATGANNNC
NNNNNGNNTNGNCTNNCNNNTCAAGNAATCANNNAGGNNNNNNNNTCNNNGNNNNNNNNCNNTCNCNN
ACTGAGGGTNNNNNNNNCATCTGCTNNNNNNNNNNTNCGCNGGNGTNNCNNNNNNGCNGTATNGNGTNA
NNCANNNNNNNNNNNNNNNNNTNAAACAANNNNNNNTNNNN
>CoLao586
GTGNNNNNNAGCCCNNNNNAGTTNGACANNNCNNNNNNNNNNNNTNNNNNNNNNNNNNNNNNNNNNN
NNNNNNNNNNNNNNNNNNNNNNNNNNNNNNNNNTNNNNNNNNNNNNNNNNNNNNNNNNNNNNNNNNNNNNN
NNNNNNNNNNNNNNNNNNNNNNNNNNNNNNNNNNNNNNNNNNNNNNNNNNNNNNNNNNNNNNNNNNNNNNNNN
NNNNNNNNNNNNNNNNNNNNNNNNNNNNNNNNNNNNNNNNNNNNNNNNNNNNNNNNNNNNNNNNNNNNNNNNN
NNNNNNNNNNNNNNNNNNNNNNNNNNNNNNNNNNNNNNNNNNNNNNNNNNNNNNNNNNTCTNNNNAAANNNTNNN
NNNNNNNNNNNNNNNNNNNNNNNNNNNNCNCNNTNNNNGTNANCNANNNGNACATGCAGTCCCCTCNNNNN
NNNGGCAGAGNNNNNNNCCNNNNNCNCCGNCCCCNNCCNNNNNNNNNNNNNNNNNNNNNNNNNNANNNNNN
NNNNNNTGGTACNNNNCGNNNNNGGTTGTTTAAGAGGTCCACAACGNGNTCNNGNNACNNNNGNNNAN
NNNCNNNNAACNNNNNNNNNNNNNNNNTNNNTANAAGGNNNNNNCCNTCNNGNNNNNNATNGCNATGANN
CANNNCNCNGGGGNNGTNCCCGGCGNGGNGACCTTGANNANNTCCGGGGNCAAGTATGGCNNNCNCCC
CGNGAAGGANNCNGCNGTANGTGCNNNNNANNNNNTCNTGNGANGTNGCCGNNATTCNNNTGGTCNNN
CNANANTGNCCNNACCANNCGNANNNTGGTNATGNNAGTNNNNNNNNNNNNNNNNNNNNNNNNNNNNN
NNNNNNNNNNNNNNNNNNNNNNNNNNNNNNNNNNNNGNNNNGNNNNNNCNANNNAAGGNNNTANNNCTNNTN
NTNNNNNNGGACNNNACNNNNCNNNNNNNNACNCNNNCNCNNNNNNANNNGGTTNNNNNNNCNNTANTNT
CCAGNACGCNGNGCNNNCNTTCANGNGAACNNNGNNACGCGNNAATCGTANNATCTACCTGCCGCNNG
NNNNNNTACNGNNNNNNCGGANNNTNNNNCGTANNNNNNNCANGCGTNNTNTNNTCNCNAGNCNNGTT
CGNNNANNNANTAGNANGTNTNNNNNNNNNNTGTNNCGTTCCGCNTGANNCNNCNNNNNGNNGNNCTTN
NCANCGGCNNTANCTNCNGANNTAAGNCATANNNTNNNTCNNTANCTNNNNNGGNGGGNNACTGTAAA
NGGNNAATTNGNNGNTNANTNCNNNCACNTNNCNNNGNNNNCTNNNTNCCTGNNTNCNNAATTGGNNC
CANTGNNTGNCNATCTCTNGNTTNGNNNNNNTNNNNNTNNCANNNNGGNNNNNNNNANNNCNATNTAA
NNTANANNNNCNNNNNNANNNCACNNNNNNNNNNAGNNNNNANNNTNANANNNGTAGNNNANTNAGTCT
CCGANNTNNNNNNNATAAGCGNNANACNNNNNNNNNNNNNANGANNNCGNNTTGGNNNTNNNNNNNNA
GNNAGACNNTNATGGNTNNNNNTCAANNNNTTNGTAGTGGTATAATNGNNNNNNNTTTCNCCAGNNTN
NNNGNNNNNNNCNATANNANNNNNNNGNGANNNNNNNNNNNNNNNNCTCNNNAGGNANCANCTACTNNA
GCANNGGNGCGNAGNNNGAGANNNNGACNNNCCTTGGGTGGCANNNACNGNAATAAGTNNNGTTNNNN
NTNNNAGTNNACATNNNNNCANNGNNNCNNNNNNANCNGANNGNNNNNNNNNNNNNNNNNNNNNNNNN
NNNNNNNNNNNNNNNNNNNNNNNNNNNNNNNNNNNNNANGGNNNGTNTNTGNCNCNTGNGTTTTTANNNGNN
NNNNNACGNNNNANCCCCGGTCGNNNNCNNATTGAANNCCTGNNGCATNNNNNTNCNAGAATGANNNC
NNNNNGNNTNGNCTNNCNNNTCAAGNAATCNANNAGNNNTNNNNNTCNNNNNNNNNNNNCNNTCNCNN

ACTGAGGGTNNNNNNNNCATCNGCTNNNNNNNNCTNCGCNGGNGTNNCNNNNNNGCNGTATNGNGTNA
NNCANNNNNNNNNNNNNNNNNTNAAACGANNNNNNTNNNN
>CoLao587
GTNNNNNNNNAGCCCNNNNNAGTTAGACANNNCNNNNNNNNNNNNNTNNNNNNNNNNNNNNNNNNNNNNN
NNNNNNNNNNNNNNNNNNNNNNNNNNNNNNNNNNNNNNNNNNNNNNNNNNNNNNNNNNNN
NNNNNNNNNNNNNNNNNNNNNNNNNNNNNNNNNNNNNNNNNNNNNNNNNNNNNNNNNNNN
NNNNNNNNNNNNNNNNNNNNNNNNNNNNNNNNNNNNNNNNNNNNNNNNNNNNNNNNNNNN
NNNNNNNNNNNNNNNNNNNNNNNNNNNNNNNNNNNNNNNNNNNNNNNCNTNNNNNAAANNNTNNN
NNNNNNNNNNNNNNNNNNNNNNNNNNNCNCNNTNNNNGTAANCNANNNGNACATGCAGTCCCCTCNNNNN
NNNNNCAGAGNNNNNNNCCNNNNNNNCCGNCCCCNNCCNNNNNNNNNNNNNNNNNNNNNNNNNANNNNNN
NNNNNNTGGTACNNNNCGNNNNNGGNNGTNTAAGAGGTCCACAACGNGGTCNNNNNACNNNNGNNNAN
NNNCNNNNAACNGNNNNNNNNNNNNNTNNNTANNAGGNCNNNNCCNTCNNNNNNNNNATNGCNATGANN
CANNNNNCGGGNGNNGTNCCCGGNGNGGCGACCTTGANNANGNCNGGGGNCAAGTATGGCNNNCNCCC
CGNGAAGGANNCNGCNGTANGTGCNNNNNANNNNNTCNTGNGANGTCGCCGNNATTNNNNTGGTCNNN
NNANAATGNNCNNACCANNCGNANNNTGGTNATGNNAGTNNNNNNNNNNNNNNNNNNNNNNNNNNNNN
NNNNNNNNNNNNNNNNNNNNNNNNNNNNNNNNGNNNNGNNNNNNCNANNNAAGGNNNTANNNCTNNTN
NTNNNNNNNNNNCNNNACNNNNNNNNNNNNNACNCNNNCNNNNNNNNNANNNGNTTNNNNNNCNNTANTNN
CCAGNACGCNGNGCNNNCNTTCANGNGAACNNNGNNACGCGNNAATCGTANNNTCTANCTGCCGCNNG
NNNNNNTNCNGNNNNNNNCGGANNNTNNCTNGTANNNNNNNNCNNGCGTNNTNTNNTCNCNAGNCNNGTT
CGNNNANANANTAGNANGTNTNNNNNNNNNNTGTNNCGTTCCGCNTGANNCNNCNNNNNGNNGNNNTTN
NCANCGGCNNTANCTGCNGANNTAAGNCANANNNTNNNTCNNTANCTNNNNNGGNGGGNNACTGTAAA
NGNNNAATTNGNNNNTNANTNCNNNNACNNNNCNNNGNNNNCTNNNNTNCCTGNNTNCNNAATTGGNNN
CANTGNNTGNCNATCTCTNGNTTNGNNNNNNTNNNNNTNNCANNNNGGNNNNNNANANNNCNATNTAA
NNTANANNNNCNNNNNNANNNCACNNNNNNNNNAGNNNCNANNNTNANANNNGTAGNNNANTNAGTCT
CCGANNTNNNNNNNATAAGCGNNANACNNNNNNNNNNNNNANGANNNCGNNTTGGNNNTNNNNNNNNA
GNNAGACNNTNATGGNTNNNNNTCAANNNNTTNNTAGTGGTATAATNGNNNNNNNTTTCNCCAGNNNN
NNNGNNNNNNNNCNATNNNANNNNNNNGNGANNNNNNNNNNNANNNNCTCNNNAGGNANCANCTACTNNA
GCANNGGNGCGNAGNNNGAGANNNNGACNNNCCTTGGGTGGCAANNACNGNAATAAGTNNNGTTNNNN
CTNNNAGTNNACATNNNNNCANNGNNNCNNNNNNANCNGANNGNNNNNNNNNNNNNNNNNNNNNNNNN
NNNNNNNNNNNNNNNNNNNNNNNNNNNNNNNNNNNNNNNANGGNNNGTNTNTGNCNCNTGNNTTTTTANNNGNN
NNNNNNNNNNNNNANCCCCGGTCGNNNNCNNATTGAANNCCTGNNGCATNNNNNTNCNAGAATGANNNC
NNNNNGNNTNNNCTNNNNNNNTNAAGNAATCANNNAGNNNNNNNNNTCNNNNNNNNNNNNNCNNNNNCNN
ACTGAGGGNNNNNNNNNCATNNGCTNNNNNNNNCTNCGCNGGNGTNNCNNNNNNGCNGTATNGNGTNN
NNCANNNNNNNNNNNNNNNNNTNAAACAANNNNNNTNNNN
>CoLao588
GTGNNNNNTAGCCCNNNNNAGTTAGACANNNCNNNNNNNNNNNNNTNNNNNNNNNNNNNNNNNNNNNNN
NNNNNNNNNNNNNNNNNNNNNNNNNNNNNNNNNNNNNNNNNNNNNNNNNNNNNNNNNNNN
NNNNNNNNNNNNNNNNNNNNNNNNNNNNNNNNNNNNNNNNNNNNNNNNNNNNNNNNNNNN
NNNNNNNNNNNNNNNNNNNNNNNNNNNNNNNNNNNNNNNNNNNNNNNNNNNNNNNNNNNN
NNNNNNNNNNNNNNNNNNNNNNNNNNNNNNNNNNNNNNNNNNNNNNNNTCTNNNNAAANNNTNNN
NNNNNNNNNNNNNNNNNNNNNNNNNNNCNCNTTNNNNGTAANCNANNNGNACATGCAGTCCCCNCNNNNN
NNNNNCAGAGNNNNNNCCCNNNNNNNCCGNCCCCGNCCNNNNNNNNNNNNNNNNNNNNNNNNNANNNNNN
NNNNNNTGGTACNNNNCGNGNNNGGTTGTNTAAGAGGTCCACAACGNGNTCNGNNNACNNNNGNNNAN
NNNCNNNNAACNNNNNNNNNNNNNNNATNNNTANNAGGNCNNNNCCNTNNNNNNNNNNAATTGCNATGANN
CANNNCNCGGGGGCNGTNCCCGGCGCGGCGACCTTGNNNACGTCCGGGGNCAAGTATGGCNNNCNCCC
CGGGAAGGANNCNGCNGTANGTGCNNNNNANNNNNTCNTGNGANGTCGCCGNNATTCNCGTGGTCNNN
CNNGAATGNNCNNACCANNCGNANNNTGGTNATGNNAGTNNNNNNNNNNNNNNNNNNNNNNNNNNNNN
NNNNNNNNNNNNNNNNNNNNNNNNNNNNNNNNGNNNNGNNNNNNCNANNNAAGGNNNTANNNCTNNTN
NTNNNNNNNGGNCNNNACNNNNCNNNNNNNACNCNNNCNCNNNNNNNNNNNGGTTTTNNNNCNNTANTNN
CCAGNANGCNGNGCNNNCATTCANGNGAACNNNGNNACGCGNNAATCGTANNATCTACCTGCCGCNNG
NNNNNNTACNGNNNNNNNCGGANNNTNNNNNGTANNNNNNNNCATGCGTNNTNTNNTCNCNAGNCNNGTT
CGNNNANANANTAGNANGTNTNNNNNNNNNNTGTNNCGTTCCGCNTGANNCNNCNNNNNGNNGNNNTTN
NCANCGGCNNTANCTGCNGANNTAAGNCATANNNTNNNTCNNTANCTNNNNNGGNGGGNNACTGTAAA
NGGNNAATTNGNNNNTNANTNCNNNCACNTNNCNNNGNNTNCTNNNTNCCTGNNTNCNNAATTGGNNC

CANTGNNTGNCNATCTCTNGNTTNGNNNNNNTNNNNNTNNCANNNNGGNNNGNNANANNNCNATNTAA
NNTANANNNNCNNNNNNANNNCACNNNNNNNNNAGNNNCNNNNNTTANANNNGTAGNNNAATAAGTCT
CCGANNTNNNNNNNATAAGCGNNANACNNNNNNNNNNNNNNANGANNNCGNNTTGGNNNTNNNNNNCNA
GNNAGACNNTNATGGNTNNNNNTCAANNNNTTNNTAGTGGTATAATNGNNNNNNNTTTCNCCAGNNTN
NNNGNNNNNNNCNATNNNANNNNNNNGNGANNNNNNNNNNNANNNNCTCNNNAGGNANCANCTACTNNA
GCANNGGNGCGNAGNNNGAGANNNNGACNNNCCTTGGGTGGCAANNACNGCAATAAGTNNNGTTNANN
CTNNNAGTNNACATNNNNNCANNGNNNCNNNNNNANCNGANNGNNNNNNNNNNNNNNNNNNNNNNNN
NNNNNNNNNNNNNNNNNNNNNNNNNNNNNANNNNANGGNNNGTNTNTGACNCATGNNTTTNTANNNGNN
NNNNNNNGNNNGANCCCCGGTCGNNNNCNNATTGAANNCCTGNNGCATNNNNNTNCNAGAATGANNNC
NNNNNGNNTNGNCTNNCNNNTCAAGNAATCANNNAGNNNNNNNNNTCNNNNNNNNNNNNCNNTCNCNN
ACTGAGGGTNNNNNNNNCATCNGCTNNNNNNNNNNTNCGCNGGNGTNNCNNNNNAGCNGTATNGNGTNA
NNCANNNNNNNNNNNNNNNNNTNAAACAANNNNNNNTNNNG
>CoLao589
GTGNNNNNTAGCCCNNNNNAGTTAGACANNNCNNNNNNNNNNNNNTNNNNNNNNNNNNNNNNNNNNNNN
NNNNNNNNNNNNNNNNNNNNNNNNNNNNNNNNNNNNNNNNNNNNNNNNNNNNNNNNNNNNNNNNNNNNNN
NNNNNNNNNNNNNNNNNNNNNNNNNNNNNNNNNNNNNNNNNNNNNNNNNNNNNNNNNNNNNNNNNNNNNN
NNNNNNNNNNNNNNNNNNNNNNNNNNNNNNNNNNNNNNNNNNNNNNNNNNNNNNNNNNNNNNNNNNNNNN
NNNNNNNNNNNNNNNNNNNNNNNNNNNNNNNNNNNNNNNNNNNNNNNNNNNNCNTCTNNNAAANNNTNNN
NNNNNNNNNNNNNNNNNNNNNNNNNNNCNCNNTGNNNGTAANCNANNNGNACATGCAGTCCCCTCNNNNN
NNNGGCAGAGNNNNNNCCCNNNNNNNNCCGNCCCCNNCCNNNNNNNNNNNNNNNNNNNNNNNNNANNNNNN
NNNNNNTGGTACNNNNCGNNNNNGGTTGTNTAAGAGGTCCACAACGNGGTCNNGNNACNNNNGNNNAN
NNNCNNNNAACNNNNNNNNNNNNNANNTNNNTANAAGGNNNNNNCCNTCNNGNNNNNNANTGCNATGACC
CANNNCNCGGNGGNNGTNCCCGGNGNGGCGACCTTGNNNACGTCCGGGGNCAAGTATGGCNNNCNCCC
CGGGAAGGANNCNGCNGTANGTGCNNNNNANNNNNTCNTGNGANGTCGCCGNNATTCNCNTGGTCNNN
CNAGAATGNCCNNACCANNCGNANNNTGGTNATGNNAGTNNNNNNNNNNNNNNNNNNNNNNNNNNNNN
NNNNNNNNNNNNNNNNNNNNNNNNNNNNNNNNNNNNNNNNGNNNNNNCNANNNAAGGNNNTANNNCTNNTN
NNNNNNNNGGACNNNACNNNNCNNNNNNNACNCNNNCNCNNNNNNNNNNNGGTTTTNNNNCNNTATTNT
CCAGNACGCNGNGCNNNCATTCANGNGAACNNNGNNACGCGNNAATCGTANNATCTACCTGCCGCNNG
NNNNNNTACNGNNNNNNCGGANNNTNNNNCGTANNNNNNNCANGCGTNNTNTNNTNNCNAGNCNNGTT
CGNNNANANANTAGNANGTNTNNNNNNNNNNTGTNNCGTTCCGCNTGANNCNNCANNNNGNNGNNNTTN
NCANCGGCNNTANCTNCNGANNTAAGNCATANNNTNNNTCNNTANCTNNNNNGGNGGGNNACTGTAAA
NGGNNAATTNGNNNNTNATTNCNNNCACNTNNCNNNGNNNNCTNNNTNCCTGNNTNCNNAATTGGNNC
CANTGNNTGNCNATCTCTGGNTTNGNNNNNNTGNNNNTNNCANNNNGGNNNGTNANANNNCNATNTAA
NNTANANNNNCNNNNNNANNNCACNNNNNNNGNAGNNNCNANNNTTANANNNGTAGNNNANTNAGTCT
CCGANNTNNNNNNNATAAGCGNNANACNNNNNNNNNNNNNNANGANNNCGNNTTGGNNNTNNNNNNCNA
GNNAGACNNTNATGGNTNNNNNTCAANNNNTTNGTAGTGGTATAATNGNNNNNNNTTTCNCCAGNNTN
NNNGNNNNNNNCNATANNANNNNNNNGNGANNNNNNNNNNNANNNNCTCNNNAGGGANCANCTACTNNA
GCAANGGNGCGNAGNNNGAGANNNNGACNNGCCTTGGGTGGCAANCACNGCAANAAGTNNNGTTNNNN
CTNNNAGTNNACATNNNNNCANNGNNNCNNNNNNANCNGANNGNNNNNNNNNNNNNNNNNNNNNNNN
NNNNNNNNNNNNNNNNNNNNNNNNNNNNNNNNNANNGNNNGTNTNTGNCNCATGTGTTTTTANNNGNN
NNNNNNNGNNNNANCCCCGGTCGNNNNCNNATTGAANNCCTGNNGCATNNNNNTNCNAGAATGANNNC
NNNNNGNNTNNNCTNNCNNNTCAAGNAATCAANNAGNNNNNNNNNTCNNNGNNNNNNNNCNNTCNCNN
ACTGAGGGTNNNNNNNNCATCTGCTNNNNNNNNNNTTCGCNGGNGTNNCNNNNNNGCNGTATNGNGTNA
NNCANNNNNNNNNNNNNNNNNTNAAACAANNNNNNNTNNNN
>CoLao590
GTGNNNNNTAGCCCNNNNNAGTTAGACANNNCNNNNNNNNNNNNNTNNNNNNNNNNNNNNNNNNNNNNN
NNNNNNNNNNNNNNNNNNNNNNNNNNNNNNNNNNNNNNNNNNNNNNNNNNNNNNNNNNNNNNNNNNNNNN
NNNNNNNNNNNNNNNNNNNNNNNNNNNNNNNNNNNNNNNNNNNNNNNNNNNNNNNNNNNNNNNNNNNNNN
NNNNNNNNNNNNNNNNNNNNNNNNNNNNNNNNNNNNNNNNNNNNNNNNNNNNNNNNNNNNNNNNNNNNNN
NNNNNNNNNNNNNNNNNNNNNNNNNNNNNNNNNNNNNNNNNNNNNNNNNNNNNTCTNNNAAANNNTNNN
NNNNNNNNNNNNNNNNNNNNNNNNNNNCNCNNTGNNNGTAANCNANNNGNACATGCAGTCCCCTCNNNNN
NNNNNCAGAGNNNNNNNCCNNNNNNNNCCGNCCCCNNCCNNNNNNNNNNNNNNNNNNGNNNNNNNANNNNNN
NNNNNNTGGTACNNNNCGNNNNNGGTTGTNTAAGAGGTCCACAACGNGGTCNNGNNACNNNNGNNNAN
NNNCNNNNAANNNNNNNNNNNNNNANNTNNNTANNAGGNNNNNNCCNTCNNGNNNNNNANNGCNATGANN

CANNTCNCGGGNGNNGTNCCCGGCGCNGCGACCTTGNTNACGTCCGGGGNCAAGTATGGCNNNCNCCC
CGGGAAGGANNCNGCNGTANGTGCNNNNNANNNNNTCNTGNGANNTNGCCGNNATTCNCGTGGTCTNN
CNAGAATGCCCNNACCANNCGNANNNTGGTNATGNNAGTNANNNNNNNNNNNNNNNNNNNNNNNNNNN
NNNNNNNNNNNNNNNNNNNNNNNNNNNNNNNNGNNNNGNNNNNNCNANNNAAGGNNNTANNNCTNNTN
NNNNNNNNGGNCNNNACNNNNCNNNNNNNACNCNNNCNCNNNNNNNNNNGNTTTTNNNNCNNTANTNT
CCAGNACGCNGNGCNNNCNTTCANGNGAACNNNGNNACGCGNNAATCGTANNATCTACCTGCCGCNNG
NNNNNNTNCNGNNNNNNCGGANNNTNNNNCGTANNNNNNNCANGCGTNNTNTNNTCNCNAGNCNNGTT
CGNNNANANANTAGNANGTNTNNNNNNNNNTGTNNCGTTCCGCNTGANNCNNCANNNNGNNGNNCTTN
NCANCGGCNNTANCTGCNGANNTAAGNCATANNNTNNNTCNNTANCTNNNNNNGGNGGGNACTGTAAA
NGGNTAATTNGNNNNTNANTNCNNNCACNTNNCTNNGNNNNCTNNNTNCCTGNNTNCNNAATTGGNNC
CANTGNNTGNCNATCTCTNGNTTNGNNNNNNTNNNNNTNNCANNNNGGNNNGTNANANNNCNATNTAA
NNTANANNNNCNNNNNNANNNCACNNNNNNNNNAGNNNNNANNNTNANANNNGTAGNNNANTAAGTCT
CCGANNTNNNNNNNATAAGCGNNANACNNNNNNNNNNNNNANGANNNCGNNTTGGNNNTNNNNNNCNA
GNNAGACNNTNATGGNTNNNNNTCAANNNNTTNGTAGTGGTATAATNGNNNNNNNTTTCNCCAGNNNN
NNNGNNNNNNNCNATANNANNNNNNNGNGANNNNNNNNNNANNNNCTCNNNAGGGANCANCTACTNNA
GCANNGGNGCGCAGNNNGAGANNNNGACNNNCCTTGGGTGGCAANCACNGNAATAAGTNNNGTTNNNN
CTNNNAGTNNACATNNNNNCANNGNNNCNNNNNNANCNGANNGNNNNNNNNNNNNNNNNNNNNNNNNN
NNNNNNNNNNNNNNNNNNNNNNNNNNNNAGNNNANGGNNNGTNTNTGACNCATGTGTTTTTANNNGNN
NNNNNNNGNNNNANCCCCGGTCGNNNNCNNATTGAANNCCTGNNGCATNNNNNTNCNAGAATGANNGC
NNNNNGNNTNNNCTNNCNNNTCAAGNAATCNANNANNNNNNNNNNNTCNNNGNNNNNNNCNNNCNCNN
ACTGAGGGNNNNNNNNNCATCTGCTNNNNNNNNCTNCGCNGGNNTNNCNNNNNAGCNGTATNNNGTNN
NNCANNNNNNNNNNNNNNNNTCAAACNANNNNNNTNNNN
>CoLao591
GTGNNNNNTAGCCCNNNNNAGTTAGACANNNCNNNNNNNNNNNNTNNNNNNNNNNNNNNNNNNNNNNN
NNNNNNNNNNNNNNNNNNNNNNNNNNNNNNTNNNNNNNNNNNNNNNNNNNNNNNNNNNNNNNNNNNNNNN
NNNNNNNNNNNNNNNNNNNNNNNNNNNNNNNNNNNNNNNNNNNNNNNNNNNNNNNNNNNNNNNNNNNN
NNNNNNNNNNNNNNNNNNNNNNNNNNNNNNNNNNNNNNNNNNNNNNNNNNNNNNNNNNNNNNNNNNNN
NNNNNNNNNNNNNNNNNNNNNNNNNNNNNNNNNNNNNNNNNNNNNNNNNNNNNCTNNNAAANNNTNNN
NNNNNNNNNNNNNNNNNNNNNNNNNCNCNNTNNNNGTAANCNANNNGNACATGCAGTCCCCTCNNNNN
NNNGGCAGAGNNNNNNNCCNNNNNCNCCGNCCCCNNCCNNNNNNNNNNNNNNNGNNNNNNNANNNNNN
NNNNNNTGGTACNNNNCGNNNNNGGTTGNNTAAGAGGTCCACAACGNGGTCNNGNNACNNNNGNNNAN
NNNCNNNNAANNNNNNNNNNNNANNTNNNTANNAGGNCNNNNCCNTNNNGNNNNNNANTGCNATGACC
CANNNCNCGGNGGCNGTNCCCGGCGNGGCGACCTTGANNANGTCCGGGGNCAAGTATGGCGCNCNCCC
CGGGAAGGANNCNGCNGTANGTGCNNNNNANNNNNTCNTGNGANGTCGCCGNNATTCNCGTGGTCNNN
NNAGAATGCNCNNACCANNCGNANNNTGGTNATGNNAGTNNNNNNNNNNNNNNNNNNNNNNNNNNNNN
NNNNNNNNNNNNNNNNNNNNNNNNNNNNNNNNGNNNNGNNNNNNCNANNNAAGGNNNTANNNCTNNTN
NNNNNNNNGGACNNNACNNNNCNNNNNNNACNCNNNCNCNNNNNNANNNGGTTNNNNNNCNNTANTNT
CCAGNANGCNGNGCNNNCATTCANGNGAACNNNGNNACGCGNNAATCGTANNATCTACCTGCCGCNNG
TNNNNNTNCNGNNNNNNCGGANNNTNNNNCGTANNNNNNNCANGCGTNNTNTNNTCNCNAGNCNNGTT
CGNNNANANANTAGNANGTNTNNNNNNNNNTGTNNCGTTCCGCNTGANNCNNCANNNNGNNGNNCTTN
NCANCGGCNNTANCTGCNGANNTAAGNCATANNNTNNNTCNNTANCTNNNNNNGGNGGGNACTGTAAA
NGGNNAATTNGNNGNTNANTNCNNNCACNTNNCTNNGNNNNCTNNNTNCCTGNNTNCNNAATTGGNNC
CANTGNNTGNCNATCTCTGGNTTNGNNNNNNTNNNNNTNNCANNNNGGNNNNNNANANNNCNATNTAA
NNTANANNNNCNNNNNNANNNCACNNNNNNNGNAGNNNNNANNNTNANANNNGTAGNNNANTAAGTCT
CCGANNTNNNNNNNATAAGCGNNANACNNNNNNNNNNNNNANGANNACGNNTTGGNNNTNNNNNNNNA
GNNAGACNNTNATGGNTNNNNNTCAANNNNTTNGTAGTGGTATAATNGNNNNNNNTTTCNCCAGNNTN
NNNGNNNNNNNCNATANNANNNNNNNGNGANNNNNNNNNNANNNNCTCNNNAGGGANCANCTACTNNA
GCANTGGNGCGNAGNNNGAGANNNNGACNNNCCTTGGGTGGCAANNACNGCAATAAGTNNNGTTNNNC
CTNNNAGTNNACATNNNNNCANNGNNNCNNNNNNANCNGANNGNNNNNNNNNNNNNNNNNNNNNNNNN
NNNNNNNNNNNNNNNNNNNNNNNNNNNNNNNNNANGGNNNGTNTNTGNCNCATGNNTTTTTANNNGNN
NNNNNACGNNNNANCCCCGGTCGNNNNCNNATTGAANNCCTGNNGCATNNNNNTNCNAGAATGANNNC
NNNNNGNNTNGNCTNNNNNNNTCAAGNAATCANNNANGTNNNNNNNTCNNNGNNNNNNNCNNNCCCNN
ACTGAGGGTNNNNNNNNCATCTGCTNNNNNNNNNTNCGCNGGNGTNNCNNNNNAGCNGTATNGNGTNA
NNCANNNNNNNNNNNNNNNNTNAAACNANNNNNNTNNNN

>CoLao592
GTGNNNNNNAGCCCNNNNNAGTTAGACANNNCNNNNNNNNNNNNTNNNNNNNNNNNNNNNNNNNNNNNNN
NNNNNNNNNNNNNNNNNNNNNNNNNNNNNNNNNNNNNNNNNNNNNNNNNNNNNNNNNNNNNNNNNNNNNN
NNNNNNNNNNNNNNNNNNNNNNNNNNNNNNNNNNNNNNNNNNNNNNNNNNNNNNNNNNNNNNNNNNNNNN
NNNNNNNNNNNNNNNNNNNNNNNNNNNNNNNNNNNNNNNNNNNNNNNNNNNNNNNNNNNNNNNNNNNNNN
NNNNNNNNNNNNNNNNNNNNNNNNNNNNNNNNNNNNNNNNNNNNNNNNNNNNCNTCTNNNAAANNNTNNN
NNNNNNNNNNNNNNNNNNNNNNNNNNNCNCNNTNNNNGTAANCNANNNGNACATGCAGTCCCCTCNNNNN
NNNGGCAGAGNNNNNNNCCNNNNNNNCCGNCCCCNNCCNNNNNNNNNNNNNNNNGNNNNNNNANNNNNNN
NNNNNNTGGTACNNNNCGNNNNNGGTTGNNTAAGAGGTCCACAACGNGGTCNNNNNACNNNNGNNNAN
NNNCNNNNAANNNNNNNNNNNNNANNTNNNTANNAGGNCNNNNCCNTCNNNNNNNTNATTGCNATGANN
CNNNNNNCNGGGGNNGTNCCCGGCGNGGCGACCTTGANNACGTCCGGGGNCAAGTATGGCGNNCNCCC
CGNGAAGGANNCNGCCGTANGTGCNNNNNANNNNNTCNTGNGANGTCGCCGNNATTNNCGTGGTCNNN
CNNGAATGNCCNNACCANNCGNANNNTGGTNATGNNAGTNNNNNNNNNNNNNNNNNNNNNNNNNNNNNN
NNNNNNNNNNNNNNNNNNNNNNNNNNNNNNNNNNGNNNNGNNNNNNCNANNNAAGGNNNTANNNCTNNTN
NTNNNNNNGGACNNNACNNNNCNNNNNNNACNCNNNCNCNNNNNNNNNNNGGTTTTTNNNCNNTANTNT
CCAGNACGCNGNGCNNNCATTCANGNGAACNNNGNNACGCGNNAATCGTANNATCTACCTGCCGCNNG
NNNNNNTACNGNNNNNNCGGANNNTNNCTCGTANNNNNNNCANGCGTNNTNTNNTNNCNAGNCNNGTT
CGNNNANANANTAGNANGTNTNNNNNNNNNNTGTNNCGTTCCGCNTGANNCNNCANNNNGNNGNNCTTN
NCANCGGCNNTANCTGCNGANNTAAGNCATANNNTNNNTCNNTANCTNNNNNGGNGGGNNACTGTAAA
NGGNNAATTNGNNNNTNANTNCNNNCACNTNNCNNNGNNNNCTNNNTNCCTGNNTNCNNAATTGGNNC
CANTGNNTGNCNATCTCTNGNTTNGNNNNNNTGNNNNTNNCAANNNGGNNNNNNNNANNNCNATNTAA
NNTANANNNNCNNNNNNANNNCACNNNNNNNNNAGNNNCNANNNTNANANNNGTAGNNNANTAAGTCT
CCGANNTNNNNNNNATAAGCGNNANACNNNNNNNNNNNNNNANGANNNCGNNTTGGNNNTNNNNNNNNA
GNNAGACNNTNATGGNTNNNNNTCAANNNNNTTNGTAGTGGTATAATNGNNNNNNNTTTCNCCAGNNTN
NNNGNNNNNNNNCNATANNANNNNNNNGNGANNNNNNNNNNANNNNCTCNNNAGGNANCANCTACTNNA
GCANTGGNGCGNAGNNNGAGANNNNGACNNNCCTTGGGTGGCAANNACNGNAATAAGTNNNGTTNANC
CTNNNAGTNNACATNNNNNCANNGNNNCNNNNNNANCNGANNGNNNNNNNNNNNNNNNNNNNNNNNNNN
NNNNNNNNNNNNNNNNNNNNNNNNNNNNNNANNNNANGGNNNGTNTNTGACNCATGNNTTTNTANNNGNN
NNNNNACGNNNNANCCCCGGTCGNNTNCNNATTGAANNCCTGNNGCATNNNNNTNCNAGAATGANNNC
NNNNNGNNTNGNCTNNCNTTTCAAGNAATCANNNAGGTNNNNNNNTCNNNGNNNNNNNNCNNTCNCNN
ACTGAGGGTNNNNNNNNCATCNGCTNNNNNNNNNNTNCGCNGNNGTNNCNNNNNNGCNGTATNGNGTNA
NNCANNNNNNNNNNNNNNNNNNTNAAACAANNNNNNNTNNNN
>CoLao593
GTGNNNNNNAGCCCNNNNNAGTTAGACANNNCNNNNNNNNNNNNTNNNNNNNNNNNNNNNNNNNNNNNNN
NNNNNNNNNNNNNNNNNNNNNNNNNNNNNNNNNNNNNNNNNNNNNNNNNNNNNNNNNNNNNNNNNNNNNN
NNNNNNNNNNNNNNNNNNNNNNNNNNNNNNNNNNNNNNNNNNNNNNNNNNNNNNNNNNNNNNNNNNNNNN
NNNNNNNNNNNNNNNNNNNNNNNCNNNNNNNNNNNNNNNNNNNNNNNNNNNNNNNNNNNNNNNNNNNNNN
NNNNNNNNNNNNNNNNNNNNNNNNNNNNNNNNNNNNNNNNNNNNNNNNNNNNNNTCTNNNAAANNNTNNN
NNNNNNNNNNNNNNNNNNNNNNNNNNNCNCNTTGNNNGTAANCNANNNGNACATGCAGTCCCCTCNNNNN
NNNGGCAGAGNNNNNNNNNNNNNNNCNCCGNCCCCNNCCNNNNNNNNNNNNNNNNGNNNNNNNANNNNNN
NNNNNNTGGTACNNNNCGNNNNNGGTTGTTTAAGAAGTCCACAACGNGGTCNNNNCACNNNNGNNNAN
NNNCNNNNAANNGNNNNNNNNNNANNTNNNTANNAGGNCNNNNCCNTCNNGNNNNNNANNGCNATGANN
CANNNCNCGGGGGCNGTNCCCGGCGCGGCGACCTTGNNNANGTCCGGGGNCAAGTATGGCGNNCNCCC
CGGGAAGGANNCNGCNGTANGTGCNNNNNANNNNNTCNTGNGANGTNGCCGNNATTNNCNTGGTCTNN
CNNGAATGNNCNNACCANNCGNANNNTGGTNATGNNAGTNNNNNNNNNNNNNNNNNNNNNNNANNNNNN
NNNNNNNNNNNNNNNNNNNNNNNNNNNNNNNNNNGNNNNGNNNNNNCNANNNAAGGNNNTANNNCTNNTN
NNNNNNNNGGNCNNNACNNNNCNNNNNNNACNCNNNCNCNNNNNNNNNNNGNTTTTNNNNCNNTANTNT
CCAGNACGCNGNGCNNNCNTTCANGNGAACNNNGNNACGCGNNAATCGTANNATCTACCTGCCGCNNG
NNNNNNTACNGNNNNNNCGGANNNTNNNNCGTANNNNNNNCANGCGTNNTNTNNTCNCNAGNCNNGTT
CGNNNANANANTAGNANGTNTNNNNNNNNNNTGTNNCGTTCCGCNTGANNCNNCANNNNGNNGNNNTTN
NCANCGGCNNTANCTGCNGACNTAAGNCATANNNTNNNTCNNTANCTNNNNNGGNGGGNNACTGTAAA
NGGNNAATTNGNNNNTNATTNCNNNNATNTNNCTNNGNNTNCTNNNTNCCTGNNTNCNNAATTGGNNN
CANTGNNTGNCNATCTCTNGNTTNGNNNNNNTNNNNNTNNCANNNNGGNNNGTNNNANNNCNATNTAA
NNTANANNNNCNNNNNNANNNCACNNNNNNNNNAGNNNCNANNNGNANANNNGTAGNNNGNTAAGTCT

CCGANNTNNNNNNNATAAGCTNNANACNNNNNNNNNNNNNNANGANNNCGNNTTTGNNNTNNNNNNNNA
GNNAGACNNTNGTGGNTNNNNNTCAANNNNTTNGTAGTGGTATAATNGNNNNNNNTTTCNCCAGNNTN
NNNGNNNNNNNCNATANNANNNNNNNGNGANNNNNNNNNNNANNNNCTCNNNAGGNANCANCTACTNNA
GCANTGGNGCGNAGNNNGAGANNNNGACNNNCCTTGGGTGGCANNNACNGNAATAAGTNNNGTTNANN
CTNNNAGTNNACACNNNNNCANNGNNNCNNNNNNANCNGANNGNNNNNNNNNNNNNNNNNNNNNNNNN
NNNNNNNNNNNNNNNNNNNNNNNNNNNNNNNNNNNANGGNNNGTNTNTGNCNCATGTGTTTTCANNNGNN
NNNNNACGNNNNANCCCCGGTCGNNNNCNNATTGAANNCCTGNNGCATNNNNNTNCNAGAATGANNNC
NNNNNGNNTNGNCTNNCNNNTCAAGNAATCANNNAGNNNNNNNNNTCNNNNNNNNNNNNCNNTCNCNN
ACTGAGGGTNNNNNNNNCATCTGCTNNNNNNNNNCTNCGCNGGNGTNNCNNNNNNGCNGTATNNNGTNN
NNCANNNNNNNNNNNNNNNNNTNAAACNANNNNNCTNNNN
>CoLao594
GTGNNNNNTAGCCCNNNNNAGTTAGACANNNCNNNNNNNNNNNNTNNNNNNNNNNNNNNNNNNNNNNN
NNNNNNNNNNNNNNNNNNNNNNNNNNNNNNNNNNNNNNNNNNNNNNNNNNNNNNNNNNNNNNNNNNNNNN
NNNNNNNNNNNNNNNNNNNNNNNNNNNNNNNNNNNNNNNNNNNNNNNNNNNNNNNNNNNNNNNNNNNNNN
NNNNNNNNNNNNNNNNNNNNNNNNNNNNNNNNNNNNNNNNNNNNNNNNNNNNNNNNNNNNNNNNNNNNNN
NNNNNNNNNNNNNNNNNNNNNNNNNNNNNNNNNNNNNNNNNNNNNNNNNNNNNCNTCTNNNAAANNNTNNN
NNNNNNNNNNNNNNNNNNNNNNNNNNCNCNNTNNNNGTAANCNANNNGNACATGCAGTCCCCTCNNNNN
NNNGGCAGAGNNNNNNCCNNNNNNNNNCCGNCCCCNNCCNNNNNNNNNNNNCNNNGNNNNNNNANNNNNN
NNNNNNTGGTACNNNNCGNNNNNGGTTGTNTAAGAGGTCCACAACANGNTCNNGNNACNNNNGNNNAN
NNNCNNNNNNNNNNNNNNNNNNNNNNTNNNTANNAGGNCNNNNCCNTNNNNNNNNNNNATTGCNATGANN
CNNNNNNCGGGGGNNGTNCCCGGNGNGGCGACCTTGANNACGTCNGGGGNCAAGTATGGCGNNCNCCC
CGGGAAGGANNCNGCCGTANGTGCNNNNNANNNNNTCNTGNGANGTCGCCGNNATTCNCGTGGTCNNN
CNAGAATGNCCNNACCANNCGNANNNTGGTNATGNNAGTNNNNNNNNNNNNNNNNNNNNNNNNNNNNN
NNNNNNNNNNNNNNNNNNNNNNNNNNNNNNNNNNGNNNNGNNNNNNCNANNNAAGGNNNTANNNCTNNTN
NTNNNNNNNGGACNNNACNNNNCNNNNNNNNACNCNNNCNCNNNNNNNNNNNGNTTTTNNNNCNNTATTNT
CCAGNACGCNGNGCNNNCNTTCANGNGAACNNNGNNACGCGNNAATCGTANNATCTACCTGCCGCNNG
NNNNNNTNCNGNNNNNNCGGANNNTNNCTCGTANNNNNNNCANGCGTNNTNTNNTCNCNAGNCNNGTT
CGNNNANANANTAGNANGTNTNNNNNNNNNNTGTNNCGTTCCGCNCGANNCNNCANNNNGNNGNNCTTN
NCANCGGCNNTANCTGCNGANNTAAGNCATANNNTNNNTCNNTANCTNNNNNGGNGGGNNACTGTAAA
NGGNNAATTNGNNNNTNATTNCNNNCACNTNNCTNNANNNNCTNNNTNCCTGNNTNCNNAATTGGNNN
CANTGNNTGNCNATCTCTGGNTTNGNNNNNNTGNNNNTNNCANNNNGGNNNNNNNNANNNCNATNTAA
NNTANANNNNCNNNNNNANNNCACNNNNNNNNNAGNNNCNANNNTTANANNNGTAGNNNANTAAGTCT
CCGANNTNNNNNNNATAAGCGNNANACNNNNNNNNNNNNNNANGANNNCGNNTTGGNNNTNNNNNNNNA
GNNAGACNNTNGTGGNTNNNNNTCAANNNNTTNGTAGTGGTATAATNGNNNNNNNTTTCNCCAGNNTN
NNNGNNNNNNNCNATANNANNNNNNNGNGANNNNNNNNNNNANNNNCTTNNNAGGNANCANTTACTNNA
GCAATGGNGCGNAGNNNGAGANNNNGACNNNCCTTGGGTGGCAANNACNGNAATAAGTNNNGTTNANC
CTNNNAGTNNACACNNNNNCANNGNNNCNNNNNNANCNGANNGNNNNNNNNNNNNNNNNNNNNNNNNN
NNNNNNNNNNNNNNNNNNNNNNNNNNNNNNANNNNANGGNNNGTNTNTGACNCATGTGTTTTTANNNGNN
NNNNNACGNNNNANCCCCGGTCGNNNNCNNATTGAANNCCTGNNGCATNNNNNTNCNAGAATGANNGC
NNNNNGNNTNGNCTNNCNTTTCAAGNAATCANNNAGGTNNNNNNNTCNNNGNNNNNNNNCNNNCNCNN
ACTGAGGGTNNNNNNNNCATNNGCTNNNNNNNNNCTNCGCNGGNGTNNCNNNNNNGCNGTATNGNGTNA
NNCANNNNNNNNNNNNNNNNNTNNAANNANNNNNNNTNNNN
>CoLao595
GTGNNNNNNAGCCCNNNNNAGTTAGACANNNCNNNNNNNNNNNNNNNNNNNNNNNNNNNNNNNNNNNNN
NNNNNNNNNNNNNNNNNNNNNNNNNNNNNNNNTNNNNNNNNNNNNNNNNNNNNNNNNNNNNNNNNNNNNN
NNNNNNNNNNNNNNNNNNNNNNNNNNNNNNNNNNNNNNNNNNNNNNNNNNNNNNNNNNNNNNNNNNNNNN
NNNNNNNNNNNNNNNNNNNNNNNNNNNNNNNNNNNNNNNNNNNNNNNNNNNNNNNNNNNNNNNNNNNNNN
NNNNNNNNNNNNNNNNNNNNNNNNNNNNNNNNNNNNNNNNNNNNNNNNNNNNNCNTCTNNNAAANNNTNNN
NNNNNNNNNNNNNNNNNNNNNNNGNNNCNCNTTNNNNGTAANCNANNNGNACATGCAGTCCCCTCNNNNN
NNNNNCAGAGNNNNNNCNCNNNNNNNNCCGGCCCCNNCCNNNNNNNNNNNNCNNNGNNNNNNNANNNNNN
NNNNNNTGGTACNNNNCGNNNNNGGTTGNTTAAGAGGTCCACCACGNGNTCNNNNCACNNNNGNNNAN
NNNCNNNNAANNNNNNNNNNNNNNNNNTNNNTANNAGGNNNNNNCCNTCNNGNNNNNNANTGCNATGANN
CANNNCNCNGGGGCNGTCCCCGGCGCGGCGACCTTGNNNANNTCCGGGGNCAAGTATGGCNNNCNCCC
CGGGAAGGANNCNGCNGTANGTGCNNNNNANANNNTCNTGNGANGTNGCCGNNATTCNCGTGGTCNNN

CNNGAATGNCCNNACCANNCGNANNNTGGTNATGNNAGTNNNNNNNNNNNNNNNNNNNNNNNNNNNNN
NNNNNNNNNNNNNNNNNNNNNNNNNNNNNNNNGNNNNGNNNNNNCNANNNAAGGNNNTANNNCTNNTN
NTNNNNNNNGACNNNACNNNNCNNNNNNNACNCNNNCNCNNNNNNANNNGNTTTTNNNNCNNTANTNT
CCAGNACGCNGNGCNNNCATTCANGNGAACNNNGNNACGCGNNAATCGTANNATCTACCTGCCGCNNG
NNNNNNTNCNGNNNNNNCGGANNNTNNNNCGTANNNNNNNTANACGTNNTNTNNTCNCNAGNCNNGTT
CGNNNANANANTAGNANGTNTNNNNNNNNNTGTNNCGTTCCGCNTGANNCNNCANNNNGNNGNNNTTN
TCANCGGCNNTANCTNCNGANNTAAGNCATANNNTNNNTCNNTANCTNNNNNGGNGGGNNACTGTAAA
NGGNNAATTNGNNNNTNANTNCNNNCACNTNNCTNNGNNTNCTNNNTNCCTGNNTNCNNAATTGGNNC
CANTGNNTGNCNATCTCTNGNTTNGNNNNNNNTNNNNNTNNCANNNNGGNNGTNNNANNNNNATNTAA
NNTANANNNNCNNNNNNANNNCACNNNNNNNNNAGNNNCNANNNTNANANNNGTAGNNNANTAAGTCT
CCGANNTNNNNNNATAAGCGNNANACNNNNNNNNNNNNNNANGANNNCGNNTTGGNNNTNNNNNNCNA
GNNAGACNNTNGTGGNTNNNNNTCAANNNNTTNGTAGTGGTATAATNGNNNNNNNTTTCNCCAGNNTN
NNNGNNNNNNNCNATANNANNNNNNNGNGANNNNNNNNNNANNNNCTCNNNAGGNANCANCTACTNNA
GCANNGGNGCGNAGNNNGAGANNNNGACNNNCCTTGGGTGGCAANNACNGNAATAAGTNNNGTTNNNN
CTNNNAGTTNACACNNNNNCANNGNNNCNNNNNNANCNGANNGNNNNNNNNNNNNNNNNNNNNNNNNN
NNNNNNNNNNNNNNNNNNNNNNNNNNNNNNNNNANGGNNNGTNTNTGACNCATNNNTTTTTANNNGNN
NNNNNACGNNNNANCCCCGGTCGNNNNCNNATTGAANNCCTGNNGCATNNNNNTNCNAGAATGANNNC
NNNNNGNNTNGNCTNNCNNNTCAAGNAGTCANNNANNNNNNNNNNTCNNNGNNNNNNNNCNNNCNCNN
ACTGAGGGTNNNNNNNNCATCTGCTNNNNNNNGCTNCGCNGGNGTNNCNNNNNNGCNGTATNGNGNNN
NNCANNNNNNNNNNNNNNNNNTNAAACNANNNNNTNNNN
>CoLao596
GTGNNNNNNAGCCCNNNNNAGTTAGACANNNCNNNNNNNNNNNNTNNNNNNNNNNNNNNNNNNNNNNN
NNNNNNNNNNNNNNNNNNNNNNNNNNNNNNTNNNNNNNNNNNNNNNNNNNNNNNNNNNNNNNNNNNNNN
NNNNNNNNNNNNNNNNNNNNNNNNNNNNNNNNNNNNNNNNNNNNNNNNNNNNNNNNNNGGNNNNNNNN
NNNNNNNNNNNNNNNNNNNNNNNNNNNNNNNNNNNNNNNNNNNNNNNNNNNNNNNNNNNNNNNNNNNN
NNNNNNNNNNNNNNNNNNNNNNNNNNNNNNNNNNNNNNNNNNNNNNNNNNNNTCTNNNAAANNNTNNN
NNNNNNNNNNNNNNNNNNNNNNNNNCNCNTTNNNNGTAANCNANNNGNACATGCAGTCCCCTCNNNNN
NNNGGCAGAGNNNNNNNCNNNNNNCNCCGNCCCCNNCCNNNNNNNNNNNNANNNNNNNNNNANNNNNN
NNNNNNTGGTACNNNNCGNNNNNGGTTGTNTAAGAGGTCCACAACGNGNTCNGGNNACNNNNGNNNAN
NNNCNNNNAACNNNNNNNNNNNANNTNNNTANNAGGNCNNNNCCNTCNNGNNNNNNANTGCNATGACC
CANNNCNCNGNGGNAGTNCCCGGNGNGGCGACCTTGANNANGTCCGGGGNCAAGTATGGCGNNCNCCC
CGGGAAGGANNCNGCNGTANGTGCNNNNNANNNNNTCNTGNGANGTCGCCGNNNTTCNNGTGGTCNNN
NNAGAATGNCCNNACCANNCGNANNNTGGTNATGNNAGTNNNNNNNNNNNNNNNNNNNNNNNNNNNNN
NNNNNNNNNNNNNNNNNNNNNNNNNNNNNNNNGNNNNGNNNNNNCNANNNAAGGNNNTANNNCTNNTN
NNNNNNNNGGACNNNACNNNNCNNNNNNNACNCNNNCNCNNNNNNNNNNGNTTNNNNNNCNNTANTNT
CCAGNACGCNGNGCNNNCATTCANGNGAACNNNGNNACGCGNNAATCGTANNATCTACCTGCCGCNNG
NNNNNNTNCNGNNNNNNCGGANNNTNNCTCGTANNNNNNNCATGCGTNNTNTNNTCNCNAGNCNNGTT
CGNNNANANANTAGNANGTNTNNNNNNNNNTGTNNCGTTCCGCNTGANNCNNCNNNNNGNNGNNNTTN
NCANCGGCNNTANCTGCNGANNTAAGNCATANNNTNNNTCNNTANCTNNNNNGGNGGGNNACTGTAAA
NGGNNAATTNGNNNNTNATTNCNNNCACNTNNCTNNGNNNNCTNNNTNCCTGNNTNCNNAATTGGNNC
CANTGNNTGNCNATCTCTNGNTTNGNNNNNNTNNNNNTNNCANNNNGGNNNGTNNNANNNCNATNTAA
NNTANANNNNCNNNNNNANNNCACNNNNNNNGNAGNNACNANNNTNANANNNGTAGNNNANTAAGTCN
CCGANNTNNNNNNATAAGCGNNANACNNNNNNNNNNNNNNANGANNACGNNTTGGNNNTNNNNNNCNA
GNNAGACNNTNATGGNTNNNNNTCAANNNNTTNGTAGTGGTATAATNGNNNNCTNTTTCNCCAGNNTN
NNNGNNNNNNNCNATANNANNNNNNNGNGANNNNNNNNNNANNNNNTCNNNAGGGANCANCTACTNNA
GCAATGGNGCGNAGNNNGAGANNNNGACNNNCCTTGGGTGGCANNCACNGCAATAAGTNNNGTTNANN
CTNNNAGTNNACATNNNNNCANNGNNNCNNNNNNANCNGANNGNNNNNNNNNNNNNNNNNNNNNNNNN
NNNNNNNNNNNNNNNNNNNNNNNNNNNNANNNNANNGNNNGTNTNTGACNCATGTGTTTTTANNNGNN
NNNNNNNGANNNANCCCCGGTCGNNNNCNNATTGAANNCCTGNNGCATNNNNNTNCNAGAATGANNNC
NNNNNGNNTNGNCTNNCNNNTCAAGNAATCANNNANGNNNNNNNNTCNNNNNNNNNNNNCNNTNCCNN
ACTGAGGGTNNNNNNNNCATCTGCTNNNNNNNNCTNCGCNNGNGTNNCNNNNNAGCNGTATNNNGTNN
NNCANNNNNNNNNNNNNNNNNTNANACNANNNNNTNNNN
>CoLao597
GTNNNNNNNAGCCCNNNNNAGTTNGACANNNCNNNNNNNNNNNNTNNNNNNNNNNNNNNNNNNNNNNN

NNNNNNNNNNNNNNNNNNNNNNNNNNNNNNNNNNNNNNNNNNNNNNNNNNNNNNNNNNNNNNNNNNNNNN
NNNNNNNNNNNNNNNNNNNNNNNNNNNNNNNNNNNNNNNNNNNNNNNNNNNNNNNNNNNNNNNNNNNNNN
NNNNNNNNNNNNNNNNNNNNNNNNNNNNNNNNNNNNNNNNNNNNNNNNNNNNNNNNNNNNNNNNNNNNNN
NNNNNNNNNNNNNNNNNNNNNNNNNNNNNNNNNNNNNNNNNNNNNNNNNNNNNNTCTNNNAAANNNTNNN
NNNNNNNNNNNNNNNNNNNNNNNNNNNCNCNNTNNNNGTAANCNANNNGNACATGCAGTCCCCTCNNNNN
NNNGGCAGAGNNNNNNCNNNNNNNNCCGNCCCCNNCCNNNNNNNNNNNNNNNNNNGNNNNNNNANNNNNN
NNNNNNTGGTACNNNNCGNNNNNNGGTTGTTTAAGAGGTCCACCACGNGGTCNNNNNACNNNNGNNNAN
NNNCNNNNAACNNNNNNNNNNNNANNTNNNTANNAGGNNNNNNCCNTCNNNNNNNNNNANTGCNATGANN
CNNNNNNCNGNGGNNGTNCCCGGNGNGGCGACCTTGNNNANGTCNGGGGNCAAGTATGGCNNNCNCCC
CGNGAAGGANNCNGCNGTANGTGCNNNNNANNNNNTCNTGNGANNTCGCCGNNATTCNCNTGGTCNNN
CNNNAATGNNCNNACCANNCGNANNNNGGTNATGNNAGTNNNNNNNNNNNNNNNNNNNNNNNNNNNNNN
NNNNNNNNNNNNNNNNNNNNNNNNNNNNNNNNNNGNNNNGNNNNNNCNANNNAAGGNNNTANNNCTNNTN
NTNNNNNNNNNNCNNNACNNNNCNNNNNNNACNCNNNCNCNNNNNNNNNNNGNTTNNNNNNCNNTANTNN
CCAGNACGCNGNGCNNNCNTTCANGNGAACNNNGNNACGCGNNAATCGTNNNATCTACCTGCCGCNNG
NNNNNNTNCNGNNNNNNCGGANNNTNNNNNGTANNNNNNNNTANACGTNNTNTNNTNNCNAGNCNNGTT
CGNNNANANANTAGNANGTNTNNNNNNNNNNTGTNNCGTTNNNCNTGANNCNNCNNNNNGNNGNNNTTN
NCANCGGCNNTANCTGCNGANNTAAGNCATANNNTNNNTCNNTANCTNNNNNGGNGGGNNACTGNAAA
NGNNNAATTNGNNNNTNANTNCCNNCACNTNNCNNNGNNNNCTNNNTNCCTGNNNNCNNAATTGGNNC
CANTGNNTGNCNATCTCTGGNTTNGNNNNNNTNNNNNTNNCANNNNGGNNNNNNNNANNNCNATNTAA
NNTANANNNNCNNNNNNANNNCACNNNNNNNNNNAGNNNCNANNNTNANANNNGTAGNNNANTNAGTCN
CCGANNTNNNNNNNATAAGCGNNANACNNNNNNNNNNNNNNANGANNNCGNNTTGGNNNTNNNNNNNNA
GNNAGACNNTNGTGGNTNNNNNTCAANNNNNTTNGTAGTGGTATAATNGNNNNNNNTTTCNCCAGNNNN
NNNGNNNNNNNNCNATANNANNNNNNNGNGANNNNNNNNNNNANNNNCTCNNNAGGGANCANCTACTNNA
GCANTGGNNCGNAGNNNGAGANNNNGACNNNCCTTGGGTGGCANNNACNGNAATAAGTNNGTTNNNN
CTNNNAGTNNACACNNNNNCANNGNNNCNNNNNNANCNGANNGNNNNNNNNNNNNNNNNNNNNNNNNN
NNNNNNNNNNNNNNNNNNNNNNNNNNNNNNNNNNNANNGNNNGTNTNTGNCNCNTNNNTTTTTANNNGNN
NNNNNACGNNNNANCCCCGGTCGNNNNCNNATTGAANNCCTGNNGCATNNNNNTNCNANAATGANNNC
NNNNNGNNTNGNCTNNCNNNTNAAGNAGTCANNTANNNNNNNNNNNTCNNNGNNNNNNNNCNNNCNCNN
ACTGAGGGNNNNNNNNNCANCNGCTNNNNNNNNNNTNCGCNGGNNTNNCNNNNNNGCNGTATNGNGNNN
NNCANNNNNNNNNNNNNNNNNNTNAAACAANNNNNNNTNNNN
>CoLao598
GTGNNNNNTAGCCCNNNNNAGTTAGACANNNCNNNNNNNNNNNNNTNNNNNNNNNNNNNNNNNNNNNNNN
NNNNNNNNNNNNNNNNNNNNNNNNNNNNNNNNNNNNNNNNNNNNNNNNNNNNNNNNNNNNNNNNNNNNNN
NNNNNNNNNNNNNNNNNNNNNNNNNNNNNNNNNNNNNNNNNNNNNNNNNNNNNNNNNNNNNNNNNNNNNN
NNNNNNNNNNNNNNNNNNNNNNNNNNNNNNNNNNNNNNNNNNNNNNNNNNNNNNNNNNNNNNNNNNNNNN
NNNNNNNNNNNNNNNNNNNNNNNNNNNNNNNNNNNNNNNNNNNNNNNNNNNNNNTCTNNNAAANNNTNNN
NNNNNNNNNNNNNNNNNNNNNNNNNNNCNCNNTGNNNGTAANCNANNNGNACATGCAGTCCCCTCNNNNN
NNNGGCAGAGNNNNNNCCCNNNNNNNNCCGGCCCCNNCCNNNNNNNNNNNNNNNNNNNNNNNNNANNNNNN
NNNNNNTGGTACNNNNCGNNNNNGGTTGTNTAAGAGGTCCACAACGNGNTCNGNNNACNNNNGNNNAN
NNNCNNNNANCNNNNNNNNNNNNNANNTNNNTANNAGGNCNNNNCCNTCNNNNNNNNNNANNGCNATGANN
CANNNCNCGGGGGCNGTNCCCGGNGCGGNGACCTTGANNACGTCCGGGNNCAAGTATGGCGNNCNCCC
CGGGAAGGANNCNGCNGTANGTGCNNNNNANNNNNTCNTGNGANGTNGCCGNNATTNNCNTGGTCNNN
CNAGANTGNCCNNACCANNCGNANNNTGGTNATGNTAGTNNNNNNNNNNNNNNNNNNNNNNNNNNNNN
NNNNNNNNNNNNNNNNNNNNNNNNNNNNNNNNNNGNNNNGNNNNNNCNANNNAAGGNNNTANNNCTNNTN
NNNNNNNNGGACNNNACNNNNCNNNNNNNACNCNNNCNCNNNNNNNANNNGNTTTTNNNNNCNNTANTNT
CCAGNACGCNGNGCNNNCATTCANGNGAACNNNGNNACGCGNNAATCGTANNATCTACCTGCCGCNNG
NNNNNNTNCNGNNNNNNCGGANNNTNNCTCGTANNNNNNNNCANGCGTNNTNTNNTCNCNAGNCNNGTT
CGNNNANANANTAGNANGTNTNNNNNNNNNNTGTNNCGTTCCGCNTGANNCNNCANNNNGNNGNNNTTN
NCANCGGCNNTANCTGCNGANNTAAGNCATANNNTNNNTCNNTANCTNNNNNGGNGGGNNACTGTAAA
NGGNNAATTNGNNNNTNATTNCNNNCACNTNNCTNNGNNTNCTNNNTNCCTGNNTNCNNAATTGGNNN
CANTGNNTGNCNATCTCTGGNTTNGNNNNNNTNNNNNTNNCANNNNGGNNNGTNANANNNCNATNTAA
NNTANANNNNCNNNNNNANNNCACNNNNNNNNNNAGNNNCNANNNTTANANNNGTAGNNNANTAAGTCT
CCGANNTNNNNNNNATAAGCGNNANACNNNNNNNNNNNNNNANGANNNCGNNTTGGNNNTNNNNNNNNA
GNNAGACNNTNATGGNTNNNNNTCAANNNNNTTNGTAGTGGTATAATNGNNNNNNNTTTCNCCAGNNTN

NNNGNNNNNNNNCNATANNANNNNNNNGNGANNNNNNNNNNNANNNNCTCNNNAGGNANCANCTACTNNA
GCANNGGNGCGNAGNNNGAGANNNNGACNNNCCTTGGGTGGCAANCACNGCAATAAGTNNNGTTNANN
NTNNNAGTNNACATNNNNNCANNGNNNCNNNNNNANCNGANNGNNNNNNNNNNNNNNNNNNNNNNNN
NNNNNNNNNNNNNNNNNNNNNNNNNNNNNNANNNNANGGNNNGTNTNTGACNCATGTGTTTTTANNNGNN
NNNNNNNGNNNNANCCCCGGTCGNNNNCNNATTGAANNCCTGNNGCATNNNNNTNCNAGAATGANNNC
NNNNNGNNTNNNCTNNCNNNTCAAGNAATCANNNAGNNNNNNNNNTCNNNGNNNNNNNNCNNNCNCNN
ACTGAGGGTNNNNNNNNCATCNGCTNNNNNNNNCTNCGCNGGNNNNNCNNNNNNGCNGTATNGNGNNA
NNCANNNNNNNNNNNNNNNNNTNAAACGANNNNNNNTNNNN
>CoLao599
GTGNNNNNTAGCCCNNNNNAGTTAGACANNNCNNNNNNNNNNNNNTNNNNNNNNNNNNNNNNNNNNNNN
NNNNNNNNNNNNNNNNNNNNNNNNNNNNNNNNTNNNNNNNNNNNNNNNNNNNNNNNNNNNNNNNNNNNNN
NNNNNNNNNNNNNNNNNNNNNNNNNNNNNNNNNNNNNNNNNNNNNNNNNNNNNNNNNNNNNNNNNNNNNN
NNNNNNNNNNNNNNNNNNNNNNNNNNNNNNNNNNNNNNNNNNNNNNNNANNNNNNNNNNNNNNNNNNNNNNNN
NNNNNNNNNNNNNNNNNNNNNNNNNNNNNNNNNNNNNNNNNNNNNNNNNNNNNCNTCTNNNAAANNNTNNN
NNNNNNNNNNNNNNNNNNNNNNNNNNNCNCNTTGNNNGTAANCNANNNGNACATGCAGTCCCCTCNNNNN
NNNGGCAGAGNNNNNNNCCNNNNNCNCCGGCCCCNNCCNNNNNNNNNNNNNNNNNGNNNNNNNANNNNNN
NNNNNNTGGTACNNNNCGNNNNNGGTTGTNTAAGAGGTCCACAACANGNTCNGNNNACNNNNGNNNAN
NNNCNNNNAACNNNNNNNNNNNNNANATNNNTANNAGGNNNNNNCCNTCNNNNNNNNNNATTGCNATGACC
CANNTNNCGGGGGCNGTNCCCGGCGCGGCGACCTTGANNACGTCCGGGGNCAAGTATGGCGNNCNCCC
CGGGAAGGANNCNGCCGTANGTGCNNNNNANNNNNTCNTGNGANGTCGCCGNNATTCNCGTGGTCNNN
CNAGAATGNCCNNACCANNCGNANNNTGGTNATGNNAGTNNNNNNNNNNNNNNNNNNNNNNNNNNNNN
NNNNNNNNNNNNNNNNNNNNNNNNNNNNNNNNNNNGNNNNGNNNNNNCNANNNAAGGNNNTANNNCTNNTN
NTNNNNNNNGGACNNNACNNNNCNNNNNNNNACNCNNNCNCNNNNNNNNNNNGNTTTNNNNNCNNTANTNT
CCAGNACGCNGNGCNNNCATTCANGNGAACNNNGNNACGCGNNAATCGTANNATCTACCTGCCGCNNG
NNNNNNTACNGNNNNNNNCGGANNNTNNNNCGTANNNNNNTCATGCGTNNNNTNNTCNCNAGNCNNGTT
CGNNNANANANTAGNANGTNTNNNNNNNNNNTGTNNCGTTCCGCNCGANNCNNCANNNNGNNGNNNTTN
NCANCGGCNNTANCTNCNGANNTAAGNCATANNNTNNNTCNNTANCTNNNNNGGNGGGNNACTGTAAA
NGGNNAATTNGNNNNTNATTNCNNNCACNTNNCNNNANNNNCTNNNTNCCTGNNTNCNNAATTGGNNC
CANTGNNTGNCNATCTCTGGNTTNGNNNNNNTNNNNNTNNCANNNNGGNNNGTNNNANNNCNATNTAA
NNTANANNNNCNNNNNNANNNCACNNNNNNNGNAGNNNCNANNNTNANANNNGTAGNNNANTAAGTCT
CCGANNTNNNNNNNNATAAGCGNNANACNNNNNNNNNNNNNNANGANNNCGNNTTGGNNNTNNNNNNNCNA
GNNAGACNNTNGTGGNTNNNNNTCAANNNNTTNGTAGTGGTATAATNGNNNNNNNTTTCNCCAGNNTN
NNNGNNNNNNNNCNATANNANNNNNNNGNGANNNNNNNNNNNANNNNCTTNNNAGGGANCANTTACTNNA
GCANTGGNGCGCAGNNNGAGANNNNGACNNGCCTTGGGTGGCAANCACNGNAATAAGTNNNGTTNANC
CTNNNAGTNNACACNNNNNCANNGNNNCNNNNNNANCNGANNGNNNNNNNNNNNNNNNNNNNNNNNN
NNNNNNNNNNNNNNNNNNNNNNNNNNNNNNANNNNANGGNNNGTNTNTGACNCATGTGTTTTTANNNGNN
NNNNNACGNNNNANCCCCGGTCGNNNNCNNATTGAANNCCTGNNGCATNNNNNTNCNAGAATGANNGC
NNNNNGNNTNGNCTNNCNTTTCAAGNAATCANNNAGNTNNNNNNNTCNNGGNNNNNNNNCNNTCNCNN
ACTGAGGGTNNNNNNNNCATCTGCTNNNNNNNNNNTNCGCNGGNGTNNCNNNNNAGCNGTATNGNGTNA
NNCANNNNNNNNNNNNNNNNNNTNAAACNANNNNNNNTNNNN
>CoLao600
GTGNNNNNNAGCCCNNNNNAGTTAGACANNNCNNNNNNNNNNNNNTNNNNNNNNNNNNNNNNNNNNNNN
NNNNNNNNNNNNNNNNNNNNNNNNNNNNNNNNTNNNNNNNNNNNNNNNNNNNNNNNNNNNNNNNNNNNNN
NNNNNNNNNNNNNNNNNNNNNNNNNNNNNNNNNNNNNNNNNNNNNNNNNNNNNNNNNNNNNNNNNNNNNN
NNNNNNNNNNNNNNNNNNNNNNNNNNNNNNNNNNNNNNNNNNNNNNNNNNNNNNNNNNNNNNNNNNNNNN
NNNNNNNNNNNNNNNNNNNNNNNNNNNNNNNNNNNNNNNNNNNNNNNNNNNNNCNNCTNNNAAANNNTNNN
NNNNNNNNNNNNNNNNNNNNNNNNNNNCNCNTTNNNNGTAANCNANNNGNACATGCAGTCCCCTCNNNNN
NNNGGCAGAGNNNNNNNCCNNNNNNNCCGNCCCCNNCCNNNNNNNNNNNNNNNNNGNNNNNNNANNNNNN
NNNNNNTGGTACNNNNCGNNNNNGGTTGNTTAAGAGGTCCACCACGNGNTCNNGNCACNNNNGNCNAN
NNNCNNNNAACNNNNNNNNNNNNNANNTNNNTANNAGGNNNNNNCCNTCNNGNNNNNNATTGCNATGACC
CANNNNNCGGNGGNNGTCCCCGGCGNGGCGACCTTGANNANGTCNGGGGNCAAGTATGGCGNNCNCCC
CGGGAAGGANNCNGCNGTANGTGCNNNNNANNNNNTCNTGNGANGTCGCCGNNATTCNCGTGGTCNNN
CNNGAATGNCCNNACCANNCGNANNNTGGTTATGNNAGTNNNNNNNNNNNNNNNNNNNNNNNNNNNNN
NNNNNNNNNNNNNNNNNNNNNNNNNNNNNNNNNNNGNNNNGNNNNNNCNANNNAAGGNNNTANNNCTNNTN

NNNNNNNNGGACNNNACNNNNCNNNNNNNACNCNNNCNCNNNNNNANNNGNTTNNNNNNCNNTNNTNT
CCAGNACGCNGNGCNNNCATTCANGNGAACNNNGNNACGCGNNAATCGTANNATCTACCTGCCGCNNG
NNNNNNTNCNGNNNNNNCGGANNNTNNNNNGTANNNNNNNTANACGTNNTNTNNTCNCNAGNCNNGTT
CGNNNANANANTAGNANGTNTNNNNNNNNNTGTNNCGTTCCGCNTGANNCNNCANNNNGNNGNNNTTN
NCANCGGCNCTANCTGCNGANNTAAGNCATANNNTNNNTCNNTANCTNNNNNGGNGGGNNACTGTAAA
NGGNNAATTNGNNNNTNATTNCNNNCACNTNNCNNNGNNNNCTNNNTNCCTGNNTNCNNAATTGGNNN
CANTGNNTGNCNATCTCTNGNTTNGNNNNNNTNNNNNTNNCANNNNGGNNNNNNNNANNNCNATNTAA
NNTANANNNNCNNNNNNANNNCACNNNNNNNGNAGNNNCNANNNTNANANNNGTAGNNNANTAAGTCN
CCGANNTNNNNNNNATAAGCGNNANACNNNNNNNNNNNNNANGANNNCGNNTTGGNNNTNNNNNNNNA
GNNAGACNNTNGTGGNTNNNNNTCAANNNNTTNGTAGTGGTATAATNGNNNNNNNTTTCNCCAGNNTN
NNNGNNNNNNNCNATANNANNNNNNNGNGANNNNNNNNNNANNNNNTCNNNAGGNANCANCTACTNNA
GCANNGGNGCGNAGNNNGAGANNNNGACNNNCCTTGGGTGGCAANNACNGNAANAAGTNNNGTTNANN
NTNNNAGTNNACACNNNNNCANNGNNNCNNNNNNANCNGANNGNNNNNNNNNNNNNNNNNNNNNNNNN
NNNNNNNNNNNNNNNNNNNNNNNNNNNNNNNNNNANGGNNNGTNTNTGNCNCNTGNNTTTTANNNGNN
NNNNNNNGNNNNANCCCCGGTCGNNNNCNNATTGAANNCCTGNNGCATNNNNNTNCNAGAATGANNNC
NNNNNGNNTNGNCTNNCNNNTCAAGNAGTCANNNANGNNNNNNNNTCNNNGNNNNNNNNCNNTCNCNN
ACTGAGGGTNNNNNNNNCATCNGCTNNNNNNNNCTNCGCNGGNGTNNCNNCNNAGCNGTATNGTGNNA
NNCANNNNNNNNNNNNNNNNNTNAAACGANNNNCNTNNNG
>CoLao601
GTGNNNNNTAGCCCNNNNNAGTTAGACANNNCNNNNNNNNNNNNTNNNNNNNNNNNNNNNNNNNNNNN
NNNNNNNNNNNNNNNNNNNNNNNNNNNNNNTNNNNNNNNNNNNNNNNNNNNNNNNNNNNNNNNNNNNNN
NNNNNNNNNNNNNNNNNNNNNNNNNNNNNNNNNNNNNNNNNNNNNNNNNNNNNNNNNNNNNNNNNNNN
NNNNNNNNNNNNNNNNNNNNNNNNNNNNNNNNNNNNNNNNNNNNNNNNNNNNNNNNNNNNNNNNNNNN
NNNNNNNNNNNNNNNNNNNNNNNNNNNNNNNNNNNNNNNNNNNNNNNNNNCNTCTNNNAAANNNTNNN
NNNNNNNNNNNNNNNNNNNNNNNNNCNCNNTNNNNGTAANCANNNGNACATGCAGTCCCCTCNNNNNN
NNNGGCAGAGNNNNNNCNCNNNNNNNCCGNCCCNNCCNNNNNNNNNNNNCNNNNNNNNNNANNNNNN
NNNNNNTGGTACNNNNCGNNNNNGGTTGTNTAAGAGGTCCACAACGNGGTCNGGNNACNNNNGNNNAN
NNNCNNNNAACNNNNNNNNNNNANATNNNTANNAGGNNNNNNCCNTCNNGNNNNTNANNGCNATGACC
CANNNNNCNGGNGNNGTNCCCGGCGCGGCGACCTTGANNANGTCCGGGGNCAAGTATGGCNNNCNCCC
CGGGAAGGANNCNGCNGTANGTGCNNNNNANNNNNTCNTGNGANNTCGCCGNNATTCNCNTGGTCNNN
CNAGAATGNNCNNACCANNCGNANNNTGGTNATGNNAGTANNNNNNNNNNNNNNNNNNNNNNNNNNNN
NNNNNNNNNNNNNNNNNNNNNNNNNNNNNNNNNGNNNNGNNNNNNCANNNAAGGNNNTANNNCTNNTN
NTNNNNNTGGNCNNNACNNNNCNNNNNNNACNCNNNCNCNNNNNNANNNGGTTTTNNNNCNNTANTNT
CCAGNACGCNGNGCNNNCNTTCANGNGAACNNNGNNACGCGNNAATCGTANNATCTACCTGCCGCNNG
NNNNNNTNCNGNNNNNNCGGANNNTNNNNCGTANNNNNNNCANGCGTNNTNTNNTNNCNAGNCANGTT
CGNNNANANANTAGNANGTNTNNNNNNNNNTGTNNCGTTCCGCNTGANNCNNCNNNNNGNNGNNCTTN
NCANCGGCNNTANCTGCNGANNTAAGNCATANNNTNNNTCNNTANCTNNNNNGGNGGGNNACTGTAAA
NGGNNAATTNGANGNTNATTNCNNNCACNTNNCNNNGNNNNCTNNNTNCCTGNNTNCNNAATTGGNNC
CANTGNNTGNCNATCTCTNGNTTNGNNNNNNTNNNNNTNNCANNNNGGNNNGNNNNANNNCNATNTAA
NNTANANNNNCNNNNNNANNNCACNNNNNNNGNAGNNNCNNNNNTNANANNNGTAGNNNANTNAGTCT
CCGANNTNNNNNNNATAAGCGNNANACNNNNNNNNNNNNNANGANNNCGNNTTGGNNNTNNNNNNNNA
GNNAGACNNTNATGGNTNNNNNTCAANNNNTTNGTAGTGGTATAATNGNNNNNNNTTTCNCCAGNNTN
NNNGNNNNNNNCNATANNANNNNNNNGNGANNNNNNNNNNANNNNCTCNNNAGGGANCANCTACTNNA
GCANTGGNGCGNAGNNNGAGANNNNGACNNNCCTTGGGTGGCAANNACNGNAATAAGTNNNGTTNNNN
CTNNNAGTNNACATNNNNNCANNGNNNCNNNNNNANCNGANNGNNNNNNNNNNNNNNNNNNNNNNNNN
NNNNNNNNNNNNNNNNNNNNNNNNNNNNNNNNNNANGGNNNGTNTNTGACNCATGTGTTTTANNNGNN
NNNNNACGNNNNANCCCCGGTCGNNNNCNNATTGAANNCCTGNNGCATNNNNNTNCNAGAATGANNNC
NNNNNGNNTNGNCTNNCNNNTCAAGNAATCNNNNAGNNNNNNNNNTCNNNNNNNNNNNNCNNTNNCNN
ACTGAGGGTNNNNNNNNCATCNGCTNNNNNNNNNTNCGCNGGNGTNNCNNNNNNGCNGTATNGNGNNA
NNCANNNNNNNNNNNNNNNNNTNANACAANNNNNNNTNNNN
>Dao50
GTGNNNNNTAGCCCNNNNNAGTCAGACANNNCNNNNNNNNNNNNTNNNNNNNNNNNNNNNNNNNNNNN
NNNNNNNNNNNNNNNNNNNNNNNNNNNNNNTNNNNNNNNNNNNNNNNNNNNNNNNNNNNNNNNNNNNNN
NNNNNNNNNNNNNNNNNNNNNNNNNNNNNNNNNNNNNNNNNNNNNNNNNNNNNNNNNNNNNNNNNNNN

NNNNNNNNNNNNNNNNNNNNNNNNNNNNNNNNNNNNNNNNNNNNNNNNNNNNNNNNNNNN
NNNNNNNNNNNNNNNNNNNNNNNNNNNNNNNNNNNNNNNNNNNCNTCTNNNAAANNNTNNN
NNNNNNNNNNNNNNNNNNNNNNNNNNNCNCNTTGNNNGTAANCNANNNGNACATGCAGTCCCCTCNNNNN
NNNGGCAGAGNNNNNNCCCNNNNNNNCCGGCCCCGNCCNNNNNNNNNNNCNNNGNNNNNNNANNNNNN
NNNNNNTGGTACNNNNCGNNNNNGGTTGTNTAAGAGGTCCACAACGNGGTCNGGNCACNNNNGNNNAN
NNNCNNNNAANNNNNNNNNNNNNANNTNNNTANNAGGNNNNNNCCNTCNNNNNNNNNAATTGCNATGACC
CANNNCNCNGGGGCNGTNCCCGGCGCGGCGACCTTGANNACGTCCGGGGNCAAGTATGGCGNNCNCCC
CGGGAAGGANNCNGCNGTANGTGCNNNNNANNNNNTCNTGNGANGTCGCCGNNATTCNCGTGGTCTNN
CNAGAATGNCCNNACCANNCGNANNNTGGTNATGNTAGTNNNNNNNNNNNNNNNNNNNNNNNNNNNNN
NNNNNNNNNNNNNNNNNNNNNNNNNNNNNNNNNNNNNNNNNGNNNNNNCNANNNAAGGNNNTANNNCTNNTN
NTNNNNNNGGACNNNACNNNNCNNNNNNNNACNCNNNCNCNNNNNNNNNNNGNTTTTNNNNCNNTATTNT
CCAGNANGCNGNGCNNNCNTTTANGNGAACNNNGNNACGCGNNAATCGTANNATCTACCTGCCGCNNG
TNNNNNTACNGNNNNNNCGGANNNTNNNNCGTANNNNNNNCATGCGTNNTNTNNTCNCNAGNCNNGTT
CGNNNANANANTAGNANGTNTNNNNNNNNNNTGTNNCGTTCCGCNTGANNCNNCANNNNGNNGNNCTTN
NCANCGGCNNTANCTGCNGANNTAAGNCATANNNTNNNTCNNTANCTNNNNNGGNGGGNNACTGTAAA
NGGNNAATTNGNNGNTNATTNCNNNCATNTNNCTNNGNNNNCTNNNTNCCTGNNTNCNNAATTGGNNC
CANTGNNTGNCNATCTCTGGNTTNGNNNNNNTNNNNNTNNCANNNNGGNNNGTNANANNNCNATNTAA
NNTANANNNNCNNNNNNANNNCACNNNNNNNGNAGNNNCNANNNGTANANNNGTAGNNNGATAAGTCT
CCGANNTNNNNNNNATAAGCGNNANACNNNNNNNNNNNNNNANGANNNCGNNTTTGNNNTNNNNNNCNA
GNNAGACNNTNGTGGNTNNNNNTCAANNNNTTNGTAGTGGTATAATNGNNNNNNNTTTCNCCAGNNTN
NNNGNNNNNNNCNATANNANNNNNNNGNGANNNNNNNNNNNANNNNCTCNNNAGGGANCANCTACTNNA
GCANNGGNGCGNAGNNNGAGANNNNGACNNNCCTTGGGTGGCAANCACNGCAATAAGTNNNGTTNANC
CTNNNAGTNNACACNNNNNCANNGNNNCNNNNNNANCNGANNGNNNNNNNNNNNNNNNNNNNNNNNN
NNNNNNNNNNNNNNNNNNNNNNNNNNNNNNNNNNNNNNANGGNNNGTNTNTGACNCATNNNTTTTTANNNGNN
NNNNNANGNNNNANCCCCGGTCGNNNNCNNATTGAANNCCTGNNGCATNNNNNTNCNAGAATGANNGC
NNNNNGNNTNGNCTNNCNNNTCAAGNAATCAANTAGGNNNNNNNNTCNNNGNNNNNNNNCNNNCNCNN
ACTGAGGGTNNNNNNNNCATCTGCTNNNNNNNNNNTNCGCNGGCGTNNCNNNNNAGCNGTATNGNGTNA
NNCANNNNNNNNNNNNNNNNNTNAANNNNNNNNNNNNTNNNN
>Dao51
GTNNNNNNNAGCCCNNNNNAGTCNNACANNNCNNNNNNNNNNNNTNNNNNNNNNNNNNNNNNNNNNN
NNNNNNNNNNNNNNNNNNNNNNNNNNNNNNNNNNNNNNNNNNNNNNNNNNNNNNNNNNNNNNNNNNNNNNN
NNNNNNNNNNNNNNNNNNNNNNNNNNNNNNNNNNNNNNNNNNNNNNNNNNNNNNNNNNNNCNNNNNN
NNNNNNNNNNNNNNNNNNNNNNNNNNNNNNNNNNNNNNNNNNNNNNNNNNNNNNNNNNNNNNNNNNNNNNN
NNNNNNNNNNNNNNNNNNNNNNNNNNNNNNNNNNNNNNNNNNNNNNNNNNNNNNNNNNNNAANNNNNNN
NNNNNNNNNNNNNNNNNNNNNNNNNNNCNCNNTNNNNGTAANCNANNNGNACATGCAGTCCCCTCNNNNN
NCNGGCAGAGNNNNNNNNCNNNNNNNCCGNCCCCNNCCNNNNNTCNNNNCNNNGNNNNNNNANNNNNN
NNNNNNTGGTACNNNNCGNNNNNGGNTGNNNAAGAGGTCCACAACGNGNTCNNNNNACNNNNGNNNAN
NNNCNNNNNNNNNNNNNNNNNNNNNNNNNNTANNAGGNNNNNNCCNNNNNNNNNNNNNANNGCNATGACC
CANNTCNCGGNGGNNGTNCCCGGCGNNGCGACCTTGNNNACGNCNGGGNNCAAGTATGGCGNNCNCCC
CGGGAAGGANNCNGCNGTANGTGCNNNNNANNNNNTCNTGNGANGTNGCCGNNATTNNCNTGGTCNNN
CNAGAATGNCCNNACCANNCGNANNNTGGTNATGTNAGTNNNNNNNNNNNNNNNNNNNNNNNNNNNNN
NNNNNNNNNNNNNNNNNNNNNNNNNNNNNNNNNNNNNNNNNGNNNNNNCNANNNAAGGNNNTANNNCTNNTN
NNNNNNNNNNNNCNNNACNNNNCNNNNNNNNACNCNNNCNCNNNNNNNANNNGGTTTTNNNNCNNTANTNT
CCAGNACGCNGNGCNNNCNTTCANGNGAACNNNGNNACGCGNNAATCGTANNATCTACCTGCCGCNNG
NNNNNNTNCNGNNNNNNNCGGANNNTNNNNCGTANNNNNNNCANGCGTNNTNTNNTCNCNAGNCNNGTT
CGNNNANANANTAGNANGTNNNNNNNNNNNNTGTNNCGTTCCGCNTGANNCNNCNNNNNGNNGNNNTTN
NCANCGGCNNTANCNNCNGANNTAAGNNATANNNTNNNTCNNTANCTNNNNNGGNGGGNNACTGNAAA
NGNNNAATTNGNNNNTNANTNCNNNCATNNNNCNNNGNNNNCTNNNTNCCTGNNNNCNNAATTGGNNC
CANTGNNTGNCNATCTCTNGNTTNGNNNNNNTNNNNNTNNCANNNNGGNNNNNNANANNNCNATNTAA
NNTANANNNNCNNNNNNNNNNCACNNNNNNNNNAGNNNNNANNNGNANANNNGTAGNNNGNTNAGTCN
CCGANNTNNNNNNNATAAGCGNNANACNNNNNNNNNNNNNNANGANNNCGNNTTTGNNNTNNNNNNNNA
GNNAGACNNTNGTGGNTNNNNNTCAANNNNTTNGTAGTGGTATAATNGNNNNNNNTTTCNCCAGNNNN
NNNGNNNNNNNCNATANNANNNNNNNGNGANNNNNNNNNNNNNNNNNTCNNNAGGGANCANCTACTNNA
GCANTGGNGCGNAGNNNGAGANNNNNACNNNCCTTGGGTGGCAANNACNGNAATAAGTNNNGTTNNNC

CTNNNAGTNNACACNNNNNCANNGNNNCNNNNNNANCNGANNGNNNNNNNNNNNNNNNNNNNNNNNNN
NNNNNNNNNNNNNNNNNNNNNNNNNNNNNNNNNNNANGGNNNGTNTNTGACNCNTGNNTTTTTANNNGNN
NNNNNNNGNNNNANCCCCGGTCGNNTNCNNATTGAANNCCTGNNGCATNNNNNTNCNANAATGANNNC
NNNNNGNNTNGNCTNNNNNNNTCAAGNAATCNNNNAGGTNNNNNNNTCNNNGNNNNNNNNCNNNNNCNN
ACTGAGGGNNNNNNNNNCATNNGCTNNNNNNNNNTNCGCNGGNGTNNCNNCNNAGCNGTATNNNGTNN
NNCANNNNNNNNNNNNNNNNNTNNANNNNNNNNNNNTNNNN
>Dao52
GTGNNNNNNAGCCCNNNNNAGTTAGACANNNCNNNNNNNNNNNNTNNNNNNNNNNNNNNNNNNNNNNN
NNNNNNNNNNNNNNNNNNNNNNNNNNNNNNNNNTNNNNNNNNNNNNNNNNNNNNNNNNNNNNNNNNNNN
NNNNNNNNGNNNNNNNNNNNNNNNNNNNNNNNNNNNNNNNNNNNNNNNNNNNNNNNNNNNNNNNNNNN
NNNNNNNNNNNNNNNNNNNNNNNNNNNNNNNNNNNNNNNNNNNNNNNNNNNNNNNNNNNNNNNNNNNNNN
NNNNNNNNNNNNNNNNNNNNNNNNNNNNNNNNNNNNNNNNNNNNNNNNNNNNNTCTNNNAAANNNTNNN
NNNNNNNNNNNNNNNNNNNNNNNNNNCNCNTTGNNNGTAANCNANNNGNACATGCAGTCCCCTCNNNNN
NNNNNCAGAGNNNNNNNCCNNNNNNNCCGNCCCCNNCCNNNNNNNNNNNNNNNNNNNNNNNNNNANNNNNN
NNNNNNTGGTACNNNNCGNNNNNGGTTGNNTAAGAGGTCCACAACANGGTCNGNNNACNNNNGNNNAN
NNNCNNNNAACNNNNNNNNNNNNANNTNNNTANNAGGNCNNNNCCNTCNNGNNNNTNATTGCNATGANN
CANNTCNCNGNGGNNGTNCCCGGNGNGGCGACCTTGNNNACGTCCGGGGNCAAGTATGGCGNNCNCCC
CGGGAAGGANNCNGCNGTANGTGCNNNNNANNNNNTCNTGNGANGTCGCCGNNATTCNCNTGGNCNNN
NNNGAATGNCCNNACCANNCGNANNNTGGTNATGNNAGTNNNNNNNNNNNNNNNNNNNNNNNNNNNNN
NNNNNNNNNNNNNNNNNNNNNNNNNNNNNNNNNNNGNNNNGNNNNNNCNANNNAAGGNNNTANNNCTNNTN
NTNNNNNNNNNNCNNNACNNNNCNNNNNNNNACNCNNNCNCNNNNNNANNNGNTTTTNNNNCNNTANTNT
CCAGNACGCNGNGCNNNCNTTCANGNGAACNNNGNNACGCGNNAATCGTANNATCTACCTGCCGCNNG
NNNNNNTNCNGNNNNNNCGGANNNTNNNNCGTANNNNNNNNCANGCGTNNTNTNNTCNCNAGNCNNGTT
CGNNNANANANTAGNANGTNTNNNNNNNNNNTGTNNCGTTCCGCNCGANNCNNCANNNNGNNGNNNTTN
NCANCGGCNNTANCTNCNGANNTAAGNCATANNNTNNNTCNNTANCTNNNNNGGNGGGNNACTGTAAA
NGGNNAATTNGNNNNTNANTNCNNNCACNTNNCTNNANNTNCTNNNTNCCTGNNTNCNNAATTGGNNN
CANTGNNTGNCNATCTCTNGNTTNGNNNNNNTNNNNNTNNCANNNNGGNNNNNNANANNNCNATNTAA
NNTANANNNNCNNNNNNANNNCACNNNNNNNNNNAGNNNNNANNNTTANANNNGTAGNNNANTNAGTCT
CCGANNTNNNNNNNATAAGCGNNANACNNNNNNNNNNNNNNANGANNNCGNNTTGGNNNTNNNNNNNNA
GNNAGACNNTNGTGGNTNNNNNTCAANNNNTTCGTAGTGGTATAATNGNNNNNNNTTTCNCCAGNNTN
NNNGNNNNNNNCNATANNANNNNNNNGNGANNNNNNNNNNNANNNNCTTNNNAGGGANCANTTACTNNA
GCANNGGNGCGNAGNNNGAGANNNNGACNNNCCTTGGGTGGCAANNACNGNAATAAGTNNNGTTNANN
NTNNNAGTNNNCACNNNNNCANNGNNNCNNNNNNANCNGANNGNNNNNNNNNNNNNNNNNNNNNNNNN
NNNNNNNNNNNNNNNNNNNNNNNNNNNNNNNANNNNANGGNNNGTNTNTGNCNCATGTGTTTNTANNNGNN
NNNNNNNGNNNNANCCCCGGTCGNNNNCNNATTGAANNCCTGNNGCATNNNANTNCNAGAATGANNNC
NNNNNGNNTNGNCTNNCNTTTNAAGNAATCANNNAGGNNNNNNNNNTCNNNGNNNNNNNNCNNTNNCNN
ACTGAGGGTNNNNNNNNCATCNGCTNNNNNNNNNNTNCGCNGGNNTNNCNNNNNNGCNGTATNGNGTNN
NNCANNNNNNNNNNNNNNNNNTNAAANNANNNNNNNTNNNN
>Dao623
GTGNNNNNTAGCCCNNNNNAGTTAGACANNNCNNNNNNNNNNNNTNNNNNNNNNNNNNNNNNNNNNNN
NNNNNNNNNNNNNNNNNNNNNNNNNNNNNNNNNNNNNNNNNNNNNNNNNNNNNNNNNNNNNNNNNNNNNN
NNNNNNNNNNNNNNNNNNNNNNNNNNNNNNNNNNNNNNNNNNNNNNNNNNNNNNNNNNNNNNNNNNNNNN
NNNNNNNNNNNNNNNNNNNNNNNNNNNNNNNNNNNNNNNNNNNNNNNNNNNNNNNNNNNNNNNNNNNNNN
NNNNNNNNNNNNNNNNNNNNNNNNNNNNNNNNNNNNNNNNNNNNNNNNNNNNNCNTCTNNNAAANNNTNNN
NNNNNNNNNNNNNNNNNNNNNNNNNNCNCNNTNNNNGTAANCNANNNGNACATGCAGTCCCCTCNNNNN
NNNGGCAGAGNNNNNNNCCNNNNNNNCCGGCCCCNNCCNNNNNNNNNNNNCANNGNNNNNNNGNNNNNN
NNNNNNTGGTACNNNNCGNNNNNGGTTGTNTAAGAGGTCCACCACGNGNTCNGNNNACNNNNGNNNAN
NNNCNNNNAACNNNNNNNNNNNNNNNTNNNCANNAGGNCNNNNCCNTCNNGNNNNNNATNGCNATGACC
CAANNCNCNGGNGCNGTNCCCGGCGCGGCGACCTTGNNNACNGCCGGGGNCAAGTATGGCNNNCNCCC
CGGGAAGGANNCNGCNGTANGTGCNNNNNANNNNNTCNTGNGANGTCGCCGNNATTCNNGTGGTCNNN
CNAGAATGNNCNNACCANNCGNANNNTGGTNATGNNAGTNNNNNNNNNNNNNNNNNNNNNNNNNNNNN
NNNNNNNNNNNNNNNNNNNNNNNNNNNNNNNNNNNNNNGNNNNNNCNANNNAAGGNNNTANNNCTNNTN
NTNNNNNNGGACNNNACNNNNCNNNNNNNNACNCNNNCNCNNNNNNANNNGNTTTTNNNNCNNTANTNT
CCAGNACGCNGNGCNNNCNTTCANGNGAACNNNGNNACGCGNNAATCGTANNATCTANCTGCCGCNNG

TNNNNNTACNGNNNNNNCGGANNNTNNCTCGTANNNNNNNCANGCGTNNTNTNNTCNCNAGNCNNGTT
CGNNNANANANTAGNANGTNTNNNNNNNNNTGTNNCGTTCCGCNTGANNCNNCNNNNNGNNGNNCTTN
NCANCGGCNNTANCTNCNGANNTAAGNCATANNNTNNNTCNNTANCTNNNNNGGNGGGNNACTGTAAA
NGGNNAATTNGNNNNTNATTNCNNNNACNTNNCNNNGNNTNCTNNNTNCCTGNNTNCNNAATTGGNNC
CANTGNNTGNCNATCTCTGGNTTNGNNNNNNTNNNNNTNNCANNNNGGNNNNTNNNANNNCNATNTAA
NNTANANNNNCNNNNNNANNNCACNNNNNNNGNAGNNNCNANNNTTANANNNGTAGNNNANTAAGTCT
CCGANNTNNNNNNNATAAGCGNNANACNNNNNNNNNNNNNANGANNNCGNNTTGGNNNTNNNNNNCNA
ANNAGACNNTNGTGGNTNNNNNTCAANNNNTTNGTAGTGGTATAACNGNNNNNNNTTTCNCCAGNNTN
NNNGNNNNNNNNCNATANNANNNNNNNGNGANNNNNNNNNNNANNNNCTCNNNAGGNANCANCTACTNNA
GCANNGGNGCGNAGNNNGAGANNNNGACNNNCCTTGGGTGGCAANNACNGNAATAAGTNNNGTTNANN
NTNNNAGTNNACACNNNNNCANNGNNNCNNNNNNANCNGANNTNNNNNNNNNNNNNNNNNNNNNNNNN
NNNNNNNNNNNNNNNNNNNNNNNNNNNNNAGNNNANGGNNNGTNTNTGACNCATGTGTTTNTANNNGNN
NNNNNACGNNNNANCCCCGGTCGNNTNCNNATTGAANNCCTGNNGCATNNNNNTNCNAGAATGANNGC
NNNNNGNNTNGNCTNNCNNNTCAAGNAGTCNANNAGGTNNNNNNNTCNNNGNNNNNNNNCNNNCCCNN
ACTGAGGGTNNNNNNNNCATCTGCTNNNNNNNNNNTNCGCNGNNGTNNCNNNNNAGCNGTATNGNGTNN
NNCANNNNNNNNNNNNNNNNNTNAAACAANNNNNTTNNNN
>Dao624
GTGNNNNNTAGCCCNNNNNAGTCAGACANNNCNNNNNNNNNNNNTNNNNNNNNNNNNNNNNNNNNNNN
NNNNNNNNNNNNNNNNNNNNNNNNNNNNNNNNNNNNNNNNNNNNNNNNNNNNNNNNNNNNNNNNNNNNNN
NNNNNNNNNNNNNNNNNNNNNNNNNNNNNNNNNNNNNNNNNNNNNNNNNNNNNNNNNNNNNNNNNNNNNN
NNNNNNNNNNNNNNNNNNNNNNNNNNNNNNNNNNNNNNNNNNNNNNNNNNNNNNNNNNNNNNNNNNNNNN
NNNNNNNNNNNNNNNNNNNNNNNNNNNNNNNNNNNNNNNNNNNNNNNNNNNNNNNNNTCTNNNAAANNNTNNN
NNNNNNNNNNNNNNNNNNNNNNNNNNCNCNNTNNNNGTAANCNANNNGNACATGCAGTCCCCTCNNNNN
NNNNNCAGAGNNNNNNCCNCNNNNCNCCGGCCCCNNCCNNNNNNNNNNNNNNNNNNCNNNNNANNNNNN
NNNNNNTGGTACNNNNCGNNNNNGGTTGTNTAAGNGGTCCACAACGNGGTCNGNNNACNNNNGNNNAN
NNNCNNNAAACNNNNNNNNNNNNANNTNNNTANNAGGNCNNNNCCNTCNNGNNNNNAANTGCNATGANN
CNNNNCNCGGGGGNNGTNCCCGGCGNGGCGACCTTGANNACGTCCGGGGNCAAGTATGGCNNNCNCCC
CGGGAAGGANNCNGCNGTANGTGCNNNNNANNNNNTCNTGNGANGTCGCCGNNATTCNCGTGGTCNNN
CNAGANTGNCCNNACCANNCGNANNNTGGTNATGNNAGTNNNNNNNNNNNNNNNNNNNNNNNNNNNNN
NNNNNNNNNNNNNNNNNNNNNNNNNNNNNNNNNNNNGNNNNGNNNNNNCNANNNAAGGNNNTANNNCTNNTN
NNNNNNNNGGACNNNACNNNNCNNNNNNNNACNCNNNCNCNNNNNNNNNNGNTTTTNNNNCNNTATTNT
CCAGNANGCNGNGCNNNCNTTCANGNGAACNNNGNNACGCGNNAATCGTANNATCTACCTGCCGCNNG
NNNNNNTNCNGNNNNNNCGGANNNTNNCTNGTANNNNNNNCANGCGTNNTNTNNTNNCNAGNCANGTT
CGNNNANANANTAGNANGTNTNNNNNNNNNTGTNNCGTTCCGCNTGANNCNNCANNNNGNNGNNNTTN
NCANCGGCNNTANCTGCNGANNTAAGNCATANNNTNNNTCNNTANCTNNNNNGGNGGGNNACTGNAAA
NGGNNAATTNGNNNNTNATTNCNNNCATNTNNCTNNGNNNNCTNNNTNCCTGNNTNCNNAATTGGNNN
CANTGNNTGNCNATCTCTGGNTTNGCNNNNNTNNNNNTNNCAANNNGGNNNGTNNNANNNCNATNTAA
NNTANANNNNCNNNNNNANNNCACNNNNNNNGNAGNNNCNANNNGNANANNNGTAGNNNGNTAAGTCT
CCGANNTNNNNNNNATAAGCGNNANACNNNNNNNNNNNNNANGANNACGNNTTTGNNNTNNNNNNCNA
GNNANACNNTNGTGGNTNNNNNTCAANNNNTTCGTAGTGGTATAATNGNNNNNNNTTTCNCCAGNNTN
NNNGNNNNNNNNCNATANNANNNNNNNGNGANNNNNNNNNNNANNNNCTCNNNAGGGANCANCTACTNNA
GCAANGGNGCGCAGNNNGAGANNNNGACNNNCCTTGGGTGGCAANNACNGCAATAAGTNNNGTTNNNN
NTNNNAGTNNACACNNNNNCANNGNNNCNNNNNNANCNGANNGNNNNNNNNNNNNNNNNNNNNNNNNN
NNNNNNNNNNNNNNNNNNNNNNNNNNNNNGNNNANGGNNNGTNTNTGACNCNTGNNTTTTTANNNGNN
NNNNNNCGNNNNANCCCCGGTCGNNTNCNNATTGAANNCCTGNNGCATNNNNNTNCNAGAATGANNGC
NNNNNGNNTNGNCTNNNNNNNTNAAGNAATCANNNAGNTNNNNNGNTCNNNNNNNNNNNNCNNTCCCNN
ACTGAGGGTNNNNNNNNCATNNGCTNNNNNNNNNNTNCGCNGGNGTNNCNNNNNNGCNGTATNNNGTNA
NNCANNNNNNNNNNNNNNNNATNNAANNNNNNNNNNNTNNNN
>Dao625
GTGNNNNNNAGCCCNNNNNAGTTAGACANNNCNNNNNNNNNNNNTNNNNNNNNNNNNNNNNNNNNNNN
NNNNNNNNNNNNNNNNNNNNNNNNNNNNNNNNNNNNNNNNNNNNNNNNNNNNNNNNNNNNNNNNNNNNNN
NNNNNNNNNNNNNNNNNNNNNNNNNNNNNNNNNNNNNNNNNNNNNNNNNNNNNNNNNNNNNNNNNNNNNN
NNNNNNNNNNNNNNNNNNNNNNNNNNNNNNNNNNNNNNNNNNNNNNNNNNNNNNNNNNNNNNNNNNNNNN
NNNNNNNNNNNNNNNNNNNNNNNNNNNNNNNNNNNNNNNNNNNNNNNNNNNNNNNNNTCTNNNAAANNNTNNN

NNNNNNNNNNNNNNNNNNNNNNNNNCNCNTTGNNNGTAANCNANNNGNACATGCAGTCCCCTCNNNNN
NNNNNCAGAGNNNNNNNNCNNNNNCNCCGNCCCCNNCCNNNNNNNNNNNCNNNGNNNNNNANNNNNNN
NNNNNNTGGTACNNNNCGNNNNNGGTTGTTTAAGAGGTCCACAACGNGGTCNGGNCACNNNNGNNNAN
NNNCNNNAAACNNNNNNNNNNNNNATNNNTANNAGGNNNNNNCCNTCNNGNNNNTNANNGCNATGACC
CAANTCACNGGGGCNGTNCCCGGNGNGGCGACCTTGANNANNTCCGGGGACAAGTATGGCGNNCNCCC
CGGGAAGGANNCNGCCGTANGTGCNNNNNANNNNNTCNTGNGANGTNGCCGNNATTNNNGTGGTCNNN
NNAGAATGNCCGNACCANNCGNANNNTGGTNATGTNAGTNNNNNNNNNNNNNNNNNNNNNNNNNNNNN
NNNNNNNNNNNNNNNNNNNNNNNNNNNNNNNNGNNNNGNNNNNNCNANNNAAGGANNTANNNCTNNTN
NNNNNNNNGGACNNNACNNNNCNNNNNNNACNCNNNCNCNNNNNNNNNNGNTTTTNNNNCNNTANTNT
CCAGNACGCNGNGCNNNCNTTCANGNGAACNNNGNNACGCGNNAATCGTANNATCTACCTGCCGCNNG
NNNNNNTNCNGNNNNNNCGGANNNTNNCTCGTANNNNNNNCATGCGTNNTNTNNTCNCNAGNCANGTT
CGNNNANANANTAGNANGTNTNNNNNNNNNTGTNNCGTTCCGCNTGANNCNNCANNNNGNNGNNNTTN
NCANCGGCNNTANCTGCNGANNTAAGNCATANNNTNNATCNNTANCTNNNNNGGNGGGNNACTGTAAA
NGGNNAATTNGNNGNTNANTNCNNNNACNTNNCTNNGNNNNCTNNNTNCCTGNNTNCNNAATTGGNNN
CANTGNNTGNCNATCTCTGGNTTNGNNNNNNTGNNNNTNNCAANNNGGNNNNNNANANCNCNATNTAA
NNTANANNNNCNNNNNNANNNCACNANNNNNNNAGNNNCNANNNTNANANNNGTAGNNNAATAAGTCT
CCGANNTNNNNNNATAAGCGNNANACNNNNNNNNNNNNNNANGANNACGNNTTGGNNNTNNNNNNNNA
GNNAGACNNTNATGGNTNNNNNTCAANNNNTTCGTAGTGGTATAATNGNNNNNNNTTTCNCCAGNNTN
NNNNNNNNNNNCNATANNANNNNNNNGNGANNNNNNNNNNANNNNCTCNNNAGGGANCANCTACTNNA
GCANTGGNGCGNAGNNNGAGANNNNGACNNNCCTTGGGTGGCAANCACNGCGATAAGTNNNGTTNANC
CTNNNAGTNNACATNNNNNCANNGNNNCNNNNNNANCNGANNGNNNNNNNNNNNNNNNNNNNNNNNNN
NNNNNNNNNNNNNNNNNNNNNNNNNNNNNGNNNANNGNNNGTNTNTGNCNCATGTGTTTNTANNNGNN
NNNNNACGNNNNANCCCCGGTCGNNNNCNNATTGAANNCCTGNNGCATNNNNNTNCNAGAATGANNNC
NNNNNGNNTNGNCTNNCNTTTCAAGNAATCANNNAGNNNNNGNTCNNNNNNNNNNNNNNCNNNCCCNN
ACTGAGGGTNNNNNNNNCATCTGCTNNNNNNNNNTNCGCNGGCGTNNCNNNNNNGCNGTATNGNGTNN
NNCANNNNNNNNNNNNNNNTNAAACNANNNNNNNTNNNN
>Dao626
GTGNNNNNTAGCCCNNNNNAGTCAGACANNNCNNNNNNNNNNNTNNNNNNNNNNNNNNNNNNNNNNNN
NNNNNNNNNNNNNNNNNNNNNNNNNNNNNNTNNNNNNNNNNNNNNNNNNNNNNNNNNNNNNNNNNNNN
NNNNNNNNNNNNNNNNNNNNNNNNNNNNNNNNNNNNNNNNNNNNNNNNNNNNNNNNNNNNNNNNNNNN
NNNNNNNNNNNNNNNNNNNNNNNNNNNNNNNNNNNNNNNNNNNNNNNNNNNNNNNNNNNNNNNNNNNN
NNNNNNNNNNNNNNNNNNNNNNNNNNNNNNNNNNNNNNNNNNNNNNNNNNNNTCTNNNAAANNNTNNN
NNNNNNNNNNNNNNNNNNNNNNNNNCNCNTTNNNNGTAANCNANNNGNACATGCAGTCCCCTCNNNNN
NNNGGCAGAGNNNNNNCCCNNNNNNNCCGNCCCCNNCCNNNNNNNNNNNNNNNGNNNNNNANNNNNNN
NNNNNNTGGTACNNNNCGNNNNNGGTTGTNTAAGAGGTCCACAACGNGNTCNGNNNACNNNNGNNTAN
NNNCNNNNAACNNNNNNNNNNNANATNNNTANNAGGNCNNNNCCNTCNNGNNNNNNANTGCNATGACC
CANNNNACGGGGGNNGTNCCCGGCGCGGCGACCTTGNNNACGTCCGGGGACAAGTATGGCGNNCNCCC
CGNGAAGGANNCNGCCGTANGTGCNNNNNANNNNNTCNTGNGANGTNGCCGNNATTNNCNTGGTCNNN
NNAGAATGCNCNNACCANNCGNANNNTGGTNATGNNAGTNNNNNNNNNNNNNNNNNNNNNNNNNNNNN
NNNNNNNNNNNNNNNNNNNNNNNNNNNNNNNNGNNNNGNNNNNNCNANNNAAGGNNNTANNNCTNNTN
NNNNNNNNGGACNNNACNNNNCNNNNNNNACNCNNNCNCNNNNNNANNNGGTTTNNNNNCNNTANTNT
CCAGNACGCNGNGCNNNCATTCANGNGAACNNNGNNACGCGNNAATCGTANNATCTACCTGCCGCNNG
NNNNNNTACNGNNNNNNCGGANNNTNNCTCGTANNNNNNNCANGCGTNNTNTNNTCNCNAGNCANGTT
CGNNNANANANTAGNANGTNTNNNNNNNNNTGTNNCGTTCCGCNTGANNCNNCANNNNGNNGNNNTTN
NCANCGGCNNTANCTGCNGANNTAAGNCATANNNTNNNTCNNTANCTNNNNNGGNGGGNNACTGTAAA
NGGNNAATTNGNNNNTNANTNCNNNCATNTNNCTNNGNNTNCTNNNTNCCTGNNTNCNNAATTGGNNC
CANTGNNTGNCNATCTCTNGNTTNGNNNNNNTNNNNNTNNCANNNNGGNNNGTNNNANNNCNATNTAA
NNTANANNNNCNNNNNNANNNCACNNNNNNNGNAGNNNCNANNNGNANANNNGTAGNNNGNTAAGTCT
CCGANNTNNNNNNATAAGCGNNANACNNNNNNNNNNNNNNANGANNACGNNTTTGNNNTNNNNNNNNA
GNNAGACNNTNGTGGNTNNNNNTCAANNNNTTNGTAGTGGTATAATNGNNNNNNNTTTCNCCAGNNNN
NNNGNNNNNNNCNATANNANNNNNNNGNGANNNNNNNNNNANNNNCTCNNNAGGGANCANCTACTTNA
GCANTGGNGCGNAGNNNGAGANNNNGACNNNCCTTGGGTGGCAANNACNGCAATAAGTNNNGTTNANC
CTNNNAGTNNACACNNNNNCANNGNNNCNNNNNNANCNGANNGNNNNNNNNNNNNNNNNNNNNNNNNN
NNNNNNNNNNNNNNNNNNNNNNNNNNNNAGNNNANGGNNNGTNTNTGNCNCANGTGTTTTTANNNGNN

NNNNNACGNNNNANCCCCGGTCGNNNNCNNATTGAANNCCTGNNGCATNNNNNTNCNAGAATGANNNC
NNNNNGNNTNGNCTNNNNTTTCAAGNAATCANNNAGGTNNNNNNNTCNNNGNNNNNNNNCNNTCNCNN
ACTGAGGGTNNNNNNNNCATNTGCTNNNNNNNNCTNCGCNGGNGTNNCNNNNNNGCNGTATNGNGTNN
NNCANNNNNNNNNNNNNNNNNNTNAANNGANNNNNCTNNNN
>Dao627
GTGNNNNNTAGCCCNNNNNAGTCAGACANNNCNNNNNNNNNNNNTNNNNNNNNNNNNNNNNNNNNNNN
NNNNNNNNNNNNNNNNNNNNNNNNNNNNNNNNNNNNNNNNNNNNNNNNNNNNNNNNNNNNNNNNNNNN
NNNNNNNNNNNNNNNNNNNNNNNNNNNNNNNNNNNNNNNNNNNNNNNNNNNNNNNNNNNNNNNNNNNN
NNNNNNNNNNNNNNNNNNNNNNNNNNNNNNNNNNNNNNNNNNNNNNNNNNNNNNNNNNNNNNNNNNNN
NNNNNNNNNNNNNNNNNNNNNNNNNNNNNNNNNNNNNNNNNNNNNNNNNNNNTCTNNNAAANNNTNNN
NNNNNNNNNNNNNNNNNNNNNNNNNCNCNTTGNNNGTAANCNANNNGNACATGCAGTCCCCTCNNNNN
NNNGGCAGAGNNNNNNCCNNNNNNNNCCGNCCCCNNCCNNNNNNNNNNNCNNNGNNNNNNNANNNNNN
NNNNNNTGGTACNNNNCGNNNNNGGTTGTTTAAGAGGTCCACAACGNGGTCNNNNCACNNNNGNNTAN
NNNCNNNNAACNNNNNNNNNNNNNNNTNNNTANNAGGNCNNNNCCNTCNNNNNNNNNANNGCNATGACC
CANNTNNCGGGGGCNGTNCCCGGCGCGGCGACCTTGANNACGTCCGGGNNCAAGTATGGCNNNCNCCC
CGGGAAGGANNCNGCCGTANGTGCNNNNNANNNNNTCNTGNGANNTCGCCGNNATTCNCGTGNNCNNN
CNAGAATGNCCNNACCANNCGNANNNTGGTNATGNNAGTNNNNNNNNNNNNNNNNNNNNNNNNNNNNN
NNNNNNNNNNNNNNNNNNNNNNNNNNNNNNNNGNNNNGNNNNNNCNANNNAAGGNNNTANNNCTNNTN
NTNNNNNNGGACNNNACNNNNCNNNNNNNACNCNNNCNCNNNNNNNNNNGNTTTTNNNNCNNTANTNT
CCAGNANGCNGNGCNNNCATTCANGNGAACNNNGNNACGCGNNAATCGTANNATCTACCTGCCGCNNG
NNNNNNTACNGNNNNNNCGGANNNTNNNNCGTANNNNNNTCANGCGTNNTNTNNTCNCNAGNCNNGTT
CGNNNANANANTAGNANGTNTNNNNNNNNNTGTNNCGTTCCGCNTGANNCNNCANNNNGNNGNNCTTN
NCANCGGCNNTANCTGCNGANNTAAGNCATANNNTNNNTCNNTANCTNNNNNGGNGGGNNACTGTAAA
NGGNNAATTNGNNGNTNANTNCNNNCATNTNNCTNNGNNNNCTNNNTNCCTGNNTNCNNAATTGGNNC
CANTGNNTGNCNATCTCTGGNTTNGCNNNNNTNNNNNTNNCANNNNGGNNNNNNNNANNNCNATNTAA
NNTATANNNNCNNNNNNANNNCACNNNNNNNGNAGNNACNANNNGNANANNNGTAGNNNGNTAAGTCT
CCGANNTNNNNNNNATAAGCGNNANACNNNNNNNNNNNNNANGANNACGNNTTTGNNNTNNNNNNNNA
GNNAGACNNTNGTGGNTNNNNNTCAANNNNTTNGTAGTGGTATAATNGNNNNNNNTTTCNCCAGNNTN
NNNGNNNNNNNCNATANNANNNNNNNGNGANNNNNNNNNNANNNNCTCNNNAGGGANCANCTACTNGA
GCANTGGNGCGNAGNNNGAGANNNNGACNNNCCTTGGGTGGCAANCACNGCAATAAGTNNNGTTNANC
CTNNNAGTNNACACNNNNNCANNGNNNCNNNNNNANCNGANNGNNNNNNNNNNNNNNNNNNNNNNNNN
NNNNNNNNNNNNNNNNNNNNNNNNNNNNAGNNNANNGNNNGTNTNTGNCNCATGTGTTTTTANNNGNN
NNNNNACGNNNNNNCCCCGGTCGNNNNCNNATTGAANNCCTGNNGCATNNNNNTNCNAGAATGANNGC
NNNNNGNNTNGNCTNNCNNNTCAAGNAATCANNNAGNNNNNGCGNTCNNNGNNNNNNNNCNNNCCCNN
ACTGAGGGTNNNNNNNNCATCTGCTNNNNNNNNNNTNCGCNGGNGTNNCNNNNNNGCNGTATNGNGTNA
NNCANNNNNNNNNNNNNNNNNNTNNAACNANNNNNNNTNNNN
>Dao628
GTNNNNNNTAGCCCNNNNNAGTCNGACANNNCNNNNNNNNNNNNTNNNNNNNNNNNNNNNNNNNNNNN
NNNNNNNNNNNNNNNNNNNNNNNNNNNNNNTNNNNNNNNNNNNNNNNNNNNNNNNNNNNNNNNNNNNN
NNNNNNNNNNNNNNNNNNNNNNNNNNNNNNNNNNNNNNNNNNNNNNNNNNNNNNNNNNNNNNNNNNNN
NNNNNNNNNNNNNNNNNNNNNNNNNNNNNNNNNNNNNNNNNNNNNNNNNNNNNNNNNNNNNNNNNNNN
NNNNNNNNNNNNNNNNNNNNNNNNNNNNNNNNNNNNNNNNNNNNNNNNNNCNNCTNNNNAANNNTNNN
NNNNNNNNNNNNNNNNNNNNNNNNNNNCNNTNNNNGTAANCNANNNGNACATGCAGTCCCCTCNNNNN
NNNGGCAGAGNNNNNNNCCNNNNNNNCCGGCCCCNNCCNNNNNNNNNNNCNNNGNNNNNNNANNNNNN
NNNNNNTGGTACNNNNCGNNNNNGGTTGNNTAAGAGGTCCACAACGNGNTCNNNNNACNNNNGNNNAN
NNNCNNNNAANNNNNNNNNNNNNNNATNNNTANNAGGNNNNNNCCNTCNNNNNNNNNANTGCNATGANN
CANNTNNCGGNGGCNGTNCCCGGCGCGGCGACCTTGNNNACGTCNGGGGNCAAGTATGGCNNNCNCCC
CGNGAAGGANNCNGCNGTANGTGCNNNNNANNNNNTCNTGNGANGTNGCCGNNATTNNCGTGNTCNNN
CNNGAATGNCCNNACCANNCGNANNNTGGTNATGNNAGTNNNNNNNNNNNNNNNNNNNNNNNNNNNNN
NNNNNNNNNNNNNNNNNNNNNNNNNNNNNNNNNNNNNGNNNNNNCNANNNAAGGNNNTANNNCTNNTN
NNNNNNNNGGNCNNNACNNNNCNNNNNNNACNCNNNCNCNNNNNNNNNNGGTTNNNNNNCNNTANTNT
CCAGNACGCNGNGCNNNCNTTCANGNGAACNNNGNNACGCGNNAATCGTANNATCTACCTGCCGCNNG
NNNNNNTNCNGNNNNNNCGGANNNTNNCTCGTANNNNNNNCANGCGTNNNNTNNTCNCNAGNCNNGTT
CGNNNANAAANTAGNANGTNTNNNNNNNNNTGTNNCGTTCCGCNTGANNCNNCNNNNNGNNGNNNTTN

NCANCGGCNNTANCTNNNGANNTAAGNCATANNNTNNNTCNNTANCTNNNNNGGNGGGNACTGTAAA
NGGNNAATTNGNNNNTNATTNCNNNCATNTNNCNNNGNNNNCTNNNTNCCTGNNTNCNNAATTGGNNN
CANTGNNTGNCNATCTCTNGNTTNGNNNNNNTNNNNNTNNCANNNNGGNNNGTNANANNNNNATNTAA
NNNANANNNNCNNNNNNANNNCACNNNNNNNNNAGNNNNNANNNGNANANNNGTAGNNNGNTAAGTCN
CCGANNTNNNNNNNATAAGCGNNANACNNNNNNNNNNNNNNANGANNNCGNNTTTGNNNTNNNNNNNNA
GNNAGACNNTNGTGGNTNNNNNTCAANNNNTTNGTAGTGGTATAATNGNNNNNNNTTTCNCCAGNNNN
NNNGNNNNNNNCNATANNANNNNNNNGNGANNNNNNNNNNNANNNNNTCNNNAGGNANCANCTACTNNA
GCANTGGNGCNNAGNNNGAGANNNNGACNNNCCTTGGGTGGCAANNACNGNANTAAGTNNNGTTNANN
CTNNNAGTNNACACNNNNNCANNGNNNCNNACNNANCNGANNGNNNNNNNNNNNNNNNNNNNNNNNNN
NNNNNNNNNNNNNNNNNNNNNNNNNNNNNNNNNNANGGNNNGTNTNTGNCNCNTNNNTTTTANNNGNN
NNNNNNNGNNNNANCCCCGGTCGNNNNCNNATTGAANNCCTGNNGCNTNNNNNTNCNAGAATGANNNC
NNNNNGNNTNGNCTNNNNNNTCANGNAATNNNNNAGGTNNNNNNNTCNNNNNNNNNNNNCNNNNNCNN
ACTGAGGGNNNNNNNNNCATNNGCTNNNNNNNNNNTNCGCNGGNGTNNCNNNNNAGCNGTATNGNGTNN
NNCANNNNNNNNNNNNNNNNNTNANACNNNNNNNNNTNNNN
>Dao629
GTGNNNNNTAGCCCNNNNNAGTCAGACANNNCNNNNNNNNNNNNTNNNNNNNNNNNNNNNNNNNNNNN
NNNNNNNNNNNNNNNNNNGNNNNNNNNNNNNNNNNNNNNNNNNNNNNNNNNNNNNNNNNNNNNNNNNN
NNNNNNNNNNNNNNNNNNNNNNNNNNNNNNNNNNNNNNNNNNNNNNNNNNNNNNNNNNNNNNNNNNNN
NNNNNNNNNNNNNNNNNNNNNNNNNNNNNNNNNNNNNNNNNNNNNNNNNNNNNNNNNNNNNNNNNNNN
NNNNNNNNNNNNNNNNNNNNNNNNNNNNNNNNNNNNNNNNNNNNNNNNNNNCNTCTNNNAAANNNTNNN
NNNNNNNNNNNNNNNNNNNNNNNNNNCNCNTTNNNNGTAANCNANNNGNACATGCAGTCCCCTCNNNNN
NNNNNCAGAGNNNNNNCCCNNNNNNNCCGNCCCCNNCCNNNNNNNNNNNNNNNNNNNNNNNNNANNNNNN
NNNNNNTGGTACNNNNCGNNNNNGGNTGTNTNAGAGGTCCACAACGNGGTCNNNNCACNNNNGNNTAN
NNNCNNNNAANNNNNNNNNNNNNNNNNTNNNTANNAGGNNNNNGCCNTCNNGNNNNNNANNGCNATGACC
CANNNCACNGNGGCNGTNCCCGGCGCGGCGACCTTGNNNANGTCCGGGGNCAAGTATGGCNNNCNCCC
CGGGAAGGANNCNGCNGTANGTGCNNNNNANNNNNTCNTGNGANGTNGCCGNNATTNNCGTGGTCNNN
CNAGAATGNCCNNACCANNCGNANNNTGGTNATGNNAGTNNNNNNNNNNNNNNNNNNNNNNNNNNNNN
NNNNNNNNNNNNNNNNNNNNNNNNNNNNNNNNNNGNNNNGNNNNNNCNANNNAAGGNNNTANNNCTNNTN
NNNNNNNNGGACNNNACNNNNCNNNNNNNACNCNNNCNCNNNNNNANNNGGTTTTNNNNCNNTANTNT
CCAGNACGCNGNGCNNNCATTCANGNGAACNNNGNNACGCGNNAATCGTANNATCTACCTGCCGCNNG
NNNNNNTACNGNNNNNNCGGANNNTNNCTCGTANNNNNNNCANGCGTNNTNTNNTCNCNAGNCNNGTT
CGNNNANANANTAGNANGTNTNNNNNNNNNTGTNNCGTTCCGCNTGANNCNNCANNNNGNCGNNCTTN
NCANCGGCNNTANCTGCNGANNTAAGNCATANNNTNNATCNNTANCTNNNNNGGNGGGNACTGTAAA
NGGNNAATTNGNNNNTNANTNCNNNCATNTNNCNNNGNNNNCTNNNTNCCTGNNTNCNNAATTGGNNC
CANTGNNTGNCNATCTCTGGNTTNGNNNNNNTGNNNNTNNCANNNNGGNNNGTNNNANNNCNATNTAA
NNTANANNNNCNNNNNNANNNCACNANNNNNGNAGNNNCNANNNGTANANNNGTAGNNNGNTAAGTCT
CCGANNTNNNNNNNATAAGCGNNANACNNNNNNNNNNNNNNANGANNNCGNNTTTGNNNTNNNNNNNCNA
GNNAGACNNTNGTGGNTNNNNNTCAANNNNTTCGTAGTGGTATAATNGNNNNNNNTTTCNCCAGNNTN
NNNGNNNNNNNCNATANNANNNNNNNGNGANNNNNNNNNNNANNNNCTCNNNAGGNANCANCTACTNNA
GCANTGGNGCGCAGNNNGAGANNNNGACNNNCCTTGGGTGGCAANCACNGCAATAAGTNNNGTTNANN
CTNNNAGTNNACACNNNNNCANNGNNNCNNNNNNANCNGANNGNNNNNNNNNNNNNNNNNNNNNNNNN
NNNNNNNNNNNNNNNNNNNNNNNNNNNNNANNNNANGGNNNGTNTNTGACNCATGTGTTTNTANNNGNN
NNNNNACGNNNNANCCCCGGTCGNNTNCNNATTGAANNCCTGNNGCATNNNNNTNCNAGAATGANNNC
NNNNNGNNTNGNCTNNCNNNTCAAGNAATCANNNAGGTNNNNNNNTCNNNNNNNNNNNNCNNTCCCNN
ACTGAGGGTNNNNNNNNCATCNGCTNNNNNNNNNCTNCGCNGGCGTNNCNNNNNAGCNGTATNGNGTNA
NNCANNNNNNNNNNNNNNNNNTNNANCAANNNNNTTNNNN
>Dao630
GTGNNNNNNAGCCCNNNNNAGTCAGACANNNCNNNNNNNNNNNNTNNNNNNNNNNNNNNNNNNNNNCNN
NNNNNNNNNNNNNNNNNNNNNNNNNNNNNNNTNNNNNNNNNNNNNNNNNNNNNNNNNNNNNNNNNNNNNN
NNNNNNNNNNNNNNNNNNNNNNNNNNNNNNNNNNNNNNNNNNNNNNNNNNNNNNNNNNNNNNNNNNNN
NNNNNNNNNNNNNNNNNNNNNNNNNNNNNNNNNNNNNNNNNNNNNNNNNNNNNNNNNNNNNNNNNNNN
NNNNNNNNNNNNNNNNNNNNNNNNNNNNNNNNNNNNNNNNNNNNNNNNNNNCNTCTNNNAAANNNTNNN
NNNNNNNNNNNNNNNNNNNNNNNNNNCNCNTTGNNNGTAANCNANNNGNACATGCAGTCCCCTCNNNNN
NNNGGCAGAGNNNNNNCNCNNNNNNNCCGNCCCCNNCCNNNNNNNNNNNNNNNNNNGNNNNNNNANNNNNN

NNNNNNTGGTACNNNNCGNNNNNGGTTGTNTAAGAGGTCCACAACGNGGTCNNGNNACNNNNGNNNAN
NNNCNNNNAANNNNNNNNNNNNNANNTNNNTANNAGGNCNNNNCCNTCNNNNNNNNNANTGCNATGACC
CANNNCACGGNNGCNGTCCCCGGCGCNGCNACCTTGNNNACGTCCGGGGNCAAGTATGGCGNNCNCCC
CGGGAAGGANNCNGCNGTANGTGCNNNNNANNNNNTCNTGNGANGTNGCCGNNATTNNCGTGGTCNNN
NNAGAATGNNCNNACCANNCGNANNNTGGTNATGNNAGTNNNNNNNNNNNNNNNNNNNNNNNNNNNNNN
NNNNNNNNNNNNNNNNNNNNNNNNNNNNNNNNNNGNNNNGNNNNNNCNANNNAAGGNNNTANNNCTNNTN
NTNNNNNNGGACNNNACNNNNCNNNNNNNACNCNNNCNCNNNNNNANNNGNTTTTNNNNCNNTATTNT
CCAGNACGCNGNGCNNNCATTCANGNGAACNNNGNNACGCGNNAATCGTANNATCTACCTGCCGCNNG
NNNNNNTNCNGNNNNNNCGGANNNTNNCTNGTANNNNNNNCANGCGTNNTNTNNTCNCNAGNCNNGTT
CGNNNANANANTAGNANGTNTNNNNNNNNNNTGTNNCGTTCCGCNTGANNCNNCNNNNNGNNGNNCTTN
NCANCGGCNNTANCTGCNGANNTAAGNCATANNNTNNNTCNNTANCTNNNNNGGNGGGNNACTGTAAA
NGGNNAATTNGNNGNTNATTNCNNNCATNTNNCNNNGNNTNCTNNNTNCCTGNNTNCNNAATTGGNNC
CANTGNNTGNCNATCTCTGGNTTNGNNNNNNTNNNNNTNNCANNNNGGNNNNNNANANNNCNATNTAA
NNTANANNNNCNNNNNNANNNCACNNNNNNNNNAGNNNNNANNNGNANANNNGTAGNNNGATAAGTCT
CCGANNTNNNNNNNATAAGCGNNANACNNNNNNNNNNNNNANGANNNCGNNTTTGNNNTNNNNNNCNA
GNNAGACNNTNGTGGNTNNNNNTCAANNNNTTCGTAGTGGTATAATNGNNNNNNNTTTCNCCAGNNTN
NNNGNNNNNNNNCNATANNANNNNNNNGNGANNNNNNNNNNANNNNCTCNNNAGGNANCANCTACTNNA
GCANTGGNGCGNAGNNNGAGANNNNGACNNNCCTTGGGTGGCAANNACNGCAATAAGTNNGTTNNNN
CTNNNAGTNNACACNNNNNCANNGNNNCNNNNNNANCNGANNGNNNNNNNNNNNNNNNNNNNNNNNNN
NNNNNNNNNNNNNNNNNNNNNNNNNNNNNNNNNNNANGGNNNGTNTNTGNCNCATNNNTTTNTANNNGNN
NNNNNNNGNNNGANCCCCGGTCGNNNNCNNATTGAANNCCTGNNGCATNNNNNNTNCNAGAATGANNNC
NNNNNGNNTNGNCTNNCNNNTNAAGNAATCANNNAGNNNNNNNNNNTCNNNGNNNNNNNNCNNNCNCNN
ACTGAGGGTNNNNNNNNCATCTGCTNNNNNNNNNNTNCGCNGGNGTNNCNNNNNAGCNGTATNNNGTNA
NNCANNNNNNNNNNNNNNNANTNAAACAANNNNNNCTNNNG
>Dao631
GTGNNNNNTAGCCCNNNNNAGTTAGACANNNCNNNNNNNNNNNNTNNNNNNNNNNNNNNNNNNNNNNN
NNNNNNNNNNNNNNNNNNNNNNNNNNNNNNNNNNNNNNNNNNNNNNNNNNNNNNNNNNNNNNNNNNNNNN
NNNNNNNNNNNNNNNNNNNNNNNNNNNNNNNNNNNNNNNNNNNNNNNNNNNNNNNNNNNNNNNNNNNNNN
NNNNNNNNNNNNNNNNNNNNNNNNNNNNNNNNNNNNNNNNNNNNNNNNNNNNNNNNNNNNNNNNNNNNNN
NNNNNNNNNNNNNNNNNNNNNNNNNNNNNNNNNNNNNNNNNNNNNNNNNNNNNNNNTCTNNNAAANNNTNNN
NNNNNNNNNNNNNNNNNNNNNNNNNNCNCNNTNNNNGTAANCNANNNGNACATGCAGTCCCCTCNNNNN
NNNNNCAGAGNNNNNNNCCNNNNNNNCCGGCCCCNNCCNNNNNNNNNNNNNNNNNGNNNNNNNANNNNNN
NNNNNNTGGTACNNNNCGNNNNNGGTTGTNTAAGAGGTCCACCACGNGGTCNNGNNACNNNNGNNNAN
NNNCNNNNANCNNNNNNNNNNNNANNTNNNTANNAGGNCNNNNCCNTCNNNNNNNNNANTGCNATGACC
CANNNCNCGGGGGCNGTNCCCGGCGCGGCGACCTTGANNANGTCCGGGGNCAAGTATGGCNNNCNCCC
CGGGAAGGANNCNGCNGTANGTGCNNNNNANNNNNTCNTGNGANGTNGCCGNNATTCNCGTGGTCNNN
CNAGAATGNCCNNACCANNCGNANNNTGGTTATGNNAGTNNNNNNNNNNNNNNNNNNNNNNNNNNNNNN
NNNNNNNNNNNNNNNNNNNNNNNNNNNNNNNNNNGNNNNGNNNNNNCNANNNAAGGNNNTANNNCTNNTN
NNNNNNNNGGACNNNACNNNNCNNNNNNNACNCNNNCNCNNNNNNANNNGGTTTTNNNNCNNTANTNN
CCAGNACGCNGNGCNNNCNTTCANGNGAACNNNGNNACGCGNNAATCGTANNATCTACCTGCCGCNNG
NNNNNNTACNGNNNNNNCGGANNNTNNCTCGTANNNNNNNTANACGTNNTNTNNTCNCNAGNCANGTT
CGNNNANANANTAGNANGTNTNNNNNNNNNNTGTNNCGTTCCGCNTGANNCNNCANNNNGNNGNNCTTN
NCANCGGCNNTANCTGCNGANNTAAGNCATANNNTNNNTCNNTANCTNNNNNGGNGGGNNACTGTAAA
NGGNNAATTNGNNNNTNANTNCNNNCACNTNNCNNNGNNTNCTNNNTNCCTGNNTNCNNAATTGGNNC
CANTGNNTGNCNATCTCTGGNTTNGNNNNNNTNNNNNTNNCANNNNGGNNNNNNNNANNNCNATNTAA
NNTANANNNNCNNNNNNANNNCACNNNNNNNGNAGNNNCNANNNTTANANNNGTAGNNNANTAAGTCT
CCGANNTNNNNNNNATAAGCGNNANACNNNNNNNNNNNNNANGANNNCGNNTTGGNNNTNNNNNNCNA
GNNAGACNNTNGTGGNTNNNNNTCAANNNNTTNGTAGTGGTATAATNGNNNNNNNTTTCNCCAGNNTN
NNNGNNNNNNNNCNATANNANNNNNNNGNGANNNNNNNNNNANNNNCTCNNNAGGGANCANCTACTNNA
GCANTGGNGCGNAGNNNGAGANNNNGACNNNCCTTGGGTGGCAANNACNGCAATAAGTNNGTTNANC
CTNNNAGTNNACACNNNNNCANNGNNNCNNNNNNANCNGANNGNNNNNNNNNNNNNNNNNNNNNNNNN
NNNNNNNNNNNNNNNNNNNNNNNNNNNNNANNNNANGGNNNGTNTNTGACNCATGTGTTTNTANNNGNN
NNNNNACGNNNNANCCCCGGTCGNNNNCNNATTGAANNCCTGNNGCATNNNNNNTNCNAGAATGANNGC
NNNNNGNNTNNNCTNNCNNNTCAAGNAGTCANNNAGGTNNNNNGNTCNNNNNNNNNNNNNCNNTCCCNN

ACTGAGGGTNNNNNNNNCATCTGCTNNNNNNNNNTNCGCNGGNNTNNCNNNNNNGCNGTATNNNGNNA
NNCANNNNNNNNNNNNNNNNNTNAAACNANNNNNNNTNNNN
>Dao632
GTGNNNNNNAGCTCNNNNNAGTTAGACANNNCNNNNNNNNNNNNNNNNNNNNNNNNNNNNNNNNNNNN
NNNNNNNNNNNNNNNNNNNNNNNNNNNNNNNNNNNNNNNNNNNNNNNNNNNNNNNNNNNNNNNNNNNN
NNNNNNNNNNNNNNNNNNNNNNNNNNNNNNNNNNNNNNNNNNNNNNNNNNNNNNNNNNNNNNNNNNNN
NNNNNNNNNNNNNNNNNNNNNNNNNNNNNNNNNNNNNNNNNNNNNNNNNNNNNNNNNNNNNNNNNNNN
NNNNNNNNNNNNNNNNNNNNNNNNNNNNNNNNNNNNNNNNNNNNNNNNNNNNTNNNNNAAANNNTNNN
NNNNNNNNNNNNNNNNNNNNNNNNNCNCNNTNNNNGTAANCNANNNGNACATGCAGTCCCCTCNNNNN
NNNNNCAGAGNNNNNNNNCNNNNNNNCCGNCCCCNNCCNNNNNNNNNNNNNNNGNNNNNNNGNNNNNN
NNNNNNTGGTACNNNNCGNNNNNGGTTGNNTAAGAGGTCCACCACGNGNTCNNNNNACNNNNGNNNAN
NNNCNNNNNNCNNNNNNNNNNNNNNTNNNCANNAGGNNNNNNCCNTCNNNNNNNNNANNGCNATGANN
CANNNCGCNGGGACNGTNACCGGCGNGGCGACCTTGANNANGGTNGGGGNCAAGTATGTCNNNCNCCC
CGGGAAGGANNCNGCNGTANGTGCNNNNNANNNNNTCNTGNGANNTNGCCGNNATTCNCNTGGTCNNN
CNAGANTGNCCNNACCANNCGNANNNTGGTNATGNNAGTNNNNNNNNNNNNNNNNNNNNNNNNNNNNN
NNNNNNNNNNNNNNNNNNNNNNNNNNNNNNNNGNNNNGNNNNNNCNANNNAAGGNNNTANNNTTNNTN
NNNNNNNNGGACNNNACNNNNCNNNNNNNACNCNNNCNCNNNNNNANNNGGTTNNNNNNCNNTATTNT
CCAGNANGCNGNGCNNNCNTTCANGNGAACNNNGNNACGCGNNAATCGTANNATCTACCTGCCGCNNG
NNNNNNTNCNGNNNNNNCGGANNNTNNCTCGTANNNNNNTCANGCGTNNTNTNNTTNCNAGNCNNGTT
CGNNNANANANTAGNANGTNTNNNNNNNNNNGTNNCGTTCCGCNTGANNCNNCANNNNGNNGNNNTTN
NCANCGGCNNTANCTNCNGANNTAAGNCATANNNTNNNTCNNTANCGNNNNNGGNGGGNACTGTAAA
NGGNNAATTNGNNNNTNANTNCNNNCACNTNNCNNNGNNTNCTNNNCNCCTGNNTNCNNAATTGGNNN
CANTGNNTGNCNATCTCTNGNTTNGNNNNNNTNNNNNTNNTANNNNGGNNNNNNANANNNNNATNTAA
NNTANANNNNCNNNNNNANNNCACNNNNNNNGNAGNNNCNANNNTNANGNNNGTAGNNNANTAAGTCT
CCGANNTNNNNNNNATAAGCGNNANACNNNNNNNNNNNNNANGANNNCGNNTTGGNNNTNNNNNNCNA
ANNANACNNCNGTGGNTNNNNNTCAANNNNTTNGTAGTGGTATAACNANNNNNNNTTTCNCCAGNNTN
NNNGNNNNNNNCNATANNANNNNNNNGNGANNNNNNNNNNANNNNNCCNNNAGGNANCANCTACTNNA
GCANTGGNGCGNAGNNNGAGANNNNGACNNNCCTTGGGTGGCAANNACNGNAATAAGTNNNGTTNANN
NCNNNAGTNNACACNNNNNCANNGNNNCNNNNNNANCNGANNTNNNNNNNNNNNNNNNNNNNNNNNNN
NNNNNNNNNNNNNNNNNNNNNNNNNNNNNNNNNANGGNNNGTNTNTGACNCATGTGTTTNTANNNGNN
NNNNNNNGNNNNANCCCCGGTCGNNNNCNNATTGAANNCCTGNNGCATNNNNNTNCNAGAATGANNGC
NNNNNGNNTNNNCTNNNNNNTCAAGNAGTCANNNAGNNNNNNNNNTCNNNGNNNNNNNNCNNTCNCNN
ACTGAGGGTNNNNNNNNCATCTGCTNNNNNNNNNTNCGCNGNNNNNNTNNNNNNGCNGTATNGNGNNN
NNCANNNNNNNNNNNNNNNNNTNAAACNANNNNNNNTNNNN
>Dao633
GTGNNNNNTAGCCCNNNNNAGTCAGACANNNCNNNNNNNNNNNNNTNNNNNNNNNNNNNNNNNNNNNN
NNNNNNNNNNNNNNNNNNNNNNNNNNNNNNNNNNNNNNNNNNNNNNNNNNNNNNNNNNNNNNNNNNNN
NNNNNNNNNNNNNNNNNNNNNNNNNNNNNNNNNNNNNNNNNNNNNNNNNNNNNNNNNNNNNNNNNNNN
NNNNNNNNNNNNNNNNNNNNNNNNNNNNNNNNNNNNNNNNNNNNNNNNNNNNNNNNNNNNNNNNNNNN
NNNNNNNNNNNNNNNNNNNNNNNNNNNNNNNNNNNNNNNNNNNNNNNNNNNNTCTNNNAAANNNTNNN
NNNNNNNNNNNNNNNNNNNNNNNNNCNCNTTGNNNGTAANCNANNNGNACATGCAGTCCCCTCNNNNN
NNNNNCAGAGNNNNNNNCCNNNNNNNCCGNCCCCNNCCNNNNNNNNNNNNNNNGNNNNNNNANNNNNN
NNNNNNTGGTACNNNNCGNNNNNGGTTGTTTAAGAGGTCCACAACGNGGTCNNGNNACNNNNGNNTAN
NNNCNNNNAACNNNNNNNNNNNANNTNNNTANNAGGNNNNNNCCNTCNNGNNNNNAATTGCNATGACC
CAANNCACGGGGGCNGTCCCCGGCGCGGCGACCTTGNNNACGTCCGGGGACAAGTATGGCGNNCNCCC
CGGGAAGGANNCNGCCGTANGTGCNNNNNANNNNNTCNTGNGANGTCGCCGNNATTCNNGTGGTCNNG
CNAGAATGCNCNNACCANNCGNANNNTGGTNATGNNAGTNNNNNNNNNNNNNNNNNNNNNNNNNNNNN
NNNNNNNNNNNNNNNNNNNNNNNNNNNNNNNNGNNNNGNNNNNNCNANNNAAGGANNTANNNCTNNTN
NTNNNNNNGGACNNNACNNNNCNNNNNNNACNCNNNCNCNNNNNNANNNGNTTTTTNNNCNNTATTNT
CCAGNACGCNGNGCNNNCATTCANGNGAACNNNGNNACGCGNNAATCGTANNATCTACCTGCCGCNGG
TNNNNNTACNGNNNNNNCGGANNNTNNCTCGTANNNNNNTCATGCGTNNTNTNNTCNCNAGNCNNGTT
CGNNNANANANTAGNANGTNTNNNNNNNNNTGTNNCGTTCCGCNTGANNCNNCANNNNGNCGNNNTTN
NCANCGGCNCTANCTGCNGANNTAAGNCATANNNTNNNTCNNTANCTNNNNNGGNGGGNACTGTAAA
NGGNTAATTNGNNNNTNANTNCCNNCATNTNNCTNNGNNTNCTNNNTNCCTGNNTNCNNAATTGGNNC

CANTGNNTGNCNATCTCTGGNTTNGNNNNNNTNNNNNNNTNGCAANNNGGNNNGTNANANNNCNATNTAA
NNTATANNNNCNNNNNNANNNCACNNNNNNNNNNNAGNNNCNANNNGTANANNNGTAGNNNGATAAGTCT
CCGANNTNNNNNNNATAAGCGNNANACNNNNNNNNNNNNNNANGANNACGNNTTTGNNNTNNNNNNNCNA
GNNAGACNNTNGTGGNTNNNNNNTCAANNNNTTCGTAGTGGTATAATNGNNNNNNNTTTCNCCAGNNTN
NNNGNNNNNNNNCNATANNANNNNNNNNGNGANNNNNNNNNNNANNNNCTCNNNAGGGANCANCTACTTNA
GCANTGGNGCGCAGNNNGAGANNNNGACNNGCCTTGGGTGGCAANCACNGCAATAAGTNNNGTTNANC
CTNNNAGTNNACACNNNNNCANNGNNNCNNNNNNANCNGANNGNNNNNNNNNNNNNNNNNNNNNNNN
NNNNNNNNNNNNNNNNNNNNNNNNNNNNNAGNNNANGGNNNGTNTNTGACNCATGTGTTTTTANNNGNN
NNNNNACGNNNNANCCCCGGTCGNNNNCNNATTGAANNCCTGNNGCATNNNNNTNCNAGAATGANNNC
NNNNNGNNTNGNCTNNCNNNTCAAGNAATCANNNAGGTNNNGCGNTCNNNGNNNNNNNNCNNTCCCNN
ACTGAGGGTNNNNNNNNCATCTGCTNNNNNNNNCTTCGCNGGCGTNNCNNNNNAGCNGTATNGNGTNA
NNCANNNNNNNNNNNNNNNNNCNAAACNANNNNNNNTNNNN
>Dao634
GTNNNNNNNAGCCCNNNNNAGTCAGACANNNCNNNNNNNNNNNNNTNNNNNNNNNNNNNNNNNNNNNNNN
NNNNNNNNNNNNNNNNNNNNNNNNNNNNNNNNNNNNNNNNNNNNNNNNNNNNNNNNNNNNNNNNNNNNNN
NNNNNNNNNNNNNNNNNNNNNNNNNNNNNNNNNNNNNNNNNNNNNNNNNNNNNNNNNNNNNNNNNNNNNN
NNNNNNNNNNNNNNNNNNNNNNNNNNNNNNNNNNNNNNNNNNNNGANNNNNNNNNNNNNNNNNNNNNNNN
NNNNNNNNNNNNNNNNNNNNNNNNNNNNNNNNNNNNNNNNNNNNNNNNNNNNNNTCTNNNAAANNNTNNN
NNNNNNNNNNNNNNNNNNNNNNNNNNCNCNNTGNNNGTAANCNANNNGNACATGCAGTCCCCTCNNNNN
NNNGGCAGAGNNNNNNNCNNNNNNNNCCGNCCCCNNCCNNNNNNNNNNNNNNNNGNNNNNNNANNNNNN
NNNNNNTGGTACNNNNCGNNNNNGGTTGTNTAAGAGGTCCACAACGNGNTCNGNNNACNNNNGNNNAN
NNNCNNNNNNCNNNNNNNNNNNNANATNNNTANNAGGNNNNNNCCNTCNNGNNNNTNATNGCNATGACC
CAANTCNCNGNGGCNGTNCCCGGCGNGGCGACCTTGNNNACGTCCGGGGNCAAGTATGGCNNNCNCCC
CGGGAAGGANNCNGCNGTANGTGCNNNNNANNNNNTCNTGNGANGTCGCCGNNATTCNCGTGGTCNNN
NNAGAATGNCCNNACCANNCGNANNNTGGTNATGNNAGTNNNNNNNNNNNNNNNNNNNNNNNNNNNNNN
NNNNNNNNNNNNNNNNNNNNNNNNNNNNNNNNNNNGNNNNGNNNNNNCNANNNAAGGNNNTANNNCTNNTN
NNNNNNNNGGACNNNACNNNNCNNNNNNNACNCNNNCNCNNNNNNANNNGNTTTTNNNNCNNTATTNT
CCAGNACGCNGNGCNNNCNTTCANGNGAACNNNGNNACGCGNNAATCGTANNATCTANCTGCCGCNNG
NNNNNNTNCNGNNNNNNNCGGANNNTNNNNCGTANNNNNNNCANGCGTNNTNTNNTCNCNAGNCNNGTT
CGNNNANANANTAGNANGTNTNNNNNNNNNNTGTNNCGTTCCGCNTGANNCNNCANNNNGNNGNNNTTN
NCANCGGCNNTANCTGCNGANNTAAGNCATANNNTNNNTCNNTANCTNNNNNGGNGGGNNACTGTAAA
NGGNNAATTNGNNNNTNATTNCNNNCATNTNNCTNNGNNNNCTNNNTNCCTGNNTNCNNAATTGGNNN
CANTGNNTGNCNATCTCTNGNTTNGNNNNNNTNNNNNTNNCANNNNGGNNNNTNANANNNCNATNTAA
NNTANANNNNCNNNNNNANNNCACNNNNNNNNNNAGNNNNNANNNGNANANNNGTAGNNNGNTAAGTCT
CCGANNTNNNNNNNATAAGCGNNANACNNNNNNNNNNNNNNANGANNNCGNNTTTGNNNTNNNNNNNNNA
GNNAGACNNTNGTGGNTNNNNNTCAANNNNTTNGTAGTGGTATAATNGNNNNNNNTTTCNCCAGNNTN
NNNGNNNNNNNNCNATANNANNNNNNNNGNGANNNNNNNNNNNANNNNCTCNNNAGGGANCANCTACTTNA
GCANTGGNGCGNAGNNNGAGANNNNGACNNNCCTTGGGTGGCAANNACNGCAATAAGTNNNGTTNNNN
CTNNNAGTNNACACNNNNNCANNGNNNCNNNNNNANCNGANNGNNNNNNNNNNNNNNNNNNNNNNNN
NNNNNNNNNNNNNNNNNNNNNNNNNNNNNNGNNNANGGNNNGTNTNTGACNCATGTNTTTTTANNNGNN
NNNNNACGNNNNANCCCCGGTCGNNTNCNNATTGAANNCCTGNNGCATNNNNNTNCNAGAATGANNNC
NNNNNGNNTNGNCTNNCNNNTCAAGNAATCANNNANGTNNNNCGNTCNNNGNNNNNNNNCNNTCCCNN
ACTGAGGGTNNNNNNNNCATCNGCTNNNNNNNNCTNCGCNGGNGTNNCNNNNNNGCNGTATNNNGTNA
NNCANNNNNNNNNNNNNNNNNCNAAACAANNNNNNNTNNNN
>Dao635
GTGNNNNNTAGCCCNNNNNAGTTAGACANNNCNNNNNNNNNNNNNTNNNNNNNNNNNNNNNNNNNNNNNN
NNNNNNNNNNNNNNNNNNNNNNNNNNNNNNNNTNNNNNNNNNNNNNNNNNNNNNNNNNNNNNNNNNNNNN
NNNNNNNNNNNNNNNNNNNNNNNNNNNNNNNNNNNNNNNNNNNNNNNNNNNNNNNNNNNNNNNNNNNNNN
NNNNNNNNNNNNNNNNNNNNNNNNNNNNNNNNNNNNNNNNNNNNNNNNNNNNNNNNNNNNNNNNNNNNNN
NNNNNNNNNNNNNNNNNNNNNNNNNNNNNNNNNNNNNNNNNNNNNNNNNNNNNNTCTNNNAAANNNTNNN
NNNNNNNNNNNNNNNNNNNNNNNNNNCNCNNTGNNNGTAANCNANNNGNACATGCAGTCCCCTCNNNNN
NNNNNCAGAGNNNNNNNCCNNNNNNNCCGGCCCCNNCCNNNNNNNNNNNNCNNNGNNNNNNNANNNNNN
NNNNNNTGGTACNNNNCGNNNNNGGTTGNNTAAGAGGTCCACCACGNGNTCNNNNNACNNNNGNNNAN
NNNCNNNNAACNNNNNNNNNNNNANNTNNNTANNAGGNCNNNNCCNTCNNGNNNNNNATTGCNATGACC

CAANNCACGGGGGNNGTNCCCGGCGNGGCGACCTTGATNACGTCCGGGGACAAGTATGGCNTNCNCCC
CGGGAAGGANNCNGCCGTANGTGCNNNNNANNNNNTCNTGNGANGTCGCCGNNATTNNCGTGGTCNNN
CNAGAATGNCCNNACCANNCGNANNNTGGTNATGNNAGTNNNNNNNNNNNNNNNNNNNNNNNNNNNNN
NNNNNNNNNNNNNNNNNNNNNNNNNNNNNNNNGNNNNGNNNNNNCNANNNAAGGANNTANNNCTNNTN
NTNNNNNNGGACNNNACNNNNCNNNNNNNACNCNNNCNCNNNNNNNNNNGNTTTTNNNNCNNTANTNT
CCAGNACGCNGNGCNNNCATTCANGNGAACNNNGNNACGCGNNAATCGTANNATCTACCTGCCGCNNG
NNNNNNTNCNGNNNNNNCGGANNNTNNNNCGTANNNNNNTTATACGTNNTNTNNTCNCNAGNCNNGTT
CGNNNANANANTAGNANGTNTNNNNNNNNNTGTNNCGTTCCGCNTGANNCNNCANNNNGNNGNNCTTN
NCANCGGCNNTANCTGCNGANNTAAGNCATANNNTNNNTCNNTANCTNNNNNGGNGGGNNACTGTAAA
NGGNNAATTNGNNNNTNATTNCNNNCACNTNNCNNNGNNNNCTNNNTNCCTGNNTNCNNAATTGGNNC
CANTGNNTGNCNATCTCTNGNTTNGNNNNNNTNNNNNTNNCANNNNGGNNNGTNNNANNNCNATNTAA
NNTANANNNNCNNNNNNANNNCACNNNNNNNGNAGNNNCNANNNTTANANNNGTAGNNNAATAAGTCT
CCGANNTNNNNNNNATAAGCGNNANACNNNNNNNNNNNNNNANGANNNCGNNTTGGNNNTNNNNNNCNA
GNNAGACNNTNGTGGNTNNNNNTCAANNNNTTNGTAGTGGTATAATNGNNNNNNNTTTCNCCAGNNTN
NNNGNNNNNNNNCNATANNANNNNNNNGNGANNNNNNNNNNNANNNNCTCNNNAGGNANCANCTACTNNA
GCANTGGNGCGNAGNNNGAGANNNNGACNNNCCTTGGGTGGCAANNACNGCAATAAGTNNNGTTNANN
CTNNNAGTNNACACNNNNNCANNGNNNCNNNNNNANCNGANNGNNNNNNNNNNNNNNNNNNNNNNNNN
NNNNNNNNNNNNNNNNNNNNNNNNNNNNANNNNANNGNNNGTNTNTGACNCATGNNTTTTTANNNGNN
NNNNNNNGNNNNANCCCCGGTCGNNNNCNNATTGAANNCCTGNNGCATNNNNNNTNCNAGAATGANNGC
NNNNNGNNTNNNCTNNCNNNTCAAGNAGTCANNNAGNNNNNNNNNNTCNNNNNNNNNNNNNCNNTCCCNN
ACTGAGGGTNNNNNNNNCATCNGCTNNNNNNNNNNTNCGCNGGCGTNNCNNNNNNGCNGTATNGNGNNN
NNCANNNNNNNNNNNNNNNNNNTNAAACNANNNNNNNTNNNN
>Dao636
GTGNNNNNTAGCCCNNNNNAGTCAGACANNNCNNNNNNNNNNNNTNNNNNNNNNNNNNNNNNNNNNNN
NNNNNNNNNNNNNNNNNNNNNNNNNNNNNNTNNNNNNNNNNNNNNNNNNNNNNNNNNNNNNNNNNNNNN
NNNNNNNNNNNNNNNNNNNNNNNNNNNNNNNNNNNNNNNNNNNNNNNNNNNNNNNNNNNNNNNNNNNN
NNNNNNNNNNNNNNNNNNNNNNNNNNNNNNNNNNNNNNNNNNNNNNNNNNNNNNNNNNNNNNNNNNNN
NNNNNNNNNNNNNNNNNNNNNNNNNNNNNNNNNNNNNNNNNNNNNNNNNNNNTCTNNNAAANNNTNNN
NNNNNNNNNNNNNNNNNNNNNNNNNCNCNNTNNNNGTAANCNANNNGNACATGCAGTCCCCTCNNNNN
NNNGGCAGAGNNNNNNNCCNNNNNNNCCGGCCCCNNCCNNNNNNNNNNNNNNNGNNNNNNNANNNNNN
NNNNNNTGGTACNNNNCGNNNNNGGTTGNNTAAGAGGTCCACAACGNGNTCNGNNCACNNNNGNNNAN
NNNCNNNAAACNNNNNNNNNNNANNTNNNTANNAGGNCNNNNCCNTCNNGNNNNNAATTGCNATGANN
CANNNNACGGGGGNNGTNCCCGGCGNGGCGACCTTGNNNACGTCCGGGGNCAAGTATNGCGNNCNCCC
CGGGAAGGANNCNGCNGTANGTGCNNNNNANNNNNTCNTGNGANGTNGCCGNNATTCNCGTGGTCNNN
CNAGAATGNCCNNACCANNCGNANNNTGGTNATGNNAGTNNNNNNNNNNNNNNNNNNNNNNNNNNNNN
NNNNNNNNNNNNNNNNNNNNNNNNNNNNNNNNNNNNNGNNNNNNCNANNNAAGGNNNTANNNCTNNTN
NTNNNNNNGGACNNNACNNNNCNNNNNNNACNCNNNCNCNNNNNNNNNNNGNTTTTNNNNCNNTANTNT
CCAGNACGCNGNGCNNNCATTTANGNGAACNNNGNNACGCGNNAATCGTANNATCTACCTGCCGCNNG
NNNNNNTNCNGNNNNNNCGGANNNTNNNNCGTANNNNNNNCANGCGTNNTNTNNTNNCNAGNCNNGTT
CGNNNANNNANTAGNANGTNTNNNNNNNNNTGTNNCGTTCCGCNTGANNCNNCANNNNGNNGNNNTTN
NCANCGGCNNTANCTGCNGANNTAAGNCATANNNTNNNTCNNTANCTNNNNNGGNGGGNNACTGTAAA
NGGNNAATTNGNNGNTNANTNCNNNCATNTNNCTNNGNNTNCTNNNTNCCTGNNTNCNNAATTGGNNN
CANTGNNTGNCNATCTCTGGNTTNGNNNNNNTNNNNNTNNCANNNNGGNNNGTNANANNNCNATNTAA
NNTANANNNNCNNNNNNANNNCACNNNNNNNGNAGNNNCNANNNGTANANNNGTAGNNNGNTNAGTCT
CCGANNTNNNNNNNATAAGCGNNANACNNNNNNNNNNNNNNANGANNNCGNNTTTGNNNTNNNNNNCNA
GNNAGACNNTNGTGGNTNNNNNTCAANNNNTTCGTAGTGGTATAATNGNNNNNNNTTTCNCCAGNNTN
NNNGNNNNNNNNCNATANNANNNNNNNGNGANNNNNNNNNNNANNNNCTCNNNAGGNANCANCTACTNNA
GCANNGGNNCGNAGNNNGAGANNNNGACNNGCCTTGGGTGGCAANCACNGCAATAAGTNNNGTTNANC
CTNNNAGTNNACACNNNNNCANNGNNNCNNNNNNANCNGANNGNNNNNNNNNNNNNNNNNNNNNNNNN
NNNNNNNNNNNNNNNNNNNNNNNNNNNNANNNNANGGNNNGTNTNTGACNCATGTGTTTTTANNNGNN
NNNNNNNGNNNNANCCCCGGTCGNNNNCNNATTGAANNCCTGNNGCATNNNNNNTNCNAGAATGANNNC
NNNNNGNNTNGNCTNNCNNNTNAAGNAATCAANTAGNNNNNNNNNNTCNNNNNNNNNNNNNCNNTCCCNN
ACTGAGGGNNNNNNNNNCATNNGCTNNNNNNNNNCTNCGCNGGNGTNNCNNNNNAGCNGTATNGNGTNN
NNCANNNNNNNNNNNNNNNNNNTNAAACAANNNNNNCTNNNN

>Dao637
GTGNNNNNTAGCCCNNNNNAGTTNGACANNNCNNNNNNNNNNNNTNNNNNNNNNNNNNNNNNNNNNNNNN
NNNNNNNNNNNNNNNNNNNNNNNNNNNNNNTNNNNNNNNNNNNNNNNNNNNNNNNNNNNNNNNNNNNNNN
NNNNNNNNNNNNNNNNNNNNNNNNNNNNNNNNNNNNNNNNNNNNNNNNNNNNNNNNNNNNNNNNNNNNNN
NNNNNNNNNNNNNNNNNNNNNNNNNNNNNNNNNNNNNNNNNNNNNNNNNNNNNNNNNNNNNNNNNNNNNN
NNNNNNNNNNGNNNNNNNNNNNNNNNNNNNNNNNNNNNNNNNNNNNNNNNNNNTCTNNNAAANNNTNNN
NNNNNNNNNNNNNNNNNNNNNNNNNNNCNCNNTGNNNGTAANCNANNNGNACATGCAGTCCCCTCNNNNN
NNNNNCAGAGNNNNNNNCCNNNNNNNCCGNCCCCNNCCNNNNNNNNNNNCNNNGNNNNNNNANNNNNN
NNNNNNTGGTACNNNNCGNNNNNGGTTGNNTAAGAGGTCCACAACGNGGTCNGNNNACNNNNGNNNAN
NNNCNNNNAACNNNNNNNNNNNNANNTNNNTANNAGGNCNNNNCCNTNNNNNNNNNNNATNGCNATGACC
CANNNCACGGGGGCNGTNCCCGGCGCGGCGACCTTGANNACGTCNGGGGNCAAGTATGGCGNNCNCCC
CGNGAAGGANNCNGCNGTANGTGCNNNNNANNNNNTCTTGNGANGTNGCCGNNATTCNCGTGGTCNNN
CNAGAATGNCCNNACCANNCGNANNNTGGTNATGNNAGTNNNNNNNNNNNNNNNNNNNNNNNNNNNNN
NNNNNNNNNNNNNNNNNNNNNNNNNNNNNNNNNNNNNNGNNNNNNCNANNNAAGGNNNTANNNCTNNTN
NTNNNNNNGGACNNNACNNNNCNNNNNNNACNCNNNCNCNNNNNNNNNNGGTTTTNNNNCNNTANTNT
CCAGNANGCNGNGCNNNCATTCANGNGAACNNNGNNACGCGNNAATCGTANNATCTACCTGCCGCNNG
NNNNNNTNCNGNNNNNNCGGANNNTNNNNCGTANNNNNNNCANGCGTNNTNTNNTCNCNAGNCNNGTT
CGNNNANANANTAGNANGTNTNNNNNNNNNTGTNNCGTTCCGCNTGANNCNNCANNNNGNNGNNNTTN
NCANCGGCNNTANCTGCNGANNTAAGNCATANNNTNNNTCNNTANCTNNNNNGGNGGGNNACTGTAAA
NGGNNAATTNGNNNNTNANTNCNNNNACNTNNCNNNGNNNNCTNNNTNCCTGNNTNCNNAATTGGNNC
CANTGNNTGNCNATCTCTGGNTTNGNNNNNNTNNNNNTNNCANNNNGGNNNGTNNNANNNCNATNTAA
NNTANANNNNCNNNNNNANNNCACNNNNNNNNNAGNNNNNANNNTNANANNNGTAGNNNANTAAGTCT
CCGANNTNNNNNNNATAAGCGNNANACNNNNNNNNNNNNNANGANNACGNNTTGGNNNTNNNNNNCNA
GNNAGACNNTNATGGNTNNNNNTCAANNNNTTNGTAGTGGTATAATNGNNNNNNNTTTCNCCAGNNTN
NNNGNNNNNNNCNATANNANNNNNNNGNGANNNNNNNNNNNANNNNCTCNNNAGGGANCANCTACTNNA
GCANNGGNGCGNAGNNNGAGANNNNGACNNNCCTTGGGTGGCAANNACNGCGATAAGTNNNGTTNNNN
NTNNNAGTNNACATNNNNNCANNGNNNCNNNNNNANCNGANNGNNNNNNNNNNNNNNNNNNNNNNNNN
NNNNNNNNNNNNNNNNNNNNNNNNNNNNNNANNNNANGGNNNGTNTNTGNCNCATGNNTTTNTANNNGNN
NNNNNNNGNNNNANCCCCGGTCGNNNNCNNATTGAANNCCTGNNGCATNNNNNTNCNAGAATGANNNC
NNNNNGNNTNGNCTNNNNNNNTCAAGNAATCANNNAGGNNNNNNNNTCNNNNNNNNNNNNCNNTCNCNN
ACTGAGGGTNNNNNNNNCATCNGCTNNNNNNNNCTNCGCNGGNGTNNCNNNNNAGCNGTATNGNGTNA
NNCANNNNNNNNNNNNNNNNNTNAAACNANNNNNNNTNNNN
>Dao638
GTGNNNNNNAGCCCNNNNNAGTCNGACANNNCNNNNNNNNNNNNTNNNNNNNNNNNNNNNNNNNNNNNNN
NNNNNNNNNNNNNNNNNNNNNNNNNNNNNNTNNNNNNNNNNNNNNNNNNNNNNNNNNNNNNNNNNNNNNN
NNNNNNNNNNNNNNNNNNNNNNNNNNNNNNNNNNNNNNNNNNNNNNNNNNNNNNNNNNNNNNNNNNNNNN
NNNNNNNNNNNNNNNNNNNNNNNNNNNNNNNNNNNNNNNNNNNNNNNNNNNNNNNNNNNNNNNNNNNNNN
NNNNNNNNNNNNNNNNNNNNNNNNNNNNNNNNNNNNNNNNNNNNNNNNNNNNNTCTNNNAAANNNTNNN
NNNNNNNNNNNNNNNNNNNNNNNNNNNCNCNNTNNNNGTAANCNANNNGNACATGCAGTCCCCTCNNNNN
NNNGGCAGAGNNNNNNNCCNNNNNNNCCGNCCCCNNCCNNNNNNNNNNNNNNNNGNNNNNNNANNNNNN
NNNNNNTGGTACNNNNCGNNNNNGGTTGTTTAAGAGGTCCACAACGNGNTCNNGNNACNNNNGNNNAN
NNNCNNNNAACNNNNNNNNNNNNANNTNNNTANNAGGNNNNNNCCNTNNNGNNNNNNATTGCNATGANN
CANNTNNCNGGGGNAGTCCCCGGCGNGGNGACCTTGNNNANGTCCGGGGNCAAGTATGGCNNNCNCCC
CGGGAAGGANNCNGCNGTANGTGCNNNNNANNNNNTCNTGNGANGTCGCCGNNATTCNCGTGGTCNNN
CNNGANTGNNCNNACCANNCGNANNNTGGTNATGNNAGTNNNNNNNNNNNNNNNNNNNNNNNNNNNNN
NNNNNNNNNNNNNNNNNNNNNNNNNNNNNNNNNGNNNNGNNNNNNCNANNNAAGGNNNTANNNCTNNTN
NNNNNNNNGGNCNNNACNNNNCNNNNNNNACNCNNNCNCNNNNNNNNNNGGTTTNNNNNCNNTNNTNT
CCAGNANGCNGNGCNNNCATTCANGNGAACNNNGNNACGCGNNAATCGTANNATCTANCTGCCGCNNG
NNNNNNTNCNGNNNNNNCGGANNNTNNNNNGTANNNNNNNCANGCGTNNTNTNNTNNCNAGNCNNGTT
CGNNNANANANTAGNANGTNTNNNNNNNNNTGTNNCGTTCCGCNTGANNCNNCANNNNGNNGNNNTTN
NCANCGGCNNTANCTNCNGANNTAAGNCATANNNTNNNTCNNTANCTNNNNNGGNGGGNNACTGTAAA
NGNNNAATTNGNNNNTNANTNCNNNCATNTNNCNNNGNNNNCTNNTTNCCTGNNTNCNNAATTGGNNN
CANTGNNTGNCNATCTCTNGNTTNGNNNNNNTNNNNNTNNCANNNNGGNNNNNNNNANNNCNATNTAA
NNTANANNNNCNNNNNNANNNCACNNNNNNNNNAGNNNNNANNNGNANANNNGTAGNNNGNTAAGTCT

CCGANNTNANNNNNATAAGCGNNANACNNNNNNNNNNNNNNANGANNNCGNNTTTGNNNTNNNNNNNNA
GNNAGACNNTNGTGGNTNNNNNTCAANNNNTTNGTAGTGGTATAATNGNNNNNNNTTTCNCCAGNNTN
NNNGNNNNNNNCNATANNANNNNNNNGNGANNNNNNNNNNANNNNCTCNNNAGGGANCANCTACNNNA
GCANNGGNNCGNAGNNNGAGANNNNGACNNNCCTTGGGTGGCAANNACNGCAATAAGTNNNGTTNNNN
NTNNNAGTNNACACNNNNNCANNGNNNCNNNNNNANCNGANNGNNNNNNNNNNNNNNNNNNNNNNNNN
NNNNNNNNNNNNNNNNNNNNNNNNNNNNNNNNNNNNANNGNNNGTNTNNGACNCATNNNTTTNTANNNGNN
NNNNNNNGNNNNANCCCCGGTCGNNNNCNNATTGAANNCCTGNNGCATNNNNNTNCNAGAATGANNNC
NNNNNGNNTNGNCTNNCNNNTNAAGNAATCANNNAGNNNNNNNNNTCNNNNNNNNNNNNCNNTCNCNN
ACTGAGGGTNNNNNNNNCATNNGCTNNNNNNNNNNTNCGCNNGNGTNNCNNCNNNGCNGTATNGNGNNA
NNCANNNNNNNNNNNNNNNNNATNAAACGANNNNNNTNNNN
>Dao639
GTGNNNNNNAGCCCNNNCNAGTCAGACANNNCNNNNNNNNNNNNNTNNNNNNNNNNNNNNNNNNNNNN
NNNNNNNNNNNNNNNNNNNNNNNNNNNNNNNNNNNNNNNNNNNNNNNNNNNNNNNNNNNNNNNNNNNN
NNNNNNNNNNNNNNNNNNNNNNNNNNNNNNNNNNNNNNNNNNNNNNNNNNNNNNNNNNNNNNNNNNNN
NNNNNNNNNNNNNNNNNNNNNNNNNNNNNNNNNNNNNNNNNNNNNNNNNNNNNNNNNNNNNNNNNNNN
NNNNNNNNNNNNNNNNNNNNNNNNNNNNNNNNNNNNNNNNNNNNNNNNNNNCNTCTNNNAAANNNTNNN
NNNNNNNNNNNNNNNNNNNNNNNNNCNCNTTGNNNGTAANCNANNNGNACATGCAGTCCCCTCNNNNN
NNNGGCAGAGNNNNNNCCNNNNNNNNNCCGNCCCCNNCCNNNNNNNNNNNNCNNNNNNCNNNNANNNNNN
NNNNNNTGGTACNNNNCGNNNNNGGTTGTTTAAGAGGTCCACAACGNGNTCNNGNNACNNNNGNNNAN
NNNCNNNAAACNNNNNNNNNNNNANNTNNNTANNAGGNNNNNNCCNTCNNGNNANTNATNGCNATGACC
CANNNCACNGGGGCNGTNCCCGGCGNGGNGACCTTGANNANGTCCGGGGNCAAGTATGGCGNNCNCCC
CGGGAAGGANNCNGCNGTANGTGCNNNNNANNNNNTCNTGNGANNTCGCCGNNATTCNCNTGGTCNNN
CNAGAATGNCCNNACCANNCGNANNNTGGTTATGNNAGTNNNNNNNNNNNNNNNNNNNNNNNNNNNNN
NNNNNNNNNNNNNNNNNNNNNNNNNNNNNNNNNNNNGNNNNGNNNNNNCNANNNAAGGNNNTANNNCTNNTN
NTNNNNNNNGGACNNNACNNNNCNNNNNNNNACNCNNNCNCNNNNNNNNNNNGGTTTTNNNNCNNTATTNT
CCAGNACGCNGNGCNNNCATTCANGNGAACNNNGNNACGCGNNAATCGTANNATCTACCTGCCGCNNG
NNNNNNTACNGNNNNNNCGGANNNTNNCNCGTANNNNNNNCATGCGTNNTNTNNTNNCNAGNCNNGTT
CGNNNANANANTAGNANGTNTNNNNNNNNNNTGTNNCGTTCCGCNTGANNCNNCANNNNGNNGNNCTTN
NCANCGGCNNTANCTGCNGANNTAAGNCATANNNTNNNTCNNTANCTNNNNNGGNGGGNNACTGTAAA
NGNNNAATTNGNNGNTNANTNCNNNCATNTNNCNNNGNNTNCTNNNTNCCTGNNTNCNNAATTGGNNC
CANTGNNTGNCNATCTCTNGNTTNGNNNNNNTNNNNNTNNCAANNNGGNNNGTNANANNNCNATNTAA
NNTANANNNNCNNNNNNANNNCACNNNNNNNNNAGNNNCNANNNGNANANNNGTAGNNNGNTAAGTCT
CCGANNTNNNNNNNATAAGCGNNANACNNNNNNNNNNNNNNANGANNACGNNTTTGNNNTNNNNNNNNA
GNNAGACNNTNGTGGNTNNNNNTCAANNNNTTCGTAGTGGTATAATNGNNNNNNNTTTCNCCAGNNTN
NNNGNNNNNNNCNATANNANNNNNNNGNGANNNNNNNNNNANNNNCTCNNNAGGGANCANCTACTNNA
GCANTGGNGCGNAGNNNGAGANNNNGACNNNCCTTGGGTGGCAANNACNGNAATAAGTNNNGTTNANC
CTNNNAGTNNACACNNNNNCANNGNNNCNNNNNNANCNGANNGNNNNNNNNNNNNNNNNNNNNNNNNN
NNNNNNNNNNNNNNNNNNNNNNNNNNNNNAGNNNANGGNNNGTNTNTGACNCATNNNTTTTTANNNGNN
NNNNNNNGNNNNANCCCCGGTCGNNNNCNNATTGAANNCCTGNNGCATNNNNNTNCNAGAATGANNNC
NNNNNGNNTNGNCTNNCNNTTCAAGNAATCANNNAGGTNNNNNNNTCNNNNNNNNNNNNCNNTCNCNN
ACTGAGGGTNNNNNNNNCATCTGCTNNNNNNNNNNTNCGCNGGCGTNNCNNCNNNGCNGTATNGNGTNN
NNCANNNNNNNNNNNNNNNNNNTNAAACGNNNNNNNTNNNN
>Dao640
GTGNNNNNTAGCCCNNNNNAGTCAGACANNNCNNNNNNNNNNNNNNNNNNNNNNNNNNNNNNNNNNNN
NNNNNNNNNNNNNNNNNNNNNNNNNNNNNNNNNNNNNNNNNNNNNNNNNNNNNNNNNNNNNNNNNNNN
NNNNNNNNNNNNNNNNNNNNNNNNNNNNNNNNNNNNNNNNNNNNNNNNNNNNNNNNNNNNNNNNNNNN
NNNNNNNNNNNNNNNNNNNNNNNNNNNNNNNNNNNNNNNNNNNNNNNNNNNNNNNNNNNNNNNNNNNN
NNNNNNNNNNNNNNNNNNNNNNNNNNNNNNNNNNNNNNNNNNNNNNNNNNNNNTCTNNNAAANNNTNNN
NNNNNNNNNNNNNNNNNNNNNNNNNCNCNNTGNNNGTAANCNANNNGNACATGCAGTCCCCTCNNNNN
NNNNNCAGAGNNNNNNNNNCNNNNNNNNCCGNCCCCNNCCNNNNNNNNNNNNNNNNNGNNNNNNNANNNNNN
NNNNNNTGGTACNNNNCGNNNNNGGTTGNTTAAGAGGTCCACAACGNGGTCNNNNNACNNNNGNNNAN
NNNCNNNNANCNNNNNNNNNNNNNNNNTNNNTANNAGGNNNNNNCCNTCNNGNNNNNAATTGCNATGACC
CANNNNNCGGNGGNNGTNCCCGGCGNGGNGACCTTGNTNACGTCCGGGGNCAAGTATGGCNNNCNCCC
CGGGAAGGANNCNGCNGTANGTGCNNNNNANNNNNTCNTGNGANGTNGCCGNNANNNNCNTGGTCNNN

CNNGAATGNCCNNACCANNCGNANNNNGGTNATGNNAGTNNNNNNNNNNNNNNNNNNNNNNNNNNNNN
NNNNNNNNNNNNNNNNNNNNNNNNNNNNNNNNNNNNNNGNNNNNNCANNNAAGGNNNTANNNCTNNTN
NNNNNNNNGGACNNNACNNNNCNNNNNNNACNCNNNCNCNNNNNNNNNNGNTTTTTNNNCNNTANTNT
CCAGNANGCNGNGCNNNCNTTCANGNGAACNNNGNNACGCGNNAATCGTANNATCTACCTGCCGCNNG
TNNNNNTNCNGNNNNNNCGGANNNTNNNNNGTANNNNNNNCANGCGTNNNNTNNTCNCNAGNCNNGTT
CGNNNANANANTAGNANGTNTNNNNNNNNNTGTNNCGTTCCGCNTGANNCNNCANNNNGNNGNNCTTN
NCANCGGCNNTANCTNCNGANNTAAGNCATANNNTNNNTCNNTANCTNNNNNGGNGGGNNACTGTAAA
NGGNNAATTNGNNNNTNANTNCNNNCATNTNNCNNNGNNTNCTNNNTNCCTGNNNNCNNAATTGGNNN
CANTGNNTGNCNATCTCTNGNTTNGNNNNNNTNNNNNTNNCANNNNGGNNNGNNANANNNCNATNTAA
NNTANANNNNCNNNNNNANNNCACNNNNNNNNNAGNNNCNANNNGNANANNNGTAGNNNGNTAAGTCT
CCGANNTNNNNNNNATAAGCGNNANACNNNNNNNNNNNNNANGANNACGNNTTTGNNNTNNNNNNNNA
GNNAGACNNTNGTGGNTNNNNNTCAANNNNTTNGTAGTGGTATAATNGNNNNNNNTTTCNCCAGNNTN
NNNGNNNNNNNCNATANNANNNNNNNGNGANNNNNNNNNNNANNNNCTCNNNAGGGANCANCTACTNNA
GCANTGGNGCGNAGNNNGAGANNNNGACNNNCCTTGGGTGGCANNNACNGCAATAAGTNNNGTTNNNN
CTNNNAGTNNACACNNNNNCANNGNNNCNNNNNNANCNGANNGNNCNNNNNNNNNNNNNNNNNNNNNN
NNNNNNNNNNNNNNNNNNNNNNNNNNNNNNNNNANGGNNNGTNTNTGACNCANGTGTTTNTANNNGNN
NNNNNNNGNNNNANCCCCGGTCGNNNNCNNATTGAANNCCTGNNGCATNNNNNTNCNAGAATGANNNC
NNNNNGNNTNGNCTNNCNNNTCAAGNAATCANNNAGNTNNNNNNNTCNNNGNNNNNNNNCNNTCNCNN
ACTGAGGGTNNNNNNNNCATCNGCTNNNNNNNNNNTNCNCNGGNNTNNCNNNNNGCNGTATNGNGTNN
NNCANNNNNNNNNNNNNNNNTNAAANNANNNNNNTNNNN
>Dao641
GTGNNNNNNAGCCCNNNNNAGTCAGACANNNCNNNNNNNNNNNNNTNNNNNNNNNNNNNNNNNNNNNN
NNNNNNNNNNNNNNNNNNNNNNNNNNNNNNNNNNNNNNNNNNNNNNNNNNNNNNNNNNNNNNNNNNNN
NNNNNNNNNNNNNNNNNNNNNNNNNNNNNNNNNNNNNNNNNNNNNNNNNNNNNNNNNNNNNNNNNNNN
NNNNNNNNNNNNNNNNNNNNNNNNNNNNNNNNNNNNNNNNNNNNNNNNNNNNNNNNNNNNNNNNNNNN
NNNNNNNNNNNNNNNNNNNNNNNNNNNNNNNNNNNNNNNNNNNNNNNNNNCNTCTNNNAAANNNTNNN
NNNNNNNNNNNNNNNNNNNNNNNNNCNCNTTGNNNGTAANCNANNNGNACATGCAGTCCCCTCNNNNN
NNNGGCAGAGNNNNNNCCCNNNNNNNCCGGCCCCNNCCNNNNNNNNNNNNNNNGNNNNNNNANNNNNN
NNNNNNTGGTACNNNNCGNNNNNGGTTGTTTAAGAGGTCCACAACGNGGTCNNGNNACNNNNGNNNAN
NNNCNNNNNACNNNNNNNNNNNANNTNNNTANNAGGNCNNNNCCNTCNNGNNNNNNATTGCNATGACC
CANNNNACGGGGGCAGTCCCCGGCGCGGCGACCTTGANNACGTCCGGGGACAAGTATGGCNNNCNCCC
CGGGAAGGANNCNGCCGTANGTGCNNNNNANNNNNTCNTGNGANGTCGCCGNNATTCNCNTGGTCNNN
CNAGANTGNCCNNACCANNCGNANNNTGGTNATGNNAGTNNNNNNNNNNNNNNNNNNNNNNNNNNNNN
NNNNNNNNNNNNNNNNNNNNNNNNNNNNNNNNNGNNNNGNNNNNNCANNNAAGGNNNTANNNCTNNTN
NTNNNNNNGGACNNNACNNNNCNNNNNNNACNCNNNCNCNNNNNNNNNNGGTTTTTNNNCNNTANTNT
CCAGNANGCNGNGCNNNCATTCANGNGAACNNNGNNACGCGNNAATCGTANNATCTACCTGCCGCNNG
NNNNNNTACNGNNNNNNCGGANNNTNNNNCGTANNNNNNTCANGCGTNNTNTNNTCNCNAGNCANGTT
CGNNNANANANTAGNANGTNTNNNNNNNNNTGTNNCGTTCCGCNTGANNCNNCANNNNGNNGNNCTTN
NCANCGGCNNTANCTGCNGANNTAAGNCATANNNTNNNTCNNTANCTNNNNNGGNGGGNNACTGTAAA
NGGNNAATTNGNNGNTNANTNCNNNCATNTNNCTNNGNNNNCTNNNTNCCTGNNTNCNNAATTGGNNC
CANTGNNTGNCNATCTCTGGNTTNGNNNNNNTNNNNNTNNCAANNNGGNNNGTNANANNNCNATNTAA
NNTANANNNNCNNNNNNANNNCACNANNNNNGNAGNNACNANNNGNANANNNGTAGNNNGATAAGTCT
CCGANNTNNNNNNNATAAGCGNNANACNNNNNNNNNNNNNANGANNACGNNTTTGNNNTNNNNNNCNA
GNNAGACNNTNGTGGNTNNNNNTCAANNNNTTCGTAGTGGTATAATNGNNNNNNNTTTCNCCAGNNTN
NNNGNNNNNNNCNATANNANNNNNNNGNGANNNNNNNNNNNANNNNCTCNNNAGGNANCANCTACTNNA
GCANNGGNGCGCAGNNNGAGANNNNGACNNNCCTTGGGTGGCAANNACNGCAATAAGTNNNGTTNNNC
CTNNNAGTNNACACNNNNNCANNGNNNCNNNNNNANCNGANNGNNNNNNNNNNNNNNNNNNNNNNNNN
NNNNNNNNNNNNNNNNNNNNNNNNNNNNANNNNANGGNNNGTNTNTGACNCATGNNTTTTTANNNGNN
NNNNNACGNNNNANCCCCGGTCGNNNNCNNATTGAANNCCTGNNGCATNNNNNTNCNAGAATGANNGC
NNNNNGNNTNGNCTNNCNTTTCAAGNAATCANNTAGNNATNGCGNTCNNNNNNNNNNNNCNNTCNCNN
ACTGAGGGTNNNNNNNNCATCTGCTNNNNNNNNNNTNCGCNGGCGTNNCNNNNNGCNGTATNGNGTNA
NNCANNNNNNNNNNNNNNNNTNAAACNANNNNNNTNNNN
>Dao642
GTGNNNNNTAGCCCNNNNNAGTTANACANNNCNNNNNNNNNNNNNNNNNNNNNNNNNNNNNNNNNNNN

NNNNNNNNNNNNNNNNNNNNNNNNNNNNNNNNNNNNNNNNNNNNNNNNNNNNNNNNNNNNNNNNNNNNNN
NNNNNNNNNNNNNNNNNNNNNNNNNNNNNNNNNNNNNNNNNNNNNNNNNNNNNNNNNNNNNNNNNNNNNN
NNNNNNNNNNNNNNNNNNNNNNNNNNNNNNNNNNNNNNNNNNNNNNNNNNNNNNNNNNNNNNNNNNNNNN
NNNNNNNNNNNNNNNNNNNNNNNNNNNNNNNNNNNNNNNNNNNNNNNNNNNNNNTCTNNNAAANNNTNNN
NNNNNNNNNNNNNNNNNNNNNNNNNNCNCNNTNNNNGTAANCNANNNGNACATGCAGTCCCCTCNNNNN
NNNNNCAGAGNNNNNNNCCNNNNNNNCCGGCCCCNNCCNNNNNNNNNNNNNNNNGNNNNNNNANNNNNN
NNNNNNTGGTACNNNNCGNNNNNGGTTGTTTNAGAGGTCCACCACGNGGTCNGGNNACNNNNGNNNAN
NNNCNNNNAACNNNNNNNNNNNNANNTNNNTANNAGGNCNNNGCCNTCNNNNNNNNNNATTGCNATGACC
CAANNCACNGGGGCAGTNCCCGGCGCGGCGACCTTGANNANNTCCGGGGNCAAGTATGGCGNNCNCCC
CGGGAAGGANNCNGCCGTANGTGCNNNNNANNNNNTCNTGNGANGTNGCCGNNATTNNCGTGGNCNNN
NNAGAATGNCCGNACCANNCGNANNNTGGTNATGTNAGTANNNNNNNNNNNNNNNNNNNNNNNNNNNNN
NNNNNNNNNNNNNNNNNNNNNNNNNNNNNNNNNNNNNNNNNGNNNNNNCNANNNAAGGNNNTANNNCTNNTN
NNNNNNNNGGACNNNACNNNNCNNNNNNNACNCNNNCNCNNNNNNNNNNNGNTTTTNNNNCNNTANTNT
CCAGNANGCNGNGCNNNCATTCANGNGAACNNNGNNACGCGNNAATCGTANNATCTACCTGCCGCNNG
NNNNNNTACNGNNNNNNCGGANNNTNNCTCGTANNNNNNNTATACGTNNNNTNNTCNCNAGNCNNGTT
CGNNNANANANTAGNANGTNTNNNNNNNNNNTGTNNCGTTCCGCNTGANNCNNCNNNNNGNNGNNNTTN
NCANCGGCNNTANCTGCNGANNTAAGNCATANNNTNNNTCNNTANCTNNNNNGGNGGGNNACTGTAAA
NGGNNAATTNGNNNNTNANTNCCNNNACNTNNCTNNGNNNNCTNNNTNCCTGNNTNCNNAATTGGNNC
CANTGNNTGNCNATCTCTGGNTTNGNNNNNNTNNNNNTNNCANNNNGGNNNNNNANANNNCNATNTAA
NNTANANNNNCNNNNNNANNNCACNNNNNNNGNAGNNNCNANNNTTANANNNGTAGNNNANTNAGTCT
CCGNNNTNNNNNNNATAAGCGNNANACNNNNNNNNNNNNNNANGANNACGNNTTGGNNNTNNNNNNCNA
GNNAGANNNTNGTGGNTNNNNNTCAANNNNNTTNGTAGTGGTATAATNGNNNNNNNTTTCNCCAGNNTN
NNNGNNNNNNNNCNATANNANNNNNNNGNGANNNNNNNNNNNANNNNCTCNNNAGGGANCANCTACTNNA
GCANNGGNGCGNAGNNNGAGANNNNGACNNNCCTTGGGTGGCAANNACNGNAATAAGTNNNGTTNANC
CTNNNAGTNNACACNNNNNCANNGNNNCNNNNNNANCNGANNGNNNNNNNNNNNNNNNNNNNNNNNN
NNNNNNNNNNNNNNNNNNNNNNNNNNNNNNNNNNNNNNNANGGNNNGTNTNTGACNCNTGTGTTTTTANNNGNN
NNNNNNNGNNNGANCCCCGGTCGNNNNCNNATTGAANNCCTGNNGCATNNNNNTNCNAGAATGANNNC
NNNNNGNNTNGNCTNNNNNNNTCAAGNAGTCNNNNAGNNNNNNNNNNTCNNNGNNNNNNNNCNNNNNCNN
ACTGAGGGTNNNNNNNNCATCNNCTNNNNNNNNNCTNCGCNGGNNTNNCNNCNNNGCNGTATNNNGNNN
NNCANNNNNNNNNNNNNNNNNNCNAAACNNNNNNNNNTNNNN
>Dao643
GTGNNNNNTAGCCCNNNNNAGTTAGACANNNCNNNNNNNNNNNNNTNNNNNNNNNNNNNNNNNNNNNNNN
NNNNNNNNNNNNNNNNNNNNNNNNNNNNNNNTNNNNNNNNNNNNNNNNNNNNNNNNNNNNNNNNNNNNNN
NNNNNNNNNNNNNNNNNNNNNNNNNNNNNNNNNNNNNNNNNNNNNNNNNNNNNNNNNNNNNNNNNNNNNN
NNNNNNNNNNNNNNNNNNNNNNNNNNNNNNNNNNNNNNNNNNNNNNNNNNNNNNNNNNNNNNNNNNNNNN
NNNNNNNNNNNNNNNNNNNNNNNNNNNNNNNNNNNNNNNNNNNNNNNNNNNNNTCTNNNAAANNNTNNN
NNNNNNNNNNNNNNNNNNNNNNNNNNCNCNTTNNNNGTAANCNANNNGNACATGCAGTCCCCTCNNNNN
NNNGGCAGAGNNNNNNNCCNNNNNNNCCGNCCCCNNCCNNNNNNNNNNNNNNNNNNNNNNNNNNANNNNNN
NNNNNNTGGTACNNNNCGNNNNNGGTTGTNTAAGAGGTCCACAACANGGTCNGGNCACNNNNGNNNAN
NNNCNNNAAACNNNNNNNNNNNNNNNNTNNNTANNAGGNCNNNNCCNTCNNNNNNNTNATTGCNATGACC
CANNNCNCGGGGGNNGTCCCCGGCGCGGCGACCTTGANNACGTCCGGGGNCAAGTATGGCGNNCNCCC
CGGGAAGGANNCNGCNGTANGTGCNNNNNANNNNNTCNTGNGANGTCGCCGNNATTCNCNTGGTCNNN
CNAGAATGNCCNNACCANNCGNANNNTGGTNATGNNAGTNNNNNNNNNNNNNNNNNNNNNNNNNNNNNN
NNNNNNNNNNNNNNNNNNNNNNNNNNNNNNNNNNNNNGNNNNGNNNNNNCNANNNAAGGNNNTANNNCTNNTN
NTNNNNNNGGACNNNACNNNNCNNNNNNNACNCNNNCNCNNNNNNANNNGNTTTTTNNNCNNTATTNT
CCAGNACGCNGNGCNNNCNTTCANGNGAACNNNGNNACGCGNNAATCGTANNATCTACCTGCCGCNNG
NNNNNNTACNGNNNNNNCGGANNNTNNNNCGTANNNNNNNCATGCGTNNTNTNNTCNCNAGNCANGTT
CGNNNANANANTAGNANGTNTNNNNNNNNNNTGTNNCGTTCCGCNCGANNCNNCANNNNGNNGNNCTTN
NCANCGGCNNTANCTGCNGANNTAAGNCATANNNTNNNTCNNTANCTNNNNNGGNGGGNNACTGTAAA
NGGNNAATTNGNNNNTNATTNCCNNCACNTNNCTNNANNTNCTNNNTNCCTGNNTNCNNAATTGGNNC
CANTGNNTGNCNATCTCTGGNTTNGNNNNNNTNNNNNTNNCANNNNGGNNNGTNANANNNCNATNTAA
NNTANANNNNCNNNNNNANNNCACNNNNNNNNNAGNNNCNANNNTTANANNNGTAGNNNANTAAGTCT
CCGANNTNNNNNNNATAAGCGNNANACNNNNNNNNNNNNNNANGANNACGNNTTGGNNNTNNNNNNNNA
GNNAGACNNTNGTGGNTNNNNNTCAANNNNNTTCGTAGTGGTATAATNGNNNNNNNTTTCNCCAGNNTN

NNNGNNNNNNNNCNATANNANNNNNNNGNGANNNNNNNNNNNANNNNCTTNNNAGGNANCANTTACTTNA
GCANTGGNGCGNAGNNNGAGANNNNGACNNNCCTTGGGTGGCAANCACNGCAATAAGTNNNGTTNANN
NTNNNAGTNNACACNNNNNCANNGNNNCNNNNNNANCNGANNGNNNNNNNNNNNNNNNNNNNNNNNNN
NNNNNNNNNNNNNNNNNNNNNNNNNNNNNAGNNNANNGNNNGTNTNTGACNCATGTGTTTTTANNNGNN
NNNNNNNGNNNNANCCCCGGTCGNNNNCNNATTGAANNCCTGNNGCATNNNNNTNCNAGAATGANNGC
NNNNNGNNTNGNCTNNCNNNTCAAGNAATCANNNAGGNNNNNNNNTCNNNGNNNNNNNNCNNTCCCNN
ACTGAGGGTNNNNNNNNCATCTGCTNNNNNNNNNTNCGCNGGNGTNNCNNNNNNGCNGTATNGNGNNN
NNCANNNNNNNNNNNNNNNNNTNAAAANANNNNNNTNNNN
>Dao645
GTGNNNNNTAGCCCNNNNNAGTCAGACANNNCNNNNNNNNNNNNNTNNNNNNNNNNNNNNNNNNNNNNN
NNNNNNNNNNNNNNNNNNNNNNNNNNNNNNNNNNNNNNNNNNNNNNNNNNNNNNNNNNNNNNNNNNNNN
NNNNNNNNNNNNNNNNNNNNNNNNNNNNNNNNNNNNNNNNNNNNNNNNNNNNNNNNNNNNNNNCNNNNNN
NNNNNNNNNNNNNNNNNNNNNNNNNNNNNNNNNNNNNNNNNNNNNNNNNNNNNNNNNNNNNNNNNNNNN
NNNNNNNNNNNNNNNNNNNNNNNNNNNNNNNNNNNNNNNNNNNNNNNNNNNCNTCTNNNAAANNNTNNN
NNNNNNNNNNNNNNNNNNNNNNNNNNCNCNNTGNNNGTAANCNANNNGNACATGCAGTCCCCTCNNNNN
NNNGGCAGAGNNNNNNNCCNNNNNNNCCGGCCCCGNCCNNNNNNNTNNNCNNNGNNNNNNNANNNNNN
NNNNNNTGGTACNNNNCGNNNNNGGTTGTNTAAGAGGTCCACAACGNGGTCNGNNNACNNNNGNNNAN
NNNCNNNAAANNNNNNNNNNNNNNANNTNNNTANNAGGNCNNNNCCNTCNNNNNNNNNNATTGCNATGACC
CANNNNACGGGGGCNGTNCCCGGNGCGGCGACCTTGNNNANGTCCGGGGNCAAGTATGGCNNNCNCCC
CGNGAAGGANNCNGCNGTANGTGCNNNNNANNNNNTCNTGNGANGTNGCCGNNATTNNCGTGGTCNNN
CNAGAATGNCCNNACCANNCGNANNNTGGTNATGTNAGTNNNNNNNNNNNNNNNNNNNNNNNNNNNNN
NNNNNNNNNNNNNNNNNNNNNNNNNNNNNNNNNNNGNNNNGNNNNNNCNANNNAAGGNNNTANNNCTNNTN
NTNNNNNNNGGACNNNACNNNNCNNNNNNNACNCNNNCNCNNNNNNNNNNNGGTTTTTNNCCNNTATTNT
CCAGNACGCNGNGCNNNCNTTCANGNGAACNNNGNNACGCGNNAATCGTANNATCTACCTGCCGCNNG
NNNNNNTACNGNNNNNNCGGANNNTNNNNCGTANNNNNNNCANGCGTNNTNTNNTCNCNAGNCNNGTT
CGNNNANANANTAGNANGTNTNNNNNNNNNNTGTNNCGTTCCGCNTGANNCNNCANNNNGNNGNNCTTN
NCANCGGCNNTANCTGCNGANNTAAGNCATANNNTNNNTCNNTANCTNNNNNGGNGGGNNACTGTAAA
NGGNNAATTNGNNNNTNANTNCNNNCATNTNNCNNNGNNTNCTNNNTNCCTGNNTNCNNAATTGGNNC
CANTGNNTGNCNATCTCTGGNTTNGNNNNNNTNNNNNTNNCANNNNGGNNNGNNNNANNNCNATNTAA
NNTANANNNNCNNNNNNANNNCACNNNNNNNGNAGNNNCNANNNGNANANNNGTAGNNNGNTAAGTCT
CCGANNTNNNNNNNATAAGCGNNANACNNNNNNNNNNNNNNANGANNNCGNNTTTGNNNTNNNNNNCNA
GNNAGACNNTNGTGGNTNNNNNTNNANNNNTTNGTAGTGGTATAATNGNNNNNNNTTTCNCCAGNNNN
NNNGNNNNNNNNCNATANNANNNNNNNGNGANNNNNNNNNNNANNNNCTCNNNAGGGANCANCTACTNNA
GCANTGGNGCGCAGNNNGAGANNNNGACNNNCCTTGGGTGGCAANNACNGCAATAAGTNNNGTTNANC
CTNNNAGTNNACACNNNNNCANNGNNNCNNNNNNANCNGANNGNNNNNNNNNNNATNNNNNNNNNNNNN
NANNNNNNNNNNNNNNNNNNNNNNNNNNNNNNNNNNANGGNNNGTNTNTGACNCATGTGTTTTTANNNGNN
NNNNNACGNNNNANCCCCGGTCGNNNNCNNATTGAANNCCTGNNGCATNNNNNTNCNAGAATGANNGC
NNNNNGNNTNGNCTNNCNTTTCAAGNAATCNNNNAGGNNNNNNNNTCNNNGNNNNNNNNCNNTCCCNN
ACTGAGGGTNNNNNNNNCATCTGCTNNNNNNNNNTNCGCNGGCGTNNCNNNNNNGCNGTATNNNGNNN
NNCANNNNNNNNNNNNNNNNNTNAAACNANNNNNNNTNNNN
>Dao646
GTGNNNNNTAGCCCNNNNNAGTTAGACANNNCNNNNNNNNNNNNNTNNNNNNNNNNNNNNNNNNNNNNN
NNNNNNNNNNNNNNNNNNNNNNNNNNNNNNNTNNNNNNNNNNNNNNNNNNNNNNNNNNNNNNNNNNNNN
NNNNNNNNNNNNNNNNNNNNNNNNNNNNNNNNNNNNNNNNNNNNNNNNNNNNNNNNNNNNNNNNNNNNN
NNNNNNNNNNNNNNNNNNNNNNNNNNNNNNNNNNNNNNNNNNNNNNNNNNNNNNNNNNNNNNNNNNNNN
NNNNNNNNNNNNNNNNNNNNNNNNNNNNNNNNNNNNNNNNNNNNNNNNNNNCNTCTNNNAAANNNTNNN
NNNNNNNNNNNNNNNNNNNNNNNNNNCNCNNTGNNNGTAANCNANNNGNACATGCAGTCCCCTCNNNNN
NNNNNCAGAGNNNNNNCCCNNNNNCNCCGGCCCCNNCCNNNNNNNTNNNCNNNGNNNNNNNANNNNNN
NNNNNNTGGTACNNNTCGNNNNNGGTTGTNTAAGAGGTCCACAACGNGGTCNGGNNACNNNNGNNNAN
NNNCNNNNAACNNNNNNNNNNNNANATNNNTANNAGGNCNNNNCCNTNNNGNNNNTNATNGCNATGACC
CAANNCNCGGGGGCNGTCCCCGGCGCGGCGACCTTGANNACGTCCGGGGNCAAGTATGGCNNNCNCCC
CGGGAAGGANNCNGCNGTANGTGCNNNNNANNNNNTCNTGNGANGTCGCCGNNATTCNCGTGGTCNNN
CNAGAATGNCCNNACCANNCGNANNNTGGTNATGNNAGTANNNNNNNNNNNNNNNNNNNNNNNNNNNN
NNNNNNNNNNNNNNNNNNNNNNNNNNNNNNNNNNNNNGNNNNNNCNANNNAAGGNNNTANNNCTNNTN

NNNNNNNNGGACNNNACNNNNCNNNNNNNACNCNNNCNCNNNNNNANNNGGTTTTNNNCNNTATTNT
CCAGNACGCNGNGCNNNCNTTCANGNGAACNNNGNNACGCGNNAATCGTANNATCTACCTGCCGCNNG
NNNNNNTACNGNNNNNNCGGANNNTNNCNCGTANNNNNNNCANGCGTNNTNTNNTCNCNAGNCNNGTT
CGNNNANANANTAGNANGTNTNNNNNNNNNNTGTNNCGTTCCGCNTGANNCNNCNNNNNGNNGNNNTTN
NCANCGGCNNTANCTGCNGANNTAAGNCATANNNTNNNTCNNTANCTNNNNNGGNGGGNNACTGTAAA
NGNNNAATTNGNNNNTNATTNCNNNCACNTNNCTNNGNNTNCTNNNTNCCTGNNTNCNNAATTGGNNC
CANTGNNTGNCNATCTCTGGNTTNGNNNNNNTNNNNNTNNCANNNNGGNNNGTNNNANNNCNATNTAA
NNTANANNNNCNNNNNNANNNCACNNNNNNNNNAGNNNCNANNNTTANANNNGTAGNNNANTNAGTCT
CCGANNTNNNNNNNNTAAGCGNNANACNNNNNNNNNNNNNNANGANNACGNNTTGGNNNTNNNNNNCNA
GNNAGACNNTNATGGNTNNNNNTCAANNNNTTNGTAGTGGTATAATNGNNNNNNNTTTCNCCAGNNTN
NNNGNNNNNNNNCNATACNANNNNNNNGNGANNNNNNNNNNNANNNNCTCNNNAGGGANCANCTACTTNA
GCANTGGNGCGCAGNNNGAGANNNNGACNNGCCTTGGGTGGCAANCACNGCAATAAGTNNNGTTNNNC
CTNNNAGTNNACATNNNNNCANNGNNNCNNNNNNANCNGANNGNNNNNNNNNNNNNNNNNNNNNNNNNN
NNNNNNNNNNNNNNNNNNNNNNNNNNNNNNNNNNNNANGGNNNGTNTNTGACNCATGNNTTTTTANNNGNN
NNNNNACGNNNNANCCCCGGTCGNNTNCNNATTGAANNCCTGNNGCATNNNNNTNCNAGAATGANNNC
NNNNNGNNTNGNCTNNCNNNTCAAGNAATCNANNAGGTNTNNNNNTCNNNGNNNNNNNNCNNTCNCNN
ACTGAGGGTNNNNNNNNCATCTGCTNNNNNNNNNCTNCGCNGGNGTNNCNNCNNNGCNGTATNGNGTNA
NNCANNNNNNNNNNNNNNNNNNCNAAACNANNNNNNNTNNNN
>Dao647
GTGNNNNNNAGCCCNNNNNAGTCANACANNNCNNNNNNNNNNNNNNNNNNNNNNNNNNNNNNNNNNNNNN
NNNNNNNNNNNNNNNNNNNNNNNNNNNNNNNTNNNNNNNNNNNNNNNNNNNNNNNNNNNNNNNNNNNNNN
NNNNNNNNNNNNNNNNNNNNNNNNNNNNNNNNNNNNNNNNNNNNNNNNNNNNNNNNNNNNNNNNNNNNNN
NNNNNNNNNNNNNNNNNNNNNNNNNNNNNNNNNNNNNNNNNNNNNNNNNNNNNNNNNNNNNNNNNNNNNN
NNNNNNNNNNNNNNNNNNNNNNNNNNNNNNNNNNNNNNNNNNNNNNNNNNCNTNNNNNAAANNNTNNN
NNNNNNNNNNNNNNNNNNNNNNNNNNCNCNTTNNNNGTAANCNANNNGNACATGCAGTCCCCTCNNNNN
NNNNNCAGAGNNNNNNNCCNNNNNCNCCGNCCCCGNCCNNNNNNNNNNNNNNNNNGNNNNNNNANNNNNN
NNNNNNTGGTACNNNNCGNNNNNGGTTGTNTAAGAGGTCCACAACGNGGTCNGGNNACNNNNGNNNAN
NNNCNNNNNNNNNNNNNNNNNNNANNTNNNTANNAGGNNNNNNCCNTNNNNNNNNNNNANNGCNATGACC
CAANNCACNGGGGNNGTNCCCGGCGNGGCGACCTTGNNNANGTCNGGGGNCAAGTATGGCNNNCNCCC
CGGGAAGGANNCNGCCGTANGTGCNNNNNANNNNNTCNTGNGANGTNGCCGNNATTCNCGTGGTCNNN
NNNGAATGNCCNNACCANNCGNANNNTGGTNATGNNAGTNNNNNNNNNNNNNNNNNNNNNNNNNNNNN
NNNNNNNNNNNNNNNNNNNNNNNNNNNNNNNNNNTNNNNGNNNNNNCNANNNANGGNNNTANNNCTNNTN
NTNNNNNNNGGNCNNNACNNNNCNNNNNNNACNCNNNCNCNNNNNNNNNNNGNTTNNNNNNNCNNTNTTNN
CCAGNANGCNGNGCNNNCNTTCANGNGAACNNNGNNACGCGNNAATCGTANNATCTACCTGCCGCNNG
NNNNNNTNCNGNNNNNNCGGANNNTNNNNCGTANNNNNNNTCANGCGTNNTNTNNTCNCNAGNCNNGTT
CGNNNANANANTAGNANGTNTNNNNNNNNNNTGTNNCGTTCCGCNTGANNCNNCANNNNGNNGNNNTTN
NCANCGGCNNTANCTGCNGANNTAAGNCATANNNTNNNTCNNTANCTNNNNNGGNGGGNNACTGTAAA
NGGNTAATTNGNNNNTNANTNCNNNCATNTNNCNNNGNNTNCTNNNTNCCTGNNTNCNNAATTGGNNN
CANTGNNTGNCNATCTCTGGNTTNGNNNNNNTNNNNNTNNCANNNNGGNNNNTNNNANNNCNATNTAA
NNTANANNNNCNNNNNNANNNCACNNNNNNNNNAGNNNCNANNGTANANNNGTAGNNNNNTAAGTCN
CCGANNTNNNNNNNATAAGCGNNANACNNNNNNNNNNNNNNANGANNNCGNNTTTGNNNTNNNNNNNNA
GNNAGACNNTNGTGGNTNNNNNTCAANNNNTTCGTAGTGGTATAATNGNNNNNNNTTTCNCCAGNNTN
NNNGNNNNNNNNCNATANNANNNNNNNGNGANNNNNNNNNNNANNNNCTCNNNAGGGANCANCTACTNNA
GCANTGGNNCGCAGNNNGAGANNNNGACNNNCCTTGGGTGGCANNCACNGNAATAAGTNNNGTTNANC
CTNNNAGTNNACACNNNNNCANNGNNNCNNNNNNANCNGANNGNNNNNNNNNNNNNNNNNNNNNNNNNN
NNNNNNNNNNNNNNNNNNNNNNNNNNNNNNNNNNNNANNGNNNGTNTNTGACNCANGTGTTNNTANNNGNN
NNNNNACGNNNNANCCCCGGTCGNNNNCNNATTGAANNCCTGNNGCATNNNNNTNCNAGAATGANNGC
NNNNNGNNTNGNCTNNCNNNTNAAGNAATCNNNNAGGNNNNNNGNTCNNNNNNNNNNNNNCNNNCCCNN
ACTGAGGGTNNNNNNNNCATCTGCTNNNNNNNNNCTNCGCNGGCGTNNCNNNNNAGCNGTATNNNGTNN
NNCANNNNNNNNNNNNNNNNNNNNAAACGANNNNNNTTNNNN
>Dao648
GTGNNNNNTAGCCCNNNNNAGTCAGACANNNCNNNNNNNNNNNNTNNNNNNNNNNNNNNNNNNNNNNNNN
NNNNNNNNNNNNNNNNNNNNNNNNNNNNNNNTNNNNNNNNNNNNNNNNNNNNNNNNNNNNNNNNNNNNNN
NNNNNNNNNNNNNNNNNNNNNNNNNNNNNNNNNNNNNNNNNNNNNNNNNNNNNNNNNNNNNNNNNNNNNN

NNNNNNNNNNNNNNNNNNNNNNNNNNNNNNNNNNNNNNNNNNNNNNNNNNNNNNNNNNNNNNNNNNNN
NNNNNNNNNNNNNNNNNNNNNNNNNNNNNNNNNNNNNNNNNNNNNNNNNNNNTCTNNNAAANNNTNNN
NNNNNNNNNNNNNNNNNNNNNNNNNCNCNNTGNNNGTAANCNANNNGNACATGCAGTCCCCTCNNNNN
NNNGGCAGAGNNNNNNNCCNNNNNNNCCGNCCCCNNCCNNNNNNNNNNNNNNNGNNNNNNANNNNNNN
NNNNNNTGGTACNNNNCGNNNNNGGTTGTNTAAGAGGTCCACAACGNGGTCNGNNNACNNNNGNNTAN
NNNCNNNNNNCNNNNNNNNNNNANNTNNNTANNAGGNCNNNNCCNTCNNGNNNNNNANTGCNATGACC
CAANNNACNGNGGNNGTNCCCGGNGCGGNGACCTTGANNANNTCCGGGNACAAGTATGGCGNNCNCCC
NGGGAAGGANNCNGCNGTANGTGCNNNNNANNNNNTCNTGNGANGTCGCCGNNATTCNNNTGGTCNNN
NNAGAATGNCCNNACCANNCGNANNNTGGTNATGTNAGTNNNNNNNNNNNNNNNNNNNNNNNNNNNNN
NNNNNNNNNNNNNNNNNNNNNNNNNNNNNNNNGNNNNGNNNNNNCNANNNAAGGNNNTANNNCTNNTN
NTNNNNNNGGACNNNACNNNNCNNNNNNNACNCNNNCNCNNNNNNNNNNGNTTTTTNNNCNNTATTNT
CCAGNACGCNGNGCNNNCNTTCANGNGAACNNNGNNACGCGNNAATCGTANNATCTACCTGCCGCNNG
NNNNNNTNCNGNNNNNNCGGANNNTNNNNCGTANNNNNNNCATGCGTNNTNTNNTNNCNAGNCNNGTT
CGNNNANANANTAGNANGTNTNNNNNNNNNTGTNNCGTTCCGCNTGANNCNNCANNNNGNNGNNCTTN
NCANCGGCNNTANNTGCNGANNTAAGNCATANNNTNNATCNNTANCTNNNNNGGNGGGNNACTGTAAA
NGGNNAATTNGNNNNTNANTNCNNNCATNTNNCTNNGNNNNCTNNNTNCCTGNNTNCNNAATTGGNNC
CANTGNNTGNCNATCTCTGGNTTNGNNNNNNTNNNNNTNNCANNNNGGNNNNNNNNANNNCNATNTAA
NNTANANNNNCNNNNNNANNNCACNNNNNNNNNAGNNNNNANNNGNANANNNGTAGNNNGNTAAGTCT
CCGANNTNNNNNNATAAGCGNNANACNNNNNNNNNNNNNNANGANNNCGNNTTTGNNNTNNNNNNNNA
GNNAGACNNTNGTGGNTNNNNNTCAANNNNTTNGTAGTGGTATAATNGNNNNNNNTTTCNCCAGNNNN
NNNGNNNNNNCNATNNNANNNNNNNGNGANNNNNNNNNNNANNNNCTCNNNAGGNANCANCTACTNNA
GCANNGGNGCGNAGNNNGAGANNNNGACNNNCCTTGGGTGGCAANNACNGCAATAAGTNNNGTTNNNC
CTNNNAGTNNACACNNNNNCANNGNNNCNNNNNNANCNGANNGNNNNNNNNNNNNNNNNNNNNNNNNN
NNNNNNNNNNNNNNNNNNNNNNNNNNNNANNNNANNGNNNGTNTNTGACNCNNNNNTTTNTANNNGNN
NNNNNACGNNNNANCCCCGGTCGNNNNCNNATTGAANNCCTGNNGCATNNNNNTNCNAGAATGANNGC
NNNNNGNNTNGNCTNNCNNNTNAAGNAATNNNNNAGGTNNNNNGNTCNNNGNNNNNNNNCNNTCNCNN
ACTGAGGGNNNNNNNNNCATCTGCTNNNNNNNNNTNCGCNGGCGTNNCNNNNNAGCNGTATNGNGTNA
NNCANNNNNNNNNNNNNNNNTNAAACAANNNNNNNTNNNN
>Dao649
GTGNNNNNTAGCCCNNNNNAGTTNGACANNNCNNNNNNNNNNNNTNNNNNNNNNNNNNNNNNNNNNNN
NNNNNNNNNNNNNNNNNNNNNNNNNNNNNNNNNNNNNNNNNNNNNNNNNNNNNNNNNNNNNNNNNNNN
NNNNNNNNNNNNNNNNNNNNNNNNNNNNNNNNNNNNNNNNNNNNNNNNNNNNNNNNNNNNNNNNNNNN
NNNNNNNNNNNNNNNNNNNNNNNNNNNNNNNNNNNNNNNNNNNNNNNNNNNNNNNNNNNNNNNNNNNN
NNNNNNNNNNNNNNNNNNNNNNNNNNNNNNNNNNNNNNNNNNNNNNNNNNNNNNNNNNNNNNNNNNNN
NNNNNNNNNNNNNNNNNNNNNNNNNNNNNNNNNNNNNNNNNNNNNNNNNNCNTCTNNNAAANNNTNNN
NNNNNNNNNNNNNNNNNNNNNNNNNCNCNTTGNNNGTAANCNANNNGNACATGCAGTCCCCTCNNNNN
NNNNNCAGAGNNNNNNNCCNNNNNNNCCGNCCCCNNCCNNNNNNNNNNNNNNNGNNNNNNANNNNNNN
NNNNNNTGGTACNNNNCGNNNNNGGTTGNNTNAGAGGTCCACAACGNGNTCNNGNNACNNNNGNNNAN
NNNCNNNNNNANNNNNNNNNNNANNTNNNTANNAGGNNNNNNCCNTCNNNNNNNNNATNGCNATGACC
CANNNCACGGNGGCAGTNCCCGGCGNGGCGACCTTGNTNANNTCCGGGGNCAAGTATGGCNNNCNCCC
CGGGAAGGANNCNGCCGTANGTGCNNNNNANNNNNTCNTGNGANGTCGCCGNNATTNNCGTNGTCNNN
NNAGANTGNNCNNACCANNCGNANNNTGGTNATGNTAGTNNNNNNNNNNNNNNNNNNNNNNNNNNNNN
NNNNNNNNNNNNNNNNNNNNNNNNNNNNNNNNGNNNNGNNNNNNCNANNNAAGGANNTANNNCTNNTN
NTNNNNNNGGACNNNACNNNNCNNNNNNNACNCNNNCNCNNNNNNNNNNGNTTNNNNNNCNNTATTNT
CCAGNACGCNGNGCNNNCATTCANGNGAACNNNGNNACGCGNNAATCGTANNATCTACCTGCCGCNNG
NNNNNNTACNGNNNNNNCGGANNNTNNNNNGTANNNNNNNCANGCGTNNTNTNNTCNCNAGNCANGTT
CGNNNANANANTAGNANGTNTNNNNNNNNNNGTNNCGTTCCGCNTGANNCNNCANNNNGNNGNNNTTN
NCANCGGCNNTANCTNCNGANNTAAGNCATANNNTNNNTCNNTANCTNNNNNGGAGGGNNACTGTAAA
NGGNNAATTNGNNNNTNATTNCNNNCACNTNNCTNNGNNTNCTNNNTNCCTGNNTNCNNAATTGGNNN
CANTGNNTGNCNATCTCTNGNTTNGNNNNNNTNNNNNTNNCANNNNGGNNNGTNANANNNNNATNTAA
NNTANANNNNCNNNNNNANNNCACNNNNNNNNNAGNNNNNANNNTNANANNNGTAGNNNANTAAGTCT
CCGANNTNNNNNNATAAGCGNNANACNNNNNNNNNNNNNNANGANNNCGNNTTGGNNNTNNNNNNCNA
GNNAGANNNTNATGGNTNNNNNTCAANNNNTTNGTAGTGGTATAANNGNNNNNNNTTTCNCCAGNNTN
NNNGNNNNNNCNATNNNANNNNNNNGNGANNNNNNNNNNNNNNNNCTCNNNAGGNANCANCTACTNNA
GCANNGGNGCGNAGNNNGAGANNNNGACNNNCCTTGGGTGGCANNNACNGNAATAAGTNNNGTTNANC

CTNNNAGTNNACATNNNNNCANNGNNNCNNNNNNANCNNANNGNNNNNNNNNNNNNNNNNNNNNNNNN
NNNNNNNNNNNNNNNNNNNNNNNNNNNNNNNNNNANGGNNNGTNTNTGACNCATNNNTTTTANNNGNN
NNNNNACGNNNNANCCCCGGTCGNNNNCNNATTGAANNCCTGNNGCATNNNNNTNCNAGAATGANNNC
NNNNNGNNTNGCTNNNNNNTCAAGNAATCNNNNAGNNNNNNNNNTCNNNNNNNNNNNNNCNNNCCCNN
ACTGAGGGTNNNNNNNNCATCTGCTNNNNNNNNNTNCGCNNNNNNNNCNNNNNAGCNGTATNNNGTNN
NNCANNNNNNNNNNNNNNNNCNAAACNANNNNNNTNNNN
>Dao650
GTGNNNNNNAGCCCNNNNNAGTCAGACANNNCNNNNNNNNNNNNTNNNNNNNNNNNNNNNNNNNNNNN
NNNNNNNNNNNNNNNNNNNNNNNNNNNNNNNNNNNNNNNNNNNNNNNNNNNNNNNNNNNNNNNNNNNN
NNNNNNNNNNNNNNNNNNNNNNNNNNNNNNNNNNNNNNNNNNNNNNNNNNNNNNNNNNNNNNNNNNNN
NNNNNNNNNNNNNNNNNNNNNNNNNNNNNNNNNNNNNNNNNNNNNNNNNNNNNNNNNNNNNNNNNNNN
NNNNNNNNNNNNNNNNNNNNNNNNNNNNNNNNNNNNNNNNNNNNNNNNNNCNTCTNNNAAANNNTNNN
NNNNNNNNNNNNNNNNNNNNNNNNNCNCNNTGNNNGTAANCNANNNGNACATGCAGTCCCCTCNNNNN
NNNGGCAGAGNNNNNNNCCNNNNNNNCCGNCCCCNNCCNNNNNNNNNNNNNNNGNNNNNNNANNNNNN
NNNNNNTGGTACNNNNCGNNNNNGGTTGTNTAAGAGGTCCACAACGNGNTCNGGNNACNNNNGNNNAN
NNNCNNNNAACNNNNNNNNNNNNNNTNNNTANNAGGNNNNNNCCNTCNNGNNNNNNATNGCNATGANN
CANNNCACGGGGGCNGTNCCCGGNGCGGNGACCTTGANNANGNCCGGGGNCAAGTATGGCNNNCNCCC
CGGGAAGGANNCNGCCGTANGTGCNNNNNANNNNNTCNTGNGANGTNGCCGNNATTNNCGTGGTCNNN
CNAGAATGNCCNNACCANNCGNANNNTGGTNATGNNAGTNNNNNNNNCNNNNNNNNNNNNNNNNNNNN
NNNNNNNNNNNNNNNNNNNNNNNNNNNNNNNNGNNNNGNNNNNNCNANNNAAGGNNNTANNNCTNNTN
NTNNNNNNGGACNNNACNNNNCNNNNNNNACNCNNNCNCNNNNNNANNNGNTTTTNNNNCNNTANTNT
CCAGNACGCNGNGCNNNCATTCANGNGAACNNNGNNACGCGNNAATCGTANNATCTACCTGCCGCNNG
TNNNNNTACNGNNNNNNCGGANNNTNNNNCGTANNNNNNNCANGCGTNNTNTNNTCNCNAGNCANGTT
CGNNNANANANTAGNANGTNTNNNNNNNCTTGTNNCGTTCCGCNTGANNCNNCANNNNGNNGNNCTTN
NCANCGGCNNTANCTGCNGANNTAAGNCATANNNTNNNTCNNTANCTNNNNNGGNGGGNNACTGTAAA
NGGNNAATTNGNNGNTNATTNCCNNCATNTNNCTNNGNNTNCTNNNTNCCTGNNTNCNNAATTGGNNC
CANTGNNTGNCNATCTCTNGNTTNGNNNNNNTNNNNNTNNCANNNNGGNNNGTNNNANNNCNATNTAA
NNTANANNNNCNNNNNNANNNCACNNNNNNNGNAGNNNCNANNNGNANANNNGTAGNNNGNTNAGTCT
CCGANNTNNNNNNNATAAGCGNNANACNNNNNNNNNNNNNNANGANNACGNNTTTGNNNTNNNNNNNA
GNNAGACNNTNGTGGNTNNNNNTCAANNNNTTNGTAGTGGTATAATNGNNNNNNNTTTCNCCAGNNTN
NNNGNNNNNNNCNATANNANNNNNNGNGANNNNNNNNNNNANNNNCTCNNNAGGGANCANCTACTNNA
GCAATGGNGCGCAGNNNGAGANNNNGACNNGCCTTGGGTGGCAANNACNGCAATAAGTNNNGTTNANC
CTNNNAGTNNACACNNNNNCANNGNNNCNNNNNNANCNGANNGNNNNNNNNNNNNNNNNNNNNNNNNN
NNNNNNNNNNNNNNNNNNNNNNNNNNNNANNNNANNGNNNGTNTNTGACNCATGTGTTTTTANNNGNN
NNNNNACGNNNNANCCCCGGTCGNNNNCNNATTGAANNCCTGNNGCATNNNNNTNCNAGAATGANNGC
NNNNNGNNTNGCTNNCNTTTCAAGNAATCANNNAGNNNNNNNGNTCNNNNNNNNNNNNNCNNTCCCNN
ACTGAGGGNNNNNNNNNCATCNGCTNNNNNNNNNTNCGCNGGNGTNNCNNNNNNGCNGTATNGNGTNN
NNCANNNNNNNNNNNNNNNNTNAAACNANNNNCNTNNNN
>Dao651
GTGNNNNNTAGCCCNNNNNAGTTAGACANNNCNNNNNNNNNNNNTNNNNNNNNNNNNNNNNNNNNNNN
NNNNNNNNNNNNNNNNNNNNNNNNNNNNNNTNNNNNNNNNNNNNNNNNNNNNNNNNNNNNNNNNNNNN
NNNNNNNNNNNNNNNNNNNNNNNNNNNNNNNNNNNNNNNNNNNNNNNNNNNNNNNNNNNNNNNNNNNN
NNNNNNNNNNNNNNNNNNNNNNNNNNNNNNNNNNNNNNNNNNNNNNNNNNNNNNNNNNNNNNNNNNNN
NNNNNNNNNNNNNNNNNNNNNNNNNNNNNNNNNNNNNNNNNNNNNNNNNNCNTCTNNNAAANNNTNNN
NNNNNNNNNNNNNNNNNNNNNNNNNCNCNTTGNNNGTAANCNANNNGNACATGCAGTCCCCTCNNNNN
NNNGGCAGAGNNNNNNCCCNNNNNNNCCGNCCCCNNCCNNNNNNNNNNNCNNNGNNNNNNNANNNNNN
NNNNNNTGGTACNNNNCGNNNNNGGTTGNNTAAGAGGTCCACAACANGGTCNNGNCACNNNNGNNNAN
NNNCNNNAAANNNNNNNNNNNNANNTNNNTANNAGGNCNNNNCCNTCNNNNNNNNAANTGCNATGACC
CANNNCACGGGGGCNGTNCCCGGCGCGGCGACCTTGANNANGTCCGGGGACAAGTATGGCNNNCNCCC
CGNGAAGGANNCNGCNGTANGTGCNNNNNANNNNNTCNTGNGANGTNGCCGNNATTNNCGTGGTCNNN
NNAGANTGNNCNNACCANNCGNANNNTGGTNATGNNAGTNNNNNNNNNNNNNNNNNNNNNNNNNNNNN
NNNNNNNNNNNNNNNNNNNNNNNNNNNNNNNNGNNNNGNNNNNNCNANNNAAGGNNNTANNNCTNNTN
NNNNNNNNGGACNNNACNNNNCNNNNNNNACNCNNNCNCNNNNNNNNNNGNTTTTNNNNCNNTANTNT
CCAGNACGCNGNGCNNNCNTTCANGNGAACNNNGNNACGCGNNAATCGTANNATCTACCTGCCGCNNG

TNNNNNTACNGNNNNNNCGGANNNTNNCTCGTANNNNNNNCATGCGTNNTNTNNTCNCNAGNCNNGTT
CGNNNANANANTAGNANGTNTNNNNNNNNNNTGTNNCGTTCCGCNCGANNCNNCANNNNGNNGNNNTTN
NCANCGGCNNTANCTGCNGANNTAAGNCATANNNTNNNTCNNTANCTNNNNNGGNGGGNNACTGTAAA
NGGNNAATTNGNNNNTNANTNCNNNCACNTNNCTNNANNNNCTNNNTNCCTGNNTNCNNAATTGGNNC
CANTGNNTGNCNATCTCTGGNTTNGNNNNNNTGNNNNTNNCANNNNGGNNNNNNANANNNCNATNTAA
NNTANANNNNCNNNNNNANNNCACNNNNNNNGNAGNNNCNANNNTTANANNNGTAGNNNANTAAGTCT
CCGANNTNNNNNNNATAAGCGNNANACNNNNNNNNNNNNNNANGANNACGNNTTGGNNNTNNNNNNCNA
GNNAGACNNTNGTGGNTNNNNNTCAANNNNTTNGTAGTGGTATAATNGNNNNNNNTTTCNCCAGNNTN
NNNGNNNNNNNNCNATANNANNNNNNNGNGANNNNNNNNNNNANNNNCTTNNNAGGNANCANTTACTNNA
GCAATGGNNCGCAGNNNGAGANNNNGACNNNCCTTGGGTGGCAANCACNGCAATAAGTNNNGTTNNNC
CTNNNAGTNNACACNNNNNCANNGNNNCNNNNNNANCNGANNGNNNNNNNNNNNNNNNNNNNNNNNNN
NNNNNNNNNNNNNNNNNNNNNNNNNNNNNNAGNNNANNGNNNGTNTNTGACNCNTGTGTTTTTANNNGNN
NNNNNACGNNNNANCCCCGGTCGNNNNCNNATTGAANNCCTGNNGCATNNNNNTNCNAGAATGANNGC
NNNNNGNNTNGNCTNNCNNNTCAAGNAATCAANNANGTANNNNNNTCNNNGNNNNNNNNCNNNCCCNN
ACTGAGGGTNNNNNNNNCATCTGCTNNNNNNNNNNTNCGCNGGCGTNNCNNNNNAGCNGTATNGNGNNN
NNCANNNNNNNNNNNNNNNNNNTNAAACAANNNNNTTNNNN
>Dao652
GTGNNNNNNAGCCCNNNNNAGTTANACANNNCNNNNNNNNNNNNTNNNNNNNNNNNNNNNNNNNNNNNNN
NNNNNNNNNNNNNNNNNNNNNNNNNNNNNNTNNNNNNNNNNNNNNNNNNNNNNNNNNNNNNNNNNNNNNNN
NNNNNNNNNNNNNNNNNNNNNNNNNNNNNNNNNNNNNNNNNNNNNNNNNNNNNNNNNNNNNNNNNNNNNN
NNNNNNNNNNNNNNNNNNNNNNNNNNNNNNNNNNNNNNNNNNNNNNNNNNNNNNNNNNNNNNNNNNNNNN
NNNNNNNNNNNNNNNNNNNNNNNNNNNNNNNNNNNNNNNNNNNNNNNNNNNNNNTCTNNNAAANNNTNNN
NNNNNNNNNNNNNNNNNNNNNNNNNNNCNCNNTGNNNGTAANCNANNNGNACATGCAGTNCCCTCNNNNN
NNNNNCAGAGNNNNNNNCCNNNNNNNCCGNCCCCNNCCNNNNNNNNNNNNNNNNNNNNNNNNNANNNNNN
NNNNNNTGGTACNNNNCGNNNNNGGTTGNNTAAGAGGTCCACAACGNGGTCNNNNNACNNNNGNNNAN
NNNCNNNAAANNNNNNNNNNNNNNNNNTNNNTANNAGGNCNNNNCCNTNNNGNNNNNNATNGCNATGACC
CANNNCNCGGGGGCNGTNCCCGGNGNGGCGACCTTGANNANGTCCGGGGNCAAGTATGGCNNNCNCCC
CGNGAAGGANNCNGCCGTANGTGCNNNNNANNNNNTCNTGNGANNTNGCCGNNATTCNCGTGGTCNNN
CNAGAATGNNCGNACCANNCGNANNNTGGTNATGNNAGTNNNNNNNNNNNNNNNNNNNNNNNNNNNNNN
NNNNNNNNNNNNNNNNNNNNNNNNNNNNNNNNNNGNNNNGNNNNNNCNANNNAAGGNNNTANNNCTNNTN
NTNNNNNNNGGACNNNACNNNNCNNNNNNNACNCNNNCNCNNNNNNANNNGGTTTNNNNNCNNTANTNT
CCAGNACGCNGNGCNNNCNTTCANGNGAACNNNGNNACGCGNNAATCGTANNATCTACCTGCCGCNNG
NNNNNNTACNGNNNNNNCGGANNNTNNNNNGTANNNNNNNCANGCGTNNTNTNNTCNCNAGNCNNGTT
CGNNNANNNANTAGNANGTNTNNNNNNNNNNTGTNNCGTTCCGCNTGANNCNNCNNNNNGNNGNNNTTN
NCANCGGCNNTANCTGCNGANNTAAGNCATANNNTNNNTCNNTANCTNNNNNGGNGGGNNACTGTAAA
NGGNNAATTNGNNGNTNANTNCNNNCACNTNNCNNNGNNTNCTNNNTNCCTGNNTNCNNAATTGGNNC
CANTGNNTGNCNATCTCTNGNTTNGNNNNNNTNNNNNTNNCANNNNGGNNNGTNANANNNCNATNTAA
NNTANANNNNCNNNNNNANNNCACNNNNNNNNNAGNNNCNANNNTNANANNNGTAGNNNANTNAGTCT
CCGANNTNNNNNNNATAAGCGNNANACNNNNNNNNNNNNNNANGANNNCGNNTTGGNNNTNNNNNNCNA
GNNAGACNNTNATGGNTNNNNNTCAANNNNTTCNTAGTGGTATAATNGNNNNNNNTTTCNCCAGNNTN
NNNGNNNNNNNNCNATANNANNNNNNNGNGANNNNNNNNNNNANNNNCTCNNNAGGNANCANCTACTNNA
GCANTGGNGCGNAGNNNGAGANNNNGACNNNCCTTGGGTGGCAANNACNGNGATAAGTNNNGTTNANN
CTNNNAGTNNACATNNNNNCANNGNNNCNNNNNNANCNGANNGNNNNNNNNNNNNNNNNNNNNNNNNN
NNNNNNNNNNNNNNNNNNNNNNNNNNNNNANNNNANNGNNNGTNTNTGACNCANNNNTTTNTANNNGNN
NNNNNNNGNNNNANCCCCGGTCGNNNNCNNATTGAANNCCTGNNGCATNNNNNTNCNAGAATGANNGC
NNNNNGNNTNGNCTNNCNNNTNAAGNAATCANNNAGNNNNNNNNNTCNNNGNNNNNNNNCNNNCCCNN
ACTGAGGGTNNNNNNNNCANCNGCTNNNNNNNNNCTNCGCNGGCGTNNCNNNNNAGCNGTATNGNGNNN
NNCANNNNNNNNNNNNNNNNNNCCAAACAANNNNNTTNNNN
>Dao653
GTGNNNNNTAGCCCNNNNNAGTTAGACANNNCNNNNNNNNNNNNNNNNNNNNNNNNNNNNNNNNNNNNNN
NNNNNNNNNNNNNNNNNNNNNNNNNNNNNNTNNNNNNNNNNNNNNNNNNNNNNNNNNNNNNNNNNNNNNNN
NNNNNNNNNNNNNNNNNNNNNNNNNNNNNNNNNNNNNNNNNNNNNNNNNNNNNNNNNNNNNNNNNNNNNN
NNNNNNNNNNNNNNNNNNNNNNNNNNNNNNNNNNNNNNNNNNNNNNNNNNNNNNNNNNNNNNNNNNNNNN
NNNNNNNNNNNNNNNNNNNNNNNNNNNNNNNNNNNNNNNNNNNNNNNNNNNNCNTCTNNNAAANNNTNNN

NNNNNNNNNNNNNNNNNNNNNNNNNCNCNNTNNNNGTAANCNANNNGNACATGCAGTNNCCTCNNNNN
NNNNNCAGAGNNNNNNCCCNNNNNCNCCGNCCCCNNCCNNNNNNNNNNNNNNNNNNNNNNNNNANNNNNN
NNNNNNTGGTACNNNNCGNNNNNGGTTGTNTNAGAGGTCCACAACGNGGTCNNGNNACNNNNGNNNAN
NNNCNNNNAANNNNNNNNNNNNNNNNNTNNNTANNAGGNCNNNNCCNTCNNNNNNNNNANNGCNATGACC
CANNNNACNGNNGCNGTNCCCGGNGCGGCNACCTTGNNNANGTCCGGGNNCAAGTATGGCNNNCNCCC
CGNGAAGGANNCNGCNGTANGTGCNNNNNANNNNNTCNTGNGANGTNNNCGNNATTNNNNTGGTCNNN
CNAGAATGNNCNNACCANNCGNANNNTGGTNATGNNAGTNNNNNNNNNNNNNNNNNNNNNNNNNNNNNN
NNNNNNNNNNNNNNNNNNNNNNNNNNNNNNNNNNGNNNNGNNNNNNCNANNNAAGGNNNTANNNCTNNTN
NNNNNNNNGGACNNNNCNNNNCNNNNNNNACNCNNNCNCNNNNNNANNNGNTTNNNNNNCNNTANTNT
CCAGNANGCNGNGCNNNCATTCANGNGAACNNNGNNACGCGNNAATCGTANNATCTACCTGCCGCNNG
NNNNNNTACNGNNNNNNCGGANNNTNNNNCGTANNNNNNTCATGCNTNNTNTNNTCNCNAGNCNNGTT
CGNNNANNNANTAGNANGTNTNNNNNNNNNNTGTNNCGTTCCGCNTGANNCNNCNNNNNGNNGNNNTTN
NCANCGGCNNTANCTGCNGANNTAAGNCATANNNTNNNTCNNTANCTNNNNNGGNGGGNNACTGTAAA
NGGNNAATTNGNNGNTNANTNCNNNNACNTNNCNNNGNNNNCTNNNTNCCTGNNTNCNNAATTGGNNN
CANTGNNTGNCNATCTCTGGNTTNGNNNNNNTGNNNNTNNCANNNNGGNNNNNNNNANNNNNATNTAA
NNTANANNNNCNNNNNNANNNCACNNNNNNNGNAGNNNCNANNNTTANANNNGTAGNNNANTAAGTCT
CCGANNTNNNNNNNATAAGCGNNANACNNNNNNNNNNNNNANGANNNCGNNTTGGNNNTNNNNNNNNA
GNNAGANNNTNATGGNTNNNNNTCAANNNNTTNGTAGTGGTATAATNGNNNNNNNTTTCNCCAGNNTN
NNNGNNNNNNNCNATANNANNNNNNNGNGANNNNNNNNNNNANNNNCTCNNNAGGGANCANCTACTNNA
GCANTGGNGCGCAGNNNGAGANNNNGACNNNCCTTGGGTGGCANNNACNGCAANAAGTNNNGTTNANC
CTNNNAGTNNACATNNNNNCANNGNNNCNNNNNNANCNGANNGNNNNNNNNNNNNNNNNNNNNNNNNNN
NNNNNNNNNNNNNNNNNNNNNNNNNNNNNNNNNNANGGNNNGTNTNTGACNCATGTNTTTNTANNNGNN
NNNNNACGNNNGANCCCCGGTCGNNNNCNNATTGAANNCCTGNNGCATNNNNNTNCNAGAATGANNNC
NNNNNGNNTNGNCTNNNNNNTCAAGNAATCANNNANNNNNNNNNNNTCNNNNNNNNNNNNCNNTCCCNN
ACTGAGGGNNNNNNNNNCANCTGCTNNNNNNNNNNTNCGCNGGNGTNNCNNCNNNGCNGTATNGNGTNN
NNCANNNNNNNNNNNNNNNNNTNANACAANNNNNNNTNNNN
>Dao654
GTGNNNNNTAGCCCNNNNNAGTTAGACANNNCNNNNNNNNNNNNNTNNNNNNNNNNNNNNNNNNNNNNN
NNNNNNNNNNNNNNNNNNNNNNNNNNNNNNNNNNNNNNNNNNNNNNNNNNNNNNNNNNNNNNNNNNNNNN
NNNNNNNNNNNNNNNNNNNNNNNNNNNNNNNNNNNNNNNNNNNNNNNNNNNNNNNNNNNNNNNNNNNNNN
NNNNNNNNNNNNNNNNNNNNNNNNNNNNNNNNNNNNNNNNNNNNNNNNNNNNNNNNNNNNNNNNNNNNNN
NNNNNNNNNNNNNNNNNNNNNNNNNNNNNNNNNNNNNNNNNNNNNNNNNNNNNNNTCTNNNAAANNNTNNN
NNNNNNNNNNNNNNNNNNNNNNNNNCNCNNTGNNNGTAANCNANNNGNACATGCAGTCCCCTCNNNNN
NNNNNCAGAGNNNNNNNNNCNNNNNNNCCGNCCCCNNCCNNNNNNNNNNNCNNNNNNNNNNNANNNNNN
NNNNNNTGGTACNNNNCGNNNNNGGTTGNNTNAGNGGTCCACAACGNGGTCNNNNNACNNNNGNNNAN
NNNCNNNNAANNNNNNNNNNNNNNNNNATNNNTANNAGGNNNNNNCCNTCNNGNNNNTNATTGCNATGANN
CANNNCACNGGGGCNGTNCCCGGNGNGGCGACCTTGANNANNTCCGGGNNCAAGTATGGCNNNCNCCC
CGGGAAGGANNCNGCCGTANGTGCNNNNNANNNNNTCNTGNGANGTCGCCGNNATTNNNNTGGNCNNN
NNAGAATGNCCNNACCANNCGNANNNTGGTNATGNNAGTNNNNNNNNNNNNNNNNNNNNNNNNNNNNNN
NNNNNNNNNNNNNNNNNNNNNNNNNNNNNNNNNNGNNNNGNNNNNNCNANNNAAGGNNNTANNNCTNNTN
NTNNNNNNGGACNNNACNNNNCNNNNNNNACNCNNNCNCNNNNNNANNNGNTTTTNNNNNCNNTATTNT
CCAGNANGCNGNGCNNNCATTCANGNGAACNNNGNNACGCGNNAATCGTANNATCTACCTGCCGCNNG
NNNNNNTNCNGNNNNNNCGGANNNTNNNTCGTANNNNNNNCATGCGTNNTNTNNTCNCNAGNCNNGTT
CGNNNANANANTAGNANGTNTNNNNNNNNNNTGTNNCGTTCCGCNTGANNCNNCANNNNGNNGNNNTTN
TCANCGGCNNTANCTGCNGANNTAAGNCATANNNTNNNTCNNTANCTNNNNNGGNGGGNNACTGTAAA
NGGNNAATTNGNNNNTNANTNCNNNNACNTNNCNNNGNNNNCTNNNTNCCTGNNTNCNNAATTGGNNC
CANTGNNTGNCNATCTCTGGNTTNGNNNNNNTGNNNNTNNCANNNNGGNNNNNNNNANNNCNATNTAA
NNTANANNNNCNNNNNNANNNCACNNNNNNNNNAGNNNCNANNNTTANANNNGTAGNNNAATNAGTCT
CCGANNTNNNNNNNATAAGCGNNANACNNNNNNNNNNNNNANGANNNCGNNTTGGNNNTNNNNNNCNA
GNNANACNNTNATGGNTNNNNNTCAANNNNTTNGTAGTGGTATAATNGNNNNNNNTTTCNCCAGNNTN
NNNGNNNNNNNCNATANNANNNNNNNGNGANNNNNNNNNNNANNNNCTCNNNAGGNANCANCTACTNNA
GCANTGGNNCGNAGNNNGAGANNNNGACNNNCCTTGGGTGGCANNNACNGNAATAAGTNNNGTTNNNN
NTNNNAGTNNACATNNNNNCANNGNNNCNNNNNNANCNGANNGNNNNNNNNNNNNNNNNNNNNNNNNNN
NNNNNNNNNNNNNNNNNNNNNNNNNNNNNNANNNNANNGNNNGTNTNTGACNCATGTGTTTNTANNNGNN

NNNNNNNGNNNNANCCCCGGTCGNNNNCNNATTGAANNCCTGNNGCATNNNNNTNCNAGAATGANNGC
NNNNNGNNTNGNCTNNCNTTTCAAGNAATNANNNANNNNNNNNNNTCNNNGNNNNNNNNCNNNCCCNN
ACTGAGGGTNNNNNNNNCATCTGCTNNNNNNNNCTNCGCNGGNGTNNCNNNNNNGCNGTATNGNGTNA
NNCATNNNNNNNNNNNNNNNNNTCAAACAANNNNNNTNNNN
>Dao655
GNGNNNNNNAGCCCNNNNNAGTTNGANANNNCNNNNNNNNNNNNNTNNNNNNNNNNNNNNNNNNNNNNNN
NNNNNNNNNNNNNNNNNNNNNNNNNNNNNNNNNNNNNNNNNNNNNNNNNNNNNNNNNNNNNNNNNNNNNN
NNNNNNNNNNNNNNNNNNNNNNNNNNNNNNNNNNNNNNNNNNNNNNNNNNNNNNNNNNNNNNNNNNNNNN
NNNNNNNNNNNNNNNNNNNNNNNNNNNNNNNNNNNNNNNNNNNNNNNNNNNNNNNNNNNNNNNNNNNNNN
NNNNNNNNNNNNNNNNNNNNNNNNNNNNNNNNNNNNNNNNNNNNNNNNNNNNNTCTNNNAAANNNTNNN
NNNNNNNNNNNNNNNNNNNNNNNNNNCNCNTTNNNNGTAANCNANNNGNACATGCAGTNCCCTCNNNNN
NNNNNCAGANNNNNNNNNNNNNNNNNNCCGGCCCCNNCCNNNNNNNNNNNNNNNNNNNNNNNNNANNNNNN
NNNNNNTGGTACNNNNCGNNNNNGGNNGNNTNAGAGGTCCACNACANGGTCNNGNNACNNNNGNNNAN
NNNCNNNNNNNNNNNNNNNNNNNANNTNNNTANNAGGNCNNNNCCNTNNNNNNNNNNNANTGCNATGANN
CNNNNNNCNGNNGNNGTNCCCGGNGNGGCNACCTTGNNNANNTCNGGNGNCAAGTATGGCNNNCNCNC
CGGGAAGGANNCNGCNGTANGTGCNNNNNANNNNNTCNTGNGANNTCGCCGNNNTTNNNNTGNNCNNN
CNAGANTGNNCNNACCANNCGNANNNTGGTNATGNNAGTNNNNNNNNNNNNNNNNNNNNNNNNNNNNNNN
NNNNNNNNNNNNNNNNNNNNNNNNNNNNNNNNNNNNNNNNGNNNNNNCNANNNAAGGNNNTANNNCTNNTN
NTNNNNNNNNNNCNNNACNNNNCNNNNNNNACNCNNNCNCNNNNNNNNNNNGNTTNNNNNNNCNNTANTNT
CNAGNANGCNGNGCNNNCNTTCANGNGAACNNNGNNACGCGNNAATCGTANNATCTACCTGCCGCNNG
NNNNNNTNCNGNNNNNNNCGGNNNNTNNCTNGTANNNNNNNNCANGCGTNNNNTNNTNNCNAGNCNNGTT
CGNNNANNNANTAGNANGTNTNNNNNNNNNNTGTNNCGTTCCGCNCGANNCNNCANNNNGNNGNNNTTN
NCANCGGCNNTANNTGCNGANNTAAGNCATNNNNTNNNTCNNTANCTNNNNNGGNGGGNNACTGNAAA
NGGNNAATTNGNNNNTNANTNCNNNCACNTNNCNNNANNNNCTNNNTNCCTGNNNNCNNAATTGGNNC
CANTGNNTGNCNATCTCTGGNTTNGNNNNNNTNNNNNTNNCANNNNGGNNNGTNNNANNNNNATNTAA
NNTANANNNNCNNNNNNANNNCACNNNNNNNNNAGNNNCNANNNTNANANNNGTAGNNNAATAAGTCT
CCGANNTNNNNNNNATAAGCGNNANACNNNNNNNNNNNNNANGANNNCGNNTTGGNNNTNNNNNNNNN
GNNAGANNNTNGTGNNTNNNNNTCAANNNNTTNNTAGTGGTATAATNGNNNNNNNTTTCNCCAGNNTN
NNNGNNNNNNNNCNATANNANNNNNNNGNGANNNNNNNNNNNNNNNCTTNNNAGGGANCANTTACTNNA
NCANNGGNNCGNAGNNNGAGANNNNGACNNNCCTTGGGTGGCANNNACNGCAATAAGNNNNGTTNNNC
CTNNNNNTNNACACNNNNNCANNGNNNCNNNNNNANCNGANNGNNNNNNNNNNNNNNNNNNNNNNNNNN
NNNNNNNNNNNNNNNNNNNNNNNNNNNNNANNNNANNGNNNGTNTNTGACNCNTGNNTTTTTANNNGNN
NNNNNNNGNNNNNNCCCCGGTCGNNNNNNNATTGAANNCCTGNNGCATNNNNNTNCNAGAATGANNGC
NNNNNGNNTNNNCTNNNNNNTCAAGNAATNNNNNANNNNNNNNNNTCNNNNNNNNNNNNCNNNCCCNN
ACTGAGGNTNNNNNNNNCANNNNCTNNNNNNNNNNTNCNCNGGNGTNNCNNNNNAGCNGTATNNNGNNN
NNCANNNNNNNNNNNNNNNNNNTNNAACNANNNNNNNTNNNG
>Dao656
GTNNNNNNNAGCCCNNNNNAGTTAGACANNNCNNNNNNNNNNNNNTNNNNNNNNNNNNNNNNNNNNNNNN
NNNNNNNNNNNNNNNNNNNNNNNNNNNNNNNNNNNNNNNNNNNNNNNNNNNNNNNNNNNNNNNNNNNNNN
NNNNNNNNNNNNNNNNNNNNNNNNNNNNNNNNNNNNNNNNNNNNNNNNNNNNNNNNNNNNNNNNNNNNNN
NNNNNNNNNNNNNNNNNNNNNNNNNNNNNNNNNNNNNNNNNNNNNNNNNNNNNNNNNNNNNCNNNNNNNNNN
NNNNNNNNNNNNNNNNNNNNNNNNNNNNNNNNNNNNNNNNNNNNNNNNNNNNNTCTNNNAAANNNTNNN
NNNNNNNNNNNNNNNNNNNNNNNNNNCNCNNTNNNNGTAANCNANNNGNACATGCAGTCCCCTCNNNNN
NNNNNCAGAGNNNNNNNCNNNNNNNNNCCGNCCCCNNCCNNNNNNNNNNNNCNNNGNNNNNNNANNNNNN
NNNNNNTGGTACNNNNCGNNNNNGGTTGTNTAAGAGGTCCACAACGNGNTCNGNNNACNNNNGNNNAN
NNNCNNNNAANNNNNNNNNNNNNANNTNNNTANNAGGNNNNNNCCNNNNNGNNNNNNANNGCNATGANN
CNNNNNNCNGGGGNNGTNCCCGGCGNGGCGACCTTGNNNACGTCCGGGNNCAAGTATGGCNNNCNCCC
CGGGAAGGANNCNGCNGTANGTGCNNNCNANNNNNTCNTGNGANGTNGCCGNNATTCNNNTGGTCNNN
CNNGAATGNNCNNACCANNCGNANNNTGGTNATGNNAGTNNNNNNNNNNNNNNNNNNNNNNNNNNNNNNN
NNNNNNNNNNNNNNNNNNNNNNNNNNNNNNNNNNNGNNNNGNNNNNNCNANNNAAGGANNTANNNCTNNTN
NNNNNNNNGGACNNNNCNNNNCNNNNNNNACNCNNNCNCNNNNNNNNNNNGNTTNNNNNNNCNNTNNTNN
CCAGNANGCNGNGCNNNCNTTCANGNGAACNNNGNNACGCGNNAATCGTNNNATCTACCTGCCGCNNG
NNNNNNTNCNGNNNNNNNCGGANNNTNNNNCGTANNNNNNNNCANGCGTNNNNTNNTNNCNAGNCNNGTT
CGNNNANANANTAGNANGTNTNNNNNNNNNNTGTNNCGTTCCGNNTGANNCNNCANNNNGNNGNNNTTN

TCANCGGCNNTANCTNCNGANNTAAGNCATANNNTNNNTCNNTANCTNNNNNGGNNGGNNACTGTAAA
NGNNNAATTNGNNNNTNANTNCNNNCACNTNNCTNNGNNNNCTNNNTNCCTGNNTNCNNAATTGGNNN
CANTGNNTGNCNATCTCTNGNTTNGNNNNNNTNNNNNTNNCANNNNGGNNNNNNNNANNNCNATNTAA
NNTANANNNNCNNNNNNANNNCACNNNNNNNNNAGNNNCNNNNNTTANANNNGTAGNNNAATNAGTCN
CCGANNTNNNNNNNATAAGCGNNANACNNNNNNNNNNNNNNANGANNNCGNNTTGGNNNTNNNNNNCNA
GNNAGACNNTNATGGNTNNNNNTCAANNNNTTNGTAGTGGTATAATNGNNNNNNNTTTCNCCAGNNTN
NNNGNNNNNNNCNATANNANNNNNNNGNGANNNNNNNNNNANNNNCTCNNNAGGGANCANCTACTNNA
GCANNGGNGCGNAGNNNGAGANNNNGACNNNCCTTGGGTGGCAANNACNGNAATAAGTNNNGTTNNNN
CTNNNAGTNNACATNNNNNCANNGNNNCNNNNNNANCNGANNGNNNNNNNNNNNNNNNNNNNNNNNNN
NNNNNNNNNNNNNNNNNNNNNNNNNNNNNNNNNNNANGGNNNGTNTNTGACNCNNGTGTTNNTANNNGNN
NNNNNNNGNNNNANCCNNGGTCGNNNNCNNATTGAANNCCTGNNGCATNNNNNTNCNAGAATGANNNC
NNNNNGNNTNNNCTNNCNNNTCAAGNAATCNNNNAGGTNNNNNGNTCNNNNNNNNNNNNCNNNNNCNN
ACTGAGGGTNNNNNNNNCATNNGCTNNNNNNNNNTNCGCNGGCGTNNCNNNNNNGCNGTATNGNGTNN
NNCANNNNNNNNNNNNNNNNNTNAAACAANNNNNNNTNNNN
>Dao657
GTGNNNNNTAGCCCNNNNNAGTTAGACANNNCNNNNNNNNNNNNNTNNNNNNNNNNNNNNNNNNNNNNN
NNNNNNNNNNNNNNNNNNNNNNNNNNNNNNNNNNNNNNNNNNNNNNNNNNNNNNNNNNNNNNNNNNNNNN
NNNNNNNNNNNNNNNNNNNNNNNNNNNNNNNNNNNNNNNNNNNNNNNNNNNNNNNNNNNNNNNNNCNNNN
NNNNNNNNNNNNNNNNNNNNNNNNNNNNNNNNNNNNNNNNNNNNNNNNNNNNNNNNNNNNNNNNNNNNNN
NNNNNNNNNNNNNNNNNNNNNNNNNNNNNNNNNNNNNNNNNNNNNNNNNNNNCNTCTNNNAAANNNTNNN
NNNNNNNNNNNNNNNNNNNNNNNNNNCNCNNTGNNNGTAANCNANNNGNACATGCAGTCCCCTCNNNNN
NNNNNCAGAGNNNNNNCCCNNNNNCNCCGNCCCCNNCCNNNNNNNTNNNCNNNGNNNNNNNANNNNNN
NNNNNNTGGTACNNNNCGNNNNNGGTTGNNTAAGAGGTCCACAACGNGNTCNNGNCACNNNNGNNNAN
NNNCNNNNAACNNNNNNNNNNNNANNTNNNTANNAGGNCNNNNCCNTCNNGNNNNNAANTGCNATGACC
CAANTCACGGGGGCAGTCCCCGGCGNGGCGACCTTGANNANGTCCGGGGACAAGTATGGCGNNCNCCC
CGGGAAGGANNCNGCCGTANGTGCNNNNNANNNNNTCNTGNGANGTNGCCGNNANNNNCGTGGTCNNN
NNAGAATGNCCGNACCANNCGNANNNTGGTNATGNNAGTANNNNNNNNNNNNNNNNNNNNNNNNNNNNN
NNNNNNNNNNNNNNNNNNNNNNNNNNNNNNNNNNGNNNNGNNNNNNCNANNNAAGGANNTANNNCTNNTN
NTNNNNNNNGGACNNNACNNNNCNNNNNNNACNCNNNCCCNNNNNNANNNGGTTTTNNNNCNNTATTNT
CCAGNACGCNGNGCNNNCATTCANGNGAACNNNGNNACGCGNNAATCGTANNATCTANCTGCCGCNNG
TNNNNNTACNGNNNNTNCGGANNNTNNCTCGTANNNNNNNCATGCGTNNTNTCNTCNCNAGNCANGTT
CGNNNANANANTAGNANGTNTNNNNNNNNNTGTNNCGTTCCGCNTGANNCNNCANNNNGNNGNNCTTN
TCANCGGCNNTANCTGCNGANNTAAGNCATANNNTNNNTCNNTANCTNNNNNGGNGGGNNACTGTAAA
NGGNNAATTNGANGNTNATTTCNNNCACNTNNCTNNGNNTNCTNNNTNCCTGNNTNCNNAATTGGNNC
CANTGNNTGNCNATCTCTGGNTTNGNNNNNNTNNNNNTNNCAANNNGGNNNNNNNNANNNCNATNTAA
NNTANANNNNCNNNNNNANNNCACNANNNNNGNAGNNNCNANNNTTANANNNGTAGNNNAATAAGTCT
CCGANNTNNNNNNNATAAGCGNNANACNNNNNNNNNNNNNNANGANNACGNNTTGGNNNTNNNNNNCNA
GNNAGACNNTNATGGNTNNNNNTCAANNNNTTCGTAGTGGTATAATNGNNNNNNNTTTCNCCAGNNTN
NNNGNNNNNNNCNATANNANNNNNNNGTGANNNNNNNNNNANNNNCTCNNNAGGGANCANCTACTTNA
GCAANGGNGCGNAGNNNGAGANNNNGACNNNCCTTGGGTGGCAANCACNGCGATAAGTNNNGTTNANN
CTNNNAGTNNACATNNNNNCANNGNNNCNNNNNNANCNGANNGNNNNNNNNNNNNNNNNNNNNNNNNN
NNNNNNNNNNNNNNNNNNNNNNNNNNNNNAGNNNANNGNNNGTNTNTGACNCATGTGTTTTTANNNGNN
NNNNNACGNNNGANCCCCGGTCGNNNNCNNATTGAANNCCTGNNGCATNNNNNTNCNAGAATGANNNC
NNNNNGNNTNNNCTNNCNNNTCAAGNAATCNNNTANGNANNGCGNTCNNNGNNNNNNNNCNNTCCCNN
ACTGAGGGTNNNNNNNNCATCTGCTNNNNNNNNNTTCGCNGGCGTNNCNNNNNNGCNGTATNGTGTNA
NNCANNNNNNNNNNNNNNNNNTNAAACNANNNNNNNTNNNN
>Dao658
GTGNNNNNTAGCCCNNNNNAGTCAGACANNNCNNNNNNNNNNNNNTNNNNNNNNNNNNNNNNNNNNNNN
NNNNNNNNNNNNNNNNNNNNNNNNNNNNNNNTNNNNNNNNNNNNNNNNNNNNNNNNNNNNNNNNNNNNNN
NNNNNNNNNNNNNNNNNNNNNNNNNNNNNNNNNNNNNNNNNNNNNNNNNNNNNNNNNNNNNNNNNNNNNN
NNNNNNNNNNNNNNNNNNNNNNNNNNNNNNNNNNNNNNNNNNNNNNNNNNNNNNNNNNNNNNNNNNNNNN
NNNNNNNNNNNNNNNNNNNNNNNNNNNNNNNNNNNNNNNNNNNNNNNNNNNNTCTNNNAAANNNTNNN
NNNNNNNNNNNNNNNNNNNNNNNNNNCNCNTTGNNNGTAANCNANNNGNACATGCAGTCCCCTCNNNNN
NNNNNCAGAGNNNNNNNCCNNNNNNNCCGGCCCCNNCCNNNNNNNNNNNNNNNNGNNNNNNNANNNNNN

NNNNNNTGGTACNNNNCGNNNNNGGTTGNTTNAGAGGTCCACAACGNGGTCNGNNCACNNNNGNNNAN
NNNCNNNNNNNNNNNNNNNNNNNNNTNNNTANNAGGNNNNNNCCNTCNNNNNNNNAANTGCNATGACC
CAANNCNCGGGGGCNGTNCCCGGCGNGGCGACCTTGANNANNTCCGGGNNCAAGTATGGCNNNCNCCC
CGGGAAGGANNCNGCCGTANGTGCNNNNNANNNNNTCNTGNGANGTNGCCGNNATTNNCGTGGTCNNN
CNAGAATGNNCNNACCANNCGNANNNTGGTNATGNNAGTNNNNNNNNNNNNNNNNNNNNNNNNNNNNN
NNNNNNNNNNNNNNNNNNNNNNNNNNNNNNNNNGNNNNGNNNNNNCNANNNAAGGNNNTANNNCTNNTN
NTNNNNNNGGACNNNACNNNNCNNNNNNNACNCNNNCNCNNNNNNANNNGNTTNNNNNNCNNTANTNN
CCAGNACGCNGNGCNNNCNTTTANGNGAACNNNGNNACGCGNNAATCGTANNATCTACCTGCCGCNNG
NNNNNNTACNGNNNNNNCGGANNNTNNCTNGTANNNNNNNCATGCGTNNNNTNNTCNCNAGNCANGTT
CGNNNANANANTAGNANGTNTNNNNNNNNNNTGTNNCGTTCCGCNTGANNCNNCANNNNGNNGNNNTTN
TCANCGGCNNTANCTGCNGANNTAAGNCATANNNTNNNTCNNTANCTNNNNNGGNGGGNNACTGTAAA
NGGNTAATTNGNNNNTNANTNCNNNNATNTNNCNNNGNNNNCTNNNTNCCTGNNTNCNNAATTGGNNN
CANTGNNTGNCNATCTCTGGNTTNGNNNNNNTGNNNNTNNCANNNNGGNNNGTNNNANNNCNATNTAA
NNTANANNNNCNNNNNNANNNCACNNNNNNNGNAGNNNNNANNNGTANANNNGTAGNNNNNTAAGTCT
CCGANNTNNNNNNNATAAGCGNNANACNNNNNNNNNNNNNNANGANNACGNNTTTGNNNTANNNNNNCNA
GNNAGACNNTNGTGGNTNNNNNTCAANNNNTTNGTAGTGGTATAATNGNNNNNNNTTTCNCCAGNNTN
NNNGNNNNNNNNCNATANNANNNNNNNGNGANNNNNNNNNNNANNNNCTCNNNAGGGANCANCTACTNNA
GCAATGGNGCGNAGNNNGAGANNNNGACNNGCCTTGGGTGGCAANCACNGCAATAAGTNNNGTTNANC
CTNNNAGTNNACACNNNNNCANNGNNNCNNNNNNANCNGANNGNNNNNNNNNNNNNNNNNNNNNNNN
NNNNNNNNNNNNNNNNNNNNNNNNNNNNNNNGNNNANGGNNNGTNTNTGACNCATGTGTTNNTANNNGNN
NNNNNACGNNNGANCCCCGGTCGNNNNCNNATTGAANNCCTGNNGCATNNNNNTNCNAGAATGANNGC
NNNNNGNNTNGNCTNNCNNNTCAAGNAATCANNNAGGNNNNNNGNTCNNNNNNNNNNNNNCNNNCCCNN
ACTGAGGGTNNNNNNNNCATCTGCTNNNNNNNNNNTNCGCNGGCGTNNCNNNNNNGCNGTATNNNGTNN
NNCANNNNNNNNNNNNNNNNNNTNAAAANANNNNNNNTNNNN
>Dao659
GTGNNNNNTAGCCCNNNNNAGTTAGACANNNCNNNNNNNNNNNNNTNNNNNNNNNNNNNNNNNNNNNNNN
NNNNNNNNNNNNNNNNNNNNNNNNNNNNNNNNTNNNNNNNNNNNNNNNNNNNNNNNNNNNNNNNNNNNNN
NNNNNNNNNNNNNNNNNNNNNNNNNNNNNNNNNNNNNNNNNNNNNNNNNNNNNNNNNNNNNNNNNNNNNN
NNNNNNNNNNNNNNNNNNNNNNNNNNNNNNNNNNNNNNNNNNNNNNNNNNNNNNNNNNNNNNNNNNNNNN
NNNNNNNNNNNNNNNNNNNNNNNNNNNNNNNNNNNNNNNNNNNNNNNNNNNNNNNCNTCTNNNAAANNNTNNN
NNNNNNNNNNNNNNNNNNNNNNNNNNNNNCNCNTTGNNNGTAANCNANNNGNACATGCAGTCCCCTCNNNNN
NNNGGCAGAGNNNNNNNCNNNNNNNNNCCGNCCCCNNCCNNNNNNNNNNNNNNNNNNNNNNNNNNNANNNNNN
NNNNNNTGGTACNNNNCGNNNNNGGTTGTNTAAGNGGTCCACAACGNGGTCNNGNNACNNNNGNNNAN
NNNCNNNAAANNNNNNNNNNNNNNNNNATNNNTANNAGGNNNNNNCCNTCNNNNNNNNNNANTGCNATGACC
CAANNCACGGGGGNAGTNCCCGGCGNGGCGACCTTGANNACGTCCGGGGACAAGTATGGCNNNCNCCC
CGGGAAGGANNCNGCCGTANGTGCNNNNNANNNNNTCNTGNGANGTCGCCGNNATTNNNGTGGTCNNN
CNAGAATGNCCNNACCANNCGNANNNTGGTNATGNNAGTNNNNNNNNNNNNNNNNNNNNNNNNNNNNN
NNNNNNNNNNNNNNNNNNNNNNNNNNNNNNNNNGNNNNGNNNNNNCNANNNAAGGANNTANNNCTNNTN
NTNNNNNNGGACNNNACNNNNCNNNNNNNACNCNNNCNCNNNNNNNNNNGGTTNNNNNCCNNTNNTNT
CCAGNACGCNGNGCNNNCATTCANGNGAACNNNGNNACGCGNNAATCGTANNATCTANCTGCCGCNNG
NNNNNNTNCNGNNNNNNCGGANNNTNNCTCGTANNNNNNNCANGCGTNNTNTNNTCNCNAGNCNNGTT
CGNNNANANANTAGNANGTNTNNNNNNNNNNTGTNNCGTTCCGCNTGANNCNNCNNNNNGNCGNNCTTN
NCANCGGCNNTANCTNCNGANNTAAGNCATANNNTNNNTCNNTANCTNNNNNGGNGGGNNACTGTAAA
NGGNNAATTNGNNNNTNANTNCNNNCACNTNNCNNNGNNTNCTNNNTNCCTGNNTNCNNAATTGGNNN
CANTGNNTGNCNATCTCTGGNTTNGNNNNNNTNNNNNTNNCANNNNGGNNNGTNNNANNNCNATNTAA
NNTANANNNNCNNNNNNANNNCACNNNNNNNGNAGNNNCNANNNTTANANNNGTAGNNNANTNAGTCT
CCGANNTNNNNNNNATAAGCGNNANACNNNNNNNNNNNNNNANGANNNCGNNTTGGNNNTNNNNNNCNA
GNNAGACNNTNATGGNTNNNNNTCAANNNNTTNGTAGTGGTATAATNGNNNNNNNTTTCNCCAGNNTN
NNNGNNNNNNNNCNATANNANNNNNNNGNGANNNNNNNNNNNANNNNCTCNNNAGGGANCANCTACTNNA
GCANTGGNGCGNAGNNNGAGANNNNGACNNNCCTTGGGTGGCAANCACNGCGATAAGTNNNGTTNANC
CTNNNAGTNNACATNNNNNCANNGNNNCNNNNNNANCNGANNGNNNNNNNNNNNNNNNNNNNNNNNN
NNNNNNNNNNNNNNNNNNNNNNNNNNNNNAGNNNANGGNNNGTNTNTGACNCATGTGTTTTTANNNGNN
NNNNNACGNNNGANCCCCGGTCGNNNNCNNATTGAANNCCTGNNGCATNNNNNTNCNAGAATGANNNC
NNNNNGNNTNGNCTNNNNNNNTCAAGNAATCAANNAGGTNNNNNNNTCNNNGNNNNNNNNCNNTCCCNN

ACTGAGGGTNNNNNNNNCATCTGCTNNNNNNNNNNNTNCGCNGGNGTNNCNNNNNNGCNGTATNNNGTNA
NNCANNNNNNNNNNNNNNNNNNAAACNANNNNNNNTNNNN
>Dao660
GTGNNNNNTAGCCCNNNNNAGTTAGACANNNCNNNNNNNNNNNNNTNNNNNNNNNNNNNNNNNNNNNNNN
NNNNNNNNNNNNNNNNNNNNNNNNNNNNNNNTNNNNNNNNNNNNNNNNNNNNNNNNNNNNNNNNNNNNNN
NNNNNNNNNNNNNNNNNNNNNNNNNNNNNNNNNNNNNNNNNNNNNNNNNNNNNNNNNNNNNNNCNNNNNN
NNNNNNNNNNNNNNNNNNNNNNNNNNNNNNNNNNNNNNNNNNNNNNNNNNNNNNNNNNNNNNNNNNNNNN
NNNNNNNNNNNNNNNNNNNNNNNNNNNNNNNNNNNNNNNNNNNNNNNNNNNNCNTCTNNNAAANNNTNNN
NNNNNNNNNNNNNNNNNNNNNNNNNNNCNCNTTGNNNGTAANCNANNNGNACATGCAGTCCCCTCNNNNN
NNNGGCAGAGNNNNNNNCCNNNNNCNCCGNCCCCNNCCNNNNNNNNNNNNNNNNNGNNNNNNNANNNNNN
NNNNNNTGGTACNNNNCGNNNNNNGGTTGNNTAAGNGGTCCACAACGNGNTCNNGNNACNNNNNGNNTAN
NNNCNNNNAANNNNNNNNNNNNNNANNTNNNTANNAGGNCNNNNCCNTNNNNNNNNNAANTGCNATGACC
CANNNNNCGGGGGCNGTNCCCGGNGNGGCGACCTTGANNANGTCCGGGNNCAAGTATGGCNNNCNCCC
CGGGAAGGANNCNGCNGTANGTGCNNNNNANNNNNTCNTGNGANNTCGCCGNNATTCNNGTGGTCNNN
CNAGAATGNCCNNACCANNCGNANNNTGGTNATGNNAGTNNNNNNNNNNNNNNNNNNNNNNNNNNNNNNN
NNNNNNNNNNNNNNNNNNNNNNNNNNNNNNNNNNGNNNNGNNNNNNCNANNNAAGGNNNTANNNCTNNTN
NTNNNNNNGGACNNNACNNNNCNNNNNNNACNCNNNCNCNNNNNNANNNGNTTNNNNNNCNNTANTNT
CCAGNACGCNGNGCNNNCNTTCANGNGAACNNNGNNACGCGNNAATCGTANNATCTACCTGCCGCNNG
TNNNNNTNCNGNANNNNCGGANNNTNNNNCGTANNNNNNTCANGCGTNNNNTNNTNNCNAGNCNNGTT
CGNNNANANANTAGNANGTNTNNNNNNNNNNTGTNNCGTTCCGCNTGANNCNNCNNNNNGNNGNNNTTN
NCANCGGCNNTANCTGCNGANNTAAGNCATANNNTNNNTCNNTANCTNNNNNGGNGGGNNACTGTAAA
NGGNNAATTNGNNNNTNANTNCNNNCACNTNNCNNNGNNNNCTNNNTNCCTGNNTNCNNAATTGGNNC
CANTGNNTGNCNATCTCTGGNTTNGNNNNNNTNNNNNTNNCANNNNGGNNNGNNANANNNCNATNTAA
NNTANANNNNCNNNNNNANNNCACNNNNNNNGNAGNNNCNANNNTNANANNNGTAGNNNANTAAGTCT
CCGANNTNNNNNNNATAAGCGNNANACNNNNNNNNNNNNNNNANGANNNCGNNTTGGNNNTNNNNNNNNA
GNNAGACNNTNATGGNTNNNNNTCAANNNNTTNGTAGTGGTATAATNGNNNNNNNTTTCNCCAGNNTN
NNNGNNNNNNNCNATANNANNNNNNNGNGANNNNNNNNNNANNNNCTCNNNAGGGANCANCTACTNNA
GCANTGGNGCGNAGNNNGAGANNNNGACNNNCCTTGGGTGGCAANNACNGCAATAAGTNNNGTTNNNC
CTNNNAGTNNACATNNNNNCANNGNNNCNNNNNNANCNGANNGNNNNNNNNNNNNNNNNNNNNNNNNN
NNNNNNNNNNNNNNNNNNNNNNNNNNNNNNANNNNANGGNNNGTNTNTGNCNCATGTGTTTTTANNNGNN
NNNNNNNGNNNGANCCCCGGTCGNNNNCNNATTGAANNCCTGNNGCATNNNNNNTNCNAGAATGANNNC
NNNNNGNNTNGNCTNNCNNNTCAAGNAATNANNNAGGTNNNNNGNTCNNNGNNNNNNNNCNNTCCCNN
ACTGAGGGTNNNNNNNNCATNNGCTNNNNNNNNNNTNCGCNGGNGTNNCNNCNNAGCNGTATNGNGTNN
NNCANNNNNNNNNNNNNNNNNNNTNAAACGANNNNNNNTNNNN
>Dao661
GTGNNNNNNAGCCCNNNNNAGTCAGACANNNCNNNNNNNNNNNNNTNNNNNNNNNNNNNNNNNNNNNNNN
NNNNNNNNNNNNNNNNNNNNNNNNNNNNNNNNNNNNNNNNNNNNNNNNNNNNNNNNNNNNNNNNNNNNNN
NNNNNNNNNNNNNNNNNNNNNNNNNNNNNNNNNNNNNNNNNNNNNNNNNNNNNNNNNNNNNNNNNNNNNN
NNNNNNNNNNNNNNNNNNNNNNNNNNNNNNNNNNNNNNNNNNNNNNNNNNNNNNNNNNNNNNNNNNNNNN
NNNNNNNNNNNNNNNNNNNNNNNNNNNNNNNNNNNNNNNNNNNNNNNNNNNNNTCTNNNAAANNNTNNN
NNNNNNNNNNNNNNNNNNNNNNNNNNNCNCNTTGNNNGTAANCNANNNGNACATGCAGTCCCCTCNNNNN
NNNNNCAGAGNNNNNNNCCNNNNNNCNCCGGCCCCNNCCNNNNNNNNNNNNCNNNGNNNNNNNANNNNNN
NNNNNNTGGTACNNNNCGNNNNNGGTTGTNTNAGAGGTCCACAACGNGGTCNGGNNACNNNNGNNNAN
NNNCNNNNAANNNNNNNNNNNNNNNNNTNNNTANNAGGNNNNNNCCNTCNNGNNNNNAATTGCNATGACC
CANNNCNCGGNGGCNGTNCCCGGCGCGGCGACCTTGANNACGTCCGGGGNCAAGTATGGCNNNCNCCC
CGGGAAGGANNCNGCNGTANGTGCNNNNNANNNNNTCNTGNGANGTCGCCGNNATTCNNGTGGTCNNN
NNAGAATGNNCNNACCANNCGNANNNTGGTNATGNNAGTANNNNNNNNNNNNNNNNNNNNNNNNNNNNNN
NNNNNNNNNNNNNNNNNNNNNNNNNNNNNNNNNNGNNNNGNNNNNNCNANNNAAGGNNNTANNNCTNNTN
NTNNNNNNGGACNNNACNNNNCNNNNNNNACNCNNNCNCNNNNNNNNNNGGTTTTNNNNCNNTANTNN
CCAGNACGCNGNGCNNNCATTCANGNGAACNNNGNNACGCGNNAATCGTANNATCTACCTGCCGCNNG
NNNNNNTACNGNNNNNNCGGANNNTNNNNCGTANNNNNNTCATGCGTNNTNTNNTCNCNAGNCANGTT
CGNNNANANANTAGNANGTNTNNNNNNNNNNTGTNNCGTTCCGCNTGANNCNNCANNNNGNNGNNCTTN
TCANCGGCNNTANCTGCNGANNTAAGNCATANNNTNNNTCNNTANCTNNNNNGGNGGGNNACTGTAAA
NGGNNAATTNGNNGNTNATTNCNNNCATNTNNCTNNGNNNNCTNNNTNCCTGNNTNCNNAATTGGNNC

CANTGNNTGNCNATCTCTNGNTTNGNNNNNNTNNNNNTNNCANNNNGGNNNNNNNNANNNCNATNTAA
NNTANANNNNCNNNNNNANNNCACNNNNNNNNNAGNNNCNANNNGTANANNNGTAGNNNGNTAAGTCN
CCGANNTNNNNNNNATAAGCGNNANACNNNNNNNNNNNNNANGANNNCGNNTTTGNNNTNNNNNNNNA
GNNAGACNNTNGTGGNTNNNNNTCAANNNNTTNGTAGTGGTATAATNGNNNNNNNTTTCNCCAGNNTN
NNNGNNNNNNNCNATANNANNNNNNNGNGANNNNNNNNNNNANNNNCTCNNNAGGNANCANCTACTNNA
GCANTGGNGCGCAGNNNGAGANNNNGACNNNCCTTGGGTGGCAANNACNGNAATAAGTNNNGTTNANC
CTNNNAGTNNACACNNNNNCANNGNNNCNNNNNNANCNGANNGNNNNNNNNNNNNNNNNNNNNNNNNN
NNNNNNNNNNNNNNNNNNNNNNNNNNNNNNNNNNANGGNNNGTNTNTGACNCATGTGTTTTTANNNGNN
NNNNNACGNNNNANCCCCGGTCGNNNNCNNATTGAANNCCTGNNGCATNNNNNTNCNAGAATGANNNC
NNNNNGNNTNGNCTNNCNNNTCAAGNAATCNNNNAGNTANNNNGNTCNNNGNNNNNNNNCNNTCCCNN
ACTGAGGGTNNNNNNNNCATNNGCTNNNNNNNNCTNCGCNGGNGTNNCNNNNNNGCNGTATNGNGTNN
NNCANNNNNNNNNNNNNNNNNTNAAACAANNNNNCTNNNN
>Dao662
GTGNNNNNNAGCCCNNNNNAGTTAGACANNNCNNNNNNNNNNNTTNNNNNNNNNNNNNNNNNNNNNNN
NNNNNNNNNNNNNNNNNNNNNNNNNNNNNNTNNNNNNNNNNNNNNNNNNNNNNNNNNNNNNNNNNNNNNN
NNNNNNNNNNNNNNNNNNNNNNNNNNNNNNNNNNNNNNNNNNNNNNNNNNNNNNNNNNNNNNNNNNNNNN
NNNNNNNNNNNNNNNNNNNNNNNNNNNNNNNNNNNNNNNNNNNNNNNNNNNNNNNNNNNNNNNNNNNNNN
NNNNNNNNNNNNNNNNNNNNNNNNNNNNNNNNNNNNNNNNNNNNNNNNNNNNNCNTCTNNNAAANNNTNNN
NNNNNNNNNNNNNNNNNNNNNNNNNNCNCNTTGNNNGTAANCNANNNGNACATGCAGTCCCCTCNNNNN
NNNGGCAGAGNNNNNNNCCNNNNNNNCCGNCCCCNNCCNNNNNNNNNNNNNNNNGNNNNNNNANNNNNN
NNNNNNTGGTACNNNNCGNNNNNGGTTGNNTAAGAGGTCCACAACGNGGTCNNGNNACNNNNGNNNAN
NNNCNNNNAACNNNNNNNNNNNNANNTNNNTANNAGGNCNNNNCCNTCNNGNNNNTNANTGCNATGACC
CAANNNNCGGNGGNNGTNCCCGGCGNGGCGACCTTGANNANGTCCGGGGNCAAGTATGGCNNNCNCCC
CGGGAAGGANNCNGCCGTANGTGCNNNNNANNNNNTCNTGNGANGTCGCCGNNATTNNCGTGGTCNNN
CNAGAATGNNCNNACCANNCGNANNNTGGTNATGNNAGTNNNNNNNNNNNNNNNNNNNNNNNNNNNNN
NNNNNNNNNNNNNNNNNNNNNNNNNNNNNNNNNNNGNNNNGNNNNNNCNANNNAAGGNNNTANNNCTNNTN
NNNNNNNNGGACNNNACNNNNCNNNNNNNACNCNNNCNCNNNNNNANNNGGTTTTNNNNCNNTANTNT
CCAGNANGCNGNGCNNNCNTTCANGNGAACNNNGNNACGCGNNAATCGTANNATCTACCTGCCGCNNG
NNNNNNTACNGNNNNNNNCGGANNNTNNNNNGTANNNNNNNCATGCGTNNTNTNNTCNCNAGNCNNGTT
CGNNNANANANTAGNANGTNTNNNNNNNNNNTGTNNCGTTCCGCNTGANNCNNCANNNNGNNGNNCTTN
NCANCGGCNNTANCTGCNGANNTAAGNCATANNNTNNNTCNNTANCTNNNNNGGNGGGNNACTGTAAA
NGGNNAATTNGNNNNTNATTNCNNNCACNTNNCNNNGNNTNCTNNNTNCCTGNNTNCNNAATTGGNNC
CANTGNNTGNCNATCTCTNGNTTNGNNNNNNTNNNNNTNNCANNNNGGNNNGTNANANNNCNATNTAA
NNTANANNNNCNNNNNNANNNCACNNNNNNNNGNAGNNNCNANNNTNANANNNGTAGNNNANTNAGTCT
CCGANNTNNNNNNNATAAGCGNNANACNNNNNNNNNNNNNANGANNNCGNNTTGGNNNTNNNNNNNCNA
GNNAGACNNTNATGGNTNNNNNTCAANNNNTTCGTAGTGGTATAATNGNNNNNNNTTTCNCCAGNNTN
NNNGNNNNNNNCNATANNANNNNNNNGNGANNNNNNNNNNNANNNNCTCNNNAGGGANCANCTACTNNA
GCANTGGNGCGNAGNNNGAGANNNNGACNNNCCTTGGGTGGCAANNACNGCAATAAGTNNNGTTNNNC
CTNNNAGTNNACATNNNNNCANNGNNNCNNNNNNANCNGANNGNNNNNNNNNNNNNNNNNNNNNNNNN
NNNNNNNNNNNNNNNNNNNNNNNNNNNNNNNNNNANGGNNNGTNTNTGACNCNTGTGTTTTTANNNGNN
NNNNNNNGNNNNANCCCCGGTCGNNTNCNNATTGAANNCCTGNNGCATNNNNNTNCNAGAATGANNNC
NNNNNGNNTNGNCTNNCNNNTCAAGNAATCNNNNAGNNNNNNNGNTCNNNNNNNNNNNNNCNNNCCCNN
ACTGAGGGTNNNNNNNNCATCNGCTNNNNNNNNNNTNCGCNGGCGTNNCNNNNNNGCNGTATNGNGTNA
NNCANNNNNNNNNNNNNNNNNTNAAACAANNNNNNNTNNNN
>Dao663
GTGNNNNNTAGCCCNNNNNAGTTAGACANNNCNNNNNNNNNNNNTNNNNNNNNNNNNNNNNNNNNNNN
NNNNNNNNNNNNNNNNNNNNNNNNNNNNNNNNNNNNNNNNNNNNNNNNNNNNNNNNNNNNNNNNNNNNNN
NNNNNNNNNNNNNNNNNNNNNNNNNNNNNNNNNNNNNNNNNNNNNNNNNNNNNNNNNNNNNNNNNNNNNN
NNNNNNNNNNNNNNNNNNNNNNNNNNNNNNNNNNNNNNNNNNNNNNNNNNNNNNNNNNNNNNNNNNNNNN
NNNNNNNNNNNNNNNNNNNNNNNNNNNNNNNNNNNNNNNNNNNNNNNNNNNNNCNTCTNNNAAANNNTNNN
NNNNNNNNNNNNNNNNNNNNNNNNNNCNCNTTGNNNGTAANCNANNNGNACATGCAGTNNCCTCNNNNN
NNNNNCAGAGNNNNNNNCCNNNNNNNCCGGCCCCNNCCNNNNNNNNNNNNNNNNNNNNNNNNNNANNNNNN
NNNNNNTGGTACNNNNCGNNNNNGGTTGNNTAAGAGGTCCACAACGNGGTCNNGNCACNNNNGNNNAN
NNNCNNNNANCNNNNNNNNNNNNNANNTNNNTANNAGGNNNNNNCCNTCNNNNNNNTAATTGCNATGACC

CAANNCACGGGGGNNGTNCCCGGCGCGGCGACCTTGNNNANGTCCGGGGACAAGTATGGCGNNCNCCC
CGGGAAGGANNCNGCCGTANGTGCNNNNNANNNNNTCNTGNGANGTCGCCGNNATTNNCGTGGTCNNN
CNAGAATGNCCGNACCANNCGNANNNTGGTNATGNTAGTNNNNNNNNNNNNNNNNNNNNNNNNNNNNN
NNNNNNNNNNNNNNNNNNNNNNNNNNNNNNNGNNNNGNNNNNNCNANNNNAAGGNNNTANNNCTNNTN
NTNNNNNNGGACNNNACNNNNCNNNNNNNACNCNNNCNCNNNNNNNNNNGNTTTTTNNNCNNTATTNT
CCAGNANGCNGNGCNNNCNTTCANGNGAACNNNGNNACGCGNNAATCGTANNATCTACCTGCCGCNNG
TNNNNNTACNGNNNNNNCGGANNNTNNNNCGTANNNNNNTCANGCGTNNTNTNNTCNCNAGNCNNGTT
CGNNNANANANTAGNANGTNTCNNNNNNCTTGTNNCGTTCCGCNTGANNCNNCANNNNGNCGNNCTTN
NCANCGGCNNTANCTGCNGANNTAAGNCATANNNTNNNTCNNTANCTNNNNNGGNGGGNNACTGTAAA
NGGNTAATTNGANNNTNANTNCNNNCACNTNNCTNNGNNTNCTNNNTNCCTGNNTNCNNAATTGGNNC
CANTGNNTGNCNATCTCTGGNTTNGNNNNNNTNNNNNTNNCAANNNGGNNNGTNNNANNNCNATNTAA
NNTANANNNNCNNNNNNANNNCACNNNNNNNGNAGNNNCNANNNTTANANNNGTAGNNNANTAAGTCT
CCGANNTNNNNNNNATAAGCGNNANACNNNNNNNNNNNNNANGANNACGNNTTGGNNNTNNNNNNCNA
GNNAGACNNTNATGGNTNNNNNTCAANNNNTTCGTAGTGGTATAATNGNNNNNNNTTTCNCCAGNNTN
NNNGNNNNNNNCNATANNANNNNNNNGNGANNNNNNNNNNANNNNCTCNNNAGGGANCANCTACTNNA
GCANTGGNGCGCAGNNNGAGANNNNGACNNGCCTTGGGTGGCANNCACNGCAATAAGTNNNGTTNANN
NTNNNAGTNNACATGNNNNCANNGNNNCNNNNNNANCNGANNGNNNNNNNNNNNNNNNNNNNNNNNNN
NNNNNNNNNNNNNNNNNNNNNNNNNNNNAGNNNANGGNNNGTNTNTGACNCATGTGTTTNTANNNGNN
NNNNNACGNNNNANCCCCGGTCGNNNNCNNATTGAANNCCTGNNGCATNNNNNTNCNAGAATGANNNC
NNNNNGNNTNGNCTNNCNNNTCAAGNAATCANNNAGGTNNNNNNNTCNNNGNNNNNNNNCNNTCCCNN
ACTGAGGGTNNNNNNNNCANCTGCTNNNNNNNNNNTNCGCNGGNGTNNCNNNNNAGCNGTATNGNGTNN
NNCANNNNNNNNNNNNNNNNTNAAACNANNNNNTTNNNN
>Ede102
GTGNNNNNTAGCCCNNNNNAGTTAGACANNNCNNNNNNNNNNNNTNNNNNNNNNNNNNNNNNNNNNNN
NNNNNNNNNNNNNNNNNNNNNNNNNNNNNNTNNNNNNNNNNNNNNNNNNNNNNNNNNNNNNNNNNNNN
NNNNNNNNNNNNNNNNNNNNNNNNNNNNNNNNNNNNNNNNNNNNNNNNNNNNNNNNNNNNNNNNNNNN
NNNNNNNNNNNNNNNNNNNNNNNNNNNNNNNNNNNNNNNNNNNNNNNNNNNNNNNNNNNNNNNNNNNN
NNNNNNNNNNNNNNNNNNNNNNNNNNNNNNNNNNNNNNNNNNNNNNNNNNCNTCTNNNAAANNNTNNN
NNNNNNNNNNNNNNNNNNNNNNNNNNCNCNTTGNNNGTAANCANNNGNACATGCAGTCCCCTCNNNNN
NNNNNCAGAGNNNNNNCCCNNNNNNNCCGGCCCCGNCCNNNNNNNNNNNNNNNGNNNNNNANNNNNNN
NNNNNNTGGTACNNNNCGNNNNNGGTTGTNTAAGAGGTCCACAACGNGGTCNGGNCACNNNNGNNNAN
NNNCNNNNAACNNNNNNNNNNNANNTNNNTANNAGGNCNNNNCCNTCNNNNNNNTNATNGCNATGACC
CAANNCACGGGGGCAGTNCCCGGCGCGGCGACCTTGATNACGTCCGGGGACAAGTATGGCNCNCNCCC
CGGGAAGGANNCNGCCGTANGTGCNNNNNANNNNNTCNTGNGACGTCGCCGNNATTCNCGTGGTCTNN
CNAGAATGNCCGNACCANNCGNANNNTGGTNATGNNAGTNNNNNNNNNNNNNNNNNNNNNNNNNNNNN
NNNNNNNNNNNNNNNNNNNNNNNNNNNNNNNNNNNNGNNNNNNCNANNNNAAGGANNTANNNCTNNTC
NTNNNNNNGGACNNNACNNNNCNNNNNNNACNCNNNCNCNNNNNNANNNGGTTTTTNNNCNNTATTNT
CCAGNACGCNGNGCNNNCATTCANGNGAACNNNGNNACGCGNNAATCGTANNATCTACCTGCCGCNNG
NNNNNNTACNGNNNNTNCGGANNNTNNCTCGTANNNNNNTCATGCGTNNTNTNNTCNCNAGNCNNGTT
CGNNNANANANTAGNANGTNTNNNNNNNNNTGTNNCGTTCCGCNTGANNCNNCANNNNGNNGNNCTTN
TCANCGGCNNTANCTGCNGANNTAAGNCATANNNTNNATCNNTANCTNNNNNGGNGGGNNACTGTAAA
NGGNNAATTGGAGGNTNATTNCNNNCACNTNNCTNNGNNNNCTNNNTNCCTGNNTNCNNAATTGGNNC
CANTGNNTGNCNATCTCTGGNTTNGNNNNNNTNNNNNTNNCAANNNGGNNNGTNNNANNNCNATNTAA
NNTATANNNNCNNNNNNANNNCACNNNNNNNGNAGNNNCNANNNTTANANNNGTAGNNNAATAAGTCT
CCGANNTNNNNNNNATAAGCGNNANACNNNNNNNNNNNNNANGANNACGTNTTGGNNNTNNNNNNCNA
GNNAGACNNTNATGGNTNNNNNTCAANNNNTTNGTAGTGGTATAATNGNNNNNNNTTTCNCCAGNNTN
NNNGNNNNNNNCNATANNANNNNNNNGTGANNNNNNNNNNANNNNCTCNNNAGGGANCANCTACTTNA
GCANTGGNGCGCAGNNNGAGANNNNGACNNGCCTTGGGTGGCAANCACNGCGATAAGTNNNGTTNANC
CTNNNAGTNNACATNNNNNCANNGNNNCNNNNNNANCNGANNGNNNNNNNNNNNNNNNNNNNNNNNNN
NNNNNNNNNNNNNNNNNNNNNNNNNNNNAGNNNANGGNNNGTNTNTGACNCATGTGTTTTTANNNGNN
NNNNNACGNNNNANCCCCGGTCGTNNNCNNATTGAANNCCTGNNGCATNNNNNTNCNAGAATGANNNC
NNNNNGNNTNGNCTGNCNTTTCAAGNAATCAANNAGGTANNNNNNTCNNNNNNNNNNNNCNNTCCCNN
ACTGAGGGTNNNNNNCGCATCTGCTNNNNNNNNNNTNCGCNGNCGTNNCNNNNNAGCNGTATNGNGTTA
NNCANNNNNNNNNNNNNNNNTNNAACNANNNNCNTNNNN

>Ede112
GTGNNNNNTAGCCCGNNNNAGTTAGACANNNCNNNNNNNNNNNNTNNNNNNNNNNNNNNNNNNNNNNN
NNNNNNNNNNNNNNNNNNNNNNNNNNNNNNTNNNNNNNNNNNNNNNNNNNNNNNNNNNNNNNNNNNNNN
NNNNNNNNNNNNNNNNNNNNNNNNNNNNNNNNNNNNNNNNNNNNNNNNNNNNNNNNNNNNNNNNNNNN
NNNNNNNNNNNNNNNNNNNNNNNNNNNNNNNNNNNNNNNNNNNNNNNNNNNNNNNNNNNNNNNNNNNN
NNNNNNNNNNNNNNNNNNNNNNNNNNNNNNNNNNNNNNNNNNNNNNNNNNCNTCTNNNAAANNNTNNN
NNNNNNNNNNNNNNNNNNNNNNNNNCNCNTTGNNNGTAANCNANNNGNACATGCAGTCCCCTCNNNNN
NNNGGCAGAGNNNNNNCCCNNNNNCNCCGGCCCCNNCCNNNNNNNNNNNNNNNGNNNNNNNANNNNNN
NNNNNNTGGTACNNNNCGNNNNNGGTTGTTTAAGAGGTCCACAACGNGGTCNGGNCACNNNNGNNTAN
NNNCNNAAAANNNNNNNNNNNNANNTNNNTANNAGGNCNNNNCCNTCNNGNNNNNAATTGCNATGACC
CAANNCACGGGGGCAGTCCCCGGCGCGGCGACCTTGATNACGTCCGGGGACAAGTATGGCGNNCNCCC
CGGGAAGGANNCNGCCGTANGTGCNNNNNANNNNNTCNTGNGACGTCGCCGNNATTCNCGTGGTCNNN
CNAGAATGNCCGNACCANNCGNANNNTGGTNATGTNAGTNNNNNNNNNNNNNNNNNNNNNNNNNNNNN
NNNNNNNNNNNNNNNNNNNNNNNNNNNNNNNNGNNNNGNNNNNNCNANNNAAGGANNTANNNCTNNTN
NTNNNGNNGGACNNNACNNNNCNNNNNNNACNCNNNCNCNNNNNNANNNGNTTTTTNNNCNNTATTNT
CCAGNACGCNGNGCNNNCATTCANGNGAACNNNGNNACGCGNNAATCGTANNATCTACCTGCCGCNGG
TNNNNNTACNGNNNNNNCGGANNNTNNCTCGTANNNNNNTCATGCGTNNTNTNNTCNCNAGNCANGTT
CGNNNANANANTAGNANGTNTNNNNNNNCTTGTNGCGTTCCGCNTGACNCNNCANNNNGNNGNNCTTN
TCANCGGCNNTANCTGCNGANNTAAGNCATANNNTNNATCNNTANCTNNNNNGGNGGGNNACTGTAAA
NGGNNAATTNGNGGNTNATTTCNNNCACNTNNCTNNGNNTNCTNNNTNCCTGNNTNCNNAATTGGNNC
CANTGNNTGNCNATCTCTGGNTTNGCNNNNNTNNNNNTNNCANNNNGGNNNGTNANANNNCNATNTAA
NNTATANNNNCNNNNNNANNNCACNANNNNNGNAGNNNCNANNNTTANANNNGTAGNNNAATAAGTCT
CCGANNTNNNNNNNATAAGCGNNANACNNNNNNNNNNNNNANGANNACGNNTTGGNNNTNNNNNNCNA
GNNAGACNNTNATGGNTNNNNNTCAANNNNTTCGTAGTGGTATAATNGNNNNNNNTTTCNCCAGNNTN
NNNGNNNNNNNCNATANNANNNNNNNGNGANNNNNNNNNNANNNNCTCNNNAGGGANCANCTACTTNA
GCAATGGNGCGCAGNNNGAGANNNNGACNNGCCTTGGGTGGCAANCACNGCAATAAGTNNNGTTNANC
CTNNNAGTNNACATNNNNNCANNGNNNCNNNNNNANCNGANNGNNNNNNNNNNNNNNNNNNNNNNNNN
NNNNNNNNNNNNNNNNNNNNNNNNNNNNAGNNNANGGNNNGTNTNTGACNCATGTGTTTTTANNNGNN
NNNNNACGNNNGANCCCCGGTCGNNNNCNNATTGAANNCCTGNNGCATNNNNNTNCNAGAATGANNGC
NNNNNGNNTNGNCTNNCNTTTCAAGNAATCANNNAGNNANCGCGNTCNNNGNNNNNNNNCNNTCCCNN
ACTGAGGGTNNNNNNNNCATCTGCTNNNNNNNNNTNCGCNGGNGTNNCNNNNNAGCNGTATNGTGTNN
NNCANNNNNNNNNNNNNNNNTNAAACNANNNNNNTNNNN
>Ede123
GTGNNNNNTAGCCCNNNNNAGTTAGACANNNCNNNNNNNNNNNNTNNNNNNNNNNNNNNNNNNNNNNN
NNNNNNNNNNNNNNNNNNNNNNNNNNNNNNTNNNNNNNNNNNNNNNNNNNNNNNNNNNNNNNNNNNNNN
NNNNNNNNNNNNNNNNNNNNNNNNNNNNNNNNNNNNNNNNNNNNNNNNNNNNNNNNNNNNNNNNNNNN
NNNNNNNNNNNNNNNNNNNNNNNNNNNNNNNNNNNNNNNNNNNNNNNNNNNNNNNNNNNNNNNNNNNN
NNNNNNNNNNNNNNNNNNNNNNNNNNNNNNNNNNNNNNNNNNNNNNNNNNCNTCTNNNAAANNNTNNN
NNNNNNNNNNNNNNNNNNNNNNNNNCNCNTTGNNNGTAANCNANNNGNACATGCAGTCCCCTCNNNNN
NNNGGCAGAGNNNNNNCCCNNNNNNNCCGGCCCCNNCCNNNNNNNNNNNNNNNGNNNNNNNANNNNNN
NNNNNNTGGTACNNNNCGNNNNNGGTTGTTTAAGAGGTCCACAACGNGGTCNGGNCACNNNNGNNNAN
NNNCNNNNAACNNNNNNNNNNNANNTNNNTANAAGGNNNNNNCCNTCNNNNNNNNNATTGCNATGACC
CAANTCACGGGGGCAGTNCCCGGCGNGGCGACCTTGATNANGTCCGGGGACAAGTATGGCGNNCNCCC
CGGGAAGGANNCNGCCGTANGTGCNNNNNANNNNNTCNTGNGACGTCGCCGNNATTCNCGTGGTCNNN
CNAGAATGNCCGNACCANNCGNANNNTGGTNATGNNAGTNNNNNNNNNNNNNNNNNNNNNNNNNNNNN
NNNNNNNNNNNNNNNNNNNNNNNNNNNNNNNNNNNNNGNNNNNNCNANNNAAGGANNTANNNCTNNTN
NTNNNNNNGGACNNNACNNNNCNNNNNNNACNCNNNCNCNNNNNNANNNGNTTTTTNNCCNNTATTNT
CCAGNACGCNGNGCNNNCATTCANGNGAACNNNGNNACGCGNNAATCGTANNATCTACCTGCCGCNGG
TNNNNNTACNGNNNNNNCGGANNNTNNCTCGTANNNNNNTCATGCGTNNTNTNNTCNCNAGNCANGTT
CGNNNANANANTAGNANGTNTNNNNNNNNNTGTNGCGTTCCGCNTGANNCNNCANNNNGNNGNNNTTN
NCANCGGCNNTANCTGCNGANNTAAGNCATANNNTNNNTCNNTANCTNNNNNGGNGGGNNACTGTAAA
NGGNNAATTNGNNGNTNANTNCNNNCACNTNNCNNNGNNTNCTNNNTNCCTGNNTNCNNAATTGGNNC
CANTGNNTGNCNATCTCTGGNTTNGCNNNNNTNNNNNTNNCANNNNGGNNNGTNANANNNCNATNTAA
NNTANANNNNCNNNNNNANNNCACNANNNNNGNAGNNACNANNNTTANANNNGTAGNNNAATAAGTCT

CCGANNTNNNNNNNATAAGCGNNANACNNNNNNNNNNNNNNANGANNACGNNTTGGNNNTNNNNNNCNA
GNNAGACNNTNATGGNTNNNNNTCAANNNNTTNGTAGTGGTATAATNGNNNNNNNTTTCNCCAGNNTN
NNNGNNNNNNNCNATANNANNNNNNNGNGANNNNNNNNNNNANNNNCTCNNNAGGGANCANCTACTNNA
GCANTGGNGCGCAGNNNGAGANNNNGACNNGCCTTGGGTGGCAANCACNGCAATAAGTNNNGTTNANC
CTNNNAGTNNACATGNNNNCANNGNNNCNNNNNNANCNGANNGNNNNNNNNNNNNNNNNNNNNNNNNN
NNNNNNNNNNNNNNNNNNNNNNNNNNNNNAGNNNANGGNNNGTNTNTGACNCATGTGTTTTTANNNGNN
NNNNNACGNNNGANCCCCGGTCGNNNNCNNATTGAANNCCTGNNGCATNNNNNTNCNAGAATGANNGC
NNNNNGNNTNGNCTNNCNTTTCAAGNAATCANNNAGNTANNGCGNTCNNNNNNNNNNNNNCNNTCCCNN
ACTGAGGGTNNNNNNCNCATCTGCTNNNNNNNNCTTCGCNGGCGTNNCNNCNNAGCNGTATNGNGTNA
NNCANNNNNNNNNNNNNNNNNNTNAAACAANNNNNNTNNNN
>Ede61
GTGNNNNNTAGCCCNNNNNAGTTAGACANNNCNNNNNNNNNNNNNTNNNNNNNNNNNNNNNNNNNNNNN
NNNNNNNNNNNNNNNNNNNNNNNNNNNNNNNTNNNNNNNNNNNNNNNNNNNNNNNNNNNNNNNNNNNNNNN
NNNNNNNNNNNNNNNNNNNNNNNNNNNNNNNNNNNNNNNNNNNNNNNNNNNNNNNNNNNNNNNNNNNNNNNN
NNNNNNNNNNNNNNNNNNNNNNNNNNNNNNNNNNNNNNNNNNNNNNNNNNNNNNNNNNNNNNNNNNNNNNNN
NNNNNNNNNNNNNNNNNNNNNNNNNNNNNNNNNNNNNNNNNNNNNNNNNNNNNNNNTCTNNNAAANNNTNNN
NNNNNNNNNNNNNNNNNNNNNNNNNNNCNCNTTGNNNGTAANCNANNNGNACATGCAGTCCCCTCNNNNN
NNNGGCAGAGNNNNNNCCCNNNNNNNCCGGCCCCNNCCNNNNNNNNNNNNCNNNGNNNNNNNANNNNNN
NNNNNNTGGTACNNNNCGNNNNNGGTTGTNTAAGAGGTCCACAACGNGGTCNNGNCACNNNNGNNNAN
NNNCNNNNAACNGNNNNNNNNNNANNTNNNTANNAGGNCNNNNCCNTCNNGNNNNTNATTGCNATGACC
CAANNNACGGGGGCAGTNCCCGGNGCGGCGACCTTGNTNANNTCCGGGGACAAGTATGGCGCNCNCCC
CGGGAAGGANNCNGCCGTANGTGCNNNNNANNNNNTCNTGNGANGTCGCCGNNATTCNCGTGGTCNNN
CNAGAATGNCCGNACCANNCGNANNNTGGTNATGNNAGTANNNNNNNNNNNNNNNNNNNNNNNNNNNN
NNNNNNNNNNNNNNNNNNNNNNNNNNNNNNNNNNNGNNNNGNNNNNNCNANNNAAGGANNTANNNCTNNTC
NTNNNNNNGGACNNNACNNNNCNNNNNNNACNCNNNCNCNNNNNNANNNGNTTTTTNNNCNNTATTNT
CCAGNACGCNGNGCNNNCATTCANGNGAACNNNGNNACGCGNNAATCGTANNATCTACCTGCCGCNNG
TNNNNNTACNGNNNNNNCGGANNNTNNCTCGTANNNNNNTCATGCGTNNTNTNNTCNCNAGNCNNGTT
CGNNNANANANTAGNANGTNTNNNNNNNCTTGTNNCGTTCCGCNTGANNCNNCANNNNGNNGNNCTTN
TCANCGGCNNTANCTGCNGANNTAAGNCATANNNTNNNTCNNTANCTNNNNNGGNGGGNNACTGTAAA
NGGNNAATTNGANGNTNATTTCNNNCACNTNNCTNNGNNTNCTNNNTNCCTGNNTNCNNAATTGGNNC
CANTGNNTGNCNATCTCTNGNTTNGNNNNNNTNNNNNTNNCAANNNGGNNNGTNANANNNCNATNTAA
NNTATANNNNCNNNNNNANNNCACNANNNNNGNAGNNNCNANNNTTANANNNGTAGNNNANTAAGTCT
CCGANNTNNNNNNNATAAGCGNNANACNNNNNNNNNNNNNNANGANNACGNNTTGGNNNTNNNNNNCNA
GNTAGACNNTNATGGNTNNNNNTCAANNNNTTNGTAGTGGTATAATNGNNNNNNNTTTCNCCAGNNTN
NNNGNNNNNNNCNATANNANNNNNNNGNGANNNNNNNNNNNANNNNCTCNNNAGGGANCANCTACTNNA
GCAATGGNGCGNAGNNNGAGANNNNGACNNGCCTTGGGTGGCAANCACNGCAATAAGTNNNGTTNANC
CTNNNAGTNNACATGNNNNCANNGNNNCNNNNNNANCNGANNGNNNNNNNNNNNNNNNNNNNNNNNNN
NNNNNNNNNNNNNNNNNNNNNNNNNNNNNANNNNANGGNNNGTNTNTGACNCATGTGTTTTTANNNGNN
NNNNNACGNNNNANCCCCGGTCGNNNNCNNATTGAANNCCTGNNGCATNNNNNTNCNAGAATGANNNC
NNNNNGNNTNGNCTNNCNNNTCAAGNAATCAANNAGGTANCGCGNTCANNGNNNNNNNNCNNTCCCNN
ACTGAGGGTNNNNNNNNCATCTGCTNNNNNNNNCTTCGCNGGNGTNNCNNNNNAGCNGTATNGTGTNA
NNCANNNNNNNNNNNNNNNNNNTNNAACGANNNNNNTNNNN
>Ede736
GTGNNNNNTAGCCCNNNNNAGTTAGACANNNCNNNNNNNNNNNNNTNNNNNNNNNNNNNNNNNNNNNNN
NNNNNNNNNNNNNNNNNNNNNNNNNNNNNNNTNNNNNNNNNNNNNNNNNNNNNNNNNNNNNNNNNNNNNNN
NNNNNNNNNNNNNNNNNNNNNNNNNNNNNNNNNNNNNNNNNNNNNNNNNNNNNNNNNNNNNNNNNNNNNNNN
NNNNNNNNNNNNNNNNNNNNNNNNNNNNNNNNNNNNNNNNNNNNNNNNNNNNNNNNNNNNNNNNNNNNNNNN
NNNNNNNNNNNNNNNNNNNNNNNNNNNNNNNNNNNNNNNNNNNNNNNNNNNNNNNCNTCTNNNAAANNNTNNN
NNNNNNNNNNNNNNNNNNNNNNNNNNNCNCNTTGNNNGTAANCNANNNGNACATGCAGTCCCCTCNNNNN
NNNGGCAGAGNNNNNNCCCNNNNNNNCCGGCCCCNNCCNNNNNNNNNNNNCNNNGNNNNNNNANNNNNN
NNNNNNTGGTACNNNNCGNNNNNGGTTGTTTAAGAGGTCCACAACGNGGTCNGGNCACNNNNGNNNAN
NNNCNNNAAACNGNNNNNNNNNNANATNNNTANNAGGNCNNNNCCNTCNNGNNNNNNATTGCNATGACC
CAANTCACGGGGGCAGTNCCCGGCGCGGCGACCTTGANNACGTCCGGGGACAAGTATGGCGCNCNCCC
CGGGAAGGANNCNGCCGTANGTGCNNNNNANNNNNTCNTGNGACGTCGCCGNNATTCNCGTGGTCNNN

CNAGAATGCCCGNACCANNCGNANNNTGGTNATGNTAGTNNNNNNNNNNNNNNNNNNNNNNNNNNNNN
NNNNNNNNNNNNNNNNNNNNNNNNNNNNNNNNGNNNNGNNNNNNCANNNNAAGGAGNTANNNCTNNTN
NTNNNNNNGGACNNNACNNNNCNNNNNNNACNCNNNCNCNNNNNNANNNGNTTTTTNNNCNNTATTNT
CCAGNACGCNGNGCNNTCATTCANGNGAACNNNGNNACGCGNNAATCGTANNATCTACCTGCCGCNNG
TNNNNNTACNGNNNNTNCGGANNNTNNCTCGTANNNNNNTCATGCGTNNTNTNNTCNCNAGNCANGTT
CGNNNANANANTAGNANGTNTNNNNNNNNNTGTNGCGTTCCGCNTGANNCNNCANNNNGNNGNNCTTN
TCANCGGCNNTANCTGCNGANNTAAGNCATANNNTNNATCNNTANCTNNNNNGGNGGGNACTGTAAA
NGGNNAATTGGNNGNTNATTNCNNNCACNTNNCNNNGNNTNCTNNNTNCCTGNNTNCNNAATTGGNNC
CANTGNNTGNCNATCTCTGGNTTNGCNNNNNTNNNNNTNNCAANNNGGNNNGTNANANNNCNATNTAA
NNTANANNNNCNNNNNNANNNCACNANNNNNGNAGNNNCNANNNTTANANNNGTAGNNNAATAAGTCT
CCGANNTNNNNNNNATAAGCGNNANACNNNNNNNNNNNNNANGANNACGNNTTGGNNNTNNNNNNCNA
GNNAGACNNTNATGGNTNNNNNTCAANNNNTTCGTAGTGGTATAATNGNNNNNNNTTTCNCCAGNNTN
NNNGNNNNNNNCNATACNANNNNNNNGTGANNNNNNNNNNANNNNCTCNNNAGGGANCANCTACTNNA
GCAATGGNGCGCAGNNNGAGANNNNGACNNGCCTTGGGTGGCAANCACNGCAATAAGTNNNGTTNANC
CTNNNAGTNNACATNNNNNCANNGNNNCNNNNNNANCNGANNGNNNNNNNNNNNNNNNNNNNNNNNN
NNNNNNNNNNNNNNNNNNNNNNNNNNNAGNNNANGGNNNGTNTNTGACNCATGTGTTTTTANNNGNN
NNNNNACGNNNGANCCCCGGTCGTNTNCNNATTGAANNCCTGNNGCATNNNNNTNCNAGAATGANNGC
NNNNNGNNTNGCTNNCNTTTCAAGNAATCANNTAGGTNNCGCGNTCNNNNNNNNNNNNNCNNTCCCNN
ACTGAGGGTNNNNNNNNCATCTGCTNNNNNNNNCTNCGCNGGCGTNNCNGNNNAGCNGTATNGTGTNA
NNCANNNNNNNNNNNNNNNNTNAAACNANNNNNCTNNNN
>Ede737
GTGNNNNNTAGCCCNNNNNAGTTAGACANNNCNNNNNNNNNNNNTNNNNNNNNNNNNNNNNNNNNNNN
NNNNNNNNNNNNNNNNNNNNNNNNNNNNNNTNNNNNNNNNNNNNNNNNNNNNNNNNNNNNNNNNNNNN
NNNNNNNNNNNNNNNNNNNNNNNNNNNNNNNNNNNNNNNNNNNNNNNNNNNNNNNNNNNNNNNNNNNN
NNNNNNNNNNNNNNNNNNNNNNNNNNNNNNNNNNNNNNNNNNNNNNNNNNNNNNNNNNNNNNNNNNNN
NNNNNNNNNNNNNNNNNNNNNNNNNNNNNNNNNNNNNNNNNNNNNNNNNNCNTCTNNNAAANNNTNNN
NNNNNNNNNNNNNNNNNNNNNNNNNCNCNTTGNNNGTAANCANNNGNACATGCAGTCCCCTCNNNNN
NNNGGCAGAGNNNNNNCCCNNNNNCNCCGGCCCCNNCCNNNNNNNTNNNCNNNGNNNNNNNANNNNNN
NNNNNNTGGTACNNNNCGNNNNNGGTTGTNTAAGAGGTCCACAACGNGGTCNNNNCACNNNNGNNNAN
NNNCNNNNAACNNNNNNNNNNNANNTNNNTANNAGGNCNNNNCCNTCNNGNNNNNNATTGCNATGACC
CAANTCACGGGGGCAGTCCCCGGCGCGGCGACCTTGATTACGTCCGGGGACAAGTATGGCGNNCNCCC
CGGGAAGGANNCNGCCGTANGTGCNNNNNANNNNNTCNTGNGACGTCGCCGNNATTCNCGTGGTCNNN
CNAGAATGNCCNNACCANNCGNANNNTGGTNATGNTAGTNNNNNNNNNNNNNNNNNNNNNNNNNNNNN
NNNNNNNNNNNNNNNNNNNNNNNNNNNNNNNNGNNNNGNNNNNNCANNNNAAGGANNTANNNCTNNTN
NTNNNNNNGGACNNNACNNNNCNNNNNNNACNCNNNCNCNNNNNNANNNGGTTTTTNNNCNNTATTNT
CCAGNACGCNGNGCNNNCATTCANGNGAACNNNGNNACGCGNNAATCGTANNATCTACCTGCCGCNNG
TNNNNNTACNGNNNNNNCGGANNNTNNCTCGTANNNNNNTCATGCGTNNTNTNNTCNCNAGNCNNGTT
CGNNNANANANTAGNANGTNTNNNNNNNNNTGTNNCGTTCCGCNTGANNCNNCANNNNGNNGNNCTTN
NCANCGGCNCTANCTGCNGANNTAAGNCATANNNTNNNTCNNTANCTNNNNNGGNGGGNACTGTAAA
NGGNNAATTNGNNGNTNATTNCNNNCACNTNNCTNNGNNTNCTNNNTNCCTGNNTNCNNAATTGGNNC
CANTGNNTGNCNATCTCTGGNTTNGCNNNNNTNNNNNTNNCANNNNGGNNNGTNANANNNCNATNTAA
NNTANANNNNCNNNNNNANNNCACNANNNNNGNAGNNACNANNNTTANANNNGTAGNNNAATAAGTCT
CCGANNTNNNNNNNATAAGCGNNANACNNNNNNNNNNNNNANGANNACGNNTTGGNNNTNNNNNNCNA
GNNAGACNNTNATGGNTNNNNNTCAANNNNTTNGTAGTGGTATAATNGNNNNNNNTTTCNCCAGNNTN
NNNGNNNNNNNCNATANNANNGNNNNGNGANNNNNNNNNNANNNNCTCNNNAGGGANCANCTACTNNA
GCAATGGNGCGCAGNNNGAGANNNNGACNNGCCTTGGGTGGCAANNACNGCAATAAGTNNNGTTNANC
CTNNNAGTNNACATNNNNNCANNGNNNCNNNNNNANCNGANNGNNNNNNNNNNNTNNNNNNNNNNNNN
NNNNNNNNNNNNNNNNNNNNNNNNNNNAGNNNANGGNNNGTNTNTGACNCATGTGTTTTTANNNGNN
NNNNNACGNNNNANCCCCGGTCGNNTNCNNATTGAANNCCTGNNGCATNNNNNTNCNAGAATGANNGC
NNNNNGNNTNGCTNNCNNNTCAAGNAATCAANNAGGTNNNGCGNTCNNNGNNNNNNNNCNNTCCCNN
ACTGAGGGTNNNNNNNNCATCTGCTNNNNNNNNCTNCGCNGGCGTNNCNNNNNNGCNGTATNGNGTNA
NNCANNNNNNNNNNNNNNNNTNNAACAANNNNNNTNNCN
>Ede738
GTGNNNNNTAGCCCNNNNNAGTCAGACANNNCNNNNNNNNNNNNTNNNNNNNNNNNNNNNNNNNNNNN

NNNNNNNNNNNNNNNNNNNNNNNNNNNNNNNTNNNNNNNNNNNNNNNNNTNNNNNNNNNNNNNNNNNNNN
NNNNNNNNNNNNNNNNNNNNNNNNNNNNNNNNNNNNNNNNNNNNNNNNNNNNNNNNNNNNNNNNNNNNNN
NNNNNNNNNNNNNNNNNNNNNNNNNNNNNNNNNNNNNNNNNNNNNNNNNNNNNNNNNNNNNNANNNNNNN
NNNNNNNNNNNNNNNNNNNNNNNNNNNNNNNNNNNNNNNNNNNNNNNNNNNNCNTCTNNNAAANNNTNNN
NNNNNNNNNNNNNNNNNNNNNNNNNNNCNCNTTGNNNGTAANCNANNNGNACATGCAGTCCCCTCNNNNN
NNNGGCAGAGNNNNNNCCCNNNNNANCCGGCCCCNNCCNNNNNNNNNNNCNNNGNNNNNNNANNNNNN
NNNNNNTGGTACNNNNCGNNNNNGGTTGTTTAAGAGGTCCACAACGNGGTCNGGNCACNNNNGNNNAN
NNNCNNNNAANNNNNNNNNNNNNNNNATNNNTANNAGGNNNNNNCCNTCNNGNNNNNNATTGCNATGACC
CAANTCACGGGGGCAGTNCCCGGCGCGGCGACCTTGANNACGTCCGGGGACAAGTATGGCGNNCNCCC
CGGGAAGGANNCNGCCGTANGTGCNNNNNANNNNNTCNTGNGACGTNGCCGNNATTCNCGTGGTCNNN
CNAGAATGNCCGNACCANNCGNANNNTGGTNATGNNAGTNNNNNNNNNNNNNNNNNNNNNNNNNNNNNNN
NNNNNNNNNNNNNNNNNNNNNNNNNNNNNNNNNNNGNNNNGNNNNNNCNANNNAAGGANNTANNNCTNNTN
NTNNNNNNGGACNNNACNNNNCNNNNNNNACNCNNNCNCNNNNNNANNNGNTTTTNNNNCNNTATTNT
CCAGNACGCNGNGCNNNCATTTANGNGAACNNNGNNACGCGNNAATCGTANNATCTACCTGCCGCNNG
TNNNNNTNCNGNNNNTNCGGANNNTNNNNCGTANNNNNGNCATGCGTNNTNTNNTCNCNAGNCANGTT
CGNNNANANANTAGNANGTNTNNNNNNNNNNTGTNNCGTTCCGCNTGANNCNNCANNNNGNNGNNCTTN
TCANCGGCNNTANCTGCNGANNTAAGNCATANNNTNNATCNNTANCTNNNNNGGNGGGNNACTGTAAA
NGGNNAATTNGNNGNTNANTNCCNNCATNTNNCTNNGNNTNCTNNNTNCCTGNNTNCNNAATTGGNNC
CANTGNNTGNCNATCTCTGGNTTNGNNNNNNTNNNNNTNNCANNNNGGNNNGTNANANNNCNATNTAA
NNTATANNNNCNNNNNNANNNCACNANNNNNGNAGNNNCNANNNGTANANNNGTAGNNNGATAAGTCT
CCGANNTNNNNNNNATAAGCGNNANACNNNNNNNNNNNNNNANGANNACGNNTTTGNNNTNNNNNNCNA
GNNAGACNNTNGTGGNTNNNNNTCAANNNNNTTCGTAGTGGTATAATNGNNNNNNNTTTCNCCAGNNTN
NNNGNNNNNNNNCNATANNANNNNNNNGNGANNNNNNNNNNNANNNNCTCNNNAGGGANCANCTACTTNA
GCANTGGNGCGCAGNNNGAGANNNNGACNNGCCTTGGGTGGCAANCACNGCAATAAGTNNNGTTNANC
CTNNNAGTNNACACNNNNNCANNGNNNCNNNNNNANCNGANNGNNNNNNNNNNNNNNNNNNNNNNNNNN
NNNNNNNNNNNNNNNNNNNNNNNNNNNNNAGNNNANGGNNNGTNTNTGACNCATGTGTTTTTANNNGNN
NNNNNACGNNNGANCCCCGGTCGTNNNCNNATTGAANNCCTGNNGCATNNNNNNTNCNAGAATGANNGC
NNNNNGNNTNGNCTGNCNTTTCAAGNAATCAANNAGGTANCGCGNTCNNNGNNNNNNNNCNNTCCCNN
ACTGAGGGTNNNNNNNNCATCTGCTNNNNNNNNNNTNCGCNGGCGTNNCNNNNNAGCNGTATNGNGTTN
NNCANNNNNNNNNNNNNNNNNNTTAAACNANNNNCNTNNNN
>Ede739
GTGNNNNNTAGCCCNNNNNAGTTAGACANNNCNNNNNNNNNNNNNTNNNNNNNNNNNNNNNNNNNNNNNN
NNNNNNNNNNNNNNNNNNNNNNNNNNNNNNNTNNNNNNNNNNNNNNNNNNNNNNNNNNNNNNNNNNNNNN
NNNNNNNNNNNNNNNNNNNNNNNNNNNNNNNNNNNNNNNNNNNNNNNNNNNNNNNNNNNNNNNNNNNNNN
NNNNNNNNNNNNNNNNNNNNNNNNNNNNNNNNNNNNNNNNNNNNNNNNNNNNNNNNNNNNNNNNNNNNNN
NNNNNNNNNNNNNNNNNNNNNNNNNNNNNNNNNNNNNNNNNNNNNNNNNNNNNTCTNNNAAANNNTNNN
NNNNNNNNNNNNNNNNNNNNNNNNNNNCNCNTTGNNNGTAANCNANNNGNACATGCAGTCCCCTCNNNNN
NNNGGCAGAGNNNNNNNCCNNNNNCNCCGGCCCCGNCCNNNNNNNNNNNNCNNNGNNNNNNNANNNNNN
NNNNNNTGGTACNNNNCGNNNNNGGTTGTTTAAGAGGTCCACAACGNGGTCNGGNCACNNNNGNNNAN
NNNCNNANAACNNNNNNNNNNNNNANNTNNNTANNAGGNCNNNNCCNTCNNNNNNNTNATTGCNATGACC
CAANTCACGGGGGCAGTNCCCGGCGCGGCGACCTTGATNACGTCCGGGGACAAGTATGGCGNNCNCCC
CGGGAAGGANNCNGCCGTANGTGCNNNNNANNNNNTCNTGNGACGTCGCCGNNATTCNCGTGGTCNAN
CNAGAATGNCCGNACCANNCGNANNNTGGTNATGNNAGTANNNNNNNNNNNNNNNNNNNNNNNNNNNNNN
NNNNNNNNNNNNNNNNNNNNNNNNNNNNNNNNNNNGNNNNGNNNNNNCNANNNAAGGANNTANNNCTNNTN
NTNNNGNNGGACNNNACNNNNCNNNNNNNACNCNNNCNCNNNNNNANNNGGTTTTTNNNCNNTATTNT
CCAGNACGCNGNGCNNNCATTCANGNGAACNNNGNNACGCGNNAATCGTANNATCTACCTGCCGCNNG
NNNNNNTACNGNNNNTNCGGANNNTNNCTCGTANNNNNNTCATGCGTNNTNTNNTCNCNAGNCANGTT
CGNNNANANANTAGNANGTNTNNNNNNNNNNTGTNNCGTTCCGCNTGANNCNNCANNNNGNCGNNCTTN
TCANCGGCNNTANCTGCNGANNTAAGNCATANNNTNNATCNNTANCTNNNNNGGNGGGNNACTGTAAA
NGGNNAATTNGNGGNTNATTNCNNNCACNTNNCTNNGNNTNCTNNNTNCCTGNNTNCNNAATTGGNNC
CANTGNNTGNCNATCTCTGGNTTNGCNNNNNTGNNNNTNNCAANNNGGNNNGTNANANNNCNATNTAA
NNTATANNNNCNNNNNNANNNCACNANNNNNGNAGNNACNANNNTTANANNNGTAGNNNAATAAGTCT
CCGANNTNNNNNNNATAAGCGNNANACNNNNNNNNNNNNNNANGANNACGNNTTGGNNNTNNNNNNCNA
GNNAGACNNTNATGGNTNNNNNTCAANNNNNTTCGTAGTGGTATAATNGNNNNNNNTTTCNCCAGNNTN

NNNGNNNNNNNNCNATANNANNNNNNNGNGANNNNNNNNNNNANNNNCTCNNNAGGGANCANCTACTNNA
GCANTGGNGCGCAGNNNGAGANNNNGACNNGCCTTGGGTGGCAANCACNGCAATAAGTNNNGTTNANC
CTNNNAGTNNACATNNNNNCANNGNNNCNNNNNNANCNGANNGNNNNNNNNNNNNNNNNNNNNNNNNN
NNNNNNNNNNNNNNNNNNNNNNNNNNNNNNNAGNNNANGGNNNGTNTNTGACNCATGTGTTTTTANNNGNN
NNNNNACGNNNGANCCCCGGTCGNNTNCNNATTGAANNCCTGNNGCATNNNNNTNCNAGAATGANNGC
NNNNNGNNTNGNCTNNCNTTTCAAGNAATCAANNAGGTNNNGCGNTCNNNGNNNNNNNNCNNTCCCNN
ACTGAGGGTNNNNNNNNCATCTGCTNNNNNNNNCTNCGCNGGCGTNNCNNNNNAGCNGTATNGTGTNA
NNCANNNNNNNNNNNNNNNNNTNANACNANNNNNNTNNNN
>Ede74
GTGNNNNNTAGCCCNNNNNAGTTAGACANNNCNNNNNNNNNNNNTNNNNNNNNNNNNNNNNNNNNNNN
NNNNNNNNNNNNNNNNNNNNNNNNNNNNNNNTNNNNNNNNNNNNNNNNNNNNNNNNNNNNNNNNNNNNNN
NNNNNNNNNNNNNNNNNNNNNNNNNNNNNNNNNNNNNNNNNNNNNNNNNNNNNNNNNNNNNNNNNNNNNNNN
NNNNNNNNNNNNNNNNNNNNNNNNNNNNNNNNNNNNNNNNNNNNNNNNNNNNNNNNNNNNNNNNNNNNNNNN
NNNNNNNNNNNNNNNNNNNNNNNNNNNNNNNNNNNNNNNNNNNNNNNNNNNNNCNTCTNNNAAANNGTNNN
NNNNNNNNNNNNNNNNNNNNNNNNNNCNCNTTGNNNGTAANCNANNNANACGTGCAGTCCCCTCNNNNN
NNNGGCAGAGNNNNNNCCCNNNNNCNCCGNCCCCNNCCNNNNNNNNNNNNCNNNGNNNNNNNANNNNNN
NNNNNNTGGTACNNNNCGNNNNNGGTTGTTTAAGAGGTCCACCACGNAGTCNGGNCACNNNNANNNAN
NNNCNNNNNACNNNNNNNNNNNNANNGNNNCANNAGGNCNNNNCCNTCNNGNNNNNNGTNGCNATGACC
CAANTCACGGGGGCNGTNCCCGGCGNAGCGACCTTGATNACGTCCGGGAACAAGTATGGCGNNTNCCC
CGGGAAGGANNCNGCCGTANGTGCNNNNNANNNNNTCTTGNGACGTCGCCGNNATTCNCGTGGTCNNN
NNAGAATGNCCGNACCANNCGNANNNTGGTTATGNNAGTNNNNNNNNNNNNNNNNNNNNNNNNNNNNN
NNNNNNNNNNNNNNNNNNNNNNNNNNNNNNNNNNNNNNGNNNNNNCNANNNAAGGANNTANNNCTNNTN
NTNNNNNNNGGACNNNACNNNNCNNNNNNNNACNCNNNCNCNNNNNNANNNGNTTTTTNNNCNNTATTNC
CCAGNACGCNGNGCNNTCATTCANGNGAACNNNGNNACGCGNNAATCGTCNNATCTACCTGCCGCNGG
NNNNNNTNCNGNNNNNNCGGANNNTNNCTCGTANNNNNNTCATGCGTNNTNTNNTCNCNAGNCANTTT
CGNNNGNANANTAGNANGTNTNNNNNNNNNNTGTNNCGTTCCGCNTGGNNCNNCANNNNGNNGNNCTTN
TCANCGGCNNTANTTGCNGANNTAAGNCATANNNCNNNTCNNCANCTNNNNNGGAGGGNNACTGTAAA
NGGNNAATTNGANGNCNATTTCCNNCACNTNNCTNNGNNTNCTNNNTNCTTANNTNCNNAGTTGGNNC
CANTANNTGNTNATCTCTGGNTTNGNNNNNNTNNNNNTNGCANNNNGGNNNGTNANGNNNCNATNTAA
NNTANANNNNCNNNNNNANNNCACNNNNNNNGNAGNNNCNANNNTTANANNNGTAGNNNACTAAGTCT
CCGANNTNNNNNNNATAGGCGNNANACNNNNNNNNNNNNNNANGANNACGNNTTGGNNNTNNNNNNNANA
ANNAGACNNTNGTGGNTNNNNNTCAANNNNCTNGTAGTGGTATAATNGNNNNNNNTTGCNCCAGNNTN
NNNGNNNNNNNNCNATANNAGNNNNNNGNGANNNNNNNNNNNANNNNCTCNNNAGGGANCANCTACTNNA
GCANTGGNGCGCAGNNNTAGANNNNGACNNACCTTGGGTGGCAANCACNGCAATAAGTNNNGTTNANC
CTNNNAGTNNACACNNNNNCANNANNNCNNNNNNANCNGANNTNNNNNNNNNNNNNNNNNNNNNNNNN
NNNNNNNNNNNNNNNNNNNNNNNNNNNNNNTNNNANGGNNNGTNTNTGACNCATGTGTTTTTANNNGNN
NNNNNACGNNNGANCCCCGGTCGTNTNCNNATTAAANNCCTGNNGCATNNNNNTNCNATAATGANNGC
NNNNNGNNTNGNCTNNCNTTTCAAGNAGTCANNNAGGTANNGCGNTCNNGGNNNNNNNNCNNTCCCNN
ACTGAGGGTNNNNNNNNCATCTGCTNNNNNNNNCTTCGCNGGCGTNNCNGNNNATCNGTATNGTGTNA
NNCANNNNNNNNNNNNNNNNNTNNNNNNNNNNNNNNTNNNN
>Ede740
GTGNNNNNTAGCCCNNNNNAGTTAGACANNNCNNNNNNNNNNNNTNNNNNNNNNNNNNNNNNNNNNNN
NNNNNNNNNNNNNNNNNNNNNNNNNNNNNNNTNNNNNNNNNNNNNNNNNNNNNNNNNNNNNNNNNNNNNN
NNNNNNNNNNNNNNNNNNNNNNNNNNNNNNNNNNNNNNNNNNNNNNNNNNNNNNNNNNNNNNNNNNNNNNNN
NNNNNNNNNNNNNNNNNNNNNNNNNNNNNNNNNNNNNNNNNNNNNNNNNNNNNNNNNNNNNNNNNNNNNNNN
NNNNNNNNNNNNNNNNNNNNNNNNNNNNNNNNNNNNNNNNNNNNNNNNNNNNNCNTCTNNNAAANNNTNNN
NNNNNNNNGCNNNNNNNNNNNNNNNNCNCNTTGNNNGTAANCNANNNGNACATGCAGTCCCCTCNNNNN
NNNGGCAGAGNNNNNNCCCNNNNNNNCCGGCCCCGNCCNNNNNNNTNNNCNNNGTNNNNNNANNNNNN
NNNNNNTGGTACNNNNCGNNNNNGGTTGTTTAAGAGGTCCACAACGNGGTCNGGNCACNNNNGNNNAN
NNNCNNNAAACNNNNNNNGNNNNNNTNNNTANAAGGNCNNNNCCNTCNNGNNNNTNATTGCNATGACC
CAANTCACGGGGGCAGTCCCCGGNGCGGCGACCTTGATNANGTCCGGGGACAAGTATGGCNCNCNCCC
CGGGAAGGANNCNGCCGTANGTGCNNNNNANNNNNTCNTGNGANGTCGCCGNNATTNNCGTGGTCNNN
CNAGAATGNCCGNACCANNCGNANNNTGGTNATGNTAGTNNNNNNNNNNNNNNNNNNNNNNNNNNNNN
NNNNNNNNNNNNNNNNNNNNNNNNNNNNNNNNNNGNNNNGNNNNNNCNANNNAAGGANNTANNNCTNNTN

NTNNNNNNNGGACNNNACNNNNCNNNNNNNACNCNNNCNCNNNNNNANNNGNTTTTTNNCCNNTATTNT
CCAGNACGCNGNGCNNTCATTCANGNGAACNNNGNNACGCGNNAATCGTANNATCTACCTGCCGCNNG
TNNNNNTNCNGNNNNNNCGGANNNTNNCTCGTANNNNNNTCATGCGTNNTNTNNTCNCNAGNCANGTT
CGNNNANANANTAGNANGTNTNNNNNNNCTTGTNNCGTTCCGCNTGACNCNNCANNNNGNCGNNCTTN
TCANCGGCNNTANCTGCNGANNTAAGTCATANNNTNNATCNNTANCTNNNNNGGAGGGNNACTGTAAA
NGGNNAATTGGNNGNTNATTNCCNNCACNTNNCTNNGNNTNCTNNNTNCCTGNNTNCNNAATTGGNNC
CANTGNNTGNCNATCTCTGGNTTNGNNNNNNTNNNNNTNNCAANNNGGNNNGTNANANNNCNATNTAA
NNTANANNNNCNNNNNNANNNCACNNNNNNNGNAGNNNCNANNNTTANANNNGTAGNNNAATAAGTCT
CCGANNTNNNNNNNATAAGCGNNANACNNNNNNNNNNNNNANGANNACGNNTTGGNNNTNNNNNNCNA
GNNAGACNNTNATGGNTNNNNNTCAANNNNTTNGTAGTGGTATAATNGNNNNNNNTTTCNCCAGNNTG
NNNGNNNNNNNCNATANTANNNNNNNGTGANNNNNNNNNNNANNNNCTCNNNAGGGANCANCTACTNNA
GCANTGGNGCGCAGNNNGAGANNNNGACNNGCCTTGGGTGGCAANCACNGCAATAAGTNNNGTTNANC
CTNNNAGTNNACATNNNNNCANNGNNNCNNNNNNANCNGANNGNNNNNNNNNNNNNNNNNNNNNNNNN
NNNNNNNNNNNNNNNNNNNNNNNNNNNNNAGNNNANGGNNNGTNTNTGACNCATGTGTTTTTANNNGNN
NNNNNACGNNNGANCCCCGGTCGTNNNCNNATTGAANNCCTGNNGCATNNNNNTNCNAGAATGANNGC
NNNNNGNNTNGNCTNNCNTTTCAAGNAATCAAANAGGTANNNNNNTCNNNGNNNNNNNNCNNTCCCNN
ACTGAGGGTNNNNNNCGCATCTGCTGNNNNNNNCTNCGCNGGCGTNNCNGNNNAGCNGTATNGTGTTA
NNCANNNNNNNNNNNNNNNNNCNAAACAANNNNCNTNNNN
>Ede741
GTGNNNNNTAGCCCNCNNNAGTTAGACANNNCNNNNNNNNNNNNTNNNNNNNNNNNNNNANNNNNNNN
NNNNNNNNNNNNNNNNNNNNNNNNNNNNNNNNTNNNNNNNNNNNNNNNNNNNNNNNNNNNNNNNNNNN
NNNNNNNNNNNNNNNNNNNNNNNNNNNNNNNNNNNNNNNNNNNNNNNNNNNNNNNNNNNNNNNNNNNN
NNNNNNNNNNNNNNNNNNNNNNNNNNNNNNNNNNNNNNNNNNNNNNNNNNNNNNNNNNNNNNNNNNNN
NNNNNNNNNNNNNNNNNNNNNNNNNNNNNNNNNNNNNNNNNNNNNNNNNNNNCNTCTNNNAAANNNTNNN
NNNNNNNNNNNNNNNNNNNNNNNNNNCNCNTTGNNNGTAANCNANNNGNACATGCAGTCCCCTCNNNNN
NNNGGCAGAGNNNNNNCCNNNNNNNNCCGGCCCCNNCCNNNNNNNNNNNCNNNGNNNNNNNANNNNNN
NNNNNNTGGTACNNNNCGNNNNNGGTTGTTTAAGAGGTCCACAACGNGGTCNGGNCACNNNNGNNNAN
NNNCNNNNANCNNNNNNNNNNNNANATNNNTANNAGGNCNNNNCCNTCNNGNNNNTAATTGCNATGACC
CAANNCNCGGGGGCAGTNCCCGGCGCGGCGACCTTGNTNACGTCCGGGGACAAGTATGGCGNNCNCCC
CGGGAAGGANNCNGCCGTANGTGCNNNNNANNNNNTCNTGNGANGTCGCCGNNATTCNCGTGGTCNNN
CNAGAATGNCCGNACCANNCGAANNNTGGTTATGNNAGTNNNNNNNNNNNNNNNNNNNNNNNNNNNNN
NNNNNNNNNNNNNNNNNNNNNNNNNNNNNNNNNNNNNNNNNGNNNNNNCNANNNAAGGANNTANNNCTNNTN
NTNNNNNNNGGACNNNACNNNNCNNNNNNNACNCNNNCNCNNNNNNANNNGNTTTTTNNCCNNTATTNT
CCAGNACGCNGNGCNNNCATTCANGNGAACNNNGNNACGCGNNAATCGTANNATCTACCTGCCGCNNG
TNNNNNTACNGNNNNTNCGGANNNTNNCTCGTANNNNNNTCANGCGTNNTNTNNTCNCNAGNCANGTT
CGNNNANANANTAGNANGTNTNNNNNNNNNNTGTNNCGTTCCGCNTGANNCNNCANNNNGNNGNNCTTN
TCANCGGCNNTANCTGCNGANNTAAGTCATANNNTNNATCNNTANCTNNNNNGGNGGGNNACTGTAAA
NGGNTAATTNGANGNTNATTNCNNNCACNTNNCNNNGNNNNCTNNNTNCCTGNNTNCNNAATTGGNNC
CANTGNNTGNCNATCTCTGGNTTNGNNNNNNTNNNNNTNNCAANNNGGNNNGTNANANNNCNATNTAA
NNTATANNNNCNNNNNNANNNCACNANNNNNGNAGNNNCNANNNTTANANNNGTAGNNNAATAAGTCT
CCGANNTNNNNNNNATAAGCGNNANACNNNNNNNNNNNNNANGANNACGNNTTGGNNNTNNNNNNCNA
GNNAGACNNTNATGGNTNNNNNTCAANNNNTTCGTAGTGGTATAATNGNNNNNNNTTTCNCCAGNNTN
NNNGNNNNNNNCNATANNANNNNNNNGNGANNNNNNNNNNNANNNNCTCNNNAGGGANCANCTACTNNA
GCANTGGNGCGCAGNNNGAGANNNNGACNNGCCTTGGGTGGCAANCACNGCAATAAGTNNNGTTNANC
CTNNNAGTNNACATNNNNNCANNGNNNCNNNNNNANCNGANNGNNNNNNNNNNNNNNNNNNNNNNNNN
NNNNNNNNNNNNNNNNNNNNNNNNNNNNNAGNNNANGGNNNGTNTNTGACNCATGTGTTTTTANNNGNN
NNNNNACGNNNGANCCCCGGTCGNNNNCNNATTGAANNCCTGNNGCATNNNNNTNCNAGAATGANNGC
NNNNNGNNTNGNCTNNCNTTTCAAGNAATCANNNAGGNANCGCGNTCNNNGNNNNNNNNCNNTCCCNN
ACTGAGGGTNNNNNNNNCATCTGCTNNNNNNNNNNTNCGCNGGNGTNNCNNCNNAGCNGTATNGNGTNN
NNCANNNNNNNNNNNNNNNNNTNAAACAANNNNNNNTNNNN
>Ede742
GTGNNNNNTAGCCCNNNNNAGTTAGACANNNCNNNNNNNNNNNNTNNNNNNNNNNNNNNNNNNNNNNN
NNNNNNNNNNNNNNNNNNNNNNNNNNNNNNNNNNNNNNNNNNNNNNNNNNNNNNNNNNNNNNNNNNNN
NNNNNNNNNNNNNNNNNNNNNNNNNNNNNNNNNNNNNNNNNNNNNNNNNNNNNNNNNNNNNNNNNNNN

NNNNNNNNNNNNNNNNNNNNNNNNNNNNNNNNNNNNNNNNNNNNNNNNNNNNNNNNNNNN
NNNNNNNNNNNNNNNNNNNNNNNNNNNNNNNNNNNNNNNNNNNNNNNCNTCTNNNAAANNNTNNN
NNNNNNNNNNNNNNNNNNNNNNNNNNCNCNTTGNNNGTAANCNANNNANACGTGCAGTCCCCTCNNNNN
NNNGGCAGAGNNNNNNCCCNNNNNNNCCGGCCCCNNCCNNNNNNNNNNNNNNNNGNNNNNNNANNNNNN
NNNNNNTGGTACNNNNCGNNNNNGGTTGTTTAAGAGGTCCACCACGNAGTCNGGNCACNNNNANNNAN
NNNCNNNAAACNNNNNNNNNNNNANNGNNNCANNAGGNCNNNNCCNTCNNGNNANNAGTTGCNATGACC
CAANTCACGGGGGCAGTNCCCGGCGCAGCGACCTTGNTNANNTCCGGGAACAAGTATGGCNNNTNCCC
CGGGAAGGANNCNGCCGTANGTGCNNNNNANNNNNTCNTGNGANGTCGCCGNNATTCNCGTGGTCTNN
NNAGAATGCCCNNACCANNCGNANNNTGGTNATGNNAGTNNNNNNNNNNNNNNNNNNNNNNNNNNNN
NNNNNNNNNNNNNNNNNNNNNNNNNNNNNNNNNNNNNNNGNNNNNNCNANNNAAGGANNTANNNCTNNTN
NTNNNNNNGGACNNNACNNNNCNNNNNNNACNCNNNCNCNNNNNNANNNGNTTTTTNNNCNNTANTNC
CCAGNACGCNGNGCNNNCATTCANGNGAACNNNGNNACGCGTAAATCGTCNNATCTACCTGCCGCNNG
NNNNNNTACNGNNNNTNCGGANNNTNNCTCGTANNNNNNNCATGCGTNNTNTNNTCNCNAGNCNNTTT
CGNNNGNANANTAGNANGTNTNNNNNNNCTTGTNNCGTTCCGCNTGGNNCNNCANNNNGNNGNNNTTN
TCANCGGCNNTANTTGCNGANNTAAGNCATANNNCNNNTCNNCANCTNNNNNGGNGGGNNACTGTAAA
NGGNNAATTNGAGGNCNATTNCNNNCACNTNNCTNNGNNNNCTNNNTNCTTANNTNCNNAGTTGGNNC
CANTANNTGNTNATCTCTGGNTTNGNNNNNNTNNNNNTNNCANNNNGGNNNGTNANGNNNCNATNTAA
NNTANANNNNCNNNNNNANNNCACNNNNNNNGNAGNNNCNANNNTTANANNNGTAGNNNACTAAGTCT
CCGANNTNNNNNNNATAGGCGNNANACNNNNNNNNNNNNNNANGANNACGNNTTGGNNNTNNNNNNANA
ANNAGACNNTNGTGGNTNNNNNTCAANNNNCTNGTAGTGGTATAATNGNNNNNNNTTGCNCCAGNNTN
NNNGNNNNNNNCNATANNANNNNNNNGNGANNNNNNNNNNNANNNNCTCNNNAGGGANCANCTACTNNA
GCANTGGNGCGCAGNNNTAGANNNNGACNNNCCTTGGGTGGCAANNACNGCAATAAGTNNNGTTNANC
CTNNNAGTNNACACGNNNNCANNANNNCNNNNNNANCNGANNTNNNNNNNNNNNNNNNNNNNNNNNNN
NNNNNNNNNNNNNNNNNNNNNNNNNNNNNATNNNANGGNNNGTNTNTGACNCATGTGTTTTTANNNGNN
NNNNNACGNNNNANCCCCGGTCGNNNNCNNATTAAANNCCTGNNGCATNNNNNTNCNATAATGANNGC
NNNNNGNNTNGNCTNNCNNNTCAAGNAGTCANNTAGNNNNNNNGNTCNNNGNNNNNNNNCNNTCCCNN
ACTGAGGGTNNNNNNNNCATCTGCTNNNNNNNNNNTNCGCNGGCGTNNCNNNNNATCNGTATNGTGTNA
NNCANNNNNNNNNNNNNNNNNTNNAACNANNNNNNNTNNNN
>Ede743
GTGNNNNNTAGCCCNNNNNAGTTAGACANNNCNNNNNNNNNNNNTNNNNNNNNNNNNNNNNNNNNNN
NNNNNNNNNNNNNNNNNNNNNNNNNNNNNNTNNNNNNNNNNNNNNNNNNNNNNNNNNNNNNNNNNNNNN
NNNNNNNNNNNNNNNNNNNNNNNNNNNNNNNNNNNNNNNNNNNNNNNNNNNNNNNNNNNNNNNNNNNN
NNNNNNNNNNNNNNNNNNNNNNNNNNNNNNNNNNNNNNNNNNNNNNNNNNNNNNNNNNNNNNNNNNNN
NNNNNNNNNNNNNNNNNNNNNNNNNNNNNNNNNNNNNNNNNNNNNNNCNTCTNNNAAANNNTNNN
NNNNNNNNNNNNNNNNNNNNNNNNNNCNCNTTGNNNGTAANCNANNNGNACATGCAGTCCCCTCNNNNN
NNNGGCAGAGNNNNNNCCCNNNNNNNCCGGCCCCNNCCNNNNNNNNNNNNCNNNGNNNNNNNANNNNNN
NNNNNNTGGTACNNNNCGNNNNNGGTTGTNTAAGAGGTCCACAACGNGGTCNGGNCACNNNNGNNNAN
NNNCNNAAAACNNNNNNNNNNGNNNNTNNNTANNAGGNCNNNNCCNTCNNGNNNNNNATTGCNATGACC
CAANTCACGGGGGCAGTNCCCGGCGCGGCGACCTTGATNACGTCCGGGGACAAGTATGGCGNNCNCCC
CGGGAAGGANNCNGCCGTANGTGCNNNNNANNGNNTCNTGNGACGTCGCCGNNATTCNCGTGGTCNNN
CNAGAATGNCCGNACCANNCGNANNNTGGTNATGNTAGTNNNNNNNNNNNNNNNNNNNNNNNNNNNN
NNNNNNNNNNNNNNNNNNNNNNNNNNNNNNNNNNGNNNNGNNNNNNCNANNNAAGGNNNTANNNCTNNTC
NTNNNNNNGGACNNNACNNNNCNNNNNNNACNCNNNCNCNNNNNNANNNGGTTTTTNNNCNNTATTNT
CCAGNACGCNGNGCNNNCATTCANGNGAACNNNGNNACGCGNNAATCGTANNATCTACCTGCCGCNNG
NNNNNNTACNGNNNNTNCGGANNNTNNCTCGTANNNNNNTCATGCGTNNTNTNNTCNCNAGNCANGTT
CGNNNANANANTAGNANGTNTNNNNNNNNNTGTNNCGTTCCGCNTGANNCNNCANNNNGNNGNNCTTN
TCANCGGCNNTANCTGCNGANNTAAGNCATANNNTNNATCNNTANCTNNNNNGGNGGGNNACTGTAAA
NGGNTAATTNGNGGNTNATTNCCNNCACNTNNCNNNGNNTNCTNNNTNCCTGNNTNCNNAATTGGNNC
CANTGNNTGNCNATCTCTGGNTTNGNNNNNNTNNNNNTNNCANNNNGGNNNNNNANANNNCNATNTAA
NNTATANNNNCNNNNNNANNNCACNANNNNNGNAGNNACNANNNTTANANNNGTAGNNNAATAAGTCT
CCGANNTNNNNNNNATAAGCGNNANACNNNNNNNNNNNNNNANGANNACGNNTTGGNNNTNNNNNNCNA
GNNAGACNNTNATGGNTNNNNNTCAANNNNTTCGTAGTGGTATAATNGNNNNNNNTTTCNCCAGNNTN
NNNGNNNNNNNCNATANNANNNNNNNGNGANNNNNNNNNNNANNNTCTCNNNAGGGANCANCTACTTNA
GCANTGGNGCGCAGNNNGAGANNNNGACNNGCCTTGGGTGGCAANCACNGCAATAAGTNNNGTTNANC

CTNNNAGTNNACATNNNNNCANNGNNNCNNNNNNANCNGANNGNNNNNNNNNNNNNNNNNNNNNNNNN
NNNNNNNNNNNNNNNNNNNNNNNNNNNNAGNNNANGGNNNGTNTNTGACNCATGTGTTTTTANNNGNN
NNNNNACGNNNNANCCCCGGTCGNNNNCNNATTGAANNCCTGNNGCATNNNNNTNCNAGAATGANNGC
NNNNNGNNTNGNCTNNCNNNTCAAGNAATCAANNAGNTNNCGCGNTCNNNGNNNNNNNNCNNTCCCNN
ACTGAGGGTNNNNNNCGCATCTGCTNNNNNNNNCTNCGCNGGNGTNNCNNNNNAGCNGTATNGTGTNA
NNCANNNNNNNNNNNNNNNNNCNAAACNANNNNNNTNNNN
>Ede744
GTGNNNNNTAGCCCGNNNNAGTTAGACANNNCNNNNNNNNNNNNTNNNNNNNNNNNNNNNNNNNNNNN
NNNNNNNNNNNNNNNNNNNNNNNNNNNNNNNNNNNNNNNNNNNNNNNNNNNNNNNNNNNNNNNNNNNN
NNNNNNNNNNNNNNNNNNNNNNNNNNNNNNNNNNNNNNNNNNNNNNNNNNNNNNNNNNNNNNNNNNNN
NNNNNNNNNNNNNNNNNNNNNNNNNNNNNNNNNNNNNNNNNNNNNNNNNNNNNNNNNNNNNNNNNNNN
NNNNNNNNNNNNNNNNNNNNNNNNNNNNNNNNNNNNNNNNNNNNNNNNNNCNTCTNNNAAANNNTNNN
NNNNNNNNNNNNNNNNNNNNNNNNNCNCNTTGNNNGTAANCNANNNGNACATGCAGTCCCCTCNNNNN
NNNGGCAGAGNNNNNNCCCNNNNNCNCCGNCCCCGNCCNNNNNNNNNNNCNNNGNNNNNNNANNNNNN
NNNNNNTGGTACNNNTCGNNNNNGGTTGTNTAAGAGGTCCACAACGNGGTCNNGNCACNNNNGNNTAN
NNNCNNNNAACNGNNNNNNNNNAANNTNNNTANNAGGNCNNNNCCNTCNNNNNNNNNAANTGCNATGACC
CAANTCACGGGGGCAGTCCCCGGCGCGGCGACCTTGANNACGTCCGGGGACAAGTATGGCNNNCNCCC
CGGGAAGGANNCNGCCGTANGTGCNNNNNANNNTNTCNTGNGANGTNGCCGNNATTCNCGTGGTCNNN
NNAGAATGNCCGNACCANNCGNANNNTGGTNATGTTAGTNNNNNNNNNNNNNNNNNNNNNNNNNNNNN
NNNNNNNNNNNNNNNNNNNNNNNNNNNNNNNNNNNNGNNNNGNNNNNNCNANNNAAGGANNTANNNCTNNTN
NTNNNNNNNGGACNNNACNNNNCNNNNNNNNACNCNNNCNCNNNNNNANNNGGTTTTTNNCCNNTATTNT
CCAGNACGCNGNGCNNNCATTCANGNGAACNNNGNNACGCGNNAATCGTANNATCTACCTGCCGCNGG
TNNNNNTACNGNNNNNNNCGGANNNTNNCTCGTANNNNNNNNCANGCGTNNTNTNNTCNCNAGNCANGTT
CGNNNANANANTAGNANGTNTCNNNNNNNNNTGTNNCGTTCCGCNTGANNCNNCANNNNGNNGNNCTTN
TCANCGGCNCTANCTGCNGANNTAAGNCATANNNTNNNTCNNTANCTNNNNNGGNGGGNNACTGTAAA
NGGNTAATTNGANGNTNATTNCNNNCACNTNNCTNNGNNTNCTNNNTNCCTGNNTNCNNAATTGGNNC
CANTGNNTGNCNATCTCTGGNTTNGNNNNNNTGNNNNTNGCAANNNGGNNNGTNANANNNCNATNTAA
NNTANANNNNCNNNNNNANNNCACNANNNNNGNAGNNNCNANNNTTANANNNGTAGNNNAATAAGTCT
CCGANNTNNNNNNNATAAGCGNNANACNNNNNNNNNNNNNNANGANNACGNNTTGGNNNTNNNNNNCNA
GNNAGACNNTNATGGNTNNNNNTCAANNNNTTCGTAGTGGTATAATNGNNNNNNNTTTCNCCAGNNTN
NNNGNNNNNNNNCNATANNANNNNNNNGTGANNNNNNNNNNNANNNNCTCNNNAGGGANCANCTACTTNA
GCAATGGNGCGCAGNNNGAGANNNNGACCAGCCTTGGGTGGCAANCACNGCAATAAGTNNNGTTNANC
CTNNNAGTNNACATNNNNNCANNGNNNCNNNNNNANCNGANNGNNNNNNNNNNNNNNNNNNNNNNNNN
NNNNNNNNNNNNNNNNNNNNNNNNNNNNNGNNNANGGNNNGTNTNTGACNCATGTGTTTTTANNNGNN
NNNNNACGNNNGANCCCCGGTCGNNTNCNNATTGAANNCCTGNNGCATNNNNNTNCNAGAATGANNGC
NNNNNGNNTNNNCTNNCNTTTCAAGNAATCANNNAGNNANNGCGNTCNNNGNNNNNNNNCNNNCCCNN
ACTGAGGGTNNNNNNCGCATCTGCTNNNNNNNNNNTNCGCNGGCGTNNCTGNNNNGCNGTATNGTGTNA
NNCANNNNNNNNNNNNNNNNNNNNAAACNANNNNNNTNNNN
>Ede745
GTGNNNNNTAGCCCNCNNNAGTTAGACANNNCNNNNNNNNNNNNTNNNNNNNNNNNNNNNNNNNNNNN
NNNNNNNNNNNNNNNNNNNNNNNNNNNNNNTNNNNNNNNNNNNNNNNNNNNNNNNNNNNNNNNNNNNN
NNNNNNNNNNNNNNNNNNNNNNNNNNNNNNNNNNNNNNNNNNNNNNNNNNNNNNNNNNNNNNNNNNNN
NNNNNNNNNNNNNNNNNNNNNNNNNNNNNNNNNNNNNNNNNNNNNNNNNNNNNNNNNNNNNNNNNNNN
NNNNNNNNNNNNNNNNNNNNNNNNNNNNNNNNNNNNNNNNNNNNNNNNNNCNTCTNNNAAANNNTNNN
NNNNNNNNNNNNNNNNNNNNNNNNNCNCNTTGNNNGTAANCNANNNGNACATGCAGTCCCCTCNNNNN
NNNGGCAGAGNNNNNNCCCNNNNNCNCCGGCCCCNNCCNNNNNNNNNNNCNNNGNNNNNNNANNNNNN
NNNNNNTGGTACNNNNCGNNNNNGGTTGTTTAAGAGGTCCACAACGNGGTCNGGNNACNNNNGNNNAN
NNNCNNNAAACNNNNNNNNNNNNANNTNNNTACAAGGNCNNNNCCNTCNNGNNNNNNATTGCNATGACC
CAANTCACGGGGGCAGTCCCCGGCGCGGCGACCTTGANNACGTCCGGGGACAAGTATGACNNNCNCCC
CGGGAAGGANNCNGCCGTANGTGCNNNNNANNNNNTCNTGNGACGTCGCCGNNATTNNCGTGGTCNNN
CNAGAATGNNCGNACCANNCGNANNNTGGTNATGTNAGTNNNNNNNNNNNNNNNNNNNNNNNNNNNNN
NNNNNNNNNNNNNNNNNNNNNNNNNNNNNNNNNNNNNNNGNNNNNNCNANNNAAGGANNTANNNCTNNTN
NTNNNNNNNGGACNNNACNNNNCNNNNNNNNACNCNNNCNCNNNNNNANNNGGTTTTNNNNCNNTATTNT
CCAGNACGCNGNGCNNNCATTCANGNGAACNNNGNNACGCGNNAATCGTANNATCTACCTGCCGCNNG

TNNNNNTACNGNNNNTNCGGANNNTNNNTCGTANNNNNNTCATGCGTNNTNTNNTCNCNAGNCANGTT
CGNNNANANANTAGNANGTNTNNNNNNNCTTGTNNCGTTCCGCNTGANNCNNCANNNNGNCGNNCTTN
TCANCGGCNNTANCTGCNGANNTAAGNCATANNNTNNATCNNTANCTNNNNNGGNGGGNNACTGTAAA
NGGNNAATTNGNNGNTNATTTCNNNCACNTNNCTNNGNNNNCTNNNTNCCTGNNTNCNNAATTGGNNC
CANTGNNTGNCNATCTCTGGNTTNGNNNNNNTNNNNNTNGCANNNNGGNNNGTNANANNNCNATNTAA
NNTANANNNNCNNNNNNANNNCACNNNNNNNNNAGNNACNANNNTTANANNNGTAGNNNAATAAGTCT
CCGANNTNNNNNNNATAAGCGNNANACNNNNNNNNNNNNNANGANNACGNNTTGGNNNTNNNNNNCNA
GNNAGACNNTNATGGNTNNNNNTCAANNNNTTNGTAGTGGTATAATNGNNNNNNNTTTCNCCAGNNTN
NNNGNNNNNNNCNATANNANNNNNNNGNGANNNNNNNNNNANNNNCTCNNNAGGGANCANCTACTNNA
GCAATGGNGCGCAGNNNGAGANNNNGACNNGCCTTGGGTGGCAANNACNGCAATAAGTNNGTTNANC
CTNNNAGTNNACATNNNNNCANNGNNNCNNNNNNANCNGANNGNNNNNNNNNNNNNNNNNNNNNNNNN
NNNNNNNNNNNNNNNNNNNNNNNNNNNNAGNNNANGGNNNGTNTNTGACNCATGTGTTTTTANNNGNN
NNNNNACGNNNGANCCCCGGTCGNNNNCNNATTGAANNCCTGNNGCATNNNNNTNCNAGAATGANNGC
NNNNNGNNTNGNCTNNCNTTTCAAGNAATCAANTAGGTNNCGCGNTCNNNNNNNNNNNNCNNTCCCNN
ACTGAGGGTNNNNNCGCATCTGCTNNNNNNNNNCTNCGCNGGNGTNNCNNNNNAGCNGTATNGTGTNA
NNCATNNNNNNNNNNNNNNNNNTNAAACNANNNNCNTNNNN
>Ede746
GTGNNNNNTAGCCCGNNNNAGTTAGACACNNCNNNNNNNNNNNNTNNNNNNNNNNNNNNNNNNNNNNN
NNNNNNNNNNNNNNNNNNNNNNNNNNNNNNTNNNNNNNNNNNNNNNNNNNNNNNNNNNNNNNNNNNNNN
NNNNNNNNNNNNNNNNNNNNNNNNNNNNNNNNNNNNNNNNNNNNNNNNNNNNNNNNNNNNNNNNNNNN
NNNNNNNNNNNNNNNNNNNNNNNNNNNNNNNNNNNNNNNNNNNNNNNNNNNNNNNNNNNNNNNNNNNN
NNNNNNNNNNNNNNNNNNNNNNNNNNNNNNNNNNNNNNNNNNNNNNNNNNCNTCTNNNAAANNNTNNN
NNNNNNNNNNNNNNNNNNNNNNNNNCNCNNTGNNNGTAANCNANNNGNACATGCAGTCCCCTCNNNNN
NNNGGCAGAGNNNNNNNCCNNNNNCNCCGGCCCCNNCCNNNNNNNNNNNCNNNGNNNNNNNANNNNNN
NNNNNNTGGTACNNNNCGNNNNNGGTTGTTTAAGAGGTCCACAACGNGGTCNGGNCACNNNNGNNTAN
NNNCNNANAACNNNNNNNNNNNANATNNNTANNAGGNCNNNGCCNTCNNGNNANTNATTGCNATGACC
CAANNCACGGGGGCAGTCCCCGGCGCGGCGACCTTGANNACGTCCGGGGACAAGTATGGCNNNCNCCC
CGGGAAGGANNCNGCCGTANGTGCNNNNNANNNNNTCTTGNGANGTCGCCGNNATTCNCGTGGTCNNN
CNAGAATGNCCGNACCANNCGNANNNTGGTNATGNTAGTNNNNNNNNNNNNNNNNNNNNNNNNNNNNN
NNNNNNNNNNNNNNNNNNNNNNNNNNNNNNNNGNNNNGNNNNNNCNANNNAAGGANNTANNNCTNNTN
NTNNNNNNGGACNNNACNNNNCNNNNNNNACNCNNNCNCNNNNNNANNNGGTTTTTNNCCNNTATTNT
CCAGNACGCNGNGCNNNCATTCANGNGAACNNNGNNACGCGNNAATCGTANNATCTACCTGCCGCNGG
NNNNNNTACNGNNNNTNCGGANNNTNNCTCGTANNNNNNTCATGCGTNNTNTCNTCNCNAGNCANGTT
CGAGAANANANTAGNANGTNTCNNNNNNCNTGTNNCGTTCCGCNTGANNCNNCANNNNGNNGNNCTTN
TCANCGGCNCTANCTGCNGANNTAAGTCATANNNTNNATCNNTANCTNNNNNGGNGGGNNACTGTAAA
NGGNTAATTGGANGNTNATTTCCNNCACNTNNCTNNGNNNNCTNNNTNCCTGNNTNCNNAATTGGNNC
CANTGNNTGNCNATCTCTGGNTTNGNNNNNNTGNNNNTNNCAANNNGGNNNGTNANANNNCNATNTAA
NNTATANNNNCNNNNNNANNNCACNANNNNNGNAGNNNCNANNNTTANANNNGTAGNNNAATAAGTCT
CCGANNTNNNNNNNATAAGCGNNANACNNNNNNNNNNNNNANGANNACGNNTTGGNNNTNNNNNNCNA
GNNAGACNNTNATGGNTNNNNNTCAANNNNTTCGTAGTGGTATAATNGNNNNNNNTTTCNCCAGNNTN
NNNGNNNNNNNCNATANTANNNNNNNGNGANNNNNNNNNNANNNNCTCNNNAGGGANCANCTACTTNA
GCAATGGNGCGCAGNNNGAGANNNNGACNAGCCTTGGGTGGCAANCACNGCAATAAGTNNGTTNANC
CTNNNAGTNNACATGNNNNCANNGNNNCNNNNNNANCNGANNGNNNNNNNNNNNNNNNNNNNNNNNNN
NNNNNNNNNNNNNNNNNNNNNNNNNNNNAGNNNANGGNNNGTNTNTGACNCATGTGTTTTTANNNGNN
NNNNNACGNNNGANCCCCGGTCGNNNNCNNATTGAANNCCTGNNGCATNNNNNTNCNAGAATGANNGC
NNNNNGNNTNGNCTNNCNNNTCAAGNAATCANNNAGNTANCGCGNTCNNGNNNNNNNNNCNNTCCCNN
ACTGAGGGTNNNNNCGCATCTGCTNNNNNNNNNCTTCGCNGGCGTNNCNGNNNAGCNGTATNGTGTNA
NNCANNNNNNNNNNNNNNNNNNTNAAACNNNNNNNNNTNNNN
>Ede747
GTGNNNNNTAGCCCNNNNNAGTTAGACANNNCNNNNNNNNNNNNTNNNNNNNNNNNNNNNNNNNNNNN
NNNNNNNNNNNNNNNNNNNNNNNNNNNNNNTNNNNNNNNNNNNNNNNNNNNNNNNNNNNNNNNNNNNNN
NNNNNNNNNNNNNNNNNNNNNNNNNNNNNNNNNNNNNNNNNNNNNNNNNNNNNNNNNNNNNNNNNNNN
NNNNNNNNNNNNNNNNNNNNNNNNNNNNNNNNNNNNNNNNNNNNNNNNNNNNNNNNNNNNNNNNNNNN
NNNNNNNNNNNNNNNNNNNNNNNNNNNNNNNNNNNNNNNNNNNNNNNNNNCNTCTNNNAAANNNTNNN

NNNNNNNNNNNNNNNNNNNNNNNNNNCNCNTTGNNNGTAGNCANNNANACATGCAGTCCCCTCNNNNN
NNNGGCAGAGNNNNNNCCCNNNNNNCNCCGNCCCCNNCCNNNNNNNTNNNCNNNGNNNNNNNNANNNNNN
NNNNNNTGGTACNNNNCGNNNNNGGTTGTTTAAGAGGTCCACCACGNAGTCNGGNCACNNNNGNNNAN
NNNCNNNNAACNNNNNNNNNNNNANNGNNNCANNAGGNCNNNNCCNTCNNNNNNNNNNGTTGCNATGACC
CAANNCNCGGGGGCAGTNCCCGGCGCGGCGACCTTGANNACGTCCGGGAACAAGTATGGCNNNTNCCC
CGGGAAGGANNCNGCCGTANGTGCNNNNNANNNNNTCNTGNGACGTCGCCGNNATTNNCGTGGTCNNN
CNAGAATGNNCGNACCANNCGNANNNTGGTNATGTNAGCANNNNNNNNNNNNNNNNNNNNNNNNNNNNNN
NNNNNNNNNNNNNNNNNNNNNNNNNNNNNNNNNNNNNNNGNNNNNNCNANNNAAGGANNTANNNCTNNTN
NTNNNGNNGGACNNNACNNNNCNNNNNNNACNCNNNCNCNNNNNNANNNGGTTTTTNNNCNNTATTNC
CCAGNACGCNGNGCNNNCATTCANGNGAACNNNGNNACGCGNNAATCGTANNATCTACCTGCCGCNNG
NNNNNNTACNGNNNNNNCGGANNNTNNCTCGTANNNNNNTCATGCGTNNTNTNNTCNCNAGNCNNGTT
CGNNNGNANANTAGNANGTNTNNNNNNNNNTGTNNCGTTCCGCNTGANNCNNCANNNNGNNGNNCTTN
TCANCGGCNNTANCTGCNGANNTAAGNCATANNNCNNNTCNNCANCTNNNNNGGNGGGNNACTGTAAA
NGGNNAATTNGNNGNCNATTNCCNNCACNTNNCTNNGNNTNCTNNNTNCTTGNNTNCNNAGTTGGNNC
CANTANNTGNTNATCTCTGGNTTNGCNNNNNTGNNNNTNNCAANNNGGNNNGTNANANNNCNATNTAA
NNTATANNNNCNNNNNNANNNCACNNNNNNNGNAGNNNCNANNNTTANANNNGTAGNNNACTAAGTCT
CCGANNTNNNNNNNATAGGCGNNANACNNNNNNNNNNNNNNANGANNACGNNTTGGNNNTNNNNNNANA
ANNAGACNNTNGTGGNTNNNNNTCAANNNNCTNGTAGTGGTATAATNGNNNNNNNTTTCNCCAGNNTN
NNNGNNNNNNNNCNATANNANNNNNNNGNGANNNNNNNNNNNANNNNCTCNNNAGGGANCANCTACTNNA
GCAATGGNGCGCAGNNNTAGANNNNGACNNNCCTTGGGTGGCAANCACNGCAATAAGTNNNGTTNANC
CTNNNAGTNNACACGNNNNCANNANNNCNNNNNNANCNGANNTNNNNNNNNNNNNNNNNNNNNNNNNNNN
NNNNNNNNNNNNNNNNNNNNNNNNNNNNNNATNNNANGGNNNGTNTNTGACNCATGTGTTTTTANNNGNN
NNNNNACGNNNNANCCCCGGTCGNNNNCNNATTAAANNCCTGNNGCATNNNNNTNCNATAATGANNGC
NNNNNGNNTNGNCTNNCNTTTCAAGNAGTCANNTAGGTNNNNCGNTCNNNGNNNNNNNNCNNTCCCNN
ACTGAGGGTNNNNNNNNCATCTGCTNNNNNNNNNCTNCGCNGGNGTNNCNNNNNATCNGTATNGTGTNA
NNCANNNNNNNNNNNNNNNNNNAANNAANNNNNNNTNNNN
>Ede748
GTGNNNNNTAGCCCNNNNNAGTTAGACANNNCNNNNNNNNNNNNTNNNNNNNNNNNNNNNNNNNNNNNNN
NNNNNNNNNNNNNNNNNNNNNNNNNNNNNNNNNNNNNNNNNNNNNNNNNNNNNNNNNNNNNNNNNNNNNN
NNNNNNNNNNNNNNNNNNNNNNNNNNNNNNNNNNNNNNNNNNNNNNNNNNNNNNNNNNNNNNNNNNNNNN
NNNNNNNNNNNNNNNNNNNNNNNNNNNNNNNNNNNNNNNNNNNNNNNNNNNNNNNNNNNNNNANNNNNNN
NNNNNNNNNNNNNNNNNNNNNNNNNNNNNNNNNNNNNNNNNNNNNNNNNNNNCNTCTNNNAAANNNTNNN
NNNNNNNNNNNNNNNNNNNNNNNNNNCNCNTTGNNNGTAANCANNNGNACATGCAGTCCCCTCNNNNN
NNNGGCAGAGNNNNNNCCCNNNNNNNNCCGGCCCCNNCCNNNNNNNNNNNNCNNNGNNNNNNNNANNNNNN
NNNNNNTGGTACNNNNCGNNNNNGGTTGTTTAAGAGGTCCACAACGNGGTCNNGNCACNNNNGNNNAN
NNNCNNNAAACNNNNNNNNNNNNANATNNNTANNAGGNCNNNNCCNTCNNNNNANNNATTGCNATGACC
CAANTCACGGGGGCAGTNCCCGGCGCGGCGACCTTGANNACGTCCGGGGACAAGTATGGCGNNCNCCC
CGGGAAGGANNCNGCCGTANGTGCNNNNNANNNNNTCNTGNGANGTCGCCGNNATTNNCGTGGTCNNN
CNAGAATGCCCGNACCANNCGNANNNTGGTNATGNNAGTNNNNNNNNNNNNNNNNNNNNNNNNNNNNNNN
NNNNNNNNNNNNNNNNNNNNNNNNNNNNNNNNNNGNNNNGNNNNNNCNANNNAAGGANNTANNNCTNNTN
NTNNNNNNGGACNNNACNNNNCNNNNNNNACNCNNNCNCNNNNNNANNNGGTTTTNNNNCNNTATTNT
CCAGNACGCNGNGCNNNCATTCAAGNGAACNNNGNNACGCGNNAATCGTANNATCTACCTGCCGCNNG
TNNNNNTNCNGNNNNNNCGGANNNTNNNNCGTANNNNNGTCATGCGTNNTNTNNTCNCNAGNCNNGTT
CGNNNANANANTAGNANGTNTNNNNNNNCTTGTNNCGTTCCGCNTGANNCNNCANNNNGNNGNNCTTN
TCANCGGCNNTANCTGCNGANNTAAGTCATANNNTNNNTCNNTANCTNNNNNGGNGGGNNACTGTAAA
NGGNNAATTNGNNNNTNATTNCNNNCACNTNNCTNNGNNNNCTNNNTNCCTGNNTNCNNAATTGGNNC
CANTGNNTGNCNATCTCTGGNTTNGCNNNNNTNNNNNTNNCANNNNGGNNNGTNANANNNCNATNTAA
NNTATANNNNCNNNNNNANNNCACNNNNNNNGNAGNNACNANNNTTANANNNGTAGNNNAATAAGTCT
CCGANNTNNNNNNNATAAGCGNNANACNNNNNNNNNNNNNNANGANNACGNNTTGGNNNTNNNNNNCNA
GNNAGACNNTNATGGNTNNNNNTCAANNNNTTNGTAGTGGTATAATNGNNNNNNNTTTCNCCAGNNTN
NNNGNNNNNNNNCNATANNANNNNNNNGNGANNNNNNNNNNNANNNNCTCNNNAGGGANCANCTACTTNA
GCANTGGNGCGCAGNNNGAGANNNNGACNNNCCTTGGGTGGCAANCACNGCGATAAGTNNNGTTNANC
CTNNNAGTNNACATNNNNNCANNGNNNCNNNNNNANCNGANNGNNNNNNNNNNNNNNNNNNNNNNNNNNN
NNNNNNNNNNNNNNNNNNNNNNNNNNNNNNAGNNNANGGNNNGTNTNTGACNCATGTGTTTTTANNNGNN

NNNNNACGNNNNANCCCCGGTCGNNTNCNNATTGAANNCCTGNNGCATNNNNNTNCNAGAATGANNNC
NNNNNGNNTNGNCTNNCNTTTCAAGNAATCAANTAGGTANNNCGNTCNNNGNNNNNNNNCNNTCCCNN
ACTGAGGGTNNNNNNCGCATCTGCTNNNNNNNNCTTCGCNGGNGTNNCNNNNNNGCNGTATNGTGTNA
NNCANNNNNNNNNNNNNNGATNNAACANNNNNNNCTNNNN
>Ede749
GTGNNNNNTAGCCCNNNNNAGTTGGACANNNCNNNNNNNNNNNNTNNNNNNNNNNNNNNNNNNNNNNNNN
NNNNNNNNNNNNNNNNNNNNNNNNNNNNNNTNNNNNNNNNNNNNNNNNNNNNNNNNNNNNNNNNNNNNNNNN
NNNNNNNNNNNNNNNNNNNNNNNNNNNNNNNNNNNNNNNNNNNNNNNNNNNNNNNNNNNNNNNNNNNNNN
NNNNNNNNNNNNNNNNNNNNNNNNNNNNNNNNNNNNNNNNNNNNNNNNNNNNNNNNNNNNNNNNNNNNNN
NNNNNNNNNNNNNNNNNNNNNNNNNNNNNNNNNNNNNNNNNNNNNNNNNNNNCNTCTNNNAAANNNTNNN
NNNNNNNNNNNNNNNNNNNNNNNNNNNCNCNTTGNNNGTAANCNANNNGNACATGCAGTCCCCTCNNNNN
NNNGGCAGAGNNNNNNCCCNNNNNCNCCGGCCCCNNCCNNNNNNNNNNNNCNNNGNNNNNNNNGNNNNNN
NNNNNNTGGTACNNNNCGNNNNNGGTTGTNTAAGAGGTCCACCACGNGGTCNGGNCACNNNNGNNNAN
NNNCNNNAAACNNNNNNNNNNNNANNTNNNCANNAGGNCNNNNCCNTCNNGNNNNTNATNGCNATGACC
CAANTCGCGGGGACAGTNACCGGCGCGGCGACCTTGATNACGGTCGGGGACAAGTATGTCNTNCNCCC
CGGGAAGGANNCNGCCGTANGTGCNNNNNANNNNNTCNTGNGACGTCGCCGNNATTCNCGTGGTCNNN
NNAGAATGNCCNNACCANNCGNANNNTGGTTATGNNAGTCNNNNNNNNNNNNNNNNNNNNNNNNNNNNNN
NNNNNNNNNNNNNNNNNNNNNNNNNNNNNNNNNNNNNNNGNNNNNNCNANNNAAGGANNTANNNTTNNTN
NTNNNGNNGGACNNNACNNNNCNNNNNNNACNCNNNCNCNNNNNNANNNGGTTTTTNNNCNNTATTNT
CCAGNACGCNGNGCNNNCATTCANGNGAACNNNGNNACGCGNNAATCGTANNATCTACCTGCCGCNNG
TNNNNNTACNGNNNNNNCGGANNNTNNCTCGTANNNNNNTCATGCGTNNTNTNNTTNCNAGNCNNGTT
CGNNNANANANTAGNANGTNTNNNNNNNCTTGTNNCGTTCCGCNTGANNCNNCANNNNGNNGNNCTTN
TCANCGGCNNTANCTGCNGANNTAAGNCATANNNTNNGTCNNTANCGNNNNNGGNGGGNNACTGTAAA
NGGNNAATTNGNNGNTNATTNCNNNCACNTNNCNNNGNNTNCTNNNCNCCTGNNTNCNNAATTGGNNC
CANTGNNTGNCNATCTCTGGNTTNGNNNNNNTNNNNNTNGTAANNNGGNNNGTNNNANNNCNATNTAA
NNTATANNNNCNNNNNNANNNCACNANNNNNGNAGNNACNANNNTGANGNNNGTAGNNNAATAAGTCT
CCGANNTNNNNNNNATAAGCGNNANACNNNNNNNNNNNNNANGANNACGNNTTGGNNNTNNNNNNCNA
ANNAGACNNCNGTGGNTNNNNNTCAANNNNTTCGTAGTGGTATAACNANNNNNNNTTTCNCCAGNNTN
NNNGNNNNNNNNCNATANNANNNNNNNGNGANNNNNNNNNNANNNNCCCNNNAGGGANCANCTATTNNA
GCAATGGNGCGCAGNNNGAGANNNNGACNNGCCTTGGGTGGCAANCACNGCAATAAGTNNNGTTNANC
CCNNNAGTNNACACGNNNNCANNGNNNCNNNNNNANCNGANNTNNNNNNNNNNNNNNNNNNNNNNNNNN
NNNNNNNNNNNNNNNNNNNNNNNNNNNNNNAGNNNANGGNNNGTNTNTGACNCATGTGTTTTTANNNGNN
NNNNNACGNNNNANCCCCGGTCGTNNNCNNATTGAANNCCTGNNGCATNNNNNTNCNAGAATGANNGC
NNNNNGNNTNGNCTNNCNTTTCAAGTAGTCAANNAGGTANNNNGNTCNNNGNNNNNNNNCNNTCCCNN
ACTGAGGGTNNNNNNNNCATCTGCTNNNNNNNNCTNCGCCGNCGTNNTNNNNNAGCNGTATNGNGTNA
NNCANNNNNNNNNNNNNNNNNTNAAACAANNNNNNTNNNN
>Ede750
GTGNNNNNTAGCCCNNNNNAGTTAGACANNNCNNNNNNNNNNNNTNNNNNNNNNNNNNNNNNNNNNNNNN
NNNNNNNNNNNNNNNNNNNNNNNNNNNNNNTNNNNNNNNNNNNNNNNNNNNNNNNNNNNNNNNNNNNNNNNN
NNNNNNNNNNNNNNNNNNNNNNNNNNNNNNNNNNNNNNNNNNNNNNNNNNNNNNNNNNNNNNNNNNNNNN
NNNNNNNNNNNNNNNNNNNNNNNNNNNNNNNNNNNNNNNNNNNNNNNNNNNNNNNNNNNNNNNNNNNNNN
NNNNNNNNNNNNNNNNNNNNNNNNNNNNNNNNNNNNNNNNNNNNNNNNNNNNNNTCTNNNAAANNNTNNN
NNNNNNNNNNNNNNNNNNNNNNNNNNNCNCNTTNNNNGTAANCNANNNGNACATGCAGTCCCCTCNNNNN
NNNGGCAGAGNNNNNNCCCNNNNNNNCCGNCCCCNNCCNNNNNNNNNNNNCNNNGNNNNNNNANNNNNN
NNNNNNTGGTACNNNNCGNNNNNGGTTGTTTAAGAGGTCCACAACGNGGTCNNGNCACNNNNGNNNAN
NNNCNNNAAACNNNNNNNNNNNNANNTNNNTANNAGGNCNNNNCCNTCNNNNNNNNNAATTGCNATGACC
CAANNCACGGGGGNNGTCCCCGGCGNGGCGACCTTGNTNANGTCCGGGGACAAGTATGACGNNCNCCC
CGGGAAGGANNCNGCCGTANGTGCNNNNNANNNNNTCNTGNGACGTNGCCGNNATTCNCGTGGTCNNN
CNAGAATGNCCNNACCANNCGNANNNTGGTNATGNNAGTNNNNNNNNNNNNNNNNNNNNNNNNNNNNNNN
NNNNNNNNNNNNNNNNNNNNNNNNNNNNNNNNNNNNNNNGNNNNNNCNANNNAAGGANNTANNNCTNNTN
NTNNNNNNGGACNNNACNNNNCNNNNNNNACNCNNNCNCNNNNNNANNNGNTTTTTNNNCNNTATTNT
CCAGNACGCNGNGCNNNCATTCANGNGAACNNNGNNACGCGNNAATCGTANNATCTACCTGCCGCNNG
TNNNNNTACNGNNNNNNCGGANNNTNNCTCGTANNNNNNTCATGCGTNNTNTNNTCNCNAGNCNNGTT
CGNNNANANANTAGNANGTNTNCNNNNNNNTGTNNCGTTCCGCNTGANNCNNCANNNNGNCGNNCTTN

TCANCGGCNNTANCTGCNGANNTAAGNCATANNNTNNNTCNNTANCTNNNNNGGNGGGNNACTGTAAA
NGGNNAATTNGNNNNTNATTNCNNNCACNTNNCNNNGNNTNCTNNNTNCCTGNNTNCNNAATTGGNNC
CANTGNNTGNCNATCTCTGGNTTNGNNNNNNTGNNNNTNNCANNNNGGNNNGTNNNANNNCNATNTAA
NNTANANNNNCNNNNNNANNNCACNANNNNNGNAGNNACNANNNTTANANNNGTAGNNNAATAAGTCT
CCGANNTNNNNNNNATAAGCGNNANACNNNNNNNNNNNNNNANGANNACGNNTTGGNNNTNNNNNNNNA
GNNAGACNNTNATGGNTNNNNNTCAANNNNTTCGTAGTGGTATAATNGNNNNNNNTTTCNCCAGNNTN
NNNGNNNNNNNNCNATANNANNNNNNNGNGANNNNNNNNNNNANNNNCTCNNNAGGGANCANCTACTNNA
GCANTGGNGCGCAGNNNGAGANNNNGACNNNCCTTGGGTGGCAANCACNGCAATAAGTNNNGTTNANC
CTNNNAGTNNACATNNNNNCANNGNNNCNNNNNNANCNGANNGNNNNNNNNNNNNNNNNNNNNNNNNN
NNNNNNNNNNNNNNNNNNNNNNNNNNNNNNAGNNNANGGNNNGTNTNTGACNCATGTGTTTTTANNNGNN
NNNNNACGNNNGANCCCCGGTCGNNNNCNNATTGAANNCCTGNNGCATNNNNNTNCNAGAATGANNGC
NNNNNGNNTNGNCTNNCNNNTCAAGNAATCANNNAGNNANCGCGNTCNNNNNNNNNNNNNCNNTCCCNN
ACTGAGGGTNNNNNNNNCATCTGCTNNNNNNNNNNTNCGCCGGNGTNNCNNNNNAGCNGTATNNNGTNA
NNCANNNNNNNNNNNNNNNGNTNAANNNANNNNNCTNNNN
>Ede88
GTGNNNNNTAGCCCNNNNNAGTCAGACANNNCNNNNNNNNNNNNTNNNNNNNNNNNNNNNNNNNNNNN
NNNNNNNNNNNNNNNNNNNNNNNNNNNNNNTNNNNNNNNNNNNNNNNNNNNNNNNNNNNNNNNNNNNN
NNNNNNNNNNNNNNNNNNNNNNNNNNNNNNNNNNNNNNNNNNNNNNNNNNNNNNNNNNNNNNNNNNNNNNN
NNNNNNNNNNNNNNNNNNNNNNNNNNNNNNNNNNNNNNNNNNNNNNNNNNNNNNNNNNNNNNNNNNNNNNN
NNNNNNNNNNNNNNNNNNNNNNNNNNNNNNNNNNNNNNNNNNNNNNNNNNNNNCNTCTNNNAAANNNTNNN
NNNNNNNNNNNNNNNNNNNNNNNNNNNCNCNTTGNNNGTAANCNANNNGNACATGCAGTCCCCTCNNNNN
NNNGGCAGAGNNNNNNCCCNNNNNNNCCGNCCCCNNCCNNNNNNNNNNNCNNNGNNNNNNNANNNNNN
NNNNNNTGGTACNNNNCGNNNNNGGTTGTNTNAGAGGTCCACAACGNGGTCNGNNNACNNNNGNNNAN
NNNCNNNNNNNNNNNNNNNNNNNNNNNTNNNTANNAGGNCNNNNCCNTCNNNNNNNNNANNGCNATGACC
CAANTCACGGGGGCAGTNCCCGGCGCGGCGACCTTGNNNACGTCCGGGGACAAGTATGGCGNNCNCCC
CGGGAAGGANNCNGCNGTANGTGCNNNNNANNNNNTCNTGNGACGTCGCCGNNATTCNCGTGGTCNNN
CNAGAATGNCCNNACCANNCGNANNNTGGTNATGNNAGTNNNNNNNNNNNNNNNNNNNNNNNNNNNNN
NNNNNNNNNNNNNNNNNNNNNNNNNNNNNNNNNNNNNNNNNGNNNNNNCNANNNAAGGNNNTANNNCTNNTN
NTNNNNNNNGGACNNNACNNNNCNNNNNNNACNCNNNCNCNNNNNNANNNGNTTTTTNNNCNNTANTNT
CCAGNACGCNGNGCNNNCATTTANGNGAACNNNGNNACGCGNNAATCGTANNATCTACCTGCCGCNGG
NNNNNNTACNGNNNNNNCGGANNNTNNNNCGTANNNNNNNNCATGCGTNNTNTNNTCNCNAGNCANGTT
CGNNNANANANTAGNANGTNTNNNNNNNNCTTGTNNCGTTCCGCNTGACNCNNCANNNNGNCGNNNTTC
TCANCGGCNNTANCTGCNGANNTAAGNCATANNNTNNNTCNNTANCTNNNNNGGNGGGNNACTGTAAA
NGGNNAATTGGANNNTNANTNCNNNCATNTNNCTNNGNNNNCTNNNTNCCTGNNTNCNNAATTGGNNC
CANTGNNTGNCNATCTCTGGNTTNGCNNNNNTNNNNNTNNCAANNNGGNNNNNNANANNNCNATNTAA
NNTANANNNNCNNNNNNANNNCACNNNNNNNGNAGNNNCNANNGTANANNNGTAGNNNGNTAAGTCT
CCGANNTNNNNNNNATAAGCGNNANACNNNNNNNNNNNNNNANGANNACGNNTTTGNNNTNNNNNNCNA
GNNAGACNNTNGTGGNTNNNNNTCAANNNNTTNGTAGTGGTATAATNGNNNNNNNTTTCNCCAGNNTN
NNNNNNNNNNNCNATANNANNNNNNNGNGANNNNNNNNNNNANNNNCTCNNNAGGGANCANCTACTTNA
GCAATGGNGCGCAGNNNGAGANNNNGACCAGCCTTGGGTGGCAANNACNGCAATAAGTNNNGTTNANC
CTNNNAGTNNACACGNNNNCANNGNNNCNNNNNNANCNGANNGNNNNNNNNNNNNNNNNNNNNNNNNN
NNNNNNNNNNNNNNNNNNNNNNNNNNNNNGNNNANGGNNNGTNTNTGACNCATGTNTTTNTANNNGNN
NNNNNACGNNNGANCCCCGGTCGNNNNCNNATTGAANNCCTGNNGCATNNNNNTNCNAGAATGANNGC
NNNNNGNNTNGNCTGNNNTTTCAAGNAATCANNNAGGTANNNNGNTCNNNNNNNNNNNNNCNNTCCCNN
ACTGAGGGTNNNNNNNNCATCTGCTNNNNNNNNNNTTCGCNGGCGTNNCNNNNNAGCCGTATNGTGTNA
NNCANNNNNNNNNNNNNNNNNNNAAACAANNNNNNNTNNNN
>Ede90
GTGNNNNNTAGCCCNNNNNAGTTAGACANNNCNNNNNNNNNNNNTNNNNNNNNNNNNNNNNNNNNNNN
NNNNNNNNNNNNNNNNNNNNNNNNNNNNNNTNNNNNNNNNNNNNNNNNNNNNNNNNNNNNNNNNNNNN
NNNNNNNNNNNNNNNNNNNNNNNNNNNNNNNNNNNNNNNNNNNNNNNNNNNNNNNNNNNNNNNNNNNNNNN
NNNNNNNNNNNNNNNNNNNNNNNNNNNNNNNNNNNNNNNNNNNNNNNNNNNNNNNNNNNNNNNNNNNNNNN
NNNNNNNNNNNNNNNNNNNNNNNNNNNNNNNNNNNNNNNNNNNNNNNNNNNNNCNTCTNNNAAANNNTNNN
NNNNNNNNNNNNNNNNNNNNNNNNNNNCNCNTTGNNNGTAANCNANNNGNACATGCAGTCCCCTCNNNNN
NNNGGCAGAGNNNNNNNCCNNNNNNNCCGGCCCCNNCCNNNNNNNNNNNCNNNGNNNNNNNANNNNNN

NNNNNNTGGTACNNNNCGNNNNNGGTTGTNTAAGAGGTCCACAACGNGGTCNGGNCACNNNNGNNTAN
NNNCNNNNAACNNNNNNNNNNNANATNNNTANNAGGNCNNNNCCNTCNNGNNNNNAATNGCNATGACC
CAANNCACGGGGGCNGTCCCCGGCGCGGCGACCTTGANNACGTCCGGGGACAAGTATGGCGNNCNCCC
CGGGAAGGANNCNGCCGTANGTGCNNNNNANNNNNTCNTGNGANGTCGCCGNNATTCNCGTGGTCNNN
CNAGAATGNCCGNACCANNCGNANNNTGGTNATGNTAGTNNNNNNNNNNNNNNNNNNNNNNNNNNNNN
NNNNNNNNNNNNNNNNNNNNNNNNNNNNNNNNGNNNNGNNNNNNCNANNNAAGGNNNTANNNCTNNTN
NTNNNNNNGGACNNNACNNNNCNNNNNNNACNCNNNCNCNNNNNNNNNNGNTTTTNNNCCNNTATTNT
CCAGNACGCNGNGCNNNCATTCANGNGAACNNNGNNACGCGNNAATCGTANNATCTACCTGCCGCNNG
TNNNNNTACNGNNNNNNCGGANNNTNNCTCGTANNNNNNTCATGCGTNNTNTNNTCNCNAGNCNNGTT
CGNNNANANANTAGNANGTNTNNNNNNNNNTGTNNCGTTCCGCNTGANNCNNCANNNNGNNGNNCTTC
TCANCGGCNNTANCTGCNGANNTAAGNCATANNNTNNATCNNTANCTNNNNNGGNGGGNNACTGTAAA
NGGNNAATTGGNNGNTNATTNCNNNCACNTNNCTNNGNNTNCTNNNTNCCTGNNTNCNNAATTGGNNC
CANTGNNTGNCNATCTCTNGNTTGNNNNNNTNNNNNNTNNCAANNNGGNNNNNNANANNNCNATNTAA
NNTATANNNNCNNNNNNANNNCACNANNNNNNNNAGNNNCANNNTTANANNNGTAGNNNAATAAGTCT
CCGANNTNNNNNNNATAAGCGNNANACNNNNNNNNNNNNNANGANNACGNNTTGGNNNTNNNNNNCNA
GNNAGACNNTNATGGNTNNNNNTCAANNNNTTNGTAGTGGTATAATNGNNNNNNNTTTCNCCAGNNTN
NNNGNNNNNNCNATANNANNNNNNNGTGANNNNNNNNNNNANNNNCTCNNNAGGGANCANCTACTNNA
GCAATGGNGCGCAGNNNGAGANNNNGACNNGCCTTGGGTGGCAANCACNGCAATAAGTNNNGTTNANC
CTNNNAGTNNACATNNNNNCANNGNNNCNNNNNNANCNGANNGNNNNNNNNNNNNNNNNNNNNNNNNN
NNNNNNNNNNNNNNNNNNNNNNNNNNNNAGNNNANGGNNNGTNTNTGACNCATGTGTTTTTANNNGNN
NNNNNACGNNNGANCCCCGGTCGNNNNCNNATTGAANNCCTGNNGCATNNNNNTNCNAGAATGANNGC
NNNNNGNNTNGNCTNNCNTTTCAAGNAATCANNNAGGTANNNNGNTCNNNGNNNNNNNNCNNTCCCNN
ACTGAGGGTNNNNNNNNCATCTGCTNNNNNNNNCTNCGCNGGCGTNNCNNNNNAGCNGTATNNNGTNA
NNCANNNNNNNNNNNNNNNNTNAANNGANNNNNNTNNNN
>Ede95
GTGNNNNNTAGCCCNNNNNAGTTAGACANNNCNNNNNNNNNNNNTNNNNNNNNNNNNNNNNNNNNNNN
NNNNNNNNNNNNNNNNNNNNNNNNNNNNNNTNNNNNNNNNNNNNNNNNNNNNNNNNNNNNNNNNNNNNNN
NNNNNNNNNNNNNNNNNNNNNNNNNNNNNNNNNNNNNNNNNNNNNNNNNNNNNNNNNNNNNNNNNNNN
NNNNNNNNNNNNNNNNNNNNNNNNNNNNNNNNNNNNNNNNNNNNNNNNNNNNNNNNNNNNNNNNNNNN
NNNNNNNNNNNNNNNNNNNNNNNNNNNNNNNNNNNNNNNNNNNNNNNNNNCNTCTNNNAAANNNTNNN
NNNNNNNNNNNNNNNNNNNNNNNNNCNCNTTGNNNGTAANCNANNNGNACATGCAGTCCCCTCNNNNN
NNNGGCAGAGNNNNNNCCCNNNNNNNCCGNCCCCGNCCNNNNNNNTNNNNNNNGNNNNNNNANNNNNN
NNNNNNTGGTACNNNNCGNNNNNGGTTGTTTAAGAGGTCCACAACGNGNTCNNNNCACNNNNGNNNAN
NNNCNNNNNANNNNNNNNNNNNNANNTNNNTANNAGGNNNNNNCCNTCNNGNNNNNNANTGCNATGACC
CAANNCNCGGGGGCAGTNCCCGGCGNGGCGACCTTGANNACGTCCGGGGACAAGTATGGCGCNCNCCC
CGGGAAGGANNCNGCCGTANGTGCNNNNNANNNNNTCNTGNGANGTCGCCGNNATTNNCNTGGTCNNN
CNAGAATGNCCGNACCANNCGNANNNTGGTNATGNTAGTNNNNNNNNNNNNNNNNNNNNNNNNNNNNN
NNNNNNNNNNNNNNNNNNNNNNNNNNNNNNNNNNNNNGNNNNNNCNANNNAAGGANNTANNNCTNNTN
NTNNNNNNGGACNNNACNNNNCNNNNNNNACNCNNNCNCNNNNNNNNNNGGTTTTTNNCCNNTATTNT
CCAGNACGCNGNGCNNNCATTCANGNGAACNNNGNNACGCGNNAATCGTANNATCTACCTGCCGCNGG
TNNNNNTACNGNNNNNNCGGANNNTNNNNCGTANNNNNNTCATGCGTNNTNTNNTCNCNAGNCANGTT
CGNNNANANANTAGNANGTNTNNNNNNNNNTGTNNCGTTCCGCNTGANNCNNCANNNNGNCGNNCTTN
TCANCGGCNNTANCTGCNGANNTAAGNCATANNNTNNNTCNNTANCTNNNNNGGNGGGNNACTGTAAA
NGGNNAATTNGNNNNTGATTTCNNNCACNTNNCTNNGNNTNCTNNNTNCCTGNNTNCNNAATTGGNNC
CANTGNNTGNCNATCTCTGGNTTGNNNNNNTNNNNNNTNNCANNNNGGNNNGTNANANNNCNATNTAA
NNTATANNNNCNNNNNNANNNCACNANNNNNGNAGNNACNANNNTTANANNNGTAGNNNAATAAGTCT
CCGANNTNNNNNNNATAAGCGNNANACNNNNNNNNNNNNNANGANNNCGNNTTGGNNNTNNNNNNCNA
GNNAGACNNTNATGGNTNNNNNTCAANNNNTTCGTAGTGGTATAATNGNNNNNNCTTTCNCCAGNNTN
NNNGNNNNNNCNATANNANNNNNNNGNGANNNNNNNNNNNANNNNCTCNNNAGGGANCANCTACTTNA
GCAATGGNGCGCAGNNNGAGANNNNGACNNGCCTTGGGTGGCAANCACNGCAATAAGTNNNGTTNANC
CTNNNAGTNNACATNNNNNCANNGNNNCNNNNNNANCNGANNGNNNNNNNNNNNNNNNNNNNNNNNNN
NNNNNNNNNNNNNNNNNNNNNNNNNNNNAGNNNANGGNNNGTNTNTGACNCATGTGTTTTTANNNGNN
NNNNNACGNNNGANCCCCGGTCGNNNNCNNATTGAANNCCTGNNGCATNNNNNTNCNAGAATGANNGC
NNNNNGNNTNGNCTGNCNTTTCAAGNAATCANNTAGNNANNGCGNTCNNNGNNNNNNNNCNNTCCCNN

ACTGAGGGTNNNNNNNNNCATCTGCTNNNNNNNNNCTTCGCNGGCGTNNCNNNNNAGCNGTATNGTGTNA
NNCANNNNNNNNNNNNNNNNNTNNANCNANNNNNNTNNCN
>Ede97
GTGNNNNNTAGCCCNNNNNAGTTAGACANNNCNNNNNNNNNNNNNTNNNNNNNNNNNNNNNNNNNNNNNN
NNNNNNNNNNNNNNNNNNNNNNNNNNNNNNNNNTNNNNNNNNNNNNNNNNNNNNNNNNNNNNNNNNNNNN
NNNNNNNNNNNNNNNNNNNNNNNNNNNNNNNNNNNNNNNNNNNNNNNNNNNNNNNNNNNNNNNNNNNNNN
CNNNNNNNNNNNNNNNNNNNNNNNNNNNNNNNNNNNNNNNNNNNNNNNNNNNNNNNNNNNNNNNNNNNNN
NNNNNNNNNNNNNNNNNNNNNNNNNNNNNNNNNNNNNNNNNNNNNNNNNNNNCNTCTNNNAAANNNTNNN
NNNNNNNNNNNNNNNNNNNNNNNNNNNCNCNTTGNNNGTAANCNANNNGNACATGCAGTCCCCTCNNNNN
NNNGGCAGAGNNNNNNCCCNNNNNNNCCGGCCCCGNCCNNNNNNNTNNNCNNNGNNNNNNNANNNNNN
NNNNNNTGGTACNNNNCGNNNNNGGTTGTTTAAGAGGTCCACAACGNGGTCNNGNCACNNNNGNNNAN
NNNCNNNNAACNNNNNNNNNNNNNNNNTNNNTANNAGGNNNNNNCCNTCNNNNNNNNNATTGCNATGACC
CAANTCACGGGGGCNGTCCCCGGCGCGGCGACCTTGANNACGTCCGGGGACAAGTATGGCGNNCNCCC
CGGGAAGGANNCNGCCGTANGTGCNNNNNANNNNNTCNTGNGANGTCGCCGNNATTCNCGTGGTCNNN
CNAGAATGNCCNNACCANNCGNANNNTGGTNATGTTAGTNNNNNNNNNNNNNNNNNNNNNNNNNNNNNN
NNNNNNNNNNNNNNNNNNNNNNNNNNNNNNNNNNNNNNGNNNNNNCNANNNAAGGANNTANNNCTNNTN
NTNNNNNNGGACNNNACNNNNCNNNNNNNACNCNNNCNCNNNNNNANNNGGTTTTTNNNCNNTATTNT
CCAGNACGCNGNGCNNNCATTCANGNGAACNNNGNNACGCGNNAATCGTANNATCTACCTGCCGCNNG
TNNNNNTACNGNNNNTNCGGANNNTNNCTCGTANNNNNNTCATGCGTNNTNTNNTCNCNAGNCANGTT
CGNNNANANANTAGNANGTNTNNNNNNNCTTGTNNCGTTCCGCNTGANNCNNCANNNNGNNGNNCTTN
NCANCGGCNNTANCTGCNGANNTAAGNCATANNNTNNNTCNNTANCTNNNNNGGNGGGNNACTGTAAA
NGGNNAATTNGNNGNTNATTTCNNNCACNTNNCTNNGNNTNCTNNNTNCCTGNNTNCNNAATTGGNNC
CANTGNNTGNCNATCTCTGGNTTNGNNNNNNTNNNNNTNNCAANNNGGNNNGTNANANNNCNATNTAA
NNTATANNNNCNNNNNNANNNCACNANNNNNNNAGNNNCNANNNTTANANNNGTAGNNNAATAAGTCT
CCGANNTNNNNNNNATAAGCGNNANACNNNNNNNNNNNNNANGANNACGNNTTGGNNNTNNNNNNCNA
GNNAGACNNTNATGGNTNNNNNTCAANNNNTTCGTAGTGGTATAATNGNNNNNNNTTTCNCCAGNNTN
NNNGNNNNNNNCNATANNANNNNNNNGNGANNNNNNNNNNANNNNCTCNNNAGGGANCANCTACTNNA
GCAATGGNGCGCAGNNNGAGANNNNGACNNNCCTTGGGTGGCAANCACNGCAATAAGTNNNGTTNANC
CTNNNAGTNNACATNNNNNCANNGNNNCNNNNNNANCNGANNGNNNNNNNNNNNNNNNNNNNNNNNNN
NNNNNNNNNNNNNNNNNNNNNNNNNNNNNNGNNNANGGNNNGTNTNTGACNCATGTGTTTTTANNNGNN
NNNNNACGNNNGANCCCCGGTCGNNTNCNNATTGAANNCCTGNNGCATNNNNNTNCNAGAATGANNGC
NNNNNGNNTNGNCTNNCNNNTCAAGNAATCANNTAGGTANCGCGNTCNNNGNNNNTNNNCNNTCCCNN
ACTGAGGGTNNNNNNNCNCATCTGCTNNNNNNNNNCTTCGCNGGNGTNNCNNCNNAGCNGTATNGNGTNA
NNCANNNNNNNNNNNNNNNNNTNAAACNANNNNNNTNNNN
>Giarai100
GNNNNNNNNNNCNNNNNNNNNTANNNNNNNCNNNNNNNNNNNNNNNNNNNNNNNNNNNNNNNNNNNNNN
NNNNNNNNNNNNNNNNNNNNNNNNNNNNNNNNNNNNNNNNNNNNNNNNNNNNNNNNNNNNNNNNNNNNNN
NNNNNNNNNNNNNNNNNNNNNNNNNNNNNNNNNNNNNNNNNNNNNNNNNNNNNNNNNNNNNNNNNNNNNN
NNNNNNNNNNNNNNNNNNNNNNNNNNNNNNNNNNNNNNNNNNNNNNNNNNNNNNNNNNNNNNNNNNNNNN
NNNNNNNNNNNNNNNNNNNNNNNNNNNNNNNNNNNNNNNNNNNNNNNNNNNNNTNNNNNNAANNNNNNN
NNNNNNNNNNNNNNNNNNNNNNNNNNNNNNNNNNNNNNNNTNNNCNANNNNNNNCNNGCANTNNNCTCNNNNN
NNNNNCAGNGNNNNNNNNNNNNNNNNNNCNNNCCCNNNCNNNNNNNNNNNNNNNNGNNNNNNNNNNNNNN
NNNNNNTGGTACNNNNCGNNNNNNNTTGNNNNAGNGGTCCANAACNNNNNNNNNNNNNNNNNNGNNNAN
NNNNNNNNNNNNNNNNNNNNNNNNNTNNNNANNNGGNNNNNNNNNNNNNNNNNNNNNNNNGCNNNNNNN
NNNNNNNNNGNNGNNGTNCCNGNNNNNNNNNNNCTNNNNNANNNNNGNGNNCNNNNNNNNNCNNNNNNNC
NNNNAAGNNNNNNNNNNNNANNTNNNNNNNNNNNNNNNNNNNNNGNNNNNNNCNNNNNNNNNCNNNNNNNNN
NNNGANNNNNNNNNNNNNNNNNNNNNNNNNNNNNNNNNNNNNNNNNNNNNNNNNNNNNNNNNNNNNNNNNN
NNNNNNNNNNNNNNNNNNNNNNNNNNNNNNNNNNNNNNNNNGNNNNNNNNNNNNNNNGANNNNNNNNNNNNN
NNNNNNNNNNNCNNNACNNNNNNNNNNNNNNANNCNNNCNCNNNNNNNNNNNNNNNNNNNNNNNNNNNNNN
NNNNNNNNNNNNGCNNNNNNNNNNNNNNNNNNNNNNNACNNNNNNNNNNNNNNNNNNNNNNCTNCNGCNNN
NNNNNNTNNNGNNNNNNNNNNNNNNNNNNCTNNNNNNNNNNNNNNNNNNNNNNNNNNNNNNCNNNNNNNNNTT
CGNNNNNANANTANNNNNTNNNNNNNNNNNNGTNNCGTTCCGCNTNNNNCNNNNNNNNNNNNNNNNNTTN
NCNNNNNNNNNNNNNNNNNNNNNNNNNNNNNNANNNTNNNNNNNNNNNCNNNNNNNNNNGGNNNCNNNNNNN
NNNNNNNNNNNNNNNNNNANNNNNNNNNNNNNNNNCNNNNNNNNNCNNNNNNNNNNNNNNNNCNNNNNNNNNNN

NANTGNNNGNNNNTCTNTGNNNNNGNNNNNNTNNNNNNNNNNNNNNNNNNNNNNNNNNNNNNNNNNNA
NNNNNNNNNNCNNNNNNNNNNNNNNNNNNNNNNAGNNNNNNNNNNNNNGNNNNNNNNNNNNNNNNNNN
CNNNNNNNNNNNNNNNNNNCNNNNNNNNNNNNNNNNNNNNNNNANNNNNNNNTNNNNNTNNNNNNNNN
NNNNGANNNNNNNNGNNNNNNNNNNNNNNNNTNNTNNNNNNATNNNNNNNNNNNNNNTNNCCNNNNTN
NNNNNNNNNNNCNNNNNNANNNNNNNNNGNNNNNNNNNNNANNNNNTCNNNNNNNANCNNCNACNNNA
GCNNNGNNNNNNNNNNNNNGANNNNNNNNNNNNTTGNGNGNNNNNNACNNNANNAANNNNNNNNNNNC
CTNNNAGTNNANATNNNNNCANNNNNNNNNNNNNNNNNNNNNNNNNNNNNNNNNNNNNNNNNNNNNNN
NNNNNNNNNNNNNNNNNNNNNNNNNNNNNNNNNNNGGNNNNTNNNNNNNNNCNNNNNNNNNNNNNNNNN
NNNNNNNGNNNNNNCCCCGGTCNNNNNNNNNTNNNNNNNCTGNNNNNTNNNNNNNNNANNNNGNNNNN
NNNNNNNNNNNNNTNNNNNNNNNNNNANTNNNNNNNNNNNNNNNNNNNNNNNNNNNNNNNNNNNNCCNNN
NNNNNNNNNNNNNNNNNNNNNNNNNNNNNNNNNNNNNNNNNNNNNNNNNNNNNNGCNGNANNNNNTNN
NNNNNNNNNNNNNNNNNNNNNNNNNAGANNNNNNTNNNN
>Giarai104
GTGNNNNNTAGCCCNNNNNAGTTAGACANNNCNNNNNNNNNNNNTNNNNNNNNNNNNNNNNNNNNNNN
NNNNNNNNNNNNNNNNNNNNNNNNNNNNNNTNNNNNNNNNNNNNNNNNNNNNNNNNNNNNNNNNNNNN
NNNNNNNNNNNNNNNNNNNNNNNNNNNNNNNNNNNNNNNNNNNNNNNNNNNNNNNNNNNNNNNNNNNN
NNNNNNNNNNNNNNNNNNNNNNNNNNNNNNNNNNNNNNNNNNNNNNNNNNNNNNNNNNNNNNNNNNNN
NNNNNNNNNNNNNNNNNNNNNNNNNNNNNNNNNNNNNNNNNNNNNNNNNNNNTCTNNNAAANNNTNNN
NNNNNNNNNNNNNNNNNNNNNNNNNCNCNTTNNNNGTAANCNANNNGNACATGCAGTCCCCTCNNNNN
NNNGGCAGAGNNNNNNNCCNNNNNCNCCGGCCCCGNCCNNNNNNNNNNNCANNNNNNNNNANNNNNNN
NNNNNNTGGTACNNNNCGNNNNNGGTTGTNTAAGAGGTCCACAACGNGGTCNGGNCACNNNNGNNTAN
NNNCNNNNAACNNNNNNNGNNNANNTNNNTANNAGGNCNNNNCCNTCNNGNNNNNAATTGCNATGACC
CAANTCNCGGGGGCNGTNCCCGGNGCGGCGACCTTGANNANGTCCGGGGNCAAGTATGGCGNNCNCCC
CGGGAAGGANNCNGCCGTANGTGCNNNNNANNNNNTCNTGNGANGTNGCCGNNATTNNCGTGGTCNNN
NNAGAATGNCCGNACCANNCGNANNNTGGTNATGTTAGTNNNNNNNNNNNNNNNNNNNNNNNNNNNNN
NNNNNNNNNNNNNNNNNNNNNNNNNNNNNNNNGNNNNGNNNNNNCNANNNAAGGANNTANNNCTNNTN
NTNNNNNNGGACNNNACNNNNCNNNNNNNACNCNNNCNCNNNNNNANNNGGTTTTTNNNCNNTATTNT
CCAGNACGCNGNGCNNNCATTCANGNGAACNNNGNNACGCGNNAATCGTANNATCTACCTGCCGCNNG
TNNNNNTACNGNNNNTNCGGANNNTNNCTCGTANNNNNNTCATGCGTNNTNTNNTCNCNAGNCANGTT
CGNNNANANANTAGNANGTNTNNNNNNNNNTGTNGCGTTCCGCNTGANNCNNCANNNNGNNGNNCTTN
TCANCGGCNNTANCTGCNGANNTAAGNCATANNNTNNNTCNNTANCTNNNNNGGNGGGNNACTGTAAA
NGGNNAATTNGAGGNTNATTNCNNNCACNTNNCTNNGNNTNCTNNNTNCCTGNNTNCNNAATTGGNNC
CANTGNNTGNCNATCTCTGGNTTNGNNNNNNTNNNNNTNNCANNNNGGNNNGTNANANNNCNATNTAA
NNTANANNNNCNNNNNNANNNCACNANNNNNGNAGNNNCNANNNTTANANNNGTAGNNNAATAAGTCT
CCGANNTNNNNNNNNATAAGCGNNANACNNNNNNNNNNNNANGANNACGNNTTGGNNNTNNNNNNCNA
GNNAGACNNTNATGGNTNNNNNTCAANNNNTTNGTAGTGGTATAATNGNNNNNNNTTTCNCCAGNNTN
NNNGNNNNNNNCNATANNANNNNNNNGNGANNNNNNNNNNANNNNCTCNNNAGGGANCANCTACTTNA
GCAATGGNGCGCAGNNNGAGANNNNGACNNNCCTTGGGTGGCAANCACNGCAATAAGTNNNGTTNANC
CTNNNAGTNNACATNNNNNCANNGNNNCNNNNNNANCNGANNGNNNNNNNNNNNNNNNNNNNNNNNNN
NNNNNNNNNNNNNNNNNNNNNNNNNNNNAGNNNANGGNNNGTNTNTGACNCATGTGTTTTTANNNGNN
NNNNNACGNNNNANCCCCGGTCGNNNNCNNATTGAANNCCTGNNGCATNNNNNTNCNAGAATGANNGC
NNNNNGNNTNGNCTNNCNNNTCAAGNAATCNNNNAGNNNNCGCGNTCNNNGNNNNNNNNCNNNCCCNN
ACTGAGGGTNNNNNNNNCATCTGCTNACNNNNNCTNCGCNGGNGTNNCNNNNNAGCNGTATNGTGTNA
NNCANNNNNNNNNNNNNNNNTNAAACAANNNNNCTNNNN
>Giarai107
GTGNNNNNTAGCCCGNNNNAGTTAGACANNNCNNNNNNNNNNNNTNNNNNNNNNNNNNNNNNNNNNNN
NNNNNNNNNNNNNNNNNNNNNNNNNNNNNNTNNNNNNNNNNNNNNNNNNNNNNNNNNNNNNNNNNNNN
NNNNNNNNNNNNNNNNNNNNNNNNNNNNNNNNNNNNNNNNNNNNNNNNNNNNNNNNNNNNNNNNNNNN
NNNNNNNNNNNNNNNNNNNNNNNNNNNNNNNNNNNNNNNNNNNNNNNNNNNNNNNNNNNNNNNNNNNN
NNNNNNNNNNNNNNNNNNNNNNNNNNNNNNNNNNNNNNNNGGNNNNNNNNCNTCTNNNAAANNNTNNN
NNNNNNNNNNNNNNNNNNNNNNNNNCNCNTTGNNNGTAANCNANNNGNACATGCAGTCCCCTCNNNNN
NNNGGCAGAGNNNNNNCCCNNNNNNNCCGGCCCCGNCCNNNNNNNNNNNCNNNGNNNNNNANNNNNNN
NNNNNNTGGTACNNNNCGNNNNNGGTTGTNTAAGAGGTCCACAACGNGGTCNNGNCACNNNNGNNNAN
NNNCNNNNAANNNNNNNNNNNNNNANATNNNTANNAGGNNNNNNCCNTCNNNNNNNTAANNGCNATGANN

CAANTCACGGGGGCAGTNCCCGGCGNGGCGACCTTGANNANGTCCGGGGACAAGTATGGCNCNCNCCC
CGGGAAGGANNCNGCCGTANGTGCNNNNNANNNNNTCNTGNGANGTNGCCGNNATTNNCGTGGTCNNN
CNAGAATGNCCNNACCANNCGNANNNTGGTTATGNTAGTNNNNNNNNNNNNNNNNNNNNNNNNNNNNN
NNNNNNNNNNNNNNNNNNNNNNNNNNNNNNNNGNNNNGNNNNNNCNANNNAAGGNNNTANNNCTNNTN
NTNNNNNNGGACNNNACNNNNCNNNNNNNACNCNNNCNCNNNNNNANNNGGTTTTTNNNCNNTANTNT
CCAGNACGCNGNGCNNNCATTCANGNGAACNNNGNNACGCGNNAATCGTANNATCTACCTGCCGCNNG
NNNNNNTACNGNNNNNNCGGANNNTNNCTCGTANNNNNNNNCANGCGTNNTNTNNTCNCAGNCNNGTT
CGNNNANANANTAGNANGTNTNNNNNNNNNNTGTNNCGTTCCGCNTGANNCNNCNNNNNGNNGNNNTTN
TCANCGGCNNTANCTGCNGANNTAAGNCATANNNTNNATCNNTANCTNNNNNGGNGGGNNACTGTAAA
NGGNNAATTNGNGGNTNATTNCNNNCACNTNNCTNNGNNTNCTNNNTNCCTGNNTNCNNAATTGGNNC
CANTGNNTGNCNATCTCTGGNTTNGNNNNNNTNNNNNTNNCANNNNGGNNNGTNNNANNNCNATNTAA
NNTATANNNNCNNNNNNANNNCACNANNNNNGNAGNNACNANNNTTANANNNGTAGNNNAATAAGTCT
CCGANNTNNNNNNNATAAGCGNNANACNNNNNNNNNNNNNNANGANNNCGNNTTGGNNNTNNNNNNCNA
GNNAGACNNTNATGGNTNNNNNTCAANNNNTTNGTAGTGGTATAATNGNNNNNNNTTTCNCCAGNNTN
NNNGNNNNNNNNCNATANNANNNNNNNGNGANNNNNNNNNNNANNNNCTCNNNAGGGANCANCTACTTNA
GCANTGGNGCGCAGNNNGAGANNNNGACNNGCCTTGGGTGGCAANCACNGCAATAAGTNNNGTTNANC
CTNNNAGTNNACATNNNNNCANNGNNNCNNNNNNANCNGANNGNNNNNNNNNNNNNNNNNNNNNNNNN
NNNNNNNNNNNNNNNNNNNNNNNNNNNNAGNNNANGGNNNGTNTNTGACNCATGTGTTTTTANNNGNN
NNNNNACGNNNNANCCCCGGTCGNNNNCNNATTGAANNCCTGNNGCATNNNNNTNCNAGAATGANNGC
NNNNNGNNTNGNCTNNCNTTTCAAGNAATCANNNAGGTNNNNNNNTCNNNGNNNNNNNNCNNTCCCNN
ACTGAGGGTNNNNNNNNCATCTGCTNNNNNNNNCTNCGCNGGNGTNNCNNCNNAGCNGTATNGNGTNA
NNCANNNNNNNNNNNNNNNNNNTNAANCAANNNNNNNTNNNG
>Giarai124
GTGNNNNNTAGCCCNNNNNAGTTAGACANNNCNNNNNNNNNNNNTNNNNNNNNNNNNNNNNNNNNNNN
NNNNNNNNNNNNNNNNNNNNNNNNNNNNNNTNNNNNNNNNNNNNNNNNNNNNNNNNNNNNNNNNNNNN
NNNNNNNNNNNNNNNNNNNNNNNNNNNNNNNNNNNNNNNNNNNNNNNNNNNNNNNNNNNNNNNNNNNN
NNNNNNNNNNNNNNNNNNNNNNNNNNNNNNNNNNNNNNNNNNNNNNNNNNNNNNNNNNNNNNNNNNNN
NNNNNNNNNNNNNNNNNNNNNNNNNNNNNNNNNNNNNNNNNNNNNNNNNNCNTCTNNNAAANNNTNNN
NNNNNNNNNNNNNNNNNNNNNNNNNCNCNTTGNNNGTAANCNANNNGNACATGCAGTCCCCTCNNNNN
NNNNNCAGAGNNNNNNCCCNNNNNNNCCGNCCCCGNCCNNNNNNNNNNNNNNNGNNNNNNNNANNNNN
NNNNNNTGGTACNNNNCGNNNNNGGTTGTNTAAGAGGTCCACCACGNGGTCNNGNCACNNNNGNNNAN
NNNCNNNAANNNNNNNNNNNNNANNTNNNCANNAGGNCNNNNCCNTNNNNNNNNNNATTGCNATGACC
CAANTCACGGNGGCNGTNCCCGGCGCGGCGACCTTGATNANNTCCGGGGACAAGTATGGCNNNCNCCC
CGGGAAGGANNCNGCCGTANGTGCNNNNNANNNNNTCNTGNGANGTCGCCGNNATTCNCGTGGTCTNG
CNAGAATGNCCGNACCANNCGNANNNTGGTNATGNTAGTNNNNNNNNNNNNNNNNNNNNNNNNNNNNN
NNNNNNNNNNNNNNNNNNNNNNNNNNNNNNNNNNNNNNGNNNNNNCNANNNAAGGANNTANNNCTNNTN
NTNNNGNNGGACNNNACNNNNCNNNNNNNACNCNNNCNCNNNNNNANNNGNTTTTTNNNCNNTATTNT
CCAGNACGCNGNGTNNTCATTCANGNGAACNNNGNNACGCGNNAATCGTANNATCTACCTGCCGCNNG
NNNNNNTNCNGNNNNTNCGGANNNTNNCTCGTANNNNNNNNCATGCGTNNTNTNNTCNCAGNCANGTT
CGNNNANANANTAGNANGTNTNNNNNNNNNNTGTNNCGTTCCGCNTGACNCNNCANNNNGNNGNNCTTN
TCANCGGCNNTANCTGCNGANNTAAGNCATANNNTNNNTCNNTANCTNNNNNGGNGGGNNACTGTAAA
NGGNNAATTNGANNNTNATTNCNNNCACNTNNCTNNGNNTNCTNNNTNCCTGNNTNCNNAATTGGNNC
CANTGNNTGNCNATCTCTNGNTTNGNNNNNNTGNNNNTNGCAANNNGGNNNGTNANANNNCNATNTAA
NNTANANNNNCNNNNNNANNNCACNANNNNNNNAGNNNCNANNNTTANANNNGTAGNNNAATAAGTCT
CCGANNTNNNNNNNATAAGCGNNANACNNNNNNNNNNNNNNANGANNACGNNTTGGNNNTNNNNNNCNA
ANNAGACNNTNGTGGNTNNNNNTCAANNNNTTCGTAGTGGTATAATNGNNNNNNNTTTCNCCAGNNTN
NNNGNNNNNNNNCNATANNANNNNNNNGTGANNNNNNNNNNNANNNNCTCNNNAGGGANCANCTACTTNA
GCANTGGNGCGCAGNNNGAGANNNNGACNNNCCTTGGGTGGCAANCACNGCAATAAGTNNNGTTNANC
CTNNNAGTNNACACNNNNNCANNGNNNCNNNNNNANCNGANNTNNNNNNNNNNNNNNNNNNNNNNNNN
NNNNNNNNNNNNNNNNNNNNNNNNNNNANNNNANGGNNNGTNTNTGACNCATGTGTTTCTANNNGNN
NNNNNACGNNNNANCCCCGGTCGTNNNCNNATTGAANNCCTGNNGCATNNNNNTNCNAGAATGANNGC
NNNNNGNNTNGNCTNNCNNNTCAAGNAGTCANNTAGGTANCGCGNTCNNNGNNNNNNNNCNNTCCCNN
ACTGAGGGTNNNNNNNGCATCTGCTNNNNNNNNCTNCGCNGNNNNNNCNGNNNAGCNGTATNGNGTNA
NNCANNNNNNNNNNNNNNNNNNNNANNNNNNNNNNNTNNNN

>Giarai134
GTGTNNNNTAGCCCNNNNNAGTCAGACANCNCNNNNNNNNNNNNTNNNNNNNNNNNNNNNNNNNNNNN
NNNNNNNNNNNNNNNNNNNNNNNNNNNNNNNNTNNNNNNNNNNNNNNNNNNNNNNNNNNNNNNNNNNNNNN
NNNNNNNNNNNNNNNNNNNNNNNNNNNNNNNNNNNNNNNNNNNNNNNNNNNNNNNNNNNNNNNNNNNNNNN
NNNNNNNNNNNNNNNNNNNNNNNNNNNNNNNNNNNNNNNNNNNNNNNNNNNNNNNNNNNNNNNNNNNNNNN
NNNNNNNNNNNNNNNNNNNNNNNNNNNNNNNNNNNNNNNNNNNNNNNNNNNNCNTCTNNNAAANNNTNNN
NNNNNNNNNNNNNNNNNNNNNNNNNNNCNCNTTGNNNGTAANCNANNNGNACATGCAGTCCCCTCNNNNN
NNNGGCAGAGNNNNNNCCCNNNNNNNCCGNCCCCNNCCNNNNNNNNNNNNCNNNGNNNNNNNANNNNNN
NNNNNNTGGTACNNNNCGNNNNNGGTTGTTTAAGAGGTCCACAACGNGGTCNGGNCACNNNNGNNNAN
NNNCNNNNNNCNNNNNNNNNNNNNNNTNNNTANNAGGNCNNNNCCNTCNNGNNNNNNANTGCNATGACC
CAANNCACGGGGGCAGTNCCCGGCGNGGCGACCTTGNTNACGTCCGGGGACAAGTATGGCNNNCNCCC
CGGGAAGGANNCNGCCGTANGTGCNNNNNANNNNNTCTTGNGACGTCGCCGNNATTCNCGTGGTCNNN
CNAGAATGNCCGNACCANNCGNANNNTGGTTATGTTAGTNNNNNNNNNNNNNNNNNNNNNNNNNNNNN
NNNNNNNNNNNNNNNNNNNNNNNNNNNNNNNNNNNNNNNNNGNNNNNNCNANNNAAGGANNTANNNCTNNTN
NTNNNNNNNGGACNNNACNNNNCNNNNNNNACNCNNNCNCNNNNNNNNNNNGNTTTTTNNNCNNTATTNT
CCAGNACGCNGNGCNNTCATTTANGNGAACNNNGNNACGCGNNAATCGTANNATCTACCTGCCGCNNG
TNNNNNTACNGNNNNTNCGGANNNTNNNNCGTANNNNNNTCATGCGTNNTNTNNTCNCNAGNCANGTT
CGAGAANANANTAGNANGTNTNNNNNNNCTTGTNNCGTTCCGCNTGANNCNNCANNNNGNNGNNCTTN
TCANCGGCNCTANCTGCNGANNTAAGTCATANNNTNNNTCNNTANCTNNNNNGGNGGGNNACTGTAAA
NGGNNAATTNGNNGNTNANTTCNNNCATNTNNCTNNGNNNNCTNNNTNCCTGNNTNCNNAATTGGNNC
CANTGNNTGNCNATCTCTGGNTTNGNNNNNNTNNNNNTNNCAANNNGGNNNNNNANANNNCNATNTAA
NNTANANNNNCNNNNNNANNNCACNNNNNNNGNAGNNNNNANNNGTANANNNGTAGNNNGATAAGTCT
CCGANNTNNNNNNNATAAGCGNNANACNNNNNNNNNNNNNANGANNNCGNNTTTGNNNTNNNNNNCNA
GNNAGACNNTNGTGGNTNNNNNTCAANNNNTTNGTAGTGGTATAATNGNNNNNNNTTTCNCCAGNNTN
NNNGNNNNNNNCNATANNANNNNNNNGNGANNNNNNNNNNNANNNNCTCNNNAGGGANCANCTACTTNA
GCANTGGNGCGCAGNNNGAGANNNNGACNNNCCTTGGGTGGCAANCACNGCAATAAGTNNGTTNANC
CTNNNAGTNNACACNNNNNCANNGNNNCNNNNNNANCNGANNGNNNNNNNNNNNNNNNNNNNNNNNNN
NNNNNNNNNNNNNNNNNNNNNNNNNNNNNNANNNNANGGNNNGTNTNTGACNCATGTGTTTTTANNNGNN
NNNNNACGNNNGANCCCCGGTCGNNTNCNNATTGAANNCCTGNNGCATNNNNNTNCNAGAATGANNGC
NNNNNGNNTNGNCTNNCNNNTCAAGNAATCANNNAGGTANCGCGNTCNNNNNNNNNNNNCNNTCCCNN
ACTGAGGGTNNNNNNNNCATCTGCTNNNNNNNNCTTCGCNGGCGTNNCNNNNNAGCNGTATNGTGTNA
NNCANNNNNNNNNNNNNNNNNNNNANNNNNNNNNNNTNNNN
>Giarai60
GTGNNNNNTAGCCCNNNNNAGTTAGACANNNCNNNNNNNNNNNNTNNNNNNNNNNNNNGNNNNNNNNN
NNNNNNNNNNNNNNNNNNNNNNNNNNNNNNNNNNNNNNNNNNNNNNNNNNNNNNNNNNNNNNNNNNNNNNN
NNNNNNNNNNNNNNNNNNNNNNNNNNNNNNNNNNNNNNNNNNNNNNNNNNNNNNNNNNNNNNNNNNNNNNN
NNNNNNNNNNNNNNNNNNNNNNNNNNNNNNNNNNNNNNNNNNNNNNNNNNNNNNNNNNNNNNNNNNNNNNN
NNNNNNNNNNNNNNNNNNNNNNNNNNNNNNNNNNNNNNNNNNNNNNNNNNNNNNTCTNNNAAANNNTNNN
NNNNNNNNNNNNNNNNNNNNNNNNNNNCNCNTTGNNNGTAANCNANNNGNACATGCAGTCCCCTCNNNNN
NNNGGCAGAGNNNNNNCCCNNNNNNNCCGGCCCCNNCCNNNNNNNNNNNNCNNNGNNNNNNNANNNNNN
NNNNNNTGGTACNNNNCGNNNNNGGTTGTTTAAGAGGTCCACAACGNGGTCNGGNNACNNNNGNNNAN
NNNCNNNNNNACNNNNNNNNNNNNANATNNNTANNAGGNCNNNNCCNTCNNGNNANNNANTGCNATGACC
CAANNCACGGGGGNAGTNCCCGGCGNGGCGACCTTGATNACGTCCGGGGACAAGTATGGCGNNCNCCC
CGGGAAGGANNCNGCCGTANGTGCNNNNNANNNNNTCNTGNGACGTCGCCGNNATTCNCGTGGTCNNN
CNAGAATGNCCGNACCANNCGNANNNTGGTNATGNNAGTNNNNNNNNNNNNNNNNNNNNNNNNNNNNN
NNNNNNNNNNNNNNNNNNNNNNNNNNNNNNNNNNNNNNNNNGNNNNNNCNANNNAAGGANNTANNNCTNNTN
NTNNNNNNNGGACNNNACNNNNCNNNNNNNACNCNNNCNCNNNNNNNNNNNGGTTTTTNNCCNNTANTNT
CCAGNACGCNGNGCNNNCATTCANGNGAACNNNGNNACGCGNNAATCGTANNATCTACCTGCCGCNGG
TNNNNNTACNGNNNNTNCGGANNNTNNCTCGTANNNNNNTCATGCGTNNTNTNNTCNCNAGNCANGTT
CGNNNANANANTAGNANGTNTNNNNNNNNNNTGTNNCGTTCCGCNTGANNCNNCANNNNGNNGNNCTTN
TCANCGGCNNTANCTGCNGANNTAAGNCATANNNTNNATCNNTANCTNNNNNGGNGGGNNACTGTAAA
NGGNNAATTNGANGNTNATTNCNNNCACNTNNCTNNGNNTNCTNNNTNCCTGNNTNCNNAATTGGNNC
CANTGNNTGNCNATCTCTGGNTTNGNNNNNNTNNNNNTNNCANNNNGGNNNGTNNNANNNCNATNTAA
NNTANANNNNCNNNNNNANNNCACNANNNNNGNAGNNNCNANNNTTANANNNGTAGNNNAATAAGTCT

CCGANNTNNNNNNNATAAGCGNNANACNNNNNNNNNNNNNANGANNACGNNTTGGNNNTNNNNNNCNA
GNNAGACNNTNATGGNTNNNNNTCAANNNNTTCGTAGTGGTATAATNGNNNNNNNTTTCNCCAGNNTN
NNNGNNNNNNNCNATANNANNNNNNNGNGANNNNNNNNNNANNNNCTCNNNAGGGANCANCTACTTNA
GCAATGGNGCGCAGNNNGAGANNNNGACNNGCCTTGGGTGGCAANCACNGCAATAAGTNNNGTTNANC
CTNNNAGTNNACATNNNNNCANNGNNNCNNNNNNANCNGANNGNNNNNNNNNTNNNNNNNNNNNNNNN
NNNNNNNNNNNNNNNNNNNNNNNNNNNANNNNNANGGNNNGTNTNTGACNCATGTGTTTTTANNNGNN
NNNNNACGNNNNANCCCCGGTCGNNTNCNNATTGAANNCCTGNNGCATNNNNNTNCNAGAATGANNGC
NNNNNGNNTNGNCTGNCNNNTCAAGNAATCANNTAGGTNNCGCGNTCNNNGNNNNNNNNCNNTCCCNN
ACTGAGGGTNNNNNNNNCATCTGCTNNNNNNNNCTNCGCNGGCGTNNCNGNNNNGCNGTATNGTGTNA
NNCANNNNNNNNNNNNNNNNTNAAACNANNNNNNTNNNN
>Giarai67
GTGNNNNNTAGCCCNNNNNAGTTAGACANNNCNNNNNNNNNNNTNNNNNNNNNNNNNNNNNNNNNNN
NNNNNNNNNNNNNNNNNNNNNNNNNNNNNNNNNNNNNNNNNNNNNNNNNNNNNNNNNNNNNNNNNNNN
NNNNNNNNNNNNNNNNNNNNNNNNNNNNNNNNNNNNNNNNNNNNNNNNNNNNNNNNNNNNNNNNNNNN
NNNNNNNNNNNNNNNNNNNNNNNNNNNNNNNNNNNNNNNNNNNNNNNNNNNNNNNNNNNNNNNNNNNN
NNNNNNNNNNNNNNNNNNNNNNNNNNNNNNNNNNNNNNNNNNNNNNNNNNCNTCTNNNAAANNNTNNN
NNNNNNNNNNNNNNNNNNNNNNNNNCNCNNTGNNNGTAANCNANNNGNACATGCAGTCCCCTCNNNNN
NNNGGCAGAGNNNNNNCCCNNNNNNCNCCGGCCCCGNCCNNNNNNNNNNCNNNGNNNNNNNANNNNNN
NNNNNNTGGTACNNNNCGNNNNNGGTTGTNTNAGAGGTCCACAACGNGGTCNNGNCACNNNNGNNNAN
NNNCNNNAAANNNNNNNNNNNNNNNTNNNTANNAGGNCNNNNCCNTCNNNNNNNNAATNGCNATGACC
CAANNCACGGNGGCAGTNCCCGGCGCGGCGACCTTGATNANGTCCGGGGACAAGTATGGCGNNCNCCC
CGGGAAGGANNCNGCCGTANGTGCNNNNNANNNNNTCNTGNGANGTCGCCGNNATTCNCGTGGTCNNN
CNAGAATGNCCNNACCANNCGNANNNTGGTNATGNTAGTNNNNNNNNNNNNNNNNNNNNNNNNNNNNN
NNNNNNNNNNNNNNNNNNNNNNNNNNNNNNNGNNNNNGNNNNNNCNANNNAAGGANNTANNNCTNNTN
NTNNNNNNGGACNNNACNNNNCNNNNNNNACNCNNNCNCNNNNNNANNNGNTTTTTNNNCNNTATTNT
CCAGNACGCNGNGCNNNCATTCANGNGAACNNNGNNACGCGNNAATCGTANNATCTACCTGCCGCNGG
TNNNNNTACNGNNNNNNCGGANNNTNNCTCGTANNNNNNTCATGCGTNNTNTNNTCNCNAGNCANGTT
CGANAANANANTAGNANGTNTNCNNNNNCTTGTNNCGTTCCGCNTGANNCNNCANNNNGNNGNNCTTN
TCANCGGCNNTANCTGCNGANNTAAGNCATANNNTNNATCNNTANCTNNNNNNGGAGGGNACTGTAAA
NGGNNAATTNGNNGNTNATTTCCNNCACNTNNCTNNGNNNNCTNNNTNCCTGNNTNCNNAATTGGNNC
CANTGNNTGNCNATCTCTGGNTTNGNNNNNNTGNNNNTNNCAANNNGGNNNGTNNNANNNCNATNTAA
NNTANANNNNCNNNNNNANNNCACNNNNNNNNNAGNNNCNANNNTTANANNNGTAGNNNAATAAGTCT
CCGANNTNNNNNNNATAAGCGNNANACNNNNNNNNNNNNNANGANNACGNNTTGGNNNTNNNNNNCNA
GNNAGACNNTNATGGNTNNNNNTCAANNNNTTCGTAGTGGTATAATNGNNNNNNNTTTCNCCAGNNTN
NNNGNNNNNNNCNATANNANNNNNNNGNGANNNNNNNNNNANNNNCTCNNNAGGGANCANCTACTNNA
GCANTGGNGCGCAGNNNGAGANNNNGACNNGCCTTGGGTGGCAANCACNGCAATAAGTNNNGTTNANC
CTNNNAGTNNACATNNNNNCANNGNNNCNNNNNNANCNGANNGNNNNNNNNNNNNNNNNNNNNNNNNN
NNNNNNNNNNNNNNNNNNNNNNNNNNNAGNNNNANGGNNNGTNTNTGACNCATGTGTTTTTANNNGNN
NNNNNACGNNNNANCCCCGGTCGNNNNCNNATTGAANNCCTGNNGCATNNNNNTNCNAGAATGANNGC
NNNNNGNNTNGNCTNNCNNNTCAAGNAATCAANNAGGTANNNNGNTCNNNNNNNNNNNNCNNTCCCNN
ACTGAGGGTNNNNNNNNCATCTGCTNNNNNNNNCTNCGCNGGCGTNNNNNNNNNNNNNNNNNNNNNNA
NNCANNNNNNNNNNNNNNNNTNAAACAANNNNNNTNNNN
>Giarai716
GTGNNNNNTAGCCCNNNNNAGTCAGACANNNCNNNNNNNNNNNTNNNNNNNNNNNNNNNNNNNNNNN
NNNNNNNNNNNNNNNNNNNNNNNNNNNNNNTNNNNNNNNNNNNNNNNNNNNNNNNNNNNNNNNNNNNNN
NNNNNNNNNNNNNNNNNNNNNNNNNNNNNNNNNNNNNNNNNNNNNNNNNNNNNNNNNNNNNNNNNNNN
NNNNNNNNNNNNNNNNNNNNNNNNNNNNNNNNNNNNNNNNNNNNNNNNNNNNNNNNNNNNNNNNNNNN
NNNNNNNNNNNNNNNNNNNNNNNNNNNNNNNNNNNNNNNNNNNNNNNNNNCNTCTNNNAAANNNTNNN
NNNNNNNNNNNNNNNNNNNNNNNNNCNCNTTGNNNGTAANCNANNNGNACATGCAGTCCCCTCNNNNN
NNNGGCAGAGNNNNNNCCCNNNNNNNCCGGCCCCNNCCNNNNNNNNNNNCNNNGNNNNNNNANNNNNN
NNNNNNTGGTACNNNNCGNNNNNGGTTGTNTAAGAGGTCCACAACGNGGTCNNGNCACNNNNGNNTAN
NNNCNNNNAACNNNNNNNNNNNNNNTNNNTANNAGGNCNNNNCCNTCNNGNNNNNNANTGCNATGACC
CAANTCNCGGNGGNNGTCCCCGGCGCGGCGACCTTGANNANGTCCGGGGACAAGTATGGCNCNCNCCC
CGGGAAGGANNCNGCCGTANGTGCNNNNNANNNNNTCNTGNGANGTNGCCGNNATTCNCGTGGTCNNN

CNAGAATGCCCNNACCANNCGNANNNTGGTNATGNNAGTANNNNNNNNNNNNNNNNNNNN
NNNNNNNNNNNNNNNNNNNNNNNNNNNNNGNNNNGNNNNNNCNANNNNAAGGANNTANNNCTNNTN
NTNNNNNNGGACNNNACNNNNCNNNNNNNACNCNNNCNCNNNNNNANNNGGTTTTTNNNCNNTANTNT
CCAGNACGCNGNGCNNNCATTCANGNGAACNNNGNNACGCGNNAATCGTANNATCTACCTGCCGCNNG
NNNNNNTNCNGNNNNNNCGGANNNTNNNNCGTANNNNNNTCATGCGTNNTNTNNTCNCNAGNCNNGTT
CGNNNANANANTAGNANGTNTNNNNNNNNNNTGTNNCGTTCCGCNTGANNCNNCANNNNGNNGNNNTTN
TCANCGGCNNTANCTGCNGANNTAAGNCATANNNTNNNTCNNTANCTNNNNNGGNGGGNNACTGTAAA
NGGNNAATTNGNGGNTNATTNCNNNCATNTNNCNNNGNNTNCTNNNTNCCTGNNTNCNNAATTGGNNC
CANTGNNTGNCNATCTCTGGNTTNGNNNNNNTNNNNNTNNCANNNNGGNNNNNNANANNNCNATNTAA
NNTANANNNNCNNNNNNANNNCACNANNNNNGNAGNNNCNANNNGTANANNNGTAGNNNGNTAAGTCT
CCGANNTNNNNNNNATAAGCGNNANACNNNNNNNNNNNNNNANGANNACGNNTTTGNNNTNNNNNNCNA
GNNAGACNNTNGTGGNTNNNNNTCAANNNNTTNGTAGTGGTATAATNGNNNNNNNTTTCNCCAGNNTN
NNNGNNNNNNNNCNATACNANNNNNNNGNGANNNNNNNNNNNANNNNCTCNNNAGGGANCANCTACTNNA
GCANTGGNGCGCAGNNNGAGANNNNGACNNGCCTTGGGTGGCAANNACNGCANTAAGTNNNGTTNANC
CTNNNAGTNNACACNNNNNCANNGNNNCNNNNNNANCNGANNGNNNNNNNNNNNNNNNNNNNNNNNNNN
NNNNNNNNNNNNNNNNNNNNNNNNNNNNNGNNNANGGNNNGTNTNTGACNCATGNNTTTTTANNNGNN
NNNNNACGNNNGANCCCCGGTCGNNNNCNNATTGAANNCCTGNNGCATNNNNNTNCNAGAATGANNGC
NNNNNGNNTNGNCTNNCNNNTCAAGNAATCANNTAGGTNNNGCGNTCNNNNNNNNNNNNCNNTCCCNN
ACTGAGGGTNNNNNNNNCATCTGCTNNNNNNNNCTNCGCNGGNGTNNCNNNNNAGCNGTATNNNGTNN
NNCANNNNNNNNNNNNNNNNNNNAANNNANNNNNNNTNNNN
>Giarai717
GTGNNNNNTAGCCCNNNNNAGTTAGACANNNCNNNNNNNNNNNNNTNNNNNNNNNNNNNNNNNNNNNNN
NNNNNNNNNNNNNNNNNNNNNNNNNNNNNNTNNNNNNNCNNNNNNNNNNNNNNNNNNNNNNNNNNNNN
NNNNNNNNNNNNNNNNNNNNNNNNNNNNNNNNNNNNNNNNNNNNNNNNNNNNNNNNNNNNNNNNNNNN
NNNNNNNNNNNNNNNNNNNNNNNNNNNNNNNNNNNNNNNNNNNNNNNNNNNNNNNNNNNNNNNNNNNN
NNNNNNNNNNNNNNNNNNNNNNNNNNNNNNNNNNNNNNNNNNNNNNNNNNNNNNNNNNTCTNNNAAANNNTNNN
NNNNNNNNNNNNNNNNNNNNNNNNNNNCNCNTTGNNNGTAANCNANNNGNACATGCAGTCCCCTCNNNNN
NNNGGCAGAGNNNNNNCCCNNNNNNNCCGGCCCCNNCCNNNNNNNNNNNNNNNNGNNNNNNNANNNNNN
NNNNNNTGGTACNNNNCGNNNNNGGTTGTTTAAGAGGTCCACAACGNGGTCNNGNCACNNNNGNNNAN
NNNCNNAAAACNNNNNNNNNNNNANNTNNNTANNAGGNCNNNNCCNTCNNGNNNNNNATNGCNATGACC
CAANNCACGGNGGCAGTNCCCGGCGCGGCGACCTTGATNANGTCCGGGGACAAGTATGGCGNNCNCCC
CGGGAAGGANNCNGCCGTANGTGCNNNNNANNNNNTCNTGNGACGTNGCCGNNATTCNCGTGGTCNNN
CNAGAATGNCCNNACCANNCGNANNNTGGTTATGNNAGTNNNNNNNNNNNNNNNNNNNNNNNNNNN
NNNNNNNNNNNNNNNNNNNNNNNNNNNNNNNNNNNNNNNGNNNNNNCNANNNNAAGGANNTANNNCTNNTC
NTNNNNNNGGACNNNACNNNNCNNNNNNNACNCNNNCNCNNNNNNANNNGNTTTTTNNCCNNTATTNT
CCAGNACGCNGNGCNNNCATTCANGNGAACNNNGNNACGCGNNAATCGTANNATCTACCTGCCGCNGG
NNNNNNTACNGNNNNNNCGGANNNTNNNNCGTANNNNNNTCATGCGTNNTNTNNTCNCNAGNCANGTT
CGNNNANANANTAGNANGTNTNNNNNNNNNNTGTNNCGTTCCGCNTGANNCNNCANNNNGNNGNNNTTN
TCANCGGCNNTANCTGCNGANNTAAGNCATANNNTNNNTCNNTANCTNNNNNGGNGGGNNACTGTAAA
NGGNNAATTGGNNGNTNATTNCNNNCACNTNNCTNNGNNTNCTNNNTNCCTGNNTNCNNAATTGGNNC
CANTGNNTGNCNATCTCTNGNTTNGNNNNNNTGNNNNTNNCAANNNGGNNNGTNNNANNNCNATNTAA
NNTANANNNNCNNNNNNANNNCACNNNNNNNGNAGNNNCNANNNTTANANNNGTAGNNNAATAAGTCT
CCGANNTNNNNNNNATAAGCGNNANACNNNNNNNNNNNNNNANGANNACGNNTTGGNNNTNNNNNNCNA
GNNAGACNNTNATGGNTNNNNNTCAANNNNTTCGTAGTGGTATAATNGNNNNNNCTTTCNCCAGNNTN
NNNGNNNNNNNNCNATANNANNNNNNNGNGANNNNNNNNNNNANNNNCTCNNNAGGGANCANCTACTNNA
GCANTGGNGCGCAGNNNGAGANNNNGACNANCCTTGGGTGGCAANCACNGCAATAAGTNNNGTTNANN
CTNNNAGTNNACATNNNNNCANNGNNNCNNNNNNANCNGANNGNNNNNNNNNNNNNNNNNNNNNNNNNN
NNNNNNNNNNNNNNNNNNNNNNNNNNNNNAGNNNANGGNNNGTNTNTGACNCATGTGTTTTTANNNGNN
NNNNNACGNNNGANCCCCGGTCGNNTNCNNATTGAANNCCTGNNGCATNNNNNTNCNAGAATGANNGC
NNNNNGNNTNGNCTNNCNNNTCAAGNAATCANNTAGNNANCGCGNTCNNNGNNNNNNNNCNNTCCCNN
ACTGAGGGTNNNNNNNNCATCTGCTNNNNNNNNCTNCGCNGGNGTNNCNNNNNAGCNGTATNGTGTNA
NNCANNNNNNNNNNNNNNNNNTNANNCNANNNNNNNTNNNN
>Giarai718
GTGNNNNNTAGCCCNNNNNAGTTAGACANNNCNNNNNNNNNNNNNTNNNNNNNNNNNNNNNNNNNNNNN

NNNNNNNNNNNNNNNNNNNNNNNNNNNNNNNNNNNNNNNNNNNNNNNNNNNNNNNNNNNNNNNNNNNNNNNN
NNNNNNNNNNNNNNNNNNNNNNNNNNNNNNNNNNNNNNNNNNNNNNNNNNNNNNNNNNNNNNNNNNNNNNNN
NNNNNNNNNNNNNNNNNNNNNNNNNNNNNNNNNNNNNNNNNNNNNNNNNNNNNNNNNNNNNNNNNNNNNNNN
NNNNNNNNNNNNNNNNNNNNNNNNNNNNNNNNNNNNNNNNNNNNNNNNNNNNNNNNTCTNNNAAANNNTNNN
NNNNNNNNNNNNNNNNNNNNNNNNNCNCNTTGNNNGTAANCNANNNGNACATGCAGTCCCCTCNNNNN
NNNGGCAGAGNNNNNNCCCNNNNNCNCCGNCCCCNNCCNNNNNNNNNNNCANNGNNNNNNNANNNNNN
NNNNNNTGGTACNNNNCGNNNNNGGTTGTTTAAGAGGTCCACAACGNGGTCNNGNCACNNNNGNNNAN
NNNCNNNAAACNNNNNNNNNNNNANNTNNNTANNAGGNNNNNNCCNTCNNGNNNNTAATTGCNATGACC
CAANNCACGGNGGNNGTCCCCGGCGCGGCGACCTTGATNACGTCCGGGGACAAGTATGGCNNNCNCCC
CGGGAAGGANNCNGCCGTANGTGCNNNNNANNNNNTCNTGNGANGTNGCCGNNATTCNCGTGGTCNNN
NNAGAATGNCCGNACCANNCGNANNNTGGTNATGNNAGTNNNNNNNNNNNNNNNNNNNNNNNNNNNNNN
NNNNNNNNNNNNNNNNNNNNNNNNNNNNNNNNNNGNNNNGNNNNNNCNANNNAAGGANNTANNNCTNNTN
NTNNNNNNGGACNNNACNNNNCNNNNNNNACNCNNNCNCNNNNNNANNNGNTTTTTNNNCNNTATTNT
CCAGNACGCNGNGCNNNCATTCANGNGAACNNNGNNACGCGNNAATCGTANNATCTACCTGCCGCNGG
TNNNNNTNCNGNNNNNNCGGANNNTNNCNCGTANNNNNGNCATGCGTNNTNTNNTCNCNAGNCANGTT
CGNNNANANANTAGNANGTNTNNNNNNNNNTGTNNCGTTCCGCNTGANNCNNCANNNNGNNGNNCTTN
TCANCGGCNNTANCTGCNGANNTAAGNCATANNNTNNNTCNNTANCTNNNNNGGNGGGNNACTGTAAA
NGGNTAATTNGANNNTNATTNCNNNCACNTNNCNNNGNNTNCTNNNTNCCTGNNTNCNNAATTGGNNC
CANTGNNTGNCNATCTCTGGNTTNGNNNNNNTNNNNNTNNCAANNNGGNNNNTNANANNNCNATNTAA
NNTATANNNNCNNNNNNANNNCACNNNNNNNGNAGNNNCNANNNTTANANNNGTAGNNNAATAAGTCT
CCGANNTNNNNNNNATAAGCGNNANACNNNNNNNNNNNNNNANGANNACGNNTTGGNNNTNNNNNNCNA
GNNAGACNNTNATGGNTNNNNNTCAANNNNTTNGTAGTGGTATAATNGNNNNNNNTTTCNCCAGNNTN
NNNGNNNNNNNCNATANNANNNNNNNGNGANNNNNNNNNNNANNNNCTCNNNAGGGANCANCTACTNNA
GCANTGGNGCGNAGNNNGAGANNNNGACNNNCCTTGGGTGGCAANCACNGCAATAAGTNNGTTNANC
CTNNNAGTNNACATNNNNNCANNGNNNCNNNNNNANCNGANNGNNNNNNNNNNNNNNNNNNNNNNNNNN
NNNNNNNNNNNNNNNNNNNNNNNNNNNNNNNANNNNANGGNNNGTNTNTGACNCATGTGTTTTTANNNGNN
NNNNNACGNNNNANCCCCGGTCGNNNNCNNATTGAANNCCTGNNGCATNNNNNNTNCNAGAATGANNNC
NNNNNGNNTNGNCTNNCTTTTCAAGNAATCANNNAGNNNNNGCGNTCNNNNNNNNNNNNNCNNTCCCNN
ACTGAGGGTNNNNNNNNCATCTGCTNNNNNNNNNNTNCGCNGGCGTNNCNNNNNAGCNGTATNGNGTNA
NNCANNNNNNNNNNNNNNNNNNCNANNNNANNNNNNNTNNNN
>Giarai719
GTGNNNNNTAGCCCNNNNNAGTTAGACANNNCNNNNNNNNNNNNTNNNNNNNNNNNNNNNNNNNNNNNN
NNNNNNNNNNNNNNNNNNNNNNNNNNNNNNTNNNNNNNNNNNNNNNNNNNNNNNNNNNNNNNNNNNNNNNN
NNNNNNNNNNNNNNNNNNNNNNNNNNNNNNNNNNNNNNNNNNNNNNNNNNNNNNNNNNNNNNNNNNNNNNNN
NNNNNNNNNNNNNNNNNNNNNNNNNNNNNNNNNNNNNNNNNNNNNNNNNNNNNNNNNNNNNNNNNNNNNNNN
NNNNNNNNNNNNNNNNNNNNNNNNNNNNNNNNNNNNNNNNNNNNNNNNNNNNNNCNTCTNNNAAANNNTNNN
NNNNNNNNNNNNNNNNNNNNNNNNNCNCNTTGNNNGTAANCNANNNGNACATGCAGTCCCCTCNNNNN
NNNGGCAGAGNNNNNNCCCNNNNNNNCCGGCCCCGNCCNNNNNNNNNNNCNNNGNNNNNNNANNNNNN
NNNNNNTGGTACNNNNCGNNNNNGGTTGTNTAAGAGGTCCACAACGNGGTCNGGNCACNNNNGNNNAN
NNNCNNNNAACNNNNNNNNNNNNANNTNNNTANNAGGNNNNNNCCNTCNNGNNNNTNATTGCNATGACC
CAANNCACGGGGGCAGTCCCCGGCGCGGCGACCTTGANNACGTCCGGGGACAAGTATGGCGNNCNCCC
CGGGAAGGANNCNGCCGTANGTGCNNNNNANNNNNTCNTGNGACGTCGCCGNNATTCNNGTGGTCNNN
CNAGAATGNCCGNACCANNCGNANNNTGGTNATGNNAGTNNNNNNNNNNNNNNNNNNNNNNNNNNNNNN
NNNNNNNNNNNNNNNNNNNNNNNNNNNNNNNNNNGNNNNGNNNNNNCNANNNAAGGANNTANNNCTNNTN
NTNNNNNNGGACNNNACNNNNCNNNNNNNACNCNNNCNCNNNNNNNNNNNGGTTTTTNNNCNNTATTNT
CCAGNACGCNGNGCNNNCATTCANGNGAACNNNGNNACGCGNNAATCGTANNATCTACCTGCCGCNNG
TNNNNNTACNGNNNNNNCGGANNNTNNCTCGTANNNNNNNCATGCGTNNTNTNNTCNCNAGNCANGTT
CGNNNANANANTAGNANGTNTNNNNNNNNNTGTNNCGTTCCGCNTGANNCNNCANNNNGNCGNNCTTN
TCANCGGCNCTANCTGCNGANNTAAGNCATANNNTNNNTCNNTANCTNNNNNGGNGGGNNACTGTAAA
NGGNNAATTNGNGGNTNANTNCNNNCACNTNNCNNNGNNTNCTNNNTNCCTGNNTNCNNAATTGGNNC
CANTGNNTGNCNATCTCTGGNTTNGNNNNNNTNNNNNTNNCAANNNGGNNNGTNANANNNCNATNTAA
NNTANANNNNCNNNNNNANNNCACNNNNNNNGNAGNNNCNANNNTTANANNNGTAGNNNAATAAGTCT
CCGANNTNNNNNNNATAAGCGNNANACNNNNNNNNNNNNNNANGANNACGNNTTGGNNNTNNNNNNCNA
GNNAGACNNTNATGGNTNNNNNTCAANNNNTTCGTAGTGGTATAATNGNNNNNNNTTTCNCCAGNNTN

NNNGNNNNNNNNCNATANNANNNNNNNGNGANNNNNNNNNNNANNNNCTCNNNAGGGANCANCTACTTNA
GCANTGGNGCGCAGNNNGAGANNNNGACNNGCCTTGGGTGGCAANCACNGCAATAAGTNNNGTTNANC
CTNNNAGTNNACATNNNNNCANNGNNNCNNNNNNANCNGANNGNNNNNNNNNNNNNNNNNNNNNNNNN
NNNNNNNNNNNNNNNNNNNNNNNNNNNNNAGNNNANGGNNNGTNTNTGACNCATGTGTTTTTANNNGNN
NNNNNACGNNNNANCCCCGGTCGNNNNCNNATTGAANNCCTGNNGCATNNNNNTNCNAGAATGANNNC
NNNNNGNNTNGNCTNNCNNNTCAAGNAATCAANNAGGTNTNNCGNTCNNNGNNNNNNNNCNNTCCCNN
ACTGAGGGTNNNNNNCGCATCTGCTNNNNNNNNCTTCGCNGGNGTNNCNGNNNAGCNGTATNGNGTNA
NNCANNNNNNNNNNNNNNNNNTNNAACNANNNNNNNTNNNN
>Giarai721
GTGNNNNNTAGCCCNNNNNAGTTAGACANNNCNNNNNNNNNNNNNTNNNNNNNNNNNNNNNNNNNNNNNN
NNNNNNNNNNNNNNNNNNNNNNNNNNNNNNTNNNNNNNNNNNNNNNNNNNNNNNNNNNNNNNNNNNNNNNN
NNNNNNNNNNNNNNNNNNNNNNNNNNNNNNNNNNNNNNNNNNNNNNNNNNNNNNNNNNNNNNNNNNNNNN
NNNNNNNNNNNNNNNNNNNNNNNNNNNNNNNNNNNNNNNNNNNNNNNNNNNNNNNNNNNNNNNNNNNNNN
NNNNNNNNNNNNNNNNNNNNNNNNNNNNNNNNNNNNNNNNNNNNNNNNNNNNCNTCTNNNAAANNNTNNN
NNNNNNNNNNNNNNNNNNNNNNNNNNNCNCNTTGNNNGTAANCNANNNGNACATGCAGTCCCCTCNNNNN
NNNGGCAGAGNNNNNNCCCNNNNNCNCCGGCCCCNNCCNNNNNNNNTNNNCNNNGNNNNNNNANNNNNN
NNNNNNTGGTACNNNNCGNNNNNGGTTGTTTAAGAGGTCCACAACGNGGTCNNGNCACNNNNGNNNAN
NNNCNNNNAACNNNNNNNNNNNNANATNNNTANNAGGNCNNNGCCNTCNNNNNNNTNATTGCNATGACC
CAANNCACGGGGGCNGTNCCCGGCGNGGCGACCTTGATNANGTCCGGGGACAAGTATGGCGNNCNCCC
CGGGAAGGANNCNGCCGTANGTGCNNNNNANNNNNTCNTGNGANGTCGCCGNNATTCNCGTGGTCNNN
CNAGAATGNCCGNACCANNCGNANNNTGGTNATGNTAGTNNNNNNNNNNNNNNNNNNNNNNNNNNNNNN
NNNNNNNNNNNNNNNNNNNNNNNNNNNNNNNNNNGNNNNGNNNNNNCNANNNAAGGANNTANNNCTNNTN
NTNNNNNNGGACNNNACNNNNCNNNNNNNACNCNNNCNCNNNNNNANNNGNTTTTTNNNCNNTATTNT
CCAGNACGCNGNGCNNNCATTCANGNGAACNNNGNNACGCGNNAATCGTANNATCTACCTGCCGCNGG
TNNNNNTACNGNNNNTNCGGANNNTNNCNCGTANNNNNNTCATGCGTNNTNTNNTCNCNAGNCNNGTT
CGNNNANANANTAGNANGTNTNNNNNNNNNNTGTNNCGTTCCGCNTGANNCNNCANNNNGNNGNNCTTN
TCANCGGCNNTANCTGCNGANNTAAGNCATANNNTNNNTCNNTANCTNNNNNGGNGGGNNACTGTAAA
NGGNNAATTNGNNGNTNANTNCNNNCACNTNNCNNNGNNTNCTNNNTNCCTGNNTNCNNAATTGGNNC
CANTGNNTGNCNATCTCTGGNTTNGNNNNNNTGNNNNTNNCAANNNGGNNNGNNANANNNCNATNTAA
NNTATANNNNCNNNNNNANNNCACNANNNNNNNAGNNNCNANNNTTANANNNGTAGNNNAATAAGTCT
CCGANNTNNNNNNNATAAGCGNNANACNNNNNNNNNNNNNANGANNACGNNTTGGNNNTNNNNNNCNA
GNNAGACNNTNATGGNTNNNNNTCAANNNNTTNGTAGTGGTATAATNGNNNNNNNTTTCNCCAGNNTN
NNNGNNNNNNNNCNATANNANNNNNNNGNGANNNNNNNNNNNANNNNCTCNNNAGGGANCANCTACTNNA
GCAATGGNGCGCAGNNNGAGANNNNGACNNNCCTTGGGTGGCAANCACNGCAATAAGTNNNGTTNANC
CTNNNAGTNNACATNNNNNCANNGNNNCNNNNNNANCNGANNGNNNNNNNNNNNNNNNNNNNNNNNNN
NNNNNNNNNNNNNNNNNNNNNNNNNNNNNAGNNNANGGNNNGTNTNTGACNCATGTGTTTTTANNNGNN
NNNNNACGNNNGANCCCCGGTCGNNNNCNNATTGAANNCCTGNNGCATNNNNNTNCNAGAATGANNGC
NNNNNGNNTNGNCTGNCNNNTCAAGNAATCAANNAGGTATCGCGNTCNNGGNNNNNNNNCNNTCCCNN
ACTGAGGGTNNNNNNCGCATCTGCTNNNNNNNNCTNCGCNGGCGTNNCNNCNNAGCNGTATNGTGTNA
NNCANNNNNNNNNNNNNNNNNTNAAACNNNNNNNNNTNNNN
>Giarai722
GTGNNNNNTAGCCCNNNNNAGTTAGACANNNCNNNNNNNNNNNNNTNNNNNNNNNNNNNNNNNNNNNNNN
NNNNNNNNNNNNNNNNNNNNNNNNNNNNNNNNNNNNNNNNNNNNNNNNNNNNNNNNNNNNNNNNNNNNNN
NNNNNNNNNNNNNNNNNNNNNNNNNNNNNNNNNNNNNNNNNNNNNNNNNNNNNNNNNNNNNNNNNNNNNN
NNNNNNNNNNNNNNNNNNNNNNNNNNNNNNNNNNNNNNNNNNNNNNNNNNNNNNNNNNNNNNNNNNNNNN
NNNNNNNNNNNNNNNNNNNNNNNNNNNNNNNNNNNNNNNNNNNNNNNNNNNNNTCTNNNAAANNNTNNN
NNNNNNNNNNNNNNNNNNNNNNNNNNNCNCNTTGNNNGTAANCNANNNGNACATGCAGTCCCCTCNNNNN
NNNGGCAGAGNNNNNNCCCNNNNNCNCCGNCCCCNNCCNNNNNNNNNNNNNNNGNNNNNNNANNNNNN
NNNNNNTGGTACNNNNCGNNNNNGGTTGTNTAAGAGGTCCACAACGNGGTCNGGNCACNNNNGNNNAN
NNNCNNNNAACNNNNNNNNNNNNANNTNNNTACNAGGNCNNNNCCNTCNNGNNNNNNATNGCNATGACC
CAANNCACGGGGGCNGTNCCCGGCGCGGCGACCTTGATNACGTCCGGGGACAAGTATGGCGNNCNCCC
CGGGAAGGANNCNGCCGTANGTGCNNNNNANNNNNTCNTGNGANGTCGCCGNNATTCNCGTGGTCNNN
CNAGAATGNCCNNACCANNCGNANNNTGGTNATGNNAGTNNNNNNNNNNNNNNNNNNNNNNNNNNNNNN
NNNNNNNNNNNNNNNNNNNNNNNNNNNNNNNNNNGNNNNGNNNNNNCNANNNAAGGANNTANNNCTNNTN

NTNNNNNNGGACNNNACNNNNCNNNNNNNACNCNNNCNCNNNNNNNNNNGNTTTTNNNCCNNTANTNT
CCAGNACGCNGNGCNNNCATTCANGNGAACNNNGNNACGCGNNAATCGTANNATCTACCTGCCGCNNG
NNNNNNTACNGNNNNNNCGGANNNTNNCTCGTANNNNNNTCATGCGTNNTNTNNTCNCNAGNCANGTT
CGNNNANANANTAGNANGTNTNNNNNNNNNNTGTNNCGTTCCGCNTGANNCNNCANNNNGNNGNNNTTN
TCANCGGCNNTANCTGCNGANNTAAGNCATANNNTNNNTCNNTANCTNNNNNGGNGGGNNACTGTAAA
NGGNNAATTGGNNGNTNATTNCNNNCACNTNNCNNNGNNTNCTNNNTNCCTGNNTNCNNAATTGGNNC
CANTGNNTGNCNATCTCTGGNTTNGCNNNNNTNNNNNTNNCAANNNGGNNNGTNNNANNNCNATNTAA
NNTANANNNNCNNNNNNANNNCACNANNNNNGNAGNNANNANNNTTANANNNGTAGNNNANTAAGTCT
CCGANNTNNNNNNNATAAGCGNNANACNNNNNNNNNNNNNNANGANNACGNNTTGGNNNTNNNNNNCNA
GNNAGACNNTNATGGNTNNNNNTCAANNNNTTCGTAGTGGTATAATNGNNNNNNNTTTCNCCAGNNTN
NNNGNNNNNNNCNATACNANNNNNNNGTGANNNNNNNNNNNANNNNCTCNNNAGGGANCANCTACTTNA
GCANTGGNGCGCAGNNNGAGANNNNGACNNGCCTTGGGTGGCAANCACNGCAATAAGTNNNGTTNANC
CTNNNAGTNNACATNNNNNCANNGNNNCNNNNNNANCNGANNGNNNNNNNNNNNNNNNNNNNNNNNNN
NNNNNNNNNNNNNNNNNNNNNNNNNNNNNAGNNNANGGNNNGTNTNTGACNCATGTGTTTTTANNNGNN
NNNNNACGNNNNANCCCCGGTCGTNNNCNNATTGAANNCCTGNNGCATNNNNNTNCNAGAATGANNNC
NNNNNGNNTNGNCTNNCNNNTCAAGNAATCAANNAGGTNNNNNGNTCNNNGNNNNNNNNCNNTCCCNN
ACTGAGGGTNNNNNNNNCATCTGCTNNNNNNNNCTNCGCNGGCGTNNCNGNNNNGCNGTATNGNGTNN
NNCANNNNNNNNNNNNNNNNNTNAAACNANNNNNNTNNNG
>Giarai723
GTGNNNNNTAGCCCNNNNNAGTTAGACANNNCNNNNNNNNNNNNTNNNNNNNNNNNNNNNNNNNNNNN
NNNNNNNNNNNNNNNNNNNNNNNNNNNNNNTNNNNNNNNNNNNNNNNNNNNNNNNNNNNNNNNNNNNNNN
NNNNNNNNNNNNNNNNNNNNNNNNNNNNNNNNNNNNNNNNNNNNNNNNNNNNNNNNNNNNNNNNNNNNNN
NNNNNNNNNNNNNNNNNNNNNNNNNNNNNNNNNNNNNNNNNNNNNNNNNNNNNNNNNNNNNNNNNNNNNN
NNNNNNNNNNNNNNNNNNNNNNNNNNNNNNNNNNNNNNNNNNNNNNNNNNNNNNTCTNNNAAANNNTNNN
NNNNNNNNNNNNNNNNNNNNNNNNNCNCNTTGNNNGTAANCNANNNGNACATGCAGTCCCCTCNNNNN
NNNGGCAGAGNNNNNNCCCNNNNNNNCCGGCCCCNNCCNNNNNNNTNNNNNNNGNNNNNNNANNNNNN
NNNNNNTGGTACNNNNCGNNNNNGGTTGTTTAAGAGGTCCACAACGNGGTCNGGNCACNNNNGNNNAN
NNNCNNNNAANNNNNNNNNNNNNANNTNNNTANAAGGNNNNNNCCNTCNNGNNNNTNATTGCNATGACC
CANNTCACGGGGGCAGTCCCCGGCGCGGCGACCTTGATNANGTCCGGGGACAAGTATGGCGCNCNCCC
CGGGAAGGANNCNGCCGTANGTGCNNNNNANNNNNTCNTGNGANGTCGCCGNNATTCNCGTGGTCNAN
CNAGAATGNCCGNACCANNCGNANNNTGGTNATGNTAGTNNNNNNNNNNNNNNNNNNNNNNNNNNNNN
NNNNNNNNNNNNNNNNNNNNNNNNNNNNNNNNNGNNNNGNNNNNNCNANNNAAGGANNTANNNCTNNTN
NTNNNNNNGGACNNNACNNNNCNNNNNNNACNCNNNCNCNNNNNNANNNGGTTTTTNNNCNNTANTNT
CCAGNACGCNGNGCNNNCATTCANGNGAACNNNGNNACGCGNNAATCGTANNATCTACCTGCCGCNNG
TNNNNNTACNGNNNNNNCGGANNNTNNCTCGTANNNNNNTCATGCGTNNTNTNNTCNCNAGNCNNGTT
CGNNNANANANTAGNANGTNTCNNNNNNCTTGTNNCGTTCCGCNTGANNCNNCANNNNGNNGNNCTTN
TCANCGGCNNTANCTGCNGANNTAAGNCATANNNTNNNTCNNTANCTNNNNNGGNGGGNNACTGTAAA
NGGNNAATTGGNGGNTNATTTCNNNCACNTNNCNNNGNNTNCTNNNTNCCTGNNTNCNNAATTGGNNC
CANTGNNTGNCNATCTCTGGNTTNGNNNNNNTNNNNNTNNCAANNNGGNNNGTNANANNNCNATNTAA
NNTANANNNNCNNNNNNANNNCACNANNNNNGNAGNNNNNANNNTTANANNNGTAGNNNAATAAGTCT
CCGANNTNNNNNNNATAAGCGNNANACNNNNNNNNNNNNNNANGANNACGNNTTGGNNNTNNNNNNCNA
GNNAGACNNTNATGGNTNNNNNTCAANNNNTTCGTAGTGGTATAATNGNNNNNNNTTTCNCCAGNNTN
NNNGNNNNNNNCNATANNANNNNNNNGNGANNNNNNNNNNNANNNNCTCNNNAGGGANCANCTACTNNA
GCAATGGNGCGCAGNNNGAGANNNNGACNNGCCTTGGGTGGCAANCACNGCAATAAGTNNNGTTNANC
CTNNNAGTNNACATNNNNNCANNGNNNCNNNNNNANCNGANNGNNNNNNNNNNNNNNNNNNNNNNNNN
NNNNNNNNNNNNNNNNNNNNNNNNNNNNNGNNNANGGNNNGTNTNTGACNCATGTGTTTTTANNNGNN
NNNNNACGNNNGANCCCCGGTCGTNNNCNNATTGAANNCCTGNNGCATNNNNNTNCNAGAATGANNGC
NNNNNGNNTNGNCTGNCNNNTCAAGNAATCAANTAGGNANNGCGNTCNNNGNNNNNNNNCNNNCCCNN
ACTGAGGGTNNNNNNCGCATCTGCTNNNNNNNNNNTTCGCNGNCGTNNCNGNNNAGCNGTATNNTGTNA
NNCANNNNNNNNNNNNNNNNNTNNNACNANNNNNNTNNNN
>Giarai724
GTGNNNNNTAGCCCNNNNNAGTTAGACANNNCNNNNNNNNNNNNTNNNNNNNNNNNNNNNNNNNNNNN
NNNNNNNNNNNNNNNNNNNNNNNNNNNNNNTNNNNNNNNNNNNNNNNNNNNNNNNNNNNNNNNNNNNNNN
NNNNNNNNNNNNNNNNNNNNNNNNNNNNNNNNNNNNNNNNNNNNNNNNNNNNNNNNNNNNNNNNNNNNNN

NNNNNNNNNNNNNNNNNNNNNNNNNNNNNNNNNNNNNNNNNNNNNNNNNNNNNNNNNNNNNNNN
NNNNNNNNNNNNNNNNNNNNNNNNNNNNNNNNNNNNNNNNNNNNNNNNNNCNTCTNNNAAANNNTNNN
NNNNNNNNNNNNNNNNNNNNNNNNNCNCNTTGNNNGTAANCNANNNGNACATGCAGTCCCCTCNNNNN
NNNGGCAGAGNNNNNNCCCNNNNNCNCCGNCCCCNNCCNNNNNNNTNNNCNNNGNNNNNNNANNNNNN
NNNNNNTGGTACNNNNCGNNNNNGGTTGTTTAAGAGGTCCACAACGNGGTCNNGNCACNNNNGNNTAN
NNNCNNNNAACNGNNNNNNNNNNNNTNNNTANNAGGNCNNNNCCNTCNNNNNNNTNATNGCNATGACC
CAANTCACGGGGGNAGTNCCCGGCGCGGCGACCTTGANNACGTCCGGGGACAAGTATGGCGCNCNCCC
CGGGAAGGANNCNGCCGTANGTGCNNNNNANNNNNTCTTGNGANGTNGCCGNNATTNNCNTGGTCTNG
CNAGAATGNNCGNACCANNCGNANNNTGGTTATGNNAGTNNNNNNNNNNNNNNNNNNNNNNNNNNNNN
NNNNNNNNNNNNNNNNNNNNNNNNNNNNNNNNNNNNNGNNNNNNCNANNNAAGGANNTANNNCTNNTN
NTNNNNNNGGACNNNACNNNNCNNNNNNNACNCNNNCNCNNNNNNNNNNGGTTTTNNNNCNNTATTNT
CCAGNACGCNGNGCNNNCATTCANGNGAACNNNGNNACGCGNNAATCGTANNATCTACCTGCCGCNGG
TNNNNNTACNGNNNNNNCGGANNNTNNNNCGTANNNNNNTCATGCGTNNTNTNNTCNCNAGNCNNGTT
CGNNAANANANTAGNANGTNTNNNNNNNCTTGTNGCGTTCCGCNTGANNCNNCANNNNGNNGNNCTTN
TCANCGGCNNTANCTGCNGANNTAAGNCATANNNTNNATCNNTANCTNNNNNGGNGGGNACTGTAAA
NGGNNAATTGGNNGNTNATTNCNNNCACNTNNCTNNGNNTNCTNNNTNCCTGNNTNCNNAATTGGNNC
CANTGNNTGNCNATCTCTGGNTTNGNNNNNNTNNNNNTNNCAANNNGGNNNGTNANANNNCNATNTAA
NNTATANNNNCNNNNNNANNNCACNANNNNNNNAGNNACNANNNTTANANNNGTAGNNNAATAAGTCT
CCGANNTNNNNNNNATAAGCGNNANACNNNNNNNNNNNNNANGANNNCGNNTTGGNNNTNNNNNNCNA
GNNAGACNNTNATGGNTNNNNNTCAANNNNTTCGTAGTGGTATAATNGNNNNNNNTTTCNCCAGNNTN
NNNGNNNNNNNCNATANNANNNNNNNGNGANNNNNNNNNNANNNNCTCNNNAGGGANCANCTACTNNA
GCANTGGNGCGCAGNNNGAGANNNNGACNNNCCTTGGGTGGCAANCACNGCAATAAGTNNNGTTNANC
CTNNNAGTNNACATNNNNNCANNGNNNCNNNNNNANCNGANNGNNNNNNNNNNNNNNNNNNNNNNNNN
NNNNNNNNNNNNNNNNNNNNNNNNNNNNANNNNANGGNNNGTNTNTGACNCATGTGTTTTTANNNGNN
NNNNNACGNNNNANCCCCGGTCGNNNNCNNATTGAANNCCTGNNGCATNNNNNTNCNAGAATGANNGC
NNNNNGNNTNGCTNNCNTNTCAAGNAATCANNNAGGTNNCGCGNTCNNNGNNNNNNNNCNNTCCCNN
ACTGAGGGTNNNNNNNNNCATCTGCTNNNNNNNNNNTNCGCNGGNGTNNCNNCNNAGCNGTATNGNGTNA
NNCANNNNNNNNNNNNNNNNNNTNAAACNANNNNNNNTNNNN
>Giarai725
GTGNNNNNTAGCCCNNNNNAGTTAGACANNNCNNNNNNNNNNNNTNNNNNNNNNNNNNNNNNNNNNNN
NNNNNNNNNNNNNNNNNNNNNNNNNNNNNNTNNNNNNNNNNNNNNNNNNNNNNNNNNNNNNNNNNNNN
NNNNNNNNNNNNNNNNNNNNNNNNNNNNNNNNNNNNNNNNNNNNNNNNNNNNNNNNNNNNNNNNNNNN
NNNNNNNNNNNNNNNNNNNNNNNNNNNNNNNNNNNNNNNNNNNNNNNNNNNNNNNNNNNNNNNNNNNN
NNNNNNNNNNNNNNNNNNNNNNNNNNNNNNNNNNNNNNNNNNNNNNNNNNCNTCTNNNAAANNNTNNN
NNNNNNNNNNNNNNNNNNNNNNNNNCNCNTTGNNNGTAANCNANNNGNACATGCAGTCCCCTCNNNNN
NNNGGCAGAGNNNNNNNCCNNNNNNNCCGNCCCCNNCCNNNNNNNNNNNNCNNNGNNNNNNNANNNNNN
NNNNNNTGGTACNNNNCGNNNNNGGTTGTTTAAGAGGTCCACAACGNGGTCNNGNCACNNNNGNNNAN
NNNCNNNNAACNNNNNNNNNNNNANNTNNNTANAAGGNCNNNNCCNTCNNGNNNNTNATTGCNATGACC
CAANNCACGGGGGCAGTCCCCGGCGCGGCGACCTTGATNANGTCCGGGGACAAGTATGGCGNNCNCCC
CGGGAAGGANNCNGCCGTANGTGCNNNNNANNNTNTCNTGNGACGTCGCCGNNATTCNCGTGGTCNNN
CNAGAATGNCCGNACCANNCGNANNNTGGTNATGNNAGTANNNNNNNNNNNNNNNNNNNNNNNNNNNN
NNNNNNNNNNNNNNNNNNNNNNNNNNNNNNNNNNNNNGNNNNNNCNANNNAAGGANNTANNNCTNNTN
NTNNNNNNGGACNNNACNNNNCNNNNNNNACNCNNNCCCNNNNNNANNNGNTTTTTNNNCNNTATTNT
CCAGNACGCNGNGCNNNCATTCANGNGAACNNNGNNACGCGNNAATCGTANNATCTACCTGCCGCNNG
TNNNNNTACNGNNNNTNCGGANNNTNNCTCGTANNNNNNNCATGCGTNNTNTNNTCNCNAGNCANGTT
CGNNNANANANTAGNANGTNTCNNNNNNNNTGTNNCGTTCCGCNTGANNCNNCANNNNGNCGNNCTTN
TCANCGGCNNTANCTGCNGANNTAAGNCATANNNTNNATCNNTANCTNNNNNGGNGGGNACTGTAAA
NGGNNAATTNGNNGNTNATTNCNNNCACNTNNCTNNGNNNNCTNNNTNCCTGNNTNCNNAATTGGNNC
CANTGNNTGNCNATCTCTGGNTTNGNNNNNNTNNNNNTNNCAANNNGGNNNGTNANANNNCNATNTAA
NNTATANNNNCNNNNNNANNNCACNANNNNNNNAGNNNCNANNNTTANANNNGTAGNNNAATAAGTCT
CCGANNTNNNNNNNATAAGCGNNANACNNNNNNNNNNNNNANGANNACGNNTTGGNNNTNNNNNNCNA
GNNAGACNNTNATGGNTNNNNNTCAANNNNTTCGTAGTGGTATAATNGNNNNNNNTTTCNCCAGNNTN
NNNGNNNNNNNCNATANNANNNNNNNGNGANNNNNNNNNNANNNNCTCNNNAGGNANCANCTACTTNA
GCANTGGNGCGNAGNNNGAGANNNNGACNNNCCTTGGGTGGCAANCACNGCAATAAGTNNNGTTNANC

CTNNNAGTNNACATNNNNNCANNGNNNCNNNNNNANCNGANNGNNNNNNNNNNNNNNNNNNNNNNNNNN
NNNNNNNNNNNNNNNNNNNNNNNNNNNNNNNAGNNNANGGNNNGTNTNTGACNCATGTGTTTNTANNNGNN
NNNNNACGNNNNANCCCCGGTCGNNNNCNNATTGAANNCCTGNNGCATNNNNNTNCNAGAATGANNGC
NNNNNGNNTNGNCTNNCNTTTCAAGNAATCANNTANGTNNCGCGNTCNNNGNNNNNNNNCNNTCCCNN
ACTGAGGGTNNNNNNNNCATCTGCTNNNNNNNNCTNCGCNGGCGTNNCNNNNNAGCNGTATNGNGTNA
NNCANNNNNNNNNNNNNNNNNCNAAACAANNNNNNTNNNN
>Giarai726
GTGNNNNNTAGCCCNNNNNAGTTAGACANNNCNNNNNNNNNNNNNTNNNNNNNNNNNNNNNNNNNNNNNN
NNNNNNNNNNNNNNNNNNNNNNNNNNNNNNNNNNNNNNNNNNNNNNNNNNNNNNNNNNNNNNNNNNNNNN
NNNNNNNNNNNNNNNNNNNNNNNNNNNNNNNNNNNNNNNNNNNNNNNNNNNNNNNNNNNNNNNNNNNNNN
NNNNNNNNNNNNNNNNNNNNNNNNNNNNNNNNNNNNNNNNNNNNNNNNNNNNNNNNNNNNNNNNNNNNNN
NNNNNNNNNNNNNNNNNNNNNNNNNNNNNNNNNNNNNNNNNNNNNNNNNNNNNNNCNTCTNNNAAANNNTNNN
NNNNNNNNNNNNNNNNNNNNNNNNNNNCNCNTTGNNNGTAANCNANNNGNACATGCAGTCCCCTCNNNNN
NNNGGCAGAGNNNNNNCCCNNNNNNNCCGNCCCCNNCCNNNNNNNTNNNNNNNGNCNNNNNANNNNNN
NNNNNNTGGTACNNNNCGNNNNNGGTTGTTTAAGAGGTCCACAACGNGGTCNGGNCACNNNNGNNNAN
NNNCNNAAAACNNNNNNNNNNNNNNTNNNTANNAGGNCNNNNCCNTCNNGNNNNNAANTGCNATGACC
CAANTCACGGGGGCAGTNCCCGGNGCGGCGACCTTGATNACGTCCGGGGACAAGTATGGCGNNCNCCC
CGGGAAGGANNCNGCCGTANGTGCNNNNNANNNNNTCNTGNGANGTCGCCGNNATTNNNGTGGTCNNN
CNAGAATGNCCNNACCANNCGNANNNTGGTNATGNNAGTNANNNNNNNNNNNNNNNNNNNNNNNNNNN
NNNNNNNNNNNNNNNNNNNNNNNNNNNNNNNNNNGNNNNGNNNNNNCNANNNAAGGANNTANNNCTNNTN
NTNNNNNNGGACNNNACNNNNCNNNNNNNACNCNNNCNCNNNNNNANNNGNTTTTTNNCCNNTATTNT
CCAGNACGCNGNGCNNNCATTCANGNGAACNNNGNNACGCGTNAATCGTANNATCTACCTGCCGCNGG
TNNNNNTACNGNNNNTNCGGANNNTNNNNCGTANNNNNNTCATGCGTNNTNTNNTCNCTAGNCANGTT
CGNNNANANANTAGNANGTNTCNNNNNNCTTGTNNCGTTCCGCNTGANNCNNCANNNNGNCGNNCTTN
TCANCGGCNNTANCTGCNGANNTAAGNCATANNNTNNNTCNNTANCTNNNNNGGNGGGNNACTGTAAA
NGGANAATTGGNNGNTNATTNCNNNCACNTNNCTNNGNNTNCTNNNTNCCTGNNTNCNNAATTGGNNC
CANTGNNTGNCNATCTCTGGNTTNGNNNNNNTNNNNNTNNCAANNNGGNNNGTNANANNNCNATNTAA
NNTATANNNNCNNNNNNANNNCACNANNNNNGNAGNNNCNANNNTTANANNNGTAGNNNANTAAGTCT
CCGANNTNNNNNNNATAAGCGNNANACNNNNNNNNNNNNNANGANNACGNNTTGGNNNTNNNNNGCNA
GNNAGACNNTNATGGNTNNNNNTCAANNNNTTNGTAGTGGTATAATNGNNNNNNNTTTCNCCAGNNTN
NNNGNNNNNNNCNATANNANNNNNNNGNGANNNANNNNNNANNNNCTCNNNAGGGANCANCTACTNNA
GCANTGGNGCGNAGNNNGAGANNNNGACNNNCCTTGGGTGGCAANCACNGCAATAAGTNNNGTTNANC
CTNNNAGTNNACATNNNNNCANNGNNNCNNNNNNANCNGANNGNNNNNNNNNNNNNNNNNNNNNNNNNN
NNNNNNNNNNNNNNNNNNNNNNNNNNNNNNNGNNNANGGNNNGTNTNTGACNCATGTGTTTTTANNNGNN
NNNNNACGNNNNANCCCCGGTCGTNNNCNNATTGAANNCCTGNNGCATNNNNNTNCNAGAATGANNNC
NNNNNGNNTNGNCTNNCNNNTCAAGNAATCANNNAGNNNNCGCGNTCNNNGNNNNNNNNCNNTCCCNN
ACTGAGGGTNNNNNNCGCATCTGCTNNNNNNNNCTNCGCNGGCGTNNCNNNNNAGCNGTATNGTGTNA
NNCANNNNNNNNNNNNNNNNNTNAAACNANNNNNNTNNNN
>Giarai727
GTGNNNNNTAGCCCNNNNNAGTCAGACANNNCNNNNNNNNNNNNNTNNNNNNNNNNNNNNNNNNNNNNNN
NNNNNNNNNNNNNNNNNNNNNNNNNNNNNNNNTNNNNNNNNNNNNNNNNNNNNNNNNNNNNNNNNNNNNN
NNNNNNNNNNNNNNNNNNNNNNNNNNNNNNNNNNNNNNNNNNNNNNNNNNNNNNNNNNNNNNNNNNNNNN
NNNNNNNNNNNNNNNNNNNNNNNNNNNNNNNNNNNNNNNNNNNNNNNNNNNNNNNNNNNNNNNNNNNNNN
NNNNNNNNNNNNNNNNNNNNNNNNNNNNNNNNNNNNNNNNNNNNNNNNNNNNNNNCNTCTNNNAAANNNTNNN
NNNNNNNNNNNNNNNNNNNNNNNNNNNCNCNTTGNNNGTAANCNANNNGNACATGCAGTCCCCTCNNNNN
NNNGGCAGAGNNNNNNCCCNNNNNNNCCGGCCCCGNCCNNNNNNNNNNNCNNNGNNNNNNNANNNNNN
NNNNNNTGGTACNNNNCGNNNNNGGTTGTNTNAGAGGTCCACAACGNGGTCNNGNCACNNNNGNNNAN
NNNCNNNNAACNNNNNNNNNNNNANNTNNNTANNAGGNCNNNNCCNTCNNGNNNNNAATTGCNATGACC
CAANTCACGGNGGCAGTNCCCGGCGCGGCGACCTTGANNACGTCCGGGGACAAGTATGGCGCNCNCCC
CGGGAAGGANNCNGCCGTANGTGCNNNNNANNNNNTCTTGNGANGTCGCCGNNATTNNCGTGGTCNNN
CNAGAATGCCCGNACCANTCGNANNNTGGTNATGNNAGTNNNNNNNNNNNNNNNNNNNNNNNNNNNNN
NNNNNNNNNNNNNNNNNNNNNNNNNNNNNNNNNNNNNNGNNNNNNCNANNNAAGGANNTANNNCTNNTN
NTNNNNNNGGACNNNACNNNNCNNNNNNNACNCNNNCNCNNNNNNNNNNNGGTTTTTNNNCNNTATTNT
CCAGNACGCNGNGCNNNCATTTANGNGAACNNNGNNACGCGNNAATCGTANNATCTACCTGCCGCNNG

TNNNNNTACNGNNNNTNCGGANNNTNNCNCGTANNNNNNNNCATGCGTNNTNTNNTCNCNAGNCANGTT
CGNNNANANANTAGNANGTNTNNNNNNNNNNTGTNNCGTTCCGCNTGANNCNNCANNNNGNNGNNNTTN
TCANCGGCNNTANCTGCNGANNTAAGNCATANNNTNNNTCNNTANCTNNNNNGGNGGGNACTGTAAA
NGGNTAATTNGANNNTNANTNCCNNCATNTNNCTNNGNNTNCTNNNTNCCTGNNTNCNNAATTGGNNC
CANTGNNTGNCNATCTCTGGNTTNGNNNNNNTGNNNNTNNCANNNNGGNNNGTNANANNNCNATNTAA
NNTATANNNNCNNNNNNANNNCACNNNNNNNGNAGNNACNANNNGTANANNNGTAGNNNGNTAAGTCT
CCGANNTNNNNNNNATAAGCGNNANACNNNNNNNNNNNNNNANGANNACGNNTTTGNNNTNNNNNNCNA
GNNAGACNNTNGTGGNTNNNNNTCAANNNNTTNGTAGTGGTATAATNGNNNNNNNTTTCNCCAGNNTN
NNNGNNNNNNNNCNATANNANNNNNNNGTGANNNNNNNNNNNANNNNCTCNNNAGGGANCANCTACTNNA
GCAATGGNGCGCAGNNNGAGANNNNGACNNNCCTTGGGTGGCAANCACNGCAATAAGTNNNGTTNANC
CTNNNAGTNNACACNNNNNCANNGNNNCNNNNNNANCNGANNGNNNNNNNNNNNNNNNNNNNNNNNNNN
NNNNNNNNNNNNNNNNNNNNNNNNNNNNNNGNNNANGGNNNGTNTNTGACNCATGTGTTTTTANNNGNN
NNNNNACGNNNGANCCCCGGTCGNNNNCNNATTGAANNCCTGNNGCATNNNNNNTNCNAGAATGANNGC
NNNNNGNNTNGNCTNNCNNNTCAAGNAATCANNNAGGTNNNNNNNTCNNNNNNNNNNNNNCNNTCCCNN
ACTGAGGGTNNNNNNCGCATCTGCTNNNNNNNNNCTNCGCNGGCGTNNCNGNNNAGCNGTATNNTGTNA
NNCANNNNNNNNNNNNNNNNNNCCAAACAANNNNNNNTNNNN
>Giarai728
GTGNNNNNTAGCCCNNNNNAGTCAGACANNNCNNNNNNNNNNNNNTNNNNNNNNNNNNNNNNNNNNNNN
NNNNNNNNNNNNNNNNNNNNNNNNNNNNNNTNNNNNNNNNNNNNNNNNNNNNNNNNNNNNNNNNNNNNNN
NNNNNNNNNNNNNNNNNNNNNNNNNNNNNNNNNNNNNNNNNNNNNNNNNNNNNNNNNNNNNNNNNNNNNN
NNNNNNNNNNNNNNNNNNNNNNNNNNNNNNNNNNNNNNNNNNNNNNNNNNNNNNNNNNNNNNNNNNNNNN
NNNNNNNNNNNNNNNNNNNNNNNNNNNNNNNNNNNNNNNNNNNNNNNNNNNNNNNTCTNNNAAANNNTNNN
NNNNNNNNNNNNNNNNNNNNNNNNNNCNCNNTGNNNGTAANCNANNNGNACATGCAGTCCCCTCNNNNN
NNNGGCAGAGNNNNNNCCCNNNNNCNCCGNCCCCGNCCNNNNNNNNNNNNCNNNGNNNNNNNANNNNNN
NNNNNNTGGTACNNNNCGNNNNNGGTTGTTTAAGAGGTCCACAACGNGGTCNGGNCACNNNNGNNNAN
NNNCNNNNAACNNNNNNNNNNNNANNTNNNTANNAGGNCNNNNCCNTCNNGNNNNNAATTGCNATGACC
CAANTCACGGGGGNAGTCCCCGGCGCGGCGACCTTGATNACGTCCGGGGACAAGTATGGCGNNCNCCC
CGGGAAGGANNCNGCCGTANGTGCNNNNNANNNNNTCNTGNGACGTCGCCGNNATTCNCGTGGTCNNN
CNAGAATGNCCGNACCANNCGNANNNTGGTNATGNNAGTNNNNNNNNNNNNNNNNNNNNNNNNNNNNN
NNNNNNNNNNNNNNNNNNNNNNNNNNNNNNNNNNNGNNNNGNNNNNNCNANNNAAGGNNNTANNNCTNNTC
NTNNNNNNGGACNNNACNNNNCNNNNNNNACNCNNNCNCNNNNNNANNNGGTTTTTNNCCNNTATTNT
CCAGNACGCNGNGCNNNCATTTANGNGAACNNNGNNACGCGNNAATCGTANNATCTACCTGCCGCNNG
TNNNNNTACNGNNNNTNCGGANNNTNNCTCGTANNNNNNTCATGCGTNNTNTNNTCNCNAGNCNNGTT
CGNNNANANANTAGNANGTNTNNNNNNNNNNTGTNNCGTTCCGCNTGANNCNNCANNNNGNNGNNCTTN
TCANCGGCNNTANCTGCNGANNTAAGNCATANNNTNNNTCNNTANCTNNNNNGGNGGGNACTGTAAA
NGGNNAATTNGNGNNTNATTTCNNNCATNTNNCTNNGNNTNCTNNNTNCCTGNNTNCNNAATTGGNNC
CANTGNNTGNCNATCTCTGGNTTNGNNNNNNTNNNNNTNNCAANNNGGNNNGNNANANNNCNATNTAA
NNTANANNNNCNNNNNNANNNCACNNNNNNNGNAGNNACNANNNGTANANNNGTAGNNNGATAAGTCT
CCGANNTNNNNNNNATAAGCGNNANACNNNNNNNNNNNNNNANGANNACGNNTTTGNNNTNNNNNNCNA
GNNAGACNNTNGTGGNTNNNNNTCAANNNNTTNGTAGTGGTATAATNGNNNNNNNTTTCNCCAGNNTN
NNNGNNNNNNNNCNATANNANNNNNNNGNGANNNNNNNNNNNANNNNCTCNNNAGGNANCANCTACTNNA
GCAATGGNGCGCAGNNNGAGANNNNGACNNGCCTTGGGTGGCAANCACNGCAATAAGTNNNGTTNANC
CTNNNAGTNNACACGNNNNCANNGNNNCNNNNNNANCNGANNGNNNNNNNNNNNNNNNNNNNNNNNNNN
NNNNNNNNNNNNNNNNNNNNNNNNNNNNNAGNNNANGGNNNGTNTNTGACNCATGTGTTTTTANNNGNN
NNNNNACGNNNNANCCCCGGTCGTNNNCNNATTGAANNCCTGNNGCATNNNNNNTNCNAGAATGANNGC
NNNNNGNNTNGNCTNNCNTTTCAAGNAATCAANNAGGTANNNNGNTCNNNNNNNNNNNNNCNNTCCCNN
ACTGAGGGTNNNNNNNNCATCTGCTNNNNNNNNNCTNCGCNGNCGTNNCNNNNNAGCNGTATNGTGTNA
NNCANNNNNNNNNNNNNNNNNANTNAAACAANNNNNNNTNNNN
>Giarai729
GTGNNNNNTAGCCCNCNNNAGTCAGACANNNCNNNNNNNNNNNNNTNNNNNNNNNNNNNNNNNNNNNNN
NNNNNNNNNNNNNNNNNNCNNNNNNNNNNNNNTNNNNNNNNNNNNNNNNNNNNNNNNNNNNNNNNNNNNN
NNNNNNNNNNNNNNNNNNNNNNNNNNNNNNNNNNNNNNNNNNNNNNNNNNNNNNNNNNNNNNNNNNNNNN
NNNNNNNNNNNNNNNNNNNNNNNNNNNNNNNNNNNNNNNNNNNNNNNNNNNNNNNNNNNNNNNNNNNNNN
NNNNNNNNNNNNNNNNNNNNNNNNNNNNNNNNNNNNNNNNNNNNNNNNNNNNNNNTCTNNNAAANNNTNNN

NNNNNNNNNNNNNNNNNNNNNNNNNNCNCNTTGNNNGTAANCNANNNGNACATGCAGTCCCCTCNNNNN
NNNGGCAGAGNNNNNNCCCNNNNNNNCCGNCCCCGNCCNNNNNNNNNNNCANNGNNNNNNNANNNNNN
NNNNNNTGGTACNNNNCGNNNNNGGTTGTNTAAGAGGTCCACAACGNGGTCNGGNCACNNNNGNNNAN
NNNCNNNNAACNNNNNNNNNNNNANATNNNTANNAGGNCNNNNCCNTCNNNNNNNNNNATTGCNATGACC
CAANTCACGGGGGCAGTCCCCGGCGCGGCGACCTTGATNACGTCCGGGGACAAGTATGGCGNNCNCCC
CGGGAAGGANNCNGCCGTANGTGCNNNNNANNNNNTCNTGNGANGTCGCCGNNATTNNCGTGGTCNNN
CNAGAATGNCCGNACCANNCGNANNNTGGTNATGNTAGTNNNNNNNNNNNNNNNNNNNNNNNNNNNNNN
NNNNNNNNNNNNNNNNNNNNNNNNNNNNNNNNNNNGNNNNGNNNNNNCNANNNAAGGANNTANNNCTNNTN
NTNNNGNNGGACNNNACNNNNCNNNNNNNACNCNNNCNCNNNNNNANNNGGTTTTTNNNCNNTATTNT
CCAGNACGCNGNGCNNNCATTTANGNGAACNNNGNNACGCGNNAATCGTANNATCTACCTGCCGCNGG
NNNNNNTACNGNNNNNNCGGANNNTNNCTCGTANNNNNNNTCATGCGTNNTNTNNTCNCNAGNCANGTT
CGNNNANANANTAGNANGTNTCNNNNNNCTTGTNNCGTTCCGCNTGANNCNNCANNNNGNNGNNCTTN
TCANCGGCNNTANCTGCNGANNTAAGNCATANNNTNNATCNNTANCTNNNNNGGNGGGNNACTGTAAA
NGGNNAATTGGANGNTNANTNCCNNCATNTNNCNNNGNNTNCTNNNTNCCTGNNTNCNNAATTGGNNC
CANTGNNTGNCNATCTCTGGNTTNGCNNNNNTGNNNNTNNCANNNNGGNNNGTNANANNNCNATNTAA
NNTATANNNNCNNNNNNANNNCACNANNNNNNNAGNNNCNANNNGTANANNNGTAGNNNGATAAGTCT
CCGANNTNNNNNNNATAAGCGNNANACNNNNNNNNNNNNNNANGANNACGNNTTTGNNNTNNNNNGCNA
GNNAGACNNTNGTGGNTNNNNNTCAANNNNTTNGTAGTGGTATAATNGNNNNNNNTTTCNCCAGNNTN
NNNGNNNNNNNCNATANNANNNNNNNGNGANNNNNNNNNNNANNNNCTCNNNAGGNANCANCTACTTNA
GCANTGGNGCGCAGNNNGAGANNNNGACCAGCCTTGGGTGGCAANCACNGCAATAAGTNNNGTTNANC
CTNNNAGTNNACACNNNNNCANNGNNNCNNNNNNANCNGANCGNNNNNNNNNNNNNNNNNNNNNNNNN
NNNNNNNNNNNNNNNNNNNNNNNNNNNNNAGNNNANGGNNNGTNTNTGACNCATGTGTTTTTANNNGNN
NNNNNACGNNNGANCCCCGGTCGTNNNCNNATTGAANNCCTGNNGCATNNNNNTNCNAGAATGANNGC
NNNNNGNNTNGNCTNNCNTTTCAAGNAATCAANNAGGTANCGCGNTCNNNGNNNNNNNNCNNTCCCNN
ACTGAGGGTNNNNNNNNCATCTGCTNNNNNNNNNNTNCGCNGGCGTNNCNGNNNAGCNGTATNGTGTNA
NNCANNNNNNNNNNNNNNNNNNTNANACAANNNNNNNTNNNG
>Giarai730
GTGNNNNNTAGCCCGNNNNAGTTAGACANNNCNNNNNNNNNNNNNTNNNNNNNNNNNNNNNNNNNNNN
NNNNNNNNNNNNNNNNNNNNNNNNNNNNNNNTNNNNNNNNNNNNNNNNNNNNNNNNNNNNNNNNNNNNN
NNNNNNNNNNNNNNNNNNNNNNNNNNNNNNNNNNNNNNNNNNNNNNNNNNNNTTNNNNNNNNNNNNNNNN
NNNNNNNNNNNNNNNNNNNNNNNNNNNNNNNNNNNNNNNNNNNNNNNNNNNNNNNNNNNNNNNNNNNNNN
NNNNNNNNNNNNNNNNNNNNNNNNNNNNNNNNNNNNNNNNNNNNNNNNNNNNNCNTCTNNNAAANNNTNNN
NNNNNNNNNNNNNNNNNNNNNNNNNNCNCNTTGNNNGTAANCNANNNGNACATGCAGTCCCCTCNNNNN
NNNGGCAGAGNNNNNNCCCNNNNNCNCCGGCCCCNNCCNNNNNNNNNNNCNNNGNNNNNNNANNNNNN
NNNNNNTGGTACNNNNCGNNNNNGGTTGTTTAAGAGGTCCACAACGNGGTCNGGNCACNNNNGNNNAN
NNNCNNNNAACNNNNNNNNNNNNANNTNNNTANNAGGNCNNNNCCNTCNNGNNNNNNATNGCNATGACC
CAANTCACGGGGGCAGTCCCCGGCGCGGCGACCTTGATNACGTCCGGGGACAAGTATGGCGNNCNCCC
CGGGAAGGANNCNGCCGTANGTGCNNNNNANNNNNTCNTGNGANGTCGCCGNNATTCNCGTGGTCNNN
NNAGAATGNCCNNACCANNCGNANNNTGGTTATGTNAGTNNNNNNNNNNNNNNNNNNNNNNNNNNNNNN
NNNNNNNNNNNNNNNNNNNNNNNNNNNNNNNNNNNGNNNNGNNNNNNCNANNNAAGGANNTANNNCTNNTN
NTNNNNNNNGGACNNNACNNNNCNNNNNNNACNCNNNCNCNNNNNNANNNGGTTTTTNNNCNNTATTNT
CCAGNACGCNGNGCNNNCATTCANGNGAACNNNGNNACGCGNNAATCGTANNATCTACCTGCCGCNGG
TNNNNNTACNGNNNNTNCGGANNNTNNCNCGTANNNNNNNTCATGCGTNNTNTNNTCNCNAGNCANGTT
CGNNNANANANTAGNANGTNTNNNNNNNNNNTGTNNCGTTCCGCNTGANNCNNCANNNNGNNGNNCTTN
TCANCGGCNNTANCTGCNGANNTAAGTCATANNNTNNATCNNTANCTNNNNNGGNGGGNNACTGTAAA
NGGNNAATTNGANGNTNATTTCNNNCACNTNNCTNNGNNTNCTNNNTNCCTGNNTNCNNAATTGGNNC
CANTGNNTGNCNATCTCTGGNTTNGNNNNNNTGNNNNTNNCAANNNGGNNNGTNANANNNCNATNTAA
NNTANANNNNCNNNNNNANNNCACNANNNNNGNAGNNACNANNNTTANANNNGTAGNNNAATAAGTCT
CCGANNTNNNNNNNATAAGCGNNANACNNNNNNNNNNNNNNANGANNACGNNTTGGNNNTNNNNNNCNA
GNNAGACNNTNATGGNTNNNNNTCAANNNNTTCGTAGTGGTATAATNGNNNNNNNTTTCNCCAGNNTN
NNNGNNNNNNNCNATANNANNNNNNNGNGANNNNNNNNNNNANNNNCTCNNNAGGGANCANCTACTNNA
GCAATGGNGCGCAGNNNGAGANNNNGACNNGCCTTGGGTGGCAANCACNGCAATAAGTNNNGTTNANC
CTNNNAGTNNACATGNNNNCANNGNNNCCNNNNNANCNGANNGNNNNNNNNNNNNNNNNNNNNNNNNN
NNNNNNNNNNNNNNNNNNNNNNNNNNNNNAGNNNANGGNNNGTNTNTGACNCATGTGTTTTTANNNGNN

NNNNNACGNNNGANCCCCGGTCGTNNNCNNATTGAANNCCTGNNGCATNNNNNTNCNAGAATGANNGC
NNNNNGNNTNGNCTNNCNNNTCAAGNAATCANNNAGGTNNCGCGNTCNNNGNNNNNNNNCNNTCCCNN
ACTGAGGGTNNNNNNNNCATCTGCTNNNNNNNNCTNCGCNGGNGTNNCNNNNNAGCNGTATNGTGTNA
NNCANNNNNNNNNNNNNNNNCNAAAANANNNNNNTNNNN
>Giarai733
GTGNNNNNTAGCTCNNNNNAGTTAGACANNNCNNNNNNNNNNNNTNNNNNNNNNNNNNNNNNNNNNNN
NNNNNNNNNNNNNNNNNNNNNNNNNNNNNNTNNNNNNNNNNNNNNNNNNNNNNNNNNNNNNNNNNNNNN
NNNNNNNNNNNNNNNNNNNNNNNNNNNNNNNNNNNNNNNNNNNNNNNNNNNNNNNNNNNNNNNNNNNN
NNNNNNNNNNNNNNNNNNNNNNNNNNNNNNNNNNNNNNNNNNNNNNNNNNNNNNNNNNNNNNNNNNNN
NNNNNNNNNNNNNNNNNNNNNNNNNNNNNNNNNNNNNNNNNNNNNNNNNNCNTCTNNNAAANNNTNNN
NNNNNNNNNNNNNNNNNNNNNNNNNCNCNTTGNNNGTAANCNANNNGNACATGCAGTCCCCTCNNNNN
NNNGGCAGAGNNNNNNCCCNNNNNNCNCCGGCCCCNNCCNNNNNNNTNNNCANNGNNNNNNNGNNNNN
NNNNNNTGGTACNNNNCGNNNNNGGTTGTTTAAGAGGTCCACCACGNGGTCNNGNCACNNNNGNNNAN
NNNCNNNNAACNNNNNNNNNNNANNTNNNCANAAGGNCNNNNCCNTCNNGNNNNNNATNGCNATGACC
CAANNCGCGGGGACAGTCACCGGCGNGGCGACCTTGNNNANNGTCGGGGACAAGTATGTCGNNCNCCC
CGGGAAGGANNCNGCCGTANGTGCNNNNNANNNNNTCNTGNGACGTCGCCGNNATTCNNGTGGTCNNN
CNAGAATGNCCNNACCANNCGNANNNTGGTNATGNNAGTNNNNNNNNNNNNNNNNNNNNNNNNNNNNN
NNNNNNNNNNNNNNNNNNNNNNNNNNNNNNNNNNNNNGNNNNNNCNANNNAAGGANNTANNNTTNNTN
NTNNNNNNGGACNNNACNNNNCNNNNNNNACNCNNNCNCNNNNNNANNNGNTTTTNNNNCNNTATTNT
CCAGNACGCNGNGCNNNCATTCANGNGAACNNNGNNACGCGNNAATCGTANNATCTACCTGCCGCNNG
TNNNNNTACNGNNNNNNCGGANNNTNNCTCGTANNNNNNTCATGCGTNNTNTNNTTNCCAGNCANGTT
CGNNNANANANTAGNANGTNTNNNNNNNNNTGTNNCGTTCCGCNTGANNCNNCANNNNGNNGNNNTTN
TCANCGGCNNTANCTGCNGANNTAAGNCATANNNTNNNTCNNTANCGNNNNNGGNGGGNNACTGTAAA
NGGNNAATTNGNNGNTNANTTCNNNCACNTNNCTNNGNNTNCTNNNCNCCTGNNTNCNNAATTGGNNC
CANTGNNTGNCNATCTCTGGNTTNGNNNNNNTNNNNNTNNTANNNNGGNNNNNNANANNNCNATNTAA
NNTATANNNNCNNNNNNANNNCACNANNNNNGNAGNNNCNANNNTTANGNNNGTAGNNNAATAAGTCT
CCGANNTNNNNNNNATAAGCGNNANACNNNNNNNNNNNNNANGANNACGNNTTGGNNNTNNNNNNCNA
ANNAGACNNCNGTGGNTNNNNNTCAANNNNTTNGTAGTGGTATAACNANNNNNNNTTTCNCCAGNNTN
NNNGNNNNNNNCNATACNANNNNNNNGTGANNNNNNNNNNANNNNCCCNNNAGGGANCANCTACTTNA
GCAATGGNGCGCAGNNNGAGANNNNGACNNNCCTTGGGTGGCAANCACNGCAATAAGTNNNGTTNANC
CCNNNAGTNNACACNNNNNCANNGNNNCNNNNNNANCNGANNTNNNNNNNNNNNNNNNNNNNNNNNNN
NNNNNNNNNNNNNNNNNNNNNNNNNNNNAGNNNANGGNNNGTNTNTGACNCATGTGTTTTTANNNGNN
NNNNNACGNNNGANCCCCGGTCGNNNNCNNATTGAANNCCTGNNGCATNNNNNTNCNAGAATGANNGC
NNNNNGNNTNGNCTNNCNTTTCAAGNAGTCAANNAGGTANNGCGNTCNNNGNNNNNNNNCNNTCCCNN
ACTGAGGGTNNNNNNNNCATCTGCTNNNNNNNNCTNCGCNGNNNNNNTNNNNNAGCNGTATNGNGTNN
NNCANNNNNNNNNNNNNNNNTNNAACAANNNNNNTNNNN
>Giarai734
GTGNNNNNTAGCCCNNNNNAGTTAGACANNNCNNNNNNNNNNNNTNNNNNNNNNNNNNNNNNNNNNNN
NNNNNNNNNNNNNNNNNNNNNNNNNNNNNNNNNNNNNNNNNNNNNNNNNNNNNNNNNNNNNNNNNNNN
NNNNNNNNNNNNNNNNNNNNNNNNNNNNNNNNNNNNNNNNNNNNNNNNNNNNNNNNNNNNNNNNNNNN
NNNNNNNNNNNNNNNNNNNNNNNNNNNNNNNNNNNNNNNNNNNNNNNNNNNNNNNNNNNNNNNNNNNN
NNNNNNNNNNNNNNNNNNNNNNNNNNNNNNNNNNNNNNNNNNNNNNNNNNCNTCTNNNAAANNNTNNN
NNNNNNNNNNNNNNNNNNNNNNNNNCNCNTTGNNNGTAANCNANNNGNACATGCAGTCCCCTCNNNNN
NNNGGCAGAGNNNNNNCCCNNNNNNNNCCGNCCCCNNCCNNNNNNNNNNNNNNNGNNNNNNNANNNNN
NNNNNNTGGTACNNNNCGNNNNNGGTTGTTTAAGAGGTCCACAACGNGGTCNGGNCACNNNNGNNNAN
NNNCNNNNAACNNNNNNNNNNNANATNNNTANNAGGNCNNNNCCNTCNNNNNNNNNATTGCNATGACC
CAANNCACGGGGGCAGTNCCCGGCGCGGCGACCTTGNTNANGTCCGGGGACAAGTATGGCGNNCNCCC
CGGGAAGGANNCNGCCGTANGTGCNNNNNANNNNNTCNTGNGANGTCGCCGNNATTCNCGTGGTCNNN
CNAGAATGNCCGNACCANNCGNANNNTGGTNATGNNAGTNNNNNNNNNNNNNNNNNNNNNNNNNNNNN
NNNNNNNNNNNNNNNNNNNNNNNNNNNNNNNNGNNNNGNNNNNNCNANNNAAGGANNTANNNCTNNTN
NTNNNNNNGGACNNNACNNNNCNNNNNNNACNCNNNCNCNNNNNNANNNGGTTTTTNNNCNNTATTNT
CCAGNACGCNGNGCNNNCATTCANGNGAACNNNGNNACGCGNNAATCGTANNATCTACCTGCCGCNNG
NNNNNNTACNGNNNNNNCGGANNNTNNCTCGTANNNNNGTCATGCGTNNTNTNNTCNCNAGNCNNGTT
CGNNNANANANTAGNANGTNTNNNNNNNNNTGTNNCGTTCCGCNTGANNCNNCANNNNGNNGNNCTTN

TCANCGGCNNTANCTGCNGANNTAAGNCATANNNTNNNTCNNTANCTNNNNNGGNGGGNNACTGTAAA
NGGNNAATTNGNNGNTNATTNCNNNCACNTNNCTNNGNNTNCTNNNTNCCTGNNTNCNNAATTGGNNC
CANTGNNTGNCNATCTCTGGNTTNGNNNNNNTNNNNNTNGCAANNNGGNNNGTNNNANNNCNATNTAA
NNTANANNNNCNNNNNNANNNCACNNNNNNNNNAGNNNCNANNNTTANANNNGTAGNNNAATAAGTCT
CCGANNTNNNNNNNATAAGCGNNANACNNNNNNNNNNNNNNANGANNACGNNTTGGNNNTNNNNNNCNA
GNNAGACNNTNATGGNTNNNNNTCAANNNNTTNGTAGTGGTATAATNGNNNNNNNTTTCNCCAGNNTN
NNNGNNNNNNNCNATANNANNNNNNNGNGANNNNNNNNNNANNNNCTCNNNAGGGANCANCTACTNNA
GCAATGGNGCGCAGNNNGAGANNNNGACNNGCCTTGGGTGGCAANCACNGCAATAAGTNNNGTTNANC
CTNNNAGTNNACATNNNNNCANNGNNNCNNNNNNANCNGANNGNNNGNNNNNNNNNNNNNNNNNNN
NNNNNNNNNNNNNNNNNNNNNNNNNNNNNAGNNNANGGNNNGTNTNTGACNCATGNNTTTTTANNNGNN
NNNNNACGNNNGANCCCCGGTCGNNNNCNNATTGAANNCCTGNNGCATNNNNNTNCNAGAATGANNGC
NNNNNGNNTNGNCTGNCNNNTCAAGNAATCAANNAGGTNNCGCGNTCNNNGNNNNNNNNCNNTCCCNN
ACTGAGGGTNNNNNNNNCATCTGCTNNNNNNNNCTTCGCNGGNGTNNCNNNNNNGCNGTATNGTGTTA
NNCANNNNNNNNNNNNNNNNNNNNNAACNANNNNNNNTNNNN
>Giarai735
GTGNNNNNTAGCCCNNNNNAGTTAGACANNNCNNNNNNNNNNNNTNNNNNNNNNNNNNNNNNNNNNN
NNNNNNNNNNNNNNNNNNNNNNNNNNNNNNNTNNNNNNNNNNNNNNNNNNNNNNNNNNNNNNNNNNNNN
NNNNNNNNNNNNNNNNNNNNNNNNNNNNNNNNNNNNNNNNNNNNNNNNNNNNNNNNNNNNNNNNNNNNNNN
NNNNNNNNNNNNNNNNNNNNNNNNNNNNNNNNNNNNNNNNNNNNNNNNNNNNNNNNNNNNNNNNNNNNNNN
NNNNNNNNNNNNNNNNNNNNNNNNNNNNNNNNNNNNNNNNNNNNNNNNNNNNNNNNTCTNNNAAANNNTNNN
NNNNNNNNNNNNNNNNNNNNNNNNNNNNCNCNTTGNNNGTAGNCNANNNANACATGCAGTCCCCTCNNNNN
NNNGGCAGAGNNNNNNNCCNNNNNNNCCGGCCCCNNCCNNNNNNNNNNNCANNGNNNNNNNANNNNNN
NNNNNNTGGTACNNNNCGNNNNNGGTTGTTTAAGAGGTCCACCACGNAGTCNGGNCACNNNNGNNNAN
NNNCNNAAAACNNCNNNNNNNNNANAGNNNCANNAGGNCNNNNCCNTCNNGNNANNAGTNGCNATGACC
CAANNCACGGGGGNAGTCCCCGGNGCGGCGACCTTGATNACGTCCGGGAACAAGTATGGCGNNTNCCC
CGGGAAGGANNCNGCCGTANGTGCNNNNNANNNNNTCNTGNGACGTCGCCGNNATTNNCGTGGTCNNN
CNAGAATGNCCNNACCANNCGNANNNTGGTTATGNNAGCNNNNNNNNNNNNNNNNNNNNNNNNNNNNNN
NNNNNNNNNNNNNNNNNNNNNNNNNNNNNNNNNNNGNNNNGNNNNNNCNANNNAAGGNNNTANNNCTNNTN
NTNNNGNNGGACNNNACNNNNCNNNNNNNACNCNNNCNCNNNNNNANNNGGTTTTTNNNCNNTATTNC
CCAGNACGCNGNGCNNNCATTCANGNGAACNNNGNNACGCGNAAATCGTANNATCTACCTGCCGCNNG
TNNNNNTACNGNNNNTNCGGANNNTNNNNCGTANNNNNNTCATGCGTNNTNTNNTCNCNAGNCANGTT
CGNNNGNANANTAGNANGTNTNNNNNNNCNTGTNGCGTTCCGCNTGANNCNNCANNNNGNNGNNCTTN
TCANCGGCNNTANCTGCNGANNTAAGNCATANNNCNNNTCNNCANCTNNNNNGGNGGGNNACTGTAAA
NGGNNAATTNGNNGNCGATTNCNNNCACNTNNCTNNGNNTNCTNNNTNCTTGNNTNCNNAGTTGGNNC
CANTANNTGNTNATCTCTGGNTTNGCNNNNNTNNNNNTNNCANNNNGGNNNGTNNNANCNCNATNTAA
NNTANANNNNCNNNNNNANNNCACNANNNNNGNAGNNNCNANNNTTANANNNGTAGNNNACTAAGTCT
CCGANNTNNNNNNNATAGGCGNNANACNNNNNNNNNNNNNNANGANNACGNNTTGGNNNTNNNNNNANA
ANNAGACNNTNGTGGNTNNNNNTCAANNNNCTCGTAGTGGTATAATNGNNNNNNNTTTCNCCAGNNTN
NNNGNNNNNNNCNATANNANNNNNNNGNGANNNNNNNNNNANNNNCTCNNNAGGGANCANCTACTNNA
GCAATGGNGCGCAGNNNTAGANNNNGACNNNCCTTGGGTGGCAANCACNGCAATAAGTNNNGTTNANC
CTNNNAGTNNACACNNNNNCANNANNNCNNNNNNANCNGANNTNNNNNNNNNNNNNNNNNNNNNNNNN
NNNNNNNNNNNNNNNNNNNNNNNNNNNNNATNNNANGGNNNGTNTNTGACNCATGTGTTTTTANNNGNN
NNNNNACGNNNGANCCCCGGTCGNNNNCNNATTAAANNCCTGNNGCATNNNNNTNCNATAATGANNGC
NNNNNGNNTNGNCTNNCNNNTCAAGNAGTCANNTAGGTNNCGCGNTCNNNGNNNNNNNNCNNTCCCNN
ACTGAGGGTNNNNNNNNCATCTGCTNNNNNNNNCTNCGCNGGCGTNNCNNNNNATCNGTATNGTGTNN
NNCANNNNNNNNNNNNNNNNNNNNAAACGANNNNNNNTNNNN
>Giarai81
GTGNNNNNTAGCCCNNNNNAGTTAGACANNNCNNNNNNNNNNNNTNNNNNNNNNNNNNNNNNNNNNN
NNNNNNNNNNNNNNNNNNNNNNNNNNNNNNNTNNNNNNNNNNNNNNNNNNNNNNNNNNNNNNNNNNNNN
NNNNNNNNNNNNNNNNNNNNNNNNNNNNNNNNNNNNNNNNNNNNNNNNNNNNNNNNNNNNNNNNNNNNNNN
NNNNNNNNNNNNNNNNNNNNNNNNNNNNNNNNNNNNNNNNNNNNNNNNNNNNNNNNNNNNNNNNNNNNNNN
NNNNNNNNNNNNNNNNNNNNNNNNNNNNNNNNNNNNNNNNNNNNNNNNNNNNNCNTCTNNNNAANNNTNNN
NNNNNNNNNNNNNNNNNNNNNNNANNCNCNTTGNNNGTAANCNANNNGNACATGCAGTCCCCTCNNNNN
NNNNNCAGAGNNNNNNNNNCNNNNNNNCCGGCCCCNNCCNNNNNNNNTNNNNNNNGNNNNNNNANNNNNN

NNNNNNTGGTACNNNNCGNNNNNGGTTGTNTNAGNGGTCCACAACGNGNTCNNGNNACNNNNGNNNAN
NNNCNNNNNNNNNNNNNNNNNNNNNTNNNTANNAGGNCNNNNCCNTCNNNNNNNNNATNGCNATGACC
CAANNCACGGGGGNNGTNCCCGGCGCGGCGACCTTGANNANNTCCGGGGACAAGTATGGCGNNCNCCC
CGGGAAGGANNCNGCCGTANGTGCNNNNNANNNNNTNNTGNGANGTCGCCGNNNTTCNCNTGGTCNNN
NNAGAATGCCCNNACCANNCGNANNNTGGTNATGNNAGTNNNNNNNNNNNNNNNNNNNNNNNNNNNNNN
NNNNNNNNNNNNNNNNNNNNNNNNNNNNNNNNNNNNNNNGNNNNNNCNANNNAAGGANNTANNNCTNNTN
NTNNNNNNGGACNNNACNNNNCNNNNNNNACNCNNNCNCNNNNNNANNNGGTTTTNNNCCNNTATTNT
CCAGNACGCNGNGCNNNCATTCANGNGAACNNNGNNACGCGNNAATCGTANNATCTACCTGCCGCNNG
NNNNNNTACNGNNNNNNCGGANNNTNNNNCGTANNNNNNTCATGCGTNNTNTNNTCNCNAGNCNNGTT
CGNNNANANANTAGNANGTNTCNNNNNNCTTGTNNCGTTCCGCNTGANNCNNCANNNNGNCGNNCTTN
NCANCGGCNNTANCTGCNGANNTAAGNCATANNNTNNNTCNNTANCTNNNNNGGNGGGNNACTGTAAA
NGNNNAATTNGNNGNTNANTTCNNNCACNTNNCTNNGNNTNCTNNNTNCCTGNNTNCNNAATTGGNNC
CANTGNNTGNCNATCTCTGGNTTNGNNNNNNTNNNNNTNNCANNNNGGNNNGNNANANNNCNATNTAA
NNTATANNNNCNNNNNNANNNCACNNNNNNNGNAGNNNCNANNNTTANANNNGTAGNNNAATAAGTCT
CCGANNTNNNNNNNATAAGCGNNANACNNNNNNNNNNNNNANGANNNCGNNTTGGNNNTNNNNNNCNA
GNNAGACNNTNATGGNTNNNNNTCAANNNNTTNGTAGTGGTATAATNGNNNNNNNTTTCNCCAGNNTN
NNNGNNNNNNNCNATANNANNNNNNNGNGANNNNNNNNNNNANNNNCTCNNNAGGGANCANCTACTNNA
GCANTGGNGCGNAGNNNGAGANNNNGACNNNCCTTGGGTGGCAANCACNGCGATAAGTNNNGTTNANC
CTNNNAGTNNACATGNNNNCANNGNNNCNNNNNNANCNGANNGNNNNNNNNNNNNNNNNNNNNNNNNN
NNNNNNNNNNNNNNNNNNNNNNNNNNNNNNNNNNNNNNANNGNNNGTNTNTGACNCATGNNTTTTTANNNGNN
NNNNNNNGNNNGANCCCCGGTCGNNNNCNNATTGAANNCCTGNNGCATNNNNNTNCNAGAATGANNGC
NNNNNGNNTNGNCTNNCNNNTCAAGNAATCANNNAGNNNNNNNNNTCNNNGNNNNNNNNCNNTCCCNN
ACTGAGGGTNNNNNNNNCATCTGCTNNNNNNNNNTNCGCNGNNGTNNCNNNNNAGCCGTATNGNGTNA
NNCANNNNNNNNNNNNNNNNNCNNAACNNNNNNNNNNNNN
>Giarai91
GTGNNNNNTAGCCCNNNNNAGTTAGACANNNCNNNNNNNNNNNNNNNNNNNNNNNNNNNNNNNNNNNN
NNNNNNNNNNNNNNNNNNNNNNNNNNNNNNNNNNNNNNNNNNNNNNNNNNNNNNNNNNNNNNNNNNNN
NNNNNNNNNNNNNNNNNNNNNNNNNNNNNNNNNNNNNNNNNNNNNNNNNNNNNNNNNNNNNNNNNNNN
NNNNNNNNNNNNNNNNNNNNNNNNNNNNNNNNNNNNNNNNNNNNNNNNNNNNNNNNNNNNNNNNNNNN
NNNNNNNNNNNNNNNNNNNNNNNNNNNNNNNNNNNNNNNNNNNNNNNNNNNNNNTCTNNNAAANGNTNNN
NNNNNNNNNNNNNNNNNNNNNNNNNNCNCNTTGNNNGTAANCNANNNANACGTGCAGTCCCCTCNNNNN
NNNGGCAGAGNNNNNNCNCNNNNNNNCCGGCCCCNNCCNNNNNNNTNNNNNNNGNNNNNNNANNNNNN
NNNNNNTGGTACNNNNCGNNNNNGGTTGTNTAAGAGGTCCACCACGNAGTCNNGNNACNNNNANNNAN
NNNCNNNNAANNNNNNNNNNNNNANAGNNNCANNAGGNNNNNNCCNTCNNGNNNNNAGTTGCNATGACC
CAANTCACGGGGGCAGTNCCCGGCGCAGCGACCTTGATNACGTCCGGGAACAAGTATGGCGNNTNCCC
CGGGAAGGANNCNGCCGTANGTGCNNNNNANNNNNTCNTGNGANGTCGCCGNNATTNNCGTGGTCNNN
CNAGAATGNCCGNACCANNCGNANNNTGGTNATGTNAGTANNNNNNNNNNNNNNNNNNNNNNNNNNNN
NNNNNNNNNNNNNNNNNNNNNNNNNNNNNNNNNNNNNNNGNNNNNNCNANNNAAGGANNTANNNCTNNTN
NTNNNNNNGGACNNNACNNNNCNNNNNNNACNCNNNCNCNNNNNNANNNGGTTTTTNNNCNNTATTNC
CCAGNACGCNGNGCNNNCATTCANGNGAACNNNGNNACGCGNNAATCGTCNNATCTACCTGCCGCNNG
NNNNNNTACNGNNNNTNCGGANNNTNNCTCGTANNNNNNTCATGCGTNNTNTCNTCNCNAGCANTTT
CGNNNGNANANTAGNANGTNTNNNNNNNNNTGTNNCGTTCCGCNTGGNNCNNCANNNNGNNGNNCTTC
TCANCGGCNNTANTTGCNGANNTAAGNCATANNNCNNATCNNCANCTNNNNNGGNGGGNNACTGTAAA
NGGNNAATTGGANGNCNATTNCNNNCACNTNNCNNNGNNNNCTNNNTNCTTANNTNCNNAGTTGGNNC
CANTANNTGNTNATCTCTGGNTTNGNNNNNNTGNNNNTNNCANNNNGGNNNGTNANGNNNCNATNTAA
NNTANANNNNCNNNNNNANNNCACNNNNNNNGNAGNNACNANNNTTANANNNGTAGNNNACTAAGTCT
CCGANNTNNNNNNNATAGGCGNNANACNNNNNNNNNNNNNANGANNACGNNTTGGNNNTNNNNNNANA
ANNAGACNNTNGTGGNTNNNNNTCAANNNNCTCGTAGTGGTATAATNGNNNNNNNTTGCNCCAGNNTN
NNNGNNNNNNNCNATANNANNNNNNNGNGANNNNNNNNNNNANNNNCTCNNNAGGGANCANCTACTTNA
GCANTGGNGCGCAGNNNTAGANNNNGACNNNCCTTGGGTGGCAANCACNGCAATAAGTNNNGTTNANC
CTNNNAGTNNACACNNNNNCANNANNNCNNNNNNANCNGANNTNNNNNNNNANNNNNNNNNNNNNNN
NNNNNNNNNNNNNNNNNNNNNNNNNNNNNATNNNANNGNNNGTNTNTGACNCATGTGTTTTTANNNGNN
NNNNNACGNNNNANCCCCGGTCGTNNNCNNATTAAANNCCTGNNGCATNNNNNTNCNATAATGANNGC
NNNNNGNNTNGNCTNNCNNTTCAAGNAGTCANNNAGNNANNNNGNTCNNNGNNNNNNNNCNNTCCCNN

ACTGAGGGTNNNNNNNNCATCTGCTNNNNNNNNCTNCGCNGGCGTNNCNNNNNATCNGTATNGNGTTA
NNCANNNNNNNNNNNNNNNNNAAACNNNNNNNNNTNNNG
>Giarai93
GTGNNNNNTAGCCCNNNNNAGTTAGACANNNCNNNNNNNNNNNNNTNNNNNNNNNNNNNNNNNNNNNNNN
NNNNNNNNNNNNNNNNNNNNNNNNNNNNNNNNNNNNNNNNNNNNNNNNNNNNNNNNNNNNNNNNNNNN
NNNNNNNNNNNNNNNNNNNNNNNNNNNNNNNNNNNNNNNNNNNNNNNNNNNNNNNNNNNNNNNNNNNN
NNNNNNNNNNNNNNNNNNNNNNNNNNNNNNNNNNNNNNNNNNNNNNNNNNNNNNNNNNNNNNNNNNNN
NNNNNNNNNNNNNNNNNNNNNNNNNNNNNNNNNNNNNNNNNNNNNNNNNNCNTCTNNAAAANNNTNNN
NNNNNNNNNNNNNNNNNNNNNNNNNCNCNTTGNNNGTAANCNANNNGNACATGCAGTCCCCTCNNNNN
NNNGGCAGAGNNNNNNCCCNNNNNNNCCGGCCCCGNCCNNNNNNNTNNNCNNNGNNNNNNNANNNNNN
NNNNNNTGGTACNNNNCGNNNNNGGTTGTTTNAGAGGTCCACAACGNGGTCNNGNNACNNNNGNNNAN
NNNCNNNNAACNNNNNNNNNNNANNTNNNTANNAGGNCNNNNCCNTCNNGNNNNTNATTGCNATGACC
CAANNCACGGGGGCAGTCCCCGGCGCGGCGACCTTGATNACGTCCGGGGACAAGTATGGCGNNCNCCC
CGGGAAGGANNCNGCCGTANGTGCNNNNNANNNNNTCNTGNGANGTCGCCGNNATTCNCGTGGTCNNN
CNAGAATGCCCGNACCANNCGNANNNTGGTNATGNNAGTNNNNNNNNNNNNNNNNNNNNNNNNNNNNN
NNNNNNNNNNNNNNNNNNNNNNNNNNNNNNNNNNNNNGNNNNNNCNANNNAAGGANNTANNNCTNNTN
NTNNNNNNGGACNNNACNNNNCNNNNNNNACNCNNNCNCNNNNNNANNNGNTTTTTNNCCNNTATTNT
CCAGNACGCNGNGCNNNCATTCANGNGAACNNNGNNACGCGNNAATCGTANNATCTACCTGCCGCNGG
TNNNNNTACNGNNNNNNCGGANNNTNNCTCGTANNNNNNNCATGCGTNNTNTNNTCNCTAGNCANGTT
CGNNNANANANTAGNANGTNTNNNNNNNCTTGTNNCGTTCCGCNTGACNCNNCANNNNGNNGNNCTTN
TCANCGGCNNTANCTGCNGANNTAAGTCATANNNTNNATCNNTANCTNNNNNGGNGGGNNACTGTAAA
NGGNNAATTNGNNNNTNATTNCCNNCACNTNNCTNNGNNTNCTNNNTNCCTGNNTNCNNAATTGGNNC
CANTGNNTGNCNATCTCTGGNTTNGNNNNNNTNNNNNTNNCANNNNGGNNNGTNANANNNCNATNTAA
NNTATANNNNCNNNNNNANNNCACNANNNNNGNAGNNACNANNNTTANANNNGTAGNNNAATAAGTCT
CCGANNTNNNNNNNATAAGCGNNANACNNNNNNNNNNNNNANGANNACGNNTTGGNNNTNNNNNNNNA
GNNAGACNNTNATGGNTNNNNNTCAANNNNTTCGTAGTGGTATAATNGNNNNNNNTTTCNCCAGNNTN
NNNGNNNNNNNCNATANNANNNNNNNGNGANNNNNNNNNNANNNNCTCNNNAGGGANCANCTACTTNA
GCAATGGNGCGCAGNNNGAGANNNNGACNNGCCTTGGGTGGCAANCACNGCAATAAGTNNNGTTNANC
CTNNNAGTNNACATNNNNNCANNGNNNCNNNNNNANCNGANNGNNNNNNNNNNNNNNNNNNNNNNNNN
NNNNNNNNNNNNNNNNNNNNNNNNNNNNAGNNNANGGNNNGTNTNTGACNCATGTGTTTTTANNNGNN
NNNNNACGNNNNANCCCCGGTCGNNNNCNNATTGAANNCCTGNNGCATNNNNNTNCNAGAATGANNGC
NNNNNGNNTNGNCTNNCNNNTCAAGNAATCAANNAGGTANNNNGNTCNNNGNNNNNNNNCNNTCCCNN
ACTGAGGGTNNNNNNCGCATCTGCTNNNNNNNNNTTCGCNGGNNTNNCNGNNNAGCNGTATNGTGTNA
NNCANNNNNNNNNNNNNNNNNANNNNANNNNNNNTNNNN
>HaNhi136
GTGNNNNNTAGCCCNNNNNAGTCAGACANNNCNNNNNNNNNNNNNTNNNNNNNNNNNNNNNNNNNNNNNN
NNNNNNNNNNNNNNNNNNNNNNNNNNNNNNNNNNNNNNNNNNNNNNNNNNNNNNNNNNNNNNNNNNNN
NNNNNNNNNNNNNNNNNNNNNNNNNNNNNNNNNNNNNNNNNNNNNNNNNNNNNNNNNNNNNNNNNNNN
NNNNNNNNNNNNNNNNNNNNNNNNNNNNNNNNNNNNNNNNNNNNNNNNNNNNNNNNNNNNNNNNNNNN
NNNNNNNNNNNNNNNNNNNNNNNNNNNNNNNNNNNNNNNNNNNNNNNNNCNTCTNNNAAANNNTNNN
NNNNNNNNNNNNNNNNNNNNNNNNNCNCNTTGNNNGTAANCNANNNGNACATGCAGTCCCCTCNNNNN
NNNGGCAGAGNNNNNNCCCNNNNNCNCCGGCCCCNNCCNNNNNNNNNNNNNNNGNNNNNNNANNNNNN
NNNNNNTGGTACNNNNCGNNNNNGGTTGTNTAAGAGGTCCACAACGNGGTCNGGNCACNNNNGNNNAN
NNNCNNNNNACNNNNNNNNNNNANNTNNNTANNAGGNCNNNNCCNTCNNGNNANNAATTGCNATGACC
CAANTCACGGGGGCAGTNCCCGGCGCGGCGACCTTGATNACGTCCGGGGACAAGTATGGCNNNCNCCC
CGGGAAGGANNCNGCCGTANGTGCNNNNNANNNNNTCNTGNGACGTCGCCGNNATTCNCGTGGTCNNN
CNAGAATGNCCGNACCANNCGNANNNTGGTNATGTNAGTANNNNNNNNNNNNNNNNNNNNNNNNNNNN
NNNNNNNNNNNNNNNNNNNNNNNNNNNNNNNNGNNNNGNNNNNNCNANNNAAGGANNTANNNCTNNTN
NTNNNNNNGGACNNNACNNNNCNNNNNNNACNCNNNCNCNNNNNNANNNGGTTTTNNNNCNNTATTNT
CCAGNACGCNGNGCNNNCATTCANGNGAACNNNGNNACGCGNNAATCGTANNATCTACCTGCCGCNNG
TNNNNNTACNGNNNNTNCGGANNNTNNNNCGTANNNNNNNCANGCGTNNTNTNNTCNCNAGNCANGTT
CGNNNANANANTAGNANGTNTCCNNNNNCTTGTNNCGTTCCGCNTGANNCNNCANNNNGNCGNNCTTC
TCANCGGCNNTANCTGCNGANNTAAGNCATANNNTNNATCNNTANCTNNNNNGGNGGGNNACTGTAAA
NGGNNAATTNGANGNTNATTTCNNNCATNTNNCTNNGNNTNCTNNNTNCCTGNNTNCNNAATTGGGNC

CANTGNNTGNCNATCTCTGGNTTNGNNNNNNTNNNNNTNNCAANNNGGNNNGTNANANCNCNATNTAA
NNTATANNNNCNNNNNNANNNCACNANNNNNGNAGNNNCNANNNGTANANNNGTAGNNNGATAAGTCT
CCGANNTNNNNNNNATAAGCGNNANACNNNNNNNNNNNNNANGANNACGNNTTTGNNNTNNNNNNCNA
GNNAGACNNTNGTGGNTNNNNNTCAANNNNTTNGTAGTGGTATAATNGNNNNNNNTTTCNCCAGNNTN
NNNGNNNNNNNCNATANNANNNNNNNGNGANNNNNNNNNNANNNNCTCNNNAGGGANCANCTACTTNA
GCANTGGNGCGCAGNNNGAGANNNNGACCAGCCTTGGGTGGCAANCACNGCAATAAGTNNNGTTNANC
CTNNNAGTNNACACNNNNNCANNGNNNCNNNNNNANCNGANNGNNNNNNNNNNNNNNNNNNNNNNNNN
NNNNNNNNNNNNNNNNNNNNNNNNNNNNNAGNNNANGGNNNGTNTNTGACNCATGTGTTTTTANNNGNN
NNNNNACGNNNNANCCCCGGTCGNNTNCNNATTGAANNCCTGNNGCATNNNNNTNCNAGAATGANNGC
NNNNNGNNTNGNCTNNCNNNTCAAGNAATCANNNAGGTANNNNNNTCNNNGANNNNNNNCNNTCCCNN
ACTGAGGGTNNNNNNCGCATCTGCTNNNNNNNNNNTTCGCNGGNGTNNCNGNNNAGCNGTATNGNGTNN
NNCANNNNNNNNNNNNNNNNNTNAAACGANNNNNNTNNNN
>HaNhi312
GTGNNNNNNAGCCCNNNNNAGTTAGACANNNCNNNNNNNNNNNNTNNNNNNNNNNNNNNNNNNNNNN
NNNNNNNNNNNNNNNNNNNNNNNNNNNNNNNTNNNNNNNNNNNNNNNNNNNNNNNNNNNNNNNNNNNN
NNNNNNNNNNNNNNNNNNNNNNNNNNNNNNNNNNNNNNNNNNNNNNNNNNNNNNNNNNNNNNNNNNNN
NNNNNNNNNNNNNNNNNNNNNNNNNNNNNNNNNNNNNNNNNNNNNNNNNNNNNNNNNNNNNNNNNNNN
NNNNNNNNNNNNNNNNNNNNNNNNNNNNNNNNNNNNNNNNNNNNNNNNNNNNNNNTCTNNNAAACNNTNNN
NNNNNNNNNNNNNNNNNNNNNNNNNNCNCNTTGNNNGTAANCNANNNGNACATGCAGTCCCCTCNNNNN
NNNGGCAGAGNNNNNNCCNNNNNNNNNCCGGCCCCNNCCNNNNNNNNNNNNNNNNNNNNNNNNNANNNNNN
NNNNNNTGGTACNNNNCGNNNNNGGTTGNNTAAGAGGTCCACAACANGGTCNNGNNACNNNNGNNNAN
NNNCNNNAAACNNNNNNNNNNNNANNTNNNTANNAGGNNNNNNCCNTCNNGNNNNTAATNGCATGACC
CAANTCACNGGGGCNGTNCCCGGCGCGGCGACCTTGANNACGTCCGGGGNCAAGTATGGCNNNCNCCC
CGGGAAGGANNCNGCNGTANGTGCNNNNNANNNNNTCNTGNGANGTCGCCGNNATTCNCNTGGTCNNN
CNAGAATGNCCNNACCANNCGNANNNTGGTNATGNNAGTNNNNNNNNNNNNNNNNNNNNNNNNNNNNN
NNNNNNNNNNNNNNNNNNNNNNNNNNNNNNNNNNNGNNNNGNNNNNNCNANNNAAGGNNNTANNNCTNNTN
NTNNNNNNNGGACNNNACNTNNCNNNNNNNNACNCNNNCNCNNNNNNNNNNNGNTTTTNNNNCNNTANTNT
CCAGNACGCNGNGCNNNCATTCANGNGAACNNNGNNACGCGNNAATCGTANNATCTANCTGCCGCNNG
NNNNNNTNCNGNNNNNNNCGGANNNTNNNNCGTANNNNNNNCANGCGTNNTNTNNTCNCNAGNCNNGTT
CGNNNANANANTAGNANGTNTNNNNNNNNNNTGTNNCGTTCCGCNCGANNCNNCANNNNGNNGNNCTTN
NCANCGGCNNTANCTNCNGANNTAAGNCATANNNTNNNTCNNTANCTNNNNNGGNGGGNNACTGTAAA
NGGNNAATTNGNNGNTNANTNCNNNCACNTNNCTNNANNTNCTNNNTNCCTGNNTNCNNAATTGGNNC
CANTGNNTGNCNATCTCTGGNTTNGCNNNNNTNNNNNTNNCANNNNGGNNNNNNNNANNNCNATNTAA
NNTANANNNNCNNNNNNANNNCACNNNNNNNGNAGNNNCNANNNTNANANNNGTAGNNNANTAAGTCT
CCGANNTNNNNNNNATAAGCGNNANACNNNNNNNNNNNNNANGANNNCGNNTTGGNNNTNNNNNNCNA
GNNAGACNNTNGTGGNTNNNNNTCAANNNNTTNGTAGTGGTATAATNGNNNNNNNTTTCNCCAGNNTN
NNNGNNNNNNNCNATANNANNNNNNNGNGANNNNNNNNNNANNNNCTTNNNAGGGANCANTTACTNNA
GCANTGGNGCGNAGNNNGAGANNNNGACNNNCCTTGGGTGGCAANNACNGNAATAAGTNNNGTTNANN
NTNNNAGTNNACACNNNNNCANNGNNNCNNNNNNANCNGANNGNNNNNNNNNNNNNNNNNNNNNNNNN
NNNNNNNNNNNNNNNNNNNNNNNNNNNNNANNNNANGGNNNGTNTNTGNCNCATGNNTTTNTANNNGNN
NNNNNACGNNNNANCCCCGGTCGNNNNCNNATTGAANNCCTGNNGCATNNNNNTNCNAGAATGANNNC
NNNNNGNNTNGNCTNNCNNNTCAAGNAATCANNTAGNNNNNNNNNNTCNNNGNNNNNNNNCNNTCNCNN
ACTGAGGGTNNNNNNNNCATCTGCTNNNNNNNNNNTNCGCNGGNGTNNCNNNNNAGCNGTATNNNGTNN
NNCANNNNNNNNNNNNNNNNNTNAAACNANNNNNNNTNNNN
>HaNhi313
GTNNNNNNNAGCCCNNNNNAGTCNGACANNNCNNNNNNNNNNNNTNNNNNNNNNNNNNNNNNNNNNN
NNNNNNNNNNNNNNNNNNNNNNNNNNNNNNNNNNNNNNNNNNNNNNNNNNNNNNNNNNNNNNNNNNNN
NNNNNNNNNNNNNNNNNNNNNNNNNNNNNNNNNNNNNNNNNNNNNNNNNNNNNNNNNNNNNNNNNNNN
NNNNNNNNNNNNNNNNNNNNNNNNNNNNNNNNNNNNNNNNNNNNNNNNNNNNNNNNNNNNNNNNNNNN
NNNNNNNNNNNNNNNNNNNNNNNNNNNNNNNNNNNNNNNNNNNNNNNNNNNNNNNTCTNNNAAANNNTNNN
NNNNNNNNNNNNNNNNNNNNNNNNNNCNCNTTGNNNGTAANCNANNNGNACATGCAGTCCCCTCNNNNN
NNNGGCAGAGNNNNNNNCNNNNNNNNNCCGGCCCCNNCCNNNNNNNNNNNCNNNNNNNNNNNANNNNNN
NNNNNNTGGTACNNNNCGNNNNNGGTTGTNTAAGAGGTCCACAACGNGGTCNNGNNACNNNNGNNNAN
NNNCNNNNANCNGNNNNNNNNNNANNTNNNTANNAGGNCNNNNCCNTCNNNNNNNNNNANTGCNATGANN

CANNTNNCGGGNGNNGTNCCCGGCGNNGCGACCTTGANNANGTCCGGNGNCAAGTATGGCNNNCNCCC
CGNGAAGGANNCNGCNGTANGTGCNNNNNANNNNNTCNTGNGANGTNGCCGNNATTCNCNTGGTCNNN
NNAGANTGNNCNNACCANNCGNANNNTGGTNATGNNAGTNNNNNNNNNNNNNNNNNNNNNNNNNNNNN
NNNNNNNNNNNNNNNNNNNNNNNNNNNNNNNNNNNNNGNNNNGNNNNNNCNANNNAAGGNNNTANNNCTNNTN
NNNNNNNNGNNCNNNACNNNNNNNNNNNNNACNCNNNCNCNNNNNNNNNNNGGTTNNNNNNCNNTANTNT
CCAGNACGCNGNGCNNNCNTTCANGNGAACNNNGNNACGCGNNAATCGTANNATCTANCTGCCGCNNG
NNNNNNTNCNGNNNNNNCGGANNNTNNNNNGTANNNNNNNCNNGCGTNNTNTNNTNNCNAGNCNNGTT
CGNNNANANANTAGNANGTNTNNNNNNNNNNTGTNNCGTTCCGCNTGANNCNNCANNNNGNNGNNNTTN
NCANCGGCNCTANCTGCNGANNTAAGNCATANNNTNNNTCNNTANCTNNNNNGGNGGGNNACTGTAAA
NGGNNAATTNGNNNNTNATTNCNNNCATNTNNCNNNGNNTNCTNNNNTNCCTGNNTNCNNAATTGGNNC
CANTGNNTGNCNATCTCTNGNTTNGNNNNNNTNNNNNTNNCANNNNGGNNNNNNNNNANNNCNATNTAA
NNTANANNNNCNNNNNNANNNCACNNNNNNNNNNAGNNNNNANNNGNANANNNGTAGNNNNNTNAGTCT
CCGANNTNNNNNNNNATAAGCGNNANACNNNNNNNNNNNNNNNANGANNNCGNNTTTGNNNTNNNNNNNNNA
GNNAGACNNTNGTGGNTNNNNNTCAANNNNTTNGTAGTGGTATAATNGNNNNNNNTTTCNCCAGNNTN
NNNGNNNNNNNNCNATANNANNNNNNNGNGANNNNNNNNNNNANNNNCTCNNNAGGGANCANCTACTTNA
GCANNGGNGCGNAGNNNGAGANNNNGACNNNCCTTGGGTGGCAANNACNGNAATAAGTNNNGTTNNNN
CTNNNAGTNNACACNNNNNCANNGNNNCNNNNNNANCNGANNGNNNNNNNNNNNNNNNNNNNNNNNNNN
NNNNNNNNNNNNNNNNNNNNNNNNNNNNNNNNNNNNANGGNNNGTNTNTGNCNCANGNNTTTNTANNNGNN
NNNNNNNGNNNNANCCCCGGTCGNNNNCNNATTGAANNCCTGNNGCATNNNNNTNCNAGAATGANNNC
NNNNNGNNTNGNCTNNCNNNTNAAGNAATCANNNAGGNNNNNNNNTCNNNNNNNNNNNNNCNNNCNCNN
ACTGAGGGTNNNNNNNNCANNNGCTNNNNNNNNNNTNCGCNGGNGTNNCNNNNNAGCNGTATNNNGTNA
NNCANNNNNNNNNNNNNNNNNTNAAACAANNNNNNNTNNNN
>HaNhi314
GTGNNNNNTAGCCCNNNNNAGTTAGACANNNCNNNNNNNNNNNNNTNNNNNNNNNNNNNNNNNNNNNNNNN
NNNNNNNNNNNNNNNNNNNNNNNNNNNNNNNNNNNNNNNNNNNNNNNNNNNNNNNNNNNNNNNN
NNNNNNNNNNNNNNNNNNNNNNNNNTNNNNNNNNNNNNNNNNNNNNNNNNNNNNNNNNNNNNNNN
NNNNNNNNNNNNNNNNNNNNNNNNNNNNNNNNNNNNNNNNNNNNNNNNNNNNNNNNNNNNNNNN
NNNNNNNNNNNNNNNNNNNNNNNNNNNNNNNNNNNNNNNNNNNNNNNNNNNNNNNTCTNNNAAANNNTNNN
NNNNNNNNNNNNNNNNNNNNNNNNNNCNCNNTNNNNGTAANCNANNNGNACATGCAGTCCCCTCNNNNN
NNNGGCAGAGNNNNNNNNNNNNNNNNNCCGNCCCCNNCCNNNNNNNNNNNNNNNNNNNNNNNNNANNNNNN
NNNNNNTGGTACNNNNCGNNNNNGGTTGTNTNAGAGGTCCACAACANGGTCNGGNNACNNNNGNNNAN
NNNCNNNNNNNCNNNNNNNNNNNNANNTNNNTANNAGGNNNNNNCCNTCNNNNNNNNNNANTGCNATGACC
CNANNCACNGGGGCNGTNCCCGGCGCGGCNACCTTGNTNANGTCCGGGGNCAAGTATGGCNNNCNCCC
CGGGAAGGANNCNGCNGTANGTGCNNNNNANNNNNTCNTGNGANGTNGCCGNNATTNNCGTGGTCNNN
CNNGAATGNCCNNACCANNCGNANNNTGGTNATGNNAGTNNNNNNNNNNNNNNNNNNNNNNNNNNNNN
NNNNNNNNNNNNNNNNNNNNNNNNNNNNNNNNNNNNNNNGNNNNNNCNANNNAAGGNNNTANNNCTNNTN
NNNNNNNNGGACNNNACNNNNCNNNNNNNNACNCNNNCNCNNNNNNNANNNGNTTTTNNNNCNNTNNTNT
CCAGNANGCNGNGCNNNCATTCANGNGAACNNNGNNACGCGNNAATCGTANNATCTACCTGCCGCNNG
TNNNNNTNCNGNNNNNNCGGANNNTNNCTNGTANNNNNNNNCANGCGTNNTNTNNTCNCNAGNCNNGTT
CGNNNANANANTAGNANGTNTNNNNNNNNNNTGTNNCGTTCCGCNCGANNCNNCNNNNNGNNGNNCTTN
NCANCGGCNNTANCTGCNGANNTAAGNCATANNNTNNNTCNNTANCTNNNNNGGNGGGNNACTGTAAA
NGGNNAATTNGNNNNTNANTNCNNNNACNNNNCTNNANNTNCTNNNNTNCCTGNNNNCNNAATTGGNNC
CANTGNNTGNCNATCTCTNGNTTNGNNNNNNTNNNNNTNNCANNNNGGNNNGTNANANNNNNATNTAA
NNTANANNNNCNNNNNNANNNCACNANNNNNNNNAGNNNCNANNNTNANANNNGTAGNNNANTAAGTCT
CCGANNTNNNNNNNNATAAGCGNNANACNNNNNNNNNNNNNNNANGANNNCGNNTTGGNNNTNNNNNNNNNA
GNNAGACNNTNGTGGNTNNNNNTCAANNNNTTNGTAGTGGTATAATNGNNNNNNNTTTCNCCAGNNTN
NNNGNNNNNNNNCNATANNANNNNNNNGNGANNNNNNNNNNNANNNNNTTNNNAGGGANCANTTACTNNA
GCANNGGNGCGNAGNNNGAGANNNNGACNNNCCTTGGGTGGCANNNACNGNAATAAGTNNNGTTNANC
CTNNNAGTNNACACNNNNNCANNGNNNCNNNNNNANCNGANNGNNNNNNNNNNNNNNNNNNNNNNNNNN
NNNNNNNNNNNNNNNNNNNNNNNNNNNNNNNNNNNNANGGNNNGTNTNTGNCNCNNGNNTTTNTANNNGNN
NNNNNANGNNNNNNCCCCGGTCGNNNNCNNATTGAANNCCTGNNGCATNNNNNTNCNAGAATGANNNN
NNNNNGNNTNGNCTNNCNTTTCANGNAATCANNNAGGNANNNNGNTCNNNNNNNNNNNNNCNNNCNCNN
ACTGAGGGTNNNNNNNNCANNNGCTNNNNNNNNNNTNCGCNGGNGTNNCNNNNNNGCNGTATNGNGTNN
NNCANNNNNNNNNNNNNNNNNNNNAAACNANNNNNNNTNNNN

>HaNhi315
GTGNNNNNTAGCCCNNNNNAGTCAGACANNNCNNNNNNNNNNNNNTNNNNNNNNNNNNNNNNNNNNNNNNNNNNN
NNNNNNNNNNNNNNNNNNNNNNNNNNNNNNTNNNNNNNNNNNNNNNNNNNNNNNNNNNNNNNNNNNNNNNNNNNNN
NNNNNNNNNNNNNNNNNNNNNNNNNNNNNNNNNNNNNNNNNNNNNNNNNNNNNNNNNNNNNNNNNNNNNNNNNNN
NNNNNNNNNNNNNNNNNNNNNNNNNNNNNNNNNNNNNNNNNNNNNNNNNNNNNNNNNNNNNNNNNNNNNNNNNNN
NNNNNNNNNNNNNNNNNNNNNNNNNNNNNNNNNNNNNNNNNNNNNNNNNNNNNNNNNCNTCTNNNAAANNNTNNN
NNNNNNNNNNNNNNNNNNNNNNNNNNNNNNNNCNCNTTGNNNGTAANCNANNNGNACATGCAGTCCCCTCNNNNN
NNNNGCAGAGNNNNNNNNNCCNNNNNNNCCGGCCCCNNCCNNNNNNNNNNNNNNCNNNGNNNNNNNANNNNNNNN
NNNNNNTGGTACNNNNCGNNNNNGGTTGTTTAAGAGGTCCACAACGNGNTCNGGNCACNNNNGNNNAN
NNNCNNNNAACNNNNNNNNNNNNANNTNNNTANNAGGNCNNNNCCNTCNNNNNNNTNATTGCNATGACC
CANNNCACGGGGGNNGTNCCCGGCGCGGCGACCTTGANNANGTCCGGGGNCAAGTATGGCNNNCNCCC
CGGGAAGGANNCNGCCGTANGTGCNNNNNANNNNNTCNTGNGANGTCGCCGNNATTCNCGTGGTCNNN
CNAGAATGNCCNNACCANNCGNANNNTGGTNATGNNAGTNNNNNNNNNNNNNNNNNNNNNNNNNNNN
NNNNNNNNNNNNNNNNNNNNNNNNNNNNNNNNNNGNNNNGNNNNNNCNANNNAAGGNNNTANNNCTNNTN
NTNNNNNNGGACNNNACNNNNCNNNNNNNACNCNNNCNCNNNNNNANNNGNTTTTNNNNCNNTATTNT
CCAGNACGCNGNGCNNNCATTCANGNGAACNNNGNNACGCGNNAATCGTANNATCTACCTGCCGCNNG
NNNNNNTACNGNNNNNNCGGANNNTNNNNCGTANNNNNNNCATGCGTNNTNTNNTCNCNAGNCNNGTT
CGNNNANANANTAGNANGTNTNNNNNNNNNNTGTNNCGTTCCGCNTGANNCNNCNNNNNGNNGNNNTTN
NCANCGGCNNTANCTGCNGANNTAAGNCATANNNTNNNTCNNTANCTNNNNNGGNGGGNNACTGTAAA
NGGNNAATTNGNNGNTNANTNCCNNCATNTNNCTNNGNNTNCTNNNTNCCTGNNTNCNNAATTGGNNC
CANTGNNTGNCNATCTCTNGNTTNGNNNNNNTGNNNNTNNCAANNNGGNNNGTNNNANNNCNATNTAA
NNTANANNNNCNNNNNNANNNCACNNNNNNNGNAGNNNCNANNNGNANANNNGTAGNNNGNTNAGTCT
CCGANNTNNNNNNNATAAGCGNNANACNNNNNNNNNNNNNNANGANNACGNNTTTGNNNTNNNNNNCNA
GNNAGACNNTNGTGGNTNNNNNTCAANNNNTTNGTAGTGGTATAATNGNNNNNNNTTTCNCCAGNNTN
NNNGNNNNNNNCNATANNANNNNNNNGNGANNNNNNNNNNANNNNCTCNNNAGGNANCANCTACTNNA
GCANTGGNGCGNAGNNNGAGANNNNGACNNNCCTTGGGTGGCAANNACNGCAATAAGTNNNGTTNANC
CTNNNAGTNNACACNNNNNCANNGNNNCNNNNNNANCNGANNGNNNNNNNNNNNNNNNNNNNNNNNN
NNNNNNNNNNNNNNNNNNNNNNNNNNNNNNNNNNNANGGNNNGTNTNTGACNCATGTGTTTTTANNNGNN
NNNNNNNGANNNANCCCCGGTCGNNNNCNNATTGAANNCCTGNNGCATNNNNNTNCNAGAATGANNNC
NNNNNGNNTNGNCTNNCNNNTNAAGNAATCANNNAGGNNNNGCGNTCNNNGNNNNNNNNCNNTCCCNN
ACTGAGGGTNNNNNNNNCATCTGCTNNNNNNNNNNTNCGCNGGNGTNNCNNNNNAGCNGTATNGNGTNA
NNCANNNNNNNNNNNNNNNNNTNAAACNANNNNNNTGNNN
>HaNhi317
GTGNNNNNTAGCCCNNNNNAGTTAGACANNNCNNNNNNNNNNNNNTNNNNNNNNNNNNNNNNNNNNNNNNNNNNN
NNNNNNNNNNNNNNNNNNNNNNNNNNNNNNNNNNNNNNNNNNNNNNNNNNNNNNNNNNNNNNNNNNNNNNNNNNN
NNNNNNNNNNNNNNNNNNNNNNNNNNNNNNNNNNNNNNNNNNNNNNNNNNNNNNNNNNNNNNNNNNNNNNNNNNN
NNNNNNNNNNNNNNNNNNNNNNNNNNNNNNNNNNNNNNNNNNNNNNNNNNNNNNNNNNNNNNNNNNNNNNNNNNN
NNNNNNNNNNNNNNNNNNNNNNNNNNNNNNNNNNNNNNNNNNNNNNNNNNNNNNNNNNNNNNCTNNNAAANNNTNNN
NNNNNNNNNNNNNNNNNNNNNNNNNNNNNNNNCNCNNTGNNNGTAANCNANNNGNACATGCAGTCCCCTCNNNNN
NNNNNCAGAGNNNNNNNNCCNNNNNNNCCGGCCCCNNCCNNNNNNNNNNNNNNNNNNGNNNNNNNGNNNNNN
NNNNNNTGGTACNNNNCGNNNNNGGTTGTNTAAGAGGTCCACCACGNGNTCNNGNNACNNNNGNNNAN
NNNCNNNNAACNNNNNNNNNNNNANNTNNNCANNAGGNNNNNNCCNTNNNGNNNNNNATTGCNATGANN
CANNNNNCGGNGGCNGTCCCCGGCGCGGNGACCTTGANNACGGCCGGGNNCAAGTATGGCNNNCNCCC
CGGGAAGGANNCNGCNGTANGTGCNNNNNANNNNNTCNTGNGANGTCGCCGNNATTCNCGTGGTCNNN
CNAGAATGNNCNNACCANNCGNANNNTGGTNATGNNAGTNNNNNNNNNNNNNNNNNNNNNNNNNNNN
NNNNNNNNNNNNNNNNNNNNNNNNNNNNNNNNNNNNNNGNNNNNNCNANNNAAGGNNNTANNNCTNNTN
NNNNGNNNGGACNNNACNNNNCNNNNNNNACNCNNNCNCNNNNNNANNNGNTTTTTNNNCNNTANTNT
CCAGNACGCNGNGCNNNCNTTCANGNGAACNNNGNNACGCGNNAATCGTANNATCTACCTGCCGCNNG
NNNNNNTNCNGNNNNNNCGGANNNTNNNNCGTANNNNNNNCATGCGTNNTNTNNTCNCNAGNCNNGTT
CGNNNANANANTAGNANGTNTNNNNNNNNNNTGTNNCGTTCCGCNTGANNCNNCANNNNGNNGNNNTTN
NCANCGGCNNTANCTNCNGANNTAAGNCATANNNTNNNTCNNTANCTNNNNNGGNGGGNNACTGTAAA
NGGNNAATTNGNNGNTNATTNCNNNCACNTNNCNNNGNNNNCTNNNTNCCTGNNTNCNNAATTGGNNC
CANTGNNTGNCNATCTCTGGNTTNGNNNNNNTNNNNNTNNCANNNNGGNNNNNNNNANNNCNATNTAA
NNTANANNNNCNNNNNNANNNCACNNNNNNNNNNAGNNNNNANNNTTANANNNGTAGNNNANTAAGTCT

CCGANNTNNNNNNNATAAGCGNNANACNNNNNNNNNNNNNNANGANNNCGNNTTGGNNNTNNNNNNNNNA
ANNAGACNNTNGTGGNTNNNNNTCAANNNNTTNGTAGTGGTATAACNGNNNNNNNTTTCNCCAGNNNN
NNNGNNNNNNNNCNATANNANNNNNNNGNGANNNNNNNNNNNANNNNCTCNNNAGGGANCANCTACTNNA
NCANTGGNGCGNAGNNNGAGANNNNGACNNNCCTTGGGTGGCAANNACNGCAATAAGTNNNGTTNNNN
NTNNNAGTNNACACNNNNNCANNGNNNCNNNNNNANCNGANNTNNNNNNNNNNNNNNNNNNNNNNNNNN
NNNNNNNNNNNNNNNNNNNNNNNNNNNNNNNANNNNANNGNNNGTNTNTGACNCNTGNNTTTTTANNNGNN
NNNNNNNGNNNNANCCCCGGTCGNNNNCNNATTGAANNCCTGNNGCATNNNNNTNCNAGAATGANNNC
NNNNNGNNTNGNCTNNNNTNTNAAGNAGTCAANNAGNNNNNNNNNTCNNNNNNNNNNNNCNNTCCCNN
ACTGAGGGTNNNNNNNNCATCNGCTNNNNNNNNNNTNCGCNGNNGTNNCNNNNNAGCNGTATNGNGTNN
NNCANNNNNNNNNNNNNNNNGNTNAAACAANNNNNNTNNNN
>HaNhi318
GTGNNNNNNAGCCCNNNNNAGTCAGACANNNCNNNNNNNNNNNNNTNNNNNNNNNNNNNNNNNNNNNNNN
NNNNNNNNNNNNNNNNNNNNNNNNNNNNNNNNNNNNNNNNNNNNNNNNNNNNNNNNNNNNNNNNNNNNNN
NNNNNNNNNNNNNNNNNNNNNNNNNNNNNNNNNNNNNNNNNNNNNNNNNNNNNNNNNNNNNNNNNNNNNN
NNNNNNNNNNNNNNNNNNNNNNNNNNNNNTNNNNNNNNNNNNNNNNNNNNNNNNNNNNNNNNNNNNNNNNN
NNNNNNNNNNNNNNNNNNNNNNNNNNNNNNNNNNNNNNNNNNNNNNNNNNNNNNNTCTNNNAAANNNTNNN
NNNNNNNNNNNNNNNNNNNNNNNNNNCNCNTTNNNNGTAANCNANNNGNACATGCAGTCCCCTCNNNNN
NNNNNCAGAGNNNNNNNNCNNNNNNNCCGGCCCCNNCCNNNNNNNNNNNNNNNNGNNNNNNNANNNNNN
NNNNNNTGGTACNNNNCGNNNNNGGTTGTNTAAGAGGTCCACAACGNGNTCNNNNNACNNNNGNNNAN
NNNCNNNNANCNNNNNNNNNNNNNNNTNNNTANNAGGNNNNNNCCNTCNNGNNNNTNANTGCNATGANN
CANNNCACGGGGGCNGTCCCCGGCGCGGCGACCTTGANNANGTCCGGGNNCAAGTATGGCNNNCNCCC
CGGGAAGGANNCNGCNGTANGTGCNNNNNANNNNNTCNTGNGANGTCGCCGNNATTNNNGTGGTCNAN
CNNGAATGNCCNNACCANNCGNANNNTGGTNATGNNAGTNNNNNNNNNNNNNNNNNNNNNNNNNNNNN
NNNNNNNNNNNNNNNNNNNNNNNNNNNNNNNNNNGNNNNGNNNNNNCNANNNAAGGNNNTANNNCTNNTN
NNNNNNNNGGACNNNACNNNNCNNNNNNNACNCNNNCNCNNNNNNANNNGNTTTNNNNNCNNTANTNN
CCAGNACGCNGNGCNNNCNTTCANGNGAACNNNGNNACGCGNNAATCGTANNATCTACCTGCCGCNNG
NNNNNNTACNGNNNNNNCGGANNNTNNNNNGTANNNNNNNCANGCGTNNTNTNNTCNCNAGNCNNGTT
CGNNNANANANTAGNANGTNTNNNNNNNNNNTGTNNCGTTCCGCNTGANNCNNCNNNNNGNNGNNNTTN
NCANCGGCNNTANCTGCCGANNTAAGNCATANNNTNNNTCNNTANCTNNNNNGGNGGGNNACTGNAAA
NGGNNAATTNGNNGNTNANTNCNNNCATNTNNCNNNGNNNNCTNNNNTNCCTGNNTNCNNAATTGGNNN
CANTGNNTGNCNATCTCTGGNTTNGNNNNNNTNNNNNTNNCANNNNGGNNNNNNNNNANNNCNATNTAA
NNTANANNNNCNNNNNNANNNCACNNNNNNNGNAGNNNCNANNNGNANANNNGTAGNNNGNTAAGTCT
CCGANNTNNNNNNNATAAGCGNNANACNNNNNNNNNNNNNNANGANNNCGNNTTTGNNNTNNNNNNNNNA
GNNAGACNNTNGTGGNTNNNNNTCAANNNNTTNGTAGTGGTATAATNGNNNNNNNTTTCNCCAGNNTN
NNNGNNNNNNNNCNATANNANNNNNNNGNGANNNNNNNNNNNANNNNCTCNNNAGGNANCANCTACTNNA
GCANTGGNGCGNAGNNNGAGANNNNGACNNNCCTTGGGTGGCAANNACNGCAATAAGTNNNGTTNNNC
CTNNNAGTNNACACNNNNNCANNGNNNCNNNNNNANCNGANNGNNNNNNNNNNNNNNNNNNNNNNNNNN
NNNNNNNNNNNNNNNNNNNNNNNNNNNNNNNANNNNANGGNNNGTNTNTGACNCNTGTGTTTTTANNNGNN
NNNNNANGNNNGANCCCCGGTCGNNNNCNNATTGAANNCCTGNNGCATNNNNNTNCNAGAATGANNNC
NNNNNGNNTNGNCTNNNNNNTCAAGNAATCNNNNAGNNNNNNNNNTCNNNNNNNNNNNNCNNTCNCNN
ACTGAGGGTNNNNNNNNCATCTGCTNNNNNNNNNNTNCGCNGGNGTNNCNNNNNAGCNGTATNGNGNNN
NNCANNNNNNNNNNNNNNNNNNTNAAACAANNNNCNTNNNN
>HaNhi319
GTGNNNNNNAGCCCNCNNNAGTCAGACANNNCNNNNNNNNNNNNNTNNNNNNNNNNNNNNNNNNNNNNNN
NNNNNNNNNNNNNNNNNNNNNNNNNNNNNNNNTNNNNNNNNNNNNNNNNNNNNNNNNNNNNNNNNNNNNN
NNNNNNNNNNNNNNNNNNNNNNNNNNNNNNNNNNNNNNNNNNNNNNNNNNNNNNNNNNNNNNNNNNNNNN
NNNNNNNNNNNNNNNNNNNNNNNNNNNNNNNNNNNNNNNNNNNNNNNNNNNNNNNNNNNNNNNNNNNNNN
NNNNNNNNNNNNNNNNNNNNNNNNNNNNNNNNNNNNNNNNNNNNNNNNNNNNNCNTCTNNNAAANNNTNNN
NNNNNNNNNNNNNNNNNNNNNNNNNNCNCNTTGNNNGTAANCNANNNGNACATGCAGTCCCCTCNNNNN
NNNNNCAGAGNNNNNNNCCNNNNNNNCCGGCCCCNNCCNNNNNNNNNNNNCNNNGNNNNNNNANNNNNN
NNNNNNTGGTACNNNNCGNNNNNGGTTGTNTNAGAGGTCCACAACGNGGTCNNNNNACNNNNGNNNAN
NNNCNNNNNNCNGNNNNNNNNNNANATNNNTANNAGGNNNNNNCCNTCNNNNNNNNTNANTGCNATGANN
CANNNCNCNGGGGNNGTNCCCGGCGCGGNGACCTTGANNANGTCNGGGGNCAAGTATGGCGNNCNCCC
CGGGAAGGANNCNGCNGTANGTGCNNNNNANNNNNTCNTGNGANGTCGCCGNNATTCNCGTGGTCNNN

CNAGAATGNCCNNACCANNCGNANNNTGGTNATGNNAGTNNNNNNNNNNNNNNNNNNNNNNNNNNNNN
NNNNNNNNNNNNNNNNNNNNNNNNNNNNNNNNGNNNNGNNNNNNCNANNNAAGGNNNTANNNCTNNTN
NTNNNNNNNNNCNNNACNNNNCNNNNNNNACNCNNNCNCNNNNNNANNNGGTTNNNNNNCNNTANTNT
CCAGNACGCNGNGCNNNCNTTCANGNGAACNNNGNNACGCGNNAATCGTANNATCTACCTGCCGCNNG
NNNNNNTNCNGNNNNNNCGGANNNTNNNNCGTANNNNNNNCANGCGTNNTNTNNTCNCNAGNCNNGTT
CGNNNANANANTAGNANGTNTNNNNNNNNNTGTNNCGTTCCGCNTGANNCNNCNNNNNGNNGNNNTTN
NCANCGGCNNTANCTNCNGANNTAAGNCATANNNTNNNTCNNTANCTNNNNNGGNGGGNNACTGTAAA
NGGNNAATTNGNNGNTNATTNCNNNCATNTNNCTNNGNNNNCTNNNTNCCTGNNTNCNNAATTGGNNC
CANTGNNTGNCNATCTCTGGNTTNGNNNNNNTNNNNNTNNCANNNNGGNNNNNNANANNNCNATNTAA
NNTANANNNNCNNNNNNANNNCACNNNNNNNGNAGNNNCNANNNGNANANNNGTAGNNNGNTAAGTCT
CCGANNTNNNNNNNATAAGCGNNANACNNNNNNNNNNNNNANGANNACGNNTTTGNNNTNNNNNNCNA
GNNAGACNNTNGTGGNTNNNNNTCAANNNNTTCGTAGTGGTATAATNGNNNNNNNTTTCNCCAGNNTN
NNNGNNNNNNNCNATANNANNNNNNNGNGANNNNNNNNNNANNNNCTCNNNAGGGANCANCTACTNNA
GCANTGGNGCGNAGNNNGAGANNNNGACNNNCCTTGGGTGGCAANNACNGNAATAAGTNNNGTTNNNC
CTNNNAGTNNACACNNNNNCANNGNNNCNNNNNNANCNGANNGNNNNNNNNNNNNNNNNNNNNNNNNN
NNNNNNNNNNNNNNNNNNNNNNNNNNNNNNNNNANGGNNNGTNTNTGNCNCATGNNTTTTTANNNGNN
NNNNNNNGNNNNANCCCCGGTCGNNNNCNNATTGAANNCCTGNNGCATNNNNNTNCNAGAATGANNNC
NNNNNGNNTNGNCTNNCNNNTNAAGNAATCANNNAGNNNTNNNNNTCNNNGNNNNNNNNCNNTCNCNN
ACTGAGGGTNNNNNNNNCATNNGCTNNNNNNNNNTNCGCNGGNGTNNCNNNNNNGCNGTATNGNGTNN
NNCANNNNNNNNNNNNNNNNTNAAACNANNNNNCTNNNN
>HaNhi320
GTNNNNNNTAGCCCNNNNNAGTTANACANNNCNNNNNNNNNNNNTNNNNNNNNNNNNNNNNNNNNNNN
NNNNNNNNNNNNNNNNNNNNNNNNNNNNNNNNNNNNNNNNNNNNNNNNNNNNNNNNNNNNNNNNNNNN
NNNNNNNNNNNNNNNNNNNNNNNNNNNNNNNNNNNNNNNNNNNNNNNNNNNNNNNNNNNNNNNNNNNN
NNNNNNNNNNNNNNNNNNNNNNNNNNNNNNNNNNNNNNNNNNNNNNNNNNNNNNNNNNNNNNNNNNNN
NNNNNNNNNNNNNNNNNNNNNNNNNNNNNNNNNNNNNNNNNNNNNNNNNNCNNCTNNNAAANNNTNNN
NNNNNNNNNNNNNNNNNNNNNNNNNCNCNNTGNNNGTAANCNANNNGNACATGCAGTCCCCTCNNNNN
NNNNNCAGAGNNNNNNNNNNNNNNNNCCGNCCCCGNCCNNNNNNNNNNNNNNNGNNNNNNNGNNNNNN
NNNNNNTGGTACNNNNCGNNNNNGGTTGTNTAAGAGGTCCACCACGNGNTCNNNNNACNNNNGNNNAN
NNNCNNAAAACNNNNNNNNNNNANNTNNNCANNAGGNNNNNNCCNTCNNGNNNNNNATTGCNATGACC
CANNNNNCNGGGGNNGTNCCCGGCGNNGCGACCTTGNNNACGGCNGGGNNCAAGTATGGCGNNCNCCC
CGGGAAGGANNCNGCNGTANGTGCNNNNNANNNNNTCNTGNGANGTCGCCGNNATTNNNGTGGTCNNN
CNAGAATGNNCNNACCANNCGNANNNTGGTNATGNNAGTNNNNNNNNNNNNNNNNNNNNNNNNNNNNN
NNNNNNNNNNNNNNNNNNNNNNNNNNNNNNNNGNNNNGNNNNNNCNANNNAAGGNNNTANNNCTNNTN
NNNNNNNNGGACNNNACNNNNCNNNNNNNACNCNNNCNCNNNNNNNNNNGNTTNNNNNNCNNTNNTNT
CCAGNANGCNGNGCNNNCNTTCANGNGAACNNNGNNACGCGNNAATCGTANNATCTACCTGCCGCNNG
NNNNNNTNCNGNNNNNNCGGANNNTNNNNCGTANNNNNNNCANGCGTNNTNTNNTCNCNAGNCNNGTT
CGNNNANANANTAGNANGTNTNNNNNNNNNTGTNNCGTTCCGCNTGANNCNNCANNNNGNNGNNNTTN
NCANCGGCNNTANCTGCNGANNTAAGNCATANNNTNNNTCNNTANCTNNNNNGGNGGGNNACTGTAAA
NGGNNAATTNGNNNNTNANTNCNNNCACNTNNCNNNGNNNNCTNNNTNCCTGNNTNCNNAATTGGNNC
CANTGNNTGNCNATCTCTNGNTTNGNNNNNNTGNNNNTNNCANNNNGGNNNNNNNNANNNCNATNTAA
NNTANANNNNCNNNNNNANNNCACNNNNNNNNNAGNNNCNANNNTNANANNNGTAGNNNANTAAGTCN
CCGANNTNNNNNNNATAAGCGNNANACNNNNNNNNNNNNNANGANNNCGNNTTGGNNNTNNNNNNNNA
ANNANACNNTNGTGGNTNNNNNTCAANNNNTTNGTAGTGGTATAACNGNNNNNNNTTTCNCCAGNNTN
NNNGNNNNNNNCNATANNANNNNNNNGNGANNNNNNNNNNANNNNNTCNNNAGGNANCANCTACTNNA
GCANTGGNGCGNAGNNNGAGANNNNGACNNNCCTTGGGTGGCAANNACNGNAATAAGTNNNGTTNNNC
CTNNNAGTNNACACNNNNNCANNGNNNCNNNNNNANCNGANNTNNNNNNNNNNNNNNNNNNNNNNNNN
NNNNNNNNNNNNNNNNNNNNNNNNNNNNNNNNNANNGNNNGTNTNTGACNCNTGTGTTTTTANNNGNN
NNNNNNNGNNNNANCCCCGGTCGNNNNCNNATTGAANNCCTGNNGCATNNNNNTNCNAGAATGANNNC
NNNNNGNNTNGNCTNNCNNNTCAAGNAGTCNNNNAGGNNNNNNNNTCNNNNNNNNNNNNCNNTCNCNN
ACTGAGGGTNNNNNNNNCANCNGCTNNNNNNNNNTNCGCNGNNGTNNCNNNNNNGCNGTATNGNGNNA
NNCANNNNNNNNNNNNNNNNTNANAANANNNNNNTNNNN
>HaNhi321
GTNNNNNNTAGCCCNNNNNAGTTAGACANNNCNNNNNNNNNNNNTNNNNNNNNNNNNNNNNNNNNNNN

NNNNNNNNNNNNNNNNNNNNNNNNNNNNNNNNNNNNNNNNNNNNNNNNNNNNNNNNNNNN
NNNNNNNNNNNNNNNNNNNNNNNNNNNNNNNNNNNNNNNNNNNNNNNNNNNNNNNNNNNN
NNNNNNNNNNNNNNNNNNNNNNNNNNNNNNNNNNNNNNNNNNNNNNNNNNNNNNNNNNNN
NNNNNNNNNNNNNNNNNNNNNNNNNNNNNNNNNNNNNNNNNNNNTNNNNNAAANNNTNNN
NNNNNNNNNNNNNNNNNNNNNNNNNCNCNTTNNNNGTAANCNANNNGNACATGCAGTCCCCTCNNNNN
NNNGGCAGAGNNNNNNNCCNNNNNNNCCGGCCCCNNCCNNNNNNNNNNNNNNNGNNNNNNNANNNNNN
NNNNNNTGGTACNNNNCGNNNNNGGTTGTTTAAGNGGTCCACAACGNGGTCNGNNNACNNNNGNNNAN
NNNCNNNNAACNNNNNNNNNNNNNNNTNNNTANNAGGNCNNNNCCNTCNNGNNNNNNATTGCNATGACC
CANNNCNCNGGGGCNGTNCCCGGCGCGGCGACCTTGANNANGTCCGGGNNCAAGTATGGCGNNCNCCC
CGGGAAGGANNCNGCNGTANGTGCNNNNNANNNTNTCNTGNGANGTNGCCGNNATTCNCGTGGTCNNN
CNNGAATGNCCNNACCANNCGNANNNTGGTNATGNNAGTNNNNNNNNNNNNNNNNNNNNNNNNNNNN
NNNNNNNNNNNNNNNNNNNNNNNNNNNNNNNNNGNNNNGNNNNNNCNANNNAAGGNNNTANNNCTNNTN
NNNNNNNNGGNCNNNACNNNNCNNNNNNNACNCNNNCNCNNNNNNANNNGGTTTTNNNNCNNTANTNN
CCAGNACGCNGNGCNNNCNTTCANGNGAACNNNGNNACGCGNNAATCGTANNATCTACCTGCCGCNNG
NNNNNNTNCNGNNNNNNCGGANNNTNNCTCGTANNNNNNNCNNGCGTNNTNTNNTNNCNAGNCNNGTT
CGNNNANANANTAGNANGTNTNNNNNNNNNTGTNNCGTTCCGCNTGANNCNNCANNNNGNNGNNCTTN
TCANCGGCNNTANCTNCNGANNTAAGNCATANNNTNNNTCNNTANCTNNNNNGGNGGGNNACTGTAAA
NGGNNAATTNGNNNNTNATTNCNNNCACNTNNCNNNGNNTNCTNNNTNCCTGNNTNCNNAATTGGNNC
CANTGNNTGNCNATCTCTNGNTTNGNNNNNNTGNNNNTNNCANNNNGGNNNGTNANANNNCNATNTAA
NNTANANNNNCNNNNNNANNNCACNNNNNNNNNAGNNNCNANNNTNANANNNGTAGNNNANTAAGTCT
CCGANNTNNNNNNNATAAGCGNNANACNNNNNNNNNNNNNNANGANNACGNNTTGGNNNTNNNNNNNNA
GNNAGACNNTNATGGNTNNNNNTCAANNNNTTNGTAGTGGTATAATNGNNNNNNNTTTCNCCAGNNTN
NNNGNNNNNNNCNATANNANNNNNNNGNGANNNNNNNNNNNANNNNCTCNNNAGGNANCANCTACTNNA
GCANNGGNGCGCAGNNNGAGANNNNGACNNNCCTTGGGTGGCAANNACNGNGATAAGTNNNGTTNNNC
CTNNNAGTNNACATNNNNNCANNGNNNCNNNNNNANCNGANNGNNNNNNNNNNNNNNNNNNNNNNNN
NNNNNNNNNNNNNNNNNNNNNNNNNNNNNNNNNNANGGNNNGTNTNTGACNCANGTGTTTNTANNNGNN
NNNNNACGNNNNANCCCCGGTCGNNNNCNNATTGAANNCCTGNNGCATNNNNNNTNCNAGAATGANNNC
NNNNNGNNTNGNCTNNCNNNTCAAGNAATCANNNAGGTNNNNNNNTCNNNNNNNNNNNNNCNNTCCCNN
ACTGAGGGTNNNNNNNNCANNNGCTNNNNNNNNCTNCGCNGGNGTNNCNNNNNNGCNGTATNGNGNNN
NNCANNNNNNNNNNNNNNNNNTNAAACNANNNNNNNTNNNN
>HaNhi322
GTNNNNNNNAGCCCNNNNNAGTTAGACANNNCNNNNNNNNNNNNTNNNNNNNNNNNNNNNNNNNNNN
NNNNNNNNNNNNNNNNNNNNNNNNNNNNNNTNNNNNNNNNNNNNNNNNNNNNNNNNNNNNNNNNNNN
NNNNNNNNNNNNNNNNNNNNNNNNNNNNNNNNNNNNNNNNNNNNNNNNNNNNNNNNNNNN
NNNNNNNNNNNNNNNNNNNNNNNNNNNNNNNNNNNNNNNNNNNNNNNNNNNNNNNNNNNN
NNNNNNNNNNNNNNNNNNNNNNNNNNNNNNNNNNNNNNNNNNNNNNNNNNNNTCTNNNAAANNNTNNN
NNNNNNNNNNNNNNNNNNNNNNNNNCNCNNTNNNNGTAANCNANNNGNACATGCAGTCCCCTCNNNNN
NNNNNCAGAGNNNNNNCCCNNNNNNNCCGNCCCCNNCCNNNNNNNNNNNNCNNNGNNNNNNNANNNNNN
NNNNNNTGGTACNNNNCGNNNNNGGTTGTNNAAGNGGTCCACAACGNGNTCNNGNNACNNNNGNNNAN
NNNCNNNNNANNNNNNNNNNNNNANATNNNTANNAGGNNNNNNCCNTCNNGNNNNNAANTGCNATGACC
CANNNNACGGNGGNNGTNCCCGGCGNGGNGACCTTGANNANNTCCGGGGNCAAGTATGGCGNNCNCCC
CGGGAAGGANNCNGCCGTANGTGCNNNNNANNNTNTCNTGNGANGTNGCCGNNANNNNNGTGGTCNNN
CNAGANTGNCCNNACCANNCGNANNNTGGTNATGNNAGTNNNNNNNNNNNNNNNNNNNNNNNNNNNN
NNNNNNNNNNNNNNNNNNNNNNNNNNNNNNNNNNNNNNGNNNNNNCNANNNAAGGNNNTANNNCTNNTN
NTNNNNNNNGGACNNNACNNNNCNNNNNNNACNCNNNCNCNNNNNNANNNGNTTNNNNNNNCNNTANTNT
CCAGNANGCNGNGCNNNCATTCANGNGAACNNNGNNACGCGNNAATCGTANNATCTACCTGCCGCNNG
NNNNNNTNCNGNNNNNNCGGANNNTNNNNNGTANNNNNNNCANGCGTNNNNNTNNTNNCNAGNCNNGTT
CGNNNANANANTAGNANGTNTNNNNNNNNNNTGTNNCGTTCCGCNTGANNCNNCANNNNGNNGNNNTTN
TCANCGGCNNTANCTNCNGANNTAAGNCATANNNTNNNTCNNTANCTNNNNNGGNGGGNNACTGTAAA
NGGNNAATTNGNNGNTNANTNCNNNCACNTNNCNNNGNNNNCTNNNTNCCTGNNTNCNNAATTGGNNC
CANTGNNTGNCNATCTCTGGNTTNGNNNNNNTGNNNNTNNCANNNNGGNNNNNNNNNANNNCNATNTAA
NNTANANNNNCNNNNNNANNNCACNNNNNNNNNAGNNNCNANNNTTANANNNGTAGNNNANTNAGTCT
CCGANNTNNNNNNNATAAGCGNNANACNNNNNNNNNNNNNNANGANNNCGNNTTGGNNNTNNNNNNNCNA
GNNAGACNNTNATGGNTNNNNNTCAANNNNTTCGTAGTGGTATAATNGNNNNNNNTTTCNCCAGNNTN

NNNGNNNNNNNCNATANNANNNNNNNGNGANNNNNNNNNNNANNNNCTCNNNAGGGANCANCTACTNNA
GCANNGGNGCGNAGNNNGAGANNNNGACNNNCCTTGGGTGGCAANNACNGCGATAAGTNNNGTTNNNN
CTNNNAGTNNACATNNNNNCANNGNNNCNNNNNNANCNGANNGNNNNNNNNNNNNNNNNNNNNNNNNNNN
NNNNNNNNNNNNNNNNNNNNNNNNNNNNNNANNNNANNGNNNGTNTNTGACNCATNNNTTTNTANNNGNN
NNNNNNNGNNNGANCCCCGGTCGNNNNCNNATTGAANNCCTGNNGCATNNNNNTNCNAGAATGANNNC
NNNNNGNNTNGNCTNNCNTTTNANGNAATCANNNAGNNNNNNNNNTCNNNGNNNNNNNNCNNNCNCNN
ACTGAGGGTNNNNNNNNCANCTGCTNNNNNNNNCTNCGCNGGNGTNNCNNNNNAGCNGTATNGNGTNA
NNCANNNNNNNNNNNNNNNNNTNAAACNANNNNNNTNNNN
>HaNhi323
GTGNNNNNTAGCCCNNNNNAGTTAGACANNNCNNNNNNNNNNNNNTNNNNNNNNNNNNNNNNNNNNNNNN
NNNNNNNNNNNNNNNNNNNNNNNNNNNNNNNNNNNNNNNNNNNNNNNNNNNNNNNNNNNNNNNNNNNNNN
NNNNNNNNNNNNNNNNNNNNNNNNNNNNNNNNNNNNNNNNNNNNNNNNNNNNNNNNNNNNNNNNNNNNNN
NNNNNNNNNNNNNNNNNNNNNNNNNNNNNNNNNNNNNNNNNNNNNNNNNNNNNNNNNNNNNNNNNNNNNN
NNNNNNNNNNNNNNNNNNNNNNNNNNNNNNNNNNNNNNNNNNNNNNNNNNNCNTCTNNNAAANNNTNNN
NNNNNNNNNNNNNNNNNNNNNNNNNNCNCNNTNNNNGTAANCNANNNGNACATGCAGTCCCCTCNNNNN
NNNGGCAGAGNNNNNNNCNNNNNNNNCCGGCCCCGNCCNNNNNNNNNNNNNNNNGNNNNNNNANNNNNN
NNNNNNTGGTACNNNNCGNNNNNGGTTGTNTNAGAGGTCCACAACGNGGTCNNNNNACNNNNGNNNAN
NNNCNNNNAANNNNNNNNNNNNNNNNNTNNNTANNAGGNNNNNNCCNTCNNNNNNNNNNANNGCNATGACC
CANNNNACNGNGGNNGTCCCCGGCGNGGNGACCTTGNTNANNTCCGGGNNCAAGTATGGCNNNCNCCC
CGGGAAGGANNCNGCCGTANGTGCNNNNNANNNNNTCNTGNGANGTNGCCGNNATTNNCGTGGTCNNN
NNAGANTGNCCGNACCANNCGNANNNTGGTNATGNNAGTNNNNNNNNNNNNNNNNNNNNNNNNNNNNNNN
NNNNNNNNNNNNNNNNNNNNNNNNNNNNNNNNNNNNNNNGNNNNNNCNANNNAAGGNNNTANNNCTNNTN
NTNNNNNNNNNNCNNNACNNNNCNNNNNNNACNCNNNCNCNNNNNNATNNGNTTTTNNNNCNNTANTNT
CCAGNACGCNGNGCNNNCATTCANGNGAACNNNGNNACGCGNNAATCGTANNATCTACCTGCCGCNNG
NNNNNNTNCNGNNNNNNCGGANNNTNNNNCGTANNNNNNNCATGCGTNNTNTNNTCNCNAGNCNNGTT
CGNNNANANANTAGNANGTNTNNNNNNNNNNTGTNNCGTTCCGCNTGANNCNNCANNNNGNNGNNNTTN
NCANCGGCNNTANCTGCNGANNTAAGNCATANNNTNNNTCNNTANCTNNNNNGGNGGGNNACTGTAAA
NGGNNAATTNGNNNNTNANTNCNNNCACNNNNCTNNGNNTNCTNNNTNCCTGNNTNCNNAATTGGNNC
CANTGNNTGNCNATCTCTGGNTTNGNNNNNNTNNNNNTNNCANNNNGGNNNGTNNNANNNCNATNTAA
NNTANANNNNCNNNNNNANNNCACNNNNNNNNNAGNNNCNANNNTNANANNNGTAGNNNANTNAGTCT
CCGANNTNNNNNNNATAAGCGNNANACNNNNNNNNNNNNNANGANNACGNNTTGGNNNTNNNNNNNNA
GNNAGACNNTNATGGNTNNNNNTCAANNNNTTNGTAGTGGTATAATNGNNNNNNNTTTCNCCAGNNTN
NNNGNNNNNNNCNATANNANNNNNNNGNGANNNNNNNNNNNANNNNCTCNNNAGGGANCANCTACTNNA
GCANTGGNNCGNAGNNNGAGANNNNGACNNNCCTTGGGTGGCAANNACNGNAATAAGTNNNGTTNANC
CTNNNAGTNNACATNNNNNCANNGNNNCNNNNNNANCNGANNGNNNNNNNNNNNNNNNNNNNNNNNNNNN
NNNNNNNNNNNNNNNNNNNNNNNNNNNNNNNNNNNANGGNNNGTNTNTGACNCATGTGTTTNTANNNGNN
NNNNNNNGNNNNANCCCCGGTCGNNNNCNNATTGAANNCCTGNNNCNTNNNNNTNCNAGAATGANNNC
NNNNNGNNTNGNCTNNCNNNTCAAGNAATCANNNAGNNNNNNNNNTCNNNGNNNNNNNNCNNTNNCNN
ACTGAGGGNNNNNNNNNCATNNGCTNNNNNNNNNTNCGCNGGNGTNNCNNNNNNGCNGTATNGNGTNA
NNCANNNNNNNNNNNNNNNNNNNNANNNNNNNNNNTNNNN
>HaNhi324
GTGNNNNNTAGCCCNNNNNAGTTAGACANNNCNNNNNNNNNNNNNNNNNNNNNNNNNNNNNNNNNNNNNN
NNNNNNNNNNNNNNNNNNNNNNNNNNNNNNNTNNNNNNNNNNNNNNNNNNNNNNNNNNNNNNNNNNNNNN
NNNNNNNNNNNNNNNNNNNNNNNNNNNNNNNNNNNNNNNNNNNNNNNNNNNNNNNNNNNNNNNNNNNNNN
NNNNNNNNNNNNNNNNNNNNNNNNNNNNNNNNNNNNNNNNNNNNNNNNNNNNNNNNNNNNNNNNNNNNNN
NNNNNNNNNNNNNNNNNNNNNNNNNNNNNNNNNNNNNNNNNNNNNNNNNNNNNNTCTNNNAAANNNTNNN
NNNNNNNNNNNNNNNNNNNNNNNNNNCNCNTTNNNNGTAANCNANNNGNACATGCAGTCCCCTCNNNNN
NNNGGCAGAGNNNNNNNCCNNNNNCNCCGGCCCCNNCCNNNNNNNNNNNNNNNNNNNNNNNNNNANNNNNN
NNNNNNTGGTACNNNNCGNNNNNGGTTGNNTAAGAGGTCCACAACGNGNTCNGNNNACNNNNGNNNAN
NNNCNNNAAACNNNNNNNNNNNNANATNNNTANNAGGNCNNNNCCNTCNNGNNNNTNATTGCNATGANN
CANNTCNCNGGGGNNGTCCCCGGCGNGGCGACCTTGNNNACGTCCGGGGNCAAGTATGGCNNNCNCCC
CGGGAAGGANNCNGCCGTANGTGCNNNNNANNNTNTCNTGNGANGTNGCCGNNATTNNCGTGGTCNNN
CNAGAATGNCCNNACCANNCGNANNNTGGTNATGNNAGTNNNNNNNNNNNNNNNNNNNNNNNNNNNNNNN
NNNNNNNNNNNNNNNNNNNNNNNNNNNNNNNNNNGNNNNGNNNNNNCNANNNAAGGNNNTANNNCTNNTN

NNNNNNNNGNNCNNNACNNNNCNNNNNNNACNCNNNCNCNNNNNNANNNGGTTNNNNNNCNNTANTNN
CCAGNACGCNGNGCNNNCNTTCANGNGAACNNNGNNACGCGNNAATCGTANNATCTACCTGCCGCNNG
NNNNNNTNCNGNNNNNNCGGANNNTNNNNCGTANNNNNNNCANGCGTNNTNTNNTCNCNAGNCNNGTT
CGNNNANANANTAGNANGTNTNNNNNNNNNTGTNNCGTTCCGCNTGANNCNNCANNNNGNNGNNNTTN
NCANCGGCNNTANCTGCNGANNTAAGNCATANNNTNNNTCNNTANCTNNNNNGGNGGGNNACTGTAAA
NGGNNAATTNGNNNNTNANTNCNNNCACNTNNCNNNGNNTNCTNNNTNCCTGNNTNCNNAATTGGNNC
CANTGNNTGNCNATCTCTNGNTTNGNNNNNNTNNNNNTNNCANNNNGGNNNNNNNNANNNCNATNTAA
NNTANANNNNCNNNNNNANNNCACNNNNNNNGNAGNNNCNANNNTTANANNNGTAGNNNANTAAGTCT
CCGANNTNNNNNNNATAAGCGNNANACNNNNNNNNNNNNNANGANNNCGNNTTGGNNNTNNNNNNCNA
GNNAGACNNTNGTGGNTNNNNNTCAANNNNTTNGTAGTGGTATAATNGNNNNNNNTTTCNCCAGNNTN
NNNGNNNNNNNCNATANNANNNNNNNGNGANNNNNNNNNNANNNNCTCNNNAGGNANCANCTACTNNA
GCANTGGNGCGNAGNNNGAGANNNNGACNNNCCTTGGGTGGCAANNACNGCAATAAGTNNNGTTNNNC
CTNNNAGTNNACACNNNNNCANNGNNNCNNNNNNANCNGANNGNNNNNNNNNNNNNNNNNNNNNNNNN
NNNNNNNNNNNNNNNNNNNNNNNNNNNNANNNNANNGNNNGTNTNTGNCNCANGNNTTTTTANNNGNN
NNNNNNNGNNNNANCCCCGGTCGNNTNCNNATTGAANNCCTGNNGCATNNNNNTNCNAGAATGANNNC
NNNNNGNNTNGNCTNNNNTTTCAAGNAATCAANNAGNNNNNNNNNTCNNNNNNNNNNNNCNNTCNCNN
ACTGAGGGTNNNNNNNNCATNNGCTNNNNNNNNNTNCGCNGGNGTNNCNNCNNNGCNGTATNGNGTNA
NNCANNNNNNNNNNNNNNNNTNAANNGANNNNNNTNNNN
>HaNhi325
GTGNNNNNNAGCCCNNNNNAGTTAGACANNNCNNNNNNNNNNNNTNNNNNNNNNNNNNNNNNNNNNNN
NNNNNNNNNNNNNNNNNNNNNNNNNNNNNNNNNNNNNNNNNNNNNNNNNNNNNNNNNNNNNNNNNNNN
NNNNNNNNNNNNNNNNNNNNNNNNNNNNNNNNNNNNNNNNNNNNNNNNNNNNNNNNNNNNNNNNNNNN
NNNNNNNNNNNNNNNNNNNNNNNNNNNNNNNNNNNNNNNNNNNNNNNNNNNNNNNNNNNNNNNNNNNN
NNNNNNNNNNNNNNNNNNNNNNNNNNNNNNNNNNNNNNNNNNNNNNNNNNNNTCTNNNAAANNNTNNN
NNNNNNNNNNNNNNNNNNNNNNNNNCNCNTTNNNNGTAANCNANNNGNACATGCAGTCCCCTCNNNNN
NNNGGCAGAGNNNNNNNNNNNNNNNNCCGNCCCCNNCCNNNNNNNNNNNNNNNGNNNNNNNANNNNNN
NNNNNNTGGTACNNNNCGNNNNNGGTTGTNTAAGAGGTCCACNACGNGNTCNNNNNACNNNNGNNNAN
NNNCNNNNNNCNNNNNNNNNNNANNTNNNTANNAGGNCNNNNCCNTCNNGNNNNTNATTGCNATGANN
CNNNNNNCGGGNGNNGTNCCCGGNGNGGCGACCTTGNNNANNTCNGGGNNCAAGTATGGCNNNCNCCC
CGGGAAGGANNCNGCNGTANGTGCNNNNNANNNNNTCNTGNGANGTNGCCGNNATTNNNNTGGTCNNN
NNNGAATGNCCNNACCANNCGNANNNTGGTNATGNNAGTNNNNNNNNNNNNNNNNNNNNNNNNNNNNN
NNNNNNNNNNNNNNNNNNNNNNNNNNNNNNNGNNNNGNNNNNNCNANNNNAAGGNNNTANNNCTNNTN
NNNNNNNNGGACNNNACNNNNCNNNNNNNACNCNNNCNCNNNNNNNNNNGGTTNNNNNNCNNTANTNT
CCAGNACGCNGNGCNNNCNTTCANGNGAACNNNGNNACGCGNNAATCGTANNATCTANCTGCCGCNNG
NNNNNNTNCNGNNNNNNCGGANNNTNNNNCGTANNNNNNNCNNGCGTNNTNTNNTCNCNAGNCNNGTT
CGNNNANANANTAGNANGTNTNNNNNNNNNTGTNNCGTTCCGCNTGANNCNNCNNNNNGNNGNNNTTN
NCANCGGCNNTANCTGCNGANNTAAGNCATANNNTNNNTCNNTANCTNNNNNGGNGGGNNACTGTAAA
NGGNNAATTNGNNNNTNANTNCCNNCACNTNNCNNNGNNNNCTNNNTNCCTGNNTNCNNAATTGGNNN
CANTGNNTGNCNATCTCTNGNTTNGNNNNNNTNNNNNTNNCANNNNGGNNNNNNNNANNNCNATNTAA
NNTANANNNNCNNNNNNANNNCACNNNNNNNNNAGNNNCNANNNTNANANNNGTAGNNNANTAAGTCN
CCGANNTNNNNNNNATAAGCGNNANACNNNNNNNNNNNNNANGANNNCGNNTTGGNNNTNNNNNNNNA
GNNAGACNNTNGTGGNTNNNNNTNNANNNNTTNGTAGTGGTATAATNGNNNNNNNTTTCNCCAGNNNN
NNNGNNNNNNNCNATANNANNNNNNNGNGANNNNNNNNNNANNNNNTCNNNAGGNANCANCTACTNNA
GCANNGGNNCGNAGNNNGAGANNNNGACNNNCCTTGGGTGGCAANNACNGNAATAAGTNNNGTTNANN
CTNNNAGTNNACACNNNNNCANNGNNNCNNNNNNANCNGANNGNNNNNNNNNNNNNNNNNNNNNNNNN
NNNNNNNNNNNNNNNNNNNNNNNNNNNNNNNNNANGGNNNGTNTNTGNCNCNTGNNTTTTTANNNGNN
NNNNNNNGNNNNANCCCCGGTCGNNTNCNNATTGAANNCCTGNNGCATNNNNNTNCNAGAATGANNNC
NNNNNGNNTNGNCTNNCNNNTCAAGNAATCNNNNAGNNNNNNNNNTCNNNGNNNNNNNNCNNTNNCNN
ACTGAGGGNNNNNNNNNCANCTGCTNNNNNNNNNTNCNCNGGNGTNNCNNNNNNGCNGTATNGNGTNN
NNCANNNNNNNNNNNNNNNNTNAAACGANNNNCCTNNNG
>HaNhi326
GTNNNNNNTAGCCCNNNNNAGTTNGACANNNCNNNNNNNNNNNNTNNNNNNNNNNNNNNNNNNNNNNN
NNNNNNNNNNNNNNNNNNNNNNNNNNNNNNNNNNNNNNNNNNNNNNNNNNNNNNNNNNNNNNNNNNNN
NNNNNNNNNNNNNNNNNNNNNNNNNNNNNNNNNNNNNNNNNNNNNNNNNNNNNNNNNNNNNNNNCNNN

NNNNNNNNNNNNNNNNNNNNNNNNNNNNNNNNNNNNNNNNNNNNNNNNNNNNNNNNNNNN
NNNNNNNNNNNNNNNNNNNNNNNNNNNNNNNNNNNNNNNNNNNNNNNCTNNNAAANNNTNNN
NNNNNNNNNNNNNNNNNNNNNNNNNCNCNTTNNNNGTAANCNANNNGNACATGCAGTCCCCTCNNNNN
NNNGGCAGAGNNNNNNNCNNNNNNNNNCCGNCCCCNNCCNNNNNNNNNNNNNNNNGNNNNNNNANNNNNN
NNNNNNTGGTACNNNNCGNNNNNGGTTGNNTAAGAGGTCCACAACGNGNTCNGNNNACNNNNGNNNAN
NNNCNNNNNNCNNNNNNNNNNNNANATNNNTANNAGGNNNNNNCCNTNNNGNNNNNNANNGCNATGANN
CANNTCNCGGGGGCNGTNCCCGGCGCGGNGACCTTGANNACGTCCGGGGNCAAGTATGGCGNNCNCCC
CGNGAAGGANNCNGCNGTANGTGCNNNNNANNNNNTCNTGNGANGTNGCCGNNATTCNNNTGNNCNNN
CNAGAATGNNCNNACCANNCGNANNNTGGTNATGNNAGTNNNNNNNNNNNNNNNNNNNNNNNNNNNNN
NNNNNNNNNNNNNNNNNNNNNNNNNNNNNNNNNNGNNNNGNNNNNNCNANNNAAGGNNNTANNNCTNNTN
NNNNNNNNNNNCNNNNCNNNNCNNNNNNNACNCNNNCNCNNNNNNNNNNNGGTTTNNNNNCNNTANTNN
CCAGNACGCNGNGCNNNCNTTCANGNGAACNNNGNNACGCGNNAATCGTANNNTCNACCTGCCGCNNG
NNNNNNTNCNGNNNNNNNCGGANNNTNNNNNGTANNNNNNNCANGCGTNNTNTNNTNNCNAGNCNNGTT
CGNNNANANANTAGNANGTNTNNNNNNNNNNTGTNNCGTTCCGCNTGANNCNNCNNNNNGNNGNNNTTN
NCANCGGCNNTANCTGCNGANNTAAGNCATANNNTNNNTCNNTANCTNNNNNGGNGGGNNACTGNAAA
NGNNNAATTNGNNGNTNANTNCNNNNACNNNNCNNNGNNNNCTNNNTNCCTGNNTNCNNAATTGGNNN
CANTGNNTGNCNATCTCTNGNTTNGNNNNNNTNNNNNTNNCANNNNGGNNNNNNNNANNNCNATNTAA
NNTANANNNNCNNNNNNANNNCACNNNNNNNNNAGNNNNNNNNNTNANANNNGTAGNNNANTNAGTCN
CCGANNTNNNNNNNNATAAGCGNNANACNNNNNNNNNNNNNNNANGANNNCGNNTTGGNNNTNNNNNNNNA
GNNAGACNNTNGTGGNTNNNNNTCAANNNNTTNGTAGTGGTATAATNGNNNNNNNTTTCNCCAGNNTN
NNNGNNNNNNNNCNATANNANNNNNNNGNGANNNNNNNNNNNANNNNCTCNNNAGGNANCANCTACTNNA
GCANNGGNGCGNAGNNNGAGANNNNGACNNNCCTTGGGTGGCAANNACNGNAATAAGTNNNGTTNNNN
NTNNNAGTNNACACNNNNNCANNGNNNCNNNNNNANCNGANNGNNNNNNNNNNNNNNNNNNNNNNNNN
NNNNNNNNNNNNNNNNNNNNNNNNNNNNNNNNNNNANNGNNNGTNTNTGACNCANNNNTTTNTANNNGNN
NNNNNNNGNNNNANCCCCGGTCGNNNNCNNATTGAANNCCNGNNGCATNNNNNTNCNAGAATGANNNC
NNNNNGNNTNNNCTNNNNNNNTNAAGNAATCANNNAGNNNNNNNNNTCNNNGNNNNNNNNNCNNTNNCNN
ACTGAGGGTNNNNNNNNCATNNGCTNNNNNNNNNNTNCGCNGGNGTNNCNNNNNNGCNGTATNGNGNNN
NNCANNNNNNNNNNNNNNNNNNTNAAACAANNNNCCTNNNN
>HaNhi327
GTGNNNNNTAGCCCNNNNNAGTTAGACANNNCNNNNNNNNNNNNTNNNNNNNNNNNNNNNNNNNNNNN
NNNNNNNNNNNNNNNNNNNNNNNNNNNNNNNNNNNNNNNNNNNNNNNNNNNNNNNNNNNNNN
NNNNNNNNNNNNNNNNNNNNNNNNNNNNNNNNNNNNNNNNNNNNNNNNNNNNNNNNNNNNNN
NNNNNNNNNNNNNNNNNNNNNNNNNNNNNNNNNNNNNNNNNNNNNNNNNNNNNNNNNNNNNN
NNNNNNNNNNNNNNNNNNNNNNNNNNNNNNNNNNNNNNNNNNNNNNNNNNCNTCTNNNAAANNNTNNN
NNNNNNNNNNNNNNNNNNNNNNNNNCNCNTTNNNNGTAANCNANNNGNACATGCAGTCCCCTCNNNNN
NNNNNCAGAGNNNNNNNCNNNNNNCNCCGNCCCCNNCCNNNNNNNNNNNNCNNNGNCNNNNNGNNNNNN
NNNNNNTGGTACNNNNCGNNNNNGGTTGTNTAAGAGGTCCACCACGNGGTCNNGNNACNNNNGNNNAN
NNNCNNAAAACNGNNNNGNNNNANNTNNNCANAAGGNNNNNNCCNTCNNNNNNNNTNANTGCNATGANN
CANNNCACGGGGGCNGTNCCCGGCGNGGCGACCTTGNNNACGGCCGGGGNCAAGTATGGCNNNCNCCC
CGGGAAGGANNCNGCCGTANGTGCNNNNNANNNTNTCNTGNGANGTCGCCGNNATTCNCGTGGTCNNN
CNAGAATGNNCGNACCANNCGNANNNTGGTNATGNNAGTNNNNNNNNNNNNNNNNNNNNNNNNNNNNN
NNNNNNNNNNNNNNNNNNNNNNNNNNNNNNNNNNGNNNNGNNNNNNCNANNNAAGGNNNTANNNCTNNTN
NNNNNNNNGGACNNNACNNNNCNNNNNNNACNCNNNCNCNNNNNNANNNGGTTTTNNNNNCNNTANTNT
CCAGNANGCNGNGCNNNCATTCANGNGAACNNNGNNACGCGNNAATCGTANNATCTANCTGCCGCNNG
TNNNNNTACNGNNNNNNNCGGANNNTNNNNNGTANNNNNNTCANGCGTNNNNTNNTCNCNANNCNNGTT
CGNNNANANANTAGNANGTNTNNNNNNNNNNTGTNNCGTTCCGCNTGANNCNNCANNNNGNNGNNNTTN
NCANCGGCNNTANCTGCNGANNTAAGNCATANNNTNNNTCNNTANCTNNNNNGGNGGGNNACTGTAAA
NGGNNAATTNGNNNNTNANTNCNNNCACNTNNCNNNGNNTNCTNNNTNCCTGNNTNCNNAATTGGNNN
CANTGNNTGNCNATCTCTGGNTTNGNNNNNNTNNNNNTNNCANNNNGGNNNNNNANANNNCNATNTAA
NNTANANNNNCNNNNNNANNNCACNNNNNNNNNAGNNNCNANNNTTANANNNGTAGNNNANTNAGTCT
CCGANNTNNNNNNNNATAAGCGNNANACNNNNNNNNNNNNNNNANGANNNCGNNTTGGNNNTNNNNNNNCNA
ANNAGACNNTNGTGGNTNNNNNTCAANNNNTTCGTAGTGGTATAACNGNNNNNNNTTTCNCCAGNNTN
NNNGNNNNNNNNCNATANNANNNNNNNGNGANNNNNNNNNNNANNNNCTCNNNAGGNANCANCTACTNNA
GCANNGGNGCGCAGNNNGAGANNNNGACNNNCCTTGGGTGGCAANNACNGNAATAAGTNNNGTTNNNN

CTNNNAGTNNACACNNNNNCANNGNNNCNNNNNNANCNGANNTNNNNNNNNNNNNNNNNNNNNNNNNN
NNNNNNNNNNNNNNNNNNNNNNNNNNNNNNNNNNNNNANGGNNNGTNTNTGACNCATGTGTTTTTANNNGNN
NNNNNACGNNNNANCCCCGGTCGNNNNCNNATTGAANNCCTGNNGCATNNNNNTNCNAGAATGANNNC
NNNNNGNNTNGNCTNNCNNNTCAAGNAGTCANNNAGGNANNNNGNTCNNNGNNNNNNNNCNNNCNCNN
ACTGAGGGTNNNNNNNNCATCTGCTNNNNNNNNNNTNCGCNGNCGTNNCNNNNNNGCNGTATNGNGTNN
NNCANNNNNNNNNNNNNNNNNNTNAAACAANNNNNNTNNNN
>HaNhi328
GTGNNNNNNAGCCCNNNNNAGTTANACANNNCNNNNNNNNNNNNTNNNNNNNNNNNNNNNNNNNNNN
NNNNNNNNNNNNNNNNNNNNNNNNNNNNNNNNNNNNNNNNNNNNNNNNNNNNNNNNNNNN
NNNNNNNNNNNNNNNNNNNNNNNNNNNNNNNNNNNNNNNNNNNNNNNNNNNNNNNNNNNN
NNNNNNNNNNNNNNNNNNNNNNNNNNNNNNNNNNNNNNNNNNNNNNNNNNNNNNNNNNNN
NNNNNNNNNNNNNNNNNNNNNNNNNNNNNNNNNNNNNNNNNNNNNNNNNNNNNNTCTNNNAAANNNTNNN
NNNNNNNNNNNNNNNNNNNNNNNNNNCNCNNTGNNNGTAANCNANNNGNACATGCAGTCCCCTCNNNNN
NNNGGCAGAGNNNNNNNCCNNNNNNNNCCGNCCCCNNCCNNNNNNNNNNNNNANNNNNNNNNNNANNNNNN
NNNNNNTGGTACNNNNCGNNNNNGGTTGTNTAAGAGGTCCACAACGNGGTCNNNNNACNNNNGNNNAN
NNNCNNNNAACNNNNNNNNNNNNANNTNNNTANNAGGNCNNNNCCNTCNNGNNNNNAATTGCNATGACC
CANNTNNCGGNGGCAGTNCCCGGCGCGGCGACCTTGANNACGTCCGGGGNCAAGTATGGCNNNCNCCC
CGGGAAGGANNCNGCNGTANGTGCNNNNNANNNNNTCNTGNGANGTCGCCGNNATTNNCNTGGTCNNN
CNAGAATGNCCNNACCANNCGNANNNTGGTNATGNNAGTNNNNNNNNNNNNNNNNNNNNNNNNNNNN
NNNNNNNNNNNNNNNNNNNNNNNNNNNNNNNNNGNNNNGNNNNNNCNANNNAAGGNNNTANNNCTNNTN
NTNNNNNNNGGACNNNACNNNNCNNNNNNNNACNCNNNCNCNNNNNNANNNGGTTTNNNNNCNNTANTNT
CCAGNACGCNGNGCNNNCATTCANGNGAACNNNGNNACGCGNNAATCGTANNATCTACCTGCCGCNNG
TNNNNNTACNGNNNNNNCGGANNNTNNCTCGTANNNNNNNCNNGCGTNNTNTNNTNNCNAGNCNNGTT
CGNNNANANANTAGNANGTNTNNNNNNNNNNTGTNNCGTTCCGCNTGANNCNNCNNNNNGNNGNNNTTN
NCANCGGCNNTANCTGCNGANNTAAGNCATANNNTNNNTCNNTANCTNNNNNGGNGGGNNACTGTAAA
NGGNNAATTNGNNGNTNATTNCNNNCACNTNNCNNNGNNTNCTNNNTNCCTGNNTNCNNAATTGGNNN
CANTGNNTGNCNATCTCTGGNTTNGNNNNNNTNNNNNTNNCANNNNGGNNNNNNANANNNCNATNTAA
NNTANANNNNCNNNNNNANNNCACNNNNNNNNNAGNNNNNANNNTNANANNNGTAGNNNANTAAGTCT
CCGANNTNNNNNNNATAAGCGNNANACNNNNNNNNNNNNNANGANNACGNNTTGGNNNTNNNNNNCNA
GNNANACNNTNATGGNTNNNNNTCAANNNNTTCGTAGTGGTATAATNGNNNNNNNTTTCNCCAGNNTN
NNNGNNNNNNNNCNATANNANNNNNNNGNGANNNNNNNNNNNANNNNCTCNNNAGGGANCANCTACTNNA
GCANNGGNNCGNAGNNNGAGANNNNGACNNNCCTTGGGTGGCAANNACTGCAATAAGTNNNGTTNNNN
NTNNNAGTNNACATNNNNNCANNGNNNCNNNNNNANCNGANNGNNNNNNNNNNNNNNNNNNNNNNNNN
NNNNNNNNNNNNNNNNNNNNNNNNNNNNNNNNNNNNNANGGNNNGTNTNTGACNCNTGTGTTTTTANNNGNN
NNNNNNNGNNNNANCCCCGGTCGNNNNCNNATTGAANNCCTGNNGCATNNNNNTNCNAGAATGANNNC
NNNNNGNNTNGNCTNNCNNNTNAAGNAATCANNNAGGTNNNNNGNTCNNNNNNNNNNNNNCNNTCNCNN
ACTGAGGGTNNNNNNNNCATNNGCTNNNNNNNNNNTNCGCNGGCGTNNCNNNNNNGCNGTATNGNGTNA
NNCANNNNNNNNNNNNNNNNNNTNAANNAANNNNNCTNNNN
>HaNhi329
GTGNNNNNTAGCCCNNNNNAGTTNGACANNNCNNNNNNNNNNNNTNNNNNNNNNNNNNNNNNNNNNN
NNNNNNNNNNNNNNNNNNNNNNNNNNNNNNNTNNNNNNNNNNNNNNNNNNNNNNNNNNNN
NNNNNNNNNNNNNNNNNNNNNNNNNNNNNNNNNNNNNNNNNNNNNNNNNNNNNNNNNNNN
NNNNNNNNNNNNNNNNNNNNNNNNNNNNNNNNNNNNNNNNNNNNNNNNNNNNNNNNNNNN
NNNNNNNNNNNNNNNNNNNNNNNNNNNNNNNNNNNNNNNNNNNNNNNNNNNNNNTCTNNNAAANNNTNNN
NNNNNNNNNNNNNNNNNNNNNNNNNNCNCNNTNNNNGTAANCNANNNGNACATGCAGTCCCCTCNNNNN
NNNGGCAGAGNNNNNNNNNCNNNNNNNNCCGNCCCCNNCCNNNNNNNNNNNNNNNNNNNNNNNNNGNNNNNN
NNNNNNTGGTACNNNNCGNNNNNGGTTGTNNAAGAGGTCCACCACGNGNTCNGNNNACNNNNGNNNAN
NNNCNNNNAACNNNNNNNNNNNNNNNATNNNCANNAGGNNNNNNCCNTNNNNNNNNNNNATNGCNATGANN
CANNNCNCGGGGGNNGTNCCCGGCGCGGCGACCTTGNNNACGGCNGGGGNCAAGTATGGCNNNCNCCC
CGGGAAGGANNCNGCNGTANGTGCNNNNNANNNNNTCNTGNGANGTNGCCGNNATTNNCGTGGTCNNN
NNAGAATGNCCNNACCANNCGNANNNTGGTNATGNNAGTNNNNNNNNNNNNNNNNNNNNNNNNNNNN
NNNNNNNNNNNNNNNNNNNNNNNNNNNNNNNNNGNNNNGNNNNNNCNANNNAAGGNNNTANNNCTNNTN
NTNNNNNNNGGACNNNACNNNNCNNNNNNNNACNCNNNCNCNNNNNNNNNNNGGTTNNNNNNCNNTANTNN
CCAGNANGCNGNGCNNNCNTTCANGNGAACNNNGNNACGCGNNAATCGTANNATCTACCTGCCGCNNG

NNNNNNTACNGNNNNNNCGGANNNTNNNNCGTANNNNNNNCANGCGTNNTNTNNTCNCNAGNCNNGTT
CGNNNANANANTAGNANGTNTNNNNNNNNNTGTNNCGTTCCGCNTGANNCNNCNNNNNGNNGNNCTTN
NCANCGGCNNTANCTGCNGANNTAAGNCATANNNTNNNTCNNTANCTNNNNNGNNGGGNNACTGTAAA
NGGNNAATTNGNNNNTNANTNCNNNCACNTNNCNNNGNNNNCTNNNTNCCTGNNTNCNNAATTGGNNC
CANTGNNTGNCNATCTCTNGNTTNGNNNNNNTNNNNNTNNCANNNNGGNNNNNNANANNNCNATNTAA
NNTANANNNNCNNNNNNANNNCACNNNNNNNNNAGNNNCNANNNTNANANNNGTAGNNNANTNAGTCT
CCGANNTNNNNNNNATAAGCGNNANACNNNNNNNNNNNNNNANGANNNCGNNTTGGNNNTNNNNNNNNA
ANNAGACNNTNGTGGNTNNNNNTCAANNNNTTNGTAGTGGTATAACNGNNNNNNNTTTCNCCAGNNTN
NNNGNNNNNNNCNATANNANNNNNNNGNGANNNNNNNNNNNANNNNCTCNNNAGGNANCANCTACTNNA
GCANNGGNGCGNAGNNNGAGANNNNGACNNNCCTTGGGTGGCANNNACNGCAATAAGTNNNGTTNANN
NTNNNAGTNNACACNNNNNCANNGNNNCNNNNNNANCNGANNTNNNNNNNNNNNNNNNNNNNNNNNNN
NNNNNNNNNNNNNNNNNNNNNNNNNNNNNNNNNNANNGNNNGTNTNTGNCNCNNGNNTTTTTANNNGNN
NNNNNNNGNNNNANCCCCGGTCGNNNNCNNATTGAANNCCTGNNGCATNNNNNTNCNANAATGANNGC
NNNNNGNNTNNNCTNNCNNNTNANGNAGTNANNNAGGTNNNNNNNTCNNNGNNNNNNNNCNNNCNCNN
ACTGAGGGTNNNNNNNNCATCNGCTNNNNNNNNNNTNCGCNGNNGTNNCNNNNNAGCNGTATNNNGTNN
NNCANNNNNNNNNNNNNNNNNTNAAACAANNNNNNNTNNNN
>HaNhi330
GTGNNNNNNAGCCCNNNNNAGTCNGACANNNCNNNNNNNNNNNNTNNNNNNNNNNNNNNNNNNNNNNN
NNNNNNNNNNNNNNNNNNNNNNNNNNNNNNNNNNNNNNNNNNNNNNNNNNNNNNNNNNNNNNNNNNNNNN
NNNNNNNNNNNNNNNNNNNNNNNNNNNNNNNNNNNNNNNNNNNNNNNNNNNNNNNNNNNNNNNNNNNNNN
NNNNNNNNNNNNNNNNNNNNCNNNNNNNNNNNNNNNNNNNNNNNNNNNNNNNNNNNNNNNNNNNNNNNNN
NNNNNNNNNNNNNNNNNNNNNNNNNNNNNNNNNNNNNNNNNNNNNNNNNNNNNNNNTCTNNNAAANNNTNNN
NNNNNNNNNNNNNNNNNNNNNNNNNNCNCNNTGNNNGTAANCNANNNGNACATGCAGTCCCCTCNNNNN
NNNGGCAGAGNNNNNNNCNNNNNNNNCCGNCCCCGNCCNNNNNNNNNNNNNNNNNGNNNNNNNANNNNNN
NNNNNNTGGTACNNNNCGNNNNNGGTTGNNTAAGAGGTCCACAACGNGNTCNGNNNACNNNNGNNNAN
NNNCNNNNAANNNNNNNNNNNNNNNNNTNNNTANNAGGNCNNNNCCNTCNNNNNNNNNNATTGCNATGACC
CANNNCNCNGGGGCAGTNCCCGGCGNGGNGACCTTGNNNANNTCNGGGNNCAAGTATGGCNNNCNCCC
CGNGAAGGANNCNGCNGTANGTGCNNNNNANNNNNTCNTGNGANGTCGCCGNNATTNNNGTGGTCNNN
CNNGAATGNCCNNACCANNCGNANNNTGGTNATGNNAGTNNNNNNNNNNNNNNNNNNNNNNNNNNNNN
NNNNNNNNNNNNNNNNNNNNNNNNNNNNNNNNNNNGNNNNGNNNNNNCNANNNANGGNNNTANNNCTNNTN
NTNNNNNNNGGACNNNACNNNNCNNNNNNNACNCNNNCNCNNNNNNNNNNGNTTNNNNNNCNNTNNTNN
CCAGNACGCNGNGCNNNCNTTCANGNGAACNNNGNNACGCGNNAATCGTANNATCTACCTGCCGCNNG
TNNNNNTNCNGNNNNNNCGGANNNTNNNNNGTANNNNNNNCANGCGTNNNNTNNTCNCNAGNCNNGTT
CGNNNANANANTAGNANGTNTNNNNNNNNNTGTNNCGTTCCGCNTGANNCNNCANNNNGNNGNNNTTN
NCANCGGCNNTANCTGCNGANNTAAGNCATANNNTNNNTCNNTANCTNNNNNGGNGGGNNACTGTAAA
NGGNNAATTNGNNNNTNANTNCNNNCATNTNNCNNNGNNNNCTNNNTNCCTGNNTNCNNAATTGGNNN
CANTGNNTGNCNATCTCTNGNTTNGNNNNNNTGNNNNTNNCANNNNGGNNNGNNNNANNNCNATNTAA
NNTANANNNNCNNNNNNANNNCACNNNNNNNNNAGNNNNNANNNGNANANNNGTAGNNNGNTNAGTCN
CCGANNTNNNNNNNATAAGCGNNANACNNNNNNNNNNNNNNANGANNNCGNNTTTGNNNTNNNNNNNNA
GNNAGACNNTNGTGGNTNNNNNTCAANNNNTTNGTAGTGGTATAATNGNNNNNNNTTTCNCCAGNNTN
NNNGNNNNNNNCNATANNANNNNNNNGNGANNNNNNNNNNNANNNNCTCNNNAGGNANCANCTACTNNA
GCANTGGNGCGNAGNNNGAGANNNNGACNNNCCTTGGGTGGCAANNACNGNAATAAGTNNNGTTNNNN
CTNNNAGTNNACACNNNNNCANNGNNNCNNNNNNANCNGANNGNNNNNNNNNNNNNNNNNNNNNNNNN
NNNNNNNNNNNNNNNNNNNNNNNNNNNNNNNNNNANGGNNNGTNTNTGNCNCATGTGTTTNTANNNGNN
NNNNNACGNNNNANCCCCGGTCGNNTNCNNATTGAANNCCTGNNNCATNNNNNTNCNAGAATGANNNN
NNNNNGNNTNNNCTNNCNNNTCAAGNAATNNNNNAGGNNNNNNNNTCNNNGNNNNNNNNCNNNCNCNN
ACTGAGGGTNNNNNNNNCATCTGCTNNNNNNNNNNTNCGCNGGNGTNNCNNNNNAGCNGTATNNNGTNA
NNCANNNNNNNNNNNNNNNNNTNAAACNANNNNNNNTNNNN
>HaNhi331
GTGNNNNNTAGCCCNNNNNAGTCAGACANNNCNNNNNNNNNNNNTNNNNNNNNNNNNNNNNNNNNNNN
NNNNNNNNNNNNNNNNNNNNNNNNNNNNNNNNNNNNNNNNNNNNNNNNNNNNNNNNNNNNNNNNNNNNNN
NNNNNNNNNNNNNNNNNNNNNNNNNNNNNNNNNNNNNNNNNNNNNNNNNNNNNNNNNNNNNNNNNNNNNN
NNNNNNNNNNNNNNNNNNNNNNNNNNNNNNNNNNNNNNNNNNNNNNNNNNNNNNNNNNNNNNNNNNNNNN
NNNNNNNNNNNNNNNNNNNNNNNNNNNNNNNNNNNNNNNNNNNNNNNNNNNNNNNNTCTNANAAANNNTNNN

NNNNNNNNNNNNNNNNNNNNNNNNNCNCNNTGNNNGTAANCNANNNGNACATGCAGTCCCCTCNNNNN
NNNNNCAGAGNNNNNNCCCNNNNNNNCCGNCCCCNNCCNNNNNNNNNNNNNNNNNNNNNNNANNNNNN
NNNNNNTGGTACNNNNCGNNNNNGGTTGTNTAAGAGGTCCACAACGNGGTCNNGNCACNNNNGNCNAN
NNNCNNNNAANNNNNNNNNNNNANNTNNNTANNAGGNCNNNNCCNTCNNNNNNNNNATTGCNATGACC
CANNTCNCGGNGGNNGTNCCCGGCGCGGCGACCTTGANNACGTCCGGGGNCAAGTATGGCNNNCNCCC
CGGGAAGGANNCNGCNGTANGTGCNNNNNANNNNNTCNTGNGANGTCGCCGNNATTCNNNTGGTCNNN
CNNGANTGNCCNNACCANNCGNANNNTGGTNATGNNAGTNNNNNNNNNNNNNNNNNNNNNNNNNNNNN
NNNNNNNNNNNNNNNNNNNNNNNNNNNNNNNNGNNNNGNNNNNNCNANNNAAGGNNNTANNNCTNNTN
NNNNNNNNGGACNNNACNNNNCNNNNNNNACNCNNNCNCNNNNNNANNNGGTTTTTNNNCNNTATTNT
CCAGNACGCNGNGCNNNCNTTCANGNGAACNNNGNNACGCGNNAATCGTANNATCTANCTGCCGCNNG
NNNNNNTNCNGNNNNNNCGGANNNTNNCNCGTANNNNNNNCNTGCGTNNTNTNNTCNCNAGNCNNGTT
CGNNNANANANTAGNANGTNTNNNNNNNNNTGTNNCGTTCCGCNTGANNCNNCANNNNGNNGNNNTTN
TCANCGGCNCTANCTGCNGANNTAAGNCATANNNTNNNTCNNTANCTNNNNNGGNGGGNNACTGTAAA
NGGNNAATTNGNNGNTNANTNCNNNCATNTNNCTNNGNNNNCTNNNTNCCTGNNTNCNNAATTGGNNN
CANTGNNTGNCNATCTCTGGNTTNGNNNNNNTNNNNNTNNCANNNNGGNNNNNNANANNNCNATNTAA
NNTANANNNNCNNNNNNANNNCACNNNNNNNGNAGNNNCNANNNGTANANNNGTAGNNNGNTNAGTCT
CCGANNTNNNNNNNATAAGCGNNANACNNNNNNNNNNNNNANGANNNCGNNTTTGNNNTNNNNNNCNA
GNNAGACNNTNGTGGNTNNNNNTCAANNNNTTNGTAGTGGTATAATNGNNNNNNNTTTCNCCAGNNTN
NNNGNNNNNNNCNATANNANNNNNNNGNGANNNNNNNNNNANNNNCTCNNNAGGGANCANCTACTTNA
NCANTGGNGCGNAGNNNGAGANNNNGACNNNCCTTGGGTGGCAANCACNGCAATAAGTNNNGTTNNNN
CTNNNAGTNNACACNNNNNCANNGNNNCNNNNNNANCNGANNGNNNNNNNNNNNNNNNNNNNNNNNNN
NNNNNNNNNNNNNNNNNNCNNNNNNNNNANNNNANGGNNNGTNTNTGNCNCATGTGTTTNTANNNGNN
NNNNNNNGNNNNANCCCCGGTCGNNNNCNNATTGAANNCCTGNNGCATNNNNNTNCNAGAATGANNNC
NNNNNGNNTNGNCTNNCNNNTCAAGNAATCNNNNAGGTNNNNNNNTCNNNGNNNNNNNNCNNTCNCNN
ACTGAGGGTNNNNNNNNCATCTGCTNNNNNNNNCTNCGCNGGCGTNNCNNNNNNGCNGTATNNNGTNN
NNCANNNNNNNNNNNNNNNNTNAAACNANNNNNCTNNNG
>HaNhi332
GNNNNNNNTAGCCCNNNNNAGTCAGACANNNCNNNNNNNNNNNNNTNNNNNNNNNNNNNNNNNNNNNN
NNNNNNNNNNNNNNNNNNNNNNNNNNNNNNNNNNNNNNNNNNNNNNNNNNNNNNNNNNNNNNNNNNNN
NNNNNNNNNNNNNNNNNNNNNNNNNNNNNNNNNNNNNNNNNNNNNNNNNNNNNNNNNNNNNNNNNNNN
NNNNNNNNNNNNNNNNNNNNNNNNNNNNNNNNNNNNNNNNNNNNNNNNNNNNNNNNNNNNNNNNNNNN
NNNNNNNNNNNNNNNNNNNNNNNNNNNNNNNNNNNNNNNNNNNNNNNNTCTNNNAAANNNTNNN
NNNNNNNNNNNNNNNNNNNNNNNNNCNCNTTGNNNGTAANCNANNNGNACATGCAGTCCCCTCNNNNN
NNNGGCAGAGNNNNNNNCCNNNNNNNCCGGCCCCNNCCNNNNNNNNNNNNNNNGNNNNNNNANNNNNN
NNNNNNTGGTACNNNNCGNNNNNGGTTGTNNAAGAGGTCCACAACGNGNTCNNGNCACNNNNGNNNAN
NNNCNNNNAACNNNNNNNNNNNANNTNNNTANNAGGNCNNNNCCNTCNNGNNNNNNATTGCNATGACC
CAANTCNCGGGGGNNGTNCCCGGCGNGGCGACCTTGANNANGTCNGGGGNCAAGTATGGCNNNCNCCC
CGGGAAGGANNCNGCNGTANGTGCNNNNNANNNNNTCNTGNGANGTCGCCGNNATTNNCNTGGTCNNN
CNAGAATGNCCNNACCANNCGNANNNTGGTNATGNNAGTNNNNNNNNNNNNNNNNNNNNNNNNNNNNN
NNNNNNNNNNNNNNNNNNNNNNNNNNNNNNNNNNNNNGNNNNNNCNANNNAAGGNNNTANNNCTNNTN
NNNNNNNNGGNCNNNACNNNNCNNNNNNNACNCNNNCNCNNNNNNANNNGGTTTNNNNNCNNTNNTNT
CCAGNANGCNGNGCNNNCNTTCANGNGAACNNNGNNACGCGNNAATCGTANNATCTACCTGCCGCNNG
NNNNNNTNCNGNNNNNNCGGANNNTNNNNCGTANNNNNNNCNTGCGTNNTNTNNNNNCNAGNCNNGTT
CGNNNANANANTAGNANGTNTNNNNNNNNNTGTNNCGTTCCGCNTGANNCNNCANNNNGNNGNNNTTN
NCANCGGCNNTANCTGCNGANNTAAGNCATANNNTNNNTCNNTANCTNNNNNGGNGGGNNACTGTAAA
NGGNNAATTNGNNNNTNANTNCNNNCATNTNNCTNNGNNTNCTNNNTNCCTGNNNNCNNAATTGGNNN
CANTGNNTGNCNATCTCTNGNTTNGNNNNNNTNNNNNTNNCANNNNGGNNNGTNNNANNNCNATNTAA
NNTANANNNNCNNNNNNANNNCACNNNNNNNNNAGNNNCNANNNGNANANNNGTAGNNNGNTNAGTCT
CCGANNTNNNNNNNATAAGCGNNANACNNNNNNNNNNNNNANGANNNCGNNTTTGNNNTNNNNNNNNA
GNNAGACNNTNGTGGNTNNNNNTCAANNNNTTNGTAGTGGTATAATNGNNNNNNNTTTCNCCAGNNTN
NNNGNNNNNNNCNATANNANNNNNNNGNGANNNNNNNNNNANNNNCTCNNNAGGNANCANCTACTNNA
GCANNGGNGCGNAGNNNGAGANNNNGACNNNCCTTGGGTGGCAANNACNGNAANAAGTNNNGTTNNNN
CTNNNAGTNNACACNNNNNCANNGNNNCNNNNNNANCNGANNGNNNNNNNNNNNNNNNNNNNNNNNNN
NNNNNNNNNNNNNNNNNNNNNNNNNNNNNNNNNANGGNNNGTNTNTGACNCANGNNTTTTTANNNGNN

NNNNNNNGNNNNANCCCCGGTCGNNNNCNNATTGAANNCCTGNNGCATNNNNNTNCNAGAATGANNNC
NNNNNGNNTNGNCTNNNNNNTCANGNAATCANNNAGNNNNNNNNNTCNNNGNNNNNNNNCNNNNNCNN
ACTGAGGGNNNNNNNNNCANCTNCTNNNNNNNNNTNCGCNGGNGTNNCNNCNNNGCNGTATNNNGTNA
NNCANNNNNNNNNNNNNNNNNTNAAACAANNNNNNTNNNN
>HaNhi333
GTGNNNNNTAGCTCNNNNNAGTTAGACANNNCNNNNNNNNNNNNTNNNNNNNNNNNNNNNNNNNNNNN
NNNNNNNNNNNNNNNNNNNNNNNNNNNNNNNNNNNNNNNNNNNNNNNNNNNNNNNNNNNNNNNNNNNN
NNNNNNNNNNNNNNNNNNNNNNNNNNNNNNNNNNNNNNNNNNNNNNNNNNNNNNNNNNNNNNNNNNNN
NNNNNNNNNNNNNNNNNNNNNNNNNNNNNNNNNNNNNNNNNNNNNNNNNNNNNNNNNNNNNNNNNNNN
NNNNNNNNNNNNNNNNNNNNNNNNNNNNNNNNNNNNNNNNNNNNNNNNNNNNCNTCTNNNAAANNNTNNN
NNNNNNNNNNNNNNNNNNNNNNNNNCNCNNTGNNNGTAANCNANNNGNACATGCAGTCCCCTCNNNNN
NNNGGCAGAGNNNNNNCCCNNNNNNNCCGGCCCCNNCCNNNNNNNNNNNNNNNGNNNNNNNGNNNNNN
NNNNNNTGGTACNNNNCGNNNNNGGTTGTTTAAGAGGTCCACCACGNGNTCNNGNNACNNNNGNNNAN
NNNCNNNNAACNNNNNNNNNNNNANATNNNCANNAGGNCNNNNCCNTCNNGNNNNTNANNGCNATGACC
CANNTCNCGGGGANAGTNACCGGCGCGGCGACCTTGANNANGGTCGGGGNCAAGTATGTCNNNCNCCC
CGGGAAGGANNCNGCNGTANGTGCNNNNNANNNNNTCNTGNGANGTCGCCGNNATTCNCGTGGTCNNN
CNNGAATGNNCNNACCANNCGNANNNTGGTNATGNNAGTNNNNNNNNNNNNNNNNNNNNNNNNNNNNN
NNNNNNNNNNNNNNNNNNNNNNNNNNNNNNNNNNNNNNNNGNNNNNNCNANNNAAGGNNNTANNNTTNNTN
NTNNNNNNGGNCNNNACNNNNCNNNNNNNACNCNNNCNCNNNNNNNNNNGNTTTNNNNNCNNTANTNT
CCAGNACGCNGNGCNNNCNTTCANGNGAACNNNGNNACGCGNNAATCGTANNATCTACCTGCCGCNNG
NNNNNNTACNGNNNNNNCGGANNNTNNNNNGTANNNNNNNCANGCGTNNTNTNNTTNCNAGNCNNGTT
CGNNNANANANTAGNANGTNTNNNNNNNNNNTGTNNCGTTCCGCNTGANNCNNCNNNNNGNNGNNCTTN
NCANCGGCNNTANCTNCNGANNTAAGNCATANNNTNNNTCNNTANCGNNNNNGGNGGGNNACTGTAAA
NGGNNAATTNGNNGNTNANTNCNNNCACNTNNCTNNGNNNNCTNNNCNCCTGNNTNCNNAATTGGNNN
CANTGNNTGNCNATCTCTGGNTTNGNNNNNNTNNNNNTNNTANNNNGGNNNGTNANANNNCNATNTAA
NNTANANNNNCNNNNNNANNNCACNNNNNNNNNAGNNNCNANNNTTANGNNNGTAGNNNANTNAGTCT
CCGANNTNNNNNNNATAAGCGNNANACNNNNNNNNNNNNNANGANNNCGNNTTGGNNNTNNNNNNCNA
ANNAGACNNCNGTGGNTNNNNNTCAANNNNTTNGTAGTGGTATAACNANNNNNNNTTTCNCCAGNNTN
NNNGNNNNNNNCNATANNANNNNNNNGNGANNNNNNNNNNANNNNCCCNNNAGGNANCANCTACTNNA
GCANTGGNGCGNAGNNNGAGANNNNGACNNNCCTTGGGTGGCAANNACNGNAATAAGTNNNGTTNNNC
CCNNNAGTNNACACNNNNNCANNGNNNCNNNNNNANCNGANNTNNNNNNNNNNNNNNNNNNNNNNNNN
NNNNNNNNNNNNNNNNNNNNNNNNNNNNNNNGNNNANGGNNNGTNTNTGACNCNNNNNTTTTTANNNGNN
NNNNNNNGNNNNANCCCCGGTCGNNNNCNNATTGAANNCCTGNNGCATNNNNNTNCNAGAATGANNNC
NNNNNGNNTNGNCTNNCNNNTCAAGNAGTCANNNAGNNNNNNNNNTCNNNGNNNNNNNNCNNTCNCNN
ACTGAGGGTNNNNNNNNCANCTGCTNNNNNNNNNTNCGCNGNNNNNNTNNNNNNGCNGTATNGNGTNN
NNCANNNNNNNNNNNNNNNNNTNAAACNANNNNNCTNNNN
>HaNhi334
GTGNNNNNTAGCCCNNNNNAGTCAGACANNNCNNNNNNNNNNNNTNNNNNNNNNNNNNNNNNNNNNNN
NNNNNNNNNNNNNNNNNNNNNNNNNNNNNNNTNNNNNNNNNNNNNNNNNNNNNNNNNNNNNNNNNNNN
NNNNNNNNNNNNNNNNNNNNNNNNNNNNNNNNNNNNNNNNNNNNNNNNNNNNNNNNNNNNNNNNNNNN
NNNNNNNNNNNNNNNNNNNNNNNNNNNNNNNNNNNNNNNNNNNNNNNNNNNNNNNNNNNNNNNNNNNN
NNNNNNNNNNNNNNNNNNNNNNNNNNNNNNNNNNNNNNNNNNNNNNNNNNNNNNTCTNNNAAANNNTNNN
NNNNNNNNNNNNNNNNNNNNNNNNNCNCNTTGNNNGTAANCNANNNGNACATGCAGTCCCCTCNNNNN
NNNGGCAGAGNNNNNNNCCNNNNNCNCCGGCCCCNNCCNNNNNNNNNNNCNNNNNNNNNNNANNNNNN
NNNNNNTGGTACNNNNCGNNNNNGGTTGTNTAAGAGGTCCACAACGNGGTCNNNNCACNNNNGNNNAN
NNNCNNNNAACNNNNNNNNNNNNANATNNNTANNAGGNCNNNNCCNTCNNGNNNNTNANNGCNATGACC
CANNNCNCGGGGGCNGTNCCCGGCGCGGCGACCTTGANNANNTCCGGGGNCAAGTATGGCGNNCNCCC
CGGGAAGGANNCNGCNGTANGTGCNNNNNANNNNNTCNTGNGANGTNGCCGNNATTNNCNTGGTCNNN
CNAGAATGNCCNNACCANNCGNANNNTGGTNATGNNAGTNNNNNNNNNNNNNNNNNNNNNNNNNNNNN
NNNNNNNNNNNNNNNNNNNNNNNNNNNNNNNNNNGNNNNGNNNNNNCNANNNAAGGNNNTANNNCTNNTN
NTNNNNNNGGACNNNACNNNNCNNNNNNNACNCNNNCNCNNNNNNANNNGGTTNNNNNNCNNTANTNT
CCAGNACGCNGNGCNNNCATTCANGNGAACNNNGNNACGCGNNAATCGTANNATCTACCTGCCGCNNG
NNNNNNTNCNGNNNNNNCGGANNNTNNNNCGTANNNNNNNCATGCGTNNTNTNNTCNCNAGNCNNGTT
CGNNNANANANTAGNANGTNTNNNNNNNNNNTGTNNCGTTCCGCNTGANNCNNCANNNNGNNGNNNTTN

NCANCGGCNNTANCTGCNGANNTAAGNCATANNNTNNNTCNNTANCTNNNNNGGNGGGNNACTGTAAA
NGGNNAATTNGNNNNTNANTNCNNNCATNTNNCNNNGNNNNCTNNNTNCCTGNNTNCNNAATTGGNNC
CANTGNNTGNCNATCTCTGGNTTNGNNNNNNTNNNNNTNNCANNNNGGNNNNNNANANNNCNATNTAA
NNTANANNNNCNNNNNNANNNCACNNNNNNNNNAGNNNCNANNNGNANANNNGTAGNNNGNTAAGTCT
CCGANNTNNNNNNNATAAGCGNNANACNNNNNNNNNNNNNANGANNNCGNNTTTGNNNTNNNNNNCNA
GNNAGACNNTNGTGGNTNNNNNTCAANNNNTTNGTAGTGGTATAATNGNNNNNNNTTTCNCCAGNNTN
NNNGNNNNNNNCNATANNANNNNNNNGNGANNNNNNNNNNANNNNCTCNNNAGGGANCANCTACTTNA
GCANTGGNGCGNAGNNNGAGANNNNGACNNNCCTTGGGTGGCAANNACNGCAATAAGTNNNGTTNNNC
CTNNNAGTNNACACNNNNNCANNGNNNCNNNNNNANCNGANNGNNNNNNNNNNNNNNNNNNNNNNNNN
NNNNNNNNNNNNNNNNNNNNNNNNNNNNNNNNNNANGGNNNGTNTNTGACNCATGTGTTTTANNNGNN
NNNNNACGNNNNANCCCCGGTCGNNNNCNNATTGAANNCCTGNNGCATNNNNNTNCNAGAATGANNNC
NNNNNGNNTNGNCTNNCNNNTNAAGNAATCANNNAGGTANNNNGNTCNNNNNNNNNNNNCNNTCCCNN
ACTGAGGGTNNNNNNNNCATCTGCTNNNNNNNNNTNCGCNGGNGTNNCNNNNNNGCNGTATNGNGTNN
NNCANNNNNNNNNNNNNNNNCNANACAANNNNNNTNNNN
>HaNhi335
GTGNNNNNTAGCTCNNNNNAGTTAGACANNNCNNNNNNNNNNNNNTNNNNNNNNNNNNNNNNNNNNNN
NNNNNNNNCNNNNNNNNNNNNNNNNNNNNNNTNNNNNNNNNNNNNNNNNNNNNNNNNNNNNNNNNNNN
NNNNNNNNNNNNNNNNNNNNNNNNNNNNNNNNNNNNNNNNNNNNNNNNNNNNNNNNNNNNNNNNNNNN
NNNNNNNNNNNNNNNNNNNNNNNNNNNNNNNNNNNNNNNNNNNNNNNNNNNNNNNNNNNNNNNNNNNN
NNNNNNNNNNNNNNNNNNNNNNNNNNNNNNNNNNNNNNNNNNNNNNNNNNNNTCTNNNAAANNNTNNN
NNNNNNNNNNNNNNNNNNNNNNNNNCNCNTTNNNNGTAANCNANNNGNACATGCAGTCCCCTCNNNNN
NNNNNCAGAGNNNNNNNCCNNNNNNNCCGNCCCCNNCCNNNNNNNNNNNNNNNGNNNNNNNGNNNNNN
NNNNNNTGGTACNNNNCGNNNNNGGTTGTTTAAGAGGTCCACCACGNGGTCNGNNNACNNNNGNNNAN
NNNCNNNNAACNNNNNNNNNNNANNTNNNCANNAGGNCNNNNCCNTCNNGNNNNNNATNGCNATGANN
CAANTNNCGGGGACNGTNACCGGCGCGGCGACCTTGANNACGGTCGGGGNCAAGTATGTCGNNCNCCC
CGGGAAGGANNCNGCNGTANGTGCNNNNNANNNNNTCNTGNGANGTCGCCGNNATTNNCNTGGTCNNN
CNAGAATGNCCNNACCANNCGNANNNTGGTNATGNNAGTNNNNNNNNNNNNNNNNNNNNNNNNNNNNN
NNNNNNNNNNNNNNNNNNNNNNNNNNNNNNNNGNNNNGNNNNNNCNANNNAAGGNNNTANNNTTNNTN
NNNNNNNNGGACNNNACNNNNCNNNNNNNACNCNNNCNCNNNNNNNNNNGNTTTTNNNNCNNTANTNT
CCAGNACGCNGNGCNNNCATTCANGNGAACNNNGNNACGCGNNAATCGTANNATCTACCTGCCGCNNG
NNNNNNTACTGNNNNNNCGGANNNTNNCTCGTANNNNNNNCATGCGTNNTNTNNTTNCNAGNCNNGTT
CGNNNANANANTAGNANGTNTNNNGNNNNNTGTNNCGTTCCGCNTGANNCNNCNNNNNGNNGNNCTTN
NCANCGGCNNTANCTGCNGANNTAAGNCATANNNTNNNTCNNTANCGNNNNNGGNGGGNNACTGTAAA
NGGNNAATTNGNNNNTNATTNCNNNCACNTNNCNNNGNNTNCTNNNCNCCTGNNTNCNNAATTGGNNN
CANTGNNTGNCNATCTCTGGNTTNGNNNNNNTNNNNNTNNTANNNNGGNNNGTNNNANNNCNATNTAA
NNTANANNNNCNNNNNNANNNCACNANNNNNGNAGNNNNNANNNTNANGNNNGTAGNNNAATNAGTCN
CCGANNTNNNNNNNATAAGCGNNANACNNNNNNNNNNNNNANGANNNCGNNTTGGNNNTNNNNNNCNA
ANNAGACNNCNGTGGNTNNNNNTCAANNNNTTCGTAGTGGTATAACNANNNNNNNTTTCNCCAGNNTN
NNNGNNNNNNNCNATANNANNNNNNNGNGANNNNNNNNNNANNNNCCCNNNAGGNANCANCTACTTNA
NCANTGGNGCGNAGNNNGAGANNNNGACNNNCCTTGGGTGGCAANNACNGCAATAAGTNNNGTTNANN
CCNNNAGTNNACACNNNNNCANNGNNNCNNNNNNANCNGANNTNNNNNNNNNNNNNNNNNNNNNNNNN
NNNNNNNNNNNNNNNNNNNNNNNNNNNNNNNNNNANGGNNNGTNTNTGNCNCANGTNTTTTANNNGNN
NNNNNACGNNNNANCCCCGGTCGNNNNCNNATTGAANNCCTGNNGCATNNNNNTNCNAGAATGANNNC
NNNNNGNNTNGNCTNNCNNNTNAAGNAGTCAANNAGNNNNNNNNNTCNNNNNNNNNNNNCNNTCNCNN
ACTGAGGGTNNNNNNNNCATCTGCTNNNNNNNNCTNCGCNGNNNNNNTNNNNNAGCNGTATNNNGTNN
NNCANNNNNNNNNNNNNNNNTNAAACNANNNNNNTNNNN
>HaNhi336
GTGNNNNNTANCCCNNGNNAGTTNGACANNNCNNNNNNNNNNNNNTNNNNNNNNNNNNNNNNNNNNNN
NNNNNNNNNNNNNNNNNNNNNNNNNNNNNNNTNNNNNNNNNNNNNNNNNNNNNNNNNNNNNNNNNNNN
NNNNNNNNNNNNNNNNNNNNNNNNNNNNNNNNNNNNNNNNNNNNNNNNNNNNNNNNNNNNNNNNNNNN
NNNNNNNNNNNNNNNNNNNNNNNNNNNNNNNNNNNNNNNNNNNNNNNNNNNNNNNNNNNNNNNNNNNN
NNNNNNNNNNNNNNNNNNNNNNNNNNNNNNNNNNNNNNNNNNNNNNNNNNNNNCTNNNAAANNNTNNN
NNNNNNNNNNNNNNNNNNNNNNNNNCNCNNTNNNNGTAANCNANNNGNACATGCAGTCCCCTCNNNNN
NNNNNCAGAGNNNNNNNCCNNNNNNNCCGNCCCCNNCCNNNNNNNNNNNNNNNNNNNNNNNANNNNNN

NNNNNNTGGTACNNNNCGNNNNNGGTTGTNTNAGAGGTCCACCACGNGNTCNGNNNACNNNNGNNNAN
NNNCNNNNAACNNNNNNNNNNNNANATNNNTANNAGGNNNNNNCCNTNNNGNNNNNNANNGCNATGANN
CANNNNNCNGNGGNNGTNCCCGGNGNGGCGACCTTGANNANGTCNGGNNNCAAGTATGGCNNNCNCCC
CGNGAAGGANNCNGCNGTANGTGCNNNNNANNNNNTCNTGNGANGTCGCCGNNATTCNNNTGGTNNNN
NNNGAATGNNCNNACCANNCGNANNNNGGTNATGTNAGTNNNNNNNNNNNNNNNNNNNNNNNNNNNNNNN
NNNNNNNNNNNNNNNNNNNNNNNNNNNNNNNNNNGNNCNGNNNNNNCNANNNANGGNNNTANNNCTNNTN
NNNNNNNNGGACNNNNCNNNNNNNNNNNNNACNCNNNCNNNNNNNNNANNNGGTTNNNNNNCNNTANTNT
CCAGNACGCNGNGCNNNCATTCANGNGAACNNNGNNACGCGNNANTCGTNNNATCTACCTGCCGCNNG
NNNNNNTNCNGNNNNNNCGGANNNTNNNNCGTANNNNNNNNNNNACGTNNTNTNNTNNCNAGNCNNGTT
CGNNNANANANTAGNANGTNTNNNNNNNNNNTGTNNCGTTCCGCNTGANNCNNNNNNNNGNNGNNNTTN
NCANCGGCNNTANCTGCNGANNTAAGNCATANNNTNNNTCNNTANCTNNNNNGGNGGGNNACTGTAAA
NGNNNAATTNGNNNNTNANTNCCNNCACNNNNCNNNGNNNNCTNNNTNCCTGNNTNCNNAATTGGNNN
CANTGNNTGNCNATCTCTNGNTTNGNNNNNNTNNNNNTNNCANNNNGGNNNNNNNNANNNCNATNTAA
NNTANANNNNCNNNNNNANNNCACNNNNNNNNNAGNNNCNNNNNTNANANNNGTAGNNNANTNAGTCT
CCGANNTNNNNNNNATAAGCGNNANACNNNNNNNNNNNNNANGANNNCGNNTTGGNNNTNNNNNNNNA
GNNAGACNNTNGTGGNTNNNNNTCAANNNNTTNGTAGTGGTATAATNGNNNNNNNTTTCNCCAGNNNN
NNNGNNNNNNNCNATANNANNNNNNNGNGANNNNNNNNNNANNNNCTCNNNAGGGANCANCTACTNNA
NCANNGGNNCGNAGNNNGAGANNNNGACNNNCCTTGGGTGGNANNNACNGNAATAAGTNNGTTNANN
CTNNNAGTNNACACNNNNNCANNGNNNCNNNNNNANCNGANNGNNNNNNNNNNNNNNNNNNNNNNNNNNN
NNNNNNNNNNNNNNNNNNNNNNNNNNNNNNNNNNNANGGNNNGTNTNTGNCNCNNNNNTTTNTANNNGNN
NNNNNNNGNNNNANCCCNGGTCGNNTNCNNATTGAANNCCTGNNGCATNNNNNNNCNANAATGANNNC
NNNNNGNNTNGNCTNNNNNNTNANGNAGTCANNNAGGNNNNNNNNTCNNNGNNNNNNNNCNNTNNCNN
ACTGAGGGTNNNNNNNNCATCNGCTNNNNNNNNNNTNCGCNGGNNNNNCNNCNNAGCNGTATNNNGNNN
NNCANNNNNNNNNNNNNNNNNTNAAACAANNNNNCTNNNN
>HaNhi337
GTGNNNNNNANCCCNNNNNAGTCNNACANNNCNNNNNNNNNNNNTNNNNNNNNNNNNNNNNNNNNNNN
NNNNNNNNNNNNNNNNNNNNNNNNNNNNNNNNNNNNNNNNNNNNNNNNNNNNNNNNNNNNNNNNNNNNNN
NNNNNNNNNNNNNNNNNNNNNNNNNNNNNNNNNNNNNNNNNNNNNNNNNNNNNNNNNNNNNNNNNNNNNN
NNNNNNNNNNNNNNNNNNNNNNNNNNNNNNNNNNNNNNNNNNNNNNNNNNNNNNNNNNNNNNNNNNNNNN
NNNNNNNNNNNNNNNNNNNNNNNNNNNNNNNNNNNNNNNNNNNNNNNNNNNNNCTNNNAAANNNTTNN
NNNNNNNNNNNNNNNNNNNNNNNNNNCNCNNTNNNNGTAANCNANNNGNACATGCAGTCCCCTCNNNNN
NNNNGCAGAGNNNNNNNNCNNNNNNNCCGNCNCCNNCCNNNNNNNNNNNNNNNNNNNNNNNNNANNNNNN
NNNNNNTGGTACNNNNCGNNNNNGGTTGNNTNAGNGGTCCACAACGNGGTCNGNNNACNNNNGNNNAN
NNNCNNNNNNCNNNNNNNNNNNNNANATNNNTANNAGGNCNNNNCCNNNNNNNNNNCNNANTGCNATGANN
CNNNNNNCNGNGGNNGTNCCCGGNGNGGNGACCTTGNNNANNTCCGGGNNCAAGTATGGCNNNCNCCC
CGGGAAGGANNCNGCNGTANGTGCNNNNNANNNNNTCNTGNGANNTCGCCGNNATTNNCNTGGTCNNN
NNAGANTGNCCNNACCANNCGNANNNTGGTNATGNNAGTNNNNNNNNNNNNNNNNNNNNNNNNNNNNNNN
NNNNNNNNNNNNNNNNNNNNNNNNNNNNNNNNNNGNNCNGNNNNNNCNANNNAAGGNNNTANNNCTNNTN
NNNNNNNNGGNCNNNACNNNNCNNNNNNNACNCNNNCNCNNNNNNNNNNNGNTTNNNNNNCNNTANNNN
CCAGNANGCNGNGCNNNCNTTCANNNGAACNNNGNNANGCGNNAATCGTANNNTCTACCTGCCGCNNG
NNNNNNTNCNGNNNNNNCGGANNNTNNNNCGTANNNNNNNNCNNGCGTNNTNTNNTNNCNAGNCNNGTT
CGNNNANANANTAGNANGTNTNNNNNNNNNNTGTNNCGTTCCGCNTGANNCNNCANNNNGNNGNNNTTN
NCANCGGCNNNANNTNCNGANNTAAGNCATANNNTNNNTCNNTANCTNNNNNNGNGGGNNACTGNAAA
NGNNNAATTNGNNGNTNATTNCNNNCATNTNNCNNNGNNNNCTNNNTNCCTNNNNNCNNAATTGGNNC
CANTGNNTGNCNATCTCTNGNTTNGNNNNNNTNNNNNTNNCANNNNGGNNNNNNNNANNNCNATNTAA
NNTANANNNNCNNNNNNANNNCACNNNNNNNNNAGNNNCNNNNNGNANANNNGTAGNNNGNTNAGTCT
CCGANNTNNNNNNNATAAGCGNNANACNNNNNNNNNNNNNANGANNNCGNNTTTGNNNTNNNNNNNNA
GNNAGACNNTNGTGGNTNNNNNTCAANNNNTTNGTAGTGGTANAATNGNNNNNNNTTTCNCCAGNNTN
NNNGNNNNNNNCNATANNANNNNNNNGNGANNNNNNNNNNANNNNCTCNNNAGGNANCANCTACTNNA
GCANNGGNGCGNAGNNNGAGANNNNGACNNNCCTTGGGTGGNAANNACNGNAATAAGTNNGTTNNNN
NTNNNAGTNNACACNNNNNCANNGNNNCNNNNNNANCNGANNGNNNNNNNNNNNTNNNNNNNNNNNNN
NNNNNNNNNNNNNNNNNNNNNNNNNNNNNNNNNNNANNGNNNGTNTNNGNCNNNNGNNTTNNTANNNGNN
NNNNNNNGNNNNANCCCCGGTCGNNNNCNNATTGAANNCCTGNNNCNTNNNNNTNCNANAATGANNNC
NNNNNGNNTNGNCTNNCNNNTNANGNAATCNNNNAGGNNNNNNNNTCNNNGNNNNNNNNCGNTCCCNN

ACTGAGGGNNNNNNNNNCATCTGCTNNNNNNNNNTNCGCNGGNNNNNCNNNNNNGCNGTATNNNGNNN
NNCANNNNNNNNNNNNNNNNNNTNAAACAANNNNNCTNNNN
>HaNhi338
GTNNNNNNTAGCTCNNNNNAGTTANACANNNCNNNNNNNNNNNNNTNNNNNNNNNNNNNNNNNNNNNNN
NNNNNNNNNNNNNNNNNNNNNNNNNNNNNNNNNNNNNNNNNNNNNNNNNNNNNNNNNNNN
NNNNNNNNNNNNNNNNNNNNNNNNNNNNNNNNNNNNNNNNNNNNNNNNNNNNNNNNNNNN
NNNNNNNNNNNNNNNNNNNNNNNNNNNNNNNNNNNNNNNNNNNNNNNNNNNNNNNNNNNN
NNNNNNNNNNNNNNNNNNNNNNNNNNNNNNNNNNNNNNNNNNNNNNNNNNTCTNNNAAANNNTNNN
NNNNNNNNNNNNNNNNNNNNNNNNNNCNCNTTNNNNGTAANCNANNNGNACATGCAGTCCCCTCNNNNN
NNNGGCAGAGNNNNNNNCCNNNNNNNCCGNCCCCNNCCNNNNNNNNNNNNNNNNGNNNNNNNGNNNNNN
NNNNNNTGGTACNNNNCGNNNNNGGTTGTNTAAGAGGTCCACNACGNGGTCNNNNCACNNNNGNNNAN
NNNCNNNNAANNNNNNNNNNNNNANATNNNCANNAGGNNNNNNCCNTNNNNNNNNNNNANTGCNATGANN
CANNTNNCNGGGANNGTNACCGGNGNGGNGACCTTGANNANGGTCGGGGNCAAGTATGTCGNNCNCCC
CGNGAAGGANNCNGCNGTANGTGCNNNNNANNNNNTCNTGNGANNTCGCCGNNATTNNCNTGGTCNNN
NNNGAATGNCCNNACCANNCGNANNNTGGTNATGNNAGTNNNNNNNNNNNNNNNNNNNNNNNNNNNN
NNNNNNNNNNNNNNNNNNNNNNNNNNNNNNNNNGNNNNGNNNNNNCNANNNAAGGNNNTANNNTTNNTN
NNNNNNNNNNNCNNNACNNNNCNNNNNNNACNCNNNCNCNNNNNNNNNNNGNTTNNNNNNCNNTNNTNT
CCAGNANNCNGNGCNNNCNTNCANNNGAACNNNGNNACGCGNNAATCGTANNATCTACCTGCCGCNNG
NNNNNNTNCNGNNNNNNCGGANNNTNNCTCGTANNNNNNNCANGCGTNNTNTNNTNNCNAGNNNNGTT
CGNNNANANANTAGNANGTNTNNNNNNNNNNTGTNNCGNNCCGCNTGANNCNNCNNNNNGNNGNNNTTN
NCANCGGCNNTANCTNCNGANNTAAGNCATANNNTNNNTCNNTANCGNNNNNGGNGGGNNACTGTAAA
NGGNNAATTNGNNNNTNANTNCNNNCACNTNNCNNNGNNNNCTNNNCNCCTGNNTNCNNAATTGGNNC
CANTGNNTGNCNATCTCTNGNTTNGNNNNNNTNNNNNTNNTANNNNGGNNNNNNNNNANNNNNATNTAA
NNTANANNNNCNNNNNNANNNCACNNNNNNNNNAGNNNCNANNNTNANGNNNGTAGNNNANTNAGTCN
CCNANNTNNNNNNNATAAGCGNNANACNNNNNNNNNNNNNNANGANNACGNNTTGGNNNTNNNNNNNNA
ANNNGACNNCNGTGGNTNNNNNTCAANNNNTTNGTAGTGGTATAACNANNNNNNNTTTCNCCAGNNTN
NNNGNNNNNNNCNATANNANNNNNNNGNGANNNNNNNNNNANNNNCCCNNNAGGNANCANCTACTTNA
GCANNGGNGCGNAGNNNGAGANNNNGACNNNCCTTGGGTGGCANNNACNGNAATAAGTNNGTTNNNN
NCNNNAGTNNACACNNNNNCANNGNNNCNNNNNNANCNGANNTNNNNNNNNNNNNNNNNNNNNNNNNN
NNNNNNNNNNNNNNNNNNNNNNNNNNNNNNNANNNNANGGNNNGTNTNTGNCNNNNNNNTTTNTANNNGNN
NNNNNNNGNNNNANCCCCGGTCGTNNNCNNATTGAANNCCTGNNGCATNNNNNTNCNAGAATGANNNC
NNNNNGNNTNNNCTNNNNNNTNAAGNAGTNNNNNAGNNNNNNNNNNCNNNNNNNNNNNNCNNNCNCNN
ACTGAGGGTNNNNNNNNCANNNGCTNNNNNNNNCTNCGCNGNNNNNNTNNNNNNGCNGTATNNNGTNN
NNCANNNNNNNNNNNNNNNNNNTNAANCAANNNNNCTNNNN
>HaNhi339
GTGNNNNNTAGCCCNNNNNAGTTAGACANNNCNNNNNNNNNNNNNTNNNNNNNNNNNNNNNNNNNNNNN
NNNNNNNNNNNNNNNNNNNNNNNNNNNNNNNNNNNNNNNNNNNNNNNNNNNNNNNNNNNN
NNNNNNNNNNNNNNNNNNNNNNNNNNNNNNNNNNNNNNNNNNNNNNNNNNNNNNNNNNNN
NNNNNNNNNNNNNNNNNNNNNNNNNNNNNNNNNNNNNNNNNNNNNNNNNNNNNNNNNNNN
NNNNNNNNNNNNNNNNNNNNNNNNNNNNNNNNNNNNNNNNNNNNNNNNNNCNTCTNNNAAANNNTNNN
NNNNNNNNNNNNNNNNNNNNNNNNNNCNCNTTGNNNGTAANCNANNNGNACATGCAGTCCCCTCNNNNN
NNNNGCAGAGNNNNNNCCNNNNNNNNCCGGCCCCNNCCNNNNNNNNNNNNNNNNGNNNNNNNANNNNNN
NNNNNNTGGTACNNNNCGNNNNNGGTTGTTTAAGAGGTCCACCACGNGGTCNNGNCACNNNNGNNTAN
NNNCNNNNANCNNNNNNNNNNNNNNATNNNTANNAGGNCNNNNCCNTCNNGNNNNNAATTGCNATGACC
CANNNCACGGNGGNNGTNCCCGGCGCGGCGACCTTGANNACGTCCGGGGNCAAGTATGGCNNNCNCCC
CGGGAAGGANNCNGCNGTANGTGCNNNNNANNNNNTCTTGNGANGTCGCCGNNATTCNCGTGGTCNNN
CNNGANTGNCCNNACCANNCGNANNNTGGTNATGNNAGTNNNNNNNNNNNNNNNNNNNNNNNNNNNN
NNNNNNNNNNNNNNNNNNNNNNNNNNNNNNNNNGNNNNGNNNNNNCNANNNAAGGANNTANNNCTNNTN
NTNNNNNNGGACNNNACNNNNCNNNNNNNACNCNNNCNCNNNNNNANNNGNTTTTTNNNCNNTANTNT
CCAGNACGCNGNGCNNNCNTTCANGNGAACNNNGNNACGCGNNAATCGTANNATCTACCTGCCGCNNG
NNNNNNTACNGNNNNNNCGGANNNTNNNNCGTANNNNNNNTANACGTNNTNTNNTNNCNAGNCNNGTT
CGNNNANANANTAGNANGTNTNNNNNNNNNNTGTNNCGTTCCGCNTGANNCNNCANNNNGNNGNNCTTN
NCANCGGCNNTANCTGCNGANNTAAGNCATANNNTNNNTCNNTANCTNNNNNGGNGGGNNACTGTAAA
NGGNNAATTNGNNNNTNANTNCNNNCACNTNNCTNNGNNTNCTNNNTNCCTGNNTNCNNAATTGGNNC

CANTGNNTGNCNATCTCTNGNTTNGNNNNNNTNNNNNTNNCANNNNGGNNNGTNANANNNCNATNTAA
NNTANANNNNCNNNNNNANNNCACNNNNNNNGNAGNNNCNANNNTTANANNNGTAGNNNANTAAGTCT
CCGANNTNNNNNNNATAAGCGNNANACNNNNNNNNNNNNNANGANNNCGNNTTGGNNNTNNNNNNCNA
GNNAGACNNTNGTGGNTNNNNNTCAANNNNTTNGTAGTGGTATAATNGNNNNNNNTTTCNCCAGNNTN
NNNGNNNNNNNCNATANNANNNNNNNGNGANNNNNNNNNNNANNNNCTCNNNAGGNANCANCTACTNNA
GCANNGGNGCGNAGNNNGAGANNNNGACNNNCCTTGGGTGGCAANNACNGCAATAAGTNNNGTTNANC
CTNNNAGTNNACACNNNNNCANNGNNNCNNNNNNANCNGANNGNNNNNNNNNNNNNNNNNNNNNNNNNN
NNNNNNNNNNNNNNNNNNNNNNNNNNNNNNNANNNNANNGNNNGTNTNTGNCNCATGTGTTTTTANNNGNN
NNNNNANGNNNNANCCCCGGTCGNNNNCNNATTGAANNCCTGNNGCATNNNNNTNCNAGAATGANNNC
NNNNNGNNTNGNCTNNCNNNTCAAGNAGTCANNNAGNNNNNNNNNTCNNNGNNNNNNNNCNNTCNCNN
ACTGAGGGNNNNNNNNNCATCNGCTNNNNNNNNNNTNCGCNGGNGTNNCNNNNNNGCNGTATNGNGNNN
NNCANNNNNNNNNNNNNNNNNTNAAACNANNNNNNNTNNNN
>HaNhi340
GTGNNNNNNAGCCCNNNNNAGTTAGACANNNCNNNNNNNNNNNNNTNNNNNNNNNNNNNNNNNNNNNN
NNNNNNNNNNNNNNNNNNNNNNNNNNNNNNNTNNNNNNNNNNNNNNNNNNNNNNNNNNNNNNNNNNNNN
NNNNNNNNNNNNNNNNNNNNNNNNNNNNNNNNNNNNNNNNNNNNNNNNNNNNNNNNNNNNNNNNNNNNNN
NNNNNNNNNNNNNNNNNNNNNNNNNNNNNNNNNNNNNNNNNNNNNNNNNNNNNNNNNNNNNNNNNNNNNN
NNNNNNNNNNNNNNNNNNNNNNNNNNNNNNNNNNNNNNNNNNNNNNNNNNNNNNNTCTNNNAAANNNTNNN
NNNNNNNNNNNNNNNNNNNNNNNNNNNCNCNNTNNNNGTAANCNANNNGNACATGCAGTCCCCTCNNNNN
NNNGGCAGAGNNNNGGCCCNNNNNNNCCGNCCCCNNCCNNNNNNNNNNNNNNNNNGNNNNNNNGNNNNNN
NNNNNNTGGTACNNNNCGNNNNNGGTTGTNTNAGAGGTCCACCACGNGGTCNNGNNACNNNNGNNNAN
NNNCNNNNAACNNNNNNNNNNNNANNTNNNCANNAGGNCNNNNCCNTCNNGNNNNNNANNGCNATGANN
CAANNNNCNGGGGNNGTCCCCGGCGCGGCGACCTTGANNANGNCNGGGNNCAAGTATGGCNNNCNCCC
CGNGAAGGANNCNGCNGTANGTGCNNNNNANNNNNTCNTGNGANGTCGCCGNNATTNNNNTGGTCNNN
NNAGAATGCCCNNACCANNCGNANNNTGGTNATGNNAGTNNNNNNNNNNNNNNNNNNNNNNNNNNNNNN
NNNNNNNNNNNNNNNNNNNNNNNNNNNNNNNNNNNNGNNNNGNNNNNNCNANNNAAGGNNNTANNNCTNNTN
NNNNNNNNGGACNNNACNNNNCNNNNNNNACNCNNNCNCNNNNNNNNNNGNTTTTNNNNCNNTANTNT
CCAGNACGCNGNGCNNNCATTCANGNGAACNNNGNNACGCGNNAATCGTANNATCTACCTGCCGCNNG
NNNNNNTNCNGNNNNNNCGGANNNTNNNNNGTANNNNNNNNANGCGTNNNNTNNTNNCNAGNCNNGTT
CGNNNANANANTAGNANGTNTNNNNNNNNNNTGTNNCGTTCCGCNTGANNCNNCANNNNGNNGNNNTTN
NCANCGGCNNTANNTGCNGANNTAAGNCATANNNTNNNTCNNTANCTNNNNNGGNGGGNNACTGTAAA
NGGNNAATTNGNNNNTNATTNCNNNNACNTNNCNNNGNNTNCTNNNTNCCTGNNTNCNNAATTGGNNN
CANTGNNTGNCNATCTCTNGNTTNGNNNNNNTGNNNNTNNCANNNNGGNNNNNNNNANNNCNATNTAA
NNTANANNNNCNNNNNNANNNCACNNNNNNNGNAGNNNCNANNNTNANANNNGTAGNNNANTAAGTCT
CCGANNTNNNNNNNATAAGCGNNANACNNNNNNNNNNNNNANGANNNCGNNTTGGNNNTNNNNNNCNA
ANNAGACNNTNGTGGNTNNNNNTCAANNNNTTNGTAGTGGTATAACNGNNNNNNNTTTCNCCAGNNTN
NNNGNNNNNNNCNATANNANNNNNNNGNGANNNNNNNNNNNANNNNCTCNNNAGGGANCANCTACTTNA
GCANTGGNNCGNAGNNNGAGANNNNGACNNNCCTTGGGTGGCAANNACNGNAATAAGTNNNGTTNNNN
NTNNNAGTNNACACNNNNNCANNGNNNCNNNNNNANCNGANNTNNNNNNNNNNNNNNNNNNNNNNNNNN
NNNNNNNNNNNNNNNNNNNNNNNNNNNNNNNANNNNANGGNNNGTNTNTGACNCNTGTGTTTTTANNNGNN
NNNNNACGNNNNANCCCCGGTCGNNNNCNNATTGAANNCCTGNNGCATNNNNNTNCNAGAATGANNNC
NNNNNGNNTNGNCTNNCNNNTNANNNAGTCANNNAGGTNNNNNNNTCNNNGNNNNNNNNCNNNCCCNN
ACTGAGGGTNNNNNNNNCANCTGCTNNNNNNNNNNTNCGCNGNNGTNNCNNNNNAGCNGTATNNNGTNN
NNCANNNNNNNNNNNNNNNNNTNAAACGANNNNNNNTNNNN
>HaNhi341
GTGNNNNNTAGCCCNNNNNAGTCNGACANNNCNNNNNNNNNNNNNTNNNNNNNNNNNNNNNNNNNNNN
NNNNNNNNNNNNNNNNNNNNNNNNNNNNNNNNNNNNNNNNNNNNNNNNNNNNNNNNNNNNNNNNNNNNNN
NNNNNNNNNNNNNNNNNNNNNNNNNNNNNNNNNNNNNNNNNNNNNNNNNNNNNNNNNNNNNNNNNNNNNN
NNNNNNNNNNNNNNNNNNNNNNNNNNNNNNNNNNNNNNNNNNNNNNNNNNNNNNNNNNNNNNNNNNNNNN
NNNNNNNNNNNNNNNNNNNNNNNNNNNNNNNNNNNNNNNNNNNNNNNNNNNNNNNCTNNNAAANNNTNNN
NNNNNNNNNNNNNNNNNNNNNNNNNNNCNCNNTGNNNGTAANCNANNNGNACATGCAGTCCCCTCNNNNN
NNNNNCAGAGNNNNNNNCNNNNNNNNNCCGNCCCCNNCCNNNNNNNTNNNCNNNNNNNNNNNNANNNNNN
NNNNNNTGGTACNNNNCGNNNNNGGTTGTNTAAGAGGTCCACAACGNGNTCNGGNNACNNNNGNNNAN
NNNCNNNNAACNNNNNNNNNNNNANNTNNNTANNAGGNCNNNNCCNTCNNGNNNNTNANTGCNATGANN

CANNNNNCNGGGGNNGTNCCCGGCGCGGNGACCTTGANNANGTCNGGGNNCAAGTATGGCNNNCNCCC
CGNGAAGGANNCNGCNGTANGTGCNNNNNANNNNNTCNTGNGANGTCGCCGNNATTCNNNTGGTCNNN
NNAGANTGNNCNNACCANNCGNANNNTGGTNATGNNAGTNNNNNNNNNNNNNNNNNNNNNNNNNNNNN
NNNNNNNNNNNNNNNNNNNNNNNNNNNNNNNNNNNNNNGNNNNGNNNNNNCNANNNAAGGNNNTANNNCTNNTN
NNNNNNNNGGACNNNACNNNNCNNNNNNNACNCNNNCNCNNNNNNANNNGNTTTTNNNNCNNTANTNT
CCAGNANGCNGNGCNNNCATTCANGNGAACNNNGNNACGCGNNAATCGTANNATCTACCTGCCGCNNG
NNNNNNTNCNGNNNNNNCGGANNNTNNNNCGTANNNNNNNCNNGCGTNNTNTNNTNNCNAGNCNNGTT
CGNNNANNNANTAGNANGTNTNNNNNNNNNNTGTNNCGTTCCGCNTGANNCNNCNNNNNGNNGNNNTTN
TCANCGGCNNTANCTNCNGANNTAAGNCATANNNTNNNTCNNTANCTNNNNNGGNGGGNNACTGTAAA
NGNNNAATTNGNNNNTNANTNCNNNNATNTNNCNNNGNNNNCTNNNTNCCTGNNTNCNNAATTGGNNN
CANTGNNTGNCNATCTCTNGNTTNGNNNNNNTGNNNNTNNCANNNNGGNNNNNNNNANNNCNATNTAA
NNTANANNNNCNNNNNNANNNCACNANNNNNNNAGNNNNNANNNGNANANNNGTAGNNNGNTNAGTCT
CCGANNTNNNNNNNATAAGCGNNANACNNNNNNNNNNNNNANGANNNCGNNTTTGNNNTNNNNNNNNA
GNNAGACNNTNGTGGNTNNNNNTCAANNNNTTNGTAGTGGTATAANNGNNNNNNNTTTCNCCAGNNTN
NNNGNNNNNNNCNATANNANNNNNNNGNGANNNNNNNNNNNANNNNCTCNNNAGGNANCANCTACTNNA
NCANNGGNGCGNAGNNNGAGANNNNGACNNNCCTTGGGTGGCANNNACNGNAATAAGTNNNGTTNNNN
CTNNNAGTNNACACNNNNNCANNGNNNCNNNNNNANCNGANNGNNNNNNNNNNNNNNNNNNNNNNNN
NNNNNNNNNNNNNNNNNNNNNNNNNNNNNNNANNNNANGGNNNGTNTNTGNCNCATGNNTTTTTANNNGNN
NNNNNNNGNNNNANCCCCGGTCGNNNNCNNATTGAANNCCTGNNGCATNNNNNTNCNAGAATGANNNC
NNNNNGNNTNGNCTNNNNNNTNAAGNAATCANNNAGGTNNNNNNNTCNNNNNNNNNNNNCNNTCNCNN
ACTGAGGGTNNNNNNNNCATNNGCTNNNNNNNNNNTNCGCNGGNNTNNCNNCNNNGCNGTATNGNGTNN
NNCANNNNNNNNNNNNNNNNNTNAANNGANNNNNNTNNNN
>HaNhi44
GTNNNNNNNAGCTCNNNNNAGTTAGACANNNCNNNNNNNNNNNNTNNNNNNNNNNNNNNNNNNNNNN
NNNNNNNNNNNNNNNNNNNNNNNNNNNNNNNTNNNNNNNNNNNNNNNNNNNNNNNNNNNNNNNNNNNN
NNNNNNNNNNNNNNNNNNNNNNNNNNNNNNNNNNNNNNNNNNNNNNNNNNNNNNNNNNNNNNNNNNNN
NNNNNNNNNNNNNNNNNNNNNNNNNNNNNNNNNNNNNNNNNNNNNNNNNNNNNNNNNNNNNNNNNNNN
NNNNNNNNNNNNNNNNNNNNNNNNNNNNNNNNNNNNNNNNNNNNNNNNNNNCNTCTNNNAAANNNTNNN
NNNNNNNNNNNNNNNNNNNNNNNNNNNCNCNTTGNNNGTAANCNANNNGNACATGCAGTCCCCTCNNNNN
NNNNNCAGAGNNNNNNNCCNNNNNNNCCGNCCCCNNCCNNNNNNNNNNNNNNNNNGNNNNNNNGNNNNNN
NNNNNNTGGTACNNNNCGNNNNNGGTTGTNTAAGAGGTCCACCACGNGNTCNNNNNACNNNNGNNNAN
NNNCNNNNAACNNNNNNNNNNNNANNTNNNCANNAGGNNNNNNCCNTCNNNNNNNTNANTGCNATGANN
CANNNNNCNGGNACNGTNACCGGCGCGGNGACCTTGNNNACGGTCGGGGNCAAGTATGTCGNNCNCCC
CGNGAAGGANNCNGCNGTANGTGCNNNNNANNNNNTCNTGNGANGTNGCCGNNATTNNCNTGGTCNNN
CNNGAATGNCCNNACCANNCGNANNNTGGTNATGNNAGTNNNNNNNNNNNNNNNNNNNNNNNNNNNNN
NNNNNNNNNNNNNNNNNNNNNNNNNNNNNNNNNNNNNNGNNNNGNNNNNNCNANNNAAGGNNNTANNNTTNNTN
NNNNNNNNGGACNNNACNNNNNNNNNNNNNACNCNNNCNCNNNNNNANNNGGTTNNNNNNCNNTATTNN
CCAGNANGCNGNGCNNNCNTTCANGNGNACNNNGNNACGCGNNAATCGTANNATCTACCTGCCGCNNG
NNNNNNTNCNGNNNNNNCGGANNNTNNNNCGTANNNNNNNCANGCGTNNTNTNNTTNCNAGNCNNGTT
CGNNNANANANTAGNANGTNTNNNNNNNNNNTGTNNCGTTCCGCNTGANNCNNCANNNNGNNGNNNTTN
NCANCGGCNNTANCTGCNGANNTAAGNCANANNNTNNNTCNNTANCGNNNNNGGNGGGNNACTGNAAA
NGGNNAATTNGNNNNTNANTNCNNNCACNTNNCNNNGNNNNCTNNNCNCCTGNNTNCNNAATTGGNNN
CANTGNNTGNCNATCTCTNGNTTNGNNNNNNTNNNNNTNNTANNNNGGNNNGTNNNANNNCNATNTAA
NNTANANNNNCNNNNNNANNNCACNANNNNNNNAGNNNCNANNNTNANGNNNGTAGNNNANTAAGTCN
CCGANNTNNNNNNNATAAGCGNNANACNNNNNNNNNNNNNANGANNNCGNNTTGGNNNTNNNNNNNNA
ANNAGACNNCNGTGGNTNNNNNTCAANNNNTTNGTAGTGGTATAACNANNNNNNNTTTCNCCAGNNTN
NNNGNNNNNNNCNATANNANNNNNNNGNGANNNNNNNNNNNANNNNCCCNNNAGGNANCANCTACNNNA
NCANNGGNGCGNAGNNNGAGANNNNGACNNNCCTTGGGTGGCAANNACNGNAATAAGTNNNGTTNNNN
CCNNNAGTNNACACNNNNNCANNGNNNCNNNNNNANCNGANNTNNNNNNNNNNNNNNNNNNNNNNNN
NNNNNNNNNNNNNNNNNNNNNNNNNNNNNNNNNANGGNNNGTNTNTGNCNCNNGTGTTTTTANNNGNN
NNNNNNNGNNNNNNCCCCGGTCGNNNNCNNATTGAANNCCTGNNGCATNNNNNTNCNAGAATGANNNC
NNNNNGNNTNGNCTNNCNNNTNAAGNAGTCANNNAGNNNNNNNNNTCNNNNNNNNNNNNCNNTNNCNN
ACTGAGGGTNNNNNNNNCATCTGCTNNNNNNNNNNTNCNCNGNNNNNNTNNNNNNGCNGTATNGNGTNN
NNCANNNNNNNNNNNNNNNNNTNAAACGANNNNNCTNNNN

>HaNhi45
GTNNNNNNNAGCCCNNNNNAGTCANACANNNCNNNNNNNNNNNNNTNNNNNNNNNNNNNNNNNNNNNNNN
NNNNNNNNNNNNNNNNNNNNNNNNNNNNNNNTNNNNNNNNNNNNNNNNNNNNNNNNNNNNNNNNNNNNNN
NNNNNNNNNNNNNNNNNNNNNNNNNNNNNNNNNNNNNNNNNNNNNNNNNNNNNNNNNNNNNNNNNNNNNN
NNNNNNNNNNNNNNNNNNNNNNNNNNNNNNNNNNNNNNNNNNNNNNNNNNNNNNNNNNNNNNNNNNNNNN
NNNNNNNNNNNNNNNNNNNNNNNNNNNNNNNNNNNNNNNNNNNNNNNNNNNNCNTCTNNNAAANNNTNNN
NNNNNNNNNNNNNNNNNNNNNNNNNNNCNCNNTNNNNGTAANCNANNNGNACATGCAGTCCCCTCNNCNN
NNNGGCAGAGNNNNNNNCCNNNNNNNCCGNCCCCNNCCNNNNNNNNNNNNNNNNNNNNNNNNNANNNNNN
NNNNNNNTGGTACNNNNCGNNNNNGGTTGTTTNAGAGGTCCACAACGNGNTCNGNNNNACNNNNGNNNAN
NNNCNNNNNANNNNNNNNNNNNNNANATNNNTANNAGGNCNNNNCCNTNNNGNNNNNNANTGCNATGACC
CANNNNNCNGGGGCNGTNCCCGGCGCGGCGACCTTGATNACGTCCGGGGNCAAGTATGGCGNNCNCCC
CGGGAAGGANNCNGCNGTANGTGCNNNNNANNNNNTCNTGNGANGTCGCCGNNATTCNCNTGGTCNNN
NNNGAATGNNCNNACCANNCGNANNNTGGTNATGNNAGTNNNNNNNNNNNNNNNNNNNNNNNNNNNNNNN
NNNNNNNNNNNNNNNNNNNNNNNNNNNNNNNNNNNGNNNNGNNNNNNCANNNAAGGNNNTANNNCTNNTN
NNNNNNNNGGACNNNACNNNNCNNNNNNNACNCNNNCNCNNNNNNNNNNNNGNTTNNNNNNCNNTANTNT
CCAGNACGCNGNGCNNNCATTCANGNGAACNNNGNNACGCGNNAATCGTANNATCTANCTGCCGCNNG
NNNNNNTNCNGNNNNNNCGGANNNTNNNNCGTANNNNNNNCANGCGTNNTNTNNTCNCNAGNCNNGTT
CGNNNANANANTAGNANGTNTNNNNNNNNNNTGTNNCGTTCCGCNTGANNCNNCANNNNGNNGNNNTTN
NCANCGGCNNTANCTGCNGANNTAAGNCATANNNTNNNTCNNTANCTNNNNNGGNGGGNNACTGTAAA
NGGNNAATTNGNNGNTNATTNCNNNCATNTNNCNNNGNNTNCTNNNTNCCTGNNTNCNNAATTGGNNN
CANTGNNTGNCNATCTCTNGNTTNGNNNNNNTNNNNNTNNCANNNNGGNNNGTNNNANNNCNATNTAA
NNTANANNNNCNNNNNNANNNCACNNNNNNNNGNAGNNNCNANNNGTANANNNGTAGNNNGNTAAGTCT
CCGANNTNNNNNNNATAAGCGNNANACNNNNNNNNNNNNNNNANGANNNCGNNTTTGNNNTNNNNNNCNA
GNNAGACNNTNGTGGNTNNNNNTCAANNNNTTCGTAGTGGTATAATNGNNNNNNNTTTCNCCAGNNTN
NNNGNNNNNNNCNATANNANNNNNNNGNGANNNNNNNNNNNANNNNCTCNNNAGGGANCANCTACTNNA
GCANNGGNGCGNAGNNNGAGANNNNGACNNNCCTTGGGTGGNAANNACNGCAATAAGTNNGTTNNNN
CTNNNAGTNNACACNNNNNCANNGNNNCNNNNNNANCNGANNGNNNNNNNNNNNNNNNNNNNNNNNNNNN
NNNNNNNNNNNNNNNNNNNNNNNNNNNNNNNNNNNANNGNNNGTNTNTGNCNCANGTGTTTTTANNNGNN
NNNNNNNGNNNNANCCCCGGTCGNNTNCNNATTGAANNCCTGNNGCATNNNNNTNCNAGAATGANNNC
NNNNNGNNTNGNCTNNCNNNTNAAGNAATCNNNNAGGTNNNNNNNTCNNNGNNNNNNNNCNNTCNCNN
ACTGAGGGNNNNNNNNNCATCNGCTNNNNNNNNNCTNCGCNGGNGTNNCNNNNNAGCNGTATNNNGTNN
NNCANNNNNNNNNNNNNNNNNNTNAAAANANNNNNNTNNNN
>HaNhi46
GNGNNNNNTAGCCCNNNNNAGTTAGACANNNCNNNNNNNNNNNNNTNNNNNNNNNNNNNNNNNNNNNNNN
NNNNNNNNNNNNNNNNNNNNNNNNNNNNNNNNNNNNNNNNNNNNNNNNNNNNNNNNNNNNNNNNNNNNNN
NNNNNNNNNNNNNNNNNNNNNNNNNNNNNNNNNNNNNNNNNNNNNNNNNNNNNNNNNNNNNNNNNNNNNN
NNNNNNNNNNNNNNNNNNNNNNNNNNNNNNNNNNNNNNNNNNNNNNNNNNNNNNNNNNNNNNNNNNNNNN
NNNNNNNNNNNNNNNNNNNNNNNNNNNNNNNNNNNNNNNNNNNNNNNNNNNNNNNCTNNNNAANNNTNNN
NNNNNNNNNNNNNNNNNNNNNNNNNNNCNCNNTGNNNGTAANCNANNNGNACATGCAGTCCCCTCNNNNN
NNNGGCAGAGNNNNNNNCCNNNNNNNCCGGCCCCNNCCNNNNNNNNNNNNNNNNNNNNNNNNNANNNNNN
NNNNNNTGGTACNNNNCGNNNNNGGTTGTTTAAGAGGTCCACCACGNGGTCNNNNNACNNNNGNNNAN
NNNCNNNAAACNNNNNNNNNNNNNNNNNTNNNTANNAGGNCNNNNCCNTCNNNNNNNNNATTGCNATGANN
CANNTNNCNGGGGNNGTNCCCGGCGNGGCGACCTTGNNNANNTCCGGGNNCAAGTATGGCGNNCNCCC
CGGGAAGGANNCNGCNGTANGTGCNNNNNANNNNNTCNTGNGANGTCGCCGNNATTNNNNTGGTCNNN
NNAGAATGNCCNNACCANNCGNANNNTGGTNATGNNAGTANNNNNNNNNNNNNNNNNNNNNNNNNNNNNN
NNNNNNNNNNNNNNNNNNNNNNNNNNNNNNNNGNNNNNNGNNNNNNCNANNNAAGGNNNTANNNCTNNTN
NNNNNNNNGGNCNNNACNNNNCNNNNNNNACNCNNNCNCNNNNNNNNNNNGGTTTNNNNNNCNNTANTNT
CCAGNANGCNGNGCNNNCATTCANGNGAACNNNGNNACGCGNNAATCGTANNATCTACCTGCCGCNNG
NNNNNNTNCNGNNNNNNCGGANNNTNNNNCGTANNNNNNNTATACGTNNTNTNNTCNCNAGNCNNGTT
CGNNNANANANTAGNANGTNTNNNNNNNNNNTGTNNCGTTCCGCNTGANNCNNCNNNNNGNNGNNNTTN
NCANCGGCNNTANCTNNNGANNTAAGNCATANNNTNNNTCNNTANCTNNNNNGGNGGGNNACTGTAAA
NGNNNAATTNGNNNNTNANTNCNNNCACNTNNCNNNGNNTNCTNNNTNCCTGNNTNCNNAATTGGNNC
CANTGNNTGNCNATCTCTGGNTTNGNNNNNNTNNNNNTNNCANNNNGGNNNNNNNNANNNCNATNTAA
NNTANANNNNCNNNNNNANNNCACNNNNNNNNNNAGNNNCNANNNTNANANNNNTAGNNNNNTAAGTCT

CCGANNTNNNNNNNATAAGCGNNANACNNNNNNNNNNNNNNANGANNNCGNNTTGGNNNTNNNNNNNNNA
GNNAGACNNTNGTGGNTNNNNNTCAANNNNTTNGTAGTGGTATAATNGNNNNNNNTTTCNCCAGNNTN
NNNGNNNNNNNNCNATANNANNNNNNNGNGANNNNNNNNNNNANNNNCTCNNNAGGNANCANCTACTNNA
NCANTGGNNCGNAGNNNGAGANNNNGACNNNCCTTGGGTGGCAANNACNGNAATAAGTNNNGTTNANC
CTNNNAGTNNACACNNNNNCANNGNNNCNNNNNNANCNGANNGNNNNNNNNNNNNNNNNNNNNNNNNNN
NNNNNNNNNNNNNNNNNNNNNNNNNNNNNNNNNNNNANGGNNNGTNTNTGNCNCNTGNNTTTTTANNNGNN
NNNNNNNGNNNNNNCCCCGGTCGNNNNCNNATTGAANNCCTGNNGCATNNNNNTNCNANAATGANNNC
NNNNNGNNTNNNCTNNNNNNTCAAGNAGTCNNNTAGGTNNNNNNNTCNNNNNNNNNNNNCNNTCNCNN
ACTGAGGGNNNNNNNNNCATCNGCTNNNNNNNNNNTNCGCNGGNNTNNCNNNNNAGCNGTATNNNGNNN
NNCANNNNNNNNNNNNNNNNNTNAAACGANNNNNNTNNNN
>HMong194
GTNNNNNNNAGCTCNNNNNAGTTANACANNNCNNNNNNNNNNNNNTNNNNNNNNNNNNNNNNNNNNNNNN
NNNNNNNNNNNNNNNNNNNNNNNNNNNNNNNNNNNNNNNNNNNNNNNNNNNNNNNNNNNNNNNNNNNNNN
NNNNNNNNNNNNNNNNNNNNNNNNNNNNNNNNNNNNNNNNNNNNNNNNNNNNNNNNNNNNNNNNNNNNNN
NNNNNNNNNNNNNNNNNNNNNNNNNNNNNNNNNNNNNNNNNNNNNNNNNNNNNNNNNNNNNNNNNNNNNN
NNNNNNNNNNNNNNNNNNNNNNNNNNNNNNNNNNNNNNNNNNNNNNNNNNNNNNNTCTNNNAAANNNTNNN
NNNNNNNNNNNNNNNNNNNNNNNNNNCNCNNTGNNNGTAANCNANNNGNACATGCAGTCCCCTCNNNNN
NNNGGCAGAGNNNNNNNNNNNNNNNNNCCGNCCCCNNCCNNNNNNNNNNNNCNNNNNNNNNNNGNNNNNN
NNNNNNTGGTACNNNNCGNNNNNGGTTGNTTNAGNGGTCCACCACGNGNTCNGNNNACNNNNGNNNAN
NNNCNNNNANCNNNNNNNNNNNNNANNTNNNCANNAGGNNNNNNCCNTNNNNNNNNNNNANNGCNATGANN
CANNNNNCNGNGANNGTNACCGGNGCGGCGACCTTGNNNANNGTCGGGGNCAAGTATGTCNNNCNCCC
CGGGAAGGANNCNGCCGTANGTGCNNNNNANNNNNTCNTGNGANNTNGCCGNNATTNNCNTGGTCNNN
CNAGAATGNNCNNACCANNCGNANNNTGGTNATGNNAGTCNNNNNNNNNNNNNNNNNNNNNNNNNNNNN
NNNNNNNNNNNNNNNNNNNNNNNNNNNNNNNNNNNNNGNNNNGNNNNNNCNANNNAAGGNNNTANNNTTNNTN
NTNNNNNNNGGACNNNACNTNNCNNNNNNNNACNCNNNCNCNNNNNNNNNNNGGTTNNNNNNNCNNTANTNT
CCAGNACGCNGNGCNNNCNTTCANGNGAACNNNGNNACGCGNNAATCGTANNATCTACCTGCCGCNNG
NNNNNNTNCNGNNNNNNCGGANNNTNNNNNGTANNNNNNNCANGCGTNNTNTNNTNNCNAGNCNNGTT
CGNNNANANANTAGNANGTNTNNNNNNNNNNTGTNNCGTTCCGCNTGANNCNNCANNNNGNNGNNNTTN
NCANCGGCNNTANNTGCNGANNTAAGNCATANNNTNNNTCNNTANCGNNNNNGGNGGGNNACTGTAAA
NGGNNAATTNGNNNNTNATTNCNNNCACNTNNCNNNGNNNNCTNNNCNCCTGNNTNCNNAATTGGNNC
CANTGNNTGNCNATCTCTNGNTTNGNNNNNNTNNNNNTNNTANNNNGGNNNGTNNNANNNCNATNTAA
NNTANANNNNCNNNNNNANNNCACNNNNNNNNNAGNNNCNANNNTNANGNNNGTAGNNNANTAAGTCT
CCGANNTNNNNNNNATAAGCGNNANACNNNNNNNNNNNNNNANGANNNCGNNTTGGNNNTNNNNNNNCNA
ANNANACNNCNGTGGNTNNNNNTCAANNNNTTCGTAGTGGTATAACNANNNNNNNTTTCNCCAGNNTN
NNNGNNNNNNNNCNATANNANNNNNNNGNGANNNNNNNNNNNANNNNNCCCNNNAGGNANCANCTACTNNA
GCANTGGNGCGNAGNNNGAGANNNNGACNNGCCTTGGGTGGCANNNACNGCAATAAGTNNNGTTNNNN
CCNNNAGTNNACACNNNNNCANNGNNNCNNNNNNANCNGANNTNNNNNNNNNNNNNNNNNNNNNNNNNN
NNNNNNNNNNNNNNNNNNNNNNNNNNNNNNNNNNNNANGGNNNGTNTNTGNCNCNTGTGTTTTTANNNGNN
NNNNNACGNNNNANCCCCGGTCGNNTNCNNATTGAANNCCTGNNGCATNNNNNTNCNAGAATGANNNC
NNNNNGNNTNGNCTNNCNNNTCAAGNAGTCANNNANNTANNNCGNTCNNNNNNNNNNNNNCNNNNNCNN
ACTGAGGGTNNNNNNNNNCATNTGCTNNNNNNNNNCTNCGCNGNNNNNNTNNNNNAGCNGTATNNNGTNN
NNCANNNNNNNNNNNNNANNNNTNAAACNANNNNNNNTNNNN
>HMong195
GTGNNNNNTAGCTCNNNNNAGTTAGACANNNCNNNNNNNNNNNNNTNNNNNNNNNNNNNNNNNNNNNNNN
NNNNNNNNNNNNNNNNNNNNNNNNNNNNNNNNTNNNNNNNNNNNNNNNNNNNNNNNNNNNNNNNNNNNNNN
NNNNNNNNNNNNNNNNNNNNNNNNNNNNNNNNNNNNNNNNNNNNNNNNNNNNNNNNNNNNNNNNNNNNNN
NNNNNNNNNNNNNNNNNNNNNNNNNNNNNNNNNNNNNNNNNNNNNNNNNNNNNNNNNNNNNNNNNNNNNN
NNNNNNNNNNNNNNNNNNNNNNNNNNNNNNNNNNNNNNNNNNNNNNNNNNNNNNNTCTNNNAAANNNTNNN
NNNNNNNNNNNNNNNNNNNNNNNNNNCNCNTTGNNNGTAANCNANNNGNACATGCAGTCCCCTCNNNNN
NNNNNCAGAGNNNNNNNCCNNNNNNNCCGNCCCCNNCCNNNNNNNNNNNNNNNNGNNNNNNNGNNNNNN
NNNNNNTGGTACNNNNCGNNNNNGGTTGTNTAAGAGGTCCACCACGNGGTCNNGNCACNNNNGNNNAN
NNNCNNNNNANNNNNNNNNNNNNNNTNNNCANNAGGNNNNNNCCNTCNNGNNANNNATNGCNATGACC
CAANNCGCNGGGACAGTNACCGGCGCGGCGACCTTGANNANNGTCGGGGNCAAGTATGTCNNNCNCCC
CGGGAAGGANNCNGCCGTANGTGCNNNNNANNNNNTCNTGNGANGTCGCCGNNATTCNCGTGGTCNNN

```
NNAGAATGNNCNNACCANNCGNANNNTGGTNATGNNAGTNNNNNNNNNNNNNNNNNNNNNNNNNNNN
NNNNNNNNNNNNNNNNNNNNNNNNNNNNNNNNNNNNNGNNNNNNCNANNNAAGGANNTACNNTTNNTN
NTNNNNNNGGACNNNACNNNNCNNNNNNNACNCNNNCNCNNNNNNNNNNGNTTTTTNNNCNNTATTNT
CCAGNACGCNGNGCNNNCATTCANGNGAACNNNGNNACGCGNNAATCGTANNATCTANCTGCCGCNNG
TNNNNNTACNGNNNNNNCGGANNNTNNNNCGTANNNNNNNCATGCGTNNTNTNNTTNCNAGNCNNGTT
CGNNNANANANTAGNANGTNTNNNNNNNNNNTGTNNCGTTCCGCNTGANNCNNCANNNNGNNGNNNTTN
NCANCGGCNNTANCTGCNGANNTAAGNCATANNNTNNNTCNNTANCGNNNNNGGNGGGNNACTGTAAA
NGGNNAATTNGNNNNTNANTNCNNNCACNTNNCTNNGNNNNCTNNNCNCCTGNNTNCNNAATTGGNNC
CANTGNNTGNCNATCTCTGGNTTNGNNNNNNTNNNNNTNNTAANNNGGNNNGTNNNANNNCNATNTAA
NNTANANNNNCNNNNNNANNNCACNNNNNNNNNAGNNNNNANNNTTANGNNNGTAGNNNNNTAAGTCT
CCGANGTNNNNNNNATAAGCGNNANACNNNNNNNNNNNNNANGANNACGNNTTGGNNNTNNNNNNNNA
ANNAGACNNCNGTGGNTNNNNNTCAANNNNTTNGTAGTGGTATAACNANNNNNNNTTTCNCCAGNNTN
NNNGNNNNNNNNCNATANNANNNNNNNGNGANNNNNNNNNNNANNNNCCCNNNAGGGANCANCTACTNNA
GCANNGGNGCGCAGNNNGAGANNNNGACNNNCCTTGGGTGGCANNCACNGCAATAAGTNNGTTNANC
CCNNNAGTNNACACNNNNNCANNGNNNCNNNNNNANCNGANNTNNNNNNNNNNNNNNNNNNNNNNNNNN
NNNNNNNNNNNNNNNNNNNNNNNNNNNNNNANNNNANGGNNNGTNTNTGACNCNTGTGTTTTTANNNGNN
NNNNNACGNNNNNNCCCCGGTCGNNNNCNNATTGAANNCCTGNNGCATNNNNNTNCNAGAATGANNNC
NNNNNGNNTNGNCTNNCNNNTCAAGNAGTCAANNANGTNNNNNGNTCNNNNNNNNNNNNCNNTCCCNN
ACTGAGGGNNNNNNCNNCATCTGCTNNNNNNNNNNTTCGCNGNNNNNNTNNNNNNGCNGTATNGNGTNN
NNCANNNNNNNNNNNNNNNNNNTNANNNGANNNNNNNTNNNN
>HMong196
GTGNNNNNNAGCTCNNNNNAGTTNGACANNNCNNNNNNNNNNNNNTNNNNNNNNNNNNNNNNNNNNNN
NNNNNNNNNNNNNNNNNNNNNNNNNNNNNNNNNNNNNNNNNNNNNNNNNNNNNNNNNNNNNNNNNNNN
NNNNNNNNNNNNNNNNNNNNNNNNNNNNNNNNNNNNNNNNNNNNNNNNNNNNNNNNNNNNNNNNNNNN
NNNNNNNNNNNNNNNNNNNNNNNNNNNNNNNNNNNNNNNNNNNNNNNNNNNNNNNNNNNNNNNNNNNN
NNNNNNNNNNNNNNNNNNNNNNNNNNNNNNNNNNNNNNNNNNNNNNNNNNNNNNNNCTNNNAAANNNTNNN
NNNNNNNNNNNNNNNNNNNNNNNNNNNCNCNNTNNNNGTAANCNANNNGNACATGCAGTCCCCTCNNNNN
NNNGGCAGAGNNNNNNNNNNNNNNNNNCCGNCCCCNGCCNNNNNNNNNNNNNNNNNGNNNNNNNGNNNNNN
NNNNNNTGGTACNNNNCGNNNNNGGTTGTNTNAGAGGTCCACCACGNGNTCNGNNNACNNNNGNNNAN
NNNCNNNNAACNNNNNNNNNNNNNANNTNNNCANAAGGNNNNNNCCNTCNNNNNNNNNNATTGCNATGANN
CANNNNNCNGNGANNGTNACCGGNGNGGNGACCTTGANNACGNTCGGGGNCAAGTATGTCGNNCNCCC
CGGGAAGGANNCNGCNGTANGTGCNNNNNANNNNNTCNTGNGANNTNGCCGNNATTNNNNTGGTCNNN
CNNGAATGNCCNNACCANNCGNANNNTGGTNATGNNAGTNNNNNNNNNNNNNNNNNNNNNNNNNNNN
NNNNNNNNNNNNNNNNNNNNNNNNNNNNNNNNNNNNGNNNTGNNNNNNCNANNNAAGGNNNTANNNTTNNTN
NNNNNNNNGGACNNNACNNNNCNNNNNNNACNCNNNCNCNNNNNNANNNGGTTTNNNNNCNNTNNTNT
CCAGNANGCNGNGCNNNCNTTCANGNGAACNNNGNNACGCGNNAATCGTANNATCTACCTGCCGCNNG
NNNNNNTNCNGNNNNNNCGGANNNTNNNNCGTANNNNNNNCNNGCGTNNNNTNNTTNCNAGNCNNGTT
CGNNNANANANTAGNANGTNTNNNNNNNNNNTGTNNCGTTCCGCNTGANNCNNCNNNNNGNNGNNCTTN
NCANCGGCNNTANCTNCNGANNTAAGNCATANNNTNNNTCNNTANCGNNNNNGGNGGGNNACTGTAAA
NGGNNAATTNGNNNNTNATTNCNNNCACNTNNCTNNGNNTNCTNNNCNCCTGNNTNCNNAATTGGNNN
CANTGNNTGNCNATCTCTNGNTTNGNNNNNNTNNNNNTNNTANNNNGGNNNNNNNNANNNNNATNTAA
NNTANANNNNCNNNNNNANNNCACNNNNNNNNNAGNNNCNNNNNTNANGNNNGTAGNNNNNTAAGTCT
CCGANNTNNNNNNNATAAGCGNNANACNNNNNNNNNNNNNANGANNNCGNNTTGGNNNTNNNNNNNNA
ANNAGACNNCNGTGGNTNNNNNTCAANNNNTTNGTAGTGGTATAACNANNNNNNNTTTCNCCAGNNTN
NNNGNNNNNNNNCNATANNANNNNNNNGNGANNNNNNNNNNNANNNNNCCNNNAGGNANCANCTACTNNA
NCANNGGNGCGNAGNNNGAGANNNNGACNNNCCTTGGGTGGCANNNACNGNAATAAGTNNGTTNANN
NCNNNAGTNNACACNNNNNCANNGNNNCNNNNNNANCNNANNTNNNNNNNNNNNNNNNNNNNNNNNNNN
NNNNNNNNNNNNNNNNNNNNNNNNNNNNNNANNNNANGGNNNGTNTNTGNCNCNNGTNTTTNTANNNGNN
NNNNNACGNNNGANCCCCGGTCGNNNNCNNATTGAANNCCTGNNGCATNNNNNTNCNANAATGANNNC
NNNNNGNNTNGNCTNNCNNNTCAAGNAGTCNNNNANNNNNNNNNNNNCNNNNNNNNNNNNCNNTNNCNN
ACTGAGGGTNNNNNNNNCANNNGCTNNNNNNNNNNTNCGCNGNNNNNNTNNNNNAGCNGTATNGNGTNN
NNCANNNNNNNNNNNNNNNNNNTNAAACGANNNNNNNTNNNN
>HMong197
GTGNNNNNTAGCTCNNNNNAGTTANACANNNCNNNNNNNNNNNNNTNNNNNNNNNNNNNNNNNNNNNN
```

NNNNNNNNNNNNNNNNNNNNNNNNNNNNNNNNNNNNNNNNNNNNNNNNNNNNNNNNNNNN
NNNNNNNNNNNNNNNNNNNNNNNNNNNNNNNNNNNNNNNNNNNNNNNNNNNNNNNNNNNN
NNNNNNNNNNNNNNNNNNNNNNNNNNNNNNNNNNNNNNNNNNNNNNNNNNNNNNNNNNNN
NNNNNNNNNNNNNNNNNNNNNNNNNNNNNNNNNNNNNNNNNNNNTCTNNNAAANNNTNNN
NNNNNNNNNNNNNNNNNNNNNNNNNCNCNTTGNNNGTAANCNANNNGNACATGCAGTCCCCTCNNNNN
NNNNNCAGAGNNNNNNNCCNNNNNNNCCGNCCCCGNCCNNNNNNNNNNNNNNNNGNNNNNNNGNNNNNN
NNNNNNTGGTACNNNNCGNNNNNGGTTGNNTAAGAGGTCCACCACGNGGTCNNGNNACNNNNGNNNAN
NNNCNNAAAANNNNNNNNNNNNNNNNATNNNCANNAGGNNNNNNCCNTCNNGNNNNNNANTGCNATGACC
CAANNCGCGGGGACNGTNACCGGCGCGGCGACCTTGANNACGGTCGGGGACAAGTATGTCNNNCNCCC
CGGGAAGGANNCNGCCGTANGTGCNNNNNANNNNNTCNTGNGANGTCGCCGNNATTCNCGTGGTCNNN
CNAGAATGNNCNNACCANNCGNANNNTGGTNATGNNAGTNNNNNNNNNNNNNNNNNNNNNNNNNNNNN
NNNNNNNNNNNNNNNNNNNNNNNNNNNNNNNNNNNNNGNNNNNNCNANNNAAGGNNNTANNNTTNNTN
NTNNNNNNGGACNNNACNNNNCNNNNNNNACNCNNNCNCNNNNNNNNNNGNTTTNNNNNCCNNTANTNT
CCAGNACGCNGNGCNNNCNTTCANGNGAACNNNGNNACGCGNNAATCGTANNATCTACCTGCCGCNNG
NNNNNNTNCNGNNNNNNCGGANNNTNNCTCGTANNNNNNNCATGCGTNNTNTNNTTNCNAGNCANGTT
CGNNNANANANTAGNANGTNTNNNNNNNNNTGTNNCGTTCCGCNTGANNCNNCANNNNGNNGNNCTTN
NCANCGGCNNTANCTNCNGANNTAAGNCATANNNTNNNTCNNTANCGNNNNNGGNGGGNNACTGTAAA
NGGNNAATTNGNNNNTNATTTCNNNCACNTNNCTNNGNNNNCTNNNCNCCTGNNTNCNNAATTGGNNC
CANTGNNTGNCNATCTCTGGNTTNGNNNNNNTGNNNNTNNTANNNNGGNNNGTNANANNNCNATNTAA
NNTANANNNNCNNNNNNANNNCACNNNNNNNGNAGNNNCNANNNTNANGNNNGTAGNNNAATNAGTCT
CCGANNTNNNNNNNATAAGCGNNANACNNNNNNNNNNNNNNANGANNACGNNTTGGNNNTNNNNNNCNA
ANNAGACNNCNGTGGNTNNNNNTCAANNNNTTCGTAGTGGTATAACNANNNNNNNNTTTCNCCAGNNTN
NNNGNNNNNNNNCNATANNANNNNNNNGNGANNNNNNNNNNNANNNNCCCNNNAGGGANCANCTACTTNA
GCANTGGNGCGNAGNNNGAGANNNNGACNNGCCTTGGGTGGCAANNACNGCAATAAGTNNNGTTNNNC
CCNNNAGTNNACACNNNNNCANNGNNNCNNNNNNANCNGANNTNNNNNNNNNNNNNNNNNNNNNNNNN
NNNNNNNNNNNNNNNNNNNNNNNNNNNNNNNAGNNNANGGNNNGTNTNTGACNCATNTGTTTTTANNNGNN
NNNNNACGNNNNANCCCCGGTCGNNNNCNNATTGAANNCCTGNNGCATNNNNNNTNCNAGAATGANNNC
NNNNNGNNTNGNCTNNNNNNNTCAAGNAGTCAANTAGNTNNNNNNNNTCNNNGNNNNNNNNCNNNCCCNN
ACTGAGGGTNNNNNNNNCATNNGCTNNNNNNNNNNTNCGCNGNNNNNNNTNNNNNNGCNGTATNNNGTNN
NNCANNNNNNNNNNNNNNNNNNTNAAACNANNNNNNNTNNNN
>HMong198
GTGNNNNNTAGCTCNNNNNAGTTAGACANNNCNNNNNNNNNNNNTNNNNNNNNNNNNNNNNNNNNNN
NNNNNNNNNNNNNNNNNNNNNNNNNNNNNNNNNNNNNNNNNNNNNNNNNNNNNNNNNNNN
NNNNNNNNNNNNNNNNNNNNNNNNNNNNNNNNNNNNNNNNNNNNNNNNNNNNNNNNNNNN
NNNNNNNNNNNNNNNNNNNNNNNNNNNNNNNNNNNNNNNNNNNNNNNNNNNNNNNNNNNN
NNNNNNNNNNNNNNNNNNNNNNNNNNNNNNNNNNNNNNNNNNNNTCTNNNAAANNNTNNN
NNNNNNNNNNNNNNNNNNNNNNNNNCNCNTTGNNNGTAANCNANNNGNACATGCAGTCCCCTCNNNNN
NNNNNCAGAGNNNNNNNCCNNNNNNNCCGNCCCCNNCCNNNNNNNNNNNNNNNNGNNNNNNNGNNNNNN
NNNNNNTGGTACNNNNCGNNNNNGGTTGTNTNAGAGGTCCACCACGNGGTCNNGNNACNNNNGNNNAN
NNNCNNNNAANNNNNNNNNNNNNNANNTNNNCANNAGGNNNNNNCCNTCNNGNNNNTNANNGCNATGACC
CANNTCGCGGNGANNGTNACCGGCGNGGCGACCTTGANNANGGTCGGGGNCAAGTATGTCNNNCNCCC
CGGGAAGGANNCNGCCGTANGTGCNNNNNANNNNNTCNTGNGANGTNGCCGNNATTNNCNTGGTCNNN
CNAGAATGNNCNNACCANNCGNANNNTGGTNATGNNAGTNNNNNNNNNNNNNNNNNNNNNNNNNNNNN
NNNNNNNNNNNNNNNNNNNNNNNNNNNNNNNNNNGNNNNGNNNNNNCNANNNAAGGNNNTANNNTTNNTN
ANNNNNNNGGACNNNNCNNNNCNNNNNNNACNCNNNCNCNNNNNNNNNNNGNTTTTNNNNNCNNTANTNT
CCAGNANGCNGNGCNNNCATTCANGNGAACNNNGNNACGCGNNAATCGTANNATCTACCTGCCGCNNG
NNNNNNTACNGNNNNNNCGGANNNTNNNNCGTANNNNNNNCANGCGTNNTNTNNTTNCNAGNCNNGTT
CGNNNANANANTAGNANGTNTNNNNNNNNNTGTNNCGTTCCGCNTGANNCNNCANNNNGNNGNNCTTN
NCANCGGCNNTANCTGCNGANNTAAGNCATANNNTNNNTCNNTANCGNNNNNGGNGGGNNACTGTAAA
NGGNNAATTNGNNNNTNANTNCNNNCACNTNNCTNNGNNNNCTNNNCNCCTGNNTNCNNAATTGGNNN
CANTGNNTGNCNATCTCTGGNTTNGNNNNNNTNNNNNTNNTAANNNGGNNNNNNNNANNNCNATNTAA
NNTANANNNNCNNNNNNANNNCACNNNNNNNGNAGNNNCNANNNTNANGNNNGTAGNNNANTAAGTCT
CCGANNTNNNNNNNATAAGCGNNANACNNNNNNNNNNNNNNANGANNNCGNNTTGGNNNTNNNNNNCNA
ANNAGACNNCNGTGGNTNNNNNTCAANNNNTTNGTAGTGGTATAACNANNNNNNNNTTTCNCCAGNNTN

NNNGNNNNNNNNCNATANNANNNNNNNGNGANNNNNNNNNNNANNNNCCCNNNAGGNANCANCTACTNNA
GCANTGGNGCGNAGNNNGAGANNNNGACNNNCCTTGGGTGGCAANNACNGCAATAAGTNNNGTTNANC
CCNNNAGTNNACACNNNNNCANNGNNNCNNNNNNANCNGANNTNNNNNNNNNNNNNNNNNNNNNNNNN
NNNNNNNNNNNNNNNNNNNNNNNNNNNNNNAGNNNANGGNNNGTNTNTGACNCNNGTGTTTTTANNNGNN
NNNNNACGNNNNANCCCCGGTCGNNNNCNNATTGAANNCCTGNNGCATNNNNNTNCNAGAATGANNNC
NNNNNGNNTNNNCTNNCNNNTNAAGNAGTCANNNAGNNNNNNNNNTCNNNGNNNNNNNNCNNNCNCNN
ACTGAGGGTNNNNNNNNCANCTGCTNNNNNNNNNNTNCGCNGNNNNNNTNNNNNNGCNGTATNGNGTNN
NNCANNNNNNNNNNNNNNNNNCNNAANAANNNNNNNTNNNN
>HMong199
GTGNNNNNTAGCCCNNNNNAGTCAGACANNNCNNNNNNNNNNNNNTNNNNNNNNNNNNNNNNNNNNNNN
NNNNNNNNNNNNNNNNNNNNNNNNNNNNNNNNTNNNNNNNNNNNNNNNNNNNNNNNNNNNNNNNNNNNNN
NNNNNNNNNNNNNNNNNNNNNNNNNNNNNNNNNNNNNNNNNNNNNNNNNNNNNNNNNNNNNNNNNNNNNN
NNNNNNNNNNNNNNNNNNNNNNNNNNNNNNNNNNNNNNNNNNNNNNNNNNNNNNNNNNNNNNNNNNNNNN
NNNNNNNNNNNNNNNNNNNNNNNNNNNNNNNNNNNNNNNNNNNNNNNNNNNNCNTCTNNNAAANNNTNNN
NNNNNNNNNNNNNNNNNNNNNNNNNNNCNCNTTGNNNGTAANCNANNNGNACATGCAGTCCCCTCNNNNN
NNNGGCAGAGNNNNNNCCCNNNNNNNNCCGNCCCCNNCCNNNNNNNNNNNNCNNNGNNNNNNNANNNNNN
NNNNNNTGGTACNNNNCGNNNNNGGTTGTTTAAGAGGTCCACAACGNGGTCNGGNCACNNNNGNNNAN
NNNCNNNNAACNNNNNNNNNNNNANATNNNTANNAGGNCNNNNCCNTCNNGNNNNTAANTGCNATGANN
CAANNCACGGGGGCNGTNCCCGGCGCGGCGACCTTGNNNACGTCCGGGGNCAAGTATGGCGNNCNCCC
CGGGAAGGANNCNGCCGTANGTGCNNNNNANNNNNTCNTGNGANGTNGCCGNNATTNNCGTGGTCNNN
CNAGAATGNCCNNACCANNCGNANNNTGGTNATGTNAGTNNNNNNNNNNNNNNNNNNNNNNNNNNNNNN
NNNNNNNNNNNNNNNNNNNNNNNNNNNNNNNNNNNNNGNNNNGNNNNNNCNANNNAAGGNNNTANNNCTNNTN
NTNNNNNNNGGACNNNACNNNNCNNNNNNNACNCNNNCNCNNNNNNANNNGGTTTTTNNNCNNTANTNT
CCAGNACGCNGNGCNNNCNTTTANGNGAACNNNGNNACGCGNNAATCGTANNATCTACCTGCCGCNNG
NNNNNNTACNGNNNNNNNCGGANNNTNNCNCGTANNNNNNNNCATGCGTNNTNTNNTCNCNAGNCNNGTT
CGNNNANANANTAGNANGTNTNNNNNNNCNNTGTNNCGTTCCGCNTGANNCNNCANNNNGNNGNNCTTN
TCANCGGCNNTANCTGCNGANNTAAGNCATANNNTNNATCNNTANCTNNNNNGGAGGGNNACTGTAAA
NGGNNAATTNGNNNNTNANTNCNNNCATNTNNCTNNGNNNNCTNNNTNCCTGNNTNCNNAATTGGNNC
CANTGNNTGNCNATCTCTGGNTTNGNNNNNNNTGNNNNTNNCANNNNGGNNNGTNANANNNCNATNTAA
NNTANANNNNCNNNNNNNANNNCACNNNNNNNNGNAGNNNCNANNNGTANANNNGTAGNNNGNTAAGTCT
CCGANNTNNNNNNNNATAAGCGNNANACNNNNNNNNNNNNNNNANGANNACGNNTTTGNNNTNNNNNNNCNA
GNNAGACNNTNGTGGNTNNNNNNTCAANNNNNTTCGTAGTGGTATAATNGNNNNNNNNTTTCNCCAGNNTN
NNNGNNNNNNNNCNATANNANNNNNNNGNGANNNNNNNNNNNANNNNCTCNNNAGGGANCANCTACTNNA
GCANTGGNGCGCAGNNNGAGANNNNGACNNNCCTTGGGTGGCAANCACNGCAATAAGTNNNGTTNANC
CTNNNAGTNNACACNNNNNCANNGNNNCNNNNNNANCNGANNGNNNNNNNNNNNNNNNNNNNNNNNNN
NNNNNNNNNNNNNNNNNNNNNNNNNNNNNNAGNNNANGGNNNGTNTNTGACNCATGTGTTTTTANNNGNN
NNNNNACGNNNGANCCCCGGTCGNNNNCNNATTGAANNCCTGNNGCATNNNNNTNCNAGAATGANNGC
NNNNNGNNTNGNCTNNCNNNTCAAGNAATCANNNAGGTANNNNNNNTCNNNGNNNNNNNNCNNTCCCNN
ACTGAGGGTNNNNNNNNCATCTGCTNNNNNNNNNCTNCGCNGGNGTNNCNNNNNNGCNGTATNGNGTNA
NNCANNNNNNNNNNNNNNNNNNTNAAACAANNNNNNNTNNNN
>HMong200
GTGNNNNNNAGCCCNNNNNAGTTAGACANNNCNNNNNNNNNNNNNTNNNNNNNNNNNNNNNNNNNNNNN
NNNNNNNNNNNNNNNNNNNNNNNNNNNNNNNNTNNNNNNNNNNNNNNNNNNNNNNNNNNNNNNNNNNNNN
NNNNNNNNNNNNNNNNNNNNNNNNNNNNNNNNNNNNNNNNNNNNNNNNNNNNNNNNNNNNNNNNCNNNNNN
NNNNNNNNNNNNNNNNNNNNNNNNNNNNNNNNNNNNNNNNNNNNNNNNNNNNNNNNNNNNNNNNNNNNNN
NNNNNNNNNNNNNNNNNNNNNNNNNNNNNNNNNNNNNNNNNNNNNNNNNNNNNTCTNNNAAANNNTNNN
NNNNNNNNNNNNNNNNNNNNNNNNNNNCNCNTTGNNNGTAANCNANNNGNACATGCAGTCCCCTCNNNNN
NNNGGCAGAGNNNNNNNCCNNNNNNNNCCGNCCCCNNCCNNNNNNNNNNNNNNNNNNNNNNNNNNANNNNNN
NNNNNNTGGTACNNNNCGNNNNNGGTTGTNTAAGAGGTCCACAACGNGGTCNNGNCACNNNNGNNNAN
NNNCNNNNAACNNNNNNNNNNNNNNNATNNNTANNAGGNNNNNNCCNTCNNGNNNNNNANTGCNATGACC
CAANNCACGGGGGCAGTNCCCGGCGCGGCGACCTTGANNACGTCCGGGGACAAGTATGGCNNNCNCCC
CGGGAAGGANNCNGCCGTANGTGCNNNNNANNNNNTCNTGNGANGTCGCCGNNATTNNCGTGGTCNNN
CNAGAATGNCCNNACCANNCGNANNNTGGTNATGNNAGTNNNNNNNNNNNNNNNNNNNNNNNNNNNNNN
NNNNNNNNNNNNNNNNNNNNNNNNNNNNNNNNNNNNNGNNNNGNNNNNNCNANNNAAGGNNNTANNNCTNNTN

NTNNNNNNGGACNNNACCNNNCNNNNNNNACNCNNNCNCNNNNNNANNNGNTTTNNNNNCNNTANNNT
CCAGNANGCNGNGCNNNCNTTCANGNGAACNNNGNNACGCGNNAATCGTANNATCTACCTGCCGCNNG
NNNNNNTACNGNNNNNNCGGANNNTNNCTCGTANNNNNNNCANGCGTNNTNTNNTCNCNAGNCNNGTT
CGNNNANANANTAGNANGTNTNNNNNNNNNNTGTNNCGTTCCGCNTGANNCNNCANNNNGNNGNNCTTN
TCANCGGCNNTANCTNCNGANNTAAGNCATANNNTNNNTCNNTANCTNNNNNGGNGGGNNACTGTAAA
NGGNNAATTNGNNNNTNATTNCCNNCACNTNNCNNNGNNNNCTNNNTNCCTGNNTNCNNAATTGGNNC
CANTGNNTGNCNATCTCTGGNTTNGNNNNNNTNNNNNTNNCANNNNGGNNNGTNANANNNCNATNTAA
NNTANANNNNCNNNNNNANNNCACNNNNNNNNNAGNNNCNANNNTTANANNNGTAGNNNANTNAGTCT
CCGANNTNNNNNNNATAAGCGNNANACNNNNNNNNNNNNNNANGANNNCGNNTTGGNNNTNNNNNNCNA
GNNAGACNNTNATGGNTNNNNNTCAANNNNTTNGTAGTGGTATAATNGNNNNNNNTTTCNCCAGNNTN
NNNGNNNNNNNCNATANNANNNNNNNGNGANNNNNNNNNNNANNNNCTCNNNAGGNANCANCTACTNNA
GCANTGGNNCGNAGNNNGAGANNNNGACNNNCCTTGGGTGGCAANNACNGCAATAAGTNNNGTTNANC
CTNNNAGTNNACATNNNNNCANNGNNNCNNNNNNANCNGANNGNNNNNNNNNNNNNNNNNNNNNNNNN
NNNNNNNNNNNNNNNNNNNNNNNNNNNNNNNNNNNNANGGNNNGTNTNTGACNCNTNNNTTTTTANNNGNN
NNNNNANGNNNNANCCCCGGTCGNNNNCNNATTGAANNCCTGNNGCATNNNNNTNCNAGAATGANNNC
NNNNNGNNTNGNCTNNCNNNTCAAGNAATCANNNAGNNNNNNNGNTCNNNGNNNNNNNNCNNNCCCNN
ACTGAGGGTNNNNNNNNCATNNGCTNNNNNNNNNTNCGCNGGNGTNNCNNNNNAGCNGTATNGNGTNN
NNCANNNNNNNNNNNNNNNNNTNAANCAANNNNNNNTNNCG
>HMong201
GNGNNNNNTAGCCCNNNNNAGTTANACANNNCNNNNNNNNNNNNNNNNNNNNNNNNNNNNNNNNNNNNNN
NNNNNNNNNNNNNNNNNNNNNNNNNNNNNNNNNNNNNNNNNNNNNNNNNNNNNNNNNNNNNNNNNNNNNN
NNNNNNNNNNNNNNNNNNNNNNNNNNNNNNNNNNNNNNNNNNNNNNNNNNNNNNNNNNNNNNNNNNNNNN
NNNNNNNNNNNNNNNNNNNNNNNNNNNNNNNNNNNNNNNNNNNNNNNNNNNNNNNNNNNNNNNNNNNNNN
NNNNNNNNNNNNNNNNNNNNNNNNNNNNNNNNNNNNNNNCNNNNNNNNNNNNNNNNNNNNNNCTNNNAAANNNTNNN
NNNNNNNNNNNNNNNNNNNNNNNNANNCNCNNTNNNNGTAANCNANNNGNACATGCAGTCCCCTCNNNNN
NNNNNCAGAGNNNNNNNNNNNNNNNNNNCCGNCCCCNNCCNNNNNNNNNNNNNNNNNGNNNNNNNANNNNNN
NNNNNNTGGTACNNNNCGNNNNNGGTTGTNTAAGNGGTCCACAACGNGNTCNNGNCACNNNNGNNNAN
NNNCNNNNNNNNNNNNNNNNNNNNNNNTNNNTANNAGGNNNNNNCCNTNNNNNNNNNNNNANNGCNATGACC
CAANNCACGGGGGNNGTNCCCGGCGNGGNGACCTTGANNANNTCCGGGGACAAGTATNGCGNNCNCNC
CGGGAAGGANNCNGCCGTANGTGCNNNNNANNNNNTCNTGNGANGTNGCCGNNATTNNCGTGGTCNNN
NNAGAATGNCCNNACCANNCGNANNNTGGTNATGNNAGTNNNNNNNNNNNNNNNNNNNNNNNNNNNNN
NNNNNNNNNNNNNNNNNNNNNNNNNNNNNNNNNNNNNNNNNGNNNNNNCNANNNAAGGNNNTANNNCTNNTN
NNNNNNNNNNNCNNNACNNNNCNNNNNNNACNCNNNCNCNNNNNNANNNGNTTTTNNNNCNNTANTNN
CCAGNANGCNGNGCNNNCNTTCANGNGAACNNNNNNACGCGNNAATCGTANNATCTACCTGCCGCNNG
NNNNNNTNCNGNNNNNNCGGANNNTNNNNCGTANNNNNNNCANGCGTNNTNTNNTNNCNAGNCNNGTT
CGNNNANANANTAGNANGTNTNNNNNNNNNNTGTNNCGTTCCGCNTGANNCNNCNNNNNGNNGNNCTTN
NCANCGGCNNTANCTGCNGANNTAAGNCATANNNTNNNTCNNTANCTNNNNNGGNGGGNNACTGTAAA
NGGNNAATTNGNNNNTNATTNCNNNNACNTNNCTNNGNNNNCTNNNTNCCTGNNNNCNNAATTGGNNC
CANTGNNTGNCNATCTCTGGNTTNGNNNNNNTNNNNNTNNCANNNNGGNNNNNNANANNNNNNATNTAA
NNTANANNNNCNNNNNNANNNCACNNNNNNNNNAGNNNCNANNNTNANANNNGTAGNNNNNTAAGTCT
CCGANNTNNNNNNNATAAGCGNNANACNNNNNNNNNNNNNNANGANNNCGNNTTGGNNNTNNNNNNCNA
GNNANACNNTNATGGNTNNNNNTCAANNNNTTNGTAGTGGTATAATNGNNNNNNNTTTCNCCAGNNNN
NNNGNNNNNNNCNATANNANNNNNNNGNGANNNNNNNNNNNANNNNCTCNNNAGGNANCANCTACTNNA
GCANTGGNGCGNAGNNNGAGANNNNGACNNNCCTTGGGTGGCANNNACNGNAATAAGTNNNGTTNANN
CTNNNNGTNNACATNNNNNCANNGNNNCNNNNNNANCNGANNGNNNNNNNNNNNNNNNNNNNNNNNNN
NNNNNNNNNNNNNNNNNNNNNNNNNNNNNNNNNANNNNANNGNNNGTNTNTGACNCNTGTGTTTNTANNNGNN
NNNNNACGNNNNANCCCCGGTCGNNNNCNNATTGAANNCCTGNNGCATNNNNNTNCNAGAATGANNNC
NNNNNGNNTNNNCTNNNNNNTCAANNAATCNNNNAGNNNNNNNNNTCNNNGNNNNNNNNCNNNCCCNN
ACTGAGGGTNNNNNNNNCATCNGCTNNNNNNNNNTNCGCNGGNGTNNCNGNNNNGCNGTATNGNGTNN
NNCANNNNNNNNNNNNNNNNNNNANACNANNNNNNNTNNNN
>HMong202
GTGNNNNNTAGCCCNNNNNAGTCANACANNNCNNNNNNNNNNNNTNNNNNNNNNNNNNNNNNNNNNNNN
NNNNNNNNNNNNNNNNNNNNNNNNNNNNNNNNNTNNNNNNNNNNNNNNNNNNNNNNNNNNNNNNNNNNN
NNNNNNNNNNNNNNNNNNNNNNNNNNNNNNNNNNNNNNNNNNNNNNNNNGNNNNNNNNNNNNNNNNNNNN

NNNNNNNNNNNNNNNNNNNNNNNNNNNNNNNNNNNNNNNNNNNNNNNNNNNNNNNNNNNNNNNNNNNN
NNNNNNNNNNNNNNNNNNNNNNNNNNNNNNNNNNNNNNNNNNNNNNNNNNCNTCTNNNAAANNNTNNN
NNNNNNNNNNNNNNNNNNNNNNNNNCNCNNTNNNNGTAANCNANNNGNACATGCAGTCCCCTCNNNNN
NNNGGCAGAGNNNNNNNCCNNNNNNNCCGGCCCCNNCCNNNNNNNNNNNNNNNNGNNNNNNNANNNNNN
NNNNNNTGGTACNNNNCGNNNNNGGTTGNNTAAGAGGTCCACAACGNGNTCNNNNCANNNNNGNNNAN
NNNCNNNNANCNNNNNNNNNNNNNNNTNNNTANNAGGNNNNNNCCNTCNNGNNNNNNANNGCNATGACC
CAANNCNCGGGGGCNGTNCCCGGNGCGGCGACCTTGNNNACGTCCGGGGACAAGTATNGCNNNCNCCC
CGGGAAGGANNCNGCNGTANGTGCNNNNNANNNNNTCNTGNGANGTNGCCGNNATTCNCGTGGTCNNN
NNAGAATGNNCNNANCANNCGNANNNTGGTNATGNNAGTNNNNNNNNNNNNNNNNNNNNNNNNNNNNN
NNNNNNNNNNNNNNNNNNNNNNNNNNNNNNNNNNNNNNNGNNNNNNCNANNNAAGGANNTANNNCTNNTN
NTNNNNNNNGGACNNNACNNNNCNNNNNNNNACNCNNNCNCNNNNNNNNNNNGNTTNNTNNNCNNTATTNN
CCAGNACGCNGNGCNNNCATTTANGNGAACNNNGNNACGCGNNAATCGTANNATCTACCTGCCGCNNG
NNNNNNTACNGNNNNNNCGGANNNTNNCTCGTANNNNNNNCANGCGTNNNNTNNTCNCNAGNCNNGTT
CGNNNANANANTAGNANGTNTNNNNNNNNNNTGTNNCGTTCCGCNTGANNCNNCANNNNGNCGNNNTTN
NCANCGGCNNTANCTGCNGANNTAAGNCATANNNTNNNTCNNTANCTNNNNNGGNGGGNNACTGTAAA
NGGNNAATTNGNNNNTNANTNCNNNCATNTNNCTNNGNNNNCTNNNTNCCTGNNTNCNNAATTGGNNN
CANTGNNTGNCNATCTCTNGNTTNGNNNNNNTNNNNNTNNCAANNNGGNNNNNNNNANNNCNATNTAA
NNTANANNNNCNNNNNNANNNCACNNNNNNNGNAGNNNCNANNNGTANANNNGTAGNNNGNTAAGTCT
CCGANNTNNNNNNNATAAGCGNNANACNNNNNNNNNNNNNNANGANNNCGNNTTTGNNNTNNNNNNCNA
GNNAGACNNTNGTGGNTNNNNNTCAANNNNTTNGTAGTGGTATAATNGNNNNNNNTTTCNCCAGNNTN
NNNGNNNNNNNCNATANNANNNNNNNGNGANNNNNNNNNNNANNNNCTCNNNAGGNANCANCTACTNNA
GCAANGGNGCGNAGNNNGAGANNNNGACNNGCCTTGGGTGGCAANNACNGCAATAAGTNNNGTTNANN
NTNNNAGTNNACACNNNNNCANNGNNNCNNNNNNANCNGANNGNNNNNNNNNNNNNNNNNNNNNNNNN
NNNNNNNNNNNNNNNNNNNNNNNNNNNNNNNNNGNNNANGGNNNGTNTNTGNCNCNTNTNTTTTTANNNGNN
NNNNNACGNNNNNNCCCCGGTCGNNNNCNNATTGAANNCCTGNNGCATNNNNNTNCNAGAATGANNGC
NNNNNGNNTNGNCTNNNNNNTCAAGNAATCNNNNAGNNNNNGCGNTCNNNGNNNNNNNNCNNTCCCNN
ACTGAGGGTNNNNNNNNCATCNGCTNNNNNNNNNNTNCGCNGGNGTNNCNNNNNAGCNGTATNGNGTNA
NNCANNNNNNNNNNNNNNNNNTNAAACNNNNNNNNNTNNNN
>HMong203
GTGNNNNNTAGCCCNNNNNAGTTGGACANNNCNNNNNNNNNNNNNNNNNNNNNNNNNNNNNNNNNNNNN
NNNNNNNNNNNNNNNNNNNNNNNNNNNNNNNNNNNNNNNNNNNNNNNNNNNNNNNNNNNNNNNNNNNN
NNNNNNNNNNNNNNNNNNNNNNNNNNNNNNNNNNNNNNNNNNNNNNNNNNNNNNNNNNNNNNNNNNNN
NNNNNNNNNNNNNNNNNNNNNNNNNNNNNNNNNNNNNNNNNNNNNNNNNNNNNNNNNNNNNNNNNNNN
NNNNNNNNNNNNNNNNNNNNNNNNNNNNNNNNNNNNNNNNNNNNNNNNNNCNTCTNNNAAANNNTNNN
NNNNNNNNNNNNNNNNNNNNNNNNNCNCNNTGNNNGTAANCNANNNGNACATGCAGTCCCCTCNNNNN
NNNGGCAGAGNNNNNNNCCNNNNNNNCCGGCCCCNNCCNNNNNNNNNNNNNCNNNNNNNNNNNNGNNNNNN
NNNNNNTGGTACNNNNCGNNNNNGGTTGTNTNAGNGGTCCACCACGNGGTCNNGNCACNNNNGNNNAN
NNNCNNNAAANNNNNNNNNNNNNNANNTNNNCANAAGGNNNNNNCCNTNNNNNNNNNNNATTGCNATGACC
CAANNCNCGGNGACNGTNACCGGCGNGGCGACCTTGATNACGGTCGGGGNCAAGTATGTCNNNCNCCC
CGGGAAGGANNCNGCCGTANGTGCNNNNNANNNNNTCNTGNGANGTNGCCGNNATTNNNGTGNTCNNN
NNAGAATGTCCNNACCANNCGNANNNTGGTNATGNNAGTNNNNNNNNNNNNNNNNNNNNNNNNNNNNN
NNNNNNNNNNNNNNNNNNNNNNNNNNNNNNNNNNNGNNNNGNNNNNNCNANNNAAGGNNNTANNNTTNNTN
NTNNNNNNNGGACNNNACNNNNCNNNNNNNNACNCNNNCNCNNNNNNNNNNNGNTTTTNNNNCNNTANTNT
CCAGNANGCNGNGCNNNCATTCANGNGAACNNNGNNACGCGNNAATCGTANNATCTACCTGCCGCNNG
TNNNNNTACNGNNNNNNCGGANNNTNNNNCGTANNNNNNNCANGCGTNNTNTNNTTNCNAGNCNNGTT
CGNNNANANANTAGNANGTNTNNNNNNNNNNTGTNNCGTTCCGCNTGANNCNNCANNNNGNNGNNCTTN
TCANCGGCNNTANCTGCNGANNTAAGNCATANNNTNNNTCNNTANCGNNNNNGGNGGGNNACTGTAAA
NGGNNAATTNGNNNNTNANTNCNNNCACNTNNCNNNGNNNNCTNNNCNCCTGNNTNCNNAATTGGNNN
CANTGNNTGNCNATCTCTNGNTTNGNNNNNNTNNNNNTNNTAANNNGGNNNGTNNNANNNCNATNTAA
NNTANANNNNCNNNNNNANNNCACNNNNNNNNNAGNNNCNANNNTNANGNNNGTAGNNNANTAAGTCT
CCGANNTNNNNNNNATAAGCGNNANACNNNNNNNNNNNNNNANGANNACGNNTTGGNNNTNNNNNNCNA
ANNAGACNNCNGTGGNTNNNNNTCAANNNNTTNGTAGTGGTATAACNANNNNNNNNTTTCNCCAGNNTN
NNNGNNNNNNNCNATANNANNNNNNNGNGANNNNNNNNNNNANNNNCCCNNNAGGNANCANCTATTNNA
GCANTGGNGCGCAGNNNGAGANNNNGACNNGCCTTGGGTGGCAANNACNGCAATAAGTNNNGTTNNNC

CCNNNAGTNNACACNNNNNCANNGNNNCNNNNNNANCNGANNTNNNNNNNNNNNNNNNNNNNNNNNN
NNNNNNNNNNNNNNNNNNNNNNNNNNNNNNNGNNNANNGNNNGTNTNTGACNCNNGTGTTTNTANNNGNN
NNNNNACGNNNNNNCCCCGGTCGNNNNCNNATTGAANNCCTGNNGCATNNNNNTNCNAGAATGANNGC
NNNNNGNNTNGNCTNNNNNNTCAAGNAGTCAANNAGGTNNNNNGNTCNNNNNNNNNNNNNCNNNCNCNN
ACTGAGGGTNNNNNNNNCATCTGCTNNNNNNNNCTNCGCNGNNGTNNTNNNNNAGCNGTATNGNGTNN
NNCANNNNNNNNNNNNNNNNNTNAAACGANNANCTTNNNN
>HMong204
GTGNNNNNTAGCCCNNNNNAGTTNNACANNNCNNNNNNNNNNNNNNNNNNNNNNNNNNNNNNNNNNNN
NNNNNNNNNNNNNNNNNNNNNNNNNNNNNNNNNNNNNNNNNNNNNNNNNNNNNNNNNNNNNNNNNNNN
NNNNNNNNNNNNNNNNNNNNNNNNNNNNNNNNNNNNNNNNNNNNNNNNNNNNNNNNNNNNNNNNNNNN
NNNNNNNNNNNNNNNNNNNNNNNNNNNNNNNNNNNNNNNNNNNNNNNNNNNNNNNNNNNNNNNNNNNN
NNNNNNNNNNNNNNNNNNNNNNNNNNNNNNNNNNNNNNNNNNNNNNNNNNNNNTCTNNNAAANNNTNNN
NNNNNNNNNNNNNNNNNNNNNNNNNNNNNCNNTNNNNGTAANCNANNNGNACATGCAGTCCCCTCNNNNN
NNNNNCAGAGNNNNNNNCCNNNNNNNCCGNCCCCNNCCNNNNNNNNNNNNNNNNNGNNNNNNNGNNNNNN
NNNNNNTGGTACNNNNCGNNNNNGGTTGNNTAAGAGGTCCACCACGNGGTCNNNNCACNNNNGNNNAN
NNNCNNNNAACNNNNNNNNNNNNANNTNNNCANNAGGNCNNNNCCNTCNNGNNNNNNANTGCNATGANN
CNNNNNNCGGGGANNGTNACCGGNGNGGCGACCTTGNNNANNGTNGGGGNCAAGTATGTCNNNCNCCC
CGGGAAGGANNCNGCCGTANGTGCNNNNNANNNNNTCNTGNGANGTCGCCGNNATTCNCNTGGTCNNN
CNNNANTGNNCNNACCANNCGNANNNTGGTNATGTNAGTNNNNNNNNNNNNNNNNNNNNNNNNNNNNNN
NNNNNNNNNNNNNNNNNNNNNNNNNNNNNNNNNNNNGNNNNGNNNNNNCNANNNAAGGNNNTANNNTTNNTN
NNNNNNNNGGNCNNNACNNNNCNNNNNNNACNCNNNCNCNNNNNNANNNGNTTTNNNNNCNNTANTNN
CCAGNANGCNGNGCNNNCNTTCANGNGAACNNNGNNACGCGNNAATCGTANNATCTACCTGCCGCNNG
TNNNNNTACNGNNNNNNCGGANNNTNNNNCGTANNNNNNNCANGCGTNNTNTNNTNNCNAGNCNNGTT
CGNNNANANANTAGNANGTNTNNNNNNNNNNTGTNNCGTTCCGCNTGANNCNNCNNNNNGNNGNNCTTN
NCANCGGCNCTANNTNCNGANNTAAGNCANANNNTNNNTCNNTANCGNNNNNGGNGGGNNACTGTAAA
NGGNNAATTNGNNNNTNANTNCNNNNACNTNNCNNNGNNNNCTNNNCNCCTGNNTNCNNAATTGGNNC
CANTGNNTGNCNATCTCTNGNTTNGCNNNNNTNNNNNTNNTANNNNGGNNNNNNANANNNCNATNTAA
NNTANANNNNCNNNNNNANNNCACNNNNNNNNNAGNNNCNANNNTNANGNNNGTAGNNNANTNAGTCT
CCGANNTNNNNNNNATAAGCGNNANACNNNNNNNNNNNNNANGANNACGNNTTGGNNNTNNNNNNNNA
ANNAGACNNCNGTGGNTNNANNTCAANNNNTTNGTAGTGGTATAACNANNNNNNNTTTCNCCAGNNTN
NNNGNNNNNNNCNATANNANNNNNNNGNGANNNNNNNNNNNANNNNCCCNNNAGGNANCANCTATTNNA
GCANNGGNNCGNAGNNNGAGANNNNGACNNNCCTTGGGTGGCAANNACNGCAATAAGTNNNGTTNANC
CCNNNAGTNNACACNNNNNCANNGNNNCNNNNNNANCNGANNTNNNNNNNNNNNNNNNNNNNNNNNN
NNNNNNNNNNNNNNNNNNNNNNNNNNNNNNNNNNNANGGNNNGTNTNTGNCNNNTGNNTTTTTANNNGNN
NNNNNNNGNNNGANCCCCGGTCGNNNNCNNATTGAANNCCTGNNGCATNNNNNTNCNAGAATGANNNC
NNNNNGNNTNGNCTNNCNNNTNAAGNAGTCNNNNAGGNNNNNNNNTCNNGGNNNNNNNNCNNNNCCNN
ACTGAGGNTNNNNNNNNCATCTGCTNNNNNNNNNNTNCGCNGNCGTNNTNNNNNNGCNGTATNNNGTNN
NNCANNNNNNNNNNNNNNNNNTNAAACAANNNNNTTNNNN
>HMong205
GTGNNNNNNAGCTCNNNNNAGTTANACANNNCNNNNNNNNNNNNTNNNNNNNNNNNNNNNNNNNNN
NNNNNNNNNNNNNNNNNNNNNNNNNNNNNNNNTNNNNNNNNNNNNNNNNNNNNNNNNNNNNNNNNNNN
NNNNNNNNNNNNNNNNNNNNNNNNNNNNNNNNNNNNNNNNNNNNNNNNNNNNNNNNNNNNNNNNNNNN
NNNNNNNNNNNNNNNNNNNNNNNNNNNNNNNNNNNNNNNNNNNNNNNNNNNNNNNNNNNNNNNNNNNN
NNNNNNNNNNNNNNNNNNNNNNNNNNNNNNNNNNNNNNNNNNNNNNNNNNNCNTCTNNNAAANNNTNNN
NNNNNNNNNNNNNNNNNNNNNNNNNNNCNCNTTGNNNGTAANCNANNNGNACATGCAGTCCCCTCNNNNN
NNNNNCAGAGNNNNNNNCCNNNNNNNCCGNCCCCNNCCNNNNNNNNNNNNNNNNNNNNNNNNNNGNNNNNN
NNNNNNTGGTACNNNNCGNNNNNGGTTGTNTAAGAGGTCCACCACGNGGTCNNNNNACNNNNGNNNAN
NNNCNNNAAACNNNNNNNNNNNNANATNNNCANNAGGNCNNNNCCNTCNNGNNNNNNANNGCNATGACC
CAANTNGCNGGGACAGTNACCGGCGCGGCGACCTTGNNNACGGTCGGGGACAAGTATGTCGNNCNCCC
CGGGAAGGANNCNGCNGTANGTGCNNNNNANNNNNTCNTGNGANGTCGCCGNNATTNNNGTGGTCNNN
CNAGANTGNNCNNACCANNCGNANNNTGGTNATGNNAGTNNNNNNNNNNNNNNNNNNNNNNNNNNNNNN
NNNNNNNNNNNNNNNNNNNNNNNNNNNNNNNNNNNNGNNNTGNNNNNNCNANNNAAGGNNNTANNNTTNNTN
NNNNNNNNGGACNNNACNNNNCNNNNNNNACNCNNNCNCNNNNNNNNNNNGNTTTTNNNNNCNNTANTNT
CCAGNANGCNGNGCNNNCATTCANGNGAACNNNGNNACGCGNNAATCGTANNATCTACCTGCCGCNNG

NNNNNNTNCNGNNNNNNCGGANNNTNNCTCGTANNNNNNNCANGCGTNNTNTNNTTNCNAGNCNNGTT
CGNNNANANANTAGNANGTNTNNNNNNNNNTGTNNCGTTCCGCNTGANNCNNCANNNNGNNGNNCTTN
NCANCGGCNCTANCTGCNGANNTAAGNCATANNNTNNNTCNNTANCGNNNNNGGNGGGNNACTGTAAA
NGGNNAATTNGNNGNTNANTNCNNNCACNTNNCNNNGNNNNCTNNNCNCCTGNNTNCNNAATTGGNNC
CANTGNNTGNCNATCTCTNGNTTNGNNNNNNTNNNNNTNNTANNNNGGNNNNTNNNANNNCNATNTAA
NNTANANNNNCNNNNNNANNNCACNNNNNNNNNAGNNNNNANNNTTANGNNNGTAGNNNANTNAGTCT
CCGANNTNNNNNNNATAAGCGNNANACNNNNNNNNNNNNNNANGANNNCGNNTTGGNNNTNNNNNNCNA
ANNAGACNNCNGTGGNTNNNNNTCAANNNNTTNGTAGTGGTATAACNANNNNNNNTTTCNCCAGNNTN
NNNGNNNNNNNCNATANNANNNNNNNGNGANNNNNNNNNNNANNNNCCCNNNAGGNANCANCTACTNNA
GCANTGGNGCGCAGNNNGAGANNNNGACNNGCCTTGGGTGGCAANNACNGCAATAAGTNNNGTTNANC
CCNNNAGTNNACACNNNNNCANNGNNNCNNNNNNANCNGANNTNNNNNNNNNNNNNNNNNNNNNNNNN
NNNNNNNNNNNNNNNNNNNNNNNNNNNNNAGNNNANGGNNNGTNTNTGACNCATGTGTTTTTANNNGNN
NNNNNACGNNNNANCCCCGGTCGNNNNCNNATTGAANNCCTGNNGCATNNNNNTNCNAGAATGANNNC
NNNNNGNNTNGNCTNNCNNNTNAAGNAGTCNNNTAGNNNNNNNNNTCNNNGNNNNNNNNCNNTCNCNN
ACTGAGGGTNNNNNNNNCANCNGCTNNNNNNNNNTNCGCNGNNNNNNTNNNNNNGCNGTATNGNGTNN
NNCANNNNNNNNNNNNNNNNNTNAAACNANNNNNNTNNNN
>HMong206
GTGNNNNNTAGCTCNNNNNAGTTAGACANNNCNNNNNNNNNNNNTNNNNNNNNNNNNNNNNNNNNNNN
NNNNNNNNNNNNNNNNNNNNNNNNNNNNNNNTNNNNNNNNNNNNNNNNNNNNNNNNNNNNNANNNNNNNNN
NNNNNNNNNNNNNNNNNNNNNNNNNNNNNNNNNNNNNNNNNNNNNNNNNNNNNNNNNNNNNNNNNNNNNN
NNNNNNCNNNNNNNNNNNNNNNNNNNNNNNNNNNNNNNNNNNNNNNNNNNNNNNNNNNNNNNNNNNNNNN
NNNNNNNNNNNNNNNNNNNNNNNNNNNNNNNNNNNNNNNNNNNNNNNNNNNNNCNTCTNNNAAANNNTNNN
NNNNNNNNNNNNNNNNNNNNNNNNNNCNCNTTGNNNGTAANCNANNNGNACATGCAGTCCCCTCNNNNN
NNNGGCAGAGNNNNNNCCNNNNNNNNCCGGCCCCNNCCNNNNNNNNNNNNNNNNNGNNNNNNNGNNNNNN
NNNNNNTGGTACNNNNCGNNNNNGGTTGTTTAAGAGGTCCACCACGNGGTCNGGNNACNNNCGNNNAN
NNNCNNNNAACNNNNNNNNNNNNANNTNNNCANNAGGNCNNNNCCNTCNNGNNNNNNANTGCNATGACC
CANNTCGCGGGGACNGTCACCGGCGNGGCGACCTTGANNANGGTCGGGGNCAAGTATGTCNNNCNCCC
CGGGAAGGANNCNGCCGTANGTGCNNNNNANNNNNTCNTGNGANGTNGCCGNNATTCNCNTGGTCNNN
CNNGAATGNCCNNACCANNCGNANNNTGGTNATGNNAGTCNNNNNNNNNNNNNNNNNNNNNNNNNNNN
NNNNNNNNNNNNNNNNNNNNNNNNNNNNNNNNNNNNGNNNNGNNNNNNCNANNNAAGGNNNTANNNTTNNTN
NTNNNNNNNGGACNNNACNNNNCNNNNNNNNACNCNNNCNCNNNNNNANNNGNTTTTTNNNCNNTANTNT
CCAGNANGCNGNGCNNNCATTCANGNGAACNNNGNNACGCGNNAATCGTANNATCTACCTGCCGCNNG
NNNNNNTACNGNNNNNNNCGGANNNTNNNNCGTANNNNNNNCANGCGTNNTNTNNTTNCNAGNCNNGTT
CGNNNANANANTAGNANGTNTNNNNNNNNNTGTNNCGTTCCGCNTGANNCNNCANNNNGNNGNNNTTN
TCANCGGCNNTANCTNCNGANNTAAGNCATANNNTNNNTCNNTANCGNNNNNGGNGGGNNACTGTAAA
NGGNNAATTNGNNNNTNANTNCNNNCACNTNNCNNNGNNNNCTNNNCNCCTGNNTNCNNAATTGGNNC
CANTGNNTGNCNATCTCTGGNTTNGNNNNNNTGNNNNTNNTANNNNGGNNNGTNANANNNCNATNTAA
NNTANANNNNCNNNNNNANNNCACNNNNNNNGNAGNNNCNANNNTTANGNNNGTAGNNNANTAAGTCT
CCGANNTNNNNNNNATAAGCGNNANACNNNNNNNNNNNNNNANGANNACGNNTTGGNNNTNNNNNNCNA
ANNAGACNNCNGTGGNTNNNNNTCAANNNNTTNGTAGTGGTATAACNANNNNNNNTTTCNCCAGNNTN
NNNGNNNNNNNCNATANNANNNGNNNGNGANNNNNNNNNNNANNNNCCCNNNAGGGANCANCTACTTNA
GCANNGGNGCGCAGNNNGAGANNNNGACNNNCCTTGGGTGGCAANCACNGCAATAAGTNNNGTTNNNN
CCNNNAGTNNACACNNNNNCANNGNNNCNNNNNNANCNGANNTNNNNNNNNNNNNNNNNNNNNNNNNN
NNNNNNNNNNNNNNNNNNNNNNNNNNNNNNNNNNNNANNGNNNGTNTNTGACNCATGTGTTTTTANNNGNN
NNNNNACGNNNNANCCCCGGTCGNNNNCNNATTGAANNCCTGNNGCATNNNNNTNCNAGAATGANNGC
NNNNTGNNTNGNCTNNCNNNTCAAGNAGTCANNTAGNNNNNNNNNTCNNNGNNNNNNNNCNNTCCCNN
ACTGAGGGTNNNNNNNNCATCTGCTNNNNNNNNNTNCGCNGNNNNNNTNNNNNNGCNGTATNGNGTNN
NNCANNNNNNNNNNNNNNNNNTNAAACGANNNNNNTNNNN
>HMong207
GTGNNNNNTAGCCCNNNNNAGTTAGACANNNCNNNNNNNNNNNNTNNNNNNNNNNNNNNNNNNNNNNN
NNNNNNNNNNNNNNNNNNNNNNNNNNNNNNNNNNNNNNNNNNNNNNNNNNNNNNNNNNNNNNNNNNNNNN
NNNNNNNNNNNNNNNNNNNNNNNNNNNNNNNNNNNNNNNNNNNNNNNNNNNNNNNNNNNNNNNNNNNNNN
NNNNNNNNNNNNNNNNNNNNNNNNNNNNNNNNNNNNNNNNNNNNNNNNNNNNNNNNNNNNNNNNNNNNNN
NNNNNNNNNNNNNNNNNNNNNNNNNNNNNNNNNNNNNNNNNNNNNNNNNNNNNNTCTNNNAAANNNTNNN

NNNNNNNNNNNNNNNNNNNNNNNNNNCNCNNTNNNNGTAANCNANNNGNACATGCAGTCCCCTCNNNNN
NNNGGCAGAGNNNNGNCCCNNNNNNNCCGGCCCCNNCCNNNNNNNNNNNNNNNNGNNNNNNNANNNNNN
NNNNNNTGGTACNNNNCGNNNNNGGTTGTTTNAGAGGTCCACAACGNGGTCNNGNCACNNNNGNNNAN
NNNCNNNNNNCNNNNNNNNNNNNANNTNNNTANNAGGNNNNNNCCNTCNNNNNNNTAATTGCNATGACC
CAANNCACGGGGGCNGTNCCCGGCGNGGCGACCTTGANNANNTCCGGGGNCAAGTATGGCGNNCNCCC
CGGGAAGGANNCNGCNGTANGTGCNNNNNANNNNNTCNTGNGANGTCGCCGNNATTCNNGTGGTCNNN
CNAGAATGNCCNNACCANNCGNANNNTGGTNATGNTAGTNNNNNNNNNNNNNNNNNNNNNNNNNNNNN
NNNNNNNNNNNNNNNNNNNNNNNNNNNNNNNNGNNNNGNNNNNNCNANNNAAGGNNNTANNNCTNNTN
NTNNNNNNGGACNNNACNNNNCNNNNNNNACNCNNNCNCNNNNNNNNNNGNTTTTTNNCCNNTATTNT
CCAGNACGCNGNGCNNNCATTCANGNGAACNNNGNNACGCGNNAATCGTANNATCTACCTGCCGCNNG
NNNNNNTACNGNNNNNNCGGANNNTNNNNCGTANNNNNNTCATGCGTNNTNTNNTCNCNAGNCNNGTT
CGNNNANANANTAGNANGTNTNNNNNNNCTTGTNNCGTTCCGCNTGANNCNNCANNNNGNNGNNCTTN
NCANCGGCNNTANCTGCNGANNTAAGNCATANNNTNNATCNNTANCTNNNNNGGNGGGNNACTGTAAA
NGGNTAATTNGNNNNTNANTNCNNNCACNTNNCTNNGNNNNCTNNNNTNCCTGNNTNCNNAATTGGNNC
CANTGNNTGNCNATCTCTGGNTTNGNNNNNNTNNNNNTNNCANNNNGGNNNGTNANANNNCNATNTAA
NNTANANNNNCNNNNNNANNNCACNANNNNNGNAGNNNCNANNNTTANANNNGTAGNNNANTAAGTCT
CCGANNTNNNNNNNATAAGCGNNANACNNNNNNNNNNNNNNANGANNNCGNNTTGGNNNTNNNNNNCNN
GNNAGACNNTNATGGNTNNNNNTCAANNNNTTCGTAGTGGTATAATNGNNNNNNNTTTCNCCAGNNTN
NNNGNNNNNNNCNATANNANNNNNNNGNGANNNNNNNNNNNANNNNCTCNNNAGGGANCANCTACTNNA
GCANTGGNGCGNAGNNNGAGANNNNGACNNGCCTTGGGTGGCAANNACNGCAATAAGTNNNGTTNANC
CTNNNAGTNNACATNNNNNCANNGNNNCNNNNNNANCNGANNGNNNNNNNNNNNNNNNNNNNNNNNNN
NNNNNNNNNNNNNNNNNNNNNNNNNNNNNNAGNNNANGGNNNGTNTNTGACNCATGTGTTTTTANNNGNN
NNNNNACGNNNNANCCCCGGTCGNNNNCNNATTGAANNCCTGNNGCATNNNNNTNCNAGAATGANNNC
NNNNNGNNTNGNCTNNCNNNTNAAGNAATCANNTAGNTNNNNNGNTCNNNGNNNNNNNNCNNTCCCNN
ACTGAGGGTNNNNNNNNCATCNGCTNNNNNNNNNNTNCGCNGGNGTNNCNNNNNNGCNGTATNGNGTNN
NNCANNNNNNNNNNNNNNNNNNCNAAACNNNNNNNNNTNNNN
>HMong208
GTGNNNNNTAGCTCNNNNNAGTTAGACANNNCNNNNNNNNNNNNNTNNNNNNNNNNNNNNNNNNNNNNNN
NNNNNNNNNNNNNNNNNNNNNNNNNNNNNNNNTNNNNNNNNNNNNNNNNNNNNNNNNNNNNNNNNNNNNN
NNNNNNNNNNNNNNNNNNNNNNNNNNNNNNNNNNNNNNNNNNNNNNNNNNNNNNNNNNNNNNNNNNNNNN
NNNNNNNNNNNNNNNNNNNNNNNNNNNNNNNNNNNNNNNNNNNNNNNNNNNNNNNNNNNNNNNNNNNNNN
NNNNNNNNNNNNNNNNNNNNNNNNNNNNNNNNNNNNNNNNNNNNNNNNNNNNNNNNNTCTNNNAAANNNTNNN
NNNNNNNNNNNNNNNNNNNNNNNNNNCNCNNTNNNNGTAANCNANNNGNACATGCAGTCCCCTCNNNNN
NNNNNCAGAGNNNNNNNCNNNNNNCNCCGGCCCCNNCCNNNNNNNNNNNNNNNNGNNNNNNNGNNNNNN
NNNNNNTGGTACNNNNCGNNNNNGGTTGNTTAAGAGGTCCACCACGNGGTCNGGNCACNNNNGNNNAN
NNNCNNNNAACNNNNNNNNNNNNNANNTNNNCANNAGGNCNNNNCCNTCNNGNNNNNAANTGCNATGACC
CAANTCGCGGGGACNGTNACCGGCGCGGCGACCTTGNNNACGGTCGGGGNCAAGTATGTCNNNCNCCC
CGGGAAGGANNCNGCCGTANGTGCNNNNNANNNNNTCNTGNGANGTCGCCGNNATTNNCGTGGTCNNN
CNAGAATGNCCGNACCANNCGNANNNTGGTNATGNNAGTNNNNNNNNNNNNNNNNNNNNNNNNNNNNN
NNNNNNNNNNNNNNNNNNNNNNNNNNNNNNNNGNNNNGNNNNNNCNANNNAAGGNNNTANNNTTNNTN
NTNNNNNNGGACNNNACNNNNCNNNNNNNACNCNNNCNCNNNNNNANNNGNTTTTNNNNCNNTATTNT
CCAGNACGCNGNGCNNNCNTTCANGNGAACNNNGNNACGCGNNAATCGTANNATCTACCTGCCGCNNG
TNNNNNTACNGNNNNNNCGGANNNTNNCTCGTANNNNNNNCATGCGTNNTNTNNTTNCNAGNCANGTT
CGNNNANANANTAGNANGTNTNNNNNNNNNNTGTNNCGTTCCGCNTGANNCNNCANNNNGNNGNNNTTN
TCANCGGCNNTANCTGCNGANNTAAGNCATANNNTNNGTCNNTANCGNNNNNGGNGGGNNACTGTAAA
NGGNNAATTNGNNNNTNATTNCNNNCACNTNNCTNNGNNNNCTNNNCNCCTGNNTNCNNAATTGGNNC
CANTGNNTGNCNATCTCTGGNTTNGNNNNNNTNNNNNTNNTANNNNGGNNNGNNANANNNCNATNTAA
NNTATANNNNCNNNNNNANNNCACNNNNNNNGNAGNNNCNANNNTNANGNNNGTAGNNNANTAAGTCT
CCGANNTNNNNNNNATAAGCGNNANACNNNNNNNNNNNNNNANGANNNCGNNTTGGNNNTNNNNNNCNA
ANNAGACNNCNGTGGNTNNNNNTCAANNNNTTNGTAGTGGTATAACNANNNNNNNTTTCNCCAGNNTN
NGNGNNNNNNNCNATANNANNNNNNNGNGANNNNNNNNNNNANNNNCCCNNNAGGGANCANCTACTNNA
GCAATGGNGCGCAGNNNGAGANNNNGACNNGCCTTGGGTGGCAANCACNGCAATAAGTNNNGTTNANC
CCNNNAGTNNACACNNNNNCANNGNNNCNNNNNNANCNGANNTNNNNNNNNNNNNNNNNNNNNNNNNN
NNNNNNNNNNNNNNNNNNNNNNNNNNNNNAGNNNANGGNNNGTNTATGACNCATGTGTTTTTANNNGNN

NNNNNNNGNNNGANCCCCGGTCGNNNNCNNATTGAANNCCTGNNGCATNNNNNTNCNAGAATGANNGC
NNNNNGNNTNGNCTNNCNNNTCAAGNAGTCANNNAGNNNNNNNGNTCNNNGNNNNNNNNCNNNCCCNN
ACTGAGGGTNNNNNNNNCATCTGCTNNNNNNNNCTNCGCNGNNNNNNTNNNNNNGCNGTATNGNGTNN
NNCANNNNNNNNNNNNNNNNNTNAAACAANNNNNNNTNNNN
>HMong209
GTGNNNNNNAGCCCNNNNNAGTTANACANNNCNNNNNNNNNNNTTNNNNNNNNNNNNNNNNNNNNNNN
NNNNNNNNNNNNNNNNNNNNNNNNNNNNNNNNNNNNNNNNNNNNNNNNNNNNNNNNNNNNANNNNNNNN
NNNNNNNNNNNNNNNNNNNNNNNNNNNNNNNNNNNNNNNNNNNNNNNNNNNNNNNNNNNNNNNNNNNN
NNNNNNNNNNNNNNNNNNNNNNNNNNNNNNNNNNNNNNNNNNNNNNNNNNNNNNNNNNNNNNNNNNNN
NNNNNNNNNNNNNNNNNNNNNNNNNNNNNNNNNNNNNNNNNNNNNNNNNNNNNTCTNNNAAANNNTNNN
NNNNNNNNNNNNNNNNNNNNNNNNNNCNCNNTNNNNGTAANCNANNNGNACATGCAGTCCCCTCNNNNN
NNNNNCAGAGNNNNNNNCNNNNNNCNCCGNCCCCNNCCNNNNNNNNNNNNNNNNNNNNNNNNANNNNNN
NNNNNNTGGTACNNNNCGNNNNNGGNTGTNNNAGAGGTCCACAACGNGGTCNNGNNACNNNNGNNNAN
NNNCNNNNANCNNNNNNNNNNNNNNATNNNTANNAGGNCNNNNCCNTNNNNNNNNNNNANNGCNATGACC
CAANNCNCGGNGGNNGTNCCCGGNGNGGNGACCTTGANNACGTCNGGGGNCAAGTATGGCNNNCNCCC
CGGGAAGGANNCNGCCGTANGTGCNNNNNANNNNNTCNTGNGANGTNGCCGNNATTNNCNTGGTCNNN
NNAGANTGNNCNNACCANNCGNANNNNGGTNATGNNAGTNNNNNNNNNNNNNNNNNNNNNNNNNNNNN
NNNNNNNNNNNNNNNNNNNNNNNNNNNNNNNNNNGNNNNGNNNNNNCNANNNANGGNNNTANNNCTNNTN
NTNNNNNNNGGNCNNNACNNNNCNNNNNNNACNCNNNCNCNNNNNNNNNNNGNTTTTNNNNCNNTNNTNT
CNAGNANGCNGNGCNNNCATTCANGNGAACNNNGNNACGCGNNAATCGTANNATCTACCTGCCGCNNG
NNNNNNTNCNGNNNNNNCGGANNNTNNNNNGTANNNNNNNCANGCGTNNTNTNNTCNCNAGNCNNGTT
CGNNNANANANTAGNANGTNTNNNNNNNNNNTGTNNCGTTCCGCNTGANNCNNCANNNNGNNGNNCTTN
NCANCGGCNNTANCTGCNGANNTAAGNCATANNNTNNNTCNNTANCTNNNNNGNNGGGNNACTGTAAA
NGNNNAATTNGNNGNTNANTNCNNNNACNNNNCTNNGNNTNCTNNNTNCCTGNNTNCNNAATTGGNNN
CANTGNNTGNCNATCTCTNGNTTNGNNNNNNTNNNNNTNNCANNNNGGNNNNNNNNANNNCNATNTAA
NNTANANNNNCNNNNNNANNNCACNNNNNNNNNAGNNNNNANNNTNANANNNGTAGNNNAATAAGTCT
CCGANNTNNNNNNNATAAGCGNNANACNNNNNNNNNNNNNANGANNNCGNNTTGGNNNTNNNNNNCNA
GNNAGACNNTNATGGNTNNNNNTCAANNNNTTNGTAGTGGTATAATNGNNNNNNNTTTCNCCAGNNNN
NNNGNNNNNNNCNATANNANNNNNNNGNGANNNNNNNNNNANNNNCTCNNNAGGNANCANCTACTNNA
GCANNGGNNCGNAGNNNGAGANNNNGACNNNCCTTGGGTGNNAANNACNGNANTAAGTNNGTTNNNN
NTNNNAGTNNACATNNNNNCANNGNNNCNNNNNNANCNGANNGNNNNNNNNNNNNNNNNNNNNNNNNN
NNNNNNNNNNNNNNNNNNNNNNNNNNNNNNNNNNNANNGNNNGTNTNTGACNCNTGTNTTTNTANNNGNN
NNNNNNCGNNNNANCCCCGGTCGNNNNCNNATTGAANNCCTGNNGCATNNNNNTNCNANAATGANNNC
NNNNNGNNTNGNCTNNCNNNTCAAGNAATCNNNNAGNNNNNNNNNTCNNNNNNNNNNNNCNNNCNCNN
ACTGAGGGTNNNNNNNNCATCTGCTNNNNNNNNNNTNCGCNGGNNNNNCNNNNNNGCNGTATNNNGNNN
NNCANNNNNNNNNNNNNNNNNTNAAACAANNNNNNNTNNNN
>HMong210
GTGNNNNNNAGCTCNNNNNAGTTAGACANNNCNNNNNNNNNNNNNTNNNNNNNNNNNNNNNNNNNNNN
NNNNNNNNNNNNNNNNNNNNNNNNNNNNNNNNNNNNNNNNNNNNNNNNNNNNNNNNNNNNNNNNNNNN
NNNNNNNNNNNNNNNNNNNNNNNNNNNNNNNNNNNNNNNNNNNNNNNNNNNNNNNNNNNNNNNNNNNN
NNNNNNNNNNNNNNNNNNNNNNNNNNNNNNNNNNNNNNNNNNNNNNNNNNNNNNNNNNNNNNNNNNNN
NNNNNNNNNNNNNNNNNNNNNNNNNNNNNNNNNNNNNNNNNNNNNNNNNNNNNNCTNNNAAANNNTNNN
NNNNNNNNNNNNNNNNNNNNNNNNNNNNCNNTNNNNGTAANCNANNNGNACATGCAGTCCCCTCNNNNN
NNNNNCAGAGNNNNNNNCCNNNNNCNCCGNCCCCNNCCNNNNNNNNNNNCNNNGNNNNNNNGNNNNNN
NNNNNNTGGTACNNNNCGNNNNNGGTTGNNNAAGAGGTCCACCACGNGGTCNNGNNACNNNNGNNNAN
NNNCNNNNAANNNNNNNNNNNNNANATNNNCANNAGGNCNNNNCCNTCNNGNNANNNATTGCNATGANN
CAANNCNCNGNGANNGTNACCGGCGNGGCGACCTTGANNANGGTCGGGNNCAAGTATGTCNNNCNCCC
CGGGAAGGANNCNGCNGTANGTGCNNNNNANNNNNTCNTGNGANNTCGCCGNNATTNNNGTGGTCNNN
NNAGANTGNNCNNACCANNCGNANNNTGGTNATGNNAGTNNNNNNNNNNNNNNNNNNNNNNNNNNNNN
NNNNNNNNNNNNNNNNNNNNNNNNNNNNNNNNNNGNNNGGNNNNNNCNANNNAAGGNNNTANNNTTNNTN
NNNNNNNNGGACNNNACNNNNCNANNNNNACNCNNNCNCNNNNNNNNNNNGGTTNNNNNNCNNTATTNT
CCAGNANGCNGNGCNNNCNTTCANGNGAACNNNGNNACGCGNNAATCGTANNATCTACCTGCCGCNNG
NNNNNNTNCNGNNNNNNCGGANNNTNNNNCGTANNNNNNNCANGCGTNNNNTNNTTNCNAGNCNNGTT
CGNNNANANANTANNANGTNTNNNNNNNNNNTGTNNCGTTCCGCNTGANNCNNCANNNNGNNGNNNTTN

NCANCGGCNNTANCTGCNGANNTAAGNCATANNNTNNNTCNNTANCGNNNNNGGNGGGNNACTGTAAA
NGGNNAATTNGNNNNTNANTNCNNNCACNTNNCNNNGNNNNCTNNNCNCCTGNNTNCNNAATTGGNNC
CANTGNNTGNCNATCTCTNGNTTNGNNNNNNTNNNNNTNNTANNNNGGNNNNNNNNANNNCNATNTAA
NNTANANNNNCNANNNNANNNCACNNNNNNNGNAGNNNCNANNNTNANGNNNGTAGNNNAATNAGTCT
CCGANNTNNNNNNNATAAGCGNNANACNNNNNNNNNNNNNNANGANNNCGNNTTGGNNNTNNNNNNNNA
ANNAGACNNCNGTGGNTNNNNNTCAANNNNTTNGTAGTGGTATAACNANNNNNNNTTTCNCCAGNNNN
NNNGNNNNNNNCNATANNANNNNNNNGNGANNNNNNNNNNNANNNNCCCNNNAGGNANCANCTACTNNA
GCANNGGNGCGNAGNNNGAGANNNNNACNNNCCTTGGGTGGCAANNACNGNAATAAGTNNNGTTNNNN
CCNNNAGTNNACACNNNNNCANNGNNNCNNNNNNANCNGANNTNNNNNNNNNNNNNNNNNNNNNNNNN
NNNNNNNNNNNNNNNNNNNNNNNNNNNNNNANNNNANNGNNNGTNTNTGACNCNTNNNNTTTNTANNNGNN
NNNNNNNGNNNNANCCCCGGTCGNNNNCNNATTGAANNCCTGNNGCATNNNNNTNCNAGAATGANNNC
NNNNNGNNTNGNCTNNCNNNTCANGNAGTCANNNAGNNNNNNNNNTCNNNNNNNNNNNNCNNTCCCNN
ACTGAGGGNNNNNNNNNCATCTGCTNNNNNNNNNTNCGCNGNNNNNNTNNNNNAGCNGTATNNNGNNN
NNCANNNNNNNNNNNNNNNNNTNAAAAAANNNNNNTNNNN
>HMong211
GTGNNNNNTAGCTCNNNNNAGTTAGACANNNCNNNNNNNNNNNNNTNNNNNNNNNNNNNNNNNNNNNN
NNNNNNNNNNNNNNNNNNNNNNNNNNNNNNNTNNNNNNNNNNNNNNNNNNNNNNNNNNNNNNNNNNNNN
NNNNNNNNNNNNNNNNNNNNNNNNNNNNNNNNNNNNNNNNNNNNNNNNNNNNNNNNNNNNNNNNNNNN
NNNNNNNNNNNNNNNNNNNNNNNNNNNNNNNNNNNNNNNNNNNNNNNNNNNNNNNNNNNNNNNNNNNN
NNNNNNNNNNNNNNNNNNNNNNNNNNNNNNNNNNNNNNNNNNNNNNNNNNNNNNCNTCTNNNAAANNNTNNN
NNNNNNNNNNNNNNNNNNNNNNNNNNNNNCNCNTTNNNNGTAACCNANNNGNACATGCAGTCCCCTCNNNNN
NNNNNCAGAGNNNNNNNCCNNNNNNNCCGGCCCCNNCCNNNNNNNNNNNNNNNNNGNNNNNNNGNNNNNN
NNNNNNTGGTACNNNNCGNNNNNGGTTGTNTNAGAGGTCCACCACGNGGTCNGGNCACNNNNGNNTAN
NNNCNNNNANCNNNNNNNNNNNNANATNNNCANNAGGNNNNNNCCNTCNNNNNNNNTNATTGCNATGACC
CAANNCGCGGNGANAGTNACCGGCGCGGNGACCTTGNNNANGGTCGGGGACAAGTATGTCGNNCNCCC
CGGGAAGGANNCNGCCGTANGTGCNNNNNANNNNNTCNTGNGANGTCGCCGNNATTCNCNTGGNCNNN
CNAGAATGNCCNNACCANNCGNANNNTGGTNATGNNAGTNNNNNNNNNNNNNNNNNNNNNNNNNNNN
NNNNNNNNNNNNNNNNNNNNNNNNNNNNNNNNNNNGNNNNGNNNNNNCNANNNAAGGNNNTANNNTTNNTN
NTNNNNNNNGGACNNNACNNNNCNNNNNNNACNCNNNCNCNNNNNNANNNGNTTTTTNNNCNNTANTNT
CCAGNACGCNGNGCNNNCATTCANGNGAACNNNGNNACGCGNNAATCGTANNATCTACCTGCCGCNNG
TNGNNNTNCNGNNNNTNCGGANNNTNNCTCGTANNNNNNNCATGCGTNNTNTNNTTNCNAGNCNNGTT
CGNNNANANANTAGNANGTNTNNNNNNNNNTGTNNCGTTCCGCNTGANNCNNCANNNNGNNGNNCTTN
TCANCGGCNNTANCTGCNGANNTAAGNCATANNNTNNNTCNNTANCGNNNNNGGNGGGNNACTGTAAA
NGGNNAATTNGNNGNTNANTTCCNNCACNTNNCNNNGNNTNCTNNNCNCCTGNNTNCNNAATTGGNNC
CANTGNNTGNCNATCTCTNGNTTNGNNNNNNTNNNNNTNNTAANNNGGNNNNNNANANNNCNATNTAA
NNTANANNNNCNNNNNNANNNCACNNNNNNNGNAGNNNNNANNNTTANGNNNGTAGNNNNNTAAGTCT
CCGANNTNNNNNNNATAAGCGNNANACNNNNNNNNNNNNNNANGANNACGNNTTGGNNNTNNNNNNCNA
ANNAGACNNCNGTGGNTNNNNNTCAANNNNTTNGTAGTGGTATAACNANNNNNNNTTTCNCCAGNNTN
NNNGNNNNNNNCNATANNANNNNNNNGNGANNNNNNNNNNNANNNNCCCNNNAGGGANCANCTACTNNA
GCANTGGNGCGNAGNNNGAGANNNNGACNNNCCTTGGGTGGCAANCACNGCAATAAGTNNNGTTNANC
CCNNNAGTNNACACNNNNNCANNGNNNCNNNNNNANCNGANNTNNNNNNNNNNNNTNNNNNNNNNNNNN
NNNNNNNNNNNNNNNNNNNNNNNNNNNNNNAGNNNANGGNNNGTNTNTGACNCNTGTGTTTTTANNNGNN
NNNNNACGNNNNANCCCCGGTCGNNNNCNNATTGAANNCCTGNNGCATNNNNNTNCNAGAATGANNNC
NNNNNGNNTNGNCTNNNNNNNTCAAGNAGTCAANNAGGTNNNNNGNTCNNNNNNNNNNNNNCNNTCCCNN
ACTGAGGGTNNNNNNNNNCATCTGCTNNNNNNNNNNTNCGCNGNNNNNNTNNNNNAGCNGTATNGNGTNN
NNCANNNNNNNNNNNNNNNNNTNAAACNANNNNNNTTNNNN
>HMong212
GTGNNNNNTAGCCCNNNNNAGTTGNACANNNCNNNNNNNNNNNNNTNNNNNNNNNNNNNNNNNNNNNN
NNNNNNNNNNNNNNNNNNNNNNNNNNNNNNNTNNNNNNNNNNNNNNNNNNNNNNNNNNNNNNNNNNNNN
NNNNNNNNNNNNNNNNNNNNNNNNNNNNNNNNNNNNNNNNNNNNNNNNNNNNNNNNNNNNNNNNNNNN
NNNNNNNNNNNNNNNNNNNNNNNNNNNNNNNNNNNNNNNNNNNNNNNNNNNNNNNNNNNNNNNNNNNN
NNNNNNNNNNNNNNNNNNNNNNNNNNNNNNNNNNNNNNNNNNNNNNNNNNNNNNNTCNNNNAAANNNTNNN
NNNNNNNNNNNNNNNNNNNNNNNNNNNNNCNCNNTGNNNGTAANCNANNNGNACATGCAGTCCCCTCNNNNN
NNNNNCAGAGNNNNNNCCNNNNNNCNCCGGCCCCNNCCNNNNNNNNNNNNNNNNNNNNNNNNNNGNNNNNN

NNNNNNTGGTACNNNNCGNNNNNGGTTGTNTNAGAGGTCCACCACGNGGTCNNGNCACNNNNGNNNAN
NNNCNNNNAANNNNNNNNNNNNANNTNNNCANAAGGNNNNNNCCNTCNNNNNNNNNATTGCNATGANC
CANNNNNCGGGGANNGTNACCGGCGNGGCGACCTTGNNNACGGTCGGGGNCAAGTATGTCGNNCNCCC
CGGGAAGGANNCNGCCGTANGTGCNNNNNANNNNNTCNTGNGANGTCGCCGNNATTNNNNTGGTCNNN
NNNGANTGNCCNNACCANNCGNANNNNGGTNATGNNAGTNNNNNNNNNNNNNNNNNNNNNNNNNNNNN
NNNNNNNNNNNNNNNNNNNNNNNNNNNNNNNNGNNNNGNNNNNNCNANNNANGGNNNTANNNTTNNTN
NTNNNNNNGGACNNNACNNNNCNNNNNNNACNCNNNCNCNNNNNNNNNNGGTTTTNNNNCNNTNNTNT
CCAGNACGCNGNGCNNNCNTTCANGNGAACNNNGNNACGCGNNAATCGTANNATCTACCTGCCGCNNG
TNNNNNTNCNGNNNNNNCGGANNNTNNCTNGTANNNNNNNCATGCGTNNTNTNNTTNCNAGNCNNGTT
CGNNNANANANTAGNANGTNTNNNNNNNNNNTGTNNCGTTCCGCNTGANNCNNCANNNNGNCGNNCTTN
NCANCGGCNNTANCTGCNGANNTAAGNCATANNNTNNNTCNNTANCGNNNNNGGAGGGNNACTGTAAA
NGGNNAATTNGNNNNTNANTTCNNNCACNTNNCNNNGNNNNCTNNNCNCCTGNNTNCNNAATTGGNNN
CANTGNNTGNCATCTCTGGNTTNGNNNNNNNTNNNNNTNNTANNNNGGNNNGTNNNANNNCNATNTAA
NNTANANNNNCNNNNNNANNNCACNNNNNNNNNAGNNANNANNNTNANGNNNGTAGNNNANTAAGTCT
CCGANNTNNNNNNATAAGCGNNANACNNNNNNNNNNNNNNANGANNACGNNTTGGNNNTNNNNNNCNA
ANNAGACNNCNGTGGNTNNNNNTCAANNNNTTNGTAGTGGTATAACNANNNNNNNTTTCNCCAGNNTN
NNNGNNNNNNNCNATANNANNNNNNNGNGANNNNNNNNNNANNNNCCCNNNAGGGANCANCTATTNNA
GCANNGGNNCGNAGNNNGAGANNNNGACNNNCCTTGGGTGGCAANNACNGNAATAAGTNNNGTTNNNN
CCNNNAGTNNACACNNNNNCANNGNNNCNNNNNNANCNGANNTNNNNNNNNNNNNNNNNNNNNNNNNN
NNNNNNNNNNNNNNNNNNNNNNNNNNNNNNNNNNANNGNNNGTNTNTGACNCNNNNNTTTTANNNGNN
NNNNNNCGNNNNANCCCCGGTCGNNNNCNNATTGAANNCCTGNNGCATNNNNNTNCNAGAATGANNNC
NNNNNGNNTNGCTNNCNNNTCAAGNAGTCANNNANNTANNNNNNNTCNNNGNNNNNNNNCNNTCCCNN
ACTGAGGGTNNNNNNNNCATNNGCTNNNNNNNNNTNCGCNGNNGTNNTNNCNNNGCNGTATNGNGNNN
NNCANNNNNNNNNNNNNNNNTNAAACAANNNNNTTNNNN
>HMong213
GTGNNNNNTAGCTCNCNNNAGTTAGACANNNCNNNNNNNNNNNNTNNNNNNNNNNNNNNNNNNNNNNN
NNNNNNNNNNNNNNNNNNNNNNNNNNNNNNNNNNNNNNNNNNNNNNNNNNNNNNNNNNNNNNNNNNNN
NNNNNNNNNNNNNNNNNNNNNNNNNNNNNNNNNNNNNNNNNNNNNNNNNNNNNNNNNNNNNNNNNNNN
NNNNNNNNNNNNNNNNNNNNNNNNNNNNNNNNNNNNNNNNNNNNNNNNNNNNNNNNNNNNNNNNNNNN
NNNNNNNNNNNNNNNNNNNNNNNNNNNNNNNNNNNNNNNNNNNNNNNNNNNTCTNNNAAANNNTNNNN
NNNNNNNNNNNNNNNNNNNNNNNNNCNCNTTNNNNGTAANCNANNNGNACATGCAGTCCCCTCNNNNN
NNNGGCAGAGNNNNNNCCCNNNNNNNNCCGNCCCCNNCCNNNNNNNNNNNNNNGNNNNNNNGNNNNNN
NNNNNNTGGTACNNNNCGNNNNNGGTTGTNTAAGAGGTCCACCACGNGGTCNNNNNACNNNNGNNNAN
NNNCNNNNAACNNNNNNNNNNNANATNNNCANNAGGNCNNNNCCNTCNNNNNANNNATTGCNATGACC
CANNNNNCGGGGACNGTNACCGGCGCGGCGACCTTGANNACNGTCGGGGACAAGTATGTCGNNCNCCC
CGGGAAGGANNCNGCCGTANGTGCNNNNNANNNNNTCNTGNGANGTCGCCGNNATTCNCGTGGTCTNN
NNAGAATGNNCNNACCANNCGNANNNTGGTNATGTNAGTNNNNNNNNNNNNNNNNNNNNNNNNNNNNN
NNNNNNNNNNNNNNNNNNNNNNNNNNNNNNNNGNNNNGNNNNNNCNANNNAAGGNNNTANNNTTNNTN
NNNNNNNNGGNCNNNACNNNNCNNNNNNNACNCNNNCNCNNNNNNNNNNGNTTTNNNNNCNNTANTNT
CCAGNACGCNGNGCNNNCNTTCANGNGAACNNNGNNACGCGNNAATCGTANNATCTACCTGCCGCNNG
TNNNNNTACNGNNNNNNCGGANNNTNNCTNGTANNNNNNNCATGCGTNNTNTNNTNNCNAGNCNNGTT
CGNNNANANANTAGNANGTNTNNNNNNNNNNTGTNNCGTTCCGCNTGANNCNNCANNNNGNNGNNNTTN
TCANCGGCNNTANCTGNNGANNTAAGNCATANNNTNNNTCNNTANCGNNNNNGGNGGGNNACTGTAAA
NGGNNAATTNGNNGNTNATTNCNNNCACNTNNCNNNGNNNNCTNNNCNCCTGNNTNCNNAATTGGNNC
CANTGNNTGNCATCTCTGGTTTNGNNNNNNNTNNNNNTNNTANNNNGGNNNNNNANANNNCNATNTAA
NNTANANNNNCNNNNNNANNNCACNNNNNNNNNAGNNNCNANNNTTANGNNNGTAGNNNANTAAGTCT
CCGANNTNNNNNNATAAGCGNNANACNNNNNNNNNNNNNNANGANNNCGNNTTGGNNNTNNNNNNCNA
ANNAGACNNCNGTGGNTNNNNNTCAANNNNTTNGTAGTGGTATAACNANNNNNNNTTTCNCCAGNNTN
NNNGNNNNNNNCNATANNANNNNNNNGNGANNNNNNNNNNANNNNCCCNNNAGGNANCANCTACTNNA
GCANTGGNGCGNAGNNNGAGANNNNGACNNGCCTTGGGTGGCAANNACNGCAATAAGTNNNGTTNNNN
NCNNNAGTNNACACNNNNNCANNGNNNCNNNNNNANCNGANNTNNNNNNNNNNNNNNNNNNNNNNNNN
NNNNNNNNNNNNNNNNNNNNNNNNNNNNAGNNNANGGNNNGTNTNTGNCNCATGNNTTTNTANNNGNN
NNNNNANGNNNNANCCCCGGTCGNNNNCNNATTGAANNCCTGNNGCATNNNNNTNCNAGAATGANNNC
NNNNNGNNTNGCTNNCNNNTCAAGNAGTCANNNAGGTNNNNNGNTCNNGGNNNNNNNNNCNNNCNCNN

ACTGAGGGTNNNNNNNNCATNNGCTNNNNNNNNNTNCGCNGNNNNNNTNNNNNNGCNGTATNGNGTNN
NNCANNNNNNNNNNNNNNNTNAAACNANNNNNNNTNNNN
>HMong214
GTGNNNNNNAGCTCNNNNNAGTTANACANNNCNNNNNNNNNNNTNNNNNNNNNNNNNNNNNNNNNNNN
NNNNNNNNNNNNNNNNNNNNNNNNNNNNNNNNTNNNNNNNNNNNNNNNNNNNNNNNNNNNNNNNNNNN
NNNNNNNNNNNNNNNNNNNNNNNNNNNNNNNNNNNNNNNNNNNNNNNNNNNNNNNNNNNNNNNNNNNN
NNNNNNNNNNNNNNNNNNNNNNNNNNNNNNNNNNNNNNNNNNNNNNNNNNNNNNNNNNNNNNNNNNNN
NNNNNNNNNNNNNNNNNNNNNNNNNNNNNNNNNNNNNNNNNNNNNNNNNNNNTCTNNNAAANNNTNNN
NNNNNNNNNNNNNNNNNNNNNNNNNCNCNTTNNNNGTAANCNANNNGNACATGCAGTCCCCTCNNNNN
NNNNNCAGAGNNNNNNNCCNNNNNNNCCGNCCCCNNCCNNNNNNNNNNNNNNNNNNNNNNNGNNNNNN
NNNNNNTGGTACNNNNCGNNNNNGGTTGTTTAAGAGGTCCACCACGNGNTCNGGNCACNNNNGNNTAN
NNNCNNNNAANNNNNNNNNNNNANNTNNNCANNAGGNCNNNNCCNTCNNGNNNNNNANNGCNATGANN
CAANTCNCNGGGANAGTNACCGGCGNGGNGACCTTGANNANGGTCGGGGNCAAGTATGTCNNNCNCCC
CGGGAAGGANNCNGCNGTANGTGCNNNNNANNNNNTCNTGNGANGTNGCCGNNATTNNNGTGGTCNNN
CNNGANTGNCCNNACCANNCGNANNNTGGTNATGNNAGTNNNNNNNNNNNNNNNNNNNNNNNNNNNNN
NNNNNNNNNNNNNNNNNNNNNNNNNNNNNNNNGNNNNGNNNNNNCNANNNAAGGNNNTANNNTTNNTN
NTNNNNNNGGACNNNACNNCNCNNNNNNNACNCNNNCNCNNNNNNNNNNGGTTTTNNNNCNNTANTNT
CCAGNACGCNGNGCNNNCATTCANGNGAACNNNGNNACGCGNNAATCGTANNATCTACCTGCCGCNNG
TNNNNNTACNGNNNNNNCGGANNNTNNCTCGTANNNNNNTCANGCGTNNNNTNNTTNCNAGNCNNGTT
CGNNNANANANTAGNANGTNTNNNNNNNNNTGTNNCGTTCCGCNTGANNCNNCANNNNGNNGNNCTTN
TCANCGGCNNTANCTGCNGANNTAAGNCATANNNTNNNTCNNTANCGNNNNNGGNGGGNNACTGTAAA
NGGNNAATTNGNNNNTNANTNCNNNCACNTNNCTNNGNNTNCTNNNCNCCTGNNTNCNNAATTGGNNC
CANTGNNTGNCNATCTCTNGNTTNGNNNNNNTNNNNNTNNTANNNNGGNNNNNNANANNNCNATNTAA
NNTANANNNNCNNNNNNANNNCACNNNNNNNNNAGNNNNNANNNTNANGNNNGTAGNNNAATAAGTCT
CCGANNTNNNNNNNATAAGCGNNANACNNNNNNNNNNNNNANGANNNCGNNTTGGNNNTNNNNNNNNA
ANNAGACNNCNGTGGNTNNNNNTCAANNNNTTNGTAGTGGTATAACNANNNNNNNTTTCNCCAGNNTN
NNNGNNNNNNNCNATANNANNNNNNNGNGANNNNNNNNNNANNNNCCCNNNAGGGANCANCTACTNNA
GCANNGGNGCGNAGNNNGAGANNNNGACNNNCCTTGGGTGGCAANNACNGCAATAAGTNNNGTTNNNN
NCNNNAGTNNACACNNNNNCANNGNNNCNNNNNNANCNGANNTNNNNNNNNNNNNNNNNNNNNNNNNN
NNNNNNNNNNNNNNNNNNNNNNNNNNNNANNNNANGGNNNGTNTNTGACNCATGTGTTTNTANNNGNN
NNNNNNNGNNNNANCCCCGGTCGNNNNCNNATTGAANNCCTGNNGCATNNNNNTNCNAGAATGANNGC
NNNNNGNNTNGNCTNNCNNNTNAAGNAGTCANNNAGGTNNNNNGNTCNNNGNNNNNNNNCNNNCCCNN
ACTGAGGGTNNNNNNNNCATNNGCTNNNNNNNNNTNCGCNGNNNNNNTNNNNNNGCNGTATNGNGNNN
NNCANNNNNNNNNNNNNNNTNAAACNANNNNNNCTNNNN
>HMong215
GTGNNNNNTAGCCCNNNNNAGTCAGACANNNCNNNNNNNNNNNTNNNNNNNNNNNNNNNNNNNNNNNN
NNNNNNNNNNNNNNNNNNNNNNNNNNNNNNNNTNNNNNNNNNNNNNNNNNNNNNNNNNNNNNNNNNNN
NNNNNNNNNNNNNNNNNNNNNNNNNNNNNNNNNNNNNNNNNNNNNNNNNNNNNNNNNNNNNNNNNNNN
NNNNNNNNNNNNNNNNNNNNNNNNNNNNNNNNNNNNNNNNNNNNNNNNNNNNNNNNNNNNNNNNNNNN
NNNNNNNNNNNNNNNNNNNNNNNNNNNNNNNNNNNNNNNNNNNNNNNNNNNNTCTNNNAAANNNTNNN
NNNNNNNNNNNNNNNNNNNNNNNNNCNCNNTGNNNGTAANCNANNNGNACATGCAGTCCCCTCNNNNN
NNNGGCAGAGNNNNNNNCNNNNNNNNCCGNCCCCNNCCNNNNNNNNNNNNNNNGNNNNNNNANNNNNN
NNNNNNTGGTACNNNNCGNNNNNGGTTGTNTNAGAGGTCCACAACGNGGTCNNGNNACNNNNGNNNAN
NNNCNNNNAACNNNNNNNNNNNNNNTNNNTANNAGGNNNNNNCCNTCNNNNNNNNNATTGCNATGANC
CAANNCNCGGGGGCNGTNCCCGGCGNGGCGACCTTGNNNANGTCCGGGGNCAAGTATGGCGNNCNCCC
CGGGAAGGANNCNGCNGTANGTGCNNNNNANNNNNTCNTGNGANGTNGCCGNNATTNNCNTGGTCNNN
NNAGAATGNNCNNACCANNCGNANNNTGGTNATGNNAGTNNNNNNNNNNNNNNNNNNNNNNNNNNNNN
NNNNNNNNNNNNNNNNNNNNNNNNNNNNNNNNGNNNNGNNNNNNCNANNNAAGGNNNTANNNCTNNTN
NTNNNNNNGGNCNNNACNNNNCNNNNNNNACNCNNNCNCNNNNNNNNNNGNTTTNNNNNCNNTANTNT
CCAGNACGCNGNGCNNNCATTTANGNGAACNNNGNNACGCGNNAATCGTANNATCTACCTGCCGCNNG
TNNNNNTNCNGNNNNNNCGGANNNTNNCTCGTANNNNNNTCANGCGTNNTNTNNTNNCNAGNCNNGTT
CGNNNANANANTAGNANGTNTNNNNNNNNNTGTNNCGTTCCGCNTGANNCNNCANNNNGNNGNNNTTN
NCANCGGCNNTANCTNCNGANNTAAGNCATANNNTNNNTCNNTANCTNNNNNGGNGGGNNACTGTAAA
NGGNNAATTNGNNNNTNANTNCNNNCATNTNNCTNNGNNTNCTNNNTNCCTGNNTNCNNAATTGGNNC

CANTGNNTGNCNATCTCTGGNTTNGNNNNNNTNNNNNTNNCAANNNGGNNNNNNNNANNNCNATNTAA
NNTANANNNNCNNNNNNANNNCACNANNNNNGNAGNNNCNANNNGNANANNNGTAGNNNGNTAAGTCN
CCGANNTNNNNNNNATAAGCGNNANACNNNNNNNNNNNNNANGANNNCGNNTTTGNNNTNNNNNNCNA
GNNAGACNNTNGTGGNTNNNNNTCAANNNNTTNGTAGTGGTATAATNGNNNNNNNTTTCNCCAGNNTN
NNNGNNNNNNNCNATANNANNNNNNNGNGANNNNNNNNNNNANNNNCTCNNNAGGNANCANCTACTNNA
GCAANGGNGCGNAGNNNGAGANNNNGACNNNCCTTGGGTGGCAANNACNGCAATAAGTNNNGTTNANC
CTNNNAGTNNACACNNNNNCANNGNNNCNNNNNNANCNGANNGNNNNNNNNNNNNNNNNNNNNNNNNNN
NNNNNNNNNNNNNNNNNNNNNNNNNNNNNAGNNNANNGNNNGTNTNTGACNCNTNNNNTTNNTANNNGNN
NNNNNACGNNNNANCCCCGGTCGNNNNCNNATTGAANNCCTGNNGCATNNNNNTNCNAGAATGANNGC
NNNNNGNNTNGNCTNNCNNNTCAAGNAATCANNNAGGTANNNNNNTCNNNGNNNNNNNNCNNNCCCNN
ACTGAGGGTNNNNNNNNCATCTGCTNNNNNNNNNNTNCGCNGGNGTNNCNNNNNNGCNGTATNGNGTNN
NNCANNNNNNNNNNNNNNNNNTNANNNGANNNNNNTNNNG
>HMong216
GTGNNNNNNAGCTCNNNNNAGTTAGACANNNCNNNNNNNNNNNNNTNNNNNNNNNNNNNNNNNNNNNNNN
NNNNNNNNNNNNNNNNNNNNNNNNNNNNNNNNNNNNNNNNNNNNNNNNNNNNNNNNNNNNNNNNNNNNNN
NNNNNNNNNNNNNNNNNNNNNNNNNNNNNNNNNNNNNNNNNNNNNNNNNNNNNNNNNNNNNNNNNNNNNN
NNNNNNNNNNNNNNNNNNNNNNNNNNNNNNNNNNNNNNNNNNNNNNNNNNNNNNNNNNNNNNNNNNNNNN
NNNNNNNNNNNNNNNNNNNNNNNNNNNNNNNNNNNNNNNNNNNNNNNNNNNNNNNNNTCTNNNAAANNNTNNN
NNNNNNNNNNNNNNNNNNNNNNNNNNNCNCNTTGNNNGTAANCNANNNGNACATGCAGTCCCCTCNNNNN
NNNGGCAGAGNNNNNNNNCNNNNNNNCCGNCCCCNNCCNNNNNNNNNNNNNNNNNNNNNNNNNNGNNNNNN
NNNNCNTGGTACNNNNCGNNNNNGGTTGTNTAAGAGGTCCACCACGNGGTCNGGNNACNNNNGNNNAN
NNNCNNNNANCNNNNNNNNNNNNANNTNNNCANNAGGNNNNNNCCNTCNNGNNNNNNATTGCNATGACC
CANNTCGCGGGGANNGTCACCGGCGNGGCGACCTTGNTNACGGTCGGGGNCAAGTATGTCGNNCNCCC
CGGGAAGGANNCNGCCGTANGTGCNNNNNANNNNNTCNTGNGANGTCGCCGNNATTNNCNTGGTCNNN
CNNGAATGNNCNNACCANNCGNANNNTGGTNATGNNAGTNNNNNNNNNNNNNNNNNNNNNNNNNNNNNN
NNNNNNNNNNNNNNNNNNNNNNNNNNNNNNNNNNNGNNNNGNNNNNNCNANNNAAGGNNNTANNNTTNNTN
NNNNNNNNGGACNNNACNNNNCNNNNNNNACNCNNNCNCNNNNNNNNNNGNTTTTNNNNCNNTATTNT
CCAGNACGCNGNGCNNNCATTCANGNGAACNNNGNNACGCGNNAATCGTANNATCTACCTGCCGCNNG
NNNNNNTNCNGNNNNNNCGGANNNTNNCTNGTANNNNNNTCANGCGTNNTNTNNTTNCNAGNCANGTT
CGNNNANANANTAGNANGTNTNNNNNNNNNNTGTNNCGTTCCGCNTGANNCNNCNNNNNGNNGNNNTTN
NCANCGGCNNTANCTGCNGANNTAAGNCATANNNTNNNTCNNTANCGNNNNNGGNGGGNNACTGTAAA
NGGNNAATTNGNNNNTNANTNCNNNCACNTNNCTNNGNNTNCTNNNCNCCTGNNTNCNNAATTGGNNC
CANTGNNTGNCNATCTCTNGNTTNGCNNNNNTGNNNNTNNTANNNNGGNNNNNNNNANNNCNATNTAA
NNTANANNNNCNNNNNNANNNCACNNNNNNNGNAGNNNCNANNNTTANGNNNGTAGNNNAATAAGTCN
CCGANNTNNNNNNNATAAGCGNNANACNNNNNNNNNNNNNANGANNNCGNNTTGGNNNTNNNNNNCNA
ANNAGACNNCNGTGGNTNNNNNTCAANNNNTTCGTAGTGGTATAACNANNNNNNNTTTCNCCAGNNTN
NNNGNNNNNNNCNATANNANNNNNNNGNGANNNNNNNNNNNANNNNCCCNNNAGGGANCANCTACTTNA
GCANTGGNGCGNAGNNNGAGANNNNGACNNGCCTTGGGTGGCAANCACNGCAATAAGTNNNGTTNNNN
NCNNNAGTNNACACNNNNNCANNGNNNCNNNNNNANCNGANNTNNNNNNNNNNNNNNNNNNNNNNNNNN
NNNNNNNNNNNNNNNNNNNNNNNNNNNNNANNNNANNGNNNGTNTNTGACNCATGTGTTTTTANNNGNN
NNNNNACGNNNNANCCCCGGTCGNNTNCNNATTGAANNCCTGNNGCATNNNNNTNCNAGAATGANNGC
NNNNNGNNTNGNCTNNCNNNTCAAGNAGTCANNNAGGNNNNGCGNTCNNNNNNNNNNNNCNNTCNCNN
ACTGAGGGTNNNNNNNNCATCTGCTNNNNNNNNNNTNCGCNGNNNNNNTNNNNNAGCNGTATNGNGTNN
NNCANNNNNNNNNNNNNNNNNTNAANNNNNNNNNNNTNNNN
>HMong217
GNGNNNNNNAGCCCNNNNNAGTCAGACANNNCNNNNNNNNNNNNNTNNNNNNNNNNNNNNNNNNNNNNNN
NNNNNNNNNNNNNNNNNNNNNNNNNNNNNNNNNNNNNNNNNNNNNNNNNNNNNNNNNNNNNNNNNNNNNN
NNNNNNNNNNNNNNNNNNNNNNNNNNNNNNNNNNNNNNNNNNNNNNNNNNNNNNNNNNNNNNNNNNNNNN
NNNNNNNNNNNNNNNNNNNNNNNNNNNNNNNNNNNNNNNNNNNNNNNNNNNNNNNNNNNNNNNNNNNNNN
NNNNNNNNNNNNNNNNNNNNNNNNNNNNNNNNNNNNNNNNNNNNNNNNNNNNNNNNNTCTNNNAAANNNTNNN
NNNNNNNNNNNNNNNNNNNNNNNNNNNCNCNNTGNNNGTAANCNANNNGNACATGCAGTCCCCTCNNNNN
NNNGGCAGAGNNNNNNNNNNNNNNNNNNCCGNCCCCNNCCNNNNNNNNNNNNNNNNNNNNNNNNNNANNNNNN
NNNNNNTGGTACNNNNCGNNNNNGGTTGNTTAAGAGGTCCACAACGNGNTCNNGNNACNNNNGNNNAN
NNNCNNNNAANNNNNNNNNNNNNNANATNNNTANNAGGNCNNNNCCNTNNNNNNNNNNAATNGCNATGANN

CANNNCNCNGGGGCNGTNCCCGGNGCGGNGACCTTGNNNACGTNNGGGNNCAAGTATGGCNNNCNCCC
CGGGAAGGANNCNGCNGTANGTGCNNNNNANNNNNTCNTGNGANGTCGCCGNNATTNNNGTGGTCNNN
NNAGANTGNNCNNACCANNCGNANNNTGGTNATGNNAGTNNNNNNNNNNNNNNNNNNNNNNNNNNNNN
NNNNNNNNNNNNNNNNNNNNNNNNNNNNNNNNGNNNNGNNNNNNCNANNNAAGGNNNTANNNCTNNTN
NNNNNNNNGNNCNNNACNNNNCNNNNNNNACNCNNNCNCNNNNNNNNNNGNTTNNNNNCCNNTANTNT
CCAGNACGCNGNGCNNNCNTTTANGNGAACNNNGNNACGCGNNAATCGTANNATCTACCTGCCGCNNG
NNNNNNTNCNGNNNNNNNCGGANNNTNNNNGTANNNNNNNCANGCGTNNNNTNNTNNCNAGNCNNGTT
CGNNNANNNANTAGNANGTNTNNNNNNNNNTGTNNCGTTCCGCNTGANNCNNCANNNNGNNGNNNTTN
NCANCGGCNNTANCTNCNGANNTAAGNCATANNNTNNNTCNNTANCTNNNNNGGNNGGNNACTGTAAA
NGNNNAATTNGNNNNTNANTNCNNNNATNTNNCTNNGNNNNCTNNNTNCCTGNNTNCNNAATTGGNNC
CANTGNNTGNCNATCTCTNGNTTNGNNNNNNTNNNNNTNNCANNNNGGNNNNNNNNANNNCNATNTAA
NNTANANNNNCNNNNNNANNNCACNNNNNNNNNAGNNNNNANNNGNANANNNGTAGNNNNNTNAGTCN
CCGANNTNNNNNNNATAAGCGNNANACNNNNNNNNNNNNNANGANNNCGNNTTTGNNNTNNNNNNNNA
GNNAGACNNTNGTGGNTNNNNNTCAANNNNTTNGTAGTGGTATAATNGNNNNNNNTTTCNCCAGNNTN
NNNNNNNNNNNCNATANNANNNNNNNGNGANNNNNNNNNNANNNNCTCNNNAGGNANCANCTACTNNA
GCANNGGNGCGNAGNNNGAGANNNNGACNNNCCTTGGGTGGCANNNACNGCAATAAGTNNNGTTNANN
NTNNNAGTNNACACNNNNNCANNGNNNCNNNNNNANCNGANNGNNNNNNNNNNNNNNNNNNNNNNNNN
NNNNNNNNNNNNNNNNNNNNNNNNNNNNNNNNNANNGNNNGTNTNTGNCNCNTGTGTTTNTANNNGNN
NNNNNNNGNNNNANCCCCGGTCGNNNNCNNATTGAANNCCTGNNGCATNNNNNTNCNAGAATGANNNC
NNNNNGNNTNGNCTNNNNNNTNAAGNAATCNNNNAGNNNNNNNNNTCNNNGNNNNNNNNCNNTNNCNN
ACTGAGGGTNNNNNNNNCANCNGCTNNNNNNNNNTNCGCNGGNGTNNCNNNNNNGCNGTATNGNGTNN
NNCANNNNNNNNNNNNNNNNTNAAACNANNNNNNNTNNNN
>HMong218
GNGNNNNNTAGCCCNNNNNAGTCNNACANNNCNNNNNNNNNNNNNNNNNNNNNNNNNNNNNNNNNNNN
NNNNNNNNNNNNNNNNNNNNNNNNNNNNNNTNNNNNNNNNNNNNNNNNNNNNNNNNNNNNNNNNNNNN
NNNNNNNNNNNNNNNNNNNNNNNNNNNNNNNNNNNNNNNNNNNNNNNNNNNNNNNNNNNNNNNNNNNN
NNNNNNNNNNNNNNNNNNNNNNNNNNNNNNNNNNNNNNNNNNNNNNNNNNNNNNNNNNNNNNNNNNNN
NNNNNNNNNNNNNNNNNNNNNNNNNNNNNNNNNNNNNNNNNNNNNNNNNNNNTNNNNNAAANNNTNNN
NNNNNNNNNNNNNNNNNNNNNNNNNCNCNTTNNNNGTAANCNANNNGNACATGCAGTNNCCTCNNNNN
NNNNNCAGAGNNNNNNNCCNNNNNNNCCGNCCCCNNCCNNNNNNNNNNNNNNNGNNNNNNANNNNNNN
NNNNNNTGGTACNNNNCGNNNNNGGNNNNNTNAGAGGTCCACNACGNGNTCNNGNNACNNNNGNNNAN
NNNNNNNNAANNNNNNNNNNNNNNNTNNNTANNAGGNNNNNCCNTNNNNNNNNNNNANTGCNATGANN
CNNNNNNCGGNNGNNGTNCCCGGNGNNGNGACCTTGNNNANNTCNGGGNNCAAGTATNGCNNNCNCCC
CGGGAAGGANNCNGCNGTANGTGCNNNNNANNNNNTCNTGNGANGTNGCCGNNANNNNNNTGGTCNNN
CNAGAATGNNCNNACCANNCGNANNNTGGTNATGNNAGTNNNNNNNNNNNNNNNNNNNNNNNNNNNNN
NNNNNNNNNNNNNNNNNNNNNNNNNNNNNNNNGNNNNGNNNNNNCNANNNAAGGNNNTANNNCTNNTN
NTNNNNNNNNNCNNNACNNNNCNNNNNNNACNCNNNCNCNNNNNNNNNNGNTTNNNNNNCNNTNNTNT
CNAGNANGCNGNGCNNNCNTTTANGNGNACNNNGNNACGCGNNANTCGTANNNTCTNNCTGCCGCNNN
NNNNNNTNCNGCNNNNNNCGGANNNTNNNNGTANNNNNNNCNNGCGTNNTNTNNTNNCNAGNCNNGTT
CGNNNANANANTAGNANGTNNNNNNNNNNNTGTNNCGTTCCGCNTGANNCNNCNNNNNGNNGNNNTTN
NCANCGGCNNTANCTNCNGANNTAAGNCANNNNNTNNNTCNNTANCTNNNNNGGNGGGNNACTGTAAA
NGNNNAATTNGNNNNTNANTNCNNNNATNTNNCNNNGNNNNCTNNNTNCCTGNNTNCNNAATTGGNNN
NANTGNNTGNCNATCTCTNGNTTNGNNNNNNTNNNNNTNNCANNNNGGNNNNNNNNANNNCNANNTAA
NNTANANNNNCNNNNNNANNNCACNNNNNNNNNAGNNNCNNNNNGTANANNNGTAGNNNNNTAAGTCN
CCNANNTNNNNNNNATAAGCGNNANACNNNNNNNNNNNNNANGANNNCGNNTTTGNNNTNNNNNNCNN
GNNANACNNTNGTGGNTNNNNNTNNANNNNTTNGTAGTGGTATAANNGNNNNNNNTTTCNCCAGNNNN
NNNGNNNNNNNCNATANNANNNNNNNGNGANNNNNNNNNNANNNNCTCNNNAGGGANCANCTACTNNA
NNANNGGNNCGNAGNNNGAGANNNNNACNNNCCTTGGGTGGCANNNNNNGNAATAAGTNNNGTTNNNN
CTNNNAGTNNACACNNNNNCANNGNNNCNNNNNNANCNGANNGNNNNNNNNNNNNNNNNNNNNNNNNN
NNNNNNNNNNNNNNNNNNNNNNNNNNNNANNNNANNGNNNGTNTNTGNCNNNNNNNTTNNTANNNGNN
NNNNNNNGNNNNNNCCCCGNTCGNNNNCNNATTGAANNCCTGNNGCATNNNNNTNCNAGAATGANNNN
NNNNNGNNTNNNCTNNNNNNTNANGNAATNNNNNAGNNNNNNNNNTCNNNGNNNNNNNNCNNTNNCNN
ACTGAGGGNNNNNNNNNCATNNGCTNNNNNNNNNTNCNCNNGNNNNNCNNNNNNGCNGTATNNNGNNA
NNCANNNNNNNNNNNNNNNNTNAAACAANNNNNNNTNNNN

>HMong219
GTGNNNNNTAGCCCNNNNNAGTTGNACANNNCNNNNNNNNNNNNTNNNNNNNNNNNNNNNNNNNNNN
NNNNNNNNNNNNNNNNNNNNNNNNNNNNNNNNNNNNNNNNNNNNNNNNNNNNNNNNNNNN
NNNNNNNNNNNNNNNNNNNNNNNNNNNNNNNNNNNNNNNNNNNNNNNNNNNNNNNNNNNN
NNNNNNNNNNNNNNNNNNNNNNNNNNNNNNNNNNNNNNNNNNNNNNNNNNNNNNNNNNNN
NNNNNNNNNNNNNNNNNNNNNNNNNNNNNNNNNNNNNNNNNNNNNNNNTCTNNNAAANNNTNNN
NNNNNNNNNNNNNNNNNNNNNNNNNNNCNCNTTNNNNGTAANCNANNNGNACATGCAGTCCCCTCNNNNN
NNNGGCAGAGNNNNNNNNNNNNNNNNCCGNCCCCNNCCNNNNNNNNNNNNNNNNNNNNNNNNNGNNNNNN
NNNNNNTGGTACNNNNCGNNNNNGGTTGTNTNAGAGGTCCACCACGNGNTCNNGNCACNNNNGNNNAN
NNNCNNNNNNCNNNNNNNNNNNNANNTNNNCANNAGGNNNNNNCCNTCNNGNNNNNNANNGCNATNANN
CAANNNGCGGGGACAGTNACCGGNGNGGNGACCTTGNNNANNGTCGGGGNCAAGTATGTCNNNCNCNC
CGGGAAGGANNCNGCCGTANGTGCNNNNNANNNNNTCNTNNGANGTNGCCGNNATTNNCGTGGTCTNN
CNAGAATGNNCNNACCANNCGNANNNTGGTNATGNNAGTNNNNNNNNNNNNNNNNNNNNNNNNNNNNN
NNNNNNNNNNNNNNNNNNNNNNNNNNNNNNNNNNNGNNNNGNNNNNNCNANNNAAGGANNTANNNTTNNTN
NNNNNNNNGGACNNNACNNNNCNNNNNNNACNCNNNCNNNNNNNNANNNGNTTTTTNNNCNNTANTNT
CCAGNACGCNGNGCNNNCNTTCANGNGAACNNNGNNACGCGNNAATCGTANNATCTACCTGCCGCNNG
TNNNNNTACNGNNNNNNCGGANNNTNNCTNGTANNNNNNNCATGCGTNNNNTNNTTNCNAGNCNNGTT
CGNNNANANANTAGNANGTNTNNNNNNNNNNTGTNNCGTTCCGCNTGANNCNNCANNNNGNNGNNNTTN
NCANCGGCNNTANCTGCNGANNTAAGNCATANNNTNNNTCNNTANCGNNNNNGGNGGGNNACTGTAAA
NGGNNAATTNGANNNTNANTNCNNNCACNTNNCNNNGNNNNCTNNNCNCCTGNNTNCNNAATTGGNNN
CANTGNNTGNCNATCTCTGGNTTNGCNNNNNTNNNNNTNNTANNNNGGNNNGTNNNANNNCNATNTAA
NNTANANNNNCNNNNNNANNNCACNNNNNNNNNAGNNNNNANNNTGANGNNNGTAGNNNANTAAGTCT
CCGANNTNNNNNNNATAAGCGNNANACNNNNNNNNNNNNNANGANNACGNNTTGGNNNTNNNNNNCNA
ANNAGACNNCNGTGGNTNNNNNTCAANNNNTTNGTAGTGGTATAACNANNNNNNNTTTCNCCAGNNTN
NNNNNNNNNNNCNATANNANNNNNNNGNGANNNNNNNNNNNANNNNCCCNNNAGGNANCANCTATTNNA
GCANNGGNGCGNAGNNNGAGANNNNGACNNGCCTTGGGTGGCAANNACNGCAATAAGTNNGTTNANC
CCNNNAGTNNACACNNNNNCANNGNNNCNNNNNNANCNGANNTNNNNNNNNNNNNNNNNNNNNNNNN
NNNNNNNNNNNNNNNNNNNNNNNNNNNNNNNNNNNANGGNNNGTNTNTGACNCNTGTGTTTTTANNNGNN
NNNNNACGNNNGNNCCCCGGTCGNNNNCNNATTGAANNCCTGNNGCATNNNNNTNCNAGAATGANNNC
NNNNNGNNTNGNCTNNCNNNTCAAGNAGTCANNNAGNNNNNNCGNTCNNNNNNNNNNNNCNNNCCCNN
ACTGAGGGTNNNNNNNNCATCNGCTNNNNNNNNNNTNCGCNGNCGTNNTNNNNNNGCNGTATNNNGTNN
NNCANNNNNNNNNNNNNNNNNNTNAAACAANNNNNNNTNNNN
>HMong220
GTGNNNNNNAGCTCNNNNNAGTTAGACANNNCNNNNNNNNNNNNTNNNNNNNNNNNNNNNNNNNNNN
NNNNNNNNNNNNNNNNNNNNNNNNNNNNNNTNNNNNNNNNNNNNNNNNNNNNNNNNNNNN
NNNNNNNNNNNNNNNNNNNNNNNNNNNNNNNNNNNNNNNNNNNNNNNNNNNNNNNNNNNN
NNNNNNNNNNNNNNNNNNNNNNNNNNNNNNNNNNNNNNNNNNNNNNNNNNNNNNNNNNNN
NNNNNNNNNNNNNNNNNNNNNNNNNNNNNNNNNNNNNNNNNNNNNNNNCNTCTNNNAAANNNTNNN
NNNNNNNNNNNNNNNNNNNNNNNNNNNNNCNTTNNNNGTAANCNANNNGNACATGCAGTCCCCTCNNNNN
NNNNNCAGANNNNNNNNCCNNNNNNNNNCCGNCCCCNNCCNNNNNNNNNNNNNNNNNNNGNNNNNNNNGNNNNNN
NNNNNNTGGTACNNNNCGNNNNNGGTTGTNTAAGAGGTCCACCACGNGGTCNNGNCACNNNNGNNTAN
NNNCNNANAACNNNNNNNNNNNNNNNNTNNNCANNAGGNCNNNNCCNTCNNNNNNNTAANTGCNATGACC
CANNNCGCGGGGACNGTNACCGGCGNGGCGACCTTGNNNANNGTCGGGGNCAAGTATGTCNNNCNCCC
CGGGAAGGANNCNGCCGTANGTGCNNNNNANNNNNTCNTGNGANGTCGCCGNNATTNNCGTGGTCNNN
NNAGAATGNCCNNACCANNCGNANNNTGGTNATGNNAGTNNNNNNNNNNNNNNNNNNNNNNNNNNNNN
NNNNNNNNNNNNNNNNNNNNNNNNNNNNNNNNNNNGNNCNGNNNNNNCNANNNAAGGNNNTANNGTTNNTN
NTNNNNNNGNNCNNNACNNNNCNNNNNNNACNCNNNCNCNNNNNNNNNNNGNTTTTNNNNCNNTANTNT
CCAGNACGCNGNGCNNNCATTCANGNGAACNNNGNNACGCGNNAATCGTANNATCTACCTGCCGCNNG
NNNNNNTNCNGNNNNNNCGGANNNTNNCTNGTANNNNNNNCATGCGTNNNNTNNTTNCNAGNNNNGTT
CGNNNANANANTAGNANGTNTNNNNNNNNNNTGTNNCGTTCCGCNTGANNCNNNNNNNNGNNGNNCTTN
NCANCGGCNNTANCTGCNGANNTAAGNCATANNNTNNNTCNNTANCGNNNNNGGNGGGNNACTGTAAA
NGGNNAATTNGNNGNTNANTNCNNNCACNTNNCTNNGNNNNCTNNNCNCCTGNNTNCNNAATTGGNNN
CANTGNNTGNCNATCTCTNGNTTNGCNNNNNTNNNNNTNNTANNNNGGNNNNNNNNANNNCNATNTAA
NNTANANNNNCNNNNNNANNNCACNNNNNNNNNAGNNNCNANNNTTANGNNNGTAGNNNANTAAGTCT

CCGANNTNNNNNNNATAAGCGNNANACNNNNNNNNNNNNNNNANGANNACGNNTTGGNNNTNNNNNNCNA
ANNAGACNNCNGTGGNTNNNNNTCAANNNNTTCGTAGTGGTATAACNANNNNNNNTTTCNCCAGNNNN
NNNGNNNNNNNNCNATNNNANNNNNNNGNGANNNNNNNNNNNANNNNCCCNNNAGGGANCANCTACTNNA
GCANNGGNNCGNAGNNNGAGANNNNGACNNNCCTTGGGTGGCAANCACNGNAATAAGTNNNGTTNNNC
CCNNNAGTNNACACNNNNNCANNGNNNCNNNNNNANCNGANNTNNNNNNNNNNNNNNNNNNNNNNNNN
NNNNNNNNNNNNNNNNNNNNNNNNNNNNNNNANNNNANGGNNNGTNTNTGACNCATNNNTTTNTATNNGNN
NNNNNACGNNNGANCCNCGGTCGNNTNCNNATTGAANNCCTGNNGCATNNNNNTNCNAGAATGANNGC
NNNNNGNNTNGNCTNNCNNNTCAAGNAGTCANNNAGNNNNNNNNNTCNNNGNNNNNNNNCNNNCCCNN
ACTGAGGGTNNNNNNNNCATNNGCTNNNNNNNNNTNCGCNGNNNNNNTNNNNNAGCNGTATNNNGTNN
NNCANNNNNNNNNNNNNNNNNTNAAACNNNNNNNNNTNNNN
>HMong221
GTNNNNNNTAGCTCNNNNNAGTTANACANNNCNNNNNNNNNNNNNNNNNNNNNNNNNNNNNNNNNNNNNN
NNNNNNNNNNNNNNNNNNNNNNNNNNNNNNNTNNNNNNNNNNNNNNNNNNNNNNNNNNNNNNNNNNNNNN
NNNNNNNNNNNNNNNNNNNNNNNNNNNNNNNNNNNNNNNNNNNNNNNNNNNNNNNNNNNNNNNNNNNNNN
NNNNNNNNNNNNNNNNNNNNNNNNNNNNNNNNNNNNNNNNNNNNNNNNNNNNNNNNNNNNNNNNNNNNNN
NNNNNNNNNNNNNNNNNNNNNNNNNNNNNNNNNNNNNNNNNNNNNNNNNNNNNNNTCTNNNAAANNNTNNN
NNNNNNNNNNNNNNNNNNNNNNNNNNCNCNNTNNNNGTAANCNANNNGNACATGCAGTCCCCTCNNNNN
NNNGGCAGAGNNNNNNNCCNNNNNNNCCGGCCCCNNCCNNCNNNNNNNNNNNNNGNNNNNNNGNNNNNN
NNNNNNTGGTACNNNNCGNNNNNGGTTGTNTNAGAGGTCCACCACGNGGTCNGNNNACNNNNGNNTAN
NNNCNNAANNCNNNNNNNNNNNNNNNNTNNNCANNAGGNNNNNNCCNTNNNNNNNNNNNATNGCNATGACC
CANNTCGCGGGGACNGTNACCGGCGNGGCGACCTTGNNNACNGTCGGGGNCAAGTATGTCNNNCNCCC
CGGGAAGGANNCNGCCGTANGTGCNNNNNANNNNNTCNTGNGANGTCGCCGNNATTNNCGTGGTCNNN
CNNGAATGNNCNNACCANNCGNANNNTGGTNATGNNAGTNNNNNNNNNNNNNNNNNNNNNNNNNNNNN
NNNNNNNNNNNNNNNNNNNNNNNNNNNNNNNNNNNNGNNNNGNNNNNNCNANNNAAGGNNNTANNNTTNNTN
NTNNNNNNNGGACNNNACNNNNCNNNNNNNNACNCNNNCNCNNNNNNANNNGNTTTTTNNNCNNTANTNT
CCAGNACGCNGNGCNNNCNTTCANGNGAACNNNGNNACGCGNNAATCGTANNATCTACCTGCCGCNNG
NNNNNNTACNGNNNNNNCGGANNNTNNCTCGTANNNNNNNCANGCGTNNNNTNNTTNCNAGNCANGTT
CGNNNANANANTAGNANGTNTNNNNNNNNNNTGTNNCGTTCCGCNTGANNCNNCNNNNNGNNGNNCTTN
NCANCGGCNNTANCTGCNGANNTAAGNCATANNNTNNNTCNNTANCGNNNNNGGNGGGNNACTGTAAA
NGGNNAATTNGNNNNTNANTNCNNNCACNTNNCNNNGNNNNCTNNNCNCCTGNNTNCNNAATTGGNNC
CANTGNNTGNCNATCTCTGGNTTNGCNNNNNTNNNNNTNNTANNNNGGNNNGTNNNANNNCNATNTAA
NNTANANNNNCNNNNNNANNNCACNNNNNNNNNNAGNNNNTANNNTTANGNNNGTAGNNNANTAAGTCN
CCGANNTNNNNNNNATAAGCGNNANACNNNNNNNNNNNNNNNANGANNNCGNNTTGGNNNTNNNNNNNNA
ANNAGACNNCNGTGGNTNNNNNTCAANNNNTTNGTAGTGGTATAACNANNNNNNNTTTCNCCAGNNTN
NNNGNNNNNNNNCNATANNANNNNNNNNGNGANNNNNNNNNNNANNNNCCCNNNAGGNANCANCTACTNNA
GCANTGGNGCGNAGNNNGAGANNNNGACNNNCCTTGGGTGGCANNCACNGCAATAAGTNNNGTTNNNC
CCNNNAGTNNACACNNNNNCANNGNNNCNNNNNNANCNGANNTNNNNNNNNNNNNNNNNNNNNNNNNN
NNNNNNNNNNNNNNNNNNNNNNNNNNNNNNNGNNNANNGNNNGTNTNTGNCNCATGTGTTTTTANNNGNN
NNNNNNNGNNNNANCCCCGGTCGNNNNCNNATTGAANNCCTGNNGCATNNNNNTNCNAGAATGANNGC
NNNNNGNNTNGNCTNNCNNNTNAAGNAGTCANNNAGNNANNNNNNTCNNNGNNNNNNNNCNNTCCCNN
ACTGAGGGTNNNNNNNNCANCNGCTNNNNNNNNNTNCGCNGNNNNNNTNNNNNNGCNGTATNNNGTNN
NNCANNNNNNNNNNNNNNNNNTNNAACNANNNNCTTNNNN
>HMong222
GNGNNNNNTAGCCCNNNNNAGTTGGACANNNCNNNNNNNNNNNNTTNNNNNNNNNNNNNNNNNNNNNNNN
NNNNNNNNNNNNNNNNNNNNNNNNNNNNNNNTNNNNNNNNNNNNNNNNNNNNNNNNNNNNNNNNNNNNNN
NNNNNNNNNNNNNNNNNNNNNNNNNNNNNNNNNNNNNNNNNNNNNNNNNNNNNNNNNNNNNNNNNNNNNN
NNNNNNNNNNNNNNNNNNNNNNNNNNNNNNNNNNNNNNNNNNNNNNNNNNNNNNNNNNNNNNNNNNNNNN
NNNNNNNNNNNNNNNNNNNNNNNNNNNNNNNNNNNNNNNNNNNNNNNNNNNNNNCNTCTNNNNAANNNTNNN
NNNNNNNNNNNNNNNNNNNNNNNNNNCNCNNTGNNNGTANNCNANNNGNACATGCAGTCCCCTCNNNNN
NNNNNCAGAGNNNNNNCCCNNNNNCNCCGGCCCCGNCCNNNNNNNNNNNNCNNNGNNNNNNNGNNNNNN
NNNNNNTGGTACNNNNCGNNNNNGGTTGTNTAAGAGGTCCACCACGNGGTCNNGNCACNNNNGNNNAN
NNNCNNAAAACNGNNNNNNNNNNNNNNTNNNCANNAGGNNNNNNCCNTCNNNNNANNNANTGCNATGANN
CAANNCGCGGNGACAGTNACCGGNGCGGCGACCTTGANNACGGTCGGGGACAAGTATGTCNTNCNCCC
CGGGAAGGANNCNGCCGTANGTGCNNNNNANNNNNNNNTGNGANGTCGCCGNNATTCNNGTGGTCNNG

CNAGAATGNCCGNACCANNCGNANNNTGGTNATGNNAGTNNNNNNNNNNNNNNNNNNNNN
NNNNNNNNNNNNNNNNNNNNNNNNGNNNTGNNNNNNCNANNNANGGANNTANNNTTNNTN
NTNNNGNNGGACNNNACNNNNCNNNNNNNACNCNNNCNCNNNNNNANANGNTTTTTNNNCNNTNNNNT
CCAGNANGCNGNGCNNNCNNTCANGNGAACNNNGNNACGCGNNAATCGTANNATCTACCTGCCGCNNG
NNNNNNTACNGNNNNNNCGGANNNTNNNNNGTANNNNNNNNNATGCGNNNNNTNNNNNCCAGNCNNGTT
CGNNNANNNANTAGNANGTNTNNNNNNNNCTTGTNNCGTTCCGCNTGANNCNNCANNNNGNNGNNCTTN
NCANCGGCNNTANCTNNNGANNTAAGNCATANNNTNNNTCNNTANCGNNNNNGNAGGGNNACTGTAAA
NGGNNAATTNGNNNNTNANTNCNNNNACNTNNCTNNGNNNNCTNNNCNCCTGNNNNCNNAATTGGNNC
CANTGNNTGNCNATCTCTGGNTTNGNNNNNNTNNNNNTNNTAANNNGGNNNNNNNNANNNCNATNTAA
NNTANANNNNCNNNNNNANNNCANNNNNNNNGNAGNNNCNNNNNTGANGNNNGTAGNNNNNTNAGTCN
CCGANNTNNNNNNNATAANCGNNANACNNNNNNNNNNNNNNANGANNACGNNTTGGNNNTNNNNNNCNA
ANNAGACNNCNGTGGNTNNNNNTCAANNNNTTCGTAGTGGTATAACNANNNNNNNTTTCNCCAGNNTN
NNNGNNNNNNNNCNATANNANNNNNNNGNGANNNNNNNNNNANNNNCCCNNNAGGGANCANCTATTNNA
GCAATGGNNCGCAGNNNGAGANNNNGACNNGCCTTGGGTGGCAANCACNGCAATAAGTNNNGTTNNNC
CCNNNNGTNNACACGNNNNCANNGNNNCNNNNNNANCNGANNTNNNNNNNNNNNNNNNNNNNNNNNNN
NNNNNNNNNNNNNNNNNNNNNNNNNNNNNNNNANNGNNNNTNTNTGACNCATGTGNNNNTNNNNGNN
NNNNNACGNNNGNNCCCCGGTCGNNNNNNNATTGAANNCCTGNNGCATNNNNNNTNCNAGAATGANNGC
NNNNNGNNTNNNCTNNNNNNTCANGNAGTNNNNTAGGTANNNNGNTCNNNGNNNNNNNNNNNNCCCNN
ACTGAGGGTNNNNNNNNCATCNGCTNNNNNNNNNNTNCGCNGNCGTNNNNNNNNNGCNGTATNNNGTNN
NNCANNNNNNNNNNNNNNNNNTTAAACAANNNNNNNTNNNN
>HMong223
GTGNNNNNTAGCCCNNNNNAGTTAGACANNNCNNNNNNNNNNNNNNNNNNNNNNNNNNNNN
NNNNNNNNNNNNNNNNNNNNNNNNNNNNNNNNNNNNNNNNNNNNNNNNNNNNNNNNNNNNNNN
NNNNNNNNNNNNNNNNNNNNNNNNNNNNNNNNNNNNNNNNNNNNNNNNNNNNNNNNNNNNNNN
NNNNNNNNNNNNNNNNNNNNNNNNNNNNNNNNNNNNNNNNNNNNNNNNNNNNNNNNNNNNNNN
NNNNNNNNNNNNNNNNNNNNNNNNNNNNNNNNNNNNNNNNNNNNNNNNNNTCTNNNAAANNNTNNN
NNNNNNNNNNNNNNNNNNNNNNNNNNCNCNNTNNNNGTAANCNANNNGNACATGCAGTCCCCTCNNNNN
NNNGGCAGAGNNNNNNCNCNNNNNNNNCCGGCCCCGNCCNNNNNNNNNNNNNNNNNGNNNNNNNANNNNNN
NNNNNNTGGTACNNNNCGNNNNNGGTTGNNTAAGAGGTCCACAACGNGGTCNNGNCACNNNNGNNNAN
NNNCNNNNAACNNNNNNNNNNNNNNNNTNNNTANNAGGNNNNNNCCNTCNNNNNNNNNAANTGCNATGACC
CAANNCACGGGGGCNGTNCCCGGCGCGGCGACCTTGANNANGTCCGGGGACAAGTATGGCGNNCNCCC
CGGGAAGGANNCNGCNGTANGTGCNNNNNANNNNNTCNTGNGANGTCGCCGNNATNNNCGTGGTCTNN
NNAGAATGNCCGNACCANNCGNANNNTGGTNATGNNAGTNNNNNNNNNNNNNNNNNNNNNNN
NNNNNNNNNNNNNNNNNNNNNNNNGNNNNGNNNNNNCNANNNAAGGNNNTANNNCTNNTN
NTNNNNNNGGACNNNACNNNNCNNNNNNNACNCNNNCNCNNNNNNANNNGNTTTTNNNCCNNTANTNN
CCAGNACGCNGNGCNNNCNTTCANGNGAACNNNGNNACGCGNNAATCGTANNATCTACCTGCCGCNNG
NNNNNNTACNGNNNNNNCGGANNNTNNNNCGTANNNNNNNNCATGCGTNNTNTNNTCNCNAGNCNNGTT
CGNNNANANANTAGNANGTNTNNNNNNNNNNTGTNNCGTTCCGCNTGANNCNNCANNNNGNNGNNNTTN
NCANCGGCNNTANCTGCNGANNTAAGNCATANNNTNNNTCNNTANCTNNNNNGGNGGGNNACTGTAAA
NGGNNAATTNGNNNNTNANTNCNNNNACNTNNCTNNGNNNNCTNNNTNCCTGNNTNCNNAATTGGNNC
CANTGNNTGNCNATCTCTGGNTTNGNNNNNNTNNNNNTNNCAANNNGGNNNGTNNNANNNCNATNTAA
NNTANANNNNCNNNNNNANNNCACNNNNNNNNNAGNNNCNANNNTNANANNNGTAGNNNANTAAGTCN
CCGANNTNNNNNNNATAAGCGNNANACNNNNNNNNNNNNNNANGANNNCGNNTTGGNNNTNNNNNNCNA
GNNAGACNNTNATGGNTNNNNNTCAANNNNTTCGTAGTGGTATAATNGNNNNNNNTTTCNCCAGNNNN
NNNGNNNNNNNNCNATNNNANNNNNNNGNGANNNNNNNNNNANNNNCTCNNNAGGGANCANCTACTNNA
GCANTGGNGCGNAGNNNGAGANNNNGACNNNCCTTGGGTGGCANNNACNGCAATAAGTNNNGTTNANC
CTNNNAGTNNACATNNNNNNCANNGNNNCNNNNNNANCNGANNGNNNNNNNNNNNNNNNNNNNNNNNNN
NNNNNNNNNNNNNNNNNNNNNNNNNNNNNGNNNANGGNNNGTNTNTGACNCATGTGTTTNTANNNGNN
NNNNNACGNNNNNNNCCCCGGTCGNNNNCNNATTGAANNCCTGNNGCATNNNNNNTNCNAGAATGANNGC
NNNNNGNNTNGNCTNNCNNNTCANGNAATCNNNNAGGTNNNNNNNTCNNNGNNNNNNNNCNNNCCCNN
ACTGAGGGTNNNNNNNNCATCTGCTNNNNNNNNNNTNCGCNGNCGTNNCNNNNNNGCNGTATNGNGTNN
NNCANNNNNNNNNNNNNNNNNTNAAACAANNNNNNNTNNNN
>HMong224
GTGNNNNNTAGCTCNNNNNAGTTAGACANNNCNNNNNNNNNNNNNTNNNNNNNNNNNNNNNNNNNNNN

NNNNNNNNNNNNNNNNNNNNNNNNNNNNNNTNNNNNNNNNNNNNNNNNNNNNNNNNNNNNNNNNNNNNN
NNNNNNNNNNNNNNNNNNNNNNNNNNNNNNNNNNNNNNNNNNNNNNNNNNNNNNNNNNNNNNNNNNNN
NNNNNNNNNNNNNNNNNNNNNNNNNNNNNNNNNNCANNNNNNNNNNNNNNNNNNNNNNNNNNNNNNNN
NNNNNNNNNNNNNNNNNNNNNNNNNNNNNNNNNNNNNNNNNNNNNNNNNNNNTCTNNNAAANNNTNNN
NNNNNNNNNNNNNNNNNNNNNNNNNCNCNTTGNNNGTAANCNANNNGNACATGCAGTCCCCTCNNNNN
NNNNNCAGAGNNNNNNNCCNNNNNCNCCGGCCCCNNCCNNNNNNNNNNNCNNNGNNNNNNNGNNNNNN
NNNNNNTGGTACNNNNCGNNNNNGGTTGNTTAAGAGGTCCACCACGNGGTCNGGNNACNNNNGNNNAA
NNNCNNNAAACNNNNNNNNNNNNNNTNNNCANNAGGNNNNNNCCNTCNNGNNANNNATTGCNATGACC
CAANNCGCGGGGACAGTNACCGGCGNGGCGACCTTGNTNANGGTCGGGGACAAGTATGTCGNNCNCCC
CGGGAAGGANNCNGCCGTANGTGCNNNNNANNNNNTCNTGNGANGTCGCCGNNATTCNCGTGGTCNNN
CNAGAATGNCCNNACCANNCGNANNNTGGTNATGNTAGTNNNNNNNNNNNNNNNNNNNNNNNNNNNNN
NNNNNNNNNNNNNNNNNNNNNNNNNNNNNNNNNNNNNGNNNNNNCNANNNAAGGNNNTANNNTTNNTN
NTNNNNNNGGACNNNACNNNNCNNNNNNNACNCNNNCNCGGNNNNANNNGNTTTTTNNNCNNTANTNT
CCAGNANGCNGNGCNNNCATTCANGNGAACNNNGNNACGCGNNAATCGTANNATCTACCTGCCGCNNG
NNNNNNTACNGNNNNNNCGGANNNTNNNNNCGTANNNNNNTCATGCGTNNTNTNNTTNCAGNCNNGTT
CGNNNATANANTAGNANGTNTNNNNNNNNNTGTNNCGTTCCGCNTGANNCNNCANNNNGNNGNNCTTN
NCANCGGCNNTANCTGCNGANNTAAGNCATANNNTNNNTCNNTANCGNNNNNGGAGGGNNACTGTAAA
NGGNNAATTNGNNNNTNATTNCNNNCACNTNNCTNNGNNTNCTNNNCNCCTGNNTNCNNAATTGGNNN
CANTGNNTGNCATCTCTNGNTTNGNNNNNNNTGNNNNTNNTAANNNGGNNNGTNANANNNCNATNTAA
NNTANANNNNCNNNNNNANNNCACNANNNNNGNAGNNNNNANNNTTANGNNNGTAGNNNANTAAGTCT
CCGANNTNNNNNNNATAAGCGNNANACNNNNNNNNNNNNNANGANNNCGNNTTGGNNNTNNNNNNCNA
ANNAGACNNCNGTGGNTNNNNNTCAANNNNTTCGTAGTGGTATAACNANNNNNNNTTTCNCCAGNNTN
NNNGNNNNNNNCNATANNANNNNNNNGNGANNNNNNNNNNANNNNCCCNNNAGGGANCANCTACTNNA
GCAATGGNGCGCAGNNNGAGANNNNGACNNNCCTTGGGTGGCAANNACNGCAATAAGTNNNGTTNANC
CCNNNAGTNNACACNNNNNCANNGNNNCNNNNNNANCNGANNTNNNNNNNNNNNNNNNNNNNNNNNNN
NNNNNNNNNNNNNNNNNNNNNNNNNNNNAGNNNANNGNNNGTNTNTGACNCATGNNTTTNTANNNGNN
NNNNNNNGNNNNANCCCCGGTCGNNNNCNNATTGAANNCCTGNNGCATNNNNNTNCNAGAATGANNGC
NNNNNGNNTNGNCTNNNNNNNTCAAGNAGTCANNNAGGTANNGCGNTCNNNNNNNNNNNCNNTCCCNN
ACTGAGGGTNNNNNNNNCATCTGCTNNNNNNNNNNTNCGCNGNNNNNTNNNNNNGCNGTATNNNGTNN
NNCANNNNNNNNNNNNNNNNTNAAACNNNNNNNNTNNNN
>HMong225
GTGNNNNNNAGCTCNNNNNAGTTAGACANNNCNNNNNNNNNNNTNNNNNNNNNNNNNNNNNNNNNNN
NNNNNNNNNNNNNNNNNNNNNNNNNNNNNNNNNNNNNNNNNNNNNNNNNNNNNNNNNNNNNNNNNNNN
NNNNNNNNNNNNNNNNNNNNNNNNNNNNNNNNNNNNNNNNNNNNNNNNNNNNNNNNNNNNNNNNNNNN
NNNNNNNNNNNNNNNNNNNNNNNNNNNNNNNNNNNNNNNNNNNNNNNNNNNNNNNNNNNNNNNNNNNN
NNNNNNNNNNNNNNNNNNNNNNNNNNNNNNNNNNNNNNNNNNNNNNNNNNNNTCTNNNAAANNNTNNN
NNNNNNNNNNNNNNNNNNNNNNNNNCNCNTTGNNNGTAANCNANNNGNACATGCAGTCCCCTCNNNNN
NNNNNCAGAGNNNNNNNCNNNNNNNNCCGNCCCCNNCCNNNNNNNNNNNCNNNNNNNNNNNGNNNNNN
NNNNNNTGGTACNNNNCGNNNNNGGTTGTNTAAGAGGTCCACCACGNGGTCNNNNNACNNNNGNNNAN
NNNCNNNNAACNNNNNNNNNNNNNNTNNNCANNAGGNCNNNNCCNTCNNNNNNNNNATNGCNATGANN
CANNNCNCNGGGANNGTNACCGGNGCGGCGACCTTGNNNACGGTCGGGGNCAAGTATNTCNNNCCCCC
CGNGAAGGANNCNGCCGTANGTGCNNNNNANNNNNTCNTGNGANGTCGCCGNNATTNNCGTGNNCNNN
NNNGAATGNCCNNACCANNCGNANNNTGGTNATGNNAGTNNNNNNNNNNNNNNNNNNNNNNNNNNNNN
NNNNNNNNNNNNNNNNNNNNNNNNNNNNNNNNGNNNNGNNNNNNCNANNNAAGGNNNTANNNTTNNTN
NNNNNNNNGGACNNNACNNNNCNNNNNANACNCNNNCNCNNNNNNNNNNGNTTTNNNNNCNNTANTNT
CCAGNANGCNGNGCNNNCATTCANGNGAACNNNGNNACGCGNNAATCGTANNNTCTANCTGCCGCNNG
NNNNNNTACNGNNNNNNCGGANNNNNNNNNGTANNNNNNNNCANGCGTNNTNTNNTTNCAGNCNNGTT
CGNNNANANANTAGNANGTNTNNNNNNNNNTGTNNCGTTCCGCNTGANNCNNCANNNNGNNGNNNTTN
NCANCGGCNNTANCTGCNGANNTAAGNCATANNNTNNNTCNNTANCGNNNNNGGNGGGNNACTGTAAA
NGGNNAATTNGNNNNTNANTNCNNNCACNTNNCNNNGNNNNCTNNNCNCCTGNNTNCNNAATTGGNNC
CANTGNNTGNCATCTCTGGNTTNGNNNNNNNTNNNNNTNNTANNNNGGNNNNNNNNANNNCNANNTAA
NNTANANNNNCNNNNNNANNNCACNNNNNNNGNAGNNNNNANNNTNANGNNNGTAGNNNANTAAGTCN
CCGANNTNNNNNNNATAAGCGNNANACNNNNNNNNNNNNNANGANNNCGNNTTGGNNNTNNNNNNCNA
ANNAGACNNCNGTGGNTNNNNNTCAANNNNTTNGTAGTGGTATAACNANNNNNNNTTTCNCCAGNNTN

NNNGNNNNNNNNCNATNNNANNNNNNNNGNGANNNNNNNNNNNNANNNNCCCNNNAGGGANCANCTACTNNA
GCANNGGNNCGNAGNNNGAGANNNNGACNNNCCTTGGGTGGNAANNACNGNAANAAGTNNNGTTNNNN
NCNNNNGTNNACACNNNNNCANNGNNNCNNNNNNANCNGANNTNNNNNNNNNNNNNNNNNNNNNNNNN
NNNNNNNNNNNNNNNNNNNNNNNNNNNNNNNNNNNNNANNGNNNGTNTNTGACNCATGTGTTTTTANNNGNN
NNNNNACGNNNNANCCCCGGTCGNNNNCNNATTGAANNCCTGNNGCATNNNNNNTNCNAGAATGANNNC
NNNNNGNNTNGNCTNNNNNNNTNAAGNAGTCAANNAGGNNNNNNNNTCNNNGNNNNNNNNCNNNNNCNN
ACTGAGGGTNNNNNNNNCANCTGCTNNNNNNNNNNTNCGCNGNNNNNNTNNNNNNGCNGTATNGNGTNN
NNCANNNNNNNNNNNNNNNNNNTNAAACGANNNNNNNTNNNN
>HMong226
GTGNNNNNNAGCTCNNNNNAGTTAGACANNNCNNNNNNNNNNNNNTNNNNNNNNNNNNNNNNNNNNNNNN
NNNNNNNNNNNNNNNNNNNNNNNNNNNNNNNNNNNNNNNNNNNNNNNNNNNNNNNNNNNN
NNNNNNNNNNNNNNNNNNNNNNNNNNNNNNNNNNNNNNNNNNNNNNNNNNNNNNNNNNNN
NNNNNNNNNNNNNNNNNNNNNNNNNNNNNNNNNNNNNNNNNNNNNNNNNNNNNNNNNNNN
NNNNNNNNNNNNNNNNNNNNNNNNNNNNNNNNNNNNNNNNNNNNNNNNNNNNTCTNNNAAANNNTNNN
NNNNNNNNNNNNNNNNNNNNNNNNNNNNCNNTGNNNGTAANCNANNNGNACATGCAGTCCCCTCNNNNN
NNNNNCAGAGNNNNNNNNCNNNNNNNCCGGCCCCNNCCNNNNNNNNNNNNCANNNNNNNNNNNGNNNNNN
NNNNNNTGGTACNNNNCGNNNNNGGNNGTNTAAGAGGTCCACCACGNGNTCNNNNNACNNNNGNNNAN
NNNCNNNNNNNCNNNNNNNNNNNNNNNNTNNNCANNAGGNNNNNNCCNTCNNGNNNNTNANTGCNATGANN
CNNNNCGCNGNGACNGTNACCGGNGCGGCGACCTTGANNANNGTCGGGGNCAAGTATGTCNNNCNCCC
NGNGAAGGANNCNGCNGTANGTGCNNNNNANNNNNTCNTGNGANNTCGCCGNNATTNNNGTGGTCNNN
NNNNAATGNNCNNACCANNCGNANNNTGGTNATGNTAGTNNNNNNNNNNNNNNNNNNNNNNNNNNNNNN
NNNNNNNNNNNNNNNNNNNNNNNNNNNNNNNNNNNNGNNNNGNNNNNNCNANNNAAGGNNNTANNNTTNNTN
NNNNNNNNGGACNNNACNNNNCNNNNNNNACNCNNNCNCNNNNNNANNNGGTTNNNNNNCNNTNNTNT
CCAGNACGCNGNGCNNNCNTTCANGNGAACNNNGNNACGCGNNAATCGTANNATCTACCTGCCGCNNG
NNNNNNTNCNGNNNNNNCGGANNNTNNNNNGTANNNNNNNCANGCGTNNNNTNNTNNCNAGNCNNGTT
CGNNNANANANTAGNANGTNTNNNNNNNNNNTGTNNCGTTCCGCNTGANNCNNCANNNNGNNGNNCTTN
NCANCGGCNNTANCTNCNGANNTAAGNCATANNNTNNNTCNNTANCGNNNNNGGNGGGNNACTGTAAA
NGGNNAATTNGNNNNTNANTNCNNNCACNTNNCNNNGNNNNCTNNNCNCCTGNNNNCNNAATTGGNNC
CANTGNNTGNCNATCTCTNGNTTNGNNNNNNTNNNNNTNNTANNNNGGNNNNNNNNANNNCNATNTAA
NNTANANNNNCNNNNNNANNNCACNNNNNNNNNNAGNNNNNANNNTNANGNNNGTAGNNNANTAAGTCT
CCGANNTNNNNNNNATAAGCGNNANACNNNNNNNNNNNNNNANGANNNCGNNTTGGNNNTNNNNNNNNA
ANNAGACNNCNGTGGNTNNNNNTCAANNNNTTNGTAGTGGTATAACNANNNNNNNTTTCNCCAGNNTN
NNNGNNNNNNNNCNATANNANNNNNNNNGNGANNNNNNNNNNNNANNNNCCCNNNAGGGANCANCTACNNNA
GCANTGGNGCGNAGNNNGAGANNNNGACNNNCCTTGGGTGGCAANNACNGNAATAAGTNNNGTTNNNN
NCNNNNGTNNACACNNNNNCANNGNNNCNNACNNANCNGANNTNNNNNNNNNNNNNNNNNNNNNNNNN
NNNNNNNNNNNNNNNNNNNNNNNNNNNNNNNANNNNANNGNNNGTNTNTGNCNCNNGNNTTNNTANNNGNN
NNNNNACGNNNNANCCCCGGTCGNNNNCNNATTGAANNCCTGNNGCATNNNNNNTNCNAGAATGANNGC
NNNNNGNNTNNNCTNNCNNNTCAAGNAGTCNNNNAGNNNNNNNNNNNCNNNNNNNNNNNNCNNNCNCNN
ACTGAGGGTNNNNNNNNCATNNGCTNNNNNNNNNNTNCGCNGNNNNNNTNNNNNAGCNGTATNNNGNNN
NNCANNNNNNNNNNNNNNNNNNTNAAACAANNNNCNTNNNN
>HMong227
GTGNNNNNNAGCCCNNNNNAGTTAGACANNNCNNNNNNNNNNNNNTNNNNNNNNNNNNNNNNNNNNNNNN
NNNNNNNNNNNNNNNNNNNNNNNNNNNNNNNTNNNNNNNNNNNNNNNNNNNNNNNNNNNN
NNNNNNNNNNNNNNNNNNNNNNNNNNNNNNNNNNNNNNNNNNNNNNNNNNNNNNNNNNNN
NNNNNNNNNNNNNNNNNNNNNNNNNNNNNNNNNNNNNNNNNNNNNNNNNNNNNNNNNNNN
NNNNNNNNNNNNNNNNNNNNNNNNNNNNNNNNNNNNNNNNNNNNNNNNNNNNTCTNNNAAANNNTNNN
NNNNNNNNNNNNNNNNNNNNNNNNNNNCNCNNTNNNNGTAANCNANNNGNACATGCAGTCCCCTCNNNNN
NNNNNCAGAGNNNNNNNNCNNNNNNNNCCGNCCCCNNCCNNNNNNNNNNNNNNNNNGNNNNNNNANNNNNN
NNNNNNTGGTACNNNNCGNNNNNGGTTGNNNAAGNGGTCCACAACGNGGTCNNGNNACNNNNGNNNAN
NNNCNNNNNNNCNNNNNNNNNNNNNANNTNNNTANNAGGNNNNNNCCNTNNNNNNNNNNNANNGCNATGANN
CANNNCACGGNGGNNGTNCCCGGNGCGGCGACCTTGANNANNTCCGGGGNCAAGTATGGCNNNCNCCC
CGGGAAGGANNCNGCCGTANGTGCNNNNNANNNNNTCNTGNGANGTNGCCGNNATTNNNNTGGTCNNN
NNAGANTGNNCNNACCANNCGNANNNTGGTNATGNNAGTNNNNNNNNNNNNNNNNNNNNNNNNNNNNNN
NNNNNNNNNNNNNNNNNNNNNNNNNNNNNNNNNNNNGNNNNGNNNNNNCNANNNAAGGNNNTANNNCTNNTN

NTNNNNNNGGACNNNACNNNNCNNNNNNNACNCNNNCNCNNNNNNNNNNGNTTTTNNNNCNNTANTNN
CCAGNANGCNGNGCNNNCNTTCANGNGAACNNNGNNACGCGNNAATCGTANNATCTACCTGCCGCNNG
NNNNNNTACNGNNNNNNCGGANNNTNNCTCGTANNNNNNNNATGCGTNNTNTNNTCNCNAGNCNNGTT
CGNNNANANANTAGNANGTNTNNNNNNNNNNNGTNNCGTTCCGCNTGANNCNNCANNNNGNNGNNCTTN
NCANCGGCNNTANCTGCNGANNTAAGNCATANNNTNNNTCNNTANCTNNNNNGGNGGGNNACTGTAAA
NGGNNAATTNGNNNNTNATTNCNNNNACNTNNCNNNGNNNNCTNNNTNCCTGNNTNCNNAATTNGNNC
CANTGNNTGNCNATCTCTGGNTTNGNNNNNNTNNNNNTNNCANNNNGGNNNGNNNNANNNCNATNTAA
NNTANANNNNCNNNNNNANNNCACNNNNNNNGNAGNNNNNANNNTTANANNNGTAGNNNANTNAGTCN
CCGANNTNNNNNNNATAAGCGNNANACNNNNNNNNNNNNNNANGANNNCGNNTTGGNNNTNNNNNNCNA
GNNAGACNNTNATGGNTNNNNNNCAANNNNTTNGTAGTGGTATAATNGNNNNNNNTTTCNCCAGNNTN
NNNGNNNNNNNCNATANNANNNNNNNGNGANNNNNNNNNNNANNNNCTCNNNAGGNANCANCTACTTNA
GCANNGGNNCGNAGNNNGAGANNNNGACNNNCCTTGGGTGGCAANNACNGCANNAAGTNNNGTTNNNN
NTNNNAGTNNACANNNNNNCANNGNNNCNNNNNNANCNGANNGNNNNNNNNNNNNNNNNNNNNNNNNN
NNNNNNNNNNNNNNNNNNNNNNNNNNNNNNNNNNNNNANNGNNNGTNTNTGACNCATNNNTTNNTATNNGNN
NNNNNNNGNNNNANCCCCGGTCGNNNNCNNATTGAANNCCTGNNGCATNNNNNTNCNAGAATGANNGC
NNNNNGNNTNNNCTNNNNNNTNAAGNAANCANNNAGNNNTNNNNNTCNNNGNNNNNNNNCNNNCNCNN
ACTGAGGGTNNNNNNNNCANNNGCTNNNNNNNNNNTNCGCNGGCGNNNCNNNNNNGCNGTATNNNGNNN
NNCANNNNNNNNNNNNNNNNNNTNAAACAANNNNNNNTNNNN
>HMong228
GTGNNNNNTAGCTCNNNNNAGTTAGACANNNCNNNNNNNNNNNNNTNNNNNNNNNNNNNNNNNNNNNNN
NNNNNNNNNNNNNNNNNNNNNNNNNNNNNNNNNNNNNNNNNNNNNNNNNNNNNNNNNNNN
NNNNNNNNNNNNNNNNNNNNNNNNNNNNNNNNNNNNNNNNNNNNNNNNNNNNNNNNNNNN
NNNNNNNNNNNNNNNNNNNNNNNNNNNNNNNNNNNNNNNNNNNNNNNNNNNNNNNNNNNN
NNNNNNNNNNNNNNNNNNNNNNNNNNNNNNNNNNNNNNNNNNNNNNNNNNTCTNNNNAAANNNTNNN
NNNNNNNNNNNNNNNNNNNNNNNNNNNCNCNTTNNNNGTAANCNANNNGNACATGCAGTCCCCTCNNNNN
NNNNNCAGAGNNNNNNCCNNNNNNNNNCCGNCCCCNNCCNNNNNNNNNNNNNNNNNNGNNNNNNNGNNNNNN
NNNNNNTGGTACNNNNCGNNNNNGGTTGTNTAAGAGGTCCACCACGNGGTCNGNNNACNNNNGNNNAN
NNNCNNNAAACNNNNNNGNNNNANNTNNNCANNAGGNNNNNNCCNTNNNGNNANNNATTGCNATGACC
CAANNCGCNGGGANNGTNACCGGNGNGGCGACCTTGANNANGGNCGGGGNCAAGTATGTCNNNCNCCC
CGGGAAGGANNCNGCCGTANGTGCNNNNNANNNNNTCNTGNGANGTCGCCGNNATTCNNGTGGTCNNN
CNNGAATGNNCGNACCANNCGNANNNTGGTNATGNNAGTNNNNNNNNNNNNNNNNNNNNNNNNNNNNN
NNNNNNNNNNNNNNNNNNNNNNNNNNNNNNNNNNNNGNNNNGNNNNNNCNANNNANGGNNNTANNNTTNNTN
NTNNNNNNGGACNNNACNNNNCNNNNNNNACNCNNNCNCNNNNNNNNNNGNTTTTTNNNCNNTANTNT
CCAGNANGCNGNGCNNNCNTTCANGNGAACNNNGNNACGCGNNAATCGTANNATCTACCTGCCGCNNG
NNNNNNTACNGNNNNNNCGGANNNTNNNNNGTANNNNNNNNCANGCGTNNTNTNNTNNCNAGNCANGTT
CGNNNANANANTAGNANGTNTNNNNNNNNNNTGTNNCGTTCCGCNTGANNCNNCANNNNGNNGNNNTTN
NCANCGGCNNTANCTGCNGANNTAAGNCATANNNTNNGTCNNTANCGNNNNNGGNGGGNNACTGTAAA
NGGNNAATTNGNNNNTNANTNCNNNNACNTNNCTNNGNNNNCTNNNCNCCTGNNTNCNNAATTGGNNC
CANTGNNTGNCNATCTCTGGNTTNGNNNNNNTNNNNNTNNTANNNNGGNNNGNNANANNNCNATNTAA
NNTANANNNNCNNNNNNANNNCACNNNNNNNGNAGNNNNNANNNTNANGNNNGTAGNNNNNTAAGTCN
CCGANNTNNNNNNNATAAGCGNNANACNNNNNNNNNNNNNNANGANNNCGNNTTGGNNNTNNNNNNCNA
ANNAGACNNCNGTGGNTNNNNNTCAANNNNTTNGTAGTGGTATAACNANNNNNNNNTTTCNCCAGNNTN
NNNGNNNNNNNCNATACNANNNNNNNNGNGANNNNNNNNNNNANNNNCCCNNNAGGNANCANCTACTNNA
GCANTGGNGCGCAGNNNGAGANNNNGACNNGCCTTGGGTGGCAANNACNGCAATAAGTNNNGTTNANN
CCNNNAGTNNACACNNNNNCANNGNNNCNNNNNNANCNGANNTNNNNNNNNNNNNNNNNNNNNNNNN
NNNNNNNNNNNNNNNNNNNNNNNNNNNNNANNNNANGGNNNGTNTNTGACNCNTGTGTTTTTANNNGNN
NNNNNNNGNNNNANCCCCGGTCGNNNNCNNATTGAANNCCTGNNGCATNNNNNTNCNAGAATGANNGC
NNNNNGNNTNGNCTNNCNTTTNAAGNAGTCANNNAGNNNNNNNNNTCNNNGNNNNNNNNCNNTCNCNN
ACTGAGGGNNNNNNNNNCATCTGCTNNNNNNNNNCTNCGCNGNNNNNNTNNNNNAGCNGTATNNNGTNN
NNCANNNCNNNNNNNNNNNNNTNNAACNANNNNNNNTNNNG
>HMong229
GTGNNNNNTAGCTCNCNNNAGTTAGACANNNCNNNNNNNNNNNNNTNNNNNNNNNNNNNNNNNNNNNNN
NNNNNNNNNNNNNNNNNNNNNNNNNNNNNNNNTNNNNNNNNNNNNNNNNNNNNNNNNNNNNNNNN
NNNNNNNNNNNNNNNNNNNNNNNNNNNNNNNNNNNNNNNNNNNNNNNNNNNNNNNNNNNN

NNNNNNNNNNNNNNNNNNNNNNNNNNNNNNNNNNNNNNNNNNNNNNNNNNNNNNNNNNNNNNNNNNNN
NNNNNNNNNNNNNNNNNNNNNNNNNNNNNNNNNNNNNNNNNNNNNNNNNNNNTCTNNNAAANNNTNNN
NNNNNNNNNNNNNNNNNNNNNNNNNCNCNNTGNNNGTAANCNANNNGNACATGCAGTCCCCTCNNNNN
NNNNNCAGAGNNNNNNNCCNNNNNNNCCGGCCCCNNCCNNNNNNNNNNNCNNNGNNNNNNNGNNNNNN
NNNNNNTGGTACNNNNCGNNNNNGGTTGTNTNAGAGGTCCACCACGNGGTCNGGNCACNNNNGNNNAN
NNNCNNNNAANNNNNNNNNNNNNNTNNNCANNAGGNNNNNNNCCNTCNNNNNNNTNATTGCNATGANN
CAANNCNCGGGGACNGTNACCGGCGNGGCGACCTTGATNANGGTCGGGGACAAGTATGTCGNNCNCCC
CGNGAAGGANNCNGCCGTANGTGCNNNNNANNNNNTCNTGNGANGTNGCCGNNATTNNCGTGGTCNNN
NNAGANTGNCCGNACCANNCGNANNNTGGTNATGTNAGTNNNNNNNNNNNNNNNNNNNNNNNNNNNNN
NNNNNNNNNNNNNNNNNNNNNNNNNNNNNNNNGNNNNGNNNNNNCNANNNAAGGNNNTANNNTTNNTN
NTNNNNNNGGACNNNACNNNNCNNNNNNNACNCNNNCNCNNNNNNANNNGNTTTTTNNNCNNTATTNN
CCAGNANGCNGNGCNNNCNTTCANGNGAACNNNGNNACGCGNNAATCGTANNATCTACCTGCCGCNNG
NNNNNNTNCNGNNNNNNCGGANNNTNNCTNGTANNNNNNTCATGCGTNNTNTNNTTNCNAGNCNNGTT
CGNNNANANANTAGNANGTNTNNNNNNNNNTGTNNCGTTCCGCNTGANNCNNCANNNNGNNGNNNTTN
TCANCGGCNNTANCTGCNGANNTAAGNCATANNNTNNNTCNNTANCGNNNNNGGNGGGNNACTGTAAA
NGGNNAATTNGNNNNTNANTTCNNNCACNTNNCTNNGNNTNCTNNNCNCCTGNNTNCNNAATTGGNNC
CANTGNNTGNCATCTCTNGNTTNGCNNNNNNTNNNNNTNNTANNNNGGNNNNNNANANNNCNATNTAA
NNTANANNNNCNNNNNNANNNCACNNNNNNNNNAGNNNCNANNNTTANGNNNGTAGNNNAATAAGTCT
CCGANNTNNNNNNATAAGCGNNANACNNNNNNNNNNNNNNANGANNNCGNNTTGGNNNTNNNNNNCNA
ANNAGACNNCNGTGGNTNNNNNTCAANNNNTTNGTAGTGGTATAACNANNNNNNNTTTCNCCAGNNTN
NNNGNNNNNNCNATANNANNNNNNNGNGANNNNNNNNNNNANNNNCCCNNNAGGNANCANCTACTNNA
GCANTGGNGCGNAGNNNGAGANNNNGACNNNCCTTGGGTGGCAANCACNGCAATAAGTNNNGTTNNNC
CCNNNAGTNNACACNNNNNCANNGNNNCNNNNNNANCNGANNTNNNNNNNNNNNNNNNNNNNNNNNNN
NNNNNNNNNNNNNNNNNNNNNNNNNNNNANNNNANGGNNNGTNTNTGACNCATGTGTTTTTANNNGNN
NNNNNACGNNNNANCCCCGGTCGNNNNCNNATTGAANNCCTGNNGCATNNNNNTNCNAGAATGANNGC
NNNNNGNNTNGNCTGNNNNTTCAAGNAGTCAANTAGGTANNNNNNTCNNGGNNNNNNNNCNNTCNCNN
ACTGAGGGTNNNNNNNNCATCTGCTNNNNNNNNNNTNCGCNGNNNNNTNNNNNAGCNGTATNNNGTNN
NNCANNNNNNNNNNNNNNNNTNNAACNANNNNNCTNNNN
>HMong230
GNGNNNNNAGCTCNNNNNAGTTANACANNNCNNNNNNNNNNNNTNNNNNNNNNNNNNNNNNNNNNNN
NNNNNNNNNNNNNNNNNNNNNNNNNNNNNNNNNNNNNNNNNNNNNNNNNNNNNNNNNNNNNNNNNNNN
NNNNNNNNNNNNNNNNNNNNNNNNNNNNNNNNNNNNNNNNNNNNNNNNNNNNNNNNNNNNNNNNNNNN
NNNNNNNNNNNNNNNNNNNNNNNNNNNNNNNNNNNNNNNNNNNNNNNNNNNNNNNNNNNNNNNNNNNN
NNNNNNNNNNNNNNNNNNNNNNNNNNNNNNNNNNNNNNNNNNNNNNNNNNNNTCTNNNAAANNNTNNN
NNNNNNNNNNNNNNNNNNNNNNNNNCNCNTTGNNNGTAANCNANNNGNACATGCAGTCCCCTCNNNNN
NNNNNCAGAGNNNNNNNNCNNNNNNNCCGNCCCCNNCCNNNNNNNNNNNNNNNGNNNNNNNGNNNNNN
NNNNNNTGGTACNNNNCGNNNNNGGTTGNNNAAGAGGTCCACCACGNGGTCNNGNNACNNNNGNNNAN
NNNCNNNAAACNNNNNNNNNNNNNTNNNCANNAGGNNNNNNNCCNTCNNNNNNNNAATTGCNATGACC
CAANNCGCGGGGACNGTNACCGGNGNGGCGACCTTGNNNACGGTNGGGGNCAAGTATGTCNNNCNCCC
CGGGAAGGANNCNGCNGTANGTGCNNNNNANNNNNTCNTGNGANGTNGCCGNNATTNNCGTGGTCNNN
NNAGAATGNNCNNACCANNCGNANNNNGGTNATGNNAGTNNNNNNNNNNNNNNNNNNNNNNNNNNNNN
NNNNNNNNNNNNNNNNNNNNNNNNNNNNNNNNGNNNNGNNNNNNCNANNNANGGNNNTANNNTTNNTN
NNNNNNNNGGACNNNACNNNNCNNNNNNNACNCNNNCNCNNNNNNANNNGGTTTNNNNNCNNTANTNT
CCAGNANGCNNGCNNNCNTTCANGNGAACNNNGNNACGCGNNAATNGTANNATCTACCTGCCGCNNG
NNNNNNTACNGNNNNNNCGGANNNTNNCTCGTANNNNNNNCNNGCGTNNTNTNNTNNCNAGNCNNGTT
CGNNNANANANTAGNANGTNTNNNNNNNNNTGTNNCGTTCCGCNTGANNCNNCNNNNNGNNGNNNTTN
NCANCGGCNNTANCTGCNGANNTAAGNCATANNNTNNNTCNNTANCGNNNNNGGNGGGNNACTGTAAA
NGGNNAATTNGNNNNTNATTNCNNNNACNTNNCNNNGNNNNCTNNNCNCCTGNNTNCNNAATTGGNNN
CANTGNNTGNCATCTCTGGNTTNGNNNNNNNTNNNNNTNNTANNNNGGNNNNNNNNANNNCNATNTAA
NNTANANNNNCNNNNNNANNNCACNNNNNNNNNAGNNNCNANNNTTANGNNNGTAGNNNANTNAGTCT
CCGANNTNNNNNNATAAGCGNNANACNNNNNNNNNNNNNNANGANNNCGNNTTGGNNNTNNNNNNCNA
ANNAGACNNCNGTGGNTNNNNNTCAANNNNTTNGTAGTGGTATAACNANNNNNNNTTTCNCCAGNNTN
NNNGNNNNNNCNATANNANNNNNNNGNGANNNNNNNNNNNANNNNCCCNNNAGGGANCANCTACTNNA
NCANTGGNGCGCAGNNNGAGANNNNGACNNGCCTTGGGTGGCAANCACNGCAATAAGTNNNGTTNNNN

CCNNNAGTNNACACNNNNNCANNGNNNCNNNNNNANCNGANNTNNNNNNNNNNNNNNNNNNNNNNNNNN
NNNNNNNNNNNNNNNNNNNNNNNNNNNNNAGNNNANNGNNNGTNTNTGACNCATNNNTTTNTANNNGNN
NNNNNACGNNNNANCCCCGGTCGNNNNCNNATTGAANNCCTGNNGCATNNNNNTNCNAGAATGANNNC
NNNNNGNNTNNNNTNNNNNNTCAAGNAGTCANNNAGNNNNNNNNNTCNNNNNNNNNNNNCNNTCCCNN
ACTGAGGGTNNNNNNNNCANNNGCTNNNNNNNNNTNCGCNGNNNNNNTNNNNNNGCNGTATNNNGTNN
NNCANNNNNNNNNNNNNNNNNTNNAACAANNNNNNTNNNN
>HMong231
GTGNNNNNNAGCCCNNNNNAGTTANACANNNCNNNNNNNNNNNNTNNNNNNNNNNNNNNNNNNNNNNN
NNNNNNNNNNNNNNNNNNNNNNNNNNNNNNNNNTNNNNNNNNNNNNNNNNNNNNNNNNNNNNNNNNNNNN
NNNNNNNNNNNNNNNNNNNNNNNNNNNNNNNNNNNNNNNNNNNNNNNNNNNNNNNNNNNNNNNNNNNNNNNN
NNNNNNNNNNNNNNNNNNNNNNNNNNNNNNNNNNNNNNNNNNNNNNNNNNNNNNNNNNNNNNNNNNNNNNNN
NNNNNNNNNNNNNNNNNNNNNNNNNNNNNNNNNNNNNNNNNNNNNNNNNNNNNTCTNNNNAANNNTNNN
NNNNNNNNNNNNNNNNNNNNNNNNNNCNCNTTNNNNGTAANCNANNNGNACATGCAGTCCCCTCNNNNN
NNNNNCAGAGNNNNNNCCCNNNNNNNCCGNCCCCNNCCNNNNNNNNNNNNCNNNGNNNNNNNANNNNNN
NNNNNNTGGTACNNNNCGNNNNNGGTTGNNNAAGAGGTCCACAACANGNTCNNGNNACNNNNGNNNAN
NNNCNNNAAACNNNNNNNNNNNNANATNNNTANNAGGNNNNNNCCNTCNNGNNNNNNATTGCNATGACC
CAANNNNCGGGGGNNGTCCCCGGCGNGGCGACCTTGANNANGTCCGGGGNCAAGTATGGCNNNCNCCC
CGGGAAGGANNCNGCCGTANGTGCNNNNNANNNNNTCNTGNGANGTNGCCGNNATNNNCGTGGTCNNN
NNAGAATGNCCNNACCANNCGNANNNTGGTNATGNNAGTNNCNNNNNNNNNNNNNNNNNNNNNNNNNN
NNNNNNNNNNNNNNNNNNNNNNNNNNNNNNNNNNNNNNNNNGNNNNNNCNANNNAAGGNNNTANNNCTNNTN
NTNNNNNNNGGACNNNACNNNNCNNNNNNNNACNCNNNCNCNNNNNNNANNNGGTTTTTNNNCNNTANTNT
CCAGNANGCNGNGCNNNCNTTCANGNGAACNNNGNNACGCGNNAATCGTANNATCTACCTGCCGCNNG
NNNNNNTNCNGNNNNNNNCGGANNNTNNNNCGTANNNNNNNCATGCGTNNTNTNNNCNCNAGNCANGTT
CGNNNANANANTAGNANGTNTCNNNNNNNNNTGTNNCGTTCCGCNCGANNCNNCANNNNGNNGNNCTTN
NCANCGGCNNTANCTGCNGANNTAAGNCATANNNTNNNTCNNTANCTNNNNNGGNGGGNNACTGTAAA
NGGNNAATTNGNNGNTNATTNCNNNCACNTNNCTNNANNTNCTNNNTNCCTGNNTNCNNAATTGGNNC
CANTGNNTGNCNATCTCTGGNTTNGNNNNNNTNNNNNTNNCAANNNGGNNNGTNANANNNNNATNTAA
NNTANANNNNCNNNNNNANNNCACNNNNNNNNNAGNNNCNANNNTTANANNNGTAGNNNANTNAGTCT
CCGANNTNNNNNNNATAAGCGNNANACNNNNNNNNNNNNNANGANNNCGNNTTGGNNNTNNNNNNCNA
GNNAGACNNTNGTGGNTNNNNNTCAANNNNTTNGTAGTGGTATAATNGNNNNNNNTTTCNCCAGNNTN
NNNGNNNNNNNCNATANNANNNNNNNGNGANNNNNNNNNNNANNNNCTTNNNAGGGANCANTTACTNNA
GCANTGGNGCGNAGNNNNAGANNNNGACNNNCCTTGGGTGGCAANCACNGNAATAAGTNNNGTTNNNC
CTNNNAGTNNACACNNNNNCANNGNNNCNNNNNNANCNGANNGNNNNNNNNNNNNNNNNNNNNNNNNNN
NNNNNNNNNNNNNNNNNNNNNNNNNNNNNNNNNNNNANGGNNNGTNTNTGACNCATGTGTTTTTANNNGNN
NNNNNACGNNNNANCCCCGGTCGNNTNCNNATTGAANNCCTGNNGCATNNNNNTNCNAGAATGANNNC
NNNNNGNNTGGNCTNNNNNNTCANGNAATCANNNAGGTANNNNNNTCNNNNNNNNNNNNCNNNCCCNN
ACTGAGGGTNNNNNNNNCATCNGCTNNNNNNNNNNTNCGCNGGNNTNNCNNNNNNGCNGTATNNNGTNN
NNCANNNNNNNNNNNNNNNNNTNAAACNANNNNNNNTNNNN
>HMong232
GTGNNNNNTAGCCCNNNNNAGTCAGACANNNCNNNNNNNNNNNNNNNNNNNNNNNNNNNNNNNNNNNNNN
NNNNNNNNNNNNNNNNNNNNNNNNNNNNNNNNNNNNNNNNNNNNNNNNNNNNNNNNNNNNNNNNNNNNNNNN
NNNNNNNNNNNNNNNNNNNNNNNNNNNNNNNNNNNNNNNNNNNNNNNNNNNNNNNNNNNNNNNNNNNNNNNN
NNNNNNNNNNNNNNNNNNNNNNNNNNNNNNNNNNNNNNNNNNNNNNNNNNNNNNNNNNNNNNNNNNNNNNNN
NNNNNNNNNNNNNNNNNNNNNNNNNNNNNNNNNNNNNNNNNNNNNNNNNNNNNTCTNNNNAAANNNNNNN
NNNNNNNNNNNNNNNNNNNNNNNNNNCNCNNTGNNNGTAANCNANNNGNACATGCAGTCCCCTCNNNNN
NNNGGCAGAGNNNNNNNCNNNNNNCNCCGNCCCCNNCCNNNNNNNNNNNNCNNNGNNNNNNNANNNNNN
NNNNNNTGGTACNNNNCGNNNNNGGTTGNNTNAGNGGTCCACAACGNGGTCNNGNCACNNNNGNNNAN
NNNCNNAAAACNNNNNNNNNNNNNNNNTNNNTANNAGGNCNNNNCCNTCNNNNNNNNNNANNGCNATGACC
CAANNCNCGGNGGNAGTNCCCGGNGCGGCNACCTTGNNNACGTCCGGGGACAAGTATGGCNNNCNCCC
CGGGAAGGANNCNGCCGTANGTGCNNNNNANNNNNTCNTGNGANGTNGCCGNNNTTNNCNTGGTCNNN
NNAGAATGNNCGNACCANNCGNANNNTGGTNATGNNAGTNNNNNNNNNNNNNNNNNNNNNNNNNNNNN
NNNNNNNNNNNNNNNNNNNNNNNNNNNNNNNNNNNNNGNNNNGNNNNNNCNANNNAAGGANNTANNNCTNNTN
NTNNNNNNNGGACNNNACNNNNCNNNNNNNNACNCNNNCNCNNNNNNNNNNNGNTTTTNNNNNCNNTATTNT
CCAGNANGCNGNGCNNNCATTTANGNGAACNNNGNNACGCGNNAATCGTANNATCTACCTGCCGCNNG

TNNNNNTACNGNNNNNNCGGANNNTNNNNNGTANNNGNNTCATGCGTNNTNTNNTCNCNAGNCANGTT
CGNNNANANANTNGNANGTNTCCNNNNNCNTGTNNCGTTCCGCNTGANNCNNCANNNNGNCGNNNTTN
NCANCGGCNNTANCTGCNGANNTAAGNCNTANNNTNNATCNNTANCTNNNNNGGNGGGNNACTGTAAA
NGGNNAATTNGANNNTNANTNCNNNCATNTNNCNNNGNNNNCTNNNTNCCTGNNTNCNNAATTGGNNN
CANTGNNTGNCNATCTCTGGNTTNGNNNNNNTNNNNNTNNCANNNNGGNNNGTNNNANNNCNATNTAA
NNTANANNNNCNNNNNNANNNCACNANNNNNNNAGNNNNNANNNGTANANNNGTAGNNNNNTAAGTCT
CCGNNNTNNNNNNNATAANCGNNANACNNNNNNNNNNNNNANGANNACGNNTTTGNNNTNNNNNNNNA
GNNANACNNTNGTGGNTNNNNNTCAANNNNTTNGTAGTGGTATAATNGNNNNNNNTTTCNCCAGNNNN
NNNGNNNNNNNNCNATANNANNNNNNNGNGANNNNNNNNNNNNNNCTCNNNAGGGANCANCTACTTNA
GCANTGGNGCGCAGNNNGAGANNNNGACCNGCCTTGGGTGGCAANCACNGCAATAAGTNNNGTTNANN
CTNNNAGTNNACACGNNNNCANNGNNNCNNNNNNANCNGANNGNNNNNNNNNNNNNNNNNNNNNNNNN
NNNNNNNNNNNNNNNNNCNNNNNNNNNNNGNNNANNGNNNGTNTNTGACNCATGTGTTTTTANNNGNN
NNNNNACGNNNNANCCCCGGTCGNNNNCNNATTGAANNCCTGNNGCATNNNNNTNCNAGAATGANNGC
NNNNNGNNTNGNCTNNNNNTTCAAGNAATCANNNAGNNANCGCGNTCNNNNNNNNNNNNCNNTCCCNN
ACTGAGGGTNNNNNNCGCATCTGCTNNNNNNNNNNTNCGCCGNNNNNNCNNNNNAGCNGTATNNTGTNN
NNCANNNNNNNNNNNNNNNNTNAAACNANNNNNNNTNNNN
>HMong233
GTGNNNNNTAGCCCNNNNNAGTTANACANNNCNNNNNNNNNNNNTNNNNNNNNNNNNNNNNNNNNNNN
NNNNNNNNNNNNNNNNNNNNNNNNNNNNNNNNNNNNNNNNNNNNNNNNNNNNNNNNNNNNNNNNNNNN
NNNNNNNNNNNNNNNNNNNNNNNNNNNNNNNNNNNNNNNNNNNNNNNNNNNNNNNNNNNNNNNNNNNN
NNNNNNNNNNNNNNNNNNNNNNNNNNNNNNNNNNNNNNNNNNNNNNNNNNNNNNNNNNNNNNNNNNNN
NNNNNNNNNNNNNNNNNNNNNNNNNNNNNNNNNNNNNNNNNNNNNNNNNNNNTCTNNNAAANNNTNNN
NNNNNNNNNNNNNNNNNNNNNNNNNCNCNNTGNNNGTAANCNANNNGNACATGCAGTCCCCTCNNNNN
NNNNNCAGAGNNNNNNNCNNNNNNNNCCGNCCCCNNCCNNNNNNNNNNNCNNNNNNNNNNANNNNNNN
NNNNNNTGGTACNNNNCGNNNNNGGTTGNNTAAGAGGTCCACAACANGGTCNGNNNACNNNNGNNNAN
NNNCNNNNAACNNNNNCNNNNNANNTNNNTANNAGGNCNNNNCCNTCNNGNNNNNNANNGCNATGANN
CNANTCACGGNGGCNGTNCCCGGNGNGGCGACCTTGNNNACGTCCGGGGNCAAGTATNGCNNNCNCCC
CGGGAAGGANNCNGCNGTANGTGCNNNNNANNNNNTCNTGNGANGTNGCCGNNATTNNCGTGGTCNNN
NNAGAATGNCCNNACCANNCGNANNNTGGTNATGTNAGTNNNNNNNNNNNNNNNNNNNNNNANNNNNN
NNNNNNNNNNNNNNNNNNNNNNNNNNNNNNNNGNNNNGNNNNNNCNANNNAAGGNNNTANNNCTNNTN
NNNNNNNNGGACNNNACNNNNCNNNNNNNACNCNNNCNCNNNNNNANNNGNTTTTNNNCCNNTATTNT
CCAGNANGCNGNGCNNNCNTTCANGNGAACNNNGNNACGCGNNAATCGTANNATCTACCTGCCGCNNG
TNNNNNTACNGNNNNNNCGGANNNTNNNNCGTANNNNNNNCNTGCGTNNTNTNNTCNCNAGNCNNGTT
CGNNNANANANTAGNANGTNTNNNNNNNNNTGTNNCGTTCCGCNCGANNCNNCNNNNNGNNGNNCTTN
NCANCGGCNNTANCTGCNGANNTAAGNCATANNNTNNNTCNNTANCTNNNNNGGNGGGNNACTGTAAA
NGGNNAATTNGNNNNTNANTNCNNNCACNTNNCNNNANNNNCTNNNTNCCTGNNTNCNNAATTGGNNN
CANTGNNTGNCNATCTCTNGNTTNGNNNNNNTNNNNNTNNCANNNNGGNNNNNNANANNNCNATNTAA
NNTANANNNNCNNNNNNANNNCACNNNNNNNNNAGNNNCNANNNTNANANNNGTAGNNNANTAAGTCT
CCGANNTNNNNNNNATAAGCGNNANACNNNNNNNNNNNNNANGANNNCGNNTTGGNNNTNNNNNNCNA
GNNAGACNNTNGTGGNTNNNNNTCAANNNNTTNGTAGTGGTATAATNGNNNNNNNTTTCNCCAGNNTN
NNNGNNNNNNNNCNATANNANNNNNNNGNGANNNNNNNNNANNNNCTTNNNAGGGANCANTTACTNNA
GCANTGGNGCGNAGNNNGAGANNNNGACNNNCCTTGGGTGGNAANNACNGNAATAAGTNNNGTTNNNC
CTNNNAGTNNACACNNNNNCANNGNNNCNNNNNNANCNGANNGNNNNNNNNNNNNCNNNNNNNNNNNN
NNNNNNNNNNNNNNNNNNNNNNNNNNNNNNNNNANNGNNNGTNTNTGACNCNTGTGTTTTTANNNGNN
NNNNNNNGNNNNNNCCCCGGTCGNNNNCNNATTGAANNCCTGNNGCATNNNNNTNCNAGAATGANNNC
NNNNNGNNTNGNCTNNCNNNTCAAGNAATCANNNAGGTNNNNNGNTCNNNGNNNNNNNNCNNTCNCNN
ACTGAGGGTNNNNNNNNCATCTGCTNNNNNNNNNNTNCGCNGGNGTNNCNNNNNNGCNGTATNGNGNNN
NNCANNNNNNNNNNNNNNNNTNAAACAANNNNCNTNNNN
>HMong234
GTGNNNNNTAGCCCNNNNNAGTTAGACANNNCNNNNNNNNNNNNTNNNNNNNNNNNNNNNNNNNNNNN
NNNNNNNNNNNNNNNNNNNNNNNNNNNNNNTNNNNNNNNNNNNNNNNNNNNNNNNNNNNNNNNNNNNN
NNNNNNNNNNNNNNNNNNNNNNNNNNNNNNNNNNNNNNNNNNNNNNNNNNNNNNNNNNNNNNNNNNNN
NNNNNNNNNNNNNNNNNNNNNNNNNNNNNNNNNNNNNNNNNNNNNNNNNNNNNNNNNNNNNNNNNNNN
NNNNNNNNNNNNNNNNNNNNNNNNNNNNNNNNNNNNNNNNNNNNNNNNNNNNTCTNNNAAANNNTNNN

NNNNNNNNNNNNNNNNNNNNNNNNNNNCNCNTTGNNNGTAANCNANNNGNACATGCAGTCCCCTCNNNNN
NNNNNCAGAGNNNNNNNNCNNNNNNNCCGNCCCCNNCCNNNNNNNNNNNCNNNNNNNNNNNANNNNNN
NNNNNNTGGTACNNNNCGNNNNNGGTTGTNTAAGAGGTCCACAACGNGGTCNGGNCACNNNNGNNNAN
NCNCNNNAAACNNNNNNNNNNNNANNTNNNTANNAGGNNNNNNCCNTCNNNNNNNNNNANTGCNATGACC
CNANNNNCGGGGGNNGTNCCCGGCGNGGCGACCTTGANNANNTCCGGGGNCAAGTATGGCNNNCNCCC
CGNGAAGGANNCNGCNGTANGTGCNNNNNANNNNNTCNTGNGANGTNGCCGNNATTCNNNTGGNCNNN
NNAGAATGNNCGNACCANNCGNANNNTGGTNATGNNAGTNNNNNNNNNNNNNNNNNNNNNNNNNNNNNN
NNNNNNNNNNNNNNNNNNNNNNNNNNNNNNNNNNNNNNNNNGNNNNNNCNANNNAAGGANNTANNNCTNNTN
NTNNNNNNNGGACNNNACNNNNCNNNNNNNACNCNNNCNCNNNNNNNNNNNGNTTTTNNNNCNNTANTNT
CCAGNANGCNGNGCNNNCATTCANGNGAACNNNGNNACGCGNNAATCGTANNATCTACCTGCCGCNNG
NNNNNNTNCNGNNNNNNCGGANNNTNNNNCGTANNNNNNNCATGCGTNNTNTNNTCNCNAGNCNNGTT
CGNNNANANANTAGNANGTNTNNNNNNNNNNTGTNNCGTTCCGCNTGANNCNNCNNNNNGNNGNNNTTN
NCANCGGCNNTANCTNCNGANNTAAGNCATANNNTNNNTCNNTANCTNNNNNGGNGGGNNACTGTAAA
NGNNNAATTNGNNNNTNATTNCNNNCACNTNNCNNNGNNNNCTNNNTNCCTGNNTNCNNAATTGGNNC
CANTGNNTGNCNATCTCTNGNTTNGNNNNNNTNNNNNTNNCANNNNGGNNNNNNANANNNCCATNTAA
NNTANANNNNCNNNNNNANNNCACNNNNNNNNNNAGNNNCNANNNTNANANNNGTAGNNNAATAAGTCT
CCGANNTNNNNNNNATAAGCGNNANACNNNNNNNNNNNNNNANGANNNCGNNTTGGNNNTNNNNNNCNA
GNNAGACNNTNATGGNTNNNNNTCAANNNNNTTNGTAGTGGTATAATNGNNNNNNNTTTCNCCAGNNTN
NNNGNNNNNNNCNATANNANNNNNNNGNGANNNNNNNNNNNANNNNCTCNNNAGGGANCANCTACTNNA
GCANNGGNGCGNAGNNNGAGANNNNGACNNNCCTTGGGTGGCAANNACNGCAATAAGTNNNGTTNNNC
CTNNNAGTNNACATNNNNNCANNGNNNCNNNNNNANCNGANNGNNNNNNNNNNNNNNNNNNNNNNNNN
NNNNNNNNNNNNNNNNNNNNNNNNNNNNNNNNNNNNANGGNNNGTNTNTGNCNCATGNNTTTTTANNNGCN
NNNNNNNGNNNNANCCCCGGTCGNNNNCNNATTGAANNCCTGNNGCATNNNNNTNCNAGAATGANNGC
NNNNNGNNTNNNCTNNNNNNNTCAAGNAATCANNNAGNNNNNNNNNNTCNNNNNNNNNNNNNCNNNNNCNN
ACTGAGGGTNNNNNNNNCATCNGCTNNNNNNNNNCTNCGCNGGNGTNNCNNNNNNGCNGTATNNNGTNN
NNCANNNNNNNNNNNNNNNNNNNTNAAACAANNNNNNNTNNNN
>Kinh01
GTGNNNNNTAGCTCNNNNNNGTTANACANNNCNNNNNNNNNNNNNNNNNNNNNNNNNNNNNNNNNNNNNN
NNNNNNNNNNNNNNNNNNNNNNNNNNNNNNNNNNNNNNNNNNNNNNNNNNNNNNNNNNNNNNNNNNNNNN
NNNNNNNNNNNNNNNNNNNNNNNNNNNNNNNNNNNNNNNNNNNNNNNNNNNNNNNNNNNNNNNNNNNNNN
NNNNNNNNNNNNNNNNNNNNNNNNNNNNNNNNNNNNNNNNNNNNNNNNNNNNNNNNNNNNNNNNNNNNNN
NNNNNNNNNNNNNNNNNNNNNNNNNNNNNNNNNNNNNNNNNNNNNNNNNNNNNNTCTNNNAAANNNTNNN
NNNNNNNNNNNNNNNNNNNNNNNNNNNCNCNTTNNNNGTAANCNANNNGNACATGCAGTNCCCTCNNNNN
NNNNNCAGAGNNNNNNNCCNNNNNNNCCGNCCCCNNCCNNNNNNNNNNNNNNNNGNNNNNNNGNNNNNN
NNNNNNTGGTACNNNNCGNNNNNGGTTGTNTAAGAGGTCCACCACGNGNTCNNNNNACNNNNGNNNAN
NNNNNNNNNNCNNNNNNNNNNNNNNNNTNNNCANAAGGNNNNNNCCNTNNNNNNNNNNNANTGCNATGACC
CANNTNNCGGNGANAGTNACCGGNGNGGNGACCTTGANNANGGTCGGGNACAAGTATGTCNNNCNCCC
CGGGAAGGANNCNGCCGTANGTGCNNNNNANNNNNTCNTNNGANGTNGCCGNNNNNNNCGTGGTCNNN
CNAGANTGTCCGNACCANNCGNANNNTGGTNATGNNAGCNNNNNNNNNNNNNNNNNNNNNNNNNNNNN
NNNNNNNNNNNNNNNNNNNNNNNNNNNNNNNNNNNNNNGNNNNGNNNNNNCNANNNAAGGANNTANNNTTNNTN
NNNNNNNNNGNNCNNNACNNNNCNNNNNNNACNCNNNCNCNNNNNNNNNNNGNTTTTNNNNCNNTANNNT
CCAGNANGCNGNGCNNNCATTCANGNGAACNNNGNNACGCGNNAATCGTANNNTCTACCTGCCGCNNG
NNNNNNTNCNGNNNNNNCGGANNNNNNCTCGTANNNNNNNNNANGCGTNNTNTNNTTNCNAGNCANGTT
CGNNNANANANTAGNANGTNNNNNNNNNNNNTGTNNCGTTCCGCNTGANNCNNCANNNNGNNGNNCTTN
NCANCGGCNNTANCTNCNGANNTAAGNCATANNNTNNGTCNNTANCGNNNNNGGNGGGNNACTGTAAA
NGNNNAATTGGNNGNTNATTNCNNNCACNTNNCTNNGNNNNCTNNNCNCCTGNNTNCNNAATTGGNNN
CANTGNNTGNCNATCTCTGGNTTNGNNNNNNTNNNNNTNNTANNNNGGNNNGTNANANNNCNATNTAA
NNTANANNNNCNNNNNNANNNCACNNNNNNNNNNAGNNNCNNNNNTTANGNNNGTAGNNNNNTAAGTCN
CCGANNTNNNNNNNATAAGCGNNANACNNNNNNNNNNNNNNANGANNNCGNNTTGGNNNTNNNNNNCNA
ANNAGACNNCNGTGGNTNNNNNTCAANNNNNTTNGTAGTGGTATAACNANNNNNNNNTTTCNCCAGNNNN
NNNNNNNNNNNCNATANNANNNNNNNGNGANNNNNNNNNNNANNNNNCCNNNAGGNANCANCTACTNNA
GCANNGGNNCGNAGNNNGAGANNNNGACNNNCCTTGGGTGGCAANNACNGCAATAAGTNNNGTTNANC
CCNNNANTNNACACNNNNNCNNNGNNNCNNNNNNANCNGANNTNNNNNNNNNNNNNNNNNNNNNNNNN
NNNNNNNNNNNNNNNNNNNNNNNNNNNNNNNNNNNNANGGNNNGTNTNTGACNCATGTGTTTTTANNNGNN

NNNNNACGNNNNANCCCCGGTCGNNNCCNNATTGAANNCCTGNNGCATNNNNNNNCNAGAATGANNNC
NNNNNGNNTNGNNTNNNNNNTCAAGNAGTCNNNNANNNNNNNNNNTCNNNNNNNNNNNNCNNNNNCNN
ACTGAGGGTNNNNNNNNCATCNGCTNNNNNNNNNTNCGCNNNNNNNNTNNNNNNGCNGTATNNNGTNN
NNCANNNNNNNNNNNNNNNNNNNNAACNNNNNNNNTNNNN
>Kinh02
GTNNNNNNTAGNTNNNNNNNGNTANACANNNCNNNNNNNNNNNNNNNNNNNNNNNNNNNNNNNNNNNNNN
NNNNNNNNNNNNNNNNNNNNNNNNNNNNNNNNNNNNNNNNNNNNNNNNNNNNNNNNNNNNNNNNNNNNNN
NNNNNNNNNNNNNNNNNNNNNNNNNNNNNNNNNNNNNNNNNNNNNNNNNNNNNNNNNNNNNNNNNNNNNN
NNNNNNNNNNNNNNNNNNNNNNNNNNNNNNNNNNNNNNNNNNNNNNNNNNNNNNNNNNNNNNNNNNNNNN
NNNNNNNNNNNNNNNNNNNNNNNNNNNNNNNNNNNNNNNNNNNNNNNNNNNNNNNNNNNNNNAANNNTNNN
NNNNNNNNNNNNNNNNNNNNNNNNNNNCNNTNNNNNTAANCNANNNGNACATGCAGTCCCCTCNNNNN
NNNNNCAGAGNNNNNNNNCNNNNNNNCCGNCCCCNNCCNNNNNNNNNNNNNNNNNNNNNNNGNNNNNN
NNNNNNTGGTACNNNNCGNNNNNGGTTGNNNNAGNGGTCCACCACGNGNTCNNGNCANNNNNGNNNAN
NNNCNNNNAANNNNNNNNNNNNNNNNTNNNCANNAGGNCNNNNCCNTCNNNNNNNNNANNGCNATGANN
CNNNNCNCGGNGACNGTNACCGGNGNGGCGACCTTGANNANNGNNGGGNNCAANTNNNTCNNNCNCCC
CGNNAAGNANNCNNCNGTANGTGCNNNNNANNNNNTNNNNNGANGTNGCCGNNNTTNNCNTGGTCNNN
NNAGAATGNCCNNACCANNCGNANNNNNGTNATGNNAGTNNNNNNNNNNNNNNNNNNNNNNNNNNNNN
NNNNNNNNNNNNNNNNNNNNNNNNNNNNNNNNNNGNNNNGNNNNNNCNANNNANGGANNTANNNTTNNTN
NTNNNNNNGGACNNNACNNNNNNNNNNNNNACNCNNNCNCNNNNNNNNNNNNTTTTNNNNCNNTNNTNN
CNAGNNNGCNNNGCNNNCANNNANGNGAACNNNGNNACGCGNNNNNCGTANNNTCTANCTGCCGCNNN
NNNNNNTNCNGNNNNNNCGGANNNNNNNNNGNANNNNNNNNNNGCNTNNTNNNNTNNCNAGNNNNGTT
CGNNNANNNANTANNANGTNTNNNNNNNNNTGTNNCGTTCCGCNTGANNCNNCNNNNNGNNGNNNTTN
NCANCGGNNNTNNCTNCNGANNTAAGNCATANNNNNNNTCNNTANCGNNNNNGNNGGGNNACTGTAAA
NGNNNNNTTNGNNNNTNANNNCNNNNACNTNNCNNNNNNNNNCNNNNCNCCTGNNTNCNNAATTGGNNN
CANTGNNTGNCNATCTCTGGNTTNGNNNNNNTNNNNNTNNTANNNNGGNNNNNNNNANNNCNANNTAA
NNTANANNNNCNNNNNNANNNCACNNNNNNNNNAGNNNNNANNNTNANGNNNGTAGNNNNNTNAGTCN
CCGNNNTNNNNNNNATAANCGNNANANNNNNNNNNNNNNNNANGANNNCGNNTTGGNNNTNNNNNNNNN
ANNNGACNNCNGTGNNTNNNNNTCAANNNNNTNGTAGTGGTATAACNANNNNNNNTTNNNCCAGNNNN
NNNGNNNNNNNCNATNNNANNNNNNNNNGANNNNNNNNNNANNNNNCCNNNAGGNANCANCNACTNNA
GCANNGGNNCGNAGNNNGAGNNNNNNACNNNCCTTGGGTGGNANNNANNGCANTAAGTNNNGTNNNNN
NCNNNNNTNNACACNNNNNCANNGNNNCNNNNNNANNNGANNTNNNNNNNNNNNNNNNNNNNNNNNN
NNNNNNNNNNNNNNNNNNNNNNNNNNNNANNNNANNGNNNGTNTNTGACNNATNNNNNTTNANNNGNN
NNNNNNNGNNNGNNCCCCGGTCGNNNNCNNATTGAANNCCTGNNGCATNNNNNTNCNAGAATGANNNC
NNNNNGNNTNGNNTNNNNNNTCANNNAGTNNNNNAGNNNNNNNNNTCNNNNNNNNNNNNNNNNNNCCNN
ACTGAGGGNNNNNNNNNCATCNNCTNNNNNNNNNNTNCGCNGNNNNNNNNNNNNNNGCNGTATNNNGTNN
NNCANNNNNNNNNNNNNNNNNNNNNNNNNNNNNNNTNNNN
>Kinh03
GTGNNNNNTAGCCCNNNNNAGTTAGACANNNCNNNNNNNNNNNNTNNNNNNNNNNNNNNNNNNNNNNN
NNNNNNNNNNNNNNNNNNNNNNNNNNNNNNTNNNNNNNNNNNNNNNNNNNNNNNNNNNNNNNNNNNNNNN
NNNNNNNNNNNNNNNNNNNNNNNNNNNNNNNNNNNNNNNNNNNNNNNNNNNNNNNNNNNNNNNNNNNNNN
NNNNNNNNNNNNNNNNNNNNNNNNNNNNNNNNNNNNNNNNNNNNNNNNNNNNNNNNNNNNNNNNNNNNNN
NNNNNNNNNNNNNNNNNNNNNNNNNNNNNNNNNNNNNNNNNNNNNNNNNNNNNNNNNNNNNNAANNNTNNN
NNNNNNNNNNNNNNNNNNNNNNNNNNNCNNTNNNNGTAANCNANNNGNACATGCAGTCCCCTCNNNNN
NNNNNCAGAGNNNNNNNCCNNNNNNNCCGNCCCCNNCCNNNNNNNNNNNNNNNNGNNNNNNNANNNNNN
NNNNNNTGGTACNNNNCNNNNNNGGTTGNNTNAGAGGTCCACAACGNGNTCNNNNNACNNNNGNNNAN
NNNCNNNNNNNNNNNNNNNNNNNNNNTNNNTANNAGGNNNNNNCCNTCNNNNNNNNNNATNGCNNTNACC
CANNNNNCNGNNGNNGTNCCCGGCGCGGCGACCTTGANNACGTCNGGGNNCAAGTATGGCNNNCNCCC
CGGGAAGGANNCNGCNGTANGTGCNNNNNANNNNNTNNTGNGANGTNGCCGNNNTTCNCGTGGTCNNN
CNAGAATGCCCNNACCANNCGNANNNTGGTNATGNNAGTNNNNNNNNNNNNNNNNNNNNNNNNNNNNN
NNNNNNNNNNNNNNNNNNNNNNNNNNNNNNNNNNNNNNGNNNNNNCNANNNAAGGNNNTANNNCTNNTN
NNNNNNNNGNNCNNNACNNNNCNNNNNNNACNCNNNCNCNNNNNNNNNNNGNTTTTTNNNCNNTANTNT
CCAGNANGCNGNGCNNNCNTTCANGNGAACNNNGNNACGCGNNAATCGTANNATCTACCTGCCGCNNG
NNNNNNTNCNGNNNNNNCGGANNNTNNNNCGTANNNNNNNCANGCGTNNNNTNNTCNCNAGNCANGTT
CGNNNANANANTAGNANGTNNNNNNNNNNNNTGTNNCGTTCCGCNTGANNCNNCNNNNNGNNGNNNTTN

NCANCGNNNNTANCTNNNGANNTAAGNCATANNNTNNNTCNNTANCTNNNNNGGNNGGNNACTGTAAA
NGGNNAATTNGNNNNTNATTNCNNNCANNNNNCNNNGNNNNCTNNNTNCCTGNNTNCNNAATTGGNNN
CANTGNNTGNCNATCTCTNGNTTNGNNNNNNTNNNNNTNNCANNNNGGNNNNNNANANNNCNATNTAA
NNTANANNNNCNNNNNNANNNCACNNNNNNNNNAGNNNNNANNNTNANANNNNTAGNNNANTNAGTCT
CCGANNTNNNNNNNATAAGCGNNANACNNNNNNNNNNNNNANGANNNCGNNTTGGNNNTNNNNNNCNA
GNNAGACNNTNATGGNTNNNNNTCAANNNNTTNGTAGTGGTATAATNGNNNNNNNTTTCNCCAGNNNN
NNNGNNNNNNNNNNATANNANNNNNNNGNGANNNNNNNNNNNANNNNNTCNNNAGGNANCANCTACTNNA
GCANTGGNGCGNAGNNNGAGANNNNGACNNNCCTTGGGTGGCAANNACNGNAATAAGTNNNGTTNANC
CTNNNAGTNNACATNNNNNCANNGNNNCNNNNNNANCNGANNGNNNNNNNNNNNNNNNNNNNNNNNNN
NNNNNNNNNNNNNNNNNNNNNNNNNNNNNNNNNNANGGNNNGTNTNNGNCNCATGTGTTTNTANNNGNN
NNNNNNNGNNNGANCCCCGGTCGNNNNCNNATTGAANNCCTGNNNCATNNNNNTNCNNGAATGANNNC
NNNNNGNNTNGNCTNNNNNNTCAAGNAATCAANNAGGNNNNNNNNTCNNNNNNNNNNNNCNNNCNCNN
ANNGAGGNTNNNNNNNNCATNNGCTNNNNNNNNNNTNCGCNGGNGTNNCNNNNNAGCNGTATNGNGNNN
NNCANNNNNNNNNNNNNNNNNTNNNNNNNNNNNNNNTNNNN
>Kinh04
GTGNNNNNTAGCCCNNNNNAGTTANACANNNCNNNNNNNNNNNNNTNNNNNNNNNNNNNNNNNNNNNNNN
NNNNNNNNNNNNNNNNNNNNNNNNNNNNNNTNNNNNNNNNNNNNNNNNNNNNNNNNNNNNNNNNNNNNNN
NNNNNNNNNNNNNNNNNNNNNNNNNNNNNNNNNNNNNNNNNNNNNNNNNNNNNNNNNNNNNNNNNNNNNN
NNNNNNNNNNNNNNNNNNNNNNNNNNNNNNNNNNNNNNNNNNNGANNNNNNNNNNNNNNNNNNNNNNNNN
NNNNNNNNNNNNNNNNNNNNNNNNNNNNNNNNNNNNNNNNNNNNNNNNNNNNNNNNNNNNNAANNNTNNN
NNNNNNNNNNNNNNNNNNNNNNNNNNCNCNNTNNNNGTNANCNANNNGNACATGCAGTCCCCTCNNNNN
NNNGGCAGAGNNNNNNNNCNNNNNNNCCGNCCCCGNCCNNNNNNNNNNNNNNNNNNNNNNNNNANNNNNN
NNNNNNTGGTACNNNNCGNNNNNGGTTGTNNNAGAGGTCCACAACGNGNTCNNNNCACNNNNGNNNAN
NNNCNNNNNNNNNNNNNNNNNNNNNNNNNNNTANNAGGNCNNNNCCNTCNNNNNNNNNANNGCNATNACC
CAANNCNCGGGGGCAGTNCCCGGCGCGGNGACCTTGANNANGTNNGGGGNCAAGTATGGCNNNCNCCC
CGGGAAGGANNCNGCCGTANGTGCNNNNNANNNNNNCNTGNGANGTNGCCGNNNTTCNCGTGGTCNNN
NNNGAATGNNCNNACCANNNGNANNNTGGTNATGNNAGTNNNNNNNNNNNNNNNNNNNNNNNNNNNNN
NNNNNNNNNNNNNNNNNNNNNNNNNNNNNNNNNNNNNNNNNGNNNNNNCNANNNAAGGANNTANNNCTNNTN
NNNNNNNNGGACNNNACNNNNCNNNNNNNACNCNNNCNCNNNNNNANNNGNTTTTNNNNCNNTATTNT
CCAGNANGCNGNGCNNNCATTCANGNGAACNNNGNNACGCGNNAATCGTANNATCTACCTGCCGCNNG
NNNNNNTNCNGNNNNNNCGGANNNTNNCTCGTANNNNNNTCANGCGTNNNNTNNTCNCNAGNNNNNTT
CGNNNANANANTAGNANGTNTNNNNNNNNNNNGTNNCGTTCCGCNTGANNCNNCNNNNNGNNGNNNTTN
NCANCGGCNNTANCTNNNGANNTAAGNNATANNNTNNNTCNNTANCTNNNNNGGNNGGNNACTGTAAA
NGGNNAATTNGNNNNTNANTNCNNNCACNTNNCNNNGNNNNCTNNNTNCCTGNNTNCNNAATTGGNNC
CANTGNNTGNCNATCTCTNGNTTNGNNNNNNNNNNNNNTNNCANNNNGGNNNNNNANANNNNNATNTAA
NNTANANNNNCNNNNNNANNNCACNNNNNNNNNAGNNNNNANNNNNANANNNGTAGNNNANTNAGTCT
CCNNNNTNNNNNNNATAAGCGNNANACNNNNNNNNNNNNNANGANNNCGNNTTGGNNNTNNNNNNNNA
GNNAGACNNTNNTGGNTNNNNNTCAANNNNTTNGTAGTGGTATAATNGNNNNNNNTTTCNCCAGNNTN
NNNGNNNNNNNNCNATANNANNNNNNNNNNNNANNNNNNNNNNNANNNNNTCNNNAGGGANCANCTACTNNA
GCANTGGNNNGNAGNNNGAGANNNNGACNNNCCTTGGGTGGCANNNACNGNAATAAGTNNNGTTNNNC
CTNNNAGTNNACATNNNNNCANNGNNNCNNNNNNANCNNNNNGNNNNNNNNNNNNNNNNNNNNNNNNN
NNNNNNNNNNNNNNNNNNNNNNNNNNNNNNNNNNANGGNNNGTNTNTGNCNCATGTGTTTTTANNNGNN
NNNNNACGNNNNANCCCCGGTCGNNNNCNNATTGAANNCCTGNNGCATNNNNNTNCNAGAATGANNNC
NNNNNGNNTNGNCTNNNNNNTCAANNAATCANNNAGGTNNNNNNNTCNNNGNNNNNNNNCNNNNNCNN
ACTGAGGGNNNNNNNNNCANNNNCTNNNNNNNNNNTNCGCNGGNGTNNCNNNNNNGCNGTATNNNGTNA
NNCANNNNNNNNNNNNNNNNNTNANNNNANNNNNNTNNNG
>Kinh05
GTGNNNNNTAGCCCNNNNNAGTTNNACANNNCNNNNNNNNNNNNNTNNNNNNNNNNNNNNNNNNNNNNNN
NNNNNNNNNNNNNNNNNNNNNNNNNNNNNNNNNNNNNNNNNNNNNNNNNNNNNNNNNNNNNNNNNNNNNN
NNNNNNNNNNNNNNNNNNNNNNNNNNNNNNNNNNNNNNNNNNNNNNNNNNNNNNNNNNNNNNNNNNNNNN
NNNNNNNNNNNNNNNNNNNNNNNNNNNNNNNNNNNNNNNNNNNNNNNNNNNNNNNNNNNNNNNNNNNNNN
NNNNNNNNNNNNNNNNNNNNNNNNNNNNNNNNNNNNNNNNNNNNNNNNNNNNCNTCTNNNAAANNNTNNN
NNNNNNNNNNNNNNNNNNNNNNNNNNNNCNNTNNNNGTAANCNANNNGNACATGCAGTNNNCNCNNNNN
NNNNNCAGAGNNNNNNNCNNNNNNNNCCGNCCCCNNCCNNNNNNNNNNNNNNNNNNNNNNNNNANNNNNN

NNNNNNTGGTACNNNNCGNNNNNGGTTGNNTNAGAAGTCCACAACGNGNTCNNNNNANNNNNGNNNAN
NNNCNNNNNNNNNNNNNNNNNNNNNNTNNNTANNAGGNNNNNNCCNTNNNNNNNNNNNANNGCNATGACC
CANNNNNCNGNGGNNGTNCCCGGNGNNGCGACCTTNNNNANNTCCGGGGNCAAGTATGGCGNNCNCCC
CGNGAAGGANNCNGCNGTANGTGCNNNNNANNNNNTNNTGNGANNTNGCCGNNATTNNCGTGGTCNAN
NNAGAATGNCCNNACCANNCGNANNNTGGTNATGNNAGTNNNNNNNNNNNNNNNNNNNNNNNNNNNNNNN
NNNNNNNNNNNNNNNNNNNNNNNNNNNNNNNNNNNGNNNNGNNNNNNCNANNNAAGGNNNTANNNCTNNNN
NNNNNNNNGGACNNNACNNNNNNNNNNNNNACNCNNNCNNNNNNNNNNNNNGNTTNNNNNNCNNTANTNT
CCAGNANGCNGNGCNNNCNTTCANGNGAACNNNGNNACGCGNNAATCGTANNATCTACCTNCCGCNNG
TNNNNNTNCNGNNNNNNCGGANNNTNNNNCGTANNNNNNNCANGCGTNNNNTNNTCNCNAGNCNNGTT
CGNNNANANANTAGNANGTNNNNNNNNNNNNTGTNNCGTTCCGCNTGANNCNNCANNNNGNNGNNCTTN
NCANCGGCNNTANCTNNNGANNTAAGNCATANNNTNNNTCNNTANCTNNNNNGGNNGGNNACTGTAAA
NGNNNAATTNGNNNNTNANTNCNNNCANNNNNCNNNGNNTNCTNNNTNCCTGNNNNCNNAATTGGNNN
CANTGNNTGNCNATCTCTNGNTTNGNNNNNNTNNNNNTNNCANNNNGGNNNNNNANANNNNNNATNTAA
NNTANANNNNCNNNNNNNNNNNCACNNNNNNNNNAGNNNNNANNNGNANANNNGTAGNNNNNNTNAGTCT
CCGANNTNNNNNNNATAAGCTNNANACNNNNNNNNNNNNNNNNGANNNCGNNTTTGNNNTNNNNNNNNNA
GNNAGACNNTNGTGGNTNNNNNTCAANNNNTTNGTAGTGGTATAATNGNNNNNNNTTTCNCCAGNNNN
NNNNNNNNNNNCNATANNANNNNNNNGNNNNNNNNNNNNNNNNNNNNTCNNNAGGGANCANCTACTTNA
GCANTGGNGCGNAGNNNGAGNNNNNGACNNNCCTTGGGTGGCAANNACNGNANNAAGTNNGTTNNNC
CTNNNNGTNNACACNNNNNCANNGNNNCNNNNNNANCNNANNGNNNNNNNNNNNNNNNNNNNNNNNNNN
NNNNNNNNNNNNNNNNNNNNNNNNNNNNNNNNNNNNNANGGNNNNNNTNTGACNCATGNNTTTTCANNNGNN
NNNNNACGNNNNANCCCCGGTCGNNTNCNNATTGAANNCCTGNNGCNTNNNNNTNCNAGAATGNNNNC
NNNNNGNNTNGNCTNNNNNNNTCAAGNAATCANNNAGNNNNNNNNNTCNNNNNNNNNNNNCNNNNNNNN
ACTGAGGNNNNNNNNNNCANNTGCTNNNNNNNNNNTNCGCNNNNGTNNCNNNNNAGCNGTATNNNGTNN
NNCANNNNNNNNNNNNNNNNNNCNAAACNNNNNNNCTNNNN
>Kinh06
GTGNNNNNNAGCCCNCNNNAGTTAGACANNNCNNNNNNNNNNNNTNNNNNNNNNNNNNNNNNNNNNNN
NNNNNNNNNNNNNNNNNNNNNNNNNNNNNNNNNNNNNNNNNNNNNNNNNNNNNNNNNNNNNNNNNNNNNN
NNNNNNNNNNNNNNNNNNNNNNNNNNNNNNNNNNNNNNNNNNNNNNNNNNNNNNNNNNNNNNNNNNNNNN
NNNNNNNNNNNNNNNNNNNNNNNNNNNNNNNNNNNNNNNNNNNNNNNNNNNNNNNNNNNNNNNNNNNNNN
NNNNNNNNNNNNNNNNNNNNNNNNNNNNNNNNNNNNNNNNNNNNNNNNNNNNNNNNTCNNNNAAANNNTNNN
NNNNNNNNNNNNNNNNNNNNNNNNNNNCNCNNTNNNNGTAANCNANNNGNACATGCAGTCCCCTCNNNNN
NNNNNCAGAGNNNNNNNCCNNNNNNNCCGNCCCCNNCCNNNNNNNNNNNNNNNNNNNNNNNNNNANNNNNN
NNNNNNTGGTACNNNNCGNNNNNGGTTGNNTNAGNGGTCCACAACGNGGTCNNGNNACNNNNGNNNAN
NNNCNNNNNNNNNNNNNNNNNNNNNNTNNNTANNAGGNNNNNNCCNTNNNNNNNNNNNANNGCNATNANN
CNANNCNCNGNGGCNGTNCCCGGNGNNGCGACCTTGNNNANNTCCGGGGACAAGTATGGCNNNCNCCC
CGGGAAGGANNCNGCCGTANGTGCNNNNNANNNNNTCNTGNGANGTNGCCGNNATTCNNGTGGTCNNN
CNAGAATGNCCNNACCANNCGNANNNTGGTNATGNNAGTNNNNNNNNNNNNNNNNNNNNNNNNNNNNNNN
NNNNNNNNNNNNNNNNNNTNNNNNNNNNNNNNNNNGNNNNGNNNNNNCNANNNAAGGNNNTANNNCTNNTN
NNNNNNNNNNNNCNNNACNNNNCNNNNNNNNACNCNNNCNNNNNNNNNANNNGNTTTTNNNCCNNTATTNT
CCAGNACGCNGNGCNNNCNTTCANGNGAACNNNGNNACGCGNNAATCGTANNATCTACCTGCCGCNNG
TNNNNNTNCNGNNNNNNNCGGANNNTNNCTNGTANNNNNNNNCATGCGTNNTNTNNTCNCNAGNCANGTT
CGNNNANNNANTAGNANGTNTNNNNNNNNNNTGTNNCGTTCCGCNTGANNCNNCNNNNNGNNGNNCTTN
NCANCGGNNNTANCTNNNGANNTAAGNCATANNNTNNNTCNNTANCTNNNNNGGNGGGNNACTGTAAA
NGGNNAATTNGNNNNTNATTNCNNNCACNNNNCTNNGNNNNCTNNNTNCCTGNNTNCNNAATTGGNNN
CANTGNNTGNCNATCTCTGGNTTNGNNNNNNTNNNNNTNNCAANNNGGNNNNNNNNANNNCNATNTAA
NNTANANNNNCNNNNNNANNNCACNNNNNNNNNAGNNNCNNNNNTNANANNNGTAGNNNANTAAGTCN
CCGANNTNNNNNNNATAAGCGNNANACNNNNNNNNNNNNNNNANGANNNCGNNTTGGNNNTNNNNNNCNA
GNNAGACNNTNATGGNTNNNNNTCAANNNNTTNGTAGTGGTATAATNGNNNNNNNTTTCNCCAGNNTN
NNNNNNNNNNNCNATANNANNNNNNNGNGANNNNNNNNNNNNNNNNCTCNNNAGGNANCANCTACTNNA
GCANTGGNNCGNAGNNNGAGANNNNGACNNGCCTTGGGTGGCAANCACNGCAATAAGTNNNGTTNANC
CTNNNAGTNNACATNNNNNCANNGNNNCNNNNNNANCNGANNGNNNNNNNNNNNNNNNNNNNNNNNNNN
NNNNNNNNNNNNNNNNNNNNNNNNNNNNNNNNNNNNNANGGNNNGTNTNTGNCNCATGTGTTTNTANNNGNN
NNNNNACGNNNGANCCCCGGTCGNNNNCNNATTGAANNCCTGNNNCNTNNNNNTNCNAGAATGANNGC
NNNNNGNNTNGNCTNNNNNNNTCAAGNAATCANNNAGGTANNNNNNTCNNNNNNNNNNNNNCNNNNNCNN

ACTGAGGGNNNNNNNNNCATCNGCTNNNNNNNNNTNCGCNGGNNTNNCNNNNNNGCNGTATNGNGNNN
NNCANNNNNNNNNNNNNNNNNTNANNNGANNNNNNNTNNNN
>Kinh07
GTGNNNNNNAGCCCNNNNNAGTTNNNCANNNCNNNNNNNNNNNNNNNNNNNNNGNNNNNNNNNNNNNN
NNNNNNNNNNNNNNNNNNNNNNNNNNNNNNNNNNNNNNNNNNNNNNNNNNNNNNNNNNNN
NNNNNNNNNNNNNNNNNNNNNNNNNNNNNNNNNNNNNNNNNNNNNNNNNNNNNNNNNNNN
NNNNNNNNNNNNNNNNNNNNNNNNNNNNNNNNNNNNNNNNNNNNNNNNNNNNNNNNNNNN
NNNNNNNNNNNNNNNNNNNNNNNNNNNNNNNNNNNNNNNNNNNNNNNNNCTNNNAAANNNTNNN
NNNNNNNNNNNNNNNNNNNNNNNNNNNNCNNTGNNNGTAANCNANNNGNACATGCAGTCCCCTCNNNNN
NNNNNCAGAGNNNNNNNCCNNNNNNNCCGNNCCCNNCCNNNNNNNNNNNNNNNNGNNNNNNNANNNNNN
NNNNNNTGGTACNNNNCGNNNNNGGTTGNNNNAGAGGTCCACAACGNGNTCNNNNNACNNNNGNNNAN
NNNCNNNNNNNNNNNNNNNNNNNNNNNTNNNTANNAGGNNNNNNCCNNNNNNNNNNNNAANNGCNATGACC
CANNNNNCNGGGGNNGTNCCCGGNGNGGNNACCTTNANNACNTCNGGGNNCAAGTATGGCNNNCNCCC
CGNGAAGGANNCNGCNGTANGTGCNNNNNANNNNNTCNTGNGANGTNGCCGNNATTNNCGTGGTCTNN
NNNGAATGNCCNNACCANNCGNANNNTGGTNATGNNAGTNNNNNNNNNNNNNNNNNNNNNNNNNNNNN
NNNNNNNNNNNNNNNNNNNNNNNNNNNNNNNNNNGNNNNGNNNNNNCNANNNAAGGNNNTANNNCTNNTN
NNNNNNNNGGACNNNACNNNNNNNNNNNNNACNCNNNCNCNNNNNNANNNGNTTNNNNNNCNNTNTNNT
CCAGNANGCNGNGCNNNCNTTCANGNGAACNNNGNNACGCGNNAATNGTANNNTCTACCTGCCGCNNG
NNNNNNTACNGNNNNNNCGGANNNTNNNNNGTANNNNNNNCNNGCGTNNNNTNNTCNCNAGNCANGTT
CGNNNANNNANTANNANGTNNNNNNNNNNNNTGTNNCGTTCCGCNTGANNCNNCNNNNNGNNGNNCTTN
NCANCGGCNNTANCTNNNGANNTAAGNCATANNNTNNATCNNTANCTNNNNNGGNGGGNNACTGTAAA
NGNNNAATTNGNNNNTNATNNCNNNCACNTNNCNNNGNNNNCTNNNNTNCCTGNNNNCNNAATTGGNNN
CANTGNNTGNCNATCTCTGGNTTNGCNNNNNTNNNNNTNNCANNNNGGNNNNNNNNNANNNNNATNTAA
NNTANANNNNCNNNNNNANNNCACNNNNNNNNNNAGNNNCNNNNNTNANANNNGTAGNNNANTNAGTCN
CCGANNTNNNNNNNATAAGCGNNANACNNNNNNNNNNNNNNANGANNNCGNNTTGGNNNTNNNNNNNNNA
GNNAGACNNTNATGGNTNNNNNTCAANNNNNTTNGTAGTGGTATAATNGNNNNNNNTTTCNCCAGNNTN
NNNNNNNNNNNCNNNNNNANNNNNNNGNGANNNNNNNNNNNNNNNNNTCNNNAGGNANCANCTACTNNA
GCANNGGNGCGNAGNNNGAGANNNNGACNNNCCTTGGGTGGCAANNACNGNGANAAGTNNGTTNNNN
CTNNNAGTNNACATNNNNNCANNGNNNCNNNNNNANCNNANNGNNNNNNNNNNNNNNNNNNNNNNNNN
NNNNNNNNNNNNNNNNNNNNNNNNNNNNNNNNNNNNNANGGNNNGTNTNNGACNCATGTGTTTTTANNNGNN
NNNNNACGNNNNANCCCCGGTCGNNNNCNNATTGAANNCCTGNNGCNTNNNNNNNCNAGAATGANNNC
NNNNNGNNTNGNCTNNNNNNTCANGNAATNNNNNAGNNNNNNNNNTCNNNNNNNNNNNNCNNTCNCNN
ACTGAGGGNNNNNNNNNCANNGNTNNNNNNNNNTNCGCNGGNNNNNCNNNNNNGCNGTATNNNGNNN
NNCANNNNNNNNNNNNNNNNNTNNANCAANNNNNNNTNNNN
>Kinh08
GNNNNNNNTAGCCCNNNNNAGNTNNACANNNCNNNNNNNNNNNNNTNNNNNNNNNNNNNNNNNNNNNN
NNNNNNNNNNNNNNNNNNNNNNNNNNNNNNNNNNNNNNNNNNNNNNNNNNNNNNNNNNNN
NNNNNNNNNNNNNNNNNNNNNNNNNNNNNNNNNNNNNNNNNNNNNNNNNNNNNNNNNNNN
NNNNNNNNNNNNNNNNNNNNNNNNNNNNNNNNNNNNNNNNNNNNNNNNNNNNNNNNNNNN
NNNNNNNNNNNNNNNNNNNNNNNNNNNNNNNNNNNNNNNNNNNNNNNNNNNNNNNNNNNNNNNAANNNNNNN
NNNNNNNNNNNNNNNNNNNNNNNNNNCNCNNTNNNNGTAANCNANNNGNACATGCAGTNCCCTCNNNNN
NNNNNCAGAGNNNNNNNCNNNNNNNNNCCGNCNCCNNCCNNNNNNNNNNNNNNNNGNNNNNNNANNNNNN
NNNNNNTGGTACNNNNCGNNNNNGGTTGNNTAAGAAGTCCACAACGNGNTCNNNNNACNNNNGNNNAN
NNNCNNNNNNNNNNNNNNNNNNNNNNNNNNTANNAGGNNNNNNCCNNCNNNNNNNNNNANNGCNATNANN
CANNNNNCNGNNGNNGTNCCCGGNGNNGNGNCCNNGNNNANNNNNGGGNNCAAGTATGGCGNNCNCNC
CGNGAAGGANNCNNCNGTANGTGCNNNNNANNNNNNNNTGNGNNGTNGCCGNNANNNNCNTGGTCNNN
CNAGAATGNCCNNACCANNCGNANNNTGGTNATGNNAGTNNNNNNNNNNNNNNNNNNNNNNNNNNNNN
NNNNNNNNNNNNNNNNNNNNNNNNNNNNNNNNNNNNNNNNNGNNNNNNCNANNNAAGGNNNTANNNCTNNTN
NNNNNNNNNNNCNNNACNNNNNNNNNNNNNACNCNNNCNNNNNNNNNNNNNNNTTNNNNNNCNNTNNNNN
CCAGNANGCNGNGCNNNCNTTCANNNGAACNNNGNNACGCGNNAATCGTANNATNNNNCTGCCGCNNG
NNNNNNTNCNNNNNNNNNCGGANNNTNNNNNGTANNNNNNNCNNGCGTNNNNTNNTNNCNAGNCANGTT
CGNNNANANANTAGNNNGTNNNNNNNNNNNNTGTNNCGTTCCGCNTGANNCNNCNNNNNGNNGNNNTTN
NCANCGGNNNTANCTNCNGNNNTANGNCANNNNNTNNNNCNNTANCTNNNNNNGNNGGNNACTGNAAA
NNNNNNNTTNGNNNNTNANNNCNNNCANNNNNCNNNGNNNNCNNNNTNCCTGNNNNCNNAATTGGNNN

CNNTGNNTGNCNATCTCTNGNNTNGNNNNNNNNNNNNNNTNNNANNNNGGNNNNNNANANNNNNATNTNA
NNTNNNNNNNCNNNNNNANNNCANNNNNNNNNNNAGNNNNNNNNNGNANANNNGTAGNNNGNTNAGTCN
CCGANNTNNNNNNNNTAAGCTNNNNACNNNNNNNNNNNNNNANGANNNCGNNTTTGNNNTNNNNNNNNNA
GNNAGACNNTNGTGGNNNNNNNNTCAANNNNTTNGNAGTGGTATAANNGNNNNNNNTTTCNCCAGNNNN
NNNGNNNNNNNNCNATANNANNNNNNNNNGANNNNNNNNNNNNNNNNNTCNNNAGGNANCANCNACTNNA
GCANNGGNNCNNAGNNNNAGNNNNNGACNNNCCTTGGGTGGCAANNACNGCANTAAGTNNNGTTNNNN
NTNNNAGTNNACACNNNNNCANNGNNNCNNNNNNANCNGANNGNNNNNNNNNNNNNNNNNNNNNNNNN
NNNNNNNNNNNNNNNNNNNNNNNNNNNNNNNNNNNANGGNNNGTNNNNGNCNCANGTGTTTNCANNNGNN
NNNNNNNGNNNNANCCCCGGTCGNNNNCNNATTGAANNCCTGNNNCATNNNNNNNCNANNNNGNNNNN
NNNNNNNNTNGNCTNNNNNNNTCAAGNAATNNNNNAGNNNNNNNNNTCNNNNNNNNNNNNCNNNNNCNN
ACNNNGGNNNNNNNNNNCANNNNNNNNNNNNNNNNTNCGCNGGNNTNNCNNNNNNGCNGTATNNNGTNN
NNCANNNNNNNNNNNNNNNNNCNNANNNANNNNNNTNNNN
>Kinh10
GTGNNNNNNAGCCCNNNNNAGTTNGACANNNCNNNNNNNNNNNNNTNNNNNNNNNNNNNNNNNNNNNNN
NNNNNNNNNNNNNNNNNNNNNNNNNNNNNNNNNNNNNNNNNNNNNNNNNNNNNNNNNNNNNNNNNNNNNN
NNNNNNNNNNNNNNNNNNNNNNNNNNNNNNNNNNNNNNNNNNNNNNNNNNNNNNNNNNNNNNNNNNNNNN
NNNNNNNNNNNNNNNNNNNNNNNNNNNNNNNNNNNNNNNNNNNNNNNNNNNNNNNNNNNNNNNNNNNNNN
NNNNNNNNNNNNNNNNNNNNNNNNNNNNNNNNNNNNNNNNNNNNNNNNNNNCNNNNNNNNAANNNTNNN
NNNNNNNNNNNNNNNNNNNNNNNNNNNNCNNTNNNNGTANNCNANNNGNNCATGCAGTNCCCNCNNNNN
NNNNNNAGAGNNNNNNNNNNNNNNNNNNCCGNCCCCNNCCNNNNNNNNNNNNNNNNNGNNNNNNNANNNNNN
NNNNNNTGGTACNNNNCGNNNNNGGNNGNNTNAGAGGTCCACAACGNGNNCNNNNNANNNNNGNNNAN
NNNCNNNNNNNNNNNNNNNNNNNNNNNNNNNTANNAGGNNNNNNCCNNNNNGNNNNNNANNGCNATGACC
CNNNNCNCNGGNGNNGNNCCCGGCGNNGNGACCTTNNNNNCGNCNGGGGNCAAGTATNGCNNNCNCCN
NGGGAAGGANNCNGCCGTANGTGCNNNNNANNNNNTNNTGNGANGTNGCCGNNNNNNNCNTGGTCNNN
NNNGAATGNCCNNACCANNCGNANNNTGGTNNNGNNAGTNNNNNNNNNNNNNNNNNNNNNNNNNNNNNN
NNNNNNNNNNNNNNNNNNNNNNNNNNNNNNNNNNNNGNNNNGNNNNNNCNANNNANGGNNNTANNNCTNNTN
NNNNNNNNGGACNNNACNNNNCNNNNNNNACNCNNNCNNNNNNNNNANNNGNTTNNNNNCCNNTNNTNT
CCAGNANGNNNNGCNNNCNTTCANGNGAACNNNGNNACGCGNNAATCGNANNATNTANCTNCNGCNNG
NNNNNNTNCNGNNNNNNCGGANNNTNNNNNGNANNNNNNNCNNGCGTNNNNTNNTCNCNAGNCANNTT
CGNNNANNNANTAGNANNTNNNNNNNNNNNTGTNNCNNNCCGCNTGANNCNNCNNNNNGNNGNNNTTN
NCNNNGGNNNNANNTNNNGANNTAANNCATANNNTNNNTCNNTNNCTNNNNNNGNNGGNNACNGTAAA
NNNNNNNTTNGNNNNTNANTNCNNNCACNNNNCNNNGNNNNNTNNNNNCCTGNNNNCNNAATTNGNNN
CANTGNNTGNCNATCTCTGGNTTNGNNNNNNNTNNNNNTNNCANNNNNGNNNNNNNNNNNNNCNANNTAA
NNTANANNNNCNNNNNNANNNCANNNNNNNNNNAGNNNNNANNNTNANANNNNTAGNNNNNTNAGTCT
CCNNNNTNNNNNNNNTAAGNNNNANANNNNNNNNNNNNNNNANGANNNCGNNTTGGNNNTNNNNNNNNNA
NNNANACNNTNATNGNTNNNNNNTNNANNNNTTNNTAGTGNNATAATNGNNNNNNNTTTCNCCAGNNNN
NNNGNNNNNNNNCNATANNANNNNNNNGNGANNNNNNNNNNNANNNNNTCNNNAGGNANCNNCTACNNNA
GNANTGGTNCGNAGNNNGAGANNNNGACNNGCCTTGGGTGGCAANNACNGNGANNAGTNNNGTTNNNC
CTNNNNNTNNACATNNNNNCANNGNNNCNNNNNNANCNNNNNGNNNNNNNNNNNNNNNNNNNNNNNNNN
NNNNNNNNNNNNNNNNNNNNNNNNNNNNNNNNNNNANNGNNNGTNTNTGNNNCNTNNNNTTNNNANNNGNN
NNNNNNNGNNTNNNCCNNGGTCGNNNNNNNATTGAANNNNTGNNNCNTNNNNNTNCNNNAATGANNNN
NNNNNGNNTNNNCTNNNNNNNTCANGNAANNNNNNANGTNNNNNNNTCNNNGNNNNNNNNCNNNCNCNN
ANNGAGGGNNNNNNNNNNCANNNNNTNNNNNNNNNNTNCGCNGGNNNNNCNNNNNNGCNGTATNNNGNNN
NNCANNNNNNNNNNNNNNNNNNTNAAACNANNNNNNNTNNNN
>Kinh11
GTGNNNNNTAGCCCNNNNNAGTTNGNCANNNCNNNNNNNNNNNNNNNNNNNNNNNNNNNNNNNNNNNNNNN
NNNNNNNNNNNNNNNNNNNNNNNNNNNNNNNNTNNNNNNNNNNNNNNNNNNNNNNNNNNNNNNNNNNNNN
NNNNNNNNNNNNNNNNNNNNNNNNNNNNNNNNNNNNNNNNNNNNNNNNNNNNNNNNNNNNNNNNNNNNNN
NNNNNNNNNNNNNNNNNNNNNNNNNNNNNNNNNNNNNNNNNNNNNNNNNNNNNNNNNNNNNNNNNNNNNN
NNNNNNNNNNNNNNNNNNNNNNNNNNNNNNNNNNNNNNNNNNNNNNNNNNNCNNNNNNNAAANNNTNNN
NNNNNNNNNNNNNNNNNNNNNNNNNNNCNCNNTGNNNGTAANCNANNNGNACATGCAGTNCCNTCNNNNN
GNNNNCAGANNNNNNNNNCCNNNNNNNNCCGGCCCCNNCCNNNNNNNNNNNNNNNNNGNNNNNNNANNNNNN
NNNNNNTGGTACNNNNCGNNNNNGGTTGNNTNAGAGGTCCACCACGNANTCNNNNNANNNNNGNNNAN
NNNCNNNNNNNNNNNNNNNNNNNNNNNGNNNCANNAGGNNNNNNCCNNNNNNNNNANNNGNNGCNATNACC

CANNNNNCNGGGGNNGTNCCCGGCNNGGCGACCTTGNNNANGTCCGGGNNCAAGTATGGCGNNTNCCC
CGNGAAGGANNCNGCNGTANGTGCNNNNNANNNNNTCNTGNGANGTNGCCGNNANNNNCGTGGTCNNN
CNNGAATGNCCNNACCANNCGNANNNTGGTNATGNNAGTANNNNNNNNNNNNNNNNNNNNNNNNNNNN
NNNNNNNNNNNNNNNNNNNNNNNNNNNNNNNNNNNNNNNGNNNNNNCNANNNAAGGNNNTANNNCTNNNN
NTNNNNNNNNNNCNNNACNNNNCNNNNNNNACNCNNNCNCNNNNNNANNNGGTTTTNNNNCNNTANTNC
CCAGNANGCNGNGCNNNCNTTCANGNGAACNNNGNNACGTGNNAATCGTANNATCTANCTNCCGCNNG
NNNNNNTNCNGNNNNNNCGGNNNNTNNNNCGTANNNNNNNCNNGCGTNNNNTNNTCNCNAGNCNNGTT
CGNNNANNNANTANNANGTNNNNNNNNNNNTGTNNCGTTCCGCNTGANNCNNCNNNNNGNNGNNNTTN
NCANCGGNNNTANCTGCNGNNNTAAGNCATANNNCNNNTCNNTANCTNNNNNGGNGGGNNACTGNAAA
NNNNNAATTNGNNNNTNATTNCNNNCACNTNNCTNNGNNNNCTNNNNTNCCTGNNTNCNNAGTTGGNNC
NANTANNTGNTNATCTCTGGNTTNGNNNNNNTNNNNNTNNCANNNNGGNNNGTNANANNNNNATNTAA
NNTANANNNNCNNNNNNANNNCACNNNNNNNNNAGNNNCNNNNNTNANANNNGTAGNNNANTNAGTCT
CCGANNTNNNNNNNATAGGCGNNANACNNNNNNNNNNNNNNANGANNNCGNNTTGGNNNTNNNNNNCNA
ANNAGACNNTNGTGGNTNNNNNTCAANNNNCTNGTAGTGGTATAATNGNNNNNNNTTTCNCCAGNNTN
NNNNNNNNNNNCNATNNNANNNNNNNGNNANNNNNNNNNNNANNNNNTCNNNAGGNANCANCTACTNNA
GCANTGGNNCGNAGNNNTANANNNNGACNNNCCTTGGGTGGCAANNACNGNAATAAGNNNNGTTNNNN
CTNNNNGTNNACACNNNNNCANNANNNCNNNNNNANCNGANNTNNNNNNNNNNNNNNNNNNNNNNNNN
NNNNNNNNNNNNNNNNNNNNNNNNNNNNNNNNNNNANNGNNNGTNTNTGNCNCATGTGTTTNTANNNGNN
NNNNNNNGNNNGANCCCCGGTCGNNNNCNNATTGAANNCCTGNNGCATNNNNNTNCNATAATGANNNC
NNNNNGNNTNGNCTNNNNNNTCAAGNAGTCANNNAGNNNNNNNNNTCNNNNNNNNNNNNCNNTNNCNN
ACTGNGGGTNNNNNNNNCANCNGNTNNNNNNNNNNTNCGCNNGCGTNNCNNNNNATCNGTATNNNGNNN
NNCANNNNNNNNNNNNNNNNNTNNAACNANNNNNNTNNNN
>Kinh12
GTGNNNNNTAGCCCNNNNNAGTTAGACANNNCNNNNNNNNNNNNNTNNNNNNNNNNNNNNNNNNNNNNN
NNNNNNNNNNNNNNNNNNNNNNNNNNNNNNNNTNNNNNNNNNNNNNNNNNNNNNNNNNNNNNNNNNNNNN
NNNNNNNNNNNNNNNNNNNNNNNNNNNNNNNNNNNNNNNNNNNNNNNNNNNNNNNNNNNNNNNNNNNNNN
NNNNNNNNNNNNNNNNNNNNNNNNNNNNNNNNNNNNNNNNNNNNNNNNNNNNNNNNNNNNNNNNNNNNNN
NNNNNNNNNNNNNNNNNNNNNNNNNNNNNNNNNNNNNNNNNNNNNNNNNNNNNNNNTCNNNNAAANNNTNNN
NNNNNNNNNNNNNNNNNNNNNNNNNNCNCNNTNNNNGTAANCNANNNGNACATGCAGTCCCCTCNNNNN
NNNNNCAGAGNNNNNNNCNNNNNNNNNCCGNCCCCNNCCNNNNNNNNNNNNCNNNGNNNNNNNANNNNNN
NNNNNNTGGTACNNNNCGNNNNNGGTTGNNTNAGAGGTCCACAACANGNTCNNNNNACNNNNGNNNAN
NNNCNNNNNNCNNNNNNNNNNNNANNTNNNTANNAGGNCNNNNCCNTCNNNNNNNNNNANTGCNATGACC
CANNNNNCNGGNGNNGTNCCCGGNGCGGCGACCTTGANNANNTCCGGGGNCAAGTATGGCNNNCNCCC
CGGGAAGGANNCNGCNGTANGTGCNNNNNANNNNNTCNTGNGANGTNGCCGNNANNCNCNTGGTCNNN
NNAGAATGNCCNNACCANNCGNANNNTGGTNATGNNAGTNNNNNNNNNNNNNNNNNNNNNNNNNNNNN
NNNNNNNNNNNNNNNNNNNNNNNNNNNNNNNNNNNGNNNNGNNNNNNCNANNNANGGNNNTANNNCTNNTN
NNNNNNNNNNNNCNNNACNNNNCNNNNNNNACNCNNNCNNNNNNNNNANNNGNTTNNNNNNNCNNTNTTNT
CCAGNANGCNGNGCNNNCNTTCANGNGAACNNNGNNACGCGNNAATCGTNNNATCTACCTGCCGCNNG
NNNNNNTACNGNNNNNNCGGANNNTNNCTCGTANNNNNNNCNNGCGTNNTNTNNTNNCNAGNCNNGTT
CGNNNANANANTAGNANGTNTNNNNNNNNNNTGTNNCGTTCCGCNCGANNCNNCANNNNGNNGNNCTTN
NCANCGGCNNTANNTNCNGANNTAAGNCATANNNTNNNTCNNTANCTNNNNNGGNGGGNNACTGTAAA
NGGNNAATTNGNNNNTNATTNCNNNCACNTNNCNNNANNNNCTNNNNTNCCTGNNTNCNNAATTGGNNN
CANTGNNTGNCNATCTCTNGNTTNGNNNNNNTNNNNNTNNCANNNNGGNNNNNNNNANNNCNATNTAA
NNTANANNNNCNNNNNNANNNCACNANNNNNGNAGNNNCNANNNTTANANNNGTAGNNNANTNAGTCT
CCGANNTNNNNNNNATAAGCGNNANACNNNNNNNNNNNNNNANGANNNCGNNTTGGNNNTNNNNNNCNA
GNNAGACNNTNGTGGNTNNNNNTCAANNNNTTNGTAGTGGTATAATNGNNNNNNNTTTCNCCAGNNTN
NNNGNNNNNNNNCNATANNANNNNNNNGNGANNNNNNNNNNNANNNNCTTNNNAGGNANCANTTACTNNA
GCANTGGNNCGNAGNNNGAGANNNNGACNNGCCTTGGGTGGCAANNACNGCAATAAGTNNNGTTNANN
CTNNNAGTNNACACNNNNNCANNGNNNCNNNNNNANCNGANNGNNNNNNNNNNNNNNNNNNNNNNNNN
NNNNNNNNNNNNNNNNNNNNNNNNNNNNNNNNNNNANNGNNNGTNTNTGNCNCATGTGTTTNTANNNGNN
NNNNNNNGNNNNANCCCCGGTCGNNNNCNNATTGAANNCCTGNNGCATNNNNNTNCNAGAATGANNGC
NNNNNGNNTNGNCTNNNNNNTNAAGNAATCNANNAGGTNNNNNNNTCNNNNNNNNNNNNCNNTCNCNN
ACTGAGGGTNNNNNNNNCATNNGCTNNNNNNNNNNTNCGCNGGCGTNNCNNNNNNGCNGTATNNNGTNN
NNCANNNNNNNNNNNNNNNNNNNANACGANNNNNNNTNNNN

>Kinh13
GTNNNNNNTAGCCCNNNNNAGTTNNACANNNCNNNNNNNNNNNNTNNNNNNNNNNNNNNNNNNNNNNN
NNNNNNNNNNNNNNNNNNNNNNNNNNNNNNNNNNNNNNNNNNNNNNNNNNNNNNNNNNNNNNNNNNNN
NNNNNNNNNNNNNNNNNNNNNNNNNNNNNNNNNNNNNNNNNNNNNNNNNNNNNNNNNNNNNNNNNNNN
NNNNNNNNNNNNNNNNNNNNNNNNNNNNNNNNNNNNNNNNNNNNNNNNNNNNNNNNNNNNNNNNNNNN
NNNNNNNNNNNNNNNNNNNNNNNNNNNNNNNNNNNNNNNNNNNNNNNNNNNNNNNNNNNAANNNTNNN
NNNNNNNNNNNNNNNNNNNNNNNNNCNCNNTNNNNGTAANCNANNNGNACATGCAGTCCCCTCNNNNN
NNNNNCAGAGNNNNNNNNNNNNNNNNCCGNCCCCNNCCNNNNNNNNNNNCNNNGNNNNNNNANNNNNN
NNNNNNTGGTACNNNNCGNNNNNGGTTGNNTNAGAGGTCCACAACGNGNTCNNNNNACNNNNGNNNAN
NNNCNNNNNNNNNNNNNNNNNNNNNTNNNTANNAGGNNNNNNCCNNCNNNNNANNNANNGCNATGACC
CANNNCACNGGGGNNGTNCCCGGNGNGGCGACCTTGNNNACGTCCGGGGNCAAGTATGGCNNNCNCCC
CGNGAAGGANNCNGCCGTANGTGCNNNNNANNNNNTCNTGNGANGTNGCCGNNNTTCNNGTGGTCNNN
CNAGAATGNCCNNACCANNCGNANNNTGGTNATGNNAGTNNNNNNNNNNNNNNNNNNNNNNNNNNNNN
NNNNNNNNNNNNNNNNNNNNNNNNNNNNNNNGNNNNGNNNNNNCNANNNNAAGGNNNTANNNCTNNTN
NNNNNNNNGGACNNNACNNNNCNNNNNNNACNCNNNCNCNNNNNNANNNGNTTTTTNNNCNNTNTTNN
CCAGNACGCNGNGCNNNCNTTCANGNGAACNNNGNNACGCGNNAATCGTANNATCTACCTGCCGCNNG
NNNNNNTNCNGNNNNNNCGGANNNTNNNNNGTANNNNNNNCANGCGTNNNNTNNTNNCNAGNCNNGTT
CGNNNANANANTAGNANGTNTNNNNNNNNNTGTNNCGTTCCGCNTGANNCNNCNNNNNGNNGNNCTTN
NCANCGGCNNTANCTGNNGANNTAAGNCATANNNTNNNTCNNTANCTNNNNNGGNGGGNNACTGTAAA
NGGNNAATTNGNNNNTNANTNCNNNCACNNNNCTNNGNNNNCTNNNTNCCTGNNNNCNNAATTGGNNN
CANTGNNTGNCNATCTCTNGNTTNGNNNNNNTNNNNNTNNCANNNNGGNNNNNNNNANNNCNATNTAA
NNTANANNNNCNNNNNNANNNCACNNNNNNNNNAGNNNNNANNNTNANANNNGTAGNNNANTNAGTCT
CCGANNTNNNNNNATAAGCGNNANACNNNNNNNNNNNNNNANGANNNCGNNTTGGNNNTNNNNNNNNA
GNNAGACNNTNATGGNTNNNNNTCAANNNNTTNGTAGTGGTATAANNGNNNNNNNTTTCNCCAGNNTN
NNNNNNNNNNNCNATANNANNNNNNNGNGANNNNNNNNNNANNNNCTCNNNAGGNANCANCTACTNNA
GCANNGGNNCNNAGNNNGAGANNNNGACNNNCCTTGGGTGGCAANCACNGCGATAAGTNNNGTTNNNC
CTNNNAGTNNACATNNNNNCANNGNNNCNNNNNNANCNGANNGNNNNNNNNNNNNNNNNNNNNNNNNN
NNNNNNNNNNNNNNNNNNNNNNNNNNNNNNNNNANGGNNNGTNTNTGNCNCATGTGTTTTTANNNGNN
NNNNNACGNNNNNNCCCCGGTCGNNNNNNNATTGAANNCCTGNNGCATNNNNNTNCNAGAATGANNGC
NNNNNGNNTNGNCTNNNNNNNTCAAGNAATCNNNNAGGTNNNNNGNTCNNNNNNNNNNNCNNNCNCNN
ACTGAGGGTNNNNNNNNCANNNGNTNNNNNNNNNTNCGCNGGCGTNNCNNNNNAGCNGTATNNNGTNA
NNCANNNNNNNNNNNNNNNNNNAAACNNNNNNNNTNNNN
>Kinh14
GTGNNNNNTAGCCCNNNNNAGTCAGACANNNCNNNNNNNNNNNNTNNNNNNNNNNNNNNNNNNNNNNN
NNNNNNNNNNNNNNNNNNNNNNNNNNNNNNTNNNNNNNNNNNNNNNNNNNNNNNNNNNNNNNNNNNNNN
NNNNNNNNNNNNNNNNNNNNNNNNNNNNNNNNNNNNNNNNNNNNNNNNNNNNNNNNNNNNNNNNNNNN
NNNNNNNNNNNNNNNNNNNNNNNNNNNNNNNNNNNNNNNNNNNNNNNNNNNNNNNNNNNNNNNNNNNN
NNNNNNNNNNNNNNNNNNNNNNNNNNNNNNNNNNNNNNNNNNNNNNNNNNNNNNNNNNNAANNNTNNN
NNNNNNNNNNNNNNNNNNNNNNNNNCNCNNTGNNNGTAANCNANNNGNACATGCAGTCCCCTCNNNNN
NNNNNCAGAGNNNNNNCCCNNNNNNNCCGNCCCCNNCCNNNNNNNNNNNNNNNGNNNNNNNANNNNNN
NNNNNNTGGTACNNNNCGNNNNNGGTTGTNTNAGAGGTCCACAACGNGGTCNNNNNACNNNNGNNNAN
NNNCNNNNNNNNNNNNNNNNNNNNNTNNNTANNAGGNCNNNNCCNTNNNNNNANNNNNNGCNNTGACC
CAANNNNCNGNGGNNGTNCCCGGCGCNGCGNCCTTGNNNACNNCCGGGGNCAAGTATGGCGNNCNCCC
CGGGAAGGANNCNGCNGTANGTGCNNNNNANNNNNTCNTGNGANGTNGCCGNNATTCNCGTGGTCNNN
CNAGAATGNCCGNACCANNCGNANNNTGGTNATGNNAGTNNNNNNNNNNNNNNNNNNNNNNNNNNNNN
NNNNNNNNNNNNNNNNNNNNNNNNNNNNNNNNNNNNGNNNNNNCNANNNNAAGGANNTANNNCTNNTN
NNNNNGNNGGACNNNACNNNNCNNNNNNNACNCNNNCNCNNNNNNNNNNGNTTTTNNNCCNNTNTTNT
CCAGNANGCNGNGCNNNCNTTTANGNGAACNNNGNNACGCGNNAATCGTANNATCTACCTGCCGCNNG
NNNNNNTNCNGNNNNNNCGGANNNTNNNNCGTANNNNNNNCATGCGTNNTNTNNTCNCNAGNCNNGTT
CGNNNANANANTAGNANGTNNNNNNNNNNNTGTNNCGTTCCGCNTGANNCNNCANNNNGNNGNNCTTN
NCANCNGNNNTANCTNCNGANNTAAGNCATANNNTNNNTCNNTANCTNNNNNGGNGGGNNACTGTAAA
NGNNNAATTNGNNNNTNANTNCNNNCATNNNNCNNNGNNNNCTNNNTNCCTGNNNNCNNAATTGGNNN
CANTGNNTGNCNATCTCTGGNTTNGNNNNNNTNNNNNTNNCAANNNGGNNNNNNANANNNCNATNTAA
NNTANNNNNNCNNNNNNANNNCANNNNNNNNNNAGNNNCNANNNGTANANNNGTAGNNNNNTNAGTCT

CCGANNTNNNNNNNATAAGCGNNANACNNNNNNNNNNNNNNANGANNNCGNNTTTGNNNTNNNNNNNCNA
GNNAGACNNTNGTGGNTNNNNNTCAANNNNNTNGTAGTGGTATAATNGNNNNNNCTTTCNCCAGNNTN
NNNGNNNNNNNNCNATANNANNNNNNNGNGANNNNNNNNNNNNNNNNNTCNNNAGGGANCANCTACTNNA
GCANTGGNGCGNAGNNNGAGANNNNGACNAGCCTTGGGTGGCAANCACNGCAATAAGTNNNGTTNNNC
CTNNNNGTNNACACNNNNNCANNGNNNCNNNNNNANCNGANNGNNNNNNNNNNNNNNNNNNNNNNNNN
NNNNNNNNNNNNNNNNNNNNNNNNNNNNNNAGNNNANNGNNNGTNTNTGACNCATGTGTTTTTANNNGNN
NNNNNACGNNNNANCCCCGGTCGNNNNCNNATTGAANNCCTGNNGCNTNNNNNTNCNANAATGANNNC
NNNNNGNNTNNNCTNNNNNNNTCAAGNAATCANNNAGGTANNGCGNTCNNNGNNNNNNNNCNNNCNCNN
ACNGAGGNNNNNNNNNNNCATNNGCTNNNNNNNNNNTNCGCNGNCGTNNCNNNNNAGCNGTATNGTGTNA
NNCANNNNNNNNNNNNNNNNNNNNAANNNNNNNNNNCTNNNN
>Kinh15
GTGNNNNNTAGCCCNNNNNAGTTNGACANNNCNNNNNNNNNNNNTNNNNNNNNNNNNNNNNNNNNNNN
NNNNNNNNNNNNNNNNNNNNNNNNNNNNNNNNNNNNNNNNNNNNNNNNNNNNNNNNNNNNNNNNNNNCNN
NNNNNNNNNNNNNNNNNNNNNNNNNNNNNNNNNNNNNNNNNNNNNNNNNNNNNNNNNNNNNNNNNNNNNN
NNNNNNNNNNNNNNNNNNNNNNNNNNNNNNNNNNNNNNNNNNNNNNNNNNNNNNNNNNNNNNNNNNNNNN
NNNNNNNNNNNNNNNNNNNNNNNNNNNNNNNNNNNNNNNNNNNNNNNNNNNNNNCNNNNAAANNNTNNN
NNNNNNNNNNNNNNNNNNNNNNNNNNCNCNTTNNNNGTAANCNANNNGNACATGCAGTCCCCTCNNNNN
NNNNNCAGAGNNNNNNCCCNNNNNNNCCGNCCCCNNCCNNNNNNNNNNNNNNNNGNNNNNNNANNNNNN
NNNNNNTGGTACNNNNCGNNNNNGGTTGTNTNAGAGGTCCACCACGNGGTCNNNNNACNNNNGNNNAN
NNNCNNNNNNNNNNNNNNNNNNNNNNTNNNTANNAGGNNNNNNCCNTNNNNNNNNNNNANNGCNATGACC
CANNNNNCNGNGGNNGTNCCCGGCGNGGCGACCTTGNNNANGNCCGGGNNCAAGTATGGCNNNCNCCC
CGGGAAGGANNCNGCNGTANGTGCNNNNNANNNNNTCNTGNGANGTNGCCGNNATTNNCGTGGTCNNN
CNAGAATGNCCNNACCANNCGNANNNTGGTNATGNNAGTNNNNNNNNNNNNNNNNNNNNNNNNNNNNN
NNNNNNNNNNNNNNNNNNNNNNNNNNNNNNNNNNNNNNNNNGNNNNNNCNANNNAAGGANNTANNNCTNNTN
NTNNNNNNNGGACNNNACNNNNCNNNNNNNNACNCNNNCNCNNNNNNNANNNGNTTTTNNNNCNNTANTNT
CCAGNANGCNGNGCNNNCATTCANGNGAACNNNGNNACGCGNNAATCGTANNATCTACCTGCCGCNNG
NNNNNNTACNGNNNNNNCGGANNNTNNCTCGTANNNNNNNTTATACGTNNTNTNNTCNCNAGNCNNGTT
CGNNNANANANTAGNANGTNNNNNNNNNNNNTGTNNCGTTCCGCNTGANNCNNCANNNNGNNGNNCTTN
NCANCGGCNNTANCTNNNGANNTAAGNCATNNNNNTNNNTCNNTANCTNNNNNGGNGGGNNACTGTAAA
NGNNNAATTNGNNNNTNANTNCNNNCACNTNNCNNNGNNNNCTNNNTNCCTGNNTNCNNAATTGGNNN
CANTGNNTGNCNATCTCTGGNTTNGNNNNNNTNNNNNTNNCANNNNGGNNNNNNANANNNCNATNNAA
NNTANANNNNCNNNNNNANNNCACNNNNNNNNNNAGNNNNNNNNNNTNANANNNGTAGNNNNNTNAGTCN
CCGANNTNNNNNNNATAAGCGNNANACNNNNNNNNNNNNNNANGANNNCGNNTTGGNNNTNNNNNNNCNA
GNNAGACNNTNGTGGNTNNNNNTCAANNNNNTTNNTAGTGGTATAATNGNNNNNNNTTTCNCCAGNNTN
NNNGNNNNNNNNCNATANNANNNNNNNGNGANNNNNNNNNNNANNNNNTCNNNAGGNANCANCTACTNNA
GCANTGGNGCGCAGNNNGAGANNNNGACCNGCCTTGGGTGGCAANCACNGCAATAAGTNNNGTTNNNC
CTNNNAGTNNACACNNNNNCANNGNNNCNNNNNNANCNGANNGNNNNNNNNNNNNNNNNNNNNNNNNN
NNNNNNNNNNNNNNNNNNNNNNNNNNNNNNNNNNNANGGNNNGTNTNTGACNCANGTGTTTTTANNNGNN
NNNNNACGNNNNANCCCCGGTCGNNNNCNNATTGAANNCCTGNNNCNTNNNNNTNCNAGAATGANNNC
NNNNNGNNTNGNCTNNNNNNNTCAAGNAGTCANNNAGGTNNNNNNNTCNNGNNNNNNNNNNCNNNCCCNN
ACTNAGNNTNNNNNNNNNCANNNNCTNNNNNNNNNNTNCGCNGNNGTNNCNNNNNAGCNGTATNNNGNNN
NNCANNNNNNNNNNNNNNNNNNTNANACNANNNNNNNTNNNN
>Kinh16
GTGNNNNNTAGCCCNNNNNAGTTANACANNNCNNNNNNNNNNNNNTNNNNNNNNNNNNNNNNNNCNNNN
NNNNNNNNNNNNNNNNNNNNNNNNNNNNNNNNNNNNNNNNNNNNNNNNNNNNNNNNNNNNNNNNNNNNNN
NNNNNNNNNNNNNNNNNNNNNNNNNNNNNNNNNNNNNNNNNNNNNNNNNNNNNNNNNNNNNNNNNNNNNN
NNNNNNNNNNNNNNNNNNNNNNNNNNNNNNNNNNNNNNNNNNNNNNNNNNNNNNNNNNNNNNNNNNNNNN
NNNNNNNNNNNNNNNNNNNNNNNNNNNNNNNNNNNNNNNNNNNNNNNNNNNNNNNNNNNNNAANNNTNNN
NNNNNNNNNNNNNNNNNNNNNNNNNNNNNNNNNTNNNNGTAANCNANNNGNACATGCAGTCCCCTCNNNNN
NNNNNCAGAGNNNNNNNNNCNNNNNNNCCGNCCCCNNCCNNNNNNNNNNNNNNNNGNNNNNNNANNNNNN
NNNNNNTGGTACNNNNCGNNNNNGGTTGNNTAAGAGGTCCACAACGNGNTCNNGNNACNNNNGNNNAN
NNNNNNNNNNNNNNNNNNNNNNNNNNTNNNTANNAGGNNNNNNCCNNNNNNNNNNNNNNNNGNNNTGANN
CNNNNNNCNGNNGNNGTNCCCGGNGNGGCGACCTNGNNNANNNCNGGGGNCAAGTATGGCNNNCNCCC
CGGGAAGGANNCNGCNGTANGTGCNNNNNANNNNNTNNTNNGANGTNGCCGNNATTNNCGTGGTCNNG

CNNGAATGCCCNNACCANNCGNANNNTGGTNATGNNAGTNNNNNNNNNNNNNNNNNNNNN
NNNNNNNNNNNNNNNNNNNNNNNNNGNNNNGNNNNNNCANNNAAGGNNNTANNNCTNNNN
NNNNNNNNGGNCNNNACNNNNCNNNNNNNACNCNNNCNCNNNNNNNNNNGNTTNNNNNNCNNTANTNT
CCAGNANGCNGNGCNNNCNTTCANGNGAACNNNGNNACGCGNNAATCGTANNATCTACCTGCCGCNNG
NNNNNNTNCNGNNNNNNCGGANNNTNNNNCGTANNNNNNTCANGCGTNNTNTNNTNNCNAGNCANGTT
CGNNNANANANTAGNANGTNNNNNNNNNNNNTGTNNCGTTCCGCNTGANNCNNCNNNNNGNNGNNNTTN
NCANCGGNNNTANNTNNNGANNTANGNCATNNNNTNNNTCNNTANCTNNNNNGGNNGGNNACTGNAAA
NGNNNNATTNGNNNNTNATTNCNNNCACNNNNCNNNGNNNNCTNNNTNCCTGNNTNCNNAATTNGNNN
NANTGNNTGNCNATCTCTNGNTTNGNNNNNNTNNNNNTNNCANNNNGNNNNNNNANANNNNNNATNTAA
NNTANANNNNCNNNNNNANNNCACNNNNNNNNNAGNNNNNNNNNTNANNNNNGTAGNNNANTNAGTCT
CCGANNTNNNNNNNNNTAAGCGNNANACNNNNNNNNNNNNNNANGANNNCGNNTTGGNNNTNNNNNNNNNA
GNNAGACNNTNATGGNTNNNNNTCAANNNNTTNGTAGTGGTATAATNGNNNNNNNTTTCNCCAGNNNN
NNNGNNNNNNNNCNATANNANNNNNNNNNNNANNNNNNNNNNNNNNNNNTCNNNAGGNANCANCTACTNNA
GCANTGGNNCGNAGNNNGAGNNNNNGACNNNCCTTGGGTGGCAANNACNGNGANAAGTNNNGTTNNNC
CTNNNAGTNNACATNNNNNCANNGNNNCNNNNNNANCNGANNGNNNNNNNNNNNNNNNNNNNNNNNNN
NNNNNNNNNNNNNNNNNNNNNNNNNNNNNNNNNNNANGGNNNGTNTNTGNCNCANGNNTTTTTANNNGNN
NNNNNNNGNNNNANCCCCGGTCGNNNNCNNATTGAANNCCTGNNNCNTNNNNNTNCNANAATGANNNC
NNNNNGNNTNGNCTNNNNNNTCAAGNAATCANNNAGGNNNNNNNNTCNNNNNNNNNNNNCNNNNNCNN
ACTGANGNTNNNNNNNNCANNNGCTNNNNNNNNNTNCGCNGGNGTNNCNNNNNAGCNGTATNNNGTNA
NNCANNNNNNNNNNNNNNNNNNNNNNNNNNNNNNTNNNN
>Kinh17
GNGNNNNNNAGCCCNNNNNAGTTANACANNNCNNNNNNNNNNNNNNNNNNNNNNNNNNNNNNNNNNN
NNNNNNNNNNNNNNNNNNNNNNNNNNNNNNNNNNNNNNNNNNNNNNNNNNNNNNNNNNNNNNNNNN
NNNNNNNNNNNNNNNNNNNNNNNNNNNNNNNNNNNNNNNNNNNNNNNNNNNNNNNNNNNNNNNNNN
NNNNNNNNNNNNNNNNNNNNNNNNNNNNNNNNNNNNNNNNNNNNNNNNNNNNNNNNNNNNNNNNNN
NNNNNNNNNNNNNNNNNNNNNNNNNNNNNNNNNNNNNNNNNNNNNNNNNNNNCNNCTNNNNAANNNTNNN
NNNNNNNNNNNNNNNNNNNNNNNNNCNNNNTNNNNGTAACCNANNNNNACATGCAGTCCCCTCNNNNN
NNNNNCAGAGNNNNNNNNNNNNNNNNNCCGGCNCCNNCCNNNNNNNNNNNNNNNNNGNNNNNNNANNNNNN
NNNNNNTGGTACNNNNCNNNNNNGGTTGNNTNAGAGGTCCACAACGNGNTCNGNNNACNNNNGNNNAN
NNNCNNNNNNNNNNNNNNNNNNNNNNNTNNNTANNAGGNNNNNNCCNTNNNNNNNNNNNANTGCNATGANN
CAANNCNCNGNGNNNGTNCCCGGNGCNGCGNCCTTGNNNANNTNNGGGNNCAAGTATNGCGNNCNCCC
CGNNAAGGNNNCNNCNGTANGTGCNNNNNANNNNNTCNTGNGANGTNGCCGNNATTNNCGTGGTCNNN
NNAGANTGNCCNNACCANNCGNANNNTGGTNATGNNAGTNNNNNNNNNNNNNNNNNNNNNNNNNNNN
NNNNNNNNNNNNNNNNNNNNNNNNNNNNNNNNNNGNNNNGNNNNNNCANNNANGGNNNTANNNCTNNTN
NNNNNNNNNNNNCNNNACNNNNCNNNNNNNACNCNNNCNCNNNNNNNNNNNGNTTNNNNNNCNNTNNTNN
CCAGNANGCNGNGCNNNCNTTCANGNGAACNNNGNNACGCNNNAATCGTANNATCTANCTNCNGCNNG
NNNNNNTNCNGNNNNNNCGGANNNTNNCTCGTANNNNNNNCATGCNNNNTNTNNTCNCNAGNCNNGTT
CGNNNANNNANTAGNANGNNNNNNNNNNNNNNGTNNCGTTCCGCNTGACNCNNCANNNNGNNGNNNTTN
NNNNCGGCNNTANCTNNNGANNTAAGNNATNNNNNNNNNTCNNTANCTNNNNNGGNGGGNNNCTGNAAA
NGNNNAATTNGNNNNTNANTNCNNNCANNTNNNNNNNNNNNNCTNNNTNCCTGNNTNCNNAATTGGNNN
CANTGNNTGNCNATCTCTGGNNTNGNNNNNNTNNNNNTNNCANNNNGGNNNNNNNNANNNCNATNNAN
NNTANANNNNCNNNNNNNNNNNNACNANNNNNNNNAGNNNNNANNNTNANNNNNGTAGNNNNNTNAGTCN
CCGANNTNNNNNNNNATAAGCGNNANACNNNNNNNNNNNNNNANGANNACGNNTTGGNNNNNNNNNNNNA
GNNAGACNNTNANGGNTNNNNNTNNANNNNTTNGTAGTGGTATAATNGNNNNNNNTTTCNCCAGNNNN
NNNNNNNNNNNCNNNNNNANNNNNNNNNGANNNNNNNNNNNNNNNNNNTCNNNAGGNANCANCTACTNNA
GCANTGGNNCGNAGNNNGNGNNNNNGACNNNCCTTGGGTGGCAANNNNNGNGNTAAGTNNNGTTNANC
CTNNNAGTNNACATNNNNNCANNGNNNCNNNNNNANCNGANNNNNNNNNNNNNNNNNNNNNNNNNNNNN
NNNNNNNNNNNNNNNNNNNNNNNNNNNNNNNNNNNNNNGNNNGTNTNTGACNCATGTGTTNNTANNNGNN
NNNNNNNNNNNNNNNCCCCGGTCGNNNNCNNATTGAANNCCTNNNGNNTNNNNNTNCNAGAATGNNNNC
NNNNNGNNTNNNCTNNNNNNTCAAGNAATCANNNAGNNNNNNNNNTCNNNGNNNNNNNNCNNNNNCNN
ACNGAGGNNNNNNNNNNCANNNGCTNNNNNNNNNTNCGCNGGNNNNNNNNNNNNNGCNGTATNGNGNNA
NNCANNNNNNNNNNNNNNNNNNTNAANNNANNNNNNTNNNN
>Kinh18
GTGNNNNNNAGCCCNNNNNAGTTAGACANNNCNNNNNNNNNNNNTNNNNNNNNNNNNNNNNNNNNNN

NNNNNNNNNNNNNNNNNNNNNNNNNNNNNNNNNNNNNNNNNNNNNNNNNNNNNNNNNNNNNNNNNNNNNN
NNNNNNNNNNNNNNNNNNNNNNNNNNNNNNNNNNNNNNNNNNNNNNNNNNNNNNNNNNNNNNNNNNNNNN
NNNNNNNNNNNNNNNNNNNNNNNNNNNNNNNNNNNNNNNNNNNNNNNNNNNNNNNNNNNNNNNNNNNNNN
NNNNNNNNNNNNNNNNNNNNNNNNNNNNNNNNNNNNNNNNNNNNNNNNNNNNNNTNNNNNAAANNNTNNN
NNNNNNNNNNNNNNNNNNNNNNNNNNCNCNNTNNNNGTAANCNANNNGNACATGCAGTCCCCTCNNNNN
GNNNNCNNAGNNNNNNNCCNNNNNCNCCGNCCCCNNCCNNNNNNNNNNNNNNNGNNNNNNNANNNNNN
NNNNNNTGGTACNNNNCGNNNNNGGTTGTNNAAGNGGTCCACAACGNGNTCNNGNNACNNNNGNNNAN
NNNCNNNNAACNNNNNNNNNNNNNNNTNNNTANNAGGNCNNNNCCNTNNNNNNNNNNNNANTGCNATGANN
CANNNNNCNGGGGNNGTNCCCGGNGNGGCGACCTTGNNNANGTCCGGGNNCAAGTATNGCNNNCNCCC
CGNGAAGGANNCNGCNGTANGTGCNNNNNANNNNNTCNTGNGANGTCGCCGNNANNNNCNTGNNCNNN
CNANANTGNNCNNACCANNCGNANNNTGGTNATGNNAGTNNNNNNNNNNNNNNNNNNNNNNNNNNNNNN
NNNNNNNNNNNNNNNNNNNNNNNNNNNNNNNNNNGNNNNGNNNNNNCNANNNAAGGNNNTANNNCTNNTN
NNNNNNNNNNNCNNNACNNNNCNNNNNNNACNCNNNCNCNNNNNNNNNNGNTTTTNNNNNNNNTNNTNT
CCAGNACGCNGNGCNNNCNTTCANGNGAACNNNGNNACGCGNNAATCGTANNATCTACCTGCCGCNNG
NNNNNNTNCNGNNNNNNCGGANNNTNNNNNGTANNNNNNNCNTGCNTNNNNTNNTNNCNAGNCNNGTT
CGNNNANANANTAGNANGTNTNNNNNNNNNNTGTNNCGTTCCGCNTGANNCNNCNNNNNGNNGNNNTTN
NCANCGGCNNNANCTNCNGANNTAAGNCATANNNTNNNTCNNTANCTNNNNNGGNGGGNNACTGTAAA
NGNNNAATTNGNNNNTNANTNCNNNCACNTNNCTNNGNNNNCTNNNTNCCTGNNTNCNNAATTGGNNN
CANTGNNTGNCNATCTCTNGNTTNGNNNNNNTNNNNNTNNCANNNNGGNNNNNNNNANNNCNATNTAA
NNTANANNNNCNNNNNNANNNCACNNNNNNNGNAGNNNCNANNNTNANANNNGTAGNNNANTNAGTCN
CCGANNTNNNNNNNATAAGCGNNANACNNNNNNNNNNNNNNANGANNNCGNNTTGGNNNTNNNNNNCNN
GNNAGACNNTNATGGNTNNNNNTNNANNNNTTNGTAGTGGTATNNTNGNNNNNNNTTTCNCCAGNNNN
NNNNNNNNNNNCNATANNANNNNNNGNGANNNNNNNNNNNANNNNCTCNNNAGGGANCANCTACTNNA
NCANNGGNNCGNAGNNNGAGANNNNGACNNNCCTTGGGTGGCANNNACNGNGATAAGTNNNGTTNNNC
CTNNNAGTNNACATNNNNNCANNGNNNCNNNNNNANCNGANNGNNNNNNNNNNNNNNNNNNNNNNNNNN
NNNNNNNNNNNNNNNNNNNNNNNNNNNNNNNNNANNGNNNGTNTNTGACNCNNGNNTTNNTANNNGNN
NNNNNNNGNNNNNNCCCCGGTCGNNNNCNNATTGAANNCCTGNNGCATNNNNNNTNCNAGAATGANNNC
NNNNNGNNTNNNCTNNNNNNNTNAANNAATNNNNNAGNNNNNNNNNTCNNNGNNNNNNNNCNNNNNCNN
ACTGAGGGTNNNNNNNNCANNNNCTNNNNNNNNNNTNCGCNGNCGNNNCNNNNNNGCNGTATNGNGNNA
NNCANNNNNNNNNNNNNNNNNTCAAACAANNNNNNTNNNN
>Kinh19
GTGNNNNNTAGCCCNNNNNAGTCAGACANNNCNNNNNNNNNNNNNTNNNNNNNNNNNNNNNNNNNNNNNNNN
NNNNNNNNNNNNNNNNNNNNNNNNNNNNNNNTNNNNNNNNNNNNNNNNNNNNNNNNNNNNNNNNNNNNNN
NNNNNNNNNNNNNNNNNNNNNNNNNNNNNNNNNNNNNNNNNNNNNNNNNNNNNNNNNNNNNNNNNNNNNN
NNNNNNNNNNNNNCNNNNNNNNNNNNNNNNNNNNNNNNNNNNNNNNNNNNNNNNNNNNNNNNNNNNNNNN
NNNNNNNNNNNNNNNNNNNNNNNNNNNNNNNNNNNNNNNNNNNNNNNNNNNNNTCTNNNAAANNNTNNN
NNNNNNNNNNNNNNNNNNNNNNNNNNCNCNTTGNNNGTAANCNANNNGNACATGCAGTCCCCTCNNNNN
NNNGGCAGAGNNNNNNNCCNNNNNNNCCGNCCCCNNCCNNNNNNNNNNNNNNANNGNNNNNNNANNNNNN
NNNNNNTGGTACNNNNCGNNNNNGGTTGTNTNAGAGGTCCACAACGNGGTCNGGNCACNNNNGNNNAN
NNNCNNNNAACNGNNNNNNNNNNANATNNNTANNAGGNCNNNNCCNTNNNGNNNNTNATTGCNATGACC
CAANTNNCGGGGGCAGTCCCCGGCGCGGCGACCTTGANNANGTCCGGGGACAAGTATGGCNNNCNCCC
CGGGAAGGANNCNGCCGTANGTGCNNNNNANNNNNTCNTGNGANGTNGCCGNNATTCNCNTGGTCNNN
NNAGAATGCNCNNACCANNCGNANNNTGGTNATGNNAGTNNNNNNNNNNNNNNNNNNNNNNNNNNNNNN
NNNNNNNNNNNNNNNNNNNNNNNNNNNNNNNNNNGNNNNGNNNNNNCNANNNAAGGNNNTANNNCTNNTN
NNNNNNNNGGACNNNACNNNNCNNNNNNNACNCNNNCNCNNNNNNANNNGGTTTTNNNNNCNNTANTNT
CCAGNACGCNGNGCNNNCNTTTANGNGAACNNNGNNACGCGNNAATCGTANNATCTACCTGCCGCNNG
NNNNNNTNCNGNNNNNNCGGANNNTGNNNCGTANNNNNNNCANGCGTNNTNTNNTCNCNAGNCNNGTT
CGNNNANANANTAGNANGTNTNNNNNNNNNNTGTNNCGTTCCGCNTGANNCNNCANNNNGNNGNNCTTN
TCANCGGCNNTANCTGCNGANNTAAGNCATANNNTNNNTCNNTANCTNNNNNGGNGGGNNACTGTAAA
NGGNNAATTNGNNNNTNATTNCNNNCATNTNNCNNNGNNNNCTNNNTNCCTGNNTNCNNAATTGGNNC
CANTGNNTGNCNATCTCTNGNTTNGNNNNNNTNNNNNTNNCANNNNGGNNNNNNNNANNNCNATNTAA
NNTANANNNNCNNNNNNANNNCACNNNNNNNNNNAGNNNCNANNNGTANANNNGTAGNNNGNTAAGTCT
CCGANNTNNNNNNNATAAGCGNNANACNNNNNNNNNNNNNNANGANNNCGNNTTTGNNNTNNNNNNCNA
GNNAGACNNTNGTGGNTNNNNNTCAANNNNTTNGTAGTGGTATAATNGNNNNNNNTTTCNCCAGNNTN

NNNGNNNNNNNNCNATANNANNNNNNNGNGANNNNNNNNNNNANNNNCTCNNNAGGNANCANCTACTNNA
GCANTGGNNCGNAGNNNGAGANNNNGACNNNCCTTGGGTGGCAANNACNGCAATAAGTNNNGTTNANN
CTNNNAGTNNACACNNNNNCANNGNNNCNNNNNNANCNGANNGNNNNNNNNNNNNNNNNNNNNNNNNN
NNNNNNNNNNNNNNNNNNNNNNNNNNNNNAGNNNANNGNNNGTNTNTGNCNCNTGTGTTTNTANNNGNN
NNNNNACGNNNNANCCCCGGTCGNNNNCNNATTGAANNCCTGNNGCATNNNNNTNCNAGAATGANNNC
NNNNNGNNTNGNCTNNCNNNTCAAGNAATCNNNNAGGTNNNNCGNTCNNNGNNNNNNNNCNNNCCCNN
ACTGAGGGTNNNNNNNNCATCTGCTNNNNNNNNNNTNCGCNGGNGTNNCNNNNNAGCNGTATNGNGTNN
NNCANNNNNNNNANNNNNNNNNTNAAACNANNNNNNNTNNNN
>Kinh20
GNGNNNNNNAGCCCNNNNNAGTTANACANNNCNNNNNNNNNNNNNNNNNNNNNNNNNNNNNNNNNNNNNNN
NNNNNNNNNNNNNNNNNNNNNNNNNNNNNNNNNNNNNNNNNNNNNNNNNNNNNNNNNNNNNNNNNNNNNN
NNNNNNNNNNNNNNNNNNNNNNNNNNNNNNNNNNNNNNNNNNNNNNNNNNNNNNNNNNNNNNNNNNNNNN
NNNNNNNNNNNNNNNNNNNNNNNNNNNNNNNNNNNNNNNNNNNNNNNNNNNNNNNNNNNNNNNNNNNNNN
NNNNNNNNNNNNNNNNNNNNNNNNNNNNNNNNNNNNNNNNNNNNNNNNNNNNNTNNNNNAAANNNTNNN
NNNNNNNNNNNNNNNNNNNNNNNNNNCNCNNTNNNNGTAANCNANNNGNACATGCAGTNCCCTCNNNNN
NNNNNCAGAGNNNNNNNCNNNNNNNNCCGNCCCCNNCCNNNNNNNNNNNNNNNNGNNNNNNNANNNNNN
NNNNNNTGGTACNNNNCNNNNNNGGTTGNNTNAGAAGTCCACAACGNGNTCNNNNNANNNNNGNNNNN
NNNCNNNNNNNNNNNNNNNNNNNNNNTNNNTANNAGGNNNNNNCCNNNNNNNNNNNNNANNGCNNTNACC
CANNNNNNNNNNGNNGNNCCCGGNNNNGNNNCCNNNNNNANNNNNGGGGNCAAGTATGGCNNNCNCCC
CGGGAAGGANNCNNCNGTANGTGCNNNNNANNNNNTNNNNNGANGTCGCCGNNATTNNCGTGGTCNNN
NNAGAATGNCCNNACCANNCGNANNNTGGTNATNNNAGTNNNNNNNNNNNNNNNNNNCNNNNNNNNNN
NNNNNNNNNNNNNNNNNNNNNNNNNNNNNNNNNNNNNNNGNNNNNNCNANNNAAGGNNNTANNNCTNNNN
NNNNNNNNNNNCNNNACNNNNNNNNNNNNNACNCNNNCNCNNNNNNNNNNNGNTTNNNNNNCNNTNNTNN
CCAGNANGCNGNGCNNNCATTCANGNGNACNNNGNNACGCGNNAATCGTANNATCTANCTGCCGCNNG
TNNNNNTNCNGNNNNNNCGGNNNNTNNNNNGTANNNNNNNNNNTGCGTNNTNTNNTCNCNAGNCNNGTT
CGNNNANNNANTAGNANNTNNNNNNNNNNNNTGTNNCGTTCCGCNTGANNCNNNNNNNNGNNGNNCTTN
NCANCGGCNNTANNTNNNGNNNTAANNCANNNNNNNNNNTCNNTANCTNNNNNGGNNGNNNANTGNAAA
NGNNNAATTNGNNNNTNANTNCNNNCANNNNNCNNNGNNNNCTNNNNNCCTGNNTNCNNAATTGGNNN
NNNTGNNTGNCNATCTCTNGNTTNGNNNNNNTNNNNNTNNCANNNNGNNNNNNNANANNNNNATNNAA
NNTANNNNNNCNNNNNNNNNNCANNNNNNNNNNAGNNNNNNNNNGNANNNNNNTAGNNNNNTNAGTCN
CCGANNTNNNNNNNNTAAGCTNNNNACNNNNNNNNNNNNNNANGANNNCGNNTTTGNNNTNNNNNNNNNA
NNNAGACNNTNGTGGNNNNNNNTCAANNNNNTNNTAGTGGTATAATNGNNNNNNNTTTCNCCAGNNNN
NNNNNNNNNNNCNATANNANNNNNNNNNNGANNNNNNNNNNNANNNNNTNNNNAGGNANCANCTACTNNA
GCANNGGNGCGNAGNNNGAGNNNNNGACNNNCCTTGGGTGGCAANNANNGNAANAAGTNNNGTTNNNN
NTNNNNNTNNANACNNNNNCANNGNNNCNNNNNNANCNNANNGNNNNNNNNNNNNNNNNNNNNNNNNNN
NNNNNNNNNNNNNNNNNNNNNNNNNNNNNANNNNANGGNNNNTNNNNGNNNCNTGTGTTNNCANNNGNN
NNNNNNNGNNNNANCCCCGGTCGNNNNCNNATTGAANNNCTGNNNCATNNNNNTNCNANAATGANNNN
NNNNNNNNTNGNCTNNNNNNTCAAGNAATNANNNANNNNNNNNNNNTCNNNGNNNNNNNNCNNNNNNNN
ANNNAGGGTNNNNNNNNCNNNNNNTNNNNNNNNNNTNCGCNNGNGTNNCNNNNNAGCNGTATNNNGNNN
NNCANNNNNNNNNNNNNNNNNNTNNAACNANNNNNNNTNNNN
>Kinh21
GTNNNNNNTAGCCCNNNNNAGTTNNACANNNCNNNNNNNNNNNNNTNNNNNNNNNNNNNNNNNNNNNNNN
NNNNNNNNNNNNNNNNNNNNNNNNNNNNNNNNNNNNNNNNNNNNNNNNNNNNNNNNNNNNNNNNNNNNNN
NNNNNNNNNNNNNNNNNNNNNNNNNNNNNNNNNNNNNNNNNNNNNNNNNNNNNNNNNNNNNNNNNNNNNN
NNNNNNNNNNNNNNNNNNNNNNNNNNNNNNNNNNNNNNNNNNNNNNNNNNNNNNNNNNNNNNNNNNNNNN
NNNNNNNNNNNNNNNNNNNNNNNNNNNNNNNNNNNNNNNNNNNNNNNNNNNNCNTCNNNNNAAANNNTNNN
NNNNNNNNNNNNNNNNNNNNNNNNNNCNCNTTNNNNGTAANCNANNNGNACATGCAGTNCCCTCNNNNN
NNNNNCAGAGNNNNNNNCCNNNNNNNCCGNCCCCNNCCNNNNNNNNNNNNNNNNGNNNNNNNANNNNNN
NNNNNNTGGTACNNNNCGNNNNNGGTTGTNTNAGAGGTCCACAACGNGNTCNNNNCANNNNNGNNNAN
NNNCNNNNAANNNNNNNNNNNNNNNNNTNNNTANNAGGNNNNNNCCNTCNNNNNNNNNNANNGCNATGACC
CANNNNACGGNNGNNGTNCCCGGNGNGGNGACCTTGNNNANNTCNGGGNNCAAGTATGGCNNNCNCCC
CGGGAAGGANNCNNCNGTANGTGCNNNNNANNNNNTCNTGNGANGTNGCCGNNNTTNNNGTGGTCNNN
NNNGAATGNCCNNACCANNCGNANNNTGGTNATGNNAGTNNNNNNNNNNNNNNNNNNNNNNNNNNNNNN
NNNNNNNNNNNNNNNNNNNNNNNNNNNNNNNNNNNNNNNGNNNNNNCNANNNAAGGNNNTANNNCTNNTN

NNNNNNNNGGNCNNNACNNNNCNNNNNNNACNCNNNCNCNNNNNNNNNNNGNTNNNNNNNCNNTNNTNN
CCAGNANGCNGNGCNNNCNTTCANGNGAACNNNGNNACGCGNNNANNGTANNATCTACCTNCCGCNNG
NNNNNNTNCNGNNNNNNCGGNNNNTNNCTNGTANNNNNNNNNANGCGTNNTNNNNNNNCNAGNCNNGTT
CGNNNANNNANTNNNANGTNNNNNNNNNNNNNGTNNCGTTCCGCNTGANNCNNCNNNNNGNNGNNCTTN
NCANCGGCNNTANNTNNNGANNTAAGNCATANNNTNNNTCNNTANCTNNNNNGGNNGGNNACTGNAAA
NGGNNAATTNGNNNNTNANTNCNNNCACNTNNCTNNGNNNNCTNNNTNCCTGNNTNCNNAATTGGNNN
CANTGNNTGNCNATCTCTGGNTTNGNNNNNNTNNNNNTNNCANNNNGGNNNNNNNNANNNNNANNTAA
NNTANANNNNCNNNNNNANNNNACNNNNNNNGNAGNNNNNNNNNTNANANNNNTAGNNNNNTAAGTCN
CCGANNTNNNNNNNATAAGCGNNANACNNNNNNNNNNNNNANGANNACGNNTTGGNNNTNNNNNNCNA
GNNANACNNTNATGGNTNNNNNTCAANNNNTTNGTAGTGGTATAATNGNNNNNNNTTTCNCCAGNNNN
NNNNNNNNNNNCNATANNANNNNNNNGNGANNNNNNNNNNNANNNNCTCNNNAGGGANCANCTACTNNA
NCANTGGNNCNNAGNNNGAGNNNNNNACNNNCCTTGGGTGGCAANNACNGNGNTAAGTNNNGTTNNNN
NTNNNNNTNNNCATNNNNNCANNGNNNCNNNNNNANCNGANNGNNNNNNNNNNNNNNNNNNNNNNNNN
NNNNNNNNNNNNNNNNNNNNNNNNNNNNNNNNNNNNANNGNNNGTNTNNGACNCATGTGTTTTTANNNGNN
NNNNNNNGNNNNANCCCCGGTCGNNNNCNNATTGAANNCCTGNNGCATNNNNNTNCNANAATGANNNN
NNNNNNNNTNGNNTNNCNNNTCAAGNAATNNNNNANNTNNNNNGNTCNNNNNNNNNNNNCNNNNNCNN
ACTGAGGNTNNNNNNNNCATNNNCTNNNNNNNNNNTNCGCNGGNGTNNNNNNNNNGCNGTATNNNGNNA
NNCANNNNNNNNNNNNNNNNNCNAAACNANNNNCCTNNNN
>Kinh22
GNGNNNNNTAGCCCNNNNNAGTCAGNCANNNCNNNNNNNNNNNNNNNNNNNNNNNNNNNNNNNNNNNNNN
NNNNNNNNNNNNNNNNNNNNNNNNNNNNNNNNNNNNNNNNNNNNNNNNNNNNNNNNNNNNNNNNNNNNNN
NNNNNNNNNNNNNNNNNNNNNNNNNNNNNNNNNNNNNNNNNNNNNNNNNNNNNNNNNNNNNNNNNNNNNN
NNNNNNNNNNNNNNNNNNNNNNNNNNNNNNNNNNNNNNCNNNNNNNNNNNNNNNNNNNNNNNNNNNNNNNN
NNNNNNNNNNNNNNNNNNNNNNNNNNNNNNNNNNNNNNNNNNNNNNNNNNNNNNNNNNNNNNNAANNNNNNN
NNNNNNNNNNNNNNNNNNNNNNNNNNNCNCNNTNNNNGTAANCNANNNGNACATGCAGTCCCCTCNNNNN
NNNNNCAGAGNNNNNNCCNNNNNNNNNCNNNCCCCNNCCNNNNNNNNNNNNCNNNGNNNNNNNANNNNNN
NNNNNNTGGTACNNNNCNNNNNNGGTTGNNTAAGAGGTCCACAACGNGNTCNNNNNANNNNNGNNNNN
NNNNNNNNNNNNNNNNNNNNNNNNNNTNNNTANNAGGNNNNNNNCNNNNNNNNNNNNNANNGCNATNACC
CANNNNNCNGNNGNNGTNCCCGGNNNNGNGNCCTNGNNNANNTCCGGGGNCAAGTATGGCGNNCNCCC
CGNGAAGGANNCNNCNGTANGTGCNNNNNANNNNNTNNNNNGANGNNGCCGNNANNNNNGTGGTCNNN
NNAGAATGNCCNNACCANNCGNANNNTGGTNATGNNAGTNNNNNNNNNNNNNNNNNNNNNNNNNNNNNN
NNNNNNNNNNNNNNNNNNNNNNNNNNNNNNNNNNNNNNNGNNNNNNCNANNNAAGGNNNTANNNCTNNTN
NNNNNNNNGGACNNNACNNNNNNNNNNNNNACNCNNNCNCNNNNNNNNNNNGNTTNNNNNNNCNNTNNTNT
CCAGNNNNCNGNGCNNNCATTTANNNGAACNNNGNNACGCGNNAATCGTANNNTCTANCTGNNGCNNG
TNNNNNTNCNGNNNNNNCGGANNNTNNNNCGTANNNNNNNCANGCGTNNTNTNNTCNCNAGNCNNGTT
CGNNNANNNANTAGNANGTNNNNNNNNNNNTGTNNCGTTCCGCNTGANNCNNCANNNNGNNGNNNTNN
NCANCGGNNNTANCTNNNGANNNAAGNCNTANNNTNNNTCNNTANCTNNNNNGGNGGGNNACTGTAAA
NGNNNNNTTNGNNNNTNANTNCNNNCATNTNNCTNNGNNNNCTNNNTNCCTGNNNNCNNAATTNGNNN
CANTGNNNGNCNATCTCTNGNTTNGNNNNNNTNNNNNTNNCANNNNGGNNNNNNNNANNNNNATNTAA
NNTNNANNNNCNNNNNNNNNNCANNNNNNNNNNAGNNNNNANNNGNANNNNNNTANNNNNNTAAGTCT
CCGANNTNNNNNNNNNTNAGCGNNANACNNNNNNNNNNNNNANGANNNNGNNTTTGNNNNNNNNNNNNA
GNNAGACNNTNGTNNNTNNNNNTCAANNNNTNNGTAGTGGTATAATNGNNNNNNNTTTCNCCAGNNTN
NNNNNNNNNNNCNNTNNNANNNNNNNGNGANNNNNNNNNNNANNNNCTCNNNAGNNANCANCTACNNNA
GCANTGGNNCGNAGNNNGANANNNNGACNNNCCTTGGGTGGCAANNACNGCANNAAGTNNNGTTNNNC
CTNNNNGTNNNNACNNNNNCANNGNNNCNNNNNNANCNNANNNNNNNNNNNNNNNNNNNNNNNNNNNN
NNNNNNNNNNNNNNNNNNNNNNNNNNNNNNANNNNANNGNNNGTNTNNGACNCATGTGNNNNTANNNGNN
NNNNNNNNNNNGNNCCCCGGTCGNNNNCNNNTTGAANNCCTGNNNCNTNNNNNTNCNANAATNANNNC
NNNNNGNNTNGNNTNNCNNNTCAAGNAATCNNNNANNTNNNNNNNTCNNNNNNNNNNNNCNNNCNNNN
ACTNANNNNNNNNNNNNNCANNNNCTNNNNNNNNNNTNCGCNGNNNNNNNNNNNNNGCNGTATNGNGTNN
NNCANNNNNNNNNNNNNNNNNNNANNNNANNNNNNNTNNNN
>Kinh23
GTGNNNNNNAGCCCNNNNNAGTTAGACANNNCNNNNNNNNNNNNTNNNNNNNNNNNNNNNNNNNNNNN
NNNNNNNNNNNNNNNNNNNNNNNNNNNNNNTNNNNNNNNNNNNNNNNNNNNNNNNNNNNNNNNNNNNNNNN
NNNNNNNNNNNNNNNNNNNNNNNNNNNNNNNNNNNNNNNNNNNNNNNNNNNNNNNNNNNNNNNNNNNNNN

NNNNNNNNNNNNNNNNNNNNNNNNNNNNNNNNNNNNNNNNNNNNNNNNNNNNNNNNNNNN
NNNNNNNNNNNNNNNNNNNNNNNNNNNNNNNNNNNNNNNNNNNNNTCTNNNAAANNNTNNN
NNNNNNNNNNNNNNNNNNNNNNNNNNCNCNNTGNNNGTAANCNANNNGNACATGCAGTCCCCTCNNNNN
NNNNNCAGAGNNNNNNNNCNNNNNNNCCGGCCCCNNCCNNNNNNNNNNNCNNNGNNNNNANANNNNNN
NNNNNNTGGTACNNNNCGNNNNNGGTTGTNTNAGAAGTCCACAACGNGGTCNGNNCACNNNNGNNNAN
NNNCNNNNAANNNNNNNNNNNNNANNTNNNTANNAGGNCNNNNCCNTNNNNNNNNNNNANNGCNATGANN
CAANNCACGGNGGNNGTNCCCGGNGNGGCGACCTTGANNACGNCCGGGGNCAAGTATGGCNCNCNCCC
CGNGAAGGANNCNGCNGTANGTGCNNNNNANNNNNTCNTGNGANGTNGCCGNNATTCNCGTGGTCNNN
CNNGAATGCCCNNACCANNCGNANNNTGGTNATGNNAGTNNNNNNNNCNNNNNNNNNNNNNNNNNNN
NNNNNNNNNNNNNNNNNNNNNNNNNNNNNNNNNNNGNNNNGNNNNNNCNANNNAAGGNNNTANNNCTNNTN
NNNNNNNNGGACNNNACNNNNCNNNNNNNACNCNNNCNCNNNNNNANNNGGTTTTTNNNCNNTANTNT
CCAGNANGCNGNGCNNNCNTTCANGNGAACNNNGNNACGCGNNAATCGTANNATCTACCTGCCGCNNG
NNNNNNTNCNGNNNNNNCGGANNNTNNNNCGTANNNNNNNCANGCGTNNNNTNNTCNCNAGNCNNGTT
CGNNNANANANTAGNANGTNTNNNNNNNNNNTGTNNCGTTCCGCNTGANNCNNCANNNNGNNGNNCTTN
NCANCGGCNNTANCTNCNGANNTAAGNCATANNNTNNNTCNNTANCTNNNNNGGNGGGNNACTGTAAA
NGGNNAATTNGNNNNTNATTNCCNNCATNTNNCNNNGNNNNCTNNNTNCCTGNNTNCNNAATTGGNNN
CANTGNNTGNCNATCTCTGGNTTNGNNNNNNTNNNNNTNNCANNNNGGNNNNNNANANNNCNATNTAA
NNTANANNNNCNNNNNNANNNCACNNNNNNNNNAGNNNCNANNNGNANANNNGTAGNNNGNTNAGTCT
CCGANNTNNNNNNNATAAGCTNNANACNNNNNNNNNNNNNANGANNNCGNNTTTGNNNTNNNNNNCNA
GNNAGACNNTNGTGGNTNNNNNTCAANNNNTTNGTAGTGGTATAATNGNNNNNNNTTTCNCCAGNNTN
NNNGNNNNNNNCNATANNANNNNNNNGNGANNNNNNNNNNNANNNNCTCNNNAGGNANCANCTACTNNA
GCANTGGNGCGNAGNNNGAGANNNNGACNNGCCTTGGGTGGCANNCACNGNAATAAGTNNNGTTNNNC
CTNNNAGTNNACACNNNNNCANNGNNNCNNNNNNANCNGANNGNNNNNNNNNNNNNNNNNNNNNNNN
NNNNNNNNNNNNNNNNNNNNNNNNNNNNNNNNNANNNNANGGNNNGTNTNTGNCNCATGNNTTTNCANNNGNN
NNNNNACGNNNNANCCCCGGTCGNNTNCNNATTGAANNCCTGNNGCATNNNNNTNCNAGAATGANNGC
NNNNNGNNTNGNCTNNCNNNTNANGNAATCANNNANNTNNNNNNNTCNNNGNNNNNNNNCNNNCCCNN
ACTGAGGGTNNNNNNNNCATNNGCTNNNNNNNNCTNCGCNGGCGTNNCTNNNNAGCNGTATNNNGTNN
NNCANNNNNNNNNNGNNNNNNTNAAACGANNNNCNTNNNN
>Kinh24
GTGNNNNNNAGCTCNNNNNAGTTNGACANNNCNNNNNNNNNNNNNNNNNNNNNNNNNNNNNNNNN
NNNNNNNNNNNNNNNNNNNNNNNNNNNNNNNNNNNNNNNNNNNNNNNNNNNNNNNNNNNN
NNNNNNNNNNNNNNNNNNNNNNNNNNNNNNNNNNNNNNNNNNNNNNNNNNNNNNNNNNNN
NNNNNNNNNNNNNNNNNNNNNNNNNNNNNNNNNNNNNNNNNNNNNNNNNNNNNNNNNNNN
NNNNNNNNNNNNNNNNNNNNNNNNNNNNNNNNNNNNNNNNNNNNNTCTNNNAAANNNTNNN
NNNNNNNNNNNNNNNNNNNNNNNNNNCNCNTTNNNNGTAANCNANNNGNACATGCAGTCCCCTCNNNNN
NNNGGCAGAGNNNNNNNCCNNNNNNNCCGGCCCCNNCCNNNNNNNNNNNNNNNNNNNNNNNNNGNNNNNN
NNNNNNTGGTACNNNNCGNNNNNGGTTGNNTAAGAGGTCCACCACGNGNTCNNGNNACNNNNGNNNAN
NNNCNNNNAANNNNNNNNNNNNNNNNNTNNNCANNAGGNCNNNNCCNTNNNGNNNNNNANNGCNATGANN
CANNNNNCNGNGANNGTNACCGGNGCGGNGACCTTGANNANNNTNGGGGNCAAGTATNTCNNNCNCCC
CGNGAAGGANNCNGCNGTANGTGCNNNNNANNNNNTCNTGNGANNTNGCCGNNATTNNCNTGGTCNNN
NNNGAATGNNCNNACCANNCGNANNNTGGTNATGNNAGTNNNNNNNNNNNNNNNNNNNNNNNNNNNNN
NNNNNNNNNNNNNNNNNNNNNNNNNNNNNNNNNNNGNNNNGNNNNNNCNANNNAAGGNNNTANNNTTNNTN
NTNNNNNNGGACNNNNCNNNNCNNNNNNNACNCNNNCNCNNNNNNANNNGNTTNNNNNNCNNTANNNT
CCAGNANGCNGNGCNNNCNTTCANGNGAACNNNGNNACGCGNNAATCGTANNATCTACCTGCCGCNNG
TNNNNNTNCNGNNNNNNCGGANNNTNNNNGTANNNNNNNCANGCGTNNTNTNNTTNCNAGNCNNGTT
CGNNNANANANTAGNANGTNTNNNNNNNNNNTGTNNCGTTCCGCNTGANNCNNCANNNNGNNGNNNTTN
NCANCGGCNNTANCTGCNGANNTAAGNCATANNNTNNNTCNNTANCGNNNNNGGNGGGNNACTGTAAA
NGGNNAATTNGNNGNTNANTTCNNNCACNTNNCNNNGNNNNCTNNNCNCCTGNNTNCNNAATTGGNNN
CANTGNNTGNCNATCTCTNGNTTNGNNNNNNTNNNNNTNNTANNNNGGNNNNNNNNANNNCNATNTAA
NNTANANNNNCNNNNNNANNNCACNNNNNNNNNAGNNNCNANNNTTANGNNNGTAGNNNAATAAGTCN
CCNANNTNNNNNNNATAAGCGNNANACNNNNNNNNNNNNNANGANNNCGNNTTGGNNNTNNNNNNNNA
ANNAGACNNCNGTGGNTNNNNNTCAANNNNTTNGTAGTGGTATAACNANNNNNNNTTTCNCCAGNNTN
NNNGNNNNNNNCNATANNANNNNNNNGTGANNNNNNNNNNNANNNNCCCNNNAGGNANCANCTACTNNA
NCANTGGNGCNNAGNNNGAGANNNNGACNNNCCTTGGGTGGCAANCACNGCANTAAGTNNNGTTNNNN

NCNNNAGTNNACACNNNNNCANNGNNNCNNNNNNANCNGANNTNNNNNNNNNNNNNNNNNNNNNNN
NNNNNNNNNNNNNNNNNNNNNNNNNNNNNANNNNANNGNNNGTNTNTGNCNCANGNNTTTNTANNNGNN
NNNNNNNGNNNNNNCCCCGGTCGNNNNCNNATTGAANNCCTGNNGCATNNNNNTNCNAGAATGANNNC
NNNNNGNNTNGNCTNNCNNNTCANGNAGTCANNNAGNNNNNNNNNTCNNNNNNNNNNNNCNNNCNCNN
ACTGAGGGTNNNNNNNNCANNTNCTNNNNNNNNCTNCGCNGNNNNNNTNNNNNNGCNGTATNGNGTNN
NNCANNNNNNNNNNNNNNNANTNAAACGANNNNCCTNNNG
>Kinh25
GTGNNNNNNAGCCCNNNNNAGTCAGACANNNCNNNNNNNNNNNNNNNNNNNNNNNNNNNNNNNNNN
NNNNNNNNNNNNNNNNNNNNNNNNNNNNNNNNNNNNNNNNNNNNNNNNNNNNNNNNNNNN
NNNNNNNNNNNNNNNNNNNNNNNNNNNNNNNNNNNNNNNNNNNNNNNNNNNNNNNNNNNN
NNNNNNNNNNNNNNNNNNNNNNNNNNNNNNNNNNNNNNNNNNNNNNNNNNNNNNNNNNNN
NNNNNNNNNNNNNNNNNNNNNNNNNNNNNNNNNNNNNNNNNNNNNNNNNNNNNTCTNNNAAANNNTNNN
NNNNNNNNNNNNNNNNNNNNNNNNNNNCNTTGNNNGTAANCNANNNGNACATGCAGTCCCCTCNNNNN
NNNNNCAGAGNNNNNNNCCNNNNNNNCCGGCCCCNNCCNNNNNNNNNNNNNNNNNNNNNNNNNANNNNNN
NNNNNNTGGTACNNNNCGNNNNNGGTTGNNTNAGAGGTCCACAACGNGNTCNGNNNACNNNNGNNNAN
NNNCNNNNNNNNNNNNNNNNNNNNNNTNNNTANNAGGNNNNNNCCNTNNNNNNNNNNNANNGCNATGANN
CNNNNNACGGGGGNAGTNCCCGGNGNGGNGACCTTGANNACGTCNGGGGACAAGTATGGCNNNCNCCC
CGNGAAGGANNCNGCNGTANGTGCNNNNNANNNNNTCNTGNGANNTNGCCGNNATTCNCGTNNNCNNN
CNNGAATGNNCNNACCANNCGNANNNTGGTNATGNNAGTNNNNNNNNNNNNNNNNNNNNNNNNNNNN
NNNNNNNNNNNNNNNNNNNNNNNNNNNNNNNNNNNGNNNNGNNNNNNCNANNNAAGGNNNTANNNCTNNTN
NNNNNNNNGGACNNNACNNNNCNNNNNNNACNCNNNCNCNNNNNNNNNNNGNTTNNNNNNCNNTANTNT
CCAGNANGCNNNGCNNNCATTCANGNGAACNNNGNNACGCGNNAATCGTANNATCTACCTGCCGCNNG
NNNNNNTNCNGNNNNNNCGGANNNTNNCTCGTANNNNNNNNCNNGCGTNNNNTNNTCNCNAGNCNNGTT
CGNNNANANANTAGNANGTNTNNNNNNNNNNTGTNNCGTTCCGCNTGANNCNNCNNNNNGNNGNNNTTN
NCANCGGCNNTANCTNCNGANNTAAGNCATANNNTNNATCNNTANCTNNNNNGGNGGGNNACTGTAAA
NGGNNAATTNGNNGNTNANTNCNNNCATNTNNCNNNGNNNNCTNNNTNCCTGNNTNCNNAATTGGNNN
CANTGNNTGNCNATCTCTGGNTTNGNNNNNNTNNNNNTNNCANNNNGGNNNNNNNNANNNCNANNTAA
NNTANANNNNCNNNNNNANNNCACNNNNNNNNNAGNNNCNANNNGNANANNNGTAGNNNGNTNAGTCT
CCGANNTNNNNNNNATAAGCGNNANACNNNNNNNNNNNNNANGANNNCGNNTTTGNNNTNNNNNNNNA
GNNANACNNTNGTGGNTNNNNNTCAANNNNTTNGTAGTGGTATAATNGNNNNNNNTTTCNCCAGNNNN
NNNGNNNNNNNNCNATANNANNNNNNNGNGANNNNNNNNNNNANNNNCTCNNNAGGNANCANCTACTNNA
GCANTGGNGCGNAGNNNGAGANNNNGACNNNCCTTGGGTGGCAANNACNGCAATAAGTNNGTTNANN
NTNNNAGTNNACACNNNNNCANNGNNNCNNNNNNANCNGANNGNNNNNNNNNNNNNNNNNNNNNNNN
NNNNNNNNNNNNNNNNNNNNNNNNNNNNNANNNNANNGNNNGTNTNTGNCNCNTGTGTTTNTANNNGNN
NNNNNNNGNNNNANCCCCGGTCGNNNNCNNATTGAANNCCTGNNGCATNNNNNTNCNAGAATGANNNC
NNNNNGNNTNGNCTNNNNNNTNANGNAATCNNNNAGNNNNNNNGNTCNNNNNNNNNNNNNCNNNCNCNN
ACTGAGGGNNNNNNNNNCATCNGCTNNNNNNNNNNTNCGCNGGNGTNNCNNNNNNGCNGTATNNNGTNN
NNCANNNNNNNNNNNNNNNNNTNAANNNNNNNNNNNNTNNNN
>Kinh26
GTNNNNNNNAGCCCNNNNNAGTTNGACANNNCNNNNNNNNNNNNNTNNNNNNNNNNNNNNNNNNNNNNN
NNNNNNNNNNNNNNNNNNNNNNNNNNNNNNNNTNNNNNNNNNNNNNNNNNNNNNNNNNNNNNNNNNNNN
NNNNNNNNNNNNNNNNNNNNNNNNNNNNNNNNNNNNNNNNNNNNNNNNNNNNNNNNNNNN
NNNNNNNNNNNNNNNNNANNNNNNNNNNNNNNNNNNNNNNNNNNNNNNNNNNNNNNNNNNNNNNNNNN
NNNNNNNNNNNNNNNNNNNNNNNNNNNNNNNNNNNNNNNNNNNNNNNNNNNNNTCTNNNAAANNNTNNN
NNNNNNNNNNNNNNNNNNNNNNNNNNNCNNTNNNNGTAANCNANNNGNACATGCAGTCCCCTCNNNNN
NNNNNCAGAGNNNNNNCNNNNNNNCNCCGNCCCCNNCCNNNNNNNNNNNNNNNNNNGNNNNNNNANNNNNN
NNNNNNTGGTACNNNNCGNNNNNGGTTGTNTNAGAGGTCCACCACGNAGTCNNNNNACNNNNGNNNAN
NNNCNNNAAANNNNNNNNNNNNNNNNNNGNNNCANAAGGNNNNNNCCNTNNNNNNNNNTNGNNGCNATGACC
CNNNNCACNGGGGCNGTNCCCGGCGNGGNGACCTTGANNANNTCNGGGANCAAGTATGGCNNNTNCCC
CGNGAAGGANNCNNCNGTANGTGCNNNNNANNNNNTCNTGNGANNTCGCCGNNANNNNNGTGGTCNNN
NNAGAATGNNCNNACCANNCGNANNNTGGTNATGNNAGTNNNNNNNNNNNNNNNNNNNNNNNNNNNN
NNNNNNNNNNNNNNNNNNNNNNNNNNNNNNNNNNNNNNGNNNNNNCNANNNAAGGNNNTANNNCTNNTN
NTNNNNNNGGACNNNACNNNNCNNNNNNNACNCNNNCNCNNNNNNNNNNNGNTTTNNNNNCNNTANTNC
CCAGNANGCNGNGCNNNCNTTCANGNGAACNNNGNNACGTGNNAATCGTANNATCTACCTGCCGCNNG

NNNNNNTACNGNNNNNNCGGANNNTNNCTCGTANNNNNNNCANGCGTNNTNTNNTCNCNAGNCNNGTT
CGNNNANANANTAGNANGTNTNNNNNNNNNTGTNNCGTTCCGCNTGANNCNNCANNNNGNNGNNNTTN
NCANCGGCNNTANCTNCNGANNTAAGNCATANNNCNNNTCNNTANCTNNNNNNGNGGGNNACTGTAAA
NGGNNAATTNGNNNNTNANTNCNNNCACNTNNCNNNGNNNNCTNNNTNCCTGNNTNCNNAGTTGGNNN
CANTANNTGNTNATCTCTNGNTTNGNNNNNNTNNNNNTNNCANNNNGGNNNNNNNNANNNNNNANNTAA
NNTANANNNNCNNNNNNANNNCACNNNNNNNNNAGNNNCNANNNTNANANNNGTAGNNNANTNAGTCT
CCGANNTNNNNNNNATAGGCGNNNNACNNNNNNNNNNNNNNANGANNNCGNNTTGGNNNTNNNNNNCNA
ANNAGACNNTNGTGGNTNNNNNTCAANNNNCTNGTAGTGGTATAATNGNNNNNNNTTTCNCCAGNNTN
NNNGNNNNNNNNCNATANNANNNNNNNGNGANNNNNNNNNNNANNNNCTCNNNAGGGANCANCTACTNNA
GCANNGGNNCGNAGNNNTAGANNNNGACNNNCCTTGGGTGGCAANNACNGNAATAAGTNNNGTTNANN
NTNNNAGTNNACACNNNNNCANNANNNCNNNNNNANCNGANNTNNNNNNNNNNNNNNNNNNNNNNNNN
NNNNNNNNNNNNNNNNNNNNNNNNNNNNNNANNNNANGGNNNGTNTNTGACNCNNGTGTTTNTANNNGNN
NNNNNNNGNNNNANCCCCGGTCGNNNNCNNATTGAANNCCTGNNGCATNNNNNNTNCNATAATGANNNC
NNNNNGNNTNGNCTNNNNNNNTCAAGNAGTCANNNAGNNNNNNNGNTCNNNGNNNNNNNNCNNNCCCNN
ACTGAGGGTNNNNNNNNCATNTNCTNNNNNNNNNNTNCGCNGNCGTNNCNNNNNNTCNGTATNGNGNNN
NNCANNNNNNNNNNNNNNNNNTNAAACAANNNNNNNTNNNG
>Kinh27
GTGNNNNNTAGCCCNNNNNAGTTNGACANNNCNNNNNNNNNNNNNTNNNNNNNNNNNNNNNNNNNNNN
NNNNNNNNNNNNNNNNNNNNNNNNNNNNNNNNNNNNNNNNNNNNNNNNNNNNNNNNNNNNNNNNNNNN
NNNNNNNNNNNNNNNNNNNNNNNNNNNNNNNNNNNNNNNNNNNNNNNNNNNNNNNNNNNNNNNCNNNN
NNNNNNNNNNNNNNNNNNNNNNNNNNNNNNNNNNNNNNNNNNNNNNNNNNNNNNNNNNNNNNNNNNNN
NNNNNNNNNNNNNNNNNNNNNNNNNNNNNNNNNNNNNNNNNNNNNNNNNNCNTCTNNNAAANNNTNNN
NNNNNNNNNNNNNNNNNNNNNNNNNCNCNNTNNNNGTAANCNANNNGNACATGCAGTCCCCTCNNNNN
NNNNNCAGAGNNNNNNNCCNNNNNNNCCGNCCCCGNCCNNNNNNNTNNNNNNNGNNNNNNNANNNNNN
NNNNNNTGGTACNNNNCGNNNNNGGTTGTATAAGAAGTCCACAACGNGNTCNNGNNACNNNNGNNNAN
NNNCNNNNAACNNNNNNNNNNNNNNNTNNNTANNAGGNNNNNNCCNTCNNNNNNNNANTGCNATGACC
CANNNCNCNGGGGNNGTNCCCGGCGCGGCGACCTTGANNANNTCCGGGGNCAAGTATGGCNNNCNCCC
CGGGAAGGANNCNGCCGTANGTGCNNNNNANNNNNTCNTGNGANNTNGCCGNNATTNNNGTGGTCNNN
CNANAATGNCCNNACCANNCGNANNNTGGTNATGNNAGTNNNNNNNNNNNNNNNNNNNNNNNNNNNNNN
NNNNNNNNNNNNNNNNNNNNNNNNNNNNNNNNNNNNGNNNNGNNNNNNCNANNNAAGGANNTANNNCTNNTN
NNNNNNNNGGACNNNACNNNNCNNNNNNNACNCNNNCNCNNNNNNNNNNGNTTTTNNNNCNNTATTNT
CCAGNACGCNGNGCNNNCNTTCANGNGAACNNNGNNACGCGNNAATCGTANNATCTANCTGCCGCNNG
NNNNNNTNCNGNNNNNNNCGGANNNTNNNNCGTANNNNNNNCANGCGTNNTNTNNTCNCNAGNCNNGTT
CGNNNANANANTAGNANGTNTNNNNNNNNNTGTNNCGTTCCGCNTGANNCNNCANNNNGNNGNNCTTN
NCANCGGCNNTANCTNCNGANNTAAGNCATANNNTNNNTCNNTANCTNNNNNGGNGGGNNACTGTAAA
NGGNNAATTNGNNNNTNANTNCCNNCATNTNNCNNNGNNNNCTNNNTNCCTGNNTNCNNAATTGGNNN
CANTGNNTGNCATCTCTGGNTTNGNNNNNNTNNNNNTNNCANNNNGGNNNNNNANANNNCNATNTAA
NNTANANNNNCNNNNNNANNNCACNNNNNNNGNAGNNNNNANNNGTANANNNGTAGNNNGNTAAGTCT
CCGANNTNNNNNNNATAAGCTNNANACNNNNNNNNNNNNNNANGANNNCGNNTTTGNNNTNNNNNNNNNA
GNNAGACNNTNGTGGNTNNNNNTCAANNNNTTNGTAGTGGTATAATNGNNNNNNNTTTCNCCAGNNTN
NNNGNNNNNNNNCNATANNANNNNNNNGNGANNNNNNNNNNNANNNNCTCNNNAGGNANCANCTACTNNA
GCANNGGNNCGNAGNNNGAGANNNNGACNNNCCTTGGGTGGCANNNACNGCAATAAGTNNNGTTNANC
CTNNNAGTNNACACNNNNNCANNGNNNCNNNNNNANCNGANNGNNNNNNNNNNNNNNNNNNNNNNNNN
NNNNNNNNNNNNNNNNNNNNNNNNNNNNNNAGNNNANGGNNNGTNTNTGACNCATGNNTTTTCANNNGNN
NNNNNACGNNNNANCCCCGGTCGNNNNCNNATTGAANNCCTGNNGCATNNNNNNTNCNAGAATGANNNC
NNNNNGNNTNGNCTNNCNNNTCAAGNAATCANNNAGGTNNNNNNNNTCNNNGNNNNNNNNCNNNCNCNN
ACTGAGGGTNNNNNNNNCATCNNCTNNNNNNNNNCTNCGCNGGNGTNNCNNNNNNGCNGTATNGNGNNN
NNCANNNNNNNNNNNNNNNNNTNAAACAANNNNNNNTNNNG
>Kinh28
GTGNNNNNNAGCTCNNNNNAGTTNNACANNNCNNNNNNNNNNNNNTNNNNNNNNNNNNNNNNNNNNNN
NNNNNNNNNNNNNNNNNNNNNNNNNNNNNNNNNNNNNNNNNNNNNNNNNNNNNNNNNNNNNNNNNNNN
NNNNNNNNNNNNNNNNNNNNNNNNNNNNNNNNNNNNNNNNNNNNNNNNNNNNNNNNNNNNNNNNNNNN
NNNNNNNNNNNNNNNNNNNNNNNNNNNNNNNNNNNNNNNNNNNNNNNNNNNNNNNNNNNNNNNNNNNN
NNNNNNNNNNNNNNNNNNNNNNNNNNNNNNNNNNNNNNNNNNNNNNNNNNNNTCTNNNAAANNNTNNN

NNNNNNNNNNNNNNNNNNNNNNNNNNCNCNNTNNNNGTAANCNANNNGNACATGCAGTCCCCTCNNCNN
NNNNNCAGAGNNNNNNNCNNNNNNNNCCGGCCCCGNCCNNNNNNNTNNNNNNNNNNNNNNNGNNNNNN
NNNNNNTGGTACNNNNCGNNNNNGGTTGNNNAAGNGGTCCACCACGNGGTCNNGNCACNNNNGNNTAN
NNNCNNNNANNNNNNNNNNNNNNNNNNNTNNNCANNAGGNNNNNNCCNTCNNNNNNNNNNANNGCNATGANN
CAANNCNCGGGGACNGTNACCGGCGNGGCNACCTTGNNNANGGTCGGGGNCAAGTATGTCNNNCNCCC
CGGGAAGGANNCNGCCGTANGTGCNNNNNANNNNNTNNTGNGANNTCGCCGNNNTTCNCNTGGTCNNN
NNAGANTGNNCNNACCANNCGNANNNNGGTNATGNNAGTNNNNNNNNNNNNNNNNNNNNNNNNNNNNNN
NNNNNNNNNNNNNNNNNNNNNNNNNNNNNNNNNNNNGNNNNGNNNNNNCNANNNANGGNNNTANNNTTNNTN
NNNNNNNNGGACNNNACNNNNCNNNNNNNACNCNNNCNCNNNNNNNNNNNGNTTTTNNNNCNNTNTTNT
CCAGNANGCNGNGCNNNCNTTCANGNGAACNNNGNNACGCGNNANTCGTANNATCTANCTGCNGCNNG
NNNNNNTNCNGNNNNNNCGGANNNTNNNNNGTANNNNNNNCANGCGTNNTNTNNTNNCNAGNCNNGTT
CGNNNANANANTAGNANGTNTNNNNNNNNNNTGTNNCGTTCCGCNTGANNCNNCANNNNGNNGNNNTTN
NCANCGGCNNTANCTGCNGANNTAAGNCATANNNTNNNTCNNTANCGNNNNNGGNGGGNNACTGTAAA
NGGNNAATTNGNNNNTNANTNCNNNCACNTNNCTNNGNNNNCTNNNCNCCTNNNNNCNNAATTGGNNC
CANTGNNTGNCNATCTCTNGNTTNGNNNNNNTNNNNNTNNTANNNNGGNANGTNNNANNNCNATNTAA
NNTANANNNNCNNNNNNANNNCACNNNNNNNNNAGNNNNNANNNTNANGNNNGTAGNNNANTNAGTCT
CCGNNNTNNNNNNNATAAGCGNNANACNNNNNNNNNNNNNNANGANNNCGNNTTGGNNNTNNNNNNNNA
ANNANACNNCNGTGGNTNNNNNTCAANNNNTTNGTAGTGGTATAACNANNNNNNNTTTCNCCAGNNTN
NNNGNNNNNNNCNATANNANNNNNNNGNGANNNNNNNNNNNANNNNCCCNNNAGGNANCANCTACTNNA
NCANNGGNGCGNAGNNNGAGANNNNGACNNNCCTTGGGTGGCANNNACNGNAATAAGTNNNGTTNNNC
CCNNNAGTNNACACNNNNNCANNGNNNCNNNNNNANCNGANNTNNNNNNNNNNNNNNNNNNNNNNNNN
NNNNNNNNNNNNNNNNNNNNNNNNNNNNNNNNNNNNNNNNANNGNNNGTNTNTGACNCNTGTNTTTTTANNNGNN
NNNNNNNGNNNNANCCCCGGTCGNNNNCNNATTGAANNCCTGNNGCATNNNNNTNCNANAATGANNGC
NNNNNGNNTNNNCTNNNNNNTCANNNAGTCANNNANNNNNNNNGNTCNNNNNNNNNNNNNCNNNCNCNN
ACTGAGGGNNNNNNNNNCANNNGCTNNNNNNNNNTNCGCNGNNNNNNNTNNNNNAGCNGTATNNNGTNN
NNCANNNNNNNNNNNNNNNNNTNAAACAANNNNCNTNNNN
>Kinh29
GTGNNNNNTAGCCCNNNNNAGTTAGACANNNCNNNNNNNNNNNNNTNNNNNNNNNNNNNNNNNNNNNNNN
NNNNNNNNNNNNNNNNNNNNNNNNNNNNNNTNNNNNNNNNNNNNNNNNNNNNNNNNNNNNNNNNNNNNNN
NNNNNNNNNNNNNNNNNNNNNNNNNNNNNNNNNNNNNNNNNNNNNNNNNNNNNNNNNNNNNNNNNNNNNN
NNNNNNNNNNNNNNNNNNNNNNNNNNNNNNNNNNNNNNNNNNNNNNNNNNNNNNNNNNNNNNNNNNNNNN
NNNNNNNNNNNNNNNNNNNNNNNNNNNNNNNNNNNNNNNNNNNNNNNNNNNNCNTCTNNNAAANNNTNNN
NNNNNNNNNNNNNNNNNNNNNNNNNNCNCNTTGNNNGTAANCNANNNGNACATGCAGTCCCCTCNNNNN
NNNGGCAGAGNNNNNNCCCNNNNNCNCCGNCCCCNNCCNNNNNNNNNNNNNNNNGNNNNNNNANNNNNN
NNNNNNTGGTACNNNNCGNNNNNGGTTGTNTAAGNAGTCCACAACGNGGTCNNNNNACNNNNGNNNAN
NNNCNNNNAACNNNNNNNNNNNNANNTNNNTANNAGGNNNNNNCCNTCNNGNNNNNNANTGCNATGACN
CNANTNNCGGGGGCNGTNCCCGGCGCGGCGACCTTGANNANNTCCGGGGNCAAGTATGGCNNNCNCCC
CGGGAAGGANNCNGCCGTANGTGCNNNNNANNNTNTCNTGNGANGTNGCCGNNATTNNNNTGGTCNNN
NNAGAATGCCCNNACCANNCGNANNNTGGTNATGNNAGTNNNNNNNNNNNNNNNNNNNNNNNNNNNNNN
NNNNNNNNNNNNNNNNNNNNNNNNNNNNNNNNNNNNGNNNNGNNNNNNCNANNNAAGGNNNTANNNCTNNTN
NNNNNNNNGGACNNNACNNNNCNNNNNNNACNCNNNCNCNNNNNNNANNNGGTTTTNNNNCNNTANTNT
CCAGNACGCNGNGCNNNCNTTCANGNGAACNNNGNNACGCGNNAATCGTANNATCTACCTGCCGCNNG
NNNNNNTACNGNNNNNNCGGANNNTNNCTNGTANNNNNNNNCATGCGTNNNNTNNTCNCNAGNCANGTT
CGNNNANANANTAGNANGTNTNNNNNNNNNNTGTNNCGTTCCGCNTGANNCNNCNNNNNGNNGNNNTTN
NCANCGGCNNTANCTGCNGANNTAAGNCATANNNTNNNTCNNTANCTNNNNNGGNGGGNNACTGTAAA
NGGNNAATTNGNNNNTNANTNCNNNCATNTNNCTNNGNNNNCTNNNTNCCTGNNTNCNNAATTGGNNN
CANTGNNTGNCNATCTCTNGNTTNGNNNNNNTNNNNNTNNCANNNNGGNNNNNNNNANNNCNATNTAA
NNTANANNNNCNNNNNNANNNCACNNNNNNNNNAGNNNCNANNNGTANANNNGTAGNNNNNTNAGTCT
CCGANNTNNNNNNNATAAGCTNNANACNNNNNNNNNNNNNNANGANNNCGNNTTTGNNNTNNNNNNNNA
GNNAGACNNTNGTGGNTNNNNNTCAANNNNTTNGTAGTGGTATAATNGNNNNNNNTTTCNCCAGNNTN
NNNGNNNNNNNCNATANNANNNNNNNGNGANNNNNNNNNNNANNNNCTCNNNAGGGANCANCTACTNNA
GCANTGGNGCGNAGNNNGAGANNNNGACNNNCCTTGGGTGGCAANNACNGNAANAAGTNNNGTTNNNN
NTNNNAGTNNACACNNNNNCANNGNNNCNNNNNNANCNGANNGNNNNNNNNNNNNNNNNNNNNNNNNNN
NNNNNNNNNNNNNNNNNNNNNNNNNNNNNNNNNNNNNNNANNGNNNGTNTNTGNCNCNNGTGTTTNCANNNGNN

NNNNNNNGNNNNANCCCCGGTCGNNNNCNNATTGAANNCCTGNNGCATNNNNNTNCNAGAATGANNGC
NNNNNGNNTNGNCTNNCNNNTCAAGNAATCNANNAGNNNNNNNNNTCNNNNNNNNNNNNCNNNNNCNN
ACTGAGGGTNNNNNNNNCANCNNCTNNNNNNNNNTNCGCNGGNGTNNCNNNNNNGCNGTATNGNGTNN
NNCANNNNNNNNNNNNANNNNTNAAACAANNNNNNTNNNG
>Kinh30
GTGNNNNNTAGCCCNNNNNAGTCAGACANNNCNNNNNNNNNNNNTNNNNNNNNNNNNNNNNNNNNNN
NNNNNNNNNNNNNNNNNNNNNNNNNNNNNNNNNNNNNNNNNNNNNNNNNNNNNNNNNNNNNNNNNNN
NNNNNNNNNNNNNNNNNNNNNNNNNNNNNNNNNNNNNNNNNNNNNNNNNNNNNNNNNNNNNNNNNNN
NNNNNNNNNNNNNNNNNNNNNNNNNNNNNNNNNNNNNNNNNNNNNNNNNNNNNNNNNNNNNNNNNNN
NNNNNNNNNNNNNNNNNNNNNNNNNNNNNNNNNNNNNNNNNNNNNNNNCNNCTNNNNAANNNTNNN
NNNNNNNNNNNNNNNNNNNNNNNNNNCNCNNTNNNNGTAANCNANNNGNACATGCAGTCCCCTCNNNNN
NNNNNCAGAGNNNNNNNCCNNNNNNNCCGGCCCCNNCCNNNNNNNNNNNNANNGNNNNNNNANNNNNN
NNNNNNTGGTACNNNNCGNNNNNGGTTGTNNAAGAGGTCCACAACGNGNTCNNGNCANNNNNGNNNAN
NNNCNNNNNNNNNNNNNNNNNNNNNNNNNTANNAGGNNNNNNCCNTNNNNNNNNNNNATNGCNATGACC
CAANNCACGGNGGNNGTNCCCGGNGCGGCGACCTTGANNANGTCCGGGGNCAAGTATGGCNNNCNCCC
CGGGAAGNANNCNGCCGTANGTGCNNNNNANNNNNTCNTGNGANGTNGCCGNNNTTNNCGTGGTCNNN
NNAGAATGNCCNNACCANNNGNANNNTGGTNATGNNAGTNNNNNNNNNNNNNNNNNNNNNNNNNNNNN
NNNNNNNNNNNNNNNNNNNNNNNNNNNNNNNNNNNNNNNGNNNNNNCNANNNAAGGANNTANNNCTNNTN
NTNNNNNNGGACNNNACNNNNCNNNNNNNACNCNNNCNCNNNNNNNNNNGGTTTTTNNNCNNTANTNT
CCAGNANGCNGNGCNNNCNTTCANGNGAACNNNGNNACGCGNNAATCGTANNATCTACCTGCCGCNNG
TNNNNNTACNGNNNNNNCGGANNNTNNNNCGTANNNNNNNCATGCGTNNTNTNNTCNCNAGNCANGTT
CGNNNANNNANTAGNANGTNTNNNNNNNNNTGTNNCGTTCCGCNTGANNCNNCANNNNGNNGNNNTTN
TCANCGGCNNTANCTNCNGANNTAAGNCATANNNTNNNTCNNTANCTNNNNNGGNGGGNNACTGTAAA
NGGNNAATTNGNNNNTNANTNCNNNCATNTNNCNNNGNNNNCTNNNTNCCTGNNTNCNNAATTGGNNC
CANTGNNTGNCNATCTCTGGNTTNGNNNNNNTNNNNNTNNCANNNNGGNNNNNNANANNNNNATNTAA
NNTANANNNNCNNNNNNANNNCACNNNNNNNNNAGNNANNANNNGNANANNNGTAGNNNGNTNAGTCT
CCGANNTNNNNNNNATAAGCGNNANACNNNNNNNNNNNNNANGANNNCGNNTTTGNNNTNNNNNNNNA
GNNAGACNNNNGTGGNTNNNNNTCAANNNNTTNGTAGTGGTATAATNGNNNNNNNTTTCNCCAGNNTN
NNNGNNNNNNNCNATANNANNNNNNNGNGANNNNNNNNNNANNNNCTCNNNAGGGANCANCTACTNNA
GCANTGGNNCGNAGNNNGAGANNNNGACNNNCCTTGGGTGGCAANNACNGCAATAAGTNNNGTTNNNC
CTNNNAGTNNACACNNNNNCANNGNNNCNNNNNNANCNGANNGNNNNNNNNNNNNNNNNNNNNNNNN
NNNNNNNNNNNNNNNNNNNNNNNNNNNNNNNNNNNNANGGNNNGNNTNTGNCNCATGTGTTTTTANNNGNN
NNNNNACGNNNNANCCCCGGTCGNNNNCNNATTGAANNCCTGNNGCATNNNNNTNCNAGAATGANNNC
NNNNNGNNTNGNCTNNCNNNTCANNNAATCANNNAGGTANNNNGNTCNNNNNNNNNNNNNCNNNCCCNN
ACTGAGGGTNNNNNNNNCANCNNCTNNNNNNNNNTNCGCNGGCGTNNCNNNNNNGCNGTATNNNGTNN
NNCANNNNNNNNNNNNNNNNNTNAAACNNNNNNNNNTNNNN
>Kinh31
GNNNNNNNTAGCCCNNNNNAGTTNGACANNNCNNNNNNNNNNNNTNNNNNNNNNNNNNNNNNNNNNN
NNNNNNNNNNNNNNNNNNNNNNNNNNNNNNNNNNNNNNNNNNNNNNNNNNNNNNNNNNNNNNNNNNN
NNNNNNNNNNNNNNNNNNNNNNNNNNNNNNNNNNNNNNNNNNNNNNNNNNNNNNNNNNNNNNNNNNN
NNNNNNNNNNNNNNNNNNNNNNNNNNNNNNNNNNNNNNNNNNNNNNNNNNNNNNNNNNNNNNNNNNN
NNNNNNNNNNNNNNNNNNNNNNNNNNNNNNNNNNNNNNNNNNNNNNNNNNNNNNNNNNAANNNNNNN
NNNNNNNNNNNNNNNNNNNNNNNNNNCNCNNTNNNNGTAANCNANNNGNACATGCAGTCCCCTCNNNNN
NNNNNCAGAGNNNNNNNNCNNNNNNNCCGNCCCCNNCCNNNNNNNNNNNNCNNNGNNNNNNNANNNNNN
NNNNNNTGGTACNNNNCGNNNNNGGTTGNNNNAGAAGTCCACAACGNGNTCNNNNNANNNNNGNNNAN
NNNCNNNNNNNNNNNNNNNNNNNANNNNANNTANNAGGNNNNNNCNNNNNNNNNNNNNNANNGCNANGANN
CNNNNNNCNGGGGNNGTNCCCGGNGNGGCGACCTTGANNANNTCNGGNGNCAAGTATGGCNNNCNCCC
CGNNAAGNNNNCNNCNGTANGTGCNNNNNANNNNNTCNTGNGANGTNGCCGNNANNNNNGTGGTCNNN
NNAGAATGNNCNNACCNNNNGNANNNTGGTNATGNNAGTNNNNNNNNNNNNNNNNNNNNNNNNNNNNN
NNNNNNNNNNNNNNNNNNNNNNNNNNNNNNNNNNNNNNNGNNNNNNCNANNNAAGGANNTANNNCTNNNN
NTNNNNNNGGNCNNNACNNNNCNNNNNNNACNCNNNCNCNNNNNNNANNNGNTTTTNNNNCNNTNNNNT
CCAGNANGCNGNGCNNNCNTTCANGNGAACNNNGNNACGCGNNAATCGTANNATCTACCTGCCGCNNG
NNNNNNTNCNGNNNNNNNCGGANNNTNNCTCGTANNNNNNNNCNNGCGTNNNNNTNNTNNCNAGNCNNGTT
CGNNNANANANTAGNANGTNTNNNNNNNNNTGTNNCGTTCCGCNTGANNCNNCNNNNNGNNGNNNTTN

NCANCGNCNNTANCNNNNGANNTAAGNNANANNNNNNNTCNNTANCTNNNNNNGNGGGNNACTGNAAA
NGGNNNNTTNGNNNNTNANTNCNNNNATNNNNCNNNGNNNNCNNNNTNCCTGNNNNCNNAATTGGNNN
CANTGNNTGNCNATCTCTNGNTTNGNNNNNNTNNNNNTNNCANNNNGGNNNNNNNNANNNNNANNTAA
NNTANANNNNCNNNNNNANNNCACNANNNNNNNAGNNNCNANNNNNANNNNNGTAGNNNGNTNAGTCN
CCGANNTNNNNNNNANAAGCTNNANACNNNNNNNNNNNNNANGANNNCGNNTTTGNNNTNNNNNNNNA
GNNAGACNNTNGTGGNTNNNNNTCAANNNNTTNGTAGTGGTATAATNGNNNNNNNTTNCNCCAGNNNN
NNNNNNNNNNNCNATANNANNNNNNNGNNANNNNNNNNNNNANNNNCTCNNNAGGNANCANCTACTNNA
GCANNGGNGNGNAGNNNGAGANNNNGACNNNCCTTGGGTGGCAANNACNGNANTAAGTNNNGTTNNNN
CTNNNAGTNNACACNNNNNCANNGNNNCNNNNNNANCNGANNGNNNNNNNNNNNNNNNNNNNNNNNNN
NNNNNNNNNNNNNNNNNNNNNNNNNNNNNNNNNNNNNGNNNNTNTNTGNCNCNNNNNNNTNCANNNGNN
NNNNNNNNNNNNNANCCCCGGTCGNNNNCNNATTGAANNCCTGNNGCATNNNNNTNCNAGAATGANNNN
NNNNNGNNTNGNCTNNNNNNNTCANNNAATCNNNNAGGTNNNNNNNTCNNNNNNNNNNNNCNNNCCCNN
ANNGNGNNTNNNNNNNNCANNNNCTNNNNNNNNNTNCGCNGGNGTNNCNNNNNNGCNGTATNNNGNNN
NNCANNNNNNNNNNNNNNNNNTNAAACNANNNNNNNTNNNN
>Kinh32
GTNNNNNNTAGCTCNNNNNAGTTANACANNNCNNNNNNNNNNNNTNNNNNNNNNNNNNNNNNNNNNNN
NNNNNNNNNNNNNNNNNNNNNNNNNNNNNNNNNNNNNNNNNNNNNNNNNNNNNNNNNNNNNNNNNNNN
NNNNNNNNNNNNNNNNNNNNNNNNNNNNNNNNNNNNNNNNNNNNNNNNNNNNNNNNNNNNNNNNNNNN
NNNNNNNNNNNNNNNNNNNNNNNNNNNNNNNNNNNNNNNNNNNNNNNNNNNNNNNNNNNNNNNNNNNN
NNNNNNNNNNNNNNNNNNNNNNNNNNNNNNNNNNNNNNNNNNNNNNNNNNNNNNNNNNNNAANNNNNNN
NNNNNNNNNNNNNNNNNNNNNNNNNNCNCNNTGNNNGTAANCNANNNGNACATGCAGTCCCCTCNNNNN
NNNGGCAGAGNNNNNNNNNNNNNNNNNCCGNCCCCNNCCNNNNNNNNNNNNNNNNGNNNNNNNGNNNNNN
NNNNNNTGGTACNNNNCGNNNNNGGTTGNNNNAGAGGTCCACCACGNGNTCNNNNNACNNNNGNNNAN
NNNCNNNNNNNNNNNNNNNNNNNANNTNNNCANNAGGNNNNNNCCNTNNNNNNNNNNNANNGCNATGACC
CANNNCGCNGNNANNGTNACCGGCGCGGCGACCTTGANNANNGTCGGGGNCAAGTATGTCGNNCNCCC
CGNGAAGGANNCNGCNGTANGTGCNNNNNANNNNNTNNTGNGANGTNGCCGNNATTNNCGTGGNCNNN
CNNGANTGNCCGNANCANNNGNANNNTGGTNATGNNAGTCNNNNNNNNNNNNNNNNNNNNNNNNNNNNN
NNNNNNNNNNNNNNNNNNNNNNNNNNNNNNNNNNNNNNNGNNNNNNCNANNNAAGGNNNTANNNTTNNTN
NNNNNNNNGGACNNNACNNNNCNNNNNNNACNCNNNCNCNNNNNNNNNNGGTTNNNNNNCNNTANTNT
CCAGNANGCNGNGCNNNCNTTCANGNGAACNNNGNNACGCGNNAATCGTANNATCTACCTGCCGCNNG
NNNNNNTNCNGNNNNNNCGGANNNTNNNNCGTANNNNNNNCANGCGTNNNNTNNTTNCNAGNCNNGTT
CGNNNANANANTAGNANGTNTNNNNNNNNNTGTNNCGTTCCGCNTGANNCNNCANNNNGNNGNNCTTN
NCANCGGCNNTANCTNNNGANNTAAGNCATANNNTNNNTCNNTANCGNNNNNGGNGGGNNACTGNAAA
NGNNNAATTNGNNNNTNANTNCNNNCACNTNNCNNNGNNNNCTNNNCNCCTGNNTNCNNAATTGGNNC
CANTGNNTGNCNATCTCTGGNTTNGNNNNNNTNNNNNTNNTANNNNGGNNNNNNNNANNNCNATNTAA
NNTANANNNNCNNNNNNANNNCACNNNNNNNNNAGNNNNNANNNTNANGNNNGTAGNNNANTAAGTCT
CCGNNNTNNNNNNNATAAGCGNNANACNNNNNNNNNNNNNANGANNNCGNNTTGGNNNTNNNNNNNNA
ANNAGACNNCNGTGGNTNNNNNTCAANNNNTTNGTAGTGGTATAACNANNNNNNNTTTCNCCAGNNNN
NNNGNNNNNNNNCNATACNANNNNNNNGNGANNNNNNNNNNANNNNNCCNNNAGGGANCANCTACTNNA
GCANNGGNNCGNNGNNNGAGANNNNGACNNNCCTTGGGTGGCAANNACNGNAATAAGTNNNGTTNANN
CCNNNAGTNNACACNNNNNCANNGNNNCNNNNNNANCNGANNTNNNNNNNNNNNNNNNNNNNNNNNNN
NNNNNNNNNNNNNNNNNNNNNNNNNNNNNNNNNNNANNGNNNGTNTNTGNCNCATNNNTTTNTANNNGNN
NNNNNNNGNNNNANCCCCGGTCGNNNNCNNATTGAANNCCTGNNGCANNNNNNTNCNAGAATGANNNC
NNNNNGNNTNGNCTNNNNNNNTCANGNAGTCNNNNAGGNNNNNNNNTCNNNGNNNNNNNNCNNTCCCNN
ACTGAGGGTNNNNNNNNCANCNNCTNNNNNNNNNNTNCGCNGNNNNNNTNNNNNNGCNGTATNNNGNNN
NNCANNNNNNNNNNNNNNNNNCNAAACGANNNNNNTNNNN
>Kinh33
GTGNNNNNNAGCCCNNNNNAGTTNGACANNNCNNNNNNNNNNNNTNNNNNNNNNNNNNNNNNNNNNNN
NNNNNNNNNNNNNNNNNNNNNNNNNNNNNNNNNNNNNNNNNNNNNNNNNNNNNNNNNNNNNNNNNNNN
NNNNNNNNNNNNNNNNNNNNNNNNNNNNNNNNNNNNNNNNNNNNNNNNNNNNNNNNNNNNNNNNNNNN
NNNNNNNNNNNNNNNNNNNNNNNNNNNNNNNNNNNNNNNNNNNNNNNNNNNNNNNNNNNNNNNNNNNN
NNNNNNNNNNNNNNNNNNNNNNNNNNNNNNNNNNNNNNNNNNNNNNNNNNNNNCTNNNNAANNNTNNN
NNNNNNNNNNNNNNNNNNNNNNNNNNNNCNTTNNNNGTAANCNANNNGNACATGCAGTCCCCTCNNNNN
NNNNNCAGAGNNNNNNNCCNNNNNNNCCGNCCCCNNCCNNNNNNNNNNNNNNNNNNNNNNNNNANNNNNN

NNNNNNTGGTACNNNNCGNNNNNGGTTGNNTAAGAGGTCCACAACGNGNTCNNNNNANNNNNGNNNAN
NNNCNNNNNNNNNNNNNNNNNNNNNNNNNNTANNAGGNNNNNNCCNTNNNNNNANNNNNNGCNATGANN
CNANNCNCNGNGGNNGTNCCCGGNGCGGCGACCTTGANNANGTCNGGGGNCAAGTATGGCNNNCNCCC
CGNGAAGGANNCNGCCGTANGTGCNNNNNANNNNNTCNTGNGANGTNGCCGNNATTCNCGTGGNCNNN
CNAGAATGNCCGNACCANNCGNANNNTGGTNATGNNAGTNNNNNNNNNNNNNNNNNNNNNNNNNNNNNN
NNNNNNNNNNNNNNNNNNNNNNNNNNNNNNNNNNNNNNNGNNNNNNCNANNNAAGGNNNTANNNCTNNTN
NNNNNNNNNNNNCNNNACNNNNCNNNNNNNACNCNNNCNCNNNNNNANNNGNTTTNTNNNCNNTANTNT
CCAGNANGCNGNGCNNNCNTTCANGNGAACNNNGNNACGCGNNAATCGTANNATCTACCTGCCGCNNN
NNNNNNTNCNGNNNNNNCGGANNNTNNCTCGTANNNNNNNCATGCGTNNNNTNNTCNCNAGNCNNGTT
CGNNNANANANTAGNANGTNTNNNNNNNNNNTGTNNCGTTCCGCNTGANNCNNCANNNNGNNGNNNTTN
NCANCGGCNNTANCTNNNGANNTAAGNCATNNNNTNNNTCNNTANCTNNNNNGGNGGGNNACTGTAAA
NGGNNAATTNGNNNNTNANTNCNNNNANNNNNCNNNGNNNNCTNNNTNCCTNNNNNCNNAATTGGNNN
CANTGNNTGNCNATCTCTGGNTTNGNNNNNNTNNNNNTNNCANNNNGGNNNGTNNNANNNCNATNTAA
NNTANANNNNCNNNNNNANNNCACNNNNNNNNNAGNNNNNNNNNTNANANNNGTAGNNNANTNAGTCT
CCGANNTNNNNNNNATAAGCGNNANACNNNNNNNNNNNNNNANGANNNCGNNTTGGNNNTNNNNNNNNA
GNNAGACNNTNATGGNTNNNNNTCAANNNNTTNGTAGTGGTATAATNGNNNNNNNTTTCNCCAGNNTN
NNNNNNNNNNNCNATANNANNNNNNNGNGANNNNNNNNNNANNNNNTCNNNAGGNANCANCTACTNNA
GCANTGGNNCGNAGNNNGAGANNNNGACNNNCCTTGGGTGGCAANNACNGNGATAAGTNNGTTNNNN
CTNNNNGTNNACATNNNNNCANNGNNNCNNNNNNANCNNANNGNNNNNNNNNNNNNNNNNNNNNNNNN
NNNNNNNNNNNNNNNNNNNNNNNNNNNNNNNNNNNNNNNGNNNGTNTNTGNCNCATGTGTTTTTANNNGNN
NNNNNNNGNNNNANCCCCGGTCGNNNNCNNATTGAANNCCTGNNGCATNNNNNTNCNANAATGANNNC
NNNNNGNNTNGNCTNNNNNNNTCAAGNAATCNNNNAGNNANNNNNNTCNNNNNNNNNNNNNCNNNCNCNN
ACTGAGGGNNNNNNNNNCANCNGCTNNNNNNNNNNTNCGCNGNNGTNNCNNNNNNGCNGTATNNNGNNN
NNCANNNNNNNNNNNNNNNNNNNNNNNNANNNNNNNNTNNNN
>Kinh34
GTGNNNNNNNAGCCCNNNNNAGTTANACANNNCNNNNNNNNNNNNNTNNNNNNNNNNNNNNNNNNNNNNN
NNNNNNNNNNNNNNNNNNNNNNNNNNNNNNNNNNNNNNNNNNNNNNNNNNNNNNNNNNNNNNNNNNNNNN
NNNNNNNNNNNNNNNNNNNNNNNNNNNNNNNNNNNNNNNNNNNNNNNNNNNNNNNNNNNNNNNNNNNNNN
NNNNNNNNNNNNNNNNNNNNNNNNNNNNNNNNNNNNNNNNNNNNNNNNNNNNNNNNNNNNNNNNNNNNNN
NNNNNNNNNNNNNNNNNNNNNNNNNNNNNNNNNNNNNNNNNNNNNNNNNNNNNNNNTCTNNNNAANNNTNNN
NNNNNNNNNNNNNNNNNNNNNNNNNNCNCNTTGNNNGTAANCNANNNGNACATGCAGTCCCCTCNNNNN
NNNNNCAGAGNNNNNNCNCNNNNNNNNCCGNCCCCNNCCNNNNNNNNNNNNNNNNNNNNNNNNNNANNNNNN
NNNNNNTGGTACNNNNCGNNNNNGGTTGNNTNAGAGGTCCACAACGNGNTCNNGNNACNNNNGNNNAN
NNNCNNNNAANNNNNNNNNNNNNNNNNTNNGTANNAGGNNNNNNCCNTCNNGNNNNNNANTGCNATGACC
CNANNCNCGGNGGCNGTNCCCGGCGCGGNGACCTTGNNNACGTCNGGGGNCAAGTATGGCNNNCNCCC
CGGGAAGGANNCNGCCGTANGTGCNNNNNANNNNNTCNTGNGANGTNGCCGNNATTNNCGTGGTCNNN
NNAGANTGNNCNNACCANNCGNANNNTGGTNATGNNAGTNNNNNNNNNNNNNNNNNNNNNNNNNNNNNN
NNNNNNNNNNNNNNNNNNNNNNNNNNNNNNNNNNNNNNNGNNNNNNCNANNNAAGGANNTANNNCTNNTN
NTNNNNNNGGNCNNNACNNNNCNNNNNNNACNCNNNCNCNNNNNNANNNGGTTTTNNNNCNNTANTNN
CCAGNANGCNGNGCNNNCNTTCANGNGAACNNNGNNACGCGNNAATCGTANNATCTACCTGCCGCNNN
NNNNNNTNCNGNNNNNNCGGANNNTNNNNNNTANNNNNNNCATGCGTNNTNTNNTCNCNAGNCNNGTT
CGNNNATANANTAGNANGTNTNNNNNNNNNNNGTNNCGTTCCGCNTGANNCNNCNNNNNGNNGNNNTTN
NCANCGGCNNTANCTGCNGANNTAAGNCATANNNTNNNTCNNTANCTNNNNNGGNGGGNNACTGTAAA
NGGNNAATTNGNNGNTNATTNCNNNCACNTNNCNNNGNNNNCTNNNTNCCTGNNTNCNNAATTGGNNN
CANTGNNTGNCNATCTCTNGNTTNGNNNNNNTNNNNNTNNCAANNNGGNNNNNNNNANNNCNATNTAA
NNTANANNNNCNNNNNNANNNCACNANNNNNNNNAGNNNCNANNNTTANANNNGTAGNNNNNTNAGTCN
CCGANNTNNNNNNNATAAGCGNNANACNNNNNNNNNNNNNNANGANNNCGNNTTGGNNNTNNNNNNNNN
GNNANACNNTNATGGNTNNNNNTCAANNNNTTNGTAGTGGTATAATNGNNNNNNNTTNCNCCAGNNTN
NNNGNNNNNNNNCNATANNANNNNNNNGNGANNNNNNNNNNANNNNCTCNNNAGGGANCANCTACTNNA
GCANTGGNGCGNAGNNNGAGANNNNGACNNGCCTTGGGTGGCAANNACNGNAATAAGTNNNGTTNANN
NTNNNAGTNNACATNNNNNCANNGNNNCNNNNNNANCNGANNGNNNNNNNNNNNNNNNNNNNNNNNNN
NNNNNNNNNNNNNNNNNNNNNNNNNNNNNNNNNNNNNANNGNNNGTNTNTGNCNCATNTGTTTTTANNNGNN
NNNNNNNGNNNNANCCCCGGTCGNNNNCNNATTGAANNCCTGNNGCATNNNNNTNCNAGAATGANNGN
NNNNNGNNTNGNCTNNNNNNNTCANGNAATCANNNAGNNNNNNNNNNTCNNNGNNNNNNNNNCNNNCCCNN

ACTGAGGGTNNNNNNNNCANCTGCTNNNNNNNNNNTNCGCNGNNGTNNCNNNNNNGCNGTATNNNGTNN
NNCANNNNNNNNNNNNNNNNNTNAAACGNNNNNNNTNNNN
>Kinh699
GTGNNNNNAGCCCNNNNNAGTTAGACANNNCNNNNNNNNNNTNNNNNNNNNNNNNNNNNN
NNNNNNNNNNNNNNNNNNNNNNNNNNNNNNNNNNNNNNNNNNTNNNNNNNNNNNNNNNNN
NNNNNNNNNNNNNNNNNNNNNNNNNNNNNNNNNNNNNNNNNNNNNNNNNNNNNNNNNNNN
NNNNNNNNNNNNNNNNNNNNNNNNNNNNNNNNNNNNNNNNNNNNNNNNNNNNNNNNNNNN
NNNNNNNNNNNNNNNNNNNNNNNNNNNNNNNNNNNNNNNNNNNNTCTNNNAAANNNTNNN
NNNNNNNNNNNNNNNNNCNCNNTGNNNGTAANCNANNNGNACATGCAGTCCCCTCNNNNN
NNNGGCAGAGNNNNNNNNCNNNNGNNCCGNCCCCNNCCNNNNNNNNNNNNNNNNNNNNNNNNNNANNNNNN
NNNNNNTGGTACNNNNCGNNNNNGGTTGNTTNAGAGGTCCACAACGNGGTCNGGNNACNNNNGNNNAN
NNNCNNNNAACNNNNNNNNNNNNANNTNNNTANNAGGNNNNNNCCNTNNNGNNNNNAANNGCNATGACN
CANNNCNCGGGNGNAGTNCCCGGNGNGGNGACCTTGNNNACGTCCGGGGNCAAGTATGGCNNNCNCCC
CGGGAAGGANNCNGCNGTANGTGCNNNNNANNNNNTCNTGNGANGTNGCCGNNATTNNCNTGGTCNNN
CNNGAATGNCCNNACCANNCGNANNNTGGTNATGNNAGTNNNNNNNNNNNNNNNNNNNNN
NNNNNNNNNNNNNNNNNNNNNNNNGNNNNGNNNNNNCNANNNAAGGNNNTANNNCTNNTN
NTNNNNNNGNNCNNNNCNNNNCNNNNNNNACNCNNNCNCNNNNNNANNNGGTTNNNNNNCNNTANTNT
CCAGNACGCNGNGCNNNCATTCANGNGAACNNNGNNACGCGNNAATCGTANNATCTANCTGCCGCNNG
NNNNNNTNCNGNNNNNNCGGANNNTNNNNCGTANNNNNNNCANGCGTNNTNTNNTCNCNAGNCNNGTT
CGNNNANANANTAGNANGTNTNNNNNNNNNNTGTNNCGTTCCGCNTGANNCNNCNNNNNGNNGNNNTTN
NCANCGGCNCTANCTGCNGANNTAAGNCATANNNTNNNTCNNTANCTNNNNNGGNGGGNNACTGTAAA
NGGNNAATTNGAGNNTNANTNCNNNCACNTNNCNNNGNNNNCTNNNTNCCTGNNNNCNNAATTGGNNC
CANTGNNTGNCNATCTCTGGNTTNGNNNNNNTNNNNNTNNCANNNNGGNNNNNNANANNNCNATNTAA
NNTANANNNNCNNNNNNANNNCACNNNNNNNGNAGNNNCNANNNTTANANNNGTAGNNNANTNAGTCT
CCGANNTNNNNNNNATAAGCGNNANACNNNNNNNNNNNNNNANGANNACGNNTTGGNNNTNNNNNNNCNA
GNNAGACNNTNATGGNTNNNNNTCAANNNNTTNGTAGTGGTATAATNGNNNNNNNTTTCNCCAGNNTN
NNNGNNNNNNNNCNATANNANNNNNNNGNGANNNNNNNNNNNANNNNCTCNNNAGGGANCANCTACTNNA
GCANNGGNNCGNAGNNNGAGANNNNGACNNNCCTTGGGTGGCAANNACNGCGANAAGTNNGTTNNNN
CTNNNAGTNNACATNNNNNCANNGNNNCNNNNNNANCNGANNGNNNNNNNNNNNNNNNNN
NNNNNNNNNNNNNNNNNNNNANNNNANNGNNNGTNTNTGNCNCNTNNNTTTTTANNNGNN
NNNNNACGNNNNNNCCCCGGTCGNNNNCNNATTGAANNCCTGNNGCATNNNNNTNCNAGAATGANNNC
NNNNNGNNTNNNCTNNCNNNTNAAGNAATCANNNAGGNNNNNNNNTCNNNGNNNNNNNNCNNNNNCNN
ACTGAGGGTNNNNNNNNCATNTGCTNNNNNNNNNNTNCGCNGGNGTNNCNNNNNAGCNGTATNNNGTNA
NNCANNNNNNNNNNNNNNNNANTNAAACNANNNNNCTNNNN
>Kinh700
GTGNNNNNAGCCCNNNNNAGTCAGACANNNCNNNNNNNNNNTNNNNNNNNNNNNNNNNNN
NNNNNNNNNNNNNNNNNNNNNNNNNNNNNNNNNNNNNNNNNNNNNNNNNNNNNNNNNNNN
NNNNNNNNNNNNNNNNNNNNNNNNNNNNNNNNNNNNNNNNNNNNNNNNNNNNNNNNNNNN
NNNNNNNNNNNNNNNNNNNNNNNNNNNNNNNNNNNNNNNNNNNNNNNNNNNNNNNNNNNN
NNNNNNNNNNNNNNNNNNNNNNNNNNNNNNNNNNNNNNNNNNNNTCTNNNAAANNGTNNN
NNNNNNNNNNNNNNNNNCNCNNTNNNNGTAANCNANNNGNACATGCAGTCCCCTCNNNNN
NNNNNCAGAGNNNNNNNNCCNNNNNNNNCCGNCCCCNNCCNNNNNNNTNNNNNNNNNNNNNNNNANNNNNN
NNNNNNTGGTACNNNNCGNNNNNGGTTGTNTNAGAGGTCCACAACGTGNTCNGNNNACNNNNGNNNAN
NNNCNNNAAACNNNNNNNNNNNNANNTNNNTANNAGGNCNNNNCCNTNNNNNNANNNANTGCNATGACC
CANNNNNCNGGGGCNGTNCCCGGCGCGGCGACCTTGATNANGTCCGGGGNCAAGTATGGCNNNCNCCC
CGGGAAGGANNCNGCCGTANGTGCNNNNNANNNNNTCNTGNGANGTNGCCGNNATTCNNNTGGTCNNN
CNAGAATGNCCNNACCANNCGNANNNTGGTNATGNNAGTANNNNNNNNNNNNNNNNNNNN
NNNNNNNNNNNNNNNNNNNNNNNNGNNNNGNNNNNNCNANNNAAGGNNNTANNNCTNNTN
NNNNNNNNGGACNNNACNNNNCNNNNNNNACNCNNNCNNNNNNNNANNNGNTTTTNNNNNCNNTANTNN
CCAGNACGCNGNGCNNNCNTTCANGNGAACNNNGNNACGCGNNAATCGTANNATCTACCTGCCGCNNG
NNNNNNTACNGNNNNNNCGGANNNTNNNNCGTANNNNNNNCANGCGTNNTNTNNTNNCNAGNCNNGTT
CGNNNANANANTAGNANGTNTNNNNNNNNNNTGTNNCGTTCCGCNTGANNCNNCANNNNGNNGNNCTTN
NCANCGGCNNTANCTGCNGANNTAAGNCATANNNTNNNTCNNTANCTNNNNNGGNGGGNNACTGTAAA
NGGNNAATTNGNNNNTNATTNCNNNCATNTNNCTNNGNNNNCTNNNTNCCTGNNTNCNNAATTGGNNC

CANTGNNTGNCNATCTCTGGNTTNGNNNNNNTNNNNNTNNCANNNNGGNNNGTNANANNNCNATNTAA
NNTANANNNNCNNNNNNANNNCACNNNNNNNNNAGNNNCNANNNGNANANNNGTAGNNNGNTAAGTCT
CCGANNTNNNNNNNATAAGCGNNANACNNNNNNNNNNNNNNANGANNNCGNNTTTGNNNTNNNNNNCNA
GNNAGACNNTNGTGGNTNNNNNTCAANNNNTTNGTAGTGGTATAATNGNNNNNNNTTTCNCCAGNNTN
NNNGNNNNNNNCNATANNANNNNNNNGNGANNNNNNNNNNANNNNCTCNNNAGGNANCANCTACTTNA
GCANTGGNGCGNAGNNNGAGANNNNGACNNNCCTTGGGTGGCAANCACNGNAATAAGTNNNGTTNNNC
CTNNNAGTNNACACNNNNNCANNGNNNCNNNNNNANCNGANNGNNNNNNNNNNNNNNNNNNNNNNNNN
NNNNNNNNNNNNNNNNNNNNNNNNNNNNNNANNNNANNGNNNGTNTNTGACNCATGTGTTTNTANNNGNN
NNNNNNNGNNNNANCCCCGGTCGNNNNCNNATTGAANNCCTGNNGCATNNNNNTNCNAGAATGANNGC
NNNNNGNNTNGNCTNNCNTTTCAAGNAATCANNNAGNNNNNNNGNTCNNNNNNNNNNNNCNNTCNCNN
ACTGAGGGTNNNNNNNNCATNNGCTNNNNNNNNNTNCGCNGGNGTNNCNNNNNAGCNGTATNGNGTNN
NNCANNNNNNNNNNNNNNNNNTNAAACAANNNNNNTNNNN
>Kinh701
GTGNNNNNTAGCCCNNNNNAGTCNGACANNNCNNNNNNNNNNNNTNNNNNNNNNNNNNNNNNNNNNNN
NNNNNNNNNNNNNNNNNNNNNNNNNNNNNNNNNNNNNNNNNNNNNNNNNNNNNNNNNNNNNNNNNNNN
NNNNNNNNNNNNNNNNNNNNNNNNNNNNNNNNNNNNNNNNNNNNNNNNNNNNNNNNNNNNNNNNNNNN
NNNNNNNNNNNNNNNNNNNNNNNNNNNNNNNNNNNNNNNNNNNNNNNNNNNNNNNNNNNNNNNNNNNN
NNNNNNNNNNNNNNNNNNNNNNNNNNNNNNNNNNNNNNNNNTNNNNNNNNNNCNTCTNNNAAANNNTNNN
NNNNNNNNNNNNNNNNNNNNNNNNNNCNCNTTGNNNGTAANCNANNNGNACATGCAGTCCCCTCNNNNN
NNNNNCAGAGNNNNNNCCCNNNNNNNCCGNCCCCNNCCNNNNNNNNNNNCNNNGNNNNNNNANNNNNN
NNNNNNTGGTACNNNNCGNNNNNGGTTGTNTAAGAGGTCCACAACGNGGTCNNGNNACNNNNGNNNAN
NNNCNNNNAACNNNNNNNNNNNANNTNNNTANNAGGNCNNNNCCNTCNNNNNANTAANTGCNATGACC
CAANNCACGGNNGCNGTNCCCGGNGNGGCGACCTTGANNANGTCCGGGGACAAGTATGGCGNNCNCCC
CGGGAAGGANNCNGCCGTANGTGCNNNNNANNNNNTCNTGNGANGTNGCCGNNATTNNCGTGGTCNNN
CNAGAATGNCCGNACCANNCGNANNNTGGTNATGNNAGTNNNNNNNNNNNNNNNNNNNNNNNNNNNNN
NNNNNNNNNNNNNNNNNNNNNNNNNNNNNNNNNNNNNNNGNNNNNNCNANNNAAGGNNNTANNNCTNNTN
NTNNNNNNNGGACNNNACNNNNCNNNNNNNACNCNNNCNCNNNNNNNNNNGNTTTTNNNNCNNTATTNN
CCAGNACGCNGNGCNNNCATTTANGNGAACNNNGNNACGCGNNAATCGTANNATCTACCTGCCGCNNG
TNNNNNTACNGNNNNNNCGGANNNTNNCNCGTANNNNNNNCANGCGTNNTNTNNTNNCNAGNCNNGTT
CGNNNANANANTAGNANGTNTNNNNNNNNNTGTNNCGTTCCGCNTGANNCNNCANNNNGNNGNNCTTN
NCANCGGCNNTANCTGCNGANNTAAGNCATANNNTNNNTCNNTANCTNNNNNGGNGGGNNACTGTAAA
NGGNNAATTNGNNNNTNANTNCNNNCATNTNNCTNNGNNTNCTNNNTNCCTGNNTNCNNAATTGGNNC
CANTGNNTGNCNATCTCTGGNTTNGNNNNNGTNNNNNTNNCANNNNGGNNNGNNNNANNNCNATNTAA
NNTANANNNNCNNNNNNANNNCACNANNNNNNNAGNNNCNANNNGNANANNNGTAGNNNGATAAGTCT
CCGANNTNNNNNNNATAAGCGNNANACNNNNNNNNNNNNNNANGANNACGNNTTTGNNNTNNNNNNCNA
GNNAGACNNTNGTGGNTNNNNNTCAANNNNTTNGTAGTGGTATAATNGNNNNNNNTTTCNCCAGNNTN
NNNGNNNNNNNCNATANNANNNNNNNGNGANNNNNNNNNNANNNNCTCNNNAGGGANCANCTACTNNA
GCANNGGNGCGNAGNNNGAGANNNNGACNNGCCTTGGGTGGCAANNACNGCAATAAGTNNNGTTNANC
CTNNNAGTNNACACNNNNNCANNGNNNCNNNNNNANCNGANNGNNNNNNNNNNNNNNNNNNNNNNNNN
NNNNNNNNNNNNNNNNNNNNNNNNNNNNNNANNNNANGGNNNGTNTNTGACNCATGNNTTTTTANNNGNN
NNNNNNNGNNNNANCCCCGGTCGNNNNCNNATTGAANNCCTGNNGCATNNNNNTNCNAGAATGANNNC
NNNNNGNNTNGNCTNNCNNNTCAAGNAATCAANNANNNNNNNNGNTCNNNGNNNNNNNNCNNTCNCNN
ACTGAGGGTNNNNNNNNCATCNGCTNNNNNNNNCTNCGCNGGNNNNNCNNNNNNGCNGTATNNTGTNN
NNCANNNNNNNNNNNNNNNNNTNAANCNANNNNNNTNNNN
>Kinh702
GTGNNNNNTAGCCCNNNNNAGTTAGACANNNCNNNNNNNNNNNNTNNNNNNNNNNNNNNNNNNNNNNN
NNNNNNNNNNNNNNNNNNNNNNNNNNNNNNNNNNNNNNNNNNNNNNNNNNNNNNNNNNNNNNNNNNNN
NNNNNNNNNNNNNNNNNNNNNNNNNNNNNNNNNNNNNNNNNNNNNNNNNNNNNNNNNNNNNNNNNNNN
NNNNNNNNNNNNNNNNNNNNNNNNNNNNNNNNNNNNNNNNNNNNNNNNNNNNNNNNNNNNNNNNNNNN
NNNNNNNNNNNNNNNNNNNNNNNNNNNNNNNNNNNNNNNNNNNNNNNNNNNNNCNTCTNNNAAANNNTNNN
NNNNNNNNNNNNNNNNNNNNNNNNNNCNCNNTNNNNGTAANCNANNNGNACATGCAGTCCCCTCNNNNN
NNNGGCAGAGNNNNNNNCCNNNNNNNCCGGCCCCNNCCNNNNNNNNNNNNNNNNGNNNNNNNANNNNNN
NNNNNNTGGTACNNNNCGNNNNNGGTTGTNTAAGAGGTCCACAACGNGNTCNGGNCACNNNNGNNNAN
NNNCNNNNAACNNNNNNNNNNAANATNNNTANNAGGNCNNNNCCNTCNNGNNNNNNATTGCNATGANN

CANNNCACGGNGGCNGTNCCCGGNGCGGCGACCTTGANNACGTCCGGGGNCAAGTATGGCNNNCNCCC
CGGGAAGGANNCNGCNGTANGTGCNNNNNANNNNNTCNTGNGANGTNGCCGNNATTNNCGTGGTCNNN
CNAGANTGNCCNNACCANNCGNANNNTGGTNATGNNAGTNNNNNNNNNNNNNNNNNNNNNNNNNNNN
NNNNNNNNNNNNNNNNNNNNNNNNNNNNNNNNNNNGNNNNGNNNNNNCNANNNAAGGNNNTANNNCTNNTN
ATNNNNNNGGACNNNACNNNNCNNNNNNNACNCNNNCNCNNNNNNANNNGGTTTTNNNNCNNTANTNT
CCAGNACGCNGNGCNNNCATTCANGNGAACNNNGNNACGCGNNAATCGTANNATCTACCTGCCGCNNG
TNNNNNTACNGNNNNNNCGGANNNTNNNTCGTANNNNNNNCATGCGTNNTNTNNTCNCNAGNCANGTT
CGNNNANANANTAGNANGTNTCNNNNNNNNTGTNNCGTTCCGCNTGANNCNNCANNNNGNNGNNNTTN
NCANCGGCNNTANCTNCNGANNTAAGNCATANNNTNNNTCNNTANCTNNNNNGGAGGGNNACTGTAAA
NGGNNAATTNGNNGNTNANTNCNNNCACNTNNCTNNGNNTNCTNNNTNCCTGNNTNCNNAATTGGNNC
CANTGNNTGNCNATCTCTNGNTTNGNNNNNNTGNNNNTNNCANNNNGGNNNGTNNNANNNCNATNTAA
NNTANANNNNCNNNNNNANNNCACNNNNNNNNNAGNNNCNANNNTNANANNNGTAGNNNAATNAGTCT
CCGANNTNNNNNNNATAAGCGNNANACNNNNNNNNNNNNNANGANNACGNNTTGGNNNTNNNNNNCNA
GNNAGACNNTNATGGNTNNNNNTCAANNNNTTNGTAGTGGTATAATNGNNNNNNNTTTCNCCAGNNTN
NNNGNNNNNNNCNATANNANNNNNNNGNGANNNNNNNNNNANNNNCTCNNNAGGGANCANCTACTNNA
GCANTGGNGCGCAGNNNGAGANNNNGACCAGCCTTGGGTGGCAANCACNGCAATAAGTNNNGTTNANC
CTNNNAGTNNACATNNNNNCANNGNNNCNNNNNNANCNGANNGNNNNNNNNNNNNNNNNNNNNNNNN
NNNNNNNNNNNNNNNNNNNNNNNNNNNNANNNNANGGNNNGTNTNTGACNCNTGTGTTTTTANNNGNN
NNNNNACGNNNNANCCCCGGTCGNNTNCNNATTGAANNCCTGNNGCATNNNNNTNCNAGAATGANNNC
NNNNNGNNTNGNCTNNCNNNTCAAGNAATCAANNAGGTNNNNNNNTCNNNNNNNNNNNNCNNTCCCNN
ACTGAGGGTNNNNNNNNCATCTGCTNNNNNNNNNNTNCGCNGGNNNNNCNNNNNAGCNGTATNGTGTNA
NNCANNNNNNNNNNNNNNNNNTNAANNGANNNNNNTNNNN
>Kinh703
GTGNNNNNTAGCCCNNNNNAGTTAGACANNNCNNNNNNNNNNNNTNNNNNNNNNNNNNNNNNNNNNN
NNNNNNNNNNNNNNNNNNNNNNNNNNNNNNNTNNNNNNNNNNNNNNNNNNNNNNNNNNNNNNNNNNNNNN
NNNNNNNNNNNNNNNNNNNNNNNNNNNNNNNNNNNNNNNNNNNNNNNNNNNNNNNNNNNNNNNNNNNN
NNNNNNNNNNNNNNNNNNNNNNNNNNNNNNNNNNNNNNNNNNNNNNNNNNNNNNNNNNNNNNNNNNNN
NNNNNNNNNNNNNNNNNNNNNNNNNNNNNNNNNNNNNNNNNNNNNNNNNNNNNNNTCTNNNAAANNNTNNN
NNNNNNNNNNNNNNNNNNNNNNNNNNNCNCNNTGNNNGTAANCNANNNGNACATGCAGTCCCCTCNNNNN
NNNNNCAGAGNNNNNNNCNNNNNNCNCCGNCCCCNNCCNNNNNNNNNNNNNNNNNNNNNNNNNANNNNNN
NNNNNNTGGTACNNNNCGNNNNNGGTTGNNTAAGAGGTCCACAACGNGNTCNNGNCACNNNNGNNNAN
NNNCNNNNAACNNNNNNNNNNNNANNTNNNTANNAGGNNNNNNCCNTCNNGNNNNTAATTGCNATGACC
CAANNCNCNGGGGCNGTNCCCGGCGCGGCGACCTTGANNACGTCCGGGGNCAAGTATGGCGNNCNCCC
CGNGAAGGANNCNGCCGTANGTGCNNNNNANNNNNTCNTGNGANGTCGCCGNNATTNNCGTGGTCNNN
CNAGAATGNCCNNACCANNCGNANNNTGGTNATGTNAGTNNNNNNNNNNNNNNNNNNNNNNNNNNNN
NNNNNNNNNNNNNNNNNNNNNNNNNNNNNNNNNNNGNNNNGNNNNNNCNANNNAAGGNNNTANNNCTNNTN
NTNNNNNNGGNCNNNACNNNNCNNNNNNNACNCNNNCNCNNNNNNANNNGNTTTTNNNNCNNTANTNT
CCAGNACGCNGNGCNNNCATTCANGNGAACNNNGNNACGCGNNAATCGTANNATCTACCTGCCGCNNG
NNNNNNTNCNGNNNNNNCGGANNNTNNNNCGTANNNNNNNCATGCGTNNTNTNNTCNCNAGNCANGTT
CGNNNANANANTAGNANGTNTNNNNNNNNNTGTNNCGTTCCGCNTGANNCNNCANNNNGNNGNNNTTN
NCANCGGCNNTANCTNCNGANNTAAGNCATANNNTNNNTCNNTANCTNNNNNGGNGGGNNACTGTAAA
NGGNNAATTNGNNNNTNATTNCNNNCACNTNNCTNNGNNNNCTNNNTNCCTGNNTNCNNAATTGGNNC
CANTGNNTGNCNATCTCTGGNTTNGNNNNNNTNNNNNTNNCANNNNGGNNNNNNNNANNNCNATNTAA
NNTANANNNNCNNNNNNANNNCACNNNNNNNGNAGNNNCNANNNTNANANNNGTAGNNNANTAAGTCT
CCGANNTNNNNNNNATAAGCGNNANACNNNNNNNNNNNNNANGANNNCGNNTTGGNNNTNNNNNNCNA
GNNAGACNNTNATGGNTNNNNNTCAANNNNTTNGTAGTGGTATAATNGNNNNNNNTTTCNCCAGNNTN
NNNGNNNNNNNCNATANNANNNNNNNGNGANNNNNNNNNNANNNNCTCNNNAGGGANCANCTACTNNA
GCANTGGNGCGNAGNNNGAGANNNNGACNNNCCTTGGGTGGCAANNACNGNGATAAGTNNNGTTNNNN
CTNNNAGTNNACATGNNNNCANNGNNNCNNNNNNANCNGANNGNNNNNNNNNNNNNNNNNNNNNNNN
NNNNNNNNNNNNNNNNNNNNNNNNNNNNANNNNANGGNNNGTNTNTGACNCANGTGTTTTTANNNGNN
NNNNNACGNNNNANCCCCGGTCGNNNNCNNATTGAANNCCTGNNGCATNNNNNTNCNAGAATGANNGC
NNNNNGNNTNGNCTNNCNNNTCAAGNAATCAANNAGNNNNNNNNNTCNNNNNNNNNNNNCNNTNNCNN
ACTGAGGGTNNNNNNNNCATCTGCTNNNNNNNNNCTNCGCNGGCGTNNCNNNNNNGCNGTATNGNGTNA
NNCANNNNNNNNNNNNNNNNNTNAAACAANNNNNCTNNNN

>Kinh704
GTGNNNNNTAGCCCNNNNNAGTTAGACANNNCNNNNNNNNNNNNTNNNNNNNNNNNNNNNNNNNNNNN
NNNNNNNNNNNNNNNNNNNNNNNNNNNNNNTNNNNNNNNNNNNNNNNNNNNNNNNNNNNNNNNNNNNN
NNNNNNNNNNNNNNNNNNNNNNNNNNNNNNNNNNNNNNNNNNNNNNNNNNNNNNNNNNNNNNNNNNNN
NNNNNNNNNNNNNNNNNNNNNNNNNNNNNNNNNNNNNNNNNNNNNNNNNNNNNNNNNNNNNNNNNNNN
NNNNNNNNNNNNNNNNNNNNNNNNNNNNNNNNNNNNNNNNNNNNNNNNNNCNTCTNNNAAANNNTNNN
NNNNNNNNNNNNNNNNNNNNNNNNNCNCNNTGNNNGTAANCNANNNGNACATGCAGTCCCCTCNNNNN
NNNNNCAGAGNNNNNNNCCNNNNNNNCCGGCCCCGNCCNNNNNNNNNNNCNNNGNNNNNNNANNNNNN
NNNNNNTGGTACNNNNCGNNNNNGGTTGNNTAAGAGGTCCACAACGNGGTCNGGNCACNNNNGNNNAN
NNNCNNNAAACNNNNNNNNNNNANNTNNNTANNAGGNNNNNNCCNTCNNGNNANNNATTGCNATGACC
CAANNCACGGGGGCNGTNCCCGGCGCGGCGACCTTGANNANNTCCGGGGACAAGTATGGCGNNCNCCC
CGGGAAGGANNCNGCCGTANGTGCNNNNNANNNNNTCNTGNGANGTNGCCGNNATTNNCGTGGTCNNN
CNAGAATGNCCGNACCANNCGNANNNTGGTNATGNNAGTNNNNNNNNNNNNNNNNNNNNNNNNNNNNN
NNNNNNNNNNNNNNNNNNNNNNNNNNNNNNNNGNNNNGNNNNNNCNANNNAAGGANNTANNNCTNNTN
NTNNNNNNGGACNNNACNNNNCNNNNNNNACNCNNNCNCNNNNNNANNNGNTTTTTNNNCNNTATTNT
CCAGNACGCNGNGCNNNCATTCANGNGAACNNNGNNACGCGNNAATCGTANNATCTACCTGCCGCNNG
TNNNNNTNCNGNNNNNNCGGANNNTNNNNCGTANNNNNNNCATGCGTNNTNTNNTCNCNAGNCNNGTT
CGNNNANANANTAGNANGTNTNNNNNNNCTTGTNNCGTTCCGCNTGANNCNNCANNNNGNNGNNCTTC
NCANCGGCNNTANCTGCNGANNTAAGNCATAANNTNNNTCNNTANCTNNNNNGGNGGGNNACTGTAAA
NGGNNAATTNGNNNNTNATTNCCNNCACNTNNCNNNGNNNNCTNNNTNCCTGNNTNCNNAATTGGNNC
CANTGNNTGNCNATCTCTGGNTTNGNNNNNNTNNNNNTNNCANNNNGGNNNGNNNNANNNCNATNTAA
NNTANANNNNCNNNNNNANNNCACNNNNNNNGNAGNNNCNANNNTNANANNNGTAGNNNANTAAGTCT
CCGANNTNNNNNNNATAAGCGNNANACNNNNNNNNNNNNNANGANNACGNNTTGGNNNTNNNNNNCNA
GNNAGACNNTNATGGNTNNNNNTCAANNNNTTNGTAGTGGTATAATNGNNNNNNNTTTCNCCAGNNNN
NNNGNNNNNNNCNATANNANNNNNNNGNGANNNNNNNNNNANNNNCTCNNNAGGGANCANCTACTNNA
GCANTGGNGCGNAGNNNGAGANNNNGACNNGCCTTGGGTGGCAANNACNGCGATAAGTNNNGTTNANC
CTNNNAGTNNACATNNNNNCANNGNNNCNNNNNNANCNGANNGNNNNNNNNNNNNNNNNNNNNNNNNN
NNNNNNNNNNNNNNNNNNNNNNNNNNNNAGNNNANGGNNNGTNTNTGACNCATGNNTTTTTANNNGNN
NNNNNACGNNNNANCCCCGGTCGNNNNCNNATTGAANNCCTGNNGCATNNNNNTNCNAGAATGANNGC
NNNNNGNNTNGNCTNNCNNNTCAAGNAATCANNTAGGTANNGCGNTCNNNGNNNNNNNNCNNTCCCNN
ACTGAGGGTNNNNNNNNCATCTGCTNNNNNNNNCTNCGCNGGNGTNNCNNNNNAGCNGTATNGTGTNA
NNCANNNNNNNNNNNNNNNNNNTNAAACGANNNNTNNNN
>Kinh705
GTGNNNNNTAGCCCNNNNNAGTCAGACANNNCNNNNNNNNNNNNTNNNNNNNNNNNNNNNNNNNNNNN
NNNNNNNNNNNNNNNNNNNNNNNNNNNNNNTNNNNNNNNNNNNNNNNNNNNNNNNNNNNNNNNNNNNN
NNNNNNNNNNNNNNNNNNNNNNNNNNNNNNNNNNNNNNNNNNNNNNNNNNNNNNNNNNNNNNNNNNNN
NNNNNNNNNNNNNNNNNNNNNNNNNNNNNNNNNNNNNNNNNNNNNNNNNNNNNNNNNNNNNNNNNNNN
NNNNNNNNNNNNNNNNNNNNNNNNNNNNNNNNNNNNNNNNNNNNNNNNNNNNTCTNNNAAANNNTNNN
NNNNNNNNNNNNNNNNNNNNNNNNNCNCNNTGNNNGTAANCNANNNGNACATGCAGTCCCCTCNNNNN
NNNNNCAGAGNNNNNNNCCNNNNNNNCCGNCCCCNNCCNNNNNNNNNNNCNNNGNNNNNNNANNNNNN
NNNNNNTGGTACNNNNCGNNNNNGGTTGNNTNAGAGGTCCACAACGNGGTCNNGNNACNNNNGNNNAN
NNNCNNNNAANNNNNNNNNNNNANNTNNNTANNAGGNNNNNNCCNTNNNNNNNNNNANTGCNATGACC
CAANNNACNGGGGNNGTNCCCGGCGNGGCGACCTTGNTNANGTCCGGGGNCAAGTATGGCNNNCNCCC
CGNGAAGGANNCNGCCGTANGTGCNNNNNANNNNNTCNTGNGANGTCGCCGNNNTTNNCGTGGTCNNN
CNAGAATGNCCGNACCANNCGNANNNTGGTNATGNNAGTNNNNNNNNNNNNNNNNNNNNNNNNNNNNN
NNNNNNNNNNNNNNNNNNNNNNNNNNNNNNNNNNNNNGNNNNNNCNANNNAAGGNNNTANNNCTNNTN
NTNNNNNNGGACNNNACNNNNCNNNNNNNACNCNNNCNCNNNNNNNNNNGGTTNNNNNNCNNTATTNT
CCAGNANGCNGNGCNNNCNTTCANGNGAACNNNGNNACGCGNNAATCGTANNATCTACCTGCCGCNNG
NNNNNNTACNGNNNNNNCGGANNNTNNNNCGTANNNNNNNCATGCGTNNTNTNNTCNCNAGNCANGTT
CGNNNANANANTAGNANGTNTNNNNNNNNNTGTNNCGTTCCGCNTGANNCNNCANNNNGNNGNNCTTN
NCANCGGCNNTANCTNCNGANNTAAGNCATANNNTNNNTCNNTANCTNNNNNGGNGGGNNACTGTAAA
NGNNNAATTNGNNNNTNANTNCNNNCATNTNNCTNNGNNTNCTNNNTNCCTGNNTNCNNAATTGGNNN
CANTGNNTGNCNATCTCTNGNTTNGNNNNNNTNNNNNTNNCANNNNGGNNNGTNNNANNNCNATNTAA
NNTANANNNNCNNNNNNANNNCACNNNNNNNNNAGNNNNNANNNGTANANNNGTAGNNNGNTNAGTCT

CCGANNTNNNNNNNATAAGCGNNANACNNNNNNNNNNNNNNANGANNACGNNTTTGNNNTNNNNNNNNNA
GNNAGACNNTNGTGGNTNNNNNTCAANNNNTTNGTAGTGGTATAATNGNNNNNNNTTTCNCCAGNNNN
NNNGNNNNNNNCNATANNANNNNNNNGNGANNNNNNNNNNNANNNNNTCNNNAGGGANCANCTACTTNA
GCANTGGNGCGNAGNNNGAGANNNNGACNNGCCTTGGGTGGCAANCACNGCAATAAGTNNNGTTNANC
CTNNNAGTNNACACNNNNNCANNGNNNCNNNNNNANCNGANNGNNNNNNNNNNNNNNNNNNNNNNNN
NNNNNNNNNNNNNNNNNNNNNNNNNNNNNNNNNNNANGGNNNGTNTNTGNCNCANGTGTTTTTANNNGNN
NNNNNNNGNNNNANCCCCGGTCGNNNNCNNATTGAANNCCTGNNGCATNNNNNTNCNAGAATGANNNC
NNNNNGNNTNGNCTNNCNTTTCAAGNAATCNNNNAGNNANNNNGNTCNNNGNNNNNNNNCNNTCCCNN
ACTGAGGGTNNNNNNNNCATCTGCTNNNNNNNNNNTNCGCNGGNGTNNCNNNNNAGCNGTATNNTGTNA
NNCANNNNNNNNNNNNNNNNNNTNNAACNNNNNNNNTTNNNN
>Kinh706
GTGNNNNNTAGCCCNNNNNAGTTAGACANNNCNNNNNNNNNNNNNTNNNNNNNNNNNNNNNNNNNNNNNN
NNNNNNNNNNNNNNNNNNNNNNNNNNNNNNNTNNNNNNNNNNNNNNNNNNNNNNNNNNNNNNNNNNNNNN
NNNNNNNNNNNNNNNNNNNNNNNNNNNNNNNNNNNNNNNNNNNNNNNNNNNNNNNNNNNNNNNNNNNNNN
NNNNNNNNNNNNNNNNNNNNNNNNNNNNNNNNNNNNNNNNNNNNNNNNNNNNNNNNNNNNNNNNNNNNNN
NNNNNNNNNNNNNNNNNNNNNNNNNNNNNNNNNNNNNNNNNNNNNNNNNNNNNNTCTNNNAAANNNTNNN
NNNNNNNNNNNNNNNNNNNNNNNNNNNCNCNNTGNNNGTAANCNANNNGNACATGCAGTCCCCTCNNNNN
NNNGGCAGAGNNNNNNCNCNNNNNNNNCCGGCCCCGNCCNNNNNNNNNNNCNNNNNNNNNNNANNNNNN
NNNNNNTGGTACNNNNCGNNNNNGGTTGTNTNAGAGGTCCACAACGNGGTCNGNNNACNNNNGNNNAN
NNNCNNNAAACNNNNNNNNNNNNANNTNNNTANNAGGNNNNNNCCNTCNNNNNNNNNAANTGCNATGACC
CANNNCNCGGGGGNNGTNCCCGGCGCGGNGACCTTGANNACGTCCGGGNNCAAGTATGGCNNNCNCCC
CGGGAAGGANNCNGCCGTANGTGCNNNNNANNNNNTCNTGNGANNTCGCCGNNATTNNNGTGGTCNNN
NNAGAATGNCCGNACCANNCGNANNNTGGTTATGNNAGTNNNNNNNNNNNNNNNNNNNNNNNNNNNNNN
NNNNNNNNNNNNNNNNNNNNNNNNNNNNNNNNNNNNGNNNNGNNNNNNCNANNNAAGGNNNTANNNCTNNTN
NTNNNNNNNGGACNNNACNNNNCNNNNNNNACNCNNNCNCNNNNNNANNNGNTTNNNNNNCNNTANTNN
CCAGNACGCNGNGCNNNCNTTCANGNGAACNNNGNNACGCGNNAATCGTANNATCTACCTGCCGCNNG
NNNNNNTACNGNNNNNNCGGANNNTNNNNCGTANNNNNNNCANGCGTNNTNTNNTCNCNAGNCANGTT
CGNNNATANANTAGNANGTNTCNNNNNNNNNTGTNNCGTTCCGCNTGANNCNNCANNNNGNNGNNNTTN
NCANCGGCNNTANCTNCNGANNTAAGNCATANNNTNNNTCNNTANCTNNNNNGGNGGGNNACTGTAAA
NGNNNAATTNGNNNNTNANTNCNNNCACNTNNCNNNGNNTNCTNNNTNCCTGNNTNCNNAATTGGNNN
CANTGNNTGNCNATCTCTNGNTTNGNNNNNNTNNNNNTNNCANNNNGGNNNGTNANANNNCNATNTAA
NNTANANNNNCNNNNNNANNNCACNNNNNNNNNAGNNNCNANNNTNANANNNGTAGNNNANTAAGTCT
CCGANNTNNNNNNNATAAGCGNNANACNNNNNNNNNNNNNNANGANNACGNNTTGGNNNTNNNNNNNCNA
GNNAGACNNTNATGGNTNNNNNTCAANNNNTTNGTAGTGGTATAATNGNNNNNNNTTTCNCCAGNNTN
NNNGNNNNNNNCNATANNANNNNNNNGNGANNNNNNNNNNNANNNNCTCNNNAGGGANCANCTACTNNA
GCANTGGNGCGCAGNNNGAGANNNNGACNNGCCTTGGGTGGCAANCACNGCAATAAGTNNNGTTNANC
CTNNNAGTNNACATNNNNNCANNGNNNCNNNNNNANCNGANNGNNNNNNNNNNNNNNNNNNNNNNNN
NNNNNNNNNNNNNNNNNNNNNNNNNNNNNNNGNNNANGGNNNGTNTNTGACNCATGTGTTTTTANNNGNN
NNNNNACGNNNGANCCCCGGTCGNNNNCNNATTGAANNCCTGNNGCATNNNNNTNCNAGAATGANNGC
NNNNNGNNTNGNCTNNCNNNTNAAGNAATCANNNAGGNNNNNNGNTCNNNGNNNNNNNNCNNTNCCNN
ACTGAGGGTNNNNNNNNCATCTGCTNNNNNNNNNNTNCGCNGGNGTNNCNNNNNNGCNGTATNNNGTNA
NNCANNNNNNNNNNNNNNNNNNTNAAANNNNNNNNNNNNTNNNN
>Kinh707
GTGNNNNNTAGCCCNNNNNAGTTAGACANNNCNNNNNNNNNNNNNTNNNNNNNNNNNNNNNNNNNNNNNN
NNNNNNNNNNNNNNNNNNNNNNNNNNNNNNNNNNNNNNNNNNNNNNNNNNNNNNNNNNNNNNNNNNNNNN
NNNNNNNNNNNNNNNNNNNNNNNNNNNNNNNNNNNNNNNNNNNNNNNNNNNNNNNNNNNNNNNNNNNNNN
NNNNNNNNNNNNNNNNNNNNNNNNNNNNNNNNNNNNNNNNNNNNNNNNNNNNNNNNNNNNNNNNNNNNNN
NNNNNNNNNNNNNNNNNNNNNNNNNNNNNNNNNNNNNNNNNNNNNNNNNNNNNNTCTNNNAAANNNTNNN
NNNNNNNNNNNNNNNNNNNNNNNNNNNCNCNNTGNNNGTAANCNANNNGNACATGCAGTCCCCTCNNNNN
NNNNNCAGAGNNNNNNCCNNNNNNNNNCCGNCCCCNNCCNNNNNNNNNNNNNNNNNNNNNNNNNANNNNNN
NNNNNNTGGTACNNNNCGNNNNNGGTTGNNTAAGAGGTCCACAACGNGGTCNGNNCACNNNNGNNNAN
NNNCNNAAAANNNNNNNNNNNNNNNNATNNNTANNAGGNNNNNNCCNTCNNGNNNNNNATTGCNATGACC
CANNNNACGGGGGCNGTNCCCGGCGNGGCGACCTTGANNANNTCCGGGGNCAAGTATGGCGNNCNCCC
CGGGAAGGANNCNGCCGTANGTGCNNNNNANNNNNTCNTGNGANGTNGCCGNNANNNNCGTGGTCNNN

NNAGAATGNNCNNACCANNCGNANNNTGGTNATGNNAGTNNNNNNNNNNNNNNNNNNNNN
NNNNNNNNNNNNNNNNNNNNNNNNNNNNNGNNNNNNCNANNNAAGGNNNTANNNCTNNTN
NTNNNNNNGGACNNNACNNNNCNNNNNNNACNCNNNCNCNNNNNNNNNNGNTTTNNNNCCNNTANTNT
CCAGNACGCNGNGCNNNCATTCANGNGAACNNNGNNACGCGNNAATCGTANNATCTACCTGCCGCNNG
TNNNNNTACNGNNNNNNCGGANNNTNNNNNGTANNNNNNNCANGCGTNNNNTNNTNNCNAGNCNNGTT
CGNNNANANANTAGNANGTNTNNNNNNNNNNTGTNNCGTTCCGCNTGANNCNNCANNNNGNNGNNCTTN
NCANCGGCNNTANCTNCNGANNTAAGTCATANNNTNNATCNNTANCTNNNNNGGNGGGNNACTGTAAA
NGGNNAATTNGNNNNTNANTNCNNNCACNTNNCNNNGNNNNCTNNNTNCCTGNNTNCNNAATTGGNNN
CANTGNNTGNCNATCTCTNGNTTNGNNNNNNTGNNNNTNNCAANNNGGNNNNNNNNANNNCNATNTAA
NNTANANNNNCNNNNNNANNNCACNNNNNNNNNAGNNNCNANNNTTANANNNGTAGNNNANTAAGTCT
CCGANNTNNNNNNNATAAGCGNNANACNNNNNNNNNNNNNNANGANNACGNNTTGGNNNTNNNNNNCNA
GNNAGACNNTNATGGNTNNNNNTCAANNNNTTCGTAGTGGTATAATNGNNNNNNNTTTCNCCAGNNTN
NNNGNNNNNNNCNATANNANNNNNNNGNGANNNNNNNNNNNANNNNCTCNNNAGGNANCANCTACTNNA
GCANTGGNGCGCAGNNNGAGANNNNGACNNNCCTTGGGTGGCAANCACNGNGATAAGTNNGTTNNNC
CTNNNAGTNNACATNNNNNCANNGNNNCNNNNNNANCNGANNGNNNNNNNNNNNNNNNNNNNNNNNNN
NNNNNNNNNNNNNNNNNNNNNNNNNNNNNGNNNANNGNNNGTNTNTGACNCATGTGTTTTTANNNGNN
NNNNNANGNNNNANCCCCGGTCGNNNNCNNATTGAANNCCTGNNGCATNNNNNTNCNAGAATGANNNC
NNNNNGNNTNGNCTNNCNNNTCAAGNAATCNANNAGNNNNNNNNNTCNNNNNNNNNNNNCNNTCCCNN
ACTGAGGGTNNNNNNNNCATCTGCTNNNNNNNNCTNCGCNGGNGTNNCNNNNNNGCNGTATNGNGNNA
NNCANNNNNNNNNNNNNNNNNTNANNCNANNNNNNTNNNN
>Kinh708
GTGNNNNNTAGCCCNNNNNAGTTAGACANNNCNNNNNNNNNNNNTNNNNNNNNNNNNNNNNNNNNNN
NNNNNNNNNNNNNNNNNNNNNNNNNNNNNNNNNNNNNNNNNNNNNNNNNNNNNGNNNNNNN
NNNNNNNNNNNNNNNNNNNNNNNNNNNNNNNNNNNNNNNNNNNNNNNNNNNNNNNNNNNN
NNNNNNNNNNNNNNNNNNNNNNNNNNNNNNNNNNNNNNNNNNNNNNNNNNNNNNNNNNNN
NNNNNNNNNNNNNNNNNNNNNNNNNNNNNNNNNNNNNNNNNNNNNNNNNNCNTCTNNNAAANNNTNNN
NNNNNNNNNNNNNNNNNNNNNNNNNNCNCNTTGNNNGTAANCNANNNGNACATGCAGTCCCCTCNNNNN
NNNGGCAGAGNNNNNNCCCNNNNNNNCCGGCCCCNNCCNNNNNNNNNNNNNNNNGNNNNNNNANNNNNN
NNNNNNTGGTACNNNNCGNNNNNGGTTGNTTNAGAGGTCCACAACGNGNTCNNGNNACNNNNGNNNAN
NNNCNNAAAACNNNNNNNNNNNNANNTNNNTANNAGGNNNNNNCCNTCNNNNNNNNNNANTGCNATGANN
CAANNCACGGNGGCNGTNCCCGGCGCGGCGACCTTGANNANNTCCGGGGACAAGTATGGCNCNCNCCC
CGGGAAGGANNCNGCCGTANGTGCNNNNNANNNNNTCNTGNGANGTNGCCGNNATTCNCGTGGTCNNN
CNAGANTGNCCNNACCANNCGNANNNTGGTNATGNNAGTNNNNNNNNNNNNNNNNNNNNNNNNNN
NNNNNNNNNNNNNNNNNNNNNNNNNNNNNNNGNNNNGNNNNNNCNANNNAAGGANNTANNNCTNNTC
NTNNNNNNGGACNNNACNNNNCNNNNNNNACNCNNNCNCNNNNNNNNNNGNTTTTTNNNCNNTANTNT
CCAGNACGCNGNGCNNNCNTTCANGNGAACNNNGNNACGCGNNAATCGTANNATCTACCTGCCGCNNG
TNNNNNTACNGNNNNNNCGGANNNTNNCTCGTANNNNNNNCNTGCGTNNTNTNNTNNCNAGNCNNGTT
CGNNNANANANTAGNANGTNNNNNNNNNNNNTGTNNCGTTCCGCNTGANNCNNCANNNNGNNGNNNTTN
NCANCGGCNNTANCTGCNGANNTAAGNCATANNNTNNNTCNNTANCTNNNNNGGNGGGNNACTGTAAA
NGGNNAATTNGNNNNTNATTNCNNNCACNTNNCNNNGNNTNCTNNNTNCCTGNNTNCNNAATTGGNNN
CANTGNNTGNCNATCTCTNGNTTNGNNNNNNTNNNNNTNNCAANNNGGNNNNNNANANNNCNATNTAA
NNTANANNNNCNNNNNNANNNCACNNNNNNNNNAGNNNNNANNNTTANANNNGTAGNNNAATAAGTCT
CCGANNTNNNNNNNATAAGCGNNANACNNNNNNNNNNNNNNANGANNACGNNTTGGNNNTNNNNNNNNA
GNNAGACNNTNATGGNTNNNNNTCAANNNNTTNGTAGTGGTATAATNGNNNNNNNTTTCNCCAGNNTN
NNNGNNNNNNNCNATANNANNNNNNNGNGANNNNNNNNNNNANNNNCTCNNNAGGGANCANCTACTNNA
GCAATGGNGCGNAGNNNGAGANNNNGACNNNCCTTGGGTGGCAANNACNGCAATAAGTNNNGTTNNNC
CTNNNAGTNNACATNNNNNCANNGNNNCNNNNNNANCNGANNGNNNNNNNNNNNNNNNNNNNNNNNNN
NNNNNNNNNNNNNNNNNNNNNNNNNNNNNGNNNANNGNNNGTNTNTGNCNCNTGTGTTTTTANNNGNN
NNNNNACGNNNNANCCCCGGTCGNNTNCNNATTGAANNCCTGNNGCATNNNNNTNCNAGAATGANNGC
NNNNNGNNTNGNCTNNCNTTTCAAGNAATCANNNAGNNNNNNNNGNTCNNNNNNNNNNNNCNNTCCCNN
ACTGAGGGTNNNNNNNNCATCTGCTNNNNNNNNNNTNCGCNGGNGTNNCNNNNNAGCNGTATNGNGTNA
NNCANNNNNNNNNNNNNNNNNCNAAACAANNNNNNTNNNN
>Kinh709
GTGNNNNNTAGCCCNNNNNAGTTAGACANNNCNNNNNNNNNNNNTNNNNNNNNNNNNNNNNNNNNNN

NNNNNNNNNNNGNNNNNNNNNNNNNNNNNNNTNNNNNNNNNNNNNNNNNNNNNNNNNNNNNNNNNNNNNN
NNNNNNNNNNNNNNNNNNNNNNNNNNNNNNNNNNNNNNNNNNNNNNNNNNNNNNNNNNNNNNNNNNNNNN
NNNNNNNNNNNNNNNNNNNNNNNNNNNNNNNNNNNNNNNNNNNNNNNNNNNNNNNNNNNNNNNNNNNNNN
NNNNNNNNNNNNNNNNNNNNNNNNNNNNNNNNNNNNNNNNNNNNNNNNNNNNCNTCTNNNAAANNNTNNN
NNNNNNNNNNNNNNNNNNNNNNNNNNCNCNTTNNNNGTAANCNANNNGNACATGCAGTCCCCTCNNNNN
NNNGGCAGAGNNNNNNCCCNNNNNNNCCGGCCCCNNCCNNNNNNNNNNNCNNNGNNNNNNNANNNNNN
NNNNNNTGGTACNNNNCGNNNNNGGTTGTNTAAGAGGTCCACCACGNGGTCNNGNNACNNNNGNNNAN
NNNCNNNNAANNNNNNNNNNNNNNANNTNNNTANNAGGNCNNNGCCNTCNNNNCNNTNATTGCNATGACC
CAANNCNCGGGGGCNGTNCCCGGCGCGGCGACCTTGNNNACGTCCGGGGACAAGTATGGCNNNCNCCC
CGGGAAGGANNCNGCCGTANGTGCNNNNNANNNNNTCNTGNGANGTCGCCGNNATTNNCGTGGTCNNN
CTAGAATGNCCGNACCANNCGNANNNTGGTNATGNNAGTNNNNNNNNNNNNNNNNNNNNNNNNNNNNNN
NNNNNNNNNNNNNNNNNNNNNNNNNNNNNNNNNGNNNNGNNNNNNCNANNNAAGGANNTANNNCTNNTN
NTNNNNNNGGACNNNACNNNNCNNNNNNNACNCNNNCNCNNNNNNANNNGNTTTTNNNNCNNTANTNT
CCAGNACGCNGNGCNNNCATTCANGNGAACNNNGNNACGCGNNAATCGTANNATCTACCTGCCGCNNG
NNNNNNTACNGNNNNNNCGGANNNTNNCTCGTANNNNNNTTANACGTNNTNTNNTCNCNAGNCNNGTT
CGNNNANANANTAGNANGTNTNNNNNNNNNTGTNNCGTTCCGCNTGANNCNNCANNNNGNNGNNCTTC
NCANCGGCNNTANCTNCNGANNTAAGNCATANNNTNNNTCNNTANCTNNNNNGGNGGGNNACTGTAAA
NGGNNAATTNGNNNNTNATTNCNNNCACNTNNCTNNGNNTNCTNNNTNCCTGNNTNCNNAATTGGNNC
CANTGNNTGNCNATCTCTGGNTTNGCNNNNNTNNNNNTNNCANNNNGGNNNNNNNNANNNCNATNTAA
NNTANANNNNCNNNNNNANNNCACNNNNNNNGNAGNNNCNANNNTNANANNNGTAGNNNANTNAGTCT
CCGANNTNNNNNNNATAAGCGNNANACNNNNNNNNNNNNNNANGANNACGNNTTGGNNNTNNNNNNCNA
GNNAGACNNTNGTGGNTNNNNNTCAANNNNTTNGTAGTGGTATAATNGNNNNNNNTTTCNCCAGNNTN
NNNGNNNNNNNNCNATANNANNNNNNNGNGATNNNNNNNNNNANNNNCTCNNNAGGGANCANCTACTNNA
GCAATGGNGCGCAGNNNGAGANNNNGACNNNCCTTGGGTGGCAANNACNGCAATAAGTNNNGTTNNNC
CTNNNAGTNNACACNNNNNCANNGNNNCNNNNNNANCNGANNGNNNNNNNNNNNNNNNNNNNNNNNNNN
NNNNNNNNNNNNNNNNNNNNNNNNNNNNANNNNANGGNNNGTNTNTGACNCATGTGTTTTTANNNGNN
NNNNNACGNNNNANCCCCGGTCGNNTNCNNATTGAANNCCTGNNGCATNNNNNTNCNAGAATGANNGC
NNNNNGNNTNGNCTNNCNTTTCAAGNAGTCAANNANNNNNNNNGNTCNNNGNNNNNNNNCNNNCCCNN
ACTGAGGGTNNNNNNNNCATCTGCTNNNNNNNNNNTNCGCNGGNGTNNCNNNNNNGCNGTATNGNGNNA
NNCANNNNNNNNNNNNNNNNNNTNAAACNANNNNNNNTNNNN
>Kinh710
GTGNNNNNTAGCCCNNNNNAGTTAGACANNNCNNNNNNNNNNNNTNNNNNNNNNNNNNNNNNNNNNNNNN
NNNNNNNNNNNNNNNNNNNNNNNNNNNNNNNTNNNNNNNNNNNNNNNNNNNNNNNNNNNNNNNNNNNNNN
NNNNNNNNNNNNNNNNNNNNNNNNNNNNNNNNNNNNNNNNNNNNNNNNNNNNNNNNNNNNNNNNNNNNNN
NNNNNNNNNNNNNNNNNNNNNNNNNNNNNNNNNNNNNNNNNNNNNNNNNNNNNNNNNNNNNNNNNNNNNN
NNNNNNNNNNNNNNNNNNNNNNNNNNNNNNNNNNNNNNNNNNNNNNNNNNNNTCTNNNAAANNNTNNN
NNNNNNNNNNNNNNNNGNNNNNNNNCNCNNTGNNNGTAANCNANNNGNACATGCAGTCCCCTCNNNNN
NNNGGCAGAGNNNNNNNCCNNNNNNNCCGNCCCCNNCCNNNNNNNTNNNCNNNGNNNNNNNANNNNNN
NNNNNNTGGTACNNNNCGNNNNNGGTTGTNTAAGAGGTCCACAACGNGNTCNGGNNACNNNNGNNNAN
NNNCNNNNAACNNNNNNNNNNNNNNNTNNNTANNAGGNCNNNNCCNTCNNGNNNNNNANNGCNATGACC
CAANNCNCGGGGGCAGTNCCCGGCGNGGCGACCTTGANNANGTCCGGGGNCAAGTATGGCGNNCNCCC
CGGGAAGGANNCNGCCGTANGTGCNNNNNANNNNNTCNTGNGANGTNGCCGNNATTCNCGTGGTCNNN
CNAGAATGNCCGNACCANNCGNANNNTGGTNATGTNAGTNNNNNNNNNNNNNNNNNNNNNNNNNNNNNN
NNNNNNNNNNNNNNNNNNNNNNNNNNNNNNNNNGNNNNGNNNNNNCNANNNAAGGNNNTANNNCTNNTN
NTNNNNNNGGACNNNACNNNNCNNNNNNNACNCNNNCNCNNNNNNANNNGNTTTTNNNNCNNTATTNT
CCAGNACGCNGNGCNNNCATTCANGNGAACNNNGNNACGCGNNAATCGTANNATCTACCTGCCGCNNG
NNNNNNTACNGNNNNNNCGGANNNTNNCTCGTANNNNNNNCANGCGTNNTNTNNTNNCNAGNCNNGTT
CGNNNANANANTAGNANGTNTNNNNNNNNNTGTNNCGTTCCGCNTGANNCNNCANNNNGNNGNNNTTN
NCANCGGCNNTANCTGCNGANNTAAGNCATANNNTNNNTCNNTANCTNNNNNGGNGGGNNACTGTAAA
NGNNNAATTNGNNNNTNATTNCNNNCACNTNNCNNNGNNNNCTNNNTNCCTGNNTNCNNAATTGGNNN
CANTGNNTGNCNATCTCTGGNTTNGNNNNNNTGNNNNTNNCANNNNGGNNNGTNNNANNNCNATNTAA
NNTANANNNNCNNNNNNANNNCACNNNNNNNGNAGNNNCNANNNTNANANNNGTAGNNNANTAAGTCT
CCGANNTNNNNNNNATAAGCGNNANACNNNNNNNNNNNNNNANGANNACGNNTTGGNNNTNNNNNNCNA
GNNAGACNNTNATGGNTNNNNNTCAANNNNTTCGTAGTGGTATAATNGNNNNNNNTTTCNCCAGNNTN

NNNGNNNNNNNNCNATANNANNNNNNNGNGANNNNNNNNNNNANNNNCTCNNNAGGGANCANCTACTNNA
GCAANGGNGCGCAGNNNGAGANNNNGACNNNCCTTGGGTGGCAANCACNGCGATAAGTNNNGTTNANC
CTNNNAGTNNACATNNNNNCANNGNNNCNNNNNNANCNGANNGNNNGNNNNNNNNNNNNNNNNNNNNNN
NNNNNNNNNNNNNNNNNNNNNNNNNNNNNANNNNANGGNNNGTNTNTGACNCATGTGTTTTTANNNGNN
NNNNNACGNNNNANCCCCGGTCGNNNNCNNATTGAANNCCTGNNGCATNNNNNTNCNAGAATGANNGC
NNNNNGNNTNGNCTNNCNTTTCAAGNAATCANNNAGNNNNNNCGNTCNNNGNNNNNNNNCNNTCCCNN
ACTGAGGGTNNNNNNNNCATCTGCTNNNNNNNNCTNCGCNGGCGTNNCNNCNNAGCNGTATNNTGTNA
NNCANNNNNNNNNNNNNNNNNCNAANCNANNNNNNNTNNNN
>Kinh711
GTGNNNNNTAGCCCNNNNNAGTTAGACANNNCNNNNNNNNNNNNNTNNNNNNNNNNNNNNNNNNNNNNN
NNNNNNNNNNNNNNNNNNNNNNNNNNNNNNNNNNNNNNNNNNNNNNNNNNNNNNNNNNNNNNNNNNNNNN
NNNNNNNNNNNNNNNNNNNNNNNNNNNNNNNNNNNNNNNNNNNNNNNNNNNNNNNNNNNNNNNNNNNNNN
NNNNNNNNNNNNNNNNNNNNNNNNNNNNNNNNNNNNNNNNNNNNNNNNNNNNNNNNNNNNNNNNNNNNNN
NNNNNNNNNNNNNNNNNNNNNNNNNNNNNNNNNNNNNNNNNNNNNNNNNNNNNNTCTNNNAAANNNTNNN
NNNNNNNNNNNNNNNNNNNNNNNNNNNCNCNTTNNNNGTAANCNANNNGNACATGCAGTCCCCTCNNNNN
NNNGGCAGAGNNNNNNNCCNNNNNNNCCGGCCCCGNCCNNNNNNNNNNNNNNNNGNNNNNNNANNNNNN
NNNNNNTGGTACNNNNCGNNNNNGGTTGTNTAAGAGGTCCACAACGNGGTCNGGNNACNNNNGNNNAN
NNNCNNNNAANNNNNNNNNNNNNNANNTNNNTANNAGGNCNNNNCCNTCNNGNNNNNNANTGCNATGACC
CNNNTCACGGGGGCNGTNCCCGGCGNGGCGACCTTGANNACGTCCGGGGACAAGTATGGCGNNCNCCC
CGGGAAGGANNCNGCNGTANGTGCNNNNNANNNNNTCNTGNGANGTCGCCGNNATTCNCNTGGTCNNN
NNAGAATGNNCNNACCANNCGNANNNTGGTNATGNNAGTNNNNNNNNNNNNNNNNNNNNNNNNNNNNNNN
NNNNNNNNNNNNNNNNNNNNNNNNNNNNNNNNNNGNNNNGNNNNNNCNANNNAAGGNNNTANNNCTNNTN
NTNNNNNNNGGACNNNACNNNNCNNNNNNNNACNCNNNCNCNNNNNNANNNGGTTTTNNNNCNNTANTNT
CCAGNACGCNGNGCNNNCATTCANGNGAACNNNGNNACGCGNNAATCGTANNATCTACCTGCCGCNNG
NNNNNNTNCNGNNNNNNNCGGANNNTNNCTCGTANNNNNNNCATGCGTNNTNTNNTCNCNAGNCANGTT
CGNNNANANANTAGNANGTNTCNNNNNNNNNTGTNNCGTTCCGCNTGANNCNNCANNNNGNNGNNCTTN
NCANCGGCNNTANCTGCNGANNTAAGNCATANNNTNNATCNNTANCTNNNNNGGNGGGNNACTGTAAA
NGGNNAATTNGNNNNTNATTNCNNNCACNTNNCNNNGNNNNCTNNNTNCCTGNNTNCNNAATTGGNNN
CANTGNNTGNCNATCTCTGGNTTNGNNNNNNTGNNNNTNNCAANNNGGNNNGTNNNANNNCNATNTAA
NNTANANNNNCNNNNNNANNNCACNNNNNNNNNNAGNNNCNANNNTNANANNNGTAGNNNANTAAGTCT
CCGANNTNNNNNNNNATAAGCGNNANACNNNNNNNNNNNNNNANGANNACGNNTTGGNNNTNNNNNNCNA
GNNAGACNNTNATGGNTNNNNNTCAANNNNTTCGTAGTGGTATAATNGNNNNNNNTTTCNCCAGNNTN
NNNGNNNNNNNNCNATANNANNNNNNNGNGANNNNNNNNNNNANNNNCTCNNNAGGGANCANCTACTTNA
GCANTGGNGCGNAGNNNGAGANNNNGACNNNCCTTGGGTGGCAANCACNGCGATAAGTNNNGTTNANN
CTNNNAGTNNACATNNNNNCANNGNNNCNNNNNNANCNGANNGNNNNNNNNNNNNNNNNNNNNNNNNNNN
NNNNNNNNNNNNNNNNNNNNNNNNNNNNNNGNNNANGGNNNGTNTNTGACNCATGTGTTTTTANNNGNN
NNNNNNNGNNNGANCCCCGGTCGNNTNCNNATTGAANNCCTGNNGCATNNNNNTNCNAGAATGANNNC
NNNNNGNNTNGNCTNNCNNNTCAAGNAATCAANNAGGTNTNNNNNTCNNNNNNNNNNNNNCNNNCNCNN
ACTGAGGGTNNNNNNNNCATCNGCTNNNNNNNNCTNCGCNGGNGTNNCNNNNNNGCNGTATNGNGTNA
NNCANNNNNNNNNNNNNNNNNNTNAANNGNNNNNNNNTNNNN
>Kinh713
GTGNNNNNTAGCCCNNNNNAGTTAGACANNNCNNNNNNNNNNNNNTNNNNNNNNNNNNNNNNNNNNNNN
NNNNNNNNNNNNNNNNNNNNNNNNNNNNNNNTNNNNNNNNNNNNNNNNNNNNNNNNNNNNNNNNNNNNNN
NNNNNNNNNNNNNNNNNNNNNNNNNNNNNNNNNNNNNNNNNNNNNNNNNNNNNNNNNNNNNNNNNNNNNN
NNNNNNNNNNNNNNNNNNNNNNNNNNNNNNNNNNNNNNNNNNNNNNNNNNNNNNNNNNNNNNNNNNNNNN
NNNNNNNNNNNNNNNNNNNNNNNNNNNNNNNNNNNNNNNNNNNNNNNNNNNNNNTCTNNNAAANNNTNNN
NNNNNNNNNNNNNNNNNNNNNNNNNNNCNCNNTGNNNGTAANCNANNNGNACATGCAGTCCCCTCNNNNN
NNNGGCAGAGNNNNNNNCCNNNNNNNCCGNCCCCNNCCNNNNNNNNNNNNCNNNGNNNNNNNANNNNNN
NNNNNNTGGTACNNNNCGNNNNNGGTTGTNTAAGAGGTCCACAACGNGGTCNGNNNACNNNNGNNNAN
NNNCNNNNAACNNNNNNNNNNNNNANNTNNNTANNAGGNCNNNNCCNTCNNGNNNNNNATTGCNATGACC
CAANNCNCGGGGGCNGTNCCCGGCGNGGCGACCTTGNTNACGTCCGGGGNCAAGTATGGCNNNCNCCC
CGGGAAGGANNCNGCNGTANGTGCNNNNNANNNNNTCNTGNGANGTNGCCGNNNTTNNCNTGGTCNNN
CNAGAATGNCCNNACCANNCGNANNNTGGTNATGNNAGTNNNNNNNNNNNNNNNNNNNNNNNNNNNNNNN
NNNNNNNNNNNNNNNNNNNNNNNNNNNNNNNNNNNNNNNGNNNNNNCNANNNAAGGANNTANNNCTNNTN

NTNNNNNNGGACNNNACNNNNCNNNNNNNACNCNNNCNCNNNNNNNNNNGGTTTNTNNNCNNTANTNT
CCAGNANGCNGNGCNNNCNTTCANGNGAACNNNGNNACGCGNNAATCGTANNATCTACCTGCCGCNNG
TNNNNNTNCNGNNNNNNCGGANNNTNNNNCGTANNNNNNNCANGCGTNNTNTNNTCNCNAGNCNNGTT
CGNNNANANANTAGNANGTNTNNNNNNNNNNTGTNNCGTTCCGCNTGANNCNNCANNNNGNNGNNNTTN
NCANCGGCNNTANCTGCNGANNTAAGNCATANNNTNNNTCNNTANCTNNNNNGGNGGGNNACTGTAAA
NGGNNAATTNGNNNNTNATTNCCNNCACNTNNCNNNGNNNNCTNNNTNCCTGNNTNCNNAATTGGNNN
CANTGNNTGNCNATCTCTGGNTTNGNNNNNNTNNNNNTNNCANNNNGGNNNNNNANANNNCNATNTAA
NNTANANNNNCNNNNNNANNNCACNNNNNNNNNAGNNNCNANNNTTANANNNGTAGNNNANTAAGTCT
CCGANNTNNNNNNNATAAGCGNNANACNNNNNNNNNNNNNNANGANNACGNNTTGGNNNTNNNNNNCNA
GNNAGACNNTNATGGNTNNNNNTCAANNNNTTCGTAGTGGTATAATNGNNNNNNNTTTCNCCAGNNTN
NNNGNNNNNNNCNATANNANNNNNNNGTGANNNNNNNNNNNANNNNCTCNNNAGGNANCANCTACTNNA
GCANTGGNGCGCAGNNNGAGANNNNGACNNNCCTTGGGTGGCAANCACNGCAATAAGTNNNGTTNANC
CTNNNAGTNNACATNNNNNCANNGNNNCNNNNNNANCNGANNGNNNNNNNNNNNNNNNNNNNNNNNNNNN
NNNNNNNNNNNNNNNNNNNNNNNNNNNCNANNNNANGGNNNGTNTNTGACNCATNNGTTTTTANNNGNN
NNNNNNNGNNNNANCCCCGGTCGNNNNCNNATTGAANNCCTGNNGCATNNNNNTNCNAGAATGANNNC
NNNNNGNNTNGNCTNNCNTTTCAAGNAATCANNNAGGTNNNNNNNTCNNNGNNNNNNNNCNNTCCCNN
ACTGAGGGTNNNNNNNNCATCTGCTNNNNNNNNNNTNCGCNGGNNNNNCNNNNNAGCNGTATNNTGTNA
NNCANNNNNNNNNNNNNNNNNTNAAACAANNNNNNNTNNNN
>Kinh714
GTGNNNNNTAGCCCNNNNNAGTTAGACANNNCNNNNNNNNNNNNNTNNNNNNNNNNNNNNNNNNNNNNNN
NNNNNNNNNNNNNNNNNNNNNNNNNNNNNNTNNNNNNNNNNNNNNNNNNNNNNNNNNNNNNNNNNNNNNNN
NNNNNNNNNNNNNNNNNNNNNNNNNNNNNNNNNNNNNNNNNNNNNNNNNNNNNNNNNNNNNNNCNNNNNN
NNNNNNNNNNNNNNNNNNNNNNNNNNNNNNNNNNNNNNNNNNNNNNNNNNNNNNNNNNNNNNNNNNNNNN
NNNNNNNNNNNNNNNNNNNNNNNNNNNNNNNNNNNNNNNNNNNNNNNNNNNCNTCTNNNAAANNNTNNN
NNNNNNNNNNNNNNNNNNNNNNNNNNCNCNTTGNNNGTAANCNANNNGNACATGCAGTCCCCTCNNNNN
NNNGGCAGAGNNNNNNCCCNNNNNNNCCGNCCCCNNCCNNNNNNNNNNNNCNNNNNNNNNNNANNNNNNN
NNNNNNTGGTACNNNNCGNNNNNGGTTGTNTAAGAGGTCCACAACANGNTCNGGNCACNNNNGNNNAN
NNNCNNNNAACNNNNNNNNNNNANNNTNNNTANNAGGNNNNNNCCNTCNNGNNNNNNANNGCNATGACC
CAANTCACGGGGGCNGTNCCCGGCGCGGCGACCTTGANNANGTCCGGGNACAAGTATGGCNCNCNCCC
CGGGAAGGANNCNGCCGTANGTGCNNNNNANNNNNTCNTGNGANNTCGCCGNNATTNNCGTGGTCNNN
CNAGAATGNCCNNACCANNCGNANNNTGGTNATGNNAGTNNNNNNNNNNNNNNNNNNNNNNNNNNNNNNN
NNNNNNNNNNNNNNNNNNNNNNNNNNNNNNNNNGNNNNGNNNNNNCNANNNAAGGANNTANNNCTNNTN
NTNNNNNNGGACNNNACNNNNCNNNNNNNACNCNNNCNCNNNNNNANNNGGTTNNTNNNCNNTANTNT
CCAGNACGCNGNGCNNNCNTTCANGNGAACNNNGNNACGCGNNAATCGTANNATCTACCTGCCGCNNG
NNNNNNTACNGNNNNNNCGGANNNTNNNNCGTANNNNNNNCANGCGTNNTNTNNTCNCNAGNCNNGTT
CGNNNANANANTAGNANGTNTNNNNNNNNNNTGTNNCGTTCCGCNCGANNCNNCANNNNGNNGNNNTTN
NCANCGGCNNTANCTGCNGANNTAAGNCATANNNTNNNTCNNTANCTNNNNNGGNGGGNNACTGTAAA
NGGNNAATTNGANNNTNANTTCNNNCACNTNNCNNNANNTNCTNNNTNCCTGNNTNCNNAATTGGNNC
CANTGNNTGNCNATCTCTNGNTTNGNNNNNNTNNNNNTNNCAANNNGGNNNGTNNNANNNCCATNTAA
NNTANANNNNCNNNNNNANNNCACNNNNNNNGNAGNNNNNANNNTNANANNNGTAGNNNANTAAGTCT
CCGANNTNNNNNNNATAAGCGTNANACNNNNNNNNNNNNNNANGANNNCGNNTTGGNNNTNNNNNNNNA
GNNAGACNNTNGTGGNTNNNNNTCAANNNNTTNGTAGTGGTATAATNGNNNNNNNTTTCNCCAGNNTN
NNNGNNNNNNNCNATANNANNNNNNNGNGANNNNNNNNNNNANNNNCTTNNNAGGGANCANTTACTNNA
GCAANGGNGCGNAGNNNGAGANNNNGACNNNCCTTGGGTGGCAANNACNGCAATAAGTNNNGTTNANC
CTNNNAGTNNACACNNNNNCANNGNNNCNNNNNNANCNGANNGNNNNNNNNNNNNNNNNNNNNNNNNNNN
NNNNNNNNNNNNNNNNNNNNNNNNNNNNNNNANNNNANGGNNNGTNTNTGACNCATGTGTTTTTANNNGNN
NNNNNNNGNNNNANCCCCGGTCGNNNNCNNATTGAANNCCTGNNGCATNNNNNTNCNAGAATGANNGC
NNNNNGNNTNGNCTNNCNNNTCAAGNAATCANNNAGNNNNNNNGNTCNNNNNNNNNNNNCNNTCCCNN
ACTGAGGGTNNNNNNNNCATCNGCTNNNNNNNNNNTNCGCNGGCGTNNCNNNNNNGCNGTATNGNGTNN
NNCANNNNNNNNNNNNNNNNNTNNCNNNANNNNNNNTNNNN
>Kinh715
GTGNNNNNTAGCCCNNNNNAGTTAGACANNNCNNNNNNNNNNNNNTNNNNNNNNNNNNNNNNNNNNNNNN
NNNNNNNNNNNNNNNNNNNNNNNNNNNNNNTNNNNNNNNNNNNNNNNNNNNNNNNNNNNNNNNNNNNNNNN
NNNNNNNNNNNNNNNNNNNNNNNNNNNNNNNNNNNNNNNNNNNNNNNNNNNNNNNNNNNNNNNNNNNNNN

NNNNNNNNNNNNNNNNNNNNNNNNNNNNNNNNNNNNNNNNNNNNNNNNNNNNNNNNNNNN
NNNNNNNNNNNNNNNNNNNNNNNNNNNNNNNNNNNNNNNNNNNNTCTNNNAAANNNTNNN
NNNNNNNNNNNNNNNNNNNNNNNNNNNCNCNTTNNNNGTAANCNANNNGNACATGCAGTCCCCTCNNNNN
NNNGGCAGAGNNNNNNNCCNNNNNNNCCGNCCCCNNCCNNNNNNNNNNNNNNNNNNNNNNNNANNNNNN
NNNNNNTGGTACNNNNCGNNNNNGGTTGTNTAAGAGGTCCACAACGNGGTCNNGNCACNNNNGNNTAN
NNNCNNNNAACNGNNNNNNNNNANNTNNNTANNAGGNNNNNNCCNTCNNGNNNNTNATTGCNATGACC
CANNTCACGGGGGNNGTNCCCGGCGCGGCGACCTTGANNACGTCCGGGGNCAAGTATGGCGNNCNCCC
CGGGAAGGANNCNGCNGTANGTGCNNNNNANNNNNTCNTGNGANGTCGCCGNNATTCNCNTGGTCTNN
CNAGAATGNCCNNACCANNCGNANNNTGGTNATGNNAGTNNNNNNNNNNNNNNNNNNNNNNNNNNNNNN
NNNNNNNNNNNNNNNNNNNNNNNNNNNNNNNNNNNNNNNNGNNNNNNCNANNNAAGGNNNTANNNCTNNTN
NTNNNNNNGGACNNNACNNNNCNNNNNNNACNCNNNCNCNNNNNNANNNGNTTTTNNNNCNNTANTNT
CCAGNACGCNGNGCNNNCNTTCANGNGAACNNNGNNACGCGNNAATCGTANNATCTACCTGCCGCNNG
NNNNNNTNCNGNNNNNNCGGANNNTNNNNCGTANNNNNNNCANGCGTNNTNTNNTNNCNAGNCNNGTT
CGNNNANANANTAGNANGTNTNNNNNNNNNNTGTNNCGTTCCGCNTGANNCNNCANNNNGNNGNNCTTN
NCANCGGCNNTANCTGCNGANNTAAGNCATANNNTNNNTCNNTANCTNNNNNGGNGGGNNACTGTAAA
NGGNNAATTNGNNNNTNATTNCNNNCACNTNNCTNNGNNTNCTNNNTNCCTGNNTNCNNAATTGGNNC
CANTGNNTGNCNATCTCTGGNTTNGNNNNNNTNNNNNTNNCANNNNGGNNNNNNNNANNNCNATNTAA
NNTATANNNNCNNNNNNANNNCACNNNNNNNNNAGNNNCNANNNTNANANNNGTAGNNNANTNAGTCT
CCGANNTNNNNNNNATAAGCGNNANACNNNNNNNNNNNNNANGANNACGNNTTGGNNNTNNNNNNCNA
GNNAGACNNTNGTGGNTNNNNNTCAANNNNTTNGTAGTGGTATAATNGNNNNNNNTTTCNCCAGNNTN
NNNGNNNNNNNCNATACNANNNNNNNGNGANNNNNNNNNNNANNNNCTCNNNAGGGANCANCTACTNNA
GCANTGGNGCGNAGNNNGAGANNNNGACNNNCCTTGGGTGGCAANNACNGCAATAAGTNNNGTTNANC
CTNNNAGTNNACACNNNNNCANNGNNNCNNNNNNANCNGANNGNNNNNNNNNNNNNNNNNNNNNNNNN
NNNNNNNNNNNNNNNNNNNNNNNNNNNNNNNNNNNNNANGGNNNGTNTNTGACNCATGTGTTTTTANNNGNN
NNNNNNNGNNNNANCCCCGGTCGNNNNCNNATTGAANNCCTGNNGCATNNNNNNTNCNAGAATGANNGC
NNNNNGNNTNGNCTNNCNTTTCAAGNAATCANNNANGTNNNNNNNNTCNNNGNNNNNNNNNCNNTCNCNN
ACTGAGGGTNNNNNNNNCATCNGCTNNNNNNNNNCTNCGCNGGCGTNNCNNNNNAGCNGTATNGNGTNA
NNCANNNNNNNNNNNNNNNNNNTNAAACAANNNNCNTNNNN
>LaChi530
GTGNNNNNTAGCCCNNNNNAGTTAGACANNNCNNNNNNNNNNNNNNNNNNNNNNNNNNNNN
NNNNNNNNNNNNNNNNNNNNNNNNNNNNNNTNNNNNNNNNNNNNNNNNNNNNNNNNNNNN
NNNNNNNNNNNNNNNNNNNNNNNNNNNNNNNNNNNNNNNNNNNNNNNNNNNNNNNNNNNN
NNNNNNNNNNNNNNNNNNNNNNNNNNNNNNNNNNNNNNNNNNNNNNNNNNNNNNNNNNNN
NNNNNNNNNNNNNNNNNNNNNNNNNNNNNNNNNNNNNNNNNNNNNNNNCNTCTNNNAAANNNTNNN
NNNNNNNNNNNNNNNNNNNNNNNNNNNCNCNNTGNNNGTAANCNANNNGNACATGCAGTCCCCTCNNNNN
NNNNNCAGAGNNNNNNNCCNNNNNNNCCGGCCCCNNCCNNNNNNNNNNNNNNNNNGNNNNNNNANNNNNN
NNNNNNTGGTACNNNNCGNNNNNGGTTGTNTAAGAGGTCCACAACANGGTCNNGNCACNNNNGNNNAN
NNNCNNNNNANNNNNNNNNNNNNNNNTNNNTANNAGGNCNNNNCCNTNNNGNNNNNNATNGCNATGACC
CNNNNCACNGNGGCNGTNCCCGGCGCGGNGACCTTGNNNANNTCCGGGGNCAAGTATGGCNNNCNCCC
CGNGAAGGANNCNGCCGTANGTGCNNNNNANNNNNTCNTGNGANGTNGCCGNNANNNNNGTGGTCNNN
NNAGAATGNCCNNACCANNCGNANNNTGGTNATGNNAGTANNNNNNNNNNNNNNNNNNNNNNNNNNNNN
NNNNNNNNNNNNNNNNNNNNNNNNNNNNNNNNNNNNNNNNGNNNNNNCNANNNAAGGNNNTANNNCTNNTN
NTNNNNNNGGACNNNACNNNNCNNNNNNNACNCNNNCNCNNNNNNNNNNNGNTTTTNNNNCNNTATTNT
CCAGNANGCNGNGCNNNCATTCANGNGAACNNNGNNACGCGNNAATCGTANNATCTACCTGCCGCNNG
NNNNNNTACNGNNNNNNCGGANNNTNNCTCGTANNNNNNNCATGCGTNNTNTNNTCNCNAGNCNNGTT
CGNNNANANANTAGNANGTNTNNNNNNNNNNTGTNNCGTTCCGCNCGANNCNNCNNNNNGNNGNNNTTN
TCANCGGCNNTANCTNCNGANNTAAGNCATANNNTNNNTCNNTANCTNNNNNGGNGGGNNACTGTAAA
NGGNNAATTNGNNGNTNANTNCCNNCACNTNNCTNNANNNNCTNNNTNCCTGNNTNCNNAATTGGNNC
CANTGNNTGNCNATCTCTNGNTTNGNNNNNNTGNNNNTNNCANNNNGGNNNNNNANANNNCNATNTAA
NNTANANNNNCNNNNNNANNNCACNNNNNNNNNAGNNNCNANNNTTANANNNGTAGNNNANTAAGTCT
CCGANNTNNNNNNNATAAGCGNNANACNNNNNNNNNNNNNANGANNNCGNNTTGGNNNTNNNNNNNNA
GNNAGACNNTNGTGGNTNNNNNTCAANNNNTTCGTAGTGGTATAATNGNNNNNNNTTTCNCCAGNNTN
NNNGNNNNNNNCNATANNANNNNNNNGNGANNNNNNNNNNNANNNNCTTNNNAGGNANCANTTACTTNA
GCANTGGNGCGNAGNNNGAGANNNNGACNNNCCTTGGGTGGCAANCACNGCAATAAGTNNNGTTNNNC

CTNNNAGTNNACACNNNNNCANNGNNNCNNNNNNANCNGANNGNNNNNNNNNNNNNNNNNNNNNNNNN
NNNNNNNNNNNNNNNNNNNNNNNNNNNNNNNNNANNGNNNGTNTNTGACNCATGTGTTTTTANNNGNN
NNNNNACGNGNNANCCCCGGTCGNNNNCNNATTGAANNCCTGNNGCATNNNNNTNCNAGAATGANNNC
NNNNNGNNTNGNCTNNCNNNTCAAGNAATCANNNAGGTNTNNNNNTCNNGNNNNNNNNNCNNTCNCNN
ACTGAGGGTNNNNNNNNCATCNGCTNNNNNNNNNNTNCGCNGGNGTNNCNNNNNGCNGTATNGNGTNN
NNCANNNNNNNNNNNNNNNNNTNAANNNANNNNNTNNNN
>LaChi531
GTGNNNNNTAGCCCNNNNNAGTTAGACANNNCNNNNNNNNNNNNTNNNNNNNNNNNNNNNNNNNNNNN
NNNNNNNNNNNNNNNNNNNNNNNNNNNNNNNNNNNNNNNNNNNNNNNNNNNNNNNNNNNNNNNNNNNN
NNNNNNNNNNNNNNNNNNNNNNNNNNNNNNNNNNNNNNNNNNNNNNNNNNNNNNNNNNNNNNNNNNNN
NNNNNNNNNNNNNNNNNNNNNNNNNNNNNNNNNNNNNNNNNNNNNNNNNNNNNNNNNNNNNNNNNNNN
NNNNNNNNNNNNNNNNNNNNNNNNNNNNNNNNNNNNNNNNNNNNNNNNNNNNTCTNNNAAANNNTNNN
NNNNNNNNNNNNNNNNNNNNNNNNNCNCNTTGNNNGTAANCNANNNGNACATGCAGTCCCCTCNNNNN
NCNNNCAGAGNNNNNNCCCNNNNNCNCCGNCCCCNNCCNNNNNNNNNNNNNNNGNNNNNNNANNNNNN
NNNNNNTGGTACNNNNCGNNNNNGGTTGNNTAAGAGGTCCACAACGNGGTCNGGNCACNNNNGNNNAN
NNNCNNNNNNCNNNNNNNNNNNANNTNNNTANNAGGNNNNNGCCNTCNNNNNNNTAANTGCNATGACC
CAANTCACNGGGGCNGTNCCCGGCGCGGCGACCTTGATNACGTCCGGGGACAAGTATGGCGNNCNCCC
CGGGAAGGANNCNGCCGTANGTGCNNNNNANNNNNTCNTGNGANGTNGCCGNNATTCNCGTGGTCNNN
CNAGAATGNCCGNACCANNCGNANNNTGGTNATGNNAGTNNNNNNNNNNNNNNNNNNNNNNNNNNNNN
NNNNNNNNNNNNNNNNNNNNNNNNNNNNNNNNGNNNNGNNNNNNCNANNNAAGGNNNTANNNCTNNTN
NTNNNNNNGGACNNNACNNNNCNNNNNNNACNCNNNCNCNNNNNNANNNGNTTTTTNNNCNNTATTNT
CCAGNACGCNGNGCNNNCATTCANGNGAACNNNGNNACGCGNNAATCGTANNATCTACCTGCCGCNNG
TNNNNNTACNGNNNNNNCGGANNNTNNNNCGTANNNNNNTCATGCGTNNTNTNNTNNCNAGNCANGTT
CGNNNANANANTAGNANGTNTCNNNNNNNNTGTNNCGTTCCGCNTGANNCNNCANNNNGNNGNNCTTN
TCANCGGCNNTANCTNCNGANNTAAGNCATANNNTNNNTCNNTANCTNNNNNGGAGGGNNACTGTAAA
NGGNNAATTNGNNNNTNATTNCNNNCACNTNNCNNNGNNNNCTNNNTNCCTGNNTNCNNAATTGGNNC
CANTGNNTGNCNATCTCTGGNTTGNNNNNNTNNNNNTNNCANNNNGGNNNNGTNANANNNCNATNTAA
NNTANANNNNCNNNNNNANNNCACNNNNNNNNNAGNNNCNANNNTTANANNNGTAGNNNANTNAGTCT
CCGANNTNNNNNNNATAAGCGNNANACNNNNNNNNNNNNNANGANNACGNNTTGGNNNTNNNNNNCNA
GNNAGACNNTNATGGNTNNNNNTCAANNNNTTNGTAGTGGTATAATNGNNNNNNNTTTCNCCAGNNTN
NNNGNNNNNNNNCNATANNANNNNNNGNGANNNNNNNNNNANNNNCTCNNNAGGGANCANCTACTNNA
GCANTGGNGCGNAGNNNGAGANNNNGACNNNCCTTGGGTGGCAANNACNGNGATAAGTNNNGTTNANC
CTNNNAGTNNACATNNNNNCANNGNNNCNNNNNNANCNGANNGNNNNNNNNNNNNNNNNNNNNNNNNN
NNNNNNNNNNNNNNNNNNNNNNNNNNNNNGNNNANGGNNNGTNTNTGACNCATGTGTTTTTANNNGNN
NNNNNACGNNNGANCCCCGGTCGNNTNCNNATTGAANNCCTGNNGCATNNNNNTNCNAGAATGANNGC
NNNNNGNNTNGNCTNNCNNNTCAAGNAATCANNNAGGTNNNNNNNTCNNNNNNNNNNNNCNNTCCCNN
ACTGAGGGTNNNNNNNNCATCTGCTNNNNNNNNNCTNCGCNGGCGTNNCNNNNNGCNGTATNGNGTNN
NNCANNNNNNNNNNNNNNNNNTNAAACNANNNNCTNNNN
>LaChi532
GTGNNNNNTAGCCCGNNNNAGTCAGACANNNCNNNNNNNNNNNNTNNNNNNNNNNNNNNNNNNNNNNN
NNNNNNNNNNNNNNNNNNNNNNNNNNNNNNNNNNNNNNNNNNNNNNNNNNNNNNNNNNNNNNNNNNNN
NNNNNNNNNNNNNNNNNNNNNNNNNNNNNNNNNNNNNNNNNNNNNNNNNNNNNNNNNNNNNNNNNNNN
NNNNNNNNNNNNNNNNNNNNNNNNNNNNNNNNNNNNNNNNNNNNNNNNNNNNNNNNNNNNNNNNNNNN
NNNNNNNNNNNNNNNNNNNNNNNNNNNNNNNNNNNNNNNNNNNNNNNNNNNNTCTNNNAAANNNTNNN
NNNNNNNNNNNNNNNNNNNNNNNNNCNCNTTGNNNGTAANCNANNNGNACATGCAGTCCCCTCNNNNN
NNNGGCAGAGNNNNNNCCCNNNNNNNCCGNCCCCNNCCNNNNNNNNNNNNNNNGNNNNNNNANNNNNN
NNNNNNTGGTACNNNNCGNNNNNGGTTGNTTAAGAGGTCCACAACGNGGTCNGGNCACNNNNGNNNAN
NNNCNNNAAACNNNNNNNNNNNANNTNNNTANNAGGNCNNNNCCNTCNNGNNANTAATTGCNATGACC
CAANNCACGGGGGCNGTNCCCGGCGCGGCGACCTTGNNNACGTCCGGGGACAAGTATGGCGNNCNCCC
CGGGAAGGANNCNGCCGTANGTGCNNNNNANNNNNTCNTGNGANGTCGCCGNNATTCNCGTGGTCNNN
CNNGAATGNNCNNACCANNCGNANNNTGGTNATGNNAGTNNNNNNNNNNNNNNNNNNNNNNNNNNNNN
NNNNNNNNNNNNNNNNNNNNNNNNNNNNNNNNGNNNNGNNNNNNCNANNNAAGGNNNTANNNCTNNTN
NTNNNNNNGGACNNNACNNNNCNNNNNNNACNCNNNCNCNNNNNNANNNGGTTTTNNNNCNNTATTNT
CCAGNANGCNGNGCNNNCATTCANGNGAACNNNGNNACGCGNNAATCGTANNATCTACCTGCCGCNNG

TNNNNNTACNGNNNNNNCGGANNNTNNNNCGTANNNNNNNCATGCGTNNTNTNNTNNCNAGNCNNGTT
CGNNNANANANTAGNANGTNTNNNNNNNNNNTGTNNCGTTCCGCNTGANNCNNCANNNNGNNGNNCTTN
NCANCGGCNCTANCTGCNGANNTAAGNCATANNNTNNNTCNNTANCTNNNNNGGNGGGNNACTGTAAA
NGGNNAATTNGNNNNTNATTNCNNNCATNTNNCTNNGNNNNCTNNNTNCCTGNNTNCNNAATTGGNNC
CANTGNNTGNCNATCTCTGGNTTNGNNNNNNTGNNNNTNNCANNNNGGNNNNNNNNANNNCNATNTAA
NNTANANNNNCNNNNNNANNNCACNNNNNNNGNAGNNNCNANNNGTANANNNGTAGNNNGNTNAGTCT
CCGANNTNNNNNNNATAAGCGNNANACNNNNNNNNNNNNNNANGANNACGNNTTTGNNNTNNNNNNCNA
GNNAGACNNTNGTGGNTNNNNNTCAANNNNTTNGTAGTGGTATAATNGNNNNNNNTTTCNCCAGNNTN
NNNGNNNNNNNCNATANNANNNNNNNGNGANNNNNNNNNNNANNNNCTCNNNAGGGANCANCTACTNNA
GCANTGGNNCGNAGNNNGAGANNNNGACNNNCCTTGGGTGGCAANCACNGCAATAAGTNNNGTTNNNC
CTNNNAGTNNACACNNNNNCANNGNNNCNNNNNNANCNGANNGNNNNNNNNNNNNNNNNNNNNNNNNN
NNNNNNNNNNNNNNNNNNNNNNNNNNNNAGNNNANNGNNNGTNTNTGACNCNTGTGTTTTTANNNGNN
NNNNNACGNNNGANCCCCGGTCGNNNNCNNATTGAANNCCTGNNGCATNNNNNTNCNAGAATGANNGC
NNNNNGNNTNGNCTNNCNTTTCAAGNAATCANNNAGGTNNNGCGNTCNNNGNNNNNNNNCNNTCCCNN
ACTGAGGGTNNNNNNNNCATCTGCTNNNNNNNNNNTNCGCNGGCGTNNCNNNNNNGCNGTATNNTGTNA
NNCANNNNNNNNNNNNNNNNNTNAAACAANNNNNNTNNNN
>LaChi533
GTGNNNNNTAGCCCNNNNNAGTTAGACANNNCNNNNNNNNNNNNTNNNNNNNNNNNNNNNNNNNNNNN
NNNNNNNNNNNNNNNNNNNNNNNNNNNNNNNNNNNNNNNNNNNNNNNNNNNNNNNNNNNN
NNNNNNNNNNNNNNNNNNNNNNNNNNNNNNNNNNNNNNNNNNNNNNNNNNNNNNNNNNNN
NNNNNNNNNNNNNNNNNNNNNNNNNNNNNNNNNNNNNNNNNNNNNNNNNNNNNNNNNNNN
NNNNNNNNNNNNNNNCCNNNNNNNNNNNNNNNNNNNNNNNNNNNNNNNNNNNNNNCNTCTNNNAAANNNTNNN
NNNNNNNNNNNNNNNNNNNNNNNNNCNCNTTGNNNGTAANCNANNNGNACATGCAGTCCCCTCNNNNN
NNNGGCAGAGNNNNNNNCCNNNNNNNCCGGCCCCNNCCNNNNNNNNNNNNCNNNGNNNNNNNANNNNNN
NNNNNNTGGTACNNNNCGNNNNNGGTTGNNTNAGAGGTCCACAACANGGTCNNGNNACNNNNGNNNAN
NNNCNNNNAANNNNNNNNNNNNNNANNTNNNTANNAGGNNNNNNCCNTCNNGNNNNNNATTGCNATGANN
CAANNCNCGGGGGCNGTNCCCGGNGCGGCGACCTTGATNANGTCCGGGGACAAGTATGGCGNNCNCCC
CGGGAAGGANNCNGCCGTANGTGCNNNNNANNNNNTCNTGNGANGTCGCCGNNATTCNCNTGGTCNNN
CNAGANTGNNCGNACCANNCGNANNNTGGTNATGNNAGTNNNNNNNNNNNNNNNNNNNNNNNNNNNN
NNNNNNNNNNNNNNNNNNNNNNNNNNNNNNNNNNGNNNNGNNNNNNCNANNNAAGGANNTANNNCTNNTN
NTNNNNNNNGGACNNNACNNNNCNNNNNNNACNCNNNCNCNNNNNNANNNGNTTNNNNNNNCNNTATTNT
CCAGNACGCNGNGCNNNCNTTCANGNGAACNNNGNNACGCGNNAATCGTANNATCTACCTGCCGCNNG
NNNNNNTACNGNNNNNNNCGGANNNTNNNTCGTANNNNNNNCATGCGTNNTNTNNTCNCNAGNCANGTT
CGNNNANANANTAGNANGTNTNNNNNNNNNNTGTNNCGTTCCGCNCGANNCNNCANNNNGNNGNNCTTN
NCANCGGCNNTANCTGCNGANNTAAGNCATANNNTNNNTCNNTANCTNNNNNGGNGGGNNACTGTAAA
NGGANAATTNGNNGNTNANTNCNNNCACNTNNCNNNANNNNCTNNNTNCCTGNNTNCNNAATTGGNNC
CANTGNNTGNCNATCTCTGGNTTNGNNNNNNTNNNNNTNNCAANNNGGNNNNNNNNANNNCNATNTAA
NNTANANNNNCNNNNNNANNNCACNNNNNNNGNAGNNNCNANNNTTANANNNGTAGNNNANTAAGTCT
CCGANNTNNNNNNNATAAGCGNNANACNNNNNNNNNNNNNNANGANNACGNNTTGGNNNTNNNNNNNNA
GNNAGACNNTNGTGGNTNNNNNTCAANNNNTTCGTAGTGGTATAATNGNNNNNNNTTTCNCCAGNNTN
NNNGNNNNNNNCNATANNANNNNNNNGNGANNNNNNNNNNNANNNNCTTNNNAGGGANCANTTACTNNA
GCANTGGNGCGCAGNNNGAGANNNNGACNNGCCTTGGGTGGCAANCACNGCAATAAGTNNNGTTNANC
CTNNNAGTNNACACNNNNNCANNGNNNCNNNNNNANCNGANNGNNNNNNNNNNNNNNNNNNNNNNNNN
NNNNNNNNNNNNNNNNNNNNNNNNNNNNANNNNANNGNNNGTNTNTGACNCATGTGTTTTTANNNGNN
NNNNNACGNNNNANCCCCGGTCGNNNNCNNATTGAANNCCTGNNGCATNNNNNTNCNAGAATGANNGC
NNNNNGNNTNGNCTNNCNNNTCAAGNAATCANNNAGNTNNNGCGNTCNNNGNNNNNNNNCNNNCCCNN
ACTGAGGGTNNNNNNNNCANNNGCTNNNNNNNNNNTNCGCNGGNNTNNCNNNNNAGCNGTATNGTGTNN
NNCANNNNNNNNNNNNNNNNNTNAAACNANNNNNCTNNNN
>LaChi534
GTGNNNNNTAGCCCNNNNNAGTCAGACANNNCNNNNNNNNNNNNTNNNNNNNNNNNNNNNNNNNNNNN
NNNNNNNNNNNNNNNNNNNNNNNNNNNNNNNNNNNNNNNNNNNNNNNNNNNNNNNNNNNN
NNNNNNNNNNNNNNNNNNNNNNNNNNNNNNNNNNNNNNNNNNNNNNNNNNNNNNNNNNNN
NNNNNNNNNNNNNNNNNNNNNNNNNNNNNNNNNNNNNNNNNNNNNNNNNNNNNNNNNNNN
NNNNNNNNNNNNNNNNNNNNNNNNNNNNNNNNNNNNNNNNNNNNNNNNNNNCNNNNNNNAAANNNTNNN

NNNNNNNNNNNNNNNNNNNNNNNNNNNNNCNNTGNNNGTAANCNANNNGNACATGCAGTCCCCTCNNNNN
NNNGGCAGAGNNNNNNNCCNNNNNNNNCCGNCCCCNNCCNNNNNNNNNNNNCNNNGNNNNNNANNNNNNN
NNNNNNTGGTACNNNNNCGNNNNNGGTTGNNTNAGAGGTCCACAACGNGGTCNNGNNANNNNNNGNNNAN
NNNCNNNNNNNNNNNNNNNNNNNNNNNTNNNTANNAGGNNNNNNCCNTNNNGNNANNNANNGCNNTGACC
CNANNNNCNGNNGCNGTNCCCGGNGCGGCNACCTTNNNNANGTCCGGGGNCAAGTATGGCGNNCNCCC
CGGGAAGGANNCNGCNGTANGTGCNNNNNANNNNNTCNTGNGANGTNGCCGNNNTTNNCGTGGTCNNN
NNAGAATGCCCGNACCANNCGNANNNTGGTNATGNNAGTANNNNNNNNNNNNNNNNNNNNNNNNNNNNN
NNNNNNNNNNNNNNNNNNNNNNNNNNNNNNNNNNNNNNGNNNNNNCNANNNAAGGNNNTANNNCTNNTN
NTNNNNNNGNNCNNNACNNNNCNNNNNNNACNCNNNCNCNNNNNNANNNGNTTTTNNNNCNNTANTNT
CCAGNANGCNGNGCNNNCATTCANGNGAACNNNGNNACGCGNNAATCGTANNATCTACCTGCCGCNGG
NNNNNNTNCNGNNNNNNCGGANNNTNNCTCGTANNNNNNNCATGCGTNNNNTNNTCNCNAGNCNNGTT
CGNNNANANANTAGNANGTNTNNNNNNNNNTGTNNCGTTCCGCNTGANNCNNCANNNNGNNGNNCTTN
NCANCGGCNNTANCTNCNGANNTAAGNCATANNNTNNNTCNNTANCTNNNNNGGNGGGNNACTGTAAA
NGNNNAATTGGNNNNTNANTNCNNNCATNNNNCNNNGNNNNCTNNNTNCCTGNNTNCNNAATTGGNNN
CANTGNNTGNCNATCTCTGGNTTNGNNNNNNTGNNNNTNNCANNNNGGNNNNNNANANNNNNNATNTAA
NNTANANNNNCNNNNNNANNNCANNNNNNNNNNAGNNNNNANNNGNANANNNGTAGNNNGNTAAGTCT
CCGANNTNNNNNNNATAAGCGNNANACNNNNNNNNNNNNNNANGANNNCGNNTTTGNNNNNNNNNNNNA
GNNAGACNNTNGTGGNTNNNNNTCAANNNNTTNGTAGTGGTATAATNGNNNNNNNTTTCNCCAGNNTN
NNNGNNNNNNNNCNATANNANNNNNNNNNGANNNNNNNNNNNANNNNCTCNNNAGGNANCANCTACTNNA
GCANTGGNGCGCAGNNNGANANNNNGACNNGCCTTGGGTGGCAANNACNGCAATAAGTNNNGTTNNNC
CTNNNAGTNNACACNNNNNCANNGNNNCNNNNNNANCNGANNGNNNNNNNNNNNNNNNNNNNNNNNNNN
NNNNNNNNNNNNNNNNNNNNNNNNNNNNNNNNNNNNNANGGNNNGTNTNTGACNCATGTGTTTTTANNNGNN
NNNNNNNGNNNGANCCCCGGTCGNNNNCNNATTGAANNCCTGNNGCATNNNNNTNCNAGAATGANNGC
NNNNNGNNTNGNCTNNNNNNNTCAAGNAATCANNNAGGTNNNNNGNTCNNNNNNNNNNNNNCNNNNNCNN
ACTGAGGGTNNNNNNNNCANNNNCTNNNNNNNNNNTNCGCNNGNGTNNCNNNNNAGCCGTATNGNGNNN
NNCANNNNNNNNNNNNNNNNNTNNANNNNNNNNNNNNTNNNN
>LaChi535
GTGNNNNNTAGCCCNNNNNAGTTAGACANNNCNNNNNNNNNNNNNNNNNNNNNNNNNNNNNNNNNNNNNNN
NNNNNNNNNNNNNNNNNNNNNNNNNNNNNNNNNNNNNNNNNNNNNNNNNNNNNNNNNNNNNNNNNNNNNN
NNNNNNNNNNNNNNNNNNNNNNNNNNNNNNNNNNNNNNNNNNNNNNNNNNNNNNNNNNNNNNNNNNNNNN
NNNNNNNNNNNNNNNNNNNNNNNNNNNNNNNNNNNNNNNNNNNNNNNNNNNNNNNNNNNNNNNNNNNNNN
NNNNNNNNNNNNNNNNNNNNNNNNNNNNNNNNNNNNNNNNNNNNNNNNNNNNNNNCTNNNAAANNNTNNN
NNNNNNNNNNNNNNNNNNNNNNNNNNNNNCNNTGNNNGTAANCNANNNGNACATGCAGTCCCCTCNNNNN
NNNGGCAGAGNNNNNNNCCCNNNNNNNNCCGNCCCCNNCCNNNNNNNNNNNNCNNNGNNNNNNANNNNNN
NNNNNNTGGTACNNNNNCGNNNNNGGTTGNNTNAGAGGTCCACAACGNGGTCNNNNNACNNNNGNNNAN
NNNCNNNNNNNCNNNNNNNNNNNNNANNTNNNTANNAGGNNNNNNCCNTNNNNNNNANNNANNGCNATGACC
CAANNCNCGGGGGNNGTNCCCGGCGNGGCGACCTTGNNNANGTCCGGGNNCAAGTATNGCNNNCNCCC
CGGGAAGGANNCNGCNGTANGTGCNNNNNANNNNNTCNTGNGANGTCGCCGNNNTTNNCGTGGTCNNN
NNAGAATGCCCGNACCANNCGNANNNTGGTNATGNNAGTANNNNNNNNNNNNNNNNNNNNNNNNNNNNN
NNNNNNNNNNNNNNNNNNNNNNNNNNNNNNNNNNNNGNNNNGNNNNNNCNANNNAAGGANNTANNNCTNNTN
NTNNNNNNGGACNNNACNNNNCNNNNNNNACNCNNNCNCNNNNNNNNNNNGNTTTTNNNNCNNTANTNT
CCAGNACGCNGNGCNNNCATTCANGNGAACNNNGNNACGCGNNAATCGTANNATCTACCTGCCGCNNG
NNNNNNTACNGNNNNNNCGGANNNTNNNNCGTANNNNNNNCATGCGTNNTNTNNTCNCNAGNCNNGTT
CGNNNANANANTAGNANGTNTNNNNNNNNNTGTNNCGTTCCGCNTGANNCNNCNNNNNGNNGNNNTTN
NCANCGGCNNTANCTNNNGANNTAAGNCATANNNTNNNTCNNTANCTNNNNNGGNGGGNNACTGTAAA
NGNNNAATTNGNNNNTNATTNCNNNCACNTNNCTNNGNNNNCTNNNTNCCTGNNTNCNNAATTGGNNN
CANTGNNTGNCNATCTCTNGNTTNGNNNNNNTNNNNNTNNCANNNNGGNNNNNNNNANNNCNATNTAA
NNTANANNNNCNNNNNNANNNCACNNNNNNNNNAGNNNCNANNNTNANANNNGTAGNNNANTNAGTCT
CCGANNTNNNNNNNATAAGCGNNANACNNNNNNNNNNNNNNANGANNACGNNTTGGNNNNNNNNNNNNA
GNNAGACNNTNATGGNTNNNNNTCAANNNNTTNGTAGTGGTATAATNGNNNNNNNTTTCNCCAGNNTN
NNNNNNNNNNNCNATANNANNNNNNNGNGANNNNNNNNNNNANNNNNTCNNNAGGGANCANCTACTNNA
GCANTGGNGCGNAGNNNGAGANNNNGACCAGCCTTGGGTGGCAANCACNGCGATAAGTNNNGTTNANC
CTNNNAGTNNACATNNNNNCANNGNNNCNNNNNNANCNGANNGNNNNNNNNNNNNNNNNNNNNNNNNNN
NNNNNNNNNNNNNNNNNNNNNNNNNNNNNAGNNNANGGNNNGTNTNTGNCNCATGTNTTTTTANNNGNN

NNNNNNNGNNNGANCCCCGGTCGNNNNCNNATTGAANNCCTGNNNCATNNNNNTNCNAGAATGANNNC
NNNNNGNNTNGNCTNNCNNNTCANGNAATCANNNANGNNNNNNNNTCNNNNNNNNNNNNCNNTCNCNN
ACTGAGGGNNNNNNNNNCATNNGCTNNNNNNNNNNTNCGCNGGNNNNNCNNNNNNGCNGTATNGNGTNA
NNCANNNNNNNNNNNNNNNNNNNNAANNNNNNNNNNCTNNNN
>LaChi536
GTGNNNNNTAGCCCNNNNNAGTTAGACANNNCNNNNNNNNNNNNNTNNNNNNNNNNNNNNNNNNNNNNNN
NNNNNNNNNNNNNNNNNNNNNNNNNNNNNNNNNNNNNNNNNNNNNNNNNNNNNNNNNNNNNNNNNNNNNN
NNNNNNNNNNNNNNNNNNNNNNNNNNNNNNNNNNNNNNNNNNNNNNNNNNNNNNNNNNNNNNNNNNNNNN
NNNNNNNNNNNNNNNNNNNNNNNNNNNNNNNNNNNNNNNNNNNNNNNNNNNNNNNNNNNNNNNNNNNNNN
NNNNNNNNNNNNNNNNNNNNNNNNNNNNNNNNNNNNNNNNNNNNNNNNNNNNCNTCTNNNAAANNNTNNN
NNNNNNNNNNNNNNNNNNNNNNNNNNNCNCNTTGNNNGTAANCNANNNGNACATGCAGTCCCCTCNNNNN
NNNGGCAGAGNNNNNNNCCNNNNNNNCCGGCCCCNNCCNNNNNNNTNNNCANNGNNNNNNNANNNNNN
NNNNNNTGGTACNNNNCGNNNNNGGTTGNNTAAGAGGTCCACAACGNGGTCNNGNCACNNNNGNNNAN
NNNCNNNNAACNNNNNNNNNNNNANNTNNNTANNAGGNCNNNNCCNTCNNGNNNNNNANTGCNATGACC
CAANTCACGGGGGCNGTNCCCGGCGNGGCGACCTTGNNNACGTCCGGGGACAAGTATGGCGNNCNCCC
CGGGAAGGANNCNGCCGTANGTGCNNNNNANNNNNTCNTGNGACGTCGCCGNNATTCNNGTGGTCNNN
CNAGAATGNCCNNACCANNCGNANNNTGGTNATGNNAGTNNNNNNNNNNNNNNNNNNNNNNNNNNNNNN
NNNNNNNNNNNNNNNNNNNNNNNNNNNNNNNNNNGNNNNGNNNNNNCNANNNAAGGNNNTANNNCTNNTN
NTNNNNNNGGACNNNACNNNNCNNNNNNNACNCNNNCNCNNNNNNNNNNGGTTTTNNNNNCNNTANTNT
CCAGNANGCNGNGCNNNCNTTCANGNGAACNNNGNNACGCGNNAATCGTANNATCTACCTGCCGCNNG
NNNNNNTACNGNNNNNNCGGANNNTNNNNCGTANNNNNNTCATGCGTNNTNTNNTCNCNAGNCANGTT
CGNNNANANANTAGNANGTNTNNNNNNNNNNTGTNNCGTTCCGCNTGANNCNNCANNNNGNNGNNCTTN
NCANCGGCNNTANCTNCNGANNTAAGNCATANNNTNNATCNNTANCTNNNNNGGNGGGNNACTGTAAA
NGGNNAATTNGNGGNTNANTNCNNNCACNTNNCTNNGNNTNCTNNNTNCCTGNNTNCNNAATTGGNNC
CANTGNNTGNCNATCTCTGGNTTNGNNNNNNTNNNNNTNNCANNNNGGNNNGTNNNANNNCNATNTAA
NNTANANNNNCNNNNNNANNNCACNNNNNNNGNAGNNNCNANNNTTANANNNGTAGNNNANTAAGTCT
CCGANNTNNNNNNNATAAGCGNNANACNNNNNNNNNNNNNANGANNNCGNNTTGGNNNTNNNNNNCNA
GNNAGACNNTNATGGNTNNNNNTCAANNNNTTNGTAGTGGTATAATNGNNNNNNNTTTCNCCAGNNTN
NNNGNNNNNNNCNATANNANNNNNNNGNGANNNNNNNNNNANNNNCTCNNNAGGGANCANCTACTNNA
GCANTGGNGCGCAGNNNGAGANNNNGACNNNCCTTGGGTGGCAANCACNGCGATAAGTNNNGTTNNNC
CTNNNAGTNNACATNNNNNCANNGNNNCNNNNNNANCNGANNGNNNNNNNNNNNNNNNNNNNNNNNNNNN
NNNNNNNNNNNNNNNNNNNNNNNNNNNNNNNANNNNANNGNNNGTNTNTGACNCATGNNTTTTANNNGNN
NNNNNACGNNNNANCCCCGGTCGNNNNCNNATTGAANNCCTGNNGCATNNNNNTNCNAGAATGANNNC
NNNNNGNNTNNNCTNNCNNNTCAAGNAATCAANTAGGTNNNNNGNTCNNNNNNNNNNNNCNNTCCCNN
ACTGAGGGTNNNNNNNNCATCTGCTNNNNNNNNNNTNCGCNGGCGTNNCNNNNNNGCNGTATNNTGTNA
NNCANNNNNNNNNNNNNNNNNNTNNANNNANNNNNNTNNNN
>LaChi537
GTGNNNNNTAGCCCNNNNNAGTTAGACANNNCNNNNNNNNNNNNNTNNNNNNNNNNNNNNNNNNNNNNNN
NNNNNNNNNNNNNNNNNNNNNNNNNNNNNNNTNNNNNNNNNNNNNNNNNNNNNNNNNNNNNNNNNNNNNN
NNNNNNNNNNNNNNNNNNNNNNNNNNNNNNNNNNNNNNNNNNNNNNNNNNNNNNNNNNNNNNNNNNNNNN
NNNNNNNNNNNNNNNNNNNNNNNNNNNNNNNNNNNNNNNNNNNNNNNNNNNNNNNNNNNNNNNNNNNNNN
NNNNNNNNNNNNNNNNNNNNNNNNNNNNNNNNNNNNNNNNNNNNNNNNNNNNCNTCTNNNAAANNNTNNN
NNNNNNNNNNNNNNNNNNNNNNNNNNNCNCNTTNNNNGTAANCNANNNGNACATGCAGTCCCCTCNNNNN
NNNNNCAGAGNNNNNNNCCNNNNNNNCCGGCCCCNNCCNNNNNNNNNNNCNNNGNNNNNNNANNNNNN
NNNNNNTGGTACNNNNCGNNNNNGGTTGTNTAAGAGGTCCACAACGNGGTCNGGNCACNNNNGNNNAN
NNNCNNNNAACNNNNNNNNNNNNANNTNNNTANNAGGNNNNNNCCNTCNNGNNNNNNATTGCNATGACC
CAANNCACGGGGGCNGTNCCCGGCGCGGCGACCTTGNNNACGTCCGGGGNCAAGTATGGCNNNCNCCC
CGGGAAGGANNCNGCCGTANGTGCNNNNNANNNTNTCNTGNGANGTCGCCGNNATTNNCGTGGTCNNN
CNAGAATGNCCNNACCANNCGNANNNTGGTNATGTNAGTNNNNNNNNNNNNNNNNNNNNNNNNNNNNNNN
NNNNNNNNNNNNNNNNNNNNNNNNNNNNNNNNNNNNNNNGNNNNNNCNANNNAAGGNNNTANNNCTNNTN
NTNNNNNNGGACNNNACNNNNCNNNNNNNACNCNNNCNCNNNNNNANNNGNTTNNNNNNCNNTATTNT
CCAGNACGCNGNGCNNNCATTCANGNGAACNNNGNNACGCGNNAATCGTANNATCTACCTGCCGCNNG
NNNNNNTACNGNNNNNNCGGANNNTNNCTCGTANNNNNNNCANGCGTNNTNTNNTCNCNAGNCANGTT
CGNNNANANANTAGNANGTNTNNNNNNNNNNTGTNNCGTTCCGCNTGANNCNNCNNNNNGNNGNNNTTN

NCANCGGCNNTANCTGCNGANNTAAGCATANNNTNNNTCNNTANCTNNNNNGGNGGGNNACTGTAAA
NGGNNAATTNGNNNNTNATTNCNNNNACNTNNCTNNGNNTNCTNNNTNCCTGNNTNCNNAATTGGNNC
CANTGNNTGNCNATCTCTGGNTTNGCNNNNNTGNNNNTNNCANNNNGGNNNNNNNNANNNCNATNTAA
NNTANANNNNCNNNNNNANNNCACNNNNNNNNNAGNNNNNANNNTNANANNNGTAGNNNANTNAGTCT
CCGANNTNNNNNNNATAAGCGNNANACNNNNNNNNNNNNNANGANNNCGNNTTGGNNNTNNNNNNNNA
GNNAGACNNTNATGGNTNNNNNTCAANNNNTTCGTAGTGGTATAATNGNNNNNNNTTTCNCCAGNNTN
NNNGNNNNNNNNCNATANNANNNNNNNGNGANNNNNNNNNNANNNNCTCNNNAGGGANCANCTACTTNA
GCANTGGNGCGCAGNNNGAGANNNNGACNNNCCTTGGGTGGCAANCACNGNGATAAGTNNNGTTNANC
CTNNNAGTNNACATNNNNNCANNGNNNCNNNNNNANCNGANNGNNNNNNNNNNNNNNNNNNNNNNNN
NNNNNNNNNNNNNNNNNNNNNNNNNNNNNNNNNNANGGNNNGTNTNTGACNCATGTGTTTTTANNNGNN
NNNNNACGNNNGANCCCCGGTCGNNNNCNNATTGAANNCCTGNNGCATNNNANTNCNAGAATGANNNC
NNNNNGNNTNGNCTGNNNNTTCAAGNAATCANNNAGGTNNNNNNNTCNNNGNNNNNNNNCNNTCCCNN
ACTGAGGGTNNNNNNNNCATCTGCTNNNNNNNNCTNCGCNGGNGTNNCNNNNNNGCNGTATNGNGTNA
NTCANNNNNNNNNNNNNNNNNTNAAACNANNNNNNNTNNNN
>LaChi538
GTGNNNNNTAGCCCNNNNNAGTTAGACANNNCNNNNNNNNNNNNNTNNNNNNNNNNNNNNNNNNNNNNN
NNNNNNNNNNNNNNNNNNNNNNNNNNNNNNNNNNNNNNNNNNNNNNNNNNNNNNNNNNNNNNNNNNNNNN
NNNNNNNNNNNNNNNNNNNNNNNNNNNNNNNNNNNNNNNNNNNNNNNNNNNNNNNNNNNNNNNNNNNNNN
NNNNNNNNNNNNNNNNNNNNNNNNNNNNNNNNNNNNNNNNNNNNNNNNNNNNNNNNNNNNNNNNNNNNNN
NNNNNNNNNNNNNNNNNNNNNNNNNNNNNNNNNNNNNNNNNNNNNNNNNNNCNTCTNNNAAANNNTNNN
NNNNNNNNNNNNNNNNNNNNNNNNNNCNCNNTNNNNGTAANCNANNNGNACATGCAGTCCCCTCNNNNN
NNNGGCAGAGNNNNNNNCCNCNNNNNCCGGCCCCGNCCNNNNNNNNNNNNNNNNNNNNNNNNANNNNNN
NNNNNNTGGTACNNNNCGNNNNNGGTTGNNTAAGAGGTCCACAACANGGTCNNNNCACNNNNGNNNAN
NNNCNNNNNNNCNNNNNNNNNNNNANNTNNNTANNAGGNNNNNNCCNTCNNNNNNNTNANTGCNATGACC
CNANNCACGGGGGCAGTNCCCGGCGCGGCGACCTTGNNNANNTCCGGGGNCAAGTATGGCNNNCNCCC
CGGGAAGGANNCNNCCGTANGTGCNNNNNANNNNNTCNTGNGANGTCGCCGNNATTNNCGTGGTCNNN
NNAGAATGNCCNNACCANNCGNANNNTGGTNATGNNAGTNNNNNNNNNNNNNNNNNNNNNNNNNNNN
NNNNNNNNNNNNNNNNNNNNNNNNNNNNNNNNNNNNNNGNNNNNNCNANNNAAGGNNNTANNNCTNNTC
NTNNNGNNGGACNNNACNNNNCNNNNNNNACNCNNNCNCNNNNNNANNNGNTTTTNNNNCNNTANTNT
CCAGNACGCNGNGCNNNCNTTCANGNGAACNNNGNNACGCGNNAATCGTANNATCTANCTGCCGCNNG
TNNNNNTACNGNNNNNNCGGANNNTNNNTCGTANNNNNNTCATGCGTNNTNTNNTCNCNAGNCNNGTT
CGNNNANANANTAGNANGTNTCNNNNNNNNNTGTNNCGTTCCGCNCGANNCNNCANNNNGNNGNNCTTN
NCANCGGCNNTANCTNCNGANNTAAGNCATANNNTNNNTCNNTANCTNNNNNGGNGGGNNACTGTAAA
NGGNNAATTNGNNGNTNANTNCNNNCACNTNNCTNNANNNNCTNNNTNCCTGNNTNCNNAATTGGNNN
CANTGNNTGNCNATCTCTGGNTTNGNNNNNNTNNNNNTNNCANNNNGGNNNNNTNNANCNCNATNTAA
NNTANANNNNCNNNNNNANNNCACNNNNNNNGNAGNNNCNANNNTNANANNNGTAGNNNANTNAGTCT
CCGANNTNNNNNNNATAAGCGNNANACNNNNNNNNCNNNNANGANNACGNNTTGGNNNTNANNNNCNA
GNNAGACNNTNGTGGNTNNNNNTCAANNNNTTCGTAGTGGTATAATNGNNNNNNNTTTCNCCAGNNNN
NNNGNNNNNNNNCNATANNANNNNNNNGNGANNNNNNNNNNANNNNCTTNNNAGGGANCANTTACTNNA
GCANTGGNGCGNAGNNNGAGANNNNGACNAGCCTTGGGTGGCAANCACNGNAATAAGTNNGTTNANC
CTNNNAGTNNACACNNNNNCANNGNNNCNNNNNNANCNGANNGNNNNNNNNNNNNNNNNNNNNNNNN
NNNNNNNNNNNNNNNNNNNNNNNNNNNNNNNGNNNANGGNNNGTNTNTGACNCATGNNTTTTTANNNGNN
NNNNNACGNNNNANCCCCGGTCGNNNNCNNATTGAANNCCTGNNGCATNNNNNTNCNAGAATGANNGC
NNNNNGNNTNGNCTNNCNNNTCAAGNAATCANNNAGGTNNNNNNNTCNNNNNNNNNNNNCNNTCCCNN
ACTGAGGGTNNNNNNNNCATCTGCTNNNNNNNNNNTNCGCNGGNGTNNCNNNNNNGCNGTATNGTGTNN
NNCANNNNNNNNNNNNNNNNNTNNANNANNNNNNNNTNNNN
>LaChi539
GTGNNNNNTAGCCCNNNNNAGTTAGACANNNCNNNNNNNNNNNNNTNNNNNNNNNNNNNNNNNNNNNNN
NNNNNNNNNNNNNNNNNNNNNNNNNNNNNNNNTNNNNNNNNNNNNNNNNNNNNNNNNNNNNNNNNNNNN
NNNNNNNNNNNNNNNNNNNNNNNNNNNNNNNNNNNNNNNNNNNNNNNNNNNNNNNNNNNNNNNNNNNNNN
NNNNNNNNNNNNNNNNNNNNNNNNNNNNNNNNNNNNNNNNNNNNNNNNNNNNNNNNNNNNNNNNNNNNNN
NNNNNNNNNNNNNNNNNNNNNNNNNNNNNNNNNNNNNNNNNNNNNNNNNNNCNTCTNNNAAANNNTNNN
NNNNNNNNNNNNNNNNNNNNNNNNNNCNCNNTGNNNGTAANCNANNNGNACATGCAGTCCCCTCNNNNN
NNNGGCAGAGNNNNNNNCCNNNNNCNCCGGCCCCNNCCNNNNNNNNNNNNCNNNGNNNNNNAANNNNNN

NNNNNNTGGTACNNNNCGNNNNNGGTTGTNTAAGAGGTCCACAACGNGGTCNNGNNACNNNNGNNNAN
NNNCNNNNNNNNNNNNNNNNNNNNANNTNNNTANNAGGNCNNNNCCNTNNNNNNNNNNNANTGCNATGACC
CAANNNNCGGNGGNNGTNCCCGGNGCGGCGACCTTGNNNANGTCCGGGGNCAAGTATGGCGNNCNCCC
CGGGAAGGANNCNGCNGTANGTGCNNNNNANNNNNTCNTGNGACGTCGCCGNNATTCNCGTGGTCNNN
CNAGAATGNNCGNACCANNCGNANNNTGGTNATGNNAGTANNNNNNNNNNNNNNNNNNNNNNNNNNN
NNNNNNNNNNNNNNNNNNNNNNNNNNNNNNNNNGNNNNGNTNNNNCNANNNAAGGANNTANNNCTNNTN
NTNNNNNNGGACNNNACNNNNCNNNNNNNACNCNNNCNCNNNNNNANNNGGTTTTTNNNCNNTANTNT
CCAGNACGCNGNGCNNNCATTCANGNGAACNNNGNNACGCGNNAATCGTANNATCTACCTGCCGCNNG
TNNNNNTACNGNNNNNNCGGANNNTNNCTCGTANNNNNNTCANGCGTNNTNTNNTCNCNAGNCANGTT
CGNNNANANANTAGNANGTNTNNNNNNNNNNTGTNNCGTTCCGCNTGANNCNNCANNNNGNNGNNNTTN
NCANCGGCNNTANCTNCNGANNTAAGNCATANNNTNNNTCNNTANCTNNNNNGGNGGGNNACTGTAAA
NGNNNAATTNGNNNNTNATTNCNNNCACNTNNCTNNGNNNNCTNNNTNCCTGNNTNCNNAATTGGNNC
CANTGNNTGNCNATCTCTGGNTTNGNNNNNNTNNNNNTNNCAANNNGGNNNGTNANANNNCNATNTAA
NNTANANNNNCNNNNNNANNNCACNNNNNNNGNAGNNNCNANNNTNANANNNGTAGNNNANTNAGTCT
CCGANNTNNNNNNNATAAGCGNNANACNNNNNNNNNNNNNNANGANNACGNNTTGGNNNTNNNNNNCNA
GNNAGACNNTNATGGNTNNNNNTCAANNNNTTNGTAGTGGTATAATNGNNNNNNNTTTCNCCAGNNTN
NNNGNNNNNNNCNATANNANNNNNNNGNGANNNNNNNNNNNANNNNCTCNNNAGGGANCANCTACTNNA
GCAATGGNGCGNAGNNNGAGANNNNGACCAGCCTTGGGTGGCAANCACNGCGATAAGTNNNGTTNNNC
CTNNNAGTNNACATNNNNNCANNGNNNCNNNNNNANCNGANNGNNNNNNNNNNNNNNNNNNNNNNNNN
NNNNNNNNNNNNNNNNNNNNNNNNNNNNNNNNNNANGGNNNGTNTNTGACNCATGTGTTTNTANNNGNN
NNNNNACGNNNGANCCCCGGTCGNNNNCNNATTGAANNCCTGNNGCATNNNNNTNCNAGAATGANNGC
NNNNNGNNTNGNCTNNCNTTTCAAGNAATNANNNAGGTANCGCGNTCNNNGNNNNNNNNCNNNCCCNN
ACTGAGGGNNNNNNNNNCATNNGCTNNNNNNNNNNTTCGCNGGCGNNNCNNNNNAGCNGTATNGNGTNN
NNCANNNNNNNNNNNNNNNNNNTNANNNNANNNNNTTNNNN
>LaChi540
GTGNNNNNTAGCCCNNNNNAGTCAGACANNNCNNNNNNNNNNNNTNNNNNNNNNNNNNNNNNNNNNN
NNNNNNNNNNNNNNNNNNNNNNNNNNNNNNNNNNNNNNNNNNNNNNNNNNNNNNNNNNNNNNNNNNNNNN
NNNNNNNNNNNNNNNNNNNNNNNNNNNNNNNNNNNNNNNNNNNNNNNNNNNNNNNNNNNNNNNNNNNNNN
NNNNNNNNNNNNNNNNNNNNNNNNNNNNNNNNNNNNNNNNNNNNNNNNNNNNNNNNNNNNNNNNNNNNNN
NNNNNNNNNNNNNNNNNNNNNNNNNNNNNNNNNNNNNNNNNNNNNNNNNNNNNNNTCTNNNAAANNNTNNN
NNNNNNNNNNNNNNNNNNNNNNNNNNCNCNTTGNNNGTAANCNANNNGNACATGCAGTCCCCTCNNNNN
NNNGGCAGAGNNNNNNNCCNNNNNCNCCGNCCCCNNCCNNNNNNNNNNNNNNNNNGNNNNNNNANNNNNN
NNNNNNTGGTACNNNNCGNNNNNGGTTGTNTAAGAGGTCCACAACGNGNTCNNGNCACNNNNGNNNAN
NNNCNNNNAACNNNNNNNNNNNNANATNNNTANAAGGNNNNNNCCNTCNNNNNNNNNNATTGCNATGACC
CANNNCNCGGNGGNNGTNCCCGGNGNGGNGACCTTGNNNANNTCCGGGGACAAGTATGGCGNNCNCCC
CGGGAAGGANNCNGCCGTANGTGCNNNNNANNNNNTCNTGNGANGTNGCCGNNATTNNCGTGGTCNNN
CNAGAATGNCCGNACCANNCGNANNNTGGTNATGNNAGTNNNNNNNNNNNNNNNNNNNNNNNNNNNNNN
NNNNNNNNNNNNNNNNNNNNNNNNNNNNNNNNNGNNNNGNNNNNNCNANNNAAGGANNTANNNCTNNTN
NTNNNNNNGGACNNNACNNNNCNNNNNNNACNCNNNCNCNNNNNNANNNGNTTTTNNNNNCNNTANTNT
CCAGNACGCNGNGCNNNCATTCANGNGAACNNNGNNACGCGNNAATCGTANNATCTACCTGCCGCNNG
TNNNNNTACNGNNNNTNCGGANNNTNNNNCGTANNNNNNTCATGCGTNNTNTNNTCNCNAGNCNNGTT
CGNNNANANANTAGNANGTNTNNNNNNNNNNTGTNNCGTTCCGCNTGANNCNNCANNNNGNNGNNNTTC
TCANCGGCNNTANCTGCNGANNTAAGNCATANNNTNNNTCNNTANCTNNNNNGGNGGGNNACTGTAAA
NGGNNAATTNGNNNNTNATTNCNNNCATNTNNCTNNGNNTNCTNNNTNCCTGNNTNCNNAATTGGNNN
CANTGNNTGNCNATCTCTGGNTTNGNNNNNNTNNNNNTNNCANNNNGGNNNGNNNNANNNCNATNTAA
NNTANANNNNCNNNNNNANNNCACNNNNNNNNNNAGNNNCNANNNGTANANNNGTAGNNNGATAAGTCT
CCGANNTNNNNNNNATAAGCGNNANACNNNNNNNNNNNNNNANGANNACGNNTTTGNNNTNNNNNNCNA
GNNAGACNNTNGTGGNTNNNNNTCAANNNNTTNGTAGTGGTATAATNGNANNNNNTTTCNCCAGNNTN
NNNGNNNNNNNCNATANNANNNNNNNGNGANNNNNNNNNNNANNNNCTCNNNAGGNANCANCTACTNNA
GCANNGGNGCGCAGNNNGAGANNNNGACNNGCCTTGGGTGGCAANCACNGCAATAAGTNNNGTTNANC
CTNNNAGTNNACACNNNNNCANNGNNNCNNNNNNANCNGANNGNNNNNNNNNNNNNNNNNNNNNNNNN
NNNNNNNNNNNNNNNNNNNNNNNNNNNNNAGNNNANNGNNNGTNTNTGACNCATGTGTTTTTANNNGNN
NNNNNACGNNNGANCCCCGGTCGTNNNCNNATTGAANNCCTGNNGCATNNNNNTNCNAGAATGANNGC
NNNNNGNNTNGNCTNNCNNNTCAAGNAATCANNTANGTANCGCGNTCNNNGNNNNNNNNCNNTCCCNN

ACTGAGGGTNNNNNNNNCATCTGCTNNNNNNNNNTNCGCNGGNGTNNCNNNNNAGCNGTATNNNGTNA
NNCANNNNNNNNNNNNNNNNNNTNAAACAANNNNNNNTNNNN
>LaChi541
GTGNNNNNTAGCCCNNNNNAGTCAGACANNNCNNNNNNNNNNNNTNNNNNNNNNNNNNNNNNNNNNNNNN
NNNNNNNNNNNNNNNNNNNNNNNNNNNNNNNNNNNNNNNNNNNNNNNNNNNNNNNNNNNNNNNNNNNNNN
NNNNNNNNNNNNNNNNNNNNNNNNNNNNNNNNNNNNNNNNNNNNNNNNNNNNNNNNNNNNNNNNNNNNNN
NNNNNNNNNNNNNNNNNNNNNNNNNNNNNNNNNNNNNNNNNNNNNNNNNNNNNNNNNNNNNNNNNNNNNN
NNNNNNNNNNNNNNNNNNNNNNNNNNNNNNNNNNNNNNNNNNNNNNNNNNNNNNTCTNNNAAANNNTNNN
NNNNNNNNNNNNNNNNNNNNNNNNNNNCNCNTTGNNNGTAANCNANNNGNACATGCAGTCCCCTCNNNNN
NNNGGCAGAGNNNNNNCCCNNNNNCNCCGGCCCCNNCCNNNNNNNNNNNNNNNNGNNNNNNNANNNNNN
NNNNNNTGGTACNNNNCGNNNNNGGTTGNNTNAGAGGTCCACAACGNGGTCNNNNNACNNNNGNNNAN
NNNCNNNNNNCNNNNNNNNNNNNANATNNNTANNAGGNCNNNNCCNTCNNGNNANNAANTGCNATGACC
CAANTCACGGGGGCNGTNCCCGGCGCGGCGACCTTGANNANGTCCGGGGACAAGTATGGCGNNCNCCC
CGGGAAGGANNCNGCCGTANGTGCNNNNNANNNNNTCNTGNGACGTCGCCGNNATTCNCGTGGTCNNN
CNAGAATGNCCNNACCANNCGNANNNTGGTNATGTNAGTNNNNNNNNNNNNNNNNNNNNNNNNNNNNNNN
NNNNNNNNNNNNNNNNNNNNNNNNNNNNNNNNNNGNNNNGNNNNNNCNANNNAAGGANNTANNNCTNNTN
NTNNNGNNGGACNNNACNNNNCNNNNNNNACNCNNNCNCNNNNNNANNNGNTTTTTNNNCNNTATTNT
CCAGNACGCNGNGCNNNCATTCANGNGAACNNNGNNACGCGNNAATCGTANNATCTACCTGCCGCNNG
NNNNNNTACNGNNNNNNCGGANNNTNNCTCGTANNNNNNNCATGCGTNNTNTNNTCNCNAGNCANGTT
CGNNNANANANTAGNANGTNTNNNNNNNNNNTGTNNCGTTCCGCNTGANNCNNCANNNNGNNGNNCTTN
NCANCGGCNNTANCTGCNGANNTAAGNCATANNNTNNNTCNNTANCTNNNNNGGNGGGNNACTGTAAA
NGGNNAATTGGANNNTNATTNCNNNCATNTNNCTNNGNNNNCTNNNTNCCTGNNTNCNNAATTGGNNC
CANTGNNTGNCNATCTCTGGNTTNGNNNNNNTNNNNNTNNCANNNNGGNNNNNNNNANNNCNATNTAA
NNTANANNNNCNNNNNNANNNCACNNNNNNNGNAGNNNCNANNNGTANANNNGTAGNNNGATAAGTCT
CCGANNTNNNNNNNATAAGCGNNANACNNNNNNNNNNNNNNANGANNACGNNTTTGNNNTNNNNNNNCNA
GNNAGACNNTNGTGGNTNNNNNTCAANNNNTTNGTAGTGGTATAATNGNNNNNNNTTTCNCCAGNNTN
NNNGNNNNNNNNCNATANNANNNNNNNGNGANNNNNNNNNNNANNNNCTCNNNAGGGANCANCTACTTNA
GCANTGGNGCGCAGNNNGAGANNNNGACNNGCCTTGGGTGGCAANCACNGCAATAAGTNNNGTTNANC
CTNNNAGTNNACACNNNNNCANNGNNNCNNNNNNANCNGANNGNNNNNNNNNNNNNNNNNNNNNNNNNNN
NNNNNNNNNNNNNNNNNNNNNNNNNNNNNNNGNNNANGGNNNGTNTNTGACNCATGTGTTTTTANNNGNN
NNNNNACGNNNGANCCCCGGTCGNNNNCNNATTGAANNCCTGNNGCATNNNNNNTNCNAGAATGANNNC
NNNNNGNNTNGNCTNNCNNNTCAAGNAATCANNNAGGTNNNNNGNTCNNNGNNNNNNNNCNNTCCCNN
ACTGAGGGTNNNNNNCGCATCTGCTNNNNNNNNNTNCGCNGGCGTNNCNNNNNAGCNGTATNGTGTNA
NNCANNNNNNNNNNNNNNNNNNTNNANCGANNNNNNNTNNNN
>LaChi542
GTGNNNNNTAGCCCNNNNNAGTTAGACANNNCNNNNNNNNNNNNTNNNNNNNNNNNNNNNNNNNNNNNNN
NNNNNNNNNNNNNNNNNNNNNNNNNNNNNNNNNNNNNNNNNNNNNNNNNNNNNNNNNNNNNNNNNNNNNN
NNNNNNNNNNNNNNNNNNNNNNNNNNNNNNNNNNNNNNNNNNNNNNNNNNNNNNNNNNNNNNNNNNNNNN
NNNNNNNNNNNNNNNNNNNNNNNNNNNNNNNNNNNNNNNNNNNNNNNNNNNNNNNNNNNNNNNNNNNNNN
NNNNNNNNNNNNNNNNNNNNNNNNNNNNNNNNNNNNNNNNNNNNNNNNNNNNNNTCTNNNAAANNNTNNN
NNNNNNNNNNNNNNNNNNNNNNNNNNNCNCNTTNNNNGTAANCNANNNGNACATGCAGTCCCCTCNNNNN
NNNGGCAGAGNNNNNNNCNNNNNNNNCCGNCCCCNNCCNNNNNNNNNNNNNNNNGNNNNNNNANNNNNN
NNNNNNTGGTACNNNNCGNNNNNGGTTGNTNAAGAGGTCCACAACANGGTCNNGNCACNNNNGNNNAN
NNNCNNNNAACNNNNNNNNNNNNNNNTNNNTANNAGGNNNNNNCCNTCNNNNNNNNNANTGCNATGACC
CNANNCACGGNGGNAGTNCCCGGCGNGGCGACCTTGNNNANNTCCGGGGACAAGTATGGCGNNCNCCC
CGGGAAGGANNCNGCCGTANGTGCNNNNNANNNNNTCNTGNGANNTNGCCGNNATTNNCGTGGTCNNN
CNAGAATGNCCGNACCANNCGNANNNTGGTNATGNNAGTNNNNNNNNNNNNNNNNNNNNNNNNNNNNNNN
NNNNNNNNNNNNNNNNNNNNNNNNNNNNNNNNNNGNNNNGNNNNNNCNANNNAAGGANNTANNNCTNNTN
NTNNNNNNNGGACNNNACNNNNCNNNNNNNACNCNNNCNCNNNNNNANNNGNTTTTTNNCCNNTATTNT
CCAGNACGCNGNGCNNNCATTCANGNGAACNNNGNNACGCGNNAATCGTANNATCTACCTGCCGCNNG
NNNNNNTACNGNNNNNNCGGANNNTNNCTCGTANNNNNNTCATGCGTNNTNTNNTCNCNAGNCANGTT
CGNNNANANANTAGNANGTNTNNNNNNNNNNTGTNNCGTTCCGCNCGANNCNNCANNNNGNNGNNCTTN
NCANCGGCNNTANCTGCNGANNTAAGTCATANNNTNNNTCNNTANCTNNNNNGGNGGGNNACTGTAAA
NGGNNAATTNGNNNNTNATTNCNNNCACNTNNCNNNANNNNCTNNNTNCCTGNNTNCNNAATTGGNNC

CANTGNNTGNCNATCTCTGGNTTNGNNNNNNNTNNNNNTNNCANNNNNGGNNNGNNNNANNNCNATNTAA
NNTANANNNNCNNNNNNANNNCACNANNNNNNNNAGNNANNANNNTTANANNNGTAGNNNAATAAGTCT
CCGANNTNNNNNNNATAAGCGNNANACNNNNNNNNNNNNNNANGANNACGNNTTGGNNNTNNNNNNCNA
GNNAGACNNTNGTGGNTNNNNNTCAANNNNTTCGTAGTGGTATAATNGNNNNNNNTTTCNCCAGNNTN
NNNGNNNNNNNNCNATANNANNNNNNNGNGANNNNNNNNNNNANNNNCTTNNNAGGGANCANTTACTTNA
GCANTGGNGCGCAGNNGGAGANNNNGACNNGCCTTGGGTGGCAANCACNGCAATAAGTNNNGTTNANC
CTNNNAGTNNACACGNNNNCANNGNNNCNNNNNNANCNGANNGNNNNNNNNNNNNNNNNNNNNNNNNNNN
NNNNNNNNNNNNNNNNNNNNNNNNNNNNNNNGNNNANNGNNNGTNTNTGACNCATGTGTTTTTANNNGNN
NNNNNNNGNNNGNNCCCCGGTCGNNNNCNNATTGAANNCCTGNNGCATNNNNNTNCNAGAATGANNGC
NNNNNGNNTNGNCTGNNNNNTCANGNAATCANNNAGGTANNNCGNTCNNNGNNNNNNNNCNNTCCCNN
ACTGAGGGTNNNNNNNNCATCTGCTNNNNNNNNNTNCGCNGGNGTNNCNNNNNNGCNGTATNGTGTNN
NNCANNNNNNNNNNNNNNNNNNNNANNNANNNNNNNTNNNN
>LaChi543
GTGNNNNNTAGCCCNNNNNAGTTAGACANNNCNNNNNNNNNNNNNTNNNNNNNNNNNNNNNNNNNNNNNNNN
NNNNNNNNNNNNNNNNNNNNNNNNNNNNNNNNNNNNNNNNNNNNNNNNNNNNNNNNNNNNNNNNNNNNNN
NNNNNNNNNNNNNNNNNNNNNNNNNNNNNNNNNNNNNNNNNNNNNNNNNNNNNNNNNNNNNNNNNNNNNN
NNNNNNNNNNNNNNNNNNNNNNNNNNNNNNNNNNNNNNNNNNNNNNNNNNNNNNNNNNNNNNNNNNNNNN
NNNNNNNNNNNNNNNNNNNNNNNNNNNNNNNNNNNNNNNNNNNNNNNNNNNNNNTCTNNNAAANNGTNNN
NNNNNNNNNNNNNNNNNNNNNNNNNNNCNCNTTGNNNGTAANCNANNNGNACATGCAGTCCCCTCNNNNN
NNNGGCAGAGNNNNNNCCCNNNNNNNNCCGGCCCCNNCCNNNNNNNNNNNNNNNNNNNNNNNNANNNNNN
NNNNNNTGGTACNNNNCGNNNNNNGGTTGTNTNAGAGGTCCACAACGNGNTCNNGNCACNNNNNGNNNAN
NNNCNNNNAACNNNNNNNNNNNNNANNTNNNTANNAGGNCNNNNCCNTCNNGNNNNNNANTGCNATGACC
CAANTNACGGNGGNNGTNCCCGGCGCGGCGACCTTGNNNACGTCCGGGGACAAGTATGGCNNNCNCCC
CGGGAAGGANNCNGCNGTANGTGCNNNNNANNNNNTCNTGNGANGTNGCCGNNATTNNCGTGGTCNNN
CNAGAATGCCCNNACCANNCGNANNNTGGTNATGNNAGTNNNNNNNNNNNNNNNNNNNNNNNNNNNNN
NNNNNNNNNNNNNNNNNNNNNNNNNNNNNNNNNNNNNNGNNNNNNCNANNNAAGGANNTANNNCTNNTC
NNNNNNNNGGACNNNACNNNNCNNNNNNNACNCNNNCNCNNNNNNANNNGGTTTTTNNNCNNTATTNT
CCAGNACGCNGNGCNNNCATTCANGNGAACNNNGNNACGCGNNAATCGTANNATCTACCTGCCGCNNG
TNNNNNTACNGNNNNTNCGGANNNTNNCTCGTANNNNNNTCATGCGTNNNNTNNTCNCNAGNCANGTT
CGNNNANANANTAGNANGTNTNNNNNNNCTTGTNNCGTTCCGCNTGANNCNNCANNNNGNNGNNCTTN
NCANCGGCNNTANCTNCNGANNTAAGNCATANNNTNNNTCNNTANCTNNNNNGGNGGGNNACTGTAAA
NGGNNAATTNGNNNNTNATTNCNNNCACNTNNCNNNGNNTNCTNNNTNCCTGNNTNCNNAATTGGNNC
CANTGNNTGNCNATCTCTNGNTTNGNNNNNNNTNNNNNTNGCANNNNGGNNNNNNANANNNCNATNTAA
NNTATANNNNCNNNNNNANNNCACNNNNNNNNNNAGNNNCNANNNTTANANNNGTAGNNNANTAAGTCT
CCGANNTNNNNNNNATAAGCGNNANACNNNNNNNNNNNNNNANGANNACGNNTTGGNNNTNNNNNNCNA
GNNAGACNNTNATGGNTNNNNNTCAANNNNTTNGTAGTGGTATAATNGNNNNNNNTTTCNCCAGNNTN
NNNGNNNNNNNNCNATANNANNNNNNNGNGANNNNNNNNNNNANNNNCTCNNNAGGGANCANCTACTTNA
GCAATGGNGCGCAGNNNGAGANNNNGACNNNCCTTGGGTGGCAANNACNGNGATAAGTNNNGTTNANC
CTNNNAGTNNACATNNNNNCANNGNNNCNNNNNNANCNGANNGNNNNNNNNNNNNNNNNNNNNNNNNNNN
NNNNNNNNNNNNNNNNNNNNNNNNNNNNNNNGNNNANNGNNNGTNTNTGACNCATGTGTTTTTANNNGNN
NNNNNACGNNNNANCCCCGGTCGNNNNCNNATTGAANNCCTGNNGCATNNNNNTNCNAGAATGANNGC
NNNNNGNNTNGNCTNNCNTTTCAAGNAATCANNNAGGNNNNNNGNTCNNNGNNNNNNNNCNNTCCCNN
ACTGAGGGTNNNNNNNNCATCTGCTNNNNNNNNNTNCGCNGGNNTNNCNNNNNNGCNGTATNGTGTNA
NNCANNNNNNNNNNNNNNNNNTNNANNNNNNNNNNNNTNNNN
>LaChi544
GTGNNNNNTAGCCCNNNNNAGTTAGACANNNCNNNNNNNNNNNNNTNNNNNNNNNNNNNNNNNNNNNNNN
NNNNNNNNNNNNNNNNNNNNNNNNNNNNNNNTNNNNNNNNNNNNNNNNNNNNNNNNNNNNNNNNNNNNNN
NNNNNNNNNNNNNNNNNNNNNNNNNNNNNNNNNNNNNNNNNNNNNNNNNNNNNNNNNNNNNNNNNNNNNN
NNNNNNNNNNNNNNNNNNNNNNNNNNNNNNNNNNNNNNNNNNNNNNNNNNNNNNNNNNNNNNNNNNNNNN
NNNNNNNNNNNNNNNNNNNNNNNNNNNNNNNNNNNNNNNNNNNNNNNNNNNCNTCTNNNAAANNNTNNN
NNNNNNNNNNNNNNNNNNNNNNNNNNNCNCNTTNNNNGTAANCNANNNGNACATGCAGTCCCCTCNNNNN
NNNGGCAGAGNNNNNNNCCNNNNNNNNCCGNCCCCGNCCNNNNNNNNNNNNNNNNNNNNNNNNANNNNNN
NNNNNNTGGTACNNNNCGNNNNNGGTTGTNTAAGAGGTCCACAACANGGTCNNGNCACNNNNGNNNAN
NNNCNNNNNACNNNNNNNNNNNNNNNTNNNTANNAGGNCNNNNCCNTNNNGNNNNNNATTGCNATGACC

CNANNCACGGNGGNAGTNCCCGGCGCGGCGACCTTGATNACGTCCGGGGNCAAGTATGGCNCNCNCCC
CGGGAAGGANNCNGCCGTANGTGCNNNNANNNNNTCNTGNGANGTNGCCGNNATNNNCGTGGTCNNNN
NTAGAATGNCCGNACCANNCGNANNNTGGTNATGNNAGTNANNNNNNNNNNNNNNNNNNNNNNNNNNN
NNNNNNNNNNNNNNNNNNNNNNNNNNNNNNNNNNNNNGNNNNNNCNANNNAAGGANNTANNNCTNNTN
NTNNNNNNGGACNNNACNNNNCNNNNNNNACNCNNNCNCNNNNNNANNNGGTTTTTNNCCNNTATTNT
CCAGNACGCNGNGCNNNCATTCANGNGAACNNNGNNACGCGNNAATCGTANNATCTACCTGCCGCNNG
NNNNNNTACNGNNNNNNCGGANNNTNNCTCGTANNNNNNTCATGCGTNNTNTNNTCNCNAGNCANGTT
CGNNNANANANTAGNANGTNTCNNNNNNNNTGTNNCGTTCCGCNCGACNCNNCANNNNGNNGNNCTTN
NCANCGGCNNTANCTGCNGANNTAAGNCATANNNTNNNTCNNTANCTNNNNNGGNGGGNNACTGTAAA
NGGNNAATTGGNNNNTNANTNCNNNCACNTNNCTNNANNNNCTNNNTNCCTGNNTNCNNAATTGGNNC
CANTGNNTGNCNATCTCTGGNTTNGNNNNNNTNNNNNTNNCAANNNGGNNNGTNANANNNCNATNTAA
NNTANANNNNCNNNNNNANNNCACNNNNNNNGNAGNNNNNANNNTTANANNNGTAGNNNANTAAGTCT
CCGANGTNNNNNNNATAAGCGNNANACNNNNNNNNNNNNNANGANNACGNNTTGGNNNTNNNNNNCNA
GNNAGACNNTNGTGGNTNNNNNTCAANNNNTTCGTAGTGGTATAATNGNNNNNNNTTTCNCCAGNNTN
NNNGNNNNNNCNATANNANNNNNNNGNGANNNNNNNNNNNANNNNCTTNNNAGGGANCANTTACTNNA
GCANTGGNGCGCAGNNNGAGANNNNGACNAGCCTTGGGTGGCAANCACNGCAATAAGTNNNGTTNANC
CTNNNAGTNNACACNNNNNCANNGNNNCNNNNNNANCNGANNGNNNNNNNNNNNNNNNNNNNNNNNNN
NNNNNNNNNNNNNNNNNNNNNNNNNNNNNGNNNANNGNNNGTNTNTGACNCATGTGTTTTTANNNGNN
NNNNNACGNNNGANCCCCGGTCGNNNNCNNATTGAANNCCTGNNGCATNNNNNTNCNAGAATGANNGC
NNNNNGNNTNGNCTGNCNNNTCAAGNAATCANNNANNTNNCGCGNTCNNNGANNNNNNNCNNTCCCNN
ACTGAGGGTNNNNNNNGCATCTGCTNNNNNNNNCTNCGCNGGCGTNNCNNNNNNGCNGTATNNTGTTN
NNCANNNNNNNNNNNNNNNNNNNNNNNNNNNNNNTNNNN
>LaChi545
GTGNNNNNNAGCCCNNNNNAGTTAGACANNNCNNNNNNNNNNNNTNNNNNNNNNNNNNNNNNNNNNNN
NNNNNNNNNNNNNNNNNNNNNNNNNNNNNNNNNNNNNNNNNNNNNNNNNNNNNNNNNNNNNNNNNNNN
NNNNNNNNNNNNNNNNNNNNNNNNNNNNNNNNNNNNNNNNNNNNNNNNNNNNNNNNNNNNNNNNNNNN
NNNNNNNNNNNNNNNNNNNNNNNNNNNNNNNNNNNNNNNNNNNNNNNNNNNNNNNNNNNNNNNNNNNN
NNNNNNNNNNNNNNNNNNNNNNNNNNNNNNNNNNNNNNNNNNNNNNNNNNNNNNNNNNNNNNNNNNNN
NNNNNNNNNNNNNNNNNNNNNNNNNNNNNNNNNNNNNNNNNNNNNNNNTCTNNNAAANNNTNNN
NNNNNNNNNNNNNNNNNNNNNNNNNCNCNTTGNNNGTAANCNANNNGNACATGCAGTCCCCTCNNNNN
NNNNNCAGAGNNNNNNNNCNNNNNNNCCGGCCCCNNCCNNNNNNNNNNNCNNNNNNNNNNNANNNNNN
NNNNNNTGGTACNNNNCGNNNNNGGTTGTNTNAGAGGTCCACAACANGGTCNNGNCACNNNNGNNNAN
NNNCNNNNAACNNNNNNNNNNNANATNNNTANNAGGNCNNNNCCNTCNNGNNNNNNATNGCNATGANN
CAANTNNCNGGGGCNGTNCCCGGCGCGGCGACCTTGANNACGTCCGGGGACAAGTATGGCNNNCNCCC
CGGGAAGGANNCNGCCGTANGTGCNNNNANNNNNTCNTGNGANGTCGCCGNNATTNNCGTGGTCNNNN
CNAGAATGNCCNNACCANNCGNANNNTGGTNATGTNAGTNNNNNNNNNNNNNNNNNNNNNNNNNNNNN
NNNNNNNNNNNNNNNNNNNNNNNNNNNNNNNNGNNNNGNNNNNNCNANNNAAGGNNNTANNNCTNNTN
NTNNNNNNGGACNNNACNNNNCNNNNNNNACNCNNNCNCNNNNNNANNNGGTTTTNNNNCNNTATTNT
CCAGNANGCNGNGCNNNCATTCANGNGAACNNNGNNACGCGNNAATCGTANNATCTACCTGCCGCNNG
NNNNNNTACNGNNNNNNCGGANNNTNNCNCGTANNNNNNNCANGCGTNNTNTNNTCNCNAGNCNNGTT
CGNNNANANANTAGNANGTNTNNNNNNNNNTGTNNCGTTCCGCNCGANNCNNCANNNNGNNGNNCTTN
NCANCGGCNNTANCTNCNGANNTAAGNCATANNNTNNNTCNNTANCTNNNNNGGNGGGNNACTGTAAA
NGGNNAATTNGNNGNTNATTNCNNNCACNTNNCTNNANNNNCTNNNTNCCTGNNTNCNNAATTGGNNC
CANTGNNTGNCNATCTCTGGNTTNGNNNNNNTNNNNNTNNCAANNNGGNNNNNNNNANNNCNATNTAA
NNTANANNNNCNNNNNNANNNCACNANNNNNGNAGNNNCNANNNTTANANNNGTAGNNNANTAAGTCT
CCGANNTNNNNNNNATAAGCGNNANACNNNNNNNNNNNNNANGANNACGNNTTGGNNNTNNNNNNCNA
GNNAGACNNTNGTGGNTNNNNNTCAANNNNTTCGTAGTGGTATAATNGNNNNNNNTTTCNCCAGNNTN
NNNGNNNNNNCNATANNANNNNNNNGNGANNNNNNNNNNNANNNNCTTNNNAGGGANCANTTACTNNA
GCANTGGNGCGNAGNNNGAGANNNNGACNNNCCTTGGGTGGCAANNACNGCAATAAGTNNNGTTNNNC
CTNNNAGTNNACACNNNNNCANNGNNNCNNNNNNANCNGANNGNNNNNNNNNNNNNNNNNNNNNNNNN
NNNNNNNNNNNNNNNNNNNNNNNNNNNNAGNNNANNGNNNGTNTNTGACNCATGTGTTTTTANNNGNN
NNNNNACGNNNGANCCCCGGTCGNNNNCNNATTGAANNCCTGNNGCATNNNNNTNCNAGAATGANNGC
NNNNNGNNTNGNCTNNNNNNTCAAGNAATCANNNAGNNANNNNNNTCNNNNNNNNNNNNCNNTCCCNN
ACTGAGGGTNNNNNNNNCATCNGCTNNNNNNNNCTNCGCNGGNGTNNCNNNNNAGCNGTATNNNGTNN
NNCANNNNNNNNNNNNNNNNTNAAACAANNNNNCTNNNN

>LaChi546
GTGNNNNNTAGCCCNNNNNAGTTAGACANNNCNNNNNNNNNNNNTNNNNNNNNNNNNNNNNNNNNNNN
NNNNNNNNNNNNNNNNNNNNNNNNNNNNNNNNNNNNNNNNNNNNNNNNNNNNNNNNNNNNNNNNNNNN
NNNNNNNNNNNNNNNNNNNNNNNNNNNNNNNNNNNNNNNNNNNNNNNNNNNNNNNNNNNNNNNNNNNN
NNNNNNNNNNNNNNNNNNNNNNNNNNNNNNNNNNNNNNNNNNNNNNNNNNNNNNNNNNNNNNNNNNNN
NNNNNNNNNNNNNNNNNNNNNNNNNNNNNNNNNNNNNNNNNNNNNNNNNNCNTCTNNNAAANNNTNNN
NNNNNNNNNNNNNNNNNNNNNNNNNCNCNTTGNNNGTAANCNANNNGNACATGCAGTCCCCTCNNNNN
NNNGGCAGAGNNNNNNNCCNNNNNNNCCGGCCCCNNCCNNNNNNNNNNNCNNNGNNNNNNANNNNNNN
NNNNNNTGGTACNNNNCGNNNNNGGTTGTNTNAGAGGTCCACAACANGGTCNGGNCANNNNNGNNNAN
NNNCNNNNNNCNNNNNNNNNNNNNNTNNNTANNAGGNCNNNNCCNTCNNNNNNNNNANTGCNATGACC
CAANNCACGGGGGCAGTCCCCGGCGCGGCNACCTTGNNNACGTCCGGGGACAAGTATGGCNNNCNCCC
CGGGAAGGANNCNGCCGTANGTGCNNNNNANNNNNTCTTGNGANGTCGCCGNNNTTNNCGTGGTCNNN
CNAGAATGNCCGNACCANNCGNANNNTGGTNATGTNAGTNNNNNNNNNNNNNNNNNNNNNNNNNNNNN
NNNNNNNNNNNNNNNNNNNNNNNNNNNNNNNNNNNNNGNNNNNCCNANNNAAGGANNTANNNCTNNTN
NTNNNNNNGGACNNNACNNNNCNNNNNNNACNCNNNCNCNNNNNNANNNGGTTTTTNNNCNNTATTNT
CCAGNANGCNGNGCNNNCATTCANGNGAACNNNGNNACGCGNNAATCGTANNATCTACCTGCCGCNNG
NNNNNNTACNGNNNNNNCGGANNNTNNCTCGTANNNNNNTCATGCGTNNTNTNNTCNCNAGNCNNGTT
CGNNNANANANTAGNANGTNTNNNNNNNNNTGTNNCGTTCCGCNCGANNCNNCANNNNGNNGNNCTTN
NCANCGGCNNTANCTGCNGANNTAAGNCATANNNTNNNTCNNTANCTNNNNNGGNGGGNNACTGTAAA
NGGNNAATTNGNNNNTNATTNCNNNNACNTNNCTNNANNTNCTNNNTNCCTGNNTNCNNAATTGGNNC
CANTGNNTGNCNATCTCTNGNTTNGNNNNNNTGNNNNTNNCAANNNGGNNNNTNANANNNCNATNTAA
NNTANANNNNCNNNNNNANNNCACNANNNNNGNAGNNNCNANNNTNANANNNGTAGNNNANTAAGTCT
CCGANNTNNNNNNNATAAGCGNNANACNNNNNNNNNNNNNANGANNACGNNTTGGNNNTNNNNNNCNA
GNNAGACNNTNGTGGNTNNNNNTCAANNNNTTCGTAGTGGTATAATNGNNNNNNNTTTCNCCAGNNTN
NNNGNNNNNNNCNATANNANNNNNNNGNGANNNNNNNNNNANNNNCTTNNNAGGGANCANTTACTNNA
GCAATGGNGCGCAGNNNGAGANNNNGACCAGCCTTGGGTGGCAANCACNGCAATAAGTNNNGTTNANC
CTNNNAGTNNACACNNNNNCANNGNNNCNNNNNNANCNGANNGNNNNNNNNNNNNNNNNNNNNNNNNN
NNNNNNNNNNNNNNNNNNNNNNNNNNNNAGNNNANGGNNNGTNTNTGACNCATGTGTTTTTANNNGNN
NNNNNACGNNNNANCCCCGGTCGNNNNCNNATTGAANNCCTGNNGCATNNNNNTNCNAGAATGANNGC
NNNNNGNNTNGCTNNCNTTTCAAGNAATCANNNAGNNNNNGCGNTCNNGNNNNNNNNNNCNNTCCCNN
ACTGAGGGTNNNNNNNNCATCTGCTNNNNNNNNNTTCGCNGGNNNNNCNNNNNAGCNGTATNGTGTNN
NNCANNNNNNNNNNNNNNNTNNAACNNNNNNNNNTNNNN
>LaChi547
GTGNNNNNTAGCCCNNNNNAGTCAGACANNNCNNNNNNNNNNNNNNNNNNNNNNNNNNNNNNNNNNNN
NNNNNNNNNNNNNNNNNNNNNNNNNNNNNNTNNNNNNNNNNNNNNNNNNNNNNNNNNNNNNNNNNNNN
NNNNNNNNNNNNNNNNNNNNNNNNNNNNNNNNNNNNNNNNNNNNNNNNNNNNNNNNNNNNNNNNNNNN
NNNNNNNNNNNNNNNNNNNNNNNNNNNNNNNNNNNNNNNNNNNNNNNNNNNNNNNNNNNNNNNNNNNN
NNNNNNNNNNNNNNNNNNNNNNNNNNNNNNNNNNNNNNNNNNNNNNNNNNNNTCTNNNAAANNNTNNN
NNNNNNNNNNNNNNNNNNNNNNNNNCNCNTTGNNNGTAANCNANNNGNACATGCAGTCCCCTCNNNNN
NNNGGCAGAGNNNNNNNCCNNNNNNNCCGNCCCCNNCCNNNNNNNNNNNNNNNGNNNNNNANNNNNNN
NNNNNNTGGTACNNNNCGNNNNNGGTTGNNTAAGAGGTCCACAACGNGGTCNGNNNACNNNNGNNNAN
NNNCNNNNNNCNNNNNNNNNNNNNNATNNNTANNAGGNCNNNNCCNTCNNGNNNNNNANNGCNATGACC
CAANNCACGGNGGCNGTNCCCGGNGNGGCGACCTTGATNANGTCCGGGGNCAAGTATGGCNNNCNCCC
CGNGAAGGANNCNGCNGTANGTGCNNNNNANNNNNTCNTGNGANGTCGCCGNNATTCNCGTGGTCNNN
CNAGAATGNCCNNACCANNCGNANNNTGGTNATGNNAGTNNNNNNNNNNNNNNNNNNNNNNNNNNNNN
NNNNNNNNNNNNNNNNNNNNNNNNNNNNNNNNGNNNNGNNNNNNCNANNNAAGGNNNTANNNCTNNTN
NNNNNNNNGGACNNNACNNNNCNNNNNNNACNCNNNCNCNNNNNNNNNNGNTTTTNNNNCNNTATTNT
CCAGNACGCNGNGCNNNCATTCANGNGAACNNNGNNACGCGNNAATCGTANNATCTACCTGCCGCNNG
NNNNNNTACNGNNNNNNCGGANNNTNNCTCGTANNNNNNNCATGCGTNNTNTNNTCNCNAGNCANGTT
CGNNNANANANTAGNANGTNTNNNNNNNNNTGTNNCGTTCCGCNTGANNCNNCANNNNGNNGNNNTTN
NCANCGGCNNTANCTGCNGANNTAAGNCATANNNTNNATCNNTANCTNNNNNGGNGGGNNACTGTAAA
NGGNNAATTNGANNNTNANTNCCNNCATNTNNCNNNGNNTNCTNNNTNCCTGNNTNCNNAATTGGNNC
CANTGNNTGNCNATCTCTGGNTTNGCNNNNNTNNNNNTNNCANNNNGGNNNNNNNNANNNCNATNTAA
NNTANANNNNCNNNNNNANNNCACNNNNNNNNNAGNNNCNANNNGTANANNNGTAGNNNGNTAAGTCT

CCGANNTNNNNNNNATAAGCGNNANACNNNNNNNNNNNNNNANGANNACGNNTTTGNNNTNNNNNNCNA
GNNAGACNNTNGTGGNTNNNNNTCAANNNNTTNGTAGTGGTATAATNGNNNNNNNTTTCNCCAGNNTN
NNNGNNNNNNNNCNATANNANNNNNNNGNGANNNNNNNNNNNANNNNCTCNNNAGGNANCANCTACTNNA
NCANTGGNGCGCAGNNNGAGANNNNGACNNNCCTTGGGTGGCAANCACNGCAATAAGTNNNGTTNANC
CTNNNAGTNNACACNNNNNCANNGNNNCNNNNNNANCNGANNGNNNNNNNNNNNNNNNNNNNNNNNNNN
NNNNNNNNNNNNNNNNNNNNNNNNNNNNNNNNNNNNNANNGNNNGTNTNTGACNCATGNNTTTTTANNNGNN
NNNNNACGNNNNANCCCCGGTCGNNNNCNNATTGAANNCCTGNNGCATNNNNNTNCNAGAATGANNGC
NNNNNGNNTNGNCTNNCNNNTCAAGNAATCANNNAGNTNNNNNNNTCNNNGNNNNNNNNCNNTNNCNN
ACTGAGGGTNNNNNNNNCATCTGCTNNNNNNNNNNTNCGCNGGCGTNNCNNNNNAGCNGTATNGTGTNA
NNCANNNNNNNNNNNNNNNNNNTNAANCNANNNNNNNTNNNN
>LaChi548
GTGNNNNNTAGCCCNNNNNAGTTAGACANNNCNNNNNNNNNNNNNTNNNNNNNNNNNNNNNANNNNNNNN
NNNNNNNNNNNNNNNNNNNNNNNNNNNNNNNNTNNNNNNNNNNNNNNNNNNNNNNNNNNNNNNNNNNNNN
NNNNNNNNNNNNNNNNNNNNNNNNNNNNNNNNNNNNNNNNNNNNNNNNNNNNNNNNNNNNNNNNNNNNNN
NNNNNNNNNNNNNNNNNNNNNNNNNNNNNNNNNNNNNNNNNNNNNNNNNNNNNNNNNNNNNNNNNNNNNN
NNNNNNNNNNNNNNNNNNNNNNNNNNNNNNNNNNNNNNNNNNNNNNNNNNNNNNNCNTCTNNNAAANNNTNNN
NNNNNNNNNNNNNNNNNNNNNNNNNNNCNCNTTGNNNGTAANCNANNNGNACATGCAGTCCCCTCNNNNN
NNNGGCAGAGNNNNNNCCCNNNNNNNCCGGCCCCGNCCNNNNNNNNNNNCNNNGNNNNNNNANNNNNN
NNNNNNTGGTACNNNNCGNNNNNGGTTGTNTAAGAGGTCCACAACGNGGTCNNGNCACNNNNGNNNAN
NNNCNNAAAACNNNNNNNNNNNAANNTNNNTANNAGGNCNNNNCCNTCNNGNNNNNAANTGCNATGACC
CAANNCACGGGGGCNGTCCCCGGCGCGGCGACCTTGANNANGTCCGGGGACAAGTATGGCNNNCNCCC
CGGGAAGGANNCNGCCGTANGTGCNNNNNANNNNNTCNTGNGANGTCGCCGNNATTCNCGTGGTCNNN
CNAGAATGNCCNNACCANNCGNANNNTGGTNATGNNAGTNNNNNNNNNNNNNNNNNNNNNNNNNNNNNN
NNNNNNNNNNNNNNNNNNNNNNNNNNNNNNNNNNNNNNNNNNGNNNNNNCNANNNAAGGANNTANNNCTNNTC
NTNNNNNNNGGACNNNACNNNNCNNNNNNNACNCNNNCNCNNNNNNANNNGNTTTTTNNNCNNTATTNT
CCAGNACGCNGNGCNNNCATTCANGNGAACNNNGNNACGCGNNAATCGTANNATCTACCTGCCGCNGG
NNNNNNTACNGNNNNTNCGGANNNTNNCTCGTANNNNNNNCATGCGTNNTNTNNTCNCNAGNCANGTT
CGNNNANANANTAGNANGTNTNNNNNNNCTTGTNNCGTTCCGCNTGANNCNNCANNNNGNNGNNCTTN
NCANCGGCNNTANCTGCNGANNTAAGNCATANNNTNNATCNNTANCTNNNNNGGNGGGNNACTGTAAA
NGGNNAATTNGANGNTNATTTCNNNCACNTNNCTNNGNNTNCTNNNTNCCTGNNTNCNNAATTGGNNC
CANTGNNTGNCNATCTCTGGNTTNGNNNNNNTGNNNNTNNCANNNNGGNNNGTNNNANNNCNATNTAA
NNTANANNNNCNNNNNNANNNCACNANNNNNNNAGNNNCNANNNTTANANNNGTAGNNNAATAAGTCT
CCGANNTNNNNNNNATAAGCGNNANACNNNNNNNNNNNNNNANGANNACGNNTTGGNNNTNNNNNNCNA
GNNAGACNNTNATGGNTNNNNNTCAANNNNTTCGTAGTGGTATAATNGNNNNNNNTTTCNCCAGNNTN
NNNGNNNNNNNNCNATANNANNNNNNNGNGANNNNNNNNNNNANNNNCTCNNNAGGGANCANCTACTTNA
GCANTGGNGCGCAGNNNGAGANNNNGACNNNCCTTGGGTGGCAANCACNGCGATAAGTNNNGTTNANC
CTNNNAGTNNACATNNNNNCANNGNNNCNNNNNNANCNGANNGNNNNNNNNNNNNNNNNNNNNNNNNNN
NNNNNNNNNNNNNNNNNNNNNNNNNNNNNNNNNAGNNNANGGNNNGTNTNTGACNCATGTGTTTTTANNNGNN
NNNNNACGNNNNANCCCCGGTCGTNTNCNNATTGAANNCCTGNNGCATNNNNNTNCNAGAATGANNGC
NNNNNGNNTNGNCTGNCNTTTCAAGNAATCANNNAGGNANNNCGNTCNNNGNNNNNNNNCNNTCCCNN
ACTGAGGGTNNNNNNNNCATCTGCTNNNNNNNNNNTTCGCNGGCGTNNCNNNNNNGCNGTATNGTGTNA
NNCANNNNNNNNNNNNNNNNNNNNNNNAACNNNNNNNNNTNNNN
>LaChi549
GTGNNNNNTAGCCCNNNNNAGTTAGACANNNCNNNNNNNNNNNNNTNNNNNNNNNNNNNNNNNNNNNNNN
NNNNNNNNNNNNNNNNNNNNNNNNNNNNNNNNNNNNNNNNNNNNNNNNNNNNNNNNNNNNNNNNNNNNNN
NNNNNNNNNNNNNNNNNNNNNNNNNNNNNNNNNNNNNNNNNNNNNNNNNNNNNNNNNNNNNNNNNNNNNN
NNNNNNNNNNNNNNNNNNNNNNNNNNNNNNNNNNNNNNNNNNNNNNNNNNNNNNNNNNNNNNNNNNNNNN
NNNNNNNNNNNNNNNNNNNNNNNNNNNNNNNNNNNNNNNNNNNNNNNNNNNNNNNCNTCTNNNAAANNNTNNN
NNNNNNNNNNNNNNNNNNNNNNNNNNNCNCNNTGNNNGTAANCNANNNGNACATGCAGTCCCCTCNNNNN
NNNGGCAGAGNNNNNNNCCNNNNNNNCCGNCCCCNNCCNNNNNNNNNNNNNNNNNNNNNNNNNNNANNNNNN
NNNNNNTGGTACNNNNCGNNNNNGGTTGNNTAAGAGGTCCACAACANGNTCNNGNNACNNNNGNNNAN
NNNCNNNNAACNNNNNNNNNNNNNANNTNNNTANNAGGNNNNNNCCNTNNNGNNNNNNATNGCNATGACC
CAANNCACGGGGGNNGTNCCCGGNGCGGNGACCTTGANNACNTCCGGGGNCAAGTATGGCGNNCNCCC
CGGGAAGGANNCNGCNGTANGTGCNNNNNANNNNNTCNTGNGANGTCGCCGNNATTNNCGTGGTCNNN

CNAGAATGNCCGNACCANNCGNANNNTGGTNATGNNAGTNNNNNNNNNNNNNNNNNNNNNNNNNNNNN
NNNNNNNNNNNNNNNNNNNNNNNNNNNNNNNNNNNNNGNNNNNNCNANNNAAGGANNTANNNCTNNTN
NNNNNNNNGGACNNNACNNNNCNNNNNNNACNCNNNCNCNNNNNNANNNGGTTTTTNNNCNNTATTNT
CCAGNANGCNGNGCNNNCNTTCANGNGAACNNNGNNACGCGNNAATCGTANNATCTACCTGCCGCNNG
NNNNNNTACNGNNNNNNCGGANNNTNNNNCGTANNNNNNTCATGCGTNNTNTNNTCNCNAGNCNNGTT
CGNNNANANANTAGNANGTNTNNNNNNNNNTGTNNCGTTCCGCNCGANNCNNCANNNNGNNGNNNTTN
NCANCGGCNNTANCTGCNGANNTAAGNCATANNNTNNNTCNNTANCTNNNNNGGNGGGNNACTGTAAA
NGNNNAATTGGNNNNTNANTNCNNNCACNTNNCNNNANNNNCTNNNTNCCTGNNTNCNNAATTGGNNN
CANTGNNTGNCNATCTCTNGNTTNGNNNNNNTNNNNNTNNCANNNNGGNNNGTNNNANNNCNATNTAA
NNTANANNNNCNNNNNNANNNCACNNNNNNNGNAGNNNCNANNNTTANANNNGTAGNNNANTAAGTCT
CCGANNTNNNNNNNATAAGCGNNANACNNNNNNNNNNNNNANGANNNCGNNTTGGNNNTNNNNNNNNA
GNNAGACNNTNGTGGNTNNNNNTCAANNNNTTCGTAGTGGTATAATNGNNNNNNNTTTCNCCAGNNNN
NNNGNNNNNNNCNATANNANNNNNNNGNGANNNNNNNNNNANNNNCTTNNNAGGNANCANTTACTNNA
GCANTGGNGCGNAGNNNGAGANNNNGACNNGCCTTGGGTGGCAANNACNGCAATAAGTNNNGTTNNNN
CTNNNAGTNNACACNNNNNCANNGNNNCNNNNNNANCNGANNGNNNNNNNNNNNNNNNNNNNNNNNNN
NNNNNNNNNNNNNNNNNNNNNNNNNNNNNGNNNANNGNNNGTNTNTGACNCATGNNTTTTTANNNGNN
NNNNNNNGNNNGANCCCCGGTCGNNNNCNNATTGAANNCCTGNNGCATNNNNNTNCNAGAATGANNNC
NNNNNGNNTNGCTNNCNTTTCAAGNAATNAANNAGNNNNNNNNNTCNNNGNNNNNNNNNCNNTNNCNN
ACTGAGGGTNNNNNNNNCATCTGCTNNNNNNNNNTNCGCNGGCGTNNCNNNNNNGCNGTATNGTGTNA
NNCANNNNNNNNNNNNNNNNTNNAACNANNNNNNTNNNN
>LaChi550
GTGNNNNNTAGCCCNNNNNAGTTAGACANNNCNNNNNNNNNNNNTNNNNNNNNNNNNNNNNNNNNNNN
NNNNNNNNNNNNNNNNNNNNNNNNNNNNNNNNNNNNNNNNNNNNNNNNNNNNNNNNNNNNNNNNNNNN
NNNNNNNNNNNNNNNNNNNNNNNNNNNNNNNNNNNNNNNNNNNNNNNNNNNNNNNNNNNNNNNNNNNN
NNNNNNNNNNNNNNNNNNNNNNNNNNNNNNNNNNNNNNNNNNNNNNNNNNNNNNNNNNNNNNNNNNNN
NNNNNNNNNNNNNNNNNNNNNNNNNNNNNNNNNNNNNNNNNNNNNNNNNNCNTCTNNNAAANNNTNNN
NNNNNNNNNNNNNNNNNNNNNNNNNCNCNTTGNNNGTAANCNANNNGNACATGCAGTCCCCTCNNNNN
NNNGGCAGAGNNNNNNCCCNNNNNNNCCGGCCCCNNCCNNNNNNNNNNNNNNNGNNNNNNNANNNNNN
NNNNNNTGGTACNNNNCGNNNNNGGTTGTTTAAGAGGTCCACAACANGGTCNNGNCACNNNNGNNNAN
NNNCNNNNANNNNNNNNNNNNNNNNTNNNTANNAGGNNNNNNCCNTCNNNNNNNTNANNGCNATGACC
CAANTCACGGGGGCAGTNCCCGGCGNGGCGACCTTGNNNANGTCCGGGGNCAAGTATGGCGCNCNCCC
CGGGAAGGANNCNGCCGTANGTGCNNNNNANNNTNTCNTGNGANGTNGCCGNNATTNNCGTGGTCNNN
CNAGAATGNCCNNACCANNCGNANNNTGGTNATGNNAGTNNNNNNNNNNNNNNNNNNNNNNNNNNNNN
NNNNNNNNNNNNNNNNNNNNNNNNNNNNNNNNNNNNNGNNNNNNCNANNNAAGGANNTANNNCTNNTN
NTNNNGNNGGACNNNACNNNNCNNNNNNNACNCNNNCNCNNNNNNANNNGNTTNNNNNCCNNTATTNT
CCAGNACGCNGNGCNNNCATTCANGNGAACNNNGNNACGCGNNAATCGTANNATCTANCTGCCGCNNG
TNNNNNTACNGNNNNNNCGGANNNTNNNTCGTANNNNNNTCATGCGTNNTNTNNTCNCNAGNCNNGTT
CGAGAANANANTAGNANGTNTCNNNNNNNNTGTNGCGTTCCGCNCGANNCNNCANNNNGNNGNNCTTN
TCANCGGCNNTANCTGCNGANNTAAGNCATANNNTNNNTCNNTANCTNNNNNGGAGGGNNACTGTAAA
NGGNNAATTNGANGNTNATTNCNNNCACNTNNCTNNANNTNCTNNNTNCCTGNNTNCNNAATTGGNNC
CANTGNNTGNCNATCTCTGGNTTNGCNNNNNTNNNNNTNNCANNNNGGNNNGTNANANNNCNATNTAA
NNTANANNNNCNNNNNNANNNCACNNNNNNNGNAGNNACNANNNTTANANNNGTAGNNNAATAAGTCT
CCGANNTNNNNNNNATAAGCGNNANACNNNNNNNNNNNNNANGANNACGNNTTGGNNNTNNNNNNCNA
GNNAGACNNTNGTGGNTNNNNNTCAANNNNTTCGTAGTGGTATAATNGNNNNNNNTTTCNCCAGNNTN
NNNGNNNNNNNCNATANNANNNNNNNGNGANNNNNNNNNNANNNNCTTNNNAGGGANCANTTACTNNA
GCAATGGNGCGNAGNNNGAGANNNNGACNNNCCTTGGGTGGCAANCACNGCAATAAGTNNNGTTNANC
CTNNNAGTNNACACNNNNNCANNGNNNCNNNNNNANCNGANNGNNNNNNNNNNNNNNNNNNNNNNNNN
NNNNNNNNNNNNNNNNNNNNNNNNNNNNAGNNNANGGNNNGTNTNTGACNCATGTGTTTTTANNNGNN
NNNNNACGNNNNANCCCCGGTCGNNNNCNNATTGAANNCCTGNNGCATNNNNNTNCNAGAATGANNGC
NNNNNGNNTNGCTNNCNNNTCAAGNAATCANNNAGGTNNNNCGNTCNNNGNNNNNNNNNCNNNCNCNN
ACTGAGGGTNNNNNNCGCATCTGCTNNNNNNNNCTNCGCNGGNGTNNCNNNNNNGCNGTATNGTGTNA
NNCANNNNNNNNNNNNNNNNTNNNACNANNNNNNTNNNN
>LaChi551
GTGNNNNNNAGCCCNNNNNAGTTAGACANNNCNNNNNNNNNNNNTNNNNNNNNNNNNNNNNNNNNNNN

NNNNNNNNNNNNNNNNNNNNNNNNNNNNNNNNNNNNNNNNNNNNNNNNNNNNNNNNNNNNNNNNNNNN
NNNNNNNNNNNNNNNNNNNNNNNNNNNNNNNNNNNNNNNNNNNNNNNNNNNNNNNNNNNNNNNNNNNN
NNNNNNNNNNNNNNNNNNNNNNNNNNNNNNNNNNNNNNNNNNNNNNNNNNNNNNNNNNNNNNNNNNNN
NNNNNNNNNNNNNNNNNNNNNNNNNNNNNNNNNNNNNNNNNNNNNNNNNNCNTCTNNNAAANNNTNNN
NNNNNNNNNNNNNNNNNNNNNNNNNCNCNTTGNNNGTAANCNANNNGNACATGCAGTCCCCTCNNNNN
NNNNNCAGAGNNNNNNCCCNNNNNNNCCGNCCCCNNCCNNNNNNNNNNNNNNNNNNNNNNNANNNNNN
NNNNNNTGGTACNNNNCGNNNNNGGTTGTTTAAGAGGTCCACAACANGGTCNNGNNACNNNNGNNNAN
NNNCNNNNANCNNNNNNNNNNNNNNTNNNTANNAGGNNNNNNCCNNCNNGNNNNNNANTGCNATGACN
CAANNCACGGNGGCNGTNCCCGGCGCGGCGACCTTGNNNACGTCCGGGGACAAGTATGGCNNNCNCCC
CGGGAAGGANNCNGCCGTANGTGCNNNNNANNNNNTCNTGNGANNTCGCCGNNATTNNCNTGGTCNNN
NNNGAATGCCCNNACCANNCGNANNNTGGTNATGNNAGTNNNNNNNNNNNNNNNNNNNNNNNNNNNNN
NNNNNNNNNNNNNNNNNNNNNNNNNNNNNNNNGNNNNGNNNNNNCNANNNAAGGANNTANNNCTNNTN
NTNNNNNNGGACNNNACNNNNCNNNNNNNACNCNNNCNCNNNNNNANNNGNTTTTNNNNCNNTATTNT
CCAGNACGCNGNGCNNNCATTCANGNGAACNNNGNNACGCGNNAATCGTANNATCTACCTGCCGCNNG
NNNNNNTNCNGNNNNNNCGGANNNTNNCTCGTANNNNNNNCATGCGTNNTNTNNTCNCNAGNCNNGTT
CGNNNANANANTAGNANGTNTNNNNNNNNNTGTNNCGTTCCGCNCGANNCNNCANNNNGNNGNNNTTN
NCANCGGCNNTANCTNCNGANNTAAGNCATANNNTNNNTCNNTANCTNNNNNGGNGGGNNACTGTAAA
NGGNNAATTNGNNNNTNATTNCNNNCACNTNNCTNNANNTNCTNNNTNCCTGNNTNCNNAATTGGNNN
CANTGNNTGNCNATCTCTNGNTTNGNNNNNNTGNNNNTNNCANNNNGGNNNGTNNNANNNCNATNTAA
NNTANANNNNCNNNNNNANNNCACTNNNNNNNNAGNNNNNANNNTTANANNNGTAGNNNAATAAGTCT
CCGANNTNNNNNNNATAAGCGNNANACNNNNNNNNNNNNNANGANNACGNNTTGGNNNTNNNNNNCNA
GNNAGACNNTNGTGGNTNNNNNTCAANNNNTTNGTAGTGGTATAATNGNNNNNNNTTTCNCCAGNNTN
NNNGNNNNNNNCNATANNANNNNNNNGNGANNNNNNNNNNANNNNCTTNNNAGGGANCANTTACTNNA
GCANTGGNGCGNAGNNNGAGANNNNGACNNNCCTTGGGTGGCANNCACNGNAATAAGTNNNGTTNANC
CTNNNAGTNNACACNNNNNCANNGNNNCNNNNNNANCNGANNGNNNNNNNNNNNNNNNNNNNNNNNNN
NNNNNNNNNNNNNNNNNNNNNNNNNNNNNNNNNANGGNNNGTNTNTGACNCNTGNNTTTTTANNNGNN
NNNNNACGNNNNANCCCCGGTCGNNNNCNNATTGAANNCCTGNNGCATNNNNNTNCNAGAATGANNNC
NNNNNGNNTNGNCTNNNNNNTCAAGNAATNANNTANGTNNNNNGNTCNNGNNNNNNNNNCNNNCNCNN
ACTGAGGGTNNNNNNNNCATNNGCTNNNNNNNNNTNCGCNGGNGTNNCNNNNNNGCNGTATNNNGTNA
NNCANNNNNNNNNNNNNNNNNTNANNCNANNNNNNNTNNNN
>LaChi552
GTGNNNNNTAGCCCNNNNNAGTTAGACANNNCNNNNNNNNNNNNTNNNNNNNNNNNNNNNNNNNNNNN
NNNNNNNNNNNNNNNNNNNNNNNNNNNNNNNNNNNNNNNNNNNNNNNNNNNNNNNNNNNNNNNNNNNN
NNNNNNNNNNNNNNNNNNNNNNNNNNNNNNNNNNNNNNNNNNNNNNNNNNNTNNNNNNNNNNNNNNNN
NNNNNNNNNNNNNNNNNNNNNNNNNNNNNNNNNNNNNNNNNNNNNNNNNNNNNNNNNNNNNNNNNNNN
NNNNNNNNNNNNNNNNNNNNNNNNNNNNNNNNNNNNNNNNNNNNNNNNNNCNTCTNNNAAANNNTNNN
NNNNNNNNNNNNNNNNNNNNNNNNNCNCNTTGNNNGTAANCNANNNGNACATGCAGTCCCCTCNNNNN
NNNGGCAGAGNNNNNNCCNNNNNCNCCGGCCCCNNCCNNNNNNNNNNNNNNNNGNNNNNNNANNNNNN
NNNNNNTGGTACNNNNCGNNNNNGGTTGTNTAAGAGGTCCACAACANGGTCNNGNNACNNNNGNNTAN
NNNCNNNNAACNNNNNNNNNNNNNNTNNNTANNAGGNCNNNNCCNTCNNGNNNNNNANTGCNATGACC
CAANTCACGGGGGCAGTNCCCGGNGNGGCGACCTTGANNACGTCCGGGGNCAAGTATGGCNNNCNCCC
CGGGAAGGANNCNGCCGTANGTGCNNNNNANNNNNTCNTGNGANGTNGCCGNNATTCNCGTGGTCNNN
CNAGAATGNCCNNACCANNCGNANNNTGGTNATGNNAGTNNNNNNNNNNNNNNNNNNNNNNNNNNNNN
NNNNNNNNNNNNNNNNNNNNNNNNNNNNNNNNNNNNNGNNNNNNCNANNNAAGGNNNTANNNCTNNTN
NTNNNNNNGGACNNNACNNNNCNNNNNNNACNCNNNCNCNNNNNNANNNGNTTTTTNNNCNNTATTNT
CCAGNACGCNGNGCNNNCATTCANGNGAACNNNGNNACGCGNNAATCGTANNATCTACCTGCCGCNNG
TNNNNNTACNGNNNNNNCGGANNNTNNCNCGTANNNNNNNCANGCGTNNTNTNNTCNCNAGNCNNGTT
CGNNNANANANTAGNANGTNTNNNNNNNNNTGTNNCGTTCCGCNCGANNCNNCANNNNGNNGNNNTTN
TCANCGGCNNTANCTGCNGANNTAAGNCATANNNTNNNTCNNTANCTNNNNNGGNGGGNNACTGTAAA
NGGNNAATTNGNNGNTNATTNCNNNCACNTNNCNNNANNTNCTNNNTNCCTGNNTNCNNAATTGGNNC
CANTGNNTGNCNATCTCTGGNTTNGNNNNNNTNNNNNTNNCANNNNGGNNNGTNANANNNCNATNTAA
NNTANANNNNCNNNNNNANNNCACNNNNNNNNNAGNNNCNANNNTTANANNNGTAGNNNANTNAGTCT
CCGANNTNNNNNNNATAAGCGNNANACNNNNNNNNNNNNNANGANNACGNNTTGGNNNTNNNNNNCNA
GNNAGACNNTNGTGGNTNNNNNTCAANNNNTTNGTAGTGGTATAATNGNNNNNNNTTTCNCCAGNNTN

NNNGNNNNNNNCNATANNANNNNNNGNGANNNNNNNNNNNANNNNCTTNNNAGGNANCANTTACTNNA
GCANTGGNGCGCAGNNNGAGANNNNGACNNGCCTTGGGTGGCAANNACNGCAATAAGTNNNGTTNNNC
CTNNNAGTNNACACNNNNNCANNGNNNCNNNNNNANCNGANNGNNNNNNNNNNNNNNNNNNNNNNNNN
NNNNNNNNNNNNNNNNNNNNNNNNNNNAGNNNNANGGNNNGTNTNTGACNCNTGTNTTTTTANNNGNN
NNNNNACGNNNNANCCCCGGTCGNNNNCNNATTGAANNCCTGNNGCATNNNNNTNCNAGAATGANNNC
NNNNNGNNTNGCTNNCNTTTCAAGNAATCANNNAGNNNNNNNNNNTCNNNGNNNNNNNNCNNTCCCNN
ACTGAGGGTNNNNNNNNCATCTGCTNNNNNNNNNTNCGCNGGNNTNNCNNNNNNGCNGTATNGNGTNA
NNCANNNNNNNNNNNNNNNNTNAAACNNNNNNNNTNNNN
>LaChi553
GTGNNNNNTAGCCCNNNNNAGTTAGACANNNCNNNNNNNNNNNNNTNNNNNNNNNNNNNNNNNNNNNN
NNNNNNNNNNNNNNNNNNNNNNNNNNNNNNNNNNNNNNNNNNNNNNNNNNNNNNNNNNNNNNNNNNNN
NNNNNNNNNNNNNNNNNNNNNNNNNNNNNNNNNNNNNNNNNNNNNNNNNNNNNNNNNNNNNNNNNNNN
NNNNNNNNNNNNNNNNNNNNNNNNNNNNNNNNNNNNNNNNNNNNNNNNNNNNNNNNNNNNNNNNNNNN
NNNNNNNNNNNNNNNNNNNNNNNNNNNNNNNNNNNNNNNNNNNNNNNNNNNTCTNNNAAANNNTNNN
NNNNNNNNNNNNNNNNNNNNNNNNNCNCNNTGNNNGTAANCNANNNGNACATGCAGTCCCCTCNNNNN
NNNNNCAGAGNNNNNNCCNNNNNNNNCCGNCCCCNNCCNNNNNNNNNNNNNNNGNNNNNNNANNNNNN
NNNNNNTGGTACNNNNCGNNNNNGGTTGTNTNAGAGGTCCACAACANGGTCNNGNCACNNNNGNNNAN
NNNCNNNNANNNNNNNNNNNNNNNNTNNNTANNAGGNCNNNNCCNTNNNGNNNNNNANTGCNATGANN
CNNNTCACGGNGGCNGTNCCCGGNNNNGCGACCTTGANNANNTCCGGGGNCAAGTATGGCNNNCNCCC
CGGGAAGGANNCNGCCGTANGTGCNNNNNANNNNNTCNTGNGANGTCGCCGNNNNNNNCGTGGTCNNN
NNAGAATGCCCNNACCANNCGNANNNTGGTNATGNNAGTNNNNNNNNNNNNNNNNNNNNNNNNNNNNN
NNNNNNNNNNNNNNNNNNNNNNNNNNNNNNNNNNNNGNNNNNNCNANNNNAAGGANNTANNNCTNNTN
NNNNNNNNGGACNNNACNNNNNNNNNNNNACNCNNNCNCNNNNNNANNNGNTTTTTNNNCNNTATTNT
CCAGNANGCNGNGCNNNCATTCANGNGAACNNNGNNACGCGNNAATCGTANNATCTACCTGCCGCNGG
NNNNNNTACNGNNNNNNCGGANNNTNNCTCGTANNNNNNNCATGCGTNNTNTNNTCNCNAGNCANGTT
CGNNNANANANTAGNANGTNTNNNNNNNNNTGTNNCGTTCCGCNCGANNCNNCANNNNGNNGNNCTTC
NCANCGNCNNTANCTNCNGANNTAAGNCATANNNTNNNTCNNTANCTNNNNNGGNGGGNNACTGTAAA
NGGNNAATTNGNNGNTNANTNCNNNCACNTNNCNNNANNNNCTNNNTNCCTGNNTNCNNAATTGGNNN
CANTGNNTGNCNATCTCTNGNTTNGNNNNNNTNNNNNTNNCAANNNGGNNNNNNNNANNNCNATNTAA
NNTANANNNNCNNNNNNANNNCACNANNNNNGNAGNNNCNANNNTTANANNNGTAGNNNANTNAGTCT
CCGANNTNNNNNNNATAAGCGNNANACNNNNNNNNNNNNNANGANNACGNNTTGGNNNTNNNNNNCNA
GNNAGACNNTNGTGGNTNNNNNTCAANNNNTTNGTAGTGGTATAATNGNNNNNNNTTTCNCCAGNNTN
NNNGNNNNNNNCNATANNANNNNNNGNGANNNNNNNNNNNANNNNCTTNNNAGGGANCANTTACTNNA
GCANTGGNGCGNAGNNNGAGANNNNGACNANCCTTGGGTGGCAANCACNGCAATAAGTNNNGTTNANC
CTNNNAGTNNACACNNNNNCANNGNNNCNNNNNNANCNGANNGNNNNNNNNNNNNNNNNNNNNNNNNN
NNNNNNNNNNNNNNNNNNNNNNNNNNNNANNNNANNGNNNGTNTNTGACNCATNNNTTTTTANNNGNN
NNNNNACNNNNGANCCNCGGTCGNNNNCNNATTGAANNCCTGNNGCATNNNNNTNCNAGAATGANNGC
NNNNNGNNTNNNCTNNNNNNTCAAGNAATNNNNNAGNNANNNNGNTCNNNGNNNNNNNNCNNNCCCNN
ACTGAGGNTNNNNNNNNCATCTGCTNNNNNNNNNTNCGCNGGNNNNNCNNNNNNGCNGTATNNTGTNN
NNCANNNNNNNNNNNNNNNNTNNNNNNANNNNNNTNNNG
>LaChi554
GTGNNNNNTAGCCCNNNNNAGTTAGACANNNCNNNNNNNNNNNNNTNNNNNNNNNNNNNNNNNNNNNN
NNNNNNNNNNNNNNNNNNNNNNNNNNNNNNNNNNNNNNNNNNNNNNNNNNNNNNNNNNNNNNNNNNNN
NNNNNNNNNNNNNNNNNNNNNNNNNNNNNNNNNNNNNNNNNNNNNNNNNNNNNNNNNNNNNNNNNNNN
NNNNNNNNNNNNNNNNNNNNNNNNNNNNNNNNNNNNNNNNNNNNNNNNNNNNNNNNNNNNNNNNNNNN
NNNNNNNNNNNNNNNNNNNNNNNNNNNNNNNNNNNNNNNNNNNNNNNNNNNTCTNNNAAANNNTNNN
NNNNNNNNNNNNNNNNNNNNNNNNNCNCNNTGNNNGTAANCNANNNGNACATGCAGTCCCCTCNNNNN
NNNNNCAGAGNNNNNNNNCNNNNNCNCCGNCCCCNNCCNNNNNNNNNNNNNNNNNNNNNNNANNNNNN
NNNNNNTGGTACNNNNCGNNNNNGGTTGNTTAAGAGGTCCACAACGNGGTCNNGNCACNNNNGNNNAN
NNNCNNNAAACNNNNNNNNNNNNNNTNNNTANNAGGNCNNNNCCNTCNNNNNNNNNANTGCNATGACC
CAANNNNCNGNGGCNGTNCCCGGCGNGGCGACCTTGANNANNTCCGGGGNCAAGTATNGCNNNCNCCC
CGGGAAGGANNCNGCCGTANGTGCNNNNNANNNNNTCNTGNGANGTCGCCGNNATTNNNNTGGTCNNN
NNAGAATGNNCNNACCANNCGNANNNTGGTNATGNNAGTNNNNNNNNNNNNNNNNNNNNNNNNNNNNN
NNNNNNNNNNNNNNNNNNNNNNNNNNNNNNNGNNNNGNNNNNNCNANNNNAAGGANNTANNNCTNNTN

NTNNNNNNGGACNNNACNNNNCNNNNNNNACNCNNNCNCNNNNNNANNNGNTTTTNNNNCNNTANTNT
CCAGNANGCNGNGCNNNCNTTCANGNGAACNNNGNNACGCGNNAATCGTANNATCTACCTGCCGCNNG
NNNNNNTACNGNNNNNNCGGANNNTNNNNCGTANNNNNNNCATGCGTNNTNTNNTCNCNAGNCANGTT
CGNNNANANANTAGNANGTNTNNNNNNNNNTGTNNCGTTCCGCNTGANNCNNCANNNNGNNGNNCTTN
NCANCGGCNNTANCTNCNGANNTAAGNCATANNNTNNNTCNNTANCTNNNNNGGNGGGNNACTGTAAA
NGGNNAATTNGANNNTNANTTCNNNNACNTNNCTNNGNNTNCTNNNTNCCTGNNTNCNNAATTGGNNN
CANTGNNTGNCNATCTCTGGNTTNGNNNNNNTNNNNNTNNCANNNNGGNNNGTNNNANNNCNATNTAA
NNTANANNNNCNNNNNNANNNCACNANNNNNGNAGNNNCNANNNTTANANNNGTAGNNNAATAAGTCT
CCGANNTNNNNNNNATAAGCGNNANACNNNNNNNNNNNNNANGANNNCGNNTTGGNNNTNNNNNNCNA
GNNAGACNNTNATGGNTNNNNNTCAANNNNTTCGTAGTGGTATAATNGNNNNNNNTTTCNCCAGNNTN
NNNGNNNNNNNCNATANNANNNNNNNGTGANNNNNNNNNNANNNNCTCNNNAGGGANCANCTACTNNA
GCANTGGNNCGNAGNNNGAGANNNNGACNNGCCTTGGGTGGCAANNACNGCGNTAAGTNNNGTTNANN
CTNNNAGTNNACATNNNNNCANNGNNNCNNNNNNANCNGANNGNNNNNNNNNNNNNNNNNNNNNNNNN
NNNNNNNNNNNNNNNNNNNNNNNNNNNNNNNNANNGNNNGTNTNTGACNCATNNNNNNTNTANNNGNN
NNNNNACGNNNGANCCCCGGTCGNNNNCNNATTGAANNCCTGNNGCATNNNNNTNCNAGAATGANNNC
NNNNNGNNTNGNCTNNCNTTTCAAGNAATCANNNAGGTANCGCGNTCNNNGNNNNNNNNCNNTCCCNN
ACTGAGGGTNNNNNNNNCATCNGCTNNNNNNNNNTNCGCNNNNNTNNCNGNNNNGCNGTATNNTGTNA
NNCANNNNNNNNNNNNNNNNTNNNNNNANNNNNCTNNNN
>LaChi555
GTGNNNNNTAGCCCNNNNNAGTTAGACANNNCNNNNNNNNNNNNTNNNNNNNNNNNNNNNNNNNNNNN
NNNNNNNNNNNNNNNNNNNNNNNNNNNNNNNNNNNNNNNNNNNNNNNNNNNNNNNNNNNNNNNNNNNN
NNNNNNNNNNNNNNNNNNNNNNNNNNNNNNNNNNNNNNNNNNNNNNNNNNNNNNNNNNNNNNNNNNNN
NNNNNNNNNNNNNNNNNNNNNNNNNNNNNNNNNNNNNNNNNNNNNNNNNNNNNNNNNNNNNNNNNNNN
NNNNNNNNNNNNNNNNNNNNNNNNNNCNNNNNNNNNNNNNNNNNNNNNNNCNTCTNNNAAANNNTNNN
NNNNNNNNNNNNNNNNNNNNNNNNNCNCNTTNNNNGTAANCNANNNGNACATGCAGTCCCCTCNNNNN
NNNGGCAGAGNNNNNNNCCCNNNNNNCCGGCCCCNNCCNNNNNNNTNNNCNNNGNNNNNNNANNNNNN
NNNNNNTGGTACNNNNCGNNNNNGGTTGNNTAAGAGGTCCACAACGNGGTCNNGNCACNNNNGNNNAN
NNNCNNNNAACNNNNNNNNNNANATNNNTANNAGGNNNNNNCCNTCNNNNNNNNNNATNGCNATGACC
CAANNCACGGGGGCAGTNCCCGGCGCGGCGACCTTGATNACGTCCGGGGACAAGTATGGCGNNCNCCC
CGGGAAGGANNCNGCCGTANGTGCNNNNNANNNNNTCTTGNGANGTCGCCGNNATTNNCGTGGTCNNN
CNAGANTGNCCGNACCANNCGNANNNTGGTNATGTNAGTNNNNNNNNNNNNNNNNNNNNNNNNNNNNN
NNNNNNNNNNNNNNNNNNNNNNNNNNNNNNNNNNNNNGNNNNNNCNANNNAAGGANNTANNNCTNNTN
NTNNNNNNGGACNNNACNNNNCNNNNNNNACNCNNNCNCNNNNNNNNNNGNTTTTTNNNCNNTATTNT
CCAGNACGCNGNGCNNNCATTCANGNGAACNNNGNNACGCGNNAATCGTANNATCTACCTGCCGCNGG
NNNNNNTACNGNNNNNNCGGANNNTNNCTCGTANNNNNNTCATGCGTNNTNTNNTCNCNAGNCANGTT
CGNNNANANANTAGNANGTNTCNNNNNNNNTGTNNCGTTCCGCNTGANNCNNCANNNNGNNGNNCTTN
TCANCGGCNNTANCTGCNGANNTAAGNCATANNNTNNNTCNNTANCTNNNNNGGAGGGNNACTGTAAA
NGGNNAATTNGNNNNTNATTNCNNNCACNTNNCTNNGNNNNCTNNNTNCCTGNNTNCNNAATTGGNNC
CANTGNNTGNCNATCTCTGGNTTNGNNNNNNTGNNNNTNNCANNNNGGNNNGTNNNANNNCNATNTAA
NNTANANNNNCNNNNNNANNNCACNNNNNNNNNAGNNNCNANNNTTANANNNGTAGNNNAATAAGTCT
CCGANNTNNNNNNNATAAGCGNNANACNNNNNNNNNNNNNANGANNACGNNTTGGNNNTNNNNNNCNA
GNNAGACNNTNATGGNTNNNNNTCAANNNNTTCGTAGTGGTATAATNGNNNNNNNTTTCNCCAGNNTN
NNNGNNNNNNNCNATANNANNNNNNNGNGANNNNNNNNNNANNNNCTCNNNAGGGANCANCTACTNNA
GCAATGGNGCGCAGNNNGAGANNNNGACNNGCCTTGGGTGGCAANCACNGCAATAAGTNNNGTTNANC
CTNNNAGTNNACATGNNNNCANNGNNNCNNNNNNANCNGANNGNNNNNNNNNNNNNNNNNNNNNNNNN
NNNNNNNNNNNNNNNNNNNNNNNNNNNNANNNNANGGNNNGTNTNTGACNCATGTGTTTTTANNNGNN
NNNNNACGNNNNANCCCCGGTCGTNNNCNNATTGAANNCCTGNNGCATNNNNNTNCNAGAATGANNGC
NNNNNGNNTNGNCTNNCNTTTCAAGNAATCANNNAGGTANNNNNNTCNNNGNNNNNNNNCNNTCCCNN
ACTGAGGGTNNNNNNCGCATCTGCTNNNNNNNNNTNCGCNGGCGTNNCNNNNNAGCNGTATNNTGTNA
NNCANNNNNNNNNNNNNNNNTNNANCNANNNNNNTNNNN
>LaChi556
GTGNNNNNTAGCCCNNNNNAGTTAGACANNNCNNNNNNNNNNNNTNNNNNNNNNNNNNNNNNNNNNNN
NNNNNNNNNNNNNNNNNNNNNNNNNNNNNNTNNNNNNNNNNNNNNNNNNNNNNNNNNNNNNNNNNNNN
NNNNNNNNNNNNNNNNNNNNNNNNNNNNNNNNNNNNNNNNNNNNNNNNNNNNNNNNNNNNNNNNNNNN

NNNNNNNNNNNNNNNNNNNNNNNNNNNNNNNNNNNNNNNNNNNNNNNNNNNNNNNNNNNNNNNNNNNN
NNNNNNNNNNNNNNNNNNNNNNNNNNNNNNNNNNNNNNNNNNNNNNNNNNNNTCTNNNAAANNNTNNN
NNNNNNNNNNNNNNNNNNNNNNNNNCNCNTTGNNNGTAANCNANNNGNACATGCAGTCCCCTCNNNNN
NNNNNCAGAGNNNNNNNCCNNNNNCNCCGNCCCCNNCCNNNNNNNNNNNCNNNGNNNNNNNANNNNNN
NNNNNNTGGTACNNNNCGNNNNNGGTTGTTTNAGAGGTCCACAACANGGTCNNGNCACNNNNGNNNAN
NNNCNNNNAACNNNNNNNNNNNNNNNTNNNTANNAGGNNNNNNCCNTCNNNNNNNNNNANNGCNATGACC
CAANNCACGGNGGNNGTNCCCGGCGCGGCGACCTTGNNNACGTCCGGGGACAAGTATGGCGNNCNCCC
CGGGAAGGANNCNGCCGTANGTGCNNNNNANNNNNTCNTGNGANGTNGCCGNNATTNNCGTGGTCNNN
NNAGAATGNCCGNACCANNCGNANNNTGGTNATGNNAGTNNNNNNNNNNNNNNNNNNNNNNNNNNNNN
NNNNNNNNNNNNNNNNNNNNNNNNNNNNNNNNNNNNNNNNNGNNNNNNCNANNNAAGGANNTANNNCTNNTN
NTNNNNNNGGACNNNACNNNNCNNNNNNNACNCNNNCNCNNNNNNANNNGGTTTTNNNNCNNTATTNN
CCAGNACGCNGNGCNNNCATTCANGNGAACNNNGNNACGCGNNAATCGTANNATCTACCTGCCGCNNG
NNNNNNTACNGNNNNNNCGGANNNTNNCTCGTANNNNNNTCATGCGTNNTNTNNTCNCNAGNCANGTT
CGNNNANANANTAGNANGTNTNNNNNNNNNNTGTNNCGTTCCGCNCGANNCNNCANNNNGNNGNNCTTN
TCANCGGCNNTANCTGCNGANNTAAGTCATANNNTNNNTCNNTANCTNNNNNGGNGGGNNACTGTAAA
NGGNNAATTNGNNNNTNATTNCNNNCACNTNNCTNNANNNNCTNNNTNCCTGNNTNCNNAATTGGNNC
CANTGNNTGNCNATCTCTGGNTTNGCNNNNNTNNNNNTNNCANNNNGGNNNGTNNNANNNCNATNTAA
NNTANANNNNCNNNNNNANNNCACNANNNNNGNAGNNNCNANNNTTANANNNGTAGNNNANTAAGTCT
CCGANNTNNNNNNNATAAGCGNNANACNNNNNNNNNNNNNNANGANNACGNNTTGGNNNTNNNNNNCNA
GNNAGACNNTNGTGGNTNNNNNTCAANNNNTTNGTAGTGGTATAATNGNNNNNNNTTTCNCCAGNNTN
NNNGNNNNNNNCNATANNANNNNNNNGNGANNNNNNNNNNNANNNNCTTNNNAGGGANCANTTACTNNA
GCAATGGNGCGNAGNNNGAGANNNNGACNNNCCTTGGGTGGCAANCACNGCAATAAGTNNNGTTNANC
CTNNNAGTNNACACNNNNNCANNGNNNCNNNNNNANCNGANNGNNNNNNNNNNNNNNNNNNNNNNNNN
NNNNNNNNNNNNNNNNNNNNNNNNNNNNNNAGNNNANGGNNNGTNTNTGACNCATNTGTTTTTANNNGNN
NNNNNACGNNNGANCCCCGGTCGTNNNCNNATTGAANNCCTGNNGCATNNNNNTNCNAGAATGANNNC
NNNNNGNNTNGNCTNNNNNNNTCAAGNAATCNANNAGGTANCGCGNTCNNNGNNNNNNNNCNNNCCCNN
ACTGAGGGTNNNNNNNNCATCTGCTNNNNNNNNCTNCGCNGGCGTNNCNNNNNAGCNGTATNGTGTNA
NNCATNNNNNNNNNNNNNNNNNNNAACNANNNNNNNTNNNN
>LaChi557
GTGNNNNNTAGCCCGNNNNAGTTAGACANNNCNNNNNNNNNNNNTNNNNNNNNNNNNNNNNNNNNNNN
NNNNNNNNNNNNNNNNNNNNNNNNNNNNNNNNNNNNNNNNNNNNNNNNNNNNNNNNNNNNNNNNNNNN
NNNNNNNNNNNNNNNNNNNNNNNNNNNNNNNNNNNNNNNNNNNNNNNNNNNNNNNNNNNNNNNNNNNN
NNNNNNNNNNNNNNNNNNNNNNNNNNNNNNNNNNNNNNNNNNNNNNNNNNNNNNNNNNNNNNNNNNNN
NNNNNNNNNNNNNNNNNNNNNNNNNNNNNNNNNNNNNNNNNNNNNNNNNNCNTCTNNNAAANNNTNNN
NNNNNNNNNNNNNNNNNNNNNNNNNCNCNNTGNNNGTAANCNANNNGNACATGCAGTCCCCTCNNNNN
NNNNNCAGAGNNNNNNNCNNNNNNCNCCGNCCCCNNCCNNNNNNNNNNNCANNNNNNNNNNANNNNNN
NNNNNNTGGTACNNNNCGNNNNNGGTTGTNTAAGAGGTCCACAACANGGTCNNGNCACNNNNGNNNAN
NNNCNNNNNNNNNNNNNNNNNNNNANNTNNNTANNAGGNNNNNNCCNTCNNGNNANNNANNGCNATGACC
CAANNCACGGNGGCAGTNCCCGGNGCGGCGACCTTGNNNANNTCCGGGGACAAGTATGGCNNNCNCCC
CGGGAAGGANNCNGCCGTANGTGCNNNNNANNNNNTCNTGNGANGTNGCCGNNNTTCNCNTGGTCNNN
CNAGAATGCNCGNACCANNCGNANNNTGGTNATGNNAGTNNNNNNNNNNNNNNNNNNNNNNNNNNNNN
NNNNNNNNNNNNNNNNNNNNNNNNNNNNNNNNNNNGNNNNGNNNNNNCNANNNAAGGANNTANNNCTNNTN
NTNNNNNNGGACNNNACNNNNCNNNNNNNACNCNNNCNCNNNNNNANNNGNTTTTNNNNCNNTATTNN
CCAGNANGCNGNGCNNNCATTCANGNGAACNNNGNNACGCGNNAATCGTANNATCTACCTNCCGCNNG
NNNNNNTACNGNNNNTNCGGANNNTNNCTCGTANNNNNNTCATGCGTNNTNTNNTCNCNAGNCNNGTT
CGNNNANANANTAGNANGTNTCNNNNNNNNNTGTNNCGTTCCGCNCGANNCNNCANNNNGNCGNNCTTN
NCANCGGCNNTANCTGCNGANNTAAGTCATANNNTNNNTCNNTANCTNNNGNGGNGGGNNACTGTAAA
NGGNNAATTNGNNNNTNATTTCNNNCACNTNNCTNNANNTNCTNNNTNCCTGNNTNCNNAATTGGNNC
CANTGNNTGNCNATCTCTGGNTTNGNNNNNNTNNNNNTNNCAANNNGGNNNGTNANANNNCNATNTAA
NNTANANNNNCNNNNNNANNNCACNNNNNNNGNAGNNNCNANNNTNANANNNGTAGNNNANTAAGTCT
CCGANNTNNNNNNNATAAGCGNNANACNNNNNNNNNNNNNNANGANNACGNNTTGGNNNTNNNNNNCNA
GNNANACNNTNGTGGNTNNNNNTCAANNNNTTNGTAGTGGTATAATNGNNNNNNNTTTCNCCAGNNTN
NNNGNNNNNNNCNATANNANNNNNNNGNGANNNNNNNNNNNANNNNCTTNNNAGGGANCANTTACTTNA
GCAATGGNNCGCAGNNNGAGANNNNGACNNGCCTTGGGTGGCAANCACNGCAATAAGTNNNGTTNANC

CTNNNAGTNNACACNNNNNCANNGNNNCNNNNNNANCNGANNGNNNNNNNNNNNNNNNNNNNNNNNNNNN
NNNNNNNNNNNNNNNNNNNNNNNNNNNNNNAGNNNANGGNNNGTNTNTGACNCANGNNTTTTTANNNGNN
NNNNNACGNNNGNNCCCCGGTCGNNNNNCNNATTGAANNCCTGNNGCATNNNNNNTNCNAGAATGANNGC
NNNNNGNNTNGNCTGNCNNNTCAAGNAATCANNNAGGTNNCGCGNTCNNNGNNNNNNNNCNNTCCCNN
ACTGAGGGTNNNNNNCGCATCTGCTNNNNNNNNNNTNCGCNGGCGTNNCNNNNNNGCCGTATNNTGNNA
NNCANNNNNNNNNNNNNNNNNNNNAANNNANNNNNNTNNNN
>LaChi558
GTGNNNNNTAGCCCNNNNNAGTTAGACANNNCNNNNNNNNNNNNNTNNNNNNNNNNNNNNNNNNNNNNNN
NNNNNNNNNNNNNNNNNNNNNNNNNNNNNNNNTNNNNNNNNNNNNNNNNNNNNNNNNNNNNNNNNNNNNN
NNNNNNNNNNNNNNNNNNNNNNNNNNNNNNNNNNNNNNNNNNNNNNNNNNNNNNNNNNNNNNNNNNNNNN
NNNNNNNNNNNNNNNNNNNNNNNNNNNNNNNNNNNNNNNNNNNNNNNNNNNNNNNNNNNNNNNNNNNNNN
NNNNNNNNNNNNNNNNNNNNNNNNNNNNNNNNNNNNNNNNNNNNNNNNNNNNNNNNNNNNNNAAANNNNNNN
NNNNNNNNNNNNNNNNNNNNNNNNNNNNNCNTTGNNNGTAANCNANNNGNACATGCAGTCCCCTCNNNNN
NNNGGCAGAGNNNNNNNNCNNNNNNNCCGNCCCCNNCCNNNNNNNNNNNNCNNNGNNNNNNNANNNNNN
NNNNNNTGGTACNNNNCGNNNNNGGTTGNNTAAGAGGTCCACAACANGGTCNNNNCACNNNNGNNNAN
NNNCNNNNNNNNNNNNNNNNNNNNNNNNTNNNTANNAGGNNNNNNCCNTNNNNNNNNNNNATNGCNATGACC
CAANNCNCNGGGGCNGTNCCCGGCGNGGCGACCTTGNNNACGTCCGGGGACAAGTATGGCGNNCNCCC
CGGGAAGGANNCNGCNGTANGTGCNNNNNANNNNNTCNTGNGANGTNGCCGNNATTNNCGTGGTCTNN
CNAGAATGNCCNNACCANNCGNANNNTGGTNATGNTAGTNNNNNNNNNNNNNNNNNNNNNNNNNNNNN
NNNNNNNNNNNNNNNNNNNNNNNNNNNNNNNNNNNNNNNNNGNNNNNNCNANNNAAGGNNNTANNNCTNNTN
NTNNNNNNNGGACNNNACNNNNCNNNNNNNACNCNNNCNCNNNNNNANNNGNTTTTTNNNCNNTATTNT
CCAGNACGCNGNGCNNNCNTTCANGNGAACNNNGNNACGCGNNAATCGTANNATCTACCTGCCGCNNG
NNNNNNTACNGNNNNNNCGGANNNTNNCTCGTANNNNNNTCATGCGTNNTNTNNTCNCNAGNCANGTT
CGNNNANANANTAGNANGTNTNNNNNNNNNNTGTNNCGTTCCGCNCGANNCNNCANNNNGNNGNNCTTN
NCANCNGNNNTANCTNCNGANNTAAGNCATANNNTNNNTCNNTANCTNNNNNGGNGGGNNACTGTAAA
NGNNNAATTNGNNNNTNATTNCNNNCACNTNNCNNNANNTNCTNNNTNCCTGNNTNCNNAATTGGNNN
CANTGNNTGNCNATCTCTGGNTTNGNNNNNNTNNNNNTNNCANNNNGGNNNNNNNNANNNCNATNTAA
NNTANANNNNCNNNNNNANNNCACNNNNNNNNNNAGNNNNNANNNTTANANNNGTAGNNNANTNAGTCT
CCGANNTNNNNNNNATAAGCGNNANACNNNNNNNNNNNNNANGANNNCGNNTTGGNNNTNNNNNNNNA
GNNAGACNNTNGTGGNTNNNNNTCAANNNNTTNGTAGTGGTATAATNGNNNNNNNTTTCNCCAGNNTN
NNNGNNNNNNNNCNATANNANNNNNNNGNGANNNNNNNNNNNANNNNCTTNNNAGGGANCANTTACTNNA
GCANTGGNGCGCAGNNNGAGANNNNGACCAGCCTTGGGTGGCAANNACNGCAATAAGTNNNGTTNANC
CTNNNAGTNNACACNNNNNCANNGNNNCNNNNNNANCNGANNGNNNNNNNNNNNNNNNNNNNNNNNNNNN
NNNNNNNNNNNNNNNNNNNNNNNNNNNNNNNNNNNANGGNNNGTNTNTGACNCATGTGTTTTTANNNGNN
NNNNNNNGNNNGANCCCCGGTCGNNTNCNNATTGAANNCCTGNNGCATNNNNNTNCNAGAATGANNNC
NNNNNGNNTNGNCTNNNNNNNTCAAGNAATCANNNAGGTNNNNNNNNTCNNNGNNNNNNNNCNNNCCCNN
ACTGAGGNNNNNNNNNNNCATCNGCTNNNNNNNNNNTNCGCNGGNGTNNCNNCNNAGCNGTATNGTGTNA
NNCANNNNNNNNNNNNNNNNNTNAANNNNNNNNNNNNTNNNN
>LaChi559
GTGNNNNNTAGCCNNNNNNAGTTAGACANNNCNNNNNNNNNNNNNTNNNNNNNNNNNNNNNNNNNNNNNN
NNNNNNNNNNNNNNNNNNNNNNNNNNNNNNNNTNNNNNNNNNNNNNNNNNNNNNNNNNNNNNNNNNNNNN
NNNNNNNNNNNNNNNNNNNNNNNNNNNNNNNNNNNNNNNNNNNNNNNNNNNNNNNNNNNNNNNNNNNNNN
NNNNNNNNNNNNNNNNNNNNNNNNNNNNNNNNNNNNNNNNNNNNNNNNNNNNNNNNNNNNNNNNNNNNNN
NNNNNNNNNNNNNNNNNNNNNNNNNNNNNNNNNNNNNNNNNNNNNNNNNNNNNNNTCTNNNAAANNGTNNN
NNNNNNNNNNNNNNNNNNNNNNNNNNCNCNNTGNNNGTAANCNANNNGNACATGCAGTCCCCTCNNNNN
NNNGGCAGAGNNNNNNNCCNNNNNNNCCGGCCCCNNCCNNNNNNNNNNNNNNNNGNNNNNNNANNNNNN
NNNNNNTGGTACNNNNCGNNNNNGGTTGTNTAAGAGGTCCACAACANGGTCNNGNCACNNNNGNCNAN
NNNCNNAANNNNNNNNNNNNNNNNNNNTNNNTANNAGGNNNNNNCCNTNNNGNNNNNNANNGCNATGACC
CAANTCACGGGGGCAGTNCCCGGNGNNGCGACCTTGNNNANNTCCGGGNACAAGTATGGCNNNCNCCC
CGGGAAGGANNCNGCCGTANGTGCNNNNNANNNNNTCNTGNGANGTCGCCGNNATTCNCGTGGTCNNN
CNAGAATGNNCGNACCANNCGNANNNTGGTNATGNNAGTNNNNNNNNNNNNNNNNNNNNNNNNNNNNN
NNNNNNNNNNNNNNNNNNNNNNNNNNNNNNNNNNNNNNNNNGNNNNNNCNANNNAAGGANNTANNNCTNNTN
NTNNNNNNNGGACNNNACNNNNCNNNNNNNACNCNNNCNCNNNNNNNNNNNGNTTTNNNNNCCNNTATTNN
CCAGNACGCNGNGCNNTCATTCANGNGAACNNNGNNACGCGNNAATCGTANNATCTACCTGCCGCNNG

TNNNNNTACNGNNNNNNCGGANNNTNNNNNGTANNNNNNTCANGCGTNNTNTNNTCNCNAGNCNNGTT
CGNNNANANANTAGNANGTNTNNNNNNNNNNTGTNNCGTTCCGCNCGANNCNNCANNNNGNNGNNNTTN
TCANCGGCNNTANCTGCNGANNTAAGTCATANNNTNNNTCNNTANCTNNNNNGGNGGGNNACTGTAAA
NGGNNAATTNGNNGNTNANTNCNNNCACNTNNCTNNANNNNCTNNNTNCCTGNNTNCNNAATTGGNNN
CANTGNNTGNCNATCTCTNGNTTNGNNNNNNTNNNNNTNNCANNNNGGNNNNNNNNANNNCNATNTAA
NNTANANNNNCNNNNNNANNNCACNNNNNNNGNAGNNNNNANNNTTANANNNGTAGNNNNNTAAGTCT
CCGANNTNNNNNNNATAAGCGNNANACNNNNNNNNNNNNNNANGANNNCGNNTTGGNNNTNNNNNNCNA
GNNAGACNNTNGTGGNTNNNNNTCAANNNNTTNGTAGTGGTATAATNGNNNNNNNTTTCNCCAGNNTN
NNNGNNNNNNNNCNATANNANNNNNNNGNGANNNNNNNNNNNANNNNCTTNNNAGGNANCANTTACTNNA
GCAATGGNGCGCAGNNNGAGANNNNGACNNNCCTTGGGTGGCAANCACNGCAATAAGTNNNGTTNANC
CTNNNAGTNNACACNNNNNCANNGNNNCNNNNNNANCNGANNGNNNNNNNNNNNNNNNNNNNNNNNNNN
NNNNNNNNNNNNNNNNNNNNNNNNNNNNNNANNNNANGGNNNGTNTNTGACNCNTNNNNTTTTTANNNGNN
NNNNNACGNNNNANCCCCGGTCGTNNNCNNATTGAANNCCTGNNGCATNNNNNTNCNAGAATGANNGC
NNNNNGNNTNGNCTNNCNNNTCAAGNAATCANNNAGGNANCGCGNTCNNNNNNNNNNNNCNNNCCCNN
ACTGAGGGTNNNNNNCGCATCTGCTNNNNNNNNNCTTCGCNGGNNNNNCNNNNNAGCNGTATNNTGTNN
NNCANNNNNNNNNNNNNNNNNNNNNAACNANNNNNNNTNNNN
>LaChi560
GTGNNNNNTAGCCCNCNNNAGTTAGACANNNCNNNNNNNNNNNNTNNNNNNNNNNNNNNNNNNNNNNN
NNNNNNNNNNNNNNNNNNNNNNNNNNNNNNNNNNNNNNNNNNNNNNNNNNNNNNNNNNNN
NNNNNNNNNNNNNNNNNNNNNNNNNNNNNNNNNNNNNNNNNNNNNNNNNNNNNNNNNNNN
NNNNNNNNNNNNNNNNNNNNNNNNNNNNNNNNNNNNNNNNNNNNNNNNNNNNNNNNNNNN
NNNNNNNNNNNNNNNNNNNNNNNNNNNNNNNNNNNNNNNNNNNNNNNNNNNNNNNCNTCTNNNAAANNNTNNN
NNNNNNNNNNNNNNNNNNNNNNNNNNCNCNTTGNNNGTAANCNANNNGNACATGCAGTCCCCTCNNNNN
NNNNNCAGAGNNNNNNNCCNNNNNNNCCGGCCCCNNCCNNNNNNNNNNNNNNNNNNNNNNNNNANNNNNN
NNNNNNTGGTACNNNNCGNNNNNGGTTGTNTNAGAGGTCCACAACGNGNTCNGGNCACNNNNGNNNAN
NNNCNNNNNNNNNNNNNNNNNNNNNNTNNNTANNAGGNCNNNNCCNTNNNNNNNNNNNANNGCNATGACC
CAANNNACGGNGGNNGTNCCCGGNGNGGCGACCTTGNNNANGTCCGGGGACAAGTATGGCNCNCNCCC
CGGGAAGGANNCNGCCGTANGTGCNNNNNANNNNNTCNTGNGANGTNGCCGNNATTNNNGTGGTCNNN
CNAGAATGNCCGNACCANNCGNANNNTGGTNATGNNAGTNNNNNNNNNNNNNNNNNNNNNNNNNNNN
NNNNNNNNNNNNNNNNNNNNNNNNNNNNNNNNNNNNNNNNGNNNNNNCNANNNAAGGANNTANNNCTNNTN
NTNNNGNNGGACNNNACNNNNCNNNNNNNACNCNNNCNCNNNNNNNNNNGNTTTTNNNCCNNTATTNT
CCAGNANGCNGNGCNNNCATTCANGNGAACNNNGNNACGCGNNAATCGTANNATCTACCTGCCGCNNG
NNNNNNTNCNGNNNNNNCGGANNNTNNNNNGTANNNNNGNCATGCGTNNTNTNNTCNCNAGNCNNGTT
CGNNNANANANTAGNANGTNTNNNNNNNNNNTGTNNCGTTCCGCNTGANNCNNCANNNNGNNGNNCTTN
NCANCGGNNNTANCTGCNGANNTAAGNCATANNNTNNNTCNNTANCTNNNNNGGNGGGNNACTGTAAA
NGGNNAATTNGANNNTNANTNCNNNCACNTNNCTNNGNNTNCTNNNTNCCTGNNTNCNNAATTGGNNC
CANTGNNTGNCNATCTCTGGNTTNGNNNNNNTNNNNNTNNCANNNNGGNNNGTNNNANNNCNATNTAA
NNTANANNNNCNNNNNNANNNCACNNNNNNNNNAGNNNCNANNNTTANANNNGTAGNNNANTAAGTCT
CCGANNTNNNNNNNATAAGCGNNANACNNNNNNNNNNNNNNANGANNNCGNNTTGGNNNTNNNNNNCNA
GNNAGACNNTNATGGNTNNNNNTCAANNNNTTCGTAGTGGTATAATNGNNNNNNNTTTCNCCAGNNTN
NNNGNNNNNNNNCNATANNANNNNNNNGNGANNNNNNNNNNNANNNNCTCNNNAGGGANCANCTACTNNA
GCANTGGNGCGNAGNNNNAGANNNNGACNNNCCTTGGGTGGCAANCACNGNANTAAGTNNNGTTNANC
CTNNNAGTNNACATNNNNNCANNGNNNCNNNNNNANCNGANNGNNNNNNNNNNNNNNNNNNNNNNNNNN
NNNNNNNNNNNNNNNNNNNNNNNNNNNNNNGNNNANNGNNNGTNTNTGACNCATGNNTTTTTANNNGNN
NNNNNACGNNNNNNCCCCGGTCGNNNNCNNATTGAANNCCTGNNGCATNNNNNTNCNAGAATGANNGC
NNNNNGNNTNNNNTGNCNTTTCANGNAATCANNNANGTANNNNNNTCNNNNNNNNNNNNCNNTCNCNN
ACTGAGGGTNNNNNNNGCATNTGCTNNNNNNNNNNTNCGCNGGNGTNNCNNNNNNGCNGTATNNNGTNN
NNCANNNNNNNNNNNNNNNNNNNNNNACNNNNNNNNNTNNNN
>LaChi561
GTGNNNNNTAGCCCGNNNNAGTTAGACANNNCNNNNNNNNNNNNTNNNNNNNNNNNNNNNNNNNNNNN
NNNNNNNNNNNNNNNNNNNNNNNNNNNNNNNNNNNNNNNNNNNNNNNNNNNNNNNNNNNN
NNNNNNNNNNNNNNNNNNNNNNNNNNNNNNNNNNNNNNNNNNNNNNNNNNNNNNNNNNNN
NNNNNNNNNNNNNNNNNNNNNNNNNNNNNNNNNNNNNNNNNNNNNNNNNNNNNNNNNNNN
NNNNNNNNNNNNNNNNNNNNNNNNNNNNNNNNNNNNNNNNNNNNNNNNNNNNNNNNTCTNNNAAANNNTNNN

NNNNNNNNNNNNNNNNNNNNNNNNNCNCNNTGNNNGTAANCNANNNGNACATGCAGTCCCCTCNNNNN
NNNGGCAGAGNNNNNNNCCNNNNNNNCCGNCCCCNNCCNNNNNNNNNNNCNNNGNNNNNNANNNNNNN
NNNNNNTGGTACNNNNCGNNNNNGGTTGTNTNAGNGGTCCACAACANGGTCNNGNCACNNNNGNNNAN
NNNCNNNNNNNNNNNNNNNNNNNNNTNNNTANNAGGNNNNNNCCNTCNNNNNNNNAANNGCNATGACC
CAANNCACGGGGGCAGTNCCCGGNGCGGCGACCTTGNTNACGTCCGGGGNCAAGTATGGCNNNCNCCC
CGGGAAGGANNCNGCCGTANGTGCNNNNNANNNNNTCNTGNGANGTNGCCGNNANNNNCGTGGTCNNN
CNAGAATGNCCGNACCANNCGNANNNTGGTNATGNNAGTNNNNNNNNNNNNNNNNNNNNNNNNNNNNN
NNNNNNNNNNNNNNNNNNNNNNNNNNNNNNNNNNNNNGNNNNNNCNANNNAAGGANNTANNNCTNNTN
NNNNNNNNGGACNNNACNNNNCNNNNNNNACNCNNNCNCNNNNNNANNNGNTTTTTNNNCNNTATTNT
CCAGNACGCNGNGCNNNCATTCANGNGAACNNNGNNACGCGNNAATCGTANNATCTACCTGCCGCNGG
NNNNNNTNCNGNNNNNNCGGANNNTNNCTCGTANNNNNNTCANGCGTNNTNTNNTCNCNAGNCANGTT
CGNNNANNNANTAGNANGTNTNNNNNNNCTTGTNNCGTTCCGCNCGANNCNNCANNNNGNCGNNCTTC
NCANCGGCNNTANCTGCNGANNTAAGTCATANNNTNNNTCNNTANCTNNNNNGGNGGGNNACTGTAAA
NGGNNAATTNGANNNTNANTNCNNNCACNTNNCTNNANNNNCTNNNTNCCTGNNTNCNNAATTGGNNN
CANTGNNTGNCNATCTCTGGNTTNGCNNNNNTNNNNNTNNCAANNNGGNNNGTNANANNNCNATNTAA
NNTANANNNNCNNNNNNANNNCACNNNNNNNGNAGNNNCNANNNTTANANNNGTAGNNNANTAAGTCT
CCGANNTNNNNNNNATAAGCGNNANACNNNNNNNNNNNNNNANGANNACGNNTTGGNNNTNNNNNCNA
GNNAGACNNTNGTGGNTNNNNNTCAANNNNTTCGTAGTGGTATAATNGNNNNNNNTTTCNCCAGNNTN
NNNGNNNNNNNCNATANNANNNNNNNGNGANNNNNNNNNNANNNNCTTNNNAGGGANCANTTACTNNA
GCAATGGNGCGCAGNNGGAGANNNNGACCANCCTTGGGTGGCAANCACNGCAATAAGTNNNGTTNANC
CTNNNAGTNNACACGNNNNCANNGNNNCNNNNNNANCNGANNGNNNNNNNNNNNNNNNNNNNNNNNNN
NNNNNNNNNNNNNNNNNNNNNNNNNNNNAGNNNANGGNNNGTNTNTGACNCATGNNTTTTTANNNGNN
NNNNNACGNNNGNNCCCCGGTCGNNNNCNNATTGAANNCCTGNNGCATNNNNNTNCNAGAATGANNGC
NNNNNGNNTNNNCTGNCNNNTCAAGNAATCANNNAGGTNNCGCGNTCNNNGNNNNNNNNCNNTCCCNN
ACTGAGGGTNNNNNCGCATCTGCTNNNNNNNNNCTTCGCNGGCNNNNCNNNNNAGCNGTATNGTGTTN
NNCANNNNNNNNNNNNNNNNNNTNNNACNANNNNNNNTNNNN
>LaChi562
GTGNNNNNTAGCCCNNNNNAGTCAGACACNNCNNNNNNNNNNNNTNNNNNNNNNNNNNNNNNNNNNNN
NNNNNNNNNNNNNNNNNNNNNNNNNNNNNNTNNNNNNNNNNNNNNNNNNNNNNNNNNNNNNNNNNNNN
NNNNNNNNNNNNNNNNNNNNNNNNNNNNNNNNNNNNNNNNNNNNNNNNNNNNNNNNNNNNNNNNNNNN
NNNNNNNNNNNNNNNNNNNNNNNNNNNNNNNNNNNNNNNNNNNNNNNNNNNNNNNNNNNNNNNNNNNN
NNNNNNNNNNNNNNNNNNNNNNNNNNNNNNNNNNNNNNNNNNNNNNNNNNCNTCTNNNAAANNNTNNN
NNNNNNNNNNNNNNNNNNNNNNNNNCNCNNTGNNNGTAANCNANNNGNACATGCAGTCCCCTCNNNNN
NNNGGCAGAGNNNNNNNCCNNNNNNNCCGGCCCCGNCCNNNNNNNNNNNCNNNGNNNNNNANNNNNNN
NNNNNNTGGTACNNNNCGNNNNNGGTTGNNTNAGAGGTCCACAACGNGNTCNNGNCACNNNNGNNNAN
NNNCNNNNAACNNNNNNNNNNNNNNTNNNTANNAGGNCNNNNCCNTCNNGNNNNNNATTGCNATGACC
CAANNCACGGNGGCNGTNCCCGGCGCGGCGACCTTGANNACGTCCGGGGACAAGTATGGCGNNCNCCC
CGGGAAGGANNCNGCCGTANGTGCNNNNNANNNNNTCTTGNGANGTNGCCGNNATTCNCGTGGTCNNN
CNAGAATGCCCGNACCANNCGNANNNTGGTNATGNNAGTNNNNNNNNNNNNNNNNNNNNNNNNNNNNN
NNNNNNNNNNNNNNNNNNNNNNNNNNNNNNNNGNNNNGNNNNNNCNANNNAAGGNNNTANNNCTNNTN
NTNNNNNNGGACNNNACNNNNCNNNNNNNACNCNNNCNCNNNNNNNNNNGNTTTTNNNCCNNTANTNT
CCAGNACGCNGNGCNNNCATTCANGNGAACNNNGNNACGCGNNAATCGTANNATCTACCTGCCGCNNG
TNNNNNTACNGNNNNNNCGGANNNTNNCTCGTANNNNNNTCATGCGTNNTNTNNTCNCNAGNCANGTT
CGNNNANANANTAGNANGTNTCNNNNNNNNTGTNNCGTTCCGCNTGANNCNNCANNNNGNNGNNCTTN
TCANCGGCNNTANCTNCNGANNTAAGNCATANNNTNNATCNNTANCTNNNNNGGAGGGNNACTGTAAA
NGGNNAATTNGNNNNTNATTNCNNNCATNTNNCTNNGNNTNCTNNNTNCCTGNNTNCNNAATTGGNNC
CANTGNNTGNCNATCTCTNGNTTNGCNNNNNTNNNNNTNNCANNNNGGNNNGTNNNANNNCNATNTAA
NNTANANNNNCNNNNNNANNNCACNANNNNNGNAGNNNCNANNNGTANANNNGTAGNNNGNTNAGTCT
CCGANNTNNNNNNNATAAGCGNNANACNNNNNNNNNNNNNNANGANNACGNNTTTGNNNTNNNNNCNA
GNNAGACNNTNGTGGNTNNNNNTCAANNNNTTCGTAGTGGTATAATNGNNNNNNNTTTCNCCAGNNTN
NNNGNNNNNNNCNATANNANNNNNNNGNGANNNNNNNNNNANNNNCTCNNNAGGGANCANCTACTNNA
GCANTGGNGCGNAGNNNGAGANNNNGACCAGCCTTGGGTGGCAANNACNGCAATAAGTNNNGTTNANC
CTNNNAGTNNACACNNNNNCANNGNNNCNNNNNNANCNGANNGNNNNNNNNNNNNNNNNNNNNNNNNN
NNNNNNNNNNNNNNNNNNNNNNNNNNNNNGNNNANGGNNNGTNTNTGNCNCATGTGTTTNTANNNGNN

NNNNNACGNNNGNNCCCCGGTCGNNNNCNNATTGAANNCCTGNNGCATNNNNNTNCNAGAATGANNGC
NNNNNGNNTNGCTNNCNNNTCAAGNAATCANNNAGGTNNNGCGNTCNNNNNNNNNNNNNCNNTCCCNN
ACTGAGGGTNNNNNNNCATCTGCTNNNNNNNNNCTNCGCNGGNGTNNCTNNNNAGCNGTATNNTGTNA
NNCANNNNNNNNNNNNNNNNNNAAACAANNNNNNTNNNN
>LaChi563
GTGNNNNNTAGCCCNNNNNAGTCAGACANNNCNNNNNNNNNNNNTNNNNNNNNNNNNNNNNNNNNNNN
NNNNNNNNNNNNNNNNNNNNNNNNNNNNNNTNNNNNNNNNNNNNNNNNNNNNNNNNNNNNNNNNNNNN
NNNNNNNNNNNNNNNNNNNNNNNNNNNNNNNNNNNNNNNNNNNNNNNNNNNNNNNNNNNNNNNNNNNN
NNNNNNNNNNNNNNNNNNNNNNNNNNNNNNNNNNNNNNNNNNNNNNNNNNNNNNNNNNNNNNNNNNNN
NNNNNNNNNNNNNNNNNNNNNNNNNNNNNNNNNNNNNNNNNNNNNNNNNNCNTCTNNNAAANNNTNNN
NNNNNNNNNNNNNNNNNNNNNNNNNCNCNTTGNNNGTAANCNANNNGNACATGCAGTCCCCTCNNNNN
NNNNNCAGAGNNNNNNCCCNNNNNCNCCGGCCCCNNCCNNNNNNNNNNNNNNNGNNNNNNNANNNNNN
NNNNNNTGGTACNNNNCGNNNNNGGTTGTNTNAGAGGTCCACAACGNGGTCNNGNCACNNNNGNNNAN
NNNCNNNNAANNNNNNNNNNNNANNTNNNTANNAGGNCNNNNCCNTCNNGNNNNNAANNGCNATGACC
CAANNCACGGNGGNNGTNCCCGGNGNGGCGACCTTGNNNACGTCCGGGGACAAGTATGGCNNNCNCCC
CGNGAAGGANNCNGCCGTANGTGCNNNNNANNNNNTCTTGNGANGTCGCCGNNATTCNCGTGGTCNNN
NNAGAATGNCCNNACCANNCGNANNNTGGTNATGNNAGTNNNNNNNNNNNNNNNNNNNNNNNNNNNNN
NNNNNNNNNNNNNNNNNNNNNNNNNNNNNNNNGNNNNGNNNNNNCNANNNAAGGANNTANNNCTNNTC
NTNNNNNNGGACNNNACNNNNCNNNNNNNACNCNNNCNCNNNNNNNNNNGGTTTTTNNNCNNTATTNT
CCAGNANGCNGNGCNNNCATTCANGNGAACNNNGNNACGCGNNAATCGTANNATCTACCTGCCGCNGG
TNNNNNTNCNGNNNNTNCGGANNNTNNCTCGTANNNNNNTCATGCGTNNTNTNNTCNCNAGNCANGTT
CGNNNANANANTAGNANGTNNNNNNNNNNNTGTNNCGTTCCGCNTGANNCNNCANNNNGNNGNNNTTN
NCANCGGCNNTANCTGCNGANNTAAGNCATANNNTNNATCNNTANCTNNNNNGGNGGGNNACTGTAAA
NGGNNAATTNGNNNNTNATTNCNNNCATNTNNCNNNGNNTNCTNNNTNCCTGNNTNCNNAATTGGNNC
CANTGNNTGNCNATCTCTNGNTTGNNNNNNNTNNNNNTNNCAANNNGGNNNGTNANANNNCNATNTAA
NNTANANNNNCNNNNNNANNNCACNNNNNNNGNAGNNNCNANNNGTANANNNGTAGNNNGNTAAGTCT
CCGANNTNNNNNNNATAAGCGNNANACNNNNNNNNNNNNNANGANNACGNNTTTGNNNTNNNNNNCNA
GNNAGACNNTNGTGGNTNNNNNTCAANNNNTTCGTAGTGGTATAATNGNNNNNNNTTTCNCCAGNNTN
NNNGNNNNNNNCNATANNANNNNNNNGNGANNNNNNNNNNANNNNCTCNNNAGGNANCANCTACTNNA
GCAATGGNGCGCAGNNNGAGANNNNGACNNGCCTTGGGTGGCAANCACNGCAATAAGTNNNGTTNANC
CTNNNAGTNNACACNNNNNCANNGNNNCNNNNNNANCNGANNGNNNNNNNNNNNNNNNNNNNNNNNNN
NNNNNNNNNNNNNNNNNNNNNNNNNNNNAGNNNANGGNNNGTNTNTGACNCATGNNTTTTTANNNGNN
NNNNNACGNNNNANCCCCGGTCGNNNNCNNATTGAANNCCTGNNGCATNNNNNTNCNAGAATGANNGC
NNNNNGNNTNGCTNNCNTTTCAAGNAATCANNTAGNNNNNNNGNTCNNNGNNNNNNNNNCNNTCCCNN
ACTGAGGGTNNNNNNCGCATCTGCTNNNNNNNNNTNCGCNGGCNNNNCNNNNNAGCNGTATNGTGTNN
NNCANNNNNNNNNNNNNNNNNNAANCANNNNNNNTNNNN
>LaChi564
GTGNNNNNTAGCCCNNNNNAGTCAGACANNNCNNNNNNNNNNNNTNNNNNNNNNNNNNNNNNNNNNNN
NNNNNNNNNNNNNNNNNNNNNNNNNNNNNNNNNNNNNNNNNNNNNNNNNNNNNNNNNNNNNNNNNNNN
NNNNNNNNNNNNNNNNNNNNNNNNNNNNNNNNNNNNNNNNNNNNNNNNNNNNNNNNNNNNNNNNNNNN
NNNNNNNNNNNNNNNNNNNNNNNNNNNNNNNNNNNNNNNNNNNNNNNNNNNNNNNNNNNNNNNNNNNN
NNNNNNNNNNNNNNNNNNNNNNNNNNNNNNNNNNNNNNNNNNNNNNNNNNNNTCTNNNAAANNNTNNN
NNNNNNNNNNNNNNNNNNNNNNNNNCNCNTTGNNNGTAANCNANNNGNACATGCAGTCCCCTCNNNNN
NNNNNCAGAGNNNNNNCCNNNNNNNNCCGGCCCCGNCCNNNNNNNNNNNCNNNGNNNNNNNANNNNNN
NNNNNNTGGTACNNNNCGNNNNNGGTTGTNTNAGAGGTCCACAACGNGGTCNGGNCACNNNNGNNNAN
NNNCNNNNNNCNNNNNNNNNNNNNNTNNNTANNAGGNCNNNNCCNTCNNGNNNNNNANNGCNATGACC
CAANTNACGGNGGCAGTNCCCGGCGCGGCGACCTTGATNANGTCCGGGNACAAGTATGGCGNNCNCCC
CGGGAAGGANNCNGCCGTANGTGCNNNNNANNNNNTCTTGNGANGTCGCCGNNATTNNCGTGGTCNNN
NNAGANTGNCCGNACCANNCGNANNNTGGTNATGNNAGTNNNNNNNNNNNNNNNNNNNNNNNNNNNNN
NNNNNNNNNNNNNNNNNNNNNNNNNNNNNNNNGNNNNGNNNNNNCNANNNAAGGANNTANNNCTNNTN
NTNNNNNNGGACNNNACNNNNCNNNNNNNACNCNNNCNCNNNNNNANNNGNTTTTTNNNCNNTATTNT
CCAGNACGCNGNGCNNNCNTTCANGNGAACNNNGNNACGCGNNAATCGTANNATCTACCTGCCGCNNG
NNNNNNTNCNGNNNNNNCGGANNNTNNCNCGTANNNNNNTCATGCGTNNTNTNNTCNCNAGNCNNGTT
CGNNNANANANTAGNANGTNTCNNNNNNCTTGTNNCGTTCCGCNTGANNCNNCANNNNGNNGNNNTTC

NCANCGGCNNTANCTGCNGANNTAAGNCATANNNTNNNTCNNTANCTNNNNNGGNGGGNNACTGTAAA
NGGNNAATTGGANNNTNANTNCNNNCATNTNNCTNNGNNNNCTNNNTNCCTGNNTNCNNAATTGGNNN
CANTGNNTGNCNATCTCTGGNTTNGNNNNNNTNNNNNTNNCAANNNGGNNNGNNANANNNCNATNTAA
NNTANANNNNCNNNNNNANNNCACNNNNNNNGNAGNNACNANNNGTANANNNGTAGNNNGNTAAGTCT
CCGANNTNNNNNNNATAAGCGNNANACNNNNNNNNNNNNNANGANNACGNNTTTGNNNTNNNNNNCNA
GNNAGACNNTNGTGGNTNNNNNTCAANNNNTTNGTAGTGGTATAATNGNNNNNNNTTTCNCCAGNNTN
NNNGNNNNNNNCNATANNANNNNNNNGNGANNNNNNNNNNANNNNCTCNNNAGGNANCANCTACTNNA
GCANTGGNGCGNAGNNNGAGANNNNGACNNNCCTTGGGTGGCAANCACNGCAATAAGTNNNGTTNANC
CTNNNAGTNNACACGNNNNCANNGNNNCNNNNNNANCNGANNGNNNNNNNNNNNNNNNNNNNNNNNNN
NNNNNNNNNNNNNNNNNNNNNNNNNNNNAGNNNANGGNNNGTNTNTGACNCATGTGTTTTTANNNGNN
NNNNNACGNNNNANCCCCGGTCGNNNNCNNATTGAANNCCTGNNGCATNNNNNTNCNAGAATGANNGC
NNNNNGNNTNGNCTNNCNNNTCAAGNAATCANNNAGNNANCGCGNTCNNNGNNNNNNNNCNNTCCCNN
ACTGAGGGTNNNNNNNNCATCTGCTNNNNNNNNNNTNCGCNGGCNTNNCNNNNNGCNGTATNNTGTNA
NNCANNNNNNNNNNNNNNNNNNNANNNANNNNNNTNNNN
>LaChi565
GTGNNNNNTAGCCCGNNNNAGTTAGACANNNCNNNNNNNNNNNNTNNNNNNNNNNNNNNNNNNNNNNN
NNNNNNNNNNNNNNNNNNNNNNNNNNNNNNTNNNNNNNNNNNNNNNNNNNNNNNNNNNNNNNNNNNNNN
NNNNNNNNNNNNNNNNNNNNNNNNNNNNNNNNNNNNNNNNNNNNNNNNNNNNNNNNNNNNNNNNNNNN
NNNNNNNNNNNNNNNNNNNNNNNNNNNNNNNNNNNNNNNNNNNNNNNNNNNNNNNNNNNNNNNNNNNN
NNNNNNNNNNNNNNNNNNNNNNNNNNNNNNNNNNNNNNNNNNNNNNNNNNNTCTNNNAAANNNTNNN
NNNNNNNNNNNNNNNNNNNNNNNNNCNCNTTGNNNGTAANCNANNNGNACATGCAGTCCCCTCNNNNN
NNNGGCAGAGNNNNNNNCCNNNNNNNCCGNCCCCNNCCNNNNNNNNNNNCNNNNNNNNNNNANNNNNN
NNNNNNTGGTACNNNNCGNNNNNGGTTGTTTAAGAGGTCCACAACGNGGTCNGGNNACNNNNGNNNAN
NNNCNNNNAACNNNNNNNNNNNNNNTNNNTANNAGGNCNNNNCCNTCNNNNNNNNNANTGCNATGACC
CAANNCACGGGGGCNGTNCCCGGCGCGGCGACCTTGNNNANNTCCGGGGACAAGTATGGCNNNCNCCC
CGGGAAGGANNCNGCCGTANGTGCNNNNNANNNNNTCNTGNGANGTCGCCGNNNTTCNCGTGGTCNNN
CNAGAATGNCCGNACCANNCGNANNNTGGTNATGNNAGTNNNNNNNNNNNNNNNNNNNNNNNNNNNNN
NNNNNNNNNNNNNNNNNNNNNNNNNNNNNNNNNNNNNGNNNNNNCNANNNAAGGANNTANNNCTNNTN
NTNNNNNNGGACNNNACNNNNCNNNNNNNACNCNNNCNCNNNNNNANNNGGTTTTTNNNCNNTANTNT
CCAGNACGCNGNGCNNNCATTCANGNGAACNNNGNNACGCGTNAATCGTANNATCTACCTGCCGCNNG
NNNNNNTACNGNNNNTNCGGANNNTNNCTCGTANNNNNNTCANGCGTNNTNTNNTCNCNAGNCNNGTT
CGNNNANANANTAGNANGTNTNNNNNNNNNTGTNNCGTTCCGCNTGANNCNNCANNNNGNNGNNNTTN
NCANCGGCNNTANCTGCNGANNTAAGNCATANNNTNNATCNNTANCTNNNNNGGNGGGNNACTGTAAA
NGGNNAATTNGANNNTNANTNCNNNCACNTNNCNNNGNNTNCTNNNTNCCTGNNTNCNNAATTGGNNN
CANTGNNTGNCNATCTCTGGNTTNGNNNNNNTNNNNNTNNCANNNNGGNNNGTNNNANNNCNATNTAA
NNTANANNNNCNNNNNNANNNCACNNNNNNNGNAGNNNNNANNNTTANANNNGTAGNNNAATAAGTCT
CCGANNTNNNNNNNATAAGCGNNANACNNNNNNNNNNNNNANGANNACGNNTTGGNNNTNNNNNNCNA
GNNAGACNNTNATGGNTNNNNNTCAANNNNTTNGTAGTGGTATAATNGNNNNNNNTTTCNCCAGNNTN
NNNGNNNNNNNCNATANNANNNNNNNGNGANNNNNNNNNNANNNNCTCNNNAGGGANCANCTACTTNA
GCANTGGNGCGNAGNNNGAGANNNNGACCAGCCTTGGGTGGCAANCACNGCAATAAGTNNNGTTNANN
CTNNNAGTNNACATNNNNNCANNGNNNCNNNNNNANCNGANNGNNNNNNNNNNNNNNNNNNNNNNNNN
NNNNNNNNNNNNNNNNNNNNNNNNNNNNAGNNNANGGNNNGTNTNTGACNCATGTGTTTTTANNNGNN
NNNNNACGNNNGANCCCCGGTCGNNNNCNNATTGAANNCCTGNNGCATNNNNNTNCNAGAATGANNGC
NNNNNGNNTNGNCTNNCNNNTCAAGNAATCAANNAGGTANNNNNNTCNNNGNNNNNNNNCNNNCCCNN
ACTGAGGGTNNNNNNNNCATCTGCTNNNNNNNNNNTNCGCNGGCNTNNCNNNNAGCNGTATNNNGTNA
NNCANNNNNNNNNNNNNNNNTNNAACAANNNNNCTNNNN
>LaHu342
GTGNNNNNTAGCCCNNNNNAGTCAGACANNNCNNNNNNNNNNNNTNNNNNNNNNNNNNNNNNNNNNNN
NNNNNNNNNNNNNNNNNNNNNNNNNNNNNNNNNNNNNNNNNNNNNNNNNNNNNNNNNNNNNNNNNNNN
NNNNNNNNNNNNNNNNNNNNNNNNNNNNNNNNNNNNNNNNNNNNNNNNNNNNNNNNNNNNNNNNNNNN
NNNNNNNNNNNNNNNNNNNNNNNNNNNNNNNNNNNNNNNNNNNNNNNNNNNNNNNNNNNNNNNNNNNN
NNNNNNNNNNNNNNNNNNNNNNNNNNNNNNNNNNNNNNNNNNNNNNNNNNNNTCTNNNAAANNNTNNN
NNNNNNNNNNNNNNNNNNNNNNNNNCNCNNTNNNNGTAANCNANNNGNACATGCAGTCCCCTCNNNNN
NNNNNCAGAGNNNNNNNCCNNNNNNNCCGNCCCCNNCCNNNNNNNNNNNNNNNGNNNNNNNANNNNNN

NNNNNNTGGTACNNNNCGNNNNNGGTTGTTTAAGAGGTCCACAACGNGGTCNNGNNACNNNNGNNNAN
NNNCNNNNAACNNNNNNNNNNNNANNTNNNTANNAGGNCNNNNCCNTCNNNNNNNNNANNGCNATGACC
CANNNNNCGGNGGCNGTNCCCGGNGCGGNGACCTTGNNNACGTCCGGGGACAAGTATGGCGNNCNCCC
CGGGAAGGANNCNGCCGTANGTGCNNNNNANNNNNTCNTGNGANGTCGCCGNNATTNNCNTGGTCNNN
CNAGAATGNCCNNACCANNCGNANNNTGGTNATGNNAGTNNNNNNNNNNNNNNNNNNNNNNNNNNNNN
NNNNNNNNNNNNNNNNNNNNNNNNNNNNNNNNNNGNNNNGNNNNNNCNANNNAAGGNNNTANNNCTNNTN
NTNNNNNNGGACNNNACNNNNCNNNNNNNACNCNNNCNCNNNNNNANNNGNTTTTTNNNCNNTATTNT
CCAGNACGCNGNGCNNNCATTCANGNGAACNNNGNNACGCGNNAATCGTANNATCTACCTGCCGCNNG
NNNNNNTACNGCNNNNNCGGANNNTNNCTCGTANNNNNNTCATGCGTNNTNTNNTCNCNAGNCANGTT
CGNNNANANANTAGNANGTNTCNNNNNNNNNTGTNNCGTTCCGCNTGANNCNNCANNNNGNNGNNNTTN
TCANCGGCNCTANCTNCNGANNTAAGNCATANNNTNNNTCNNTANCTNNNNNGGNGGGNNACTGTAAA
NGGNNAATTNGNNNNTNATTTCNNNCATNTNNCTNNGNNNNCTNNNTNCCTGNNTNCNNAATTGGNNC
CANTGNNTGNCNATCTCTGGNTTNGNNNNNNTNNNNNTNNCANNNNGGNNNGTNANANNNCNATNTAA
NNTANANNNNCNNNNNNANNNCACNNNNNNNNNAGNNNCNANNNGTANANNNGTAGNNNGNTNAGTCT
CCGANNTNNNNNNNATAAGCGNNANACNNNNNNNNNNNNNNANGANNACGNNTTTGNNNTNNNNNNCNA
GNNANACNNTNGTGGNTNNNNNTCAANNNNTTCGTAGTGGTATAATNGNNNNNNNTTTCNCCAGNNTN
NNNGNNNNNNNNCNATANNANNNNNNNGNGANNNNNNNNNNNANNNNCTCNNNAGGGANCANCTACTTNA
GCANTGGNGCGNAGNNNGAGANNNNGACNNNCCTTGGGTGGCAANNACNGCAATAAGTNNNGTTNANC
CTNNNAGTNNACACNNNNNCANNGNNNCNNNNNNANCNGANNGNNNNNNNNNNNNNNNNNNNNNNNNN
NNNNNNNNNNNNNNNNNNNNNNNNNNNNNNNNGNNNANGGNNNGTNTNTGACNCATGTGTTTTTANNNGNN
NNNNNACGNNNNANCCCCGGTCGNNNNCNNATTGAANNCCTGNNGCATNNNNNNTNCNAGAATGANNNC
NNNNNGNNTNGNCTNNNNNNNTCAAGNAATCNNNNANNNNNNNNNGNTCNNNGNNNNNNNNNCNNNCCCNN
ACTGAGGGTNNNNNNNNCATCTGCTNNNNNNNNNNTNCGCNGGNGTNNCNNNNNNGCNGTATNGNGTNN
NNCANNNNNNNNNNNNNNNNNNTNAAACNNNNNNNNNTNNNN
>LaHu343
GTGNNNNNTAGCCCNNNNNAGTCAGACANNNCNNNNNNNNNNNNNTNNNNNNNNNNNNNNNNNNNNNNNN
NNNNNNNNNNNNNNNNNNNNNNNNNNNNNNNNNTNNNNNNNNNNNNNNNNNNNNNNNNNNNNNNNNNNNN
NNNNNNNNNNNNNNNNNNNNNNNNNNNNNNNNNNNNNNNNNNNNNNNNNNNNNNNNNNNNNNNNNNNNNN
NNNNNNNNNNNNNNNNNNNNNNNNNNNNNNNNNNNNNNNNNNNNNNNNNNNNNNNNNNNNNNNNNNNNNN
NNNNNNNNNNNNNNNNNNNNNNNNNNNNNNNNNNNNNNNNNNNNNNNNNNNNNNTCTNNNAAANNNTNNN
NNNNNNNNNNNNNNNNNNNNNNNNNNNCNCNTTGNNNGTAANCNANNNGNACATGCAGTCCCCTCNNNNN
NNNGGCAGAGNNNNNNNCCNNNNNCNCCGGCCCCNNCCNNNNNNNTNNNNNNNGNNNNNNNANNNNNN
NNNNNNTGGTACNNNNCGNNNNNGGTTGTNTAAGAGGTCCACAACGNGGTCNGGNNACNNNNGNNTAN
NNNCNNNNAACNNNNNNNNNNNNANNTNNNTANNAGGNCNNNNCCNTCNNNNNANTAANTGCNATGACC
CAANNCNCGGGGGCNGTNCCCGGCGNGGCGACCTTGANNANNTCCGGGGNCAAGTATNGCNNNCNCCC
CGNGAAGGANNCNGCCGTANGTGCNNNNNANNNNNTCNTGNGANGTCGCCGNNATTCNNGTGGTCNNN
CNAGAATGNCCNNACCANNCGNANNNTGGTNATGNNAGTNNNNNNNNNNNNNNNNNNNNNNNNNNNNN
NNNNNNNNNNNNNNNNNNNNNNNNNNNNNNNNNNGNNNNGNNNNNNCNANNNAAGGNNNTANNNCTNNTN
NTNNNNNNGGACNNNACNNNNCNNNNNNNACNCNNNCNCNNNNNNNNNNNGNTTTTNNNNCNNTANTNT
CCAGNANGCNGNGCNNNCNTTCANGNGAACNNNGNNACGCGNNAATCGTANNATCTACCTGCCGCNNG
NNNNNNTACNGNNNNNNCGGANNNTNNCTCGTANNNNNNTCATGCGTNNTNTNNTCNCNAGNCNNGTT
CGNNNANANANTAGNANGTNTNNNNNNNNNNTGTNNCGTTCCGCNTGANNCNNCANNNNGNNGNNNTTN
TCANCGGCNNTANCTGCNGANNTAAGNCATANNNTNNNTCNNTANCTNNNNNGGNGGGNNACTGTAAA
NGGNNAATTGGNNNNTNATTNCNNNCATNTNNCTNNGNNTNCTNNNTNCCTGNNTNCNNAATTGGNNC
CANTGNNTGNCNATCTCTNGNTTNGNNNNNNTNNNNNTNNCANNNNGGNNNNNNANANNNCNATNTAA
NNTANANNNNCNNNNNNANNNCACNNNNNNNNNAGNNNCNANNNGTANANNNGTAGNNNGNTAAGTCT
CCGANNTNNNNNNNATAAGCGNNANACNNNNNNNNNNNNNNANGANNACGNNTTTGNNNTNNNNNNCNA
GNNAGACNNTNGTGGNTNNNNNTCAANNNNTTNGTAGTGGTATAATNGNNNNNNNTTTCNCCAGNNTN
NNNGNNNNNNNNCNATANNANNNNNNNGNGANNNNNNNNNNNANNNNCTCNNNAGGGANCANCTACTNNA
GCANTGGNNCGCAGNNNGAGANNNNGACNNGCCTTGGGTGGCAANNACNGCAATAAGTNNNGTTNANC
CTNNNAGTNNACACNNNNNCANNGNNNCNNNNNNANCNGANNGNNNNNNNNNNNNNNNNNNNNNNNNN
NNNNNNNNNNNNNNNNNNNNNNNNNNNNNNNNNNNNANGGNNNGTNTNTGACNCATGTGTTTTTANNNGNN
NNNNNACGNNNNANCCCCGGTCGNNNNCNNATTGAANNCCTGNNGCATNNNNNNTNCNAGAATGANNNC
NNNNNGNNTNGNCTNNCNNNTCAAGNAATCANNTAGNNNNNNNNNNTCNNNGNNNNNNNNNCNNNCCCNN

ACTGAGGGTNNNNNNNNCATCTGCTNNNNNNNNNNCTNCGCNGGNGTNNCNNNNNAGCNGTATNNTGTNA
NNCANNNNNNNNNNNNNNNNNNTNAAACAANNNNNCTGNNN
>LaHu344
GTGNNNNNTAGCCCNNNNNAGTTAGACANNNCNNNNNNNNNNNNNTNNNNNNNNNNNNNNNNNNNNNNNN
NNNNNNNNNNNNNNNNNNNNNNNNNNNNNNNNNNNNNNNNNNNNNNNNNNNNNNNNNNNNNNNNNNNNNN
NNNNNNNNNNNNNNNNNNNNNNNNNNNNNNNNNNNNNNNNNNNNNNNNNNNNNNNNNNNNNNNNNNNNNN
NNNNNNNNNNNNNNNNNNNNNNNNNNNNNNNNNNNNNNNNNNNNNNNNNNNNNNNNNNNNNNNNNNNNNN
NNNNNNNNNNNNNNNNNNNNNNNNNNNNNNNNNNNNNNNNNNNNNNNNNNNNNNCNTCTNNNAAANNNTNNN
NNNNNNNNNNNNNNNNNNNNNNNNNNNCNCNTTGNNNGTAANCNANNNGNACATGCAGTCCCCTCNNNNN
NNAGGCAGAGNNNNNNCNCNNNNNNNNCCGGCCCCNNCCNNNNNNNNNNNNNNNNNGNNNNNNNANNNNNN
NNNNNNTGGTACNNNNCGNNNNNGGTTGTNTNAGAGGTCCACCACGNGGTCNNGNNACNNNNGNNNAN
NNNCNNNNAACNNNNNNNNNNNNNANATNNNTANNAGGNCNNNNCCNTCNNGNNNNTAATNGCNATGACC
CAANNNACGNNNGCNGTNCCCGGCGNGGCGACCTTGANNANGTCCGGGNNCAAGTATGGCGNNCNCCC
CGGGAAGGANNCNGCCGTANGTGCNNNNNANNNNNTCNTGNGANGTNGCCGNNATTNNCGTGGTCNNN
CNAGAATGNCCGNACCANNCGNANNNTGGTNATGTNAGTNNNNNNNNNNNNNNNNNNNNNNNNNNNNNN
NNNNNNNNNNNNNNNNNNNNNNNNNNNNNNNNNNNGNNNNGNNNNNNCNANNNAAGGANNTANNNCTNNTN
NTNNNNNNNGGACNNNACNNNNCNNNNNNNACNCNNNCNCNNNNNNNNNNNGNTTTTTNNNCNNTATTNT
CCAGNACGCNGNGCNNNCNTTCANGNGAACNNNGNNACGCGNNAATCGTANNATCTACCTGCCGCNNG
NNNNNNTNCNGNNNNNNCGGANNNTNNNNCGTANNNNNNNTATACGTNNTNTNNTCNCNAGNCANGTT
CGNNNANANANTAGNANGTNTNNNNNNNNNNTGTNNCGTTCCGCNTGANNCNNCANNNNGNNGNNCTTN
TCANCGGCNNTANCTGCNGANNTAAGNCATANNNTNNNTCNNTANCTNNNNNGGNGGGNNACTGTAAA
NGGNNAATTGGANGNTNANTNCNNNCACNTNNCNNNGNNTNCTNNNTNCCTGNNTNCNNAATTGGNNC
CANTGNNTGNCNATCTCTNGNTTNGNNNNNNTNNNNNTNNCANNNNGGNNNNNNNNANNNCNANNTAA
NNTANANNNNCNNNNNNANNNCACNNNNNNNGNAGNNNCNANNNTTANANNNGTAGNNNANTAAGTCT
CCGANNTNNNNNNNATAAGCGNNANACNNNNNNNNNNNNNNANGANNNCGNNTTGGNNNTNNNNNNCNA
GNNAGACNNTNGTGGNTNNNNNTCAANNNNNTTCGTAGTGGTATAATNGNNNNNNNTTTCNCCAGNNTN
NNNGNNNNNNNCNATANNANNNNNNNGNGANNNNNNNNNNNANNNNCTCNNNAGGGANCANCTACTNNA
GCAATGGNGCGCAGNNNGAGANNNNGACNNNCCTTGGGTGGCAANNACNGNAATAAGTNNNGTTNNNC
CTNNNAGTNNACACNNNNNCANNGNNNCNNNNNNANCNGANNGNNNNNNNNNNNNNNNNNNNNNNNNNN
NNNNNNNNNNNNNNNNNNNNNNNNNNNNNNAGNNNANGGNNNGTNTNTGACNCATGNNTTTTTANNNGNN
NNNNNNNGNNNNANCCCCGGTCGNNNNCNNATTGAANNCCTGNNGCATNNNNNNTNCNAGAATGANNNC
NNNNNGNNTNGNCTNNCNTTTCAAGNAGTNANNTAGGTNNNNNGNTCNNNGNNNNNNNNCNNNCCCNN
ACTGAGGGTNNNNNNNNCATCNGCTNNNNNNNNNNTNCGCNGGNGTNNCNNNNNAGCNGTATNGNGNNA
NNCANNNNNNNNNNNNNNNNNNNNNAAACNANNNNNNNTNNNN
>LaHu345
GTGNNNNNTAGCCCNNNNNAGTTAGACANNNCNNNNNNNNNNNNNTNNNNNNNNNNNNNNNNNNNNNNNN
NNNNNNNNNNNNNNNNNNNNNNNNNNNNNNNTNNNNNNNNNNNNNNNNNNNNNNNNNNNNNNNNNNNNNN
NNNNNNNNNNNNNNNNNNNNNNNNNNNNNNNNNNNNNNNNNNNNNNNNNNNNNNNNNNNNNNNNNNNNNN
NNNNNNNNNNNNNNNNNNNNNNNNNNNNNNNNNNNNNNNNNNNNNNNNNNNNNNNNNNNNNNNNNNNNNN
NNNNNNNNNNNNNNNNNNNNNNNNNNNNNNNNNNNNNNNNNNNNNNNNNNNNNNNCNTCTNNNAAANNNTNNN
NNNNNNNNNNNNNNNNNNNNNNNNNNNCNCNNTGNNNGTAANCNANNNGNACATGCAGTCCCCTCNNNNN
NNNGGCAGAGNNNNNNNNCCNNNNNNNNCCGGCCCCNNCCNNNNNNNNNNNNNNNNNNNNNNNNNNNANNNNNN
NNNNNNTGGTACNNNNCGNNNNNGGTTGNNTAAGAGGTCCACAACGNGGTCNNGNCACNNNNGNNNAN
NNNCNNNNAANNNNNNNNNNNNNNANNTNNNTANNAGGNCNNNNCCNTCNNGNNANNNATNGCNATGACC
CAANNNACGGGGGCNGTNCCCGGCGCGGCGACCTTGANNACGTCCGGGGACAAGTATGGCGCNCNCCC
CGGGAAGGANNCNGCCGTANGTGCNNNNNANNNNNTCNTGNGANGTCGCCGNNATTNNCGTGGTCNNN
NNAGAATGNNCGNACCANNCGNANNNTGGTNATGNNAGTNNNNNNNNNNNNNNNNNNNNNNNNNNNNNN
NNNNNNNNNNNNNNNNNNNNNNNNNNNNNNNNNNNNNNNGNNNNNNCNANNNAAGGNNNTANNNCTNNTN
NTNNNNNNNGGACNNNACNNNNCNNNNNNNACNCNNNCNCNNNNNNANNNGNTTTTTNNNCNNTANTNT
CCAGNACGCNGNGCNNNCATTCANGNGAACNNNGNNACGCGNNAATCGTANNATCTACCTGCCGCNNG
NNNNNNTACNGNNNNNNCGGANNNTNNNNCGTANNNNNNTCATGCGTNNTNTNNTCNCNAGNCANGTT
CGNNNANANANTAGNANGTNTNNNNNNNNNNTGTNNCGTTCCGCNTGANNCNNCANNNNGNNGNNCTTN
TCANCGGCNNTANCTGCNGANNTAAGNCATANNNTNNNTCNNTANCTNNNNNGGNGGGNNACTGTAAA
NGGNNAATTNGNNNNTNANTNCNNNCACNTNNCTNNGNNTNCTNNNTNCCTGNNTNCNNAATTGGNNC

CANTGNNTGNCNATCTCTGGNTTNGNNNNNNTNNNNNTNNCAANNNGGNNNGTNANANNNCNATNTAA
NNTATANNNNCNNNNNNANNNCACNANNNNNGNAGNNNCNANNNTNANANNNGTAGNNNANTAAGTCT
CCGANNTNNNNNNNATAAGCGNNANACNNNNNNNNNNNNNNANGANNACGNNTTGGNNNTNNNNNNCNA
GNNANACNNTNATGGNTNNNNNTCAANNNNTTCGTAGTGGTATAATNGNNNNNNNTTTCNCCAGNNTN
NNNGNNNNNNNNCNATANNANNNNNNNGNGANNNNNNNNNNNANNNNCTCNNNAGGGANCANCTACTNNA
GCANTGGNGCGCAGNNNGAGANNNNGACNNNCCTTGGGTGGCAANCACNGCGATAAGTNNNGTTNANC
CTNNNAGTNNACATNNNNNCANNGNNNCNNNNNNANCNGANNGNNNNNNNNNNNNNNNNNNNNNNNNN
NNNNNNNNNNNNNNNNNNNNNNNNNNNNNNAGNNNANGGNNNGTNTNTGACNCATGTGTTTTTANNNGNN
NNNNNACGNNNNANCCCCGGTCGNNNNCNNATTGAANNCCTGNNGCATNNNNNTNCNAGAATGANNGC
NNNNNGNNTNGNCTNNCNNNTCAAGNAATCAANNAGGTNNNGCGNTCNNNNNNNNNNNNCNNTCCCNN
ACTGAGGGTNNNNNNNNCATCNGCTNNNNNNNNCTTCGCNGGCGTNNCNNNNNAGCNGTATNGNGTNN
NNCANNNNNNNNNNNNNNNNNTNAAACGANNNNNCTNNNN

>LaHu346

GTGNNNNNTAGCCCNNNNNAGTTAGACANNNCNNNNNNNNNNNNNTNNNNNNNNNNNNNNNNNNNNNNNN
NNNNNNNNNNNNNNNNNNNNNNNNNNNNNNNNNNNNNNNNNNNNNNNNNNNNNNNNNNNNNNNNNNNNNN
NNNNNNNNNNNNNNNNNNNNNNNNNNNNNNNNNNNNNNNNNNNNNNNNNNNNNNNNNNNNNNNNNNNNNN
NNNNNNNNNNNNNNNNNNNNNNNNNNNNNNNNNNNNNNNNNNNNNNNNNNNNNNNNNNNNNNNNNNNNNN
NNNNNNNNNNNNNNNNNNNNNNNNNNNNNNNNNNNNNNNNNNNNNNNNNNNNNCNTCTNNNAAANNNTNNN
NNNNNNNNNNNNNNNNNNNNNNNNNNCNCNNTGNNNGTAANCNANNNGNACATGCAGTCNCCTCNNNNN
NNNGGCAGAGNNNNNNNCNNNNNNNNCCGGCCCCGNCCNNNNNNNNNNNNNNNNNGNNNNNNNGNNNNNN
NNNNNNTGGTACNNNNCGNNNNNGGTTGTNTAAGAGGTCCACCACGNGGTCNNGNCACNNNNGNNNAN
NNNCNNNAAACNNNNNNNNNNNNNNNATNNNCANNAGGNCNNNNCCNTCNNNNNNNNNAATNGCNATGACC
CAANNCACGGGGGCNGTNCCCGGCGNGGCGACCTTGNNNANNGCCGGGGACAAGTATGGCGNNCNCCC
CGGGAAGGANNCNGCCGTANGTGCNNNNNANNNNNTCNTGNGANGTCGCCGNNATTCNCGTGGNCNNN
CNNGAATGNNCGNACCANNCGNANNNTGGTTATGNNAGTNNNNNNNNNNNNNNNNNNNNNNNNNNNNNN
NNNNNNNNNNNNNNNNNNNNNNNNNNNNNNNNNNGNNNNGNNNNNNCNANNNAAGGNNNTANNNCTNNTN
NTNNNNNNNGGACNNNACNNNNCNNNNNNNACNCNNNCNCNNNNNNANNNGNTTTTTNNNCNNTANTNT
CCAGNACGCNGNGCNNNCATTCANGNGAACNNNGNNACGCGNNAATCGTANNATCTACCTGCCGCNNG
TNNNNNTACNGNNNNNNCGGANNNTNNCTCGTANNNNNNNCATGCGTNNTNTNNTNNCNAGNCANGTT
CGNNNANANANTAGNANGTNTNNNNNNNNNNTGTNNCGTTCCGCNTGANNCNNCANNNNGNNGNNNTTN
TCANCGGCNNTANCTGCNGANNTAAGNCATANNNTNNATCNNTANCTNNNNNGGNGGGNNACTGTAAA
NGGNNAATTNGNNNNTNANTTCNNNCACNTNNCTNNGNNNNCTNNNTNCCTGNNTNCNNAATTGGNNC
CANTGNNTGNCNATCTCTGGNTTNGNNNNNNTNNNNNTNNCAANNNGGNNNGTNANANNNCNATNTAA
NNTANANNNNCNNNNNNANNNCACNNNNNNNGNAGNNNCNANNNTTANANNNGTAGNNNANTAAGTCT
CCGANNTNNNNNNNATAAGCGNNANACNNNNNNNNNNNNNNANGANNACGNNTTGGNNNTNNNNNNCNA
ANNAGACNNTNGTGGNTNNNNNTCAANNNNTTNGTAGTGGTATAACNGNNNNNNNTTTCNCCAGNNTN
NNNGNNNNNNNNCNATANNANNNNNNNGNGANNNNNNNNNNNANNNNCTCNNNAGGGANCANCTACTTNA
GNANTGGNGCGNAGNNNGAGANNNNGACNNGCCTTGGGTGGCAANCACNGCAATAAGTNNNGTTNANC
CTNNNAGTNNACACNNNNNCANNGNNNCNNNNNNANCNGANNTNNNNNNNNNNNNNNNNNNNNNNNNN
NNNNNNNNNNNNNNNNNNNNNNNNNNNNNNAGNNNANNGNNNGTNTNTGACNCATNNNTTTTTANNNGNN
NNNNNACGNNNGANCCCCGGTCGNNNNCNNATTGAANNCCTGNNGCATNNNNNTNCNAGAATGANNNC
NNNNNGNNTNGNCTNNCNNNTCAAGNAGTCANNTAGGTANNNNGNTCNNNGNNNNNNNNCNNNCCCNN
ACTGAGGGTNNNNNNNNCATCTGCTNNNNNNNNNNTNCGCNGNCGTNNCNNNNNAGCNGTATNGTGNNN
NNCANNNNNNNNNNNNNNNNNTNANACGANNNNNNNTNNNN

>LaHu347

GTGNNNNNTAGCCCNNNNNAGTTAGACANNNCNNNNNNNNNNNNNTNNNNNNNNNNNNNNNNNNNNNNNN
NNNNNNNNNNNNNNNNNNNNNNNNNNNNNNNNTNNNNNNNNNNNNNNNNNNNNNNNNNNNNNNNNNNNNN
NNNNNNNNNNNNNNNNNNNNNNNNNNNNNNNNNNNNNNNNNNNNNNNNNNNNNNNNNNNNNNNNNNNNNN
NNNNNNNNNNNNNNNNNNNNNNNNNNNNNNNNNNNNNNNNNNNNNNNNNNNNNNNNNNNNNNNNNNNNNN
NNNNNNNNNNNNNNNNNNNNNNNNNNNNNNNNNNNNNNNNNNNNNNNNNNNNNCNTCTNNNAAANNNTNNN
NNNNNNNNNNNNNNNNNNNNNNNNNNCNCNTTGNNNGTAANCNANNNGNACATGCAGTCCCCTCNNNNN
NNNGGCAGAGNNNNNNCCCNNNNNCNCCGGCCCCNNCCNNNNNNNNNNNNNNNNNNNNNNNNNNANNNNNN
NNNNNNTGGTACNNNNCGNNNNNGGTTGTNTAAGAGGTCCACAACGNGGTCNNGNCACNNNNGNNNAN
NNNCNNNAAACNNNNNNNNNNNNNANNTNNNTANNAGGNCNNNNCCNTCNNGNNNNNAATTGCNATGACC

CAANNCACGGGGGCAGTCCCCGGCGCGGCGACCTTGANNACGTCCGGGGACAAGTATGGCGNNCNCCC
CGGGAAGGANNCNGCCGTANGTGCNNNNNANNNTNTCNTGNGANGTCGCCGNNATTNNCGTGGTCNNN
NNAGAATGCCCGNACCANNCGNANNNTGGTNATGNNAGTNNNNNNNNNNNNNNNNNNNNNNNNNNNNN
NNNNNNNNNNNNNNNNNNNNNNNNNNNNNNNGNNNNGNNNNNNCNANNNNAAGGANNTANNNCTNNTN
NTNNNNNNGGACNNNACNNNNCNNNGNNNACNCNNNCNCNNNNNNANNNGGTTTTTNNCCNNTATTNT
CCAGNACGCNGNGCNNNCATTCANGNGAACNNNGNNACGCGNNAATCGTANNATCTACCTGCCGCNNG
TNNNNNTACNGNNNNNNCGGANNNTNNNNCGTANNNNNNTCATGCGTNNTNTNNTCNCNAGNCANGTT
CGNNNANANANTAGNANGTNTNNNNNNNNNNTGTNNCGTTCCGCNTGANNCNNCANNNNGNNGNNCTTC
NCANCGGCNNTANCTGCNGANNTAAGNCATANNNTNNATCNNTANCTNNNNNGGNGGGNNACTGTAAA
NGGNNAATTNGNNNNTNANTTCNNNCACNTNNCTNNGNNTNCTNNNTNCCTGNNTNCNNAATTGGNNC
CANTGNNTGNCNATCTCTGGNTTNGNNNNNNTNNNNNTNNCANNNNGGNNNGTNANANNNCNATNTAA
NNTANANNNNCNNNNNNANNNCACNNNNNNNGNAGNNNCNANNNTTANANNNGTAGNNNAATAAGTCT
CCGANNTNNNNNNNATAAGCGNNANACNNNNNNNNNNNNNANGANNACGNNTTGGNNNTNNNNNNCNA
GNNAGACNNTNATGGNTNNNNNTCAANNNNTTNGTAGTGGTATAATNGNNNNNNNTTTCNCCAGNNTN
NNNGNNNNNNNCNATANNANNNNNNNGNGANNNNNNNNNNNANNNNCTCNNNAGGGANCANCTACTTNA
GCAATGGNGCGCAGNNNGAGANNNNGACNNGCCTTGGGTGGCAANCACNGCGATAAGTNNNGTTNANC
CTNNNAGTNNACATNNNNNCANNGNNNCNNNNNNANCNGANNGNNNNNNNNNNNNNNNNNNNNNNNNN
NNNNNNNNNNNNNNNNNNNNNNNNNNNNCAGNNNANGGNNNGTNTNTGACNCATGTGTTTTANNNGNN
NNNNNACGNNNNANCCCCGGTCGTNNNCNNATTGAANNCCTGNNGCATNNNNNTNCNAGAATGANNGC
NNNNNGNNTNGNCTNNCNTTTCAAGNAATCANNNAGGTANNNNGNTCNNNGNNNNNNNNCNNTCCCNN
ACTGAGGGTNNNNNNNNCATCTGCTNNNNNNNNNCTNCGCNGGNGTNNCTNNNNAGCNGTATNGTGTNA
NNCANNNNNNNNNNNNNNNNNTNAAACAANNNNNCTNNNN
>LaHu348
GTGNNNNNTAGCCCNNNNNAGTTAGACANNNCNNNNNNNNNNNNTNNNNNNNNNNNNNNNNNNNNNNN
NNNNNNNNNNNNNNNNNNNNNNNNNNNNNNTNNNNNNNNNNNNNNNNNNNNNNNNNNNNNNNNNNNNNN
NNNNNNNNNNNNNNNNNNNNNNNNNNNNNNNNNNNNNNNNNNNNNNNNNNNNNNNNNNNNNNNNNNNN
NNNNNNNNNNNNNNNNNNNNNNNNNNNNNNNNNNNNNNNNNNNNNNNNNNNNNNNNNNNNNNNNNNNN
NNNNNNNNNNNNNNNNNNNNNNNNNNNNNNNNNNNNNNNNNNNNNNNNNNNNTCTNNNAAANNNTNNN
NNNNNNNNNNNNNNNNNNNNNNNNNCNCNTTGNNNGTAANCNANNNGNACATGCAGTCCCCTCNNNNN
NNNNGCAGAGNNNNNNCCCNNNNNCNCCGGCCCCGNCCNNNNNNNNNNNCNNNGNNNNNNNGNNNNNN
NNNNNNTGGTACNNNNCGNNNNNGGTTGTNTAAGAGGTCCACCACGNGGTCNNGNNACNNNNGNNTAN
NNNCNNNNAACNNNNNNNNNNNANNTNNNCANNAGGNCNNNGCCNTCNNGNNNNTAATTGCNATGACC
CAANNCACGGGGGCNGTNCCCGGCGCGGCGACCTTGNTNACGGCCGGGGNCAAGTATGGCGNNCNCCC
CGGGAAGGANNCNGCCGTANGTGCNNNNNANNNNNTCNTGNGACGTCGCCGNNATTNNCGTGGTCNNN
CNAGAATGCNCGNACCANNCGNANNNTGGTNATGNNAGTNNNNNNNNNNNNNNNNNNNNNNNNNNNNN
NNNNNNNNNNNNNNNNNNNNNNNNNNNNNNNGNNNNGNNNNNNCNANNNNAAGGNNNTANNNCTNNTN
NTNNNNNNGGACNNNACNNNNCNNNNNNNACNCNNNCNCNNNNNNNNNNGNTTTTTNNNCNNTANTNT
CCAGNACGCNGNGCNNNCATTCANGNGAACNNNGNNACGCGNNAATCGTANNATCTACCTGCCGCNNG
NNNNNNTACNGNNNNNNCGGANNNTNNCTCGTANNNNNNNCATGCGTNNTNTNNTCNCNAGNCANGTT
CGNNNANANANTAGNANGTNTCNNNNNNNNNTGTNNCGTTCCGCNTGACNCNNCANNNNGNNGNNNTTC
TCANCGGCNNTANCTGCNGANNTAAGNCATANNNTNNATCNNTANCTNNNNNGGNGGGNNACTGTAAA
NGGNNAATTNGNNGNTNATTNCNNNCACNTNNCTNNGNNTNCTNNNTNCCTGNNTNCNNAATTGGNNC
CANTGNNTGNCNATCTCTGGNTTNGNNNNNNTGNNNNTNNCAANNNGGNNNGTNNNANNNCNATNTAA
NNTANANNNNCNNNNNNANNNCACNANNNNNNNAGNNNCNANNNTTANANNNGTAGNNNNATAAGTCT
CCGANNTNNNNNNNATAAGCGNNANACNNNNNNNNNNNNNANGANNACGNNTTGGNNNTNNNNNNCNA
ANNAGACNNTNGTGGNTNNNNNTCAANNNNTTNGTAGTGGTATAACNGNNNNNNNTTTCNCCAGNNTN
NNNGNNNNNNNCNATANNANNNNNNNGNGANNNNNNNNNNNANNNNCTCNNNAGGGANCANCTACTNNA
GCANTGGNGCGNAGNNNGAGANNNNGACNNGCCTTGGGTGGCAANCACNGCAATAAGTNNNGTTNNNC
CTNNNAGTNNACACNNNNNCANNGNNNCNNNNNNANCNGANNTNNNNNNNNNNNNNNNNNNNNNNNNN
NNNNNNNNNNNNNNNNNNNNNNNNNNNNNAGNNNANGGNNNGTNTNTGACNCATGTGTTTTANNNGNN
NNNNNACGNNNNANCCCCGGTCGNNNNCNNATTGAANNCCTGNNGCATNNNNNTNCNAGAATGANNNC
NNNNNGNNTNGNCTNNCNNTTCAAGNAGTCANNNAGGTANNNNGNTCNNNGNNNNNNNNCNNTCCCNN
ACTGAGGGTNNNNNNNNCATCTGCTNNNNNNNNNNTTCGCNGNCGTNNCNNCNNAGCNGTATNGTGTNN
NNCANNNNNNNNNNNNNNNNNTNAAACNANNNNNNNTNNNN

>LaHu349
GTGNNNNNTAGCCCNNNNNAGTTAGACANNNCNNNNNNNNNNNNTNNNNNNNNNNNNNNNNNNNNNNN
NNNNNNNNNNNNNNNNNNNNNNNNNNNNNNTNNNNNNNNNNNNNNNNNNNNNNNNNNNNNNNNNNNNN
NNNNNNNNNNNNNNNNNNNNNNNNNNNNNNNNNNNNNNNNNNNNNNNNNNNNNNNNNNNNNNNNNNNN
NNNNNNNNNNNNNNNNNNNNNNNNNNNNNNNNNNNNNNNNNNNNNNNNNNNNNNNNNNNNNNNNNNNN
NNNNNNNNNNNNNNNNNNNNNNNNNNNNNNNNNNNNNNNNNNNNNNNNNNCNTCTNNNAAANNNTNNN
NNNNNNNNNNNNNNNNNNNNNNNNNCNCNTTGNNNGTAANCNANNNGNACATGCAGTCCCCTCNNNNN
NNNGGCAGAGNNNNNNCCNNNNNNCNCCGGCCCCNNCCNNNNNNNNNNNCNNNNNNNNNNNGNNNNNN
NNNNNNTGGTACNNNNCGNNNNNGGTTGTNTNAGAGGTCCACCACGNGGTCNNGNCACNNNNGNNNAN
NNNCNNNAAANNNNNNNNNNNNNNATNNNCANNAGGNCNNNNCCNTNNNNNNNNNNANTGCNATGACC
CAANNCACGGNGGCAGTCCCCGGCGNGGCGACCTTGATNACGGCCGGGGNCAAGTATGGCGTNCNCCC
CGGGAAGGANNCNGCCGTANGTGCNNNNNANNNNNTNNTGNGACGTNGCCGNNNNNNNCGTGGTCNNN
CNAGAATGNCCNNACCANNCGNANNNTGGTNATGNTAGTNNNNNNNNNNNNNNNNNNNNNNNNNNNNN
NNNNNNNNNNNNNNNNNNNNNNNNNNNNNNNNNNNNNGNNNNNNCNANNNAAGGANNTANNNCTNNTN
NTNNNNNNGGACNNNACNNNNCNNNNNNNACNCNNNCNCNNNNNNANNNGNTTTTTNNNCNNTATTNT
CCAGNANGCNGNGCNNNCATTCANGNGAACNNNGNNACGCGNNAATCGTANNNTCTACCTGCCGCNNG
NNNNNNTACNGNNNNNNCGGANNNTNNNNCGTANNNNNNNCATGCGTNNTNTNNTCNCNAGNCANGTT
CGNNNANANANTAGNANGTNTCNNNNNNNNTGTNNCGTTCCGCNTGANNCNNCANNNNGNNGNNNTTN
NCANCGGCNNTANCTGCNGANNTAAGTCATANNNTNNNTCNNTANCTNNNNNGGNGGGNNACTGTAAA
NGGNNAATTNGNNNNTNANTNCNNNCACNTNNCNNNGNNNNCTNNNTNCCTGNNTNCNNAATTGGNNC
CANTGNNTGNCNATCTCTGGNTTNGNNNNNNTNNNNNTNNCANNNNGGNNNNNNNNANNNCNATNTAA
NNTANANNNNCNNNNNNANNNCACNNNNNNNGNAGNNNCNANNNTTANANNNGTAGNNNANTAAGTCT
CCGANNTNNNNNNATAAGCGNNANACNNNNNNNNNNNNNNANGANNACGNNTTGGNNNTNNNNNNCNA
ANNAGACNNTNGTGGNTNNNNNTCAANNNNTTNGTAGTGGTATAACNGNNNNNNNTTTCNCCAGNNTN
NNNGNNNNNNNCNATANNANNNNNNNGNGANNNNNNNNNNANNNNCTCNNNAGGNANCANCTACTNNA
GCANTGGNGCGNAGNNGGAGANNNNGACNNGCCTTGGGTGGCAANCACNGCANTAAGTNNTGTTNANC
CTNNNAGTNNACACNNNNNCANNGNNNCNNNNNANCNGANNTNNNNNNNNNNNNNNNNNNNNNNNNNN
NNNNNNNNNNNNNNNNNNNNNNNNNNNNNAGNNNANGGNNNGTNTNTGACNCNTGNNTTTTTANNNGNN
NNNNNACGNNNGNNCCCCGGTCGNNNNCNNATTGAANNCCTGNNGCATNNNNNTNCNAGAATGANNNC
NNNNNGNNTNGNCTNNNNTTTCAAGNAGTCNNNNAGNNNNNNNNNTCNNNGNNNNNNNNCNNNCCCNN
ACTGAGGGTNNNNNNNNCATCTGCTNNNNNNNNCTTCGCNGNCGTNNCNNNNNNGCNGTATNNNGTNN
NNCANNNNNNNNNNNNNNNNTNAANNNANNNNNNTNNNN
>LaHu350
GTGNNNNNTAGCCCNNNNNAGTCAGACANNNCNNNNNNNNNNNNTNNNNNNNNNNNNNNNNNNNNNNN
NNNNNNNNNNNNNNNNNNNNNNNNNNNNNNTNNNNNNNNNNNNNNNNNNNNNNNNNNNNNNNNNNNNN
NNNNNNNNNNNNNNNNNNNNNNNNNNNNNNNNNNNNNNNNNNNNNNNNNNNNNNNNNNNNNNNNNNNN
NNNNNNNNNNNNNNNNNNNNNNNNNNNNNNNNNNNNNNNNNNNNNNNNNNNNNNNNNNNNNNNNNNNN
NNNNNNNNNNNNNNNNNNNNNNNNNNNNNNNNNNNNNNNNNNNNNNNNNNNNTCTNNNAAANNNTNNN
NNNNNNNNNNNNNNNNNNNNNNNNNCNCNTTNNNNGTAANCNANNNGNACATGCAGTCCCCTCNNNNN
NNNGNCAGAGNNNNNNNCCNNNNNNNCCGNCCCCNNCCNNNNNNNNNNNNNNNNNNNNNNNANNNNNN
NNNNNNTGGTACNNNNCGNNNNNGGTTGTNTAAGAGGTCCACAACGNGGTCNNGNNACNNNNGNNNAN
NNNCNNAAAANNNNNNNNNNNNANATNNNTANNAGGNNNNNNCCNTCNNNNNNNNNATTGCNATGACC
CAANNCACGGGGGCAGTNCCCGGCGCGGCGACCTTGANNACGTCCGGGGACAAGTATGGCGNNCNCCC
CGGGAAGGANNCNGCCGTANGTGCNNNNNANNNNNTCNTGNGANGTNGCCGNNATTNNCGTGGTCNNN
CNAGAATGNCCNNACCANNCGNANNNTGGTNATGNNAGTNNNNNNNNNNNNNNNNNNNNNNNNNNNNN
NNNNNNNNNNNNNNNNNNNNNNNNNNNNNNNNGNNNNGNNNNNNCNANNNAAGGANNTANNNCTNNTN
NTNNNNNNGGACNNNACNNNNCNNNNNNNACNCNNNCNCNNNNNNANNNGNTTTTTNNCCNNTATTNT
CCAGNACGCNGNGCNNNCATTCANGNGAACNNNGNNACGCGNNAATCGTANNATCTACCTGCCGCNNG
NNNNNNTACNGNNNNNNCGGANNNTNNNNCGTANNNNNNTCATGCGTNNTNTNNTCNCNAGNCNNGTT
CGNNNANANANTAGNANGTNTNNNNNNNNNTGTNNCGTTCCGCNTGANNCNNCANNNNGNNGNNNTTN
NCANCGGCNNTANCTGCNGANNTAAGNCATANNNTNNNTCNNTANCTNNNNNGGNGGGNNACTGTAAA
NGGNNAATTNGANNNTNATTNCCNNCATNTNNCTNNGNNNNCTNNNTNCCTGNNTNCNNAATTGGNNC
CANTGNNTGNCNATCTCTGGNTTNGNNNNNNTNNNNNTNNCAANNNGGNNNGTNNNANNNCNATNTAA
NNTANANNNNCNNNNNNANNNCACNANNNNNGNAGNNNCNANNNGTANANNNGTAGNNNGATAAGTCT

CCGANNTNNNNNNNATAAGCGNNANACNNNANNNNNNNNNNANGANNNCGNNTTTGNNNTNNNNNNNCNA
GNNAGACNNTNGTGGNTNNNNNTCAANNNNTTCGTAGTGGTATAATNGNNNNNNNTTTCNCCAGNNTN
NNNGNNNNNNNNCNATANNANNNNNNNNGNGANNNNNNNNNNNANNNNCTCNNNAGGGANCANCTACTNNA
GCANTGGNGCGNAGNNNGAGANNNNGACNNNCCTTGGGTGGCAANNACNGCAATAAGTNNNGTTNANC
CTNNNAGTNNACACNNNNNCANNGNNNCNNNNNNANCNGANNGNNNNNNNNNNNNNTNNNNNNNNNNNN
NNNNNNNNNNNNNNNNNNNNNNNNNNNNNNNNNGNNNANGGNNNGTNTNTGACNCATGTGTTTTTANNNGNN
NNNNNACGNNNNANCCCCGGTCGNNNNCNNATTGAANNCCTGNNGCATNNNNNTNCNAGAATGANNNC
NNNNNGNNTNGNCTNNCNTTTCAAGNAATCANNTAGGTANNNNNNTCNNNNNNNNNNNNCNNTCCCNN
ACTGAGGGTNNNNNNNNCATCTGCTNNNNNNNNNNTTCGCNGGNGTNNCNGNNNAGCNGTATNGNGTNN
NNCANNNNNNNNNNNNNNNNNNTNAAACNANNNNNNNTNNNN
>LaHu351
GTGNNNNNTAGCCCNNNNNAGTTAGACANNNCNNNNNNNNNNNNNTNNNNNNNNNNNNNNNNNNNNNNNN
NNNNNNNNNNNNNNNNNNNNNNNNNNNNNNNNTNNNNNNNNNNNNNNNNNNNNNNNNNNNNNNNNNNNNN
NNNNNNNNNNNNNNNNNNNNNNNNNNNNNNNNNNNNNNNNNNNNNNNNNNNNNNNNNNNNNNNNNNNNNN
NNNNNNNNNNNNNNNNNNNNNNNNNNNNNNNNNNNNNNNNNNNNNNNNNNNNNNNNNNNNNNNNNNNNNN
NNNNNNNNNNNNNNNNNNNNNNNNNNNNNNNNNNNNNNNNNNNNNNNNNNNNNNCNTCTNNNAAANNNTNNN
NNNNNNNNNNNNNNNNNNNNNNNNNNNCNCNTTGNNNGTAANCNANNNGNACATGCAGTCCCCTCNNNNN
NNNGGCAGAGNNNNNNNCCNNNNNNNCCGNCCCCNNCCNNNNNNNNNNNNNNNNNNNNNNNNNANNNNNN
NNNNNNTGGTACNNNNCGNNNNNGGTTGTTTAAGAGGTCCACAACGNGGTCNGGNCACNNNNGNNNAN
NNNCNNNNAANNNNNNNNNNNNNNANNTNNNTANNAGGNCNNNNCCNTCNNGNNNNNNANTGCNATGACC
CANNTCNCGGGGGCNGTNCCCGGCGNGGCGACCTTGNNNACGTCCGGGGACAAGTATGGCGNNCNCCC
CGGGAAGGANNCNGCCGTANGTGCNNNNNANNNNNTCNTGNGANGTCGCCGNNATTNNCGTGGTCNNN
CNAGAATGCCCNNACCANNCGNANNNTGGTNATGNNAGTANNNNNNNNNNNNNNNNNNNNNNNNNNNNN
NNNNNNNNNNNNNNNNNNNNNNNNNNNNNNNNNNNNGNNNNGNNNNNNCNANNNAAGGNNNTANNNCTNNTN
NTNNNNNNNGGNCNNNACNNNNCNNNNNNNNACNCNNNCNCNNNNNNANNNGNTTTTNNNNCNNTATTNT
CCAGNANGCNGNGCNNNCATTCANGNGAACNNNGNNACGCGNNAATCGTANNATCTACCTGCCGCNNG
NNNNNNTACNGNNNNTNCGGANNNTNNNNCGTANNNNNNTCATGCGTNNTNTNNTCNCNAGNCNNGTT
CGNNNANANANTAGNANGTNTNNNNNNNNNNTGTNNCGTTCCGCNTGANNCNNCANNNNGNNGNNCTTN
NCANCGGCNNTANCTGCNGANNTAAGNCATANNNTNNATCNNTANCTNNNNNGGNGGGNNACTGTAAA
NGGNNAATTNGNNNNTNANTNCCNNCACNTNNCTNNGNNNNCTNNNTNCCTGNNTNCNNAATTGGNNC
CANTGNNTGNCNATCTCTNGNTTNGNNNNNNTNNNNNTNNCANNNNGGNNNGTNANANNNCNATNTAA
NNTANANNNNCNNNNNNANNNCACNNNNNNNGNAGNNNCNANNNTNANANNNGTAGNNNAATAAGTCT
CCGANNTNNNNNNNATAAGCGNNANACNNNNNNNNNNNNNNNANGANNACGNNTTGGNNNTNNNNNNNCNA
GNNAGACNNTNATGGNTNNNNNTCAANNNNTTNGTAGTGGTATAATNGNNNNNNNTTTCNCCAGNNTN
NNNGNNNNNNNNCNATANNANNNNNNNNGNGANNNNNNNNNNNANNNNCTCNNNAGGGANCANCTACTNNA
GCANTGGNGCGNAGNNNGAGANNNNGACNNNCCTTGGGTGGCAANCACNGCAATAAGTNNNGTTNANC
CTNNNAGTNNACATNNNNNCANNGANNCNNNNNNANCNGANNGNNNNNNNNNNNNNNNNNNNNNNNNN
NNNNNNNNNNNNNNNNNNNNNNNNNNNNNNNNNANNNNANGGNNNGTNTNTGACNCATGTGTTTTTANNNGNN
NNNNNACGNNNNANCCCCGGTCGNNNNCNNATTGAANNCCTGNNGCATNNNNNTNCNAGAATGANNNC
NNNNNGNNTNGNCTGNCNNNTCAAGNAATCAANNAGGTNNNGCGNTCNNNGNNNNNNNNCNNNCCCNN
ACTGAGGGTNNNNNNNNCATCTGCTNNNNNNNNNNTTCGCNGGNGTNNCNNNNNAGCNGTATNGNGTNA
NNCANNNNNNNNNNNNNNNNNNCNAAACNANNNNNNNTNNNN
>LaHu352
GTGNNNNNTAGCCCNCNNNAGTTAGACANNNCNNNNNNNNNNNNNTNNNNNNNNNNNNNNNNNNNNNNNN
NNNNNNNNNNNNNNNNNNNNNNNNNNNNNNNNNNNNNNNNNNNNNNNNNNNNNNNNNNNNNNNNNNNNNN
NNNNNNNNNNNNNNNNNNNNNNNNNNNNNNNNNNNNNNNNNNNNNNNNNNNNNNNNNNNNNNNNNNNNNN
NNNNNNNNNNNNNNNNNNNNNNNNNNNNNNNNNNNNNNNNNNNNNNNNNNNNNNNNNNNNNNNNNNNNNN
NNNNNNNNNNNNNNNNNNNNNNNNNNNNNNNNNNNNNNNNNNNNNNNNNNNNNNNNTCTNNNAAANNNTNNN
NNNNNNNNNNNNNNNNNNNNNNNNNNNCNCNNTGNNNGTAANCNANNNGNACATGCAGTCCCCTCNNNNN
NNNNNCAGAGNNNNNNNNNCNNNNNNNCCGNCCCCNNCCNNNNNNNNNNNNNNNNNNNNNNNNNNGNNNNNN
NNNNNNTGGTACNNNNCGNNNNNGGTTGNNTAAGAGGTCCACCACGNGGTCNNGNNACNNNNGNNNAN
NNNCNNNNAANNNNNNNNNNNNNNANNTNNNCANNAGGNNNNNNCCNTNNNNNNNNNNNNATTGCNATGANN
CNNNNCNCGGGGGNNGTNCCCGGCGCGGCGACCTTGNNNACNGCCGGGGACAAGTATGGCNNNCNCCC
CGGGAAGGANNCNGCCGTANGTGCNNNNNANNNNNTCNTGNGANGTNGCCGNNATTNNCGTGGNCNNN

NNAGAATGNCCNNACCANNCGNANNNTGGTNATGNNAGTNNNNNNNNNNNNNNNNNNNNNNNNNNNNN
NNNNNNNNNNNNNNNNNNNNNNNNNNNNNNNNNNNNNNGNNNNNNCANNNAAGGNNNTANNNCTNNTN
NTNNNNNNGGACNNNACNNNNCNNNNNNNACNCNNNCNCNNNNNNANNNGGTTTTTNNNCNNTANTNT
CCAGNACGCNGNGCNNNCNTTCANGNGAACNNNGNNACGCGNNAATCGTANNATCTACCTGCCGCNNG
NNNNNNTNCNGNNNNNNCGGANNNTNNNNCGTANNNNNNNCATGCGTNNTNTNNTCNCNAGNCANGTT
CGNNNANANANTAGNANGTNTNNNNNNNNNTGTNNCGTTCCGCNTGANNCNNCNNNNNGNNGNNCTTN
TCANCGGCNNTANCTGCNGANNTAAGNCATANNNTNNNTCNNTANCTNNNNNGGNGGGNNACTGTAAA
NGGNNAATTNGNNGNTNANTNCNNNCACNTNNCNNNGNNNNCTNNNTNCCTGNNTNCNNAATTGGNNC
CANTGNNTGNCNATCTCTNGNTTNGNNNNNNTNNNNNTNNCANNNNGGNNNGTNNNANNNCNATNTAA
NNTANANNNNCNNNNNNANNNCACNNNNNNNGNAGNNNNNANNNTTANANNNGTAGNNNAATAAGTCT
CCGANNTNNNNNNNATAAGCGNNANACNNNNNNNNNNNNNANGANNACGNNTTGGNNNTNNNNNNCNA
ANNAGACNNTNGTGGNTNNNNNTCAANNNNTTCGTAGTGGTATAACNGNNNNNNNTTTCNCCAGNNTN
NNNGNNNNNNNCNATANNANNNNNNNGNGANNNNNNNNNNANNNNCTCNNNAGGGANCANCTACTNNA
GCANTGGNGCGNAGNNNGAGANNNNGACNNNCCTTGGGTGGCAANNACNGCAATAAGTNNNGTTNANN
NTNNNAGTNNACACNNNNNCANNGNNNCNNNNNNANCNGANNTNNNNNNNNNNNNNNNNNNNNNNNNN
NNNNNNNNNNNNNNNNNNNNNNNNNNNNAGNNNANNGNNNGTNTNTGACNCATNTGTTTTTANNNGNN
NNNNNACGNNNNANCCCCGGTCGNNNNCNNATTGAANNCCTGNNGCATNNNNNTNCNAGAATGANNNC
NNNNNGNNTNGNCTNNCNNNTCAAGNAGTCNANNANNTNNNNNNNTCNNNGNNNNNNNNCNNTCNCNN
ACTGAGGGTNNNNNNNCATCNGCTNNNNNNNNNNTNCGCNGNCGTNNCNNNNNNGCNGTATNNNGTNN
NNCANNNNNNNNNNNNNNNNTNAAACGANNNNNTTNNNN
>LaHu353
GTGNNNNNTAGCCCNNNNNAGTTANACANNNCNNNNNNNNNNNNNTNNNNNNNNNNNNNNNNNNNNNN
NNNNNNNNNNNNNNNNNNNNNNNNNNNNNNNNNNNNNNNNNNNNNNNNNNNNNNNNNNNNNNNNNNNN
NNNNNNNNNNNNNNNNNNNNNNNNNNNNNNNNNNNNNNNNNNNNNNNNNNNNNNNNNNNNNNNNNNNN
NNNNNNNNNNNNNNNNNNNNNNNNNNNNNNNNNNNNNNNNNNNNNNNNNNNNNNNNNNNNNNNNNNNN
NNNNNNNNNNNNNNNNNNNNNNNNNNNNNNNNNNNNNNNNNNNNNNNNNNNNNNNNNNNNNNNNNNNN
NNNNNNNNNNNNNNNNNNNNNNNNNNNNNNNNNNNNNNNNNNNNNNNNNNCNTCTNNNAAANNNTNNN
NNNNNNNNNNNNNNNNNNNNNNNNNCNCNNTGNNNGTAANCNANNNGNACATGCAGTCCCCTCNNNNN
NNNGGCAGAGNNNNNNNCCNNNNNCNCCGNCCCCNNCCNNNNNNNNNNNNNNNNNNNNNNNANNNNNN
NNNNNNTGGTACNNNNCGNNNNNGGTTGTTTAAGAGGTCCACCACGNGGTCNNGNCACNNNNGNNNAN
NNNCNNNNAACNNNNNNNNNNNANNTNNNTANNAGGNCNNNNCCNTCNNGNNNNNNANTGCNATGACC
CAANTCACGNNNGCNGTCCCCGGCGCGGCGACCTTGANNANGTCCGGGGNCAAGTATGGCNNNCNCCC
CGGGAAGGANNCNGCCGTANGTGCNNNNNANNNNNTCCTGNGACNTCGCCGNNATTNNCGTGGTCNNN
CNAGANTGNCCGNACCANNCGNANNNTGGTNATGTNAGTNNNNNNNNNNNNNNNNNNNNNNNNNNNNN
NNNNNNNNNNNNNNNNNNNNNNNNNNNNNNNNNGNNNNGNNNNNNCANNNAAGGNNNTANNNCTNNTN
NNNNNNNNGGACNNNACNNNNCNNNNNNNACNCNNNCNCNNNNNNANNNGNTTTTNNNCCNNTANTNT
CCAGNACGCNGNGCNNNCATTCANGNGAACNNNGNNACGCGNNAATCGTANNATCTACCTGCCGCNNG
TNNNNNTACNGNNNNNNCGGANNNTNNCTCGTANNNNNNNTATACGTNNTNTNNTCNCNAGNCNNGTT
CGNNNANANANTAGNANGTNTNNNNNNNCTTGTNNCGTTCCGCNTGANTCNNCANNNNGNNGNNCTTN
NCANCGGCNNTANCTGCNGANNTAAGNCATANNNTNNNTCNNTANCTNNNNNGGNGGGNNACTGTAAA
NGGNNAATTNGANNNTNANTNCNNNCACNTNNCTNNGNNNNCTNNNTNCCTGNNTNCNNAATTGGNNC
CANTGNNTGNCNATCTCTNGNTTNGNNNNNNTNNNNNTNNCANNNNGGNNNGTNNNANNNCNATNTAA
NNTANANNNNCNNNNNNANNNCACNNNNNNNGNAGNNNCNANNNTTANANNNGTAGNNNANTAAGTCT
CCGANNTNNNNNNNATAAGCGNNANACNNNNNNNNNNNNNANGANNACGNNTTGGNNNTNNNNNNCNA
GNNAGACNNTNGTGGNTNNNNNTCAANNNNTTNGTAGTGGTATAATNGNNNNNNNTTTCNCCAGNNTN
NNNGNNNNNNNCNATANNANNNNNNNGNGANNNNNNNNNNANNNNCTCNNNAGGGANCANCTACTNNA
GCAATGGNGCGCAGNNNGAGANNNNGACNNNCCTTGGGTGGCAANNACNGCAATAAGTNNNGTTNANC
CTNNNAGTNNACACNNNNNCANNGNNNCNNNNNNANCNGANNGNNNNNNNNNNNNNNNNNNNNNNNNN
NNNNNNNNNNNNNNNNNNNNNNNNNNNNAGNNNANGGNNNGTNTNTGACNCNTGTGTTTTTANNNGNN
NNNNNACGNNNNANCCCCGGTCGNNNNCNNATTGAANNCCTGNNGCATNNNNNTNCNAGAATGANNNC
NNNNNGNNTNGNCTNNCNNNTCAAGNAGTCAANNAGGTNNNNNGNTCNNNGNNNNNNNNCNNTCCCNN
ACTGAGGGTNNNNNNNCATCTGCTNNNNNNNNNCTNCGCNGGNGTNNCNNNNNNGCNGTATNNTGNNA
NNCANNNNNNNNNNNNNNNCNAAACNANNNNNNNTNNNN
>LaHu354
GTGNNNNNTAGCCCNNNNNAGTCAGACANNNCNNNNNNNNNNNNNTNNNNNNNNNNNNNNNNNNNNNN

NNNNNNNNNNNNNNNNNNNNNNNNNNNNNNNNNNNNNNNNNNNNNNNNNNNNNNNNNNNNNNNNNNNNNN
NNNNNNNNNNNNNNNNNNNNNNNNNNNNNNNNNNNNNNNNNNNNNNNNNNNNNNNNNNNNNNNNNNNNNN
NNNNNNNNNNNNNNNNNNNNNNNNNNNNNNNNNNNNNNNNNNNNNNNNNNNNNNNNNNNNNNNNNNNNNN
NNNNNNNNNNNNNNNNNNNNNNNNNNNNNNNNNNNNNNNNNNNNNNNNNNNNNNTCTNNNAAANNNTNNN
NNNNNNNNNNNNNNNNNNNNNNNNNNNCNCNNTGNNNGTAANCNANNNGNACATGCAGTCCCCTCNNNNN
NNNGGCAGAGNNNNNNNCCCNNNNCNCCGNCCCCNNCCNNNNNNNNNNNNNNNNNGNNNNNNNANNNNNN
NNNNNNTGGTACNNNNCGNNNNNGGTTGTNTAAGAGGTCCACAACGNGNTCNGNNNACNNNNGNNNAN
NNNCNNNNAACNNNNNNNNNNNNNNNNTNNNTANNAGGNNNNNNCCNTCNNGNNNNNNANNGCNATGANN
CAANNCACGGNGGCNGTNCCCGGCGNGGCGACCTTGNNNANNTCCGGGGNCAAGTATGGCGNNCNCCC
CGGGAAGGANNCNGCCGTANGTGCNNNNNANNNNNTCNTGNGANGTNGCCGNNATTNNCNTGGTCNNN
NNAGAATGNNCNNACCANNCGNANNNTGGTNATGNNAGTNNNNNNNNNNNNNNNNNNNNNNNNNNNNNNN
NNNNNNNNNNNNNNNNNNNNNNNNNNNNNNNNNNGNNNNGNNNNNNCNANNNAAGGNNNTANNNCTNNTN
NTNNNNNNGGACNNNACNNNNCNNNNNNNACNCNNNCNCNNNNNNANNNGNTTTTNNNNCNNTATTNT
CCAGNANGCNGNGCNNNCATTCANGNGAACNNNGNNACGCGNNAATCGTANNATCTACCTGCCGCNNG
NNNNNNTACNGNNNNNNCGGANNNTNNNNCGTANNNNNNNCANGCGTNNTNTNNTCNCNAGNCNNGTT
CGNNNANANANTAGNANGTNTNNNNNNNNNTGTNNCGTTCCGCNTGANNCNNCANNNNGNNGNNNTTN
NCANCGGCNNTANCTGCNGANNTAAGNCATANNNTNNNTCNNTANCTNNNNNGGNGGGNACTGTAAA
NGGNNAATTNGNNNNTNANTNCNNNCATNTNNCTNNGNNNNCTNNNTNCCTGNNTNCNNAATTGGNNN
CANTGNNTGNCNATCTCTNGNTTNGCNNNNNTNNNNNTNNCANNNNGGNNNNNNANANNNCNATNTAA
NNTANANNNNCNNNNNNANNNCACNANNNNNGNAGNNNNNANNNGTANANNNGTAGNNNGNTAAGTCT
CCGANNTNNNNNNNATAAGCGNNANACNNNNNNNNNNNNNNANGANNNCGNNTTTGNNNTNNNNNNCNA
GNNAGACNNTNGTGGNTNNNNNTCAANNNNTTNGTAGTGGTATAATNGNNNNNNNTTTCNCCAGNNTN
NNNGNNNNNNNCNATANNANNNNNNNGNGANNNNNNNNNNNANNNNCTCNNNAGGNANCANCTACTNNA
GCANTGGNGCGNAGNNNGAGANNNNGACNNNCCTTGGGTGGCAANCACNGCAATAAGTNNGTTNNNC
CTNNNAGTNNACACNNNNNCANNGNNNCNNNNNNANCNGANNGNNNNNNNNNNNNNNNNNNNNNNNNNNN
NNNNNNNNNNNNNNNNNNNNNNNNNNNNNNNGNNNANGGNNNGTNTNTGACNCATGNNTTTTTANNNGNN
NNNNNACGNNNNANCCCCGGTCGNNNNCNNATTGAANNCCTGNNGCATNNNNNTNCNAGAATGANNNC
NNNNNGNNTNGNCTNNCNNNTCAAGNAATCANNNAGGNNNNNNNNNTCNNNGNNNNNNNNNCNNTCCCNN
ACTGAGGGTNNNNNNNNCATCTGCTNNNNNNNNNTNCGCNGGNGTNNCNNNNNAGCNGTATNNTGTNN
NNCANNNNNNNNNNNNNNNNNNTTAAACAANNNNNNNTNNNN
>LaHu355
GTGNNNNNNAGCCCNNNNNAGTTAGACANNNCNNNNNNNNNNNNNTNNNNNNNNNNNNNNNNNNNNNNNN
NNNNNNNNNNNNNNNNNNNNNNNNNNNNNNNNNNNNNNNNNNNNNNNNNNNNNNNNNNNNNNNNNNNNNN
NNNNNNNNNNNNNNNNNNNNNNNNNNNNNNNNNNNNNNNNNNNNNNNNNNNNNNNNNNNNNNNNNNNNNN
NNNNNNNNNNNNNNNNNNNNNNNNNNNNNNNNNNNNNNNNNNNNNNNNNNNNNNNNNNNNNNNNNNNNNN
NNNNNNNNNNNNNNNNNNNNNNNNNNNNNNNNNNNNNNNNNNNNNNNNNNNNNNTCTNNNAAANNNTNNN
NNNNNNNNNNNNNNNNNNNNNNNNNNNCNCNTTGNNNGTAANCNANNNGNACATGCAGTCCCCTCNNNNN
NNNGGCAGAGNNNNNNCCCNNNNNCNCCGNCCCCNNCCNNNNNNNNNNNNCNNNNTNNNNNNGNNNNNN
NNNNNNTGGTACNNNNCGNNNNNGGTTGNNTNAGAGGTCCACCACGNGGTCNNNNCACNNNNGNNNAN
NNNCNNNAAACNNNNNNNNNNNNNNNNTNNNCANNAGGNCNNNNCCNTCNNGNNNNNNANTGCNATGACC
CANNNCNCGGNGGCNGTNCCCGGNGCGGCGACCTTGANNACGGCCGGGGNCAAGTATGGCNNNCNCCC
CGNGAAGGANNCNGCCGTANGTGCNNNNNANNNNNTCNTGNGANGTCGCCGNNATTNNCGTGGTCNNN
NNAGAATGCNCGNACCANNCGNANNNTGGTNATGNNAGTNNNNNNNNNNNNNNNNNNNNNNNNNNNNNNN
NNNNNNNNNNNNNNNNNNNNNNNNNNNNNNNNNNNNNNNGNNNNNNCNANNNAAGGANNTANNNCTNNTN
NTNNNNNNGGACNNNACNNNNCNNNNNNNACNCNNNCNCNNNNNNANNNGGTTTNNNNNCNNTATTNT
CCAGNACGCNGNGCNNNCNTTCANGNGAACNNNGNNACGCGNNAATCGTANNATCTACCTGCCGCNNG
TNNNNNTACNGNNNNNNCGGANNNTNNNNCGTANNNNNNNCANGCGTNNNNTNNTCNCNAGNCANGTT
CGNNNANANANTAGNANGTNTNNNNNNNNNTGTNNCGTTCCGCNTGANNCNNCANNNNGNNGNNNTTN
NCANCGGCNNTANCTGCNGANNTAAGNCATANNNTNNNTCNNTANCTNNNNNGGNGGGNACTGTAAA
NGGNNAATTNGNNNNTNANTNCNNNCACNTNNCNNNGNNNNCTNNNTNCCTGNNTNCNNAATTGGNNC
CANTGNNTGNCNATCTCTGGNTTNGNNNNNNTNNNNNTNNCANNNNGGNNNNNNNNANNNCNATNTAA
NNTANANNNNCNNNNNNANNNCACNNNNNNNGNAGNNNCNANNNTNANANNNGTAGNNNANTNAGTCT
CCGANNTNNNNNNNATAAGCGNNANACNNNNNNNNNNNNNNANGANNNCGNNTTGGNNNTNNNNNNCNA
ANNAGACNNTNGTGGNTNNNNNTCAANNNNTTNGTAGTGGTATAACNGNNNNNNNTTTCNCCAGNNTN

NNNGNNNNNNNNCNATANNANNNNNNNGNGANNNNNNNNNNNANNNNCTCNNNAGGGANCANCTACTNNA
GCANNGGNGCGCAGNNNGAGANNNNGACNNNCCTTGGGTGGCAANNACNGNAANAAGTNNNGTTNANN
CTNNNAGTNNACACNNNNNCANNGNNNCNNNNNNANCNGANNTNNNNNNNNNNNNNNNNNNNNNNNNNN
NNNNNNNNNNNNNNNNNNNNNNNNNNNNNANNNNANNGNNNGTNTNTGACNCATGNNTTTTTANNNGNN
NNNNNACGNNNNANCCCCGGTCGNTNNCNNATTGAANNCCTGNNGCATNNNNNTNCNAGAATGANNNC
NNNNNGNNTNGNCTNNCNTTTCAAGNAGTCANNNAGNNNNNNNNNTCNNNGNNNNNNNNCNNTCNCNN
ACTGAGGGTNNNNNNNNCATCTGCTNNNNNNNNCTNCGCNGNCGTNNCNNNNNNGCNGTATNNTGTNN
NNCANNNNNNNNNNNNNNNNNTNAANNGANNNNNNTNNNN
>LaHu356
GTGNNNNNTAGCCCNNNNNAGTTAGACANNNCNNNNNNNNNNNNNTNNNNNNNNNNNNNNNNNNNNNNNN
NNNNNNNNNNNNNNNNNNNNNNNNNNNNNNNTNNNNNNNNNNNNNNNNNNNNNNNNNNNNNNNNNNNNNN
NNNNNNNNNNNNNNNNNNNNNNNNNNNNNNNNNNNNNNNNNNNNNNNNNNNNNNNNNNNNNNNNNNNNNN
NNNNNNNNNNNNNNNNNNNNNNNNNNNNNNNNNNNNNNNNNNNNNNNNNNNNNNNNNNNNNNNNNNNNNN
NNNNNNNNNNNNNNNNNNNNNNNNNNNNNNNNNNNNNNNNNNNNNNNNNNNNNNTCTNNNAAANNNTNNN
NNNNNNNNNNNNNNNNNNNNNNNNNNCNCNTTGNNNGTAANCNANNNGNACATGCAGTCCCCTCNNNNN
NNNGGCAGAGNNNNNNNCCNNNNNCNCCGNCCCCNNCCNNNNNNNNNNNNNNNNNNNNNNNNNANNNNNN
NNNNNNTGGTACNNNNCGNNNNNGGTTGTNTAAGAGGTCCACAACGNGGTCNGNNCACNNNNGNNNAN
NNNCNNNNNNNCNNNNNNNNNNNNANNTNNNTANAAGGNNNNNNCCNTCNNNNNNNNNNATNGCNATGACC
CAANNCACGGGGGCAGTNCCCGGCGCGGCGACCTTGANNANNTCCGGGGACAAGTATGGCGNNCNCCC
CGGGAAGGANNCNGCCGTANGTGCNNNNNANNNNNTCNTGNGANGTCGCCGNNANNNNCGTGGTCNAN
CNAGAATGCNCNNACCANNCGNANNNTGGTNATGNNAGTNNNNNNNNNNNNNNNNNNNNNNNNNNNNN
NNNNNNNNNNNNNNNNNNNNNNNNNNNNNNNNNNNNGNNNNGNNNNNNCNANNNAAGGNNNTANNNCTNNTN
NTNNNNNNNGGACNNNACNNNNCNNNNNNNACNCNNNCNCNNNNNNANNNGGTTNNNNNNCNNTANTNT
CCAGNACGCNGNGCNNNCATTCANGNGAACNNNGNNACGCGNNAATCGTANNATCTACCTGCCGCNNG
TNNNNNTNCNGNNNNNNCGGANNNTNNCTCGTANNNNNNNCATGCGTNNTNTNNTCNCNAGNCANGTT
CGNNNANANANTAGNANGTNTNNNNNNNNNNTGTNNCGTTCCGCNTGANNCNNCANNNNGNNGNNCTTN
NCANCGGCNNTANCTGCNGANNTAAGNCATANNNTNNNTCNNTANCTNNNNNGGNGGGNNACTGTAAA
NGGNNAATTGGNNNNTNANTNCCNNCACNTNNCTNNGNNNNCTNNNTNCCTGNNTNCNNAATTGGNNC
CANTGNNTGNCNATCTCTNGNTTNGNNNNNNTGNNNNTNNCANNNNGGNNNGTNANANNNCNATNTAA
NNTANANNNNCNNNNNNANNNCACNNNNNNNNNAGNNACNANNNTNANANNNGTAGNNNANTAAGTCT
CCGANNTNNNNNNNATAAGCGNNANACNNNNNNNNNNNNNANGANNACGNNTTGGNNNTNNNNNNCNA
GNNAGACNNTNGTGGNTNNNNNTCAANNNNTTNGTAGTGGTATAATNGNNNNNNNTTTCNCCAGNNTN
NNNGNNNNNNNNCNATACNANNNNNNNGNGANNNNNNNNNNNANNNNCTCNNNAGGGANCANCTACTTNA
GCANTGGNGCGNAGNNNGAGANNNNGACNNGCCTTGGGTGGCAANNACNGCAATAAGTNNNGTTNANC
CTNNNAGTNNACACNNNNNCANNGNNNCNNNNNNANCNGANNGNNNNNNNNNNNNNNNNNNNNNNNNNN
NNNNNNNNNNNNNNNNNNNNNNNNNNNNNANNNNANGGNNNGTNTNTGACNCATNNNTTTTTANNNGNN
NNNNNACGNNNNANCCCCGGTCGNNNNCNNATTGAANNCCTGNNGCATNNNNNTNCNAGAATGANNNC
NNNNNGNNTNGNCTNNCNNNTCAAGNAATCANNNAGNNNNNNNGNTCNNNNNNNNNNNNNCNNNCCCNN
ACTGAGGGTNNNNNNNNCANCTGCTNNNNNNNNCTNCGCNGGCGTNNCNNNNNNGCNGTATNNNGTNA
NNCANNNNNNNNNNNNNNNNNCNAAACNANNNNNNNTNNNN
>LaHu357
GTGNNNNNTAGCCCNNNNNAGTTAGACANNNCNNNNNNNNNNNNNTNNNNNNNNNNNNNNNNNNNNNNNN
NNNNNNNNNNNNNNNNNNNNNNNNNNNNNNNTNNNNNNNNNNNNNNNNNNNNNNNNNNNNNNNNNNNNNN
NNNNNNNNNNNNNNNNNNNNNNNNNNNNNNNNNNNNNNNNNNNNNNNNNNNNNNNNNNNNNNNNNNNNNN
NNNNNNNNNNNNNNNNNNNNNNNNNNNNNNNNNNNNNNNNNNNNNNNNNNNNNNNNNNNNNNNNNNNNNN
NNNNNNNNNNNNNNNNNNNNNNNNNNNNNNNNNNNNNNNNNNNNNNNNNNNNNNTCTNNNAAANNNTNNN
NNNNNNNNNNNNNNNNNNNNNNNNNNCNCNNTGNNNGTAANCNANNNGNACATGCAGTCCCCTCNNNNN
NNNGGCAGAGNNNNNNNCCNNNNNNNCCGNCCCCNNCCNNNNNNNNNNNNNNNNNGNNNNNNNGNNNNNN
NNNNNNTGGTACNNNNCGNNNNNGGTTGNNTAAGAGGTCCACCACGNGGTCNNGNNACNNNNGNNNAN
NNNCNNNNNAACNNNNNNNNNNNNANNTNNNCANNAGGNCNNNNCCNTCNNGNNNNNNANNGCNATGACC
CAANNCACGGNGGCNGTNCCCGGCGNGGCGACCTTGANNACGGCCGGGGACAAGTATGGCGNNCNCCC
CGGGAAGGANNCNGCCGTANGTGCNNNNNANNNNNTCNTGNGANGTCGCCGNNATTCNCGTGGTCNNN
NNAGAATGNCCNNACCANNCGNANNNTGGTNATGNNAGTNNNNNNNNNNNNNNNNNNNNNNNNNNNNN
NNNNNNNNNNNNNNNNNNNNNNNNNNNNNNNNNNNNGNNNNGNNNNNNCNANNNAAGGNNNTANNNCTNNTN

NTNNNNNNNGGACNNNACNNNNCNNNNNNNNACNCNNNCNCNNNNNNANNNGNTTTTTNNNCNNTATTNT
CCAGNACGCNGNGCNNNCNTTCANGNGAACNNNGNNACGCGNNAATCGTANNATCTANCTGCCGCNNG
TNNNNNTACNGNNNNNNCGGANNNTNNNNNGTANNNNNNTCATGCGTNNTNTNNTNNCNAGNCNNGTT
CGNNNANANANTAGNANGTNTNNNNNNNNNTGTNNCGTTCCGCNTGANNCNNCANNNNGNNGNNNTTN
NCANCGGCNNTANCTGCNGANNTAAGNCATANNNTNNNTCNNTANCTNNNNNGGNGGGNNACTGTAAA
NGGNNAATTNGNGNNTNATTNCNNNCACNTNNCTNNGNNNNCTNNNTNCCTGNNTNCNNAATTGGNNC
CANTGNNTGNCNATCTCTNGNTTNGNNNNNNTNNNNNTNNCANNNNGGNNNNNNANANNNCNATNTAA
NNTANANNNNCNNNNNNANNNCACNANNNNNNNAGNNNCNANNNTNANANNNGTAGNNNANTAAGTCT
CCGANNTNNNGNNNATAAGCGNNANACNNNNNNNNNNNNNANGANNACGNNTTGGNNNTNNNNNNCNA
ANNAGACNNTNGTGGNTNNNNNTCAANNNNTTCGTAGTGGTATAACNGNNNNNNNTTTCNCCAGNNTN
NNNGNNNNNNNNCNATANNANNNNNNNGNGANNNNNNNNNNNANNNNCTCNNNAGGGANCANCTACTNNA
GCANTGGNGCGCAGNNNGAGANNNNGACNNNCCTTGGGTGGCAANNACNGCAATAAGTNNNGTTNANC
CTNNNAGTNNACACNNNNNCANNGNNNCNNNNNNANCNGANNTNNNNNNNNNNNNNNNNNNNNNNNNN
NNNNNNNNNNNNNNNNNNNNNNNNNNNNNNNNANNNNANGGNNNGTNTNTGACNCATGTGTTTTTANNNGNN
NNNNNNNGNNNNANCCCCGGTCGNNNNCNNATTGAANNCCTGNNGCATNNNNNTNCNAGAATGANNGC
NNNNNGNNTNGNCTNNCNNNTNAAGNAGTCANNTAGNNNTNGCGNTCNNNNNNNNNNNNNCNNTCCCNN
ACTGAGGGTNNNNNNNNCATCTGCTNNNNNNNNNNTNCGCNGNCGTNNCNNNNNNGCNGTATNNTGTNA
NNCANNNNNNNNNNNNNNNNNNTNAAACGANNNNNNNTNNNG
>LaHu361
GTGNNNNNTAGCCCNNNNNAGTTAGACANNNCNNNNNNNNNNNNNTNNNNNNNNNNNNNNNNNNNNNNNNN
NNNNNNNNNNNNNNNNNNNNNNNNNNNNNNNNNTNNNNNNNNNNNNNNNNNNNNNNNNNNNNNNNNNNNNNN
NNNNNNNNNNNNNNNNNNNNNNNNNNNNNNNNNNNNNNNNNNNNNNNNNNNNNNNNNNNNNNNNNNNNNNNN
NNNNNNNNNNNNNNNNNNNNNNNNNNNNNNNNNNNNNNNNNNNNNNNNNNNNNNNNNNNNNNNNNNNNNNNN
NNNNNNNNNNNNNNNNNNNNNNNNNNNNNNNNNNNNNNNNNNNNNNNNNNNNNNNCNTCTNNNAAANNNTNNN
NNNNNNNNNNNNNNNNNNNNNNNNNNNNCNCNNTNNNNGTAANCNANNNGNACATGCAGTCCCCTCNNNNN
NNNGGCAGAGNNNNNNNCCNNNNNNNCCGGCCCCNNCCNNNNNNNNNNNNNNNNGNNNNNNNGNNNNNN
NNNNNNTGGTACNNNNCGNNNNNGGTTGTNTNAGAGGTCCACCACGNGGTCNGGNCACNNNNGNCTAN
NNNCNNNNAACNNNNNNNNNNNNANATNNNCANNAGGNCNNNNCCNTCNNGNNNNNNANNGCNATGACC
CAANNCACGGGGGCAGTNCCCGGCGCGGCGACCTTGANNACGGCCGGGGACAAGTATGGCNTNCNCCC
CGGGAAGGANNCNGCCGTANGTGCNNNNNANNNNNTCNTGNGANGTNGCCGNNATTCNCGTGGTCNNN
CNAGAATGNCCNNACCANNCGNANNNTGGTNATGNNAGTANNNNNNNNNNNNNNNNNNNNNNNNNNNNNN
NNNNNNNNNNNNNNNNNNNNNNNNNNNNNNNNNNNNNNNNNGNNNNNNCNANNNAAGGNNNTANNNCTNNTN
NTNNNNNNNGGACNNNACNNNNCNNNNNNNNACNCNNNCNCNNNNNNANNNGNTTNTNNNNCNNTATTNT
CCAGNACGCNGNGCNNNCATTCANGNGAACNNNGNNACGCGNNAATCGTANNATCTACCTGCCGCNNG
NNNNNNTACNGNNNNNNCGGANNNTNNNNCGTANNNNNNNCANGCGTNNTNTNNTCNCNAGNCNNGTT
CGNNNANANANTAGNANGTNTNNNNNNNNNTGTNNCGTTCCGCNTGANNCNNCANNNNGNNGNNNTTN
TCANCGGCNNTANCTGCNGANNTAAGNCATANNNTNNATCNNTANCTNNNNNGGNGGGNNACTGTAAA
NGGNNAATTNGNNNNTNANTNCNNNCACNTNNCNNNGNNNNCTNNNTNCCTGNNTNCNNAATTGGNNN
CANTGNNTGNCNATCTCTGGNTTNGNNNNNNTNNNNNTNNCANNNNGGNNNGTNNNANNNCNATNTAA
NNTANANNNNCNNNNNNANNNCACNNNNNNNGNAGNNNCNANNNTNANANNNGTAGNNNANTAAGTCT
CCGANNTNNNNNNNATAAGCGNNANACNNNNNNNNNNNNNANGANNACGNNTTGGNNNTNNNNNNCNA
ANNAGACNNTNGTGGNTNNNNNTCAANNNNTTNGTAGTGGTATAACNGNNNNNNNTTTCNCCAGNNTN
NNNGNNNNNNNNCNATANNANNNNNNNGNGANNNNNNNNNNNANNNNCTCNNNAGGGANCANCTACTNNA
GCANNGGNGCGNAGNNNGAGANNNNGACNNGCCTTGGGTGGCAANNACNGCAATAAGTNNNGTTNANN
CTNNNAGTNNACACNNNNNCANNGNNNCNNNNNNANCNGANNTNNNNNNNNNNNNNNNNNNNNNNNNN
NNGNNNNNNNNNNNNNNNNNNNNNNNNNNNNGNNNANGGNNNGTNTNTGNCNCATNNNNTTTTTANNNGNN
NNNNNACGNNNGANCCCCGGTCGNNNNCNNATTGAANNCCTGNNGCATNNNNNTNCNAGAATGANNGC
NNNNNGNNTNGNCTGNCNNNTNAAGNAGTCAANNAGGTNNNNNGNTCNNNGNNNNNNNNCNNTCCCNN
ACTGAGGGTNNNNNNNNCATCTGCTNNNNNNNNNNTNCGCCGNCGTNNCNNNNNAGCNGTATNGNGTNN
NNCANNNNNNNNNNNNNNNNNNTNAANCNANNNNNNNTNNNN
>LaHu362
GTGNNNNNTAGCCCNNNNNAGTTAGACANNNCNNNNNNNNNNNNNTNNNNNNNNNNNNNNNNNNNNNNNNN
NNNNNNNNNNNNNNNNNNNNNNNNNNNNNNNNNNNNNNNNNNNNNNNNNNNNNNNNNNNNNNNNNNNNNNNN
NNNNNNNNNNNNNNNNNNNNNNNNNNNNNNNNNNNNNNNNNNNNNNNNNNNNNNNNNNNNNNNNNNNNNNNN

NNNNNNNNNNNNNNNNNNNNNNNNNNNNNNNNNNNNNNNNNNNNNNNNNNNNNNNNNNNN
NNNNNNNNNNNNNNNNNNNNNNNNNNNNNNNNNNNNNNNNNNNNNNCNTCTNNNAAANNNTNNN
NNNNNNNNNNNNNNNNNNNNNNNNNCNCNTTNNNNGTAANCNANNNGNACATGCAGTCCCCTCNNNNN
NNNNNCAGAGNNNNNNNCCNNNNNNNCCGNCCCCNNCCNNNNNNNNNNNNNNNNGNNNNNNNGNNNNNN
NNNNNNTGGTACNNNNCGNNNNNGGTTGNNTAAGAGGTCCACCACGNGGTCNNGNCACNNNNGNNNAN
NNNCNNNNAACNGNNNNNNNNNNANNTNNNCANNAGGNCNNNGCCNTCNNNNNNNNNAANTGCNATGACC
CAANNCACGGNGGNNGTNCCCGGCGNGGCGACCTTGNNNANGGCCGGGGACAAGTATGGCGNNCNCCC
CGGGAAGGANNCNGCCGTANGTGCNNNNNANNNNNTCNTGNGANGTCGCCGNNATTNNCGTGGTCNNN
NNAGAATGNCCNNACCANNCGNANNNTGGTNATGNNAGTNNNNNNNNNNNNNNNNNNNNNNNNNNNNN
NNNNNNNNNNNNNNNNNNNNNNNNNNNNNNNNNNGNNNNGNNNNNNCNANNNAAGGANNTANNNCTNNTN
NTNNNNNNNGGACNNNACNNNNCNNNNNNNNACNCNNNCNCNNNNNNNNNNNGNTTTTTNNNCNNTATTNT
CCAGNACGCNGNGCNNNCNTTCANGNGAACNNNGNNACGCGNNAATCGTANNATCTACCTGCCGCNNG
NNNNNNTNCNGNNNNNNNCGGANNNTNNCTCGTANNNNNNNTCANGCGTNNTNTNNTCNCNAGNCNNGTT
CGNNNANANANTAGNANGTNTNNNNNNNNCNTGTNNCGTTCCGCNTGANNCNNCANNNNGNNGNNCTTN
NCANCGGCNNTANCTGCNGANNTAAGNCATANNNTNNNTCNNTANCTNNNNNGGNGGGNNACTGTAAA
NGGNNAATTNGNNGNTNANTNCNNNCACNTNNCTNNGNNTNCTNNNTNCCTGNNTNCNNAATTGGNNN
CANTGNNTGNCNATCTCTGGNTTNGNNNNNNTNNNNNTNNCAANNNGGNNNGTNNNANNNCNATNTAA
NNTANANNNNCNNNNNNANNNCACNNNNNNNNNAGNNACNANNNTNANANNNGTAGNNNAATAAGTCT
CCGANNTNNNNNNNNATAAGCGNNANACNNNNNNNNNNNNNNANGANNACGNNTTGGNNNTNNNNNNNNA
ANNAGACNNTNGTGGNTNNNNNTCAANNNNTTNGTAGTGGTATAACNGNNNNNNNTTTCNCCAGNNTN
NNNGNNNNNNNNCNATANNANNNNNNNGNGANNNNNNNNNNNANNNNCTCNNNAGGGANCANCTACTNNA
GCANTGGNGCGCAGNNNGAGANNNNGACNNNCCTTGGGTGGCAANNACNGCAATAAGTNNNGTTNNNC
CTNNNAGTNNACACNNNNNCANNGNNNCNNNNNNANCNGANNTNNNNNNNNNNNNNNNNNNNNNNNNN
NNNNNNNNNNNNNNNNNNNNNNNNNNNNNNANNNNANGGNNNGTNTNTGACNCNTGTGTTTTTANNNGNN
NNNNNNNGNNNGANCCCCGGTCGNNNNCNNATTGAANNCCTGNNGCATNNNNNNTNCNAGAATGANNGC
NNNNNGNNTNGNCTNNCNNNTCAAGNAGTCANNNANGTNNNNNGNTCNNNGNNNNNNNNCNNTCCCNN
ACTGAGGGTNNNNNNNNCATCTGCTNNNNNNNNNNTNCGCNGNCGTNNCNNNNNNGCNGTATNNNGTNA
NNCANNNNNNNNNNNNNNNNNNTNAAACNANNNNNNNTNNNN
>LaHu363
GTGNNNNNTAGCCCNCNNNAGTTAGACANNNCNNNNNNNNNNNNNTNNNNNNNNNNNNNNNNNNNNNNN
NNNNNNNNNNNNNNNNNNNNNNNNNNNNNNNNNNNNNNNNNNNNNNNNNNNNNNNNNNNNNNNNNNNN
NNNNNNNNNNNNNNNNNNNNNNNNNNNNNNNNNNNNNNNNNNNNNNNNNNNNNNNNNNNNNNNNNNNN
NNNNNNNNNNNNNNNNNNNNNNNNNNNNNNNNNNNNNNNNNNNNNNNNNNNNNNNNNNNNNNNNNNNN
NNNNNNNNNNNNNNNNNNNNNNNNNNNNNNNNNNNNNNNNNNNNNNCNTCTNNNAAANNNTNNN
NNNNNNNNNNNNNNNNNNNNNNNNNCNCNTTGNNNGTAANCNANNNGNACATGCAGTCCCCTCCNNNN
NNNGGCAGAGNNNNNNNCCNNNNNCNCCGGCCCCNNCCNNNNNNNNTNNNNNNNNGNNNNNNNANNNNNN
NNNNNNTGGTACNNNNCGNNNNNGGTTGNNTAAGAGGTCCACAACGNGGTCNGGNCACNNNNGNNNAN
NNNCNNNNAACNNNNNNNNNNNNNANNTNNNTANAAGGNCNNNNCCNTCNNGNNNNNNATTGCNATGACC
CNANNCNCGGGGGCAGTNCCCGGCGCGGCGACCTTGNTNACGTCCGGGGNCAAGTATGGCNNNCNCCC
CGGGAAGGANNCNGCCGTANGTGCNNNNNANNNNNTCNTGNGANGTNGCCGNNATTNNCGTGGTCNNN
CNAGAATGNCCGNACCANNCGNANNNTGGTNATGNNAGTNNNNNNNNNNNNNNNNNNNNNNNNNNNNN
NNNNNNNNNNNNNNNNNNNNNNNNNNNNNNNNNNGNNNNGNNNNNNCNANNNAAGGNNNTANNNCTNNTN
NTNNNNNNNGGACNNNACNNNNCNNNNNNNNACNCNNNCNCNNNNNNNANNNGNTTNNNNNNNCNNTATTNT
CCAGNANGCNGNGCNNNCATTCANGNGAACNNNGNNACGCGNNAATCGTANNATCTACCTGCCGCNNG
NNNNNNTACNGNNNNNNNCGGANNNTNNNNCGTANNNNNNNTCATGCGTNNTNTNNTCNCNAGNCNNGTT
CGNNNANANANTAGNANGTNTNNNNNNNNNNNTGTNNCGTTCCGCNTGANNCNNCANNNNGNNGNNCTTN
NCANCGGCNNTANCTGCNGANNTAAGNCATANNNTNNNTCNNTANCTNNNNNGGNGGGNNACTGTAAA
NGGNNAATTNGNNNNTNATTNCCNNCACNTNNCTNNGNNNNCTNNNTNCCTGNNTNCNNAATTGGNNN
CANTGNNTGNCNATCTCTGGNTTNGNNNNNNTNNNNNTNNCANNNNGGNNNGTNNNANNNCNATNTAA
NNTANANNNNCNNNNNNANNNCACNNNNNNNNGNAGNNNNNANNNTTANANNNGTAGNNNAATNAGTCT
CCGANNTNNNNNNNNATAAGCGNNANACNNNNNNNNNNNNNNANGANNNCGNNTTGGNNNTNNNNNNNNA
GNNAGACNNTNGTGGNTNNNNNTCAANNNNTTNGTAGTGGTATAATNGNNNNNNNTTTCNCCAGNNTN
NNNGNNNNNNNNCNATANNANNNNNNNGNGANNNNNNNNNNNANNNNCTCNNNAGGGANCANCTACTTNA
GCANTGGNNCGCAGNNNGAGANNNNGACNNNCCTTGGGTGGCAANCACNGCAATAAGTNNNGTTNANC

CTNNNAGTNNACACNNNNNCANNGNNNCNNNNNNANCNGANNGNNNNNNNNNNNNNNNNNNNNNNNN
NNNNNNNNNNNNNNNNNNNNNNNNNNNNNNNNNNANGGNNNGTNTNTGACNCATGTGTTTNTANNNGNN
NNNNNACGNNNGANCCCCGGTCGNNNNCNNATTGAANNCCTGNNGCATNNNNNTNCNAGAATGANNGC
NNNNNGNNTNGNCTNNCNTTTCAAGNAATCANNNAGGTANNNNGNTCNNNGNNNNNNNNCNNTCCCNN
ACTGAGGGTNNNNNNNNCATCTGCTNNNNNNNNCTNCGCNGGCGTNNCNNNNNNGCNGTATNNNGTNA
NNCANNNNNNNNNNNNNNNNNTNAAACANNNNNNNNTNNNN
>LaHu364
GTGNNNNNTAGCCCNNNNNAGTCAGACANNNCNNNNNNNNNNTNNNNNNNNNNNNNNNNNNNN
NNNNNNNNNNNNNNNNNNNNNNNNNNNNNNNNNNTNNNNNNNNNNNNNNNNNNNNNNNNNNNNNNNNNNNNNNN
NNNNNNNNNNNNNNNNNNNNNNNNNNNNNNNNNNNNNNNNNNNNNNNNNNNNNNNNNNNNNNNNNNNNNNNNNNNNNNNNNNN
NNNNNNNNNNNNNNNNNNNNNNNNNNNNNNNNNNNNNNNNNNNNNNNNNNNNNNNNNNNNNNNNNNNNNNNNNNNNNNNNNNN
NNNNNNNNNNNNNNNNNNNNNNNNNNNNNNNNNNNNNNNNNNNNNNNNNNNNNNNNNNNNNNNNTCTNNNAAANNNTNNN
NNNNNNNNNNNNNNNNNNNNNNNNNNNNNNNCNCNNTGNNNGTAANCNANNNGNACATGCAGTCCCCTCNNNNN
NNNGGCAGAGNNNNNNNCCNNNNNNNNCCGGCCCCNNCCNNNNNNNNNNNNNNNNNNNNNNNNNNNNNANNNNNN
NNNNNNTGGTACNNNNCGNNNNNGGTTGTNTAAGAGGTCCACAACGNGGTCNGGNNACNNNNGNNNAN
NNNCNNNNAACNNNNNNNNNNNNNNNNNTNNNTANNAGGNCNNNNCCNTNNNNNNNNNNNNATTGCNATGACC
CAANNNACGGNGGCNGTNCCCGGCGNGGCGACCTTGANNACGTCCGGGGNCAAGTATGGCGNNCNCCC
CGNGAAGGANNCNGCCGTANGTGCNNNNNANNNNNTCNTGNGANGTCGCCGNNATTCNCGTGGTCNNN
NNAGAATGNCCGNACCANNCGNANNNTGGTNATGNNAGTNNNNNNNNNNNNNNNNNNNNNNNNNNNNNNN
NNNNNNNNNNNNNNNNNNNNNNNNNNNNNNNNNNNNNNGNNNNGNNNNNNCNANNNAAGGNNNTANNNCTNNTN
NTNNNNNNNGGACNNNACNNNNCNNNNNNNNACNCNNNCNCNNNNNNANNNGNTTTTTNNNCNNTANTNT
CCAGNACGCNGNGCNNNCNTTCANGNGAACNNNGNNACGCGNNAATCGTANNATCTACCTGCCGCNNG
TNNNNNTACNGNNNNNNCGGANNNTNNNNNGTANNNNNNNCANGCGTNNTNTNNTCNCNAGNCNNGTT
CGNNNANANANTAGNANGTNTNNNNNNNCTTGTNNCGTTCCGCNTGANNCNNCANNNNGNNGNNCTTN
NCANCGGCNNTANCTGCNGANNTAAGNCATANNNTNNNTCNNTANCTNNNNNGGNGGGNNACTGTAAA
NGGNNAATTNGNNGNTNANTNCNNNCATNTNNCTNNGNNNNCTNNNTNCCTGNNTNCNNAATTGGNNC
CANTGNNTGNCNATCTCTGGNTTNGNNNNNNTGNNNNTNNCANNNNGGNNNNNNNNNANNNCNATNTAA
NNTANANNNNCNNNNNNANNNCACNNNNNNNGNAGNNNCNANNNGTANANNNGTAGNNNGNTNAGTCT
CCGANNTNNNNNNNATAAGCGNNANACNNNNNNNNNNNNNANGANNACGNNTTTGNNNTNNNNNNNNA
GNNAGACNNTNGTGGNTNNNNNTCAANNNNTTNGTAGTGGTATAATNGNNNNNNNTTTCNCCAGNCTN
NNNGNNNNNNNNCNATANNANNNNNNNGNGANNNNNNNNNNNANNNNCTCNNNAGGGANCANCTACTTNA
GCANNGGNGCGNAGNNNGAGANNNNGACNNGCCTTGGGTGGCANNNACNGCAATAAGTNNNGTTNANC
CTNNNAGTNNACACNNNNNCANNGNNNCNNNNNNANCNGANNGNNNNNNNNNNNNNNNNNNNNNNNN
NNNNNNNNNNNNNNNNNNNNNNNNNNNNNNNNANNNNANGGNNNGTNTNTGACNCATGTGTTTTTANNNGNN
NNNNNACGNNNNANCCCCGGTCGNNNNCNNATTGAANNCCTGNNGCATNNNNNTNCNAGAATGANNNC
NNNNNGNNTNGNCTNNCNNNTCAAGNAATCANNNAGGTNNNNNNNNTCNNNGNNNNNNNNCNNTCCCNN
ACTGAGGGTNNNNNNNNCATCTGCTNNNNNNNNNNTNCGCNGGCGTNNCNNNNNNGCNGTATNGNGTNN
NNCANNNNNNNNNNNNNNNNNANTNAAACNANNNNNNNTNNNN
>LaHu365
GTGNNNNNTAGCCCNNNNNAGTTAGACANNNCNNNNNNNNNNTNNNNNNNNNNNNNNNNNNNN
NNNNNNNNNNNNNNNNNNNNNNNNNNNNNNNNNNNNNNNNNNNNNNNNNNNNNNNNNNNNNNNNNNNNNNNNNNNNNNNNNNN
NNNNNNNNNNNNNNNNNNNNNNNNNNNNNNNNNNNNNNNNNNNNNNNNNNNNNNNNNNNNNNNNNNNNNNNNNNNNNNNNNNN
NNNNNNNNNNNNNNNNNNNNNNNNNNNNNNNNNNNNNNNNNNNNNNNNNNNNNNNNNNNNNNNNNNNNNNNNNNNNNNNNNNN
NNNNNNNNNNNNNNNNNNNNNNNNNNNNNNNNNNNNNNNNNNNNNNNNNNNNNNNNNNNNNNNCNTCTNNNAAANNGTNNN
NNNNNNNNNNNNNNNNNNNNNNNNNNNNNNNCNCNTTGNNNGTAANCNANNNGNACATGCAGTCCCCTCNNNNN
NNNGGCAGAGNNNNNNNCCNNNNNCNCCGGCCCCNNCCNNNNNNNNNNNNNNNNNNNNNGNNNNNNNNANNNNNN
NNNNNNTGGTACNNNNCGNNNNNGGTTGTNTAAGAGGTCCACCACGNGGTCNGGNCACNNNNGNNNAN
NNNCNNNNAACNNNNNNNNNNNNNNANNTNNNTANNAGGNNNNNNCCNTCNNNNNANNNATTGCNATGACC
CAANNCACGGGGGCAGTNCCCGGCGCGGCGACCTTGATNANGTCCGGGGACAAGTATGGCNNNCNCCC
CGGGAAGGANNCNGCCGTANGTGCNNNNNANNNNNTCNTGNGANGTCGCCGNNATTCNCGTGGTCNNN
CNAGAATGNCCGNACCANNCGNANNNTGGTTATGTNAGTANNNNNNNNNNNNNNNNNNNNNNNNNNNNN
NNNNNNNNNNNNNNNNNNNNNNNNNNNNNNNNNNNNNNGNNNNGNNNNNNCNANNNAAGGANNTANNNCTNNTN
NTNNNNNNNGGACNNNACNNNNCNNNNNNNNACNCNNNCNCNNNNNNANNNGGTTTTNNNCCNNTATTNT
CCAGNACGCNGNGCNNNCATTCANGNGAACNNNGNNACGCGNNAATCGTANNATCTACCTGCCGCNGG

TNNNNNTACNGNNNNNNCGGANNNTNNCTCGTANNNNNNTTATACGTNNTNTNNTCNCNAGNCANGTT
CGNNNANANANTAGNANGTNTNNNNNNNNNTGTNNCGTTCCGCNTGACNCNNCANNNNGNNGNNCTTC
TCANCGGCNNTANCTGCNGANNTAAGNCATANNNTNNNTCNNTANCTNNNNNGGAGGGNNACTGTAAA
NGGNNAATTNGANNNTNATTNCCNNCACNTNNCTNNGNNTNCTNNNTNCCTGNNTNCNNAATTGGNNC
CANTGNNTGNCNATCTCTGGNTTGNNNNNNTNNNNNNTNNCANNNNGGNNNGTNANANNNCNATNTAA
NNTANANNNNCNNNNNNANNNCACNNNNNNNGNAGNNNCNANNNTTANANNNGTAGNNNANTAAGTCT
CCGANNTNNNNNNNATAAGCGNNANACNNNNNNNNNNNNNANGANNACGNNTTGGNNNTNNNNNGCNA
GNNAGACNNTNGTGGNTNNNNNTCAANNNNTTCGTAGTGGTATAATNGNNNNNNNTTTCNCCAGNNTN
NNNGNNNNNNNCNATANNANNNNNNNGTGANNNNNNNNNNANNNNCTCNNNAGGGANCANCTACTNNA
GCAATGGNGCGCAGNNNGAGANNNNGACCAGCCTTGGGTGGCAANCACNGCAATAAGTNNNGTTNANC
CTNNNAGTNNACACGNNNNCANNGNNNCNNNNNNANCNGANNGNNNNNNNNNNNNNNNNNNNNNNNNN
NNNNNNNNNNNNNNNNNNNNNNNNNNNNAGNNNANGGNNNGTNTNTGACNCATGTGTTTTTANNNGNN
NNNNNACGNNNGANCCCCGGTCGTNNNCNNATTGAANNCCTGNNGCATNNNNNTNCNAGAATGANNGC
NNNNNGNNTNGNCTNNCNTTTCAAGNAGTCAANNAGGTANCGCGNTCNNNGNNNNNNNNCNNTCCCNN
ACTGAGGGTNNNNNNNNCATCTGCTNNNNNNNNCTNCGCNGGCGTNNCNGNNNAGCNGTATNGNGNNN
NNCANNNNNNNNNNNNNNNNTNAAACAANNNNNNNTNNNN
>LaHu366
GTGNNNNNTAGCCCNNNNNAGTTAGACANNNCNNNNNNNNNNNNNTNNNNNNNNNNNNNNNNNNNNNN
NNNNNNNNNNNNNNNNNNNNNNNNNNNNNNNNNNNNNNNNNNNNNNNNNNNNNNNNNNNNNNNNNNNN
NNNNNNNNNNNNNNNNNNNNNNNNNNNNNNNNNNNNNNNNNNNNNNNNNNNNNNNNNNNNNNNNNNNN
NNNNNNNNNNNNNNNNNNNNNNNNNNNNNNNNNNNNNNNNNNNNNNNNNNNNNNNNNNNNNNNNNNNN
NNNNNNNNNNNNNNNNNNNNNNNNNNNNNNNNNNNNNNNNNNNNNNNNNNCNTCTNNNAAANNNTNNN
NNNNNNNNNNNNNNNNNNNNNNNNNCNCNTTGNNNGTAANCNANNNGNACATGCAGTCCCCTCNNNNN
NNNGGCAGAGNNNNNNCCCNNNNNNNCCGNCCCCNNCCNNNNNNNNNNNNNNNNNNNNNNNGNNNNNN
NNNNNNTGGTACNNNNCGNNNNNGGTTGTNTAAGAGGTCCACCACGNGGTCNGGNCACNNNNGANNAN
NNNCNNAAAACNNNNNNNNNNNANATNNNCANNAGGNCNNNNCCNTCNNGNNNNTNANTGCNATGACC
CANNTCNCGGGGGCNGTNCCCGGCGNGGCGACCTTGATNANGGCCGGGGACAAGTATGGCNNNCNCCC
CGGGAAGGANNCNGCNGTANGTGCNNNNNANNNNNTCNTGNGANGTCGCCGNNATTCNCGTGGTCNNN
CNAGAATGNCCNNACCANNCGNANNNTGGTNATGNNAGTNNNNNNNNNNNNNNNNNNNNNNNNNNNNN
NNNNNNNNNNNNNNNNNNNNNNNNNNNNNNNNGNNNNGNNNNNNCNANNNAAGGANNTANNGCTNNTN
NTNNNNNNGGACNNNACNNNNCNNNNNNNACNCNNNCNCNNNNNNANNNGGTTTTTNNNCNNTATTNT
CCAGNACGCNGNGCNNNCNTTCANGNGAACNNNGNNACGCGNNAATCGTANNATCTACCTGCCGCNNG
NNNNNNTACNGNNNNNNCGGANNNTNNCTCGTANNNNNNNCATGCGTNNTNTNNTCNCNAGNCNNGTT
CGNNNANANANTAGNANGTNTNNNNNNNNNTGTNNCGTTCCGCNTGANNCNNCANNNNGNNGNNNTTN
TCANCGGCNCTANCTGCNGANNTAAGNCATANNNTNNNTCNNTANCTNNNNNGGNGGGNNACTGTAAA
NGGNNAATTNGNNNNTNANTTCNNNCACNTNNCTNNGNNNNCTNNNTNCCTGNNTNCNNAATTGGNNC
CANTGNNTGNCNATCTCTGGNTTGNNNNNNTNNNNNNTNNCAANNNGGNNNGTNANANNNCNATNTAA
NNTANANNNNCNNNNNNANNNCACNNNNNNNNNAGNNNCNANNNTTANANNNGTAGNNNAATAAGTCT
CCGANNTNNNNNNNATAAGCGNNANACNNNNNNNNNNNNNANGANNACGNNTTGGNNNTNNNNNNCNA
ANNAGACNNTNGTGGNTNNNNNTCAANNNNTTCGTAGTGGTATAACNGNNNNNNNTTTCNCCAGNNTN
NNNGNNNNNNNCNATANNANNNNNNNGNGANNNNNNNNNNANNNNCTCNNNAGGGANCANCTACTNNA
GCAATGGNGCGCAGNNNGAGANNNNGACNNNCCTTGGGTGGCAANCACNGCAATAAGTNNNGTTNANC
CTNNNAGTNNACACNNNNNCANNGNNNCNNNNNNANCNGANNTNNNNNNNNNNGNNNNNNNNNNNNNN
NNNNNNNNNNNNNNNNNNNNNNNNNNNNANNNNANGGNNNGTNTNTGACNCATGTGTTTTTATNNGNN
NNNNNNNGNNNNANCCCCGGTCGNNNNCNNATTGAANNCCTGNNGCATNNNNNTNCNAGAATGANNGC
NNNNNGNNTNGNCTNNCNNNTCAAGNAGTCANNNAGGTNNNNNNNTCNNNNNNNNNNNNCNNTCCCNN
ACTGAGGGTNNNNNNNNCATCTGCTNNNNNNNNNTNCGCNGNCGTNNCNNNNNNGCNGTATNGNGTNN
NNCANNNNNNNNNNNNNNNNTNAAACAANNNNNNNTNNNN
>LaHu367
GTGNNNNNTAGCCCNNNNNAGTTAGACANNNCNNNNNNNNNNNNNTNNNNNNNNNNNNNNNNNNNNNN
NNNNNNNNNNNNNNNNNNNNNNNNNNNNNNNNNNNNNNNNNNNNNNNNNNNNNNNNNNNNNNNNNNNN
NNNNNNNNNNNNNNNNNNNNNNNNNNNNNNNNNNNNNNNNNNNNNNNNNNNNNNNNNNNNNNNNNNNN
NNNNNNNNNNNNNNNNNNNNNNNNNNNNNNNNNNNNNNNNNNNNNNNNNNNNNNNNNNNNNNNNNNNN
NNNNNNNNNNNNNNNNNNNNNNNNNNNNNNNNNNNNNNNNNNNNNNNNNNCNTCTNNNAAANNNTNNN

NNNNNNNNNNNNNNNNNNNNNNNNNNCNCNTTGNNNGTAANCNANNNGNACATGCAGTCCCCTCNNNNN
NNNGGCAGAGNNNNNNCCCNNNNNNNCCGNCCCCNNCCNNNNNNNTNNNNANNGNNNNNNNANNNNNN
NNNNNNTGGTACNNNNCGNNNNNGGTTGNTTAAGAGGTCCACAACGNGGTCNGGNCACNNNNGNNNAN
NNNCNNAAAANNNNNNNNNNNNNANNTNNNTANNAGGNCNNNNCCNTCNNGNNANNNATTGCNATGACC
CAANNCACGGGGGCNGTCCCCGGCGCGGCGACCTTGANNANGTCCGGGGNCAAGTATGGCNNNCNCCC
CGGGAAGGANNCNGCCGTANGTGCNNNNNANNNNNTCNTGNGANNTCGCCGNNATTCNCGTGGTCNNN
NNAGAATGNCCNNACCANNCGNANNNTGGTNATGNNAGTNNNNNNNNNNNNNNNNNNNNNNNNNNNN
NNNNNNNNNNNNNNNNNNNNNNNNNNNNNNNNNNNGNNNNGNNNNNNCNANNNAAGGANNTANNNCTNNTN
NTNNNNNNGGACNNNACNNNNCNNNNNNNACNCNNNCNCNNNNNNANNNGGTTTTTNNCCNNTATTNT
CCAGNACGCNGNGCNNNCNTTCANGNGAACNNNGNNACGCGNNAATCGTANNATCTACCTGCCGCNNG
TNNNNNTACNGNNNNNNCGGANNNTNNCTCGTANNNNNNNCATGCGTNNTNTNNTCNCNAGNCANGTT
CGNNNANANANTAGNANGTNTNNNNNNNNNTGTNNCGTTCCGCNTGANNCNNCANNNNGNNGNNCTTN
NCANCGGCNNTANCTGCNGANNTAAGNCATANNNTNNATCNNTANCTNNNNNGGAGGGNNACTGTAAA
NGGNNAATTNGNNGNTNANTNCCNNCACNTNNCNNNGNNNNCTNNNTNCCTGNNTNCNNAATTGGNNC
CANTGNNTGNCNATCTCTGGNTTNGNNNNNNTNNNNNTNNCAANNNGGNNNGTNANANNNCNATNTAA
NNTANANNNNCNNNNNNANNNCACNNNNNNNGNAGNNACNANNNTTANANNNGTAGNNNAATAAGTCT
CCGANNTNNNNNNNATAAGCGNNANACNNNNNNNNNNNNNNANGANNACGNNTTGGNNNTNNNNNNCNA
GNNAGACNNTNATGGNTNNNNNTCAANNNNTTNGTAGTGGTATAATNGNNNNNNNTTTCNCCAGNNTN
NNNGNNNNNNNCNATANNANNNNNNNGTGANNNNNNNNNNANNNNCTCNNNAGGGANCANCTACTTNA
GCANTGGNGCGCAGNNNGAGANNNNGACNNNCCTTGGGTGGCAANCACNGCGATAAGTNNNGTTNANC
CTNNNAGTNNACATNNNNNCANNGNNNCNNNNNNANCNGANNGNNNNNNNNNNNNNNNNNNNNNNNNN
NNNNNNNNNNNNNNNNNNNNNNNNNNNNNNNAGNNNANGGNNNGTNTNTGACNCATGTGTTTTTANNNGNN
NNNNNACGNNNGANCCCCGGTCGNNNNCNNATTGAANNCCTGNNGCATNNNNNTNCNAGAATGANNGC
NNNNNGNNTNGNCTNNCNTTTCAAGNAATCAANTAGGTNNNNNGNTCNNNGNNNNNNNNCNNTCCCNN
ACTGAGGGTNNNNNNNNCATCTGCTNNNNNNNNNCTNCGCNGGNGTNNCNNNNNAGCNGTATNGTGTNA
NNCANNNNNNNNNNNNNNNNNNNNAAACNANNNNNNNTNNNN
>LaHu368
GTGNNNNNTAGCCCNNNNNAGTTAGACANNNCNNNNNNNNNNNNNTNNNNNNNNNNNNNNNNNNNNNNNN
NNNNNNNNNNNNNNNNNNNNNNNNNNNNNNNNNNNNNNNNNNNNNNNNNNNNNNNNNNNNNNNNNNNNN
NNNNNNNNNNNNNNNNNNNNNNNNNNNNNNNNNNNNNNNNNNNNNNNNNNNNNNNNNNNNNNNNNNNNN
NNNNNNNNNNNNNNNNNNNNNNNNNNNNNNNNNNNNNNNNNNNNNNNNNNNNNNNNNNNNNNNNNNNNN
NNNNNNNNNNNNNNNNNNNNNNNNNNNNNNNNNNNNNNNNNNNNNNNNNNNNNNNNTCTNNNAAANNNTNNN
NNNNNNNNNNNNNNNNNNNNNNNNNNCNCNNTGNNNGTAANCNANNNGNACATGCAGTCCCCTCNNNNN
NNNNNCAGAGNNNNNNNCCNNNNNNNCCGGCCCCNNCCNNNNNNNNNNNNNNNNNNNNNNNNNANNNNNN
NNNNNNTGGTACNNNNCGNNNNNGGTTGTNTAAGAGGTCCACAACGNGGTCNNNNCACNNNNGNNNAN
NNNCNNNNANCNNNNNNNNNNNNNANNTNNNTANNAGGNCNNNNCCNTCNNNNNNNNNAATTGCNATGACC
CAANNCACGGNGGNAGTNCCCGGCGCGGCGACCTTGANNACGTCCGGGGACAAGTATGGCNNNCNCCC
CGGGAAGGANNCNGCCGTANGTGCNNNNNANNNNNTCNTGNGANGTCGCCGNNNTTCNCGTGGTCNNN
NNAGAATGNNCNNACCANNCGNANNNTGGTNATGNNAGTANNNNNNNNNNNNNNNNNNNNTNNNNNNNN
NNNNNNNNNNNNNNNNNNNNNNNNNNNNNNNNNNNGNNNNGNNNNNNCNANNNAAGGANNTANNNCTNNTN
NTNNNNNNGGACNNNACNNNNCNNNNNNNACNCNNNCNCNNNNNNNNNNNGNTTTTTNNNCNNTANTNT
CCAGNACGCNGNGCNNNCNTTCANGNGAACNNNGNNACGCGNNAATCGTANNATCTACCTGCCGCNNG
NNNNNNTACNGNNNNNNCGGANNNTNNCTCGTANNNNNNTCANGCGTNNTNTNNTCNCNAGNCANGTT
CGNNNANANANTAGNANGTNTNNNNNNNCTTGTNNCGTTCCGCNTGANNCNNCANNNNGNNGNNNTTN
NCANCGGCNNTANCTGCNGANNTAAGNCATANNNTNNNTCNNTANCTNNNNNGGNGGGNNACTGTAAA
NGGNNAATTNGNNGNTNANTNCNNNCACNTNNCNNNGNNNNCTNNNTNCCTGNNTNCNNAATTGGNNC
CANTGNNTGNCNATCTCTGGNTTNGNNNNNNTNNNNNTNNCANNNNGGNNNGTNNNANNNCNATNTAA
NNTANANNNNCNNNNNNANNNCACNNNNNNNGNAGNNNCNANNNTTANANNNGTAGNNNANTNAGTCT
CCGANNTNNNNNNNATAAGCGNNANACNNNNNNNNNNNNNNANGANNACGNNTTGGNNNTNNNNNNNNA
GNNAGACNNTNATGGNTNNNNNTCAANNNNTTNGTAGTGGTATAATNGNNNNNNNTTTCNCCAGNNTN
NNNGNNNNNNNCNATANNANNNNNNNGNGANNNNNNNNNNANNNNCTCNNNAGGGANCANCTACTTNA
GCAATGGNGCGNAGNNNGAGANNNNGACNNNCCTTGGGTGGCAANNACNGCGATAAGTNNNGTTNANC
CTNNNAGTNNACATNNNNNCANNGNNNCNNNNNNANCNGANNGNNNNNNNNNNNNNNNNNNNNNNNNN
NNNNNNNNNNNNNNNNNNNNNNNNNNNNNNGNNNANGGNNNGTNTNTGACNCATGTGTTTTTANNNGNN

NNNNNACGNNNNANCCCCGGTCGNNNNCNNATTGAANNCCTGNNGCATNNNNNTNCNAGAATGANNGC
NNNNNGNNTNGNCTNNCNTTTCAAGNAATCANNTAGGNNNNNNNNNTCNNNGNNNNNNNNCNNTCCCNN
ACTGAGGGTNNNNNNNNCATCTGCTNNNNNNNNNNTNCGCNGGNGTNNCNNNNNNGCNGTATNNNGTNA
NNCANNNNNNNNNNNNNNNNNNTNAAACANNNNNNNNTNNNN
>LaHu369
GTGNNNNNTAGCCCNNNNNAGTTAGACANNNCNNNNNNNNNNNNNTNNNNNNNNNNNNNNNNNNNNNNNN
NNNNNNNNNNNNNNNNNNNNNNNNNNNNNNNNTNNNNNNNNNNNNNNNNNNNNNNNNNNNNNNNNNNNNN
NNNNNNNNNNNNNNNNNNNNNNNNNNNNNNNNNNNNNNNNNNNNNNNNNNNNNNNNNNNNNNNNNNNNNN
NNNNNNNNNNNNNNNNNNNNNNNNNNNNNNNNNNNNNNNNNNNNNNNNNNNNNNNNNNNNNNNNNNNNNN
NNNNNNNNNNNNNNNNNNNNNNNNNNNNNNNNNNNNNNNNNNNNNNNNNNNNCNTCTNNNAAANNNTNNN
NNNNNNNNNNNNNNNNNNNNNNNNNNNCNCNNTGNNNGTAANCNANNNGNACATGCAGTCCCCTCNNNNN
NNNGGCAGAGNNNNNNCCNNNNNNNCCGNCCCCNNCCNNNNNNNNNNNNNNNNNNNNNNNNNNANNNNNN
NNNNNNTGGTACNNNNCGNNNNNGGTTGTNTAAGAGGTCCACAACGNGGTCNNGNCACNNNNGNNNAN
NNNCNNNNAACNNNNNNNNNNNNANNTNNNTANNAGGNCNNNNCCNTCNNGNNNNTNATTGCNATGACC
CAANTCACGGGGGNNGTCCCCGGNGCGGCGACCTTGANNACGTCCGGGGACAAGTATGGCNCNCNCCC
CGGGAAGGANNCNGCCGTANGTGCNNNNNANNNTNTCNTGNGANGTCGCCGNNATTNNCNTGGTCTAN
CNAGAATGNCCNNACCANNCGNANNNTGGTNATGNNAGTANNNNNNNNNNNNNNNNNNNNNNNNANNNNNN
NNNNNNNNNNNNNNNNNNNNNNNNNNNNNNNNNNNNNNGNNNNNNCNANNNAAGGANNTANNNCTNNTN
NTNNNNNNGGACNNNACNNNNCNNNNNNNACNCNNNCNCNNNNNNANNNGGTTTTNNNNCNNTANTNT
CCAGNACGCNGNGCNNNCATTCANGNGAACNNNGNNACGCGNNAATCGTANNATCTACCTGCCGCNNG
TNNNNNTACNGNNNNNNCGGANNNTNNNNCGTANNNNNNNNCATGCGTNNTNTNNTCNCNAGNCANGTT
CGNNNANANANTAGNANGTNTNNNNNNNNNNTGTNNCGTTCCGCNTGANNCNNCANNNNGNNGNNNTTN
TCANCGGCNNTANCTGCNGANNTAAGNCATANNNTNNNTCNNTANCTNNNNNGGNGGGNNACTGTAAA
NGGNNAATTNGANNNTNATTNCNNNCACNTNNCTNNGNNTNCTNNNTNCCTGNNTNCNNAATTGGNNC
CANTGNNTGNCNATCTCTNGNTTNGCNNNNNTGNNNNTNNCAANNNGGNNNGTNNNANNNCNATNTAA
NNTATANNNNCNNNNNNANNNCACNNNNNNNNNNAGNNNCNANNNTTANANNNGTAGNNNANTAAGTCT
CCGANNTNNNNNNNATAAGCGNNANACNNNNNNNNNNNNNNANGANNACGNNTTGGNNNTNNNNNNCNA
GNNAGACNNTNATGGNTNNNNNTCAANNNNTTNGTAGTGGTATAATNGNNNNNNNTTTCNCCAGNNTN
NNNGNNNNNNNCNATANNANNNNNNNGNGANNNNNNNNNNNANNNNCTCNNNAGGGANCANCTACTTNA
GCAATGGNGCGCAGNNNGAGANNNNGACNNGCCTTGGGTGGCAANCACNGCAATAAGTNNNGTTNANC
CTNNNAGTNNACATNNNNNCANNGNNNCNNNNNNANCNGANNGNNNNNNNNNNNNNNNNNNNNNNNNNN
NNNNNNNNNNNNNNNNNNNNNNNNNNNNAGNNNANGGNNNGTNTNTGACNCATGNNTTTTTANNNGNN
NNNNNACGNNNNANCCCCGGTCGTNNNCNNATTGAANNCCTGNNGCATNNNNNTNCNAGAATGANNGC
NNNNNGNNTNGNCTNNCNTTTCAAGNAATCANNTAGGNNNNNNGNTCNNNNNNNNNNNNNCNNTCCCNN
ACTGAGGGTNNNNNNNNCATCTGCTNNNNNNNNNCTNCGCNGGCGTNNCNNCNNAGCNGTATNGTGTNA
NNCANNNNNNNNNNNNNNNNNNTNANACAANNNNNNNTNNNN
>LaHu370
GTGNNNNNTAGCCCNNNNNAGTTAGACANNNCNNNNNNNNNNNNNTNNNNNNNNNNNNNNNNNNNNNNNN
NNNNNNNNNNNNNNNNNNNNNNNNNNNNNNNNNNNNNNNNNNNNNNNNNNNNNNNNNNNNNNNNNNNNNN
NNNNNNNNNNNNNNNNNNNNNNNNNNNNNNNNNNNNNNNNNNNNNNNNNNNNNNNNNNNNNNNNNNNNNN
NNNNNNNNNNNNNNNNNNNNNNNNNNNNNNNNNNNNNNNNNNNNNNNNNNNNNNNNNNNNNNNNNNNNNN
NNNNNNNNNNNNNNNNNNNNNNNNNNNNNNNNNNNNNNNNNNNNNNNNNNNNCNTCTNNNAAANNNTNNN
NNNNNNNNNNNNNNNNNNNNNNNNNNNCNCNNTGNNNGTAANCNANNNGNACATGCAGTCCCCTCNNNNN
NNNGGCAGAGNNNNNNCCCNNNNNNNCCGGCCCCNNCCNNNNNNNNNNNNNNNNNNNNNNNNNANNNNNN
NNNNNNTGGTACNNNNCGNNNNNGGTTGNNTNAGAGGTCCACCACGNGGTCNGGNCACNNNNGNNTAN
NNNCNNNNAAANNNNNNNNNNNNNNANATNNNTANNAGGNNNNNNCCNTNNNGNNANNAANTGCNATGANN
CAANNCNCGNNNGCNGTNCCCGGCGCGGNGACCTTGANNACGTCCGGGGNCAAGTATGGCNNNCNCCC
CGGGAAGGANNCNGCCGTANGTGCNNNNNANNNNNTCNTGNGANGTNGCCGNNANNNNCNTGGTCNNN
CNAGAATGNCCGNACCANNCGNANNNTGGTNATGNNAGTNNNNNNNNNNNNNNNNNNNNNNNNNNNNNN
NNNNNNNNNNNNNNNNNNNNNNNNNNNNNNNNGNNNNGNNNNNNCNANNNAAGGNNNTANNNCTNNTN
NTNNNNNNGGACNNNACNNNNCNNNNNNNACNCNNNCNCNNNNNNNNNNNGGTTTTNNNNCNNTANTNT
CCAGNANGCNGNGCNNNCATTCANGNGAACNNNGNNACGCGNNAATCGTANNATCTACCTGCCGCNNG
NNNNNNTACNGNNNNNNCGGANNNTNNCTCGTANNNNNNNTANACGTNNTNTNNTCNCNAGNCANGTT
CGNNNANANANTAGNANGTNTNNNNNNNNNNTGTNNCGTTCCGCNTGANNCNNCANNNNGNNGNNCTTN

NCANCGGCNNTANCTGCNGANNTAAGNCATANNNTNNNTCNNTANCTNNNNNGGNGGGNNACTGTAAA
NGGNNAATTGGNNNNTNATTNCNNNCACNTNNCNNNGNNNNCTNNNTNCCTGNNTNCNNAATTGGNNC
CANTGNNTGNCNATCTCTGGNTTNGNNNNNNTNNNNNTNNCAANNNGGNNNGTNNNANNNCNATNTAA
NNTANANNNNCNNNNNNANNNCACNNNNNNNNNAGNNNCNANNNTNANANNNGTAGNNNANTNAGTCT
CCGANNTNNNNNNNATAAGCGNNANACNNNNNNNNNNNNNANGANNACGNNTTGGNNNTNNNNNNCNA
GNNAGACNNTNGTGGNTNNNNNTCAANNNNTTCGTAGTGGTATAATNGNNNNNNNTTTCNCCAGNNTN
NNNGNNNNNNNCNATANNANNNNNNNGNGANNNNNNNNNNANNNNCTCNNNAGGGANCANCTACTNNA
GCAANGGNGCGNAGNNNGAGANNNNGACNNNCCTTGGGTGGCAANCACNGNAATAAGTNNNGTTNANN
NTNNNAGTNNACACNNNNNCANNGNNNCNNNNNNANCNGANNGNNNNNNNNNNNNNNNNNNNNNNNNN
NNNNNNNNNNNNNNNNNNNNNNNNNNNNANNNNANGGNNNGTNTNTGACNCATGTGTTTTTANNNGNN
NNNNNACGNNNNANCCCCGGTCGNNNNCNNATTGAANNCCTGNNGCATNNNNNTNCNAGAATGANNGC
NNNNNGNNTNGNCTNNNNNNTNANGNAGTCAANNAGGTNNNNNNNTCNNNNNNNNNNNNCNNNCNCNN
ACTGAGGGTNNNNNNNNCATCTGCTNNNNNNNNNTNCGCNGGNGTNNCNNNNNAGCNGTATNNNGNNN
NNCANNNNNNNNNNNNNNNNTNAAACAANNNNNNTNNNN
>LaHu371
GTGNNNNNTAGCCCNNNNNAGTTAGACANNNCNNNNNNNNNNNNNTNNNNNNNNNNNNNNNNNNNNNN
NNNNNNNNNNNNNNNNNNNNNNNNNNNNNNNNNNNNNNNNNNNNNNNNNNNNNNNNNNNNNNNNNNNN
NNNNNNNNNNNNNNNNNNNNNNNNNNNNNNNNNNNNNNNNNNNNNNNNNNNNNNNNNNNNNNNNNNNN
NNNNNNNNNNNNNNNNNNNNNNNNNNNNNNNNNNNNNNNNNNNNNNNNNNNNNNNNNNNNNNNNNNNN
NNNNNNNNNNNNNNNNNNNNNNNNNNNNNNNNNNNNNNNNNNNNNNNNTCTNNNAAANNNTNNN
NNNNNNNNNNNNNNNNNNNNNNNNNCNCNNTNNNNGTAANCNANNNGNACATGCAGTCCCCTCNNNNN
NNNNNCAGAGNNNNNNCCCNNNNNCNCCGGCCCCNNCCNNNNNNNNNNNNNNNGNNNNNNNANNNNNN
NNNNNNTGGTACNNNNCGNNNNNGGTTGTNTNAGAGGTCCACCACGNGGTCNGGNNACNNNNGNNTAN
NNNCNNAAAANNGNNNNNNNNNANNTNNNTANNAGGNCNNNNCCNTCNNGNNNNNNATTGCNATGACC
CAANNCACGGGGGCAGTNCCCGGCGCGGCGACCTTGATNANGTCCGGGGACAAGTATGGCNNNCNCCC
CGGGAAGGANNCNGCCGTANGTGCNNNNNANNNNNTCNTGNGANGTCGCCGNNATTNNCGTGGTCNNN
NNAGAATGNNCNNACCANNCGNANNNTGGTNATGNNAGTANNNNNNNNNNNNNNNNNNNNNNNNNNNN
NNNNNNNNNNNNNNNNNNNNNNNNNNNNNNNGNNNNGNNNNNNCNANNNNAAGGANNTANNNCTNNTN
NTNNNNNNGGACNNNACNNNNCNNNNNNNACNCNNNCNCNNNNNNANNNGGTTTTNNNNCNNTANTNT
CCAGNACGCNGNGCNNTCATTCANGNGAACNNNGNNACGCGNNAATCGTANNATCTACCTGCCGCNNG
NNNNNNTACNGNNNNNNCGGANNNTNNCTCGTANNNNNNNTATACGTNNTNTNNTCNCNAGNCNNGTT
CGNNNANANANTAGNANGTNTCNNNNNNNNTGTNNCGTTCCGCNTGANNCNNCANNNNGNNGNNNTTN
NCANCGGCNNTANCTGCNGANNTAAGNCATANNNTNNATCNNTANCTNNNNNGGNGGGNNACTGTAAA
NGGNNAATTNGANGNTNATTNCNNNCACNTNNCNNNGNNTNCTNNNTNCCTGNNTNCNNAATTGGNNN
CANTGNNTGNCNATCTCTNGNTTNGNNNNNNTNNNNNTNNCANNNNGGNNNGTNNNANNNCNATNTAA
NNTANANNNNCNNNNNNANNNCACNNNNNNNNGNAGNNACNANNNTTANANNNGTAGNNNANTAAGTCT
CCGANNTNNNNNNNATAAGCGNNANACNNNNNNNNNNNNNANGANNACGNNTTGGNNNTNNNNNNCNA
GNNANACNNTNGTGGNTNNNNNTCAANNNNTTNGTAGTGGTATAATNGNNNNNNNTTTCNCCAGNNTN
NNNGNNNNNNNCNATANNANNNNNNNGNGANNNNNNNNNNANNNNCTCNNNAGGGANCANCTACTNNA
GCANTGGNGCGCAGNNNGAGANNNNGACNNNCCTTGGGTGGCAANNACNGCAATAAGTNNNGTTNANN
CTNNNAGTNNACACNNNNNCANNGNNNCNNNNNNANCNGANNGNNNNNNNNNNNNNNNNNNNNNNNNN
NNNNNNNNNNNNNNNNNNNNNNNNNNNNNNNNNANNGNNNGTNTNTGACNCATGTGTTTTTANNNGNN
NNNNNACGNNNGANCCCCGGTCGNNTNCNNATTGAANNCCTGNNGCATNNNNNTNCNAGAATGANNNC
NNNNNGNNTNGNCTNNCNTTTCAAGNAGTCNNNTAGGTNNNNNNNTCNNNGNNNNNNNNCNNNCCCNN
ACTGAGGGTNNNNNNNNCATCTGCTNNNNNNNNNTTCGCNGGCGTNNCNNNNNAGCNGTATNGTGNNN
NNCANNNNNNNNNNNNNNNNCNAAANGANNNNNNTNNNN
>LaHu372
GTGNNNNNTAGCCCNNNNNAGTTAGACANNNCNNNNNNNNNNNNNTNNNNNNNNNNNNNNNNNNNNNN
NNNNNNNNNNNNNNNNNNNNNNNNNNNNNNTNNNNNNNNNNNNNNNNNNNNNNNNNNNNNNNNNNNNNN
NNNNNNNNNNNNNNNNNNNNNNNNNNNNNNNNNNNNNNNNNNNNNNNNNNNNNNNNNNNNNNNNNNNN
NNNNNNNNNNNNNNNNNNNNNNNNNNNNNNNNNNNNNNNNNNNNNNNNNNNNNNNNNNNNNNNNNNNN
NNNNNNNNNNNNNNNNNNNNNNNNNNNNNNNNNNNNNNNNNNNNNNNNNNCNTCTNNNAAANNNTNNN
NNNNNNNNNNNNNNNNNNNNNNNNNCNCNTTGNNNGTAANCNANNNGNACATGCAGTCCCCTCNNNNN
NNNNNCAGAGNNNNNNCCCNNNNNNNCCGGCCCCNNCCNNNNNNNNNNNNNNNGNNNNNNNGNNNNNN

NNNNNNTGGTACNNNNCGNNNNNGGTTGTNTAAGAGGTCCACCACGNGGTCNGGNNACNNNNGNNNAN
NNNCNNNAAACNNNNNNNNNNNNANNTNNNCANNAGGNCNNNNCCNTCNNGNNNNNNATTGCNATGACC
CAANNCACGGGGGCNGTCCCCGGCGNGGCGACCTTGNNNACGGCCGGGGNCAAGTATGGCNNNCNCCC
CGGGAAGGANNCNGCCGTANGTGCNNNNNANNNNNTCNTGNGACGTCGCCGNNATTCNCGTGGTCNNN
CNAGAATGNCCGNACCANNCGNANNNTGGTNATGNNAGTNNNNNNNNNNNNNNNNNNNNNNNNNNNNN
NNNNNNNNNNNNNNNNNNNNNNNNNNNNNNNNNNNNNGNNNNNNCNANNNAAGGANNTANNNCTNNTN
NTNNNNNNGGACNNNACNNNNCNNNNNNNACNCNNNCNCNNNNNNANNNGGTTTTNNNNCNNTANTNT
CCAGNACGCNGNGCNNNCATTCANGNGAACNNNGNNACGCGNNAATCGTANNATCTACCTGCCGCNNG
NNNNNNTACNGNNNNNNCGGANNNTNNCTCGTANNNNNNNCATGCGTNNTNTNNTCNCNAGNCANGTT
CGNNNANANANTAGNANGTNTNNNNNNNNNNTGTNNCGTTCCGCNTGANNCNNCANNNNGNNGNNCTTN
TCANCGGCNNTANCTGCNGANNTAAGNCATANNNTNNATCNNTANCTNNNNNGGNGGGNNACTGTAAA
NGGNNAATTNGNNGNTNATTNCNNNCACNTNNCTNNGNNNNCTNNNTNCCTGNNTNCNNAATTGGNNN
CANTGNNTGNCNATCTCTGGNTTNGCNNNNNTNNNNNTNNCANNNNGGNNNGTNNNANNNCNATNTAA
NNTANANNNNCNNNNNNANNNCACNANNNNNGNAGNNNCNANNNTTANANNNGTAGNNNANTAAGTCT
CCGANNTNNNNNNNATAAGCGNNANACNNNNNNNNNNNNNNANGANNACGNNTTGGNNNTNNNNNNCNA
ANNAGACNNTNGTGGNTNNNNNTCAANNNNTTCGTAGTGGTATAACNGNNNNNNNTTTCNCCAGNNTN
NNNGNNNNNNNNCNATANNANNNNNNNGNGANNNNNNNNNNNANNNNCTCNNNAGGNANCANCTACTTNA
GCAATGGNGCGCAGNNNGAGANNNNGACCAGCCTTGGGTGGCAANCACNGCAATAAGTNNNGTTNANC
CTNNNAGTNNACACNNNNNCANNGNNNCNNNNNNANCNGANNTNNNNNNNNNNNNNNNNNNNNNNNNN
NNNNNNNNNNNNNNNNNNNNNNNNNNNNAGNNNANGGNNNGTNTNTGACNCATGNNTTTTTANNNGNN
NNNNNACGNNNNANCCCCGGTCGNNNNCNNATTGAANNCCTGNNGCATNNNNNTNCNAGAATGANNNC
NNNNNGNNTNGNCTNNCNTTTCAAGNAGTCANNNAGGTANCGCGNTCNNNGNNNNNNNNCNNTCCCNN
ACTGAGGGTNNNNNNNNCATCTGCTNNNNNNNNNNTNCGCNGNCGTNNCNNNNNNGCNGTATNGNGTNA
NNCANNNNNNNNNNNNNNNNNCNAAACAANNNNNNNTNNNN
>LaHu373
GTGNNNNNTAGCCCNNNNNAGTTAGACANNNCNNNNNNNNNNNNTNNNNNNNNNNNNNNNNNNNNNNN
NNNNNNNNNNNNNNNNNNNNNNNNNNNNNNTNNNNNNNNNNNNNNNNNNNNNNNNNNNNNNNNNNNNN
NNNNNNNNNNNNNNNNNNNNNNNNNNNNNNNNNNNNNNNNNNNNNNNNNNNNNNNNNNNNNNNNNNNN
NNNNNNNNNNNNNNNNNNNNNNNNNNNNNNNNNNNNNNNNNNNNNNNNNNNNNNNNNNNNNNNNNNNN
NNNNNNNNNNNNNNNNNNNNNNNNNNNNNNNNNNNNNNNNNNNNNNNNNNCNTCTNNNAAANNNTNNN
NNNNNNNNNNNNNNNNNNNNNNNNNCNCNTTGNNNGTAANCNANNNGNACATGCAGTCCCCTCNNNNN
NNNGGCAGAGNNNNNNCCNNNNNCNCCGGCCCCGNCCNNNNNNNNNNNNNNNNNNNNNNNNANNNNNN
NNNNNNTGGTACNNNNCGNNNNNGGTTGTNTAAGAGGTCCACCACGNGGTCNNGNCACNNNNGNNTAN
NNNCNNNNAACNGNNNNNNNNNNANNTNNNTANNAGGNCNNNNCCNTCNNGNNNNNNATTGCNATGACC
CAANNCACGNNNGCNGTNCCCGGCGCGGCGACCTTGNTNACGTCCGGGGACAAGTATGGCGNNCNCCC
CGGGAAGGANNCNGCCGTANGTGCNNNNNANNNNNTCTTGNGACGTNGCCGNNATTCNCGTGGTCNNN
CNAGAATGNCCGNACCANNCGNANNNTGGTNATGNTAGTNNNNNNNNNNNNNNNNNNNNNNNNNNNNN
NNNNNNNNNNNNNNNNNNNNNNNNNNNNNNNNGNNNNGNNNNNNCNANNNAAGGANNTANNNCTNNTN
NTNNNNNNGGACNNNACNNNNCNNNNNNNACNCNNNCNCNNNNNNANNNGGTTTTNNNNCNNTATTNT
CCAGNACGCNGNGCNNNCATTCANGNGAACNNNGNNACGCGNNAATCGTANNATCTACCTGCCGCNGG
TNNNNNTACNGNNNNNNCGGANNNTNNCTCGTANNNNNNNTATACGTNNTNTNNTCNCNAGNCNNGTT
CGNNNANANANTAGNANGTNTNNNNNNNNCTTGTNNCGTTCCGCNTGANNCNNCANNNNGNNGNNCTTC
TCANCGGCNCTANCTGCNGANNTAAGNCATANNNTNNATCNNTANCTNNNNNGGAGGGNNACTGTAAA
NGGNNAATTNGANGNTNATTNCNNNCACNTNNCTNNGNNTNCTNNNTNCCTGNNTNCNNAATTGGGNC
CANTGNNTGNCNATCTCTGGNTTNGNNNNNNTNNNNNTNNCAANNNGGNNNGTNANANNNCNATNTAA
NNTANANNNNCNNNNNNANNNCACNNNNNNNGNAGNNNCNANNNTTANANNNGTAGNNNAATAAGTCT
CCGANNTNNNNNNNATAAGCGNNANACNNNNNNNNNNNNNNANGANNACGNNTTGGNNNTNNNNNNCNA
GNNAGACNNTNGTGGNTNNNNNTCAANNNNTTCGTAGTGGTATAATNGNNNNNNNTTTCNCCAGNNTN
NNNGNNNNNNNNCNATANNANNNNNNNGNGANNNNNNNNNNNANNNNCTCNNNAGGGANCANCTACTNNA
GCAATGGNGCGCAGNNNGAGANNNNGACCAGCCTTGGGTGGCAANCACNGCAATAAGTNNNGTTNANC
CTTNNAGTNNACACNNNNNCANNGNNNCNNNNNNANCNGANNGNNNNNNNNNNNNNNNNNNNNNNNNN
NNNNNNNNNNNNNNNNNNNNNNNNNNNNNGNNNANGGNNNGTNTNTGACNCATGTGTTTTTANNNGNN
NNNNNACGNNNGANCCCCGGTCGTNNNCNNATTGAANNCCTGNNGCATNNNNNTNCNAGAATGANNGC
NNNNNGNNTNGNCTNNCNNNTCAAGNAGTCAANNAGGTATNGCGNTCNNNGNNNNNNNNCNNTCCCNN

ACTGAGGGTNNNNNNNNCATCNGCTNNNNNNNNNNNTNCGCNGGCGTNNCNNNNNAGCNGTATNNNGNNA
NNCANNNNNNNNNNNNNNNNNTNAAACNNNNNNNNNTNNNN
>LaHu374
GTGNNNNNTAGCCCNNNNNAGTTAGACANNNCNNNNNNNNNNNNNTNNNNNNNNNNNNNNNNNNNNNNNN
NNNNNNNNNNNNNNNNNNNNNNNNNNNNNNNNNNNNNNNNNNNNNNNNNNNNNNNNNNNNNNNNNNNNNN
NNNNNNNNNNNNNNNNNNNNNNNNNNNNNNNNNNNNNNNNNNNNNNNNNNNNNNNNNNNNNNNNNNNNNN
NNNNNNNNNNNNNNNNNNNNNNNNNNNNNNNNNNNNNNNNNNNNNNNNNNNNNNNNNNNNNNNNNNNNNN
NNNNNNNNNNNNNNNNNNNNNNNNNNNNNNNNNNNNNNNNNNNNNNNNNNNNNNTCTNNNAAANNNTNNN
NNNNNNNNNNNNNNNNNNNNNNNNNNNCNCNTTGNNNGTAANCNANNNGNACATGCAGTCCCCTCNNNNN
NNNGGCAGAGNNNNNNNCCNNNNNNNCCGGCCCCNNCCNNNNNNNNNNNNNNNNNNNNNNNNANNNNNN
NNNNNNTGGTACNNNNCGNNNNNGGTTGTNTAAGAGGTCCACAACGNGGTCNGGNNACNNNNGNNNAN
NNNCNNNAAACNNNNNNNNNNNNNNNTNNNTANNAGGNCNNNNCCNTCNNNNNNNNNNANTGCNATGACC
CNANNCNCGGGGGCNGTCCCCGGCGNGGCGACCTTGNNNANGTCCGGGGACAAGTATGGCNNNCNCCC
CGGGAAGGANNCNGCCGTANGTGCNNNNNANNNTNTCNTGNGANGTNGCCGNNATTCNCGTGGTCNNN
CNAGAATGNCCNNACCANNCGNANNNTGGTNATGTNAGTNNNNNNNNNNNNNNNNNNNNNNNNNNNNNN
NNNNNNNNNNNNNNNNNNNNNNNNNNNNNNNNNNNGNNNNGNNNNNNCNANNNAAGGNNNTANNNCTNNTN
NTNNNNNNNGGACNNNACNNNNCNNNNNNNACNCNNNCNCNNNNNNNNNNNGNTTTTNNNNCNNTANTNT
CCAGNANGCNGNGCNNNCATTCANGNGAACNNNGNNACGCGNNAATCGTANNATCTACCTGCCGCNNG
NNNNNNTNCNGNNNNNNCGGANNNTNNNNCGTANNNNNNNCANGCGTNNTNTNNTCNCNAGNCNNGTT
CGNNNANANANTAGNANGTNTNNNNNNNNNNTGTNNCGTTCCGCNTGANNCNNCNNNNNGNNGNNCTTN
NCANCGGCNNTANCTGCNGANNTAAGNCATANNNTNNNTCNNTANCTNNNNNGGNGGGNNACTGTAAA
NGGNNAATTNGNNNNTNANTNCNNNCACNTNNCTNNGNNNNCTNNNTNCCTGNNTNCNNAATTGGNNC
CANTGNNTGNCNATCTCTNGNTTNGNNNNNNTNNNNNTNNCAANNNGGNNNGTNANANNNCNATNTAA
NNTANANNNNCNNNNNNANNNCACNNNNNNNNNNAGNNNCNANNNTTANANNNGTAGNNNANTAAGTCT
CCGANNTNNNNNNNATAAGCGNNANACNNNNNNNNNNNNNNANGANNACGNNTTGGNNNTNNNNNNCNA
GNNAGACNNTNATGGNTNNNNNTCAANNNNTTNGTAGTGGTATAATNGNNNNNNNTTTCNCCAGNNTN
NNNGNNNNNNNNCNATANNANNNNNNNGNGANNNNNNNNNNNANNNNCTCNNNAGGNANCANCTACTNNA
GCANTGGNGCGCAGNNNGAGANNNNGACNNNCCTTGGGTGGCAANCACNGCAATAAGTNNNGTTNANC
CTNNNAGTNNACATNNNNNCANNGNNNCNNNNNNANCNGANNGNNNNNNNNNNNNNNNNNNNNNNNNNN
NNNNNNNNNNNNNNNNNNNNNNNNNNNNNNNNNGNNNANGGNNNGTNTNTGACNCATGNNTTTTTANNNGNN
NNNNNACGNNNNANCCCCGGTCGNNNNCNNATTGAANNCCTGNNGCATNNNNNNTNCNAGAATGANNNC
NNNNNGNNTNGNCTNNCNNNTCAAGNAATCNNNNAGGTNNNNNGNTCNNNNNNNNNNNNNCNNTCCCNN
ACTGAGGGTNNNNNNNNCATCNGCTNNNNNNNNNNCTTCGCNGGNGTNNCNNNNNAGCNGTATNNNGTNA
NNCANNNNNNNNNNNNNNNNNCNAAACGANNNNNNTNNNN
>LaHu375
GTGNNNNNTAGCCCNNNNNAGTTAGACANNNCNNNNNNNNNNNNNTNNNNNNNNNNNNNNNNNNNNNNNN
NNNNNNNNNNNNNNNNNNNNNNNNNNNNNNNNTNNNNNNNNNNNNNNNNNNNNNNNNNNNNNNNNNNNNN
NNNNNNNNNNNNNNNNNNNNNNNNNNNNNNNNNNNNNNNNNNNNNNNNNNNNNTTNNNNNNNNNNNNNNN
NNNNNNNNNNNNNNNNNNNNNNNNNNNNNNNNNNNNNNNNNNNNNNNNNNNNNNNNNNNNNNNNNNNNNN
NNNNNNNNNNNNNNNNNNNNNNNNNNNNNNNNNNNNNNNNNNNNNNNNNNNNNNTCTNNNAAANNNTNNN
NNNNNNNNNNNNNNNNNNNNNNNNNNNCNCNNTGNNNGTAANCNANNNGNACATGCAGTCCCCTCNNNNN
NNNGGCAGAGNNNNNNCCNNNNNNCNCCGNCCCCNNCCNNNNNNNNNNNNCANNGNNNNNNNANNNNNN
NNNNNNTGGTACNNNNCGNNNNNGGTTGTNTNAGAGGTCCACCACGNGGTCNNGNCACNNNNGNNNAN
NNNCNNNNAACNNNNNNNNNNNNNANNTNNNTANNAGGNCNNNNCCNTCNNNNNNNNNNATTGCNATGACC
CAANNCACGNNNGCAGTNCCCGGNGCGGCGACCTTGANNACGTCCGGGGACAAGTATGGCGNNCNCCC
CGGGAAGGANNCNGCCGTANGTGCNNNNNANNNNNTCNTGNGANGTCGCCGNNNTTNNCGTGGTCNNN
NNAGAATGNCCGNACCANNCGNANNNTGGTNATGTTAGTNNNNNNNNNNNNNNNNNNNNNNNNNNNNNN
NNNNNNNNNNNNNNNNNNNNNNNNNNNNNNNNNNNGNNNNGNNNNNNCNANNNAAGGANNTANNNCTNNTN
NTNNNGNNGGACNNNACNNNNCNNNNNNNACNCNNNCNCNNNNNNANNNGNTTTTTNNNCNNTANTNT
CCAGNACGCNGNGCNNNCATTCANGNGAACNNNGNNACGCGNNAATCGTANNATCTACCTGCCGCNNG
NNNNNNTNCNGNNNNNNCGGANNNTNNCTCGTANNNNNNTTATACGTNNTNTNNTCNCNAGNCNNGTT
CGNNNANANANTAGNANGTNTNNNNNNNNNNTGTNNCGTTCCGCNTGANNCNNCANNNNGNNGNNCTTN
TCANCGGCNNTANCTGCNGANNTAAGNCATANNNTNNNTCNNTANCTNNNNNGGNGGGNNACTGTAAA
NGGNNAATTNGANGNTNANTNCNNNCACNTNNCTNNGNNTNCTNNNTNCCTGNNTNCNNAATTGGNNC

CANTGNNTGNCNATCTCTGGNTTNGNNNNNNTGNNNNTNNCANNNNGGNNNGTNNNANCNCNATNTAA
NNTANANNNNCNNNNNNANNNCACNNNNNNNGNAGNNNNNANNNTTANANNNGTAGNNNAATNAGTCT
CCGANNTNNNNNNNNATAAGCGNNANACNNNNNNNNNNNNNNANGANNACGNNTTGGNNNTNNNNNNCNA
GNNAGACNNTNGTGGNTNNNNNTCAANNNNTTNGTAGTGGTATAATNGNNTNNNNTTTCNCCAGNNTN
NNNGNNNNNNNNCNATANNANNNNNNNGNGANNNNNNNNNNNANNNNCTCNNNAGGNANCANCTACTNNA
GCANTGGNGCGCAGNNNGAGANNNNGACNNGCCTTGGGTGGCAANNACNGCAATAAGTNNNGTTNANC
CTNNNAGTNNACACNNNNNCANNGNNNCNNNNNNANCNGANNGNNNNNNNNNNNNNNNNNNNNNNNNNN
NNNNNNNNNNNNNNNNNNNNNNNNNNNNNNNNNNNANGGNNNGTNTNTGACNCATGTGTTTTTANNNGNN
NNNNNACGNNNNANCCCCGGTCGTNNNCNNATTGAANNCCTGNNGCATNNNNNTNCNAGAATGANNGC
NNNNNGNNTNGNCTGNCNNNTCAAGNAGTCANNNAGNNANNNNNNTCNNNNNNNNNNNNCNNTCCCNN
ACTGAGGGTNNNNNNNGCATCTGCTNNNNNNNNNCTNCGCNGGCGTNNCNNNNNNGCNGTATNGTGNNN
NNCANNNNNNNNNNNNNNNNNTNAAACNANNNNNNNTNNNN
>LoLo458
GTGNNNNNTAGCCCNNNNNAGTTAGACANNNCNNNNNNNNNNNNNTNNNNNNNNNNNNNNNNNNNNNNNN
NNNNNNNNNNNNNNNNNNNNNNNNNNNNNNNNTNNNNNNNNNNNNNNNNNNNNNNNNNNNNNNNNNNNNNNN
NNNNNNNNNNNNNNNNNNNNNNNNNNNNNNNNNNNNNNNNNNNNNNNNNNNNNNNNNNNNNNNNNNNNNNNN
NNNNNNNNNNNNNNNNNNNNNNNNNNNNNNNNNNNNNNNNNNNNNNNNNNNNNNNNNNNNNNNNNNNNNNNN
NNNNNNNNNNNNNNNNNNNNNNNNNNNNNNNNNNNNNNNNNNNNNNNNNNNNNNNNNTCTNNNAAANNNTNNN
NNNNNNNNNNNNNNNNNNNNNNNNNNNCNCNTTNNNNGTAANCNANNNGNACATGCAGTCCCCTCNNNNN
NNNGGCAGAGNNNNNNNCCNNNNNNNCCGNCCCCNNCCNNNNNNNNNNNNNNNNNGNNNNNNNANNNNNN
NNNNNNTGGTACNNNNCGNNNNNGGTTGTNTNAGAGGTCCACAACGNGGTCNNGNNACNNNNGNNNAN
NNNCNNNNAACNNNNNNNNNNNNANATNNNTANNAGGNCNNNNCCNTCNNNNNNNNNNANTGCNATGACC
CNNNTCNCGGGGGNNGTNCCCGGCGCGGCGACCTTGNNNANGTCCGGGNACAAGTATGGCNNNCNCCC
CGGGAAGGANNCNGCCGTANGTGCNNNNNANNNNNTCNTGNGACGTNGCCGNNNNNNNCGTGGTCNNN
NNAGAATGNCCNNACCANNCGNANNNTGGTNATGNNAGTNNNNNNNNNNNNNNNNNNNNNNNNNNNNNN
NNNNNNNNNNNNNNNNNNNNNNNNNNNNNNNNNNNNNNNNNNNGNNNNNNCNANNNAAGGNNNTANNNCTNNTN
NTNNNNNNNGGACNNNACNNNNCNNNNNNNNACNCNNNCNCNNNNNNNNNNNGNTTTTTNNNCNNTATTNT
CCAGNACGCNGNGCNNNCNTTCANGNGAACNNNGNNACGCGNNAATCGTANNATCTACCTGCCGCNNG
TNNNNNTACNGNNNNNNCGGANNNTNNCTCGTANNNNNNTCATGCGTNNTNTNNTCNCNAGNCNNGTT
CGNNNANANANTAGNANGTNTNNNNNNNNNNTGTNNCGTTCCGCNTGANNCNNCANNNNGNNGNNNTTN
TCANCGGCNNTANCTGCNGANNTAAGNCATANNNTNNNTCNNTANCTNNNNNGGNGGGNNACTGTAAA
NGGNNAATTNGNNNNTNATTNCNNNCACNTNNCNNNGNNNNCTNNNTNCCTGNNTNCNNAATTGGNNC
CANTGNNTGNCNATCTCTNGNTTNGCNNNNNTNNNNNTNNCANNNNGGNNNNNNNNANNNCNATNTAA
NNTANANNNNCNNNNNNANNNCACNNNNNNNNNAGNNNCNANNNTTANANNNGTAGNNNAATNAGTCT
CCGANNTNNNNNNNNATAAGCGNNANACNNNNNNNNNNNNNNANGANNNCGNNTTGGNNNTNNNNNNNNA
GNNAGACNNTNATGGNTNNNNNTCAANNNNTTNGTAGTGGTATAATNGNNNNNNNTTTCNCCAGNNTN
NNNGNNNNNNNNCNATANNANNNNNNNGNGANNNNNNNNNNNANNNNCTCNNNAGGGANCANCTACTNNA
GCANTGGNNCGNAGNNNGAGANNNNGACNNGCCTTGGGTGGCAANCACNGCGATAAGTNNNGTTNNNC
CTNNNAGTNNACATNNNNNCANNGNNNCNNNNNNANCNGANNGNNNNNNNNNNNNNNNNNNNNNNNNN
NNNNNNNNNNNNNNNNNNNNNNNNNNNNNNNAGNNNANGGNNNGTNTNTGACNCATGTGTTTTTANNNGNN
NNNNNACGNNNNANCCCCGGTCGTNTNCNNATTGAANNCCTGNNGCATNNNNNTNCNAGAATGANNNC
NNNNNGNNTNGNCTNNCNTTTCAAGNAATCANNNAGGNANNNNNNTCNNNGNNNNNNNNCNNNCCCNN
ACTGAGGGTNNNNNNNNCATCTGCTNNNNNNNNNNTNCGCNGGNGTNNCNNNNNNGCNGTATNGNGTNA
NNCANNNNNNNNNNNNNNNNNNNNAAACNANNNNNNCTNNNN
>LoLo459
GTGNNNNNTAGCCCNNNNNAGTTGGACANNNCNNNNNNNNNNNNNTNNNNNNNNNNNNNNNNNNNNNNNN
NNNNNNNNNNNNNNNNNNNNNNNNNNNNNNNNTNNNNNNNNNNNNNNNNNNNNNNNNNNNNNNNNNNNNNNN
NNNNNNNNNNNNNNNNNNNNNNNNNNNNNNNNNNNNNNNNNNNNNNNNNNNNNNNNNNNNNNNNNNNNNNNN
NNNNNNNNNNNNNNNNNNNNNNNNNNNNNNNNNNNNNNNNNNNNNNNNNNNNNNNNNNNNNNNNNNNNNNNN
NNNNNNNNNNNNNNNNNNNNNNNNNNNNNNNNNNNNNNNNNNNNNNNNNNNNNNNNNCNTCTNNNAAANNNTNNN
NNNNNNNNNNNNNNNNNNNNNNNNNNNCNCNTTGNNNGTAANCNANNNGNACATGCAGTCCCCTCNNNNN
NNNGGCAGAGNNNNNNCCCNNNNNNNCCGNCCCCGNCCNNNNNNNNNNNNNNNNNGNNNNNNNGNNNNNN
NNNNNNTGGTACNNNNCGNNNNNGGTTGNTTAAGAGGTCCACCACGNGGTCNGGNCACNNNNGNNNAN
NNNCNNNAAACNNNNNNNNNNNNANNTNNNCANNAGGNCNNNNCCNTCNNGNNNNNNATTGCNATGACC

CAANTCGCGGGGACAGTCACCGGCGCGGCGACCTTGANNACGGTCGGGGACAAGTATGTCGNNCNCCC
CGGGAAGGANNCNGCCGTANGTGCNNNNNANNNNNTCNTGNGANGTCGCCGNNANNCNCGTGGTCTNN
CNAGAATGNCCGNACCANNCGNANNNTGGTNATGNNAGTCNNNNNNNNNNNNNNNNNNNNNNNNNNNN
NNNNNNNNNNNNNNNNNNNNNNNNNNNNNNNNGNNNNGNNNNNNCNANNNAAGGNNNTANNNTTNNTN
NTNNNNNNGGACNNNACNNNNCNNNNNNNACNCNNNCNCNNNNNNANNNGGTTTTNNNCCNNTATTNT
CCAGNACGCNGNGCNNNCATTCANGNGAACNNNGNNACGCGNNAATCGTANNATCTACCTGCCGCNNG
TNNNNNTACNGNNNNTNCGGANNNTNNNNCGTANNNNNNTCATGCGTNNTNTNNTTNCNAGNCNNGTT
CGNNNANANANTAGNANGTNTNNNNNNNNNNTGTNNCGTTCCGCNTGANNCNNCANNNNGNNGNNCTTN
NCANCGGCNNTANCTGCNGANNTAAGCCATANNNTNNNTCNNTANCGNNNNNGGNGGGNNACTGTAAA
NGGNNAATTNGANNNTNATTTCNNNCACNTNNCTNNGNNNNCTNNNCNCCTGNNTNCNNAATTGGNNC
CANTGNNTGNCNATCTCTGGNTTNGCNNNNNTNNNNNTNNTAANNNGGNNNNNNNNANNNCNATNTAA
NNTANANNNNCNNNNNNANNNCACNNNNNNNGNAGNNNCNANNNTGANGNNNGTAGNNNAATAAGTCT
CCGANNTNNNNNNNATAAGCGNNANACNNNNNNNNNNNNNANGANNACGNNTTGGNNNTNNNNNNCNA
ANNAGACNNCNGTGGNTNNNNNTCAANNNNTTCGTAGTGGTATAACNANNNNNNNTTTCNCCAGNNTN
NNNGNNNNNNNNCNATANNANNNNNNNGNGANNNNNNNNNNNANNNNCCCNNNAGGGANCANCTATTNNA
GCANTGGNGCGCAGNNNGAGANNNNGACNNNCCTTGGGTGGCAANCACNGCAATAAGTNNNGTTNANC
CCNNNAGTNNACACNNNNNCANNGNNNCNNNNNNANCNGANNTNNNNNNNNNNNNNNNNNNNNNNNNN
NNNNNNNNNNNNNNNNNNNNNNNNNNNNAGNNNANGGNNNGTNTNTGACNCATGTNTTTTTANNNGNN
NNNNNACGNNNNANCCCCGGTCGNNNNCNNATTGAANNCCTGNNGCATNNNNNTNCNAGAATGANNNC
NNNNNGNNTNGNCTNNCNTTTCAAGNAGTNAANNAGGTNNNNNGNTCNNNNNNNNNNNNCNNTCNCNN
ACTGAGGGTNNNNNNCGCATCTGCTNNNNNNNNNNTNCGCNGNCGTNNTNNNNNAGCNGTATNGTGTNN
NNCANNNNNNNNNNNNNNNNNTNAAACAANNNNNNNTNNNN
>LoLo460
GTGNNNNNTAGCCCNNNNNAGTTAGACANNNCNNNNNNNNNNNTNNNNNNNNNNNNNNNNNNNNNNNN
NNNNNNNNNNNNNNNNNNNNNNNNNNNNNNNNNNNNNNNNNNNNNNNNNNNNNNNNNNNNNNNNNNNN
NNNNNNNNNNNNNNNNNNNNNNNNNNNNNNNNNNNNNNNNNNNNNNNNNNNNNNNNNNNNNNNNNNNN
NNNNNNNNNNNNNNNNNNNNNNNNNNNNNNNNNNNNNNNNNNNNNNNNNNNNNNNNNNNNNNNNNNNN
NNNNNNNNNNNNNNNNNNNNNNNNNNNNNNNNNNNNNNNNNNNNNNNNNNNNTCTNNNAAANNNTNNN
NNNNNNNNNNNNNNNNNNNNNNNNNCNCNTTNNNNGTAANCNANNNGNACATGCAGTCCCCTCNNNNN
NNNGGCAGAGNNNNNNCCCNNNNNCNCCGGCCCCNNCCNNNNNNNNNNNNNNNNNNNNNNNANNNNNN
NNNNNNTGGTACNNNNCGNNNNNGGTTGNNTAAGAGGTCCACAACGNGGTCNNNNNACNNNNGNNNAN
NNNCNNNNAACNNNNNNNNNNNNNNNTNNNTANNAGGNCNNNNCCNTCNNGNNNNNNANNGCNATGACC
CNANTCNCNGGGGCNGTNCCCGGCGCGGCGACCTTGNNNANGTCCGGGGACAAGTATGGCGNNCNCCC
CGGGAAGGANNCNGCNGTANGTGCNNNNNANNNNNTCNTGNGANGTCGCCGNNATTCNCGTGGTCNNN
CNAGAATGNCCGNACCANNCGNANNNTGGTNATGTNAGTNNNNNNNNNNNNNNNNNNNNNNNNNNNNN
NNNNNNNNNNNNNNNNNNNNNNNNNNNNNNNNNNNNNGNNNNNNCNANNNAAGGNNNTANNNCTNNTN
NTNNNNNNGGACNNNACNNNNCNNNNNNNACNCNNNCNCNNNNNNNNNNGGTTTTTNNNCNNTANTNT
CCAGNACGCNGNGCNNNCATTCANGNGAACNNNGNNACGCGNNAATCGTANNATCTACCTGCCGCNNG
TNNNNNTACNGNNNNNNNCGGANNNTNNNNNGTANNNNNNNCATGCGTNNNNTNNTCNCNAGNCNNGTT
CGNNNANNNANTAGNANGTNTNNNNNNNNNNTGTNNCGTTCCGCNTGANNCNNCANNNNGNNGNNNTTN
NCANCGGCNNTANCTNCNGANNTAAGNCATANNNTNNNTCNNTANCTNNNNNGGNGGGNNACTGTAAA
NGGNNAATTNGNNNNTNANTNCNNNCACNTNNCNNNGNNNNCTNNNTNCCTGNNTNCNNAATTGGNNC
CANTGNNTGNCNATCTCTGGNTTNGNNNNNNTNNNNNTNNCANNNNGGNNNGTNNNANNNCNATNTAA
NNTANANNNNCNNNNNNANNNCACNNNNNNNNNNAGNNNCNANNNTTANANNNGTAGNNNANTNAGTCT
CCGANNTNNNNNNNATAAGCGNNANACNNNNNNNNNNNNNANGANNACGNNTTGGNNNTNNNNNNNNNA
GNNAGACNNTNATGGNTNNNNNTCAANNNNTTNGTAGTGGTATAATNGNNNNNNNTTTCNCCAGNNTN
NNNGNNNNNNNNCNATANNANNNNNNNGNGANNNNNNNNNNNANNNNCTCNNNAGGGANCANCTACTNNA
GCAATGGNGCGCAGNNNGAGANNNNGACNNGCCTTGGGTGGCAANCACNGCGATAAGTNNNGTTNANN
CTNNNAGTNNACATNNNNNCANNGNNNCNNNNNNANCNGANNGNNNNNNNNNNNNNNNNNNNNNNNNN
NNNNNNNNNNNNNNNNNNNNNNNNNNNNNNNNNANGGNNNGTNTNTGACNCATNNNTTTNTANNNGNN
NNNNNNNGNNNNANCCCCGGTCGNNNNCNNATTGAANNCCTGNNGCATNNNNNTNCNAGAATGANNNC
NNNNNGNNTNGNCTNNCNTTTCAAGNAATCANNNAGGTNNNNNNNTCNNNNNNNNNNNNCNNTCCCNN
ACTGAGGGTNNNNNNNNCATCNGCTNNNNNNNNNNTNCGCNGGNGTNNCNGNNNAGCNGTATNGTGNNA
NNCANNNNNNNNNNNNNNNNNTNAAACGANNNNNNNTNNNN

>LoLo461
GTGNNNNNTAGCCCGNNNNAGTTGGACANNNCNNNNNNNNNNNNTNNNNNNNNNNNNNNNNNNNNNNN
NNNNNNNNNNNNNNNNNNNNNNNNNNNNNNTNNNNNNNNNNNNNNNNNNNNNNNNNNNNNNNNNNNNN
NNNNNNNNNNNNNNNNNNNNNNNNNNNNNNNNNNNNNNNNNNNNNNNNNNNNNNNNNNNNNNNNNNNN
NNNNNNNNNNNNNNNNNNNNNNNNNNNNNNNNNNNNNNNNNNNNNNNNNNNNNNNNNNNNNNNNNNNN
NNNNNNNNNNNNNNNNNNNNNNNNNNNNNNNNNNNNNNNNNNNNNNNNNNNCNTCTNNNAAANNNTNNN
NNNNNNNNNNNNNNNNNNNNNNNNNNNCNCNTTGNNNGTAANCNANNNGNACATGCAGTCCCCTCNNNNN
NNNGGCAGAGNNNNNNCCCNNNNNNNCCGGCCCCGNCCNNNNNNNTNNNCNNNGNNNNNNNGNNNNNN
NNNNNNTGGTACNNNNCGNNNNNGGTTGTNTNAGAGGTCCACCACGNGGTCNGGNNACNNNNGNNNAN
NNNCNNNNAACNNNNNNNNNNNNNNATNNNCANNAGGNCNNNNCCNTCNNNNNNNNNANNGCNATGACC
CAANTCGCGGGGACNGTNACCGGCGNGGCGACCTTGATNACGGTCGGGNACAAGTATGTCGNNCNCCC
CGGGAAGGANNCNGCCGTANGTGCNNNNNANNNNNTCNTGNGANGTCGCCGNNNTTCNCGTGGTCNNN
CNAGAATGNCCGNACCANNCGNANNNTGGTNATGTNAGTCNNNNNNNNNNNNNNNNNNNNNNNNNNNN
NNNNNNNNNNNNNNNNNNNNNNNNNNNNNNNNNNGNNNNGNNNNNNCNANNNAAGGANNTANNNTTNNTN
NTNNNNNNGGACNNNACNNNNCNNNNNNNACNCNNNCNCNNNNNNANNNGGTTTTTNNNCNNTATTNT
CCAGNACGCNGNGCNNNCATTCANGNGAACNNNGNNACGCGNNAATCGTANNATCTACCTGCCGCNNG
TNNNNNTACNGNNNNNNCGGANNNTNNNNCGTANNNNNNTCATGCGTNNTNTNNTTNCNAGNCNNGTT
CGNNNANANANTAGNANGTNTCNNNNNNNNTGTNNCGTTCCGCNTGANNCNNCANNNNGNNGNNCTTN
NCANCGGCNNTANCTGCNGANNTAAGNCATANNNTNNGTCNNTANCGNNNNNGGNGGGNNACTGTAAA
NGGNNAATTNGANGNTNATTNCNNNCACNTNNCTNNGNNTNCTNNNCNCCTGNNTNCNNAATTGGNNC
CANTGNNTGNCNATCTCTGGNTTNGNNNNNNTNNNNNTNNTAANNNGGNNNGTNANANNNCNATNTAA
NNTANANNNNCNNNNNNANNNCACNNNNNNNGNAGNNNCNANNNTGANGNNNGTAGNNNANTAAGTCT
CCGANNTNNNNNNNATAAGCGNNANACNNNNNNNNNNNNNNANGANNACGNNTTGGNNNTNNNNNNCNA
ANNAGACNNCNGTGGNTNNNNNTCAANNNNTTCGTAGTGGTATAACNANNNNNNNTTTCNCCAGNNTN
NNNGNNNNNNNCNATANNANNNNNNNGNGANNNNNNNNNNANNNNCCCNNNAGGGANCANCTATTNNA
GCANTGGNGCGCAGNNNGAGANNNNGACNNGCCTTGGGTGGCAANCACNGCAATAAGTNNGTTNANC
CCNNNAGTNNACACNNNNNCANNGNNNCNNNNNNANCNGANNTNNNNNNNNNNNNNNNNNNNNNNNN
NNNNNNNNNNNNNNNNNNNNNNNNNNNNNGNNNANGGNNNGTNTNTGACNCATGTGTTTTTANNNGNN
NNNNNNNGNNNGANCCCCGGTCGNNNNCNNATTGAANNCCTGNNGCATNNNNNTNCNAGAATGANNGC
NNNNNGNNTNGNCTNNCNNNTCAAGNAGTCANNNAGGTNTNNNGNTCNNNGNNNNNNNNCNNTCCCNN
ACTGAGGGTNNNNNNNNCATCNGCTNNNNNNNNNNTNCGCNGNCGTNNTNNNNNNGCNGTATNGNGTNA
NNCANNNNNNNNNNNNNNNNNTNAAACGANNNNNCTNNNN
>LoLo462
GTGNNNNNTAGCCCNNNNNAGTTAGACANNNCNNNNNNNNNNNNTNNNNNNNNNNNNNNNNNNNNNNN
NNNNNNNNNNNNNNNNNNNNNNNNNNNNNNNNNNNNNNNNNNNNNNNNNNNNNNNNNNNNNNNNNNNN
NNNNNNNNNNNNNNNNNNNNNNNNNNNNNNNNNNNNNNNNNNNNNNNNNNNNNNNNNNNNNNNNNNNN
NNNNNNNNNNNNNNNNNNGNNNNNNNNNNNNNNNNNNNNNNNNNNNNNNNNNNNNNNNNNNNNNNNNN
NNNNNNNNNNNNNNNNNNNNNNNNNNNNNNNNNNNNNNNNNNNNNNNNNNNNTCTNNNAAANNNTNNN
NNNNNNNNNNNNNNNNNNNNNNNNNNNCNCNNTGNNNGTAANCNANNNGNACATGCAGTCCCCTCNNNNN
NNNGGCAGAGNNNNNNCCCNNNNNNNCCGGCCCCNNCCNNNNNNNNNNNNNNNNGNNNNNNNANNNNNN
NNNNNNTGGTACNNNNCGNNNNNGGTTGTTTAAGAGGTCCACAACGNGGTCNGGNCACNNNNGNNNAN
NNNCNNNNAACNNNNNNNNNNNNANNTNNNTANNAGGNCNNNNCCNTCNNGNNNNNNATTGCNATGACC
CAANNCACNGGGGNNGTNCCCGGCGCGGCGACCTTGNNNACGTCCGGGGACAAGTATGGCNNNCNCCC
CGNGAAGGANNCNGCNGTANGTGCNNNNNANNNTNTCNTGNGANGTCGCCGNNATTNNCGTGGNCNNN
NNAGAATGCNCNNACCANNCGNANNNTGGTTATGNNAGTNNNNNNNNNNNNNNNNNNNNNNNNNNNNN
NNNNNNNNNNNNNNNNNNNNNNNNNNNNNNNNNNGNNNNGNNNNNNCNANNNAAGGNNNTANNNCTNNTN
NTNNNNNNGGACNNNACNNNNCNNNNNNNACNCNNNCNCNNNNNNNNNNNGGTTTTNNNNCNNTATTNT
CCAGNACGCNGNGCNNNCNTTCANGNGAACNNNGNNACGCGNNAATCGTANNATCTACCTGCCGCNNG
TNNNNNTACNGNNNNNNCGGANNNTNNNTCGTANNNNNNNCANGCGTNNTNTNNTCNCNAGNCANGTT
CGNNNANANANTAGNANGTNTNNNNNNNNNNTGTNNCGTTCCGCNTGANNCNNCANNNNGNNGNNCTTN
NCANCGGCNNTANCTGCNGANNTAAGTCATANNNTNNNTCNNTANCTNNNNNGGNGGGNNACTGNAAA
NGGNNAATTNGNNNNTNATTNCNNNCACNTNNCTNNGNNTNCTNNNTNCCTGNNTNCNNAATTGGNNN
CANTGNNTGNCNATCTCTGGNTTNGNNNNNNTNNNNNTNNCANNNNGGNNNGTNANANNNCNATNTAA
NNTANANNNNCNNNNNNANNNCACNNNNNNNGNAGNNNCNANNNTNANANNNGTAGNNNANTAAGTCT

CCGANNTNNNNNNNATAAGCGNNANACNNNNNNNNNNNNNNNANGANNACGNNTTGGNNNTNNNNNNCNA
GNNAGACNNTNATGGNTNNNNNTCAANNNNTTNGTAGTGGTATAATNGNNNNNNNTTTCNCCAGNNTN
NNNGNNNNNNNCNATANNANNNNNNNGNGANNNNNNNNNNNANNNNCTCNNNAGGGANCANCTACTNNA
GCANTGGNGCGNAGNNNGAGANNNNGACNNNCCTTGGGTGGCAANCACNGCGATAAGTNNNGTTNNNC
CTNNNAGTNNACATNNNNNCANNGNNNCNNNNNNANCNGANNGNNNNNNNNNNNNNNNNNNNNNNNNNN
NNNNNNNNNNNNNNNNNNNNNNNNNNNNNNGNNNANGGNNNGTNTNTGACNCNTGNGTTTTTANNNGNN
NNNNNACGNNNNANCCCCGGTCGNNTNCNNATTGAANNCCTGNNGCATNNNNNTNCNAGAATGANNGC
NNNNNGNNTNGNCTNNCNNNTCANGNAATNANNTANGTANNNNGNTCNNNGNNNNNNNNCNNTNNCNN
ACTGAGGGTNNNNNNNNCATCTGCTNNNNNNNNNNTNCGCNGGNGTNNCNNNNNAGCNGTATNGNGTNA
NNCANNNNNNNNNNNNNNNNNNTNAAACAANNNNNNTNNNN
>LoLo463
GTGNNNNNTAGCCCNNNNNAGTTAGACANNNCNNNNNNNNNNNNNTNNNNNNNNNNNNNNNNNNNNNNNN
NNNNNNNNNNNNNNNNNNNNNNNNNNNNNNNNNNNNNNNNNNNNNNNNNNNNNNNNNNNNNNNNNNNNNN
NNNNNNNNNNNNNNNNNNNNNNNNNNNNNNNNNNNNNNNNNNNNNNNNNNNNNNNNNNNNNNNNNNNNNN
NNNNNNNNNNNNNNNNNNNNNNNNNNNNNNNNNNNNNNNNNNNNNNNNNNNNNNNNNNNNNNNNNNNNNN
NNNNNNNNNNNNNNNNNNNNNNNNNNNNNNNNNNNNNNNNNNNNNNNNNNNNNTCTNNNAAANNNTNNN
NNNNNNNNNNNNNNNNNNNNNNNNNNCNCNNTGNNNGTAANCNANNNGNACATGCAGTCCCCTCNNNNN
NNNNNCAGAGNNNNNNCCCNNNNNNNCCGNCCCCNNCCNNNNNNNNNNNNCNNNNNNNNNNNANNNNNN
NNNNNNTGGTACNNNNCGNNNNNGGTTGNTTAAGAGGTCCACAACGNGGTCNNGNCACNNNNGNNNAN
NNNCNNNNAACNNNNNNNNNNNNNNNNTNNNTANNAGGNNNNNNCCNTCNNNNNNNNTNATTGCNATGACC
CAANNCNCGGGGGNNGTNCCCGGCGCGGCGACCTTGANNACGTCCGGGGNCAAGTATGGCGNNCNCCC
CGGGAAGGANNCNGCCGTANGTGCNNNNNANNNNNTCNTGNGANGTCGCCGNNATTNNCGTGGTCNNN
CNAGAATGNNCGNACCANNCGNANNNTGGTNATGNTAGTNNNNNNNNNNNNNNNNNNNNNNNNNNNNNN
NNNNNNNNNNNNNNNNNNNNNNNNNNNNNNNNNNGNNNNGNNNNNNCNANNNAAGGNNNTANNNCTNNTN
NTNNNNNNGGACNNNACNNNNCNNNNNNNACNCNNNCNCNNNNNNANNNGNTTTTTNNNCNNTANTNT
CCAGNACGCNGNGCNNNCNTTCANGNGAACNNNGNNACGCGNNAATCGTANNATCTANCTGCCGCNNG
NNNNNNTACNGNNNNNNCGGANNNTNNNNCGTANNNNNNNCATGCGTNNTNTNNTCNCNAGNCANGTT
CGNNNANANANTAGNANGTNTNNNNNNNNNNTGTNNCGTTCCGCNTGANNCNNCANNNNGNNGNNCTTN
TCANCGGCNNTANCTGCNGANNTAAGNCATANNNTNNNTCNNTANCTNNNNNGGNGGGNNACTGTAAA
NGGNNAATTNGNNGNTNATTNCNNNCACNTNNCTNNGNNTNCTNNNTGCCTGNNTNCNNAATTGGNNN
CANTGNNTGNCNATCTCTNGNTTNGNNNNNNTNNNNNTNNCANNNNGGNNNGTNANANNNCNATNTAA
NNTANANNNNCNNNNNNANNNCACNNNNNNNGNAGNNNCNANNNTTANANNNGTAGNNNANTAAGTCT
CCGANNTNNNNNNNATAAGCGNNANACNNNNNNNNNNNNNNNANGANNACGNNTTGGNNNTNNNNNNCNA
GNNAGACNNTNATGGNTNNNNNTCAANNNNTTNGTAGTGGTATAATNGNNNNNNNTTTCNCCAGNNTN
NNNGNNNNNNNCNATANNANNNNNNNGNGANNNNNNNNNNNANNNNNTCNNNAGGNANCANCTACTNNA
GCAANGGNNCGNAGNNNGAGANNNNGACNNNCCTTGGGTGGCAANCACNGCGATAAGTNNNGTTNANC
CTNNNAGTNNACATNNNNNCANNGNNNCNNNNNNANCNGANNGNNNNNNNNNNNNNNNNNNNNNNNNNN
NNNNNNNNNNNNNNNNNNNNNNNNNNNNNNGNNNANNGNNNGTNTNTGACNCATGTGTTTTTANNNGNN
NNNNNACGNNNNANCCCCGGTCGNNNNCNNATTGAANNCCTGNNGCATNNNNNTNCNAGAATGANNNC
NNNNNGNNTNGNCTNNCNNNTCAAGNAATCNNNNAGNNNNNNNGNTCNNNGNNNNNNNNCNNNCCCNN
ACTGAGGGTNNNNNNNNCATCTGCTNNNNNNNNNCTNCGCNGGNGTNNCNNNNNAGCNGTATNGNGNNN
NNCANNNNNNNNNNNNNNNNNNNNAAACNANNNNNNNTNNNN
>LoLo464
GTGNNNNNTAGCCCGNNNNAGTTAGACANNNCNNNNNNNNNNNNNTNNNNNNNNNNNNNNNNNNNNNNNN
NNNNNNNNNNNNNNNNNNNNNNNNNNNNNNTNNNNNNNNNNNNNNNNNNNNNNNNNNNNNNNNNNNNNNN
NNNNNNNNNNNNNNNNNNNNNNNNNNNNNNNNNNNNNNNNNNNNNNNNNNNNNNNNNNNNNNNNNNNNNN
NNNNNNNNNNNNNNNNNNNNNNNNNNNNNNNNNNNNNNNNNNNNNNNNNNNNNNNNNNNNNNNNNNNNNN
NNNNNNNNNNNNNNNNNNNNNNNNNNNNNNNNNNNNNNNNNNNNNNNNNNNNNCNTCTNNNAAANNNTNNN
NNNNNNNNNNNNNNNNNNNNNNNNNNCNCNNTGNNNGTAANCNANNNGNACATGCAGTCCCCTCNNNNN
NNNGGCAGAGNNNNNNCCCNNNNNNNCCGGCCCCNNCCNNNNNNNNNNNNNNNNNNGNNNNNNNANNNNNN
NNNNNNTGGTACNNNNCGNNNNNGGTTGTTTAAGAGGTCCACAACGNGGTCNGGNCACNNNNGNNNAN
NNNCNNNNAANNGNNNNNNNNNNANATNNNTANNAGGNNNNNNCCNTCNNNNNNNNNNANTGCNATGACC
CAANNCACNGNGGNNGTNCCCGGCGCGGCGACCTTGANNANGTCCGGGGNCAAGTATGGCGNNCNCCC
CGGGAAGGANNCNGCCGTANGTGCNNNNNANNNNNTCNTGNGANGTCGCCGNNATTCNCGTGGTCNNN

CNAGAATGNCCGNACCANNCGNANNNTGGTNATGNNAGTNNNNNNNNNNNNNNNNNNNNN
NNNNNNNNNNNNNNNNNNNNNNNNGNNNNGNNNNNNCNANNNAAGGNNNTANNNCTNNTN
NTNNNNNNGGACNNNACNNNNCNNNNNNNACNCNNNCNCNNNNNNANNNGNTTTTNNNCNNTATTNT
CCAGNACGCNGNGCNNNCATTCANGNGAACNNNGNNACGCGNNAATCGTANNATCTACCTGCCGCNNG
NNNNNNTACNGNNNNNNCGGANNNTNNCTCGTANNNNNNTCATGCGTNNTNTNNTCNCNAGNCANGTT
CGNNNANANANTAGNANGTNTNNNNNNNNNNTGTNNCGTTCCGCNTGANNCNNCANNNNGNNGNNCTTC
TCANCGGCNNTANCTNCNGANNTAAGTCATANNNTNNNTCNNTANCTNNNNNGGNGGGNNACTGTAAA
NGGNTAATTNGANGNTNATTNCNNNCACNTNNCTNNGNNTNCTNNNTNCCTGNNTNCNNAATTGGNNC
CANTGNNTGNCNATCTCTNGNTTNGNNNNNNTNNNNNTNNCAANNNGGNNNGTNANANNNCNATNTAA
NNTANANNNNCNNNNNNANNNCACNNNNNNNGNAGNNACNANNNTTANANNNGTAGNNNANTAAGTCT
CCGANNTNNNNNNNATAAGCGNNANACNNNNNNNNNNNNNNANGANNACGNNTTGGNNNTNNNNNNCNA
GNNAGACNNTNATGGNTNNNNNTCAANNNNTTCGTAGTGGTATAATNGNNNNNNNTTTCNCCAGNNTN
NNNGNNNNNNNCNATANNANNNNNNNGNGANNNNNNNNNNNANNNNCTCNNNAGGGANCANCTACTTNA
GCANTGGNGCGNAGNNNGAGANNNNGACNNGCCTTGGGTGGCAANCACNGCGATAAGTNNNGTTNANC
CTNNNAGTNNACATNNNNNCANNGNNNCNNNNNNANCNGANNGNNNNNNNNNNNNNNNNNNNNNNNNNN
NNNNNNNNNNNNNNNNNNNNNNNNNNNNNANNNNANNGNNNGTNTNTGACNCATGTNTTTTTANNNGNN
NNNNNACGNNNNANCCCCGGTCGTNNNCNNATTGAANNCCTGNNGCATNNNNNTNCNAGAATGANNGC
NNNNNGNNTNGNCTNNCNNNTCAAGNAATCANNNANGTNNNNNGNTCNNNGNNNNNNNNCNNNCCCNN
ACTGAGGGTNNNNNNNNCANCTGCTNNNNNNNNNNTNCGCNGNCGTNNCNNNNNNGCNGTATNGTGTNA
NNCANNNNNNNNNNNNNNNNNNTNANNNNANNNNNCTNNNN
>LoLo465
GTGNNNNNTAGCCCNCNNNAGTCAGACANNNCNNNNNNNNNNNNNTNNNNNNNNNNNNNNNNNNNNNN
NNNNNNNNNNNNNNNNNNNNNNNNNNNNNNTNNNNNNNNNNNNNNNNNNNNNNNNNNNNN
NNNNNNNNNNNNNNNNNNNNNNNNNNNNNNNNNNNNNNNNNNNNNNNNNNNNNNNNNNNN
NNNNNNNNNNNNNNNNNNNNNNNNNNNNNNNNNNNNNNNNNNNNNNNNNNNNNNNNNNNN
NNNNNNNNNNNNNNNNNNNNNNNNNNNNNNNNNNNNNNNNNNNNNNNNNNNNNNCNTCTNNNAAANNNTNNN
NNNNNNNNNNNNNNNNNNNNNNNNNNCNCNTTGNNNGTAANCNANNNGNACATGCAGTCCCCTCNNNNN
NNNGGCAGAGNNNNNNCCCNNNNNCNCCGGCCCCNNCCNNNNNNNTNNNNNNNGNNNNNNNANNNNNN
NNNNNNTGGTACNNNNCGNNNNNGGTTGTTTAAGAGGTCCACAACGNGGTCNNGNCACNNNNGNNNAN
NNNCNNAAAACNNNNNNNNNNNAANNTNNNTANNAGGNNNNNNCCNTCNNNNNANTNATTGCNATGACC
CAANTCNCGGGGGNNGTNCCCGGCGCGGCGACCTTGANNACGTCCGGGGNCAAGTATGGCGNNCNCCC
CGGGAAGGANNCNGCCGTANGTGCNNNNNANNNNNTCNTGNGANGTCGCCGNNATTCNCGTGGTCNNN
CNAGAATGNNCGNACCANTCGNANNNTGGTNATGNNAGTNNNNNNNNNNNNNNNNNNNNN
NNNNNNNNNNNNNNNNNNNNNNNNNNNNNNNNNNNNNNNNGNNNNNNCNANNNAAGGANNTANNNCTNNTN
NNNNNNNNGGACNNNACNNNNCNNNNNNNACNCNNNCNCNNNNNNANNNGNTTTTTNNNCNNTATTNT
CCAGNACGCNGNGCNNNCATTTANGNGAACNNNGNNACGCGNNAATCGTANNATCTACCTGCCGCNNG
TNNNNNTACNGNNNNNNCGGANNNTNNCTCGTANNNNNNTCATGCGTNNTNTNNTCNCNAGNCANGTT
CGNNNANANANTAGNANGTNTNNNNNNNNNNTGTNNCGTTCCGCNTGANNCNNCANNNNGNNGNNNTTN
TCANCGGCNNTANCTGCNGANNTAAGTCATANNNTNNNTCNNTANCTNNNNNGGNGGGNNACTGTAAA
NGGNNAATTNGAGGNTNATTNCNNNCATNTNNCNNNGNNTNCTNNNTNCCTGNNTNCNNAATTGGNNC
CANTGNNTGNCNATCTCTGGNTTNGNNNNNNTGNNNNTNNCAANNNGGNNNGTNANANNNCNATNTAA
NNTANANNNNCNNNNNNANNNCACNNNNNNNGNAGNNNCNANNNGTANANNNGTAGNNNGATAAGTCT
CCGANNTNNNNNNNATAAGCGNNANACNNNNNNNNNNNNNNANGANNACGNNTTTGNNNTNNNNNNCNA
GNNAGACNNTNGTGGNTNNNNNTCAANNNNTTCGTAGTGGTATAATNGNNNNNNNTTTCNCCAGNNTN
NNNGNNNNNNNCNATANNANNNNNNNGNGANNNNNNNNNNNANNNNCTCNNNAGGGANCANCTACTTNA
GCANTGGNGCGCAGNNNGAGANNNNGACNNGCCTTGGGTGGCAANNACNGCAATAAGTNNNGTTNANC
CTNNNAGTNNACACNNNNNCANNGNNNCNNNNNNANCNGANNGNNNNNNNNNNNNNTNNNNNNNNNNNN
NNNNNNNNNNNNNNNNNNNNNNNNNNNNNAGNNNANGGNNNGTNTNTGACNCATGTGTTTTTANNNGNN
NNNNNACGNNNNANCCCCGGTCGNNNNCNNATTGAANNCCTGNNGCATNNNNNTNCNAGAATGANNGC
NNNNNGNNTNGNCTNNCNTTTCAAGNAATCANNNAGGTANCGCGNTCNNNGNNNNNNNNCNNTCCCNN
ACTGAGGGTNNNNNNNNCATCTGCTNNNNNNNNNNTNCGCNGGNNTNNCNNNNNNGCNGTATNGTGTNA
NNCANNNNNNNNNNNNNNNNNNTNAAACANNNNNNNNTNNNN
>LoLo466
GTGNNNNNTAGCCCNNNNNAGTTGGACANNNCNNNNNNNNNNNNNTNNNNNNNNNNNNNNNNNNNNNN

NNNNNNNNNNNNNNNNNNNNNNNNNNNNNNTNNNNNNNNNNNNNNNNNNNNNNNNNNNNNNNNNNNNN
NNNNNNNNNNNNNNNNNNNNNNNNNNNNNNNNNNNNNNNNNNNNNNNNNNNNNNNNNNNNNNNNNNNN
NNNNNNNNNNNNNNNNNNNNNNNNNNNNNNNNNNNNNNNNNNNNNNNNNNNNNNNNNNNNNNNNNNNN
NNNNNNNNNNNNNNNNNNNNNNNNNNNNNNNNNNNNNNNNNNNNNNNNNNNNTCTNNNAAANNNTNNN
NNNNNNNNNNNNNNNNNNNNNNNNNCNCNTTGNNNGTAANCNANNNGNACATGCAGTCCCCTCNNNNN
NNNGGCAGAGNNNNNNCCCNNNNNNNCCGGCCCCNNCCNNNNNNNTNNNCNNNGNNNNNNNGNNNNNN
NNNNNNTGGTACNNNNCGNNNNNGGTTGTNTAAGAGGTCCACCACGNGGTCNNGNCACNNNNGNNNAN
NNNCNNNNNACNNNNNNNNNNNNNATNNNCANNAGGNCNNNNCCNTCNNGNNNNNNATTGCNATGACC
CNANNCGCGGGGACAGTNACCGGCGCGGCGACCTTGANNANNGTCGGGGACAAGTATGTCGNNCNCCC
CGGGAAGGANNCNGCCGTANGTGCNNNNNANNNNNTCTTGNGANGTCGCCGNNATTNNCGTGGTCNNN
CNNGAATGNCCGNACCANNCGNANNNTGGTNATGNNAGTNNNNNNNNNNNNNNNNNNNNNNNNNNNNN
NNNNNNNNNNNNNNNNNNNNNNNNNNNNNNNNGNNNNGNNNNNNCNANNNAAGGANNTANNNTTNNTN
NTNNNNNNGGACNNNACNNNNCNNNNNNNACNCNNNCNCNNNNNNANNNGNTTTTTNNNCNNTATTNT
CCAGNACGCNGNGCNNNCATTCANGNGAACNNNGNNACGCGNNAATCGTANNATCTACCTGCCGCNNG
TNNNNNTACNGNNNNNNCGGANNNTNNCTCGTANNNNNGNCATGCGTNNTNTNNTTNCNAGNCANGTT
CGNNNANANANTAGNANGTNTNNNNNNNCTTGTNNCGTTCCGCNTGANNCNNCANNNNGNNGNNNTTN
TCANCGGCNNTANCTGCNGANNTAAGNCATANNNTNNGTCNNTANCGNNNNNGGNGGGNNACTGTAAA
NGGNNAATTNGNNNNTNANTNCNNNCACNTNNCTNNGNNTNCTNNNCNCCTGNNTNCNNAATTGGNNC
CANTGNNTGNCATCTCTGGNTTNGCNNNNNTNNNNNTNNTANNNNGGNNNGTNANANNNCNATNTAA
NNTATANNNNCNNNNNNANNNCACNANNNNNGNAGNNNCNANNNTGANGNNNGTAGNNNANTAAGTCT
CCGANNTNNNNNNNATAAGCGNNANACNNNNNNNNNNNNNANGANNACGNNTTGGNNNTNNNNNNCNA
ANNAGACNNCNGTGGNTNNNNNTCAANNNNTTCGTAGTGGTATAACNANNNNNNNTTTCNCCAGNNTN
NNNGNNNNNNNNCNATANNANNNNNNNGNGANNNNNNNNNNNANNNNCCCNNNAGGGANCANCTATTNNA
GCAATGGNGCGCAGNNNGAGANNNNGACNNGCCTTGGGTGGCAANCACNGCAATAAGTNNNGTTNANC
CCNNNAGTNNACACNNNNNCANNGNNNCNNNNNNANCNGANNTNNNNNNNNNNNNNNNNNNNNNNNNN
NNNNNNNNNNNNNNNNNNNNNNNNNNNNAGNNNANGGNNNGTNTNTGACNCATGTGTTTTTANNNGNN
NNNNNACGNNNNANCCCCGGTCGNNNNCNNATTGAANNCCTGNNGCATNNNNNTNCNAGAATGANNGC
NNNNNGNNTNGNCTNNCNTTTCAAGNAGTCANNNAGGTNTCGCGNTCNNNGNNNNNNNNCNNTCCCNN
ACTGAGGGTNNNNNNNNCATCTGCTNNNNNNNNCTNCGCNGNCGTNNTNNNNNAGCNGTATNGTGTNA
NNCANNNNNNNNNNNNNNNTNAAACNANNNNNNNTNNNN
>LoLo467
GTGNNNNNTAGCCCNNNNNAGTTAGACANNNCNNNNNNNNNNNNNTNNNNNNNNNNNNNNNNNNNNNN
NNNNNNNNNNNNNNNNNNNNNNNNNNNNNNNNNNNNNNNNNNNNNNNNNNNNNNNNNNNNNNNNNNNN
NNNNNNNNNNNNNNNNNNNNNNNNNNNNNNNNNNNNNNNNNNNNNNNNNNNNNNNNNNNNNNNNNNNN
NNNANNNNNNNNNNNNNNNNNNNNNNNNNNNNNNNNNNNNNNNNNNNNNNNNNNNNNNNNNNNNNNNN
NNNNNNNNNNNNNNNNNNNNNNNNNNNNNNNNNNNNNNNNNNNNNNNNNNNNTCTNNNAAANNNTNNN
NNNNNNNNNNNNNNNNNNNNNNNNNCNCNTTGNNNGTAANCNANNNGNACATGCAGTCCCCTCNNNNN
NNNGGCAGAGNNNNNNNCCNNNNNNNCCGGCCCCNNCCNNNNNNNNNNNNNNNNNNNNNNNANNNNNN
NNNNNNTGGTACNNNNCGNNNNNGGTTGTNTAAGAGGTCCACAACGNGNTCNGGNCACNNNNGNNNAN
NNNCNNNNAACNGNNNNNNNNNANNTNNNTANNAGGNCNNNNCCNTCNNGNNNNNNATTGCNATGACC
CANNTCACGGGGGCNGTCCCCGGCGNGGCGACCTTGATNANNTCCGGGGNCAAGTATGGCGNNCNCCC
CGGGAAGGANNCNGCCGTANGTGCNNNNNANNNNNTCNTGNGANGTNGCCGNNATTCNCGTGGTCNNN
CNAGAATGCCCGNACCANNCGNANNNTGGTNATGNNAGTNNNNNNNNNNNNNNNNNNNNNNNNNNNNN
NNNNNNNNNNNNNNNNNNNNNNNNNNNNNNNNNNNNNGNNNNNNCNANNNAAGGNNNTANNNCTNNTN
NTNNNNNNGGACNNNACNNNNCNNNNNNNACNCNNNCNCNNNNNNANNNGGTTTTNNNNCNNTATTNT
CCAGNACGCNGNGCNNNCNTTCANGNGAACNNNGNNACGCGNNAATCGTANNATCTACCTGCCGCNNG
NNNNNNTACNGNNNNNNCGGANNNTNNCNCGTANNNNNNTCNNGCGTNNTNTNNTCNCNAGNCNNGTT
CGNNNANANANTAGNANGTNTCNNNNNNCTTGTNNCGTTCCGCNTGANNCNNCANNNNGNNGNNCTTN
TCANCGGCNNTANCTGCNGANNTAAGNCATANNNTNNNTCNNTANCTNNNNNGGNGGGNNACTGTAAA
NGGNNAATTGGNNGNTNANTNCCNNCACNTNNCTNNGNNNNCTNNNTNCCTGNNTNCNNAATTGGNNC
CANTGNNTGNCATCTCTNGNTTNGCNNNNNTNNNNNTNNCAANNNGGNNNGTNNNANNNCNATNTAA
NNTANANNNNCNNNNNNANNNCACNNNNNNNGNAGNNNCNANNNTTANANNNGTAGNNNANTAAGTCT
CCGANNTNNNNNNNATAAGCGNNANACNNNNNNNNNNNNNANGANNACGNNTTGGNNNTNNNNNNCNA
GNNAGACNNTNATGGNTNNNNNTCAANNNNTTNGTAGTGGTATAATNGNNNNNNNTTTCNCCAGNNTN

NNNGNNNNNNNNCNATANNANNNNNNNGNGANNNNNNNNNNNANNNNCTCNNNAGGGANCANCTACTNNA
GCANTGGNGCGCAGNNNGAGANNNNGACNNGCCTTGGGTGGCAANNACNGCGATAAGTNNNGTTNANC
CTNNNAGTNNACATNNNNNCANNGNNNCNNNNNNANCNGANNGNNNNNNNNNNNNNNNNNNNNNNNN
NNNNNNNNNNNNNNNNNNNNNNNNNNNNNNGNNNANGGNNNGTNTNTGACNCATGTNTTTTTANNNGNN
NNNNNACGNNNGANCCCCGGTCGNNTNCNNATTGAANNCCTGNNGCATNNNNNTNCNAGAATGANNGC
NNNNNGNNTNGNCTNNCNTTTCAAGNAATCAANTAGGTNNNNNNNTCNNNGNNNNNNNNCNNTCCCNN
ACTGAGGGTNNNNNNNNCATCTGCTNNNNNNNNNNTNCGCNGGCGTNNCNNCNNAGCNGTATNNNGTNA
NNCANNNNNNNNNNNNNNNNNCNAAACAANNNNCNTNNNN
>LoLo468
GTGNNNNNTAGCCCNNNNNAGTTAGACANNNCNNNNNNNNNNNNNTNNNNNNNNNNNNNNNNNNNNNNNN
NNNNNNNNNNNNNNNNNNNNNNNNNNNNNNNNTNNNNNNNNNNNNNNNNNNNNNNNNNNNNNNNNNNNNNN
NNNNNNNNNNNNNNNNNNNNNNNNNNNNNNNNNNNNNNNNNNNNNNNNNNNNNNNNNNNNNNNNNNNNNNN
NNNNNNNNNNNNNNNNNNNNNNNNNNNNNNNNNNNNNNNNNNNNNNNNNNNNNNNNNNNNNNNNNNNNNNN
NNNNNNNNNNNNNNNNNNNNNNNNNNNNNNNNNNNNNNNNNNNNNNNNNNNNNCNTCTNNNAAANNNTNNN
NNNNNNNNNNNNNNNNNNNNNNNNNNNCNCNTTNNNNGTAANCNANNNGNACATGCAGTCCCCTCNNNNN
NNNGGCAGAGNNNNNNNCCNNNNNNNCCGGCCCCGNCCNNNNNNNNNNNCANNNNNNNNNNANNNNNN
NNNNNNTGGTACNNNNCGNNNNNGGTTGTNTAAGAGGTCCACAACGNGGTCNNGNCACNNNNGNNNAN
NNNCNNNAAACNNNNNNNNNNNNANNTNNNTANNAGGNCNNNNCCNTCNNGNNNNTNATTGCNATGACC
CAANNCACNGNGGCNGTNCCCGGCGCGGNGACCTTGANNANNTCCGGGGNCAAGTATGGCGNNCNCCC
CGGGAAGGANNCNGCCGTANGTGCNNNNNANNNNNTCNTGNGANGTNGCCGNNANNNNCGTGGTCNNN
NNAGAATGNCCGNACCANNCGNANNNTGGTNATGTTAGTNNNNNNNNNNNNNNNNNNNNNNNNNNNNN
NNNNNNNNNNNNNNNNNNNNNNNNNNNNNNNNNNNGNNNNGNNNNNNCNANNNAAGGANNTANNNCTNNTN
NTNNNNNNNGGACNNNACNNNNCNNNNNNNACNCNNNCNCNNNNNNANNNGNTTTTNNNNCNNTANTNT
CCAGNACGCNGNGCNNNCATTCANGNGAACNNNGNNACGCGNNAATCGTANNATCTACCTGCCGCNNG
NNNNNNTNCNGNNNNNNCGGANNNTNNCTCGTANNNNNNNCATGCGTNNTNTNNTNNCNAGNCNNGTT
CGNNNANANANTAGNANGTNTNNNNNNNNNTGTNNCGTTCCGCNTGANNCNNCANNNNGNNGNNNTTN
NCANCGGCNNTANCTGCNGANNTAAGNCATANNNTNNNTCNNTANCTNNNNNGGNGGGNNACTGTAAA
NGGNNAATTNGNNNNTNANTNCNNNCACNTNNCNNNGNNTNCTNNNTNCCTGNNTNCNNAATTGGNNC
CANTGNNTGNCNATCTCTNGNTTNGNNNNNNTNNNNNTNNCANNNNGGNNNGTNNNANNNCNATNTAA
NNTANANNNNCNNNNNNANNNCACNNNNNNNNNAGNNACNANNNTNANANNNGTAGNNNAATAAGTCT
CCGANNTNNNNNNNATAAGCGNNANACNNNNNNNNNNNNNANGANNACGNNTTGGNNNTNNNNNGCNA
GNNAGACNNTNATGGNTNNNNNTCAANNNNTTNGTAGTGGTATAATNGNNNNNNNTTTCNCCAGNNTN
NNNGNNNNNNNNCNATANNANNNNNNNGNGANNNNNNNNNNNANNNNCTCNNNAGGGANCANCTACTNNA
GCAANGGNGCGCAGNNNGAGANNNNGACNNGCCTTGGGTGGCAANCACNGCGATAAGTNNNGTTNANC
CTNNNAGTNNACATNNNNNCANNGNNNCNNNNNNANCNGANNGNNNNNNNNNNNNNNNNNNNNNNNN
NNNNNNNNNNNNNNNNNNNNNNNNNNNNNNGNNNANNGNNNGTNTNTGACNCATGTGTTTTTANNNGNN
NNNNNACGNNNGANCCCCGGTCGNNNNCNNATTGAANNCCTGNNGCATNNNNNTNCNAGAATGANNGC
NNNNNGNNTNGNCTNNCNNNTCAAGNAATCANNNAGNNANNNNGNTCNNNGNNNNNNNNCNNTCCCNN
ACTGAGGGTNNNNNNNNCATNTGCTNNNNNNNNNCTNCGCNGGNGTNNCNNNNNAGCNGTATNNNGTNN
NNCANNNNNNNNNNNNNNNNNNTNAAACNANNNNNNNTNNNN
>LoLo469
GTGNNNNNTAGCCCGNNNNAGTTAGACANNNCNNNNNNNNNNNNNTNNNNNNNNNNNNNNNNNNNNNNNN
NNNNNNNNNNNNNNNNNNNNNNNNNNNNNNNNTNNNNNNNNNNNNNNNNNNNNNNNNNNNNNNNNNNNNNN
NNNNNNNNNNNNNNNNNNNNNNNNNNNNNNNNNNNNNNNNNNNNNNNNNNNNNNNNNNNNNNNNNNNNNNN
NNNNNNNNNNNNNNNNNNNNNNNNNNNNNNNNNNNNNNNNNNNNNNNNNNNNNNNNNNNNNNNNNNNNNNN
NNNNNNNNNNNNNNNNNNNNNNNNNNNNNNNNNNNNNNNNNNNNNNNNNNNNNNTCTNNNAAANNNTNNN
NNNNNNNNNNNNNNNNNNNNNNNNNNNCNCNTTGNNNGTAANCNANNNGNACATGCAGTNCCCTCNNNNN
NNNNNCAGAGNNNNNNNCCCNNNNNNNCCGNCCCCNNCCNNNNNNNNNNNNNNNNNNGNNNNNNNNANNNNNN
NNNNNNTGGTACNNNNCGNNNNNGGTTGNNTAAGAGGTCCACAACGNGGTCNGGNCACNNNNGNNTAN
NNNCNNNAAACNNNNNNNNNNNNANATNNNTANNAGGNNNNNNCCNTCNNGNNANNNATNGCNATGACC
CAANNCACGGNGGNNGTCCCCGGCGCGGCGACCTTGANNANGTCCGGGGNCAAGTATGGCGNNCNCCC
CGNGAAGGANNCNGCCGTANGTGCNNNNNANNNNNTCNTGNGANGTCGCCGNNATTCNCGTGGTCNNN
CNAGANTGNNCNNACCANNCGNANNNTGGTNATGNNAGTNNNNNNNNNNNNNNNNNNNNNNNNNNNNN
NNNNNNNNNNNNNNNNNNNNNNNNNNNNNNNNNNNGNNNNGNNNNNNCNANNNAAGGNNNTANNNCTNNTN

```
NNNNNNNNGGACNNNACNNNNCAANNNNNACNCNNNCNCNNNNNNNNNNGGTTTTNNNNCNNTANTNT
CCAGNACGCNGNGCNNNCNTTCANGNGAACNNNGNNACGCGNNAATCGTANNATCTACCTGCCGCNNG
NNNNNNTNCNGNNNNNNCGGANNNTNNNNCGTANNNNNNNCANGCGTNNTNTNNTCNCNAGNCNNGTT
CGNNNANANANTAGNANGTNTNNNNNNNNNNTGTNNCGTTCCGCNTGANNCNNCANNNNGNNGNNNTTN
NCANCGGCNNTANCTGCNGANNTAAGNCATANNNTNNATCNNTANCTNNNNNGGNGGGNNACTGTAAA
NGGNNAATTNGNNNNTNATTNCNNNCACNTNNCTNNGNNNNCTNNNTNCCTGNNTNCNNAATTGGNNN
CANTGNNTGNCNATCTCTNGNTTNGCNNNNNTGNNNNTNNCANNNNGGNNNNNNNNANNNCNATNTAA
NNTANANNNNCNNNNNNANNNCACNNNNNNNNNAGNNNCNANNNTTANANNNGTAGNNNAATAAGTCT
CCGANNTNNNNNNNATAAGCGNNANACNNNNNNNNNNNNNANGANNACGNNTTGGNNNTNNNNNNCNA
GNNAGACNNTNATGGNTNNNNNTCAANNNNTTNGTAGTGGTATAATNGNNNNNNNTTTCNCCAGNNTN
NNNGNNNNNNNCNATANNANNNNNNNGNGANNNNNNNNNNNANNNNCTCNNNAGGNANCANCTACTNNA
GCANTGGNGCGNAGNNNGAGANNNNGACNNNCCTTGGGTGGCAANCACNGCGATAAGTNNNGTTNANC
CTNNNAGTNNACATNNNNNCANNGNNNCNNNNNNANCNGANNGNNNNNNNNNNNNNNNNNNNNNNNNN
NNNNNNNNNNNNNNNNNNNNNNNNNNNNNAGNNNANGGNNNGTNTNTGACNCATGNNTTTTTANNNGNN
NNNNNACGNNNNANCCCCGGTCGNNNNCNNATTGAANNCCTGNNGCATNNNNNTNCNANAATGANNGC
NNNNNGNNTNGNCTNNCNNNTCAAGNAATCANNNANGTANNNNGNTCNNNNNNNNNNNNCNNTCNCNN
ACTGAGGGTNNNNNNNNCATCNGCTNNNNNNNNCTNCGCNGGCGTNNCNNNNNAGCNGTATNGNGNNN
NNCANNNNNNNNNNNNNNNNNNTNAAANGANNNNNCTNNNN
>LoLo470
GTGNNNNNTAGCCCNNNNNAGTTAGACANNNCNNNNNNNNNNNNNTNNNNNNNNNNNNNNNNNNNNNNN
NNNNNNNNNNNNNNNNNNNNNNNNNNNNNNNNNNNNNNNNNNNNNNNNNNNNNNNNNNNNNNNNNNNNNN
NNNNNNNNNNNNNNNNNNNNNNNNNNNNNNNNNNNNNNNNNNNNNNNNNNNNNNNNNNNNNNNNNNNNNN
NNNNNNNNNNNNNNNNNNNNNNNNNNNNNNNNNNNNNNNNNNNNNNNNNNNNNNNNNNNNNNNNNNNNNN
NNNNNNNNNNNNNNNNNNNNNNNNNNNNNNNNNNNNNNNNNNNNNNNNNNNNCNTCNNNNAAANNNTNNN
NNNNNNNNNNNNNNNNNNNNNNNNNNCNCNTTGNNNGTAANCNANNNGNACATGCAGTCCCCTCNNNNN
NNNGGCAGAGNNNNNNNCCNNNNNNNCCGGCCCCNNCCNNNNNNNNNNNNNNNNNGNNNNNNNANNNNNN
NNNNNNTGGTACNNNNCGNNNNNGGTTGTNTAAGAGGTCCACAACGNGGTCNNGNNACNNNNGNNNAN
NNNCNNNNAACNNNNNNNNNNNNANNTNNNTANNAGGNCNNNNCCNTCNNGNNNNTNANTGCNATGACC
CANNNCNCGGGGGNNGTNCCCGGNGNGGNGACCTTGNNNACGTCCGGGGACAAGTATGGCGNNCNCCC
CGGGAAGGANNCNGCNGTANGTGCNNNNNANNNNNTCNTGNGANGTNGCCGNNATTNNCGTGGTCNNN
CNAGAATGNNCNNACCANNCGNANNNTGGTNATGNNAGTNNNNNNNNNNNNNNNNNNNNNNNNNNNNNN
NNNNNNNNNNNNNNNNNNNNNNNNNNNNNNNNNNNGNNNNGNNNNNNCNANNNAAGGNNNTANNNCTNNTN
NTNNNNNNNGGACNNNACNNNNCNNNNNNNACNCNNNCNCNNNNNNANNNGNTTTTNNNNCNNTANTNT
CCAGNANGCNGNGCNNNCNTTCANGNGAACNNNGNNACGCGNNAATCGTANNATCTACCTGCCGCNNG
NNNNNNTACNGNNNNNNCGGANNNTNNCTNGTANNNNNNNCANGCGTNNTNTNNTCNCNAGNCNNGTT
CGNNNANANANTAGNANGTNTNNNNNNNNNNTGTNNCGTTCCGCNTGANNCNNCANNNNGNNGNNNTTN
NCANCGGCNNTANCTGCNGANNTAAGNCATANNNTNNNTCNNTANCTNNNNNGGNGGGNNACTGTAAA
NGGNNAATTNGNNNNTNATTNCNNNCACNTNNCNNNGNNTNCTNNNTNCCTGNNTNCNNAATTGGNNN
CANTGNNTGNCNATCTCTGGNTTNGNNNNNNTGNNNNTNNCANNNNGGNNNNNNNNANNNCNATNTAA
NNTANANNNNCNNNNNNANNNCACNNNNNNNNNAGNNNCNANNNTNANANNNGTAGNNNAATAAGTCT
CCGANNTNNNNNNNATAAGCGNNANACNNNNNNNNNNNNNANGANNACGNNTTGGNNNTNNNNNNNNA
GNNAGACNNTNATGGNTNNNNNTCAANNNNTTNGTAGTGGTATAATNGNNNNNNNTTTCNCCAGNNTN
NNNGNNNNNNNCNATANNANNNNNNNGNGANNNNNNNNNNNANNNNCTCNNNAGGNANCANCTACTNNA
GCANTGGNGCGNAGNNNGAGANNNNGACNNNCCTTGGGTGGCAANCACNGCGATAAGTNNNGTTNANC
CTNNNAGTNNACATNNNNNCANNGNNNCCNNNNNANCNGANNGNNNNNNNNNNNNNNNNNNNNNNNNN
NNNNNNNNNNNNNNNNNNNNNNNNNNNNNGNNNANGGNNNGTNTNTGNCNCATGTGTTTTTANNNGNN
NNNNNACGNNNGANCCCCGGTCGNNNNCNNATTGAANNCCTGNNGCATNNNNNTNCNAGAATGANNNC
NNNNNGNNTNGNCTNNNNTTTNAAGNAATCANNNAGNNNNNNNNNTCNNNGNNNNNNNNCNNTCCCNN
ACTGAGGGTNNNNNNNNCATCTGCTNNNNNNNNNNTNCGCNGGCGTNNCNNNNNAGCNGTATNNNGNNA
NNCANNNNNNNNNNNNNNNNNNTNAAACNANNNNNTTNNNN
>LoLo471
GTGNNNNNTAGCCCNNNNNAGTTAGACANNNCNNNNNNNNNNNNNTNNNNNNNNNNNNNNNNNNNNNNN
NNNNNNNNNNNNNNNNNNNNNNNNNNNNNNNNNTNNNNNNNNNNNNNNNNNNNNNNNNNNNNNNNNNNNN
NNNNNNNNNNNNNNNNNNNNNNNNNNNNNNNNNNNNNNNNNNNNNNNNNNNNNNNNNNNNNNNNNNNNNN
```

NNNNNNNNNNNNNNNNNNNNNNNNNNNNNNNNNNNNNNNNNNNNNNNNNNNNNNNNNNNNNNNNNNNN
NNNNNNNNNNNNNNNNNNNNNNNNNNNNNNNNNNNNNNNNNNNNNNNNNNNNTCTNNNAAANNNTNNN
NNNNNNNNNNNNNNNNNNNNNNNNNCNCNNTGNNNGTAANCNANNNGNACATGCAGTCCCCTCNNNNN
NNNNNCAGAGNNNNNNCCCNNNNNCNCCGNCCCCNNCCCNNNNNNNNNNNNNNGNNNNNNNANNNNNN
NNNNNNTGGTACNNNNCGNNNNNGGTTGTTTAAGAGGTCCACAACGNGGTCNNGNNACNNNNGNNNAN
NNNCNNNNAACNNNNNNNNNNNANATNNNTANNAGGNCNNNNCCNTNNNGNNNNTAATTGCNATGACC
CANNNCACGGGGGNNGTNCCCGGNGNGGCGACCTTGANNANGTCCGGGGNCAAGTATGGCNNNCNCCC
CGGGAAGGANNCNGCCGTANGTGCNNNNNANNNNNTCNTGNGANGTNGCCGNNATTNNCGTGGTCTNN
CNAGAATGNNCGNACCANNCGNANNNTGGTNATGNTAGTNNNNNNNNNNNNNNNNNNNNNNNNNNNNN
NNNNNNNNNNNNNNNNNNNNNNNNNNNNNNNNGNNNNGNNNNNNCNANNNAAGGANNTANNNCTNNTN
NTNNNNNNGGACNNNACNNNNCNNNNNNNACNCNNNCNCNNNNNNNNNNGNTTTTNNNNCNNTATTNT
CCAGNACGCNGNGCNNNCATTCANGNGAACNNNGNNACGCGNNAATCGTANNATCTACCTGCCGCNNG
NNNNNNTACNGNNNNNNCGGANNNTNNCTCGTANNNNNNNCATGCGTNNTNTNNTCNCNAGNCNNGTT
CGNNNANANANTAGNANGTNTNNNNNNNNNTGTNNCGTTCCGCNTGANNCNNCANNNNGNNGNNNTTN
NCANCGGCNNTANCTGCNGANNTAAGNCATANNNTNNATCNNTANCTNNNNNGGNGGGNNACTGTAAA
NGGNNAATTNGNNNNTNATTNCNNNCACNTNNCTNNGNNTNCTNNNTNCCTGNNTNCNNAATTGGNNC
CANTGNATGNCNATCTCTGGNTTNGNNNNNNTNNNNNTNNCANNNNGGNNNNNNANANNNCNATNTAA
NNTANANNNNCNNNNNNANNNCACNNNNNNNGNAGNNNCNANNNTTANANNNGTAGNNNAATAAGTCT
CCGANNTNNNNNNNATAAGCGNNANACNNNNNNNNNNNNNANGANNNCGNNTTGGNNNTNNNNNNCNA
GNNAGACNNTNATGGNTNNNNNTCAANNNNTTCGTAGTGGTATAATNGNNNNNNNTTTCNCCAGNNTN
NNNGNNNNNNNNCNATANNANNNNNNGNGANNNNNNNNNNANNNNCTCNNNAGGGANCANCTACTNNA
GCANTGGNGCGCAGNNNGAGANNNNGACNNNCCTTGGGTGGCAANCACNGCGATAAGTNNNGTTNANC
CTNNNAGTNNACATNNNNNCANNGNNNCNNNNNNANCNGANNGNNNNNNNNNNNNNNNNNNNNNNNNN
NNNNNNNNNNNNNNNNNNNNNNNNNNNNAGNNNANNGNNNGTNTNTGACNCATGTGTTTTTANNNGNN
NNNNNACGNNNNANCCCCGGTCGNNNNCNNATTGAANNCCTGNNGCATNNNNNTNCNAGAATGANNGC
NNNNNGNNTNGNCTNNNNTTTCAAGNAATCANNNAGGTNNNNCGNTCNNNNNNNNNNNNCNNTCCCNN
ACTGAGGGTNNNNNNNNCATCTGCTNNNNNNNNNNTNCGCNGGNNTNNCNNNNNGCNGTATNGNGNNA
NNCANNNNNNNNNNNNNNNNTNAAACAANNNNCNTNNNN
>LoLo472
GTGNNNNNTAGCCCNNNNNAGTTAGACANNNCNNNNNNNNNNNNTNNNNNNNNNNNNNNANNNNNNNN
NNNNNNNNNNNNNNNNNNNNNNNNNNNNNTNNNNNNNNNNNNNNNNNNNNNNNNNNNNNNNNNNNNNNNN
NNNNNNNNNNNNNNNNNNNNNNNNNNNNNNNNNNNNNNNNNNNNNNNNNNNNNNNNNNNNNNNNNNNN
NNNNNNNNNNNNNNNNNNNNNNNNNNNNNNNNNNNNNNNNNNNNNNNNNNNNNNNNNNNNNNNNNNNN
NNNNNNNNNNNNNNNNNNNNNNNNNNNNNNNNNNNNNNNNNNNNNNNNNNNNTCTNNNAAANNNTNNN
NNNNNNNNNNNNNNNNNNNNNNNNNCNCNTTGNNNGTAANCNANNNGNACATGCAGTCCCCTCNNNNN
NNNGGCAGAGNNNNNNCNCNNNNNNNCCGNCCCCNNCCNNNNNNNNNNNCNNNGNNNNNNNANNNNNN
NNNNNNTGGTACNNNNCGNNNNNGGTTGTNTAAGAGGTCCACAACGNGGTCNGGNNACNNNNGNNNAN
NNNCNNAAAANNNNNNNNNNNNANATNNNTANNAGGNCNNNNCCNTCNNGNNNNNNATTGCNATGACC
CANNNCACGGGGGCNGTNCCCGGNGCGGCGACCTTGANNACGTCCGGGGNCAAGTATGGCNNNCNCCC
CGGGAAGGANNCNGCCGTANGTGCNNNNNANNNNNTCNTGNGANGTNGCCGNNATNCNCGTGGTCNNN
CNAGAATGNCCNNACCANNCGNANNNTGGTNATGNNAGTNNNNNNNNNNNNNNNNNNNNNNNNNNNNN
NNNNNNNNNNNNNNNNNNNNNNNNNNNNNNNNTNNNNGNNNNNNCNANNNAAGGNNNTANNNCTNNTN
NTNNNNNNGGACNNNACNNNNCNNNNNNNACNCNNNCNCNNNNNNANNNGGTTTTTNNNCNNTATTNT
CCAGNACGCNGNGCNNNCATTCANGNGAACNNNGNNACGCGNNAATCGTANNATCTACCTGCCGCNNG
NNNNNNTNCNGNNNNNNCGGANNNTNNNNCGTANNNNNNNCATGCGTNNTNTNNTCNCNAGNCNNGTT
CGNNNANANANTAGNANGTNTNNNNNNNNNTGTNNCGTTCCGCNTGANNCNNCANNNNGNNGNNNTTN
TCANCGGCNNTANCTGCNGANNTAAGNCATANNNTNNNTCNNTANCTNNNNNGGNGGGNNACTGTAAA
NGGNNAATTNGANNNTNATTNCNNNCACNTNNCTNNGNNTNCTNNNTNCCTGNNTNCNNAATTGGNNC
CANTGNNTGNCNATCTCTNGNTTNGNNNNNNTGNNNNTNNCANNNNGGNNNGTNANANNNCNATNTAA
NNTANANNNNCNNNNNNANNNCACNNNNNNNNNAGNNNCNANNNTTANANNNGTAGNNNANTAAGTCT
CCGANNTNNNNNNNATAAGCGNNANACNNNNNNNNNNNNNANGANNACGNNTTGGNNNTNNNNNNCNA
GNNAGACNNTNATGGNTNNNNNTCAANNNNTTNGTAGTGGTATAATNGNNNNNNNTTTCNCCAGNNTN
NNNGNNNNNNNNCNATANNANNNNNNGNGANNNNNNNNNNANNNNCTCNNNAGGNANCANCTACTNNA
GCANTGGNGCGNAGNNNGAGANNNNGACNNNCCTTGGGTGGCAANCACNGCGATAAGTNNNGTTNANC

CTNNNAGTNNACATNNNNNCANNGNNNCNNNNNNANCNGANNGNNNNNNNNNNNNNNNNNNNNNNNNNN
NNNNNNNNNNNNNNNNNNNNNNNNNNNNNNANNNNANGGNNNGTNTNTGACNCATGTGTTTTTANNNGNN
NNNNNACGNNNNANCCCCGGTCGNNNNCNNATTGAANNCCTGNNGCATNNNNNTNCNAGAATGANNNC
NNNNNGNNTNGNCTNNCNTTTCAAGNAATCNNNNAGGNNNNNNNNTCNNNNNNNNNNNNCNNTCCCNN
ACTGAGGGTNNNNNNNNCATCTGCTNNNNNNNNNNTNCGCNGGNGTNNCNNCNNAGCNGTATNNNGTNA
NNCANNNNNNNNNNNNNNNNNNNAAACAANNNNNCTNNNN
>LoLo473
GTGNNNNNTAGCCCNNNNNAGTTAGACANNNCNNNNNNNNNNNNNTNNNNNNNNNNNNNNNNNNNNNNNN
NNNNNNNNNNNNNNNNNNNNNNNNNNNNNNNNNNNNNNNNNNNNNNNNNNNNNNNNNNNNNNNNNNNNNN
NNNNNNNNNNNNNNNNNNNNNNNNNNNNNNNNNNNNNNNNNNNNNNNNNNNNNNNNNNNNNNNNNNNNNN
NNNNNNNNNNNNNNNNNNNNNNNNNNNNNNNNNNNNNNNNNNNNNNNNNNNNNNNNNNNNNNNNNNNNNN
NNNNNNNNNNNNNNNNNNNNNNNNNNNNNNNNNNNNNNNNNNNNNNNNNNNNNNCNTCTNNNAAANNNTNNN
NNNNNNNNNNNNNNNNNNNNNNNNNNCNCNTTGNNNGTAANCNANNNGNACATGCAGTCCCCTCNNNNN
NNNGGCAGAGNNNNNNNCNNNNNNCNCCGNCCCCNNCCNNNNNNNNNNNNNNNNGNNNNNNNANNNNNN
NNNNNNTGGTACNNNNCGNNNNNGGTTGNNTAAGAGGTCCACAACGNGNTCNNGNCACNNNNGNNNAN
NNNCNNNAAACNNNNNNNNNNNNNNNTNNNTANNAGGNCNNNNCCNTCNNNNNNNNNNATTGCNATGACC
CNANTCNCNGNGGCNGTNCCCGGNGNGGCGACCTTGANNANGTCCGGGGNCAAGTATGGCNNNCNCCC
CGGGAAGGANNCNGCCGTANGTGCNNNNNANNNNNTCNTGNGANGTCGCCGNNATTNNCNTGGTCNNN
NNAGAATGNNCNNACCANNCGNANNNTGGTNATGNNAGTNNNNNNNNNNNNNNNNNNNNNNNNNNNNNN
NNNNNNNNNNNNNNNNNNNNNNNNNNNNNNNNNNNNGNNNNGNNNNNNCNANNNAAGGANNTANNNCTNNTN
NTNNNNNNNGGACNNNACNNNNCNNNNNNNACNCNNNCNCNNNNNNANNNGGTTTTNNNNCNNTANTNT
CCAGNACGCNGNGCNNNCATTCANGNGAACNNNGNNACGCGNNAATCGTANNATCTACCTGCCGCNNG
TNNNNNTACNGNNNNNNCGGANNNTNNCTCGTANNNNNNNCATGCGTNNTNTNNTCNCNAGNCNNGTT
CGNNNANANANTAGNANGTNTNNNNNNNCTTGTNNCGTTCCGCNTGANNCNNCANNNNGNNGNNCTTN
TCANCGGCNNTANCTGCNGANNTAAGNCATANNNTNNATCNNTANCTNNNNNGGNGGGNNACTGTAAA
NGGNNAATTNGNNGNTNANTNCCNNCACNTNNCTNNGNNNNCTNNNTNCCTGNNTNCNNAATTGGNNC
CANTGNNTGNCNATCTCTGGNTTNGNNNNNNTGNNNNTNNCANNNNGGNNNGTNNNANNNCNATNTAA
NNTANANNNNCNNNNNNANNNCACNANNNNNNNAGNNNCNANNNTTANANNNGTAGNNNANTAAGTCT
CCGANNTNNNNNNNATAAGCGNNANACNNNNNNNNNNNNNANGANNNCGNNTTGGNNNTNNNNNNCNA
GNNAGACNNTNATGGNTNNNNNTCAANNNNTTCGTAGTGGTATAATNGNNNNNNNTTTCNCCAGNNTN
NNNGNNNNNNNNCNATANNANNNNNNNGNGANNNNNNNNNNNANNNNCTCNNNAGGGANCANCTACTNNA
GCANTGGNGCGCAGNNNGAGANNNNGACNNNCCTTGGGTGGCAANCACNGCGATAAGTNNNGTTNANC
CTNNNAGTNNACATNNNNNCANNGNNNCNNNNNNANCNGANNGNNNNNNNNANNNNNNNNNNNNNNNN
NNNNNNNNNNNNNNNNNNNNNNNNNNNNNNAGNNNANNGNNNGTNTNTGACNCNTGTGTTTTTANNNGNN
NNNNNNNGNNNNANCCCCGGTCGNNTNCNNATTGAANNCCTGNNGCATNNNNNTNCNAGAATGANNGC
NNNNNGNNTNGNCTNNCNTTTCAAGNAATCAANNAGNNANNNNNNTCNNNGNNNNNNNNCNNNCCCNN
ACTGAGGGTNNNNNNNNCATCTGCTNNNNNNNNNNTNCGCNGGNGTNNCNNNNNNGCNGTATNGNGNNN
NNCANNNNNNNNNNNNNNNNNNTNAAACNANNNNNNNTNNNN
>LoLo474
GTGNNNNNTAGCCCNNNNNAGTTAGACANNNCNNNNNNNNNNNNNTNNNNNNNNNNNNNNNNNNNNNNNN
NNNNNNNNNNNNNNNNNNNNNNNNNNNNNNNNTNNNNNNNNNNNNNNNNNNNNNNNNNNNNNNNNNNNNN
NNNNNNNNNNNNNNNNNNNNNNNNNNNNNNNNNNNNNNNNNNNNNNNNNNNNNNNNNNNNNNNNNNNNNN
NNNNNNNNNNNNNNNNNNNNNNNNNNNNNNNNNNNNNNNNNNNNNNNNNNNNNNNNNNNNNNNNNNNNNN
NNNNNNNNNNNNNNNNNNNNNNNNNNNNNNNNNNNNNNNNNNNNNNNNNNNNNNCNTCTNNNAAANNNTNNN
NNNNNNNNNNNNNNNNNNNNNNNNNNCNCNTTGNNNGTAANCNANNNGNACATGCAGTCCCCTCNNNNN
NNNGGCAGAGNNNNNNNCNNNNNNNNCCGNCCCCNNCCNNNNNNNTNNNCNNNGNNNNNNNANNNNNN
NNNNNNTGGTACNNNNCGNNNNNGGTTGTNTAAGAGGTCCACAACGNGGTCNGGNCACNNNNGNNNAN
NNNCNNNAAANNNNNNNNNNNNNANNATNNNTANNAGGNNNNNNCCNTCNNGNNNNNAATTGCNATGACC
CAANTCACGGGGGCAGTCCCCGGCGNGGCGACCTTGATNACGTCCGGGGNCAAGTATGGCGNNCNCCC
CGGGAAGGANNCNGCCGTANGTGCNNNNNANNNNNTCNTGNGANGTNGCCGNNATTNNCGTGGTCNNN
CNAGAATGNCCNNACCANNCGNANNNTGGTNATGNNAGTNNNNNNNNNNNNNNNNNNNNNNNNNNNNNN
NNNNNNNNNNNNNNNNNNNNNNNNNNNNNNNNNNNNGNNNGGNNNNNNCNANNNAAGGNNNTANNNCTNNTN
NTNNNNNNNGGNCNNNACNNNNCNNNNNNNACNCNNNCNCNNNNNNANNNGGTTTTTNNNCNNTATTNT
CCAGNACGCNGNGCNNNCATTCANGNGAACNNNGNNACGCGNNAATCGTANNATCTACCTGCCGCNNG

NNNNNNTACNGNNNNNNCGGANNNTNNNNCGTANNNNNNNNCATGCGTNNTNTNNTCNCNAGNCANGTT
CGNNNANANANTAGNANGTNTNNNNNNNNNNTGTNNCGTTCCGCNTGACNCNNCANNNNGNNGNNCTTN
NCANCGGCNNTANCTGCNGANNTAAGNCATANNNTNNNTCNNTANCTNNNNNGGNGGGNNACTGTAAA
NGGNTAATTNGNNNNTNATTNCNNNCACNTNNCTNNGNNNNCTNNNTNCCTGNNTNCNNAATTGGNNC
CANTGNNTGNCNATCTCTGGNTTNGCNNNNNTNNNNNTNNCAANNNGGNNNGTNANANNNCNATNTAA
NNTANANNNNCNNNNNNANNNCACNNNNNNNGNAGNNNCNANNNTNANANNNGTAGNNNANTAAGTCT
CCGANNTNNNNNNNATAAGCGNNANACNNNNNNNNNNNNNNANGANNACGNNTTGGNNNTNNNNNNCNA
GNNAGACNNTNATGGNTNNNNNTCAANNNNTTNGTAGTGGTATAATNGNNNNNNNTTTCNCCAGNNTN
NNNGNNNNNNNCNATANNANNNNNNNGNGANNNNNNNNNNNANNNNCTCNNNAGGGANCANCTACTNNA
GCANTGGNGCGCAGNNNGAGANNNNGACNNGCCTTGGGTGGCAANCACNGCGATAAGTNNNGTTNANC
CTNNNAGTNNACATNNNNNCANNGNNNCNNNNNNANCNGANNGNNNNNNNNNNNNNNNNNNNNNNNNN
NNNNNNNNNNNNNNNNNNNNNNNNNNNNNAGNNNANGGNNNGTNTNTGACNCATGTGTTTNTANNNGNN
NNNNNACGNNNNANCCCCGGTCGNNTNCNNATTGAANNCCTGNNGCATNNNNNTNCNAGAATGANNGC
NNNNNGNNTNGNCTNNCNTTTCAAGNAATCANNNAGGTNNNNCGNTCNNNGNNNNNNNNCNNTCCCNN
ACTGAGGGTNNNNNNNNCATNNGCTNNNNNNNNCTNCGCNGGCGTNNCNNNNNAGCNGTATNGTGTNN
NNCANNNNNNNNNNNNNNNNNTNAAACANNNNNNNNTNNNN
>LoLo475
GTGNNNNNTAGCCCNNNNNAGTTGGACANNNCNNNNNNNNNNNNTNNNNNNNNNNNNNNNNNNNNNNN
NNNNNNNNNNNNNNNNNNNNNNNNNNNNNNTNNNNNNNNNNNNNNNNNNNNNNNNNNNNNNNNNNNNNNN
NNNNNNNNNNNNNNNNNNNNNNNNNNNNNNNNNNNNNNNNNNNNNNNNNNNNNNNNNNNNNNNNNNNNNN
NNNNNNNNNNNNNNNNNNNNNNNNNNNNNNNNNNNNNNNNNNNNNNNNNNNNNNNNNNNNNNNNNNNNNN
NNNNNNNNNNNNNNNNNNNNNNNNNNNNNNNNNNNNNNNNNNNNNNNNNNNNNNCNTCTNNNAAANNNTNNN
NNNNNNNNNNNNNNNNNNNNNNNNNNCNCNNTGNNNGTAANCNANNNGNACATGCAGTCCCCTCNNNNN
NNNNNCAGAGNNNNNNNNCNNNNNNNCCGGCCCCNNCCNNNNNNNNNNNNCNNNGNNNNNNNGNNNNNN
NNNNNNTGGTACNNNNCGNNNNNGGTTGTNTNAGAGGTCCACCACGNGGTCNGGNCACNNNNGNNNAN
NNNCNNNNANNNNNNNNNNNNNNNNNTNNNCANNAGGNNNNNNCCNTNNNNNNNNNNNANTGCNATGACC
CAANNCGCGGNGACAGTNACCGGNGCGGCGACCTTGANNANGGTCGGGGNCAAGTATGTCNNNCNCCC
CGGGAAGGANNCNGCCGTANGTGCNNNNNANNNNNTCNTGNGANGTCGCCGNNATTNNNNTGGNCNNN
CNAGAATGNNCNNACCANNCGNANNNTGGTNATGNTAGTNNNNNNNNNNNNNNNNNNNNNNNNNNNNN
NNNNNNNNNNNNNNNNNNNNNNNNNNNNNNNNNGNNNNGNNNNNNCNANNNAAGGANNTANNNTTNNTN
NTNNNNNNGGACNNNACNNNNCNNNNNNNACNCNNNCNCNNNNNNANNNGNTTNNNNNNCNNTATTNT
CCAGNACGCNGNGCNNNCATTCANGNGAACNNNGNNACGCGNNAATCGTANNATCTACCTGCCGCNNG
TNNNNNTNCNGNNNNNNCGGANNNTNNNNCGTANNNNNNTCANGCGTNNTNTNNTTNCNAGNCNNGTT
CGNGAANANANTAGNANGTNTNNNNNNNNNNTGTNNCGTTCCGCNTGANNCNNCANNNNGNCGNNNTTC
TCANCGGCNNTANCTGCNGANNTAAGNCATANNNTNNNTCNNTANCGNNNNNGGNGGGNNACTGTAAA
NGGNNAATTNGNNNNTNANTNCNNNCACNTNNCNNNGNNTNCTNNNCNCCTGNNTNCNNAATTGGNNN
CANTGNNTGNCNATCTCTNGNTTNGNNNNNNTNNNNNTNNTANNNNGGNNNGTNNNANNNCNATNTAA
NNTANANNNNCNNNNNNANNNCACNNNNNNNNNAGNNNCNANNNTGANGNNNGTAGNNNANTAAGTCT
CCGANNTNNNNNNNATAAGCGNNANACNNNNNNNNNNNNNNANGANNNCGNNTTGGNNNTNNNNNNCNA
ANNAGACNNCNGTGGNTNNNNNTCAANNNNTTNGTAGTGGTATAACNANNNNNNNTTTCNCCAGNNTN
NNNGNNNNNNNCNATANNANNNNNNNGNGANNNNNNNNNNNANNNNCCCNNNAGGGANCANCTATTNNA
GCANTGGNGCGNAGNNNGAGANNNNGACCAGCCTTGGGTGGCAANCACNGCAATAAGTNNNGTTNNNC
CCNNNAGTNNACACNNNNNCANNGNNNCNNNNNNANCNGANNTNNNNNNNNNNNNNNNNNNNNNNNNN
NNNNNNNNNNNNNNNNNNNNNNNNNNNNNAGNNNANGGNNNGTNTNTGACNCATNNNTTTTTANNNGNN
NNNNNACGNNNNANCCCCGGTCGNNNNCNNATTGAANNCCTGNNGCATNNNNNTNCNAGAATGANNGC
NNNNNGNNTNGNCTNNCNNNTCANGNAGTCANNNAGNNANNNNNNTCNNNGNNNNNNNNCNNNCCCNN
ACTGAGGGTNNNNNNNNCATCTGCTNNNNNNNNNNTNCGCNGNCGTNNTNNNNNAGCNGTATNNTGTNN
NNCANNNNNNNNNNNNNNNNNTNAAACAANNNNNTTNNNN
>LoLo476
GTGNNNNNTAGCCCNNNNNAGTTGGACANNNCNNNNNNNNNNNNTNNNNNNNNNNNNNNNNNNNNNNN
NNNNNNNNNNNNNNNNNNNNNNNNNNNNNNNNNNNNNNNNNNNNNNNNNNNNNNNNNNNNNNNNNNNNNN
NNNNNNNNNNNNNNNNNNNNNNNNNNNNNNNNNNNNNNNNNNNNNNNNNNNNNNNNNNNNNNNNNNNNNN
NNNNNNNNNNNNNNNNNNNNNNNNNNNNNNNNNNNNNNNNNNNNNNNNNNNNNNNNNNNNNNNNNNNNNN
NNNNNNNNNNNNNNNNNNNNNNNNNNNNNNNNNNNNNNNNNNNNNNNNNNNNNNNTCTNNNAAANNNTNNN

NNNNNNNNNNNNNNNNNNNNNNNNNNNCNCNTTGNNNGTAANCNANNNGNACATGCAGTCCCCTCNNNNN
NNNNNCAGAGNNNNNNNCCNNNNNCNCCGNCCCCNNCCNNNNNNNTNNNCNNNNNNNNNNNGNNNNNN
NNNNNNTGGTACNNNNCGNNNNNGGTTGTNTAAGAGGTCCACCACGNGGTCNNGNCACNNNNGNNNAN
NNNCNNNNAACNNNNNNNNNNNNNNNTNNNCANNAGGNCNNNNCCNTCNNNNNNNNNANNGCNATGACC
CAANNCGCGGGGACNGTNACCGGCGNGGCNACCTTGNNNANGGTCGGGGACAAGTATGTCGNNCNCCC
CGGGAAGGANNCNGCCGTANGTGCNNNNNANNNNNTCNTGNGANGTCGCCGNNATTNNCGTGGTCNNN
NNAGAATGNNCNNACCANNCGNANNNTGGTNATGNNAGTNNNNNNNNNNNNNNNNNNNNNNNNNNNNN
NNNNNNNNNNNNNNNNNNNNNNNNNNNNNNNNNGNNNNGNNNNNNCNANNNAAGGANNTANNNTTNNTN
NTNNNNNNGGACNNNACNNNNCNNNNNNNACNCNNNCNCNNNNNNANNNGNTTTTNNNNCNNTANTNT
CCAGNACGCNGNGCNNNCATTCANGNGAACNNNGNNACGCGNNAATCGTANNATCTACCTGCCGCNNG
NNNNNNTNCNGNNNNNNCGGANNNTNNCTCGTANNNNNNNCATGCGTNNTNTNNTTNCNAGNCNNGTT
CGNNNANANANTAGNANGTNTNNNNNNNCTTGTNNCGTTCCGCNTGANNCNNCANNNNGNNGNNNTTN
NCANCGGCNNTANCTGCNGANNTAAGNCATANNNTNNNTCNNTANCGNNNNNGGNGGGNNACTGTAAA
NGGNNAATTNGNNNNTNANTNCNNNCACNTNNCNNNGNNNNCTNNNCNCCTGNNTNCNNAATTGGNNC
CANTGNNTGNCNATCTCTGGNTTNGNNNNNNTNNNNNTNNTANNNNGGNNNNNNNNANNNCNATNTAA
NNTANANNNNCNNNNNNANNNCACNANNNNNGNAGNNNCNANNNTGANGNNNGTAGNNNANTAAGTCT
CCGANNTNNNNNNNATAAGCGNNANACNNNNNNNNNNNNNNANGANNNCGNNTTGGNNNTNNNNNNNNA
ANNAGACNNCNGTGGNTNNNNNTCAANNNNTTCGTAGTGGTATAACNANNNNNNNTTTCNCCAGNNTN
NNNGNNNNNNNNCNATANNANNNNNNNGNGANNNNNNNNNNANNNNCCCNNNAGGGANCANCTATTNNA
GCAATGGNGCGNAGNNNGAGANNNNGACNNNCCTTGGGTGGCAANCACNGNAATAAGTNNNGTTNANC
CCNNNAGTNNACACNNNNNCANNGNNNCNNNNNNANCNGANNTNNNNNNNNNNNNNNNNNNNNNNNNN
NNNNNNNNNNNNNNNNNNNNNNNNNNNNNNAGNNNANNGNNNGTNTNTGACNCANGTGTTTTTANNNGNN
NNNNNACGNNNNANCCCCGGTCGNNNNCNNATTGAANNCCTGNNGCATNNNNNTNCNAGAATGANNNC
NNNNNGNNTNGNCTNNCNNNTNAAGNAGTCAANNANGTNNNGNGNTCNNNGNNNNNNNNCNNNCCCNN
ACTGAGGGTNNNNNNNNCATCNGCTNNNNNNNNNNTNCGCNGNNGTNNTNNNNNAGCNGTATNNNGTNN
NNCANNNNNNNNNNNNNNNNNTNANACGANNNNNNTNNNN
>LoLo477
GTGNNNNNTAGCCCNNNNNAGTTAGACANNNCNNNNNNNNNNNNTNNNNNNNNNNNNNNNNNNNNNNN
NNNNNNNNNNNNNNNNNNNNNNNNNNNNNNNNNNNNNNNNNNNNNNNNNNNNNNNNNNNNNNNNNNNN
NNNNNNNNNNNNNNNNNNNNNNNNNNNNNNNNNNNNNNNNNNNNNNNNNNNNNNNNNNNNNNNNNNNN
NNNNNNNNNNNNNNNNNNNNNNNNNNNNNNNNNNNNNNNNNNNNNNNNNNNNNNNNNNNNNNNNNNNN
NNNNNNNNNNNNNNNNNNNNNNNNNNNNNNNNNNNNNNNNNNNNNNNNNNNNCNTCTNNNAAANNNTNNN
NNNNNNNNNNNNNNNNNNNNNNNNNNNCNCNTTGNNNGTAANCNANNNGNACATGCAGTCCCCTCNNNNN
NNNGGCAGAGNNNNNNCCCNNNNNCNCCGNCCCCNNCCNNNNNNNTNNNNNNNGNNNNNNNANNNNNN
NNNNNNTGGTACNNNNCGNNNNNGGTTGTNTAAGAGGTCCACAACGNGGTCNGNNCACNNNNGNNNAN
NNNCNNAAAACNNNNNNNNNNNNANNTNNNTANNAGGNCNNNNCCNTCNNGNNNNTNATTGCNATGACC
CAANTCACGGGGGCAGTNCCCGGCGCGGCGACCTTGNNNANGTCCGGGGACAAGTATGGCGNNCNCCC
CGGGAAGGANNCNGCCGTANGTGCNNNNNANNNNNTCTTGNGANGTCGCCGNNATTCNCGTGGTCNNN
NNAGAATGCNCNNACCANNCGNANNNTGGTNATGNNAGTNNNNNNNNNNNNNNNNNNNNNNNNNNNNN
NNNNNNNNNNNNNNNNNNNNNNNNNNNNNNNNNGNNNNGNNNNNNCNANNNAAGGNNNTANNNCTNNTN
NTNNNNNNGGACNNNACNNNNCNNNNNNNACNCNNNCNCNNNNNNANNNGNTTTTNNNNCNNTANTNT
CCAGNACGCNGNGCNNNCNTTCANGNGAACNNNGNNACGCGNNAATCGTANNATCTACCTGCCGCNNG
TNNNNNTACNGNNNNNNCGGANNNTNNNNNGTANNNNNNTCANGCGTNNTNTNNTCNCNAGNCNNGTT
CGNNNANANANTAGNANGTNTNNNNNNNNNNTGTNNCGTTCCGCNTGANNCNNCANNNNGNNGNNCTTN
TCANCGGCNNTANCTGCNGANNTAAGNCATANNNTNNNTCNNTANCTNNNNNGGNGGGNNACTGTAAA
NGGNNAATTNGNNGNTNANTNCNNNCACNTNNCNNNGNNTNCTNNNTNCCTGNNTNCNNAATTGGNNC
CANTGNNTGNCNATCTCTGGNTTNGNNNNNNTNNNNNTNNCAANNNGGNNNGTNNNANNNCNATNTAA
NNTANANNNNCNNNNNNANNNCACNNNNNNNNNAGNNACNANNNTNANANNNGTAGNNNANTAAGTCT
CCGANNTNNNNNNNATAAGCGNNANACNNNNNNNNNNNNNNANGANNACGNNTTGGNNNTNNNNNNCNA
GNNAGACNNTNATGGNTNNNNNTCAANNNNTTCGTAGTGGTATAATNGNNNNNNNTTTCNCCAGNNTN
NNNGNNNNNNNNCNATACNANNNNNNNGNGANNNNNNNNNNANNNNCTCNNNAGGGANCANCTACTNNA
GCANTGGNGCGCAGNNNGAGANNNNGACNNNCCTTGGGTGGCAANNACNGCGATAAGTNNNGTTNANC
CTNNNAGTNNACATNNNNNCANNGNNNCNNNNNNANCNGANNGNNNNNNNNNNNNNNNNNNNNNNNNN
NNNNNNNNNNNNNNNNNNNNNNNNNNNNNNAGNNNANGGNNNGTNTNTGACNCATGNNTTTTTANNNGNN

NNNNNNNGNNNNANCCCCGGTCGNNNNCNNATTGAANNCCTGNNGCATNNNNNTNCNAGAATGANNGC
NNNNNGNNTNGNCTNNCNNNTCAAGNAATCANNNAGGTANNNNGNTCNNNGNNNNNNNNCNNTCCCNN
ACTGAGGGTNNNNNNNNCATCTGCTNNNNNNNNNNTNCGCNGGNGTNNCNNCNNNGCNGTATNNTGNNA
NNCANNNNNNNNNNNNNNNNNTNAAACNANNNNNNNTNNNN
>LoLo478
GTGNNNNNTAGCCCNNNNNAGTTAGACANNNCNNNNNNNNNNNNTNNNNNNNNNNNNNNNNNNNNNNN
NNNNNNNNNNNNNNNNNNNNNNNNNNNNNNNNTNNNNNNNNNNNNNNNNNNNNNNNNNNNNNNNNNNNNN
NNNNNNNNNNNNNNNNNNNNNNNNNNNNNNNNNNNNNNNNNNNNNNNNNNNNNNNNNNNNNNNNNNNNNN
NNNNNNNNNNNNNNNNNNNNNNNNNNNNNNNNNNNNNNNNNNNNNNNNNNNNNNNNNNNNNNNNNNNNNN
NNNNNNNNNNNNNNNNNNNNNNNNNNNNNNNNNNNNNNNNNNNNNNNNNNNNNNNNTCTNNNAAANNNTNNN
NNNNNNNNNNNNNNNNNNNNNNNNNNCNCNTTGNNNGTAANCNANNNGNACATGCAGTCCCCTCNNNNN
NNNGGCAGAGNNNNNNCCCNNNNNNNCCGGCCCCNNCCNNNNNNNNNNNCNNNGNNNNNNNANNNNNN
NNNNNNTGGTACNNNNCGNNNNNGGTTGTNTAAGAGGTCCACAACGNGGTCNNGNCACNNNNGNNNAN
NNNCNCNNAACNNNNNNNNNNNNANNTNNNTANNAGGNNNNNNCCNTCNNGNNNNNNATTGCNATGACC
CAANNCACGGGGGNNGTNCCCGGNGNGGCGACCTTGNNNACNTCCGGGGNCAAGTATGGCGNNCNCCC
CGGGAAGGANNCNGCNGTANGTGCNNNNNANNNNNTCNTGNGANGTCGCCGNNATTCNCGTGGTCNNN
CNAGAATGNCCNNACCANNCGNANNNTGGTNATGTNAGTNNNNNNNNNNNNNNNNNNNNNNNNNNNNN
NNNNNNNNNNNNNNNNNNNNNNNNNNNNNNNNNNNGNNNNGNNNNNNCNANNNAAGGNNNTANNNCTNNTN
NTNNNNNNGGACNNNACNNNNCNNNNNNNACNCNNNCNCNNNNNNNNNNNGGTTTTNNNCCNNTANTNT
CCAGNACGCNGNGCNNNCATTCANGNGAACNNNGNNACGCGNNAATCGTANNATCTACCTGCCGCNNG
TNNNNNTACNGNNNNNNCGGANNNTNNCTCGTANNNNNNNCANGCGTNNTNTNNTCNCNAGNCNNGTT
CGNNNANANANTAGNANGTNTNNNNNNNNNNTGTNNCGTTCCGCNTGANNCNNCANNNNGNNGNNCTTN
NCANCGGCNNTANCTGCNGANNTAAGNCATANNNTNNNTCNNTANCTNNNNNGGNGGGNNACTGTAAA
NGGNNAATTNGANGNTNATTNCNNNCACNTNNCNNNGNNNNCTNNNTNCCTGNNTNCNNAATTGGNNC
CANTGNNTGNCNATCTCTGGNTTNGNNNNNNTNNNNNTNNCANNNNGGNNNGTNNNANNNCNATNTAA
NNTANANNNNCNNNNNNANNNCACNNNNNNNNNNAGNNNCNANNNTTANANNNGTAGNNNAATAAGTCT
CCGANNTNNNNNNNATAAGCGNNANACNNNNNNNNNNNNNNANGANNNCGNNTTGGNNNTNNNNNNCNA
GNNAGANNNTNATGGNTNNNNNTCAANNNNTTNGTAGTGGTATAATNGNNNNNNNTTTCNCCAGNNTN
NNNGNNNNNNNNCNATANNANNNNNNNGNGANNNNNNNNNNNANNNNCTCNNNAGGGANCANCTACTNNA
GCANTGGNGCGCAGNNNGAGANNNNGACNNGCCTTGGGTGGCAANCACNGNGATAAGTNNNGTTNNNC
CTNNNAGTNNACATNNNNNCANNGNNNCNNNNNNANCNGANNGNNNNNNNNNNNNNNNNNNNNNNNNN
NNNNNNNNNNNNNNNNNNNNNNNNNNNNNNNAGNNNANGGNNNGTNTNTGACNCATGTGTTTTTANNNGNN
NNNNNACGNNNNANCCCCGGTCGNNNNCNNATTGAANNCCTGNNGCATNNNNNTNCNAGAATGANNGC
NNNNNGNNTNGNCTNNCNNNTCAAGNAATCANNNAGNNNNNNNNNNTCNNNNNNNNNNNNNCNNTCCCNN
ACTGAGGGTNNNNNNNNCATCTGCTNNNNNNNNNNTNCGCNGNNGTNNCNNCNNNGCNGTATNNNGTNA
NNCANNNNNNNNNNNNNNNNNTNAAANAANNNNNCTNNNN
>LoLo479
GTGNNNNNTAGCCCNNNNNAGTTAGACANNNCNNNNNNNNNNNNTNNNNNNNNNNNNNNNNNNNNNNN
NNNNNNNNNNNNNNNNNNNNNNNNNNNNNNNNTNNNNNNNNNNNNNNNNNNNNNNNNNNNNNNNNNNNNN
NNNNNNNNNNNNNNNNNNNNNNNNNNNNNNNNNNNNNNNNNNNNNNNNNNNNNNNNNNNNNNNNNNNNNN
NNNNNNNNNNNNNNNNNNNNNNNNNNNNNNNNNNNNNNNNNNNNNNNNNNNNNNNNNNNNNNNNNNNNNN
NNNNNNNNNNNNNNNNNNNNNNNNNNNNNNNNNNNNNNNNNNNNNNNNNNNNNNNNTCTNNNAAANNNTNNN
NNNNNNNNNNNNNNNNNNNNNNNNNNCNCNTTNNNNGTAANCNANNNGNACATGCAGTCCCCTCNNNNN
NNNGGCAGAGNNNNNNNCCNNNNNNNCCGGCCCCNNCCNNNNNNNNNNNCNNNGNNNNNNNANNNNNN
NNNNNNTGGTACNNNNCGNNNNNGGTTGTTTNAGAGGTCCACAACGNGGTCNGGNNACNNNNGNNNAN
NNNCNNNAAANNNNNNNNNNNNNANNTNNNTANNAGGNNNNNNCCNTCNNGNNNNNNANNGCNATGACC
CAANTCNCNGNGGCNGTNCCCGGCGCNGCGACCTTGANNANGTCCGGGGNCAAGTATGGCNNNCNCCC
CGNGAAGGANNCNGCCGTANGTGCNNNNNANNNNNTCNTGNGANGTCGCCGNNATTNNNGTGGTCNNN
CNAGAATGNCCGNACCANNCGNANNNTGGTTATGNNAGTNNNNNNNNNNNNNNNNNNNNNNNNNNNNN
NNNNNNNNNNNNNNNNNNNNNNNNNNNNNNNNNNNNNGNNNNNNCNANNNAAGGANNTANNNCTNNTN
NTNNNNNNGGACNNNACNNNNCNNNNNNNACNCNNNCNCNNNNNNANNNGGTTTTNNNNCNNTANTNT
CCAGNACGCNGNGCNNNCATTCANGNGAACNNNGNNACGCGNNAATCGTANNATCTACCTGCCGCNNG
NNNNNNTACNGNNNNNNCGGANNNTNNCTCGTANNNNNNTCATGCGTNNTNTNNTCNCNAGNCANGTT
CGNNNANANANTAGNANGTNTNNNNNNNNNNTGTNNCGTTCCGCNTGANNCNNCANNNNGNNGNNCTTN

NCANCGGCNNTANCTGCNGANNTAAGNCATANNNTNNNTCNNTANCTNNNNNGGNGGGNNACTGTAAA
NGGNNAATTNGNNGNTNATTNCNNNCACNTNNCNNNGNNNNCTNNNTNCCTGNNTNCNNAATTGGNNC
CANTGNNTGNCNATCTCTNGNTTGNNNNNNTNNNNNTNNCANNNNGGNNGTNANANNNCNATNTAA
NNTANANNNNCNNNNNNANNNCACNNNNNNNNNAGNNNCNANNNTTANANNGTAGNNNANTNAGTCT
CCGANNTNNNNNNNATAAGCGNNANACNNNNNNNNNNNNNANGANNACGNNTTGGNNNTNNNNNNNNA
GNNAGACNNTNATGGNTNNNNNTCAANNNNTTNGTAGTGGTATAATNGNNNNNNNTTTCNCCAGNNTN
NNNGNNNNNNNCNATANNANNNNNNGNGANNNNNNNNNNANNNNCTCNNNAGGGANCANCTACTNNA
GCAATGGNGCGCAGNNNGAGANNNNGACNNGCCTTGGGTGGCAANCACNGCGNTAAGTNNNGTTNANN
NTNNNAGTNNACATNNNNNCANNGNNNCNNNNNNANCNGANNGNNNNNNNNNNNNNNNNNNNNNNNNN
NNNNNNNNNNNNNNNNNNNNNNNNNNNNNNNNNANGGNNNGTNTNTGACNCATGTGTTTTTANNNGNN
NNNNNACGNNNNANCCCCGGTCGNNNNCNNATTGAANNCCTGNNGCATNNNNNTNCNAGAATGANNNC
NNNNNGNNTNGNCTNNCNNNTNAAGNAATCAANNAGNNNNCGCGNTCNNNGNNNNNNNNCNNTCCCNN
ACTGAGGGNNNNNNNNNCATCTGCTNNNNNNNNNNTNCGCNGGNNTNNCNNNNNAGCNGTATNGTGTNA
NNCANNNNNNNNNNNNNNNNNTNAAACNANNNNNCTNNNN
>LoLo480
GTGNNNNNTAGCCCNNNNNAGTTAGACANNNCNNNNNNNNNNNNNTNNNNNNNNNNNNNNNNNNNNNN
NNNNNNNNNNNNNNNNNNNNNNNNNNNNNNNNNNNNNNNNNNNNNNNNNNNNNNNNNNNNNNNNNNNN
NNNNNNNNNNNNNNNNNNNNNNNNNNNNNNNNNNNNNNNNNNNNNNNNNNNNNNNNNNNNNNNNNNNN
NNNNNNNNNNNNNNNNNNNNNNNNNNNNNNNNNNNNNNNNNNNNNNNNNNNNNNNNNNNNNNNNNNNN
NNNNNNNNNNNNNNNNNNNNNNNNNNNNNNNNNNNNNNNNNNNNNNNNNNCNTCTNNNAAANNNTNNN
NNNNNNNNNNNNNNNNNNNNNNNNNCNCNNTNNNNGTAANCNANNNGNACATGCAGTCCCCTCNNNNN
NNNNNCAGAGNNNNNNCCCNNNNNNNCCGNCCCCNNCCNNNNNNNNNNNCNNNGNNNNNNNANNNNNN
NNNNNNTGGTACNNNNCGNNNNNGGTTGTNTAAGAGGTCCACAACGNGGTCNGGNNACNNNNGNNNAN
NNNCNNNAAANNNNNNNNNNNNNNNNTNNNTANNAGGNCNNNNCCNTCNNNNNNNNNATTGCNATGACC
CANNNCNCGGNGGCNGTNCCCGGCGCGGCNACCTTGANNANGTCCGGGGNCAAGTATGGCGNNCNCCC
CGGGAAGGANNCNGCCGTANGTGCNNNNNANNNNNTCNTGNGANGTNGCCGNNATTNNCNTGGTCNNN
NNAGAATGNCCNNACCANNCGNANNNTGGTNATGNNAGTNNNNNNNNNNNNNNNNNNNNNNNNNNNNN
NNNNNNNNNNNNNNNNNNNNNNNNNNNNNNNNNNNNNGNNNNNNCNANNNAAGGNNNTANNNCTNNTN
NTNNNNNNGGACNNNACNNNNCNNNNNNNACNCNNNCNCNNNNNNNNNNGNTTTTTNNNCNNTANTNT
CCAGNANGCNGNGCNNNCATTCANGNGAACNNNGNNACGCGNNAATCGTANNATCTACCTGCCGCNNG
NNNNNNTNCNGNNNNNNCGGANNNTNNCNCGTANNNNNNNCATGCGTNNTNTNNTCNCNAGNCANGTT
CGNNNANANANTAGNANGTNTNNNNNNNNNNTGTNNCGTTCCGCNTGANNCNNCANNNNGNNGNNNTTN
NCANCGGCNNTANCTGCNGANNTAAGNCATANNNTNNNTCNNTANCTNNNNNGGNGGGNNACTGTAAA
NGGNNAATTNGNNNNTNANTNCNNNCACNTNNCTNNGNNTNCTNNNTNCCTGNNTNCNNAATTGGNNC
CANTGNNTGNCNATCTCTNGNTTGNNNNNNTNNNNNTNNCANNNNGGNNGTNANANNNCNATNTAA
NNTANANNNNCNNNNNNANNNCACNNNNNNNGNAGNNNCNANNNTTANANNGTAGNNNAATAAGTCT
CCGANNTNNNNNNNATAAGCGNNANACNNNNNNNNNNNNNANGANNACGNNTTGGNNNTNNNNNNCNA
GNNAGACNNTNATGGNTNNNNNTCAANNNNTTNGTAGTGGTATAATNGNNNNNNNTTTCNCCAGNNTN
NNNGNNNNNNNCNATANNANNNNNNGNGANNNNNNNNNNANNNNCTCNNNAGGNANCANCTACTNNA
GCANTGGNGCGNAGNNNGAGANNNNGACNNNCCTTGGGTGGCAANCACNGCGATAAGTNNNGTTNNNC
CTNNNAGTNNACATNNNNNCANNGNNNCNNNNNNANCNGANNGNNNNNNNNNNNNNNNNNNNNNNNNN
NNNNNNNNNNNNNNNNNNNNNNNNNNNNANNNNANGGNNNGTNTNTGACNCATGNNTTTTTANNNGNN
NNNNNACGNNNNANCCCCGGTCGNNNNCNNATTGAANNCCTGNNGCATNNNNNTNCNAGAATGANNNC
NNNNNGNNTNGNCTNNCNTTTCAAGNAATCANNTAGNNNNNNNGNTCNNNGNNNNNNNNCNNTCCCNN
ACTGAGGGTNNNNNNNNCATCNNCTNNNNNNNNNCTTCGCNGGNGTNNCNGNNNNGCNGTATNGNGTNN
NNCANNNNNNNNNNNNNNNNNNNANNNAANNNNNNNTNNNN
>LoLo481
GTGNNNNNTAGCCCNNNNNAGTTAGACANNNCNNNNNNNNNNNNNTNNNNNNNNNNNNNNNNNNNNNN
NNNNNNNNNNNNNNNNNNNNNNNNNNNNNNNNNNNNNNNNNNNNNNNNNNNNNNNNNNNNNNNNNNNN
NNNNNNNNNNNNNNNNNNNNNNNNNNNNNNNNNNNNNNNNNNNNNNNNNNNNNNNNNNNNNNNNNNNN
NNNNNNNNNNNNNNNNNNNNNNNNNNNNNNNNNNNNNNNNNNNNNNNNNNNNNNNNNNNNNNNNNNNN
NNNNNNNNNNNNNNNNNNNNNNNNNNNNNNNNNNNNNNNNNNNNNNNNNNNNTNTNNNAAANNNTNNN
NNNNNNNNNNNNNNNNNNNNNNNNNCNCNNTGNNNGTAANCNANNNGNACATGCAGTCCCCTCNNNNN
NNNNNCAGAGNNNNNNCCCNNNNNNNCCGNCCCCNNCCNNNNNNNNNNNNNNNNGNNNNNNNANNNNNN

NNNNNNTGGTACNNNNCGNNNNNGGTTGTNTNAGAGGTCCACAACGNGGTCNNNNCACNNNNGNNNAN
NNNCNNNNAACNNNNNNNNNNNNNNNNTNNNTANNAGGNNNNNNCCNTCNNNNNNNNNNANTGCNATGACC
CNANNCACGGGGGCNGTNCCCGGNGNGGCGACCTTGNNNANNTCCGGGGNCAAGTATGGCNNNCNCCC
CGGGAAGGANNCNGCNGTANGTGCNNNNNANNNNNTCNTGNGANGTNGCCGNNATTCNCGTGGTCNAN
CNAGAATGNNCNNACCANNCGNANNNTGGTNATGNNAGTNNNNNNNNNNNNNNNNNNNNNNNNNNNNN
NNNNNNNNNNNNNNNNNNNNNNNNNNNNNNNNNNNNGNNNNGNNNNNNCNANNNAAGGNNNTANNNCTNNTN
NTNNNNNNGGACNNNACNNNNCNNNNNNNACNCNNNCNCNNNNNNANNNGGTTTTNNNNCNNTANTNT
CCAGNACGCNGNGCNNNCATTCANGNGAACNNNGNNACGCGNNAATCGTANNATCTACCTGCCGCNNG
NNNNNNTACNGNNNNNNCGGANNNTNNNNCGTANNNNNNNCATGCGTNNTNTNNTCNCNAGNCNNGTT
CGNNNANANANTAGNANGTNTNNNNNNNNNNTGTNNCGTTCCGCNTGANNCNNCANNNNGNNGNNCTTN
TCANCGGCNNTANCTGCNGANNTAAGNCATANNNTNNATCNNTANCTNNNNNGGNGGGNNACTGTAAA
NGGNNAATTNGNNNNTNATTNCNNNCACNTNNCNNNGNNTNCTNNNTNCCTGNNTNCNNAATTGGNNC
CANTGNNTGNCNATCTCTGGNTTNGNNNNNNTNNNNNTNNCAANNNGGNNNGTNANANNNCNATNTAA
NNTANANNNNCNNNNNNANNNCACNNNNNNNNNAGNNNCNANNNTTANANNNGTAGNNNANTAAGTCT
CCGANNTNNNNNNNATAAGCGNNANACNNNNNNNNNNNNNNANGANNACGNNTTGGNNNTNNNNNNCNA
GNNAGACNNTNATGGNTNNNNNTCAANNNNTTNGTAGTGGTATAATNGNNNNNNNTTTCNCCAGNNTN
NNNGNNNNNNNNCNATANNANNNNNNNGNGANNNNNNNNNNNANNNNCTCNNNAGGGANCANCTACTTNA
GCANTGGNGCGCAGNNNGAGANNNNGACNNNCCTTGGGTGGCAANNACNGCGATAAGTNNNGTTNNNC
CTNNNAGTNNACATNNNNNCANNGNNNCNNNNNNANCNGANNGNNNNNNNNNNNNNNNNNNNNNNNNN
NNNNNNNNNNNNNNNNNNNNNNNNNNNNNNNAGNNNANGGNNNGTNTNTGACNCATGNNTTTTTANNNGNN
NNNNNACGNNNNNNCCCCGGTCGNNTNCNNATTGAANNCCTGNNGCATNNNNNTNCNAGAATGANNNC
NNNNNGNNTNGNCTNNCNNNTCAAGNAATCAANNAGGNNNNNNNNTCNNNGNNNNNNNNCNNTCCCNN
ACTGAGGGTNNNNNNNNCATCTGCTNNNNNNNNNNTNCGCNGGNGTNNCNNNNNNGCNGTATNGTGNNA
NNCANNNNNNNNNNNNNNNNNNTNAAACNANNNNNNNTNNNN
>LoLo482
GTGNNNNNTAGCCCNNNNNAGTTAGACANNNCNNNNNNNNNNNNNTNNNNNNNNNNNNNNNNNNNNNN
NNNNNNNNNNNNNNNNNNNNNNNNNNNNNNNNNNNNNNNNNNNNNNNNNNNNNNNNNNNNNNNNNNNN
NNNNNNNNNNNNNNNNNNNNNNNNNNNNNNNNNNNNNNNNNNNNNNNNNNNNNNNNNNNNNNNNNNNN
NNNNNNNNNNNNNNNNNNNNNNNNNNNNNNNNNNNNNNNNNNNNNNNNNNNNNNNNNNNNNNNNNNNN
NNNNNNNNNNNNNNNNNNNNNNNNNNNNNNNNNNNNNNNNNNNNNNNNNNNNNNTCTNNNAAANNNTNNN
NNNNNNNNNNNNNNNNNNNNNNNNNNNCNCNTTGNNNGTAANCNANNNGNACATGCAGTCCCCTCNNNNN
NNNGGCAGAGNNNNNNCCCNNNNNCNCCGGCCCCNNCCNNNNNNNTNNNCNNNGNNNNNNNANNNNNN
NNNNNNTGGTACNNNNCGNNNNTGGTTGTTTAAGAGGTCCACAACGNGGTCNGGNCACNNNNGNNNAN
NNNCNNNNAACNNNNNNNNNNNNANNTNNNTANNAGGNCNNNNCCNTCNNGNNNNTAATTGCNATGACC
CANNTCNCGGGGGNAGTCCCCGGCGCGGCGACCTTGATNACGTCCGGGGACAAGTATGGCNNNCNCCC
CGGGAAGGANNCNGCCGTANGTGCNNNNNANNNNNTCNTGNGANGTCGCCGNNATTCNCGTGGTCNNN
CNAGAATGCCCNNACCANNCGNANNNTGGTTATGTNAGTNNNNNNNNNNNNNNNNNNNNNNNNNNNNN
NNNNNNNNNNNNNNNNNNNNNNNNNNNNNNNNNNNNGNNNNGNNNNNNCNANNNAAGGNNNTANNNCTNNTN
NTNNNNNNGGACNNNACNNNNCNNNNNNNACNCNNNCNCNNNNNNANNNGGTTTTTNNCCNNTATTNT
CCAGNACGCNGNGCNNNCATTCANGNGAACNNNGNNACGCGNNAATCGTANNATCTACCTGCCGCNNG
TNNNNNTACNGNNNNNNCGGANNNTNNNNCGTANNNNNNNCATGCGTNNTNTNNTCNCNAGNCANGTT
CGNNNANANANTAGNANGTNTNNNNNNNNNNTGTNNCGTTCCGCNTGANNCNNCANNNNGNNGNNCTTN
NCANCGGCNNTANCTGCNGANNTAAGNCATANNNTNNNTCNNTANCTNNNNNGGNGGGNNACTGTAAA
NGGNTAATTNGNNNNTNATTNCNNNCACNTNNCNNNGNNTNCTNNNTNCCTGNNTNCNNAATTGGNNC
CANTGNATGNCNATCTCTGGNTTNGCNNNNNTGNNNNTNNCANNNNGGNNNGTNANANNNCNATNTAA
NNTANANNNNCNNNNNNANNNCACNNNNNNNNNAGNNACNANNNTNANANNNGTAGNNNAATAAGTCT
CCGANNTNNNNNNNATAAGCGNNANACNNNNNNNNNNNNNNANGANNACGNNTTGGNNNTNNNNNNCNA
GNNAGACNNTNATGGNTNNNNNTCAANNNNTTCGTAGTGGTATAATNGNNNNNNNTTTCNCCAGNNTN
NNNGNNNNNNNNCNATANNANNNNNNNGNGANNNNNNNANNNANNNNCTCNNNAGGGANCANCTACTNNA
GCANTGGNGCGNAGNNNGAGANNNNGACNNGCCTTGGGTGGCAANNACNGCGATAAGTNNNGTTNANC
CTNNNAGTNNACATNNNNNCANNGNNNCNNNNNNANCNGANNGNNNNNNNNNNNNNNNNNNNNNNNNN
NNNNNNNNNNNNNNNNNNNNNNNNNNNNNNNANNNNANGGNNNGTNTNTGACNCATGTGTTTTTANNNGNN
NNNNNACGNNNGANCCCCGGTCGNNTNCNNATTGAANNCCTGNNGCATNNNNNTNCNAGAATGANNGC
NNNNNGNNTNGNCTNNCNTTTCAAGNAATCAANTAGGTNNNNNNNTCNNNGNNNNNNNNCNNTCCCNN

ACTGAGGGTNNNNNNNNCATCTGCTNNNNNNNNNNNTNCGCNGGNGTNNCNNNNNAGCNGTATNGNGTNA
NNCANNNNNNNNNNNNNNNNNTNAANNNANNNNNCTNNNN
>LoLo483
GTGNNNNNTAGCCCNNNNNAGTTAGACANNNCNNNNNNNNNNNNNTNNNNNNNNNNNNNNNNNNNNNNNN
NNNNNNNNNNNNNNNNNNNNNNNNNNNNNNNTNNNNNNNNNNNNNNNNNNNNNNNNNNNNNNNNNNNNNN
NNNNNNNNNNNNNNNNNNNNNNNNNNNNNNNNNNNNNNNNNNNNNNNNNNNNNNNNNNNNNNNNNNNNNN
NNNNNNNNNNNNNNNNNNNNNNNNNNNNNNNNNNNNNNNNNNNNNNNNNNNNNNNNNNNNNNNNNNNNNN
NNNNNNNNNNNNNNNNNNNNNNNNNNNNNNNNNNNNNNNNNNNNNNNNNNNNNNTCTNNNAAANNNTNNN
NNNNNNNNNNNNNNNNNNNNNNNNNNNCNCNTTNNNNGTAANCNANNNGNACATGCAGTCCCCTCNNNNN
NNNNNCAGAGNNNNNNNCNNNNNNCNCCGGCCCCNNCCNNNNNNNNNNNNNNNNNGNNNNNNNANNNNNN
NNNNNNTGGTACNNNNNCGNNNNNGGTTGNNNTNAGAGGTCCACAACGNGGTCNGGNCACNNNNGNNNAN
NNNCNNNNNANNNNNNNNNNNNNNANNTNNNTANNAGGNCNNNNCCNTCNNNNNNNNNANTGCNATGACC
CAANNCACGGNGGNAGTNCCCGGNGNGGCGACCTTGANNACGTCCGGGNACAAGTATGGCNNNCNCCC
CGGGAAGGANNCNGCCGTANGTGCNNNNNANNNNNTCNTGNGANGTNGCCGNNATTCNCGTGGTCNNN
CNNGANTGNCCNNACCANNCGNANNNTGGTNATGNNAGTNNNNNNNNNNNNNNNNNNNNNNNNNNNNN
NNNNNNNNNNNNNNNNNNNNNNNNNNNNNNNNNNNNNNGNNNNNNCNANNNAAGGNNNTANNNCTNNTN
NTNNNNNNNGGACNNNACNNNNCNNNNNNNACNCNNNCNCNNNNNNNNNNNGNTTTTNNNNCNNTATTNT
CCAGNACGCNGNGCNNNCATTCANGNGAACNNNGNNACGCGNNAATCGTANNATCTANCTGCCGCNGG
NNNNNNTNCNGNNNNNNCGGANNNTNNNNCGTANNNNNNNCANGCGTNNTNTNNTCNCNAGNCNNGTT
CGNNNANANANTAGNANGTNTNNNNNNNNNNTGTNNCGTTCCGCNTGANNCNNCANNNNGNNGNNNTTN
NCANCGGCNNTANCTNCNGANNTAAGNCATANNNTNNNTCNNTANCTNNNNNGGNGGGNNACTGTAAA
NGGNNAATTNGNNNNTNANTNCNNNCACNTNNCNNNGNNTNCTNNNTNCCTGNNTNCNNAATTGGNNC
CANTGNNTGNCNATCTCTGGNTTNGNNNNNNTNNNNNTNNCAANNNGGNNNNNNNNANNNCNATNTAA
NNTANANNNNCNNNNNNANNNCACNNNNNNNGNAGNNNNNANNNTTANANNNGTAGNNNANTNAGTCT
CCGANNTNNNNNNNATAAGCGNNANACNNNNNNNNNNNNNNANGANNNCGNNTTGGNNNTNNNNNNNNA
GNNAGACNNTNATGGNTNNNNNTCAANNNNTTNGTAGTGGTATAATNGNNNNNNNTTTCNCCAGNNTN
NNNGNNNNNNNCNATANNANNNNNNNGNGANNNNNNNNNNNANNNNCTCNNNAGGGANCANCTACTNNA
GCANNGGNGCGCAGNNNGAGANNNNGACNNNCCTTGGGTGGCAANCACNGNGATAAGTNNNGTTNANC
CTNNNAGTNNACATNNNNNCANNGNNNCNNNNNNANCNGANNGNNNNNNNNNNNNNNNNNNNNNNNNNN
NNNNNNNNNNNNNNNNNNNNNNNNNNNNNNAGNNNANGGNNNGTNTNTGACNCNTGTGTTTTTANNNGNN
NNNNNACGNNNNANCCCCGGTCGNNNNCNNATTGAANNCCTGNNGCATNNNNNNTNCNAGAATGANNNC
NNNNNGNNTNGNCTNNCNNNTCAAGNAATCANNNAGGNNNNNNGNTCNNNGNNNNNNNNCNNNCCCNN
ACTGAGGGTNNNNNNNNCATCNGCTNNNNNNNNNNNTNCGCNGGNGTNNCNNNNNAGCNGTATNNNGTNA
NNCANNNNNNNNNNNNNNNNNNNAAACAANNNNNCTNNNN
>LoLo484
GTGNNNNNTAGCCCNNNNNAGTTAGACANNNCNNNNNNNNNNNNNTNNNNNNNNNNNNNNNNNNNNNNNN
NNNNNNNNNNNNNNNNNNNNNNNNNNNNNNNNNNNNNNNNNNNNNNNNNNNNNNNNNNNNNNNNNNNNNNNN
NNNNNNNNNNNNNNNNNNNNNNNNNNNNNNNNNNNNNNNNNNNNNNNNNNNNNNNNNNNNNNNNNNNNNN
NNNNNNNNNNNNNNNNNNNNNNNNNNNNNNNNNNNNNNNNNNNNNNNNNNNNNNNNNNNNNNNNNNNNNN
NNNNNNNNNNNNNNNNNNNNNNNNNNNNNNNNNNNNNNNNNNNNNNNNNNNNNNTCTNNNAAANNNTNNN
NNNNNNNNNNNNNNNNNNNNNNNNNNNCNCNTTGNNNGTAANCNANNNGNACATGCAGTCCCCTCNNNNN
NNNGGCAGAGNNNNNNNCCNNNNNNNCCGGCCCCNNCCNNNNNNNTNNNNANNGNNNNNNNANNNNNN
NNNNNNTGGTACNNNNNCGNNNNNGGTTGTNTAAGAGGTCCACAACGNGGTCNNGNCACNNNNGNNNAN
NNNCNNNNAACNNNNNNNNNNNNNNATNNNTANNAGGNNNNNNCCNTCNNGNNNNTNATTGCNATGACC
CAANNCNCGGGGGNAGTNCCCGGCGNGGCGACCTTGNNNACGTCCGGGGACAAGTATGGCGNNCNCCC
CGGGAAGGANNCNGCCGTANGTGCNNNNNANNNNNTCNTGNGANGTNGCCGNNATTNNCGTGGTCNNN
CNAGAATGNNCNNACCANNCGNANNNTGGTNATGNNAGTNNNNNNNNNNNNNNNNNNNNNNNNNNNNN
NNNNNNNNNNNNNNNNNNNNNNNNNNNNNNNNNNGNNNNGNNNNNNCNANNNAAGGNNNTANNNCTNNTN
NTNNNNNNNGGACNNNACNNNNCNNNNNNNACNCNNNCNCNNNNNNNNNNNGGTTTTTNNNCNNTATTNT
CCAGNACGCNGNGCNNNCATTCANGNGAACNNNGNNACGCGNNAATCGTANNATCTACCTGCCGCNNG
TNNNNNTACNGNNNNNNCGGANNNTNNNNCGTANNNNNNNCANGCGTNNTNTNNTCNCNAGNCNNGTT
CGNNNANANANTAGNANGTNTNNNNNNNNNNTGTNNCGTTCCGCNTGANNCNNCANNNNGNNGNNNTTN
TCANCGGCNNTANCTNCNGANNTAAGNCATANNNTNNNTCNNTANCTNNNNNGGNGGGNNACTGTAAA
NGGNNAATTNGAGGNTNANTNCNNNCACNTNNCNNNGNNNNCTNNNTNCCTGNNTNCNNAATTGGNNC

CANTGNNTGNCNATCTCTNGNTTNGNNNNNNTNNNNNTNNCANNNNGGNNNNNNNNANNNCNATNTAA
NNTATANNNNCNNNNNNANNNCACNANNNNNNNAGNNNCNANNNTNANANNNGTAGNNNANTAAGTCT
CCGANNTNNNNNNNATAAGCGNNANACNNNNNNNNNNNNNNANGANNNCGNNTTGGNNNTNNNNNNNNA
GNNAGACNNTNATGGNTNNNNNTCAANNNNTTCGTAGTGGTATAATNGNNNNNNNTTTCNCCAGNNTN
NNNGNNNNNNNCNATANNANNNNNNNGNGANNNNNNNNNNNANNNNCTCNNNAGGNANCANCTACTTNA
GCAANGGNGCGNAGNNNGAGANNNNGACNNNCCTTGGGTGGCAANNACNGCGATAAGTNNNGTTNANN
CTNNNAGTNNACATNNNNNCANNGNNNCNNNNNNANCNGANNGNNNNNNNNNNNNNNNNNNNNNNNNN
NNNNNNNNNNNNNNNNNNNNNNNNNNNNNNAGNNNANNGNNNGTNTNTGACNCATGTGTTTTTANNNGNN
NNNNNNNGNNNGANCCCCGGTCGNNNNCNNATTGAANNCCTGNNGCATNNNNNTNCNAGAATGANNGC
NNNNNGNNTNGNCTNNCNNNTCAAGNAATCAANNAGNNNNCGCGNTCNNNNNNNNNNNNNCNNTCCCNN
ACTGAGGGTNNNNNNNNCATNNGCTNNNNNNNNNNTTCGCNGGNGTNNCNNNNNNGCCGTATNGNGTNA
NNCANNNNNNNNNNNNNNNNNTNNAACNANNNNNNNTNNNN
>LoLo485
GTGNNNNNTAGCCCNNNNNAGTTAGACANNNCNNNNNNNNNNNNNTNNNNNNNNNNNNNNNNNNNNNNN
NNNNNNNNNNNNNNNNNNNNNNNNNNNNNNNNNNNNNNNNNNNNNNNNNNNNNNNNNNNNNNNNNNNNNN
NNNNNNNNNNNNNNNNNNNNNNNNNNNNNNNNNNNNNNNNNNNNNNNNNNNNNNNNNNNNNNNNNNNNNN
NNNNNNNNNNNNNNNNNNNNNNNNNNNNNNNNNNNNNNNNNNNNNNNNNNNNNNNNNNNNNNNNNNNNNN
NNNNNNNNNNNNNNNNNNNNNNNNNNNNNNNNNNNNNNNNNNNNNNNNNNNNNNNTCTNNNAAANNNTNNN
NNNNNNNNNNNNNNNNNNNNNNNNNNNCNCNNTGNNNGTAANCNANNNGNACATGCAGTNCCCTCNNNNN
NNNGGCAGAGNNNNNNNCCNNNNNCNCCGNCCCCNNCCNNNNNNNNNNNNCNNNNNNNNNNNANNNNNN
NNNNNNTGGTACNNNNCGNNNNNGGTTGTNTAAGAGGTCCACAACGNGGTCNGNNCACNNNNGNNNAN
NNNCNNNNAACNNNNNNNNNNNNNNATNNNTANNAGGNNNNNNCCNTCNNNNNNNTNATNGCNATGACC
CANNNNACGGGGGCNGTNCCCGGCGCGGCGACCTTGANNANGTCCGGGGNCAAGTATGGCNNNCNCCC
CGGGAAGGANNCNGCCGTANGTGCNNNNNANNNNNTCNTGNGANGTCGCCGNNATTNNNGTGGTCNNN
CNAGAATGNCCNNACCANNCGNANNNTGGTNATGNNAGTNNNNNNNNNNNNNNNNNNNNNNNNNNNNN
NNNNNNNNNNNNNNNNNNNNNNNNNNNNNNNNNNNGNNNNGNNNNNNCNANNNAAGGNNNTANNNCTNNTN
NTNNNNNNNGGACNNNACNNNNCNNNNNNNACNCNNNCNCNNNNNNANNNGNTTNNNNNNCNNTATTNT
CCAGNACGCNGNGCNNNCATTCANGNGAACNNNGNNACGCGNNAATCGTANNATCTACCTGCCGCNNG
NNNNNNTNCNGNNNNNNCGGANNNTNNNNCGTANNNNNNNCANGCGTNNTNTNNTCNCNAGNCNNGTT
CGNNNANANANTAGNANGTNTNNNNNNNNNNTGTNNCGTTCCGCNTGANNCNNCANNNNGNNGNNCTTN
NCANCGGCNNTANCTGCNGANNTAAGNCATANNNTNNNTCNNTANCTNNNNNGGNGGGNNACTGTAAA
NGGNNAATTNGANGNTNANTNCNNNCACNTNNCNNNGNNNNCTNNNTNCCTGNNTNCNNAATTGGNNC
CANTGNNTGNCNATCTCTGGNTTNGNNNNNNTGNNNNTNNCANNNNGGNNNGTNANANNNCNATNTAA
NNTANANNNNCNNNNNNANNNCACNANNNNNGNAGNNNCNANNNTTANANNNGTAGNNNANTNAGTCT
CCGANNTNNNNNNNATAAGCGNNANACNNNNNNNNNNNNNNANGANNACGNNTTGGNNNTNNNNNNCNA
GNNAGACNNTNATGGNTNNNNNTCAANNNNTTCGTAGTGGTATAATNGNNNNNNNTTTCNCCAGNNTN
NNNGNNNNNNNCNATANNANNNNNNNGNGANNNNNNNNNNNANNNNCTCNNNAGGGANCANCTACTNNA
GCANTGGNGCGCAGNNNGAGANNNNGACNNNCCTTGGGTGGCAANCACNGNGATAAGTNNNGTTNANC
CTNNNAGTNNACATNNNNNCANNGNNNCNNNNNNANCNGANNGNNNNNNNNNNNNNNNNNNNNNNNNN
NNNNNNNNNNNNNNNNNNNNNNNNNNNNNNAGNNNANGGNNNGTNTNTGNCNCATGNNTTTTTANNNGNN
NNNNNACGNNNNANCCCCGGTCGNNTNCNNATTGAANNCCTGNNGCATNNNNNTNCNAGAATGANNNC
NNNNNGNNTNGNCTNNCNNNTCAAGNAATCANNTAGNNNNNNNNNNTCNNNGNNNNNNNNCNNTCCCNN
ACTGAGGGTNNNNNNNNCATNNGCTNNNNNNNNNNTNCGCNGNNGTNNCNNNNNNGCNGTATNGNGNNA
NNCANNNNNNNNNNNNNNNNNCNAAACNANNNNNNNTNNNG
>LoLo486
GTGNNNNNTAGCCCNNNNNAGTTAGACANNNCNNNNNNNNNNNNNTNNNNNNNNNNNNNNNNNNNNNNN
NNNNNNNNNNNNNNNNNNNNNNNNNNNNNNNNTNNNNNNNNNNNNNNNNNNNNNNNNNNNNNNNNNNNNN
NNNNNNNNNNNNNNNNNNNNNNNNNNNNNNNNNNNNNNNNNNNNNNNNNNNNNNNNNNNNNNNNNNNNNN
NNNNNNNNNNNNNNNNNNNNNNNNNNNNNNNNNNNNNNNNNNNNNGANNNNNNNNNNNNNNNNNNNNNNNN
NNNNNNNNNNNNNNNNNNNNNNNNNNNNNNNNNNNNNNNNNNNNNNNNNNNCNTCTNNNAAANNNTNNN
NNNNNNNNNNNNNNNNNNNNNNNNNNNCNCNNTGNNNGTAANCNANNNGNACATGCAGTCCCCTCNNNNN
NNNNGCAGAGNNNNNNNCCNNNNNCNCCGGCCCCNNCCNNNNNNNNNNNNNNNNGNNNNNNNANNNNNN
NNNNNNTGGTACNNNNCGNNNNNGGTTGTNTAAGAAGTCCACAACGNGGTCNNGNCACNNNNGNNNAN
NNNCNNNNAANNNNNNNNNNNNNNNNNTNNNTANNAGGNCNNNNCCNTCNNNNNNNTNANTGCNATGACC

CNNNNCNCNGNGGNNGTNCCCGGCGCGGCGACCTTGANNANGTCNGGGGNCAAGTATGGCNNNCNCCC
CGGGAAGGANNCNGCNGTANGTGCNNNNNANNNNNTCTTGNGANGTCGCCGNNATTCNCGTGGTCNNN
CNNGAATGNCCGNACCANNCGNANNNTGGTNATGNNAGTNNNNNNNNNNNNNNNNNNNNNNNNNNNNN
NNNNNNNNNNNNNNNNNNNNNNNNNNNNNNNNGNNNNGNNNNNNCNANNNAAGGNNNTANNNCTNNTN
NNNNNNNNGGACNNNACNNNNCNNNNNNNACNCNNNCNCNNNNNNANNNGNTTTTNNNNCNNTANTNN
CCAGNACGCNGNGCNNNCATTCANGNGAACNNNGNNACGCGNNAATCGTANNATCTACCTGCCGCNNG
TNNNNNTACNGNNNNNNCGGANNNTNNCTCGTANNNNNNNCATGCGTNNTNTNNTCNCNAGNCANGTT
CGNNNANANANTAGNANGTNTNNNNNNNNNNTGTNNCGTTCCGCNTGANNCNNCANNNNGNNGNNCTTN
NCANCGGCNNTANCTNCNGANNTAAGNCATANNNTNNNTCNNTANCTNNNNNGGNGGGNNACTGTAAA
NGGNNAATTNGNNGNTNATTNCNNNCATNTNNCTNNGNNTNCTNNNTNCCTGNNTNCNNAATTGGNNC
CANTGNNTGNCNATCTCTNGNTTNGNNNNNNTNNNNNTNNCANNNNGGNNNNNNANANNNCNATNTAA
NNTANANNNNCNNNNNNANNNCACNNNNNNNNNAGNNNCNANNNGTANANNNGTAGNNNGNTAAGTCT
CCGANNTNNNNNNNATAAGCTNNANACNNNNNNNNNNNNNANGANNNCGNNTTTGNNNTNNNNNNCNA
GNNAGACNNTNGTGGNTNNNNNTCAANNNNTTNGTAGTGGTATAATNGNNNNNNNTTTCNCCAGNNTN
NNNGNNNNNNNCNATANNANNNNNNNGNGANNNNNNNNNNNANNNNCTCNNNAGGGANCANCTACTNNA
GCANTGGNGCGCAGNNNGAGANNNNGACNNNCCTTGGGTGGCAANNACNGNAATAAGTNNNGTTNANC
CTNNNAGTNNACACNNNNNCANNGNNNCNNNNNNANCNGANNGNNNNNNNNNNNNNNNNNNNNNNNNN
NNNNNNNNNNNNNNNNNNNNNNNNNNNNANNNNANNGNNNGTNTNTGACNCATGNGTTTTCANNNGNN
NNNNNACGNNNNANCCCCGGTCGNNNNCNNATTGAANNCCTGNNGCATNNNNNTNCNAGAATGANNGC
NNNNNGNNTNGNCTNNCNNNTCAAGNAATCAANNAGGTNNNNCGNTCNNNNNNNNNNNNNCNNNCNCNN
ACTGAGGGTNNNNNNNNCATCNGCTNNNNNNNNNCTNCGCNGGCGTNNCNNNNNAGCNGTATNGNGTNN
NNCANNNNNNNNNNNNNNNNNTNAAACNANNNNNTTNNNN
>LoLo487
GTGNNNNNTAGCCCNNNNNAGTTAGACANNNCNNNNNNNNNNNNTNNNNNNNNNNNNNNNNNNNNNNN
NNNNNNNNNNNNNNNNNNNNNNNNNNNNNNNNNNNNNNNNNNNNNNNNNNNNNNNNNNNNNNNNNNNN
NNNNNNNNNNNNNNNNNNNNNNNNNNNNNNNNNNNNNNNNNNNNNNNNNNNNNNNNNNNNNNNNNNNN
NNNNNNNNNNNNNNNNNNNNNNNNNNNNNNNNNNNNNNNNNNNNNNNNNNNNNNNNNNNNNNNNNNNN
NNNNNNNNNNNNNNNNNNNNNNNNNNNNGNNNNNNNNNNNNNNNNNNNNNCNTCTNNNAAANNNTNNN
NNNNNNNNNNNNNNNNNNNNNNNNNCNCNTTNNNNGTAANCNANNNGNACATGCAGTCCCCTCNNNNN
NNNGGCAGAGNNNNNNCCCNNNNNNNCCGNCCCCNNCCNNNNNNNNNNNNNNNNNNNNNNNANNNNNN
NNNNNNTGGTACNNNNCGNNNNNGGTTGTNTAAGNGGTCCACAACGNGGTCNNNNCACNNNNGNNNAN
NNNCNNNNAACNNNNNNNNNNNNANNTNNNTANNAGGNCNNNNCCNTCNNNNNNNNAATTGCNATGACC
CAANNCNCGGGGGCNGTNCCCGGCGCGGNGACCTTGANNANNTCCGGGGACAAGTATGGCGNNCNCCC
CGGGAAGGANNCNGCCGTANGTGCNNNNNANNNNNTCNTGNGANGTCGCCGNNATTNNNGTGGTCNNN
NNAGAATGCCCGNACCANNCGNANNNTGGTNATGNNAGTNNNNNNNNNNNNNNNNNNNNNNNNNNNNN
NNNNNNNNNNNNNNNNNNNNNNNNNNNNNNNNGNNNNGNNNNNNCNANNNAAGGNNNTANNNCTNNTN
NTNNNNNNGGACNNNACNNNNCNNNNNNNACNCNNNCNCNNNNNNNNNNGNTTNNNNNNCNNTATTNT
CCAGNACGCNGNGCNNNCATTCANGNGAACNNNGNNACGCGNNAATCGTANNATCTACCTGCCGCNNG
NNNNNNTACNGNNNNNNCGGANNNTNNCTCGTANNNNNNTCATGCGTNNTNTNNTCNCNAGNCNNGTT
CGNNNANANANTAGNANGTNTNNNNNNNNNNTGTNNCGTTCCGCNTGANNCNNCANNNNGNNGNNNTTN
TCANCGGCNNTANCTGCNGANNTAAGTCATANNNTNNNTCNNTANCTNNNNNGGNGGGNNACTGTAAA
NGGNNAATTNGNNNNTNATTTCNNNCACNTNNCTNNGNNTNCTNNNTNCCTGNNTNCNNAATTGGNNC
CANTGNNTGNCNATCTCTNGNTTNGNNNNNNTNNNNNTNNCANNNNGGNNNNTNANANNNCNATNTAA
NNTANANNNNCNNNNNNANNNCACNANNNNNNNAGNNNCNANNNTTANANNNGTAGNNNAATNAGTCN
CCGANNTNNNNNNNATAAGCGNNANACNNNNNNNNNNNNNANGANNACGNNTTGGNNNTNNNNNNCNA
GNNAGACNNTNATGGNTNNNNNTCAANNNNTTNGTAGTGGTATAATNGNNNNNNNTTTCNCCAGNNTN
NNNGNNNNNNNCNATANNANNNNNNNGNGANNNNNNNNNNNANNNNCTCNNNAGGNANCANCTACTTNA
GCANTGGNGCGCAGNNNGAGANNNNGACNNNCCTTGGGTGGCAANCACNGCGATAAGTNNNGTTNANC
CTNNNAGTNNACATNNNNNCANNGNNNCNNNNNNANCNGANNGNNNNNNNNNNNNNNNNNNNNNNNNN
NNNNNNNNNNNNNNNNNNNNNNNNNNNNAGNNNANGGNNNGTNTNTGACNCATGTGTTTTTANNNGNN
NNNNNACGNNNGANCCCCGGTCGNNNNCNNATTGAANNCCTGNNGCATNNNNNTNCNAGAATGANNNC
NNNNNGNNTNGNCTNNNNTTTCAAGNAATCANNNAGNNNNCGCGNTCNNNGNNNNNNNNCNNNCCCNN
ACTGAGGGTNNNNNNNNCATCTGCTNNNNNNNNNCTTCGCNGGNGTNNCNNNNNAGCNGTATNNNGTNA
NNCANNNNNNNNNNNNNNNNNNTNAANNNNNNNNNNNNTNNNN

>LoLo488
GTGNNNNNTAGCCCNNNNNAGTCAGACANNNCNNNNNNNNNNNNTNNNNNNNNNNNNNNNNNNNNNNN
NNNNNNNNNNNNNNNNNNNNNNNNNNNNNTNNNNNNNNNNNNNNNNNNNNNNNNNNNNNNNNNNNNNNNN
NNNNNNNNNNNNNNNNNNNNNNNNNNNNNNNNNNNNNNNNNNNNNNNNNNNNNNNNNNNNNNNNNNNN
NNNNNNNNNNNNNNNNNNNNNNNNNNNNNNNNNNNNNNNNNNNNNNNNNNNNNNNNNNNNNNNNNNNN
NNNNNNNNNNNNNNNNNNNNNNNNNNNNNNNNNNNNNNNNNNNNNNNNNNCNTCTNNNAAANNNTNNN
NNNNNNNNNNNNNNNNNNNNNNNNNCNCNNTNNNNGTAANCNANNNGNACATGCAGTCCCCTCNNNNN
NNNGGCAGAGNNNNNNCCNNNNNNNNCCGNCCCCNNCCNNNNNNNNNNNNNNNGNNNNNNANNNNNNN
NNNNNNTGGTACNNNNCGNNNNNGGTTGTTTNAGAGGTCCACAACGNGGTCNNGNCACNNNNGNNNAN
NNNCNNNNAACNNNNNNNNNNNNNNTNNNTANNAGGNNNNNNCCNTCNNGNNNNNAATNGCNATGACC
CAANTCACNGGGGCNGTNCCCGGCGCGGCGACCTTGANNANGTCCGGGGNCAAGTATGGCNNNCNCCC
CGGGAAGGANNCNGCCGTANGTGCNNNNNANNNNNTCNTGNGACGTNGCCGNNNTTNNCNTGGTCNNN
CNAGANTGNCCGNACCANNCGNANNNTGGTNATGNNAGTNNNNNNNNNNNNNNNNNNNNNNNNNNNNN
NNNNNNNNNNNNNNNNNNNNNNNNNNNNNNNNGNNNNGNNNNNNCNANNNANGGNNNTANNNCTNNTN
NTNNNNNNGNNCNNNACNNNNCNNNNNNNACNCNNNCNCNNNNNNNNNNGNTTTTTNNNCNNTANTNT
CCAGNACGCNGNGCNNNCATTTANGNGAACNNNGNNACGCGNNAATCGTANNATCTACCTGCCGCNNG
NNNNNNTNCNGNNNNNNCGGANNNTNNNNCGTANNNNNNTCANGCGTNNTNTNNTCNCNAGNCNNGTT
CGNNNANANANTAGNANGTNTNNNNNNNNNTGTNNCGTTCCGCNTGANNCNNCNNNNNGNNGNNNTTN
NCANCGGCNNTANCTNCNGANNTAAGNCATANNNTNNNTCNNTANCTNNNNNGGNGGGNNACTGTAAA
NGGNNAATTNGNNNNTNANTNCNNNCATNTNNCNNNGNNNNCTNNNTNCCTGNNTNCNNAATTGGNNC
CANTGNNTGNCNATCTCTGGNTTNGNNNNNNTNNNNNTNNCAANNNGGNNNNNNNNANNNCNATNTAA
NNTANANNNNCNNNNNNANNNCACNNNNNNNNNAGNNACNANNNGTANANNNGTAGNNNGNTAAGTCT
CCGANNTNNNNNNNATAAGCGNNANACNNNNNNNNNNNNNNANGANNACGNNTTTGNNNTNNNNNCNA
GNNAGACNNTNGTGGNTNNNNNTCAANNNNTTNGTAGTGGTATAATNGNNNNNNNTTTCNCCAGNNTN
NNNGNNNNNNCNATANNANNNNNNNGNGANNNNNNNNNNNANNNNCTCNNNAGGNANCANCTACTNNA
GCANNGGNGCGCAGNNNGAGANNNNGACNNNCCTTGGGTGGCAANCACNGCAATAAGTNNNGTTNANC
CTNNNAGTNNACACNNNNNCANNGNNNCNNNNNNANCNGANNGNNNNNNNNNNNNNNNNNNNNNNNNN
NNNNNNNNNNNNNNNNNNNNNNNNNNNNAGNNNANNGNNNGTNTNTGACNCATGTNTTTNTANNNGNN
NNNNNACGNNNNANCCCCGGTCGNNNNCNNATTGAANNCCTGNNGCATNNNNNTNCNAGAATGANNNC
NNNNNGNNTNGNCTNNCNNNTNAAGNAATCANNTANGTNNNNNNNTCNNNGNNNNNNNNCNNTCCCNN
ACTGAGGGTNNNNNNNNCATNNGCTNNNNNNNNCTNCGCNGGCGTNNCNNNNNNGCNGTATNGNGTNA
NNCANNNNNNNNNNNNNNNNTNAAACAANNNNNNTNNNN
>LoLo489
GTGNNNNNTAGCCCNNNNNAGTTGGACANNNCNNNNNNNNNNNNTNNNNNNNNNNNNNNNNNNNNNNN
NNNNNNNNNNNNNNNNNNNNNNNNNNNNNNNNNNNNNNNNNNNNNNNNNNNNNNNNNNNNNNNNNNNN
NNNNNNNNNNNNNNNNNNNNNNNNNNNNNNNNNNNNNNNNNNNNNNNNNNNNNNNNNNNNNNNNNNNN
NNNNNNNNNNNNNNNNNNNNNNNNNNNNNNNNNNNNNNNNNNNNNNNNNNNNNNNNNNNNNNNNNNNN
NNNNNNNNNNNNNNNNNNNNNNNNNNNNNNNNNNNNNNNNNNNNNNNNNNCNTCTNNNAAANNNTNNN
NNNNNNNNNNNNNNNNNNNNNNNNNCNCNTTNNNNGTAANCNANNNGNACATGCAGTCCCCTCNNNNN
NNNNNCAGAGNNNNNNCCCNNNNNNNCCGNCCCCNNCCNNNNNNNNNNNCNNNNNNNNNNNGNNNNNN
NNNNNNTGGTACNNNNCGNNNNNGGTTGTNTAAGAGGTCCACCACGNGGTCNNGNNACNNNNGNNNAN
NNNCNNNNNNCNNNNNNNNNNNNNNTNNNCANNAGGNNNNNNCCNTCNNGNNNNNAANTGCNATGACC
CAANTCGCGGNGACAGTNACCGGNGCGGCGACCTTGNTNACGGTCGGGGACAAGTATGTCNTNCNCCC
CGGGAAGGANNCNGCCGTANGTGCNNNNNANNNNNTCNTGNGANGTCGCCGNNATTCNNGTGGTCNNN
NNAGANTGNCCGNACCANNCGNANNNTGGTNATGNNAGTNNNNNNNNNNNNNNNNNNNNNNNNNNNNN
NNNNNNNNNNNNNNNNNNNNNNNNNNNNNNNNNNNNNGNNNNNNCNANNNAAGGANNTANNNTTNNTN
NTNNNNNNGGACNNNACNNNNCNNNNNNNACNCNNNCNCNNNNNNNNNNGNTTTTTNNNCNNTATTNT
CCAGNANGCNGNGCNNNCATTCANGNGAACNNNGNNACGCGNNAATCGTANNATCTACCTGCCGCNNG
TNNNNNTACNGNNNNNNCGGANNNTNNCTCGTANNNNNNNCATGCGTNNTNTNNTTNCNAGNCNNGTT
CGNNNANANANTAGNANGTNTNNNNNNNNNTGTNNCGTTCCGCNTGANNCNNCANNNNGNNGNNCTTN
TCANCGGCNNTANCTGCNGANNTAAGNCATANNNTNNGTCNNTANCGNNNNNGGNGGGNNACTGTAAA
NGGNNAATTNGANGNTNATTNCNNNCACNTNNCTNNGNNTNCTNNNCNCCTGNNTNCNNAATTGGNNC
CANTGNNTGNCNATCTCTNGNTTNGNNNNNNTNNNNNTNNTAANNNGGNNNGNNANANNNCNATNTAA
NNTANANNNNCNNNNNNANNNCACNANNNNNNNAGNNNNNANNNTGANGNNNGTAGNNNANTAAGTCT

CCGANNTNNNNNNNATAAGCGNNANACNCNNNNNNNNNNNNANGANNNCGNNTTGGNNNTNNNNNNCNA
ANNAGACNNCNGTGGNTNNNNNTCAANNNNTTCGTAGTGGTATAACNANNNNNNNTTTCNCCAGNNTN
NNNGNNNNNNNCNATANNANNNNNNNGNGANNNNNNNNNNNANNNNCCCNNNAGGGANCANCTATTNNA
GCAATGGNGCGNAGNNNGAGANNNNGACNNNCCTTGGGTGGCAANCACNGCAATAAGTNNNGTTNANC
CCNNNAGTNNACACGNNNNCANNGNNNCNNNNNNANCNGANNTNNNNNNNNNNNNNNNNNNNNNNNNN
NNNNNNNNNNNNNNNNNNNNNNNNNNNNNAGNNNANGGNNNGTNTNTGACNCATGTGTTTTTANNNGNN
NNNNNNNGNNNNANCCCCGGTCGTNNNCNNATTGAANNCCTGNNGCATNNNNNTNCNAGAATGANNGC
NNNNNGNNTNGNCTNNCNNNTCAAGNAGTCAANNAGNNNNNNNNNTCNNNNNNNNNNNNCNNNCCCNN
ACTGAGGGTNNNNNNNNCATCTGCTNNNNNNNNNNTNCGCNGNNGTNNTNNCNNNGCNGTATNNNGTNA
NNCANNNNNNNNNNNNNNNNNTNANACGNNNNNNNNTNNNN
>LoLo490
GTGNNNNNTAGCCCNNNNNAGTTAGACANNNCNNNNNNNNNNNNTNNNNNNNNNNNNNNNNNNNNNN
NNNNNNNNNNNNNNNNNNNNNNNNNNNNNNNTNNNNNNNNNNNNNNNNNNNNNNNNNNNNNNNNNNNN
NNNNNNNNNNNNNNNNNNNNNNNNNNNNNNNNNNNNNNNNNNNNNNNNNNNNNNNNNNNNNNNNNNNN
NNNNNNNNNNNNNNNNNNNNNNNNNNNNNNNNNNNNNNNNNNNNNNNNNNNNNNNNNNNNNNNNNNNN
NNNNNNNNNNNNNNNNNNNNNNNNNNNNNNNNNNNNNNNNNNNNNNNNNNNNNNNCNTCTNNNAAANNNTNNN
NNNNNNNNNNNNNNNNNNNNNNNNNNCNCNTTGNNNGTAANCNANNNGNACATGCAGTCCCCTCNNNNN
NNNGGCAGAGNNNNNNCCCNNNNNCNCCGGCCCCNNCCNNNNNNNNNNNCNNNGNNNNNNNGNNNNNN
NNNNNNTGGTACNNNNCGNNNNNGGTTGTTTAAGAGGTCCACCACGNGGTCNNGNCACNNNNGNNNAN
NNNCNNNNAACNGNNNNNNNNNNANNTNNNCANNAGGNCNNNNCCNTCNNGNNNNTNATTGCGATGACC
CAANTCACGGGGGCNGTNCCCGGCGCGGCGACCTTGANNACGGCCGGGGNCAAGTATGGCGTNCNCCC
CGGGAAGGANNCNGCCGTANGTGCNNNNNANNNNNTCNTGNGANGTCGCCGNNATTCNCGTGGTCNNN
CNAGAATGCCCNNACCANNCGNANNNTGGTNATGNNAGTANNNNNNNNNNNNNNNNNNNNNNNNNNNN
NNNNNNNNNNNNNNNNNNNNNNNNNNNNNNNNNNGNNNNGNNNNNNCNANNNAAGGNNNTANNNCTNNTN
NTNNNNNNNGGACNNNACNNNNCNNNNNNNACNCNNNCNCNNNNNNANNNGGTTTTNNNNCNNTATTNT
CCAGNACGCNGNGCNNNCATTCANGNGAACNNNGNNACGCGNNAATCGTANNATCTACCTGCCGCNNG
NNNNNNTACNGNNNNNNCGGANNNTNNNNCGTANNNNNNTCANGCGTNNTNTNNTCNCNAGNCNNGTT
CGNNNANANANTAGNANGTNTNNNNNNNNNNTGTNNCGTTCCGCNTGANNCNNCANNNNGNNGNNNTTN
NCANCGGCNNTANCTGCNGANNTAAGNCATANNNTNNNTCNNTANCTNNNNNGGNGGGNNACTGTAAA
NGGNNAATTNGNNGNTNATTNCNNNCACNTNNCTNNGNNNNCTNNNTNCCTGNNTNCNNAATTGGNNC
CANTGNNTGNCNATCTCTNGNTTNGNNNNNNTNNNNNTNNCAANNNGGNNNGTNANANNNCNATNTAA
NNTANANNNNCNNNNNNANNNCACNNNNNNNGNAGNNNCNANNNTTANANNNGTAGNNNAATAAGTCT
CCGANNTNNNNNNNATAAGCGNNANACNNNNNNNNNNNNNNANGANNACGNNTTGGNNNTNNNNNNCNA
ANNAGACNNTNGTGGNTNNNNNTCAANNNNTTCGTAGTGGTATAACNGNNNNNNNTTTCNCCAGNNTN
NNNGNNNNNNNCNATANNANNNNNNNGNGANNNNNNNNNNNANNNNCTCNNNAGGGANCANCTACTNNA
GCANTGGNGCGCAGNNNGAGANNNNGACNNNCCTTGGGTGGCAANCACNGCAATAAGTNNNGTTNANC
CTNNNAGTNNACACNNNNNCANNGNNNCNNNNNNANCNGANNTNNNNNNNNNNNNNNNNNNNNNNNNN
NNNNNNNNNNNNNNNNNNNNNNNNNNNNNAGNNNANGGNNNGTNTNTGACNCATGTGTTTTTANNNGNN
NNNNNACGNNNNANCCCCGGTCGNNTNCNNATTGAANNCCTGNNGCATNNNNNTNCNAGAATGANNGC
NNNNNGNNTNGNCTNNCNTTTCAAGNAGTCANNNAGNTANNNNGNTCNNNGNNNNNNNNCNNTCCCNN
ACTGAGGGTNNNNNNNNCATCTGCTNNNNNNNNNNTNCGCNGNCGTNNCNNCNNAGCNGTATNNNGTNN
NNCANNNNNNNNNNNNNNNNNTNAAACAANNNNNCTNNNN
>LoLo491
GTGNNNNNTAGCCCNNNNNAGTTAGACANNNCNNNNNNNNNNNNTNNNNNNNNNNNNNNNNNNNNNN
NNNNNNNNNNNNNNNNNNNNNNNNNNNNNNNNNNNNNNNNNNNNNNNNNNNNNNNNNNNNNNNNNNNN
NNNNNNNNNNNNNNNNNNNNNNNNNNNNNNNNNNNNNNNNNNNNNNNNNNNNNNNNNNNNNNNNNNNN
NNNNNNNNNNNNNNNNNNNNNNNNNNNNNNNNNNNNNNNNNNNNNNNNNNNNNNNNNNNNNNNNNNNN
NNNNNNNNNNNNNNNNNNNNNNNNNNNNNNNNNNNNNNNNNNNNNNNNNNNNNNNNTCTNNNAAANNNTNNN
NNNNNNNNNNNNNNNNNNNNNNNNNNCNCNTTGNNNGTAANCNANNNGNACATGCAGTCCCCTCNNNNN
NNNNNCAGAGNNNNNNNCNNNNNNCNCCGGCCCCNNCCNNNNNNNNNNNCNNNGNNNNNNNANNNNNN
NNNNNNTGGTACNNNNCGNNNNNGGTTGTNTNAGAGGTCCACAACGNGGTCNNGNNACNNNNGNNNAN
NNNCNNNNAANNNNNNNNNNCNNNNNTNNNTANNAGGNNNNNNCCNTCNNNNNNNNNNATNGCNATGACC
CAANNCACGGGGGCNGTNCCCGGNGNGGCGACCTTGNTNANNTCCGGGGACAAGTATGGCGCNCNCCC
CGGGAAGGANNCNGCCGTANGTGCNNNNNANNNNNTCNTGNGANGTCGCCGNNANNNNCNTGGTCNNN

CNAGAATGNCCGNACCANNCGNANNNTGGTNATGNNAGTNNNNNNNNNNNNNNNNNNNNNNNNNNNN
NNNNNNNNNNNNNNNNNNNNNNNNNNNNNNNNNNGNNNNGNNNNNNCNANNNAAGGANNTANNNCTNNTN
NTNNNNNNGGACNNNACNNNNCNNNNNNNACNCNNNCNCNNNNNNANNNGNTTTTTNNNCNNTATTNT
CCAGNANGCNGNGCNNNCATTCANGNGAACNNNGNNACGCGNNAATCGTANNATCTACCTGCCGCNGG
TNNNNNTACNGNNNNNNCGGANNNTNNCTCGTANNNNNNTCATGCGTNNTNTNNTCNCNAGNCANGTT
CGNNNANANANTAGNANGTNTNNNNNNNNNNTGTNNCGTTCCGCNTGANNCNNCANNNNGNNGNNCTTC
TCANCGGCNNTANCTGCNGANNTAAGTCATANNNTNNNTCNNTANCTNNNNNGGNGGGNNACTGTAAA
NGGNNAATTNGNNNNTNANTTCNNNCACNTNNCTNNGNNNNCTNNNTNCCTGNNTNCNNAATTGGNNC
CANTGNNTGNCNATCTCTNGNTTNGNNNNNNTNNNNNTNNCAANNNGGNNNGTNNNANNNCNATNTAA
NNTANANNNNCNNNNNNANNNCACNNNNNNNGNAGNNNCNANNNTTANANNNGTAGNNNAATAAGTCT
CCGANNTNNNNNNNATAAGCGNNANACNNNNNNNNNNNNNNANGANNNCGNNTTGGNNNTNNNNNNCNA
GNNANACNNTNATGGNTNNNNNTCAANNNNTTNGTAGTGGTATAATNGNNNNNNNTTTCNCCAGNNTN
NNNGNNNNNNNNCNATANNANNNNNNNGTGANNNNNNNNNNNANNNNCTCNNNAGGGANCANCTACTNNA
GCAATGGNNCGNAGNNNGAGANNNNGACNANCCTTGGGTGGCAANCACNGCGATAAGTNNNGTTNANC
CTTNNAGTNNACATNNNNNCANNGNNNCNNNNNNANCNGANNGNNNNNNNNNNNNNNNNNNNNNNNNN
NNNNNNNNNNNNNNNNNNNNNNNNNNNNNAGNNNANGGNNNGTNTNTGACNCANNNNTTTTTANNNGNN
NNNNNACGNNNNANCCCCGGTCGNNNNCNNATTGAANNCCTGNNGCATNNNNNTNCNAGAATGANNNC
NNNNNGNNTNGNNTNNCNNNTCAAGNAATCANNNAGNNNNCGCGNTCNNNNNNNNNNNNNCNNNCCCNN
ACTGAGGGTNNNNNNNGCATCTGCTNNNNNNNNNNTNCGCNGGNNNNNCNGNNNAGCNGTATNNTGTNN
NNCANNNNNNNNNNNNNNNNNNNNNAANNNNNNNNNNTNNNN
>LoLo492
GTGNNNNNTAGCCCNNNNNAGTTAGACANNNCNNNNNNNNNNNNNTNNNNNNNNNNNNNNNNNNNNNNN
NNNNNNNNNNNNNNNNNNNNNNNNNNNNNNNNTNNNNNNNNNNNNNNNNNNNNNNNNNNNNNNNNNNNNNN
NNNNNNNNNNNNNNNNNNNNNNNNNNNNNNNNNNNNNNNNNNNNNNNNNNNNNNNNNNNNNNNNNNNNNNN
NNNNNNNNNNNNNNNNNNNNNNNNNNNNNNNNNNNNNNNNNNNNNNNNNNNNNNNNNNNNNNNNNNNNNNN
NNNNNNNNNNNNNNNNNNNNNNNNNNNNNNNNNNNNNNNNNNNNNNNNNNNNNNNCNTCTNNNAAANNNTNNN
NNNNNNNNNNNNNNNNNNNNNNNNNNNCNCNTTGNNNGTAANCNANNNGNACATGCAGTCCCCTCNNNNN
NNNGGCAGAGNNNNNNCCCNNNNNCNCCGGCCCCNNCCNNNNNNNNNNNNCNNNGNNNNNNNANNNNNN
NNNNNNTGGTACNNNNCGNNNNNGGTTGTNTAAGAGGTCCACCACGNGGTCNGGNNACNNNNGNNNAN
NNNCNNNNAACNNNNNNNNNNNNNNNNTNNNTANAAGGNCNNNNCCNTCNNGNNNNNAANNGCNATGANN
CAANNNACGGGGGNNGTNCCCGGCGCGGCGACCTTGNNNACGTCCGGGNACAAGTATGGCGTNCNCCC
CGGGAAGGANNCNGCCGTANGTGCNNNNNANNNNNTCNTGNGANGTCGCCGNNATTNNCGTGGTCNNN
NNAGAATGNCCGNACCANNCGNANNNTGGTNATGNNAGTNNNNNNNNNNNNNNNNNNNNNNNNNNNNN
NNNNNNNNNNNNNNNNNNNNNNNNNNNNNNNNNNGNNNNGNNNNNNCNANNNAAGGANNTANNNCTNNTN
NTNNNNNNGGACNNNACNTNNCNNNNNNNACNCNNNCNCNNNNNNANNNGGTTTTNNNCCNNTANTNT
CCAGNACGCNGNGCNNNCATTCANGNGAACNNNGNNACGCGNNAATCGTANNATCTACCTGCCGCNNG
TNNNNNTACNGNNNNNNCGGANNNTNNNNCGTANNNNNNNTATACGTNNTNTNNTCNCNAGNCNNGTT
CGNNNANANANTAGNANGTNTNNNNNNNNNNTGTNNCGTTCCGCNTGANNCNNCANNNNGNNGNNNTTN
NCANCGGCNNTANCTGCNGANNTAAGTCATANNNTNNNTCNNTANCTNNNNNGGNGGGNNACTGTAAA
NGGNNAATTNGANNNTNATTNCNNNCACNTNNCTNNGNNTNCTNNNTNCCTGNNTNCNNAATTGGNNC
CANTGNNTGNCNATCTCTGGNTTNGNNNNNNTNNNNNTNNCAANNNGGNNNGTNANANNNCNATNTAA
NNTANANNNNCNNNNNNANNNCACNNNNNNNGNAGNNNCNANNNTTANANNNGTAGNNNAATAAGTCT
CCGANNTNNNNNNNATAAGCGNNANACNNNNNNNNNNNNNNANGANNACGNNTTGGNNNTNNNNNNCNA
GNNAGACNNTNGTGGNTNNNNNTCAANNNNTTNGTAGTGGTATAATNGNNNNNNNTTTCNCCAGNNTN
NNNGNNNNNNNNCNATANNANNNNNNNGNGANNNNNNNNNNNANNNNCTCNNNAGGGANCANCTACTNNA
GCANTGGNGCGCAGNNNGAGANNNNGACNNNCCTTGGGTGGCAANNACNGCAATAAGTNNNGTTNANC
CTNNNAGTNNACACNNNNNCANNGNNNCNNNNNNANCNGANNGNNNNNNNNNNNNNNNNNNNNNNNNN
NNNNNNNNNNNNNNNNNNNNNNNNNNNNNAGNNNANGGNNNGTNTNTGACNCATGTGTTTTTANNNGNN
NNNNNACGNNNNANCCCCGGTCGNNNNCNNATTGAANNCCTGNNGCATNNNNNTNCNAGAATGANNNC
NNNNNGNNTNGNCTNNCNNTTCAAGNAGTCANNNAGGTNNNNNGNTCNNNGNNNNNNNNCNNTCCCNN
ACTGAGGGTNNNNNNNNCATCTGCTNNNNNNNNNNTNCGCNGGNNNNNCNNNNNNGCNGTATNNNGNNA
NNCANNNNNNNNNNNNNNNNNNTCNAACAANNNNNCTNNNN
>LoLo493
GTGNNNNNTAGCCCNNNNNAGTTAGACANNNCNNNNNNNNNNNNNTNNNNNNNNNNNNNNNNNNNNNNN

NNNNNNNNNNNNNNNNNNNNNNNNNNNNNNNNNNNNNNNNNNNNNNNNNNNNNNNNNNNNNNNNNNNNNN
NNNNNNNNNNNNNNNNNNNNNNNNNNNNNNNNNNNNNNNNNNNNNNNNNNNNNNNNNNNNNNNCNNNNNN
NNNNNNNNNNNNNNNNNNNNNNNNNNNNNNNNNNNNNNNNNNNNNNNNNNNNNNNNNNNNNNNNNNNNNN
NNNNNNNNNNNNNNNNNNNNNNNNNNNNNNNNNNNNNNNNNNNNNNNNNNNNCNTCTNNNAAANNNTNNN
NNNNNNNNNNNNNNNNNNNNNNNNNNCNCNNTGNNNGTAANCNANNNGNACATGCAGTCCCCTCNNNNN
NNNGGCAGAGNNNNNNNCCNNNNNNNNCCGNCCCCNNCCNNNNNNNNNNNNNNNNGNNNNNNNANNNNNN
NNNNNNTGGTACNNNNCGNNNNNGGTTGNTTNAGAGGTCCACAACGNGNTCNGGNNACNNNNGNNNAN
NNNCNNNNNANNNNNNNNNNNNNNNNNTNNNTANNAGGNNNNNNCCNTCNNNNNNNNNNANTGCNATGACC
CANNNCNCGGGGGCNGTNCCCGGCGCGGCGACCTTGANNACGTCCGGGGACAAGTATGGCNNNCNCCC
CGGGAAGGANNCNGCCGTANGTGCNNNNNANNNNNTCNTGNGANGTNGCCGNNATTNNCGTGGTCNNN
NNAGAATGNNCNNACCANNCGNANNNTGGTNATGTNAGTNNNNNNNNNNNNNNNNNNNNNNNNNNNNNNN
NNNNNNNNNNNNNNNNNNNNNNNNNNNNNNNNNGNNNNGNNNNNNCNANNNAAGGNNNTANNNCTNNTN
NTNNNNNNGGACNNNACNNNNCNNNNNNNACNCNNNCNCNNNNNNNNNNGNTTNNTNNNCNNTATTNT
CCAGNACGCNGNGCNNNCATTCANGNGAACNNNGNNACGCGNNAATCGTANNATCTACCTGCCGCNNG
NNNNNNTNCNGNNNNNNCGGANNNTNNCTCGTANNNNNNTCANGCGTNNTNTNNTCNCNAGNCANGTT
CGNNNANANANTAGNANGTNTNNNNNNNNNTGTNNCGTTCCGCNTGANNCNNCANNNNGNNGNNNTTN
NCANCGGCNNTANCTGCNGANNTAAGNCATANNNTNNNTCNNTANCTNNNNNGGNGGGNNACTGTAAA
NGGNNAATTNGANGNTNANTTCNNNCACNTNNCNNNGNNNNCTNNNTNCCTGNNTNCNNAATTGGNNN
CANTGNNTGNCNATCTCTGGNTTNGNNNNNNTNNNNNTNNCAANNNGGNNNGTNNNANNNCNATNTAA
NNTANANNNNCNNNNNNANNNCACNANNNNNGNAGNNNCNANNNTTANANNNGTAGNNNANTAAGTCT
CCGANNTNNNNNNNATAAGCGNNANACNNNNNNNNNNNNNANGANNACGNNTTGGNNNTNNNNNNCNA
GNNAGACNNTNATGGNTNNNNNTCAANNNNTTNGTAGTGGTATAATNGNNNNNNNTTTCNCCAGNNTN
NNNGNNNNNNNCNATANNANNNNNNNGNGANNNNNNNNNNNANNNNCTCNNNAGGNANCANCTACTTNA
GCANNGGNGCGNAGNNNGAGANNNNGACNNNCCTTGGGTGGCAANNACNGCGATAAGTNNNGTTNANC
CTNNNAGTNNACATNNNNNCANNGNNNCNNNNNNANCNGANNGNNNNNNNNNNNNNNNNNNNNNNNNN
NNNNNNNNNNNNNNNNNNNNNNNNNNNNNAGNNNANNGNNNGTNTNTGACNCATNNNTTTTTANNNGNN
NNNNNACGNNNNANCCCCGGTCGNNNNCNNATTGAANNCCTGNNGCATNNNNNNTNCNAGAATGANNGC
NNNNNGNNTNNNCTNNCNNNTCAAGNAATCANNNAGGTANNNNGNTCNNNNNNNNNNNNNCNNTCCCNN
ACTGAGGGTNNNNNNNNCATCTGCTNNNNNNNNNNTNCGCNGNCGTNNCNNNNNAGCNGTATNGNGTNA
NNCANNNNNNNNNNNNNNNNNNCNNNACNNNNNNNNNTNNNN
>Mang273
GTGNNNNNTAGCCCNNNNNAGTTAGACANNNCNNNNNNNNNNNNNTNNNNNNNNNNNNNNNNNNNNNN
NNNNNNNNNNNNNNNNNNNNNNNNNNNNNNNNNNNNNNNNNNNNNNNNNNNNNNNNNNNNNNNNNNNNNN
NNNNNNNNNNNNNNNNNNNNNNNNNNNNNNNNNNNNNNNNNNNNNNNNNNNNNNNNNNNNNNNNNNNNNN
NNNNNNNNNNNNNNNNNNNNNNNNNNNNNNNNNNNNNNNNNNNNNNNNNNNNNNNNNNNNNNNNNNNNNN
NNNNNNNNNNNNNNNNNNNNNNNNNNNNNNNNNNNNNNNNNNNNNNNNNNNNNNTCTNNNAAANNNTNNN
NNNNNNNNNNNNNNNNNNNNNNNNNNCNCNNTGNNNGTAANCNANNNGNACATGCAGTCCCCTCNNNNN
NNNNNCAGAGNNNNNNNCCCNNNNNNNCCGNCCCCNNCCNNNNNNNNNNNNNNNNNNNNNNNNNANNNNNN
NNNNNNTGGTACNNNNCGNNNNNGGTTGTNTAAGAGGTCCACAACGNGNTCNGGNNACNNNNGNNNAN
NNNCNNNAAACNGNNNNNNNNNNNNNTNNNTANNAGGNCNNNNCCNTCNNGNNNNNNATTGCNATGANC
CAANNCACGGGGGCAGTNCCCGGNGNGGCGACCTTGANNANNTCCGGGGACAAGTATGGCNNNCNCCC
CGGGAAGGANNCNGCCGTANGTGCNNNNNANNNNNTCNTGNGANGTCGCCGNNATTCNCGTGGTCNNN
NNNGAATGNCCNNACCANNCGNANNNTGGTNATGNNAGTNNNNNNNNNNNNNNNNNNNNNNNNNNNNNN
NNNNNNNNNNNNNNNNNNNNNNNNNNNNNNNNNGNNNNGNNNNNNCNANNNAAGGANNTANNNCTNNTN
NTNNNNNNGGACNNNACNNNNCNNNNNNNACNCNNNCNCNNNNNNANNNGNTTTTNNNNNCNNTANTNT
CCAGNACGCNGNGCNNNCATTCANGNGAACNNNGNNACGCGNNAATCGTANNATCTACCTGCCGCNNG
NNNNNNTACNGNNNNNNCGGANNNTNNNNCGTANNNNNNNNCATGCGTNNTNTNNTCNCNAGNCNNGTT
CGNNNANANANTAGNANGTNTNNNNNNNNNNTGTNNCGTTCCGCNTGANNCNNCANNNNGNNGNNNTTN
TCANCGGCNNTANCTNCNGANNTAAGNCATANNNTNNNTCNNTANCTNNGNNGGNGGGNNACTGTAAA
NGGNNAATTNGNNNNTNANTNCNNNCACNTNNCTNNGNNNNCTNNNTNCCTGNNTNCNNAATTGGNNC
CANTGNNTGNCNATCTCTNGNTTNGCNNNNNTNNNNNTNNCANNNNGGNNNNNNNNANNNCNATNTAA
NNTANANNNNCNNNNNNANNNCACNANNNNNNNAGNNNCNANNNTNANANNNGTAGNNNANTAAGTCT
CCGANNTNNNNNNNATAAGCGNNANACNNNNNNNNNNNNNANGANNACGNNTTGGNNNTNNNNNNCNA
GNNAGACNNTNATGGNTNNNNNTCAANNNNTTNGTAGTGGTATAATNGNNNNNNNTTTCNCCAGNNTN

NNNGNNNNNNNNCNATANNANNNNNNNNGNGANNNNNNNNNNNANNNNCTCNNNAGGGANCANCTACTNNA
GCAATGGNGCGNAGNNNGAGANNNNGACNNNCCTTGGGTGGCAANCACNGCAATAAGTNNNGTTNNNN
CTNNNAGTNNACATNNNNNCANNGNNNCNNNNNNANCNGANNGNNNNNNNNNNNNNNNNNNNNNNNN
NNNNNNNNNNNNNNNNNNNNNNNNNNNNNANNNNANGGNNNGTNTNTGACNCATGTGTTTTTANNNGNN
NNNNNACGNNNNANCCCCGGTCGNNNNCNNATTGAANNCCTGNNGCATNNNNNTNCNAGAATGANNGC
NNNNNGNNTNGNCTNNCNNNTNAAGNAATCANNNAGNTNNNGCNNTCNNNGNNNNNNNNCNNNCCCNN
ACTGAGGGTNNNNNNNNCATCNGCTNNNNNNNNNNTNCGCNGGNGTNNCNNNNNAGCNGTATNGNGTNA
NNCANNNNNNNNNNNNNNNNNCNANNNGANNNNNNTNNNN
>Mang274
GTGNNNNNTAGCCCNNNNNAGTTAGACACNNCNNNNNNNNNNNNTNNNNNNNNNNNNNNNNNNNNNN
NNNNNNNNNNNNNNNNNNNNNNNNNNNNNNNNTNNNNNNNNNNNNNNNNNNNNNNNNNNNNNNNNNNNNNN
NNNNNNNNNNNNNNNNNNNNNNNNNNNNNNNNNNNNNNNNNNNNNNNNNNNNNNNNNNNNNNNNNNNNNNN
NNNNNNNNNNNNNNNNNNNNNNNNNNNNNNNNNNNNNNNNNNNNNNNNNNNNNNNNNNNNNNNNNNNNNNN
NNNNNNNNNNNNNNNNNNNNNNNNNNNNNNNNNNNNNNNNNNNNNNNNNNNNNCNTCTNNNAAANNNTNNN
NNNNNNNNNNNNNNNNNNNNNNNNNNCNCNTTGNNNGTAANCNANNNGNACATGCAGTCCCCTCNNNNN
NNNGGCAGAGNNNNNNCCCNNNNNNNCCGGCCCCNNCCNNNNNNNNNNNCNNNGNNNNNNNANNNNNN
NNNNNNTGGTACNNNNCGNNNNNGGTTGTNTAAGAGGTCCACAACGNGGTCNGGNCACNNNNGNNNAN
NNNCNNAAAACNNNNNNNNNNNNANNTNNNTANNAGGNCNNNNCCNTCNNGNNANNAATTGCNATGACC
CAANNCACGGGGGCAGTCCCCGGCGCGGCGACCTTGANNACGTCCGGGGACAAGTATGGCGCNCNCCC
CGGGAAGGANNCNGCCGTANGTGCNNNNNANNNNNTCTTGNGANGTNGCCGNNATTCNCGTGGTCNNN
NNAGAATGNCCGNACCANNCGNANNNTGGTNATGNNAGTNNNNNNNNNNNNNNNNNNNNNNNNNNNNN
NNNNNNNNNNNNNNNNNNNNNNNNNNNNNNNNNNNNNNNNNGNNNNNNCNANNNAAGGANNTANNNCTNNTC
NTNNNNNNNGGACNNNACNNNNCNNNNNNNNACNCNNNCNCNNNNNNNNNNNGNTTTTTNNNCNNTATTNT
CCAGNACGCNGNGCNNNCATTCANGNGAACNNNGNNACGCGNNAATCGTANNATCTACCTGCCGCNNG
TNNNNNTACNGNNNNNNNCGGANNNTNNCNCGTANNNNNNNTCATGCGTNNTNTNNTCNCNAGNCNNGTT
CGNNNANANANTAGNANGTNTNNNNNNNNNNTGTNNCGTTCCGCNTGANNCNNCANNNNGNNGNNNTTN
TCANCGGCNNTANCTGCNGANNTAAGNCATANNNTNNATCNNTANCTNNNNNGGNGGGNNACTGTAAA
NGGNTAATTNGANGNTNATTNCNNNCACNTNNCTNNGNNTNCTNNNTNCCTGNNTNCNNAATTGGNNC
CANTGNNTGNCNATCTCTNGNTTNGNNNNNNTGNNNNTNNCAANNNGGNNNGTNANANNNCNATNTAA
NNTANANNNNCNNNNNNANNNCACNANNNNNGNAGNNACNANNNTTANANNNGTAGNNNANTAAGTCT
CCGANNTNNNNNNNNATAAGCGNNANACNNNNNNNNNNNNNNNANGANNACGNNTTGGNNNTNANNNNCNA
GNNAGACNNTNATGGNTNNNNNTCAANNNNTTCGTAGTGGTATAATNGNNNNNNNTTTCNCCAGNNTN
NNNGNNNNNNNNCNATANNANNNNNNNNGTGANNNNNNNNNNNANNNNCTCNNNAGGNANCANCTACTTNA
GCAATGGNGCGCAGNNNGAGANNNNGACNNGCCTTGGGTGGCAANCACNGCAATAAGTNNNGTTNANC
CTNNNAGTNNACATNNNNNCANNGNNNCNNNNNNANCNGANNGNNNNNNNNNNNNNNNNNNNNNNNN
NNNNNNNNNNNNNNNNNNNNNNNNNNNNNAGNNNANGGNNNGTNTNTGACNCATGTGTTTTTANNNGNN
NNNNNACGNNNNANCCCCGGTCGNNNNCNNATTGAANNCCTGNNGCATNNNNNTNCNAGAATGANNGC
NNNNNGNNTNGNCTNNCNTTTCAAGNAATCANNTAGGTANNNNGNTCNNGGNNNNNNNNCNNTCCCNN
ACTGAGGGTNNNNNNNCGCATCTGCTNNNNNNNNNNTNCGCNGGCGTNNCNNNNNAGCNGTATNGTGTNA
NNCANNNNNNNNNNNNNNNNNNTNNAACGANNNNNNNTNNNN
>Mang275
GTGNNNNNTAGCCCNNNNNAGTTAGACANNNCNNNNNNNNNNNNTNNNNNNNNNNNNNNNNNNNNNN
NNNNNNNNNNNNNNNNNNNNNNNNNNNNNNNNNNNNNNNNNNNNNNNNNNNNNNNNNNNNNNNNNNNNNNN
NNNNNNNNNNNNNNNNNNNNNNNNNNNNNNNNNNNNNNNNNNNNNNNNNNNNNNNNNNNNNNNNNNNNNNN
NNNNNNNNNNNNNNNNNNNNNNNNNNNNNNNNNNNNNNNNNNNNNNNNNNNNNNNNNNNNNNNNNNNNNNN
NNNNNNNNNNNNNNNNNNNNNNNNNNNNNNNNNNNNNNNNNNNNNNNNNNNNNCNTCTNNNAAANNNTNNN
NNNNNNNNNNNNNNNNNNNNNNNNNNCNCNTTGNNNGTAANCNANNNGNACATGCAGTCCCCTCNNNNN
NNNGGCAGAGNNNNNNNCCNNNNNNNCCGGCCCCNNCCNNNNNNNNNNNNNNNNGNNNNNNNANNNNNN
NNNNNNTGGTACNNNNCGNNNNNGGTTGTTTAAGAGGTCCACAACGNGGTCNNGNCACNNNNGNNNAN
NNNCNNNNAACNNNNNNNNNNNNNNNATNNNTANNAGGNCNNNNCCNTCNNNNNNNNNNANTGCNATGACC
CAANNCACGGNGGCNGTNCCCGGCGCGGCGACCTTGNTNANNTCCGGGGACAAGTATGGCNNNCNCCC
CGGGAAGGANNCNGCCGTANGTGCNNNNNANNNNNTCNTGNGANGTCGCCGNNATTNNCNTGGTCNNN
CNAGAATGNCCGNACCANNCGNANNNTGGTNATGNTAGTNNNNNNNNNNNNNNNNNNNNNNNNNNNNN
NNNNNNNNNNNNNNNNNNNNNNNNNNNNNNNNNNNNNNNNNGNNNNNNCNANNNAAGGNNNTANNNCTNNTN

NTNNNNNNNGGACNNNACNNNNCNNNNNNNACNCNNNCNCNNNNNNANNNGGTTTTTNNNCNNTATTNT
CCAGNACGCNGNGCNNNCATTCANGNGAACNNNGNNACGCGNNAATCGTANNATCTACCTGCCGCNNG
TNNNNNTACNGNNNNNNCGGANNNTNNCTCGTANNNNNNNCATGCGTNNTNTNNTCNCNAGNCNNGTT
CGNNNANANANTAGNANGTNTNNNNNNNNNNTGTNNCGTTCCGCNTGANNCNNCANNNNGNNGNNCTTN
NCANCGGCNNTANCTGCNGANGTAAGNCATANNNTNNATCNNTANCTNNNNNGGNGGGNNACTGTAAA
NGGNNAATTNGNNGNTNANTNCNNNCACNTNNCTNNGNNNNCTNNNTNCCTGNNTNCNNAATTGGNNN
CANTGNNTGNCNATCTCTGGNTTNGNNNNNNTNNNNNTNNCANNNNGGNNNNNNNNANNNCNATNTAA
NNTANANNNNCNNNNNNANNNCACNNNNNNNGNAGNNNCNANNNTTANANNNGTAGNNNAATAAGTCT
CCGANNTNNNNNNNATAAGCGNNANACNNNNNNNNNNNNNNANGANNACGNNTTGGNNNTNNNNNNNNA
GNNAGACNNTNATGGNTNNNNNTCAANNNNTTNGTAGTGGTATAATNGNNNNNNNTTTCNCCAGNNTN
NNNGNNNNNNNCNATANNANNNNNNNGNGANNNNNNNNNNNANNNNCTCNNNAGGNANCANCTACTNNA
GCAATGGNGCGCAGNNNGAGANNNNGACNNNCCTTGGGTGGCAANCACNGCAATAAGTNNNGTTNANC
CTNNNAGTNNACATNNNNNCANNGNNNCNNNNNNANCNGANNGNNNNNNNNNNNNNNNNNNNNNNNNN
NNNNNNNNNNNNNNNNNNNNNNNNNNNNNAGNNNANNGNNNGTNTNTGACNCATGTGTTTTTANNNGNN
NNNNNACGNNNNANCCCCGGTCGNNNNCNNATTGAANNCCTGNNGCATNNNNNTNCNAGAATGANNNC
NNNNNGNNTNGNCTNNCNNNTCAAGNAATCANNNAGNNNNNGCGNTCNNNNNNNNNNNNCNNTCCCNN
ACTGAGGGTNNNNNNNNCATCTGCTNNNNNNNNNNTNCGCNGGCNTNNCNNNNNAGCNGTATNGNGTNA
NNCANNNNNNNNNNNNNNNNNTNAAACNANNNNNNTNNNN
>Mang276
GTGNNNNNTAGCCCNNNNNAGTTAGACANNNCNNNNNNNNNNNNTNNNNNNNNNNNNNNNNNNNNNNN
NNNNNNNNNNNNNNNNNNNNNNNNNNNNNNNTNNNNNNNNNNNNNNNNNNNNNNNNNNNNNNNNNNNNNN
NNNNNNNNNNNNNNNNNNNNNNNNNNNNNNNNNNNNNNNNNNNNNNNNNNNNNNNNNNNNNNNNNNNNNN
NNNNNNNNNNNNNNNNNNNNNNNNNNNNNNNNNNNNNNNNNNNNNNNNNNNNNNNNNNNNNNNNNNNNNN
NNNNNNNNNNNNNNNNNNNNNNNNNNNNNNNNNNNNNNNNNNNNNNNNNNNNNNNTCTNNNAAANNNTNNN
NNNNNNNNNNNNNNNNNNNNNNNNNNCNCNNTGNNNGTAANCNANNNGNACATGCAGTCCCCTCNNNNN
NNNGGCAGAGNNNNNNNCCNNNNNNNCCGGCCCCNNCCNNNNNNNNNNNNNNNNNNNNNNNNNANNNNNN
NNNNNNTGGTACNNNNCGNNNNNGGTTGTNTNAGAGGTCCACAACGNGGTCNNGNNACNNNNGNNNAN
NNNCNNNAAACNGNNNNNNNNNNANNTNNNTANNAGGNCNNNNCCNTCNNGNNNNNNATTGCNATGACC
CAANNCACGGGGGCNGTNCCCGGCGCGGCGACCTTGANNANNTCCGGGNNCAAGTATGGCNNNCNCCC
CGGGAAGGANNCNGCCGTANGTGCNNNNNANNNNNTCNTGNGANGTCGCCGNNATTCNCGTGGTCNNN
CNAGAATGNCCNNACCANNCGNANNNTGGTNATGNNAGTNNNNNNNNNNNNNNNNNNNNNNNNNNNNN
NNNNNNNNNNNNNNNNNNNNNNNNNNNNNNNNNNNNNNNNNGNNNNNNCNANNNAAGGANNTANNNCTNNTN
NTNNNNNNNGGACNNNACNNNNCNNNNNNNACNCNNNCNCNNNNNNNNNNGGTTTTNNNNCNNTNTTNT
CCAGNACGCNGNGCNNNCATTCANGNGAACNNNGNNACGCGNNAATCGTANNATCTACCTGCCGCNNG
NNNNNNTNCNGNNNNTNCGGANNNTNNNNCGTANNNNNNNCATGCGTNNTNTNNTCNCNAGNCNNGTT
CGNNNANANANTAGNANGTNTNNNNNNNNNNTGTNNCGTTCCGCNTGANNCNNCANNNNGNNGNNNTTN
NCANCGGCNNTANCTNCNGANNTAAGNCATANNNTNNNTCNNTANCTNNNNNGGNGGGNNACTGTAAA
NGGNNAATTNGNNNNTNATTNCNNNCACNTNNCTNNGNNNNCTNNNTNCCTGNNTNCNNAATTGGNNC
CANTGNNTGNCNATCTCTGGNTTNGCNNNNNTGNNNNTNNCAANNNGGNNNNNNNNANNNCNATNTAA
NNTANANNNNCNNNNNNANNNCACNANNNNNNNAGNNANNANNNTTANANNNGTAGNNNANTAAGTCT
CCGANNTNNNNNNNATAAGCGNNANACNNNNNNNNNNNNNNANGANNNCGNNTTGGNNNTNNNNNNCNA
GNNAGACNNTNATGGNTNNNNNTCAANNNNTTCGTAGTGGTATAATNGNNNNNNNTTTCNCCAGNNTN
NNNGNNNNNNNCNATANNANNNNNNNGNGANNNNNNNNNNNANNNNCTCNNNAGGGANCANCTACTNNA
GCAATGGNGCGCAGNNNGAGANNNNGACNNNCCTTGGGTGGCANNNACNGCAATAAGTNNNGTTNANC
CTNNNAGTNNACATNNNNNCANNGNNNCNNNNNNANCNGANNGNNNNNNNNNNNNNNNNNNNNNNNNN
NNNNNNNNNNNNNNNNNNNNNNNNNNNNNANNNNNANGGNNNGTNTNTGACNCNTGTGTTTTTANNNGNN
NNNNNNNGNNNGANCCCCGGTCGNNNNCNNATTGAANNCCTGNNGCATNNNNNTNCNAGAATGANNNC
NNNNNGNNTNGNCTGNCNNNTCAAGNAATCANNNANGTNNNGCGNTCNNNNNNNNNNNNCNNNCCCNN
ACTGAGGGTNNNNNNNNCATCTGCTNNNNNNNNNNTNCGCNGNNGNNNCNNNNNNGCNGTATNNNGTNA
NNCANNNNNNNNNNNNNNNNNTNAANNNANNNNNNNTNNNN
>Mang277
GTGNNNNNTAGCCCNCNNNAGTTAGACANNNCNNNNNNNNNNNNTNNNNNNNNNNNNNNNNNNNNNNN
NNNNNNNNNNNNNNNNNNNNNNNNNNNNNNNTNNNNNNNNNNNNNNNNNNNNNNNNNNNNNNNNNNNNNN
NNNNNNNNNNNNNNNNNNNNNNNNNNNNNNNNNNNNNNNNNNNNNNNNNNNNNNNNNNNNNNNNNNNNNN

NNNNNNNNNNNNNNNNNNNNNNNNNNNNNNNNNNNNNNNNNNNNNNNNNNNNNNNNNNNNNNNNNNNNNN
NNNNNNNNNNNNNNNNNNNNNNNNNNNNNNNNNNNNNNNNNNNNNNNNNNNNCNTCTNNNAAANNNTNNN
NNNNNNNNNNNNNNNNNNNNNNNNNNNCNCNNTGNNNGTAANCNANNNGNACATGCAGTCCCCTCNNNNN
NNNGGCAGAGNNNNNNNCCNNNNNNNCCGNCCCCNNCCNNNNNNNNNNNNNNNNNNNNNNNNNANNNNNN
NNNNNNTGGTACNNNNCGNNNNNGGTTGTNTNAGAGGTCCACAACGNGGTCNNGNNACNNNNGNNNAN
NNNCNNNNAANNNNNNNNNNNNNANNTNNNTANNAGGNCNNNNCCNTCNNGNNNNNNANNGCNATGACC
CAANNCNCGGNGGNNGTNCCCGGNGNGGCGACCTTGANNACNTCCGGGGNCAAGTATGGCNNNCNCCC
CGGGAAGGANNCNGCCGTANGTGCNNNNNANNNNNTCNTGNGANGTCGCCGNNNTTNNCNTGGTCNNN
NNAGAATGNNCGNACCANNCGNANNNTGGTNATGNNAGTNNNNNNNNNNNNNNNNNNNNNNNNNNNNNN
NNNNNNNNNNNNNNNNNNNNNNNNNNNNNNNNNNNNNGNNNNGNNNNNNCNANNNAAGGNNNTANNNCTNNTN
NTNNNNNNGGACNNNACNNNNCNNNNNNNACNCNNNCNCNNNNNNANNNGGTTTTNNNNCNNTANTNT
CCAGNACGCNGNGCNNNCATTCANGNGAACNNNGNNACGCGNNAATCGTANNATCTACCTGCCGCNNG
NNNNNNTACNGNNNNNNCGGANNNTNNCTCGTANNNNNNTCANGCGTNNTNTNNTCNCNAGNCANGTT
CGNNNANANANTAGNANGTNTNNNNNNNNNNTGTNNCGTTCCGCNTGANNCNNCANNNNGNNGNNNTTN
NCANCGGCNNTANCTGCNGANNTAAGNCATANNNTNNNTCNNTANCTNNNNNGGNGGGNNACTGTAAA
NGGNNAATTNGNNNNTNANTNCNNNCACNTNNCNNNGNNNNCTNNNTNCCTGNNTNCNNAATTGGNNC
CANTGNNTGNCNATCTCTGGNTTNGNNNNNNTGNNNNTNNCAANNNGGNNNNNNANANNNCNATNTAA
NNTANANNNNCNNNNNNANNNCACNNNNNNNNNAGNNNNNANNNTTANANNNGTAGNNNANTAAGTCT
CCGANNTNNNNNNNATAAGCGNNANACNNNNNNNNNNNNNANGANNNCGNNTTGGNNNTNNNNNNNNA
GNNANACNNTNATGGNTNNNNNTCAANNNNTTNGTAGTGGTATAATNGNNNNNNNTTTCNCCAGNNTN
NNNGNNNNNNNNCNATANNANNNNNNNGNGANNNNNNNNNNNANNNNCTCNNNAGGGANCANCTACTTNA
GCANTGGNGCGCAGNNNGAGANNNNGACNNNCCTTGGGTGGCAANCACNGCAATAAGTNNNGTTNNNC
CTNNNAGTNNACATNNNNNCANNGNNNCNNNNNNANCNGANNGNNNNNNNNNNNNNNNNNNNNNNNNN
NNNNNNNNNNNNNNNNNNNNNNNNNNNNNNNGNNNANGGNNNGTNTNTGACNCNTGTNTTTTTANNNGNN
NNNNNACGNNNNANCCCCGGTCGNNNNCNNATTGAANNCCTGNNGCATNNNNNTNCNAGAATGANNNC
NNNNNGNNTNGNCTNNCNNNTCAAGNAATCANNNANGTNNNNNNNTCNNNGNNNNNNNNCNNNCCCNN
ACTGAGGGTNNNNNNNNCATCTGCTNNNNNNNNNNTNCGCNGGNGTNNCNNNNNAGCNGTATNNTGTNA
NNCANNNNNNNNNNNNNNNNNNTNAANNNANNNNNNTNNNN
>Mang278
GTGNNNNNNAGCCCNNNNNAGTTAGACANNNCNNNNNNNNNNNNTNNNNNNNNNNNNNNNNNNNNNNNN
NNNNNNNNNNNNNNNNNNNNNNNNNNNNNNNNNNNNNNNNNNNNNNNNNNNNNNNNNNNNNNNNNNNNNN
NNNNNNNNNNNNNNNNNNNNNNNNNNNNNNNNNNNNNNNNNNNNNNNNNNNNNNNNNNNNNNNNNNNNNN
NNNNNNNNNNNNNNNNNNNNNNNNNNNNNNNNNNNNNNNNNNNNNNNNNNNNNNNNNNNNNNNNNNNNNN
NNNNNNNNNNNNNNNNNNNNNNNNNNNNNNNNNNNNNNNNNNNNNNNNNNNNNTCTNNNAAANNNTNNN
NNNNNNNNNNNNNNNNNNNNNNNNNNNCNCNNTGNNNGTAANCNANNNGNACATGCAGTCCCCTCNNNNN
NNNNNCAGAGNNNNNNCNNNNNNNNNCCGNCCCCNNCCNNNNNNNNNNNNNNNNNNNNNNNNNANNNNNN
NNNNNNTGGTACNNNNCGNNNNNGGTTGTNTNAGAGGTCCACAACGNGGTCNGGNCACNNNNGNNNAN
NNNCNNNNAANNNNNNNNNNNNNANNTNNNTANNAGGNCNNGCCNTCNNGNNNNNNANTGCNATGACC
CAANNCACGGGGGNNGTNCCCGGCGNGGCGACCTTGANNANNTCCGGGGNCAAGTATGGCNNNCNCCC
CGGGAAGGANNCNGCCGTANGTGCNNNNNANNNNNTCNTGNGANGTNGCCGNNATTNNCNTGGTCNNN
CNNGAATGNNCGNACCANNCGNANNNTGGTNATGNNAGTANNNNNNNNNNNNNNNNNNNNNNNNNNNNN
NNNNNNNNNNNNNNNNNNNNNNNNNNNNNNNNNNNNNNNNNGNNNNNNCNANNNAAGGNNNTANNNCTNNTN
NTNNNNNNGGACNNNACNNNNCNNNNNNNACNCNNNCNCNNNNNNANNNGGTTTTTNNCCNNTATTNT
CCAGNACGCNGNGCNNNCNTTCANGNGAACNNNGNNACGCGNNAATCGTANNATCTACCTGCCGCNNG
NNNNNNTACNGNNNNNNCGGANNNTNNNNCGTANNNNNNTCATGCGTNNTNTNNTCNCNAGNCNNGTT
CGNNNANANANTAGNANGTNTNNNNNNNNNNTGTNNCGTTCCGCNTGANNCNNCANNNNGNNGNNCTTN
NCANCGGCNNTANCTGCNGANNTAAGNCATANNNTNNATCNNTANCTNNNNNGGNGGGNNACTGTAAA
NGGNNAATTGGNNNNTNATTNCNNACACNTNNCTNNGNNNNCTNNNTNCCTGNNTNCNNAATTGGNNC
CANTGNNTGNCNATCTCTGGTTTNGNNNNNNTNNNNNTNNCAANNNGGNNNGTNNNANNNCNATNTAA
NNTANANNNNCNNNNNNANNNCACNANNNNNGNAGNNNNNANNNTTANANNNGTAGNNNAATAAGTCT
CCGANNTNNNNNNNATAAGCGNNANACNNNNNNNNNNNNNANGANNNCGNNTTGGNNNTNNNNNNNCNA
GNNAGACNNTNATGGNTNNNNNTCAANNNNTTCGTAGTGGTATAATNGNNNNNNNTTTCNCCAGNNTN
NNNGNNNNNNNNCNATANNANNNNNNNGNGANNNNNNNNNNNANNNNCTCNNNAGGGANCANCTACTTNA
GCANTGGNGCGNAGNNNGAGANNNNGACNNNCCTTGGGTGGCAANCACNGNAATAAGTNNNGTTNANC

CTNNNAGTNNACATNNNNNCANNGNNNCNNNNNNANCNGANNGNNNNNNNNNNNNNNNNNNNNNNNNNNN
NNNNNNNNNNNNNNNNNNNNNNNNNNNNNANNNNANNGNNNGTNTNTGACNCATGTGTTTTTANNNGNN
NNNNNACGNNNNANCCCCGGTCGNNNNCNNATTGAANNCCTGNNGCATNNNNNTNCNAGAATGANNGC
NNNNNGNNTNGNCTNNCNNNTCAAGNAATCANNNANGTANNGCGNTCNNNGNNNNNNNNCNNTCCCNN
ACTGAGGGTNNNNNNNNCATCTGCTNNNNNNNNCTNCGCNGGNNNNNCNNNNNNGCNGTATNGTGTNA
NNCANNNNNNNNNNNNNNNNNNTNNAACNANNNNNNNNNNNN
>Mang279
GTGNNNNTAGCCCNNNNNAGTTAGACANNNCNNNNNNNNNNNNNNTNNNNNNNNNNNNNNNNNNNNNNNN
NNNNNNNNNNNNNNNNNNNNNNNNNNNNNNNNNNNNNNNNNNNNNNNNNNNNNNNNNNNNNNNNNNNNNN
NNNNNNNNNNNNNNNNNNNNNNNNNNNNNNNNNNNNNNNNNNNNNNNNNNNNNNNNNNNNNNNNNNNNNN
NNNNNNNNNNNNNNNNNNNNNNNNNNNNNNNNNNNNNNNNNNNNNNNNNNNNNNNNNNNNNNNNNNNNNN
NNNNNNNNNNNNNNNNNNNNNNNNNNNNNNNNNNNNNNNNNNNNNNNNNNNCNTCTNNNAAANNNTNNN
NNNNNNNNNNNNNNNNNNNNNNNNNNCNCNTTGNNNGTAANCNANNNGNACATGCAGTCCCCTCNNNNN
NNNNNCAGAGNNNNNNNCCNNNNNCNCCGNCCCCNNCCNNNNNNNNNNNCNNNGNNNNNNNANNNNNN
NNNNNNTGGTACNNNNCGNNNNNGGTTGTNTNAGAGGTCCACAACGNGGTCNNGNCACNNNNGNNNAN
NNNCNNNNANNNNNNNNNNNNNNNNNTNNNTANNAGGNNNNNNCCNTCNNNNNNNNNNATTGCNATGACC
CAANNCACGGNGGNAGTNCCCGGCGCGGCGACCTTGANNACGTCCGGGGNCAAGTATGGCNNNCNCCC
CGGGAAGGANNCNGCCGTANGTGCNNNNNANNNNNTCNTGNGANGTCGCCGNNATTNNCGTGGTCNNN
NNAGAATGNNCGNACCANNCGNANNNTGGTNATGNNAGTNNNNNNNNNNNNNNNNNNNNNNNNNNNNNN
NNNNNNNNNNNNNNNNNNNNNNNNNNNNNNNNNNGNNNNGNNNNNNCNANNNAAGGANNTANNNCTNNTN
NTNNNNNNGGACNNNACNNNNCNNNNNNNACNCNNNCNCNNNNNNANNNGNTTTTTNNNCNNTATTNT
CCAGNANGCNGNGCNNNCATTCANGNGAACNNNGNNACGCGNNAATCGTANNATCTACCTGCCGCNNG
NNNNNNTACNGNNNNNNCGGANNNTNNNNCGTANNNNNNTCATGCGTNNTNTNNTCNCNAGNCANGTT
CGNNNANANANTAGNANGTNTNNNNNNNNNNTGTNNCGTTCCGCNTGACNCNNCANNNNGNNGNNNTTN
TCANCGGCNNTANCTGCNGANNTAAGNCATANNNTNNNTCNNTANCTNNNNNGGNGGGNNACTGTAAA
NGGNNAATTNGNNNNTNANTNCNNNCACNTNNCTNNGNNTNCTNNNTNCCTGNNTNCNNAATTGGNNC
CANTGNNTGNCNATCTCTGGNTTNGCNNNNNTNNNNNTNNCANNNNGGNNNGTNANANNNCNATNTAA
NNTANANNNNCNNNNNNANNNCACNNNNNNNGNAGNNNCNANNNTTANANNNGTAGNNNANTAAGTCN
CCGANNTNNNNNNNATAAGCGNNANACNNNNNNNNNNNNNANGANNACGNNTTGGNNNTNNNNNNCNA
GNNAGACNNTNATGGNTNNNNNTCAANNNNTTCGTAGTGGTATAATNGNNNNNNNTTTCNCCAGNNTN
NNNGNNNNNNNCNATANNANNNNNNNGNGANNNNNNNNNNNANNNNCTCNNNAGGGANCANCTACTNNA
GCANTGGNGCGCAGNNNGAGANNNNGACCAGCCTTGGGTGGCAANCACNGCAATAAGTNNNGTTNANN
CTNNNAGTNNACATGNNNNCANNGNNNCNNNNNNANCNGANNGNNNNNNNNNNNNNNNNNNNNNNNNNNN
NNNNNNNNNNNNNNNNNNNNNNNNNNNNNANNNNANGGNNNGTNTNTGNCNCATGTGTTTTTANNNGNN
NNNNNACGNNNNANCCCCGGTCGNNNNCNNATTGAANNCCTGNNGCATNNNNNTNCNAGAATGANNGC
NNNNNGNNTNGNCTNNCNNNTNAAGNAATCAANNAGNNNNCGCGNTCNNNNNNNNNNNNNCNNTCCCNN
ACTGAGGGTNNNNNNNNCATCTGCTNNNNNNNNCTNCGCNGGCGTNNCNNNNNNGCNGTATNNNGTNA
NNCANNNNNNNNNNNNNNNNNNNNNNNCNANNNNNNNTNNNN
>Mang280
GTGNNNNTAGCCCNNNNNAGTTAGACANNNCNNNNNNNNNNNNNNTNNNNNNNNNNNNNNNNNNNNNNNN
NNNNNNNNNNNNNNNNNNNNNNNNNNNNNNNNNTNNNNNNNNNNNNNNNNNNNNNNNNNNNNNNNNNNNN
NNNNNNNNNNNNNNNNNNNNNNNNNNNNNNNNNNNNNNNNNNNNNNNNNNNNNNNNNNNNNNNNNNNNNN
NNNNNNNNNNNNNNNNNNNNNNNNNNNNNNNNNNNNNNNNNNNNNNNNNNNNNNNNNNNNNNNNNNNNNN
NNNNNNNNNNNNNNNNNNNNNNNNNNNNNNNNNNNNNNNNNNNNNNNNNNNNTCTNNNAAANNNTNNN
NNNNNNNNNNNNNNNNNNNNNNNNNNCNCNTTGNNNGTAANCNANNNGNACATGCAGTCCCCTCNNNNN
NNNGGCAGAGNNNNNNNCCNNNNNNNCCGNCCCCNNCCNNNNNNNNNNNNNNNNNNTNNNNNNANNNNNN
NNNNNNTGGTACNNNNCGNNNNNGGTTGTTTNAGAGGTCCACAACGNGGTCNNGNNACNNNNGNNNAN
NNNCNNNAAANNNNNNNNNNNNNANNTNNNTANNAGGNCNNNNCCNTCNNNNNANNNATNGCNATGACC
CAANTCACGGGGGNNGTCCCCGGCGNNGNGACCTTGANNANGTCCGGGGNCAAGTATGGCGNNCNCCC
CGGGAAGGANNCNGCCGTANGTGCNNNNNANNNNNTCNTGNGANGTNGCCGNNATTNNCGTGGTCNNN
CNAGAATGNCCGNACCANNCGNANNNTGGTNATGNNAGTNNNNNNNNNNNNNNNNNNNNNNNNNNNNNN
NNNNNNNNNNNNNNNNNNNNNNNNNNNNNNNNNNGNNNNGNNNNNNCNANNNAAGGNNNTANNNCTNNTN
NTNNNNNNGGACNNNACNNNNCNNNNNNNACNCNNNCNCNNNNNNNNNNNGNTTTTNNNNCNNTANTNT
CCAGNACGCNGNGCNNNCATTCANGNGAACNNNGNNACGCGNNAATCGTANNATCTACCTGCCGCNNG

TNNNNNTACNGNNNNNNCGGANNNTNNCTCGTANNNNNNTCATGCGTNNTNTNNTCNCNAGNCNNGTT
CGNNNANANANTAGNANGTNTCNNNNNNCTTGTNNCGTTCCGCNTGACNCNNCANNNNGNNGNNCTTN
TCANCGGCNNTANCTGCNGANNTAAGNCATANNNTNNATCNNTANCTNNNNNGGNGGGNNACTGTAAA
NGGNNAATTNGNNNNTNATTNCCNNCACNTNNCTNNGNNNNCTNNNTNCCTGNNTNCNNAATTGGNNC
CANTGNNTGNCNATCTCTGGNTTNGNNNNNNTNNNNNTNNCANNNNGGNNNGTNANANNNCNATNTAA
NNTATANNNNCNNNNNNANNNCACNNNNNNNGNAGNNNCNANNNTTANANNNGTAGNNNAATAAGTCT
CCGANNTNNNNNNNATAAGCGNNANACNNNNNNNNNNNNNANGANNACGNNTTGGNNNTNNNNNNNNA
GNNAGACNNTNATGGNTNNNNNTCAANNNNTTCGTAGTGGTATAATNGNNNNNNNTTTCNCCAGNNTN
NNNGNNNNNNNNCNATANNANNNNNNNGNGANNNNNNNNNNNANNNNCTCNNNAGGGANCANCTACTNNA
GCAATGGNGCGCAGNNNGAGANNNNGACNNNCCTTGGGTGGCAANCACNGCAATAAGTNNNGTTNANC
CTNNNAGTNNACATNNNNNCANNGNNNCNNNNNNANCNGANNGNNNNNNNNNNNNNNNNNNNNNNNNN
NNNNNNNNNNNNNNNNNNNNNNNNNNNNNANNNNANGGNNNGTNTNTGACNCATGNNTTTTTANNNGNN
NNNNNNNGNNNNANCCCCGGTCGNNNNCNNATTGAANNCCTGNNGCATNNNNNTNCNAGAATGANNNC
NNNNNGNNTNGNCTGNCNTTTCAAGNAATCANNNAGNTANNNNGNTCNNNNNNNNNNNNCNNNCCCNN
ACTGAGGGTNNNNNNNNCATCTGCTNNNNNNNNCTNCGCNGGCGTNNCNNNNNNGCNGTATNGNGTNN
NNCANNNNNNNNNNNNNNNNNCNNAACNNNNNNNNNTNNNN
>Mang281
GTGNNNNNTAGCCCNNNNNAGTTAGACANNNCNNNNNNNNNNNNNTNNNNNNNNNNNNNNANNNNNNNN
NNNNNNNNNNNNNNNNNNNNNNNNNNNNNNNNTNNNNNNNNNNNNNNNNNNNNNNNNNNNNNNNNNNNN
NNNNNNNNNNNNNNNNNNNNNNNNNNNNNNNNNNNNNNNNNNNNNNNNNNNNNNNNNNNNNNNNNNNNN
NNNNNNNNNNNNNNNNNNNNNNNNNNNNNNNNNNNNNNNNNNNNNNNNNNNNNNNNNNNNNNNNNNNNN
NNNNNNNNNNNNNNNNNNNNNNNNNNNNNNNNNNNNNNNNNNNNNNNNNNNNNNCNTCTNNNAAANNNTNNN
NNNNNNNNNNNNNNNNNNNNNNNNNNNNCNCNTTGNNNGTAANCNANNNGNACATGCAGTCCCCTCNNNNN
NNNNNCAGAGNNNNNNNCCNNNNNNNCCGNCCCCNNCCNNNNNNNNNNNNCNNNGNNNNNNNANNNNNN
NNNNNNTGGTACNNNNCGNNNNNGGTTGTNTAAGAGGTCCACAACGNGGTCNGGNCACNNNNGNNNAN
NNNCNNNNANNNNNNNNNNNNNNNANNTNNNTANNAGGNCNNNNCCNTCNNGNNNNNAANTGCNATGACC
CAANNCACGGGGGCNGTNCCCGGCGCGGCGACCTTGNNNANGTCCGGGGACAAGTATGGCNNNCNCCC
CGGGAAGGANNCNGCNGTANGTGCNNNNNANNNTNTCNTGNGANGTCGCCGNNATTNNNNTGGTCNNN
CNAGAATGNCCGNACCANNCGNANNNTGGTNATGNNAGTNNNNNNNNNNNNNNNNNNNNNNNNNNNNN
NNNNNNNNNNNNNNNNNNNNNNNNNNNNNNNNNNNNNNNNNCNGNNNNNNCNANNNAAGGNNNTANNNCTNNTN
NTNNNNNNGGACNNNACNNNNCNNNNNNNACNCNNNCNCNNNNNNNNNNGNTTTTTNNNCNNTANTNT
CCAGNANGCNGNGCNNNCATTCANGNGAACNNNGNNACGCGNNAATCGTANNATCTACCTGCCGCNNG
TNNNNNTACNGNNNNNNCGGANNNTNNNNCGTANNNNNNTCATGCGTNNTNTNNTCNCNAGNCANGTT
CGNNNANANANTAGNANGTNTNNNNNNNNNNTGTNNCGTTCCGCNTGANNCNNCANNNNGNNGNNCTTN
TCANCGGCNNTANCTGCNGANNTAAGNCATANNNTNNNTCNNTANCTNNNNNGGNGGGNNACTGTAAA
NGGNNAATTNGNNNNTNANTNCNNNCACNTNNCNNNGNNNNCTNNNTNCCTGNNTNCNNAATTGGNNC
CANTGNNTGNCNATCTCTNGNTTNGNNNNNNTNNNNNTNNCANNNNGGNNNGTNNNANNNCNATNTAA
NNTANANNNNCNNNNNNANNNCACNNNNNNNGNAGNNACNANNNTTANANNNGTAGNNNANTAAGTCT
CCGANNTNNNNNNNATAAGCGNNANACNNNNNNNNNNNNNANGANNACGNNTTGGNNNTNNNNNNNCNA
GNNAGACNNTNATGGNTNNNNNTCAANNNNTTNGTAGTGGTATAATNGNNNNNNNTTTCNCCAGNNTN
NNNGNNNNNNNNCNATANTANNNNNNNGNGANNNNNNNNNNNANNNNCTCNNNAGGNANCANCTACTNNA
GCANTGGNGCGCAGNNNGAGANNNNGACNNGCCTTGGGTGGCAANNACNGCAATAAGTNNNGTTNANC
CTNNNAGTNNACATNNNNNCANNGNNNCNNNNNNANCNGANNGNNNNNNNNNNNNNNNNNNNNNNNNN
NNNNNNNNNNNNNNNNNNNNNNNNNNNNNAGNNNANNGNNNGTNTNTGACNCATGTGTTTTTANNNGNN
NNNNNACGNNNNANCCCCGGTCGNNNNCNNATTGAANNCCTGNNGCATNNNNNTNCNAGAATGANNGC
NNNNNGNNTNGNCTNNCNTTTCAAGNAATCANNNAGNNNNNNNNNNTCNNNGNNNNNNNNCNNTCCCNN
ACTGAGGGTNNNNNNNNCATCTGCTNNNNNNNNNTNCGCNGGNGTNNCNNNNNAGCNGTATNGNGTNA
NNCANNNNNNNNNNNNNNNNNNTNAAACAANNNNNNNTNNNN
>Mang283
GTGNNNNNTAGCCCNNNNNAGTTAGACANNNCNNNNNNNNNNNNNTNNNNNNNNNNNNNNNNNNNNNNN
NNNNNNNNNNNNNNNNNNNNNNNNNNNNNNNNTNNNNNNNNNNNNNNNNNNNNNNNNNNNNNNNNNNNN
NNNNNNNNNNNNNNNNNNNNNNNNNNNNNNNNNNNNNNNNNNNNNNNNNNNNNNNNNNNNNNNNNNNNN
NNNNNNNNNNNNNNNNNNNNNNNNNNNNNNNNNNNNNNNNNNNNNNNNNNNNNNNNNNNNNNNNNNNNN
NNNNNNNNNNNNNNNNNNNNNNNNNNNNNNNNNNNNNNNNNNNNNNNNNNNNNNCNTCTNNNAAANNNTNNN

NNNNNNNNNNNNNNNNNNNNNNNNNCNCNTTGNNNGTAANCNANNNGNACATGCAGTCCCCTCNNNNN
NNNGGCAGAGNNNNNNCCCNNNNNCNCCGGCCCCNNCCNNNNNNNNNNNCNNNNNNNNNNNANNNNNN
NNNNNNTGGTACNNNNCGNNNNNGGTTGTTTNAGAGGTCCACAACGNGGTCNNGNCACNNNNGNNNAN
NNNCNNAAAACNGNNNNNNNNNANNTNNNTANNAGGNCNNNNCCNTCNNGNNNNTNATTGCNATGACC
CAANTCACGGGGGCAGTCCCCGGCGCGGCGACCTTGANNACGTCCGGGGACAAGTATGGCGCNCNCCC
CGGGAAGGANNCNGCCGTANGTGCNNNNNANNNNNTCNTGNGACGTCGCCGNNATTCNCGTGGTCNAN
CNAGAATGCCCGNACCANNCGNANNNTGGTNATGNNAGTNNNNNNNNNNNNNNNNNNNNNNNNNNNNN
NNNNNNNNNNNNNNNNNNNNNNNNNNNNNNNNGNNNNGNNNNNNCNANNNAAGGANNTANNNCTNNTN
NTNNNNNNGGACNNNACNNNNCNNNNNNNACNCNNNCNCNNNNNNANNNGGTTTTTNNNCNNTATTNT
CCAGNACGCNGNGCNNNCATTCANGNGAACNNNGNNACGCGNNAATCGTANNATCTACCTGCCGCNNG
TNNNNNTACNGNNNNNNCGGANNNTNNCTCGTANNNNNNTCATGCGTNNTNTNNTCNCNAGNCANGTT
CGNNNANANANTAGNANGTNTNNNNNNNNNTGTNNCGTTCCGCNTGANNCNNCANNNNGNNGNNCTTN
TCANCGGCNNTANCTGCNGANNTAAGNCATANNNTNNATCNNTANCTNNNNNGGNGGGNNACTGTAAA
NGGNNAATTNGAGGNTNATTNCNNNCACNTNNCTNNGNNTNCTNNNTNCCTGNNTNCNNAATTGGNNC
CANTGNNTGNCNATCTCTGGNTTNGCNNNNNTNNNNNTNNCAANNNGGNNNGTNNNANNNCNATNTAA
NNTANANNNNCNNNNNNANNNCACNANNNNNNAGNNNCNANNNTTANANNNGTAGNNNAATAAGTCT
CCGANNTNNNNNNNATAAGCGNNANACNNNNNNNNNNNNNANGANNACGNNTTGGNNNTNNNNNNCNA
GNNAGACNNTNATGGNTNNNNNTCAANNNNTTCGTAGTGGTATAATNGNNNNNNNTTTCNCCAGNNTN
NNNGNNNNNNNCNATANNANNNNNNNGNGANNNNNNNNNNANNNNCTCNNNAGGGANCANCTACTTNA
GCAANGGNGCGCAGNNNGAGANNNNGACNNGCCTTGGGTGGCAANCACNGCAATAAGTNNNGTTNANC
CTNNNAGTNNACATNNNNNCANNGNNNCNNNNNNANCNGANNGNNNNNNNNNNNNNNNNNNNNNNNN
NNNNNNNNNNNNNNNNNNNNNNNNNNNNAGNNNANGGNNNGTNTNTGACNCATGTGTTTTTANNNGNN
NNNNNACGNNNGANCCCCGGTCGNNNNCNNATTGAANNCCTGNNGCATNNNNNTNCNAGAATGANNGC
NNNNNGNNTNGNCTNNCNNNTCAAGNAATCANNNAGGTNNCGCGNTCNNNGNNNNNNNNCNNTCCCNN
ACTGAGGGTNNNNNNCGCATCTGCTNNNNNNNNCTTCGCNGGCGTNNCNNCNNAGCNGTATNGNGTNA
NNCANNNNNNNNNNNNNNNTNAAACNANNNNNNNTNNNN
>Mang284
GTGNNNNNTAGCCCNNNNNAGTTAGACANNNCNNNNNNNNNNNNTNNNNNNNNNNNNNNNNNNNNNNN
NNNNNNNNNNNNNNNNNNNNNNNNNNNNNNTNNNNNNNNNNNNNNNNNNNNNNNNNNNNNNNNNNNNN
NNNNNNNNNNNNNNNNNNNNNNNNNNNNNNNNNNNNNNNNNNNNNNNNNNNNNNNNNNNNNNNNNNNN
NNNNNNNNNNNNNNNNNNNNNNNNNNNNNNNNNNNNNNNNNNNNNNNNNNNNNNNNNNNNNNNNNNNN
NNNNNNNNNNNNNNNNNNNNNNNNNNNNNNNNNNNNNNNNNNNNNNNNNNCNTCTNNNAAANNNTNNN
NNNNNNNNNNNNNNNNNNNNNNNNNCNCNTTNNNNGTAANCNANNNGNACATGCAGTCCCCTCNNNNN
NNNGGCAGAGNNNNNNNCCNNNNNNNCCGNCCCCNNCCNNNNNNNNNNNNNNNNNNNNNNNANNNNNN
NNNNNNTGGTACNNNNCGNNNNNGGTTGTTTAAGAGGTCCACAACGNGGTCNNNNCACNNNNGNNTAN
NNNCNNNNAACNNNNNNNNNNNANNTNNGTANNAGGNNNNNNCCNTCNNGNNNNNNATTGCNATGACC
CAANTCACGGGGGNAGTNCCCGGCGNGGCGACCTTGANNACGTCCGGGGACAAGTATGGCNNNCNCCC
CGGGAAGGANNCNGCCGTANGTGCNNNNNANNNNNTCNTGNGANGTNGCCGNNATTCNCGTGGTCNNN
CNAGAATGNCCNNACCANNCGNANNNTGGTNATGNNAGTNNNNNNNNNNNNNNNNNNNNNNNNNNNNN
NNNNNNNNNNNNNNNNNNNNNNNNNNNNNNNNGNNNNGNNNNNNCNANNNAAGGNNNTANNNCTNNTN
NTNNNNNNGGACNNNACNNNNCNNNNNNNACNCNNNCNCNNNNNNANNNGNTTTTNNNNCNNTANTNT
CCAGNACGCNGNGCNNNCATTCANGNGAACNNNGNNACGCGNNAATCGTANNATCTANCTGCCGCNGG
TNNNNNTACNGNNNNNNCGGANNNTNNNNCGTANNNNNNTCATGCGTNNTNTNNTCNCNAGNCANGTT
CGNNNANANANTAGNANGTNTNNNNNNNNNTGTNNCGTTCCGCNTGANNCNNCANNNNGNNGNNCTTN
TCANCGGCNNTANCTGCNGANNTAAGNCATANNNTNNNTCNNTANCTNNNNNGGNGGGNNACTGTAAA
NGGNNAATTNGNNGNTNANTNCCNNCACNTNNCNNNGNNTNCTNNNTNCCTGNNTNCNNAATTGGNNC
CANTGNNTGNCNATCTCTNGNTTNGNNNNNNTGNNNNTNNCANNNNGGNNNGTNNNANNNCNATNTAA
NNTANANNNNCNNNNNNANNNCACNNNNNNNNAGNNNCNANNNTTANANNNGTAGNNNAATAAGTCT
CCGANNTNNNNNNNATAAGCGNNANACNNNNNNNNNNNNNANGANNACGNNTTGGNNNTNNNNNNCNA
GNNAGACNNTNATGGNTNNNNNTCAANNNNTTCGTAGTGGTATAATNGNNNNNNNTTTCNCCAGNNTN
NNNGNNNNNNNCNATANNANNNNNNNGNGANNNNNNNNNNANNNNCTCNNNAGGNANCANCTACTTNA
GCAATGGNGCGNAGNNNGAGANNNNGACNNNCCTTGGGTGGCAANCACNGCAATAAGTNNNGTTNANC
CTNNNAGTNNACATNNNNNCANNGNNNCNNNNNNANCNGANNGNNNNNNNNNNNNNNNNNNNNNNNN
NNNNNNNNNNNNNNNNNNNNNNNNNNNNAGNNNANNGNNNGTNTNTGACNCATGTGTTTNTANNNGNN

NNNNNACGNNNNANCCCCGGTCGNNNNCNNATTGAANNCCTGNNGCATNNNNNTNCNAGAATGANNGC
NNNNNGNNTNGNCTNNCNNNTCAAGNAATCAANNAGGTANNNNGNTCNNNNNNNNNNNNCNNTCCCNN
ACTGAGGGTNNNNNNCNCANCTGCTNNNNNNNNNNTNCGCNGGCGTNNCNNNNNNGCNGTATNGTGTNA
NNCANNNNNNNNNNNNNNNNNTNNAACAANNNNNNNTNNNN
>Mang286
GTGNNNNNTAGCCCNNNNNAGTTAGACANNNCNNNNNNNNNNNNTNNNNNNNNNNNNNNNNNNNNNNN
NNNNNNNNNNNNNNNNNNNNNNNNNNNNNNNTNNNNNNNNNNNNNNNNNNNNNNNNNNNNNNNNNNNNN
NNNNNNNNNNNNNNNNNNNNNNNNNNNNNNNNNNNNNNNNNNNNNNNNNNNNNNNNNNNNNNNNNNNNN
NNNNNNNNNNNNNNNNNNNNNNNNNNNNNNNNNNNNNNNNNNNNNNNNNNNNNNNNNNNNNNNNNNNNN
NNNNNNNNNNNNNNNNNNNNNNNNNNNNNNNNNNNNNNNNNNNNNNNNNNNNNNTCTNNNAAANNNTNNN
NNNNNNNNNNNNNNNNNNNNNNNNNNNCNCNTTGNNNGTAANCNANNNGNACATGCAGTCCCCTCNNNNN
NNNGGCAGAGNNNNNNNCCNNNNNNNNCCGGCCCCNNCCNNNNNNNNNNNNCANNGNNNNNNNANNNNNN
NNNNNNTGGTACNNNNCGNNNNNGGTTGNTTAAGAGGTCCACAACGNGGTCNGGNNACNNNNGNNNAN
NNNCNNNNAACNGNNNNNNNNNNANNTNNNTANNAGGNCNNNNCCCTCNNGNNNNTNANTGCNATGACC
CAANTCACGGGGGCAGTNCCCGGCGCGGCGACCTTGNNNACGTCCGGGGACAAGTATGGCGNNCNCCC
CGNGAAGGANNCNGCCGTANGTGCNNNNNANNNNNTCNTGNGANGTCGCCGNNATTCNCGTGGTCNNN
CNAGAATGNCCNNACCANNCGNANNNTGGTNATGNNAGTNNNNNNNNNNNNNNNNNNNNNNNNNNNNN
NNNNNNNNNNNNNNNNNNNNNNNNNNNNNNNNNNNGNNNNGNNNNNNCNANNNAAGGNNNTANNNCTNNTN
NTNNNNNNNGGACNNNACNNNNCNNNNNNNNACNCNNNCNCNNNNNNNNNNNGNTTTTNNNNCNNTANTNT
CCAGNACGCNGNGCNNNCATTCANGNGAACNNNGNNACGCGNNAATCGTANNATCTACCTGCCGCNNG
TNNNNNTNCNGNNNNNNCGGANNNTNNNNCGTANNNNNNNCATGCGTNNTNTNNTCNCNAGNCNNGTT
CGNNNANANANTAGNANGTNTNNNNNNNNNNTGTNNCGTTCCGCNTGANNCNNCANNNNGNNGNNCTTN
NCANCGGCNNTANCTGCNGANNTAAGNCATANNNTNNNTCNNTANCTNNNNNGGNGGGNNACTGTAAA
NGGNNAATTNGNNGNTNANTNCCNNCACNTNNCTNNGNNNNCTNNNTNCCTGNNTNCNNAATTGGNNC
CANTGNNTGNCNATCTCTNGNTTNGNNNNNNTGNNNNTNNCAANNNGGNNNGTNANANNNCNATNTAA
NNTATANNNNCNNNNNNANNNCACNNNNNNNNNAGNNNCNANNNTTANANNNGTAGNNNANTAAGTCT
CCGANNTNNNNNNNATAAGCGNNANACNNNNNNNNNNNNNANGANNACGNNTTGGNNNTNNNNNNCNA
GNNAGACNNTNATGGNTNNNNNTCAANNNNTTNGTAGTGGTATAATNGNNNNNNNTTTCNCCAGNNTN
NNNGNNNNNNNCNATANNANNNNNNNGNGANNNNNNNNNNANNNNCTCNNNAGGGANCANCTACTNNA
GCAANGGNGCGCAGNNNGAGANNNNGACNNNCCTTGGGTGGCAANCACNGCAATAAGTNNNGTTNANC
CTNNNAGTNNACATNNNNNCANNGNNNCNNNNNNANCNGANNGNNNNNNNNNNNNNNNNNNNNNNNNN
NNNNNNNNNNNNNNNNNNNNNNNNNNNNNNANNNNANNGNNNGTNTNTGACNCATGTGTTTTTANNNGNN
NNNNNACGNNNNANCCCCGGTCGNNTNCNNATTGAANNCCTGNNGCATNNNNNTNCNAGAATGANNGC
NNNNNGNNTNGNCTNNCNNNTCAAGNAATCANNNAGNNANNNNGNTCNNNGNNNNNNNNNCNNTCCCNN
ACTGAGGGTNNNNNNNNCATCTGCTNNNNNNNNNNTNCGCNGGNGTNNCNNNNNAGCNGTATNGNGTNA
NNCANNNNNNNNNNNNNNNNNNTNAAACGANNNNNNNTNNNN
>Mang287
GTGNNNNNTAGCCCNNNNNAGTTAGACANNNCNNNNNNNNNNNNTNNNNNNNNNNNNNNNNNNNNNNN
NNNNNNNNNNNNNNNNNNNNNNNNNNNNNNNTNNNNNNNNNNNNNNNNNNNNNNNNNNNNNNNNNNNNN
NNNNNNNNNNNNNNNNNNNNNNNNNNNNNNNNNNNNNNNNNNNNNNNNNNNNNNNNNNNNNNNNNNNNN
NNNNNNNNNNNNNNNNNNNNNNNNNNNNNNNNNNNNNNNNNNNNNNNNNNNNNNNNNNNNNNNNNNNNN
NNNNNNNNNNNNNNNNNNNNNNNNNNNNNNNNNNNNNNNNNNNNNNNNNNNNNCNTCTNNNAAANNNTNNN
NNNNNNNNNNNNNNNNNNNNNNNNNNNCNCNTTGNNNGTAANCNANNNGNACATGCAGTCCCCTCNNNNN
NNNGGCAGAGNNNNNNNCCNNNNNNNNCCGGCCCCNNCCNNNNNNNNNNNNNNNNNGNNNNNNNANNNNNN
NNNNNNTGGTACNNNNCGNNNNNGGTTGTNTAAGAGGTCCACAACGNGGTCNGGNNACNNNNGNNNAN
NNNCNNNNAACNNNNNNNNNNNNNANNTNNNTANNAGGNCNNNNCCNTCNNNNNNNNNAANTGCNATGACC
CAANNCACGGGGGCNGTNCCCGGCGCGGCGACCTTGANNANGTCCGGGGNCAAGTATGGCGNNCNCCC
CGGGAAGGANNCNGCNGTANGTGCNNNNNANNNNNTCNTGNGANGTCGCCGNNATTNNCGTGGNCNNN
CNAGAATGNCCNNACCANNCGNANNNTGGTTATGNNAGTNNNNNNNNNNNNNNNNNNNNNNNNNNNNN
NNNNNNNNNNNNNNNNNNNNNNNNNNNNNNNNNNNNNGNNNNNNCNANNNAAGGNNNTANNNCTNNTN
NTNNNNNNNGGACNNNACNNNNCNNNNNNNNACNCNNNCNCNNNNNNNANNNGNTTTTTNNNCNNTATTNT
CCAGNACGCNGNGCNNNCNTTCANGNGAACNNNGNNACGCGNNAATCGTANNATCTACCTGCCGCNGG
NNNNNNTACNGNNNNNNCGGANNNTNNCTNGTANNNNNNTCANGCGTNNTNTNNTCNCNAGNCNNGTT
CGNNNANANANTAGNANGTNTNNNNNNNNNNTGTNNCGTTCCGCNTGANNCNNCANNNNGNCGNNCTTN

NCANCGGCNNTANCTGCNGANNTAAGNCATANNNTNNNTCNNTANCTNNNNNGGNGGGNNACTGTAAA
NGGNNAATTNGNNGNTNANTNCNNNCACNTNNCNNNGNNNNCTNNNTNCCTGNNTNCNNAATTGGNNC
CANTGNNTGNCNATCTCTGGNTTNGNNNNNNTNNNNNTNNCANNNNGGNNNGTNANANNNCNATNTAA
NNTANANNNNCNNNNNNANNNCACNNNNNNNGNAGNNACNANNNTTANANNNGTAGNNNANTAAGTCT
CCGANNTNNNNNNNATAAGCGNNANACNNNNNNNNNNNNNNANGANNNCGNNTTGGNNNTNNNNNNCNA
GNNAGACNNTNATGGNTNNNNNTCAANNNNTTCGTAGTGGTATAATNGNNNNNNNTTTCNCCAGNNTN
NNNGNNNNNNNCNATANNANNNNNNNGNGANNNNNNNNNNNANNNNCTCNNNAGGGANCANCTACTNNA
GCANTGGNGCGNAGNNNGAGANNNNGACNNNCCTTGGGTGGCAANNACNGCAATAAGTNNNGTTNANC
CTNNNAGTNNACATNNNNNCANNGNNNCNNNNNNANCNGANNGNNNNNNNNNNNNNNNNNNNNNNNNNN
NNNNNNNNNNNNNNNNNNNNNNNNNNNNNNNAGNNNANGGNNNGTNTNTGACNCATGNNTTTTANNNGNN
NNNNNACGNNNNANCCCCGGTCGNNNNCNNATTGAANNCCTGNNGCATNNNNNNTNCNAGAATGANNGC
NNNNNGNNTNGNCTNNCNTTTCAAGNAANCANNNAGNNANNNNGNTCNNNGNNNNNNNNCNNTCCCNN
ACTGAGGGTNNNNNNNNCATCTGCTNNNNNNNNNNTNCGCNGGCGTNNCNNNNNAGCNGTATNGNGTNA
NNCANNNNNNNNNNNNNNNNNNNNNANNNANNNNNNNTNNNN
>Mang288
GTGNNNNNTAGCCCNNNNNAGTTAGACANNNCNNNNNNNNNNNNTNNNNNNNNNNNNNNNNNNNNNNNNN
NNNNNNNNNNNNNNNNNNNNNNNNNNNNNNNNNNNNNNNNNNNNNNNNNNNNNNNNNNNNNNNNNNNNNN
NNNNNNNNNNNNNNNNNNNNNNNNNNNNNNNNNNNNNNNNNNNNNNNNNNNNNNNNNNNNNNNNNNNNNN
NNNNNNNNNNNNNNNNNNNNNNNNNNNNNNNNNNNNNNNNNNNNNNNNNNNNNNNNNNNNNNNGNNNNNN
NNNNNNNNNNNNNNNNNNNNNNNNNNNNNNNNNNNNNNNNNNNNNNNNNNNNNNTCTNNNAAANNNTNNN
NNNNNNNNNNNNNNNNNNNNNNNNNNNCNCNNTNNNNGTAANCNANNNGNACATGCAGTCCCCTCNNNNN
NNNNNCAGAGNNNNNNCCCNNNNNCNCCGNCCCCGNCCNNNNNNNNNNNCNNNGNNNNNNNANNNNNN
NNNNNNTGGTACNNNNCGNNNNNGGNTGTNTNAGAGGTCCACAACGNGGTCNNGNCACNNNNGNNNAN
NNNCNNNNAACNNNNNNNNNNNNANNTNNNTANNAGGNNNNNNCCNTCNNNNNNNNNNANNGCNATGACC
CNANNCACGGGGGCAGTNCCCGGNGCGGNGACCTTGNNNACGTCCGGGGNCAAGTATGGCNNNCNCCC
CGGGAAGGANNCNGCCGTANGTGCNNNNNANNNNNTCTTGNGANGTCGCCGNNATTNNCGTGGTCTNN
NNAGAATGNNCGNACCANNCGNANNNTGGTNATGNNAGTNNNNNNNNNNNNNNNNNNNNNNNNNNNNNNN
NNNNNNNNNNNNNNNNNNNNNNNNNNNNNNNNNNNNNNNGNNNNNNCNANNNAAGGNNNTANNNCTNNTN
NTNNNNNNNGGACNNNACNNNNCNNNNNNNACNCNNNCNCNNNNNNNNNNNGNTTTTTNNNCNNTANTNT
CCAGNANGCNGNGCNNNCATTCANGNGAACNNNGNNACGCGNNAATCGTANNATCTACCTGCCGCNNG
TNNNNNTNCNGNNNNNNCGGANNNTNNCTCGTANNNNNGTCATGCGTNNTNTNNTCNCTAGNCNNGTT
CGNNNANANANTAGNANGTNTNNNNNNNNCTTGTNNCGTTCCGCNTGANNCNNCANNNNGNNGNNNTTN
NCANCGGCNNTANCTGCNGANNTAAGNCATANNNTNNNTCNNTANCTNNNNNGGAGGGNNACTGTAAA
NGNNTAATTGGNNNNTNANTNCNNNNACNTNNCNNNGNNNNCTNNNTNCCTGNNTNCNNAATTGGNNC
CANTGNNTGNCNATCTCTNGNTTNGNNNNNNTNNNNNTNNCANNNNGGNNNGTNANANNNCNATNTAA
NNTATANNNNCNNNNNNANNNCACNANNNNNNNNAGNNNNNANNNTTANANNNGTAGNNNAATAAGTCT
CCGANNTNNNNNNNATAAGCGNNANACNNNNNNNNNNNNNNANGANNACGNNTTGGNNNTNNNNNNCNA
GNNAGACNNTNATGGNTNNNNNTCAANNNNTTNGTAGTGGTATAATNGNNNNNNNTTTCNCCAGNNTN
NNNGNNNNNNNCNATANNANNNNNNNGNGANNNNNNNNNNNANNNNCTCNNNAGGNANCANCTACTNNA
GCANTGGNGCGCAGNNNGAGANNNNGACNNNCCTTGGGTGGCAANCACNGCAATAAGTNNNGTTNNNC
CTNNNAGTNNACATGNNNNCANNGNNNCNNNNNNANCNGANNGNNNNNNNNNNNNNNNNNNNNNNNNNN
NNNNNNNNNNNNNNNNNNNNNNNNNNNNNNNAGNNNANGGNNNGTNTNTGACNCATNTNTTNNTANNNGNN
NNNNNACGNNNNANCCCCGGTCGNNNNCNNATTGAANNCCTGNNGCATNNNNNNTNCNAGAATGANNNC
NNNNNGNNTNGNCTNNCNTTTCAAGNAATCANNNAGNNNNCGCGNTCNNNGNNNNNNNNCNNNCCCNN
ACTGAGGGTNNNNNNNNCATCTGCTNNNNNNNNNNTNCGCNGGCGTNNCNNNNNAGCNGTATNNNGNNA
NNCANNNNNNNNNNNNNNNNNNCNAAACAANNNNNNNTNANN
>Mang289
GTGNNNNNTAGCCCNNNNNAGTTAGACANNNCNNNNNNNNNNNNTNNNNNNNNNNNNNNNNNNNNNNNNN
NNNNNNNNNNNNNNNNNNNNNNNNNNNNNNNTNNNNNNNNNNNNNNNNNNNNNNNNNNNNNNNNNNNNNN
NNNNNNNNNNNNNNNNNNNNNNNNNNNNNNNNNNNNNNNNNNNNNNNNNNNNNNNNNNNNNNNNNNNNNN
NNNNNNNNNNNNNNNNNNNNNNNNNNNNNNNNNNNNNNNNNNNNNNNNNNNNNNNNNNNNNNNNNNNNNN
NNNNNNNNNNNNNNNNNNNNNNNNNNNNNNNNNNNNNNNNNNNNNNNNNNNNNNTCTNNNAAANNNTNNN
NNNNNNNNNNNNNNNNNNNNNNNNNNNCNCNTTGNNNGTAANCNANNNGNACATGCAGTCCCCTCNNNNN
NNNGGCAGAGNNNNNNNCNNNNNNNNCCGNCCCCNNCCNNNNNNNNNNNNCNNNGNNNNNNNANNNNNN

NNNNNNTGGTACNNNNCGNNNNNGGTTGTNTAAGAGGTCCACAACGNGGTCNGGNCACNNNNGNNNAN
NNNCNNNNAACNNNNNNNNNNNNANNTNNNTANNAGGNNNNNNCCNTCNNGNNNNTNATTGCNATGACC
CAANNNACGGGGGCNGTNCCCGGCGNGGCGACCTTGATNANNTCCGGGGACAAGTATGGCGNNCNCCC
CGGGAAGGANNCNGCCGTANGTGCNNNNNANNNNNTCNTGNGANGTNGCCGNNATTCNCNTGGTCNNN
CNAGAATGNCCGNACCANNCGNANNNTGGTNATGNNAGTNNNNNNNNNNNNNNNNNNNNNNNNNNNNN
NNNNNNNNNNNNNNNNNNNNNNNNNNNNNNNNNNGNNNNGNNNNNNCNANNNAAGGNNNTANNNCTNNTN
NTNNNNNNGGACNNNACNTNNCNNNNNNNACNCNNNCNCNNNNNNANNNGNTTTTTNNNCNNTATTNT
CCAGNACGCNGNGCNNNCATTCANGNGAACNNNGNNACGCGNNAATCGTANNATCTACCTGCCGCNNG
TNNNNNTACNGNNNNNNCGGANNNTNNCTCGTANNNNNNTCATGCGTNNTNTNNTCNCNAGNCANGTT
CGNNNANANANTAGNANGTNTNNNNNNNNNTGTNNCGTTCCGCNTGANNCNNCANNNNGNCGNNCTTN
NCANCGGCNNTANCTGCNGANNTAAGNCATANNNTNNNTCNNTANCTNNNNNGGAGGGNNACTGTAAA
NGGNNAATTNGAGGNTNANTNCNNNCACNTNNCTNNGNNNNCTNNNTNCCTGNNTNCNNAATTGGNNC
CANTGNNTGNCNATCTCTGGNTTNGNNNNNNTNNNNNTNNCAANNNGGNNNGTNNNANNNCNATNTAA
NNTANANNNNCNNNNNNANNNCACNNNNNNNNNAGNNNCNANNNTTANANNNGTAGNNNANTAAGTCT
CCGANNTNNNNNNNATAAGCGNNANACNNNNNNNNNNNNNANGANNACGNNTTGGNNNTNNNNNNNNA
GNNAGACNNTNATGGNTNNNNNTCAANNNNTTCGTAGTGGTATAATNGNNNNNNNTTTCNCCAGNNTN
NNNGNNNNNNNCNATANNANNNNNNNGNGANNNNNNNNNNNANNNNCTCNNNAGGGANCANCTACTNNA
GCANTGGNGCGNAGNNNGAGANNNNGACNNNCCTTGGGTGGCAANCACNGCAATAAGTNNNGTTNANC
CTNNNAGTNNACATNNNNNCANNGNNNCNNNNNNANCNGANNGNNNNNNNNNNNNNNNNNNNNNNNN
NNNNNNNNNNNNNNNNNNNNNNNNNNNNNNAGNNNANGGNNNGTNTNTGACNCNTGNNTTTTTANNNGNN
NNNNNACGNNNNANCCCCGGTCGNNNNCNNATTGAANNCCTGNNGCATNNNNNNTNCNAGAATGANNGC
NNNNNGNNTNGNCTGNNNNNTCAAGNAATCANNNANNNNNNNNNGNTCNNNGNNNNNNNNCNNTCCCNN
ACTGAGGGTNNNNNNNNCATCTGCTNNNNNNNNNNTNCGCNGGNGTNNCNNNNNAGCNGTATNNNGTNA
NNCANNNNNNNNNNNNNNNNNNNAANCNANNNNNNNTNNNG
>Mang290
GTGNNNNNTAGCCCNNNNNAGTTAGACANNNCNNNNNNNNNNNNNTNNNNNNNNNNNNNNNANNNNNNNNN
NNNNNNNNNNNNNNNNNNNNNNNNNNNNNNNNNNNNNNNNNNNNNNNNNNNNNNNNNNNNNNNNN
NNNNNNNNNNNNNNNNNNNNNNNNNNNNNNNNNNNNNNNNNNNNNNNNNNNNNNNNNNNNNNNNN
NNNNNNNNNNNNNNNNNNNNNNNNNNNNNNNNNNNNNNNNNNNNNNNNNNNNNNNNNNNNNNNNN
NNNNNNNNNNNNNNNNNNNNNNNNNNNNNNNNNNNNNNNNNNNNNNNNNNNNCNTCTNNNAAANNNTNNN
NNNNNNNNNNNNNNNNNNNNNNNNNNCNCNTTGNNNGTAANCNANNNGNACATGCAGTCCCCTCNNNNN
NNNGGCAGAGNNNNNNNCCNNNNNNNCCGGCCCCNNCCNNNNNNNNNNNNNNNNNGNNNNNNNANNNNNN
NNNNNNTGGTACNNNNCGNNNNNGGTTGTNTAAGAGGTCCACAACGNGGTCNNGNCACNNNNGNNTAN
NNNCNNNAAACNGNNNNNNNNNNANNTNNNTANNAGGNCNNNNCCNTCNNNNNNNNAATTGCNATGACC
CAANNCACGGGGGCAGTNCCCGGCGCGGCGACCTTGATNACGTCCGGGGACAAGTATGGCNNNCNCCC
CGGGAAGGANNCNGCCGTANGTGCNNNNNANNNNNTCNTGNGACGTCGCCGNNATTNNCGTGGTCNNN
CNAGAATGNCCNNACCANNCGNANNNTGGTNATGNTAGTANNNNNNNNNNNNNNNNNNNNNNNNNNNN
NNNNNNNNNNNNNNNNNNNNNNNNNNNNNNNNNNNNNGNNNNNNCNANNNAAGGNNNTANNNCTNNTN
NTNNNNNNGGACNNNACNNNNCNNNNNNNACNCNNNCNCNNNNNNANNNGGTTTTTNNCCNNTANTNT
CCAGNACGCNGNGCNNNCATTCANGNGAACNNNGNNACGCGNNAATCGTANNATCTACCTGCCGCNNG
TNNNNNTACNGNNNNNNCGGANNNTNNCTCGTANNNNNNTCATGCGTNNTNTNNTCNCNAGNCNNGTT
CGNNNANANANTAGNANGTNTCCNNNNNNNTGTNNCGTTCCGCNTGANNCNNCANNNNGNNGNNCTTC
TCANCGGCNNTANCTGCNGANNTAAGNCATANNNTNNATCNNTANCTNNNNNGGNGGGNNACTGTAAA
NGGNNAATTGGNNGNTNANTNCNNNCACNTNNCNNNGNNTNCTNNNTNCCTGNNTNCNNAATTGGNNC
CANTGNNTGNCNATCTCTGGNTTNGNNNNNNTGNNNNTNNCAANNNGGNNNGTNANANNNCNATNTAA
NNTANANNNNCNNNNNNANNNCACNNNNNNNGNAGNNNCNANNNTTANANNNGTAGNNNAATAAGTCT
CCGANNTNNNNNNNATAAGCGNNANACNNNNNNNNNNNNNANGANNACGNNTTGGNNNTNNNNNNCNA
GNNAGACNNTNATGGNTNNNNNTCAANNNNTTNGTAGTGGTATAATNGNNNNNNNTTTCNCCAGNNTN
NNNGNNNNNNNCNATANNANNNNNNNGTGANNNNNNNNNNNANNNNCTCNNNAGGGANCANCTACTNNA
GCAATGGNGCGCAGNNNGAGANNNNGACNNGCCTTGGGTGGCAANCACNGCAATAAGTNNNGTTNANC
CTNNNAGTNNACATNNNNNCANNGNNNCNNNNNNANCNGANNGNNNNNNNNNNNNNNNNNNNNNNNN
NNNNNNNNNNNNNNNNNNNNNNNNNNNNNNAGNNNANNGNNNGTNTNTGACNCATGTGTTTTTANNNGNN
NNNNNACGNNNGANCCCCGGTCGNNNNCNNATTGAANNCCTGNNGCATNNNNNNTNCNAGAATGANNGC
NNNNNGNNTNGNCTNNCNTTTCAAGNAATCANNTAGGNNTNNNNNNTCNNNGANNNNNNNCNNTCCCNN

ACTGAGGGTNNNNNNNNCATCTGCTNNNNNNNNCTNCGCNGGCGTNNCNNNNNAGCNGTATNGTGTNA
NNCANNNNNNNNNNNNNNNNNTNNANNAANNNNNNTNNNN
>Mang291
GTGNNNNNNAGCCCNNNNNAGTTAGACANNNCNNNNNNNNNNNNTNNNNNNNNNNNNNNNNNNNNNNN
NNNNNNNNNNNNNNNNNNNNNNNNNNNNNNNNNNNNNNNNNNNNNNNNNNNNNNNNNNNNNNNNNNNNNN
NNNNNNNNNNNNNNNNNNNNNNNNNNNNNNNNNNNNNNNNNNNNNNNNNNNNNNNNNNNNNNNNNNNNNN
NNNNNNNNNNNNNNNNNNNNNNNNNNNNNNNNNNNNNNNNNNNNNNNNNNNNNNNNNNNNNNNNNNNNNN
NNNNNNNNNNNNNNNNNNNNNNNNNNNNNNNNNNNNNNNNNNNNNNNNNNNNNNTCTNNNAAANNNTNNN
NNNNNNNNNNNNNNNNNNNNNNNNNNNCNCNTTGNNNGTAANCNANNNGNACATGCAGTCCCCTCNNNNN
NNNNNCAGAGNNNNNNCCCNNNNNNNCCGGCCCCNNCCNNNNNNNNNNNNNNNNNNNNNNNNNANNNNNN
NNNNNNTGGTACNNNNCGNNNNNGGTTGNTTNAGAGGTCCACAACGNGGTCNNGNCACNNNNGNNNAN
NNNCNNNNAACNNNNNNNNNNNANNTNNNTANNAGGNNNNNGCCNTNNNGNNNNNNATTGCNATGACC
CNNNNCACNGGGGCNGTNCCCGGNGNGGCGACCTTGNNNANGTCCGGGGNCAAGTATGGCNNNCNCCC
CGGGAAGGANNCNGCCGTANGTGCNNNNNANNNNNTCNTGNGANGTCGCCGNNATTNNCNTGGTCNNN
NNAGAATGNCCNNACCANNCGNANNNTGGTTATGNNAGTNNNNNNNNNNNNNNNNNNNNNNNNNNNNN
NNNNNNNNNNNNNNNNNNNNNNNNNNNNNNNNGNNNNGNNNNNNCNANNNANGGNNNTANNNCTNNTN
NTNNNNNNNGGACNNNACNNNNCNNNNNNNACNCNNNCNCNNNNNNNNNNGNTTTTNNNNCNNTATTNT
CCAGNACGCNGNGCNNNCNTTCANGNGAACNNNGNNACGCGNNAATCGTANNATCTANCTGCCGCNNG
NNNNNNTACNGNNNNTNCGGANNNTNNNNCGTANNNNNNNCATGCGTNNTNTNNTCNCNAGNCNNGTT
CGNNNANANANTAGNANGTNTCNNNNNNNNTGTNNCGTTCCGCNTGANNCNNCANNNNGNNGNNNTTN
NCANCGGCNNTANCTGCNGANNTAAGNCATANNNTNNNTCNNTANCTNNNNNGGNGGGNNACTGTAAA
NGGNNAATTNGNNGNTNATTNCNNNCACNTNNCTNNGNNNNCTNNNTNCCTGNNTNCNNAATTGGNNC
CANTGNNTGNCNATCTCTNGNTTNGNNNNNNTNNNNNTNNCAANNNGGNNNGTNNNANNTCNATNTAA
NNTANANNNNCNNNNNNANNNCACNNNNNNNNNAGNNNCNANNNTTANANNNGTAGNNNANTAAGTCT
CCGANNTNNNNNNNATAAGCGNNANACNNNNNNNNNNNNNANGANNACGNNTTGGNNNTNNNNNNCNA
GNNAGACNNTNATGGNTNNNNNTCAANNNNTTNGTAGTGGTATAATNGNNNNNNNTTTCNCCAGNNTN
NNNGNNNNNNNCNATANNANNNNNNNGNGANNNNNNNNNNANNNNCTCNNNAGGNANCANCTACTNNA
GCANTGGNGCGNAGNNNGAGANNNNGACNNGCCTTGGGTGGCAANNACNGCAATAAGTNNNGTTNANC
CTNNNAGTNNACATNNNNNCANNGNNNCNNNNNNANCNGANNGNNNNNNNNNNNNNNNNNNNNNNNNN
NNNNNNNNNNNNNNNNNNNNNNNNNNNNNNNGNNNANGGNNNGTNTNTGACNCATGTGTTTTTANNNGNN
NNNNNACGNNNGANCCCCGGTCGNNNNCNNATTGAANNCCTGNNGCATNNNNNNTNCNAGAATGANNNC
NNNNNGNNTNGNCTGNCNNNTCAAGNAATCAANNAGNNNNNNNGNTCNNNGNNNNNNNNCNNTCCCNN
ACTGAGGGTNNNNNNNNCATCTGCTNNNNNNNNNNTNCGCNGGNGTNNCNNNNNNGCNGTATNGNGTNA
NNCANNNNNNNNNNNNNNNNNTNAANNAANNNNNNTNNNN
>Mang292
GTGNNNNNTAGCCCNNNNNAGTTAGACANNNCNNNNNNNNNNNNTNNNNNNNNNNNNNNNNNNNNNNN
NNNNNNNNNNNNNNNNNNNNNNNNNNNNNNNTNNNNNNNNNNNNNNNNNNNNNNNNNNNNNNNNNNNNNNN
NNNNNNNNNNNNNNNNNNNNNNNNNNNNNNNNNNNNNNNNNNNNNNNNNNNNNNNNNNNNNNNNNNNNNN
NNNNNNNNNNNNNNNNNNNNNNNNNNNNNNNNNNNNNNNNNNNNNNNNNNNNNNNNNNNNNNNNNNNNNN
NNNNNNNNNNNNNNNNNNNNNNNNNNNNNNNNNNNNNNNNNNNNNNNNNNNNNNTCTNNNAAANNGTNNN
NNNNNNNNNNNNNNNNNNNNNNNNNNNCNCNNTGNNNGTAANCNANNNGNACATGCAGTCCCCTCNNNNN
NNNNNCAGAGNNNNNNCCCNNNNNNNCCGGCCCCNNCCNNNNNNNNTNNNNNNNGTNNNNNNANNNNNN
NNNNNNTGGTACNNNNCGNNNNNGGTTGTTTAAGAGGTCCACAACGNGGTCNGGNCACNNNNGNNNAN
NNNCNNNAAACNNNNNNNNNCNNANATNNNTANNAGGNCNNNNCCNTCNNGNNANNAATTGCNATGACC
CAANNCACGGGGGCAGTCCCCGGCGCGGCGACCTTGANNACGTCCGGGGACAAGTATGGCNNNCNCCC
CGGGAAGGANNCNGCCGTANGTGCNNNNNANNNNNTCNTGNGACGTCGCCGNNATTCNCGTGGTCNNG
CNAGAATGNCCGNACCANNCGNANNNTGGTNATGTNAGTNNNNNNNNNNNNNNNNNNNNNNNNNNNNN
NNNNNNNNNNNNNNNNNNNNNNNNNNNNNNNNGNNNNGNNNNNNCNANNNAAGGANNTANNNCTNNTN
NTNNNNNNNGGACNNNACNNNNCNNNNNNNACNCNNNCNCNNNNNNNANNNGGTTTTTNNNCNNTATTNT
CCAGNACGCNGNGCNNNCATTCANGNGAACNNNGNNACGCGNNAATCGTANNATCTACCTGCCGCNNG
TNNNNNTACNGNNNNNNCGGANNNTNNCTCGTANNNNNNTCATGCGTNNTNTNNTCNCTAGNCANGTT
CGNNNANANANTAGNANGTNTCNNNNNNNNTGTNNCGTTCCGCNTGACNCNNCANNNNGNNGNNNTTN
TCANCGGCNNTANCTGCNGANNTAAGNCATANNNTNNATCNNTANCTNNNNNGGAGGGNNACTGTAAA
GGGNNAATTNGNNNNTNATTTCNNNCACNTNNCTNNGNNNNCTNNNTNCCTGNNTNCNNAATTGGNNC

CANTGNNTGNCNATCTCTGGNTTNGNNNNNNTGNNNNTNNCAANNNGGNNNGTNANANNNCNATNTAA
NNTANANNNNCNNNNNNANNNCACNNNNNNNGNAGNNNCNANNNTTANANNNGTAGNNNAATAAGTCT
CCGANNTNNNNNNNATAAGCGNNANACNNNNNNNNNNNNNANGANNACGNNTTGGNNNTNNNNNNCNA
GNNAGACNNTNATGGNTNNNNNTCAANNNNTTCGTAGTGGTATAATNGNNNNNNNTTTCNCCAGNNTN
NNNGNNNNNNNCTATANNANNNNNNNGTGANNNNNNNNNNANNNNCTCNNNAGGGANCANCTACTNNA
GCAATGGNGCGCAGNNNGAGANNNNGACNANCCTTGGGTGGCAANCACNGCAATAAGTNNNGTTNANC
CTNNNAGTNNACATGNNNNCANNGNNNCNNNNNNANCNGANNGNNNNNNNNNNNNNNNNNNNNNNNNN
NNNNNNNNNNNNNNNNNNNNNNNNNNNNNAGNNNANGGNNNGTNTNTGACNCATGTGTTTTTANNNGNN
NNNNNACGNNNGANCCCCGGTCGNNNNCNNATTGAANNCCTGNNGCATNNNNNTNCNAGAATGANNGC
NNNNNGNNTNGNCTGNCNNNTCAAGNAATCAANNAGGTATNNNGNTCNNNGNNNNNNNNCNNTCCCNN
ACTGAGGGTNNNNNNNNCATCTGCTNNNNNNNNCTNCGCNGGCGTNNCNNNNNAGCNGTATNGTGTTA
NNCANNNNNNNNNNNNNNNNNTNAAACGANNNNNNTNNNN
>Mang293
GTGNNNNNTAGCCCNNNNNAGTTAGACANNNCNNNNNNNNNNNNNTNNNNNNNNNNNNNNNNNNNNNNN
NNNNNNNNNNNNNNNNNNNNNNNNNNNNNNNNTNNNNNNNNNNNNNNNNNNNNNNNNNNNNNNNNNNNNN
NNNNNNNNNNNNNNNNNNNNNNNNNNNNNNNNNNNNNNNNNNNNNNNNNNNNNNNNNNNNNNNNNNNNN
NNNNNNNNNNNNNNNNNNNNNNNNNNNNNNNNNNNNNNNNNNNNNNNNNNNNNNNNNNNNNNNNNNNNN
NNNNNNNNNNNNNNNNNNNNNNNNNNNNNNNNNNNNNNNNNNNNNNNNNNNNNNNTCTNNNAAANNNTNNN
NNNNNNNNNNNNNNNNNNNNNNNNNNCNCNTTGNNNGTAANCNANNNGNACATGCAGTCCCCTCNNNNN
NNNGGCAGAGNNNNNNCNCNNNNNNNCCGNCCCCNNCCNNNNNNNNNNNNNNNNGNNNNNNNANNNNNN
NNNNNNTGGTACNNNNCGNNNNNGGTTGTNTAAGAGGTCCACAACGNGGTCNGGNCACNNNNGNNNAN
NNNCNNNNANCNNNNNNNNNNNNNNNTNNNTANNAGGNCNNNNCCNTCNNNNNNNTNATTGCNATGACC
CAANNCNCGGNGGNNGTNCCCGGCGCGGNGACCTTGATNANGTCCGGGGACAAGTATGGCGCNCNCCC
CGGGAAGGANNCNGCCGTANGTGCNNNNNANNNNNTCNTGNGANGTCGCCGNNATTCNCGTGGTCNNN
CNAGAATGNNCGNACCANNCGNANNNTGGTNATGNNAGTNNNNNNNNNNNNNNNNNNNNNNNNNNNNN
NNNNNNNNNNNNNNNNNNNNNNNNNNNNNNNNNNNNNNNGNNNNNNCNANNNAAGGNNNTANNNCTNNTN
NTNNNNNNNGGACNNNACNNNNCNNNNNNNACNCNNNCNCNNNNNNANNNGGTTTTNNNNCNNTATTNT
CCAGNACGCNGNGCNNNCATTCANGNGAACNNNGNNACGCGNNAATCGTANNATCTACCTGCCGCNGG
TNNNNNTACNGNNNNNNCGGANNNTNNCTCGTANNNNNNTCATGCGTNNTNTNNTCNCNAGNCANGTT
CGNNNANANANTAGNANGTNTNNNNNNNNNNTGTNNCGTTCCGCNTGANNCNNCANNNNGNNGNNCTTN
TCANCGGCNNTANCTGCNGANNTAAGNCATANNNTNNNTCNNTANCTNNNNNGGNGGGNACTGTAAA
NGGNNAATTNGNNNNTNATTNCNNNCACNTNNCTNNGNNTNCTNNNTNCCTGNNTNCNNAATTGGNNC
CANTGNNTGNCNATCTCTGGNTTNGNNNNNNTGNNNNTNNCAANNNGGNNNGTNANANNNCNATNTAA
NNTANANNNNCNNNNNNANNNCACNANNNNNNNAGNNNNNANNNTTANANNNGTAGNNNANTAAGTCT
CCGANNTNNNNNNNATAAGCGNNANACNNNNNNNNNNNNNANGANNACGNNTTGGNNNTNNNNNNCNA
GNNAGACNNTNATGGNTNNNNNTCAANNNNTTNGTAGTGGTATAATNGNNNNNNNTTTCNCCAGNNTN
NNNGNNNNNNNCNATANNANNNNNNNGNGANNNNNNNNNNANNNNCTCNNNAGGGANCANCTACTNNA
GCAATGGNGCGCAGNNNGAGANNNNGACNNNCCTTGGGTGGCAANCACNGCAATAAGTNNNGTTNANC
CTNNNAGTNNACATGNNNNCANNGNNNCNNNNNNANCNGANNGNNNNNNNNNNNNNNNNNNNNNNNNN
NNNNNNNNNNNNNNNNNNNNNNNNNNNNNNGNNNANNGNNNGTNTNTGACNCATGTGTTTTTANNNGNN
NNNNNNNGNNNNANCCCCGGTCGNNNNCNNATTGAANNCCTGNNGCATNNNNNTNCNAGAATGANNNC
NNNNNGNNTNGNCTNNCNTTTCAAGNAATCANNNAGGTNNCGCGNTCNNGGNNNNNNNNCNNTCCCNN
ACTGAGGGTNNNNNNNNCATCTGCTNNNNNNNNCTNCGCNGGCGTNNCNNNNNAGCNGTATNGNGTNA
NNCANNNNNNNNNNNNNNNNNTNNNACNANNNNNNNTNNNN
>Mang294
GTGNNNNNTAGCCCNNNNNAGTTANACANNNCNNNNNNNNNNNNNTNNNNNNNNNNNNNNNNNNNNNNN
NNNNNNNNNNNNNNNNNNNNNNNNNNNNNNNNNNNNNNNNNNNNNNNNNNNNNNNNNGNNNNNNNNNNNN
NNNNNNNNNNNNNNNNNNNNNNNNNNNNNNNNNNNNNNNNNNNNNNNNNNNNNNNNNNNNNNNNNNNNN
NNNNNNNNNNNNNNNNNNNNNNNNNNNNNNNNNNNNNNNNNNNNNNNNNNNNNNNNNNNNNNNNNNNNN
NNNNNNNNNNNNNNNNNNNNNNNNNNNNNNNNNNNNNNNNNNNNNNNNNNNNNNNTCTNNNAAANNNTNNN
NNNNNNNNNNNNNNNNNNNNNNNNNNCNCNTTGNNNGTAANCNANNNGNACATGCAGTCCCCTCNNNNN
NNNGGCAGAGNNNNNNNNCNNNNNCNCCGGCCCCGNCCNNNNNNNNNNNNNNNNGNNNNNNNANNNNNN
NNNNNNTGGTACNNNNCGNNNNNGGTTGTTTAAGAGGTCCACAACGNGGTCNGGNCACNNNNGNNNAN
NNNCNNNNAANNNNNNNNNNNNNNNNATNNNTANNAGGNNNNNNCCNTCNNGNNNNNNATTGCNATGACC

CAANNCACGGGGGCNGTNCCCGGCGCGGCGACCTTGANNANNTCCGGGGNCAAGTATGGCNNNCNCCC
CGGGAAGGANNCNGCCGTANGTGCNNNNNANNNNNTCNTGNGANGTCGCCGNNATTCNCGTGGTCNNNN
NNAGAATGNCCGNACCANNCGNANNNTGGTNATGNNAGTNNNNNNNNNNNNNNNNNNNNNNNNNNNNNNN
NNNNNNNNNNNNNNNNNNNNNNNNNNNNNNNNNNGNNNNGNNNNNNCNANNNAAGGANNTANNNCTNNTN
NTNNNNNNGGACNNNACNNNNCNNNNNNNACNCNNNCNCNNNNNNNNNNGNTTTTTNNNCNNTATTNT
CCAGNANGCNGNGCNNNCATTCANGNGAACNNNGNNACGCGNNAATCGTANNATCTACCTGCCGCNNG
TNNNNNTNCNGNNNNNNCGGANNNTNNNNCGTANNNNNNTCATGCGTNNTNTNNTCNCNAGNCNNGTT
CGNNNANANANTAGNANGTNTCNNNNNNNNTGTNNCGTTCCGCNTGANNCNNCANNNNGNCGNNCTTN
NCANCGGCNNTANCTGCNGANNTAAGTCATANNNTNNATCNNTANCTNNNNNGGNGGGNNACTGTAAA
NGGNTAATTNGNNGNTNANTNCNNNCACNTNNCTNNGNNTNCTNNNTNCCTGNNTNCNNAATTGGNNC
CANTGNNTGNCNATCTCTGGNTTNGNNNNNNTGNNNNTNNCANNNNGGNNNGTNANANNNCNATNTAA
NNTANANNNNCNNNNNNANNNCACNNNNNNNGNAGNNNCNANNNTTANANNNGTAGNNNAATAAGTCT
CCGANNTNNNNNNNATAAGCGNNANACNNNNNNNNNNNNNNANGANNACGNNTTGGNNNTNNNNNNCNA
GNNAGACNNTNATGGNTNNNNNTCAANNNNTTNGTAGTGGTATAATNGNNNNNNNTTTCNCCAGNNTN
NNNGNNNNNNNCNATANNANNNNNNNGNGANNNNNNNNNNNANNNNCTCNNNAGGGANCANCTACTNNA
GCANTGGNGCGCAGNNNGAGANNNNGACNNNCCTTGGGTGGCAANCACNGCAATAAGTNNNGTTNANC
CTNNNAGTNNACATNNNNNCANNGNNNCNNNNNNANCNGANNGNNNNNNNNNNNNNNNNNNNNNNNNNNN
NNNNNNNNNNNNNNNNNNNNNNNNNNNNNNAGNNNANGGNNNGTNTNTGACNCATGTGTTTTTANNNGNN
NNNNNNCGNNNNANCCCCGGTCGNNNNCNNATTGAANNCCTGNNGCATNNNNNTNCNAGAATGANNGC
NNNNNGNNTNGNCTGNCNNNTCAAGNAATCANNNAGGTANNNNGNTCNNNGNNNNNNNNCNNTCCCNN
ACTGAGGGTNNNNNNNNCATCTGCTNNNNNNNNNTNCGCNGGNGTNNCNNNNNAGCNGTATNNNGTNA
NNCANNNNNNNNNNNNNNNNTNAAAAAANNNNNNTNNNN
>Mang295
GTGNNNNNTAGCCCNNNNNAGTTAGACANNNCNNNNNNNNNNNNNNNNNNNNNNNNNNNNNNNNNNNNNN
NNNNNNNNNNNNNNNNNNNNNNNNNNNNNNNNNNNNNNNNNNNNNNNNNNNNNNNNNNNNNNNNNNNNNN
NNNNNNNNNNNNNNNNNNNNNNNNNNNNNNNNNNNNNNNNNNNNNNNNNNNNNNNNNNNNNNNNNNNNNN
NNNNNNNNNNNNNNNNNNNNNNNNNNNNNNNNNNNNNNNNNNNNNNNNNNNNNNNNNNNNNNNNNNNNNN
NNNNNNNNNNNNNNNNNNNNNNNNNNNNNNNNNNNNNNNNNNNNNNNNNNNNNNTCTNNNAAANNNTNNN
NNNNNNNNNNNNNNNNNNNNNNNNNNNCNCNNTNNNNGTAANCNANNNGNACATGCAGTCCCCTCNNNNN
NNNNNCAGAGNNNNNNNNNCNNNNNCNCCGGCCCCNNCCNNNNNNNNNNNNNNNNGNNNNNNNANNNNNN
NNNNNNTGGTACNNNNCGNNNNNGGNTGNNTNAGAGGTCCACAACGNGGTCNNGNNACNNNNGNNNAN
NNNCNNNNAACNNNNNNNNNNNNANNTNNNTANNAGGNNNNNNCCNTCNNNNNNNNNNANNGCNATGANN
CANNNNACNGGGGNNGTNCCCGGNGCGGCNACCTTGANNANNTCCGGGGNCAAGTATGGCGNNCNCCC
CGGGAAGGANNCNGCCGTANGTGCNNNNNANNNNNTCNTGNGANGTNGCCGNNATTNNCNTGGTCNNN
CNAGAATGNCCNNACCANNCGNANNNTGGTNATGNNAGTNNNNNNNNNNNNNNNNNNNNNNNNNNNNNNN
NNNNNNNNNNNNNNNNNNNNNNNNNNNNNNNNNNGNNNNGNNNNNNCNANNNAAGGNNNTANNNCTNNTN
NNNNNNNNGGACNNNACNNNNCNNNNNNNACNCNNNCNCNNNNNNNNNNNGGTTTTNNNNCNNTANTNT
CCAGNACGCNGNGCNNNCNTTCANGNGAACNNNGNNACGCGNNAATCGTANNATCTACCTGCCGCNNG
NNNNNNTNCNGNNNNNNCGGANNNTNNNNNGTANNNNNNNCANGCGTNNNNTNNTNNCNAGNCANGTT
CGNNNANANANTAGNANGTNTNNNNNNNNNTGTNNCGTTCCGCNTGANNCNNCANNNNGNNGNNNTTN
NCANCGGCNNTANCTNCNGANNTAAGNCATANNNTNNNTCNNTANCTNNNNNGGNGGGNNACTGTAAA
NGGNNAATTNGNNGNTNATTNCNNNCACNTNNCNNNGNNNNCTNNNTNCCTGNNTNCNNAATTGGNNC
CANTGNNTGNCNATCTCTNGNTTNGNNNNNNTNNNNNTNNCANNNNGGNNNGTNNNANNNCNATNTAA
NNTANANNNNCNNNNNNANNNCACNNNNNNNNNAGNNNNNANNNTNANANNNGTAGNNNANTNAGTCT
CCNANNTNNNNNNNATAAGCGNNANACNNNNNNNNNNNNNNANGANNNCGNNTTGGNNNTNNNNNNNNA
GNNAGACNNTNATGGNTNNNNNTCAANNNNTTNGTAGTGGTATAATNGNNNNNNNTTTCNCCAGNNTN
NNNGNNNNNNNCNATANNANNNNNNNGNGANNNNNNNNNNNANNNNCTCNNNAGGNANCANCTACTNNA
GCANNGGNGCGNAGNNNGAGANNNNGACNNNCCTTGGGTGGCAANCACNGNANTAAGTNNNGTTNANN
CTNNNAGTNNACATNNNNNCANNGNNNCNNNNNNANCNGANNGNNNNNNNNNNNNNNNNNNNNNNNNNNN
NNNNNNNNNNNNNNNNNNNNNNNNNNNNNNANNNNANNGNNNGTNTNTGACNCNTGTGTTTTTANNNGNN
NNNNNNNGNNNGANCCCCGGTCGNNNNCNNATTGAANNCCTGNNGCATNNNNNTNCNAGAATGANNNC
NNNNNGNNTNGNCTNNCNNNTCAAGNAATCANNNAGGTNNNNNNNTCNNNGNNNNNNNNCNNNCCCNN
ACTGAGGGTNNNNNNNNCANCNNCTNNNNNNNNCTNCGCNGGNNNNNCNNNNNNGCNGTATNNNGTNN
NNCANNNNNNNNNNNNNNNNCNANNNNANNNNNNNTNNNG

>Mang296
GTGNNNNNTAGCCCNNNNNAGTTANACANNNCNNNNNNNNNNNNTNNNNNNNNNNNNNNNNNNNNNN
NNNNNNNNNNNNNNNNNNNNNNNNNNNNNNNNNNNNNNNNNNNNNNNNNNNNNNNNNNNN
NNNNNNNNNNNNNNNNNNNNNNNNNNNNNNNNNNNNNNNNNNNNNNNNNNNNNNNNNNNN
NNNNNNNNNNNNNNNNNNNNNNNNNNNNNNNNNNNNNNNNNNNNNNNNNNNNNNNNNNNN
NNNNNNNNNNNNNNNNNNNNNNNNNNNNNNNNNNNNNNNNNNNNNNNNTCTNNNNAANNNTNNN
NNNNNNNNNNNNNNNNNNNNNNNNNNCNCNTTGNNNGTAANCNANNNGNACATGCAGTCCCCTCNNNNN
NNNNNCAGAGNNNNNNNCCNNNNNNNCCGGCCCCNNCCNNNNNNNNNNNCNNNGNNNNNNNANNNNNN
NNNNNNTGGTACNNNNCGNNNNNGGTTGTNTAAGAGGTCCACAACGNGGTCNNGNCACNNNNGNNNAN
NNNCNNNNNNNNNNNNNNNNNNNANNTNNNTANNAGGNNNNNNCCNTCNNNNNNNTNATTGCNATGACC
CNNNTCACGGGGGNNGTNCCCGGCGCGGCGACCTTGNNNANGTCCGGGGACAAGTATGGCNNNCNCCC
CGGGAAGGANNCNGCNGTANGTGCNNNNNANNNNNTCNTGNGANGTNGCCGNNANNNNCGTGGTCNNN
CNNGAATGNCCGNACCANNCGNANNNTGGTNATGNTAGTNNNNNNNNNNNNNNNNNNNNNNNNNNNN
NNNNNNNNNNNNNNNNNNNNNNNNNNNNNNNNNNGNNNNGNNNNNNCNANNNAAGGNNNTANNNCTNNTN
NTNNNNNNNGGACNNNACNNNNCNNNNNNNACNCNNNCNCNNNNNNANNNGNTTNTTNNNCNNTANTNT
CCAGNANGCNGNGCNNNCATTCANGNGAACNNNGNNACGCGNNAATCGTANNATCTACCTGCCGCNNG
NNNNNNTNCNGNNNNNNCGGANNNTNNNNNGTANNNNNNNCATGCGTNNTNTNNTNNCNAGNCANGTT
CGNNNANANANTAGNANGTNTNNNNNNNCNTGTNNCGTTCCGCNTGANNCNNCANNNNGNNGNNNTTC
NCANCGGCNNTANCTNCNGANNTAAGNCATANNNTNNATCNNTANCTNNNNNGGNGGGNNACTGTAAA
NGGNTAATTNGNNNNTNANTNCNNNCACNTNNCTNNGNNTNCTNNNTNCCTGNNTNCNNAATTGGNNN
CANTGNNTGNCNATCTCTGGNTTNGNNNNNNNTNNNNNTNNCANNNNGGNNNGTNANANNNCNATNTAA
NNTANANNNNCNNNNNNANNNCACNNNNNNNNGNAGNNNCNANNNTTANANNNGTAGNNNANTAAGTCN
CCGANNTNNNNNNNATAAGCGNNANACNNNNNNNNNNNNNANGANNNCGNNTTGGNNNTNNNNNNCNA
GNNANANNNTNATGGNTNNNNNTCAANNNNTTNGTAGTGGTATAATNGNNNNNNNTTTCNCCAGNNTN
NNNGNNNNNNNCNATANNANNNNNNNGNGANNNNNNNNNNNANNNNCTCNNNAGGGANCANCTACTNNA
GCANTGGNGCGNAGNNNGAGANNNNGACNNGCCTTGGGTGGCAANCACNGCAATAAGTNNNGTTNANC
CTNNNAGTNNACATNNNNNCANNGNNNCNNNNNNANCNGANNGNNNNNNNNNNNNNNNNNNNNNNNN
NNNNNNNNNNNNNNNNNNNNNNNNNNNNNNNGNNNANGGNNNGTNTNTGACNCNTNNNTTNNTANNNGNN
NNNNNACGNNNNANCCCCGGTCGNNNNNNNATTGAANNCCTGNNGCATNNNNNTNCNAGAATGANNGC
NNNNNGNNTNNNCTNNCNNNTCAAGNAATCAANNAGGTNNNNNNNTCNNNNNNNNNNNNCNNTCCCNN
ACTGAGGGTNNNNNNNNCANCTGCTNNNNNNNNNTTCGCNGGNGTNNCNGCNNNGCNGTATNNNGTNA
NNCANNNNNNNNNNNNNNNNNTNAAACNANNNNNNNTNNNN
>Mang297
GTGNNNNNTAGCCCGNNNNAGTTAGACANNNCNNNNNNNNNNNNTNNNNNNNNNNNNNNNNNNNNNN
NNNNNNNNNNNNNNNNNNNNNNNNNNNNNTNNNNNNNNNNNNNNNNNNNNNNNNNNNNNNNN
NNNNNNNNNNNNNNNNNNNNNNNNNNNNNNNNNNNNNNNNNNNNNNNNNNNNNNNNNNNN
NNNNNNNNNNNNNNNNNNNNNNNNNNNNNNNNNNNNNNNNNNNNNNNNNNNNNNNNNNNN
NNNNNNNNNNNNNNNNNNNNNNNNNNNNNNNNNNNNNNNNNNNNNNNNCNTCTNNNAAANNNTNNN
NNNNNNNNNNNNNNNNNNNNNNNNNNCNCNTTGNNNGTAANCNANNNGNACATGCAGTCCCCTCNNNNN
NNNGGCAGAGNNNNNNCCCCNNNNCNCCGGCCCCGNCCNNNNNNNNNNNCNNNGNNNNNNNANNNNNN
NNNNNNTGGTACNNNNCGNNNNNGGTTGTTTAAGAGGTCCACAACGNGGTCNGGNCACNNNNGNNTAN
NNNCNNNNAACNGNNNNNNNNNAANNTNNNTANNAGGNCNNNNCCNTCNNGNNANNAATTGCNATGACC
CAANTCACGGGGGNNGTNCCCGGCGCGGCGACCTTGANNACGTCCGGGGACAAGTATGGCGNNCNCCC
CGGGAAGGANNCNGCCGTANGTGCNNNNNANNNNNTCTTGNGANGTCGCCGNNATTCNCGTGGTCNNN
CNAGAATGNCCGNACCANNCGNANNNTGGTNATGNNAGTNNNNNNNNNNNNNNNNNNNNNNNNNNN
NNNNNNNNNNNNNNNNNNNNNNNNNNNNNNNNNNGNNNNGNNNNNNCNANNNAAGGANNTANNNCTNNTN
NTNNNNNNNGGACNNNACNNNNCNNNNNNNACNCNNNCNCNNNNNNANNNGGTTTTTNNCCNNTATTNT
CCAGNACGCNGNGCNNTCATTCANGNGAACNNNGNNACGCGNNAATCGTANNATCTACCTGCCGCNGG
TNNNNNTACNGNNNNNNCGGANNNTNNCTCGTANNNNNNTCATGCGTNNTNTNNTCNCNAGNCANGTT
CGNNNANANANTAGNANGTNTCNNNNNNNNTGTNNCGTTCCGCNTGANNCNNCANNNNGNCGNNCTTC
TCANCGGCNNTANCTGCNGANNTAAGNCATANNNTNNATCNNTANCTNNNNNGGAGGGTNACTGTAAA
NGGNNAATTNGANGNTNATTTCNNNCACNTNNCTNNGNNTNCTNNNTNCCTGNNTNCNNAATTGGGNC
CANTGNNTGNCNATCTCTGGNTTNGCNNNNNTGNNNNTNGCAANNNGGNNNGTNANANCNCNATNTAA
NNTATANNNNCNNNNNNANNNCACNANNNNNGNAGNNNCNANNNTTANANNNGTAGNNNANTAAGTCT

CCGANNTNNNNNNNATAAGCGNNANACNNNNNNNNNNNNNNANGANNACGNNTTGGNNNTNNNNNNNCNA
GNNAGACNNTNATGGNTNNNNNTCAANNNNTTCGTAGTGGTATAATNGNNNNNNNTTTCNCCAGNNTN
NNNGNNNNNNNCNATACNANNNNNNNGNGANNNNNNNNNNNANNNNCTCNNNAGGGANCANCTACTNNA
GCAATGGNGCGCAGNNNGAGANNNNGACCAGCCTTGGGTGGCAANCACNGCAATAAGTNNNGTTTANC
CTNNNAGTNNACATGNNNNCANNGNNNCNNNNNNANCNGANNGNNNNNNNNNNNNNNNNNNNNNNNNNN
NNNNNNNNNNNNNNNNNNNNNNNNNNNNNAGNNNANGGNNNGTNTNTGACNCATGTGTTTTTANNNGNN
NNNNNACGNNNGANCCCCGGTCGTNNNCNNATTGAANNCCTGNNGCATNNNNNTNCNAGAATGANNGC
NNNNNGNNTNGNCTNNCNTTTCAAGNAATCANNNAGGTANCGCGNTCNNGGNNNNNNNNCNNTCCCNN
ACTGAGGGTNNNNNNCGCATCTGCTNNNNNNNNCTTCGCNGGCGTNNCNGNNNAGCNGTATNGTGTNA
NNCANNNNNNNNNNNNNNNNNTNAAACAANNNNNNNTNNNN
>Mang298
GTGNNNNNTAGCCCNNNNNAGTTAGACANNNCNNNNNNNNNNNNNTNNNNNNNNNNNNNNNNNNNNNNNN
NNNNNNNNNNNNNNNNNNNNNNNNNNNNNNNTNNNNNNNNNNNNNNNNNNNNNNNNNNNNNNNNNNNNNN
NNNNNNNNNNNNNNNNNNNNNNNNNNNNNNNNNNNNNNNNNNNNNNNNNNNNNNNNNNNNNNNNNNNNNN
NNNNNNNNNNNNNNNNNNNNNNNNNNNNNNNNNNNNNNNNNNNNNNNNNNNNNNNNNNNNNNNNNNNNNN
NNNNNNNNNNNNNNNNNNNNNNNNNNNNNNNNNNNNNNNNNNNNNNNNNNNNCNTCTNNNAAANNNTNNN
NNNNNNNNNNNNNNNNNNNNNNNNNNCNCNTTGNNNGTAANCNANNNGNACATGCAGTCCCCTCNNNNN
NNNNNCAGAGNNNNNNCCCNNNNNNNCCGGCCCCNNCCNNNNNNNTNNNCNNNGNNNNNNNANNNNNN
NNNNNNTGGTACNNNNCGNNNNNGGTTGTNTAAGAGGTCCACAACGNGGTCNGGNCACNNNNGNNNAN
NNNCNNAAAANNNNNNNNNNNNNNANNTNNNTANNAGGNCNNNNCCNTCNNNNNNCTNATTGCNATGACC
CAANNCNCGGGGGNNGTNCCCGGCGNGGCGACCTTGANNACGTCCGGGGACAAGTATGGCNNNCNCCC
CGGGAAGGANNCNGCCGTANGTGCNNNNNANNNNNTCNTGNGANGTCGCCGNNATTCNCGTGGTCNNN
CNAGAATGNNCGNACCANNCGNANNNTGGTNATGNNAGTNNNNNNNNNNNNNNNNNNNNNNNNNNNNN
NNNNNNNNNNNNNNNNNNNNNNNNNNNNNNNNNNNNGNNNNGNNNNNNCNANNNAAGGNNNTANNNCTNNTN
NTNNNNNNNGGACNNNACNNNNCNNNNNNNACNCNNNCNCNNNNNNANNNGNTTTTTNNNCNNTANTNT
CCAGNACGCNGNGCNNNCNTTCANGNGAACNNNGNNACGCGNNAATCGTANNATCTACCTGCCGCNNG
TNNNNNTNCNGNNNNNNCGGANNNTNNCTCGTANNNNNNTCATGCGTNNTNTNNTNNCNAGNCNNGTT
CGNNNANANANTAGNANGTNTNNNNNNNNNTGTNNCGTTCCGCNTGANNCNNCANNNNGNNGNNCTTN
NCANCGGCNNTANCTGCNGANNTAAGNCATANNNTNNATCNNTANCTNNNNNGGNGGGNNACTGTAAA
NGGNNAATTNGNNNNTNANTNCCNNCACNTNNCTNNGNNNNCTNNNTNCCTGNNTNCNNAATTGGNNN
CANTGNNTGNCNATCTCTNGNTTNGNNNNNNTGNNNNTNNCANNNNGGNNNGTNNNANNNCNATNTAA
NNTANANNNNCNNNNNNANNNCACNNNNNNNGNAGNNNNNANNNTTANANNNGTAGNNNAATNAGTCT
CCGANNTNNNNNNNATAAGCGNNANACNNNNNNNNNNNNNNANGANNACGNNTTGGNNNTNNNNNNNCNA
GNNAGACNNTNATGGNTNNNNNTCAANNNNTTCGTAGTGGTATAATNGNNNNNNNTTTCNCCAGNNTN
NNNGNNNNNNNCNATACNANNNNNNNGNGANNNNNNNNNNNANNNNCTCNNNAGGGANCANCTACTNNA
GCANTGGNGCGCAGNNNGAGANNNNGACNNGCCTTGGGTGGCAANNACNGCAATAAGTNNNGTTNANC
CTNNNAGTNNACATNNNNNNCANNGNNNCNNNNNNANCNGANNGNNNNNNNNNNNNNNNNNNNNNNNNNN
NNNNNNNNNNNNNNNNNNNNNNNNNNNNNANNNNANGGNNNGTNTNTGACNCATGTGTTTTTANNNGNN
NNNNNACGNNNNANCCCCGGTCGNNTNCNNATTGAANNCCTGNNGCATNNNNNTNCNAGAATGANNGC
NNNNNGNNTNGNCTNNCNTTTCAAGNAATCANNNAGNTATNNCGNTCNNGNNNNNNNNNCNNNCCCNN
ACTGAGGGTNNNNNNNNCATCTGCTNNNNNNNNCTNCGCNGNNGTNNCNNNNNNGCNGTATNGTGTNA
NNCANNNNNNNNNNNNNNNNNTNANACAANNNNNNNTNNNN
>Mang300
GTGNNNNNTAGCCCNNNNNAGTTAGACANNNCNNNNNNNNNNNNNTNNNNNNNNNNNNNNNNNNNNNNNN
NNNNNNNNNNNNNNNNNNNNNNNNNNNNNNNTNNNNNNNNNNNNNNNNNNNNNNNNNNNNNNNNNNNNNN
NNNNNNNNNNNNNNNNNNNNNNNNNNNNNNNNNNNNNNNNNNNNNNNNNNNNNNNNNNNNNNNNNNNNNN
NNNNNNNNNNNNNNNNNNNNNNNNNNNNNNNNNNNNNNNNNNNNNNNNNNNNNNNNNNNNNNNNNNNNNN
NNNNNNNNNNNNNNNNNNNNNNNNNNNNNNNNNNNNNNNNNNNNNNNNNNNNNTCTNNNAAANNNTNNN
NNNNNNNNNNNNNNNNNNNNNNNNNNCNCNTTGNNNGTAANCNANNNGNACATGCAGTCCCCTCNNNNN
NNNGGCAGAGNNNNNNCCCNNNNNNCNCCGNCCCCNNCCNNNNNNNNNNNNCNNNNNNNNNNNNANNNNNN
NNNNNNTGGTACNNNNCGNNNNNGGTTGNNTAAGAGGTCCACAACGNGGTCNNGNNACNNNNGNNNAN
NNNCNNNNAACNNNNNNNNNNNNNNNTNNNTANNAGGNCNNNNCCNTCNNGNANNNATTGCNATGACC
CAANNCACGGNGGCAGTNCCCGGNGCGGCGACCTTGANNACGTCCGGGGACAAGTATGGCNNNCNCCC
CGGGAAGGANNCNGCCGTANGTGCNNNNNANNNNNTCNTGNGANGTCGCCGNNATTNNCGTGGTCNNN

CNAGAATGCCCNNACCANNCGNANNNTGGTNATGNNAGTNNNNNNNNNNNNNNNNNNNNNNNNNNNNN
NNNNNNNNNNNNNNNNNNNNNNNNNNNNNNNNGNNNNGNNNNNNCANNNNAAGGANNTANNNCTNNTN
NTNNNNNNGGACNNNACNNNNCNNNNNNNACNCNNNCNCNNNNNNANNNGNTTTTTNNNCNNTATTNT
CCAGNACGCNGNGCNNNCNTTCANGNGAACNNNGNNACGCGNNAATCGTANNATCTACCTGCCGCNNG
NNNNNNTACNGNNNNNNCGGANNNTNNCTCGTANNNNNNTCANGCGTNNTNTNNTCNCNAGNCANGTT
CGNNNANANANTAGNANGTNTNNNNNNNNNTGTNNCGTTCCGCNTGANNCNNCANNNNGNNGNNNTTN
TCANCGGCNNTANCTGCNGANNTAAGNCATANNNTNNATCNNTANCTNNNNNGGNGGGNNACTGTAAA
NGGNNAATTNGNNGNTNATTNCNNNCACNTNNCNNNGNNNNCTNNNTNCCTGNNTNCNNAATTGGNNC
CANTGNNTGNCATCTCTGGNTTNGNNNNNNTNNNNNNTNNCANNNNGGNNNGTNANANNNCNATNTAA
NNTANANNNNCNNNNNNANNNCACNNNNNNNNNAGNNACNANNNTNANANNNGTAGNNNANTAAGTCT
CCGANNTNNNNNNATAAGCGNNANACNNNNNNNNNNNNNNANGANNACGNNTTGGNNNTNNNNNNNNA
GNNAGACNNTNATGGNTNNNNNTCAANNNNTTCGTAGTGGTATAATNGNNNNNNNTTTCNCCAGNNTN
NNNGNNNNNNCNATANNANNNNNNNGNGANNNNNNNNNNNANNNNCTCNNNAGGGANCANCTACTNNA
GCAANGGNGCGCAGNNNGAGANNNNGACNNGCCTTGGGTGGCAANCACNGCAATAAGTNNNGTTNNNC
CTNNNAGTNNACATNNNNNCANNGNNNCNNNNNNANCNGANNGNNNNNNNNNNNNNNNNNNNNCNNNN
NNNNNNNNNNNNNNNNNNNNNNNNNNNNANNNNANGGNNNGTNTNTGACNCATGTGTTTTTANNNGNN
NNNNNACGNNNNANCCCCGGTCGNNNNCNNATTGAANNCCTGNNGCATNNNNNTNCNAGAATGANNGC
NNNNNGNNTNGCTNNCNTTTCANGNAATCANNNAGGTNNNNNNNNTCNNGGNNNNNNNNCNNTCCCNN
ACTGAGGGTNNNNNNNCATCNNCTNNNNNNNNNNTNCGCNGGNGTNNCNNNNNNGCNGTATNNNGTNA
NNCANNNNNNNNNNNNNNNNTNAANNNANNNNNNTNNNN
>Mang301
GTGNNNNNTAGCCCNNNNNAGTTAGACANNNCNNNNNNNNNNNNTNNNNNNNNNNNNNNNNNNNNNNN
NNNNNNNNNNNNNNNNNNNNNNNNNNNNNNTNNNNNNNNNNNNNNNNNNNNNNNNNNNNNNNNNNNNN
NNNNNNNNNNNNNNNNNNNNNNNNNNNNNNNNNNNNNNNNNNNNNNNNNNNNNNNNNNNNNNNNNNNN
NNNNNNNNNNNNNNNNNNNNNNNNNNNNNNNNNNNNNNNNNNNNNNNNNNNNNNNNNNNNNNNNNNNN
NNNNNNNNNNNNNNNNNNNNNNNNNNNNNNNNNNNNNNNNNNNNNNNNNNNNTCTNNNAAANNNTNNN
NNNNNNNNNNNNNNNNNNNNNNNNNCNCNTTGNNNGTAANCNANNNGNACATGCAGTCCCCTCNNNNN
NNNGGCAGAGNNNNNNCCCNNNNNNNCCGNCCCCNNCCNNNNNNNNNNNCNNNGNNNNNNNANNNNNN
NNNNNNTGGTACNNNNCGNNNNNGGTTGTTTAAGAGGTCCACAACGNGGTCNNGNCACNNNNGNNNAN
NNNCNNNAAANNNNNNNNNNNNNNNTNNNTANNAGGNCNNNNCCNTCNNNNNNNNNATTGCNATGACC
CNANNNACGGGGGNNGTNCCCGGCGNGGCGACCTTGANNANNTCCGGGGACAAGTATGGCGNNCNCCC
CGGGAAGGANNCNGCNGTANGTGCNNNNNANNNNNTCNTGNGANGTCGCCGNNATTCNNGTNNNCNNN
CNAGAATGNCCNNACCANNCGNANNNTGGTNATGNNAGTNNNNNNNNNNNNNNNNNNNNNNNNNNNNN
NNNNNNNNNNNNNNNNNNNNNNNNNNNNNNNNNGNNNNGNNNNNNCANNNAAGGNNNTANNNCTANTN
NTNNNNNNGGACNNNACNNNNCNNNNNNNACNCNNNCNCNNNNNNANNNGNTTTTTNNNCNNTANTNT
CCAGNANGCNGNGCNNNCATTCANGNGAACNNNGNNACGCGNNAATCGTANNATCTACCTGCCGCNNG
TNNNNNTNCNGNNNNNNCGGANNNTNNNNCGTANNNNNNTCATGCGTNNTNTNNTCNCNAGNCNNGTT
CGNNNANANANTAGNANGTNTNNNNNNNNNTGTNGCGTTCCGCNTGANNCNNCANNNNGNNGNNCTTN
TCANCGGCNNTANCTGCNGANNTAAGNCATANNNTNNNTCNNTANCTNNNNNGGNGGGNNACTGTAAA
NGGNNAATTNGNNNNTNATTNCCNNCACNTNNCTNNGNNNNCTNNNTNCCTGNNTNCNNAATTGGNNC
CANTGNNTGNCATCTCTNGNTTNGNNNNNNTNNNNNNTNNCANNNNGGNNNNNNNNANNNCNATNTAA
NNTANANNNNCNNNNNNANNNCACNNNNNNNNNAGNNNCNANNNTTANANNNGTAGNNNANTAAGTCT
CCGANNTNNNNNNATAAGCGNNANACNNNNNNNNNNNNNNANGANNNCGNNTTGGNNNTNNNNNNCNA
GNNAGACNNTNATGGNTNNNNNTCAANNNNTTNGTAGTGGTATAATNGNNNNNNNTTTCNCCAGNNTN
NNNGNNNNNNCNATANNANNNNNNNGNGANNNNNNNNNNNANNNNCTCNNNAGGGANCANCTACTNNA
GCANNGGNGCGCAGNNNGAGANNNNGACNANCCTTGGGTGGCAANCACNGCAATAAGTNNNGTTNANC
CTNNNAGTNNACATNNNNNCANNGNNNCNNNNNNANCNGANNGNNNNNNNNNNNNNNNNNNNNNNNNN
NNNNNNNNNNNNNNNNNNNNNNNNNNNNNGNNNANNGNNNGTNTNTGACNCATGTGTTTTTANNNGNN
NNNNNACGNNNNANCCCCGGTCGNNNNCNNATTGAANNCCTGNNGCATNNNNNTNCNAGAATGANNGC
NNNNNGNNTNGCTNNCNNNTCAAGNAATCAANNAGNNNNNNCGNTCNNNGNNNNNNNNNCNNNCCCNN
ACTGAGGGTNNNNNNNCATCTGCTNNNNNNNNNCTNCGCNGGCGTNNCNGCNNAGCNGTATNGTGTNA
NNCANNNNNNNNNNNNNNNNTNAAACNANNNNNCTNNNN
>Mang302
GTGNNNNNTAGCCCNNNNNAGTTAGACANNNCNNNNNNNNNNNNTNNNNNNNNNNNNNNNNNNNNNNN

NNNNNNNNNNNNNNNNNNNNNNNNNNNNNNNNNNNNNNNNNNNNNNNNNNNNNNNNNNNNNNNNNNNN
NNNNNNNNNNNNNNNNNNNNNNNNNNNNNNNNNNNNNNNNNNNNNNNNNNNNNNNNNNNNNNNNNNNN
NNNNNNNNNNNNNNNNNNNNNNNNNNNNNNNNNNNNNNNNNNNNNNNNNNNNNNNNNNNNNNNNNNNN
NNNNNNNNNNNNNNNNNNNNNNNNNNNNNNNNNNNNNNNNNNNNNNNNNNCNTCTNNNAAANNNTNNN
NNNNNNNNNNNNNNNNNNNNNNNNNCNCNNTGNNNGTAANCNANNNGNACATGCAGTCCCCTCNNNNN
NNNGGCAGAGNNNNNNNCCNNNNNNNCCGNCCCCNNCCNNNNNNNNNNNNNNNGNNNNNNANNNNNNN
NNNNNNTGGTACNNNNCGNNNNNGGTTGTTTAAGAGGTCCACAACGNGGTCNNGNCACNNNNGNNNAN
NNNCNNANAANNNNNNNNNNNNNATNNNTANNAGGNNNNNNNCCNTCNNGNNNNNNANTGCNATGACC
CAANNCACGGGGGNAGTCCCCGGNGCGGCGACCTTGANNACGTCCGGGGNCAAGTATGGCNNNCNCCC
CGGGAAGGANNCNGCCGTANGTGCNNNNNANNNNNTCNTGNGACGTCGCCGNNATTCNCNTGGNCNNN
CNAGAATGNNCGNACCANNCGNANNNTGGTNATGNNAGTNNNNNNNNNNNNNNNNNNNNNNNNNNNNN
NNNNNNNNNNNNNNNNNNNNNNNNNNNNNNNNGNNNNGNNNNNNCNANNNAAGGANNTANNNCTNNTN
NTNNNNNNGGACNNNACNNNNCNNNNNNNACNCNNNCNCNNNNNNANNNGGTTTTTNNNCNNTATTNT
CCAGNACGCNGNGCNNNCATTCANGNGAACNNNGNNACGCGNNAATCGTANNATCTACCTGCCGCNNG
TNNNNNTACNGNNNNNNCGGANNNTNNNNCGTANNNNNNTCATGCGTNNTNTNNTCNCNAGNCNNGTT
CGNNNANANANTAGNANGTNTCNNNNNNNNTGTNNCGTTCCGCNTGANNCNNCANNNNGNNGNNCTTN
TCANCGGCNNTANCTGCNGANNTAAGNCATANNNTNNNTCNNTANCTNNNNNGGNGGGNACTGTAAA
NGGNTAATTNGANGNTNANTNCNNNCACNTNNCTNNGNNTNCTNNNTNCCTGNNTNCNNAATTGGNNC
CANTGNNTGNCATCTCTGGNTTNGNNNNNNTNNNNNTNNCANNNNGGNNNGTNANANNNCNATNTAA
NNTATANNNNCNNNNNNANNNCACNNNNNNNGNAGNNNCNANNNTTANANNNGTAGNNNAATAAGTCT
CCGANNTNNNNNNNATAAGCGNNANACNNNNNNNNNNNNNANGANNACGNNTTGGNNNTNNNNNNCNA
GNNAGACNNTNATGGNTNNNNNTCAANNNNTTNGTAGTGGTATAATNGNNNNNNNTTTCNCCAGNNTN
NNNGNNNNNNNCNATANNANNNNNNNGNGANNNNNNNNNNNANNNNCTCNNNAGGGANCANCTACTNNA
GCANTGGNGCGCAGNNNGAGANNNNGACNNNCCTTGGGTGGCAANCACNGCAATAAGTNNNGTTNANC
CTNNNAGTNNACATNNNNNCANNGNNNCNNNNNNANCNGANNGNNNNNNNNNNNNNNNNNNNNNNNNN
NNGNNNNNNNNNNNNNNNNNNNNNNNNNAGNNNANGGNNNGTNTNTGACNCATGTGTTTTTANNNGNN
NNNNNANGNNNNANCCCCGGTCGNNNNCNNATTGAANNCCTGNNGCATNNNNNTNCNAGAATGANNNC
NNNNNGNNTNGNCTNNCNNNTCAAGNAATCANNNAGGTNNNNNNNTCNNNGNNNNNNNNCNNTCCCNN
ACTGAGGGTNNNNNNNNCATCTGCTNNNNNNNNCTTCGCNGGNGTNNCNNNNNNGCNGTATNGNGTNN
NNCANNNNNNNNNNNNNNNNTNAAACNNNNNNNNTNNNN
>Mang303
GTGNNNNNTAGCCCNNNNNAGTTAGACANNNCNNNNNNNNNNNTNNNNNNNNNNNNNNNNNNNNNNN
NNNNNNNNNNNNNNNNNNNNNNNNNNNNNNTNNNNNNNNNNNNNNNNNNNNNNNNNNNNNNNNNNNNNN
NNNNNNNNNNNNNNNNNNNNNNNNNNNNNNNNNNNNNNNNNNNNNNNNNNNNNNNNNNNNNNNNNNNN
NNNNNNNNNNNNNNNNNNNNNNNNNNNNNNNNNNNNNNNNNNNNNNNNNNNNNNNNNNNNNNNNNNNN
NNNNNNNNNNNNNNNNNNNNNNNNNNNNNNNNNNNNNNNNNNNNNNNNNNNNTCTNNNAAANNNTNNN
NNNNNNNNNNNNNNNNNNNNNNNNNCNCNTTGNNNGTAANCNANNNGNACATGCAGTCCCCTCNNNNN
NNNGGCAGAGNNNNNNCCCNNNNNCNCCGGCCCCGNCCNNNNNNNNNNNCNNNGNNNNNNANNNNNNN
NNNNNNTGGTACNNNNCGNNNNNGGTTGTTTNAGAGGTCCACAACGNGGTCNGGNCACNNNNGNNNAN
NNNCNNNNAACNNNNNNNNNNNANATNNNTANNAGGNCNNNGCCNTCNNGNNNNTAATTGCNATGACC
CNANTCACGGGGGCAGTNCCCGGCGCGGCGACCTTGATNACGTCCGGGGACAAGTATGGCGNNCNCCC
CGGGAAGGANNCNGCCGTANGTGCNNNNNANNNNNTCNTGNGANGTCGCCGNNATTCNCGTGGTCNNN
CNAGAATGNCCGNACCANNCGNANNNTGGTNATGNNAGTNNNNNNNNNNNNNNNNNNNNNNNNNNNNN
NNNNNNNNNNNNNNNNNNNNNNNNNNNNNNNNGNNNNGNNNNNNCNANNNAAGGANNTANNNCTNNTC
NTNNNNNNGGACNNNACNNNNCNNNNNNNACNCNNNCNCNNNNNNANNNGGTTTTTNNCCNNTATTNT
CCAGNANGCNGNGCNNNCATTCANGNGAACNNNGNNACGCGNNAATCGTANNATCTACCTGCCGCNGG
TNNNNNTNCNGNNNNNNCGGANNNTNNCTCGTANNNNNNTCATGCGTNNTNTNNTCNCNAGNCANGTT
CGNNNANANANTAGNANGTNTNNNNNNNNNTGTNNCGTTCCGCNTGACNCNNCANNNNGNCGNNCTTN
TCANCGGCNNTANCTGCNGANNTAAGNCATANNNTNNATCNNTANCTNNNNNGGNGGGNACTGTAAA
NGGNNAATTNGANGNTNATTNCNNNCACNTNNCTNNGNNTNCTNNNTNCCTGNNTNCNNAATTGGNNC
CANTGNNTGNCATCTCTNGNTTNGNNNNNNTNNNNNTNNCAANNNGGNNNGTNANANNNCNATNTAA
NNTATANNNNCNNNNNNANNNCACNANNNNNGNAGNNANNANNNTTANANNNGTAGNNNANTAAGTCT
CCGANNTNNNNNNNATAAGCGNNANACNNNNNNNNNNNNNANGANNACGNNTTGGNNNTNNNNNNCNA
GNNAGACNNTNATGGNTNNNNNTCAANNNNTTCGTAGTGGTATAATNGNNNNNNNTTTCNCCAGNNTN

NNNGNNNNNNNNCNATANNANNNNNNNGNGANNNNNNNNNNNANNNNCTCNNNAGGGANCANCTACTNNA
GCANTGGNGCGCAGNNNGAGANNNNGACNNGCCTTGGGTGGCAANCACNGCAATAAGTNNNGTTNANC
CTNNNAGTNNACATNNNNNCANNGNNNCNNNNNNANCNGANNGNNNNNNNNNNNNNNNNNNNNNNNNNNN
NNNNNNNNNNNNNNNNNNNNNNNNNNNNNNAGNNNANGGNNNGTNTNTGACNCATGTGTTTTTANNNGNN
NNNNNACGNNNNANCCCCGGTCGNNNNCNNATTGAANNCCTGNNGCATNNNNNTNCNAGAATGANNGC
NNNNNGNNTNGNCTGNCNTTTCAAGNAATCANNNAGGTANNGCGNTCNNNGNNNNNNNNCGNTCCCNN
ACTGAGGGTNNNNNNNNCATCTGCTNNNNNNNNCTTCGCNGGCGTNNCTNNNNNGCNGTATNGNGTTA
NNCANNNNNNNNNNNNNNNNNTNAAACAANNNNNNNTNNNN
>Mang305
GTGNNNNNTAGCCCNNNNNAGTTAGACANNNCNNNNNNNNNNNNNTNNNNNNNNNNNNNNNNNNNNNNNN
NNNNNNNNNNNNNNNNNNNNNNNNNNNNNNNTNNNNNNNNNNNNNNNNNNNNNNNNNNNNNNNNNNNNNN
NNNNNNNNNNNNNNNNNNNNNNNNNNNNNNNNNNNNNNNNNNNNNNNNNNNNNNNNNNNNNNNNNNNNNN
NNNNNNNNNNNNNNNNNNNNNNNNNNNNNNNNNNNNNNNNNNNNNNNNNNNNNNNNNNNNNNNNNNNNNN
NNNNNNNNNNNNNNNNNNNNNNNNNNNNNNNNNNNNNNNNNNNNNNNNNNNNNNNTCTNNNAAANNNTNNN
NNNNNNNNNNNNNNNNNNNNNNNNNNNCNCNTTGNNNGTAANCNANNNGNACATGCAGTCCCCTCNNNNN
NNNNNCAGAGNNNNNNNCCNNNNNNNCCGNCCCCNNCCNNNNNNNNNNNNNNNNNNNNNNNNNANNNNNN
NNNNNNTGGTACNNNNCGNNNNNGGTTGTTTNAGAGGTCCACAACGNGGTCNNGNNACNNNNGNNNAN
NNNCNNNNAACNNNNNNNNNNNANNATNNNTANNAGGNCNNNNCCNTCNNGNNNNNAATNGCNATGACC
CAANNCACGGNGGCAGTCCCCGGNGNGGCGACCTTGANNANNTCCGGGGACAAGTATGGCNNNCNCCC
CGGGAAGGANNCNGCCGTANGTGCNNNNNANNNNNTCNTGNGANGTCGCCGNNATTCNCGTGGTCNNN
NNAGAATGCNCGNACCANNCGNANNNTGGTNATGNNAGTNNNNNNNNNNNNNNNNNNNNNNNNNNNNNNN
NNNNNNNNNNNNNNNNNNNNNNNNNNNNNNNNNNGNNNNGNNNNNNCNANNNAAGGNNNTANNNCTNNTC
NTNNNNNNGGACNNNACNNNNCNNNNNNNACNCNNNCNCNNNNNNNNNNNGGTTTTNNNNCNNTATTNT
CCAGNACGCNGNGCNNNCNTTCANGNGAACNNNGNNACGCGNNAATCGTANNATCTACCTGCCGCNNG
TNNNNNTNCNGNNNNNNCGGANNNTNNCTCGTANNNNNNTCATGCGTNNTNTNNTCNCNAGNCNNGTT
CGNNNANANANTAGNANGTNTNNNNNNNNNNTGTNNCGTTCCGCNTGANNCNNCANNNNGNNGNNNTTN
NCANCGGCNNTANCTGCNGANNTAAGNCATANNNTNNNTCNNTANCTNNNNNGGNGGGNNACTGTAAA
NGGNTAATTNGNNNNTNANTNCNNNCACNTNNCTNNGNNNNCTNNNTNCCTGNNTNCNNAATTGGNNN
CANTGNNTGNCNATCTCTGGNTTNGNNNNNNTNNNNNTNNCANNNNGGNNNGTNANANNNCNATNTAA
NNTANANNNNCNNNNNNANNNCACNANNNNNGNAGNNNCNANNNTTANANNNGTAGNNNANTAAGTCT
CCGANNTNNNNNNNATAAGCGNNANACNNNNNNNNNNNNNTANGANNACGNNTTGGNNNTNNNNNNNNA
GNNAGACNNTNATGGNTNNNNNTCAANNNNTTCGTAGTGGTATAATNGNNNNNNNTTTCNCCAGNNTN
NNNGNNNNNNNNCNATACNANNNNNNNGNGANNNNNNNNNNNANNNNCTCNNNAGGGANCANCTACTNNA
GCANTGGNNCGCAGNNNGAGANNNNGACNNNCCTTGGGTGGCAANNACNGCAATAAGTNNNGTTNANC
CTNNNAGTNNACATNNNNNCANNGNNNCNNNNNNANCNGANNGNNNNNNNNNNNNNNNNNNNNNNNNNNN
NNNNNNNNNNNNNNNNNNNNNNNNNNNNNNAGNNNANNGNNNGTNTNTGACNCATNNNTTTTTANNNGNN
NNNNNACGNNNNANCCCCGGTCGNNNNCNNATTGAANNCCTGNNGCATNNNNNTNCNAGAATGANNGC
NNNNNGNNTNGNCTNNCNNNTCAAGNAATCANNNANNNNNNNCNNTCNNNGNNNNNNNNCNNTCCCNN
ACTGAGGGTNNNNNNNNCATCNGCTNNNNNNNNNNTNCGCNGNNGTNNCNNNNNAGCCGTATNGTGTNA
NNCANNNNNNNNNNNNNNNNNNNNAAAANANNNNNNNTNNNN
>Mang306
GTGNNNNNTAGCCCNNNNNAGTTAGACANNNCNNNNNNNNNNNNNTNNNNNNNNNNNNNNNNNNNNNNNN
NNNNNNNNNNNNNNNNNNNNNNNNNNNNNNNNNNNNNNNNNNNNNNNNNNNNNNNNNNNNNNNNNNNNNN
NNNNNNNNNNNNNNNNNNNNNNNNNNNNNNNNNNNNNNNNNNNNNNNNNNNNNNNNNNNNNNNNNNNNNN
NNNNNNNNNNNNNNNNNNNNNNNNNNNNNNNNNNNNNNNNNNNNNNNNNNNNNNNNNNNNNNNNNNNNNN
NNNNNNNNNNNNNNNNNNNNNNNNNNNNNNNNNNNNNNNNNNNNNNNGNNNNNNTCTNNNAAANNGTNNN
NNNNNNNNNNNNNNNNNNNNNNNNNNNCNCNTTGNNNGTAANCNANNNGNACATGCAGTCCCCTCNNNNN
NNNGGCAGAGNNNNNNNNNNNNNNCNCCGNCCCCNNCCNNNNNNNNNNNNNNNNNGNNNNNNNANNNNNN
NNNNNNTGGTACNNNNCGNNNNNGGTTGTNTAAGAGGTCCACAACGNGGTCNNGNCACNNNNGNNNAN
NNNCNNNNAACNNNNNNNNNNNNANNTNNNTANNAGGNCNNNNCCNTCNNGNNNNNNATTGCNATGACC
CAANNNACGGGGGCNGTNCCCGGCGCGGCGACCTTGANNACGTCCGGGGNCAAGTATGGCGNNCNCCC
CGGGAAGGANNCNGCCGTANGTGCNNNNNANNNNNTCNTGNGANGTCGCCGNNATTCNCGTGGTCNNN
CNAGAATGNNCNNACCANNCGNANNNTGGTNATGNNAGTNNNNNNNNNNNNNNNNNNNNNNNNNNNNNNN
NNNNNNNNNNNNNNNNNNNNNNNNNNNNNNNNNNGNNNNGNNNNNNCNANNNAAGGANNTANNNCTNNTN

NTNNNNNNGGACNNNACNNNNCNNNNNNNACNCNNNCNCNNNNNNANNNGNTTTTNNNCNNTATTNT
CCAGNACGCNGNGCNNNCATTCANGNGAACNNNGNNACGCGNNAATCGTANNATCTACCTGCCGCNNG
TNNNNNTACNGNNNNTNCGGANNNTNNCTCGTANNNNNNTCATGCGTNNTNTNNTCNCNAGNCNNGTT
CGNNNANANANTAGNANGTNTNNNNNNNNNNTGTNNCGTTCCGCNTGANNCNNCANNNNGNNGNNCTTC
TCANCGGCNNTANCTGCNGANNTAAGNCATANNNTNNATCNNTANCTNNNNNGGAGGGNNACTGTAAA
NGGNNAATTGGANNNTNATTNCNNNCACNTNNCTNNGNNTNCTNNNTNCCTGNNTNCNNAATTGGNNC
CANTGNNTGNCNATCTCTGGNTTNGCNNNNNTNNNNNTNNCAANNNGGNNNGTNNNANNNCNATNTAA
NNTANANNNNCNNNNNNANNNCACNANNNNNGNAGNNNCNANNNTTANANNNGTAGNNNANTAAGTCT
CCGANNTNNNNNNNATAAGCGNNANACNNNNNNNNNNNNNNANGANNACGNNTTGGNNNTNNNNNNCNA
GNNAGACNNTNATGGNTNNNNNTCAANNNNTTCGTAGTGGTATAATNGNNNNNNNTTTCNCCAGNNTN
NNNGNNNNNNNCNATANNANNNNNNNGNGANNNNNNNNNNNANNNNCTCNNNAGGGANCANCTACTNNA
GCANTGGNGCGNAGNNNGAGANNNNGACCAGCCTTGGGTGGCAANCACNGCAATAAGTNNNGTTNANC
CTNNNAGTNNACATNNNNNCANNGNNNCNNNNNNANCNGANNGNNNNNNNNNNNNNNNNNNNNNNNNN
NNNNNNNNNNNNNNNNNNNNNNNNNNNNNNGNNNANGGNNNGTNTNTGACNCATGTGTTTTTANNNGNN
NNNNNACGNNNNANCCCCGGTCGNNNNCNNATTGAANNCCTGNNGCATNNNNNTNCNAGAATGANNNC
NNNNNGNNTNGNCTGNCNNNTCAAGNAATNAANNAGNTNNCGCGNTCNNGGNNNNNNNNCNNTCCCNN
ACTGAGGGTNNNNNNNNCATCTGCTNNNNNNNNCTNCGCNGGNGTNNCNNNNNNGCNGTATNGNGTNN
NNCANNNNNNNNNNNNNNNNNTNAAACAANNNNNNTNNNN
>Mang307
GTGNNNNNTAGCCCNNNNNAGTTAGACANNNCNNNNNNNNNNNNNTNNNNNNNNNNNNNNNNNNNNNNN
NNNNNNNNNNNNNNNNNNNNNNNNNNNNNNNNTNNNNNNNNNNNNNNNNNNNNNNNNNNNNNNNNNNNNN
NNNNNNNNNNNNNNNNNNNNNNNNNNNNNNNNNNNNNNNNNNNNNNNNNNNNNNNNNNNNNNNNNNNNNN
NNNNNNNNNNNNNNNNNNNNNNNNNNNNNNNNNNNNNNNNNNNNNNNNNNNNNNNNNNNNNNNNNNNNNN
NNNNNNNNNNNNNNNNNNNNNNNNNNNNNNNNNNNNNNNNNNNNNNNNNNNNNCNTCTNNNAAANNNTNNN
NNNNNNNNNNNNNNNNNNNNNNNNNNNCNCNTTGNNNGTAANCNANNNGNACATGCAGTCCCCTCNNNNN
NNNGGCAGAGNNNNNNCCCNNNNNCNCCGNCCCCNNCCNNNNNNNNNNNNCNNNGNNNNNNNANNNNNN
NNNNNNTGGTACNNNNCGNNNNNGGTTGTTTAAGAGGTCCACAACGNGGTCNNGNNACNNNNGNNNAN
NNNCNNNNAACNNNNNNNNNNNNANNTNNNTANNAGGNCNNNNCCNTCNNNNNANNNATTGCNATGACC
CAANTCACGGGGGCAGTCCCCGGCGCGGCGACCTTGANNANGTCCGGGGACAAGTATGGCNNNCNCCC
CGGGAAGGANNCNGCCGTANGTGCNNNNNANNNNNTCNTGNGANGTCGCCGNNATTCNCGTGGTCNNN
CNAGAATGNCCNNACCANNCGNANNNTGGTTATGNNAGTNNNNNNNNNNNNNNNNNNNNNNNNNNNNNNN
NNNNNNNNNNNNNNNNNNNNNNNNNNNNNNNNNNNNNNNGNTNNNNCNANNNAAGGANNTANNNCTNNTN
NTNNNNNNGGACNNNACNNNNCNNNNNNNACNCNNNCNCNNNNNNNNNNGNTTTTNNNNCNNTATTNT
CCAGNACGCNGNGCNNNCATTCANGNGAACNNNGNNACGCGNNAATCGTANNATCTACCTGCCGCNNG
NNNNNNTACNGNNNNNNCGGANNNTNNCTCGTANNNNNNNCATGCGTNNTNTNNTCNCNAGNCNNGTT
CGNNNANANANTAGNANGTNTNNNNNNNNNNTGTNNCGTTCCGCNTGANNCNNCANNNNGNCGNNNTTN
TCANCGGCNNTANCTGCNGANNTAAGNCATANNNTNNNTCNNTANCTNNNNNGGNGGGNNACTGTAAA
NGGNNAATTNGNNGNTNATTNCNNNCACNTNNCNNNGNNTNCTNNNTNCCTGNNTNCNNAATTGGNNC
CANTGNNTGNCNATCTCTGGNTTNGNNNNNNTNNNNNTNNCAANNNGGNNNGTNNNANNNCNATNTAA
NNTATANNNNCNNNNNNANNNCACNNNNNNNGNAGNNACNANNNTTANANNNGTAGNNNANTAAGTCT
CCGANNTNNNNNNNATAAGCGNNANACNNNNNNNNNNNNNNANGANNACGNNTTGGNNNTNNNNNNCNA
GNNAGACNNTNATGGNTNNNNNTCAANNNNTTCGTAGTGGTATAATNGNNNNNNNTTTCNCCAGNNTN
NNNGNNNNNNNCNATANNANNNNNNNGNGANNNNNNNNNNNANNNNCTCNNNAGGNANCANCTACTNNA
GCANTGGNGCGNAGNNNGAGANNNNGACNNNCCTTGGGTGGCAANCACNGCAATAAGTNNNGTTNANC
CTNNNAGTNNACATNNNNNCANNGNNNCNNNNNNANCNGANNGNNNNNNNNNNNNNNNNNNNNNNNNN
NNNNNNNNNNNNNNNNNNNNNNNNNNNNNNGNNNANGGNNNGTNTNTGACNCATGNNTTTTTANNNGNN
NNNNNACGNNNNANCCCCGGTCGNNNNCNNATTGAANNCCTGNNGCATNNNNNTNCNAGAATGANNGC
NNNNNGNNTNGNCTNNCNNTTCAAGNAATCANNNAGNNNNNNCGNTCNNNGNNNNNNNNCNNTCCCNN
ACTGAGGGTNNNNNNNNCATCTGCTNNNNNNNNNNTNCGCNGGCGTNNCNNNNNAGCNGTATNGNGTNA
NNCANNNNNNNNNNNNNNNNNTNAANNNANNNNNNTNNNN
>Mang308
GTGNNNNNTAGCCCNNNNNAGTTAGACANNNCNNNNNNNNNNNNNTNNNNNNNNNNNNNNNNNNNNNNN
NNNNNNNNNNNNNNNNNNNNNNNNNNNNNNNNNNNNNNNNNNNGNNNNNNNNNNNNNNNNNNNNNNNNNN
NNNNNNNNNNNNNNNNNNNNNNNNNNNNNNNNNNNNNNNNNNNNNNNNNNNNNNNNNNNNNNNNNNNNNN

NNNNNNNNNNNNNNNNNNNNNNNNNNNNNNNNNNNNNNNNNNNNNNNNNNNNNNNNNNNNNNNNNNNN
NNNNNNNNNNNNNNNNNNNNNNNNNNNNNNNNNNNNNNNNNNNNNNNNNNCNTCTNNNAAANNNTNNN
NNNNNNNNNNNNNNNNNNNNNNNNNCNCNTTNNNNGTAANCNANNNGNACATGCAGTCCCCTCNNNNN
NNNGGCAGAGNNNNNNNCCNNNNNNNCCGNCCCCNNCCNNNNNNNNNNNNNNNGNNNNNNNANNNNNN
NNNNNNTGGTACNNNNCGNNNNNGGTTGTTTNAGAGGTCCACAACGNGGTCNGGNNACNNNNGNNNAN
NNNCNNNNAACNNNNNNNNNNNNANNTNNNTANNAGGNCNNNNCCNTCNNNNNNNNNNATNGCNATGACC
CAANTCNCGGGGGNNGTNCCCGGCGCGGCGACCTTGANNANGTCCGGGGNCAAGTATGGCNNNCNCCC
CGNGAAGGANNCNGCCGTANGTGCNNNNNANNNNNTCNTGNGANGTCGCCGNNATTCNCGTGGTCNNN
CNAGANTGNCCNNACCANNCGNANNNTGGTNATGNNAGTNNNNNNNNNNNNNNNNNNNNNNNNNNNNN
NNNNNNNNNNNNNNNNNNNNNNNNNNNNNNNNNNNGNNNNGNNNNNNCNANNNAAGGNNNTANNNCTNNTN
NTNNNNNNGGACNNNACNNNNCNNNNNNNACNCNNNCNCNNNNNNANNNGGTTTNNNNCCNNTANTNT
CCAGNACGCNGNGCNNNCNTTCANGNGAACNNNGNNACGCGNNAATCGTANNATCTACCTGCCGCNNG
NNNNNNTACNGNNNNNNCGGANNNTNNCTCGTANNNNNNTCANGCGTNNTNTNNTCNCNAGNCANGTT
CGNNNANANANTAGNANGTNTNNNNNNNNNNTGTNNCGTTCCGCNTGANNCNNCANNNNGNNGNNCTTN
TCANCGGCNNTANCTGCNGANNTAAGNCATANNNTNNNTCNNTANCTNNNNNGGNGGGNNACTGTAAA
NGGNNAATTNGNNNNTNATTNCNNNCACNTNNCNNNGNNNNCTNNNTNCCTGNNTNCNNAATTGGNNC
CANTGNNTGNCNATCTCTGGNTTNGNNNNNNTNNNNNTNNCANNNNGGNNNGTNNNANNNCNATNTAA
NNTANANNNNCNNNNNNANNNCACNNNNNNNNNAGNNNCNANNNTTANANNNGTAGNNNANTNAGTCT
CCGANNTNNNNNNNATAAGCGNNANACNNNNNNNNNNNNNANGANNACGNNTTGGNNNTNNNNNNNNA
GNNAGACNNTNATGGNTNNNNNTCAANNNNTTNGTAGTGGTATAATNGNNNNNNNTTTCNCCAGNNTN
NNNGNNNNNNNNCNATANNANNNNNNNGNGANNNNNNNNNNNANNNNCTCNNNAGGNANCANCTACTNNA
GCANTGGNGCGNAGNNNGAGANNNNGACNNGCCTTGGGTGGCAANNACNGCAATAAGTNNNGTTNANN
CTNNNAGTNNACATNNNNNCANNGNNNCNNNNNNANCNGANNGNNNNNNNNNNNNNNNNNNNNNNNNN
NNNNNNNNNNNNNNNNNNNNNNNNNNNNNNNNNNNANGGNNNGTNTNTGACNCATGTGTTTTTANNNGNN
NNNNNACGNNNGANCCCCGGTCGNNTNCNNATTGAANNCCTGNNGCATNNNNNNTNCNAGAATGANNGC
NNNNNGNNTNGNCTNNNNNTTCAAGNAATCANNNAGGNANNNNGNTCNNNGNNNNNNNNCNNTCCCNN
ACTGAGGGTNNNNNNNNCANCNGCTNNNNNNNNCTNCGCNGGNGTNNCNNNNNAGCNGTATNGNGTNA
NNCANNNNNNNNNNNNNNNNNNTNNAACAANNNNNNTNNNN
>Mang309
GTGNNNNNTAGCCCNNNNNAGTTAGACANNNCNNNNNNNNNNNNNTNNNNNNNNNNNNNNNNNNNNNN
NNNNNNNNNNNNNNNNNNNNNNNNNNNNNNNNTNNNNNNNNNNNNNNNNNNNNNNNNNNNNNNNNNNN
NNNNNNNNNNNNNNNNNNNNNNNNNNNNNNNNNNNNNNNNNNNNNNNNNNNNNNNNNNNNNNNNNNNN
NNNNNNNNNNNNNNNNNNNNNNNNNNNNNNNNNNNNNNNNNNNNNNNNNNNNNNNNNNNNNNNNNNNN
NNNNNNNNNNNNNNNNNNNNNNNNNNNNNNNNNNNNNNNNNNNNNNNNNNCNTCTNNNAAANNNTNNN
NNNNNNGNNNNNNNNNNNNNNNNNNCNCNTTNNNNGTAANCNANNNGNACATGCAGTCCCCTCNNNNN
NNNGGCAGAGNNNNNNCCCCNNNNNNNCCGNCCCCNNCCNNNNNNNNNNNNCNNNGNNNNNNNANNNNNN
NNNNNNTGGTACNNNNCGNNNNNGGTTGTTTAAGAGGTCCACAACGNGGTCNGGNNACNNNNGNNNAN
NNNCNNNNAACNNNNNNNNNNNNANNTNNNTANNAGGNCNNNNCCNTCNNNNNNNNNNATTGCNATGACC
CAANTCACGGGGGCAGTNCCCGGCGCGGCGACCTTGATNANNTCCGGGGACAAGTATGGCNNNCNCCC
CGGGAAGGANNCNGCCGTANGTGCNNNNNANNNNNTCNTGNGANGTCGCCGNNATTCNCNTGGTCNNN
CNAGAATGNNCNNACCANNCGNANNNTGGTNATGNNAGTNNNNNNNNNNNNNNNNNNNNNNNNNNNNN
NNNNNNNNNNNNNNNNNNNNNNNNNNNNNNNNNNNGNNNNGNNNNNNCNANNNAAGGANNTANNNCTNNTN
NTNNNNNNGGACNNNACNNNNCNNNNNNNACNCNNNCNCNNNNNNANNNGGTTTTTNNNCNNTATTNT
CCAGNANGCNGNGCNNNCATTCANGNGAACNNNGNNACGCGNNAATCGTANNATCTACCTGCCGCNNG
TNNNNNTNCNGNNNNNNCGGANNNTNNCTCGTANNNNNNNCATGCGTNNTNTNNTCNCNAGNCANGTT
CGNNNANANANTAGNANGTNTCNNNNNNNNNTGTNNCGTTCCGCNTGANNCNNCANNNNGNNGNNNTTN
NCANCGGCNNTANCTGCNGANNTAAGNCATANNNTNNNTCNNTANCTNNNNNGGNGGGNNACTGTAAA
NGGNNAATTNGNNNNTNATTTCCNNCACNTNNCTNNGNNNNCTNNNTNCCTGNNTNCNNAATTGGNNC
CANTGNNTGNCNATCTCTGGNTTNGNNNNNNTNNNNNTNNCAANNNGGNNNGTNNNANNTCNATNTAA
NNTANANNNNCNNNNNNANNNCACNNNNNNNGNAGNNNCNANNNTTANANNNGTAGNNNAATAAGTCT
CCGANNTNNNNNNNATAAGCGNNANACNNNNNNNNNNNNNANGANNACGNNTTGGNNNTNNNNNNNCNA
GNNAGACNNTNATGGNTNNNNNTCAANNNNTTNGTAGTGGTATAATNGNNNNNNNTTTCNCCAGNNTN
NNNGNNNNNNNNCNATANNANNNNNNNGNGANNNNNNNNNNNANNNNCTCNNNAGGGANCANCTACTTNA
GCAATGGNGCGCAGNNNGAGANNNNGACNAGCCTTGGGTGGCAANCACNGCAATAAGTNNNGTTNANC

CTNNNAGTNNACATNNNNNCANNGNNNCNNNNNNANCNGANNGNNNNNNNNNNNNNNNNNNNNNNNNN
NNNNNNGNNNNNNNNNNNNNNNNNNNNNNNAGNNNANGGNNNGTNTNTGACNCATGNNTTTTTANNNGNN
NNNNNACGNNNNANCCCCGGTCGNNNNCNNATTGAANNCCTGNNGCATNNNNNTNCNAGAATGANNNC
NNNNNGNNTNGNCTNNCNTTTCAAGNAATCANNNAGGTNTNNNGNTCNNNGNNNNNNNNCNNTCCCNN
ACTGAGGGTNNNNNNNNCATCTGCTNNNNNNNNNTNCGCNGGNGTNNCNNNNNAGCNGTATNGTGTNA
NNCANNNNNNNNNNNNNNNNNTNAAACNANNNNNNTNNNN
>Mang310
GTGNNNNNTAGCCCNNNNNAGTTAGACANNNCNNNNNNNNNNNNNTNNNNNNNNNNNNNNNNNNNNNNNN
NNNNNNNNNNNNNNNNNNNNNNNNNNNNNNNNNNNNNNNNNNNNNNNNNNNNNNNNNNNNNNNNNNNNNN
NNNNNNNNNNNNNNNNNNNNNNNNNNNNNNNNNNNNNNNNNNNNNNNNNNNNNNNNNNNNNNNNNNNNNN
NNNNNNNNNNNNNNNNNNNNNNNNNNNNNNNNNNNNNNNNNNNNNNNNNNNNNNNNNNNNNNNNNNNNNN
NNNNNNNNNNNNNNNNNNNNNNNNNNNNNNNNNNNNNNNNNNNNNNNNNNNCNTCTNNNAAANNNTNNN
NNNNNNNNNNNNNNNNNNNNNNNNNNNCNCNNTGNNNGTAANCNANNNGNACATGCAGTCCCCTCNNNNN
NNNGGCAGAGNNNNNNNCCNNNNNCNCCGNCCCCNNCCNNNNNNNNNNNCNNNGNNNNNNNANNNNNN
NNNNNNTGGTACNNNNCGNNNNNGGTTGNNTAAGAGGTCCACAACGNGGTCNGGNCACNNNNGNNNAN
NNNCNNNAAACNNNNNNNNNNNANNTNNNTANNAGGNCNNNNCCNTCNNGNNNNTAATNGCNATGACC
CAANTCNCGGNGGCNGTNCCCGGCGCGGCGACCTTGANNANNTCCGGGGACAAGTATGGCGNNCNCCC
CGGGAAGGANNCNGCCGTANGTGCNNNNNANNNNNTCNTGNGANGTCGCCGNNATTCNCGTGGTCNNN
NNAGAATGNCCNNACCANNCGNANNNTGGTNATGNNAGTANNNNNNNNNNNNNNNNNNNNNNNNNNNNN
NNNNNNNNNNNNNNNNNNNNNNNNNNNNNNNNNNNGNNNNGNNNNNNCNANNNAAGGANNTANNNCTNNTN
NTNNNNNNNGGNCNNNACNNNNCNNNNNNNNACNCNNNCNCNNNNNNNNNNNGGTTTTNNNNCNNTATTNT
CCAGNACGCNGNGCNNNCATTCANGNGAACNNNGNNACGCGNNAATCGTANNATCTACCTGCCGCNGG
NNNNNNTACNGNNNNNNCGGANNNTNNCNCGTANNNNNNNCANGCGTNNTNTNNTCNCNAGNCNNGTT
CGNNNANANANTAGNANGTNTNNNNNNNNNNTGTNNCGTTCCGCNTGANNCNNCANNNNGNNGNNCTTN
NCANCGGCNNTANCTGCNGANNTAAGNCATANNNTNNNTCNNTANCTNNNNNGGNGGGNNACTGTAAA
NGGNNAATTNGNGNNTNATTNCNNNCACNTNNCNNNGNNTNCTNNNTNCCTGNNTNCNNAATTGGNNC
CANTGNNTGNCNATCTCTGGNTTNGNNNNNNTNNNNNTNNCANNNNGGNNNGTNANANNNCNATNTAA
NNTANANNNNCNNNNNNANNNCACNANNNNNGNAGNNNCNANNNTTANANNNGTAGNNNAATAAGTCT
CCGANNTNNNNNNNATAAGCGNNANACNNNNNNNNNNNNNANGANNACGNNTTGGNNNTNNNNNNCNA
GNNAGACNNTNATGGNTNNNNNTCAANNNNTTNGTAGTGGTATAATNGNNNNNNNTTTCNCCAGNNTN
NNNGNNNNNNNCNATACNANNNNNNNGNGANNNNNNNNNNNANNNNCTCNNNAGGNANCANCTACTNNA
GCANTGGNGCGCAGNNNGAGANNNNGACNNNCCTTGGGTGGCAANCACNGCAATAAGTNNNGTTNANC
CTNNNAGTNNACATNNNNNCANNGNNNCNNNNNNANCNGANNGNNNNNNNNNNNNNNNNNNNNNNNNN
NNNNNNNNNNNNNNNNNNNNNNNNNNNNNNAGNNNANGGNNNGTNTNTGACNCATGNNTTTTTANNNGNN
NNNNNNNGNNNNANCCCCGGTCGNNNNCNNATTGAANNCCTGNNGCATNNNNNTNCNAGAATGANNGC
NNNNNGNNTNGNCTNNCNTTTCAAGNAATCANNTAGGTNTNNNGNTCNNNGNNNNNNNNCNNTCCCNN
ACTGAGGGTNNNNNNNNCATCTGCTNNNNNNNNNTNCGCNGGNGTNNCNNNNNAGCNGTATNGTGTNA
NNCANNNNNNNNNNNNNNNNNTNAAAAAANNNNNNTNNNN
>Mang311
GTGNNNNNTAGCCCNNNNNAGTTAGACANNNCNNNNNNNNNNNNNTNNNNNNNNNNNNNNNNNNNNNNNN
NNNNNNNNNNNNNNNNNNNNNNNNNNNNNNNTNNNNNNNNNNNNNNNNNNNNNNNNNNNNNNNNNNNNNN
NNNNNNNNNNNNNNNNNNNNNNNNNNNNNNNNNNNNNNNNNNNNNNNNNNNNNNNNNNNNNNNNNNNNNN
NNNNNNNNNNNNNNNNNNNNNNNNNNNNNNNNNNNNNNNNNNNNNNNNNNNNNNNNNNNNNNNNNNNNNN
NNNNNNNNNNNNNNNNNNNNNNNNNNNNNNNNNNNNNNNNNNNNNNNNNNNCNTCTNNNAAANNNTNNN
NNNNNNNNNNNNNNNNNNNNNNNNNNNCNCNTTGNNNGTAANCNANNNGNACATGCAGTCCCCTCNNNNN
NNNGGCAGAGNNNNNNNCCNNNNNNNNCCGGCCCCNNCCNNNNNNNNNNNCNNNGNNNNNNNANNNNNN
NNNNNNTGGTACNNNNCGNNNNNGGTTGTTTAAGNGGTCCACAACGNGGTCNGGNCACNNNNGNNNAN
NNNCNNNAAACNGNNNNNNNNNNANATNNNTANNAGGNCNNNNCCNTCNNGNNNNTNATTGCNATGACC
CAANTCACGGGGGCAGTNCCCGGCGCGGCGACCTTGATNACGTCCGGGGACAAGTATGGCNNNCNCCC
CGGGAAGGANNCNGCCGTANGTGCNNNNNANNNNNTCNTGNGANGTCGCCGNNATTNNNGTGGTCNNN
NNAGAATGNCCNNACCANNCGNANNNTGGTNATGNTAGTNNNNNNNNNNNNNNNNNNNNNNNNNNNNNN
NNNNNNNNNNNNNNNNNNNNNNNNNNNNNNNNNNNNNNGNNNNNNCNANNNAAGGANNTANNNCTNNTN
NTNNNNNNNGGACNNNACNNNNCNNNNNNNNACNCNNNCNCNNNNNNNANNNGNTTTTNNNCCNNTATTNT
CCAGNANGCNGNGCNNNCATTCANGNGAACNNNGNNACGCGNNAATCGTANNATCTACCTGCCGCNNG

TNNNNNTACNGNNNNNNCGGANNNTNNCTCGTANNNNNNNNTCATGCGTNNTNTNNTCNCNAGNCANGTT
CGNNNANANANTAGNANGTNTNNNNNNNNNNNTGTNNCGTTCCGCNTGANNCNNCANNNNGNNGNNNTTN
NCANCGGCNNTANCTGCNGANNTAAGNCATANNNTNNATCNNTANCTNNNNNGGNGGGNNACTGTAAA
NGGNNAATTNGNNGNTNATTNCNNNCACNTNNCNNNGNNNNCTNNNTNCCTGNNTNCNNAATTGGNNC
CANTGNNTGNCNATCTCTGGNTTNGNNNNNNTNNNNNTNNCANNNNGGNNNGTNANANNNCNATNTAA
NNTANANNNNCNNNNNNANNNCACNNNNNNNGNAGNNNCNANNNTTANANNNGTAGNNNANTNAGTCT
CCGANNTNNNNNNNATAAGCGNNANACNNNNNNNNNNNNNNANGANNACGNNTTGGNNNTNNNNNNCNA
GNNAGACNNTNATGGNTNNANNTCAANNNNTTNGTAGTGGTATAATNGNNNNNNNTTTCNCCAGNNTN
NNNGNNNNNNNCNATANNANNNNNNNGNGANNNNNNNNNNNANNNNCTCNNNAGGGANCANCTACTNNA
GCANTGGNGCGNAGNNNGAGANNNNGACNNNCCTTGGGTGGCAANCACNGCAATAAGTNNNGTTNANC
CTNNNAGTNNACATNNNNNCANNGNNNCNNNNNNANCNGANNGNNNNNNNNNNNNNNNNNNNNNNNNNNN
NNNNNNNNNNNNNNNNNNNNNNNNNNNNNNAGNNNANGGNNNGTNTNTGACNCATGTGTTTTTANNNGNN
NNNNNACGNNNGANCCCCGGTCGNNTNCNNATTGAANNCCTGNNGCATNNNNNTNCNAGAATGANNNC
NNNNNGNNTNGNCTNNCNTTTCAAGNAATCAANTAGGTNNNGCGNTCNNNGNNNNNNNNCNNTCCCNN
ACTGAGGGTNNNNNNNNCATCTGCTNNNNNNNNNCTNCGCNGGCGTNNCNNNNNAGCNGTATNNTGTNA
NNCANNNNNNNNNNNNNNNNNNTNAAACGANNNNNNNTNNNN
>Mang399
GTGNNNNNTAGCCCNNNNNAGTTAGACANNNCNNNNNNNNNNNNNTNNNNNNNNNNNNNNNNNNNNNNNN
NNNNNNNNNNNNNNNNNNNNNNNNNNNNNNNNNNNNNNNNNNNNNNNNNNNNNNNNNNNNNNNNNNNNNN
NNNNNNNNNNNNNNNNNNNNNNNNNNNNNNNNNNNNNNNNNNNNNNNNNNNNNNNNNNNNNNNNNNNNNN
NNNNNNNNNNNNNNNNNNNNNNNNNNNNNNNNNNNNNNNNNNNNNNNNNNNNNNNNNNNNNNNNNNNNNN
NNNNNNNNNNNNNNNNNNNNNNNNNNNNNNNNNNNNNNNNNNNNNNNNNNNNCNTCTNNNAAANNNTNNN
NNNNNNNNNNNNNNNNNNNNNNNNNNCNCNTTGNNNGTAANCNANNNGNACATGCAGTCCCCTCNNNNN
NNNGGCAGAGNNNNNNCNCNNNNNNNCCGNCCCCNNCCNNNNNNNNNNNNNNNNNNNNNANNNNNNNNNN
NNNNNNTGGTACNNNNCGNNNNNGGTTGTNTAAGAGGTCCACAACGNGGTCNGGNCACNNNNGNNNAN
NNNCNNNNAACNNNNNNNNNNNNANNTNNNTANNAGGNNNNNNCCNTCNNGNNNNNNATTGCNATGACC
CAANNCACGGGGGCNGTCCCCGGCGNGGCGACCTTGNTNANGTCCGGGGNCAAGTATGGCNNNCNCCC
CGGGAAGGANNCNGCCGTANGTGCNNNNNANNNNNTCNTGNGANGTCGCCGNNATTNNCNTGGTCNNN
CNAGAATGNCCNNACCANNCGNANNNTGGTNATGNNAGTNNNNNNNNNNNNNNNNNNNNNNNNNNNNNNN
NNNNNNNNNNNGNNNNNNNNNNNNNNNNNNNNNNNNNNNGNNNNNNCNANNNAAGGANNTANNNCTNNTN
NTNNNGNNGGACNNNACNNNNCNNNNNNNACNCNNNCNCNNNNNNNNNNNGNTTTTTNNNCNNTATTNT
CCAGNACGCNGNGCNNNCATTCANGNGAACNNNGNNACGCGNNAATCGTANNATCTACCTGCCGCNNG
TNNNNNTACNGNNNNTNCGGANNNTNNCTCGTANNNNNNNNTCATGCGTNNTNTNNTCNCNAGNCANGTT
CGNNNANANANTAGNANGTNTNNNNNNNNNNNTGTNNCGTTCCGCNTGANNCNNCANNNNGNNGNNCTTN
NCANCGGCNNTANCTNCNGANNTAAGTCATANNNTNNNTCNNTANCTNNNNNGGNGGGNNACTGTAAA
NGGNNAATTNGNNGNTNATTNCNNNCACNTNNCTNNGNNTNCTNNNTNCCTGNNTNCNNAATTGGNNC
CANTGNNTGNCNATCTCTGGNTTNGCNNNNNTNNNNNTNNCAANNNGGNNNGTNANANNNCNATNTAA
NNTANANNNNCNNNNNNANNNCACNANNNNNGNAGNNNNNANNNTTANANNNGTAGNNNANTAAGTCT
CCGANNTNNNNNNNATAAGCGNNANACNNNNNNNNNNNNNNANGANNACGNNTTGGNNNTNNNNNNCNA
GNNAGACNNTNATGGNTNNNNNTCAANNNNTTCGTAGTGGTATAATNGNNNNNNNTTTCNCCAGNNTN
NNNGNNNNNNNCNATANNANNNNNNNGNGANNNNNNNNNNNANNNNCTCNNNAGGGANCANCTACTNNA
GCAATGGNGCGCAGNNNGAGANNNNGACNANCCTTGGGTGGCAANCACNGCAATAAGTNNNGTTNANC
CTNNNAGTNNACATNNNNNCANNGNNNCNNNNNNANCNGANNGNNNNNNNNNNNNNNNNNNNNNNNNNNN
NNNNNNNNNNNNNNNNNNNNNNNNNNNNNNAGNNNANGGNNNGTNTNTGACNCATGTGTTTTTANNNGNN
NNNNNACGNNNNANCCCCGGTCGNNNNCNNATTGAANNCCTGNNGCATNNNNNTNCNAGAATGANNGC
NNNNNGNNTNGNCTNNCNNNTCAAGNAATCAANNAGGTNNNNNNNTCNNNGNNNNNNNNCNNTCCCNN
ACTGAGGGTNNNNNNNNCATCTGCTNNNNNNNNNNTNCGCNGGNGTNNCNNNNNAGCNGTATNGTGTNA
NNCANNNNNNNNNNNNNNNNNNNNAAANNNNNNNNNNNTNNNNN
>Nung47
GTGNNNNNNAGCCCNNNNNAGTTAGACANNNCNNNNNNNNNNNNNNNNNNNNNNNNNNNNNNNNNNNNNN
NNNNNNNNNNNNNNNNNNNNNNNNNNNNNNTNNNNNNNNNNNNNNNNNNNNNNNNNNNNNNNNNNNNNNNN
NNNNNNNNNNNNNNNNNNNNNNNNNNNNNNNNNNNNNNNNNNNNNNNNNNNNNNNNNNNNNNNNNNNNNN
NNNNNNNNNNNNNNNNNNNNNNNNNNNNNNNNNNNNNNNNNNNNNNNNNNNNNNNNNNNNNNNNNNNNNN
NNNNNNNNNNNNNNNNNNNNNNNNNNNNNNNNNNNNNNNNNNNNNNNNNNNNNNTCTNNNAAANNNTNNN

NNNNNNNNNNNNNNNNNNNNNNNNNCNCNTTGNNNGTAANCNANNNGNACATGCAGTCCCCTCNNNNN
NNNGGCAGAGNNNNNNCCCNNNNNCNCCGNCCCCGNCCNNNNNNNNNNNNCNNNNNNNNNNNNANNNNNN
NNNNNNTGGTACNNNNCGNNNNNGGTTGTNTAAGAGGTCCACAACANGGTCNNNNCACNNNNGNNNAN
NNNCNNNAAACNNNNNNNNNNNNNANNTNNNTANNAGGNNNNNNCCNTCNNGNNANNNANTGCNATGACC
CAANTNACGGNGGCNGTNCCCGGCGCGGCGACCTTGATNACGTCCGGGGNCAAGTATGGCGNNCNCCC
CGGGAAGGANNCNGCCGTANGTGCNNNNNANNNNNTCNTGNGANGTCGCCGNNATTNNCGTGGTCTNN
NNAGANTGNCCNNACCANNCGNANNNTGGTNATGTTAGTNNNNNNNNNNNNNNNNNNNNNNNNNNNN
NNNNNNNNNNNNNNNNNNNNNNNNNNNNNNNNNNNNNNNNNGNNNNNNCNANNNAAGGANNTANNNCTNNTN
NNNNNNNNGGACNNNACNNNNCNNNNNNNACNCNNNCNCNNNNNNANNNGNTTTTTNNNCNNTATTNT
CCAGNACGCNGNGCNNNCATTCANGNGAACNNNGNNACGCGNNAATCGTANNATCTACCTGCCGCNNG
TNNNNNTACNGNNNNNNCGGANNNTNNCTCGTANNNNNNNCANGCGTNNTNTNNTCNCTAGNCNNGTT
CGNNNANANANTAGNANGTNTNNNNNNNNNNTGTNNCGTTCCGCNCGANNCNNCANNNNGNNGNNCTTN
TCANCGGCNNTANCTGCNGANNTAAGTCATANNNTNNNTCNNTANCTNNNNNGGNGGGNNACTGTAAA
NGGNNAATTGGNNNNTNATTNCNNNCACNTNNCNNNANNNNCTNNNTNCCTGNNTNCNNAATTGGNNC
CANTGNNTGNCNATCTCTGGNTTNGCNNNNNTNNNNNTNNCANNNNGGNNNGTNANANNNCNATNTAA
NNTANANNNNCNNNNNNANNNCACNNNNNNNGNAGNNNCNANNNTNANANNNGTAGNNNANTAAGTCT
CCGANNTNNNNNNNATAAGCGNNANACNNNNNNNNNNNNNNANGANNNCGNNTTGGNNNTNNNNNNCNA
GNNAGAANNTNGTGGNTNNNNNTCAANNNNTTNGTAGTGGTATAATNGNNNNNNNTTTCNCCAGNNTN
NNNGNNNNNNNCNATANNANNNNNNNGNGANNNNNNNNNNNANNNNCTTNNNAGGGANCANTTACTNNA
GCAATGGNGCGCAGNNNGAGANNNNGACNNNCCTTGGGTGGCAANCACNGCAATAAGTNNNGTTNANC
CTNNNAGTNNACACNNNNNCANNGNNNCNNNNNNANCNGANNGNNNNNNNNNNNNNNNNNNNNNNNNN
NNNNNNNNNNNNNNNNNNNNNNNNNNNNNNNAGNNNANGGNNNGTNTNTGACNCATGTGTTTTTANNNGNN
NNNNNACGNNNNANCCCCGGTCGNNNNCNNATTGAANNCCTGNNGCATNNNNNTNCNAGAATGANNNC
NNNNNGNNTNGNCTNNCNTTTCAAGNAATCANNNAGGTNNNNNNNTCNNNGNNNNNNNNCNNTCACNN
ACTGAGGGTNNNNNNNNCATCTGCTNNNNNNNNNCTNCGCNGGNGTNNCNNNNNAGCNGTATNGNGTNN
NNCANNNNNNNNNNNNNNNNNNTNAAACNANNNNNNNTNNNN
>Nung48
GTGNNNNNTAGCTCNNNNNAGTTAGACANNNCNNNNNNNNNNNNNTNNNNNNNNNNNNNNNNNNNNNNNN
NNNNNNNNNNNNNNNNNNNNNNNNNNNNNNNTNNNNNNNNNNNNNNNNNNNNNNNNNNNNNNNNNNNNNNN
NNNNNNNNNNNNNNNNNNNNNNNNNNNNNNNNNNNNNNNNNNNNNNNNNNNNNNNNNNNNNNNNNNNNNNNNN
NNNNNNNNNNNNNNNNNNNNNNNNNNNNNNNNNNNNNNNNNNNNNNNNNNNNNNNNNNNNNNNNNNNNNNNNN
NNNNNNNNNNNNNNNNNNNNNNNNNNNNNNNNNNNNNNNNNNNNNNNNNNNNNNNTNNNNNAAANNNTNNN
NNNNNNNNNNNNNNNNNNNNNNNNNNCNCNNTGNNNGTAANCNANNNGNACATGCAGTCCCCTCNNNNN
NNNGGCAGAGNNNNNNCCCNNNNNNNCCGNCCCCNNCCNNNNNNNNNNNNNNNNNGNNNNNNNGNNNNNN
NNNNNNTGGTACNNNNCGNNNNNGGTTGNTNAAGAGGTCCACCACGNGGTCNNGNNACNNNNGNNNAN
NNNCNNNNAANNNNNNNNNNNNNNNNNTNNNCANNAGGNNNNNNCCNTNNNNNNNNNNNANNGCNATGANC
CAANNCNCGGGGANNGTNACCGGCGNGGCGACCTTGANNANNGTCGGGGNCAAGTATGTCNNNCNCCC
CGGGAAGGANNCNGCCGTANGTGCNNNNNANNNNNTCNTGNGANGTNGCCGNNATTCNCGTGGTCNNN
CNAGAATGNCCGNANCANNCGNANNNTGGTNATGNNAGTNNNNNNNNNNNNNNNNNNNNNNNNNNNN
NNNNNNNNNCNNNNNNNNNNNNNNNNNNNNNNNNNNNNNGNNNNNNCNANNNAAGGNNNTANNNTTNNTN
NTNNNNNNNNNNCNNNACNNNNCNNNNNNNACNCNNNCNCNNNNNNANNNGNTTTTTNNNCNNTATTNT
CCAGNACGCNGNGCNNNCATTCANGNGAACNNNGNNACGCGNNAATCGTANNATCTACCTGCCGCNNG
NNNNNNTNCNGNNNNNNCGGANNNTNNCTCGTANNNNNNTCATGCGTNNNNTNNTTNCNAGNCANGTT
CGNNNANANANTAGNANGTNTNNNNNNNNNNTGTNGCGTTCCGCNTGANNCNNCANNNNGNNGNNCTTN
NCANCGGCNNTANCTNCNGANNTAAGNCATANNNTNNNTCNNTANCGNNNNNGGNGGGNNACTGTAAA
NGNNNAATTNGNNGNTNANTNCNNNCACNNNNCTNNGNNNNCTNNNCNCCTGNNTNCNNAATTGGNNN
CANTGNNTGNCNATCTCTGGNTTNGNNNNNNTNNNNNTNNTANNNNGGNNNGTNANANNNCNATNTAA
NNTANANNNNCNNNNNNANNNCACNNNNNNNNNAGNNNNNANNNTNANGNNNNTAGNNNANTAAGTCT
CCGANNTNNNNNNNATAAGCGNNANACNNNNNNNNNNNNNNANGANNNCGNNTTGGNNNTNNNNNNCNA
ANNAGACNNCNGTGGNTNNNNNTCAANNNNTTNGTAGTGGTATAACNANNNNNNNTTTCNCCAGNNTN
NNNGNNNNNNNCNATANNANNNNNNNGNGANNNNNNNNNNNANNNNCCCNNNAGGGANCANCTACTNNA
GCAATGGNGCGNAGNNNGAGANNNNGACNNGCCTTGGGTGGCAANCACNGCAATAAGTNNTGTTNANC
CCNNNAGTNNACACNNNNNCANNGNNNCNNNNNNANCNGANNTNNNNNNNNNNNNNNNNNNNNNNNNN
NNNNNNNNNNNNNNNNNNNNNNNNNNNNNNNNNNNANGGNNNGTNTNTGACNCATGNNTTTTTANNNGNN

NNNNNACGNNNGANCCCCGGTCGTNNNCNNATTGAANNCCTGNNGCATNNNNNTNCNAGAATGANNNC
NNNNNGNNTNGNCTNNCNTTTCAAGNAGTCANNTAGGTANNNNNNTCNNNGNNNNNNNNCNNNCCCNN
ACTGAGGGNNNNNNNNNCANCTGCTNNNNNNNNCTNCGCNGNNNNNNTNNNNNAGCNGTATNGNGTNN
NNCANNNNNNNNNNNNNNNNNNANNNNNNNNNNNTNNNN
>Nung49
GTGNNNNNTAGCCCGNNNNAGTCAGACANCNCNNNNNNNNNNNNTNNNNNNNNNNNNNNNNNNNNNNN
NNNNNNNNNNNNNNNNNNNNNNNNNNNNNNTNNNNNNNNNNNNNNNNNNNNNNNNNNNNNNNNNNNNNN
NNNNNNNNNNNNNNNNNNNNNNNNNNNNNNNNNNNNNNNNNNNNNNNNNNNNNNNNNNNNNNNNNNNN
NNNNNNNNNNNNNNNNNNNNNNNNNNNNNNNNNNNNNNNNNNNNNNNNNNNNNNNNNNNNNNNNNNNN
NNNNNNNNNNNNNNNNNNNNNNNNNNNNNNNNNNNNNNNNNNNNNNNNNNCNTCTNNNAAANNNTNNN
NNNNNNNNNNNNNNNNNNNNNNNNNCNCNTTGNNNGTAANCNANNNGNACATGCAGTCCCCTCNNNNN
NNNGGCAGAGNNNNNNCCCNNNNNNNCCGNCCCCNNCCNNNNNNNNNNNCANNGNNNNNNNANNNNNNN
NNNNNNTGGTACNNNNCGNNNNNGGTTGTNTNAGAGGTCCACAACGNGGTCNNGNCACNNNNGNNNAN
NNNCNNAAAACNNNNNNNNNNNANNTNNNTANNAGGNCNNNNCCNTCNNGNNNNNAATNGCNATGACC
CAANNCACGGGGGNAGTNCCCGGCGNGGCGACCTTGANNACGTCCGGGGNCAAGTATGGCGNNCNCCC
CGGGAAGGANNCNGCCGTANGTGCNNNNNANNNNNTCNTGNGANGTCGCCGNNATTCNCGTGGTCNNN
NNAGAATGNCCGNACCANNCGNANNNTGGTNATGNNAGTNNNNNNNNNNNNNNNNNNNNNNNNNNNNN
NNNNNNNNNNNNNNNNNNNNNNNNNNNNNNNNGNNNNGNNNNNNCNANNNAAGGANNTANNNCTNNTC
NTNNNNNNGGACNNNACNNNNCNNNNNNNACNCNNNCNCNNNNNNNNNNGNTTTTTNNNCNNTATTNT
CCAGNACGCNGNGCNNNCATTCANGNGAACNNNGNNACGCGNNAATCGTANNATCTACCTGCCGCNGG
TNNNNNTACNGNNNNNNCGGANNNTNNCTCGTANNNNNNNCATGCGTNNTNTNNTCNCNAGNCANGTT
CGNNNANANANTAGNANGTNTCNNNNNNNNTGTNNCGTTCCGCNTGANNCNNCANNNNGNNGNNCTTN
NCANCGGCNNTANCTGCNGANNTAAGNCATANNNTNNATCNNTANCTNNNNNGGNGGGNNACTGTAAA
NGGNNAATTNGANGNTNATTTCCNNCATNTNNCTNNGNNTNCTNNNTNCCTGNNTNCNNAATTGGNNC
CANTGNNTGNCNATCTCTGGNTTNGNNNNNNTGNNNNTNNCANNNNGGNNNGTNNNANNNCNATNTAA
NNTATANNNNCNNNNNNANNNCACNNNNNNNNNAGNNNCNANNNGTANANNNGTAGNNNGNTNAGTCT
CCGANNTNNNNNNNATAAGCGNNANACNNNNNNNNNNNNNANGANNACGNNTTTGNNNTNNNNNNCNA
GNNAGACNNTNGTGGNTNNNNNTCAANNNNTTNGTAGTGGTATAATNGNNNNNNNTTTCNCCAGNNTN
NNNGNNNNNNNCNATANNANNNNNNGNGANNNNNNNNNNNANNNNCTCNNNAGGGANCANCTACTNNA
GCAATGGNGCGNAGNNNGAGANNNNGACNNNCCTTGGGTGGCAANCACNGCAATAAGTNNNGTTNANC
CTNNNAGTNNACACNNNNNCANNGNNNCNNNNNNANCNGANNGNNNNNNNNNNNNNNNNNNNNNNNNN
NNNNNNNNNNNNNNNNNNNNNNNNNNNNAGNNNANGGNNNGTNTNTGACNCATGNGTTTTTANNNGNN
NNNNNACGNNNNANCCCCGGTCGNNNNCNNATTGAANNCCTGNNGCATNNNNNTNCNAGAATGANNNC
NNNNNGNNTNGNCTNNCNNNTCAAGNAATCANNNAGGTANNGCGNTCNNNGNNNNNNNNCNNTCCCNN
ACTGAGGGTNNNNNNCGCATCTGCTNNNNNNNNNTNCGCNGGCGTNNCNNCNNAGCNGTATNNTGTNA
NNCANNNNNNNNNNNNNNNTNAAACNANNNNNNNTNNNN
>Nung664
GTGNNNNNTAGCCCNNNNNAGTTAGACANNNCNNNNNNNNNNNNTNNNNNNNNNNNNNNNNNNNNNNN
NNNNNNNNNNNNNNNNNNNNNNNNNNNNNNNNNNNNNNNNNNNNNNNNNNNNNNNNNNNNNNNNNNNN
NNNNNNNNNNNNNNNNNNNNNNNNNNNNNNNNNNNNNNNNNNNNNNNNNNNNNNNNNNNNNNNNNNNN
NNNNNNNNNNNNNNNNNNNNNNNNNNNNNNNNNNNNNNNNNNNNNNNNNNNNNNNNNNNNNNNNNNNN
NNNNNNNNNNNNNNNNNNNNNNNNNNNNNNNNNNNNNNNNNNNNNNNNNNNNTCTNNNAAANNNTNNN
NNNNNNNNNNNNNNNNNNNNNNNNNCNCNNTGNNNGTAANCNANNNGNACATGCAGTCCCCTCNNNNN
NNNNNCAGAGNNNNNNCCCNNNNNNNCCGGCCCCNNCCNNNNNNNTNNNCNNNGNNNNNNNANNNNNN
NNNNNNTGGTACNNNNCGNNNNNGGTTGNNTAAGAGGTCCACAACGNGNTCNNNNCACNNNNGNNNAN
NNNCNNNNAANNNNNNNNNNNNNNATNNNTANNAGGNNNNNNCCNTCNNGNNNNNNATTGCNATGACN
CANNNNACGGNGGNNGTNCCCGGCGNGGCGACCTTGANNANGTCCGGGGNCAAGTATGGCGNNCNCCC
CGGGAAGGANNCNGCNGTANGTGCNNNNNANNNNNTCNTGNGANGTNGCCGNNATTNNCGTGGTCNNN
CNAGAATGNCCNNACCANNCGNANNNTGGTNATGNNAGTNNNNNNNNNNNNNNNNNNNNNNNNNNNNN
NNNNNNNNNNNNNNNNNNNNNNNNNNNNNNNNNNNNNGNNNNNNCNANNNAAGGNNNTANNNCTNNTN
NNNNNNNNGGACNNNACNNNNNNNNNNNNACNCNNNCNCNNNNNNNNNNGNTTTTNNNNCNNTANTNT
CCAGNANGCNGNGCNNNCATTCANGNGAACNNNGNNACGCGNNAATCGTANNATCTACCTGCCGCNNG
NNNNNNTACNGNNNNNNCGGANNNTNNCTCGTANNNNNNNCANGCGTNNTNTNNTCNCNAGNCNNGTT
CGNNNANANANTAGNANGTNTNNNNNNNNNTGTNNCGTTCCGCNTGANNCNNCANNNNGNNGNNCTTN

NCANCGGCNNTANCTNCNGANNTAAGNCATANNNTNNNTCNNTANCTNNNNNGGNGGGNNACTGTAAA
NGGNTAATTNGNNGNTNANTNCNNNCACNTNNCTNNGNNNNCTNNNTNCCTGNNTNCNNAATTGGNNC
CANTGNNTGNCNATCTCTNGNTTNGNNNNNNTNNNNNTNNCANNNNGGNNNGTNANANNNCNATNTAA
NNTANANNNNCNNNNNNANNNCACNNNNNNNNNAGNNNCNANNNTNANANNNGTAGNNNANTAAGTCT
CCGANNTNNNNNNNATAAGCGNNANACNNNNNNNNNNNNNNANGANNACGNNTTGGNNNTNNNNNNCNA
GNNAGACNNTNATGGNTNNNNNTCAANNNNTTNGTAGTGGTATAATNGNNNNNNNTTTCNCCAGNNTN
NNNGNNNNNNNCNATANNANNNNNNNGNGANNNNNNNNNNNANNNNCTCNNNAGGGANCANCTACTNNA
GCANTGGNNCGCAGNNNGAGANNNNGACNNNCCTTGGGTGGCAANNACNGCGATAAGTNNNGTTNANC
CTNNNAGTNNACATNNNNNCANNGNNNCNNNNNNANCNGANNGNNNNNNNNNNNNNNNNNNNNNNNNNNN
NNNNNNNNNNNNNNNNNNNNNNNNNNNNNNNNNNNANGGNNNGTNTNTGACNCATGTGTTTTTANNNGNN
NNNNNACGNNNNANCCCCGGTCGNNNNCNNATTGAANNCCTGNNGCATNNNNNTNCNAGAATGANNGC
NNNNNGNNTNGNCTGNCNNNTCAAGNAATCANNNAGNTANNNNNNTCNNNNNNNNNNNNCNNTCCCNN
ACTGAGGGTNNNNNNNNCATCTGCTNNNNNNNNNNTTCGCNGGNGTNNCNNNNNAGCNGTATNGNGTNA
NNCANNNNNNNNNNNNNNNNNNTNAAACGANNNNNNTNNNN
>Nung665
GTGNNNNNTAGCCCNNNNNAGTTAGACANNNCNNNNNNNNNNNNNTNNNNNNNNNNNNNNNNNNNNNNNN
NNNNNNNNNNNNNNNNNNNNNNNNNNNNNNNNNNNNNNNNNNNNNNNNNNNNNNNNNNNNNNNNNNNNNN
NNNNNNNNNNNNNNNNNNNNNNNNNNNNNNNNNNNNNNNNNNNNNNNNNNNNNNNNNNNNNNNNNNNNNN
NNNNNNNNNNNNNNNNNNNNNNNNNNNNNNNNNNNNNNNNNNNNNNNNNNNNNNNNNNNNNNNNNNNNNN
NNNNNNNNNNNNNNNNNNNNNNNNNNNNNNNNNNNNNNNNNNNNNNNNNNNNCNTCTNNNAAANNNTNNN
NNNNNNNNNNNNNNNNNNNNNNNNNNNCNCNNTGNNNGTAANCNANNNGNACATGCAGTCCCCTCNNNNN
NNNGGCAGAGNNNNNNNCCNNNNNNNCCGGCCCCNNCCNNNNNNNNNNNCNNNGNNNNNNNANNNNNN
NNNNNNTGGTACNNNNCGNNNNNGGTTGTNTAAGAGGTCCACAACGNGGTCNGGNCACNNNNGNNNAN
NNNCNNNAAACNNNNNNNNNNNNNNNNTNNNTANNAGGNCNNNNCCNTCNNNNNNNNNATNGCNATGACC
CAANNCACGGGGGCNGTNCCCGGCGCGGCGACCTTGATNACGTCCGGGGACAAGTATGGCGNNCNCCC
CGGGAAGGANNCNGCCGTANGTGCNNNNNANNNNNTCNTGNGANGTCGCCGNNATTCNCGTGGTCNNG
CNAGAATGNCCNNACCANNCGNANNNTGGTNATGNNAGTNNNNNNNNNNNNNNNNNNNNNNNNNNNNNNN
NNNNNNNNNNNNNNNNNNNNNNNNNNNNNNNNNNGNNNNGNNNNNNCNANNNAAGGANNTANNNCTNNTN
NTNNNNNNNGGACNNNACNNNNCNNNNNNNACNCNNNCNCNNNNNNNNNNNGGTTTTTNNNCNNTANTNT
CCAGNACGCNGNGCNNNCATTCANGNGAACNNNGNNACGCGNNAATCGTANNATCTACCTGCCGCNNG
NNNNNNTNCNGNNNNNNCGGANNNTNNCTCGTANNNNNNTCATGCGTNNTNTNNTCNCNAGNCANGTT
CGNNNANANANTAGNANGTNTNNNNNNNCTTGTNNCGTTCCGCNTGANNCNNCANNNNGNNGNNCTTN
NCANCGGCNNTANCTGCNGANNTAAGNCATAANNTNNNTCNNTANCTNNNNNGGNGGGNNACTGTAAA
NGGNNAATTNGNNGNTNANTNCNNNCACNTNNCTNNGNNTNCTNNNTNCCTGNNTNCNNAATTGGNNC
CANTGNNTGNCNATCTCTGGNTTNGNNNNNNTNNNNNTNNCAANNNGGNNNNNNANANNNCNATNTAA
NNTANANNNNCNNNNNNANNNCACNANNNNNGNAGNNNCNANNNTTANANNNGTAGNNNAATAAGTCT
CCGANNTNNNNNNNATAAGCGNNANACNNNNNNNNNNNNNNANGANNACGNNTTGGNNNTNNNNNNCNA
GNNAGACNNTNATGGNTNNNNNTCAANNNNTTNGTAGTGGTATAATNGNNNNNNNTTTCNCCAGNNTN
NNNGNNNNNNNCNATANNANNNNNNNGNGANNNNNNNNNNNANNNNCTCNNNAGGGANCANCTACTNNA
GCAANGGNGCGNAGNNNGAGANNNNGACCANCCTTGGGTGGCAANCACNGCAATAAGTNNNGTTNANC
CTNNNAGTNNACATNNNNNCANNGNNNCNNNNNNANCNGANNGNNNNNNNNNNNNNNNNNNNNNNNNNNN
NNNNNNNNNNNNNNNNNNNNNNNNNNNNNNAGNNNANGGNNNGTNTNTGACNCATGTGTTTNTANNNGNN
NNNNNACGNNNGANCCCCGGTCGNNNNCNNATTGAANNCCTGNNGCATNNNNNTNCNAGAATGANNGC
NNNNNGNNTNGNCTNNCNNNTCAAGNAATCANNNAGGTANNNNGNTCNNNGNNNNNNNNCNNNCCCNN
ACTGAGGGTNNNNNNNNCATCTGCTNNNNNNNNCTNCGCNGNNGTNNCNNNNNNGCNGTATNGNGTNA
NNCANNNNNNNNNNNNNNNNNNNNAANNAANNNNNNNTNNNN
>Nung666
GTGNNNNNNAGCCCNNNNNAGTTAGACANNNCNNNNNNNNNNNNNTNNNNNNNNNNNNNNNNNNNNNNNN
NNNNNNNNNNNNNNNNNNNNNNNNNNNNNNNNNNNNNNNNNNNNNNNNNNNNNNNNNNNNNNNNNNNNNN
NNNNNNNNNNNNNNNNNNNNNNNNNNNNNNNNNNNNNNNNNNNNNNNNNNNNNNNNNNNNNNNNNNNNNN
NNNNNNNNNNNNNNNNNNNNNNNNNNNNNNNNNNNNNNNNNNNNNNNNNNNNNNNNNNNNNNNNNNNNNN
NNNNNNNNNNNNNNNNNNNNNNNNNNNNNNNNNNNNNNNNNNNNNNNNNNNNNNTCTNNAAAANNNTNNN
NNNNNNNNNNNNNNNNNNNNNNNNNNNCNCNNTGNNNGTAANCNANNNGNACATGCAGTCCCCTCNNNNN
NNNNNCAGAGNNNNNNNCNNNNNNCNCCGNCCCCNNCCNNNNNNNNNNNNNNNNNGNNNNNNNANNNNNN

NNNNNNTGGTACNNNNCGNNNNNGGTTGTNTAAGAGGTCCACAACGNGGTCNGGNCACNNNNGNNNAN
NNNCNNNNANCNNNNNNNNNNNNNNNTNNNTANNAGGNCNNNNCCNTCNNGNNNNNNATNGCNATGACC
CANNTNACNGGGGCNGTCCCCGGNGCGGCGACCTTGATNANNTCCGGGGNCAAGTATGGCNNNCNCCC
CGGGAAGGANNCNGCCGTANGTGCNNNNNANNNNNTCNTGNGANGTNGCCGNNATTNNNGTNGTCNNN
NNAGANTGNNCNNACCANNCGNANNNTGGTNATGTNAGTNNNNNNNNNNNNNNNNNNNNNNNNNNNNN
NNNNNNNNNNNNNNNNNNNNNNNNNNNNNNNNGNNNNGNNNNNNCNANNNAAGGNNNTANNNCTNNTN
NNNNNNNNGGACNNNACNNNNCNNNNNNNACNCNNNCNCNNNNNNNNNNGGTTTTNNNNCNNTATTNT
CCAGNACGCNGNGCNNNCATTCANGNGAACNNNGNNACGCGNNAATCGTANNATCTACCTGCCGCNNG
TNNNNNTACNGNNNNNNCGGANNNTNNNNNGTANNNNNNNCANGCGTNNTNTNNTNNCNAGNCNNGTT
CGNNNANANANTAGNANGTNTNNNNNNNCTTGTNNCGTTCCGCNTGANNCNNCNNNNNGNNGNNNTTN
NCANCGGCNNTANCTNCNGANNTAAGNCATANNNTNNATCNNTANCTNNNNNGGNGGGNNACTGTAAA
NGGNNAATTNGNNNNTNATTNCNNNCACNTNNCNNNGNNTNCTNNNTNCCTGNNTNCNNAATTGGNNC
CANTGNNTGNCNATCTCTGGNTTNGNNNNNNTNNNNNTNNCANNNNGGNNNNNNANANNNCNATNTAA
NNTANANNNNCNNNNNNANNNCACNNNNNNNNNNAGNNNCNANNNTNANANNNGTAGNNNAATNAGTCN
CCGANNTNNNNNNNNATAAGCGNNANACNNNNNNNNNNNNNNANGANNACGNNTTGGNNNTNNNNNNNNA
GNNAGACNNTNATGGNTNNNNNTCAANNNNTTNGTAGTGGTATAATNGNNNNNNNTTTCNCCAGNNTN
NNNGNNNNNNNNCNATANNANNNNNNNGNGANNNNNNNNNNNANNNNCTCNNNAGGNANCANCTACTNNA
GCAANGGNGCGCAGNNNGAGANNNNGACNNNCCTTGGGTGGCAANCACNGCGATAAGTNNNGTTNANN
CTNNNAGTNNACATNNNNNCANNGNNNCNNNNNNANCNGANNGNNNNNNNNNNNNNNNNNNNNNNNNN
NNNNNNNNNNNNNNNNNNNNNNNNNNNNNNNNNNNANGGNNNGTNTNTGACNCATGTGTTTNTANNNGNN
NNNNNACGNNNNANCCCCGGTCGNNNNCNNATTGAANNCCTGNNGCATNNNNNNTNCNAGAATGANNNC
NNNNNGNNTNGNCTNNNNNNTCAAGNAATCANNNAGGTNTNGCGNTCNNNGNNNNNNNNCNNTCCCNN
ACTGAGGGTNNNNNNNNCATCNGCTNNNNNNNNCTNCGCNGGNGTNNCNNNNNAGCNGTATNNNGTNA
NNCANNNNNNNNNNNNNNNNNTNAANNAANNNNNNTNNNN
>Nung667
GTGNNNNNTAGCCCNNNNNAGTTAGACANNNCNNNNNNNNNNNNTNNNNNNNNNNNNNNNNNNNNNNN
NNNNNNNNNNNNNNNNNNNNNNNNNNNNNNNTNNNNNNNNNNNNNNNNNNNNNNNNNNNNNNNNNNNN
NNNNNNNNNNNNNNNNNNNNNNNNNNNNNNNNNNNNNNNNNNNNNNNNNNNNNNNNNNNNNNNNNNNN
NNNNNNNNNNNNNNNNNNNNNNNNNNNNNNNNNNNNNNNNNNNNNNNNNNNNNNNNNNNNNNNNNNNN
NNNNNNNNNNNNNNNNNNNNNNNNNNNNNNNNNNNNNNNNNNNNNNNNNNNNNTCTNNNAAANGNTNNN
NNNNNNNNNNNNNNNNNNNNNNNNNCNCNTTGNNNGTAANCNANNNGNACATGCAGTCCCCTCNNNNN
NNNGGCAGAGNNNNNNCCCNNNNNCNCCGGCCCCGNCCNNNNNNNNNNNNCNNNGNNNNNNNANNNNNN
NNNNNNTGGTACNNNNCGNNNNNGGTTGTTTAAGAGGTCCACAACGNGGTCNGGNCACNNNNGNNTAN
NNNCNNNAAACNNNNNNNNNNNNANNTNNNTANNAGGNCNNNNCCNTCNNGNNNNTNATTGCNATGACC
CAANTCACGGGGGCAGTCCCCGGCGCGGCGACCTTGATNACGTCCGGGGACAAGTATGGCNNNCNCCC
CGGGAAGGANNCNGCCGTANGTGCNNNNNANNNNNTCNTGNGANGTCGCCGNNATTCNCGTGGTCNAN
CNAGAATGNCCGNACCANNCGNANNNTGGTNATGNNAGTNNNNNNNNNNNNNNNNNNNNNNNNNNNNN
NNNNNNNNNNNNNNNNNNNNNNNNNNNNNNNNGNNNNGNNNNNNCNANNNAAGGNNNTANNNCTNNTN
NTNNNNNNGGACNNNACNNNNCNNNNNNNACNCNNNCNCNNNNNNANNNGGTTTTTNNNCNNTATTNT
CCAGNACGCNGNGCNNNCATTCANGNGAACNNNGNNACGCGNNAATCGTANNATCTACCTGCCGCNNG
TNNNNNTNCNGNNNNNNCGGANNNTNNNTCGTANNNNNNTCATGCGTNNTNTNNTCNCNAGNCANGTT
CGNNNANANANTAGNANGTNTNNNNNNNNNNTGTNNCGTTCCGCNTGANNCNNCANNNNGNNGNNCTTN
TCANCGGCNNTANCTGCNGANNTAAGNCATANNNTNNATCNNTANCTNNNNNGGNGGGNNACTGTAAA
NGGNNAATTNGNNGNTNATTTCNNNCACNTNNCTNNGNNNNCTNNNTNCCTGNNTNCNNAATTGGNNC
CANTGNNTGNCNATCTCTGGNTTNGNNNNNNTGNNNNTNNCANNNNGGNNNGTNANANNNCNATNTAA
NNTANANNNNCNNNNNNANNNCACNANNNNNGNAGNNACNANNNTTANANNNGTAGNNNAATAAGTCT
CCGANNTNNNNNNNNATAAGCGNNANACNNNNNNNNNNNNNNANGANNACGNNTTGGNNNTNNNNNNNCNA
GNNAGACNNTNATGGNTNNNNNTCAANNNNTTCGTAGTGGTATAATNGNNNNNNNTTTCNCCAGNNTN
NNNGNNNNNNNNCNATANNANNNNNNNGNGANNNNNNNNNNNANNNNCTCNNNAGGGANCANCTACTNNA
GCANTGGNGCGCAGNNNGAGANNNNGACCANCCTTGGGTGGCAANCACNGCGATAAGTNNNGTTNANC
CTNNNAGTNNACATNNNNNCANNGNNNCNNNNNNANCNGANNGNNNNNNNNNNNNNNNNNNNNNNNNN
NNNNNNNNNNNNNNNNNNNNNNNNNNNNNNNAGNNNANGGNANGTNTNTGACNCATGTGTTTTTANNNGNN
NNNNNACGNNNNANCCCCGGTCGNNNNCNNATTGAANNCCTGNNGCATNNNNNNTNCNAGAATGANNGC
NNNNNGNNTNGNCTNNCNTTTCAAGNAATCANNNAGGTNNNGCGNTCNNNGNNNNNNNNCNNTCCCNN

ACTGAGGGTNNNNNNNNCATCTGCTNNNNNNNNCTNCGCNGGCGTNNCNNNNNAGCNGTATNGTGTNA
NNCANNNNNNNNNNNNNNNNNNTNAAACNANNNNNNNTNNNN
>Nung668
GTGNNNNNTAGCCCGNNNNAGTTAGACANNNCNNNNNNNNNNNNTNNNNNNNNNNNNNNNNNNNNNNN
NNNNNNNNNNNNNNNNNNNNNNNNNNNNNNTNNNNNNNNNNNNNNNNNNNNNNNNNNNNNNNNNNN
NNNNNNNNNNNNNNNNNNNNNNNNNNNNNNNNNNNNNNNNNNNNNNNNNNNNNNNNNNNNNNNNNN
NNNNNNNNNNNNNNNNNNNNNNNNNNNNNNNNNNNNNNNNNNNNNNNNNNNNNNNNNNNNNNNNNN
NNNNNNNNNNNNNNNNNNNNNNNNNNNNNNNNNNNNNNNNNNNNNNNNNNNNCNTCTNNNAAANNNTNNN
NNNNNNNNNNNNNNNNNNNNNNNNNNNNCNCNNTNNNNGTAANCNANNNGNACATGCAGTCCCCTCNNNNN
NNNNNCAGAGNNNNNNNCCNNNNNNNCCGNCCCCNNCCNNNNNNNNNNNCNNNNNNNNNNNANNNNNN
NNNNNNTGGTACNNNNCGNNNNNGGTTGTNTAAGAGGTCCACCACGNGGTCNGNNCACNNNNGNNNAN
NNNCNNNNAACNNNNNNNNNNNNANNTNNNTANNAGGNCNNNNCCNTCNNGNNNNNNATNGCNATGACC
CANNNCACGGGGGCNGTNCCCGGCGNGGCGACCTTGATNACGTCCGGGGNCAAGTATGGCGNNCNCCC
CGGGAAGGANNCNGCCGTANGTGCNNNNNANNNNNTCNTGNGANGTCGCCGNNATTNNCGTGGTCNNN
CNAGAATGNCCNNACCANNCGNANNNTGGTNATGNNAGTNNNNNNNNNNNNNNNNNNNNNNNNNNNN
NNNNNNNNNNNNNNNNNNNNNNNNNNNNNNNNNGNNNNGNNNNNNCNANNNAAGGNNNTANNNCTNNTN
NNNNNNNNGGACNNNACNNNNCNNNNNNNACNCNNNCNCNNNNNNNNNNGGTTTNNNNNCNNTANTNT
CCAGNACGCNGNGCNNNCATTCANGNGAACNNNGNNACGCGNNAATCGTANNATCTACCTGCCGCNNG
TNNNNNTACNGNNNNNNCGGANNNTNNCTCGTANNNNNNNTANACGTNNTNTNNTCNCNAGNCNNGTT
CGNNNANANANTAGNANGTNTNNNNNNNNNNTGTNNCGTTCCGCNTGANNCNNCNNNNNGNNGNNCTTN
TCANCGGCNNTANCTGCNGANNTAAGNCATANNNTNNNTCNNTANCTNNNNNGGNGGGNNACTGTAAA
NGGNNAATTNGNNNNTNANTTCCNNCACNTNNCTNNGNNNNCTNNNTNCCTGNNTNCNNAATTGGNNC
CANTGNNTGNCNATCTCTGGNTTNGNNNNNNTNNNNNTNNCANNNNGGNNNNNNANANNNCNATNTAA
NNTANANNNNCNNNNNNANNNCACNNNNNNNGNAGNNNCNANNNTTANANNNGTAGNNNANTAAGTCT
CCGANNTNNNNNNNATAAGCGNNANACNNNNNNNNNNNNNANGANNNCGNNTTGGNNNTNNNNNNCNA
GNNAGACNNTNGTGGNTNNNNNTCAANNNNTTNGTAGTGGTATAATNGNNNNNNNTTTCNCCAGNNTN
NNNGNNNNNNNCNATANNANNNNNNNGNGANNNNNNNNNNANNNNCTCNNNAGGNANCANCTACTNNA
GCAATGGNNCGCAGNNNGAGANNNNGACNNNCCTTGGGTGGCAANCACNGCAATAAGTNNNGTTNANC
CTNNNAGTNNACACNNNNNCANNGNNNCNNNNNNANCNGANNGNNNNNNNNNNNNNNNNNNNNNNNN
NNNNNNNNNNNNNNNNNNNNNNNNNNNNNNNANNNNANNGNNNGTNTNTGACNCATGTGTTTTTANNNGNN
NNNNNACGNNNNANCCCCGGTCGNNNNCNNATTGAANNCCTGNNGCATNNNNNTNCNAGAATGANNGC
NNNNNGNNTNGNCTNNCNNNTCAAGNAGTCANNNAGGTNNNNNGNTCNNNGNNNNNNNNCNNTCCCNN
ACTGAGGGTNNNNNNNNCATCTGCTNNNNNNNNCTNCGCNGGNGTNNCNNNNNNGCNGTATNNNGNNA
NNCANNNNNNNNNNNNNNNNNNTNAAACNANNNNNNNTNNNG
>Nung669
GTGNNNNNTAGCCCNNNNNAGTTGGACANNNCNNNNNNNNNNNNNNNNNNNNNNNNNNNNNNNNNNN
NNNNNNNNNNNNNNNNNNNNNNNNNNNNNNNNNNNNNNNNNNNNNNNNNNNNNNNNNNNNNNNNNN
NNNNNNNNNNNNNNNNNNNNNNNNNNNNNNNNNNNNNNNNNNNNNNNNNNNNNNNNNNNNNNNNNN
NNNNNNNNNNNNNNNNNNNNNNNNNNNNNNNNNNNNNNNNNNNNNNNNNNNNNNNNNNNNNNNNNN
NNNNNNNNNNNNNNNNNNNNNNNNNNNNNNNNNNNNNNNNNNNNNNNNNNNNNTCTNNNAAANNNTNNN
NNNNNNNNNNNNNNNNNNNNNNNNNNNNCNCNTTGNNNGTAANCNANNNGNACATGCAGTCCCCTCNNNNN
NNNNNCAGAGNNNNNNNCNNNNNNCNCCGGCCCCNNCCNNNNNNNNNNNNNNNNNNNNNNNNNNGNNNNNN
NNNNNNTGGTACNNNNCGNNNNNGGNTGNTTNAGAGGTCCACCACGNGGTCNGGNCACNNNNGNNNAN
NNNCNNNAAACNNNNNNNNNNNNNNNNTNNNCANNAGGNCNNNNCCNTCNNNNNNNNNAATTGCNATGACC
CAANNCNCGGGGANNGTNACCGGNGNGGCGACCTTGNNNACGGTCGGGGACAAGTATGTCNTNCNCCC
CGGGAAGGANNCNGCCGTANGTGCNNNNNANNNNNTCNTGNGANGTCGCCGNNATTCNNGTGGTCNNN
CNAGAATGNCCNNACCANNCGNANNNTGGTNATGTNAGTNNNNNNNNNNNNNNNNNNNNNNNNNNNN
NNNNNNNNNNNNNNNNNNNNNNNNNNNNNNNNNNNNNNNGNNNNNNCNANNNAAGGNNNTANNNTTNNTN
NTNNNNNNGGACNNNACNNNNCNNNNNNNACNCNNNCNCNNNNNNNNNNGNTTTTTNNNCNNTATTNT
CCAGNACGCNGNGCNNNCATTCANGNGAACNNNGNNACGCGNNAATCGTANNATCTACCTGCCGCNNG
TNNNNNTNCNGNNNNNNCGGANNNTNNNNCGTANNNNNNNTCATGCGTNNTNTNNTTNCNAGNCNNGTT
CGNNNANNNANTNGNANGTNTNNNNNNNNNNTGTNNCGTTCCGCNTGANNCNNCANNNNGNNGNNCTTN
NCANCGGCNCTANCTGCNGANNTAAGNCATANNNTNNNTCNNTANCGNNNNNGGNGGGNNACTGTAAA
NGGNNAATTNGANNNTNATTTCCNNCACNTNNCTNNGNNNNCTNNNCNCCTGNNTNCNNAATTGGNNC

CANTGNNTGNCNATCTCTGGNTTNGNNNNNNTNNNNNTNNTAANNNGGNNNGTNANANNNCNATNTAA
NNTANANNNNCNNNNNNANNNCACNNNNNNNGNAGNNNNNANNNTGANGNNNGTAGNNNANTAAGTCT
CCGANNTNNNNNNNATAAGCGNNANACNNNNNNNNNNNNNANGANNACGNNTTGGNNNTNNNNNNCNA
ANNAGACNNCNGTGGNTNNNNNTCAANNNNTTCGTAGTGGTATAACNANNNNNNNTTTCNCCAGNNTN
NNNGNNNNNNNCNATANNANNNNNNNGNGANNNNNNNNNNANNNNCCCNNNAGGGANCANCTATTNNA
GCAATGGNNCGCAGNNNGAGANNNNGACCANCCTTGGGTGGCAANCACNGCAATAAGTNNNGTTNANC
CCNNNAGTNNACACGNNNNCANNGNNNCNNNNNNANCNGANNTNNNNNNNNNNNNNNNNNNNNNNNNN
NNNNNNNNNNNNNNNNNNNNNNNNNNNNAGNNNANNGNNNGTNTNTGACNCATGTGNNTNTANNNGNN
NNNNNACGNNNNANCCCCGGTCGNNNNCNNATTGAANNCCTGNNGCATNNNNNTNCNAGAATGANNGC
NNNNNGNNTNGNCTNNCNNNTCAAGNAGTCANNNANNNNNNNNNNTCNNNGNNNNNNNNCNNTCCCNN
ACTGAGGGTNNNNNNNNCATCNGCTNNNNNNNNNTTCGCNGNCGTNNTNNNNNAGCNGTATNNTGTNA
NNCANNNNNNNNNNNNNNNNNTNAAACAANNNNNNTNNNN
>Nung670
GTGNNNNNTAGCCCNNNNNAGTTAGACANNNCNNNNNNNNNNNNNTNNNNNNNNNNNNNNNNNNNNNNNN
NNNNNNNNNNNNNNNNNNNNNNNNNNNNNNNNNNNNNNNNNNNNNNNNNNNNNNNNNNNNNNNNNNNNNN
NNNNNNNNNNNNNNNNNNNNNNNNNNNNNNNNNNNNNNNNNNNNNNNNNNNNNNNNNNNNNNNNNNNNNN
NNNNNNNNNNNNNNNNNNNNNNNNNNNNNNNNNNNNNNNNNNNNNNNNNNNNNNNNNNNNNNNNNNNNNN
NNNNNNNNNNNNNNNNNNNNNNNNNNNNNNNNNNNNNNNNNNNNNNNNNNNNNNTCTNNNAAANNNTNNN
NNNNNNNNNNNNNNNNNNNNNNNNNNNCNCNNTGNNNGTAANCNANNNGNACATGCAGTCCCCTCNNNNN
NNNGGCAGAGNNNNNNNNCNNNNNCNCCGNCCCCGNCCNNNNNNNNNNNNNNNNNNNNNNNNNANNNNNN
NNNNNNTGGTACNNNNCGNNNNNGGTTGTNTAAGAGGTCCACAACGNGGTCNGGNCACNNNNGNNNAN
NNNCNNAAAACNNNNNNNNNNNNANNTNNNTANNAGGNNNNNNCCNTCNNGNNNNNNATTGCNATGACC
CAANNCACNGGGGCAGTNCCCGGNGCGGCGACCTTGATNACGTCCGGGGNCAAGTATGGCNNNCNCCC
CGGGAAGGANNCNGCCGTANGTGCNNNNNANNNNNTCNTGNGANGTCGCCGNNATTCNCGTGGTCNNN
CNAGAATGNNCNNACCANNCGNANNNTGGTNATGNNAGTNNNNNNNNNNNNNNNNNNNNNNNNNNNNNN
NNNNNNNNNNNNNNNNNNNNNNNNNNNNNNNNNNNNGNNNNGNNNNNNCNANNNAAGGNNNTANNNCTNNTN
NTNNNNNNNGGACNNNACNNNNCNNNNNNNACNCNNNCNCNNNNNNANNNGNTTTTNNNNCNNTATTNT
CCAGNACGCNGNGCNNNCATTCANGNGAACNNNGNNACGCGNNAATCGTANNATCTACCTGCCGCNNG
TNNNNNTNCNGNNNNNNCGGANNNTNNCTCGTANNNNNNTCANGCGTNNTNTNNTCNCNAGNCANGTT
CGNNNANANANTAGNANGTNTNNNNNNNNNNTGTNNCGTTCCGCNTGANNCNNCANNNNGNNGNNCTTN
NCANCGGCNNTANCTGCNGANNTAAGNCATANNNTNNATCNNTANCTNNNNNGGAGGGNNACTGTAAA
NGGNNAATTNGANGNTNATTNCNNNCACNTNNCNNNGNNTNCTNNNTNCCTGNNTNCNNAATTGGNNN
CANTGNNTGNCNATCTCTGGNTTNGNNNNNNTNNNNNTNNCAANNNGGNNNGTNNNANNNCNATNTAA
NNTANANNNNCNNNNNNANNNCACNNNNNNNGNAGNNNCNANNNTTANANNNGTAGNNNAATNAGTCT
CCGANNTNNNNNNNATAAGCGNNANACNNNNNNNNNNNNNANGANNACGNNTTGGNNNTNNNNNNCNA
GNNAGACNNTNATGGNTNNNNNTCAANNNNTTCGTAGTGGTATAATNGNNNNNNNTTTCNCCAGNNTN
NNNGNNNNNNNCNATANNANNNNNNNGNGANNNNNNNNNNANNNNCTCNNNAGGNANCANCTACTNNA
GCANNGGNGCGNAGNNNGAGANNNNGACNNGCCTTGGGTGGCAANCACNGCAATAAGTNNNGTTNANC
CTNNNAGTNNACATNNNNNCANNGNNNCNNNNNNANCNGANNGNNNNNNNNNNNNNNNNNNNNNNNNN
NNNNNNNNNNNNNNNNNNNNNNNNNNNNNNGNNNANGGNNNGTNTNTGACNCATGNNTTTTTANNNGNN
NNNNNACGNNNNANCCCCGGTCGNNNNCNNATTGAANNCCTGNNGCATNNNNNTNCNAGAATGANNGC
NNNNNGNNTNGNCTNNCNTTTCAAGNAATCAANNAGGTNTNNNGNTCNNGNNNNNNNNNCNNTCCCNN
ACTGAGGGTNNNNNNNNCATCTGCTNNNNNNNNCTNCGCNGGNGTNNCNNNNNAGCNGTATNGNGTNN
NNCANNNNNNNNNNNNNNNNNNNNAACNANNNNNNNTNNCN
>Nung671
GTGNNNNNTAGCCCNNNNNAGTTGGACANNNCNNNNNNNNNNNNNTNNNNNNNNNNNNNNNNNNNNNNNN
NNNNNNNNNNNNNNNNNNNNNNNNNNNNNNNNNNNNNNNNNNNNNNNNNNNNNNNNNNNNNNNNNNNNNN
NNNNNNNNNNNNNNNNNNNNNNNNNNNNNNNNNNNNNNNNNNNNNNNNNNNNNNNNNNNNNNNNNNNNNN
NNNNNNNNNNNNNNNNNNNNNNNNNNNNNNNNNNNNNNNNNNNNNNNNNNNNNNNNNNNNNNNNNNNNNN
NNNNNNNNNNNNNNNNNNNNNNNNNNNNNNNNNNNNNNNNNNNNNNNNNNNNNCNTCTNNNAAANNNTNNN
NNNNNNNNNNNNNNNNNNNNNNNNNNNCNCNTTNNNNGTAANCNANNNGNACATGCAGTCCCCTCNNNNN
NNNNNCAGAGNNNNNNCCCNNNNNNNCCGGCCCCNGCCNNNNNNNNNNNNCNNNGNNCNNNNGNNNNNN
NNNNNNTGGTACNNNNCGNNNNNGGTTGTNTAAGAGGTCCACCACGNGGTCNGGNCACNNNNGNNNAN
NNNCNNAAAACNNNNNNNNNNNNANNTNNNCANNAGGNCNNNNCCNTCNNNNNANNAATTGCNATGACC

CAANNCGCGGGGACAGTNACCGGCGCGGCGACCTTGATNANNGTCGGGGACAAGTATGTCGNNCNCCC
CGGGAAGGANNCNGCCGTANGTGCNNNNNANNNNNTCNTGNGANGTCGCCGNNATTCNCGTGGTCNNN
CNAGAATGNCCGNACCANNCGNANNNTGGTNATGNNAGTNNNNNNNNNNNNNNNNNNNNNNNNNNNN
NNNNNNNNNNNNNNNNNNNNNNNNNNNNNNNNNNGNNNNGNNNNNNCNANNNAAGGNNNTANNNTTNNTN
NTNNNNNNGGACNNNACNNNNCNNNNNNNACNCNNNCNCNNNNNNNNNNGNTTTTTNNNCNNTATTNT
CCAGNACGCNGNGCNNNCATTCANGNGAACNNNGNNACGCGNNAATCGTANNATCTACCTGCCGCNNG
TNNNNNTACNGNNNNNNCGGANNNTNNCTCGTANNNNNNTCATGCGTNNTNTNNTTNCNAGNCANGTT
CGNNNANANANTAGNANGTNTNNNNNNNNNTGTNNCGTTCCGCNTGANNCNNCANNNNGNNGNNCTTN
NCANCGGCNNTANCTNCNGANNTAAGNCATANNNTNNGTCNNTANCGNNNNNGGNGGGNNACTGTAAA
NGGNNAATTNGNNGNTNANTNCNNNCACNTNNCTNNGNNNNCTNNNCNCCTGNNTNCNNAATTGGNNC
CANTGNNTGNCNATCTCTGGNTTNGNNNNNNTNNNNNTNNTAANNNGGNNNGTNANANNNCNATNTAA
NNTANANNNNCNNNNNNANNNCACNNNNNNNGNAGNNNCNANNNTGANGNNNGTAGNNNANTAAGTCT
CCGANNTNNNNNNNATAAGCGNNANACNNNNNNNNNNNNNANGANNACGNNTTGGNNNTNNNNNNNNA
ANNAGACNNCNGTGGNTNNNNNTCAANNNNTTNGTAGTGGTATAACNANNNNNNNTTTCNCCAGNNTN
NNNGNNNNNNNCNATANNANNNNNNNGNGANNNNNNNNNNNANNNNCCCNNNAGGGANCANCTATTNNA
GCAATGGNGCGCAGNNNGAGANNNNGACNNNCCTTGGGTGGCAANCACNGCAATAAGTNNNGTTNANC
CCNNNAGTNNACACNNNNNCANNGNNNCNNNNNNANCNGANNTNNNNNNNNNNNNNNNNNNNNNNNNN
NNNNNNNNNNNNNNNNNNNNNNNNNNNNNNNNGNNNANGGNNNGTNTNTGACNCATGTGTTTTTANNNGNN
NNNNNACGNNNGANCCCCGGTCGNNNNCNNATTGAANNCCTGNNGCATNNNNNTNCNAGAATGANNGC
NNNNNGNNTNGNCTNNCNNNTCAAGNAGTCAANNAGGTANCGCGNTCNNNNNNNNNNNNNCNNTCCCNN
ACTGAGGGTNNNNNNNNCATNTGCTNNNNNNNNNCTNCGCNGNCGTNNTNNNNNNGCNGTATNNTGTNN
NNCANNNNNNNNNNNNNNNNNNTNAANNAANNNNNNNTNNNN
>Nung672
GTGNNNNNTAGCCCNNNNNAGTTAGACANNNCNNNNNNNNNNNNNTNNNNNNNNNNNNNNNNNNNNNNN
NNNNNNNNNNNNNNNNNNNNNNNNNNNNNNNNNNNNNNNNNNNNNNNNNNNNNNNNNNNNNNNNNNNN
NNNNNNNNNNNNNNNNNNNNNNNNNNNNNNNNNNNNNNNNNNNNNNNNNNNNNNNNNNNNNNNNNNNN
NNNNNNNNNNNNNNNNNNNNNNNNNNNNNNNNNNNNNNNNNNNNNNNNNNNNNNNNNNNNNNNNNNNN
NNNNNNNNNNNNNNNNNNNNNNNNNNNNNNNNNNNNNNNNNNNNNNNNNNNNNNNTCTNNNNAAANNNTNNN
NNNNNNNNNNNNNNNNNNNNNNNNNNNCNCNTTGNNNGTAANCNANNNGNACATGCAGTCCCCTCNNNNN
NNNGGCAGAGNNNNNNNCCNNNNNNNCCGGCCCCNNCCNNNNNNNNNNNNNNNNGNNNNNNNANNNNNN
NNNNNNTGGTACNNNNCGNNNNNGGTTGTTTAAGAGGTCCACAACGNGGTCNNGNNACNNNNGNNNAN
NNNCNNAAAACNNNNNNNNNNNNNNATNNNTANNAGGNCNNNNCCNTCNNGNNNNNNANTGCNATGACC
CANNNCACGGGGGCNGTNCCCGGNGCGGCGACCTTGATNACGTCCGGGGNCAAGTATGGCNNNCNCCC
CGGGAAGGANNCNGCCGTANGTGCNNNNNANNNNNTCNTGNGANGTNGCCGNNATTCNNGTGGTCNNN
CNAGAATGNNCGNACCANNCGNANNNTGGTNATGNNAGTNNNNNNNNNNNNNNNNNNNNNNNNNNNN
NNNNNNNNNNNNNNNNNNNNNNNNNNNNNNNNNNGNNNNGNNNNNNCNANNNAAGGNNNTANNNCTNNTN
NTNNNNNNGGACNNNACNNNNCNNNNNNNACNCNNNCNCNNNNNNANNNGGTTTTTNNNCNNTANTNT
CCAGNACGCNGNGCNNNCATTCANGNGAACNNNGNNACGCGNNAATCGTANNATCTACCTGCCGCNNG
NNNNNNTACNGNNNNNNCGGANNNTNNCTCGTANNNNNNTCATGCGTNNTNTNNTCNCNAGNCNNGTT
CGNNNANANANTAGNANGTNTNNNNNNNNNTGTNNCGTTCCGCNTGANNCNNCANNNNGNNGNNCTTN
TCANCGGCNNTANCTGCNGANNTAAGTCATANNNTNNNTCNNTANCTNNNNNGGNGGGNNACTGTAAA
NGGNNAATTNGNNGNTNANTNCNNNCACNTNNCNNNGNNNNCTNNNTNCCTGNNTNCNNAATTGGNNC
CANTGNNTGNCNATCTCTNGNTTNGNNNNNNTNNNNNTNNCAANNNGGNNNGTNNNANNNCNATNTAA
NNTANANNNNCNNNNNNANNNCACNANNNNNNNAGNNNCNANNNTTANANNNGTAGNNNAATAAGTCT
CCGANNTNNNNNNNATAAGCGNNANACNNNNNNNNNNNNNANGANNACGNNTTGGNNNTNNNNNNNCNA
GNNAGACNNTNATGGNTNNNNNTCAANNNNTTNGTAGTGGTATAATNGNNNNNNNTTTCNCCAGNNTN
NNNGNNNNNNNCNATANNANNNNNNNGNGANNNNNNNNNNNANNNNCTCNNNAGGGANCANCTACTNNA
GCAATGGNGCGCAGNNNGAGANNNNGACNANCCTTGGGTGGCAANNACNGCGATAAGTNNNGTTNANC
CTNNNAGTNNACATNNNNNCANNGNNNCNNNNNNANCNGANNGNNNNNNNNNNNNNNNNNNNNNNNNN
NNNNNNNNNNNNNNNNNNNNNNNNNNNNNAGNNNANNGNNNGTNTNTGACNCATGTGTTTTTANNNGNN
NNNNNACGNNNGANCCCCGGTCGNNNNCNNATTGAANNCCTGNNGCATNNNNNTNCNAGAATGANNNC
NNNNNGNNTNGNCTNNCNTTTCAAGNAATCANNTAGNTNNNGCGNTCNNNGNNNNNNNNCNNTCCCNN
ACTGAGGGTNNNNNNNNCATCTGCTNNNNNNNNNNTNCGCNGGNGTNNCNNNNNAGCNGTATNGTGTNA
NNCANNNNNNNNNNNNNNNNNNTNAAACNANNNNNNNTNNNN

>Nung673
GTGNNNNNTAGCCCNNNNNAGTTAGACANNNCNNNNNNNNNNNNNTNNNNNNNNNNNNNNNNNNNNNNNN
NNNNNNNNNNNNNNNNNNNNNNNNNNNNNNNNNNNNNNNNNNNNNNNNNNNNNNNNNNNNNNNNNNNNNN
NNNNNNNNNNNNNNNNNNNNNNNNNNNNNNNNNNNNNNNNNNNNNNNNNNNNNNNNNNNNNNNNNNNNNN
NNNNNNNNNNNNNNNNNNNNNNNNNNNNNNNNNNNNNNNNNNNNNNNNNNNNNNNNNNNNNNNNNNNNNN
NNNNNNNNNNNNNNNNNNNNNNNNNNNNNNNNNNNNNNNNNNNNNNNNNNNNNNTCTNNNAAANNNTNNN
NNNNNNNNNNNNNNNNNNNNNNNNNNNCNCNTTGNNNGTAANCNANNNGNACATGCAGTCCCCTCNNNNN
NNNGGCAGAGNNNNNNCCCNNNNNNNCCGNCCCCNNCCNNNNNNNNNNNNNNNNNGNNNNNNNANNNNNN
NNNNNNTGGTACNNNNCGNNNNNGGTTGTNTAAGAGGTCCACAACGNGNTCNNGNCACNNNNGNNNAN
NNNCNNNNAACNNNNNNNNNNNNANNTNNNTANNAGGNCNNNNCCNTCNNGNNNNNNANTGCNATGACC
CANNTNACGGGGGNNGTNCCCGGCGCGGCGACCTTGNNNACGTCCGGGGNCAAGTATGGCGNNCNCCC
CGGGAAGGANNCNGCCGTANGTGCNNNNNANNNNNTCNTGNGANGTCGCCGNNATTCNCGTGGTCNNN
CNAGAATGNNCGNACCANNCGNANNNTGGTNATGNNAGTNNNNNNNNNNNNNNNNNNNNNNNNNNNNNNN
NNNNNNNNNNNNNNNNNNNNNNNNNNNNNNNNNNGNNNNGNNNNNNCNANNNAAGGNNNTANNNCTNNTN
NTNNNNNNGGACNNNACNNNNCNNNNNNNACNCNNNCNCNNNNNNANNNGGTTTTNNNNCNNTANTNT
CCAGNACGCNGNGCNNTCNTTCANGNGAACNNNGNNACGCGNNAATCGTANNATCTACCTGCCGCNNG
NNNNNNTNCNGNNNNNNCGGANNNTNNCTCGTANNNNNNTCATGCGTNNTNTNNTCNCNAGNCANGTT
CGNNNANANANTAGNANGTNTNNNNNNNNNNTGTNNCGTTCCGCNTGANNCNNCANNNNGNNGNNNTTN
NCANCGGCNNTANCTGCNGANNTAAGNCATANNNTNNNTCNNTANCTNNNNNGGAGGGNNACTGTAAA
NGGNNAATTNGNNNNTNANTNCNNNCACNTNNCTNNGNNTNCTNNNTNCCTGNNTNCNNAATTGGNNC
CANTGNNTGNCNATCTCTNGNTTNGNNNNNNTNNNNNTNNCANNNNGGNNNGNNNNANNNCNATNTAA
NNTANANNNNCNNNNNNANNNCACNNNNNNNNNNAGNNNCNANNNTNANANNNGTAGNNNANTAAGTCT
CCGANNTNNNNNNNATAAGCGNNANACNNNNNNNNNNNNNNANGANNACGNNTTGGNNNTNNNNNNCNA
GNNAGACNNTNATGGNTNNNNNTCAANNNNTTCGTAGTGGTATAATNGNNNNNNNTTTCNCCAGNNTN
NNNGNNNNNNNCNATANNANNNNNNNGNGANNNNNNNNNNNANNNNCTCNNNAGGNANCANCTACTNNA
GCANNGGNGCGNAGNNNGAGANNNNGACNNNCCTTGGGTGGCAANCACNGCAATAAGTNNNGTTNNNC
CTNNNAGTNNACATNNNNNCANNGNNNCNNNNNNANCNGANNGNNNNNNNNANNNNNNNNNNNNNNNNNN
NNNNNNNNNNNNNNNNNNNNNNNNNNNNNNNGNNNANGGNNNGTNTNTGACNCATGNNTTTTTANNNGNN
NNNNNNNGNNNGANCCCCGGTCGNNNNCNNATTGAANNCCTGNNGCATNNNNNTNCNAGAATGANNNC
NNNNNGNNTNGNCTNNCNNNTCAAGNAATCANNNAGGNNNNNNGNTCNNNGANNNNNNNCNNTCCCNN
ACTGAGGGTNNNNNNNNCATCTGCTNNNNNNNNCTNCGCNGNNGTNNCNNNNNAGCNGTATNGNGTNN
NNCANNNNNNNNNNNNNNNNNNTNAAACNNNNNNNNCTNNNN
>Nung674
GTGNNNNNTAGCCCNNNNNAGTTAGACANNNCNNNNNNNNNNNNNTNNNNNNNNNNNNNNNNNNNNNNNN
NNNNNNNNNNNNNNNNNNNNNNNNNNNNNNNNNNNNNNNNNNNNNNNNNNNNNNNNNNNNNNNNNNNNNN
NNNNNNNNNNNNNNNNNNNNNNNNNNNNNNNNNNNNNNNNNNNNNNNNNNNNNNNNNNNNNNNNNNNNNN
NNNNNNNNNNNNNNNNNNNNNNNNNNNNNNNNNNNNNNNNNNNNNNNNNNNNNNNNNNNNNNNNNNNNNN
NNNNNNNNNNNNNNNNNNNNNNNNNNNNNNNNNNNNNNNNNNNNNNNNNNNNCNTCTNNNAAANNNTNNN
NNNNNNNNNNNNNNNNNNNNNNNNNNNCNCNTTGNNNGTAANCNANNNGNACATGCAGTCCCCTCNNNNN
NNNNNCAGAGNNNNNNNCCNNNNNNNCCGGCCCCNNCCNNNNNNNNNNNNNNNNNGNNNNNNNANNNNNN
NNNNNNTGGTACNNNNCGNNNNNGGTTGTNTNAGAGGTCCACAACGNGGTCNGGNCACNNNNGNNNAN
NNNCNNNNAACNNNNNNNNNNNNANNTNNNTANNAGGNNNNNNCCNTCNNGNNNNNNATTGCNATGANN
CANNTNACGGGGGCNGTNCCCGGCGCGGCGACCTTGANNANGTCCGGGGACAAGTATGGCGNNCNCCC
CGGGAAGGANNCNGCCGTANGTGCNNNNNANNNNNTCNTGNGANGTCGCCGNNATTCNCGTGGTCNNN
CNAGAATGNNCNNACCANNCGNANNNTGGTNATGNNAGTNNNNNNNNNNNNNNNNNNNNNNNNNNNNNNN
NNNNNNNNNNNNNNNNNNNNNNNNNNNNNNNNNNNNNNNGNNNNNNCNANNNAAGGNNNTANNNCTNNTN
NTNNNNNNGGACNNNACNNNNCNNNNNNNACNCNNNCNCNNNNNNANNNGNTTTTTNNNCNNTANTNT
CCAGNACGCNGNGCNNNCNTTCANGNGAACNNNGNNACGCGNNAATCGTANNATCTACCTGCCGCNNG
TNNNNNTNCNGNNNNNNCGGANNNTNNCTCGTANNNNNNNCATGCGTNNTNTNNTNNCNAGNCNNGTT
CGNNNANANANTAGNANGTNTNNNNNNNNNNTGTNNCGTTCCGCNTGANNCNNCANNNNGNNGNNNTTN
NCANCGGCNNTANCTGCNGANNTAAGNCATANNNTNNNTCNNTANCTNNNNNGGNGGGNNACTGTAAA
NGGNNAATTNGNNNNTNANTNCNNNCACNTNNCTNNGNNNNCTNNNTNCCTGNNTNCNNAATTGGNNC
CANTGNNTGNCNATCTCTGGNTTNGNNNNNNTGNNNNTNNCANNNNGGNNNGTNANANNNCNATNTAA
NNTANANNNNCNNNNNNANNNCACNNNNNNNNGNAGNNNCNANNNTTANANNNGTAGNNNAATAAGTCT

CCGANNTNNNNNNNATAAGCGNNANACNNNNNNNNNNNNNNANGANNACGNNTTGGNNNTNNNNNNCNA
GNNANACNNTNATGGNTNNNNNTCAANNNNTTCGTAGTGGTATAATNGNNNNNNNTTTCNCCAGNNTN
NNNGNNNNNNNCNATANNANNNNNNNGNGANNNNNNNNNNNANNNNCTCNNNAGGNANCANCTACTTNA
GCANNGGNGCGNAGNNNGAGANNNNGACNNNCCTTGGGTGGCAANNACNGCGATAAGTNNNGTTNANC
CTNNNAGTNNACATNNNNNCANNGNNNCNNNNNNANCNGANNGNNNNNNNNNNNNNNNNNNNNNNNNN
NNNNNNNNNNNNNNNNNNNNNNNNNNNNNNNNNNNNNANNGNNNGTNTNTGACNCATGTGTTTTTANNNGNN
NNNNNNNGNNNNANCCCCGGTCGNNNNCNNATTGAANNCCTGNNGCATNNNNNTNCNAGAATGANNNC
NNNNNGNNTNGNCTNNCNNNTCAAGNAATCANNNAGGNATNNNGNTCNCNNNNNNNNNNCNNNCCCNN
ACTGAGGGTNNNNNNNNCATCNGCTNNNNNNNNNNTNCGCNGGNGTNNCNNNNNNGCNGTATNGNGTNA
NNCANNNNNNNNNNNNNNNNNNTNAAACGANNNNNNNTNNNN
>Nung675
GTGNNNNNTAGCCCNNNNNAGTTAGACANNNCNNNNNNNNNNNNNTNNNNNNNNNNNNNNNNNNNNNNNN
NNNNNNNNNNNNNNNNNNNNNNNNNNNNNNTNNNNNNNNNNNNNNNNNNNNNNNNNNNNNNNNNNNNNNNN
NNNNNNNNNNNNNNNNNNNNNNNNNNNNNNNNNNNNNNNNNNNNNNNNNNNNNNNNNNNNNNNNNNNNNN
NNNNNNNNNNNNNNNNNNNNNNNNNNNNNNNNNNNNNNNNNNNGANNNNNNNNNNNNNNNNNNNNNNNNN
NNNNNNNNNNNNNNNNNNNNNNNNNNNNNNNNNNNNNNNNNNNNNNNNNNNNNNTCTNNNAAANNNTNNN
NNNNNNNNNNNNNNNNNNNNNNNNNNNCNCNTTGNNNGTAANCNANNNGNACATGCAGTCCCCTCNNNNN
NNNGGCAGAGNNNNNNCCCNNNNNNNCCGGCCCCGNCCNNNNNNNNNNNCNNNNNNNNNNNANNNNNN
NNNNNNTGGTACNNNNCGNNNNNGGTTGTNTAAGAGGTCCACAACGNGGTCNGGNCACNNNNGNNTAN
NNNCNNAAAACNGNNNNNNNNNNANATNNNTANNAGGNCNNNNCCNTCNNGNNNNTAATTGCNATGACC
CAANNCACGGGGGCAGTNCCCGGCGCGGCGACCTTGATNACGTCCGGGGACAAGTATGGCNCNCNCCC
CGGGAAGGANNCNGCCGTANGTGCNNNNNANNNNNTCNTGNGANGTCGCCGNNATTCNCGTGGTCNNN
CNAGAATGNCCGNACCANNCGNANNNTGGTNATGNNAGTANNNNNNNCNNNNNNNNNNNNNNNNNNNN
NNNNNNNNNNNNNNNNNNNNNNNNCNNNNNNNNGNNNNGNNNNNNCNANNNAAGGANNTANNNCTNNTN
NTNNNNNNNGGACNNNACNNNNCNNNNNNNACNCNNNCNCNNNNNNANNNGGTTTTTNNNCNNTATTNT
CCAGNACGCNGNGCNNNCATTCANGNGAACNNNGNNACGCGNNAATCGTANNATCTACCTGCCGCNNG
TNNNNNTACNGNNNNTNCGGANNNTNNNNCGTANNNNNNTCATGCGTNNTNTNNTCNCNAGNCANGTT
CGNNNANANANTAGNANGTNTNNNNNNNCTTGTNNCGTTCCGCNTGANNCNNCANNNNGNNGNNCTTN
TCANCGGCNCTANCTGCNGANNTAAGNCATANNNTNNATCNNTANCTNNNNNGGNGGGNNACTGTAAA
NGGNNAATTNGNNGNTNATTNCCNNCACNTNNCTNNGNNNNCTNNNTNCCTGNNTNCNNAATTGGNNC
CANTGNNTGNCNATCTCTGGNTTNGNNNNNNTGNNNNTNGCAANNNGGNNNGTNANANNNCNATNTAA
NNTATANNNNCNNNNNNANNGCACNNNNNNNGNAGNNNCNANNNTTANANNNGTAGNNNAATAAGTCT
CCGANNTNNNNNNNATAAGCGNNANACNNNNNNNNNNNNNNANGANNACGNNTTGGNNNTNNNNNNCNA
GNNAGACNNTNATGGNTNNNNNTCAANNNNTTCGTAGTGGTATAATNGNNNNNNNTTTCNCCAGNNTN
NNNGNNNNNNNCNATANNANNNNNNNGNGANNNNNNNNNNNANNNNCTCNNNAGGGANCANCTACTTNA
GCAATGGNGCGCAGNNNGAGANNNNGACNNGCCTTGGGTGGCAANCACNGCAATAAGTNNNGTTNANC
CTNNNAGTNNACATNNNNNCANNGNNNCNNNNNNANCNGANNGNNNNNNNNNNNNNNNNNNNNNNNNN
NNNNNNNNNNNNNNNNNNNNNNNNNNNNNNAGNNNANGGNNNGTNTNTGACNCATGTGTTTTTANNNGNN
NNNNNACGNNNNANCCCCGGTCGTNTNCNNATTGAANNCCTGNNGCATNNNNNTNCNAGAATGANNGC
NNNNNGNNTNGNCTNNCNNTTCAAGNAATCANNTAGGTANCGCGNTCNNNGNNNNNNNNCNNTCCCNN
ACTGAGGGTNNNNNNNGCATCTGCTNNNNNNNNCTTCGCNGGCGTNNCNNNNNAGCNGTATNGTGTNA
NNCANNNNNNNNNNNNNNNNNNTNAAACNANNNNNCTNNNG
>Nung676
GTGNNNNNTAGCCCNNNNNAGTTAGACANNNCNNNNNNNNNNNNNTNNNNNNNNNNNNNNNNNNNNNNNN
NNNNNNNNNNNNNNNNNNNNNNNNNNNNNNTNNNNNNNNNNNNNNNNNNNNNNNNNNNNNNNNNNNNNNNN
NNNNNNNNNNNNNNNNNNNNNNNNNNNNNNNNNNNNNNNNNNNNNNNNNNNNNNNNNNNNNNNNNNNNNN
NNNNNNNNNNNNNNNNNNNNNNNNNNNNNNNNNNNNNNNNNNNNNNNNNNNNNNNNNNNNNNNNNNNNNN
NNNNNNNNNNNNNNNNNNNNNNNNNNNNNNNNNNNNNNNNNNNNNNNNNNNNNNTCTNNNAAANNNTNNN
NNNNNNNNNNNNNNNNNNNNNNNNNNNCNCNTTGNNNGTAANCNANNNGNACATGCAGTCCCCTCNNNNN
NNNGGCAGAGNNNNNNCCCNNNNNCNCCGNCCCCGNCCNNNNNNNNNNNNNNNNGNNNNNNNANNNNNN
NNNNNNTGGTACNNNNCGNNNNNGGTTGTTTAAGAGGTCCACAACGNGGTCNGGNCACNNNNGNNNAN
NNNCNNNAAACNNNNNNNNNNNNNNNATNNNTANAAGGNCNNNNCCNTCNNGNNANTAATTGCNATGACC
CAANNCACGGGGGCNGTNCCCGGNGNGGCGACCTTGANNACGTCCGGGGACAAGTATGGCNNNCNCCC
CGGGAAGGANNCNGCCGTANGTGCNNNNNANNNNNTCNTGNGANGTCGCCGNNATTCNCGTGGTCTNN

CNAGAATGNNCGNACCANNCGNANNNTGGTNATGNTAGTNNNNNNNNNNNNNNNNNNNNNNNNNNNNN
NNNNNNNNNNNNNNNNNNNNNNNNNNNNNNNNNNNNNGNNNNNNCNANNNAAGGANNTANNNCTNNTN
NTNNNNNNGGACNNNACNNNNCNNNNNNNACNCNNNCNCNNNNNNANNNGNTTTTTNNNCNNTATTNT
CCAGNACGCNGNGCNNNCATTCANGNGAACNNNGNNACGCGNNAATCGTANNATCTACCTGCCGCNNG
NNNANNTACNGNNNNTNCGGANNNTNNCTCGTANNNNNNNCATGCGTNNTNTNNTCNCNAGNCNNGTT
CGNNNANANANTAGNANGTNTCNNNNNNCTTGTNNCGTTCCGCNTGANNCNNCANNNNGNNGNNCTTC
TCANCGGCNNTANCTGCNGANNTAAGTCATANNNTNNNTCNNTANCTNNNNNGGNGGGNNACTGTAAA
NGGNTAATTGGANGNTNANTTCNNNCACNTNNCTNNGNNTNCTNNNTNCCTGNNTNCNNAATTGGNNC
CANTGNNTGNCNATCTCTGGNTTNGNNNNNNTGNNNNTNNCAANNNGGNNNGTNANANNNCNATNTAA
NNTANANNNNCNNNNNNANNNCACNANNNNNNNAGNNNCNANNNTTANANNNGTAGNNNAATAAGTCT
CCGANNTNNNNNNNATAAGCGNNANACNNNNNNNNNNNNNANGANNACGNNTTGGNNNTNNNNNNCNA
GNNAGACNNTNATGGNTNNNNNTCAANNNNTTCGTAGTGGTATAATNGNNNNNNNTTTCNCCAGNNTN
NNNGNNNNNNNCNATANNANNNNNNNGNGANNNNNNNNNNANNNNCTCNNNAGGGANCANCTACTNNA
GCAATGGNGCGCAGNNNGAGANNNNGACNNGCCTTGGGTGGCAANCACNGCGATAAGTNNNGTTNANC
CTNNNAGTNNACATNNNNNCANNGNNNCNNNNNNANCNGANNGNNNNNNNNNNNNNNNNNNNNNNNNN
NNNNNNNNNNNNNNNNNNNNNNNNNNNNNNNNNANGGNNNGTNTNTGACNCATGTGTTTTTANNNGNN
NNNNNACGNNNNANCCCCGGTCGNNNNCNNATTGAANNCCTGNNGCATNNNNNTNCNAGAATGANNGC
NNNNNGNNTNGNCTGNCNTTTCAAGNAATCANNNAGGTANCGCGNTCNNNGNNNNNNNNCNNNCCCNN
ACTGAGGGTNNNNNNNNCATCTGCTNNNNNNNNCTNCGCCGGCGTNNCNGNNNAGCNGTATNGTGTNA
NNCANNNNNNNNNNNNNNNTNAAACNNNNNNNNNTNNNN
>Nung677
GTGNNNNNTAGCCCNNNNNAGTTGGACANNNCNNNNNNNNNNNTNNNNNNNNNNNNNNNNNNNNNNNN
NNNNNNNNNNNNNNNNNNNNNNNNNNNNNNNNNNNNNNNNNNNNNNNNNNNNNNNNNNNNNNNNNNNN
NNNNNNNNNNNNNNNNNNNNNNNNNNNNNNNNNNNNNNNNNNNNNNNNNNNNNNNNNNNNNNNNNNNN
NNNNNNNNNNNNNNNNNNNNNNNNNNNNNNNNNNNNNNNNNNNNNNNNNNNNNNNNNNNNNNNNNNNN
NNNNNNNNNNNNNNNNNNNNNNNNNNNNNNNNNNNNNNNNNNNNNNNNNNNNTCTNNNAAANNNTNNN
NNNNNNNNNNNNNNNNNNNNNNNNNCNCNTTGNNNGTAANCNANNNGNACATGCAGTCCCCTCNNNNN
NNNNNCAGAGNNNNNNNCCNNNNNCNCCGGCCCCGNCCNNNNNNNNNNNNNNNNNNNNNNNGNNNNNN
NNNNNNTGGTACNNNNCGNNNNNGGTTGNNTAAGAGGTCCACCACGNGGTCNGGNCACNNNNGNNNAN
NNNCNNNNNNNNNNNNNNNNNNANNTNNNCANNAGGNNNNNNCCNTCNNNNNNNNAATTGCNATGACC
CAANNCGCGGGGACNGTNACCGGCGNGGCGACCTTGNTNANNGTCGGGGACAAGTATGTCNNNCNCCC
CGGGAAGGANNCNGCCGTANGTGCNNNNNANNNNNTCTTGNGANGTNGCCGNNATTCNCGTGGTCNNN
NNAGAATGNNCGNACCANNCGNANNNTGGTNATGNTAGTNNNNNNNNNNNNNNNNNNNNNNNNNNNNN
NNNNNNNNNNNNNNNNNNNNNNNNNNNNNNNNNNNNNGNNNNNNCNANNNAAGGANNTANNNTTNNTN
NTNNNNNNGGACNNNACNNNNCNNNNNNNACNCNNNCNCNNNNNNANNNGNTTTTTNNNCNNTANTNT
CCAGNACGCNGNGCNNNCATTCANGNGAACNNNGNNACGCGNNAATCGTANNATCTACCTGCCGCNAG
TNNNNNTNCNGNNNNNNCGGANNNTNNNNCGTANNNNNNTCATGCGTNNTNTNNTTNCNAGNCANGTT
CGNNNANNNANTAGNANGTNTNNNNNNNNNTGTNNCGTTCCGCNTGANNCNNCANNNNGNNGNNCTTN
TCANCGGCNNTANCTGCNGANNTAAGCCATANNNTNNGTCNNTANCGNNNNNGGAGGGNNACTGTAAA
NGGNNAATTGGNNNNTNATTTCNNNCACNTNNCTNNGNNNNCTNNNCNCCTGNNTNCNNAATTGGNNC
CANTGNATGNCNATCTCTGGNTTNGNNNNNNTNNNNNTNNTANNNNGGNNNGTNANANCNCNATNTAA
NNTANANNNNCNNNNNNANNNCACNANNNNNNNAGNNNCNANNNTGANGNNNGTAGNNNANTAAGTCT
CCGANNTNNNNNNNATAAGCGNNANACNNNNNNNNNNNNNANGANNNCGNNTTGGNNNTNNNNNNCNA
ANNAGACNNCNGTGGNTNNNNNTCAANNNNTTCGTAGTGGTATAACNANNNNNNNTTTCNCCAGNNTN
NNNGNNNNNNNCNATANNANNNNNNNGNGANNNNNNNNNNANNNNCCCNNNAGGGANCANCTATTNNA
GCANTGGNGCGCAGNNNGAGANNNNGACNNNCCTTGGGTGGCAANCACNGCAATAAGTNNNGTTNANC
CCNNNAGTNNACACGNNNNCANNGNNNCNNNNNNANCNGANNTNNNNNNNNNNNNNNNNNNNNNNNNN
NNNNNNNNNNNNNNNNNNNNNNNNNNNNAGNNNANNGNNNGTNTNTGACNCATGTGTTTTTANNNGNN
NNNNNACGNNNGANCCCCGGTCGNNNNCNNATTGAANNCCTGNNGCATNNNNNTNCNAGAATGANNGC
NNNNNGNNTNNNCTNNNNNNTCAAGNAGTCAANNAGGTNNNGCGNTCNNNGNNNNNNNNCNNTCCCNN
ACTGAGGGTNNNNNNNNCATCTGCTNNNNNNNNNTNCGCNGNCGTNNTNNNNNNGCNGTATNNTGTNN
NNCANNNNNNNNNNNNNNNTNAAACAANNNNNNNTNNNN
>Nung678
GTGNNNNNTAGCCCNNNNNAGTTAGACANNNCNNNNNNNNNNNTNNNNNNNNNNNNNNNNNNNNNNNN

NNNNNNNNNNNNNNNNNNNNNNNNNNNNNNNNNNNNNNNNNNNNNNNNNNNNNNNNNNNN
NNNNNNNNNNNNNNNNNNNNNNNNNNNNNNNNNNNNNNNNNNNNNNNNNNNNNNNNNNNN
NNNNNNNNNNNNNNNNNNNNNNNNNNNNNNNNNNNNNNNNNNNNNNNNNNNNNNNNNNNN
NNNNNNNNNNNNNNNNNNNNNNNNNNNNNNNNNNNNNNNNNNNNTCTNNNAAANNNTNNN
NNNNNNNNNNNNNNNNNCNCNTTGNNNGTAANCNANNNGNACATGCAGTCCCCTCNNNNN
NNNGGCAGAGNNNNNNNCNNNNNNNNCCGNCCCCNNCCNNNNNNNNNNNNNNNNGNNNNNNNANNNNNN
NNNNNNTGGTACNNNNCGNNNNNGGTTGTNTAAGAGGTCCACAACGNGGTCNNGNNACNNNNGNNNAN
NNNCNNNAAACNNNNNNNNNNNNANATNNNTANNAGGNCNNNNCCNTCNNGNNNNNNATTGCNATGACC
CAANNCACGGGGGCAGTNCCCGGCGCGGCGACCTTGANNANGTCCGGGGNCAAGTATGGCGNNCNCCC
CGGGAAGGANNCNGCCGTANGTGCNNNNNANNNNNTCNTGNGANGTCGCCGNNATTNNCGTGGNCNNN
CNAGAATGNNCGNACCANNCGNANNNTGGTNATGNNAGTNNNNNNNNNNNNNNNNNNNNNNNNNNNNN
NNNNNNNNNNNNNNNNNNNNNNNNNNNNNNNNGNNNNGNNNNNNCNANNNAAGGNNNTANNNCTNNTN
NTNNNNNNNGGACNNNACNNNNCNNNNNNNACNCNNNCNCNNNNNNNNNNNGNTTTTNNNCCNNTATTNT
CCAGNACGCNGNGCNNNCATTCANGNGAACNNNGNNACGCGNNAATCGTANNATCTACCTGCCGCNNG
TNNNNNTACNGNNNNNNCGGANNNTNNNNCGTANNNNNNTCATGCGTNNTNTNNTCNCNAGNCNNGTT
CGNNNANANANTAGNANGTNTNNNNNNNNNTGTNNCGTTCCGCNTGANNCNNCANNNNGNNGNNCTTN
TCANCGGCNNTANCTGCNGANNTAAGNCATANNNTNNATCNNTANCTNNNNNGGNGGGNNACTGTAAA
NGGNNAATTNGNNGNTNANTNCNNNCACNTNNCNNNGNNTNCTNNNTNCCTGNNTNCNNAATTGGNNC
CANTGNNTGNCNATCTCTNGNTTNGNNNNNNTNNNNNTNNCAANNNGGNNNGTNANANNNCNATNTAA
NNTANANNNNCNNNNNNANNNCACNNNNNNNGNAGNNNCNANNNTNANANNNGTAGNNNAATAAGTCT
CCGANNTNNNNNNNATAAGCGNNANACNNNNNNNNNNNNNNANGANNNCGNNTTGGNNNTNNNNNNNNA
GNNAGACNNTNATGGNTNNNNNTCAANNNNTTNGTAGTGGTATAATNGNNNNNNNTTTCNCCAGNNTN
NNNGNNNNNNNCNATANNANNNNNNNGNGANNNNNNNNNNNANNNNCTCNNNAGGGANCANCTACTNNA
GCAATGGNGCGCAGNNNGAGANNNNGACNNGCCTTGGGTGGCAANCACNGCAATAAGTNNNGTTNANC
CTNNNAGTNNACATNNNNNCANNGNNNCNNNNNNANCNGANNGNNNNNNNNNNNNNNNNNNNNNNNNN
NNNNNNNNNNNNNNNNNNNNNNNNNNNNNNANNNNANGGNNNGTNTNTGACNCATGTGTTTTTANNNGNN
NNNNNACGNNNGANCCCCGGTCGNNNNCNNATTGAANNCCTGNNGCATNNNNNTNCNAGAATGANNGC
NNNNNGNNTNGNCTNNCNNTTCAAGNAATCANNTAGGTNNNGCGNTCNNNGNNNNNNNNCNNTCCCNN
ACTGAGGGTNNNNNNCGCATCTGCTNNNNNNNNNNTNCGCNGGCGTNNCNNCNNNGCNGTATNGNGTNA
NNCANNNNNNNNNNNNNNNNNNNNANNNANNNNNCTNNNN
>Nung679
GTGNNNNNTAGCCCNNNNNAGTTAGACANNNCNNNNNNNNNNNNNTNNNNNNNNNNNNNNNNNNNNNN
NNNNNNNNNNNNNNNNNNNNNNNNNNNNNNNNNNNNNNNNNNNNNNNNNNNNNNNNNNNN
NNNNNNNNNNNNNNNNNNNNNNNNNNNNNNNNNNNNNNNNNNNNNNNNNNNNNNNNNNNN
NNNNNNNNNNNNNNNNNNNNNNNNNNNNNNNNNNNNNNNNNNNNNNNNNNNNNNNNNNNN
NNNNNNNNNNNNNNNNNNNNNNNNNNNNNNNNNNNNNNNNNNNNTCTNNNAAANNNTNNN
NNNNNNNNNNNNNNNNNCNCNNTGNNNGTAANCNANNNGNACATGCAGTCCCCTCNNNNN
NNNGGCAGAGNNNNNNNCCNNNNNNNCCGGCCCCNNCCNNNNNNNNNNNNNNNNGNNNNNNNAANNNNNN
NNNNNNTGGTACNNNNCGNNNNNGGTTGTNTAAGAGGTCCACAACGNGGTCNGGNCACNNNNGNNNAN
NNNCNNNNANCNNNNNNNNNNNNNNNTNNNTANNAGGNNNNNNCCNTCNNGNNNNTNATTGCNATGACC
CAANNNNCGGGGGCNGTNCCCGGCGNGGCGACCTTGANNANGTCCGGGGNCAAGTATGGCNNNCNCCC
CGGGAAGGANNCNGCCGTANGTGCNNNNNANNNNNTCNTGNGANGTNGCCGNNATTCNCNTGGTCNNN
NNAGAATGNCCGNACCANNCGNANNNTGGTNATGNNAGTNNNNNNNNNNNNNNNNNNNNNNNNNNNNN
NNNNNNNNNNNNNNNNNNNNNNNNNNNNNNNNGNNNNGNNNNNNCNANNNAAGGNNNTANNNCTNNTN
NTNNNNNNNGGACNNNACNNNNCNNNNNNNACNCNNNCNCNNNNNNNNNNNGNTTTTTNNCCNNTANTNT
CCAGNACGCNGNGCNNNCATTCANGNGAACNNNGNNACGCGNNAATCGTANNATCTACCTGCCGCNNG
TNNNNNTACNGNNNNNNCGGANNNTNNNNCGTANNNNNNTCATGCGTNNTNTNNTCNCNAGNCANGTT
CGNNNANANANTAGNANGTNTNNNNNNNNCTTGTNNCGTTCCGCNTGANNCNNCANNNNGNNGNNCTTN
TCANCGGCNCTANCTGCNGANNTAAGNCATANNNTNNATCNNTANCTNNNNNGGNGGGNNACTGTAAA
NGGNNAATTNGNNNNTNATTNCNNNCACNTNNCNNNGNNNNCTNNNTNCCTGNNTNCNNAATTGGNNC
CANTGNNTGNCNATCTCTGGNTTNGCNNNNNTNNNNNTNNCANNNNGGNNNNNNNNANNNCNATNTAA
NNTANANNNNCNNNNNNANNNCACNNNNNNNNNAGNNACNANNNTTANANNNGTAGNNNANTAAGTCT
CCGANNTNNNNNNNATAAGCGNNANACNNNNNNNNNNNNNNANGANNNCGNNTTGGNNNTNNNNNNNCNA
GNNAGACNNTNATGGNTNNNNNTCAANNNNTTNGTAGTGGTATAATNGNNNNNNNTTTCNCCAGNNTN

NNNGNNNNNNNCNATANNANNNNNNNGNGANNNNNNNNNNANNNNCTCNNNAGGGANCANCTACTTNA
GCANTGGNGCGCAGNNNGAGANNNNGACNNNCCTTGGGTGGCAANCACNGCGATAAGTNNNGTTNANC
CTNNNAGTNNACATNNNNNCANNGNNNCNNNNNNANCNGANNGNNNNNNNNNNNNNNNNNNNNNNNNN
NNNNNNNNNNNNNNNNNNNNNNNNNNNNAGNNNANGGNNNGTNTNTGACNCATGNNTTTTTANNNGNN
NNNNNACGNNNNANCCCCGGTCGNNNNCNNATTGAANNCCTGNNGCATNNNNNTNCNAGAATGANNNC
NNNNNGNNTNGCTNNCNTTTCAAGNAATCANNNAGNTNNNNNGNTCNNNNNNNNNNNNNCNNTCNCNN
ACTGAGGGTNNNNNNNNCATCTGCTNNNNNNNNNTNCGCNGNNNNNNCTNNNNAGCNGTATNGNGNNA
NNCANNNNNNNNNNNNNNNNTNAAACAANNNNNNNTNNNN
>Nung68
GTGNNNNNTAGCCCNNNNNAGTTAGACANNNCNNNNNNNNNNNTNNNNNNNNNNNNNNNNNNNNNNNN
NNNNNNNNNNNNNNNNNNNNNNNNNNNNNNTNNNNNNNNNNNNNNNNNNNNNNNNNNNNNNNNNNNNN
NNNNNNNNNNNNNNNNNNNNNNNNNNNNNNNNNNNNNNNNNNNNNNNNNNNNNNNNNNNNNNNNNNNN
NNNNNNNNNNNNNNNNNNNNNNNNNNNNNNNNNNNNNNNNNNNNNNNNNNNNNNNNNNNNNNNNNNNN
NNNNNNNNNNNNNNNNNNNNNNNNNNNNNNNNNNNNNNNNNNNNNNNNNNNNTCTNNNAAANNNTNNN
NNNNNNNNNNNNNNNNNNNNNNNNNCNCNTTGNNNGTAANCNANNNGNACATGCAGTCCCCTCNNNNN
NNNNNCAGAGNNNNNNNCCNNNNNNNCCGNCCCCNNCCNNNNNNNNNNNNNNNGNNNNNNNANNNNNN
NNNNNNTGGTACNNNNCGNNNNNGGTTGTTTNAGAGGTCCACAACGNGGTCNGGNCACNNNNGNNNAN
NNNCNNNNAACNNNNNNNNNNNANATNNNTANNAGGNCNNNNCCNTCNNGNNNNNNATTGCNATGACC
CAANNCACNGGGGCNGTNCCCGGCGCGGCGACCTTGANNANNTCCGGGGACAAGTATGGCNNNCNCCC
CGGGAAGGANNCNGCCGTANGTGCNNNNNANNNNNTCNTGNGANGTCGCCGNNATTCNCNTGGTCNNN
NNAGAATGNNCNNACCANNCGNANNNTGGTNATGNTAGNNNNNNNNNNNNNNNNNNNNNNNNNNNNNN
NNNNNNNNNNNNNNNNNNNNNNNNNNNNNNNNNNNNNGNNNNNNCNANNNAAGGNNNTANNNCTNNTN
NTNNNNNNGGACNNNACNNNNCNNNNNNNACNCNNNCNCNNNNNNNNNNGNTTTTTNNCCNNTANTNT
CCAGNACGCNGNGCNNNCATTCANGNGAACNNNGNNACGCGNNAATCGTANNATCTACCTGCCGCNNG
NNNNNNTNCNGNNNNNNCGGANNNTNNNNCGTANNNNNNTCANGCGTNNTNTNNTCNCNAGNCNNGTT
CGNNNANANANTAGNANGTNTNNNNNNNNNTGTNNCGTTCCGCNTGANNCNNCANNNNGNNGNNNTTN
TCANCGGCNNTANCTGCNGANNTAAGNCATANNNTNNNTCNNTANCTNNNNNGGNGGGNNACTGTAAA
NGGNNAATTNGNNGNTNANTTCNNNCACNTNNCTNNGNNTNCTNNNTNCCTGNNTNCNNAATTGGNNC
CANTGNNTGNCATCTCTNGNTTNGNNNNNNTNNNNNTNNCANNNNGGNNNGTNNNANNNCNATNTAA
NNTANANNNNCNNNNNNANNNCACNNNNNNNNNAGNNACNANNNTTANANNNGTAGNNNANTAAGTCT
CCGANNTNNNNNNNATAAGCGNNANACNNNNNNNNNNNNNANGANNACGNNTTGGNNNTNNNNNNCNA
GNNAGACNNTNATGGNTNNNNNTCAANNNNTTNGTAGTGGTATAATNGNNNNNNNTTTCNCCAGNNTN
NNNGNNNNNNNCNATANNANNNNNNNGNGANNNNNNNNNNANNNNCTCNNNAGGGANCANCTACTTNA
GCANTGGNGCGNAGNNNGAGANNNNGACNNNCCTTGGGTGGCAANCACNGCGATAAGTNNNGTTNANC
CTNNNAGTNNACATNNNNNCANNGNNNCNNNNNNANCNGANNGNNNNNNNNNNNNNNNNNNNNNNNNN
NNNNNNNNNNNNNNNNNNNNNNNNNNNNNGNNNANNGNNNGTNTNTGACNCATGTGTTTTTANNNGNN
NNNNNACGNNNGANCCCCGGTCGNNNNCNNATTGAANNCCTGNNGCATNNNNNTNCNAGAATGANNNC
NNNNNGNNTNGCTNNCNTTTCAAGNAATCANNNAGGTNNCGCGNTCNNNGNNNNNNNNNCNNTCCCNN
ACTGAGGGTNNNNNNNNCATCTGCTNNNNNNNNNCTNCGCNGGNGTNNCNNNNNGCNGTATNGNGTNN
NNCANNNNNNNNNNNNNNNNTNAANNGNNNNNNTTNNNN
>Nung680
GTGNNNNNTAGCCCNNNNNAGTTAGACANNNCNNNNNNNNNNNTNNNNNNNNNNNNNNNNNNNNNNNN
NNNNNNNNNNNNNNNNNNNNNNNNNNNNNNTNNNNNNNNNNNNNNNNNNNNNNNNNNNNNNNNNNNNN
NNNNNNNNNNNNNNNNNNNNNNNNNNNNNNNNNNNNNNNNNNNNNNNNNNNNNNNNNNNNNNNNNNNN
NNNNNNNNNNNNNNNNNNNNNNNNNNNNNNNNNNNNNNNNNNNNNNNNNNNNNNNNNNNNNNNNNNNN
NNNNNNNNNNNNNNNNNNNNNNNNNNNNNNNNNNNNNNNNNNNNNNNNNNCNTCTNNNAAANGNTNNN
NNNNNNNNNNNNNNNNNNNNNNNNNCNCNTTGNNNGTAANCNANNNGNACATGCAGTCCCCTCNNNNN
NNNGGCAGAGNNNNNNCCCNNNNNCNCCGGCCCCGNCCNNNNNNNNNNNNNNNGNNNNNNNANNNNNN
NNNNNNTGGTACNNNNCGNNNNNGGTTGNNTAAGAGGTCCACAACGNGGTCNGGNNACNNNNGNNNAN
NNNCNNNNAACNNNNNNNNNNNANNTNNNTANNAGGNCNNNNCCNTCNNGNNNNNNATTGCNATGACC
CAANNCACGGGGGCNGTNCCCGGCGCGGCGACCTTGANNACGTCCGGGGNCAAGTATGGCGNNCNCCC
CGGGAAGGANNCNGCCGTANGTGCNNNNNANNNNNTCNTGNGANGTCGCCGNNATTCNCGTGGTCNNN
CNAGAATGNNCGNACCANNCGNANNNTGGTNATGNNAGNNNNNNNNNNNNNNNNNNNNNNNNNNNNNN
NNNNNNNNNNNNNNNNNNNNNNNNNNNNNNNNGNNNNGNNNNNNCNANNNAAGGNNNTANNNCTNNTN

NTNNNNNNGGACNNNACNNNNCNNNNNNNACNCNNNCNCNNNNNNANNNGNTTTTNNNCNNTATTNT
CCAGNACGCNGNGCNNNCATTCANGNGAACNNNGNNACGCGNNAATCGTANNATCTACCTGCCGCNNG
TNNNNNTACNGNNNNNNCGGANNNTNNNNCGTANNNNNNNCANGCGTNNTNTNNTCNCNAGNCANGTT
CGNNNANANANTAGNANGTNTNNNNNNNNNTGTNNCGTTCCGCNTGANNCNNCANNNNGNNGNNCTTN
TCANCGGCNNTANCTGCNGANNTAAGNCATANNNTNNNTCNNTANCTNNNNNGGNGGGNNACTGTAAA
NGGNNAATTNGNNGNTNANTNCNNNCACNTNNCTNNGNNNNCTNNNTNCCTGNNTNCNNAATTGGNNC
CANTGNNTGNCNATCTCTGGNTTNGCNNNNNTNNNNNTNNCANNNNGGNNNGTNANANNNCNATNTAA
NNTANANNNNCNNNNNNANNNCACNNNNNNNGNAGNNACNANNNTNANANNNGTAGNNNAATAAGTCT
CCGANNTNNNNNNNATAAGCGNNANACNNNNNNNNNNNNNNANGANNNCGNNTTGGNNNTNNNNNNNNA
GNNAGACNNTNATGGNTNNNNNTCAANNNNTTNGTAGTGGTATAATNGNNNNNNNTTTCNCCAGNNTN
NGNGNNNNNNNCNATANNANNNNNNNGNGANNNNNNNNNNNANNNNCTCNNNAGGNANCANCTACTNNA
GCAATGGNGCGNAGNNNGAGANNNNGACNNGCCTTGGGTGGCAANCACNGCGATAAGTNNNGTTNANC
CTNNNAGTNNACATNNNNNCANNGNNNCNNNNNNANCNGANNGNNNNNNNNNNNNNNNNNNNNNNNN
NNNNNNNNNNNNNNNNNNNNNNNNNNNNNAGNNNANGGNNNGTNTNTGACNCNTGTGTTTTTANNNGNN
NNNNNACGNNNGANCCCCGGTCGNNNNCNNATTGAANNCCTGNNGCATNNNNNTNCNAGAATGANNGC
NNNNNGNNTNGNCTNNCNNTTCAAGNAATCANNNAGNNNNNNNGNTCNNNNNNNNNNNNCNNTCCCNN
ACTGAGGGTNNNNNNNNCATCTGCTNNNNNNNNNNTNCGCNGGNGTNNCNNNNNNGCNGTATNNNGTNA
NNCANNNNNNNNNNNNNNNNNNNAANCNANNNNNNNTNNNN
>Nung683
GTGNNNNNTAGCCCNNNNNAGTCAGACANNNCNNNNNNNNNNNNTNNNNNNNNNNNNNNNNNNNNNNN
NNNNNNNNNNNNNNNNNNNNNNNNNNNNNNNTNNNNNNNNNNNNNNNNNNNNNNNNNNNNNNNNNNNN
NNNNNNNNNNNNNNNNNNNNNNNNNNNNNNNNNNNNNNNNNNNNNNNNNNNNNNNNNNNNNNNNNNN
NNNNNNNNNNNNNNNNNNNNNNNNNNNNNNNNNNNNNNNNNNNNNNNNNNNNNNNNNNNNNNNNNNN
NNNNNNNNNNNNNNNNNNNNNNNNNNNNNNNNNNNNNNNNNNNNNNNNNNNNNNNNTCTNNNNAAANNNTNNN
NNNNNNNNNNNNNNNNNNNNNNNNNNCNCNTTNNNNGTAANCNANNNGNACATGCAGTCCCCTCNNNNN
NNNGGCAGAGNNNNNNNCCNNNNNNNCCGGCCCCGNCCNNNNNNNNNNNNNNNNNNNNNNNNNANNNNNN
NNNNNNTGGTACNNNNCGNNNNNGGTTGTNTNAGAGGTCCACAACGNGGTCNNGNNACNNNNGNNNAN
NNNCNNNNAACNNNNNNNNNNNNANNTNNNTANNAGGNCNNNNCCNTCNNNNNNNNNANNGCNATGACC
CAANNCACGGGGGCAGTNCCCGGCGCGGCGACCTTGNNNANGTCCGGGGACAAGTATGGCGNNCNCCC
CGGGAAGGANNCNGCCGTANGTGCNNNNNANNNNNTCNTGNGANGTCGCCGNNATTNNCGTGGNCNNN
NNAGAATGNCCGNACCANNCGNANNNTGGTNATGNNAGTNNNNNNNNNNNNNNNNNNNNNNNNNNNNN
NNNNNNNNNNNNNNNNNNNNNNNNNNNNNNNNNNNGNNNNGNNNNNNCNANNNAAGGANNTANNNCTNNTN
NTNNNNNNGGACNNNACNNNNCNNNNNNNACNCNNNCNCNNNNNNNNNNGNTTTTNNNNCNNTATTNT
CCAGNACGCNGNGCNNNCATTTANGNGAACNNNGNNACGCGNNAATCGTANNATCTACCTGCCGCNNG
NNNNNNTACNGNNNNNNCGGANNNTNNCTCGTANNNNNNNCATGCGTNNTNTNNTCNCNAGNCNNGTT
CGNNNANNNANTAGNANGTNTNNNNNNNNNTGTNNCGTTCCGCNTGANNCNNCANNNNGNNGNNNTTN
TCANCGGCNNTANCTGCNGANNTAAGNCATANNNTNNNTCNNTANCTNNNNNGGNGGGNNACTGTAAA
NGGNNAATTNGNNNNTNANTNCCNNCATNTNNCTNNGNNNNCTNNNTNCCTGNNTNCNNAATTGGNNC
CANTGNNTGNCNATCTCTNGNTTNGNNNNNNTGNNNNTNNCANNNNGGNNNNNNANANNNCNATNTAA
NNTANANNNNCNNNNNNANNNCACNNNNNNNGNAGNNNNNANNNGTANANNNGTAGNNNGNTAAGTCT
CCGANNTNNNNNNNATAAGCGNNANACNNNNNNNNNNNNNNANGANNNCGNNTTTGNNNTNNNNNNNCNA
GNNAGACNNTNGTGGNTANNNNTCAANNNNTTNGTAGTGGTATAATNGNNNNNNNTTTCNCCAGNNTN
NNNGNNNNNNNCNATANNANNNNNNNGNGANNNNNNNNNNNANNNNCTCNNNAGGNANCANCTACTNNA
GCAANGGNGCGNAGNNNGAGANNNNGACNNNCCTTGGGTGGCAANCACNGCAATAAGTNNNGTTNNNC
CTNNNAGTNNACACNNNNNCANNGNNNCNNNNNNANCNGANNGNNNNNNNNNNNNNNNNNNNNNNNN
NNNNNNNNNNNNNNNNNNNNNNNNNNNNNAGNNNANNGNNNGTNTNTGACNCATGNNTTTTTANNNGNN
NNNNNACGNNNNANCCCCGGTCGNNNNCNNATTGAANNCCTGNNGCATNNNNNTNCNAGAATGANNNC
NNNNNGNNTNGNCTNNCNNNTCAAGNAATCNANNAGNTNNNNNGNTCNNNGNNNNNNNNCNNTCCCNN
ACTGAGGGTNNNNNNNNCATNNGCTNNNNNNNNNNTNCGCNGGCGTNNCNNNNNNGCNGTATNGNGTNN
NNCANNNNNNNNNNNNNNNNNNTNNANNAANNNNNNNTNNNN
>Nung684
GTGNNNNNTAGCCCNNNNNAGTCAGACANNNCNNNNNNNNNNNNTNNNNNNNNNNNNNNNNNNNNNNN
NNNNNNNNNNNNNNNNNNNNNNNNNNNNNNNTNNNNNNNNNNNNNNNNNNNNNNNNNNNNNNNNNNNN
NNNNNNNNNNNNNNNNNNNNNNNNNNNNNNNNNNNNNNNNNNNNNNNNNNNNNNNNNNNNNNNNNNN

NNNNNNNNNNNNNNNNNNNNNNNNNNNNNNNNNNNNNNNNNNNNNNNNNNNNNNNNNNNN
NNNNNNNNNNNNNNNNNNNNNNNNNNNNNNNNNNNNNNNNNNCNTCTNNNAAANNNTNNN
NNNNNNNNNNNNNNNNNCNCNTTGNNNGTAANCNANNNGNACATGCAGTCCCCTCNNNNN
NNNGGCAGAGNNNNNNCCCNNNNNNNCCGGCCCCGNCCNNNNNNNTNNNCANNGNNNNNNNANNNNNN
NNNNNNTGGTACNNNNCGNNNNNGGTTGTTTAAGAGGTCCACAACGNGGTCNGGNCACNNNNGNNNAN
NNNCNNAAAACNNNNNNNNNNNANNTNNNTANNAGGNCNNNNCCNTCNNGNNNNNAATTGCNATGACC
CAANTCACGGGGGCAGTNCCCGGCGCGGCGACCTTGATNACGTCCGGGGACAAGTATGGCGNNCNCCC
CGGGAAGGANNCNGCCGTANGTGCNNNNNANANNNTCNTGNGACGTCGCCGNNATTCNCGTGGTCNNN
CNAGAATGCCCGNACCANNCGNANNNTGGTNATGNNAGTNNNNNNNNNNNNNNNNNNNNN
NNNNNNNNNNNNNNNNNNNNNNNNGNNNNGNNNNNNCNANNNAAGGANNTANNNCTNNTN
NTNNNNNNGGACNNNACNNNNCNNNNNNNACNCNNNCNCNNNNNNANNNGGTTTTTNNNCNNTATTNT
CCAGNACGCNGNGCNNNCATTTANGNGAACNNNGNNACGCGNNAATCGTANNATCTACCTGCCGCNNG
TNNNNNTACNGNNNNNNCGGANNNTNNCTCGTANNNNNNTCATGCGTNNTNTNNTCNCNAGNCNNGTT
CGNNNANANANTAGNANGTNTNNNNNNNCTTGTNNCGTTCCGCNTGANNCNNCANNNNGNNGNNCTTN
TCANCGGCNNTANCTGCNGANNTAAGNCATANNNTNNATCNNTANCTNNNNNGGAGGGNNACTGTAAA
NGGNNAATTNGNGGNTNATTNCNNNCATNTNNCTNNGNNTNCTNNNTNCCTGNNTNCNNAATTGGNNC
CANTGNNTGNCNATCTCTGGNTTNGNNNNNNTGNNNNTNNCAANNNGGNNNGTNANANNNCNATNTAA
NNTANANNNNCNNNNNNANNNCACNNNNNNNGNAGNNNCNANNNGTANANNNGTAGNNNGATAAGTCT
CCGANNTNNNNNNNATAAGCGNNANACNNNNNNNNNNNNNNANGANNACGNNTTTGNNNTNNNNNNCNA
GNNAGACNNTNGTGGNTNNNNNTCAANNNNNTTCGTAGTGGTATAATNGNNNNNNNTTTCNCCAGNNTN
NNNGNNNNNNNNCNATANNANNNNNNNGNGANNNNNNNNNNNANNNNCTCNNNAGGGANCANCTACTTNA
GCAATGGNGCGCAGNNNGAGANNNNGACNNGCCTTGGGTGGCAANCACNGCAATAAGTNNNGTTTANC
CTNNNAGTNNACACNNNNNCANNGNNNCNNNNNNANCNGANNGNNNNNNNNANGTNNNNNNNNNNNNN
NNNNNNNNNNNNNNNNNNNNNNNNNAGNNNANGGNNNGTNTNTGACNCATGTGTTTTTANNNGNN
NNNNNACGNNNGANCCCCGGTCGTNTNCNNATTGAANNCCTGNNGCATNNNNNNTNCNAGAATGANNGC
NNNNNGNNTNGNCTNNCNTTTCAAGNAATCANNNAGGTANCGCGNTCNNNGNNNNNNNNCNNTCCCNN
ACTGAGGGTNNNNNNNGCATCTGCTNNNNNNNNNCTTCGCNGGCGTNNCNNNNNAGCNGTATNGTGTNA
NNCANNNNNNNNNNNNNNNNNNTNAAACAANNNNNNNTNNNN
>Nung685
GTGNNNNNTAGCCCNNNNNAGTTAGACANNNCNNNNNNNNNNNNTNNNNNNNNNNNNNNN
NNNNNNNNNNNNNNNNNNNNNNNNNNNNNNNNNNNNNNNNNNNNNNNNNNNNNNNNNNNN
NNNNNNNNNNNNNNNNNNNNNNNNNNNNNNNNNNNNNNNNNNNNNNNNNNNNNNNNNNNN
NNNNNNNNNNNNNNNNNNNNNNNNNNNNNNNNNNNNNNNNNNNNNNNNNNNNNNNNNNNN
NNNNNNNNNNNNNNNNNNNNNNNNNNNNNNNNNNNNNNNNNNCNTCTNNNAAANNNTNNN
NNNNNNNNNNNNNNNNNCNCNTTGNNNGTAANCNANNNGNACATGCAGTCCCCTCNNNNN
NNNNNCAGAGNNNNNNNNCNNNNNNCNCCGGCCCCNNCCNNNNNNNNNNNNCNNNGNNNNNNNANNNNNN
NNNNNNTGGTACNNNNCGNNNNNGGTTGTTTNAGAGGTCCACAACGNGGTCNNGNCACNNNNGNNNAN
NNNCNNNNAACNNNNNNNNNNNNNNNNTNNNTANNAGGNNNNNNCCNTCNNGNNNNNNATTGCNATGACC
CAANTCACGGNGGCNGTNCCCGGCGNGGCGACCTTGNTNANGTCCGGGGACAAGTATGGCNNNCNCCC
CGGGAAGGANNCNGCCGTANGTGCNNNNNANNNNNTCNTGNGANGTNGCCGNNATTCNCGTGGTCNNN
CNAGAATGNNCNNACCANNCGNANNNTGGTNATGNNAGTNNNNNNNNNNNNNNNNNNNNN
NNNNNNNNNNNNNNNNNNNNNNNNNNNNNGNNNNNNCNANNNAAGGANNTANNNCTNNTN
NTNNNNNNGGACNNNACNNNNCNNNNNNNACNCNNNCNCNNNNNNNNNNNGNTTTTNNNNCNNTANTNT
CCAGNACGCNGNGCNNNCATTCANGNGAACNNNGNNACGCGNNAATCGTANNATCTACCTGCCGCNNG
TNNNNNTACNGNNNNNNCGGANNNTNNNNCGTANNNNNNTCATGCGTNNTNTNNTCNCNAGNCANGTT
CGNNNANANANTAGNANGTNTNNNNNNNNNNTGTNNCGTTCCGCNTGANNCNNCANNNNGNNGNNCTTN
TCANCGGCNNTANCTGCNGANNTAAGNCATANNNTNNATCNNTANCTNNNNNGGNGGGNNACTGTAAA
NGGNNAATTNGNNGNTNATTNCCNNCACNTNNCNNNGNNTNCTNNNTNCCTGNNTNCNNAATTGGNNC
CANTGNNTGNCNATCTCTGGNTTNGNNNNNNTNNNNNTNNCANNNNGGNNNGNNNNANNNCNATNTAA
NNTANANNNNCNNNNNNANNNCACNNNNNNNNNNAGNNNCNANNNTTANANNNGTAGNNNAATAAGTCT
CCGANNTNNNNNNNATAAGCGNNANACNNNNNNNNNNNNNNANGANNACGNNTTGGNNNTNNNNNNCNA
GNNAGACNNTNATGGNTNNNNNTCAANNNNNTTCGTAGTGGTATAATNGNNNNNNNTTTCNCCAGNNTN
NNNGNNNNNNNNCNATANNANNNNNNNGNGANNNNNNNNNNNANNNNCTCNNNAGGGANCANCTACTNNA
GCAATGGNGCGCAGNNNGAGANNNNGACNNNCCTTGGGTGGCAANNACNGCGATAAGTNNNGTTNANN

CTNNNAGTNNACATNNNNNCANNGNNNCNNNNNNANCNGANNGNNNNNNNNNNNNNNNNNNNNNNNN
NNNNNNNNNNNNNNNNNNNNNNNNNNNNNAGNNNANGGNNNGTNTNTGACNCATNNNTTTTANNNGNN
NNNNNACGNNNNANCCCCGGTCGNNNNCNNATTGAANNCCTGNNGCATNNNNNTNCNAGAATGANNNC
NNNNNGNNTNGNCTGNCNNNTCAAGNAATCANNTAGGTANNNNGNTCNNNGNNNNNNNNCNNTCCCNN
ACTGAGGGTNNNNNNNNCATCTGCTNNNNNNNNCTNCGCNGGCGTNNCNNNNNAGCNGTATNGNGTNA
NNCANNNNNNNNNNNNNNNNNNTNAAACNANNNNNNTNNNN
>Nung686
GTGNNNNNTAGCCCNNNNNAGTTAGACANNNCNNNNNNNNNNNNTNNNNNNNNNNNNNNNNNNNNNN
NNNNNNNNNNNNNNNNNNNNNNNNNNNNNNNNTNNNNNNNCNNNNNNNNNNNNNNNNNNNNNNNNNNNN
NNNNNNNNNNNNNNNNNNNNNNNNNNNNNNNNNNNNNNNNNNNNNNNNNNNNNNNNNNNNNNNNNNNN
NNNNNNNNNNNNNNNNNNNNNNNNNNNNNNNNNNNNNNNNNNNNNNNNNNNNNNNNNNNNNNNNNNNN
NNNNNNNNNNNNNNNNNNNNNNNNNNNNNNNNNNNNNNNNNNNNNNNNNNNNNNTCTNNNAAANNNTNNN
NNNNNNNNNNNNNNNNNNNNNNNNNNNCNCNTTGNNNGTAANCNANNNGNACATGCAGTCCCCTCNNNNN
NNNGGCAGAGNNNNNNNCCNNNNNCNCCGNCCCCNNCCNNNNNNNNNNNNNNNNNNNNNNNNNNANNNNNN
NNNNNNTGGTACNNNNCGNNNNNGGTTGNNTNAGAGGTCCACAACGNGGTCNGGNCACNNNNGNNNAN
NNNCNNNAAANNNNNNNNNNNNNANNTNNNTANNAGGNNNNNNCCNTCNNNNNNNNNNANTGCNATGACC
CAANNCNCGGGGGCNGTNCCCGGNGNGGCGACCTTGNNNANNTCCGGGGNCAAGTATGGCNNNCNCCC
CGGGAAGGANNCNGCCGTANGTGCNNNNNANNNNNTCNTGNGANGTCGCCGNNNTTNNNNTGGTCNNN
NNAGAATGNCCNNACCANNCGNANNNTGGTNATGNNAGTNNNNNNNNNNNNNNNNNNNNNNNNNNNNN
NNNNNNNNNNNNNNNNNNNNNNNNNNNNNNNNNNNNNNNGNNNNNNCNANNNAAGGNNNTANNNCTNNTC
NTNNNNNNNGGACNNNACNNNNCNNNNNNNACNCNNNCNCNNNNNNANNNGNTTNNNNNNCNNTATTNT
CCAGNACGCNGNGCNNNCATTCANGNGAACNNNGNNACGCGNNAATCGTANNATCTACCTGCCGCNNG
TNNNNNTACNGNNNNNNCGGANNNTNNCTCGTANNNNNNTCANGCGTNNTNTNNTCNCNAGNCNNGTT
CGNNNANANANTAGNANGTNTNNNNNNNNNNTGTNNCGTTCCGCNTGANNCNNCNNNNNGNNGNNCTTN
TCANCGGCNNTANCTGCNGANNTAAGNCATANNNTNNATCNNTANCTNNNNNGGAGGGNNACTGTAAA
NGGNNAATTNGNNNNTNATTNCNNNCACNTNNCTNNGNNNNCTNNNTNCCTGNNTNCNNAATTGGNNN
CANTGNNTGNCNATCTCTGGNTTNGCNNNNNTNNNNNTNNCAANNNGGNNNNNNNNANNNCNATNTAA
NNTANANNNNCNNNNNNANNNCACNNNNNNNNNAGNNACNANNNTTANANNNGTAGNNNAATAAGTCT
CCGANNTNNNNNNNATAAGCGNNANACNNNNNNNNNNNNNANGANNNCGNNTTGGNNNTNNNNNNCNA
GNNAGACNNTNGTGGNTNNNNNTCAANNNNTTNGTAGTGGTATAATNGNNNNNNNTTTCNCCAGNNTN
NNNGNNNNNNNCNATANTANNNNNNNGNGANNNNNNNNNNNANNNNCTCNNNAGGNANCANCTACTNNA
GCANTGGNGCGCAGNNNGAGANNNNGACNNGCCTTGGGTGGCAANCACNGCAATAAGTNNNGTTNANC
CTNNNAGTNNACACNNNNNCANNGNNNCNNNNNNANCNGANNGNNNNNNNNNNNNNNNNNNNNNNNN
NNNNNNNNNNNNNNNNNNNNNNNNNNNNNAGNNNANGGNNNGTNTNTGACNCATGTGTTNNTANNNGNN
NNNNNACGNNNGANCCCCGGTCGNNNNCNNATTGAANNCCTGNNGCATNNNNNTNCNAGAATGANNNC
NNNNNGNNTNNNCTNNCNNTTCAAGNAATCAANNAGGTANNNNNNTCNNNGNNNNNNNNCNNTCCCNN
ACTGAGGGTNNNNNNNNCATCTGCTNNNNNNNNNTNCGCNGGNNTNNCNNNNNAGCNGTATNNTGTNN
NNCANNNNNNNNNNNNNNNNNNNNANNNANNNNNNTNNNN
>Nung687
GTGNNNNNTAGCCCNNNNNAGTTAGACANNNCNNNNNNNNNNNNTNNNNNNNNNNNNNNNNNNNNNN
NNNNNNNNNNNNNNNNNNNNNNNNNNNNNNNNTNNNNNNNNNNNNNNNNNTNNNNNNNNNNNNNNNNNNN
NNNNNNNNNNNNNNNNNNNNNNNNNNNNNNNNNNNNNNNNNNNNNNNNNNNNNNNNNNNNNNNNNNNN
NNNNNNNNNNNNNNNNNNNNNNNNNNNNNNNNNNNNNNNNNNNNNNNNNNNNNNNNNNNNNNNNNNNN
NNNNNNNNNNNNNNNNNNNNNNNNNNNNNNNNNNNNNNNNNNNNNNNNNNNNNNTCTNNNAAANNNTNNN
NNNNNNNNNNNNNNNNNNNNNNNNNNNCNCNTTGNNNGTAANCNANNNGNACATGCAGTCCCCTCNNNNN
NNNGGCAGAGNNNNNNNCCNNNNNNNCCGGCCCCNNCCNNNNNNNTNNNNNNNNNNNNNNNNNANNNNNN
NNNNNNTGGTACNNNNCGNNNNNGGTTGNNTNAGAGGTCCACAACGNGGTCNNGNCACNNNNGNNNAN
NNNCNNAANACNNNNNNNNNNNNNANNTNNNTANNAGGNCNNNNCCNTCNNNNNNNNNNATNGCNATGACC
CAANNCACGGGGGCAGTCCCCGGCGNGGCGACCTTGANNACGTCCGGGGNCAAGTATGGCNNNCNCCC
CGGGAAGGANNCNGCCGTANGTGCNNNNNANNNNNTCNTGNGANGTCGCCGNNATTNNNGTGGTCNNN
NNAGAATGNCCGNACCANNCGNANNNTGGTNATGNTAGTANNNNNNNNNNNNNNNNNNNNNNNNNNNN
NNNNNNNNNNNNNNNNNNNNNNNNNNNNNNNNNNNGNNNNGNNNNNNCNANNNAAGGANNTANNNCTNNTN
NTNNNNNNNGGACNNNACNNNNCNNNNNNNACNCNNNCNCNNNNNNANNNGNTTTTTNNNCNNTATTNT
CCAGNACGCNGNGCNNNCATTCANGNGAACNNNGNNACGCGNNAATCGTANNATCTACCTGCCGCNNG

TNNNNNTNCNGNNNNNNCGGANNNTNNCTCGTANNNNNNTCATGCGTNNTNTNNTCNCNAGNCNNGTT
CGNNNANANANTAGNANGTNTNNNNNNNNNNTGTNNCGTTCCGCNTGANNCNNCANNNNGNNGNNCTTN
TCANCGGCNNTANCTGCNGANNTAAGNCATANNNTNNNTCNNTANCTNNNNNGGNGGGNNACTGTAAA
NGGNNAATTNGNNGNTNANTNCNNNCACNTNNCNNNGNNTNCTNNNTNCCTGNNTNCNNAATTGGNNN
CANTGNNTGNCNATCTCTGGNTTNGNNNNNNTNNNNNTNNCANNNNGGNNNGTNNNANNNCNATNTAA
NNTANANNNNCNNNNNNANNNCACNNNNNNNGNAGNNNCNANNNTTANANNNGTAGNNNANTAAGTCT
CCGANNTNNNNNNNATAAGCGNNANACNNNNNNNNNNNNNNANGANNACGNNTTGGNNNTNNNNNNCNA
GNNAGACNNTNGTGGNTNNNNNTCAANNNNTTCGTAGTGGTATAATNGNNNNNNNTTTCNCCAGNNTN
NNNGNNNNNNNCNATANNANNNNNNNGNGANNNNNNNNNNNANNNNCTCNNNAGGGANCANCTACTNNA
GCANTGGNGCGNAGNNNGAGANNNNGACNNGCCTTGGGTGGCAANCACNGCAATAAGTNNNGTTNANC
CTNNNAGTNNACACNNNNNCANNGNNNCNNNNNNANCNGANNGNNNNNNNNNNNNNNNNNNNNNNNNNNN
NNNNNNNNNNNNNNNNNNNNNNNNNNNNNNNGNNNANNGNNNGTNTNTGACNCATNTGTTTTTANNNGNN
NNNNNACGNNNGANCCCCGGTCGNNNNCNNATTGAANNCCTGNNGCATNNNNNTNCNAGAATGANNGC
NNNNNGNNTNGNCTGNCNNNTCANGNAATCANNNAGNNANCGCGNTCNNNGNNNNNNNNCNNTCCCNN
ACTGAGGGTNNNNNNNNCATCTGCTNNNNNNNNNNTNCGCNGGNGTNNCNNNNNNGCNGTATNGTGNNN
NNCANNNNNNNNNNNNNNNNNTNNAACNNNNNNNNNTNNNN
>Nung688
GTGNNNNNTAGCCCNNNNNAGTTAGACANNNCNNNNNNNNNNNNNTNNNNNNNNNNNNNNNNNNNNNNNN
NNNNNNNNNNNNNNNNNNNNNNNNNNNNNNNNNNNNNNNNNNNNNNNNNNNNNNNNNNNNNNNNNNNNNN
NNNNNNNNNNNNNNNNNNNNNNNNNNNNNNNNNNNNNNNNNNNNNNNNNNNNNNNNNNNNNNNNNNNNNN
NNNNNNNNNNNNNNNNNNNNNNNNNNNNNNNNNNNNNNNNNNNNNNNNNNNNNNNNNNNNNNNNNNNNNN
NNNNNNNNNNNNNNNNNNNNNNNNNNNNNNNNNNNNNNNNNNNNNNNNNNNNNNTCTNNNAAANNNTNNN
NNNNNNNNNNNNNNNNNNNNNNNNNNNCNCNTTNNNNGTAANCNANNNGNACATGCAGTCCCCTCNNNNN
NNNGGCAGAGNNNNNNNCCNNNNNCNCCGGCCCCNNCCNNNNNNNNNNNNCNNNGNNNNNNNANNNNNN
NNNNNNTGGTACNNNNCGNNNNNGGTTGTTTAAGAGGTCCACAACGNGGTCNGGNCACNNNNGNNNAN
NNNCNNNNAACNNNNNNNNNNNNANNTNNNTANNAGGNCNNNNCCNTCNNNNNNNNAATTGCNATGACC
CAANTCACGGGGGNNGTCCCCGGCGCGGCGACCTTGATNACGTCCGGGGNCAAGTATGGCGNNCNCCC
CGGGAAGGANNCNGCCGTANGTGCNNNNNANNNNNTCNTGNGANGTCGCCGNNATTNNCGTGGTCNNN
CNAGAATGNNCNNACCANNCGNANNNTGGTNATGNNAGTNNNNNNNNNNNNNNNNNNNNNNNNNNNNNNN
NNNNNNNNNNNNNNNNNNNNNNNNNNNNNNNNNNGNNNNGNNNNNNCNANNNAAGGNNNTANNNCTNNTN
NTNNNNNNGGNCNNNACNNNNCNNNNNNNACNCNNNCNCNNNNNNANNNGNTTTTTNNCCNNTATTNT
CCAGNACGCNGNGCNNNCATTCANGNGAACNNNGNNACGCGNNAATCGTANNATCTACCTGCCGCNNG
TNNNNNTACNGNNNNNNCGGANNNTNNNNCGTANNNNNNNCATGCGTNNTNTNNTNNCTAGNCANGTT
CGNNNANANANTAGNANGTNTNNNNNNNNNNTGTNNCGTTCCGCNTGANNCNNCANNNNGNNGNNCTTC
NCANCGGCNNTANCTGCNGANNTAAGNCATANNNTNNNTCNNTANCTNNNNNGGAGGGNNACTGTAAA
NGGNNAATTNGNNNNTNATTNCCNNCACNTNNCTNNGNNNNCTNNNTNCCTGNNTNCNNAATTGGNNC
CANTGNNTGNCNATCTCTGGNTTNGNNNNNNTNNNNNTNNCANNNNGGNNNGTNANANNNCNATNTAA
NNTANANNNNCNNNNNNANNNCACNANNNNNGNAGNNNNNANNNTTANANNNGTAGNNNAATAAGTCT
CCGANNTNNNNNNNATAAGCGNNANACNNNNNNNNNNNNNNANGANNACGNNTTGGNNNTNNNNNNCNA
GNNAGACNNTNGTGGNTNNNNNTCAANNNNTTNGTAGTGGTATAATNGNNNNNNNTTTCNCCAGNNTN
NNNGNNNNNNNCNATANNANNNNNNNGNGANNNNNNNNNNNANNNNCTCNNNAGGGANCANCTACTNNA
GCAATGGNGCGCAGNNNGAGANNNNGACNNGCCTTGGGTGGCAANCACNGCAATAAGTNNNGTTNANC
CTNNNAGTNNACACNNNNNCANNGNNNCNNNNNNANCNGANNGNNNNNNNNNNGNNNNNNNNNNNNNNNN
NNNNNNNNNNNNNNNNNNNNNNNNNNNNNNAGNNNANGGNNNGTNTNTGACNCATNNNTTTNTANNNGNN
NNNNNNNGNNNNANCCCCGGTCGNNNNCNNATTGAANNCCTGNNGCATNNNNNTNCNAGAATGANNNC
NNNNNGNNTNGNCTNNNNNTTTCAAGNAATCANNNAGGTANNNCGNTCNNNGNNNNNNNNCNNTCCCNN
ACTGAGGGTNNNNNNNNCATCTGCTNNNNNNNNNCTNCGCNGGNGTNNCNNNNNAGCCGTATNGTGTNA
NNCANNNNNNNNNNNNNNNNNTNAAACNANNNNNNNTNNNN
>Nung689
GTGNNNNNTAGCCCNNNNNAGTCAGACANNNCNNNNNNNNNNNNNTNNNNNNNNNNNNNNNNNNNNNNNN
NNNNNNNNNNNNNNNNNNNNNNNNNNNNNNNTNNNNNNNNNNNNNNNNNNNNNNNNNNNNNNNNNNNNNN
NNNNNNNNNNNNNNNNNNNNNNNNNNNNNNNNNNNNNNNNNNNNNNNNNNNNNNNNNNNNNNNNNNNNNN
NNNNNNNNNNNNNNNNNNNNNNNNNNNNNNNNNNNNNNNNNNNNNNNNNNNNNNNNNNNNNNNNNNNNNN
NNNNNNNNNNNNNNNNNNNNNNNNNNNNNNNNNNNNNNNNNNNNNNNNNNNNCNTCTNNNAAANNNTNNN

NNNNNNNNNNNNNNNNNNNNNNNNNCNCNTTGNNNGTAANCNANNNGNACATGCAGTCCCCTCNNNNN
NNNGGCAGAGNNNNNNCCCNNNNNCNCCGGCCCCNNCCNNNNNNNNNNNNNNNGNNNNNNANNNNNNN
NNNNNNTGGTACNNNNCGNNNNNGGTTGTNTAAGAGGTCCACAACGNGGTCNGNNCACNNNNGNNNAN
NNNCNNNNAACNNNNNNNNNNNANATNNNTANAAGGNCNNNNCCNTCNNNNNNNNAATTGCNATGACC
CAANNCNCGGGGGCAGTCCCCGGCGCGGCGACCTTGANNACGTCCGGGGNCAAGTATGGCGNNCNCCC
CGGGAAGGANNCNGCCGTANGTGCNNNNNANNNNNTCNTGNGANGTCGCCGNNATTCNCGTGGTCNNN
CNAGAATGNNCNNACCANNCGNANNNTGGTNATGNNAGTNNNNNNNNNNNNNNNNNNNNNNNNNNNNN
NNNNNNNNNNNNNNNNNNNNNNNNNNNNNNNNGNNNNGNNNNNNCNANNNAAGGNNNTANNNCTNNTN
NTNNNNNNGGACNNNACNNNNCNNNNNNNACNCNNNCNCNNNNNNANNNGGTTTTTNNNCNNTANTNT
CCAGNACGCNGNGCNNNCATTTANGNGAACNNNGNNACGCGNNAATCGTANNATCTACCTGCCGCNNG
NNNNNNTACNGNNNNTNCGGANNNTNNCTCGTANNNNNNNCATGCGTNNTNTNNTCNCNAGNCANGTT
CGNNNANANANTAGNANGTNTNNNNNNNCNTGTNNCGTTCCGCNTGANNCNNCANNNNGNNGNNCTTN
NCANCGGCNNTANCTGCNGANNTAAGNCATANNNTNNNTCNNTANCTNNNNNGGNGGGNNACTGTAAA
NGGNNAATTNGNNNNTNATTNCCNNCATNTNNCTNNGNNTNCTNNNTNCCTGNNTNCNNAATTGGNNC
CANTGNNTGNCATCTCTGGNTTNGCNNNNNNTNNNNNTNNCANNNNGGNNNGTNANANNNCNATNTAA
NNTANANNNNCNNNNNNANNNCACNNNNNNNNNAGNNNCNANNNGNANANNNGTAGNNNGATAAGTCT
CCGANNTNNNNNNNATAAGCGNNANACNNNNNNNNNNNNNANGANNACGNNTTTGNNNTNNNNNNNNA
GNNAGACNNTNGTGGNTNNNNNTCAANNNNTTNGTAGTGGTATAATNGNNNNNNNTTTCNCCAGNNTN
NNNGNNNNNNCNATANNANNNNNNNNGNGANNNNNNNNNNANNNNCTCNNNAGGGANCANCTACTNNA
GCAATGGNGCGCAGNNNGAGANNNNGACNNNCCTTGGGTGGCAANCACNGCAATAAGTNNNGTTNANC
CTNNNAGTNNACACNNNNNCANNGNNNCNNNNNNANCNGANNGNNNNNNNNNNNNNNNNNNNNNNNNN
NNNNNNNNNNNNNNNNNNNNNNNNNNNNAGNNNANGGNNNGTNTNTGACNCATGTGTTTTTANNNGNN
NNNNNACGNNNNANCCCCGGTCGNNNNCNNATTGAANNCCTGNNGCATNNNNNTNCNAGAATGANNGC
NNNNNGNNTNGCTNNCNNTTCAAGNAATCAANNAGGTNNCGCGNTCNNNNNNNNNNNNNCNNTCCCNN
ACTGAGGGTNNNNNNNNCATCTGCTNNNNNNNNNNTNCGCNGGNGTNNCNNNNNGCNGTATNGNGTNA
NNCANNNNNNNNNNNNNNNNNNAAACAANNNNNCTNNNG
>Nung690
GTGNNNNTAGCCCNNNNNAGTTAGACANNNCNNNNNNNNNNNNNTNNNNNNNNNNNNNNNNNNNNNNN
NNNNNNNNNNNNNNNNNNNNNNNNNNNNNNTNNNNNNNNNNNNNNNNNNNNNNNNNNNNNNNNNNNNNN
NNNNNNNNNNNNNNNNNNNNNNNNNNNNNNNNNNNNNNNNNNNNNNNNNNNNNNNNNNNNNNNNNNNN
NNNNNNNNNNNNNNNNNNNNNNNNNNNNNNNNNNNNNNNNNNNNNNNNNNNNNNNNNNNNNNNNNNNN
NNNNNNNNNNNNNNNNNNNNNNNNNNNNNNNNNNNNNNNNNNNNNNNNNNNNTCTNNNAAANNNTNNN
NNNNNNNNNNNNNNNNNNNNNNNNNCNCNNTGNNNGTAANCNANNNGNACATGCAGTCCCCTCNNNNN
NNNGGCAGAGNNNNNNNCCNNNNNCNCCGNCCCCNNCCNNNNNNNNNNNCNNNGNNNNNNANNNNNNN
NNNNNNTGGTACNNNNCGNNNNNGGTTGTNTAAGAGGTCCACAACGNGGTCNGGNNACNNNNGNNTAN
NNNCNNNAAACNNNNNNNNNNNANNTNNNTANNAGGNCNNNNCCNTCNNGNNNNNNATTGCNATGACC
CAANNCACGGGGGCNGTNCCCGGCGNGGCGACCTTGANNACGTCCGGGGACAAGTATGGCGNNCNCCC
CGGGAAGGANNCNGCCGTANGTGCNNNNNANNNTNTCNTGNGANGTNGCCGNNATTCNCGTGGTCNNN
NNAGAATGNCCGNACCANNCGNANNNTGGTNATGNNAGTNNNNNNNNNNNNNNNNNNNNNNNNNNNNN
NNNNNNNNNNNNNNNNNNNNNNNNNNNNNNNNNNNNNGNNNNNNCNANNNAAGGNNNTANNNCTNNTC
NTNNNNNNGGACNNNACNNNNCNNNNNNNACNCNNNCNCNNNNTNANNNGGTTTTTNNNCNNTANTNT
CCAGNACGCNGNGCNNNCATTCANGNGAACNNNGNNACGCGNNAATCGTANNATCTACCTGCCGCNNG
TNNNNNTACNGNNNNNNCGGANNNTNNCTCGTANNNNNNTCANGCGTNNTNTNNTCNCNAGNCANGTT
CGNNNANANANTAGNANGTNTNNNNNNNNNTGTNNCGTTCCGCNTGANNCNNCANNNNGNNGNNCTTN
NCANCGGCNNTANCTGCNGANNTAAGNCATANNNTNNNTCNNTANCTNNNNNGGNGGGNNACTGTAAA
NGGNNAATTNGANGNTNATTNCNNNCACNTNNCNNNGNNNNCTNNNTNCCTGNNTNCNNAATTGGNNC
CANTGNNTGNCATCTCTGGNTTNGNNNNNNNTGNNNNTNNCANNNNGGNNNGTNNNANNNCNATNTAA
NNTANANNNNCNNNNNNANNNCACNNNNNNNGNAGNNNCNANNNTTANANNNGTAGNNNAATAAGTCT
CCGANNTNNNNNNNATAAGCGNNANACNNNNNNNNNNNNNANGANNACGNNTTGGNNNTNNNNNNNNA
GNNAGACNNTNATGGNTNNNNNTCAANNNNTTNGTAGTGGTATAATNGNNNNNNNTTTCNCCAGNNTN
NNNGNNNNNNCNATANNANTNNNNNNGNGANNNNNNNNNNANNNNCTCNNNAGGNANCANCTACTTNA
GCAANGGNGCGNAGNNNGAGANNNNGACNNNCCTTGGGTGGCAANNACNGCAATAAGTNNNGTTNANC
CTNNNAGTNNACATNNNNNCANNGNNNCNNNNNNANCNGANNGNNNNNNNNNNNNNNNNNNNNNNNNN
NNNNNNNNNNNNNNNNNNNNNNNNNNNNAGNNNANGGNNNGTNTNTGACNCATGNNTTTTTANNNGNN

NNNNNACGNNNGANCCCCGGTCGNNNNCNNATTGAANNCCTGNNGCATNNNNNNTNCNAGAATGANNGC
NNNNNGNNTNGNCTNNCNNNTCAAGNAATCAANNAGGTNNNNNNNTCNNNGNNNNNNNNCNNNCCCNN
ACTGAGGGTNNNNNNCGCATCNGCTNNNNNNNNCTNCGCNGGCGTNNCNNNNNAGCNGTATNGNGTNA
NNCANGNNNNNNNNNNNNNNNNTNAAACGANNNNNCTNNNN
>Nung691
GTGNNNNNTAGCCCNNNNNAGTTAGACANNCCNNNNNNNNNNNNNTNNNNNNNNNNNNNNNNNNNNNNNN
NNNNNNNNNNNNNNNNNNNNNNNNNNNNNNTNNNNNNNNNNNNNNNNNNNNNNNNNNNNNNNNNNNNNNN
NNNNNNNNNNNNNNNNNNNNNNNNNNNNNNNNNNNNNNNNNNNNNNNNNNNNNNNNNNNNNNNNNNNNNN
NNNNNNNNNNNNNNNNNNNNNNNNNNNNNNNNNNNNNNNNNNNNNNNNNNNNNNNNNNNNNNNNNNNNNN
NNNNNNNNNNNNNNNNNNNNNNNNNNNNNNNNNNNNNNNNNNNNNNNNNNNCNTCTNNNAAANNNTNNN
NNNNNNNNNNNNNNNNNNNNNNNNNNNCNCNNTGNNNGTAANCNANNNGNACATGCAGTCCCCTCNNNNN
NNNNNCAGAGNNNNNNCCCNNNNNNNCCGGCCCCGNCCNNNNNNNNNNNNCANNNNNNNNNANNNNNNNN
NNNNNNTGGTACNNNNCGNNNNNGGTTGTNTNAGAAGTCCACAACGNGGTCNGGNNACNNNNGNNNAN
NNNCNNNNAACNNNNNNNNNNNNANATNNNTANNAGGNNNNNNCCNTCNNNNNNNNNNANTGCNATGACC
CAANNCNCGGNGGCNGTNCCCGGCGCGGCGACCTTGATNACGTCCGGGGNCAAGTATGGCNNNCNCCC
CGGGAAGGANNCNGCCGTANGTGCNNNNNANNNNNTCNTGNGACGTNGCCGNNATTNNCGTGGTCNNN
CNAGAATGNCCGNACCANNCGNANNNTGGTNATGTNAGTNNNNNNNNNNNNNNNNNNNNNNNNNNNNNNN
NNNNNNNNNNNNNNNNNNNNNNNNNNNNNNNNNNGNNNNGNNNNNNCNANNNAAGGANNTANNNCTNNTN
NTNNNNNNGGACNNNACNNNNCNNNNNNNACNCNNNCNCNNNNNNANNNGNTTTTNNNNCNNTANTNT
CCAGNANGCNGNGCNNNCNTTCANGNGAACNNNGNNACGCGNNAATCGTANNATCTACCTGCCGCNNG
TNNNNNTACNGNNNNNNCGGANNNTNNNNCGTANNNNNNTCATGCGTNNTNTNNTCNCNAGNCNNGTT
CGNNNANANANTAGNANGTNTNNNNNNNNNNTGTNNCGTTCCGCNTGANNCNNCANNNNGNNGNNNTTN
TCANCGGCNNTANCTGCNGANNTAAGNCATANNNTNNNTCNNTANCTNNNNNGGNGGGNNACTGTAAA
NGGNTAATTNGNNGNTNATTTCNNNCATNTNNCNNNGNNNNCTNNNTNCCTGNNTNCNNAATTGGNNC
CANTGNNTGNCNATCTCTNGNTTNGNNNNNNTNNNNNTNNCANNNNGGNNNGTNANANNNCNATNTAA
NNTANANNNNCNNNNNNANNNCACNNNNNNNGNAGNNNCNANNNGNANANNNGTAGNNNGNTNAGTCT
CCGANNTNNNNNNNNATAAGCTNNANACNNNNNNNNNNNNNNANGANNNCGNNTTTGNNNTNNNNNNNNA
GNNAGACNNTNGTGGNTNNNNNTCAANNNNTTCGTAGTGGTATAATNGNNNNNNNTTTCNCCAGNNTN
NNNGNNNNNNNCNATANNANNNNNNNGNGANNNNNNNNNNNANNNNCTCNNNAGGNANCANCTACTNNA
GCANTGGNGCGNAGNNNGAGANNNNGACNNGCCTTGGGTGGCAANCACNGCAATAAGTNNNGTTNANC
CTNNNAGTNNACACNNNNNCANNGNNNCNNNNNNANCNGANNGNNNNNNNNNNNNNNNNNNNNNNNNNNN
NNNNNNNNNNNNNNNNNNNNNNNNNNNNNNAGNNNANGGNNNGTNTNTGACNCATGTGTTTTCANNNGNN
NNNNNACGNNNNANCCCCGGTCGNNNNCNNATTGAANNCCTGNNGCATNNNNNNTNCNAGAATGANNNC
NNNNNGNNTNGNCTNNCNTTTCAAGNAATCANNNAGNNNNNNNNNTCNNNGNNNNNNNNCNNTCCCNN
ACTGAGGGTNNNNNNNNCATCTGCTNNNNNNNNNNTNCGCNGGNGTNNCNNNNNAGCNGTATNGNGTNN
NNCANNNNNNNNNNNNNNNNNNTNAAACNANNNNNCTNNNN
>Nung692
GTGNNNNNTAGCCCNNNNNAGTTAGACANNNCNNNNNNNNNNNNNTNNNNNNNNNNNNNNNANNNNNNNN
NNNNNNNNNNNNNNNNNNNNNNNNNNNNNNTNNNNNNNNNNNNNNNNNNNNNNNNNNNNNNNNNNNNNNN
NNNNNNNNNNNNNNNNNNNNNNNNNNNNNNNNNNNNNNNNNNNNNNNNNNNNNNNNNNNNNNNNNNNNNC
NNNNNNNNNNNNNNNNNNNNNNNNNNNNNNNNNNNNNNNNNNNNNNNNNNNNNNNNNNNNNNNNNNNNNN
NNNNNNNNNNNNNNNNNNNNNNNNNNNNNNNNNNNNNNNNNNNNNNNNNNNNCNTCTNNNAAANNNTNNN
NNNNNNNNNNNNNNNNNNNNNNNNNNNCNCNTTGNNNGTAANCNANNNGNACATGCAGTCCCCTCNNNNN
NNNGGCAGAGNNNNNNCCCNNNNNNNCCGNCCCCNNCCNNNNNNNTNNNNNNNGNNNNNNNANNNNCC
NNNNNNTGGTACNNNNCGNNNNNGGTTGTNTAAGAGGTCCACAACGNGGTCNGGNCACNGNNGNNNAN
NNNCNNNNAACNNNNNNNNNNNNATATNNNTANNAGGNCNNNGCCNTCNNGNNNNTAATTGCNATGACC
CAANTCACGGGGGCNGTNCCCGGCGCGGCGACCTTGATNACGTCCGGGGACAAGTATGGCGNNCNCCC
CGGGAAGGANNCNGCCGTANGTGCNNNNNANNNNNTCNTGNGANGTCGCCGNNATTCNCGTGGTCNNN
CNAGAATGNCCNNACCANNCGNANNNTGGTNATGNNAGTNNNNNNNNNNNNNNNNNNNNNNNNNNNNNNN
NNNNNNNNNNNNNNNNNNNNNNNNNNNNNNNNNNGNNNNGNNNNNNCNANNNAAGGNNNTANNNCTNNTN
ATNNNNNNGGACNNNACNNNNCNNNNNNNACNCNNNCNCNNNNNNANNNGGTTTTTNNNCNNTATTNT
CCAGNACGCNGNGCNNNCATTCANGNGAACNNNGNNACGCGNNAATCGTANNATCTACCTGCCGCNNG
TNNNNNTACNGNNNNNNCGGANNNTNNCTCGTANNNNNNTCATGCGTNNTNTNNTCNCNAGNCANGTT
CGNNNANANANTAGNANGTNTCNNNNNNNNTTGTNNCGTTCCGCNTGACNCNNCANNNNGNNGNNCTTN

NCANCGGCNNTANCTGCNGANNTAAGNCATANNNTNNATCNNTANCTNNNNNGGAGGGNNACTGTAAA
NGGNNAATTGGANGNTNATTTCCNNCACNTNNCTNNGNNTNCTNNNTNCCTGNNTNCNNAATTGGNNC
CANTGNNTGNCNATCTCTGGNTTNGNNNNNNTNNNNNTNNCANNNNGGNNNNNNANANNNCNATNTAA
NNTANANNNNCNNNNNNANNNCACNNNNNNNGNAGNNACNANNNTTANANNNGTAGNNNAATAAGTCT
CCGANNTNNNNNNNATAAGCGNNANACNNNNNNNNNNNNNNANGANNACGNNTTGGNNNTNNNNNNCNA
GNNAGACNNTNGTGGNTNNNNNTCAANNNNTTNGTAGTGGTATAATNGNNNNNNNTTTCNCCAGNNTN
NNNGNNNNNNNCNATANNANNNNNNNGTGANNNNNNNNNNNANNNNCTCNNNAGGGANCANCTACTNNA
GCAATGGNGCGNAGNNNGAGANNNNGACCAGCCTTGGGTGGCAANCACNGCAATAAGTNNNGTTNANC
CTNNNAGTNNACACNNNNNCANNGNNNCNNNNNNANCNGANNGNNNNNTNNNNNNNNNNNNNNNNNNN
NNNNNNNNNNNNNNNNNNNNNNNNNNNNNAGNNNANGGGNNGTNTNTGACNCATGTGTTTTTANNNGNN
NNNNNACGNNNNANCCCCGGTCGNNNNCNNATTGAANNCCTGNNGCATNNNNNNTNCNAGAATGANNGC
NNNNNGNNTNGNCTNNCNTTTCAAGNAATCANNTAGGTANCGNGNTCNNNGNNNNNNNNCNNTCCCNN
ACTGAGGGTNNNNNNNNCATCTGCTNNNNNNNNNCTTCGCNGGCGTNNCNNNNNAGCNGTATNGTGTTA
NNCANNNNNNNNNNNNNNNNNNTNAAACAANNNNNNNTNNNN
>Nung693
GTGNNNNNTAGCCCNNNNNAGTTAGACANNNCNNNNNNNNNNNNNNNNNNNNNNNNNNNNNNNNNNNNN
NNNNNNNNNNNNNNNNNNNNNNNNNNNNNNNNNNNNNNNNNNNNNNNNNNNNNNNNNNNNNNNNNNNNNN
NNNNNNNNNNNNNNNNNNNNNNNNNNNNNNNNNNNNNNNNNNNNNNNNNNNNNNNNNNNNNNNNNNNNNN
NNNNNNNNNNNNNNNNNNNNNNNNNNNNNNNNNNNNNNNNNNNNNNNNNNNNNNNNNNNNNNNNNNNNNN
NNNNNNNNNNNNNNNNNNNNNNNNNNNNNNNNNNNNNNNNNNNNNNNNNNNNNTCTNNNAAANNNTNNN
NNNNNNNNNNNNNNNNNNNNNNNNNNNCNCNNTNNNNGTAANCNANNNGNACATGCAGTNCCCTCNNNNN
NNNNNCAGAGNNNNNNNNCNNNNNNNCCGNCCCCNNCCNNNNNNNNNNNNNNNNGNNNNNNNANNNNNN
NNNNNNTGGTACNNNNCGNNNNNGGTTGTTTAAGAGGTCCACAACGNGGTCNNGNNACNNNNGNNNAN
NNNCNNNNANCNNNNNNNNNNNNNNNNTNNNTANNAGGNCNNNNCCNTCNNNNNNNNNNATTGCNATGACC
CAANNCACGGNGGNNGTNCCCGGCGNGGCGACCTTGNNNACNTCCGGGNNCAAGTATNGCNNNCNCCC
CGGGAAGGANNCNGCCGTANGTGCNNNNNANNNNNTCNTGNGANGTCGCCGNNATTCNCGTGGTCNNN
CNAGAATGNCCNNACCANNCGNANNNTGGTNATGNNAGTNNNNNNNNNNNNNNNNNNNNNNNNNNNNN
NNNNNNNNNNNNNNNNNNNNNNNNNNNNNNNNNNGNNNNGNNNNNNCNANNNAAGGNNNTANNNCTNNTN
NTNNNNNNNGGACNNNACNNNNCNNNNNNNACNCNNNCNCNNNNNNANNNGNTTTTTNNNCNNTATTNT
CCAGNACGCNGNGCNNNCNTTCANGNGAACNNNGNNACGCGNNAATCGTANNATCTACCTGCCGCNGG
TNNNNNTACNGNNNNNNCGGANNNTNNNNCGTANNNNNNTCATGCGTNNTNTNNTCNCNAGNCANGTT
CGNNNATANANTAGNANGTNTNNNNNNNNNNTGTNNCGTTCCGCNTGANNCNNCANNNNGNNGNNCTTN
NCANCGGCNNTANCTGCNGANNTAAGTCATANNNTNNNTCNNTANCTNNNNNGGNGGGNNACTGTAAA
NGGNNAATTNGANNNTNANTNCCNNCACNTNNCTNNGNNNNCTNNNTNCCTGNNTNCNNAATTGGNNN
CANTGNNTGNCNATCTCTNGNTTNGNNNNNNTGNNNNTNNCAANNNGGNNNGTNANANNNCNATNTAA
NNTATANNNNCNNNNNNANNNCACNANNNNNNNAGNNNCNNNNNTTANANNNGTAGNNNANTAAGTCT
CCGANNTNNNNNNNATAAGCGNNANACNNNNNNNNNNNNNNANGANNACGNNTTGGNNNTNNNNNNNNA
GNNAGACNNTNGTGGNTNNNNNTCAANNNNTTNGTAGTGGTATAATNGNNNNNNNTTTCNCCAGNNTN
NNNGNNNNNNNCNATANNANNNNNNNGNGANNNNNNNNNNNANNNNCTCNNNAGGGANCANCTACTNNA
GCAATGGNGCGCAGNNNGAGANNNNGACNNNCCTTGGGTGGCAANCACNGCAATAAGTNNNGTTNANC
CTNNNAGTNNACACNNNNNCANNGNNNCNNNNNNANCNGANNGNNNNNNNNNNNNNNNNNNNNNNNNN
NNNNNNNNNNNNNNNNNNNNNNNNNNNNNNNNNNNNNANNGNNNGTNTNTGACNCATGTGTTTTTANNNGNN
NNNNNACGNNNNANCCCCGGTCGNNNNCNNATTGAANNCCTGNNGCATNNNNNNTNCNAGAATGANNNC
NNNNNGNNTNGNCTNNCNNNTCAAGNAATCNNNNANGTANNNNGNTCNNNNNNNNNNNNNCNNTCCCNN
ACTGAGGGTNNNNNNNNCATCNGCTNNNNNNNNNNTNCGCNGGCGTNNCNNNNNAGCNGTATNNTGTNN
NNCANNNNNNNNNNNNNNNNNNTNNAACNANNNNNNNTNNNN
>Nung694
GTGNNNNNTAGCCCNNNNNAGTTAGACANNNCNNNNNNNNNNNNNNNNNNNANNNNNNNNNNNNNNNNNN
NNNNNNNNNNNNNNNNNNNNNNNNNNNNNNNNNNNNNNNNNNNNNNNNNNNNNNNNNNNNNNNNNNNNNN
NNNNNNNNNNNNNNNNNNNNNNNNNNNNNNNNNNNNNNNNNNNNNNNNNNNNNNNNNNNNNNNNNNNNNN
NNNNNNNNNNNNNNNNNNNNNNNNNNNNNNNNNNNNNNNNNNNNNNNNNNNNNNNNNNNNNNNNNNNNNN
NNNNNNNNNNNNNNNNNNNNNNNNNNNNNNNNNNNNNNNNNNNNNNNNNNNNNTCTNNNAAANNNTNNN
NNNNNNNNNNNNNNNNNNNNNNNNNNNCNCNTTGNNNGTAANCNANNNGNACATGCAGTCCCCTCNNNNN
NNNGGCAGAGNNNNNNCCCNNNNNNNCCGGCCCCNNCCNNNNNNNNNNNNNNNNNNNNNNNNNNANNNNNN

NNNNNNTGGTACNNNNCGNNNNNGGTTGTNTAAGAGGTCCACAACGNGGTCNGGNCACNNNNGNNNAN
NNNCNNNNAANNNNNNNNNNNNNNNNNTNNNTANNAGGNNNNNNCCNTCNNGNNNNNNATTGCNATGACC
CAANNNACGGGGGCNGTNCCCGGCGNGGCGACCTTGNNNACGTCCGGGGACAAGTATGGCNCNCNCCC
CGGGAAGGANNCNGCCGTANGTGCNNNNNANNNNNTCNTGNGANGTNGCCGNNATTNNCNTGGTCNNN
NNAGANTGNCCGNACCANNCGNANNNTGGTNATGNNAGTNNNNNNNNNNNNNNNNNNNNNNNNNNNNN
NNNNNNNNNNNNNNNNNNNNNNNNNNNNNNNNNNGNNNNGNNNNNNCNANNNAAGGNNNTANNNCTNNTN
NTNNNNNNGGACNNNACNNNNCNNNNNNNACNCNNNCNCNNNNNNANNNGNTTTTTNNNCNNTATTNT
CCAGNACGCNGNGCNNNCATTCANGNGAACNNNGNNACGCGNNAATCGTANNATCTACCTGCCGCNNG
NNNNNNTACNGNNNNTNCGGANNNTNNNNCGTANNNNNNTCATGCGTNNTNTNNTCNCNAGNCANGTT
CGNNNANANANTAGNANGTNTNNNNNNNNNNTGTNNCGTTCCGCNTGANNCNNCANNNNGNNGNNCTTN
TCANCGGCNNTANCTGCNGANNTAAGNCATANNNTNNATCNNTANCTNNNNNGGNGGGNNACTGTAAA
NGGNNAATTNGNNGNTNANTTCNNNCACNTNNCTNNGNNTNCTNNNTNCCTGNNTNCNNAATTGGNNC
CANTGNNTGNCNATCTCTGGNTTNGCNNNNNTNNNNNTNNCANNNNGGNNNGTNANANNNCNATNTAA
NNTANANNNNCNNNNNNANNNCACNNNNNNNGNAGNNNCNANNNTTANANNNGTAGNNNAATAAGTCT
CCGANNTNNNNNNNATAAGCGNNANACNNNNNNNNNNNNNANGANNACGNNTTGGNNNTNNNNNNCNA
GNNAGACNNTNGTGGNTNNNNNTCAANNNNTTCGTAGTGGTATAATNGNNNNNNNTTTCNCCAGNNTN
NNNGNNNNNNNCNATANNANNNNNNNGTGANNNNNNNNNNANNNNCTCNNNAGGNANCANCTACTNNA
GCANTGGNGCGNAGNNNGAGANNNNGACNNNCCTTGGGTGGCAANCACNGCAATAAGTNNNGTTNANC
CTNNNAGTNNACACNNNNNCANNGNNNCNNNNNNANCNGANNGNNNNNNNNNNNNNNNNNNNNNNNNN
NNNNNNNNNNNNNNNNNNNNNNNNNNNNNAGNNNANGGNNNGTNTNTGACNCATGTGTTTTTANNNGNN
NNNNNNNGNNNNANCCCCGGTCGNNNNCNNATTGAANNCCTGNNGCATNNNNNTNCNAGAATGANNNC
NNNNNGNNTNGNCTNNCNNNTNAAGNAATCANNNAGGTNNCGCGNTCNNNGNNNNNNNNCNNTCCCNN
ACTGAGGGTNNNNNNNNCATCTGCTNNNNNNNNNNTNCGCNGGNGTNNCNNNNNAGCNGTATNGTGNNA
NNCANNNNNNNNNNNNNNNNNTNAAACGANNNNNTTNNNN
>Nung695
GTGNNNNNTAGCCCNNNNNAGTTAGACANNNCNNNNNNNNNNNNNNNNNNNNNNNNNNNNNNNNNNN
NNNNNNNNNNNNNNNNNNNNNNNNNNNNNNNNNNNNNNNNNNNNNNNNNNNNNNNNNNNNNNNNNNNN
NNNNNNNNNNNNNNNNNNNNNNNNNNNNNNNNNNNNNNNNNNNNNNNNNNNNNNNNNNNNNNNNNNNN
NNNNNNNNNNNNNNNNNNNNNNNNNNNNNNNNNNNNNNNNNNNNNNNNNNNNNNNNNNNNNNNNNNNN
NNNNNNNNNNNNNNNNNNNNNNNNNNNNNNNNNNNNNNNNNNNNNNNNNNNNCNTCTNNNAAANNNTNNN
NNNNNNNNNNNNNNNNNNNNNNNNNNCNCNNTGNNNGTAANCNANNNGNACATGCAGTCCCCTCNNNNN
NNNGGCAGAGNNNNNNCCCNNNNNNNCCGGCCCCNNCCNNNNNNNNNNNNNNNNNNNNNNNNNANNNNNN
NNNNNNTGGTACNNNNCGNNNNNGGNNNNNTNAGAGGTCCACAACGNGGTCNNGNCANNNNNGNNNAN
NNNCNNNNAACNNNNNNNNNNNNNNNNTNNNTANNAGGNCNNNNCCNTCNNGNNNNNAATTGCNATGACC
CANNTNACGGGGGCNGTNCCCGGCGCGGNGACCTTGNNNACGTCCGGGGNCAAGTATNGCNNNCNCCC
CGGGAAGGANNCNGCNGTANGTGCNNNNNANNNNNTCNTGNGANGTCGCCGNNATTCNCGNGGTCNNN
NNAGAATGNNCGNACCANNCGNANNNTGGTNATGNNAGTNNNNNNNNNNNNNNNNNNNNNNNNNNNNN
NNNNNNNNNNNNNNNNNNNNNNNNNNNNNNNNNNGNNNNGNNNNNNCNANNNAAGGNNNTANNNCTNNTN
NTNNNGNNGGACNNNACNNNNCNNNNNNNACNCNNNCNCNNNNNNNNNNNGGTTTTNNNCCNNTATTNN
CCAGNANGCNGNGCNNNCATTCANGNGAACNNNGNNACGCGNNAATCGTANNATCTACCTGCCGCNNG
NNNNNNTACNGNNNNNNNCGGANNNTNNNNCGTANNNNNNTCANGCGTNNTNTNNTCNCNAGNCNNGTT
CGNNNANANANTAGNANGTNTNNNNNNNNNNTGTNNCGTTCCGCNTGANNCNNCANNNNGNNGNNCTTN
TCANCGGCNNTANCTGCNGANNTAAGNCATANNNTNNNTCNNTANCTNNNNNGGNGGGNNACTGTAAA
NGNNNAATTNGANNNTNATTNCNNNNACNTNNCTNNGNNNNCTNNNTNCCTGNNTNCNNAATTGGNNN
CANTGNNTGNCNATCTCTGGNTTNGNNNNNNTNNNNNTNNCANNNNGGNNNGTNNNANNNCNATNTAA
NNTANANNNNCNNNNNNANNNCACNNNNNNNNNAGNNNCNNNNNTTANANNNGTAGNNNANTNAGTCT
CCGANNTNNNNNNNATAAGCGNNANACNNNNNNNNNNNNNANGANNNCGNNTTGGNNNTNNNNNNCNA
GNNAGACNNTNATGGNTNNNNNTCAANNNNTTNGTAGTGGTATAATNGNNNNNNNTTTCNCCAGNNTN
NNNGNNNNNNNCNATANNANNNNNNNGTGANNNNNNNNNNANNNNCTCNNNAGGGANCANCTACTTNA
GCANTGGNGCGNAGNNNGAGANNNNGACNNNCCTTGGGTGGCAANCACNGCGATAAGTNNNGTTNANN
NTNNNAGTNNACATNNNNNCANNGNNNCNNNNNNANCNGANNGNNNNNNNNNNNNNNNNNNNNNNNNN
NNNNNNNNNNNNNNNNNNNNNNNNNNNNNNNNNNANNGNNNGTNTNTGNCNCNTGTGTTTTTANNNGNN
NNNNNACGNNNGANCCCCGGTCGNNNNCNNATTGAANNCCTGNNGCATNNNNNTNCNAGAATGANNNC
NNNNNGNNTNNNCTNNCNNNTCAAGNAATCANNNAGNNNNNGCGNTCNNNNNNNNNNNNNCNNTCCCNN

ACTGAGGGTNNNNNNCATCTGCTNNNNNNCTNCGCNGNCNNNNCNNNNNNGCNGTATNGNGTNA
NNCANNNNNNNNNNNNNNNNNNNAANNNANNNNNNTNNNN
>Nung696
GTGNNNNNTAGCCCGNNNNAGTTANACANNNCNNNNNNNNNNNNNTNNNNNNNNNNNNNNNNNNNNNN
NNNNNNNNNNNNNNNNNNNNNNNNNNNNNNNNNNNNNNNNNNNNNNNNNNNNNNNNNNNN
NNNNNNNNNNNNNNNNNNNNNNNNNNNNNNNNNNNNNNNNNNNNNNNNNNNNNNNNNNNN
NNNNNNNNNNNNNNNNNNNNNNNNNNNNNNNNNNNNNNNNNNNNNNNNNNNNNNNNNNNN
NNNNNNNNNNNNNNNNNNNNNNNNTNNNNNNNNNNNNNNNNNNNNNNNNNNNNNNNNNNNNNTCTNNNAAANNNTNNN
NNNNNNNNNNNNNNNNNNNNNNNNNNNNNCNCNTTGNNNGTAANCNANNNGNACATGCAGTNCCCTCNNNNN
NNNNNCAGAGNNNNNNNCNNNNNNNNNCCGNCCCCNNCCNNNNNNNNNNNNNNNNNNNNNNNNNNNANNNNNN
NNNNNNTGGTACNNNNCGNNNNNGGTTGTNTNAGNGGTCCACAACGNGGTCNNGNCACNNNNGNNNAN
NNNCNNNNNNNNNNNNNNNNNNNNANNTNNNTANNAGGNNNNNNCCNTCNNNNNNNNNNANNGCNATGACC
CAANNNACGGGGGCNGTNCCCGGNGNGGCGACCTTGNNNANNTCCGGGGNCAAGTATGGCGNNCNCCC
CGGGAAGGANNCNGCCGTANGTGCNNNNNANNNNNTCNTGNGACNTCGCCGNNATTNNCGTGGTCNNN
NNAGAATGNNCGNACCANNCGNANNNTGGTNATGTTAGTNNNNNNNNNNNNNNNNNNNNNNNNNNNNNNN
NNNNNNNNNNNNNNNNNNNNNNNNNNNNNNNNNNNNNNNNNGNNNNNNCNANNNAAGGNNNTANNNCTNNTC
NTNNNNNNGGACNNNACNNNNCNNNNNNNACNCNNNCNCNNNNNNNNNNGNTTTTNNNNCNNTANTNT
CCAGNACGCNGNGCNNNCATTCANGNGAACNNNGNNACGCGNNAATCGTANNATCTACCTGCCGCNNG
NNNNNNTNCNGNNNNNNCGGANNNTNNNNCGTANNNNNNTCATGCGTNNTNTNNTCNCNAGNCANGTT
CGNNNANANANTAGNANGTNTNNNNNNNNNNTGTNNCGTTCCGCNTGANNCNNCANNNNGNNGNNNTTN
TCANCGGCNNTANCTGCNGANNTAAGNCATANNNTNNNTCNNTANCTNNNNNGGNGGGNNACTGTAAA
NGGNNAATTNGNNNNTNANTNCNNNCACNTNNCTNNGNNNNCTNNNTNCCTGNNTNCNNAATTGGNNC
CANTGNNTGNCNATCTCTGGNTTNGNNNNNNTNNNNNTNNCAANNNGGNNNNNNANANNNCNATNTAA
NNTANANNNNCNNNNNNANNNCACNANNNNNGNAGNNNNNANNNTTANANNNGTAGNNNNNTAAGTCT
CCGANNTNNNNNNNATAAGCGNNANACNNNNNNNNNNNNNNANGANNNCGNNTTGGNNNTNNNNNNCNA
GNNAGACNNTNATGGNTNNNNNTCAANNNNTTCGTAGTGGTATAATNGNNNNNNNTTTCNCCAGNNTN
NNNGNNNNNNNNCNATANNANNNNNNNGNGANNNNNNNNNNNANNNNCTCNNNAGGGANCANCTACTTNA
GCANTGGNGCGNAGNNNGAGANNNNGACCAGCCTTGGGTGGCAANCACNGCGNTAAGTNNGTTNANN
CTNNNAGTNNACATNNNNNCANNGNNNCNNNNNNANCNGANNGNNNNNNNNNNNCNNNNNNNNNNNNN
NNNNNNNNNNNNNNNNNNNNNNNNNNNNNNNNNNNNNANNGNNNGTNTNTGNCNCANGTGTTTTTANNNGNN
NNNNNACGNNNNANCCCCGGTCGNNNNCNNATTGAANNCCTGNNGCATNNNNNNTNCNAGAATGANNGC
NNNNNGNNTNGNCTNNCNNNTCAAGNAATCAANNANNNNNCGCGNTCNNNNNNNNNNNNNCNNTCCCNN
ACTGAGGGTNNNNNNNNCATCTGCTNNNNNNNNCTNCGCNGNNGTNNCNNNNNAGCNGTATNGNGTNA
NNCANNNNNNNNNNNNNNNNNTNAAACAANNNNNNNTNNNN
>Nung697
GTGNNNNNTAGCCCNNNNNAGTTAGACANNNCNNNNNNNNNNNNNTNNNNNNNNNNNNNNNNNNNNNN
NNNNNNNNNNNNNNNNNNNNNNNNNNNNNNNTNNNNNNNNNNNNNNNNNNNNNNNNNNNNNNNNNNNN
NNNNNNNNNNNNNNNNNNNNNNNNNNNNNNNNNNNNNNNNNNNNNNNNNNNNNNNNNNNN
NNNNNNNNNNNNNNNNNNNNNNNNNNNNNNNNNNNNNNNNNNNNNNNNNNNNNNNNNNNN
NNNNNNNNNNNNNNNNNNNNNNNNNNNNNNNNNNNNNNNNNNNNNNNNNNNNNNNNNNNNNCNTCTNNNAAANNNTNNN
NNNNNNNNNNNNNNNNNNNNNNNNNNNNNCNCNTTGNNNGTAANCNANNNGNACATGCAGTCCCCTCNNNNN
NNNGGCAGAGNNNNNNNCCNNNNNNNNCCGGCCCCNNCCNNNNNNNNNNNNNCNNNNNNNNNNNNNANNNNNN
NNNNNNTGGTACNNNNCGNNNNNGGTTGNNTNAGAGGTCCACAACGNGGTCNNNNCACNNNNGNNNAN
NNNCNNNNAANNNNNNNNNNNNNNNANNTNNNTANNAGGNNNNNNCCNTNNNGNNNNNNANTGCNATGACC
CANNNCACGGGGGNAGTNCCCGGCGCGGCGACCTTGANNANNTCCGGGGACAAGTATNGCNNNCNCCC
CGGGAAGGANNCNGCCGTANGTGCNNNNNANNNNNTCNTGNGANGTCGCCGNNANNNNNNTGGTCNNN
CNAGAATGNCCNNACCANNCGNANNNTGGTNATGNNAGTNNNNNNNNNNNNNNNNNNNNNNNNNNNNNNN
NNNNNNNNNNNNNNNNNNNNNNNNNNNNNNNNNNNGNNNNGNNNNNNCNANNNAAGGANNTANNNCTNNTN
NTNNNNNNGGNCNNNACNNNNCNNNNNNNACNCNNNCNCNNNNNNNNNNGNTTTTTNNCCNNTANTNT
CCAGNACGCNGNGCNNNCATTCANGNGAACNNNGNNACGCGNNAATCGTANNATCTACCTGCCGCNNG
TNNNNNTNCNGNNNNNNCGGANNNTNNNNCGTANNNNNNNCANGCGTNNTNTNNTCNCNAGNCNNGTT
CGNNNANANANTAGNANGTNNNNNNNNNNNNTGTNNCGNTCCGCNTGANNCNNCANNNNGNNGNNCTTN
NCANCGGCNNTANCTGCNGANNTAAGNCATANNNTNNNTCNNTANCTNNNNNGGNGGGNNACTGTAAA
NGGNNAATTNGNNNNTNANTNCNNNCACNTNNCTNNGNNNNCTNNNTNCCTGNNTNCNNAATTGGNNN

CANTGNNTGNCNATCTCTNGNTTNGNNNNNNTNNNNNTNNCANNNNGGNNNGNNNNANNNCNATNTAA
NNTANANNNNCNNNNNNANNNCACNNNNNNNGNAGNNNCNANNNTTANANNNGTAGNNNNNNTNAGTCT
CCGANNTNNNNNNNATAAGCGNNANACNNNNNNNNNNNNNNANGANNNCGNNTTGGNNNTNNNNNNCNA
GNNAGACNNTNATGGNTNNNNNTCAANNNNTTNGTAGTGGTATAATNGNNNNNNNTTTCNCCAGNNNN
NNNGNNNNNNNCNATANNANNNNNNNGNGANNNNNNNNNNNANNNNCTCNNNAGGGANCANCTACTNNA
NCANTGGNGCGNAGNNNGAGANNNNGACCANCCTTGGGTGGCAANNACNGNAATAAGTNNNGTTNANC
CTNNNAGTNNACATNNNNNCANNGNNNCNNNNNNANCNGANNGNNNNNNNNNNNNNNNNNNNNNNNNN
NNNNNNNNNNNNNNNNNNNNNNNNNNNNNNNNNNNANGGNNNGTNTNTGACNCATNNNTTTNTANNNGNN
NNNNNNNGNNNNANCCCCGGTCGTNNNCNNATTGAANNCCTGNNGCATNNNNNTNCNAGAATGANNNC
NNNNNGNNTNNNNTNNNNNNTCAAGNAATCNNNNAGGNNNNNCGNTCNNNNNNNNNANNCNNTCCCNN
ACTGAGGGTNNNNNNNNCATNTGCTNNNNNNNNCTNCGCNGGCGNNNCNNNNNNGCNGTATNGTGNNA
NNCANNNNNNNNNNNNNNNNNTNAAACNANNNNNNTNNNN
>Nung698
GTGNNNNNTAGCCCNNNNNAGTTAGACANNNCNNNNNNNNNNNNNTNNNNNNNNNNNNNNNNNNNNNN
NNNNNNNNNNNNNNNNNNNNNNNNNNNNNNNNNNNNNNNNNNNNNNNNNNNNNNNNNNNNNNNNNNNN
NNNNNNNNNNNNNNNNNNNNNNNNNNNNNNNNNNNNNNNNNNNNNNNNNNNNNNNNNNNNNNNNNNNN
NNNNNNNNNNNNNNNNNNNNNNNNNNNNNNNNNNNNNNNNNNNNNNNNNNNNNNNNNNNNNNNNNNNN
NNNNNNNNNNNNNNNNNNNNNNNNNNNNNNNNNNNNNNNNNNNNNNNNNNNNCNTCTNNNAAANNNTNNN
NNNNNNNNNNNNNNNNNNNNNNNNNNNCNCNTTGNNNGTAANCNANNNGNACATGCAGTCCCCTCNNNNN
NNNNGCAGAGNNNNNNCCNNNNNNNNNCCGNCCCCNNCCNNNNNNNNNNNNCNNNNNNNNNNNANNNNNN
NNNNNNTGGTACNNNNCGNNNNNGGTTGTNTNAGAGGTCCACAACGNGGTCNGGNCACNNNNGNNNAN
NNNCNNANAACNNNNNNNNNNNNNNNTNNNTANNAGGNNNNNNCCNTCNNGNNNNTNATNGCNATGACC
CNNNNCACGGNGGCNGTNCCCGGNGCGGCNACCTTGATNACGTCCGGGGNCAAGTATGGCGNNCNCCC
CGGGAAGGANNCNGCCGTANGTGCNNNNNANNNNNTCNTGNGANGTNGCCGNNATTNNCGTGGTCNNN
NNAGANTGNNCGNACCANNCGNANNNTGGTNATGNNAGTNNNNNNNNNNNNNNNNNNNNNNNNNNNNN
NNNNNNNNNNNNNNNNNNNNNNNNNNNNNNNNNNNGNNNNGNNNNNNCNANNNAAGGNNNTANNNCTNNTN
NNNNNNNNGGACNNNACNNNNCNNNNNNNACNCNNNCNCNNNNNNANNNGNTTTTTNNNCNNTANTNT
CCAGNACGCNGNGCNNNCATTCANGNGAACNNNGNNACGCGNNAATCGTANNATCTACCTGCCGCNGG
NNNNNNTNCNGNNNNNNCGGANNNTNNNNNGTANNNNNNTCATGCGTNNTNTNNTCNCNAGNCANGTT
CGNNNANANANTAGNANGTNTNNNNNNNNNNTGTNNCGTTCCGCNTGANNCNNCANNNNGNNGNNCTTN
NCANCGGCNNTANCTGCNGANNTAAGNCATANNNTNNNTCNNTANCTNNNNNGGNGGGNNACTGTAAA
NGGNNAATTNGNNGNTNATTNCNNNCACNTNNCNNNGNNNNCTNNNTNCCTGNNTNCNNAATTGGNNC
CANTGNNTGNCNATCTCTNGNTTNGNNNNNNTNNNNNTNNCANNNNGGNNNGTNNNANNNCNATNTAA
NNTANANNNNCNNNNNNANNNCACNNNNNNNNNAGNNNCNANNNTTANANNNGTAGNNNANTAAGTCT
CCGANNTNNNNNNNATAAGCGNNANACNNNNNNNNNNNNNNANGANNNCGNNTTGGNNNTNNNNNNCNA
GNNAGACNNTNATGGNTNNNNNTCAANNNNTTNGTAGTGGTATAATNGNNNNNNCTTTCNCCAGNNTN
NNNGNNNNNNNCNATANNANNNNNNNNNGANNNNNNNNNNNANNNNCTCNNNAGGNANCANCTACTNNA
GCANNGGNGCGNAGNNNGAGANNNNGACNNGCCTTGGGTGGCAANCACNGCANTAAGTNNNGTTNANC
CTNNNAGTNNACATNNNNNCANNGNNNCNNNNNNANCNGANNGNNNNNNNNNNNNNNNNNNNNNNNNN
NNNNNNNNNNNNNNNNNNNNNNNNNNNNNNNANNNNANNGNNNGTNTNTGACNCNTGTGTTTTTANNNGNN
NNNNNACGNNNNANCCCCGGTCGNNNNCNNATTGAANNCCTGNNGCATNNNNNTNCNAGAATGANNNC
NNNNNGNNTNGNCTNNCNTTTCAAGNAATCANNNAGNNANNNNNNTCNNNGNNNNNNNNCNNTCCCNN
ACTGAGGGTNNNNNNNNCATCTGCTNNNNNNNNCTNCGCNGNNGTNNCNNNNNAGCNGTATNGTGTNA
NNCANNNNNNNNNNNNNNNNNTNAANCNANNNNNNTNNNN
>PaThen494
GTGNNNNNTAGCCCNNNNNAGTCAGACANNNCNNNNNNNNNNNNNNNNNNNNNNNNNNNNNNNNNNNN
NNNNNNNNNNNNNNNNNNNNNNNNNNNNNNNNNNNNNNNNNNNNNNNNNNNNNNNNNNNNNNNNNNNN
NNNNNNNNNNNNNNNNNNNNNNNNNNNNNNNNNNNNNNNNNNNNNNNNNNNNNNNNNNNNNNNNNNNN
NNNNNNNNNNNNNNNNNNNNNNNNNNNNNNNNNNNNNNNNNNNNNNNNNNNNNNNNNNNNNNNNNNNN
NNNNNNNNNNNNNNNNNNNNNNNNNNNNNNNNNNNNNNNNNNNNNNNNNNNNNTCTNNNAAANNNTNNN
NNNNNNNNNNNNNNNNNNNNNNNNNNNCNCNTTGNNNGTAANCNANNNGNACATGCAGTCCCCTCNNNNN
NNNNNCAGAGNNNNNNNCNNNNNNNNNCCGNCCCCNNCCNNNNNNNNNNNNNNNNNNNNNNNNNANNNNNN
NNNNNNTGGTACNNNNCGNNNNNGGTTGTTTNAGAGGTCCACAACGNGGTCNNGNNACNNNNGNCNAN
NNNCNNNNNNNNNNNNNNNNNNNNNNNTNNNTANNAGGNCNNNNCCNTCNNNNNANNAANNGCNATGACC

CAANNNACGGNGGNNGTNCCCGGCGCGGCNACCTTGNNNANGTCCGGGGACAAGTATGGCNCNCNCCC
CGGGAAGGANNCNGCCGTANGTGCNNNNNANNNNNTCNTGNGANGTNGCCGNNATTCNCGTGGTCNNN
NNAGAATGNNCGNACCANNCGNANNNTGGTNATGNTAGTNNNNNNNNNNNNNNNNNNNNNNNNNNNNN
NNNNNNNNNNNNNNNNNNNNNNNNNNNNNNNNGNNNNGNNNNNNCNANNNAAGGANNTANNNCTNNTN
NTNNNNNNGGNCNNNACNNNNCNNNNNNNACNCNNNCNCNNNNNNANNNGNTTTTNNNNCNNTATTNT
CCAGNACGCNGNGCNNNCATTTANGNGAACNNNGNNACGCGNNAATCGTANNATCTACCTGCCGCNNG
TNNNNNTACNGNNNNNNCGGANNNTNNNNCGTANNNNNNTCANGCGTNNTNTNNTCNCNAGNCNNGTT
CGNNNANNNANTAGNANGTNTNNNNNNNNNTGTNNCGTTCCGCNTGANNCNNCANNNNGNNGNNCTTN
TCANCGGCNNTANCTGCNGANNTAAGNCATANNNTNNATCNNTANCTNNNNNGGAGGGNNACTGTAAA
NGGNNAATTNGANNNTNANTTCNNNCATNTNNCTNNGNNTNCTNNNTNCCTGNNTNCNNAATTGGNNC
CANTGNNTGNCNATCTCTGGNTTNGNNNNNNTNNNNNTNNCANNNNGGNNNGTNANANNNCNATNTAA
NNTANANNNNCNNNNNNANNNCACNANNNNNGNAGNNNCNANNNGTANANNNGTAGNNNGNTAAGTCT
CCGANNTNNNNNNATAAGCGNNANACNNNNNNNNNNNNNNANGANNACGNNTTTGNNNTNNNNNNCNA
GNNAGACNNTNGTGGNTNNNNNTNNANNNNTTNGTAGTGGTATAATNGNNNNNNNTTTCNCCAGNNTN
NNNGNNNNNNNCNATANNANNNNNNNGNGANNNNNNNNNNANNNNCTCNNNAGGGANCANCTACTNNA
GCANTGGNGCGNAGNNNNAGANNNNGACNNGCCTTGGGTGGCAANCACNGCAATAAGTNNNGTTNANC
CTNNNAGTNNACACNNNNNCANNGNNNCNNNNNNANCNGANNGNNNNNNNNNNNNNNNNNNNNNNNNN
NNNNNNNNNNNNNNNNNNNNNNNNNNNNANNNNANGGNNNGTNTNTGACNCATNNNTTTTTANNNGNN
NNNNNNNGNNNNANCCCCGGTCGNNNNCNNATTGAANNCCTGNNGCATNNNNNTNCNAGAATGANNGC
NNNNNGNNTNGNCTGNCNNNTCAAGNAATCNNNNANNTNNNNNNNNTCNNNNNNNNNNNCNNTCCCNN
ACTGAGGGTNNNNNNNNCATNTGCTNNNNNNNNNTNCGCNGNNGTNNCNNNNNNGCNGTATNGTGTNA
NNCANNNNNNNNNNNNNNNNCNAAACNANNNNNNTNNNN
>PaThen495
GTGNNNNNTAGCCCNNNNNAGTTAGACANNNCNNNNNNNNNNNNNNNNNNNNNNNNNNNNNNNNNNNN
NNNNNNNNNNNNNNNNNNNNNNNNNNNNNNTNNNNNNNNNNNNNNNNNNNNNNNNNNNNNNNNNNNNNN
NNNNNNNNNNNNNNNNNNNNNNNNNNNNNNNNNNNNNNNNNNNNNNNNNNNNNNNNNNNNNNNNNNNN
NNNNNNNNNNNNNNNNNNNNNNNNNNNNNNNNNNNNNNNNNNNNNNNNNNNNNNNNNNNNNNNNNNNN
NNNNNNNNNNNNNNNNNNNNNNNNNNNNNNNNNNNNNNNNNNNNNNNNNNNNTCTNNNNAANNNTNNN
NNNNNNNNNNNNNNNNNNNNNNNNNCNCNNTGNNNGTAANCNANNNGNACATGCAGTNNCCTCNNNNN
NNNGGCAGAGNNNNNNNNNNNNNNCNCCGNCCCCNNCCNNNNNNNNNNNNNNNNNNNNNNNANNNNNN
NNNNNNTGGTACNNNNCGNNNNNGGNNNTNTNAGAAGTCCACAACGNGNTCNNGNNACNNNNGNNNAN
NNNCNNNNNNNNNNNNNNNNNNNNNNTNNNTANNAGGNCNNNNCCNTCNNNNNNNNANNGCNATGACC
CNANTNACNGNGGNNGTNCCCGGNGNGGCGACCTTGNNNANNTCCGGGGACAAGTATNGCNNNCNCCC
CGGGAAGGANNCNGCCGTANGTGCNNNNNANNNNNTCNTGNGANGTNGCCGNNATTNNNNTGNNCTNN
CNAGANTNNNCNNACCANNCGNANNNNGGTNATGNNAGTANNNNNNNNNNNNNNNNNNNNNNNNNNNN
NNNNNNNNNNNNNNNNNNNNNNNNNNNNNNNNGNNNNGNNNNNNCNANNNAAGGNNNTANNNCTNNTN
NTNNNNNNGGACNNNACNNNNCNNNNNNNACNCNNNCNCNNNNNNNNNNGGTTNNNNNNCNNTATNNT
CCAGNACGCNNNGCNNNCATTCANGNGAACNNNGNNACGCGNNAATCGTANNATCTACCTGCCGCNGG
TNNNNNTNCNGNNNNNNCGGANNNTNNNNCGTANNNNNGNCATGCGTNNTNTNNTCNCNAGNCNNGTT
CGAGAANANANTAGNANGTNNNNNNNNNNNTGTNNCGTTCCGCNTGANNCNNCANNNNGNNGNNNTTN
TCANCGGCNNTNNCTGCNGANNTAAGNCATANNNTNNNTCNNTANCTNNNNNGGNGGGNNACTGTAAA
NGGNNAATTNGNNNNTNANTNCNNNCATNTNNCNNNGNNNNCTNNNTNCCTGNNTNCNNAATTGGNNC
CANTGNNTGNCNATCTCTNGNTTNGNNNNNNTNNNNNTNNCANNNNGGNNNNNNNNANNNCNATNTAA
NNTANANNNNCNNNNNNANNNCACNANNNNNNNAGNNANNANNNGTANANNNGTAGNNNGNTAAGTCT
CCGANNTNNNNNNATAAGCTNNANACNNNNNNNNNNNNNNANGANNACGNNTTTGNNNTNNNNNNCNA
GNNANACNNTNGTGGNTNNNNNTCAANNNNTTNGTAGTGGTATAATNGNNNNNNNTTTCNCCAGNNTN
NNNGNNNNNNNCNATANNANNNNNNNNNGANNNNNNNNNNANNNNCTCNNNAGGGANCANCTACTNNA
GCANNGGNGCGNAGNNNGAGANNNNGACNNGCCTTGGGTGGCAANNACNGCANTAAGTNNNGTTNNNC
CTNNNAGTNNANACNNNNNCANNGNNNCNNNNNNANCNGANNGNNNNNNNNNNNNNNNNNNNNNNNNN
NNNNNNNNNNNNNNNNNNNNNNNNNNNNNNNNNANNGNNNGTNTNTGACNCANNNNTTTNCANNNGNN
NNNNNACGNNNGNNCCCCGGTCGNNNNCNNATTGAANNCCTGNNGCATNNNNNTNCNAGAATGANNGC
NNNNNGNNTNGNCTNNCNNNTNAAGNAANCANNNAGNNNNCGCGNTCNNNNNNNNNNNNCNNNNNCNN
ACTGAGGGTNNNNNNNNCATCTGCTNNNNNNNNCTNCGCNGNNNNNNCNNNNNNGCNGTATNGTGTNN
NNCANNNNNNNNNNNNNNNNTNAAACNANNNNNNTNNNN

>PaThen496
GTGNNNNNTAGCCCNNNNNAGTCAGACANNNCNNNNNNNNNNNNTNNNNNNNNNNNNNNNNNNNNNNN
NNNNNNNNNNNNNNNNNNNNNNNNNNNNNTNNNNNNNNNNNNNNNNNNNNNCNNNNNNNNNNNNNNNN
NNNNNNNNNNNNNNNNNNNNNNNNNNNNNNNNNNNNNNNNNNNNNNNNNNNNNNNNNNNNNNNNNNNN
NNNNNNNNNNNNNNNNNNNNNNNNNNNNNNNNNNNNNNNNNNNNNNNNNNNNNNNNNNNNNNNNNNNN
NNNNNNNNNNNNNNNNNNNNNNNNNNNNNNNNNNNNNNNNNNNNNNNNNNNNTCTNNNAAANNNTNNN
NNNNNNNNNNNNNNNNNNNNNNNNNCNCNTTGNNNGTAANCNANNNGNACATGCAGTCCCCTCNNNNN
NNNGGCAGAGNNNNNNCCCNNNNNNNCCGNCCCCNNCCNNNNNNNNNNNNNNNGNNNNNNNANNNNNN
NNNNNNTGGTACNNNNCGNNNNNGGTTGTTTAAGAGGTCCACAACGNGGTCNNGNCACNNNNGNNNAN
NNNCNNNNAACNNNNNNNNNNNANNTNNNTANNAGGNNNNNNCCNTNNNNNNNNNNANNGCNATGACC
CNANNCACGGGGGNNGTCCCCGGCGCGGCGACCTTGANNACGTCCGGGGACAAGTATGGCNNNCNCCC
CGGGAAGGANNCNGCCGTANGTGCNNNNNANNNNNTCNTGNGANGTNGCCGNNATTCNNNTGGNCNNN
NNAGAATGNCCNNACCANNCGNANNNTGGTNATGNNAGTANNNNNNNNNNNNNNNNNNNNNNNNNNNN
NNNNNNNNNNNNNNNNNNNNNNNNNNNNNNNNGNNNNGNNNNNNCNANNNAAGGANNTANNNCTNNTN
NNNNNGNNGGACNNNACNNNNCNNNNNNNACNCNNNCNCNNNNNNNNNNGGTTTTNNNNCNNTATTNT
CCAGNACGCNGNGCNNNCATTTANGNGAACNNNGNNACGCGNNAATCGTANNATCTACCTGCCGCNNG
TNNNNNTACNGNNNNNNCGGANNNTNNCTCGTANNNNNNTCATGCGTNNTNTNNTCNCNAGNCANGTT
CGNNNANANANTAGNANGTNTNNNNNNNCNTGTNNCGTTCCGCNTGANNCNNCANNNNGNNGNNCTTN
TCANCGGCNNTANCTGCNGANNTAAGTCATANNNTNNNTCNNTANCTNNNNNGGNGGGNNACTGTAAA
NGGNNAATTGGNNGNTNATTNCNNNCATNTNNCNNNGNNTNCTNNNTNCCTGNNTNCNNAATTGGNNC
CANTGNNTGNCNATCTCTNGNTTNGNNNNNNTNNNNNTNNCAANNNGGNNNGTNNNANNNCNATNTAA
NNTANANNNNCNNNNNNANNNCACNNNNNNNNNAGNNNCNANNNGNANANNNGTAGNNNGNTAAGTCN
CCGANNTNNNNNNNATAAGCGNNANACNNNNNNNNNNNNNANGANNNCGNNTTTGNNNTNNNNNNCNA
GNNAGACNNTNGTGGNTNNNNNTCAANNNNTTCGTAGTGGTATAATNGNNNNNNNTTTCNCCAGNNTN
NNNGNNNNNNNCNATANNANNNNNNNGNGANNNNNNNNNNANNNNCTCNNNAGGGANCANCTACTNNA
GCANTGGNGCGCAGNNNGAGANNNNGACNNNCCTTGGGTGGCAANCACNGCAATAAGTNNTGTTNANC
CTNNNAGTNNACACNNNNNCANNGNNNCNNNNNNANCNGANNGNNNNNNNNNNNNNNNNNNNNNNNNN
NNNNNNNNNNNNNNNNNNNNNNNNNNNNANNNNANNGNNNGTNTNTGACNCATGNNTTTNTANNNGNN
NNNNNACGNNNNANCCCCGGTCGNNNNCNNATTGAANNCCTGNNGCATNNNNNTNCNAGAATGANNNC
NNNNNGNNTNGCTNNCNNNTCAAGNAATCANNNAGGTNNNNNNNTCNNNGNNNNNNNNCNNTCCCNN
ACTGAGGGTNNNNNNNNCATCTGCTNNNNNNNNNTNCGCNGNNGTNNCTNNNNNGCNGTATNGTGTNA
NNCANNNNNNNNNNNNNNNNTNAAACNANNNNNNNTNNNN
>PaThen497
GTGNNNNNTAGCCCNNNNNAGTCAGACANNNCNNNNNNNNNNNNTNNNNNNNNNNNNNNNNNNNNNNN
NNNNNNNNNNNNNNNNNNNNNNNNNNNNNNNNNNNNNNNNNNNNNNNNNNNNNNNNNNNNNNNNNNNN
NNNNNNNNNNNNNNNNNNNNNNNNNNNNNNNNNNNNNNNNNNNNNNNNNNNNNNNNNNNNNNNNNNNN
NNNNNNNNNNNNNNNNNNNNNNNNNNNNNNNNNNNNNNNNNNNNNNNNNNNNNNNNNNNNNNNNNNNN
NNNNNNNNNNNNNNNNNNNNNNNNNNNNNNNNNNNNNNNNNNNNNNNNNNNNTCTNNNAAANNNTNNN
NNNNNNNNNNNNNNNNNNNNNNNNNCNCNTTGNNNGTAANCNANNNGNACATGCAGTNNCCTCNNNNN
NNNNNCAGAGNNNNNNNCNNNNNNNNCCGNCCCCNNCCNNNNNNNNNNNNNNNNNNNNNNNANNNNNN
NNNNNNTGGTACNNNNCGNNNNNGGTTGTNTNAGNGGTCCACAACGNGNTCNNGNCACNNNNGNNNAN
NNNCNNNNAANNNNNNNNNNNNNNNTNNNTANNAGGNNNNNNCCNTCNNNNNNNNNANNGCNATGACC
CNANNCACNGGGGNNGTNCCCGGCGCGGCGACCTTGNNNANNTCCGGGGACAAGTATGGCNNNCNCCC
CGNGAAGGANNCNGCCGTANGTGCNNNNNANNNNNTCNTGNGANGTNGCCGNNATTCNCGTNGNCNNN
CNAGANTGNNCGNACCANNCGNANNNTGGTNATGNNAGTNNNNNNNNNNNNNNNNNNNNNNNNNNNNN
NNNNNNNNNNNNNNNNNNNNNNNNNNNNNNNNGNNNNGNNNNNNCNANNNAAGGANNTANNNCTNNTN
NTNNNNNNGGACNNNACNNNNCNNNNNNNACNCNNNCNCNNNNNNNNNNGNTTTTTNNCCNNTATTNT
CCAGNANGCNGNGCNNNCATTTANGNGAACNNNGNNACGCGNNAATCGTANNATCTACCTGNCGCNGG
NNNNNNTACNGNNNNNNCGGANNNTNNCTCGTANNNNNNTCANGCGTNNTNTNNTCNCNAGNCANGTT
CGNNNANNNANTAGNANGTNTCCNNNNNCTTGTNNCGTTCCGCNTGANNCNNCANNNNGNNGNNNTTN
TCANCGGCNNTANCTGCNGANNTAAGNCATANNNTNNNTCNNTANCTNNNNNGGNGGGNNACTGTAAA
NGGNNAATTGGANNNTNANTNCNNNCATNTNNCTNNGNNTNCTNNNTNCCTGNNTNCNNAATTGGNNN
CANTGNNTGNCNATCTCTGGNTTNGNNNNNNTNNNNNTNNCAANNNGGNNNGTNNNANNNCNATNTAA
NNTATANNNNCNNNNNNANNNCACNNNNNNNNNAGNNNCNANNNGTANANNNGTAGNNNGNTNAGTCN

CCGANNTNNNNNNNATAAGCGNNANACNNNNNNNNNNNNNNANGANNACGNNTTTGNNNTNNNNNNCNA
GNNAGACNNTNGTGGNTNNNNNTCAANNNNTTCGTAGTGGTATAATNGNNNNNNNTTTCNCCAGNNTN
NNNGNNNNNNNCNATANNANNNNNNNGNGANNNNNNNNNNNANNNNCTCNNNAGGNANCANCTACTTNA
GCANTGGNGCGCAGNNNGAGANNNNGACCAGCCTTGGGTGGCAANCACNGCAATAAGTNNNGTTNANC
NTNNNAGTNNACACNNNNNCANNGNNNCNNNNNNANCNGANNGNNNNNNNNNNNNNNNNNNNNNNNNN
NNNNNNNNNNNNNNNNNNNNNNNNNNNNNGNNNANGGNNNGTNTNTGACNCATGNNTTTTTANNNGNN
NNNNNACGNNNNNNCCCCGGTCGNNNNCNNATTGAANNCCTGNNGCATNNNNNTNCNAGAATGANNGC
NNNNNGNNTNGNCTNNCNNNTCAAGNAATCANNNANGTANNGCGNTCNNNNNNNNNNNNCNNTCCCNN
ACTGAGGGTNNNNNNCGCATCTGCTNNNNNNNNNTNCGCNGGCNNNNCNNNNNAGCNGTATNNTGNNN
NNCANNNNNNNNNNNNNNNNNTNAAACAANNNNNNTNNNN
>PaThen498
GTGNNNNNTNGCCNNNNNNAGTCAGACANNNCNNNNNNNNNNNNNNNNNNNNNNNNNNNNNNNNNNNNN
NNNNNNNNNNNNNNNNNNNNNNNNNNNNNNNNNNNNNNNNNNNNNNNNNNNNNNNNNNNNNNNNNNNNN
NNNNNNNNNNNNNNNNNNNNNNNNNNNNNNNNNNNNNNNNNNNNNNNNNNNNNNNNNNNNNNNNNNNNN
NNNNNNNNNNNNNNNNNNNNNNNNNNNNNNNNNNNNNNNNNNNNNNNNNNNNNNNNNNNNNNNNNNNNN
NNNNNNNNNNNNNNNNNNNNNNNNNNNNNNNNNNNNNNNNNNNNNNNNNNNNNTCTNNNAAANNNTNNN
NNNNNNNNNNNNNNNNNNNNNNNNNNNCNCNNTGNNNGTAANCNANNNGNACATGCAGTCCCCTCNNNNN
NNNNNCAGAGNNNNNNNNNNNNNNNCNCCGNCCCCNNCCNNNNNNNNNNNNNNNNNNNNNNNNNANNNNNN
NNNNNNTGGTACNNNNCGNNNNNGGNTGNNTNAGNGGTCCACAACGNGNTCNNGNNACNNNNGNNNAN
NNNCNNAAAANNNNNNNNNNNNNNNNTNNNTANNAGGNNNNNNCCNTNNNGNNNNNNANNGCNATGACC
CNANNCACGGNGGNNGTNCCCGGNGNNGCGNCCTTGNNNANNTCCGGGGNCAAGTATNGCNNNCNCCC
CGNGAAGGANNCNGCCGTANGTGCNNNNNANNNNNTCNTGNGANGTNGCCGNNATTNNNNTGGTCNNN
NNAGAATGNNCNNACCANNCGNANNNTGGTNATGNTAGTNNNNNNNNNNNNNNNNNNNNNNNNNNNNN
NNNNNNNNNNNNNNNNNNNNNNNNNNNNNNNNNNNGNNNNGNNNNNNCNANNNAAGGANNTANNNCTNNTN
NTNNNNNNGGACNNNACNNNNCNNNNNNNACNCNNNCNCNNNNNNNNNNGNTTTTTNNNCNNTANTNT
CNAGNACGCNNNGCNNNCATTTANGNGAACNNNGNNACGCGNNAATCGTANNNTCTACCTGCCGCNNG
NNNNNNTACNGNNNNNNCGGANNNTNNNNCGTANNNNNNTCATGCGTNNTNTNNTCNCNAGNCNNGTT
CGNNNANANANTAGNANGTNTNNNNNNNNNNTGTNNCGTTCCGCNTGACNCNNCANNNNGNNGNNNTTN
NCANCGGNNNTANCTGCNGANNTANGNCATANNNTNNATCNNTANCTNNNNNGGNGGGNNACTGTAAA
NGGNNAATTNGNNNNTNANTTCNNNCATNTNNCNNNGNNNNCTNNNTNCCTGNNTTCNNAATTNGNNC
CANTGNNTGNCNATCTCTGGNTTNGCNNNNNTNNNNNTNNCANNNNGGNNNNNNNNANNNCNATNTAA
NNTANANNNNCNNNNNNANNNCACNNNNNNNNNAGNNNCNANNNGTANANNNGTAGNNNNNTNAGTCN
CCGANNTNNNNNNNATAAGCGNNANACNNNANNNNNNNNNNANGANNNCGNNTTTGNNNTNNNNNNCNA
GNNAGANNNTNGTGGNTNNNNNTCAANNNNTTCGTAGTGGTATAATNGNNNNNNNTTTCNCCAGNNNN
NNNNNNNNNNNCNATANNANNNNNNNGTGANNNNNNNNNNNANNNNCTCNNNAGGNANCANCTACTNNA
GCANNGGNGCGNAGNNNGAGANNNNGACNNGCCTTGGGTGGCAANCACNGCAATAAGTNNNGTTNANC
CTNNNAGTNNACACNNNNNCANNGNNNCNNNNNNANCNGANNGNNNNNNNNNNNNNNNNNNNNNNNNN
NNNNNNNNNNNNNNNNNNNNNNNNNNNNNNAGNNNANGGNNNGTNTNTGACNNATGTNTTTTTANNNGNN
NNNNNACGNNNNANCCCCGGTCGNNNNCNNATTGAANNCCTGNNGCATNNNNNTNCNAGAATGANNNC
NNNNNGNNTNNNCTNNNNNNTCAAGNAATCNNNNANNNNNNNNGNTCNNNNNNNNNNNNCNNNCCCNN
ACTGAGGGNNNNNNNNNCATCNGCTNNNNNNNNCTNCGCNGNNNNNNCTGNNNNGCNGTATNGTGNNN
NNCANNNNNNNNNNNNNNNNNTNNAACNNNNNNNNNTNNNN
>PaThen499
GTGNNNNNTAGCTCNNNNNAGTTAGACANNNCNNNNNNNNNNNNNTNNNNNNNNNNNNNNNNNNNNNNN
NNNNNNNNNNNNNNNNNNNNNNNNNNNNNNNNNNNNNNNNNNNNNNNNNNNNNNNNNNNNNNNNNNNNN
NNNNNNNNNNNNNNNNNNNNNNNNNNNNNNNNNNNNNNNNNNNNNNNNNNNNNNNNNNNNNNNNNNNNN
NNNNNNNNNNNNNNNNNNNNNNNNNNNNNNNNNNNNNNNNNNNNNNNNNNNNNNNNNNNNNNNNNNNNN
NNNNNNNNNNNNNNNNNNNNNNNNNNNNNNNNNNNNNNNNNNNNNNNNNNNCNTCTNNNAAANNNTNNN
NNNNNNNNNNNNNNNNNNNNNNNNNNNCNCNTTGNNNGTAANCNANNNGNACATGCAGTNCCCTCNNNNN
NNNNNCAGAGNNGNNNNCCNNNNNCNCCGGCCCCNNCCNNNNNNNNNNNNCNNNGNNNNNNNGNNNNNN
NNNNNNTGGTACNNNNCGNNNNNGGTTGTNTAAGNGGTCCACCACGNGGTCNNNNNACNNNNGNNNAN
NNNCNNNNAACNNNNNNNNNNNNANNTNNNCANNAGGNNNNNNCCNTCNNNNNNNNNNATNGCNATGANN
CNANNCNCGGGGACAGTNACCGGCGNGGCGACCTTGNTNACGGTCGGGGNCAAGTATGTCNNNCNCNC
CGGGAAGGANNCNGCCGTANGTGCNNNNNANNNNNTCNTGNGANGTNGCCGNNANNNNCNTGGTCNNN

NNAGAATGNCCNNACCANNCGNANNNTGGTNATGNNAGTNNNNNNNNNNNNNNNNNNNNNNNNNNNNN
NNNNNNNNNNNNNNNNNNNNNNNNNNNNNNNNGNNNNGNNNNNNCANNNNAAGGANNTANNNTTNNTN
NTNNNGNNGGACNNNACNNNNCNNNNNNNACNCNNNCNCNNNNNNNNNNGNTTTTTNNCCNNTATTNT
CCAGNACGCNGNGCNNNCATTCANGNGAACNNNGNNACGCGNNAATCGTANNATCTACCTGCCGCNNG
NNNNNNTACNGNNNNNNCGGANNNTNNCTCGTANNNNNNTCATGCGTNNTNTNNTTNCNAGNCANGTT
CGNNNANANANTAGNANGTNTNNNNNNNNNTGTNNCGTTCCGCNTGANNCNNCANNNNGNNGNNNTTN
TCANCGGCNNTANCTGCNGANNTAAGNCATANNNTNNNTCNNTANCGNNNNNGGNGGGNNACTGTAAA
NGGNNAATTNGNNGNTNANTNCNNNCACNTNNCNNNGNNNNCTNNNCNCCTGNNTNCNNAATTGGNNC
CANTGNNTGNCATCTCTNGNTTNGNNNNNNNTGNNNNTNNTAANNNGGNNNNNNANANCNCNATNTAA
NNTANANNNNCNNNNNNANNNCACNNNNNNNGNAGNNNNNANNNTTANGNNNGTAGNNNANTAAGTCT
CCGANGTNNNNNNNATAAGCGNNANACNNNNNNNNNNNNNANGANNACGNNTTGGNNNTNNNNNNCNA
ANNAGACNNCNGTGGNTNNNNNTCAANNNNTTCGTAGTGGTATAACNANNNNNNNTTTCNCCAGNNTN
NNNGNNNNNNNCNATANNANNNNNNNGNGANNNNNNNNNNANNNNCCCNNNAGGGANCANCTACTNNA
GCAATGGNGCGNAGNNNGAGANNNNGACNNNCCTTGGGTGGCAANCACNGCAATAAGTNNNGTTNNNC
CCNNNAGTNNACACNNNNNCANNGNNNCNNNNNNANCNGANNTNNNNNNNNNNNNNNNNNNNNNNNNN
NNNNNNNNNNNNNNNNNNNNNNNNNNNNAGNNNANNGNNNGTNTNTGACNCATGTGTTTTTANNNGNN
NNNNNACGNNNNANCCCCGGTCGNNNNCNNATTGAANNCCTGNNGCATNNNNNTNCNAGAATGANNNC
NNNNNGNNTNNNCTNNCNNNTCAAGNAGTCANNTAGGNANCGCGNTCNNGNNNNNNNNNCNNTCCCNN
ACTGAGGGTNNNNNNNNCATCTGCTNNNNNNNNNTNCGCNGNNNNNNTNNNNNNGCNGTATNGTGTNN
NNCANNNNNNNNNNNNNNNNTNNAACNNNNNNNNNTNNNN
>PaThen500
GTGNNNNNTAGCCCNNNNNAGTTAGACANNNCNNNNNNNNNNNNNNNNNNNNNNNNNNNNNNNNNNNN
NNNNNNNNNNNNNNNNNNNNNNNNNNNNNNTNNNNNNNNNNNNNNNNNNNNNNNNNNNNNNNNNNNNN
NNNNNNNNNNNNNNNNNNNNNNNNNNNNNNNNNNNNNNGNNNNNNNNNNNNNNNNNNNNNNNNNNNNN
NNNNNNNNNNNNNNNNNNNNNNNNNNNNNNNNNNNNNNNNNNNNNNNNNNNNNNNNNNNNNNNNNNNN
NNNNNNNNNNNNNNNNNNNNNNNNNNNNNNNNNNNNNNNNNNNNNNNNNNNNTCTNNNAAANNNTNNN
NNNNNNNNNNNNNNNNNNNNNNNNNCNCNTTGNNNGTAANCNANNNGNACATGCAGTCCCCTCNNNNN
NNNNNCAGAGNNNNNNCCCNNNNNNNCCGGCCCCNNCCNNNNNNNNNNNNNNNGNNNNNNNANNNNNN
NNNNNNTGGTACNNNNCGNNNNNGGTTGTNTNAGAAGTCCACAACGNGGTCNNGNCACNNNNGNNNAN
NNNCNNNNAACNNNNNNNNNNNNNNTNNNTANNAGGNCNNNNCCNTCNNNNNNNNNATNGCNATGACC
CAANTCACGGGGGCAGTNCCCGGNGNGGCGACCTTGNNNACGTCCGGGGACAAGTATGGCNNNCNCCC
CGGGAAGGANNCNGCCGTANGTGCNNNNNANNNNNTCNTGNGANGTCGCCGNNATTCNNGTGGTCTNN
NNAGAATGNCCNNACCANNCGNANNNTGGTNATGNNAGTNNNNNNNNNNNNNNNNNNNNNNNNNNNNN
NNNNNNNNNNNNNNNNNNNNNNNNNNNNNNNNGNNNNGNNNNNNCANNNNAAGGANNTANNNCTNNTN
NTNNNNNNGGACNNNACNNNNCNNNNNNNACNCNNNCNCNNNNNNNNNNGNTTTTNNNNCNNTATTNT
CCAGNACGCNGNGCNNNCATTCANGNGAACNNNGNNACGCGNNAATCGTANNATCTACCTGCCGCNNG
TNNNNNTACNGNNNNNNCGGANNNTNNCTCGTANNNNNNTCATGCGTNNTNTNNTCNCNAGNCNNGTT
CGNNNANANANTAGNANGTNTNNNNNNNNNTGTNNCGTTCCGCNTGANNCNNCANNNNGNNGNNCTTN
NCANCGGCNNTANCTGCNGANNTAAGNCATANNNTNNATCNNTANCTNNNNNGGNGGGNNACTGTAAA
NGGNNAATTNGANNNTNATTTCCNNCATNTNNCTNNGNNTNCTNNNTNCCTGNNTNCNNAATTGGNNC
CANTGNNTGNCATCTCTGGNTTNGCNNNNNNTNNNNNTNNCANNNNGGNNNGTNNNANNNCNATNTAA
NNTANANNNNCNNNNNNANNNCACNANNNNNNNAGNNNCNANNNGTANANNNGTAGNNNGNTAAGTCT
CCGANNTNNNNNNNATAAGCTNNANACNNNNNNNNNNNNNANGANNACGNNTTTGNNNTNNNNNNCNA
GNNAGACNNTNGTGGNTNNNNNTCAANNNNTTCGTAGTGGTATAATNGNNNNNNNTTTCNCCAGNNTN
NNNGNNNNNNNCNATANNANNNNNNNGNGANNNNNNNNNNANNNNCTCNNNAGGNANCANCTACTNNA
GCAANGGNGCGCAGNNNGAGANNNNGACNNNCCTTGGGTGGCAANCACNGCAATAAGTNNNGTTNANC
CTNNNAGTNNACACNNNNNCANNGNNNCNNNNNNANCNGANNGNNNNNNNNNNNNNNNNNNNNNNNNN
NNNNNNNNNNNNNNNNNNNNNNNNNNNNAGNNNANGGNNNGTNTNTGACNCATGTGTTTTCANNNGNN
NNNNNNNGNNNNANCCCCGGTCGNNNNCNNATTGAANNCCTGNNGCATNNNNNTNCNAGAATGANNNC
NNNNNGNNTNGNCTNNCNTTTCAAGNAATCANNNAGGTNTNNCGNTCNNNNNNNNNNNNCNNTCCCNN
ACTGAGGGTNNNNNNNNCATCNGCTNNNNNNNNNTNCGCNGNNNNNNCNNNNNAGCNGTATNGTGTNN
NNCANNNNNNNNNNNNNNNNTNAAACANNNNNNNNTNNNN
>PaThen501
GTGNNNNNTAGCTCNNNNNAGTTAGACANNNCNNNNNNNNNNNNTNNNNNNNNNNNNNNNNNNNNNNN

NNNNNNNNNNNNNNNNNNNNNNNNNNNNNNNNNNNNNNNNNNNNNNNNNNNNNNNNNNNNNNNNNNNN
NNNNNNNNNNNNNNNNNNNNNNNNNNNNNNNNNNNNNNNNNNNNNNNNNNNNNNNNNNNNNNNNNNNN
NNNNNNNNNNNNNNNNNNNNNNNNNNNNNNNNNNNNNNNNNNNNNNNNNNNNNNNNNNNNNNNNNNNN
NNNNNNNNNNNNNNNNNNNNNNNNNNNNNNNNNNNNNNNNNNNNNNNNNNCNTCTNNNAAANNNTNNN
NNNNNNNNNNNNNNNNNNNNNNNNNCNCNTTNNNNGTAANCNANNNGNACATGCAGTCCNCTCNNNNN
NNNNNCAGAGNNNNNNNCCNNNNNNNCCGNCCCCNNCCNNNNNNNNNNNNNNNGNNNNNNNGNNNNNN
NNNNNNTGGTACNNNNCGNNNNNGGTTGNNTNAGAGGTCCACCACGNGGTCNNNNCACNNNNGNNNAN
NNNCNNNNAACNNNNNNNNNNNNNNTNNNCANNAGGNCNNNNCCNTNNNGNNNNNNANNGCNATGACC
CNANNCGCGGNGACNGTNACCGGNGCGGCGACCTTGNNNANNGTCGGGNACAAGTATGTCNNNCNCCC
CGNGAAGGANNCNGCCGTANGTGCNNNNNANNNNNTCNTGNGANGTNGCCGNNANNNNCNTGGTCNNN
NNAGAATGNCCGNACCANNCGNANNNTGGTNATGNNAGTNNNNNNNNNNNNNNNNNNNNNNNNNNNNN
NNNNNNNNNNNNNNNNNNNNNNNNNNNNNNNNNNNNNGNNNNNNCNANNNAAGGNNNTANNNTTNNTN
NTNNNGNNGGACNNNACNNNNCNNNNNNACNCNNNCNCNNNNNNNNNNNGNTTNNNNNNCNNTANNNT
CCAGNACGCNGNGCNNNCATTCANGNGAACNNNGNNACGCGNNAATCGTANNATCTACCTGCCGCNNG
NNNNNNTNCNGNNNNNNCGGANNNTNNNNCGTANNNNNNNCANGCGTNNTNTNNTTNCNAGNCNNGTT
CGNNNANNNANTAGNANGTNTNNNNNNNNNTGTNNCGTTCCGCNTGANNCNNCANNNNGNNGNNNTTN
TCANCGGNNNTNNCTGCNGANNTAAGNCATANNNTNNGTCNNTANCGNNNNNGGNGGGNNACTGTAAA
NGNNNAATTNGNNNNTNANTTCNNNCACNTNNCTNNGNNNNCTNNNCNCCTGNNTNCNNAATTGGNNN
CANTGNNTGNCATCTCTGGNTTNGNNNNNNNTNNNNNTNNTANNNNGGNNNNNNNNANNNCNATNTAA
NNTANANNNNCNNNNNNANNNCACNANNNNNNNAGNNNNNANNNTTANGNNNGTAGNNNANTAAGTCT
CCGANNTNNNNNNNATAAGCGNNANACNNNNNNNNNNNNNANGANNNCGNNTTGGNNNTNNNNNNNNA
ANNAGANNNCNGTGGNTNNNNNTCAANNNNTTNGTAGTGGTATAACNANNNNNNNTTTCNCCAGNNNN
NNNGNNNNNNNCNATANNANNNNNNNGNGANNNNNNNNNNANNNNCCCNNNAGGNANCANCTACTNNA
GCAANGGNGCGCAGNNNGAGANNNNGACNNNCCTTGGGTGGCAANNACNGCAATAAGTNNGTTNNNNC
CCNNNAGTNNACACNNNNNCANNGNNNCNNNNNNANCNGANNTNNNNNNNNNNNNNNNNNNNNNNNNN
NNNNNNNNNNNNNNNNNNNNNNNNNNNNNNNNNANGGNNNGTNTNTGACNNATNNNTTTTTANNNGNN
NNNNNNNGNNNNNNCCCCGGTCGTNNNCNNATTGAANNCCTGNNGCATNNNNNTNCNAGAATGANNNC
NNNNNGNNTNNNCTNNCNNNTNANGNAGTCANNNAGNTNNNNNNNNTCNNNNNNNNNNNCNNNCCCNN
ACTGAGGGTNNNNNNNNCATCTGCTNNNNNNNNNTNCGCNGNNNNNTNNNNNNAGCNGTATNNTGTNN
NNCANNNNNNNNNNNNNNNNTNNAACAANNNNNNNTNNNN
>PaThen502
GTGNNNNTAGCTCNNNNNAGTTAGACANNNCNNNNNNNNNNNNNNNNNNNNNNNNNNNNNNNNNNNNN
NNNNNNNNNNNNNNNNNNNNNNNNNNNNNNNNNNNNNNNNNNNNNNNNNNNNNNNNNNNNNNNNNNNN
NNNNNNNNNNNNNNNNNNNNNNNNNNNNNNNNNNNNNNNNNNNNNNNNNNNNNNNNNNNNNNNNNNNN
NNNNNNNNNNNNNNNNNNNNNNNNNNNNNNNNNNNNNNNNNNNNNNNNNNNNNNNNNNNNNNNNNNNN
NNNNNNNNNNNNNNNNNNNNNNNNNNNNNNNNNNNNNNNNNNNNNNNNNNNNTCTNNNAAANNNTNNN
NNNNNNNNNNNNNNNNNNNNNNNNNCNCNTTGNNNGTAANCNANNNGNACATGCAGTNNCCTCNNNNN
NNNGGCAGAGNNNNNNNNNNNNNNNNCCGGCCCCNNCCNNNNNNNNNNNNNNNGNNNNNNNGNNNNNN
NNNNNNTGGTACNNNNCGNNNNNGGTTGTNTNAGAGGTCCACCACGNGGTCNNGNCACNNNNGNNNAN
NNNCNNNNANCNNNNNNNNNNNNNNTNNNCANNAGGNCNNNNCCNTCNNNNNANNNATTGCNATGACC
CAANTCGCGGGGACNGTNACCGGCGCGGCGACCTTGANNANNGTCGGGGACAAGTATGTCNNNCNCCC
CGNGAAGGANNCNGCCGTANGTGCNNNNNANNNNNTCTTGNGANGTCGCCGNNATTNNCNTGGNCNNN
NNAGAATGNCCNNACCANNCGNANNNTGGTNATGNNAGTNNNNNNNNNNNNNNNNNNNNNNNNNNNNN
NNNNNNNNNNNNNNNNNNNNNNNNNNNNNNNNGNNNNGNNNNNNCNANNNAAGGANNTANNNTTNNTN
NTNNNNNNGGACNNNACNNNNCNNNNNNACNCNNNCNCNNNNNNNANNNGNTTTTTNNNCNNTATTNT
CCAGNACGCNGNGCNNNCATTCANGNGAACNNNGNNACGCGNNAATCGTANNATCTACCTGCCGCNAG
TNNNNNTACNGNNNNNNCGGANNNTNNCTCGTANNNNNNNCATGCGTNNTNTCNTTNCNAGNCNNGTT
CGNNNANANANTAGNANGTNTCNNNNNNNNTGTNNCGTTCCGCNTGANNCNNCANNNNGNCGNNCTTN
TCANCGGCNNTANCTGCNGANNTAAGNCATANNNTNNGTCNNTANCGNNNNNGGNGGGNNACTGTAAA
NGGNNAATTNGNNNNTNATTTCNNNCACNTNNCTNNGNNTNCTNNNCNCCTGNNTNCNNAATTGGNNC
CANTGNNTGNCATCTCTGGNTTNGNNNNNNNTNNNNNTNNTAANNNGGNNNGTNANANNNCNATNTAA
NNTANANNNNCNNNNNNANNNCACNANNNNNGNAGNNANNANNNTTANGNNNGTAGNNNAATAAGTCT
CCGANNTNNNNNNNATAAGCGNNANACNNNNNNNNNNNNNANGANNACGNNTTGGNNNTNNNNNNCNA
ANNAGACNNCNGTGGNTNNNNNTCAANNNNTTNGTAGTGGTATAACNANNNNNNNTTTCNCCAGNNTN

NNNGNNNNNNNNCNATANNANNNNNNNGNGANNNNNNNNNNNANNNNCCCNNNAGGGANCANCTACTNNA
GCANTGGNGCGNAGNNNGAGANNNNGACCAGCCTTGGGTGGCAANNACNGCAANAAGTNNNGTTNANC
CCNNNAGTNNACACGNNNNCANNGNNNCNNNNNNANCNGANNTNNNNNNNNNNNNNNNNNNNNNNNN
NNNNNNNNNNNNNNNNNNNNNNNNNNNNNNAGNNNANNGNNNGTNTNTGACNCATGTGTTTTTANNNGNN
NNNNNACGNNNGNNCCCCGGTCGNNNNCNNATTGAANNCCTGNNGCATNNNNNNTNCNAGAATGANNNC
NNNNNGNNTNGNCTNNCNNNTCAAGNAGTCANNNANNNANCGCGNTCNNNNNNNNNNNNNCNNTCCCNN
ACTGAGGGTNNNNNNCGCATCTGCTNNNNNNNNNNTNCGCNGNNNNNNTNNNNNAGCNGTATNGNGTNN
NNCANNNNNNNNNNNNNNNNNCNANACGANNNNNNNTNNNN
>PaThen503
GTGNNNNNTAGCCCNNNNNAGTCAGACANNNCNNNNNNNNNNNNNNNNNNNNNNNNNNNNNNNNNNN
NNNNNNNNNNNNNNNNNNNNNNNNNNNNNNNNNNNNNNNNNNNNNNNNNNNNNNNNNNNNNNNN
NNNNNNNNNNNNNNNNNNNNNNNNNNNNNNNNNNNNNNNNNNNNNNNNNNNNNNNNNNNNNNNN
NNNNNNNNNNNNNNNNNNNNNNNNNNNNNNNNNNNNNNNNNNNNNNNNNNNNNNNNNNNNNNNN
NNNNNNNNNNNNNNNNNNNNNNNNNNNNNNNNNNNNNNNNNNNNNNNNNNNNNNTCTNNNAAANNNTNNN
NNNNNNNNNNNNNNNNNNNNNNNNNNNNCNCNTTGNNNGTAANCNANNNGNACATGCAGTCCCCTCNNNNN
NNNGGCAGAGNNNNNNCCCNNNNNCNCCGGCCCCGNCCNNNNNNNNNNNNCNNNNNNNNNNNANNNNNN
NNNNNNTGGTACNNNNCGNNNNNGGTTGTTTAAGAGGTCCACAACGNGGTCNNGNCACNNNNGNNNAN
NNNCNNNAAACNNNNNNNNNNNNANNTNNNTANNAGGNCNNNNCCNTNNNNNNANNNATNGCNATGACC
CAANNNACGGGGGCAGTNCCCGGNGNGGCGACCTTGNNNANNTCCGGGNACAAGTATGGCNNNCNCCC
CGGGAAGGANNCNGCCGTANGTGCNNNNNANNNNNTCNTGNGANGTNGCCGNNATTCNNNTNGTCNNN
CNAGANTGNCCGNACCANNCGNANNNTGGTNATGNNAGTNNNNNNNNNNNNNNNNNNNNNNNNNNNN
NNNNNNNNNNNNNNNNNNNNNNNNNNNNNNNNNNNNGNNNNGNNNNNNCNANNNAAGGNNNTANNNCTNNTN
NTNNNNNNGGACNNNACNNNNCNNNNNNNACNCNNNCNCNNNNNNANNNGNTTTTNNNCCNNTANTNT
CCAGNACGCNGNGCNNNCATTTANGNGAACNNNGNNACGCGNNAATCGTANNATCTACCTGCCGCNNG
NNNNNNTACNGNNNNNNCGGANNNTNNCTCGTANNNNNNNCATGCGTNNTNTNNTCNCNAGNCANGTT
CGNNNANANANTAGNANGTNTNNNNNNNNNNGTNNCGTTCCGCNTGANNCNNCANNNNGNNGNNCTTN
TCANCGGCNNTANCTGCNGANNTAAGNCATANNNTNNATCNNTANCTNNNNNGGAGGGNNACTGTAAA
NGGNNAATTNGNNGNTNANTTCNNNCATNTNNCTNNGNNTNCTNNNTNCCTGNNTNCNNAATTGGNNC
CANTGNNTGNCNATCTCTGGNTTNGNNNNNNTNNNNNTNNCANNNNGGNNNGTNANANNNCNATNTAA
NNTANANNNNCNNNNNNANNNCACNANNNNNNNAGNNNCNANNNGTANANNNGTAGNNNGATAAGTCT
CCGANNTNNNNNNNATAAGCGNNANACNNNNNNNNNNNNNNANGANNACGNNTTTGNNNTNNNNNNNCNA
GNNAGACNNTNGTGGNTNNNNNTCAANNNNTTNGTAGTGGTATAATNGNNNNNNNTTTCNCCAGNNTN
NNNGNNNNNNNNCNATANNANNNNNNNGNGANNNNNNNNNNNANNNNCTCNNNAGGGANCANCTACTNNA
GCAANGGNGCGCAGNNGGAGANNNNGACNNNCCTTGGGTGGCAANCACNGCAATAAGTNNNGTTNANC
CTNNNAGTNNACACNNNNNCANNGNNNCNNNNNNANCNGANNGNNNNNNNNNNNNNNNNNNNNNNNNN
NNNNNNNNNNNNNNNNNNNNNNNNNNNNNNNNNNANNGNNNGTNTNTGACNCATGTGTTTTTANNNGNN
NNNNNACGNNNNANCCCCGGTCGNNNNCNNATTGAANNCCTGNNGCATNNNNNNTNCNAGAATGANNNC
NNNNNGNNTNNNCTNNCNNNTCAAGNAATCANNNAGGNNNNNCGNTCNNNNNNNNNNNNNCNNTCCCNN
ACTGAGGGNNNNNTCCGCATCTGCTNNNNNNNNNNTNCGCNNNNNNNNNCNNNNNAGCNGTATNGNGTNN
NNCANNNNNNNNNNNNNNNNNNCNNAACAANNNNNNNTNNNN
>PaThen504
GTGNNNNNTAGCCCNNNNNAGTTAGACANNNCNNNNNNNNNNNNNNNNNNNNNNNNNNNNNNNNNNN
NNNNNNNNNNNNNNNNNNNNNNNNNNNNNNNTNNNNNNNNNNNNNNNNNNNNNNNNNNNNNNNN
NNNNNNNNNNNNNNNNNNNNNNNNNNNNNNNNNNNNNNNNNNNNNNNNNNNNNNNNNNNNNNNN
NNNNNNNNNNNNNNNNNNNNNNNNNNNNNNNNNNNNNNNNNNNNNNNNNNNNNNNNNNNNNNNN
NNNNNNNNNNNNNNNNNNNNNNNNNNNNNNNNNNNNNNNNNNNNNNNNNNNNNCNTCTNNNAAANNGTNNN
NNNNNNNNNNNNNNNNNNNNNNNNNNNNCNCNNTGNNNGTAANCNANNNGNACATGCAGTCCCCTCNNNNN
NNNGGCAGAGNNNNNNCCNNNNNNCNCCGGCCCCGNCCNNNNNNNNNNNNCNNNGNNNNNNNANNNNNN
NNNNNNTGGTACNNNNCGNNNNNGGTTGTNTAAGAGGTCCACAACGNGGTCNGGNCACNNNNGNNNAN
NNNCNNNAAACNNNNNNNNNNNNANATNNNTANNAGGNCNNNNCCNTCNNGNNNNTAATTGCNATGACC
CAANNCACGGGGGCNGTNCCCGGNGCGGCGACCTTGNNNANGTCCGGGGACAAGTATGGCNCNCNCCC
CGGGAAGGANNCNGCCGTANGTGCNNNNNANNNNNTCNTGNGANGTCGCCGNNATTCNNGTGGTCNNN
CNAGAATGNNCGNACCANNCGNANNNTGGTNATGNTAGTNNNNNNNNNNNNNNNNNNNNNNNNNNNN
NNNNNNNNNNNNNNNNNNNNNNNNNNNNNNNNNNNNGNNNNGNNNNNNCNANNNAAGGANNTANNNCTNNTN

NTNNNNNNNGGACNNNACNNNNCNNNNNNNACNCNNNCNCNNNNNNANNNGGTTTTTNNNCNNTATTNT
CCAGNACGCNGNGCNNNCATTCANGNGAACNNNGNNACGCGNNAATCGTANNATCTACCTGCCGCNGG
TNNNNNTACNGNNNNNNCGGANNNTNNCTCGTANNNNNNNCATGCGTNNTNTNNTCNCNAGNCANGTT
CGNNNANANANTAGNANGTNTNNNNNNNCTTGTNNCGTTCCGCNTGANNCNNCANNNNGNCGNNCTTN
NCANCGGCNNTANCTGCNGANNTAAGNCATANNNTNNATCNNTANCTNNNNNGGNGGGNNACTGTAAA
NGGNNAATTGGANGNTNANTNCNNNCACNTNNCTNNGNNTNCTNNNTNCCTGNNTNCNNAATTGGNNC
CANTGNNTGNCNATCTCTGGNTTNGCNNNNNTNNNNNTNNCAANNNGGNNNGTNNNANNNCNATNTAA
NNTATANNNNCNNNNNNANNNCACNANNNNNGNAGNNNNNANNNTTANANNNGTAGNNNAATAAGTCT
CCGANNTNNNNNNNATAAGCGNNANACNNNNNNNNNNNNNANGANNACGNNTTGGNNNTNNNNNNCNA
GNNANACNNTNATGGNTNNNNNTCAANNNNTTNGTAGTGGTATAATNGNNNNNNNTTTCNCCAGNNTN
NNNGNNNNNNNNCNATANNANNNNNNNGNGANNNNNNNNNNNANNNNCTCNNNAGGGANCANCTACTNNA
GCAATGGNGCGCAGNNNGAGANNNNGACNAGCCTTGGGTGGCAANCACNGCGATAAGTNNNGTTNNNC
CTNNNAGTNNACATNNNNNCANNGNNNCNNNNNNANCNGANNGNNNNNNNNNNNNNNNNNNNNNNNNNNN
NNNNNNNNNNNNNNNNNNNNNNNNNNNNNNAGNNNANNGNNNGTNTNTGACNCATGTGTTTTTANNNGNN
NNNNNACGNNNNANCCCCGGTCGNNNNCNNATTGAANNCCTGNNGCATNNNNNTNCNAGAATGANNGC
NNNNNGNNTNGNCTGNCNNNTCAAGNAATCANNNAGNNNNNNNGNTCNNNGNNNNNNNNCNNTCCCNN
ACTGAGGGTNNNNNNCGCATCTGCTNNNNNNNNNCTNCGCNGNNGTNNCNGNNNAGCNGTATNNTGTNA
NNCANNNNNNNNNNNNNNNNNNTNNAACGANNNNNNTNNNN
>PaThen505
GTGNNNNNTAGCTCNNNNNAGTTAGACANNNCNNNNNNNNNNNNNTNNNNNNNNNNNNNNNNNNNNNNNN
NNNNNNNNNNNNNNNNNNNNNNNNNNNNNNNNNNNNNNNNNNNNNNNNNNNNNNNNNNNNANNNNNNNNN
NNNNNNNNNNNNNNNNNNNNNNNNNNNNNNNNNNNNNNNNNNNNNNNNNNNNNNNNNNNNNNNNNNNNNN
NNNNNNNNNNNNNNNNNNNNNNNNNNNNNNNNNNNNNNNNNNNNNNNNNNNNNNNNNNNNNNNNNNNNNN
NNNNNNNNNNNNNNNNNNNNNNNNNNNNNNNNNNNNNNNNNNNNNNNNNNNNNNTCTNNNAAANNNTNNN
NNNNNNNNNNNNNNNNNNNNNNNNNCNCNTTGNNNGTAANCNANNNGNACATGCAGTCCCCTCNNNNN
NNNGGCAGAGNNNNNNCNNNNNNNNNCCGNCCCCNNCCNNNNNNNNNNNNNNNNNNNNNNNNNGNNNNNN
NNNNNNTGGTACNNNNCGNNNNNGGTTGTTTNAGAGGTCCACCACGNGGTCNNGNCACNNNNGNNNAN
NNNCNNAAAACNNNNNNNNNNNANNNTNNNCANNAGGNCNNNACCNTCNNNNNNNNNNANNGCNATGACC
CANNTNGCNGGGANNGTNACCGGCGNGGCGACCTTGNNNACGGTCGGGGACAAGTATGTCNNNCNCCC
CGGGAAGGANNCNGCCGTANGTGCNNNNNANNNNNTCNTGNGANGTNGCCGNNATTCNNGTGGTCNNN
CNAGAATGNNCNNACCANNCGNANNNTGGTNATGNNAGTNNNNNNNNNNNNNNNNNNNNNNNNNNNNNN
NNNNNNNNNNNNNNNNNNNNNNNNNNNNNNNNNNGNNNNGNNNNNNCNANNNAAGGNNNTANNNTTNNTN
NNNNNNNNGGACNNNACNNNNCNNNNNNNACNCNNNCNCNNNNNNNNNNNGNTTTTTNNNCNNTATTNT
CCAGNACGCNGNGCNNNCATTCANGNGAACNNNGNNACGCGNNAATCGTANNATCTACCTGCCGCNAG
TNNNNNTACNGNNNNNNCGGANNNTNNNNCGTANNNNNNNCATGCGTNNTNTNNTTNCNAGNCNNGTT
CGNNNANNNANTAGNANGTNTNNNNNNNNNNTGTNNCGTTCCGCNTGANNCNNCANNNNGNNGNNCTTN
TCANCGGCNNTANCTGCNGANNTAAGNCATANNNTNNNTCNNTANCGNNNNNGGNGGGNNACTGTAAA
NGGNNAATTGGNNGNTNANTTCNNNCACNTNNCTNNGNNTNCTNNNCNCCTGNNTNCNNAATTGGNNC
CANTGNNTGNCNATCTCTNGNTTNGNNNNNNTNNNNNTNNTAANNNGGNNNGTNNNANNNCNATNTAA
NNTANANNNNCNNNNNNANNNCACNNNNNNNNNAGNNNCNANNNTTANGNNNGTAGNNNAATAAGTCT
CCGANNTNNNNNNNATAAGCGNNANACNNNNNNNNNNNNNANGANNACGNNTTGGNNNTNNNNNNCNA
ANNAGACNNCNGTGGNTNNNNNTCAANNNNTTCGTAGTGGTATAACNANNNNNNNTTTCNCCAGNNTN
NNNGNNNNNNNNCNATANNANNNNNNNGNGANNNNNNNNNNNANNNNCCCNNNAGGNANCANCTACTNNA
GCAATGGNGCGCAGNNNGAGANNNNGACCANCCTTGGGTGGCAANCACNGCAATAAGTNNNGTTNANC
CCNNNAGTNNACACNNNNNCANNGNNNCNNNNNNANCNGANNTNNNNNNNNNNNNNNNNNNNNNNNNNNN
NNNNNNNNNNNNNNNNNNNNNNNNNNNNNNAGNNNANNGNNNGTNTNTGACNCATGTGTTTTTANNNGNN
NNNNNACGNNNNANCCCCGGTCGNNNNCNNATTGAANNCCTGNNGCATNNNNNTNCNAGAATGANNGC
NNNNNGNNTNGNCTNNNNNNNTCAAGNAGTCNNNNAGNNNNNNNNNTCNNNNNNNNNNNNCNNTCCCNN
ACTGAGGGTNNNNNNCGCATCTGCTNNNNNNNNNNTNCGCNNNNNNNNTTNNNNAGCNGTATNGNGTNN
NNCANNNNNNNNNNNNNNNNNNNAAACNANNNNNNNTNNNN
>PaThen506
GTGNNNNNTAGCCCNNNNNAGTCAGACANNNCNNNNNNNNNNNNNTNNNNNNNNNNNNNNNNNNNNNNNN
NNNNNNNNNNNNNNNNNNNNNNNNNNNNNNNNTNNNNNNNNNNNNNNNNNNNNNNNNNNNNNNNNNNNNN
NNNNNNNNNNCGNNNNNNNNNNNNNNNNNNNNNNNNNNNNNNNNNNNNNNNNNNNNNNNNNNNNNNNNNN

NNNNNNNNNNNNNNNNNNNNNNNNNNNNNNNNNNNNNNNNNNNNNNNNNNNNNNNNNNNNNNNNNNNNNN
NNNNNNNNNNNNNNNNNNNNNNNNNNNNNNNNNNNNNNNNNNNNNNNNNNNNNNTCTNNNAAANNNTNNN
NNNNNNNNNNNNNNNNNNNNNNNNNNNCNCNTTGNNNGTAANCNANNNGNACATGCAGTCCCCTCNNNNN
NNNGGCAGAGNNNNNNNNCNNNNNNNCCGGCCCCNNCCNNNNNNNTNNNNNNNNNNNNNNNANNNNNN
NNNNNNTGGTACNNNNCGNNNNNGGTTGNNTNAGAGGTCCACAACGNGGTCNGGNNACNNNNGNNNAN
NNNCNNNNANCNNNNNNNNNNNNANNTNNNTANNAGGNNNNNNCCNTNNNGNNANNNATTGCNATGACC
CAANNCNCGGGGGCAGTNCCCGGNGCGGCGACCTTGNNNACGTCCGGGGACAAGTATGGCNCNCNCCC
CGGGAAGGANNCNGCCGTANGTGCNNNNNANNNNNTCTTGNGANGTNGCCGNNATTCNCGTGGTCNNN
CNAGAATGNNCGNACCANNCGNANNNTGGTNATGNTAGTNNNNNNNNNNNNNNNNNNNNNNNNNNNNN
NNNNNNNNNNNNNNNNNNNNNNNNNNNNNNNNNNGNNNNGNNNNNNCNANNNAAGGANNTANNNCTNNTC
NTNNNNNNGGACNNNACNNNNCNNNNNNNACNCNNNCNCNNNNNNNNNNNGNTTTTTNNNCNNTATTNT
CCAGNACGCNGNGCNNNCATTTANGNGAACNNNGNNACGCGNNAATCGTANNATCTACCTGCCGCNNG
TNNANNTNCNGNNNNNNCGGANNNTNNCTCGTANNNNNNTCATGCGTNNTNTNNTCNCNAGNCNNGTT
CGNNNANNNANTAGNANGTNTCNNNNNNNNTGTNNCGTTCCGCNTGANNCNNCANNNNGNCGNNCTTN
TCANCGGCNNTANCTGCNGANNTAAGTCATANNNTNNNTCNNTANCTNNNNNGGNGGGNNACTGTAAA
NGGNNAATTNGANGNTNANTTCNNNCATNTNNCTNNGNNNNCTNNNTNCCTGNNTNCNNAATTGGNNC
CANTGNNTGNCNATCTCTGGNTTNGNNNNNNTNNNNNTNNCANNNNGGNNNGTNANANNNCNATNTAA
NNTANANNNNCNNNNNNANNNCACNANNNNNGNAGNNNCNANNNGTANANNNGTAGNNNGNTAAGTCT
CCGANNTNNNNNNNATAAGCGNNANACNNNNNNNNNNNNNANGANNACGNNTTTGNNNTNNNNNGCNA
GNNAGACNNTNGTGGNTNNNNNTCAANNNNTTNGTAGTGGTATAATNGNNNNNNNTTTCNCCAGNNTN
NNNGNNNNNNNCNATANNANNNNNNNGTGANNNNNNNNNNANNNNCTCNNNAGGGANCANCTACTNNA
GCAATGGNGCGCAGNNNGAGANNNNGACNNGCCTTGGGTGGCAANCACNGCAATAAGTNNNGTTNANC
CTNNNAGTNNACACGNNNNCANNGNNNCNNNNNNANCNGANNGNNNNNNNNNNNNNNNNNNNNNNNNN
NNNNNNNNNNNNNNNNNNNNNNNNNNNNNNNAGNNNANNGNNNGTNTNTGACNCATGTGTTTTTANNNGNN
NNNNNACGNNNNANCCCCGGTCGNNNNCNNATTGAANNCCTGNNGCATNNNNNTNCNAGAATGANNGC
NNNNNGNNTNGNCTNNCTTTTCAAGNAATCAANNAGGTANCGCGNTCNNGGNNNNNNNNCNNTCCCNN
ACTGAGGGTNNNNNNCGCATCTGCTNNNNNNNNNNTNCGCNGNCGTNNCTNNNNNGCNGTATNGTGTNN
NNCANNNNNNNNNNNNNNNNNNCNAAACNANNNNNNNTNNNG
>PaThen507
GTGNNNNNTAGCCCNNNNNAGTTAGACANNNCNNNNNNNNNNNNNTNNNNNNNNNNNNNNNNNNNNNNNN
NNNNNNNNNNNNNNNNNNNNNNNNNNNNNNNNNNNNNNNNNNNNNNNNNNNNNNNNNNNNNNNNNNNNNN
NNNNNNNNNNNNNNNNNNNNNNNNNNNNNNNNNNNNNNNNNNNNNNNNNNNNNNNNNNNNNNNNNNNNNN
NNNNNNNNNNNNNNNNNNNNNNNNNNNNNNNNNNNNNNNNNNNNNNNNNNNNNNNNNNNNNNNNNNNNNN
NNNNNNNNNNNNNNNNNNNNNNNNNNNNNNNNNNNNNNNNNNNNNNNNNNNNNNTCTNNNAAANNNTNNN
NNNNNNNNNNNNNNNNNNNNNNNNNNNCNCNNTGNNNGTAANCNANNNGNACATGCAGTCCCCTCNNNNN
NNNGGCAGAGNNNNNNCCNNNNNNNNNCCGNCCCCGNCCNNNNNNNNNNNNCNNNGNNNNNNNANNNNNN
NNNNNNTGGTACNNNNCGNNNNNGGTTGTNTAAGNGGTCCACAACGNGGTCNNNNNACNNNNGNNNAN
NNNCNNNNAANNNNNNNNNNNNNNNNNNTNNNTANNAGGNNNNNNCCNTCNNNNNANNNANNGCNATGACC
CAANNCACGGGGGCAGTNCCCGGNGCGGCGACCTTGANNACGTCCGGGNACAAGTATNGCNNNCNCCC
CGGGAAGGANNCNGCCGTANGTGCNNNNNANNNNNTCNTGNGANGTNGCCGNNATTCNCGTGGNCNNN
NNAGANTGNNCNNACCANNCGNANNNTGGTNATGNTAGTNNNNNNNNNNNNNNNNNNNNNNNNNNNNN
NNNNNNNNNNNNNNNNNNNNNNNNNNNNNNNNNNGNNNNGNNNNNNCNANNNAAGGANNTANNNCTNNTN
NTNNNNNNGGACNNNACNNNNCNNNNNNNACNCNNNCNCNNNNNNNNNNNGNTTTTNNNNCNNTATTNT
CCAGNACGCNGNGCNNNCATTCANGNGAACNNNGNNACGCGNNAATCGTANNATCTACCTGCCGCNNG
TNNNNNTACNGNNNNNNCGGANNNTNNCTCGTANNNNNNNCATGCGTNNTNTNNTCNCNAGNCNNGTT
CGAGAANANANTAGNANGTNTCNNNNNNNNTGTNNCGTTCCGCNTGANNCNNCANNNNGNNGNNNTTN
NCANCGGCNNTANCTGCNGANNTAAGNCATANNNTNNNTCNNTANCTNNNNNGGNGGGNNACTGTAAA
NGGNNAATTNGNNNNTNATTNCNNNCACNTNNCNNNGNNTNCTNNNTNCCTGNNTNCNNAATTGGNNC
CANTGNNTGNCNATCTCTNGNNTNGNNNNNNTNNNNNTNNCAANNNGGNNNGTNNNANNNCNATNTAA
NNTANANNNNCNNNNNNANNNCACNANNNNNGNAGNNNNNANNNTTANANNNGTAGNNNANTAAGTCT
CCGANNTNNNNNNNATAAGCGNNANACNNNNNNNNNNNNNANGANNACGNNTTGGNNNTNNNNNNNNA
GNNAGACNNTNATGGNTNNNNNTCAANNNNTTNGTAGTGGTATAATNGNNNNNNNTTTCNCCAGNNTN
NNNGNNNNNNNCNATANNANNNNNNNGNGANNNNNNNNNNANNNNCTCNNNAGGNANCANCTACTNNA
GCANTGGNGCGCAGNNNGAGANNNNGACCAGCCTTGGGTGGCAANCACNGCGATAAGTNNNGTTNNNC

CTNNNAGTNNACATNNNNNCANNGNNNCNNNNNNANCNGANNGNNNNNNNNNNNNNNNNNNNNNNNNN
NNNNNNNNNNNNNNNNNNNNNNNNNNNNNNAGNNNANNGNNNGTNTNTGACNCATGTGTTTTTANNNGNN
NNNNNACGNNNNANCCCCGGTCGNNNNCNNATTGANNNCCTGNNGCATNNNNNTNCNAGAATGANNGC
NNNNNGNNTNGNCTNNCNNNTCAAGNAATCNNNNAGGTANNNCGNTCNNNNNNNNNNNNNCNNNCCCNN
ACTGAGGGTNNNNNNNNCATCNGCTNNNNNNNNNNTNCGCNGNNNTNNCNNNNNNGCNGTATNGTGTNN
NNCANNNNNNNNNNNNNNNNNNTNAAACAANNNNNNTNNNN
>PaThen508
GTGNNNNNTAGCCCNNNNNAGTTAGACANNNCNNNNNNNNNNNNTNNNNNNNNNNNNNNNNNNNNNNNN
NNNNNNNNNNNNNNNNNNNNNNNNNNNNNNNNNNNNNNNNNNNNNNNNNNNNNNNNNNNNNNNNNNNNNN
NNNNNNNNNNNNNNNNNNNNNNNNNNNNNNNNNNNNNNNNNNNNNNNNNNNNNNNNNNNNNNNNNNNNNN
NNNNNNNNNNNNNNNNNNNNNNNNNNNNNNNNNNNNNNNNNNNNNNNNNNNNNNNNNNNNNNNNNNNNNN
NNNNNNNNNNNNNNNNNNNNNNNNNNNNNNNNNNNNNNNNNNNNNNNNNNNTNNNNNAAANNNTNNN
NNNNNNNNNNNNNNNNNNNNNNNNNNCNCNTTGNNNGTAANCNANNNGNACATGCAGTCCCCTCNNNNN
NNNGGCAGAGNNNNNNNCNNNNNNNNCCGGCCCCNNCCNNNNNNNNNNNNNNNNNNNNNNNNNANNNNNN
NNNNNNTGGTACNNNNCGNNNNNGGTTGTTTAAGAGGTCCACAACANGGTCNNGNCACNNNNGNNNAN
NNNCNNNNNNNNNNNNNNNNNNNNNNTNNNTANNAGGNCNNNNCCNTNNNNNNNNNNNANTGCNATGANN
CNANNCNCGGNGGNAGTNCCCGGCGNGGCGACCTTGNTNANGTCCGGGGACAAGTATGGCNNNCNCCC
CGGGAAGGANNCNGCCGTANGTGCNNNNNANNNNNTCNTGNGANGTNGCCGNNANTCNNGTGGTCNNN
NNAGAATGNNCNNACCANNCGNANNNTGGTNATGNNAGTNNNNNNNNNNNNNNNNNNNNNNNNNNNNN
NNNNNNNNNNNNNNNNNNNNNNNNNNNNNNNNNNGNNNNGNNNNNNCNANNNAAGGANNTANNNCTNNTN
NNNNNNNNGGACNNNACNNNNNNNNNNNNNACNCNNNCNCNNNNNNNNNNNGNTTNNNNNNCNNTANTNT
CCAGNACGCNGNGCNNTCATTCANGNGAACNNNGNNACGCGNNAATCGTANNATCTACCTGCNGCNNG
NNNNNNTACNGNNNNNNCGGANNNTNNNNCGTANNNNNNNCATGCGTNNTNTNNTCNCNAGNCANGTT
CGNNNANNNANTAGNANGTNTNNNNNNNNNTGTNNCGTTCCGCNCGANNCNNCANNNNGNNGNNNTTN
NCANCGGCNNTANCTGCNGANNTAAGNCATANNNTANNTCNNTANCTNNNNNGGNGGGNNACTGTAAA
NGGNNAATTNGANGNTNANTNCNNNCACNTNNCNNNANNNNCTNNNTNCCTGNNTNCNNAATTGGNNN
CANTGNNTGNCNATCTCTGGNNTNGNNNNNNTNNNNNTNNCANNNNGGNNNNNNANANNNCNATNTAA
NNTANANNNNCNNNNNNANNNCACNANNNNNNNAGNNNCNANNNTTANANNNGTAGNNNNATAAGTCT
CCGANNTNNNNNNNATAAGCGNNANACNNNNNNNNNNNNNANGANNACGNNTTGGNNNTNNNNNNCNA
GNNANACNNTNGTGGNTNNNNNTCAANNNNTTNGTAGTGGTATAATNGNNNNNNNTTTCNCCAGNNTN
NNNGNNNNNNNCNATANNANNNNNNNGNGANNNNNNNNNNNANNNNNTTNNNAGGNANCANTTACTNNA
GNAATGGNGCGNAGNNNGAGANNNNGACNNGCCTTGGGTGGCAANCACNGCAATAAGTNNNGTTNANC
CTNNNAGTNNACACNNNNNCANNGNNNCNNNNNNANCNGANNGNNNNNNNNNNNNNNNNNNNNNNNNN
NNNNNNNNNNNNNNNNNNNNNNNNNNNNNNAGNNNANNGNNNGTNTNTGACNCATGTNTTTTTANNNGNN
NNNNNACGNNNGANCCCCGGTCGNNNNCNNATTGAANNCCTGNNGCATNNNNNTNCNAGAATGANNNC
NNNNNGNNTNGNCTNNCNNNTCAAGNAATCNNNNAGGTNNNNNNNTCNNNNNNNNNNNNNCNNTCCCNN
ACTGAGGGTNNNNNNNNCATCTGCTNNNNNNNNNNTNCGCNGNNNNNNCNNNNNNGCNGTATNGNGTNA
NNCANNNNNNNNNNNNNNNNNNNNAAACNANNNNNNNTNNNN
>PaThen509
GTGNNNNNTAGCCCNNNNNAGTCAGACANNNCNNNNNNNNNNNNTNNNNNNNNNNNNNNNNNNNNNNNN
NNNNNNNNNNNNNNNNNNNNNNNNNNNNNNNNNNNNNNNNNNNNNNNNNNNNNNNNNNNNNNNNNNNNNN
NNNNNNNNNNNNNNNNNNNNNNNNNNNNNNNNNNNNNNNNNNNNNNNNNNNNNNNNNNNNNNNNNNNNNN
NNNNNNNNNNNNNNNNNNNNNNNNNNNNNNNNNNNNNNNNNNNNNNNNNNNNNNNNNNNNNNNNNNNNNN
NNNNNNNNNNNNNNNNNNNNNNNNNNNNNNNNNNNNNNNNNNNNNNNNNNNTCNNNNAAANNNTNNN
NNNNNNNNNNNNNNNNNNNNNNNNNNCNCATTGNNNGTAANCNANNNGNACATGCAGTCCCCTCNNNNN
NNNGGCAGAGNNNNNNCNNNNNNNNNCCGNCCCCNNCCNNNNNNNNNNNNNNNNNNNNNNNNNANNNNNN
NNNNNNTGGTACNNNNCGNNNNNGGTTGTTTAAGAGGTCCACAACGNGGTCNNNNCACNNNNGNNNAN
NNNCNNNNAANNNNNNNNNNNNNNNNNTNNNTANNAGGNNNNNNCCNTCNNNNNNNNNNANNGCNATGANC
CNANNCACGGGGGCAGTNCCCGGCGCGGNNACCTTGNTNACGTCCGGGGNCAAGTATGGCNNNCNCCC
CGNGAAGGANNCNGCCGTANGTGCNNNNNANNNNNTCNTGNGANGTNGCCGNNANNNNCGTGGTCNNN
CNAGAATGNNCGNACCANNCGNANNNTGGTNATGNTAGTNNNNNNNNNNNNNNNNNNNNNNNNNNNNN
NNNNNNNNNNNNNNNNNNNNNNNNNNNNNNNNNNGNNNNGNNNNNNCNANNNAAGGANNTANNNCTNNTN
NTNNNNNNNGGACNNNACNNNNCNNNNNNNNACNCNNNCNCNNNNNNNNNNNGGTTNNNNNCCNNTATTNT
CCAGNANGCNGNGCNNTCATTTANGNGAACNNNGNNACGCGNNAATCGTANNATCTACCTGCCGCNNG

NNNNNNTACNGNNNNTNCGGANNNTNNCTCGTANNNNNNTCATGCGTNNTNTNNTCNCNAGNCANGTT
CGNNNANANANTAGNANGTNTNNNNNNNNNNTGTNNCGTTCCGCNTGANNCNNCANNNNGNNGNNNTTN
TCANCGGCNNTANCTGCNGANNTAAGNCATANNNTNNNTCNNTANCTNNNNNGGNGGGNNACTGTAAA
NGGNNAATTNGNNNNTNANTTCNNNCATNTNNCTNNGNNNNCTNNNTNCCTGNNTNCNNAATTGGNNC
CANTGNNTGNCNATCTCTNGNTTNGNNNNNNTNNNNNTNNCAANNNGGNNNGTNANANNNCNATNTAA
NNTANANNNNCNANNNNANNNCACNANNNNNGNAGNNNNNANNNGTANANNNGTAGNNNGNTAAGTCT
CCGANNTNNNNNNNATAAGCGNNANACNNNNNNNNNNNNNNANGANNNCGNNTTTGNNNTNNNNNNCNA
GNNAGACNNTNGTGGNTNNNNNTCAANNNNTTCGTAGTGGTATAATNGNNNNNNNTTTCNCCAGNNTN
NNNGNNNNNNNNCNATANNANNNNNNNGNGANNNNNNNNNNNANNNNCTCNNNAGGGANCANCTACTNNA
GCANTGGNGCGNAGNNNGAGANNNNGACNNNCCTTGGGTGGCAANCACNGCAATAAGTNNNGTTNNNN
NTNNNAGTNNACACNNNNNCANNGNNNCNNNNNNANCNGANNGNNNNNNNNNNNNNNNNNNNNNNNNNN
NNNNNNNNNNNNNNNNNNNNNNNNNNNNNAGNNNANNGNNNGTNTNTGACNCATGNNTTTTTANNNGNN
NNNNNACGNNNNNNCCCCGGTCGNNNNCNNATTGAANNCCTGNNGCATNNNNNNTNCNAGAATGANNNC
NNNNNGNNTNNNCTNNCNNNTNANGNAATCANNNANNNNNNNNNNNTCNNNNNNNNNNNNCNNNCCCNN
ACTGAGGGTNNNNNNNGCATCTGCTNNNNNNNNNCTNCGCNGNNNNNNCTNCNNNGCCGTATNGNGNNN
NNCANNNNNNNNNNNNNNNNNNNAAACNANNNNNNNTNNNN
>PaThen510
GTGNNNNNTAGCCCNNNNNAGTTAGACANNNNNNNNNNNNNNNNNTNNNNNNNNNNNNNNNNNNNNNNNN
NNNNNNNNNNNNNNNNNNNNNNNNNNNNNNTNNNNNNNNNNNNNNNNNNNNNNNNNNNNNNNNNNNNNNNN
NNNNNNNNNNNNNNNNNNNNNNNNNNNNNNNNNNNNNNNNNNNNNNNNNNNNNNNNNNNNNNNNNNNNNN
NNNNNNNNNNNNNNNNNNNNNNNNNNNNNNNNNNNNNNNNNNNNNNNNNNNNNNNNNNNNNNNNNNNNNN
NNNNNNNNNNNNNNNNNNNNNNNNNNNNNNNNNNNNNNNNNNNNNNNNNNNNNNNNTCTNNNAAANNNTNNN
NNNNNNNNNNNNNNNNNNNNNNNNNNNCNCNNTGNNNGTAANCNANNNGNACATGCAGTCCCCTCNNNNN
NNNGGCAGAGNNNNNNNNCNNNNNNNCCGGCCCCNNCCNNNNNNNNNNNNNNNNGNNNNNNNANNNNNN
NNNNNNTGGTACNNNNCGNNNNNGGTTGTNTNAGAAGTCCACAACGNGGTCNNGNNACNNNNGNNNAN
NNNCNNNNANNNNNNNNNNNNNNANNTNNNTANNAGGNNNNNNCCNTCNNNNNNNNAATTGCNATGACC
CNANNCACGGGGGNAGTNCCCGGCGCGGCGACCTTGATNANGTCCGGGGACAAGTATGGCNNNCNCCC
CGGGAAGGANNCNGCCGTANGTGCNNNNNANNNNNTCTTGNGANGTCGCCGNNATTCNNGTGGTCNNN
NNAGAATGNNCGNACCANNCGNANNNTGGTNATGNNAGTANNNNNNNNNNNNNNNNNNNNNNNNNNNN
NNNNNNNNNNNNNNNNNNNNNNNNNNNNNNNNNGNNNNGNNNNNNCNANNNAAGGANNTANNNCTNNTN
NTNNNNNNGGACNNNACNNNNCNNNNNNNACNCNNNCNCNNNNNNANNNGNTTTTTNNNCNNTATTNT
CCAGNACGCNGNGCNNNCATTCANGAGAACNNNGNNACGCGNNAATCGTANNATCTACCTGCCGCNNG
NNNNNNTNCNGNNNNNNCGGANNNTNNCTCGTANNNNNNTCANGCGTNNTNTNNTCNCNAGNCNNGTT
CGNNNANANANTAGNANGTNTNNNNNNNNNNTGTNNCGTTCCGCNTGANNCNNCANNNNGNNGNNNTTN
TCANCGGCNNTANCTNCNGANNTAAGNCATANNNTNNATCNNTANCTNNNNNGGNGGGNNACTGTAAA
NGGNNAATTNGANNNTNANTNCCNNCATNTNNCTNNGNNTNCTNNNTNCCTGNNTNCNNAATTGGNNC
CANTGNNTGNCNATCTCTNGNTTNGNNNNNNTGNNNNTNNCAANNNGGNNNGTNANANNNCNATNTAA
NNTANANNNNCNNNNNNANNNCACNNNNNNNNNAGNNNCNANNNGTANANNNGTAGNNNNNTAAGTCT
CCGANNTNNNNNNNATAAGCTNNANACNNNNNNNNNNNNNNANGANNACGNNTTTGNNNTNNNNNNCNA
GNNAGACNNTNGTGGNTNNNNNTCAANNNNTTNGTAGTGGTATAATNGNNNNNNNTTTCNCCAGNNTN
NNNGNNNNNNNNCNATANNANNNNNNNGNGANNNNNNNNNNNANNNNCTCNNNAGGGANCANCTACTNNA
GCANTGGNGCGCAGNNNGAGANNNNGACNNNCCTTGGGTGGCAANCACNGCAATAAGTNNNGTTNNNC
CTNNNAGTNNACACNNNNNCANNGNNNCNNNNNNANCNNANNGNNNNNNNNNNNNNNNNNNNNNNNNNN
NNNNNNNNNNNNNNNNNNNNNNNNNNNNNAGNNNANGGNNNGTNTNTGACNCATGTGNNNNCANNNGNN
NNNNNACGNNNNANCCCCGGTCGNNNNCNNATTGAANNCCTGNNGCATNNNNNNTNCNAGAATGANNGC
NNNNNGNNTNGNCTNNCNTTTCAAGNAATCANNNAGNNNNCGCGNTCNNNGNNNNNNNNCNNNCCCNN
ACTGAGGGTNNNNNNCGCATCTGCTNNNNNNNNNNTNCGCNGNCGNNNCNNNNNNGCNGTATNGTGTNN
NNCANNNNNNNNNNNNNNNNNNTNAAACAANNNNNNNTNNNN
>PaThen511
GTGNNNNNNAGCCCNNNNNAGTTGGACANNNNNNNNNNNNNNNNNNNNNNNNNNNNNNNNNNNNNNNNNNNN
NNNNNNNNNNNNNNNNNNNNNNNNNNNNNNNNNNNNNNNNNNNNNNNNNNNNNNNNNNNNNNNNNNNNNN
NNNNNNNNNNNNNNNNNNNNNNNNNNNNNNNNNNNNNNNNNNNNNNNNNNNNNNNNNNNNNNNNNNNNNN
NNNNNNNNNNNNNNNNNNNNNNNNNNNNNNNNNNNNNNNNNNNNNNNNNNNNNNNNNNNNNNNNNNNNNN
NNNNNNNNNNNNNNNNNNNNNNNNNNNNNNNNNNNNNNNNNNNNNNNNNNNNNNNNNCTNNNNAANNNTNNN

NNNNNNNNNNNNNNNNNNNNNNNNNCNCNTTGNNNGTAANCNANNNGNACATGCAGTNNCCTCNNNNN
NNNGGCAGAGNNNNNNCCNNNNNNNNNCCGNCCCCNNCCNNNNNNNNNNNNNNNNNNNNNNNNNGNNNNNN
NNNNNNTGGTACNNNNCGNNNNNGGTTGTNTNAGAGGTNCACCACGNGNTCNNGNNACNNNNGNNNAN
NNNCNNNNAANNNNNNNNNNNNNANNTNNNCANNAGGNNNNNNCCNTCNNNNNNNNNAANNGCNATNANN
CAANNCNCGGNGACAGTNACCGGNGNGGNGACCTTGANNACNGTCGGGGACAAGTATGTCNNNCNCCC
CGNGAAGGANNCNGCCGTANGTGCNNNNNANNNNNTNNTGNGANGTNGCCGNNATNCNCNNGGNCNNN
NNAGANTGNCCGNACCANNCGNANNNNGGTNATGNNAGTNNNNNNNNNNNNNNNNNNNNNNNNNNNNNN
NNNNNNNNNNNNNNNNNNNNNNNNNNNNNNNNNNNNNNNNGNNNNNNCNANNNAAGGNNNTANNNTTNNTN
NNNNNNNNGGACNNNACNNNNCNNNNNNNACNCNNNCNCNNNNNNNNNNGGTTTTTNNNCNNTATTNT
CCAGNACGCNGNGCNNNCATTCANGNGAACNNNGNNACGCGNNAATCGTANNATCTACCTGCCGCNAG
NNNNNNTNCNGNNNNNNCGGANNNTNNCNCGTANNNNNNTCANGCGTNNTNTNNTTNCNAGNNANGTT
CGNNNANNNANTAGNANGTNTCNNNNNNNNNGTNNCGTTCCGCNTGACNCNNCANNNNGNNGNNCTTN
NCANCGGCNNTANCTNCNGANNTAAGNCATANNNTNNNTCNNTANCGNNNNNGGNGGGNNACTGTAAA
NGGNNAATTNGANGNTNANTNCNNNCACNTNNCTNNGNNNNCTNNNCNCCTGNNTNCNNAATTGGNNN
CANTGNNTNNCNATCTCTGGNTTNGNNNNNNTNNNNNTNNTANNNNGGNNNNNNANANNNCNATNTAA
NNTANANNNNCNNNNNNANNNCACNNNNNNNNNAGNNNCNANNNTGANGNNNGTAGNNNNNTNAGTCT
CCGANNTNNNNNNNATAANCGNNANACNNNNNNNNNNNNNNANGANNACGNNTTGGNNNTNNNNNGNNA
ANNAGANNNCNGTGGNTNNNNNTCNANNNNTTNGTAGTGGTATAACNANNNNNNNTTTCNCCAGNNTN
NNNNNNNNNNNCNATANNANNNNNNNGNGANNNNNNNNNNNANNNNCCCNNNAGGNANCANCTATTNNA
GCANTGGNGCGCAGNNNGAGANNNNGACNNNCCTTGGGTGGCAANCACNGCANTAAGTNNTGTTNNNC
CCNNNNGTNNACACNNNNNCANNGNNNCNNNNNNANCNGANNTNNNNNNNNNNNNNNNNNNNNNNNNN
NNNNNNNNNNNNNNNNNNNNNNNNNNNNNNNANNNNANGGNNNGTNTNTGACNCATNNNNNNNTTANNNGNN
NNNNNACGNNNNANCCCCGGTCGNNNNCNNATTGAANNCCTGNNGCATNNNNNNTNCNAGAATGANNNC
NNNNNGNNTNNNCTGNCNNNTNANGNAGTCANNNANNNNNNGCGNTCNNNGNNNNNNNNCNNTCCCNN
ACTGAGGGTNNNNNNNNCATNNGCTNNNNNNNNNNTNCGCNGNCGTNNTNNNNNAGCNGTATNNTGTNN
NNCANNNNNNNNNNNNNNNNNNNNNAAACAANNNNNNNTNNNN
>PaThen512
GTGNNNNNTAGCCCNNNNNAGTTAGACANNNCNNNNNNNNNNNNNTNNNNNNNNNNNNNNNNNNNNNNNN
NNNNNNNNNNNNNNNNNNNNNNNNNNNNNNNNNTNNNNNNNNNNNNNNNNNNNNNNNNNNNNNNNNNNNNN
NNNNNNNNNNNNNNNNNNNNNNNNNNNNNNNNNNNNNNNNNNNNNNNNNNNNNNNNNNNNNNNNNNNNNN
NNNNNNNNNNNNNNNNNNNNNNNNNNNNNNNNNNNNNNNNNNNNNNNNNNNNNNNNNNNNNNNNNNNNNN
NNNNNNNNNNNNNNNNNNNNNNNNNNNNNNNNNNNNNNNNNNNNNNNNNNNNNCNTCTNNNAAANNGTNNN
NNNNNNNNNNNNNNNNNNNNNNNNNCNCNTTGNNNGTAANCNANNNGNACATGCAGTCCCCTCNNNNN
NNNGGCAGAGNNNNNNCCNNNNNNNNNCCGNCCCCGNCCNNNNNNNNNNNNNNNNNNNNNNNNNANNNNNN
NNNNNNTGGTACNNNNCGNNNNNGGTTGTTTAAGAGGTCCACAACGNGGTCNNGNCACNNNNGNNNAN
NNNCNNNNAANNNNNNNNNNNNNAANNTNNNTANNAGGNCNNNNCCNTCNNNNNANTAATNGCNATGANC
CAANNCACGGGGGCNGTNCCCGGNGCGGCGACCTTGNNNANNTCCGGGGACAAGTATGGCNNNCNCCC
CGGGAAGGANNCNGCCGTANGTGCNNNNNANNNNNTCNTGNGANGTCGCCGNNATTCNCNTGGTCNNN
NTAGAATGNCCGNACCANNCGNANNNTGGTNATGNTAGTNNNNNNNNNNNNNNNNNNNNNNNNNNNNNN
NNNNNNNNNNNNNNNNNNNNNNNNNNNNNNNNNNNNGNNNNGNNNNNNCNANNNAAGGANNTANNNCTNNTN
NTNNNNNNNGGACNNNACNNNNCNNNNNNNACNCNNNCNCNNNNNNNNNNNGNTTTTNNNNCNNTATTNT
CCAGNACGCNGNGCNNNCATTCANGNGAACNNNGNNACGCGNNAATCGTANNATCTACCTGCCGCNNG
NNNNNNTACNGNNNNNNCGGANNNTNNCTCGTANNNNNNTCATGCGTNNTNTNNTCNCNAGNCNNGTT
CGNNNANANANTAGNANGTNTNNNNNNNNCTTGTNNCGTTCCGCNTGANNCNNCANNNNGNNGNNCTTN
TCANCGGCNNTANCTGCNGANNTAAGNCATANNNTNNNTCNNTANCTNNNNNGGAGGGNNACTGTAAA
NGGNTAATTGGNNGNTNATTTCNNNCACNTNNCTNNGNNTNCTNNNTNCCTGNNTNCNNAATTGGNNC
CANTGNNTGNCNATCTCTGGNTTNGNNNNNNTNNNNNTNNCAANNNGGNNNGTNANANNNCNATNTAA
NNTATANNNNCNNNNNNANNNCACNNNNNNNNGNAGNNNCNANNNTTANANNNGTAGNNNAATAAGTCT
CCGANNTNNNNNNNATAAGCGNNANACNNNNNNNNNNNNNNANGANNACGNNTTGGNNNTNNNNNNNCNA
GNNAGACNNTNATGGNTNNNNNTCAANNNNTTCGTAGTGGTATAATNGNNNNNNNTTTCNCCAGNNTN
NNNGNNNNNNNNCNATANNAGNNNNNNGNGANNNNNNNNNNNANNNNCTCNNNAGGGANCANCTACTTNA
GCAATGGNGCGCAGNNNGAGANNNNGACNNNCCTTGGGTGGCAANCACNGCAATAAGTNNNGTTNNNC
CTNNNAGTNNACATGNNNNCANNGNNNCNNNNNNANCNGANNGNNNNNNNNNNNNNNNNNNNNNNNNN
NNNNNNNNNNNNNNNNNNNNNNNNNNNNNNANNNNANGGNNNGTNTNTGACNCATGNGTTTTTANNNGNN

NNNNNACGNNNNANCCCCGGTCGNNNNCNNATTGAANNCCTGNNGCATNNNNNTNCNAGAATGANNGC
NNNNNGNNTNGNCTNNCNNTTCAAGNAATCAANNAGGNATNNNGNTCNNNGNNNNNNNNCNNTCCCNN
ACTGAGGGTNNNNNNNNCATCTGCTNNNNNNNNNNTNCGCNGGNGTNNCNNNNNAGCNGTATNGTGTNA
NNCANNNNNNNNNNNNNNNNNNTNAAACAANNNNNNNTNNNN
>PaThen513
GTGNNNNNTAGCCCNNNNNAGTTAGACANNNCNNNNNNNNNNNNNNNNNNNNNNNNNNNNNNNNNNNNNNN
NNNNNNNNNNNNNNNNNNNNNNNNNNNNNNNNNNNNNNNNNNNNNNNNNNNNNNNNNNNNNNNNNNNNNN
NNNNNNNNNNNNNNNNNNNNNNNNNNNNNNNNNNNNNNNNNNNNNNNNNNNNNNNNNNNNNNNNNNNNNN
NNNNNNNNNNNNNNNNNNNNNNNNNNNNNNNNNNNNNNNNNNNNNNNNNNNNNNNNNNNNNNNNNNNNNN
NNNNNNNNNNNNNNNNNNNNNNNNNNNNNNNNNNNNNNNNNNNNNNNNNNNNNNTCTNNNAAANNNTNNN
NNNNNNNNNNNNNNNNNNNNNNNNNNNCNCNTTGNNNGTAANCNANNNGNACATGCAGTNNNCNCNNNNN
NNNNNCAGAGNNNNNNNNCNNNNNNNCCGNCCCCNNCCNNNNNNNNNNNCNNNNNNNNNNNNNANNNNNN
NNNNNNTGGTACNNNNCGNNNNNGGTTGNNTNAGAAGTCCACAACGNGGTCNNGNCACNNNNGNNNAN
NNNCNNNNNNNNNNNNNNNNNNNNNNTNNNTANNAGGNNNNNNCCNTNNNNNNNNNNNATNGCNATGACC
CAANNNACGGNGGNAGTNCCCGGCGCGGCGACCTTGANNANNTCCGGGGACAAGTATGGCNCNCNCCC
CGGGAAGGANNCNGCCGTANGTGCNNNNNANNNNNTCNTGNGANGTNGCCGNNATTCNCNTGGTCNNN
NNAGAATGNNCGNACCANNCGNANNNTGGTNATGNNAGTNNNNNNNNNNNNNNNNNNNNNNNNNNNNNNN
NNNNNNNNNNNNNNNNNNNNNNNNNNNNNNNNNNNNNNNGNNNNNNCNANNNAAGGANNTANNNCTNNTN
NTNNNNNNGGACNNNACNNNNCNNNNNNNACNCNNNCNCNNNNNNNNNNGGTTTTNNNCCNNTATTNT
CCAGNACGCNGNGCNNNCATTCANGNGAACNNNGNNACGCGNNAATCGTANNATCTACCTGCCGCNNG
NNNNNNTNCNGNNNNNNCGGANNNTNNCTCGTANNNNNNNCATGCGTNNTNTNNTCNCTAGNCNNGTT
CGNNNANANANTAGNANGTNTNNNNNNNCNTGTNNCGTTCCGCNTGANNCNNCANNNNGNCGNNCTTN
TCANCGGCNNTANCTGCNGANNTAAGNCATANNNTNNATCNNTANCTNNNNNGGAGGGNNACTGTAAA
NGGNNAATTNGNNNNTNATTNCNNNCATNTNNCTNNGNNNNCTNNNTNCCTGNNTNCNNAATTGGNNC
CANTGNNTGNCNATCTCTNGNTTNGNNNNNNTGNNNNTNNCAANNNGGNNNNNNANANNNCNATNTAA
NNTATANNNNCNNNNNNANNNCACNANNNNNNNAGNNNNNANNNGTANANNNGTAGNNNNNTAAGTCT
CCGANNTNNNNNNNATAAGCTNNANACNNNNNNNNNNNNNNANGANNACGNNTTTGNNNTNNNNNNCNA
GNNAGACNNTNGTGGNTNNNNNTCAANNNNTTCGTAGTGGTATAATNGNNNNNNNTTTCNCCAGNNNN
NNNGNNNNNNNNCNATANNANNNNNNNGNGANNNNNNNNNNNANNNNCTCNNNAGGGANCANCTACTTNA
GCANTGGNGCGNAGNNNGAGANNNNGACCANCCTTGGGTGGCAANCACNGCANTAAGTNNNGTTNNNC
CTNNNAGTNNACACNNNNNCANNGNNNCNNNNNNANCNGANNGNNNNNNNNNNNNNNNNNNNNNNNNNNN
NNNNNNNNNNNNNNNNNNNNNNNNNNNNNNANNNNANNGNNNGTNTNTGACNCATGTNTTTTCANNNGNN
NNNNNACGNNNNANCCCCGGTCGNNNNCNNATTGAANNCCTGNNGCATNNNNNTNCNAGAATGANNGC
NNNNNGNNTNGNCTGNCNNNTCAAGNAATCANNNAGGTNTCGCGNTCNNNGNNNNNNNNCNNTCCCNN
ACTGAGGGTNNNNNNNNCATCNGCTNNNNNNNNNNTNCGCNGNCGTNNCNNCNNAGCNGTATNNTGTNN
NNCANNNNNNNNNNNNNNNNNNCNAANNANNNNNNNNTNANN
>PaThen514
GTGNNNNNTAGCCCNNNNNAGTCAGACANNNCNNNNNNNNNNNNNNNNNNNNNNNNNNNNNNNNNNNNNNN
NNNNNNNNNNNNNNNNNNNNNNNNNNNNNNNNNNNNNNNNNNNNNNNNNNNNNNNNNNNNNNNNNNNNNN
NNNNNNNNNNNNNNNNNNNNNNNNNNNNNNNNNNNNNNNNNNNNNNNNNNNNNNNNNNNNNNNNNNNNNN
NNNNNNNNNNNNNNNNNNNNNNNNNNNNNNNNNNNNNNNNNNNNNNNNNNNNNNNNNNNNNNNNNNNNNN
NNNNNNNNNNNNNNNNNNNNNNNNNNNNNNNNNNNNNNNNNNNNNNNNNNNNNNTCTNNNAAANNNTNNN
NNNNNNNNNNNNNNNNNNNNNNNNNNNNNCNTTGNNNGTAANCNANNNGNACATGCAGTCCCCTCNNNNN
NNNNNCAGAGNNNNNNNNCNNNNNNNNCCGNCCCCNNCCNNNNNNNNNNNNNNNNNNNNNNNNANNNNNN
NNNNNNTGGTACNNNNCGNNNNNGGNTGNNTAAGAGGTCCACAACGNGGTCNNNNNACNNNNGNNNAN
NNNCNNNNNACNNNNNNNNNNNNNNNNTNNNTANNAGGNNNNNNCCNTCNNGNNNNNAANNGCNATGANN
CNANNCNCGGGGGCNGTNCCCGGNGNGGNNACCTTNNNNANNTCCGGGGNCAAGTATNGCNNNCNCCC
CGNGAAGGANNCNGCNGTANGTGCNNNNNANNNNNTNNTGNGANGTNGCCGNNNTTCNNGTGGTCNNN
CNAGAATGNCCNNACCANNCGNANNNTGGTNATGNNAGTNNNNNNNNNNNNNNNNNNNNNNNNNNNNNNN
NNNNNNNNNNNNNNNNNNNNNNNNNNNNNNNNNNNNNNNGNNNNNNCNANNNAAGGANNTANNNCTNNTN
NNNNNNNNGGACNNNNCNNNNCNNNNNNNACNCNNNCNCNNNNNNNNNNGNTTNNNNNCCNNTANTNN
CCAGNACGCNGNGCNNNCATTTANGNGAACNNNGNNACGCGNNAATCGTANNATCTACCTGCNGCNNG
NNNNNNTNCNGNNNNNNCGGANNNTNNCTCGTANNNNNNNCATGCGTNNTNTNNTCNCNAGNCNNGTT
CGNNNANNNANTAGNANGTNTNNNNNNNNNNTGTNNCGTTCCGCNTGANNCNNCANNNNNNNGNNNTTN

TCANCGGCNNTANNTNCNGANNTAAGNCATANNNTNNNTCNNTANCTNNNNNGGNGGGNNACTGTAAA
NGGNNAATTNGNNNNTNANTNCNNNCATNTNNCTNNGNNTNCTNNNTNCCTGNNTNCNNAATTGGNNC
CANTGNNTGNCNATCTCTNGNTTNGNNNNNNTNNNNNTNNCANNNNGGNNNGTNANANNNCNATNTAA
NNTANANNNNCNNNNNNANNNCACNNNNNNNNNAGNNNNNANNNGTANANNNGTAGNNNGNTAAGTCT
CCGANNTNNNNNNNATAAGCGNNANACNNNNNNNNNNNNNNANGANNACGNNTTTGNNNTNNNNNNCNA
GNNAGACNNTNGTGGNTNNNNNTCAANNNNTTNNTAGTGGTATAATNGNNNNNNNTTTCNCCAGNNTN
NNNGNNNNNNNCNATANNANNNNNNNGNGANNNNNNNNNNNANNNNCTCNNNAGGNANCANCTACTNNA
GNAATGGNNCGCAGNNNGAGANNNNGACNNNCCTTGGGTGGCAANCACNGCAATAAGTNNNGTTNANC
CTNNNNGTNNACACNNNNNCANNGNNNCNNNNNNANCNGANNGNNNNNNNNNNNNNNNNNNNNNNNNN
NNNNNNNNNNNNNNNNNNNNNNNNNNNNNNAGNNNANNGNNNGTNTNTGACNCATGTGTTTTTANNNGNN
NNNNNACGNNNNANCCCCGGTCGNNNNCNNATTGAANNCCTGNNGCATNNNNNTNCNAGAATGANNNC
NNNNNGNNTNNNCTGNCNNNTCANGNAATCAANNANNNNNNGCGNTCNNNNNNNNNNNNNCNNNCCCNN
ACTGAGGGTNNNNNNNNCATCTGCTNNNNNNNNNNTNCGCNGNNNNNNCNNNNNNGCNGTATNGTGNNN
NNCANNNNNNNNNNNNNNNNNNNNNANNNNNNNNNNNTNNNN
>PaThen515
GTGNNNNNTAGCCCNNNNNAGTCAGACANNNCNNNNNNNNNNNNNTNNNNNNNNNNNNNNNNNNNNNNN
NNNNNNNNNNNNNNNNNNNNNNNNNNNNNNNNNNNNNNNNNNNNNNNNNNNNNNNNNNNNNNNNNNNNNN
NNNNNNNNNNNNNNNNNNNNNNNNNNNNNNNNNNNNNNNNNNNNNNNNNNNNNNNNNNNNNNNNNNNNNN
NNNNNNNNNNNNNNNNNNNNNNNNNNNNNNNNNNNNNNNNNNNNNNNNNNNNNNNNNNNNNNNNNNNNNN
NNNNNNNNNNNNNNNNNNNNNNNNNNNNNNNNNNNNNNNNNNNNNNNNNNNNNCNTCTNNNAAANNNTNNN
NNNNNNNNNNNNNNNNNNNNNNNNNNNNCNCNTTGNNNGTAANCNANNNGNACATGCAGTCCCCTCNNNNN
NNNGGCAGAGNNNNNNCCNNNNNNNNNCCGGCCCCNNCCNNNNNNNNNNNNCNNNGNNNNNNNANNNNNN
NNNNNNTGGTACNNNNCGNNNNNGGTTGTNTAAGAGGTCCACAACGNGGTCNGGNCACNNNNGNNNAN
NNNCNNAAAANNNNNNNNNNNNNNANNTNNNTANNAGGNCNNNNCCNTCNNGNNNNNAANNGCNATGACC
CNANTCACGGGGGCAGTCCCCGGCGNGGCGACCTTGNTNANGTCCGGGGACAAGTATGGCGCNCNCCC
CGNGAAGGANNCNGCCGTANGTGCNNNNNANNNNNTCNTGNGANGTCGCCGNNATTCNCNTGGTCNNN
CNAGAATGNCCNNACCANNCGNANNNTGGTNATGTNAGTNNNNNNNNNNNNNNNNNNNNNNNNNNNNN
NNNNNNNNNNNNNNNNNNNCNNNNNNNNNNNNNNNNNNNNNGNNNNNNCNANNNAAGGANNTANNNCTNNTN
NTNNNNNNNGGACNNNACNNNNCNNNNNNNNACNCNNNCNCNNNNNNNNNNNGGTTTTTNNNCNNTATTNT
CCAGNACGCNGNGCNNNCATTTANGNGAACNNNGNNACGCGNNAATCGTANNATCTACCTGCCGCNNG
TNNNNNTNCNGNNNNTNCGGANNNTNNNNCGTANNNNNNNTCATGCGTNNTNTNNTCNCNAGNCANGTT
CGNNNANANANTAGNANGTNTNNNNNNNCTTGTNNCGTTCCGCNTGANNCNNCANNNNGNNGNNCTTC
TCANCGGCNNTANCTGCNGANNTAAGNCATANNNTNNNTCNNTANCTNNNNNGGNGGGNNACTGTAAA
NGGNNAATTNGANGNTNATTNCCNNCATNTNNCTNNGNNNNCTNNNTNCCTGNNTNCNNAATTGGNNC
CANTGNNTGNCNATCTCTNGNTTNGNNNNNNTNNNNNTNNCAANNNGGNNNGTNANANCNCNATNTAA
NNTATANNNNCNNNNNNANNNCACNANNNNNGNAGNNNCNANNNGTANANNNGTAGNNNGATAAGTCT
CCGANNTNNNNNNNATAAGCGNNANACNNNNNNNNNNNNNNANGANNACGNNTTTGNNNTNNNNNNCNA
GNNAGACNNTNGTGGNTNNNNNTCAANNNNTTCGTAGTGGTATAATNGNNNNNNNTTTCNCCAGNNTN
NNNGNNNNNNNCNATANNANNNNNNNGTGANNNNNNNNNNNANNNNCTCNNNAGGGANCANCTACTTNA
GCAATGGNGCGCAGNNNGAGANNNNGACNNGCCTTGGGTGGCAANCACNGCAATAAGTNNNGTTNANC
CTNNNAGTNNACACNNNNNCANNGNNNCNNNNNNANCNGANNGNNNNNNNNNNNNNNNNNNNNNNNNN
NNNNNNNNNNNNNNNNNNNNNNNNNNNNNNAGNNNANGGNNNGTNTNTGACNCATGTGTTTTTANNNGNN
NNNNNACGNNNGANCCCCGGTCGNNNNCNNATTGAANNCCTGNNGCATNNNNNTNCNAGAATGANNGC
NNNNNGNNTNGNCTGNCNNNTCAAGNAATCANNNAGNNNNNGCGNTCNNNNNNNNNNNNNCNNTCCCNN
ACTGAGGGTNNNNNNCNCATCTGCTNNNNNNNNNNTNCGCNGGNGTNNCNNNNNAGCNGTATNNTGTNN
NNCANNNNNNNNNNNNNNNNNNNNNAACGANNNNNNNTNNNN
>PaThen516
GTGNNNNNTAGCCCNNNNNAGTTAGACANNNCNNNNNNNNNNNNNTNNNNNNNNNNNNNNNNNNNNNNN
NNNNNNNNNNNNNNNNNNNNNNNNNNNNNNNNNNNNNNNNNNNNNNCNNNNNNNNNNNNNNNNNNNNNNNN
NNNNNNNNNNNNNNNNNNNNNNNNNNNNNNNNNNNNNNNNNNNNNNNNNNNNNNNNNNNNNNNNNNNNNN
NNNNNNNNNNNNNNNNNNNNNNNNNNNNNNNNNNNNNNNNNNNNNNNNNNNNNNNNNNNNNNNNNNNNNN
NNNNNNNNNNNNNNNNNNNNNNNNNNNNNNNNNNNNNNNNNNNNNNNNNNNNNCNTCTNNNAAANNNTNNN
NNNNNNNNNNNNNNNNNNNNNNNNNNNNCNCNTTGNNNGTAANCNANNNGNACATGCAGTCCCCTCNNNNN
NNNNGCAGAGNNNNNNNCNNNNNNCNCCGGCCCCNNCCNNNNNNNNNNNNCNNNNNNNNNNNANNNNNN

NNNNNNTGGTACNNNNCGNNNNNGGTTGTTTAAGAGGTCCACAACANGGTCNNGNCACNNNNGNNNAN
NNNCNNNNANNNGNNNNNNNNNNANNTNNNTANNAGGNNNNNNCCNTNNNGNNNNNNANNGCNATGACC
CAANTCACGGGGGCNGTNCCCGGNGNGGCGACCTTGNTNACGTCCGGGGACAAGTATGGCNNNCNCCC
CGGGAAGGANNCNGCCGTANGTGCNNNNNANNNNNTCNTGNGANGTCGCCGNNATTCNCGTGGTCNNN
CNAGAATGNNCGNACCANNCGNANNNTGGTNATGNNAGTNNNNNNNNNNNNNNNNNNNNNNNNNNNNN
NNNNNNNNNNNNNNNNNNNNNNNNNNNNNNNNNNNNGNNNNGNNNNNNCNANNNAAGGNNNTANNNCTNNTN
NTNNNNNNGGACNNNACNNNNCNNNNNNNACNCNNNCNCNNNNNNANNNGNTTTTNNNNCNNTATTNT
CCAGNACGCNGNGCNNNCATTCANGNGAACNNNGNNACGCGNNAATCGTANNATCTACCTGCCGCNNG
TNNNNNTACNGNNNNNNCGGANNNTNNNNCGTANNNNNNTCATGCGTNNTNTNNTCNCNAGNCNNGTT
CGNNNANANANTAGNANGTNTNNNNNNNNNNTGTNNCGTTCCGCNCGANNCNNCANNNNGNNGNNCTTN
NCANCGGCNNTANCTGCNGANNTAAGNCATANNNTNNNTCNNTANCTNNNNNGGNGGGNNACTGTAAA
NGGNNAATTNGNNNNTNATTTCNNNCACNTNNCTNNANNNNCTNNNTNCCTGNNTNCNNAATTGGNNC
CANTGNNTGNCNATCTCTGGNTTNGNNNNNNTGNNNNTNNCANNNNGGNNNGTNANANNNCNATNTAA
NNTANANNNNCNNNNNNANNNCACNANNNNNNNNAGNNACNANNNTTANANNNGTAGNNNAATAAGTCT
CCGANNTNNNNNNNATAAGCGNNANACNNNNNNNNNNNNNNANGANNACGNNTTGGNNNTNNNNNNCNA
GNNAGACNNTNGTGGNTNNNNNTCAANNNNTTCGTAGTGGTATAATNGNNNNNNNTTTCNCCAGNNTN
NNNGNNNNNNNNCNATANNANNNNNNNGNGANNNNNNNNNNNANNNNCTTNNNAGGNANCANTTACTNNA
GCAATGGNGCGCAGNNNGAGANNNNGACNNNCCTTGGGTGGCAANCACNGCAATAAGTNNGTTNANN
CTNNNAGTNNACACNNNNNCANNGNNNCNNNNNNANCNGANNGNNNNNNNNNNNNNNNNNNNNNNNNN
NNNNNNNNNNNNNNNNNNNNNNNNNNNNNNANNNNANNGNNNGTNTNTGACNCATGTGTTTTTANNNGNN
NNNNNACGNNNGANCCCCGGTCGNNNNCNNATTGAANNCCTGNNGCATNNNNNTNCNAGAATGANNGC
NNNNNGNNTNGNCTGNCNNNTCAAGNAATCANNNAGNNNNNNCGNTCNNNNNNNNNNNNCNNTCCCNN
ACTGAGGGTNNNNNNNNCATCTGCTNNNNNNNNCTTCGCNGGCGTNNCNNNNNAGCNGTATNGTGNNN
NNCANNNNNNNNNNNNNNNNNTNAAACAANNNNNNNTNNNN
>PaThen517
GTGNNNNNTNGCCCNNNNNAGTCAGACANNNCNNNNNNNNNNNNNNNNNNNNNNNNNNNNNNNNNNNNN
NNNNNNNNNNNNNNNNNNNNNNNNNNNNNNNNNNNNNNNNNNNNNNNNNNNNNNNNNNNNNNNNNNNN
NNNNNNNNNNNNNNNNNNNNNNNNNNNNNNNNNNNNNNNNNNNNNNNNNNNNNNNNNNNNNNNNNNNN
NNNNNNNNNNNNNNNNNNNNNNNNNNNNNNNNNNNNNNNNNNNNNNNNNNNNNNNNNNNNNNNNNNNN
NNNNNNNNNNNNNNNNNNNNNNNNNNNNNNNNNNNNNNNNNNNNNNNNNNNNNNCNTCTNNNAAANNNTNNN
NNNNNNNNNNNNNNNNNNNNNNNNNNNCNCNTTGNNNGTAANCNANNNGNACATGCAGTCCCCTCNNNNN
NNNGGCAGAGNNNNNNCCCNNNNNNNCCGGCCCCNNCCNNNNNNNNNNNNNNNNNGNNNNNNNANNNNNN
NNNNNNTGGTACNNNNCGNNNNNGGTTGTNNAAGAGGTCCACAACGNGGTCNNGNCACNNNNGNNNAN
NNNCNNNNAANNNNNNNNNNNNNNNNNTNNNTANNAGGNNNNNNCCNTCNNGNNNNNNANNGCNATGACC
CAANNNACGGGGGNNGTNCCCGGCGNGGCGACCTTGNNNACNTCCGGGGNCAAGTATGGCNNNCNCCC
CGGGAAGGANNCNGCCGTANGTGCNNNNNANNNNNTCNTGNGANGTNGCCGNNATTNNNGTGGTCNNN
NNAGANTGNCCGNACCANNCGNANNNTGGTNATGNNAGTNNNNNNNNNNNNNNNNNNNNNNNNNNNNN
NNNNNNNNNNNNNNNNNNNNNNNNNNNNNNNNNNNNGNNNNGNNNNNNCNANNNAAGGANNTANNNCTNNTN
NTNNNNNNGGACNNNNCNNNNNNNNNNNNNNACNCNNNCNCNNNNNNANNNGNTTNNNNNNNCNNTATTNT
CCAGNACGCNGNGCNNNCATTCANGNGAACNNNGNNACGCGNNAATCGTANNATCTACCTGCCGCNNG
NNNNNNTACNGNNNNNNCGGANNNTNNCTCGTANNNNNNNNCATGCGTNNTNTNNTCNCNAGNNNNGTT
CGANNANANANTAGNANGTNTNNNNNNNNNNTGTNNCGTTCCGCNTGANNCNNCANNNNGNNGNNNTTN
NCANCGGCNNTANCTGCNGANNTAAGNCATANNNTNNNTCNNTANCTNNNNNGGNGGGNNACTGTAAA
NGGNNAATTNGNNNNTNATTNCNNNCATNTNNCNNNGNNNNCTNNNTNCCTGNNTNCNNAATTGGNNC
CANTGNNTGNCNATCTCTNGNTTNGNNNNNNTNNNNNTNNCAANNNGGNNNGTNNNANNNCNATNTAA
NNTANANNNNCNNNNNNANNNCACNNNNNNNNNNAGNNNCNANNNGTANANNNGTAGNNNGATAAGTCT
CCGANNTNNNNNNNATAAGCGNNANACNNNNNNNNNNNNNNANGANNACGNNTTTGNNNTNNNNNNCNA
GNNANACNNTNGTGGNTNNNNNTCAANNNNTTNGTAGTGGTATAATNGNNNNNNNTTTCNCCAGNNTN
NNNGNNNNNNNNCNATANNANNNNNNNGNGANNNNNNNNNNNNNNNNNCTCNNNAGGGANCANCTACTNNA
GCANTGGNGCGNAGNNNGAGANNNNGACNNNCCTTGGGTGGCAANNACNGCAATAAGTNNNGTTNNNN
NTNNNAGTNNACACNNNNNCANNGNNNCNNNNNNANCNGANNGNNNNNNNNNNNNNNNNNNNNNNNNN
NNNNNNNNNNNNNNNNNNNNNNNNNNNNNNAGNNNANGGNNNGTNTNTGACNCATGTNTTTNTANNNGNN
NNNNNACGNNNNANCCCCGGTCGNNNNCNNATTGAANNCCTGNNGCATNNNNNTNCNAGAATGANNNC
NNNNNGNNTNGNCTNNNNNTTTCAAGNAATCANNNAGGTNNCGCGNTCNNNGNNNNNNNNCNNTCCCNN

ACTGAGGGTNNNNNNNGCATCTGCTNNNNNNNNNTTCGCNGGNGTNNCNNNNNNGCNGTATNGTGTNN
NNCANNNNNNNNNNNNANNNNTNAAACNANNNNNNTNNNN
>PaThen518
GTGNNNNNTAGCTCNNNNNAGTTAGACANNNCNNNNNNNNNNNNTNNNNNNNNNNNNNNNNNNNNNN
NNNNNNNNNNNNNNNNNNNNNNNNNNNNNNTNNNNNNNNNNNNNNNNNNNNNNNNNNNNNNNNNNN
NNNNNNNNNNNNNNNNNNNNNNNNNNNNNNNNNNNNNNNNNNNNNNNNNNNNNNNNNNNNNNNNNN
NNNNNNNNNNNNNNNNNNNNNNNNNNNNNNNNNNNNNNNNNNNNNNNNNNNNNNNNNNNNNNNNNN
NNNNNNNNNNNNNNNNNNNNNNNNNNNNNNNNNNNNNNNNNNNNNNNNNTCTNNNAAANNNTNNN
NNNNNNNNNNNNNNNNNNNNNNNNNNCNCNTTGNNNGTAANCNANNNGNACATGCAGTCCCCTCNNNNN
NNNGGCAGAGNNNNNNNCCNNNNNCNCCGNCCCCGNCCNNNNNNNNNNNNNNNNNNNNNNNNGNNNNN
NNNNNNTGGTACNNNNCGNNNNNGGTTGTTTNAGAGGTCCACCACGNGGTCNGGNCACNNNNGNNNAN
NNNCNNAAANCNNNNNNNNNNNNANATNNNCANNAGGNNNNNACCNTCNNNNNNNNAATNGCNATGACC
CAANNNGCGGGGANNGTNACCGGNGCGGCGACCTTGATNANNGTCGGGGACAAGTATGTCNNNCNCCC
CGGGAAGGANNCNGCCGTANGTGCNNNNNANNNNNTCNTGNGANGTNGCCGNNATTCNNGTGGTCNNN
NNAGAATGNCCGNACCANNCGNANNNTGGTNATGNTAGTNNNNNNNNNNNNNNNNNNNNNNNNNNNNN
NNNNNNNNNNNNNNNNNNNNNNNNNNNNNNNNNTNNNNGNNNNNNCNANNNAAGGANNTANNNTTNNTN
NTNNNNNNNGGACNNNACNNNNCNNNNNNNACNCNNNCNCNNNNNNNNNNGNTTTTTNNNCNNTATTNT
CCAGNACGCNGNGCNNNCATTCANGNGAACNNNGNNACGCGNNAATCGTANNATCTACCTGCCGCNNG
TNNNNNTACNGNNNNNNCGGANNNTNNCTCGTANNNNNNNCATGCGTNNTNTNNTTNCNAGNCNNGTT
CGAGAANANANTAGNANGTNTNNNNNNNNNNTGTNNCGTTCCGCNTGANNCNNCANNNNGNNGNNNTTC
TCANCGGCNNTANCTGCNGANNTAAGNCATANNNTNNGTCNNTANCGNNNNNGGNGGGNNACTGTAAA
NGGNNAATTGGNNNNTNATTNCNNNCACNTNNCTNNGNNNNCTNNNCNCCTGNNTNCNNAATTGGGNC
CANTGNNTGNCNATCTCTNGNTTNGNNNNNNTNNNNNTNGTANNNNGGNNNGNNANANCNCNATNTAA
NNTANANNNNCNNNNNNANNNCACNNNNNNNGNAGNNNCNANNNTTANGNNNGTAGNNNAATAAGTCN
CCGANNTNNNNNNNATAAGCGNNANACNNNNNNNNNNNNNANGANNACGNNTTGGNNNTNNNNNNCNA
ANNAGACNNCNGTGGNTNNNNNTCAANNNNTTNGTAGTGGTATAACNANNNNNNNTTTCNCCAGNNTN
NNNGNNNNNNNCNATANNANNNNNNNGTGANNNNNNNNNNANNNNCCCNNNAGGGANCANCTACTNNA
GCANTGGNGCGCAGNNNGAGANNNNGACNNGCCTTGGGTGGCAANCACNGCAATAAGTNNNGTTNANC
CCNNNAGTNNACACGNNNNCANNGANNCNNNNNNANCNGANNTNNNNNNNNNNNNNNNNNNNNNNNNN
NNNNNNNNNNNNNNNNNNNNNNNNNNNNNNNAGNNNANGGNNNGTNTNTGACNCATGTGTTTTTANNNGNN
NNNNNACGNNNNANCCCCGGTCGNNNNCNNATTGAANNCCTGNNGCATNNNNNTNCNAGAATGANNNC
NNNNNGNNTNGNCTNNCNTTTCAAGNAGTCAANNAGNNNNCGCGNTCNNNGNNNNNNNNCNNNCCCNN
ACTGAGGGTNNNNNNNNCATCTGCTNNNNNNNNNCTNCGCNGNNNNNNTTGNNNNGCNGTATNGTGTNN
NNCANNNNNNNNNNNNNNNNNTNAAACAANNNNCCTNNNN
>PaThen519
GTGNNNNNTAGCCCNNNNNAGTTAGACANNNCNNNNNNNNNNNNTNNNNNNNNNNNNNNNNNNNNNN
NNNNNNNNNNNNNNNNNNNNNNNNNNNNNNNNNNNNNNNNNNNNNNNNNNNNNNNNNNNNNNNNNNNN
NNNNNNNNNNNNNNNNNNNNNNNNNNNNNNNNNNNNNNNNNNNNNNNNNNNNNNNNNNNNNNNNNNNN
NNNNNNNNNNNNNNNNNNNNNNNNNNNNNNNNNNNNNNNNNNNNNNNNNNNNNNNNNNNNNNNNNNNN
NNNNNNNNNNNNNNNNNNNNNNNNNNNNNNNNNNNNNNNNNNNNNNNNNCNTCTNNNAAANNNTNNN
NNNNNNNNNNNNNNNNNNNNNNNNNNCNCNTTGNNNGTAANCNANNNGNACATGCAGTCCCCTCNNNNN
NNNGGCAGAGNNNNNNCNCNNNNNCNCCGGCCCCNNCCNNNNNNNNNNNNCNNNGNNNNNNNANNNNNN
NNNNNNTGGTACNNNNCGNNNNNGGTTGTTTAAGAGGTCCACAACGNGGTCNGGNCACNNNNGNNNAN
NNNCNNNNNNNNNNNNNNNNNNNANNTNNNTANNAGGNCNNNNCCNTNNNNNNNNNNNATTGCNATGACC
CANNNNACNGGGGCNGTNCCCGGCGCGGCGACCTTGANNANNTCCGGGGACAAGTATGGCNNNCNCCC
CGGGAAGGANNCNGCCGTANGTGCNNNNNANNNNNTCNTGNGANGTCGCCGNNATTNNCGTGGTCNNN
NNAGAATGNNCGNACCANNCGNANNNTGGTNATGNTAGTNNNNNNNNNNNNNNNNNNNNNNNNNNNNN
NNNNNNNNNNNNNNNNNNNNNNNNNNNNNNNNNNNNNNGNNNNNNCNANNNAAGGNNNTANNNCTNNTN
NTNNNNNNNGGACNNNACNNNNCNNNNNNNACNCNNNCNCNNNNNNNNNNGGTTNNNNNNCNNTATTNT
CCAGNACGCNGNGCNNNCATTCANGNGAACNNNGNNACGCGNNAATCGTANNATCTACCTGCCGCNNG
TNNNNNTACNGNNNNNNCGGANNNTNNCTCGTANNNNNNTCATGCGTNNTNTNNTCNCNAGNCANGTT
CGNNNANANANTAGNANGTNTNNNNNNNNNNTGTNNCGTTCCGCNTGANNCNNCANNNNGNNGNNCTTC
TCANCGGCNNTANCTGCNGANNTAAGTCATANNNTNNATCNNTANCTNNNNNGGNGGGNNACTGTAAA
NGGNNAATTNGNNGNTNATTTCNNNCACNTNNCTNNGNNTNCTNNNTNCCTGNNTNCNNAATTGGNNC

CANTGNNTGNCNATCTCTNGNTTNGNNNNNNTGNNNNTNNCAANNNGGNNNGTNNNANNNCNATNTAA
NNTANANNNNCNNNNNNANNNCACNANNNNNNNAGNNNCNANNNTTANANNNGTAGNNNAATAAGTCT
CCGANNTNNNNNNNATAAGCGNNANACNNNNNNNNNNNNNANGANNACGNNTTGGNNNTNNNNNNNNA
GNNAGACNNTNATGGNTNNNNNTCAANNNNTTCGTAGTGGTATAATNGNNNNNNNTTTCNCCAGNNTN
NNNGNNNNNNNCNATANNANNNNNNNGNGANNNNNNNNNNNANNNNCTCNNNAGGGANCANCTACTTNA
GCANTGGNGCGCAGNNNGAGANNNNGACNNNCCTTGGGTGGCAANCACNGCAATAAGTNNNGTTNANC
CTNNNAGTNNACATNNNNNCANNGNNNCNNNNNNANCNGANNGNNNNNNNNNNNNNNNNNNNNNNNNN
NNNNNNNNNNNNNNNNNNNNNNNNNNNNNANNNNANGGNNNGTNTNTGACNCATGTGTTTTTANNNGNN
NNNNNACGNNNNANCCCCGGTCGNNTNCNNATTGAANNCCTGNNGCATNNNNNTNCNAGAATGANNNC
NNNNNGNNTNGNCTNNCTTTTCAAGNAATCAANNAGNNNNCGCGNTCNNNNNNNNNNNNCNNTCCCNN
ACTGAGGGTNNNNNNNNCATCTGCTNNNNNNNNNNTTCGCNGNNNTNNCNNNNNNGCNGTATNGTGTNA
NNCANNNNNNNNNNNNNNNNNCNNAACNNNNNNNNNTNNNN
>PaThen520
GTGNNNNNTAGCCCNNNNNAGTCAGACANNNCNNNNNNNNNNNNTNNNNNNNNNNNNNNNNNNNNNNN
NNNNNNNNNNNNNNNNNNNNNNNNNNNNNNNNNNNNNNNNNNNNNNNNNNNNNNNNNNNNNNNNNNNN
NNNNNNNNNNNNNNNNNNNNNNNNNNNNNNNNNNNNNNNNNNNNNNNNNNNNNNNNNNNNNNNNNNNN
NNNNNNNNNNNNNNNNNNNNNNNNNNNNNNNNNNNNNNNNNNNNNNNNNNNNNNNNNNNNNNNNNNNN
NNNNNNNNNNNNNNNNNNNNNNNNNNNNNNNNNNNNNNNNNNNNNNNNNNNNNNTCTNNNAAANNNTNNN
NNNNNNNNNNNNNNNNNNNNNNNNNNCNCNTTGNNNGTAANCNANNNGNACATGCAGTCCCCTCNNNNN
NNNNNCAGAGNNNNNNCCCNNNNNNNCCGGCCCCGNCCNNNNNNNNNNNNNNNNNNNNNNNNNANNNNNN
NNNNNNTGGTACNNNNCGNNNNNGGTTGNTTAAGAGGTCCACAACGNGGTCNNGNCACNNNNGNNNAN
NNNCNNAAAANNNNNNNNNNNNNANATNNNTANNAGGNCNNNNCCNTCNNGNNANNAATTGCNATGACC
CAANNNNCGGNGGNNGTNCCCGGCGNGGCGACCTTGATNACGTCCGGGGACAAGTATGGCGNNCNCCC
CGGGAAGGANNCNGCCGTANGTGCNNNNNANNNNNTCNTGNGANGTNGCCGNNATTCNCNTGGTCNNN
CNAGAATGNNCGNACCANNCGNANNNTGGTNATGTNAGTNNNNNNNNNNNNNNNNNNNNNNNNNNNNN
NNNNNNNNNNNNNNNNNNNNNNNNNNNNNNNNNNNGNNNNGNNNNNNCNANNNAAGGANNTANNNCTNNTN
NTNNNNNNGGACNNNACNNNNCNNNNNNNACNCNNNCNCNNNNNNANNNGGTTTTNNNNCNNTATTNT
CCAGNACGCNGNGCNNNCATTTANGNGAACNNNGNNACGCGTNAATCGTANNATCTACCTGCCGCNNG
TNNNNNTACNGNNNNNNCGGANNNTNNNNCGTANNNNNNTCATGCGTNNTNTNNTCNCNAGNCNNGTT
CGNNNANANANTAGNANGTNTNNNNNNNNNNTGTNNCGTTCCGCNTGANNCNNCANNNNGNNGNNCTTN
TCANCGGCNNTANCTGCNGANNTAAGNCATANNNTNNNTCNNTANCTNNNNNGGNGGGNNACTGTAAA
NGGNNAATTNGNNNNTNANTTCNNNCATNTNNCTNNGNNNNCTNNNTNCCTGNNTNCNNAATTGGNNC
CANTGNNTGNCNATCTCTNGNTTNGCNNNNNTNNNNNTNNCAANNNGGNNNGTNANANNNCNATNTAA
NNTATANNNNCNNNNNNANNNCACNNNNNNNNNAGNNNCNANNNGTANANNNGTAGNNNGNTAAGTCT
CCGANNTNNNNNNNATAAGCGNNANACNNNNNNNNNNNNNANGANNACGNNTTTGNNNTNNNNNNCNA
GNNAGACNNTNGTGGNTNNNNNTCAANNNNTTCGTAGTGGTATAATNGNNNNNNNTTTCNCCAGNNTN
NNNGNNNNNNNCNATANNANNNNNNNGNGANNNNNNNNNNNANNNNCTCNNNAGGNANCANCTACTNNA
GCANTGGNGCGCAGNNNGAGANNNNGACNNGCCTTGGGTGGCAANCACNGCAATAAGTNNNGTTNNGC
CTNNNAGTNNACACNNNNNCANNGNNNCNNNNNNANCNGANNGNNNNNNNNNNNNNNNNNNNNNNNNN
NNNNNNNNNNNNNNNNNNNNNNNNNNNNNANNNNANGGNNNGTNTNTGACNCANGTGTTTTTANNNGNN
NNNNNACGNNNNANCCCCGGTCGNNNNCNNATTGAANNCCTGNNGCATNNNNNTNCNAGAATGANNGC
NNNNNGNNTNGNCTNNCNTTTNAAGNAATCNNNNAGGTANNNNGNTCNNNGNNNNNNNNCNNTCCCNN
ACTGAGGGTNNNNNNNNCATCTGCTNNNNNNNNNCTNCGCNNGNGTNNCNNNNNAGCNGTATNGTGTNN
NNCANNNNNNNNNNNNNNNNNTNAAACAANNNNNNNTNNNN
>PaThen521
GTGNNNNNTAGCCCGNNNNAGTCAGACANNNCNNNNNNNNNNNNTNNNNNNNNNNNNNNNNNNNNNNN
NNNNNNNNNNNNNNNNNNNNNNNNNNNNNNNNNNNNNNNNNNNNNNNNNNNNNNNNNNNNNNNNNNNN
NNNNNNNNNNNNNNNNNNNNNNNNNNNNNNNNNNNNNNNNNNNNNNNNNNNNNNNNNNNNNNNNNNNN
NNNNNNNNNNNNNNNNNNNNNNNNNNNNNNNNNNNNNNNNNNNNNNNNNNNNNNNNNNNNNNNNNNNN
NNNNNNNNNNNNNNNNNNNNNNNNNNNNNNNNNNNNNNNNNNNNNNNNNNNNNNTCTNNNAAANNNTNNN
NNNNNNNNNNNNNNNNNNNNNNNNNNCNCNTTGNNNGTAANCNANNNGNACATGCAGTCCCCTCNNNNN
NNNGGCAGAGNNNNNNCCCNNNNNNNCCGNCCCCNNCCNNNNNNNNNNNNNNNNNGNNNNNNNANNNNNN
NNNNNNTGGTACNNNNCGNNNNNGGTTGTTTAAGAGGTCCACAACGNGGTCNNGNCACNNNNGNNNAN
NNNCNNNNANCNNNNNNNNNNNNNNNNTNNNTANNAGGNCNNNNCCNTCNNNNNNNNNNANTGCNATGACC

CNANNCACGGGGGCNGTNCCCGGNGCGGCGACCTTGNTNANNTCCGGGGACAAGTATGGCNNNCNCCC
CGGGAAGGANNCNGCCGTANGTGCNNNNNANNNNNTCNTGNGANGTNGCCGNNATTCNNGTGGTCTNN
NNAGANTGNNCGNACCANNCGNANNNTGGTNATGTNAGTNNNNNNNNNNNNNNNNNNNNNNNNNNNNN
NNNNNNNNNNNNNNNNNNNNNNNNNNNNNNNNNNNNNGNNNNNNCNANNNAAGGANNTANNNCTNNTN
NTNNNNNNGGACNNNACNNNNCNNNNNNNACNCNNNCNCNNNNNNNNNNGNTTTTTNNNCNNTATTNT
CCAGNACGCNGNGCNNNCATTTANGNGAACNNNGNNACGCGNNAATCGTANNATCTACCTGCCGCNNG
TNNNNNTACNGNNNNNNCGGANNNTNNNNCGTANNNNNNTCATGCGTNNTNTNNTCNCAGNCNNGTT
CGNNNANANANTAGNANGTNTCNNNNNNNNTGTNNCGTTCCGCNTGANNCNNCANNNNGNCGNNCTTN
TCANCGGCNNTANCTGCNGANNTAAGTCATANNNTNNNTCNNTANCTNNNNNGGNGGGNNACTGTAAA
NGGNNAATTGGANNNTNATTTCCNNCATNTNNCTNNGNNNNCTNNNTNCCTGNNTNCNNAATTGGNNC
CANTGNNTGNCNATCTCTGGNTTNGNNNNNNTNNNNNTNNCANNNNGGNNNGTNANANNNCNATNTAA
NNTATANNNNCNNNNNNANNNCACNANNNNNGNAGNNNCNANNNGTANANNNGTAGNNNGNTAAGTCT
CCGANNTNNNNNNNATAAGCGNNANACNNNNNNNNNNNNNANGANNACGNNTTTGNNNTNNNNNNCNA
GNNAGACNNTNGTGGNTNNNNNTCAANNNNTTCGTAGTGGTATAATNGNNNNNNNTTTCNCCAGNNTN
NNNGNNNNNNNCNATANTANNNNNNNGNGANNNNNNNNNNANNNNCTCNNNAGGGANCANCTACTNNA
GCAATGGNGCGCAGNNNGAGANNNNGACNNNCCTTGGGTGGCAANCACNGCAATAAGTNNNGTTNANC
CTNNNAGTNNACACNNNNNCANNGNNNCNNNNNNANCNGANNGNNNNNNNNNNNNNNNNNNNNNNNNN
NNNNNNNNNNNNNNNNNNNNNNNNNNNNAGNNNANGGNNNGTNTNTGACNCATGTGTTTTTANNNGNN
NNNNNACGNNNNANCCCCGGTCGTNNNCNNATTGAANNCCTGNNGCATNNNNNTNCNAGAATGANNGC
NNNNNGNNTNGNCTGNCNNNTCAAGNAATCANNNAGNNANCGCGNTCNNNNNNNNNNNNCNNTCCCNN
ACTGAGGGTNNNNNNGCATCTGCTNNNNNNNNNNTNCGCNGGNNNNNCNGNNNAGCNGTATNGTGNNN
NNCANNNNNNNNNNNNNNNNTNAAACAANNNNNNTNNNN
>PaThen522
GTGNNNNNTAGCCCNNNNNAGTCAGACANNNCNNNNNNNNNNNNTNNNNNNNNNNNNNNNNNNNNNNN
NNNNNNNNNNNNNNNNNNNNNNNNNNNNNNNNNNNNNNNNNNNNNNNNNNNNNNNNNNNNNNNNNNNN
NNNNNNNNNNNNNNNNNNNNNNNNNNNNNNNNNNNNNNNNNNNNNNNNNNNNNNNNNNNNNNNNNNNN
NNNNNNNNNNNNNNNNNNNNNNNNNNNNNNNNNNNNNNNNNNNNNNNNNNNNNNNNNNNNNNNNNNNN
NNNNNNNNNNNNNNNNNNNNNNNNNNNNNNNNNNNNNNNNNNNNNNNNNNNNNNNNNNNNNNNNNNNN
NNNNNNNNNNNNNNNNNNNNNNNNNNNNNNNNNNNNNNNNNNNNNNNNNNNTCTNNNAAANNNTNNN
NNNNNNNNNNNNNNNNNNNNNNNNNCNCNNTNNNNGTAANCNANNNGNACATGCAGTCCCCTCNNNNN
NNNNNCAGAGNNNNNNNCNNNNNNCNCCGNCCCCNNCCNNNNNNNNNNNNNNNGNNNNNNANNNNNNN
NNNNNNTGGTACNNNNCGNNNNNGGTTGTNTNAGAGGTCCACAACGNGGTCNNNNNACNNNNGNNNAN
NNNCNNNNNACNNNNNNNNNNNANNTNNNTANNAGGNCNNNNCCNTCNNNNNNNNNANNGCNATGANN
CANNNCACGGNGGCNGTNCCCGGNGNGGCGACCTTGANNANNTCCGGGNACAAGTATGGCNNNCNCNC
CGGGAAGGANNCNGCCGTANGTGCNNNNNANNNNNTCNTGNGANGTCNNCGNNATTNNNGNGGTCNNN
NNNGAATGNCCNNACCANNCGNNNNNTGGTNATGNNAGTNNNNNNNNNNNNNNNNNNNNNNNNNNNNN
NNNNNNNNNNNNNNNNNNNNNNNNNNNNNNNNGNNNNGNNNNNNCNANNNAAGGNNNTANNNCTNNTN
NTNNNNNNGGACNNNACNNNNCNNNNNNNACNCNNNCNCNNNNNNNNNNGNTTTNNNNCCNNTANTNT
CCAGNANGCNGNGCNNNCATTTANGNGAACNNNGNNACGCGNNAATCGTANNATCTACCTGCCGCNGG
NNNNNNTACNGNNNNNNCGGANNNTNNCTCGTANNNNNNTCATGCGTNNTNTNNTCNCAGNCNNGTT
CGNNNANANANTAGNANGTNTNNNNNNNNNTGTNNCNNNCCGCNTGANNCNNCANNNNGNNGNNNTTN
TCANCGGCNNTANCTGCNGANNTAAGNCATANNNTNNNTCNNTANCTNNNNNGGNGGGNNACTGTAAA
NGGNNAATTNGNNNNTNANTNCNNNCATNTNNCTNNGNNTNCTNNNTNCCTGNNTNCNNAATTGGNNC
CANTGNNTGNCNATCTCTNGNTTNGNNNNNNTNNNNNTNNCAANNNGGNNNNNNANANNNCNATNTAA
NNTANANNNNCNNNNNNANNNCACNNNNNNNNNAGNNNNNANNNGTANANNNGTAGNNNGNTNAGTCT
CCGANNTNNNNNNNATAAGCGNNANACNNNNNNNNNNNNNANGANNACGNNTTTGNNNTNNNNNNCNA
GNNAGACNNTNGTGGNTNNNNNTCAANNNNTTCGTAGTGGTATAATNGNNNNNNNTTTCNCCAGNNNN
NNNGNNNNNNNCNATANNANNNNNNNGNGANNNNNNNNNNANNNNCTCNNNAGGNANCANCTACTNNA
GCANTGGNGCGNAGNNNGAGANNNNGACNNNCCTTGGGTGGCAANCACNGCAATAAGTNNNGTTNNNC
NTNNNAGTNNACACNNNNNCANNGNNNCNNNNNNANCNGANNGNNNNNNNNNNNNNNNNNNNNNNNNN
NNNNNNNNNNNNNNNNNNNNNNNNNNNNNGNNNANNGNNNGTNTNTGACNCNTGTGTTNNTANNNGNN
NNNNNACGNNNNANCCCCGGTCGNNNNCNNATTGAANNCCTGNNGCATNNNNNTNCNAGAATGANNNC
NNNNNGNNTNGNCTNNNNNNNTNAAGNAATCANNTAGNTANNNNGNTCNNNNNNNNNNNCNNTCCCNN
ACTGAGGGTNNNNNNNNCATCTGCTNNNNNNNNNTNCGCNGNCGTNNCNNNNNNGCNGTATNNTGTNN
NNCANNNNNNNNNNNNNNNNTNAAACNANNNNNNTNNNN

>PaThen523
GTGNNNNNTAGCCCNNNNNAGTCAGACANNNCNNNNNNNNNNNNTNNNNNNNNNNNNNNNNNNNNNNN
NNNNNNNNNNNNNNNNNNNNNNNNNNNNNNNNNNNNNNNNNNNNNNNNNNNNNNNNNNNNNNNNNNNN
NNNNNNNNNNNNNNNNNNNNNNNNNNNNNNNNNNNNNNNNNNNNNNNNNNNNNNNNNNNNNNNNNNNN
NNNNNNNNNNNNNNNNNNNNNNNNNNNNNNNNNNNNNNNNNNNNNNNNNNNNNNNNNNNNNNNNNNNN
NNNNNNNNNNNNNNNNNNNNNNNNNNNNNNNNNNNNNNNNNNNNNNNNNNTCTNNNNAAANNNNTNNN
NNNNNNNNNNNNNNNNNNNNNNNNNCNCNTTGNNNGTAANCNANNNGNACATGCAGTCCCCTCNNNNN
NNNGGCAGAGNNNNNNNCNNNNNNNCCGGCCCCNNCCNNNNNNNNNNNCNNNGNNNNNNNANNNNNNN
NNNNNNTGGTACNNNNCGNNNNNGGTTGTNTAAGAGGTCCACAACGNGGTCNGGNCACNNNNGNNNAN
NNNCNNNNAACNGNNNNNNNNNNNATNNNTANNAGGNNNNNNCCNTCNNGNNNNNNANNGCNATGACC
CAANNCNCGGGGGCAGTNCCCGGCGNGGCGACCTTGNTNANGTCCGGGGACAAGTATGGCNCNCNCCC
CGGGAAGGANNCNGCCGTANGTGCNNNNNANNNNNTCTTGNGANGTCGCCGNNATTCNCGTGNTCTNN
NNAGAATGNNCGNACCANNCGNANNNTGGTTATGNTAGTNNNNNNNNNNNNNNNNNNNNNNNNNNNNN
NNNNNNNNNNNNNNNNNNNNNNNNNNNNNNNNNNNNNGNNNNNNCNANNNAAGGANNTANNNCTNNTN
NTNNNGNNGGACNNNACNNNNCNNNNNNNACNCNNNCNCNNNNNNNNNNGGTTTTTNNNCNNTATTNT
CCAGNACGCNGNGCNNNCATTTANGNGAACNNNGNNACGCGNNAATCGTANNATCTACCTGCCGCNNG
NNNNNNTACNGNNNNNNCGGANNNTNNCTCGTANNNNNNTCATGCGTNNTNTNNTCNCNAGNCANGTT
CGNNNANANANTAGNANGTNTNNNNNNNNNTGTNNCGTTCCGCNTGANNCNNCANNNNGNNGNNNTTN
TCANCGGCNNTANCTGCNGANNTAAGNCATANNNTNNNTCNNTANCTNNNNNGGNGGGNNACTGTAAA
NGGNNAATTGGNNGNTNANTNCNNNCATNTNNCTNNGNNNNCTNNNTNCCTGNNTNCNNAATTGGNNC
CANTGNNTGNCNATCTCTGGNTTNGNNNNNNTNNNNNTNGCAANNNGGNNNNNNANANNNCNATNTAA
NNTATANNNNCNNNNNNANNNCACNANNNNNGNAGNNACNANNNGTANANNNGTAGNNNGNTAAGTCT
CCGANNTNNNNNNNATAAGCGNNANACNNNNNNNNNNNNNANGANNACGNNTTTGNNNTNNNNNNCNA
GNNAGACNNTNGTGGNTNNNNNTCAANNNNTTNGTAGTGGTATAATNGNNNNNNNTTTCNCCAGNNNN
NNNGNNNNNNNCNATANNANNNNNNNGTGANNNNNNNNNNANNNNCTCNNNAGGGANCANCTACTNNA
GCAATGGNGCGCAGNNNGAGANNNNGACNNGCCTTGGGTGGCAANCACNGCAATAAGTNNTGTTNANC
CTNNNAGTNNACACNNNNNCANNGNNNCNNNNNNANCNGANNGNNNNNNNNNNNNNNNNNNNNNNNNN
NNNNNNNNNNNNNNNNNNNNNNNNNNNNANNNNANGGNNNGTNTNTGACNCATGTGTTTNTANNNGNN
NNNNNACGNNNNANCCCCGGTCGNNNNCNNATTGAANNCCTGNNGCATNNNNNTNCNAGAATGANNNC
NNNNNGNNTNGNCTGNCNNNTCAAGNAATCAANNAGNNANNNNNNTCNNNGNNNNNNNNCNNTCCCNN
ACTGAGGGTNNNNNNNNCATCTGCTNNNNNNNNNTNCGCNGGNGTNNCNNNNNAGCNGTATNGTGTNN
NNCANNNNNNNNNNNNNNNTNANACANNNNNNNNTNNNN
>PaThen524
GTGNNNNNTAGCCCNNNNNAGTTAGACANNNCNNNNNNNNNNNNNNNNNNNNNNNNNNNNNNNNNNNN
NNNNNNNNNNNNNNNNNNNNNNNNNNNNNNNNNNNNNNNNNNNNNNNNNNNNNNNNNNNNNNNNNNNN
NNNNNNNNNNNNNNNNNNNNNNNNNNNNNNNNNNNNNNNNNNNNNNNNNNNNNNNNNNNNNNNNNNNN
NNNNNNNNNNNNNNNNNNNNNNNNNNNNNNNNNNNNNNNNNNNNNNNNNNNNNNNNNNNNNNNNNNNN
NNNNNNNNNNNNNNNNNNNNNNNNNNNNNNNNNNNNNNNNNNNNNNNNNNTCTNNNNAAANNNNTNNN
NNNNNNNNNNNNNNNNNNNNNNNNNCNCNTTGNNNGTAANCNANNNGNACATGCAGTNNCCTCNNNNN
NNNGGCAGAGNNNNNNNCCNNNNNNCCGGCCCCNNCCNNNNNNNNNNNNNNNGNNNNNNNANNNNNNN
NNNNNNTGGTACNNNNCGNNNNNGGTTGTNTAAGAGGTCCACAACANGGTCNGGNNACNNNNGNNNAN
NNNCNNNNNNNNNNNNNNNNNNNNNTNNNTANNAGGNNNNNNCCNTCNNGNNNNNNATNGCNATGACC
CNANTCACNGGGGNAGTNCCCGGCGCGGCNACCTTGANNANNTCCGGGGNCAAGTATGGCNNNCNCCC
CGGGAAGGANNCNGCCGTANGTGCNNNNNANNNNNTCNTGNGANGTNGCCGNNATTCNCNTGGTCNNN
CNAGAATGNCCNNACCANNCGNANNNTGGTNATGNNAGTNNNNNNNNNNNNNNNNNNNNNNNNNNNNN
NNNNNNNNNNNNNNNNNNNNNNNNNNNNNNNNGNNNNGNNNNNNCNANNNAAGGNNNTANNNCTNNTN
NTNNNNNNGGACNNNACNNNNCNNNNNNNACNCNNNCNCNNNNNNNNNNGNTTTTTNNNCNNTATTNT
CCAGNACGCNGNGCNNNCATTCANGNGAACNNNGNNACGCGNNAATCGTANNATCTANCTGCCGCNNG
NNNNNNTACNGNNNNNNCGGANNNTNNCTCGTANNNNNNTCATGCGTNNTNTNNTCNCNAGNCANGTT
CGNNNANANANTAGNANGTNTNNNNNNNNNTGTNNCGTTCCGCNCGANNCNNCANNNNGNNGNNNTTN
TCANCGGCNNTANCTGCNGANNTAAGNCATANNNTNNNTCNNTANCTNNNNNGGNGGGNNACTGTAAA
NGGNNAATTNGNNNNTNATTTCNNNCACNTNNCTNNANNTNCTNNNTNCCTGNNTNCNNAATTGGNNC
CANTGNNTGNCNATCTCTNGNTTNGNNNNNNTGNNNNTNNCANNNNGGNNNNNNNNANNNCNATNTAA
NNTANANNNNCNNNNNNANNNCACNANNNNNGNAGNNNNNANNNTTANANNNGTAGNNNANTAAGTCT

CCGANNTNNNNNNNATAAGCGNNANACNNNNNNNNNNNNNNANGANNACGNNTTGGNNNTNNNNNNNNNA
GNNAGACNNTNGTGGNTNNNNNTCAANNNNTTCGTAGTGGTATAATNGNNNNNNNTTTCNCCAGNNTN
NNNNNNNNNNNCNATANNANNNNNNNGNGANNNNNNNNNNNANNNNCTTNNNAGGGANCANTTACTNNA
GCAATGGNGCGNAGNNNGAGANNNNGACNNGCCTTGGGTGGCAANCACNGCAATAAGTNNNGTTNANN
CTNNNAGTNNACACNNNNNCANNGNNNCNNNNNNANCNGANNGNNNNNNNNNNNNNNNNNNNNNNNNN
NNNNNNNNNNNNNNNNNNNNNNNNNNNNNAGNNNANNGNNNGTNTNTGACNCANGTGTTTNTANNNGNN
NNNNNACGNNNNANCCCCGGTCGNNNNCNNATTGAANNCCTGNNGCATNNNNNTNCNAGAATGANNGC
NNNNNGNNTNNNCTNNCNNNTCAAGNAATCANNNAGNNANNGCGNTCNNNNNNNNNNNNCNNTCCCNN
ACTGAGGGTNNNNNNNNCATCTGCTNNNNNNNNCTNCGCNGGNGTNNCNNNNNAGCNGTATNNTGTNN
NNCANNNNNNNNNNNNNNNNNNTNNNACNANNNNNNNTNNNN
>PaThen525
GTGNNNNNTAGCTCNNNNNAGTTAGACANNNCNNNNNNNNNNNNNTNNNNNNNNNNNNNNNNNNNNNNN
NNNNNNNNNNNNNNNNNNNNNNNNNNNNNNNNNNNNNNNNNNNNNNNNNNNNNNNNNNNNNNNNNNNNN
NNNNNNNNNNNNNNNNNNNNNNNNNNNNNNNNNNNNNNNNNNNNNNNNNNNNNNNNNNNNNNNNNNNNN
NNNNNNNNNNNNNNNNNNNNNNNNNNNNNNNNNNNNNNNNNNNNNNNNNNNNNNNNNNNNNNNNNNNNN
NNNNNNNNNNNNNNNNNNNNNNNNNNNNNNNNNNNNNNNNNNNNNNNNNNNCNTCTNNNAAANNNTNNN
NNNNNNNNNNNNNNNNNNNNNNNNNNNCNCNTTGNNNGTAANCNANNNGNACATGCAGTNCCCTCNNNNN
NNNGGCAGAGNNNNNNCCCNNNNNNNNCCGNCCCCNNCCNNNNNNNNNNNNCNNNNNNNNNNNGNNNNNN
NNNNNNTGGTACNNNNCGNNNNNGGTTGTTTAAGAGGTCCACCACGNGGTCNNGNCACNNNNGNCNAN
NNNCNNNNAANNNNNNNNNNNNNNNNNNTNNNCANNAGGNNNNNACCNTCNNNNNNNNNNATTGCNATGACC
CNANNCGCGGGGACNGTNACCGGCGNGGCGACCTTGANNANNGTCGGGGACAAGTATGTCNNNCNCCC
CGGGAAGGANNCNGCCGTANGTGCNNNNNANNNNNTCNTGNGANGTNGCCGNNATTCNCGTGGTCNNN
CNAGAATGNCCGNACCANNCGNANNNTGGTNATGNNAGTNNNNNNNNNNNNNNNNNNNNNNNNNNNN
NNNNNNNNNNNNNNNNNNNNNNNNNNNNNTNNNNGNNNNGNNNNNNCNANNNAAGGANNTANNNTTNNTN
NTNNNNNNNGGACNNNACNNNNCNNNNNNNNACNCNNNCNCNNNNNNANNNGNTTTTTNNNCNNTATTNT
CCAGNACGCNGNGCNNNCATTCANGNGAACNNNGNNACGCGTAAATCGTANNATCTACCTGCCGCNAG
TNNNNNTACNGNNNNTNCGGANNNTNNNNCGTANNNNNNTCATGCGTNNTNTNNTTNCNAGNCNNGTT
CGNNNANANANTAGNANGTNTCNNNNNNNNTGTNNCGTTCCGCNTGACNCNNCANNNNGNNGNNCTTN
NCANCGGCNNTANCTGCNGANNTAAGNCATANNNTNNNTCNNTANCGNNNNNGGNGGGNNACTGTAAA
NGGNNAATTNGNNGNTNANTTCNNNCACNTNNCTNNGNNNNCTNNNCNCCTGNNTNCNNAATTGGNNC
CANTGNNTGNCNATCTCTGGNTTNGNNNNNNTGNNNNTNNTANNNNGGNNNGTNANANNNCNATNTAA
NNTATANNNNCNNNNNNANNNCACNANNNNNNNNAGNNNCNANNNTTANGNNNGTAGNNNAATAAGTCT
CCGANNTNNNNNNNATAAGCGNNANACNNNNNNNNNNNNNNANGANNACGNNTTGGNNNTNNNNNGCNA
ANNAGACNNCNGTGGNTNNNNNTCAANNNNTTCGTAGTGGTATAACNANNNNNNNTTTCNCCAGNNTN
NNNGNNNNNNNCNATANNANNNNNNNGNGANNNNNNNNNNNANNNNCCCNNNAGGGANCANCTACTNNA
GCANTGGNGCGCAGNNNGAGANNNNGACNNGCCTTGGGTGGCAANCACNGCAATAAGTNNNGTTNANN
NCNNNAGTNNACACNNNNNCANNGNNNCNNNNNNANCNGANNTNNNNNNNNNNNNNNNNNNNNNNNNN
NNNNNNNNNNNNNNNNNNNNNNNNNNNNNAGNNNANGGNNNGTNTNTGACNCATGTGTTTTTANNNGNN
NNNNNACGNNNNANCCCCGGTCGNNNNCNNATTGAANNCCTGNNGCATNNNNNTNCNAGAATGANNGC
NNNNNGNNTNGNCTNNCNNNTCAAGNAGTCANNNAGNNNNCGCGNTCNNNGNNNNNNNNCNNTCCCNN
ACTGAGGGTNNNNNNNNCATCTGCTNNNNNNNNNNTTCGCNGNNNNNNTNNNNNAGCNGTATNGTGTNN
NNCANNNNNNNNNNNNNNNNNNTNAAACNNNNNNNNNTNNNN
>PaThen526
GTGNNNNNTAGCCCNNNNNAGTCAGACANNNCNNNNNNNNNNNNNTNNNNNNNNNNNNNNNNNNNNNNN
NNNNNNNNNNNNNNNNNNNNNNNNNNNNNNNNNNNNNNNNNNNNNNNNNNNNNNNNNNNNNNNNNNNNN
NNNNNNNNNNNNNNNNNNNNNNNNNNNNNNNNNNNNNNNNNNNNNNNNNNNNNNNNNNNNNNNNNNNNN
NNNNNNNNNNNNNNNNNNNNNNNNNNNNNNNNNNNNNNNNNNNNNNNNNNNNNNNNNNNNNNNNNNNNN
NNNNNNNNNNNNNNNNNNNNNNNNNNNNNNNNNNNNNNNNNNNNNNNNNNNNNNTCTNNNAAANNNTNNN
NNNNNNNNNNNNNNNNNNNNNNNNNNNCNCNTTGNNNGTAANCNANNNGNACATGCAGTCCCCTCNNNNN
NNNGGCAGAGNNNNNNCCNNNNNNNNNCCGNCCCCNNCCNNNNNNNNNNNNNNNNNNNNNNNNNNANNNNNN
NNNNNNTGGTACNNNNCGNNNNNGGTTGTNTNAGAGGTCCACAACGNGGTCNNGNCACNNNNGNNNAN
NNNCNNNNANNNNNNNNNNNNNNNNNNTNNNTANNAGGNNNNNNCCNTNNNGNNNNNNATNGCNATGACN
CAANNNACGGGGGCNGTNCCCGGNGCGGCNACCTTGANNACGTCCGGGGACAAGTATGGCGNNCNCCC
CGGGAAGGANNCNGCCGTANGTGCNNNNNANNNNNTCNTGNGANGTNGCCGNNNTTCNNNTGGNCNNN

CNAGAATGCCCNNACCANNCGNANNNTGGTNATGNNAGTANNNNNNNNNNNNNNNNNNNNNNNNNN
NNNNNNNNNNNNGCNNNNNNNNNNNNNNNNNNNGNNNNGNNNNNNCNANNNAAGGNNNTANNNCTNNTN
NTNNNNNNGGACNNNACNNNNCNNNNNNNACNCNNNCNCNNNNNNNNNNGNTTTTNNNNCNNTANTNT
CCAGNANGCNGNGCNNNCATTTANGNGAACNNNGNNACGCGNNAATCGTANNATCTACCTGCCGCNGG
NNNNNNTNCNGNNNNTNCGGANNNTNNNNCGTANNNNNNTCATGCGTNNTNTNNTCNCNAGNCNNGTT
CGNNNANANANTAGNANGTNTNNNNNNNNNNTGTNNCGTTCCGCNTGANNCNNCANNNNGNNGNNCTTN
TCANCGGCNNTANCTNCNGANNTAAGNCATANNNTNNATCNNTANCTNNNNNGGNGGGNNACTGTAAA
NGGNTAATTGGNNNNTNANTTCNNNCATNTNNCTNNGNNNNCTNNNTNCCTGNNTNCNNAATTGGNNC
CANTGNNTGNCNATCTCTGGNTTNGCNNNNNTNNNNNTNNCAANNNGGNNNNNNANANNNCNATNTAA
NNTANANNNNCNNNNNNANNNCACNNNNNNNGNAGNNNCNANNNGTANANNNGTAGNNNGNTAAGTCT
CCGANNTNNNNNNNATAAGCGNNANACNNNNNNNNNNNNNNANGANNACGNNTTTGNNNTNNNNNNCNA
GNNANACNNTNGTGGNTNNNNNTCAANNNNTTCGTAGTGGTATAATNGNNNNNNNTTTCNCCAGNNTN
NNNGNNNNNNNNCNATANNANNNNNNNGTGANNNNNNNNNNNANNNNCTCNNNAGGGANCANCTACTTNA
GCANTGGNGCGCAGNNNGAGANNNNGACNNNCCTTGGGTGGCAANCACNGCAATAAGTNNNGTTNANC
CTNNNAGTNNACACNNNNNCANNGNNNCNNNNNNANCNGANNGNNNNNNNNNNNNNNNNNNNNNNNNN
NNNNNNNNNNNNNNNNNNNNNNNNNNNNNAGNNNANGGNNNGTNTNTGACNCATGTGTTTTTANNNGNN
NNNNNACGNNNNANCCCCGGTCGNNNNCNNATTGAANNCCTGNNGCATNNNNNNTNCNAGAATGANNGC
NNNNNGNNTNGNCTNNCNNNTCAAGNAATCANNNAGGTNNNNCGNTCNNNGNNNNNNNNCNNTCCCNN
ACTGAGGGTNNNNNNCGCATCTGCTNNNNNNNNNNTNCGCNGGCGTNNCNNNNNAGCNGTATNGTGTNA
NNCANNNNNNNNNNNNNNNNNNTNAAACNANNNNNNNTNNNN
>PaThen527
GTGNNNNNTAGCCCNNNNNAGTCAGACANNNCNNNNNNNNNNNNNTNNNNNNNNNNNNNNNNNNNNNNNN
NNNNNNNNNNNNNNNNNNNNNNNNNNNNNNNNTNNNNNNNNNNNNNNNNNNNNNNNNNNNNNNNNNNNNNN
NNNNNNNNNNNNNNNNNNNNNNNNNNNNNNNNNNNNNNNNNNNNNNNNNNNNNNNNNNNNNNNNNNNNNNN
NNNNNNNNNNNNNNNNNNNNNNNNNNNNNNNNNNNNNNNNNNNNNNNNNNNNNNNNNNNNNNNNNNNNNNN
NNNNNNNNNNNNNNNNNNNNNNNNNNNNNNNNNNNNNNNNNNNNNNNNNNNNNNNCNTCTNNNAAANNNTNNN
NNNNNNNNNNNNNNNNNNNNNNNNNNNCNCNNTGNNNGTAANCNANNNGNACATGCAGTCCCCTCNNNNN
NNNGGCAGAGNNNNNNNCCNNNNNNNCCGGCCCCNNCCNNNNNNNNNNNCNNNNNNNNNNNANNNNNN
NNNNCNTGGTACNNNNCGNNNNNGGTTGTNTNAGAGGTCCACAACGNGGTCNGGNNACNNNNGNNNAN
NNNCNNNNAANNNNNNNNNNNNNNNNNTNNNTANNAGGNCNNNNCCNTCNNGNNANNNATTGCNATGACC
CAANNCACGGNGGCNGTNCCCGGCGCGGCGACCTTGNTNANGTCCGGGGNCAAGTATGGCNNNCNCCC
CGGGAAGGANNCNGCCGTANGTGCNNNNNANNNNNTCNTGNGANGTNGCCGNNATTCNCGTGGTCTNN
NNAGAATGNNCNNACCANNCGNANNNTGGTNATGNTAGTANNNNNNNNNNNNNNNNNNNNNNNNNNNN
NNNNNNNNNNNNNNNNNNNNNNNNNNNNNNNNNNNGNNNNGNNNNNNCNANNNAAGGNNNTANNNCTNNTN
NTNNNNNNGGACNNNACNNNNCNNNNNNNACNCNNNCNCNNNNNNNNNNGGTTTTTNNNCNNTANTNT
CCAGNACGCNGNGCNNNCATTTANGNGAACNNNGNNACGCGNNAATCGTANNATCTACCTGCCGCNGG
TNNNNNTACNGNNNNNNCGGANNNTNNNNCGTANNNNNNNCATGCGTNNTNTNNTCNCNAGNCNNGTT
CGNNNANANANTAGNANGTNTNNNNNNNNNNTGTNGCGTTCCGCNTGANNCNNCANNNNGNNGNNNTTN
TCANCGGCNNTANCTNCNGANNTAAGNCATANNNTNNNTCNNTANCTNNNNNGGNGGGNNACTGTAAA
NGGNNAATTNGNGNNTNANTNCNNNCATNTNNCTNNGNNNNCTNNNTNCCTGNNTNCNNAATTGGNNC
CANTGNNTGNCNATCTCTGGNTTNGNNNNNNTNNNNNTNNCAANNNGGNNNGTNNNANNNCNATNTAA
NNTANANNNNCNNNNNNANNNCACNANNNNNNNAGNNNCNANNNGTANANNNGTAGNNNGATNAGTCT
CCGANNTNNNNNNNATAAGCGNNANACNNNNNNNNNNNNNNANGANNACGNNTTTGNNNTNNNNNNCNA
GNNAGACNNTNGTGGNTNNNNNTCAANNNNTTCGTAGTGGTATAATNGNNNNNNNTTTCNCCAGNNTN
NNNGNNNNNNNNCNATANNANNNNNNNGNGANNNNNNNNNNNANNNNCTCNNNAGGNANCANCTACTNNA
GCANNGGNGCGNAGNNNGAGANNNNGACNNGCCTTGGGTGGCAANCACNGCAATAAGTNNNGTTNANC
CTNNNAGTNNACACNNNNNCANNGNNNCNNNNNNANCNGANNGNNNNNNNNNNNNNNNNNNNNNNNNN
NNNNNNNNNNNNNNNNNNNNNNNNNNNNNAGNNNANNGNNNGTNTNTGACNCATGTGTTTNTANNNGNN
NNNNNACGNNNNANCCCCGGTCGNNNNCNNATTGAANNCCTGNNGCATNNNNNNTNCNAGAATGANNNC
NNNNNGNNTNGNCTNNNNTTTCAAGNAATCANNNAGNTNNCGCGNTCNNNNNNNNNNNNNCNNTCCCNN
ACTGAGGGNNNNNNNCGCATCTGCTNNNNNNNNNCTNCGCNGGNNNNNCNGNNNAGCNGTATNGTGNNN
NNCANNNNNNNNNNNNNNNNNNTNAANCNNNNNNNNNTNNNN
>PaThen528
GTGNNNNNTAGCCCNNNNNAGTTAGACANNNCNNNNNNNNNNNNNTNNNNNNNNNNNNNNNNNNNNNNNN

NNNNNNNNNNNNNNNNNNNNNNNNNNNNNNTNNNNNNNNNNNNNNNNNNNNNNNNNNNNNNN
NNNNNNNNNNNNNNNNNNNNNNNNNNNNNNNNNNNNNNNNNNNNNNNNNNNNNNNNNNNNNN
NNNNNNNNNNNNNNNNNNNNNNNNNNNNNNNNNNNNNNNNNNNNNNNNNNNNNNNNNNNNNN
NNNNNNNNNNNNNNNNNNNNNNNNNNNNNNNNNNNNNNNNNNNNNNNNNCNTCTNNNAAANNNTNNN
NNNNNNNNNNNNNNNNNNNNNNNNNNCNCNTTGNNNGTAANCNANNNGNACATGCAGTCCCCTCNNNNN
NNNGGCAGAGNNNNNNNNCNNNNNCNCCGGCCCCGNCCNNNNNNNNNNNCNNNGNNNNNNNANNNNNN
NNNNNNTGGTACNNNNCGNNNNNGGTTGTTTAAGAGGTCCACAACGNGGTCNNGNCACNNNNGANNAN
NNNCNNNNAACNNNNNNNNNNNNANATNNNTANAAGGNCNNNNCCNTCNNGNNNNTNATTGCNATGACC
CAANNCACGGGGGCNGTNCCCGGCGCGGCGACCTTGATNACGTCCGGGGACAAGTATGGCNCNCNCCC
CGGGAAGGANNCNGCCGTANGTGCNNNNNANNNNNTCNTGNGANGTCGCCGNNATTCNCGTGGTCNNN
NNAGAATGNCCNNACCANNCGNANNNTGGTNATGNNAGTANNNNNNNNNNNNNNNNNNNNNNNNNNNN
NNNNNNNNNNNNNNNNNNNNNNNNNNNNNNNNNGNNNNGNNNNNNCNANNNAAGGANNTANNNCTNNTC
NTNNNNNNGGACNNNACNNNNCNNNNNNNACNCNNNCNCNNNNNNANNNGNTTTTNNNNCNNTATTNT
CCAGNACGCNGNGCNNNCATTCANGNGAACNNNGNNACGCGTNAATCGTANNATCTACCTGCCGCNGG
TNNNNNTACNGNNNNTNCGGANNNTNNNNCGTANNNNNNTCATGCGTNNTNTNNTCNCNAGNCNNGTT
CGNNNANANANTAGNANGTNTCNNNNNNNNTGTNNCGTTCCGCNTGANNCNNCANNNNGNNGNNCTTN
TCANCGGCNNTANCTGCNGANNTAAGNCATANNNTNNATCNNTANCTNNNNNGGNGGGNNACTGTAAA
NGGNNAATTNGNNGNTNATTNCNNNCACNTNNCTNNGNNTNCTNNNTNCCTGNNTNCNNAATTGGNNC
CANTGNNTGNCNATCTCTGGNTTNGNNNNNNTNNNNNTNNCAANNNGGNNNGTNANANNNCNATNTAA
NNTATANNNNCNNNNNNANNNCACNANNNNNNNAGNNNCNANNNTTANANNNGTAGNNNAATAAGTCT
CCGANNTNNNNNNNATAAGCGNNANACNNNNNNNNNNNNNANGANNACGNNTTGGNNNTNNNNNNCNA
GNNAGACNNTNATGGNTNNNNNTCAANNNNTTCGTAGTGGTATAATNGNNNNNNNTTTCNCCAGNNTN
NNNGNNNNNNNCNATANNANNNNNNNGTGANNNNNNNNNNANNNNCTCNNNAGGGANCANCTACTTNA
GCANTGGNGCGCAGNNNGAGANNNNGACCAGCCTTGGGTGGCAANCACNGCGATAAGTNNNGTTNANC
CTNNNAGTNNACATNNNNNCANNGNNNCNNNNNNANCNGANNGNNNNNNNNNNNNNNNNNNNNNNNN
NNNNNNNNNNNNNNNNNNNNNNNNNNNNNAGNNNANGGNNNGTNTNTGACNCATGTGTTTTTANNNGNN
NNNNNACGNNNNANCCCCGGTCGNNNNCNNATTGAANNCCTGNNGCATNNNNNNTNCNAGAATGANNGC
NNNNNGNNTNGNCTNNCNTTTCAAGNAATCANNNAGGTANCGCGNTCNNNGNNNNNNNNCNNTCCCNN
ACTGAGGGTNNNNNNCGCATCTGCTNNNNNNNNCTNCGCNGGCGTNNCNNNNNAGCNGTATNGNGTNA
NNCANNNNNNNNNNNNNNNNNTNAAACGANNNNCCTNNNN
>PaThen529
GTGNNNNNTAGCCCNNNNNAGTCAGACANNNCNNNNNNNNNNNNTNNNNNNNNNNNNNNNNNNNNNN
NNNNNNNNNNNNNNNNNNNNNNNNNNNNNNNNNNNNNNNNNNNNNNNNNNNNNNNNNNNNNNNN
NNNNNNNNNNNNNNNNNNNNNNNNNNNNNNNNNNNNNNNNNNNNNNNNNNNNNNNNNNNNNNNN
NNNNNNNNNNNNNNNNNNNNNNNNNNNNNNNNNNNNNNNNNNNNNNNNNNNNNNNNNNNNNNNN
NNNNNNNNNNNNNNNNNNNNNNNNNNNNNNNNNNNNNNNNNNNNNNNNNNTCTNNNAAANNNTNNN
NNNNNNNNNNNNNNNNNNNNNNNNNNCNCNTTGNNNGTAANCNANNNGNACATGCAGTCCCCTCNNNNN
NNNNNCAGAGNNNNNNNNNCNNNNNNNCCGGCCCCNNCCNNNNNNNNNNNCNNNNNNNNNNNANNNNNN
NNNNNNTGGTACNNNNCGNNNNNGGTTGNNTAAGAGGTCCACAACGNGGTCNNGNCACNNNNGNNNAN
NNNCNNNNNNNNNNNNNNNNNNNNNTNNNTANNAGGNNNNNNCCNTCNNNNNNNTNANNGCNATGACC
CAANNCACGGGGGNAGTNCCCGGCGNGGNGACCTTGNNNANNTCCGGGGACAAGTATGGCNNNCNCCC
CGGGAAGGANNCNGCCGTANGTGCNNNNNANNNNNTCNTGNGANGTNGCCGNNATTCNNGTGGTCNNN
CNAGAATGNNCGNACCANNCGNANNNTGGTNATGNNAGTNNNNNNNNNNNNNNNNNNNNNNNNNNNN
NNNNNNNNNNNNNNNNNNNNNNNNNNNNNNNNNGNNNNGNNNNNNCNANNNAAGGANNTANNNCTNNTC
NTNNNNNNGGACNNNACNNNNCNNNNNNNACNCNNNCNCNNNNNNANNNGNTTTNNNNNCNNTATTNT
CCAGNACGCNGNGCNNNCATTTANGNGAACNNNGNNACGCGNNAATCGTANNATCTACCTGCCGCNNG
NNNNNNTACNGNNNNNNCGGANNNTNNCNCGTANNNNNNNCATGCGTNNTNTNNTCNCNAGNCNNGTT
CGNNNANANANTAGNANGTNTNNNNNNNCTTGTNNCGTTCCGCNTGANNCNNCANNNNGNNGNNCTTN
TCANCGGCNNTANCTGCNGANNTAAGNCATANNNTNNNTCNNTANCTNNNNNGGAGGGNNACTGTAAA
NGGNTAATTNGANGNTNANTTCNNNCATNTNNCTNNGNNNNCTNNNTNCCTGNNTNCNNAATTGGNNC
CANTGNNTGNCNATCTCTNGNTTNGNNNNNNTNNNNNTNNCAANNNGGNNNGTNANANNNCNATNTAA
NNTANANNNNCNNNNNNANNNCACNANNNNNNNAGNNNNNANNNGTANANNNGTAGNNNGATAAGTCT
CCGANNTNNNNNNNNATAAGCGNNANACNNNNNNNNNNNNNANGANNACGNNTTTGNNNTNNNNNNNNA
GNNAGACNNTNGTGGNTNNNNNTCAANNNNTTCGTAGTGGTATAATNGNNNNNNNTTTCNCCAGNNTN

NNNGNNNNNNNNCNATANNANNNNNNNGNGANNNNNNNNNNNNANNNNCTCNNNAGGGANCANCTACTNNA
GCANTGGNGCGCAGNNNGAGANNNNGACCAGCCTTGGGTGGCAANCACNGCAATAAGTNNNGTTNANC
CTNNNAGTNNACACNNNNNCANNGNNNCNNNNNNANCNGANNGNNNNNNNNNNNNNNNNNNNNNNNN
NNNNNNNNNNNNNNNNNNNNNNNNNNNNNNNNNNNAGNNNANGGNNNGTNTNTGACNCATGTGTTTTTANNNGNN
NNNNNACGNNNNANCCCCGGTCGNNNNCNNATTGAANNCCTGNNGCATNNNNNTNCNAGAATGANNNC
NNNNNGNNTNGNCTNNNNNNNTCAAGNAATCANNNAGGTNNCGCGNTCNNNNNNNNNNNNNCNNTCCCNN
ACTGAGGGTNNNNNNCGCATCTGCTNNNNNNNNNCTTCGCNGNCGTNNCNNNNNNGCNGTATNGNGTNN
NNCANNNNNNNNNNNNNNNNNNTNAAACNANNNNNNNTNNNN
>PhuLa141
GTGNNNNNTAGCCCGNNNNAGTTAGACANNNCNNNNNNNNNNNNNTNNNNNNNNNNNNNNNNNNNNNNNN
NNNNNNNNNNNNNNNNNNNNNNNNNNNNNNNNNNNNNNNNNNNNNNNNNNNNNNNNNNNNNNNNNNNNNNNNNNNNNNNN
NNNNNNNNNNNNNNNNNNNNNNNNNNNNNNNNNNNNNNNNNNNNNNNNNNNNNNNNNNNNNNNNNNNNNNNNNNNNNNNN
NNNNNNNNNNNNNNNNNNNNNNNNNNNNNNNNNNNNNNNNNNNNNNNNNNNNNNNNNNNNNNNNNNNNNNNNNNNNNNNN
NNNNNNNNNNNNNNNNNNNNNNNNNNNNNNNNNNNNNNNNNNNNNNNNNNNNNNNNNNNNCNTCTNNNAAANNNTNNN
NNNNNNNNNNNNNNNNNNNNNNNNNNNNNNNCNCNNTNNNNGTAANCNANNNGNACATGCAGTCCCCNCNNNNN
NNNNNCAGAGNNNNNNCCNNNNNNNNNCCGNCCCCNNCCNNNNNNNNNNNNCANNGNNNNNNNANNNNNN
NNNNNNTGGTACNNNNCGNNNNNGGTTGNNTAAGAGGTCCACAACGNGGTCNNGNCACNNNNGNNNAN
NNNCNNNNNANNNNNNNNNNNNNNNNNNTNNNTANNAGGNNNNNNCCNTCNNGNNNNNNANNGCNATGACC
CAANTCACGGGGGCNGTNCCCGGCGNGGCGACCTTGNTNACGTCCGGGGACAAGTATGGCNNNCNCCC
CGGGAAGGANNCNGCCGTANGTGCNNNNNANNNNNTCTTGNGANGTCGCCGNNANNNNCGTGGTCNNN
NNAGAATGNNCGNACCANNCGNANNNTGGTTATGNNAGTNNNNNNNNNNNNNNNNNNNNNNNNNNNNNNN
NNNNNNNNNNNNNNNNNNNNNNNNNNNNNNNNNNNNNNNNNNNGNNNNNNCNANNNAAGGANNTANNNCTNNTN
NTNNNNNNNGGACNNNACNNNNCNNNNNNNNACNCNNNCNCNNNNNNANNNGNTTTTNNNCCNNTATTNT
CCAGNANGCNGNGCNNNCATTCANGNGAACNNNGNNACGCGNNAATCGTANNATCTACCTGCCGCNNG
NNNNNNTACNGNNNNNNNCGGANNNTNNCTCGTANNNNNNNNCATGCGTNNTNTNNTCNCNAGNNANGTT
CGNNNANANANTAGNANGTNTNNNNNNNNCTTGTNGCGTTCCGCNTGANNCNNCANNNNGNNGNNCTTC
TCANCGGCNNTANCTGCNGANNTAAGNCATANNNTNNNTCNNTANCTNNNNNGGNGGGNNACTGTAAA
NGGNNAATTNGNNNNTNANTNCNNNCATNTNNCTNNGNNNNCTNNNTNCCTGNNTNCNNAATTGGNNC
CANTGNNTGNCNATCTCTNGNTTNGNNNNNNNTNNNNNTNNCANNNNGGNNNGTNANANNNCNATNTAA
NNTANANNNNCNNNNNNANNNCACNNNNNNNGNAGNNNCNANNNNNANANNNGTAGNNNANTAAGTCT
CCGANNTNNNNNNNNATAAGCGNNANACNNNNNNNNNNNNNNANGANNACGNNTTGGNNNTNNNNNNNNNA
GNNAGACNNTNGTGGNTNNNNNTCAANNNNTTCGTAGTGGTATAATNGNNNNNNNTTTCNCCAGNNTN
NNNGNNNNNNNNCNATANNANNNNNNNGNGANNNNNNNNNNNNANNNNCTCNNNAGGGANCANCTACTNNA
GCAATGGNGCGCAGNNNGAGANNNNGACNNGCCTTGGGTGGCAANCACNGCAATAAGTNNTGTTNANC
CTNNNAGTNNACACGNNNNCANNGNNNCNNNNNNANCNGANNGNNNNNNNNNNNNNNNNNNNNNNNN
NNNNNNNNNNNNNNNNNNNNNNNNNNNNNNNNNNNNNNANNGNNNGTNTNTGACNCATGTGTTTTTANNNGNN
NNNNNACGNNNNANCCCCGGTCGNNNNCNNATTGAANNCCTGNNGCATNNNNNTNCNAGAATGANNGC
NNNNNGNNTNGNCTNNCNNNTCANGNAATCNANNAGGTANNNNGNTCNNNNNNNNNNNNNCNNNCCCNN
ACTGAGGGTNNNNNNNNCATCNNCTNNNNNNNNNCTNCGCNGNCGNNNCNNNNNNGCNGTATNNTGTNN
NNCANNNNNNNNNNNNNNNNNNTNAANNNANNNNNNNTNNNN
>PhuLa142
GTGNNNNNTAGCCCNNNNNAGTTNGACANNNCNNNNNNNNNNNNNTNNNNNNNNNNNNNNNNNNNNNNNN
NNNNNNNNNNNNNNNNNNNNNNNNNNNNNNNNNNNNNNNNNNNNNNNNNNNNNNNNNNNNNNNNNNNNNNNNNNNNNNNN
NNNNNNNNNNNNNNNNNNNNNNNNNNNNNNNNNNNNNNNNNNNNNNNNNNNNNNNNNNNNNNNNNNNNNNNNNNNNNNNN
NNNNNNNNNNNNNNNNNNNNNNNNNNNNNNNNNNNNNNNNNNNNNNNNNNNNNNNNNNNNNNNNNNNNNNNNNNNCNNNNN
NNNNNNNNNNNNNNNNNNNNNNNNNNNNNNNNNNNNNNNNNNNNNNNNNNNNNNNNNNNNNNNTCTNNNAAANNNTNNN
NNNNNNNNNNNNNNNNNNNNNNNNNNNNNNNCNCNNTGNNNGTAANCNANNNGNACATGCAGTNNNCTCNNNNN
NNNNNCAGAGNNNNNNNCNNNNNNNNNCCGNCCCCNNCCNNNNNNNNNNNNCNNNNNNNNNNNNANNNNNN
NNNNNNTGGTACNNNNCGNNNNNGGTTGNNTNAGAGGTCCACAACGNGGTCNNGNCACNNNNGNNNAN
NNNCNNNNNANNNNNNNNNNNNNNNNNNTNNNTANNAGGNNNNNNCCNTNNNNNNNNNNNNATTGCNATGACC
CNANNCACGGGGGCAGTNCCCGGCGCNGCGACCTTGNTNANGTCCGGGGACAAGTATGGCNNNCNCCC
CGGGAAGGANNCNGCCGTANGTGCNNNNNANNNNNTCNTGNGANGTCGCCGNNANNNNNGTGGTCNNN
NNAGAATGNNCNNACCANNCGNANNNTGGTNATGNNAGTNNNNNNNNNNNNNNNNNNNNNNNNNNNNNNN
NNNNNNNNNNNNNNNNNNNNNNNNNNNNNNNNNNNNNNNGNNNNGNNNNNNCNANNNAAGGANNTANNNCTNNTN

NTNNNNNNGGACNNNACNNNNCNNNNNNNACNCNNNCNCNNNNNNNNNNGNTTTNNNNNCNNTATTNT
CCAGNACGCNGNGCNNNCATTCANGNGAACNNNGNNACGCGNNAATCGTANNATCTACCTGCCGCNGG
NNNNNNTACNGNNNNNNCGGANNNTNNNNCGTANNNNNNTCATGCGTNNTNTNNTCNCNAGNCNNGTT
CGNNNANNNANTAGNANGTNTNNNNNNNNNNTGTNNCGTTCCGCNTGACNCNNCANNNNGNNGNNNTTN
TCANCGGCNNTANCTGCNGANNTAAGNCATANNNTNNATCNNTANCTNNNNNGGNGGGNNACTGTAAA
NGGNNAATTNGNNNNTNANTNCNNNCACNTNNCNNNGNNNNCTNNNTNCCTGNNTNCNNAATTGGNNC
CANTGNNTGNCNATCTCTGGNTTNGNNNNNNTNNNNNTNGCANNNNGGNNNGTNNNANNTCNATNTAA
NNTANANNNNCNNNNNNANNNCACNANNNNNGNAGNNNNNANNNTTANANNNGTAGNNNANTNAGTCT
CCGANNTNNNNNNNATAAGCGNNANACNNNNNNNNNNNNNNANGANNACGNNTTGGNNNTNNNNNNNNA
GNNAGACNNTNATGGNTNNNNNTCAANNNNTTNGTAGTGGTATAATNGNNNNNNNTTTCNCCAGNNTN
NNNGNCNNNNNCNATANNANNNNNNNGNGANNNNNNNNNNNANNNNCTCNNNAGGNANCANCTACTTNA
GCANNGGNGCGNAGNNNGAGANNNNGACNNGCCTTGGGTGGCANNCACNGCAATAAGTNNNGTTNNNN
NTNNNAGTNNACATNNNNNCANNGNNNCNNNNNNANCNGANNGNNNNNNNNNNNNNNNNNNNNNNNNN
NNNNNNNNNNNNNNNNNNNNNNNNNNNNNNNNNNNNNANNGNNNGTNTNTGACNCATNNNTTTTTANNNGNN
NNNNNACGNNNNANCCCCGGTCGNNNNCNNATTGAANNCCTGNNGCATNNNNNTNCNAGAATGANNGC
NNNNNGNNTNNNCTNNCNNNTCAAGNAATCANNNAGGTANNNNNNTCNNNGNNNNNNNNCNNNCCCNN
ACTGAGGGTNNNNNNNNCATNTGCTNNNNNNNNNNTNCGCNGGNNNNNCNNNNNAGCNGTATNGNGTNN
NNCANNNNNNNNNNNNNNNNNNTNNAACAANNNNNNTNNNN
>PhuLa143
GTGNNNNNTAGCCCGNNNNAGTCAGACANNNCNNNNNNNNNNNNTNNNNNNNNNNNNNNNNNNNNNNN
NNNNNNNNNNNNNNNNNNNNNNNNNNNNNNNNNNNNNNNNNNNNNNNNNNNNNNNNNNNNNNNNNNNNN
NNNNNNNNNNNNNNNNNNNNNNNNNNNNNNNNNNNNNNNNNNNNNNNNNNNNNNNNNNNNNNNNNNNNN
NNNNNNNNNNNNNNNNNNNNNNNNNNNNNNNNNNNNNNNNNNNNNNNNNNNNNNNNNNNNNNNNNNNNN
NNNNNNNNNNNNNNNNNNNNNNNNNNNNNNNNNNNNNNNNNNNNNNNNNNNNCNTCTNNNAAANNNTNNN
NNNNNNNNNNNNNNNNNNNNNNNNNNCNCNTTGNNNGTAANCNANNNGNACATGCAGTNCCCTCNNNNN
NNNNNCAGAGNNNNNNNNCNNNNNCNCCGGCCCCNNCCNNNNNNNNNNNNNNNNNGNNNNNNNANNNNNN
NNNNNNTGGTACNNNNCGNNNNNGGTTGTNTNAGAGGTCCACAACGNGGTCNNGNCACNNNNGNNNAN
NNNCNNNNAACNNNNNNNNNNNNNNNNTNNNTANNAGGNNNNNNCCNTCNNNNNANNNATTGCNATGACC
CAANNCACGGGGGCAGTCCCCGGNGCGGCGACCTTGNTNANNTCCGGGGACAAGTATGGCGNNCNCCC
CGGGAAGGANNCNGCCGTANGTGCNNNNNANNNNNTCNTGNGANGTNGCCGNNATTCNCGTGGTCNNN
CNAGAATGCCCGNACCANNCGNANNNTGGTNATGNTAGTNNNNNNNNNNNNNNNNNNNNNNNNNNNNN
NNNNNNNNNNNNNNNNNNNNNNNNNNNNNNNNNNNGNNNNGNNNNNNCNANNNAAGGANNTANNNCTNNTN
NTNNNGNNGGACNNNACNNNNCNNNNNNNACNCNNNCNCNNNNNNNNNNGNTTTTTNNCCNNTATTNT
CCAGNANGCNGNGCNNTCATTTANGNGAACNNNGNNACGCGNNAATCGTANNATCTACCTGCCGCNNG
NNNNNNTACNGNNNNNNCGGANNNTNNCTCGTANNNNNNTCATGCGTNNTNTNNTCNCNAGNCANGTT
CGNNNANANANTAGNANGTNTCNNNNNNNNTGTNNCGTTCCGCNTGANNCNNCANNNNGNCGNNCTTN
TCANCGGCNNTANCTGCNGANNTAAGTCATANNNTNNATCNNTANCTNNNNNGGAGGGNNACTGTAAA
NGGNTAATTNGNGGNTNANTNCNNNCATNTNNCNNNGNNTNCTNNNTNCCTGNNTNCNNAATTGGNNC
CANTGNNTGNCNATCTCTGGNTTNGCNNNNNTNNNNNTNGCAANNNGGNNNGTNANANNNCNATNTAA
NNTATANNNNCNNNNNNANNNCACNANNNNNGNAGNNNCNANNNGTANANNNGTAGNNNNATAAGTCT
CCGANNTNNNNNNNATAAGCGNNANACNNNNNNNNNNNNNNANGANNACGNNTTTGNNNTNNNNNNCNA
GNNAGANNNTNGTGGNTNNNNNTCAANNNNTTNGTAGTGGTATAATNGNNNNNNNTTTCNCCAGNNTN
NNNNNNNNNNNCNATANNANNNGNNNGNGANNNNNNNNNNNANNNNCTCNNNAGGGANCANCTACTTNA
GCANTGGNGCGCAGNNNGAGANNNNGACNNGCCTTGGGTGGCAANCACNGCAATAAGTNNNGTTNANC
CTTNNAGTNNACACGNNNNCANNGNNNCNNNNNNANCNGANNGNNNNNNNNNCNNNNNNNNNNNNNNN
NNNNNNNNNNNNNNNNNNNNNNNNNNNNNNANNNNANGGNNNGTNTNTGACNCATNNNTTTTTANNNGNN
NNNNNACGNNNNANCCCCGGTCGNNNNCNNATTGAANNCCTGNNGCATNNNNNTNCNAGAATGANNGC
NNNNNGNNTNGNCTGNCNNNTCAAGNAATCANNTANGTANNGCGNTCNNNGNNNNNNNNCNNTCCCNN
ACTGAGGGTNNNNNNCGCATCTGCTNNNNNNNNNNTNCGCNGGNNNNNCNNNNNAGCNGTATNGTGTNN
NNCANNNNNNNNNNNNNNNNNNTNAAACNANNNNNNNTNNNG
>PhuLa144
GTGNNNNNTAGCCCNNNNNAGTTAGACANNNCNNNNNNNNNNNTTNNNNNNNNNNNNNNNNNNNNNNN
NNNNNNNNNNNNNNNNNNNNNNNNNNNNNNNNNNNNNNNNNNNNNNNNNNNNNNNNNNNNNNNNNNNNN
NNNNNNNNNNNNNNNNNNNNNNNNNNNNNNNNNNNNNNNNNNNNNNNNNNNNNNNNNNNNNNNNNNNNN

NNNNNNNNNNNNNNNNNNNNNNNNNNNNNNNNNNNNNNNNNNNNNNNNNNNNNNNNNNNN
NNNNNNNNNNNNNNNNNNNNNNNNNNNNNNNNNNNNNNNNNNNNNNNNTCTNNNAAANNNTNNN
NNNNNNNNNNNNNNNNNNNNNNNNNNCNCNNTNNNNGTAANCNANNNGNACATGCAGTCCCCTCNNNNN
NNNNNCAGAGNNNNNNNCNNNNNNNNCCGGCCCCGNCCNNNNNNNNNNNCNNNGNNNNNNNGNNNNNN
NNNNNNTGGTACNNNNCGNNNNNGGTTGTNTAAGAGGTCCACCACGNGGTCNNGNCACNNNNGNNNAN
NNNCNNNNNNNNNNNNNNNNNNNNNTNNNCANNAGGNNNNNNCCNTCNNGNNNNNNATNGCNATGACC
CNANNCACGGGGGCNGTNCCCGGNGCGGNGACCTTGNNNANNGCCGGGGACAAGTATGGCNNNCNCCC
CGGGAAGGANNCNGCCGTANGTGCNNNNNANNNNNTCNTNNGANNTNGCCGNNATTNNNNTGGTCNNN
CNAGANTGNNCGNACCANNCGNANNNTGGTNATGNTAGTNNNNNNNNNNNNNNNNNNNNNNNNNNN
NNNNNNNNNNNNNNNNNNNNNNNNNNNNNNNNNNNNNNNGNNNNNNCNANNNAAGGANNTANNNCTNNTN
NTNNNNNNNGGACNNNACNNNNCNNNNNNNACNCNNNCNCNNNNNNNNNNNGNTTTTNNNNCNNTATTNT
CCAGNANGCNGNGCNNNCATTCANGNGAACNNNGNNACGCGNNAATCGTANNATCTACCTGCCGCNNG
NNNNNNTACNGNNNNNNCGGANNNTNNNTCGTANNNNNNNTCATGCGTNNTNTNNTCNCNAGNNNNGTT
CGAGAANANANTAGNANGTNTCNNNNNNNNTGTNNCGTTCCGCNTGANNCNNCANNNNGNNGNNCTTN
NCANCGGCNNTANCTNCNGANNTAAGNCATANNNTNNNTCNNTANCTNNNNNGGNGGGNNACTGTAAA
NGGNNAATTNGANNNTNATTNCNNNCACNTNNCTNNGNNTNCTNNNTNCCTGNNTNCNNAATTGGNNC
CANTGNNTGNCNATCTCTGGNTTNGNNNNNNTNNNNNTNNCANNNNGGNNNNNNANANCNCNATNTAA
NNTANANNNNCNNNNNNANNNCACNANNNNNNNAGNNNNNANNNTTANANNNGTAGNNNNNTAAGTCT
CCGNNNTNNNNNNNATAAGCGNNANACNNNNNNNNNNNNNNANGANNACGNNTTGGNNNTNNNNNNCNA
ANNAGACNNTNGTGGNTNNNNNTCAANNNNTTCGTAGTGGTATAACNGNNNNNNNTTTCNCCAGNNTN
NNNGNNNNNNNNCNATANNANNNNNNNGNGANNNNNNNNNNNANNNNCTCNNNAGGGANCANCTACTNNA
NCANNGGNGCGNAGNNNGAGANNNNGACNAGCCTTGGGTGGCAANNACNGCAATAAGTNNNGTTNANC
CTNNNAGTNNACACNNNNNCANNGNNNCNNNNNNANCNGANNTNNNNNNNNNNNNNNNNNNNNNNN
NNNNNNNNNNNNNNNNNNNNNNNNNNNNNNANNNNANGGNNNGTNTNTGACNCATNNNTTTTTANNNGNN
NNNNNACGNNNNANCCCCGGTCGNNNNCNNATTGAANNCCTGNNGCATNNNNNTNCNAGAATGANNGC
NNNNNGNNTNGNCTGNCNNNTCAAGNAGTCANNNAGNNNNNNCGNTCNNNNNNNNNNNNCNNNCCCNN
ACTGAGGGTNNNNNNNNCATCNGCTNNNNNNNNNNTNCGCNGNCGTNNCNNNNNNGCNGTATNNNGTTA
NNCANNNNNNNNNNNNNNNNNTNAAACNANNNNNNNTNNNN

>PhuLa145

GTGNNNNNTAGCCCGNNNNAGTTAGACANNNCNNNNNNNNNNNNTNNNNNNNNNNNNNNNNNNNNNN
NNNNNNNNNNNNNNNNNNNNNNNNNNNNNNNNNNNNNNNNNNNNNNNNNNNNNNNNNNNNNNNN
NNNNNNNNNNNNNNNNNNNNNNNNNNNNNNNNNNNNNNNNNNNNNNNNNNNNNNNNNNNNNNNN
NNNNNNNNNNNNNNNNNNNNNNNNNNNNNNNNNNNNNNNNNNNNNNNNNNNNNNNNNNNNNNNN
NNNNNNNNNNNNNNNNNNNNNNNNNNNNNNNNNNNNNNNNNNNNNNNNTCTNNNAAANNNTNNN
NNNNNNNNNNNNNNNNNNNNNNNNNNCNCNTTGNNNGTAANCNANNNGNACATGCAGTCCCCTCNNNNN
NNNNNCAGAGNNNNNNNCCNNNNNNNCCGNCCCCNNCCNNNNNNNNNNNNNNNNNNNNNNNNNGNNNNNN
NNNNNNTGGTACNNNNCGNNNNNGGTTGNNTNAGAGGTCCACCACGNGGTCNNGNCACNNNNGNNNAN
NNNCNNNNNANNNNNNNNNNNNNNNNTNNNCANNAGGNNNNNNCCNTNNNNNNANNNANNGCNATGACC
CAANNNACGGGGGCAGTNCCCGGCGCGGNGACCTTGATNACGGCCGGGGACAAGTATGGCNTNCNCCC
CGGGAAGGANNCNGCCGTANGTGCNNNNNANNNNNTCNTGNGACGTNGCCGNNATTCNNGTGGTCNNN
CNAGANTGNCCGNACCANNCGNANNNTGGTNATGNNAGTNNNNNNNNNNNNNNNNNNNNNNNNNNN
NNNNNNNNNNNNNNNNNNNNNNNNNNNNNNNNNNNNNNNGNNNNNNCNANNNAAGGNNNTANNNCTNNTN
NTNNNNNNNGGACNNNACNNNNCNNNNNNNACNCNNNCNCNNNNNNNNNNNGNTTTTTNNNCNNTATTNT
CCAGNACGCNGNGCNNNCATTCANGNGAACNNNGNNACGCGNNAATCGTANNATCTACCTGCCGCNGG
TNNNNNTACNGNNNNTNCGGANNNTNNCTCGTANNNNNNNNCATGCGTNNTNTNNTCNCNAGNCNNGTT
CGNNNANANANTAGNANGTNTNNNNNNNNCNTGTNNCGTTCCGCNTGANNCNNCANNNNGNNGNNNTTN
NCANCGGCNNTANCTGCNGANNTAAGNCATANNNTNNNTCNNTANCTNNNNNGGNGGGNNACTGTAAA
NGGNNAATTGGNNGNTNANTTCNNNCACNTNNCTNNGNNTNCTNNNTNCCTGNNTNCNNAATTGGGNC
CANTGNNTGNCNATCTCTGGNTTNGCNNNNNTNNNNNTNNCAANNNGGNNNGTNNNANNNCNATNTAA
NNTANANNNNCNNNNNNANNNCACNNNNNNNNNNAGNNNCNANNNTTANANNNGTAGNNNANTAAGTCT
CCGANNTNNNNNNNATAAGCGNNANACNNNNNNNNNNNNNNANGANNACGNNTTGGNNNTNNNNNNCNA
ANNAGACNNTNGTGGNTNNNNNTCAANNNNTTCGTAGTGGTATAACNGNNNNNNNTTTCNCCAGNNTN
NNNGNNNNNNNNCNATANNANNNNNNNGNGANNNNNNNNNNNANNNNCTCNNNAGGNANCANCTACTNNA
GCAATGGNGCGCAGNNNGAGANNNNGACNNGCCTTGGGTGGNAANCACNGCAATAAGTNNTGTTNANC

CTNNNAGTNNACACNNNNNCANNGNNNCNNNNNNANCNGANNTNNNNNNNNNNNNNNNNNNNNNNNNNNN
NNNNNNNNNNNNNNNNNNNNNNNNNNNNNNAGNNNANGGNNNGTNTNTGACNCATGTNTTTTTANNNGNN
NNNNNNNGNNNNANCCCCGGTCGTNNNCNNATTGAANNCCTGNNGCATNNNNNTNCNAGAATGANNGC
NNNNNGNNTNGNCTNNCNTTTCAAGNAGNCAANTAGGNANNNCGNTCNNNNNNNNNNNNNCNNTCCCNN
ACTGAGGGTNNNNNNNNCATCTGCTNNNNNNNNNNTNCGCNGNCGTNNCNNNNNAGCNGTATNNTGTNN
NNCANNNNNNNNNNNNNNNNNNTNAAACNNNNNNNNNTNNNN
>PhuLa146
GTGNNNNNTAGCCCNNNNNAGTTAGACANNNCNNNNNNNNNNNNNNNNNNNNNNNNNNNNNNNNNNNNNN
NNNNNNNNNNNNNNNNNNNNNNNNNNNNNNNNNNNNNNNNNNNNNNNNNNNNNNNNNNNNNNNNNNNNNN
NNNNNNNNNNNNNNNNNNNNNNNNNNNNNNNNNNNNNNNNNNNNNNNNNNNNNNNNNNNNNNNNNNNNNN
NNNNNNNNNNNNNNNNNNNNNNNNNNNNNNNNNNNNNNNNNNNNNNNNNNNNNNNNNNNNNNNNNNNNNN
NNNNNNNNNNNNNNNNNNNNNNNNNNNNNNNNNNNNNNNNNNNNNNNNNNNNNNTCTNNNAAANNNTNNN
NNNNNNNNNNNNNNNNNNNNNNNNNNNCNCNTTGNNNGTAANCNANNNGNACATGCAGTCCCCTCNNNNN
NNNGGCAGAGNNNNNNCCCNNNNNNNCCGGCCCCNNCCNNNNNNNNNNNNNNNNNNNNNNNNNGNNNNNN
NNNNNNTGGTACNNNNCGNNNNNGGTTGTNTAAGAGGTCCACCACGNGGTCNGGNCACNNNNGNNNAN
NNNCNNAAAACNNNNNNNNNNNNANNTNNNCANNAGGNCNNNNCCNTCNNGNNNNNAATNGCNATGACC
CAANNNACGGGGGNNGTNCCCGGCGCGGCGACCTTGATNACGGCCGGGGACAAGTATGGCNNNCNCCC
CGGGAAGGANNCNGCCGTANGTGCNNNNNANNNNNTCNTGNGANGTCGCCGNNATTCNCGTGGTCNNN
NNNGAATGNNCNNACCANNCGNANNNTGGTNATGNTAGTNNNNNNNNNNNNNNNNNNNNNNNNNNNNNNN
NNNNNNNNNNNNNNNNNNNNNNNNNNNNNNNNNNNNNNNGNNNNNNCNANNNAAGGNNNTANNNCTNNTN
NTNNNNNNGGACNNNACNNNNCNNNNNNNACNCNNNCNCNNNNNNANNNGNTTNTTNNNCNNTANTNT
CCAGNACGCNGNGCNNNCATTCANGNGAACNNNGNNACGCGNNAATCGTANNATCTACCTGCCGCNNG
NNNNNNTACNGNNNNNNCGGANNNTNNCNCGTANNNNNNNCATGCGTNNTNTNNTCNCNAGNCNNGTT
CGNNNANANANTAGNANGTNTCNNNNNNNNTGTNNCGTTCCGCNTGANNCNNCANNNNGNNGNNNTTC
TCANCGGCNNTANCTGCNGANNTAAGNCATANNNTNNATCNNTANCTNNNNNGGNGGGNNACTGTAAA
NGGNNAATTGGNNNNTNATTNCNNNCACNTNNCTNNGNNNNCTNNNTNCCTGNNTNCNNAATTGGNNC
CANTGNNTGNCNATCTCTNGNTTNGNNNNNNTNNNNNTNNCAANNNGGNNNGTNANANCNCNATNTAA
NNTATANNNNCNNNNNNANNNCACNNNNNNNGNAGNNNCNANNNTTANANNNGTAGNNNANTAAGTCT
CCGANNTNNNNNNNATAAGCGNNANACNNNNNNNNNNNNNANGANNACGNNTTGGNNNTNNNNNNCNA
ANNAGACNNTNGTGGNTNNNNNTCAANNNNTTNGTAGTGGTATAACNGNNNNNNNTTTCNCCAGNNNN
NNNGNNNNNNNCNATANNANNNNNNNGNGANNNNNNNNNNNANNNNCTCNNNAGGNANCANCTACTNNA
GCANTGGNGCGCAGNNNGAGANNNNGACNNGCCTTGGGTGGCAANCACNGCAATAAGTNNGTTNNNN
NTNNNAGTNNACACNNNNNCANNGNNNCNNNNNNANCNGANNTNNNNNNNNNNNNNNNNNNNNNNNNNNN
NNNNNNNNNNNNNNNNNNNNNNNNNNNNNNAGNNNANNGNNNGTNTNTGACNCATGTGTTTTTANNNGNN
NNNNNACGNNNNANCCCCGGTCGNNNNCNNATTGAANNCCTGNNGCATNNNNNTNCNAGAATGANNGC
NNNNNGNNTNGNCTNNCNNTTCANGNAGTCANNNAGGNANNGCGNTCNNNGNNNNNNNNCNNTCCCNN
ACTGAGGGTNNNNNNNNCATCTGCTNNNNNNNNNNTNCGCCGNCGTNNCNNNNNAGCNGTATNGNGTNA
NNCANNNNNNNNNNNNNNNNNNTNNAACAANNNNNNNTNNNN
>PhuLa147
GTGNNNNNTAGCCCNNNNNAGTTAGACANNNCNNNNNNNNNNNNNTNNNNNNNNNNNNNNNNNNNNNNNN
NNNNNNNNNNNNNNNNNNNNNNNNNNNNNNNNNNNNNNNNNNNNNNNNNNNNNNNNNNNNNNNNNNNNNN
NNNNNNNNNNNNNNNNNNNNNNNNNNNNNNNNNNNNNNNNNNNNNNNNNNNNNNNNNNNNNNNNNNNNNN
NNNNNNNNNNNNNNNNNNNNNNNNNNNNNNNNNNNNNNNNNNNNNNNNNNNNNNNNNNNNNNNNNNNNNN
NNNNNNNNNNNNNNNNNNNNNNNNNNNNNNNNNNNNNNNNNNNNNNNNNNNNNNTCTNNNAAANNNTNNN
NNNNNNNNNNNNNNNNNNNNNNNNNNNCNCNTTGNNNGTAANCNANNNGNACATGCAGTCCCCTCNNNNN
NNNNNCAGAGNNNNNNCCCNNNNNNNCCGNCCCCNNCCNNNNNNNNNNNNNCNNNGNNNNNNNGNNNNNN
NNNNNNTGGTACNNNNCGNNNNNGGTTGNNTNAGAGGTCCACCACGNGGTCNGGNCACNNNNGNNNAN
NNNCNNNNAACNNNNNNNNNNNNANATNNNCANNAGGNCNNNNCCNTCNNNNNNNNNNANTGCNATGANN
CAANNCACGGNGGCAGTNCCCGGCGCGGCGACCTTGNNNACGGCCGGGGACAAGTATGGCNNNCNCCC
CGGGAAGGANNCNGCCGTANGTGCNNNNNANNNNNTCNTGNGANGTCGCCGNNATTCNCNTGGTCNNN
CNAGANTGNNCGNACCANNCGNANNNTGGTNATGTTAGTNNNNNNNNNNNNNNNNNNNNNNNNNNNNNNN
NNNNNNNNNNNNNNNNNNNNNNNNNNNNNNNNNNNNNNNGNNNNNNCNANNNAAGGANNTANNNCTNNTN
NTNNNNNNGGACNNNACNNNNCNNNNNNNACNCNNNCNCNNNNNNANNNGGTTTTTNNCCNNTATTNT
CCAGNACGCNGNGCNNNCATTCANGNGAACNNNGNNACGCGNNAATCGTANNATCTACCTGCCGCNNG

NNNNNNTACNGNNNNNNCGGANNNTNNNNCGTANNNNNNNNCATGCGTNNTNTNNTCNCNAGNCNNGTT
CGNNNANANANTAGNANGTNTNNNNNNNNNNTGTNNCGTTCCGCNTGANNCNNCANNNNGNNGNNCTTN
NCANCGGCNNTANCTGCNGANNTAAGTCATANNNTNNATCNNTANCTNNNNNGGAGGGNNACTGTAAA
NGGNNAATTNGNNNNTNANTTCNNNCACNTNNCTNNGNNTNCTNNNTNCCTGNNTNCNNAATTGGNNC
CANTGNNTGNCNATCTCTGGNTTNGNNNNNNTGNNNNTNNCAANNNGGNNNGTNANANNNCNATNTAA
NNTANANNNNCNNNNNNANNNCACNANNNNNGNAGNNNCNANNNTTANANNNGTAGNNNAATAAGTCT
CCGANNTNNNNNNNATAAGCGNNANACNNNNNNNNNNNNNNANGANNACGNNTTGGNNNTNNNNNNCNA
ANNAGACNNTNGTGGNTNNNNNTCAANNNNTTCGTAGTGGTATAACNGNNNNNNNTTTCNCCAGNNTN
NNNGNNNNNNNNCNATANNANNNNNNNGNGANNNNNNNNNNNANNNNCTCNNNAGGNANCANCTACTTNA
GCANTGGNGCGCAGNNNGAGANNNNGACCAGCCTTGGGTGGCAANCACNGCAATAAGTNNNGTTNANC
CTNNNAGTNNACACNNNNNCANNGNNNCNNNNNNANCNGANNTNNNNNNNNNNNNNNNNNNNNNNNNN
NNNNNNNNNNNNNNNNNNNNNNNNNNNNNANNNNANGGNNNGTNTNTGACNCATGTGTTTTTANNNGNN
NNNNNACGNNNNANCCCCGGTCGNNTNCNNATTGAANNCCTGNNGCATNNNNNTNCNAGAATGANNGC
NNNNNGNNTNNNCTNNCNNTTCAAGNAGTCAANTAGGTNNCGCGNTCNNNNNNNTNNNNCNNTCCCNN
ACTGAGGGTNNNNNNCGCATCTGCTNNNNNNNNNNTTCGCNGNCGTNNCNNNNNAGCNGTATNGTGTNA
NNCANNNNNNNNNNNNNNNNNTNAAACNANNNNNNTNNNN
>PhuLa148
GTGNNNNNNAGCCCNNNNNAGTTAGACANNNCNNNNNNNNNNNNTNNNNNNNNNNNNNNNNNNNNNN
NNNNNNNNNNNNNNNNNNNNNNNNNNNNNNTNNNNNNNNNNNNNNNNNNNNNNNNNNNNNNNNNNNNN
NNNNNNNNNNNNNNNNNNNNNNNNNNNNNNNNNNNNNNNNNNNNNNNNNNNNNNNNNNNNNNNNNNNN
NNNNNNNNNNNNNNNNNNNNNNNNNNNNNNNNNNNNNNNNNNNNNNNNNNNNNNNNNNNNNNNNNNNN
NNNNNNNNNNNNNNNNNNNNNNNNNNNNNNNNNNNNNNNNNNNNNNNNNNNNNNTCTNNNAAANNNTNNN
NNNNNNNNNNNNNNNNNNNNNNNNNNCNCNTTGNNNGTAANCNANNNGNACATGCAGTCCCCTCNNNNN
NNNNNCAGAGNNNNNNNNCNNNNNCNCCGGCCCCNNCCNNNNNNNNNNNNCNNNNNNNNNNNANNNNNN
NNNNNNTGGTACNNNNCGNNNNNGGTTGTTTAAGAGGTCCACAACGNGGTCNNGNCACNNNNGNNNAN
NNNCNNNNNNNCNNNNNNNNNNNNNNNTNNNTANNAGGNCNNNNCCNTCNNNNNANTNANTGCNATGACC
CAANTCACGGNGGNNGTNCCCGGCGNNGCGACCTTGANNANGTCCGGGGACAAGTATGGCNCNCNCCC
CGGGAAGGANNCNGCCGTANGTGCNNNNNANNNNNTCNTGNGANGTNGCCGNNATTCNCGTGGTCNNN
NNAGAATGNCCGNACCANNCGNANNNTGGTNATGNTAGTNNNNNNNNNNNNNNNNNNNNNNNNNNNNN
NNNNNNNNNNNNNNNNNNNNNNNNNNNNNNNNNNGNNNNGNNNNNNCNANNNAAGGNNNTANNNCTNNTN
NTNNNNNNNGGACNNNACNNNNCNNNNNNNNACNCNNNCNCNNNNNNNNNNNGGTTTTTNNNCNNTATTNT
CCAGNANGCNGNGCNNNCATTCANGNGAACNNNGNNACGCGNNAATCGTANNATCTACCTGCCGCNNG
NNNNNNTACNGNNNNNNCGGANNNTNNCTCGTANNNNNNNTCATGCGTNNTNTNNTCNCNAGNCANGTT
CGNNNANANANTAGNANGTNTCNNNNNNCTTGTNNCGTTCCGCNTGANNCNNCANNNNGNCGNNCTTC
TCANCGGCNCTANCTGCNGANNTAAGNCATANNNTNNATCNNTANCTNNNNNGGNGGGNNACTGTAAA
NGGNNAATTNGNNNNTNATTTCNNNCACNTNNCTNNGNNNNCTNNNTNCCTGNNTNCNNAATTGGNNN
CANTGNNTGNCNATCTCTGGNTTNGNNNNNNTGNNNNTNNCAANNNGGNNNGTNANANCNCNATNTAA
NNTANANNNNCNNNNNNANNNCACNNNNNNNNNNAGNNNCNANNNTTANANNNGTAGNNNAATAAGTCT
CCGANNTNNNNNNNATAAGCGNNANACNNNNNNNNNNNNNNANGANNACGNNTTGGNNNTNNNNNNCNA
GNNAGACNNTNATGGNTNNNNNTCAANNNNTTNGTAGTGGTATAATNGNNNNNNNTTTCNCCAGNNTN
NNNGNNNNNNNNCNATANNANNNNNNNGNGANNNNNNNNNNNANNNNCTCNNNAGGGANCANCTACTTNA
GCAATGGNGCGCAGNNNGAGANNNNGACCAGCCTTGGGTGGCAANCACNGCAATAAGTNNNGTTNANC
NTNNNAGTNNACATNNNNNCANNGNNNCNNNNNNANCNGANNGNNNNNNNNNNNNNNNNNNNNNNNNN
NNNNNNNNNNNNNNNNNNNNNNNNNNNNNAGNNNANNGNNNGTNTNTGACNCATGTGTTTTTANNNGNN
NNNNNACGNNNNANCCCCGGTCGNNNNCNNATTGAANNCCTGNNGCATNNNNNTNCNAGAATGANNNC
NNNNNGNNTNGNCTNNCNNNTCAAGNAATCANNNAGGNNNNNNGNTCNNNGNNNNNNNNCNNTCCCNN
ACTGAGGGTNNNNNNNNCATCTGCTNNNNNNNNCTNCGCNGGNNNNNCNNNNNAGCNGTATNNTGTNA
NNCANNNNNNNNNNNNNNNNNTNAANNAANNNNNNTNNNN
>PhuLa149
GTGNNNNNTAGCCCGNNNNAGTTAGACANNNCNNNNNNNNNNNNTNNNNNNNNNNNNNNNNNNNNNN
NNNNNNNNNNNNNNNNNNNNNNNNNNNNNNTNNNNNNNNNNNNNNNNNNNNNNNNNNNNNNNNNNNNN
NNNNNNNNNNNNNNNNNNNNNNNNNNNNNNNNNNNNNNNNNNNNNNNNNNNNNNNNNNNNNNNNNNNN
NNNNNNNNNNNNNNNNNNNNNNNNNNNNNNNNNNNNNNNNNNNNNNNNNNNNNNNNNNNNNNNNNNNN
NNNNNNNNNNNNNNNNNNNNNNNNNNNNNNNNNNNNNNNNNNNNNNNNNNNNNNCNTCTNNNAAANNNTNNN

NNNNNNNNNNNNNNNNNNNNNNNNNCNCNNTGNNNGTAANCNANNNGNACATGCAGTCCCCTCNNNNN
NNNGGCAGAGNNNNNNCCCNNNNNNNCCGGCCCCNNCCNNNNNNNNNNNNNNNNNNNNNNNANNNNNN
NNNNNNTGGTACNNNNCGNNNNNGGTTGTNTNAGAGGTCCACAACGNGGTCNGGNCACNNNNGNNNAN
NNNCNNAAAANNNCNNNNNNNNNNNTNNNTANNAGGNCNNNNCCNTCNNNNNANNAATTGCNATGACC
CAANNCACGGGGGCAGTNCCCGGCGCGGCGACCTTGANNANNTCCGGGGACAAGTATGGCGNNCNCCC
CGGGAAGGANNCNGCCGTANGTGCNNNNNANNNNNTCNTGNGANGTNGCCGNNATTNNCGTGGNCNNN
CNAGANTGNNCGNACCANNCGNNNNNTGGTNATGNNAGTANNNNNNNNNNNNNNNNNNNNNNNNNNNN
NNNNNNNNNNNNNNNNNNNNNNNNNNNNNNNNGNNNNGNNNNNNCNANNNAAGGANNTANNNCTNNTN
NNNNNNNNGGACNNNACNNNNCNNNNNNNACNCNNNCNCNNNNNNNNNNGNTTTTTNNNCNNTATTNT
CCAGNANGCNGNGCNNNCATTCANGNGAACNNNGNNACGCGNNAATCGTANNATCTACCTGCCGCNNG
NNNNNNTNCNGNNNNTNCGGANNNTNNCTNGTANNNNNNTCATGCGTNNTNTNNTCNCNAGNCANGTT
CGNNNANANANTAGNANGTNTNNNNNNNNNTGTNNCGTTCCGCNTGANNCNNCANNNNGNNGNNCTTN
TCANCGGCNNTANCTGCNGANNTAAGNCATANNNTNNATCNNTANCTNNNNNGGAGGGNNACTGTAAA
NGGNNAATTNGNNNNTNANTNCNNNCACNTNNCNNNGNNNNCTNNNTNCCTGNNTNCNNAATTGGGNC
CANTGNNTGNCATCTCTGGNTTNGNNNNNNTNNNNNNTNNCAANNNGGNNNGTNANANNNCNATNTAA
NNTANANNNNCNNNNNNANNNCACNANNNNNNNAGNNNNNANNNTNANANNNGTAGNNNANTAAGTCT
CCGANNTNNNNNNATAAGCGNNANACNNNNNNNNNNNNNNANGANNNCGNNTTGGNNNTNNNNNNCNA
GNNAGACNNTNATGGNTNNNNNTCAANNNNTTCGTAGTGGTATAATNGNNNNNNNTTTCNCCAGNNTN
NNNGNNNNNNNCNATANNANNNNNNNGNGANNNNNNNNNNNNNNNCTCNNNAGGGANCANCTACTNNA
GCANTGGNNCGNAGNNNGAGANNNNGACNNNCCTTGGGTGGCAANCACNGCAATAAGTNNNGTTNANC
CTNNNAGTNNACATNNNNNCANNGNNNCNNNNNNANCNGANNGNNNNNNNNNNNNNNNNNNNNNNNNN
NNNNNNNNNNNNNNNNNNNNNNNNNNNNAGNNNANGGNNNGTNTNTGACNCANGTGTTTNTANNNGNN
NNNNNNCGNNNNANCCCCGGTCGNNNNCNNATTGAANNCCTGNNGCATNNNNNTNCNAGAATGANNGC
NNNNNGNNTNGNCTNNCNTTTCAAGNAATCANNNAGGTANNNNNNTCNNNNNNNNNNNNCNNTCCCNN
ACTGAGGGTNNNNNNNCATCNGCTNNNNNNNNNNTNCGCNGGCGTNNCNNNNNAGCNGTATNNNGTNA
NNCANNNNNNNNNNNNNNNNTNAAACNANNNNNNTNNNN
>PhuLa150
GTGNNNNNTAGCCCNNNNNAGTTAGACANNNCNNNNNNNNNNNNTNNNNNNNNNNNNNNNNNNNNNNN
NNNNNNNNNNNNNNNNNNNNNNNNNNNNNNNNNNNNNNNNNNNNNNNNNNNNNNNNNNNNNNNNNNNN
NNNNNNNNNNNNNNNNNNNNNNNNNNNNNNNNNNNNNNNNNNNNNNNNNNNNNNNNNNNNNNNNNNNN
NNNNNNNNNNNNNNNNNNNNNNNNNNNNNNNNNNNNNNNNNNNNNNNNNNNNNNNNNNNNNNNNNNNN
NNNNNNNNNNNNNNNNNNNNNNNNNNNNNNNNNNNNNNNNNNNNNNNNNNNNTCTNNNAAANNNTNNN
NNNNNNNNNNNNNNNNNNNNNNNNNCNCNTTGNNNGTAANCNANNGGNACATGCAGTCCCCTCNNNNN
NNNGNCAGAGNNNNNNNCCNNNNNNNCCGNCCCCNNCCNNNNNNNNNNNNNNNNNNNNNNNANNNNNN
NNNNNNTGGTACNNNNCGNNNNNGGTTGTNTAAGAGGTCCACAACGNGGTCNNGNCACNNNNGNNNAN
NNNCNNNNANNNNNNNNNNNNNNNNTNNNTANNAGGNCNNNNCCNTNNNNNNNNNNANNGCNATGANN
CAANNCNCNGGGGCAGTCCCCGGNGCGGCGACCTTGNNNACNTCCGGGGACAAGTATGGCNCNCNCCC
CGGGAAGGANNCNGCCGTANGTGCNNNNNANNNNNTCNTGNGACGTNGCCGNNATNNNNGTGGTCNNN
CNAGAATGNNCGNACCANNCGNANNNTGGTNATGNTAGTNNNNNNNNNNNNNNNNNNNNNNNNNNNNN
NNNNNNNNNNNNNNNNNNNNNNNNNNNNNNNNNNNNNGNNNNNNCNANNCAAGGNNNTANNNCTNNTN
NTNNNNNNGGACNNNACNNNNCNNNNNNNACNCNNNCNCNNNNNNNNNNGNTTTTTNNNCNNTANTNN
CCAGNACGCNGNGCNNTCATTCANGNGAACNNNGNNACGCGNNAATCGTANNATCTACCTGCCGCNNG
NNNNNNTACNGNNNNNNCGGANNNTNNNTNGTANNNNNNTCATGCGTNNTNTNNTCNCNAGNCNNGTT
CGNNNANANANTAGNANGTNTNNNNNNNNNTGTNNCNTNNCGCNTGANNCNNCANNNNGNNGNNCTTC
TCANCGGCNNTANCTNCNGANNTAAGNCATANNNTNNNTCNNTANCTNNNNNGGAGGGNNACTGTAAA
NGGNNAATTNGNNGNTNANTNCNNNCACNTNNCNNNGNNNNCTNNNTNCCTGNNTNCNNAATTGGNNC
CANTGNNTGNCATCTCTGGNTTNGNNNNNNTNNNNNNTNNCAANNNGGNNNNNNNNANNNCNATNTAA
NNTATANNNNCNNNNNNANNNCACNANNNNNNNAGNNNNNANNNTTANANNNGTAGNNNANTAAGTCN
CCGANNTNNNNNNATAAGCGNNANACNNNNNNNNNNNNNNANGANNACGNNTTGGNNNTNNNNNNCNA
GNNAGACNNTNATGGNTNNNNNTCAANNNNTTNGTAGTGGTATAATNGNNNNNNNTTTCNCCAGNNTN
NNNGNNNNNNNCNATANNANNNNNNNGNGANNNNNNNNNNANNNNCTCNNNAGGGANCANCTACTNNA
GCANNGGNGCGCAGNNNGAGANNNNGACNNNCCTTGGGTGGCAANCACNGCANTAAGTNNNGTTNANC
CTNNNAGTNNACATNNNNNCANNGNNNCNNNNNNANCNGANNGNNNNNNNNNNNNNNNNNNNNNNNNN
NNNNNNNNNNNNNNNNNNNNNNNNNNNNAGNNNANNGNNNGTNTNTGACNCATGNNTTTTTANNNGNN

NNNNNACGNNNGANCCCCGGTCGNNNNCNNATTGAANNCCTGNNGCATNNNNNTNCNAGAATGANNGC
NNNNNGNNTNGNCTGNCTTTTCAAGNAATCNNNNANNNNNNNNNNTCNNNNNNNNNNNNCNNTCCCNN
ACTGAGGGTNNNNNNNNCATCNGCTNNNNNNNNCTTCGCNGNCNNNNCNNNNNAGCNGTATNNTGTNA
NNCANNNNNNNNNNNNNNNNNTNAAACNANNNNNNNTNNNN
>PhuLa151
GTGNNNNNTAGCCCNNNNNAGTTAGACANNNCNNNNNNNNNNNNTNNNNNNNNNNNNNNNNNNNNNN
NNNNNNNNNNNNNNNNNNNNNNNNNNNNNNNNNNNNNNNNNNNNNNNNNNNNNNNNNNNNNNNNNNNN
NNNNNNNNNNNNNNNNNNNNNNNNNNNNNNNNNNNNNNNNNNNNNNNNNNNNNNNNNNNNNNNNNNNN
NNNNNNNNNNNNNNNNNNNNNNNNNNNNNNNNNNNNNNNNNNNNNNNNNNNNNNNNNNNNNNNNNNNN
NNNNNNNNNNNNNNNNNNNNNNNNNNNNNNNNNNNNNNNNNNNNNNNNNNNNCNTCTNNNAAANNGTNNN
NNNNNNNNNNNNNNNNNNNNNNNNNNCNCNNTGNNNGTAANCNANNNGNACATGCAGTCCCCTCNNNNN
NNNNNCAGAGNNNNNNNCNNNNNNNNCCGGCCCCGNCCNNNNNNNNNNNNNNNNNNNNNNNNGNNNNNN
NNNNNNTGGTACNNNNCGNNNNNGGTTGNNTNAGAGGTCCACCACGNGGTCNNGNCACNNNNGNCNAN
NNNCNNNAAANNNNNNNNNNNNNANNTNNNCANNAGGNNNNNNCCNNCNNGNNNNNNANTGCNATGANN
CANNTNNCGGGGGNNGTCCCCGGCGCGGCGACCTTGNTNANGGCCGGGGACAAGTATGGCNTNCNCCC
CGNGAAGGANNCNGCCGTANGTGCNNNNNANNNNNTCNTGNGANGTCGCCGNNATTCNNGTGGTCNNN
CNAGANTGNNCGNACCANNCGNANNNTGGTNATGNNAGTANNNNNNNNNNNNNNNNNNNNNNNNNNNNN
NNNNNNNNNNNNNNNNNNNNNNNNNNNNNNNNNNNNNNGNNNNNNCNANNNAAGGANNTANNNCTNNTN
NTNNNNNNGGACNNNACNNNNCNNNNNNNACNCNNNCNCNNNNNNNNNNGGTTTTNNNNCNNTANTNT
CCAGNACGCNGNGCNNNCATTCANGNGAACNNNGNNACGCGNNAATCGTANNATCTACCTGCCGCNNG
TNNNNNTACNGNNNNNACGGANNNTNNCTCGTANNNNNNTCATGCGTNNTNTNNTCNCNAGNCNNGTT
CGNNNANANANTAGNANGTNTCNNNNNNNNTGTNNCGTTCCGCNTGANNCNNCANNNNGNNGNNCTTC
TCANCGGCNNTANCTGCNGANNTAAGNCATANNNTNNNTCNNTANCTNNNNNGGNGGGNNACTGTAAA
NGGNNAATTNGANNNTNANTTCNNNCACNTNNCTNNGNNTNCTNNNTNCCTGNNTNCNNAATTGGNNC
CANTGNNTGNCNATCTCTGGNTTNGNNNNNNTNNNNNTNNCANNNNGGNNNGTNANANNNCNATNTAA
NNTATANNNNCNNNNNNANNNCACNANNNNNGNAGNNNCNANNNTTANANNNGTAGNNNANTNAGTCT
CCGANNTNNNNNNNATAAGCGNNANACNNNNNNNNNNNNNANGANNACGNNTTGGNNNTNNNNNGNNA
ANNAGACNNTNGTGGNTNNNNNTCAANNNNTTNGTAGTGGTATAACNGNNNNNNNTTTCNCCAGNNTN
NNNGNNNNANNCNATANNANNNNNNNGTGANNNNNNNNNNANNNNCTCNNNAGGGANCANCTACTTNA
GCAATGGNGCGCAGNNNGAGANNNNGACCAGCCTTGGGTGGCAANCACNGCAATAAGTNNNGTTNANC
CTNNNAGTNNACACNNNNNCANNGNNNCNNNNNNANCNGANNTNNNNNNNNNCNNNNNNNNNNNNNNN
NNNNNNNNNNNNNNNNNNNNNNNNNNNNNAGNNNANNGNNNGTNTNTGNCNCATNNNTTTTTANNNGNN
NNNNNACGNNNNANCCCCGGTCGNNNNCNNATTGAANNCCTGNNGCATNNNNNTNCNAGAATGANNGC
NNNNNGNNTNGNCTNNCNNNTCNAGNAGNCANNNAGGTNNCGCGNTCNNNNANNNNNNNCNNTCCCNN
ACTGAGGGTNNNNNNNNCATCTGCTNNNNNNNNNNTNCGCCGNCGTNNCNNNNNAGCNGTATNGTGTNN
NNCANNNNNNNNNNNNNNNNNNCNAAACNANNNNNNNTNNNN
>PhuLa152
GTGNNNNNTAGCCCNNNNNAGTTAGACANNNCNNNNNNNNNNNNTNNNNNNNNNNNNNNNNNNNNNN
NNNNNNNNNNNNNNNNNNNGNNNNNNNNNNNNNNNNNNNNNNNNNNNNNNNNNNNNNNNNNNNNNNNN
NNNNNNNNNNNNNNNNNNNNNNNNNNNNNNNNNNNNNNNNNNNNNNNNNNNNNNNNNNNNNNNNNNNN
NNNNNNNNNNNNNNNNNNNNNNNNNNNNNNNNNNNNNNNNNNNNNNNNNNNNNNNNNNNNNNNNNNNN
NNNNNNNNNNNNNNNNNNNNNNNNNNNNNNNNNNNNNNNNNNNNNNNNNNNNCNTCTNNNAAANNNTNNN
NNNNNNNNNNNNNNNNNNNNNNNNNNCNCNNTNNNNGTAANCNANNNGNACATGCAGTCCCCTCNNNNN
NNNGGCAGAGNNNNNNCCCNNNNNNNCCGNCCCCNNCCNNNNNNNNNNNNNNNNNGNNNNNNNGNNNNNN
NNNNNNTGGTACNNNNCGNNNNNGGNNGTNTAAGAGGTCCACCACGNGGTCNGGNCACNNNNGNNNAN
NNNCNNNNAACNNNNNNNNNNNNANATNNNCANNAGGNNNNNNCCNTCNNNNNNNNNNANTGCNATGANC
CNANNCACGGNGGNAGTNCCCGGCGCGGNGACCTTGANNANGGCCGGGGNCAAGTATGGCNTNCNCCC
CGGGAAGGANNCNGCCGTANGTGCNNNNNANNNNNTCNTGNGANGTNGCCGNNATTCNNGTGGTCNNN
NNAGAATGNNCGNACCANNCGNANNNTGGTNATGNNAGTNNNNNNNNNNNNNNNNNNNNNNNNNNNNN
NNNNNNNNNNNNNNNNNNNNNNNNNNNNNNNNNNNNNNGNNNNNNCNANNNAAGGANNTANNNCTNNTN
NTNNNNNNGGACNNNACNNNNCNNNNNNNACNCNNNCNCNNNNNNNNNNGNTTTTTNNNCNNTATTNT
CCAGNACGCNGNGCNNNCATTCANGNGAACNNNGNNACGCGNNAATCGTANNATCTACCTGCCGCNNG
TNNNNNTACNGNNNNTNCGGANNNTNNNNCGTANNNNNNNCANGCGTNNTNTNNTCNCNAGNCNNGTT
CGNNNANANANTAGNANGTNTNNNNNNNNNNTGTNNCGTTCCGCNTGANNCNNCANNNNGNNGNNCTTN

TCANCGGCNNTANCTNCNGANNTAAGNCATANNNTNNNTCNNTANCTNNNNNGGNGGGNNACTGTAAA
NGGNNAATTNGNGNNTNANTNCNNNCACNTNNCTNNGNNTNCTNNNTNCCTGNNTNCNNAATTGGNNC
CANTGNNTGNCNATCTCTGGNTTNGNNNNNNTNNNNNTNNCAANNNGGNNNGTNNNANNNCNATNTAA
NNTANANNNNCNNNNNNANNNCACNANNNNNGNAGNNNCNANNNTTANANNNGTAGNNNAATAAGTCT
CCGANNTNNNNNNNATAAGCGNNANACNNNNNNNNNNNNNANGANNACGNNTTGGNNNTNNNNNNCNA
ANNAGACNNTNGTGGNTNNNNNTCAANNNNTTNGTAGTGGTATAACNGNNNNNNNTTTCNCCAGNNTN
NNNGNNNNNNNCNATANNANNNNNNNGNGANNNNNNNNNNANNNNCTCNNNAGGGANCANCTACTNNA
NCANTGGNNCGCAGNNNGAGANNNNGACNNNCCTTGGGTGGCAANNACNGCAATAAGTNNNGTTNNNC
CTNNNAGTNNACACNNNNNCANNGNNNCNNNNNNANCNGANNTNNNNNNNNNNNNNNNNNNNNNNNNN
NNNNNNNNNNNNNNNNNNNNNNNNNNNNNNNNNANNGNNNGTNTNTGACNCATGNNTTTTTANNNGNN
NNNNNACGNNNNANCCCCGGTCGNNNNCNNATTGAANNCCTGNNGCATNNNNNTNCNAGAATGANNGC
NNNNNGNNTNGNCTNNNNNNTNAAGNAGTCANNNAGGNNNNNCGNTCNNGNNNNNNNNNCNNTCCCNN
ACTGAGGGTNNNNNNNNCATCTGCTNNNNNNNNNTNCGCNGNCGTNNCNNNNNAGCNGTATNNNGNNN
NNCANNNNNNNNNNNNNNNNTNAAACAANNNNNNNTNNNN
>PhuLa153
GTGNNNNNTAGCCCNNNNNAGTTAGNCACNNCNNNNNNNNNNNNTNNNNNNNNNNNNNNNNNNNNNNN
NNNNNNNNNNNNNNNNNNNNNNNNNNNNNNNNNNNNNNNNNNNNNNNNNNNNNNNNNNNNNNNNNNNN
NNNNNNNNNNNNNNNNNNNNNNNNNNNNNNNNNNNNNNNNNNNNNNNNNNNNNNNNNNNNNNNNNNNN
NNNNNNNNNNNNNNNNNNNNNNNNNNNNNNNNNNNNNNNNNNNNNNNNNNNNNNNNNNNNNNNNNNNN
NNNNNNNNNNNNNNNNNNNNNNNNNNNNNNNNNNNNNNNNNNNNNNNNNNNNTCTNNNAAANNNTNNN
NNNNNNNNNNNNNNNNNNNNNNNNNCNCNNTGNNNGTAANCNANNNGNACATGCAGTCCCCTCNNNNN
NNNNNCAGAGNNNNNNNCNNNNNNNNCCGNCCCCNNCCNNNNNNNNNNNNNNGNNNNNNNGNNNNNN
NNNNNNTGGTACNNNNCGNNNNNGGNTGNNTNAGAGGTCCACCACGNGGTCNNGNCACNNNNGNNNAN
NNNCNNNNNNNCNNNNNNNNNNNNNNTNNNCANNAGGNNNNNCCNTCNNNNNNNNNATNGCNATGACC
CAANTNACGGNGGCAGTNCCCGGCGNGGCGACCTTGNTNACGGCCGGGGACAAGTATGGCNTNCNCCC
CGGGAAGGANNCNGCCGTANGTGCNNNNNANNNNNTCTTGNGANGTCGCCGNNATTCNCNTGGTCNNN
NNAGANTGNCCGNACCANNCGNANNNTGGTNATGTNAGTNNNNNNNNNNNNNNNNNNNNNNNNNNNNN
NNNNNNNNNNNNNNNNNNNNNNNNNNNNNNNNNNNNNGNNNNNNCNANNNAAGGANNTANNNCTNNTN
NTNNNNNNGGACNNNACNNNNCNNNNNNNACNCNNNCNCNNNNNNANNNGGTTTTTNNCCNNTATTNT
CCAGNACGCNGNGCNNTCATTCANGNGAACNNNGNNACGCGNNAATCGTANNATCTACCTGCCGCNGG
NNNNNNTACNGNNNNTNCGGANNNTNNNNCGTANNNNNNNCANGCGTNNTNTNNTCNCNAGNCANGTT
CGNNNANANANTAGNANGTNTCNNNNNNNNTGTNGCGTTCCGCNTGANNCNNCANNNNGNNGNNNTTC
NCANCGGCNNTANCTGCNGANNTAAGNCATANNNTNNNTCNNTANCTNNNNNGGNGGGNNACTGTAAA
NGGNNAATTGGNNNNTNANTTCNNNCACNTNNCTNNGNNNNCTNNNTNCCTGNNTNCNNAATTGGNNC
CANTGNNTGNCNATCTCTNGNTTNGNNNNNNTNNNNNTNNCAANNNGGNNNGTNNNANNNCNATNTAA
NNTATANNNNCNNNNNNANNNCACNNNNNNNNNAGNNNCNANNNTNANANNNGTAGNNNANTAAGTCT
CCGANNTNNNNNNNATAAGCGNNANACNNNNNNNNNNNNNANGANNACGNNTTGGNNNTNNNNNNCNA
ANNAGACNNTNGTGGNTNNNNNTCAANNNNTTCGTAGTGGTATAACNGNNNNNNNTTTCNCCAGNNTN
NNNGNNNNNNNCNATANNANNNNNNNGNGANNNNNNNNNNANNNNCTCNNNAGGNANCANCTACTNNA
GCANTGGNGCGCAGNNNGAGANNNNGACNNGCCTTGGGTGGCAANCACNGCAATAAGTNNNGTTNANC
CTNNNAGTNNACACNNNNNCANNGNNNCNNNNNNANCNGANNTNNNNNNNNNNNNNNNNNNNNNNNNN
NNNNNNNNNNNNNNNNNNNNNNNNNNNNAGNNNANNGNNNGTNTNTGACNCATGTGTTTTTANNNGNN
NNNNNACGNNNNNNCCCCGGTCGNNNNCNNATTGAANNCCTGNNGCATNNNNNTNCNAGAATGANNGC
NNNNNGNNTNNNCTNNCNNNTNAAGNAGTCANNNAGNTANCGCGNTCNNNNNNNNNNNNCNNTCCCNN
ACTGAGGGTNNNNNNCGCANCTGCTNNNNNNNNNTTCGCNGNCGTNNCNNNNNAGCNGTATNNTGTNN
NNCANNNNNNNNNNNNNNNNNNANACGANNNNNNNTNNNN
>PhuLa154
GTGNNNNNTAGCCCNNNNNAGTTAGACANNNCNNNNNNNNNNNNTNNNNNNNNNNNNNNNNNNNNNNN
NNNNNNNNNNNNNNNNNNNNNNNNNNNNNNTNNNNNNNNNNNNNNNNNNNNNNNNNNNNNNNNNNNNN
NNNNNNNNNNNNNNNNNNNNNNNNNNNNNNNNNNNNNNNNNNNNNNNNNNNNNNNNNNNNNNNNNNNN
NNNNNNNNNNNNNNNNNNNNNNNNNNNNNNNNNNNNNNNNNNNNNNNNNNNNNNNNNNNNNNNNNNNN
NNNNNNNNNNNNNNNNNNNNNNNNNNNNNNNNNNNNNNNNNNNNNNNNNNNNTCTNNNAAANNNTNNN
NNNNNNNNNNNNNNNNNNNNNNNNNCNCNTTGNNNGTAANCNANNNGNACATGCAGTNCCCTCNNNNN
NNNNGCAGAGNNNNNNNCCNNNNNCNCCGGCCCCNNCCNNNNNNNNNNNNNNNNNNNNNNGNNNNNN

NNNNNNTGGTACNNNNCGNNNNNGGTTGTNTAAGAGGTCCACCACGNGGTCNNGNCACNNNNGNNNAN
NNNCNNNNANNNNNNNNNNNNNNANNTNNNCANNAGGNNNNNNCCNTCNNNNNNNNNNATNGCNATGACC
CAANNCNCGGGGGNAGTCCCCGGCGNGGNGACCTTGNNNANNGCCGGGNACAAGTATGGCNNNCNCCC
CGGGAAGGANNCNGCCGTANGTGCNNNNNANNNNNTCNTGNGANGTCGCCGNNATTCNCGTGGTCNNN
CNAGAATGNCCGNACCANNCGNANNNTGGTNATGNTAGTNNNNNNNNNNNNNNNNNNNNNNNNNNNNN
NNNNNNNNNNNNNNNNNNNNNNNNNNNNNNNNNNNGNNNNGNNNNNNCNANNNAAGGNNNTANNNCTNNTN
NTNNNNNNGGACNNNACNNNNCNNNNNNNACNCNNNCNCNNNNNNNNNNGNTTTTTNNNCNNTANTNT
CCAGNACGCNGNGCNNNCATTCANGNGAACNNNGNNACGCGNNAATCGTANNATCTACCTGCCGCNGG
TNNNNNTACNGNNNNNNCGGANNNTNNNNNGTANNNNNNNCANGCGTNNTNTNNTCNCNAGNCNNGTT
CGNNNANANANTAGNANGTNTNNNNNNNNNNTGTNNCGTTCCGCNTGANNCNNCANNNNGNNGNNNTTN
TCANCGGCNNTANCTGCNGANNTAAGNCATANNNTNNNTCNNTANCTNNNNNGGNGGGNNACTGTAAA
NGGANAATTGGNNNNTNATTNCNNNCACNTNNCTNNGNNTNCTNNNTNCCTGNNTNCNNAATTGGNNN
CANTGNNTGNCNATCTCTNGNTTNGNNNNNNTNNNNNTNNCAANNNGGNNNGTNANANNNCNATNTAA
NNTANANNNNCNNNNNNANNNCACNANNNNNGNAGNNNCNANNNTTANANNNGTAGNNNANTAAGTCT
CCGANNTNNNNNNNATAAGCGNNANACNNNNNNNNNNNNNNANGANNACGNNTTGGNNNTNNNNNNCNA
ANNANACNNTNGTGGNTNNNNNTCAANNNNTTCGTAGTGGTATAACNGNNNNNNNTTTCNCCAGNNTN
NNNNNNNNNNNCNATANNANNNNNNNGNGANNNNNNNNNNNNNNNNCTCNNNAGGGANCANCTACTNNA
GCANTGGNGCGCAGNNNGAGANNNNGACNNNCCTTGGGTGGCAANCACNGCAATAAGTNNNGTTNANC
CTNNNAGTNNACACNNNNNCANNGNNNCNNNNNNANCNGANNTNNNNNNNNNNNNNNNNNNNNNNNNN
NNNNNNNNNNNNNNNNNNNNNNNNNNNNNNNNGNNNANGGNNNGTNTNTGNCNCATGTGTTTTTANNNGNN
NNNNNACGNNNNANCCCCGGTCGNNNNCNNATTGAANNCCTGNNGCATNNNNNTNCNAGAATGANNGC
NNNNNGNNTNGNCTGNCNNNTCANGNAGTCANNTAGNTANNNNGNTCNNNGNNNNNNNNCNNTCCCNN
ACTGAGGGTNNNNNNNNCATCTGCTNNNNNNNNNNTNCGCNGNCGTNNCNNNNNNGCNGTATNGTGNNN
NNCANNNNNNNNNNNNNNNNNTNAAACNANNNNNNNTNNNN
>PhuLa602
GTGNNNNNTAGCCCNNNNNAGTTAGACANNNCNNNNNNNNNNNNTNNNNNNNNNNNNNNNNNNNNNN
NNNNNNNNNNNNNNNNNNNNNNNNNNNNNNNNNNNNNNNNNNNNNNNNNNNNNNNNNNNNNNNNNNNN
NNNNNNNNNNNNNNNNNNNNNNNNNNNNNNNNNNNNNNNNNNNNNNNNNNNNNNNNNNNNNNNNNNNN
NNNNNNNNNNNNNNNNNNNNNNNNNNNNNNNNNNNNNNNNNNNNNNNNNNNNNNNNNNNNNNNNNNNN
NNNNNNNNNNNNNNNNNNNNNNNNNNNNNNNNNNNNNNNNNNNNNNNNNNNNNTCTNNNAAANNNTNNN
NNNNNNNNNNNNNNNNNNNNNNNNNNCNCNNTGNNNGTAANCNANNNGNACATGCAGTCCCCTCNNNNN
NNNGGCAGAGNNNNNNCCCNNNNNNNCCGGCCCCNNCCNNNNNNNNNNNNNNNNNGNNNNNNNGNNNNNN
NNNNNNTGGTACNNNNCGNNNNNGGTTGNTTNAGAGGTCCACCACGNGGTCNNGNCACNNNNGNNNAN
NNNCNNAAAACNNNNNNNNNNNNNNNNTNNNCANNAGGNCNNNNCCNTCNNGNNNNTNANTGCNATGACC
CAANNCACGGGGGCAGTNCCCGGCGCGGCGACCTTGANNANGGCCGGGGACAAGTATGGCGNNCNCCC
CGGGAAGGANNCNGCCGTANGTGCNNNNNANNNNNTCNTGNGANGTCGCCGNNANNNNNGTGGTCNNN
NNAGAATGNNCNNACCANNCGNANNNTGGTNATGNTAGTNNNNNNNNNNNNNNNNNNNNNNNNNNNNN
NNNNNNNNNNNNNNNNNNNNNNNNNNNNNNNNNNNGNNNNGNNNNNNCNANNNAAGGANNTANNNCTNNTN
NTNNNNNNGGACNNNACNNNNCNNNNNNNACNCNNNCNCNNNNNNANNNGNTTTTTNNCCNNTANTNT
CCAGNACGCNGNGCNNNCATTCANGNGAACNNNGNNACGCGNNAATCGTANNATCTACCTGCCGCNNG
NNNNNNTACNGNNNNTNCGGANNNTNNCTCGTANNNNNNNCATGCGTNNTNTNNTCNCNAGNCNNGTT
CGNNNANANANTAGNANGTNTNNNNNNNNNNTGTNNCGTTCCGCNTGANNCNNCANNNNGNNGNNCTTN
NCANCGGCNNTANCTGCNGANNTAAGNCATANNNTNNNTCNNTANCTNNNNNGGNGGGNNACTGTAAA
NGGNNAATTNGANNNTNANTTCCNNCACNTNNCTNNGNNNNCTNNNTNCCTGNNTNCNNAATTGGNNC
CANTGNNTGNCNATCTCTNGNTTNGNNNNNNTNNNNNTNNCAANNNGGNNNGTNANANNNCNATNTAA
NNTANANNNNCNNNNNNANNNCACNANNNNNNNNAGNNNCNANNNTTANANNNGTAGNNNAATAAGTCT
CCGANNTNNNNNNNATAAGCGNNANACNNNNNNNNNNNNNNANGANNACGNNTTGGNNNTNNNNNNCNA
ANNAGACNNTNGTGGNTNNNNNTCAANNNNTTNGTAGTGGTATAACNGNNNNNNNTTTCNCCAGNNTN
NNNGNNNNNNNNCNATANNANNNNNNNGNGANNNNNNNNNNNANNNNCTCNNNAGGNANCANCTACTTNA
GCANTGGNGCGCAGNNNGAGANNNNGACNNGCCTTGGGTGGCAANCACNGCAATAAGTNNNGTTNANC
CTNNNAGTNNACACNNNNNCANNGNNNCNNNNNNANCNGANNTNNNNNNNNNNNNNNNNNNNNNNNNN
NNNNNNNNNNNNNNNNNNNNNNNNNNNNNNNNGNNNANNGNNNGTNTNTGACNCATGNNTTTTTANNNGNN
NNNNNNNGNNNGANCCCCGGTCGTNNNCNNATTGAANNCCTGNNGCATNNNNNTNCNAGAATGANNGC
NNNNNGNNTNNNCTNNCNNNTCAAGNAGTCAANNAGNNNNNNNGNTCNNNGNNNNNNNNCNNTCCCNN

ACTGAGGGTNNNNNNNGCATCTGCTNNNNNNNNCTTCGCNGNCGTNNCNNNNNAGCNGTATNGTGTNA
NNCANNNNNNNNNNNNNNNNNNNTNAAACAANNNNNNNTNNNN
>PhuLa603
GTGNNNNNTAGCCCNNNNNAGTTAGACANNNCNNNNNNNNNNNNNTNNNNNNNNNNNNNNNNNNNNNN
NNNNNNNNNNNNNNNNNNNNNNNNNNNNNNTNNNNNNNNNNNNNNNNNNNNNNNNNNNNNNNNNNNNN
NNNNNNNNNNNNNNNNNNNNNNNNNNNNNNNNNNNNNNNNNNNNNNNNNNNNNNNNNNNNNNNNNNNN
NNNNNNNNNNNNNNNNNNNNNNNNNNNNNNNNNNNNNNNNNNNNNNNNNNNNNNNNNNNNNNNNNNNN
NNNNNNNNNNNNNNNNNNNNNNNNNNNNNNNNNNNNNNNNNNNNNNNNNNNNTCTNNNAAANNNTNNN
NNNNNNNNNNNNNNNNNNNNNNNNNCNCNTTGNNNGTAANCNANNNGNACATGCAGTCCCCTCNNNNN
NNNGGCAGAGNNNNNNCCCNNNNNCNCCGNCCCCNNCCNNNNNNNNNNNCNNNNNNNNNNNGNNNNNN
NNNNNNTGGTACNNNNCGNNNNNGGTTGTNTAAGAGGTCCACCACGNGGTCNNGNCACNNNNGNNNAN
NNNCNNNNAACNNNNNNNNNNNANNTNNNCANNAGGNCNNNNCCNTCNNGNNNNTNANTGCNATGACC
CAANNCACGGGGGCAGTCCCCGGCGCGGCGACCTTGANNACNGCCGGGGACAAGTATGGCGNNCNCCC
CGGGAAGGANNCNGCCGTANGTGCNNNNNANNNNNTCNTGNGANGTCGCCGNNATTCNCGTGGTCNNN
CNAGAATGNNCNNACCANNCGNANNNTGGTTATGNNAGTANNNNNNNNNNNNNNNNNNNNNNNNNNNN
NNNNNNNNNNNNNNNNNNNNNNNNNNNNNNNNGNNNNGNNNNNNCNANNNAAGGNNNTANNNCTNNTN
NTNNNNNNGGACNNNACNNNNCNNNNNNNACNCNNNCNCNNNNNGANNNGNTTTTTNNNCNNTATTNN
CCAGNACGCNGNGCNNNCATTCANGNGAACNNNGNNACGCGNNAATCGTANNATCTACCTGCCGCNGG
NNNNNNTACNGNNNNNNCGGANNNTNNCTCGTANNNNNNTCATGCGTNNTNTNNTCNCNAGNCANGTT
CGNNNANANANTAGNANGTNTNNNNNNNNNTGTNNCGTTCCGCNTGANNCNNCANNNNGNNGNNNTTN
TCANCGGCNNTANCTGCNGANNTAAGTCATANNNTNNNTCNNTANCTNNNNNGGNGGGNACTGTAAA
NGGNNAATTNGNNNNTNATTNCNNNCACNTNNCTNNGNNNNCTNNNTNCCTGNNTNCNNAATTGGNNC
CANTGNNTGNCNATCTCTGGNTTNGNNNNNNTNNNNNTNGCAANNNGGNNNGTNANANNNCNATNTAA
NNTANANNNNCNNNNNNANNNCACNNNNNNNNNNAGNNNCNANNNTTANANNNGTAGNNNAATAAGTCT
CCGANNTNNNNNNNATAAGCGNNANACNNNNNNNNNNNNNNANGANNACGNNTTGGNNNTNNNNNNCNA
ANNAGACNNTNGTGGNTNNNNNTCAANNNNTTCGTAGTGGTATAACNGNNNNNNNTTTCNCCAGNNTN
NNNGNNNNNNNCNATANNANNNNNNNGNGANNNNNNNNNNNANNNNCTCNNNAGGGANCANCTACTNNA
GCAATGGNGCGNAGNNNGAGANNNNGACCAGCCTTGGGTGGCAANNACNGCAATAAGTNNNGTTNANC
CTNNNAGTNNACACNNNNNCANNGNNNCNNNNNNANCNGANNTNNNNNNNNNNNNNNNNNNNNNNNNN
NNNNNNNNNNNNNNNNNNNNNNNNNNNNANNNNANGGNNNGTNTNTGACNCATGTGTTTTTANNNGNN
NNNNNACGNNNNANCCCCGGTCGNNNNCNNATTGAANCCCTGNNGCATNNNNNTNCNAGAATGANNNC
NNNNNGNNTNGNCTGNCNNNTCAAGNAGTCANNNAGGTNNNNNNNTCNNNGNNNNNNNNCNNTCCCNN
ACTGAGGGTNNNNNNNNCATCTGCTNNNNNNNNCTNCGCNGNCGTNNCNNNNNAGCNGTATNNNGTNA
NNCANNNNNNNNNNNNNNNNNNNTNAAACAANNNNNNNTNNNG
>PhuLa604
GTGNNNNNTAGCCCNNNNNAGTTNGACANNNCNNNNNNNNNNNNNNNNNNNNNNNNNNNNNNNNNNNN
NNNNNNNNNNNNNNNNNNNNNNNNNNNNNNNNNNNNNNNNNNNNNNNNNNNNNNNNNNNNNNNNNNNN
NNNNNNNNNNNNNNNNNNNNNNNNNNNNNNNNNNNNNNNNNNNNNNNNNNNNNNNNNNNNNNNNNNNN
NNNNNNNNNNNNNNNNNNNNNNNNNNNNNNNNNNNNNNNNNNNNNNNNNNNNNNNNNNNNNNNNNNNN
NNNNNNNNNNNNNNNNNNNNNNNNNNNNNNNNNNNNNNNNNNNNNNNNNNNCNTCTNNNAAANNNTNNN
NNNNNNNNNNNNNNNNNNNNNNNNNCNCNTTGNNNGTAANCNANNNGNACATGCAGTCCCCTCNNNNN
NNNGGCAGAGNNNNNNNCNNNNNNNNCCGNCCCCGNCCNNNNNNNNNNNNNNNNNNNNNNNGNNNNNN
NNNNNNTGGTACNNNNCGNNNNNGGTTGTNTAAGAGGTCCACCACGNGNTCNNGNNACNNNNGNNNAN
NNNCNNNNAACNNNNNNNNNNNANATNNNCANNAGGNNNNNNCCNTCNNNNNNNTNANTGCNATGACC
CAANNCACGGGGGNNGTNCCCGGNGNGGCGACCTTGANNANNGCCGGGGACAAGTATGGCNNNCNCCC
CGGGAAGGANNCNGCCGTANGTGCNNNNNANNNNNTCNTGNGANGTCGCCGNNATTNNNGTGGTCNNN
CNAGAATGNCCNNACCANNCGNANNNTGGTNATGNNAGTNNNNNNNNNNNNNNNNNNNNNNNNNNNNN
NNNNNNNNNNNNNNNNNNNNNNNNNNNNNNNNGNNNNGNNNNNNCNANNNAAGGNNNTANNNCTNNTC
NTNNNNNNGGACNNNACNNNNCNNNNNNNACNCNNNCNCNNNNNNANNNGNTTTTTNNNCNNTATTNT
CCAGNACGCNGNGCNNNCATTCANGNGAACNNNGNNACGCGNNAATCGTANNATCTACCTGCCGCNGG
NNNNNNTNCNGNNNNTNCGGANNNTNNNNCGTANNNNNNTCATGCGTNNTNTNNTCNCNAGNCNNGTT
CGNNNANANANTAGNANGTNTNNNNNNNNNTGTNNCGTTCCGCNTGANNCNNCANNNNGNNGNNNTTN
TCANCGGCNNTANCTGCNGANNTAAGNCATANNNTNNNTCNNTANCTNNNNNGGNGGGNACTGTAAA
NGGNNAATTNGNNGNTNATTTCNNNCACNTNNCNNNGNNTNCTNNNTNCCTGNNTNCNNAATTGGNNC

CANTGNNTGNCNATCTCTNGNTTNGNNNNNNTNNNNNTNNCANNNNGGNNNGNNNNANCNCNATNTAA
NNTATANNNNCNNNNNNANNNCACNNNNNNNGNAGNNNCNANNNTTANANNNGTAGNNNAATAAGTCT
CCGANNTNNNNNNNATAAGCGNNANACNNNNNNNNNNNNNANGANNACGNNTTGGNNNTNNNNNNCNA
ANNAGACNNTNGTGGNTNNNNNTCAANNNNTTNGTAGTGGTATAACNGNNNNNNNTTTCNCCAGNNTN
NNNGNNNNNNNCNATANNANNNNNNNGNGANNNNNNNNNNANNNNCTCNNNAGGNANCANCTACTNNA
GCANTGGNGCGNAGNNNGAGANNNNGACNNNCCTTGGGTGGCAANCACNGCAATAAGTNNNGTTNANC
CTNNNAGTNNACACNNNNNCANNGNNNCNNNNNNANCNGANNTNNNNNNNNNNNNNNNNNNNNNNN
NNNNNNNNNNNNNNNNNNNNNNNNNNNANNNNANGGNNNGTNTNTGNCNCATGTGTTTNTANNNGNN
NNNNNACGNNNNANCCCCGGTCGNNNNCNNATTGAANNCCTGNNGCATNNNNNTNCNAGAATGANNGC
NNNNNGNNTNNNCTNNCNNNTCAAGNAGTCAANTAGNNNTNGCGNTCNNNGNNNNNNNNCNNTCCCNN
ACTGAGGGTNNNNNNNNCATCTGCTNNNNNNNNCTNCGCNGNCGTNNCNGNNNNGCNGTATNNTGTNA
NNCANNNNNNNNNNNNNNNNTNANACGANNNNNTTNNNN
>PhuLa605
GTGNNNNNTAGCCCNNNNNAGTTAGACANNNCNNNNNNNNNNNNTNNNNNNNNNNNNNNNNNNNNN
NNNNNNNNNNNNNNNNNNNNNNNNNNNNNNNNNNNNNNNNCNNNNNNNNNNNNNNNNNNNNNNNNN
NNNNNNNNNNNNNNNNNNNNNNNNNNNNNNNNNNNNNNNNNNNNNNNNNNNNNNNNNNNNNNNNNN
NNNNNNNNNNNNNNNNNNNNNNNNNNNNNNNNNNNNNNNNNNNNNNNNNNNNNNNNNNNNNNNNNN
NNNNNNNNNNNNNNNNNNNNNNNNNNNNNNNNNNNNNNNNNNNNNNNNCNTCTNNNNAANNNTNNN
NNNNNNNNNNNNNNNNNNNNNNNNNCNCNTTGNNNGTAANCNANNNGNACATGCAGTCCCCTCNNNNN
NNNNGCAGAGNNNNNNNCCNNNNNNNCCGNCCCCNNCCNNNNNNNNNNNNNNNNNGNNNNNNNGNNNNNN
NNNNNNTGGTACNNNNCGNNNNNGGTTGTTTAAGAGGTCCACCACGNGGTCCGGNCACNNNNGNNNAN
NNNCNNNNNNCNNNNNNNNNNNNNNNTNNNCANNAGGNCNNNGCCNTCNNNNNNNNNATNGCNATGACC
CANNNCACGGNGGNNGTNCCCGGNGNGGNGACCTTGNNNANNGCCGGGGACAAGTATGGCNNNCNCCC
CGGGAAGGANNCNGCCGTANGTGCNNNNNANNNNNTCNTGNGANGTNGCCGNNATTNNCNTGGTCNNN
CNAGANTGNNCGNACCANNCGNANNNTGGTNATGTTAGTNNNNNNNNNNNNNNNNNNNNNNNNNNN
NNNNNNNNNNNNNNNNNNNNNNNNNNNNNNNNNNNNNNGNNNNNNCNANNNAAGGANNTANNNCTNNTN
NTNNNNNNGGACNNNNCNNNNCNNNNNNNACNCNNNCNCNNNNNNANNNGNTTTTTNNCCNNTATTNT
CCAGNACGCNGNGCNNTCATTCANGNGAACNNNGNNACGCGNNAATCGTANNATCTACCTGCCGCNNG
TNNNNNTACNGNNNNNNCGGANNNTNNNNNGTANNNNNNTCATGCGTNNTNTNNTCNCNAGNCANGTT
CGNNNANANANTAGNANGTNTNNNNNNNNNTGTNNCGTTCCGCNTGANNCNNCANNNNGNNGNNNTTC
TCANCGGCNNTANCTGCNGANNTAAGNCATANNNTNNNTCNNTANCTNNNNNGGNGGGNNACTGTAAA
NGGNNAATTNGNNNNTNATTNCNNNCACNTNNCTNNGNNTNCTNNNTNCCTGNNTNCNNAATTGGNNC
CANTGNNTGNCNATCTCTNGNTTNGNNNNNNTNNNNNTNNCANNNNGGNNNGTNNNANNNCNATNTAA
NNTANANNNNCNNNNNNANNNCACNNNNNNNGNAGNNNCNANNNTTANANNNGTAGNNNAATAAGTCT
CCGANNTNNNNNNNATAAGCGNNANACNNNNNNNNNNNNNANGANNACGNNTTGGNNNTNNNNNNCNA
ANNAGACNNTNGTGGNTNNNNNTCAANNNNTTNGTAGTGGTATAACNGNNNNNNNTTTCNCCAGNNTN
NNNNNNNNNNNCNATANNANNNNNNNGTGANNNNNNNNNNANNNNCTCNNNAGGNANCANCTACTNNA
GCAATGGNGCGCAGNNNGAGANNNNGACNNGCCTTGGGTGGCAANCACNGCAATAAGTNNNGTTNANC
NTNNNAGTNNACACNNNNNCANNGNNNCNNNNNNANCNGANNTNNNNNNNNNNNTNNNNNNNNNNNN
NNNNNNNNNNNNNNNNNNNNNNNNNNNNNNGNNNANNGNNNGTNTNTGACNCATGTGTTTTTANNNGNN
NNNNNACGNNNGANCCCCGGTCGNNNNCNNATTGAANNCCTGNNGCATNNNNNTNCNAGAATGANNGC
NNNNNGNNTNGNCTNNCNNNTCAAGNAGTCANNNANGTNNCNCGNTCNNNGNNNNNNNNCNNTNNCNN
ACTGAGGGTNNNNNNCGCATCTGCTNNNNNNNNNNTNCGCNGNCGTNNCNNNNNAGCNGTATNGTGTNN
NNCANNNNNNNNNNNNNNNNNCNAAACGANNNNNNNTNNNN
>PhuLa606
GTGNNNNNTAGCCCNNNNNAGTTAGACANNNCNNNNNNNNNNNNNNNNNNNNNNNNNNNNNNNNNNNN
NNNNNNNNNNNNNNNNNNNNNNNNNNNNNNNNNNNNNNNNNNNNNNNNNNNNNNNNNNNNNNNNNN
NNNNNNNNNNNNNNNNNNNNNNNNNNNNNNNNNNNNNNNNNNNNNNNNNNNNNNNNNNNNNNNNNN
NNNNNNNNNNNNNNNNNNNNNNNNNNNNNNNNNNNNNNNNNNNNNNNNNNNNNNNNNNNNNNNNNN
NNNNNNNNNNNNNNNNNNNNNNNNNNNNNNNNNNNNNNNNNNNNNNNNNCNTCTNNNAAANNNTNNN
NNNNNNNNNNNNNNNNNNNNNNNNNCNCNTTGNNNGTAANCNANNNGNACATGCAGTCCCCTCNNNNN
NNNGGCAGAGNNNNNNNCCNNNNNNNCCGNCCCCGNCCNNNNNNNNNNNNNNNNNNNNNNNNGNNNNNN
NNNNNNTGGTACNNNNCGNNNNNGGTTGTNTNAGAGGTCCACCACGNGGTCNNGNCACNNNNGNNNAN
NNNCNNAAAACNNNNNNNGNNNANATNNNCANNAGGNCNNNGCCNTCNNNNNNNNNAATTGCNATGACC

CAANNCACGGGGGCAGTNCCCGGCGCGGCGACCTTGNTNANGGCCGGGGACAAGTATGGCNNNCNCCC
CGGGAAGGANNCNGCCGTANGTGCNNNNNANNNNNTCNTGNGANGTCGCCGNNATTCNCGTGGTCNNN
NNAGAATGNCCGNACCANNCGNANNNTGGTNATGTTAGTANNNNNNNNNNNNNNNNNNNNNNNNNNNN
NNNNNNNNNNNNNNNNNNNNNNNNNNNNNNNNGNNNNGNNNNNNCNANNNAAGGANNTANNNCTNNTN
NTNNNGNNGGACNNNACNNNNCNNNNNNNACNCNNNCNCNNNNNNANNNGGTTTTNNNNCNNTATTNT
CCAGNANGCNGNGCNNNCATTCANGNGAACNNNGNNACGCGNNAATCGTANNATCTACCTGCCGCNNG
TNNNNNTNCNGNNNNTNCGGANNNTNNNNCGTANNNNNNTCANGCGTNNTNTNNTCNCNAGNCNNGTT
CGNNNANANANTAGNANGTNTCNNNNNNNNTGTNGCGTTCCGCNTGANNCNNCANNNNGNNGNNCTTN
NCANCGGCNNTANCTGCNGANNTAAGNCATANNNTNNATCNNTANCTNNNNNGGNGGGNNACTGTAAA
NGGNNAATTGGANGNTNATTNCCNNCACNTNNCTNNGNNNNCTNNNTNCCTGNNTNCNNAATTGGNNC
CANTGNNTGNCNATCTCTGGNTTNGNNNNNNTNNNNNTNNCAANNNGGNNNGTNANANNNCNATNTAA
NNTATANNNNCNNNNNNANNNCACNANNNNNGNAGNNNCNANNNTTANANNNGTAGNNNNNTAAGTCT
CCGANNTNNNNNNNATAAGCGNNANACNNNNNNNNNNNNNANGANNACGNNTTGGNNNTNNNNNNCNA
ANNAGACNNTNGTGGNTNNNNNTCAANNNNTTNGTAGTGGTATAACNGNNNNNNCTTTCNCCAGNNTN
NNNGNNNNNNNCNATANNANNNNNNNGTGANNNNNNNNNNANNNNCTCNNNAGGGANCANCTACTTNA
GCAATGGNGCGCAGNNNGAGANNNNGACNNNCCTTGGGTGGCAANCACNGCAATAAGTNNNGTTNANC
CTNNNAGTNNACACNNNNNCANNGNNNCNNNNNNANCNGANNTNNNNNNNNNNNNNNNNNNNNNNNNN
NNNNNNNNNNNNNNNNNNNNNNNNNNNNAGNNNANGGNNNGTNTNTGACNCATGTGTTTTTANNNGNN
NNNNNACGNNNNANCCCCGGTCGNNNNCNNATTGAANNCCTGNNGCATNNNNNTNCNAGAATGANNGC
NNNNNGNNTNGNCTGNCNTTTCAAGNAGTCANNNAGGTNNCGCGNTCNNNNNNNNNNNNCNNTCCCNN
ACTGAGGGTNNNNNNCGCATCTGCTNNNNNNNNCTNCGCNGNCGTNNCNNNNNNGCCGTATNGTGNTA
NNCANNNNNNNNNNNNNNNTNAAACAANNNNNNNTNNNN
>PhuLa607
GTGNNNNNTAGCCCGNNNNAGTTAGACANNNCNNNNNNNNNNNNTNNNNNNNNNNNNNNNNNNNNNNN
NNNNNNNNNNNNNNNNNNNNNNNNNNNNNNNNNNNNNNNNNNNNNNNNNNNNNNNNNNNNNNNNNNNN
NNNNNNNNNNNNNNNNNNNNNNNNNNNNNNNNNNNNNNNNNNNNNNNNNNNNNNNNNNNNNNNNNNNN
NNNNNNNNNNNNNNNNNNNNNNNNNNNNNNNNNNNNNNNNNNNNNNNNNNNNNNNNNNNNNNNNNNNN
NNNNNNNNNNNNNNNNNNNNNNNNNNNNNNNNNNNNNNNNNNNNNNNNNNNNTCTNNNAAANNNTNNN
NNNNNNNNNNNNNNNNNNNNNNNNNCNCNTTGNNNGTAANCNANNNGNACATGCAGTCNCCTCNNNNN
NNNNNCAGAGNNNNNNNNCNNNNNNNCCGGCCCCGNCCNNNNNNNNNNNNNNNNNNNNNNNNGNNNNN
NNNNNNTGGTACNNNNCGNNNNNGGTTGNNTNAGNGGTCCACCACGNGGTCNGNNCACNNNNGNNNAN
NNNCNNNNAACNNNNNNNNNNNANNTNNNCANNAGGNCNNNNCCNTCNNNNNNNNNATNGCNATGACC
CAANNCACGGGGGNNGTNCCCGGNGCNGCGACCTTGATNANNGCCGGGGNCAAGTATGGCNTNCNCCC
CGGGAAGGANNCNGCCGTANGTGCNNNNNANNNNNTCNTGNGANGTNGCCGNNATTNNCGTGGTCNNN
CNAGAATGCCCGNACCANNCGNANNNTGGTNATGTTAGTNNNNNNNNNNNNNNNNNNNNNNNNNNNNN
NNNNNNNNNNNNNNNNNNNNNNNNNNNNNNNNGNNNNGNNNNNNCNANNNAAGGANNTANNNCTNNTN
NTNNNNNNGGACNNNACNNNNCNNNNNNNACNCNNNCNCNNNNNNNNNNGNTTTTTNNNCNNTANTNT
CCAGNACGCNGNGCNNNCATTCANGNGAACNNNGNNACGCGNNAATCGTANNATCTACCTGCCGCNNG
TNNNNNTNCNGNNNNNNCGGANNNTNNNNNGTANNNNNNNCATGCGTNNTNTNNTCNCNAGNCNNGTT
CGNNNANANANTAGNANGTNTNNNNNNNNNTGTNNCGTTCCGCNTGANNCNNCANNNNGNNGNNCTTN
TCANCGGCNNTANCTGCNGANNTAAGNCATANNNTNNNTCNNTANCTNNNNNGGNGGGTNACTGTAAA
NGGNNAATTNGNNNNTNANTNCNNNCACNTNNCTNNGNNNNCTNNNTNCCTGNNTNCNNAATTGGNNC
CANTGNNTGNCNATCTCTNGNTTNGNNNNNNTNNNNNTNNCANNNNGGNNNNNNNNANNNCNATNTAA
NNTATANNNNCNNNNNNANNNCACNNNNNNNGNAGNNNCNANNNTTANANNNGTAGNNNAATAAGTCT
CCGANNTNNNNNNNATAAGCGNNANACNNNNNNNNNNNNNANGANNACGNNTTGGNNNTNNNNNNCNA
ANNAGACNNTNGTGGNTNNNNNTCAANNNNTTNGTAGTGGTATAACNGNNNNNNNTTTCNCCAGNNTN
NNNGNNNNNNNCNATANNANNNNNNNGNGANNNNNNNNNNANNNNCTCNNNAGGNANCANCTACTNNA
GCANTGGNGCGCAGNNNGAGANNNNGACNNGCCTTGGGTGGCAANCACNGCAATAAGTNNNGTTNANC
CTNNNAGTNNACACNNNNNCANNGNNNCNNNNNNANCNGANNTNNNNNNNNNNNNNNNNNNNNNNNNN
NNNNNNNNNNNNNNNNNNNNNNNNNNNNANNNNANNGNNNGTNTNTGACNCATGTGTTTTTANNNGNN
NNNNNACGNNNNANCCCCGGTCGNNNNCNNATTGAANNCCTGNNGCATNNNNNTNCNAGAATGANNGC
NNNNNGNNTNNNCTNNCTTTTCAAGNAGTCANNNANNNNNNNCGNTCNNNNNNNNNNNNCNNNCCCNN
ACTGAGGGTNNNNNNNGCATCTGCTNNNNNNNNNTTCGCNGNCGTNNCNNNNNAGCNGTATNNNGTNA
NNCANNNNNNNNNNNNNNNTNAACCNANNNNNNNTNNNN

>PhuLa608
GTGNNNNNTAGCCCNNNNNAGTTAGACANNNCNNNNNNNNNNNNTNNNNNNNNNNNNNNNNNNNNNNN
NNNNNNNNNNNNNNNNNNNNNNNNNNNNNNTNNNNNNNNNNNNNNNNNNNNNNNNNNNNNNNNNNNNN
NNNNNNNNNNNNNNNNNNNNNNNNNNNNNNNNNNNNNNNNNNNNNNNNNNNNNNNNNNNNNNNNNNNN
NNNNNNNNNNNNNNNNNNNNNNNNNNNNNNNNNNNNNNNNNNNNNNNNNNNNNNNNNNNNNNNNNNNN
NNNNNNNNNNNNNNNNNNNNNNNNNNNNNNNNNNNNNNNNNNNNNNNNNNNNTCTNNNAAANNNTNNN
NNNNNNNNNNNNNNNNNNNNNNNNNCNCNTTGNNNGTAANCNANNNGNACATGCAGTCCCCTCNNNNN
NNNGGCAGAGNNNNNNNCCNNNNNCNCCGGCCCCGNCCNNNNNNNNNNNNNNNGNNNNNNNGNNNNNN
NNNNNNTGGTACNNNNCGNNNNNGGTTGTTTNAGAGGTCCACCACGNGGTCNGGNCACNNNNGNNTAN
NNNCNNNAAACNNNNNNNNNNNANNTNNNCANNAGGNCNNNGCCNTCNNGNNNNNNATTGCNATGACC
CAANNCACGGGGGCAGTNCCCGGCGCGGCGACCTTGNTNACGGCCGGGGACAAGTATGGCGNNCNCCC
CGGGAAGGANNCNGCCGTANGTGCNNNNNANNNNNTCNTGNGANGTCGCCGNNATTCNCNTGGTCNNN
CNAGANTGNCCGNACCANNCGNANNNTGGTNATGNNAGTNNNNNNNNNNNNNNNNNNNNNNNNNNNNN
NNNNNNNNNNNNNNNNNNNNNNNNNNNNNNNNGNNNNGNNNNNNCNANNNAAGGANNTANNNCTNNTC
NTNNNNNNGGACNNNACNNNNCNNNNNNNACNCNNNCNCNNNNNNANNNGGTTTTTNNCCNNTATTNT
CCAGNACGCNGNGCNNNCATTCANGNGAACNNNGNNACGCGNNAATCGTANNATCTACCTGCCGCNGG
TNNNNNTACNGNNNNNNCGGANNNTNNCTCGTANNNNNNTCATGCGTNNTNTNNTCNCNAGNCANGTT
CGNNNATANANTAGNANGTNTNNNNNNNNNTGTNNCGTTCCGCNTGANNCNNCANNNNGNNGNNCTTN
TCANCGGCNNTANCTGCNGANNTAAGTCATANNNTNNATCNNTANCTNNNNNGGNGGGNNACTGTAAA
NGGNNAATTNGNNGNTNANTNCNNNCACNTNNCTNNGNNTNCTNNNTNCCTGNNTNCNNAATTGGNNC
CANTGNNTGNCNATCTCTGGNTTNGCNNNNNTNNNNNTNNCAANNNGGNNNGTNANANNNCNATNTAA
NNTANANNNNCNNNNNNANNNCACNANNNNNGNAGNNNCNANNNTTANANNNGTAGNNNANTAAGTCT
CCGANNTNNNNNNNATAAGCGNNANACNNNNNNNNNNNNNANGANNACGNNTTGGNNNTNNNNNNCNA
ANNAGACNNTNGTGGNTNNNNNTCAANNNNTTCGTAGTGGTATAACNGNNNNNNNTTTCNCCAGNNTN
NNNGNNNNNNNCNATANNANNNNNNNGNGANNNNNNNNNNANNNNCTCNNNAGGGANCANCTACTNNA
GCANTGGNGCGCAGNNNGAGANNNNGACNNNCCTTGGGTGGCAANCACNGCAATAAGTNNNGTTNANC
CTNNNAGTNNACACNNNNNCANNGNNNCNNNNNNANCNGANNTNNNNNNNNNNNNNNNNNNNNNNNNN
NNNNNNNNNNNNNNNNNNNNNNANNNNNAGNNNANGGNNNGTNTNTGACNCATGTGTTTTTANNNGNN
NNNNNACGNNNNANCCCCGGTCGTNNNCNNATTGAANNCCTGNNGCATNNNNNTNCNAGAATGANNNC
NNNNNGNNTNGCTNNCNNNTCAAGNAGTCAANNAGGTANCGCGNTCNNNNNNNNNNNNNCNNNCCCNN
ACTGAGGGTNNNNNNNNCATCNGCTNNNNNNNNCTNCGCNGNCGTNNCNNCNNAGCNGTATNNTGTTA
NNCANNNNNNNNNNNNNNNNTNAAACGANNNNNNTNNNN
>PhuLa609
GTGNNNNNTAGCCCNNNNNAGTTAGACANNNCNNNNNNNNNNNTTNNNNNNNNNNNNNNNNNNNNNNN
NNNNNNNNNNNNNNNNNNNNNNNNNNNNNNNNNNNNNNNNNNNNNNNNNNNNNNNNNNNNNNNNNNNN
NNNNNNNNNNNNNNNNNNNNNNNNNNNNNNNNNNNNNNNNNNNNNNNNNNNNNNNNNNNNNNNNNNNN
NNNNNNNNNNNNNNNNNNNNNNNNNNNNNNNNNNNNNNNNNNNNNNNNNNNNNNNNNNNNNNNNNNNN
NNNNNNNNNNNNNNNNNNNNNNNNNNNNNNNNCNNNNNNNNNNNNNNNNNNNTCTNNNAAANNNTNNN
NNNNNNNNNNNNNNNNNNNNNNNNNCNCNTTGNNNGTAANCNANNNGNACATGCAGTCCCCTCNNNNN
NNNGGCAGAGNNNNNNNCCNNNNNNNCCGGCCCCNNCCNNNNNNNNNNNNNNNGNNNNNNNGNNNNNN
NNNNNNTGGTACNNNNCGNNNNNGGTTGTNTAAGAGGTCCACCACGNGGTCNNGNCACNNNNGNNNAN
NNNCNNAAAACNGNNNNNNNNNANNTNNNCANNAGGNNNNNNCCNTCNNNNNNNNNATNGCNATGACC
CAANNCACGGNGGCNGTNCCCGGCGCGGCGACCTTGNNNANGGCCGGGGACAAGTATGGCNNNCNCCC
CGGGAAGGANNCNGCCGTANGTGCNNNNNANNNNNTCNTGNGACGTCGCCGNNATTCNCGTGGTCNNN
CNAGAATGNCCGNACCANNCGNANNNTGGTNATGNNAGTNNNNNNNNNNNNNNNNNNNNNNNNNNNNN
NNNTNNNNNNNNNNNNNNNNNNNNNNNNNNNNGNNNNGNNNNNNCNANNNAAGGNNNTANNNCTNNTN
NTNNNNNNGGACNNNACNNNNCNNNNNNNACNCNNNCNCNNNNNNNNNNGNTTTTNNNNCNNTATTNT
CCAGNACGCNGNGCNNNCATTCANGNGAACNNNGNNACGCGNNAATCGTANNATCTACCTGCCGCNNG
NNNNNNTACNGNNNNNNCGGANNNTNNNNCGTANNNNNNTCANGCGTNNTNTNNTCNCNAGNCANGTT
CGNNNANANANTAGNANGTNTNNNNNNNNNTGTNNCGTTCCGCNTGACNCNNCANNNNGNNGNNCTTN
NCANCGGCNNTANCTGCNGANNTAAGNCATANNNTNNATCNNTANCTNNNNNGGNGGGNNACTGTAAA
NGGNNAATTNGANNNTNANTNCNNNCACNTNNCTNNGNNNNCTNNNTNCCTGNNTNCNNAATTGGGNC
CANTGNNTGNCNATCTCTNGNTTNGNNNNNNTNNNNNTNNCANNNNGGNNNNNNANANNNCNATNTAA
NNTANANNNNCNNNNNNANNNCACNNNNNNNGNAGNNNCNANNNTTANANNNGTAGNNNANTAAGTCT

CCGANNTNNNNNNNATAAGCGNNANACNNNNNNNNNNNNNNNANGANNACGNNTTGGNNNTNNNNNNCNA
ANNAGACNNTNGTGGNTNNNNNTCAANNNNTTNGTAGTGGTATAACNGNNNNNNNTTTCNCCAGNNTN
NNNGNNNNNNNNCNATANNANNNNNNNGTGANNNNNNNNNNNANNNNCTCNNNAGGGANCANCTACTNNA
GCAANGGNGCGCAGNNNGAGANNNNGACNNNCCTTGGGTGGCAANCACNGCAATAAGTNNNGTTNANC
CTNNNAGTNNACACNNNNNCANNGNNNCNNNNNNANCNGANNTNNNNNNNNNNNNNNNNNNNNNNNNNN
NNNNNNNNNNNNNNNNNNNNNNNNNNNNNNNNNNNANGGNNNGTNTNTGACNCATGNNTTTNTANNNGNN
NNNNNACGNNNNANCCCCGGTCGNNNNCNNATTGAANNCCTGNNGCATNNNNNTNCNAGAATGANNGC
NNNNNGNNTNGNCTNNCNNNTCAAGNAGTCANNNAGNNANCNCGNTCNNNNNNNNNNNNCNNTCCCNN
ACTGAGGGTNNNNNNNNCATCTGCTNNNNNNNNCTNCGCNGNCGTNNCNNNNNAGCNGTATNGNGTNA
NNCANNNNNNNNNNNNNNNNNTNAAACNANNNNNNTNNNN
>PhuLa610
GTGNNNNNTAGCCCGNNNNAGTTAGACANNNCNNNNNNNNNNNNTNNNNNNNNNNNNNNNNNNNNNNN
NNNNNNNNNNNNNNNNNNNNNNNNNNNNNNNNNNNNNNNNNNNNNNNNNNNNNNNNNNNNNNNNNNNNNN
NNNNNNNNNNNNNNNNNNNNNNNNNNNNNNNNNNNNNNNNNNNNNNNNNNNNNNNNNNNNNNNNNNNNNN
NNNNNNNNNNNNNNNNNNNNNNNNNNNNNNNNNNNNNNNNNNNNNNNNNNNNNNNNNNNNNNNNNNNNNN
NNNNNNNNNNNNNNNNNNNNNNNNNNNNNNNNNNNNNNNNNNNNNNNNNNNNNCNTCTNNNAAANNNTNNN
NNNNNNNNNNNNNNNNNNNNNNNNNNCNCNNTNNNNGTAANCNANNNGNACATGCAGTCCCCTCNNNNN
NNNNNCAGAGNNNNNNNCNNNNNNNNNCCGGCCCCNNCCNNNNNNNNNNNNNNNNGNNNNNNNGNNNNNN
NNNNNNTGGTACNNNNCGNNNNNGGTTGNNTAAGAGGTCCACCACGNGGTCNNNNCACNNNNGNNNAN
NNNCNNNNAACNNNNNNNNNNNNNNATNNNCANNAGGNNNNNNCCNTNNNNNNANNAATTGCNATGACC
CAANNCACGGGGGCNGTCCCCGGCGCGGCGACCTTGATNANNGCCGGGGACAAGTATGGCNNNCNCNC
CGGGAAGGANNCNGCCGTANGTGCNNNNNANNNNNTCNTGNGANGTNGCCGNNATTCNCGTGGTCNNN
NNAGAATGNNCGNACCANNCGNANNNTGGTNATGTNAGTNNNNNNNNNNNNNNNNNNNNNNNNNNNNN
NNNNNNNNNNNNNNNNNNNNNNNNNNNNNNNNNNNNNNNNGNNNNNNCNANNNAAGGANNTANNNCTNNTN
NTNNNNNNNGGACNNNACNNNNCNNNNNNNACNCNNNCNCNNNNNNANNNGNTTNNNNNNCNNTATTNN
CCAGNANGCNGNGCNNNCATTCANGNGAACNNNGNNACGCGNNAATCGTANNATCTACCTGCCGCNNG
TNNNNNTACNGNNNNNNCGGANNNTNNNNCGTANNNNNNNTCATGCGTNNTNTNNTCNCNAGNCANGTT
CGNNNANANANTAGNANGTNTNNNNNNNNNNTGTNNCGTTCCGCNTGANNCNNCANNNNGNNGNNCTTN
NCANCGGCNNTANCTNCNGANNTAAGNCATANNNTNNNTCNNTANCTNNNNNGGNGGGNNACTGTAAA
NGGNNAATTNGNNNNTNATTNCNNNCACNTNNCTNNGNNNNCTNNNTNCCTGNNTNCNNAATTGGNNC
CANTGNNTGNCNATCTCTGGNTTNGNNNNNNTNNNNNTNNCAANNNGGNNNGTNNNANNNCNATNTAA
NNTANANNNNCNNNNNNANNNCACNANNNNNNNNAGNNNCNANNNTTANANNNGTAGNNNAATAAGTCT
CCGANNTNNNNNNNATAAGCGNNANACNNNNNNNNNNNNNNNANGANNNCGNNTTGGNNNTNNNNNNCNA
ANNAGACNNTNGTGGNTNNNNNTCAANNNNTTNGTAGTGGTATAACNGNNNNNNNTTTCNCCAGNNTN
NNNGNNNNNNNNCNATANNANNNNNNNGNGANNNNNNNNNNNANNNNCTCNNNAGGGANCANCTACTNNA
NCANTGGNGCGNAGNNNGAGANNNNGACNNGCCTTGGGTGGCAANCACNGCAATAAGTNNNGTTNANC
CTNNNAGTNNACACNNNNNCANNGNNNCNNNNNNANCNGANNTNNNNNNNNNNNNNNNNNNNNNNNNNN
NNNNNNNNNNNNNNNNNNNNNNNNNNNNNNNNNNNANGGNNNGTNTNTGACNCATNNNTTTTTANNNGNN
NNNNNACGNNNNANCCCCGGTCGNNNNCNNATTGAANNCCTGNNGCATNNNNNTNCNAGAATGANNGC
NNNNNGNNTNNNCTNNCNNNTCAAGNAGTCNNNNAGGTANCGCGNTCNNNGNNNNNNNNCNNNCCCNN
ACTGAGGGTNNNNNNNNCATCTGCTNNNNNNNNCTNCGCNGNCGTNNCNNNNNNGCNGTATNGNGTNA
NNCANNNNNNNNNNNNNNNNNTNAANNNNNNNNNNNNTNNNN
>PhuLa611
GTGNNNNNTAGCCCNNNNNAGTTAGACANNNCNNNNNNNNNNNNTNNNNNNNNNNNNNNNNNNNNNNN
NNNNNNNNNNNNNNNNNNNNNNNNNNNNNNNNNNNNNNNNNNNNNNNNNNNNNNNNNNNNNNNNNNNNNN
NNNNNNNNNNNNNNNNNNNNNNNNNNNNNNNNNNNNNNNNNNNNNNNNNNNNNNNNNNNNNNNNNNNNNN
NNNNNNNNNNNNNNNNNNNNNNNNNNNNNNNNNNNNNNNNNNNNNNNNNNNNNNNNNNNNNNNNNNNNNN
NNNNNNNNNNNNNNNNNNNNNNNNNNNNNNNNNNNNNNNNNNNNNNNNNNNNNNTCTNNNAAANNNTNNN
NNNNNNNNNNNNNNNNNNNNNNNNNNCNCNTTGNNNGTAANCNANNNGNACATGCAGTCCCCTCNNNNN
NNNGGCAGAGNNNNNNCCCNNNNNNNNCCGNCCCCNNCCNNNNNNNNNNNNCNNNGNNNNNNNGNNNNNN
NNNNNNTGGTACNNNNCGNNNNNGGTTGTNTAAGAGGTCCACCACGNGGTCNNGNCACNNNNGNNNAN
NNNCNNNNNNNCNNNNNNNNNNNNANNTNNNCANNAGGNNNNNNCCNTCNNNNNANTAATTGCNATGACC
CAANNCACGGGGGCNGTNCCCGGCGCGGCGACCTTGATNANGGCCGGGGACAAGTATGGCGNNCNCCC
CGGGAAGGANNCNGCCGTANGTGCNNNNNANNNNNTCNTGNGACGTCGCCGNNATTCNCGTGGTCNNN

CNNGAATGNCCGNACCANNCGNANNNTGGTNATGNNAGTNNNNNNNNNNNNNNNNNNNNNNNNNNN
NNNNNNNNNNNNNNNNNNNNNNNNNNNNNNNNNGNNNNGNNNNNNCNANNNAAGGNNNTANNNCTNNTN
NTNNNNNNGGACNNNACNNNNCNNNNNNNACNCNNNCNCNNNNNNANNNGGTTTTTNNNCNNTATTNT
CCAGNACGCNGNGCNNNCATTCANGNGAACNNNGNNACGCGTAAATCGTANNATCTACCTGCCGCNNG
TNNNNNTACNGNNNNNNCGGANNNTNNCTCGTANNNNNNNCATGCGTNNTNTNNTCNCTAGNCANGTT
CGNNNANANANTAGNANGTNTNNNNNNNNNNTGTNNCGTTCCGCNTGANNCNNCANNNNGNNGNNNTTN
TCANCGGCNNTANCTGCNGANNTAAGNCATANNNTNNNTCNNTANCTNNNNNGGNGGGNNACTGTAAA
NGGNNAATTNGANGNTNATTNCNNNCACNTNNCTNNGNNNNCTNNNTNCCTGNNTNCNNAATTGGNNC
CANTGNNTGNCNATCTCTNGNTTNGCNNNNNTNNNNNTNNCAANNNGGNNNGNNNNANNNCNATNTAA
NNTATANNNNCNNNNNNANNNCACNANNNNNNNAGNNNCNANNNTTANANNNGTAGNNNAATAAGTCT
CCGANNTNNNNNNNATAAGCGNNANACNNNNNNNNNNNNNNANGANNACGNNTTGGNNNTNNNNNNNNA
ANNAGACNNTNGTGGNTNNNNNTCAANNNNTTNGTAGTGGTATAACNGNNNNNNNTTTCNCCAGNNTN
NNNGNNNNNNNCNATACNANNNNNNNGNGANNNNNNNNNNNANNNNCTCNNNAGGGANCANCTACTNNA
GCAATGGNGCGNAGNNNGAGANNNNGACNNGCCTTGGGTGGCAANCACNGCAATAAGTNNNGTTNNNC
CTNNNAGTNNACACNNNNNCANNGNNNCNNNNNNANCNGANNTNNNNNNNNNNNNNNNNNNNNNNNNN
NNNNNNNNNNNNNNNNNNNNNNNNNNNNNNAGNNNANNGNNNGTNTNTGACNCATGTGTTTTTANNNGNN
NNNNNACGNNNNANCCCCGGTCGNNNNCNNATTGAANNCCTGNNGCATNNNNNTNCNAGAATGANNGC
NNNNNGNNTNGNCTNNCNNNTCAAGNAGTCANNTAGGTANNGCGNTCNNNNNNNNNNNNNCNNNCCCNN
ACTGAGGGTNNNNNNNNCATCTGCTNNNNNNNNCTNCGCNGNCGTNNCNNNNNNGCNGTATNGTGTNA
NNCANNNNNNNNNNNNNNNNNTNAAACNANNNNNNTNNNN
>PhuLa612
GTGNNNNNTAGCCCNNNNNAGTCAGACANNNCNNNNNNNNNNNNNNNNNNNNNNNNNNNNNNNNNNN
NNNNNNNNNNNNNNNNNNNNNNNNNNNNNNNNNNNNNNNNNNNNNNNNNNNNNNNNNNNNNNNNNNNNNNN
NNNNNNNNNNNNNNNNNNNNNNNNNNNNNNNNNNNNNNNNNNNNNNNNNNNNNNNNNNNNNNNNNNNNNNN
NNNNNNNNNNNNNNNNNNNNNNNNNNNNNNNNNNNNNNNNNNNNNNNNNNNNNNNNNNNNNNNNNNNNNNN
NNNNNNNNNNNNNNNNNNNNNNNNNNNNNNNNNNNNNNNNNNNNNNNNNNNNNNNCNTCTNNNAAANNNTNNN
NNNNNNNNNNNNNNNNNNNNNNNNNNNCNCNTTNNNNGTAANCNANNNGNACATGCAGTCCCCTCNNNNN
NNNGGCAGAGNNNNNNNCCNNNNNNNCCGNCCCCNNCCNNNNNNNNNNNNNNNNNGNNNNNNNANNNNNN
NNNNNNTGGTACNNNNCGNNNNNGGTTGTNTNAGNGGTCCACAACGNGGTCNNGNCACNNNNGNNNAN
NNNCNNNNAACNNNNNNNNNNNNNANNTNNNTANNAGGNCNNNNCCNTCNNNNNNNNTNATTGCNATGACC
CAANTCACGGGGGCNGTNCCCGGCGCGGCGACCTTGNNNACGTCCGGGGACAAGTATGGCNNNCNCCC
CGGGAAGGANNCNGCCGTANGTGCNNNNNANNNNNTCNTGNGANGTCGCCGNNATTNNCGTGGTCNNN
NNAGAATGNNCNNACCANNCGNANNNTGGTNATGTTAGTNNNNNNNNNNNNNNNNNNNNNNNNNNNN
NNNNNNNNNNNNNNNNNNNNNNNNNNNNNNNNNGNNNNGNNNNNNCNANNNAAGGNNNTANNNCTNNTN
NTNNNNNNGGACNNNACNNNNCNNNNNNNACNCNNNCNCNNNNNNANNNGGTTTTTNNNCNNTATTNT
CCAGNACGCNGNGCNNNCATTCANGNGAACNNNGNNACGCGNNAATCGTANNATCTACCTGCCGCNNG
TNNNNNTACNGNNNNTNCGGANNNTNNCTCGTANNNNNNTCATGCGTNNTNTNNTCNCNAGNCNNGTT
CGNNNANANANTAGNANGTNTNNNNNNNNNNTGTNNCGTTCCGCNTGANNCNNCANNNNGNNGNNCTTN
TCANCGGCNNTANCTGCNGANNTAAGNCATANNNTNNATCNNTANCTNNNNNGGAGGGNNACTGTAAA
NGGNNAATTNGNNGNTNATTNCNNNCATNTNNCNNNGNNTNCTNNNTNCCTGNNTNCNNAATTGGNNC
CANTGNNTGNCNATCTCTGGNTTNGNNNNNNTNNNNNTNNCAANNNGGNNNGNNANANNNCNATNTAA
NNTANANNNNCNNNNNNANNNCACNANNNNNGNAGNNNCNANNNGTANANNNGTAGNNNGATAAGTCT
CCGANNTNNNNNNNATAAGCGNNANACNNNNNNNNNNNNNNANGANNACGNNTTTGNNNTNNNNNNNNA
GNNAGACNNTNGTGGNTNNNNNTCAANNNNTTCGTAGTGGTATAATNGNNNNNNNTTTCNCCAGNNTN
NNNGNNNNNNNCNATACNANNNNNNNGNGANNNNNNNNNNNANNNNCTCNNNAGGNANCANCTACTNNA
GCANTGGNGCGCAGNNNGAGANNNNGACNNNCCTTGGGTGGCAANCACNGCAATAAGTNNNGTTNANC
CTNNNAGTNNACACNNNNNCANNGNNNCNNNNNNANCNGANNGNNNNNNNNNNNNNNNNNNNNNNNNN
NNNNNNNNNNNNNNNNNNNNNNNNNNNNNANNNNANGGNNNGTNTNTGACNCATGTGTTTTTANNNGNN
NNNNNNNGNNNNANCCCCGGTCGNNTNCNNATTGAANNCCTGNNGCATNNNNNTNCNAGAATGANNGC
NNNNNGNNTNNNCTNNCNTTTCAAGNAATCANNNAGGTANNGCGNTCNNNNNNNNNNNNNCNNTCCCNN
ACTGAGGGTNNNNNNNNCATCTGCTNNNNNNNNCTNCGCNGGCGTNNCNNNNNAGCNGTATNGNGNNA
NNCANNNNNNNNNNNNNNNNNTNNAACNANNNNNNTNANN
>PhuLa613
GTGNNNNNTAGCCCNNNNNAGTCAGACANNNCNNNNNNNNNNNNNTNNNNNNNNNNNNNNNNNNNNNNN

NNNNNNNNNNNNNNNNNNNNNNNNNNNNNNNNNNNNNNNNNNNNNNNNNNNNNNNNNNNNNNNNNNNNNNNN
NNNNNNNNNNNNNNNNNNNNNNNNNNNNNNNNNNNNNNNNNNNNNNNNNNNNNNNNNNNNNNNNNNNNNNNN
NNNNNNNNNNNNNNNNNNNNNNNNNNNNNNNNNNNNNNNNNNNNNNNNNNNNNNNNNNNNNNNNNNNNNNNN
NNNNNNNNNNNNNNNNNNNNNNNNNNNNNNNNNNNNNNNNNNNNNNNNNNNNNNCNTCTNNNAAANNNTNNN
NNNNNNNNNNNNNNNNNNNNNNNNNNCNCNTTNNNNGTAANCNANNNGNACATGCAGTCCCCTCNNNNN
NNNNNCAGAGNNNNNNNNNNNNNNNNNCCGNCCCCGNCCNNNNNNNNNNNNNNNNNNNNNNNNNANNNNNN
NNNNNNTGGTACNNNNCGNNNNNGGTTGTNTAAGNGGTCCACAACGNGGTCNNGNCACNNNNGNNNAN
NNNCNNAAAANNGNNNNNNNNNNANNTNNNTANNAGGNCNNNNCCNTCNNGNNNNNAATTGCNATGANN
CAANTCNCGGGGGCAGTNCCCGGCGCGGCGACCTTGNTNANGTCCGGGGNCAAGTATGGCGNNCNCCC
CGGGAAGGANNCNGCCGTANGTGCNNNNNANNNNNTCNTGNGANGTNGCCGNNATTCNCNTGGTCNNN
NNAGAATGNCCGNACCANNCGNANNNTGGTNATGNNAGTNNNNNNNNNNNNNNNNNNNNNNNNNNNN
NNNNNNNNNNNNNNNNNNNNNNNNNNNNNNNNNGNNNNGNNNNNNCNANNNANGGANNTANNNCTNNTN
NTNNNNNNNGGACNNNACNNNNCNNNNNNNACNCNNNCNCNNNNNNNNNNNGNTTTTNNNNCNNTATTNN
CCAGNANGCNGNGCNNNCATTCANGNGAACNNNGNNACGCGNNAATCGTANNATCTACCTGCCGCNNG
TNNNNNTACNGNNNNNNCGGANNNTNNNNCGTANNNNNNTCATGCGTNNTNTNNTCNCNAGNCNNGTT
CGNNNANANANTAGNANGTNTNNNNNNNNNNGTNNCGTTCCGCNTGANNCNNCANNNNGNNGNNCTTN
NCANCGGCNNTANCTGCNGANNTAAGNCATANNNTNNNTCNNTANCTNNNNNGGNGGGNNACTGTAAA
NGGNNAATTNGNNNNTNANTNCNNNCATNTNNCNNNGNNTNCTNNNTNCCTGNNTNCNNAATTGGNNC
CANTGNNTGNCNATCTCTGGNTTNGNNNNNNTNNNNNTNGCAANNNGGNNNNNNNNANNNCNATNTAA
NNTANANNNNCNNNNNNANNNCACNNNNNNNGNAGNNNCNANNNGNANANNNGTAGNNNGATNAGTCN
CCGANNTNNNNNNNATAAGCGNNANACNNNNNNNNNNNNNNANGANNACGNNTTTGNNNTNNNNNNCNA
GNNAGACNNTNGTGGNTNNNNNTCAANNNNTTNGTAGTGGTATAATNGNNNNNNNTTTCNCCAGNNTN
NNNGNNNNNNNCNATANNANNNNNNNGTGANNNNNNNNNNNANNNNCTCNNNAGGGANCANCTACTNNA
GCANTGGNGCGCAGNNNGAGANNNNGACNNNCCTTGGGTGGCAANCACNGCAATAAGTNNNGTTNNNC
CTNNNAGTNNACACNNNNNCANNGNNNCNNNNNNANCNGANNGNNNNNNNNNNNNNNNNNNNNNNNNN
NNNNNNNNNNNNNNNNNNNNNNNNNNNNNNNANNNNANGGNNNGTNTNTGACNCNTGTGTTTNTANNNGNN
NNNNNACGNNNNNNCCCCGGTCGNNNNCNNATTGAANNCCTGNNGCATNNNNNTNCNAGAATGANNNC
NNNNNGNNTNNNCTGNCNTTTNAAGNAATCANNNAGGNNNNNNGNTCNNNNNNNNNNNNNCNNNCCCNN
ACTGAGGGTNNNNNNNNCATCNGCTNNNNNNNNNNTTCGCNGGCGTNNCNNNNNAGCNGTATNGNGTNA
NNCANNNNNNNNNNNNNNNNNNTNAAACNANNNNNNNTNNNN
>PhuLa614
GTGNNNNNNTAGCCCNNNNNAGTTAGACANNNCNNNNNNNNNNNNTNNNNNNNNNNNNNNNNNNNNNNNN
NNNNNNNNNNNNNNNNNNNNNNNNNNNNNNNNNNNNNNNNNNNNNNNNNNNNNNNNNNNNNNNNNNNNNNNN
NNNNNNNNNNNNNNNNNNNNNNNNNNNNNNNNNGNNNNNNNNNNNNNNNNNNNNNNNNNNNNNNNNNNNNNN
NNNNNNNNNNNNNNNNNNNNNNNNNNNNNNNNNNNNNNNNNNNNNNNNNNNNNNNNNNNNNNNNNNNNNNNN
NNNNNNNNNNNNNNNNNNNNNNNNNNNNNNNNNNNNNNNNNNNNNNNNNNNNNNCNTCTNNNAAANNNTNNN
NNNNNNNNNNNNNNNNNNNNNNNNNNCNCNTTNNNNGTAANCNANNNGNACATGCAGTCCCCTCNNNNN
NNNGGCAGAGNNNNNNNCNNNNNNNNNCCGGCCCCNNCCNNNNNNNNNNNNNNNNNNNNNNNNNNGNNNNNN
NNNNNNTGGTACNNNNCGNNNNNGGTTGNNTNAGAGGTCCACCACGNGGTCNNGNCACNNNNGNNNAN
NNNCNNNNAACNGNNNNNNNNNNNNNTNNNCANNAGGNNNNNNCCNTCNNGNNNNTNATTGCNATGACC
CAANNCACGGNGGCAGTNCCCGGCGCGGCGACCTTGANNACGGCCGGGGNCAAGTATGGCNNNCNCCC
CGGGAAGGANNCNGCCGTANGTGCNNNNNANNNNNTCNTGNGANGTNGCCGNNATTCNCGTGGTCNNN
CNAGANTGNCCNNACCANNCGNANNNTGGTNATGNNAGTNNNNNNNNNNNNNNNNNNNNNNNNNNNN
NNNNNNNNNNNNNNNNNNNNNNNNNNNNNNNNNNNNNNNGNNNNNNCNANNNAAGGANNTANNNCTNNTN
NTNNNNNNNGGACNNNACNNNNCNNNNNNNACNCNNNCNCNNNNNNANNNGNTTTTNNNNCNNTATTNN
CCAGNACGCNGNGCNNNCATTCANGNGAACNNNGNNACGCGNNAATCGTANNATCTACCTGCCGCNNG
NNNNNNTNCNGNNNNTNCGGANNNTNNCTCGTANNNNNNTCATGCGTNNTNTNNTCNCNAGNCNNGTT
CGNNNANANANTAGNANGTNTNNNNNNNNNNTGTNNCGTTCCGCNTGANNCNNCANNNNGNNGNNCTTN
TCANCGGCNNTANCTGCNGANNTAAGNCATANNNTNNNTCNNTANCTNNNNNGGNGGGNNACTGTAAA
NGNNNAATTNGNNGNTNANTNCNNNCACNTNNCTNNGNNNNCTNNNTNCCTGNNTNCNNAATTGGNNC
CANTGNNTGNCNATCTCTNGNTTNGNNNNNNTGNNNNTNNCANNNNGGNNNGTNANANNNCNATNTAA
NNTATANNNNCNNNNNNANNNCACNNNNNNNNNAGNNNCNANNNTTANANNNGTAGNNNANTNAGTCN
CCGANNTNNNNNNNATAAGCGNNANACNNNNNNNNNNNNNNANGANNACGNNTTGGNNNTNNNNNNCNA
ANNAGACNNTNGTGGNTNNNNNTCAANNNNTTCGTAGTGGTATAACNGNNNNNNNTTTCNCCAGNNTN

NNNGNNNNNNNNCNATACNANNNNNNNNGNGANNNNNNNNNNANNNNCTCNNNAGGGANCANCTACTNNA
GCANNGGNGCGNAGNNNGAGANNNNGACNNNCCTTGGGTGGCAANCACNGCAATAAGTNNNGTTNANC
CTNNNAGTNNACACNNNNNCANNGNNNCNNNNNNANCNGANNTNNNNNNNNNNNNNNNNNNNNNNNNN
NNNNNNNNNNNNNNNNNNTNNNNNNNNNNGNNNANGGNNNGTNTNTGACNCNTGTGTTTNTANNNGNN
NNNNNACGNNNNANCCCCGGTCGNNNNCNNATTGAANNCCTGNNGCATNNNNNTNCNAGAATGANNGC
NNNNNGNNTNGNCTNNCNNNTCAAGNAGTCNNNTAGNNNNNNCGNTCNNNNNNNNNNNNCNNTCCCNN
ACTGAGGGTNNNNNNNNCATCTGCTNNNNNNNNNTNCGCNGNCGTNNCNNNNNAGCNGTATNNNGTNN
NNCANNNNNNNNNNNNNNNNNTNAAACAANNNNNNTNNNN
>PhuLa615
GTGNNNNNTAGCCCNNNNNAGTTAGACANNNCNNNNNNNNNNNNTNNNNNNNNNNNNNNNNNNNNNN
NNNNNNNNNNNNNNNNNNNNNNNNNNNNNNNNNNNNNNNNNNNNNNNNNNNNNNNNNNNNNNNNNNNNNN
NNNNNNNNNNNNNNNNNNNNNNNNNNNNNNNNNNNNNNNNNNNNNNNNNNNNNNNNNNNNNNNNNNNNNN
NNNNNNNNNNNNNNNNNNNNNNNNNNNNNNNNNNNNNNNNNNNNNNNNNNNNNNNNNNNNNNNNNNNNNN
NNNNNNNNNNNNNNNNNNNNNNNNNNNNNNNNNNNNNNNNNNNNNNNNNNNNNCNTCTNNNAAANNNTNNN
NNNNNNNNNNNNNNNNNNNNNNNNNNCNCNTTGNNNGTAANCNANNNGNACATGCAGTNNCCTCNNNNN
NNNGGCAGAGNNNNNNCCCNNNNNNNNCCGGCCCCNNCCNNNNNNNNNNNNCNNNGNNNNNNNGNNNNNN
NNNNNNTGGTACNNNNCGNNNNNGGTTGTNTAAGAGGTCCACCACGNGGTCNNGNCACNNNNGNNNAN
NNNCNNNNAANNNNNNNNNNNNNNNNNTNNNCANNAGGNNNNNNCCNTCNNGNNNNNAANNGCNATGACC
CAANTCACGGGGGCAGTCCCCGGCGCGGCGACCTTGATNANNGCCGGGGACAAGTATGGCNTNCNCCC
CGGGAAGGANNCNGCCGTANGTGCNNNNNANNNNNTCTTGNGANGTCGCCGNNATTCNCGTGGTCNNN
CNAGAATGNCCGNACCANNCGNANNNTGGTNATGNTAGTNNNNNNNNNNNNNNNNNNNNNNNNNNNNNN
NNNNNNNNNNNNNNNNNNNNNNNNNNNNNNNNNNNNNNNNGNNNNNNCNANNNAAGGANNTANNNCTNNTN
NTNNNNGNGGACNNNACNNNNCNNNNNNNACNCNNNCNCNNNNNNNNNNNGGTTTTTNNNCNNTANTNT
CCAGNACGCNGNGCNNNCATTCANGNGAACNNNGNNACGCGNNAATCGTANNATCTACCTGCCGCNGG
TNNNNNTACNGNNNNNNCGGANNNTNNCTCGTANNNNNNNCATGCGTNNTNTNNTCNCNAGNCNNGTT
CGNNNANANANTAGNANGTNTCCNNNNNCTTGTNNCGTTCCGCNTGANNCNNCANNNNGNNGNNCTTN
TCANCGGCNNTANCTGCNGANNTAAGTCATANNNTNNNTCNNTANCTNNNNNGGNGGGNNACTGTAAA
NGGNNAATTGGANNNTNATTNCNNNCACNTNNCTNNGNNNNCTNNNTNCCTGNNTNCNNAATTGGNNC
CANTGNNTGNCNATCTCTGGNTTNGNNNNNNTNNNNNTNNCAANNNGGNNNGTNANANNNCNATNTAA
NNTATANNNNCNNNNNNANNNCACNANNNNNNNNAGNNNCNANNNTTANANNNGTAGNNNANTAAGTCT
CCGANNTNNNNNNNATAAGCGNNANACNNNNNNNNNNNNNNANGANNACGNNTTGGNNNTNNNNNNNCNA
ANNAGACNNTNGTGGNTNNNNNTCAANNNNTTNGTAGTGGTATAACNGNNNNNNNTTTCNCCAGNNTN
NNNGNNNNNNNNCNATANNANNNNNNNNGNGANNNNNNNNNNNNNNNNCTCNNNAGGGANCANCTACTNNA
GCANTGGNGCGNAGNNNGAGANNNNGACNNGCCTTGGGTGGCAANCACNGCAATAAGTNNNGTTNNNC
CTNNNAGTNNACACNNNNNCANNGNNNCNNNNNNANCNGANNTNNNNNNNNNNNNNNNNNNNNNNNNN
NNNNNNNNNNNNNNNNNNNNNNNNNNNNNNAGNNNANGGNNNGTNTNTGACNCATGTGTTTTTANNNGNN
NNNNNACGNNNNNNNCCCCGGTCGNNNNCNNATTGAANNCCTGNNGCATNNNNNTNCNAGAATGANNGC
NNNNNGNNTNGNCTNNCNNTTCAAGNAGTCANNTAGGTNNNNCGNTCNNNGNNNNNNNNCNNTCCCNN
ACTGAGGGTNNNNNNCGCATCTGCTNNNNNNNNNCTTCGCNGNCGTNNCNNNNNAGCCGTATNNTGTNN
NNCANNNNNNNNNNNNNNNNNTNNNACGANNNNNNTNNNN
>PhuLa616
GTGNNNNNTAGCCCNNNNNAGTCAGACANNNCNNNNNNNNNNNNTNNNNNNNNNNNNNNNNNNNNNN
NNNNNNNNNNNNNNNNNNNNNNNNNNNNNNNNNNNNNNNNNNNNNNNNNNNNNNNNNNNNNNNNNNNNNN
NNNNNNNNNNNNNNNNNNNNNNNNNNNNNNNNNNNNNNNNNGNNNNNNNNNNNNNNNNNNNNNNNNNNNN
NNNNNNNNNNNNNNNNNNNNNNNNNNNNNNNNNNNNNNNNNNNNNNNNNNNNNNNNNNNNNNNNNNNNNN
NNNNNNNNNNNNNNNNNNNNNNNNNNNNNNNNNNNNNNNNNNNNNNNNNNNNCNTCTNNNAAANNNTNNN
NNNNNNNNNNNNNNNNNNNNNNNNNNCNCNTTGNNNGTAANCNANNNGNACATGCAGTCCCCTCNNNNN
NNNGGCAGAGNNNNNNNCCNNNNNCNCCGNCCCCGNCCNNNNNNNNNNNNNNNNGNNNNNNNANNNNNN
NNNNNNTGGTACNNNNCGNNNNNGGTTGTNTAAGAGGTCCACAACGNGGTCNGGNCACNNNNGNNNAN
NNNCNNNNAACNNNNNNNNNNNNANNTNNNTANNAGGNCNNNNCCNTNNNGNNNNNNANTGCNATGACC
CAANNCACGGGGGCNGTNCCCGGCGCGGCGACCTTGATNACGTCCGGGGACAAGTATGGCNCNCNCCC
CGGGAAGGANNCNGCCGTANGTGCNNNNNANNNNNTCTTGNGANGTNGCCGNNATTCNCGTGGTCNNN
CNAGAATGNCCGNACCANNCGNANNNTGGTNATGNNAGTANNNNNNNNNNNNNNNNNNNNNNNNNNNNN
NNNNNNNNNNNNNNNNNNNNNNNNNNNNNNNNNNGNNNNGNNNNNNCNANNNAAGGANNTANNNCTNNTN

NTNNNNNNGGACNNNACNNNNCNNNNNNNACNCNNNCNCNNNNNNANNNGNTTTTTNNNCNNTATTNT
CCAGNACGCNGNGCNNNCATTCANGNGAACNNNGNNACGCGNNAATCGTANNATCTACCTGCCGCNNG
TNNNNNTACNGNNNNNNCGGANNNTNNCTCGTANNNNNNTCATGCGTNNTNTNNTCNCNAGNCNNGTT
CGNNNANANANTAGNANGTNTCNNNNNNNNTGTNNCGTTCCGCNTGANNCNNCANNNNGNNGNNCTTN
NCANCGGCNNTANCTGCNGANNTAAGNCATANNNTNNATCNNTANCTNNNNNGGNGGGNNACTGTAAA
NGGNNAATTNGNNNNTNATTNCNNNCATNTNNCTNNGNNTNCTNNNTNCCTGNNTNCNNAATTGGNNC
CANTGNNTGNCNATCTCTNGNTTNGNNNNNNTGNNNNTNNCAANNNGGNNNGNNANANNNCNATNTAA
NNTATANNNNCNNNNNNANNNCACNANNNNNGNAGNNNCNANNNGTANANNNGTAGNNNGATAAGTCT
CCGANNTNNNNNNNATAAGCGNNANACNNNNNNNNNNNNNNANGANNACGNNTTTGNNNTNNNNNNCNA
GNNAGACNNTNGTGGNTNNNNNTCAANNNNTTCGTAGTGGTATAATNGNNNNNNNTTTCNCCAGNNTN
NNNGNNNNNNNCNATACNANNNNNNNGNGANNNNNNNNNNNANNNNCTCNNNAGGGANCANCTACTNNA
GCANTGGNGCGCAGNNNGAGANNNNGACNNNCCTTGGGTGGCAANCACNGCAATAAGTNNNGTTNANC
CTNNNAGTNNACACNNNNNCANNGNNNCNNNNNNANCNGANNGNNNNNNNNNNNNNNNNNNNNNNNNN
NNNNNNNNNNNNNNNNNNNNNNNNNNNNNAGNNNANGGNNNGTNTNTGACNCATGTGTTTTTANNNGNN
NNNNNACGNNNNANCCCCGGTCGNNNNCNNATTGAANNCCTGNNGCATNNNNNTNCNAGAATGANNGC
NNNNNGNNTNGNCTGNCNNNTNAAGNAATCANNNAGGTANNGCGNTCNNNGNNNNNNNNCNNTCCCNN
ACTGAGGGTNNNNNNCGCATCTGCTNNNNNNNNCTTCGCNGGCGTNNCNNNNNAGCNGTATNGTGTTA
NNCANNNNNNNNNNNNNNNNNTNAAACNANNNNNNTNNNN
>PhuLa617
GTGNNNNNTAGCCCNNNNNAGTTAGACANNNCNNNNNNNTNNNNNNNNNNNNNNNNNNNN
NNNNNNNNNNNNNNNNNNNNNNNNNNNNNNNNNNNNNNNNNNNNNNNNNNNNNNNNNNNN
NNNNNNNNNNNNNNNNNNNNNNNNNNNNNNNNNNNNNNNNNNNNNNNNNNNNNNNNNNNN
NNNNNNNNNNNNNNNNNNNNNNNNNNNNNNNNNNNNNNNNNNNNNNNNNNNNNNNNNNNN
NNNNNNNNNNNNNNNNNNNNNNNNNNNNNNNNNNNNNNNNNNNNNNNNNNTCTNNNAAANNNTNNN
NNNNNNNNNNNNNNNNNNNNNNNNNNCNCNNTGNNNGTAANCNANNNGNACATGCAGTCCCCTCNNNNN
NNNGGCAGAGNNNNNNCCNNNNNNNNCCGGCCCCGNCCNNNNNNNNNNNNNNNNGNNNNNNNGNNNNNN
NNNNNNTGGTACNNNNCGNNNNNGGTTGTNTAAGAGGTCCACCACGNGGTCNNGNNACNNNNGNNNAN
NNNCNNNNAACNNNNNNNNNNNNANNTNNNCANNAGGNNNNNNCCNTCNNNNNNNNNANNGCNATGANN
CANNNCACGGGGGCAGTNCCCGGNGCGGCGACCTTGNNNANNGCCGGGGACAAGTATGGCNNNCNCCC
CGGGAAGGANNCNGCCGTANGTGCNNNNNANNNNNTCNTGNGANGTCGCCGNNATTNNNGTGGTCNNN
NNAGAATGNNCGNACCANNCGNANNNTGGTNATGNNAGTNNNNNNNNNNNNNNNNNNNNNNNNNNNN
NNNNNNNNNNNNNNNNNNNNNNNNNNNNNNNNNNNNNNNGNNNNNNCNANNNAAGGNNNTANNNCTNNTN
NTNNNGNNGGACNNNACNNNNCNNNNNNNACNCNNNCNCNNNNNNNNNNNGGTTTTTNNCCNNTANTNT
CCAGNACGCNGNGCNNNCATTCANGNGAACNNNGNNACGCGNNAATCGTANNATCTACCTGCCGCNNG
TNNNNNTACNGNNNNNNCGGANNNTNNCTCGTANNNNNNNCATGCGTNNTNTNNTCNCNAGNCNNGTT
CGNNNANANANTAGNANGTNTCNNNNNNNTTGTNNCGTTCCGCNTGANNCNNCANNNNGNNGNNCTTC
TCANCGGCNNTANCTGCNGANNTAAGNCATANNNTNNNTCNNTANCTNNNNNGGNGGGNNACTGTAAA
NGGNNAATTGGNNGNTNANTNCNNNNACNTNNCNNNGNNNNCTNNNTNCCTGNNTNCNNAATTGGNNC
CANTGNNTGNCNATCTCTGGNTTNGNNNNNNTNNNNNTNGCAANNNGGNNNNNNNNANNNCNATNTAA
NNTATANNNNCNNNNNNANNNCACNANNNNNGNAGNNNCNANNNTTANANNNGTAGNNNANTAAGTCT
CCGANNTNNNNNNNATAAGCGNNANACNNNNNNNNNNNNNNANGANNACGNNTTGGNNNTNNNNNNCNA
ANNAGACNNTNGTGGNTNNNNNTCAANNNNTTNGTAGTGGTATAACNGNNNNNNNTTTCNCCAGNNTN
NNNGNNNNNNNCNATANNANNNNNNNGNGANNNNNNNNNNNANNNNCTCNNNAGGNANCANCTACTNNA
GCAATGGNGCGCAGNNNGAGANNNNGACNNNCCTTGGGTGGCAANCACNGCAATAAGTNNNGTTNANC
CTTNNAGTNNACACNNNNNCANNGNNNCNNNNNNANCNGANNTNNNNNNNNNNNNNNNNNNNNNNNNN
NNNNNNNNNNNNNNNNNNNNNNNNNNNNNANNNNANGGNNNGTNTNTGACNCANGTGTTTTTANNNGNN
NNNNNNCGNNNNANCCCCGGTCGNNNNCNNATTGAANNCCTGNNGCATNNNNNTNCNAGAATGANNGC
NNNNNGNNTNGNCTNNNNNNNTCAAGNAGTCANNNAGGTATNGCGNTCNNNGNNNNNNNNCNNTCCCNN
ACTGAGGGTNNNNNNCGCATCNGCTNNNNNNNNNNTNCGCNGNCGTNNCNNNNNAGCCGTATNGTGTNN
NNCANNNNNNNNNNNNNNNNNTNAAACANNNNNNNTNNNN
>PhuLa618
GTGNNNNNTAGCCCNNNNNAGTTAGACANNNCNNNNNNNTNNNNNNNNNNNNNNNNNNNN
NNNNNNNNNNNNNNNNNNNNNNNNNNTNNNNNNNNNNNNNNNNNNNNNNNNNNNNNNNNN
NNNNNNNNNNNNNNNNNNNNNNNNNNNNNNNNNNNNNNNNNNNNNNNNNNNNNNNNNNNN

NNNNNNNNNNNNNNNNNNNNNNNNNNNNNNNNNNNNNNNNNNNNNNNNNNNNNNNNNNNNNNNNNNNTNN
NNNNNNNNNNNNNNNNNNNNNNNNNNNNNNNNNNNNNNNNNNNNNNNNNNNNNNTCTNNNAAANGNTNNN
NNNNNNNNNNNNNNNNNNNNNNNNNNNCNCNTTGNNNGTAANCNANNNGNACATGCAGTCCCCTCNNNNN
NNNGGCAGAGNNNNNNCCCNNNNNNNNCCGGCCCCNNCCNNNNNNNNNNNNNNNNGNNNNNNNGNNNNNN
NNNNNNTGGTACNNNNCGNNNNNGGTTGTTTAAGAGGTCCACCACGNGGTCNGGNCACNNNNGNNNAN
NNNCNNAAAACNNNNNNNNNNNNANNTNNNCANNAGGNCNNNNCCNTCNNNNNNNTAATTGCNATGACC
CAANNNNCGGGGGCAGTCCCCGGCGCGGCGACCTTGATNACGGCCGGGGACAAGTATGGCNTNCNCCC
CGGGAAGGANNCNGCCGTANGTGCNNNNNANNNTNTCNTGNGANGTCGCCGNNATTCNCGTGGTCNNN
CNAGAATGNCCGNACCANNCGNANNNTGGTNATGNTAGTNNNNNNNNNNNNNNNNNNNNNNNNNNNNNN
NNNNNNNNNNNNNNNNNNNNNNNNNNNNNNNNNNNNNNNNNGNNNNNNCNANNNAAGGANNTANNNCTNNTN
NTNNNNNNGGACNNNACNNNNCNNNNNNNACNCNNNCNCNNNNNNANNNGGTTTTTNNNCNNTANTNT
CCAGNACGCNGNGCNNNCATTCANGNGAACNNNGNNACGCGNNAATCGTANNATCTACCTGCCGCNNG
NNNNNNTNCNGNNNNNNCGGANNNTNNCNCGTANNNNNNTCATGCGTNNTNTNNTCNCNAGNCNNGTT
CGNNNANANANTAGNANGTNTCNNNNNNNNTGTNNCGTTCCGCNTGANNCNNCANNNNGNNGNNCTTC
TCANCGGCNNTANCTGCNGANNTAAGNCATANNNTNNATCNNTANCTNNNNNGGNGGGNNACTGTAAA
NGGNNAATTNGANGNTNANTNCCNNCACNTNNCTNNGNNTNCTNNNTNCCTGNNTNCNNAATTGGNNC
CANTGNNTGNCNATCTCTGGNTTNGCNNNNNTNNNNNTNNCAANNNGGNNNGTNANANNNCNATNTAA
NNTANANNNNCNNNNNNANNNCACNANNNNNGNAGNNNCNANNNTTANANNNGTAGNNNANTAAGTCT
CCGANNTNNNNNNNATAAGCGNNANACNNNNNNNNNNNNNANGANNACGNNTTGGNNNTNNNNNNNNA
ANNAGACNNTNGTGGNTNNNNNTCAANNNNTTCGTAGTGGTATAACNGNNNNNNNTTTCNCCAGNNTN
NNNGNNNNNNNNCNATANNANNNNNNNGNGANNNNNNNNNNNANNNNCTCNNNAGGGANCANCTACTTNA
GCANTGGNGCGCAGNNNGAGANNNNGACNNGCCTTGGGTGGCAANCACNGCAATAAGTNNNGTTNANC
CTNNNAGTNNACACNNNNNCANNGNNNCNNNNNNANCNGANNTNNNNNNNNNNNNNNNNNNNNNNNNN
NNNNNNNNNNNNNNNNNNNNNNNNNNNNNNNAGNNNANGGNNNGTNTNTGACNCATGTGTTTTTANNNGNN
NNNNNACGNNNGANCCCCGGTCGNNNNCNNATTGAANNCCTGNNGCATNNNNNNTNCNAGAATGANNGC
NNNNNGNNTNGNCTNNCNTTTCAAGNAGTCANNNAGGTNNNGCGNTCNNNGNNNNNNNNCNNTCCCNN
ACTGAGGGTNNNNNNNNCATCTGCTNNNNNNNNNNTTCGCNGNCGTNNCNNNNNAGCNGTATNGTGTTA
NNCANNNNNNNNNNNNNNNNNNTNAAACAANNNNNNTTNNNN
>PhuLa619
GTGNNNNNTAGCCCNNNNNAGTTAGACANNNCNNNNNNNNNNNNNNNNNNNNNNNNNNNNNNNNNNNNNN
NNNNNNNNNNNNNNNNNNNNNNNNNNNNNNNNNNNNNNNNNNNNNNNNNNNNNNNNNNNNNNNNNNNNNN
NNNNNNNNNNNNNNNNNNNNNNNNNNNNNNNNNNNNNNNNNNNNNNNNNNNNNNNNNNNNNNNNNNNNNN
NNNNNNNNNNNNNNNNNNNNNNNNNNNNNNNNNNNNNNNNNNNNNNNNNNNNNNNNNNNNNNNNNNNNNN
NNNNNNNNNNNNNNNNNNNNNNNNNNNNNNNNNNNNNNNNNNNNNNNNNNNNNNNTCTNNNAAANNNTNNN
NNNNNNNNNNNNNNNNNNNNNNNNNNNCNCNTTGNNNGTAANCNANNNGNACATGCAGTNCCCTCNNNNN
NNNNNCAGAGNNNNNNNCNNNNNNNNCCGNCCCCNNCCNNNNNNNNNNNNNNNNNNNNNNNNNGNNNNNN
NNNNNNTGGTACNNNNCGNNNNNGGTTGTTTAAGAGGTCCACCACGNGGTCNNGNNACNNNNGNNNAN
NNNCNNNNNNCNNNNNNNNNNNNANNTNNNCANNAGGNNNNNNCCNTCNNNNNNNNNAANNGCNATGACC
CAANTCACGGNGGCAGTNCCCGGNGCGGCGACCTTGNNNANGGCCGGGGACAAGTATGGCNNNCNCCC
CGNGAAGGANNCNGCCGTANGTGCNNNNNANNNNNTCNTGNGANGTNGCCGNNATTNNCGTNGTCNNN
CNAGAATGNCCGNACCANNCGNANNNTGGTNATGNNAGTNNNNNNNNNNNNNNNNNNNNNNNNNNNNNN
NNNNNNNNNNNNNNNNNNNNNNNNNNNNNNNNNNNNGNNNNGNNNNNNCNANNNAAGGNNNTANNNCTNNTN
NTNNNNNNGGACNNNACNNNNCNNNNNNNACNCNNNCNCNNNNNNANNNGGTTTTNNNNCNNTATTNT
CCAGNACGCNGNGCNNNCATTCANGNGAACNNNGNNACGCGNNAATCGTANNATCTACCTGCCGCNNG
TNNNNNTACNGNNNNNNCGGANNNTNNNNCGTANNNNNNTCATGCGTNNTNTNNTCNCNAGNCANGTT
CGNNNANANANTAGNANGTNTNNNNNNNNNNTGTNNCGTTCCGCNTGANNCNNCANNNNGNNGNNCTTN
TCANCGGCNNTANCTGCNGANNTAAGTCATANNNTNNATCNNTANCTNNNNNGGNGGGNNACTGTAAA
NGGNNAATTNGANGNTNANTTCNNNCACNTNNCTNNGNNTNCTNNNTNCCTGNNTNCNNAATTGGNNC
CANTGNNTGNCNATCTCTGGNTTNGNNNNNNTNNNNNTNNCANNNNGGNNNGTNANANNNCNATNTAA
NNTANANNNNCNNNNNNANNNCACNANNNNNGNAGNNACNANNNTTANANNNGTAGNNNAATAAGTCT
CCGANNTNNNNNNNATAAGCGNNANACNNNNNNNNNNNNNANGANNACGNNTTGGNNNTNNNNNNCNA
ANNAGACNNTNGTGGNTNNNNNTCAANNNNTTCGTAGTGGTATAACNGNNNNNNNTTTCNCCAGNNTN
NNNGNNNNNNNNCNATANNANNNNNNNGTGANNNNNNNNNNNANNNNCTCNNNAGGGANCANCTACTNNA
GCAATGGNGCGNAGNNNGAGANNNNGACNNNCCTTGGGTGGCAANCACNGCAATAAGTNNNGTTNANC

CTNNNAGTNNACACNNNNNCANNGNNNCNNNNNNANCNGANNTNNNNNNNNNNNNNNNNNNNNNNNNNNN
NNNNNNNNNNNNNNNNNNNNNNNNNNNNNNANNNNANNGNNNGTNTNTGACNCATGTGTTTTTANNNGNN
NNNNNACGNNNNANCCCCGGTCGNNNNCNNATTGAANNCCTGNNGCATNNNNNTNCNAGAATGANNGC
NNNNNGNNTNGNCTNNCNNNTCAAGNAGTCAANNAGGTNNNNNGNTCNNNNNNNNNNNNNNCNNNCCCNN
ACTGAGGGTNNNNNNNNCATCTGCTNNNNNNNNNNTTCGCNGNCGTNNCNNNNNNAGCNGTATNGNGTNA
NNCANNNNNNNNNNNNNNNNNTNAAACAANNNNNNTNNCN
>PhuLa620
GTGNNNNNTAGCCCNNNNNAGTTAGACANNNCNNNNNNNNNNNNNTNNNNNNNNNNNNNNNNNNNNNNNN
NNNNNNNNNNNNNNNNNNNNNNNNNNNNNNNTNNNNNNNNNNNNNNNNNNNNNNNNNNNNNNNNNNNNNN
NNNNNNNNNNNNNNNNNNNNNNNNNNNNNNNNNNNNNNNNNNNNNNNNNNNNNNNNNNNNNNNNNNNNNN
NNNNNNNNNNNNNNNNNNNNNNNNNNNNNNNNNNNNNNNNNNNNNNNNNNNNNNNNNNNNNNNNNNNNNN
NNNNNNNNNNNNNNNNNNNNNNNNNNNNNNNNNNNNNNNNNNNNNNNNNNNNNNTCTNNNAAANNNTNNN
NNNNNNNNNNNNNNNNNNNNNNNNNNNCNCNTTGNNNGTAANCNANNNGNACATGCAGTCCCCTCNNNNN
NNNNNCAGAGNNNNNNCCCNNNNNCNCCGGCCCCNNCCNNNNNNNNNNNNNNNNNNNNNNNNNGNNNNNN
NNNNNNTGGTACNNNNNCGNNNNNGGTTGTTTAAGAGGTCCACCACGNGGTCNNGNCACNNNNGNNNAN
NNNCNNANAACNNNNNNNNNNNNNNNNTNNNCANNAGGNCNNNNCCNTCNNGNNNNNAATTGCNATGACC
CAANTCACGGGGGCAGTNCCCGGCGCGGCNACCTTGATNANGGCCGGGGACAAGTATGGCGNNCNCCC
CGGGAAGGANNCNGCCGTANGTGCNNNNNANNNNNTCNTGNGACGTNGCCGNNATTCNNGTGGTCNNN
CNAGAATGNNCGNACCANNCGNANNNTGGTNATGTTAGTNNNNNNNNNNNNNNNNNNNNNNNNNNNNNNN
NNNNNNNNNNNNNNNNNNNNNNNNNNNNNNNNNNNNNNNGNNNNNNCNANNNAAGGANNTANNNCTNNTC
NTNNNNNNGGACNNNACNNNNCNNNNNNNACNCNNNCNCNNNNNNANNNGNTTNNNNNCCNNTATTNT
CCAGNACGCNGNGCNNNCATTCANGNGAACNNNGNNACGCGNNAATCGTANNATCTACCTGCCGCNGG
TNNNNNTACNGNNNNTNCGGANNNTNNCTCGTANNNNNNTCATGCGTNNTNTNNTCNCNAGNCANGTT
CGNNNANANANTAGNANGTNTNNNNNNNNNTGTNNCGTTCCGCNTGANNCNNCANNNNGNNGNNCTTN
TCANCGGCNNTANCTNCNGANNTAAGTCATANNNTNNATCNNTANCTNNNNNGGNGGGNACTGTAAA
NGGNNAATTNGNNGNTNATTTCCNNCACNTNNCTNNGNNTNCTNNNTNCCTGNNTNCNNAATTGGNNC
CANTGNNTGNCNATCTCTGGNTTNGCNNNNNTGNNNNTNNCAANNNGGNNNGTNANANNNCNATNTAA
NNTANANNNNCNNNNNNANNNCACNANNNNNGNAGNNNCNANNNTTANANNNGTAGNNNANTAAGTCT
CCGANNTNNNNNNNATAAGCGNNANACNNNNNNNNNNNNNANGANNACGNNTTGGNNNTNNNNNGCNA
ANNAGACNNTNGTGGNTNNNNNTCAANNNNTTCGTAGTGGTATAACNGNNNNNNNTTTCNCCAGNNTN
NNNGNNNNNNNCNATANNANNNNNNNGNGANNNNNNNNNNNANNNNCTCNNNAGGGANCANCTACTTNA
GCANTGGNGCGCAGNNNGAGANNNNGACCAGCCTTGGGTGGCAANCACNGCAATAAGTNNGTTNANN
CTNNNAGTNNACACNNNNNCANNGNNNCNNNNNNANCNGANNTNNNNNNNNNNNNNNNNNNNNNNNNNNN
NNNNNNNNNNNNNNNNNNNNNNNNNNNNNNANNNNANGGNNNGTNTNTGACNCATGTGTTTTTANNNGNN
NNNNNNNGANNNANCCCCGGTCGNNNNCNNATTGAANNCCTGNNGCATNNNNNTNCNAGAATGANNGC
NNNNNGNNTNGNCTNNCNNNTCAAGNAGTCANNNAGGTATNGCGNTCNNNNNNNNNNNNNCNNTCCCNN
ACTGAGGGTNNNNNNNNNCATCTGCTNNNNNNNNNCTTCGCNGNCGTNNCNNNNNNGCNGTATNGTGTNA
NNCANNNNNNNNNNNNNNNNNTNAAACAANNNNNNTNNNN
>PhuLa621
GTGNNNNNTAGCCCGNNNNAGTTAGACANNNCNNNNNNNNNNNNNTNNNNNNNNNNNNNNNNNNNNNNNN
NNNNNNNNNNNNNNNNNNNNNNNNNNNNNNNTNNNNNNNNNNNNNNNNNNNNNNNNNNNNNNNNNNNNNN
NNNNNNNNNNNNNNNNNNNNNNNNNNNNNNNNNNNNNNNNNNNNNNNNNNNNNNNNNNNNNNNNNNNNNN
NNNNNNNNNNNNNNNNNNNNNNNNNNNNNNNNNNNNNNNNNNNNNNNNNNNNNNNNNNNNNNNNNNNNNN
NNNNNNNNNNNNNNNNNNNNNNNNNNNNNNNNNNNNNNNNNNNNNNNNNNNNCNTCTNNNAAANNNTNNN
NNNNNNNNNNNNNNNNNNNNNNNNNNNCNCNTTGNNNGTAANCNANNNGNACATGCAGTCCCCTCNNNNN
NNNNNCAGAGNNNNNNCCCNNNNNCNCCGGCCCCGNCCNNNNNNNNNTNNNANNNNNNNNNNNGNNNNNN
NNNNNNTGGTACNNNNNCGNNNNNGGTTGTTTAAGAGGTCCACCACGNGGTCNGGNCACNNNNGNNTAN
NNNCNNNNAANNNNNNNNNNNNNAANNTNNNCANNAGGNCNNNGCCNTCNNGNNANTNATTGCNATGACC
CAANNCACGGGGGCAGTNCCCGGCGCGGCGACCTTGATNACGGCCGGGGACAAGTATGGCNNNCNCCC
CGGGAAGGANNCNGCCGTANGTGCNNNNNANNNNNTCTTGNGACGTCGCCGNNATTCNCGTGGTCNNN
CNAGAATGNCCGNACCANNCGNANNNTGGTNATGNNAGTNNNNNNNNNNNNNNNNNNNNNNNNNNNNNNN
NNNNNNNNNNNNNNNNNNNNNNNNNNNNNNNNNNGNNNNGNNNNNNCNANNNAAGGANNTANNNCTNNTN
NTNNNNNNGGACNNNACNNNNCNNNNNNNACNCNNNCNCNNNNNNNNNNGNTTTTNNNCCNNTATTNT
CCAGNACGCNGNGCNNNCATTCANGNGAACNNNGNNACGCGNNAATCGTANNATCTACCTGCCGCNNG

TNNNNNTACNGNNNNTNCGGANNNTNNNNCGTANNNNNNNTCATGCGTNNTNTCNTCNCNAGNCNNGTT
CGNNNANANANTAGNANGTNTNNNNNNNNNNTGTNNCGTTCCGCNTGACNCNNCANNNNGNNGNNCTTN
TCANCGGCNNTANCTGCNGANNTAAGNCATANNNTNNNTCNNTANCTNNNNNGGNGGGNNACTGTAAA
NGGNTAATTNGNNGNTNANTTCNNNCACNTNNCTNNGNNTNCTNNNTNCCTGNNTNCNNAATTGGNNC
CANTGNNTGNCNATCTCTGGNTTNGCNNNNNTNNNNNTNGCAANNNGGNNNGTNANANNNCNATNTAA
NNTANANNNNCNNNNNNANNNCACNANNNNNGNAGNNNCNANNNTTANANNNGTAGNNNANTAAGTCT
CCGANNTNNNNNNNATAAGCGNNANACNNNNNNNNNNNNNNANGANNACGNNTTGGNNNTNNNNNNCNA
ANNAGACNNTNGTGGNTNNNNNTCAANNNNTTCGTAGTGGTATAACNGNNNNNNNTTTCNCCAGNNTN
NNNGNNNNNNNNCNATANNANNNNNNNGTGANNNNNNNNNNNANNNNCTCNNNAGGGANCANCTACTNNA
GCAATGGNGCGNAGNNNGAGANNNNGACNNNCCTTGGGTGGCAANCACNGCAATAAGTNNNGTTNANC
CTNNNAGTNNACACNNNNNCANNGNNNCNNNNNNANCNGANNTNNNNNNNNNNNNNNNNNNNNNNNNNNN
NNNNNNNNNNNNNNNNNNNNNNNNNNNNNNNGNNNANGGNNNGTNTNTGACNCATGTGTTTTTANNNGNN
NNNNNACGNNNNANCCCCGGTCGNNNNCNNATTGAANNCCTGNNGCATNNNNNTNCNAGAATGANNGC
NNNNNGNNTNGNNTNNCNTTTCAAGNAGTCANNNAGGNNTCGCGNTCNNNGNNNNNNNNCNNTCCCNN
ACTGAGGGTNNNNNNNNCATCTGCTNNNNNNNNCTTCGCNGNCGTNNCNNNNNAGCNGTATNGTGTNA
NNCANNNNNNNNNNNNNNNNNTNAAACAANNNNNNTNNNN
>PhuLa622
GTGNNNNNTAGCCCNNNNNAGTTAGACANNNCNNNNNNNNNNNNTNNNNNNNNNNNNNNNNNNNNNNNNN
NNNNNNNNNNNNNNNNNNNNNNNNNNNNNNTNNNNNNNNNNNNNNNNNNNNNNNNNNNNNNNNNNNNNNN
NNNNNNNNNNNNNNNNNNNNNNNNNNNNNNNNNNNNNNNNNNNNNNNNNNNNNNNNNNNNNNNNNNNNNN
NNNNNNNNNNNNNNNNNNNNNNNNNNNNNNNNNNNNNNNNNNNNNNNNNNNNNNNNNNNNNNNNNNNNNN
NNNNNNNNNNTNNNNNNNNNNNNNNNNNNNNNNNNNNNNNNNNNNNNNNNNNNNNTCTNNNAAANNNTNNN
NNNNNNNNNNNNNNNNNNNNNNNNNNNCNCNTTGNNNGTAANCNANNNGNACATGCAGTCCCCTCNNNNN
NNNNNCAGAGNNNNNNCCCNNNNNNNCCGNCCCCGNCCNNNNNNNNNNNCNNNGNNNNNNNGNNNNNN
NNNNNNTGGTACNNNNCGNNNNNGGTTGTNTAAGAGGTCCACCACGNGGTCNGGNCACNNNNGNNNAN
NNNCNNNNAACNGNNNNNNNNNNNNNTNNNCANNAGGNNNNNNCCNTCNNNNNNNTNATTGCNATGACC
CAANNCACGGGGGCNGTNCCCGGCGCGGCGACCTTGATNANGGCCGGGGACAAGTATGGCNNNCNCCC
CGGGAAGGANNCNGCCGTANGTGCNNNNNANNNNNTCNTGNGANGTNGCCGNNATTNNNGTGGTCNNN
NNAGAATGNNCNNACCANNCGNANNNTGGTNATGNTAGTNNNNNNNNNNNNNNNNNNNNNNNNNNNNNNN
NNNNNNNNNNNNNNNNNNNNNNNNNNNNNNNNNNGNNNNGNNNNNNCNANNNAAGGNNNTANNNCTNNTN
NTNNNNNNGGACNNNACNNNNCNNNNNNNACNCNNNCNCNNNNNNANNNGNTTTTTNNNCNNTATTNT
CCAGNANGCNGNGCNNNCATTCANGNGAACNNNGNNACGCGNNAATCGTANNATCTACCTGCCGCNNG
TNNNNNTACNGNNNNNNCGGANNNTNNNNCGTANNNNNNNTCATGCGTNNTNTNNTCNCNAGNCANGTT
CGNNNANANANTAGNANGTNTNNNNNNNNNNTGTNNCGTTCCGCNTGANNCNNCANNNNGNCGNNCTTN
TCANCGGCNCTANCTGCNGANNTAAGNCATANNNTNNNTCNNTANCTNNNNNGGNGGGNNACTGTAAA
NGGNNAATTNGNNNNTNANTNCCNNCACNTNNCTNNGNNNNCTNNNTNCCTGNNTNCNNAATTGGNNC
CANTGNNTGNCNATCTCTGGNTTNGCNNNNNTGNNNNTNNCAANNNGGNNNGTNANANNNCNATNTAA
NNTANANNNNCNNNNNNANNNCACNANNNNNGNAGNNNCNANNNTTANANNNGTAGNNNAATAAGTCT
CCGANNTNNNNNNNATAAGCGNNANACNNNNNNNNNNNNNNANGANNACGNNTTGGNNNTNNNNNNCNA
ANNAGACNNTNGTGGNTNNNNNTCAANNNNTTCGTAGTGGTATAACNGNNNNNNNTTTCNCCAGNNTN
NNNGNNNNNNNNCNATANNANNNNNNNGNGANNNNNNNNNNNANNNNCTCNNNAGGGANCANCTACTNNA
GCANTGGNGCGNAGNNNGAGANNNNGACNNGCCTTGGGTGGCAANCACNGCAATAAGTNNNGTTNANC
CTNNNAGTNNACACNNNNNCANNGNNNCNNNNNNANCNGANNTNNNNNNNNNNNNNNNNNNNNNNNNNNN
NNNNNNNNNNNNNNNNNNNNNNNNNNNNNNAGNNNANGGNNNGTNTNTGACNCATGTGTTTTTANNNGNN
NNNNNACGNNNGANCCCCGGTCGNNNNCNNATTGAANNCCTGNNGCATNNNNNTNCNAGAATGANNGC
NNNNNGNNTNGNCTNNCNNNTCAAGNAGTCAANNAGGTNNNNNNNTCNNNGNNNNNNNNCNNTCCCNN
ACTGAGGGTNNNNNNNNCATCTGCTNNNNNNNNCTNCGCNGNCGTNNCNNNNNAGCNGTATNGTGTNA
NNCANNNNNNNNNNNNNNNNNTNAAACNANNNNNNTNNNN
>SiLa427
GTGNNNNNTAGCCCNNNNNAGTTAGACANNNCNNNNNNNNNNNNNNNNNNNNNNNNNNNNNNNNNNNNNN
NNNNNNNNNNNNNNNNNNNNNNNNNNNNNNTNNNNNNNNNNNNNNNNNNNNNNNNNNNNNNNNNNNNNNN
NNNNNNNNNNNNNNNNNNNNNNNNNNNNNNNNNNNNNNNNNNNNNNNNNNNNNNNNNNNNNNNNNNNNNN
NNNNNNNNNNNNNNNNNNNNNNNNNNNNNNNNNNNNNNNNNNNNNNNNNNNNNNNNNNNNNNNNNNNNNN
NNNNNNNNNNNNNNNNNNNNNNNNNNNNNNNNNNNNNNNNNNNNNNNNNNNNCNTCTNNNAAANNNTNNN

NNNNNNNNNNNNNNNNNNNNNNNNNNNCNCNTTGNNNGTAANCNANNNGNACATGCAGTCCCCTCNNNNN
NNNGGCAGAGNNNNNNCCCNNNNNCNCCGGCCCCNNCCNNNNNNNNNNNNNNNNNNNNNNNNANNNNNN
NNNNNNTGGTACNNNNCGNNNNNGGTTGNNTAAGAGGTCCACAACGNGGTCNGGNCACNNNNGNNNAN
NNNCNNNNNNCNNNNNNNNNNNNANATNNNTANAAGGNNNNNNCCNTNNNGNNNNNAATTGCNATGACC
CAANTCACGGGGGCNGTNCCCGGCGCGGCGACCTTGATNANNTCCGGGGNCAAGTATGGCNNNCNCCC
CGGGAAGGANNCNGCCGTANGTGCNNNNNANNNNNTCNTGNGANGTNGCCGNNATTNNCGTGGTCTNN
CNAGAATGNCCNNACCANNCGNANNNTGGTTATGNTAGTNNNNNNNNNNNNNNNNNNNNNNNNNNNNNNN
NNNNNNNNNNNNNNNNNNNNNNNNNNNNNNNNNNGNNNNGNNNNNNCNANNNAAGGNNNTANNNCTNNTN
NNNNNNNNGGACNNNACNNNNCNNNNNNNACNCNNNCNCNNNNNNNNNNNGNTTTTTNNNCNNTATTNT
CCAGNACGCNGNGCNNNCATTCANGNGAACNNNGNNACGCGNNAATCGTANNATCTACCTGCCGCNGG
NNNNNNTACNGNNNNTNCGGANNNTNNCTCGTANNNNNNNTCATGCGTNNTNTNNTCNCNAGNCANGTT
CGNNNANANANTAGNANGTNTNNNNNNNNCNTGTNNCGTTCCGCNTGANNCNNCANNNNGNNGNNCTTC
TCANCGGCNNTANCTGCNGANNTAAGNCATANNNTNNNTCNNTANCTNNNNNGGNGGGNNACTGTAAA
NGGNNAATTNGNNNNTNANTTCNNNCACNTNNCTNNGNNTNCTNNNTNCCTGNNTNCNNAATTGGNNC
CANTGNNTGNCNATCTCTNGNTTNGNNNNNNTGNNNNTNNCANNNNGGNNNGTNANANCNCNATNTAA
NNTATANNNNCNNNNNNANNNCACNNNNNNNGNAGNNNNNANNNTTANANNNGTAGNNNANTAAGTCT
CCGANNTNNNNNNNATAAGCGNNANACNNNNNNNNNNNNNNANGANNACGNNTTGGNNNTNNNNNNCNA
GNNAGACNNTNGTGGNTNNNNNTCAANNNNTTCGTAGTGGTATAATNGNNNNNNNTTTCNCCAGNNTN
NNNGNNNNNNNCNATANNANNNNNNNGNGANNNNNNNNNNNANNNNCTCNNNAGGGANCANCTACTNNA
NCANTGGNGCGCAGNNNGAGANNNNGACCANCCTTGGGTGGCAANCACNGCAATAAGTNNNGTTNANC
CTNNNAGTNNACACNNNNNCANNGNNNCNNNNNNANCNGANNGNNNNNNNNNNNNNNNNNNNNNNNNNNN
NNNNNNNNNNNNNNNNNNNNNNNNNNNNNNAGNNNANGGNNNGTNTNTGACNCATGTGTTTTTANNNGNN
NNNNNACGNNNNANCCCCGGTCGNNNNCNNATTGAANNCCTGNNGCATNNNNNTNCNAGAATGANNGC
NNNNNGNNTNGNCTNNCNNNTCAAGNAATCANNNAGGTNNNNNGNTCNNGGNNNNNNNNCNNTCCCNN
ACTGAGGGTNNNNNNNNCATCTGCTNNNNNNNNCTNCGCNGGCGTNNCNNNNNNGCNGTATNNTGNNN
NNCANNNNNNNNNNNNNNNNNNTNNAACNANNNNNTTNNNG
>SiLa428
GTGNNNNNTAGCTCNNNNNAGTTAGACANNNCNNNNNNNNNNNNNTNNNNNNNNNNNNNNNNNNNNNNNN
NNNNNNNNNNNNNNNNNNNNNNNNNNNNNNNNNNNNNNNNNNNNNNNNNNNNNNNNNNNNNNNNNNNNNN
NNNNNNNNNNNNNNNNNNNNNNNNNNNNNNNNNNNNNNNNNNNNNNNNNNNNNNNNNNNNNNNNNNNNNN
NNNNNNNNNNNNNNNNNNNNNNNNNNNNNNNNNNNNNNNNNNNNNNNNNNNNNNNNNNNNNNNNNNNNNN
NNNNNNNNNNNNNNNNNNNNNNNNNNNNNNNNNNNNNNNNNNNNNNNNNNNNNNTCTNNNAAANNNTNNN
NNNNNNNNNNNNNNNNNNNNNNNNNNNCNCNTTGNNNGTAANCNANNNGNACATGCAGTNCCCTCNNNNN
NNNGGCAGAGNNNNNNCCCNNNNNCNCCGNCCCCNNCCNNNNNNNNNNNNNNNNNGNNNNNNNGNNNNNN
NNNNNNTGGTACNNNNCGNNNNNGGTTGTNTAAGAGGTCCACCACGNGGTCNNGNCACNNNNGNNNAN
NNNCNNAAAANNNNNNNNNNNNNANATNNNCANNAGGNNNNNNCCNTCNNGNNANNAATTGCNATGACC
CAANNCGCGGGGACAGTNACCGGCGCGGCGACCTTGATNANGGTCGGGGACAAGTATGTCGTNCNCCC
CGGGAAGGANNCNGCCGTANGTGCNNNNNANNNNNTCNTGNGANGTCGCCGNNATTCNNGTGGTCNNN
CNAGANTGNNCGNACCANNCGNANNNTGGTNATGTNAGTNNNNNNNNNNNNNNNNNNNNNNNNNNNNNNN
NNNNNNNNNNNNNNNNNNNNNNNNNNNNNNNNNNNNNNNGNNNNNNCNANNNAAGGNNNTANNNTTNNTN
NTNNNNNNGGACNNNACNNNNCNNNNNNNACNCNNNCNCNNNNNNNANNNGGTTTTTNNNCNNTATTNT
CCAGNACGCNGNGCNANCATTCANGAGAACNNNGNNACGCGTNAATCGTANNATCTACCTGCCGCNNG
TNNNNNTACNGNNNNTNCGGANNNTNNNNCGTANNNNNNNTCATGCGTNNTNTNNTTNCCAGNCANGTT
CGNNNANANANTAGNANGTNTCNNNNNNNNNTGTNNCGTTCCGCNTGANNCNNCANNNNGNNGNNCTTN
NCANCGGCNNTANCTGCNGANNTAAGCCATANNNTNNGTCNNTANCGNNNNNGGNGGGNNACTGTAAA
NGGNNAATTNGANNNTNATTTCNNNCACNTNNCTNNGNNTNCTNNNCNCCTGNNTNCNNAATTGGNNC
CANTGNNTGNCNATCTCTGGNTTNGCNNNNNTNNNNNTNNTANNNNGGNNNGTNANANNNCNATNTAA
NNTANANNNNCNNNNNNANNNCACNNNNNNNGNAGNNNCNANNNTTANGNNNGTAGNNNAATAAGTCT
CCGANGTNNNNNNNATAAGCGNNANACNNNNNNNNNNNNNNANGANNACGNNTTGGNNNTNNNNNNCNA
ANNAGACNNCNGTGGNTNNNNNTCAANNNNTTCGTAGTGGTATAACNANNNNNNNTTTCNCCAGNNTN
NNNGNNNNNNNCNATANNANNNNNNNGTGANNNNNNNNNNNANNNNCCCNNNAGGGANCANCTACTNNA
GCAATGGNGCGCAGNNNGAGANNNNGACNAGCCTTGGGTGGCAANCACNGCAATAAGTNNNGTTNANC
CCNNNAGTNNACACNNNNNCANNGNNNCNNNNNNANCNGANNTNNNNNNNNNNNNNNNNNNNNNNNNNNN
NNNNNNNNNNNNNNNNNNNNNNNNNNNNNNAGNNNANGGNNNGTNTNTGACNCATGTGTTTTTANNNGNN

NNNNNACGNNNNANCCCCGGTCGTNNNCNNATTGAANNCCTGNNGCATNNNNNTNCNAGAATGANNGC
NNNNNGNNTNGNCTGNCNNNTCAAGNAGTCANNNAGGTANCGCGNTCNNGNNNNNNNNNCNNTCCCNN
ACTGAGGGTNNNNNNNNCATCTGCTNNNNNNNNCTNCGCNGNNNNNNTNNNNNAGCCGTATNGTGTNN
NNCANNNNNNNNNNNNNNNNTNAAACNANNNNNCTNNNN
>SiLa429
GTGNNNNNTAGCCCNCNNNAGTTAGACANNNCNNNNNNNNNNNNTNNNNNNNNNNNNNNNNNNNNNNN
NNNNNNNNNNNNNNNNNNNNNNNNNNNNNNNNNNNNNNNNNNNNNNNNNNNNNNNNNNNNNNNNNNNN
NNNNNNNNNNNNNNNNNNNNNNNNNNNNNNNNNNNNNNNNNNNNNNNNNNNNNNNNNNNNNNNNNNNN
NNNNNNNNNNNNNNNNNNNNNNNNNNNNNNNNNNNNNNNNNNNNNNNNNNNNNNNNNNNNNNNNNNNN
NNNNNNNNNNNNNNNNNNNNNNNNNNNNNNNNNNNNNNNNNNNNNNNNNNCNTCTNNNAAANNNTNNN
NNNNNNNNNNNNNNNNNNNNNNNNNCNCNTTNNNNGTAANCNANNNGNACATGCAGTCCCCTCNNNNN
NNNGGCAGAGNNNNNNCCCNNNNNNNCCGNCCCCNNCCNNNNNNNNNNNCNNNGNCNNNNNANNNNNN
NNNNNNTGGTACNNNNCGNNNNNGGTTGNNTNAGAGGTCCACAACGNGGTCNGGNNACNNNNGNNNAN
NNNCNNNNAANNNNNNNNNNNNNNNTNNNTANNAGGNCNNNNCCNTNNNNNNNNNAATTGCNATGACC
CAANTCACGGGGGCNGTNCCCGGCGCGGCGACCTTGNNNANNTCCGGGGNCAAGTATGGCGNNCNCCC
CGGGAAGGANNCNGCCGTANGTGCNNNNNANNNNNTCNTGNGANNTCGCCGNNATTNNCNTGGTCNNN
CNAGANTGNCCNNACCANNCGNANNNTGGTNATGNNAGTNNNNNNNNNNNNNNNNNNNNNNNNNNNNN
NNNNNNNNNNNNNNNNNNNNNNNNNNNNNNNNNNNNNGNNNNNNCNANNNAAGGNNNTANNNCTNNTN
NTNNNNNNGGACNNNACNNNNCNNNNNNNACNCNNNCNCNNNNNNNNNNGNTTTTTNNNCNNTATTNT
CCAGNACGCNGNGCNNNCATTCANGNGAACNNNGNNACGCGNNAATCGTANNATCTACCTGCCGCNNG
NNNNNNTACNGNNNNTNCGGANNNTNNNNCGTANNNNNNNCATGCGTNNTNTNNTCNCNAGNCNNGTT
CGNNNANANANTAGNANGTNTCNNNNNNNNTGTNNCGTTCCGCNTGANNCNNCANNNNGNNGNNNTTN
TCANCGGCNNTANCTGCNGANNTAAGNCATANNNTNNNTCNNTANCTNNNNNGGNGGGNNACTGTAAA
NGGNNAATTNGNNNNTNANTNCNNNCACNTNNCTNNGNNNNCTNNNTNCCTGNNTNCNNAATTGGNNC
CANTGNNTGNCNATCTCTGGNTTNGNNNNNNTNNNNNTNNCAANNNGGNNNNNNNNANNNCNATNTAA
NNTANACNNNCNNNNNNANNNCACNANNNNNNNAGNNNCNANNNTTANANNNGTAGNNNANTAAGTCT
CCGANNTNNNNNNNATAAGCGNNANACNNNNNNNNNNNNNANGANNACGNNTTGGNNNTNNNNNNCNA
GNNAGACNNTNGTGGNTNNNNNTCAANNNNTTNGTAGTGGTATAATNGNNNNNNNTTTCNCCAGNNTN
NNNGNNNNNNNCNATANNANNNNNNNGNGANNNNNNNNNNANNNNCTCNNNAGGGANCANCTACTNNA
GCANNGGNGCGCAGNNNGAGANNNNGACNNNCCTTGGGTGGCAANNACNGCAATAAGTNNNGTTNANC
CTNNNAGTNNACACNNNNNCANNGNNNCNNNNNNANCNGANNGNNNNNNNNNNNNNNNNNNNNNNNNN
NNNNNNNNNNNNNNNNNNNNNNNNNNNNANNNNANGGNNNGTNTNTGACNCATGNNTTTTTANNNGNN
NNNNNACGNNNNANCCCCGGTCGNNNNCNNATTGAANNCCTGNNGCATNNNNNTNCNAGAATGANNGC
NNNNNGNNTNGNCTNNCNNNTCAAGNAATCAANNAGGTNNNNNGNTCNNNGNNNNNNNNCNNTCCCNN
ACTGAGGGTNNNNNNNNCATCTGCTNNNNNNNNCTNCGCNGGNGTNNCNNNNNAGCNGTATNGNGTNN
NNCANNNNNNNNNNNNNNNNCNAAACNANNNNCNTNNNN
>SiLa430
GTGNNNNNTAGCTCNNNNNAGTTAGACANNNCNNNNNNNNNNNNTNNNNNNNNNNNNNNNNNNNNNNN
NNNNNNNNNNNNNNNNNNNNNNNNNNNNNNNNNNNNNNNNNNNNNNNNNNNNNNNNNNNNNNNNNNNN
NNNNNNNNNNNNNNNNNNNNNNNNNNNNNNNNNNNNNNNNNNNNNNNNNNNNNNNNNNNNNNNNNNNN
NNNNNNNNNNNNNNNNNNNNNNNNNNNNNNNNNNNNNNNNNNNNNNNNNNNNNNNNNNNNNNNNNNNN
NNNNNNNNNNNNNNNNNNNNNNNNNNNNNNNNNNNNNNNNNNNNNNNNNNNNTCTNNNAAANNNTNNN
NNNNNNNNNNNNNNNNNNNNNNNNNCNCNTTGNNNGTAANCNANNNGNACATGCAGTCCCCTCNNNNN
NNNGGCAGAGNNNNNNCCCNNNNNNNCCGGCCCCGNCCNNNNNNNNNNNNNNNGNNNNNNNGNNNNNN
NNNNNNTGGTACNNNNCGNNNNNGGTTGTNTAAGAGGTCCACCACGNGGTCNGGNCACNNNNGNNNAN
NNNCNNNNAACNNNNNNNNNNNANATNNNCANNAGGNCNNNNCCNTCNNGNNNNNNANTGCNATGACC
CAANTCGCGGGGACAGTNACCGGCGCGGCGACCTTGANNACGGTCGGGGACAAGTATGTCNNNCNCCC
CGGGAAGGANNCNGCCGTANGTGCNNNNNANNNNNTCNTGNGANGTCGCCGNNATTCNNNTGGTCNNN
CNAGAATGNCCNNACCANNCGNANNNTGGTNATGNNAGTNNNNNNNNNNNNNNNNNNNNNNNNNNNNN
NNNNNNNNNNNNNNNNNNNNNNNNNNNNNNNNGNNNNGNNNNNNCNANNNAAGGANNTANNNTTNNTN
NTNNNNNNGGACNNNACNNNNCNNNNNNNACNCNNNCNCNNNNNNANNNGNTTTTNNNNCNNTATTNT
CCAGNACGCNGNGCNNNCATTCANGNGAACNNNGNNACGCGNNAATCGTANNATCTACCTGCCGCNNG
TNNNNNTACNGNNNNTNCGGANNNTNNNNCGTANNNNNNTCATGCGTNNTNTNNTTNCNAGNCANGTT
CGNNNANANANTAGNANGTNTNNNNNNNCTTGTNGCGTTCCGCNTGANNCNNCANNNNGNNGNNCTTN

TCANCGGCNNTANCTGCNGANNTAAGCCATANNNTNNGTCNNTANCGNNNNNGGNGGGNNACTGTAAA
NGGNNAATTNGNGNNTNATTTCNNNCACNTNNCTNNGNNTNCTNNNCNCCTGNNTNCNNAATTGGNNC
CANTGNNTGNCNATCTCTGGNTTNGNNNNNNTGNNNNTNNTANNNNGGNNNGTNANANNNCNATNTAA
NNTATANNNNCNNNNNNANNNCACNANNNNNNNAGNNNCNANNNTTANGNNNGTAGNNNANTAAGTCT
CCGANNTNNNNNNNATAAGCGNNANACNNNNNNNNNNNNNANGANNACGNNTTGGNNNTNNNNNNCNA
ANNAGACNNCNGTGGNTNNNNNTCAANNNNTTCGTAGTGGTATAACNANNNNNNNTTTCNCCAGNNTN
NNNGNNNNNNNCNATANNANNNNNNNGNGANNNNNNNNNNANNNNCCCNNNAGGGANCANCTACTNNA
GCANTGGNGCGCAGNNNGAGANNNNGACNNGCCTTGGGTGGCAANNACNGCAATAAGTNNNGTTNANC
CCNNNAGTNNACACNNNNNCANNGNNNCNNNNNNANCNGANNTNNNNNNNNNNNNNNNNNNNNNNNNN
NNNNNNNNNNNNNNNNNNNNNNNNNNNNNAGNNNANNGNNNGTNTNTGACNCATNNNTTTTTANNNGNN
NNNNNACGNNNNANCCCCGGTCGNNNNCNNATTGAANNCCTGNNGCATNNNNNTNCNAGAATGANNGC
NNNNNGNNTNGNCTNNCNTTTCAAGNAGTCANNTAGGTNNCGCGNTCNNNGNNNNNNNNCNNTCCCNN
ACTGAGGGTNNNNNNNNCATCTGCTNNNNNNNNCTTCGCNGNNNNNNTNNNNNAGCNGTATNGTGTNN
NNCANNNNNNNNNNNNNNNNNTNAAANNANNNNNCTNNNN
>SiLa431
GTGNNNNNTAGCCCNNNNNAGTTAGACANNNCNNNNNNNNNNNNTNNNNNNNNNNNNNNNNNNNNNNN
NNNNNNNNNNNNNNNNNNNNNNNNNNNNNNTNNNNNNNNNNNNNNNNNNNNNNNNNNNNNNNNNNNNN
NNNNNNNNNNNNNNNNNNNNNGGNNNNNNNNNNNNNNNNNNNNNNNNNNNNNNNNNNNNNNNNNNNNN
NNNNNNNNNNNNNNNNNNNNNNNNNNNNNNNNNNNNNNNNNNNNNNNNNNNNNNNNNNNNNNNNNNNN
NNNNNNNNNNNNNNNNNNNNNNNNNNNNNNNNNNNNNNNNNNNNNNNNNNNNNTCTNNNAAANNNTNNN
NNNNNNNNNNNNNNNNNNNNNNNNNNCNCNTTGNNNGTAANCNANNNGNACATGCAGTCCCCTCNNNNN
NNNGGCAGAGNNNNNNNCCNNNNNCNCCGNCCCCNNCCNNNNNNNNNNNCNNNGNNNNNNNANNNNNN
NNNNNNTGGTACNNNNCGNNNNNGGTTGTNTAAGAGGTCCACAACGNGGTCNNNNCACNNNNGNNTAN
NNNCNNNNAACNNNNNNNNNNNNANNTNNNTANNAGGNCNNNNCCNTNNNGNNNNTAANTGCNATGACC
CAANNCNCGGGGGNAGTNCCCGGCGCGGCGACCTTGATNANGTCCGGGGACAAGTATGGCNNNCNCCC
CGGGAAGGANNCNGCCGTANGTGCNNNNNANNNNNTCNTGNGANGTCGCCGNNATTNNCNTGGCNNN
CNAGAATGNCCNNACCANNCGNANNNTGGTNATGNNAGTANNNNNNNNNNNNNNNNNNNNNNNNNNNN
NNNNNNNNNNNNNNNNNNNNNNNNNNNNNNNNNNGNNNNGNNNNNNCNANNNAAGGANNTANNNCTNNTN
NTNNNNNNNGGACNNNACNNNNCNNNNNNNACNCNNNCNCNNNNNNANNNGNTTTTTNNNCNNTATTNT
CCAGNACGCNGNGCNNNCATTCANGNGAACNNNGNNACGCGNNAATCGTANNATCTACCTGCCGCNNG
TNNNNNTACNGNNNNNNCGGANNNTNNCTCGTANNNNNNNCATGCGTNNTNTNNTCNCNAGNCNNGTT
CGNNNANANANTAGNANGTNTNNNNNNNNNTGTNNCGTTCCGCNTGANNCNNCANNNNGNNGNNCTTN
TCANCGGCNNTANCTGCNGANNTAAGNCATANNNTNNNTCNNTANCTNNNNNGGNGGGNNACTGTAAA
NGGNNAATTGGNNGNTNANTNCNNACACNTNNCTNNGNNNNCTNNNTNCCTGNNTNCNNAATTGGNNC
CANTGNNTGNCNATCTCTNGNTTNGNNNNNNTGNNNNTNGCANNNNGGNNNNNNANANNNCNATNTAA
NNTATANNNNCNNNNNNANNNCACNANNNNNGNAGNNNCNANNNTTANANNNGTAGNNNAATAAGTCT
CCGANNTNNNNNNNATAAGCGNNANACNNNNNNNNNNNNNANGANNACGNNTTGGNNNTNNNNNNCNA
GNNAGACNNTNGTGGNTNNNNNTCAANNNNTTCGTAGTGGTATAATNGNNNNNNNTTTCNCCAGNNTN
NNNGNNNNNNNCNATANNANNNNNNNGNGANNNNNNNNNNANNNNCTCNNNAGGNANCANCTACTNNA
GCAANGGNGCGCAGNNNGAGANNNNGACNNGCCTTGGGTGGCAANCACNGCAATAAGTNNNGTTNANC
CTNNNAGTNNACACNNNNNCANNGNNNCNNNNNNANCNGANNGNNNNNNNNNNNNNNNNNNNNNNNNN
NNNNNNNNNNNNNNNNNNNNNNNNNNNNNAGNNNANNGNNNGTNTNTGACNCATGTGTTTTTANNNGNN
NNNNNACGNNNNANCCCCGGTCGNNTNCNNATTGAANNCCTGNNGCATNNNNNTNCNAGAATGANNGC
NNNNNGNNTNGNCTNNCNNNTCAAGNAATCANNNAGNNNNCGCNNTCNNNGNNNNNNNNCNNNCCCNN
ACTGAGGGTNNNNNNNNCATCTGCTNNNNNNNNCTNCGCNGGNGTNNCNNNNNAGCNGTATNGNGTNA
NNCANNNNNNNNNNNNNNNNNTNAAACNANNNNCTTNNNN
>SiLa432
GTGNNNNNTAGCCCNNNNNAGTTAGACANNNCNNNNNNNNNNNNTNNNNNNNNNNNNNNNNNNNNNNN
NNNNNNNNNNNNNNNNNNNNNNNNNNNNNNTNNNNNNNNNNNNNNNNNNNNNNNNNNNNNNNNNNNNN
NNNNNNNNNNNNNNNNNNNNNNNNNNNNNNNNNNNNNNNNNNNNNNNNNNNNNNNNNNNNNNNNNNNN
NNNNNNNNNNNNNNNNNNNNNNNNNNNNNNNNNNNNNNNNNNNNNNNNNNNNNNNNNNNNNNNNNNNN
NNNNNNNNNNNNNNNNNNNNNNNNNNNNNNNNNNNNNNNNNNNNNNNNNNNNNTCTNNNAAANNNTNNN
NNNNNNNNNNNNNNNNNNNNNNNNNNCNCNTTGNNNGTAANCNANNNGNACATGCAGTCCCCTCNNNNN
NNNGGCAGAGNNNNNNNCCNNNNNNNCCGNCCCCGNCCNNNNNNNNNNNCNNNNNNNNNNNANNNNNN

NNNNNNTGGTACNNNNCGNNNNNGGTTGTNTNAGAGGTCCACAACGNGGTCNGGNCACNNNNGNNNAN
NNNCNNNAAACNGNNNNNNNNNNANNTNNNTANNAGGNCNNNNCCNTNNNGNNNNNNATTGCNATGACC
CAANNCACGGGGGCAGTNCCCGGCGCGGCGACCTTGATNACGTCCGGGGACAAGTATGGCGCNCNCCC
CGGGAAGGANNCNGCCGTANGTGCNNNNNANNNNNTCNTGNGANGTNGCCGNNATTNNNGTGGTCNNN
CNAGAATGNCCGNACCANNCGNANNNTGGTNATGNNAGTNNNNNNNNNNNNNNNNNNNNNNNNNNNNN
NNNNNNNNNNNNNNNNNNNNNNNNNNNNNNNNNNGNNNNGNNNNNNCNANNNAAGGANNTANNNCTNNTN
NTNNNNNNGGACNNNACNNNNCNNNNNNNACNCNNNCNCNNNNNNNNNNNGNTTTTNNNNCNNTATTNT
CCAGNACGCNGNGCNNNCATTCANGNGAACNNNGNNACGCGNNAATCGTANNATCTACCTGCCGCNGG
TNNNNNTACNGNNNNTNCGGANNNTNNNNCGTANNNNNNNCATGCGTNNTNTNNTCNCNAGNCANGTT
CGNNNATANANTAGNANGTNTNNNNNNNNNNTGTNNCGTTCCGCNTGANNCNNCANNNNGNCGNNCTTN
TCANCGGCNNTANCTGCNGANNTAAGNCATANNNTNNNTCNNTANCTNNNNNGGNGGGNNACTGTAAA
NGGNTAATTNGNNGNTNATTNCCNNCACNTNNCTNNGNNNNCTNNNTNCCTGNNTNCNNAATTGGNNC
CANTGNNTGNCNATCTCTNGNTTNGNNNNNNTGNNNNTNNCANNNNGGNNNGTNANANNNCNATNTAA
NNTATANNNNCNNNNNNANNNCACNANNNNNGNAGNNACNANNNTTANANNNGTAGNNNANTAAGTCT
CCGANNTNNNNNNNATAAGCGNNANACNNNNNNNNNNNNNNANGANNACGNNTTGGNNNTNNNNNNCNA
GNNAGACNNTNGTGGNTNNNNNTCAANNNNTTNGTAGTGGTATAATNGNNNNNNNTTTCNCCAGNNTN
NNNGNNNNNNNCNATACNANNNNNNNGNGANNNNNNNNNNNANNNNCTCNNNAGGGANCANCTACTTNA
GCANTGGNGCGCAGNNNGAGANNNNGACNNNCCTTGGGTGGCAANCACNGCAATAAGTNNNGTTNANC
CTNNNAGTNNACACNNNNNCANNGNNNCNNNNNNANCNGANNGNNNNNNNNNNNNNNNNNNNNNNNNN
NNNNNNNNNNNNNNNNNNNNNNNNNNNNNNNAGNNNANGGNNNGTNTNTGACNCATGTGTTTTTANNNGNN
NNNNNACGNNNGANCCCCGGTCGTNNNCNNATTGAANNCCTGNNGCATNNNNNNTNCNAGAATGANNGC
NNNNNGNNTNGNCTNNCTTTTCAAGNAATCANNNAGGTNNNGCGGTCNNNNNNNNNNNNNCNNTCCCNN
ACTGAGGGTNNNNNNNNCATCTGCTNNNNNNNNNCTTCGCNGGCGTNNCNNNNNAGCNGTATNGTGNNN
NNCANNNNNNNNNNNNNNNNNTNAAACAANNNNNNNTNNNN
>SiLa433
GTGNNNNNTAGCCCGNNNNAGTTAGACANNNCNNNNNNNNNNNNNTNNNNNNNNNNNNNNNNNNNNNNN
NNNNNNNNNNNNNNNNNNNNNNNNNNNNNNNNNNNNNNNNNNNNNNNNNNNNNNNNNNNNNNNNNNN
NNNNNNNNNNNNNNNNNNNNNNNNNNNNNNNNNNNNNNNNNNNNNNNNNNNNNNNNNNNNNNNNNNN
NNNNNNNNNNNNNNNNNNNNNNNNNNNNNNNNNNNNNNNNNNNNNNNNNNNNNNNNNNNNNNNNNNN
NNNNNNNNNNNNNNNNNNNNNNNNNNNNNNNNNNNNNNNNNNNNNNNNNNNNNNNNTCTNNNAAANNNTNNN
NNNNNNNNNNNNNNNNNNNNNNNNNNNCNCNNTNNNNGTAANCNANNNGNACATGCAGTCCCCTCNNNNN
NNNNNCAGAGNNNNNNCCCNNNNNNNCCGGCCCCNNCCNNNNNNNNNNNNNNNNNGNNNNNNNANNNNNN
NNNNNNTGGTACNNNNCGNNNNNGGTTGTNTNAGAGGTCCACAACGNGGTCNGGNCANNNNNGNNNAN
NNNCNNNNNNNNNNNNNNNNNNNNNNTNNNTANNAGGNNNNNNCCNTCNNNNNNNNNNATNGCNATGANC
CANNTCACGGGGGCAGTNCCCGGCGCGGCGACCTTGNTNANGTCCGGGGACAAGTATGGCNNNCNCCC
CGGGAAGGANNCNGCCGTANGTGCNNNNNANNNNNTCNTGNGACGTCGCCGNNATTNNCGTGGTCTNN
CNAGAATGNCCNNACCANNCGNANNNTGGTNATGNNAGTNNNNNNNNNNNNNNNNNNNNNNNNNNNNN
NNNNNNNNNNNNNNNNNNNNNNNNNNNNNNNNNNNNNNNGNNNNNNCNANNNAAGGNNNTANNNCTNNTN
NTNNNNNNGGACNNNACNNNNCNNNNNNNACNCNNNCNCNNNNNNNANNNGNTTTTTNNNCNNTATTNT
CCAGNANGCNGNGCNNNCATTCANGNGAACNNNGNNACGCGNNAATCGTANNATCTACCTGCCGCNNG
TNNNNNTNCNGNNNNNNNCGGANNNTNNCTCGTANNNNNNNNCATGCGTNNTNTNNTCNCNAGNCANGTT
CGNNNANANANTAGNANGTNTNNNNNNNNNNTGTNNCGTTCCGCNTGANNCNNCANNNNGNNGNNCTTN
NCANCGGCNNTANCTGCNGANNTAAGNCATANNNTNNNTCNNTANCTNNNNNGGNGGGNNACTGTAAA
NGGNNAATTGGNNGNTNATTNCNNNCACNTNNCTNNGNNTNCTNNNTNCCTGNNTNCNNAATTGGNNC
CANTGNNTGNCNATCTCTGGNTTNGCNNNNNTNNNNNTNNCANNNNGGNNNNNNANANNNCNATNTAA
NNTANANNNNCNNNNNNANNNCACNANNNNNNNAGNNNCNANNNTTANANNNGTAGNNNANTAAGTCT
CCGANNTNNNNNNNATAAGCGNNANACNNNNNNNNNNNNNNANGANNNCGNNTTGGNNNTNNNNNNCNA
GNNAGACNNTNGTGGNTNNNNNTCAANNNNTTNGTAGTGGTATAATNGNNNNNNNTTTCNCCAGNNTN
NNNGNNNNNNNCNATANNANNNNNNNGNGANNNNNNNNNNNANNNNCTCNNNAGGNANCANCTACTTNA
GCAATGGNGCGCAGNNNGAGANNNNGACNNNCCTTGGGTGGCAANCACNGCAATAAGTNNNGTTNANC
CTNNNAGTNNACACNNNNNCANNGNNNCNNNNNNANCNGANNGNNNNNNNNNNNNNNNNNNNNNNNNN
NNNNNNNNNNNNNNNNNNNNNNNNNNNNNNNANNNNANGGNNNGTNTNTGACNCATGTGTTTTTANNNGNN
NNNNNACGNNNNNNCCCCGGTCGNNNNCNNATTGAANNCCTGNNGCATNNNNNNTNCNAGAATGANNNC
NNNNNGNNTNGNCTNNCNTTTCAAGNAATCAANNAGGTANNNNGNTCNNNNNNNNNNNNNCNNTCCCNN

ACTGAGGGTNNNNNNNNCATCTGCTNNNNNNNNNNTNCGCNGGCGTNNCTNNNNAGCNGTATNGTGNNN
NNCANNNNNNNNNNNNNNNNNNNAAANNANNNNNNTNNNN
>SiLa434
GTGNNNNNTAGCCCNNNNNAGTTAGACANNNCNNNNNNNNNNNNNTNNNNNNNNNNNNNNNNNNNNNNN
NNNNNNNNNNNNNNNNNNNNNNNNNNNNNNNTNNNNNNNNNNNNNNNNNNNNNNNNNNNNNNNNNNNNN
NNNNNNNNNNNNNNNNNNNNNNNNNNNNNNNNNNNNNNNNNNNNNNNNNNNNNNNNNNNNNNNNNNNNN
NNNNNNNNNNNNNNNNNNNNNNNNNNNNNNNNNNNNNNNGNNNNNNNNNNNNNNNNNNNNNNNNNNNNN
NNNNNNNNNNNNNNNNNNNNNNNTNNNNNNNNNNNNNNNNNNNNNNNNNNNNNNNNNTCTNNNAAANNNTNNN
NNNNNNNNNNNNNNNNNNNNNNNNNNCNCNTTGNNNGTAANCNANNNGNACATGCAGTCCCCTCNNNNN
NNNGGCAGAGNNNNNNCCCNNNNNNNCCGNCCCCNNCCNNNNNNNNNNNNNANNNNNNNNNNNANNNNNN
NNNNNNTGGTACNNNNCGNNNNNGGTTGTNTAAGAGGTCCACAACGNGGTCNGNNCACNNNNGNNNAN
NNNCNNNAAANNNNNNNNNNNNNNNNNTNNNTANNAGGNCNNNNCCNTNNNGNNNNNNATTGCNATGACN
CAANNCACGGGGGNAGTNCCCGGNGCGGCGACCTTGNNNANGTCCGGGGACAAGTATGGCGNNCNCCC
CGGGAAGGANNCNGCCGTANGTGCNNNNNANNNNNTCNTGNGANGTCGCCGNNATTNNCGTGGTCNNN
CNAGAATGNCCGNACCANNCGNANNNTGGTNATGNNAGTNNNNNNNNNNNNNNNNNNNNNNNNNNNNN
NNNNNNNNNNNNNNNNNNNNNNNNNNNNNNNNGNNNNGNNNNNNCNANNNAAGGANNTANNNCTNNTN
NTNNNNNNGGACNNNACNNNNCNNNNNNNACNCNNNCNCNNNNNNANNNGNTTTTTNNNCNNTATTNT
CCAGNACGCNGNGCNNNCATTCANGNGAACNNNGNNACGCGNNAATCGTANNATCTACCTGCCGCNNG
NNNNNNTACNGNNNNTNCGGANNNTNNNTCGTANNNNNNNCATGCGTNNTNTNNTCNCNAGNCANGTT
CGNNNANANANTAGNANGTNTNNNNNNNNNTGTNNCGTTCCGCNTGANNCNNCANNNNGNNGNNCTTN
TCANCGGCNNTANCTGCNGANNTAAGNCATANNNTNNNTCNNTANCTNNNNNGGNGGGNNACTGTAAA
NGGNNAATTNGNNGNTNANTNCNNNCACNTNNCTNNGNNTNCTNNNTNCCTGNNTNCNNAATTGGNNC
CANTGNNTGNCNATCTCTGGNTTNGNNNNNNTGNNNNTNNCAANNNGGNNNGTNNNANNNCNATNTAA
NNTANANNNNCNNNNNNANNNCACNNNNNNNNNAGNNNCNANNNTTANANNNGTAGNNNANTAAGTCT
CCGANNTNNNNNNNATAAGCGNNANACNNNNNNNNNNNNNNANGANNACGNNTTGGNNNTNNNNNNCNA
GNNANACNNTNGTGGNTNNNNNTCAANNNNTTNGTAGTGGTATAATNGNNNNNNNTTTCNCCAGNNTN
NNNGNNNNNNNCNATACNANNNNNNNGNGANNNNNNNNNNANNNNCTCNNNAGGNANCANCTACTNNA
GCANTGGNGCGCAGNNNGAGANNNNGACNNGCCTTGGGTGGCAANCACNGCAATAAGTNNNGTTNANN
CTNNNAGTNNACACNNNNNCANNGNNNCNNNNNNANCNGANNGNNNNNNNNNNNNNNNNNNNNNNNNN
NNNNNNNNNNNNNNNNNNNNNNNNNNNNNNANNNNANGGNNNGTNTNTGACNCATGTGTTTTTANNNGNN
NNNNNNNGNNNGANCCCCGGTCGNNNNCNNATTGAANNCCTGNNGCATNNNNNTNCNAGAATGANNGC
NNNNNGNNTNGNCTNNCNNNTCAAGNAATCANNNAGGTATNNCGNTCNNNNNNNNNNNNCNNTCCCNN
ACTGAGGGTNNNNNNNNCATCTGCTNNNNNNNNNNTNCGCNGGNGTNNCNNNNNNGCNGTATNGNGNNN
NNCANNNNNNNNNNNNNNNNNTNAAACAANNNNCNTNNNN
>SiLa435
GTGNNNNNTAGCCCNNNNNAGTTAGACANNNCNNNNNNNNNNNNNTNNNNNNNNNNNNNNNNNNNNNNN
NNNNNNNNNNNNNNNNNNNNNNNNNNNNNNNTNNNNNNNNNNNNNNNNNNNNNNNNNNNNNNNNNNNNN
NNNNNNNNNNNNNNNNNNNNNNNNNNNNNNNNNNNNNNNNNNNNNNNNNNNNNNNNNNNNNNNNNNNNN
NNNNNNNNNNNNNNNNNNNNNNNNNNNNNNNNNNNNNNNNNNNNNNNNNNNNNNNNNNNNNNNNNNNNN
NNNNNNNNNNNNNNNNNNNNNNNNNNNNNNNNNNNNNNNNNNNNNNNNNNNNNNNNNTCTNNNAAANNNTNNN
NNNNNNNNNNNNNNNNNNNNNNNNNNCNCNTTGNNNGTAANCNANNNGNACATGCAGTCCCCTCNNNNN
NNNNNCAGAGNNNNNNNNCNNNNNNCNCCGNCCCCNNCCNNNNNNNNTNNNNNNNNNNNNNNNNANNNNNN
NNNNNNTGGTACNNNNCGNNNNNGGTTGTNTAAGAGGTCCACAACGNGGTCNNGNNACNNNNGNNNAN
NNNCNNNNNNCNNNNNNNNNNNNNNNNTNNNTANNAGGNCNNNNCCNTNNNGNNNNNNATNGCNATGACC
CAANTCACGGGGGCAGTNCCCGGNGCGGCGACCTTGATNACGTCCGGGGACAAGTATGGCNCNCNCCC
CGGGAAGGANNCNGCCGTANGTGCNNNNNANNNNNTCNTGNGANGTNGCCGNNATTCNCGTGGTCTNN
CNAGAATGNCCGNACCANNCGNANNNTGGTNATGNNAGTNNNNNNNNNNNNNNNNNNNNNNNNNNNNN
NNNNNNNNNNNNNNNNNNNNNNNNNNNNNNNNGNNNNGNNNNNNCNANNNAAGGNNNTANNNCTNNTN
NTNNNNNNGGACNNNACNNNNCNNNNNNNACNCNNNCNCNNNNNNNNNNGNTTTTTTNNCNNTATTNT
CCAGNACGCNGNGCNNTCATTCANGNGAACNNNGNNACGCGNNAATCGTANNATCTACCTGCCGCNNG
TNNNNNTNCNGNNNNNNCGGANNNTNNCTCGTANNNNNNTCATGCGTNNTNTNNTCNCNAGNCNNGTT
CGNNNANANANTAGNANGTNTNNNNNNNCTTGTNNCGTTCCGCNTGANNCNNCANNNNGNNGNNCTTN
NCANCGGCNNTANCTGCNGANNTAAGNCATANNNTNNNTCNNTANCTNNNNNGGAGGGNNACTGTAAA
NGGNTAATTNGNNNNTNATTTCNNNCACNTNNCTNNGNNTNCTNNNTNCCTGNNTNCNNAATTGGNNC

CANTGNNTGNCNATCTCTGGNTTNGNNNNNNTNNNNNTNNCAANNNGGNNNGTNANANNNCNATNTAA
NNTANANNNNCNNNNNNANNNCACNANNNNNGNAGNNNCNANNNTTANANNNGTAGNNNANTAAGTCT
CCGANNTNNNNNNNATAAGCGNNANACNNNNNNNNNNNNNNANGANNACGNNTTGGNNNTNNNNNNCNA
GNNAGACNNTNGTGGNTNNNNNTCAANNNNTTNGTAGTGGTATAATNGNNNNNNNTTTCNCCAGNNTN
NNNGNNNNNNNCNATANNANNNNNNNGTGANNNNNNNNNNNANNNNCTCNNNAGGGANCANCTACTNNA
GCANTGGNGCGCAGNNNGAGANNNNGACNNNCCTTGGGTGGCAANCACNGCAATAAGTNNNGTTNANC
CTNNNAGTNNACACNNNNNCANNGNNNCNNNNNNANCNGANNGNNNNNNNNNNNNNNNNNNNNNNNNN
NNNNNNNNNNNNNNNNNNNNNNNNNNNNNNNNANNNNANNGNNNGTNTNTGACNCATGNNTTTNTANNNGNN
NNNNNNCGNNNNANCCCCGGTCGNNNNCNNATTGAANNCCTGNNGCATNNNNNTNCNAGAATGANNNC
NNNNNGNNTNGNCTNNCNNNTCAAGNAATCANNNAGGNNNCGCGNTCNNNGNNNNNNNNCNNTCCCNN
ACTGAGGGTNNNNNNNGCATCTGCTNNNNNNNNCTNCGCNGNCGTNNCNNNNNAGCNGTATNGTGNNN
NNCANNNNNNNNNNNNNNNNNNNAAACNANNNNNNNTNNNN
>SiLa436
GTGNNNNNTAGCTCNNNNNAGTTAGACANNNCNNNNNNNNNNNNTNNNNNNNNNNNNNNNNNNNNNNNNN
NNNNNNNNNNNNNNNNNNNNNNNNNNNNNNNTNNNNNNNNNNNNNNNNNNNNNNNNNNNNNNNNNNNNNN
NNNNNNNNNNNNNNNNNNNNNNNNNNNNNNNNNNNNNNNNNNNNNNNNNNNNNNNNNNNNNNNNNNNNNN
NNANNNNNNNNNNNNNNNNNNNNNNNNNNNNNNNNNNNNNNNNNNNNNNNNNNNNNNNNNNNNNNNNNNN
NNNNNNNNNNNNNNNNNNNNNNNNNNNNNNNNNNNNNNNNNNNNNNNNNNNNCNTCTNNNAAANNNTNNN
NNNNNNNNNNNNNNNNNNNNNNNNNTCNCNTTGNNNGTAANCNANNNGNACATGCAGTCCCCTCNNNNN
NNNNNCAGAGNNNNNNCCCNNNNNNNCCGNCCCCNNCCNNNNNNNTNNNCNNNNNNNNNNNGNNNNNN
NNNNNNTGGTACNNNNCGNNNNNGGTTGTTTNAGAGGTCCACCACGNGGTCNGGNNACNNNNGNNTAN
NNNCNNNNAACNNNNNNNNNNNNANATNNNCANNAGGNNNNNNCCNTCNNGNNNNNNATTGCNATGACC
CAANTCGCGGGGACAGTNACCGGCGCGGCGACCTTGATNACGGTCGGGGACAAGTATGTCGTNCNCCC
CGGGAAGGANNCNGCCGTANGTGCNNNNNANNNNNTCNTGNGANGTCGCCGNNATTCNCGTGGTCNNN
CNAGAATGNCCGNACCANNCGNANNNTGGTNATGTTAGTNNNNNNNNNNNNNNNNNNNNNNNNNNNNN
NNNNNNNNNNNNNNNNNNNNNNNNNNNNNNNNNNNGNNNCGNNNNNNCNANNNAAGGANNTANNNTTNNTN
NTNNNNNNNGGACNNNACNNNNCNNNNNNNACNCNNNCNCNNNNNNANNNGGTTTTTNNNCNNTATTNT
CCAGNACGCNGNGCNNNCATTCANGNGAACNNNGNNACGCGTAAATCGTANNATCTACCTGCCGCNNG
TNNNNNTACNGNNNNTNCGGANNNTNNCNCGTANNNNNNTCATGCGTNNTNTNNTTNCNAGNCANGTT
CGNNNANANANTAGNANGTNTCNNNNNNNNTGTNNCGTTCCGCNTGANNCNNCANNNNGNNGNNCTTN
TCANCGGCNNTANCTNCNGANNTAAGNCATANNNTNNNTCNNTANCGNNNNNGGNGGGNNACTGTAAA
NGGNNAATTGGANGNTNATTNCNNNCACNTNNCTNNGNNTNCTNNNCNCCTGNNTNCNNAATTGGNNC
CANTGNNTGNCNATCTCTGGNTTNGNNNNNNTNNNNNTNNTAANNNGGNNNGTNANANNNCNATNTAA
NNTANANNNNCNNNNNNANNNCACNNNNNNNNNNAGNNNCNANNNTTANGNNNGTAGNNNAATAAGTCT
CCGANNTNNNNNNNATAAGCGNNANACNNNNNNNNNNNNNNANGANNACGNNTTGGNNNTNNNNNNCNA
ANNAGACNNCNGTGGNTNNNNNTCAANNNNTTCGTAGTGGTATAACNANNNNNNNTTTCNCCAGNNTN
NNNGNNNNNNNCNATACNANNNNNNNGNGANNNNNNNNNNNANNNNCCCNNNAGGGANCANCTACTTNA
GCAATGGNGCGCAGNNNGAGANNNNGACCANCCTTGGGTGGCAANCACNGCAATAAGTNNNGTTNANC
CCNNNAGTNNACACNNNNNCANNGNNNCNNNNNNANCNGANNTNNNNNNNNNNNNNNNNNNNNNNNNN
NNNNNNNNNNNNNNNNNNNNNNNNNNNNNNNAGNNNANGGNNNGTNTNTGACNCATGTGTTTTTANNNGNN
NNNNNACGNNNNANCCCCGGTCGNNNNCNNATTGAANNCCTGNNGCATNNNNNTNCNAGAATGANNGC
NNNNNGNNTNGNCTNNCNNNTCAAGNAGTCAANNAGGTNNNNNNNTCNNNGNNNNNNNNCNNTCCCNN
ACTGAGGGTNNNNNNNNCATCTGCTNNNNNNNNCTNCGCNGNNNNNNTTGCNNAGCNGTATNGTGTNN
NNCANNNNNNNNNNNNNNNNNTNAAACAANNNNNNNTNNNN
>SiLa437
GTGNNNNNTAGCCCNNNNNAGTTAGACANNNCNNNNNNNNNNNNTNNNNNNNNNNNNNNNNNNNNNNNNN
NNNNNNNNNNNNNNNNNNNNNNNNNNNNNNNTNNNNNNNNNNNNNNNNNNNNNNNNNNNNNNNNNNNNNN
NNNNNNNNNNNNNNNNNNNNNNNNNNNNNNNNNNNNNNNNNNNNNNNNNNNNNNNNNNNNNNNNNNNNNN
NNNNNNNNNNNNNNNNNNNNNNNNNNNNNNNNNNNNNNNNNNNNNNNNNNNNNNNNNNNNNNNNNNNNNN
NNNNNNNNNNNNNNNNNNNNNNNNNNNNNNNNNNNNNNNNNNNNNNNNNNNNCNTCTNNNAAANNNTNNN
NNNNNNNNNNNNNNNNNNNNNNNNNNCNCNTTGNNNGTAANCNANNNGNACATGCAGTCCCCTCNNNNN
NNNGGCAGAGNNNNNNNCCNNNNNNNCCGNCCCCNNCCNNNNNNNNNNNCNNNGNNNNNNNANNNNNN
NNNNNNTGGTACNNNNCGNNNNNGGTTGTTTNAGAGGTCCACAACGNGGTCNGGNCACNNNNGNNTAN
NNNCNNNNAACNGNNNNNNNNNNANNTNNNTANNAGGNNNNNNCCNTCNNNNNNNNTNATTGCNATGACC

CANNNCACGGGGGCAGTNCCCGGCGCGGCGACCTTGATNANNTCCGGGGNCAAGTATGGCNNNCNCCC
CGGGAAGGANNCNGCCGTANGTGCNNNNNANNNNNTCNTGNGANGTCGCCGNNATTNNCGTGGTCNNN
CNAGANTGNCCNNACCANNCGNANNNTGGTNATGNNAGTANNNNNNNNNNNNNNNNNNNNNNNNNNNNN
NNNNNNNNNNNNNNNNNNNNNNNNNNNNNNNNNGNNNNGNNNNNNCNANNNNAAGGNNNTANNNCTNNTN
NTNNNNNNGGACNNNACNNNNCNNNNNNNACNCNNNCNCNNNNNNNNNNGNTTTTTNNNCNNTATTNT
CCAGNACGCNGNGCNNNCATTCANGNGAACNNNGNNACGCGNNAATCGTANNATCTACCTGCCGCNGG
NNNNNNTACNGNNNNTNCGGANNNTNNNNCGTANNNNNNTCATGCGTNNTNTNNTCNCNAGNCNNGTT
CGNNNANANANTAGNANGTNTCNNNNNNNNTGTNNCGTTCCGCNTGANNCNNCANNNNGNNGNNCTTC
TCANCGGCNNTANCTGCNGANNTAAGNCATANNNTNNATCNNTANCTNNNNNGGNGGGNNACTGTAAA
NGGNNAATTNGNNNNTNANTNCNNNCACNTNNCTNNGNNNNCTNNNTNCCTGNNTNCNNAATTGGNNC
CANTGNNTGNCNATCTCTNGNTTNGNNNNNNTNNNNNTNNCAANNNGGNNNGTNANANNNCNATNTAA
NNTATANNNGCNNNNNNANNNCACNANNNNNGNAGNNACNANNNTTANANNNGTAGNNNANTAAGTCT
CCGANNTNNNNNNNATAAGCGNNANACNNNNNNNNNNNNNNANGANNACGNNTTGGNNNTNNNNNNNNA
GNNAGACNNTNGTGGNTNNNNNTCAANNNNTTCGTAGTGGTATAATNGNNNNNNNTTTCNCCAGNNTN
NNNGNNNNNNNNCNATANNANNNNNNNGTGANNNNNNNNNNNANNNNCTCNNNAGGGANCANCTACTNNA
GCAATGGNGCGCAGNNNGAGANNNNGACNNNCCTTGGGTGGCAANCACNGCAATAAGTNNNGTTNANC
CTNNNAGTNNACACNNNNNCANNGNNNCNNNNNNANCNGANNGNNNNNNNNNNNNNNNNNNNNNNNNN
NNNNNNNNNNNNNNNNNNNNNNNNNNNNNNGNNNANNGNNNGTNTNTGACNCATGTGTTTTTANNNGNN
NNNNNACGNNNNANCCCCGGTCGNNNNCNNATTGAANNCCTGNNGCATNNNNNNTNCNAGAATGANNGC
NNNNNGNNTNGNCTNNCNNNTCAAGNAATCAANTAGGTNNCGCGNTCNNNGNNNNNNNNNCNATCCCNN
ACTGAGGGTNNNNNNNNCATCTGCTNNNNNNNNNNTNCGCNGGNGTNNCNNNNNAGCNGTATNGNGNNN
NNCANNNNNNNNNNNNNNNNANTNAAACAANNNNNNNTNNNN
>SiLa438
GTGNNNNNTAGCCCNNNNNAGTTAGACANNNCNNNNNNNNNNNNNTNNNNNNNNNNNNNNNNNNNNNNNN
NNNNNNNNNNNNNNNNNNNNNNNNNNNNNNNNNNNNNNNNNNNNNNNNNNNNNNNNNNNNNNNNNNNNNN
NNNNNNNNNNNNNNNNNNNNNNNNNNNNNNNNNNNNNNNNNNNNNNNNNNNNNNNNNNNNNNNNCNNNNNN
NNNNNNNNNNNNNNNNNNNNNNNNNNNNNNNNNNNNNNNNNNNNNNNNNNNNNNNNNNNNNNNNNNNNNN
NNNNNNNNNNNNNNNNNNNNNNNNNNNNNNNNNNNNNNNNNNNNNNNNNNNNNCNTCTNNNAAANNGTNNN
NNNNNNNNNNNNNNNNNNNNNNNNNNCNCNTTGNNNGTAANCNANNNGNACATGCAGTCCCCTCNNNNN
NNNGGCAGAGNNNNNNCCCNNNNNCNCCGNCCCCNNCCNNNNNNNNNNNNNNNNGNNNNNNNANNNNNN
NNNNNNTGGTACNNNNCGNNNNNGGTTGTNTNAGAGGTCCACAACGNGGTCNGGNCACNNNNGNNNAN
NNNCNNNNAACNNNNNNNNNNNNANATNNNTANNAGGNCNNNNCCNTCNNNNNANTNATTGCNATGACC
CAANNCACGGGGGCAGTNCCCGGCGCGGCGACCTTGNNNACGTCCGGGGACAAGTATGGCNNNCNCCC
CGGGAAGGANNCNGCCGTANGTGCNNNNNANNNNNTCCTGNGANGTCGCCGNNATTNNCGTGGTCTNN
CNAGAATGNCCGNACCANNCGNANNNTGGTNATGNNAGTNNNNNNNNNNNNNNNNNNNNNNNNNNNNN
NNNNNNNNNNNNNNNNNNNNNNNNNNNNNNNNNGNNNNGNNNNNNCNANNNNAAGGNNNTANNNCTNNTN
NTNNNNGNGGACNNNACNNNNCNNNNNNNACNCNNNCNCNNNNNNANNNGNTTTTNNNNCNNTANTNT
CCAGNACGCNGNGCNNNCATTCANGNGAACNNNGNNACGCGNNAATCGTANNATCTACCTGCCGCNNG
TNNNNNTACNGNNNNNNCGGANNNTNNCNCGTANNNNNNTCATGCGTNNTNTNNTCNCNAGNCANGTT
CGNNNANANANTAGNANGTNTNNNNNNNNNNTGTNNCGTTCCGCNTGANNCNNCANNNNGNNGNNNTTN
TCANCGGCNNTANCTGCNGANNTAAGNCATANNNTNNATCNNTANCTNNNNNGGNGGGNNACTGTAAA
NGGNNAATTNGNGGNTNANTTCNNNCACNTNNCNNNGNNNNCTNNNTNCCTGNNTNCNNAATTGGNNC
CANTGNNTGNCNATCTCTGGNTTNGNNNNNNTNNNNNTNNCAANNNGGNNNGTNANANNNCNATNTAA
NNTANANNNNCNNNNNNANNNCACNNNNNNNGNAGNNNCNANNNTTANANNNGTAGNNNAATAAGTCT
CCGANNTNNNNNNNATAAGCGNNANACNNNNNNNNNNNNNNANGANNNCGNNTTGGNNNTNNNNNNCNA
GNNAGACNNTNGTGGNTNNNNNTCAANNNNTTCGTAGTGGTATAATNGNNNNNNNTTTCNCCAGNNTN
NNNGNNNNNNNNCNATANNANNNNNNNGNGANNNNNNNNNNNANNNNCTCNNNAGGGANCANCTACTTNA
GCAATGGNGCGNAGNNNGAGANNNNGACNNNCCTTGGGTGGCAANCACNGCAATAAGTNNNGTTNANC
CTNNNAGTNNACACNNNNNCANNGNNNCNNNNNNANCNGANNGNNNNNNNNNNNNNNNNNNNNNNNNN
NNNNNNNNNNNNNNNNNNNNNNNNNNNNNNAGNNNANGGNNNGTNTNTGACNCATGTGTTTTTANNNGNN
NNNNNACGNNNNANCCCCGGTCGNNNNCNNATTGAANNCCTGNNGCATNNNNNNTNCNAGAATGANNNC
NNNNNGNNTNNNCTNNCNNNTCAAGNAATCAANNAGGNANNNNNNTCNNNNNNNNNNNNNCNNTCCCNN
ACTGAGGGTNNNNNNNNCATCTGCTNNNNNNNNNCTTCGCNGGNGTNNCNNNNNAGCNGTATNGTGTNA
NNCANNNNNNNNNNNNNNNNNNTNNAACANNNNNCNTNNNN

>SiLa439
GTGNNNNNTAGCCCNNNNNAGTTAGACANNNCNNNNNNNNNNNNTNNNNNNNNNNNNNNNNNNNNNNNNN
NNNNNNNNNNNNNNNNNNNNNNNNNNNNNNNNNNNNNNNNNNNNNNNNNNNNNNNNNNNNNNNNNNNNNN
NNNNNNNNNNNNNNNNNNNNNNNNNNNNNNNNNNNNNNNNNNNNNNNNNNNNNNNNNNNNNNNNNNNNNN
NNNNNNNNNNNNNNNNNNNNNNNNNNNNNNNNNNNNNNNNNNNNNNNNNNNNNNNNNNNNNNNNNNNNNN
NNNNNNNNNNNNNNNNNNNNNNNNNNNNNNNNNNNNNNNNNNNNNNNNNNNNNNTCTNNNAAANNNTNNN
NNNNNNNNNNNNNNNNNNNNNNNNNNNCNCNTTGNNNGTAANCNANNNGNACATGCAGTCCCCTCNNNNN
NNNGGCAGAGNNNNNNCNCNNNNNNNCCGNCCCCNNCCNNNNNNNNNNNNNNNNNGNNNNNNNANNNNNN
NNNNNNTGGTACNNNNCGNNNNNGGTTGTTTAAGAGGTCCACAACGNGGTCNGGNCACNNNNGNNNAN
NNNCNNNNAANNNNNNNNNNNNNANNTNNNTANNAGGNCNNNNCCNTNNNGNNNNNAATTGCNATGACC
CAANNCACGGGGGCNGTCCCCGGCGCGGCGACCTTGATNANGTCCGGGGNCAAGTATGGCNNNCNCCC
CGGGAAGGANNCNGCCGTANGTGCNNNNNANNNNNTCNTGNGANGTCGCCGNNATTCNCGTGGTCNNN
NNAGAATGNNCNNACCANNCGNANNNTGGTNATGNTAGTNNNNNNNNNNNNNNNNNNNNNNNNNNNNNN
NNNNNNNNNNNNNNNNNNNNNNNNNNNNNNNNNNGNNNNGNNNNNNCNANNNAAGGNNNTANNNCTNNTN
NTNNNNNNGGACNNNACNNNNCNNNNNNNACNCNNNCNCNNNNNNNNNNNGNTTTTTNNCCNNTATTNT
CCAGNACGCNGNGCNNNCATTCANGNGAACNNNGNNACGCGNNAATCGTANNATCTACCTGCCGCNNG
TNNNNNTACNGNNNNNNCGGANNNTNNCTCGTANNNNNNNCANGCGTNNTNTNNTCNCNAGNCNNGTT
CGNNNANANANTAGNANGTNTNNNNNNNNNNTGTNNCGTTCCGCNTGANNCNNCANNNNGNNGNNCTTN
TCANCGGCNNTANCTGCNGANNTAAGNCATANNNTNNATCNNTANCTNNNNNGGNGGGNNACTGTAAA
NGGNNAATTNGNNGNTNANTNCNNNCACNTNNCTNNGNNNNCTNNNTNCCTGNNTNCNNAATTGGNNC
CANTGNNTGNCNATCTCTGGNTTNGNNNNNNTNNNNNTNNCANNNNGGNNNGTNANANNNCNATNTAA
NNTANANNNNCNNNNNNANNNCACNNNNNNNNNAGNNNCNANNNTTANANNNGTAGNNNNNTAAGTCT
CCGANNTNNNNNNNATAAGCGNNANACNNNNNNNNNNNNNANGANNACGNNTTGGNNNTNNNNNNCNA
GNNAGACNNTNGTGGNTNNNNNTCAANNNNTTNGTAGTGGTATAATNGNNNNNNNTTTCNCCAGNNTN
NNNGNNNNNNNCNATANNANNNNNNNGNGANNNNNNNNNNANNNNCTCNNNAGGNANCANCTACTNNA
GCANTGGNGCGCAGNNNGAGANNNNGACNNNCCTTGGGTGGCAANCACNGCAATAAGTNNNGTTNANC
CTNNNAGTNNACACNNNNNCANNGNNNCNNNNNNANCNGANNGNNNNNNNNNNNNNNNNNNNNNNNNN
NNNNNNNNNNNNNNNNNNNNNNNNNNNNNANNNNANNGNNNGTNTNTGACNCATGTGTTTTTANNNGNN
NNNNNACGNNNNANCCCCGGTCGTNNNCNNATTGAANNCCTGNNGCATNNNNNTNCNAGAATGANNNC
NNNNNGNNTNGNCTNNCNNNTCAAGNAATCAANNAGGTANCGCGNTCNNNGNNNNNNNNCNNNCCCNN
ACTGAGGGTNNNNNNCGCATCTGCTNNNNNNNNNCTNCGCCGNCGTNNCNNNNNAGCNGTATNGNGTNN
NNCANNNNNNNNNNNNNNNNNNTNAAACAANNNNNNTTNNNN
>SiLa440
GTGNNNNNTAGCCCNNNNNAGTTAGACANNNCNNNNNNNNNNNNTNNNNNNNNNNNNNNNNNNNNNNNNN
NNNNNNNNNNNNNNNNNNNNNNNNNNNNNNTNNNNNNNNNNNNNNNNNNNNNNNNNNNNNNNNNNNNNNN
NNNNNNNNNNNNNNNNNNNNNNNNNNNNNNNNNNNNNNNNNNNNNNNNNNNNNNNNNNNNNNNNNNNNNN
NNNNNNNNNNNNNNNNNNNNNNNNNNNNNNNNNNNNNNNNNNNNNNNNNNNNNNNNNNNNNNNNNNNNNN
NNNNNNNNNNNNNNNNNNNNNNNNNNNNNNNNNNNNNNNNNNNNNNNNNNNNNNTCTNNNAAANNNTNNN
NNNNNNNNNNNNNNNNNNNNNNNNNNNCNCNTTGNNNGTAANCNANNNGNACATGCAGTCCCCTCNNNNN
NNNNNCAGAGNNNNNNCCCNNNNNNNCCGGCCCCGNCCNNNNNNNNNNNNNNNNNGNNNNNNNANNNNNN
NNNNNNTGGTACNNNNCGNNNNNGGTTGTTTAAGAGGTCCACAACGNGGTCNGNNCACNNNNGNNNAN
NNNCNNNNAACNNNNNNNNNNNNNNATNNNTANNAGGNCNNNNCCNTCNNGNNNNNNANTGCNATGACC
CAANNCACGGNGGNNGTNCCCGGCGNGGCGACCTTGANNACGTCCGGGGACAAGTATGGCNCNCNCCC
CGGGAAGGANNCNGCCGTANGTGCNNNNNANNNNNTCNTGNGANGTNGCCGNNATTCNCGTGGTCNNN
CNAGAATGNCCNNACCANNCGNANNNTGGTNATGNNAGTNNNNNNNNNNNNNNNNNNNNNNNNNNNNNN
NNNNNNNNNNNNNNNNNNNNNNNNNNNNNNNNNNGNNNNGNNNNNNCNANNNAAGGANNTANNNCTNNTN
NTNNNNNNGGACNNNACNNNNCNNNNNNNACNCNNNCNCNNNNNNANNNGNTTTNNNNNCNNTATTNT
CCAGNANGCNGNGCNNNCATTCANGNGAACNNNGNNACGCGNNAATCGTANNATCTACCTGCCGCNGG
TNNNNNTNCNGNNNNNNCGGANNNTNNNNCGTANNNGNNTCATGCGTNNTNTNNTCNCNAGNCNNGTT
CGNNNATANANTAGNANGTNTNNNNNNNNNNTGTNNCGTTCCGCNTGANNCNNCANNNNGNNGNNNTTN
TCANCGGCNNTANCTGCNGANNTAAGNCATANNNTNNNTCNNTANCTNNNNNGGNGGGNNACTGTAAA
NGGNNAATTNGNNGNTNANTNCNNNCACNTNNCTNNGNNTNCTNNNTNCCTGNNTNCNNAATTGGNNC
CANTGNNTGNCNATCTCTGGNTTNGNNNNNNTNNNNNTNNCANNNNGGNNNGTNANANNNCNATNTAA
NNTATANNNNCNNNNNNANNNCACNNNNNNNGNAGNNNNNANNNTNANANNNGTAGNNNAATAAGTCT

CCGANNTNNNNNNNATAAGCGNNANACNCNNNNNNNNNNNNANGANNACGNNTTGGNNNTNNNNNNNCNA
GNNANACNNTNGTGGNTNNNNNTCAANNNNTTNGTAGTGGTATAATNGNNNNNNNTTTCNCCAGNNTN
NNNGNNNNNNNNCNATANNANNNNNNNGNGANNNNNNNNNNNANNNNCTCNNNAGGNANCANCTACTNNA
GCANTGGNGCGCAGNNNGAGANNNNGACNNNCCTTGGGTGGCAANCACNGCAATAAGTNNNGTTNANC
CTNNNAGTNNACACNNNNNCANNGNNNCNNNNNNANCNGANNGNNNNNNNNNNNNNNNNNNNNNNNNNN
NNNNNNNNNNNNNNNNNNNNNNNNNNNNNNNAGNNNANGGNNNGTNTNTGACNCATGTGTTTTTANNNGNN
NNNNNACGNNNNANCCCCGGTCGNNNNCNNATTGAANNCCTGNNGCATNNNNNTNCNAGAATGANNNC
NNNNNGNNTNGNCTNNCNNNTCAAGNAATCNNNTAGGTNNNNCGNTCNNNNNNNNNNNNCNNTCCCNN
ACTGAGGGTNNNNNNNNCATCTGCTNNNNANNNNTNCGCNGGNGTNNCNNNNNAGCNGTATNGNGNNN
NNCANNNNNNNNNNNNNNNNNTNAAACAANNNNCNTNNNN
>SiLa441
GTGNNNNNTAGCCCNNNNNAGTTAGACANNNCNNNNNNNNNNNNNNNNNNNNNNNNNNNNNNNNNNNNNN
NNNNNNNNNNNNNNNNNNNNNNNNNNNNNNNNNNNNNNNNNNNNNNNNNNNNNNNNNNNNNNNNNNNNNN
NNNNNNNNNNNNNNNNNNNNNNNNNNNNNNNNNNNNNNNNNNNNNNNNNNNNNNNNNNNNNNNNNNNNNN
NNNNNNNNNNNNNNNNNNNNNNNNNNNNNNNNNNNNNNNNNNNNNNNNNNNNNNNNNNNNNNNNNNNNNN
NNNNNNNNNNNNNNNNNNNNNNNNNNNNNNNNNNNNNNNNCNNNNNNNNNNNNNNCNTCTNNNAAANNNTNNN
NNNNNNNNNNNNNNNNNNNNNNNNNNNCNCNTTGNNNGTAANCNANNNGNACATGCAGTCCCCTCNNNNN
NNNNNCAGAGNNNNNNNCCNNNNNCNCCGGCCCCNNCCNNNNNNNNNNNNNNNNNNNNNNNNNANNNNNN
NNNNNNTGGTACNNNNCGNNNNNGGTTGTNTAAGAGGTCCACAACGNGGTCNGGNCACNNNNGNNNAN
NNNCNNNAAANNNNNNNNNNNNNNNNNNTNNNTANNAGGNCNNNGCCNTCNNGNNNNNAANNGCNATGACC
CNANNCACGGNGGCAGTNCCCGGCGCGGCGACCTTGNTNANNTCCGGGGACAAGTATGGCNCNCNCCC
CGGGAAGGANNCNGCCGTANGTGCNNNNNANNNNNTCNTGNGANGTCGCCGNNATTCNNGTGGTCNNN
NNAGANTGNCCGNACCANNCGNANNNTGGTNATGTTAGTNNNNNNNNNNNNNNNNNNNNNNNNNNNNN
NNNNNNNNNNNNNNNNNNNNNNNNNNNNNNNNNNNNGNNNNGNNNNNNCNANNNAAGGANNTANNNCTNNTN
NTNNNNNNNGGACNNNACNNNNCNNNNNNNNACNCNNNCNCNNNNNNNNNNNGNTTTTTNNNCNNTATTNT
CCAGNACGCNGNGCNNNCATTCANGNGAACNNNGNNACGCGNNAATCGTANNATCTACCTGCCGCNGG
NNNNNNTACNGNNNNNNCGGANNNTNNCTCGTANNNNNNTCATGCGTNNTNTNNTCNCNAGNCANGTT
CGNNNANANANTAGNANGTNTCNNNNNNNNNTGTNNCGTTCCGCNTGANNCNNCANNNNGNNGNNCTTN
NCANCGGCNNTANCTGCNGANNTAAGNCATANNNTNNATCNNTANCTNNNNNGGNGGGNNACTGTAAA
NGGNNAATTNGANNNTNATTNCNNNCACNTNNCNNNGNNTNCTNNNTNCCTGNNTNCNNAATTGGNNC
CANTGNNTGNCNATCTCTNGNTTNGNNNNNNNTNNNNNTNNCANNNNGGNNNGNNANANNNCNATNTAA
NNTATANNNNCNNNNNNANNNCACNANNNNNNNNAGNNNCNANNNTTANANNNGTAGNNNAATAAGTCT
CCGANNTNNNNNNNATAAGCGNNANACNNNNNNNNNNNNNNANGANNNCGNNTTGGNNNTNNNNNNNCNA
GNNANACNNTNGTGGNTNNNNNTCAANNNNTTCGTAGTGGTATAATNGNNNNNNNTTTCNCCAGNNTN
NNNGNNNNNNNNCNATANNANNNNNNNGNGANNNNNNNNNNNANNNNCTCNNNAGGNANCANCTACTTNA
GCAATGGNGCGCAGNNNGAGANNNNGACNNGCCTTGGGTGGCAANCACNGCAATAAGTNNNGTTNNNC
CTNNNAGTNNACACNNNNNCANNGNNNCNNNNNNANCNGANNGNNNNNNNNNNNNNNNNNNNNNNNNNN
NNNNNNNNNNNNNNNNNNNNNNNNNNNNNNNAGNNNANGGNNNGTNTNTGACNCATGTGTTTTTANNNGNN
NNNNNACGNNNNANCCCCGGTCGNNNNCNNATTGAANNCCTGNNGCATNNNNNTNCNAGAATGANNNC
NNNNNGNNTNGNCTNNCNNTTCAAGNAATCAANNAGGNNNNNCGNTCNNNNNNNNNNNNCNNTCCCNN
ACTGAGGGTNNNNNNCGCATCTGCTNNNNNNNNNNTNCGCCGGNGTNNCNNNNNAGCNGTATNNNGTNA
NNCANNNNNNNNNNNNNNNNNTNAAACAANNNNNNNTNNNN
>SiLa442
GTGNNNNNTAGCCCNNNNNAGTTAGACANNNCNNNNNNNNNNNNNTNNNNNNNNNNNNNNNNNNNNNNNN
NNNNNNNNNNNNNNNNNNNNNNNNNNNNNNNNNNNNNNNNNNNNNNNNNNNNNNNNNNNNNNNNNNNNNN
NNNNNNNNNNNNNNNNNNNNNNNNNNNNNNNNNNNNNNNNNNNNNNNNNNNNNNNNNNNNNNNNNNNNNN
NNNNNNNNNNNNNNNNNNNNNNNNNNNNNNNNNNNNNNNNNNNNNNNNNNNNNNNNNNNNNNNNNNNNNN
NNNNNNNNNNNNNNNNNNNNNNNNNNNNNNNNNNNNNNNNNNNNNNNNNNNNNNNNNTCTNNNAAANNNTNNN
NNNNNNNNNNNNNNNNNNNNNNNNNNNCNCNTTGNNNGTAANCNANNNGNACATGCAGTCCCCTCNNNNN
NNNNNCAGAGNNNNNNNCCCNNNNNNNNCCGGCCCCNNCCNNNNNNNNNNNNNNANNGNNNNNNNNANNNNNN
NNNNNNTGGTACNNNNCGNNNNNGGTTGTNTAAGAGGTCCACAACGNGGTCNGGNCACNNNNGNNNAA
NNNCNNNNAACNNNNNNNNNNNNANNTNNNTANAAGGNCNNNGCCNTCNNNNNNNNNNATTGCNATGACC
CAANNCACGGGGGCAGTNCCCGGCGCGGCGACCTTGATNACGTCCGGGGACAAGTATGGCNNNCNCCC
CGGGAAGGANNCNGCCGTANGTGCNNNNNANNNTNTCNTGNGACGTCGCCGNNATTCNCGTGGTCNNN

CNAGAATGNCCGNACCANNCGNANNNTGGTNATGTNAGTANNNNNNNNNNNNNNNNNNNN
NNNNNNNNNNNNNNNNNNNNNNNNGNNNNGNNNNNNCNANNNAAGGANNTANNNCTNNTN
NTNNNNNNGGACNNNACNNNNCNNNNNNNACNCNNNCCCNNNNNNANNNGGTTTTTNNNCNNTATTNT
CCAGNACGCNGNGCNNNCATTCANGNGAACNNNGNNACGCGNNAATCGTANNATCTACCTGCCGCNGG
TNNNNNTACNGNNNNNNCGGANNNTNNCTCGTANNNNNNTCATGCGTNNTNTNNTCNCNAGNCANGTT
CGNNNANANANTAGNANGTNTCNNNNNNNNTGTNNCGTTCCGCNTGANNCNNCANNNNGNNGNNCTTN
TCANCGGCNNTANCTGCNGANNTAAGTCATANNNTNNNTCNNTANCTNNNNNGGNGGGNNACTGTAAA
NGGNNAATTGGANGNTNANTNCCNNCACNTNNCTNNGNNTNCTNNNTNCCTGNNTNCNNAATTGGNNC
CANTGNNTGNCNATCTCTGGNTTNGCNNNNNTGNNNNTNGCANNNNGGNNNGTNANANNNCNATNTAA
NNTANANNNNCNNNNNNANNNCACNNNNNNNGNAGNNNCNANNNTTANANNNGTAGNNNAATAAGTCT
CCGANNTNNNNNNNATAAGCGNNANACNNNNNNNNNNNNNNANGANNACGNNTTGGNNNTNNNNNNCNA
GNNAGACNNTNGTGGNTNNNNNTCAANNNNTTCGTAGTGGTATAATNGNNNNNNNTTTCNCCAGNNTN
NNNGNNNNNNNNCNATANNANNNNNNNGTGANNNNNNNNNNNANNNNCTCNNNAGGGANCANCTACTNNA
GCANTGGNGCGCAGNNNGAGANNNNGACCANCCTTGGGTGGCAANCACNGCAATAAGTNNNGTTNANC
CTNNNAGTNNACACNNNNNCANNGNNNCNNNNNNANCNGANNGNNNNNNNNNNNNNNNNNNNNNNN
NNNNNNNNNNNNNNNNNNNNNNNNNNNNNANNNNANGGNNNGTNTNTGACNCATGTGTTTTTANNNGNN
NNNNNACGNNNNANCCCCGGTCGTNNNCNNATTGAANNCCTGNNGCATNNNNNTNCNAGAATGANNGC
NNNNNGNNTNGNCTNNCTNNTCAAGNAATCAANTAGGTATNGCGNTCNNNNNNNNNNNNCNNTCCCNN
ACTGAGGGTNNNNNNCGCATCTGCTNNNNNNNNCTNCGCCGGCGTNNCNNNNNAGCNGTATNGTGNNN
NNCANNNNNNNNNNNNNNNNNTNAAACNANNNNNNTNNNN
>SiLa443
GTGNNNNNTAGCCCNNNNNAGTTAGACANNNCNNNNNNNNNNNNTNNNNNNNNNNNNNNNNNNNNNNN
NNNNNNNNNNNNNNNNNNNNNNNNNNNNNNNNCNNNNNNNNNNNNNNNNNNNNNNNNNNNNNNNNNNN
NNNNNNNNNNNNNNNNNNNNNNNNNNNNNNNNNNNNNNNNNNNNNNNNNNNNNNNNNNNNNNNNNNNNN
NNNNNNNNNNNNNNNNNNNNNNNNNNNNNNNNNNNNNNNNNNNNNNNNNNNNNNNNNNNNNNNNNNNNN
NNNNNNNNNNNNNNNNNNNNNNNNNNNNNNNNNNNNNNNNNNNNNNNNNNNNNCNTCTNNNAAANNNTNNN
NNNNNNNNNNNNNNNNNNNNNNNNNNCNCNTTGNNNGTAANCNANNNGNACATGCAGTCCCCTCNNNNN
NNNGGCAGAGNNNNNNCNCNNNNNNNNCCGGCCCCNNCCNNNNNNNNNNNNNNNNNGNNNNNNNANNNNNN
NNNNNNTGGTACNNNNCGNNNNNGGTTGTNTNAGAGGTCCACAACGNGGTCNNGNCACNNNNGNNTAN
NNNCNNNNAANNNNNNNNNNNNNNNNNNTNNNTANNAGGNCNNNNCCNTCNNGNNANNNATTGCNATGACC
CANNNCACGGGGGCAGTNCCCGGCGCGGCGACCTTGNTNACGTCCGGGGACAAGTATGGCNCNCNCCC
CGGGAAGGANNCNGCCGTANGTGCNNNNNANNNNNTCNTGNGANGTCGCCGNNATTCNCNTGGTCTNN
CNAGANTGNCCNNACCANNCGNANNNTGGTNATGNTAGTANNNNNNNNNNNNNNNNNNNNNNNNNNNN
NNNNNNNNNNNNNNNNNNNNNNNNNNNNNNNNNNGNNNNGNNNNNNCNANNNAAGGNNNTANNNCTNNTN
NTNNNNNNGGACNNNACNNNNCNNNNNNNACNCNNNCNCNNNNNNNNNNGNTTTTTNNNCNNTATTNT
CCAGNACGCNGNGCNNNCATTCANGNGAACNNNGNNACGCGNNAATCGTANNATCTACCTGCCGCNGG
TNNNNNTACNGNNNNTACGGANNNTNNCTCGTANNNNNNTCATGCGTNNTNTNNTCNCNAGNCNNGTT
CGNNNANANANTAGNANGTNTNNNNNNNNNNTGTNNCGTTCCGCNTGANNCNNCANNNNGNNGNNNTTN
TCANCGGCNCTANCTGCNGANNTAAGNCATANNNTNNNTCNNTANCTNNNNNGGNGGGNNACTGTAAA
NGGNNAATTNGNGGNTNATTTCNNNCACNTNNCTNNGNNTNCTNNNTNCCTGNNTNCNNAATTGGNNC
CANTGNNTGNCNATCTCTNGNTTNGNNNNNNTNNNNNTNNCANNNNGGNNNGTNANANNNCNATNTAA
NNTANANNNNCNNNNNNANNNCACNANNNNNGNAGNNNCNANNNTTANANNNGTAGNNNAATAAGTCT
CCGANNTNNNNNNNATAAGCGNNANACNNNNNNNNNNNNNNANGANNACGNNTTGGNNNTNNNNNNCNA
GNNAGACNNTNGTGGNTNNNNNTCAANNNNTTNGTAGTGGTATAATNGNNNNNNNTTTCNCCAGNNTN
NNNGNNNNNNNNCNATANNANNNNNNNGTGANNNNNNNNNNNANNNNCTCNNNAGGGANCANCTACTTNA
GCAATGGNGCGCAGNNNGAGANNNNGACNNGCCTTGGGTGGCAANCACNGCAATAAGTNNNGTTNANC
CTNNNAGTNNACACNNNNNCANNGNNNCNNNNNNANCNGANNGNNNNNNNNNNNNNNNNNNNNNNN
NNNNNNNNNNNNNNNNNNNNNNNNNNNNNNGNNNANGGNNNGTNTNTGACNCATGTGTTTTTANNNGNN
NNNNNACGNNNNANCCCCGGTCGTNNNCNNATTGAANNCCTGNNGCATNNNNNTNCNAGAATGANNGC
NNNNNGNNTNNNCTGNCNTTTCAAGNAATCANNNAGNNNNCGCGNTCNNNGNNNNNNNNCNNNCCCNN
ACTGAGGGTNNNNNNNNCATCNGCTNNNNNNNNNNTTCGCNGGCGNNNCNNNNNAGCNGTATNGTGNNN
NNCANNNNNNNNNNNNNNNNNTNAAANNANNNNCNTNNNN
>SiLa444
GTGNNNNNTAGCCCNNNNNAGTTAGACANNNCNNNNNNNNNNNNNNNNNNNNNNNNNNNNNNNNNNNN

NNNNNNNNNNNNNNNNNNNNNNNNNNNNNNNNTNNNNNNNNNNNNNNNNNNNNNNNNNNNNNNNNNNNNNNN
NNNNNNNNNNNNNNNNNNNNNNNNNNNNNNNNNNNNNNNNNNNNNNNNNNNNNNNNNNNNNNNNNNNNNNNN
NNNNNNNNNNNNNNNNNNNNNNNNNNNNNNNNNNNNNNNNNNNNNNNNNNNNNNNNNNNNNNNNNNNNNNNN
NNNNNNNNNNNNNNNNNNNNNNNNNNNNNNNNNNNNNNNNNNNNNNNNNNNNNNTCTNNNAAANNNTNNN
NNNNNNNNNNNNNNNNNNNNNNNNNNCNCNTTGNNNGTAANCNANNNGNACATGCAGTCCCCTCNNNNN
NNNNNCAGAGNNNNNNCCCNNNNNNNCCGGCCCCNNCCNNNNNNNNNNNNNNNNNNNNNNNNANNNNNN
NNNNNNTGGTACNNNNCGNNNNNGGTTGTTTNAGAGGTCCACAACGNGGTCNGGNCACNNNNGNNNAN
NNNCNNNNAACNNNNNNNNNNNNANNTNNNTANNAGGNNNNNNCCNTCNNNNNNNNAATTGCNATGACC
CAANNCNCGGGGGCAGTNCCCGGNGCGGCGACCTTGNTNANNTCCGGGNACAAGTATGGCNCNCNCCC
CGGGAAGGANNCNGCCGTANGTGCNNNNNANNNNNTCNTGNGANGTNGCCGNNATTCNCGTGGNCNNN
NNAGAATGNNCGNACCANNCGNANNNTGGTNATGNNAGTNANNNNNNNNNNNNNNNNNNNNNNNNNNN
NNNNNNNNNNNNNNNNNNNNNNNNNNNNNNNNNGNNNNGNNNNNNCNANNNAAGGANNTANNNCTNNTN
NTNNNNNNGGACNNNACNNNNCNNNNNNNACNCNNNCNCNNNNNNNNNNGNTTTTTNNNCNNTATTNT
CCAGNACGCNGNGCNNNCATTCANGNGAACNNNGNNACGCGTNAATCGTANNATCTACCTGCCGCNGG
TNNNNNTACNGNNNNTNCGGANNNTNNCTCGTANNNNNGTCATGCGTNNTNTNNTCNCNAGNCNNGTT
CGNNNANANANTAGNANGTNTNNNNNNNCTTGTNNCGTTCCGCNTGANNCNNCANNNNGNNGNNCTTN
TCANCGGCNNTANCTGCNGANNTAAGNCATANNNTNNNTCNNTANCTNNNNNGGNGGGNNACTGTAAA
NGGNNAATTNGAGGNTNATTNCNNNCACNTNNCTNNGNNNNCTNNNTNCCTGNNTNCNNAATTGGNNC
CANTGNNTGNCNATCTCTNGNNTNGNNNNNNTNNNNNTNGCANNNNGGNNNNNNANANNNCNANNTAA
NNTATANNNNCNNNNNNANNNCACNANNNNNGNAGNNNCNANNNTTANANNNGTAGNNNAATAAGTCT
CCGANNTNNNNNNNATAAGCGNNANACNNNNNNNNNNNNNANGANNACGNNTTGGNNNTNNNNNNCNA
GNNAGACNNTNGTGGNTNNNNNTCAANNNNTTCGTAGTGGTATAATNGNNNNNNNTTTCNCCAGNNTN
NNNGNNNNNNNCNATANNANNNNNNNGTGANNNNNNNNNNANNNNCTCNNNAGGNANCANCTACTNNA
GCANTGGNGCGNAGNNNGAGANNNNGACNNNCCTTGGGTGGCAANCACNGCAATAAGTNNNGTTNANC
CTNNNAGTNNACACNNNNNCANNGNNNCNNNNNNACCNGANNGNNNNNNNNNNNNNNNNNNNNNNNNN
NNNNNNNNNNNNNNNNNNNNNNNNNNNNNNAGNNNANNGNNNGTNTNTGACNCATGTGTTTTTANNNGNN
NNNNNACGNNNNANCCCCGGTCGNNNNCNNATTGAANNCCTGNNGCATNNNNNTNCNAGAATGANNNC
NNNNNGNNTNGNCTNNCNNNTNAAGNAANCANNTANNNNNCGCGNTCNNNNNNNNNNNNCNNTCCCNN
ACTGAGGGTNNNNNNNNCATCTGCTNNNNNNNNNCTNCGCNGNNGTNNCNNNNNAGCCGTATNGNGNNN
NNCANNNNNNNNNNNNNNNNNNNAANNNANNNNCNTNNNN
>SiLa445
GTGNNNNNTAGCCCGNNNNAGTTAGACANNNCNNNNNNNNNNNNTNNNNNNNNNNNNNNNNNNNNNNNN
NNNNNNNNNNNNNNNNNNNNNNNNNNNNNNNNNNNNNNNNNNNNNNNNNNNNNNNNNNNNNNNNNNNNNNNN
NNNNNNNNNNNNNNNNNNNNNNNNNNNNNNNNNNNNNNNNNNNNNNNNNNNNNNNNNNNNNNNNNNNNNNNN
NNNNNNNNNNNNNNNNNNNNNNNNNNNNNNNNNNNNNNNNNNNNNNNNNNNNNNNNNNNNNNNNNNNNNNNN
NNNNNNNNNNNNNNNNNNNNNNNNNNNNNNNNNNNNNNNNNNNNNNNNNNNNNNTCTNNNAAANNNTNNN
NNNNNNNNNNNNNNNNNNNNNNNNNNCNCNTTNNNNGTAANCNANNNGNACATGCAGTCCCCTCNNNNN
NNNGGCAGAGNNNNNNCCNNNNNNNNNCCGNCCCCNNCCNNNNNNNNNNNCNNNGNNNNNNNANNNNNN
NNNNNNTGGTACNNNNCGNNNNNGGTTGTTTAAGAGGTCCACAACGNGGTCNNGNCACNNNNGNNNAN
NNNCNNNNAANNNNNNNNNNNNNNNNNTNNNTANNAGGNCNNNNCCNTCNNNNNANNNATNGCNATGACC
CAANTCACGGGGGCAGTNCCCGGNGNGGCGACCTTGATNACGTCCGGGGACAAGTATGGCGCNCNCCC
CGGGAAGGANNCNGCCGTANGTGCNNNNNANNNNNTCTTGNGANGTCGCCGNNATNNNCGTGGTCNNN
NNAGAATGNCCGNACCANNCGNANNNTGGTNATGTTAGTNNNNNNNNNNNNNNNNNNNNNNNNNNNNN
NNNNNNNNNNNNNNNNNNNNNNNNNNNNNNNNNGNNNNGNNNNNNCNANNNAAGGANNTANNNCTNNTN
NTNNNNNNGGACNNNACNNNNCNNNNNNNACNCNNNCNCNNNNNNNNNNGNTTTTTNNNCNNTATTNT
CCAGNACGCNGNGCNNNCATTCANGNGAACNNNGNNACGCGTAAATCGTANNATCTACCTGCCGCNNG
TNNNNNTACNGNNNNTNCGGANNNTNNCTCGTANNNNNGTCATGCGTNNTNTNNTCNCNAGNCANGTT
CGNNNANANANTAGNANGTNTCNNNNNNNNTGTNNCGTTCCGCNTGANNCNNCANNNNGNNGNNCTTN
TCANCGGCNNTANCTGCNGANNTAAGNCATANNNTNNNTCNNTANCTNNNNNGGNGGGNNACTGTAAA
NGGNTAATTNGNGGNTNATTNCCNNCACNTNNCTNNGNNTNCTNNNTNCCTGNNTNCNNAATTGGNNC
CANTGNNTGNCNATCTCTGGNTTNGNNNNNNTNNNNNTNGCAANNNGGNNNGTNANANNNCNATNTAA
NNTATANNNNCNNNNNNANNNCACNANNNNNNNAGNNANNANNNTTANANNNGTAGNNNAATAAGTCT
NCGANNTNNNNNNNATAAGCGNNANACNNNNNNNNNNNNNANGANNACGNNTTGGNNNTNNNNNNCNA
GNNAGACNNTNGTGGNTNNNNNTCAANNNNTTCGTAGTGGTATAATNGNNNNNNNTTTCNCCAGNNTN

NNNGNNNNNNNNCNATANNANNNNNNNGTGANNNNNNNNNNNANNNNCTCNNNAGGGANCANCTACTTNA
GCAANGGNGCGNAGNNNGAGANNNNGACCAGCCTTGGGTGGCAANCACNGCAATAAGTNNNGTTNANC
CTNNNAGTNNACACNNNNNCANNGNNNCNNNNNNANCNGANNGNNNNNNNNNNNNNNNNNNNNNNNN
NNNNNNNNNNNNNNNNNNNNNNNNNNNNNAGNNNANGGNNNGTNTNTGACNCATGTGTTTTTANNNGNN
NNNNNACGNNNNANCCCCGGTCGNNNNCNNATTGAANNCCTGNNGCATNNNNNTNCNAGAATGANNGC
NNNNNGNNTNGNCTNNCNTTTCAAGNAATCANNNAGGTATCGCGNTCNNNGNNNNNNNNCNNTCCCNN
ACTGAGGGTNNNNNNCGCATNTGCTNNNNNNNNNNTTCGCNGGCNNNNCNNNNNAGCNGTATNGNGTNN
NNCANNNNNNNNNNNNNNNNNNNNNACNANNNNNNNTNNNN
>SiLa446
GTGNNNNNTAGCCCNNNNNAGTTAGACANNNCNNNNNNNNNNNNNTNNNNNNNNNNNNNNNNNNNNNNN
NNNNNNNNNNNNNNNNNNNNNNNNNNNNNNNNNNNNNNNNNNNNNNNNNNNNNNNNNNNNNNNNNNNNNN
NNNNNNNNNNNNNNNNNNNNNNNNNNNNNNNNNNNNNNNNNNNNNNNNNNNNNNNNNNNNNNNNNNNNNN
NNNNNNNNNNNNNNNNNNNNNNNNNNNNNNNNNNNNNNNNNNNNNNNNNNNNNNNNNNNNNNNNNNNNNN
NNNNNNNNNNNNNNNNNNNNNNNNNNNNNNNNNNNNNNNNNNNNNNNNNNNNNNNTCTNNNAAANNNTNNN
NNNNNNNNNNNNNNNNNNNNNNNNNNNCNCNTTGNNNGTAANCNANNNGNACATGCAGTCCCCTCNNNNN
NNNNNCAGAGNNNNNNNCCNNNNNNNCCGGCCCCNNCCNNNNNNNNNNNNNNNNGNNNNNNNANNNNNN
NNNNNNTGGTACNNNNCGNNNNNGGTTGTNTNAGAGGTCCACAACGNGGTCNNGNCACNNNNGNNNAN
NNNCNNNNNNNNNNNNNNNNNNNNNATNNNTANNAGGNCNNNNCCNTCNNGNNNNTAATTGCNATGACC
CNANNCACGGGGGCNGTNCCCGGCGCGGCGACCTTGATNANNTCCGGGGACAAGTATGGCNNNCNCCC
CGGGAAGGANNCNGCCGTANGTGCNNNNNANNNNNTCNTGNGANGTCGCCGNNATTCNCGTGGTCTNN
CNAGAATGNNCNNACCANNCGNANNNTGGTNATGNNAGTANNNNNNNNNNNNNNNNNNNNNNNNNNNN
NNNNNNNNNNNNNNNNNNNNNNNNNNNNNNNNNNGNNNNGNNNNNNCNANNNAAGGANNTANNNCTNNTN
NTNNNNNNNGGACNNNACNNNNCNNNNNNNACNCNNNCNCNNNNNNANNNGNTTTTTNNNCNNTATTNT
CCAGNACGCNGNGCNNTCATTCANGNGAACNNNGNNACGCGNNAATCGTANNATCTACCTGCCGCNNG
TNNNNNTNCNGNNNNTNCGGANNNTNNNNCGTANNNNNNTCATGCGTNNTNTNNTCNCTAGNCNNGTT
CGNNNANANANTAGNANGTNTNNNNNNNNNNTGTNNCGTTCCGCNTGANNCNNCANNNNGNNGNNCTTN
TCANCGGCNNTANCTGCNGANNTAAGTCATANNNTNNATCNNTANCTNNNNNGGNGGGNNACTGTAAA
NGGNNAATTGGNGNNTNANTTCNNNCACNTNNCTNNGNNTNCTNNNTNCCTGNNTNCNNAATTGGNNC
CANTGNNTGNCNATCTCTGGNTTNGNNNNNNTNNNNNTNGCANNNNGGNNNGTNANANNNCNATNTAA
NNTATANNNNCNNNNNNANNNCACNANNNNNGNAGNNANNANNNTTANANNNGTAGNNNAATAAGTCT
CCGANNTNNNNNNNATAAGCGNNANACNNNNNNNNNNNNNANGANNACGNNTTGGNNNTNNNNNNCNA
GNNAGACNNTNGTGGNTNNNNNTCAANNNNTTNGTAGTGGTATAATNGNNNNNNNTTTCNCCAGNNTN
NNNGNNNNNNNNCNATANNANNNNNNNGNGANNNNNNNNNNNANNNNCTCNNNAGGGANCANCTACTTNA
GCANTGGNGCGNAGNNNGAGANNNNGACNNNCCTTGGGTGGCAANCACNGCAATAAGTNNNGTTNANC
CTNNNAGTNNACACNNNNNCANNGNNNCNNNNNNANCNGANNGNNNNNNNNNNNNNNNNNNNNNNNN
NNNNNNNNNNNNNNNNNNNNNNNNNNNNNNGNNNANGGNNNGTNTNTGACNCATGTGTTTTTANNNGNN
NNNNNACGNNNGANCCCCGGTCGNNNNCNNATTGAANNCCTGNNGCATNNNNNTNCNAGAATGANNGC
NNNNNGNNTNGNCTNNCNTTTCAAGNAATCANNNAGGNANNNCGNTCNNNNNNNNNNNNNCNNTCCCNN
ACTGAGGGTNNNNNNNNCATCTGCTNNNNNNNNNNTTCGCNGGCGTNNCNNNNNNGCNGTATNGTGTNN
NNCANNNNNNNNNNNNNNNNNNNNAAACNANNNNNNNTNNNN
>SiLa447
GTGNNNNNTAGCCCNNNNNAGTTAGACANNNCNNNNNNNNNNNNNTNNNNNNNNNNNNNNNNNNNNNNN
NNNNNNNNNNNNNNNNNNNNNNNNNNNNNNNNNNNNNNNNNNNNNNNNNNNNNNNNNNNNNNNNNNNNNN
NNNNNNNNNNNNNNNNNNNNNNNNNNNNNNNNNNNNNNNNNNNNNNNNNNNNNNNNNNNNNNNNNNNNNN
NNNNNNNNNNNNNNNNNNNNNNNNNNNNNNNNNNNNNNNNNNNNNNNNNNNNNNNNNNNNNNNNNNNNNN
NNNNNGNNNNNNNNNNNNNNNNNNNNNNNNNNNNNNNNNNNNNNNNNNNNNNNCNTCTNNNAAANNNTNNN
NNNNNNNNNNNNNNNNNNNNNNNNNNNCNCNTTGNNNGTAANCNANNNGNACATGCAGTCCCCTCNNNNN
NNNGGCAGAGNNNNNNNCCNNNNNCNCCGGCCCCNNCCNNNNNNNNNNNNNNNNGNNNNNNNANNNNNN
NNNNNNTGGTACNNNNCGNNNNNGGTTGTTTNAGAGGTCCACAACGNGGTCNGGNCACNNNNGNNNAN
NNNCNNNNANCNNNNNNNNNNNNANNTNNNTANNAGGNCNNNNCCNTCNNNNNNNNNNATNGCNATGACC
CAANNCACGGGGGCAGTCCCCGGCGCGGCGACCTTGNTNANNTCCGGGGACAAGTATGGCNCNCNCCC
CGGGAAGGANNCNGCCGTANGTGCNNNNNANNNNNTCNTGNGACGTNGCCGNNATTCNCGTGGTCTNN
NNAGAATGNCCNNACCANNCGNANNNTGGTTATGNTAGTNNNNNNNNNNNNNNNNNNNNNNNNNNNNN
NNNNNNNNNNNNNNNNNNNNNNNNNNNNNNNNNNGNNNNGNNNNNNCNANNNAAGGANNTANNNCTNNTN

NTNNNNNNGGACNNNACNNNNCNNNNNNNACNCNNNCNCNNNNNNANNTGGTTTTTNNNCNNTATTNT
CCAGNACGCNGNGCNNNCATTCANGNGAACNNNGNNACGCGNNAATCGTANNATCTACCTGCCGCNGG
TNNNNNTACNGNNNNTNCGGANNNTNNCTCGTANNNNNNTCATGCGTNNTNTNNTCNCNAGNCNNGTT
CGNNNANANANTAGNANGTNTCCNNNNNCTTGTNNCGTTCCGCNTGANNCNNCANNNNGNNGNNCTTN
TCANCGGCNNTANCTGCNGANNTAAGTCATANNNTNNNTCNNTANCTNNNNNGGAGGGNNACTGTAAA
NGGNNAATTGGAGGNTNATTTCNNNCACNTNNCTNNGNNTNCTNNNTNCCTGNNTNCNNAATTGGNNC
CANTGNNTGNCNATCTCTGGNTTNGNNNNNNTNNNNNTNGCAANNNGGNNNGTNANANCNCNATNTAA
NNTATANNNNCNNNNNNANNNCACNANNNNNGNAGNNNCNANNNTTANANNNGTAGNNNANTAAGTCT
CCGANNTNNNNNNNATAAGCGNNANACNNNNNNNNNNNNNANGANNACGNNTTGGNNNTNNNNNNCNA
GNNAGACNNTNGTGGNTNNNNNTCAANNNNTTCGTAGTGGTATAATNGNNNNNNNTTTCNCCAGNNTN
NNNGNNNNNNNCNATANNANNNNNNNGTGANNNNNNNNNNANNNNCTCNNNAGGGANCANCTACTTNA
GCAATGGNGCGCAGNNNGAGANNNNGACNNNCCTTGGGTGGCAANCACNGCAATAAGTNNNGTTNANC
CTNNNAGTNNACACNNNNNCANNGNNNCNNNNNNANCNGANNGNNNNNNNNNNNNNNNNNNNNNNNNN
NNNNNNNNNNNNNNNNNNNNNNNNNNNNAGNNNANGGNNNGTNTNTGACNCATGTGTTTTTANNNGNN
NNNNNACGNNNGANCCCCGGTCGNNNNCNNATTGAANNCCTGNNGCATNNNNNTNCNAGAATGANNGC
NNNNNGNNTNNNCTNNCNTTTCAAGNAATCANNNAGGTANCGCGNTCNNNNNNNNNNNNCNNTCCCNN
ACTGAGGGTNNNNNNNGCATCTGCTNNNNNNNNCTTCGCNGNNGTNNCNNNNNAGCNGTATNGTGTTA
NNCANNNNNNNNNNNNNNNNTNAAACNANNNNCNTNNNG
>SiLa448
GTGNNNNNTAGCCCNNNNNAGTTAGACANNNCNNNNNNNNNNNNTNNNNNNNNNNNNNNNNNNNNNNN
NNNNNNNNNNNNNNNNNNNNNNNNNNNNNNTNNNNNNNNNNNNNNNNNCNNNNNNNGNNNNNNNNNNN
NNNNNNNNNNNNNNNNNNNNNNNNNNNNNNNNNNNNNNNNNNNNNNNNNNNNNNNNNNNNNNNNNNNN
NNNNNNNNNNNNNNNNNNNNNNNNNNNNNNNNNNNNNNNNNNNNNNNNNNNNNNNNNNNNNNNNNNNN
NNNNNNNNNNNNNNNNNNNNNNNNNNNNNNNNNNNNNNNNNNNNNNNNNNNNTCTNNNAAANNGTNNN
NNNNNNNNNNNNNNNNNNNNNNNNNCNCNTTGNNNGTAANCNANNNGNACATGCAGTCCCCTCNNNNN
NNNGGCAGAGNNNNNNCCCNNNNNNNCCGNCCCCNNCCNNNNNNNNNNNCNNNGNNNNNNNANNNNNN
NNNNNNTGGTACNNNNCGNNNNNGGTTGTNTAAGAGGTCCACAACGNGGTCNNGNCACNNNNGNNTAN
NNNCNNNNAANNNNNNNNNNNNNNNTNNNTANNAGGNNNNNNCCNTCNNNNNNNNAATTGCNATGACC
CAANTCACGGGGGCAGTNCCCGGNGCGGCGACCTTGATNACGTCCGGGGACAAGTATGGCGCNCNCCC
CGGGAAGGANNCNGCCGTANGTGCNNNNNANNNNNTCNTGNGACGTCGCCGNNATTCNCGTGGTCNNN
CNAGAATGNNCGNACCANNCGNANNNTGGTNATGTTAGTNNNNNNNNNNNNNNNNNNNNNNNNNNNNN
NNNNNNNNNNNNNNNNNNNNNNNNNNNNNNNNNNNNNGNNNNNNCNANNNAAGGNNATANNNCTNNTN
NTNNNNNNGGACNNNACNNNNCNNNNNNNACNCNNNCNCNNNNNNANNNGNTTTTTNNNCNNTATTNT
CCAGNACGCNGNGCNNNCATTCANGNGAACNNNGNNACGCGNNAATCGTANNATCTACCTGCCGCNGG
TNNNNNTACNGNNNNNNCGGANNNTNNNNCGTANNNNNNTCATGCGTNNTNTNNTCNCNAGNCANGTT
CGNNNANANANTAGNANGTNTNNNNNNNNNTGTNNCGTTCCGCNTGANNCNNCANNNNGNNGNNCTTN
TCANCGGCNNTANCTGCNGANNTAAGTCATANNNTNNNTCNNTANCTNNNNNGGNGGGNNACTGTAAA
NGNNNAATTGGNNNNTNATTTCNNNCACNTNNCTNNGNNTNCTNNNTNCCTGNNTNCNNAATTGGNNC
CANTGNNTGNCNATCTCTGGNTTNGNNNNNNTNNNNNTNNCAANNNGGNNNGTNANANNNCNATNTAA
NNTATANNNNCNNNNNNANNNCACNANNNNNGNAGNNACNANNNTTANANNNGTAGNNNANTAAGTCT
CCGANNTNNNNNNNATAAGCGNNANACNNNNNNNNNNNNNANGANNACGNNTTGGNNNTNNNNNNCNA
GNNAGACNNTNGTGGNTNNNNNTCAANNNNTTCGTAGTGGTATAATNGNNNNNNNTTTCNCCAGNNTN
NNNGNNNNNNNCNATANNANNNNNNNGNGANNNNNNNNNNANNNNCTCNNNAGGGANCANCTACTNNA
GCANTGGNGCGNAGNNNGAGANNNNGACNNGCCTTGGGTGGCAANCACNGCAATAAGTNNNGTTNANC
CTNNNAGTNNACACNNNNNCANNGNNNCNNNNNNANCNGANNGNNNNNNNNNNNNNNNNNNNNNNNNN
NNNNNNNNNNNNNNNNNNNNNNNNNNNNAGNNNANGGNNNGTNTNTGACNCATGNNTTTTTANNNGNN
NNNNNACGNNNNANCCCCGGTCGTNNNCNNATTGAANNCCTGNNGCATNNNNNTNCNAGAATGANNGC
NNNNNGNNTNGNCTNNCNNTTCAAGNAATCANNNANGTANCGCGNTCNNNGNNNNNNNNCNNTCCCNN
ACTGAGGGTNNNNNNNGCATCTGCTNNNNNNNNNTNCGCNGNCGTNNCNNNNNNGCNGTATNGNGTNN
NNCANNNNNNNNNNNNNNNNTNAAANNNNNNNNNTNNNN
>SiLa449
GTGNNNNNTAGCCCNNNNNAGTTAGACANNNCNNNNNNNNNNNNNNNNNNNNNNNNNNNNNNNNNNNN
NNNNNNNNNNNNNNNNNNNNNNNNNNNNNNNNNNNNNNNNNNNNNNNNNNNNNNNNNNNNNNNNNNNN
NNNNNNNNNNNNNNNNNNNNNNNNNNNNNNNNNNNNNNNNNNNNNNNNNNNNNNNNNNNNNNNNNNNN

NNNNNNNNNNNNNNNNNNNNNNNNNNNNNNNNNNNNNNNNNNNNNNNNNNNNNNNNNNNNNNCNNNNN
NNNNNNNNNNNNNNNNNNNNNNNNNNNNNNNNNNNNNNNNNNNNNNNNNNNNTCTNNNAAANNNTNNN
NNNNNNNNNNNNNNNNNNNNNNNNNCNCNTTGNNNGTAANCNANNNGNACATGCAGTCCCCTCNNNNN
NNNNNCAGAGNNNNNNNCCCNNNNNNNCCGNCCCCNNCCNNNNNNNNNNNNNNNNNNNNNNNANNNNNN
NNNNNNTGGTACNNNNCGNNNNNGGTTGTNTNAGAGGTCCACAACGNGGTCNNGNCACNNNNGNNNAN
NNNCNNNNAACNNNNNNNNNNNNNANNTNNNTANNAGGNCNNNNCCNTCNNGNNNNNNANNGCNATGACC
CAANNCACGGGGGCNGTCCCCGGCGCGGCGACCTTGATNANGTCCGGGGACAAGTATGGCNNNCNCCC
CGGGAAGGANNCNGCCGTANGTGCNNNNNANNNNNTCNTGNGANGTCGCCGNNATTCNCGTGGTCNNN
CNAGAATGNCCGNACCANNCGNANNNTGGTNATGNTAGTNNNNNNNNNNNNNNNNNNNNNNNNNNNNN
NNNNNNNNNNNNNNNNNNNNNNNNNNNNNNNNNNGNNNNGNNNNNNCNANNNAAGGANNTANNNCTNNTC
NTNNNNNNGGACNNNACNNNNCNNNNNNNACNCNNNCNCNNNNNNNNNNNGGTTTTNNNCCNNTATTNT
CCAGNACGCNGNGCNNNCATTCANGNGAACNNNGNNACGCGNNAATCGTANNATCTACCTGCCGCNGG
TNNNNNTACNGNNNNTNCGGANNNTNNCTCGTANNNNNNTCATGCGTNNTNTNNTCNCNAGNCANGTT
CGNNNANANANTAGNANGTNTCNNNNNNNNTGTNNCGTTCCGCNTGANNCNNCANNNNGNCGNNCTTN
TCANCGGCNNTANCTGCNGANNTAAGNCATANNNTNNATCNNTANCTNNNNNGGNGGGNNACTGTAAA
NGGNTAATTNGAGGNTNANTTCNNNCACNTNNCTNAGNNTNCTNNNTNCCTGNNTNCNNAATTGGNNC
CANTGNNTGNCNATCTCTGGNTTNGNNNNNNTNNNNNTNGCANNNNGGNNNGTNANANNNCNATNTAA
NNTATANNNNCNNNNNNANNNCACNANNNNNGNAGNNNCNANNNTTANANNNGTAGNNNAATAAGTCT
CCGANNTNNNNNNNATAAGCGNNANACNNNNNNNNNNNNNNANGANNACGNNTTGGNNNTNNNNNNCNA
GNNANACNNTNGTGGNTNNNNNTCAANNNNTTCGTAGTGGTATAATNGNNNNNNNTTTCNCCAGNNTN
NNNGNNNNNNNNCNATANNANNNNNNNGNGANNNNNNNNNNNANNNNCTCNNNAGGGANCANCTACTNNA
GCANNGGNGCGCAGNNNGAGANNNNGACNNNCCTTGGGTGGCAANCACNGCAATAAGTNNNGTTNANN
NTNNNAGTNNACACNNNNNCANNGNNNCNNNNNNANCNGANNGNNNNNNNNNNNNNNNNNNNNNNNNN
NNNNNNNNNNNNNNNNNNNNNNNNNNNNNNANNNNANNGNNNGTNTNTGACNCATGTGTTTTTANNNGNN
NNNNNACGNNNNANCCCCGGTCGTNNNCNNATTGAANNCCTGNNGCATNNNNNNTNCNAGAATGANNGC
NNNNNGNNTNGNCTGNCNNNTCAAGNAATCAANNAGNNANCGCGNTCNNNNANNNNNNNNCNNTCCCNN
ACTGAGGGTNNNNNNNNCATCTGCTNNNNNNNNNNTNCGCNGGCGTNNCNGNNNAGCNGTATNGNGNNN
NNCANNNNNNNNNNNNNNNNNNNNAAACAANNNNCCTNNNN
>SiLa450
GTGNNNNNTAGCCCGNNNNAGTTAGACANNNCNNNNNNNNNNNNNTNNNNNNNNNNNNNNNNNNNNNN
NNNNNNNNNNNNNNNNGNNNNNNNNNNNNNNNNNNNNNNNNNTNNNNNNNNNNNNNNNNNNNNNNNNN
NNNNNNNNNNNNNNNNNNNNNNNNNNNNNNNNNNNNNNNNNNNNNNNNNNNNNNNNNNNNNNNNNNNN
NNNNNNNNNNNNNNNNNNNNNNNNNNNNNNNNNNNNNNNNNNNNNNNNNNNNNNNNNNNNNNNNNNNN
NNNNNNNNNNNNNNNNNNNNNNNNNNNNNNNNNNNNNNNNNNNNNNNNNNNNCNTCTNNNAAANNNTNNN
NNNNNNNNNNNNNNNNNNNNNNNNNCNCNTTGNNNGTAANCNANNNGNACATGCAGTCCCCTCNNNNN
NNNGGCAGAGNNNNNNNCCNNNNNNNNCCGNCCCCNNCCNNNNNNNNNNNNNCNNNGNNNNNNNANNNNNN
NNNNNNTGGTACNNNNCGNNNNNGGTTGTNTAAGAGGTCCACAACANGGTCNGGNCACNNNNGNNNAN
NNNCNNNNAACNNNNNNNNNNNNNANATNNNTANNAGGNCNNNNCCNTCNNGNNANNNATTGCNATGACC
CANNNCACGGGGGCAGTNCCCGGCGCGGCGACCTTGNTNANNTCCGGGGACAAGTATGGCNCNCNCCC
CGGGAAGGANNCNGCCGTANGTGCNNNNNANNNNNTCNTGNGANGTCGCCGNNATTCNCGTGGTCNNN
NNAGAATGNNCNNACCANNCGNANNNTGGTNATGNNAGTANNNNNNNNNNNNNNNNNNNNNNNNNNNN
NNNNNNNNNNNNNNNNNNNNNNNNNNNNNNNNNNGNNNNGNNNNNNCNANNNAAGGNNNTANNNCTNNTN
NTNNNNNNGGACNNNACNNNNCNNNNNNNACNCNNNCNCNNNNNNNNNNNGNTTTTTNNCCNNTATTNT
CCAGNACGCNGNGCNNTCATTCANGNGAACNNNGNNACGCGNNAATCGTANNATCTACCTGCCGCNGG
NNNNNNTACNGNNNNTNCGGANNNTNNCNCGTANNNNNNTCATGCGTNNTNTNNTCNCNAGNCNNGTT
CGNNNANANANTAGNANGTNTNNNNNNNNNNTGTNNCGTTCCGCNCGANNCNNCANNNNGNNGNNCTTC
TCANCGGCNNTANCTGCNGANNTAAGNCATANNNTNNNTCNNTANCTNNNNNGGNGGGNNACTGTAAA
NGGATAATTNGNNNNTNANTTCNNNCACNTNNCNNNANNNNCTNNNTNCCTGNNTNCNNAATTGGNNC
CANTGNNTGNCNATCTCTNGNTTNGNNNNNNTNNNNNTNGCAANNNGGNNNGTNANANNNCNATNTAA
NNTATANNNNCNNNNNNANNNCACNANNNNNNNAGNNACNANNNTTANANNNGTAGNNNAATAAGTCT
CCGANNTNNNNNNNATAAGCGNNANACNNNNNNNNNNNNNNANGANNACGNNTTGGNNNTNNNNNNCNA
GNNAGACNNTNGTGGNTNNNNNTCAANNNNTTNGTAGTGGTATAATNGNNNNNNNTTTCNCCAGNNTN
NNNGNNNNNNNNCNATANNANNNNNNNGTGANNNNNNNNNNNANNNNCTTNNNAGGGANCANTTACTNNA
GCAATGGNGCGNAGNNNGAGANNNNGACNNNCCTTGGGTGGCAANCACNGCAATAAGTNNNGTTNANN

CTNNNAGTNNACACNNNNNCANNGNNNCNNNNNNANCNGANNGNNNNNNNNNNNNNNNNNNNNNNNNN
NNNNNNNNNNNNNNNNNNNNNNNNNNNNANNNNANNGNNNGTNTNTGACNCATGTGTTTTTANNNGNN
NNNNNACGNNNNANCCCCGGTCGNNNNCNNATTGAANNCCTGNNGCATNNNNNTNCNAGAATGANNNC
NNNNNGNNTNGNCTNNCNNTTCAAGNAATCANNNAGNNNTCGCGNTCNNNGNNNNNNNNCNNTCCCNN
ACTGAGGGTNNNNNNCGCATCTGCTNNNNNNNNNTNCGCNGGNGTNNCNNNNNAGCNGTATNGTGTNN
NNCANNNNNNNNNNNNNNNNTNAANNAANNNNNNTNNNN
>SiLa451
GTGNNNNNTAGCCCNNNNNAGTTAGACANNNCNNNNNNNNNNNNTNNNNNNNNNNNNNNNNNNNNNNN
NNNNNNNNNNNNNNNNNNNNNNNNNNNNNNTNNNNNNNNNNNNNNNNNNNNNNNNNNNNNNNNNNNNNN
NNNNNNNNNNNNNNNNNNNNNNNNNNNNNNNNNNNNNNNNNNNNNNNNNNNNNNNNNNNNNNNNNNNN
NNNNNNNNNNNNNNNNNNNNNNNNNNNNNNNNNNNNNNNNNNNNNNNNNNNNNNNNNNNNNNNNNNNN
NNNNNNNNNNNNNNNNNNNNNNNNNNNNNNNNNNNNNNNNNNNNNNNNNNCNTCTNNNAAANNGTNNN
NNNNNNNNNNNNNNNNNNNNNNNNNCNCNTTGNNNGTAANCNANNNGNACATGCAGTCCCCTCNNNNN
NNNGGCAGAGNNNNNNCCNNNNNNCNCCGNCCCCGNCCNNNNNNNTNNNNNNNGNNNNNNNANNNNNN
NNNNNNTGGTACNNNNCGNNNNNGGTTGTNTAAGAGGTCCACCACGNGGTCNGGNCACNNNNGNNNAN
NNNCNNNNANCNNNNNNNNNNANATNNNTANNAGGNNNNNNCCNTNNNNNNNNNNNATNGCNATGACC
CAANNCACGGNGGCAGTCCCCGGCGCGGCGACCTTGATNACGTCCGGGGNCAAGTATGGCGNNCNCCC
CGGGAAGGANNCNGCCGTANGTGCNNNNNANNNNNTCNTGNGACGTCGCCGNNATTCNCGTGGTCNNN
CNAGAATGNCCGNACCANNCGNANNNTGGTNATGNTAGTNNNNNNNNNNNNNNNNNNNNNNNNNNNNN
NNNNNNNNNNNNNNNNNNNNNNNNNNNNNNNNNNNNNGNNNNNNCCANNNAAGGANNTANNNCTNNTN
NTNNNGNNGGACNNNACNNNNCNNNNNNNACNCNNNCNCNNNNNNNNNNGNTTTNTNNNCNNTATTNT
CCAGNACGCNGNGCNNNCATTCANGNGAACNNNGNNACGCGNNAATCGTANNATCTACCTGCCGCNNG
TNNNNNTACNGNNNNTNCGGANNNTNNNNCGTANNNNNNTTATACGTNNTNTNNTCNCNAGNCANGTT
CGNNNANANANTAGNANGTNTNNNNNNNCTTGTNNCGTTCCGCNTGANNCNNCANNNNGNNGNNNTTC
TCANCGGCNNTANCTGCNGANNTAAGNCATANNNTNNATCNNTANCTNNNNNGGAGGGNNACTGTAAA
NGGNTAATTNGNGGNTNANTTCNNNCACNTNNCTNNGNNTNCTNNNTNCCTGNNTNCNNAATTGGNNC
CANTGNNTGNCNATCTCTGGNTTNGNNNNNNTNNNNNTNNCAANNNGGNNNNNNANANNNCNATNTAA
NNTATANNNNCNNNNNNANNNCACNANNNNNGNAGNNNCNANNNTTANANNNGTAGNNNANTAAGTCT
CCGANNTNNNNNNNATAAGCGNNANACNNNNNNNNNNNNNANGANNACGNNTTGGNNNTNNNNNNCNA
GNNAGACNNTNGTGGNTNNNNNTCAANNNNTTCGTAGTGGTATAATNGNNNNNNNTTTCNCCAGNNTN
NNNGNNNNNNNCNATANNANNNNNNNGNGANNNNNNNNNNANNNNCTCNNNAGGGANCANCTACTNNA
GCANTGGNGCGCAGNNNGAGANNNNGACNNNCCTTGGGTGGCAANCACNGCAATAAGTNNNGTTNANC
CTNNNAGTNNACACNNNNNCANNGNNNCNNNNNNANCNGANNGNNNNNNNNNNNNNNNNNNNNNNNNN
NNNNNNNNNNNNNNNNNNNNNNNNNNNNNGNNNANGGNNNGTNTNTGACNCATGTGTTTTTANNNGNN
NNNNNACGNNNGANCCCCGGTCGTNNNCNNATTGAANNCCTGNNGCATNNNNNTNCNAGAATGANNGC
NNNNNGNNTNGNCTNNCTNNTCAAGNAGTCANNNAGNTATNGCGNTCNNNGNNNNNNNNCNNNCCCNN
ACTGAGGGTNNNNNNCGCATCTGCTNNNNNNNNNTTCGCCGGNGTNNCTNNNNAGCNGTATNNTGNNA
NNCANNNNNNNNNNNNNNNNTNAAACAANNNNNNTNNNN
>SiLa452
GTGNNNNNTAGCCCNNNNNAGTTAGACANNNCNNNNNNNNNNNNTNNNNNNNNNNNNNNNNNNNNNNN
NNNNNNNNNNNNNNNNNNNNNNNNNNNNNNNNNNNNNNNNNNNNNNNNNNNNNNNNNNNNNNNNNNNN
NNNNNNNNNNNNNNNNNNNNNNNNNNNNNNNNNNNNNNNNNNNNNNNNNNNNNNNNNNNNNNNNNNNN
NNNNNNNNNNNNNNNNNNNNNNNNNNNNNNNNNNNNNNNNNNNNNNNNNNNNNNNNNNNNNNNNNNNN
NNNNNNNNNNNNNNNNNNNNNNNNNNNNNNNNNNNNNNNNNNNNNNNNNNNNTCTNNNAAANNNTNNN
NNNNNNNNNNNNNNNNNNNNNNNNNCNCNTTGNNNGTAANCNANNNGNACATGCAGTCCCCTCNNNNN
NNNNNCAGAGNNNNNNCCNNNNNNNNCCGNCCCCNNCCNNNNNNNNNNNCNNNGNNNNNNNANNNNNN
NNNNNNTGGTACNNNNCGNNNNNGGTTGTTTAAGAGGTCCACAACGNGGTCNNGNNACNNNNGNNNAN
NNNCNNNNAANNNNNNNNNNNANNTNNNTANNAGGNCNNNNCCNTNNNNNNNNNNNATTGCNATGACC
CAANNCACGGGGGCAGTNCCCGGCGCGGCGACCTTGNTNANNTCCGGGGACAAGTATGGCGCNCNCCC
CGGGAAGGANNCNGCCGTANGTGCNNNNNANNNNNTCNTGNGANGTNGCCGNNATTCNCGTGGTCNNN
CNAGAATGNNCNNACCANNCGNANNNTGGTNATGNNAGTANNNNNNNNNNNNNNNNNNNNNNNNNNNN
NNNNNNNNNNNNNNNNNNNNNNNNNNNNNNNNNNNNNGNNNNNNCNANNNAAGGANNTANNNCTNNTN
NTNNNNNNGGACNNNACNNNNCNNNNNNNACNCNNNCNCNNNNNNANNNGNTTTTTNNNCNNTATTNT
CCAGNACGCNGNGCNNNCATTCANGNGAACNNNGNNACGCGNNAATCGTANNATCTACCTGCCGCNGG

TNNNNNTACNGNNNNTNCGGANNNTNNNNCGTANNNNNNNTCATGCGTNNTNTNNTCNCNAGNCNNGTT
CGNNNANANANTAGNANGTNTNNNNNNNNNNTGTNNCGTTCCGCNTGANNCNNCANNNNGNNGNNNTTC
TCANCGGCNNTANCTGCNGANNTAAGNCATANNNTNNNTCNNTANCTNNNNNGGNGGGNNACTGTAAA
NGGNTAATTNGNNNNTNANTNCNNNCACNTNNCTNNGNNNNCTNNNTNCCTGNNTNCNNAATTGGNNC
CANTGNNTGNCNATCTCTGGNTTNGNNNNNNTNNNNNTNNCAANNNGGNNNNNNANANNNCNATNTAA
NNTATANNNNCNNNNNNANNNCACNANNNNNNNAGNNNCNANNNTTANANNNGTAGNNNAATAAGTCT
CCGANNTNNNNNNNATAAGCGNNANACNNNNNNNNNNNNNNANGANNNCGNNTTGGNNNTNNNNNNCNA
GNNAGACNNTNGTGGNTNNNNNTCAANNNNTTNGTAGTGGTATAATNGNNNNNNNTTTCNCCAGNNTN
NNNGNNNNNNNNCNATANNANNNNNNNGNGANNNNNNNNNNNANNNNCTCNNNAGGGANCANCTACTTNA
GCANTGGNGCGNAGNNNGAGANNNNGACNNGCCTTGGGTGGCAANCACNGCAATAAGTNNNGTTNNNC
CTNNNAGTNNACACNNNNNCANNGNNNCNNNNNNANCNGANNGNNNNNNNNNNNNNNNNNNNNNNNNN
NNNNNNNNNNNNNNNNNNNNNNNNNNNNNNGNNNANNGNNNGTNTNTGACNCATGTGTTTTTANNNGNN
NNNNNACGNNNNANCCCCGGTCGNNNNCNNATTGAANNCCTGNNGCATNNNNNTNCNAGAATGANNGC
NNNNNGNNTNGNCTGNCTNNTCAAGNAATCANNNAGNTNNCGCGNTCNNNNNNNNNNNNCNNTCCCNN
ACTGAGGGTNNNNNNNNCATCTGCTNNNNNNNNNTNCGCNGGCGTNNCNGNNNAGCNGTATNGTGNNN
NNCANNNNNNNNNNNNNNNNNTNAANCNANNNNNNNTNNNN
>SiLa455
GTGNNNNNTAGCCCNNNNNAGTTAGACANNNCNNNNNNNNNNNNNTNNNNNNNNNNNNNNNNNNNNNNN
NNNNNNNNNNNNNNNNNNNNNNNNNNNNNNNNNNNNNNNNNNNNNNNNNNNNNNNNNNNNNNNNNNNNN
NNNNNNNNNNNNNNNNNNNNNNNNNNNNNNNNNNNNNNNNNNNNNNNNNNNNNNNNNNNNNNNNNNNNN
NNNNNNNNNNNNNNNNNNNNNNNNNNNNNNNNNNNNNNNNNNNNNNNNNNNNNNNNNNNNNNNNNNNNN
NNNNNNNNNNNNNNNNNNNNNNNNNNNNNNNNNNNNNNNNNNNNNNNNNNNNCNTCTNNNAAANNNTNNN
NNNNNNNNNNNNNNNNNNNNNNNNNNNCNCNTTGNNNGTAANCNANNNGNACATGCAGTCCCCTCNNNNN
NNNGGCAGAGNNNNNNNCCNNNNNNNCCGGCCCCNNCCNNNNNNNNNNNNCNNNGNNNNNNNANNNNNN
NNNNNNTGGTACNNNNCGNNNNNGGTTGTTTAAGAGGTCCACAACGNGGTCNGGNCACNNNNGNNTAN
NNNCNNNAAACNNNNNNNNNNNNNANNTNNNTANNAGGNCNNNNCCNTCNNNNNNNNNAANTGCNATGACC
CAANNCACGGGGGCAGTCCCCGGCGCGGCGACCTTGATNACGTCCGGGGACAAGTATGGCNNNCNCCC
CGGGAAGGANNCNGCCGTANGTGCNNNNNANNNNNTCNTGNGANGTCGCCGNNATTCNCGTGGTCNNN
NNAGAATGNCCNNACCANNCGNANNNTGGTNATGNTAGTNNNNNNNNNNNNNNNNNNNNNNNNNNNNN
NNNNNNNNNNNNNNNNNNNNNNNNNNNNNNNNNNNNNNNNNGNNNNNNCNANNNAAGGANNTANNNCTNNTN
NTNNNNNNNGGACNNNACNNNNCNNNNNNNACNCNNNCNCNNNNNNANNNGNTTTTTNNNCNNTANTNT
CCAGNACGCNGNGCNNNCATTCANGNGAACNNNGNNACGCGNNAATCGTANNATCTACCTGCCGCNNG
TNNNNNTACNGNNNNNNNCGGANNNTNNNNCGTANNNNNNNNCATGCGTNNTNTNNTCNCNAGNCNNGTT
CGNNNANANANTAGNANGTNTCNNNNNNNNNTGTNNCGTTCCGCNTGANNCNNCANNNNGNNGNNNTTN
TCANCGGCNNTANCTGCNGANNTAAGNCATANNNTNNATCNNTANCTNNNNNGGNGGGNNACTGTAAA
NGGNNAATTNGNNNNTNATTNCCNNCACNTNNCTNNGNNNNCTNNNTNCCTGNNTNCNNAATTGGGNC
CANTGNNTGNCNATCTCTGGNTTNGNNNNNNTGNNNNTNNCAANNNGGNNNGTNANANNNCNATNTAA
NNTANANNNNCNNNNNNANNNCACNNNNNNNNNNAGNNNCNANNNTTANANNNGTAGNNNANTAAGTCT
CCGANNTNNNNNNNATAAGCGNNANACNNNNNNNNNNNNNNANGANNACGNNTTGGNNNTNNNNNNCNA
GNNAGACNNTNGTGGNTNNNNNTCAANNNNTTCGTAGTGGTATAATNGNNNNNNNTTTCNCCAGNNTN
NNNGNNNNNNNNCNATANNANNNNNNNGNGANNNNNNNNNNNANNNNCTCNNNAGGNANCANCTACTTNA
GCANTGGNGCGCAGNNNGAGANNNNGACNNNCCTTGGGTGGCAANCACNGCAATAAGTNNNGTTNANC
CTNNNAGTNNACACNNNNNCANNGNNNCNNNNNNANCNGANNGNNNNNNNNNNNNNNNNNNNNNNNNN
NNNNNNNNNNNNNNNNNNNNNNNNNNNNNNAGNNNANGGNNNGTNTNTGACNCATGTGTTTTTANNNGNN
NNNNNACGNNNGANCCCCGGTCGNNNNCNNATTGAANNCCTGNNGCATNNNNNTNCNAGAATGANNGC
NNNNNGNNTNGNCTNNCNTTTCAAGNAATCANNTAGGTANNNCGNTCNNNGNNNNNNNNCNNNCCCNN
ACTGAGGGTNNNNNNNNCATCTGCTNNNNNNNNNCTTCGCNGGNGTNNCNNNNNAGCNGTATNGTGNNN
NNCANNNNNNNNNNNNNNNNNNNNNAACAANNNNNNNTNNNN
>SiLa456
GTGNNNNNTAGCCCNNNNNAGTTAGACANNNCNNNNNNNNNNNNNTNNNNNNNNNNNNNNNNNNNNNNN
NNNNNNNNNNNNNNNNNNNNNNNNNNNNNNNNNNNNNNNNNNNNNNNNNNNNNNNNNNNNNNNNNNNNN
NNNNNNNNNNNNNNNNNNNNNNNNNNNNNNNNNNNNNNNNNNNNNNNNNNNNNNNNNNNNNNNNNNNNN
NNNNNNNNNNNNNNNNNNNNNNNNNNNNNNNNNNNNNNNNNNNNNNNNNNNNNNNNNNNNNNNNNNNNN
NNNNNNNNNNNNNNNNNNNNNNNNNNNNNNNNNNNNNNNNNNNNNNNNNNNNNTCTNNNAAANNNTNNN

NNNNNNNNNNNNNNNNNNNNNNNNNNCNCNTTGNNNGTAANCNANNNGNACATGCAGTCCCCTCNNNNN
NNNNNCAGAGNNNNNNCCCNNNNNNNCCGGCCCCNNCCNNNNNNNNNNNNNNNNGNNNNNNNANNNNNN
NNNNNNTGGTACNNNNCGNNNNNGGTTGTTTAAGAGGTCCACAACGNGGTCNNGNCACNGNNGNNNAN
NNNCNNNNANCNGNNNNNNNNNNNNNTNNNTANNAGGNCNNNGCCNTCNNGNNNNNNATTGCNATGACC
CAANNCACGGGGGNAGTNCCCGGCGCGGCGACCTTGATNACGTCCGGGGACAAGTATGGCNNNCNCCC
CGGGAAGGANNCNGCCGTANGTGCNNNNNANNNNNTCNTGNGANGTNGCCGNNATTCNCGTGGTCNNN
CNAGAATGNCCGNACCANNCGNANNNTGGTNATGNNAGTNNNNNNNNNNNNNNNNNNNNNNNNNNNNN
NNNNNNNNNNNNNNNNNNNNNNNNNNNNNNNGNNNNNNGNNNNNNCNANNNAAGGANNTANNNCTNNTC
NTNNNNNNNGGACNNNACNNNNCNNNNNNNACNCNNNCNCNNNNNNANNNGNTTTTTNNCCNNTATTNT
CCAGNACGCNGNGCNNNCATTCANGNGAACNNNGNNACGCGNNAATCGTANNATCTACCTGCCGCNGG
TNNNNNTACNGNNNNTNCGGANNNTNNNNCGTANNNNNGTCATGCGTNNTNTNNTCNCNAGNCANGTT
CGNNNANANANTAGNANGTNTCNNNNNNCTTGTNNCGTTCCGCNTGANNCNNCANNNNGNNGNNCTTN
TCANCGGCNNTANCTGCNGANNTAAGNCATANNNTNNATCNNTANCTNNNNNGGNGGGNNACTGTAAA
NGGNNAATTNGNNGNTNATTTCNNNCACNTNNCNNNGNNTNCTNNNTNCCTGNNTNCNNAATTGGNNC
CANTGNNTGNCNATCTCTGGNTTNGNNNNNNTNNNNNTNNCAANNNGGNNNGTNANANNNCNATNTAA
NNTATANNNNCNNNNNNANNNCACNANNNNNGNAGNNACNANNNTTANAGGNGTAGNNNAATAAGTCT
CCGANNTNNNNNNNATAAGCGNNANACNNNNNNNNNNNNNANGANNACGNNTTGGNNNTNNNNNNCNA
GNNAGACNNTNGTGGNTNNNNNTCAANNNNTTNGTAGTGGTATAATNGNNNNNNNTTTCNCCAGNNTN
NNNGNNNNNNNNCNATANNANNNNNNNGTGANNNNNNNNNNNANNNNCTCNNNAGGNANCANCTACTNNA
GCANTGGNGCGCAGNNNGAGANNNNGACNAGCCTTGGGTGGCAANCACNGCAATAAGTNNNGTTNANC
CTNNNAGTNNACACNNNNNCANNGNNNCNNNNNNANCNGANNGNNNNNNNNNNNNNNNNNNNNNNNNN
NNNNNNNNNNNNNNNNNNNNNNNNNNNNNNGNNNANGGNNNGTNTNTGACNCATGTGTTTTTANNNGNN
NNNNNACGNNNNNNCCCCGGTCGNNNNCNNATTGAANNCCTGNNGCATNNNNNTNCNAGAATGANNGC
NNNNNGNNTNGNCTNNCTTTTCAAGNAATCANNNAGNTANCGCGNTCNNNGNNNNNNNNCNNTCCCNN
ACTGAGGGTNNNNNNNNCATCTGCTNNNNNNNNNCTTCGCNGGNGTNNCNGNNNAGCNGTATNGTGTNN
NNCANNNNNNNNNNNNNNNNNTNAAACNNNNNNNNNTNNNN
>SiLa457
GTGNNNNNTAGCCCNNNNNAGTTAGACANNNCNNNNNNNNNNNNNTNNNTNNNNNNNNNNNNNNNNNNN
NNNNNNNNNNNNNNNNNNNNNNNNNNNNNNNNNTNNNNNNNNNNNNNNNNNNNNNNNNNNNNNNNNNNN
NNNNNNNNNNNNNNNNNNNNNNNNNNNNNNNNNNNNNNNNNNNNNNNNNNNNNNNNNNNNNNNNNNNNN
NNNNNNNNNNNNNNNNNNNNNNNNNNNNNNNNNNNNNNNNNNNNNNNNNNNNNNNNNNNNNNNNNNNNN
NNNNNNNNNNNNNNNNNCNNNNNNNNNNNNNNNNNNNNNNNNNNNNNNNNNNNNNNNCNTCTNNNAAANNNTNNN
NNNATTNNNNNNNNNNNNNNNNNNNNCNCNTTGNNNGTAANCNANNNGNACATGCAGTCCCCTCNNNNN
NNNGGCAGAGNCNNNNNCCNNNNNNNCCGGCCCCNNCCNNNNNNNNNNNNNNNNGNNNNNNNANNNNNN
NNNNNNTGGTACNNNNCGNNNNNGGTTGTTTAAGAGGTCCACAACGNGGTCNNGNCACNNNNGNNNAN
NNNCNNAAAACNNNNNNNNNNNNNNNNTNNNTANNAGGNCNNNGCCNTCNNNNNANNAATTGCNATGANN
CAANNCACGGGGGCAGTNCCCGGCGCGGCGACCTTGATNANNTCCGGGGACAAGTATGGCGCNCNCCC
CGGGAAGGANNCNGCCGTANGTGCNNNNNANNNNNTCNTGNGANGTNGCCGNNATTCNNGTGGNCNNN
NNAGAATGNCCGNACCANNCGNANNNTGGTNATGNNAGTAANNNNNNNNNNNNNNNNNNNNNNNNNNN
NNNNNNNNNNNNNNNNNNNNNNNNNNNNNNNNNNGNNNNGNNNNNNCNANNNAAGGNNNTANNNCTNNTN
NTNNNNNNNGGACNNNACNNNNCNNNNNNNACNCNNNCNCNNNNNNNNNNNGNTTTTTNNCCNNTATTNT
CCAGNACGCNGNGCNNNCATTCANGNGAACNNNGNNACGCGNNAATCGTANNATCTACCTGCCGCNGG
TNNNNNTACNGNNNNTNCGGANNNTNNNNCGTANNNNNGTCATGCGTNNTNTNNTCNCNAGNCNNGTT
CGNNNANANANTAGNANGTNTNNNNNNNNCNTGTNNCGTTCCGCNTGANNCNNCANNNNGNNGNNNTTN
TCANCGGCNNTANCTGCNGANNTAAGTCATANNNTNNATCNNTANCTNNNNNGGNGGGNNACTGTAAA
NGGNNAATTNGAGGNTNATTTCNNNCACNTNNCTNNGNNNNCTNNNTNCCTGNNTNCNNAATTGGNNC
CANTGNNTGNCNATCTCTGGNTTNGNNNNNNTNNNNNTNNCAANNNGGNNNGTNANANNNCNATNTAA
NNTATANNNNCNNNNNNANNNCACNANNNNNGNAGNNNCNANNNTTANANNNGTAGNNNAATAAGTCT
CCGANNTNNNNNNNATAAGCGNNANACNNNNNNNNNNNNNANGANNACGNNTTGGNNNTNNNNNNCNA
GNNAGACNNTNGTGGNTNNNNNTCAANNNNTTCGTAGTGGTATAATNGNNNNNNNTTTCNCCAGNNTN
NNNGNNNNNNNNCNATANNANNNNNNNGTGANNNNNNNNNNNANNNNCTCNNNAGGGANCANCTACTTNA
GCAATGGNGCGCAGNNNGAGANNNNGACNNNCCTTGGGTGGCAANCACNGCAATAAGTNNNGTTNANC
CTNNNAGTNNACACNNNNNCANNGNNNCNNNNNNANCNGANNGNNNNNNNNNNNNNNNNNNNNNNNNN
NNNNNNNNNNNNNNNNNNNNNNNNNNNNNAGNNNANGGNNNGTNTNTGACNCATGTGTTTTTANNNGNN

NNNNNACGNNNNANCCCCGGTCGNNNNCNNATTGAANNCCTGNNGCATNNNNNTNCNAGAATGANNNC
NNNNNGNNTGNNCTNNCTTTTCAAGNAATCAANNAGNNATNNCGNTCNNNGANNNNNNNCNNTCCCNN
ACTGAGGGTNNNNNNNNCATCTGCTNNNNNNNNNTTCGCNGGCGTNNCNNNNNAGCCGTATNGTGNNN
NNCANNNNNNNNNNNNNNNNNTNAANNNANNNNNNTNNNN
>Tay103
GTGNNNNNTAGCCCNNNNNAGTTAGACANNNCNNNNNNNNNNNNTNNNNNNNNNNNNNNNNNNNNNNN
NNNNNNNNNNNNNNNNNNNNNNNNNNNNNNNNNNNNNNNNNNNNNNNNNNNNNNNNNNNNNNNNNNNNNN
NNNNNNNNNNCNNNNNNNNNNNNNNNNNNNNNNNNNNNNNNNNNNNNNNNNNNNNNNNNNNNNNNNNNNN
NNNNNNNNNNNNNNNNNNNNNNNNNNNNNNNNNNNNNNNNNNNNNNNNNNNNNNNNNNNNNNNNNNNNNN
NNNNNNNNNNNNNNNNNNNNNNNNNNNNNNNNNNNNNNNNNNNNNNNNNNNNNCNTCTNNNAAANNNTNNN
NNNNNNNNNNNNNNNNNNNNNNNNNNCNCNTTGNNNGTAANCNANNNGNACATGCAGTCCCCTCNNNNN
NNNGGCAGAGNNNNNNNCCNNNNNNNNCCGGCCCCNNCCNNNNNNNNNNNNCNNNGNNNNNNNANNNNNN
NNNNNNTGGTACNNNNCGNNNNNGGTTGTNTAAGAGGTCCACAACGNGGTCNNGNCACNNNNGNNNAN
NNNCNNNNNNNCNNNNNNNNNNNNNNNTNNNTANNAGGNCNNNNCCNTCNNGNNNNNAATTGCNATGANN
CAANNCACNGNGGCNGTNCCCGGCGCGGCGACCTTGATNACGTCCGGGGACAAGTATGGCNCNCNCCC
CGGGAAGGANNCNGCCGTANGTGCNNNNNANNNNNTCNTGNGACGTCGCCGNNATTCNCGTGGTCNNN
CNAGAATGNCCGNACCANNCGNANNNTGGTNATGNTAGTNNNNNNNNNNNNNNNNNNNNNNNNNNNNN
NNNNNNNNNNNNNNNNNNNNNNNNNNNNNNNNGNNNNGNNNNNNCNANNNAAGGANNTANNNCTNNTN
NTNNNNNNGGACNNNACNNNNCNNNNNNNACNCNNNCNCNNNNNNNNNNGNTTTTNNNCCNNTATTNT
CCAGNACGCNGNGCNNNCATTCANGNGAACNNNGNNACGCGNNAATCGTANNATCTACCTGCCGCNGG
TNNNNNTACNGNNNNTNCGGANNNTNNCTCGTANNNNNGTCATGCGTNNTNTNNTCNCNAGNCNNGTT
CGNNNANANANTAGNANGTNTCCNNNNNNNTGTNNCGTTCCGCNTGACNCNNCANNNNGNNGNNCTTN
TCANCGGCNNTANCTGCNGANNTAAGTCATANNNTNNNTCNNTANCTNNNNNGGNGGGNNACTGTAAA
NGGNNAATTNGNGGNTNATTTCNNNCACNTNNCTNNGNNTNCTNNNTNCCTGNNTNCNNAATTGGGNC
CANTGNNTGNCNATCTCTGGNTTNGNNNNNNTGNNNNTNNCAANNNGGNNNGTNANANNNCNATNTAA
NNTATANNNNCNNNNNNANNNCACNANNNNNGNAGNNNCNANNNTTANANNNGTAGNNNAATAAGTCT
CCGANNTNNNNNNNATAAGCGNNANACNNNNNNNNNNNNNANGANNACGNNTTGGNNNTNNNNNGCNA
GNNAGACNNTNATGGNTNNNNNTCAANNNNTTCGTAGTGGTATAATNGNNNNNNNTTTCNCCAGNNTN
NNNGNNNNNNNCNATANTANNNNNNNGTGANNNNNNNNNNNANNNNCTCNNNAGGGANCANCTACTTNA
GCANTGGNGCGCAGNNNGAGANNNNGACCNNCCTTGGGTGGCAANCACNGCAATAAGTNNNGTTNANC
CTNNNAGTNNACATGNNNNCANNGNNNCNNNNNNANCNGANNGNNNNNNNNNNNNNNNNNNNNNNNNNN
NNNNNNNNNNNNNNNNNNNNNNNNNNNNNAGNNNANGGNNNGTNTNTGACNCATGTGTTTTTANNNGNN
NNNNNACGNNNGANCCCCGGTCGNNNNCNNATTGAANNCCTGNNGCATNNNNNTNCNAGAATGANNGC
NNNNNGNNTNGNCTNNCTTTTCAAGNAATCANNNAGGTNNCGCGNTCNNNNNNNNNNNNCNNTCCCNN
ACTGAGGGTNNNNNNCGCATCTGCTNNNNNNNNNNTTCGCNGGNGTNNCTNCNNAGCCGTATNGTGTTA
NNCANNNNNNNNNANNNNNNNNTNAAACNANNNNNNNTNNNN
>Tay129
GTGNNNNNTAGCCCGNNNNAGTTAGACANNNCNNNNNNNNNNNNTNNNNNNNNNNNNNNNNNNNNNNN
NNNNNNNNNNNNNNNNNNNNNNNNNNNNNNNNNNNNNNNNNNNNNNNNNNNNNNNNNNNNNNNNNNNNNN
NNNNNNNNNNNNNNNNNNNNNNNNNNNNNNNNNNNNNNNNNNNNNNNNNNNNNNNNNNNNNNNNNNNNNN
NNNNNNNNNNNNNNNNNNNNNNNNNNNNNNNNNNNNNNNNNNNNNNNNNNNNNNNNNNNNNNNNNNNNNN
NNNNNNNNNNNNNNNNNNNNNNNNNNNNNNNNNNNNNNNNNNNNNNNNNNNNNNTCTNNNAAANNNTNNN
NNNNNNNNNNNNNNNNNNNNNNNNNNCNCNTTGNNNGTAANCNANNNGNACATGCAGTCCCCTCNNNNN
NNNNNCAGAGNNNNNNNCCCNNNNNNNNCCGGCCCCNNCCNNNNNNNNTNNNCNNNGNNNNNNNANNNNNN
NNNNNNTGGTACNNNNCGNNNNNGGTTGTTTNAGAGGTCCACAACGNGGTCNGGNCACNNNNGNNNAN
NNNCNNNNAACNNNNNNNNNNNNNANNTNNNTANNAGGNCNNNNCCNTNNNGNNNNNNATTGCNATGACC
CAANNCACGGGGGCAGTNCCCGGCGNGGCGACCTTGATNANGTCCGGGGACAAGTATGGCGCTCNCCC
CGGGAAGGANNCNGCCGTANGTGCNNNNNANNNNNTCNTGNGACGTCGCCGNNATTCNCGTGGTCNNN
CNAGAATGNNCGNACCANNCGNANNNTGGTNATGTTAGTNNNNNNNNNNNNNNNNNNNNNNNNNNNNN
NNNNNNNNNNNNNNNNNNNNNNNNNNNNNNNNGNNNNGNNNNNNCNANNNAAGGANNTANNNCTNNTC
NTNNNNNNGGACNNNACNNNNCNNNNNNNACNCNNNCNCNNNNNNNNNNGNTTTTTNNCCNNTATTNT
CCAGNACGCNGNGCNNNCATTCANGNGAACNNNGNNACGCGTAAATCGTANNATCTACCTGCCGCNGG
TNNNNNTACNGNNNNTNCGGANNNTNNCNCGTANNNNNGTCATGCGTNNTNTNNTCNCNAGNCANGTT
CGNNNANANANTAGNANGTNTCCNNNNNNNTGTNNCGTTCCGCNTGANNCNNCANNNNGNNGNNNTTC

TCANCGGCNNTANCTGCNGANNTAAGNCATANNNTNNATCNNTANCTNNNNNGGAGGGNNACTGTAAA
NGGATAATTNGNNGNTNATTTCNNNCACNTNNCTNNGNNTNCTNNNTNCCTGNNTNCNNAATTGGNNC
CANTGNNTGNCNATCTCTGGNTTNGNNNNNNTNNNNNTNGCAANNNGGNNNGTNANANNNCNATNTAA
NNTANANNNNCNNNNNNANNNCACNANNNNNGNAGNNACNANNNTTANANNNGTAGNNNAATAAGTCT
CCGANNTNNNNNNNATAAGCGNNANACNNNNNNNNNNNNNNANGANNACGNNTTGGNNNTNNNNNNCNA
GNNAGACNNTNATGGNTNNNNNTCAANNNNTTCGTAGTGGTATAATNGNNNNNNNTTTCNCCAGNNTN
NNNGNNNNNNNCNATANNANNNNNNNGTGANNNANNNNNNANNNNCTCNNNAGGGANCANCTACTNNA
GCANTGGNGCGCAGNNNGAGANNNNGACNAGCCTTGGGTGGCAANCACNGCAATAAGTNNTGTTNANC
CTNNNAGTNNACATNNNNNCANNGNNNCNNNNNNANCNGANNGNNNNNNNNNNNNTNNNNNNNNNNN
NNNNNNNNNNNNNNNNNNNNNNNNNNNNNAGNNNANGGNNNGTNTNTGACNCATGTGTTTTTANNNGNN
NNNNNNCGNNNNANCCCCGGTCGNNTNCNNATTGAANNCCTGNNGCATNNNANTNCNAGAATGANNGC
NNNNNGNNTNNNCTGNCNNNTCAAGNAATCANNNAGGTNTCGCGNTCNNNGNNNNNNNNCNNTCCCNN
ACTGAGGGTNNNNNNCGCATCTGCTNNNNNNNNNCTTCGCNGGCGTNNCTNNNNAGCCGTATNGTGTTA
NNCANNNNNNNNNNNNNNNNNNTNNAACNANNNNNNNTNNNN
>Tay135
GTGNNNNNTAGCCCGNNNNAGTTAGACANNNCNNNNNNNNNNNNTNNNNNNNNNNNNNNNNNNNNNNN
NNNNNNNNNNNNNNNNNNNNNNNNNNNNNNNNNNNNNNNNNNNNNNNNNNNNNNNNNNNNNNNNNNNNNN
NNNNNNNNNNNNNNNNNNNNNNNNNNNNNNNNNNNNNNNNNNNNNNNNNNNNNNNNNNNNNNNNNNNNNN
NNNNNNNNNNNNNNNNNNNNNNNNNNNNNNNNNNNNNNNNNNNNNNNNNNNNNNNNNNNNNNNNNNNNNN
NNNNNNNNNNNNNNNNNNNNNNNNNNNNNNNNNNNNNNNNNNNNNNNNNNNNNNNTCTNNNAAANNNTNNN
NNNNNNNNNNNNNNNNNNNNNNNNNNCNCNTTGNNNGTAANCNANNNGNACATGCAGTCCCCTCNNNNN
NNNGGCAGAGNNNNNNCCCNNNNNNNCCGGCCCCGNCCNNNNNNNTNNNNNNNGNNNNNNNANNNNNN
NNNNNNTGGTACNNNNCGNNNNNGGTTGTTTAAGAGGTCCACAACGNGGTCNGGNCACNNNNGNNNAN
NNNCNNAAAACNNNNNNNNNNNAANNTNNNTANNAGGNNNNNGCCNTCNNGNNNNTAATNGCNATGACC
CAANTCACGGGGGCAGTNCCCGGCGCGGCGACCTTGNTNACGTCCGGGGACAAGTATGGCNCNCNCCC
CGGGAAGGANNCNGCCGTANGTGCNNNNNANNNNNTCCTGNGANGTCGCCGNNATTCNCGTGGTCTNN
CNAGAATGNCCGNACCANNCGNANNNTGGTNATGTTAGTANNNNNNNNNNNNNNNNNNNNNNNNNNN
NNNNNNNNNNNNNNNNNNNNNNNNNNNNNNNNNNNNNNNNGNNNNNNCNANNNAAGGANNTANNNCTNNTC
NTNNNNNNNGGACNNNACNNNNCNNNNNNNACNCNNNCNCNNNNNNANNNGNTTTTTTNNCNNTATTNT
CCAGNACGCNGNGCNNTCATTCANGNGAACNNNGNNACGCGNNAATCGTANNATCTACCTGCCGCNGG
TNNNNNTNCNGNNNNTNCGGANNNTNNCTCGTANNNNNGTCATGCGTNNTNTNNTCNCNAGNCANGTT
CGAGAANANANTAGNANGTNTCNNNNNNNNNTGTNNCGTTCCGCNTGACNCNNCANNNNGNNGNNCTTN
TCANCGGCNCTANCTGCNGANNTAAGTCATANNNTNNNTCNNTANCTNNNNNGGAGGGNNACTGTAAA
NGGNTAATTGGNNGNTNANTTCNNNCACNTNNCTNNGNNTNCTNNNTNCCTGNNTNCNNAATTGGNNC
CANTGNNTGNCNATCTCTGGNTTNGCNNNNNTNNNNNTNGCAANNNGGNNNGTNANANNNCNATNTAA
NNTATANNNNCNNNNNNANNNCACNANNNNNNNNAGNNNCNANNNTTANANNNGTAGNNNAATAAGTCT
CCGANNTNNNNNNNATAAGCGNNANACNNNNNNNNNNNNNNANGANNACGNNTTGGNNNTNNNNNNCNA
GNNAGACNNTNGTGGNTNNNNNTCAANNNNTTCGTAGTGGTATAATNGNNNNNNNTTTCNCCAGNNTN
NNNGNNNNNNNCNATANNANNNNNNNGTGANNNNNNNNNNNANNNNCTCNNNAGGGANCANCTACTTNA
GCAATGGNGCGCAGNNNGAGANNNNGACNNGCCTTGGGTGGCAANCACNGCAATAAGTNNNGTTNANC
CTNNNAGTNNACACGNNNNCANNGNNNCNNNNNNANCNGANNGNNNNNNNNNNNNNNNNNNNNNNNNN
NNNNNNNNNNNNNNNNNNNNNNNNNNNNNAGNNNANGGNNNGTNTNTGACNCATGTGTTTTTANNNGNN
NNNNNACGNNNGANCCCCGGTCGNNNNCNNATTGAANNCCTGNNGCATNNNNNTNCNAGAATGANNGC
NNNNNGNNTNGNCTGNCNTNTCAAGNAATCAANTAGGTANCGCGNTCNNNNNNNNNNNNNCNNTCCCNN
ACTGAGGGTNNNNNNCGCATCTGCTNNNNNNNNNNTNCGCNGGNGTNNCNNNNNAGCNGTATNGNGTNA
NNCANNNNNNNNNNNNNNNNNCNNANNNNNNNNNNNNTNNNN
>Tay138
GTGNNNNNTAGCCCNNNNNAGTTAGACANNNCNNNNNNNNNNNNTNNNNNNNNNNNNNNNNNNNNNNN
NNNNNNNNNNNNNNNNNNNNNNNNNNNNNNNNNNNNNNNNNNNNNNNNNNNNNNNNNNNNNNNNNNNNNN
NNNNNNNNNNNNNNNNNNNNNNNNNNNNNNNNNNNNNNNNNNNNNNNNNNNNNNNNNNNNNNNNNNNNNN
NNNNNNNNNNNNNNNNNNNNNNNNNNNNNNNNNNNNNCNNNNNNNNNNNNNNNNNNNNNNNNNNNNNNNN
NNNNNNNNNNNNNNNNNNNNNNNNNNNNNNNNNNNNNNNNNNNNNNNNNNNNNNNCNTCTNNNAAANNNTNNN
NNNNNNNNNNNNNNNNNNNNNNNNNNCNCNTTGNNNGTAANCNANNNGNACATGCAGTCCCCTCNNNNN
NNNGGCAGAGNNNNNNCCCNNNNNNNCCGNCCCCGNCCNNNNNNNTNNNNNNNGNNNNNNNANNNNNN

NNNNNNTGGTACNNNNCGNNNNNGGTTGTTTAAGAGGTCCACAACGNGGTCNNGNCACNNNNGNNNAN
NNNCNNNNNACNNNNNNNNNNNNANATNNNTANNAGGNCNNNNCCNTCNNNNNNNNNNATNGCNATGANN
CAANTNACGGNGGNAGTNCCCGGCGNGGCGACCTTGNTNACGTCCGGGGACAAGTATGGCGCNCNCCC
CGGGAAGGANNCNGCCGTANGTGCNNNNNANNNTNTCTTGNGANGTNGCCGNNATTNNNNTGGTCNNN
CNAGANTGNNCGNACCANNCGNANNNTGGTNATGNNAGTNNNNNNNNNNNNNNNNNNNNNNNNNNNNNN
NNNNNNNNNNNNNNNNNNNNNNNNNNNNNNNNNNNGNNNNGNNNNNNCNANNNAAGGNNNTANNNCTNNTN
NTNNNNNNGGACNNNACNNNNCNNNNNNNACNCNNNCNCNNNNNNNNNNNGNTTTTTNNNCNNTANTNT
CCAGNANGCNGNGCNNNCATTCANGNGAACNNNGNNACGCGNNAATCGTANNATCTACCTGCCGCNNG
TNNNNNTACNGNNNNNNCGGANNNTNNNNCGTANNNNNNTCATGCGTNNTNTNNTCNCNAGNCNNGTT
CGNNNATANANTAGNANGTNTNNNNNNNNNNTGTNNCGTTCCGCNTGANNCNNCANNNNGNNGNNCTTN
NCANCGGCNNTANCTGCNGANNTAAGNCATANNNTNNNTCNNTANCTNNNNNGGNGGGNNACTGTAAA
NGGNNAATTNGNNGNTNANTTCNNNCACNTNNCTNNGNNNNCTNNNTNCCTGNNTNCNNAATTGGNNC
CANTGNNTGNCNATCTCTNGNTTNGNNNNNNTNNNNNTNNCAANNNGGNNNNNNANANNNCNATNTAA
NNTATANNNNCNNNNNNANNNCACNNNNNNNNNNAGNNNNNANNNTTANANNNGTAGNNNAATAAGTCT
CCGANNTNNNNNNNATAAGCGNNANACNNNNNNNNNNNNNNANGANNACGNNTTGGNNNTNNNNNNNNA
GNNAGACNNTNATGGNTNNNNNTCAANNNNTTNGTAGTGGTATAATNGNNNNNNNTTTCNCCAGNNTN
NNNGNNNNNNNNCNATANNANNNNNNNGNGANNNNNNNNNNNANNNNCTCNNNAGGGANCANCTACTTNA
GCANTGGNGCGCAGNNNGAGANNNNGACNNNCCTTGGGTGGCANNCACNGCAATAAGTNNNGTTNANC
CTNNNAGTNNACATNNNNNCANNGNNNCNNNNNNANCNGANNGNNNNNNNNNNNNNNNNNNNNNNNNNN
NNNNNNNNNNNNNNNNNNNNNNNNNNNNNNANNNNANNGNNNGTNTNTGACNCATNNNTTTTTANNNGNN
NNNNNACGNNNNANCCCCGGTCGNNNNCNNATTGAANNCCTGNNGCATNNNNNTNCNAGAATGANNNC
NNNNNGNNTNGNCTNNCNTNTCAAGNAATCANNNAGGTNNCGCGNTCNNNGNNNNNNNNCNNTCCCNN
ACTGAGGGTNNNNNNNNCATCTGCTNNNNNNNNCTNCGCNGGNGTNNCNNNNNAGCNGTATNNTGTNN
NNCANNNNNNNNNNNNNNNNNTNAAACNANNNNNNNTNNNN
>Tay155
GTGNNNNNTAGCCCNNNNNAGTTAGACANNNCNNNNNNNNNNNNNTNNNNNNNNNNNNNNNNNNNNNNN
NNNNNNNNNNNNNNNNNNNNNNNNNNNNNNNNNNNNNNNNNNNNNNNNNNNNNNNNNNNNNNNNNNNNNN
NNTNNNNNNNNNNNNNNNNNNNNNNNNNNNNNNNNNNNNNNNNNNNNNNNNNNNNNNNNNNNNNNNNNN
NNNNNNNNNNNNNNNNNNNNNNNNNNNNNNNNNNNNNNNNNNNNNNNNNNNNNNNNNNNNNNNNNNNNNN
NNNNNNNNNNNNNNNNNNNNNNNNNNNNNNNNNNNNNNNNNNNNNNNNNNNNNCNTCTNNNAAANNNTNNN
NNNNNNNNNNNNNNNNNNNNNNNNNNNCNCNTTGNNNGTAANCNANNNGNACATGCAGTCCCCTCNNNNN
NNNGGCAGAGNNNNNNNCCNNNNNCNCCGNCCCCNNCCNNNNNNNNNNNNNNNNNGNNNNNNNANNNNNN
NNNNNNTGGTACNNNNCGNNNNNGGTTGTTTAAGAGGTCCACAACGNGGTCNGGNCACNNNNGNNNAN
NNNCNNNNAANNGNNNNNNNNNNNNNTNNNTANNAGGNCNNNNCCNTCNNGNNNNNNATNGCNATGACC
CAANNCACGGGGGCNGTNCCCGGCGNGGCGACCTTGNNNANGTCCGGGGNCAAGTATGGCNNNCNCCC
CGGGAAGGANNCNGCCGTANGTGCNNNNNANNNNNTCNTGNGANGTNGCCGNNATTCNCNTGGTCNNN
NNAGAATGNCCGNACCANNCGNANNNTGGTNATGNNAGTNNNNNNNNNNNNNNNNNNNNNNNNNNNNNN
NNNNNNNNNNNNNNNNNNNNNNNNNNNNNNNNNNNGNNNNGNNNNNNCNANNNAAGGNNNTANNNCTNNTN
NTNNNNNNGGACNNNACNNNNCNNNNNNNACNCNNNCNCNNNNNNANNNGNTTTTTNNCCNNTATTNT
CCAGNACGCNGNGCNNNCATTCANGNGAACNNNGNNACGCGNNAATCGTANNATCTACCTGCCGCNNG
TNNNNNTNCNGNNNNTNCGGANNNTNNNNCGTANNNNNNTCATGCGTNNTNTNNTCNCNAGNCNNGTT
CGNNNATANANTAGNANGTNTNNNNNNNNNNTGTNNCGTTCCGCNTGANNCNNCANNNNGNNGNNCTTC
TCANCGGCNNTANCTGCNGANNTAAGNCATANNNTANNTCNNTANCTNNNNNGGNGGGNNACTGTAAA
NGGNNAATTNGNNGNTNATTTCNNNCACNTNNCTNNGNNTNCTNNNTNCCTGNNTNCNNAATTGGGNC
CANTGNNTGNCNATCTCTGGNTTNGNNNNNNTGNNNNTNGCAANNNGGNNNGTNANANCNCNATNTAA
NNTATANNNNCNNNNNNANNNCACNNNNNNNNGNAGNNNCNANNNTTANANNNGTAGNNNAATAAGTCT
CCGANNTNNNNNNNATAAGCGNNANACNNNNNNNNNNNNNNANGANNACGNNTTGGNNNTNNNNNNNCNA
GNNAGACNNTNATGGNTNNNNNTCAANNNNTTNGTAGTGGTATAATNGNNNNNNNTTTCNCCAGNNTN
NNNGNNNNNNNNCNATANNANNNNNNNGNGANNNNNNNNNNNANNNNCTCNNNAGGGANCANCTACTTNA
GCANTGGNGCGCAGNNNGAGANNNNGACNNNCCTTGGGTGGCAANCACNGCAATAAGTNNNGTTNANC
CTNNNAGTNNACATNNNNNCANNGNNNCNNNNNNANCNGANNGNNNNNNNNNNNNNNNNNNNNNNNNNN
NNNNNNNNNNNNNNNNNNNNNNNNNNNNNNAGNNNANNGNNNGTNTNTGACNCATGTGTTTTTANNNGNN
NNNNNACGNNNNANCCCCGGTCGNNNNCNNATTGAANNCCTGNNGCATNNNNNTNCNAGAATGANNGC
NNNNNGNNTNGNCTGNCNTTTCAAGNAATCANNNAGGTNNCGCGNTCNNNNNNNNNNNNNCNNTCCCNN

ACTGAGGGTNNNNNNNNNCATCTGCTNNNNNNNNNCTNCGCNGNCGTNNCNNNNNAGCCGTATNNTGTNA
NNCANNNNNNNNNNNNNNNNNCNAANNNANNNNNNTNNNN
>Tay156
GTGNNNNNTAGCCCNNNNNAGTTAGACANNNCNNNNNNNNNNNNNTNNNNNNNNNNNNNNNNNNNNNNNN
NNNNNNNNNNNNNNNNNNNNNNNNNNNNNNNNNNNNNNNNNNNNNNNNNNNNNNNNNNNNNNNNNNNNNN
NNNNNNNNNNNNNNNNNNNNNNNNNNNNNNNNNNNNNNNNNNNNNNNNNNNNNNNNNNNNNNNNNNNNNN
NNNNNNNNNNNNNNNNNNNNNNNNNNNNNNNNNNNNNNNNNNNNNNNNNNNNNNNNNNNNNNNNNNNNNN
NNNNNNNNNNNNNNNNNNNNNNNNNNNNNNNNNNNNNNNNNNNNNNNNNNNNNNTCTNNNAAANNNTNNN
NNNNNNNNNNNNNNNNNNNNNNNNNNNCNCNTTGNNNGTAANCNANNNGNACATGCAGTCCCCTCNNNNN
NNNGGCAGAGNNNNNNCCCNNNNNNNCCGGCCCCGNCCNNNNNNNNNNNNNNNNNGNNNNNNNANNNNNN
NNNNNNTGGTACNNNNNCGNNNNNGGTTGTTTNAGAGGTCCACAACGNGGTCNGGNCACNNNNNGNNNAN
NNNCNNAAAACNGNNNNNNGNNNANNTNNNTANNAGGNCNNNNCCNTCNNGNNNNNNNATTGCNATGACC
CAANTCACGGGGGCNGTNCCCGGCGCGGCGACCTTGATNANGTCCGGGGACAAGTATGGCGNNCNCCC
CGGGAAGGANNCNGCCGTANGTGCNNNNNANNNNNTCNTGNGANGTCGCCGNNATTCNCGTGGTCTNN
NNAGAATGNCCGNACCANNCGNANNNTGGTNATGNNAGTNNNNNNNNNNNNNNNNNNNNNNNNNNNNNNN
NNNNNNNNNNNNNNNNNNNNNNNNNNNNNNNNNNNNNNNGNNNNNNCNANNNAAGGANNTANNNCTNNTN
NTNNNNNNGGACNNNACNNNNCNNNNNNNACNCNNNCNCNNNNNNANNNGNTTTTTNNNCNNTATTNT
CCAGNACGCNGNGCNNNCATTCANGNGAACNNNGNNACGCGNNAATCGTANNATCTACCTGCCGCNGG
TNNNNNTACNGNNNNNACGGANNNTNNCTCGTANNNNNGTCATGCGTNNTNTNNTCNCNAGNCANGTT
CGNGAANANANTAGNANGTNTCNNNNNNNNTGTNNCGTTCCGCNTGANNCNNCANNNNGNNGNNCTTN
TCANCGGCNNTANCTGCNGANNTAAGNCATANNNTNNATCNNTANCTNNNNNGGNGGGNNACTGTAAA
NGGNTAATTNGNGGNTNATTTCNNNCACNTNNCTNNGNNTNCTNNNTNCCTGNNTNCNNAATTGGNNC
CANTGNNTGNCNATCTCTGGNTTNGNNNNNNTNNNNNTNGCAANNNGGNNNGTNANANNNCNATNTAA
NNTATANNNNCNNNNNNANNNCACNANNNNNGNAGNNNCNANNNTTANANNNGTAGNNNAATAAGTCT
CCGANNTNNNNNNNATAAGCGNNANACNNNNNNNNNNNNNANGANNACGNNTTGGNNNTNNNNNNCNA
GNNAGACNNTNATGGNTNNNNNTCAANNNNTTNGTAGTGGTATAATNGNNNNNNNTTTCNCCAGNNTN
NNNGNNNNNNNCNATANNANNNNNNNGNGANNNNNNNNNNNANNNNCTCNNNAGGGANCANCTACTTNA
GCAATGGNGCGCAGNNNGAGANNNNGACNNGCCTTGGGTGGCAANCACNGCGATAAGTNNNGTTNANC
CTNNNAGTNNACATGNNNNCANNGNNNCNNNNNNANCNGANNGNNNNNNNNNNNNNNNNNNNNNNNNN
NNNNNNNNNNNNNNNNNNNNNNNNNNNNNNNAGNNNANGGNNNGTNTNTGACNCATGTGTTTTTANNNGNN
NNNNNACGNNNNANCCCCGGTCGTNNNCNNATTGAANNCCTGNNGCATNNNNNTNCNAGAATGANNGC
NNNNNGNNTNGNCTNNCNTTTCAAGNAATCANNNAGGTNNCGCGNTCNNNGNNNNTNNNCNNTCCCNN
ACTGAGGGTNNNNNNNNNCATCTGCTNNNNNNNNNCTTCGCNGGCGTNNCTNNNNAGCNGTATNGTGTNA
NNCANNNNNNNNNNNNNNNNNNNANACGANNNNNNNTNNNN
>Tay157
GTGNNNNNTAGCCCNNNNNAGTTAGACANNNCNNNNNNNNNNNNNTNNNNNNNNNNNNNNNNNNNNNNNN
NNNNNNNNNNNNNNNNNNNNNNNNNNNNNNTNNNNNNNNNNNNNNNNNNNNNNNNNNNNNNNNNNNNNNNNN
NNNNNNNNNNNNNNNNNNNNNNNNNNNNNNNNNNNNNNNNNNNNNNNNNNNNNNNNNNNNNNNNNNNNNN
NNNNNNNNNNNNNNNNNNNNNNNNNNNNNNNNNNNNNNNNNNNNNNNNNNNNNNNNNNNNNNNNNNNNNN
NNNNNNNNNNNNNNNNNNNNNNNNNNNNNNNNNNNNNNNNNNNNNNNNNNNNCNTCTNNNAAANNNTNNN
NNNNNNNNNNNNNNNNNNNNNNNNNNNCNCNTTGNNNGTAANCNANNNGNACATGCAGTCCCCTCNNNNN
NNNGGCAGAGNNNNNNCCCNNNNNNNCCGGCCCCNNCCNNNNNNNNNNNNNCNNNGNNNNNNNANNNNNN
NNNNNNTGGTACNNNNNCGNNNNNGGTTGTTTAAGAGGTCCACAACGNGGTCNGGNCACNNNNNGNNNAN
NNNCNNNNAACNNNNNNNNNNNNNANNTNNNTANNAGGNNNNNNCCNTCNNGNNNNNNNATTGCNATGACC
CAANTCACGGGGGCAGTNCCCGGCGCGGCGACCTTGATNANNTCCGGGGACAAGTATGGCGCNCNCCC
CGGGAAGGANNCNGCCGTANGTGCNNNNNANNNNNTCNTGNGACGTCGCCGNNATTCNCGTGGTCNNN
CNAGAATGNCCGNACCANNCGNANNNTGGTTATGTNAGTNNNNNNNNNNNNNNNNNNNNNNNNNNNNNN
NNNNNNNNNNNNNNNNNNNNNNNNNNNNNNNNNNGNNNNGNNNNNNCNANNNAAGGAGNTANNNCTNNTN
NTNNNNNNGGACNNNACNNNNCNNNNNNNACNCNNNCNCNNNNNNANNNGGTTTTTNNNCNNTATTNT
CCAGNACGCNGNGCNNNCATTCANGNGAACNNNGNNACGCGNNAATCGTANNATCTACCTGCCGCNGG
TNNNNNTACNGNNNNTNCGGANNNTNNCTCGTANNNNNGTCATGCGTNNTNTNNTCNCNAGNCANGTT
CGNNNANANANTAGNANGTNTNNNNNNNCTTGTNNCGTTCCGCNTGANNCNNCANNNNGNNGNNCTTN
TCANCGGCNNTANCTGCNGANNTAAGNCATANNNTNNATCNNTANCTNNNNNGGNGGGNNACTGTAAA
NGGNTAATTGGNGGNTNATTNCNNNCACNTNNCTNNGNNTNCTNNNTNCCTGNNTNCNNAATTGGNNC

CANTGNNTGNCNATCTCTGGNTTNGNNNNNNTGNNNNTNGCAANNNGGNNNGTNANANNNCNATNTAA
NNTATANNNNCNNNNNNANNNCACNANNNNNGNAGNNNCNANNNTTANANNNGTAGNNNAATAAGTCT
CCGANNTNNNNNNNATAAGCGNNANACNNNNNNNNNNNNNNANGANNACGNNTTGGNNNTNNNNNNCNA
GNNAGACNNTNATGGNTNNNNNTCAANNNNTTCGTAGTGGTATAATNGNNNNNNNTTTCNCCAGNNTN
NNNGNNNNNNNCNATANNANNNNNNNGNGANNNNNNNNNNNANNNNCTCNNNAGGGANCANCTACTTNA
GCAATGGNGCGCAGNNNGAGANNNNGACNNNCCTTGGGTGGCAANCACNGCGATAAGTNNNGTTNANC
CTNNNAGTNNACATNNNNNCANNGNNNCNNNNNNANCNGANNGNNNNNNNNNNNNNNNNNNNNNNNNN
NNNNNNNNNNNNNNNNNNNNNNNNNNNNNAGNNNANGGNNNGTNTNTGACNCATGTGTTTTTANNNGNN
NNNNNACGNNNGANCCCCGGTCGNNNNCNNATTGAANNCCTGNNGCATCNNNNTNCNAGAATGANNGC
NNNNNGNNTNGNCTNNCNTTTCAAGNAATCAANTAGGTNNCGCGNTCNNGGNNNNNNNNCNNTCCCNN
ACTGAGGGTNNNNNNNNCATCTGCTNNNNNNNNNNTNCGCNGGCGTNNCNNNNNAGCNGTATNGTGTNA
NNCANNNNNNNNNNNNNNNNNTNNNNNNNNNNNNNNTNNNN
>Tay158
GTGNNNNNTAGCCCNNNNNAGTTAGACANNNCNNNNNNNNNNNNNTNNNNNNNNNNNNNNNNNNNNNNN
NNNNNNNNNNNNNNNNNNNNNNNNNNNNNNNNNNNNNNNNNNNNNNNNNNNNNNNNNNNNNNNNNNNN
NNNNNNNNNNNNNNNNNNNNNNNNNNNNNNNNNNNNNNNNNNNNNNNNNNNNNNNNNNNNNNNNNNNN
NNNNNNNNNNNNNNNNNNNNNNNNNNNNNNNNNNNNNNNNNNNNNNNNNNNNNNNNNNNNNNNNNNNN
NNNNNNNNNNNNNNNNNNNNNNNNNNNNNNNNNNNNNNNNNNNNNNNGNNCNTCTNNNAAANNNTNNN
NNNNNNNNNNNNNNNNNNNNNNNNNNCNCNTTGNNNGTAANCNANNNGNACATGCAGTCCCCTCNNNNN
NNNGGCAGAGNNNNNNNCCNNNNNNNCCGGCCCCNNCCNNNNNNNNNNNNNNNNNNNNNNNNNANNNNNN
NNNNNNTGGTACNNNNCGNNNNNGGTTGTTTAAGAGGTCCACAACGNGGTCNNGNCACNNNNGNNTAN
NNNCNNNAAACNNNNNNNNNNNNNNNTNNNTANNAGGNCNNNNCCNTCNNNNNNNNNATTGCNATGACC
CAANNCACGGGGGCNGTNCCCGGCGCGGCGACCTTGNTNACNTCCGGGGACAAGTATGGCNNNCNCCC
CGGGAAGGANNCNGCCGTANGTGCNNNNNANNNNNTCNTGNGANGTCGCCGNNATTCNNNTGGTCNNN
CNAGAATGNCCGNACCANNCGNANNNTGGTNATGNNAGTANNNNNNNNNNNNNNNNNNNNNNNNNNNN
NNNNNNNNNNNNNNNNNNNNNNNNNNNNNNNNNNGNNNNGNNNNNNCNANNNAAGGANNTANNNCTNNTN
NTNNNNNNNGGACNNNACNNNNCNNNNNNNACNCNNNCNCNNNNNNNNNNNGNTTTTNNNNCNNTATTNT
CCAGNACGCNGNGCNNNCATTCANGNGAACNNNGNNACGCGNNAATCGTANNATCTACCTGCCGCNNG
TNNNNNTACNGNNNNTACGGANNNTNNNNCGTANNNNNNTCATGCGTNNTNTNNTCNCNAGNCNNGTT
CGNNNANANANTAGNANGTNTNNNNNNNNNNTGTNNCGTTCCGCNTGANNCNNCANNNNGNNGNNCTTN
TCANCGGCNNTANCTGCNGANNTAAGNCATANNNTNNATCNNTANCTNNNNNGGAGGGNNACTGTAAA
NGGNNAATTNGAGGNTNANTTCCNNCACNTNNCTNNGNNNNCTNNNTNCCTGNNTNCNNAATTGGNNC
CANTGNNTGNCNATCTCTGGNTTNGNNNNNNTGNNNNTNNCAANNNGGNNNGTNANANNNCNATNTAA
NNTATANNNNCNNNNNNANNNCACNANNNNNGNAGNNNCNANNNTTANANNNGTAGNNNAATAAGTCT
CCGANNTNNNNNNNATAAGCGNNANACNNNNNNNNNNNNNNANGANNACGNNTTGGNNNTNNNNNNCNA
GNNAGACNNTNATGGNTNNNNNTCAANNNNTTCGTAGTGGTATAATNGNNNNNNNTTTCNCCAGNNTN
NNNGNNNNNNNCNATANNANNNNNNNGNGANNNNNNNNNNNANNNNCTCNNNAGGGANCANCTACTTNA
GCAANGGNGCGCAGNNNGAGANNNNGACNNGCCTTGGGTGGCAANCACNGCAATAAGTNNNGTTNANC
CTNNNAGTNNACATNNNNNCANNGNNNCNNNNNNANCNGANNGNNNNNNNNNNNNNNNNNNNNNNNNN
NNNNNNNNNNNNNNNNNNNNNNNNNNNNNAGNNNANGGNNNGTNTNTGACNCATGTGTTTTTANNNGNN
NNNNNACGNNNNANCCCCGGTCGNNTNCNNATTGAANNCCTGNNGCATNNNNNTNCNAGAATGANNGC
NNNNNGNNTNGNCTNNCNTTTCAAGNAATCAANNAGGTANCGCGNTCNNNGNNNNNNNNCNNTCCCNN
ACTGAGGGTNNNNNNNNCATCTGCTNNNNNNNNNNTNCGCNGGCGTNCCNNNNNAGCNGTATNGNGTNA
NNCANNNNNNNNNNNNNNNNNNNAAACNANNNNNNNTNNNN
>Tay159
GTGNNNNNTAGCCCNNNNNAGTTAGACANNNCNNNNNNNNNNNNNTNNNNNNNNNNNNNNNNNNNNNNN
NNNNNNNNNNNNNNNNNNNNNNNNNNNNNNNNTNNNNNNNNNNNNNNNNNNNNNNNNNNNNNNNNNNNN
NNNNNNNNNNNNNNNNNNNNNNNNNNNNNNNNNNNNNNNNNNNNNNNNNNNNNNNNNNNNNNNNNNNN
NNNNNNNNNNNNNNNNNNNNNNNNNNNNNNNNNNNNNNNNNNNNNNNNNNNNNNNNNNNNNNNNNNNN
NNNNNNNNNNNNNNNNNNNNNNNNNNNNNNNNNNNNNNNNNNNNNNNNNNNTCTNNNAAANNNTNNN
NNNNNNNNNNNNNNNNNNNNNNNNNNCNCNTTGNNNGTAANCNANNNGNACATGCAGTCCCCTCNNNNN
NNNGGCAGAGNNNNNNCCCNNNNNNNCCGGCCCCGNCCNNNNNNNTNNNCANNGNNNNNNNANNNNNN
NNNNNNTGGTACNNNNCGNNNNNGGTTGTTTAAGAGGTCCACAACGNGGTCNNGNCACNNNNGNNNAN
NNNCNNNNAACNNNNNNNNNNNNNANNTNNNTANNAGGNCNNNNCCNTCNNGNNNNNAATTGCNATGACC

CAANTCACGGGGGGCNGTCCCCGGCGNGGCGACCTTGATNACGTCCGGGGACAAGTATGGCGNNCNCCC
CGGGAAGGANNCNGCCGTANGTGCNNNNNANNNNNTCNTGNGANGTCGCCGNNATTNNNGTGGTCNNN
CNAGAATGNCCGNACCANNCGNANNNTGGTNATGNTAGTANNNNNNNNNNNNNNNNNNNNNNNNNNNN
NNNNNNNNNNNNNNNNNNNNNNNNNNNNNNNNNNGNNNNGNNNNNNCNANNNAAGGANNTANNNCTNNTC
NTNNNNNNGGACNNNACNNNNCNNNNNNNACNCNNNCNCNNNNNNNNNNGNTTTTTNNNCNNTATTNT
CCAGNACGCNGNGCNNNCATTCANGNGAACNNNGNNACGCGNNAATCGTANNATCTACCTGCCGCNNG
TNNNNNTACNGNNNNTNCGGANNNTNNNNCGTANNNNNNTCATGCGTNNTNTNNTCNCNAGNCANGTT
CGNNNANANANTAGNANGTNTCNNNNNNNNTGTNNCGTTCCGCNTGACNCNNCANNNNGNNGNNCTTC
TCANCGGCNNTANCTGCNGANNTAAGTCATANNNTNNATCNNTANCTNNNNNGGNGGGNNACTGTAAA
NGGATAATTGGNNGNTNATTTCNNNCACNTNNCTNNGNNNNCTNNNTNCCTGNNTNCNNAATTGGNNC
CANTGNNTGNCNATCTCTGGNTTNGNNNNNNTNNNNNTNGCAANNNGGNNNGTTANANCNCNATNTAA
NNTATANNNNCNNNNNNANNNCACNANNNNNGNAGNNACNANNNTTANANNNGTAGNNNANTAAGTCT
CCGANNTNNNNNNNATAAGCGNNANACNNNNNNNNNNNNNANGANNACGNNTTGGNNNTNNNNNNCNA
GNNAGACNNTNATGGNTNNNNNTCAANNNNTTCGTAGTGGTATAATNGNNNNNNNTTTCNCCAGNNTN
NNNGNNNNNNNCNATANNANNNNNNNGTGANNNNNNNNNNNANNNNCTCNNNAGGGANCANCTACTTNA
GCANTGGNGCGCAGNNNGAGANNNNGACNNNCCTTGGGTGGCAANCACNGCAATAAGTNNNGTTNANC
CTNNNAGTNNACATNNNNNCANNGNNNCNNNNNNANCNGANNGNNNNNNNNNNNNNNNNNNNNNNNNN
NNNNNNNNNNNNNNNNNNNNNNNNNNNNNAGNNNANGGNNNGTNTNTGACNCATGTGTTTTTANNNGNN
NNNNNACGNNNGANCCCCGGTCGNNNNCNNATTGAANNCCTGNNGCATNNNNNTNCNAGAATGANNGC
NNNNNGNNTNGNCTNNCNNNTCAAGNAATCANNTAGNTNNCGCGNTCNNNGANNNNNNNCNNTCCCNN
ACTGAGGGTNNNNNNCGCATCTGCTNNNNNNNNNNTTCGCNGNCGTNNCNNNNNAGCNGTATNGTGTNA
NNCANNNNNNNNNNNNNNNNNNTNNNNNNNANNNNNNNTNNNN
>Tay160
GTGNNNNNTAGCCCNNNNNAGTTAGACANNNCNNNNNNNNNNNNNTNNNNNNNNNNNNNNNNNNNNNNN
NNNNNNNNNNNNNNNNNNNNNNNNNNNNNNNNTNNNNNNNNNNNNNNNNNNNNNNNNNNNNNNNNNNNNN
NNNNNNNNNNNNNNNNNNNNNNNNNNNNNNNNNNNNNNNNNNNNNNNNNNNNNNNNNNNNNNNNNNNNNN
NNNNNNNNNNNNNNNNNNNNNNNNNNNNNNNNNNNNNNNNNNNNNNNNNNNNNNNNNNNNNNNNNNNNNN
NNNNNNNNNNNNNNNNNNNNNNNNNNNNNNNNNNNNNNNNNNNNNNNNNNNNNNNTCTNNNAAANNNTNNN
NNNNNNNNNNNNNNNNNNNNNNNNNNCNCNTTGNNNGTAANCNANNNGNACATGCAGTCCCCTCNNNNN
NNNGGCAGAGNNNNNNCNCNNNNNNNNCCGNCCCCGNCCNNNNNNNTNNNCNNNNNNNNNNNGNNNNNN
NNNNNNTGGTACNNNNCGNNNNNGGTTGTTTAAGAGGTCCACCACGNGGTCNNGNCACNNNNGNNNAN
NNNCNNANAANNNNNNNNNNNNNNNNATNNNCANNAGGNCNNNNCCNTCNNNNNNNNNAATTGCNATGACC
CAANNCACGGGGGGCAGTNCCCGGNGCGGCGACCTTGNTNANNGCCGGGGACAAGTATGGCNTNCNCCC
CGGGAAGGANNCNGCCGTANGTGCNNNNNANNNNNTCCTGNGANGTNGCCGNNATTCNCGTGGTCNNN
CNAGAATGNCCGNACCANNCGNANNNTGGTTATGTTAGTNNNNNNNNNNNNNNNNNNNNNNNNNNNNN
NNNNNNNNNNNNNNNNNNNNNNNNNNNNNNNNNNNNNNNNNGNNNNNNCNANNNAAGGAGNTANNNCTNNTC
NTNNNNNNGGACNNNACNNNNCNNNNNNNACNCNNNCNCNNNNNNNNNNNGNTTTTNNNCCNNTATTNT
CCAGNACGCNGNGCNNTCATTCAAGNGAACNNNGNNACGCGTAAATCGTANNATCTACCTGCCGCNGG
TNNNNNTACNGNNNNTNCGGANNNTNNNNCGTANNNNNGTCATGCGTNNTNTNNTCNCNAGNCANGTT
CGNNNANANANTAGNANGTNTCNNNNNNNNTGTNGCGTTCCGCNTGANNCNNCANNNNGNNGNNCTTC
TCANCGGCNNTANCTGCNGANNTAAGNCATANNNTNNNTCNNTANCTNNNNNGGNGGGNNACTGTAAA
NGGANAATTGGAGGNTNATTTCNNNCACNTNNCTNNGNNNNCTNNNTNCCTGNNTNCNNAATTGGNNC
CANTGNNTGNCNATCTCTGGNTTNGCNNNNNTNNNNNTNGCANNNNGGNNNGTNANANNNCNATNTAA
NNTATANNNNCNANNNNANNNCACNANNNNNGNAGNNNCNANNNTTANANNNGTAGNNNAATAAGTCT
CCGANNTNNNNNNNATAAGCGNNANACNCNNNNNNNNNNNNANGANNACGNNTTGGNNNTNNNNNGCNA
ANNAGACNNTNGTGGNTNNNNNTCAANNNNTTCGTAGTGGTATAACNGNNNNNNNTTTCNCCAGNNTN
NNNGNNNNNNNCNATANNANNNNNNNGTGANNNNNNNNNNNANNNNCTCNNNAGGGANCANCTACTTNA
GCANTGGNGCGCAGNNNGAGANNNNGACCNGCCTTGGGTGGCAANCACNGCAATAAGTNNNGTTNANC
CTNNNAGTNNACACNNNNNCANNGNNNCNNNNNNANCNGANNTNNNNNNNNNNNNNNNNNNNNNNNNN
NNNNNNNNNNNNNNNNNNNNNNNNNNNNNAGNNNANNGNNNGTNTNTGACNCATGTGTTTTTANNNGNN
TNNNNACGNNNNANCCCCGGTCGTNNNCNNATTGAANNCCTGNNGCATNNNNNTNCNAGAATGANNGC
NNNNNGNNTNGNCTGNCNTTTCAAGNAGTCAANTAGNTANCGCGNTCNNNGNNNNNNNNCNNTCCCNN
ACTGAGGGTNNNNNNCGCATCTGCTNNNNNNNNNNTNCGCNGNCGTNNCTNNNNAGCNGTATNGTGTNN
NNCANNNNNNNNNNNNNNNNNNNNAACNNNNNNNNNTNNNN

>Tay161
GTGNNNNNTAGCCCNNNNNAGTTAGACANNNCNNNNNNNNNNNNTNNNNNNNNNNNNNNNNNNNNNNN
NNNNNNNNNNNNNNNNNNNNNNNNNNNNNNTNNNNNNNNNNNNNNNNNNNNNNNNNNNNNNNNNNNNN
NNNNNNNNNNNNNNNNNNNNNNNNNNNNNNNNNNNNNNNNNNNNNNNNNNNNNNNNNNNNNNNNNNNN
NNNNNNNNNNNNNNNNNNNNNNNNNNNNNNNNNNNNNNNNNNNNNNNNNNNNNNNNNNNNNNNNNNNN
NNNNNNNNNNNNNNNNNNNNNNNNNNNNNNNNNNNNNNNNNNNNNNNNNNCNTCTNNNAAANNNTNNN
NNNNNNNNNNNNNNNNNNNNNNNNNCNCNTTGNNNGTAANCNANNNGNACATGCAGTCCCCTCNNNNN
NNNGGCAGAGNNNNNNCCCNNNNNCNCCGGCCCCGNCCNNNNNNNNNNNCNNNGNNNNNNANNNNNNN
NNNNNNTGGTACNNNNCGNNNNNGGTTGTTTAAGAGGTCCACAACGNGGTCNNGNCACNNNNGNNTAN
NNNCNCNNAACNNNNNNNNNNNANNTNNNTANNAGGNCNNNNCCNTCNNGNNNNTAATNGCNATGACC
CAANNCACGGGGGCAGTNCCCGGNGCGGCGACCTTGATNANGTCCGGGGACAAGTATGGCNCNCNCCC
CGGGAAGGANNCNGCCGTANGTGCNNNNNANNNNNTCNTGNGANGTCGCCGNNATTCNCGTGGTCNNN
CNAGAATGNCCGNACCANNCGNANNNTGGTNATGNNAGTNNNNNNNNNNNNNNNNNNNNNNNNNNNNN
NNNNNNNNNNNNNNNNNNNNNNNNNNNNNNNNNGNNNNGNNNNNNCANNNAAGGANNTANNNCTNNTN
NTNNNNNNGGACNNNACNNNNCNNNNNNNACNCNNNCNCNNNNNNANNNGNTTTTNNNNCNNTATTNT
CCAGNACGCNGNGCNNNCATTCANGNGAACNNNGNNACGCGNNAATCGTANNATCTACCTGCCGCNGG
TNNNNNTNCNGNNNNTNCGGANNNTNNCTCGTANNNNNGTCATGCGTNNTNTNNTCNCNAGNCANGTT
CGNNNANANANTAGNANGTNTCNNNNNNCNTGTNNCGTTCCGCNTGANNCNNCANNNNGNNGNNCTTN
TCANCGGCNNTANCTGCNGANNTAAGTCATANNNTNNATCNNTANCTNNNNNGGNGGGNNACTGTAAA
NGGNTAATTNGAGGNTNATTTCNNNCACNTNNCTNNGNNTNCTNNNTNCCTGNNTNCNNAATTGGNNC
CANTGNNTGNCNATCTCTGGNTTNGNNNNNNTGNNNNTNGCAANNNGGNNNGTNANANNNCNATNTAA
NNTATANNNNCNNNNNNANNNCACNANNNNNGNAGNNNCNANNNTTANANNNGTAGNNNAATAAGTCT
CCGANNTNNNNNNNATAAGCGNNANACNNNNNNNNNNNNNANGANNACGNNTTGGNNNTNNNNNGCNA
GNNAGACNNTNATGGNTNNNNNTCAANNNNTTCGTAGTGGTATAATNGNNNNNNNTTTCNCCAGNNTN
NNNGNNNNNNNCNATANNANNNNNNNGTGANNNNNNNNNNANNNNCTCNNNAGGGANCANCTACTTNA
GCAATGGNGCGCAGNNNGAGANNNNGACNNGCCTTGGGTGGCAANCACNGCAATAAGTNNNGTTNANC
CTNNNAGTNNACATNNNNNCANNGNNNCNNNNNNANCTGANNGNNNNNNNNNNNNNNNNNNNNNNNNN
NNNNNNNNNNNNNNNNNNNNNNNNCTNNAGNNNANGGNNNGTNTNTGACNCATGTGTTTTTANNNGNN
NNNNNACGNNNNANCCCCGGTCGTNNNCNNATTGAANNCCTGNNGCATNNNNNTNCNAGAATGANNGC
NNNNNGNNTNGCTNNCTTTTCAAGNAATCANNNAGNNNNCGCGNTCNNGNNNNNNNNNNCNNTCCCNN
ACTGAGGGTNNNNNNCGCATCTGCTNNNNNNNNCTTCGCNGGNGTNNCTGNNNAGCNGTATNGTGTTA
NNCANNNNNNNNNNNNNNNNTNNANNNNNNNNNTNNNNN
>Tay162
GTGNNNNNTAGCCCNNNNNAGTTAGACANNNCNNNNNNNNNNNNTNNNNNNNNNNNNNNNNNNNNNNN
NNNNNNNNNNNNNNNNNNNNNNNNNNNNNNNNNNNNNNNNNNNNNNNNNNNNNNNNNNNNNNNNNNNN
NNNNNNNNNNNNNNNNNNNNNNNNNNNNNNNNNNNNNNNNNNNNNNNNNNNNNNNNNNNNNNNNNNNN
NNNNNNNNNNNNNNNNNNNNNNNNNNNNNNNNNNNNNNNNNNNNNNNNNNNNNNNNNNAGNNNNNNNN
NNNNNNNNNNNNNNNNNNNNNNNNNNNNNNNNNNNNNNNNNNNNNNNNNNNNTCTNNNAAANNNTNNN
NNNNNNNNNNNNNNNNNNNNNNNNNCNCNTTGNNNGTAANCNANNNGNACATGCAGTCCCCTCNNNNN
NNNGGCAGAGNNNNNNCCCNNNNNNNCCGNCCCCNNCCNNNNNNNTNNNNNNNGNNNNNNANNNNNNN
NNNNNNTGGTACNNNNCGNNNNNGGTTGTTTNAGAGGTCCACAACGNGGTCNGGNCACNNNNGNNNAN
NNNCNNNNAACNNNNNNNNNNNNATNNNTANNAGGNNNNNNGCCNTCNNNNNNNNNATTGCNATGACC
CANNNCNCGGGGGCNGTNCCCGGNGCGGCGACCTTGATNACGTCCGGGGACAAGTATGGCGCNCNCCC
CGGGAAGGANNCNGCCGTANGTGCNNNNNANNNNNTCNTGNGACGTCGCCGNNATTCNCGTGGTCNNN
CNAGAATGNNCGNACCANNCGNAGNNTGGTNATGNTAGTNNNNNNNNNNNNNNNNNNNNNNNNNNNNN
NNNNNNNNNNNNNNNNNNNNNNNNNNNNNNNNNGNNNNGNNNNNNCANNNAAGGANNTANNNCTNNTN
NTNNNNNNGGACNNNACNNNNCNNNNNNNACNCNNNCNCNNNNNNANNNGGTTTTNNNNCNNTATTNT
CCAGNACGCNGNGCNNNCATTCANGNGAACNNNGNNACGCGTNAATCGTANNATCTACCTGCCGCNGG
TNNNNNTACNGNNNNTNCGGANNNTNNCNCGTANNNNNNTCATGCGTNNTNTNNTCNCNAGNCANGTT
CGNNNANANANTAGNANGTNTCNNNNNNNNTGTNNCGTTCCGCNTGANNCNNCANNNNGNNGNNCTTN
TCANCGGCNNTANCTGCNGANNTAAGNCATANNNTNNATCNNTANCTNNNNNGGNGGGNNACTGTAAA
NGGNNAATTGGNNGNTNATTTCNNNCACNTNNCTNNGNNNNCTNNNTNCCTGNNTNCNNAATTGGNNC
CANTGNNTGNCNATCTCTGGNTTNGNNNNNNTNNNNNTNNCAANNNGGNNNNNNANANNNCNATNTAA
NNTATANNNNCNNNNNNANNNCACNNNNNNNNNAGNNNCNANNNTTANANNNGTAGNNNAATAAGTCT

CCGANNTNNNNNNNATAAGCGNNANACNNNNNNNNNNNNNNANGANNACGNNTTGGNNNTNNNNNGCNA
GNNAGACNNTNATGGNTNNNNNTCAANNNNTTNGTAGTGGTATAATNGNNNNNNNTTTCNCCAGNNTN
NNNGNNNNNNNNCNATANNANNNNNNNGTGANNNNNNNNNNNANNNNCTCNNNAGGGANCANCTACTTNA
GCANTGGNGCGCAGNNNGAGANNNNGACNNNCCTTGGGTGGCAANCACNGCAATAAGTNNNGTTNANC
CTNNNAGTNNACATNNNNNCANNGNNNCNNNNNNANCNGANNGNNNNNNNNNNNNNNNNNNNNNNNNN
NNNNNNNNNNNNNNNNNNNNNNNNNNNNNAGNNNANGGNNNGTNTNTGACNCATGTGTTTTTANNNGNN
NNNNNACGNNNNANCCCCGGTCGNNNNCNNATTGAANNCCTGNNGCATNNNNNTNCNAGAATGANNNC
NNNNNGNNTNGNCTGNCTTTTCAAGNAATCANNNAGGTNNCGCGNTCNNNNNNNNNNNNNCNNTCCCNN
ACTGAGGGTNNNNNNCGCATCTGCTNNNNNNNNNCTNCGCNGGNGTNNCNNNNNAGCNGTATNNTGTNA
NNCANNNNNNNNNNNNNNNNNNTNAANNGNNNNNNCTNNNN
>Tay163
GTGNNNNNTAGCCCNNNNNAGTTAGACANNNCNNNNNNNNNNNNNTNNNNNNNNNNNNNNNNNNNNNN
NNNNNNNNNNNNNNNNNNNNNNNNNNNNNNNNNNNNNNNNNNNNNNNNNNNNNNNNNNNNNNNNNNNNNN
NNNNNNNNNNNNNNNNNNNNNNNNNNNNNNNNNNNNNNNNNNNNNNNNNNNNNNNNNNNNNNNNNNNNNN
NNNNNNNNNNNNNNNNNNNNNNNNNNNNNNNNNNNNNNNNNNNNNNNNNNNNNNNNNNNNNNNNNNNNNN
NNNNNNNNNNNNNNNNNNNNNNNNNNNNNNNNNNNNNNNNNNNNNNNNNNNCNTCTNNNAAANNNTNNN
NNNNNNNNNNNNNNNNNNNNNNNNNNCNCATTGNNNGTAANCNANNNGNACATGCAGTCCCCTCNNNNN
NNNGGCAGAGNNNNNNNCCNNNNNNNCCGNCCCCGNCCNNNNNNNNNNNCANNGNNNNNNNANNNNNN
NNNNNNTGGTACNNNNCGNNNNNGGTTGTNTAAGAGGTCCACAACGNGGTCNNGNCACNNNNGNNNAN
NNNCNNNNAACNNNNNNNNNNNNANNTNNNTANNAGGNCNNNNCCNTCNNGNNNNTAATTGCNATGACC
CAANNCACGGGGGCNGTCCCCGGCGCGGCGACCTTGATNANGTCCGGGGACAAGTATGGCGCNCNCCC
CGGGAAGGANNCNGCCGTANGTGCNNNNNANNNTNTCNTGNGANGTCGCCGNNATTCNCGTGGTCNNN
CNAGAATGNCCGNACCANNCGNANNNTGGTNATGTNAGTNNNNNNNNNNNNNNNNNNNNNNNNNNNNN
NNNNNNNNNNNNNNNNNNNNNNNNNNNNNNNNNNNNNNNGNNNNNNCNANNNAAGGANNTANNNCTNNTN
NTNNNNNNNGGACNNNACNNNNCNNNNNNNACNCNNNCNCNNNNNNNNNNNGNTTTTTNNCCNNTATTNT
CCAGNACGCNGNGCNNNCATTCANGNGAACNNNGNNACGCGNNAATCGTANNATCTACCTGCCGCNGG
TNNNNNTACTGNNNNNNCGGANNNTNNCTCGTANNNGNNTCATGCGTNNTNTNNTCNCNAGNCANGTT
CGNNNANANANTAGNANGTNTCNNNNNNCTTGTNNCGTTCCGCNTGACNCNNCANNNNGNNGNNCTTN
TCANCGGCNNTANCTGCNGANNTAAGNCATANNNTNNNTCNNTANCTNNNNNGGAGGGNNACTGTAAA
NGGNNAATTGGNNNNTGATTTCNNNCACNTNNCTNNGNNTNCTNNNTNCCTGNNTNCNNAATTGGNNC
CANTGNNTGNCNATCTCTGGNTTNGNNNNNNTNNNNNTNGCAANNNGGNNNGTNANANCNCNATNTAA
NNTATANNNNCNNNNNNANNNCACNANNNNNNNAGNNNCNANNNTTANANNNGTAGNNNANTAAGTCT
CCGANNTNNNNNNNATAAGCGNNANACNNNNNNNNNNNNNNANGANNACGNNTTGGNNNTNNNNNNCNA
GNNAGACNNTNATGGNTNNNNNTCAANNNNTTNGTAGTGGTATAATNGNNNNNNNTTTCNCCAGNNTN
NNNGNNNNNNNNCNATANNANNNNNNNGNGANNNNNNNNNNNANNNNCTCNNNAGGGANCANCTACTNNA
GCAATGGNGCGCAGNNNGAGANNNNGACNNGCCTTGGGTGGCAANCACNGCAATAAGTNNNGTTNANC
CTNNNAGTNNACATNNNNNCANNGNNNCNNNNNNANCNGANNGNNNNNNNNNNNNNNNNNNNNNNNNN
NNNNNNNNNNNNNNNNNNNNNNNNNNNNNAGNNNANGGNNNGTNTNTGACNCATGTGTTTTTANNNGNN
NNNNNACGNNNGANCCCCGGTCGTNNNCNNATTGAANNCCTGNNGCATNNNNNTNCNAGAATGANNGC
NNNNNGNNTNGNCTGNCNTTTCAAGNAATCAANNAGGTANNGCGNTCNNNNNNNNNNNNNCNNTCCCNN
ACTGAGGGTNNNNNNNNCATCTGCTNNNNNNNNNCTTCGCNGNCGTNNCNNNNNAGCCGTATNGNGTNA
NNCANNNNNNNNNNNNNNNNNNNAAANNANNNNNNTNANN
>Tay164
GTGNNNNNTAGCCCGNNNNAGTTAGACANNNCNNNNNNNNNNNNNTNNNNNNNNNNNNNNNNNNNNNN
NNNNNNNNNNNNNNNNNNNNNNNNNNNNNNNTNNNNNNNNNNNNNNNNNNNNNNNNNNNNNNNNNNNNNN
NNNNNNNNNNNNNNNNNNNNNNNNNNNNNNNNNNNNNNNNNNNNNNNNNNNNNNNNNNNNNNNNNNNNNN
NNNNNNNNNNNNNNNNNNNNNNNNNNNNNNNNNNNNNNNNNNNNNNNNNNNNNNNNNNNNNNNNNNNNNN
NNNNNNNNNNNNNNNNNNNNNNNNNNNNNNNNNNNNNNNNNNNNNNNNNNNCNTCTNNNAAANNGTNNN
NNNNNNNNNNNNNNNNNNNNNNNNNNCNCNTTGNNNGTAANCNANNNGNACATGCAGTCCCCTCNNNNN
NNNNNCAGAGNNNNNNNNNCNNNNNNNCCGNCCCCGNCCNNNNNNNNNNNCNNNGNNNNNNNANNNNNN
NNNNNNTGGTACNNNNCGNNNNNGGTTGTTTAAGAGGTCCACAACGNGGTCNNGNCACNNNNGNNNAN
NNNCNNNNAACNNNNNNNNNNNNANNTNNNTANNAGGNNNNNNCCNTNNNGNNNNNNATTGCNATGACC
CAANNCACGGGGGCAGTCCCCGGNGCGGCGACCTTGATNANGTCCGGGGACAAGTATGGCNCNCNCCC
CGGGAAGGANNCNGCCGTANGTGCNNNNNANNNNNTCNTGNGANGTNGCCGNNATTCNCGTGGTCNNG

CNAGANTGNNCGNACCANNCGNANNNTGGTNATGNNAGTNNNNNNNNNNNNNNNNNNNNNNNNNNNNN
NNNNNNNNNNNNNNNNNNNNNNNNNNNNNNNNGNNNNGNNNNNNCNANNNAAGGANNTANNNCTNNTN
NTNNNNNNGGACNNNACNNNNCNNNNNNNACNCNNNCNCNNNNNNNNNNGNTTTTNNNNCNNTATTNT
CCAGNACGCNGNGCNNNCATTCANGNGAACNNNGNNACGCGNNAATCGTANNATCTACCTGCCGCNGG
TNNNNNTACNGNNNNTNCGGANNNTNNCTCGTANNNNNGTCATGCGTNNTNTNNTCNCNAGNCNNGTT
CGNNNANANANTAGNANGTNTCNNNNNNNNTGTNNCGTTCCGCNTGANNCNNCANNNNGNNGNNCTTN
TCANCGGCNNTANCTGCNGANNTAAGNCATANNNTNNNTCNNTANCTNNNNNGGNGGGNNACTGTAAA
NGGNNAATTNGNNGNTNATTTCNNNCACNTNNCTNNGNNNNCTNNNTNCCTGNNTNCNNAATTGGNNC
CANTGNNTGNCNATCTCTNGNTTNGNNNNNNTGNNNNTNGCAANNNGGNNNGTNANANNNCNATNTAA
NNTATANNNNCNNNNNNANNNCACNANNNNNGNAGNNNCNANNNTTANANNNGTAGNNNAATAAGTCT
CCGANNTNNNNNNNATAAGCGNNANACNNNNNNNNNNNNNANGANNACGNNTTGGNNNTNNNNNNCNA
GNNAGACNNTNATGGNTNNNNNTCAANNNNTTCGTAGTGGTATAATNGNNNNNNNTTTCNCCAGNNTN
NNNGNNNNNNNCNATANNANNNNNNNGTGANNNNNNNNNNANNNNCTCNNNAGGGANCANCTACTTNA
GCAATGGNGCGCAGNNNGAGANNNNGACNNNCCTTGGGTGGCAANCACNGCAATAAGTNNTGTTNANC
CTNNNAGTNNACATGNNNNCANNGNNNCNNNNNNANCNGANNGNNNNNNNNNNNNNNNNNNNNNNNNN
NNNNNNNNNNNNNNNNNNNNNNNNNNNNAGNNNANNGNNNGTNTNTGACNCATGTGTTTTTANNNGNN
NNNNNACGNNNNANCCCCGGTCGNNNNCNNATTGAANNCCTGNNGCATNNNNNTNCNAGAATGANNNC
NNNNNGNNTNGNCTNNCNNNTCAAGNAATCNNNNAGGTNNCGCGNTCNNNNNNNNNNNNCNNTCCCNN
ACTGAGGGTNNNNNNCGCATCTGCTNNNNNNNNCTNCGCNGGCGTNNCTNNNNAGCNGTATNGTGTNA
NNCANNNNNNNNNNNNNNNTNAAACAANNNNNNTTNNNN
>Tay165
GTGNNNNNTAGCCCGNNNNAGTTAGACANNNCNNNNNNNNNNNNTNNNNNNNNNNNNNNNNNNNNNNN
NNNNNNNNNNNNNNNNNNNNNNNNNNNNNNTNNNNNNNNNNNNNNNNNNNNNNNNNNNNNNNNNNNNNN
NNNNNNNNNNNNNNNNNNNNNNNNNNNNNNNNNNNNNNNNNNNNNNNNNNNNNNNNNNNNNNNNNNNN
NNNNNNNNNNNNNNNNNNNNNNNNNNNNNNNNNNNNNNNNNNNNNNNNNNNNNNNNNNNNNNNNNNNN
NNNNNNNNNNNNNNNNNNNNNNNNNNNNNNNNNNNNNNNNNNNNNNNNNNCNTCTNNNAAANNNTNNN
NNNNNNNNNNNNNNNNNNNNNNNNNCNCNTTGNNNGTAANCNANNNGNACATGCAGTCCCCTCNNNNN
NNNGGCAGAGNNNNNNCCCNNNNNNNCCGGCCCCGNCCNNNNNNNNNNNCNNNGNNNNNNNANNNNNN
NNNNNNTGGTACNNNNCGNNNNNGGTTGTNTAAGAGGTCCACAACGNGGTCNGGNNACNNNNGNNNAN
NNNCNNAAAACNGNNNNNNNNANNNTNNNTANNAGGNCNNNNCCNTCNNGNNNNNNATTGCNATGACC
CAANTCACGGGGGCAGTNCCCGGCGCGGCGACCTTGATNANGTCCGGGGACAAGTATGGCGNNCNCCC
CGGGAAGGANNCNGCCGTANGTGCNNNNNANNNNNTCNTGNGACGTCGCCGNNATTCNNGTGGTCNNN
CNAGAATGNCCNNACCANNCGNANNNTGGTNATGTNAGTNNNNNNNNNNNNNNNNNNNNNNNNNNNNN
NNNNNNNNNNNNNNNNNNNNNNNNNNNNNNNNGNNNNGNNNNNNCNANNNAAGGANNTANNNCTNNTC
NTNNNGNNGGACNNNACNNNNCNNNNNNNACNCNNNCNCNNNNNNANNNGGTTTNNNNNCNNTATTNT
CCAGNACGCNGNGCNNNCATTCANGNGAACNNNGNNACGCGNNAATCGTANNATCTACCTGCCGCNGG
TNNNNNTACNGNNNNTACGGANNNTNNNNCGTANNNNNNTCATGCGTNNTNTNNTCNCNAGNCANGTT
CGNNNANANANTAGNANGTNTCNNNNNNCTTGTNNCGTTCCGCNTGANNCNNCANNNNGNNGNNCTTN
TCANCGGCNNTANCTGCNGANNTAAGNCATANNNTNNNTCNNTANCTNNNNNGGNGGGNNACTGTAAA
NGGNNAATTNGNGGNTNATTNCNNNCACNTNNCTNNGNNTNCTNNNTNCCTGNNTNCNNAATTGGNNC
CANTGNNTGNCNATCTCTNGNTTNGCNNNNNTGNNNNTNNCAANNNGGNNNGTNANANNNCNATNTAA
NNTANANNNNCNNNNNNANNNCACNNNNNNNGNAGNNNCNANNNTTANANNNGTAGNNNAATAAGTCT
CCGANNTNNNNNNNATAAGCGNNANACNNNNNNNNNNNNNANGANNACGNNTTGGNNNTNNNNNNCNA
GNNAGACNNTNATGGNTNNNNNTCAANNNNTTCGTAGTGGTATAATNGNNNNNNNTTTCNCCAGNNTN
NNNGNNNNNNNCNATANNANNNNNNNGNGANNNNNNNNNNANNNNCTCNNNAGGNANCANCTACTTNA
GCAATGGNGCGCAGNNNGAGANNNNGACCNGCCTTGGGTGGCAANCACNGCAATAAGTNNNGTTNANC
CTNNNAGTNNACATGNNNNCANNGNNNCNNNNNNANCNGANNGNNNNNNNNNNNNNNNNNNNNNNNNN
NNNNNNNNNNNNNNNNNNNNNNNNNNNNAGNNNANGGNNNGTNTNTGACNCATGTGTTTTTANNNGNN
NNNNNACGNNNNANCCCCGGTCGTNNNCNNATTGAANNCCTGNNGCATNNNNNTNCNAGAATGANNGC
NNNNNGNNTNNNCTGNCNTTTCAAGNAATCANNNAGGTANNGCGNTCNNNGANNNNNNNCNNTCCCNN
ACTGAGGGTNNNNNNNNCATCTGCTNNNNNNNNCTNCGCNGGNGTNNCNNNNNAGCNGTATNGTGTNA
NNCANNNNNNNNNNNNNNNTNANACNANNNNNNNTNNNN
>Tay166
GTGNNNNNTAGCCCNNNNNAGTTAGACANNNCNNNNNNNNNNNNTNNNNNNNNNNNNNNNNNNNNNNN

NNNNNNNNNNNNNNNNNNNNNNNNNNNNNNNNNNNNNNNNNNNNNNNNNNNNNNNNNNNNNNNNNNNNNN
NNNNNNNNNNNNNNNNNNNNNNNNNNNNNNNNNNNNNNNNNNNNNNNNNNNNNNTNNNNNNNNNNNNNNN
NNNNNNNNNNNNNNNNNNNNNNNNNNNNNNNNNNNNNNNNNNNNNNNNNNNNNNNNNNNNNNNNNNNNNN
NNNNNNNNNNNNNNNNNNNNNNNNNNNNNNNNNNNNNNNNNNNNNNNNNNNNCNTCTNNNAAANNGTNNN
NNNNNNNNNNNANNNNNNNNNNNNNNNNCNCNTTGNNNGTAANCNANNNGNACATGCAGTCCCCTCNNNNN
NNNGGCAGAGNNNNNNCCCNNNNNNNCCGGCCCCGNCCNNNNNNNNNNNNNNNNGNNNNNNNANNNNNN
NNNNNNTGGTACNNNNCGNNNNNGGTTGTNTAAGAGGTCCACAACGNGGTCNNGNCACNNNNGNNNAN
NNNCNNNAAACNNNNNNNNNNNNNNATNNNTANNAGGNNNNNNCCNTCNNGNNNNNNATNGCNATGACC
CAANNCACGGGGGCAGTNCCCGGCGCGGCGACCTTGATNACGTCCGGGGACAAGTATGGCNCNCNCCC
CGGGAAGGANNCNGCCGTANGTGCNNNNNANNNNNTCNTGNGACGTCGCCGNNATTCNNGTGGTCNNN
CNAGAATGNCCGNACCANNCGNANNNTGGTNATGNTAGTNNNNNNNNNNNNNNNNNNNNNNNNNNNNNNN
NNNNNNNNNNNNNNNNNNNNNNNNNNNNNNNNNNNNNNNGNNNNNNCNANNNAAGGNNNTANNNCTNNTN
NTNNNGNNGGACNNNACNNNNCNNNNNNNACNCNNNCNCNNNNNNANNNGNTTTTNNNNCNNTATTNT
CCAGNACGCNGNGCNNTCATTCANGNGAACNNNGNNACGCGNNAATCGTANNATCTACCTGCCGCNNG
TNNNNNTNCNGNNNNTACGGANNNTNNCTCGTANNNNNNTCATGCGTNNTNTNNTCNCNAGNCANGTT
CGNNNANANANTAGNANGTNTNNNNNNNCTTGTNNCGTTCCGCNTGANNCNNCANNNNGNNGNNCTTN
TCANCGGCNCTANCTGCNGANNTAAGNCATANNNTNNNTCNNTANCTNNNNNGGAGGGNNACTGTAAA
NGGNTAATTNGNGGNTNATTTCNNNCACNTNNCTNNGNNTNCTNNNTNCCTGNNTNCNNAATTGGNNC
CANTGNNTGNCNATCTCTGGNTTNGNNNNNNTGNNNNTNGCANNNNGGNNNGTNANANCNCNATNTAA
NNTATANNNNCNNNNNNANNNCACNANNNNNGNAGNNACNANNNTTANANNNGTAGNNNAATAAGTCT
CCGANNTNNNNNNNATAAGCGNNANACNNNNNNNNNNNNNNANGANNACGNNTTGGNNNTNNNNNNCNA
GNNAGACNNTNATGGNTNNNNNTCAANNNNTTCGTAGTGGTATAATNGNNNNNNNTTTCNCCAGNNTN
NNNGNNNNNNNCNATANNANNNNNNNGNGANNNNNNNNNNNANNNNCTCNNNAGGGANCANCTACTNNA
GCANTGGNGCGCAGNNNGAGANNNNGACNAGCCTTGGGTGGCAANCACNGCAATAAGTNNNGTTNANC
CTNNNAGTNNACATNNNNNCANNGNNNCNNNNNNANCNGANNGNNNNNNNNNNNNNNNNNNNNNNNNNNN
NNNNNNNNNNNNNNNNNNNNNNNNNNNNNAGNNNANGGNNNGTNTNTGACNCATGTGTTTTTANNNGNN
NNNNNACGNNNGANCCCCGGTCGTNNNCNNATTGAANNCCTGNNGCATNNNNNTNCNAGAATGANNGC
NNNNNGNNTNGNCTNNCNTTTCAAGNAATCAANNAGNTATNGCGNTCNNNNNNNNNNNNNCNNTCCCNN
ACTGAGGGTNNNNNNCGCATCTGCTNNNNNNNNNNTTCGCNGGNGTNNCNGNNNAGCNGTATNGTGTNA
NNCANNNNNNNNNNNNNNNNNNNNAANNNNNNNNNNNNTNNNN
>Tay167
GTGNNNNNTAGCCCNNNNNAGTCAGACANNNCNNNNNNNNNNNNNTNNNNNNNNNNNNNNNNNNNNNNNN
NNNNNNNNNNNNNNNNNNNNNNNNNNNNNNNNNNNNNNNNNNNNNNNNNNNNNNNNNNNNNNNNNNNNNN
NNNNNNNNNNNNNNNNNNNNNNNNNNNNNNNNNNNNNNNNNNNNNNNNNNNNNNNNNNNNNNNNNNNNNN
NNNNNNNNNNNNNNNNNNNNNNNNNNNNNNNNNNNNNNNNNNNNNNNNNNNNNNNNNNNNNNNNNNNNNN
NNNNNNNNNNNNNNNNNNNNNNNNNNNNNNNNNNNNNNNNNNNNNNNNNNNNCNTCTNNNAAANNNTNNN
NNNNNNNNNNNNNNNNNNNNNNNNNNNCNCNTTGNNNGTAANCNANNNGNACATGCAGTCCCCTCNNNNN
NNNNNCAGAGNNNNNNNCCNNNNNCNCCGGCCCCNNCCNNNNNNNNNNNNCNNNGNNNNNNNANNNNNN
NNNNNNTGGTACNNNNCGNNNNNGGTTGTNTAAGAGGTCCACAACGNGGTCNNGNCACNNNNGNNNAN
NNNCNNAAAANNNNNNNNNNNNNNNNTNNNTANNAGGNNNNNNCCNTCNNNNNNNNNAATTGCNATGACC
CAANNCACGGGGGCAGTNCCCGGCGCGGCGACCTTGATNANNTCCGGGGACAAGTATGGCNNNCNCCC
CGGGAAGGANNCNGCCGTANGTGCNNNNNANNNNNTCNTGNGANGTNGCCGNNATTCNCGTGGTCNNN
CNAGAATGNCCGNACCANNCGNANNNTGGTTATGNTAGTNNNNNNNNNNNNNNNNNNNNNNNNNNNNNNN
NNNNNNNNNNNNNNNNNNNNNNNNNNNNNNNNNNGNNNNGNNNNNNCNANNNAAGGNNNTANNNCTNNTN
NTNNNNNNGGACNNNACNNNNCNNNNNNNACNCNNNCNCNNNNNNANNNGNTTTTNNNNCNNTANTNT
CCAGNACGCNGNGCNNNCATTCANGNGAACNNNGNNACGCGNNAATCGTANNATCTACCTGCCGCNGG
TNNNNNTACNGNNNNNNCGGANNNTNNNNCGTANNNNNGNCATGCGTNNTNTNNTCNCNAGNCNNGTT
CGNNNANANANTAGNANGTNTCNNNNNNNNTGTNGCGTTCCGCNTGANNCNNCANNNNGNNGNNCTTN
TCANCGGCNNTANCTGCNGANNTAAGNCATANNNTNNNTCNNTANCTNNNNNGGNGGGNNACTGTAAA
NGGNNAATTNGNGGNTNATTTCNNNCATNTNNCTNNGNNTNCTNNNTNCCTGNNTNCNNAATTGGNNC
CANTGNNTGNCNATCTCTGGNTTNGNNNNNNTNNNNNTNNCANNNNGGNNNGTNANANNNCNATNTAA
NNTANANNNNCNNNNNNANNNCACNNNNNNNNNAGNNNNNANNNGTANANNNGTAGNNNGATAAGTCT
CCGANNTNNNNNNNATAAGCGNNANACNNNNNNNNNNNNNNANGANNACGNNTTTGNNNTNNNNNNCNA
GNNAGACNNTNGTGGNTNNNNNTCAANNNNTTCGTAGTGGTATAATNGNNNNNNNTTTCNCCAGNNTN

NNNGNNNNNNNCNATANNANNNNNNNNGTGANNNNNNNNNNANNNNNCTCNNNAGGNANCANCTACTNNA
GCAANGGNGCGCAGNNNGAGANNNNNGACCAGCCTTGGGTGGCAANCACNGCAATAAGTNNNGTTNANC
CTNNNAGTNNACACNNNNNNCANNGNNNCNNNNNNANCNGANNGNNNNNNNNNNNNNNNNNNNNNNNNNN
NNNNNNNNNNNNNNNNNNNNNNNNNNNNNNNNNNNANGGNNNGTNTNTGACNCATGNNTTTTTANNNGNN
TNNNNACGNNNNANCCCCGGTCGTNNNCNNATTGAANNCCTGNNGCATNNNNNTNCNAGAATGANNNC
NNNNNGNNTNGNCTNNCNNNTCAAGNAATCANNNAGGNNNNNNNNTCNNNGNNNNNNNNCNNTCCCNN
ACTGAGGGTNNNNNNNGCATCTGCTNNNNNNNNNNTNCGCNGGNGTNNCTNNNNAGCCGTATNNNGTNN
NNCANNNNNNNNNNNNNNNNNNNNNNNNNNNNNNNNNTNNNN
>Tay168
GTGNNNNNTAGCCCGNNNNAGTTAGACANNNCNNNNNNNNNNNNNTNNNNNNNNNNNNNNNNNNNNNNNN
NNNNNNNNNNNNNNNNNNNNNNNNNNNNNNNNNNNNNNNNNNNNNNNNNNNNNNNNNNNNNNNNNNNNNN
NNNNNNNNNNNNNNNNNNNNNNNNNNNNNNNNNNNNNNNNNNNNNNNNNNNNNNNNNNNNNNNNNNNNNN
NNNNNNNNNNNNNNNNNNNNNNNNNNNNNNNNNNNNNNNNNNNNNNNNNNNNNNNNNNNNNNNNNNNNNN
NNNNNNNNNNNNNNNNNNNNNNNNNNNNNNNNNNNNNNNNNNNNNNNNNNNCNTCTNNNAAANNGTNNN
NNNNNNNNNNNNNNNNNNNNNNNNNNCNCNTTGNNNGTAANCNANNNGNACATGCAGTCCCCTCNNNNN
NNNGGCAGAGNNNNNNNNCNNNNNCNCCGNCCCCGNCCNNNNNNNTNNNNNNGGNNNNNNNGNNNNNN
NNNNNNTGGTACNNNNCGNNNNNGGTTGTTTAAGAGGTCCACCACGNGGTCNNGNCACNNNNGNCNAN
NNNCNNNNNACNNNNNNNNNNNNNNATNNNCANNAGGNCNNNNCCNTNNNGNNNNNAATTGCNATGACC
CAANNCACGGGGGCNGTNCCCGGCGCGGCGACCTTGATNACGGCCGGGGACAAGTATGGCNTNCNCCC
CGGGAAGGANNCNGCCGTANGTGCNNNNNANNNNNTCNTGNGANGTCGCCGNNATTCNCGTGGTCNNN
NNAGAATGNCCGNACCANNCGNANNNTGGTNATGTTAGTANNNNNNNNNNNNNNNNNNNNNNNNNNNNNN
NNNNNNNNNNNNNNNNNNNNNNNNNNNNNNNNNNNGNNNNGNNNNNNCNANNNAAGGANNTANNNCTNNTN
NTNNNGNNGGACNNNACNNNNCNNNNNNNACNCNNNCNCNNNNNNNNNNNGNTTTTTNNNCNNTATTNT
CCAGNACGCNGNGCNNNCATTCANGNGAACNNNGNNACGCGNNAATCGTANNATCTACCTGCCGCNGG
TNNNNNTACNGNNNNTNCGGANNNTNNCTCGTANNNNNNTCATGCGTNNTNTNNTCNCTAGNCANGTT
CGNNNANANANTAGNANGTNTNNNNNNNCTTGTNNCGTTCCGCNTGANNCNNCANNNNGNNGNNCTTN
TCANCGGCNCTANCTGCNGANNTAAGTCATANNNTNNATCNNTANCTNNNNNGGAGGGNNACTGTAAA
NGGATAATTGGAGGNTNATTTCNNNCACNTNNCTNNGNNNNCTNNNTNCCTGNNTNCNNAATTGGNNC
CANTGNNTGNCNATCTCTGGNTTNGNNNNNNTNNNNNTNNCAANNNGGNNNGTNANANNNCNATNTAA
NNTATANNNNCNNNNNNANNNCACNANNNNNGNAGNNNCNANNNTTANANNNGTAGNNNAATAAGTCT
CCGANNTNNNNNNNATAAGCGNNANACNNNNNNNNNNNNNNANGANNACGNNTTGGNNNTNNNNNNCNA
ANNAGACNNTNGTGGNTNNNNNTCAANNNNTTCGTAGTGGTATAACNGNNNNNNNTTTCNCCAGNNTN
NNNGNNNNNNNCNATANNANNNNNNNNGTGANNNNNNNNNNANNNNNCTCNNNAGGGANCANCTACTNNA
GCAATGGNGCGCAGNNNGAGANNNNNGACNAGCCTTGGGTGGCAANCACNGCAATAAGTNNNGTTNANC
CTNNNAGTNNACACNNNNNNCANNGNNNCNNNNNNANCNGANNTNNNNNNNNNNNNNNNNNNNNNNNNNN
NNNNNNNNNNNNNNNNNNNNNNNNNNNNNAGNNNANGGNNNGTNTNTGACNCATGTGTTTTTANNNGNN
NNNNNACGNNNNANCCCCGGTCGNNNNCNNATTGAANNCCTGNNGCATNNTNNTNCNAGAATGANNGC
NNNNNGNNTNGNCTGNCNTTTCAAGNAGTCNNNNAGGTANCGCGNTCNNNNNNNNNNNNNCNNTCCCAN
ACTGAGGGTNNNNNNCGCATCTGCTNNNNNNNNNCTTCGCNGNCGTNNCTNNNNAGCCGTATNGTGTNN
NNCANNNNNNNNNNNNNNNNNNNTNNAACAANNNNNNNTNNNN
>Tay169
GTGNNNNNTAGCCCNNNNNAGTCAGACANNNCNNNNNNNNNNNNNTNNNNNNNNNNNNNNNNNNNNNNNN
NNNNNNNNNNNNNNNNNNNNNNNNNNNNNNNNNNNNNNNNNNNNNNNNNNNNNNNNNNNNNNNNNNNNNN
NNNNNNNNNNNNNNNNNNNNNNNNNNNNNNNNNNNNNNNNNNNNNNNNNNNNNNNNNNNNNNNNNNNNNN
NNNNNNNNNNNNNNNNNNNNNNNNNNNNNNNNNNNNNNNNNNNNNNNNNNNNNNNNNNNNNNNNNNNNNN
NNNNNNNNNNNNNNNNNNNNNNNNNNNNNNNNNNNNNNNNNNNNNNNNNNNCNTCTNNNAAANNNTNNN
NNNNNNNNNNNANNNNNNNNNNNNNNNCNCNTTGNNNGTAANCNANNNGNACATGCAGTCCCCTCNNNNN
NNNNNCAGAGNNNNNNNCNCNNNNNNNNCCGGCCCCNNCCNNNNNNNTNNNNNNNGNNNNNNNANNNNNN
NNNNNNTGGTACNNNNCGNNNNNGGTTGTNTAAGAGGTCCACAACGNGGTCNGGNCACNNNNGNNNAN
NNNCNNNNAACNNNNNNNNNNNNNANATNNNTANNAGGNCNNNNCCNTCNNNNNNNNNNATNGCNATGACC
CANNTCACGGGGGNNGTNCCCGGCGCGGCGACCTTGATNANNTCCGGGGACAAGTATGGCGCNCNCCC
CGGGAAGGANNCNGCCGTANGTGCNNNNNANNNNNTCTTGNGANGTNGCCGNNATTCNNGTGGTCNNN
CNAGANTGNCCGNACCANNCGNANNNTGGTNATGNNAGCNNNNNNNNNNNNNNNNNNNNNNNNNNNNNN
NNNNNNNNNNNNNNNNNNNNNNNNNNNNNNNNNNNGNNNNGNNNNNNCNANNNAAGGANNTANNNCTNNTN

NTNNNNNNGGACNNNACNNNNCNNNNNNNACNCNNNCNCNNNNNNNNNNGNTTTTTNNNCNNTATTNT
CCAGNACGCNGNGCNNNCATTTANGNGAACNNNGNNACGCGTAAATCGTANNATCTACCTGCCGCNGG
TNNNNNTACNGNNNNTNCGGANNNTNNCTCGTANNNNNGTCATGCGTNNTNTNNTCNCNAGNCANGTT
CGAGAANANANTAGNANGTNTNNNNNNNCTTGTNNCGTTCCGCNTGANNCNNCANNNNGNNGNNCTTC
TCANCGGCNNTANCTGCNGANNTAAGTCATANNNTNNATCNNTANCTNNNNNGGNGGGNNACTGTAAA
NGGNTAATTNGNNGNTNANTTCCNNCATNTNNCTNNGNNTNCTNNNTNCCTGNNTNCNNAATTGGNNC
CANTGNNTGTCNATCTCTNGNTTNGCNNNNNTGNNNNTNGCAANNNGGNNNNTNANANNNCNATNTAA
NNTANANNNNCNNNNNNANNNCACNANNNNNGNAGNNNCNANNNGTANANNNGTAGNNNGATAAGTCT
CCGANNTNNNNNNNATAAGCGNNANACNNNNNNNNNNNNNANGANNACGNNTTTGNNNTNNNNNNCNA
GNNAGACNNTNGTGGNTNNNNNTCAANNNNTTCGTAGTGGTATAATNGNNNNNNCTTTCNCCAGNNTN
NNNGNNNNNNNCNATANNANNNNNNNGTGANNNNNNNNNNANNNNCTCNNNAGGGANCANCTACTNNA
GCAATGGNGCGCAGNNNGAGANNNNGACCNGCCTTGGGTGGCAANCACNGCAATAAGTNNNGTTNANC
CTTNNAGTNNACACNNNNNCANNGNNNCNNNNNNANCNGANNGNNNNNNNNNNNNNNNNNNNNNNNNN
NNNNNNNNNNNNNNNNNNNNNNNNNNNNAGNNNANGGNNNGTNTNTGACNCATGTGTTTTTANNNGNN
NNNNNNNGNNNNANCCCCGGTCGTNNNCNNATTGAANNCCTGNNGCATNNNNNTNCNAGAATGANNGC
NNNNNGNNTNGNCTNNCNTTTCAAGNAATCAANTAGGTNTNGCGNTCNNNGNNNNNNNNCNNTCCCNN
ACTGAGGGTNNNNNNCGCATCTGCTNNNNNNNNNTNCGCNGGCGTNNCNNCNNAGCNGTATNGTGTTN
NNCANNNNNNNNNNNNNNNNTNNAACNANNNNNNTNNNN
>Tay170
GTGNNNNNTAGCCCNNNNNAGTCAGACANNNCNNNNNNNNNNNNTNNNNNNNNNNNNNNNNNNNNNNN
NNNNNNNNNNNNNNNNNNNNNNNNNNNNNNTNNNNNNNNNNNNNNNNNNNNNNNNNNNNNNNNNNNNN
NNNNNNNNNNNNNNNNNNNNNNNNNNNNNNNNNNNNNNNNNNNNNNNNNNNNNNNNNNNNNNNNNNNN
NNNNNNNNNNNNNNNNNNNNNNNNNNNNNNNNNNNNNNNNNNNNNNNNNNNNNNNNNNNNNNNNNNNN
NNNNNNNNNNNNNNNNNNNNNNNNNNNNNNNNNNNNNNNNNNNNNNNNNNNTCTNNNAAANNNTNNN
NNNNNNNNNNNNNNNNNNNNNNNNNCNCNTTGNNNGTAANCNANNNGNACATGCAGTCCCCTCNNNNN
NNNGGCAGAGNNNNNNNCCNNNNNCNCCGGCCCCNNCCNNNNNNNTNNNNNNNGNNNNNNNANNNNNN
NNNNNNTGGTACNNNNCGNNNNNNGGTTGTTTAAGAGGTCCACAACGNGGTCNNGNCACNNTGNNNAN
NNNCNNANAANNNNNNNNNNNNNNNTNNNTANNAGGNCNNNNCCNTCNNGNNANNNANTGCNATGANN
CAANTCACGGGGGCAGTCCCCGGCGCGGCGACCTTGATNACGTCCGGGGACAAGTATGGCGNNCNCCC
CGGGAAGGANNCNGCCGTANGTGCNNNNNANNNNNTCNTGNGACGTCGCCGNNATTCNCGTGGNCNNN
CNAGAATGCCCGNACCANNCGNANNNTGGTNATGNNAGTANNNNNNNNNNNNNNNNNNNNNNNNNNNN
NNNNNNNNNNNNNNNNNNNNNNNNNNNNNNNNGNNNNGNNNNNNCNANNNAAGGANNTANNNCTNNTC
NTNNNNNNGGACNNNACNNNNCNNNNNNNACNCNNNCNCNNNNNNANNNGNTTTTTNNNCNNTATTNT
CCAGNACGCNGNGCNNNCATTCANGNGAACNNNGNNACGCGNNAATCGTANNATCTACCTGCCGCNGG
TNNNNNTACNGNNANTNCGGANNNTNNCTCGTANNNNNNTCATGCGTNNTNTNNTCNCNAGNCNNGTT
CGNNNANANANTAGNANGTNTNNNNNNNNNTGTNNCGTTCCGCNTGANNCNNCANNNNGNNGNNCTTN
TCANCGGCNNTANCTGCNGANNTAAGTCATANNNTNNNTCNNTANCTNNNNNGGNGGGNNACTGTAAA
NGGNNAATTNGANGNTNATTTCNNNCATNTNNCTNNGNNTNCTNNNTNCCTGNNTNCNNAATTGGNNC
CANTGNNTGNCNATCTCTGGNTTNGNNNNNNTGNNNNTNNCAANNNGGNNNGTNANANNNCNATNTAA
NNTATANNNNCNNNNNNANNNCACNANNNNNGNAGNNNCNANNNGTANANNNGTAGNNNGATAAGTCT
CCGANNTNNNNNNNATAAGCGNNANACNNNNNNNNNNNNNANGANNACGNNTTTGNNNTNNNNNNCNA
GNNAGACNNTNGTGGNTNNNNNTCAANNNNTTNGTAGTGGTATAATNGNNNNNNNTTTCNCCAGNNTN
NNNGNNNNNNNCNATANNANNNNNNNGTGANNNNNNNNNNANNNNCTCNNNAGGNANCANCTACTTNA
GCAATGGNGCGCAGNNNGAGANNNNGACNNGCCTTGGGTGGCAANCACNGCAATAAGTNNNGTTNANC
CTNNNAGTNNACACNNNNNCANNGNNNCNNNNNNANCNGANNGNNNNNNNNNNNNNNNNNNNNNNNNN
NNNNNNNNNNNNNNNNNNNNNNNNNNNNAGNNNANNGNNNGTNTNTGACNCATGTGTTTTTANNNGNN
NNNNNACGNNNNANCCCCGGTCGNNNNCNNATTGAANNCCTGNNGCATNNNNNTNCNAGAATGANNNC
NNNNNGNNTNGNCTNNCNTTTCAAGNAATCAANNAGNNATCGCGNTCNNNGNNNNNNNNCNNTCCCNN
ACTGAGGGTNNNNNNNCATCTGCTNNNNNNNNNCTNCGCNGGNGTNNCNNNNNAGCNGTATNGTGTTA
NNCANNNNNNNNNNNNNNNNNNAAACNANNNNNNTNNNG
>Tay171
GTGNNNNNTAGCCCNNNNNAGTCAGACANNNCNNNNNNNNNNNNNNNNNNNNNNNNNNNNNNNNNNNN
NNNNNNNNNNNNNNNNNNNNNNNNNNNNNNTNNNNNNNNNNNNNNNNNNNNNNNNNNNNNNNNNNNNN
NNNNNNNNNNNNNNNNNNNNNNNNNNNNNNNNNNNNNNNNNNNNNNNNNNNNNNNNNNNNNNNNNNNN

NNNNNNNNNNNNNNNNNNNNNNNNNNNNNNNNNNNNNNNNNNNNNNNNNNNNNNNNNNNN
NNNNNNNNNNNNNNNNNNNNCNNNNNNNNNNNNNNNNNNNNNNNNNTCTNNNAAANNNTNNN
NNNNNNNNNNNNNNNNNNNNNNNNNNCNCNTTGNNNGTAANCNANNNGNACATGCAGTCCCCTCNCNNN
NNNGGCAGAGNNNNNNCCCNNNNNCNCCGGCCCCGNCCNNNNNNNNNNNCANNGNNNNNNNANNNNNN
NNNNNNTGGTACNNNNCGNNNNNGGTTGTTTAAGAGGTCCACAACGNGGTCNNGNNACNNNNGNNNAN
NNNCNNNNAACNNNNNNNNNNNNNNNTNNNTANNAGGNCNNNNCCNTCNNGNNNNNAANTGCNATGACC
CAANNCACGGGGGCAGTNCCCGGNGCGGCGACCTTGATNACGTCCGGGGACAAGTATGGCGCNCNCCC
CGGGAAGGANNCNGCCGTANGTGCNNNNNANNNNNTCNTGNGACGTCGCCGNNATTNNCGTGGTCNNN
CNAGAATGNCCNNACCANNCGNANNNTGGTNATGNNAGTNNNNNNNNNNNNNNNNNNNNNNNNNNNN
NNNNNNNNNNNNNNNNNNNNNNNNNNNNNNNNNNNGNNNNGNNNNNNCNANNNAAGGANNTANNNCTNNTC
NTNNNNNNNGGACNNNACNNNNCNNNNNNNNACNCNNNCNCNNNNNNNNNNNGNTTTTTNNNCNNTATTNT
CCAGNACGCNGNGCNNNCATTCANGNGAACNNNGNNACGCGTNAATCGTANNATCTACCTGCCGCNGG
NNNNNNTACNGNNNNTNCGGANNNTNNCTCGTANNNNNNTCATGCGTNNTNTNNTCNCNAGNCANGTT
CGNNNANANANTAGNANGTNTCNNNNNNNNNTGTNNCGTTCCGCNTGANNCNNCANNNNGNNGNNCTTN
TCANCGGCNNTANCTGCNGANNTAAGTCATANNNTNNNTCNNTANCTNNNNNGGAGGGNNACTGTAAA
NGGNNAATTNGNNGNTNATTTCNNNCATNTNNCNNNGNNNNCTNNNTNCCTGNNTNCNNAATTGGNNC
CANTGNNTGNCNATCTCTNGNTTNGNNNNNNTNNNNNTNNCANNNNGGNNNGTNANANNTCNATNTAA
NNTATANNNNCNNNNNNANNNCACNNNNNNNGNAGNNANNANNNGTANANNNGTAGNNNGATAAGTCT
CCGANNTNNNNNNNATAAGCGNNANACNNNNNNNNNNNNNNANGANNACGNNTTTGNNNTNNNNNNCNA
GNNAGACNNTNGTGGNTNNNNNTCAANNNNTTNGTAGTGGTATAATNGNNNNNNNTTTCNCCAGNNTN
NNNGNNNNNNNNCNATANNANNNNNNNGNGANNNNNNNNNNNANNNNCTCNNNAGGGANCANCTACTTNA
GCAATGGNGCGCAGNNNGAGANNNNGACNNGCCTTGGGTGGCAANCACNGCAATAAGTNNTGTTNANC
CTNNNAGTNNACACNNNNNCANNGNNNCNNNNNNANCNGANNGNNNNNNNNNNNNNNNNNNNNNNN
NNNNNNNNNNNNNNNNNNNNNNNNNNNNNAGNNNANGGNNNGTNTNTGACNCATGTGTTTTTANNNGNN
NNNNNACGNNNNANCCCCGGTCGNNNNCNNATTGAANNCCTGNNGCATNNNNNNTNCNAGAATGANNNC
NNNNNGNNTNGNCTGNCNNNTCAAGNAATCNNNTANGTANNNCGNTCNNNNANNNNNNNCNNTCCCNN
ACTGAGGGTNNNNNNNNCATCTGCTNNNNNNNNCTNCGCNGGCGTNNCNNNNNNGCNGTATNGTGTTA
NNCANNNNNNNNNNNNNNNNNNNNAAACNANNNNNNCTNNNN
>Tay172
GTGNNNNNTAGCCCGNNNNAGTTAGACANNNCNNNNNNNNNNNNTNNNNNNNNNNNNNNNNNNNNNNN
NNNNNNNNNNNNNNNNNNNNNNNNNNNNNNNNNNNNNNNNNNNNNNNNNNNNNNNNNNNNNNNNN
NNNNNNNNNNNNNNNNNNNNNNNNNNNNNNNNNNNNNNNNNNNNNNNNNNNNNNNNNNNNNNNNN
NNNNNNNNNNNNNNNNNNNNNNNNNNNNNNNNNNNNNNNNNNNNNNNNNNNNNNNNNNNNNNNNN
NNNNNNNNNNNNNNNNNNNNNNNNNNNNNNNNNNNNNNNNNNNNNNNNNNNNNNNNTCTNNNAAANNNTNNN
NNNNNNNNNNNNNNNNNNNNNNNNNNCNCNTTGNNNGTAANCNANNNGNACATGCAGTCCCCTCNNNNN
NNNGGCAGAGNNNNNNNCCNNNNNNNCCGGCCCCNNCCNNNNNNNNNNNNNNNNNNNNNNNNNNANNNNNN
NNNNNNTGGTACNNNNCGNNNNNGGTTGTTTAAGAGGTCCACAACANGGTCNNGNCACNNNNGNNNAN
NNNCNNNNAACNNNNNNNNNNNNANNTNNNTANNAGGNNNNNGCCNTCNNNNNNNNNNANNGCNATGACC
CNANNCACGGGGGCAGTCCCCGGCGCGGCGACCTTGNTNACGTCCGGGGACAAGTATGGCNCNCNCCC
CGGGAAGGANNCNGCCGTANGTGCNNNNNANNNNNTCNTGNGACGTNGCCGNNATTCNCGTGGTCNNN
CNAGAATGNNCGNACCANNCGNANNTTGGTNATGNTAGTANNNNNNNNNNNNNNNNNNNNNNNNNNNN
NNNNNNNNNNNNNNNNNNNNNNNNNNNNNNNNNNNTNNNNGNNNNNNCNANNNAAGGNNNTANNNCTNNTN
NTNNNNNNNGGACNNNACNNNNCNNNNNNNNACNCNNNCNCNNNNNNNNNNNGNTTTTTNNCCNNTATTNT
CCAGNACGCNGNGCNNNCATTCANGNGAACNNNGNNACGCGNNAATCGTANNATCTACCTGCCGCNGG
TNNNNNTNCNGNNNNTNCGGANNNTNNCTCGTANNNNNNTCATGCGTNNTNTNNTCNCNAGNCANGTT
CGNNNATANANTAGNANGTNTCNNNNNNNNNTGTNNCGTTCCGCNCGANNCNNCANNNNGNNGNNCTTN
TCANCGGCNNTANCTGCNGANNTAAGTCATANNNTNNNTCNNTANCTNNNNNGGNGGGNNACTGTAAA
NGGNTAATTGGNNGNTNATTNCNNNCACNTNNCTNNANNTNCTNNNTNCCTGNNTNCNNAATTGGNNC
CANTGNNTGNCNATCTCTNGNTTNGNNNNNNTGNNNNTNGCAANNNGGNNNGTNANANNNCNATNTAA
NNTATANNNNCNNNNNNANNNCACNANNNNNGNAGNNNCNANNNTTANANNNGTAGNNNANTAAGTCT
CCGANNTNNNNNNNATAAGCGNNANACNNNNNNNNNNNNNNANGANNACGNNTTGGNNNTNNNNNGCNA
GNNAGACNNTNGTGGNTNNNNNTCAANNNNTTNGTAGTGGTATAATNGNNNNNNNTTTCNCCAGNNTN
NNNGNNNNNNNNCNATANNANNNNNNNGNGANNNNNNNNNNNANNNNCTTNNNAGGGANCANTTACTTNA
GCAATGGNGCGCAGNNNGAGANNNNGACNNGCCTTGGGTGGCAANCACNGCAATAAGTNNTGTTNANC

CTNNNAGTNNACACNNNNNCANNGNNNCNNNNNNANCNGANNGNNNNNNNNNNNNNNNNNNNNNNNNN
NNNNNNNNNNNNNNNNNNNNNNNNNNNNNAGNNNANGGNNNGTNTNTGACNCATGTGTTTTANNNGNN
TNNNNACGNNNNANCCCCGGTCGNNNNCNNATTGAANNCCTGNNGCATNNNNNTNCNAGAATGANNGC
NNNNNGNNTNGCTGNCNTTTCAAGNAATCANNNANGTNNCGCGNTCNNGGNNNNNNNNNCNNTCCCNN
ACTGAGGGTNNNNNNNNCATCTGCTNNNNNNNNCTTCGCNGGCGTNNCNNNNNAGCNGTATNGTGTTA
NNCANNNNNNNNNNNNNNNNNNNNNNNNNANNNNNNNTNNNN
>Tay173
GTGNNNNNTAGCCCNNNNNAGTTAGACANNNCNNNNNNNNNNNNNTNNNNNNNNNNNNNNNNNNNNNNNN
NNNNNNNNNNNNNNNNNNNNNNNNNNNNNNNTNNNNNNNNNNNNNNNNNNNNNNNNNNNNNNNNNNNN
NNNNNNNNNNNNNNNNNNNNNNNNNNNNNNNNNNNNNNNNNNNNNNNNNNNNNNNNNNNNNNNNNNNN
NNNNNNNNNNNNNNNNNNNNNNNNNNNNNNNNNNNNNNNNNNNNNNNNNNNNNNNNNNNNNNNNNNNN
NNNNNNNNNNNNNNNNNNNNNNNNNNNNNNNNNNNNNNNNNNNNNNNNNNCNTCTNNNAAANNNTNNN
NNNNNNNNNNNNNNNNNNNNNNNNNCNCNTTGNNNGTAANCNANNNGNACATGCAGTCCCCTCNNNNN
NNNGGCAGAGNNNNNNCCCNNNNCNCCGGCCCCNNCCNNNNNNNNNNNNCANNGNNNNNNNANNNNNN
NNNNNNTGGTACNNNNCGNNNNNGGTTGTTTAAGAGGTCCACAACANGGTCNGGNCACNNNNGNNNAN
NNNCNNNAAACNGNNNNNNNNNANNTNNNTANNAGGNNNNNNCCNTCNNNNNNNNAATTGCNATGACC
CAANTCACGGGGGCAGTCCCCGGCGCGGCGACCTTGATNANNTCCGGGGACAAGTATGGCGCNCNCCC
CGGGAAGGANNCNGCCGTANGTGCNNNNNANNNNNTCNTGNGACGTNGCCGNNATTCNCGTGGTCNNN
CNAGAATGNNCGNACCANNCGNANNNTGGTNATGNTAGTANNNNNNNNNNNNNNNNNNNNNNNNNNNN
NNNNNNNNNNNNNNNNNNNNNNNNNNNNNNNNGNNNNGNNNNNNCNANNNAAGGANNTANNNCTNNTN
NTNNNNNNGGACNNNACNNNNCNNNNNNNACNCNNNCNCNNNNNNNNNNGNTTTTTNNNCNNTATTNT
CCAGNACGCNGNGCNNNCATTCANGNGAACNNNGNNACGCGNNAATCGTANNATCTACCTGCCGCNGG
TNNNNNTACNGNNNNTNCGGANNNTNNNNCGTANNNNNNTCATGCGTNNTNTNNTCNCNAGNCANGTT
CGNNNANANANTAGNANGTNTNNNNNNNCNTGTNNCGTTCCGCNCGANNCNNCANNNNGNNGNNNTTN
TCANCGGCNNTANCTGCNGANNTAAGTCATANNNTNNNTCNNTANCTNNNNNGGNGGGNNACTGTAAA
NGGNNAATTNGNNGNTNANTTCNNACACNTNNCNNNANNTNCTNNNTNCCTGNNTNCNNAATTGGNNC
CANTGNNTGNCNATCTCTGGNTTNGNNNNNNTGNNNNTNGCANNNNGGNNNNNNANANNNCNATNTAA
NNTATANNNNCNNNNNNANNNCACNANNNNNGNAGNNNCNANNNTTANANNNGTAGNNNAATAAGTCT
CCGANNTNNNNNNNATAAGCGNNANACNNNNNNNNNNNNNANGANNACGNNTTGGNNNTNNNNNNCNA
GNNAGACNNTNGTGGNTNNNNNTCAANNNNTTNGTAGTGGTATAATNGNNNNNNNTTTCNCCAGNNTN
NNNGNNNNNNNCNATANNANNNNNNNGTGANNNNNNNNNNANNNNCTTNNNAGGGANCANTTACTTNA
GCAATGGNGCGNAGNNNGAGANNNNGACNNNCCTTGGGTGGCAANCACNGCAATAAGTNNNGTTNANC
CTNNNAGTNNACACNNNNNCANNGNNNCNNNNNNANCNGANNGNNNNNNNNNNNNNNNNNNNNNNNNN
NNNNNNNNNNNNNNNNNNNNNNNNNNNNNAGNNNANGGNNNGTNTNTGACNCATGTGTTTTANNNGNN
NNNNNACGNNNNANCCCCGGTCGNNNNCNNATTGAANNCCTGNNGCATNNNNNTNCNAGAATGANNNC
NNNNNGNNTNGCTNNCTTTTCAAGNAATCAANNAGGNNTCGCGNTCNNGGNNNNNNNNNCNNTCCCNN
ACTGAGGGTNNNNNNNNCATCTGCTNNNNNNNNCTTCGCNGGCGTNNCNNNNNAGCNGTATNGTGTNN
NNCANNNNNNNNNNNNNNNNNNNNNAAAAANNNNNNTNNNN
>Tay174
GTGNNNNNTAGCCCGNNNNAGTCAGNCANNNCNNNNNNNNNNNNNTNNNNNNNNNNNNNNNNNNNNNNNN
NNNNNNNNNNNNNNNNNNNNNNNNNNNNNNNNNNNNNNNNNNNNNNNNNNNNNNNNNNNNNNNNNNNN
NNNNNNNNNNNNNNNNNNNNNNNNNNNNNNNNNNNNNNNNNNNNNNNNNNNNNNNNNNNNNNNNNNNN
NNNNNNNNNNNNNNNNNNNNNNNNNNNNNNNNNNNNNNNNNNNNNNNNNNNNNNNNNNNNNNNNNNNN
NNNNNNNNNNNNNNNNNNNNNNNNNNNNNNNNNNNNNNNNNNNNNNNNNNCNTCTNNNAAANNNTNNN
NNNNNNNNNNNNNNNNNNNNNNNNNCNCNTTGNNNGTAANCNANNNGNACATGCAGTCCCCTCNNNNN
NNNGGCAGAGNNNNNNCCNNNNNNNCCGGCCCCGNCCNNNNNNNNNNNNCANNGNNNNNNNANNNNNN
NNNNNNTGGTACNNNNCGNNNNNGGTTGTNTAAGAGGTCCACAACGNGGTCNNGNCACNNNNGNNNAN
NNNCNNAAAACNNNNNNNNNNNANNTNNNTANNAGGNCNNNNCCNTCNNGNNNNNAANTGCNATGACC
CAANTCACGGGGGCAGTCCCCGGCGCGGCGACCTTGNTNACNTCCGGGGNCAAGTATGGCNCNCNCCC
CGGGAAGGANNCNGCCGTANGTGCNNNNNANNNNNTCNTGNGANGTCGCCGNNANTCNNGTGGTCNNN
NNAGAATGNCCNNACCANNCGNANNNTGGTNATGNNAGTNNNNNNNNNNNNNNNNNNNNNNNNNNNNN
NNNNNNNNNNNNNNNNNNNNNNNNNNNNNNNNGNNNNGNNNNNNCNANNNAAGGANNTANNNCTNNTN
NTNNNGNTGGACNNNACNNNNCNNNNNNNACNCNNNCNCNNNNNNNNNNGNTTTTNNNCCNNTATTNT
CCAGNACGCNGNGCNNNCATTCANGNGAACNNNGNNACGCGNNAATCGTANNATCTACCTGCCGCNNG

TNNNNNTACNGNNNNTACGGANNNTNNNNCGTANNNNNNNCATGCGTNNTNTNNTCNCNAGNCANGTT
CGNNNANANANTAGNANGTNTNNNNNNNNNNTGTNNCGTTCCGCNTGANNCNNCANNNNGNCGNNCTTN
TCANCGGCNNTANCTGCNGANNTAAGNCATANNNTNNNTCNNTANCTNNNNNGGAGGGNNACTGTAAA
NGGNNAATTGGANGNTNATTNCNNNCATNTNNCTNNGNNNNCTNNNTNCCTGNNTNCNNAATTGGNNC
CANTGNNTGNCNATCTCTGGNTTNGNNNNNNTNNNNNTNNCAANNNGGNNNGTNNNANNNCNATNTAA
NNTATANNNNCNNNNNNANNNCACNNNNNNNGNAGNNNCNANNNGTANANNNGTAGNNNGNTAAGTCT
CCGANNTNNNNNNNATAAGCGNNANACNNNNNNNNNNNNNNANGANNACGNNTTTGNNNTNNNNNNCNA
GNNAGACNNTNGTGGNTNNNNNTCAANNNNTTNGTAGTGGTATAATNGNNNNNNNTTTCNCCAGNNTN
NNNGNNNNNNNNCNATANNANNNNNNNGTGANNNNNNNNNNNANNNNCTCNNNAGGGANCANCTACTNNA
GCANTGGNGCGCAGNNNGAGANNNNGACCAGCCTTGGGTGGCAANCACNGCAATAAGTNNNGTTNANC
CTNNNAGTNNACACNNNNNCANNGNNNCNNNNNNANCNGANNGNNNNNNNNNNNNNNNNNNNNNNNNNN
NNNNNNNNNNNNNNNNNNNNNNNNNNNNNNNGNNNANNGNNNGTNTNTGACNCATGTNTTTTTANNNGNN
NNNNNACGNNNGNNCCCCGGTCGTNNNCNNATTGAANNCCTGNNGCATNNNNNTNCNAGAATGANNNC
NNNNNGNNTNGNCTNNCNNNTCANGNAATCANNNAGGTANNGCGNTCNNNGNNNNNNNNCNNTCCCNN
ACTGAGGGTNNNNNNCGCATCTGCTNNNNNNNNNNTTCGCNGGNGTNNCNNNNNAGCNGTATNGTGTNA
NNCANNNNNNNNNNNNNNNNNNNNAACNNNNNNNNNTNNNN
>Tay175
GTGNNNNNTAGCCCNNNNNAGTCAGACANNNCNNNNNNNNNNNNTNNNNNNNNNNNNNNNNNNNNNNN
NNNNNNNNNNNNNNNNNNNNNNNNNNNNNNNNTNNNNNNNNNNNNNNNNNNNNNNNNNNNNNNNNNNNNN
NNNNNNNNNNNNNNNNNNNNNNNNNNNNNNNNNNNNNNNNNNNNNNNNNNNNNNNNNNNNNNNNNNNNNN
NNNNNNNNNNNNNNNNNNNNNNNNNNNNNNNNNNNNNNNNNNNNGNNNNNNNNNNNNNNNNNNNNNNNNN
NNNNNNNNNNNNNNNNNNNNNNNNNNNNNNNNNNNNNNNNNNNNNNNNNNNNNCNTCTNNNAAANNGTNNN
NNNNNNNNNNNNNNNNNNNNNNNNNNCNCNTTGNNNGTAANCNANNNGNACATGCAGTCCCCTCNNNNN
NNNGGCAGAGNNNNNNCNCNNNNNCNCCGGCCCCNNCCNNNNNNNNNNNNNNNNGNNNNNNNANNNNNN
NNNNNNTGGTACNNNNCGNNNNNGGTTGTTTAAGAGGTCCACAACGNGGTCNNGNCACNNNNGNNNAN
NNNCNNANAACNNNNNNNNNNNNANNTNNNTACNAGGNCNNNNCCNTCNNGNNNNNAATNGCNATGACC
CAANNCACGGGGGCAGTNCCCGGCGCGGCGACCTTGATNANGTCCGGGGACAAGTATGGCNCNCNCCC
CGGGAAGGANNCNGCCGTANGTGCNNNNNANNNNNTCNTGNGACGTNGCCGNNATTCNCNTGGNCNNN
CNAGAATGCNCGNACCANNCGNANNNTGGTNATGNTAGCAANNNNNNNNNNNNNNNNNNNNNNNNNNN
NNNNNNNNNNNNNNNNNNNNNNNNNNNNNNNNNNNNGNNNNGNNNNNNCNANNNAAGGANNTANNNCTNNTN
NTNNNGNNGGACNNNACNNNNCNNNNNNNACNCNNNCNCNNNNNNNNNNNGNTTTTTNNCCNNTATTNT
CCAGNACGCNGNGCNNTCATTTANGNGAACNNNGNNACGCGNNAATCGTANNATCTACCTGCCGCNGG
TNNNNNTACNGNNNNTNCGGANNNTNNCTCGTANNNNNGTCATGCGTNNTNTNNTCNCNAGNCNNGTT
CGNNNANANANTAGNANGTNTCNNNNNNNNNTGTNNCGTTCCGCNTGACNCNNCANNNNGNNGNNCTTN
TCANCGGCNNTANCTGCNGANNTAAGTCATANNNTNNATCNNTANCTNNNNNGGNGGGNNACTGTAAA
NGGNTAATTNGNGGNTNATTTCNNNCATNTNNCTNNGNNTNCTNNNTNCCTGNNTNCNNAATTGGNNC
CANTGNNTGNCNATCTCTNGNTTNGCNNNNNTNNNNNTNGCAANNNGGNNNGTNANANNNCNATNTAA
NNTATANNNNCNNNNNNANNNCACNANNNNNNNNAGNNNCNANNNGTANANNNGTAGNNNGATAAGTCT
CCGANNTNNNNNNNATAAGCGNNANACNNNNNNNNNNNNNNANGANNACGNNTTTGNNNTNNNNNGCNA
GNNAGACNNTNGTGGNTNNNNNTCAANNNNTTCGTAGTGGTATAATNGNNNNNNNTTTCNCCAGNNTN
NNNGNNNNNNNNCNATANNANNNNNNNGTGANNNNNNNNNNNANNNNCTCNNNAGGGANCANCTACTTNA
GCAATGGNGCGCAGNNNGAGANNNNGACNNNCCTTGGGTGGCAANCACNGCAATAAGTNNTGTTNANC
CTNNNAGTNNACACNNNNNCANNGNNNCNNNNNNANCNGANNGNNNNNNNNNNNNNNNNNNNNNNNNNN
NNNNNNNNNNNNNNNNNNNNNNNNNNNNNNNAGNNNANGGNNNGTNTNTGACNCATGTGTTTTTATNNGNN
NNNNNACGNNNNANCCCCGGTCGNNNNCNNATTGAANNCCTGNNGCATNNTNNTNCNAGAATGANNGC
NNNNNGNNTNGNCTNNCTTTTCAAGNAATCANNNAGGTNTCGCGNTCNNNNNNNNNNNNNCNNTCCCNN
ACTGAGGGTNNNNNNNNCATCTGCTNNNNNNNNNNTNCGCNGGCGNNNCNNNNNAGCNGTATNGNGTNA
NNCANNNNNNNNNNNNNNNNNNTTNNNNANNNNNNNNTNNNN
>Tay176
GTGNNNNNTAGCCCNNNNNAGTCAGACANNNCNNNNNNNNNNNNTNNNNNNNNNNNNNNNNNNNNNNN
NNNNNNNNNNNNNNNNNNNNNNNNNNNNNNNNNNNNNNNNNNNNNNNNNNNNNNNNNNNNNNNNNNNNNN
NNNNNNNNNNNNNNNNNNNNNNNNNNNNNNNNNNNNNNNNNNNNNNNNNNNNNNNNNNNNNNNNNNNNNN
NNNNNNNNNNNNNNNNNNNNNNNNNNNNNNNNNNNNNNNNNNNNNNNNNNNNNNNNNNNNNNNNNNNNNN
NNNNNNNNNNNNNNNNNNNNNNNNNNNNNNNNNNNNNNNNNNNNNNNNNNNNNCNTCTNNNAAANNNTNNN

NNNNNNNNNNNNNNNNNNNNNNNNNCNCNTTGNNNGTAANCNANNNGNACATGCAGTCCCCTCNNNNN
NNNNGCAGAGNNNNNNCCCNNNNNNNCCGGCCCCNNCCNNNNNNNNNNNCNNNGNNNNNNANNNNNNN
NNNNNNTGGTACNNNNCGNNNNNGGTTGTTTNAGAGGTCCACAACGNGGTCNNGNCACNNNNGNNNAN
NNNCNNNAAACNNNNNNNNNNNNNANNTNNNTANNAGGNNNNNNCCNTCNNGNNANNAANTGCNATGACC
CAANNCACGGGGGCAGTCCCCGGCGCGGCGACCTTGATNANGTCCGGGGACAAGTATGGCNCNCNCCC
CGGGAAGGANNCNGCCGTANGTGCNNNNNANNNNNTCNTGNGANNTCGCCGNNATTCNCGTGGTCTNN
CNAGAATGNCCGNACCANNCGNANNNTGGTNATGNNAGTANNNNNNNNNNNNNNNNNNNNNNNNNNNN
NNNNNNNNNNNNNNNNNNNNNNNNNNNNNNNNNNNNNGNNNNNNCNANNNAAGGNNNTANNNCTNNTN
NTNNNNNNGGACNNNACNNNNCNNNNNNNACNCNNNCNCNNNNNNANNNGGTTTTTNNNCNNTATTNT
CCAGNACGCNGNGCNNNCATTCANGNGAACNNNGNNACGCGNNAATCGTANNATCTACCTGCCGCNNG
TNNNNNTNCNGNNNNTNCGGANNNTNNCTCGTANNNNNNTCATGCGTNNTNTNNTCNCNAGNCANGTT
CGNNNANANANTAGNANGTNTCNNNNNNNNTGTNNCGTTCCGCNTGANNCNNCANNNNGNNGNNNTTN
TCANCGGCNNTANCTGCNGANNTAAGTCATANNNTNNNTCNNTANCTNNNNNGGNGGGNNACTGTAAA
NGGNNAATTNGANGNTNANTTCNNNCATNTNNCTNNGNNTNCTNNNTNCCTGNNTNCNNAATTGGNNC
CANTGNNTGNCNATCTCTGGNTTNGNNNNNNTNNNNNTNGCANNNNGGNNNGTNANANNNCNATNTAA
NNTATANNNNCNNNNNNANNNCACNANNNNNGNAGNNACNANNNGTANANNNGTAGNNNGATAAGTCT
CCGANNTNNNNNNNATAAGCGNNANACNNNNNNNNNNNNNNANGANNACGNNTTTGNNNTNNNNNNCNA
GNNAGACNNTNGTGGNTNNNNNTCAANNNNTTNGTAGTGGTATAATNGNNNNNNNTTTCNCCAGNNTN
NNNGNNNNNNNNCNATANNANNNNNNNGNGANNNNNNNNNNNANNNNCTCNNNAGGGANCANCTACTNNA
GCANTGGNGCGNAGNNNGAGANNNNGACNNNCCTTGGGTGGCAANCACNGCAATAAGTNNNGTTNANC
CTNNNAGTNNACACNNNNNCANNGNNNCNNNNNNANCNGANNGNNNNNNNNNNNNNNNNNNNNNNNNN
NNNNNNNNNNNNNNNNNNNNNNNNNNNNNNNNNNNANGGNNNGTNTNTGACNCATGTGTTTTTANNNGNN
NNNNNACGNNNNANCCCCGGTCGNNTNCNNATTGAANNCCTGNNGCATNNNNNNTNCNAGAATGANNGC
NNNNNGNNTNNNCTNNCNTTTCAAGNAATCANNNAGGTNNCGCGNTCNNNGNNNNNNNNNCNNTCCCNN
ACTGAGGGTNNNNNNNNCATCTGCTNNNNNNNNNCTTCGCNGGCGTNNCNNNNNAGCNGTATNGTGTNA
NNCANNNNNNNNNNNNNNNNNNNTNAAACNANNNNNNNTNNNG
>Tay177
GTGNNNNNTAGCCCGNNNNAGTCAGACANNNCNNNNNNNNNNNNNNNNNNNNNNNNNNNNNNNNNNNN
NNNNNNNNNNNNNNNNNNNNNNNNNNNNNNNNNNNNNNNNNNNNNNNNNNNNNNNNNNNNNNNNNNNN
NNNNNNNNNNNNNNNNNNNNNNNNNNNNNNNNNNNNNNNNNNNNNNNNNNNNNNNNNNNNNNNNNNNN
NNNNNNNNNNNNNNNNNNNNNNNNNNNNNNNNNNNNNNNNNNNNNNNNNNNNNNNNNNNNNNNNNNNN
NNNNNNNNNNNNNNNNNNNNNNNNNNNNNNNNNNNNNNNNNNNNNNNNNNNNNTCTNNNAAACNGTNNN
NNNNNNNNNNNNNNNNNNNNNNNNNCNCNTTGNNNGTAANCNANNNGNACATGCAGTCCCCTCNNNNN
NNNGGCAGAGNNNNNNCCCNNNNNNCNCCGGCCCCNNCCNNNNNNNNNNNCNNNGNNNNNNNNANNNNNN
NNNNNNTGGTACNNNNCGNNNNNGGTTGTNTAAGAGGTCCACAACGNGGTCNNGNCACNNNNGNNNAN
ANNCNNNNNNCNNNNNNNNNNNNNNNNTNNNTANNAGGNCNNNNCCNTCNNGNNNNNAANNGCNATGACC
CNANNCACGGNGGCAGTCCCCGGCGCGGCGACCTTGNTNANGTCCGGGGACAAGTATGGCNCNCNCCC
CGGGAAGGANNCNGCCGTANGTGCNNNNNANNNNNTCNTGNGANGTNGCCGNNATTCNCGTGGTCNNN
NNAGAATGNNCNNACCANNCGNANNNTGGTNATGNNAGTNANNNNNNNNNNNNNNNNNNNNNNNNNNN
NNNNNNNNNNNNNNNNNNNNNNNNNNNNNNNNNNNNNGNNNNNNCNANNNAAGGANNTANNNCTNNTN
NTNNNNNNGGACNNNACNNNNCNNNNNNNACNCNNNCNCNNNNNNNNNNNGNTTTTTNNNCNNTATTNT
CCAGNACGCNGNGCNNNCATTCANGNGAACNNNGNNACGCGNNAATCGTANNATCTACCTGCCGCNNG
TNNNNNTACNGNNNNTNCGGANNNTNNNNCGTANNNNNGTCATGCGTNNTNTNNTCNCNAGNCNNGTT
CGNNNANANANTAGNANGTNTNNNNNNNNNNTGTNNCGTTCCGCNTGANNCNNCANNNNGNCGNNNTTN
TCANCGGCNNTANCTGCNGANNTAAGNCATANNNTNNNTCNNTANCTNNNNNGGNGGGNNACTGTAAA
NGGNNAATTNGNNGNTNATTTCNNNCATNTNCCNNNGNNNNCTNNNTNCCTGNNTNCNNAATTGGNNC
CANTGNNTGNCNATCTCTGGNTTNGNNNNNNTNNNNNTNGCAANNNGGNNNNNNNNANCNCNATNTAA
NNTATANNNNCNNNNNNANNNCACNANNNNNGNAGNNNCNANNNGTANANNNGTAGNNNGATAAGTCT
CCGANNTNNNNNNNATAAGCGNNANACNNNNNNNNNNNNNNANGANNACGNNTTTGNNNTNNNNNNCNA
GNNAGACNNTNGTGGNTNNNNNTCAANNNNTTCGTAGTGGTATAATNGNNNNNNNTTTCNCCAGNNTN
NNNGNNNNNNNNCNATANNANNNNNNNGTGANNNNNNNNNNNANNNNCTCNNNAGGNANCANCTACTTNA
GCANTGGNGCGCAGNNNGAGANNNNGACNNNCCTTGGGTGGCAANCACNGCAATAAGTNNNGTTNANC
CTNNNAGTNNACACNNNNNCANNGNNNCNNNNNNANCNGANNGNNNNNNNNNNNNNNNNNNNNNNNNN
NNNNNNNNNNNNNNNNNNNNNNNNNNNNNAGNNNANNGNNNGTNTNTGACNCATGTGTTTTTANNNGNN

NNNNNACGNNNNNNNCCCCGGTCGNNNNCNNATTGAANNCCTGNNGCATNNNNNTNCNAGAATGANNGC
NNNNNGNNTNGCTNNCTTTTCAAGNAATCNNNNAGGTANNNCGNTCNNNNNNNNNNNNNNNCNNTCCCNN
ACTGAGGGTNNNNNNNNCATCTGCTNNNNNNNNNNNTNCGCNGNNGTNNCNNNNNAGCNGTATNGTGTNN
NNCANNNNNNNNNNNNNNNNTNNNNNNNNNNNNNNTNNNN
>Tay178
GTGNNNNNTAGCCCNNNNNAGTTAGACANNNCNNNNNNNNNNNNNTNNNNNNNNNNNNNNNNNNNNNNNN
NNNNNNNNNNNNNNNNNNNNNNNNNNNNNNNNNNNNNNNNNNNNNNNNNNNNNNNNNNNNNNNNNNNNNN
NNNNNNNNNNNNNNNNNNNNNNNNNNNNNNNNNNNNNNNNNNNNNNNNNNNNNNNNNNNNNNNNNNNNNN
NNNNNNNNNNNNNNNNNNNNNNNNNNNNNNNNNNNNNNNNNNNNNNNNNNNNNNNNNNNNNNNNNNNNNN
NNNNNNNNNNNNNNNNNNNNNNNNNNNNNNNNNNNNNNNNNNNNNNNNNNNNCNTCTNNNAAANNNTNNN
NNNNNNNNNNNNNNNNNNNNNNNNNNNCNCNTTGNNNGTAANCNANNNGNACATGCAGTCCCCTCNNNNN
NNNNNCAGAGNNNNNNCCCNNNNNNNCCGNCCCCGNCCNNNNNNNNNNNNNNNNNGNNNNNNNGNNNNNN
NNNNNNTGGTACNNNNCGNNNNNGGTTGTTTAAGAGGTCCACCACGNGGTCNNGNCACNNNNGNNNAN
NNNCNNNNNNNCNNNNNNNNNNNNNANNTNNNCANNAGGNCNNNNCCNTCNNNNNNNNNAATNGCNATGACC
CAANNCACGGGGGNNGTCCCCGGCGCGGCGACCTTGNTNANNGCCGGGGACAAGTATGGCNTNCNCCC
CGGGAAGGANNCNGCCGTANGTGCNNNNNANNNNNTCNTGNGACGTCGCCGNNATTCNCGTGGTCNNN
CNAGAATGNCCGNACCANNCGNANNNTGGTNATGNNAGTANNNNNNNNNNNNNNNNNNNNNNNNNNNNNN
NNNNNNNNNNNNNNNNNNNNNNNNNNNNNNNNNNNNNNNNNGNNNNNNCNANNNAAGGANNTANNNCTNNTC
NTNNNNNNNGGACNNNACNNNNCNNNNNNNACNCNNNCNCNNNNNNANNNGNTTTTTNNCCNNTATTNT
CCAGNACGCNGNGCNNNCATTCANGNGAACNNNGNNACGCGTAAATCGTANNATCTACCTGCCGCNGG
TNNNNNTNCNGNNNNTNCGGANNNTNNNNCGTANNNNNGTCATGCGTNNTNTNNTCNCNAGNCANGTT
CGNNNANANANTAGNANGTNTNNNNNNNNNNTGTNNCGTTCCGCNTGANNCNNCANNNNGNNGNNCTTC
TCANCGGCNNTANCTGCNGANNTAAGNCATANNNTNNNTCNNTANCTNNNNNGGNGGGNNACTGTAAA
NGGNNAATTNGNGGNTNANTTCNNNCACNTNNCTNNGNNNNCTNNNTNCCTGNNTNCNNAATTGGNNC
CANTGNNTGNCNATCTCTGGNTTNGNNNNNNTNNNNNTNNCANNNNGGNNNGTNANANNNCNATNTAA
NNTATANNNNCNNNNNNANNNCACNANNNNNGNAGNNNCNANNNTTANANNNGTAGNNNAATAAGTCT
CCGANNTNNNNNNNATAAGCGNNANACNNNNNNNNNNNNNNANGANNACGNNTTGGNNNTNNNNNNCNA
ANNANACNNTNGTGGNTNNNNNTCAANNNNTTCGTAGTGGTATAACNGNNNNNNNTTTCNCCAGNNTN
NNNGNNNNNNNNCNATANNANNNNNNNGTGANNNNNNNNNNNANNNNCTCNNNAGGGANCANCTACTTNA
GCANNGGNGCGCAGNNNGAGANNNAGACNNGCCTTGGGTGGCAANCACNGCAATAAGTNNNGTTTANC
CTNNNAGTNNACACGNNNNCANNGNNNCNNNNNNANCNGANNTNNNNNNNNNNNNNNNNNNNNNNNNNN
NNNNNNNNNNNNNNNNNNNNNNNNNNNNNNAGNNNANGGNNNGTNTNTGACNCATGTGTTTTTANNNGNN
NNNNNACGNNNGANCCCCGGTCGNNNNCNNATTGAANNCCTGNNGCATNNNNNTNCNAGAATGANNGC
NNNNNGNNTNGCTNNCNTTTCAAGNAGTCANNTAGGTANCGCGNTCNNNGNNNNNNNNCNNTCCCNN
ACTGAGGGTNNNNNNNGCATCTGCTNNNNNNNNNNNTNCGCNGNCGTNNCNNNNNAGCNGTATNGTGTNA
NNCANNNNNNNNNNNNNNNNNNNNAAACNANNNNNNNTNNNN
>Tay378
GTGNNNNNTAGCCCNNNNNAGTTAGACANNNCNNNNNNNNNNNNNTNNNNNNNNNNNNNNNNNNNNNNNN
NNNNNNNNNNNNNNNNNNNNNNNNNNNNNNNNTNNNNNNNNNNNNNNNNNNNNNNNNNNNNNNNNNNNNN
NNNNNNNNNNNNNNNNNNNNNNNNNNNNNNNNNNNNNNNNNNNNNNNNNNNNNNNNNNNNNNNNNNNNNN
NNNNNNNNNNNNNNNNNNNNNNNNNNNNNNNNNNNNNNNNNNNNNNNNNNNNNNNNNNNNNNNNNNNNNN
NNNNNNNNNNNNNNNNNNNNNNNNNNNNNNNNNNNNNNNNNNNNNNNNNNNNCNTCTNNNAAANNNTNNN
NNNNNNNNNNNNNNNNNNNNNNNNNNNCNCNTTGNNNGTAANCNANNNGNACATGCAGTCCCCTCNNNNN
NNNNNCAGAGNNNNNNCCCNNNNNNNCCGNCCCCNNCCNNNNNNNNNNNNCNNNNNNNNNNNNANNNNNN
NNNNNNTGGTACNNNNCGNNNNNGGTTGTTTAAGAGGTCCACAACGNGGTCNGGNCACNNNNGNNNAN
NNNCNNNNNNNCNNNNNNNNNNNNNANNTNNNTANNAGGNCNNNNCCNTCNNNNNANNNATTGCNATGACC
CAANNCACGGGGGCAGTNCCCGGNGNGGCGACCTTGANNANNTCCGGGGNCAAGTATGGCNCNCNCCC
CGGGAAGGANNCNGCCGTANGTGCNNNNNANNNNNTCNTGNGACGTCGCCGNNATTCNCGTGGTCNNN
CNAGAATGNCCGNACCANNCGNANNNTGGTNATGTNAGTNNNNNNNNNNNNNNNNNNNNNNNNNNNNNN
NNNNNNNNNNNNNNNNNNNNNNNNNNNNNNNNNNNNNNNNNGNNNNNNCNANNNAAGGANNTANNNCTNNTN
NTNNNGNNGGACNNNACNNNNCNNNNNNNACNCNNNCNCNNNNNNNNNNNGNTTTTTNNCCNNTATTNT
CCAGNACGCNGNGCNNNCATTCANGNGAACNNNGNNACGCGTNAATCGTANNATCTACCTGCCGCNNG
NNNNNNTNCNGNNNNNNCGGANNNTNNNNCGTANNNNNNTCATGCGTNNTNTNNTCNCNAGNCANGTT
CGNNNANANANTAGNANGTNTCNNNNNNNNNTGTNNCGTTCCGCNTGANNCNNCANNNNGNNGNNCTTN

TCANCGGCNNTANCTGCNGANNTAAGNCATANNNTNNATCNNTANCTNNNNNGGAGGGNNACTGTAAA
NGGNNAATTNGNNGNTNATTNCNNNCACNTNNCNNNGNNNNCTNNNTNCCTGNNTNCNNAATTGGNNC
CANTGNNTGNCNATCTCTGGNTTNGNNNNNNTNNNNNTNNCAANNNGGNNNGTNANANNNCNATNTAA
NNTANANNNNCNNNNNNANNNCACNANNNNNGNAGNNNCNANNNTTANANNNGTAGNNNAATAAGTCT
CCGANNTNNNNNNNATAAGCGNNANACNNNNNNNNNNNNNANGANNACGNNTTGGNNNTNNNNNNCNA
GNNAGACNNTNATGGNTNNNNNTCAANNNNTTNGTAGTGGTATAATNGNNNNNNNTTTCNCCAGNNTN
NNNGNNNNNNNNCNATANTANNNNNNNGTGANNNNNNNNNNNANNNNCTCNNNAGGGANCANCTACTNNA
GCANTGGNGCGCAGNNNGAGANNNNGACNNGCCTTGGGTGGCAANCACNGCAATAAGTNNNGTTNANN
CTNNNAGTNNACATNNNNNCANNGNNNCNNNNNNANCNGANNGNNNNNNNNNNNNNNNNNNNNNNNN
NNNNNNNNNNNNNNNNNNNNNNNNNNNNNAGNNNANGGNNNGTNTNTGACNCATGTGTTTTTANNNGNN
NNNNNACGNNNNANCCCCGGTCGNNNNCNNATTGAANNCCTGNNGCATNNNNNTNCNAGAATGANNGC
NNNNNGNNTNGNCTNNCNTTTCAAGNAATCANNNAGGTNNNNNGNTCNNNGNNNNNNNNCNNTCCCNN
ACTGAGGGTNNNNNNNNCATCTGCTNNNNNNNNCTTCGCNGGCNTNNCNGNNNAGCNGTATNGTGTNN
NNCANNNNNNNNNNNNNNNNNCNANNNNANNNNNNTNNNN
>Tay379
GTGNNNNNTAGCCCNNNNNAGTTAGACANNNCNNNNNNNNNNNNTNNNNNNNNNNNNNNNNNNNNNN
NNNNNNNNNNNNNNNNNNNNNNNNNGNNNNNTNNNNNNNNNNNNNNNNNNNNNNNNNNNNNNNNNNNNNN
NNNNNNNNNNNNNNNNNNNNNNNNNNNNNNNNNNNNNNNNNNNNNNNNNNNNNNNNNNNNNNNNNNNNNN
NNNNNNNNNNNNNNNNNNNNNNNNNNNNNNNNNNNNNNNNNNNNNNNNNNNNNNNNNNNNNNNNNNNNNN
NNNNNNNNNNNNNNNNNNNNNNNNNNNNNNNNNNNNNNNNNNNNNNNNNNNCNTCTNNNAAANNNTNNN
NNNNNNNNNNNNNNNNNNNNNNNNNNCNCNTTGNNNGTAANCNANNNGNACATGCAGTCCCCTCNNNNN
NNNGGCAGAGNNNNNNCCNNNNNNNNCCGNCCCCNNCCNNNNNNNNNNNNNNNNNNNNNNNNNNANNNNNN
NNNNNNTGGTACNNNNCGNNNNNGGTTGTTTAAGAGGTCCACAACGNGGTCNNGNCACNNNNGNNNAN
NNNCNNNNAACNNNNNNNNNNNNANNTNNNTANNAGGNCNNNNCCNTNNNGNNNNNAANTGCNATGANC
CNNNNCACGGGGGCAGTNCCCGGCGCGGCGACCTTGANNACGTCCGGGGACAAGTATGGCGNNCNCCC
CGGGAAGGANNCNGCCGTANGTGCNNNNNANNNNNTCNTGNGANGTCGCCGNNATTCNCGTGGTCNNN
CNAGAATGNNCNNACCANNCGNANNNTGGTTATGNNAGTNNNNNNNNNNNNNNNNNNNNNNNNNNNNN
NNNNNNNNNNNNNNNNNNNNNNNNNNNNNNNNGNGNNNNGNNNNNNCNANNNAAGGANNTANNNCTNNTC
NTNNNNNNGGACNNNACNNNNCNNNNNNNACNCNNNCNCNNNNNNNNNNNGNTTTTTNNCCNNTATTNT
CCAGNANGCNGNGCNNTCATTCANGNGAACNNNGNNACGCGNNAATCGTANNATCTACCTGCCGCNNG
TNNNNNTACNGNNNNTNCGGANNNTNNNNCGTANNNNNNTCATGCGTNNTNTNNTCNCNAGNCNNGTT
CGNNNANANANTAGNANGTNTNNNNNNNNNNTGTNNCGTTCCGCNTGANNCNNCANNNNGNNGNNCTTN
TCANCGGCNNTANCTGCNGANNTAAGTCATANNNTNNNTCNNTANCTNNNNNGGNGGGNNACTGTAAA
NGGNNAATTGGNNGNTNATTTCNNNCACNTNNCTNNGNNNNCTNNNTNCCTGNNTNCNNAATTGGNNC
CANTGNNTGNCNATCTCTGGNTTNGNNNNNNTNNNNNTNNCANNNNGGNNNGTNANANCNCNATNTAA
NNTATANNNNCNNNNNNANNNCACNANNNNNGNAGNNNCNANNNTTANANNNGTAGNNNAATAAGTCT
CCGANNTNNNNNNNATAAGCGNNANACNNNNNNNNNNNNNANGANNNCGNNTTGGNNNTNNNNNNCNA
GNNAGACNNTNATGGNTNNNNNTCAANNNNTTNGTAGTGGTATAATNGNNNNNNNTTTCNCCAGNNTN
NNNGNNNNNNNNCNATANNANNNNNNNGTGANNNNNNNNNNNANNNNCTCNNNAGGGANCANCTACTNNA
GCAATGGNGCGCAGNNNGAGANNNNGACNNNCCTTGGGTGGCAANCACNGCAATAAGTNNNGTTNANC
CTNNNAGTNNACATNNNNNCANNGNNNCNNNNNNANCNGANNGNNNNNNNNNNNNNNNNNNNNNNNN
NNNNNNNNNNNNNNNNNNNNNNNNNNNNNAGNNNANNGNNNGTNTNTGACNCATGTGTTTTTANNNGNN
NNNNNACGNNNNANCCCCGGTCGTNNNCNNATTGAANNCCTGNNGCATNNNNNTNCNAGAATGANNGC
NNNNNGNNTNGNCTNNCNTTTCAAGNAATCANANAGGTATNNNGNTCNNNGANNNNNNNCNNTCCCNN
ACTGAGGGTNNNNNNNGCATCTGCTNNNNNNNNCTTCGCNGGCGTNNCNNNNNAGCNGTATNGTGTNN
NNCANNNNNNNNNNNNNNNNNTNAAACNANNNNNNNTNNNN
>Tay380
GTGNNNNNTAGCCCNNNNNAGTTAGACANNNCNNNNNNNNNNNNTNNNNNNNNNNNNNNANNNNNNNN
NNNNNNNNNNNNNNNNNNNNNNNNNNNNNNNNTNNNNNNNNNNNNNNNNNNNNNNNNNNNNNNNNNNNNN
NNNNNNNNNNNNNNNNNNNNNNNNNNNNNNNNNNNNNNNNNNNNNNNNNNNNNNNNNNNNNNNNNNNNNN
NNNNNNNNNNNNNNNNNNNNNNNNNNNNNNNNNNNNNNNNNNNNNNNNNNNNNNNNNNNNNNGNNNNNN
NNNNNNNNNNNNNNNNNNNNNNNNNNNNNNNNNNNNNNNNNNNNNNNNNNNNTCTNNNAAANNNTNNN
NNNNNNNNNNNNNNNNNNNNNNNNNNCNCNTTGNNNGTAANCNANNNGNACATGCAGTCCCCTCNNNNN
NNNGGCAGAGNNNNNNNCCNNNNNNNCCGGCCCCNNCCNNNNNNNNNNNNCNNNGNNNNNNNANNNNNN

NNNNNNTGGTACNNNNCGNNNNNGGTTGTNTAAGAGGTCCACAACGNGGTCNNGNNACNNNNGNNNAN
NNNCNNNNANNNNNNNNNNNNNNNANNTNNNTANNAGGNCNNNNCCNTCNNGNNNNTAATTGCNATGACC
CAANTCNCGGGGGCAGTNCCCGGCGCGGCGACCTTGNTNACGTCCGGGGACAAGTATGGCGNNCNCCC
CGGGAAGGANNCNGCCGTANGTGCNNNNNANNNNNTCNTGNGANGTCGCCGNNATTCNCGTGGTCNNN
CNAGANTGNNCGNACCANNCGNANNNTGGTNATGTNAGTANNNNNNNNNNNNNNNNNNNNNNNNNNNNNN
NNNNNNNNNNNNNNNNNNNNNNNNNNNNNNNNNNGNNNNGNNNNNNCNANNNAAGGANNTANNNCTNNTC
NTNNNGNNGGACNNNACNNNNCNNNNNNNACNCNNNCNCNNNNNNANNNGGTTTTTNNNCNNTATTNT
CCAGNACGCNGNGCNNNCATTCANGNGAACNNNGNNACGCGTNAATCGTANNATCTACCTGCCGCNGG
TNNNNNTACNGNNNNNNCGGANNNTNNNNCGTANNNNNNTCATGCGTNNTNTNNTCNCNAGNCANGTT
CGNNNANANANTAGNANGTNTCNNNNNNNNTGTNNCGTTCCGCNTGANNCNNCANNNNGNNGNNCTTN
TCANCGGCNNTANCTGCNGANNTAAGTCATANNNTNNNTCNNTANCTNNNNNGGNGGGNNACTGTAAA
NGGNTAATTGGANGNTNATTTCNNNCACNTNNCTNAGNNNNCTNNNTNCCTGNNTNCNNAATTGGNNC
CANTGNNTGNCNATCTCTNGNTTNGNNNNNNTGNNNNTNNCAANNNGGNNNGTNANANNNCNATNTAA
NNTANANNNNCNNNNNNANNNCACNNNNNNNGNAGNNNCNANNNTTANANNNGTAGNNNAATAAGTCT
CCGANNTNNNNNNNATAAGCGNNANACNNNNNNNNNNNNNNANGANNACGNNTTGGNNNTNANNNNCNA
GNNAGACNNTNATGGNTNNNNNTCAANNNNTTCGTAGTGGTATAATNGNNNNNNNTTTCNCCAGNNTN
NNNGNNNNNNNNCNATANNANNNNNNNGTGANNNNNNNNNNNANNNNCTCNNNAGGGANCANCTACTTNA
GCAATGGNGCGCAGNNNGAGANNNNGACNNGCCTTGGGTGGCAANCACNGCAATAAGTNNNGTTNANC
CTNNNAGTNNACATNNNNNCANNGNNNCNNNNNNANCNGANNGNNNNNNNNNNNNNNNNNNNNNNNNNNN
NNNNNNNNNNNNNNNNNNNNNNNNNNNNNNAGNNNANGGNNNGTNTNTGACNCATGTGTTTTTANNNGNN
NNNNNACGNNNGANCCCCGGTCGNNNNCNNATTGAANNCCTGNNGCATNNNNNNTNCNAGAATGANNGC
NNNNNGNNTNGNCTNNCNTTTCAAGNAATCAANNAGGTANCGCGNTCNNNGANNNNNNNCNNTCCCNN
ACTGAGGGTNNNNNNNNCATCTGCTNNNNNNNNCTTCGCNGGNGTNNCNNNNNAGCNGTATNGTGTTA
NNCANNNNNNNNNNNNNNNNNNNNNNANNNANNNNNNNTNNNN
>Tay387
GTGNNNNNTAGCCCNNNNNAGTTAGACANNNCNNNNNNNNNNNNNTNNNNNNNNNNNNNNNNNNNNNNNN
NNNNNNNNNNNNNNNNNNNNNNNNNNNNNNTNNNNNNNNNNNNNNNNNNNNNNNNNNNNNNNNNNNNNNN
NNNNNNNNNNNNNNNNNNNNNNNNNNNNNNNNNNNNNNNNNNNNNNNNNNNNNNNNNNNNNNNNNNNNNN
NNNNNNNNNNNNNNNNNNNNNNNNNNNNNNNNNNNNNNNNNNNNNNNNNNNNNNNNNNNNNNNNNNNNNN
NNNNNNNNNNNNNNNNNNNNNNNNNNNNNNNNNNNNNNNNNNNNNNNNNNNNNNTCTNNNAAANNNTNNN
NNNNNNNNNNNNNNNNNNNNNNNNNNNCNCNTTGNNNGTAANCNANNNGNACATGCAGTCCCCTCNNNNN
NNNGGCAGAGNNNNNNCNCNNNNNNNCCGGCCCCGNCCNNNNNNNTNNNNNNNNNNNNNNNNNNANNNNN
NNNNNNTGGTACNNNNCGNNNNNGGTTGTTTAAGAGGTCCACAACANGGTCNGGNCACNNNNGNNNAN
NNNCNNNNAACNNNNNNNNNNNNNNNNTNNNTANNAGGNNNNNNCCNTCNNGNNNNTNATTGCNATGACC
CAANNCACGGGGGCAGTNCCCGGCGCGGCGACCTTGANNACGTCCGGGGACAAGTATGGCGCNCNCCC
CGGGAAGGANNCNGCCGTANGTGCNNNNNANNNNNTCNTGNGANGTCGCCGNNATTNNNNTGGTCNNN
CNAGAATGNCCGNACCANNCGNANNNTGGTNATGNTAGTANNNNNNNNNNNNNNNNNNNNNNNNNNNNNN
NNNNNNNNNNNNNNNNNNNNNNNNNNNNNNNNNNNNNNNGNNNNNNCNANNNAAGGANNTANNNCTNNTN
NTNNNNNNGGACNNNACNNNNCNNNNNNNACNCNNNCNCNNNNNNNNNNGGTTTTTNNNCNNTATTNT
CCAGNACGCNGNGCNNNCATTCANGNGAACNNNGNNACGCGNNAATCGTANNATCTACCTGCCGCNNG
TNNNNNTACNGNNNNNNCGGANNNTNNNNCGTANNNNNNTCATGCGTNNTNTNNTCNCNAGNCNNGTT
CGNNNANANANTAGNANGTNTCNNNNNNNNTGTNGCGTTCCGCNCGANNCNNCANNNNGNNGNNCTTC
TCANCGGCNCTANCTGCNGANNTAAGNCATANNNTNNATCNNTANCTNNNNNGGAGGGNNACTGTAAA
NGGNNAATTNGANGNTNATTTCNNNCACNTNNCTNNANNTNCTNNNTNCCTGNNTNCNNAATTGGNNC
CANTGNNTGNCNATCTCTGGNTTNGCNNNNNTGNNNNTNNCAANNNGGNNNGTNANANNNCNATNTAA
NNTATANNNNCNNNNNNANNNCACNNNNNNNGNAGNNNCNANNNTTANANNNGTAGNNNAATAAGTCT
CCGANNTNNNNNNNATAAGCGNNANACNNNNNNNNNNNNNNANGANNACGNNTTGGNNNTNNNNNNNNA
GNNAGACNNTNGTGGNTNNNNNTCAANNNNTTCGTAGTGGTATAATNGNNNNNNNTTTCNCCAGNNTN
NNNGNNNNNNNNCNATANNANNNNNNNGTGANNNNNNNNNNNANNNNCTTNNNAGGGANCANTTACTNNA
GCAATGGNGCGCAGNNNGAGANNNNGACNNGCCTTGGGTGGCAANCACNGCAATAAGTNNNGTTNANC
CTNNNAGTNNACACGNNNNCANNGNNNCNNNNNNANCNGANNGNNNNNNNNNNNNNNNNNNNNNNNNNNN
NNNNNNNNNNNNNNNNNNNNNNNNNNNNNNAGNNNANGGNNNGTNTNTGACNCATGTGTTTTTANNNGNN
NNNNNACGNNNGANCCCCGGTCGNNNNCNNATTGAANNCCTGNNGCATNNTNNTNCNAGAATGANNGC
NNNNNGNNTNGNCTGNCNNNTCAAGNAATCANNNAGGTANNNGNTCNNNNNNNNNNNNCNNTCCCNN

ACTGAGGGTNNNNNNCGCATCTGCTNNNNNNNNCTTCGCNGGCGTNNCTGNNNAGCNGTATNNTGTNN
NNCANNNNNNNNNNNNNNNNNTNAAACGNNNNNNNTNNNN
>Tay388
GTGNNNNNTAGCCCGNNNNAGTTAGACANNNCNNNNNNNNNNNNTNNNNNNNNNNNNNNNNNNNNNNN
NNNNNNNNNNNNNNNNNNNNNNNNNNNNNNNTNNNNNNNNNNNNNNNNNNNNNNNNNNNNNNNNNNNN
NNNNNNNNNNNNNNNNNNNNNNNNNNNNNNNNNNNNNNNNNNNNNNNNNNNNNNNNNNNNNNNNNNNN
NNNNNNNNNNNNNNNNNNNNNNNNNNNNNNNNNNNNNNNNNNNNNNNNGTNNNNNNNNNNNNNNNNNNN
NNNNNNNNNNNNNNNNNNNNNNNNNNNNNNNNNNNNNNNNNNNNNNNNNNNNNTCTNNNAAANNNTNNN
NNNNNNNNNNNNNNNNNNNNNNNNNNCNCNTTGNNNGTAANCNANNNGNACATGCAGTCCCCTCNNNNN
NNNGGCAGAGNNNNNNNCCNNNNNNNCCGNCCCCNNCCNNNNNNNNNNNCANNNNNNNNNNANNNNNN
NNNNNNTGGTACNNNNCGNNNNNGGTTGTTTAAGAGGTCCACAACGNGGTCNNGNCACNNNNGNNNAN
NNNCNNNNAACNNNNNNNNNNNNANNTNNNTANNAGGNCNNNNCCNTCNNGNNNNNAATTGCNATGACC
CAANNCACGGNGGCAGTNCCCGGCGNGGCGACCTTGNTNANNTCCGGGGACAAGTATGGCNCNCNCCC
CGGGAAGGANNCNGCCGTANGTGCNNNNNANNNNNTCNTGNGACGTCGCCGNNATTCNCGTGGTCNNN
CNAGAATGNCCNNACCANNCGNANNNTGGTTATGNNAGTAANNNNNNNNNNNNNNNNNNNNNNNNNNN
NNNNNNNNNNNNNNNNNNNNNNNNNNNNNNNNGNNNNGNNNNNNCNANNNAAGGANNTANNNCTNNTN
NTNNNGNNGGACNNNACNNNNCNNNNNNNACNCNNNCNCNNNNNNNNNNGNTTTTTNNNCNNTATTNT
CCAGNACGCNGNGCNNNCATTCANGNGAACNNNGNNACGCGNNAATCGTANNATCTACCTGCCGCNGG
TNNNNNTACNGNNNNTNCGGANNNTNNNNCGTANNNNNNTCATGCGTNNTNTNNTCNCNAGNCNNGTT
CGNNNANANANTAGNANGTNTCNNNNNNNNTGTNNCGTTCCGCNTGANNCNNCANNNNGNNGNNNTTN
TCANCGGCNNTANCTGCNGANNTAAGTCATANNNTNNATCNNTANCTNNNNNGGNGGGNNACTGTAAA
NGGNTAATTNGNNGNTNANTTCNNNCACNTNNCTNNGNNTNCTNNNTNCCTGNNTNCNNAATTGGNNC
CANTGNNTGNCNATCTCTGGNTTNGNNNNNNTGNNNNTNGCAANNNGGNNNGTNANANNNCNATNTAA
NNTATANNNNCNNNNNNANNNCACNANNNNNNNNAGNNNCNANNNTTANANNNGTAGNNNAATAAGTCT
CCGANNTNNNNNNNATAAGCGNNANACNNNNNNNNNNNNNANGANNACGNNTTGGNNNTNNNNNNCNA
GNNAGACNNTNATGGNTNNNNNTCAANNNNTTCGTAGTGGTATAATNGNNNNNNNTTTCNCCAGNNTN
NNNGNNNNNNNCNATANNANNNNNNNGTGANNNNNNNNNNANNNNCTCNNNAGGGANCANCTACTTNA
GCAATGGNGCGNAGNNNGAGANNNNGACNNGCCTTGGGTGGCAANCACNGCGATAAGTNNNGTTNANC
CTNNNAGTNNACATNNNNNCANNGNNNCNNNNNNANCNGANNGNNNNNNNNNNNNNNNNNNNNNNNNNN
TNNNNNNNNNNNNNNNNNNNNNNNNNNNNNNAGNNNANGGNNNGTNTNTGACNCNTGTGTTTTTANNNGNN
NNNNNACGNNNGANCCCCGGTCGNNNNCNNATTGAANNCCTGNNGCATNNNNNTNCNAGAATGANNGC
NNNNNGNNTNGNCTNNCNNTTCAAGNAATCAANNAGGTANCGCGNTCNNNGNNNNNNNNCNNTCCCNN
ACTGAGGGTNNNNNNCGCATCTGCTNNNNNNNNNNTNCGCNGGNGTNNCTNNNNAGCNGTATNGNGTNN
NNCANNNNNNNNNNNNNNNNNTNAAACAANNNNNCTNNNG
>Tay390
GTGNNNNNTAGCCCNNNNNAGTTAGACANNNCNNNNNNNNNNNNTNNNNNNNNNNNNNNNNNNNNNNN
NNNNNNNNNNNNNNNNNNNNNNNNNNNNNNNNNNNNNNNNNNNNNNNNNNNNNNNNNNNNNNNNNNNN
NNNNNNNNNNNNNNNNNNNNNNNNNNNNNNNNNNNNNNNNNNNNNNNNNNNNNNNNNNNNNNNNNNNN
NNNNNNNNNNNNNNNNNNNNNNNNNNNNNNNNNNNNNNNNNNNNNNNNNNNNNNNNNNNNNNNNNNNN
NNNNNNNNNNNNNNNNNNNNNNNNNNNNNNNNNNNNNNNNNNNNNNNNNNNNNTCTNNNAAANNNTNNN
NNNNNNNNNNNNNNNNNNNNNNNNNNCNCNTTGNNNGTAANCNANNNGNACATGCAGTCCCCTCNNNNN
NNNNNCAGAGNNNNNNCCCNNNNNNNCCGNCCCCNNCCNNNNNNNNNNNNNNNNNNNNNNNNNANNNNNN
NNNNNNTGGTACNNNNCGNNNNNGGTTGTNTAAGAGGTCCACAACGNGGTCNGGNCACNNNNGNNNAN
NNNCNNNNAACNNNNNNNNNNNNANATNNNTANNAGGNCNNNNCCNTNNNNNNNANNAANNGCNATGACC
CNANTCACGGGGGCNGTNCCCGGCGCGGCGACCTTGATNANNTCCGGGGACAAGTATGGCGNNCNCCC
CGGGAAGGANNCNGCCGTANGTGCNNNNNANNNNNTCNTGNGANGTCGCCGNNATTCNCGTGGNCNNN
CNNGAATGCCCGNACCANNCGNANNNTGGTNATGTNAGTNNNNNNNNNNNNNNNNNNNNNNNNNNNNN
NNNNNNNNNNNNNNNNNNNNNNNNNNNNNNNNGNNNNGNNNNNNCNANNNAAGGNNNTANNNCTNNTC
NTNNNNNNGGACNNNACNNNNCNNNNNNNACNCNNNCNCNNNNNNNNNNNGGTTTTNNNNCNNTATTNT
CCAGNANGCNGNGCNNNCATTCANGNGAACNNNGNNACGCGNNAATCGTANNATCTACCTGCCGCNNG
NNNNNNTACNGNNNNNNCGGANNNTNNCNCGTANNNNNNTCATGCGTNNTNTNNTCNCNAGNCNNGTT
CGNNNATANANTAGNANGTNTNNNNNNNCTTGTNNCGTTCCGCNTGANNCNNCANNNNGNNGNNCTTN
TCANCGGCNCTANCTGCNGANNTAAGNCATANNNTNNNTCNNTANCTNNNNNGGNGGGNNACTGTAAA
NGGNNAATTNGNNGNTNATTNCNNNCACNTNNCTNNGNNTNCTNNNTNCCTGNNTNCNNAATTGGNNC

CANTGNNTGNCNATCTCTNGNTTNGNNNNNNTNNNNNTNNCAANNNGGNNNGTNNNANNNCNATNTAA
NNTATANNNNCNNNNNNANNNCACNANNNNNNNAGNNNCNANNNTTANANNNGTAGNNNAATAAGTCT
CCGANNTNNNNNNNATAAGCGNNANACNNNNNNNNNNNNNANGANNACGNNTTGGNNNTNNNNNNCNA
GNNANACNNTNATGGNTNNNNNTCAANNNNTTNGTAGTGGTATAATNGNNNNNNNTTTCNCCAGNNTN
NNNGNNNNNNNCNATANNANNNNNNNGNGANNNNNNNNNNANNNNCTCNNNAGGGANCANCTACTNNA
NCANTGGNGCGCAGNNNGAGANNNNGACCANCCTTGGGTGGCAANCACNGCAATAAGTNNNGTTNANN
CTNNNAGTNNACATGNNNNCANNGNNNCNNNNNNANCNGANNGNNNNNNNNNNNNNNNNNNNNNNNNN
NNNNNNNNNNNNNNNNNNNNNNNNNNNNNANNNNANGGNNNGTNTNTGACNCNTGTGTTTTTANNNGNN
NNNNNACGNNNNANCCCCGGTCGNNNNCNNATTGAANNCCTGNNGCATNNNNNTNCNAGAATGANNGC
NNNNNGNNTNGNCTNNCNNTTCAAGNAATCANNNAGGTANNNNGNTCNNNGNNNNNNNNCNNTCCCNN
ACTGAGGGTNNNNNNNNCATCTGCTNNNNNNNNNTNCGCNGGCATNNCNGNNNAGCNGTATNGTGTNA
NNCANNNNNNNNNNNNNNNNNCNAAACNANNNNNNTNNNN
>Tay391
GTGNNNNNTAGCCCGNNNNAGTTAGACANNNCNNNNNNNNNNNNNNNNNNNNNNNNNNNNNNNNNNNNN
NNNNNNNNNNNNNNNNNNNNNNNNNNNNNNNNNNNNNNNNNNNNNNNNNNNNNNNNNNNNNNNNNNNNNN
NNNNNNNNNNNNNNNNNNNNNNNNNNNNNNNNNNNNNNNNNNNNNNNNNNNNNNNNNNNNNNNNNNNNNN
NNNNNNNNNNNNNNNNNNNNNNNNNNNNNNNNNNNNGNNNNNNNNNNNNNNNNNNNNNNNNNNNNNNNN
NNNNNNNNNNNNNNNNNNNNNNNNNNNNNNNNNNNNNNNNNNNNNNNNNNNNNNTCTNNNAAANNNTNNN
NNNNNNNNNNNNNNNNNNNNNNNNNCNCNTTGNNNGTAANCNANNNGNACATGCAGTCCCCTCNNNNN
NNNGGCAGAGNNNNNNNCCNNNNNNNCCGGCCCCNNCCNNNNNNNNNNNCNNNNNNNNNNNANNNNNN
NNNNNNTGGTACNNNNCGNNNNNGGTTGTNTAAGAGGTCCACCACGNGGTCNGGNNACNNNNGNNNAN
NNNCNNANAACNNNGNNNNNNNANNTNNNTANNAGGNCNNNNCCNTCNNGNNNNNNANNGCNATGACC
CAANNCACGGGGGCAGTNCCCGGCGCGGCGACCTTGANNACGTCCGGGGACAAGTATGGCNTNCNCCC
CGNGAAGGANNCNGCNGTANGTGCNNNNNANNNNNTCNTGNGANGTNGCCGNNATTCNNGTGGTCTNN
CNAGANTGNCCGNACCANNCGNANNNTGGTNATGNNAGTANNNNNNNNNNNNNNNNNNNNNNNNNNNN
NNNNNNNNNNNNNNNNNNNNNNNNNNNNNNNNNNNGNNNNGNNNNNNCNANNNAAGGNNNTANNNCTNNTN
NTNNNNNNNGGACNNNACNNNNCNNNNNNNACNCNNNCNCNNNNNNANNNGNTTTTTNNCCNNTATTNT
CCAGNACGCNGNGCNNNCATTCANGNGAACNNNGNNACGCGNNAATCGTANNATCTACCTGCCGCNNG
NNNNNNTACNGNNNNTNCGGANNNTNNNNCGTANNNNNNTTATACGTNNTNTNNTCNCNAGNCNNGTT
CGNNNANANANTAGNANGTNTNNNNNNNNNNTGTNNCGTTCCGCNTGANNCNNCANNNNGNNGNNCTTN
TCANCGGCNNTANCTGCNGANNTAAGNCATANNNTNNNTCNNTANCTNNNNNGGNGGGTNACTGTAAA
NGGNNAATTNGNNGNTNATTNCNNNCACNTNNCNNNGNNTNCTNNNTNCCTGNNTNCNNAATTGGNNC
CANTGNNTGNCNATCTCTGGNTTNGNNNNNNTNNNNNTNNCANNNNGGNNNNNNNNANNNCNATNTAA
NNTANANNNNCNNNNNNANNNCACNNNNNNNGNAGNNNCNANNNTTANANNNGTAGNNNAATAAGTCT
CCGANNTNNNNNNNATAAGCGNNANACNNNNNNNNNNNNNANGANNACGNNTTGGNNNTNANNNNCNA
GNNAGACNNTNGTGGNTNNNNNTCAANNNNTTCGTAGTGGTATAATNGNNNNNNNTTTCNCCAGNNTN
NNNGNNNNNNNCNATANNANNNNNNNGNGANNNNNNNNNNANNNNCTCNNNAGGGANCANCTACTNNA
GCAANGGNGCGCAGNNNGAGANNNNGACNNGCCTTGGGTGGCAANCACNGCAATAAGTNNNGTTNANC
CTNNNAGTNNACACNNNNNCANNGNNNCNNNNNNANCNGANNGNNNNNNNNNNNNNNNNNNNNNNNNN
NNNNNNNNNNNNNNNNNNNNNNNNNNNNNAGNNNANGGNNNGTNTNTGACNCATGTGTTTTTANNNGNN
NNNNNACGNNNNANCCCCGGTCGNNNNCNNATTGAANNCCTGNNGCATNNNNNTNCNAGAATGANNGC
NNNNNGNNTNGNCTNNCNNTTCAAGNAGTCANNTAGNNNNNGCGNTCNNNGNNNNNNNNCNNTCCCNN
ACTGAGGGTNNNNNNNNCATCTGCTNNNNNNNNCTNCGCNGGNGTNNCNNNNNAGCNGTATNGTGNNN
NNCANNNNNNNNNNNNNNNNNNTNNAACNANNNNNNTNNNN
>Tay392
GTGNNNNNTAGCCCNNNNNAGTTAGACANNNCNNNNNNNNNNNNNTNNNNNNNNNNNNNNNNNNNNNNN
NNNNNNNNNNNNNNNNNNNNNNNNNNNNNNNNNNNNNNNNNNNNNNNNNNNNNNNNNNNNNNNNNNNNNN
NNNNNNNNNNNNNNNNNNNNNNNNNNNNNNNNNNNNNNNNNNNNNNNNNNNNNNNNNNNNNNNNNNNNNN
NNNNNNNNNNNNNNNNNNNNNNNNNNNNNNNNNNNNNNNNNNNNNNNNNNNNNNNNNNNNNNNNNNNNNN
NNNNNNNNNNNNNNNNNNNNNNNNNNNNNNNNNNNNNNNNNNNNNNNNNNNNNNNTCTNNNAAANNNTNNN
NNNNNNNNNNNNNNNNNNNNNNNNNCNCNTTGNNNGTAANCNANNNGNACATGCAGTCCCCTCNNNNN
NNNGGCAGAGNNNNNNCCCNNNNNNNCCGGCCCCNNCCNNNNNNNNNNNCNNNGNNNNNNNANNNNNN
NNNNNNTGGTACNNNNCGNNNNNGGTTGTTTAAGAGGTCCACAACGNGGTCNNGNCACNNNNGNNNAN
NNNCNNAAAACNNNNNNNNNNNNNNNATNNNTANNAGGNNNNNNCCNTCNNGNNNNNNATTGCNATGACC

CANNNCACGGGGGCNGTNCCCGGCGNGGCGACCTTGATNANGTCCGGGGACAAGTATGGCGNNCNCCC
CGGGAAGGANNCNGCCGTANGTGCNNNNNANNNNNTCNTGNGANGTCGCCGNNATTNNCGTGGTCNNN
NNAGAATGNNCGNACCANNCGNANNNTGGTNATGTNAGTNNNNNNNNNNNNNNNNNNNNNNNNNNNNN
NNNNNNNNNNNNNNNNNNNNNNNNNNNNNNNNGNNNNGNNNNNNCNANNNAAGGANNTANNNCTNNTN
NTNNNNNNGGACNNNACNNNNCNNNNNNNACNCNNNCNCNNNNNNNNNNGNTTTTTNNCCNNTATTNT
CCAGNACGCNGNGCNNNCATTCANGNGAACNNNGNNACGCGNNAATCGTANNATCTACCTGCCGCNGG
TNNNNNTNCNGNNNNTNCGGANNNTNNNNCGTANNNNNGTCATGCGTNNTNTNNTCNCNAGNCANGTT
CGNNNANANANTAGNANGTNTCNNNNNNNNTGTNNCGTTCCGCNTGANNCNNCANNNNGNNGNNCTTN
TCANCGGCNNTANCTGCNGANNTAAGNCATANNNTNNNTCNNTANCTNNNNNGGNGGGNNACTGTAAA
NGGNTAATTNGNGGNTNANTNCNNNCACNTNNCTNNGNNNNCTNNNTNCCTGNNTNCNNAATTGGNNC
CANTGNNTGNCNATCTCTGGNTTNGNNNNNNTNNNNNTNNCANNNNGGNNNGTNANANNNCNATNTAA
NNTANANNNNCNNNNNNANNNCACNANNNNNGNAGNNACNANNNTTANANNNGTAGNNNAATAAGTCT
CCGANNTNNNNNNNATAAGCGNNANACNNNNNNNNNNNNNANGANNACGNNTTGGNNNTNNNNNNCNA
GNNAGACNNTNATGGNTNNNNNTCAANNNNTTCGTAGTGGTATAATNGNNNNNNNTTTCNCCAGNNTN
NNNGNNNNNNCNATANNANNNNNNNGNGANNNNNNNNNNANNNNNCTCNNNAGGGANCANCTACTTNA
GCANTGGNGCGCAGNNNGAGANNNNGACNNGCCTTGGGTGGCAANCACNGCAATAAGTNNNGTTNANC
CTNNNAGTNNACATNNNNNCANNGNNNCNNNNNNANCNGANNGNNNNNNNNANNNNNNNNNNNNNNNN
NNNNNNNNNNNNNNNNNNNNNNNNNNNNAGNNNANGGNNNGTNTNTGACNCATGNNTTTTTANNNGNN
NNNNNACGNNNNANCCCCGGTCGTNNNCNNATTGAANNCCTGNNGCATNNNNNTNCNAGAATGANNNC
NNNNNGNNTNNNCTNNCNTTTCAAGNAATCANNNAGNNNTNNNGNTCNNGGNNNNNNNNCNNTCCCNN
ACTGAGGGTNNNNNNCGCATCTGCTNNNNNNNNTNCGCNGGCGTNNCTNNNNNAGCNGTATNGTGTNA
NNCANNNNNNNNNNNNNNNTNAAACNANNNNNNNTNNNN
>Tay394
GTGNNNNNTAGCCCNNNNNAGTTAGACANNNCNNNNNNNNNNNTNNNNNNNNNNNNNNNNNNNNNNNN
NNNNNNNNNNNNNNNNNNNNNNNNNNNNNNNNNNNNNNNNNNCNNNNNNNNNNNNNNNNNNNNNNNNN
NNNNNNNNNNNNNNNNNNNNNNNNNNNNNNNNNNNNNNNNNNNNNNNNNNNNNNNNNNNNNNNNNNNN
NNNNNNNNNNNNNNNNNNNNNNNNNNNNNNNNNNNNNNNNNNNNNNNNNNNNNNNNNNNNNNNNNNNN
NNNNNNNNNNNNNNNNNNNNNNNNNNNNNNNNNNNNNNNNNNNNNNNNNNCNTCTNNNAAANNNTNNN
NNNNNNNNNNNNNNNNNNNNNNNNNNCNCNTTGNNNGTAANCANNNGNACATGCAGTCCCCTCNNNNN
NNNGGCAGAGNNNNNNCCCNNNNNNNCCGNCCCCGNCCNNNNNNNNNNNNANNGNNNNNNNANNNNNN
NNNNNNTGGTACNNNNCGNNNNNGGTTGTTTNAGAGGTCCACAACGNGGTCNNNNCACNNNNGNNNAN
NNNCNNAANANNNNNNNNNNNNNNNTNNNTANNAGGNCNNNNCCNTCNNNNNNNNNATTGCNATGACC
CAANNCACGGGGGCAGTNCCCGGCGCGGCGACCTTGATNANNTCCGGGGACAAGTATGGCNNNCNCCC
CGGGAAGGANNCNGCCGTANGTGCNNNNNANNNNNTCNTGNGANGTCGCCGNNATTCNCGTGGTCNNN
NNAGAATGNCCGNACCANNCGNANNNTGGTTATGNNAGTNNNNNNNNNNNNNNNNNNNNNNNNNNNNN
NNNNNNNNNNNNNNNNNNNNNNNNNNNNNNNNTNNNNGNNNNNNCNANNNAAGGANNTANNNCTNNTN
NTNNNNNNGGACNNNACNNNNCNNNNNNNACNCNNNCNCNNNNNNANNNGGTTTTTNNCCNNTATTNT
CCAGNACGCNGNGCNNNCATTCANGNGAACNNNGNNACGCGNNAATCGTANNATCTACCTGCCGCNNG
TNNNNNTACNGNNNNTNCGGANNNTNNCTCGTANNNNNNTCATGCGTNNTNTNNTCNCNAGNCNNGTT
CGNNNANANANTAGNANGTNTNNNNNNNNNTGTNNCGTTCCGCNTGANNCNNCANNNNGNNGNNCTTN
TCANCGGCNNTANCTGCNGANNTAAGNCATANNNTNNNTCNNTANCTNNNNNGGNGGGNNACTGTAAA
NGGNNAATTNGNNGNTNATTNCNNNCACNTNNCNNNGNNTNCTNNNTNCCTGNNTNCNNAATTGGNNC
CANTGNNTGNCNATCTCTGGNTTNGCNNNNNTNNNNNTNNCAANNNGGNNNGTNANANNNCNATNTAA
NNTATANNNNCNNNNNNANNNCACNANNNNNGNAGNNNCNANNNTTANANNNGTAGNNNAATAAGTCT
CCGANNTNNNNNNNATAAGCGNNANACNNNNNNNNNNNNNANGANNACGNNTTGGNNNTNNNNNNCNA
GNNAGACNNTNATGGNTNNNNNTCAANNNNTTCGTAGTGGTATAATNGNNNNNNNTTTCNCCAGNNTN
NNNGNNNNNNCNATANNANNNNNNNGNGANNNNNNNNNNANNNNNNTCNNNAGGGANCANCTACTTNA
GCAATGGNGCGNAGNNNGAGANNNNGACNNNCCTTGGGTGGCAANCACNGCAATAAGTNNNGTTNANC
CTNNNAGTNNACATNNNNNCANNGNNNCNNNNNNANCNGANNGNNNNNNNNNNNNNNNNNNNNNNNN
NNNNNNNNNNNNNNNNNNNNNNNNNNNNNGNNNANGGNNNGTNTNTGACNCATGTGTTTTTANNNGNN
NNNNNACGNNNGANCCCCGGTCGNNTNCNNATTGAANNCCTGNNGCATNNNNNTNCNAGAATGANNGC
NNNNNGNNTNGNCTGNCNNNTCAAGNAATCAANNAGGTANNNNGNTCNNGGNNNNNNNNCNNTCCCNN
ACTGAGGGTNNNNNNCGCATCTGCTNNNNNNNNCTNCGCNGGNGTNNCNNNNNAGCNGTATNGTGTNA
NNCANNNNNNNNNNNNNNNCNAAANNANNNNNNNTNNNN

>Tay395
GTGNNNNNTAGCCCNNNNNAGTTAGACANNNCNNNNNNNNNNNNNNNNNNNNNNNNNNNNNNNNNNNNNN
NNNNNNNNNNNNNNNNNNNNNNNNNNNNNNNNNNNNNNNNNNNNNNNNNNNNNNNNNNNNNNNNNNNNNN
NNNNNNNNNNNNNNNNNNNNNNNNNNNNNNNNNNNNNNNNNNNNNNNNNNNNNNNNNNNNNNNNNNNNNN
NNNNNNNNNNNNNNNNNNNNNNNNNNNNNNNNNNNNNNNNNNNNNNNNNNNNNNNNNNNNNNNNNNNNNN
NNNNNNNNNNNNNNNNNNNNNNNNNNNNNNNNNNNNNNNNNNNNNNNNNNNNNNTCTNNNAAANNNTNNN
NNNNNNNNNNNNNNNNNNNNNNNNNNNCNCNTTGNNNGTAANCNANNNGNACATGCAGTCCCCTCNNNNN
NNNNNCNNAGNNNNNNNCCNNNNNNNCCGNCCCCNNCCNNNNNNNNNNNNNNNNNNNNNNNNNANNNNNN
NNNNNNTGGTACNNNNNCGNNNNNGGTTGTNTNAGAGGTCCACAACGNGGTCNNNNNNACNNNNGNNNAN
NNNCNNNNAACNNNNNNNNNNNNNNNNTNNNTANNAGGNCNNNNCCNTNNNNNNNNNNANNGCNATGANN
CANNNNACGGNGGCAGTNCCCGGCNCGGCGACCTTGANNANNTCCGGGGNCAAGTATGGCGNNCNCCC
CGNGAAGGANNCNGCNGTANGTGCNNNNNANNNNNTCNTGNGANNTNGCCGNNATTNNNGTGGNCNNN
CNAGANTGNNCNNACNANNCGNNNNNTGGTNATGNNAGTNNNNNNNNNNNNNNNNNNNNNNNNNNNNNNN
NNNNNNNNNNNNNNNNNNNNNNNNNNNNNNNNNNNNNNNGNNNNNNCNANNNAAGGNNNTANNNCTNNTN
NTNNNNNNGGACNNNACNNNNCNNNNNNNACNCNNNCNNNNNNNNNNNNNGNTTTTNNNNCNNTATTNT
CCAGNACGCNGNGCNNNCATTCANGNGAACNNNGNNACGCGNNAATCGTANNATCTACCTGCCGCNNG
NNNNNNTNCNGNNNNNNCGGANNNTNNNNCGTANNNNNNNCATGCGTNNTNTNNTCNCNAGNCNNGTT
CGNNNANANANTAGNANGTNTNNNNNNNNNNTGTNNCGTTCCGCNTGANNCNNCANNNNGNNGNNNTTN
NCANCGGCNNTANCTGCNGANNTAAGNCATNNNNTNNNTCNNTANCTNNNNNGGNGGGNNACTGTAAA
NGGNNAATTNGNNNNTNATTTCNNNCACNTNNCNNNGNNNNCTNNNTNCCTGNNTNCNNAATTGGNNC
CANTGNNTGNCNATCTCTGGNTTNGNNNNNNTNNNNNTNNCAANNNGGNNNGTNANANNNCNATNTAA
NNTATANNNNCNNNNNNANNNCACNNNNNNNGNAGNNNNNANNNTTANANNNGTAGNNNAATAAGTCT
CCGANNTNNNNNNNATAAGCGNNANACNNNNNNNNNNNNNNANGANNACGNNTTGGNNNTNNNNNNNNA
GNNANACNNTNATGGNTNNNNNTCAANNNNTTNGTAGTGGTATAATNGNNNNNNNTTTCNCCAGNNTN
NNNGNNNNNNNCNATANNANNNNNNNGNGANNNNNNNNNNNANNNNCTCNNNAGGNANCANCTACTNNA
NCANTGGNGCGCAGNNNGAGANNNNGACNNNCCTTGGGTGGCAANCACNGCGATAAGTNNNGTTNANC
CTNNNNGTNNACATNNNNNCANNGNNNCNNNNNNANCNGANNGNNNNNNNNNNNNNNNNNNNNNNNNNNN
NNNNNNNNNNNNNNNNNNNNNNNNNNNNNNANNNNANGGNNNGTNTNTGACNCNTGTGTTTTTANNNGNN
NNNNNACGNNNNANCCCCGGTCGNNNNCNNATTGAANNCCTGNNGCATNNNNNTNCNAGAATGANNGC
NNNNNGNNTNGNCTNNCNTTTCAAGNAATCANNNAGGTNNNGCGNTCNNNNNNNNNNNNNCNNNCCCNN
ACTGAGGGTNNNNNNNNCATCTGCTNNNNNNNNNNTNCGCNGGNGTNNCNNNNNAGCNGTATNGNGNNA
NNCANNNNNNNNNNNNNNNNNNNACACNNNNNNNNNTNNNN
>Tay396
GTGNNNNNTAGCCCNNNNNAGTTAGACANNNCNNNNNNNNNNNTTNNNNNNNNNNNNNNNNNNNNNNNNN
NNNNNNNNNNNNNNNNNNNNNNNNNNNNNNNTNNNNNNNNNNNNNNNNNNNNNNNNNNNNNNNNNNNNNN
NNNNNNNNNNNNNNNNNNNNNNNNNNNNNNNNNNNNNNNNNNNNNNNNNNNNNNNNNNNNNNNNNNNNNN
NNNNNNNNNNNNNNNNNNNNNNNNNNNNNNNNNNNNNNNNNNNNNNNNNNNNNNNNNNNNNNNNNNNNNN
NNNNNNNNNNNNNNNNNNNNNNNNNNNNNNNNNNNNNNNNNNNNNNNGNNNNCNTCTNNNAAANNNTNNN
NNNNNNNNNNNNNNNNNNNNNNNNNNNCNCNTTGNNNGTAANCNANNNGNACATGCAGTCCCCTCNNNNN
NNNGGCAGAGNNNNNNCNCNNNNNNNCCGNCCCCGNCCNNNNNNNNNNNNCNNNGNNNNNNNANNNNNN
NNNNNNTGGTACNNNNCGNNNNNGGTTGTTTAAGAGGTCCACAACGNGGTCNNGNCACNNNNGNNNAN
NNNCNNNAAANNNNNNNNNNNNNNANNTNNNTANNAGGNNNNNNCCNTCNNNNNANTNATTGCNATGACC
CAANTCACGGGGGCAGTCCCCGGCGCGGCGACCTTGATNANGTCCGGGGACAAGTATGGCGNNCNCCC
CGGGAAGGANNCNGCCGTANGTGCNNNNNANNNNNTCNTGNGACGTCGCCGNNATTCNCGTGGTCTNN
CNAGAATGCCCGNACCANNCGNANNNTGGTNATGNTAGTANNNNNNNNNNNNNNNNNNNNNNNNNNNNNN
NNNNNNNNNNNNNNNNNNNNNNNNNNNNNNNNNNNNNNNGNNNNNNCNANNNAAGGAGNTANNNCTNNTN
NTNNNNNNGGACNNNACNNNNCNNNNNNNACNCNNNCNCNNNNNNANNNGGTTTTTNNCCNNTATTNT
CCAGNACGCNGNGCNNNCATTCANGNGAACNNNGNNACGCGNNAATCGTANNATCTACCTGCCGCNNG
TNNNNNTACNGNNNNNNCGGANNNTNNNNCGTANNNNNNTCATGCGTNNTNTNNTCNCNAGNCNNGTT
CGNNNANANANTAGNANGTNTCNNNNNNNNNTGTNNCGTTCCGCNTGANNCNNCANNNNGNNGNNNTTN
TCANCGGCNCTANCTGCNGANNTAAGTCATANNNTNNATCNNTANCTNNNNNGGNGGGNNACTGTAAA
NGGNNAATTNGNGGNTNATTTCNNNCACNTNNCTNNGNNTNCTNNNTNCCTGNNTNCNNAATTGGNNC
CANTGNNTGNCNATCTCTGGNTTNGNNNNNNTNNNNNTNNCANNNNGGNNNGTNANANNNCNATNTAA
NNTATANNNNCNNNNNNANNNCACNANNNNNNGNAGNNNCNANNNTTANANNNGTAGNNNAATAAGTCT

CCGANNTNNNNNNNATAAGCGNCANACNNNNNNNNNNNNNNANGANNNCGNNTTGGNNNTNNNNNNCNA
GNNAGACNNTNATGGNTNNNNNTCAANNNNTTNGTAGTGGTATAATNGNNNNNNNNTTTCNCCAGNNTN
NNNGNNNNNNNNCNATANNANNNNNNNNGNGANNNNNNNNNNNANNNNCTCNNNAGGGANCANCTACTNNA
GCAATGGNGCGCAGNNNGAGANNNNGACNAGCCTTGGGTGGCAANCACNGCAATAAGTNNNGTTNANC
CTNNNAGTNNACATNNNNNCANNGNNNCNNNNNNANCNGANNGNNNNNNNNNNNNNNNNNNNNNNNNN
NNNNNNNNNNNNNNNNNNNNNNNNNNNNNANNNNANGGNNNGTNTNTGACNCATGTGTTTTTANNNGNN
NNNNNACGNNNGANCCCCGGTCGNNNNCNNATTGAANNCCTGNNGCATNNNNNTNCNAGAATGANNNC
NNNNNGNNTNGNCTNNCTTTTCAAGNAATCANNNAGGTNNCGCGNTCNNNGNNNNNNNNCNNTCCCNN
ACTGAGGGTNNNNNNNNCATCTGCTNNNNNNNNCTNCGCNGGCGTNNCNNNNNAGCNGTATNGTGTNA
NNCANNNNNNNNNNNNNNNNNTNANNCNANNNNNNNTNNNN
>Tay397
GTGNNNNNTAGCCCNNNNNAGTTAGACANNNCNNNNNNNNNNNNNNNNNNNNNNNNNNNNNNNNNNNNN
NNNNNNNNNNNNNNNNNNNNNNNNNNNNNNNTNNNNNNNNNNNNNNNNNNNNNNNNNNNNNNNNNNNNNN
NNNNNNNNNNNNNNNNNNNNNNNNNNNNNNNNNNNNNNNNNNNNNNNNNNNNNNNNNNNNNNNNNNNNNN
NNNNNNNNNNNNNNNNNNNNNNNNNNNNNNNNNNNNNNNNNNNNNNNNNNNNNNNNNNNNNNNNNNNNNN
NNNNNNNNNNNNNNNNNNNNNNNNNNNNNNNNNNNNNNNNNNNNNNNNNNNNNTCTNNNAAANNNTNNN
NNNNNNNNNNNNNNNNNNNNNNNNNNCNCNTTGNNNGTAANCNANNNGNACATGCAGTCCCCTCNNNNN
NNNGGCAGAGNNNNNNNCCNNNNNNNNCCGGCCCCGNCCNNNNNNNNNNNNNNNNGNNNNNNNANNNNNN
NNNNNNTGGTACNNNNCGNNNNNGGTTGTTTAAGAGGTCCACAACGNGGTCNNGNCACNNNNGNNNAN
NNNCNNNNNNACNNNNNNNNNNNNNNNNTNNNTANNAGGNCNNNNCCNTCNNGNNNNNNATTGCNATGACC
CAANNCACGGGGGCAGTNCCCGGCGCGGCGACCTTGNTNACGTCCGGGGACAAGTATGGCGCNCNCCC
CGGGAAGGANNCNGCCGTANGTGCNNNNNANNNNNTCNTGNGANGTCGCCGNNATTCNNGTGGTCNNN
CNAGAATGNNCGNACCANNCGNANNNTGGTNATGNTAGTNNNNNNNNNNNNNNNNNNNNNNNNNNNNN
NNNNNNNNNNNNNNNNNNNNNNNNNNNNNNNNNNNNGNNNNGNNNNNNCNANNNAAGGANNTANNNCTNNTN
NTNNNGNNGGACNNNACNNNNCNNNNNNNNACNCNNNCNCNNNNNNNANNNGNTTTTTNNNCNNTATTNT
CCAGNANGCNGNGCNNNCATTCANGNGAACNNNGNNACGCGTNAATCGTANNATCTACCTGCCGCNGG
TNNNNNTACNGNNNNTNCGGANNNTNNNNCGTANNNNNNTCATGCGTNNTNTNNTCNCNAGNCNNGTT
CGNNNANANANTAGNANGTNTNNNNNNNNNNTGTNNCGTTCCGCNTGANNCNNCANNNNGNNGNNCTTN
TCANCGGCNNTANCTGCNGANNTAAGNCATANNNTNNATCNNTANCTNNNNNGGNGGGNNACTGTAAA
NGGNNAATTNGNNGNTNATTNCNNNCACNTNNCNNNGNNNNCTNNNTNCCTGNNTNCNNAATTGGNNC
CANTGNNTGNCNATCTCTGGNTTNGNNNNNNTNNNNNTNGCANNNNGGNNNGTNANANCNCNATNTAA
NNTATANNNNCNNNNNNANNNCACNANNNNNGNAGNNNCNANNNTTANANNNGTAGNNNAATAAGTCT
CCGANNTNNNNNNNATAAGCGNNANACNNNNNNNNNNNNNNANGANNACGNNTTGGNNNTNNNNNNNNA
GNNAGACNNTNATGGNTNNNNNTCAANNNNTTNGTAGTGGTATAATNGNNNNNNNNTTTCNCCAGNNTN
NNNGNNNNNNNNCNATANNANNNNNNNNGNGANNNNNNNNNNNANNNNCTCNNNAGGGANCANCTACTTNA
GCAANGGNGCGCAGNNNGAGANNNNGACNNGCCTTGGGTGGCAANCACNGCAATAAGTNNNGTTNANC
CTNNNAGTNNACATNNNNNCANNGNNNCNNNNNNANCNGANNGNNNNNNNNNNNNNNNNNNNNNNNNN
NNNNNNNNNNNNNNNNNNNNNNNNNNNNNAGNNNANNGNNNGTNTNTGACNCATGNNTTTTTANNNGNN
NNNNNACGNNNNANCCCCGGTCGNNNNCNNATTGAANNCCTGNNGCATNNNNNTNCNAGAATGANNNC
NNNNNGNNTNGNCTNNCNTTTCAAGNAATCNNNNAGNTNNCGCGNTCNNNNNNNNNNNNCNNTCCCNN
ACTGAGGGTNNNNNNNNCATCTGCTNNNNNNNNCTNCGCNGGNNTNCCNNNNNAGCNGTATNGTGTNA
NNCANNNNNNNNNNNNNNNNNTNAAACAANNNNNNNTNNNN
>Tay401
GTGNNNNNTAGCCCNNNNNAGTCAGACANNNCNNNNNNNNNNNNNNNNNNNNNNNNNNNNNNNNNNNNN
NNNNNNNNNNNNNNNNNNNNNNNNNNNNNNNNNNNNNNNNNNNNNNNNNNNNNNNNNNNNNNNNNNNNNN
NNNNNNNNNNNNNNNNNNNNNNNNNNNNNNNNNNNNNNNNNCNNNNNNNNNNNNNNNNNNNNNNNNNNNNN
NNNNNNNNNNNNNNNNNNNNNNNNNNNNNNNNNNNNNNNNNNNNNNNNNNNNNNNNNNNNNNNNNNNNNN
NNNNNNNNNNNNNNNNNNNNNNNNNNNNNNNNNNNNNNNNNNNNNNNNNNNNCNTCTNNNAAANNNTNNN
NNNNNNNNNNNNNNNNNNNNNNNNNNCNCNTTGNNNGTAANCNANNNGNACATGCAGTCCCCTCNNNNN
NNNGGCAGAGNNNNNNNCCCNNNNNNNCCGNCCCCNNCCNNNNNNNNNNNNNNNNGNNNNNNNANNNNNN
NNNNNNTGGTACNNNNCGNNNNNGGTTGTTTNAGAGGTCCACAACGNGGTCNGGNCACNNNNGNNNAN
NNNCNNAAAACNNNNNNNNNNNNANNTNNNTANNAGGNCNNNNCCNTCNNGNNNNNNANTGCNATGACC
CAANNNACGGGGGCAGTCCCCGGCGCGGCGACCTTGATNACGTCCGGGGACAAGTATGGCGCNCNCCC
CGGGAAGGANNCNGCCGTANGTGCNNNNNANNNNNTCNTGNGANGTCGCCGNNATTCNCGTGGTCNNN

CNAGAATGNCCGNACCANNCGNANNNTGGTNATGNTAGTANNNNNNNNNNNNNNNNNNNNNNNNNNNN
NNNNNNNNNNNNNNNNNNNNNNNNNNNNNNNNGNNNNGNNNNNNCNANNNAAGGANNTANNNCTNNTN
NTNNNNNNGGACNNNACNNNNCNNNNNNNACNCNNNCNCNNNNNNNNNNGNTTTTTNNNCNNTATTNT
CCAGNACGCNGNGCNNTCATTCANGNGAACNNNGNNACGCGNNAATCGTANNATCTACCTGCCGCNGG
TNNNNNTACNGNNNNTACGGANNNTNNCTCGTANNNNNGTCATGCGTNNTNTNNTCNCNAGNCANGTT
CGNNNANANANTAGNANGTNTCCNNNNNNNTGTNNCGTTCCGCNTGACNCNNCANNNNGNNGNNCTTN
TCANCGGCNNTANCTGCNGANNTAAGNCATANNNTNNATCNNTANCTNNNNNGGNGGGNNACTGTAAA
NGGNNAATTNGNGGNTNATTTCNNNCATNTNNCTNNGNNNNCTNNNTNCCTGNNTNCNNAATTGGNNC
CANTGNNTGNCNATCTCTGGNTTNGNNNNNNTNNNNNTNGCAANNNGGNNNGTNANANNNCNATNTAA
NNTATANNNNCNANNNNANNNCACNANNNNNGNAGNNACNANNNGTANANNNGTAGNNNGATAAGTCT
CCGANNTNNNNNNNATAAGCGNNANACNNNNNNNNNNNNNANGANNACGNNTTTGNNNTNNNNNNCNA
GNNAGACNNTNGTGGNTNNNNNTCAANNNNTTCGTAGTGGTATAATNGNNNNNNNTTTCNCCAGNNTN
NNNGNNNNNNNNCNATANNANNNNNNNGTGANNNNNNNNNANNNNCTCNNNAGGGANCANCTACTTNA
GCAATGGNGCGCAGNNNGAGANNNNGACNNNCCTTGGGTGGCAANCACNGCAATAAGTNNNGTTNANC
CTNNNAGTNNACACNNNNNCANNGNNNCNNNNNNANCNGANNGNNNNNNNNNNNNNNNNNNNNNNNNN
NNNNNNNNNNNNNNNNNNNNNNNNNNNNAGNNNANGGNNNGTNTNTGACNCATGTGTTTTTANNNGNN
NNNNNACGNNNNANCCCCGGTCGNNNNCNNATTGAANNCCTGNNGCATNNNNNTNCNAGAATGANNGC
NNNNNGNNTNGNCTGNCNTTTCAAGNAATCANNNAGGTNTNGCGNTCNCNGNNNNNNNNCNNTCCCNN
ACTGAGGGTNNNNNNNNCATCTGCTNNNNNNNNNTNCGCNGGNGTNNCNGNNNAGCNGTATNGTGTTN
NNCANNNNNNNNNNNNNNNNNNNAACNNNNNNNNTNNNN
>Tay406
GTGNNNNNTAGCCCNNNNNAGTTAGACANNNCNNNNNNNNNNNNTNNNNNNNNNNNNNNNNNNNNNNN
NNNNNNNNNNNNNNNNNNNNNNNNNNNNNNNNNNTNNNNNNNNNNNNNNNNNNNNNNNNNNNNNNNNN
NNNNNNNNNNNNNNNNNNNNNNNNNNNNNNNNNNNNNNNNNNNNNNNNNNNNNNNNNNNNNNNNNNNN
NNNNNNNNNNNNNNNNNNNNNNNNNNNNNNNNNNNNNNNNNNNNNNNNNNNNNNNNNNNNNNNNNNNN
NNNNNNNNNNNNNNNNNNNNNNNNNNNNNNNNNNNNNNNNNNNNNNNNNNCNTCTNNNAAANNNTNNN
NNNNNNNNNNNNNNNNNNNNNNNNNNCNCNTTGNNNGTAANCANNNGNACATGCAGTCCCCTCNNNNN
NNNGGCAGAGNNNNNNCCCNNNNNNNCCGGCCCCNNCCNNNNNNNNNNNCANNGNNNNNNNANNNNNN
NNNNNNTGGTACNNNNCGNNNNNGGTTGTTTAAGAGGTCCACAACGNGGTCNGGNCACNNNNGNNNAN
NNNCNNNNAANNNNNNNNNNNNANNTNNNTANNAGGNCNNNNCCNTCNNGNNNNNNATNGCNATGACC
CAANNCACNGGGGCAGTCCCCGGCGCGGCGACCTTGATNANGTCCGGGGACAAGTATGGCGNNCNCCC
CGGGAAGGANNCNGCCGTANGTGCNNNNNANNNNNTCNTGNGACGTCGCCGNNATTCNNGTGGTCNNN
CNAGAATGNCCGNACCANNCGNANNNTGGTNATGNTAGTNNNNNNNNNNNNNNNNNNNNNNNNNNNNN
NNNNNNTNNNNNNNNNNNNNNNNNNGNNNNGNNNNNNCNANNNAAGGANNTANNNCTNNTC
NTNNNGNNGGACNNNACNNNNCNNNNNNNACNCNNNCNCNNNNNNNNNNGNTTTTTNNNCNNTANTNT
CCAGNANGCNGNGCNNNCATTCANGNGAACNNNGNNACGCGNNAATCGTANNATCTACCTGCCGCNGG
NNNNNNTACNGNANNNNCGGANNNTNNCTCGTANNNNNNTCATGCGTNNTNTNNTCNCNAGNCANGTT
CGNNNANANANTAGNANGTNTNNNNNNNNNTGTNNCGTTCCGCNTGANNCNNCANNNNGNCGNNCTTC
TCANCGGCNNTANCTGCNGANNTAAGTCATANNNTNNNTCNNTANCTNNNNNGGAGGGNNACTGTAAA
NGGNNAATTGGNNNNTNATTTCNNNCACNTNNCTNNGNNNNCTNNNTNCCTGNNTNCNNAATTGGNNC
CANTGNNTGNCNATCTCTGGNTTNGNNNNNNTNNNNNTNNCAANNNGGNNNGTNANANNNCNATNTAA
NNTATANNNNCNNNNNNANNNCACNANNNNNNNAGNNACNANNNTTANANNNGTAGNNNAATAAGTCT
CCGANNTNNNNNNNATAAGCGNNANACNNNNNNNNNNNNNANGANNACGNNTTGGNNNTNNNNNNNNA
GNNAGACNNTNATGGNTNNNNNTCAANNNNTTCGTAGTGGTATAATNGNNNNNNNTTTCNCCAGNNTN
NNNGNNNNNNNNCNATANNANNNNNNNGNGANNNNNNNNNANNNNCTCNNNAGGNANCANCTACTTNA
GCAANGGNGCGCAGNNNGAGANNNNGACNNNCCTTGGGTGGCAANCACNGCAATAAGTNNNGTTNANC
CTNNNAGTNNACATNNNNNCANNGNNNCCNNNNNANCNGANNGNNNNNNNNNNNNNNNNNNNNNNNNN
NNNNNNNNNNNNNNNNNNNNNNNNNNNNAGNNNANGGNNNGTNTNTGACNCATGTGTTTTTANNNGNN
NNNNNACGNNNGANCCCCGGTCGNNNNCNNATTGAANNCCTGNNGCATNNNNNTNCNAGAATGANNGC
NNNNNGNNTNGNCTNNCNNNTCAAGNAATCANNTAGGNNNNGNGNTCNNGGNNNNNNNNCNNTCCCNN
ACTGAGGGTNNNNNNNNCATCTGCTNNNNNNNNCTNCGCNGGCGTNNCNNNNNAGCNGTATNNNGTNA
NNCANNNNNNNNNNNNNNNNNTNAAACNANNNNNTNNNN
>Tay72
GTGNNNNNTAGCCCGNNNNAGTTAGACANNNCNNNNNNNNNNNNTNNNNNNNNNNNNNNNNNNNNNNN

NNNNNNNNNNNNNNNNNNNNNNNNNNNNNNNNNNNNNNNNNNNNNNNNNNNNNNNNNNNNNNNNNNNNNN
NNNNNNNNNNNNNNNNNNNNNNNNNNNNNNNNNNNNNNNNNNNNNNNNNNNNNNNNNNNNNNNNNNNNNN
NNNNNNNNNNNNNNNNNNNNNNNNNNNNNNNNNNNNNNNNNNNNNNNNNNNNNNNNNNNNNNNNNNNNNN
NNNNNNNNNNNNNNNNNNNNNNNNNNNNNNNNNNNNNNNNNNNNNNNNNNNNNNNTCTNNNAAANNNTNNN
NNNNNNNNNNNNNNNNNNNNNNNNNCNCNTTGNNNGTAANCNANNNGNACATGCAGTNNNCTCNNNNN
NNNNNCAGAGNNNNNNNCCNNNNNCNCCGNCCCCNNCCNNNNNNNNNNNNNNNNNNNNNNNNNANNNNNN
NNNNNNTGGTACNNNNCGNNNNNGGTTGTNTNAGAGGTCCACAACGNGGTCNNGNNACNNNNGNNNAN
NNNCNNNNANCNGNNNNNNNNNNNNNTNNNTACNAGGNNNNNNCCNTCNNNNNNNNNNATNGCNATGACC
CAANNCACGGGGGCAGTNCCCGGNGCGGCGACCTTGATNANNTCCGGGGACAAGTATGGCNCNCNCCC
CGGGAAGGANNCNGCCGTANGTGCNNNNNANNNNNTCNTGNGANGTCGCCGNNATTCNNGTGGTCNNN
CNAGAATGNCCGNACCANNCGNANNNTGGTNATGNNAGTNNNNNNNNNNNNNNNNNNNNNNNNNNNNNN
NNGTNNNNNNNNNNNNNNNNNNNNNNNNNNNNNNGNNNGGNNNNNNCCANNNAAGGNNNTANNNCTNNTN
NTNNNNNNGGACNNNACNNNNCNNNNNNNACNCNNNCNCNNNNNNANNNGNTTTTTNNNCNNTATTNT
CCAGNACGCNGNGCNNNCATTCANGNGAACNNNGNNACGCGNNAATCGTANNATCTACCTGCCGCNNG
TNNNNNTNCNGNNNNTNCGGANNNTNNNNCGTANNNNNNTCATGCGTNNTNTNNTCNCNAGNCNNGTT
CGNNNANANANTAGNANGTNTNNNNNNNNNNTGTNNCGTTCCGCNTGANNCNNCANNNNGNNGNNNTTC
TCANCGGCNNTANCTGCNGANNTAAGNCATANNNTNNNTCNNTANCTNNNNNGGNGGGNNACTGTAAA
NGGNTAATTNGNNGNTNATTNCNNNCACNTNNCTNNGNNTNCTNNNTNCCTGNNTNCNNAATTGGNNC
CANTGNNTGNCNATCTCTGGNTTNGNNNNNNTGNNNNTNGCAANNNGGNNNGTNANANNNCNATNTAA
NNTANANNNNCNNNNNNANNNCACNANNNNNNNAGNNNCNANNNTTANANNNGTAGNNNAATAAGTCT
CCGANNTNNNNNNNATAAGCGNNANACNNNNNNNNNNNNNNANGANNACGNNTTGGNNNTNNNNNGCNA
GNNAGACNNTNATGGNTNNNNNTCAANNNNTTCGTAGTGGTATAATNGNNNNNNNTTTCNCCAGNNTN
NNNGNNNNNNNNCNATANNANNNNNNNGTGANNNNNNNNNNNANNNNCTCNNNAGGNANCANCTACTTNA
GCAANGGNGCGCAGNNNGAGANNNNGACCAGCCTTGGGTGGCAANCACNGCAATAAGTNNNGTTNANC
CTNNNAGTNNACATNNNNNCANNGNNNCNNNNNNANCNGANNGNNNNNNNNNNNNNNNNNNNNNNNNNN
NNNNNNNNNNNNNNNNNNNNNNNNNNNNNNNANNNNANNGNNNGTNTNTGACNCATGTGTTTTTANNNGNN
NNNNNNNGNNNGANCCCCGGTCGNNNNCNNATTGAANNCCTGNNGCATNNNNNTNCNAGAATGANNGC
NNNNNGNNTNGNCTNNCNTTTCAAGNAATCANNNAGGTNNCGCGNTCNNNNNNNNNNNNNCNNTCCCNN
ACTGAGGGTNNNNNNNNCATCTGCTNNNNNNNNNCTNCGCCGGNGTNNCNNNNNAGCNGTATNGTGNTA
NNCANNNNNNNNNNNNNNNNNNTNAAACNANNNNNNNTNNNN
>Tay78
GTGNNNNNTAGCCCNNNNNAGTTAGACANNNCNNNNNNNNNNNNNNNNNNNNNNNNNNNNNNNNNNNNNN
NNNNNNNNNNNNNNNNNNNNNNNNNNNNNNNTNNNNNNNNNNNNNNNNNNNNNNNNNNNNNNNNNNNNNN
NNNNNNNNNNNNNNNNNNNNNNNNNNNNNNNNNNNNNNNNNNNNNNNNNNNNNNNNNNNNNNNNNNNNNN
NNNNNNNNNNNNNNNNNNNNNNNNNNNNNNNNNNNNNNNNNNNNNNNNNNNNNNNNNNNNNNNNNNNNNN
NNNNNNNNNNNNNNNNNNNNNNNNNNNNNNNNNNNNNNNNNNNNNNNNNNNNNNNTCTNNNAAANNNTNNN
NNNNNNNNNNNNNNNNNNNNNNNNNCNCNTTGNNNGTAANCNANNNGNACATGCAGTCCCCTCNNNNN
NNNGGCAGAGNNNNNNCCCNNNNNNNCCGGCCCCNNCCNNNNNNNNNNNNNNNNNGNNNNNNNANNNNNN
NNNNNNTGGTACNNNNCGNNNNNGGTTGTTTAAGAGGTCCACAACANGGTCNGGNNACNNNNGNNNAN
NNNCNNAAAACNNNNNNNNNNNNNANNTNNNTANNAGGNCNNNGCCNTNNNGNNNNNAANNGCNATGACC
CAANNCACGGNGGCAGTNCCCGGCGNGGCGACCTTGNTNACGTCCGGGGACAAGTATGGCGNNCNCCC
CGGGAAGGANNCNGCCGTANGTGCNNNNNANNNNNTCNTGNGANGTCGCCGNNATTNNCGTGGTCTNN
CNAGAATGNCCGNACCANNCGNANNNTGGTNATGNTAGTANNNNNNNNNNNNNNNNNNNNNNNNNNNNN
NNNNNNNNNNNNNNNNNNNNNNNNNNNNNNNNNNNNNNNNNGNNNNNNCNANNNAAGGANNTANNNCTNNTN
NTNNNNNNGGACNNNACNNNNCNNNNNNNACNCNNNCNCNNNNNNANNNGNTTTTNNNNNCNNTATTNT
CCAGNACGCNGNGCNNNCATTCANGNGAACNNNGNNACGCGNNAATCGTANNATCTACCTGCCGCNGG
TNNANNTACNGNNNNTNCGGANNNTNNNNCGTANNNNNNTCATGCGTNNTNTNNTCNCNAGNCANGTT
CGNNNANANANTAGNANGTNTNNNNNNNNNNTGTNNCGTTCCGCNCGACNCNNCANNNNGNNGNNCTTN
TCANCGGCNNTANCTGCNGANNTAAGNCATANNNTNNATCNNTANCTNNNNNGGNGGGNNACTGTAAA
NGGNNAATTNGANGNTNATTTCNNNCACNTNNCTNNANNTNCTNNNTNCCTGNNTNCNNAATTGGNNC
CANTGNNTGNCNATCTCTGGNTTNGNNNNNNTNNNNNTNNCAANNNGGNNNGTNANANNNCNATNTAA
NNTANANNNNCNNNNNNANNNCACNANNNNNGNAGNNACNANNNTTANANNNGTAGNNNAATAAGTCT
CCGANNTNNNNNNNATAAGCGNNANACNNNNNNNNNNNNNNANGANNACGNNTTGGNNNTNNNNNNCNA
GNNAGACNNTNGTGGNTNNNNNTCAANNNNTTCGTAGTGGTATAATNGNNNNNNNTTTCNCCAGNNTN

NNNGNNNNNNNNCNATANNANNNNNNNGNGANNNNNNNNNNNANNNNCTTNNNAGGNANCANTTACTNNA
GCANTGGNGCGCAGNNNGAGANNNNGACNNNCCTTGGGTGGCAANCACNGCAATAAGTNNNGTTNANN
NTNNNAGTNNACACNNNNNCANNGNNNCNNNNNNANCNGANNGTACNNNNNNNNNNNNNNNNNNNNNN
NNNNNNNNNNNNNNNNNNNNNNNNNNNNNNNGNNNANGGNNNGTNTNTGACNCATGTGTTTTTANNNGNN
NNNNNACGNNNNANCCCCGGTCGNNNNCNNATTGAANNCCTGNNGCATNNNNNTNCNAGAATGANNGC
NNNNNGNNTNGNCTNNCNTTTCAAGNAATCAANNAGGTNNNNNGNTCNNNGNNNNNNNNCNNTCCCNN
ACTGAGGGTNNNNNNNNCATCTGCTNNNNNNNNCTNCGCNGGCGTNNCNNNNNAGCNGTATNGTGTNA
NNCANNNNNNNNNNNNNNNNNNNNAANNNANNNNNNNTNNNN
>Tay82
GTGNNNNNTAGCCCNNNNNAGTTAGACANNNCNNNNNNNNNNNNNTNNNNNNNNNNNNNNNNNNNNNNN
NNNNNNNNNNNNNNNNNNNNNNNNNNNNNNNNTNNNNNNNNNNNNNNNNNNNNNNNNNNNNNNNNNNNNNNN
NNNNNNNNNNNNNNNNNNNNNNNNNNNNNNNNNNNNNNNNNNNNNNNNNNNNNNNNNNNNNNNNNNNNNNNNNNNN
NNNNNNNNNNNNNNNNNNNNNNNNNNNNNNNNNNNNNNNNNNNNNNNNNNNNNNNNNNNNNNNNNNNNNNNNNNNN
NNNNNNNNNNNNNNNNNNNNNNNNNNNNNNNNNNNNNNNNNNNNNNNNNNNNNNNCNTCTNNNAAANNNTNNN
NNNNNNNNNNNNNNNNNNNNNNNNNNNNCNCNTTGNNNGTAANCNANNNGNACATGCAGTCCCCTCNNNNN
NNNNGCAGAGNNNNNNNCCNNNNNCNCCGNCCCCNNCCNNNNNNNNNNNNNNNNGNNNNNNNANNNNNN
NNNNNNTGGTACNNNNCGNNNNNGGTTGTNTNAGAGGTCCACAACGNGGTCNNGNCACNNNNGNNNAN
NNNCNNNNAACNNNNNNNNNNNNANNTNNNTANNAGGNCNNNNCCNTCNNNNNNNNNNATTGCNATGACC
CAANNCACGGGGGCNGTNCCCGGNGCGGCGACCTTGNTNANNTCCGGGGACAAGTATGGCGNNCNCCC
CGGGAAGGANNCNGCCGTANGTGCNNNNNANNNNNTCNTGNGANGTCGCCGNNATTCNCGTGGTCNNN
CNAGAATGNCCNNACCANNCGNANNNTGGTNATGNNAGTNNNNNNNNNNNNNNNNNNNNNNNNNNNNN
NNNNNNNNNNNNNNNNNNNNNNNNNNNNNNNNNNNNGNNNNGNNNNNNCNANNNAAGGANNTANNNCTNNTN
NTNNNNNNNGGACNNNACNNNNCNNNNNNNACNCNNNCNCNNNNNNANNNGNTTTTNNNNCNNTATTNT
CCAGNANGCNGNGCNNNCATTCANGNGAACNNNGNNACGCGNNAATCGTANNATCTACCTGCCGCNGG
TNNNNNTACNGNNNNTNCGGANNNTNNNNCGTANNNNNNTCATGCGTNNTNTNNTCNCNAGNCANGTT
CGNNNANANANTAGNANGTNTNNNNNNNNNTGTNNCGTTCCGCNTGANNCNNCANNNNGNNGNNCTTN
TCANCGGCNNTANCTGCNGANNTAAGNCATANNNTNNATCNNTANCTNNNNNGGNGGGNNACTGTAAA
NGGNNAATTNGNGGNTNATTTCNNNCACNTNNCNNNGNNTNCTNNNTNCCTGNNTNCNNAATTGGNNC
CANTGNNTGNCNATCTCTGGNTTNGNNNNNNTGNNNNTNNCANNNNGGNNNNNNANANNNCNATNTAA
NNTATANNNNCNNNNNNANNNCACNNNNNNNNNAGNNNCNANNNTTANANNNGTAGNNNAATAAGTCT
CCGANNTNNNNNNNATAAGCGNNANACNNNNNNNNNNNNNNANGANNACGNNTTGGNNNTNNNNNNCNA
GNNAGACNNTNATGGNTNNNNNTCAANNNNTTNGTAGTGGTATAATNGNNNNNNNTTTCNCCAGNNNN
NNNGNNNNNNNNCNATANNANNNNNNNGTGANNNNNNNNNNNANNNNCTCNNNAGGNANCANCTACTTNA
GCAANGGNGCGNAGNNNGAGANNNNGACNAGCCTTGGGTGGCAANCACNGCGATAAGTNNNGTTNANN
NTNNNAGTNNACATNNNNNCANNGNNNCNNNNNNANCNGANNGNNNNNNNNNNNNNNNNNNNNNNNN
NNNNNNNNNNNNNNNNNNNNNNNNNNNNNANNNNANNGNNNGTNTNTGACNCATGTGTTTTTANNNGNN
NNNNNACGNNNGANCCCCGGTCGTNNNCNNATTGAANNCCTGNNGCATNNNNNTNCNAGAATGANNNC
NNNNNGNNTNGNCTNNCNTTTCAAGNAATCANNNAGGTANNGCGNTCNNNNNNNNNNNNNCNNTCCCAN
ACTGAGGGTNNNNNNNNCATCTGCTNNNNNNNNNTNCGCNGNCGTNNCNNNNNAGCNGTATNGNGTNA
NNCANNNNNNNNNNNNNNNNNNNNNNNNGANNNNNNNTNNNN
>Tay84
GTGNNNNNTAGCCCNNNNNAGTCAGACANNNCNNNNNNNNNNNNNTNNNNNNNNNNNNNNNNNNNNNNN
NNNNNNNNNNNNNNNNNNNNNNNNNNNNNNNNTNNNNNNNNNNNNNNNNNNNNNNNNNNNNNNNNNNNNNNN
NNNNNNNNNNNNNNNNNNNNNNNNNNNNNNNNNNNNNNNNNNNNNNNNNNNNNNNNNNNNNNNNNNNNNNNNNNNN
NNNNNNNNNNNNNNNNNNNNNNNNNNNNNNNNNNNNNNNNNNNNNNNNNNNNNNNNNNNNNNNNNNNNNNNNNNNN
NNNNNNNNNNNNNNNNNNNNNNNNNNNNNNNNNNNNNNNNNNNNNNNNNNNNNNNNNTCTNNNAAANNNTNNN
NNNNNNNNNNNNNNNNNNNNNNNNNNNNCNCNTTGNNNGTAANCNANNNGNACATGCAGTCCCCTCNNNNN
NNNGGCAGAGNNNNNNNNCNNNNNNNCCGNCCCCNNCCNNNNNNNNNNNNNNNNGNNNNNNNANNNNNN
NNNNNNTGGTACNNNNCGNNNNNGGTTGTNTNAGAGGTCCACAACGNGGTCNNGNCACNNNNGNNNAN
NNNCNNANAACNNNNNNNNNNNNNNNNTNNNTANNAGGNCNNNNCCNTCNNGNNNNNNANNGCNATGANN
CAANNCACGGGGGCAGTCCCCGGCGCGGCGACCTTGATNANGTCCGGGGACAAGTATGGCGCNCNCCC
CGGGAAGGANNCNGCCGTANGTGCNNNNNANNNNNTCNTGNGANGTNGCCGNNATTCNCGTGGTCTNN
NNAGAATGNCCGNACCANNCGNANNNTGGTNATGNNAGTANNNNNNNNNNNNNNNNNNNNNNNNNNNN
NNNNNNNNNNNNNNNNNNNNNNNNNNNNNNNNNNNNGNNNNGNNNNNNCNANNNAAGGANNTANNNCTNNTC

NTNNNNNNGGACNNNACNNNNCNNNNNNNACNCNNNCNCNNNNNNANNNGNTTTTTNNNCNNTATTNT
CCAGNACGCNGNGCNNNCATTCANGNGAACNNNGNNACGCGTAAATCGTANNATCTACCTGCCGCNGG
NNNNNNTNCNGNNNNTNCGGANNNTNNNNCGTANNNNNNTCATGCGTNNTNTNNTCNCTAGNCNNGTT
CGNNNANANANTAGNANGTNTCNNNNNNCTTGTNNCGTTCCGCNTGANNCNNCANNNNGNCGNNCTTN
TCANCGGCNNTANCTGCNGANNTAAGTCATANNNTNNATCNNTANCTNNNNNGGNGGGNNACTGTAAA
NGGNTAATTGGNNGNTNATTTCCNNCATNTNNCTNNGNNNNCTNNNTNCCTGNNTNCNNAATTGGNNC
CANTGNNTGNCNATCTCTGGNTTNGNNNNNNTNNNNNTNNCANNNNGGNNNGTNANANNNCNATNTAA
NNTATANNNNCNNNNNNANNNCACNANNNNNGNAGNNANNANNNGTANANNNGTAGNNNGATAAGTCT
CCGANNTNNNNNNNATAAGCGNNANACNNNNNNNNNNNNANGANNACGNNTTTGNNNTNNNNNNCNA
GNNAGACNNTNGTGGNTNNNNNTCAANNNNTTCGTAGTGGTATAATNGNNNNNNCTTTCNCCAGNNTN
NNNGNNNNNNNCNATANNANNNNNNNGNGANNNNNNNNNNNANNNNCTCNNNAGGNANCANCTACTNNA
GCANNGGNGCGCAGNNNGAGANNNNGACNNNCCTTGGGTGGCAANCACNGCAATAAGTNNNGTTNANC
CTNNNAGTNNACACGNNNNCANNGNNNCNNNNNNANCNGANNGNNNNNNNNNNNNNNNNNNNNNNNNN
NNNNNNNNNNNNNNNNNNNNNNNNNNNNAGNNNANNGNNNGTNTNTGACNCATGTGTTTTTANNNGNN
NNNNNACGNNNGANCCCCGGTCGNNNNCNNATTGAANNCCTGNNGCATNNTNNTNCNAGAATGANNGC
NNNNNGNNTNGNCTGNCNNTTCAAGNAATCANNNAGNNNNCGCGNTCNNNGNNNNNNNNCNNTCCCNN
ACTGAGGGTNNNNNNCGCATCTGCTNNNNNNNNNNTNCGCCGNNGTNNCNNNNNAGCNGTATNGNGTNN
NNCANNNNNNNNNNNNNNNNTNNNNNANNNNNNNTNNNN
>Tay87
GTGNNNNNTAGCCCNNNNNAGTCAGACANNNCNNNNNNNNNNNNTNNNNNNNNNNNNNNNNNNNNNNN
NNNNNNNNNNNNNNNNNNNNNNNNNNNNNNNNNNNNNNNNNNNNNNNNNNNNNNNNNNNNNNNNNNNN
NNNNNNNNNNNNNNNNNNNNNNNNNNNNNNNNNNNNNNNNNNNNNNNNNNNNNNNNNNNNNNNNNNNN
NNNNNNNNNNNNNNNNNNNNNNNNNNNNNNNNNNNNNNNNNNNNNNNNNNNNNNNNNNNNNNNNNNNN
NNNNNNNNNNNNNNNNNNNNNNNNNNNNNNNNNNNNNNNNNNNNNNNNNNCNTCTNNNAAANNNTNNN
NNNNNNNNNNNNNNNNNNNNNNNNNCNCNTTGNNNGTAANCNANNNGNACATGCAGTCCCCTCNNNNN
NNNNNCAGAGNNNNNNCCCNNNNNNNCCGNCCCCNNCCNNNNNNNNNNNNNNNGNNNNNNNANNNNNN
NNNNNNTGGTACNNNNCGNNNNNGGTTGTTTAAGAGGTCCACAACGNGGTCNNGNNACNNNNGNNNAN
NNNCNNNNAANNGNNNNNNNNNANNTNNNTACNAGGNCNNNNCCNTCNNGNNNNNAATNGCNATGACC
CAANNCACGGGGGCAGTNCCCGGNGNGGCGACCTTGANNACGTCCGGGGACAAGTATGGCNCNCNCCC
CGGGAAGGANNCNGCCGTANGTGCNNNNNANNNNNTCNTGNGANGTNGCCGNNATTNNCGTGGTCNNG
NNAGANTGNNCNNACCANNCGNANNNTGGTNATGNTAGTNNNNNNNNNNNNNNNNNNNNNNNNNNNNN
NNNNNNNNNNNNNNNNNNNNNNNNNNNNNNNNNNNNNGNNNNNNCNANNNAAGGANNTANNNCTNNTN
NTNNNNNNGGACNNNACNNNNCNNNNNNNACNCNNNCNCNNNNNNANNNGNTTTTTNNCCNNTANTNT
CCAGNACGCNGNGCNNNCATTTANGNGAACNNNGNNACGCGNNAATCGTANNATCTACCTGCCGCNNG
TNNNNNTACNGNNNNNNCGGANNNTNNNNCGTANNNNNNTCATGCGTNNTNTNNTCNCNAGNCNNGTT
CGNNNANANANTAGNANGTNTNNNNNNNNNTGTNNCGTTCCGCNTGANNCNNCANNNNGNNGNNCTTN
TCANCGGCNNTANCTGCNGANNTAAGNCATANNNTNNNTCNNTANCTNNNNNGGNGGGNNACTGTAAA
NGGNNAATTNGANGNTNATTTCNNNCATNTNNCNNNGNNTNCTNNNTNCCTGNNTNCNNAATTGGNNC
CANTGNNTGNCNATCTCTGGNTTNGNNNNNNTNNNNNTNNCAANNNGGNNNGTNANANNNCNATNTAA
NNTATANNNNCNNNNNNANNNCACNANNNNNGNAGNNNCNANNNGTANANNNGTAGNNNGATAAGTCT
CCGANNTNNNNNNNATAAGCGNNANACNNNNNNNNNNNNANGANNACGNNTTTGNNNTNNNNNNCNA
GNNAGACNNTNGTGGNTNNNNNTCAANNNNTTCGTAGTGGTATAATNGNNNNNNNTTTCNCCAGNNTN
NNNGNNNNNNNCNATANNANNNNNNNGTGANNNNNNNNNNNANNNNCTCNNNAGGGANCANCTACTTNA
GCANTGGNGCGCAGNNNGAGANNNNGACNNGCCTTGGGTGGCAANCACNGCAATAAGTNNNGTTNANC
CTNNNAGTNNACACNNNNNCANNGNNNCNNNNNNANCNGANNGNNNGNNNNNNNTNNNNNNNNNNNNN
NNNNNNNNNNNNNNNNNNNNNNNNNNNNAGNNNANGGNNNGTNTNTGACNCNTGTGTTTTTATNNGNN
NNNNNACGNNNNANCCCCGGTCGNNNNCNNATTGAANNCCTGNNGCATNNNNNTNCNAGAATGANNGC
NNNNNGNNTNGNCTNNCNTTTCAAGNAATCAANNAGGTANCGCGNTCNNNGNNNNNNNNCNNTCCCNN
ACTGAGGGTNNNNNNCGCATCTGCTNNNNNNNNNNTTCGCNGGCGTNNCNNNNNAGCNGTATNGTGTNA
NNCANNNNNNNNNNNNNNNNTNNNNCNNNNNNNNTNNNN
>Thai130
GTGNNNNNTAGCCCNNNNNAGTTAGACANNNCNNNNNNNNNNNNTNNNNNNNNNNNNNNANNNNNNNN
NNNNNNNNNNNNNNNNNNNNNNNNNNNNNNNNNNNNNNNNNNNNNNGNNNNNNNNNNNNNNNNNNNNN
NNNNNNNNNNNNNNNNNNNNNNNNNNNNNNNNNNNNNNNNNNNNNNNNNNNNNNNNNNNNNNNNNNNN

NNNNNNNNNNNNNNNNNNNNNNNNNNNNNNNNNNNNNNNNNNNNNNNNNNNNNNNNNNNNNNNNNNNNNNNN
NNNNNNNNNNNNNNNNNNNNNNNNNNNNNNNNNNNNNNNNNNNNNNNNNNNNNNNNTCTNNNAAANNNTNNN
NNNNNNNNNNNNNNNNNNNNNNNNNNCNCNTTGNNNGTAANCNANNNGNACATGCAGTCCCCTCNNNNN
NNNNNCAGAGNNNNNNCCCNNNNNCNCCGGCCCCNNCCNNNNNNNNNNNNNNNNGNNNNNNNANNNNNN
NNNNNNTGGTACNNNNCGNNNNNGGTTGTNTAAGAGGTCCACAACGNGGTCNNGNNACNNNNGNNNAN
NNNCNNNNAANNNNNNNNNNNNNNNNNTNNNTANNAGGNNNNNNCCNTCNNNNNNNNAATNGCNATGACC
CAANTCACGGNGGNAGTNCCCGGNGCGGCGACCTTGATNACGTCCGGGGACAAGTATGGCNCNCNCCC
CGGGAAGGANNCNGCCGTANGTGCNNNNNANNNNNTCNTGNGANGTCGCCGNNATTCNCGTGGTCNNN
CNAGANTGNNCNNACCANNCGNANNNTGGTNATGNNAGTNNNNNNNNNNNNNNNNNNNNNNNNNNNNN
NNNNNNNNNNNNNNNNNNNNNNNNNNNNNNNNNNGNNNNGNNNNNNCNANNNAAGGNNNTANNNCTNNTN
NTNNNNNNGGACNNNACNNNNCNNNNNNNACNCNNNCNCNNNNNNANNNGNTTTTTNNNCNNTATTNT
CCAGNACGCNGNGCNNNCATTCANGNGAACNNNGNNACGCGNNAATCGTANNATCTACCTGCCGCNGG
TNNNNNTACNGNNNNTNCGGANNNTNNCTCGTANNNNNNTCATGCGTNNTNTNNTCNCNAGNCNNGTT
CGNNNANANANTAGNANGTNTCNNNNNNNNTGTNNCGTTCCGCNTGANNCNNCANNNNGNCGNNCTTC
TCANCGGCNNTANCTGCNGANNTAAGNCATANNNTNNNTCNNTANCTNNNNNGGNGGGNNACTGTAAA
NGGNTAATTNGNNGNTNATTTCNNNCACNTNNCTNNGNNTNCTNNNTNCCTGNNTNCNNAATTGGNNC
CANTGNNTGNCNATCTCTNGNTTNGNNNNNNTGNNNNTNGCAANNNGGNNNNNNANANNNCNATNTAA
NNTATANNNNCNNNNNNANNNCACNANNNNNGNAGNNNCNANNNTTANANNNGTAGNNNAATAAGTCT
CCGANNTNNNNNNNATAAGCGNNANACNNNNNNNNNNNNNANGANNACGNNTTGGNNNTNNNNNNCNA
GNNAGACNNTNATGGNTNNNNNTCAANNNNTTCGTAGTGGTATAATNGNNNNNNNTTTCNCCAGNNTN
NNNGNNNNNNNCNATANNANNNNNNNGNGANNNNNNNNNNNANNNNCTCNNNAGGNANCANCTACTTNA
GCANTGGNGCGNAGNNNGAGANNNNGACNNGCCTTGGGTGGCAANCACNGCGATAAGTNNGTTNANC
CTNNNAGTNNACATNNNNNCANNGNNNCNNNNNNANCNGANNGNNNNNNNNNNNNNNNNNNNNNNNNN
NNNNNNNNNNNNNNNNNNNNNNNNNNNNNNAGNNNANGGNNNGTNTNTGACNCATGTGTTTTTANNNGNN
NNNNNACGNNNNANCCCCGGTCGNNNNCNNATTGAANNCCTGNNGCATNNNNNTNCNAGAATGANNGC
NNNNNGNNTNGNCTNNCNTTTCAAGNAATCANNNAGNTNNCGCGNTCNNNNNNNNNNNNNCNNTCCCNN
ACTGAGGGTNNNNNNNNCATCTGCTNNNNNNNNNCTNCGCNGGCGTNNCNNNNNAGCNGTATNNTGTNA
NNCANNNNNNNNNNNNNNNNNNTNANNCNANNNNNNNTNNNN
>Thai136
GTGNNNNNTAGCCCNNNNNAGTTANNCANNNCNNNNNNNNNNNNNNNNNNNNNNNNNNNNNNNNNNNNNN
NNNNNNNNNNNNNNNNNNNNNNNNNNNNNNNNNNNNNNNNNNNNNNNNNNNNNNNNNNNNNNNNNNNNNNNN
NNNNNNNNNNNNNNNNNNNNNNNNNNNNNNNNNNNNNNNNNNNNNNNNNNNNNNNNNNNNNNNNNNNNNNNN
NNNNNNNNNNNNNNNNNNNNNNNNNNNNNNNNNNNNNNNNNNNNNNNNNNNNNNNNNNNNNNNNNNNNNNNN
NNNNNNNNNNNNNNNNNNNNNNNNNNNNNNNNNNNNNNNNNNNNNNNNNNNNNNNNNNNNNNNNAANNNNNNN
NNNNNNNNNNNNNNNNNNNNNNNNNNNNNCNNTNNNNGTNANCNANNNGNACATGCAGTNNNCTCNNNNN
NNNNNCAGAGNNNNNNNNNNNNNNNNNNCCGNCCCCNNCCNNNNNNNNNNNNNNNNNNNNNNNNNNNANNNNNN
NNNNNNTGGTACNNNNCGNNNNNGGNNGTNTNAGAGGTCCACAACGNGGTCNNGNNANNNNNGNNNAN
NNNNNNNNNNNNNNNNNNNNNNNNNNNTNNNTNNNAGGNNNNNNCCNNCNNNNNNNNAANNGCNATNANN
CNNNNNNCNGNGGNNGTNCCCGGNGNNGNNACCTTGNTNACGTCNGGGNNCAAGTATNGCNNNCNCCC
CGNGAAGGANNCNGCNGTANGTGCNNNNNANNNNNTCNTGNNANNTNGCCGCANTTNNNNTGGNCNNN
NNNNANTNNNCNNACNANNNGNNNNNTGGTNATGNNAGTNNNNNNNNNNNNNNNNNNNNNNNNNNNNNN
NNNNNNNNNNNNNNNNNNNNNNNNNNNNNNNNNNGNNNNGNNNNNNCNANNNAAGGANNTANNNCTNNTN
NNNNNNNNGGACNNNACNNNNCNNNNNNNACNCNNNCNCNNNNNNNNNNNGNTTNNNNNNCNNTANTNT
CCAGNACGCNGNGCNNNCATTCANNNGAANNNNGNNACGCGNNAATCGTANNATCTACCTGNCGCNNG
NNNNNNTNCTGNNNNNNNCGGANNNTNNNNCGTANNNNNNNCANGCGTNNTNTNNTCNCNNGNNNNNTT
CGNNNANNNANTANNANGTNNNNNNNNNNNNNNTNNCGTTCCGCNTGANNCNNCANNNNGNNGNNNTTN
NCANCGGCNNTANCTNCNGANNTAAGNCATANNNTNNNTCNNTANCTNNNNNGGNNGGNNACTGTAAA
NGNNNAATTNGNNNNTNANTNCNNNCACNTNNCTNNGNNNNCTNNNTNCCTGNNTNCNNANTTGGNNN
CANTGNNTGNCNATCTCTNNNTTNGNNNNNNTNNNNNTNNCANNNNGGNNNNNNNNANNNCNATNTAA
NNNATANNNNCNNNNNNANNNCACNNNNNNNGNAGNNNCNANNNTNANANNNGTAGNNNANTAAGTCN
CCGNNNTNNNNNNNATAAGNGNNANACNNNNNNNNNNNNNANGANNNCNNNTTGGNNNTNNNNNNNNA
GNNAGNCNNTNATGGNTNNNNNTNNANNNNTTCGTAGTGGTATAATNGNNNNNNNTTTCNCCAGNNTN
NNNNNCNNNNNCNATANNANNNNNNNGNNANNNNNNNNNNNANNNNNTCNNNAGGNANNANCTACTNNA
NCANNGGNGCGNAGNNNNAGNNNNNGACNNNCCTTGGGTGNNNANCACNGNANNAAGTNNNGTTNNNN

NTNNNAGTNNANATNNNNNCANNGNNNNNNNNNNNANCNGANNGNNNNNNNNNNNNNNNNNNNNNNNNNN
NNNNNNNNNNNNNNNNNNNNNNNNNNNNNNNNNNNANGGNNNGTNTNNGNCNCNTGTGTTNNTANNNGNN
NNNNNACNNNNNNNCCCCGGTCGNNNNCNNATTGAANNCCTGNNNCATNNNNNTNCNAGAATGANNNC
NNNNNGNNTNNNCTNNNNNNNNNNNNNAATCNNNNAGNNNNNNNNNTCNNNNNNNNNNNNCNNNNNNNN
ACTGAGGGTNNNNNNNNCATNNGCTNNNNNNNNNNTNCNCNNNNNNNNNCNNNNNNGCNGTATNNNGNNN
NNCANNNNNNNNNNNNNNNNNTTAAACAANNNNCTTNNNG
>Thai248
GTGNNNNNTAGCCCGNNNNAGTTAGACANNNCNNNNNNNNNNNNNTNNNNNNNNNNNNNNNNNNNNNNNN
NNNNNNNNNNNNNNNNNNNNNNNNNNNNNNNNNNNNNNNNNNNNNNNNNNNNNNNNNNNNNNNNNNNNNN
NNNNNNNNNNNNNNNNNNNNNNNNNNNNNNNNNNNNNNNNNNNNNNNNNNNNNNNNNNNNNNNNNNNNNN
NNNNNNNNNNNNNNNNNNNNNNNNNNNNNNNNNNNNNNNNNNNNNNNNNNNNNNNNNNNNNNNNNNNNNN
NNNNNNNNNNNNNNNNNNNNNNNNNNNNNNNNNNNNNNNNNNNNNNNNNNNCNTCTNNNAAANNNTNNN
NNNNNNNNNNNNNNNNNNNNNNNNNNNCNCNTTGNNNGTAANCNANNNGNACATGCAGTCCCCTCNNNNN
NNNGGCAGAGNNNNNNNCCNNNNNCNCCGNCCCCGNCCNNNNNNNTNNNCNNNNNNNNNNNNANNNNNN
NNNNNNTGGTACNNNNCGNNNNNGGTTGTNTNAGAGGTCCACAACGNGGTCNNGNCACNNNNGNNNAN
NNNCNNNNAANNNNNNNNNNNNNNNATNNNTANNAGGNCNNNNCCNTNNNNNNNNNNAATNGCNATGACC
CAANTCACGGNGGCAGTCCCCGGCGCGGCGACCTTGATNACGTCCGGGGACAAGTATGGCGNNCNCCC
CGGGAAGGANNCNGCCGTANGTGCNNNNNANNNNNTCNTGNGANGTCGCCGNNATTCNCGTGGTCNNN
NNAGANTGNCCGNACCANNCGNANNNTGGTTATGTTAGTNNNNNNNNNNNNNNNNNNNNNNNNNNNNNNN
NNNNNNNNNNNNNNNNNNNNNNNNNNNNNNNNNNGNNNNGNNNNNNCNANNNAAGGANNTANNNCTNNTN
NTNNNGNNGGACNNNACNNNNCNNNNNNNACNCNNNCNCNNNNNNANNNGNTTTTTNNNCNNTANTNT
CCAGNACGCNGNGCNNNCATTCANGNGAACNNNGNNACGCGNNAATCGTANNATCTACCTGCCGCNNG
TNNNNNTACNGNNNNTNCGGANNNTNNNNCGTANNNNNNTCATGCGTNNTNTNNTCNCNAGNCANGTT
CGNNNATANANTAGNANGTNTNNNNNNNNCNTGTNNCGTTCCGCNTGANNCNNCANNNNGNNGNNCTTN
TCANCGGCNNTANCTGCNGANNTAAGNCATANNNTNNNTCNNTANCTNNNNNGGNGGGNNACTGTAAA
NGGNNAATTNGAGGNTNATTTCNNNCACNTNNCTNNGNNTNCTNNNTNCCTGNNTNCNNAATTGGNNN
CANTGNNTGNCNATCTCTGGNTTNGCNNNNNTNNNNNTNNCANNNNGGNNNGTNNNANNNCNATNTAA
NNTANANNNNCNNNNNNANNNCACNANNNNNGNAGNNNCNANNNTTANANNNGTAGNNNAATAAGTCT
CCGANNTNNNNNNNATAAGCGNNANACNNNNNNNNNNNNNNANGANNACGNNTTGGNNNTNNNNNNCNA
GNNAGACNNTNATGGNTNNNNNTCAANNNNTTCGTAGTGGTATAATNGNNNNNNNTTTCNCCAGNNTN
NNNGNNNNNNNCNATANNANNNNNNNGNGANNNNNNNNNNNANNNNCTCNNNAGGGANCANCTACTNNA
GCAATGGNGCGCAGNNNGAGANNNNGACCAGCCTTGGGTGGCAANCACNGCAATAAGTNNNGTTNANC
CTNNNAGTNNACATNNNNNCANNGNNNCNNNNNNANCNGANNGNNNNNNNNNNNNNNNNNNNNNNNNNN
NNNNNNNNNNNNNNNNNNNNNNNNNNNNNNAGNNNANNGNNNGTNTNTGACNCATGTGTTTTTANNNGNN
NNNNNACGNNNNANCCCCGGTCGNNNNCNNATTGAANNCCTGNNGCATNNNNNTNCNAGAATGANNGC
NNNNNGNNTNNNCTNNCNNNTCAAGNAATCANNTAGGTANNNNGNTCNNNGANNNNNNNCNNTCCCNN
ACTGAGGGTNNNNNNCGCATCTGCTNNNNNNNNNNTTCGCNGGNGTNNCNNNNNNGCCGTATNGTGTNN
NNCANNNNNNNNNNNNNNNNNNNANNNNNNNNNNNNNNNN
>Thai249
GTGNNNNNTAGCCCNCNNNAGTCAGACANNNCNNNNNNNNGNNNNTNNNNNNNNNNNNNNNNNNNNNNNN
NNNNNNNNNNNNNNNNNNNNNNNNNNNNNNTNNNNNNNNNNNNNNNNNNNNNNNNNNNNNNNNNNNNNNNN
NNNNNNNNNNNNNNNNNNNNNNNNNNNNNNNNNNNNNNNNNNNNNNNNNNNNNNNNNNNNNNNNNNNNNN
NNNNNNNNNNNNNNNNNNNNNNNNNNNNNNNNNNNNNNNNNNNNNNNNNNNNNNNNNNNNNNNNNNNNNN
NNNNNNNNNNNNNNNNNNNNNNNNNNNNNNNNNNNNNNNNNNNNNNNNNNNNNNTCTNNNAAANNNTNNN
NNNNNNNNNNNNNNNNNNNNNNNNNNNCNCNTTGNNNGTAANCNANNNGNACATGCAGTCCCCTCNNNNN
NNNGGCAGAGNNNNNNCNNNNNNNCNCCGNCCCCGNCCNNNNNNNNNNNNNNNGNNNNNNNANNNNNN
NNNNNNTGGTACNNNNCGNNNNNGGTTGTTTNAGAGGTCCACAACGNGGTCNGGNCACNNNNGNNNAN
NNNCNNAAAACNNNNNNNNNNNNNNNTNNNTANNAGGNNNNNNCCNTCNNGNNNNNNANNGCNATGACC
CNANTCACGGGGGCAGTNCCCGGCGCGGCGACCTTGNTNACGTCCGGGGACAAGTATGGCNCNCNCCC
CGGGAAGGANNCNGCCGTANGTGCNNNNNANNNNNTCTTGNGANGTNGCCGNNATTCNNGTGGTCNNN
CNAGAATGNNCGNACCANNCGNANNNTGGTNATGNTAGTANNNNNNNNNNNNNNNNNNNNNNNNNNNNNN
NNNNNNNNNNNNNNNNNNNNNNNNNNNNNNNNNNGNNNNGNNNNNNCNANNNAAGGANNTANNNCTNNTC
NTNNNGNNGGACNNNACNNNNCNNNNNNNACNCNNNCNCNNNNNNANNNGNTTTTTTNCCGNTATTNT
CCAGNACGCNGNGCNNNCATTTANGNGAACNNNGNNACGCGNNAATCGTANNATCTACCTGCCGCNGG

TNNNNNTACNGNNNNTNCGGANNNTNNCTCGTANNNNNGTCATGCGTNNTNTNNTCNCNAGNCNNGTT
CGNNNANANANTAGNANGTNTCNNNNNNCTTGTNNCGTTCCGCNTGANNCNNCANNNNGNNGNNCTTN
TCANCGGCNNTANCTGCNGANNTAAGTCATANNNTNNNTCNNTANCTNNNNNGGNGGGNNACTGTAAA
NGGNNAATTNGNGGNTNATTTCNNNCATNTNNCTNNGNNNNCTNNNTNCCTGNNTNCNNAATTGGGNC
CANTGNNTGNCNATCTCTGGNTTNGCNNNNNTNNNNNTNGCANNNNGGNNNGTNANANNNCNATNTAA
NNTATANNNNCNNNNNNANNNCACNANNNNNGNAGNNNCNANNNGTANANNNGTAGNNNGATAAGTCT
CCGANNTNNNNNNNATAAGCGNNANACNNNNNNNNNNNNNANGANNACGNNTTTGNNNTNNNNNGCNA
GNNAGACNNTNGTGGNTNNNNNTCAANNNNTTCGTAGTGGTATAATNGNNNNNNNTTTCNCCAGNNTN
NNNGNNNNNNNCNATANNANNNNNNNGTGANNNNNNNNNNNANNNNCTCNNNAGGGANCANCTACTNNA
GCAATGGNGCGCAGNNGGAGANNNNGACCAGCCTTGGGTGGCAANCACNGCAATAAGTNNNGTTTANC
CTNNNAGTNNACACNNNNNCANNGNNNCNNNNNNANCNGANNGNNNNNNNNNNNNNNNNNNNNNNNNNN
NNNNNNNNNNNNNNNNNNNNNNNNNNNNNAGNNNANNGNNNGTNTNTGACNCATGTGTTTTTANNNGNN
TNNNNACGNNNGNNCCCCGGTCGTNNNCNNATTGAANNCCTGNNGCATNNNNNNTNCNAGAATGANNGC
NNNNNGNNTNGNCTNNCNTTTCAAGNAATCNNNNAGGTNTCGCGNTCNNNGNNNNNNNNCNNTCCCNN
ACTGAGGGTNNNNTCNNCATCTGCTNNNNNNNNNNCTTCGCNGGNGTNNCTNNNNAGCNGTATNGTGTNN
NNCANNNNNNNNNNNNNNNNNNTNNNACNNNNNNNNNTNNNN

>Thai250

GTGNNNNNTAGCCCNNNNNAGTTAGACANNNCNNNNNNNNNNNNNNNTNNNNNNNNNNNNNNNNNNNNNN
NNNNNNNNNNNNNNNNNNNNNNNNNNNNNNTNNNNNNNNNNNNNNNNNNNNNNNNNNNNNNNNNNNNNNN
NNNNNNNNNNNNNNNNNNNNNNNNNNNNNNNNNNNNNNNNNNNNNNNNNNNNNNNNNNNNNNNNNNNNNN
NNNNNNNNNNNNNNNNNNNNNNNNNNNNNNNNNNNNNNNNNNNNNNNNNNNNNNNNNNNNNNNNNNNNNN
NNNNNNNNNNNNNNNNNNNNNNNNNNNNNNNNNNNNNNNNNNNNNNNNNNNNNNTCTNNNAAANNNTNNN
NNNNNNNNNNNNNNNNNNNNNNNNNCNCNTTNNNNNNGTAANCNANNNGNACATGCAGTCCCCTCNNNNN
NNNGGCAGAGNNNNNNNNCNNNNNNNCCGGCCCCGNCCNNNNNNNNNNNNCNNNGNNNNNNNANNNNNN
NNNNNNTGGTACNNNNCGNNNNNGGTTGTTTAAGAGGTCCACAACGNGGTCNNNNCACNNNNGNNTAN
NNNCNNNAAANNNNNNNNNNNNNNNNNTNNNTANNAGGNCNNNNCCNTCNNNNNNNNNAANTGCNATGACC
CAANTNACGGGGGCAGTNCCCGGCGCGGCGACCTTGATNANGTCCGGGGACAAGTATGGCNCNCNCCC
CGGGAAGGANNCNGCCGTANGTGCNNNNNANNNNNTCNTGNGANGTCGCCGNNATTCNCGTGGTCNNN
CNAGANTGNCCGNACCANNCGNANNNTGGTNATGTTAGTANNNNNNNNNNNNNNNNNNNNNNNNNNNNN
NNNNNNNNNNNNNNNNNNNNNNNNNNNNNNNNNGNNNNGNNNNNNCNANNNAAGGANNTANNNCTNNTN
NTNNNNNNNGGACNNNACNNNNCNNNNNNNACNCNNNCNCNNNNTGNNNNGNTTTTNNNCCNNTATTNT
CCAGNACGCNGNGCNNNCATTCANGNGAACNNNGNNACGCGNNAATCGTANNATCTACCTGCCGCNNG
TNNNNNTACNGNNNNTNCGGANNNTNNNNCGTANNNNNNNTCATGCGTNNTNTNNTCNCNAGNCANGTT
CGNNNANANANTAGNANGTNTCNNNNNNNNTGTNNCGTTCCGCNTGANNCNNCANNNNGNNGNNNTTN
TCANCGGCNNTANCTGCNGANNTAAGTCATANNNTNNNTCNNTANCTNNNNNGGNGGGNNACTGTAAA
NGGNNAATTNGNNGNTNATTTCNNNCACNTNNCTNNGNNTNCTNNNTNCCTGNNTNCNNAATTGGNNC
CANTGNNTGNCNATCTCTNGNTTNGNNNNNNTGNNNNTNGCANNNNGGNNNGTNANANNNCNATNTAA
NNTATANNNNCNNNNNNANNNCACNANNNNNNNAGNNNCNANNNTTANANNNGTAGNNNANTAAGTCT
CCGANNTNNNNNNNATAAGCGNNANACNNNNNNNNNNNNNANGANNACGNNTTGGNNNTNNNNNNCNA
GNNAGACNNTNATGGNTNNNNNTCAANNNNTTNGTAGTGGTATAATNGNNNNNNNTTTCNCCAGNNTN
NNNGNNNNNNNCNATANNANNNNNNNGNGANNNNNNNNNNNANNNTCTCNNNAGGNANCANCTACTTNA
GCANNGGNGCGCAGNNNGAGANNNNGACNNNCCTTGGGTGGCAANCACNGCAATAAGTNNNGTTNANC
CTNNNAGTNNACATNNNNNCANNGNNNCNNNNNNANCNGANNGNNNNNNNNNNNNNNNNNNNNNNNNNN
NNNNNNNNNNNNNNNNNNNNNNNNNNNNNAGNNNANGGNNNGTNTNTGACNCATGTNTTTTTANNNGNN
NNNNNNNGNNNNANCCCCGGTCGNNNNCNNATTGAANNCCTGNNGCATNNNNNNTNCNAGAATGANNNC
NNNNNGNNTNGNCTNNCNTTTCAAGNAATCAANTAGGTNNCGCGNTCNNNGNNNNNNNNCNNTCCCNN
ACTGAGGGTNNNNNNNNCATCTGCTNNNNNNNNNNTNCGCNGGNGTNNCTNNNNAGCNGTATNGNGTNA
NNCANNNNNNNNNNNNNNNNACNNANCNANNNNNNNTNNNN

>Thai251

GTGNNNNNTAGCCCNNNNNAGTTNGACANNNCNNNNNNNNNNNNNNNTNNNNNNNNNNNNNNNNNNNNNN
NNNNNNNNNNNNNNNNNNNNNNNNNNNNNNNNNNNNNNNNNNNNNNNNNNNNNNNNNNNNNNNNNNNNNN
NNNNNNNNNNNNNNNNNNNNNNNNNNNNNNNNNNNNNNNNNNNNNNNNNNNNNNNNNNNNNNNNNNNNNN
NNNNNNNNNNNNNNNNNNNNNNNNNNNNNNNNNNNNNNNNNNNNNNNNNNNNNNNNNNNNNNNNNNNNNN
NNNNNNNNNNNNNNNNNNNNNNNNNNNNNNNNNNNNNNNNNNNNNNNNNNNNNNNNNNNNNNAANNNNNNN

NNNNNNNNNNNNNNNNNNNNNNNNNNNCNNTNNNNGTAANCNANNNGNACATGCAGTCCCCTCNNNNN
NNNNNCAGAGNNNNNNNNNNNNNNNNCCGGCCCCNNCCNNNNNNNNNNNCANNGNNNNNNNANNNNNN
NNNNNNTGGTACNNNNCGNNNNNGGNNGNNNNAGAGGTCCACAACGNGGTCNNGNNACNNNNGNNNAN
NNNCNNNNNNNNNNNNNNNNNNNNNNNNNTANNAGGNNNNNNCCNTNNNNNNNNNNANNGCNNNGACC
CANNNCNCGGNGGNNGTNCCCGGNGNNGCNACCTTGNNNANNTCCGGGNNCAAGTATGGCGCNCNCCC
CGGGAAGGANNCNGCCGTANGTGCNNNNNANNNNNTCNTGNGANGTNGCCGNNNTTNNCGTGGTCNNN
NNAGAATGNCCNNACCANNNGNANNNTGGTNATGNNAGTNNNNNNNNNNNNNNNNNNNNNNNNNNNNN
NNNNNNNNNNNNNNNNNNNNNNNNNNNNNNNNNNNNNGNNNNNNCNANNNAAGGNNNTANNNCTNNTN
NNNNNNNNNNNCNNNACNNNNCNNNNNNNACNCNNNCNCNNNNNNNNNNGNTTTTTNNCCNNTATTNT
CCAGNANGCNGNGCNNNCATTCANGNGAACNNNGNNACGCGNNAATCGTANNATCTACCTNCCGCNNG
TNNNNNTACNGNNNNNNCGGANNNTNNCTCGTANNNNNNNCATGCGTNNTNTNNNCNCNAGNCNNGTT
CGNNNANANANTAGNANGTNNNNNNNNNNNNGTNNCGTTCCGCNTGACNCNNCANNNNGNNGNNCTTN
TCANNGGNNNTANCTNCNNANNTAAGNNATANNNNNNNTCNNTANCTNNNNNGGNGGGTNACNGNAAA
NGNNNAATTNGNNNNTGANTNCNNNCACNTNNCNNNGNNNNNTNNNTNCCTGNNTNCNNAATTGGNNN
CANTGNNTGNCNATCTCTGGNTTNGNNNNNNTGNNNNTNNCAANNNGGNNNNNNANANNNCNATNTAA
NNTANANNNNCNNNNNNNNNNNACNANNNNNNNAGNNNNNANNNTTANANNNGNAGNNNAATNAGTCT
CCGANNTNNNNNNNNANAAGCGNNANANNNNNNNNNNNNNANGANNNCGNNTTGGNNNNNNNNNNCNA
GNNAGACNNTNATGGNTNNNNNTCAANNNNTTNGTAGTGGTATAATNGNNNNNNNTTTCNCCAGNNTN
NNNNNNNNNNNCNATANNANNNNNNNGNGANNNNNNNNNNANNNNCTCNNNAGGGANCANCTACTNNA
GCAATGGNGCGNAGNNNGAGANNNNGACNNGCCTTGGGTGGCAANCACNGCANTAAGTNNNGTTTANC
CTNNNAGTNNACATNNNNNCANNGNNNCNNNNNNANCNNANNGNNNNNNNNNNNNNNNNNNNNNNNNN
NNNNNNNNNNNNNNNNNNNNNNNNNNNNNNNNNANGGNNNGTNNNNGACNCATGNNTTTTTANNNGNN
NNNNNNNGNNNNANCCCCGGTCGTNNNCNNATTGAANNCCTGNNGCATNNNNNTNCNANAATGANNNC
NNNNNGNNTNGNCTNNNNNNTCAANNAATNNNNNAGGTANNNNNNTCNNNGNNNNNNNNCNNNCCCNN
ACTGAGGGNNNNNNNNNCANNTGCTNNNNNNNNNNTTCNCGNNNNNNCNNNNNNGCNGTATNNNGTNN
NNCANNANNNNNNNNNNNNNNNNTNNNNNNNNNNNNNNN
>Thai252
GTGNNNNNTAGCCCNNNNNAGTTAGACANNNCNNNNNNNNNNNNNNNNNNNNNNNNNNNNNNNNNNNN
NNNNNNNNNNNNNNNNNNNNNNNNNNNNNNNNNNNNNNNNNNNNNNNNNNNNNNNNNNNNNNNNNNNN
NNNNNNNNNNNNNNNNNNNNNNNNNNNNNNNNNNNNNNNNNNNNNNNNNNNNNTNNNNNNNNNNNNNN
NNNNNNNNNNNNNNNNNNNNNNNNNNNNNNNNNNNNNNNNNNNNNNNNNNNNNNNNNNNNNNNNNNNN
NNNNNNNNNNNNNNNNNNNNNNNNNNNNNNNNNNNNNNNNNNNNNNNNNNNNTCTNNNAAANNNTNNN
NNNNNNNNNNNNNNNNNNNNNNNNNCNCNTTGNNNGTAANCNANNNGNACATGCAGTCCCCTCNNNNN
NNNGGCAGAGNNNNNNCCCNNNNNNNCCGGCCCCNNCCNNNNNNNNNNNNNNNGNNNNNNNGNNNNNN
NNNNNNTGGTACNNNNCGNNNNNGGTTGTNTAAGAGGTCCACCACGNGGTCNNGNCANNNNNGNNNAN
NNNCNNNNNNNNNNNNNNNNNNNNNTNNNCANNAGGNNNNNNCCNTNNNNNNNNNNANNGCNATGACC
CAANNCACGGGGGCNGTNCCCGGCGNGGCGACCTTGATNANNGCCGGGGACAAGTATGGCGTNCNCCC
CGNGAAGGANNCNGCCGTANGTGCNNNNNANNNNNTCNTGNGANGTNGCCGNNATTNNCGTGGTCNNN
CNAGAATGNCCGNACCANNCGNANNNTGGTNATGNNAGTANNNNNNNNNNNNNNNNNNNNNNNNNNNN
NNNNNNNNNNNNNNNNNNNNNNNNNNNNNNNNNNNNNGNNNNNNCNANNNAAGGNNNTANNNCTNNTC
NTNNNNNNGGACNNNACNNNNCNNNNNNNACNCNNNCNCNNNNNNANNNGNTTTTTNNNCNNTATTNT
CCAGNACGCNGNGCNNNCATTCANGNGAACNNNGNNACGCGNNAATCGTANNATCTACCTGCCGCNGG
TNNNNNTACNGNNNNTNCGGANNNTNNCTCGTANNNNNNTCATGCGTNNTNTNNTCNCNAGNCANGTT
CGNNNANNNANTAGNANGTNTNNNNNNNNNTGTNNCGTTCCGCNTGANNCNNCANNNNGNNGNNCTTN
TCANCGGCNNTANCTGCNGANNTAAGNCATANNNTNNNTCNNTANCTNNNNNGGNGGGNNACTGTAAA
NGGNNAATTNGNGGNTNATTTCNNNCACNTNNCTNNGNNNNCTNNNTNCCTGNNTNCNNAATTGGNNC
CANTGNNTGNCNATCTCTGGNTTNGNNNNNNTNNNNNTNNCAANNNGGNNNGTNANANNNCNATNTAA
NNTATANNNNCNNNNNNANNNCACNANNNNNGNAGNNNCNANNNTTANANNNGTAGNNNAATAAGTCT
CCGANNTNNNNNNNNATAAGCGNNANACNNNNNNNNNNNNANGANNACGNNTTGGNNNTNNNNNNCNA
ANNAGACNNTNGTGGNTNNNNNTCAANNNNTTNGTAGTGGTATAACNGNNNNNNNTTTCNCCAGNNTN
NNNGNNNNNNNCNATANNANNNNNNNGTGANNNNNNNNNNANNNNCTCNNNAGGGANCANCTACTNNA
GCANTGGNGCGNAGNNNGAGANNNNGACNNGCCTTGGGTGGCAANCACNGCAATAAGTNNNGTTNANC
CTNNNAGTNNACACNNNNNCANNGNNNCNNNNNNANCNGANNTNNNNNNNNNNNNNNNNNNNNNNNNN
NNNNNNNNNNNNNNNNNNNNNNNNNNNNAGNNNANGGNNNGTNTNTGACNCATGTGTTTTTANNNGNN

NNNNNACGNNNGANCCCCGGTCGNNNNCCNATTGAANNCCTGNNGCATNNNNNTNCNAGAATGANNGC
NNNNNGNNTNGNNTNNCTTTTCAAGNAGTCANNNAGGTANCGCGNTCNNNGNNNNNNNNCNNTCCCNN
ACTGAGGGTNNNNNNNNCATNNGCTNNNNNNNNNTNCGCNGNCGTNNCNNNNNAGCNGTATNGTGTTA
NNCANNNNNNNNNNNNNNNNNNNNNNNANNNNNNTNNNN
>Thai253
GTGNNNNNTAGCCCNNNNNAGTTAGACANNNCNNNNNNNNNNNNNNNNNNNNNNNNNNNNNNNNNNNN
NNNNNNNNNNNNNNNNNNNNNNNNNNNGNNTNNNNNNNNNNNNNNNNNNNNNNNNNNNNNNNNNNNNN
NNNNNNNNNNNNNNNNNNNNNNNNNNNNNNNNNNNNNNNNNNNNNNNNNNNNNNNNNNNNNNNNNNNN
NNNNNNNNNNNNNNNNNNNNNNNNNNNNNNNNNNNNNNNNNNNNNNNNNNNNNNNNNNNNNNNNNNNN
NNNNNNNNNNNNNNNNNNNNNNNNNNNNNNNNNNNNNNNNNNNNNNNNNNCNTCTNNNAAANNNTNNN
NNNNNNNNNNNNNNNNNNNNNNNNNCNCNTTGNNNGTAANCNANNNGNACATGCAGTCCCCTCNNNNN
NNNGGCAGAGNNNNNNNCNNNNNNNNCCGGCCCCNNCCNNNNNNNNNNNNNNNGNNNNNNNANNNNNN
NNNNNNTGGTACNNNNCGNNNNNGGTTGTTTAAGAGGTCCACAACGNGGTCNGGNNACNNNNGNNNAN
NNNCNNANNNCNNNNNNNNNNNNNTNNNNTANNAGGNNNNNNCCNTCNNGNNNNNNATNGCNATGACC
CAANNCACGGGGGNNGTCCCCGGCGCGGCGACCTTGATNACGTCCGGGGACAAGTATGGCNCNCNCCC
CGGGAAGGANNCNGCCGTANGTGCNNNNNANNNNNTCNTGNGANGTCGCCGNNATTCNNNTGGTCNNN
CNAGAATGNNCNNACCANNCGNANNNTGGTNATGNTAGTANNNNNNNNNNNNNNNNNNNNNNNNNNNN
NNNNNNNNNNNNNNNNNNNNNNNNNNNNNNNNGNNNNGNNNNNNCNANNNAAGGANNTANNNCTNNTN
NTNNNNNNGGACNNNACNNNNCNNNNNNNACNCNNNCNCNNNNNNNNNNGNTTTTTNNCCNNTATTNT
CCAGNACGCNGNGCNNTCATTCANGNGAACNNNGNNACGCGNNAATCGTANNATCTACCTGCCGCNGG
TNNNNNTACNGNNNNTACGGANNNTNNCTCGTANNNNNGTCATGCGTNNTNTNNTCNCNAGNCANGTT
CGNNNANANANTAGNANGTNTNNNNNNNNNTGTNNCGTTCCGCNTGANNCNNCANNNNGNNGNNCTTN
TCANCGGCNNTANCTGCNGANNTAAGTCATANNNTNNNTCNNTANCTNNNNNGGNGGGNNACTGTAAA
NGGATAATTGGNNGNTNATTTCNNNCACNTNNCTNNGNNNNCTNNNTNCCTGNNTNCNNAATTGGNNC
CANTGNNTGNCNATCTCTNGNTTNGNNNNNNTNNNNNTNGCANNNNGGNNNNTNANANNNCNATNTAA
NNTATANNNNCNNNNNNANNNCACNANNNNNGNAGNNNCNANNNTTANANNNGTAGNNNAATAAGTCT
CCGANNTNNNNNNNATAAGCGNNANACNNNNNNNNNNNNNANGANNACGNNTTGGNNNTNNNNNGCNA
GNNAGACNNTNATGGNTNNNNNTCAANNNNTTCGTAGTGGTATAATNGNNNNNNNTTTCNCCAGNNTN
NNNGNNNNNNNCNATANNANNNNNNNGNGANNNNNNNNNNANNNNCTCNNNAGGGANCANCTACTTNA
GCAATGGNGCGCAGNNNGAGANNNNGACNAGCCTTGGGTGGCAANCACNGCAATAAGTNNNGTTNANC
CTTNNAGTNNACATGNNNNCANNGNNNCNNNNNNANCNGANNGNNNNNNNNNNNNNNNNNNNNNNNNN
NNNNNNNNNNNNNNNNNNNNNNNNNNNNAGNNNANGGNNNGTNTNTGACNCATGTGTTTTTANNNGNN
TNNNNACGNNNNNNCCCCGGTCGNNNNCNNATTGAANNCCTGNNGCATNNNNNTNCNAGAATGANNGC
NNNNNGNNTNNNCTNNCTTTTCAAGNAATCANNNAGNNATCGCGNTCNNNGNNNNNNNNCNNTCCCNN
ACTGAGGGTNNNNNNNNCATCTGCTNNNNNNNNCTTCGCNGGNGTNCCNNNNNAGCNGTATNGTGTTA
NNCANNNNNNNNNNNNNNNNTNNANNNANNNNNNTNNNN
>Thai254
GTGNNNNNTAGCCCGNNNNAGTCAGACANNNCNNNNNNNNNNNNTNNNNNNNNNNNNNNNNNNNNNNN
NNNNNNNNNNNNNNNNNNNNNNNNNANNNNTNNNNNNNNNNNNNNNNNNNNNNNNNNNNNNNNNNNNNN
NNNNNNNNNNNNNNNNNNNNNNNNNNNNNNNNNNNNNNNNNNNNNNNNNNNNNNNNNNNNNNNNNNNN
NNNNNNNNNNNNNNNNNNNNNNNNNNNNNNNNNNNNNNNNNNNNNNNNNNNNNNNNNNNNNNNNNNNN
NNNNNNNNNNNNNNNNNNNNNNNNNNNNNNNNNNTNNNNNNNNNNNNNNNCNTCTNNNAAANNNTNNN
NNNNNNNNNNNNNNNNNNNNNNNNNCNCNTTGNNNGTAANCNANNNGNACATGCAGTCCCCTCNNNNN
NNNGGCAGAGNNNNNNCCNNNNNNCNCCGGCCCCGNCCNNNNNNNNNNNNANNGNNNNNNNANNNNNN
NNNNNNTGGTACNNNNCGNNNNNGGTTGTTTNAGAGGTCCACAACGNGGTCNNGNCACNNNNGNNNAN
NNNCNNNNNNCNNNNNNNNNNNANNTNNNTANNAGGNCNNNNCCNTCNNGNNNNNNATNGCNATGACC
CAANNCACGGGGGCNGTCCCCGGCGCGGCGACCTTGATNACGTCCGGGGACAAGTATGGCNCNCNCCC
CGGGAAGGANNCNGCCGTANGTGCNNNNNANNNNNTCNTGNGANGTCGCCGNNATTCNCNTGGTCTNN
CNAGANTGNNCGNACCANNCGNANNNTGGTNATGNTAGTNNNNNNNNNNNNNNNNNNNNNNNNNNNNN
NNNNNNNNNNNNNNNNNNNNNNNNNNNNNNNNGNNNNGNNNNNNCNANNNAAGGANNTANNNCTNNTN
NTNNNNNNGGACNNNACNNNNCNNNNNNNACNCNNNCNCNNNNNNNNNNGNTTTTTNNNCNNTATTNT
CCAGNACGCNGNGCNNNCATTTANGNGAACNNNGNNACGCGNNAATCGTANNATCTACCTGCCGCNGG
TNNNNNTACNGNNNNTNCGGANNNTNNNNCGTANNNNNGTCATGCGTNNTNTNNTCNCNAGNCNNGTT
CGNNNANANANTAGNANGTNTCCNNNNNNNTGTNGCGTTCCGCNTGANNCNNCANNNNGNNGNNCTTN

TCANCGGCNNTANCTGCNGANNTAAGTCATANNNTNNNTCNNTANCTNNNNNGGNGGGNNACTGTAAA
NGGNTAATTNGNNGNTNATTTCNNNCATNTNNCTNNGNNNNCTNNNTNCCTGNNTNCNNAATTGGNNC
CANTGNNTGNCNATCTCTGGNTTNGCNNNNNTGNNNNTNGCAANNNGGNNNGTNANANNNCNATNTAA
NNTATANNNNCNNNNNNANNNCACNANNNNNNNAGNNNCNANNNGTANANNNGTAGNNNGATAAGTCT
CCGANNTNNNNNNNATAAGCGNNANACNNNNNNNNNNNNNANGANNACGNNTTTGNNNTNNNNNNCNA
GNNAGACNNTNGTGGNTNNNNNTCAANNNNTTNGTAGTGGTATAATNGNNNNNNNTTTCNCCAGNNTN
NNNGNNNNNNNCNATANNANNNNNNNGTGANNNNNNNNNNANNNNCTCNNNAGGGANCANCTACTTNA
GCAATGGNGCGCAGNNNGAGANNNNGACNNGCCTTGGGTGGCAANCACNGCAATAAGTNNNGTTNANC
CTNNNAGTNNACACNNNNNCANNGNNNCNNNNNNANCNGANNGNNNNNNNNNNNNNNNNNNNNNNNNN
NNNNNNNNNNNNNNNNNNNNNNNNNNNNNAGNNNANGGNNNGTNTNTGACNCATGTGTTTTTANNNGNN
NNNNNACGNNNGANCCCCGGTCGNNNNCNNATTGAANNCCTGNNGCATNNNNNTNCNAGAATGANNGC
NNNNNGNNTNGNCTNNCTTTTCAAGNAATCAANNAGNTNNCGCGNTCNNNGANNNNNNNCNNTCCCNN
ACTGAGGGTNNNNNNCGCATCTGCTNNNNNNNNCTNCGCNGGNNNNNCNNNNNAGCNGTATNGTGTNN
NNCANNNNNNNNNNNNNNNNNNNNNNNNNANNNNNNNTNNNN
>Thai255
GTGNNNNNTAGCCCNNNNNAGTCAGACANNNCNNNNNNNNNNNNTNNNNNNNNNNNNNNNNNNNNNNN
NNNNNNNNNNNNNNNNNNNNNNNNNNNNNNNNNNNNNNNNNNNNNNNNNNNNNNNNNNNNNNNNNNNN
NNNNNNNNNNNNNNNNNNNNNNNNNNNNNNNNNNNNNNNNNNNNNNNNNNNNNNNNNNNNNNNNNNNN
NNNNNNNNNNNNNNNNNNNNNNNNNNNNNNNNNNNNNNNNNNNNNNNNNNNNNNNNNNNNNNNNNNNN
NNNNNNNNNNNNNNNNNNNNNNNNNNNNNNNNNNNNNNNNNNNNNNNNNNCNTCTNNNAAANNNTNNN
NNNNNNNNNNNNNNNNNNNNNNNNNNNCNCNTTGNNNGTAANCNANNNGNACATGCAGTCCCCTCNNNNN
NNNGGCAGAGNNNNNNNCCNNNNNCNCCGGCCCCNNCCNNNNNNNTNNNNNNNGNNNNNNNANNNNNN
NNNNNNTGGTACNNNNCGNNNNNGGTTGTTTAAGAGGTCCACAACGNGGTCNGGNCACNNNNGNNNAN
NNNCNNNNAANNNNNNNNNNNNNNNNNTNNNTANNAGGNCNNNNCCNTCNNGNNNNNNATTGCNATGACC
CNANNCACGGNGGCAGTNCCCGGCGCGGCGACCTTGATNANNTCCGGGGACAAGTATGGCGCNCNCCC
CGGGAAGGANNCNGCCGTANGTGCNNNNNANNNNNTCNTGNGACGTNGCCGNNATTCNCGTGGTCNNN
CNAGAATGNNCGNACCANNCGNANNNTGGTNATGNNAGTNNNNNNNNNNNNNNNNNNNNNNNNNNNN
NNNNNNNNNNNNNNNNNNNNNNNNNNNNNNNNNGNNNNGNNNNNNCNANNNAAGGANNTANNNCTNNTN
NTNNNNNNNGGACNNNACNNNNCNNNNNNNNACNCNNNCNCNNNNNNANNNGNTTTTTNNCCNNTATTNT
CCAGNACGCNGNGCNNTCATTCANGNGAACNNNGNNACGCGNNAATCGTANNATCTACCTGCCGCNNG
TNNNNNTNCNGNNNNTNCGGANNNTNNCTCGTANNNNNGTCATGCGTNNTNTNNTCNCNAGNCANGTT
CGNNNANANANTAGNANGTNTCCNNNNNNNNTGTNGCGTTCCGCNTGANNCNNCANNNNGNNGNNCTTN
TCANCGGCNNTANCTGCNGANNTAAGNCATANNNTNNATCNNTANCTNNNNNGGNGGGNNACTGTAAA
NGGNTAATTNGNNGNTNATTTCCNNCATNTNNCTNNGNNNNCTNNNTNCCTGNNTNCNNAATTGGNNC
CANTGNNTGNCNATCTCTNGNTTNGNNNNNNNTNNNNNTNNCAANNNGGNNNGTNANANNNCNATNTAA
NNTANANNNNCNNNNNNANNNCACNANNNNNGNAGNNANNANNNGTANANNNGTAGNNNGATAAGTCT
CCGANNTNNNNNNNATAAGCGNNANACNNNNNNNNNNNNNANGANNACGNNTTTGNNNTNNNNNNNNA
GNNAGACNNTNGTGGNTNNNNNTCAANNNNTTNGTAGTGGTATAATNGNNNNNNNTTTCNCCAGNNTN
NNNGNNNNNNNCNATANNANNNNNNNGTGANNNNNNNNNNANNNNCTCNNNAGGGANCANCTACTTNA
GCANTGGNGCGCAGNNNGAGANNNNGACNNGCCTTGGGTGGCAANCACNGCAATAAGTNNNGTTNANC
CTNNNAGTNNACACNNNNNCANNGNNNCNNNNNNANCNGANNGNNNNNNNNNNNNNNNNNNNNNNNNN
NNNNNNNNNNNNNNNNNNNNNNNNNNNNNAGNNNANGGNNNGTNTNTGACNCATGTGTTTTTANNNGNN
NNNNNACGNNNNNNCCCCGGTCGNNNNCNNATTGAANNCCTGNNGCATNNNNNTNCNAGAATGANNGC
NNNNNGNNTNGNCTGNCNTTTCAAGNAATCANNNAGNTANCGCGNTCNNNGNNNNNNNNCNNTCCCNN
ACTGAGGGTNNNNNNCGCATCTGCTNNNNNNNNCTNCGCNGGCGNNNCNNNNNAGCNGTATNGTGTTN
NNCANNNNNNNNNNNNNNNNNTNAAANNNNNNNNNNNTNNNN
>Thai257
GTGNNNNNTAGCCCNNNNNAGTTAGACANNNCNNNNNNNNNNNNNNNNNNNNNNNNNNNNNNNNNNNN
NNNNNNNNNNNNNNNNNNNNNNNNNNNNNNNNTNNNNNNNNNNNNNNNNNNNNNNNNNNNNNNNNNNN
NNNNNNNNNNNNNNNNNNNNNNNNNNNNNNNNNNNNNNNNNNNNNNNNNNNNNNNNNNNNNNNNNNNN
NNNNNNNNNNNNNNNNNNNNNNNNNNNNNNNNNNNNNNNNNNNNNNNNNNNNNNNNNNNNNNNNNNNN
NNNNNNNNNNNNNNNNNNNNNNNNNNNNNNNNNNNNNNNNNNNNNNNNNNNTCTNNNAAANNNTNNN
NNNNNNNNNNNNNNNNNNNNNNNNNNNCNCNTTGNNNGTAANCNANNNGNACATGCAGTCCCCTCNNNNN
NNNGGCAGAGNNNNNNNNNNNNNNNNNCCGNCCCCNNCCNNNNNNNNNNNNCNNNGNNNNNNNANNNNNN

NNNNNNTGGTACNNNNCGNNNNNGGTTGTTTNAGAGGTCCACAACGNGGTCNGGNCACNNNNGNNNAN
NNNCNNNNNACNGNNNNNNNNNNANNTNNNTANNAGGNCNNNNCCNTCNNGNNNNNAANTGCNATGACC
CAANNCACGGGGGCNGTNCCCGGNGCGGCGACCTTGATNANNTCCGGGGACAAGTATGGCNCNCNCCC
CGGGAAGGANNCNGCCGTANGTGCNNNNNANNNNNTCNTGNGANGTCGCCGNNATTCNNGTGGTCNNN
NNAGAATGNCCGNACCANNCGNANNNTGGTNATGNTAGTANNNNNNNNNNNNNNNNNNNNNNNNNNNNNN
NNNNNNNNNNNNNNNNNNNNNNNNNNNNNNNNNNGNNNNGNNNNNNCANNNNAAGGNNNTANNNCTNNTC
NTNNNNNNGGACNNNACNNNNCNNNNNNNACNCNNNCNCNNNNNNANNNGNTTTTTNNNCNNTATTNT
CCAGNACGCNGNGCNNNCATTCANGNGAACNNNGNNACGCGNNAATCGTANNATCTACCTGCCGCNGG
TNNNNNTACNGNNNNTNCGGANNNTNNNNCGTANNNNNNTCATGCGTNNTNTNNTCNCNAGNCNNGTT
CGNNNANANANTAGNANGTNTNNNNNNNCTTGTNGCGTTCCGCNTGANNCNNCANNNNGNNGNNCTTC
TCANCGGCNNTANCTGCNGANNTAAGTCATANNNTNNATCNNTANCTNNNNNGGNGGGNNACTGTAAA
NGGNNAATTGGAGGNTNATTTCNNNCACNTNNCTNNGNNTNCTNNNTNCCTGNNTNCNNAATTGGNNC
CANTGNNTGNCNATCTCTGGNTTNGCNNNNNTNNNNNTNGCAANNNGGNNNGTNANANCNCNATNTAA
NNTATANNNNCNNNNNNANNNCACNANNNNNGNAGNNNCNANNNTTANANNNGTAGNNNAATAAGTCT
CCGANNTNNNNNNNATAAGCGNNANACNNNNNNNNNNNNNNANGANNACGNNTTGGNNNTNNNNNGCNA
GNNAGACNNTNATGGNTNNNNNTCAANNNNTTCGTAGTGGTATAATNGNNNNNNNTTTCNCCAGNNTN
NNNGNNNNNNNCNATANNANNNNNNNGTGANNNNNNNNNNNANNNNCTCNNNAGGGANCANCTACTTNA
GCAANGGNGCGCAGNNNGAGANNNNGACNNNCCTTGGGTGGCAANCACNGCAATAAGTNNNGTTNANC
CTNNNAGTNNACATGNNNNCANNGNNNCNNNNNNANCNGANNGNNNNNNNNNNNNNNNNNNNNNNNNNNN
NNNNNNNNNNNNNNNNNNNNNNNNNNNNNNAGNNNANGGNNNGTNTNTGACNCATGTGTTTTTANNNGNN
TNNNNACGNNNNANCCCCGGTCGNNNNCNNATTGAANNCCTGNNGCATNNNNNTNCNAGAATGANNGC
NNNNNGNNTNGNCTGNCNTTTCAAGNAATCANNNAGGTNTCGCGNTCNNGGNNNNNNNNCNNTCCCNN
ACTGAGGGTNNNNNNNNCATCTGCTNNNNNNNNCTTCGCCGGCNNNNCNGNNNAGCCGTATNGTGTTA
NNCANNNNNNNNNNNNNNNNNNNAACAANNNNNNNTNNNN
>Thai258
GTGNNNNNTAGCCCGNNNNAGTTAGACANNNCNNNNNNNNNNNNTNNNNNNNNNNNNNNNNNNNNNNNNN
NNNNNNNNNNNNNNNNNNNNNNNNNNNNNNNNNNNNNNNNNNNNNNNNNNNNNNNNNNNNNNNNNNNNNN
NNNNNNNNNNNNNNNNNNNNNNNNNNNNNNNNNNNNNNNNNNNNNNNNNNNNNNNNNNNNNNNNNNNNNN
NCNNNNNNNNNNNNNNNNNNNNNNNNNNNNNNNNNNNNNNNNNNNNNNNNNNNNNNNNNNNNNNNNNNNN
NNNNNNNNNNNNNNNNNNNNNNNNNNNNNNNNNNNNNNNNNNNNNNNNNNNNCNTCTNNNAAANNNTNNN
NNNNNNNNNNNNNNNNNNNNNNNNNNNCNCNTTGNNNGTAANCNANNNGNACATGCAGTCCCCTCNNNNN
NNNGGCAGAGNNNNNNCCCNNNNNCNCCGGCCCCGNCCNNNNNNNNNNNNCNNNGNNNNNNNANNNNNN
NNNNNNTGGTACNNNNCGNNNNNGGTTGTTTAAGAGGTCCACAACGNGGTCNNGNCACNNNNGNNNAN
NNNCNNNNAACNGNNNNNNNNNNNNATNNGTANNAGGNCNNNNCCNTNNNGNNNNTNATTGCNATGACC
CAANTCACGGGGGCAGTNCCCGGCGCGGCGACCTTGATNACGTCCGGGGACAAGTATGGCNCNCNCCC
CGGGAAGGANNCNGCCGTANGTGCNNNNNANNNNNTCNTGNGANGTNGCCGNNATTCNCNTGGTCNNN
NNAGAATGNCCNNACCANNCGNANNNTGGTNATGTNAGTANNNNNNNNNNNNNNNNNNNNNNNNNNNNNN
NNNNNNNNNNNNNNNNNNNNNNNNNNNNNNNNNNGNNNNGNNNNNNCANNNNAAGGANNTANNNCTNNTC
NTNNNNNNGGACNNNACNNNNCNNNNNNNACNCNNNCNCNNNNNNANNNGNTTTTTNNNCNNTATTNT
CCAGNACGCNGNGCNNNCATTCANGNGAACNNNGNNACGCGNNAATCGTANNATCTACCTGCCGCNGG
TNNNNNTACNGNNNNTNCGGANNNTNNCTCGTANNNNNGTCATGCGTNNTNTNNTCNCTAGNCNNGTT
CGNNNANANANTAGNANGTNTNNNNNNNNNTGTNNCGTTCCGCNTGANNCNNCANNNNGNNGNNNTTN
TCANCGGCNNTANCTGCNGANNTAAGTCATANNNTNNNTCNNTANCTNNNNNGGNGGGNNACTGTAAA
NGGNNAATTNGNNGNTNANTTCCNNCACNTNNCTNNGNNTNCTNNNTNCCTGNNTNCNNAATTGGNNC
CANTGNNTGNCNATCTCTGGNTTNGNNNNNNTNNNNNTNNCAANNNGGNNNGTNNNANNNCNATNTAA
NNTATANNNNCNNNNNNANNNCACNANNNNNGNAGNNACNANNNTTANANNNGTAGNNNAATAAGTCT
CCGANNTNNNNNNNATAAGCGNNANACNNNNNNNNNNNNNNANGANNACGNNTTGGNNNTNNNNNNCNA
GNNAGACNNTNATGGNTNNNNNTCAANNNNTTNGTAGTGGTATAATNGNNNNNNCTTTCNCCAGNNTN
NNNGNNNNNNNCNATANNANNNNNNNGTGANNNNNNNNNNNANNNNCTCNNNAGGGANCANCTACTTNA
GCAATGGNGCGNAGNNNGAGANNNNGACNNNCCTTGGGTGGCAANCACNGCAATAAGTNNNGTTNANN
CTNNNAGTNNACATNNNNNCANNGNNNCNNNNNNANCNGANNGNNNNNNNNNNNNNNNNNNNNNNNNNNN
NNNNNNNNNNNNNNNNNNNNNNNNNNNNNNAGNNNANGGNNNGTNTNTGACNCATGTGTTTTTANNNGNN
NNNNNACGNNNNANCCCCGGTCGNNNNCNNATTGAANNCCTGNNGCATNNNNNTNCNAGAATGANNGC
NNNNNGNNTNGNCTGNCTTTTCAAGNAATCANNNAGNNANCGCGNTCNNNGNNNNNNNNCNNTCCCNN

ACTGAGGGTNNNNNNNNCATCTGCTNNNNNNNNNTNCGCNGNCGTNNCNNNNNAGCNGTATNGNGTNA
NNCANNNNNNNNNNNNNNNNTNAAACNNNNNNNNNNNNN
>Thai262
GTGNNNNNTAGCCCNNNNNAGTCAGACANNNCNNNNNNNNNNNNNTNNNNNNNNNNNNNNNNNNNNNNNN
NNNNNNNNNNNNNNNNNNNNNNNNNNNNNNNNNNNNNNNNNNNNNNNNNNNNNNNNNNNNNNNNNNNNNN
NNNNNNNNNNNNNNNNNNNNNNNNNNNNNNNNNNNNNNNNNNNNNNNNNNNNNNNNNNNNNNNNNNNNNN
NNNNNNNNNNNNNNNNNNNNNNNNNNNNNNNNNNNNNNNNNNNNNNNNNNNNNNNNNNNNNNNNNNNNNN
NNNNNNNNNNNNNNNNNNNNNNNNNNNNNNNNNNNNNNNNNNNNNNNNNNNNNNTCTNNNAAANNNTNNN
NNNNNNNNNNNNNNNNNNNNNNNNNNNCNCNTTGNNNGTAANCNANNNGNACATGCAGTCCCCTCNNNNN
NNNGGCAGAGNNNNNNNNCNNNNNCNCCGGCCCCNNCCNNNNNNNNNNNNNNNNNGNNNNNNNANNNNNN
NNNNNNTGGTACNNNNCGNNNNNGGTTGTNTAAGAGGTCCACAACGNGGTCNGGNCACNNNNGNNNAN
NNNCNNNAAANNNNNNNNNNNNNNANNTNNNTANAAGGNNNNNNCCNTCNNNNNNNNNAATNGCNATGACC
CAANTCACGGGGGCAGTNCCCGGCGCGGCGACCTTGNTNANNTCCGGGGACAAGTATGGCGNNCNCCC
CGNGAAGGANNCNGCCGTANGTGCNNNNNANNNNNTCNTGNGACGTCGCCGNNATTCNCGTGGTCNNN
CNAGAATGNNCNNACCANNCGNANNNTGGTTATGNTAGTANNNNNNNNNNNNNNNNNNNNNNNNNNNNN
NNNNNNNNNNNNNNNNNNNNNNNNNNNNNNNNNNGNNNNGNNNNNNCNANNNAAGGANNTANNNCTNNTN
NTNNNNNNGGACNNNACNNNNCNNNNNNNACNCNNNCNCNNNNNNNNNNGGTTTTTNNNCNNTATTNT
CCAGNACGCNGNGCNNNCATTTANGNGAACNNNGNNACGCGNNAATCGTANNATCTACCTGCCGCNNG
TNNNNNTACNGNNNNNNCGGANNNTNNCTCGTANNNNNNTCATGCGTNNTNTNNTCNCNAGNCANGTT
CGNNNANANANTAGNANGTNTCNNNNNNNNTGTNNCGTTCCGCNTGACNCNNCANNNNGNNGNNCTTC
TCANCGGCNNTANCTGCNGANNTAAGNCATANNNTNNNTCNNTANCTNNNNNGGAGGGNNACTGTAAA
NGGNNAATTNGNGGNTNANTNCNNNCATNTNNCNNNGNNTNCTNNNTNCCTGNNTNCNNAATTGGNNC
CANTGNNTGNCNATCTCTNGNTTNGNNNNNNTGNNNNTNNCAANNNGGNNNGNNANANNNCNATNTAA
NNTANANNNNCNNNNNNANNNCACNANNNNNGNAGNNNCNANNNGTANANNNGTAGNNNGATAAGTCT
CCGANNTNNNNNNNATAAGCGNNANACNNNNNNNNNNNNNNANGANNACGNNTTTGNNNTNNNNNNCNA
GNNAGACNNTNGTGGNTNNNNNTCAANNNNTTNGTAGTGGTATAATNGNNNNNNNTTTCNCCAGNNTN
NNNGNNNNNNNNCNATANNANNNNNNNGTGANNNNNNNNNNNANNNNCTCNNNAGGGANCANCTACTTNA
GCAATGGNGCGCAGNNNGAGANNNNGACCAGCCTTGGGTGGCAANCACNGCAATAAGTNNNGTTNANC
CTNNNAGTNNACACNNNNNCANNGNNNCNNNNNNANCNGANNGNNNNNNNNNNNNNNNNNNNNNNNNN
NNNNNNNNNNNNNNNNNNNNNNNNNNNNNNAGNNNANGGNNNGTNTNTGACNCATGTGTTTTTANNGGNN
NNNNNACGNNNGANCCCCGGTCGNNNNCNNATTGAANNCCTGNNGCATNNNNNNTNCNAGAATGANNNC
NNNNNGNNTNGNCTNNCNNNTCAAGNAATCANNNAGGNNNNNNGNTCNNGGNNNNNNNNCNNNCCCNN
ACTGAGGGTNNNNNNCGCATCTGCTNNNNNNNNNCTNCGCNGGNGTNNCTNNNNAGCNGTATNGNGTNN
NNCANNNNNNNNNNNNNNNNTNAAACGANNNNNNNTNNNN
>Thai263
GTGNNNNNTAGCCCNNNNNAGTTAGACANNNCNNNNNNNNNNNNNTNNNNNNNNNNNNNNNNNNNNNNNN
NNNNNNNNNNNNNNNNNNNNNNNNNNNNNNNNNNNNNNNNNNNNNNNNNNNNNNNNNNNNNNNNNNNNNG
NNNNNNNNNNNNNNNNNNNNNNNNNNNNNNNNNNNNNNNNNNNNNNNNNNNNNNNNNNNNNNNNNNNNNN
NNNNNNNNNNNNNNNNNNNNNNNNNNNNNNNNNNNNNNNNNNNNNNNNNNNNNNNNNNNNNNNNNNNNNN
NNNNNNNNNNNNNNNNNNNNNNNNNNNNNNNNNNNNNNNNNNNNNNNNNNNNCNTCTNNNAAANNNTNNN
NNNNNNNNNNNNNNNNNNNNNNNNNNNCNCNTTGNNNGTAANCNANNNGNACATGCAGTCCCCTCNNNNN
NNNGGCAGAGNNNNNNNCCNNNNNNNCCGNCCCCNNCCNNNNNNNNNNNNNNNNNNNNNNNNNANNNNNN
NNNNNNTGGTACNNNNCGNNNNNGGTTGTTTAAGAGGTCCACAACGNGGTCNNGNCACNNNNGNNNAN
NNNCNNNNANCNNNNNNNNNNNNNANNTNNNTANNAGGNNNNNNCCNTNNNGNNNNNNANTGCNATGACC
CAANTCACGGGGGNNGTCCCCGGCGCGGCGACCTTGATNACGTCCGGGGACAAGTATGGCNNNCNCCC
CGGGAAGGANNCNGCCGTANGTGCNNNNNANNNNNTCNTGNGANGTCGCCGNNATTCNCNTGGTCNNN
CNAGAATGNCCGNACCANNCGNANNNTAGTNATGTNAGTNNNNNNNNNNNNNNNNNNNNNNNNNNNNN
NNNNNNNNNNNNNNNNNNNNNNNNNNNNNNNNNNGNNNNGNNNNNNCNANNNAAGGNNNTANNNCTNNTN
NTNNNNNNGGACNNNACNNNNCNNNNNNNACNCNNNCNCNNNNNNANNNGNTTTTTNNNCNNTATTNT
CCAGNACGCNGNGCNNNCATTCANGNGAACNNNGNNACGCGNNAATCGTANNATCTACCTGCCGCNNG
TNNNNNTACNGNNNNNTNCGGANNNTNNNNCGTANNNNNNTCATGCGTNNTNTNNTCNCNAGNCANGTT
CGNNNANANANTAGNANGTNTCNNNNNNNNTGTNNCGTTCCGCNTGANNCNNCANNNNGNNGNNCTTN
TCANCGGCNNTANCTGCNGANNTAAGNCATANNNTNNATCNNTANCTNNNNNGGNGGGNNACTGTAAA
NGGNNAATTNGNNGNTNATTTCNNNCATNTNNCNNNGNNTNCTNNNTNCCTGNNTNCNNAATTGGNNC

CANTGNNTGNCNATCTCTNGNTTNGNNNNNNTNNNNNTNNCAANNNGGNNNNNNANANNNCNATNTAA
NNTATANNNNCNNNNNNANNNCACNNNNNNNGNAGNNNNNANNNTTANANNNGTAGNNNAATAAGTCT
CCGANNTNNNNNNNATAAGCGNNANACNNNNNNNNNNNNNANGANNACGNNTTGGNNNTNNNNNNCNA
GNNAGACNNTNGTGGNTNNNNNTCAANNNNTTCGTAGTGGTATAATNGNNNNNNNTTTCNCCAGNNTN
NNNGNNNNNNNCNATANNANNNNNNNGNGANNNNNNNNNNANNNNCTCNNNAGGNANCANCTACTNNA
GCAATGGNGCGCAGNNNGAGANNNNGACNNNCCTTGGGTGGCAANCACNGCAATAAGTNNNGTTNANC
CTNNNAGTNNACACNNNNNCANNGNNNCNNNNNNANCNGANNGNNNNNNNNNNNNNNNNNNNNNNNNNNN
NNNNNNNNNNNNNNNNNNNNNNNNNNNNNNAGNNNANGGNNNGTNTNTGACNCATGTGTTTTTANNNGNN
NNNNNACGNNNNANCCCCGGTCGTNNNCNNATTGAANNCCTGNNGCATNNNNNTNCNAGAATGANNGC
NNNNNGNNTNGCTNNCNNNTCAAGNAATCANNNAGGNNTCGCGNTCNNNNNNNNNNNNNCNNTCCCNN
ACTGAGGGTNNNNTCNNCATCTGCTNNNNNNNNNNTTCGCNGGCGTNNCNNNNNAGCNGTATNGTGTNN
NNCANNNNNNNNNNNNNNNNNTNAAACAANNNNNNNTNNNN
>Thai268
GTGNNNNNTAGCCCNNNNNAGTTAGACANNNCNNNNNNNNNNNNNNNNNTNNNNNNNNNNNNNNNNNNNN
NNNNNNNNNNNNNNNNNNNNNNNNNNNNNNNNNNNNNNNNNNNNNNNNNNNNNNNNNNNNNNNNNNNNNN
NNNNNNNNNNNNNNNNNNNNNNNNNNNNNNNNNNNNNNNNNNNNNNNNNNNNNNNNNNNNNNNNNNNNNN
NNNNNNNNNNNNNNNNNNNNNNNNNNNNNNNNNNNNNNNNNNNNNNNNNNNNNNNNNNNNNNNNNNNNNN
NNNNNNNNNNNNNNNNNNNNNNNNNNNNNNNNNNNNNNNNNNNNNNNNNNNNCNTCTNNNAAANNGTNNN
NNNNNNNNNNNNNNNNNNNNNNNNNNNCNCNTTGNNNGTAANCNANNNGNACATGCAGTCCCCTCNNNNN
NNNGGCAGAGNNNNNNCCNNNNNNCNCCGNCCCCNNCCNNNNNNNTNNNNNNNGNNNNNNNANNNNNN
NNNNNNTGGTACNNNNCGNNNNNGGTTGTNTAAGAGGTCCACAACGNGGTCNNGNCACNNNNGNNNAN
NNNCNNNNAACNNNNNNNNNNNNNANNTNNNTANNAGGNCNNNNCCNTCNNNNNNNNNAANNGCNATGANN
CAANNCACGGNGGCNGTCCCCGGCGCGGCGACCTTGANNANGTCCGGGGACAAGTATGGCNCNCNCCC
CGGGAAGGANNCNGCCGTANGTGCNNNNNANNNNNTCNTGNGACGTCGCCGNNATTCNNGTGGTCNNN
CNAGAATGNNCGNACCANNCGNANNNTGGTNATGNTAGTNNNNNNNNNNNNNNNNNNNNNNNNNNNNNNN
NNNNNNNNNNNNNNNNNNNNNNNNNNNNNNNNNNGNNNNGNNNNNNCNANNNAAGGANNTANNNCTNNTC
NTNNNNNNNGGACNNNACNNNNCNNNNNNNACNCNNNCNCNNNNNNANNNGGTTTTTNNCCNNTATTNT
CCAGNACGCNGNGCNNTCATTCANGNGAACNNNGNNACGCGNAAATCGTANNATCTACCTGCCGCNGG
TNNNNNTACNGNNNNTNCGGANNNTNNNNCGTAANNNNGTCATGCGTNNTNTNNTCNCNAGNCANGTT
CGNNNANANANTAGNANGTNTCNNNNNNNNTGTNNCGTTCCGCNTGANNCNNCANNNNGNNGNNNTTN
TCANCGGCNNTANCTGCNGANNTAAGNCATANNNTNNATCNNTANCTNNNNNGGAGGGNNACTGTAAA
NGGNNAATTGGNNNNTNATTTCNNNCACNTNNCTNNGNNTNCTNNNTNCCTGNNTNCNNAATTGGNNC
CANTGNNTGNCNATCTCTGGNTTNGNNNNNNTNNNNNTNNCAANNNGGNNNGTNANANNNCNATNTAA
NNTATANNNNCNNNNNNANNNCACNANNNNNNNAGNNNNNANNNTTANANNNGTAGNNNAATAAGTCT
CCGANNTNNNNNNNATAAGCGNNANACNNNNNNNNNNNNNANGANNACGNNTTGGNNNTNNNNNNCNA
GNNAGACNNTNATGGNTNNNNNTCAANNNNTTCGTAGTGGTATAATNGNNNNNNNTTTCNCCAGNNTN
NNNGNNNNNNNCNATANNANNNNNNNGNGANNNNNNNNNNANNNNCTCNNNAGGGANCANCTACTTNA
GCANTGGNGCGCAGNNNGAGANNNNGACCNNCCTTGGGTGGCAANCACNGCAATAAGTNNNGTTNANC
CTNNNAGTNNACATNNNNNCANNGNNNCNNNNNNANCNGANNGNNNNNNNNNNNNNNNNNNNNNNNNNNN
NNNNNNNNNNNNNNNNNNNNNNNNNNNNNNAGNNNANGGNNNGTNTNTGACNCATGTGTTTTTANNNGNN
NNNNNACGNNNGANCCCCGGTCGNNNNCNNATTGAANNCCTGNNGCATNNNNNTNCNAGAATGANNGC
NNNNNGNNTNGCTNNCNTTTCAAGNAATCNNNNAGGNNNCGCGNTCNNNNNNNNNNNNNCNNTCCCNN
ACTGAGGGTNNNNNNCGCATCTGCTNNNNNNNNNNTNCGCNGNNGTNNCTNNNNAGCNGTATNGTGTNA
NNCANNNNNNNNNNNNNNNNNNCNAAACGANNNNNNNTNNNN
>Thai269
GTGNNNNNTAGCCNNNNNNAGTTAGACANNNCNNNNNNNNNNNNNNNNNTNNNNNNNNNNNNNNNNNNNN
NNNNNNNNNNNNNNNNNNNNNNNNNNNNNNNNNNNNNNNNNNNNNNNNNNNNNNNNNNNNNNNNNNNNNN
NNNNNNNNNNNNNNNNNNNNNNNNNNNNNNNNNNNNNNNNNNNNNNNNNNNNNNNNNNNNNNNNNNNNNN
NNNNNNNNNNNNNNNNNNNNNNNNNNNNNNNNNNNNNNNNNNNNNNNNNNNNNNNNNNNNNNNNNNNNNN
NNNNNNNNNNNNNNNNNNNNNNNNNNNNNNNNNNNNNNNNNNNNNNNNNNNNCNTCTNNNAAANNNTNNN
NNNNNNNNNNNNNNNNNNNNNNNNNNNCNCNTTGNNNGTAANCNANNNGNACATGCAGTCCCCTCNNNNN
NNNGGCAGAGNNNNNNCNCNNNNNNNCCGGCCCCNNCCNNNNNNNNNNNNNNNNNGNNNNNNNANNNNNN
NNNNNNTGGTACNNNNCGNNNNNGGTTGTNTNAGAGGTCCACAACGNGGTCNNGNCACNNNNGNNNAN
NNNCNNNNNNNNNNNNNNNNNNNNNNNTNNNTANNAGGNNNNNNCCNTCNNGNNNNNNATNGCNATGACC

CNANNCACGGGGGCAGTCCCCGGNGNGGCGACCTTGATNANNTCCGGGGNCAAGTATGGCNCNCNCCC
CGGGAAGGANNCNGCCGTANGTGCNNNNNANNNNNTCNTGNGACGTNGCCGNNATTNNCGTGGTCNNG
NNAGAATGNNCNNACCANNCGNANNNTGGTNATGNTAGTNNNNNNNNNNNNNNNNNNNNNNNNNNNN
NNNNNNNNNNNNNNNNNNNNNNNNNNNNNNNNNNNNNNNNGNNNNNNCNANNNAAGGNNNTANNNCTNNTN
NTNNNGNNGGACANNNCNNNNCNNNNNNNACNCNNNCNCNNNNNNNNNNGNTTTTNNNCCNNTATTNT
CCAGNACGCNGNGCNNNCATTCANGNGAACNNNGNNACGCGNNAATCGTANNATCTACCTGCCGCNGG
TNNNNNTNCNGNNNNTACGGANNNTNNNNCGTANNNNNGTCATGCGTNNTNTNNTCNCNAGNCANGTT
CGNNNANNNANTAGNANGTNTNNNNNNNNNTGTNNCGTTCCGCNTGANNCNNCANNNNGNNGNNNTTN
TCANCGGCNCTANCTGCNGANNTAAGNCATANNNTNNATCNNTANCTNNNNNGGNGGGNNACTGTAAA
NGGNNAATTGGAGGNTNATTNCNNNCACNTNNCNNNGNNTNCTNNNTNCCTGNNTNCNNAATTGGNNC
CANTGNNTGNCNATCTCTGGNTTNGNNNNNNTNNNNNTNGCANNNNGGNNNGTNANANNNCNATNTAA
NNTATANNNNCNNNNNNANNNCACNANNNNNGNAGNNNNNANNNTTANANNNGTAGNCNANTAAGTCT
CCGANNTNNNNNNNATAAGCGNNANACNNNNNNNNNNNNNNANGANNACGNNTTGGNNNTNNNNNGCNA
GNNAGACNNTNATGGNTNNNNNTCAANNNNTTNGTAGTGGTATAATNGNNNNNNNTTTCNCCAGNNTN
NNNGNNNNNNNNCNATANNANNNNNNNGNGANNNNNNNNNNNANNNNCTCNNNAGGGANCANCTACTTNA
NCAATGGNGCGCAGNNNGAGANNNNGACNNNCCTTGGGTGGCAANCACNGCANTAAGTCCTGTTNANC
CTNNNAGTNNACATNNNNNCANNGNNNCNNNNNNANCNGANNGNNNNNNNNNNNNNNNNNNNNNNNNN
NNNNNNNNNNNNNNNNNNNNNNNNNNNNNAGNNNANGGNNNGTNTNTGACNCATGTGTTTTTANNNGNN
NNNNNACGNNNNNNCCCCGGTCGNNNNCNNATTGAANNCCTGNNGCATNNNNNNTNCNAGAATGANNNC
NNNNNGNNTNNNNTNNCTTTTCAAGNAATCAANNAGGTANCGCGNTCNNNGNNNNNNNNCNNTCCCNN
ACTGAGGGTNNNNNNNNCATCTGCTNNNNNNNNNNTNCGCNGGNGTNNCNNNNNAGCNGTATNGTGTNA
NNCANNNNNNNNNNNNNNNNNNNAAACNANNNNNNNNNNNN
>Thai270
GTGNNNNNTAGCCCNNNNNAGTTAGACANNNCNNNNNNNNNNNNNNCNNNNNNNNNNNNNNNNNNNN
NNNNNNNNNNNNNNNNNNNNNNNNNNNNNCNTNNNNNNNNNNNNNNNNNNNNNNNNNNNNNNNNNNNNNNNN
NNNNNNNNNNNNNNNNNNNNNNNNNNNNNNNNNNNNNNNNNNNNNNNNNNNNNNNNNNNNNNNNNNNNNNN
NNNNNNNNNNNNNNNNNNNNNNNNNNNNNNNNNNNNNNNNNNNNNNNNNNNNNNNNNNNNNNNNNNNNNNN
NNNNNNNNNNNNNNNNNNNNNNNNNNNNNNNNNNNNNNNNNNNNNNNNNNNNNCNTCTNNNAAANNNTNNN
NNNNNNNNNNNNNNNNNNNNNNNNNNCNCNTTGNNNGTAANCNANNNGNACATGCAGTCCCCTCNNNNN
NNNGGCAGAGNNNNNNCCCNNNNNCNCCGNCCCCGNCCNNNNNNNNNNNNNNNNNNNNNNNNNANNNNNN
NNNNNNTGGTACNNNNCGNNNNNGGTTGTNTNAGAGGTCCACAACGNGGTCNGGNCACNNNNGNNNAN
NNNCNNNNAACNGNNNNNNNNNNANNTNNNTANNAGGNCNNNNCCNTNNNGNNNNTAATNGCNATGACC
CAANNCACGGNGGNNGTNCCCGGNGNGGCGACCTTGANNANGTCCGGGGACAAGTATGGCNCNCNCCC
CGGGAAGGANNCNGCCGTANGTGCNNNNNANNNNNTCNTGNGACGTNGCCGNNATTCNNGTGGTCNNN
CNAGAATGNCCGNACCANNCGNANNNTGGTNATGTNAGTNNNNNNNNNNNNNNNNNNNNNNNNNNNNN
NNNNNNNNNNNNNNNNNNNNNNNNNNNNNNNNNNNGNNNNGNNNNNNCNANNNAAGGNNNTANNNCTNNTC
NTNNNNNNNGGACNNNACNNNNCNNNNNNNACNCNNNCNCNNNNNNNNNNNGGTTTTNNNNNCNNTATTNT
CCAGNACGCNGNGCNNTCATTCANGNGAACNNNGNNACGCGNNAATCGTANNATCTACCTGCCGCNNG
TNNNNNTNCNGNNNNTNCGGANNNTNNNNCGTANNNNNNNTCATGCGTNNTNTNNTCNCNAGNCANGTT
CGNNNANANANTAGNANGTNTNNNNNNNNNTGTNNCGTTCCGCNTGANNCNNCANNNNGNCGNNCTTC
TCANCGGCNNTANCTGCNGANNTAAGNCATANNNTNNNTCNNTANCTNNNNNGGNGGGNNACTGTAAA
NGGNNAATTNGNNNNTNANTTCNNNCACNTNNCTNNGNNNNCTNNNTNCCTGNNTNCNNAATTGGNNC
CANTGNNTGNCNATCTCTNGNTTNGNNNNNNTGNNNNTNNCANNNNGGNNNGTNANANCNCNATNTAA
NNTATANNNNCNNNNNNANNNCACNANNNNNNNAGNNNNNANNNTTANANNNGTAGNNNAATAAGTCT
CCGANGTNNNNNNNATAAGCGNNANACNNNNNNNNNNNNNNANGANNACGNNTTGGNNNTNNNNNNCNA
GNNAGACNNTNATGGNTNNNNNTCAANNNNTTNGTAGTGGTATAATNGNNNNNNNTTTCNCCAGNNTN
NNNGNNNNNNNNCNATANNANNNNNNNGTGANNNNNNNNNNNANNNNCTCNNNAGGNANCANCTACTNNA
GCANTGGNGCGCAGNNNGAGANNNNGACNNGCCTTGGGTGGCAANCACNGCAATAAGTNNNGTTNANN
CTNNNAGTNNACATNNNNNCANNGNNNCNNNNNNANCNGANNGNNNNNNNNNNNNNNNNNNNNNNNNN
NNNNNNNNNNNNNNNNNNNNNNNNNNNNNAGNNNANGGNNNGTNTNTGACNCATGTGTTTTTANNNGNN
NNNNNACGNNNNNANCCCCGGTCGNNNNCNNATTGAANNCCTGNNGCATNNTNNTNCNAGAATGANNGC
NNNNNGNNTNGNCTNNCNNTTCAAGNAATCANNNAGNNNTNNNGNTCNNNGNNNNNNNNCNNTCCCNN
ACTGAGGGTNNNNNNCGCATCTGCTNNNNNNNNNCTTCGCNGGNGTNNCNNNNNAGCNGTATNGNGTNN
NNCANNNNNNNNNNNNNNNNNCNAAACAANNNNNCTNNNN

```
>Thai271
GTGNNNNNTAGCCCNNNNNAGTTAGACANNNCNNNNNNNNNNNNTNNNNNNNNNNNNNNNNNNNNNNNNN
NNNNNNNNNNNNNNNNNNNNNNNNNNNNNNNNNNNNNNNNNNNNNNNNNNNNNNNNNNNNNNNNNNNNNN
NNNNNNNNNNNNNNNNNNNNNNNNNNNNNNNNNNNNNNNNNNNNNNNNNNNNNNNNNNNNNNNNNNNNNN
NNNNNNNNNNNNNNNNNNNNNNNNNNNNNNNNNNNNNNNNNNNNNNNNNNNNNNNNNNNNNNNNNNNNNN
NNNNNNNNNNNNNNNNNNNNNNNNNNNNNNNNNNNNNNNNNNNNNNNNNNNNNNTCTNNNAAANNNTNNN
NNNNNNNNNNNNNNNNNNNNNNNNNNNCNCNTTGNNNGTAANCNANNNGNACATGCAGTCCCCTCNNNNN
NNNGGCAGAGNNNNNNNNCNNNNNNNCCGGCCCCNNCCNNNNNNNNNNNNCNNNNNNNNNNNNNANNNNN
NNNNNNTGGTACNNNNCGNNNNNNGGTTGTTTAAGAGGTCCACAACGNGGTCNNGNCACNNNNGNNNAN
NNNCNNAAAACNNNNNNNNNNNNANNTNNNTANNAGGNCNNNNCCNTCNNGNNNNTNATNGCNATGANN
CANNNNNCGGGGGCAGTNCCCGGNGCGGNGACCTTGNTNACGTCCGGGGACAAGTATGGCNCNCNCCC
CGGGAAGGANNCNGCCGTANGTGCNNNNNANNNNNTCNTGNGANGTCGCCGNNATTCNCNTGGTCNNN
CNNGAATGNCCGNACCANNCGNANNNTGGTTATGNNAGTNANNNNNNNNNNNNNNNNNNNNNNNNNNNNN
NNNNNNNNNNNNNNNNNNNNNNNNNNNNNNNNNNGTNNNGNNNNNNCNANNNAAGGNNNTANNGCTNNTN
NTNNGNNNGGACNNNACNNNNCNNNNNNNACNCNNNCNCNNNNNNANNNGNTTTTNNNNCNNTATTNT
CCAGNACGCNGNGCNNNCATTCANGNGAACNNNGNNACGCGNNAATCGTANNATCTACCTGCCGCNGG
TNNNNNTACNGNNNNTNCGGANNNTNNNNCGTANNNNNNTCATGCGTNNTNTNNTCNCNAGNCANGTT
CGNNNANANANTAGNANGTNTNNNNNNNNNNTGTNNCGTTCCGCNTGANNCNNCANNNNGNNGNNCTTN
TCANCGGCNNTANCTGCNGANNTAAGNCATANNNTNNNTCNNTANCTNNNNNGGAGGGNNACTGTAAA
NGGNNAATTGGANGNTNATTNCNNNCACNTNNCTNNGNNTNCTNNNTNCCTGNNTNCNNAATTGGNNC
CANTGNNTGNCNATCTCTNGNTTNGNNNNNNTGNNNNTNGCAANNNGGNNNNNNANANNNCNATNTAA
NNTATANNNNCNNNNNNANNNCACNANNNNNGNAGNNNCNANNNTTANANNNGTAGNNNANTAAGTCT
CCGANNTNNNNNNNATAAGCGNNANACNNNNNNNNNNNNNNANGANNACGNNTTGGNNNTNNNNNNCNA
GNNAGACNNTNATGGNTNNNNNTCAANNNNTTNGTAGTGGTATAATNGNNNNNNNTTTCNCCAGNNTN
NNNGNNNNNNNCNATANNANTNNNNNGNGANNNNNNNNNNNANNNNCTCNNNAGGGANCANCTACTNNA
GCAATGGNGCGCAGNNNGAGANNNNGACNNNCCTTGGGTGGCAANCACNGCAATAAGTNNNGTTNANC
CTNNNAGTNNACATNNNNNCANNGNNNCNNNNNNANCNGANNGNNNNNNNNNNNNNNNNNNNNNNNNNNN
NNNNNNNNNNNNNNNNNNNNNNNNNNNNNNAGNNNANGGNNNGTNTNTGACNCATGTGTTTTTANNNGNN
NNNNNACGNNNNANCCCCGGTCGNNNNCNNATTGAANNCCTGNNGCATNNNNNTNCNAGAATGANNGC
NNNNNGNNTNGNCTNNCNNNTCAAGNAATCANNNAGNNANCGCGNTCNNNNNNNNNNNNNCNNTCCCNN
ACTGAGGGTNNNNNNNNCATCTGCTNNNNNNNNNNTNCGCNGGNGTNNCNNNNNAGCNGTATNGTGTNA
NNCANNNNNNNNNNNNNNNNNCNAAACAANNNNNNNTNNNN
>Thai272
GTGNNNNNTAGCCCNNNNNAGTTAGACANNNCNNNNNNNNNNNNNNNNNNNNNNNNNNNNNNNNNNNNNN
NNNNNNNNNNNNNNNNNNNNNNNNNNNNNNNNNNNNNNNNNNNNNNNNNNNNNNNNNNNNNNNNNNNNNN
NNNNNNNNNNNNNNNNNNNNNNNNNNNNNNNNNNNNNNNNNNNNNNNNNNNNNNNNNNNNNNNNNNNNNN
NNNNNNNNNNNNNNNNNNNNNNNNNNNNNNNNNNNNNNNNNNNNNNNNNNNNNNNNNNNNNNNNNNNNNN
NNNNNNNNNNNNNNNNNNNNNNNNNNNNNNNNNNNNNNNNNNNNNNNNNNNCNTCTNNNAAANGGTNNN
NNNNNNNNNNNNNNNNNNNNNNNNNNNCNCNTTGNNNGTAANCNANNNGNACATGCAGTCCCCTCNNNNN
NNNGNCAGAGNNNNNNCNCNNNNNNNNCCGGCCCCGNCCNNNNNNNNNNNNNNNNNNNNNNNNNNANNNNNN
NNNNNNTGGTACNNNNCGNNNNNGGTTGTTTAAGAGGTCCACAACGNGGTCNNGNCACNNNNGNNTAN
NNNCNNNNAANNNNNNNNNNNNNNANNTNNNTANNAGGNCNNNNCCNTCNNGNNNNNNATTGCNATGACN
CAANNNACGGNGGCNGTNCCCGGCGNGGCGACCTTGNTNANNTCCGGGGACAAGTATGGCGCNCNCCC
CGGGAAGGANNCNGCCGTANGTGCNNNNNANNNNNTCNTGNGANGTCGCCGNNATTCNNGTGNNCNNN
NNAGANTGNCCGNACCANNCGNANNNTAGTTATGNTAGTANNNNNNNNNNNNNNNNNNNNNNNNNNNNNN
NNNNNNNNNNNNNNNNNNNNNNNNNNNNNNNNNNGNNNNGNNNNNNCNANNNAAGGANNTANNNCTNNTN
NTNNNGGNGGACNNNACNNNNCNNNNNNNACNCNNNCNCNNNNNNNNNNNGGTTTTTNNNCNNTATTNT
CCAGNACGCNGNGCNNNCATTCANGNGAACNNNGNNACGCGNAAATCGTANNATCTACCTGCCGCNGG
TNNNNNTACNGNNNNTACGGANNNTNNNNCGTANNNNNNTCATGCGTNNTNTNNTCNCNAGNCANGTT
CGNNNANANANTAGNANGTNTNCNNNNNNNNTGTNNCGTTCCGCNTGANNCNNCANNNNGNNGNNCTTN
TCANCGGCNNTANCTGCNGANNTAAGNCATANNNTNNNTCNNTANCTNNNNNGGNGGGNNACTGTAAA
NGGNTAATTGGNGGNTNANTTCNNNCATNTNNCTNNGNNTNCTNNNTNCCTGGNTNCNNAATTGGNNC
CANTGNNTGNCNATCTCTGGNTTNGCNNNNNTNNNNNTNGCAANNNGGNNNGTNANANNNCNATNTAA
NNTATANNNNCNNNNNNANNNCACNANNNNNNNAGNNNNNANNNTTANANNNGTAGNNNAATAAGTCT
```

CCGANNTNNNNNNNATAAGCGNNANACNNNNNNNNNNNNNNANGANNACGNNTTGGNNNTNNNNNNCNA
GNNAGACNNTNGTGGNTNNNNNTCAANNNNTTCGTAGTGGTATAATNGNNNNNNNTTTCNCCAGNNTN
NNNGNNNNNNNCNATANNANNNNNNNGTGANNNNNNNNNNANNNNCTCNNNAGGGANCANCTACTTNA
GCAATGGNGCGCAGNNNGAGANNNNGACCAGCCTTGGGTGGCAANCACNGCAATAAGTNNNGTTNANC
CTNNNAGTNNACACNNNNNCANNGNNNCNNNNNNANCNGANNGNNNNNNNNNNNNNNNNNNNNNNNNN
NNNNNNNNNNNNNNNNNNNNNNNNNNNNNNAGNNNANGGNNNGTNTNTGACNCATGTGTTTTTANNNGNN
NNNNNACGNNNNANCCCCGGTCGNNNNCNNATTGAANNCCTGNNGCATNNNNNTNCNAGAATGANNNC
NNNNNGNNTNGNCTNNCNTTTCAAGNAATCANNNANNNNTNGCGNTCNNNGNNNNNNNNCNNTCCCNN
ACTGAGGGTNNNNNNCNCATCTGCTNNNNNNNNCTTCGCNGGNGTNNCNGNNNAGCNGTATNGTGTTN
NNCANNNNNNNNNNNNNNNNNNTNNANCNANNNNNNTNNNG
>Thai393
GTGNNNNNTNGCCCNNNNNAGTTANACANNNNNNNNNNNNNNNNNNNNNNNNNNNNNNNNNNNNNNNNNN
NNNNNNNNNNNNNNNNNNNNNNNNNNNNNNNNNNNNNNNNNNNNNNNNNNNNNNNNNNNNNNNNNNNNNN
NNNNNNNNNNNNNNNNNNNNNNNNNNNNNNNNNNNNNNNNNNNNNNNNNNNNNNNNNNNNNNNNNNNNNN
NNNNNNNNNNNNNNNNNNNNNNNNNNNNNNNNNNNNNNNNNNNNNNNNNNNNNNNNNNNNNNNNNNNNNN
NNNNNNNNNNNNNNNNNNNNNNNNNNNNNNNNNNNNNNNNNNNNNNNNNNNNNTCNNNNNAANNNTNNN
NNNNNNNNNNNNNNNNNNNNNNNNNNNNCNTTNNNNGTAANCNANNNGNACATGCAGTCCNCNNNNNNN
NNNNNCAGAGNNNNNNNNNNNNNNNNNCCGNCCCCNNCCNNNNNNNNNNNNNNNNNNNNNNNNNNANNNNNN
NNNNNNTGGTACNNNNCGNNNNNGGNNNNNTNAGNGGTNCACNACGNGGTCNNGNNANNNNNGNNNAN
NNNCNNNNNNNNNNNNNNNNNNNNNNNTNNNTANNAGGNNNNNNCCNNNNNNNNNNNNNANNGNNATGANN
CNNNNNNCNGNNGNNGTNCCCGNNGNNGCNACCTTGANNANNTCNGGGNNCAAGTATNGCNNNCNCNN
CGGGAAGGANNCNNCCGTANGTGCNNNNNANNGNNTCNTGNGANGNNGNCNNNNNNNNCNNGGNCNNN
NNAGANTGNNCNNACCANNCGNANNNNGGTNATGNNAGTNNNNNNNNNNNNNNNNNNNNNNNNNNNNNN
NNNNNNNNNNNNNNNNNNNNNNNNNNNNNNNNNNNNNNNNNNNGNNNNNNCNANNNAAGGNNNTANNNCTNNNN
NTNNNNNNNGGACNNNNCNNNNCNNNNNNNACNCNNNCNNNNNNNNNNNNNGNTTTNNNNNCNNTANTNT
CNAGNANGCNNNGCNNNCNTTCANGNGAACNNNGNNACGNGNNNATCGTANNATNTACCTGCNGCNNG
NNNNNNTNCNGNNNNNNCGGANNNTNNNNCGTANNNNNNNCANGCGTNNTNTNNTCNCNAGNNNNNTT
CGNNNANNNANTAGNANGTNTNNNNNNNNNNGTNNCGTTNNNNNTGANNNNNNANNNNGNNGNNNTTN
NCANCGGNNNTNNCTGNNGANNTAAGNCNTNNNNTNNNTCNNTANCTNNNNNNGNNGGNNACTGTAAA
NGNNNAATTNGNNNNTNANTNCNNNNACNTNNCNNNGNNNNCTNNNNTNCCTGNNTNCNNAATTGGNNC
CANTGNNTGNCNATCTCTNNNNTNGNNNNNNTNNNNNTNNCAANNNGGNNNNNNNNANNNCNATNTAA
NNTANNNNNNCNNNNNNANNNCACNNNNNNNNNAGNNNNNANNNTNANANNNGTAGNNNANTNAGTCT
NCNANNTNNNNNNNATAAGCGNNANACNNNNNNNNNNNNNNNGANNNCGNNTTGNNNNTNNNNNNNNA
GNNNNANNNTNATGGNTNNNNNNCAANNNNTTNNNAGTGGTATAATNGNNNNNNNTTTCNCCAGNNNN
NNNNNNNNNNNCNATNNNANNNNNNNGNGANNNNNNNNNNNNNNNNNTCNNNAGGNANCANCTACNNNA
NCANNGGTGCGNAGNNNNAGANNNNGACNNNCCTTGGGTGGCANNCACNGNANNAAGTNNNGTTNANN
NTNNNAGTNNANATNNNNNCANNGNNNCNNNNNNANCNGANNGNNNNNNNNNNNNNNNNNNNNNNNNN
NNNNNNNNNNNNNNNNNNNNNNNNNNNNNNANNNNNNNNNNNNNGTNTNNGNCNCNNNNNNNNNNNANNNGNN
NNNNNACGNNNNNNCCCCGGTCGNNNNCNNATTGAANNCCTGNNGCATNNNNNTNCNAGAATNANNNC
NNNNNGNNTNNNCTNNNNNNTCANGNAANNNNNNANNNNNNNNNNNTCNNNGNNNNNNNNCNNNNNCNN
ACTGAGGNNNNNNNNNNCANNNNCTNNNNNNNNNNTNCNNNGNNNNNNCNNNNNAGCNGTATNNNGTNN
NNCANNNNNNNNNNNNNNNNNNNNNANNNNNNNNNNNTNNNN
>Thai55
GTGNNNNNTAGCCCGNNNNAGTTAGACANNNCNNNNNNNNNNNNNTNNNNNNNNNNNNNNNNNNNNNNNN
NNNNNNNNNNNNNNNNNNNNNNNNNNNNNNNTNNNNNNNNNNNNNNNNNNNNNNNNNNNNNNNNNNNNNN
NNNNNNNNNNNNNNNNNNNNNNNNNNNNNNNGNNNNNNNNNNNNNNNNNNNNNNNNNNNNNNNNNNNNNN
NNNNNNNNNNNNNNNNNNNNNNNNNNNNNNNNNNNNNNNNNNNNNNNNNNNNNNNNNNNNNNNNNNNNNN
NNNNNNNNNNNNNNNNNNNNNNNNNNNNNNNNNNNNNNNNNNNNNNNNNNNNNTCTNNNAAANNNTNNN
NNNNNNNNNNNNNNNNNNNNNNNNNNCNCNTTGNNNGTAANCNANNNGNACATGCAGTCCCCTCNNNNN
NNNGGCAGAGNNNNNNNCCNNNNNCNCCGGCCCCNNCCNNNNNNNNNNNNNNNNNNNNNNNNNANNNNNN
NNNNNNTGGTACNNNNCGNNNNNGGTTGTTTAAGAGGTCCACAACGNGGTCNNGNCACNNNNGNNNAN
NNNCNNNAAACNNNNNNNNNNNNANATNNNTANNAGGNCNNNGCCNTCNNGNNNNTAATNGCNATGACN
CAANNCACGGGGGNNGTNCCCGGCGNGGCGACCTTGNTNACGTCCGGGGACAAGTATGGCGCNCNCCC
CGGGAAGGANNCNGCCGTANGTGCNNNNNANNNNNTCNTGNGACGTNGCCGNNATTCNCGTGGTCTNN

NNAGAATGNCCGNACCANNCGNANNNTGGTNATGNNAGTANNNNNNNNNNNNNNNNNNNNNNNNNNNN
NNNNNNNNNNNNNNNNNNNNNNNNNNNNNNNNNNNNNNNGNNNNNNCNANNNAAGGANNTANNNCTNNTC
NTNNNNNNGGACNNNACNNNNCNNNNNNNACNCNNNCNCNNNNNNANNNGGTTTTTNNNCNNTATTNT
CCAGNACGCNGNGCNNNCATTCANGNGAACNNNGNNACGCGNNAATCGTANNATCTACCTGCCGCNGG
TNNNNNTACNGNNNNTNCGGANNNTNNNTCGTANNNNNNTCATGCGTNNTNTNNTCNCNAGNCNNGTT
CGNNNATANANTAGNANGTNTCNNNNNNCNTGTNNCGTTCCGCNTGANNCNNCANNNNGNNGNNCTTN
TCANCGGCNNTANCTGCNGANNTAAGTCATANNNTNNNTCNNTANCTNNNNNGGNGGGNNACTGTAAA
NGGNTAATTNGNGGNTNATTTCNNNCACNTNNCTNNGNNTNCTNNNTNCCTGNNTNCNNAATTGGNNC
CANTGNNTGNCNATCTCTGGNTTNGNNNNNNTNNNNNTNGCAANNNGGNNNGTNANANNNCNATNTAA
NNTATANNNNCNNNNNNANNNCACNANNNNNGNAGNNACNANNNTTANANNNGTAGNNNAATAAGTCT
CCGANNTNNNNNNNATAAGCGNNANACNNNNNNNNNNNNNANGANNACGNNTTGGNNNTNNNNNNCNA
GNNAGACNNTNATGGNTNNNNNTCAANNNNTTNGTAGTGGTATAATNGNNNNNNNTTTCNCCAGNNTN
NNNGNNNNNNNCNATANNANNNNNNNGTGANNNNNNNNNNANNNNCTCNNNAGGGANCANCTACTNNA
GCAATGGNGCGCAGNNNGAGANNNNGACNAGCCTTGGGTGGCAANCACNGCAATAAGTNNNGTTNANC
CTNNNAGTNNACATGNNNNCANNGNNNCNNNNNNANCNGANNGNNNNNNNNNNNNNNNNNNNNNNNNNN
NNNNNNNNNNNNNNNNNNNNNNNNNNNNNAGNNNANNGNNNGTNTNTGACNCATGTGTTTTTANNNGNN
NNNNCACGNNNNANCCCCGGTCGNNNNCNNATTGAANNCCTGNNGCATNNNNNTNCNAGAATGANNGC
NNNNNGNNTNGNCTGNCNTTTCAAGNAATCAANTAGGTNNCGCGNTCNNNNNNNNNNNNCNNTCCCNN
ACTGAGGGTNNNNNNCGCATCTGCTNNNNNNNNCTTCGCNGGCGTNNCTNNNNAGCNGTATNGTGTTA
NNCANNNNNNNNNNNNNNNNNNNANNNNANNNNNNNTNNNN
>Thai56
GTGNNNNNTAGCCCNNNNNAGTCAGACANNNCNNNNNNNNNNNNTNNNNNNNNNNNNNNNNNNNNNNN
NNNNNNNNNNNNNNNNNNNNNNNNNNNNNNNNNNNNNNNNNNNNNNNNNNNNNNNNNNNNNNNNNNNNNN
NNNNNNNNNNNNNNNNNNNNNNNNNNNNNNNNNNNNNNNNNNNNNNNNNNNNNNNNNNNNNNNNNNNNNN
NNNNNNNNNNNNNNNNNNNNNNNNNNNNNNNNNNNNNNNNNNNNNNNNNNNNNNNNNNNNNNNNNNNNNN
NNNNNNNNNNNNNNNNNNNNNNNNNNNNNNNNNNNNNNNNNNNNNNNNNNNNNNNNNTCTNNNAAANNNTNNN
NNNNNNNNNNNNNNNNNNNNNNNNNNCNCNTTGNNNGTAANCNANNNGNACATGCAGTCCCCTCNNNNN
NNNNNCAGAGNNNNNNNNNCNNNNNNNCCGNCCCCNNCCNNNNNNNNNNNNNNNNNNNNNNNNNANNNNNN
NNNNNNTGGTACNNNNCGNNNNNGGTTGTTTAAGAGGTCCACAACGNGGTCNNGNNACNNNNGNNNAN
NNNCNNNNAANNNNNNNNNNNNNANNTNNNTANNAGGNNNNNNCCNTNNNGNNNNNAANNGCNATGACC
CAANNNACGGGGGNNGTCCCCGGCGNGGCGACCTTGATNANNTCCGGGGACAAGTATGGCNCNCNCCC
CGGGAAGGANNCNGCCGTANGTGCNNNNNANNNNNTCNTGNGANGTCGCCGNNATTCNCGTGGTCNNN
NNAGAATGNNCNNACCANNCGNANNNTGGTNATGNTAGTANNNNNNNNNNNNNNNNNNNNNNNNNNNNN
NNNNNNNNNNNNNNNNNNNNNNNNNNNNNNNNNNNGNNNNGNNNNNNCNANNNAAGGNNNTANNNCTNNTN
NTNNNNNNGGACNNNACNNNNCNNNNNNNACNCNNNCNCNNNNNNANNNGGTTTTTNNNCNNTATTNT
CCAGNACGCNGNGCNNTCATTCANGNGAACNNNGNNACGCGNNAATCGTANNATCTACCTGCCGCNNG
TNNNNNTACNGNNNNTNCGGANNNTNNCTCGTANNNNNNTCATGCGTNNTNTNNTCNCNAGNCNNGTT
CGNNNANANANTAGNANGTNTNNNNNNNNNNTGTNNCGTTCCGCNTGANNCNNCANNNNGNNGNNCTTN
TCANCGGCNNTANCTGCNGANNTAAGNCATANNNTNNATCNNTANCTNNNNNGGNGGGNNACTGTAAA
NGGNTAATTNGNNGNTNATTNCNNNCATNTNNCTNNGNNTNCTNNNTNCCTGNNTNCNNAATTGGNNC
CANTGNNTGNCNATCTCTNGNTTNGNNNNNNTGNNNNTNNCAANNNGGNNNGTNANANNNCNATNTAA
NNTATANNNNCNNNNNNANNNCACNANNNNNNNAGNNNCNANNNGTANANNNGTAGNNNGATAAGTCT
CCGANNTNNNNNNNATAAGCGNNANACNNNNNNNNNNNNNANGANNACGNNTTTGNNNTNNNNNNCNA
GNNAGACNNTNGTGGNTNNNNNTCAANNNNTTCGTAGTGGTATAATNGNNNNNNNTTTCNCCAGNNTN
NNNGNNNNNNNCNATACNANNNNNNNGTGANNNNNNNNNNANNNNCTCNNNAGGGANCANCTACTNNA
GCANTGGNGCGCAGNNNGAGANNNNGACNNNCCTTGGGTGGCAANCACNGCAATAAGTNNNGTTNANN
CTNNNAGTNNACACNNNNNCANNGNNNCNNNNNNANCNGANNGNNNNNNNNNNNNNNNNNNNNNNNNN
NNNNNNNNNNNNNNNNNNNNNNNNNNNNNAGNNNANGGNNNGTNTNTGACNCATGTGTTTTTANNNGNN
NNNNNACGNNNGANCCCCGGTCGNNNNCNNATTGAANNCCTGNNGCATNNNNNTNCNAGAATGANNNC
NNNNNGNNTNNNCTNNCNNNTCAAGNAATCANNNAGGTNNCGCGNTCNNNGNNNNNNNNCNNTCCCNN
ACTGAGGGTNNNNNNNNCATCNGCTNNNNNNNNNNTTCGCNGNNGTNNCNNNNNAGCNGTATNGTGTNN
NNCANNNNNNNNNNNNNNNNNCNNAACNNNNNNNNNTNNNN
>Thai57
GTGNNNNNTAGCCCNNNNNAGTTAGACANNNCNNNNNNNNNNNNTNNNNNNNNNNNNNNNNNNNNNNN

NNNNNNNNNNNNNNNNNNNNNNNNNNNNNNNTNNNNNNNNNNNNNNNNNNNNNNNNNNNNNNNNNNNNNN
NNNNNNNNNNNNNNNNNNNNNNNNNNNNNNNNNNNNNNNNNNNNNNNNNNNNNNNNGCNNNNNNNNNNNN
NNNNNNNNNNNNNNNNNNNNNNNNNNNNNNNNNNNNNNNNNNNNNNNNNNNNNNNNNNNNNNNNNNNNNN
NNNNNNNNNNNNNNNNNNNNNNNNNNNNNNNNNNNNNNNNNNNNNNNNNNNNNNTCTNNNAAANNNTNNN
NNNNNNNNNNNNNNNNNNNNNNNNNNNCNCNTTGNNNGTAANCNANNNGNACATGCAGTCCCCTCNNNNN
NNNGGCAGAGNNNNNNCNCNNNNNNNNCCGGCCCCGNCCNNNNNNNNNNNNCNNNGNNNNNNNANNNNNN
NNNNNNTGGTACNNNNCGNNNNNGGTNGTTTNAGAGGTCCACAACGNGGTCNNGNNACNNNNGNNNAN
NNNCNNNNNNNNNNNNNNNNNNNANNTNNNTANNAGGNNNNNNCCNTCNNNNNNNNNNANTGCNATGACC
CAANNCACGGNGGCAGTNCCCGGCGCGGCGACCTTGATNANGTCCGGGNACAAGTATGGCNCNCNCCC
CGGGAAGGANNCNGCCGTANGTGCNNNNNANANNNTCNTGNGANGTCGCCGNNATTCNCNTGGTCNNN
NNAGAATGNNCGNACCANNCGNANNNTGGTTATGNTAGTNNNNANNNNNNNNNNNNNNNNNNNNNNNNNN
NNNNNNNNNNNNNNNNNNNNNNNNNNNNNNNNNNNNNNNGNNNNNNCNANNNAAGGANNTANNNCTNNTN
NTNNNNNNNGGACNNNACNNNNCNNNNNNNNACNCNNNCNCNNNNNNNNNNNGNTTTTTNNNCNNTATTNT
CCAGNACGCNGNGCNNNCATTCANGNGAACNNNGNNACGCGNNAATCGTANNATCTACCTGCCGCNNG
TNNNNNTNCNGNNNNTNCGGANNNTNNNNCGTANNNNNNTCATGCGTNNTNTNNTCNCNAGNCNNGTT
CGNNNANANANTAGNANGTNTNNNNNNNNNNTGTNNCGTTCCGCNTGANNCNNCANNNNGNNGNNCTTN
TCANCGGCNNTANCTGCNGANNTAAGNCATANNNTNNNTCNNTANCTNNNNNGGNGGGNNACTGTAAA
NGGNNAATTNGNNGNTNATTTCNNNCACNTNNCTNNGNNNNCTNNNTNCCTGNNTNCNNAATTGGNNC
CANTGNNTGNCNATCTCTGGNTTNGNNNNNNTNNNNNTNNCAANNNGGNNNGTNANANNNCNATNTAA
NNTATANNNNCNANNNNANNNCACNANNNNNNNAGNNNCNANNNTTANANNNGTAGNNNAATAAGTCT
CCGANNTNNNNNNNATAAGCGNNANACNNNNNNNNNNNNNNANGANNACGNNTTGGNNNTNNNNNNCNA
GNNAGACNNTNATGGNTNNNNNTCAANNNNTTCGTAGTGGTATAATNGNNNNNNNTTTCNCCAGNNTN
NNNGNNNNNNNNCNATANNANNNNNNNGTGANNNNNNNNNNNANNNNCTCNNNAGGGANCANCTACTTNA
GCANTGGNGCGCAGNNNGAGANNNNGACNNGCCTTGGGTGGCAANCACNGCAATAAGTNNNGTTNANC
CTNNNAGTNNACATNNNNNCANNGNNNCNNNNNNANCNGANNGNNNNNNNNNNNNNNNNNNNNNNNNNNN
NNNNNNNNNNNNNNNNNNNNNNNNNNNNNNAGNNNANGGNNNGTNTNTGACNCATGTGTTTTTANNNGNN
NNNNNACGNNNNNNNCCCCGGTCGNNNNCNNATTGAANNCCTGNNGCATNNNNNNTNCNAGAATGANNNC
NNNNNGNNTNGNCTNNCNNNTCAAGNAATCANNNAGGNANCGCGNTCNNNNNNNNNNNNNCNNTCCCNN
ACTGAGGGTNNNNNNNGCATCTGCTNNNNNNNNNNTNCGCNGGNNNNNCNNNNNAGCNGTATNGNGTNA
NNCANNNNNNNNNNNNNNNNNTNNAANNNNNNNNNNNTNNNN
>Thai99
GTGNNNNNTAGCCCNNNNNAGTTAGACANNNCNNNNNNNNNNNNTNNNNNNNNNNNNNNNNNNNNNNNNN
NNNNNNNNNNNNNNNNNNNNNNNNNNNNNNNNNNNNNNNNNNNNNNNNNNNNNNNNNNNNNNNNNNNNNN
NNNNNNNNNNNNNNNNNNNNNNNNNNNNNNNNNNNNNNNNNNNNNNNNNNNNNNNNNNNGNNNCNNNNNN
NNNNNNNNNNNNNNNNNNNNNNNNNNNNNNNNNNNNNNNNNNNNNNNNNNNNNNNNNNNNNNNNNNNNNN
NNNNNNNNNNNNNNNNNNNNNNNNNNNNNNNNNNNNNNNNNNNNNNNNNNNNCNTCTNNNAAANNNTNNN
NNNNNNNNNNNNNNNNNNNNNNNNNNNCNCNTTGNNNGTAANCNANNNGNACATGCAGTCCCCTCNNNNN
NNNGGCAGAGNNNNNNCCCNNNNNNCNCCGNCCCCNNCCNNNNNNNNNNNNCNNNNNNNNNNNNANNNNNN
NNNNNNTGGTACNNNNCGNNNNNGGTTGTTTAAGAGGTCCACAACGNGGTCNGGNCACNNNNGNNNAN
NNNCNNAAAACNNNNNNNNNNNNNNNNTNNNTANNAGGNCNNNNCCNTCNNGNNNNNNATTGCNATGACC
CAANNCACGGGGGCAGTCCCCGGNGCGGCGACCTTGNNNACGTCCGGGGACAAGTATGGCGCNCNCCC
CGGGAAGGANNCNGCCGTANGTGCNNNNNANNNNNTCNTGNGACGTCGCCGNNATTCNCGTGGTCTNN
CNAGAATGNCCGNACCANNCGNANNNTGGTNATGNNAGTANNNNNNNNNNNNNNNNNNNNNNNNNNNNNN
NNNNNNNNNNNNNNNNNNNNNNNNNNNNNNNNNNGNNNNGNNNNNNCNANNNAAGGANNTANNNCTNNTC
NTNNNGNNGGACNNNACNNNNCNNNNNNNNACNCNNNCNCNNNNNNNNNNNGNTTTTTNNNCNNTATTNT
CCAGNACGCNGNGCNNNCATTCANGNGAACNNNGNNACGCGNNAATCGTANNATCTACCTGCCGCNGG
TNNNNNTACNGNNNNTACGGANNNTNNNNCGTANNNNNNTCATGCGTNNTNTNNTCNCNAGNCANGTT
CGNNNANANANTAGNANGTNTCNNNNNNCTTGTNNCGTTCCGCNTGANNCNNCANNNNGNCGNNCTTN
TCANCGGCNNTANCTGCNGANNTAAGNCATANNNTNNNTCNNTANCTNNNNNGGNGGGNNACTGTAAA
NGGNTAATTNGANGNTNATTTCNNNCACNTNNCTNNGNNTNCTNNNTNCCTGNNTNCNNAATTGGNNC
CANTGNNTGNCNATCTCTNGNTTNGNNNNNNTGNNNNTNGCAANNNGGNNNGTNANANNNCNATNTAA
NNTATANNNNCNNNNNNANNNCACNANNNNNNNAGNNNCNANNNTTANANNNGTAGNNNAATAAGTCT
CCGANNTNNNNNNNATAAGCGNNANACNNNNNNNNNNNNNNANGANNACGNNTTGGNNNTNNNNNNCNA
GNNAGACNNTNATGGNTNNNNNTCAANNNNTTCGTAGTGGTATAATNGNNNNNNNTTTCNCCAGNNTN

NNNGNNNNNNNNCNATANNANNNNNNNNGNGANNNNNNNNNNNANNNNCTCNNNAGGGANCANCTACTTNA
GCAATGGNGCGCAGNNNGAGANNNNGACNNGCCTTGGGTGGCAANCACNGCAATAAGTNNNGTTNANC
CTNNNAGTNNACATGNNNNCANNGNNNCNNNNNNANCNGANNGNNNNNNNNNNNNNNNNNNNNNNNNN
NNNNNNNNNNNNNNNNNNNNNNNNNNNNAGNNNANNGNNNGTNTNTGACNCATGTGTTTTTANNNGNN
NNNNNACGNNNNANCCCCGGTCGTNNNCNNATTGAANNCCTGNNGCATNNNNNTNCNAGAATGANNNC
NNNNNGNNTNGNCTNNCNNNTCAAGNAATCAANTAGGTNNCGCGNTCNNGGNNNNNNNNCNNTCCCNN
ACTGAGGGTNNNNNNCGCATCTGCTNNNNNNNNCTNCGCNGGNNNNNCNNNNNAGCNGTATNGTGTTA
NNCANNNNNNNNNNNNNNNNNNNAAACNANNNNNNNTNNNN
