# Supplementary material for: Investigating demic versus cultural diffusion and sex bias in the spread of Austronesian languages in Vietnam
Source: PLoS One. 2024 Jun 17;19(6):e0304964. doi: 10.1371/journal.pone.0304964 (PMC11182502; doi:10.1371/journal.pone.0304964)
Supplement: S5 Dataset — (PDF) [file pone.0304964.s018.pdf]

#Chromosomal positions on hg19 for 847 SNPs
Y:2655180
Y:2661306
Y:2661694
Y:2710309
Y:2712237
Y:2722252
Y:2731887
Y:2734854
Y:2739468
Y:2815425
Y:2818883
Y:2825845
Y:2828196
Y:2828425
Y:2831112
Y:2843538
Y:2846401
Y:2847910
Y:2887824
Y:3108652
Y:6190103
Y:6190184
Y:6190215
Y:6370460
Y:6372705
Y:6372857
Y:6400042
Y:6636899
Y:6659638
Y:6662168
Y:6677619
Y:6697604
Y:6736812
Y:6737845
Y:6740510
Y:6740704
Y:6812031
Y:6845657
Y:6868118
Y:6893093
Y:6894741
Y:6906074
Y:6932546
Y:6938119
Y:6938186
Y:6938665
Y:6941151
Y:6943622
Y:6951960
Y:6951995
Y:6952023
Y:6954470
Y:6961386

Y:6965215
Y:6989173
Y:6989176
Y:6992073
Y:6992130
Y:6992134
Y:6995523
Y:6995947
Y:7032024
Y:7034770
Y:7057319
Y:7057795
Y:7057887
Y:7065032
Y:7067665
Y:7069017
Y:7069410
Y:7072035
Y:7072339
Y:7143650
Y:7173143
Y:7245239
Y:7279986
Y:7279988
Y:7280064
Y:7280070
Y:7280083
Y:7317911
Y:7331425
Y:7401585
Y:7401836
Y:7420744
Y:7420756
Y:7420794
Y:7501465
Y:7513676
Y:7518782
Y:7528917
Y:7533750
Y:7543038
Y:7546726
Y:7548915
Y:7555789
Y:7558795
Y:7559671
Y:7559753
Y:7565996
Y:7568568
Y:7571122
Y:7586919
Y:7602738
Y:7628900
Y:7635648
Y:7638823

Y:7643967
Y:7690471
Y:7707135
Y:7716262
Y:7730226
Y:7771131
Y:7828081
Y:7855759
Y:7900560
Y:7900625
Y:7900883
Y:7926775
Y:7963031
Y:8021340
Y:8033198
Y:8086815
Y:8148869
Y:8149729
Y:8231698
Y:8240725
Y:8281294
Y:8285596
Y:8334875
Y:8348932
Y:8352384
Y:8353707
Y:8363884
Y:8364460
Y:8365549
Y:8380220
Y:8383516
Y:8383517
Y:8389216
Y:8392350
Y:8398483
Y:8422121
Y:8424089
Y:8424741
Y:8426277
Y:8428087
Y:8430685
Y:8432095
Y:8442341
Y:8442742
Y:8467053
Y:8467095
Y:8474189
Y:8483228
Y:8485542
Y:8490706
Y:8499467
Y:8501357
Y:8502236
Y:8532844

Y:8533735
Y:8538392
Y:8551166
Y:8568831
Y:8575495
Y:8590752
Y:8602518
Y:8614138
Y:8635928
Y:8637791
Y:8643763
Y:8667179
Y:8675467
Y:8679843
Y:8680661
Y:8681209
Y:8684795
Y:8684890
Y:8694916
Y:8696594
Y:8696975
Y:8700380
Y:8714022
Y:8714163
Y:8727033
Y:8728974
Y:8758629
Y:8758632
Y:8759325
Y:8793919
Y:8796078
Y:8845380
Y:8871613
Y:8884016
Y:8890588
Y:8895656
Y:8898284
Y:8908270
Y:8965961
Y:8966028
Y:8966055
Y:8967006
Y:8967242
Y:8968890
Y:8980200
Y:8989382
Y:8993466
Y:8998486
Y:8998738
Y:8998941
Y:9000384
Y:9003725
Y:9004143
Y:9004925

Y:9005135
Y:9027915
Y:9060591
Y:9060781
Y:9083228
Y:9083261
Y:9098400
Y:9104370
Y:9107406
Y:9115037
Y:9142173
Y:9142204
Y:9144043
Y:9145499
Y:9148716
Y:9153466
Y:9153472
Y:9163445
Y:9171887
Y:9172309
Y:9172552
Y:9172643
Y:9172709
Y:9193040
Y:9376415
Y:9381487
Y:9381672
Y:9381846
Y:9382303
Y:9382917
Y:9383100
Y:9385266
Y:9391033
Y:9448354
Y:9448625
Y:9464078
Y:9466878
Y:9493957
Y:9500700
Y:9744845
Y:9787327
Y:9791250
Y:9792145
Y:9792147
Y:9819205
Y:9819227
Y:9819254
Y:9840350
Y:9840371
Y:9850515
Y:13687186
Y:13896332
Y:13990448
Y:14003516

Y:14040500
Y:14060308
Y:14063609
Y:14092982
Y:14140277
Y:14144593
Y:14199284
Y:14199508
Y:14243581
Y:14247835
Y:14342918
Y:14342926
Y:14342945
Y:14404954
Y:14461702
Y:14469411
Y:14548082
Y:14640715
Y:14641193
Y:14666569
Y:14698928
Y:14710265
Y:14804077
Y:14813717
Y:14813991
Y:14814540
Y:14814550
Y:14838700
Y:14846030
Y:14847494
Y:14850341
Y:14851554
Y:14864191
Y:14869076
Y:14871976
Y:14873551
Y:14874103
Y:14888783
Y:14889974
Y:14890987
Y:14891532
Y:14898570
Y:14902414
Y:14904859
Y:14920900
Y:14922396
Y:14922583
Y:14922817
Y:14924869
Y:14930316
Y:14945705
Y:14968331
Y:14968449
Y:14971330

Y:14976204
Y:15008677
Y:15016536
Y:15017505
Y:15018459
Y:15018582
Y:15018696
Y:15021104
Y:15022005
Y:15022465
Y:15022707
Y:15025506
Y:15025605
Y:15026424
Y:15029433
Y:15030767
Y:15030878
Y:15030915
Y:15031385
Y:15033712
Y:15039955
Y:15042327
Y:15043822
Y:15053676
Y:15064733
Y:15067573
Y:15069836
Y:15095580
Y:15194083
Y:15204710
Y:15206662
Y:15228609
Y:15265913
Y:15267960
Y:15273084
Y:15277795
Y:15315047
Y:15360621
Y:15371669
Y:15371841
Y:15372130
Y:15437152
Y:15437410
Y:15467364
Y:15469724
Y:15469740
Y:15470057
Y:15471258
Y:15506055
Y:15510064
Y:15517851
Y:15526751
Y:15544614
Y:15562290

Y:15564625
Y:15576203
Y:15590342
Y:15591201
Y:15591445
Y:15591447
Y:15592033
Y:15594523
Y:15601452
Y:15625242
Y:15633954
Y:15635497
Y:15635573
Y:15668070
Y:15704602
Y:15705458
Y:15725184
Y:15800326
Y:15809326
Y:15814487
Y:15816093
Y:15816094
Y:15816112
Y:15816113
Y:15816140
Y:15816142
Y:15816157
Y:15816206
Y:15877654
Y:15877995
Y:15879017
Y:15939485
Y:15970265
Y:15977973
Y:16060785
Y:16172915
Y:16190764
Y:16199051
Y:16202267
Y:16213100
Y:16213163
Y:16230262
Y:16251357
Y:16253694
Y:16263675
Y:16283856
Y:16291778
Y:16315153
Y:16326080
Y:16338537
Y:16339916
Y:16347425
Y:16348910
Y:16364417

Y:16371619
Y:16377198
Y:16388815
Y:16404607
Y:16407250
Y:16417756
Y:16478520
Y:16497020
Y:16578072
Y:16596916
Y:16629782
Y:16638804
Y:16660759
Y:16661107
Y:16715315
Y:16735413
Y:16751001
Y:16751825
Y:16758450
Y:16773870
Y:16774007
Y:16777011
Y:16779407
Y:16788383
Y:16788539
Y:16788664
Y:16788665
Y:16792142
Y:16804852
Y:16817026
Y:16817030
Y:16817923
Y:16824270
Y:16839499
Y:16856357
Y:16903692
Y:16922783
Y:16925100
Y:17004094
Y:17007767
Y:17053771
Y:17117331
Y:17135154
Y:17135618
Y:17156422
Y:17236796
Y:17236990
Y:17256018
Y:17280486
Y:17285993
Y:17286288
Y:17301401
Y:17312195
Y:17327936

Y:17340373
Y:17341676
Y:17358410
Y:17394830
Y:17396329
Y:17398083
Y:17420297
Y:17423320
Y:17446588
Y:17461478
Y:17470112
Y:17493630
Y:17495914
Y:17508337
Y:17533325
Y:17536190
Y:17536230
Y:17550661
Y:17570599
Y:17571473
Y:17571692
Y:17583053
Y:17589815
Y:17595842
Y:17614366
Y:17696109
Y:17742219
Y:17762668
Y:17765230
Y:17814087
Y:17821258
Y:17871905
Y:17871912
Y:17891241
Y:17907131
Y:17921495
Y:17922248
Y:17937048
Y:17937365
Y:17938904
Y:17958770
Y:17958994
Y:17974575
Y:18032753
Y:18099341
Y:18167403
Y:18167479
Y:18170078
Y:18248698
Y:18257568
Y:18387494
Y:18405504
Y:18560005
Y:18561042

Y:18563482
Y:18578476
Y:18601274
Y:18608096
Y:18608276
Y:18615346
Y:18647042
Y:18667642
Y:18679818
Y:18684926
Y:18694500
Y:18706036
Y:18712055
Y:18719565
Y:18731810
Y:18736860
Y:18739828
Y:18742969
Y:18759669
Y:18821912
Y:18823187
Y:18827301
Y:18831084
Y:18835911
Y:18839375
Y:18842841
Y:18881548
Y:18907236
Y:18914441
Y:19068036
Y:19096363
Y:19105726
Y:19114679
Y:19114835
Y:19118027
Y:19166861
Y:19179335
Y:19179463
Y:19179540
Y:19213252
Y:19267344
Y:19272435
Y:19279765
Y:19292547
Y:19304761
Y:19305126
Y:19306942
Y:19349615
Y:19372700
Y:19380619
Y:19413335
Y:19414474
Y:19482416
Y:19510566

Y:19515784
Y:19515794
Y:19550740
Y:20812228
Y:20812848
Y:20828795
Y:20832034
Y:20832062
Y:20832084
Y:20832092
Y:20832125
Y:20832136
Y:20832137
Y:20836447
Y:20836471
Y:20836551
Y:20836567
Y:20837553
Y:21080707
Y:21150837
Y:21157531
Y:21166358
Y:21186263
Y:21229621
Y:21229652
Y:21229653
Y:21229680
Y:21250252
Y:21264006
Y:21312064
Y:21327172
Y:21327234
Y:21327293
Y:21332123
Y:21461202
Y:21466597
Y:21480095
Y:21593470
Y:21610301
Y:21618856
Y:21622006
Y:21641094
Y:21642296
Y:21643364
Y:21645133
Y:21646196
Y:21658207
Y:21659451
Y:21674068
Y:21674378
Y:21688300
Y:21717208
Y:21721419
Y:21728755

Y:21729056
Y:21729491
Y:21733133
Y:21739006
Y:21740760
Y:21741920
Y:21752037
Y:21754097
Y:21759161
Y:21766113
Y:21777403
Y:21811108
Y:21811903
Y:21841289
Y:21844896
Y:21865821
Y:21866424
Y:21866491
Y:21868006
Y:21868068
Y:21868672
Y:21868726
Y:21868863
Y:21870638
Y:21872337
Y:21872738
Y:21878708
Y:21878762
Y:21878825
Y:21881573
Y:21881825
Y:21892572
Y:21894407
Y:21894447
Y:21896261
Y:21898279
Y:21900849
Y:21901035
Y:21902969
Y:21903202
Y:21903383
Y:21903853
Y:21904023
Y:21905416
Y:21905917
Y:21906109
Y:21907648
Y:21916516
Y:21916548
Y:21917313
Y:21925276
Y:21925314
Y:21930287
Y:21935753

Y:21936138
Y:21938158
Y:21938444
Y:21978583
Y:21978600
Y:22003770
Y:22042987
Y:22149593
Y:22158010
Y:22178569
Y:22181421
Y:22214221
Y:22513726
Y:22514081
Y:22574028
Y:22596423
Y:22597106
Y:22646728
Y:22648375
Y:22677409
Y:22702594
Y:22715814
Y:22718513
Y:22725379
Y:22737801
Y:22738775
Y:22739367
Y:22741799
Y:22741818
Y:22741821
Y:22744939
Y:22744945
Y:22744993
Y:22745051
Y:22746786
Y:22749853
Y:22754570
Y:22822229
Y:22822230
Y:22822346
Y:22893888
Y:22917995
Y:22918577
Y:22919819
Y:22919840
Y:22922129
Y:22923019
Y:22924348
Y:22928067
Y:22928699
Y:22973793
Y:23021978
Y:23030717
Y:23031377

Y:23035132
Y:23035483
Y:23035504
Y:23116369
Y:23121708
Y:23123790
Y:23124367
Y:23148103
Y:23148323
Y:23156865
Y:23165895
Y:23208693
Y:23235373
Y:23248930
Y:23257819
Y:23265885
Y:23265887
Y:23265910
Y:23292782
Y:23300109
Y:23353901
Y:23403749
Y:23454394
Y:23476936
Y:23483755
Y:23490117
Y:23533521
Y:23571026
Y:23571646
Y:23571841
Y:23577373
Y:23580343
Y:23626712
Y:23634362
Y:23638549
Y:23654049
Y:23655582
Y:23744218
Y:23748997
Y:23785316
Y:23799139
Y:23801231
Y:23839740
Y:23853320
Y:23865297
Y:23865327
Y:23865472
Y:23873042
Y:23873446
Y:23873465
Y:23873538
Y:23873680
Y:23873761
Y:23874186

Y:23874255
Y:23874448
Y:23883612
Y:23883634
Y:23891444
Y:23894015
Y:23894099
Y:23894215
Y:23894867
Y:23895012
Y:23959349
Y:23980011
Y:23984056
Y:23987612
Y:24007524
Y:24046004
Y:24070035
Y:24070054
Y:24359931
Y:24439196
Y:24443836
Y:24444622
Y:24452096
Y:24464547
Y:24478403
Y:24497922
Y:24505075
Y:24522333
Y:24880863
Y:28498442
Y:28498446
Y:28815656
Y:28817000
Y:28817368
Y:28817442
Y:28817458
Y:28817799
Y:58883690
