# Supplementary material for: Investigating demic versus cultural diffusion and sex bias in the spread of Austronesian languages in Vietnam
Source: PLoS One. 2024 Jun 17;19(6):e0304964. doi: 10.1371/journal.pone.0304964 (PMC11182502; doi:10.1371/journal.pone.0304964)
Supplement: S6 Dataset — (PDF) [file pone.0304964.s019.pdf]

#Genotypes of 847 SNPs for 768 individuals (170 newly genotyped here individuals and 598 previously published)
>BANAN10
GTGTAGCCCAGTTAGACACTTCTAAGTCCTTGGTAACAGACATGCAGTCCCCTCGGCAGAGCCCCGCC
CCCCGATGGTACCGGGTTGCTAAGAGGTCCACAACGGGTCGCACGACAACATTAAGGCCTCATGCATG
ACCCAACACGGGGGCGTCCCGGCGCGGCGACCTTGAAGTCCGGGGACAAGTATGTCCCCCCGGGAGGG
GCGCCACAGGGAATCCGGAGTGTTGATCTGCAGCCCGAGACTCCACTACGATGGTATGAACGGCAAAG
GTACTTTGGACACCACCCCGTTTTCTATTTCCAGACGCGGCCATTCAGGAACGACGCGAATCGTAATC
TACCTGCCGCGTACGCGGATCGTACATGCGTTTTCCAGCGTTCGAAATAGAGTTTGTCGTTCCGCTGA
CCAGGTTTCACGGCTACTGCGATAAGCATATTCTACTGGGGGACTGTAAAGGAATTGTATCCACTCTG
CTTCCTGTCAATTGGCCATGTGCATCTCTGGTTGTTCAGGGTACATTAATAACACACAGCATTAAGTA
GATAAGTCTCCGATATAAGCGAACAGAACGTTGGTCAGAGACTATGGTTCAATTGTAGTGGTATAATG
TTTCCCAGTGCATAAGGAACTCAGGGACACTACTAGCATGGGCGAGGAGAGACCCTTGGGTGGCAACA
CGCAATAAGTGTTACCTAGTACATCAGCACGAGAGAGGGTTTGACCATGTGTTTTTAGACGACCCCGG
TCGCATTGAACCTGGCATTCAGAATGACGTGCTCTCAAGAATCGGGGTCATCCCCCTTAGTGTCACCT
GCTTCGCGGGTCAGCGTATGGTCATAAACAT
>BANAN13
GTGTAGCCCAGTTAGACACTTCTAAGTCCTTGGTAACAGACATGCAGTCCCCTCGGCAGAGCCCCGCC
CCCCGATGGTACCGGGTTGCTAAGAGGTCCACAACGGGTCGCACGACAACATTAAGGCCTCATGCATG
ACCCAACACGGGGGCGTCCCGGCGCGGCGACCTTGAAGTCCGGGGACAAGTATGTCCCCCCGGGAGGG
GCGCCACAGGGAATCCGGAGTGTTGATCTGCAGCCCGAGACTCCACTACGATGGTATGAACGGCAAAG
GTACTTTGGACACCACCCCGTTTTCTATTTCCAGACGCGGCCATTCAGGAACGACGCGAATCGTAATC
TACCTGCCGCGTACGCGGATCGTACATGCGTTTTCCAGCGTTCGAAATAGAGTTTGTCGTTCCGCTGA
CCAGGTTTCACGGCTACTGCGATAAGCATATTCTACTGGGGGACTGTAAAGGAATTGTATCCATTCTG
CTTCCTGTCAATTGGCCATGTGCATCTCTGGTTGTTCAGGGTACATTAATAACACACAGCATTAAGTA
GATAAGTCTCCGATATAAGCGAACAGAACGTTGGTCAGAGACTGTGGTTCAATTGTAGTGGTATAATG
TTTCCCAGTGCATAAGGAACTCAGGGACACTACTAGCATGGGCGAGGAGAGACCCTTGGGTGGCAACA
CGCAATAAGTGTTACCTAGTACACCAGCACGAGAGAGGGTTTGACCATGTGTTTTTAGACGACCCCGG
TCGCATTGAACCTGGCATTCAGAATGACGTGCTCTCAAGAATCGGGGTCATCCCCCTTAGTGTCACCT
GCTTCGCGGGTCAGCGTATGGTCATAAACAT
>BANAN14
GTGTAGCCCAGTTAGACACTTCTAAGTCCTTGGTAACAGACATGCAGTCCCCTCGGCAGAGCCCCGCC
CCCCGATGGTACCGGGTTGCTAAGAGGTCCACCACGGGTCGCACGACAACATTAAGGCCTCATGCATG
ACCCAACACGGGGGCGTCCCGGCGCGGCGACCTTGAAGTCCGGGGACAAGTATGTCCCCCCGGGAGGG
GCGCCACAGGGAATCCGGAGTGTTGATCTGCAGCCCGAGACTCCACTACGATGGTATGAACGGCAAAG
GTACTTTGGACACCACCCCGTTTTCTATTTCCAGACGCGGCCATTCAGGAACGACGCGAATCGTAATC
TACCTGCCGCGTACGCGGATCGTATATNCGTTTTCCAGCGTTCGAAATAGAGTTTGTCGTTCCGCTGA
CCAGGTTTCACGGCTACTGCGATAAGCATATTCTACTGGGGGACTGTAAAGGAATTGTATCCACTCTG
CTTCCTGTCAATTGGCCATGTGCATCTCTGGTTGTTCAGGGTACATTAATAACACACAGCATTAAGTA
GATAAGTCTCCGATATAAGCGAACAGAACGTTGGTCAGAGACTGTGGTTCAATTGTAGTGGTATAATG
TTTCCCAGTGCATAAGGAACTCAGGGACACTACTAGCATGGGCGAGGAGAGACCCTTGGGTGGCAACA
CGCAATAAGTGTTACCTAGTACACCAGCACGAGAGAGGGTTTGACCATGTGTTTTTAGACGACCCCGG
TCGCATTGAACCTGGCATTCAGAATGACGTGCTCTCAAGAGTCGGGGTCATCCCCCTTAGTGTCACCT
GCTTCGCGGGTCAGCGTATGGTCATAAACAT
>BANAN15
GTGTAGCCCAGTTAGACACTTCTAAGTCCTTGGTAGCAAACATGCAGTCCCCTCGGCAGAGCCCCGCC
CCCCGATGGTACCGGGTTGCTAAGAGGTCCACCACGAGTCGCACGACAACAGCAAGGCCTCGTGCATG
ACCCAACACGGGGGCGTCCCGGCGCGGCGACCTTGAAGTCCGGGAACAAGTATGTCTCCCCGGGAGGG
GCGCCACAGGGAATCCGGAGTGTTGATCTGCAGCCCGAGACTCCACTACGATGGTATGAACGGCAAAG
GTACTTTGGACACCACCCCGTTTTCTATTCCCAGACGCGGCCATTCAGGAACGACGCGAATCGTAATC
TACCTGCCGCGTACGCGGATCGTACATGCGTTTTCCAGCGTTCGGAATAGAGTTTGTCGTTCCGCTGA
CCAGGTTTCACGGCTACTGCGATAAGCATACTCCACTGGGGGACTGTAAAGGAATTGCATCCACTCTG
CTTCTTGTCAGTTGGCCATATGTATCTCTGGTTGTTCAGGGTACATTAATAACACACAGCATTAAGTA
GATAAGTCTCCGATATAGGCGAACAGAACGTTGGTAAAAGACTGTGGTTCAACTGTAGTGGTATAATG

TTTCCCAGTGCATAAGGAACTCAGGGACACTACTAGCATGGGCGAGTAGAGACCCTTGGGTGGCAACA
CGCAATAAGTGTTACCTAGTACACCAACACGATATAGGGTTTGACCATGTGTTTTTAGACGACCCCGG
TCGCATTAAACCTGGCATTCATAATGACGTGCTCTCAAGAGTCGGGGTCATCCCCCTTAGTGTCACCT
GCTTCGCGGGTCATCGTATGGTCATAAACAT
>BANAN18
GTGTAGCCCAGTCAGACACTTCTAAGTCCTTGGTAACAGACATGCAGTCCCCTCGGCAGAGCCCCGCC
CCCCGATGGTACCGGGTTGCTAAGAGGTCCACAACGGGTCGCACGACAACATTAAGGCCTCATGCATG
ACCCAACACGGGGGCGTCCCGGCGCGGCGACCTTGAAGTCCGGGGACAAGTATGTCCCCCCGGGAGGG
GCGCCACAGGGAATCCGGAGTGTTGATCTGCAGCCCGAGACTCCACTACGATGGTATGAACGGCAAAG
GTACTTTGGACACCACCCCGTTTTCTATTTCCAGACGCGGCCATTTAGGAACGACGCGAATCGTAATC
TACCTGCCGCGTACGCGGATCGTACATGCGTTTTCCAGCGTTCGAAATAGAGTTTGTCGTTCCGCTGA
CCAGGTTTCACGGCTACTGCGATAAGCATATTCTACTGGGGGACTGTAAAGGAATTGTATCCATTCTG
CTTCCTGTCAATTGGCCATGTGCATCTCTGGTTGTTCAGGGTACATTAATAACACACAGCAGTAAGTA
GGTAAGTCTCCGATATAAGCGAACAGAACGTTTGTCAGAGACTGTGGTTCAATTGTAGTGGTATAATG
TTTCCCAGTGCATAAGGAACTCAGGGACACTACTAGCATGGGCGAGGAGAGACCCTTGGGTGGCAACA
CGCAATAAGTGTTACCTAGTACACCAGCACGAGAGAGGGTTTGACCATGTGTTTTTAGACGACCCCGG
TCGCATTGAACCTGGCATTCAGAATGACGTGCTCTCAAGAATCGGGGTCATCCCCCTTAGTGTCACCT
GCTTCGCGGGTCAGCGTATGGTCATAAACAT
>BANAN19
GTGTAGCCCAGTCAGACACTTCTAAGTCCTTGGTAACAGACATGCAGTCCCCTCGGCAGAGCCCCGCC
CCCCGATGGTACCGGGTTGCTAAGAGGTCCACAACGGGTCGCACGACAACATTAAGGCCTCATGCATG
ACCCAACACGGGGGCGTCCCGGCGCGGCGACCTTGAAGTCCGGGGACAAGTATGTCCCCCCGGGAGGG
GCGCCACAGGGAATCCGGAGTGTTGATCTGCAGCCCGAGACTCCACTACGATGGTATGAACGGCAAAG
GTACTTTGGACACCACCCCGTTTTCTATTTCCAGACGCGGCCATTTAGGAACGACGCGAATCGTAATC
TACCTGCCGCGTACGCGGATCGTACATGCGTTTTCCAGCGTTCGAAATAGAGTTTGTCGTTCCGCTGA
CCAGGTTTCACGGCTACTGCGATAAGCATATTCTACTGGGGGACTGTAAAGGAATTGTATCCATTCTG
CTTCCTGTCAATTGGCCATGTGCATCTCTGGTTGTTCAGGGTACATTAATAACACACAGCAGTAAGTA
GGTAAGTCTCCGATATAAGCGAACAGAACGTTTGTCAGAGACTGTGGTTCAATTGTAGTGGTATAATG
TTTCCCAGTGCATAAGGAACTCAGGGACACTACTAGCATGGGCGAGGAGAGACCCTTGGGTGGCAACA
CGCAATAAGTGTTACCTAGTACACCAGCACGAGAGAGGGTTTGACCATGTGTTTTTAGACGACCCCGG
TCGCATTGAACCTGGCATTCAGAATGACGTGCTCTCAAGAATCGGGGTCATCCCCCTTAGTGTCACCT
GCTTCGCGGGTCAGCGTATGGTCATAAACAT
>BANAN20
GTGTAGCCCAGTTAGACACTTCTAAGTCCTTGGTAACAGACATGCAGTCCCCTCGGCAGAGCCCCGCC
CCCCGATGGTACCGGGTTGCTAAGAGGTCCACAACGGGTCGCACGACAACATTAAGGCCTCATGCATG
ACCCAACACGGGGGCGTCCCGGCGCGGCGACCTTGAAGTCCGGGGACAAGTATGTCCCCCCGGGAGGG
GCGCCACAGGGAATCCGGAGTGTTGATCTGCAGCCCGAGACTCCACTACGATGGTATGAACGGCAAAG
GTACTTTGGACACCACCCCGTTTTCTATTTCCAGACGCGGCCATTCAGGAACGACGCGAATCGTAATC
TACCTGCCGCGTACGCGGATCGTACATGCGTTTTCCAGCGTTCGAAATAGAGTTTGTCGTTCCGCTGA
CCAGGTTTCACGGCTACTGCGATAAGCATATTCTACTGGGGGACTGTAAAGGAATTGTATCCACTCTG
CTTCCTGTCAATTGGCCATGTGCATCTCTGGTTGTTCAGGGTACATTAATAACACACAGCATTAAGTA
GATAAGTCTCCGATATAAGCGAACAGAACGTTGGTCAGAGACTATGGTTCAATTGTAGTGGTATAATG
TTTCCCAGTGCATAAGGAACTCAGGGACACTACTAGCATGGGCGAGGAGAGACCCTTGGGTGGCAACA
CGCAATAAGTGTTACCTAGTACATCAGCACGAGAGAGGGTTTGACCATGTGTTTTTAGACGACCCCGG
TCGCATTGAACCTGGCATTCAGAATGACGTGCTCTCAAGAATCGGGGTCATCCCCCTTAGTGTCACCT
GCTTCGCGGGTCAGCGTATGGTCATAAACAT
>BANAN21
GTGTAGCCCAGTTAGACACTTCTAAGTCCTTGGTAACAGACATGCAGTCCCCTCGGCAGAGCCCCGCC
CCCCGATGGTACCGGGTTGCTAAGAGGTCCACAACGGGTCGCACGACAACATTAAGGCCTCATGCATG
ACCCAACACGGGGGCGTCCCGGCGCGGCGACCTTGAAGTCCGGGGACAAGTATGTCCCCCCGGGAGGG
GCGCCACAGGGAATCCGGAGTGTTGATCTGCAGCCCGAGACTCCACTACGATGGTATGAACGGCAAAG
GTACTTTGGACACCACCCCGTTTTCTATTTCCAGACGCGGCCATTCAGGAACGACGCGAATCGTAATC
TACCTGCCGCGTACGCGGATCGTACATGCGTTTTCCAGCGTTCGAAATAGAGTTTGTCGTTCCGCTGA
CCAGGTTTCACGGCTACTGCGATAAGCATATTCTACTGGGGGACTGTAAAGGAATTGTATCCATTCTG

CTTCCTGTCAATTGGCCATGTGCATCTCTGGTTGTTCAGGGTACATTAATAACACACAGCATTAAGTA
GATAAGTCTCCGATATAAGCGAACAGAACGTTGGTCAGAGACTGTGGTTCAATTGTAGTGGTATAATG
TTTCCCAGTGCATAAGGAACTCAGGGACACTACTAGCATGGGCGAGGAGAGACCCTTGGGTGGCAACA
CGCAATAAGTGTTACCTAGTACACCAGCACGAGAGAGGGTTTGACCATGTGTTTTTAGACGACCCCGG
TCGCATTGAACCTGGCATTCAGAATGACGTGCTCTCAAGAATCGGGGTCATCCCCCTTAGTGTCACCT
GCTTCGCGGGTCAGCGTATGGTCATAAACAT
>BANAN22
GTGTAGCCCAGTCAGACACTTCTAAGTCCTTGGTAACAGACATGCAGTCCCCTCGGCAGAGCCCCGCC
CCCCGATGGTACCGGGTTGCTAAGAGGTCCACAACGGGTCGCACGACAACATTAAGGCCTCATGCATG
ACCCAACACGGGGGCGTCCCGGCGCGGCGACCTTGAAGTCCGGGGACAAGTATGTCCCCCCGGGAGGG
GCGCCACAGGGAATCCGGAGTGTTGATCTGCAGCCCGAGACTCCACTACGATGGTATGAACGGCAAAG
GTACTTTGGACACCACCCCGTTTTCTATTTCCAGACGCGGCCATTTAGGAACGACGCGAATCGTAATC
TACCTGCCGCGTACGCGGATCGTACATGCGTTTTCCAGCGTTCGAAATAGAGTTTGTCGTTCCGCTGA
CCAGGTTTCACGGCTACTGCGATAAGCATATTCTACTGGGGGACTGTAAAGGAATTGTATCCATTCTG
CTTCCTGTCAATTGGCCATGTGCATCTCTGGTTGTTCAGGGTACATTAATAACACACAGCAGTAAGTA
GGTAAGTCTCCGATATAAGCGAACAGAACGTTTGTCAGAGACTGTGGTTCAATTGTAGTGGTATAATG
TTTCCCAGTGCATAAGGAACTCAGGGACACTACTAGCATGGGCGAGGAGAGACCCTTGGGTGGCAACA
CGCAATAAGTGTTACCTAGTACACCAGCACGAGAGAGGGTTTGACCATGTGTTTTTAGACGACCCCGG
TCGCATTGAACCTGGCATTCAGAATGACGTGCTCTCAAGAATCGGGGTCATCCCCCTTAGTGTCACCT
GCTTCGCGGGTCAGCGTATGGTCATAAACAT
>BANAN23
GTGTAGCCCAGTTAGACACTTCTAAGTCCTTGGTAACAGACATGCAGTCCCCTCGGCAGAGCCCCGCC
CCCCGATGGTACCGGGTTGCTAAGAGGTCCACAACGGGTCGCACGACAACATTAAGGCCTCATGCATG
ACCCAACACGGGGGCGTCCCGGCGCGGCGACCTTGAAGTCCGGGGACAAGTATGTCCCCCCGGGAGGG
GCGCCACAGGGAATCCGGAGTGTTGATCTGCAGCCCGAGACTCCACTACGATGGTATGAACGGCAAAG
GTACTTTGGACACCACCCCGTTTTCTATTTCCAGACGCGGCCATTCAGGAACGACGCGAATCGTAATC
TACCTGCCGCGTACGCGGATCGTACATGCGTTTTCCAGCGTTCGAAATAGAGTTTGTCGTTCCGCTGA
CCAGGTTTCACGGCTACTGCGATAAGCATATTCTACTGGGGGACTGTAAAGGAATTGTATCCACTCTG
CTTCCTGTCAATTGGCCATGTGCATCTCTGGTTGTTCAGGGTACATTAATAACACACAGCATTAAGTA
GATAAGTCTCCGATATAAGCGAACAGAACGTTGGTCAGAGACTATGGTTCAATTGTAGTGGTATAATG
TTTCCCAGTGCATAAGGAACTCAGGGACACTACTAGCATGGGCGAGGAGAGACCCTTGGGTGGCAACA
CGCAATAAGTGTTACCTAGTACATCAGCACGAGAGAGGGTTTGACCATGTGTTTTTAGACGACCCCGG
TCGCATTGAACCTGGCATTCAGAATGACGTGCTCTCAAGAATCGGGGTCATCCCCCTTAGTGTCACCT
GCTTCGCGGGTCAGCGTATGGTCATAAACAT
>BANAN24
GTGTAGCCCAGTTAGACACTTCTAAGTCCTTGGTAGCAAACATGCAGTCCCCTCGGCAGAGCCCCGCC
CCCCGATGGTACCGGGTTGCTAAGAGGTCCACCACGAGTCGCACGACAACAGCAAGGCCTCGTGCATG
ACCCAACACGGGGGCGTCCCGGCGCGGCGACCTTGAAGTCCGGGAACAAGTATGTCTCCCCGGGAGGG
GCGCCACAGGGAATCCGGAGTGTTGATCTGCAGCCCGAGACTCCACTACGATGGTATGAACGGCAAAG
GTACTTTGGACACCACCCCGTTTTCTATTCCCAGACGCGGCCATTCAGGAACGACGCGAATCGTAATC
TACCTGCCGCGTACGCGGATCGTACATGCGTTTTCCAGCGTTCGGAATAGAGTTTGTCGTTCCGCTGA
CCAGGTTTCACGGCTACTGCGATAAGCATACTCCACTGGGGGACTGTAAAGGAATTGCATCCACTCTG
CTTCTTGTCAGTTGGCCATATGTATCTCTGGTTGTTCAGGGTACATTAATAACACACAGCATTAAGTA
GATAAGTCTCCGATATAGGCGAACAGAACGTTGGTAAAAGACTGTGGTTCAACTGTAGTGGTATAATG
TTTCCCAGTGCATAAGGAACTCAGGGACACTACTAGCATGGGCGAGTAGAGACCCTTGGGTGGCAACA
CGCAATAAGTGTTACCTAGTACACCAACACGATATAGGGTTTGACCATGTGTTTTTAGACGACCCCGG
TCGCATTAAACCTGGCATTCATAATGACGTGCTCTCAAGAGTCGGGGTCATCCCCCTTAGTGTCACCT
GCTTCGCGGGTCATCGTATGGTCATAAACAT
>BANAN25
GTGTAGCCCAGTTAGACACTTCTAAGTCCTTGGTAACAGACATGCAGTCCCCTCGGCAGAGCCCCGCC
CCCCGATGGTACCGGGTTGCTAAGAGGTCCACAACGGGTCGCACGACAACATTAAGGCCTCATGCATG
ACCCAACACGGGGGCGTCCCGGCGCGGCGACCTTGAAGTCCGGGGACAAGTATGTCCCCCCGGGAGGG
GCGCCACAGGGAATCCGGAGTGTTGATCTGCAGCCCGAGACTCCACTACGATGGTATGAACGGCAAAG
GTACTTTGGACACCACCCCGTTTTCTATTTCCAGACGCGGCCATTCAGGAACGACGCGAATCGTAATC

TACCTGCCGCGTACGCGGATCGTACATGCGTTTTCCAGCGTTCGAAATAGAGTTTGTCGTTCCGCTGA
CCAGGTTTCACGGCTACTGCGATAAGCATATTCTACTGGGGGACTGTAAAGGAATTGTATCCACTCTG
CTTCCTGTCAATTGGCCATGTGCATCTCTGGTTGTTCAGGGTACATTAATAACACACAGCATTAAGTA
GATAAGTCTCCGATATAAGCGAACAGAACGTTGGTCAGAGACTATGGTTCAATTGTAGTGGTATAATG
TTTCCCAGTGCATAAGGAACTCAGGGACACTACTAGCATGGGCGAGGAGAGACCCTTGGGTGGCAACA
CGCAATAAGTGTTACCTAGTACATCAGCACGAGAGAGGGTTTGACCATGTGTTTTTAGACGACCCCGG
TCGCATTGAACCTGGCATTCAGAATGACGTGCTCTCAAGAATCGGGGTCATCCCCCTTAGTGTCACCT
GCTTCGCGGGTCAGCGTATGGTCATAAACAT
>BANAN26
GTGTAGCCCAGTTAGACACTTCTAAGTCCTTGGTAACAGACATGCAGTCCCCTCGGCAGAGCCCCGCC
CCCCGATGGTACCGGGTTGCTAAGAGGTCCACAACGGGTCGCACGACAACATTAAGGCCTCATGCATG
ACCCAACACGGGGGCGTCCCGGCGCGGCGACCTTGAAGTCCGGGGACAAGTATGTCCCCCCGGGAGGG
GCGCCACAGGGAATCCGGAGTGTTGATCTGCAGCCCGAGACTCCACTACGATGGTATGAACGGCAAAG
GTACTTTGGACACCACCCCGTTTTCTATTTCCAGACGCGGCCATTCAGGAACGACGCGAATCGTAATC
TACCTGCCGCGTACGCGGATCGTACATGCGTTTTCCAGCGTTCGAAATAGAGTTTGTCGTTCCGCTGA
CCAGGTTTCACGGCTACTGCGATAAGCATATTCTACTGGGGGACTGTAAAGGAATTGTATCCACTCTG
CTTCCTGTCAATTGGCCATGTGCATCTCTGGTTGTTCAGGGTACATTAATAACACACAGCATTAAGTA
GATAAGTCTCCGATATAAGCGAACAGAACGTTGGTCAGAGACTATGGTTCAATTGTAGTGGTATAATG
TTTCCCAGTGCATAAGGAACTCAGGGACACTACTAGCATGGGCGAGGAGAGACCCTTGGGTGGCAACA
CGCAATAAGTGTTACCTAGTACATCAGCACGAGAGAGGGTTTGACCATGTGTTTTTAGACGACCCCGG
TCGCATTGAACCTGGCATTCAGAATGACGTGCTCTCAAGAATCGGGGTCATCCCCCTTAGTGTCACCT
GCTTCGCGGGTCAGCGTATGGTCATAAACAT
>BANAN27
GTGTAGCCCAGTTAGACACTTCTAAGTCCTTGGTAACAGACATGCAGTCCCCTCGGCAGAGCCCCGCC
CCCCGATGGTACCGGGTTGCTAAGAGGTCCACAACGGGTCGCACGACAACATTAAGGCCTCATGCATG
ACCCAACACGGGGGCGTCCCGGCGCGGCGACCTTGAAGTCCGGGGACAAGTATGTCCCCCCGGGAGGG
GCGCCACAGGGAATCCGGAGTGTTGATCTGCAGCCCGAGACTCCACTACGATGGTATGAACGGCAAAG
GTACTTTGGACACCACCCCGTTTTCTATTTCCAGACGCGGCCATTCAGGAACGACGCGAATCGTAATC
TACCTGCCGCGTACGCGGATCGTACATGCGTTTTCCAGCGTTCGAAATAGAGTTTGTCGTTCCGCTGA
CCAGGTTTCACGGCTACTGCGATAAGCATATTCTACTGGGGGACTGTAAAGGAATTGTATCCACTCTG
CTTCCTGTCAATTGGCCATGTGCATCTCTGGTTGTTCAGGGTACATTAATAACACACAGCATTAAGTA
GATAAGTCTCCGATATAAGCGAACAGAACGTTGGTCAGAGACTATGGTTCAATTGTAGTGGTATAATG
TTTCCCAGTGCATAAGGAACTCAGGGACACTACTAGCATGGGCGAGGAGAGACCCTTGGGTGGCAACA
CGCAATAAGTGTTACCTAGTACATCAGCACGAGAGAGGGTTTGACCATGTGTTTTTAGACGACCCCGG
TCGCATTGAACCTGGCATTCAGAATGACGTGCTCTCAAGAATCGGGGTCATCCCCCTTAGTGTCACCT
GCTTCGCGGGTCAGCGTATGGTCATAAACAT
>BANAN28
GTGTAGCCCAGTTAGACACTTCTAAGTCCTTGGTAACAGACATGCAGTCCCCTCGGCAGAGCCCCGCC
CCCCGATGGTACCGGGTTGCTAAGAGGTCCACAACGGGTCGCACGACAACATTAAGGCCTCATGCATG
ACCCAACACGGGGGCGTCCCGGCGCGGCGACCTTGAAGTCCGGGGACAAGTATGTCCCCCCGGGAGGG
GCGCCACAGGGAATCCGGAGTGTTGATCTGCAGCCCGAGACTCCACTACGATGGTATGAACGGCAAAG
GTACTTTGGACACCACCCCGTTTTCTATTTCCAGACGCGGCCATTCAGGAACGACGCGAATCGTAATC
TACCTGCCGCGTACGCGGATCGTACATGCGTTTTCCAGCGTTCGAAATAGAGTTTGTCGTTCCGCTGA
CCAGGTTTCACGGCTACTGCGATAAGCATATTCTACTGGGGGACTGTAAAGGAATTGTATCCACTCTG
CTTCCTGTCAATTGGCCATGTGCATCTCTGGTTGTTCAGGGTACATTAATAACACACAGCATTAAGTA
GATAAGTCTCCGATATAAGCGAACAGAACGTTGGTCAGAGACTATGGTTCAATTGTAGTGGTATAATG
TTTCCCAGTGCATAAGGAACTCAGGGACACTACTAGCATGGGCGAGGAGAGACCCTTGGGTGGCAACA
CGCAATAAGTGTTACCTAGTACATCAGCACGAGAGAGGGTTTGACCATGTGTTTTTAGACGACCCCGG
TCGCATTGAACCTGGCATTCAGAATGACGTGCTCTCAAGAATCGGGGTCATCCCCCTTAGTGTCACCT
GCTTCGCGGGTCAGCGTATGGTCATAAACAT
>BANAN29
GTGTAGCCCAGTTAGACACTTCTAAGTCCTTGGTAACAAACGTGCAGTCCCCTCGGCAGAGCCCCGCC
CCCCGATGGTACCGGGTTGCTAAGAGGTCCACCACGAGTCGCACAACAACAGCAAGGCCTCGTGCATG
ACCCAACACGGGGGCGTCCCGGCGCAGCGACCTTGAAGTCCGGGAACAAGTATGTCTCCCCGGGAGGG

GCGCCACAGGGAATCCGGAGTGTTGATCTGCAGCCCGAGACTCCACTACGATGGTATGAACGGCAAAG
GTACTTTGGACACCACCCCGTTTTCTATTCCCAGACGCGGCCATTCAGGAACGACGCGAATCGTCATC
TACCTGCCGCGTACGCGGATCGTACATGCGTTTTCCAGCTTTCGGAATAGAGTTTGTCGTTCCGCTGG
CCAGGTTTCACGGCTATTGCGATAAGCATACTCCACTGGGGGACTGTAAAGGAATTGCATCCACTCTG
CTTCTTATCAGTTGGCCATATGTATCTCTGGTTGTTCAGGGTGCATTAATAACACACAGCATTAAGTA
GATAAGTCTCCGATATAGGCGAACAGAACGTTGGTAAAAGACTGTGGTTCAACTGTAGTGGTATAATG
TTGCCCAGTGCATAAGGAACTCAGGGACACTACTAGCATGGGCGAGTAGAGACCCTTGGGTGGCAACA
CGCAATAAGTGTTACCTAGTACACCAACACGATATAGGGTTTGACCATGTGTTTTTAGACGACCCCGG
TCGCATTAAACCTGGCATTCATAATGACGTGCTCTCAAGAGTCGGGGTCATCCCCCTTAGTGTCACCT
GCTTCGCGGGTCATCGTATGGTCATAAACAT
>BANAN30
GTGTAGCCCAGTTAGACACTTCTAAGTCCTTGGTAACAGACATGCAGTCCCCTCGGCAGAGCCCCGCC
CCCCGATGGTACCGGGTTGCTAAGAGGTCCACAACGGGTCGCACGACAACATTAAGGCCTCATGCATG
ACCCAACACGGGGGCGTCCCGGCGCGGCGACCTTGAAGTCCGGGGACAAGTATGTCCCCCCGGGAGGG
GCGCCACAGGGAATCCGGAGTGTTGATCTGCAGCCCGAGACTCCACTACGATGGTATGAACGGCAAAG
GTACTTTGGACACCACCCCGTTTTCTATTTCCAGACGCGGCCATTCAGGAACGACGCGAATCGTAATC
TACCTGCCGCGTACGCGGATCGTACATGCGTTTTCCAGCGTTCGAAATAGAGTTTGTCGTTCCGCTGA
CCAGGTTTCACGGCTACTGCGATAAGCATATTCTACTGGGGGACTGTAAAGGAATTGTATCCACTCTG
CTTCCTGTCAATTGGCCATGTGCATCTCTGGTTGTTCAGGGTACATTAATAACACACAGCATTAAGTA
GATAAGTCTCCGATATAAGCGAACAGAACGTTGGTCAGAGACTATGGTTCAATTGTAGTGGTATAATG
TTTCCCAGTGCATAAGGAACTCAGGGACACTACTAGCATGGGCGAGGAGAGACCCTTGGGTGGCAACA
CGCAATAAGTGTTACCTAGTACATCAGCACGAGAGAGGGTTTGACCATGTGTTTTTAGACGACCCCGG
TCGCATTGAACCTGGCATTCAGAATGACGTGCTCTCAAGAATCGGGGTCATCCCCCTTAGTGTCACCT
GCTTCGCGGGTCAGCGTATGGTCATAAACAT
>BANAN31
GTGTAGCCCAGTTAGACACTTCTAAGTCCTTGGTAACAGACATGCAGTCCCCTCGGCAGAGCCCCGCC
CCCCGATGGTACCGGGTTGCTAAGAGGTCCACAACGGGTCGCACGACAACATTAAGGCCTCATGCATG
ACCCAACACGGGGGCGTCCCGGCGCGGCGACCTTGAAGTCCGGGGACAAGTATGTCCCCCCGGGAGGG
GCGCCACAGGGAATCCGGAGTGTTGATCTGCAGCCCGAGACTCCACTACGATGGTATGAACGGCAAAG
GTACTTTGGACACCACCCCGTTTTCTATTTCCAGACGCGGCCATTCAGGAACGACGCGAATCGTAATC
TACCTGCCGCGTACGCGGATCGTACATGCGTTTTCCAGCGTTCGAAATAGAGTTTGTCGTTCCGCTGA
CCAGGTTTCACGGCTACTGCGATAAGCATATTCTACTGGGGGACTGTAAAGGAATTGTATCCACTCTG
CTTCCTGTCAATTGGCCATGTGCATCTCTGGTTGTTCAGGGTACATTAATAACACACAGCATTAAGTA
GATAAGTCTCCGATATAAGCGAACAGAACGTTGGTCAGAGACTATGGTTCAATTGTAGTGGTATAATG
TTTCCCAGTGCATAAGGAACTCAGGGACACTACTAGCATGGGCGAGGAGAGACCCTTGGGTGGCAACA
CGCAATAAGTGTTACCTAGTACATCAGCACGAGAGAGGGTTTGACCATGTGTTTTTAGACGACCCCGG
TCGCATTGAACCTGGCATTCAGAATGACGTGCTCTCAAGAATCGGGGTCATCCCCCTTAGTGTCACCT
GCTTCGCGGGTCAGCGTATGGTCATAAACAT
>BANAN34
GTGTAGCCCAGTTAGACACTTCTAAGTCCTTGGTAACAAACGTGCAGTCCCCTCGGCAGAGCCCCGCC
CCCCGATGGTACCGGGTTGCTAAGAGGTCCACCACGAGTCGCACAACAACAGCAAGGCCTCGTGCATG
ACCCAACACGGGGGCGTCCCGGCGCAGCGACCTTGAAGTCCGGGAACAAGTATGTCTCCCCGGGAGGG
GCGCCACAGGGAATCCGGAGTGTTGATCTGCAGCCCGAGACTCCACTACGATGGTATGAACGGCAAAG
GTACTTTGGACACCACCCCGTTTTCTATTCCCAGACGCGGCCATTCAGGAACGACGCGAATCGTCATC
TACCTGCCGCGTACGCGGATCGTACATGCGTTTTCCAGCTTTCGGAATAGAGTTTGTCGTTCCGCTGG
CCAGGTTTCACGGCTATTGCGATAAGCATACTCCACTGGGGGACTGTAAAGGAATTGCATCCACTCTG
CTTCTTATCAGTTGGCCATATGTATCTCTGGTTGTTCAGGGTGCATTAATAACACACAGCATTAAGTA
GATAAGTCTCCGATATAGGCGAACAGAACGTTGGTAAAAGACTGTGGTTCAACTGTAGTGGTATAATG
TTGCCCAGTGCATAAGGAACTCAGGGACACTACTAGCATGGGCGAGTAGAGACCCTTGGGTGGCAACA
CGCAATAAGTGTTACCTAGTACACCAACACGATATAGGGTTTGACCATGTGTTTTTAGACGACCCCGG
TCGCATTAAACCTGGCATTCATAATGACGTGCTCTCAAGAGTCGGGGTCATCCCCCTTAGTGTCACCT
GCTTCGCGGGTCATCGTATGGTCATAAACAT
>BANAN35
GTGTAGCCCAGTCAGACACTTCTAAGTCCTTGGTAACAGACATGCAGTCCCCTCGGCAGAGCCCCGCC

CCCCGATGGTACCGGGTTGCTAAGAGGTCCACAACGGGTCGCACGACAACATTAAGGCCTCATGCATG
ACCCAACACGGGGGCGTCCCGGCGCGGCGACCTTGAAGTCCGGGGACAAGTATGTCCCCCCGGGAGGG
GCGCCACAGGGAATCCGGAGTGTTGATCTGCAGCCCGAGACTCCACTACGATGGTATGAACGGCAAAG
GTACTTTGGACACCACCCCGTTTTCTATTTCCAGACGCGGCCATTCAGGAACGACGCGAATCGTAATC
TACCTGCCGCGTACGCGGATCGTACATGCGTTTTCCAGCGTTCGAAATAGAGTTTGTCGTTCCGCTGA
CCAGGTTTCACGGCTACTGCGATAAGCATATTCTACTGGGGGACTGTAAAGGAATTGTATCCATTCTG
CTTCCTGTCAATTGGCCATGTGCATCTCTGGTTGTTCAGGGTACATTAATAACACACAGCAGTAAGTA
GGTAAGTCTCCGATATAAGCGAACAGAACGTTTGTCAGAGACTGTGGTTCAATTGTAGTGGTATAATG
TTTCCCAGTGCATAAGGAACTCAGGGACACTACTAGCATGGGCGAGGAGAGACCCTTGGGTGGCAACA
CGCAATAAGTGTTACCTAGTACACCAGCACGAGAGAGGGTTTGACCATGTGTTTTTAGACGACCCCGG
TCGCATTGAACCTGGCATTCAGAATGACGTGCTCTCAAGAATCGGGGTCATCCCCCTTAGTGTCACCT
GCTTCGCGGGTCAGCGTATGGTCATAAACAT
>GIRAN01
GTGTAGCCCAGTTAGACACTTCTAAGTCCTTGGTAACAGACATGCAGTCCCCTCGGCAGAGCCCCGCC
CCCCGATGGTACCGGGTTGCTAAGAGGTCCACAACGGGTCGCACGACAACATTAAGGCCTCATGCATG
ACCCAACACGGGGGCGTCCCGGCGCGGCGACCTTGAAGTCCGGGGACAAGTATGTCCCCCCGGGAGGG
GCGCCACAGGGAATCCGGAGTGTTGATCTGCAGCCCGAGACTCCACTACGATGGTATGAACGGCAAAG
GTACTTTGGACACCACCCCGTTTTCTATTTCCAGACGCGGCCATTCAGGAACGACGCGAATCGTAATC
TACCTGCCGCGTACGCGGATCGTACATGCGTTTTCCAGCGTTCGAAATAGAGTTTGTCGTTCCGCTGA
CCAGGTTTCACGGCTACTGCGATAAGCATATTCTACTGGGGGACTGTAAAGGAATTGTATCCACTCTG
CTTCCTGTCAATTGGCCATGTGCATCTCTGGTTGTTCAGGGTACATTAATAACACACAGCATTAAGTA
GATAAGTCTCCGATATAAGCGAACAGAACGTTGGTCAGAGACTATGGTTCAATTGTAGTGGTATAATG
TTTCCCAGTGCATAAGGAACTCAGGGACACTACTAGCATGGGCGAGGAGAGACCCTTGGGTGGCAACA
CGCAATAAGTGTTACCTAGTACATCAGCACGAGAGAGGGTTTGACCATGTGTTTTTAGACGACCCCGG
TCGCATTGAACCTGGCATTCAGAATGACGTGCTCTCAAGAATCGGGGTCATCCCCCTTAGTGTCACCT
GCTTCGCGGGTCAGCGTATGGTCATAAACAT
>GIRAN02
GTGTAGCCCAGTTAGACACTTCTAAGTCCTTGGTAACAGACATGCAGTCCCCTCGGCAGAGCCCCGCC
CCCCGATGGTACCGGGTTGCTAAGAGGTCCACAACGGGTCGCACGACAACATTAAGGCCTCATGCATG
ACCCAACACGGGGGCGTCCCGGCGCGGCGACCTTGAAGTCCGGGGACAAGTATGTCCCCCCGGGAGGG
GCGCCACAGGGAATCCGGAGTGTTGATCTGCAGCCCGAGACTCCACTACGATGGTATGAACGGCAAAG
GTACTTTGGACACCACCCCGTTTTCTATTTCCAGACGCGGCCATTCAGGAACGACGCGAATCGTAATC
TACCTGCCGCGTACGCGGATCGTACATGCGTTTTCCAGCGTTCGAAATNGAGTTTGTCGTTCCGCTGA
CCAGGTTTCACGGCTACTGCGATAAGCATATTCTACTGGGGGACTGTAAAGGAATTGTATCCACTCTG
CTTCCTGTCAATTGGCCATGTGCATCTCTGGTTGTTCAGGGTACATTAATAACACACAGCATTAAGTA
GATAAGTCTCCGATATAAGCGAACAGAACGTTGGTCAGAGACTATGGTTCAATTGTAGTGGTATAATG
TTTCCCAGTGCATAAGGAACTCAGGGACACTACTAGCATGGGCGAGGAGAGACCCTTGGGTGGCAACA
CGCAATAAGTGTTACCTAGTACATCAGCACGAGAGAGGGTTTGACCATGTGTTTTTAGACGACCCCGG
TCGCATTGAACCTGGCATTCAGAATGACGTGCTCTCAAGAATCGGGGTCATCCCCCTTAGTGTCACCT
GCTTCGCGGGTCAGCGTATGGTCATAAACAT
>GIRAN03
GTGTAGCCCAGTTAGACACTTCTAAGTCCTTGGTAACAGACATGCAGTCCCCTCGGCAGAGCCCCGCC
CCCCGATGGTACCGGGTTGCTAAGAGGTCCACAACGGGTCGCACGACAACATTAAGGCCTCATGCATG
ACCCAACACGGGGGCGTCCCGGCGCGGCGACCTTGAAGTCCGGGGACAAGTATGTCCCCCCGGGAGGG
GCGCCACAGGGAATCCGGAGTGTTGATCTGCAGCCCGAGACTCCACTACGATGGTATGAACGGCAAAG
GTACTTTGGACACCACCCCGTTTTCTATTTCCAGACGCGGCCATTCAGGAACGACGCGAATCGTAATC
TACCTGCCGCGTACGCGGATCGTACATGCGTTTTCCAGCGTTCGAAATAGAGTTTGTCGTTCCGCTGA
CCAGGTTTCACGGCTACTGCGATAAGCATATTCTACTGGGGGACTGTAAAGGAATTGTATCCACTCTG
CTTCCTGTCAATTGGCCATGTGCATCTCTGGTTGTTCAGGGTACATTAATAACACACAGCATTAAGTA
GATAAGTCTCCGATATAAGCGAACAGAACGTTGGTCAGAGACTATGGTTCAATTGTAGTGGTATAATG
TTTCCCAGTGCATAAGGAACTCAGGGACACTACTAGCATGGGCGAGGAGAGACCCTTGGGTGGCAACA
CGCAATAAGTGTTACCTAGTACATCAGCACGAGAGAGGGTTTGACCATGTGTTTTTAGACGACCCCGG
TCGCATTGAACCTGGCATTCAGAATGACGTGCTCTCAAGAATCGGGGTCATCCCCCTTAGTGTCACCT
GCTTCGCGGGTCAGCGTATGGTCATAAACAT

>GIRAN04
GTGTAGCCCAGTTAGACACTTCTAAGTCCTTGGTAACAGACATGCAGTCCCCTCGGCAGAGCCCCGCC
CCCCGATGGTACCGGGTTGCTAAGAGGTCCACAACGGGTCGCACGACAACATTAAGGCCTCATGCATG
ACCCAACACGGGGGCGTCCCGGCGCGGCGACCTTGAAGTCCGGGGACAAGTATGTCCCCCCGGGAGGG
GCGCCACAGGGAATCCGGAGTGTTGATCTGCAGCCCGAGACTCCACTACGATGGTATGAACGGCAAAG
GTACTTTGGACACCACCCCGTTTTCTATTTCCAGACGCGGCCATTCAGGAACGACGCGAATCGTAATC
TACCTGCCGCGTACGCGGATCGTACATGCGTTTTCCAGCGTTCGAAATAGAGTTTGTCGTTCCGCTGA
CCAGGTTTCACGGCTACTGCGATAAGCATATTCTACTGGGGGACTGTAAAGGAATTGTATCCACTCTG
CTTCCTGTCAATTGGCCATGTGCATCTCTGGTTGTTCAGGGTACATTAATAACACACAGCATTAAGTA
GATAAGTCTCCGATATAAGCGAACAGAACGTTGGTCAGAGACTATGGTTCAATTGTAGTGGTATAATG
TTTCCCAGTGCATAAGGAACTCAGGGACACTACTAGCATGGGCGAGGAGAGACCCTTGGGTGGCAACA
CGCAATAAGTGTTACCTAGTACATCAGCACGAGAGAGGGTTTGACCATGTGTTTTTAGACGACCCCGG
TCGCATTGAACCTGGCATTCAGAATGACGTGCTCTCAAGAATCGGGGTCATCCCCCTTAGTGTCACCT
GCTTCGCGGGTCAGCGTATGGTCATAAACAT
>GIRAN05
GTGTAGCCCAGTTAGACACTTCTAAGTCCTTGGTAACAGACATGCAGTCCCCTCGGCAGAGCCCCGCC
CCCCGGTGGTACCGGGTTGCTAAGAGGTCCACCACGGGTCGCACGACAACATCAAGGCCTCATGCATG
ACCCAACACGGGGGCGTCCCGGCGCGGCGACCTTGAAGGCCGGGGACAAGTATGTCCCCCCGGGAGGG
GCGCCACAGGGAATCCGGAGTGTTGATCTGCAGCCCGAGACTCCACTACGATGGTATGAACGGCAAAG
GTACTTTGGACACCACCCCGTTTTCTATTTCCAGACGCGGCCATTCAGGAACGACGCGAATCGTAATC
TACCTGCCGCGTACGCGGATCGTACATGCGTTTTCCAGCGTTCGAAATAGAGTTTGTCGTTCCGCTGA
CCAGGTTTCACGGCTACTGCGATAAGCATATTCTACTGGGGGACTGTAAAGGAATTGTATCCACTCTG
CTTCCTGTCAATTGGCCATGTGCATCTCTGGTTGTTCAGGGTACATTAATAACACACAGCATTAAGTA
GATAAGTCTCCGATATAAGCGAACAGAACGTTGGTCAAAGACTGTGGTTCAATTGTAGTGGTATAACG
TTTCCCAGTGCATAAGGAACTCAGGGACACTACTAGCATGGGCGAGGAGAGACCCTTGGGTGGCAACA
CGCAATAAGTGTTACCTAGTACACCAGCACGATAGAGGGTTTGACCATGTGTTTTTAGACGACCCCGG
TCGCATTGAACCTGGCATTCAGAATGACGTGCTCTCAAGAGTCGGGGTCATCCCCCTTAGTGTCACCT
GCTTCGCGGGTCAGCGTATGGTCATAAACAT
>GIRAN06
GTGTAGCCCAGTTAGACACTTCTAAGTCCTTGGTAGCAAACATGCAGTCCCCTCGGCAGAGCCCCGCC
CCCCGATGGTACCGGGTTGCTAAGAGGTCCACCACGAGTCGCACGACAACAGCAAGGCCTCGTGCATG
ACCCAACACGGGGGCGTCCCGGCGCGGCGACCTTGAAGTCCGGGAACAAGTATGTCTCCCCGGGAGGG
GCGCCACAGGGAATCCGGAGTGTTGATCTGCAGCCCGAGACTCCACTACGATGGTATGAACGGCAAAG
GTACTTTGGACACCACCCCGTTTTCTATTCCCAGACGCGGCCATTCAGGAACGACGCGAATCGTAATC
TACCTGCCGCGTACGCGGATCGTACATGCGTTTTCCAGCGTTCGGAATAGAGTTTGTCGTTCCGCTGA
CCAGGTTTCACGGCTACTGCGATAAGCATACTCCACTGGGGGACTGTAAAGGAATTGCATCCACTCTG
CTTCTTGTCAGTTGGCCATATGTATCTCTGGTTGTTCAGGGTACATTAATAACACACAGCATTAAGTA
GATAAGTCTCCGATATAGGCGAACAGAACGTTGGTAAAAGACTGTGGTTCAACTGTAGTGGTATAATG
TTTCCCAGTGCATAAGGAACTCAGGGACACTACTAGCATGGGCGAGTAGAGACCCTTGGGTGGCAACA
CGCAATAAGTGTTACCTAGTACACCAACACGATATAGGGTTTGACCATGTGTTTTTAGACGACCCCGG
TCGCATTAAACCTGGCATTCATAATGACGTGCTCTCAAGAGTCGGGGTCATCCCCCTTAGTGTCACCT
GCTTCGCGGGTCATCGTATGGTCATAAACAT
>GIRAN08
GTGTAGCCCAGTTAGACACTTCTAAGTCCTTGGTAGCAAACATGCAGTCCCCTCGGCAGAGCCCCGCC
CCCCGATGGTACCGGGTTGCTAAGAGGTCCACCACGAGTCGCACGACAACAGCAAGGCCTCGTGCATG
ACCCAACACGGGGGCGTCCCGGCGCGGCGACCTTGAAGTCCGGGAACAAGTATGTCTCCCCGGGAGGG
GCGCCACAGGGAATCCGGAGTGTTGATCTGCAGCCCGAGACTCCACTACGATGGTATGAACGGCAAAG
GTACTTTGGACACCACCCCGTTTTCTATTCCCAGACGCGGCCATTCAGGAACGACGCGAATCGTAATC
TACCTGCCGCGTACGCGGATCGTACATGCGTTTTCCAGCGTTCGGAATAGAGTTTGTCGTTCCGCTGA
CCAGGTTTCACGGCTACTGCGATAAGCATACTCCACTGGGGGACTGTAAAGGAATTGCATCCACTCTG
CTTCTTGTCAGTTGGCCATATGTATCTCTGGTTGTTCAGGGTACATTAATAACACACAGCATTAAGTA
GATAAGTCTCCGATATAGGCGAACAGAACGTTGGTAAAAGACTGTGGTTCAACTGTAGTGGTATAATG
TTTCCCAGTGCATAAGGAACTCAGGGACACTACTAGCATGGGCGAGTAGAGACCCTTGGGTGGCAACA
CGCAATAAGTGTTACCTAGTACACCAACACGATATAGGGTTTGACCATGTGTTTTTAGACGACCCCGG

TCGCATTAAACCTGGCATTCATAATGACGTGCTCTCAAGAGTCGGGGTCATCCCCCTTAGTGTCACCT
GCTTCGCGGGTCATCGTATGGTCATAAACAT
>GIRAN12
GTGTAGCCCAGTCAGACACTTCTAAGTCCTTGGTAACAGACATGCAGTCCCCTCGGCAGAGCCCCGCC
CCCCGATGGTACCGGGTTGCTAAGAGGTCCACAACGGGTCGCACGACAACATTAAGGCCTCATGCATG
ACCCAACACGGGGGCGTCCCGGCGCGGCGACCTTGAAGTCCGGGGACAAGTATGTCCCCCCGGGAGGG
GCGCCACAGGGAATCCGGAGTGTTGATCTGCAGCCCGAGACTCCACTACGATGGTATGAACGGCAAAG
GTACTTTGGACACCACCCCGTTTTCTATTTCCAGACGCGGCCATTTAGGAACGACGCGAATCGTAATC
TACCTGCCGCGTACGCGGATCGTACATGCGTTTTCCAGCGTTCGAAATAGAGTTTGTCGTTCCGCTGA
CCAGGTTTCACGGCTACTGCGATAAGCATATTCTACTGGGGGACTGTAAAGGAATTGTATCCATTCTG
CTTCCTGTCAATTGGCCATGTGCATCTCTGGTTGTTCAGGGTACATTAATAACACACAGCAGTAAGTA
GGTAAGTCTCCGATATAAGCGAACAGAACGTTTGTCAGAGACTGTGGTTCAATTGTAGTGGTATAATG
TTTCCCAGTGCATAAGGAACTCAGGGACACTACTAGCATGGGCGAGGAGAGACCCTTGGGTGGCAACA
CGCAATAAGTGTTACCTAGTACACCAGCACGAGAGAGGGTTTGACCATGTGTTTTTAGACGACCCCGG
TCGCATTGAACCTGGCATTCAGAATGACGTGCTCTCAAGAATCGGGGTCATCCCCCTTAGTGTCACCT
GCTTCGCGGGTCAGCGTATGGTCATAAACAT
>GIRAN13
GTGTAGCCCAGTTAGACACTTCTAAGTCCTTGGTAACAGACATGCAGTCCCCTCGGCAGAGCCCCGCC
CCCCGATGGTACCGGGTTGCTAAGAGGTCCACAACGGGTCGCACGACAACATTAAGGCCTCATGCATG
ACCCAACACGGGGGCGTCCCGGCGCGGCGACCTTGAAGTCCGGGGACAAGTATGTCCCCCCGGGAGGG
GCGCCACAGGGAATCCGGAGTGTTGATCTGCAGCCCGAGACTCCACTACGATGGTATGAACGGCAAAG
GTACTTTGGACACCACCCCGTTTTCTATTTCCAGACGCGGCCATTCAGGAACGACGCGAATCGTAATC
TACCTGCCGCGTACGCGGATCGTACATGCGTTTTCCAGCGTTCGAAATAGAGTTTGTCGTTCCGCTGA
CCAGGTTTCACGGCTACTGCGATAAGCATATTCTACTGGGGGACTGTAAAGGAATTGTATCCACTCTG
CTTCCTGTCAATTGGCCATGTGCATCTCTGGTTGTTCAGGGTACATTAATAACACACAGCATTAAGTA
GATAAGTCTCCGATATAAGCGAACAGAACGTTGGTCAGAGACTATGGTTCAATTGTAGTGGTATAATG
TTTCCCAGTGCATAAGGAACTCAGGGACACTACTAGCATGGGCGAGGAGAGACCCTTGGGTGGCAACA
CGCAATAAGTGTTACCTAGTACATCAGCACGAGAGAGGGTTTGACCATGTGTTTTTAGACGACCCCGG
TCGCATTGAACCTGGCATTCAGAATGACGTGCTCTCAAGAATCGGGGTCATCCCCCTTAGTGTCACCT
GCTTCGCGGGTCAGCGTATGGTCATAAACAT
>GIRAN14
GTGTAGCCCAGTCAGACACTTCTAAGTCCTTGGTAACAGACATGCAGTCCCCTCGGCAGAGCCCCGCC
CCCCGATGGTACCGGGTTGCTAAGAGGTCCACAACGGGTCGCACGACAACATTAAGGCCTCATGCATG
ACCCAACACGGGGGCGTCCCGGCGCGGCGACCTTGAAGTCCGGGGACAAGTATGTCCCCCCGGGAGGG
GCGCCACAGGGAATCCGGAGTGTTGATCTGCAGCCCGAGACTCCACTACGATGGTATGAACGGCAAAG
GTACTTTGGACACCACCCCGTTTTCTATTTCCAGACGCGGCCATTTAGGAACGACGCGAATCGTAATC
TACCTGCCGCGTACGCGGATCGTACATGCGTTTTCCAGCGTTCGAAATAGAGTTTGTCGTTCCGCTGA
CCAGGTTTCACGGCTACTGCGATAAGCATATTCTACTGGGGGACTGTAAAGGAATTGTATCCATTCTG
CTTCCTGTCAATTGGCCATGTGCATCTCTGGTTGTTCAGGGTACATTAATAACACACAGCAGTAAGTA
GGTAAGTCTCCGATATAAGCGAACAGAACGTTTGTCAGAGACTGTGGTTCAATTGTAGTGGTATAATG
TTTCCCAGTGCATAAGGAACTCAGGGACACTACTAGCATGGGCGAGGAGAGACCCTTGGGTGGCAACA
CGCAATAAGTGTTACCTAGTACACCAGCACGAGAGAGGGTTTGACCATGTGTTTTTAGACGACCCCGG
TCGCATTGAACCTGGCATTCAGAATGACGTGCTCTCAAGAATCGGGGTCATCCCCCTTAGTGTCACCT
GCTTCGCGGGTCAGCGTATGGTCATAAACAT
>GIRAN15
GTGTAGCCCAGTTAGACACTTCTAAGTCCTTGGTAACAGACATGCAGTCCCCTCGGCAGAGCCCCGCC
CCCCGATGGTACCGGGTTGCTAAGAAGTCCACAACGGGTCGCACGACAACATTAAGGCCTCATGCATG
ACCCAACACGGGGGCGTCCCGGCGCGGCGACCTTGAAGTCCGGGGACAAGTATGTCCCCCCGGGAGGG
GCGCCACAGGGAATCCGGAGTGTTGATCTGCAGCCCGAGACTCCACTACGATGGTATGAACGGCAAAG
GTACTTTGGACACCACCCCGTTTTCTATTTCCAGACGCGGCCATTCAGGAACGACGCGAATCGTAATC
TACCTGCCGCGTACGCGGATCGTACATGCGTTTTCCAGCGTTCGAAATAGAGTTTGTCGTTCCGCTGA
CCAGGTTTCACGGCTACTGCGATAAGCATATTCTACTGGGGGACTGTAAAGGAATTGTATCCATTCTG
CTTCCTGTCAATTGGCCATGTGCATCTCTGGTTGTTCAGGGTACATTAATAACACACAGCAGTAAGTA
GGTAAGTCTCCGATATAAGCTAACAGAACGTTTGTCAGAGACTGTGGTTCAATTGTAGTGGTATAATG

TTTCCCAGTGCATAAGGAACTCAGGGACACTACTAGCATGGGCGAGGAGAGACCCTTGGGTGGCAACA
CGCAATAAGTGTTACCTAGTACACCAGCACGAGAGAGGGTTTGACCATGTGTTTTCAGACGACCCCGG
TCGCATTGAACCTGGCATTCAGAATGACGTGCTCTCAAGAATCGGGGTCATCCCCCTTAGTGTCACCT
GCTTCGCGGGTCAGCGTATGGTCATAAACAT
>GIRAN16
GTGTAGCCCAGTTAGACACTTCTAAGTCCTTGGTAACAGACATGCAGTCCCCTCGGCAGAGCCCCGCC
CCCCGATGGTACCGGGTTGCTAAGAGGTCCACAACGGGTCGCACGACAACATTAAGGCCTCATGCATG
ACCCAACACGGGGGCGTCCCGGCGCGGCGACCTTGAAGTCCGGGGACAAGTATGTCCCCCCGGGAGGG
GCGCCACAGGGAATCCGGAGTGTTGATCTGCAGCCCGAGACTCCACTACGATGGTATGAACGGCAAAG
GTACTTTGGACACCACCCCGTTTTCTATTTCCAGACGCGGCCATTCAGGAACGACGCGAATCGTAATC
TACCTGCCGCGTACGCGGATCGTACATGCGTTTTCCAGCGTTCGAAATAGAGTTTGTCGTTCCGCTGA
CCAGGTTTCACGGCTACTGCGATAAGCATATTCTACTGGGGGACTGTAAAGGAATTGTATCCACTCTG
CTTCCTGTCAATTGGCCATGTGCATCTCTGGTTGTTCAGGGTACATTAATAACACACAGCATTAAGTA
GATAAGTCTCCGATATAAGCGAACAGAACGTTGGTCAGAGACTATGGTTCAATTGTAGTGGTATAATG
TTTCCCAGTGCATAAGGAACTCAGGGACACTACTAGCATGGGCGAGGAGAGACCCTTGGGTGGCAACA
CGCAATAAGTGTTACCTAGTACATCAGCACGAGAGAGGGTTTGACCATGTGTTTTTAGACGACCCCGG
TCGCATTGAACCTGGCATTCAGAATGACGTGCTCTCAAGAATCGGGGTCATCCCCCTTAGTGTCACCT
GCTTCGCGGGTCAGCGTATGGTCATAAACAT
>GIRAN17
GTGTAGCCCAGTCAGACACTTCTAAGTCCTTGGTAACAGACATGCAGTCCCCTCGGCAGAGCCCCGCC
CCCCGATGGTACCGGGTTGCTAAGAGGTCCACAACGGGTCGCACGACAACATTAAGGCCTCATGCATG
ACCCAACACGGGGGCGTCCCGGCGCGGCGACCTTGAAGTCCGGGGACAAGTATGTCCCCCCGGGAGGG
GCGCCACAGGGAATCCGGAGTGTTGATCTGCAGCCCGAGACTCCACTACGATGGTATGAACGGCAAAG
GTACTTTGGACACCACCCCGTTTTCTATTTCCAGACGCGGCCATTTAGGAACGACGCGAATCGTAATC
TACCTGCCGCGTACGCGGATCGTACATGCGTTTTCCAGCGTTCGAAATAGAGTTTGTCGTTCCGCTGA
CCAGGTTTCACGGCTACTGCGATAAGCATATTCTACTGGGGGACTGTAAAGGAATTGTATCCATTCTG
CTTCCTGTCAATTGGCCATGTGCATCTCTGGTTGTTCAGGGTACATTAATAACACACAGCAGTAAGTA
GGTAAGTCTCCGATATAAGCGAACAGAACGTTTGTCAGAGACTGTGGTTCAATTGTAGTGGTATAATG
TTTCCCAGTGCATAAGGAACTCAGGGACACTACTAGCATGGGCGAGGAGAGACCCTTGGGTGGCAACA
CGCAATAAGTGTTACCTAGTACACCAGCACGAGAGAGGGTTTGACCATGTGTTTTTAGACGACCCCGG
TCGCATTGAACCTGGCATTCAGAATGACGTGCTCTCAAGAATCGGGGTCATCCCCCTTAGTGTCACCT
GCTTCGCGGGTCAGCGTATGGTCATAAACAT
>GIRAN19
GTGTAGCCCAGTTAGACACTTCTAAGTCCTTGGTAACAGACATGCAGTCCCCTCGGCAGAGCCCCGCC
CCCCGATGGTACCGGGTTGCTAAGAGGTCCACAACGGGTCGCACGACAACATTAAGGCCTCATGCATG
ACCCAACACGGGGGCGTCCCGGCGCGGCGACCTTGAAGTCCGGGGACAAGTATGTCCCCCCGGGAGGG
GCGCCACAGGGAATCCGGAGTGTTGATCTGCAGCCCGAGACTCCACTACGATGGTATGAACGGCAAAG
GTACTTTGGACACCACCCCGTTTTCTATTTCCAGACGCGGCCATTCAGGAACGACGCGAATCGTAATC
TACCTGCCGCGTACGCGGATCGTACATGCGTTTTCCAGCGTTCGAAATAGAGTTTGTCGTTCCGCTGA
CCAGGTTTCACGGCTACTGCGATAAGCATATTCTACTGGGGGACTGTAAAGGAATTGTATCCACTCTG
CTTCCTGTCAATTGGCCATGTGCATCTCTGGTTGTTCAGGGTACATTAATAACACACAGCATTAAGTA
GATAAGTCTCCGATATAAGCGAACAGAACGTTGGTCAGAGACTATGGTTCAATTGTAGTGGTATAATG
TTTCCCAGTGCATAAGGAACTCAGGGACACTACTAGCATGGGCGAGGAGAGACCCTTGGGTGGCAACA
CGCAATAAGTGTTACCTAGTACATCAGCACGAGAGAGGGTTTGACCATGTGTTTTTAGACGACCCCGG
TCGCATTGAACCTGGCATTCAGAATGACGTGCTCTCAAGAATCGGGGTCATCCCCCTTAGTGTCACCT
GCTTCGCGGGTCAGCGTATGGTCATAAACAT
>GIRAN23
GTGTAGCCCAGTCAGACACTTCTAAGTCCTTGGTAACAGACATGCAGTCCCCTCGGCAGAGCCCCGCC
CCCCGATGGTACCGGGTTGCTAAGAGGTCCACAACGGGTCGCACGACAACATTAAGGCCTCATGCATG
ACCCAACACGGGGGCGTCCCGGCGCGGCGACCTTGAAGTCCGGGGACAAGTATGTCCCCCCGGGAGGG
GCGCCACAGGGAATCCGGAGTGTTGATCTGCAGCCCGAGACTCCACTACGATGGTATGAACGGCAAAG
GTACTTTGGACACCACCCCGTTTTCTATTTCCAGACGCGGCCATTCAGGAACGACGCGAATCGTAATC
TACCTGCCGCGTACGCGGATCGTACATGCGTTTTCCAGCGTTCGAAATAGAGTTTGTCGTTCCGCTGA
CCAGGTTTCACGGCTACTGCGATAAGCATATTCTACTGGGGGACTGTAAAGGAATTGTATCCATTCTG

CTTCCTGTCAATTGGCCATGTGCATCTCTGGTTGTTCAGGGTACATTAATAACACACAGCAGTAAGTA
GGTAAGTCTCCGATATAAGCGAACAGAACGTTTGTCAGAGACTGTGGTTCAATTGTAGTGGTATAATG
TTTCCCAGTGCATAAGGAACTCAGGGACACTACTAGCATGGGCGAGGAGAGACCCTTGGGTGGCAACA
CGCAATAAGTGTTACCTAGTACACCAGCACGAGAGAGGGTTTGACCATGTGTTTTTAGACGACCCCGG
TCGCATTGAACCTGGCATTCAGAATGACGTGCTCTCAAGAATCGGGGTCATCCCCCTTAGTGTCACCT
GCTTCGCGGGTCAGCGTATGGTCATAAACAT
>GIRAN26
GTGTAGCCCAGTTAGACACTTCTAAGTCCTTGGTAACAGACATGCAGTCCCCTCGGCAGAGCCCCGCC
CCCCGATGGTACCGGGTTGCTAAGAGGTCCACAACGGGTCGCACGACAACATTAAGGCCTCATGCATG
ACCCAACACGGGGGCGTCCCGGCGCGGCGACCTTGAAGTCCGGGGACAAGTATGTCCCCCCGGGAGGG
GCGCCACAGGGAATCCGGAGTGTTGATCTGCAGCCCGAGACTCCACTACGATGGTATGAACGGCAAAG
GTACTTTGGACACCACCCCGTTTTCTATTTCCAGACGCGGCCATTCAGGAACGACGCGAATCGTAATC
TACCTGCCGCGTACGCGGATCGTACATGCGTTTTCCAGCGTTCGAAATAGAGTTTGTCGTTCCGCTGA
CCAGGTTTCACGGCTACTGCGATAAGCATATTCTACTGGGGGACTGTAAAGGAATTGTATCCACTCTG
CTTCCTGTCAATTGGCCATGTGCATCTCTGGTTGTTCAGGGTACATTAATAACACACAGCATTAAGTA
GATAAGTCTCCGATATAAGCGAACAGAACGTTGGTCAGAGACTATGGTTCAATTGTAGTGGTATAATG
TTTCCCAGTGCATAAGGAACTCAGGGACACTACTAGCATGGGCGAGGAGAGACCCTTGGGTGGCAACA
CGCAATAAGTGTTACCTAGTACATCAGCACGAGAGAGGGTTTGACCATGTGTTTTTAGACGACCCCGG
TCGCATTGAACCTGGCATTCAGAATGACGTGCTCTCAAGAATCGGGGTCATCCCCCTTAGTGTCACCT
GCTTCGCGGGTCAGCGTATGGTCATAAACAT
>GIRAN27
GTGTAGCCCAGTTAGACACTTCTAAGTCCTTGGTAACAGACATGCAGTCCCCTCGGCAGAGCCCCGCC
CCCCGATGGTACCGGGTTGCTAAGAGGTCCACAACGGGTCGCACGACAACATTAAGGCCTCATGCATG
ACCCAACACGGGGGCGTCCCGGCGCGGCGACCTTGAAGTCCGGGGACAAGTATGTCCCCCCGGGAGGG
GCGCCACAGGGAATCCGGAGTGTTGATCTGCAGCCCGAGACTCCACTACGATGGTATGAACGGCAAAG
GTACTTTGGACACCACCCCGTTTTCTATTTCCAGACGCGGCCATTCAGGAACGACGCGAATCGTAATC
TACCTGCCGCGTACGCGGATCGTACATGCGTTTTCCAGCGTTCGAAATAGAGTTTGTCGTTCCGCTGA
CCAGGTTTCACGGCTACTGCGATAAGCATATTCTACTGGGGGACTGTAAAGGAATTGTATCCACTCTG
CTTCCTGTCAATTGGCCATGTGCATCTCTGGTTGTTCAGGGTACATTAATAACACACAGCATTAAGTA
GATAAGTCTCCGATATAAGCGAACAGAACGTTGGTCAGAGACTATGGTTCAATTGTAGTGGTATAATG
TTTCCCAGTGCATAAGGAACTCAGGGACACTACTAGCATGGGCGAGGAGAGACCCTTGGGTGGCAACA
CGCAATAAGTGTTACCTAGTACATCAGCACGAGAGAGGGTTTGACCATGTGTTTTTAGACGACCCCGG
TCGCATTGAACCTGGCATTCAGAATGACGTGCTCTCAAGAATCGGGGTCATCCCCCTTAGTGTCACCT
GCTTCGCGGGTCAGCGTATGGTCATAAACAT
>GIRAN29
GTGTAGCCCAGTTAGACACTTCTAAGTCCTTGGTAACAGACATGCAGTCCCCTCGGCAGAGCCCCGCC
CCCCGATGGTACCGGGTTGCTAAGAGGTCCACAACGGGTCGCACGACAACATTAAGGCCTCATGCATG
ACCCAACACGGGGGCGTCCCGGCGCGGCGACCTTGAAGTCCGGGGACAAGTATGTCCCCCCGGGAGGG
GCGCCACAGGGAATCCGGAGTGTTGATCTGCAGCCCGAGACTCCACTACGATGGTATGAACGGCAAAG
GTACTTTGGACACCACCCCGTTTTCTATTTCCAGACGCGGCCATTCAGGAACGACGCGAATCGTAATC
TACCTGCCGCGTACGCGGATCGTACATGCGTTTTCCAGCGTTCGAAATAGAGTTTGTCGTTCCGCTGA
CCAGGTTTCACGGCTACTGCGATAAGCATATTCTACTGGGGGACTGTAAAGGAATTGTATCCACTCTG
CTTCCTGTCAATTGGCCATGTGCATCTCTGGTTGTTCAGGGTACATTAATAACACACAGCATTAAGTA
GATAAGTCTCCGATATAAGCGAACAGAACGTTGGTCAGAGACTATGGTTCAATTGTAGTGGTATAATG
TTTCCCAGTGCATAAGGAACTCAGGGACACTACTAGCATGGGCGAGGAGAGACCCTTGGGTGGCAACA
CGCAATAAGTGTTACCTAGTACATCAGCACGAGAGAGGGTTTGACCATGTGTTTTTAGACGACCCCGG
TCGCATTGAACCTGGCATTCAGAATGACGTGCTCTCAAGAATCGGGGTCATCCCCCTTAGTGTCACCT
GCTTCGCGGGTCAGCGTATGGTCATAAACAT
>GIRAN30
GTGTAGCCCAGTTAGACACTTCTAAGTCCTTGGTAACAGACATGCAGTCCCCTCGGCAGAGCCCCGCC
CCCCGATGGTACCGGGTTGCTAAGAGGTCCACAACGGGTCGCACGACAACATTAAGGCCTCATGCATG
ACCCAACACGGGGGCGTCCCGGCGCGGCGACCTTGAAGTCCGGGGACAAGTATGTCCCCCCGGGAGGG
GCGCCACAGGGAATCCGGAGTGTTGATCTGCAGCCCGAGACTCCACTACGATGGTATGAACGGCAAAG
GTACTTTGGACACCACCCCGTTTTCTATTTCCAGACGCGGCCATTCAGGAACGACGCGAATCGTAATC

TACCTGCCGCGTACGCGGATCGTACATGCGTTTTCCAGCGTTCGAAATAGAGTTTGTCGTTCCGCTGA
CCAGGTTTCACGGCTACTGCGATAAGCATATTCTACTGGGGGACTGTAAAGGAATTGTATCCACTCTG
CTTCCTGTCAATTGGCCATGTGCATCTCTGGTTGTTCAGGGTACATTAATAACACACAGCATTAAGTA
GATAAGTCTCCGATATAAGCGAACAGAACGTTGGTCAGAGACTATGGTTCAATTGTAGTGGTATAATG
TTTCCCAGTGCATAAGGAACTCAGGGACACTACTAGCATGGGCGAGGAGAGACCCTTGGGTGGCAACA
CGCAATAAGTGTTACCTAGTACATCAGCACGAGAGAGGGTTTGACCATGTGTTTTTAGACGACCCCGG
TCGCATTGAACCTGGCATTCAGAATGACGTGCTCTCAAGAATCGGGGTCATCCCCCTTAGTGTCACCT
GCTTCGCGGGTCAGCGTATGGTCATAAACAT
>GIRAN31
GTGTAGCCCAGTTAGACACTTCTAAGTCCTTGGTAACAGACATGCAGTCCCCTCGGCAGAGCCCCGCC
CCCCGATGGTACCGGGTTGCTAAGAGGTCCACAACGGGTCGCACGACAACATTAAGGCCTCATGCATG
ACCCAACACGGGGGCGTCCCGGCGCGGCGACCTTGAAGTCCGGGGACAAGTATGTCCCCCCGGGAGGG
GCGCCACAGGGAATCCGGAGTGTTGATCTGCAGCCCGAGACTCCACTACGATGGTATGAACGGCAAAG
GTACTTTGGACACCACCCCGTTTTCTATTTCCAGACGCGGCCATTCAGGAACGACGCGAATCGTAATC
TACCTGCCGCGTACGCGGATCGTACATGCGTTTTCCAGCGTTCGAAATAGAGTTTGTCGTTCCGCTGA
CCAGGTTTCACGGCTACTGCGATAAGCATATTCTACTGGGGGACTGTAAAGGAATTGTATCCACTCTG
CTTCCTGTCAATTGGCCATGTGCATCTCTGGTTGTTCAGGGTACATTAATAACACACAGCATTAAGTA
GATAAGTCTCCGATATAAGCGAACAGAACGTTGGTCAGAGACTATGGTTCAATTGTAGTGGTATAATG
TTTCCCAGTGCATAAGGAACTCAGGGACACTACTAGCATGGGCGAGGAGAGACCCTTGGGTGGCAACA
CGCAATAAGTGTTACCTAGTACATCAGCACGAGAGAGGGTTTGACCATGTGTTTTTAGACGACCCCGG
TCGCATTGAACCTGGCATTCAGAATGACGTGCTCTCAAGAATCGGGGTCATCCCCCTTAGTGTCACCT
GCTTCGCGGGTCAGCGTATGGTCATAAACAT
>HREN03
GTGTAGCCCAGTTAGACACTTCTAAGTCCTTGGTAACAGACATGCAGTCCCCTCGGCAGAGCCCCGCC
CCCCGATGGTACCGGGTTGCTAAGAGGTCCACAACGGGTCGCACGACAACATTAAGGCCTCATGCATG
ACCCAACACGGGGGCGTCCCGGCGCGGCGACCTTGAAGTCCGGGGACAAGTATGTCCCCCCGGGAGGG
GCGCCACAGGGAATCCGGAGTGTTGATCTGCAGCCCGAGACTCCACTACGATGGTATGAACGGCAAAG
GTACTTTGGACACCACCCCGTTTTCTATTTCCAGACGCGGCCATTCAGGAACGACGCGAATCGTAATC
TACCTGCCGCGTACGCGGATCGTACATGCGTTTTCCAGCGTTCGAAATAGAGTTTGTCGTTCCGCTGA
CCAGGTTTCACGGCTACTGCGATAAGCATATTCTACTGGGGGACTGTAAAGGAATTGTATCCACTCTG
CTTCCTGTCAATTGGCCATGTGCATCTCTGGTTGTTCAGGGTACATTAATAACACACAGCATTAAGTA
GATAAGTCTCCGATATAAGCGAACAGAACGTTGGTCAGAGACTATGGTTCAATTGTAGTGGTATAATG
TTTCCCAGTGCATAAGGAACTCAGGGACACTACTAGCATGGGCGAGGAGAGACCCTTGGGTGGCAACA
CGCAATAAGTGTTACCTAGTACATCAGCACGAGAGAGGGTTTGACCATGTGTTTTTAGACGACCCCGG
TCGCATTGAACCTGGCATTCAGAATGACGTGCTCTCAAGAATCGGGGTCATCCCCCTTAGTGTCACCT
GCTTCGCGGGTCAGCGTATGGTCATAAACAT
>HREN05
GTGTAGCCCAGTTAGACACTTCTAAGTCCTTGGTAACAGACATGCAGTCCCCTCGGCAGAGCCCCGCC
CCCCGATGGTACCGGGTTGCTAAGAGGTCCACAACGGGTCGCACGACAACATTAAGGCCTCATGCATG
ACCCAACACGGGGGCGTCCCGGCGCGGCGACCTTGAAGTCCGGGGACAAGTATGTCCCCCCGGGAGGG
GCGCCACAGGGAATCCGGAGTGTTGATCTGCAGCCCGAGACTCCACTACGATGGTATGAACGGCAAAG
GTACTTTGGACACCACCCCGTTTTCTATTTCCAGACGCGGCCATTCAGGAACGACGCGAATCGTAATC
TACCTGCCGCGTACGCGGATCGTACATGCGTTTTCCAGCGTTCGAAATAGAGTTTGTCGTTCCGCTGA
CCAGGTTTCACGGCTACTGCGATAAGCATATTCTACTGGGGGACTGTAAAGGAATTGTATCCACTCTG
CTTCCTGTCAATTGGCCATGTGCATCTCTGGTTGTTCAGGGTACATTAATAACACACAGCATTAAGTA
GATAAGTCTCCGATATAAGCGAACAGAACGTTGGTCAGAGACTATGGTTCAATTGTAGTGGTATAATG
TTTCCCAGTGCATAAGGAACTCAGGGACACTACTAGCATGGGCGAGGAGAGACCCTTGGGTGGCAACA
CGCAATAAGTGTTACCTAGTACATCAGCACGAGAGAGGGTTTGACCATGTGTTTTTAGACGACCCCGG
TCGCATTGAACCTGGCATTCAGAATGACGTGCTCTCAAGAATCGGGGTCATCCCCCTTAGTGTCACCT
GCTTCGCGGGTCAGCGTATGGTCATAAACAT
>HREN07
GTGTAGCCCAGTTAGACACTTCTAAGTCCTTGGTAACAGACATGCAGTCCCCTCGGCAGAGCCCCGCC
CCCCGATGGTACCGGGTTGCTAAGAGGTCCACAACGGGTCGCACGACAACATTAAGGCCTCATGCATG
ACCCAACACGGGGGCGTCCCGGCGCGGCGACCTTGAAGTCCGGGGACAAGTATGTCCCCCCGGGAGGG

GCGCCACAGGGAATCCGGAGTGTTGATCTGCAGCCCGAGACTCCACTACGATGGTATGAACGGCAAAG
GTACTTTGGACACCACCCCGTTTTCTATTTCCAGACGCGGCCATTCAGGAACGACGCGAATCGTAATC
TACCTGCCGCGTACGCGGATCGTACATGCGTTTTCCAGCGTTCGAAATAGAGTTTGTCGTTCCGCTGA
CCAGGTTTCACGGCTACTGCGATAAGCATATTCTACTGGGGGACTGTAAAGGAATTGTATCCACTCTG
CTTCCTGTCAATTGGCCATGTGCATCTCTGGTTGTTCAGGGTACATTAATAACACACAGCATTAAGTA
GATAAGTCTCCGATATAAGCGAACAGAACGTTGGTCAGAGACTATGGTTCAATTGTAGTGGTATAATG
TTTCCCAGTGCATAAGGAACTCAGGGACACTACTAGCATGGGCGAGGAGAGACCCTTGGGTGGCAACA
CGCAATAAGTGTTACCTAGTACATCAGCACGAGAGAGGGTTTGACCATGTGTTTTTAGACGACCCCGG
TCGCATTGAACCTGGCATTCAGAATGACGTGCTCTCAAGAATCGGGGTCATCCCCCTTAGTGTCACCT
GCTTCGCGGGTCAGCGTATGGTCATAAACAT
>HREN08
GTGTAGCCCAGTTAGACACTTCTAAGTCCTTGGTAACAGACATGCAGTCCCCTCGGCAGAGCCCCGCC
CCCCGATGGTACCGGGTTGCTAAGAGGTCCACAACGGGTCGCACGACAACATTAAGGCCTCATGCATG
ACCCAACACGGGGGCGTCCCGGCGCGGCGACCTTGAAGTCCGGGGACAAGTATGTCCCCCCGGGAGGG
GCGCCACAGGGAATCCGGAGTGTTGATCTGCAGCCCGAGACTCCACTACGATGGTATGAACGGCAAAG
GTACTTTGGACACCACCCCGTTTTCTATTTCCAGACGCGGCCATTCAGGAACGACGCGAATCGTAATC
TACCTGCCGCGTACGCGGATCGTACATGCGTTTTCCAGCGTTCGAAATAGAGTTTGTCGTTCCGCTGA
CCAGGTTTCACGGCTACTGCGATAAGCATATTCTACTGGGGGACTGTAAAGGAATTGTATCCACTCTG
CTTCCTGTCAATTGGCCATGTGCATCTCTGGTTGTTCAGGGTACATTAATAACACACAGCATTAAGTA
GATAAGTCTCCGATATAAGCGAACAGAACGTTGGTCAGAGACTATGGTTCAATTGTAGTGGTATAATG
TTTCCCAGTGCATAAGGAACTCAGGGACACTACTAGCATGGGCGAGGAGAGACCCTTGGGTGGCAACA
CGCAATAAGTGTTACCTAGTACATCAGCACGAGAGAGGGTTTGACCATGTGTTTTTAGACGACCCCGG
TCGCATTGAACCTGGCATTCAGAATGACGTGCTCTCAAGAATCGGGGTCATCCCCCTTAGTGTCACCT
GCTTCGCGGGTCAGCGTATGGTCATAAACAT
>HREN09
GTGTAGCCCAGTTAGACACTTCTAAGTCCTTGGTAACAGACATGCAGTCCCCTCGGCAGAGCCCCGCC
CCCCGATGGTACCGGGTTGCTAAGAGGTCCACAACGGGTCGCACGACAACATTAAGGCCTCATGCATG
ACCCAACACGGGGGCGTCCCGGCGCGGCGACCTTGAAGTCCGGGGACAAGTATGTCCCCCCGGGAGGG
GCGCCACAGGGAATCCGGAGTGTTGATCTGCAGCCCGAGACTCCACTACGATGGTATGAACGGCAAAG
GTACTTTGGACACCACCCCGTTTTCTATTTCCAGACGCGGCCATTCAGGAACGACGCGAATCGTAATC
TACCTGCCGCGTACGCGGATCGTACATGCGTTTTCCAGCGTTCGAAATAGAGTTTGTCGTTCCGCTGA
CCAGGTTTCACGGCTACTGCGATAAGCATATTCTACTGGGGGACTGTAAAGGAATTGTATCCACTCTG
CTTCCTGTCAATTGGCCATGTGCATCTCTGGTTGTTCAGGGTACATTAATAACACACAGCATTAAGTA
GATAAGTCTCCGATATAAGCGAACAGAACGTTGGTCAGAGACTATGGTTCAATTGTAGTGGTATAATG
TTTCCCAGTGCATAAGGAACTCAGGGACACTACTAGCATGGGCGAGGAGAGACCCTTGGGTGGCAACA
CGCAATAAGTGTTACCTAGTACATCAGCACGAGAGAGGGTTTGACCATGTGTTTTTAGACGACCCCGG
TCGCATTGAACCTGGCATTCAGAATGACGTGCTCTCAAGAATCGGGGTCATCCCCCTTAGTGTCACCT
GCTTCGCGGGTCAGCGTATGGTCATAAACAT
>HREN11
GTGTAGCCCAGTTAGACACTTCTAAGTCCTTGGTAACAGACATGCAGTCCCCTCGGCAGAGCCCCGCC
CCCCGATGGTACCGGGTTGCTAAGAGGTCCACAACGGGTCGCACGACAACATTAAGGCCTCATGCATG
ACCCAACACGGGGGCGTCCCGGCGCGGCGACCTTGAAGTCCGGGGACAAGTATGTCCCCCCGGGAGGG
GCGCCACAGGGAATCCGGAGTGTTGATCTGCAGCCCGAGACTCCACTACGATGGTATGAACGGCAAAG
GTACTTTGGACACCACCCCGTTTTCTATTTCCAGACGCGGCCATTCAGGAACGACGCGAATCGTAATC
TACCTGCCGCGTACGCGGATCGTACATGCGTTTTCCAGCGTTCGAAATAGAGTTTGTCGTTCCGCTGA
CCAGGTTTCACGGCTACTGCGATAAGCATATTCTACTGGGGGACTGTAAAGGAATTGTATCCACTCTG
CTTCCTGTCAATTGGCCATGTGCATCTCTGGTTGTTCAGGGTACATTAATAACACACAGCATTAAGTA
GATAAGTCTCCGATATAAGCGAACAGAACGTTGGTCAGAGACTATGGTTCAATTGTAGTGGTATAATG
TTTCCCAGTGCATAAGGAACTCAGGGACACTACTAGCATGGGCGAGGAGAGACCCTTGGGTGGCAACA
CGCAATAAGTGTTACCTAGTACATCAGCACGAGAGAGGGTTTGACCATGTGTTTTTAGACGACCCCGG
TCGCATTGAACCTGGCATTCAGAATGACGTGCTCTCAAGAATCGGGGTCATCCCCCTTAGTGTCACCT
GCTTCGCGGGTCAGCGTATGGTCATAAACAT
>HREN12
GTGTAGCCCAGTTAGACACTTCTAAGTCCTTGGTAACAGACATGCAGTCCCCTCGGCAGAGCCCCGCC

CCCCGATGGTACCGGGTTGCTAAGAGGTCCACAACGGGTCGCACGACAACATTAAGGCCTCATGCATG
ACCCAACACGGGGGCGTCCCGGCGCGGCGACCTTGAAGTCCGGGGACAAGTATGTCCCCCCGGGAGGG
GCGCCACAGGGAATCCGGAGTGTTGATCTGCAGCCCGAGACTCCACTACGATGGTATGAACGGCAAAG
GTACTTTGGACACCACCCCGTTTTCTATTTCCAGACGCGGCCATTCAGGAACGACGCGAATCGTAATC
TACCTGCCGCGTACGCGGATCGTACATGCGTTTTCCAGCGTTCGAAATAGAGTTTGTCGTTCCGCTGA
CCAGGTTTCACGGCTACTGCGATAAGCATATTCTACTGGGGGACTGTAAAGGAATTGTATCCACTCTG
CTTCCTGTCAATTGGCCATGTGCATCTCTGGTTGTTCAGGGTACATTAATAACACACAGCATTAAGTA
GATAAGTCTCCGATATAAGCGAACAGAACGTTGGTCAGAGACTATGGTTCAATTGTAGTGGTATAATG
TTTCCCAGTGCATAAGGAACTCAGGGACACTACTAGCATGGGCGAGGAGAGACCCTTGGGTGGCAACA
CGCAATAAGTGTTACCTAGTACATCAGCACGAGAGAGGGTTTGACCATGTGTTTTTAGACGACCCCGG
TCGCATTGAACCTGGCATTCAGAATGACGTGCTCTCAAGAATCGGGGTCATCCCCCTTAGTGTCACCT
GCTTCGCGGGTCAGCGTATGGTCATAAACAT
>HREN13
GTGTAGCCCAGTTAGACACTTCTAAGTCCTTGGTAACAGACATGCAGTCCCCTCGGCAGAGCCCCGCC
CCCCGATGGTACCGGGTTGCTAAGAGGTCCACAACGGGTCGCACGACAACATTAAGGCCTCATGCATG
ACCCAACACGGGGGCGTCCCGGCGCGGCGACCTTGAAGTCCGGGGACAAGTATGTCCCCCCGGGAGGG
GCGCCACAGGGAATCCGGAGTGTTGATCTGCAGCCCGAGACTCCACTACGATGGTATGAACGGCAAAG
GTACTTTGGACACCACCCCGTTTTCTATTTCCAGACGCGGCCATTCAGGAACGACGCGAATCGTAATC
TACCTGCCGCGTACGCGGATCGTACATGCGTTTTCCAGCGTTCGAAATAGAGTTTGTCGTTCCGCTGA
CCAGGTTTCACGGCTACTGCGATAAGCATATTCTACTGGGGGACTGTAAAGGAATTGTATCCACTCTG
CTTCCTGTCAATTGGCCATGTGCATCTCTGGTTGTTCAGGGTACATTAATAACACACAGCATTAAGTA
GATAAGTCTCCGATATAAGCGAACAGAACGTTGGTCAGAGACTATGGTTCAATTGTAGTGGTATAATG
TTTCCCAGTGCATAAGGAACTCAGGGACACTACTAGCATGGGCGAGGAGAGACCCTTGGGTGGCAACA
CGCAATAAGTGTTACCTAGTACATCAGCACGAGAGAGGGTTTGACCATGTGTTTTTAGACGACCCCGG
TCGCATTGAACCTGGCATTCAGAATGACGTGCTCTCAAGAATCGGGGTCATCCCCCTTAGTGTCACCT
GCTTCGCGGGTCAGCGTATGGTCATAAACAT
>HREN15
GTGTAGCCCAGTTAGACACTTCTAAGTCCTTGGTAACAGACATGCAGTCCCCTCGGCAGAGCCCCGCC
CCCCGATGGTACCGGGTTGCTAAGAGGTCCACAACGGGTCGCACGACAACATTAAGGCCTCATGCATG
ACCCAACACGGGGGCGTCCCGGCGCGGCGACCTTGNAGTCCGGGGACAAGTATGTCCCCCCGGGAGGG
GCGCCACAGGGAATCCGGAGTGTTGATCTGCAGCCCGAGACTCCACTACGATGGTATGAACGGCAAAG
GTACTTTNGACACCACCCCGTTTTCTATTTCCAGACGCGGCCATTCAGGAACGACGCGAATCGTAATC
TACCTGCCGCGTACGCGGATCGTACATGCGTTTTCCAGCGTTCGAAATAGAGTTTGTCGTTCCGCTGA
CCAGGTTTCACGGCTACTGCGATAAGCATATTCTACTGGGGGACTGTNAAGGAATTGTATCCACTCTG
CTTCCTGTCAATTGGCCATGTGCATCTCTGGTTGTTCAGGGTACATTAATAACACACAGCATTAAGTA
GATAAGTCTCCGATATAAGCGAACAGAACGTTGGTCAGAGACTATGGTTCAATTGTAGTGGTATAATG
TTTCCCAGTGCATAAGGAACTCAGGGACACTACTAGCATGGGCGAGGAGAGACCCTTGGGTGGCAACA
CGCAATAAGTGTTACCTAGTACATCAGCACGAGAGAGGGTTTGACCATGTGTTTTTAGACGACCCCGG
TCGCATTGAACCTGGCATTCAGAATGACGTGCTCTCAAGAATCGGGGTCATCCCCCTTAGTGTCACCT
GCTTCGCGGGTCAGCGTATGGTCATAAACAT
>HREN16
GTGTAGCCCAGTTAGACACTTCTAAGTCCTTGGTAACAGACATGCAGTCCCCTCGGCAGAGCCCCGCC
CCCCGATGGTACCGGGTTGCTAAGAGGTCCACAACGGGTCGCACGACAACATTAAGGCCTCATGCATG
ACCCAACACGGGGGCGTCCCGGCGCGGCGACCTTGAAGTCCGGGGACAAGTATGTCCCCCCGGGAGGG
GCGCCACAGGGAATCCGGAGTGTTGATCTGCAGCCCGAGACTCCACTACGATGGTATGAACGGCAAAG
GTACTTTGGACACCACCCCGTTTTCTATTTCCAGACGCGGCCATTCAGGAACGACGCGAATCGTAATC
TACCTGCCGCGTACGCGGATCGTACATGCGTTTTCCAGCGTTCGAAATAGAGTTTGTCGTTCCGCTGA
CCAGGTTTCACGGCTACTGCGATAAGCATATTCTACTGGGGGACTGTAAAGGAATTGTATCCACTCTG
CTTCCTGTCAATTGGCCATGTGCATCTCTGGTTGTTCAGGGTACATTAATAACACACAGCATTAAGTA
GATAAGTCTCCGATATAAGCGAACAGAACGTTGGTCAGAGACTATGGTTCAATTGTAGTGGTATAATG
TTTCCCAGTGCATAAGGAACTCAGGGACACTACTAGCATGGGCGAGGAGAGACCCTTGGGTGGCAACA
CGCAATAAGTGTTACCTAGTACATCAGCACGAGAGAGGGTTTGACCATGTGTTTTTAGACGACCCCGG
TCGCATTGAACCTGGCATTCAGAATGACGTGCTCTCAAGAATCGGGGTCATCCCCCTTAGTGTCACCT
GCTTCGCGGGTCAGCGTATGGTCATAAACAT

>HREN17
GTGTAGCCCAGTTAGACACTTCTAAGTCCTTGGTAACAGACATGCAGTCCCCTCGGCAGAGCCCCGCC
CCCCGATGGTACCGGGTTGCTAAGAGGTCCACAACGGGTCGCACGACAACATTAAGGCCTCATGCATG
ACCCAACACGGGGGCGTCCCGGCGCGGCGACCTTGAAGTCCGGGGACAAGTATGTCCCCCCGGGAGGG
GCGCCACAGGGAATCCGGAGTGTTGATCTGCAGCCCGAGACTCCACTACGATGGTATGAACGGCAAAG
GTACTTTGGACACCACCCCGTTTTCTATTTCCAGACGCGGCCATTCAGGAACGACGCGAATCGTAATC
TACCTGCCGCGTACGCGGATCGTACATGCGTTTTCCAGCGTTCGAAATAGAGTTTGTCGTTCCGCTGA
CCAGGTTTCACGGCTACTGCGATAAGCATATTCTACTGGGGGACTGTAAAGGAATTGTATCCACTCTG
CTTCCTGTCAATTGGCCATGTGCATCTCTGGTTGTTCAGGGTACATTAATAACACACAGCATTAAGTA
GATAAGTCTCCGATATAAGCGAACAGAACGTTGGTCAGAGACTATGGTTCAATTGTAGTGGTATAATG
TTTCCCAGTGCATAAGGAACTCAGGGACACTACTAGCATGGGCGAGGAGAGACCCTTGGGTGGCAACA
CGCAATAAGTGTTACCTAGTACATCAGCACGAGAGAGGGTTTGACCATGTGTTTTTAGACGACCCCGG
TCGCATTGAACCTGGCATTCAGAATGACGTGCTCTCAAGAATCGGGGTCATCCCCCTTAGTGTCACCT
GCTTCGCGGGTCAGCGTATGGTCATAAACAT
>HREN18
GTGTAGCCCAGTTAGACACTTCTAAGTCCTTGGTAACAGACATGCAGTCCCCTCGGCAGAGCCCCGCC
CCCCGATGGTACCGGGTTGCTAAGAGGTCCACAACGGGTCGCACGACAACATTAAGGCCTCATGCATG
ACCCAACACGGGGGCGTCCCGGCGCGGCGACCTTGAAGTCCGGGGACAAGTATGTCCCCCCGGGAGGG
GCGCCACAGGGAATCCGGAGTGTTGATCTGCAGCCCGAGACTCCACTACGATGGTATGAACGGCAAAG
GTACTTTGGACACCACCCCGTTTTCTATTTCCAGACGCGGCCATTCAGGAACGACGCGAATCGTAATC
TACCTGCCGCGTACGCGGATCGTACATGCGTTTTCCAGCGTTCGAAATAGAGTTTGTCGTTCCGCTGA
CCAGGTTTCACGGCTACTGCGATAAGCATATTCTACTGGGGGACTGTAAAGGAATTGTATCCACTCTG
CTTCCTGTCAATTGGCCATGTGCATCTCTGGTTGTTCAGGGTACATTAATAACACACAGCATTAAGTA
GATAAGTCTCCGATATAAGCGAACAGAACGTTGGTCAGAGACTATGGTTCAATTGTAGTGGTATAATG
TTTCCCAGTGCATAAGGAACTCAGGGACACTACTAGCATGGGCGAGGAGAGACCCTTGGGTGGCAACA
CGCAATAAGTGTTACCTAGTACATCAGCACGAGAGAGGGTTTGACCATGTGTTTTTAGACGACCCCGG
TCGCATTGAACCTGGCATTCAGAATGACGTGCTCTCAAGAATCGGGGTCATCCCCCTTAGTGTCACCT
GCTTCGCGGGTCAGCGTATGGTCATAAACAT
>HREN19
GTGTAGCCCAGTTAGACACTTCTAAGTCCTTGGTAGCAAACATGCAGTCCCCTCGGCAGAGCCCCGCC
CCCCGATGGTACCGGGTTGCTAAGAGGTCCACCACGAGTCGCACGACAACAGCAAGGCCTCGTGCATG
ACCCAACACGGGGGCGTCCCGGCGCGGCGACCTTGAAGTCCGGGAACAAGTATGTCTCCCCGGGAGGG
GCGCCACAGGGAATCCGGAGTGTTGATCTGCAGCCCGAGACTCCACTACGATGGTATGAACGGCAAAG
GTACTTTGGACACCACCCCGTTTTCTATTCCCAGACGCGGCCATTCAGGAACGACGCGAATCGTAATC
TACCTGCCGCGTACGCGGATCGTACATGCGTTTTCCAGCGTTCGGAATAGAGTTTGTCGTTCCGCTGA
CCAGGTTTCACGGCTACTGCGATAAGCATACTCCACTGGGGGACTGTAAAGGAATTGCATCCACTCTG
CTTCTTGTCAGTTGGCCATATGTATCTCTGGTTGTTCAGGGTACATTAATAACACACAGCATTAAGTA
GATAAGTCTCCGATATAGGCGAACAGAACGTTGGTAAAAGACTGTGGTTCAACTGTAGTGGTATAATG
TTTCCCAGTGCATAAGGAACTCAGGGACACTACTAGCATGGGCGAGTAGAGACCCTTGGGTGGCAACA
CGCAATAAGTGTTACCTAGTACACCAACACGATATAGGGTTTGACCATGTGTTTTTAGACGACCCCGG
TCGCATTAAACCTGGCATTCATAATGACGTGCTCTCAAGAGTCGGGGTCATCCCCCTTAGTGTCACCT
GCTTCGCGGGTCATCGTATGGTCATAAACAT
>HREN20
GTGTAGCCCAGTTAGACACTTCTAAGTCCTTGGTAACAGACATGCAGTCCCCTCGGCAGAGCCCCGCC
CCCCGATGGTACCGGGTTGCTAAGAGGTCCACAACGGGTCGCACGACAACATTAAGGCCTCATGCATG
ACCCAACACGGGGGCGTCCCGGCGCGGCGACCTTGAAGTCCGGGGACAAGTATGTCCCCCCGGGAGGG
GCGCCACAGGGAATCCGGAGTGTTGATCTGCAGCCCGAGACTCCACTACGATGGTATGAACGGCAAAG
GTACTTTGGACACCACCCCGTTTTCTATTTCCAGACGCGGCCATTCAGGAACGACGCGAATCGTAATC
TACCTGCCGCGTACGCGGATCGTACATGCGTTTTCCAGCGTTCGAAATAGAGTTTGTCGTTCCGCTGA
CCAGGTTTCACGGCTACTGCGATAAGCATATTCTACTGGGGGACTGTAAAGGAATTGTATCCACTCTG
CTTCCTGTCAATTGGCCATGTGCATCTCTGGTTGTTCAGGGTACATTAATAACACACAGCATTAAGTA
GATAAGTCTCCGATATAAGCGAACAGAACGTTGGTCAGAGACTATGGTTCAATTGTAGTGGTATAATG
TTTCCCAGTGCATAAGGAACTCAGGGACACTACTAGCATGGGCGAGGAGAGACCCTTGGGTGGCAACA
CGCAATAAGTGTTACCTAGTACATCAGCACGAGAGAGGGTTTGACCATGTGTTTTTAGACGACCCCGG

TCGCATTGAACCTGGCATTCAGAATGACGTGCTCTCAAGAATCGGGGTCATCCCCCTTAGTGTCACCT
GCTTCGCGGGTCAGCGTATGGTCATAAACAT
>HREN22
GTGTAGCCCAGTTAGACACTTCTAAGTCCTTGGTAACAGACATGCAGTCCCCTCGGCAGAGCCCCGCC
CCCCGATGGTACCGGGTTGCTAAGAGGTCCACAACGGGTCGCACGACAACATTAAGGCCTCATGCATG
ACCCAACACGGGGGCGTCCCGGCGCGGCGACCTTGAAGTCCGGGGACAAGTATGTCCCCCCGGGAGGG
GCGCCACAGGGAATCCGGAGTGTTGATCTGCAGCCCGAGACTCCACTACGATGGTATGAACGGCAAAG
GTACTTTGGACACCACCCCGTTTTCTATTTCCAGACGCGGCCATTCAGGAACGACGCGAATCGTAATC
TACCTGCCGCGTACGCGGATCGTACATGCGTTTTCCAGCGTTCGAAATAGAGTTTGTCGTTCCGCTGA
CCAGGTTTCACGGCTACTGCGATAAGCATATTCTACTGGGGGACTGTAAAGGAATTGTATCCACTCTG
CTTCCTGTCAATTGGCCATGTGCATCTCTGGTTGTTCAGGGTACATTAATAACACACAGCATTAAGTA
GATAAGTCTCCGATATAAGCGAACAGAACGTTGGTCAGAGACTATGGTTCAATTGTAGTGGTATAATG
TTTCCCAGTGCATAAGGAACTCAGGGACACTACTAGCATGGGCGAGGAGAGACCCTTGGGTGGCAACA
CGCAATAAGTGTTACCTAGTACATCAGCACGAGAGAGGGTTTGACCATGTGTTTTTAGACGACCCCGG
TCGCATTGAACCTGGCATTCAGAATGACGTGCTCTCAAGAATCGGGGTCATCCCCCTTAGTGTCACCT
GCTTCGCGGGTCAGCGTATGGTCATAAACAT
>HREN23
GTGTAGCCCAGTTAGACACTTCTAAGTCCTTGGTAACAGACATGCAGTCCCCTCGGCAGAGCCCCGCC
CCCCGATGGTACCGGGTTGCTAAGAGGTCCACAACGGGTCGCACGACAACATTAAGGCCTCATGCATG
ACCCAACACGGGGGCGTCCCGGCGCGGCGACCTTGAAGTCCGGGGACAAGTATGTCCCCCCGGGAGGG
GCGCCACAGGGAATCCGGAGTGTTGATCTGCAGCCCGAGACTCCACTACGATGGTATGAACGGCAAAG
GTACTTTGGACACCACCCCGTTTTCTATTTCCAGACGCGGCCATTCAGGAACGACGCGAATCGTAATC
TACCTGCCGCGTACGCGGATCGTACATGCGTTTTCCAGCGTTCGAAATAGAGTTTGTCGTTCCGCTGA
CCAGGTTTCACGGCTACTGCGATAAGCATATTCTACTGGGGGACTGTAAAGGAATTGTATCCACTCTG
CTTCCTGTCAATTGGCCATGTGCATCTCTGGTTGTTCAGGGTACATTAATAACACACAGCATTAAGTA
GATAAGTCTCCGATATAAGCGAACAGAACGTTGGTCAGAGACTATGGTTCAATTGTAGTGGTATAATG
TTTCCCAGTGCATAAGGAACTCAGGGACACTACTAGCATGGGCGAGGAGAGACCCTTGGGTGGCAACA
CGCAATAAGTGTTACCTAGTACATCAGCACGAGAGAGGGTTTGACCATGTGTTTTTAGACGACCCCGG
TCGCATTGAACCTGGCATTCAGAATGACGTGCTCTCAAGAATCGGGGTCATCCCCCTTAGTGTCACCT
GCTTCGCGGGTCAGCGTATGGTCATAAACAT
>HREN24
GTGTAGCCCAGTTAGACACTTCTAAGTCCTTGGTAACAGACATGCAGTCCCCTCGGCAGAGCCCCGCC
CCCCGATGGTACCGGGTTGCTAAGAGGTCCACAACGGGTCGCACGACAACATTAAGGCCTCATGCATG
ACCCAACACGGGGGCGTCCCGGCGCGGCGACCTTGAAGTCCGGGGACAAGTATGTCCCCCCGGGAGGG
GCGCCACAGGGAATCCGGAGTGTTGATCTGCAGCCCGAGACTCCACTACGATGGTATGAACGGCAAAG
GTACTTTGGACACCACCCCGTTTTCTATTTCCAGACGCGGCCATTCAGGAACGACGCGAATCGTAATC
TACCTGCCGCGTACGCGGATCGTACATGCGTTTTCCAGCGTTCGAAATAGAGTTTGTCGTTCCGCTGA
CCAGGTTTCACGGCTACTGCGATAAGCATATTCTACTGGGGGACTGTAAAGGAATTGTATCCACTCTG
CTTCCTGTCAATTGGCCATGTGCATCTCTGGTTGTTCAGGGTACATTAATAACACACAGCATTAAGTA
GATAAGTCTCCGATATAAGCGAACAGAACGTTGGTCAGAGACTATGGTTCAATTGTAGTGGTATAATG
TTTCCCAGTGCATAAGGAACTCAGGGACACTACTAGCATGGGCGAGGAGAGACCCTTGGGTGGCAACA
CGCAATAAGTGTTACCTAGTACATCAGCACGAGAGAGGGTTTGACCATGTGTTTTTAGACGACCCCGG
TCGCATTGAACCTGGCATTCAGAATGACGTGCTCTCAAGAATCGGGGTCATCCCCCTTAGTGTCACCT
GCTTCGCGGGTCAGCGTATGGTCATAAACAT
>HREN25
GTGTAGCCCAGTTAGACACTTCTAAGTCCTTGGTAACAGACATGCAGTCCCCTCGGCAGAGCCCCGCC
CCCCGATGGTACCGGGTTGCTAAGAGGTCCACAACGGGTCGCACGACAACATTAAGGCCTCATGCATG
ACCCAACACGGGGGCGTCCCGGCGCGGCGACCTTGAAGTCCGGGGACAAGTATGTCCCCCCGGGAGGG
GCGCCACAGGGAATCCGGAGTGTTGATCTGCAGCCCGAGACTCCACTACGATGGTATGAACGGCAAAG
GTACTTTGGACACCACCCCGTTTTCTATTTCCAGACGCGGCCATTCAGGAACGACGCGAATCGTAATC
TACCTGCCGCGTACGCGGATCGTACATGCGTTTTCCAGCGTTCGAAATAGAGTTTGTCGTTCCGCTGA
CCAGGTTTCACGGCTACTGCGATAAGCATATTCTACTGGGGGACTGTAAAGGAATTGTATCCACTCTG
CTTCCTGTCAATTGGCCATGTGCATCTCTGGTTGTTCAGGGTACATTAATAACACACAGCATTAAGTA
GATAAGTCTCCGATATAAGCGAACAGAACGTTGGTCAGAGACTATGGTTCAATTGTAGTGGTATAATG

TTTCCCAGTGCATAAGGAACTCAGGGACACTACTAGCATGGGCGAGGAGAGACCCTTGGGTGGCAACA
CGCAATAAGTGTTACCTAGTACATCAGCACGAGAGAGGGTTTGACCATGTGTTTTTAGACGACCCCGG
TCGCATTGAACCTGGCATTCAGAATGACGTGCTCTCAAGAATCGGGGTCATCCCCCTTAGTGTCACCT
GCTTCGCGGGTCAGCGTATGGTCATAAACAT
>HREN26
GTGTAGCCCAGTTAGACACTTCTAAGTCCTTGGTAACAGACATGCAGTCCCCTCGGCAGAGCCCCGCC
CCCCGATGGTACCGGGTTGCTAAGAGGTCCACAACGGGTCGCACGACAACATTAAGGCCTCATGCATG
ACCCAACACGGGGGCGTCCCGGCGCGGCGACCTTGAAGTCCGGGGACAAGTATGTCCCCCCGGGAGGG
GCGCCACAGGGAATCCGGAGTGTTGATCTGCAGCCCGAGACTCCACTACGATGGTATGAACGGCAAAG
GTACTTTGGACACCACCCCGTTTTCTATTTCCAGACGCGGCCATTCAGGAACGACGCGAATCGTAATC
TACCTGCCGCGTACGCGGATCGTACATGCGTTTTCCAGCGTTCGAAATAGAGTTTGTCGTTCCGCTGA
CCAGGTTTCACGGCTACTGCGATAAGCATATTCTACTGGGGGACTGTAAAGGAATTGTATCCACTCTG
CTTCCTGTCAATTGGCCATGTGCATCTCTGGTTGTTCAGGGTACATTAATAACACACAGCATTAAGTA
GATAAGTCTCCGATATAAGCGAACAGAACGTTGGTCAGAGACTATGGTTCAATTGTAGTGGTATAATG
TTTCCCAGTGCATAAGGAACTCAGGGACACTACTAGCATGGGCGAGGAGAGACCCTTGGGTGGCAACA
CGCAATAAGTGTTACCTAGTACATCAGCACGAGAGAGGGTTTGACCATGTGTTTTTAGACGACCCCGG
TCGCATTGAACCTGGCATTCAGAATGACGTGCTCTCAAGAATCGGGGTCATCCCCCTTAGTGTCACCT
GCTTCGCGGGTCAGCGTATGGTCATAAACAT
>HREN27
GTGTAGCCCAGTTAGACACTTCTAAGTCCTTGGTAACAGACATGCAGTCCCCTCGGCAGAGCCCCGCC
CCCCGATGGTACCGGGTTGCTAAGAGGTCCACAACGGGTCGCACGACAACATTAAGGCCTCATGCATG
ACCCAACACGGGGGCGTCCCGGCGCGGCGACCTTGAAGTCCGGGGACAAGTATGTCCCCCCGGGAGGG
GCGCCACAGGGAATCCGGAGTGTTGATCTGCAGCCCGAGACTCCACTACGATGGTATGAACGGCAAAG
GTACTTTGGACACCACCCCGTTTTCTATTTCCAGACGCGGCCATTCAGGAACGACGCGAATCGTAATC
TACCTGCCGCGTACGCGGATCGTACATGCGTTTTCCAGCGTTCGAAATAGAGTTTGTCGTTCCGCTGA
CCAGGTTTCACGGCTACTGCGATAAGCATATTCTACTGGGGGACTGTAAAGGAATTGTATCCACTCTG
CTTCCTGTCAATTGGCCATGTGCATCTCTGGTTGTTCAGGGTACATTAATAACACACAGCATTAAGTA
GATAAGTCTCCGATATAAGCGAACAGAACGTTGGTCAGAGACTATGGTTCAATTGTAGTGGTATAATG
TTTCCCAGTGCATAAGGAACTCAGGGACACTACTAGCATGGGCGAGGAGAGACCCTTGGGTGGCAACA
CGCAATAAGTGTTACCTAGTACATCAGCACGAGAGAGGGTTTGACCATGTGTTTTTAGACGACCCCGG
TCGCATTGAACCTGGCATTCAGAATGACGTGCTCTCAAGAATCGGGGTCATCCCCCTTAGTGTCACCT
GCTTCGCGGGTCAGCGTATGGTCATAAACAT
>MAN21
GTGTAGCCCAGTTAGACACTTCTAAGTCCTTGGTAACAGACATGCAGTCCCCTCGGCAGAGCCCCGCC
CCCCGATGGTACCGGGTTGCTAAGAGGTCCACAACGGGTCGCACGACAACATTAAGGCCTCATGCATG
ACCCAACACGGGGGCGTCCCGGCGCGGCGACCTTGAAGTCCGGGGACAAGTATGTCCCCCCGGGAGGG
GCGCCACAGGGAATCCGGAGTGTTGATCTGCAGCCCGAGACTCCACTACGATGGTATGAACGGCAAAG
GTACTTTGGACACCACCCCGTTTTCTATTTCCAGACGCGGCCATTCAGGAACGACGCGAATCGTAATC
TACCTGCCGCGTACGCGGATCGTACATGCGTTTTCCAGCGTTCGAAATAGAGTTTGTCGTTCCGCTGA
CCAGGTTTCACGGCTACTGCGATAAGCATATTCTACTGGGGGACTGTAAAGGAATTGTATCCACTCTG
CTTCCTGTCAATTGGCCATGTGCATCTCTGGTTGTTCAGGGTACATTAATAACACACAGCATTAAGTA
GATAAGTCTCCGATATAAGCGAACAGAACGTTGGTCAGAGACTATGGTTCAATTGTAGTGGTATAATG
TTTCCCAGTGCATAAGGAACTCAGGGACACTACTAGCATGGGCGAGGAGAGACCCTTGGGTGGCAACA
CGCAATAAGTGTTACCTAGTACATCAGCACGAGAGAGGGTTTGACCATGTGTTTTTAGACGACCCCGG
TCGCATTGAACCTGGCATTCAGAATGACGTGCTCTCAAGAATCGGGGTCATCCCCCTTAGTGTCACCT
GCTTCGCGGGTCAGCGTATGGTCATAAACAT
>MAN23
GTGTAGCCCAGTTAGACACTTCTAAGTCCTTGGTAACAGACATGCAGTCCCCTCGGCAGAGCCCCGCC
CCCCGATGGTACCGGGTTGCTAAGAGGTCCACAACGGGTCGCACGACAACATTAAGGCCTCATGCATG
ACCCAACACGGGGGCGTCCCGGCGCGGCGACCTTGAAGTCCGGGGACAAGTATGTCCCCCCGGGAGGG
GCGCCACAGGGAATCCGGAGTGTTGATCTGCAGCCCGAGACTCCACTACGATGGTATGAACGGCAAAG
GTACTTTGGACACCACCCCGTTTTCTATTTCCAGACGCGGCCATTCAGGAACGACGCGAATCGTAATC
TACCTGCCGCGTACGCGGATCGTACATGCGTTTTCCAGCGTTCGAAATAGAGTTTGTCGTTCCGCTGA
CCAGGTTTCACGGCTACTGCGATAAGCATATTCTACTGGGGGACTGTAAAGGAATTGTATCCACTCTG

CTTCCTGTCAATTGGCCATGTGCATCTCTGGTTGTTCAGGGTACATTAATAACACACAGCATTAAGTA
GATAAGTCTCCGATATAAGCGAACAGAACGTTGGTCAGAGACTATGGTTCAATTGTAGTGGTATAATG
TTTCCCAGTGCATAAGGAACTCAGGGACACTACTAGCATGGGCGAGGAGAGACCCTTGGGTGGCAACA
CGCAATAAGTGTTACCTAGTACATCAGCACGAGAGAGGGTTTGACCATGTGTTTTTAGACGACCCCGG
TCGCATTGAACCTGGCATTCAGAATGACGTGCTCTCAAGAATCGGGGTCATCCCCCTTAGTGTCACCT
GCTTCGCGGGTCAGCGTATGGTCATAAACAT
>MAN28
GTGTAGCCCAGTTAGACACTTCTAAGTCCTTGGTAACAGACATGCAGTCCCCTCGGCAGAGCCCCGCC
CCCCGATGGTACCGGGTTGCTAAGAGGTCCACAACGGGTCGCACGACAACATTAAGGCCTCATGCATG
ACCCAACACGGGGGCGTCCCGGCGCGGCGACCTTGAAGTCCGGGGACAAGTATGTCCCCCCGGGAGGG
GCGCCACAGGGAATCCGGAGTGTTGATCTGCAGCCCGAGACTCCACTACGATGGTATGAACGGCAAAG
GTACTTTGGACACCACCCCGTTTTCTATTTCCAGACGCGGCCATTCAGGAACGACGCGAATCGTAATC
TACCTGCCGCGTACGCGGATCGTACATGCGTTTTCCAGCGTTCGAAATAGAGTTTGTCGTTCCGCTGA
CCAGGTTTCACGGCTACTGCGATAAGCATATTCTACTGGGGGACTGTAAAGGAATTGTATCCACTCTG
CTTCCTGTCAATTGGCCATGTGCATCTCTGGTTGTTCAGGGTACATTAATAACACACAGCATTAAGTA
GATAAGTCTCCGATATAAGCGAACAGAACGTTGGTCAGAGACTATGGTTCAATTGTAGTGGTATAATG
TTTCCCAGTGCATAAGGAACTCAGGGACACTACTAGCATGGGCGAGGAGAGACCCTTGGGTGGCAACA
CGCAATAAGTGTTACCTAGTACATCAGCACGAGAGAGGGTTTGACCATGTGTTTTTAGANGACCCCGG
TCGCATTGAACCTGGCATTCAGAATGACGTGCTCTCAAGAATCGGGGTCATCCCCCTTAGTGTCACCT
GCTTCGCGGGTCAGCGTATGGTCATAAACAT
>MAN30
GTGTAGCCCAGTTAGACACTTCTAAGTCCTTGGTAACAGACATGCAGTCCCCTCGGCAGAGCCCCGCC
CCCCGATGGTACCGGGTTGCTAAGAGGTCCACAACGGGTCGCACGACAACATTAAGGCCTCATGCATG
ACCCAACACGGGGGCGTCCCGGCGCGGCGACCTTGAAGTCCGGGGACAAGTATGTCCCCCCGGGAGGG
GCGCCACAGGGAATCCGGAGTGTTGATCTGCAGCCCGAGACTCCACTACGATGGTATGAACGGCAAAG
GTACTTTGGACACCACCCCGTTTTCTATTTCCAGACGCGGCCATTCAGGAACGACGCGAATCGTAATC
TACCTGCCGCGTACGCGGATCGTACATGCGTTTTCCAGCGTTCGAAATAGAGTTTGTCGTTCCGCTGA
CCAGGTTTCACGGCTACTGCGATAAGCATATTCTACTGGGGGACTGTAAAGGAATTGTATCCACTCTG
CTTCCTGTCAATTGGCCATGTGCATCTCTGGTTGTTCAGGGTACATTAATAACACACAGCATTAAGTA
GATAAGTCTCCGATATAAGCGAACAGAACGTTGGTCAGAGACTATGGTTCAATTGTAGTGGTATAATG
TTTCCCAGTGCATAAGGAACTCAGGGACACTACTAGCATGGGCGAGGAGAGACCCTTGGGTGGCAACA
CGCAATAAGTGTTACCTAGTACATCAGCACGAGAGAGGGTTTGACCATGTGTTTTTAGACGACCCCGG
TCGCATTGAACCTGGCATTCAGAATGACGTGCTCTCAAGAATCGGGGTCATCCCCCTTAGTGTCACCT
GCTTCGCGGGTCAGCGTATGGTCATAAACAT
>MAN34
GTGTAGCCCAGTTAGACACTTCTAAGTCCTTGGTAACAGACATGCAGTCCCCTCGGCAGAGCCCCGCC
CCCCGATGGTACCGGGTTGCTAAGAGGTCCACAACGGGTCGCACGACAACATTAAGGCCTCATGCATG
ACCCAACACGGGGGCGTCCCGGCGCGGCGACCTTGAAGTCCGGGGACAAGTATGTCCCCCCGGGAGGG
GCGCCACAGGGAATCCGGAGTGTTGATCTGCAGCCCGAGACTCCACTACGATGGTATGAACGGCAAAG
GTACTTTGGACACCACCCCGTTTTCTATTTCCAGACGCGGCCATTCAGGAACGACGCGAATCGTAATC
TACCTGCCGCGTACGCGGATCGTACATGCGTTTTCCAGCGTTCGAAATAGAGTTTGTCGTTCCGCTGA
CCAGGTTTCACGGCTACTGCGATAAGCATATTCTACTGGGGGACTGTAAAGGAATTGTATCCACTCTG
CTTCCTGTCAATTGGCCATGTGCATCTCTGGTTGTTCAGGGTACATTAATAACACACAGCATTAAGTA
GATAAGTCTCCGATATAAGCGAACAGAACGTTGGTCAGAGACTATGGTTCAATTGTAGTGGTATAATG
TTTCCCAGTGCATAAGGAACTCAGGGACACTACTAGCATGGGCGAGGAGAGACCCTTGGGTGGCAACA
CGCAATAAGTGTTACCTAGTACATCAGCACGAGAGAGGGTTTGACCATGTGTTTTTAGACGACCCCGG
TCGCATTGAACCTGGCATTCAGAATGACGTGCTCTCAAGAATCGGGGTCATCCCCCTTAGTGTCACCT
GCTTCGCGGGTCAGCGTATGGTCATAAACAT
>MAN31
GTGTAGCCCAGTTAGACACTTCTAAGTCCTTGGTAACAGACATGCAGTCCCCTCGGCAGAGCCCCGCC
CCCCGATGGTACCGGGTTGCTAAGAGGTCCACAACGGGTCGCACGACAACATTAAGGCCTCATGCATG
ACCCAACACGGGGGCGTCCCGGCGCGGCGACCTTGAAGTCCGGGGACAAGTATGTCCCCCCGGGAGGG
GCGCCACAGGGAATCCGGAGTGTTGATCTGCAGCCCGAGACTCCACTACGATGGTATGAACGGCAAAG
GTACTTTGGACACCACCCCGTTTTCTATTTCCAGACGCGGCCATTCAGGAACGACGCGAATCGTAATC

TACCTGCCGCGTACGCGGATCGTACATGCGTTTTCCAGCGTTCGAAATAGAGTTTGTCGTTCCGCTGA
CCAGGTTTCACGGCTACTGCGATAAGCATATTCTACTGGGGGACTGTAAAGGAATTGTATCCACTCTG
CTTCCTGTCAATTGGCCATGTGCATCTCTGGTTGTTCAGGGTACATTAATAACACACAGCATTAAGTA
GATAAGTCTCCGATATAAGCGAACAGAACGTTGGTCAGAGACTATGGTTCAATTGTAGTGGTATAATG
TTTCCCAGTGCATAAGGAACTCAGGGACACTACTAGCATGGGCGAGGAGAGACCCTTGGGTGGCAACA
CGCAATAAGTGTTACCTAGTACATCAGCACGAGAGAGGGTTTGACCATGTGTTTTTAGACGACCCCGG
TCGCATTGAACCTGGCATTCAGAATGACGTGCTCTCAAGAATCGGGGTCATCCCCCTTAGTGTCACCT
GCTTCGCGGGTCAGCGTATGGTCATAAACAT
>EDEN27
GTGTAGCCCAGTTAGACACTTCTAAGTCCTTGGTAACAGACATGCAGTCCCCTCGGCAGAGCCCCGCC
CCCCGATGGTACCGGGTTGCTAAGAGGTCCACAACGGGTCGCACGACAACATTAAGGCCTCATGCATG
ACCCAACACGGGGGCGTCCCGGCGCGGCGACCTTGAAGTCCGGGGACAAGTATGTCCCCCCGGGAGGG
GCGCCACAGGGAATCCGGAGTGTTGATCTGCAGCCCGAGACTCCACTACGATGGTATGAACGGCAAAG
GTACTTTGGACACCACCCCGTTTTCTATTTCCAGACGCGGCCATTCAGGAACGACGCGAATCGTAATC
TACCTGCCGCGTACGCGGATCGTACATGCGTTTTCCAGCGTTCGAAATAGAGTTTGTCGTTCCGCTGA
CCAGGTTTCACGGCTACTGCGATAAGCATATTCTACTGGGGGACTGTAAAGGAATTGTATCCACTCTG
CTTCCTGTCAATTGGCCATGTGCATCTCTGGTTGTTCAGGGTACATTAATAACACACAGCATTAAGTA
GATAAGTCTCCGATATAAGCGAACAGAACGTTGGTCAGAGACTATGGTTCAATTGTAGTGGTATAATG
TTTCCCAGTGCATAAGGAACTCAGGGACACTACTAGCATGGGCGAGGAGAGACCCTTGGGTGGCAACA
CGCAATAAGTGTTACCTAGTACATCAGCACGAGAGAGGGTTTGACCATGTGTTTTTAGACGACCCCGG
TCGCATTGAACCTGGCATTCAGAATGACGTGCTCTCAAGAATCGGGGTCATCCCCCTTAGTGTCACCT
GCTTCGCGGGTCAGCGTATGGTCATAAACAT
>EDEN29
GTGTAGCCCAGTTAGACACTTCTAAGTCCTTGGTAACAGACATGCAGTCCCCTCGGCAGAGCCCCGCC
CCCCGATGGTACCGGGTTGCTAAGAGGTCCACAACGGGTCGCACGACAACATTAAGGCCTCATGCATG
ACCCAACACGGGGGCGTCCCGGCGCGGCGACCTTGAAGTCCGGGGACAAGTATGTCCCCCCGGGAGGG
GCGCCACAGGGAATCCGGAGTGTTGATCTGCAGCCCGAGACTCCACTACGATGGTATGAACGGCAAAG
GTACTTTGGACACCACCCCGTTTTCTATTTCCAGACGCGGCCATTCAGGAACGACGCGAATCGTAATC
TACCTGCCGCGTACGCGGATCGTACATGCGTTTTCCAGCGTTCGAAATAGAGTTTGTCGTTCCGCTGA
CCAGGTTTCACGGCTACTGCGATAAGCATATTCTACTGGGGGACTGTAAAGGAATTGTATCCACTCTG
CTTCCTGTCAATTGGCCATGTGCATCTCTGGTTGTTCAGGGTACATTAATAACACACAGCATTAAGTA
GATAAGTCTCCGATATAAGCGAACAGAACGTTGGTCAGAGACTATGGTTCAATTGTAGTGGTATAATG
TTTCCCAGTGCATAAGGAACTCAGGGACACTACTAGCATGGGCGAGGAGAGACCCTTGGGTGGCAACA
CGCAATAAGTGTTACCTAGTACATCAGCACGAGAGAGGGTTTGACCATGTGTTTTTAGACGACCCCGG
TCGCATTGAACCTGGCATTCAGAATGACGTGCTCTCAAGAATCGGGGTCATCCCCCTTAGTGTCACCT
GCTTCGCGGGTCAGCGTATGGTCATAAACAT
>EDEN30
GTGTAGCCCAGTTAGACACTTCTAAGTCCTTGGTAACAGACATGCAGTCCCCTCNGCAGAGCCCCGCC
CCCCGATGGTACCGGGTTGCTAAGAGGTCCACAACGGGTCGCACGACAACATTAAGGCCTCATGCATG
ACCCAACACGGGGGCGTCCCGGCGCGGCGACCTTGAAGTCCGGGGACAAGTATGTCCCCCCGGGAGGG
GCGCCACAGGGAATCCGGAGTGTTGATCTGCAGCCCGAGACTCCACTACGATGGTATGAACGGCAAAG
GTACTTTGGACACCACCCCGTTTTCTATTTCCAGACGCGGCCATTCAGGAACGACGCGAATCGTAATC
TACCTGCCGCGTACGCGGATCGTACATGCGTTTTCCAGCGTTCGAAATAGAGTTTGTCGTTCCGCTGA
CCAGGTTTCACGGCTACTGCGATAAGCATATTCTACTGGGGGACTGTAAAGGAATTGTATCCACTCTG
CTTCCTGTCAATTGGCCATGTGCATCTCTGGTTGTTCAGGGTACATTAATAACACACAGCATTAAGTA
GATAAGTCTCCGATATAAGCGAACAGAACGTTGGTCAGAGACTATGGTTCAATTGTAGTGGTATAATG
TTTCCCAGTGCATAAGGAACTCAGGGACACTACTAGCATGGGCGAGGAGAGACCCTTGGGTGGCAACA
CGCAATAAGTGTTACCTAGTACATCAGCACGAGAGAGGGTTTGACCATGTGTTTTTAGACGACCCCGG
TCGCATTGAACCTGGCATTCAGAATGACGTGCTCTCAAGAATCGGGGTCATCCCCCTTAGTGTCACCT
GCTTCGCGGGTCAGCGTATGGTCATAAACAT
>EDEN31
GTGTAGCCCAGTTAGACACTTCTAAGTCCTTGGTAACAGACATGCAGTCCCCTCGGCAGAGCCCCGCC
CCCCGATGGTACCGGGTTGCTAAGAGGTCCACAACGGGTCGCACGACAACATTAAGGCCTCATGCATG
ACCCAACACGGGGGCGTCCCGGCGCGGCGACCTTGAAGTCCGGGGACAAGTATGTCCCCCCGGGAGGG

GCGCCACAGGGAATCCGGAGTGTTGATCTGCAGCCCGAGACTCCACTACGATGGTATGAACGGCAAAG
GTACTTTGGACACCACCCCGTTTTCTATTTCCAGACGCGGCCATTCAGGAACGACGCGAATCGTAATC
TACCTGCCGCGTACGCGGATCGTACATGCGTTTTCCAGCGTTCGAAATAGAGTTTGTCGTTCCGCTGA
CCAGGTTTCACGGCTACTGCGATAAGCATATTCTACTGGGGGACTGTAAAGGAATTGTATCCACTCTG
CTTCCTGTCAATTGGCCATGTGCATCTCTGGTTGTTCAGGGTACATTAATAACACACAGCATTAAGTA
GATAAGTCTCCGATATAAGCGAACAGAACGTTGGTCAGAGACTATGGTTCAATTGTAGTGGTATAATG
TTTCCCAGTGCATAAGGAACTCAGGGACACTACTAGCATGGGCGAGGAGAGACCCTTGGGTGGCAACA
CGCAATAAGTGTTACCTAGTACATCAGCACGAGAGAGGGTTTGACCATGTGTTTTTAGACGACCCCGG
TCGCATTGAACCTGGCATTCAGAATGACGTGCTCTCAAGAATCGGGGTCATCCCCCTTAGTGTCACCT
GCTTCGCGGGTCAGCGTATGGTCATAAACAT
>EDEN32
GTGTAGCCCAGTTAGACACTTCTAAGTCCTTGGTAACAGACATGCAGTCCCCTCGGCAGAGCCCCGCC
CCCCGATGGTACCGGGTTGCTAAGAGGTCCACAACGGGTCGCACGACAACATTAAGGCCTCATGCATG
ACCCAACACGGGGGCGTCCCGGCGCGGCGACCTTGAAGTCCGGGGACAAGTATGTCCCCCCGGGAGGG
GCGCCACAGGGAATCCGGAGTGTTGATCTGCAGCCCGAGACTCCACTACGATGGTATGAACGGCAAAG
GTACTTTGGACACCACCCCGTTTTCTATTTCCAGACGCGGCCATTCAGGAACGACGCGAATCGTAATC
TACCTGCCGCGTACGCGGATCGTACATGCGTTTTCCAGCGTTCGAAATAGAGTTTGTCGTTCCGCTGA
CCAGGTTTCACGGCTACTGCGATAAGCATATTCTACTGGGGGACTGTAAAGGAATTGTATCCACTCTG
CTTCCTGTCAATTGGCCATGTGCATCTCTGGTTGTTCAGGGTACATTAATAACACACAGCATTAAGTA
GATAAGTCTCCGATATAAGCGAACAGAACGTTGGTCAGAGACTATGGTTCAATTGTAGTGGTATAATG
TTTCCCAGTGCATAAGGAACTCAGGGACACTACTAGCATGGGCGAGGAGAGACCCTTGGGTGGCAACA
CGCAATAAGTGTTACCTAGTACATCAGCACGAGAGAGGGTTTGACCATGTGTTTTTAGACGACCCCGG
TCGCATTGAACCTGGCATTCAGAATGACGTGCTCTCAAGAATCGGGGTCATCCCCCTTAGTGTCACCT
GCTTCGCGGGTCAGCGTATGGTCATAAACAT
>EDEN33
GTGTAGCCCAGTTAGACACTTCTAAGTCCTTGGTAACAGACATGCAGTCCCCTCGGCAGAGCCCCGCC
CCCCGATGGTACCGGGTTGCTAAGAGGTCCACAACGGGTCGCACGACAACATTAAGGCCTCATGCATG
ACCCAACACGGGGGCGTCCCGGCGCGGCGACCTNGAAGTCCGGGGACAAGTATGTCCCCCCGGGAGGG
GCGCCACAGGGAATCCGGAGTGTTGATCTGCAGCCCGAGACTCCACTACGATGGTATGAACGGCAAAG
GTACTTTGGACACCACCCCGTTTTCTATTTCCAGACGCGGCCATTCAGGAACGACGCGAATCGTAATC
TACCTGCCGCGTACGCGGATCGTACATGCGTTTTCCAGCGTTCGAAATAGAGTTTGTCGTTCCGCTGA
CCAGGTTTCACGGCTACTGCGATAAGCATATTCTACTGGGGGACTGTAAAGGAATTGTATCCACTCTG
CTTCCTGTCAATTGGCCATGTGCATCTCTGGTTGTTCAGGGTACATTAATAACACACAGCATTAAGTA
GATAAGTCTCCGATATAAGCGAACAGAACGTTGGTCAGAGACTATGGTTCAATTGTAGTGGTATAATG
TTTCCCAGTGCATAAGGAACTCAGGGACACTACTAGCATGGGCGAGGAGAGACCCTTGGGTGGCAACA
CGCAATAAGTGTTACCTAGTACATCAGCACGAGAGAGGGTTTGACCATGTGTTTTTAGACGACCCCGG
TCGCATTGAACCTGGCATTCAGAATGACGTGCTCTCAAGAATCGGGGTCATCCCCCTTAGTGTCACCT
GCTTCGCGGGTCAGCGTATGGTCATAAACAT
>EDEN34
GTGTAGCCCAGTCAGACACTTCTAAGTCCTTGGTAACAGACATGCAGTCCCCTCGGCAGAGCCCCGCC
CCCCGATGGTACCGGGTTGCTAAGAGGTCCACAACGGGTCGCACGACAACATTAAGGCCTCATGCATG
ACCCAACACGGGGGCGTCCCGGCGCGGCGACCTTGAAGTCCGGGGACAAGTATGTCCCCCCGGGAGGG
GCGCCACAGGGAATCCGGAGTGTTGATCTGCAGCCCGAGACTCCACTACGATGGTATGAACGGCAAAG
GTACTTTGGACACCACCCCGTTTTCTATTTCCAGACGCGGCCATTCAGGAACGACGCGAATCGTAATC
TACCTGCCGCGTACGCGGATCGTACATGCGTTTTCCAGCGTTCGAAATAGAGTTTGTCGTTCCGCTGA
CCAGGTTTCACGGCTACTGCGATAAGCATATTCTACTGGGGGACTGTAAAGGAATTGTATCCATTCTG
CTTCCTGTCAATTGGCCATGTGCATCTCTGGTTGTTCAGGGTACATTAATAACACACAGCAGTAAGTA
GGTAAGTCTCCGATATAAGCGAACAGAACGTTTGTCAGAGACTGTGGTTCAATTGTAGTGGTATAATG
TTTCCCAGTGCATAAGGAACTCAGGGACACTACTAGCATGGGCGAGGAGAGACCCTTGGGTGGCAACA
CGCAATAAGTGTTACCTAGTACACCAGCACGAGAGAGGGTTTGACCATGTGTTTTTAGACGACCCCGG
TCGCATTGAACCTGGCATTCAGAATGACGTGCTCTCAAGAATCGGGGTCATCCCCCTTAGTGTCACCT
GCTTCGCGGGTCAGCGTATGGTCATAAACAT
>EDEN37
GTGTAGCCCAGTTAGANACTTCTAAGTCCTTGGTAACAAACGTGCAGTCCCCTCGGCAGAGCCCCGCC

CCCCGATGGTACCGGGTTGCTAAGAGGTCCACCACGAGTCGCACAACAACAGCAAGGCCTCGTGCATG
ACCCAACACGGGGGCGTCCCGGCGCAGCGACCTTGAAGTCCGGGAACAAGTATGTCTCCCCGGGAGGG
GCGCCACAGGGAATCCGGAGTGTTGATCTGCAGCCCGAGACTCCACTACGATGGTATGAACGGCAAAG
GTACTTTGGACACCACCCCGTTTTCTATTCCCAGACGCGGCCATTCAGGAACGACGCGAATCGTCATC
TACCTGCCGCGTACGCGGATCGTACATGCGTTTTCCAGCTTTCGGAATAGAGTTTGTCGTTCCGCTGG
CCAGGTTTCACGGCTATTGCGATAAGCATACTCCACTGGGGGACTGTAAAGGAATTGCATCCACTCTG
CTTCTTATCAGTTGGCCATATGTATCTCTGGTTGTTCAGGGTGCATTAATAACACACAGCATTAAGTA
GATAAGTCTCCGATATAGGCGAACAGAACGTTGGTAAAAGACTGTGGTTCAACTGTAGTGGTATAATG
TTGCCCAGTGCATAAGGAACTCAGGGACACTACTAGCATGGGCGAGTAGAGACCCTTGGGTGGCAACA
CGCAATAAGTGTTACCTAGTACACCAACACGATATAGGGTTTGACCATGTGTTTTTAGACGACCCCGG
TCGCATTAAACCTGGCATTCATAATGACGTGCTCTCAAGAGTCGGGGTCATCCCCCTTAGTGTCACCT
GCTTCGCGGGTCATCGTATGGTCATAAACAT
>EDEN39
GTGTAGCCCAGTTAGACACTTCTAAGTCCTTGGTAACAGACATGCAGTCCCCTCGGCAGAGCCCCGCC
CCCCGATGGTACCGGGTTGCTAAGAGGTCCACAACGGGTCGCACGACAACATTAAGGCCTCATGCATG
ACCCAACACGGGGGCGTCCCGGCGCGGCGACCTTGAAGTCCGGGGACAAGTATGTCCCCCCGGGAGGG
GCGCCACAGGGAATCCGGAGTGTTGATCTGCAGCCCGAGACTCCACTACGATGGTATGAACGGCAAAG
GTACTTTGGACACCACCCCGTTTTCTATTTCCAGACGCNGCCATTCAGGAACGACGCGAATCGTAATC
TACCTGCCGCGTACGNNGATCGTACATGCGTTTTCCAGCGTTCGAAATAGAGTTTGTCGTTCCGCTGA
CCAGGTTTCACGGCTACTGCGATAAGCATATTCTACTGGGGGACTGTAAAGGAATTGTATCCACTCTG
CTTCCTGTCAATTGGCCATGTGCATCTCTGGTTGTTCAGGGTACATTAATAACACACAGCATTAAGTA
GATAAGTCTCCGATATAAGCGAACAGAACGTTGGTCAGAGACTATGGTTCAATTGTAGTGGTATAATG
TTTCCCAGTGCATAAGGAACTCAGGGACACTACTAGCATGGGCGAGGAGAGACCCTTGGGTGGCAACA
CGCAATAAGTGTTACCTAGTACATCAGCACGAGAGAGGGTTTGACCATGTGTTTTTAGACGACCCCGG
TCGCATTGAACCTGGCATTCAGAATGACGTGCTCTCAAGAATCGGGGTCATCCCCCTTAGTGTCACCT
GCTTCGCGGGTCAGCGTATGGTCATAAACAT
>EDEN40
GTGTAGCCCAGTTAGACACTTCTAAGTCCTTGGTAACAAACGTGCAGTCCCCTCGGCAGAGCCCCGCC
CCCCGATGGTACCGGGTTGCTAAGAGGTCCACCACGAGTCGCACAACAACAGCAAGGCCTCGTGCATG
ACCCAACACGGGGGCGTCCCGGCGCAGCGACCTTGAAGTCCGGGAACAAGTATGTCTCCCCGGGAGGG
GCGCCACAGGGAATCCGGAGTGTTGATCTGCAGCCCGAGACTCCACTACGATGGTATGAACGGCAAAG
GTACTTTGGACACCACCCCGTTTTCTATTCCCAGACGCGGCCATTCAGGAACGACGCGAATCGTCATC
TACCTGCCGCGTACGCGGATCGTACATGCGTTTTCCAGCTTTCGGAATAGAGTTTGTCGTTCCGCTGG
CCAGGTTTCACGGCTATTGCGATAAGCATACTCCACTGGGGGACTGTAAAGGAATTGCATCCACTCTG
CTTCTTATCAGTTGGCCATATGTATCTCTGGTTGTTCAGGGTGCATTAATAACACACAGCATTAAGTA
GATAAGTCTCCGATATAGGCGAACAGAACGTTGGTAAAAGACTGTGGTTCAACTGTAGTGGTATAATG
TTGCCCAGTGCATAAGGAACTCAGGGACACTACTAGCATGGGCGAGTAGAGACCCTTGGGTGGCAACA
CGCAATAAGTGTTACCTAGTACACCAACACGATATAGGGTTTGACCATGTGTTTTTAGACGACNCCGG
TCGCATTAAACCTGGCATTCATAATGACGTGCTCTCAAGAGTCGGGGTCATCCCCCTTAGTGTCACCT
GCTTCGCGGGTCATCGTATGGTCATAAACAT
>EDEN43
GTGTAGCCCAGTTAGACACTTCTAAGTCCTTGGTAACAGACATGCAGTCCCCTCGGCAGAGCCCCGCC
CCCCGATGGTACCGGGTTGCTAAGAGGTCCACAACGGGTCGCACGACAACATTAAGGCCTCATGCATG
ACCCAACACGGGGGCGTCCCGGCGCGGCGACCTTGAAGTCCGGGGACAAGTATGTCCCCCCGGGAGGG
GCGCCACAGGGAATCCGGAGTGTTGATCTGCAGCCCGAGACTCCACTACGATGGTATGAACGGCAAAG
GTACTTTGGACACCACCCCGTTTTCTATTTCCAGACGCGGCCATTCAGGAACGACGCGAATCGTAATC
TACCTGCCGCGTACGCGGATCGTACATGCGTTTTCCAGCGTTCGAAATAGAGTTTGTCGTTCCGCTGA
CCAGGTTTCACGGCTACTGCGATAAGCATATTCTACTGGGGGACTGTAAAGGAATTGTATCCACTCTG
CTTCCTGTCAATTGGCCATGTGCATCTCTGGTTGTTCAGGGTACATTAATAACACACAGCATTAAGTA
GATAAGTCTCCGATATAAGCGAACAGAACGTTGGTCAGAGACTATGGTTCAATTGTAGTGGTATAATG
TTTCCCAGTGCATAAGGAACTCAGGGACACTACTAGCATGGGCGAGGAGAGACCCTTGGGTGGCAACA
CGCAATAAGTGTTACCTAGTACATCAGCACGAGAGAGGGTTTGACCATGTGTTTTTAGACGACCCCGG
TCGCATTGAACCTGGCATTCAGAATGACGTGCTCTCAAGAATCGGGGTCATCCCCCTTAGTGTCACCT
GCTTCGCGGGTCAGCGTATGGTCATAAACAT

>EDEN52
GTGTAGCCCAGTTAGACACTTCTAAGTCCTTGGTAACAGACATGCAGTCCCCTCGGCAGAGCCCCGCC
CCCCGATGGTACCGGGTTGCTAAGAGGTCCACCACGAGTCGCACGACAACAGCAAAGCCTCGTGCATG
ACCCAACACGGGGGCGTCCCGGCGCGGCGACCTTGAAGTCCGGGAACAAGTATGTCTCCCCGGGAGGG
GCGCCACAGGGAATCCGGAGTGTTGATCTGCAGCCCGAGACTCCACTACGATGGTATGAACGGCAAAG
GTACTTTGGACACCACCCCGTTTTCTATTCCCAGACGCGGCCATTCAGGAACGACGTGAATCGTAATC
TATCTGCCGCGTACGCGGATCGTACATGCGTTTTCCAGCGTTCGAAATAGAGTTTGTCGTTCCGCTGA
CCAGGTTTCACGGCTACTGCGATAAGCATACTCTACTGGGGGACTGTAAAGGAATTGTATCCACTCTG
CTTCCTGTCAGTTGGCCATATGTATCTCTGGTTGTTCAGGGTACATTAATAACACACAGCATTAAGTA
GATAAGTCTCCGATATAGGCGAACAGAACGTTGGTCAAAGACTGTGGTTCAACTGTAGTGGTATAATG
TTTCCCAGTGCATAAGGAACTCAGGGACACTACTAGCATGGGCGAGTAGAGACCCTTGGGTGGCAACA
CGCAATAAGTGTTACCTAGTACACCAACACGATATAGGGTTTGACCATGTGTTTTTAGACGACCCCGG
TCGCATTGAACCTGGCATTCATAATGACGTGCTCTCAAGAGTCGGGGTCATCCCCCTTAGTGTCACCT
GCTTCGCGGGTCATCGTATGGTCATAAACAT
>EDEN55
GTGTAGCCCAGTTAGACACTTCTAAGTCCTTGGTAACAGACATGCAGTCCCCTCGGCAGAGCCCCGCC
CCCCGATGGTACCGGGTTGCTAAGAGGTCCACAACGGGTCGCACGACAACATTAAGGCCTCATGCATG
ACCCAACACGGGGGCGTCCCGGCGCGGCGACCTTGAAGTCCGGGGACAAGTATGTCCCCCCGGGAGGG
GCGCCACAGGGAATCCGGAGTGTTGATCTGCAGCCCGAGACTCCACTACGATGGTATGAACGGCAAAG
GTACTTTGGACACCACCCCGTTTTCTATTTCCAGACGCGGCCATTCAGGAACGACGCGAATCGTAATC
TACCTGCCGCGTACGCGGATCGTACATGCGTTTTCCAGCGTTCGAAATAGAGTTTGTCGTTCCGCTGA
CCAGGTTTCACGGCTACTGCGATAAGCATATTCTACTGGGGGACTGTAAAGGAATTGTATCCACTCTG
CTTCCTGTCAATTGGCCATGTGCATCTCTGGTTGTTCAGGGTACATTAATAACACACAGCATTAAGTA
GATAAGTCTCCGATATAAGCGAACAGAACGTTGGTCAGAGACTATGGTTCAATTGTAGTGGTATAATG
TTTCCCAGTGCATAAGGAACTCAGGGACACTACTAGCATGGGCGAGGAGAGACCCTTGGGTGGCAACA
CGCAATAAGTGTTACCTAGTACATCAGCACGAGAGAGGGTTTGACCATGTGTTTTTAGACGACCCCGG
TCGCATTGAACCTGGCATTCAGAATGACGTGCTCTCAAGAATCGGGGTCATCCCCCTTAGTGTCACCT
GCTTCGCGGGTCAGCGTATGGTCATAAACAT
>CHAN01
GTGTAGCCCAGTTAGACACTTCTAAGTCCTTGGTAACAGACATGCAGTCCCCTCGGCAGAGCCCCGCC
CCCCGATGGTACCGGGTTGCTAAGAGGTCCACAACGGGTCGCACGACAACATTAAGGCCTCATGCATG
ACCCAACACGGGGGCGTCCCGGCGCGGCGACCTTGAAGTCCGGGGACAAGTATGTCCCCCCGGGAGGG
GCGCCACAGGGAATCCGGAGTGTTGATCTGCAGCCCGAGACTCCACTACGATGGTATGAACGGCAAAG
GTACTTTGGACACCACCCCGTTTTCTATTTCCAGACGCGGCCATTCAGGAACGACGCGAATCGTAATC
TACCTGCCGCGTACGCGGATCGTACATGCGTTTTCCAGCGTTCGAAATAGAGTTTGTCGTTCCGCTGA
CCAGGTTTCACGGCTACTGCGATAAGCATATTCTACTGGGGGACTGTAAAGGAATTGTATCCACTCTG
CTTCCTGTCAATTGGCCATGTGCATCTCTGGTTGTTCAGGGTACATTAATAACACACAGCATTAAGTA
GATAAGTCTCCGATATAAGCGAACAGAACGTTGGTCAGAGACTATGGTTCAATTGTAGTGGTATAATG
TTTCCCAGTGCATAAGGAACTCAGGGACACTACTAGCATGGGCGAGGAGAGACCCTTGGGTGGCAACA
CGCAATAAGTGTTACCTAGTACATCAGCACGAGAGAGGGTTTGACCATGTGTTTTTAGACGACCCCGG
TCGCATTGAACCTGGCATTCAGAATGACGTGCTCTCAAGAATCGGGGTCATCCCCCTTAGTGTCACCT
GCTTCGCGGGTCAGCGTATGGTCATAAACAT
>CHAN03
GTGTAGCCCAGTTAGACACTTCTAAGTCCTTGGTAACAGACATGCAGTCCCCTCGGCAGAGCCCCGCC
CCCCGATGGTACCGGGTTGCTAAGAGGTCCACAACGGGTCGCACGACAACATTAAGGCCTCATGCATG
ACCCAACACGGGGGCGTCCCGGCGCGGCGACCTTGAAGTCCGGGGACAAGTATGTCCCCCCGGGAGGG
GCGCCACAGGGAATCCGGAGTGTTGATCTGCAGCCCGAGACTCCACTACGATGGTATGAACGGCAAAG
GTACTTTGGACACCACCCCGTTTTCTATTTCCAGACGCGGCCATTCAGGAACGACGCGAATCGTAATC
TACCTGCCGCGTACGCGGATCGTACATGCGTTTTCCAGCGTTCGAAATAGAGTTTGTCGTTCCGCTGA
CCAGGTTTCACGGCTACTGCGATAAGCATATTCTACTGGGGGACTGTAAAGGAATTGTATCCACTCTG
CTTCCTGTCAATTGGCCATGTGCATCTCTGGTTGTTCAGGGTACATTAATAACACACAGCATTAAGTA
GATAAGTCTCCGATATAAGCGAACAGAACGTTGGTCAGAGACTATGGTTCAATTGTAGTGGTATAATG
TTTCCCAGTGCATAAGGAACTCAGGGACACTACTAGCATGGGCGAGGAGAGACCCTTGGGTGGCAACA
CGCAATAAGTGTTACCTAGTACATCAGCACGAGAGAGGGTTTGACCATGTGTTTTTAGACGACCCCGG

TCGCATTGAACCTGGCATTCAGAATGACGTGCTCTCAAGAATCGGGGTCATCCCCCTTAGTGTCACCT
GCTTCGCGGGTCAGCGTATGGTCATAAACAT
>CHAN05
GTGTAGCCCAGTTAGACACTTCTAAGTCCTTGGTAACAGACATGCAGTCCCCTCGGCAGAGCCCCGCC
CCCCGATGGTACCGGGTTGCTAAGAGGTCCACAACGGGTCGCACGACAACATTAAGGCCTCATGCATG
ACCCAACACGGGGGCGTCCCGGCGCGGCGACCTTGAAGTCCGGGGACAAGTATGTCCCCCCGGGAGGG
GCGCCACAGGGAATCCGGAGTGTTGATCTGCAGCCCGAGACTCCACTACGATGGTATGAACGGCAAAG
GTACTTTGGACACCACCCCGTTTTCTATTTCCAGACGCGGCCATTCAGGAACGACGCGAATCGTAATC
TACCTGCCGCGTACGCGGATCGTACATGCGTTTTCCAGCGTTCGAAATAGAGTTTGTCGTTCCGCTGA
CCAGGTTTCACGGCTACTGCGATAAGCATATTCTACTGGGGGACTGTAAAGGAATTGTATCCACTCTG
CTTCCTGTCAATTGGCCATGTGCATCTCTGGTTGTTCAGGGTACATTAATAACACACAGCATTAAGTA
GATAAGTCTCCGATATAAGCGAACAGAACGTTGGTCAGAGACTATGGTTCAATTGTAGTGGTATAATG
TTTCCCAGTGCATAAGGAACTCAGGGACACTACTAGCATGGGCGAGGAGAGACCCTTGGGTGGCAACA
CGCAATAAGTGTTACCTAGTACATCAGCACGAGAGAGGGTTTGACCATGTGTTTTTAGACGACCCCGG
TCGCATTGAACCTGGCATTCAGAATGACGTGCTCTCAAGAATCGGGGTCATCCCCCTTAGTGTCACCT
GCTTCGCGGGTCAGCGTATGGTCATAAACAT
>CHAN06
GTGTAGCCCAGTTAGACACTTCTAAGTCCTTGGTAACAGACATGCAGTCCCCTCGGCAGAGCCCCGCC
CCCCGATGGTACCGGGTTGCTAAGAGGTCCACCACGGGTCGCACGACAACATCAAGGCCTCATGCATG
ACCCAACACGGGGGCGTCCCGGCGCGGCGATCTTGAAGGCCGGGGACAAGTATGTCCCCCCGGGAGGG
GCGCCACAGGGAATCCGGAGTGTTGATCTGCAGCCCGAGACTCCACTACGATGGTATGAACGGCAAAG
GTGCTTTGGACACCACCCCGTTTTCTATTTCCAGACGCGGCCATTCAGGAACGACGCGAATCGTAATC
TACCTGCCGCGTACGCGGATCGTACATGCGTTTTCCAGCGTTCGAAATAGAGTTTGTCGTTCCGCTGA
CCAGGTTTCACGGCTACTGCGATAAGCATATTCTACTGGGGGACTGTAAAGGAATTGTATCCACTCTG
CTTCCTGTCAATTGGCCATGTACATCTCTGGTTATTCAGGGTACATTAATAACACACAGCATTAAGTA
GATAAGTCTCCGATATAAGCGAACAGAACGCCGGTCAAAGACTGTGGTTCAATTGTAGTGGTATAACG
TTTCCCAGTGCATAAAGAACTCAGGGACACTACTAGCATGGGCGAGGAGAGACCCTTGGGTNGCAACA
CGCAATAAGTGTTACCTAGTACACCAGCACGATAGAGGGTTTGACCATGTGTTTTTCGACGACCCCGG
TCGCGTTGAACCTGGCATTCAGAATGACGTGCTCTCAAGAGTCGGGGTCATCCCCCTTAGTGTCACCT
GCTTCGCGGGTCAGCGTATGGTCATAAACAT
>CHAN07
GTGTAGCCCAGTTAGACACTTCTAAGTCCTTGGTAACAGACATGCAGTCCCCTCGGCAGAGCCCCGCC
CCCCGATGGTACCGGGTTGCTAAGAGGTCCACCACGGGTCGCACGACAACATCAAGGCCTCATGCATG
ACCCAACACGGGGGCGTCCCGGCGCGGCGANCTTGAAGGCCGGGGACAAGTATGTCCCCCCGGGAGGG
GCGCCACAGGGAATCCGGAGTGTTGATCTGCAGCCCGAGACTCCACTACGATGGTATGAACGGCAAAG
GTGCTTTGGACACCACCCCGTTTTCTATTTCCAGACGCGGCCATTCAGGAACGACGCGAATCGTAATC
TACCTGCCGCGTACGCGGATCGTACATGCGTTTTCCAGCGTTCGAAATAGAGTTTGTCGTTCCGCTGA
CCAGGTTTCACGGCTACTGCGATAAGCATATTCTACTGGGGGACTGTAAAGGAATTGTATCCACTCTG
CTTCCTGTCAATTGGCCATGTACATCTCTGGTTATTCAGGGTACATTAATAACACACAGCATTAAGTA
GATAAGTCTCCGATATAAGCGAACAGAACGCCGGTCAAAGACTGTGGTTCAATTGTAGTGGTATAACG
TTTCCCAGTGCATAAAGAACTCAGGGACACTACTAGCATGGGCGAGGAGAGACCCTTGGGTNGCAACA
CGCAATAAGTGTTACCTAGTACACCAGCACGATAGAGGGTTTGACCATGTGTTTTTCGACGACCCCGG
TCGCGTTGAACCTGGCATTCAGAATGACGTGCTCTCAAGAGTCGGGGTCATCCCCCTTAGTGTCACCT
GCTTCGCGGGTCAGCGTATGGTCATAAACAT
>CHAN08
GTGTAGCCCAGTTAGACACTTCTAAGTCCTTGGTAACAGACATGCAGTCCCCTCGGCAGAGCCCCGCC
CCCCGATGGTACCGGGTTGCTAAGAGGTCCACCACGGGTCGCACGACAACATCAAGGCCTCATGCATG
ACCCAACACGGGGGCGTCCCGGCGCGGCGATCTTGAAGGCCGGGGACAAGTATGTCCCCCCGGGAGGG
GCGCCACAGGGAATCCGGAGTGTTGATCTGCAGCCCGAGACTCCACTACGATGGTATGAACGGCAAAG
GTGCTTTGGACACCACCCCGTTTTCTATTTCCAGACGCGGCCATTCAGGAACGACGCGAATCGTAATC
TACCTGCCGCGTACGCGGATCGTACATGCGTTTTCCAGCGTTCGAAATAGAGTTTGTCGTTCCGCTGA
CCAGGTTTCACGGCTACTGCGATAAGCATATTCTACTGGGGGACTGTAAAGGAATTGTATCCACTCTG
CTTCCTGTCAATTGGCCATGTACATCTCTGGTTATTCAGGGTACATTAATAACACACAGCATTAAGTA
GATAAGTCTCCGATATAAGCGAACAGAACGCCGGTCAAAGACTGTGGTTCAATTGTAGTGGTATAACG

TTTCCCAGTGCATAAAGAACTCAGGGACACTACTAGCATGGGCGAGGAGAGACCCTTGGGTNGCAACA
CGCAATAAGTGTTACCTAGTACACCAGCACGATAGAGGGTTTGACCATGTGTTTTTCGACGACCCCGG
TCGCGTTGAACCTGGCATTCAGAATGACGTGCTCTCAAGAGTCGGGGTCATCCCCCTTAGTGTCACCT
GCTTCGCGGGTCAGCGTATGGTCATAAACAT
>CHAN09
GTGTAGCCCAGTTAGACACTTCTAAGTCCTTGGTAACAGACATGCAGTCCCCTCGGCAGAGCCCCGCC
CCCCGATGGTACCGGGTTGCTAAGAGGTCCACAACGGGTCGCACGACAACATTAAGGCCTCATGCATG
ACCCAACACGGGGGCGTCCCGGCGCGGCGACCTTGAAGTCCGGGGACAAGTATGTCCCCCCGGGAGGG
GCGCCACAGGGAATCCGGAGTGTTGATCTGCAGCCCGAGACTCCACTACGATGGTATGAACGGCAAAG
GTACTTTGGACACCACCCCGTTTTCTATTTCCAGACGCGGCCATTCAGGAACGACGCGAATCGTAATC
TACCTGCCGCGTACGCGGATCGTACATGCGTTTTCCAGCGTTCGAAATAGAGTTTGTCGTTCCGCTGA
CCAGGTTTCACGGCTACTGCGATAAGCATATTCTACTGGGGGACTGTAAAGGAATTGTATCCACTCTG
CTTCCTGTCAATTGGCCATGTGCATCTCTGGTTGTTCAGGGTACATTAATAACACACAGCATTAAGTA
GATAAGTCTCCGATATAAGCGAACAGAACGTTGGTCAGAGACTATGGTTCAATTGTAGTGGTATAATG
TTTCCCAGTGCATAAGGAACTCAGGGACACTACTAGCATGGGCGAGGAGAGACCCTTGGGTGGCAACA
CGCAATAAGTGTTACCTAGTACATCAGCACGAGAGAGGGTTTGACCATGTGTTTTTAGACGACCCCGG
TCGCATTGAACCTGGCATTCAGAATGACGTGCTCTCAAGAATCGGGGTCATCCCCCTTAGTGTCACCT
GCTTCGCGGGTCAGCGTATGGTCATAAACAT
>CHAN10
GTGTAGCCCAGTTAGACACTTCTAAGTCCTTGGTAACAGACATGCAGTCCCCTCGGCAGAGCCCCGCC
CCCCGATGGTACCGGGTTGCTAAGAGGTCCACAACGGGTCGCACGACAACATTAAGGCCTCATGCATG
ACCCAACACGGGGGCGTCCCGGCGCGGCGACCTTGAAGTCCGGGGACAAGTATGTCCCCCCGGGAGGG
GCGCCACAGGGAATCCGGAGTGTTGATCTGCAGCCCGAGACTCCACTACGATGGTATGAACGGCAAAG
GTACTTTGGACACCACCCCGTTTTCTATTTCCAGACGCGGCCATTCAGGAACGACGCGAATCGTAATC
TACCTGCCGCGTACGCGGATCGTACATGCGTTTTCCAGCGTTCGAAATAGAGTTTGTCGTTCCGCTGA
CCAGGTTTCACGGCTACTGCGATAAGCATATTCTACTGGGGGACTGTAAAGGAATTGTATCCACTCTG
CTTCCTGTCAATTGGCCATGTGCATCTCTGGTTGTTCAGGGTACATTAATAACACACAGCATTAAGTA
GATAAGTCTCCGATATAAGCGAACAGAACGTTGGTCAGAGACTATGGTTCAATTGTAGTGGTATAATG
TTTCCCAGTGCATAAGGAACTCAGGGACACTACTAGCATGGGCGAGGAGAGACCCTTGGGTGGCAACA
CGCAATAAGTGTTACCTAGTACATCAGCACGAGAGAGGGTTTGACCATGTGTTTTTAGACGACCCCGG
TCGCATTGAACCTGGCATTCAGAATGACGTGCTCTCAAGAATCGGGGTCATCCCCCTTAGTGTCACCT
GCTTCGCGGGTCAGCGTATGGTCATAAACAT
>CHAN11
GTGTAGCCCAGTTAGACACTTCTAAGTCCTTGGTAACAGACATGCAGTCCCCTCGGCAGAGCCCCGCC
CCCCGATGGTACCGGGTTGCTAAGAAGTCCACAACGGGTCGCACGACAACATTAAGGCCTCATGCATG
ACCCAACACGGGGGCGTCCCGGCGCGGCGACCTTGAAGTCCGGGGACAAGTATGTCCCCCCGGGAGGG
GCGCCACAGGGAATCCGGAGTGTTGATCTGCAGCCCGAGACTCCACTACGATGGTATGAACGGCAAAG
GTACTTTGGACACCACCCCGTTTTCTATTTCCAGACGCGGCCATTCAGGAACGACGCGAATCGTAATC
TACCTGCCGCGTACGCGGATCGTACATGCGTTTTCCAGCGTTCGAAATAGAGTTTGTCGTTCCGCTGA
CCAGGTTTCACGGCTACTGCGATAAGCATATTCTACTGGGGGACTGTAAAGGAATTGTATCCATTCTG
CTTCCTGTCAATTGGCCATGTGCATCTCTGGTTGTTCAGGGTACATTAATAACACACAGCAGTAAGTA
GGTAAGTCTCCGATATAAGCTAACAGAACGTTTGTCAGAGACTGTGGTTCAATTGTAGTGGTATAATG
TTTCCCAGTGCATAAGGAACTCAGGGACACTACTAGCATGGGCGAGGAGAGACCCTTGGGTGGCAACA
CGCAATAAGTGTTACCTAGTACACCAGCACGAGAGAGGGTTTGACCATGTGTTTTCAGACGACCCCGG
TCGCATTGAACCTGGCATTCAGAATGACGTGCTCTCAAGAATCGGGGTCATCCCCCTTAGTGTCACCT
GCTTCGCGGGTCAGCGTATGGTCATAAACAT
>CHAN12
GTGTAGCCCAGTTAGACACTTCTAAGTCCTTGGTAACAGACATGCAGTCCCCTCGGCAGAGCCCCGCC
CCCCGATGGTACCGGGTTGCTAAGAGGTCCACCACGGGTCGCACGACAACATCAAGGCCTCATGCATG
ACCCAACACGGGGGCGTCCCGGCGCGGCGATCTTGAAGGCCGGGGACAAGTATGTCCCCCCGGGAGGG
GCGCCACAGGGAATCCGGAGTGTTGATCTGCAGCCCGAGACTCCACTACGATGGTATGAACGGCAAAG
GTGCTTTGGACACCACCCCGTTTTCTATTTCCAGACGCGGCCATTCAGGAACGACGCGAATCGTAATC
TACCTGCCGCGTACGCGGATCGTACATGCGTTTTCCAGCGTTCGAAATAGAGTTTGTCGTTCCGCTGA
CCAGGTTTCACGGCTACTGCGATAAGCATATTCTACTGGGGGACTGTAAAGGAATTGTATCCACTCTG

CTTCCTGTCAATTGGCCATGTACATCTCTGGTTATTCAGGGTACATTAATAACACACAGCATTAAGTA
GATAAGTCTCCGATATAAGCGAACAGAACGCCGGTCAAAGACTGTGGTTCAATTGTAGTGGTATAACG
TTTCCCAGTGCATAAAGAACTCAGGGACACTACTAGCATGGGCGAGGAGAGACCCTTGGGTNGCAACA
CGCAATAAGTGTTACCTAGTACACCAGCACGATAGAGGGTTTGACCATGTGTTTTTCGACGACCCCGG
TCGCGTTGAACCTGGCATTCAGAATGACGTGCTCTCAAGAGTCGGGGTCATCCCCCTTAGTGTCACCT
GCTTCGCGGGTCAGCGTATGGTCATAAACAT
>CHAN15
GTGTAGCCCAGTTAGACACTTCTAAGTCCTTGGTAACAGACATGCAGTCCCCTCGGCAGAGCCCCGCC
CCCCGATGGTACCGGGTTGCTAAGAGGTCCACAACGGGTCGCACGACAACATTAAGGCCTCATGCATG
ACCCAACACGGGGGCGTCCCGGCGCGGCGACCTTGAAGTCCGGGGACAAGTATGTCCCCCCGGGAGGG
GCGCCACAGGGAATCCGGAGTGTTGATCTGCAGCCCGAGACTCCACTACGATGGTATGAACGGCAAAG
GTACTTTGGACACCACCCCGTTTTCTATTTCCAGACGCGGCCATTCAGGAACGACGCGAATCGTAATC
TACCTGCCGCGTACGCGGATCGTACATGCGTTTTCCAGCGTTCGAAATAGAGTTTGTCGTTCCGCTGA
CCAGGTTTCACGGCTACTGCGATAAGCATATTCTACTGGGGGACTGTAAAGGAATTGTATCCACTCTG
CTTCCTNTCAATTGGCCATGTGCATCTCTGGTTGTTCAGGGTACATTAATAACACACAGCATTAAGTA
GATAAGTCTCCGATATAAGCGAACAGAACGTTGGTCAGAGACTATGGTTCAATTGTAGTGGTATAATG
TTTCCCAGTGCATAAGGAACTCAGGGACACTACTAGCATGGGCGAGGAGAGACCCTTGGGTGGCAACA
CGCAATAAGTGTTACCTAGTACATCAGCACGAGAGAGGGTTTGACCATGTGTTTTTAGACGACCCCGG
TCGCATTGAACCTGGCATTCAGAATGACGTGCTCTCAAGAATCGGGGTCATCCCCCTTAGTGTCACCT
GCTTCGCGGGTCAGCGTATGGTCATAAACAT
>CHAN17
GTGTAGCCCAGTTAGACACTTCTAAGTCCTTGGTAACAGACATGCAGTCCCCTCGGCAGAGCCCCGCC
CCCCGATGGTACCGGGTTGCTAAGAGGTCCACAACGGGTCGCACGACAACATTAAGGCCTCATGCATG
ACCCAACACGGGGGCGTCCCGGCGCGGCGACCTTGAAGTCCGGGGACAAGTATGTCCCCCCGGGAGGG
GCGCCACAGGGAATCCGGAGTGTTGATCTGCAGCCCGAGACTCCACTACGATGGTATGAACGGCAAAG
GTACTTTGGACACCACCCCGTTTTCTATTTCCAGACGCGGCCATTCAGGAACGACGCGAATCGTAATC
TACCTGCCGCGTACGCGGATCGTACATGCGTTTTCCAGCGTTCGAAATAGAGTTTGTCGTTCCGCTGA
CCAGGTTTCACGGCTACTGCGATAAGCATATTCTACTGGGGGACTGTAAAGGAATTGTATCCACTCTG
CTTCCTGTCAATTGGCCATGTGCATCTCTGGTTGTTCAGGGTACATTAATAACACACAGCATTAAGTA
GATAAGTCTCCGATATAAGCGAACAGAACGTTGGTCAGAGACTATGGTTCAATTGTAGTGGTATAATG
TTTCCCAGTGCATAAGGAACTCAGGGACACTACTAGCATGGGCGAGGAGAGACCCTTGGGTGGCAACA
CGCAATAAGTGTTACCTAGTACATCAGCACGAGAGAGGGTTTGACCATGTGTTTTTAGACGACCCCGG
TCGCATTGAACCTGGCATTCAGAATGACGTGCTCTCAAGAATCGGGGTCATCCCCCTTAGTGTCACCT
GCTTCGCGGGTCAGCGTATGGTCATAAACAT
>CHAN18
GTGTAGCCCAGTTAGACACTTCTAAGTCCTTGGTAACAGACATGCAGTCCCCTCGGCAGAGCCCCGCC
CCCCGATGGTACCGGGTTGCTAAGAGGTCCACAACGGGTCGCACGACAACATTAAGGCCTCATGCATG
ACCCAACACGGGGGCGTCCCGGCGCGGCGACCTTGAAGTCCGGGGACAAGTATGTCCCCCCGGGAGGG
GCGCCACAGGGAATCCGGAGTGTTGATCTGCAGCCCGAGACTCCACTACGATGGTATGAACGGCAAAG
GTACTTTGGACACCACCCCGTTTTCTATTTCCAGACGCGGCCATTCAGGAACGACGCGAATCGTAATC
TACCTGCCGCGTACGCGGATCGTACATGCGTTTTCCAGCGTTCGAAATAGAGTTTGTCGTTCCGCTGA
CCAGGTTTCACGGCTACTGCGATAAGCATATTCTACTGGGGGACTGTAAAGGAATTGTATCCACTCTG
CTTCCTGTCAATTGGCCATGTGCATCTCTGGTTGTTCAGGGTACATTAATAACACACAGCATTAAGTA
GATAAGTCTCCGATATAAGCGAACAGAACGTTGGTCAGAGACTATGGTTCAATTGTAGTGGTATAATG
TTTCCCAGTGCATAAGGAACTCAGGGACACTACTAGCATGGGCGAGGAGAGACCCTTGGGTGGCAACA
CGCAATAAGTGTTACCTAGTACATCAGCACGAGAGAGGGTTTGACCATGTGTTTTTAGACGACCCCGG
TCGCATTGAACCTGGCATTCAGAATGACGTGCTCTCAAGAATCGGGGTCATCCCCCTTAGTGTCACCT
GCTTCGCGGGTCAGCGTATGGTCATAAACAT
>MNON07
GTGTAGCCCAGTTAGACACTTCTAAGTCCTTGGTAACAGACATGCAGTCCCCTCGGCAGAGCCCCGCC
CCCCGATGGTACCGGGTTGCTAAGAGGTCCACAACGGGTCGCACGACAACATTAAGGCCTCATGCATG
ACCCAACACGGGGGCGTCCCGGCGCGGCGACCTTGAAGTCCGGGGACAAGTATGTCCCCCCGGGAGGG
GCGCCACAGGGAATCCGGAGTGTTGATCTGCAGCCCGAGACTCCACTACGATGGTATGAACGGCAAAG
GTACTTTGGACACCACCCCGTTTTCTATTTCCAGACGCGGCCATTCAGGAACGACGCGAATCGTAATC

TACCTGCCGCGTACGCGGATCGTACATGCGTTTTCCAGCGTTCGAAATAGAGTTTGTCGTTCCGCTGA
CCAGGTTTCACGGCTACTGCGATAAGCATATTCTACTGGGGGACTGTAAAGGAATTGTATCCACTCTG
CTTCCTGTCAATTGGCCATGTGCATCTCTGGTTGTTCAGGGTACATTAATAACACACAGCATTAAGTA
GATAAGTCTCCGATATAAGCGAACAGAACGTTGGTCAGAGACTATGGTTCAATTGTAGTGGTATAATG
TTTCCCAGTGCATAAGGAACTCAGGGACACTACTAGCATGGGCGAGGAGAGACCCTTGGGTGGCAACA
CGCAATAAGTGTTACCTAGTACATCAGCACGAGAGAGGGTTTGACCATGTGTTTTTAGACGACCCCGG
TCGCATTGAACCTGGCATTCAGAATGACGTGCTCTCAAGAATCGGGGTCATCCCCCTTAGTGTCACCT
GCTTCGCGGGTCAGCGTATGGTCATAAACAT
>MNON09
GTGTAGCCCAGTTAGACACTTCTAAGTCCTTGGTAACAGACATGCAGTCCCCTCGGCAGAGCCCCGCC
CCCCGATGGTACCGGGTTGCTAAGAGGTCCACAACGGGTCGCACGACAACATTAAGGCCTCATGCATG
ACCCAACACGGGGGCGTCCCGGCGCGGCGACCTTGAAGTCCGGGGACAAGTATGTCCCCCCGGGAGGG
GCGCCACAGGGAATCCGGAGTGTTGATCTGCAGCCCGAGACTCCACTACGATGGTATGAACGGCAAAG
GTACTTTGGACACCACCCCGTTTTCTATTTCCAGACGCGGCCATTCAGGAACGACGCGAATCGTAATC
TACCTGCCGCGTACGCGGATCGTACATGCGTTTTCCAGCGTTCGAAATAGAGTTTGTCGTTCCGCTGA
CCAGGTTTCACGGCTACTGCGATAAGCATATTCTACTGGGGGACTGTAAAGGAATTGTATCCACTCTG
CTTCCTGTCAATTGGCCATGTGCATCTCTGGTTGTTCAGGGTACATTAATAACACACAGCATTAAGTA
GATAAGTCTCCGATATAAGCGAACAGAACGTTGGTCAGAGACTATGGTTCAATTGTAGTGGTATAATG
TTTCCCAGTGCATAAGGAACTCAGGGACACTACTAGCATGGGCGAGGAGAGACCCTTGGGTGGCAACA
CGCAATAAGTGTTACCTAGTACATCAGCACGAGAGAGGGTTTGACCATGTGTTTTTAGACGACCCCGG
TCGCATTGAACCTGGCATTCAGAATGACGTGCTCTCAAGAATCGGGGTCATCCCCCTTAGTGTCACCT
GCTTCGCGGGTCAGCGTATGGTCATAAACAT
>MNON11
GTGTAGCCCAGTTAGACACTTCTAAGTCCTTGGTAACAGACATGCAGTCCCCTCGGCAGAGCCCCGCC
CCCCGATGGTACCGGGTTGCTAAGAGGTCCACAACGGGTCGCACGACAACATTAAGGCCTCATGCATG
ACCCAACACGGGGGCGTCCCGGCGCGGCGACCTTGAAGTCCGGGGACAAGTATGTCCCCCCGGGAGGG
GCGCCACAGGGAATCCGGAGTGTTGATCTGCAGCCCGAGACTCCACTACGATGGTATGANCGGCAAAG
GTACTTTGGACACCACCCCGTTTTCTATTTCCAGACGCGGCCATTCAGGAACGACGCGAATCGTAATC
TACCTGCCGCGTACGCGGATCGTACATGCGTTTTCCAGCGTTCGAAATAGAGTTTGTCGTTCCGCTGA
CCAGGTTTCACGGCTACTGCGATAAGCATATTCTACTGGGGGACTGTAAAGGAATTGTATCCACTCTG
CTTCCTGTCAATTGGCCATGTGCATCTCTGGTTGTTCAGGGTACATTAATAACACACAGCATTAAGTA
GATAAGTCTCCGATATAAGCGAACAGAACGTTGGTCAGAGACTATGGTTCAATTGTAGTGGTATAATG
TTTCCCAGTGCATAAGGAACTCAGGGACACTACTAGCATGGGCGAGGAGAGACCCTTGGGTGGCAACA
CGCAATAAGTGTTACCTAGTACATCAGCACGAGAGAGGGTTTGACCATGTGTTTTTAGACGACCCCGG
TCGCATTGAACCTGGCATTCAGAATGACGTGCTCTCAAGAATCGGGGTCATCCCCCTTAGTGTCACCT
GCTTCGCGGGTCAGCGTATGGTCATAAACAT
>MNON12
GTGTAGCCCAGTTAGACACTTCTAAGTCCTTGGTAACAGACATGCAGTCCCCTCGGCAGAGCCCCGCC
CCCCGATGGTACCGGGTTGCTAAGAGGTCCACAACGGGTCGCACGACAACATTAAGGCCTCATGCATG
ACCCAACACGGGGGCGTCCCGGCGCGGCGACCTTGAAGTCCGGGGACAAGTATGTCCCCCCGGGAGGG
GCGCCACAGGGAATCCGGAGTGTTGATCTGCAGCCCGAGACTCCACTACGATGGTATGAACGGCAAAG
GTACTTTGGACACCACCCCGTTTTCTATTTCCAGACGCGGCCATTCAGGAACGACGCGAATCGTAATC
TACCTGCCGCGTACGCGGATCGTACATGCGTTTTCCAGCGTTCGAAATAGAGTTTGTCGTTCCGCTGA
CCAGGTTTCACGGCTACTGCGATAAGCATATTCTACTGGGGGACTGTAAAGGAATTGTATCCACTCTG
CTTCCTGTCAATTGGCCATGTGCATCTCTGGTTGTTCAGGGTACATTAATAACACACAGCATTAAGTA
GATAAGTCTCCGATATAAGCGAACAGAACGTTGGTCAGAGACTATGGTTCAATTGTAGTGGTATAATG
TTTCCCAGTGCATAAGGAACTCAGGGACACTACTAGCATGGGCGAGGAGAGACCCTTGGGTGGCAACA
CGCAATAAGTGTTACCTAGTACATCAGCACGAGAGAGGGTTTGACCATGTGTTTTTAGACGACCCCGG
TCGCATTGAACCTGGCATTCAGAATGACGTGCTCTCAAGAATCGGGGTCATCCCCCTTAGTGTCACCT
GCTTCGCGGGTCAGCGTATGGTCATAAACAT
>MNON15
GTGTAGCTCAGTTAGACACTTCTAAGTCCTTGGTAACAGACATGCAGTCCCCTCGGCAGAGCCCCGCC
CCCCGGTGGTACCGGGTTGCTAAGAGGTCCACCACGGGTCGCACGACAACATCAAGGCCTCATGCATG
ACCCAACGCGGGGACGTACCGGCGCGGCGACCTTGAAGGTCGGGGACAAGTATGTCCCCCCGGGAGGG

GCGCCACAGGGAATCCGGAGTGTTGATCTGCAGCCCGAGACTCCACTACGATGGTATGAACGGCAAAG
GTATTTTGGACACCACCCCGTTTTCTATTTCCAGACGCGGCCATTCAGGAACGACGCGAATCGTAATC
TACCTGCCGCGTACGCGGATCGTACATGCGTTTTTCAGCGTTCGAAATAGAGTTTGTCGTTCCGCTGA
CCAGGTTTCACGGCTACTGCGATAAGCATATTCTACGGGGGGACTGTAAAGGAATTGTATCCACTCTG
CTCCCTGTCAATTGGCCATGTGCATCTCTGGTTGTTTAGGGTACATTAATANCACACAGCATTAGGTA
GATAAGTCTCCGATATAAGCGAACAGAACGTTGGTCAAAGACCGTGGTTCAATTGTAGTGGTATAACA
TTTCCCAGTGCATAAGGAACCCAGGGACACTACTAGCATGGGCGAGGAGAGACCCTTGGGTGGCAACA
CGCAATAAGTGTTACCCAGTACACCAGCACGATAGAGGGTTTGACCATGTGTTTTTAGACGACCCCGG
TCGCATTGAACCTGGCATTCAGAATGACGTGCTCTCAAGAGTCGGGGTCATCCCCCTTAGTGTCACCT
GCTTCGCGGGTTAGCGTATGGTCATAAACAT
>MNON16
GTGTAGCCCAGTTAGACACTTCTAAGTCCTTGGTAACAGACATGCAGTCCCCTCGGCAGAGCCCCGCC
CCCCGATGGTACCGGGTTGCTAAGAGGTCCACAACGGGTCGCACGACAACATTAAGGCCTCATGCATG
ACCCAACACGGGGGCGTCCCGGCGCGGCGACCTTGAAGTCCGGGGACAAGTATGTCCCCCCGGGAGGG
GCGCCACAGGGAATCCGGAGTGTTGATCTGCAGCCCGAGACTCCACTACGATGGTATGAACGGCAAAG
GTACTTTGGACACCACCCCGTTTTCTATTTCCAGACGCGGCCATTCAGGAACGACGCGAATCGTAATC
TACCTGCCGCGTACGCGGATCGTACATGCGTTTTCCAGCGTTCGAAATAGAGTTTGTCGTTCCGCTGA
CCAGGTTTCACGGCTACTGCGATAAGCATATTCTACTGGGGGACTGTAAAGGAATTGTATCCACTCTG
CTTCCTGTCAATTGGCCATGTGCATCTCTGGTTGTTCAGGGTACATTAATAACACACAGCATTAAGTA
GATAAGTCTCCGATATAAGCGAACAGAACGTTGGTCAGAGACTATGGTTCAATTGTAGTGGTATAATG
TTTCCCAGTGCATAAGGAACTCAGGGACACTACTAGCATGGGCGAGGAGAGACCCTTGGGTGGCAACA
CGCAATAAGTGTTACCTAGTACATCAGCACGAGAGAGGGTTTGACCATGTGTTTTTAGACGACCCCGG
TCGCATTGAACCTGGCATTCAGAATGACGTGCTCTCAAGAATCGGGGTCATCCCCCTTAGTGTCACCT
GCTTCGCGGGTCAGCGTATGGTCATAAACAT
>MNON21
GTGTAGCCCAGTTAGACACTTCTAAGTCCTTGGTAACAGACATGCAGTCCCCTCGGCAGAGCCCCGCC
CCCCGATGGTACCGGGTTGCTAAGAGGTCCACAACGGGTCGCACGACAACATTAAGGCCTCATGCATG
ACCCAACACGGGGGCGTCCCGGCGCGGCGACCTTGAAGTCCGGGGACAAGTATGTCCCCCCGGGAGGG
GCGCCACAGGGAATCCGGAGTGTTGATCTGCAGCCCGAGACTCCACTACGATGGTATGAACGGCAAAG
GTACTTTGGACACCACCCCGTTTTCTATTTCCAGACGCGGCCATTCAGGAACGACGCGAATCGTAATC
TACCTGCCGCGTACGCGGATCGTACATGCGTTTTCCAGCGTTCGAAATAGAGTTTGTCGTTCCGCTGA
CCAGGTTTCACGGCTACTGCGATAAGCATATTCTACTGGGGGACTGTAAAGGAATTGTATCCACTCTG
CTTCCTGTCAATTGGCCATGTGCATCTCTGGTTGTTCAGGGTACATTAATAACACACAGCATTAAGTA
GATAAGTCTCCGATATAAGCGAACAGAACGTTGGTCAGAGACTATGGTTCAATTGTAGTGGTATAATG
TTTCCCAGTGCATAAGGAACTCAGGGACACTACTAGCATGGGCGAGGAGAGACCCTTGGGTGGCAACA
CGCAATAAGTGTTACCTAGTACATCAGCACGAGAGAGGGTTTGACCATGTGTTTTTAGACGACCCCGG
TCGCATTGAACCTGGCATTCAGAATGACGTGCTCTCAAGAATCGGGGTCATCCCCCTTAGTGTCACCT
GCTTCGCGGGTCAGCGTATGGTCATAAACAT
>MNON29
GTGTAGCCCAGTTAGACACTTCTAAGTCCTTGGTAACAGACATGCAGTCCCCTCGGCAGAGCCCCGCC
CCCCGATGGTACCGGGTTGCTAAGAGGTCCACAACGGGTCGCACGACAACATTAAGGCCTCATGCATG
ACCCAACACGGGGGCGTCCCGGCGCGGCGACCTTGAAGTCCGGGGACAAGTATGTCCCCCCGGGAGGG
GCGCCACAGGGAATCCGGAGTGTTGATCTGCAGCCCGAGACTCCACTACGATGGTATGAACGGCAAAG
GTACTTTGGACACCACCCCGTTTTCTATTTCCAGACGCGGCCATTCAGGAACGACGCGAATCGTAATC
TACCTGCCGCGTACGCGGATCGTACATGCGTTTTCCAGCGTTCGAAATAGAGTTTGTCGTTCCGCTGA
CCAGGTTTCACGGCTACTGCGATAAGCATATTCTACTGGGGGACTGTAAAGGAATTGTATCCACTCTG
CTTCCTGTCAATTGGCCATGTGCATCTCTGGTTGTTCAGGGTACATTAATAACACACAGCATTAAGTA
GATAAGTCTCCGATATAAGCGAACAGAACGTTGGTCAGAGACTATGGTTCAATTGTAGTGGTATAATG
TTTCCCAGTGCATAAGGAACTCAGGGACACTACTAGCATGGGCGAGGAGAGACCCTTGGGTGGCAACA
CGCAATAAGTGTTACCTAGTACATCAGCACGAGAGAGGGTTTGACCATGTGTTTTTAGACGACCCCGG
TCGCATTGAACCTGGCATTCAGAATGACGTGCTCTCAAGAATCGGGGTCATCCCCCTTAGTGTCACCT
GCTTCGCGGGTCAGCGTATGGTCATAAACAT
>MNON32
GTGTAGCCCAGTTAGACACTTCTAAGTCCTTGGTAACAGACATGCAGTCCCCTCGGCAGAGCCCCGCC

CCCCGATGGTACCGGGTTGCTAAGAGGTCCACAACGGGTCGCACGACAACATTAAGGCCTCATGCATG
ACCCAACACGGGGGCGTCCCGGCGCGGCGACCTTGAAGTCCGGGGACAAGTATGTCCCCCCGGGAGGG
GCGCCACAGGGAATCCGGAGTGTTGATCTGCAGCCCGAGACTCCACTACGATGGTATGAACGGCAAAG
GTACTTTGGACACCACCCCGTTTTCTATTTCCAGACGCGGCCATTCAGGAACGACGCGAATCGTAATC
TACCTGCCGCGTACGCGGATCGTACATGCGTTTTCCAGCGTTCGAAATAGAGTTTGTCGTTCCGCTGA
CCAGGTTTCACGGCTACTGCGATAAGCATATTCTACTGGGGGACTGTAAAGGAATTGTATCCACTCTG
CTTCCTGTCAATTGGCCATGTGCATCTCTGGTTGTTCAGGGTACATTAATAACACACAGCATTAAGTA
GATAAGTCTCCGATATAAGCGAACAGAACGTTGGTCAGAGACTATGGTTCAATTGTAGTGGTATAATG
TTTCCCAGTGCATAAGGAACTCAGGGACACTACTAGCATGGGCGAGGAGAGACCCTTGGGTGGCAACA
CGCAATAAGTGTTACCTAGTACATCAGCACGAGAGAGGGTTTGACCATGTGTTTTTAGACGACCCCGG
TCGCATTGAACCTGGCATTCAGAATGACGTGCTCTCAAGAATCGGGGTCATCCCCCTTAGTGTCACCT
GCTTCGCGGGTCAGCGTATGGTCATAAACAT
>MNON33
GTGTAGCCCAGTTAGACACTTCTAAGTCCTTGGTAACAGACATGCAGTCCCCTCGGCAGAGCCCCGCC
CCCCGATGGTACCGGGTTGCTAAGAGGTCCACAACGGGTCGCACGACAACATTAAGGCCTCATGCATG
ACCCAACACGGGGGCGTCCCGGCGCGGCGACCTTGAAGTCCGGGGACAAGTATGTCCCCCCGGGAGGG
GCGCCACAGGGAATCCGGAGTGTTGATCTGCAGCCCGAGACTCCACTACGATGGTATGAACGGCAAAG
GTACTTTGGACACCACCCCGTTTTCTATTTCCAGACGCGGCCATTCAGGAACGACGCGAATCGTAATC
TACCTGCCGCGTACGCGGATCGTACATGCGTTTTCCAGCGTTCGAAATAGAGTTTGTCGTTCCGCTGA
CCAGGTTTCACGGCTACTGCGATAAGCATATTCTACTGGGGGACTGTAAAGGAATTGTATCCACTCTG
CTTCCTGTCAATTGGCCATGTGCATCTCTGGTTGTTCAGGGTACATTAATAACACACAGCATTAAGTA
GATAAGTCTCCGATATAAGCGAACAGAACGTTGGTCAGAGACTATGGTTCAATTGTAGTGGTATAATG
TTTCCCAGTGCATAAGGAACTCAGGGACACTACTAGCATGGGCGAGGAGAGACCCTTGGGTGGCAACA
CGCAATAAGTGTTACCTAGTACATCAGCACGAGAGAGGGTTTGACCATGTGTTTTTAGACGACCCCGG
TCGCATTGAACCTGGCATTCAGAATGACGTGCTCTCAAGAATCGGGGTCATCCCCCTTAGTGTCACCT
GCTTCGCGGGTCAGCGTATGGTCATAAACAT
>MNON36
GTGTAGCCCAGTTAGACACTTCTAAGTCCTTGGTAACAGACATGCAGTCCCCTCGGCAGAGCCCCGCC
CCCCGATGGTACCGGGTTGCTAAGAGGTCCACAACGGGTCGCACGACAACATTAAGGCCTCATGCATG
ACCCAACACGGGGGCGTCCCGGCGCGGCGACCTTGAAGTCCGGGGACAAGTATGTCCCCCCGGGAGGG
GCGCCACAGGGAATCCGGAGTGTTGATCTGCAGCCCGAGACTCCACTACGATGGTATGAACGGCAAAG
GTACTTTGGACACCACCCCGTTTTCTATTTCCAGACGCGGCCATTCAGGAACGACGCGAATCGTAATC
TACCTGCCGCGTACGCGGATCGTACATGCGTTTTCCAGCGTTCGAAATAGAGTTTGTCGTTCCGCTGA
CCAGGTTTCACGGCTACTGCGATAAGCATATTCTACTGGGGGACTGTAAAGGAATTGTATCCACTCTG
CTTCCTGTCAATTGGCCATGTGCATCTCTGGTTGTTCAGGGTACATTAATAACACACAGCATTAAGTA
GATAAGTCTCCGATATAAGCGAACAGAACGTTGGTCAGAGACTATGGTTCAATTGTAGTGGTATAATG
TTTCCCAGTGCATAAGGAACTCAGGGACACTACTAGCATGGGCGAGGAGAGACCCTTGGGTGGCAACA
CGCAATAAGTGTTACCTAGTACATCAGCACGAGAGAGGGTTTGACCATGTGTTTTTAGACGACCCCGG
TCGCATTGAACCTGGCATTCAGAATGACGTGCTCTCAAGAATCGGGGTCATCCCCCTTAGTGTCACCT
GCTTCGCGGGTCAGCGTATGGTCATAAACAT
>MNON37
GTGTAGCCCAGTTAGACACTTCTAAGTCCTTGGTAACAGACATGCAGTCCCCTCGGCAGAGCCCCGCC
CCCCGATGGTACCGGGTTGCTAAGAGGTCCACAACGGGTCGCACGACAACATTAAGGCCTCATGCATG
ACCCAACACGGGGGCGTCCCGGCGCGGCGACCTTGAAGTCCGGGGACAAGTATGTCCCCCCGGGAGGG
GCGCCACAGGGAATCCGGAGTGTTGATCTGCAGCCCGAGACTCCACTACGATGGTATGAACGGCAAAG
GTACTTTGGACACCACCCCGTTTTCTATTTCCAGACGCGGCCATTCAGGAACGACGCGAATCGTAATC
TACCTGCCGCGTACGCGGATCGTACATGCGTTTTCCAGCGTTCGAAATAGAGTTTGTCGTTCCGCTGA
CCAGGTTTCACGGCTACTGCGATAAGCATATTCTACTGGGGGACTGTAAAGGAATTGTATCCACTCTG
CTTCCTGTCAATTGGCCATGTGCATCTCTGGTTGTTCAGGGTACATTAATAACACACAGCATTAAGTA
GATAAGTCTCCGATATAAGCGAACAGAACGTTGGTCAGAGACTATGGTTCAATTGTAGTGGTATAATG
TTTCCCAGTGCATAAGGAACTCAGGGACACTACTAGCATGGGCGAGGAGAGACCCTTGGGTGGCAACA
CGCAATAAGTGTTACCTAGTACATCAGCACGAGAGAGGGTTTGACCATGTGTTTTTAGACGACCCCGG
TCGCATTGAACCTGGCATTCAGAATGACGTGCTCTCAAGAATCGGGGTCATCCCCCTTAGTGTCACCT
GCTTCGCGGGTCAGCGTATGGTCATAAACAT

>MNON39
GTGTAGCCCAGTTAGACACTTCTAAGTCCTTGGTAACAAACGTGCAGTCCCCTCGGCAGAGCCCCGCC
CCCCGATGGTACCGGGTTGCTAAGAGGTCCACCACGAGTCGCACAACAACAGCAAGGCCTCGTGCATG
ACCCAACACGGGGGCGTCCCGGCGCAGCGACCTTGAAGTCCGGGAACAAGTATGTCTCCCCGGGAGGG
GCGCCACAGGGAATCCGGAGTGTTGATCTGCAGCCCGAGACTCCACTACGATGGTATGAACGGCAAAG
GTACTTTGGACACCACCCCGTTTTCTATTCCCAGACGCGGCCATTCAGGAACGACGCGAATCGTCATC
TACCTGCCGCGTACGCGGATCGTACATGCGTTTTCCAGCTTTCGGAATAGAGTTTGTCGTTCCGCTGG
CCAGGTTTCACGGCTATTGCGATAAGCATACTCCACTGGGGGACTGTAAAGGAATTGCATCCACTCTG
CTTCTTATCAGTTGGCCATATGTATCTCTGGTTGTTCAGGGTGCATTAATAACACACAGCATTAAGTA
GATAAGTCTCCGATATAGGCGAACAGAACGTTGGTAAAAGACTGTGGTTCAACTGTAGTGGTATAATG
TTGCCCAGTGCATAAGGAACTCAGGGACACTACTAGCATGGGCGAGTAGAGACCCTTGGGTGGCAACA
CGCAATAAGTGTTACCTAGTACACCAACACGATATAGGGTTTGACCATGTGTTTTTAGACGACCCCGG
TCGCATTAAACCTGGCATTCATAATGACGTGCTCTCAAGAGTCGGGGTCATCCCCCTTAGTGTCACCT
GCTTCGCGGGTCATCGTATGGTCATAAACAT
>MNON40
GTGTAGCCCAGTTAGACACTTCTAAGTCCTTGGTAACAGACATGCAGTCCCCTCGGCAGAGCCCCGCC
CCCCGATGGTACCGGGTTGCTAAGAGGTCCACAACGGGTCGCACGACAACATTAAGGCCTCATGCATG
ACCCAACACGGGGGCGTCCCGGCGCGGCGACCTTGAAGTCCGGGGACAAGTATGTCCCCCCGGGAGGG
GCGCCACAGGGAATCCGGAGTGTTGATCTGCAGCCCGAGACTCCACTACGATGGTATGAACGGCAAAG
GTACTTTGGACACCACCCCGTTTTCTATTTCCAGACGCGGCCATTCAGGAACGACGCGAATCGTAATC
TACCTGCCGCGTACGCGGATCGTACATGCGTTTTCCAGCGTTCGAAATAGAGTTTGTCGTTCCGCTGA
CCAGGTTTCACGGCTACTGCGATAAGCATATTCTACTGGGGGACTGTAAAGGAATTGTATCCACTCTG
CTTCCTGTCAATTGGCCATGTGCATCTCTGGTTGTTCAGGGTACATTAATAACACACAGCATTAAGTA
GATAAGTCTCCGATATAAGCGAACAGAACGTTGGTCAGAGACTATGGTTCAATTGTAGTGGTNTAATG
TTTCCCAGTGCATAAGGAACTCAGGGACACTACTAGCATGGGCGAGGAGAGACCCTTGGGTGGCAACA
CGCAATAAGTGTTACCTAGTACATCAGCACGAGAGAGGGTTTGACCATGTGTTTTTAGACGACCCCGG
TCGCATTGAACCTGGCATTCAGAATGACGTGCTCTCAAGAATCGGGGTCATCCCCCTTAGTGTCACCT
GCTTCGCGGGTCAGCGTATGGTCATAAACAT
>MNON48
GTGTAGCCCAGTTAGACACTTCTAAGTCCTTGGTAACAGACATGCAGTCCCCTCGGCAGAGCCCCGCC
CCCCGATGGTACCGGGTTGCTAAGAGGTCCACAACGGGTCGCACGACAACATTAAGGCCTCATGCATG
ACCCAACACGGGGGCGTCCCGGCGCGGCGACCTTGAAGTCCGGGGACAAGTATGTCCCCCCGGGAGGG
GCGCCACAGGGAATCCGGAGTGTTGATCTGCAGCCCGAGACTCCACTACGATGGTATGAACGGCAAAG
GTACTTTGGACACCACCCCGTTTTCTATTTCCAGACGCGGCCATTCAGGAACGACGCGAATCGTAATC
TACCTGCCGCGTACGCGGATCGTACATGCGTTTTCCAGCGTTCGAAATAGAGTTTGTCGTTCCGCTGA
CCAGGTTTCACGGCTACTGCGATAAGCATATTCTACTGGGGGACTGTAAAGGAATTGTATCCACTCTG
CTTCCTGTCAATTGGCCATGTGCATCTCTGGTTGTTCAGGGTACATTAATAACACACAGCATTAAGTA
GATAAGTCTCCGATATAAGCGAACAGAACGTTGGTCAGAGACTATGGTTCAATTGTAGTGGTATAATG
TTTCCCAGTGCATAAGGAACTCAGGGACACTACTAGCATGGGCGAGGAGAGACCCTTGGGTGGCAACA
CGCAATAAGTGTTACCTAGTACATCAGCACGAGAGAGGGTTTGACCATGTGTTTTTAGACGACCCCGG
TCGCATTGAACCTGGCATTCAGAATGACGTGCTCTCAAGAATCGGGGTCATCCCCCTTAGTGTCACCT
GCTTCGCGGGTCAGCGTATGGTCATAAACAT
>MNON49
GTGTAGCCCAGTTAGACACTTCTAAGTCCTTGGTAACAGACATGCAGTCCCCTCGGCAGAGCCCCGCC
CCCCGATGGTACCGGGTTGCTAAGAGGTCCACAACGGGTCGCACGACAACATTAAGGCCTCATGCATG
ACCCAACACGGGGGCGTCCCGGCGCGGCGACCTTGAAGTCCGGGGACAAGTATGTCCCCCCGGGAGGG
GCGCCACAGGGAATCCGGAGTGTTGATCTGCAGCCCGAGACTCCACTACGATGGTATGAACGGCAAAG
GTACTTTGGACACCACCCCGTTTTCTATTTCCAGACGCGGCCATTCAGGAACGACGCGAATCGTAATC
TACCTGCCGCGTACGCGGATCGTACATGCGTTTTCCAGCGTTCGAAATAGAGTTTGTCGTTCCGCTGA
CCAGGTTTCACGGCTACTGCGATAAGCATATTCTACTGGGGGACTGTAAAGGAATTGTATCCACTCTG
CTTCCTGTCAATTGGCCATGTGCATCTCTGGTTGTTCAGGGTACATTAATAACACACAGCATTAAGTA
GATAAGTCTCCGATATAAGCGAACAGAACGTTGGTCAGAGACTATGGTTCAATTGTAGTGGTATAATG
TTTCCCAGTGCATAAGGAACTCAGGGACACTACTAGCATGGGCGAGGAGAGACCCTTGGGTGGCAACA
CGCAATAAGTGTTACCTAGTACATCAGCACGAGAGAGGGTTTGACCATGTGTTTTTAGACGACCCCGG

TCGCATTGAACCTGGCATTCAGAATGACGTGCTCTCAAGAATCGGGGTCATCCCCCTTAGTGTCACCT
GCTTCGCGGGTCAGCGTATGGTCATAAACAT
>MNON50
GTGTAGCCCAGTTAGACACTTCTAAGTCCTTGGTAACAAACGTGCAGTCCCCTCGGCAGAGCCCCGCC
CCCCGATGGTACCGGGTTGCTAAGAGGTCCACCACGAGTCGCACAACAACAGCAAGGCCTCGTGCATG
ACCCAACACGGGGGCGTCCCGGCGCAGCGACCTTGAAGTCCGGGAACAAGTATGTCTCCCCGGGAGGG
GCGCCACAGGGAATCCGGAGTGTTGATCTGCAGCCCGAGACTCCACTACGATGGTATGAACGGCAAAG
GTACTTTGGACACCACCCCGTTTTCTATTCCCAGACGCGGCCATTCAGGAACGACGCGAATCGTCATC
TACCTGCCGCGTACGCGGATCGTACATGCGTTTTCCAGCTTTCGGAATAGAGTTTGTCGTTCCGCTGG
CCAGGTTTCACGGCTATTGCGATAAGCATACTCCACTGGGGGACTGTAAAGGAATTGCATCCACTCTG
CTTCTTATCAGTTGGCCATATGTATCTCTGGTTGTTCAGGGTGCATTAATAACACACAGCATTAAGTA
GATAAGTCTCCGATATAGGCGAACAGAACGTTGGTAAAAGACTGTGGTTCAACTGTAGTGGTATAATG
TTGCCCAGTGCATAAGGAACTCAGGGACACTACTAGCATGGGCGAGTAGAGACCCTTGGGTGGCAACA
CGCAATAAGTGTTACCTAGTACACCAACACGATATAGGGTTTGACCATGTGTTTTTAGACGACCCCGG
TCGCATTAAACCTGGCATTCATAATGACGTGCTCTCAAGAGTCGGGGTCATCCCCCTTAGTGTCACCT
GCTTCGCGGGTCATCGTATGGTCATAAACAT
>MNON51
GTGTAGCCCAGTTAGACACTTCTAAGTCCTTGGTAACAGACATGCAGTCCCCTCGGCAGAGCCCCGCC
CCCCGATGGTACCGGGTTGCTAAGAGGTCCACAACGGGTCGCACGACAACATTAAGGCCTCATGCATG
ACCCAACACGGGGGCGTCCCGGCGCGGCGACCTTGAAGTCCGGGGACAAGTATGTCCCCCCGGGAGGG
GCGCCACAGGGAATCCGGAGTGTTGATCTGCAGCCCGAGACTCCACTACGATGGTATGAACGGCAAAG
GTACTTTGGACACCACCCCGTTTTCTATTTCCAGACGCGGCCATTCAGGAACGACGCGAATCGTAATC
TACCTGCCGCGTACGCGGATCGTACATGCGTTTTCCAGCGTTCGAAATAGAGTTTGTCGTTCCGCTGA
CCAGGTTTCACGGCTACTGCGATAAGCATATTCTACTGGGGGACTGTAAAGGAATTGTATCCACTCTG
CTTCCTGTCAATTGGCCATGTGCATCTCTGGTTGTTCAGGGTACATTAATAACACACAGCATTAAGTA
GATAAGTCTCCGATATAAGCGAACAGAACGTTGGTCAGAGACTATGGTTCAATTGTAGTGGTATAATG
TTTCCCAGTGCATAAGGAACTCAGGGACACTACTAGCATGGGCGAGGAGAGACCCTTGGGTGGCAACA
CGCAATAAGTGTTACCTAGTACATCAGCACGAGAGAGGGTTTGACCATGTGTTTTTAGACGACCCCGG
TCGCATTGAACCTGGCATTCAGAATGACGTGCTCTCAAGAATCGGGGTCATCCCCCTTAGTGTCACCT
GCTTCGCGGGTCAGCGTATGGTCATAAACAT
>MNON53
GTGTAGCCCAGTTAGACACTTCTAAGTCCTTGGTAACAGACATGCAGTCCCCTCGGCAGAGCCCCGCC
CCCCGATGGTACCGGGTTGCTAAGAGGTCCACAACGGGTCGCACGACAACATTAAGGCCTCATGCATG
ACCCAACACGGGGGCGTCCCGGCGCGGCGACCTTGAAGTCCGGGGACAAGTATGTCCCCCCGGGAGGG
GCGCCACAGGGAATCCGGAGTGTTGATCTGCAGCCCGAGACTCCACTACGATGGTATGAACGGCAAAG
GTACTTTGGACACCACCCCGTTTTCTATTTCCAGACGCGGCCATTCAGGAACGACGCGAATCGTAATC
TACCTGCCGCGTACGCGGATCGTACATGCGTTTTCCAGCGTTCGAAATAGAGTTTGTCGTTCCGCTGA
CCAGGTTTCACGGCTACTGCGATAAGCATATTCTACTGGGGGACTGTAAAGGAATTGTATCCACTCTG
CTTCCTGTCAATTGGCCATGTGCATCTCTGGTTGTTCAGGGTACATTAATAACACACAGCATTAAGTA
GATAAGTCTCCGATATAAGCGAACAGAACGTTGGTCAGAGACTATGGTTCAATTGTAGTGGTATAATG
TTTCCCAGTGCATAAGGAACTCAGGGACACTACTAGCATGGGCGAGGAGAGACCCTTGGGTGGCAACA
CGCAATAAGTGTTACCTAGTACATCAGCACGAGAGAGGGTTTGACCATGTGTTTTTAGACGACCCCGG
TCGCATTGAACCTGGCATTCAGAATGACGTGCTCTCAAGAATCGGGGTCATCCCCCTTAGTGTCACCT
GCTTCGCGGGTCAGCGTATGGTCATAAACAT
>CHRUN1
GTGTAGCCCAGTTAGACACTTCTAAGTCCTTGGTAACAGACATGCAGTCCCCTCGGCAGAGCCCCGCC
CCCCGATGGTACCGGGTTGCTAAGAAGTCCACAACGGGTCGCACGACAACATTAAGGCCTCATGCATG
ACCCAACACGGGGGCGTCCCGGCGCGGCGACCTTGAAGTCCGGGGACAAGTATGTCCCCCCGGGAGGG
GCGCCACAGGGAATCCGGAGTGTTGATCTGCAGCCCGAGACTCCACTACGATGGTATGAACGGCAAAG
GTACTTTGGACACCACCCCGTTTTCTATTTCCAGACGCGGCCATTCAGGAACGACGCGAATCGTAATC
TACCTGCCGCGTACGCGGATCGTACATGCGTTTTCCAGCGTTCGAAATAGAGTTTGTCGTTCCGCTGA
CCAGGTTTCACGGCTACTGCGATAAGCATATTCTACTGGGGGACTGTAAAGGAATTGTATCCATTCTG
CTTCCTGTCAATTGGCCATGTGCATCTCTGGTTGTTCAGGGTACATTAATAACACACAGCAGTAAGTA
GGTAAGTCTCCGATATAAGCTAACAGAACGTTTGTCAGAGACTGTGGTTCAATTGTAGTGGTATAATG

TTTCCCAGTGCATAAGGAACTCAGGGACACTACTAGCATGGGCGAGGAGAGACCCTTGGGTGGCAACA
CGCAATAAGTGTTACCTAGTACACCAGCACGAGAGAGGGTTTGACCATGTGTTTTCAGACGACCCCGG
TNGCATTGAACCTGGCATTCAGAATGACGTGCTCTCAAGAATCGGGGTCATCCCCCTTAGTGTCACCT
GCTTCGCGGGTCAGCGTATGGTCATAAACAT
>CHRUN2
GTGTAGCCCAGTTAGACACTTCTAAGTCCTTGGTAACAGACATGCAGTCCCCTCGGCAGAGCCCCGCC
CCCCGATGGTACCGGGTTGCTAAGAGGTCCACAACGGGTCGCACGACAACATTAAGGCCTCATGCATG
ACCCAACACGGGGGCGTCCCGGCGCGGCGACCTTGAAGTCCGGGGACAAGTATGTCCCCCCGGGAGGG
GCGCCACAGGGAATCCGGAGTGTTGATCTGCAGCCCGAGACTCCACTACGATGGTATGAACGGCAAAG
GTACTTTGGACACCACCCCGTTTTCTATTTCCAGACGCGGCCATTCAGGAACGACGCGAATCGTAATC
TACCTGCCGCGTACGCGGATCGTACATGCGTTTTCCAGCGTTCGAAATAGAGTTTGTCGTTCCGCTGA
CCAGGTTTCACGGCTACTGCGATAAGCATATTCTACTGGGGGACTGTAAAGGAATTGTATCCACTCTG
CTTCCTGTCAATTGGCCATGTGCATCTCTGGTTGTTCAGGGTACATTAATAACACACAGCATTAAGTA
GATAAGTCTCCGATATAAGCGAACAGAACGTTGGTCAGAGACTATGGTTCAATTGTAGTGGTATAATG
TTTCCCAGTGCATAAGGAACTCAGGGACACTACTAGCATGGGCGAGGAGAGACCCTTGGGTGGCAACA
CGCAATAAGTGTTACCTAGTACATCAGCACGAGAGAGGGTTTGACCATGTGTTTTTAGACGACCCCGG
TCGCATTGAACCTGGCATTCAGAATGACGTGCTCTCAAGAATCGGGGTCATCCCCCTTAGTGTCACCT
GCTTCGCGGGTCAGCGTATGGTCATAAACAT
>CHRUN3
GTGTAGCCCAGTTAGACACTTCTAAGTCCTTGGTAACAGACATGCAGTCCCCTCGGCAGAGCCCCGCC
CCCCGATGGTACCGGGTTGCTAAGAGGTCCACAACGGGTCGCACGACAACATTAAGGCCTCATGCATG
ACCCAACACGGGGGCGTCCCGGCGCGGCGACCTTGAAGTCCGGGGACAAGTATGTCCCCCCGGGAGGG
GCGCCACAGGGAATCCGGAGTGTTGATCTGCAGCCCGAGACTCCACTACGATGGTATGAACGGCAAAG
GTACTTTGGACACCACCCCGTTTTCTATTTCCAGACGCGGCCATTCAGGAACGACGCGAATCGTAATC
TACCTGCCGCGTACGCGGATCGTACATGCGTTTTCCAGCGTTCGAAATAGAGTTTGTCGTTCCGCTGA
CCAGGTTTCACGGCTACTGCGATAAGCATATTCTACTGGGGGACTGTAAAGGAATTGTATCCACTCTG
CTTCCTGTCAATTGGCCATGTGCATCTCTGGTTGTTCAGGGTACATTAATAACACACAGCATTAAGTA
GATAAGTCTCCGATATAAGCGAACAGAACGTTGGTCAGAGACTATGGTTCAATTGTAGTGGTATAATG
TTTCCCAGTGCATAAGGAACTCAGGGACACTACTAGCATGGGCGAGGAGAGACCCTTGGGTGGCAACA
CGCAATAAGTGTTACCTAGTACATCAGCACGAGAGAGGGTTTGACCATGTGTTTTTAGACGACCCCGG
TCGCATTGAACCTGGCATTCAGAATGACGTGCTCTCAAGAATCGGGGTCATCCCCCTTAGTGTCACCT
GCTTCGCGGGTCAGCGTATGGTCATAAACAT
>CHRUN4
GTGTAGCCCAGTTAGACACTTCTAAGTCCTTGGTAACAGACATGCAGTCCCCTCGGCAGAGCCCCGCC
CCCCGATGGTACCGGGTTGCTAAGAGGTCCACAACGGGTCGCACGACAACATTAAGGCCTCATGCATG
ACCCAACACGGGGGCGTCCCGGCGCGGCGACCTTGAAGTCCGGGGACAAGTATGTCCCCCCGGGAGGG
GCGCCACAGGGAATCCGGAGTGTTGATCTGCAGCCCGAGACTCCACTACGATGGTATGAACGGCAAAG
GTACTTTGGACACCACCCCGTTTTCTATTTCCAGACGCGGCCATTCAGGAACGACGCGAATCGTAATC
TACCTGCCGCGTACGCGGATCGTACATGCGTTTTCCAGCGTTCGAAATAGAGTTTGTCGTTCCGCTGA
CCAGGTTTCACGGCTACTGCGATAAGCATATTCTACTGGGGGACTGTAAAGGAATTGTATCCACTCTG
CTTCCTGTCAATTGGCCATGTGCATCTCTGGTTGTTCAGGGTACATTAATAACACACAGCATTAAGTA
GATAAGTCTCCGATATAAGCGAACAGAACGTTGGTCAGAGACTATGGTTCAATTGTAGTGGTATAATG
TTTCCCAGTGCATAAGGAACTCAGGGACACTACTAGCATGGGCGAGGAGAGACCCTTGGGTGGCAACA
CGCAATAAGTGTTACCTAGTACATCAGCACGAGAGAGGGTTTGACCATGTGTTTTTAGACGACCCCGG
TCGCATTGAACCTGGCATTCAGAATGACGTGCTCTCAAGAATCGGGGTCATCCCCCTTAGTGTCACCT
GCTTCGCGGGTCAGCGTATGGTCATAAACAT
>CHRUN6
GTGTAGCCCAGTTAGACACTTCTAAGTCCTTGGTAACAGACATGCAGTCCCCTCGGCAGAGCCCCGCC
CCCCGATGGTACCGGGTTGCTAAGAGGTCCACAACGGGTCGCACGACAACATTAAGGCCTCATGCATG
ACCCAACACGGGGGCGTCCCGGCGCGGCGACCTTGAAGTCCGGGGACAAGTATGTCCCCCCGGGAGGG
GCGCCACAGGGAATCCGGAGTGTTGATCTGCAGCCCGAGACTCCACTACGATGGTATGAACGGCAAAG
GTACTTTGGACACCACCCCGTTTTCTATTTCCAGACGCGGCCATTCAGGAACGACGCGAATCGTAATC
TACCTGCCGCGTACGCGGATCGTACATGCGTTTTCCAGCGTTCGAAATAGAGTTTGTCGTTCCGCTGA
CCAGGTTTCACGGCTACTGCGATAAGCATATTCTACTGGGGGACTGTAAAGGAATTGTATCCACTCTG

CTTCCTGTCAATTGGCCATGTGCATCTCTGGTTGTTCAGGGTACATTAATAACACNCAGCATTAAGTA
GATAAGTCTCCGATATAAGCGAACAGAACGTTGGTCAGAGACTATGGTTCAATTGTAGTGGTATAATG
TTTCCCAGTGCATAAGGAACTCAGGGACACTACTAGCATGGGCGAGGAGAGACCCTTGGGTGGCAACA
CGCAATAAGTGTTACCTAGTACATCAGCACGAGAGAGGGTTTGACCATGTGTTTTTAGACGACCCCGG
TCGCATTGAACCTGGCATTCAGAATGACGTGCTCTCAAGAATCGGGGTCATCCCCCTTAGTGTCACCT
GCTTCGCGGGTCAGCGTATGGTCATAAACAT
>CHRUN7
GTGTAGCCCAGTTAGACACTTCTAAGTCCTTGGTAACAGACATGCAGTCCCCTCGGCAGAGCCCCGCC
CCCCGATGGTACCGGGTTGCTAAGAGGTCCACAACGGGTCGCACGACAACATTAAGGCCTCATGCATG
ACCCAACACGGGGGCGTCCCGGCGCGGCGACCTTGAAGTCCGGGGACAAGTATGTCCCCCCGGGAGGG
GCGCCACAGGGAATCCGGAGTGTTGATCTGCAGCCCGAGACTCCACTACGATGGTATGAACGGCAAAG
GTACTTTGGACACCACCCCGTTTTCTATTTCCAGACGCGGCCATTCAGGAACGACGCGAATCGTAATC
TACCTGCCGCGTACGCGGATCGTACATGCGTTTTCCAGCGTTCGAAATAGAGTTTGTCGTTCCGCTGA
CCAGGTTTCACGGCTACTGCGATAAGCATATTCTACTGGGGGACTGTAAAGGAATTGTATCCACTCTG
CTTCCTGTCAATTGGCCATGTGCATCTCTGGTTGTTCAGGGTACATTAATAACACACAGCATTAAGTA
GATAAGTCTCCGATATAAGCGAACAGAACGTTGGTCAGAGACTATGGTTCAATTGTAGTGGTATAATG
TTTCCCAGTGCATAAGGAACTCAGGGACACTACTAGCATGGGCGAGGAGAGACCCTTGGGTGGCAACA
CGCAATAAGTGTTACCTAGTACATCAGCACGAGAGAGGGTTTGACCATGTGTTTTTAGACGACCCCGG
TCGCATTGAACCTGGCATTCAGAATGACGTGCTCTCAAGAATCGGGGTCATCCCCCTTAGTGTCACCT
GCTTCGCGGGTCAGCGTATGGTCATAAACAT
>CHRUN8
GTGTAGCCCAGTTAGACACTTCTAAGTCCTTGGTAACAGACATGCAGTCCCCTCGGCAGAGCCCCGCC
CCCCGATGGTACCGGGTTGCTAAGAGGTCCACAACGGGTCGCACGACAACATTAAGGCCTCATGCATG
ACCCAACACGGGGGCGTCCCGGCGCGGCGACCTTGAAGTCCGGGGACAAGTATGTCCCCCCGGGAGGG
GCGCCACAGGGAATCCGGAGTGTTGATCTGCAGCCCGAGACTCCACTACGATGGTATGAACGGCAAAG
GTACTTTGGACACCACCCCGTTTTCTATTTCCAGACGCGGCCATTCAGGAACGACGCGAATCGTAATC
TACCTGCCGCGTACGCGGATCGTACATGCGTTTTCCAGCGTTCGAAATAGAGTTTGTCGTTCCGCTGA
CCAGGTTTCACGGCTACTGCGATAAGCATATTCTACTGGGGGACTGTAAAGGAATTGTATCCACTCTG
CTTCCTGTCAATTGGCCATGTGCATCTCTGGTTGTTCAGGGTACATTAATAACACACAGCATTAAGTA
GATAAGTCTCCGATATAAGCGAACAGAACGTTGGTCAGAGACTATGGTTCAATTGTAGTGGTATAATG
TTTCCCAGTGCATAAGGAACTCAGGGACACTACTAGCATGGGCGAGGAGAGACCCTTGGGTGGCAACA
CGCAATAAGTGTTACCTAGTACATCAGCACGAGAGAGGGTTTGACCATGTGTTTTTAGACGACCCCGG
TCGCATTGAACCTGGCATTCAGAATGACGTGCTCTCAAGAATCGGGGTCATCCCCCTTAGTGTCACCT
GCTTCGCGGGTCAGCGTATGGTCATAAACAT
>CHRUN9
GTGTAGCCCAGTTAGACACTTCTAAGTCCTTGGTAACAGACATGCAGTCCCCTCGGCAGAGCCCCGCC
CCCCGATGGTACCGGGTTGCTAAGAGGTCCACAACGGGTCGCACGACAACATTAAGGCCTCATGCATG
ACCCAACACGGGGGCGTCCCGGCGCGGCGACCTTGAAGTCCGGGGACAAGTATGTCCCCCCGGGAGGG
ACGCCACAGGGAATCCGGAGTGTTGATCTGCAGCCCGAGACTCCACTACGATGGTATGAACGGCAAAG
GTACTTTGGACACCACCCCGTTTTCTATTTCCAGACGCGGCCATTCAGGAACGACGCGAATCGTAATC
TACCTGCCGCGTACGCGGATCGTACATGCGTTTTCCAGCGTTCGAAATAGAGTTTGTCGTTCCGCTGA
CCAGGTTTCACGGCTACTGCGATAAGCATATTCTACTGGGGGACTGTAAAGGAATTGTATCCACTCTG
CTTCCTGTCAATTGGCCATGTGCATCTCTGGTTGTTCAGGGTACATTAATAACACACAGCATTAAGTA
GATAAGTCTCCGATATAAGCGAACAGAACGTTGGTCAGAGACTATGGTTCAATTGTAGTGGTATAATG
TTTCCCAGTGCATAAGGAACTCAGGGACACTACTAGCATGGGCGAGGAGAGACCCTTGGGTGGCAACA
CGCAATAAGTGTTACCTAGTACATCAGCACGAGAGAGGGTTTGACCATGTGTTTTTAGACGACCCCGG
TCGCATTGAACCTGGCATTCAGAATGACGTGCTCTCAAGAATCGGGGTCATCCCCCTTAGTGTCACCT
GCTTCGCGGGTCAGCGTATGGTCATAAACAT
>CHRUN10
GTGTAGCCCAGTTAGACACTTCTAAGTCCTTGGTAACAGACATGCAGTCCCCTCGGCAGAGCCCCGCC
CCCCGATGGTACCGGGTTGCTAAGAGGTCCACAACGGGTCGCACGACAACATTAAGGCCTCATGCATG
ACCCAACACGGGGGCGTCCCGGCGCGGCGACCTTGAAGTCCGGGGACAAGTATGTCCCCCCGGGAGGG
GCGCCACAGGGAATCCGGAGTGTTGATCTGCAGCCCGAGACTCCACTACGATGGTATGAACGGCAAAG
GTACTTTGGACACCACCCCGTTTTCTATTTCCAGACGCGGCCATTCAGGAACGACGCGAATCGTAATC

TACCTGCCGCGTACGCGGATCGTACATGCGTTTTCCAGCGTTCGAAATAGAGTTTGTCGTTCCGCTGA
CCAGGTTTCACGGCTACTGCGATAAGCATATTCTACTGGGGGACTGTAAAGGAATTGTATCCACTCTG
CTTCCTGTCAATTGGCCATGTGCATCTCTGGTTGTTCAGGGTACATTAATAACACACAGCATTAAGTA
GATAAGTCTCCGATATAAGCGAACAGAACGTTGGTCAGAGACTATGGTTCAATTGTAGTGGTATAATG
TTTCCCAGTGCATAAGGAACTCAGGGACACTACTAGCATGGGCGAGGAGAGACCCTTGGGTGGCAACA
CGCAATAAGTGTTACCTAGTACATCAGCACGAGAGAGGGTTTGACCATGTGTTTTTAGACGACCCCGG
TCGCATTGAACCTGGCATTCAGAATGACGTGCTCTCAAGAATCGGGGTCATCCCCCTTAGTGTCACCT
GCTTCGCGGGTCAGCGTATGGTCATAAACAT
>CHRUN11
GTGTAGCCCAGTTAGACACTTCTAAGTCCTTGGTAACAGACATGCAGTCCCCTCGGCAGAGCCCCGCC
CCCCGATGGTACCGGGTTGCTAAGAGGTCCACAACGGGTCGCACGACAACATTAAGGCCTCATGCATG
ACCCAACACGGGGGCGTCCCGGCGCGGCGACCTTGAAGTCCGGGGACAAGTATGTCCCCCCGGGAGGG
GCGCCACAGGGAATCCGGAGTGTTGATCTGCAGCCCGAGACTCCACTACGATGGTATGAACGGCAAAG
GTACTTTGGACACCACCCCGTTTTCTATTTCCAGACGCGGCCATTCAGGAACGACGCGAATCGTAATC
TACCTGCCGCGTACGCGGATCGTACATGCGTTTTCCAGCGTTCGAAATAGAGTTTGTCGTTCCGCTGA
CCAGGTTTCACGGCTACTGCGATAAGCATATTCTACTGGGGGACTGTAAAGGAATTGTATCCACTCTG
CTTCCTGTCAATTGGCCATGTGCATCTCTGGTTGTTCAGGGTACATTAATAACACACAGCATTAAGTA
GATAAGTCTCCGATATAAGCGAACAGAACGTTGGTCAGAGACTATGGTTCAATTGTAGTGGTATAATG
TTTCCCAGTGCATAAGGAACTCAGGGACACTACTAGCATGGGCGAGGAGAGACCCTTGGGTGGCAACA
CGCAATAAGTGTTACCTAGTACATCAGCACGAGAGAGGGTTTGACCATGTGTTTTTAGACGACCCCGG
TCGCATTGAACCTGGCATTCAGAATGACGTGCTCTCAAGAATCGGGGTCATCCCCCTTAGTGTCACCT
GCTTCGCGGGTCAGCGTATGGTCATAAACAT
>CHRUN12
GTGTAGCCCAGTTAGACACTTCTAAGTCCTTGGTAACAGACATGCAGTCCCCTCGGCAGAGCCCCGCC
CCCCGATGGTACCGGGTTGCTAAGAGGTCCACAACGGGTCGCACGACAACATTAAGGCCTCATGCATG
ACCCAACACGGGGGCGTCCCGGCGCGGCGACCTTGAAGTCCGGGGACAAGTATGTCCCCCCGGGAGGG
GCGCCACAAGGAATCCGGAGTGTTGATCTGCAGCCCGAGACTCCACTACGATGGTATGAACGGCAAAG
GTACTTTGGACACCACCCCGTTTTCTATTTCCAGACGCGGCCATTCAGGAACGACGCGAATCGTAATC
TACCTGCCGCGTACGCGGATCGTACATGCGTTTTCCAGCGTTCGAAATAGAGTTTGTCGTTCCGCTGA
CCAGGTTTCACGGCTACTGCGATAAGCATATTCTACTGGGGGACTGTAAAGGAATTGTATCCACTCTG
CTTCCTGTCAATTGGCCATGTGCATCTCTGGTTGTTCAGGGTACATTAATAACACACAGCATTAAGTA
GATAAGTCTCCGATATAAGCGAACAGAACGTTGGTCAGAGACTATGGTTCAATTGTAGTGGTATAATG
TTTCCCAGTGCATAAGGAACTCAGGGACACTACTAGCATGGGCGAGGAGAGACCCTTGGGTGGCAACA
CGCAATAAGTGTTACCTAGTACATCAGCACGAGAGAGGGTTTGACCATGTGTTTTTAGACGACCCCGG
TCGCATTGAACCTGGCATTCAGAATGACGTGCTCTCAAGAATCGGGGTCATCCCCCTTAGTGTCACCT
GCTTCGCGGGTCAGCGTATGGTCATAAACAT
>CHRUN13
GTGTAGCCCAGTTAGACACTTCTAAGTCCTTGGTAACAGACATGCAGTCCCCTCGGCAGAGCCCCGCC
CCCCGATGGTACCGGGTTGCTAAGAGGTCCACAACGGGTCGCACGACAACATTAAGGCCTCATGCATG
ACCCAACACGGGGGCGTCCCGGCGCGGCGACCTTGAAGTCCGGGGACAAGTATGTCCCCCCGGGAGGG
GCGCCACAGGGAATCCGGAGTGTTGATCTGCAGCCCGAGACTCCACTACGATGGTATGAACGGCAAAG
GTACTTTGGACACCACCCCGTTTTCTATTTCCAGACGCGGCCATTCAGGAACGACGCGAATCGTAATC
TACCTGCCGCGTACGCGGATCGTACATGCGTTTTCCAGCGTTCGAAATAGAGTTTGTCGTTCCGCTGA
CCAGGTTTCACGGCTACTGCGATAAGCATATTCTACTGGGGGACTGTAAAGGAATTGTATCCACTCTG
CTTCCTGTCAATTGGCCATGTGCATCTCTGGTTGTTCAGGGTACATTAATAACACACAGCATTAAGTA
GATAAGTCTCCGATATAAGCGAACAGAACGTTGGTCAGAGACTATGGTTCAATTGTAGTGGTATAATG
TTTCCCAGTGCATAAGGAACTCAGGGACACTACTAGCATGGGCGAGGAGAGACCCTTGGGTGGCAACA
CGCAATAAGTGTTACCTAGTACATCAGCACGAGAGAGGGTTTGACCATGTGTTTTTAGACGACCCCGG
TCGCATTGAACCTGGCATTCAGAATGACGTGCTCTCAAGAATCGGGGTCATCCCCCTTAGTGTCACCT
GCTTCGCGGGTCAGCGTATGGTCATAAACAT
>CHRUN14
GTGNAGCCCAGTTAGACACTTCTAAGTCCTTGGTAACAGACATGCAGTCCCCTCGGCAGAGCCCCGCC
CCCCGATGGTACCGGGTTGCTAAGAGGTCCACAACGGGTCGCACGACAACATTAAGGCCTCATGCATG
ACCCAACACGGNGGCGTCCCGGCGCGGCGACCTTGAAGTCCGGGGACAAGTATGTCCCCCCGGGAGGG

GCGCCACAGGGAATCCGGAGTGTTGATCTGCAGCCCGAGACTCCACTACGATGGTATGAACGGCAAAG
GTACTTTGGACACCACCCCGTTTTCTATTTCCAGACGCGGCCATTCAGGAACGACGCGAATCGTAATC
TACCTGCCGCGTACGCGGATCGTACATGCGTTTTCCAGCGTTCGAAATAGAGTTTGTCGTTCCGCTGA
CCAGGTTTCACGGCTACTGCGATAAGCATATTCTACTGGGGGACTGTAAAGGAATTGTATCCACTCTG
CTTCCTGTCAATTGGCCATGTGCATCTCTGGTTGTTCAGGGTACNTTAATAACACACAGCATTAAGTA
GATAAGTCTCCGATATAAGCGAACAGAACGTTGGTCAGAGACTATGGTTCAATTGTAGTGGTATAATG
TTTCCCAGTGCATAAGGAACTCAGGGACACTACTAGCATGGGCGAGGAGAGACCCTTGGGTGGCAACA
CGCAATAAGTGTTACCTAGTACATCAGCACGAGAGAGGGTTTGACCATGTGTTTTTAGACGACCCCGG
TCGCATTGAACCTGGCATTCAGAATGACGTGCTCTCAAGAATCGGGGTCATCCCCCTTAGTGTCACCT
GCTTCGCGGGTCAGCGTATGGTCATAAACAT
>CHRUN15
GTGTAGCCCAGTTAGACACTTCTAAGTCCTTGGTAACAGACATGCAGTCCCCTCGGCAGAGCCCCGCC
CCCCGATGGTACCGGGTTGCTAAGAGGTCCACAACGGGTCGCACGACAACATTAAGGCCTCATGCATG
ACCCAACACGGGGGCGTCCCGGCGCGGCGACCTTGAAGTCCGGGGACAAGTATGTCCCCCCGGGAGGG
GCGCCACAGGGAATCCGGAGTGTTGATCTGCAGCCCGAGACTCCACTACGATGGTATGAACGGCAAAG
GTACTTTGGACACCACCCCGTTTTCTATTTCCAGACGCGGCCATTCAGGAACGACGCGAATCGTAATC
TACCTGCCGCGTACGCGGATCGTACATGCGTTTTCCAGCGTTCGAAATAGAGTTTGTCGTTCCGCTGA
CCAGGTTTCACGGCTACTGCGATAAGCATATTCTACTGGGGGACTGTAAAGGAATTGTATCCACTCTG
CTTCCTGTCAATTGGCCATGTGCATCTCTGGTTGTTCAGGGTACATTAATAACACACAGCATTAAGTA
GATAAGTCTCCGATATAAGCGAACAGAACGTTGGTCAGAGACTATGGTTCAATTGTAGTGGTATAATG
TTTCCCAGTGCATAAGGAACTCAGGGACACTACTAGCATGGGCGAGGAGAGACCCTTGGGTGGCAACA
CGCAATAAGTGTTACCTAGTACATCAGCACGAGAGAGGGTTTGACCATGTGTTTTTAGACGACCCCGG
TCGCATTGAACCTGGCATTCAGAATGACGTGCTCTCAAGAATCGGGGTCATCCCCCTTAGTGTCACCT
GCTTCGCGGGTCAGCGTATGGTCATAAACAT
>CHRUN16
GTGTAGCCCAGTTAGACACTTCTAAGTCCTTGGTAACAGACATGCAGTCCCCTCGGCAGAGCCCCGCC
CCCCGATGGTACCGGGTTGCTAAGAGGTCCACAACGGGTCGCACGACAACATTAAGGCCTCATGCATG
ACCCAACACGGGGGCGTCCCGGCGCGGCGACCTTGAAGTCCGGGGACAAGTATGTCCCCCCGGGAGGG
GCGCCACAGGGAATCCGGAGTGTTGATCTGCAGCCCGAGACTCCACTACGATGGTATGAACGGCAAAG
GTACTTTGGACACCACCCCGTTTTCTATTTCCAGACGCGGCCATTCAGGAACGACGCGAATCGTAATC
TACCTGCCGCGTACGCGGATCGTACATGCGTTTTCCAGCGTTCGAAATAGAGTTTGTCGTTCCGCTGA
CCAGGTTTCACGGCTACTGCGATAAGCATATTCTACTGGGGGACTGTAAAGGAATTGTATCCACTCTG
CTTCCTGTCAATTGGCCATGTGCATCTCTGGTTGTTCAGGGTACATTAATAACACACAGCATTAAGTA
GATAAGTCTCCGATATAAGCGAACAGAACGTTGGTCAGAGACTATGGTTCAATTGTAGTGGTATAATG
TTTCCCAGTGCATAAGGAACTCAGGGACACTACTAGCATGGGCGAGGAGAGACCCTTGGGTGGCAACA
CGCAATAAGTGTTACCTAGTACACCAGCACGAGAGAGGGTTTGACCATGTGTTTTTAGACGACCCCGG
TCGCATTGAACCTGGCATTCAGAATGACGTGCTCTCAAGAATCGGGGTCATCCCCCTTAGTGTCACCT
GCTTCGCGGGTCAGCGTATGGTCATAAACAT
>CHRUN31
GTGTAGCCCAGTTAGACACTTCTAAGTCCTTGGTAACAGACATGCAGTCCCCTCGGCAGAGCCCCGCC
CCCCGATGGTACCGGGTTGCTAAGAGGTCCACAACGGGTCGCACGACAACATTAAGGCCTCATGCATG
ACCCAACACGGGGGCGTCCCGGCGCGGCGACCTTGAAGTCCGGGGACAAGTATGTCCCCCCGGGAGGG
GCGCCACAGGGAATCCGGAGTGTTGATCTGCAGCCCGAGACTCCACTACGATGGTATGAACGGCAAAG
GTACTTTGGACACCACCCCGTTTTCTATTTCCAGACGCGGCCATTCAGGAACGACGCGAATCGTAATC
TACCTGCCGCGTACGCGGATCGTACATGCGTTTTCCAGCGTTCGAAATAGAGTTTGTCGTTCCGCTGA
CCAGGTTTCACGGCTACTGCGATAAGCATATTCTACTGGGGGACTGTAAAGGAATTGTATCCACTCTG
CTTCCTGTCAATTGGCCATGTGCATCTCTGGTTGTTCAGGGTACNTTAATAACACACAGCATTAAGTA
GATAAGTCTCCGATATAAGCGAACAGAACGTTGGTCAGAGACTATGGTTCAATTGTAGTGGTATAATG
TTTCCCAGTGCATAAGGAACTCAGGGACACTACTAGCATGGGCGAGGAGAGACCCTTGGGTGGCAACA
CGCAATAAGTGTTACCTAGTACATCAGCACGAGAGAGGGTTTGACCATGTGTTTTTAGACGACCCCGG
TCGCATTGAACCTGGCATTCAGAATGACGTGCTCTCAAGAATCGGGGTCATCCCCCTTAGTGTCACCT
GCTTCGCGGGTCAGCGTATGGTCATAAACAT
>CHRUN33
GTGTAGCCCAGTTAGACACTTCTAAGTCCTTGGTAACAGACATGCAGTCCCCTCGGCAGAGCCCCGCC

CCCCGATGGTACCGGGTTGCTAAGAGGTCCACAACGGGTCGCACGACAACATTAAGGCCTCATGCATG
ACCCAACACGGGGGCGTCCCGGCGCGGCGACCTTGAAGTCCGGGGACAAGTATGTCCCCCCGGGAGGG
GCGCCACAGGGAATCCGGAGTGTTGATCTGCAGCCCGAGACTCCACTACGATGGTATGAACGGCAAAG
GTACTTTGGACACCACCCCGTTTTCTATTTCCAGACGCGGCCATTCAGGAACGACGCGAATCGTAATC
TACCTGCCGCGTACGCGGATCGTACATGCGTTTTCCAGCGTTCGAAATAGAGTTTGTCGTTCCGCTGA
CCAGGTTTCACGGCTACTGCGATAAGCATATTCTACTGGGGGACTGTAAAGGAATTGTATCCACTCTG
CTTCCTGTCAATTGGCCATGTGCATCTCTGGTTGTTCAGGGTACATTAATAACACACAGCATTAAGTA
GATAAGTCTCCGATATAAGCGAACAGAACGTTGGTCAGAGACTATGGTTCAATTGTAGTGGTATAATG
TTTCCCAGTGCATAAGGAACTCAGGGACACTACTAGCATGGGCGAGGAGAGACCCTTGGGTGGCAACA
CGCAATAAGTGTTACCTAGTACATCAGCACGAGAGAGGGTTTGACCATGTGTTTTTAGACGACCCCGG
TCGCATTGAACCTGGCATTCAGAATGACGTGCTCTCAAGAATCGGGGTCATCCCCCTTAGTGTCACCT
GCTTCGCGGGTCAGCGTATGGTCATAAACAT
>CHRUN37
GTGTAGCCCAGTTAGACACTTCTAAGTCCTTGGTAACAGACATGCAGTCCCCTCGGCAGAGCCCCGCC
CCCCGATGGTACCGGGTTGCTAAGAGGTCCACAACGGGTCGCACGACAACATTAAGGCCTCATGCATG
ACCCAACACGGGGGCGTCCCGGCGCGGCGACCTTGAAGTCCGGGGACAAGTATGTCCCCCCGGGAGGG
GCGCCACAGGGAATCCGGAGTGTTGATCTGCAGCCCGAGACTCCACTACGATGGTATGAACGGCAAAG
GTACTTTGGACACCACCCCGTTTTCTATTTCCAGACGCGGCCATTCAGGAACGACGCGAATCGTAATC
TACCTGCCGCGTACGCGGATCGTACATGCGTTTTCCAGCGTTCGAAATAGAGTTTGTCGTTCCGCTGA
CCAGGTTTCACGGCTACTGCGATAAGCATATTCTACTGGGGGACTGTAAAGGAATTGTATCCACTCTG
CTTCCTGTCAATTGGCCATGTGCATCTCTGGTTGTTCAGGGTACATTAATAACACACAGCATTAAGTA
GATAAGTCTCCGATATAAGCGAACAGAACGTTGGTCAGAGACTATGGTTCAATTGTAGTGGTATAATG
TTTCCCAGTGCATAAGGAACTCAGGGACACTACTAGCATGGGCGAGGAGAGACCCTTGGGTGGCAACA
CGCAATAAGTGTTACCTAGTACATCAGCACGAGAGAGGGTTTGACCATGTGTTTTTAGACGACCCCGG
TCGCGTTGAACCTGGCATTCAGAATGACGTGCTCTCAAGAATCGGGGTCATCCCCCTTAGTGTCACCT
GCTTCGCGGGTCAGCGTATGGTCATAAACAT
>CHRUN41
GTGTAGCCCAGTTAGACACTTCTAAGTCCTTGGTAACAGACATGCAGTCCCCTCGGCAGAGCCCCGCC
CCCCGATGGTACCGGGTTGCTAAGAGGTCCACAACGGGTCGCACGACAACATTAAGGCCTCATGCATG
ACCCAACACGGGGGCGTCCCGGCGCGGCGACCTTGAAGTCCGGGGACAAGTATGTCCCCCCGGGAGGG
ACGCCACAGGGAATCCGGAGTGTTGATCTGCAGCCCGAGACTCCACTACGATGGTATGAACGGCAAAG
GTACTTTGGACACCACCCCGTTTTCTATTTCCAGACGCGGCCATTCAGGAACGACGCGAATCGTAATC
TACCTGCCGCGTACGCGGATCGTACATGCGTTTTCCAGCGTTCGAAATAGAGTTTGTCGTTCCGCTGA
CCAGGTTTCACGGCTACTGCGATAAGCATATTCTACTGGGGGACTGTAAAGGAATTGTATCCACTCTG
CTTCCTGTCAATTGGCCATGTGCATCTCTGGTTGTTCAGGGTACATTAATAACACACAGCATTAAGTA
GATAAGTCTCCGATATAAGCGAACAGAACGTTGGTCAGAGACTATGGTTCAATTGTAGTGGTATAATG
TTTCCCAGTGCATAAGGAACTCAGGGACACTACTAGCATGGGCGAGGAGAGACCCTTGGGTGGCAACA
CGCAATAAGTGTTACCTAGTACATCAGCACGAGAGAGGGTTTGACCATGTGTTTTTAGACGACCCCGG
TCGCATTGAACCTGGCATTCAGAATGACGTGCTCTCAAGAATCGGGGTCATCCCCCTTAGTGTCACCT
GCTTCGCGGGTCAGCGTATGGTCATAAACAT
>CHRUN42
GTGTAGCCCAGTTAGACACTTCTAAGTCCTTGGTAACAGACATGCAGTCCCCTCGGCAGAGCCCCGCC
CCCCGATGGTACCGGGTTGCTAAGAGGTCCACAACGGGTCGCACGACAACATTAAGGCCTCATGCATG
ACCCAACACGGGGGCGTCCCGGCGCGGCGACCTTGAAGTCCGGGGACAAGTATGTCCCCCCGGGAGGG
GCGCCACAGGGAATCCGGAGTGTTGATCTGCAGCCCGAGACTCCACTACGATGGTATGAACGGCAAAG
GTACTTTGGACACCACCCCGTTTTCTATTTCCAGACGCGGCCATTCAGGAACGACGCGAATCGTAATC
TACCTGCCGCGTACGCGGATCGTACATGCGTTTTCCAGCGTTCGAAATAGAGTTTGTCGTTCCGCTGA
CCAGGTTTCACGGCTACTGCGATAAGCATATTCTACTGGGGGACTGTAAAGGAATTGTATCCACTCTG
CTTCCTGTCAATTGGCCATGTGCATCTCTGGTTGTTCAGGGTACATTAATAACACACAGCATTAAGTA
GATAAGTCTCCGATATAAGCGAACAGAACGTTGGTCAGAGACTATGGTTCAATTGTAGTGGTATAATG
TTTCCCAGTGCATAAGGAACTCAGGGACACTACTAGCATGGGCGAGGAGAGACCCTTGGGTGGCAACA
CGCAATAAGTGTTACCTAGTACATCAGCACGAGAGAGGGTTTGACCATGTGTTTTTAGACGACCCCGG
TCGCATTGAACCTGGCATTCAGAATGACGTGCTCTCAAGAATCGGGGTCATCCCCCTTAGTGTCACCT
GCTTCGCGGGTCAGCGTATGGTCATAAACAT

>COHON8
GTGTAGCCCAGTTAGACACTTCTAAGTCCTTGGTAACAGACATGCAGTCCCCTCGGCAGAGCCCCGCC
CCCCGATGGTACCGGGTTGCTAAGAGGTCCACAACGGGTCGCACGACAACATTAAGGCCTCATGCATG
ACCCAACACGGGGGCGTCCCGGCGCGGCGACCTTGAAGTCCGGGGACAAGTATGTCCCCCCGGGAGGG
GCGCCACAGGGAATCCGGAGTGTTGATCTGCAGCCCGAGACTCCACTACGATGGTATGAACGGCAAAG
GTACTTTGGACACCACCCCGTTTTCTATTTCCAGACGCGGCCATTCAGGAACGACGCGAATCGTAATC
TACCTGCCGCGTACGCGGATCGTACATGCGTTTTCCAGCGTTCGAAATAGAGTTTGTCGTTCCGCTGA
CCAGGTTTCACGGCTACTGCGATAAGCATATTCTACTGGGGGACTGTAAAGGAATTGTATCCACTCTG
CTTCCTGTCAATTGGCCATGTGCATCTCTGGTTGTTCAGGGTACATTAATAACACACAGCATTAAGTA
GATAAGTCTCCGATATAAGCGAACAGAACGTTGGTCAGAGACTATGGTTCAATTGTAGTGGTATAATG
TTTCCCAGTGCATAAGGAACTCAGGGACACTACTAGCATGGGCGAGGAGAGACCCTTGGGTGGCAACA
CGCAATAAGTGTTACCTAGTACATCAGCACGAGAGAGGGTTTGACCATGTGTTTTTAGACGACCCCGG
TCGCATTGAACCTGGCATTCAGAATGACGTGCTCTCAAGAATCGGGGTCATCCCCCTTAGTGTCACCT
GCTTCGCGGGTCAGCGTATGGTCATAAACAT
>COHON9
GTGTAGCCCAGTTAGACACTTCTAAGTCCTTGGTAACAGACATGCAGTCCCCTCGGCAGAGCCCCGCC
CCCCGATGGTACCGGGTTGCTAAGAGGTCCACAACGGGTCGCACGACAACATTAAGGCCTCATGCATG
ACCCAACACGGGGGCGTCCCGGCGCGGCGACCTTGAAGTCCGGGGACAAGTATGTCCCCCCGGGAGGG
GCGCCACAGGGAATCCGGAGTGTTGATCTGCAGCCCGAGACTCCACTACGATGGTATGAACGGCAAAG
GTACTTTGGACACCACCCCGTTTTCTATTTCCAGACGCGGCCATTCAGGAACGACGCGAATCGTAATC
TACCTGCCGCGTACGCGGATCGTACATGCGTTTTCCAGCGTTCGAAATAGAGTTTGTCGTTCCGCTGA
CCAGGTTTCACGGCTACTGCGATAAGCATATTCTACTGGGGGACTGTAAAGGAATTGTATCCACTCTG
CTTCCTGTCAATTGGCCATGTGCATCTCTGGTTGTTCAGGGTACATTAATAACACACAGCATTAAGTA
GATAAGTCTCCGATATAAGCGAACAGAACGTTGGTCAGAGACTATGGTTCAATTGTAGTGGTATAATG
TTTCCCAGTGCATAAGGAACTCAGGGACACTACTAGCATGGGCGAGGAGAGACCCTTGGGTGGCAACA
CGCAATAAGTGTTACCTAGTACATCAGCACGAGAGAGGGTTTGACCATGTGTTTTTAGACGACCCCGG
TCGCATTGAACCTGGCATTCAGAATGACGTGCTCTCAAGAATCGGGGTCATCCCCCTTAGTGTCACCT
GCTTCGCGGGTCAGCGTATGGTCATAAACAT
>COHON10
GTGTAGCCCAGTTAGACACTTCTAAGTCCTTGGTAACAGACATGCAGTCCCCTCGGCAGAGCCCCGCC
CCCCGATGGTACCGGGTTGCTAAGAGGTCCACAACGGGTCGCACGACAACATTAAGGCCTCATGCATG
ACCCAACACGGGGGCGTCCCGGCGCGGCGACCTTGAAGTCCGGGGACAAGTATGTCCCCCCGGGAGGG
GCGCCACAGGGAATCCGGAGTGTTGATCTGCAGCCCGAGACTCCACTACGATGGTATGAACGGCAAAG
GTACTTTGGACACCACCCCGTTTTCTATTTCCAGACGCGGCCATTCAGGAACGACGCGAATCGTAATC
TACCTGCCGCGTACGCGGATCGTACATGCGTTTTCCAGCGTTCGAAATAGAGTTTGTCGTTCCGCTGA
CCAGGTTTCACGGCTACTGCGATAAGCATATTCTACTGGGGGACTGTAAAGGAATTGTATCCACTCTG
CTTCCTGTCAATTGGCCATGTGCATCTCTGGTTGTTCAGGGTACATTAATAACACACAGCATTAAGTA
GATAAGTCTCCGATATAAGCGAACAGAACGTTGGTCAGAGACTATGGTTCAATTGTAGTGGTATAATG
TTTCCCAGTGCATAAGGAACTCAGGGACACTACTAGCATGGGCGAGGAGAGACCCTTGGGTGGCAACA
CGCAATAAGTGTTACCTAGTACATCAGCACGAGAGAGGGTTTGACCATGTGTTTTTAGACGACCCCGG
TCGCATTGAACCTGGCATTCAGAATGACGTGCTCTCAAGAATCGGGGTCATCCCCCTTAGTGTCACCT
GCTTCGCGGGTCAGCGTATGGTCATAAACAT
>COHON11
GTGTAGCCCAGTTAGACACTTCTAAGTCCTTGGTAACAGACATGCAGTCCCCTCGGCAGAGCCCCGCC
CCCCGATGGTACCGGGTTGCTAAGNGGTCCACAACGGGTCGCACGACAACATTAAGGCCTCATGCATG
ACCCAACACGGGGGCGTCCCGGCGCGGCGACCTTGAAGTCCGGGGACAAGTATGTCCCCCCGGGAGGG
GCGCCACAGGGAATCCGGAGTGTTGATCTGCAGCCCGAGACTCCACTACGATGGTATGAACGGCAAAG
GTACTTTGGACACCACCCCGTTTTCTATTTCCAGACGCGGCCATTCAGGAACGACGCGAATCGTAATC
TACCTGCCGCGTACGCGGATCGTACATGCGTTTTCCAGCGTTCGAAATAGAGTTTGTCGTTCCGCTGA
CCAGGTTTCACGGCTACTGCGATAAGCATATTCTACTGGGGGACTGTAAAGGAATTGTATCCACTCTG
CTTCCTGTCAATTGGCCATGTGCATCTCTGGTTGTTCAGGGTACATTAATAACACACAGCATTAAGTA
GATAAGTCTCCGATATAAGCGAACAGAACGTTGGTCAGAGACTATGGTTCAATTGTAGTGGTATAATG
TTTCCCAGTGCATAAGGAACTCAGGGACACTACTAGCATGGGCGAGGAGAGACCCTTGGGTGGCAACA
CGCAATAAGTGTTACCTAGTACATCAGCACGAGAGAGGGTTTGACCATGTGTTTTTAGACGACCCCGG

TCGCATTGAACCTGGCATTCAGAATGACGTGCTCTCAAGAATCGGGGTCATCCCCCTTAGTGTCACCT
GCTTCGCGGGTCAGCGTATGGTCATAAACAT
>COHON12
GTGTAGCCCAGTTAGACACTTCTAAGTCCTTGGTAACAGACATGCAGTCCCCTCGGCAGAGCCCCGCC
CCCCGATGGTACCGGGTTGCTAAGAGGTCCACAACGGGTCGCACGACAACATTAAGGCCTCATGCATG
ACCCAACACGGGGGCGTCCCGGCGCGGCGACCTTGAAGTCCGGGGACAAGTATGTCCCCCCGGGAGGG
GCGCCACAGGGAATCCGGAGTGTTGATCTGCAGCCCGAGACTCCACTACGATGGTATGAACGGCAAAG
GTACTTTGGACACCACCCCGTTTTCTATTTCCAGACGCGGCCATTCAGGAACGACGCGAATCGTAATC
TACCTGCCGCGTACGCGGATCGTACATGCGTTTTCCAGCGTTCGAAATAGAGTTTGTCGTTCCGCTGA
CCAGGTTTCACGGCTACTGCGATAAGCATATTCTACTGGGGGACTGTAAAGGAATTGTATCCACTCTG
CTTCCTGTCAATTGGCCATGTGCATCTCTGGTTGTTCAGGGTACATTAATAACACACAGCATTAAGTA
GATAAGTCTCCGATATAAGCGAACAGAACGTTGGTCAGAGACTATGGTTCAATTGTAGTGGTATAATG
TTTCCCAGTGCATAAGGAACTCAGGGACACTACTAGCATGGGCGAGGAGAGACCCTTGGGTGGCAACA
CGCAATAAGTGTTACCTAGTACATCAGCACGAGAGAGGGTTTGACCATGTGTTTTTAGACGACCCCGG
TCGCATTGAACCTGGCATTCAGAATGACGTGCTCTCAAGAATCGGGGTCATCCCCCTTAGTGTCACCT
GCTTCGCGGGTCAGCGTATGGTCATAAACAT
>COHON19
GTGTAGCCCAGTTAGACACTTCTAAGTCCTTGGTAACAGACATGCAGTCCCCTCGGCAGAGCCCCGCC
CCCCGATGGTACCGGGTTGCTAAGAGGTCCACAACGGGTCGCACGACAACATTAAGGCCTCATGCATG
ACCCAACACGGGGGCGTCCCGGCGCGGCGACCTTGAAGTCCGGGGACAAGTATGTCCCCCCGGGAGGG
GCGCCACAGGGAATCCGGAGTGTTGATCTGCAGCCCGAGACTCCACTACGATGGTATGAACGGCAAAG
GTACTTTGGACACCACCCCGTTTTCTATTTCCAGACGCGGCCATTCAGGAACGACGCGAATCGTAATC
TACCTGCCGCGTACGCGGATCGTACATGCGTTTTCCAGCGTTCGAAATAGAGTTTGTCGTTCCGCTGA
CCAGGTTTCACGGCTACTGCGATAAGCATATTCTACTGGGGGACTGTAAAGGAATTGTATCCACTCTG
CTTCCTGTCAATTGGCCATGTGCATCTCTGGTTGTTCAGGGTACATTAATAACACACAGCATTAAGTA
GATAAGTCTCCGATATAAGCGAACAGAACGTTGGTCAGAGACTATGGTTCAATTGTAGTGGTATAATG
TTTCCCAGTGCATAAGGAACTCAGGGACACTACTAGCATGGGCGAGGAGAGACCCTTGGGTGGCAACA
CGCAATAAGTGTTACCTAGTACATCAGCACGAGAGAGGGTTTGACCATGTGTTTTTAGACGACCCCGG
TCGCATTGAACCTGGCATTCAGAATGACGTGCTCTCAAGAATCGGGGTCATCCCCCTTAGTGTCACCT
GCTTCGCGGGTCAGCGTATGGTCATAAACAT
>COHON23
GTGTAGCCCAGTTAGACACTTCTAAGTCCTTGGTAACAGACATGCAGTCCCCTCGGCAGAGCCCCGCC
CCCCGATGGTACCGGGTTGCTAAGAGGTCCACAACGGGTCGCACGACAACATTAAGGCCTCATGCATG
ACCCAACACGGGGGCGTCCCGGCGCGGCGACCTTGAAGTCCGGGGACAAGTATGTCCCCCCGGGAGGG
GCGCCACAGGGAATCCGGAGTGTTGATCTGCAGCCCGAGACTCCACTACGATGGTATGAACGGCAAAG
GTACTTTGGACACCACCCCGTTTTCTATTTCCAGACGCGGCCATTCAGGAACGACGCGAATCGTAATC
TACCTGCCGCGTACGCGGATCGTACATGCGTTTTCCAGCGTTCGAAATAGAGTTTGTCGTTCCGCTGA
CCAGGTTTCACGGCTACTGCGATAAGCATATTCTACTGGGGGACTGTAAAGGAATTGTATCCACTCTG
CTTCCTGTCAATTGGCCATGTGCATCTCTGGTTGTTCAGGGTACATTAATAACACACAGCATTAAGTA
GATAAGTCTCCGATATAAGCGAACAGAACGTTGGTCAGAGACTATGGTTCAATTGTAGTGGTATAATG
TTTCCCAGTGCATAAGGAACTCAGGGACACTACTAGCATGGGCGAGGAGAGACCCTTGGGTGGCAACA
CGCAATAAGTGTTACCTAGTACATCAGCACGAGAGAGGGTTTGACCATGTGTTTTTAGACGACCCCGG
TCGCATTGAACCTGGCATTCAGAATGACGTGCTCTCAAGAATCGGGGTCATCCCCCTTAGTGTCACCT
GCTTCGCGGGTCAGCGTATGGTCATAAACAT
>COHON27
GTGTAGCCCAGTTAGACACTTCTAAGTCCTTGGTAACAGACATGCAGTCCCCTCGGCAGAGCCCCGCC
CCCCGATGGTACCGGGTTGCTAAGAGGTCCACAACGGGTCGCACGACAACATTAAGGCCTCATGCATG
ACCCAACACGGGGGCGTCCCGGCGCGGCGACCTTGAAGTCCGGGGACAAGTATGTCCCCCCGGGAGGG
GCGCCACAGGGAATCCGGAGTGTTGATCTGCAGCCCGAGACTCCACTACGATGGTATGAACGGCAAAG
GTACTTTGGACACCACCCCGTTTTCTATTTCCAGACGCGGCCATTCAGGAACGACGCGAATCGTAATC
TACCTGCCGCGTACGCGGATCGTACATGCGTTTTCCAGCGTTCGAAATAGAGTTTGTCGTTCCGCTGA
CCAGGTTTCACGGCTACTGCGATAAGCATATTCTACTGGGGGACTGTAAAGGAATTGTATCCACTCTG
CTTCCTGTCAATTGGCCATGTGCATCTCTGGTTGTTCAGGGTACATTAATAACACACAGCATTAAGTA
GATAAGTCTCCGATATAAGCGAACAGAACGTTGGTCAGAGACTATGGTTCAATTGTAGTGGTATAATG

TTTCCCAGTGCATAAGGAACTCAGGGACACTACTAGCATGGGCGAGGAGAGACCCTTGGGTGGCAACA
CGCAATAAGTGTTACCTAGTACATCAGCACGAGAGAGGGTTTGACCATGTGTTTTTAGACGACCCCGG
TCGCATTGAACCTGGCATTCAGAATGACGTGCTCTCAAGAATCGGGGTCATCCCCCTTAGTGTCACCT
GCTTCGCGGGTCAGCGTATGGTCATAAACAT
>COHON29
GTGTAGCCCAGTTAGACACTTCTAAGTCCTTGGTAACAGACATGCAGTCCCCTCGGCAGAGCCCCGCC
CCCCGATGGTACCGGGTTGCTAAGAGGTCCACAACGGGTCGCACGACAACATTAAGGCCTCATGCATG
ACCCAACACGGGGGCGTCCCGGCGCGGCGACCTTGAAGTCCGGGGACAAGTATGTCCCCCCGGGAGGG
GCGCCACAGGGAATCCGGAGTGTTGATCTGCAGCCCGAGACTCCACTACGATGGTATGAACGGCAAAG
GTACTTTGGACACCACCCCGTTTTCTATTTCCAGACGCGGCCATTCAGGAACGACGCGAATCGTAATC
TACCTGCCGCGTACGCGGATCGTACATGCGTTTTCCAGCGTTCGAAATAGAGTTTGTCGTTCCGCTGA
CCAGGTTTCACGGCTACTGCGATAAGCATATTCTACTGGGGGACTGTAAAGGAATTGTATCCACTCTG
CTTCCTGTCAATTGGCCATGTGCATCTCTGGTTGTTCAGGGTACATTAATAACACACAGCATTAAGTA
GATAAGTCTCCGATATAAGCGAACAGAACGTTGGTCAGAGACTATGGTTCAATTGTAGTGGTATAATG
TTTCCCAGTGCATAAGGAACTCAGGGACACTACTAGCATGGGCGAGGAGAGACCCTTGGGTGGCAACA
CGCAATAAGTGTTACCTAGTACATCAGCACGAGAGAGGGTTTGACCATGTGTTTTTAGACGACCCCGG
TCGCATTGAACCTGGCATTCAGAATGACGTGCTCTCAAGAATCGGGGTCATCCCCCTTAGTGTCACCT
GCTTCGCGGGTCAGCGTATGGTCATAAACAT
>COHON32
GTGTAGCCCAGTTAGACACTTCTAAGTCCTTGGTAACAGACATGCAGTCCCCTCGGCAGAGCCCCGCC
CCCCGATGGTACCGGGTTGCTAAGAGGTCCACAACGGGTCGCACGACAACATTAAGGCCTCATGCATG
ACCCAACACGGGGGCGTCCCGGCGCGGCGACCTTGAAGTCCGGGGACAAGTATGTCCCCCCGGGAGGG
GCGCCACAGGGAATCCGGAGTGTTGATCTGCAGCCCGAGACTCCACTACGATGGTATGAACGGCAAAG
GTACTTTGGACACCACCCCGTTTTCTATTTCCAGACGCGGCCATTCAGGAACGACGCGAATCGTAATC
TACCTGCCGCGTACGCGGATCGTACATGCGTTTTCCAGCGTTCGAAATAGAGTTTGTCGTTCCGCTGA
CCAGGTTTCACGGCTACTGCGATAAGCATATTCTACTGGGGGACTGTAAAGGAATTGTATCCACTCTG
CTTCCTGTCAATTGGCCATGTGCATCTCTGGTTGTTCAGGGTACNTTAATAACACACAGCATTAAGTA
GATAAGTCTCCGATATAAGCGAACAGAACGTTGGTCAGAGACTATGGTTCAATTGTAGTGGTATAATG
TTTCCCAGTGCATAAGGAACTCAGGGACACTACTAGCATGGGCGAGGAGAGACCCTTGGGTGGCAACA
CGCAATAAGTGTTACCTAGTACATCAGCACGAGAGAGGGTTTGACCATGTGTTTTTAGACGACCCCGG
TCGCATTGAACCTGGCATTCAGAATGACGTGCTCTCAAGAATCGGGGTCATCCCCCTTAGTGTCACCT
GCTTCGCGGGTCAGCGTATGGTCATAAACAT
>COHON33
GTGTAGCCCAGTTAGACACTTCTAAGTCCTTGGTAACAGACATGCAGTCCCCTCGGCAGAGCCCCGCC
CCCCGATGGTACCGGGTTGCTAAGAGGTCCACAACGGGTCGCACGACAACATTAAGGCCTCATGCATG
ACCCAACACGGGGGCGTCCCGGCGCGGCGACCTTGAAGTCCGGGGACAAGTATGTCCCCCCGGGAGGG
GCGCCACAGGGAATCCGGAGTGTTGATCTGCAGCCCGAGACTCCACTACGATGGTATGAACGGCAAAG
GTACTTTGGACACCACCCCGTTTTCTATTTCCAGACGCGGCCATTCAGGAACGACGCGAATCGTAATC
TACCTGCCGCGTACGCGGATCGTACATGCGTTTTCCAGCGTTCGAAATAGAGTTTGTCGTTCCGCTGA
CCAGGTTTCACGGCTACTGCGATAAGCATATTCTACTGGGGGACTGTAAAGGAATTGTATCCACTCTG
CTTCCTGTCAATTGGCCATGTGCATCTCTGGTTGTTCAGGGTACATTAATAACACACAGCATTAAGTA
GATAAGTCTCCGATATAAGCGAACAGAACGTTGGTCAGAGACTATGGTTCAATTGTAGTGGTATAATG
TTTCCCAGTGCATAAGGAACTCAGGGACACTACTAGCATGGGCGAGGAGAGACCCTTGGGTGGCAACA
CGCAATAAGTGTTACCTAGTACATCAGCACGAGAGAGGGTTTGACCATGTGTTTTTAGACGACCCCGG
TCGCATTGAACCTGGCATTCAGAATGACGTGCTCTCAAGAATCGGGGTCATCCCCCTTAGTGTCACCT
GCTTCGCGGGTCAGCGTATGGTCATAAACAT
>COHON34
GTGTAGCCCAGTTAGACACTTCTAAGTCCTTGGTAACAGACATGCAGTCCCCTCGGCAGAGCCCCGCC
CCCCGATGGTACCGGGTTGCTAAGAGGTCCACAACGGGTCGCACGACAACATTAAGGCCTCATGCATG
ACCCAACACGGGGGCGTCCCGGCGCGGCGACCTTGAAGTCCGGGGACAAGTATGTCCCCCCGGGAGGG
GCGCCACAGGGAATCCGGAGTGTTGATCTGCAGCCCGAGACTCCACTACGATGGTATGAACGGCAAAG
GTACTTTGGACACCACCCCGTTTTCTATTTCCAGACGCGGCCATTCAGGAACGACGCGAATCGTAATC
TACCTGCCGCGTACGCGGATCGTACATGCGTTTTCCAGCGTTCGAAATAGAGTTTGTCGTTCCGCTGA
CCAGGTTTCACGGCTACTGCGATAAGCATATTCTACTGGGGGACTGTAAAGGAATTGTATCCACTCTG

CTTCCTGTCAATTGGCCATGTGCATCTCTGGTTGTTCAGGGTACATTAATAACACACAGCATTAAGTA
GATAAGTCTCCGATATAAGCGAACAGAACGTTGGTCAGAGACTATGGTTCAATTGTAGTGGTATAATG
TTTCCCAGTGCATAAGGAACTCAGGGACACTACTAGCATGGGCGAGGAGAGACCCTTGGGTGGCAACA
CGCAATAAGTGTTACCTAGTACATCAGCACGAGAGAGGGTTTGACCATGTGTTTTTAGACGACCCCGG
TCGCATTGAACCTGGCATTCAGAATGACGTGCTCTCAAGAATCGGGGTCATCCCCCTTAGTGTCACCT
GCTTCGCGGGTCAGCGTATGGTCATAAACAT
>COHON36
GTGTAGCCCAGTTAGACACTTCTAAGTCCTTGGTAACAGACATGCAGTCCCCTCGGCAGAGCCCCGCC
CCCCGATGGTACCGGGTTGCTAAGAGGTCCACAACGGGTCGCACGACAACATTAAGGCCTCATGCATG
ACCCAACACGGGGGCGTCCCGGCGCGGCGACCTTGAAGTCCGGGGACAAGTATGTCCCCCCGGGAGGG
GCGCCACAGGGAATCCGGAGTGTTGATCTGCAGCCCGAGACTCCACTACGATGGTATGAACGGCAAAG
GTACTTTGGACACCACCCCGTTTTCTATTTCCAGACGCGGCCATTCAGGAACGACGCGAATCGTAATC
TACCTGCCGCGTACGCGGATCGTACATGCGTTTTCCAGCGTTCGAAATAGAGTTTGTCGTTCCGCTGA
CCAGGTTTCACGGCTACTGCGATAAGCATATTCTACTGGGGGACTGTAAAGGAATTGTATCCACTCTG
CTTCCTGTCAATTGGCCATGTGCATCTCTGGTTGTTCAGGGTACATTAATAACACACAGCATTAAGTA
GATAAGTCTCCGATATAAGCGAACAGAACGTTGGTCAGAGACTATGGTTCAATTGTAGTGGTATAATG
TTTCCCAGTGCATAAGGAACTCAGGGACACTACTAGCATGGGCGAGGAGAGACCCTTGGGTGGCAACA
CGCAATAAGTGTTACCTAGTACATCAGCACGAGAGAGGGTTTGACCATGTGTTTTTAGACGACCCCGG
TCGCATTGAACCTGGCATTCAGAATGACGTGCTCTCAAGAATCGGGGTCATCCCCCTTAGTGTCACCT
GCTTCGCGGGTCAGCGTATGGTCATAAACAT
>COHON37
GTGTAGCCCAGTTAGACACTTCTAAGTCCTTGGTAACAGACATGCAGTCCCCTCGGCAGAGCCCCGCC
CCCCGATGGTACCGGGTTGCTAAGAGGTCCACAACGGGTCGCACGACAACATTAAGGCCTCATGCATG
ACCCAACACGGGGGCGTCCCGGCGCGGCGACCTTGAAGTCCGGGGACAAGTATGTCCCCCCGGGAGGG
GCGCCACAGGGAATCCGGAGTGTTGATCTGCAGCCCGAGACTCCACTACGATGGTATGAACGGCAAAG
GTACTTTGGACACCACCCCGTTTTCTATTTCCAGACGCGGCCATTCAGGAACGACGCGAATCGTAATC
TACCTGCCGCGTACGCGGATCGTACATGCGTTTTCCAGCGTTCGAAATAGAGTTTGTCGTTCCGCTGA
CCAGGTTTCACGGCTACTGCGATAAGCATATTCTACTGGGGGACTGTAAAGGAATTGTATCCACTCTG
CTTCCTGTCAATTGGCCATGTGCATCTCTGGTTGTTCAGGGTACATTAATAACACACAGCATTAAGTA
GATAAGTCTCCGATATAAGCGAACAGAACGTTGGTCAGAGACTATGGTTCAATTGTAGTGGTATAATG
TTTCCCAGTGCATAAGGAACTCAGGGACACTACTAGCATGGGCGAGGAGAGACCCTTGGGTGGCAACA
CGCAATAAGTGTTACCTAGTACATCAGCACGAGAGAGGGTTTGACCATGTGTTTTTAGACGACCCCGG
TCGCATTGAACCTGGCATTCAGAATGACGTGCTCTCAAGAATCGGGGTCATCCCCCTTAGTGTCACCT
GCTTCGCGGGTCAGCGTATGGTCATAAACAT
>COHON38
GTGTAGCCCAGTTAGACACTTCTAAGTCCTTGGTAACAGACATGCAGTCCCCTCGGCAGAGCCCCGCC
CCCCGATGGTACCGGGTTGCTAAGAGGTCCACAACGGGTCGCACGACAACATTAAGGCCTCATGCATG
ACCCAACACGGGGGCGTCCCGGCGCGGCGACCTTGAAGTCCGGGGACAAGTATGTCCCCCCGGGAGGG
GCGCCACAGGGAATCCGGAGTGTTGATCTGCAGCCCGAGACTCCACTACGATGGTATGAACGGCAAAG
GTACTTTGGACACCACCCCGTTTTCTATTTCCAGACGCGGCCATTCAGGAACGACGCGAATCGTAATC
TACCTGCCGCGTACGCGGATCGTACATGCGTTTTCCAGCGTTCGAAATAGAGTTTGTCGTTCCGCTGA
CCAGGTTTCACGGCTACTGCGATAAGCATATTCTACTGGGGGACTGTAAAGGAATTGTATCCACTCTG
CTTCCTGTCAATTGGCCATGTGCATCTCTGGTTGTTCAGGGTACATTAATAACACACAGCATTAAGTA
GATAAGTCTCCGATATAAGCGAACAGAACGTTGGTCAGAGACTATGGTTCAATTGTAGTGGTATAATG
TTTCCCAGTGCATAAGGAACTCAGGGACACTACTAGCATGGGCGAGGAGAGACCCTTGGGTGGCAACA
CGCAATAAGTGTTACCTAGTACATCAGCACGAGAGAGGGTTTGACCATGTGTTTTTAGACGACCCCGG
TCGCATTGAACCTGGCATTCAGAATGACGTGCTCTCAAGAATCGGGGTCATCCCCCTTAGTGTCACCT
GCTTCGCGGGTCAGCGTATGGTCATAAACAT
>COHON39
GTGTAGCCCAGTTAGACACTTCTAAGTCCTTGGTAACAGACATGCAGTCCCCTCGGCAGAGCCCCGCC
CCCCGATGGTACCGGGTTGCTAAGAGGTCCACAACGGGTCGCACGACAACATTAAGGCCTCATGCATG
ACCCAACACGGGGGCGTCCCGGCGCGGCGACCTTGAAGTCCGGGGACAAGTATGTCCCCCCGGGAGGG
GCGCCACAGGGAATCCGGAGTGTTGATCTGCAGCCCGAGACTCCACTACGATGGTATGAACGGCAAAG
GTACTTTGGACACCACCCCGTTTTCTATTTCCAGACGCGGCCATTCAGGAACGACGCGAATCGTAATC

TACCTGCCGCGTACGCGGATCGTACATGCGTTTTCCAGCGTTCGAAATAGAGTTTGTCGTTCCGCTGA
CCAGGTTTCACGGCTACTGCGATAAGCATATTCTACTGGGGGACTGTAAAGGAATTGTATCCACTCTG
CTTCCTGTCAATTGGCCATGTGCATCTCTGGTTGTTCAGGGTACATTAATAACACACAGCATTAAGTA
GATAAGTCTCCGATATAAGCGAACAGAACGTTGGTCAGAGACTATGGTTCAATTGTAGTGGTATAATG
TTTCCCAGTGCATAAGGAACTCAGGGACACTACTAGCATGGGCGAGGAGAGACCCTTGGGTGGCAACA
CGCAATAAGTGTTACCTAGTACATCAGCACGAGAGAGGGTTTGACCATGTGTTTTTAGACGACCCCGG
TCGCATTGAACCTGGCATTCAGAATGACGTGCTCTCAAGAATCGGGGTCATCCCCCTTAGTGTCACCT
GCTTCGCGGGTCAGCGTATGGTCATAAACAT
>COHON40
GTGTAGCTCAGTTAGACACTTCTAAGTCCTTGGTAACAGACATGCAGTCCCCTCGGCAGAGCCCCGCC
CCCCGGTGGTACCGGGTTGCTAAGAGGTCCACCACGGGTCGCACGACAACATCAAGGCCTCATGCATG
ACCCAACGCGGGGACGTACCGGCGCGGCGACCTTGAAGGTCGGGGACAAGTATGTCCCCCCGGGAGGG
GCGCCACAGGGAATCCGGAGTGTTGATCTGCAGCCCGAGACTCCACTACGATGGTATGAACGGCAAAG
GTATTTTGGACACCACCCCGTTTTCTATTTCCAGACGCGGCCATTCAGGAACGACGCGAATCGTAATC
TACCTGCCGCGTACGCGGATCGTACATGCGTTTTTCAGCGTTCGAAATAGAGTTTGTCGTTCCGCTGA
CCAGGTTTCACGGCTACTGCGATAAGCATATTCTACGGGGGGACTGTAAAGGAATTGTATCCACTCTG
CTCCCTGTCAATTGGCCATGTGCATCTCTGGTTGTTTAGGGTACATTAATAACACACAGCATTAGGTA
GATAAGTCTCCGATATAAGCGAACAGAACGTTGGTCAAAGACCGTGGTTCAATTGTAGTGGTATAACA
TTTCCCAGTGCATAAGGAACCCAGGGACACTACTAGCATGGGCGAGGAGAGACCCTTGGGTGGCAACA
CGCAATAAGTGTTACCCAGTACACCAGCACGATAGAGGGTTTGACCATGTGTTTTTAGACGACCCCGG
TCGCATTGAACCTGGCATTCAGAATGACGTGCTCTCAAGAGTCGGGGTCATCCCCCTTAGTGTCACCT
GCTTCGCGGGTTAGCGTATGGTCATAAACAT
>COHON41
GTGTAGCCCAGTTAGACACTTCTAAGTCCTTGGTAACAGACATGCAGTCCCCTCGGCAGAGCCCCGCC
CCCCGATGGTACCGGGTTGCTAAGAGGTCCACCACGAGTCGCACGACAACAGCAAGGCCTCGTGCATG
ACCCAACACGGGGGCGTCCCGGCGCGGCGACCTTGAAGTCCGGGAACAAGTATGTCTCCCCGGGAGGG
GCGCCACAGGGAATCCGGAGTGTTGATCTGCAGCCCGAGACTCCACTACGATGGTATGAACGGCAAAG
GTACTTTGGACACCACCCCGTTTTCTATTCCCAGATGCGGCCATTCAGGAACGACGTGAATCGTAATC
TACCTGCCGCGTACGCGGATCGTACATGCGTTTTCCAGCGTTCGAAATAGAGTTTGTCGTTCCGCTGA
CCAGGTTTCACGGCTACTGCGATAAGCATACTCTACTGGGGGACTGTAAAGGAATTGTATCCACTCTG
CTTCCTGTCAGTTGGCCATATGTATCTCTGGTTGTTCAGGGTACATTAATAACACACAGCATTAAGTA
GATAAGTCTCCGATATAGGCGAACAGAACGTTGGTCAAAGACTGTGGTTCAACTGTAGTGGTATAATG
TTTCCCAGTGCATAAGGAACTCAGGGACACTACTAGCATGGGCGAGTAGAGACCCTTGGGTGGCAACA
CGCAATAAGTGTTACCTAGTACACCAACACGATATAGGGTTTGACCATGTGTTTTTAGACGACCCCGG
TCGCATTGAACCTGGCATTCATAATGACGTGCTCTCAAGAGTCGGGGTCATCCCCCTTAGTGTCACCT
GCTTCGCNGGTCATCGTATGGTCATAAACAT
>COHON44
GTGTAGCCCAGTTAGACACTTCTAAGTCCTTGGTAACAGACATGCAGTCCCCTCGGCAGAGCCCCGCC
CCCCGATGGTACCGGGTTGCTAAGAGGTCCACAACGGGTCGCACGACAACATTAAGGCCTCATGCATG
ACCCAACACGGGGGCGTCCCGGCGCGGCGACCTTGAAGTCCGGGGACAAGTATGTCCCCCCGGGAGGG
GCGCCACAGGGAATCCGGAGTGTTGATCTGCAGCCCGAGACTCCACTACGATGGTATGAACGGCAAAG
GTACTTTGGACACCACCCCGTTTTCTATTTCCAGACGCGGCCATTCAGGAACGACGCGAATCGTAATC
TACCTGCCGCGTACGCGGATCGTACATGCGTTTTCCAGCGTTCGAAATAGAGTTTGTCGTTCCGCTGA
CCAGGTTTCACGGCTACTGCGATAAGCATATTCTACTGGGGGACTGTAAAGGAATTGTATCCACTCTG
CTTCCTGTCAATTGGCCATGTGCATCTCTGGTTGTTCAGGGTACATTAATAACACACAGCATTAAGTA
GATAAGTCTCCGATATAAGCGAACAGAACGTTGGTCAGAGACTATGGTTCAATTGTAGTGGTATAATG
TTTCCCAGTGCATAAGGAACTCAGGGACACTACTAGCATGGGCGAGGAGAGACCCTTGGGTGGCAACA
CGCAATAAGTGTTACCTAGTACATCAGCACGAGAGAGGGTTTGACCATGTGTTTTTAGACGACCCCGG
TCGCATTGAACCTGGCATTCAGAATGACGTGCTCTCAAGAATCGGGGTCATCCCCCTTAGTGTCACCT
GCTTCGCGGGTCAGCGTATGGTCATAAACAT
>COHON45
GTGTAGCCCAGTTAGACACTTCTAAGTCCTTGGTAACAGACATGCAGTCCCCTCGGCAGAGCCCCGCC
CCCCGATGGTACCGGGTTGCTAAGAGGTCCACAACGGGTCGCACGACAACATTAAGGCCTCATGCATG
ACCCAACACGGGGGCGTCCCGGCGCGGCGACCTTGAAGTCCGGGGACAAGTATGTCCCCCCGGGAGGG

GCGCCACAGGGAATCCGGAGTGTTGATCTGCAGCCCGAGACTCCACTACGATGGTATGAACGGCAAAG
GTACTTTGGACACCACCCCGTTTTCTATTTCCAGACGCGGCCATTCAGGAACGACGCGAATCGTAATC
TACCTGCCGCGTACGCGGATCGTACATGCGTTTTCCAGCGTTCGAAATAGAGTTTGTCGTTCCGCTGA
CCAGGTTTCACGGCTACTGCGATAAGCATATTCTACTGGGGGACTGTAAAGGAATTGTATCCACTCTG
CTTCCTGTCAATTGGCCATGTGCATCTCTGGTTGTTCAGGGTACATTAATAACACACAGCATTAAGTA
GATAAGTCTCCGATATAAGCGAACAGAACGTTGGTCAGAGACTATGGTTCAATTGTAGTGGTATAATG
TTTCCCAGTGCATAAGGAACTCAGGGACACTACTAGCATGGGCGAGGAGAGACCCTTGGGTGGCAACA
CGCAATAAGTGTTACCTAGTACATCAGCACGAGAGAGGGTTTGACCATGTGTTTTTAGACGACCCCGG
TCGCATTGAACCTGGCATTCAGAATGACGTGCTCTCAAGAATCGGGGTCATCCCCCTTAGTGTCACCT
GCTTCGCGGGTCAGCGTATGGTCATAAACAT
>RAGLN01
GTGTAGCCCAGTCAGACACTTCTAAGTCCTTGGTAACAGACATGCAGTCCCCTCGGCAGAGCCCCGCC
CCCCGATGGTACCGGGTTGCTAAGAGGTCCACAACGGGTCGCACGACAACATTAAGGCCTCATGCATG
ACCCAACACGGGGGCGTCCCGGCGCGGCGACCTTGAAGTCCGGGGACAAGTATGTCCCCCCGGGAGGG
GCGCCACAGGGAATCCGGAGTGTTGATCTGCAGCCCGAGACTCCACTACGATGGTATGAACGGCAAAG
GTACTTTGGACACCACCCCGTTTTCTATNTCCAGACGCGGCCATTTAGGAACGACGCGAATCGTAATC
TACCTGCCGCGTACGCGGATCGTACATGCGTTTTCCAGCGTTCGAAATAGAGTTTGTCGTTCCGCTGA
CCAGGTTTCACGGCTACTGCGATAAGCATATTCTACTGGGGGACTGTAAAGGAATTGTATCCATTCTG
CTTCCTGTCAATTGGCCATGTGCATCTCTGGTTGTTCAGGGTACATTAATAACACACAGCAGTAAGTA
GGTAAGTCTCCGATATAAGCGAACAGAACGTTTGTCAGAGACTGTGGTTCAATTGTAGTGGTATAATG
TTTCCCAGTGCATAAGGAACTCAGGGACACTACTAGCATGGGCGAGGAGAGACCCTTGGGTGGCAACA
CGCAATAAGTGTTACCTAGTACACCAGCACGAGAGAGGGTTTGACCATGTGTTTTTAGACGACCCCGG
TCGCATTGAACCTGGCATTCAGAATGACGTGCTCTCAAGAATCGGGGTCATCCCCCTTAGTGTCACCT
GCTTCGCGGGTCAGCGTATGGTCATAAACAT
>RAGLN02
GTGTAGCCCAGTTAGACACTTCTAAGTCCTTGGTAACAGACATGCAGTCCCCTCGGCAGAGCCCCGCC
CCCCGATGGTACCGGGTTGCTAAGAGGTCCACAACGGGTCGCACGACAACATTAAGGCCTCATGCATG
ACCCAACACGGGGGCGTCCCGGCGCGGCGACCTTGAAGTCCGGGGACAAGTATGTCCCCCCGGGAGGG
GCGCCACAGGGAATCCGGAGTGTTGATCTGCAGCCCGAGACTCCACTACGATGGTATGAACGGCAAAG
GTACTTTGGACACCACCCCGTTTTCTATTTCCAGACGCGGCCATTCAGGAACGACGCGAATCGTAATC
TACCTGCCGCGTACGCGGATCGTACATGCGTTTTCCAGCGTTCGAAATAGAGTTTGTCGTTCCGCTGA
CCAGGTTTCACGGCTACTGCGATAAGCATATTCTACTGGGGGACTGTAAAGGAATTGTATCCACTCTG
CTTCCTGTCAATTGGCCATGTGCATCTCTGGTTGTTCAGGGTACATTAATAACACACAGCATTAAGTA
GATAAGTCTCCGATATAAGCGAACAGAACGTTGGTCAGAGACTATGGTTCAATTGTAGTGGTATAATG
TTTCCCAGTGCATAAGGAACTCAGGGACACTACTAGCATGGGCGAGGAGAGACCCTTGGGTGGCAACA
CGCAATAAGTGTTACCTAGTACATCAGCACGAGAGAGGGTTTGACCATGTGTTTTTAGACGACCCCGG
TCGCATTGAACCTGGCATTCAGAATGACGTGCTCTCAAGAATCGGGGTCATCCCCCTTAGTGTCACCT
GCTTCGCGGGTCAGCGTATGGTCATAAACAT
>RAGLN03
GTGTAGCCCAGTTAGACACTTCTAAGTCCTTGGTAACAGACATGCAGTCCCCTCGGCAGAGCCCCGCC
CCCCGATGGTACCGGGTTGCTAAGAGGTCCACAACGGGTCGCACGACAACATTAAGGCCTCATGCATG
ACCCAACACGGGGGCGTCCCGGCGCGGCGACCTTGAAGTCCGGGGACAAGTATGTCCCCCCGGGAGGG
GCGCCACAGGGAATCCGGAGTGTTGATCTGCAGCCCGAGACTCCACTACGATGGTATGAACGGCAAAG
GTACTTTGGACACCACCCCGTTTTCTATTTCCAGACGCGGCCATTCAGGAACGACGCGAATCGTAATC
TACCTGCCGCGTACGCGGATCGTACATGCGTTTTCCAGCGTTCGAAATAGAGTTTGTCGTTCCGCTGA
CCAGGTTTCACGGCTACTGCGATAAGCATATTCTACTGGGGGACTGTAAAGGAATTGTATCCACTCTG
CTTCCTGTCAATTGGCCATGTGCATCTCTGGTTGTTCAGGGTACATTAATAACACACAGCATTAAGTA
GATAAGTCTCCGATATAAGCGAACAGAACGTTGGTCAGAGACTATGGTTCAATTGTAGTGGTATAATG
TTTCCCAGTGCATAAGGAACTCAGGGACACTACTAGCATGGGCGAGGAGAGACCCTTGGGTGGCAACA
CGCAATAAGTGTTACCTAGTACATCAGCACGAGAGAGGGTTTGANCATGTGTTTTTAGACGACCCCGG
TCGCATTGAACCTGGCATTCAGAATGACGTGCTCTCAAGAATCGGGGTCATCCCCCTTAGTGTCACCT
GCTTCGCGGGTCAGCGTATGGTCATAAACAT
>RAGLN04
GTGTAGCCCAGTTAGACACTTCTAAGTCCTTGGTAACAGACATGCAGTCCCCTCGGCAGAGCCCCGCC

CCCCGATGGTACCGGGTTGCTAAGAGGTCCACAACGGGTCGCACGACAACATTAAGGCCTCATGCATG
ACCCAACACGGGGGCGTCCCGGCGCGGCGACCTTGAAGTCCGGGGACAAGTATGTCCCCCCGGGAGGG
GCGCCACAGGGAATCCGGAGTGTTGATCTGCAGCCCGAGACTCCACTACGATGGTATGAACGGCAAAG
GTACTTTGGACACCACCCCGTTTTCTATTTCCAGACGCGGCCNTTCAGGAACGACGCGAATCGTAATC
TACCTGCCGCGTACGCGGATCGTACATGCGTTTTCCAGCGTTCGAAATAGAGTTTGTCGTTCCGCTGA
CCAGGTTTCACGGCTACTGCGATAAGCATATTCTACTGGGGGACTGTAAAGGAATTGTATCCACTCTG
CTTCCTGTCAATTGGCCATGTGCATCTCTGGTTGTTCAGGGTACATTAATAACACACAGCATTAAGTA
GATAAGTCTCCGATATAAGCGAACAGAACGTTGGTCAGAGACTATGGTTCAATTGTAGTGGTATAATG
TTTCCCAGTGCATAAGGAACTCAGGGACACTACTAGCATGGGCGAGGAGAGACCCTTGGGTGGCAACA
CGCAATAAGTGTTACCTAGTACATCAGCACGAGAGAGGGTTTGACCATGTGTTTTTAGACGACCCCGG
TCGCATTGAACCTGGCATTCAGAATGACGTGCTCTCAAGAATCGGGGTCATCCCCCTTAGTGTCACCT
GCTTCGCGGGTCAGCGTATGGTCATAAACAT
>RAGLN05
GTGTAGCCCAGTTAGACACTTCTAAGTCCTTGGTAACAGACATGCAGTCCCCTCGGCAGAGCCCCGCC
CCCCGATGGTACCGGGTTGCTAAGAGGTCCACAACGGGTCGCACGACAACATTAAGGCCTCATGCATG
ACCCAACACGGGGGCGTCCCGGCGCGGCGACCTTGAAGTCCGGGGACAAGTATGTCCCCCCGGGAGGG
GCGCCACAGGGAATCCGGAGTGTTGATCTGCAGCCCGAGACTCCACTACGATGGTATGAACGGCAAAG
GTACTTTGGACACCACCCCGTTTTCTATTTCCAGACGCGGCCATTCAGGAACGACGCGAATCGTAATC
TACCTGCCGCGTACGCGGATCGTACATGCGTTTTCCAGCGTTCGAAATAGAGTTTGTCGTTCCGCTGA
CCAGGTTTCACGGCTACTGCGATAAGCATATTCTACTGGGGGACTGTAAAGGAATTGTATCCACTCTG
CTTCCTGTCAATTGGCCATGTGCATCTCTGGTTGTTCAGGGTACATTAATAACACACAGCATTAAGTA
GATAAGTCTCCGATATAAGCGAACAGAACGTTGGTCAGAGACTATGGTTCAATTGTAGTGGTATAATG
TTTCCCAGTGCATAAGGAACTCAGGGACACTACTAGCATGGGCGAGGAGAGACCCTTGGGTGGCAACA
CGCGATAAGTGTTACCTAGTACATCAGCACGAGAGAGGGTTTGACCATGTGTTTTTAGACGACCCCGG
TCGCATTGAACCTGGCATTCAGAATGACGTGCTCTCAAGAATCGGGGTCATCCCCCTTAGTGTCACCT
GCTTCGCGGGTCAGCGTATGGTCATAAACAT
>RAGLN06
GTGTAGCCCAGTTAGACACTTCTAAGTCCTTGGTAGCAAACATGCAGTCCCCTCGGCAGAGCCCCGCC
CCCCGATGGTACCGGGTTGCTAAGAGGTCCACCACGAGTCGCACGACAACAGCAAGGCCTCGTGCATG
ACCCAACACGGGGGCGTCCCGGCGCGGCGACCTTGAAGTCCGGGAACAAGTATGTCTCCCCGGGAGGG
GCGCCACAGGGAATCCGGAGTGTTGATCTGCAGCCCGAGACTCCACTACGATGGTATGAACGGCAAAG
GTACTTTGGACACCACCCCGTTTTCTATTCCCAGACGCGGCCATTCAGGAACGACGCGAATCGTAATC
TACCTGCCGCGTACGCGGATCGTACATGCGTTTTCCAGCGTTCGGAATAGAGTTTGTCGTTCCGCTGA
CCAGGTTTCACGGCTACTGCGATAAGCATACTCCACTGGGGGACTGTAAAGGAATTGCATCCACTCTG
CTTCTTGTCAGTTGGCCATATGTATCTCTGGTTGTTCAGGGTACATTAATAACACACAGCATTAAGTA
GATAAGTCTCCGATATAGGCGAACAGAACGTTGGTAAAAGACTGTGGTTCAACTGTAGTGGTATAATG
TTTCCCAGTGCATAAGGAACTCAGGGACACTACTAGCATGGGCGAGTAGAGACCCTTGGGTGGCAACA
CGCAATAAGTGTTACCTAGTACACCAACACGATATAGGGTTTGACCATGTGTTTTTAGACGACCCCGG
TCGCATTAAACCTGGCATTCATAATGACGTGCTCTCAAGAGTCGGGGTCATCCCCCTTAGTGTCACCT
GCTTCGCGGGTCATCGTATGGTCATAAACAT
>RAGLN07
GTGTAGCCCAGTCAGACACTTCTAAGTCCTTGGTAACAGACATGCAGTCCCCTCGGCAGAGCCCCGCC
CCCCGATGGTACCGGGTTGCTAAGAGGTCCACAACGGGTCGCACGACAACATTAAGGCCTCATGCATG
ACCCAACACGGGGGCGTCCCGGCGCGGCGACCTTGAAGTCCGGGGACAAGTATGTCCCCCCGGGAGGG
GCGCCACAGGGAATCCGGAGTGTTGATCTGCAGCCCGAGACTCCACTACGATGGTATGAACGGCAAAG
GTACTTTGGACACCACCCCGTTTTCTATTTCCAGACGCGGCCATTTAGGAACGACGCGAATCGTAATC
TACCTGCCGCGTACGCGGATCGTACATGCGTTTTCCAGCGTTCGAAATAGAGTTTGTCGTTCCGCTGA
CCAGGTTTCACGGCTACTGCGATAAGCATATTCTACTGGGGGACTGTAAAGGAATTGTATCCATTCTG
CTTCCTGTCAATTGGCCATGTGCATCTCTGGTTGTTCAGGGTACATTAATAACACACAGCAGTAAGTA
GGTAAGTCTCCGATATAAGCGAACAGAACGTTTGTCAGAGACTGTGGTTCAATTGTAGTGGTATAATG
TTTCCCAGTGCATAAGGAACTCAGGGACACTACTAGCATGGGCGAGGAGAGACCCTTGGGTGGCAACA
CGCAATAAGTGTTACCTAGTACACCAGCACGAGAGAGGGTTTGACCATGTGTTTTTAGACGACCCCGG
TCGCATTGAACCTGGCATTCAGAATGACGTGCTCTCAAGAATCGGGGTCATCCCCCTTAGTGTCACCT
GCTTCGCGGGTCAGCGTATGGTCATAAACAT

>RAGLN08
GTGTAGCCCAGTTAGACACTTCTAAGTCCTTGGTAACAAACGTGCAGTCCCCTCGGCAGAGCCCCGCC
CCCCGATGGTACCGGGTTGCTAAGAGGTCCACCACGAGTCGCACAACAACAGCAAGGCCTCGTGCATG
ACCCAACACGGGGGCGTCCCGGCGCAGCGACCTTGAAGTCCGGGAACAAGTATGTCTCCCCGGGAGGG
GCGCCACAGGGAATCCGGAGTGTTGATCTGCAGCCCGAGACTCCACTACGATGGTATGAACGGCAAAG
GTACTTTGGACACCACCCCGTTTTCTATTCCCAGACGCGGCCATTCAGGAACGACGCGAATCGTCATC
TACCTGCCGCGTACGCGGATCGTACATGCGTTTTCCAGCTTTCGGAATAGAGTTTGTCGTTCCGCTGG
CCAGGTTTCACGGCTATTGCGATAAGCATACTCCACTGGGGGACTGTAAAGGAATTGCATCCACTCTG
CTTCTTATCAGTTGGCCATATGTATCTCTGGTTGTTCAGGGTGCATTAATAACACACAGCATTAAGTA
GATAAGTCTCCGATATAGGCGAACAGAACGTTGGTAAAAGACTGTGGTTCAACTGTAGTGGTATAATG
TTGCCCAGTGCATAAGGAACTCAGGGACACTACTAGCATGGGCGAGTAGAGACCCTTGGGTGGCAACA
CGCAATAAGTGTTACCTAGTACACCAACACGATATAGGGTTTGACCATGTGTTTTTAGACGACCCCGG
TCGCATTAAACCTGGCATTCATAATGACGTGCTCTCAAGAGTCGGGGTCATCCCCCTTAGTGTCACCT
GCTTCGCGGGTCATCGTATGGTCATAAACAT
>RAGLN09
GTGTAGCCCAGTTAGACACTTCTAAGTCCTTGGTAACAGACATGCAGTCCCCTCGGCAGAGCCCCGCC
CCCCGATGGTACCGGGTTGCTAAGAGGTCCACAACGGGTCGCACGACAACATTAAGGCCTCATGCATG
ACCCAACACGGGGGCGTCCCGGCGCGGCGACCTTGAAGTCCGGGGACAAGTATGTCCCCCCGGGAGGG
GCGCCACAGGGAATCCGGAGTGTTGATCTGCAGCCCGAGACTCCACTACGATGGTATGAACGGCAAAG
GTACTTTGGACACCACCCCGTTTTCTATTTCCAGACGCGGCCATTCAGGAACGACGCGAATCGTAATC
TACCTGCCGCGTACGCGGATCGTACATGCGTTTTCCAGCGTTCGAAATAGAGTTTGTCGTTCCGCTGA
CCAGGTTTCACGGCTACTGCGATAAGCATATTCTACTGGGGGACTGTAAAGGAATTGTATCCACTCTG
CTTCCTGTCAATTGGCCATGTGCATCTCTGGTTGTTCAGGGTACATTAATAACACACAGCATTAAGTA
GATAAGTCTCCGATATAAGCGAACAGAACGTTGGTCAGAGACTATGGTTCAATTGTAGTGGTATAATG
TTTCCCAGTGCATAAGGAACTCAGGGACACTACTAGCATGGGCGAGGAGAGACCCTTGGGTGGCAACA
CGCGATAAGTGTTACCTAGTACATCAGCACGAGAGAGGGTTTGACCATGTGTTTTTAGACGACCCCGG
TCGCATTGAACCTGGCATTCAGAATGACGTGCTCTCAAGAATCGGGGTCATCCCCCTTAGTGTCACCT
GCTTCGCGGGTCAGCGTATGGTCATAAACAT
>RAGLN10
GTGTAGCCCAGTTAGATACTNNTAAGTCCTTGGTAACAGACATGCAGTCCCCTCGGCAGAGCCCCGCC
CCCCGGTGGTACCGGGTTGCTAAGAGGTCCACCACGGGTCGCACGACAACATCAAGGCTTCATGCATG
ACCCAACACGGGGGCGTCCCGGCGCGGCGACCGTGAAGGCCGGGGATAAGTATGTCCCCCCGGGAGGG
GCGCCACAGGGAATCCGGAGTGTTGATCTGCAGCCCGAGACTCCGCTACGATGGTATGAACGGCAAAG
GTACTTTGGACAACACCCCGTTTTCTATTTCCAGACGCGGCCATTCAGGAACGACGCGAATCGTAATC
TACCTGCCGCGTCCGCGGATCGTACATGCGTTTTCCAGCGTTCGAAATAGAGTTTGTCGTTCCGCTGA
CCAGGTTTCACGGCTACTGCGATAAGCATATTCTACTGGGGGACTGTAAAGGAATTGTATCCACTCTG
CTTCCTGTCAATTGGCCATGTGCCTCTCTGGTTGTTCAGGGTACATTAATAACACACAGCATTAAGTA
GATNAGTCTCCGANATAAGCGAACAGAACGTTGGTCAAAGACTGTGGTTCAATTGTAGTGGTATAACG
TTTCCCAGTGCATAAGGAACTCAGGGACACTACTAGCATGGGCGAGGAGAGACCCTTGGGTGGCAACA
CGCAATAAGTGTTACCTAGTACACCAGCACGATAGAGGGTTTGACCATGTGTTTTTAGACGACCCCGG
TCGCATTGAACCTGGCATTCAGAATGACGTGCTCTCAAGAGTCGGGGTCATCCCCCTTAGTGTCACCT
GCTTCGCGGGTCAGCGTATGGTCATAAACAT
>RAGLN11
GTGTAGCCCAGTTAGACACTTCTAAGTCCTTGGTAACAGACATGCAGTCCCCTCGGCAGAGCCCCGCC
CCCCGATGGTACCGGGTTGCTAAGAGGTCCACAACGGGTCGCACGACAACATTAAGGCCTCATGCATG
ACCCAACACGGGGGCGTCCCGGCGCGGCGACCTTGAAGTCCGGGGACAAGTATGTCCCCCCGGGAGGG
GCGCCACAGGGAATCCGGAGTGTTGATCTGCAGCCCGAGACTCCACTACGATGGTATGAACGGCAAAG
GTACTTTGGACACCACCCCGTTTTCTATTTCCAGACGCGGCCATTCAGGAACGACGCGAATCGTAATC
TACCTGCCGCGTACGCGGATCGTACATGCGTTTTCCAGCGTTCGAAATAGAGTTTGTCGTTCCGCTGA
CCAGGTTTCACGGCTACTGCGATAAGCATATTCTACTGGGGGACTGTAAAGGAATTGTATCCACTCTG
CTTCCTGTCAATTGGCCATGTGCATCTCTGGTTGTTCAGGGTACATTAATAACACACAGCATTAAGTA
GATAAGTCTCCGATATAAGCGAACAGAACGTTGGTCAGAGACTATGGTTCAATTGTAGTGGTATAATG
TTTCCCAGTGCATAAGGAACTCAGGGACACTACTAGCATGGGCGAGGAGAGACCCTTGGGTGGCAACA
CGCAATAAGTGTTACCTAGTACATCAGCACGAGAGAGGGTTTGACCATGTGTTTTTAGACGACCCCGG

TCGCATTGAACCTGGCATTCAGAATGACGTGCTCTCAAGAATCGGGGTCATCCCCCTTAGTGTCACCT
GCTTCGCGGGTCAGCGTATGGTCATAAACAT
>RAGLN12
GTGTAGCCCAGTTAGACACTTCTAAGTCCTTGGTAACAGACATGCAGTCCCCTCGGCAGAGCCCCGCC
CCCCGATGGTACCGGGTTGCTAAGAGGTCCACAACGGGTCGCACGACAACATTAAGGCCTCATGCATG
ACCCAACACGGGGGCGTCCCGGCGCGGCGACCTTGAAGTCCGGGGACAAGTATGTCCCCCCGGGAGGG
GCGCCACAGGGAATCCGGAGTGTTGATCTGCAGCCCGAGACTCCACTACGATGGTATGAACGGCAAAG
GTACTTTGGACACCACCCCGTTTTCTATTTCCAGACGCGGCCATTCAGGAACGACGCGAATCGTAATC
TACCTGCCGCGTACGCGGATCGTACATGCGTTTTCCAGCGTTCGAAATAGAGTTTGTCGTTCCGCTGA
CCAGGTTTCACGGCTACTGCGATAAGCATATTCTACTGGGGGACTGTAAAGGAATTGTATCCACTCTG
CTTCCTGTCAATTGGCCATGTGCATCTCTGGTTGTTCAGGGTACATTAATAACACACAGCATTAAGTA
GATAAGTCTCCGATATAAGCGAACAGAACGTTGGTCAGAGACTATGGTTCAATTGTAGTGGTATAATG
TTTCCCAGTGCATAAGGAACTCAGGGACACTACTAGCATGGGCGAGGAGAGACCCTTGGGTGGCAACA
CGCAATAAGTGTTACCTAGTACATCAGCACGAGAGAGGGTTTGACCATGTGTTTTTAGACGACCCCGG
TCGCATTGAACCTGGCATTCAGAATGACGTGCTCTCAAGAATCGGGGTCATCCCCCTTAGTGTCACCT
GCTTCGCGGGTCAGCGTATGGTCATAAACAT
>RAGLN14
GTGTAGCCCAGTTAGACACTTCTAAGTCCTTGGTAGCAAACATGCAGTCCCCTCGGCAGAGCCCCGCC
CCCCGATGGTACCGGGTTGCTAAGAGGTCCACCACGAGTCGCACGACAACAGCAAGGCCTCGTGCATG
ACCCAACACGGGGGCGTCCCGGCGCGGCGACCTTGAAGTCCGGGAACAAGTATGTCTCCCCGGGAGGG
GCGCCACAGGGAATCCGGAGTGTTGATCTGCAGCCCGAGACTCCACTACGATGGTATGAACGGCAAAG
GTACTTTGGACACCACCCCGTTTTCTATTCCCAGACGCGGCCATTCAGGAACGACGCGAATCGTAATC
TACCTGCCGCGTACGCGGATCGTACATGCGTTTTCCAGCGTTCGGAATAGAGTTTGTCGTTCCGCTGA
CCAGGTTTCACGGCTACTGCGATAAGCATACTCCACTGGGGGACTGTAAAGGAATTGCATCCACTCTG
CTTCTTGTCAGTTGGCCATATGTATCTCTGGTTGTTCAGGGTACATTAATAACACACAGCATTAAGTA
GATAAGTCTCCGATATAGGCGAACAGAACGTTGGTAAAAGACTGTGGTTCAACTGTAGTGGTATAATG
TTTCCCAGTGCATAAGGAACTCAGGGACACTACTAGCATGGGCGAGTAGAGACCCTTGGGTGGCAACA
CGCAATAAGTGTTACCTAGTACACCAACACGATATAGGGTTTGACCATGTGTTTTTAGACGACCCCGG
TCGCATTAAACCTGGCATTCATAATGACGTGCTCTCAAGAGTCGGGGTCATCCCCCTTAGTGTCACCT
GCTTCGCGGGTCATCGTATGGTCATAAACAT
>RAGLN16
GTGTAGCCCAGTTAGACACTTCTAAGTCCTTGGTAACAGACATGCAGTCCCCTCGGCAGAGCCCCGCC
CCCCGATGGTACCGGGTTGCTAAGAGGTCCACAACGGGTCGCACGACAACATTAAGGCCTCATGCATG
ACCCAACACGGGGGCGTCCCGGCGCGGCGACCTTGAAGTCCGGGGACAAGTATGTCCCCCCGGGAGGG
GCGCCACAGGGAATCCGGAGTGTTGATCTGCAGCCCGAGACTCCACTACGATGGTATGAACGGCAAAG
GTACTTTGGACACCACCCCGTTTTCTATTTCCAGACGCGGCCATTCAGGAACGACGCGAATCGTAATC
TACCTGCCGCGTACGCGGATCGTACATGCGTTTTCCAGCGTTCGAAATAGAGTTTGTCGTTCCGCTGA
CCAGGTTTCACGGCTACTGCGATAAGCATATTCTACTGGGGGACTGTAAAGGAATTGTATCCACTCTG
CTTCCTGTCAATTGGCCATGTGCATCTCTGGTTGTTCAGGGTACATTAATAACACACAGCATTAAGTA
GATAAGTCTCCGATATAAGCGAACAGAACGTTGGTCAGAGACTATGGTTCAATTGTAGTGGTATAATG
TTTCCCAGTGCATAAGGAACTCAGGGACACTACTAGCATGGGCGAGGAGAGACCCTTGGGTGGCAACA
CGCGATAAGTGTTACCTAGTACATCAGCACGAGAGAGGGTTTGACCATGTGTTTTTAGACGACCCCGG
TCGCATTGAACCTGGCATTCAGAATGACGTGCTCTCAAGAATCGGGGTCATCCCCCTTAGTGTCACCT
GCTTCGCGGGTCAGCGTATGGTCATAAACAT
>RAGLN17
GTGTAGCCCAGTCAGACACTTCTAAGTCCTTGGTAACAGACATGCAGTCCCCTCGGCAGAGCCCCGCC
CCCCGATGGTACCGGGTTGCTAAGAGGTCCACAACGGGTCGCACGACAACATTAAGGCCTCATGCATG
ACCCAACACGGGGGCGTCCCGGCGCGGCGACCTTGAAGTCCGGGGACAAGTATGTCCCCCCGGGAGGG
GCGCCACAGGGAATCCGGAGTGTTGATCTGCAGCCCGAGACTCCACTACGATGGTATGAACGGCAAAG
GTACTTTGGACACCACCCCGTTTTCTATTTCCAGACGCGGCCATTTAGGAACGACGCGAATCGTAATC
TACCTGCCGCGTACGCGGATCGTACATGCGTTTTCCAGCGTTCGAAATAGAGTTTGTCGTTCCGCTGA
CCAGGTTTCACGGCTACTGCGATAAGCATATTCTACTGGGGGACTGTAAAGGAATTGTATCCATTCTG
CTTCCTGTCAATTGGCCATGTGCATCTCTGGTTGTTCAGGGTACATTAATAACACACAGCAGTAAGTA
GGTAAGTCTCCGATATAAGCGAACAGAACGTTTGTCAGAGACTGTGGTTCAATTGTAGTGGTATAATG

TTTCCCAGTGCATAAGGAACTCAGGGACACTACTAGCATGGGCGAGGAGAGACCCTTGGGTGGCAACA
CGCAATAAGTGTTACCTAGTACACCAGCACGAGAGAGGGTTTGACCATGTGTTTTTAGACGACCCCGG
TCGCATTGAACCTGGCATTCAGAATGACGTGCTCTCAAGAATCGGGGTCATCCCCCTTAGTGTCACCT
GCTTCGCGGGTCAGCGTATGGTCATAAACAT
>RAGLN18
GTGTAGCCCAGTTAGACACTTCTAAGTCCTTGGTAACAGACATGCAGTCCCCTCGGCAGAGCCCCGCC
CCCCGATGGTACCGGGTTGCTAAGAGGTCCACAACGGGTCGCACGACAACATTAAGGCCTCATGCATG
ACCCAACACGGGGGCGTCCCGGCGCGGCGACCTTGAAGTCCGGGGACAAGTATGTCCCCCCGGGAGGG
GCGCCACAGGGAATCCGGAGTGTTGATCTGCAGCCCGAGACTCCACTACGATGGTATGAACGGCAAAG
GTACTTTGGACACCACCCCGTTTTCTATTTCCAGACGCGGCCATTCAGGAACGACGCGAATCGTAATC
TACCTGCCGCGTACGCGGATCGTACATGCGTTTTCCAGCGTTCGAAATAGAGTTTGTCGTTCCGCTGA
CCAGGTTTCACGGCTACTGCGATAAGCATATTCTACTGGGGGACTGTAAAGGAATTGTATCCACTCTG
CTTCCTGTCAATTGGCCATGTGCATCTCTGGTTGTTCAGGGTACATTAATAACACACAGCATTAAGTA
GATAAGTCTCCGATATAAGCGAACAGAACGTTGGTCAGAGACTATGGTTCAATTGTAGTGGTATAATG
TTTCCCAGTGCATAAGGAACTCAGGGACACTACTAGCATGGGCGAGGAGAGACCCTTGGGTGGCAACA
CGCAATAAGTGTTACCTAGTACATCAGCACGAGAGAGGGNTTGACCATGTGTTTTTAGACGACCCCGG
TCGCATTGAACCTGGCATTCAGAATGACGTGCTCTCAAGAATCGGGGTCATCCCCCTTAGTGTCACCT
GCTTCGCGGGTCAGCGTATGGTCATAAACAT
>RAGLN19
GTGTAGCCCAGTTAGACACTTCTAAGTCCTTGGTAACAGACATGCAGTCCCCTCGGCAGAGCCCCGCC
CCCCGATGGTACCGGGTTGCTAAGAGGTCCACAACGGGTCGCACGACAACATTAAGGCCTCATGCATG
ACCCAACACGGGGGCGTCCCGGCGCGGCGACCTTGAAGTCCGGGGACAAGTATGTCCCCCCGGGAGGG
GCGCCACAGGGAATCCGGAGTGTTGATCTGCAGCCCGAGACTCCACTACGATGGTATGAACGGCAAAG
GTACTTTGGACACCACCCCGTTTTCTATTTCCAGACGCGGCCATTCAGGAACGACGCGAATCGTAATC
TACCTGCCGCGTACGCGGATCGTACATGCGTTTTCCAGCGTTCGAAATAGAGTTTGTCGTTCCGCTGA
CCAGGTTTCACGGCTACTGCGATAAGCATATTCTACTGGGGGACTGTAAAGGAATTGTATCCACTCTG
CTTCCTGTCAATTGGCCATGTGCATCTCTGGTTGTTCAGGGTACATTAATAACACACAGCATTAAGTA
GATAAGTCTCCGATATAAGCGAACAGAACGTTGGTCAGAGACTATGGTTCAATTGTAGTGGTATAATG
TTTCCCAGTGCATAAGGAACTCAGGGACACTACTAGCATGGGCGAGGAGAGACCCTTGGGTGGCAACA
CGCAATAAGTGTTACCTAGTACATCAGCACGAGAGAGGGTTTGACCATGTGTTTTTAGACGACCCCGG
TCGCATTGAACCTGGCATTCAGAATGACGTGCTCTCAAGAATCGGGGTCATCCCCCTTAGTGTCACCT
GCTTCGCGGGTCAGCGTATGGTCATAAACAT
>RAGLN20
GTGTAGCCCAGTTAGATACTNNTAAGTCCTTGGTAACAGACATGCAGTCCCCTCGGCAGAGCCCCGCC
CCCCGGTGGTACCGGGTTGCTAAGAGGTCCACCACGGGTCGCACGACAACATCAAGGCTTCATGCATG
ACCCAACACGGGGGCGTCCCGGCGCGGCGACCGTGAAGGCCGGGGATAAGTATGTCCCCCCGGGAGGG
GCGCCACAGGGAATCCGGAGTGTTGATCTGCAGCCCGAGACTCCGCTACGATGGTATGAACGGCAAAG
GTACTTTGGACAACACCCCGTTTTCTATTTCCAGACGCGGCCATTCAGGAACGACGCGAATCGTAATC
TACCTGCCGCGTCCGCGGATCGTACATGCGTTTTCCAGCGTTCGAAATAGAGTTTGTCGTTCCGCTGA
CCAGGTTTCACGGCTACTGCGATAAGCATATTCTACTGGGGGACTGTAAAGGAATTGTATCCACTCTG
CTTCCTGTCAATTGGCCATGTGCCTCTCTGGTTGTTCAGGGTACATTAATAACACACAGCATTAAGTA
GATAAGTCTCCGANATAAGCGAACAGAACGTTGGTCAAAGACTGTGGTTCAATTGTAGTGGTATAACG
TTTCCCAGTGCATAAGGAACTCAGGGACACTACTAGCATGGGCGAGGAGAGACCCTTGGGTGGCAACA
CGCAATAAGTGTTACCTAGTACACCAGCACGATAGAGGGTTTGACCATGTGTTTTTAGACGACCCCGG
TCGCATTGAACCTGGCATTCAGAATGACGTGCTCTCAAGAGTCGGGGTCATCCCCCTTAGTGTCACCT
GCTTCGCGGGTCAGCGTATGGTCATAAACAT
>RAGLN21
GTGTAGCCCAGTTAGACACTTCTAAGTCCTTGGTAACAGACATGCAGTCCCCTCGGCAGAGCCCCGCC
CCCCGATGGTACCGGGTTGCTAAGAGGTCCACAACGGGTCGCACGACAACATTAAGGCCTCATGCATG
ACCCAACACGGGGGCGTCCCGGCGCGGCGACCTTGAAGTCCGGGGACAAGTATGTCCCCCCGGGAGGG
GCGCCACAGGGAATCCGGAGTGTTGATCTGCAGCCCGAGACTCCACTACGATGGTATGAACGGCAAAG
GTACTTTGGACACCACCCCGTTTTCTATTTCCAGACGCGGCCATTCAGGAACGACGCGAATCGTAATC
TACCTGCCGCGTACGCGGATCGTACATGCGTTTTCCAGCGTTCGAAATAGAGTTTGTCGTTCCGCTGA
CCAGGTTTCACGGCTACTGCGATAAGCATATTCTACTGGGGGACTGTAAAGGAATTGTATCCACTCTG

CTTCCTGTCAATTGGCCATGTGCATCTCTGGTTGTTCAGGGTACATTAATAACACACAGCATTAAGTA
GATAAGTCTCCGATATAAGCGAACAGAACGTTGGTCAGAGACTATGGTTCAATTGTAGTGGTATAATG
TTTCCCAGTGCATAAGGAACTCAGGGACACTACTAGCATGGGCGAGGAGAGACCCTTGGGTGGCAACA
CGCAATAAGTGTTACCTAGTACATCAGCACGAGAGAGGGTTTGACCATGTGTTTTTAGACGACCCCGG
TCGCATTGAACCTGGCATTCAGAATGACGTGCTCTCAAGAATCGGGGTCATCCCCCTTAGTGTCACCT
GCTTCGCGGGTCAGCGTATGGTCATAAACAT
>CoLao566
GTNTAGCCCAGTTAGACACTNCTAAATCCNTGGTAACAGACATGCAGTCCCCTCNNCAGAGCCCCGCC
CCCCGATGGTACCGGGTTGNTAAGNGGTCCACAACGGNTCGNACGACAACATTAAGGCCTNATGCATG
ANNCANNNCGGNGGNGTCCCGGNGCGGCGACCTTGAAGTCNGGGGNCAAGTATGGCCCCCCGNGAAGG
ACGCNGTAGTGCATCTGGAGTGCCGATTNNTGGTCCNGANTGCCACCACGATGGTATGAGTGGCAAAG
GTACTTNGGACACCACCCCGTTNNCTATTTCCAGACGCGGCCNTTCAGGAACGACGCGAATCGTAATC
TACCTGCCGCGTNCGCGGATCGTACANGCGTTTTCCAGCGTTCGAAATAGAGTTTGTCGTTCCGCTGA
CCNGGTTNCACGGCTACTGCGATAAGCATATTCTACTGGGGGACTGTAAAGGAATTGTATCNACTCNG
CTTCCTGTCAATTGGCCATGTGCATCTCTGGTTGTTCAGGNNACATTAATAACACACAGCATNAAGTA
GATNAGTCTCCGATATAAGCGAACAGANCGTTGGTCAGANACTATGGTTCAATTGTAGTGGTATAATG
TTTCCCAGTGCATAAGGAACTCAGGNACACTACTAGCANGGGCGAGGAGAGACCCTTGGGTGGCAACA
CGNAATAAGTGTTACCTAGTACATCAGCACGAGANAGGGTTTGNCCNTGTGTTTTTAGNNGACCCCGG
TCGCATTGAACCTGGCATTCAGAATGACGTGCTCTCAAGAATCAAGNTCCTCCCACTGAGGGTCATNN
GCTTCGCGGGTCNGCGTATNGTCATAAACAT
>CoLao567
GNNNAGCCCAGNNNGNCACNNNNAAATNCNNNGTAACAGNCATGCAGTNNNCTCNNCAGAGNNCCGCN
CCCCGATGGTACCGGGNNGNNNNGAGGTCCACAACGGNTCNNACGANAANNTTNAGGCCNNANGCATN
ANNCANNNCNGNNNCGTCCCGGNNNNGNNACCNTNNANNCNGGNGNCAAGTATNGCCCNCNGNGAAGG
ACNCNGTAGTGCANCTGGANTGCNGNNNNNTNNNCCNNNNTGNCACCACGNTGGTATGAGTGGCAANG
GTACTNNNNNNCNCNNCNCNGTTNNCTNNNNCNANANGCGGNCNNTNANGAACGANGNGNNNNNTNNNC
TNNCTGCNNCGTNCGCGGNNCGTACNNGCNNNNNNCAGCGTTCGANATAGNGTNNGTCGTTNNNNTGA
CCNGGTTNCACGGNTACTNCGATAAGCANATTCTNCTNGNGGACTGNAAAGNAATTGTATCCATTCNG
CTTCNNGNCNNTTGGNNATGTGNATCTCTNGTTGTTCAGGNNANATNAATANCACACAGNNNNAANTA
GNTNAGTCNCCGNTNTANGCGAACANANCGTTTGNNNGNNACTGTGGTTNNATTNNAGTGGTATAATG
TTNCCCAGTNCNNNAGNANNTCAGGNACACNANNANCANGGNCNNNNNGAGACCCTTGGGTGGNANNA
CGNANNAAGNNTTNNNTNNTNNACNAGCACGNGNNANGGTTNGNCNNNNNNNNNNTNGNNGNCCCCGG
TCNCATTGAACCNGGCNNNNANNNNNANGTNNTNNNANGAATNAANNNCCTNNNACTGAGNNNCANNN
GCTTCGCNGNTNNGCGTATNGNCANNNNNNNT
>CoLao568
GTGTAGCCCAGTTAGACACTTCTAAATCCTTGGTAACAGACATGCAGTCCCCTCGGCAGAGCCCCGCC
CCCCNATGGTACCGGGTTGTTAAGAGGTCCACAACGGNTCGCACGACAACATTAAGGCCTCATGCATG
ANNCANCNCGGNGGNGTCCCGGCGCGGNGACCTTGNANTCCGGGGNCAAGTATGGCCCCCCGGGAAGG
ACGCNGTAGTGCATCTGGAGTGCCGATTNNTGGTCCAGAATGCCACCACGATGGTATGAGTGGCAAAG
GTACTTTGGACACCACCCCGTTTTCTANTTCCAGACGCGGCCNTTCAGGAACGACGCGAATCGTAATC
TACCTGCCGCGTNCGCGGATNGTACANGCGTTTTNCAGCGTTCGAAATAGAGTTTGTCGTTCCGCTGA
CCAGGTTNCACGGCTACTGCGATAAGCATATTCTACTGGGGGACTGTAAAGGAATTGTATCNACTCTG
CTTCCTGTCAATTGGCCATGTGCATCTCTGGTTGTTCAGGNNACATTAATAACACACAGCATTAAGTA
GATNAGTCTCCGATATAAGCGAACAGANCGTTGGTNAGANACTATGGTTCAATTGTAGTGGTATAATG
TTTCCCAGTGCATAAGGAACTCAGGGACACTACTAGCANGGGCGAGGAGAGACCCTTGGGTGGCAANA
CGCAATAAGTGTTNNCTAGTACATCAGCACGAGANAGGGTTTGNCCATNNNTTTTTAGNNGACCCCGG
TCGCATTGAACCTGGCATTCAGAATGACGTGCTCTNAAGAATCAAGNTCCNNNCACTGAGGGNCATCN
GCTTCGCGGGTCNGCGTATGGNCATAAACAT
>CoLao569
GTGNAGCCCAGTTAGACACTTCTAAATCCNTGGTAACAGACATGCAGTNCCCTCGGCAGAGNNCCGCC
CCCCGATGGTACCGGGTTGTTAAGNGGTCCACAACGGNTCNNACGACANNATTAAGGCCTNATGCATG
ANNCANNNCNGNNGNGTCCCGGCGNGGCGACCTTGAAGTCCGGGNNCAAGTATGGCCCCCCGGGAAGG
ACGCNGTAGTGCATCTGGAGTGCCGANNNGTGNNCNNGAATGNCACCACGATGGTATGAGTGGCAAAG
GTACTTNGGACACCACCCCGTTTTCTANTTCCAGACGCGGCCNTTCAGGAACGACGCGAATCGTAATC

TACCTGCCGCGTACGCGGATCGTACATGCGTTTTNCAGCGTTCGAAATAGAGTTTGTCGTTCCGNTGA
CCAGGTTTCACGGCTACTGCGATAAGCATATTCTACTGGGGGACTGTAAAGGAATTGTATCCACTCNG
CTTCCTGTCAATTGGNCATGTGCATCTCTNGTTGTTCAGGNNACATTAATAACACACAGCATNAAGTA
GNTAAGTCNCCGATATAAGCGAACAGANCGTTGGTNAGAGACTATGGTTCAATTGTAGTGGTATAATG
TTTCCCAGTGCATAAGGAACTCAGGNACACTACTAGCANGGGCGAGGAGAGACCCTTGGGTGGCAANA
CGNAATAAGTGTTNNCTAGTACATCAGCACGAGNNANGGTTTGNCCNTGTGTTTTTAGNNGACCCCGG
TCGCATTGAACCTGGCATTCAGAATGACGTGCTCTCAAGAATCAAGNTCCTCNCACTGAGGGNCATNN
GCTTCGCGGNNCNGCGTATGGNCATAAACAT
>CoLao570
GTGNAGCCCAGTTAGACACTTCTAAATCCTTGGTAACAGACATGCAGTNCCCTCNNCAGAGCNCCGCC
CCCCGATGGTACCGGGTTGTNAAGAGGTCCACAACGGGTCGCACGACAANNTTAAGGCCTCATGCATG
ACCCAANNCGGGGGCGTCCCGGNGNGGCGACCTTGNAGTCCGGGGNCAAGTATGGCCCCCCGNGAAGG
ACGCCGTAGTGCATCTGGAGTGCCGATTNNTGGTCCAGAATGCCACCACGATGGTATGAGTGGCAAAG
GTACTTTGGACACCACCCCGTTTTCTATTTCCAGACGCGGCCATTCAGGAACGACGCGAATCGTAATC
TACCTGCCGCGTNCGCGGATCGTACATGCGTTTTCCAGCGTTCGAAATAGAGTTNGTCGTTCCGCTGA
CCAGGTTNCACGGCTACTGCGATAAGCATATTCTACTGGGGGACTGTAAAGGAATTGTATCCACTCTG
CTTCCTGTCAATTGGCCATGTGCATCTCTGGTTGTTCAGGNNACATTAATAACACACAGNATNAAGTA
GATAAGTCTCCGATATAAGCGAACAGAACGTTGGTNAGAGACTATGGTTCAATTGTAGTGGTATAATG
TTTCCCAGTGCATAAGGAANTCAGGNACACTACTAGCANGGGCGAGGAGAGACCCTTGGGTGGCAANA
CGCAATAAGTGTTACCTAGTACATCAGCACGAGANAGGGTTTGACCNTGTGTTTTTAGNNGACCCCGG
TCGCATTGAACCTGGCATTCAGAATGACGTGCTCTCAAGAATCAAGNTCCTCNCACTGAGGGTCATNT
GCTTCGCGGNNCAGCGTATNGTCATNAACAT
>CoLao571
GTGTAGCCCAGTTAGACACNTCTAAATNCNTGGTAACAGACATGCAGTCCCCTCNNCAGAGCCCCGCC
CCCCGATGGTACCGGGTTGTTNAGAGGTCCACAACGGGTCGNACGACAACATTAAGGCCTCATGCATG
ANNCANCACGGGGGCGTCCCGGCGCGGCGACCTTGAAGTCCGGGGNCAAGTATGGCCCCCCGNGAAGG
ACGCNGTAGTGCATCTGGAGTGCCGATTCGTGNTCNAGAATGCCACCACGATGGTATGAGTGGCAAAG
GTACTTTGGACACCACCCCGTTTTCTANTTCCAGANGCGGCCNTTCAGGAACGACGCGAATCGTAATC
TACCTGCCGCGTACGCGGATCGTACATGCGTTTTCCAGCGTTCGAAATAGAGTTTGTCGTTCCGCTGA
CCNGGTTNCACGGCTACTGCGATAAGCATATTCTACTGGGGGACTGTAAAGGAATTGTATCCACTCNG
CTTCCTGTCAATTGGCCATGTGCATCTCTGGTTGTTCAGGGTACATTAATAACACACAGCATTAAGTA
GATAAGTCTCCGATATAAGCGAACAGANCGTTGGTCAGAGACTATGGTTCAATTGTAGTGGTATAATG
TTTCCCAGNGCATAAGGAACTCAGGNACACTACTAGCATGGGCGAGGAGAGACCCTTGGGTGGCAANA
CGCAATAAGTGTTNNCTAGTACATCAGCACGAGANAGGGTTTGACCNTNNNTTTNTAGNNGACCCCGG
TCGCATTGAACCTGGCATTCAGAATGACGTGCTCTCAAGAATCAANNTCCTCCCACTGAGGGTCATCN
GCTTCGCGGGTCNGCGTATNGTCATAAACAT
>CoLao572
GTGTAGCCCAGTTAGACACTTCNAAATCCNTNGTAACAGACATGCAGTCCCCTCGGCAGAGCCCCGCC
CCCCGATGGTACCGGGTTGNTAAGAGGTCCACAACGGGTCGNACGACAACATTAAGGCCTCANGCATG
ACCCNNNNCNGGGGCGTCCCGGNGNGGCNACCTTGNANTCCGGGGACAAGTATGGCCCCCCGGGAAGG
ACGCNGTAGTGCATCTGGAGTGCCGATTNNTGGTCNAGAATGCCACCACGATGGTATGAGTGGCAAAG
GTACTTTGGACACNACCCCGTTTTCTANTTCCAGACGCGGCCATTCAGGAACGACGCGAATCGTAATC
TACCTGCCGCGTNCGCGGATCGTACATGCGTTTTCCAGCGTTCGAAATAGAGTTTGTCGTTCCGCTGA
CCAGGTTNCACGGCTACTNCGATAAGCATATTCTACTGGGGGACTGTAAAGGAATTGTATCCACTCTG
CTTCCTGTCAATTGGNCATGTGCATCTCTNGTTGTTCAGGNNACATTAATAACACACAGCATTAAGTA
GATAAGTCTCCGATATAAGCGAACAGANCGTTGGTCAGAGACTATGGTTCAATTGTAGTGGTATAATG
TTTCCCAGTGCATAAGGAACTCAGGGACACTACTAGCATGGNCGAGGAGAGACCCTTGGGTGGCAANA
CGCAATAAGTGTTANNTAGTACATCAGCACGAGNNANGGTTTGACCATGTGTTTTTAGNNGACCCNGG
TCGCATTGAACCTGGCATTCAGAATGACGTGCTCTCAAGAATCAANGTCCNCNCACTGAGGGTCATNT
GCTTCGCGGGTCAGCGTATNGTCATAAACAT
>CoLao573
GTGNAGCCCAGTTAGACACTTCTAAATCCTTNGTAACAGACATGCAGTCCCCTCGGCAGAGCCCCGCC
CCCCGATGGTACCGGGTTGTTNAGAGGTCCACAACGGGTCGNACGACANCNTTAAGGCCTCANGCATG
ANNCANCNCGGGGGCGTCCCGGCGCGGNGACCTTGAANTCCGGGGNCAAGTATGGCCCCCCGGGAAGG

ACGCCGTAGTGCATCTGGAGTGCCGATTCNTGGTCNAGAATGNCACCACGATGGTATGAGTGGCAAAG
GTACTTTGGACACCACCCCGTTTTCTATTTCCAGACGCGGCCATTCAGGAACGACGCGAATCGTAATC
TACCTGCCGCGTACGCGGATCGTACATGCGTTTTCCAGCGTTCGAAATAGAGTTTGTCGTTCCGCTGA
CCAGGTTNCACGGCTACTGCGATAAGCATATTCTACTGGGGGACTGTAAAGGAATTGTATCCACTCNG
CTTCCTGTCAATTGGCCATGTGCATCTCTGGTTGTTCAGGGTACATTAATAACACACAGCATTAAGTA
GATAAGTCTCCGATATAAGCGAACAGANCGTTGGTCAGAGACTATGGTTCAATTGTAGTGGTATAATG
TTTCCCAGTGCATAAGGAACTCAGGGACACTACTAGCANGGGCGAGGAGAGACCCTTGGGTGGCAANA
CGNAATAAGTGTTNCCTAGTACATCAGCACGAGNNANGGTTTGACCATGTGTTTTTAGACGACCCCGG
TCGCATTGAACCTGGCATTCAGAATGACGTNCTNTCAAGAATCAAGNTCCTCNCACTGAGGGTCATCN
NCTTCGCGGGTCNGCGTATGGTCATAAACNT
>CoLao574
GTGNAGCCCAGTTAGACACTTCTAAATCCNTGGTAACAGACATGCAGTCCCCTCNNCAGAGCCCCGCC
CCCCGATGGTACCGGGTTGTTNAGAGGTCCACAACGGNTCNNACGACAANATTAAGGCCTCATGCATG
ANNCAACNCNGGGGNGTCCCGGNNCGGCGACCTTGAAGTCCGGGGNCAAGTATGGCCCCCCGGGAAGG
ACGCNGTAGTGCATCTGGAGTGCCGANNCGTGGTCCNGANTGNCACCACGATGGTATGAGTNGCAAAG
GTACTTNGGACACCACCCCGTTNNCTATTTCCAGACGCGGCCNTTCAGGAACGACGCGAATCGTAATC
TACCTGCCGCGTACGCGGATCGTACANGCGTTTTCCAGCGTTCGAAATANAGTTTGTCGTTCCGCTGA
CCNGGTTNCACGGCTACTGCGATAAGCATATTCTACTGGGGGACTGTAAAGGAATTGTATCNACTCTG
CTTCCTGTCAATTGGNCATGTGCATCTCTNGTTGTTCAGGGTACATTAATAACACACAGCATNAAGTA
GATAAGTCTCCGATATAAGCGAACAGANCGTTGGTCAGAGACTATGGTTCAATTGTAGTGGTATAATG
TTTCCCAGTGCATAAGGAACTCAGGNACACTACTAGCATGGGCGAGGAGAGACCCTTGGGTGGCAANA
CGCAATAAGTGTTNCCTAGTACATCAGCACGAGANANGGTTTGNCCATNNNTTTTTAGNNGNCCCCGG
TCGCATTGAACCTGGCATTCAGAATGACGTNCTCTNAAGAATCAAGNTCCTCNCACTGAGGGTCANCN
GCTTCGCGGGTCNGCGTATNGTCATAAACAT
>CoLao575
GTGNAGCTCAGTTAGACACTTCTAAATCCNTNGTAACAGACATGCAGTCCCCTCNNCAGAGCCCCGCC
CCCCNGTGGTACCGGGTTGNTNAGAGGTCCACCACGGNTCNNACGACANCATCAAGGCCTCANGCATG
ACNCANNNCNGGGACGTACCGGCGNGGCGACCTTGAAGGTCGGGNACAAGTATGTCCCCCCGGGAAGG
ACGCNGTAGTGCATCTGGAGTGCCGATTCNTGGTCCAGAATGCCACCACGATGGTATGAGTNGCAAAG
GTATTTTGGACACCACCCCGTTNNCTANTTCCAGANGCGGCCNTTCAGGAACGACGCGAATCGTANTC
TACCTGCCGCGTNCGCGGATCGTACANGCGTNTTNCAGCGTTCGAAATAGAGTTTGTCGTTCCGCTGA
CCNGGTTNCACGGCTACTGCGATAAGCATATTCTACGGGGGGACTGTAAAGGAATTGTATCCACTCTG
CTCCCTGTCAATTGGNCATGTGCATCTCTNGTTGTTTAGGNNACATTAATAACACACAGCATNAGGTA
GATAAGTCTCCGATATAAGCGAACAGANCGTTGGTNAAAGACCGTGGTTCAATTGTAGTGGTATAACA
TTTCCCAGTGCATAAGGAACCCAGGGACACTACTANCANGGGCGAGGAGAGACCCTTGGGTGGCANNA
CGNAATAAGTGTTNNNCAGTACACCAGCACGATANAGGGTTTGNCCATGNNTTTNTAGNNGACCCCGG
TCGCATTGAACCTGGCATTCAGAATGACGTGCTCTNAAGAGTCNAGGTCCTCNCACTGAGGGTCATCT
GCTTCGCGNNNTAGCGTATGGNCATNAACAT
>CoLao576
GTGNAGCCCAGTTAGACACTNNTAAATCCNTNGTAACAGACATGCAGTNCCCTCGGCAGAGCNCCGCC
CCCCGATGGTACCGGGTTGTTNAGAGGTCCACAACGGNTCNNACGACAANNTTAAGGCCTNANGCATG
ANNCANNNCGGGGGNGTCCCGGCGNGGCGACCTTGNAGTCNGGGNNCAAGTATGGCCCCCCGNGAAGG
ACGCNGTAGTGCATCTGGAGTGCCGATTCGTGGTCCAGAATGCCACCACGATGGTATGAGTGGCAAAG
GTACTTTGNNCACCACCCCGTTNNCTANTTCCAGACGCGGCCATTCAGGAACGACGCGAATCGTAATC
TACCTGCCGCGTNCGCGGATCGTACANGCGTTTTNCAGCGTTCGAAATAGAGTTTGTCGTTCCGCTGA
CCNGGTTNCACGGCTACTNCGATAAGCATATTCTACTGGGGGACTGTAAAGGAATTGTATCCACTCNG
CTTCCTGTCAATTGGCCATGTGCATCTCTNGTTGTTCAGGGTACATTAATAACACACAGCATNAAGTA
GATAAGTCTCCGATATAAGCGAACAGAACGTTGGTNAGAGACTATGGTTCAATTGTAGTGGTATAATG
TTTCCCAGTGCATAAGGAACTCAGGGACACTACTAGCANGGGCGAGGAGAGACCCTTGGGTGGCAANA
CGNAATAAGTGTTNNCTAGTACATCAGCACGAGNNANGGTTTGACCATGTGTTTTTAGNNGACCCCGG
TCGCATTGAACCTGGCATTCAGAATGACGTNCTCTCAAGAATCNAGNTCCNNNCACTGAGGGTCATNN
GCTTCGCGGGTCAGCGTATGGTCATAAACAT
>CoLao577
GTGTAGCCCAGTTAGACACTTCTAAATCCNTGGTAACAGACATGCAGTCCCCTCNNCAGAGCCCCGCC

CCCCGATGGTACCGGGTTGTTAAGAGGTCCACAACGGNTCGNACGACNANATTAAGGCCTCANGCATG
ACCCANNNCGGNGGCGTCCCGGCGCGGCGACCTTGNAGTCCGGGGNCAAGTATGGCCCCCCGNGAAGG
ACGCNGTAGTGCATCTGGAGTGCCGATTCNTGGTCCAGAATGCCACCACGATGGTATGAGTNGCAAAG
GTACTTTGGACACCACCCCGTTTTCTANTTCCAGACGCGGCCNTTCAGGAACGACGCGAATCGTAATC
TACCTGCCGCGTNCGCGGATCGTACANGCGTTTTCCAGCGTTCGAAATAGAGTTTGTCGTTCCGCTGA
CCAGGTTNCACGGCTACTGCGATAAGCATATTCTACTGGGGGACTGTAAAGNAATTGTATCCACTCNG
CTTCCTGTCAATTGGNCATGTGCATCTCTGGTTGTTCAGGNNACATTAATAACACACAGNATTAAGTA
GATAAGTCTCCGATATAAGCGAACAGANCGTTGGTNAGAGACTATGGTTCAATTGTAGTGGTATAATG
TTTCCCAGTGCATAAGGAACTCAGGNACACTACTAGCANGGGCGAGGAGAGACCCTTGGGTGGCAANA
CGCAANAAGTGTTNNCTAGTACATCAGCACGAGNNANGGTTTGNCCATGTGTTTTTAGACGACCCCGG
TCGCATTGAACCTGGCATTCAGAATGACGTGCTCTCAAGAATCAAGNTCCTNNCACTGAGGGTCATCT
GCTTCGCGGGTCNGCGTATGGTCATAAACAT
>CoLao578
GTGTAGCCCAGTTAGACACTTCTAAATCCTTGGTAACAGACATGCAGTCCCCTCGGCAGAGCNCCGCC
CCCCNATGGTACCGGGTTGNTAAGAGGTCCACAACGGGTCGNACGACNNCATTAAGGCCTCANGCATG
ACCCANCACGGGGGNGTCCCGGCGNGGCGACCTTGAAGTCCGGGGNCAAGTATGGCCCCCCGGGAAGG
ACGCCGTAGTGCATCTGGAGTGCCGATTCGTGGTCCAGAATGCCACCACGATGGTATGAGTGGCAAAG
GTACTTNGGACACCACCCCGTTTTCTANTTCCAGACGCGGCCATTCAGGAACGACGCGAATCGTAATC
TACCTGCCGCGTACGCGGATCGTACATGCGTTTTCCAGCGTTCGAAATAGAGTTTGTCGTTCCGCTGA
CCAGGTTNCACGGCTACTGCGATAAGCATATTCTACTGGGGGACTGTAAAGGAATTGTATCCACTCTG
CTTCCTGTCAATTGGNCATGTGCATCTCTNGTTGTTCAGGNNACATTAATAACACACAGCATTAAGTA
GATAAGTCTCCGATATAAGCGAACAGAACGTTGGTNAGAGACTATGGTTCAATTGTAGTGGTATAATG
TTTCCCAGTGCATAAGGAACTCAGGGACACTACTAGCANGGGCGAGGAGAGACCCTTGGGTGGCAANA
CGCAATAAGTGTTACCTAGTACATCAGCACGAGANAGGGTTTGNCCATGTGTTTTTAGACGACCCCGG
TCGCATTGAACCTGGCATTCAGAATGACGTGCTCTCAAGAATCAAGGTCCTCCCACTGAGGGTCATCT
GCTTCGCGGGTCNGCGTATGGTCATAAACAT
>CoLao580
GTGTAGCCCAGTTAGACACTTCTAAATCCTTNGTAACAGACATGCAGTCCCCTCGGCAGAGNCCCGCC
CCCCGATGGTACCGGGTTGTTNAGAGGTCCACAACGGNTCGNACGACAANATTAAGGCCTCANGCATG
ACCCANCNCNGNGGNGTCCCGGCGCGGCGACCTTGNAGTCCGGGGNCAAGTATGGCCCCCCGGGAAGG
ACGCNGTAGTGCATCTGGAGTGCCGATTNGTGGTCCAGAATGNCACCACGATGGTATGAGTNGCAAAG
GTACTTNGNNCACCACCCCGTTTTCTANTTCCAGACGCGGCCATTCAGGAACGACGCGAATCGTAATC
TACCTGCCGCGTNCGCGGATCGTACATGCGTTTTCCAGCGTTCGAAATAGAGTTTGTCGTTCCGCTGA
CCNGGTTTCACGGCTACTGCGATAAGCATATTCTACTGGGGGACTGTAAAGGAATTGTATCCACTCTG
CTTCCTGTCAATTGGCCATGTGCATCTCTGGTTGTTCAGGNNACATTAATAACACACAGCATNAAGTA
GATAAGTCTCCGATATAAGCGAACAGAACGTTGGTCAGAGACTATGGTTCAATTGTAGTGGTATAATG
TTTCCCAGTGCATAAGGAACTCAGGNACACTACTAGCATGGGCGAGGAGAGACCCTTGGGTGGCAANA
CGCAATAAGTGTTNCCTAGTACATCAGCACGAGANAGGGTTTGACCATGTGTTTTTAGACGACCCCGG
TCGCATTGAACCTGGCATTCAGAATGACGTGCTCTCAAGAATCAAGNTCCTCNCACTGAGGGTCATNN
GCTTCGCGGGTCAGCGTATGGTCATAAACAT
>CoLao581
GTNTAGCCCAGTTNGACACNTCTAAATCCTTNGTAACAGACATGCAGTCCCCTCGGCAGAGNCCCGCC
CCCCNATGGTACCGGGTTGTTAAGAGGTCCACAACGGGTCNNACGACAANATTAAGGCCTCANGCATG
ANNCANCNCNGNGGNGTCCCGGCGNGGCGACCTTGNAGTCCGGGNNCAAGTATGGCCCCCCGGGAAGG
ACGCNGTAGTGCATCTGGAGTGCCGATTCGTGGNCCAGANTGNCACCACGATGGTATGAGTGGCAAAG
GTACTNNNNNCACCACCCCGTTNNCTANTNCCAGANGCGGCCNTTCAGGAACGACGCGAATCGTAATC
TACCTGCCGCGTNCGCGGATNGTACATGCGTNTTCCAGCGTTCGAAATAGAGTTTGTCGTTCCGCTGA
CCAGGTTNCACGGCTACTGCGATAAGCATATTCTACTGGGGGACTGTAAAGGAATTGTATCCACTCNG
CTTCCTGTCAATTGGNCATGTGCATCTCTNGTTGTTCAGGGTACATTAATAACACACAGCATNAAGTA
GATAAGTCNCCGATATAAGCGAACAGAACGTTGGTNAGAGACTATGGTTNNATTGTAGTGGTATAATG
TTNCCCAGTGCATAAGGAANTCAGGNACACTACTAGCANGGNCGAGGAGAGACCCTTGGGTGGCANNA
CGNANTAAGTGTTNNCTAGTACATCAGCACGAGNNANGGTTTGACCNTNNNTTTTTAGNNGACCCCGG
TCGCATTGAACCTGGCATTCAGAATGACGTGCTNTCAAGAATCNANNTCCNCNCACTGAGGGNCATCT
GCTTCNCGNNNCNGCGTATNGNCATAAACAT

>CoLao582
GTGTAGCCCAGTTAGACACTTCTAAATCCTTGGTAACAGACATGCAGTCCCCTCNNCAGAGCCCCGCC
CCCCGATGGTACCGGGTTGTTAAGAGGTCCACAACGGGTCGCACGACAANATTAAGGCCTCANGCATG
ACCCANCACGGNGGCGTCCCGGNGCGGCGACCTTGAAGTCCGGGGNCAAGTATGGCCCCCCGGGAAGG
ACGCCGTAGTGCATCTGGAGTGCCGATTCGTGGTCCAGAATGCCACCACGATGGTATGAGTGGCAAAG
GTACTTTGGACACCACCCCGTTTTCTANTTCCAGACGCGGCCNTTCAGGAACGACGCGAATCGTAATC
TACCTGCCGCGTACGCGGATCGTACATGCGTTTTNCAGCGTTCGAAATAGAGTTTGTCGTTCCGCTGA
CCAGGTTTCACGGCTACTGCGATAAGCATATTCTACTGGGGGACTGTAAAGGAATTGTATCCACTCNG
CTTCCTGTCAATTGGCCATGTGCATCTCTNGTTGTTCAGGGTACATTAATAACACACAGCATTAAGTA
GATAAGTCTCCGATATAAGCGAACAGAACGTTGGTCAGAGACTATGGTTCAATTGTAGTGGTATAATG
TTTCCCAGTGCATAAGGAACTCAGGGACACTACTANCANGGGCGAGGAGAGACCCTTGGGTGGCAANA
CGNAATAAGTGTTNCCTAGTACATCAGCACGAGAGANGGTTTGACCNTGTGTTTTTAGACGACCCCGG
TCGCATTGAACCTGGCATTCAGAATGACGTGCTNTCAAGAATCAAGNTCCTCCCACTGAGGGTCATCT
GCTTCGCGGGTCNGCGTATGGTCATNAACAT
>CoLao584
GTGTAGCCCAGTTAGACACTNCTAAATCCTTNGTAACAGACATGCAGTCCCCTCNNCAGAGCCCCGCC
CCCCGATGGTACCGGGTTGTTNAGAGGTCCACAACGGNTCNNACGACANNNTTAAGGCCTNANGCATG
ACCCANCNCNGGGGCGTCCCGGNGCGGNGACCTTGNAGTCCGGGNNCAAGTATGGCCCCCCGGGAAGG
ACGCNGTAGTGCATCTGGAGTGCCGATTCGTGGTCCAGAATGNCACCACGATGGTATGAGTGGCAAAG
GTACTTTGGACACCACCCCGTTTTCTANTTCCAGACGCGGCCNTTCAGGAACGACGCGAATCGTAATC
TACCTGCCGCGTACGCGGATNGTACANGCGTTTTCCAGCGTTCGAAATAGAGTTTGTCGTTCCGCTGA
CCAGGTTTCACGGCTACTNNGATAAGCATATTCTACTGGGGGACTGTAAAGGAATTGTATCCACTCTG
CTTCCTGTCAATTGGNCATGTGCATCTCTNGTTGTTCAGGGTACATTAATAACACACAGCATNAAGTA
GATAAGTCTCCGATATAAGCGAACAGAACGTTGGTNAGANACTATGGTTCAATTGTAGTGGTATAATG
TTTCCCAGTGCATAAGGAACTCAGGGACACTACTANCATGGGCGAGGAGAGACCCTTGGGTGGCAANA
CGNAATAAGTGTTNCCTAGTACATCAGCACGAGNNAGGGTTTGNCCATGTGTTTTTAGACGACCCCGG
TCGCATTGAACCTGGCATTCAGAATGACGTGCTCTCAAGAATCAAGGTCCNCNCACTGAGGGTCATNN
GCTTCGCGNGTCAGCGTATGGTCATAAACAT
>CoLao585
GTGTAGCCCAGTTAGACACTTCTAAATCCTTNGTAACAGACATGCAGTCCCCTCNNCAGAGCCCCGCC
CCCCNATGGTACCGGGTTGTTAAGAGGTCCACAACGGGTCNNACGACNNCATTAAGGCCTCANGCATG
ACCCANCNCGGGGGGCGTCCCGGCGNGGCGACCTTGAAGTCNGGGGNCAAGTATGGCCCCCCGGGAAGG
ACGCNGTAGTGCATCTGGAGTGCCGATTNNTGGTCCAGAATGNCACCACGATGGTATGAGTNGCAAAG
GTACTTTNNNCACNACCCCGTTTTCTANTTCCAGANGCGGCCNTTCAGGAACGACGCGAATCGTAATC
TACCTGCCGCGTACGCGGATCGTACANGCGTTTTCCAGCGTTCGAAATAGAGTTTGTCGTTCCGCTGA
CCAGGTTTCACGGCTACTNCGATAAGCATATTCTACTGGGGGACTGTAAAGGAATTGTATCCACTCNG
CTTCCTGTCAATTGGCCATGTGCATCTCTNGTTGTTCAGGNNACANTAATAACACACAGCATTAAGTA
GATAAGTCTCCGATATAAGCGAACAGANCGTTGGTNAGAGACTATGGTTCAATTGTAGTGGTATAATG
TTTCCCAGTGCATAAGGAACTCAGGGACACTACTAGCATGGGCGAGGAGAGACCCTTGGGTGGCAANA
CGCAATAAGTGTTNNCTAGTACATCAGCACGAGNNAGGGTTTGNCCATGTGTTTTTAGNNGACCCCGG
TCGCATTGAACCTGGCATTCAGAATGACGTGCTCTCAAGAATCAAGNTCCTCNCACTGAGGGTCATCT
GCTTCGCGGGTCNGCGTATGGTCATAAACAT
>CoLao586
GTGNAGCCCAGTTNGACACTTCTAAATCCNTNGTNACAGACATGCAGTCCCCTCGGCAGAGCCCCGCC
CCCCNATGGTACCGGGTTGTTAAGAGGTCCACAACGGNTCGNACGACAACNTTAAGGCCTCANGCATG
ANNCANCNCNGGGGNGTCCCGGCGNGGNGACCTTGAANTCCGGGGNCAAGTATGGCCCCCCGNGAAGG
ACGCNGTAGTGCATCTGGAGTGCCGATTNNTGGTCCANANTGCCACCACGATGGTATGAGTGGCAAAG
GTACTTTGGACACCACCCCGTTNNCTANTTCCAGACGCGGCCNTTCAGGAACGACGCGAATCGTAATC
TACCTGCCGCGTACGCGGATCGTACANGCGTTTTCCAGCGTTCGANATAGAGTTTGTCGTTCCGCTGA
CCNGGTTNCACGGCTACTNCGATAAGCATATTCTACTGGGGGACTGTAAAGGAATTGTATCCACTCNG
CTTCCTGTCAATTGGCCATGTGCATCTCTNGTTGTTCAGGNNACATTAATAACACACAGNATNAAGTA
GATNAGTCTCCGATATAAGCGAACAGANCGTTGGTNAGAGACTATGGTTCAATTGTAGTGGTATAATG
TTTCCCAGTGCATAAGGANCTCAGGNACACTACTAGCANGGGCGAGGAGAGACCCTTGGGTGGCANNA
CGNAATAAGTGTTNNNTAGTACATCAGCACGAGNNAGGGTTTGNCCNTGNGTTTTTAGACGACCCCGG

TCGCATTGAACCTGGCATTCAGAATGACGTGCTCTCAAGAATCNAGNTCCTCNCACTGAGGGTCATCN
GCTTCGCGGGTCNGCGTATGGTCATAAACAT
>CoLao587
GTNNAGCCCAGTTAGACACTTNNAAATCCNTNGTAACAGACATGCAGTCCCCTCNNCAGAGCCCCGCC
CCCCNATGGTACCGGGNNGTTAAGAGGTCCACAACGGGTCNNACGACAACNTTAAGGCCTCANGCATG
ANNCANNNCGGGNGNGTCCCGGNGNGGCGACCTTGAAGNCNGGGGNCAAGTATGGCCCCCCGNGAAGG
ACGCNGTAGTGCATCTGGAGTGCCGATTNNTGGTCNANAATGNCACCACGATGGTATGAGTGGCAAAG
GTACTTTNNNCACNACCCNGTTNNCTANTNCCAGACGCGGCCNTTCAGGAACGACGCGAATCGTANTC
TANCTGCCGCGTNCGCGGATNGTACNNGCGTTTTCCAGCGTTCGAAATAGAGTTTGTCGTTCCGCTGA
CCNGGTTNCACGGCTACTGCGATAAGCANATTCTACTGGGGGACTGTAAAGNAATTGTATCNACNCNG
CTTCCTGTCAATTGGNCATGTGCATCTCTNGTTGTTCAGGNNACATTAATAACACACAGCATNAAGTA
GATNAGTCTCCGATATAAGCGAACAGANCGTTGGTNAGAGACTATGGTTCAATTNTAGTGGTATAATG
TTTCCCAGNGCATNAGGAACTCAGGNACACTACTAGCANGGGCGAGGAGAGACCCTTGGGTGGCAANA
CGNAATAAGTGTTNNCTAGTACATCAGCACGAGNNAGGGTTTGNCCNTGNNTTTTAGNNNACCCCGG
TCGCATTGAACCTGGCATTCAGAATGACGTNCTNTNAAGAATCAAGNTCCNNNCACTGAGGGNCATNN
GCTTCGCGGGTCNGCGTATGGTCATAAACAT
>CoLao588
GTGTAGCCCAGTTAGACACTTCTAAATCCTTNGTAACAGACATGCAGTCCCCNCNNCAGAGCCCCGCC
CCCCNATGGTACCGGGTTGTTAAGAGGTCCACAACGGNTCNNACGACAACNTTAAGGCCTNATGCATG
ANNCANCNCGGGGGCGTCCCGGCGCGGCGACCTTGNAGTCCGGGGNCAAGTATGGCCCCCCGGGAAGG
ACGCNGTAGTGCATCTGGAGTGCCGATTCGTGGTCCNGAATGNCACCACGATGGTATGAGTGGCAAAG
GTACTTTGGNCACCACCCCGTTTTCTANTNCCAGANGCGGCCATTCAGGAACGACGCGAATCGTAATC
TACCTGCCGCGTACGCGGATNGTACATGCGTTTTCCAGCGTTCGAAATAGAGTTTGTCGTTCCGCTGA
CCNGGTTNCACGGCTACTGCGATAAGCATATTCTACTGGGGGACTGTAAAGGAATTGTATCCACTCNG
CTTCCTGTCAATTGGCCATGTGCATCTCTNGTTGTTCAGGGNACATTAATAACACACAGCNTTAAGTA
GATAAGTCTCCGATATAAGCGAACAGANCGTTGGTCAGAGACTATGGTTCAATTNTAGTGGTATAATG
TTTCCCAGTGCATNAGGAACTCAGGNACACTACTAGCANGGGCGAGGAGAGACCCTTGGGTGGCAANA
CGCAATAAGTGTTANCTAGTACATCAGCACGAGANAGGGTTTGACCATGNNTTTNTAGNNGACCCCGG
TCGCATTGAACCTGGCATTCAGAATGACGTGCTCTCAAGAATCAAGNTCCTCNCACTGAGGGTCATCN
GCTTCGCGGGTCAGCGTATGGTCATAAACAT
>CoLao589
GTGTAGCCCAGTTAGACACTTCTAAATCCNTGGTAACAGACATGCAGTCCCCTCGGCAGAGCCCCGCC
CCCCNATGGTACCGGGTTGTTAAGAGGTCCACAACGGGTCGNACGACAACATTAAGGCCTCATGCATG
ACCCANCNCGGNGGNGTCCCGGNGNGGCGACCTTGNAGTCCGGGGNCAAGTATGGCCCCCCGGGAAGG
ACGCNGTAGTGCATCTGGAGTGCCGATTCNTGGTCCAGAATGCCACCACGATGGTATGAGTNGCAAAG
GTACTTNGGACACCACCCCGTTTTCTATTTCCAGACGCGGCCATTCAGGAACGACGCGAATCGTAATC
TACCTGCCGCGTACGCGGATCGTACANGCGTTTTNCAGCGTTCGAAATAGAGTTTGTCGTTCCGCTGA
CCAGGTTNCACGGCTACTNCGATAAGCATATTCTACTGGGGGACTGTAAAGGAATTGTATCCACTCNG
CTTCCTGTCAATTGGCCATGTGCATCTCTGGTTGTTCAGGGTACATTAATAACACACAGCATTAAGTA
GATNAGTCTCCGATATAAGCGAACAGANCGTTGGTCAGAGACTATGGTTCAATTGTAGTGGTATAATG
TTTCCCAGTGCATAAGGAACTCAGGGACACTACTAGCANGGGCGAGGAGAGACCCTTGGGTGGCAACA
CGCAANAAGTGTTNNCTAGTACATCAGCACGAGNNANGGTTTGNCCATGTGTTTTTAGNNGACCCCGG
TCGCATTGAACCTGGCATTCAGAATGACGTNCTCTCAAGAATCAAGNTCCTCNCACTGAGGGTCATCT
GCTTCGCGGGTCNGCGTATGGTCATAAACAT
>CoLao590
GTGTAGCCCAGTTAGACACTTCTAAATCCNTGGTAACAGACATGCAGTCCCCTCNNCAGAGCCCCGCC
CCCCGATGGTACCGGGTTGTTAAGAGGTCCACAACGGGTCGNACGACAANATTAAGGCCTCANGCATG
ANNCANCNCGGGNGNGTCCCGGCGCNGCGACCTTGNAGTCCGGGGNCAAGTATGGCCCCCCGGGAAGG
ACGCNGTAGTGCATCTGGANTGCCGATTCGTGGTCCAGAATGCCACCACGATGGTATGAGTGGCAAAG
GTACTTNGGNCACCACCCCGTTTTCTANTTCCAGACGCGGCCNTTCAGGAACGACGCGAATCGTAATC
TACCTGCCGCGTNCGCGGATCGTACANGCGTTTTCCAGCGTTCGAAATAGAGTTTGTCGTTCCGCTGA
CCAGGTTNCACGGCTACTGCGATAAGCATATTCTACTGGGGGACTGTAAAGGAATTGTATCCACTCTG
CTTCCTGTCAATTGGCCATGTGCATCTCTNGTTGTTCAGGGTACATTAATAACACACAGNATNAAGTA
GATAAGTCTCCGATATAAGCGAACAGANCGTTGGTCAGAGACTATGGTTCAATTGTAGTGGTATAATG

TTTCCCAGNGCATAAGGAACTCAGGGACACTACTAGCANGGGCGAGGAGAGACCCTTGGGTGGCAACA
CGNAATAAGTGTTNNCTAGTACATCAGCACGAGAGAGGGTTTGACCATGTGTTTTAGNNGACCCCGG
TCGCATTGAACCTGGCATTCAGAATGACGTNCTCTCAAGAATCNANNTCCNCNCACTGAGGGNCATCT
GCTTCGCGGNTCAGCGTATNGTCATAAACAT
>CoLao591
GTGTAGCCCAGTTAGACACTNCTAAATCCNTNGTAACAGACATGCAGTCCCCTCGGCAGAGCCCCGCC
CCCCGATGGTACCGGGTTGNTAAGAGGTCCACAACGGGTCGNACGACAANATTAAGGCCTNATGCATG
ACCCANCNCGGNGGCGTCCCGGCGNGGCGACCTTGAAGTCCGGGGNCAAGTATGGCCCCCCGGGAAGG
ACGCNGTAGTGCATCTGGAGTGCCGATTCGTGGTCNAGAATGNCACCACGATGGTATGAGTGGCAAAG
GTACTTNGGACACCACCCCGTTNNCTANTTCCAGANGCGGCCATTCAGGAACGACGCGAATCGTAATC
TACCTGCCGCGTNCGCGGATCGTACANGCGTTTTCCAGCGTTCGAAATAGAGTTTGTCGTTCCGCTGA
CCAGGTTNCACGGCTACTGCGATAAGCATATTCTACTGGGGGACTGTAAAGGAATTGTATCCACTCTG
CTTCCTGTCAATTGGCCATGTGCATCTCTGGTTGTTCAGGNNACATTAATAACACACAGNATNAAGTA
GATAAGTCTCCGATATAAGCGAACAGAACGTTGGTNAGAGACTATGGTTCAATTGTAGTGGTATAATG
TTTCCCAGTGCATAAGGAACTCAGGGACACTACTAGCATGGGCGAGGAGAGACCCTTGGGTGGCAANA
CGCAATAAGTGTTNCCTAGTACATCAGCACGAGNNAGGGTTTGNCCATGNNTTTTAGACGACCCCGG
TCGCATTGAACCTGGCATTCAGAATGACGTGCTNTCAAGAATCAANNTCCNCCCACTGAGGGTCATCT
GCTTCGCGGGTCAGCGTATGGTCATAAACAT
>CoLao592
GTGNAGCCCAGTTAGACACTTCTAAATCCNTNGTAACAGACATGCAGTCCCCTCGGCAGAGCCCCGCC
CCCCGATGGTACCGGGTTGNTAAGAGGTCCACAACGGGTCNNACGACAANATTAAGGCCTCATGCATG
ANNCNNNNCNGGGGNGTCCCGGCGNGGCGACCTTGAAGTCCGGGGNCAAGTATGGCCCCCCGNGAAGG
ACGCCGTAGTGCATCTGGAGTGCCGATTCGTGGTCCNGAATGCCACCACGATGGTATGAGTGGCAAAG
GTACTTTGGACACCACCCCGTTTTCTANTTCCAGACGCGGCCATTCAGGAACGACGCGAATCGTAATC
TACCTGCCGCGTACGCGGATCGTACANGCGTTTTNCAGCGTTCGAAATAGAGTTTGTCGTTCCGCTGA
CCAGGTTNCACGGCTACTGCGATAAGCATATTCTACTGGGGGACTGTAAAGGAATTGTATCCACTCNG
CTTCCTGTCAATTGGCCATGTGCATCTCTNGTTGTTCAGGNNACATTAATAACACACAGCATNAAGTA
GATAAGTCTCCGATATAAGCGAACAGANCGTTGGTNAGAGACTATGGTTCAATTGTAGTGGTATAATG
TTTCCCAGTGCATAAGGAACTCAGGNACACTACTAGCATGGGCGAGGAGAGACCCTTGGGTGGCAANA
CGNAATAAGTGTTACCTAGTACATCAGCACGAGANAGGGTTTGACCATGNNTTTNTAGACGACCCCGG
TCGCATTGAACCTGGCATTCAGAATGACGTGCTCTCAAGAATCAAGNTCCTCNCACTGAGGGTCATCN
GCTTCGCGNGTCNGCGTATGGTCATAAACAT
>CoLao593
GTGNAGCCCAGTTAGACACTTCTAAATCCTTGGTAACAGACATGCAGTCCCCTCGGCAGAGNNCCGCC
CCCCGATGGTACCGGGTTGTTAAGAAGTCCACAACGGGTCNCACGACAANATTAAGGCCTCANGCATG
ANNCANCNCGGGGGCGTCCCGGCGCGGCGACCTTGNAGTCCGGGGNCAAGTATGGCCCCCCGGGAAGG
ACGCNGTAGTGCATCTGGAGTGCCGATTCNTGGTCCNGAATGNCACCACGATGGTATGAGTGGCAAAG
GTACTTNGGNCACCACCCCGTTTTCTANTTCCAGACGCGGCCNTTCAGGAACGACGCGAATCGTAATC
TACCTGCCGCGTACGCGGATCGTACANGCGTTTTCCAGCGTTCGAAATAGAGTTTGTCGTTCCGCTGA
CCAGGTTNCACGGCTACTGCGATAAGCATATTCTACTGGGGGACTGTAAAGGAATTGTATCNATTCTG
CTTCCTGTCAATTGGNCATGTGCATCTCTNGTTGTTCAGGGTACATTAATAACACACAGCAGNAAGTA
GGTAAGTCTCCGATATAAGCTAACAGANCGTTTGTNAGAGACTGTGGTTCAATTGTAGTGGTATAATG
TTTCCCAGTGCATAAGGAACTCAGGNACACTACTAGCATGGGCGAGGAGAGACCCTTGGGTGGCANNA
CGNAATAAGTGTTANCTAGTACACCAGCACGAGNNAGGGTTTGNCCATGTGTTTTCAGACGACCCCGG
TCGCATTGAACCTGGCATTCAGAATGACGTGCTCTCAAGAATCAAGNTCCTCNCACTGAGGGTCATCT
GCTTCGCGGGTCNGCGTATNGTCATAAACAT
>CoLao594
GTGTAGCCCAGTTAGACACTTCTAAATCCNTNGTAACAGACATGCAGTCCCCTCGGCAGAGCNCCGCC
CCCCGATGGTACCGGGTTGTTAAGAGGTCCACAACAGNTCGNACGACNNNNTTAAGGCCTNATGCATG
ANNCNNNNCGGGGGNGTCCCGGNGNGGCGACCTTGAAGTCNGGGGNCAAGTATGGCCCCCCGGGAAGG
ACGCCGTAGTGCATCTGGAGTGCCGATTCGTGGTCCAGAATGCCACCACGATGGTATGAGTGGCAAAG
GTACTTTGGACACCACCCCGTTTTCTATTTCCAGACGCGGCCNTTCAGGAACGACGCGAATCGTAATC
TACCTGCCGCGTNCGCGGATCGTACANGCGTTTTCCAGCGTTCGAAATAGAGTTTGTCGTTCCGCCGA
CCAGGTTNCACGGCTACTGCGATAAGCATATTCTACTGGGGGACTGTAAAGGAATTGTATCCACTCTA

CTTCCTGTCAATTGGNCATGTGCATCTCTGGTTGTTCAGGNNACATTAATAACACACAGCATTAAGTA
GATAAGTCTCCGATATAAGCGAACAGANCGTTGGTNAGAGACTGTGGTTCAATTGTAGTGGTATAATG
TTTCCCAGTGCATAAGGAACTTAGGNACATTACTAGCATGGGCGAGGAGAGACCCTTGGGTGGCAANA
CGNAATAAGTGTTACCTAGTACACCAGCACGAGANAGGGTTTGACCATGTGTTTTTAGACGACCCCGG
TCGCATTGAACCTGGCATTCAGAATGACGTGCTCTCAAGAATCAAGNTCCNCNCACTGAGGGTCATNN
GCTTCGCGGGTCNGCGTATGGTCATNAANAT
>CoLao595
GTGNAGCCCAGTTAGACACNTCTAAATCCTTNGTAACAGACATGCAGTCCCCTCNNCAGAGNCCCGCC
CCCCGATGGTACCGGGTTGNTAAGAGGTCCACCACGGNTCNCACGACAANNTTAAGGCCTCATGCATG
ANNCANCNCNGGGGCGTCCCGGCGCGGCGACCTTGNANTCCGGGGNCAAGTATGGCCCCCCGGGAAGG
ACGCNGTAGTGCATCTGGAGTGCCGATTCGTGGTCCNGAATGCCACCACGATGGTATGAGTGGCAAAG
GTACTTTNGACACCACCCCGTTTTCTANTTCCAGACGCGGCCATTCAGGAACGACGCGAATCGTAATC
TACCTGCCGCGTNCGCGGATCGTATANACGTTTTCCAGCGTTCGAAATAGAGTTTGTCGTTCCGCTGA
CCAGGTTTCACGGCTACTNCGATAAGCATATTCTACTGGGGGACTGTAAAGGAATTGTATCCACTCTG
CTTCCTGTCAATTGGCCATGTGCATCTCTNGTTGTTCAGGGTANATTAATAACACACAGCATNAAGTA
GATAAGTCTCCGATATAAGCGAACAGANCGTTGGTCAGAGACTGTGGTTCAATTGTAGTGGTATAATG
TTTCCCAGTGCATAAGGAACTCAGGNACACTACTAGCANGGGCGAGGAGAGACCCTTGGGTGGCAANA
CGNAATAAGTGTTNNCTAGTACACCAGCACGAGNNAGGGTTTGACCATNNNTTTTTAGACGACCCCGG
TCGCATTGAACCTGGCATTCAGAATGACGTGCTCTCAAGAGTCAANNTCCNCNCACTGAGGGTCATCT
GCTTCGCGGGTCNGCGTATGGNCATAAACAT
>CoLao596
GTGNAGCCCAGTTAGACACTTCTAAATCCTTNGTAACAGACATGCAGTCCCCTCGGCAGAGCNCCGCC
CCCCNATGGTACCGGGTTGTTAAGAGGTCCACAACGGNTCGNACGACAACATTAAGGCCTCATGCATG
ACCCANCNCNGNGGNGTCCCGGNGNGGCGACCTTGAAGTCCGGGGNCAAGTATGGCCCCCCGGGAAGG
ACGCNGTAGTGCATCTGGAGTGCCGNTTNGTGGTCNAGAATGCCACCACGATGGTATGAGTGGCAAAG
GTACTTNGGACACCACCCCGTTNNCTANTTCCAGACGCGGCCATTCAGGAACGACGCGAATCGTAATC
TACCTGCCGCGTNCGCGGATCGTACATGCGTTTTCCAGCGTTCGAAATAGAGTTTGTCGTTCCGCTGA
CCNGGTTNCACGGCTACTGCGATAAGCATATTCTACTGGGGGACTGTAAAGGAATTGTATCCACTCTG
CTTCCTGTCAATTGGCCATGTGCATCTCTNGTTGTTCAGGGTACATTAATAACACACAGCATNAAGTA
GATAAGTCNCCGATATAAGCGAACAGAACGTTGGTCAGAGACTATGGTTCAATTGTAGTGGTATAATG
TTTCCCAGTGCATAAGGAANTCAGGGACACTACTAGCATGGGCGAGGAGAGACCCTTGGGTGGCANCA
CGCAATAAGTGTTANCTAGTACATCAGCACGAGANANGGTTTGACCATGTGTTTTTAGNNGACCCCGG
TCGCATTGAACCTGGCATTCAGAATGACGTGCTCTCAAGAATCAANNTCCTNCCACTGAGGGTCATCT
GCTTCGCNGGTCAGCGTATNGTCATANACAT
>CoLao597
GTNNAGCCCAGTTNGACACTTCTAAATCCNTNGTAACAGACATGCAGTCCCCTCGGCAGAGCNCCGCC
CCCCGATGGTACCGGGTTGTTAAGAGGTCCACCACGGGTCNNACGACAACATTAAGGCCTCATGCATG
ANNCNNNNCNGNGGNGTCCCGGNGNGGCGACCTTGNAGTCNGGGGNCAAGTATGGCCCCCCGNGAAGG
ACGCNGTAGTGCATCTGGANTGCCGATTCNTGGTCCNNAATGNCACCACGANGGTATGAGTGGCAAAG
GTACTTTNNNCACCACCCCGTTNNCTANTNCCAGACGCGGCCNTTCAGGAACGACGCGAATCGTNATC
TACCTGCCGCGTNCGCGGATNGTATANACGTTTTNCAGCGTTCGAAATAGAGTTTGTCGTTNNNCTGA
CCNGGTTNCACGGCTACTGCGATAAGCATATTCTACTGGGGGACTGNAAAGNAATTGTATCCACTCNG
CTTCCTGNCAATTGGCCATGTGCATCTCTGGTTGTTCAGGNNACATTAATAACACACAGCATNAAGTA
GATNAGTCNCCGATATAAGCGAACAGANCGTTGGTNAGAGACTGTGGTTCAATTGTAGTGGTATAATG
TTTCCCAGNGCATAAGGAACTCAGGGACACTACTAGCATGGNCGAGGAGAGACCCTTGGGTGGCANNA
CGNAATAAGTGTTNNCTAGTACACCAGCACGAGNNANGGTTTGNCCNTNNNTTTTTAGACGACCCCGG
TCGCATTGAACCTGGCATTCANAATGACGTGCTCTNAAGAGTCAANNTCCNCNCACTGAGGGNCANCN
GCTTCGCGGNTCNGCGTATGGNCATAAACAT
>CoLao598
GTGTAGCCCAGTTAGACACTTCTAAATCCNTGGTAACAGACATGCAGTCCCCTCGGCAGAGCCCCGCC
CCCCNATGGTACCGGGTTGTTAAGAGGTCCACAACGGNTCNNACGACANCATTAAGGCCTCANGCATG
ANNCANCNCGGGGGCGTCCCGGNGCGGNGACCTTGAAGTCCGGGNNCAAGTATGGCCCCCCGGGAAGG
ACGCNGTAGTGCATCTGGAGTGCCGATTCNTGGTCCAGANTGCCACCACGATGGTATGAGTGGCAAAG
GTACTTNGGACACCACCCCGTTTTCTANTTCCAGACGCGGCCATTCAGGAACGACGCGAATCGTAATC

TACCTGCCGCGTNCGCGGATCGTACANGCGTTTTCCAGCGTTCGAAATAGAGTTTGTCGTTCCGCTGA
CCAGGTTNCACGGCTACTGCGATAAGCATATTCTACTGGGGGACTGTAAAGGAATTGTATCCACTCTG
CTTCCTGTCAATTGGNCATGTGCATCTCTGGTTGTTCAGGGTACATTAATAACACACAGCATTAAGTA
GATAAGTCTCCGATATAAGCGAACAGANCGTTGGTNAGAGACTATGGTTCAATTGTAGTGGTATAATG
TTTCCCAGTGCATAAGGAACTCAGGNACACTACTAGCANGGGCGAGGAGAGACCCTTGGGTGGCAACA
CGCAATAAGTGTTANNTAGTACATCAGCACGAGANAGGGTTTGACCATGTGTTTTTAGNNGACCCCGG
TCGCATTGAACCTGGCATTCAGAATGACGTNCTCTCAAGAATCAAGNTCCNCNCACTGAGGGTCATCN
GCTTCGCGGNNCNGCGTATGGNCATAAACAT
>CoLao599
GTGTAGCCCAGTTAGACACTTCTAAATCCTTGGTAACAGACATGCAGTCCCCTCGGCAGAGCCCCGCC
CCCCGATGGTACCGGGTTGTTAAGAGGTCCACAACAGNTCNNACGACAACATTAAGGCCTCATGCATG
ACCCANNNCGGGGGCGTCCCGGCGCGGCGACCTTGAAGTCCGGGGNCAAGTATGGCCCCCCGGGAAGG
ACGCCGTAGTGCATCTGGAGTGCCGATTCGTGGTCCAGAATGCCACCACGATGGTATGAGTGGCAAAG
GTACTTTGGACACCACCCCGTTTNCTANTTCCAGACGCGGCCATTCAGGAACGACGCGAATCGTAATC
TACCTGCCGCGTACGCGGATCGTACATGCGTNTTCCAGCGTTCGAAATAGAGTTTGTCGTTCCGCCGA
CCAGGTTNCACGGCTACTNCGATAAGCATATTCTACTGGGGGACTGTAAAGGAATTGTATCCACTCNA
CTTCCTGTCAATTGGCCATGTGCATCTCTGGTTGTTCAGGGTACATTAATAACACACAGCATNAAGTA
GATAAGTCTCCGATATAAGCGAACAGANCGTTGGTCAGAGACTGTGGTTCAATTGTAGTGGTATAATG
TTTCCCAGTGCATAAGGAACTTAGGGACATTACTAGCATGGGCGAGGAGAGACCCTTGGGTGGCAACA
CGNAATAAGTGTTACCTAGTACACCAGCACGAGANAGGGTTTGACCATGTGTTTTTAGACGACCCCGG
TCGCATTGAACCTGGCATTCAGAATGACGTGCTCTCAAGAATCAAGNTCCTCNCACTGAGGGTCATCT
GCTTCGCGGGTCAGCGTATGGTCATAAACAT
>CoLao600
GTGNAGCCCAGTTAGACACTNCTAAATCCTTNGTAACAGACATGCAGTCCCCTCGGCAGAGCCCCGCC
CCCCGATGGTACCGGGTTGNTAAGAGGTCCACCACGGNTCGCACGACAACATTAAGGCCTCATGCATG
ACCCANNNCGGNGGNGTCCCGGCGNGGCGACCTTGAAGTCNGGGGNCAAGTATGGCCCCCCGGGAAGG
ACGCNGTAGTGCATCTGGAGTGCCGATTCGTGGTCCNGAATGCCACCACGATGGTATGAGTGGCAAAG
GTACTTNGGACACCACCCCGTTNNCTNNTTCCAGACGCGGCCATTCAGGAACGACGCGAATCGTAATC
TACCTGCCGCGTNCGCGGATNGTATANACGTTTTCCAGCGTTCGAAATAGAGTTTGTCGTTCCGCTGA
CCAGGTTNCACGGCTACTGCGATAAGCATATTCTACTGGGGGACTGTAAAGGAATTGTATCCACTCNG
CTTCCTGTCAATTGGNCATGTGCATCTCTNGTTGTTCAGGNNACATTAATAACACACAGCATNAAGTA
GATAAGTCNCCGATATAAGCGAACAGANCGTTGGTNAGAGACTGTGGTTCAATTGTAGTGGTATAATG
TTTCCCAGTGCATAAGGAANTCAGGNACACTACTAGCANGGGCGAGGAGAGACCCTTGGGTGGCAANA
CGNAANAAGTGTTANNTAGTACACCAGCACGAGNNAGGGTTTGNCCNTGNNTTTTTAGNNGACCCCGG
TCGCATTGAACCTGGCATTCAGAATGACGTGCTCTCAAGAGTCAANNTCCTCNCACTGAGGGTCATCN
GCTTCGCGGGTCAGCGTATGGNCATAAACAT
>CoLao601
GTGTAGCCCAGTTAGACACTTCTAAATCCNTNGTAACAGACATGCAGTCCCCTCGGCAGAGNCCCGCC
CCCCNATGGTACCGGGTTGTTAAGAGGTCCACAACGGGTCGNACGACAACATTAAGGCCTCANGCATG
ACCCANNNCNGGNGNGTCCCGGCGCGGCGACCTTGAAGTCCGGGGNCAAGTATGGCCCCCCGGGAAGG
ACGCNGTAGTGCATCTGGANTGCCGATTCNTGGTCCAGAATGNCACCACGATGGTATGAGTGGCAAAG
GTACTTTGGNCACCACCCCGTTTTCTANTTCCAGACGCGGCCNTTCAGGAACGACGCGAATCGTAATC
TACCTGCCGCGTNCGCGGATCGTACANGCGTTTTNCAGCGTTCGAAATAGAGTTTGTCGTTCCGCTGA
CCNGGTTNCACGGCTACTGCGATAAGCATATTCTACTGGGGGACTGTAAAGGAATTGTATCCACTCNG
CTTCCTGTCAATTGGCCATGTGCATCTCTNGTTGTTCAGGGNACATTAATAACACACAGCNTNAAGTA
GATNAGTCTCCGATATAAGCGAACAGANCGTTGGTNAGAGACTATGGTTCAATTGTAGTGGTATAATG
TTTCCCAGTGCATAAGGAACTCAGGGACACTACTAGCATGGGCGAGGAGAGACCCTTGGGTGGCAANA
CGNAATAAGTGTTNNCTAGTACATCAGCACGAGNNAGGGTTTGACCATGTGTTTTTAGACGACCCCGG
TCGCATTGAACCTGGCATTCAGAATGACGTGCTCTCAAGAATCNAGNTCCTNNCACTGAGGGTCATCN
GCTTCGCGGGTCNGCGTATGGNCATANACAT
>Dao50
GTGTAGCCCAGTCAGACACTTCTAAATCCTTGGTAACAGACATGCAGTCCCCTCGGCAGAGCCCCGCC
CCCCGATGGTACCGGGTTGTTAAGAGGTCCACAACGGGTCGCACGACAANATTAAGGCCTCATGCATG
ACCCANCNCNGGGGCGTCCCGGCGCGGCGACCTTGAAGTCCGGGGNCAAGTATGGCCCCCCGGGAAGG

ACGCNGTAGTGCATCTGGAGTGCCGATTCGTGGTCCAGAATGCCACCACGATGGTATGAGTNGCAAAG
GTACTTTGGACACCACCCCGTTTTCTATTTCCAGANGCGGCCNTTTAGGAACGACGCGAATCGTAATC
TACCTGCCGCGTACGCGGATCGTACATGCGTTTTCCAGCGTTCGAAATAGAGTTTGTCGTTCCGCTGA
CCAGGTTNCACGGCTACTGCGATAAGCATATTCTACTGGGGGACTGTAAAGGAATTGTATCCATTCTG
CTTCCTGTCAATTGGCCATGTGCATCTCTGGTTGTTCAGGGTACATTAATAACACACAGCAGTAAGTA
GGTAAGTCTCCGATATAAGCGAACAGANCGTTTGTCAGAGACTGTGGTTCAATTGTAGTGGTATAATG
TTTCCCAGTGCATAAGGAACTCAGGGACACTACTAGCANGGGCGAGGAGAGACCCTTGGGTGGCAACA
CGCAATAAGTGTTACCTAGTACACCAGCACGAGNNAGGGTTTGACCATNNNTTTTAGANGACCCCGG
TCGCATTGAACCTGGCATTCAGAATGACGTGCTCTCAAGAATCAAGNTCCNCNCACTGAGGGTCATCT
GCTTCGCGGGTCAGCGTATGGTCATAANNNT
>Dao51
GTNNAGCCCAGTCNNACACTNNNNAANCCNTNGTAACAGACATGCAGTCCCCTCGGCAGAGNCCCGCC
CCCCGATGGTACCGGGNTGNNAAGAGGTCCACAACGGNTCNNACGACNNNNNTAAGGCCNNANGCATG
ACCCANCNCGGNGGNGTCCCGGCGNNGCGACCTTGNAGNCNGGGNNCAAGTATGGCCCCCCGGGAAGG
ACGCNGTAGTGCATCTGGAGTGCCGATTCNTGGTCCAGAATGCCACCACGATGGTATGAGTNGCAAAG
GTACTTNNNNCACCACCCCGTTTTCTANTTCCAGACGCGGCCNTTCAGGAACGACGCGAATCGTAATC
TACCTGCCGCGTNCGCGGATCGTACANGCGTTTTCCAGCGTTCGAAATAGAGTNTGTCGTTCCGCTGA
CCNGGTTNCACGGCTACNNCGATAAGNATATTCTACTGGGGGACTGNAAAGNAATTGTATCCATNCNG
CTTCCTGNCAATTGGCCATGTGCATCTCTNGTTGTTCAGGNNACATTAATAACNCACAGNAGNAAGTA
GGTNAGTCNCCGATATAAGCGAACAGANCGTTTGTNAGAGACTGTGGTTCAATTGTAGTGGTATAATG
TTTCCCAGNGCATAAGGANNTCAGGGACACTACTAGCATGGGCGAGGAGANACCCTTGGGTGGCAANA
CGNAATAAGTGTTNCCTAGTACACCAGCACGAGNNAGGGTTTGACCNTGNNTTTTAGNNGACCCCGG
TCGCATTGAACCTGGCATTCANAATGACGTGCTNTCAAGAATCNAGNTCCNNNCACTGAGGGNCATNN
GCTTCGCGGGTCAGCGTATNGTCATNANNNT
>Dao52
GTGNAGCCCAGTTAGACACTTCTAAATCCTTGGTAACAGACATGCAGTCCCCTCNNCAGAGCCCCGCC
CCCCNATGGTACCGGGTTGNTAAGAGGTCCACAACAGGTCNNACGACAACATTAAGGCCTCATGCATG
ANNCANCNCNGNGGNGTCCCGGNGNGGCGACCTTGNAGTCCGGGGNCAAGTATGGCCCCCCGGGAAGG
ACGCNGTAGTGCATCTGGAGTGCCGATTCNTGGNCNNGAATGCCACCACGATGGTATGAGTGGCAAAG
GTACTTTNNNCACCACCCCGTTTTCTANTTCCAGACGCGGCCNTTCAGGAACGACGCGAATCGTAATC
TACCTGCCGCGTNCGCGGATCGTACANGCGTTTTCCAGCGTTCGAAATAGAGTTTGTCGTTCCGCCGA
CCAGGTTNCACGGCTACTNCGATAAGCATATTCTACTGGGGGACTGTAAAGGAATTGTATCCACTCTA
CTTCCTGTCAATTGGNCATGTGCATCTCTNGTTGTTCAGGNNACATTAATAACACACAGNATTAAGTA
GATNAGTCTCCGATATAAGCGAACAGANCGTTGGTNAGAGACTGTGGTTCAATTGTAGTGGTATAATG
TTTCCCAGTGCATAAGGAACTTAGGGACATTACTAGCANGGGCGAGGAGAGACCCTTGGGTGGCAANA
CGNAATAAGTGTTANNTAGTNCACCAGCACGAGANAGGGTTTGNCCATGTGTTTNTAGNNGACCCCGG
TCGCATTGAACCTGGCATTCAGAATGACGTGCTCTNAAGAATCAAGNTCCTNNCACTGAGGGTCATCN
GCTTCGCGGNTCNGCGTATGGTCATAAANAT
>Dao623
GTGTAGCCCAGTTAGACACTTCTAAATCCNTNGTAACAGACATGCAGTCCCCTCGGCAGAGCCCCGCC
CCCCGGTGGTACCGGGTTGTTAAGAGGTCCACCACGGNTCNNACGACAACNTCAAGGCCTCANGCATG
ACCCAACNCNGGNGCGTCCCGGCGCGGCGACCTTGNANGCCGGGGNCAAGTATGGCCCCCCGGGAAGG
ACGCNGTAGTGCATCTGGAGTGCCGATTNGTGGTCCAGAATGNCACCACGATGGTATGAGTNGCAAAG
GTACTTTGGACACCACCCCGTTTTCTANTTCCAGACGCGGCCNTTCAGGAACGACGCGAATCGTAATC
TANCTGCCGCGTACGCGGATCGTACANGCGTTTTCCAGCGTTCGAAATAGAGTTTGTCGTTCCGCTGA
CCNGGTTNCACGGCTACTNCGATAAGCATATTCTACTGGGGGACTGTAAAGGAATTGTATCNACTCNG
CTTCCTGTCAATTGGCCATGTGCATCTCTGGTTGTTCAGGNTACATTAATAACACACAGCATTAAGTA
GATAAGTCTCCGATATAAGCGAACAGANCGTTGGTCAAAGACTGTGGTTCAATTGTAGTGGTATAACG
TTTCCCAGTGCATAAGGAACTCAGGNACACTACTAGCANGGGCGAGGAGAGACCCTTGGGTGGCAANA
CGNAATAAGTGTTANNTAGTACACCAGCACGATAGAGGGTTTGACCATGTGTTTNTAGACGACCCCGG
TCGCATTGAACCTGGCATTCAGAATGACGTGCTCTCAAGAGTCNAGNTCCNCCCACTGAGGGTCATCT
GCTTCGCGNGTCAGCGTATGGTCATAAACAT
>Dao624
GTGTAGCCCAGTCAGACACTTCTAAATCCNTNGTAACAGACATGCAGTCCCCTCNNCAGAGCNCCGCC

CCCCNATGGTACCGGGTTGTTAAGNGGTCCACAACGGGTCNNACGACAACATTAAGGCCTCATGCATG
ANNCNNCNCGGGGGNGTCCCGGCGNGGCGACCTTGAAGTCCGGGGNCAAGTATGGCCCCCCGGGAAGG
ACGCNGTAGTGCATCTGGAGTGCCGATTCGTGGTCCAGANTGCCACCACGATGGTATGAGTGGCAAAG
GTACTTNGGACACCACCCCGTTTTCTATTTCCAGANGCGGCCNTTCAGGAACGACGCGAATCGTAATC
TACCTGCCGCGTNCGCGGATNGTACANGCGTTTTNCAGCGTTCGAAATAGAGTTTGTCGTTCCGCTGA
CCAGGTTNCACGGCTACTGCGATAAGCATATTCTACTGGGGGACTGNAAAGGAATTGTATCCATTCTG
CTTCCTGTCAATTGGNCATGTGCATCTCTGGTTGTTCAGGGTACATTAATAACACACAGCAGNAAGTA
GGTAAGTCTCCGATATAAGCGAACAGAACGTTTGTCAGANACTGTGGTTCAATTGTAGTGGTATAATG
TTTCCCAGTGCATAAGGAACTCAGGGACACTACTAGCANGGGCGAGGAGAGACCCTTGGGTGGCAANA
CGCAATAAGTGTTNNNTAGTACACCAGCACGAGNGAGGGTTTGACCNTGNNTTTTAGNCGACCCCGG
TCGCATTGAACCTGGCATTCAGAATGACGTGCTNTNAAGAATCAAGGTCCTCCCACTGAGGGTCATNN
GCTTCGCGGGTCNGCGTATNGTCATNAANNT
>Dao625
GTGNAGCCCAGTTAGACACTTCTAAATCCTTGGTAACAGACATGCAGTCCCCTCNNCAGAGNCCCGCC
CCCCGATGGTACCGGGTTGTTAAGAGGTCCACAACGGGTCGCACGACAACNTTAAGGCCTCANGCATG
ACCCAACACNGGGGCGTCCCGGNGNGGCGACCTTGAANTCCGGGGACAAGTATGGCCCCCCGGGAAGG
ACGCCGTAGTGCATCTGGAGTGCCGATTNGTGGTCNAGAATGCCACCACGATGGTATGAGTGGCAAAG
GTACTTNGGACACCACCCCGTTTTCTANTTCCAGACGCGGCCNTTCAGGAACGACGCGAATCGTAATC
TACCTGCCGCGTNCGCGGATCGTACATGCGTTTTCCAGCGTTCGAAATAGAGTTTGTCGTTCCGCTGA
CCAGGTTNCACGGCTACTGCGATAAGCATATTCTACTGGGGGACTGTAAAGGAATTGTATCNACTCTG
CTTCCTGTCAATTGGNCATGTGCATCTCTGGTTGTTCAGGNNACATTAATAACACACAGCATNAAGTA
GATAAGTCTCCGATATAAGCGAACAGAACGTTGGTNAGAGACTATGGTTCAATTGTAGTGGTATAATG
TTTCCCAGTNCATAAGGAACTCAGGGACACTACTAGCATGGGCGAGGAGAGACCCTTGGGTGGCAACA
CGCGATAAGTGTTACCTAGTACATCAGCACGAGNGANGGTTTGNCCATGTGTTTNTAGACGACCCCGG
TCGCATTGAACCTGGCATTCAGAATGACGTGCTCTCAAGAATCAAGGTCCNCCCACTGAGGGTCATCT
GCTTCGCGGGTCNGCGTATGGTCATAAACAT
>Dao626
GTGTAGCCCAGTCAGACACTTCTAAATCCTTNGTAACAGACATGCAGTCCCCTCGGCAGAGCCCCGCC
CCCCGATGGTACCGGGTTGTTAAGAGGTCCACAACGGNTCNNACGACAACATTAAGGCCTCATGCATG
ACCCANNACGGGGGNGTCCCGGCGCGGCGACCTTGNAGTCCGGGGACAAGTATGGCCCCCCGNGAAGG
ACGCCGTAGTGCATCTGGAGTGCCGATTCNTGGTCNAGAATGNCACCACGATGGTATGAGTGGCAAAG
GTACTTNGGACACCACCCCGTTTNCTANTTCCAGACGCGGCCATTCAGGAACGACGCGAATCGTAATC
TACCTGCCGCGTACGCGGATCGTACANGCGTTTTCCAGCGTTCGAAATAGAGTTTGTCGTTCCGCTGA
CCAGGTTNCACGGCTACTGCGATAAGCATATTCTACTGGGGGACTGTAAAGGAATTGTATCCATTCTG
CTTCCTGTCAATTGGCCATGTGCATCTCTNGTTGTTCAGGGTACATTAATAACACACAGCAGNAAGTA
GGTAAGTCTCCGATATAAGCGAACAGAACGTTTGTNAGAGACTGTGGTTCAATTGTAGTGGTATAATG
TTTCCCAGNGCATAAGGAACTCAGGGACACTACTAGCATGGGCGAGGAGAGACCCTTGGGTGGCAANA
CGCAATAAGTGTTACCTAGTACACCAGCACGAGAGAGGGTTTGNCCANGTGTTTTTAGACGACCCCGG
TCGCATTGAACCTGGCATTCAGAATGACGTGCTNTCAAGAATCAAGNTCCTCNCACTGAGGGTCATNT
GCTTCGCGGGTCNGCGTATGGTCATAANNAT
>Dao627
GTGTAGCCCAGTCAGACACTTCTAAATCCTTGGTAACAGACATGCAGTCCCCTCGGCAGAGCNCCGCC
CCCCGATGGTACCGGGTTGTTAAGAGGTCCACAACGGGTCNCACGACAACNTTAAGGCCTCANGCATG
ACCCANNNCGGGGGCGTCCCGGCGCGGCGACCTTGAAGTCCGGGNNCAAGTATGGCCCCCCGGGAAGG
ACGCCGTAGTGCATCTGGANTGCCGATTCGTGNNCCAGAATGCCACCACGATGGTATGAGTGGCAAAG
GTACTTTGGACACCACCCCGTTTTCTANTTCCAGANGCGGCCATTCAGGAACGACGCGAATCGTAATC
TACCTGCCGCGTACGCGGATCGTACANGCGTTTTCCAGCGTTCGAAATAGAGTTTGTCGTTCCGCTGA
CCAGGTTNCACGGCTACTGCGATAAGCATATTCTACTGGGGGACTGTAAAGGAATTGTATCCATTCTG
CTTCCTGTCAATTGGCCATGTGCATCTCTGGTTGTTCAGGNNACATTAATAACACACAGCAGNAAGTA
GGTAAGTCTCCGATATAAGCGAACAGAACGTTTGTNAGAGACTGTGGTTCAATTGTAGTGGTATAATG
TTTCCCAGTGCATAAGGAACTCAGGGACACTACTAGCATGGGCGAGGAGAGACCCTTGGGTGGCAACA
CGCAATAAGTGTTACCTAGTACACCAGCACGAGAGANGGTTTGNCCATGTGTTTTTAGACGNCCCCGG
TCGCATTGAACCTGGCATTCAGAATGACGTGCTCTCAAGAATCAAGGTCCNCCCACTGAGGGTCATCT
GCTTCGCGGGTCNGCGTATGGTCATNAACAT

>Dao628
GTNTAGCCCAGTCNGACACTNCTNAATNCNTNGTAACAGACATGCAGTCCCCTCGGCAGAGCCCCGCC
CCCCGATGGTACCGGGTTGNTAAGAGGTCCACAACGGNTCNNACGACAANNTTAAGGCCTCATGCATG
ANNCANNNCGGNGGCGTCCCGGCGCGGCGACCTTGNAGTCNGGGGNCAAGTATGGCCCCCCGNGAAGG
ACGCNGTAGTGCATCTGGAGTGCCGATTCGTGNTCCNGAATGCCACCACGATGGTATGAGTNGCAAAG
GTACTTNGGNCACCACCCCGTTNNCTANTTCCAGACGCGGCCNTTCAGGAACGACGCGAATCGTAATC
TACCTGCCGCGTNCGCGGATCGTACANGCGTNTTCCAGCGTTCGAAATAGAGTTTGTCGTTCCGCTGA
CCNGGTTNCACGGCTACTNNGATAAGCATATTCTACTGGGGGACTGTAAAGGAATTGTATCCATTCNG
CTTCCTGTCAATTGGNCATGTGCATCTCTNGTTGTTCAGGGTANATTAANAACACACAGNAGNAAGTA
GGTAAGTCNCCGATATAAGCGAACAGANCGTTTGTNAGAGACTGTGGTTCAATTGTAGTGGTATAATG
TTTCCCAGNGCATAAGGAANTCAGGNACACTACTAGCATGGGCNAGGAGAGACCCTTGGGTGGCAANA
CGNANTAAGTGTTANCTAGTACACCAGCACGAGNNAGGGTTTGNCCNTNNNTTTTAGNNGACCCCGG
TCGCATTGAACCTGGCNTTCAGAATGACGTGCTNTCANGAATNNAGNTCCNNNCACTGAGGGNCATNN
GCTTCGCGGGTCAGCGTATGGTCATANACNT
>Dao629
GTGTAGCCCAGTCAGACACTTCTAAATCCTTNGTAACAGACATGCAGTCCCCTCNNCAGAGCCCCGCC
CCCCNATGGTACCGGGNTGTTNAGAGGTCCACAACGGGTCNCACGACAANNTTAAGGCCTCANGCATG
ACCCANCACNGNGGCGTCCCGGCGCGGCGACCTTGNAGTCCGGGGNCAAGTATGGCCCCCCGGGAAGG
ACGCNGTAGTGCATCTGGAGTGCCGATTCGTGGTCCAGAATGCCACCACGATGGTATGAGTGGCAAAG
GTACTTNGGACACCACCCCGTTTTCTANTTCCAGACGCGGCCATTCAGGAACGACGCGAATCGTAATC
TACCTGCCGCGTACGCGGATCGTACANGCGTTTTCCAGCGTTCGAAATAGAGTTTGTCGTTCCGCTGA
CCAGGTTNCACGGCTACTGCGATAAGCATATTCTACTGGGGGACTGTAAAGGAATTGTATCCATTCNG
CTTCCTGTCAATTGGCCATGTGCATCTCTGGTTGTTCAGGGTACATTAATAACACACAGCAGTAAGTA
GGTAAGTCTCCGATATAAGCGAACAGANCGTTTGTCAGAGACTGTGGTTCAATTGTAGTGGTATAATG
TTTCCCAGTGCATAAGGAACTCAGGNACACTACTAGCATGGGCGAGGAGAGACCCTTGGGTGGCAACA
CGCAATAAGTGTTANCTAGTACACCAGCACGAGANAGGGTTTGACCATGTGTTTNTAGACGACCCCGG
TCGCATTGAACCTGGCATTCAGAATGACGTGCTCTCAAGAATCAAGNTCCTCCCACTGAGGGTCATCN
GCTTCGCGGGTCAGCGTATGGTCATNANCAT
>Dao630
GTGNAGCCCAGTCAGACACTTCTAAATCCTTGGTAACAGACATGCAGTCCCCTCGGCAGAGNCCCGCC
CCCCGATGGTACCGGGTTGTTAAGAGGTCCACAACGGGTCGNACGACAANATTAAGGCCTCATGCATG
ACCCANCACGGNNGCGTCCCGGCGCNGCNACCTTGNAGTCCGGGGNCAAGTATGGCCCCCCGGGAAGG
ACGCNGTAGTGCATCTGGAGTGCCGATTCGTGGTCNAGAATGNCACCACGATGGTATGAGTGGCAAAG
GTACTTTGGACACCACCCCGTTTTCTATTTCCAGACGCGGCCATTCAGGAACGACGCGAATCGTAATC
TACCTGCCGCGTNCGCGGATNGTACANGCGTTTTCCAGCGTTCGAAATAGAGTTTGTCGTTCCGCTGA
CCNGGTTNCACGGCTACTGCGATAAGCATATTCTACTGGGGGACTGTAAAGGAATTGTATCCATTCNG
CTTCCTGTCAATTGGCCATGTGCATCTCTGGTTGTTCAGGNNACATTAATAACACACAGNAGNAAGTA
GGTAAGTCTCCGATATAAGCGAACAGANCGTTTGTCAGAGACTGTGGTTCAATTGTAGTGGTATAATG
TTTCCCAGTGCATAAGGAACTCAGGNACACTACTAGCATGGGCGAGGAGAGACCCTTGGGTGGCAANA
CGCAATAAGTGTTNNCTAGTACACCAGCACGAGNNAGGGTTTGNCCATNNNTTTNTAGNNGACCCCGG
TCGCATTGAACCTGGCATTCAGAATGACGTGCTCTNAAGAATCAAGNTCCNCNCACTGAGGGTCATCT
GCTTCGCGGGTCAGCGTATNGTCATAAACAT
>Dao631
GTGTAGCCCAGTTAGACACTTCTAAATCCNTNGTAACAGACATGCAGTCCCCTCNNCAGAGCCCCGCC
CCCCGATGGTACCGGGTTGTTAAGAGGTCCACCACGGGTCGNACGACANCATTAAGGCCTCATGCATG
ACCCANCNCGGGGGCGTCCCGGCGCGGCGACCTTGAAGTCCGGGGNCAAGTATGGCCCCCCGGGAAGG
ACGCNGTAGTGCATCTGGAGTGCCGATTCGTGGTCCAGAATGCCACCACGATGGTATGAGTGGCAAAG
GTACTTNGGACACCACCCCGTTTTCTANTNCCAGACGCGGCCNTTCAGGAACGACGCGAATCGTAATC
TACCTGCCGCGTACGCGGATCGTATANACGTTTTCCAGCGTTCGAAATAGAGTTTGTCGTTCCGCTGA
CCAGGTTNCACGGCTACTGCGATAAGCATATTCTACTGGGGGACTGTAAAGGAATTGTATCCACTCNG
CTTCCTGTCAATTGGCCATGTGCATCTCTGGTTGTTCAGGNNACATTAATAACACACAGCATTAAGTA
GATAAGTCTCCGATATAAGCGAACAGANCGTTGGTCAGAGACTGTGGTTCAATTGTAGTGGTATAATG
TTTCCCAGTGCATAAGGAACTCAGGGACACTACTAGCATGGGCGAGGAGAGACCCTTGGGTGGCAANA
CGCAATAAGTGTTACCTAGTACACCAGCACGAGANAGGGTTTGACCATGTGTTTNTAGACGACCCCGG

TCGCATTGAACCTGGCATTCAGAATGACGTNCTCTCAAGAGTCAAGGTCCTCCCACTGAGGGTCATCT
GCTTCGCGGNTCNGCGTATNGNCATAAACAT
>Dao632
GTGNAGCTCAGTTAGACACNTNNAAATCCNTNGTAACAGACATGCAGTCCCCTCNNCAGAGNCCCGCC
CCCCGGTGGTACCGGGTTGNTAAGAGGTCCACCACGGNTCNNACGACNNCNTCAAGGCCTCANGCATG
ANNCANCGCNGGGACGTACCGGCGNGGCGACCTTGAAGGTNGGGGNCAAGTATGTCCCCCCGGGAAGG
ACGCNGTAGTGCATCTGGANTGCCGATTCNTGGTCCAGANTGCCACCACGATGGTATGAGTGGCAAAG
GTATTTNGGACACCACCCCGTTNNCTATTTCCAGANGCGGCCNTTCAGGAACGACGCGAATCGTAATC
TACCTGCCGCGTNCGCGGATCGTACANGCGTTTTTCAGCGTTCGAAATAGAGTTNGTCGTTCCGCTGA
CCAGGTTNCACGGCTACTNCGATAAGCATATTCTACGGGGGGACTGTAAAGGAATTGTATCCACTCNG
CTCCCTGTCAATTGGNCATGTGCATCTCTNGTTGTTTAGGNNANATTAATAACACACAGCATNAGGTA
GATAAGTCTCCGATATAAGCGAACAGANCGTTGGTCAAANACCGTGGTTCAATTGTAGTGGTATAACA
TTTCCCAGTGCATAAGGAANCCAGGNACACTACTAGCATGGGCGAGGAGAGACCCTTGGGTGGCAANA
CGNAATAAGTGTTANNCAGTACACCAGCACGATNNAGGGTTTGACCATGTGTTTNTAGNNGACCCCGG
TCGCATTGAACCTGGCATTCAGAATGACGTNCTNTCAAGAGTCAAGNTCCTCNCACTGAGGGTCATCT
GCTTCGCGNNNTNGCGTATGGNCATAAACAT
>Dao633
GTGTAGCCCAGTCAGACACTTCTAAATCCTTGGTAACAGACATGCAGTCCCCTCNNCAGAGCCCCGCC
CCCCGATGGTACCGGGTTGTTAAGAGGTCCACAACGGGTCGNACGACAACATTAAGGCCTCATGCATG
ACCCAACACGGGGGCGTCCCGGCGCGGCGACCTTGNAGTCCGGGGACAAGTATGGCCCCCCGGGAAGG
ACGCCGTAGTGCATCTGGAGTGCCGATTNGTGGTCCAGAATGNCACCACGATGGTATGAGTGGCAAAG
GTACTTTGGACACCACCCCGTTTTCTATTTCCAGACGCGGCCATTCAGGAACGACGCGAATCGTAATC
TACCTGCCGCGTACGCGGATCGTACATGCGTTTTCCAGCGTTCGAAATAGAGTTTGTCGTTCCGCTGA
CCAGGTTNCACGGCTACTGCGATAAGCATATTCTACTGGGGGACTGTAAAGGAATTGTATCCATTCTG
CTTCCTGTCAATTGGCCATGTGCATCTCTGGTTGTTCAGGGTACATTAATAACACACAGCAGTAAGTA
GGTAAGTCTCCGATATAAGCGAACAGAACGTTTGTCAGAGACTGTGGTTCAATTGTAGTGGTATAATG
TTTCCCAGTGCATAAGGAACTCAGGGACACTACTAGCATGGGCGAGGAGAGACCCTTGGGTGGCAACA
CGCAATAAGTGTTACCTAGTACACCAGCACGAGAGAGGGTTTGACCATGTGTTTTTAGACGACCCCGG
TCGCATTGAACCTGGCATTCAGAATGACGTGCTCTCAAGAATCAAGGTCCTCCCACTGAGGGTCATCT
GCTTCGCGGGTCAGCGTATGGTCACAAACAT
>Dao634
GTNNAGCCCAGTCAGACACTTCTAAATCCNTGGTAACAGACATGCAGTCCCCTCGGCAGAGCNCCGCC
CCCCGATGGTACCGGGTTGTTAAGAGGTCCACAACGGNTCNNACGACNNCATTAAGGCCTCANGCATG
ACCCAACNCNGNGGCGTCCCGGCGNGGCGACCTTGNAGTCCGGGGNCAAGTATGGCCCCCCGGGAAGG
ACGCNGTAGTGCATCTGGAGTGCCGATTCGTGGTCNAGAATGCCACCACGATGGTATGAGTGGCAAAG
GTACTTNGGACACCACCCCGTTTTCTATTTCCAGACGCGGCCNTTCAGGAACGACGCGAATCGTAATC
TANCTGCCGCGTNCGCGGATCGTACANGCGTTTTCCAGCGTTCGAAATAGAGTTTGTCGTTCCGCTGA
CCAGGTTNCACGGCTACTGCGATAAGCATATTCTACTGGGGGACTGTAAAGGAATTGTATCCATTCTG
CTTCCTGTCAATTGGNCATGTGCATCTCTNGTTGTTCAGGNTACATTAATAACACACAGNAGNAAGTA
GGTAAGTCTCCGATATAAGCGAACAGANCGTTTGTNAGAGACTGTGGTTCAATTGTAGTGGTATAATG
TTTCCCAGTGCATAAGGAACTCAGGGACACTACTAGCATGGGCGAGGAGAGACCCTTGGGTGGCAANA
CGCAATAAGTGTTNNCTAGTACACCAGCACGAGNGAGGGTTTGACCATGTNTTTTTAGACGACCCCGG
TCGCATTGAACCTGGCATTCAGAATGACGTGCTCTCAAGAATCAANGTCCTCCCACTGAGGGTCATCN
GCTTCGCGGGTCNGCGTATNGTCACAAACAT
>Dao635
GTGTAGCCCAGTTAGACACTTCTAAATCCNTGGTAACAGACATGCAGTCCCCTCNNCAGAGCCCCGCC
CCCCGATGGTACCGGGTTGNTAAGAGGTCCACCACGGNTCNNACGACAACATTAAGGCCTCATGCATG
ACCCAACACGGGGGNGTCCCGGCGNGGCGACCTTGAAGTCCGGGGACAAGTATGGCCCCCCGGGAAGG
ACGCCGTAGTGCATCTGGAGTGCCGATTCGTGGTCCAGAATGCCACCACGATGGTATGAGTGGCAAAG
GTACTTTGGACACCACCCCGTTTTCTANTTCCAGACGCGGCCATTCAGGAACGACGCGAATCGTAATC
TACCTGCCGCGTNCGCGGATCGTATATACGTTTTCCAGCGTTCGAAATAGAGTTTGTCGTTCCGCTGA
CCAGGTTNCACGGCTACTGCGATAAGCATATTCTACTGGGGGACTGTAAAGGAATTGTATCCACTCNG
CTTCCTGTCAATTGGCCATGTGCATCTCTNGTTGTTCAGGGTACATTAATAACACACAGCATTAAGTA
GATAAGTCTCCGATATAAGCGAACAGANCGTTGGTCAGAGACTGTGGTTCAATTGTAGTGGTATAATG

TTTCCCAGTGCATAAGGAACTCAGGNACACTACTAGCATGGGCGAGGAGAGACCCTTGGGTGGCAANA
CGCAATAAGTGTTANCTAGTACACCAGCACGAGANANGGTTTGACCATGNNTTTTAGNNGACCCCGG
TCGCATTGAACCTGGCATTCAGAATGACGTNCTCTCAAGAGTCAAGNTCCTCCCACTGAGGGTCATCN
GCTTCGCGGGTCNGCGTATGGNCATAAACAT
>Dao636
GTGTAGCCCAGTCAGACACTTCTAAATCCNTNGTAACAGACATGCAGTCCCCTCGGCAGAGCCCCGCC
CCCCGATGGTACCGGGTTGNTAAGAGGTCCACAACGGNTCNCACGACAACATTAAGGCCTCATGCATG
ANNCANNACGGGGGNGTCCCGGCGNGGCGACCTTGNAGTCCGGGGNCAAGTATNGCCCCCCGGGAAGG
ACGCNGTAGTGCATCTGGAGTGCCGATTCGTGGTCCAGAATGCCACCACGATGGTATGAGTNGCAAAG
GTACTTTGGACACCACCCCGTTTTCTANTTCCAGACGCGGCCATTTAGGAACGACGCGAATCGTAATC
TACCTGCCGCGTNCGCGGATCGTACANGCGTTTTNCAGCGTTCGANATAGAGTTTGTCGTTCCGCTGA
CCAGGTTNCACGGCTACTGCGATAAGCATATTCTACTGGGGGACTGTAAAGGAATTGTATCCATTCTG
CTTCCTGTCAATTGGNCATGTGCATCTCTGGTTGTTCAGGGTACATTAATAACACACAGCAGTAAGTA
GGTNAGTCTCCGATATAAGCGAACAGANCGTTTGTCAGAGACTGTGGTTCAATTGTAGTGGTATAATG
TTTCCCAGTGCATAAGGAACTCAGGNACACTACTAGCANGGNCGAGGAGAGACCCTTGGGTGGCAACA
CGCAATAAGTGTTACCTAGTACACCAGCACGAGANAGGGTTTGACCATGTGTTTTTAGNNGACCCCGG
TCGCATTGAACCTGGCATTCAGAATGACGTGCTCTNAAGAATCAAGNTCCTCCCACTGAGGGNCATNN
GCTTCGCGGGTCAGCGTATGGTCATAAACAT
>Dao637
GTGTAGCCCAGTTNGACACTTCTAAATCCNTGGTAACAGACATGCAGTCCCCTCNNCAGAGCCCCGCC
CCCCGATGGTACCGGGTTGNTAAGAGGTCCACAACGGGTCNNACGACAACATTAAGGCCTNANGCATG
ACCCANCACGGGGGCGTCCCGGCGCGGCGACCTTGAAGTCNGGGGNCAAGTATGGCCCCCCGNGAAGG
ACGCNGTAGTGCATCTGGAGTGCCGATTCGTGGTCCAGAATGCCACCACGATGGTATGAGTNGCAAAG
GTACTTTGGACACCACCCCGTTTTCTANTTCCAGANGCGGCCATTCAGGAACGACGCGAATCGTAATC
TACCTGCCGCGTNCGCGGATCGTACANGCGTTTTCCAGCGTTCGAAATAGAGTTTGTCGTTCCGCTGA
CCAGGTTNCACGGCTACTGCGATAAGCATATTCTACTGGGGGACTGTAAAGGAATTGTATCNACTCNG
CTTCCTGTCAATTGGCCATGTGCATCTCTGGTTGTTCAGGGTACATTAATAACACACAGNATNAAGTA
GATAAGTCTCCGATATAAGCGAACAGAACGTTGGTCAGAGACTATGGTTCAATTGTAGTGGTATAATG
TTTCCCAGTGCATAAGGAACTCAGGGACACTACTAGCANGGGCGAGGAGAGACCCTTGGGTGGCAANA
CGCGATAAGTGTTNNNTAGTACATCAGCACGAGANAGGGTTTGNCCATGNNTTTNTAGNNGACCCCGG
TCGCATTGAACCTGGCATTCAGAATGACGTGCTNTCAAGAATCAAGNTCCTCNCACTGAGGGTCATCN
GCTTCGCGGGTCAGCGTATGGTCATAAACAT
>Dao638
GTGNAGCCCAGTCNGACACTTCTAAATCCNTNGTAACAGACATGCAGTCCCCTCGGCAGAGCCCCGCC
CCCCGATGGTACCGGGTTGTTAAGAGGTCCACAACGGNTCGNACGACAACATTAAGGCCTNATGCATG
ANNCANNNCNGGGGNGTCCCGGCGNGGNGACCTTGNAGTCCGGGGNCAAGTATGGCCCCCCGGGAAGG
ACGCNGTAGTGCATCTGGAGTGCCGATTCGTGGTCCNGANTGNCACCACGATGGTATGAGTGGCAAAG
GTACTTNGGNCACCACCCCGTTTNCTNNTTCCAGANGCGGCCATTCAGGAACGACGCGAATCGTAATC
TANCTGCCGCGTNCGCGGATNGTACANGCGTTTTNCAGCGTTCGAAATAGAGTTTGTCGTTCCGCTGA
CCAGGTTNCACGGCTACTNCGATAAGCATATTCTACTGGGGGACTGTAAAGNAATTGTATCCATTCNG
CTTCCTGTCAATTGGNCATGTGCATCTCTNGTTGTTCAGGNNACATTAATAACACACAGNAGNAAGTA
GGTAAGTCTCCGATATAAGCGAACAGANCGTTTGTNAGAGACTGTGGTTCAATTGTAGTGGTATAATG
TTTCCCAGTGCATAAGGAACTCAGGGACACTACNAGCANGGNCGAGGAGAGACCCTTGGGTGGCAANA
CGCAATAAGTGTTNNNTAGTACACCAGCACGAGNNANGGTTNGACCATNNNTTTNTAGNNGACCCCGG
TCGCATTGAACCTGGCATTCAGAATGACGTGCTCTNAAGAATCAAGNTCCTCNCACTGAGGGTCATNN
GCTTCGCNGGTCNGCGTATGGNCATAAACAT
>Dao639
GTGNAGCCCAGTCAGACACTTCTAAATCCTTGGTAACAGACATGCAGTCCCCTCGGCAGAGCNCCGCC
CCCCNATGGTACCGGGTTGTTAAGAGGTCCACAACGGNTCGNACGACAACATTAAGGCCTCANGCATG
ACCCANCACNGGGGCGTCCCGGCGNGGNGACCTTGAAGTCCGGGGNCAAGTATGGCCCCCCGGGAAGG
ACGCNGTAGTGCATCTGGANTGCCGATTCNTGGTCCAGAATGCCACCACGATGGTATGAGTGGCAAAG
GTACTTTGGACACCACCCCGTTTTCTATTTCCAGACGCGGCCATTCAGGAACGACGCGAATCGTAATC
TACCTGCCGCGTACGCGGATCGTACATGCGTTTTNCAGCGTTCGAAATAGAGTTTGTCGTTCCGCTGA
CCAGGTTNCACGGCTACTGCGATAAGCATATTCTACTGGGGGACTGTAAAGNAATTGTATCCATTCNG

CTTCCTGTCAATTGGCCATGTGCATCTCTNGTTGTTCAGGGTACATTAATAACACACAGCAGNAAGTA
GGTAAGTCTCCGATATAAGCGAACAGAACGTTTGTNAGAGACTGTGGTTCAATTGTAGTGGTATAATG
TTTCCCAGTGCATAAGGAACTCAGGGACACTACTAGCATGGGCGAGGAGAGACCCTTGGGTGGCAANA
CGNAATAAGTGTTACCTAGTACACCAGCACGAGAGAGGGTTTGACCATNNNTTTTAGNNGACCCCGG
TCGCATTGAACCTGGCATTCAGAATGACGTGCTCTCAAGAATCAAGNTCCTCNCACTGAGGGTCATCT
GCTTCGCGGGTCNGCGTATGGTCATAAACNT
>Dao640
GTGTAGCCCAGTCAGACACNTCTAAATCCNTGGTAACAGACATGCAGTCCCCTCNNCAGAGNCCCGCC
CCCCGATGGTACCGGGTTGNTAAGAGGTCCACAACGGGTCNNACGACANCNTTAAGGCCTCATGCATG
ACCCANNNCGGNGGNGTCCCGGCGNGGNGACCTTGNAGTCCGGGGNCAAGTATGGCCCCCCGGGAAGG
ACGCNGTAGTGCATCTGGAGTGCCGANNCNTGGTCCNGAATGCCACCACGANGGTATGAGTNGCAAAG
GTACTTNGGACACCACCCCGTTTTCTANTTCCAGANGCGGCCNTTCAGGAACGACGCGAATCGTAATC
TACCTGCCGCGTNCGCGGATNGTACANGCGTNTTCCAGCGTTCGAAATAGAGTTTGTCGTTCCGCTGA
CCAGGTTNCACGGCTACTNCGATAAGCATATTCTACTGGGGGACTGTAAAGGAATTGTATCCATTCNG
CTTCCTGNCAATTGGNCATGTGCATCTCTNGTTGTTCAGGGNACATTAATAACACACAGCAGNAAGTA
GGTAAGTCTCCGATATAAGCGAACAGAACGTTTGTNAGAGACTGTGGTTCAATTGTAGTGGTATAATG
TTTCCCAGTGCATAAGGAACTCAGGGACACTACTAGCATGGGCGAGGAGAGACCCTTGGGTGGCANNA
CGCAATAAGTGTTNNCTAGTACACCAGCACGAGNNAGGGTTTGACCANGTGTTTNTAGNNGACCCCGG
TCGCATTGAACCTGGCATTCAGAATGACGTGCTCTCAAGAATCAAGNTCCTCNCACTGAGGGTCATCN
GCTTCNCGGNTCNGCGTATGGTCATAAANAT
>Dao641
GTGNAGCCCAGTCAGACACTTCTAAATCCTTGGTAACAGACATGCAGTCCCCTCGGCAGAGCCCCGCC
CCCCGATGGTACCGGGTTGTTAAGAGGTCCACAACGGGTCGNACGACNACATTAAGGCCTCATGCATG
ACCCANNACGGGGGCGTCCCGGCGCGGCGACCTTGAAGTCCGGGGACAAGTATGGCCCCCCGGGAAGG
ACGCCGTAGTGCATCTGGAGTGCCGATTCNTGGTCCAGANTGCCACCACGATGGTATGAGTGGCAAAG
GTACTTTGGACACCACCCCGTTTTCTANTTCCAGANGCGGCCATTCAGGAACGACGCGAATCGTAATC
TACCTGCCGCGTACGCGGATCGTACANGCGTTTTCCAGCGTTCGAAATAGAGTTTGTCGTTCCGCTGA
CCAGGTTNCACGGCTACTGCGATAAGCATATTCTACTGGGGGACTGTAAAGGAATTGTATCCATTCTG
CTTCCTGTCAATTGGCCATGTGCATCTCTGGTTGTTCAGGGTACATTAATAACACACAGCAGNAAGTA
GGTAAGTCTCCGATATAAGCGAACAGAACGTTTGTCAGAGACTGTGGTTCAATTGTAGTGGTATAATG
TTTCCCAGTGCATAAGGAACTCAGGNACACTACTAGCANGGGCGAGGAGAGACCCTTGGGTGGCAANA
CGCAATAAGTGTTNCCTAGTACACCAGCACGAGANAGGGTTTGACCATGNNTTTTTAGACGACCCCGG
TCGCATTGAACCTGGCATTCAGAATGACGTGCTCTCAAGAATCAAGGTCCTCNCACTGAGGGTCATCT
GCTTCGCGGGTCNGCGTATGGTCATAAACAT
>Dao642
GTGTAGCCCAGTTANACACNTCTAAATCCNTNGTAACAGACATGCAGTCCCCTCNNCAGAGCCCCGCC
CCCCGATGGTACCGGGTTGTTNAGAGGTCCACCACGGGTCGNACGACAACATTAAGGCCTCATGCATG
ACCCAACACNGGGGCGTCCCGGCGCGGCGACCTTGAANTCCGGGGNCAAGTATGGCCCCCCGGGAAGG
ACGCCGTAGTGCATCTGGAGTGCCGATTCGTGGNCNAGAATGCCACCACGATGGTATGAGTNGCAAAG
GTACTTNGGACACCACCCCGTTTTCTANTTCCAGANGCGGCCATTCAGGAACGACGCGAATCGTAATC
TACCTGCCGCGTACGCGGATCGTATATACGTNTTCCAGCGTTCGAAATAGAGTTTGTCGTTCCGCTGA
CCNGGTTNCACGGCTACTGCGATAAGCATATTCTACTGGGGGACTGTAAAGGAATTGTATCNACTCTG
CTTCCTGTCAATTGGCCATGTGCATCTCTGGTTGTTCAGGNNACATTAATAACACACAGCATTAAGTA
GATNAGTCTCCGNTATAAGCGAACAGAACGTTGGTCAGAGANTGTGGTTCAATTGTAGTGGTATAATG
TTTCCCAGTGCATAAGGAACTCAGGGACACTACTAGCANGGGCGAGGAGAGACCCTTGGGTGGCAANA
CGNAATAAGTGTTACCTAGTACACCAGCACGAGNNAGGGTTTGACCNTGTGTTTTTAGNNGACCCCGG
TCGCATTGAACCTGGCATTCAGAATGACGTGCTNTCAAGAGTCNAGNTCCNNNCACTGAGGGTCATCN
NCTTCGCGGNTCNGCGTATNGNCACAAACNT
>Dao643
GTGTAGCCCAGTTAGACACTTCTAAATCCTTNGTAACAGACATGCAGTCCCCTCGGCAGAGCCCCGCC
CCCCNATGGTACCGGGTTGTTAAGAGGTCCACAACAGGTCGCACGACAACNTTAAGGCCTCATGCATG
ACCCANCNCGGGGGNGTCCCGGCGCGGCGACCTTGAAGTCCGGGGNCAAGTATGGCCCCCCGGGAAGG
ACGCNGTAGTGCATCTGGAGTGCCGATTCNTGGTCCAGAATGCCACCACGATGGTATGAGTGGCAAAG
GTACTTTGGACACCACCCCGTTTTCTATTTCCAGACGCGGCCNTTCAGGAACGACGCGAATCGTAATC

TACCTGCCGCGTACGCGGATCGTACATGCGTTTTCCAGCGTTCGAAATAGAGTTTGTCGTTCCGCCGA
CCAGGTTNCACGGCTACTGCGATAAGCATATTCTACTGGGGGACTGTAAAGGAATTGTATCCACTCTA
CTTCCTGTCAATTGGCCATGTGCATCTCTGGTTGTTCAGGGTACATTAATAACACACAGCATTAAGTA
GATAAGTCTCCGATATAAGCGAACAGAACGTTGGTNAGAGACTGTGGTTCAATTGTAGTGGTATAATG
TTTCCCAGTGCATAAGGAACTTAGGNACATTACTAGCATGGGCGAGGAGAGACCCTTGGGTGGCAACA
CGCAATAAGTGTTANNTAGTACACCAGCACGAGAGANGGTTTGACCATGTGTTTTTAGNNGACCCCGG
TCGCATTGAACCTGGCATTCAGAATGACGTGCTCTCAAGAATCAAGNTCCTCCCACTGAGGGTCATCT
GCTTCGCGGGTCNGCGTATGGNCATAAAAAT
>Dao645
GTGTAGCCCAGTCAGACACTTCTAAATCCNTGGTAACAGACATGCAGTCCCCTCGGCAGAGCCCCGCC
CCCCGATGGTACCGGGTTGTTAAGAGGTCCACAACGGGTCNNACGACAANATTAAGGCCTCATGCATG
ACCCANNACGGGGGCGTCCCGGNGCGGCGACCTTGNAGTCCGGGGNCAAGTATGGCCCCCCGNGAAGG
ACGCNGTAGTGCATCTGGAGTGCCGATTCGTGGTCCAGAATGCCACCACGATGGTATGAGTGGCAAAG
GTACTTTGGACACCACCCCGTTTTCTATTTCCAGACGCGGCCNTTCAGGAACGACGCGAATCGTAATC
TACCTGCCGCGTACGCGGATCGTACANGCGTTTTCCAGCGTTCGAAATAGAGTTTGTCGTTCCGCTGA
CCAGGTTNCACGGCTACTGCGATAAGCATATTCTACTGGGGGACTGTAAAGGAATTGTATCCATTCNG
CTTCCTGTCAATTGGCCATGTGCATCTCTGGTTGTTCAGGGNACATTAATAACACACAGCAGNAAGTA
GGTAAGTCTCCGATATAAGCGAACAGANCGTTTGTCAGAGACTGTGGTTNNATTGTAGTGGTATAATG
TTTCCCAGNGCATAAGGAACTCAGGGACACTACTAGCATGGGCGAGGAGAGACCCTTGGGTGGCAANA
CGCAATAAGTGTTACCTAGTACACCAGCACGAGNNAGGGTTTGACCATGTGTTTTTAGACGACCCCGG
TCGCATTGAACCTGGCATTCAGAATGACGTGCTCTCAAGAATCNAGNTCCTCCCACTGAGGGTCATCT
GCTTCGCGGGTCNGCGTATNGNCATAAACAT
>Dao646
GTGTAGCCCAGTTAGACACTTCTAAATCCNTGGTAACAGACATGCAGTCCCCTCNNCAGAGCCCCGCC
CCCCGATGGTACCGGGTTGTTAAGAGGTCCACAACGGGTCGNACGACAACATTAAGGCCTNANGCATG
ACCCAACNCGGGGGCGTCCCGGCGCGGCGACCTTGAAGTCCGGGGNCAAGTATGGCCCCCCGGGAAGG
ACGCNGTAGTGCATCTGGAGTGCCGATTCGTGGTCCAGAATGCCACCACGATGGTATGAGTNGCAAAG
GTACTTNGGACACCACCCCGTTTTCTATTTCCAGACGCGGCCNTTCAGGAACGACGCGAATCGTAATC
TACCTGCCGCGTACGCGGATCGTACANGCGTTTTCCAGCGTTCGAAATAGAGTTTGTCGTTCCGCTGA
CCNGGTTNCACGGCTACTGCGATAAGCATATTCTACTGGGGGACTGTAAAGNAATTGTATCCACTCTG
CTTCCTGTCAATTGGCCATGTGCATCTCTGGTTGTTCAGGGTACATTAATAACACACAGCATTAAGTA
GATNAGTCTCCGATNTAAGCGAACAGAACGTTGGTCAGAGACTATGGTTCAATTGTAGTGGTATAATG
TTTCCCAGTGCATAAGGAACTCAGGGACACTACTAGCATGGGCGAGGAGAGACCCTTGGGTGGCAACA
CGCAATAAGTGTTNCCTAGTACATCAGCACGAGNNAGGGTTTGACCATGNNTTTTTAGACGACCCCGG
TCGCATTGAACCTGGCATTCAGAATGACGTGCTCTCAAGAATCNAGNTCCTCNCACTGAGGGTCATCT
GCTTCGCGGGTCNGCGTATGGTCACAAACAT
>Dao647
GTGNAGCCCAGTCANACACNTNNAAATCCTTNGTAACAGACATGCAGTCCCCTCNNCAGAGCCCCGCC
CCCCGATGGTACCGGGTTGTTAAGAGGTCCACAACGGGTCGNACGACNNNATTAAGGCCTNANGCATG
ACCCAACACNGGGGNGTCCCGGCGNGGCGACCTTGNAGTCNGGGGNCAAGTATGGCCCCCCGGGAAGG
ACGCCGTAGTGCATCTGGAGTGCCGATTCGTGGTCNNGAATGCCACCACGATGGTATGAGTTGCAANG
GTACTTTGGNCACCACCCCGTTNNCTNTTNCCAGANGCGGCCNTTCAGGAACGACGCGAATCGTAATC
TACCTGCCGCGTNCGCGGATCGTACANGCGTTTTCCAGCGTTCGAAATAGAGTTTGTCGTTCCGCTGA
CCAGGTTNCACGGCTACTGCGATAAGCATATTCTACTGGGGGACTGTAAAGGAATTGTATCCATTCNG
CTTCCTGTCAATTGGNCATGTGCATCTCTGGTTGTTCAGGNTACATTAATAACACACAGCAGTAAGTA
GNTAAGTCNCCGATATAAGCGAACAGANCGTTTGTNAGAGACTGTGGTTCAATTGTAGTGGTATAATG
TTTCCCAGTGCATAAGGAACTCAGGGACACTACTAGCATGGNCGAGGAGAGACCCTTGGGTGGCANCA
CGNAATAAGTGTTACCTAGTACACCAGCACGAGNNANGGTTTGACCANGTGTTNNTAGACGACCCCGG
TCGCATTGAACCTGGCATTCAGAATGACGTGCTCTNAAGAATCNAGGTCCNCCCACTGAGGGTCATCT
GCTTCGCGGGTCAGCGTATNGTCANAAACAT
>Dao648
GTGTAGCCCAGTCAGACACTTCTAAATCCNTGGTAACAGACATGCAGTCCCCTCGGCAGAGCCCCGCC
CCCCGATGGTACCGGGTTGTTAAGAGGTCCACAACGGGTCNNACGACNNCATTAAGGCCTCATGCATG
ACCCAANACNGNGGNGTCCCGGNGCGGNGACCTTGAANTCCGGGNACAAGTATGGCCCCCNGGGAAGG

ACGCNGTAGTGCATCTGGAGTGCCGATTNNTGGTCNAGAATGCCACCACGATGGTATGAGTGGCAAAG
GTACTTTGGACACCACCCCGTTTTCTATTTCCAGACGCGGCCNTTCAGGAACGACGCGAATCGTAATC
TACCTGCCGCGTNCGCGGATCGTACATGCGTTTTNCAGCGTTCGAAATAGAGTTTGTCGTTCCGCTGA
CCAGGTTNCACGGCTANTGCGATAAGCATATTCTACTGGGGGACTGTAAAGGAATTGTATCCATTCTG
CTTCCTGTCAATTGGCCATGTGCATCTCTGGTTGTTCAGGNNACATTAATAACACACAGNAGNAAGTA
GGTAAGTCTCCGATATAAGCGAACAGANCGTTTGTNAGAGACTGTGGTTCAATTGTAGTGGTATAATG
TTTCCCAGNGCATNAGGAACTCAGGNACACTACTAGCANGGGCGAGGAGAGACCCTTGGGTGGCAANA
CGCAATAAGTGTTNCCTAGTACACCAGCACGAGANANGGTTTGACCNNNNNTTTNTAGACGACCCCGG
TCGCATTGAACCTGGCATTCAGAATGACGTGCTCTNAAGAATNNAGGTCCTCNCACTGAGGGNCATCT
GCTTCGCGGGTCAGCGTATGGTCATAAACAT
>Dao649
GTGTAGCCCAGTTNGACACTTCTAAATCCTTGGTAACAGACATGCAGTCCCCTCNNCAGAGCCCCGCC
CCCCGATGGTACCGGGTTGNTNAGAGGTCCACAACGGNTCGNACGACNANATTAAGGCCTCANGCATG
ACCCANCACGGNGGCGTCCCGGCGNGGCGACCTTGNANTCCGGGGNCAAGTATGGCCCCCCGGGAAGG
ACGCCGTAGTGCATCTGGAGTGCCGATTCGTNGTCNAGANTGNCACCACGATGGTATGAGTGGCAAAG
GTACTTTGGACACCACCCCGTTNNCTATTTCCAGACGCGGCCATTCAGGAACGACGCGAATCGTAATC
TACCTGCCGCGTACGCGGATNGTACANGCGTTTTCCAGCGTTCGAAATAGAGTTNGTCGTTCCGCTGA
CCAGGTTNCACGGCTACTNCGATAAGCATATTCTACTGGGGGACTGTAAAGGAATTGTATCCACTCTG
CTTCCTGTCAATTGGNCATGTGCATCTCTNGTTGTTCAGGGTANATTAATAACACACAGNATNAAGTA
GATAAGTCTCCGATATAAGCGAACAGANCGTTGGTCAGAGANTATGGTTCAATTGTAGTGGTATAANG
TTTCCCAGTGCATNAGGANCTCAGGNACACTACTAGCANGGGCGAGGAGAGACCCTTGGGTGGCANNA
CGNAATAAGTGTTACCTAGTACATCAGCACNAGNNAGGGTTTGACCATNNNTTTTTAGACGACCCCGG
TCGCATTGAACCTGGCATTCAGAATGACGTGCTNTCAAGAATCNAGNTCCNCCCACTGAGGGTCATCT
GCTTCGCNNNNCAGCGTATNGTCACAAACAT
>Dao650
GTGNAGCCCAGTCAGACACTTCTAAATCCNTGGTAACAGACATGCAGTCCCCTCGGCAGAGCCCCGCC
CCCCGATGGTACCGGGTTGTTAAGAGGTCCACAACGGNTCGNACGACAACNTTAAGGCCTCANGCATG
ANNCANCACGGGGGCGTCCCGGNGCGGNGACCTTGAAGNCCGGGGNCAAGTATGGCCCCCCGGGAAGG
ACGCCGTAGTGCATCTGGAGTGCCGATTCGTGGTCCAGAATGCCACCACGATGGTATGAGTGGCAAAG
GTACTTTGGACACCACCCCGTTTTCTANTTCCAGACGCGGCCATTCAGGAACGACGCGAATCGTAATC
TACCTGCCGCGTACGCGGATCGTACANGCGTTTTCCAGCGTTCGAAATAGAGTTTGTCGTTCCGCTGA
CCAGGTTNCACGGCTACTGCGATAAGCATATTCTACTGGGGGACTGTAAAGGAATTGTATCCATTCTG
CTTCCTGTCAATTGGCCATGTGCATCTCTNGTTGTTCAGGGTACATTAATAACACACAGCAGNAAGTA
GGTNAGTCTCCGATATAAGCGAACAGAACGTTTGTNAGAGACTGTGGTTCAATTGTAGTGGTATAATG
TTTCCCAGTGCATAAGGAACTCAGGGACACTACTAGCATGGGCGAGGAGAGACCCTTGGGTGGCAANA
CGCAATAAGTGTTACCTAGTACACCAGCACGAGANANGGTTTGACCATGTGTTTTTAGACGACCCCGG
TCGCATTGAACCTGGCATTCAGAATGACGTGCTCTCAAGAATCAAGGTCCTCCCACTGAGGGNCATCN
GCTTCGCGGGTCNGCGTATGGTCATAAACAT
>Dao651
GTGTAGCCCAGTTAGACACTTCTAAATCCTTGGTAACAGACATGCAGTCCCCTCGGCAGAGCCCCGCC
CCCCGATGGTACCGGGTTGNTAAGAGGTCCACAACAGGTCGCACGACAANATTAAGGCCTCATGCATG
ACCCANCACGGGGGCGTCCCGGCGCGGCGACCTTGAAGTCCGGGGACAAGTATGGCCCCCCGNGAAGG
ACGCNGTAGTGCATCTGGAGTGCCGATTCGTGGTCNAGANTGNCACCACGATGGTATGAGTGGCAAAG
GTACTTNGGACACCACCCCGTTTTCTANTTCCAGACGCGGCCNTTCAGGAACGACGCGAATCGTAATC
TACCTGCCGCGTACGCGGATCGTACATGCGTTTTCCAGCGTTCGAAATAGAGTTTGTCGTTCCGCCGA
CCAGGTTNCACGGCTACTGCGATAAGCATATTCTACTGGGGGACTGTAAAGGAATTGTATCCACTCTA
CTTCCTGTCAATTGGCCATGTGCATCTCTGGTTGTTCAGGNNACATTAATAACACACAGCATTAAGTA
GATAAGTCTCCGATATAAGCGAACAGAACGTTGGTCAGAGACTGTGGTTCAATTGTAGTGGTATAATG
TTTCCCAGTGCATAAGGAACTTAGGNACATTACTAGCATGGNCGAGGAGAGACCCTTGGGTGGCAACA
CGCAATAAGTGTTNCCTAGTACACCAGCACGAGAGANGGTTTGACCNTGTGTTTTTAGACGACCCCGG
TCGCATTGAACCTGGCATTCAGAATGACGTGCTCTCAAGAATCAANNTCCNCCCACTGAGGGTCATCT
GCTTCGCGGGTCAGCGTATGGNCATAAACAT
>Dao652
GTGNAGCCCAGTTANACACTTCTAAATCCNTGGTAACAGACATGCAGTNCCCTCNNCAGAGCCCCGCC

CCCCNATGGTACCGGGTTGNTAAGAGGTCCACAACGGGTCNNACGACAANNTTAAGGCCTNANGCATG
ACCCANCNCGGGGGCGTCCCGGNGNGGCGACCTTGAAGTCCGGGGNCAAGTATGGCCCCCCGNGAAGG
ACGCCGTAGTGCATCTGGANTGCCGATTCGTGGTCCAGAATGNCACCACGATGGTATGAGTGGCAAAG
GTACTTTGGACACCACCCCGTTTNCTANTTCCAGACGCGGCCNTTCAGGAACGACGCGAATCGTAATC
TACCTGCCGCGTACGCGGATNGTACANGCGTTTTCCAGCGTTCGANATAGAGTTTGTCGTTCCGCTGA
CCNGGTTNCACGGCTACTGCGATAAGCATATTCTACTGGGGGACTGTAAAGGAATTGTATCCACTCNG
CTTCCTGTCAATTGGCCATGTGCATCTCTNGTTGTTCAGGGTACATTAATAACACACAGCATNAAGTA
GATNAGTCTCCGATATAAGCGAACAGANCGTTGGTCAGAGACTATGGTTCAATTNTAGTGGTATAATG
TTTCCCAGTGCATAAGGAACTCAGGNACACTACTAGCATGGGCGAGGAGAGACCCTTGGGTGGCAANA
CGNGATAAGTGTTANCTAGTACATCAGCACGAGANANGGTTTGACCANNNNTTTNTAGNNGACCCCGG
TCGCATTGAACCTGGCATTCAGAATGACGTGCTCTNAAGAATCAAGNTCCNCCCACTGAGGGTCANCN
GCTTCGCGGGTCAGCGTATGGNCACAAACAT
>Dao653
GTGTAGCCCAGTTAGACACNTCTAAATCCNTNGTAACAGACATGCAGTNNCCTCNNCAGAGCCCCGCC
CCCCNATGGTACCGGGTTGTTNAGAGGTCCACAACGGGTCGNACGACAANNTTAAGGCCTCANGCATG
ACCCANNACNGNNGCGTCCCGGNGCGGCNACCTTGNAGTCCGGGNNCAAGTATGGCCCCCCGNGAAGG
ACGCNGTAGTGCATCTGGAGTNNCGATTNNTGGTCCAGAATGNCACCACGATGGTATGAGTGGCAAAG
GTACTTNGGACNCCACCCCGTTNNCTANTTCCAGANGCGGCCATTCAGGAACGACGCGAATCGTAATC
TACCTGCCGCGTACGCGGATCGTACATGCNTTTTCCAGCGTTCGANATAGAGTTTGTCGTTCCGCTGA
CCNGGTTNCACGGCTACTGCGATAAGCATATTCTACTGGGGGACTGTAAAGGAATTGTATCNACTCNG
CTTCCTGTCAATTGGNCATGTGCATCTCTGGTTGTTCAGGNNANATTAATAACACACAGCATTAAGTA
GATAAGTCTCCGATATAAGCGAACAGANCGTTGGTNAGAGANTATGGTTCAATTGTAGTGGTATAATG
TTTCCCAGTGCATAAGGAACTCAGGGACACTACTAGCATGGGCGAGGAGAGACCCTTGGGTGGCANNA
CGCAANAAGTGTTACCTAGTACATCAGCACGAGNNAGGGTTTGACCATGTNTTTNTAGACGACCCCGG
TCGCATTGAACCTGGCATTCAGAATGACGTGCTNTCAAGAATCAANNTCCTCCCACTGAGGGNCANCT
GCTTCGCGGGTCNGCGTATGGTCATANACAT
>Dao654
GTGTAGCCCAGTTAGACACTTCTAAATCCNTGGTAACAGACATGCAGTCCCCTCNNCAGAGNCCCGCC
CCCCNATGGTACCGGGTTGNTNAGNGGTCCACAACGGGTCNNACGACAANNTTAAGGCCTCATGCATG
ANNCANCACNGGGGCGTCCCGGNGNGGCGACCTTGAANTCCGGGNNCAAGTATGGCCCCCCGGGAAGG
ACGCCGTAGTGCATCTGGAGTGCCGATTNNTGGNCNAGAATGCCACCACGATGGTATGAGTGGCAAAG
GTACTTTGGACACCACCCCGTTTTCTATTTCCAGANGCGGCCATTCAGGAACGACGCGAATCGTAATC
TACCTGCCGCGTNCGCGGATCGTACATGCGTTTTCCAGCGTTCGAAATAGAGTTTGTCGTTCCGCTGA
CCAGGTTTCACGGCTACTGCGATAAGCATATTCTACTGGGGGACTGTAAAGGAATTGTATCNACTCNG
CTTCCTGTCAATTGGCCATGTGCATCTCTGGTTGTTCAGGNNACATTAATAACACACAGCATTAAGTA
GATNAGTCTCCGATATAAGCGAACAGANCGTTGGTCAGANACTATGGTTCAATTGTAGTGGTATAATG
TTTCCCAGTGCATAAGGAACTCAGGNACACTACTAGCATGGNCGAGGAGAGACCCTTGGGTGGCANNA
CGNAATAAGTGTTNNNTAGTACATCAGCACGAGANANGGTTTGACCATGTGTTTNTAGNNGACCCCGG
TCGCATTGAACCTGGCATTCAGAATGACGTGCTCTCAAGAATNAANNTCCNCCCACTGAGGGTCATCT
GCTTCGCGGGTCNGCGTATGGTCATAAACAT
>Dao655
GNGNAGCCCAGTTNGANACTTCTAAATCCTTNGTAACAGACATGCAGTNCCCTCNNCAGANNNCCGCC
CCCCNATGGTACCGGGNNGNTNAGAGGTCCACNACAGGTCGNACGACNNNATTAAGGCCTNATGCATG
ANNCNNNNCNGNNGNGTCCCGGNGNGGCNACCTTGNANTCNGGGNGNCAAGTATGGCCCNCCGGGAAGG
ACGCNGTAGTGCATCTGGANTGCCGNTTNNTGNNCCAGANTGNCACCACGATGGTATGAGTNGCAAAG
GTACTTTNNNCACCACCCCGTTNNCTANTTCNAGANGCGGCCNTTCAGGAACGACGCGAATCGTAATC
TACCTGCCGCGTNCGCGGNTNGTACANGCGTNTTNCAGCGTTCGANATAGAGTTTGTCGTTCCGCCGA
CCAGGTTNCACGGCTANTGCGATAAGCATNTTCTACTGGGGGACTGNAAAGGAATTGTATCCACTCNA
CTTCCTGNCAATTGGCCATGTGCATCTCTGGTTGTTCAGGGTANATTAATAACACACAGCATNAAGTA
GATAAGTCTCCGATATAAGCGAACAGANCGTTGGTNNGAGANTGTGNTTCAATTNTAGTGGTATAATG
TTTCCCAGTGCATAAGGANCTTAGGGACATTACTANCANGGNCGAGGAGAGACCCTTGGGTGGCANNA
CGCAATAAGNGTTNCCTNNTACACCAGCACGAGANANGGTTTGACCNTGNNTTTTTAGNNGNCCCCGG
TCGNATTGAACCTGGCATTCAGAATGACGTNCTNTCAAGAATNNANNTCCNCCCACTGAGGNTCANNN
NCTTCNCGGGTCAGCGTATNGNCATNAACAT

>Dao656
GTNNAGCCCAGTTAGACACTTCTAAATCCNTNGTAACAGACATGCAGTCCCCTCNNCAGAGCNCCGCC
CCCCGATGGTACCGGGTTGTTAAGAGGTCCACAACGGNTCNNACGACAANATTAAGGCCNNANGCATG
ANNCNNNNCNGGGGNGTCCCGGCGNGGCGACCTTGNAGTCCGGGNNCAAGTATGGCCCCCCGGGAAGG
ACGCNGTAGTGCATCTGGAGTGCCGATTNNTGGTCCNGAATGNCACCACGATGGTATGAGTGGCAAAG
GTACTTNGGACNCCACCCCGTTNNCTNNTNCCAGANGCGGCCNTTCAGGAACGACGCGAATCGTNATC
TACCTGCCGCGTNCGCGGATCGTACANGCGTNTTNCAGCGTTCGAAATAGAGTTTGTCGTTCCGNTGA
CCAGGTTTCACGGCTACTNCGATAAGCATATTCTACTGGNGGACTGTAAAGNAATTGTATCCACTCTG
CTTCCTGTCAATTGGNCATGTGCATCTCTNGTTGTTCAGGNNACATTAATAACACACAGCNTTAAGTA
GATNAGTCNCCGATATAAGCGAACAGANCGTTGGTCAGAGACTATGGTTCAATTGTAGTGGTATAATG
TTTCCCAGTGCATAAGGAACTCAGGGACACTACTAGCANGGGCGAGGAGAGACCCTTGGGTGGCAANA
CGNAATAAGTGTTNNCTAGTACATCAGCACGAGNNAGGGTTTGACCNNGTGTTNNTAGNNGACCNNGG
TCGCATTGAACCTGGCATTCAGAATGACGTNCTCTCAAGAATCNAGGTCCNNNCACTGAGGGTCATNN
GCTTCGCGGGTCNGCGTATGGTCATAAACAT
>Dao657
GTGTAGCCCAGTTAGACACTTCTAAATCCNTGGTAACAGACATGCAGTCCCCTCNNCAGAGCCCCGCC
CCCCGATGGTACCGGGTTGNTAAGAGGTCCACAACGGNTCGCACGACAACATTAAGGCCTCATGCATG
ACCCAACACGGGGGCGTCCCGGCGNGGCGACCTTGAAGTCCGGGGACAAGTATGGCCCCCCGGGAAGG
ACGCCGTAGTGCATCTGGAGTGCCGANNCGTGGTCNAGAATGCCACCACGATGGTATGAGTGGCAAAG
GTACTTTGGACACCACCCCGTTTTCTATTTCCAGACGCGGCCATTCAGGAACGACGCGAATCGTAATC
TANCTGCCGCGTACGCGGATCGTACATGCGTTTTCCAGCGTTCGAAATAGAGTTTGTCGTTCCGCTGA
CCAGGTTTCACGGCTACTGCGATAAGCATATTCTACTGGGGGACTGTAAAGGAATTGTATCCACTCTG
CTTCCTGTCAATTGGCCATGTGCATCTCTGGTTGTTCAGGNNACATTAATAACACACAGCATTAAGTA
GATAAGTCTCCGATATAAGCGAACAGAACGTTGGTCAGAGACTATGGTTCAATTGTAGTGGTATAATG
TTTCCCAGTGCATAAGGAACTCAGGGACACTACTAGCANGGGCGAGGAGAGACCCTTGGGTGGCAACA
CGCGATAAGTGTTANCTAGTACATCAGCACGAGAGANGGTTTGACCATGTGTTTTTAGACGACCCCGG
TCGCATTGAACCTGGCATTCAGAATGACGTNCTCTCAAGAATCNANGTCCTCCCACTGAGGGTCATCT
GCTTCGCGGGTCNGCGTATGGTCATAAACAT
>Dao658
GTGTAGCCCAGTCAGACACTTCTAAATCCTTGGTAACAGACATGCAGTCCCCTCNNCAGAGCCCCGCC
CCCCGATGGTACCGGGTTGNTNAGAGGTCCACAACGGGTCNCACGACNNNNTTAAGGCCTCATGCATG
ACCCAACNCGGGGGCGTCCCGGCGNGGCGACCTTGAANTCCGGGNNCAAGTATGGCCCCCCGGGAAGG
ACGCCGTAGTGCATCTGGAGTGCCGATTCGTGGTCCAGAATGNCACCACGATGGTATGAGTGGCAAAG
GTACTTTGGACACCACCCCGTTNNCTANTNCCAGACGCGGCCNTTTAGGAACGACGCGAATCGTAATC
TACCTGCCGCGTACGCGGATNGTACATGCGTNTTCCAGCGTTCGAAATAGAGTTTGTCGTTCCGCTGA
CCAGGTTTCACGGCTACTGCGATAAGCATATTCTACTGGGGGACTGTAAAGGAATTGTATCNATTCNG
CTTCCTGTCAATTGGNCATGTGCATCTCTGGTTGTTCAGGGTACATTAATAACACACAGNAGTAAGTA
GNTAAGTCTCCGATATAAGCGAACAGAACGTTTGTCAGAGACTGTGGTTCAATTGTAGTGGTATAATG
TTTCCCAGTGCATAAGGAACTCAGGGACACTACTAGCATGGGCGAGGAGAGACCCTTGGGTGGCAACA
CGCAATAAGTGTTACCTAGTACACCAGCACGAGNGAGGGTTTGACCATGTGTTNNTAGACGACCCCGG
TCGCATTGAACCTGGCATTCAGAATGACGTGCTCTCAAGAATCAAGGTCCNCCCACTGAGGGTCATCT
GCTTCGCGGGTCNGCGTATNGTCATAAAAAT
>Dao659
GTGTAGCCCAGTTAGACACTTCTAAATCCTTGGTAACAGACATGCAGTCCCCTCGGCAGAGCNCCGCC
CCCCNATGGTACCGGGTTGTTAAGNGGTCCACAACGGGTCGNACGACAANNTTAAGGCCTCATGCATG
ACCCAACACGGGGGNGTCCCGGCGNGGCGACCTTGAAGTCCGGGGACAAGTATGGCCCCCCGGGAAGG
ACGCCGTAGTGCATCTGGAGTGCCGATTNGTGGTCCAGAATGCCACCACGATGGTATGAGTGGCAAAG
GTACTTTGGACACCACCCCGTTNNCTNNTTCCAGACGCGGCCATTCAGGAACGACGCGAATCGTAATC
TANCTGCCGCGTNCGCGGATCGTACANGCGTTTTCCAGCGTTCGAAATAGAGTTTGTCGTTCCGCTGA
CCNGGTTNCACGGCTACTNCGATAAGCATATTCTACTGGGGGACTGTAAAGGAATTGTATCCACTCNG
CTTCCTGTCAATTGGNCATGTGCATCTCTGGTTGTTCAGGGTACATTAATAACACACAGCATTAAGTA
GATNAGTCTCCGATATAAGCGAACAGANCGTTGGTCAGAGACTATGGTTCAATTGTAGTGGTATAATG
TTTCCCAGTGCATAAGGAACTCAGGGACACTACTAGCATGGGCGAGGAGAGACCCTTGGGTGGCAACA
CGCGATAAGTGTTACCTAGTACATCAGCACGAGAGAGGGTTTGACCATGTGTTTTTAGACGACCCCGG

TCGCATTGAACCTGGCATTCAGAATGACGTGCTNTCAAGAATCAAGNTCCTCCCACTGAGGGTCATCT
GCTTCGCGGGTCNGCGTATNGTCANAAACAT
>Dao660
GTGTAGCCCAGTTAGACACTTCTAAATCCTTGGTAACAGACATGCAGTCCCCTCGGCAGAGCCCCGCC
CCCCGATGGTACCGGGTTGNTAAGNGGTCCACAACGGNTCGNACGACAANATTAAGGCCTNATGCATG
ACCCANNNCGGGGGCGTCCCGGNGNGGCGACCTTGAAGTCCGGGNNCAAGTATGGCCCCCCGGGAAGG
ACGCNGTAGTGCATCTGGANTGCCGATTNGTGGTCCAGAATGCCACCACGATGGTATGAGTGGCAAAG
GTACTTTGGACACCACCCCGTTNNCTANTTCCAGACGCGGCCNTTCAGGAACGACGCGAATCGTAATC
TACCTGCCGCGTNCGCGGATCGTACANGCGTNTTNCAGCGTTCGAAATAGAGTTTGTCGTTCCGCTGA
CCNGGTTNCACGGCTACTGCGATAAGCATATTCTACTGGGGGACTGTAAAGGAATTGTATCCACTCNG
CTTCCTGTCAATTGGCCATGTGCATCTCTGGTTGTTCAGGGNACATTAATAACACACAGCATNAAGTA
GATAAGTCTCCGATATAAGCGAACAGANCGTTGGTNAGAGACTATGGTTCAATTGTAGTGGTATAATG
TTTCCCAGTGCATAAGGAACTCAGGGACACTACTAGCATGGGCGAGGAGAGACCCTTGGGTGGCAANA
CGCAATAAGTGTTNCCTAGTACATCAGCACGAGANAGGGTTTGNCCATGTGTTTTTAGNNGACCCCGG
TCGCATTGAACCTGGCATTCAGAATGACGTGCTCTCAAGAATNAAGGTCCTCCCACTGAGGGTCATNN
GCTTCGCGGGTCAGCGTATGGTCATAAACAT
>Dao661
GTGNAGCCCAGTCAGACACTTCTAAATCCTTGGTAACAGACATGCAGTCCCCTCNNCAGAGCNCCGCC
CCCCGATGGTACCGGGTTGTTNAGAGGTCCACAACGGGTCGNACGACAANNTTAAGGCCTCATGCATG
ACCCANCNCGGNGGCGTCCCGGCGCGGCGACCTTGAAGTCCGGGGNCAAGTATGGCCCCCCGGGAAGG
ACGCNGTAGTGCATCTGGAGTGCCGATTNGTGGTCNAGAATGNCACCACGATGGTATGAGTGGCAAAG
GTACTTTGGACACCACCCCGTTTTCTANTNCCAGACGCGGCCATTCAGGAACGACGCGAATCGTAATC
TACCTGCCGCGTACGCGGATCGTACATGCGTTTTCCAGCGTTCGAAATAGAGTTTGTCGTTCCGCTGA
CCAGGTTTCACGGCTACTGCGATAAGCATATTCTACTGGGGGACTGTAAAGGAATTGTATCCATTCTG
CTTCCTGTCAATTGGCCATGTGCATCTCTNGTTGTTCAGGNNACATTAATAACACACAGCAGTAAGTA
GGTAAGTCNCCGATATAAGCGAACAGANCGTTTGTNAGAGACTGTGGTTCAATTGTAGTGGTATAATG
TTTCCCAGTGCATAAGGAACTCAGGNACACTACTAGCATGGGCGAGGAGAGACCCTTGGGTGGCAANA
CGNAATAAGTGTTACCTAGTACACCAGCACGAGNNAGGGTTTGACCATGTGTTTTTAGACGACCCCGG
TCGCATTGAACCTGGCATTCAGAATGACGTGCTCTCAAGAATCNAGGTCCTCCCACTGAGGGTCATNN
GCTTCGCGGGTCNGCGTATGGTCATAAACAT
>Dao662
GTGNAGCCCAGTTAGACACTTCTAAATCCTTGGTAACAGACATGCAGTCCCCTCGGCAGAGCCCCGCC
CCCCGATGGTACCGGGTTGNTAAGAGGTCCACAACGGGTCGNACGACAACATTAAGGCCTCATGCATG
ACCCAANNCGGNGGNGTCCCGGCGNGGCGACCTTGAAGTCCGGGGNCAAGTATGGCCCCCCGGGAAGG
ACGCCGTAGTGCATCTGGAGTGCCGATTCGTGGTCCAGAATGNCACCACGATGGTATGAGTGGCAAAG
GTACTTNGGACACCACCCCGTTTTCTANTTCCAGANGCGGCCNTTCAGGAACGACGCGAATCGTAATC
TACCTGCCGCGTACGCGGATNGTACATGCGTTTTCCAGCGTTCGAAATAGAGTTTGTCGTTCCGCTGA
CCAGGTTNCACGGCTACTGCGATAAGCATATTCTACTGGGGGACTGTAAAGGAATTGTATCCACTCNG
CTTCCTGTCAATTGGCCATGTGCATCTCTNGTTGTTCAGGGTACATTAATAACACACAGCATNAAGTA
GATNAGTCTCCGATATAAGCGAACAGANCGTTGGTCAGAGACTATGGTTCAATTGTAGTGGTATAATG
TTTCCCAGTGCATAAGGAACTCAGGGACACTACTAGCATGGGCGAGGAGAGACCCTTGGGTGGCAANA
CGCAATAAGTGTTNCCTAGTACATCAGCACGAGNNAGGGTTTGACCNTGTGTTTTTAGNNGACCCCGG
TCGCATTGAACCTGGCATTCAGAATGACGTGCTCTCAAGAATCNAGGTCCNCCCACTGAGGGTCATCN
GCTTCGCGGGTCNGCGTATGGTCATAAACAT
>Dao663
GTGTAGCCCAGTTAGACACTTCTAAATCCTTGGTAACAGACATGCAGTNNCCTCNNCAGAGCCCCGCC
CCCCNATGGTACCGGGTTGNTAAGAGGTCCACAACGGGTCGCACGACANCATTAAGGCCTCATGCATG
ACCCAACACGGGGGNGTCCCGGCGCGGCGACCTTGNAGTCCGGGGACAAGTATGGCCCCCCGGGAAGG
ACGCCGTAGTGCATCTGGAGTGCCGATTCGTGGTCCAGAATGCCACCACGATGGTATGAGTGGCAAAG
GTACTTTGGACACCACCCCGTTTTCTATTTCCAGANGCGGCCNTTCAGGAACGACGCGAATCGTAATC
TACCTGCCGCGTACGCGGATCGTACANGCGTTTTCCAGCGTTCGAAATAGAGTTTGTCGTTCCGCTGA
CCAGGTTNCACGGCTACTGCGATAAGCATATTCTACTGGGGGACTGTAAAGGAATTGTATCCACTCTG
CTTCCTGTCAATTGGCCATGTGCATCTCTGGTTGTTCAGGGTACATTAATAACACACAGCATTAAGTA
GATAAGTCTCCGATATAAGCGAACAGAACGTTGGTCAGAGACTATGGTTCAATTGTAGTGGTATAATG

TTTCCCAGTGCATAAGGAACTCAGGGACACTACTAGCATGGGCGAGGAGAGACCCTTGGGTGGCANCA
CGCAATAAGTGTTANNTAGTACATCAGCACGAGAGAGGGTTTGACCATGTGTTTNTAGACGACCCCGG
TCGCATTGAACCTGGCATTCAGAATGACGTGCTCTCAAGAATCAAGNTCCTCCCACTGAGGGTCANCT
GCTTCGCGGGTCAGCGTATGGTCATAAACAT
>Ede102
GTGTAGCCCAGTTAGACACTTCTAAATCCTTGGTAACAGACATGCAGTCCCCTCNNCAGAGCCCCGCC
CCCCGATGGTACCGGGTTGTTAAGAGGTCCACAACGGGTCGCACGACAACATTAAGGCCTCANGCATG
ACCCAACACGGGGGCGTCCCGGCGCGGCGACCTTGAAGTCCGGGGACAAGTATGGCCCCCCGGGAAGG
ACGCCGTAGTGCATCTGGAGTGCCGATTCGTGGTCCAGAATGCCACCACGATGGTATGAGTNGCAAAG
GTACTTTGGACACCACCCCGTTTTCTATTTCCAGACGCGGCCATTCAGGAACGACGCGAATCGTAATC
TACCTGCCGCGTACGCGGATCGTACATGCGTTTTCCAGCGTTCGAAATAGAGTTTGTCGTTCCGCTGA
CCAGGTTTCACGGCTACTGCGATAAGCATATTCTACTGGGGGACTGTAAAGGAATTGTATCCACTCTG
CTTCCTGTCAATTGGCCATGTGCATCTCTGGTTGTTCAGGGTACATTAATAACACACAGCATTAAGTA
GATAAGTCTCCGATATAAGCGAACAGAACGTTGGTCAGAGACTATGGTTCAATTGTAGTGGTATAATG
TTTCCCAGTGCATAAGGAACTCAGGGACACTACTAGCATGGGCGAGGAGAGACCCTTGGGTGGCAACA
CGCGATAAGTGTTACCTAGTACATCAGCACGAGAGAGGGTTTGACCATGTGTTTTTAGACGACCCCGG
TCGCATTGAACCTGGCATTCAGAATGACGTGCTCTCAAGAATCAAGNTCCTCCCACTGAGGGTCATCT
GCTTCGCGNGTCAGCGTATGGTCATNAACAT
>Ede112
GTGTAGCCCAGTTAGACACTTCTAAATCCTTGGTAACAGACATGCAGTCCCCTCGGCAGAGCCCCGCC
CCCCGATGGTACCGGGTTGTTAAGAGGTCCACAACGGGTCGCACGACAANATTAAGGCCTCATGCATG
ACCCAACACGGGGGCGTCCCGGCGCGGCGACCTTGAAGTCCGGGGACAAGTATGGCCCCCCGGGAAGG
ACGCCGTAGTGCATCTGGAGTGCCGATTCGTGGTCCAGAATGCCACCACGATGGTATGAGTGGCAAAG
GTACTTTGGACACCACCCCGTTTTCTATTTCCAGACGCGGCCATTCAGGAACGACGCGAATCGTAATC
TACCTGCCGCGTACGCGGATCGTACATGCGTTTTCCAGCGTTCGAAATAGAGTTTGTCGTTCCGCTGA
CCAGGTTTCACGGCTACTGCGATAAGCATATTCTACTGGGGGACTGTAAAGGAATTGTATCCACTCTG
CTTCCTGTCAATTGGCCATGTGCATCTCTGGTTGTTCAGGGTACATTAATAACACACAGCATTAAGTA
GATAAGTCTCCGATATAAGCGAACAGAACGTTGGTCAGAGACTATGGTTCAATTGTAGTGGTATAATG
TTTCCCAGTGCATAAGGAACTCAGGGACACTACTAGCATGGGCGAGGAGAGACCCTTGGGTGGCAACA
CGCAATAAGTGTTACCTAGTACATCAGCACGAGAGAGGGTTTGACCATGTGTTTTTAGACGACCCCGG
TCGCATTGAACCTGGCATTCAGAATGACGTGCTCTCAAGAATCAAGGTCCTCCCACTGAGGGTCATCT
GCTTCGCGGGTCAGCGTATGGTCATAAACAT
>Ede123
GTGTAGCCCAGTTAGACACTTCTAAATCCTTGGTAACAGACATGCAGTCCCCTCGGCAGAGCCCCGCC
CCCCGATGGTACCGGGTTGTTAAGAGGTCCACAACGGGTCGCACGACAACATTAAGGCCTCATGCATG
ACCCAACACGGGGGCGTCCCGGCGNGGCGACCTTGAAGTCCGGGGACAAGTATGGCCCCCCGGGAAGG
ACGCCGTAGTGCATCTGGAGTGCCGATTCGTGGTCCAGAATGCCACCACGATGGTATGAGTNGCAAAG
GTACTTTGGACACCACCCCGTTTTCTATTTCCAGACGCGGCCATTCAGGAACGACGCGAATCGTAATC
TACCTGCCGCGTACGCGGATCGTACATGCGTTTTCCAGCGTTCGAAATAGAGTTTGTCGTTCCGCTGA
CCAGGTTNCACGGCTACTGCGATAAGCATATTCTACTGGGGGACTGTAAAGGAATTGTATCCACTCNG
CTTCCTGTCAATTGGCCATGTGCATCTCTGGTTGTTCAGGGTACATTAATAACACACAGCATTAAGTA
GATAAGTCTCCGATATAAGCGAACAGAACGTTGGTCAGAGACTATGGTTCAATTGTAGTGGTATAATG
TTTCCCAGTGCATAAGGAACTCAGGGACACTACTAGCATGGGCGAGGAGAGACCCTTGGGTGGCAACA
CGCAATAAGTGTTACCTAGTACATCAGCACGAGAGAGGGTTTGACCATGTGTTTTTAGACGACCCCGG
TCGCATTGAACCTGGCATTCAGAATGACGTGCTCTCAAGAATCAAGGTCCTCCCACTGAGGGTCATCT
GCTTCGCGGGTCAGCGTATGGTCATAAACAT
>Ede61
GTGTAGCCCAGTTAGACACTTCTAAATCCTTGGTAACAGACATGCAGTCCCCTCGGCAGAGCCCCGCC
CCCCGATGGTACCGGGTTGTTAAGAGGTCCACAACGGGTCGCACGACAACATTAAGGCCTCATGCATG
ACCCAANACGGGGGCGTCCCGGNGCGGCGACCTTGNANTCCGGGGACAAGTATGGCCCCCCGGGAAGG
ACGCCGTAGTGCATCTGGAGTGCCGATTCGTGGTCCAGAATGCCACCACGATGGTATGAGTGGCAAAG
GTACTTTGGACACCACCCCGTTTTCTATTTCCAGACGCGGCCATTCAGGAACGACGCGAATCGTAATC
TACCTGCCGCGTACGCGGATCGTACATGCGTTTTCCAGCGTTCGAAATAGAGTTTGTCGTTCCGCTGA
CCAGGTTTCACGGCTACTGCGATAAGCATATTCTACTGGGGGACTGTAAAGGAATTGTATCCACTCTG

CTTCCTGTCAATTGGCCATGTGCATCTCTNGTTGTTCAGGGTACATTAATAACACACAGCATTAAGTA
GATAAGTCTCCGATATAAGCGAACAGAACGTTGGTCAGAGACTATGGTTCAATTGTAGTGGTATAATG
TTTCCCAGTGCATAAGGAACTCAGGGACACTACTAGCATGGGCGAGGAGAGACCCTTGGGTGGCAACA
CGCAATAAGTGTTACCTAGTACATCAGCACGAGANAGGGTTTGACCATGTGTTTTTAGACGACCCCGG
TCGCATTGAACCTGGCATTCAGAATGACGTGCTCTCAAGAATCAAGGTCCTCCCACTGAGGGTCATCT
GCTTCGCGGGTCAGCGTATGGTCATNAACAT
>Ede736
GTGTAGCCCAGTTAGACACTTCTAAATCCTTGGTAACAGACATGCAGTCCCCTCGGCAGAGCCCCGCC
CCCCGATGGTACCGGGTTGTTAAGAGGTCCACAACGGGTCGCACGACAACATTAAGGCCTCATGCATG
ACCCAACACGGGGGCGTCCCGGCGCGGCGACCTTGAAGTCCGGGGACAAGTATGGCCCCCCGGGAAGG
ACGCCGTAGTGCATCTGGAGTGCCGATTCGTGGTCCAGAATGCCACCACGATGGTATGAGTGGCAAAG
GTACTTTGGACACCACCCCGTTTTCTATTTCCAGACGCGGCCATTCAGGAACGACGCGAATCGTAATC
TACCTGCCGCGTACGCGGATCGTACATGCGTTTTCCAGCGTTCGAAATAGAGTTTGTCGTTCCGCTGA
CCAGGTTTCACGGCTACTGCGATAAGCATATTCTACTGGGGGACTGTAAAGGAATTGTATCCACTCNG
CTTCCTGTCAATTGGCCATGTGCATCTCTGGTTGTTCAGGGTACATTAATAACACACAGCATTAAGTA
GATAAGTCTCCGATATAAGCGAACAGAACGTTGGTCAGAGACTATGGTTCAATTGTAGTGGTATAATG
TTTCCCAGTGCATAAGGAACTCAGGGACACTACTAGCATGGGCGAGGAGAGACCCTTGGGTGGCAACA
CGCAATAAGTGTTACCTAGTACATCAGCACGAGAGAGGGTTTGACCATGTGTTTTTAGACGACCCCGG
TCGCATTGAACCTGGCATTCAGAATGACGTGCTCTCAAGAATCAAGGTCCTCCCACTGAGGGTCATCT
GCTTCGCGGGTCAGCGTATGGTCATAAACAT
>Ede737
GTGTAGCCCAGTTAGACACTTCTAAATCCTTGGTAACAGACATGCAGTCCCCTCGGCAGAGCCCCGCC
CCCCGATGGTACCGGGTTGTTAAGAGGTCCACAACGGGTCNCACGACAACATTAAGGCCTCATGCATG
ACCCAACACGGGGGCGTCCCGGCGCGGCGACCTTGAAGTCCGGGGACAAGTATGGCCCCCCGGGAAGG
ACGCCGTAGTGCATCTGGAGTGCCGATTCGTGGTCCAGAATGCCACCACGATGGTATGAGTGGCAAAG
GTACTTTGGACACCACCCCGTTTTCTATTTCCAGACGCGGCCATTCAGGAACGACGCGAATCGTAATC
TACCTGCCGCGTACGCGGATCGTACATGCGTTTTCCAGCGTTCGAAATAGAGTTTGTCGTTCCGCTGA
CCAGGTTNCACGGCTACTGCGATAAGCATATTCTACTGGGGGACTGTAAAGGAATTGTATCCACTCTG
CTTCCTGTCAATTGGCCATGTGCATCTCTGGTTGTTCAGGGTACATTAATAACACACAGCATTAAGTA
GATAAGTCTCCGATATAAGCGAACAGAACGTTGGTCAGAGACTATGGTTCAATTGTAGTGGTATAATG
TTTCCCAGTGCATAAGGAACTCAGGGACACTACTAGCATGGGCGAGGAGAGACCCTTGGGTGGCAANA
CGCAATAAGTGTTACCTAGTACATCAGCACGAGAGAGGGTTTGACCATGTGTTTTTAGACGACCCCGG
TCGCATTGAACCTGGCATTCAGAATGACGTGCTCTCAAGAATCAAGGTCCTCCCACTGAGGGTCATCT
GCTTCGCGGGTCNGCGTATGGTCATNAACAT
>Ede738
GTGTAGCCCAGTCAGACACTTCTAAATCCTTGGTAACAGACATGCAGTCCCCTCGGCAGAGCCCCGCC
CCCCGATGGTACCGGGTTGTTAAGAGGTCCACAACGGGTCGCACGACAANNTTAAGGCCTCATGCATG
ACCCAACACGGGGGCGTCCCGGCGCGGCGACCTTGAAGTCCGGGGACAAGTATGGCCCCCCGGGAAGG
ACGCCGTAGTGCATCTGGAGTGCCGATTCGTGGTCCAGAATGCCACCACGATGGTATGAGTGGCAAAG
GTACTTTGGACACCACCCCGTTTTCTATTTCCAGACGCGGCCATTTAGGAACGACGCGAATCGTAATC
TACCTGCCGCGTNCGCGGATCGTACATGCGTTTTCCAGCGTTCGAAATAGAGTTTGTCGTTCCGCTGA
CCAGGTTTCACGGCTACTGCGATAAGCATATTCTACTGGGGGACTGTAAAGGAATTGTATCCATTCTG
CTTCCTGTCAATTGGCCATGTGCATCTCTGGTTGTTCAGGGTACATTAATAACACACAGCAGTAAGTA
GGTAAGTCTCCGATATAAGCGAACAGAACGTTTGTCAGAGACTGTGGTTCAATTGTAGTGGTATAATG
TTTCCCAGTGCATAAGGAACTCAGGGACACTACTAGCATGGGCGAGGAGAGACCCTTGGGTGGCAACA
CGCAATAAGTGTTACCTAGTACACCAGCACGAGAGAGGGTTTGACCATGTGTTTTTAGACGACCCCGG
TCGCATTGAACCTGGCATTCAGAATGACGTGCTCTCAAGAATCAAGGTCCTCCCACTGAGGGTCATCT
GCTTCGCGGGTCAGCGTATGGTCATAAACAT
>Ede739
GTGTAGCCCAGTTAGACACTTCTAAATCCTTGGTAACAGACATGCAGTCCCCTCGGCAGAGCCCCGCC
CCCCGATGGTACCGGGTTGTTAAGAGGTCCACAACGGGTCGCACGACAACATTAAGGCCTCATGCATG
ACCCAACACGGGGGCGTCCCGGCGCGGCGACCTTGAAGTCCGGGGACAAGTATGGCCCCCCGGGAAGG
ACGCCGTAGTGCATCTGGAGTGCCGATTCGTGGTCCAGAATGCCACCACGATGGTATGAGTGGCAAAG
GTACTTTGGACACCACCCCGTTTTCTATTTCCAGACGCGGCCATTCAGGAACGACGCGAATCGTAATC

TACCTGCCGCGTACGCGGATCGTACATGCGTTTTCCAGCGTTCGAAATAGAGTTTGTCGTTCCGCTGA
CCAGGTTTCACGGCTACTGCGATAAGCATATTCTACTGGGGGACTGTAAAGGAATTGTATCCACTCTG
CTTCCTGTCAATTGGCCATGTGCATCTCTGGTTGTTCAGGGTACATTAATAACACACAGCATTAAGTA
GATAAGTCTCCGATATAAGCGAACAGAACGTTGGTCAGAGACTATGGTTCAATTGTAGTGGTATAATG
TTTCCCAGTGCATAAGGAACTCAGGGACACTACTAGCATGGGCGAGGAGAGACCCTTGGGTGGCAACA
CGCAATAAGTGTTACCTAGTACATCAGCACGAGAGAGGGTTTGACCATGTGTTTTTAGACGACCCCGG
TCGCATTGAACCTGGCATTCAGAATGACGTGCTCTCAAGAATCAAGGTCCTCCCACTGAGGGTCATCT
GCTTCGCGGGTCAGCGTATGGTCATANACAT
>Ede74
GTGTAGCCCAGTTAGACACTTCTAAATCCTTGGTAACAAACGTGCAGTCCCCTCGGCAGAGCCCCGCC
CCCCGATGGTACCGGGTTGTTAAGAGGTCCACCACGAGTCGCACAACNACAGCAAGGCCTCGNGCATG
ACCCAACACGGGGGCGTCCCGGCGNAGCGACCTTGAAGTCCGGGAACAAGTATGGCTCCCCGGGAAGG
ACGCCGTAGTGCATCTGGAGTGCCGATTCGTGGTCNAGAATGCCACCACGATGGTATGAGTNGCAAAG
GTACTTTGGACACCACCCCGTTTTCTATTCCCAGACGCGGCCATTCAGGAACGACGCGAATCGTCATC
TACCTGCCGCGTNCGCGGATCGTACATGCGTTTTCCAGCTTTCGGAATAGAGTTTGTCGTTCCGCTGG
CCAGGTTTCACGGCTATTGCGATAAGCATACTCCACTGGGGGACTGTAAAGGAATTGCATCCACTCTG
CTTCTTATCAGTTGGCCATATGTATCTCTGGTTGTTCAGGGTGCATTAATAACACACAGCATTAAGTA
GATAAGTCTCCGATATAGGCGAACAGAACGTTGGTAAAAGACTGTGGTTCAACTGTAGTGGTATAATG
TTGCCCAGTGCATAAGGAACTCAGGGACACTACTAGCATGGGCGAGTAGAGACCCTTGGGTGGCAACA
CGCAATAAGTGTTACCTAGTACACCAACACGATNTAGGGTTTGACCATGTGTTTTTAGACGACCCCGG
TCGCATTAAACCTGGCATTCATAATGACGTGCTCTCAAGAGTCAAGGTCCTCCCACTGAGGGTCATCT
GCTTCGCGGGTCATCGTATGGTCATNNNNNT
>Ede740
GTGTAGCCCAGTTAGACACTTCTAAATCCTTGGTAACAGACATGCAGTCCCCTCGGCAGAGCCCCGCC
CCCCGATGGTACCGGGTTGTTAAGAGGTCCACAACGGGTCGCACGACAACNTTAAGGCCTCATGCATG
ACCCAACACGGGGGCGTCCCGGNGCGGCGACCTTGAAGTCCGGGGACAAGTATGGCCCCCCGGGAAGG
ACGCCGTAGTGCATCTGGAGTGCCGATTCGTGGTCCAGAATGCCACCACGATGGTATGAGTGGCAAAG
GTACTTTGGACACCACCCCGTTTTCTATTTCCAGACGCGGCCATTCAGGAACGACGCGAATCGTAATC
TACCTGCCGCGTNCGCGGATCGTACATGCGTTTTCCAGCGTTCGAAATAGAGTTTGTCGTTCCGCTGA
CCAGGTTTCACGGCTACTGCGATAAGCATATTCTACTGGGGGACTGTAAAGGAATTGTATCCACTCTG
CTTCCTGTCAATTGGCCATGTGCATCTCTGGTTGTTCAGGGTACATTAATAACACACAGCATTAAGTA
GATAAGTCTCCGATATAAGCGAACAGAACGTTGGTCAGAGACTATGGTTCAATTGTAGTGGTATAATG
TTTCCCAGTGCATAAGGAACTCAGGGACACTACTAGCATGGGCGAGGAGAGACCCTTGGGTGGCAACA
CGCAATAAGTGTTACCTAGTACATCAGCACGAGAGAGGGTTTGACCATGTGTTTTTAGACGACCCCGG
TCGCATTGAACCTGGCATTCAGAATGACGTGCTCTCAAGAATCAAGNTCCTCCCACTGAGGGTCATCT
GCTTCGCGGGTCAGCGTATGGTCACAAACAT
>Ede741
GTGTAGCCCAGTTAGACACTTCTAAATCCTTGGTAACAGACATGCAGTCCCCTCGGCAGAGCNCCGCC
CCCCGATGGTACCGGGTTGTTAAGAGGTCCACAACGGGTCGCACGACANCATTAAGGCCTCATGCATG
ACCCAACNCGGGGGCGTCCCGGCGCGGCGACCTTGNAGTCCGGGGACAAGTATGGCCCCCCGGGAAGG
ACGCCGTAGTGCATCTGGAGTGCCGATTCGTGGTCCAGAATGCCACCACGATGGTATGAGTNGCAAAG
GTACTTTGGACACCACCCCGTTTTCTATTTCCAGACGCGGCCATTCAGGAACGACGCGAATCGTAATC
TACCTGCCGCGTACGCGGATCGTACANGCGTTTTCCAGCGTTCGAAATAGAGTTTGTCGTTCCGCTGA
CCAGGTTTCACGGCTACTGCGATAAGCATATTCTACTGGGGGACTGTAAAGGAATTGTATCCACTCNG
CTTCCTGTCAATTGGCCATGTGCATCTCTGGTTGTTCAGGGTACATTAATAACACACAGCATTAAGTA
GATAAGTCTCCGATATAAGCGAACAGAACGTTGGTCAGAGACTATGGTTCAATTGTAGTGGTATAATG
TTTCCCAGTGCATAAGGAACTCAGGGACACTACTAGCATGGGCGAGGAGAGACCCTTGGGTGGCAACA
CGCAATAAGTGTTACCTAGTACATCAGCACGAGAGAGGGTTTGACCATGTGTTTTTAGACGACCCCGG
TCGCATTGAACCTGGCATTCAGAATGACGTGCTCTCAAGAATCAAGGTCCTCCCACTGAGGGTCATCT
GCTTCGCGGGTCAGCGTATGGTCATAAACAT
>Ede742
GTGTAGCCCAGTTAGACACTTCTAAATCCTTGGTAACAAACGTGCAGTCCCCTCGGCAGAGCCCCGCC
CCCCGATGGTACCGGGTTGTTAAGAGGTCCACCACGAGTCGCACAACAACAGCAAGGCCTCGTGCATG
ACCCAACACGGGGGCGTCCCGGCGCAGCGACCTTGNANTCCGGGAACAAGTATGGCTCCCCGGGAAGG

ACGCCGTAGTGCATCTGGAGTGCCGATTCGTGGTCNAGAATGCCACCACGATGGTATGAGTNGCAAAG
GTACTTTGGACACCACCCCGTTTTCTANTCCCAGACGCGGCCATTCAGGAACGACGCGAATCGTCATC
TACCTGCCGCGTACGCGGATCGTACATGCGTTTTCCAGCTTTCGGAATAGAGTTTGTCGTTCCGCTGG
CCAGGTTTCACGGCTATTGCGATAAGCATACTCCACTGGGGGACTGTAAAGGAATTGCATCCACTCTG
CTTCTTATCAGTTGGCCATATGTATCTCTGGTTGTTCAGGGTGCATTAATAACACACAGCATTAAGTA
GATAAGTCTCCGATATAGGCGAACAGAACGTTGGTAAAAGACTGTGGTTCAACTGTAGTGGTATAATG
TTGCCCAGTGCATAAGGAACTCAGGGACACTACTAGCATGGGCGAGTAGAGACCCTTGGGTGGCAANA
CGCAATAAGTGTTACCTAGTACACCAACACGATATAGGGTTTGACCATGTGTTTTTAGACGACCCCGG
TCGCATTAAACCTGGCATTCATAATGACGTGCTCTCAAGAGTCAAGGTCCTCCCACTGAGGGTCATCT
GCTTCGCGGGTCATCGTATGGTCATNAACAT
>Ede743
GTGTAGCCCAGTTAGACACTTCTAAATCCTTGGTAACAGACATGCAGTCCCCTCGGCAGAGCCCCGCC
CCCCGATGGTACCGGGTTGTTAAGAGGTCCACAACGGGTCGCACGACAACNTTAAGGCCTCATGCATG
ACCCAACACGGGGGCGTCCCGGCGCGGCGACCTTGAAGTCCGGGGACAAGTATGGCCCCCCGGGAAGG
ACGCCGTAGTGCATCTGGAGTGCCGATTCGTGGTCCAGAATGCCACCACGATGGTATGAGTGGCAAAG
GTACTTTGGACACCACCCCGTTTTCTATTTCCAGACGCGGCCATTCAGGAACGACGCGAATCGTAATC
TACCTGCCGCGTACGCGGATCGTACATGCGTTTTCCAGCGTTCGAAATAGAGTTTGTCGTTCCGCTGA
CCAGGTTTCACGGCTACTGCGATAAGCATATTCTACTGGGGGACTGTAAAGGAATTGTATCCACTCNG
CTTCCTGTCAATTGGCCATGTGCATCTCTGGTTGTTCAGGNNACATTAATAACACACAGCATTAAGTA
GATAAGTCTCCGATATAAGCGAACAGAACGTTGGTCAGAGACTATGGTTCAATTGTAGTGGTATAATG
TTTCCCAGTGCATAAGGAACTCAGGGACACTACTAGCATGGGCGAGGAGAGACCCTTGGGTGGCAACA
CGCAATAAGTGTTACCTAGTACATCAGCACGAGAGAGGGTTTGACCATGTGTTTTTAGACGACCCCGG
TCGCATTGAACCTGGCATTCAGAATGACGTGCTCTCAAGAATCAAGGTCCTCCCACTGAGGGTCATCT
GCTTCGCGGGTCAGCGTATGGTCACAAACAT
>Ede744
GTGTAGCCCAGTTAGACACTTCTAAATCCTTGGTAACAGACATGCAGTCCCCTCGGCAGAGCCCCGCC
CCCCGATGGTACCGGGTTGTTAAGAGGTCCACAACGGGTCGCACGACAACATTAAGGCCTCATGCATG
ACCCAACACGGGGGCGTCCCGGCGCGGCGACCTTGAAGTCCGGGGACAAGTATGGCCCCCCGGGAAGG
ACGCCGTAGTGCATCTGGAGTGCCGATTCGTGGTCNAGAATGCCACCACGATGGTATGAGTGGCAAAG
GTACTTTGGACACCACCCCGTTTTCTATTTCCAGACGCGGCCATTCAGGAACGACGCGAATCGTAATC
TACCTGCCGCGTACGCGGATCGTACANGCGTTTTCCAGCGTTCGAAATAGAGTTTGTCGTTCCGCTGA
CCAGGTTTCACGGCTACTGCGATAAGCATATTCTACTGGGGGACTGTAAAGGAATTGTATCCACTCTG
CTTCCTGTCAATTGGCCATGTGCATCTCTGGTTGTTCAGGGTACATTAATAACACACAGCATTAAGTA
GATAAGTCTCCGATATAAGCGAACAGAACGTTGGTCAGAGACTATGGTTCAATTGTAGTGGTATAATG
TTTCCCAGTGCATAAGGAACTCAGGGACACTACTAGCATGGGCGAGGAGAGACCCTTGGGTGGCAACA
CGCAATAAGTGTTACCTAGTACATCAGCACGAGNGAGGGTTTGACCATGTGTTTTTAGACGACCCCGG
TCGCATTGAACCTGGCATTCAGAATGACGTNCTCTCAAGAATCAAGGTCCNCCCACTGAGGGTCATCT
GCTTCGCGGGTCNGCGTATGGTCANAAACAT
>Ede745
GTGTAGCCCAGTTAGACACTTCTAAATCCTTGGTAACAGACATGCAGTCCCCTCGGCAGAGCCCCGCC
CCCCGATGGTACCGGGTTGTTAAGAGGTCCACAACGGGTCGNACGACAACATTAAGGCCTCATGCATG
ACCCAACACGGGGGCGTCCCGGCGCGGCGACCTTGAAGTCCGGGGACAAGTATGACCCCCCGGGAAGG
ACGCCGTAGTGCATCTGGAGTGCCGATTCGTGGTCCAGAATGNCACCACGATGGTATGAGTNGCAAAG
GTACTTTGGACACCACCCCGTTTTCTATTTCCAGACGCGGCCATTCAGGAACGACGCGAATCGTAATC
TACCTGCCGCGTACGCGGATCGTACATGCGTTTTCCAGCGTTCGAAATAGAGTTTGTCGTTCCGCTGA
CCAGGTTTCACGGCTACTGCGATAAGCATATTCTACTGGGGGACTGTAAAGGAATTGTATCCACTCTG
CTTCCTGTCAATTGGCCATGTGCATCTCTGGTTGTTCAGGGTACATTAATAACACACAGCATTAAGTA
GATAAGTCTCCGATATAAGCGAACAGAACGTTGGTCAGAGACTATGGTTCAATTGTAGTGGTATAATG
TTTCCCAGTGCATAAGGAACTCAGGGACACTACTAGCATGGGCGAGGAGAGACCCTTGGGTGGCAANA
CGCAATAAGTGTTACCTAGTACATCAGCACGAGAGAGGGTTTGACCATGTGTTTTTAGACGACCCCGG
TCGCATTGAACCTGGCATTCAGAATGACGTGCTCTCAAGAATCAAGGTCCTCCCACTGAGGGTCATCT
GCTTCGCGGGTCAGCGTATGGTCATAAACAT
>Ede746
GTGTAGCCCAGTTAGACACTTCTAAATCCNTGGTAACAGACATGCAGTCCCCTCGGCAGAGCCCCGCC

CCCCGATGGTACCGGGTTGTTAAGAGGTCCACAACGGGTCGCACGACAACATTAAGGCCTCATGCATG
ACCCAACACGGGGGCGTCCCGGCGCGGCGACCTTGAAGTCCGGGGACAAGTATGGCCCCCCGGGAAGG
ACGCCGTAGTGCATCTGGAGTGCCGATTCGTGGTCCAGAATGCCACCACGATGGTATGAGTGGCAAAG
GTACTTTGGACACCACCCCGTTTTCTATTTCCAGACGCGGCCATTCAGGAACGACGCGAATCGTAATC
TACCTGCCGCGTACGCGGATCGTACATGCGTTTTCCAGCGTTCGAAATAGAGTTTGTCGTTCCGCTGA
CCAGGTTTCACGGCTACTGCGATAAGCATATTCTACTGGGGGACTGTAAAGGAATTGTATCCACTCTG
CTTCCTGTCAATTGGCCATGTGCATCTCTGGTTGTTCAGGGTACATTAATAACACACAGCATTAAGTA
GATAAGTCTCCGATATAAGCGAACAGAACGTTGGTCAGAGACTATGGTTCAATTGTAGTGGTATAATG
TTTCCCAGTGCATAAGGAACTCAGGGACACTACTAGCATGGGCGAGGAGAGACCCTTGGGTGGCAACA
CGCAATAAGTGTTACCTAGTACATCAGCACGAGAGAGGGTTTGACCATGTGTTTTTAGACGACCCCGG
TCGCATTGAACCTGGCATTCAGAATGACGTGCTCTCAAGAATCAAGGTCCTCCCACTGAGGGTCATCT
GCTTCGCGGGTCAGCGTATGGTCATAAACNT
>Ede747
GTGTAGCCCAGTTAGACACTTCTAAATCCTTGGTAGCAAACATGCAGTCCCCTCGGCAGAGCCCCGCC
CCCCGATGGTACCGGGTTGTTAAGAGGTCCACCACGAGTCGCACGACAACAGCAAGGCCTCGTGCATG
ACCCAACNCGGGGGCGTCCCGGCGCGGCGACCTTGAAGTCCGGGAACAAGTATGGCTCCCCGGGAAGG
ACGCCGTAGTGCATCTGGAGTGCCGATTCGTGGTCCAGAATGNCACCACGATGGTATGAGCNGCAAAG
GTACTTTGGACACCACCCCGTTTTCTATTCCCAGACGCGGCCATTCAGGAACGACGCGAATCGTAATC
TACCTGCCGCGTACGCGGATCGTACATGCGTTTTCCAGCGTTCGGAATAGAGTTTGTCGTTCCGCTGA
CCAGGTTTCACGGCTACTGCGATAAGCATACTCCACTGGGGGACTGTAAAGGAATTGCATCCACTCTG
CTTCTTGTCAGTTGGCCATATGTATCTCTGGTTGTTCAGGGTACATTAATAACACACAGCATTAAGTA
GATAAGTCTCCGATATAGGCGAACAGAACGTTGGTAAAAGACTGTGGTTCAACTGTAGTGGTATAATG
TTTCCCAGTGCATAAGGAACTCAGGGACACTACTAGCATGGGCGAGTAGAGACCCTTGGGTGGCAACA
CGCAATAAGTGTTACCTAGTACACCAACACGATATAGGGTTTGACCATGTGTTTTTAGACGACCCCGG
TCGCATTAAACCTGGCATTCATAATGACGTGCTCTCAAGAGTCAAGGTCCTCCCACTGAGGGTCATCT
GCTTCGCGGGTCATCGTATGGTCANAANNAT
>Ede748
GTGTAGCCCAGTTAGACACTTCTAAATCCTTGGTAACAGACATGCAGTCCCCTCGGCAGAGCCCCGCC
CCCCGATGGTACCGGGTTGTTAAGAGGTCCACAACGGGTCGCACGACAACATTAAGGCCTCATGCATG
ACCCAACACGGGGGCGTCCCGGCGCGGCGACCTTGAAGTCCGGGGACAAGTATGGCCCCCCGGGAAGG
ACGCCGTAGTGCATCTGGAGTGCCGATTCGTGGTCCAGAATGCCACCACGATGGTATGAGTGGCAAAG
GTACTTTGGACACCACCCCGTTTTCTATTTCCAGACGCGGCCATTCAGGAACGACGCGAATCGTAATC
TACCTGCCGCGTNCGCGGATCGTACATGCGTTTTCCAGCGTTCGAAATAGAGTTTGTCGTTCCGCTGA
CCAGGTTTCACGGCTACTGCGATAAGCATATTCTACTGGGGGACTGTAAAGGAATTGTATCCACTCTG
CTTCCTGTCAATTGGCCATGTGCATCTCTGGTTGTTCAGGGTACATTAATAACACACAGCATTAAGTA
GATAAGTCTCCGATATAAGCGAACAGAACGTTGGTCAGAGACTATGGTTCAATTGTAGTGGTATAATG
TTTCCCAGTGCATAAGGAACTCAGGGACACTACTAGCATGGGCGAGGAGAGACCCTTGGGTGGCAACA
CGCGATAAGTGTTACCTAGTACATCAGCACGAGAGAGGGTTTGACCATGTGTTTTTAGACGACCCCGG
TCGCATTGAACCTGGCATTCAGAATGACGTGCTCTCAAGAATCAAGGTCCTCCCACTGAGGGTCATCT
GCTTCGCGGGTCNGCGTATGGTCATNAACNT
>Ede749
GTGTAGCCCAGTTGGACACTTCTAAATCCTTGGTAACAGACATGCAGTCCCCTCGGCAGAGCCCCGCC
CCCCGGTGGTACCGGGTTGTTAAGAGGTCCACCACGGGTCGCACGACAACATCAAGGCCTCANGCATG
ACCCAACGCGGGGACGTACCGGCGCGGCGACCTTGAAGGTCGGGGACAAGTATGTCCCCCCGGGAAGG
ACGCCGTAGTGCATCTGGAGTGCCGATTCGTGGTCNAGAATGCCACCACGATGGTATGAGTNGCAAAG
GTATTTTGGACACCACCCCGTTTTCTATTTCCAGACGCGGCCATTCAGGAACGACGCGAATCGTAATC
TACCTGCCGCGTACGCGGATCGTACATGCGTTTTTCAGCGTTCGAAATAGAGTTTGTCGTTCCGCTGA
CCAGGTTTCACGGCTACTGCGATAAGCATATTCTACGGGGGGACTGTAAAGGAATTGTATCCACTCNG
CTCCCTGTCAATTGGCCATGTGCATCTCTGGTTGTTTAGGGTACATTAATAACACACAGCATGAGGTA
GATAAGTCTCCGATATAAGCGAACAGAACGTTGGTCAAAGACCGTGGTTCAATTGTAGTGGTATAACA
TTTCCCAGTGCATAAGGAACCCAGGGACACTATTAGCATGGGCGAGGAGAGACCCTTGGGTGGCAACA
CGCAATAAGTGTTACCCAGTACACCAGCACGATAGAGGGTTTGACCATGTGTTTTTAGACGACCCCGG
TCGCATTGAACCTGGCATTCAGAATGACGTGCTCTCAAGAGTCAAGGTCCTCCCACTGAGGGTCATCT
GCTTCGCGNGTTAGCGTATGGTCATAAACAT

>Ede750
GTGTAGCCCAGTTAGACACTTCTAAATCCTTNGTAACAGACATGCAGTCCCCTCGGCAGAGCCCCGCC
CCCCGATGGTACCGGGTTGTTAAGAGGTCCACAACGGGTCGCACGACAACATTAAGGCCTCATGCATG
ACCCAACACGGGGGNGTCCCGGCGNGGCGACCTTGNAGTCCGGGGACAAGTATGACCCCCCGGGAAGG
ACGCCGTAGTGCATCTGGAGTGCCGATTCGTGGTCCAGAATGCCACCACGATGGTATGAGTNGCAAAG
GTACTTTGGACACCACCCCGTTTTCTATTTCCAGACGCGGCCATTCAGGAACGACGCGAATCGTAATC
TACCTGCCGCGTACGCGGATCGTACATGCGTTTTCCAGCGTTCGAAATAGAGTTTGTCGTTCCGCTGA
CCAGGTTTCACGGCTACTGCGATAAGCATATTCTACTGGGGGACTGTAAAGGAATTGTATCCACTCNG
CTTCCTGTCAATTGGCCATGTGCATCTCTGGTTGTTCAGGGTACATTAATAACACACAGCATTAAGTA
GATAAGTCTCCGATATAAGCGAACAGAACGTTGGTNAGAGACTATGGTTCAATTGTAGTGGTATAATG
TTTCCCAGTGCATAAGGAACTCAGGGACACTACTAGCATGGGCGAGGAGAGACCCTTGGGTGGCAACA
CGCAATAAGTGTTACCTAGTACATCAGCACGAGAGAGGGTTTGACCATGTGTTTTTAGACGACCCCGG
TCGCATTGAACCTGGCATTCAGAATGACGTGCTCTCAAGAATCAAGGTCCTCCCACTGAGGGTCATCT
GCTTCGCGGGTCAGCGTATNGTCATAANNAT
>Ede88
GTGTAGCCCAGTCAGACACTTCTAAATCCTTGGTAACAGACATGCAGTCCCCTCGGCAGAGCCCCGCC
CCCCGATGGTACCGGGTTGTTNAGAGGTCCACAACGGGTCNNACGACNNNNTTAAGGCCTCANGCATG
ACCCAACACGGGGGCGTCCCGGCGCGGCGACCTTGNAGTCCGGGGACAAGTATGGCCCCCCGGGAAGG
ACGCNGTAGTGCATCTGGAGTGCCGATTCGTGGTCCAGAATGCCACCACGATGGTATGAGTNGCAAAG
GTACTTTGGACACCACCCCGTTTTCTANTTCCAGACGCGGCCATTTAGGAACGACGCGAATCGTAATC
TACCTGCCGCGTACGCGGATCGTACATGCGTTTTCCAGCGTTCGAAATAGAGTTTGTCGTTCCGCTGA
CCAGGTTTCACGGCTACTGCGATAAGCATATTCTACTGGGGGACTGTAAAGGAATTGTATCCATTCTG
CTTCCTGTCAATTGGCCATGTGCATCTCTGGTTGTTCAGGNNACATTAATAACACACAGCAGTAAGTA
GGTAAGTCTCCGATATAAGCGAACAGAACGTTTGTCAGAGACTGTGGTTCAATTGTAGTGGTATAATG
TTTCCCAGTNCATAAGGAACTCAGGGACACTACTAGCATGGGCGAGGAGAGACCCTTGGGTGGCAANA
CGCAATAAGTGTTACCTAGTACACCAGCACGAGNGAGGGTTTGACCATGTNTTTNTAGACGACCCCGG
TCGCATTGAACCTGGCATTCAGAATGACGTGCTNTCAAGAATCAAGGTCCTCCCACTGAGGGTCATCT
GCTTCGCGGGTCAGCGTATGGTCANAAACAT
>Ede90
GTGTAGCCCAGTTAGACACTTCTAAATCCTTGGTAACAGACATGCAGTCCCCTCGGCAGAGCCCCGCC
CCCCGATGGTACCGGGTTGTTAAGAGGTCCACAACGGGTCGCACGACAACATTAAGGCCTCANGCATG
ACCCAACACGGGGGCGTCCCGGCGCGGCGACCTTGAAGTCCGGGGACAAGTATGGCCCCCCGGGAAGG
ACGCCGTAGTGCATCTGGAGTGCCGATTCGTGGTCCAGAATGCCACCACGATGGTATGAGTGGCAAAG
GTACTTTGGACACCACCCCGTTTTCTATTTCCAGACGCGGCCATTCAGGAACGACGCGAATCGTAATC
TACCTGCCGCGTACGCGGATCGTACATGCGTTTTCCAGCGTTCGAAATAGAGTTTGTCGTTCCGCTGA
CCAGGTTTCACGGCTACTGCGATAAGCATATTCTACTGGGGGACTGTAAAGGAATTGTATCCACTCTG
CTTCCTGTCAATTGGCCATGTGCATCTCTNGTTGTTCAGGNNACATTAATAACACACAGCATTAAGTA
GATAAGTCTCCGATATAAGCGAACAGAACGTTGGTCAGAGACTATGGTTCAATTGTAGTGGTATAATG
TTTCCCAGTGCATAAGGAACTCAGGGACACTACTAGCATGGGCGAGGAGAGACCCTTGGGTGGCAACA
CGCAATAAGTGTTACCTAGTACATCAGCACGAGAGAGGGTTTGACCATGTGTTTTTAGACGACCCCGG
TCGCATTGAACCTGGCATTCAGAATGACGTGCTCTCAAGAATCAAGGTCCTCCCACTGAGGGTCATCT
GCTTCGCGGGTCAGCGTATNGTCATAANNAT
>Ede95
GTGTAGCCCAGTTAGACACTTCTAAATCCTTGGTAACAGACATGCAGTCCCCTCGGCAGAGCCCCGCC
CCCCGATGGTACCGGGTTGTTAAGAGGTCCACAACGGNTCNCACGACNANATTAAGGCCTCATGCATG
ACCCAACNCGGGGGCGTCCCGGCGNGGCGACCTTGAAGTCCGGGGACAAGTATGGCCCCCCGGGAAGG
ACGCCGTAGTGCATCTGGAGTGCCGATTCNTGGTCCAGAATGCCACCACGATGGTATGAGTNGCAAAG
GTACTTTGGACACCACCCCGTTTTCTATTTCCAGACGCGGCCATTCAGGAACGACGCGAATCGTAATC
TACCTGCCGCGTACGCGGATCGTACATGCGTTTTCCAGCGTTCGAAATAGAGTTTGTCGTTCCGCTGA
CCAGGTTTCACGGCTACTGCGATAAGCATATTCTACTGGGGGACTGTAAAGGAATTGTATCCACTCTG
CTTCCTGTCAATTGGCCATGTGCATCTCTGGTTGTTCAGGGTACATTAATAACACACAGCATTAAGTA
GATAAGTCTCCGATATAAGCGAACAGANCGTTGGTCAGAGACTATGGTTCAATTGTAGTGGTATAATG
TTTCCCAGTGCATAAGGAACTCAGGGACACTACTAGCATGGGCGAGGAGAGACCCTTGGGTGGCAACA
CGCAATAAGTGTTACCTAGTACATCAGCACGAGAGAGGGTTTGACCATGTGTTTTTAGACGACCCCGG

TCGCATTGAACCTGGCATTCAGAATGACGTGCTCTCAAGAATCAAGGTCCTCCCACTGAGGGTCATCT
GCTTCGCGGGTCAGCGTATGGTCATNANCAT
>Ede97
GTGTAGCCCAGTTAGACACTTCTAAATCCTTGGTAACAGACATGCAGTCCCCTCGGCAGAGCCCCGCC
CCCCGATGGTACCGGGTTGTTAAGAGGTCCACAACGGGTCGCACGACAACNTTAAGGCCTCATGCATG
ACCCAACACGGGGGCGTCCCGGCGCGGCGACCTTGAAGTCCGGGGACAAGTATGGCCCCCCGGGAAGG
ACGCCGTAGTGCATCTGGAGTGCCGATTCGTGGTCCAGAATGCCACCACGATGGTATGAGTNGCAAAG
GTACTTTGGACACCACCCCGTTTTCTATTTCCAGACGCGGCCATTCAGGAACGACGCGAATCGTAATC
TACCTGCCGCGTACGCGGATCGTACATGCGTTTTCCAGCGTTCGAAATAGAGTTTGTCGTTCCGCTGA
CCAGGTTNCACGGCTACTGCGATAAGCATATTCTACTGGGGGACTGTAAAGGAATTGTATCCACTCTG
CTTCCTGTCAATTGGCCATGTGCATCTCTGGTTGTTCAGGGTACATTAATAACACACAGCATTAAGTA
GATAAGTCTCCGATATAAGCGAACAGAACGTTGGTCAGAGACTATGGTTCAATTGTAGTGGTATAATG
TTTCCCAGTGCATAAGGAACTCAGGGACACTACTAGCATGGGCGAGGAGAGACCCTTGGGTGGCAACA
CGCAATAAGTGTTACCTAGTACATCAGCACGAGNGAGGGTTTGACCATGTGTTTTTAGACGACCCCGG
TCGCATTGAACCTGGCATTCAGAATGACGTGCTCTCAAGAATCAAGGTCCTCCCACTGAGGGTCATCT
GCTTCGCGGGTCAGCGTATGGTCATAAACAT
>Giarai100
GNNNNNNCNNNNTANNNNCNTNNNAANNNNNNNTNNCANNCNNGCANTNNNCTCNNCAGNGNNNCNNC
CCNCGNTGGTACCGNNTTGNNNAGNGGTCCANAACNNNNNNNNNGANNNNNTNANGGNNNNNNGCNNN
NNNNNNNNNNGNNGNGTCCNGNNNNNNNNNNNCTNNNANNNNGNGNNCNNNNNNNNNCNNNCNNNNAAGN
NNNNNNNANTNNNNNNNNGNNNNNCNNNNCNNNNNNNNGANNNNNNNNNNNNNNNNNNNNNNNNGNNNNN
GNNNNNNNNNCACNANCCCNNNNNNNNNNNNNNNNNNNNNGCNNNNNNNNNNNNNACNNNNNNNNNNNNN
NNNCTNCNGCNTNNGNNNNNNNNNNNNNNNNNNNNNNNCNNNNTTCGNAATANNNTNNGTCGTTCCGCTNN
CNNNNTTNCNNNNNNNNNNNNNNNNNNNNNNATNNNNCNNNNGGNCNNNNNNNNNNNNNNNANNNNNNCNN
CNNNNNNNCNNNNNNNNNATGNGNNTCTNTGNNNGTNNNNNNNNNNNNNANNNCNNNNAGNNNNNGNNN
NNNNNNNNNCNNNNNNNNNNCNNNNNNANNNNTNNTNNNNGANNNNNGNNNNNNTNTNNNNNNATNNNN
NNTNCCNNTNCNNNANGNANTCNNNNACNCNACNAGCNNGNNNNNNNNGANNNNNTTGNGNGNNNNNA
CNNANNAANNNNNNCCTAGTANATCANNNNNNNNNNNGGNTNNNNNCNNNNNNNNNNNNNNNGNCCCCGG
TCNNNTNNNNNCTGNNNTNNANNNNGNNNNNNTNNNNNNANTNNNNNNNNNCCNNNNNNNNNNNNNNN
NNNNNNNNNNNNNNGCGNANNNTNNNNNNAAT
>Giarai104
GTGTAGCCCAGTTAGACACTTCTAAATCCTTNGTAACAGACATGCAGTCCCCTCGGCAGAGCCCCGCC
CCCCNATGGTACCGGGTTGTTAAGAGGTCCACAACGGGTCGCACGACAACATTAAGGCCTCATGCATG
ACCCAACNCGGGGGCGTCCCGGNGCGGCGACCTTGAAGTCCGGGGNCAAGTATGGCCCCCCGGGAAGG
ACGCCGTAGTGCATCTGGAGTGCCGATTCGTGGTCNAGAATGCCACCACGATGGTATGAGTGGCAAAG
GTACTTTGGACACCACCCCGTTTTCTATTTCCAGACGCGGCCATTCAGGAACGACGCGAATCGTAATC
TACCTGCCGCGTACGCGGATCGTACATGCGTTTTCCAGCGTTCGAAATAGAGTTTGTCGTTCCGCTGA
CCAGGTTTCACGGCTACTGCGATAAGCATATTCTACTGGGGGACTGTAAAGGAATTGTATCCACTCTG
CTTCCTGTCAATTGGCCATGTGCATCTCTGGTTGTTCAGGGTACATTAATAACACACAGCATTAAGTA
GATAAGTCTCCGATATAAGCGAACAGAACGTTGGTCAGAGACTATGGTTCAATTGTAGTGGTATAATG
TTTCCCAGTGCATAAGGAACTCAGGGACACTACTAGCATGGGCGAGGAGAGACCCTTGGGTGGCAACA
CGCAATAAGTGTTACCTAGTACATCAGCACGAGAGAGGGTTTGACCATGTGTTTTTAGACGACCCCGG
TCGCATTGAACCTGGCATTCAGAATGACGTGCTCTCAAGAATCNAGGTCCNCCCACTGAGGGTCATCT
GCTTCGCGGGTCAGCGTATGGTCATAAACAT
>Giarai107
GTGTAGCCCAGTTAGACACTTCTAAATCCTTGGTAACAGACATGCAGTCCCCTCGGCAGAGCCCCGCC
CCCCGATGGTACCGGGTTGTTAAGAGGTCCACAACGGGTCGCACGACAANATTAAGGCCTCANGCATG
ANNCAACACGGGGGCGTCCCGGCGNGGCGACCTTGAAGTCCGGGGACAAGTATGGCCCCCCGGGAAGG
ACGCCGTAGTGCATCTGGAGTGCCGATTCGTGGTCCAGAATGCCACCACGATGGTATGAGTGGCAAAG
GTACTTTGGACACCACCCCGTTTTCTANTTCCAGACGCGGCCATTCAGGAACGACGCGAATCGTAATC
TACCTGCCGCGTACGCGGATCGTACANGCGTTTTCCAGCGTTCGAAATAGAGTTTGTCGTTCCGCTGA
CCNGGTTTCACGGCTACTGCGATAAGCATATTCTACTGGGGGACTGTAAAGGAATTGTATCCACTCTG
CTTCCTGTCAATTGGCCATGTGCATCTCTGGTTGTTCAGGGTACATTAATAACACACAGCATTAAGTA
GATAAGTCTCCGATATAAGCGAACAGANCGTTGGTCAGAGACTATGGTTCAATTGTAGTGGTATAATG

TTTCCCAGTGCATAAGGAACTCAGGGACACTACTAGCATGGGCGAGGAGAGACCCTTGGGTGGCAACA
CGCAATAAGTGTTACCTAGTACATCAGCACGAGAGAGGGTTTGACCATGTGTTTTTAGACGACCCCGG
TCGCATTGAACCTGGCATTCAGAATGACGTGCTCTCAAGAATCAAGNTCCTCCCACTGAGGGTCATCT
GCTTCGCGGGTCAGCGTATGGTCATAANCAT
>Giarai124
GTGTAGCCCAGTTAGACACTTCTAAATCCTTGGTAACAGACATGCAGTCCCCTCNNCAGAGCCCCGCC
CCCCGATGGTACCGGGTTGTTAAGAGGTCCACCACGGGTCGCACGACANNATCAAGGCCTNATGCATG
ACCCAACACGGNGGCGTCCCGGCGCGGCGACCTTGAANTCCGGGGACAAGTATGGCCCCCCGGGAAGG
ACGCCGTAGTGCATCTGGAGTGCCGATTCGTGGTCCAGAATGCCACCACGATGGTATGAGTNGCAAAG
GTACTTTGGACACCACCCCGTTTTCTATTTCCAGACGCGGTCATTCAGGAACGACGCGAATCGTAATC
TACCTGCCGCGTNCGCGGATCGTACATGCGTTTTCCAGCGTTCGAAATAGAGTTTGTCGTTCCGCTGA
CCAGGTTTCACGGCTACTGCGATAAGCATATTCTACTGGGGGACTGTAAAGGAATTGTATCCACTCTG
CTTCCTGTCAATTGGCCATGTGCATCTCTNGTTGTTCAGGGTACATTAATAACACACAGCATTAAGTA
GATAAGTCTCCGATATAAGCGAACAGAACGTTGGTCAAAGACTGTGGTTCAATTGTAGTGGTATAATG
TTTCCCAGTGCATAAGGAACTCAGGGACACTACTAGCATGGGCGAGGAGAGACCCTTGGGTGGCAACA
CGCAATAAGTGTTACCTAGTACACCAGCACGATANAGGGTTTGACCATGTGTTTCTAGACGACCCCGG
TCGCATTGAACCTGGCATTCAGAATGACGTGCTCTCAAGAGTCAAGGTCCTCCCACTGAGGGTCATCT
GCTTCGCGNNNCAGCGTATGGTCANNANNNT
>Giarai134
GTGTAGCCCAGTCAGACACTTCTAAATCCTTGGTAACAGACATGCAGTCCCCTCGGCAGAGCCCCGCC
CCCCGATGGTACCGGGTTGTTAAGAGGTCCACAACGGGTCGCACGACNNCNTTAAGGCCTCATGCATG
ACCCAACACGGGGGCGTCCCGGCGNGGCGACCTTGNAGTCCGGGGACAAGTATGGCCCCCCGGGAAGG
ACGCCGTAGTGCATCTGGAGTGCCGATTCGTGGTCCAGAATGCCACCACGATGGTATGAGTNGCAAAG
GTACTTTGGACACCACCCCGTTTTCTATTTCCAGACGCGGCCATTTAGGAACGACGCGAATCGTAATC
TACCTGCCGCGTACGCGGATCGTACATGCGTTTTCCAGCGTTCGAAATAGAGTTTGTCGTTCCGCTGA
CCAGGTTTCACGGCTACTGCGATAAGCATATTCTACTGGGGGACTGTAAAGGAATTGTATCCATTCTG
CTTCCTGTCAATTGGCCATGTGCATCTCTGGTTGTTCAGGNNACATTAATAACACACAGNAGTAAGTA
GGTAAGTCTCCGATATAAGCGAACAGANCGTTTGTCAGAGACTGTGGTTCAATTGTAGTGGTATAATG
TTTCCCAGTGCATAAGGAACTCAGGGACACTACTAGCATGGGCGAGGAGAGACCCTTGGGTGGCAACA
CGCAATAAGTGTTACCTAGTACACCAGCACGAGANAGGGTTTGACCATGTGTTTTTAGACGACCCCGG
TCGCATTGAACCTGGCATTCAGAATGACGTGCTCTCAAGAATCAAGGTCCTCCCACTGAGGGTCATCT
GCTTCGCGGGTCAGCGTATGGTCANNANNNT
>Giarai60
GTGTAGCCCAGTTAGACACTTCTAAATCCTTGGTAACAGACATGCAGTCCCCTCGGCAGAGCCCCGCC
CCCCGATGGTACCGGGTTGTTAAGAGGTCCACAACGGGTCGNACGACNACATTAAGGCCTCATGCATG
ACCCAACACGGGGGNGTCCCGGCGNGGCGACCTTGAAGTCCGGGGACAAGTATGGCCCCCCGGGAAGG
ACGCCGTAGTGCATCTGGAGTGCCGATTCGTGGTCCAGAATGCCACCACGATGGTATGAGTNGCAAAG
GTACTTTGGACACCACCCCGTTTTCTANTTCCAGACGCGGCCATTCAGGAACGACGCGAATCGTAATC
TACCTGCCGCGTACGCGGATCGTACATGCGTTTTCCAGCGTTCGAAATAGAGTTTGTCGTTCCGCTGA
CCAGGTTTCACGGCTACTGCGATAAGCATATTCTACTGGGGGACTGTAAAGGAATTGTATCCACTCTG
CTTCCTGTCAATTGGCCATGTGCATCTCTGGTTGTTCAGGGTACATTAATAACACACAGCATTAAGTA
GATAAGTCTCCGATATAAGCGAACAGAACGTTGGTCAGAGACTATGGTTCAATTGTAGTGGTATAATG
TTTCCCAGTGCATAAGGAACTCAGGGACACTACTAGCATGGGCGAGGAGAGACCCTTGGGTGGCAACA
CGCAATAAGTGTTACCTAGTACATCAGCACGAGANAGGGTTTGACCATGTGTTTTTAGACGACCCCGG
TCGCATTGAACCTGGCATTCAGAATGACGTGCTCTCAAGAATCAAGGTCCTCCCACTGAGGGTCATCT
GCTTCGCGGGTCNGCGTATGGTCATAAACAT
>Giarai67
GTGTAGCCCAGTTAGACACTTCTAAATCCNTGGTAACAGACATGCAGTCCCCTCGGCAGAGCCCCGCC
CCCCGATGGTACCGGGTTGTTNAGAGGTCCACAACGGGTCGCACGACAANNTTAAGGCCTCANGCATG
ACCCAACACGGNGGCGTCCCGGCGCGGCGACCTTGAAGTCCGGGGACAAGTATGGCCCCCCGGGAAGG
ACGCCGTAGTGCATCTGGAGTGCCGATTCGTGGTCCAGAATGCCACCACGATGGTATGAGTGGCAAAG
GTACTTTGGACACCACCCCGTTTTCTATTTCCAGACGCGGCCATTCAGGAACGACGCGAATCGTAATC
TACCTGCCGCGTACGCGGATCGTACATGCGTTTTCCAGCGTTCGAAATAGAGTTTGTCGTTCCGCTGA
CCAGGTTTCACGGCTACTGCGATAAGCATATTCTACTGGGGGACTGTAAAGGAATTGTATCCACTCTG

CTTCCTGTCAATTGGCCATGTGCATCTCTGGTTGTTCAGGGTACATTAATAACACACAGCATTAAGTA
GATAAGTCTCCGATATAAGCGAACAGAACGTTGGTCAGAGACTATGGTTCAATTGTAGTGGTATAATG
TTTCCCAGTGCATAAGGAACTCAGGGACACTACTAGCATGGGCGAGGAGAGACCCTTGGGTGGCAACA
CGCAATAAGTGTTACCTAGTACATCAGCACGAGAGAGGGTTTGACCATGTGTTTTTAGACGACCCCGG
TCGCATTGAACCTGGCATTCAGAATGACGTGCTCTCAAGAATCAAGGTCCTCCCACTGAGGGTCATCT
GCTTCGCGGGTNNNNNNNNNNNCATAAACAT
>Giarai716
GTGTAGCCCAGTCAGACACTTCTAAATCCTTGGTAACAGACATGCAGTCCCCTCGGCAGAGCCCCGCC
CCCCGATGGTACCGGGTTGTTAAGAGGTCCACAACGGGTCGCACGACAACNTTAAGGCCTCATGCATG
ACCCAACNCGGNGGNGTCCCGGCGCGGCGACCTTGAAGTCCGGGGACAAGTATGGCCCCCCGGGAAGG
ACGCCGTAGTGCATCTGGAGTGCCGATTCGTGGTCCAGAATGCCACCACGATGGTATGAGTGGCAAAG
GTACTTTGGACACCACCCCGTTTTCTANTTCCAGACGCGGCCATTCAGGAACGACGCGAATCGTAATC
TACCTGCCGCGTNCGCGGATCGTACATGCGTTTTCCAGCGTTCGAAATAGAGTTTGTCGTTCCGCTGA
CCAGGTTTCACGGCTACTGCGATAAGCATATTCTACTGGGGGACTGTAAAGGAATTGTATCCATTCNG
CTTCCTGTCAATTGGCCATGTGCATCTCTGGTTGTTCAGGNNACATTAATAACACACAGCAGTAAGTA
GGTAAGTCTCCGATATAAGCGAACAGAACGTTTGTCAGAGACTGTGGTTCAATTGTAGTGGTATAATG
TTTCCCAGTGCATAAGGAACTCAGGGACACTACTAGCATGGGCGAGGAGAGACCCTTGGGTGGCAANA
CGCANTAAGTGTTACCTAGTACACCAGCACGAGNGAGGGTTTGACCATGNNTTTTTAGACGACCCCGG
TCGCATTGAACCTGGCATTCAGAATGACGTGCTCTCAAGAATCAAGGTCCTCCCACTGAGGGTCATCT
GCTTCGCGGGTCAGCGTATNGTCANAANNAT
>Giarai717
GTGTAGCCCAGTTAGACACTTCTAAATCCTTGGTAACAGACATGCAGTCCCCTCGGCAGAGCCCCGCC
CCCCGATGGTACCGGGTTGTTAAGAGGTCCACAACGGGTCGCACGACAACATTAAGGCCTCANGCATG
ACCCAACACGGNGGCGTCCCGGCGCGGCGACCTTGAAGTCCGGGGACAAGTATGGCCCCCCGGGAAGG
ACGCCGTAGTGCATCTGGAGTGCCGATTCGTGGTCCAGAATGCCACCACGATGGTATGAGTNGCAAAG
GTACTTTGGACACCACCCCGTTTTCTATTTCCAGACGCGGCCATTCAGGAACGACGCGAATCGTAATC
TACCTGCCGCGTACGCGGATCGTACATGCGTTTTCCAGCGTTCGAAATAGAGTTTGTCGTTCCGCTGA
CCAGGTTTCACGGCTACTGCGATAAGCATATTCTACTGGGGGACTGTAAAGGAATTGTATCCACTCTG
CTTCCTGTCAATTGGCCATGTGCATCTCTNGTTGTTCAGGGTACATTAATAACACACAGCATTAAGTA
GATAAGTCTCCGATATAAGCGAACAGAACGTTGGTCAGAGACTATGGTTCAATTGTAGTGGTATAATG
TTTCCCAGTGCATAAGGAACTCAGGGACACTACTAGCATGGGCGAGGAGAGACCCTTGGGTGGCAACA
CGCAATAAGTGTTANCTAGTACATCAGCACGAGAGAGGGTTTGACCATGTGTTTTTAGACGACCCCGG
TCGCATTGAACCTGGCATTCAGAATGACGTGCTCTCAAGAATCAAGGTCCTCCCACTGAGGGTCATCT
GCTTCGCGGGTCAGCGTATGGTCATANNCAT
>Giarai718
GTGTAGCCCAGTTAGACACTTCTAAATCCTTGGTAACAGACATGCAGTCCCCTCGGCAGAGCCCCGCC
CCCCGATGGTACCGGGTTGTTAAGAGGTCCACAACGGGTCGCACGACAACATTAAGGCCTCATGCATG
ACCCAACACGGNGGNGTCCCGGCGCGGCGACCTTGAAGTCCGGGGACAAGTATGGCCCCCCGGGAAGG
ACGCCGTAGTGCATCTGGAGTGCCGATTCGTGGTCNAGAATGCCACCACGATGGTATGAGTGGCAAAG
GTACTTTGGACACCACCCCGTTTTCTATTTCCAGACGCGGCCATTCAGGAACGACGCGAATCGTAATC
TACCTGCCGCGTNCGCGGATCGTACATGCGTTTTCCAGCGTTCGAAATAGAGTTTGTCGTTCCGCTGA
CCAGGTTTCACGGCTACTGCGATAAGCATATTCTACTGGGGGACTGTAAAGGAATTGTATCCACTCNG
CTTCCTGTCAATTGGCCATGTGCATCTCTGGTTGTTCAGGNTACATTAATAACACACAGCATTAAGTA
GATAAGTCTCCGATATAAGCGAACAGAACGTTGGTCAGAGACTATGGTTCAATTGTAGTGGTATAATG
TTTCCCAGTGCATAAGGAACTCAGGGACACTACTAGCATGGGCGAGGAGAGACCCTTGGGTGGCAACA
CGCAATAAGTGTTACCTAGTACATCAGCACGAGANAGGGTTTGACCATGTGTTTTTAGACGACCCCGG
TCGCATTGAACCTGGCATTCAGAATGACGTGCTCTCAAGAATCAAGGTCCTCCCACTGAGGGTCATCT
GCTTCGCGGGTCAGCGTATGGTCACANNNAT
>Giarai719
GTGTAGCCCAGTTAGACACTTCTAAATCCTTGGTAACAGACATGCAGTCCCCTCGGCAGAGCCCCGCC
CCCCGATGGTACCGGGTTGTTAAGAGGTCCACAACGGGTCGCACGACAACATTAAGGCCTCATGCATG
ACCCAACACGGGGGCGTCCCGGCGCGGCGACCTTGAAGTCCGGGGACAAGTATGGCCCCCCGGGAAGG
ACGCCGTAGTGCATCTGGAGTGCCGATTNGTGGTCCAGAATGCCACCACGATGGTATGAGTGGCAAAG
GTACTTTGGACACCACCCCGTTTTCTATTTCCAGACGCGGCCATTCAGGAACGACGCGAATCGTAATC

TACCTGCCGCGTACGCGGATCGTACATGCGTTTTCCAGCGTTCGAAATAGAGTTTGTCGTTCCGCTGA
CCAGGTTTCACGGCTACTGCGATAAGCATATTCTACTGGGGGACTGTAAAGGAATTGTATCCACTCNG
CTTCCTGTCAATTGGCCATGTGCATCTCTGGTTGTTCAGGGTACATTAATAACACACAGCATTAAGTA
GATAAGTCTCCGATATAAGCGAACAGAACGTTGGTCAGAGACTATGGTTCAATTGTAGTGGTATAATG
TTTCCCAGTGCATAAGGAACTCAGGGACACTACTAGCATGGGCGAGGAGAGACCCTTGGGTGGCAACA
CGCAATAAGTGTTACCTAGTACATCAGCACGAGAGAGGGTTTGACCATGTGTTTTTAGACGACCCCGG
TCGCATTGAACCTGGCATTCAGAATGACGTGCTCTCAAGAATCAAGGTCCTCCCACTGAGGGTCATCT
GCTTCGCGGGTCAGCGTATGGTCATNAACAT
>Giarai721
GTGTAGCCCAGTTAGACACTTCTAAATCCTTGGTAACAGACATGCAGTCCCCTCGGCAGAGCCCCGCC
CCCCGATGGTACCGGGTTGTTAAGAGGTCCACAACGGGTCGCACGACAACATTAAGGCCTCATGCATG
ACCCAACACGGGGGCGTCCCGGCGNGGCGACCTTGAAGTCCGGGGACAAGTATGGCCCCCCGGGAAGG
ACGCCGTAGTGCATCTGGAGTGCCGATTCGTGGTCCAGAATGCCACCACGATGGTATGAGTGGCAAAG
GTACTTTGGACACCACCCCGTTTTCTATTTCCAGACGCGGCCATTCAGGAACGACGCGAATCGTAATC
TACCTGCCGCGTACGCGGATCGTACATGCGTTTTCCAGCGTTCGAAATAGAGTTTGTCGTTCCGCTGA
CCAGGTTTCACGGCTACTGCGATAAGCATATTCTACTGGGGGACTGTAAAGGAATTGTATCCACTCNG
CTTCCTGTCAATTGGCCATGTGCATCTCTGGTTGTTCAGGGNACATTAATAACACACAGCATTAAGTA
GATAAGTCTCCGATATAAGCGAACAGAACGTTGGTCAGAGACTATGGTTCAATTGTAGTGGTATAATG
TTTCCCAGTGCATAAGGAACTCAGGGACACTACTAGCATGGGCGAGGAGAGACCCTTGGGTGGCAACA
CGCAATAAGTGTTACCTAGTACATCAGCACGAGAGAGGGTTTGACCATGTGTTTTTAGACGACCCCGG
TCGCATTGAACCTGGCATTCAGAATGACGTGCTCTCAAGAATCAAGGTCCTCCCACTGAGGGTCATCT
GCTTCGCGGGTCAGCGTATGGTCATAAACNT
>Giarai722
GTGTAGCCCAGTTAGACACTTCTAAATCCTTGGTAACAGACATGCAGTCCCCTCGGCAGAGCCCCGCC
CCCCGATGGTACCGGGTTGTTAAGAGGTCCACAACGGGTCGCACGACAACATTAAGGCCTCANGCATG
ACCCAACACGGGGGCGTCCCGGCGCGGCGACCTTGAAGTCCGGGGACAAGTATGGCCCCCCGGGAAGG
ACGCCGTAGTGCATCTGGAGTGCCGATTCGTGGTCCAGAATGCCACCACGATGGTATGAGTGGCAAAG
GTACTTTGGACACCACCCCGTTTTCTANTTCCAGACGCGGCCATTCAGGAACGACGCGAATCGTAATC
TACCTGCCGCGTACGCGGATCGTACATGCGTTTTCCAGCGTTCGAAATAGAGTTTGTCGTTCCGCTGA
CCAGGTTTCACGGCTACTGCGATAAGCATATTCTACTGGGGGACTGTAAAGGAATTGTATCCACTCNG
CTTCCTGTCAATTGGCCATGTGCATCTCTGGTTGTTCAGGGTACATTAATAACACACAGNATTAAGTA
GATAAGTCTCCGATATAAGCGAACAGAACGTTGGTCAGAGACTATGGTTCAATTGTAGTGGTATAATG
TTTCCCAGTGCATAAGGAACTCAGGGACACTACTAGCATGGGCGAGGAGAGACCCTTGGGTGGCAACA
CGCAATAAGTGTTACCTAGTACATCAGCACGAGAGAGGGTTTGACCATGTGTTTTTAGACGACCCCGG
TCGCATTGAACCTGGCATTCAGAATGACGTGCTCTCAAGAATCAAGGTCCTCCCACTGAGGGTCATCT
GCTTCGCGGGTCNGCGTATGGTCATAAACAT
>Giarai723
GTGTAGCCCAGTTAGACACTTCTAAATCCTTGGTAACAGACATGCAGTCCCCTCGGCAGAGCCCCGCC
CCCCGATGGTACCGGGTTGTTAAGAGGTCCACAACGGGTCGCACGACAANATTAAGGCCTCATGCATG
ACCCANCACGGGGGCGTCCCGGCGCGGCGACCTTGAAGTCCGGGGACAAGTATGGCCCCCCGGGAAGG
ACGCCGTAGTGCATCTGGAGTGCCGATTCGTGGTCCAGAATGCCACCACGATGGTATGAGTGGCAAAG
GTACTTTGGACACCACCCCGTTTTCTANTTCCAGACGCGGCCATTCAGGAACGACGCGAATCGTAATC
TACCTGCCGCGTACGCGGATCGTACATGCGTTTTCCAGCGTTCGAAATAGAGTTTGTCGTTCCGCTGA
CCAGGTTTCACGGCTACTGCGATAAGCATATTCTACTGGGGGACTGTAAAGGAATTGTATCCACTCNG
CTTCCTGTCAATTGGCCATGTGCATCTCTGGTTGTTCAGGGTACATTAATAACACACAGNATTAAGTA
GATAAGTCTCCGATATAAGCGAACAGAACGTTGGTCAGAGACTATGGTTCAATTGTAGTGGTATAATG
TTTCCCAGTGCATAAGGAACTCAGGGACACTACTAGCATGGGCGAGGAGAGACCCTTGGGTGGCAACA
CGCAATAAGTGTTACCTAGTACATCAGCACGAGNGAGGGTTTGACCATGTGTTTTTAGACGACCCCGG
TCGCATTGAACCTGGCATTCAGAATGACGTGCTCTCAAGAATCAAGGTCCNCCCACTGAGGGTCATCT
GCTTCGCGNGTCAGCGTATNGTCATNNACAT
>Giarai724
GTGTAGCCCAGTTAGACACTTCTAAATCCTTGGTAACAGACATGCAGTCCCCTCGGCAGAGCCCCGCC
CCCCGATGGTACCGGGTTGTTAAGAGGTCCACAACGGGTCGCACGACAACNTTAAGGCCTCANGCATG
ACCCAACACGGGGGNGTCCCGGCGCGGCGACCTTGAAGTCCGGGGACAAGTATGGCCCCCCGGGAAGG

ACGCCGTAGTGCATCTGGAGTGCCGATTCNTGGTCCAGAATGNCACCACGATGGTATGAGTNGCAAAG
GTACTTTGGACACCACCCCGTTTTCTATTTCCAGACGCGGCCATTCAGGAACGACGCGAATCGTAATC
TACCTGCCGCGTACGCGGATCGTACATGCGTTTTCCAGCGTTCGAAATAGAGTTTGTCGTTCCGCTGA
CCAGGTTTCACGGCTACTGCGATAAGCATATTCTACTGGGGGACTGTAAAGGAATTGTATCCACTCTG
CTTCCTGTCAATTGGCCATGTGCATCTCTGGTTGTTCAGGGTACATTAATAACACACAGCATTAAGTA
GATAAGTCTCCGATATAAGCGAACAGANCGTTGGTCAGAGACTATGGTTCAATTGTAGTGGTATAATG
TTTCCCAGTGCATAAGGAACTCAGGGACACTACTAGCATGGGCGAGGAGAGACCCTTGGGTGGCAACA
CGCAATAAGTGTTACCTAGTACATCAGCACGAGANAGGGTTTGACCATGTGTTTTTAGACGACCCCGG
TCGCATTGAACCTGGCATTCAGAATGACGTGCTCTCAAGAATCAAGGTCCTCCCACTGAGGGTCATCT
GCTTCGCGGGTCAGCGTATGGTCATAAACAT
>Giarai725
GTGTAGCCCAGTTAGACACTTCTAAATCCTTGGTAACAGACATGCAGTCCCCTCGGCAGAGCCCCGCC
CCCCGATGGTACCGGGTTGTTAAGAGGTCCACAACGGGTCGCACGACAACATTAAGGCCTCATGCATG
ACCCAACACGGGGGCGTCCCGGCGCGGCGACCTTGAAGTCCGGGGACAAGTATGGCCCCCCGGGAAGG
ACGCCGTAGTGCATCTGGAGTGCCGATTCGTGGTCCAGAATGCCACCACGATGGTATGAGTNGCAAAG
GTACTTTGGACACCACCCCGTTTTCTATTTCCAGACGCGGCCATTCAGGAACGACGCGAATCGTAATC
TACCTGCCGCGTACGCGGATCGTACATGCGTTTTCCAGCGTTCGAAATAGAGTTTGTCGTTCCGCTGA
CCAGGTTTCACGGCTACTGCGATAAGCATATTCTACTGGGGGACTGTAAAGGAATTGTATCCACTCTG
CTTCCTGTCAATTGGCCATGTGCATCTCTGGTTGTTCAGGGTACATTAATAACACACAGCATTAAGTA
GATAAGTCTCCGATATAAGCGAACAGAACGTTGGTCAGAGACTATGGTTCAATTGTAGTGGTATAATG
TTTCCCAGTGCATAAGGAACTCAGGNACACTACTAGCATGGGCGAGGAGAGACCCTTGGGTGGCAACA
CGCAATAAGTGTTACCTAGTACATCAGCACGAGAGAGGGTTTGACCATGTGTTTNTAGACGACCCCGG
TCGCATTGAACCTGGCATTCAGAATGACGTGCTCTCAAGAATCAANGTCCTCCCACTGAGGGTCATCT
GCTTCGCGGGTCAGCGTATGGTCACAAACAT
>Giarai726
GTGTAGCCCAGTTAGACACTTCTAAATCCTTGGTAACAGACATGCAGTCCCCTCGGCAGAGCCCCGCC
CCCCGATGGTACCGGGTTGTTAAGAGGTCCACAACGGGTCGCACGACAACNTTAAGGCCTCATGCATG
ACCCAACACGGGGGCGTCCCGGNGCGGCGACCTTGAAGTCCGGGGACAAGTATGGCCCCCCGGGAAGG
ACGCCGTAGTGCATCTGGAGTGCCGATTNGTGGTCCAGAATGCCACCACGATGGTATGAGTGGCAAAG
GTACTTTGGACACCACCCCGTTTTCTATTTCCAGACGCGGCCATTCAGGAACGACGCGAATCGTAATC
TACCTGCCGCGTACGCGGATCGTACATGCGTTTTCCAGCGTTCGAAATAGAGTTTGTCGTTCCGCTGA
CCAGGTTTCACGGCTACTGCGATAAGCATATTCTACTGGGGGACTGTAAAGGAATTGTATCCACTCTG
CTTCCTGTCAATTGGCCATGTGCATCTCTGGTTGTTCAGGGTACATTAATAACACACAGCATTAAGTA
GATAAGTCTCCGATATAAGCGAACAGAACGTTGGTCAGAGACTATGGTTCAATTGTAGTGGTATAATG
TTTCCCAGTGCATAAGGAACTCAGGGACACTACTAGCATGGGCGAGGAGAGACCCTTGGGTGGCAACA
CGCAATAAGTGTTACCTAGTACATCAGCACGAGNGAGGGTTTGACCATGTGTTTTTAGACGACCCCGG
TCGCATTGAACCTGGCATTCAGAATGACGTGCTCTCAAGAATCAAGGTCCTCCCACTGAGGGTCATCT
GCTTCGCGGGTCAGCGTATGGTCATAAACAT
>Giarai727
GTGTAGCCCAGTCAGACACTTCTAAATCCTTGGTAACAGACATGCAGTCCCCTCGGCAGAGCCCCGCC
CCCCGATGGTACCGGGTTGTTNAGAGGTCCACAACGGGTCGCACGACAACATTAAGGCCTCATGCATG
ACCCAACACGGNGGCGTCCCGGCGCGGCGACCTTGAAGTCCGGGGACAAGTATGGCCCCCCGGGAAGG
ACGCCGTAGTGCATCTGGAGTGCCGATTCGTGGTCCAGAATGCCACCACGATGGTATGAGTNGCAAAG
GTACTTTGGACACCACCCCGTTTTCTATTTCCAGACGCGGCCATTTAGGAACGACGCGAATCGTAATC
TACCTGCCGCGTACGCGGATCGTACATGCGTTTTCCAGCGTTCGAAATAGAGTTTGTCGTTCCGCTGA
CCAGGTTTCACGGCTACTGCGATAAGCATATTCTACTGGGGGACTGTAAAGGAATTGTATCCATTCTG
CTTCCTGTCAATTGGCCATGTGCATCTCTGGTTGTTCAGGGTACATTAATAACACACAGCAGTAAGTA
GGTAAGTCTCCGATATAAGCGAACAGAACGTTTGTCAGAGACTGTGGTTCAATTGTAGTGGTATAATG
TTTCCCAGTGCATAAGGAACTCAGGGACACTACTAGCATGGGCGAGGAGAGACCCTTGGGTGGCAACA
CGCAATAAGTGTTACCTAGTACACCAGCACGAGNGAGGGTTTGACCATGTGTTTTTAGACGACCCCGG
TCGCATTGAACCTGGCATTCAGAATGACGTGCTCTCAAGAATCAAGNTCCTCCCACTGAGGGTCATCT
GCTTCGCGGGTCAGCGTATNGTCACAAACAT
>Giarai728
GTGTAGCCCAGTCAGACACTTCTAAATCCNTGGTAACAGACATGCAGTCCCCTCGGCAGAGCCCCGCC

CCCCGATGGTACCGGGTTGTTAAGAGGTCCACAACGGGTCGCACGACAACATTAAGGCCTCATGCATG
ACCCAACACGGGGGNGTCCCGGCGCGGCGACCTTGAAGTCCGGGGACAAGTATGGCCCCCCGGGAAGG
ACGCCGTAGTGCATCTGGAGTGCCGATTCGTGGTCCAGAATGCCACCACGATGGTATGAGTGGCAAAG
GTACTTTGGACACCACCCCGTTTTCTATTTCCAGACGCGGCCATTTAGGAACGACGCGAATCGTAATC
TACCTGCCGCGTACGCGGATCGTACATGCGTTTTCCAGCGTTCGAAATAGAGTTTGTCGTTCCGCTGA
CCAGGTTTCACGGCTACTGCGATAAGCATATTCTACTGGGGGACTGTAAAGGAATTGTATCCATTCTG
CTTCCTGTCAATTGGCCATGTGCATCTCTGGTTGTTCAGGGNACATTAATAACACACAGCAGTAAGTA
GGTAAGTCTCCGATATAAGCGAACAGAACGTTTGTCAGAGACTGTGGTTCAATTGTAGTGGTATAATG
TTTCCCAGTGCATAAGGAACTCAGGNACACTACTAGCATGGGCGAGGAGAGACCCTTGGGTGGCAACA
CGCAATAAGTGTTACCTAGTACACCAGCACGAGAGAGGGTTTGACCATGTGTTTTTAGACGACCCCGG
TCGCATTGAACCTGGCATTCAGAATGACGTGCTCTCAAGAATCAAGGTCCTCCCACTGAGGGTCATCT
GCTTCGCGNGTCAGCGTATGGTCATAAACAT
>Giarai729
GTGTAGCCCAGTCAGACACTTCTAAATCCTTGGTAACAGACATGCAGTCCCCTCGGCAGAGCCCCGCC
CCCCGATGGTACCGGGTTGTTAAGAGGTCCACAACGGGTCGCACGACAACATTAAGGCCTCATGCATG
ACCCAACACGGGGGCGTCCCGGCGCGGCGACCTTGAAGTCCGGGGACAAGTATGGCCCCCCGGGAAGG
ACGCCGTAGTGCATCTGGAGTGCCGATTCGTGGTCCAGAATGCCACCACGATGGTATGAGTGGCAAAG
GTACTTTGGACACCACCCCGTTTTCTATTTCCAGACGCGGCCATTTAGGAACGACGCGAATCGTAATC
TACCTGCCGCGTACGCGGATCGTACATGCGTTTTCCAGCGTTCGAAATAGAGTTTGTCGTTCCGCTGA
CCAGGTTTCACGGCTACTGCGATAAGCATATTCTACTGGGGGACTGTAAAGGAATTGTATCCATTCNG
CTTCCTGTCAATTGGCCATGTGCATCTCTGGTTGTTCAGGGTACATTAATAACACACAGCAGTAAGTA
GGTAAGTCTCCGATATAAGCGAACAGAACGTTTGTCAGAGACTGTGGTTCAATTGTAGTGGTATAATG
TTTCCCAGTGCATAAGGAACTCAGGNACACTACTAGCATGGGCGAGGAGAGACCCTTGGGTGGCAACA
CGCAATAAGTGTTACCTAGTACACCAGCACGAGAGAGGGTTTGACCATGTGTTTTTAGACGACCCCGG
TCGCATTGAACCTGGCATTCAGAATGACGTGCTCTCAAGAATCAAGGTCCTCCCACTGAGGGTCATCT
GCTTCGCGGGTCAGCGTATGGTCATANACAT
>Giarai730
GTGTAGCCCAGTTAGACACTTCTAAATCCTTGGTAACAGACATGCAGTCCCCTCGGCAGAGCCCCGCC
CCCCGATGGTACCGGGTTGTTAAGAGGTCCACAACGGGTCGCACGACAACATTAAGGCCTCANGCATG
ACCCAACACGGGGGCGTCCCGGCGCGGCGACCTTGAAGTCCGGGGACAAGTATGGCCCCCCGGGAAGG
ACGCCGTAGTGCATCTGGAGTGCCGATTCGTGGTCNAGAATGCCACCACGATGGTATGAGTGGCAAAG
GTACTTTGGACACCACCCCGTTTTCTATTTCCAGACGCGGCCATTCAGGAACGACGCGAATCGTAATC
TACCTGCCGCGTACGCGGATCGTACATGCGTTTTCCAGCGTTCGAAATAGAGTTTGTCGTTCCGCTGA
CCAGGTTTCACGGCTACTGCGATAAGCATATTCTACTGGGGGACTGTAAAGGAATTGTATCCACTCTG
CTTCCTGTCAATTGGCCATGTGCATCTCTGGTTGTTCAGGGTACATTAATAACACACAGCATTAAGTA
GATAAGTCTCCGATATAAGCGAACAGAACGTTGGTCAGAGACTATGGTTCAATTGTAGTGGTATAATG
TTTCCCAGTGCATAAGGAACTCAGGGACACTACTAGCATGGGCGAGGAGAGACCCTTGGGTGGCAACA
CGCAATAAGTGTTACCTAGTACATCAGCACGAGAGAGGGTTTGACCATGTGTTTTTAGACGACCCCGG
TCGCATTGAACCTGGCATTCAGAATGACGTGCTCTCAAGAATCAAGGTCCTCCCACTGAGGGTCATCT
GCTTCGCGGGTCAGCGTATGGTCACAAAAAT
>Giarai733
GTGTAGCTCAGTTAGACACTTCTAAATCCTTGGTAACAGACATGCAGTCCCCTCGGCAGAGCCCCGCC
CCCCGGTGGTACCGGGTTGTTAAGAGGTCCACCACGGGTCGCACGACAACATCAAGGCCTCANGCATG
ACCCAACGCGGGGACGTACCGGCGNGGCGACCTTGNANGTCGGGGACAAGTATGTCCCCCCGGGAAGG
ACGCCGTAGTGCATCTGGAGTGCCGATTNGTGGTCCAGAATGCCACCACGATGGTATGAGTNGCAAAG
GTATTTTGGACACCACCCCGTTTTCTATTTCCAGACGCGGCCATTCAGGAACGACGCGAATCGTAATC
TACCTGCCGCGTACGCGGATCGTACATGCGTTTTTCAGCGTTCGAAATAGAGTTTGTCGTTCCGCTGA
CCAGGTTTCACGGCTACTGCGATAAGCATATTCTACGGGGGGACTGTAAAGGAATTGTATCCACTCTG
CTCCCTGTCAATTGGCCATGTGCATCTCTGGTTGTTTAGGNNACATTAATAACACACAGCATTAGGTA
GATAAGTCTCCGATATAAGCGAACAGAACGTTGGTCAAAGACCGTGGTTCAATTGTAGTGGTATAACA
TTTCCCAGTGCATAAGGAACCCAGGGACACTACTAGCATGGGCGAGGAGAGACCCTTGGGTGGCAACA
CGCAATAAGTGTTACCCAGTACACCAGCACGATAGAGGGTTTGACCATGTGTTTTTAGACGACCCCGG
TCGCATTGAACCTGGCATTCAGAATGACGTGCTCTCAAGAGTCAAGGTCCTCCCACTGAGGGTCATCT
GCTTCGCGNNNTAGCGTATGGTCATNAACAT

>Giarai734
GTGTAGCCCAGTTAGACACTTCTAAATCCTTGGTAACAGACATGCAGTCCCCTCGGCAGAGCCCCGCC
CCCCGATGGTACCGGGTTGTTAAGAGGTCCACAACGGGTCGCACGACAACATTAAGGCCTCATGCATG
ACCCAACACGGGGGCGTCCCGGCGCGGCGACCTTGNAGTCCGGGGACAAGTATGGCCCCCCGGGAAGG
ACGCCGTAGTGCATCTGGAGTGCCGATTCGTGGTCCAGAATGCCACCACGATGGTATGAGTGGCAAAG
GTACTTTGGACACCACCCCGTTTTCTATTTCCAGACGCGGCCATTCAGGAACGACGCGAATCGTAATC
TACCTGCCGCGTACGCGGATCGTACATGCGTTTTCCAGCGTTCGAAATAGAGTTTGTCGTTCCGCTGA
CCAGGTTTCACGGCTACTGCGATAAGCATATTCTACTGGGGGACTGTAAAGGAATTGTATCCACTCTG
CTTCCTGTCAATTGGCCATGTGCATCTCTGGTTGTTCAGGGTACATTAATAACACACAGCATTAAGTA
GATAAGTCTCCGATATAAGCGAACAGAACGTTGGTCAGAGACTATGGTTCAATTGTAGTGGTATAATG
TTTCCCAGTGCATAAGGAACTCAGGGACACTACTAGCATGGGCGAGGAGAGACCCTTGGGTGGCAACA
CGCAATAAGTGTTACCTAGTACATCAGCACGAGAGAGGGTTTGACCATGNNTTTTAGACGACCCCGG
TCGCATTGAACCTGGCATTCAGAATGACGTGCTCTCAAGAATCAAGGTCCTCCCACTGAGGGTCATCT
GCTTCGCGGGTCNGCGTATGGTCANNAACAT
>Giarai735
GTGTAGCCCAGTTAGACACTTCTAAATCCTTGGTAGCAAACATGCAGTCCCCTCGGCAGAGCCCCGCC
CCCCGATGGTACCGGGTTGTTAAGAGGTCCACCACGAGTCGCACGACAACAGCAAGGCCTCGNGCATG
ACCCAACACGGGGGNGTCCCGGNGCGGCGACCTTGAAGTCCGGGAACAAGTATGGCTCCCCGGGAAGG
ACGCCGTAGTGCATCTGGAGTGCCGATTCGTGGTCCAGAATGCCACCACGATGGTATGAGCGGCAAAG
GTACTTTGGACACCACCCCGTTTTCTATTCCCAGACGCGGCCATTCAGGAACGACGCGAATCGTAATC
TACCTGCCGCGTACGCGGATCGTACATGCGTTTTCCAGCGTTCGGAATAGAGTTTGTCGTTCCGCTGA
CCAGGTTTCACGGCTACTGCGATAAGCATACTCCACTGGGGGACTGTAAAGGAATTGCATCCACTCTG
CTTCTTGTCAGTTGGCCATATGTATCTCTGGTTGTTCAGGGTACATTAATAACACACAGCATTAAGTA
GATAAGTCTCCGATATAGGCGAACAGAACGTTGGTAAAAGACTGTGGTTCAACTGTAGTGGTATAATG
TTTCCCAGTGCATAAGGAACTCAGGGACACTACTAGCATGGGCGAGTAGAGACCCTTGGGTGGCAACA
CGCAATAAGTGTTACCTAGTACACCAACACGATATAGGGTTTGACCATGTGTTTTTAGACGACCCCGG
TCGCATTAAACCTGGCATTCATAATGACGTGCTCTCAAGAGTCAAGGTCCTCCCACTGAGGGTCATCT
GCTTCGCGGGTCATCGTATGGTCANAAACAT
>Giarai81
GTGTAGCCCAGTTAGACACTTCTNAATCCTTGGTAACAGACATGCAGTCCCCTCNNCAGAGNCCCGCC
CCCCGATGGTACCGGGTTGTTNAGNGGTCCACAACGGNTCGNACGACNNNNTTAAGGCCTCANGCATG
ACCCAACACGGGGGNGTCCCGGCGCGGCGACCTTGAANTCCGGGGACAAGTATGGCCCCCCGGGAAGG
ACGCCGTAGTGCATNTGGAGTGCCGNTTCNTGGTCNAGAATGCCACCACGATGGTATGAGTNGCAAAG
GTACTTTGGACACCACCCCGTTTTCTATTTCCAGACGCGGCCATTCAGGAACGACGCGAATCGTAATC
TACCTGCCGCGTACGCGGATCGTACATGCGTTTTCCAGCGTTCGAAATAGAGTTTGTCGTTCCGCTGA
CCAGGTTNCACGGCTACTGCGATAAGCATATTCTACTGGGGGACTGTAAAGNAATTGTATCCACTCTG
CTTCCTGTCAATTGGCCATGTGCATCTCTGGTTGTTCAGGGNACATTAATAACACACAGCATTAAGTA
GATAAGTCTCCGATATAAGCGAACAGANCGTTGGTCAGAGACTATGGTTCAATTGTAGTGGTATAATG
TTTCCCAGTGCATAAGGAACTCAGGGACACTACTAGCATGGGCGAGGAGAGACCCTTGGGTGGCAACA
CGCGATAAGTGTTACCTAGTACATCAGCACGAGNNANGGTTTGACCATGNNTTTTAGNNGACCCCGG
TCGCATTGAACCTGGCATTCAGAATGACGTGCTCTCAAGAATCAAGNTCCTCCCACTGAGGGTCATCT
GCTTCGCGNGTCAGCGTATGGTCACNAACNN
>Giarai91
GTGTAGCCCAGTTAGACACNTCTAAATCCTTGGTAACAAACGTGCAGTCCCCTCGGCAGAGNCCCGCC
CCCCGATGGTACCGGGTTGTTAAGAGGTCCACCACGAGTCGNACAACAANAGCAAGGCCTCGTGCATG
ACCCAACACGGGGGCGTCCCGGCGCAGCGACCTTGAAGTCCGGGAACAAGTATGGCTCCCCGGGAAGG
ACGCCGTAGTGCATCTGGAGTGCCGATTCGTGGTCCAGAATGCCACCACGATGGTATGAGTNGCAAAG
GTACTTTGGACACCACCCCGTTTTCTATTCCCAGACGCGGCCATTCAGGAACGACGCGAATCGTCATC
TACCTGCCGCGTACGCGGATCGTACATGCGTTTTCCAGCTTTCGGAATAGAGTTTGTCGTTCCGCTGG
CCAGGTTTCACGGCTATTGCGATAAGCATACTCCACTGGGGGACTGTAAAGGAATTGCATCCACTCNG
CTTCTTATCAGTTGGCCATATGTATCTCTGGTTGTTCAGGGTGCATTAATAACACACAGCATTAAGTA
GATAAGTCTCCGATATAGGCGAACAGAACGTTGGTAAAAGACTGTGGTTCAACTGTAGTGGTATAATG
TTGCCCAGTGCATAAGGAACTCAGGGACACTACTAGCATGGGCGAGTAGAGACCCTTGGGTGGCAACA
CGCAATAAGTGTTACCTAGTACACCAACACGATATANGGTTTGACCATGTGTTTTTAGACGACCCCGG

TCGCATTAAACCTGGCATTCATAATGACGTGCTCTCAAGAGTCAAGGTCCTCCCACTGAGGGTCATCT
GCTTCGCGGGTCATCGTATGGTCANAAACNT
>Giarai93
GTGTAGCCCAGTTAGACACTTCTAAATCCTTGGTAACAGACATGCAGTCCCCTCGGCAGAGCCCCGCC
CCCCGATGGTACCGGGTTGTTNAGAGGTCCACAACGGGTCGNACGACAACATTAAGGCCTCATGCATG
ACCCAACACGGGGGCGTCCCGGCGCGGCGACCTTGAAGTCCGGGGACAAGTATGGCCCCCCGGGAAGG
ACGCCGTAGTGCATCTGGAGTGCCGATTCGTGGTCCAGAATGCCACCACGATGGTATGAGTNGCAAAG
GTACTTTGGACACCACCCCGTTTTCTATTTCCAGACGCGGCCATTCAGGAACGACGCGAATCGTAATC
TACCTGCCGCGTACGCGGATCGTACATGCGTTTTCCAGCGTTCGAAATAGAGTTTGTCGTTCCGCTGA
CCAGGTTTCACGGCTACTGCGATAAGCATATTCTACTGGGGGACTGTAAAGGAATTGTATCCACTCTG
CTTCCTGTCAATTGGCCATGTGCATCTCTGGTTGTTCAGGGTACATTAATAACACACAGCATTAAGTA
GATAAGTCTCCGATATAAGCGAACAGAACGTTGGTNAGAGACTATGGTTCAATTGTAGTGGTATAATG
TTTCCCAGTGCATAAGGAACTCAGGGACACTACTAGCATGGGCGAGGAGAGACCCTTGGGTGGCAACA
CGCAATAAGTGTTACCTAGTACATCAGCACGAGAGAGGGTTTGACCATGTGTTTTTAGACGACCCCGG
TCGCATTGAACCTGGCATTCAGAATGACGTGCTCTCAAGAATCAAGGTCCTCCCACTGAGGGTCATCT
GCTTCGCGGNTCAGCGTATGGTCANANNNAT
>HaNhi136
GTGTAGCCCAGTCAGACACTTCTAAATCCTTGGTAACAGACATGCAGTCCCCTCGGCAGAGCCCCGCC
CCCCGATGGTACCGGGTTGTTAAGAGGTCCACAACGGGTCGCACGACNACATTAAGGCCTCATGCATG
ACCCAACACGGGGGCGTCCCGGCGCGGCGACCTTGAAGTCCGGGGACAAGTATGGCCCCCCGGGAAGG
ACGCCGTAGTGCATCTGGAGTGCCGATTCGTGGTCCAGAATGCCACCACGATGGTATGAGTGGCAAAG
GTACTTTGGACACCACCCCGTTTTCTATTTCCAGACGCGGCCATTCAGGAACGACGCGAATCGTAATC
TACCTGCCGCGTACGCGGATCGTACANGCGTTTTCCAGCGTTCGAAATAGAGTTTGTCGTTCCGCTGA
CCAGGTTTCACGGCTACTGCGATAAGCATATTCTACTGGGGGACTGTAAAGGAATTGTATCCATTCTG
CTTCCTGTCAATTGGCCATGTGCATCTCTGGTTGTTCAGGGTACATTAATAACACACAGCAGTAAGTA
GGTAAGTCTCCGATATAAGCGAACAGAACGTTTGTCAGAGACTGTGGTTCAATTGTAGTGGTATAATG
TTTCCCAGTGCATAAGGAACTCAGGGACACTACTAGCATGGGCGAGGAGAGACCCTTGGGTGGCAACA
CGCAATAAGTGTTACCTAGTACACCAGCACGAGAGAGGGTTTGACCATGTGTTTTTAGACGACCCCGG
TCGCATTGAACCTGGCATTCAGAATGACGTGCTCTCAAGAATCAAGNTCCTCCCACTGAGGGTCATCT
GCTTCGCGGGTCAGCGTATGGTCATAAACAT
>HaNhi312
GTGNAGCCCAGTTAGACACTTCTAAATCCTTGGTAACAGACATGCAGTCCCCTCGGCAGAGCNCCGCC
CCCCNATGGTACCGGGTTGNTAAGAGGTCCACAACAGGTCGNACGACAACATTAAGGCCTCANGCATG
ACCCAACACNGGGGCGTCCCGGCGCGGCGACCTTGAAGTCCGGGGNCAAGTATGGCCCCCCGGGAAGG
ACGCNGTAGTGCATCTGGAGTGCCGATTCNTGGTCCAGAATGCCACCACGATGGTATGAGTGGCAAAG
GTACTTTGGACACCACCCCGTTTTCTANTTCCAGACGCGGCCATTCAGGAACGACGCGAATCGTAATC
TANCTGCCGCGTNCGCGGATCGTACANGCGTTTTCCAGCGTTCGAAATAGAGTTTGTCGTTCCGCCGA
CCAGGTTNCACGGCTACTNCGATAAGCATATTCTACTGGGGGACTGTAAAGGAATTGTATCCACTCTA
CTTCCTGTCAATTGGCCATGTGCATCTCTGGTTGTTCAGGNNACATTAATAACACACAGCATNAAGTA
GATAAGTCTCCGATATAAGCGAACAGANCGTTGGTCAGAGACTGTGGTTCAATTGTAGTGGTATAATG
TTTCCCAGTGCATAAGGAACTTAGGGACATTACTAGCATGGGCGAGGAGAGACCCTTGGGTGGCAANA
CGNAATAAGTGTTANNTAGTACACCAGCACGAGANAGGGTTTGNCCATGNNTTTNTAGACGACCCCGG
TCGCATTGAACCTGGCATTCAGAATGACGTGCTCTCAAGAATCAAGNTCCTCNCACTGAGGGTCATCT
GCTTCGCGGGTCAGCGTATNGTCATAAACAT
>HaNhi313
GTNNAGCCCAGTCNGACACTTCTAAATCCTTGGTAACAGACATGCAGTCCCCTCGGCAGAGCNCCGCC
CCCCNATGGTACCGGGTTGTTAAGAGGTCCACAACGGGTCGNACGACANCATTAAGGCCTCATGCATG
ANNCANNNCGGGNGNGTCCCGGCGNNGCGACCTTGAAGTCCGGNGNCAAGTATGGCCCCCCGNGAAGG
ACGCNGTAGTGCATCTGGAGTGCCGATTCNTGGTCNAGANTGNCACCACGATGGTATGAGTGGCAAAG
GTACTTNGNNCACNACCCCGTTNNCTANTTCCAGACGCGGCCNTTCAGGAACGACGCGAATCGTAATC
TANCTGCCGCGTNCGCGGATNGTACNNGCGTTTTNCAGCGTTCGAAATAGAGTTTGTCGTTCCGCTGA
CCAGGTTNCACGGCTACTGCGATAAGCATATTCTACTGGGGGACTGTAAAGGAATTGTATCCATTCNG
CTTCCTGTCAATTGGCCATGTGCATCTCTNGTTGTTCAGGNNACATTAATAACACACAGNAGNAAGTA
GNTNAGTCTCCGATATAAGCGAACAGANCGTTTGTNAGAGACTGTGGTTCAATTGTAGTGGTATAATG

TTTCCCAGTGCATAAGGAACTCAGGGACACTACTAGCANGGGCGAGGAGAGACCCTTGGGTGGCAANA
CGNAATAAGTGTTNNCTAGTACACCAGCACGAGNNAGGGTTTGNCCANGNNTTTNTAGNNGACCCCGG
TCGCATTGAACCTGGCATTCAGAATGACGTGCTCTNAAGAATCAAGNTCCNCNCACTGAGGGTCANNN
GCTTCGCGGGTCAGCGTATNGTCATAAACAT
>HaNhi314
GTGTAGCCCAGTTAGACACTTCTAAATCCNTNGTAACAGACATGCAGTCCCCTCGGCAGAGNNCCGCC
CCCCNATGGTACCGGGTTGTTNAGAGGTCCACAACAGGTCGNACGACNNCATTAAGGCCTCATGCATG
ACCCNACACNGGGGCGTCCCGGCGCGGCNACCTTGNAGTCCGGGGNCAAGTATGGCCCCCCGGGAAGG
ACGCNGTAGTGCATCTGGAGTGCCGATTCGTGGTCCNGAATGCCACCACGATGGTATGAGTNGCAAAG
GTACTTNGGACACCACCCCGTTTTCTNNTTCCAGANGCGGCCATTCAGGAACGACGCGAATCGTAATC
TACCTGCCGCGTNCGCGGATNGTACANGCGTTTTCCAGCGTTCGAAATAGAGTTTGTCGTTCCGCCGA
CCNGGTTNCACGGCTACTGCGATAAGCATATTCTACTGGGGGACTGTAAAGGAATTGTATCNACNCTA
CTTCCTGNCAATTGGCCATGTGCATCTCTNGTTGTTCAGGGTANATTAATAACACACAGCATNAAGTA
GATAAGTCTCCGATATAAGCGAACAGANCGTTGGTNAGAGACTGTGGTTCAATTGTAGTGGTATAATG
TTTCCCAGTGCATAAGGAANTTAGGGACATTACTAGCANGGGCGAGGAGAGACCCTTGGGTGGCANNA
CGNAATAAGTGTTACCTAGTACACCAGCACGAGNNAGGGTTTGNCCNNGNNTTTNTAGANGNCCCCGG
TCGCATTGAACCTGGCATTCAGAATGANGTGCTCTCANGAATCAAGGTCCNCNCACTGAGGGTCANNN
GCTTCGCGGGTCNGCGTATGGTCANAAACAT
>HaNhi315
GTGTAGCCCAGTCAGACACTTCTAAATCCTTGGTAACAGACATGCAGTCCCCTCNGCAGAGCCCCGCC
CCCCGATGGTACCGGGTTGTTAAGAGGTCCACAACGGNTCGCACGACAACATTAAGGCCTCATGCATG
ACCCANCACGGGGGNGTCCCGGCGCGGCGACCTTGAAGTCCGGGGNCAAGTATGGCCCCCCGGGAAGG
ACGCCGTAGTGCATCTGGAGTGCCGATTCGTGGTCCAGAATGCCACCACGATGGTATGAGTGGCAAAG
GTACTTTGGACACCACCCCGTTTTCTATTTCCAGACGCGGCCATTCAGGAACGACGCGAATCGTAATC
TACCTGCCGCGTACGCGGATCGTACATGCGTTTTCCAGCGTTCGAAATAGAGTTTGTCGTTCCGCTGA
CCNGGTTNCACGGCTACTGCGATAAGCATATTCTACTGGGGGACTGTAAAGGAATTGTATCCATTCTG
CTTCCTGTCAATTGGCCATGTGCATCTCTNGTTGTTCAGGGTACATTAATAACACACAGCAGNAAGTA
GGTNAGTCTCCGATATAAGCGAACAGAACGTTTGTCAGAGACTGTGGTTCAATTGTAGTGGTATAATG
TTTCCCAGTGCATAAGGAACTCAGGNACACTACTAGCATGGGCGAGGAGAGACCCTTGGGTGGCAANA
CGCAATAAGTGTTACCTAGTACACCAGCACGAGNNAGGGTTTGACCATGTGTTTTTAGNNGACCCCGG
TCGCATTGAACCTGGCATTCAGAATGACGTGCTCTNAAGAATCAAGGTCCTCCCACTGAGGGTCATCT
GCTTCGCGGGTCAGCGTATGGTCATAAACAT
>HaNhi317
GTGTAGCCCAGTTAGACACTNCTAAATCCNTGGTAACAGACATGCAGTCCCCTCNNCAGAGCCCCGCC
CCCCGGTGGTACCGGGTTGTTAAGAGGTCCACCACGGNTCGNACGACAACATCAAGGCCTNATGCATG
ANNCANNNCGGNGGCGTCCCGGCGCGGNGACCTTGAAGGCCGGGNNCAAGTATGGCCCCCCGGGAAGG
ACGCNGTAGTGCATCTGGAGTGCCGATTCGTGGTCCAGAATGNCACCACGATGGTATGAGTNGCAAAG
GTACTTNGGACACCACCCCGTTTTCTANTTCCAGACGCGGCCNTTCAGGAACGACGCGAATCGTAATC
TACCTGCCGCGTNCGCGGATCGTACATGCGTTTTCCAGCGTTCGAAATAGAGTTTGTCGTTCCGCTGA
CCAGGTTNCACGGCTACTNCGATAAGCATATTCTACTGGGGGACTGTAAAGGAATTGTATCCACTCNG
CTTCCTGTCAATTGGCCATGTGCATCTCTGGTTGTTCAGGNNACATTAATAACACACAGNATTAAGTA
GATAAGTCTCCGATATAAGCGAACAGANCGTTGGTNAAAGACTGTGGTTCAATTGTAGTGGTATAACG
TTTCCCAGNGCATAAGGAACTCAGGGACACTACTANCATGGGCGAGGAGAGACCCTTGGGTGGCAANA
CGCAATAAGTGTTNNNTAGTACACCAGCACGATANANGGTTTGACCNTGNNTTTTTAGNNGACCCCGG
TCGCATTGAACCTGGCATTCAGAATGACGTGCTNTNAAGAGTCAAGNTCCTCCCACTGAGGGTCATCN
GCTTCGCGNGTCAGCGTATGGTCATAAACAT
>HaNhi318
GTGNAGCCCAGTCAGACACTTCTAAATCCTTNGTAACAGACATGCAGTCCCCTCNNCAGAGNCCCGCC
CCCCGATGGTACCGGGTTGTTAAGAGGTCCACAACGGNTCNNACGACANCNTTAAGGCCTCATGCATG
ANNCANCACGGGGGCGTCCCGGCGCGGCGACCTTGAAGTCCGGGNNCAAGTATGGCCCCCCGGGAAGG
ACGCNGTAGTGCATCTGGAGTGCCGATTNGTGGTCCNGAATGCCACCACGATGGTATGAGTGGCAAAG
GTACTTNGGACACCACCCCGTTTNCTANTNCCAGACGCGGCCNTTCAGGAACGACGCGAATCGTAATC
TACCTGCCGCGTACGCGGATNGTACANGCGTTTTCCAGCGTTCGAAATAGAGTTTGTCGTTCCGCTGA
CCNGGTTNCACGGCTACTGCGATAAGCATATTCTACTGGGGGACTGNAAAGGAATTGTATCCATTCNG

CTTCCTGTCAATTGGNCATGTGCATCTCTGGTTGTTCAGGNNACATTAATAACACACAGCAGNAAGTA
GGTAAGTCTCCGATATAAGCGAACAGANCGTTTGTNAGAGACTGTGGTTCAATTGTAGTGGTATAATG
TTTCCCAGTGCATAAGGAACTCAGGNACACTACTAGCATGGGCGAGGAGAGACCCTTGGGTGGCAANA
CGCAATAAGTGTTNCCTAGTACACCAGCACGAGANAGGGTTTGACCNTGTGTTTTTAGANGACCCCGG
TCGCATTGAACCTGGCATTCAGAATGACGTGCTNTCAAGAATCNAGNTCCTCNCACTGAGGGTCATCT
GCTTCGCGGGTCAGCGTATGGNCATAAACAT
>HaNhi319
GTGNAGCCCAGTCAGACACTTCTAAATCCTTGGTAACAGACATGCAGTCCCCTCNNCAGAGCCCCGCC
CCCCGATGGTACCGGGTTGTTNAGAGGTCCACAACGGGTCNNACGACNNCATTAAGGCCTCATGCATG
ANNCANCNCNGGGGNGTCCCGGCGCGGNGACCTTGAAGTCNGGGGNCAAGTATGGCCCCCCGGGAAGG
ACGCNGTAGTGCATCTGGAGTGCCGATTCGTGGTCCAGAATGCCACCACGATGGTATGAGTGGCAAAG
GTACTTTNNNCACCACCCCGTTNNCTANTTCCAGACGCGGCCNTTCAGGAACGACGCGAATCGTAATC
TACCTGCCGCGTNCGCGGATCGTACANGCGTTTTCCAGCGTTCGAAATAGAGTTTGTCGTTCCGCTGA
CCNGGTTNCACGGCTACTNCGATAAGCATATTCTACTGGGGGACTGTAAAGGAATTGTATCCATTCTG
CTTCCTGTCAATTGGCCATGTGCATCTCTGGTTGTTCAGGNNACATTAATAACACACAGCAGNAAGTA
GGTAAGTCTCCGATATAAGCGAACAGAACGTTTGTCAGAGACTGTGGTTCAATTGTAGTGGTATAATG
TTTCCCAGTGCATAAGGAACTCAGGGACACTACTAGCATGGGCGAGGAGAGACCCTTGGGTGGCAANA
CGNAATAAGTGTTNCCTAGTACACCAGCACGAGNNAGGGTTTGNCCATGNNTTTTTAGNNGACCCCGG
TCGCATTGAACCTGGCATTCAGAATGACGTGCTCTNAAGAATCAAGNTCCTCNCACTGAGGGTCATNN
GCTTCGCGGGTCNGCGTATGGTCATAAACAT
>HaNhi320
GTNTAGCCCAGTTANACACTNCTAAATCCNTGGTAACAGACATGCAGTCCCCTCNNCAGAGNNCCGCC
CCCCGGTGGTACCGGGTTGTTAAGAGGTCCACCACGGNTCNNACGACAACATCAAGGCCTCATGCATG
ACCCANNNCNGGGGNGTCCCGGCGNNGCGACCTTGNAGGCNGGGNNCAAGTATGGCCCCCCGGGAAGG
ACGCNGTAGTGCATCTGGAGTGCCGATTNGTGGTCCAGAATGNCACCACGATGGTATGAGTGGCAAAG
GTACTTNGGACACCACCCCGTTNNCTNNTTCCAGANGCGGCCNTTCAGGAACGACGCGAATCGTAATC
TACCTGCCGCGTNCGCGGATCGTACANGCGTTTTCCAGCGTTCGAAATAGAGTTTGTCGTTCCGCTGA
CCAGGTTNCACGGCTACTGCGATAAGCATATTCTACTGGGGGACTGTAAAGGAATTGTATCCACTCNG
CTTCCTGTCAATTGGCCATGTGCATCTCTNGTTGTTCAGGNNACATTAATAACACACAGCATNAAGTA
GATAAGTCNCCGATATAAGCGAACAGANCGTTGGTNAAANACTGTGGTTCAATTGTAGTGGTATAACG
TTTCCCAGTGCATAAGGAANTCAGGNACACTACTAGCATGGGCGAGGAGAGACCCTTGGGTGGCAANA
CGNAATAAGTGTTNCCTAGTACACCAGCACGATNNANGGTTTGACCNTGTGTTTTTAGNNGACCCCGG
TCGCATTGAACCTGGCATTCAGAATGACGTGCTCTCAAGAGTCNAGNTCCTCNCACTGAGGGTCANCN
GCTTCGCGNGTCNGCGTATGGNCATANAAAT
>HaNhi321
GTNTAGCCCAGTTAGACACTTNNAAATCCTTNGTAACAGACATGCAGTCCCCTCGGCAGAGCCCCGCC
CCCCGATGGTACCGGGTTGTTAAGNGGTCCACAACGGGTCNNACGACAACNTTAAGGCCTCATGCATG
ACCCANCNCNGGGGCGTCCCGGCGCGGCGACCTTGAAGTCCGGGNNCAAGTATGGCCCCCCGGGAAGG
ACGCNGTAGTGCATCTGGAGTGCCGATTCGTGGTCCNGAATGCCACCACGATGGTATGAGTGGCAAAG
GTACTTNGGNCACCACCCCGTTTTCTANTNCCAGACGCGGCCNTTCAGGAACGACGCGAATCGTAATC
TACCTGCCGCGTNCGCGGATCGTACNNGCGTTTTNCAGCGTTCGAAATAGAGTTTGTCGTTCCGCTGA
CCAGGTTTCACGGCTACTNCGATAAGCATATTCTACTGGGGGACTGTAAAGGAATTGTATCCACTCNG
CTTCCTGTCAATTGGCCATGTGCATCTCTNGTTGTTCAGGGTACATTAATAACACACAGCATNAAGTA
GATAAGTCTCCGATATAAGCGAACAGAACGTTGGTNAGAGACTATGGTTCAATTGTAGTGGTATAATG
TTTCCCAGTGCATAAGGAACTCAGGNACACTACTAGCANGGGCGAGGAGAGACCCTTGGGTGGCAANA
CGNGATAAGTGTTNCCTAGTACATCAGCACGAGNNAGGGTTTGACCANGTGTTTNTAGACGACCCCGG
TCGCATTGAACCTGGCATTCAGAATGACGTGCTCTCAAGAATCAAGNTCCTCCCACTGAGGGTCANNN
GCTTCGCGGGTCNGCGTATGGNCATAAACAT
>HaNhi322
GTNNAGCCCAGTTAGACACTTCTAAATCCNTNGTAACAGACATGCAGTCCCCTCNNCAGAGCCCCGCC
CCCCGATGGTACCGGGTTGTNAAGNGGTCCACAACGGNTCGNACGACNANATTAAGGCCTCATGCATG
ACCCANNACGGNGGNGTCCCGGCGNGGNGACCTTGAANTCCGGGGNCAAGTATGGCCCCCCGGGAAGG
ACGCCGTAGTGCATCTGGAGTGCCGANNNGTGGTCCAGANTGCCACCACGATGGTATGAGTNGCAAAG
GTACTTTGGACACCACCCCGTTNNCTANTTCCAGANGCGGCCATTCAGGAACGACGCGAATCGTAATC

TACCTGCCGCGTNCGCGGATNGTACANGCGTNTTNCAGCGTTCGAAATAGAGTTTGTCGTTCCGCTGA
CCAGGTTTCACGGCTACTNCGATAAGCATATTCTACTGGGGGACTGTAAAGGAATTGTATCCACTCNG
CTTCCTGTCAATTGGCCATGTGCATCTCTGGTTGTTCAGGNNACATTAATAACACACAGCATTAAGTA
GATNAGTCTCCGATATAAGCGAACAGANCGTTGGTCAGAGACTATGGTTCAATTGTAGTGGTATAATG
TTTCCCAGTGCATAAGGAACTCAGGGACACTACTAGCANGGGCGAGGAGAGACCCTTGGGTGGCAANA
CGCGATAAGTGTTNNCTAGTACATCAGCACGAGANANGGTTTGACCATNNNTTTNTAGNNGACCCCGG
TCGCATTGAACCTGGCATTCAGAATGACGTGCTCTNANGAATCAAGNTCCNCNCACTGAGGGTCANCT
GCTTCGCGGGTCAGCGTATGGTCATAAACAT
>HaNhi323
GTGTAGCCCAGTTAGACACTTCTAAATCCNTNGTAACAGACATGCAGTCCCCTCGGCAGAGCNCCGCC
CCCCGATGGTACCGGGTTGTTNAGAGGTCCACAACGGGTCNNACGACAANNTTAAGGCCTCANGCATG
ACCCANNACNGNGGNGTCCCGGCGNGGNGACCTTGNANTCCGGGNNCAAGTATGGCCCCCCGGGAAGG
ACGCCGTAGTGCATCTGGAGTGCCGATTCGTGGTCNAGANTGCCACCACGATGGTATGAGTNGCAAAG
GTACTTTNNNCACCACCCCGTTTTCTANTTCCAGACGCGGCCATTCAGGAACGACGCGAATCGTAATC
TACCTGCCGCGTNCGCGGATCGTACATGCGTTTTCCAGCGTTCGAAATAGAGTTTGTCGTTCCGCTGA
CCAGGTTNCACGGCTACTGCGATAAGCATATTCTACTGGGGGACTGTAAAGGAATTGTATCCACNCTG
CTTCCTGTCAATTGGCCATGTGCATCTCTGGTTGTTCAGGGTACATTAATAACACACAGCATNAAGTA
GATNAGTCTCCGATATAAGCGAACAGAACGTTGGTNAGAGACTATGGTTCAATTGTAGTGGTATAATG
TTTCCCAGTGCATAAGGAACTCAGGGACACTACTAGCATGGNCGAGGAGAGACCCTTGGGTGGCAANA
CGNAATAAGTGTTACCTAGTACATCAGCACGAGNNAGGGTTTGACCATGTGTTTNTAGNNGACCCCGG
TCGCATTGAACCTGNCNTTCAGAATGACGTGCTCTCAAGAATCAAGNTCCTNNCACTGAGGGNCATNN
GCTTCGCGGGTCNGCGTATGGTCANNANNNT
>HaNhi324
GTGTAGCCCAGTTAGACACNTCTAAATCCTTNGTAACAGACATGCAGTCCCCTCGGCAGAGCCCCGCC
CCCCNATGGTACCGGGTTGNTAAGAGGTCCACAACGGNTCNNACGACAACATTAAGGCCTCATGCATG
ANNCANCNCNGGGGNGTCCCGGCGNGGCGACCTTGNAGTCCGGGGNCAAGTATGGCCCCCCGGGAAGG
ACGCCGTAGTGCATCTGGAGTGCCGATTCGTGGTCCAGAATGCCACCACGATGGTATGAGTGGCAAAG
GTACTTNGNNCACCACCCCGTTNNCTANTNCCAGACGCGGCCNTTCAGGAACGACGCGAATCGTAATC
TACCTGCCGCGTNCGCGGATCGTACANGCGTTTTCCAGCGTTCGAAATAGAGTTTGTCGTTCCGCTGA
CCAGGTTNCACGGCTACTGCGATAAGCATATTCTACTGGGGGACTGTAAAGGAATTGTATCCACTCNG
CTTCCTGTCAATTGGCCATGTGCATCTCTNGTTGTTCAGGNNACATTAATAACACACAGCATTAAGTA
GATAAGTCTCCGATATAAGCGAACAGANCGTTGGTCAGAGACTGTGGTTCAATTGTAGTGGTATAATG
TTTCCCAGTGCATAAGGAACTCAGGNACACTACTAGCATGGGCGAGGAGAGACCCTTGGGTGGCAANA
CGCAATAAGTGTTNCCTAGTACACCAGCACGAGANANGGTTTGNCCANGNNTTTTTAGNNGACCCCGG
TCGCATTGAACCTGGCATTCAGAATGACGTGCTNTCAAGAATCAAGNTCCTCNCACTGAGGGTCATNN
GCTTCGCGGGTCNGCGTATGGTCATAANNAT
>HaNhi325
GTGNAGCCCAGTTAGACACTTCTAAATCCTTNGTAACAGACATGCAGTCCCCTCGGCAGAGNNCCGCC
CCCCGATGGTACCGGGTTGTTAAGAGGTCCACNACGGNTCNNACGACNNCATTAAGGCCTCATGCATG
ANNCNNNNCGGGNGNGTCCCGGNGNGGCGACCTTGNANTCNGGGNNCAAGTATGGCCCCCCGGGAAGG
ACGCNGTAGTGCATCTGGAGTGCCGATTNNTGGTCNNGAATGCCACCACGATGGTATGAGTGGCAAAG
GTACTTNGGACACCACCCCGTTNNCTANTTCCAGACGCGGCCNTTCAGGAACGACGCGAATCGTAATC
TANCTGCCGCGTNCGCGGATCGTACNNGCGTTTTCCAGCGTTCGAAATAGAGTTTGTCGTTCCGCTGA
CCNGGTTNCACGGCTACTGCGATAAGCATATTCTACTGGGGGACTGTAAAGGAATTGTATCCACTCNG
CTTCCTGTCAATTGGNCATGTGCATCTCTNGTTGTTCAGGNNACATTAATAACACACAGCATNAAGTA
GATAAGTCNCCGATATAAGCGAACAGANCGTTGGTNAGAGACTGTGGTTNNATTGTAGTGGTATAATG
TTTCCCAGNGCATAAGGAANTCAGGNACACTACTAGCANGGNCGAGGAGAGACCCTTGGGTGGCAANA
CGNAATAAGTGTTANCTAGTACACCAGCACGAGNNAGGGTTTGNCCNTGNNTTTTTAGNNGACCCCGG
TCGCATTGAACCTGGCATTCAGAATGACGTGCTCTCAAGAATCNAGNTCCTNNCACTGAGGGNCANCT
GCTTCNCGGGTCNGCGTATGGTCATAAACAT
>HaNhi326
GTNTAGCCCAGTTNGACACTNCTAAATCCTTNGTAACAGACATGCAGTCCCCTCGGCAGAGCNCCGCC
CCCCGATGGTACCGGGTTGNTAAGAGGTCCACAACGGNTCNNACGACNNCATTAAGGCCTNANGCATG
ANNCANCNCGGGGGCGTCCCGGCGCGGNGACCTTGAAGTCCGGGGNCAAGTATGGCCCCCCGNGAAGG

ACGCNGTAGTGCATCTGGAGTGCCGATTNNTGNNCCAGAATGNCACCACGATGGTATGAGTGGCAAAG
GTACTTNNNNCNCCACCCCGTTTNCTANTNCCAGACGCGGCCNTTCAGGAACGACGCGAATCGTANTC
NACCTGCCGCGTNCGCGGATNGTACANGCGTTTTNCAGCGTTCGAAATAGAGTTTGTCGTTCCGCTGA
CCNGGTTNCACGGCTACTGCGATAAGCATATTCTACTGGGGGACTGNAAAGNAATTGTATCNACNCNG
CTTCCTGTCAATTGGNCATGTGCATCTCTNGTTGTTCAGGNNACATTAATAACACACAGNNTNAAGTA
GATNAGTCNCCGATATAAGCGAACAGANCGTTGGTNAGAGACTGTGGTTCAATTGTAGTGGTATAATG
TTTCCCAGTGCATAAGGAACTCAGGNACACTACTAGCANGGGCGAGGAGAGACCCTTGGGTGGCAANA
CGNAATAAGTGTTNNNTAGTACACCAGCACGAGNNANGGTTTGACCANNNNTTTNTAGNNGACCCCGG
TCGCATTGAACCNGGCATTCAGAATGACGTNCTNTNAAGAATCAAGNTCCTNNCACTGAGGGTCATNN
GCTTCGCGGGTCNGCGTATGGNCATAAACAT
>HaNhi327
GTGTAGCCCAGTTAGACACTTCTAAATCCTTNGTAACAGACATGCAGTCCCCTCNNCAGAGCNCCGCC
CCCCGGTGGTACCGGGTTGTTAAGAGGTCCACCACGGGTCGNACGACAACATCAAGGCCTCATGCATG
ANNCANCACGGGGGCGTCCCGGCGNGGCGACCTTGNAGGCCGGGGNCAAGTATGGCCCCCCGGGAAGG
ACGCCGTAGTGCATCTGGAGTGCCGATTCGTGGTCCAGAATGNCACCACGATGGTATGAGTGGCAAAG
GTACTTNGGACACCACCCCGTTTTCTANTTCCAGANGCGGCCATTCAGGAACGACGCGAATCGTAATC
TANCTGCCGCGTACGCGGATNGTACANGCGTNTTCCANCGTTCGAAATAGAGTTTGTCGTTCCGCTGA
CCAGGTTNCACGGCTACTGCGATAAGCATATTCTACTGGGGGACTGTAAAGGAATTGTATCCACTCNG
CTTCCTGTCAATTGGNCATGTGCATCTCTGGTTGTTCAGGNNACATTAATAACACACAGCATTAAGTA
GATNAGTCTCCGATATAAGCGAACAGANCGTTGGTCAAAGACTGTGGTTCAATTGTAGTGGTATAACG
TTTCCCAGTGCATAAGGAACTCAGGNACACTACTAGCANGGGCGAGGAGAGACCCTTGGGTGGCAANA
CGNAATAAGTGTTNNCTAGTACACCAGCACGATNNAGGGTTTGACCATGTGTTTTTAGACGACCCCGG
TCGCATTGAACCTGGCATTCAGAATGACGTGCTCTCAAGAGTCAAGGTCCNCNCACTGAGGGTCATCT
GCTTCGCGNGTCNGCGTATGGTCATAAACAT
>HaNhi328
GTGNAGCCCAGTTANACACTTCTAAATCCNTGGTAACAGACATGCAGTCCCCTCGGCAGAGCCCCGCC
CCCCNATGGTACCGGGTTGTTAAGAGGTCCACAACGGGTCNNACGACAACATTAAGGCCTCATGCATG
ACCCANNNCGGNGGCGTCCCGGCGCGGCGACCTTGAAGTCCGGGGNCAAGTATGGCCCCCCGGGAAGG
ACGCNGTAGTGCATCTGGAGTGCCGATTCNTGGTCCAGAATGCCACCACGATGGTATGAGTGGCAAAG
GTACTTTGGACACCACCCCGTTTNCTANTTCCAGACGCGGCCATTCAGGAACGACGCGAATCGTAATC
TACCTGCCGCGTACGCGGATCGTACNNGCGTTTTNCAGCGTTCGAAATAGAGTTTGTCGTTCCGCTGA
CCNGGTTNCACGGCTACTGCGATAAGCATATTCTACTGGGGGACTGTAAAGGAATTGTATCCACTCNG
CTTCCTGTCAATTGGNCATGTGCATCTCTGGTTGTTCAGGNNACATTAATAACACACAGNATNAAGTA
GATAAGTCTCCGATATAAGCGAACAGAACGTTGGTCAGANACTATGGTTCAATTGTAGTGGTATAATG
TTTCCCAGTGCATAAGGAACTCAGGGACACTACTAGCANGGNCGAGGAGAGACCCTTGGGTGGCAANA
CGCAATAAGTGTTNNNTAGTACATCAGCACGAGNNAGGGTTTGACCNTGTGTTTTTAGNNGACCCCGG
TCGCATTGAACCTGGCATTCAGAATGACGTGCTCTNAAGAATCAAGGTCCTCNCACTGAGGGTCATNN
GCTTCGCGGGTCNGCGTATGGTCATAANNAT
>HaNhi329
GTGTAGCCCAGTTNGACACTTCTAAATCCNTNGTAACAGACATGCAGTCCCCTCGGCAGAGNCCCGCC
CCCCNGTGGTACCGGGTTGTNAAGAGGTCCACCACGGNTCNNACGACAACNTCAAGGCCTNANGCATG
ANNCANCNCGGGGGNGTCCCGGCGCGGCGACCTTGNAGGCNGGGGNCAAGTATGGCCCCCCGGGAAGG
ACGCNGTAGTGCATCTGGAGTGCCGATTCGTGGTCNAGAATGCCACCACGATGGTATGAGTGGCAAAG
GTACTTTGGACACCACCCCGTTNNCTANTNCCAGANGCGGCCNTTCAGGAACGACGCGAATCGTAATC
TACCTGCCGCGTACGCGGATCGTACANGCGTTTTCCAGCGTTCGAAATAGAGTTTGTCGTTCCGCTGA
CCNGGTTNCACGGCTACTGCGATAAGCATATTCTACTGNGGGACTGTAAAGGAATTGTATCCACTCNG
CTTCCTGTCAATTGGCCATGTGCATCTCTNGTTGTTCAGGNNACATTAATAACACACAGCATNAAGTA
GATNAGTCTCCGATATAAGCGAACAGANCGTTGGTNAAAGACTGTGGTTCAATTGTAGTGGTATAACG
TTTCCCAGTGCATAAGGAACTCAGGNACACTACTAGCANGGGCGAGGAGAGACCCTTGGGTGGCANNA
CGCAATAAGTGTTANNTAGTACACCAGCACGATNNANGGTTTGNCCNNGNNTTTTTAGNNGACCCCGG
TCGCATTGAACCTGGCATTCANAATGACGTNCTCTNANGAGTNAAGNTCCNCNCACTGAGGGTCATCN
GCTTCGCGNGTCAGCGTATNGTCATAAACAT
>HaNhi330
GTGNAGCCCAGTCNGACACTTCTAAATCCNTGGTAACAGACATGCAGTCCCCTCGGCAGAGCNCCGCC

CCCCGATGGTACCGGGTTGNTAAGAGGTCCACAACGGNTCNNACGACAANNTTAAGGCCTCATGCATG
ACCCANCNCNGGGGCGTCCCGGCGNGGNGACCTTGNANTCNGGGNNCAAGTATGGCCCCCCGNGAAGG
ACGCNGTAGTGCATCTGGAGTGCCGATTNGTGGTCCNGAATGCCACCACGATGGTATGAGTGGCAANG
GTACTTTGGACACCACCCCGTTNNCTNNTNCCAGACGCGGCCNTTCAGGAACGACGCGAATCGTAATC
TACCTGCCGCGTNCGCGGATNGTACANGCGTNTTCCAGCGTTCGAAATAGAGTTTGTCGTTCCGCTGA
CCAGGTTNCACGGCTACTGCGATAAGCATATTCTACTGGGGGACTGTAAAGGAATTGTATCCATTCNG
CTTCCTGTCAATTGGNCATGTGCATCTCTNGTTGTTCAGGGNACATTAATAACACACAGNAGNAAGTA
GGTNAGTCNCCGATATAAGCGAACAGANCGTTTGTNAGAGACTGTGGTTCAATTGTAGTGGTATAATG
TTTCCCAGTGCATAAGGAACTCAGGNACACTACTAGCATGGGCGAGGAGAGACCCTTGGGTGGCAANA
CGNAATAAGTGTTNNCTAGTACACCAGCACGAGNNAGGGTTTGNCCATGTGTTTNTAGACGACCCCGG
TCGCATTGAACCTGNCATTCAGAATGANGTNCTCTCAAGAATNNAGNTCCNCNCACTGAGGGTCATCT
GCTTCGCGGGTCAGCGTATNGTCATAAACAT
>HaNhi331
GTGTAGCCCAGTCAGACACTTCTAAATCCNTGGTAACAGACATGCAGTCCCCTCNNCAGAGCCCCGCC
CCCCNATGGTACCGGGTTGTTAAGAGGTCCACAACGGGTCGCACGACAANATTAAGGCCTCATGCATG
ACCCANCNCGGNGGNGTCCCGGCGCGGCGACCTTGAAGTCCGGGGNCAAGTATGGCCCCCCGGGAAGG
ACGCNGTAGTGCATCTGGAGTGCCGATTNNTGGTCCNGANTGCCACCACGATGGTATGAGTGGCAAAG
GTACTTNGGACACCACCCCGTTTTCTATTTCCAGACGCGGCCNTTCAGGAACGACGCGAATCGTAATC
TANCTGCCGCGTNCGCGGATCGTACNTGCGTTTTCCAGCGTTCGAAATAGAGTTTGTCGTTCCGCTGA
CCAGGTTTCACGGCTACTGCGATAAGCATATTCTACTGGGGGACTGTAAAGGAATTGTATCCATTCTG
CTTCCTGTCAATTGGNCATGTGCATCTCTGGTTGTTCAGGNNACATTAATAACACACAGCAGTAAGTA
GGTNAGTCTCCGATATAAGCGAACAGANCGTTTGTCAGAGACTGTGGTTCAATTGTAGTGGTATAATG
TTTCCCAGTGCATAAGGAACTCAGGGACACTACTANCATGGGCGAGGAGAGACCCTTGGGTGGCAACA
CGCAATAAGTGTTNNCTAGTACACCAGCACGAGANAGGGTTTGNCCATGTGTTTNTAGNNGACCCCGG
TCGCATTGAACCTGGCATTCAGAATGACGTGCTCTCAAGAATCNAGNTCCTCNCACTGAGGGTCATCT
GCTTCGCGGGTCNGCGTATNGTCATAAACAT
>HaNhi332
GNNTAGCCCAGTCAGACACTTCTAAATCCTTGGTAACAGACATGCAGTCCCCTCGGCAGAGCCCCGCC
CCCCGATGGTACCGGGTTGTNAAGAGGTCCACAACGGNTCGCACGACAACATTAAGGCCTCATGCATG
ACCCAACNCGGGGGNGTCCCGGCGNGGCGACCTTGAAGTCNGGGGNCAAGTATGGCCCCCCGGGAAGG
ACGCNGTAGTGCATCTGGAGTGCCGATTCNTGGTCCAGAATGCCACCACGATGGTATGAGTNGCAAAG
GTACTTNGGNCACCACCCCGTTTNCTNNTTCCAGANGCGGCCNTTCAGGAACGACGCGAATCGTAATC
TACCTGCCGCGTNCGCGGATCGTACNTGCGTTTNNCAGCGTTCGAAATAGAGTTTGTCGTTCCGCTGA
CCAGGTTNCACGGCTACTGCGATAAGCATATTCTACTGGGGGACTGTAAAGGAATTGTATCCATTCTG
CTTCCTGNCAATTGGNCATGTGCATCTCTNGTTGTTCAGGGTACATTAATAACACACAGCAGNAAGTA
GGTNAGTCTCCGATATAAGCGAACAGANCGTTTGTNAGAGACTGTGGTTCAATTGTAGTGGTATAATG
TTTCCCAGTGCATAAGGAACTCAGGNACACTACTAGCANGGGCGAGGAGAGACCCTTGGGTGGCAANA
CGNAANAAGTGTTNNCTAGTACACCAGCACGAGNNAGGGTTTGACCANGNNTTTTTAGNNGACCCCGG
TCGCATTGAACCTGGCATTCAGAATGACGTGCTNTCANGAATCAAGNTCCNNNCACTGAGGGNCANCT
NCTTCGCGGGTCNGCGTATNGTCATAAACAT
>HaNhi333
GTGTAGCTCAGTTAGACACTTCTAAATCCNTGGTAACAGACATGCAGTCCCCTCGGCAGAGCCCCGCC
CCCCGGTGGTACCGGGTTGTTAAGAGGTCCACCACGGNTCGNACGACAACATCAAGGCCTCANGCATG
ACCCANCNCGGGGANGTACCGGCGCGGCGACCTTGAAGGTCGGGGNCAAGTATGTCCCCCCGGGAAGG
ACGCNGTAGTGCATCTGGAGTGCCGATTCGTGGTCCNGAATGNCACCACGATGGTATGAGTNGCAAAG
GTATTTTGGNCACCACCCCGTTTNCTANTTCCAGACGCGGCCNTTCAGGAACGACGCGAATCGTAATC
TACCTGCCGCGTACGCGGATNGTACANGCGTTTTTCAGCGTTCGAAATAGAGTTTGTCGTTCCGCTGA
CCNGGTTNCACGGCTACTNCGATAAGCATATTCTACGGGGGGACTGTAAAGGAATTGTATCCACTCTG
CTCCCTGTCAATTGGNCATGTGCATCTCTGGTTGTTTAGGGTACATTAATAACACACAGCATTAGGTA
GATNAGTCTCCGATATAAGCGAACAGANCGTTGGTCAAAGACCGTGGTTCAATTGTAGTGGTATAACA
TTTCCCAGTGCATAAGGAACCCAGGNACACTACTAGCATGGGCGAGGAGAGACCCTTGGGTGGCAANA
CGNAATAAGTGTTNCCCAGTACACCAGCACGATNGAGGGTTTGACCNNNNNTTTTTAGNNGACCCCGG
TCGCATTGAACCTGGCATTCAGAATGACGTGCTCTCAAGAGTCAAGNTCCTCNCACTGAGGGTCANCT
GCTTCGCGNNNTNGCGTATGGTCATAAACAT

>HaNhi334
GTGTAGCCCAGTCAGACACTTCTAAATCCTTGGTAACAGACATGCAGTCCCCTCGGCAGAGCCCCGCC
CCCCNATGGTACCGGGTTGTTAAGAGGTCCACAACGGGTCNCACGACAACATTAAGGCCTCANGCATG
ACCCANCNCGGGGGCGTCCCGGCGCGGCGACCTTGAANTCCGGGGNCAAGTATGGCCCCCCGGGAAGG
ACGCNGTAGTGCATCTGGAGTGCCGATTCNTGGTCCAGAATGCCACCACGATGGTATGAGTGGCAAAG
GTACTTTGGACACCACCCCGTTNNCTANTTCCAGACGCGGCCATTCAGGAACGACGCGAATCGTAATC
TACCTGCCGCGTNCGCGGATCGTACATGCGTTTTCCAGCGTTCGAAATAGAGTTTGTCGTTCCGCTGA
CCAGGTTNCACGGCTACTGCGATAAGCATATTCTACTGGGGGACTGTAAAGGAATTGTATCCATTCNG
CTTCCTGTCAATTGGCCATGTGCATCTCTGGTTGTTCAGGNNACATTAATAACACACAGCAGNAAGTA
GGTAAGTCTCCGATATAAGCGAACAGANCGTTTGTCAGAGACTGTGGTTCAATTGTAGTGGTATAATG
TTTCCCAGTGCATAAGGAACTCAGGGACACTACTAGCATGGGCGAGGAGAGACCCTTGGGTGGCAANA
CGCAATAAGTGTTNCCTAGTACACCAGCACGAGNNAGGGTTTGACCATGTGTTTTTAGACGACCCCGG
TCGCATTGAACCTGGCATTCAGAATGACGTGCTCTNAAGAATCAAGGTCCTCCCACTGAGGGTCATCT
GCTTCGCGGGTCNGCGTATGGTCACANACAT
>HaNhi335
GTGTAGCTCAGTTAGACACTTCTAAATCCTTNGTAACAGACATGCAGTCCCCTCNNCAGAGCCCCGCC
CCCCGGTGGTACCGGGTTGTTAAGAGGTCCACCACGGGTCNNACGACAACATCAAGGCCTCANGCATG
ANNCAANNCGGGGACGTACCGGCGCGGCGACCTTGAAGGTCGGGGNCAAGTATGTCCCCCCGGGAAGG
ACGCNGTAGTGCATCTGGAGTGCCGATTCNTGGTCCAGAATGCCACCACGATGGTATGAGTGGCAAAG
GTATTTNGGACACCACCCCGTTTTCTANTTCCAGACGCGGCCATTCAGGAACGACGCGAATCGTAATC
TACCTGCCGCGTACGCGGATCGTACATGCGTTTTTCAGCGTTCGAAATAGAGTTTGTCGTTCCGCTGA
CCNGGTTNCACGGCTACTGCGATAAGCATATTCTACGGGGGGACTGTAAAGGAATTGTATCCACTCNG
CTCCCTGTCAATTGGNCATGTGCATCTCTGGTTGTTTAGGGTACATTAATAACACACAGNATNAGGTA
GATNAGTCNCCGATATAAGCGAACAGANCGTTGGTCAAAGACCGTGGTTCAATTGTAGTGGTATAACA
TTTCCCAGTGCATAAGGAACCCAGGNACACTACTANCATGGGCGAGGAGAGACCCTTGGGTGGCAANA
CGCAATAAGTGTTANCCAGTACACCAGCACGATNNAGGGTTTGNCCANGTNTTTTTAGACGACCCCGG
TCGCATTGAACCTGGCATTCAGAATGACGTGCTCTNAAGAGTCAAGNTCCTCNCACTGAGGGTCATCT
GCTTCGCGNNNTAGCGTATNGTCATAAACAT
>HaNhi336
GTGTANCCCAGTTNGACACTNCTAAATCCNTNGTAACAGACATGCAGTCCCCTCNNCAGAGCCCCGCC
CCCCNATGGTACCGGGTTGTTNAGAGGTCCACCACGGNTCNNACGACAACATTAAGGCCTNANGCATG
ANNCANNNCNGNGGNGTCCCGGNGNGGCGACCTTGAAGTCNGGNNNCAAGTATGGCCCCCCGNGAAGG
ACGCNGTAGTGCATCTGGAGTGCCGATTNNTGGTNNNGAATGNCACCACGANGGTATGAGTGGCAANG
GTACTTNGGACNCNACCCNGTTNNCTANTTCCAGACGCGGCCATTCAGGAACGACGCGANTCGTNATC
TACCTGCCGCGTNCGCGGATCGTANNNACGTTTTNCAGCGTTCGAAATAGAGTTTGTCGTTCCGCTGA
CNNGGTTNCACGGCTACTGCGATAAGCATATTCTACTGGGGGACTGTAAAGNAATTGTATCCACNCNG
CTTCCTGTCAATTGGNCATGTGCATCTCTNGTTGTTCAGGNNACATTAATAACACACAGCNTNAAGTA
GATNAGTCTCCGATATAAGCGAACAGANCGTTGGTNAGAGACTGTGGTTCAATTGTAGTGGTATAATG
TTTCCCAGNGCATAAGGAACTCAGGGACACTACTANCANGGNCGAGGAGAGACCCTTGGGTGGNANNA
CGNAATAAGTGTTANCTAGTACACCAGCACGAGNNAGGGTTTGNCCNNNNNTTTNTAGNNGACCCNGG
TCGCATTGAACCTGGCATNCANAATGACGTGCTNTNANGAGTCAAGNTCCTNNCACTGAGGGTCATCN
GCTTCGCGGNNCAGCGTATNGNCATAAACAT
>HaNhi337
GTGNANCCCAGTCNNACACTNCTAAATCCNTNGTAACAGACATGCAGTCCCCTCNGCAGAGNCCCGCN
CCCCNATGGTACCGGGTTGNTNAGNGGTCCACAACGGGTCNNACGACNNCATTAAGGCCNNATGCATG
ANNCNNNNCNGNGGNGTCCCGGNGNGGNGACCTTGNANTCCGGGNNCAAGTATGGCCCCCCGGGAAGG
ACGCNGTAGTGCATCTGGANTGCCGATTCNTGGTCNAGANTGCCACCACGATGGTATGAGTGGCAAAG
GTACTTNGGNCACCACCCCGTTNNCTANNNCCAGANGCGGCCNTTCANGAACGANGCGAATCGTANTC
TACCTGCCGCGTNCGCGGATCGTACNNGCGTTTTNCAGCGTTCGAAATAGAGTTTGTCGTTCCGCTGA
CCAGGTTNCACGGCNANTNCGATAAGCATATTCTACTNGGGGACTGNAAAGNAATTGTATCCATTCNG
CTTCCTNNCAATTGGCCATGTGCATCTCTNGTTGTTCAGGNNACATTAATAACACACAGCNGNAAGTA
GGTNAGTCTCCGATATAAGCGAACAGANCGTTTGTNAGAGACTGTGGTTCAATTGTAGTGGTANAATG
TTTCCCAGTGCATAAGGAACTCAGGNACACTACTAGCANGGGCGAGGAGAGACCCTTGGGTGGNAANA
CGNAATAAGTGTTNNNTAGTACACCAGCACGAGNNANGGTTNGNCNNNGNNTTNNTAGNNGACCCCGG

TCGCATTGAACCTGNCNTTCANAATGACGTGCTCTNANGAATCNAGNTCCTCCCACTGAGGGNCATCT
GCTTCGCGGNNCNGCGTATNGNCATAAACAT
>HaNhi338
GTNTAGCTCAGTTANACACTTCTAAATCCTTNGTAACAGACATGCAGTCCCCTCGGCAGAGCCCCGCC
CCCCGGTGGTACCGGGTTGTTAAGAGGTCCACNACGGGTCNCACGACAANATCAAGGCCTNATGCATG
ANNCANNNCNGGGANGTACCGGNGNGGNGACCTTGAAGGTCGGGGNCAAGTATGTCCCCCCGNGAAGG
ACGCNGTAGTGCATCTGGANTGCCGATTCNTGGTCNNGAATGCCACCACGATGGTATGAGTGGCAAAG
GTATTTNNNNCACCACCCCGTTNNCTNNTTCCAGANNCGGCCNTNCANGAACGACGCGAATCGTAATC
TACCTGCCGCGTNCGCGGATCGTACANGCGTTTTNCAGNGTTCGAAATAGAGTTTGTCGNNCCGCTGA
CCNGGTTNCACGGCTACTNCGATAAGCATATTCTACGGGGGGACTGTAAAGGAATTGTATCCACTCNG
CTCCCTGTCAATTGGCCATGTGCATCTCTNGTTGTTTAGGNNANATTAATAACACACAGCATNAGGTA
GATNAGTCNCCNATATAAGCGAACAGAACGTTGGTNAANGACCGTGGTTCAATTGTAGTGGTATAACA
TTTCCCAGTGCATAAGGAACCCAGGNACACTACTAGCANGGGCGAGGAGAGACCCTTGGGTGGCANNA
CGNAATAAGTGTTNNNCAGTACACCAGCACGATANAGGGTTTGNCNNNNNNTTTNTAGNNGACCCCGG
TCGCATTGAACCTGGCATTCAGAATGACGTNCTNTNAAGAGTNNAGNNCCNCNCACTGAGGGTCANNN
GCTTCGCGNNNTNGCGTATNGTCATAANCAT
>HaNhi339
GTGTAGCCCAGTTAGACACTTCTAAATCCTTGGTAACAGACATGCAGTCCCCTCNGCAGAGCNCCGCC
CCCCGATGGTACCGGGTTGTTAAGAGGTCCACCACGGGTCGCACGACANCNTTAAGGCCTCATGCATG
ACCCANCACGGNGGNGTCCCGGCGCGGCGACCTTGAAGTCCGGGGNCAAGTATGGCCCCCCGGGAAGG
ACGCNGTAGTGCATCTGGAGTGCCGATTCGTGGTCCNGANTGCCACCACGATGGTATGAGTGGCAAAG
GTACTTTGGACACCACCCCGTTTTCTANTTCCAGACGCGGCCNTTCAGGAACGACGCGAATCGTAATC
TACCTGCCGCGTACGCGGATCGTATANACGTTTTNCAGCGTTCGAAATAGAGTTTGTCGTTCCGCTGA
CCAGGTTNCACGGCTACTGCGATAAGCATATTCTACTGGGGGACTGTAAAGGAATTGTATCCACTCTG
CTTCCTGTCAATTGGCCATGTGCATCTCTNGTTGTTCAGGGTACATTAATAACACACAGCATTAAGTA
GATAAGTCTCCGATATAAGCGAACAGANCGTTGGTCAGAGACTGTGGTTCAATTGTAGTGGTATAATG
TTTCCCAGTGCATAAGGAACTCAGGNACACTACTAGCANGGGCGAGGAGAGACCCTTGGGTGGCAANA
CGCAATAAGTGTTACCTAGTACACCAGCACGAGANANGGTTTGNCCATGTGTTTTTAGANGACCCCGG
TCGCATTGAACCTGGCATTCAGAATGACGTGCTCTCAAGAGTCAAGNTCCTCNCACTGAGGGNCATCN
GCTTCGCGGGTCNGCGTATGGNCATAAACAT
>HaNhi340
GTGNAGCCCAGTTAGACACTTCTAAATCCNTNGTAACAGACATGCAGTCCCCTCGGCAGAGCCCCGCC
CCCCGGTGGTACCGGGTTGTTNAGAGGTCCACCACGGGTCGNACGACAACATCAAGGCCTCANGCATG
ANNCAANNCNGGGGNGTCCCGGCGCGGCGACCTTGAAGNCNGGGNNCAAGTATGGCCCCCCGNGAAGG
ACGCNGTAGTGCATCTGGAGTGCCGATTNNTGGTCNAGAATGCCACCACGATGGTATGAGTGGCAAAG
GTACTTNGGACACCACCCCGTTTTCTANTTCCAGACGCGGCCATTCAGGAACGACGCGAATCGTAATC
TACCTGCCGCGTNCGCGGATNGTANANGCGTNTTNCAGCGTTCGAAATAGAGTTTGTCGTTCCGCTGA
CCAGGTTNCACGGCTANTGCGATAAGCATATTCTACTGGGGGACTGTAAAGGAATTGTATCNACTCNG
CTTCCTGTCAATTGGNCATGTGCATCTCTNGTTGTTCAGGNNACATTAATAACACACAGCATNAAGTA
GATAAGTCTCCGATATAAGCGAACAGANCGTTGGTCAAAGACTGTGGTTCAATTGTAGTGGTATAACG
TTTCCCAGTGCATAAGGAACTCAGGGACACTACTAGCATGGNCGAGGAGAGACCCTTGGGTGGCAANA
CGNAATAAGTGTTNNNTAGTACACCAGCACGATANAGGGTTTGACCNTGTGTTTTTAGACGACCCCGG
TCGCATTGAACCTGGCATTCAGAATGACGTGCTCTNANNAGTCAAGNTCCNCCCACTGAGGGTCANCT
GCTTCGCGNGTCAGCGTATNGTCATAAACAT
>HaNhi341
GTGTAGCCCAGTCNGACACTNCTAAATCCNTGGTAACAGACATGCAGTCCCCTCNNCAGAGCNCCGCC
CCCCNATGGTACCGGGTTGTTAAGAGGTCCACAACGGNTCGNACGACAACATTAAGGCCTCATGCATG
ANNCANNNCNGGGGNGTCCCGGCGCGGNGACCTTGAAGTCNGGGNNCAAGTATGGCCCCCCGNGAAGG
ACGCNGTAGTGCATCTGGAGTGCCGATTNNTGGTCNAGANTGNCACCACGATGGTATGAGTGGCAAAG
GTACTTNGGACACCACCCCGTTTTCTANTTCCAGANGCGGCCATTCAGGAACGACGCGAATCGTAATC
TACCTGCCGCGTNCGCGGATCGTACNNGCGTTTTNCAGCGTTCGANATAGAGTTTGTCGTTCCGCTGA
CCNGGTTTCACGGCTACTNCGATAAGCATATTCTACTGGGGGACTGTAAAGNAATTGTATCNATTCNG
CTTCCTGTCAATTGGNCATGTGCATCTCTNGTTGTTCAGGNNACATTAATAACACACAGNAGNAAGTA
GGTNAGTCTCCGATATAAGCGAACAGANCGTTTGTNAGAGACTGTGGTTCAATTGTAGTGGTATAANG

TTTCCCAGTGCATAAGGAACTCAGGNACACTACTANCANGGGCGAGGAGAGACCCTTGGGTGGCANNA
CGNAATAAGTGTTNNCTAGTACACCAGCACGAGANAGGGTTTGNCCATGNNTTTTAGNNGACCCCGG
TCGCATTGAACCTGGCATTCAGAATGACGTGCTNTNAAGAATCAAGNTCCTCNCACTGAGGGTCATNN
GCTTCGCGGNTCNGCGTATGGTCATAANNAT
>HaNhi44
GTNNAGCTCAGTTAGACACTTCTAAATCCTTGGTAACAGACATGCAGTCCCCTCNNCAGAGCCCCGCC
CCCCGGTGGTACCGGGTTGTTAAGAGGTCCACCACGGNTCNNACGACAACATCAAGGCCTCATGCATG
ANNCANNNCNGGNACGTACCGGCGCGGNGACCTTGNAGGTCGGGGNCAAGTATGTCCCCCCGNGAAGG
ACGCNGTAGTGCATCTGGAGTGCCGATTCNTGGTCCNGAATGCCACCACGATGGTATGAGTGGCAAAG
GTATTTNGGACACNACCCCGTTNNCTATTNCCAGANGCGGCCNTTCAGGNACGACGCGAATCGTAATC
TACCTGCCGCGTNCGCGGATCGTACANGCGTTTTTCAGCGTTCGAAATAGAGTTTGTCGTTCCGCTGA
CCAGGTTNCACGGCTACTGCGATAAGCANATTCTACGGGGGGACTGNAAAGGAATTGTATCCACTCNG
CTCCCTGTCAATTGGNCATGTGCATCTCTNGTTGTTTAGGGTACATTAATAACACACAGCATNAGGTA
GATAAGTCNCCGATATAAGCGAACAGANCGTTGGTNAAAGACCGTGGTTCAATTGTAGTGGTATAACA
TTTCCCAGTGCATAAGGAACCCAGGNACACTACNANCANGGGCGAGGAGAGACCCTTGGGTGGCAANA
CGNAATAAGTGTTNNCCAGTACACCAGCACGATNNAGGGTTTGNCCNNGTGTTTTTAGNNGNCCCCGG
TCGCATTGAACCTGGCATTCAGAATGACGTGCTCTNAAGAGTCAAGNTCCTNNCACTGAGGGTCATCT
GCTTCNCGNNNTNGCGTATGGTCATAAACAT
>HaNhi45
GTNNAGCCCAGTCANACACTTCTAAATCCNTNGTAACAGACATGCAGTCCCCTCGGCAGAGCCCCGCC
CCCCNATGGTACCGGGTTGTTNAGAGGTCCACAACGGNTCNNACGACNANATTAAGGCCTNATGCATG
ACCCANNNCNGGGGCGTCCCGGCGCGGCGACCTTGAAGTCCGGGGNCAAGTATGGCCCCCCGGGAAGG
ACGCNGTAGTGCATCTGGAGTGCCGATTCNTGGTCNNGAATGNCACCACGATGGTATGAGTGGCAAAG
GTACTTNGGACACCACCCCGTTNNCTANTTCCAGACGCGGCCATTCAGGAACGACGCGAATCGTAATC
TANCTGCCGCGTNCGCGGATCGTACANGCGTTTTCCAGCGTTCGAAATAGAGTTTGTCGTTCCGCTGA
CCAGGTTNCACGGCTACTGCGATAAGCATATTCTACTGGGGGACTGTAAAGGAATTGTATCCATTCNG
CTTCCTGTCAATTGGNCATGTGCATCTCTNGTTGTTCAGGGTACATTAATAACACACAGCAGTAAGTA
GGTAAGTCTCCGATATAAGCGAACAGANCGTTTGTCAGAGACTGTGGTTCAATTGTAGTGGTATAATG
TTTCCCAGTGCATAAGGAACTCAGGGACACTACTAGCANGGGCGAGGAGAGACCCTTGGGTGGNAANA
CGCAATAAGTGTTNNCTAGTACACCAGCACGAGNNANGGTTTGNCCANGTGTTTTTAGNNGACCCCGG
TCGCATTGAACCTGGCATTCAGAATGACGTGCTCTNAAGAATCNAGNTCCTCNCACTGAGGGNCATCN
GCTTCGCGGGTCAGCGTATNGTCATAAAAAT
>HaNhi46
GNGTAGCCCAGTTAGACACTNCTNAATCCNTGGTAACAGACATGCAGTCCCCTCGGCAGAGCCCCGCC
CCCCNATGGTACCGGGTTGTTAAGAGGTCCACCACGGGTCNNACGACAACNTTAAGGCCTCATGCATG
ANNCANNNCNGGGGNGTCCCGGCGNGGCGACCTTGNANTCCGGGNNCAAGTATGGCCCCCCGGGAAGG
ACGCNGTAGTGCATCTGGAGTGCCGATTNNTGGTCNAGAATGCCACCACGATGGTATGAGTNGCAAAG
GTACTTNGGNCACCACCCCGTTTNCTANTTCCAGANGCGGCCATTCAGGAACGACGCGAATCGTAATC
TACCTGCCGCGTNCGCGGATCGTATATACGTTTTCCAGCGTTCGAAATAGAGTTTGTCGTTCCGCTGA
CCNGGTTNCACGGCTACTNNGATAAGCATATTCTACTGGGGGACTGTAAAGNAATTGTATCCACTCNG
CTTCCTGTCAATTGGCCATGTGCATCTCTGGTTGTTCAGGNNACATTAATAACACACAGCATNAANTA
GNTAAGTCTCCGATATAAGCGAACAGANCGTTGGTNAGAGACTGTGGTTCAATTGTAGTGGTATAATG
TTTCCCAGTGCATAAGGAACTCAGGNACACTACTANCATGGNCGAGGAGAGACCCTTGGGTGGCAANA
CGNAATAAGTGTTACCTAGTACACCAGCACGAGNNAGGGTTTGNCCNTGNNTTTTAGNNGNCCCCGG
TCGCATTGAACCTGGCATTCANAATGACGTNCTNTCAAGAGTCNAGNTCCTCNCACTGAGGGNCATCN
GCTTCGCGGNTCAGCGTATNGNCATAAACAT
>HMong194
GTNNAGCTCAGTTANACACTTCTAAATCCNTGGTAACAGACATGCAGTCCCCTCGGCAGAGNNCCGCC
CCCCNGTGGTACCGGGTTGNTNAGNGGTCCACCACGGNTCNNACGACANCATCAAGGCCTNANGCATG
ANNCANNNCNGNGANGTACCGGNGCGGCGACCTTGNANGTCGGGGNCAAGTATGTCCCCCCGGGAAGG
ACGCCGTAGTGCATCTGGANTGCCGATTCNTGGTCCAGAATGNCACCACGATGGTATGAGTGGCAAAG
GTATTTTGGACACCACCCCGTTNNCTANTTCCAGACGCGGCCNTTCAGGAACGACGCGAATCGTAATC
TACCTGCCGCGTNCGCGGATNGTACANGCGTTTTNCAGCGTTCGAAATAGAGTTTGTCGTTCCGCTGA
CCAGGTTNCACGGCTANTGCGATAAGCATATTCTACGGGGGGACTGTAAAGGAATTGTATCCACTCNG

CTCCCTGTCAATTGGCCATGTGCATCTCTNGTTGTTTAGGGTACATTAATAACACACAGCATNAGGTA
GATAAGTCTCCGATATAAGCGAACAGANCGTTGGTCAAANACCGTGGTTCAATTGTAGTGGTATAACA
TTTCCCAGTGCATAAGGAACCCAGGNACACTACTAGCATGGGCGAGGAGAGACCCTTGGGTGGCANNA
CGCAATAAGTGTTNNCCAGTACACCAGCACGATNNAGGGTTTGNCCNTGTGTTTTTAGACGACCCCGG
TCGCATTGAACCTGGCATTCAGAATGACGTGCTCTCAAGAGTCAANGTCCNNNCACTGAGGGTCATNT
GCTTCGCGNNNTAGCGTATNGTCATAAACAT
>HMong195
GTGTAGCTCAGTTAGACACTTCTAAATCCTTGGTAACAGACATGCAGTCCCCTCNNCAGAGCCCCGCC
CCCCGGTGGTACCGGGTTGTTAAGAGGTCCACCACGGGTCGCACGACNANNTCAAGGCCTCANGCATG
ACCCAACGCNGGGACGTACCGGCGCGGCGACCTTGAANGTCGGGGNCAAGTATGTCCCCCCGGGAAGG
ACGCCGTAGTGCATCTGGAGTGCCGATTCGTGGTCNAGAATGNCACCACGATGGTATGAGTNGCAAAG
GTATTTTGGACACCACCCCGTTTTCTATTTCCAGACGCGGCCATTCAGGAACGACGCGAATCGTAATC
TANCTGCCGCGTACGCGGATCGTACATGCGTTTTTCAGCGTTCGAAATAGAGTTTGTCGTTCCGCTGA
CCAGGTTNCACGGCTACTGCGATAAGCATATTCTACGGGGGGACTGTAAAGGAATTGTATCCACTCTG
CTCCCTGTCAATTGGCCATGTGCATCTCTGGTTGTTTAGGGTACATTAATAACACACAGNATTAGGTA
GNTAAGTCTCCGATATAAGCGAACAGAACGTTGGTNAAAGACCGTGGTTCAATTGTAGTGGTATAACA
TTTCCCAGTGCATAAGGAACCCAGGGACACTACTAGCANGGGCGAGGAGAGACCCTTGGGTGGCANCA
CGCAATAAGTGTTACCCAGTACACCAGCACGATANAGGGTTTGACCNTGTGTTTTTAGACGNCCCCGG
TCGCATTGAACCTGGCATTCAGAATGACGTGCTCTCAAGAGTCAANGTCCTCCCACTGAGGGNCATCT
GCTTCGCGNNNTNGCGTATGGTCATANNNAT
>HMong196
GTGNAGCTCAGTTNGACACTNCTAAATCCNTNGTAACAGACATGCAGTCCCCTCGGCAGAGNNCCGCC
CCCCGGTGGTACCGGGTTGTTNAGAGGTCCACCACGGNTCNNACGACAACATCAAGGCCTCATGCATG
ANNCANNNCNGNGANGTACCGGNGNGGNGACCTTGAAGNTCGGGGNCAAGTATGTCCCCCCGGGAAGG
ACGCNGTAGTGCATCTGGANTGCCGATTNNTGGTCCNGAATGCCACCACGATGGTATGAGTGGCAAAG
GTATTTNGGACACCACCCCGTTTNCTNNTTCCAGANGCGGCCNTTCAGGAACGACGCGAATCGTAATC
TACCTGCCGCGTNCGCGGATCGTACNNGCGTNTTTCAGCGTTCGAAATAGAGTTTGTCGTTCCGCTGA
CCNGGTTNCACGGCTACTNCGATAAGCATATTCTACGGGGGGACTGTAAAGGAATTGTATCCACTCTG
CTCCCTGTCAATTGGNCATGTGCATCTCTNGTTGTTTAGGNNANATTAATAACACACAGCNTNAGGTA
GNTAAGTCTCCGATATAAGCGAACAGANCGTTGGTNAAAGACCGTGGTTCAATTGTAGTGGTATAACA
TTTCCCAGTGCATAAGGAANCCAGGNACACTACTANCANGGGCGAGGAGAGACCCTTGGGTGGCANNA
CGNAATAAGTGTTANNCAGTACACCAGCACNATANAGGGTTTGNCCNNGTNTTTNTAGACGACCCCGG
TCGCATTGAACCTGGCATTCANAATGACGTGCTCTCAAGAGTCNANNNCCTNNCACTGAGGGTCANNN
GCTTCGCGNNNTAGCGTATGGTCATAAACAT
>HMong197
GTGTAGCTCAGTTANACACTTCTAAATCCTTGGTAACAGACATGCAGTCCCCTCNNCAGAGCCCCGCC
CCCCGGTGGTACCGGGTTGNTAAGAGGTCCACCACGGGTCGNACGACAANNTCAAGGCCTCATGCATG
ACCCAACGCGGGGACGTACCGGCGCGGCGACCTTGAAGGTCGGGGACAAGTATGTCCCCCCGGGAAGG
ACGCCGTAGTGCATCTGGAGTGCCGATTCGTGGTCCAGAATGNCACCACGATGGTATGAGTNGCAAAG
GTATTTTGGACACCACCCCGTTTNCTANTTCCAGACGCGGCCNTTCAGGAACGACGCGAATCGTAATC
TACCTGCCGCGTNCGCGGATCGTACATGCGTTTTTCAGCGTTCGAAATAGAGTTTGTCGTTCCGCTGA
CCAGGTTNCACGGCTACTNCGATAAGCATATTCTACGGGGGGACTGTAAAGGAATTGTATCCACTCTG
CTCCCTGTCAATTGGCCATGTGCATCTCTGGTTGTTTAGGGTACATTAATAACACACAGCATNAGGTA
GATNAGTCTCCGATATAAGCGAACAGAACGTTGGTCAAAGACCGTGGTTCAATTGTAGTGGTATAACA
TTTCCCAGTGCATAAGGAACCCAGGGACACTACTAGCATGGGCGAGGAGAGACCCTTGGGTGGCAANA
CGCAATAAGTGTTNCCCAGTACACCAGCACGATAGAGGGTTTGACCATNTGTTTTTAGACGACCCCGG
TCGCATTGAACCTGGCATTCAGAATGACGTGCTNTCAAGAGTCAAGNTCCNCCCACTGAGGGTCATNN
GCTTCGCGNNNTNGCGTATNGTCATAAACAT
>HMong198
GTGTAGCTCAGTTAGACACTTCTAAATCCTTGGTAACAGACATGCAGTCCCCTCNNCAGAGCCCCGCC
CCCCGGTGGTACCGGGTTGTTNAGAGGTCCACCACGGGTCGNACGACAANATCAAGGCCTCANGCATG
ACCCANCGCGGNGANGTACCGGCGNGGCGACCTTGAAGGTCGGGGNCAAGTATGTCCCCCCGGGAAGG
ACGCCGTAGTGCATCTGGAGTGCCGATTCNTGGTCCAGAATGNCACCACGATGGTATGAGTGGCAAAG
GTATTTNGGACNCCACCCCGTTTTCTANTTCCAGANGCGGCCATTCAGGAACGACGCGAATCGTAATC

TACCTGCCGCGTACGCGGATCGTACANGCGTTTTTCAGCGTTCGAAATAGAGTTTGTCGTTCCGCTGA
CCAGGTTNCACGGCTACTGCGATAAGCATATTCTACGGGGGGACTGTAAAGGAATTGTATCCACTCTG
CTCCCTGTCAATTGGNCATGTGCATCTCTGGTTGTTTAGGNNACATTAATAACACACAGCATNAGGTA
GATAAGTCTCCGATATAAGCGAACAGANCGTTGGTCAAAGACCGTGGTTCAATTGTAGTGGTATAACA
TTTCCCAGTGCATAAGGAACCCAGGNACACTACTAGCATGGGCGAGGAGAGACCCTTGGGTGGCAANA
CGCAATAAGTGTTACCCAGTACACCAGCACGATAGAGGGTTTGACCNNGTGTTTTAGACGACCCCGG
TCGCATTGAACCTGGCATTCAGAATGACGTNCTCTNAAGAGTCAAGNTCCNCNCACTGAGGGTCANCT
GCTTCGCGNNNTNGCGTATGGTCACNAANAT
>HMong199
GTGTAGCCCAGTCAGACACTTCTAAATCCTTGGTAACAGACATGCAGTCCCCTCGGCAGAGCCCCGCC
CCCCGATGGTACCGGGTTGTTAAGAGGTCCACAACGGGTCGCACGACAACATTAAGGCCTCATGCATG
ANNCAACACGGGGGCGTCCCGGCGCGGCGACCTTGNAGTCCGGGGNCAAGTATGGCCCCCCGGGAAGG
ACGCCGTAGTGCATCTGGAGTGCCGATTCGTGGTCCAGAATGCCACCACGATGGTATGAGTGGCAAAG
GTACTTTGGACACCACCCCGTTTTCTANTTCCAGACGCGGCCNTTTAGGAACGACGCGAATCGTAATC
TACCTGCCGCGTACGCGGATCGTACATGCGTTTTCCAGCGTTCGAAATAGAGTTTGTCGTTCCGCTGA
CCAGGTTTCACGGCTACTGCGATAAGCATATTCTACTGGGGGACTGTAAAGGAATTGTATCCATTCTG
CTTCCTGTCAATTGGCCATGTGCATCTCTGGTTGTTCAGGGTACATTAATAACACACAGCAGTAAGTA
GGTAAGTCTCCGATATAAGCGAACAGAACGTTTGTCAGAGACTGTGGTTCAATTGTAGTGGTATAATG
TTTCCCAGTGCATAAGGAACTCAGGGACACTACTAGCATGGGCGAGGAGAGACCCTTGGGTGGCAACA
CGCAATAAGTGTTACCTAGTACACCAGCACGAGAGAGGGTTTGACCATGTGTTTTTAGACGACCCCGG
TCGCATTGAACCTGGCATTCAGAATGACGTGCTCTCAAGAATCAAGNTCCTCCCACTGAGGGTCATCT
GCTTCGCGGGTCNGCGTATGGTCATAAACAT
>HMong200
GTGNAGCCCAGTTAGACACTTCTAAATCCTTGGTAACAGACATGCAGTCCCCTCGGCAGAGCCCCGCC
CCCCNATGGTACCGGGTTGTTAAGAGGTCCACAACGGGTCGCACGACAACNTTAAGGCCTCATGCATG
ACCCAACACGGGGGCGTCCCGGCGCGGCGACCTTGAAGTCCGGGGACAAGTATGGCCCCCCGGGAAGG
ACGCCGTAGTGCATCTGGAGTGCCGATTCGTGGTCCAGAATGCCACCACGATGGTATGAGTGGCAAAG
GTACTTTGGACACCACCCCGTTTNCTANNTCCAGANGCGGCCNTTCAGGAACGACGCGAATCGTAATC
TACCTGCCGCGTACGCGGATCGTACANGCGTTTTCCAGCGTTCGAAATAGAGTTTGTCGTTCCGCTGA
CCAGGTTTCACGGCTACTNCGATAAGCATATTCTACTGGGGGACTGTAAAGGAATTGTATCCACTCNG
CTTCCTGTCAATTGGCCATGTGCATCTCTGGTTGTTCAGGGTACATTAATAACACACAGCATTAAGTA
GATNAGTCTCCGATATAAGCGAACAGANCGTTGGTCAGAGACTATGGTTCAATTGTAGTGGTATAATG
TTTCCCAGTGCATAAGGAACTCAGGNACACTACTAGCATGGNCGAGGAGAGACCCTTGGGTGGCAANA
CGCAATAAGTGTTACCTAGTACATCAGCACGAGNNAGGGTTTGACCNTNNNTTTTAGANGACCCCGG
TCGCATTGAACCTGGCATTCAGAATGACGTGCTCTCAAGAATCAAGGTCCNCCCACTGAGGGTCATNN
GCTTCGCGGGTCAGCGTATGGTCATAANCAT
>HMong201
GNGTAGCCCAGTTANACACNNCTAAATCCNTNGTAACAGACATGCAGTCCCCTCNNCAGAGNNCCGCC
CCCCGATGGTACCGGGTTGTTAAGNGGTCCACAACGGNTCGCACGACNNNNTTAAGGCCTNANGCATG
ACCCAACACGGGGGNGTCCCGGCGNGGNGACCTTGAANTCCGGGGACAAGTATNGCCCNCCGGGAAGG
ACGCCGTAGTGCATCTGGAGTGCCGATTCGTGGTCNAGAATGCCACCACGATGGTATGAGTNGCAAAG
GTACTTNNNNCACCACCCCGTTTTCTANTNCCAGANGCGGCCNTTCAGGAACNACGCGAATCGTAATC
TACCTGCCGCGTNCGCGGATCGTACANGCGTTTTNCAGCGTTCGAAATAGAGTTTGTCGTTCCGCTGA
CCNGGTTNCACGGCTACTGCGATAAGCATATTCTACTGGGGGACTGTAAAGGAATTGTATCNACTCTG
CTTCCTGNCAATTGGCCATGTGCATCTCTGGTTGTTCAGGNNANATTAATAACACACAGCATNAAGTA
GNTAAGTCTCCGATATAAGCGAACAGANCGTTGGTCAGANACTATGGTTCAATTGTAGTGGTATAATG
TTTCCCAGNGCATAAGGAACTCAGGNACACTACTAGCATGGGCGAGGAGAGACCCTTGGGTGGCANNA
CGNAATAAGTGTTANCTNGTACATCAGCACGAGANANGGTTTGACCNTGTGTTTNTAGACGACCCCGG
TCGCATTGAACCTGGCATTCAGAATGACGTNCTNTCAANAATCNAGNTCCNCCCACTGAGGGTCATCN
GCTTCGCGGGTCNGCGTATGGTCANANACAT
>HMong202
GTGTAGCCCAGTCANACACTTCTAAATCCNTNGTAACAGACATGCAGTCCCCTCGGCAGAGCCCCGCC
CCCCGATGGTACCGGGTTGNTAAGAGGTCCACAACGGNTCNCANGACANCNTTAAGGCCTCANGCATG
ACCCAACNCGGGGGCGTCCCGGNGCGGCGACCTTGNAGTCCGGGGACAAGTATNGCCCCCCGGGAAGG

ACGCNGTAGTGCATCTGGAGTGCCGATTCGTGGTCNAGAATGNCANCACGATGGTATGAGTNGCAAAG
GTACTTTGGACACCACCCCGTTNNCTATTNCCAGACGCGGCCATTTAGGAACGACGCGAATCGTAATC
TACCTGCCGCGTACGCGGATCGTACANGCGTNTTCCAGCGTTCGAAATAGAGTTTGTCGTTCCGCTGA
CCAGGTTNCACGGCTACTGCGATAAGCATATTCTACTGGGGGACTGTAAAGGAATTGTATCCATTCTG
CTTCCTGTCAATTGGNCATGTGCATCTCTNGTTGTTCAGGNNACATTAATAACACACAGCAGTAAGTA
GGTAAGTCTCCGATATAAGCGAACAGANCGTTTGTCAGAGACTGTGGTTCAATTGTAGTGGTATAATG
TTTCCCAGTGCATAAGGAACTCAGGNACACTACTAGCANGGGCGAGGAGAGACCCTTGGGTGGCAANA
CGCAATAAGTGTTANNTAGTACACCAGCACGAGNGAGGGTTTGNCCNTNTNTTTTAGACGNCCCCGG
TCGCATTGAACCTGGCATTCAGAATGACGTGCTNTCAAGAATCNAGGTCCTCCCACTGAGGGTCATCN
GCTTCGCGGGTCAGCGTATGGTCATAAACNT
>HMong203
GTGTAGCCCAGTTGGACACNTCTAAATCCNTGGTAACAGACATGCAGTCCCCTCGGCAGAGCCCCGCC
CCCCNGTGGTACCGGGTTGTTNAGNGGTCCACCACGGGTCGCACGACAANATCAAGGCCTNATGCATG
ACCCAACNCGGNGACGTACCGGCGNGGCGACCTTGAAGGTCGGGGNCAAGTATGTCCCCCCGGGAAGG
ACGCCGTAGTGCATCTGGAGTGCCGATTNGTGNTCNAGAATGCCACCACGATGGTATGAGTGGCAAAG
GTATTTTGGACACCACCCCGTTTTCTANTTCCAGANGCGGCCATTCAGGAACGACGCGAATCGTAATC
TACCTGCCGCGTACGCGGATCGTACANGCGTTTTTCAGCGTTCGAAATAGAGTTTGTCGTTCCGCTGA
CCAGGTTTCACGGCTACTGCGATAAGCATATTCTACGGGGGGACTGTAAAGGAATTGTATCCACTCNG
CTCCCTGTCAATTGGNCATGTGCATCTCTNGTTGTTTAGGGTACATTAATAACACACAGCATNAGGTA
GATAAGTCTCCGATATAAGCGAACAGAACGTTGGTCAAAGACCGTGGTTCAATTGTAGTGGTATAACA
TTTCCCAGTGCATAAGGAACCCAGGNACACTATTAGCATGGGCGAGGAGAGACCCTTGGGTGGCAANA
CGCAATAAGTGTTNCCCAGTACACCAGCACGATNGANGGTTTGACCNNGTGTTTNTAGACGNCCCCGG
TCGCATTGAACCTGGCATTCAGAATGACGTGCTNTCAAGAGTCAAGGTCCNCNCACTGAGGGTCATCT
GCTTCGCGNGTTAGCGTATGGTCATAAACAT
>HMong204
GTGTAGCCCAGTTNNACACNTCTAAATNCNTNGTAACAGACATGCAGTCCCCTCNNCAGAGCCCCGCC
CCCCGGTGGTACCGGGTTGNTAAGAGGTCCACCACGGGTCNCACGACAACATCAAGGCCTCATGCATG
ANNCNNNNCGGGGANGTACCGGNGNGGCGACCTTGNANGTNGGGGNCAAGTATGTCCCCCCGGGAAGG
ACGCCGTAGTGCATCTGGAGTGCCGATTCNTGGTCCNNANTGNCACCACGATGGTATGAGTGGCAAAG
GTATTTNGGNCACCACCCCGTTTNCTANTNCCAGANGCGGCCNTTCAGGAACGACGCGAATCGTAATC
TACCTGCCGCGTACGCGGATCGTACANGCGTTTTNCAGCGTTCGAAATAGAGTTTGTCGTTCCGCTGA
CCNGGTTNCACGGCTANTNCGATAAGCANATTCTACGGGGGGACTGTAAAGGAATTGTATCNACTCNG
CTCCCTGTCAATTGGCCATGTGCATCTCTNGTTGTTTAGGNNACATTAATAACACACAGCATNAGGTA
GATNAGTCTCCGATATAAGCGAACAGAACGTTGGTNAAAGACCGTGGTTCAATTGTAGTGGTATAACA
TTTCCCAGTGCATAAGGAACCCAGGNACACTATTAGCANGGNCGAGGAGAGACCCTTGGGTGGCAANA
CGCAATAAGTGTTACCCAGTACACCAGCACGATNNAGGGTTTGNCNNTGNNTTTTAGNNGACCCCGG
TCGCATTGAACCTGGCATTCAGAATGACGTGCTCTNAAGAGTCNAGNTCCNNCCACTGAGGNTCATCT
GCTTCGCGNGTTNGCGTATNGTCATAAACAT
>HMong205
GTGNAGCTCAGTTANACACTTCTAAATCCTTGGTAACAGACATGCAGTCCCCTCNNCAGAGCCCCGCC
CCCCNGTGGTACCGGGTTGTTAAGAGGTCCACCACGGGTCNNACGACAACATCAAGGCCTCANGCATG
ACCCAANGCNGGGACGTACCGGCGCGGCGACCTTGNAGGTCGGGGACAAGTATGTCCCCCCGGGAAGG
ACGCNGTAGTGCATCTGGAGTGCCGATTNGTGGTCCAGANTGNCACCACGATGGTATGAGTGGCAAAG
GTATTTNGGACACCACCCCGTTTTCTANTTCCAGANGCGGCCATTCAGGAACGACGCGAATCGTAATC
TACCTGCCGCGTNCGCGGATCGTACANGCGTTTTTCAGCGTTCGAAATAGAGTTTGTCGTTCCGCTGA
CCAGGTTNCACGGCTACTGCGATAAGCATATTCTACGGGGGGACTGTAAAGGAATTGTATCCACTCNG
CTCCCTGTCAATTGGCCATGTGCATCTCTNGTTGTTTAGGNTACATTAATAACACACAGNATTAGGTA
GATNAGTCTCCGATATAAGCGAACAGANCGTTGGTCAAAGACCGTGGTTCAATTGTAGTGGTATAACA
TTTCCCAGTGCATAAGGAACCCAGGNACACTACTAGCATGGGCGAGGAGAGACCCTTGGGTGGCAANA
CGCAATAAGTGTTACCCAGTACACCAGCACGATAGAGGGTTTGACCATGTGTTTTTAGACGACCCCGG
TCGCATTGAACCTGGCATTCAGAATGACGTGCTCTNAAGAGTCNAGNTCCTCNCACTGAGGGTCANCN
GCTTCGCGNNNTNGCGTATGGTCATAAACAT
>HMong206
GTGTAGCTCAGTTAGACACTTCTAAATCCTTGGTAACAGACATGCAGTCCCCTCGGCAGAGCNCCGCC

CCCCGGTGGTACCGGGTTGTTAAGAGGTCCACCACGGGTCGNACGACAACATCAAGGCCTCATGCATG
ACCCANCGCGGGGACGTACCGGCGNGGCGACCTTGAAGGTCGGGGNCAAGTATGTCCCCCCGGGAAGG
ACGCCGTAGTGCATCTGGAGTGCCGATTCNTGGTCCNGAATGCCACCACGATGGTATGAGTGGCAAAG
GTATTTTGGACACCACCCCGTTTTCTANTTCCAGANGCGGCCATTCAGGAACGACGCGAATCGTAATC
TACCTGCCGCGTACGCGGATCGTACANGCGTTTTTCAGCGTTCGAAATAGAGTTTGTCGTTCCGCTGA
CCAGGTTTCACGGCTACTNCGATAAGCATATTCTACGGGGGGACTGTAAAGGAATTGTATCCACTCNG
CTCCCTGTCAATTGGCCATGTGCATCTCTGGTTGTTTAGGGTACATTAATAACACACAGCATTAGGTA
GATAAGTCTCCGATATAAGCGAACAGAACGTTGGTCAAAGACCGTGGTTCAATTGTAGTGGTATAACA
TTTCCCAGTGCATAAGGAACCCAGGGACACTACTAGCANGGGCGAGGAGAGACCCTTGGGTGGCAACA
CGCAATAAGTGTTNNCCAGTACACCAGCACGATNNANGGTTTGACCATGTGTTTTAGACGACCCCGG
TCGCATTGAACCTGGCATTCAGAATGACGTGCTCTCAAGAGTCAAGNTCCTCCCACTGAGGGTCATCT
GCTTCGCGNNNTNGCGTATGGTCATAAACAT
>HMong207
GTGTAGCCCAGTTAGACACTTCTAAATCCNTNGTAACAGACATGCAGTCCCCTCGGCAGAGCCCCGCC
CCCCGATGGTACCGGGTTGTTNAGAGGTCCACAACGGGTCGCACGACNNCATTAAGGCCTCATGCATG
ACCCAACACGGGGGCGTCCCGGCGNGGCGACCTTGAANTCCGGGGNCAAGTATGGCCCCCCGGGAAGG
ACGCNGTAGTGCATCTGGAGTGCCGATTNGTGGTCCAGAATGCCACCACGATGGTATGAGTGGCAAAG
GTACTTTGGACACCACCCCGTTTTCTATTTCCAGACGCGGCCATTCAGGAACGACGCGAATCGTAATC
TACCTGCCGCGTACGCGGATCGTACATGCGTTTTCCAGCGTTCGAAATAGAGTTTGTCGTTCCGCTGA
CCAGGTTNCACGGCTACTGCGATAAGCATATTCTACTGGGGGACTGTAAAGGAATTGTATCCACTCTG
CTTCCTGTCAATTGGCCATGTGCATCTCTGGTTGTTCAGGGTACATTAATAACACACAGCATTAAGTA
GATAAGTCTCCGATATAAGCGAACAGANCGTTGGTCNGAGACTATGGTTCAATTGTAGTGGTATAATG
TTTCCCAGTGCATAAGGAACTCAGGGACACTACTAGCATGGGCGAGGAGAGACCCTTGGGTGGCAANA
CGCAATAAGTGTTACCTAGTACATCAGCACGAGAGAGGGTTTGACCATGTGTTTTAGACGACCCCGG
TCGCATTGAACCTGGCATTCAGAATGACGTGCTCTNAAGAATCAAGGTCCTCCCACTGAGGGTCATCN
GCTTCGCGGGTCNGCGTATGGTCACAAACNT
>HMong208
GTGTAGCTCAGTTAGACACTTCTAAATCCNTNGTAACAGACATGCAGTCCCCTCNNCAGAGCNCCGCC
CCCCGGTGGTACCGGGTTGNTAAGAGGTCCACCACGGGTCGCACGACAACATCAAGGCCTCATGCATG
ACCCAACGCGGGGACGTACCGGCGCGGCGACCTTGNAGGTCGGGGNCAAGTATGTCCCCCCGGGAAGG
ACGCCGTAGTGCATCTGGAGTGCCGATTCGTGGTCCAGAATGCCACCACGATGGTATGAGTGGCAAAG
GTATTTTGGACACCACCCCGTTTTCTATTTCCAGACGCGGCCNTTCAGGAACGACGCGAATCGTAATC
TACCTGCCGCGTACGCGGATCGTACATGCGTTTTTCAGCGTTCGAAATAGAGTTTGTCGTTCCGCTGA
CCAGGTTTCACGGCTACTGCGATAAGCATATTCTACGGGGGGACTGTAAAGGAATTGTATCCACTCTG
CTCCCTGTCAATTGGCCATGTGCATCTCTGGTTGTTTAGGGNACATTAATAACACACAGCATNAGGTA
GATAAGTCTCCGATATAAGCGAACAGANCGTTGGTCAAAGACCGTGGTTCAATTGTAGTGGTATAACA
TTTCCCAGTGCATAAGGAACCCAGGGACACTACTAGCATGGGCGAGGAGAGACCCTTGGGTGGCAACA
CGCAATAAGTGTTACCCAGTACACCAGCACGATAGAGGGTTTGACCATGTGTTTTAGNNGACCCCGG
TCGCATTGAACCTGGCATTCAGAATGACGTGCTCTCAAGAGTCAAGGTCCNCCCACTGAGGGTCATCT
GCTTCGCGNNNTNGCGTATGGTCATAAACAT
>HMong209
GTGNAGCCCAGTTANACACTTCTAAATCCNTNGTAACAGACATGCAGTCCCCTCNNCAGAGCNCCGCC
CCCCNATGGTACCGGGNTGTNNAGAGGTCCACAACGGGTCGNACGACANCNTTAAGGCCTNANGCATG
ACCCAACNCGGNGGNGTCCCGGNGNGGNGACCTTGAAGTCNGGGGNCAAGTATGGCCCCCCGGGAAGG
ACGCCGTAGTGCATCTGGAGTGCCGATTCNTGGTCNAGANTGNCACCACGANGGTATGAGTGGCAANG
GTACTTTGGNCACCACCCCGTTTTCTNNTTCNAGANGCGGCCATTCAGGAACGACGCGAATCGTAATC
TACCTGCCGCGTNCGCGGATNGTACANGCGTTTTCCAGCGTTCGAAATAGAGTTTGTCGTTCCGCTGA
CCAGGTTNCACGGCTACTGCGATAAGCATATTCTACTGNGGGACTGTAAAGNAATTGTATCNACNCTG
CTTCCTGTCAATTGGNCATGTGCATCTCTNGTTGTTCAGGNNACATTAATAACACACAGNATNAAGTA
GATAAGTCTCCGATATAAGCGAACAGANCGTTGGTCAGAGACTATGGTTCAATTGTAGTGGTATAATG
TTTCCCAGNGCATAAGGAACTCAGGNACACTACTAGCANGGNCGAGGAGAGACCCTTGGGTGNNAANA
CGNANTAAGTGTTNNNTAGTACATCAGCACGAGNNANGGTTTGACCNTGTNTTTNTAGNCGACCCCGG
TCGCATTGAACCTGGCATTCANAATGACGTGCTCTCAAGAATCNAGNTCCNCNCACTGAGGGTCATCT
GCTTCGCGGNNCNGCGTATNGNCATAAACAT

>HMong210
GTGNAGCTCAGTTAGACACTNCTAAATNCNTNGTAACAGACATGCAGTCCCCTCNNCAGAGCCCCGCC
CCCCGGTGGTACCGGGTTGNNAAGAGGTCCACCACGGGTCGNACGACAANATCAAGGCCTCATGCATG
ANNCAACNCNGNGANGTACCGGCGNGGCGACCTTGAAGGTCGGGNNCAAGTATGTCCCCCCGGGAAGG
ACGCNGTAGTGCATCTGGANTGCCGATTNGTGGTCNAGANTGNCACCACGATGGTATGAGTGGCAAAG
GTATTTNGGACACCACCCCGTTNNCTATTTCCAGANGCGGCCNTTCAGGAACGACGCGAATCGTAATC
TACCTGCCGCGTNCGCGGATCGTACANGCGTNTTTCAGCGTTCGAAATANAGTTTGTCGTTCCGCTGA
CCAGGTTNCACGGCTACTGCGATAAGCATATTCTACGGGGGGACTGTAAAGGAATTGTATCCACTCNG
CTCCCTGTCAATTGGCCATGTGCATCTCTNGTTGTTTAGGNNACATTAATAACACACAGCATNAGGTA
GATNAGTCTCCGATATAAGCGAACAGANCGTTGGTNAAAGACCGTGGTTCAATTGTAGTGGTATAACA
TTTCCCAGNGCATAAGGAACCCAGGNACACTACTAGCANGGGCGAGGAGANACCCTTGGGTGGCAANA
CGNAATAAGTGTTNNCCAGTACACCAGCACGATANANGGTTTGACCNTNNNTTTNTAGNNGACCCCGG
TCGCATTGAACCTGGCATTCAGAATGACGTGCTCTCANGAGTCAAGNTCCTCCCACTGAGGGNCATCT
GCTTCGCGNNNTAGCGTATNGNCATAAAAAT
>HMong211
GTGTAGCTCAGTTAGACACTTCTAAATCCTTNGTAACAGACATGCAGTCCCCTCNNCAGAGCCCCGCC
CCCCGGTGGTACCGGGTTGTTNAGAGGTCCACCACGGGTCGCACGACANCATCAAGGCCTCATGCATG
ACCCAACGCGGNGANGTACCGGCGCGGNGACCTTGNAGGTCGGGGACAAGTATGTCCCCCCGGGAAGG
ACGCCGTAGTGCATCTGGAGTGCCGATTCNTGGNCCAGAATGCCACCACGATGGTATGAGTGGCAAAG
GTATTTTGGACACCACCCCGTTTTCTANTTCCAGACGCGGCCATTCAGGAACGACGCGAATCGTAATC
TACCTGCCGCGTNCGCGGATCGTACATGCGTTTTTCAGCGTTCGAAATAGAGTTTGTCGTTCCGCTGA
CCAGGTTTCACGGCTACTGCGATAAGCATATTCTACGGGGGGACTGTAAAGGAATTGTATCCACTCNG
CTCCCTGTCAATTGGCCATGTGCATCTCTNGTTGTTTAGGNNACATTAATAACACACAGNATTAGGTA
GNTAAGTCTCCGATATAAGCGAACAGAACGTTGGTCAAAGACCGTGGTTCAATTGTAGTGGTATAACA
TTTCCCAGTGCATAAGGAACCCAGGGACACTACTAGCATGGGCGAGGAGAGACCCTTGGGTGGCAACA
CGCAATAAGTGTTACCCAGTACACCAGCACGATAGAGGGTTTGACCNTGTGTTTTTAGACGACCCCGG
TCGCATTGAACCTGGCATTCAGAATGACGTGCTNTCAAGAGTCAAGGTCCTCCCACTGAGGGTCATCT
GCTTCGCGNNNTAGCGTATGGTCATAAACAT
>HMong212
GTGTAGCCCAGTTGNACACTTCNAAATCCNTGGTAACAGACATGCAGTCCCCTCNNCAGAGCNCCGCC
CCCCNGTGGTACCGGGTTGTTNAGAGGTCCACCACGGGTCGCACGACAANATCAAGGCCTCATGCATG
ANCCANNNCGGGGANGTACCGGCGNGGCGACCTTGNAGGTCGGGGNCAAGTATGTCCCCCCGGGAAGG
ACGCCGTAGTGCATCTGGAGTGCCGATTNNTGGTCNNGANTGCCACCACGANGGTATGAGTGGCAANG
GTATTTTGGACACCACCCCGTTTTCTNNTTCCAGACGCGGCCNTTCAGGAACGACGCGAATCGTAATC
TACCTGCCGCGTNCGCGGATNGTACATGCGTTTTTCAGCGTTCGAAATAGAGTTTGTCGTTCCGCTGA
CCAGGTTNCACGGCTACTGCGATAAGCATATTCTACGGGGGGACTGTAAAGGAATTGTATCCACTCNG
CTCCCTGTCAATTGGNCATGTGCATCTCTGGTTGTTTAGGGTACATTAATAACACACAGNATNAGGTA
GATAAGTCTCCGATATAAGCGAACAGAACGTTGGTCAAAGACCGTGGTTCAATTGTAGTGGTATAACA
TTTCCCAGTGCATAAGGAACCCAGGGACACTATTAGCANGGNCGAGGAGAGACCCTTGGGTGGCAANA
CGNAATAAGTGTTNNCCAGTACACCAGCACGATNNANGGTTTGACCNNNNNTTTTTAGNCGACCCCGG
TCGCATTGAACCTGGCATTCAGAATGACGTGCTCTCAAGAGTCAANNTCCTCCCACTGAGGGTCATNN
GCTTCGCGNGTTNGCGTATGGNCATAAACAT
>HMong213
GTGTAGCTCAGTTAGACACTTCTAAATCCTTNGTAACAGACATGCAGTCCCCTCGGCAGAGCCCCGCC
CCCCGGTGGTACCGGGTTGTTAAGAGGTCCACCACGGGTCNNACGACAACATCAAGGCCTCATGCATG
ACCCANNNCGGGGACGTACCGGCGCGGCGACCTTGAANGTCGGGGACAAGTATGTCCCCCCGGGAAGG
ACGCCGTAGTGCATCTGGAGTGCCGATTCGTGGTCNAGAATGNCACCACGATGGTATGAGTGGCAAAG
GTATTTNGGNCACCACCCCGTTTNCTANTTCCAGACGCGGCCNTTCAGGAACGACGCGAATCGTAATC
TACCTGCCGCGTACGCGGATNGTACATGCGTTTTNCAGCGTTCGAAATAGAGTTTGTCGTTCCGCTGA
CCAGGTTTCACGGCTACTGNGATAAGCATATTCTACGGGGGGACTGTAAAGGAATTGTATCCACTCNG
CTCCCTGTCAATTGGCCATGTGCATCTCTGGTTGTTTAGGNNACATTAATAACACACAGCATTAGGTA
GATAAGTCTCCGATATAAGCGAACAGANCGTTGGTCAAAGACCGTGGTTCAATTGTAGTGGTATAACA
TTTCCCAGTGCATAAGGAACCCAGGNACACTACTAGCATGGGCGAGGAGAGACCCTTGGGTGGCAANA
CGCAATAAGTGTTNNNCAGTACACCAGCACGATAGAGGGTTTGNCCATGNNTTTNTAGANGACCCCGG

TCGCATTGAACCTGGCATTCAGAATGACGTGCTCTCAAGAGTCAAGGTCCNCNCACTGAGGGTCATNN
GCTTCGCGNNNTNGCGTATGGTCATAAACAT
>HMong214
GTGNAGCTCAGTTANACACTTCTAAATCCTTNGTAACAGACATGCAGTCCCCTCNNCAGAGCCCCGCC
CCCCNGTGGTACCGGGTTGTTAAGAGGTCCACCACGGNTCGCACGACAANATCAAGGCCTCANGCATG
ANNCAACNCNGGGANGTACCGGCGNGGNGACCTTGAAGGTCGGGGNCAAGTATGTCCCCCCGGGAAGG
ACGCNGTAGTGCATCTGGAGTGCCGATTNGTGGTCCNGANTGCCACCACGATGGTATGAGTGGCAAAG
GTATTTTGGACACCACCCCGTTTTCTANTTCCAGACGCGGCCATTCAGGAACGACGCGAATCGTAATC
TACCTGCCGCGTACGCGGATCGTACANGCGTNTTTCAGCGTTCGAAATAGAGTTTGTCGTTCCGCTGA
CCAGGTTTCACGGCTACTGCGATAAGCATATTCTACGGGGGGACTGTAAAGGAATTGTATCCACTCTG
CTCCCTGTCAATTGGCCATGTGCATCTCTNGTTGTTTAGGNNACATTAATAACACACAGNATNAGGTA
GATAAGTCTCCGATATAAGCGAACAGANCGTTGGTNAAAGACCGTGGTTCAATTGTAGTGGTATAACA
TTTCCCAGTGCATAAGGAACCCAGGGACACTACTAGCANGGGCGAGGAGAGACCCTTGGGTGGCAANA
CGCAATAAGTGTTNNNCAGTACACCAGCACGATANAGGGTTTGACCATGTGTTTNTAGNNGACCCCGG
TCGCATTGAACCTGGCATTCAGAATGACGTGCTCTNAAGAGTCAAGGTCCNCCCACTGAGGGTCATNN
GCTTCGCGNNNTNGCGTATGGNCATAAACAT
>HMong215
GTGTAGCCCAGTCAGACACTTCTAAATCCNTGGTAACAGACATGCAGTCCCCTCGGCAGAGCNCCGCC
CCCCGATGGTACCGGGTTGTTNAGAGGTCCACAACGGGTCGNACGACAACNTTAAGGCCTCATGCATG
ANCCAACNCGGGGGCGTCCCGGCGNGGCGACCTTGNAGTCCGGGGNCAAGTATGGCCCCCCGGGAAGG
ACGCNGTAGTGCATCTGGAGTGCCGATTCNTGGTCNAGAATGNCACCACGATGGTATGAGTGGCAAAG
GTACTTTGGNCACCACCCCGTTTNCTANTTCCAGACGCGGCCATTTAGGAACGACGCGAATCGTAATC
TACCTGCCGCGTNCGCGGATCGTACANGCGTTTTNCAGCGTTCGAAATAGAGTTTGTCGTTCCGCTGA
CCAGGTTNCACGGCTACTNCGATAAGCATATTCTACTGGGGGACTGTAAAGGAATTGTATCCATTCTG
CTTCCTGTCAATTGGCCATGTGCATCTCTGGTTGTTCAGGNNACATTAATAACACACAGCAGNAAGTA
GGTAAGTCNCCGATATAAGCGAACAGANCGTTTGTCAGAGACTGTGGTTCAATTGTAGTGGTATAATG
TTTCCCAGTGCATAAGGAACTCAGGNACACTACTAGCANGGGCGAGGAGAGACCCTTGGGTGGCAANA
CGCAATAAGTGTTACCTAGTACACCAGCACGAGAGANGGTTTGACCNTNNNTTNNTAGACGACCCCGG
TCGCATTGAACCTGGCATTCAGAATGACGTGCTCTCAAGAATCAAGNTCCNCCCACTGAGGGTCATCT
GCTTCGCGGGTCNGCGTATGGTCATANNNAT
>HMong216
GTGNAGCTCAGTTAGACACTTCTAAATCCTTGGTAACAGACATGCAGTCCCCTCGGCAGAGNCCCGCC
CCCCNGTGGTACCGGGTTGTTAAGAGGTCCACCACGGGTCGNACGACANCATCAAGGCCTCATGCATG
ACCCANCGCGGGGANGTACCGGCGNGGCGACCTTGNAGGTCGGGGNCAAGTATGTCCCCCCGGGAAGG
ACGCCGTAGTGCATCTGGAGTGCCGATTCNTGGTCCNGAATGNCACCACGATGGTATGAGTGGCAAAG
GTATTTNGGACACCACCCCGTTTTCTATTTCCAGACGCGGCCATTCAGGAACGACGCGAATCGTAATC
TACCTGCCGCGTNCGCGGATNGTACANGCGTTTTTCAGCGTTCGAAATAGAGTTTGTCGTTCCGCTGA
CCNGGTTNCACGGCTACTGCGATAAGCATATTCTACGGGGGGACTGTAAAGGAATTGTATCCACTCTG
CTCCCTGTCAATTGGCCATGTGCATCTCTNGTTGTTTAGGNNACATTAATAACACACAGCATTAGGTA
GATAAGTCNCCGATATAAGCGAACAGANCGTTGGTCAAAGACCGTGGTTCAATTGTAGTGGTATAACA
TTTCCCAGTGCATAAGGAACCCAGGGACACTACTAGCATGGGCGAGGAGAGACCCTTGGGTGGCAACA
CGCAATAAGTGTTNNNCAGTACACCAGCACGATANANGGTTTGACCATGTGTTTTTAGACGACCCCGG
TCGCATTGAACCTGGCATTCAGAATGACGTGCTCTCAAGAGTCAAGGTCCTCNCACTGAGGGTCATCT
GCTTCGCGNNNTAGCGTATGGTCATAANNNT
>HMong217
GNGNAGCCCAGTCAGACACTTCTAAATCCNTGGTAACAGACATGCAGTCCCCTCGGCAGAGNNCCGCC
CCCCNATGGTACCGGGTTGNTAAGAGGTCCACAACGGNTCGNACGACAANATTAAGGCCTNANGCATG
ANNCANCNCNGGGGCGTCCCGGNGCGGNGACCTTGNAGTNNGGGNNCAAGTATGGCCCCCCGGGAAGG
ACGCNGTAGTGCATCTGGAGTGCCGATTNGTGGTCNAGANTGNCACCACGATGGTATGAGTGGCAAAG
GTACTTNGNNCACCACCCCGTTNNCTANTTCCAGACGCGGCCNTTTAGGAACGACGCGAATCGTAATC
TACCTGCCGCGTNCGCGGATNGTACANGCGTNTTNCAGCGTTCGANATAGAGTTTGTCGTTCCGCTGA
CCAGGTTNCACGGCTACTNCGATAAGCATATTCTACTGGNGGACTGTAAAGNAATTGTATCNATTCTG
CTTCCTGTCAATTGGCCATGTGCATCTCTNGTTGTTCAGGNNACATTAATAACACACAGNAGNAAGTA
GNTNAGTCNCCGATATAAGCGAACAGANCGTTTGTNAGAGACTGTGGTTCAATTGTAGTGGTATAATG

TTTCCCAGTNCATAAGGAACTCAGGNACACTACTAGCANGGGCGAGGAGAGACCCTTGGGTGGCANNA
CGCAATAAGTGTTANNTAGTACACCAGCACGAGNNANGGTTTGNCCNTGTGTTTNTAGNNGACCCCGG
TCGCATTGAACCTGGCATTCAGAATGACGTGCTNTNAAGAATCNAGNTCCTNNCACTGAGGGTCANCN
GCTTCGCGGGTCNGCGTATGGTCATAAACAT
>HMong218
GNGTAGCCCAGTCNNACACNTNNAAATCCTTNGTAACAGACATGCAGTNNCCTCNNCAGAGCCCCGCC
CCCCGATGGTACCGGGNNNNTNAGAGGTCCACNACGGNTCGNACGANAANNTTAAGGCCTNATGCATG
ANNCNNNNCGGNNGNGTCCCGGNGNNGNGACCTTGNANTCNGGGNNCAAGTATNGCCCCCCGGGAAGG
ACGCNGTAGTGCATCTGGAGTGCCGANNNNTGGTCCAGAATGNCACCACGATGGTATGAGTGGCAAAG
GTACTTTNNNCACCACCCCGTTNNCTNNTTCNAGANGCGGCCNTTTAGGNACGACGCGANTCGTANTC
TNNCTGCCGCNTNCGCGGATNGTACNNGCGTTTTNCAGCGTTCGAAATAGAGTNTGTCGTTCCGCTGA
CCNGGTTNCACGGCTACTNCGATAAGCANNTTCTACTGGGGGACTGTAAAGNAATTGTATCNATTCNG
CTTCCTGTCAATTGGNNATGTGCATCTCTNGTTGTTCAGGNNACANTAATAACACACAGCNGTAAGTA
GNTAAGTCNCCNATATAAGCGAACAGANCGTTTGTCNGANACTGTGGTTNNATTGTAGTGGTATAANG
TTTCCCAGNGCATAAGGAACTCAGGGACACTACTANNANGGNCGAGGAGANACCCTTGGGTGGCANNN
NGNAATAAGTGTTNNCTAGTACACCAGCACGAGANANGGTTTGNCNNNNNNTTNNTAGNNGNCCCCGN
TCGCATTGAACCTGGCATTCAGAATGANGTNCTNTNANGAATNNAGNTCCTNNCACTGAGGGNCATNN
GCTTCNCNGNNCNGCGTATNGNCATAAACAT
>HMong219
GTGTAGCCCAGTTGNACACTTCTAAATCCTTNGTAACAGACATGCAGTCCCCTCGGCAGAGNNCCGCC
CCCCNGTGGTACCGGGTTGTTNAGAGGTCCACCACGGNTCGCACGACNNCATCAAGGCCTCANGCATN
ANNCAANGCGGGGACGTACCGGNGNGGNGACCTTGNANGTCGGGGNCAAGTATGTCCCNCCGGGAAGG
ACGCCGTAGTGCATCTNGAGTGCCGATTCGTGGTCCAGAATGNCACCACGATGGTATGAGTGGCAAAG
GTATTTNGGACACCACCCNGTTTTCTANTTCCAGACGCGGCCNTTCAGGAACGACGCGAATCGTAATC
TACCTGCCGCGTACGCGGATNGTACATGCGTNTTTCAGCGTTCGAAATAGAGTTTGTCGTTCCGCTGA
CCAGGTTNCACGGCTACTGCGATAAGCATATTCTACGGGGGGACTGTAAAGGAATTGTATCCACTCNG
CTCCCTGTCAATTGGNCATGTGCATCTCTGGTTGTTTAGGGTACATTAATAACACACAGNATGAGGTA
GATAAGTCTCCGATATAAGCGAACAGAACGTTGGTCAAAGACCGTGGTTCAATTGTAGTGGTATAACA
TTTCCCAGTNCATAAGGAACCCAGGNACACTATTAGCANGGGCGAGGAGAGACCCTTGGGTGGCAANA
CGCAATAAGTGTTACCCAGTACACCAGCACGATNNAGGGTTTGACCNTGTGTTTTTAGACGNCCCCGG
TCGCATTGAACCTGGCATTCAGAATGACGTGCTCTCAAGAGTCAAGGTCCNCCCACTGAGGGTCATCN
GCTTCGCGNGTTNGCGTATNGTCATAAACAT
>HMong220
GTGNAGCTCAGTTAGACACTTCTAAATNCTTNGTAACAGACATGCAGTCCCCTCNNCAGANCNCCGCC
CCCCGGTGGTACCGGGTTGTTAAGAGGTCCACCACGGGTCGCACGACAACNTCAAGGCCTCATGCATG
ACCCANCGCGGGGACGTACCGGCGNGGCGACCTTGNANGTCGGGGNCAAGTATGTCCCCCCGGGAAGG
ACGCCGTAGTGCATCTGGAGTGCCGATTCGTGGTCNAGAATGCCACCACGATGGTATGAGTGGCAAAG
GTATTTTGNNCACCACCCCGTTTTCTANTTCCAGACGCGGCCATTCAGGAACGACGCGAATCGTAATC
TACCTGCCGCGTNCGCGGATNGTACATGCGTNTTTCAGNGTTCGAAATAGAGTTTGTCGTTCCGCTGA
CNNGGTTNCACGGCTACTGCGATAAGCATATTCTACGGGGGGACTGTAAAGGAATTGTATCCACTCTG
CTCCCTGTCAATTGGNCATGTGCATCTCTNGTTGTTTAGGNNACATTAATAACACACAGCATTAGGTA
GATAAGTCTCCGATATAAGCGAACAGAACGTTGGTCAAAGACCGTGGTTCAATTGTAGTGGTATAACA
TTTCCCAGNGCATNAGGAACCCAGGGACACTACTAGCANGGNCGAGGAGAGACCCTTGGGTGGCAACA
CGNAATAAGTGTTNCCCAGTACACCAGCACGATANAGGGTTTGACCATNNNTTTNTAGACGACCNCGG
TCGCATTGAACCTGGCATTCAGAATGACGTGCTCTCAAGAGTCAAGNTCCNCCCACTGAGGGTCATNN
GCTTCGCGNNNTAGCGTATNGTCATAAACNT
>HMong221
GTNTAGCTCAGTTANACACNTCTAAATCCNTNGTAACAGACATGCAGTCCCCTCGGCAGAGCCCCGCC
CCCCGGTGGTACCGGGTTGTTNAGAGGTCCACCACGGGTCNNACGACNNCNTCAAGGCCTNANGCATG
ACCCANCGCGGGGACGTACCGGCGNGGCGACCTTGNANGTCGGGGNCAAGTATGTCCCCCCGGGAAGG
ACGCCGTAGTGCATCTGGAGTGCCGATTCGTGGTCCNGAATGNCACCACGATGGTATGAGTGGCAAAG
GTATTTTGGACACCACCCCGTTTTCTANTTCCAGACGCGGCCNTTCAGGAACGACGCGAATCGTAATC
TACCTGCCGCGTACGCGGATCGTACANGCGTNTTTCAGCGTTCGAAATAGAGTTTGTCGTTCCGCTGA
CCNGGTTNCACGGCTACTGCGATAAGCATATTCTACGGGGGGACTGTAAAGGAATTGTATCCACTCNG

CTCCCTGTCAATTGGCCATGTGCATCTCTGGTTGTTTAGGGTACATTAATAACACACAGNATTAGGTA
GATAAGTCNCCGATATAAGCGAACAGANCGTTGGTNAAAGACCGTGGTTCAATTGTAGTGGTATAACA
TTTCCCAGTGCATAAGGAACCCAGGNACACTACTAGCATGGGCGAGGAGAGACCCTTGGGTGGCANCA
CGCAATAAGTGTTNCCCAGTACACCAGCACGATNGANGGTTTGNCCATGTGTTTTTAGNNGACCCCGG
TCGCATTGAACCTGGCATTCAGAATGACGTGCTCTNAAGAGTCAAGNTCCTCCCACTGAGGGTCANCN
GCTTCGCGNNNTNGCGTATNGTCATNAACAT
>HMong222
GNGTAGCCCAGTTGGACACTTCTNAATCCNTGGTANCAGACATGCAGTCCCCTCNNCAGAGCCCCGCC
CCCCGGTGGTACCGGGTTGTTAAGAGGTCCACCACGGGTCGCACGACAACNTCAAGGCCTCATGCATG
ANNCAACGCGGNGACGTACCGGNGCGGCGACCTTGAAGGTCGGGGACAAGTATGTCCCCCCGGGAAGG
ACGCCGTAGTGCANNTGGAGTGCCGATTNGTGGTCCAGAATGCCACCACGATGGTATGAGTGGCAANG
GTATTTTGGACACCACCCCGTTTTCTNNNTCCAGANGCGGCCNNTCAGGAACGACGCGAATCGTAATC
TACCTGCCGCGTACGCGGATNGTANATGCGNNTNNCAGCGTTCGANATAGAGTTTGTCGTTCCGCTGA
CCAGGTTNCACGGCTACTNNGATAAGCATATTCTACGGNGGGACTGTAAAGGAATTGTATCNACTCTG
CTCCCTGNCAATTGGCCATGTGCATCTCTGGTTGTTTAGGNNACATTAATAACACANAGCNTGAGGTA
GNTNAGTCNCCGATATAANCGAACAGAACGTTGGTCAAAGACCGTGGTTCAATTGTAGTGGTATAACA
TTTCCCAGTGCATAAGGAACCCAGGGACACTATTAGCATGGNCGAGGAGAGACCCTTGGGTGGCAACA
CGCAATAAGTGTTNCCCNGTACACCAGCACGATNNANGNTTTGACCATGTGNNNNTNGACGNCCCCGG
TCGNATTGAACCTGGCATTCAGAATGACGTNCTNTCANGAGTNNAGGTCNNCCCACTGAGGGTCATCN
GCTTCGCGNGTNNGCGTATNGTCATAAACAT
>HMong223
GTGTAGCCCAGTTAGACACNTCTAAATCCNTNGTAACAGACATGCAGTCCCCTCGGCAGAGNCCCGCC
CCCCGATGGTACCGGGTTGNTAAGAGGTCCACAACGGGTCGCACGACAACNTTAAGGCCTCATGCATG
ACCCAACACGGGGGCGTCCCGGCGCGGCGACCTTGAAGTCCGGGGACAAGTATGGCCCCCCGGGAAGG
ACGCNGTAGTGCATCTGGAGTGCCGATNCGTGGTCNAGAATGCCACCACGATGGTATGAGTGGCAAAG
GTACTTTGGACACCACCCCGTTTTCTANTNCCAGACGCGGCCNTTCAGGAACGACGCGAATCGTAATC
TACCTGCCGCGTACGCGGATCGTACATGCGTTTTCCAGCGTTCGAAATAGAGTTTGTCGTTCCGCTGA
CCAGGTTNCACGGCTACTGCGATAAGCATATTCTACTGGGGGACTGTAAAGGAATTGTATCNACTCTG
CTTCCTGTCAATTGGCCATGTGCATCTCTGGTTGTTCAGGGTACATTAATAACACACAGCATNAAGTA
GATAAGTCNCCGATATAAGCGAACAGANCGTTGGTCAGAGACTATGGTTCAATTGTAGTGGTATAATG
TTTCCCAGNGCATNAGGAACTCAGGGACACTACTAGCATGGGCGAGGAGAGACCCTTGGGTGGCANNA
CGCAATAAGTGTTACCTAGTACATCAGCACGAGNGAGGGTTTGACCATGTGTTTNTAGACGNCCCCGG
TCGCATTGAACCTGGCATTCAGAATGACGTGCTCTCANGAATCNAGNTCCNCCCACTGAGGGTCATCT
GCTTCGCGNGTCNGCGTATGGTCATAAACAT
>HMong224
GTGTAGCTCAGTTAGACACTTCTAAATCCTTGGTAACAGACATGCAGTCCCCTCNNCAGAGCCCCGCC
CCCCGGTGGTACCGGGTTGNTAAGAGGTCCACCACGGGTCGNACGACAACNTCAAGGCCTCATGCATG
ACCCAACGCGGGGACGTACCGGCGNGGCGACCTTGNAGGTCGGGGACAAGTATGTCCCCCCGGGAAGG
ACGCCGTAGTGCATCTGGAGTGCCGATTCGTGGTCCAGAATGCCACCACGATGGTATGAGTNGCAAAG
GTATTTTGGACACCACCCCGTTTTCTANTTCCAGANGCGGCCATTCAGGAACGACGCGAATCGTAATC
TACCTGCCGCGTACGCGGATCGTACATGCGTTTTTCAGCGTTCGAAATAGAGTTTGTCGTTCCGCTGA
CCAGGTTNCACGGCTACTGCGATAAGCATATTCTACGGGGGGACTGTAAAGGAATTGTATCCACTCTG
CTCCCTGTCAATTGGNCATGTGCATCTCTNGTTGTTTAGGGTACATTAATAACACACAGNATTAGGTA
GATAAGTCTCCGATATAAGCGAACAGANCGTTGGTCAAAGACCGTGGTTCAATTGTAGTGGTATAACA
TTTCCCAGTGCATAAGGAACCCAGGGACACTACTAGCATGGGCGAGGAGAGACCCTTGGGTGGCAANA
CGCAATAAGTGTTACCCAGTACACCAGCACGATAGANGGTTTGACCATGNNTTTNTAGNNGACCCCGG
TCGCATTGAACCTGGCATTCAGAATGACGTGCTNTCAAGAGTCAAGGTCCTCCCACTGAGGGTCATCT
GCTTCGCGNNNTNGCGTATNGTCATAAACNT
>HMong225
GTGNAGCTCAGTTAGACACTTCTAAATCCTTGGTAACAGACATGCAGTCCCCTCNNCAGAGCNCCGCC
CCCCNGTGGTACCGGGTTGTTAAGAGGTCCACCACGGGTCNNACGACAACNTCAAGGCCTCANGCATG
ANNCANCNCNGGGANGTACCGGNGCGGCGACCTTGNAGGTCGGGGNCAAGTATNTCCCCCCGNGAAGG
ACGCCGTAGTGCATCTGGAGTGCCGATTCGTGNNCNNGAATGCCACCACGATGGTATGAGTGGCAAAG
GTATTTNGGACACCACCCCGTTTNCTANTTCCAGANGCGGCCATTCAGGAACGACGCGAATCGTANTC

TANCTGCCGCGTACGCGGANNGTACANGCGTTTTTCAGCGTTCGAAATAGAGTTTGTCGTTCCGCTGA
CCAGGTTNCACGGCTACTGCGATAAGCATATTCTACGGGGGGACTGTAAAGGAATTGTATCCACTCNG
CTCCCTGTCAATTGGCCATGTGCATCTCTGGTTGTTTAGGNNACANTAATAACACACAGNATNAGGTA
GATAAGTCNCCGATATAAGCGAACAGANCGTTGGTCAAAGACCGTGGTTCAATTGTAGTGGTATAACA
TTTCCCAGTGCATNAGGAACCCAGGGACACTACTAGCANGGNCGAGGAGAGACCCTTGGGTGGNAANA
CGNAANAAGTGTTNNNCNGTACACCAGCACGATNNANGGTTTGACCATGTGTTTTTAGACGACCCCGG
TCGCATTGAACCTGGCATTCAGAATGACGTGCTNTNAAGAGTCAAGNTCCNNNCACTGAGGGTCANCT
GCTTCGCGNNNTNGCGTATGGTCATAAACAT
>HMong226
GTGNAGCTCAGTTAGACACTTCTAAATNCNTGGTAACAGACATGCAGTCCCCTCNNCAGAGNCCCGCC
CCCCNGTGGTACCGGGNNGTTAAGAGGTCCACCACGGNTCNNACGACNNCNTCAAGGCCTCATGCATG
ANNCNNCGCNGNGACGTACCGGNGCGGCGACCTTGAANGTCGGGGNCAAGTATGTCCCCCNGNGAAGG
ACGCNGTAGTGCATCTGGANTGCCGATTNGTGGTCNNNAATGNCACCACGATGGTATGAGTGGCAAAG
GTATTTNGGACACCACCCCGTTNNCTNNTTCCAGACGCGGCCNTTCAGGAACGACGCGAATCGTAATC
TACCTGCCGCGTNCGCGGATNGTACANGCGTNTTNCAGCGTTCGAAATAGAGTTTGTCGTTCCGCTGA
CCAGGTTNCACGGCTACTNCGATAAGCATATTCTACGGGGGGACTGTAAAGGAATTGTATCCACTCNG
CTCCCTGNCAATTGGCCATGTGCATCTCTNGTTGTTTAGGNNACATTAATAACACACAGNATNAGGTA
GATAAGTCTCCGATATAAGCGAACAGANCGTTGGTNAAAGACCGTGGTTCAATTGTAGTGGTATAACA
TTTCCCAGTGCATAAGGAACCCAGGGACACTACNAGCATGGGCGAGGAGAGACCCTTGGGTGGCAANA
CGNAATAAGTGTTNNNCNGTACACCAGCACGATANANGGTTTGNCCNNGNNTTNNTAGACGACCCCGG
TCGCATTGAACCTGGCATTCAGAATGACGTNCTCTCAAGAGTCNAGNNCCNCNCACTGAGGGTCATNN
GCTTCGCGNNNTAGCGTATNGNCATAAACAT
>HMong227
GTGNAGCCCAGTTAGACACTTCTAAATCCNTNGTAACAGACATGCAGTCCCCTCNNCAGAGCNCCGCC
CCCCGATGGTACCGGGTTGNNAAGNGGTCCACAACGGGTCGNACGACNNCATTAAGGCCTNANGCATG
ANNCANCACGGNGGNGTCCCGGNGCGGCGACCTTGAANTCCGGGGNCAAGTATGGCCCCCCGGGAAGG
ACGCCGTAGTGCATCTGGAGTGCCGATTNNTGGTCNAGANTGNCACCACGATGGTATGAGTGGCAAAG
GTACTTTGGACACCACCCCGTTTTCTANTNCCAGANGCGGCCNTTCAGGAACGACGCGAATCGTAATC
TACCTGCCGCGTACGCGGATCGTANATGCGTTTTCCAGCGTTCGAAATAGAGTTNGTCGTTCCGCTGA
CCAGGTTNCACGGCTACTGCGATAAGCATATTCTACTGGGGGACTGTAAAGGAATTGTATCNACTCNG
CTTCCTGTCAATTNGCCATGTGCATCTCTGGTTGTTCAGGGNACATTAATAACACACAGNATTAAGTA
GATNAGTCNCCGATATAAGCGAACAGANCGTTGGTCAGAGACTATGGTNCAATTGTAGTGGTATAATG
TTTCCCAGTGCATAAGGAACTCAGGNACACTACTAGCANGGNCGAGGAGAGACCCTTGGGTGGCAANA
CGCANNAAGTGTTNNNTAGTACANCAGCACGAGNNANGGTTTGACCATNNNTTNNTAGNNGACCCCGG
TCGCATTGAACCTGGCATTCAGAATGACGTNCTNTNAAGAANCAAGNTCCNCNCACTGAGGGTCANNN
GCTTCGCGGGNCNGCGTATNGNCATAAACAT
>HMong228
GTGTAGCTCAGTTAGACACTTCTAAATCCTTNGTAACAGACATGCAGTCCCCTCNNCAGAGCNCCGCC
CCCCGGTGGTACCGGGTTGTTAAGAGGTCCACCACGGGTCNNACGACAACATCAAGGCCTNATGCATG
ACCCAACGCNGGGANGTACCGGNGNGGCGACCTTGAAGGNCGGGGNCAAGTATGTCCCCCCGGGAAGG
ACGCCGTAGTGCATCTGGAGTGCCGATTNGTGGTCCNGAATGNCACCACGATGGTATGAGTGGCAANG
GTATTTTGGACACCACCCCGTTTTCTANTTCCAGANGCGGCCNTTCAGGAACGACGCGAATCGTAATC
TACCTGCCGCGTACGCGGATNGTACANGCGTTTTNCAGCGTTCGAAATAGAGTTTGTCGTTCCGCTGA
CCAGGTTNCACGGCTACTGCGATAAGCATATTCTACGGGGGGACTGTAAAGGAATTGTATCNACTCTG
CTCCCTGTCAATTGGCCATGTGCATCTCTGGTTGTTTAGGGNACATTAATAACACACAGNATNAGGTA
GNTAAGTCNCCGATATAAGCGAACAGANCGTTGGTCAAAGACCGTGGTTCAATTGTAGTGGTATAACA
TTTCCCAGTGCATAAGGAACCCAGGNACACTACTAGCATGGGCGAGGAGAGACCCTTGGGTGGCAANA
CGCAATAAGTGTTANCCAGTACACCAGCACGATANAGGGTTTGACCNTGTGTTTTTAGNNGACCCCGG
TCGCATTGAACCTGGCATTCAGAATGACGTGCTCTNAAGAGTCAAGNTCCTCNCACTGAGGGNCATCT
GCTTCGCGNNNTAGCGTATNGTCATNAACAT
>HMong229
GTGTAGCTCAGTTAGACACTTCTAAATCCNTGGTAACAGACATGCAGTCCCCTCNNCAGAGCCCCGCC
CCCCGGTGGTACCGGGTTGTTNAGAGGTCCACCACGGGTCGCACGACAANNTCAAGGCCTCATGCATG
ANNCAACNCGGGGACGTACCGGCGNGGCGACCTTGAAGGTCGGGGACAAGTATGTCCCCCCGNGAAGG

ACGCCGTAGTGCATCTGGAGTGCCGATTCGTGGTCNAGANTGCCACCACGATGGTATGAGTGGCAAAG
GTATTTTGGACACCACCCCGTTTTCTATTNCCAGANGCGGCCNTTCAGGAACGACGCGAATCGTAATC
TACCTGCCGCGTNCGCGGATNGTACATGCGTTTTTCAGCGTTCGAAATAGAGTTTGTCGTTCCGCTGA
CCAGGTTTCACGGCTACTGCGATAAGCATATTCTACGGGGGGACTGTAAAGGAATTGTATCCACTCTG
CTCCCTGTCAATTGGCCATGTGCATCTCTNGTTGTTTAGGNNACATTAATAACACACAGCATTAGGTA
GATAAGTCTCCGATATAAGCGAACAGANCGTTGGTCAAAGACCGTGGTTCAATTGTAGTGGTATAACA
TTTCCCAGTGCATAAGGAACCCAGGNACACTACTAGCATGGGCGAGGAGAGACCCTTGGGTGGCAACA
CGCAATAAGTGTTNCCCAGTACACCAGCACGATANAGGGTTTGACCATGTGTTTTTAGACGACCCCGG
TCGCATTGAACCTGGCATTCAGAATGACGTGCTNTCAAGAGTCAAGNTCCTCNCACTGAGGGTCATCT
GCTTCGCGNNNTAGCGTATNGTCATNAACAT
>HMong230
GNGNAGCTCAGTTANACACTTCTAAATCCTTGGTAACAGACATGCAGTCCCCTCNNCAGAGNCCCGCC
CCCCGGTGGTACCGGGTTGNNAAGAGGTCCACCACGGGTCGNACGACAACNTCAAGGCCTCATGCATG
ACCCAACGCGGGGACGTACCGGNGNGGCGACCTTGNAGGTNGGGGNCAAGTATGTCCCCCCGGGAAGG
ACGCNGTAGTGCATCTGGAGTGCCGATTCGTGGTCNAGAATGNCACCACGANGGTATGAGTGGCAANG
GTATTTNGGACACCACCCCGTTTNCTANTTCCAGANGCNGCCNTTCAGGAACGACGCGAATNGTAATC
TACCTGCCGCGTACGCGGATCGTACNNGCGTTTTNCAGCGTTCGAAATAGAGTTTGTCGTTCCGCTGA
CCNGGTTNCACGGCTACTGCGATAAGCATATTCTACGGGGGGACTGTAAAGGAATTGTATCNACTCNG
CTCCCTGTCAATTGGNCATGTGCATCTCTGGTTGTTTAGGNNACATTAATAACACACAGCATTAGGTA
GATNAGTCTCCGATATAAGCGAACAGANCGTTGGTCAAAGACCGTGGTTCAATTGTAGTGGTATAACA
TTTCCCAGTGCATAAGGAACCCAGGGACACTACTANCATGGGCGAGGAGAGACCCTTGGGTGGCAACA
CGCAATAAGTGTTNNCCAGTACACCAGCACGATAGANGGTTTGACCATNNNTTTNTAGACGACCCCGG
TCGCATTGAACCTGGCATTCAGAATGACGTNNTNTCAAGAGTCAAGNTCCTCCCACTGAGGGTCANNN
GCTTCGCGNNNTNGCGTATNGTCATNAACAT
>HMong231
GTGNAGCCCAGTTANACACTTCTNAATCCTTNGTAACAGACATGCAGTCCCCTCNNCAGAGCCCCGCC
CCCCGATGGTACCGGGTTGNNAAGAGGTCCACAACAGNTCGNACGACAACATTAAGGCCTCATGCATG
ACCCAANNCGGGGGNGTCCCGGCGNGGCGACCTTGAAGTCCGGGGNCAAGTATGGCCCCCCGGGAAGG
ACGCCGTAGTGCATCTGGAGTGCCGATNCGTGGTCNAGAATGCCACCACGATGGTATGAGTNGCAAAG
GTACTTTGGACACCACCCCGTTTTCTANTTCCAGANGCGGCCNTTCAGGAACGACGCGAATCGTAATC
TACCTGCCGCGTNCGCGGATCGTACATGCGTTTNCCAGCGTTCGAAATAGAGTTTGTCGTTCCGCCGA
CCAGGTTNCACGGCTACTGCGATAAGCATATTCTACTGGGGGACTGTAAAGGAATTGTATCCACTCTA
CTTCCTGTCAATTGGCCATGTGCATCTCTGGTTGTTCAGGGTANATTAATAACACACAGCATTAAGTA
GATNAGTCTCCGATATAAGCGAACAGANCGTTGGTCAGAGACTGTGGTTCAATTGTAGTGGTATAATG
TTTCCCAGTGCATAAGGAACTTAGGGACATTACTAGCATGGGCGAGNAGAGACCCTTGGGTGGCAACA
CGNAATAAGTGTTNCCTAGTACACCAGCACGAGNNAGGGTTTGACCATGTGTTTTTAGACGACCCCGG
TCGCATTGAACCTGGCATTCAGAATGACGTGCTNTCANGAATCAAGNTCCNCCCACTGAGGGTCATCN
GCTTCGCGGNTCNGCGTATNGTCATAAACAT
>HMong232
GTGTAGCCCAGTCAGACACNTCTAAANCCNTGGTAACAGACATGCAGTCCCCTCGGCAGAGCNCCGCC
CCCCGATGGTACCGGGTTGNTNAGNGGTCCACAACGGGTCGCACGACAACNTTAAGGCCTCANGCATG
ACCCAACNCGGNGGNGTCCCGGNGCGGCNACCTTGNAGTCCGGGGACAAGTATGGCCCCCCGGGAAGG
ACGCCGTAGTGCATCTGGAGTGCCGNTTCNTGGTCNAGAATGNCACCACGATGGTATGAGTGGCAAAG
GTACTTTGGACACCACCCCGTTTTCTATTTCCAGANGCGGCCATTTAGGAACGACGCGAATCGTAATC
TACCTGCCGCGTACGCGGATNGTACATGCGTTTTCCAGCGTTCGAAATNGAGTTTGTCGTTCCGCTGA
CCAGGTTNCACGGCTACTGCGATAAGCNTATTCTACTGGGGGACTGTAAAGGAATTGTATCCATTCNG
CTTCCTGTCAATTGGNCATGTGCATCTCTGGTTGTTCAGGGTACATTAATAACACACAGNAGTAAGTA
GNTAAGTCTCCGNTATAANCGAACAGAACGTTTGTNAGANACTGTGGTTCAATTGTAGTGGTATAATG
TTTCCCAGNGCATAAGGANCTCAGGGACACTACTAGCATGGGCGAGGAGAGACCCTTGGGTGGCAACA
CGCAATAAGTGTTANCTAGTACACCAGCACGAGNGANGGTTTGACCATGTGTTTTTAGACGACCCCGG
TCGCATTGAACCTGGCATTCAGAATGACGTGCTNTCAAGAATCAAGGTCCTCCCACTGAGGGTCATCT
GCTTCGCGNNNCAGCGTATNGTCATAAACAT
>HMong233
GTGTAGCCCAGTTANACACTTCTAAATCCNTGGTAACAGACATGCAGTCCCCTCNNCAGAGCNCCGCC

CCCCNATGGTACCGGGTTGNTAAGAGGTCCACAACAGGTCNNACGACAACATTAAGGCCTCANGCATG
ANNCNACACGGNGGCGTCCCGGNGNGGCGACCTTGNAGTCCGGGGNCAAGTATNGCCCCCCGGGAAGG
ACGCNGTAGTGCATCTGGAGTGCCGATTCGTGGTCNAGAATGCCACCACGATGGTATGAGTGGCAAAG
GTACTTNGGACACCACCCCGTTTTCTATTTCCAGANGCGGCCNTTCAGGAACGACGCGAATCGTAATC
TACCTGCCGCGTACGCGGATCGTACNTGCGTTTTCCAGCGTTCGAAATAGAGTTTGTCGTTCCGCCGA
CCNGGTTNCACGGCTACTGCGATAAGCATATTCTACTGGGGGACTGTAAAGGAATTGTATCCACTCNA
CTTCCTGTCAATTGGNCATGTGCATCTCTNGTTGTTCAGGNNACATTAATAACACACAGCATNAAGTA
GATAAGTCTCCGATATAAGCGAACAGANCGTTGGTCAGAGACTGTGGTTCAATTGTAGTGGTATAATG
TTTCCCAGTGCATAAGGAACTTAGGGACATTACTAGCATGGGCGAGGAGAGACCCTTGGGTGGNAANA
CGNAATAAGTGTTNCCTAGTACACCAGCACGAGNNANGGTTTGACCNTGTGTTTTTAGNNGNCCCCGG
TCGCATTGAACCTGGCATTCAGAATGACGTGCTCTCAAGAATCAAGGTCCTCNCACTGAGGGTCATCT
GCTTCGCGGGTCNGCGTATGGNCATAAACAT
>HMong234
GTGTAGCCCAGTTAGACACTTCTAAATCCTTGGTAACAGACATGCAGTCCCCTCNNCAGAGNCCCGCC
CCCCNATGGTACCGGGTTGTTAAGAGGTCCACAACGGGTCGCACGACAACATTAAGGCCTCATGCATG
ACCCNANNCGGGGGNGTCCCGGCGNGGCGACCTTGAANTCCGGGGNCAAGTATGGCCCCCCGNGAAGG
ACGCNGTAGTGCATCTGGAGTGCCGATTNNTGGNCNAGAATGNCACCACGATGGTATGAGTNGCAAAG
GTACTTTGGACACCACCCCGTTTTCTANTTCCAGANGCGGCCATTCAGGAACGACGCGAATCGTAATC
TACCTGCCGCGTNCGCGGATCGTACATGCGTTTTCCAGCGTTCGAAATAGAGTTTGTCGTTCCGCTGA
CCNGGTTNCACGGCTACTNCGATAAGCATATTCTACTGGGGGACTGTAAAGNAATTGTATCCACTCNG
CTTCCTGTCAATTGGCCATGTGCATCTCTNGTTGTTCAGGNNACATTAATAACACACAGCATNAAGTA
GATAAGTCTCCGATATAAGCGAACAGANCGTTGGTCAGAGACTATGGTTCAATTGTAGTGGTATAATG
TTTCCCAGTGCATAAGGAACTCAGGGACACTACTAGCANGGGCGAGGAGAGACCCTTGGGTGGCAANA
CGCAATAAGTGTTNCCTAGTACATCAGCACGAGNNAGGGTTTGNCCATGNNTTTTTAGNNGACCCCGG
TCGCATTGAACCTGGCATTCAGAATGACGTNCTNTCAAGAATCAAGNTCCNNNCACTGAGGGTCATCN
GCTTCGCGGGTCNGCGTATNGTCATAAACAT
>Kinh01
GTGTAGCTCNGTTANACACNTCTAAATCCTTNGTAACAGACATGCAGTNCCCTCNNCAGAGCCCCGCC
CCCCGGTGGTACCGGGTTGTTAAGAGGTCCACCACGGNTCNNACGANNNCNTCAAGGCCTNATGCATG
ACCCANNNCGGNGANGTACCGGNGNGGNGACCTTGAAGGTCGGGNACAAGTATGTCCCCCCGGGAAGG
ACGCCGTAGTGCATCTNGAGTGCCGNNNCGTGGTCCAGANTGCCACCACGATGGTATGAGCGGCAAAG
GTATTTNGNNCACCACCCCGTTTTCTANNTCCAGANGCGGCCATTCAGGAACGACGCGAATCGTANTC
TACCTGCCGCGTNCGCGGANCGTANANGCGTTTTTCAGCGTTCGAAATAGAGTNTGTCGTTCCGCTGA
CCAGGTTNCACGGCTACTNCGATAAGCATATTCTACGGGGGGACTGTAAAGNAATTGTATCCACTCTG
CTCCCTGTCAATTGGNCATGTGCATCTCTGGTTGTTTAGGGTACATTAATAACACACAGCNTTAGGTA
GNTAAGTCNCCGATATAAGCGAACAGANCGTTGGTCAAAGACCGTGGTTCAATTGTAGTGGTATAACA
TTTCCCAGNNCATAAGGAANCCAGGNACACTACTAGCANGGNCGAGGAGAGACCCTTGGGTGGCAANA
CGCAATAAGTGTTACCCANTACACCNGCACGATNNAGGGTTTGACCATGTGTTTTTAGACGACCCCGG
TCGCATTGAACCTGGCATNCAGAATGACGTGNTNTCAAGAGTCNANNTCCNNNCACTGAGGGTCATCN
GCTTCGCNNNNNTNGCGTATNGTCANNAACNT
>Kinh02
GTNTAGNTNNGNTANACACNNNNNAATNCNTNNTAACAGACATGCAGTCCCCTCNNCAGAGNCCCGCC
CCCCNGTGGTACCGGGTTGNNNAGNGGTCCACCACGGNTCGCANGACAANNTCAAGGCCTCANGCATG
ANNCNNCNCGGNGACGTACCGGNGNGGCGACCTTGAANGNNGGGNNCAANTNNNTCCCCCCGNNAAGN
ACNCNGTAGTGCATNNNGAGTGCCGNTTCNTGGTCNAGAATGCCACCACGANNGTATGAGTGGCAANG
GTATTTTGGACACNACCCCNTTTTCTNNTNCNAGNNGCNGCCANNNAGGAACGACGCGNNNCGTANTC
TANCTGCCGCNTNCGCGGANNGNANNNGCNTTNTNCAGNGTTCGANATANAGTTTGTCGTTCCGCTGA
CCNGGTTNCACGGNTNCTNCGATAAGCATANTCTACGGNGGGACTGTAAAGNNNTTGTANCNACTCNN
CNCCCTGTCAATTGGNCATGTGCATCTCTGGTTGTTTAGGNNACANTAATAACACACAGNATNAGGTA
GNTNAGTCNCCGNTATAANCGAANAGANCGTTGGTNNANGACCGTGNTTCAANTGTAGTGGTATAACA
TTNNCCAGNGCATNANGAANCCAGGNACACNACTAGCANGGNCGAGGAGNNACCCTTGGGTGGNANNA
NGCANTAAGTGTNNNNCNNTACACCAGCANGATANANGGTTTGACNATNNNNNTTNAGNNGNCCCCGG
TCGCATTGAACCTGGCATTCAGAATGACGTGNTNTCANNAGTNNAGNTCNNNCCACTGAGGGNCATCN
NCTTCGCGNNNNNGCGTATNGTCANNNNNNT

>Kinh03
GTGTAGCCCAGTTAGACACTNNNNAATNCNTNGTAACAGACATGCAGTCCCCTCNNCAGAGCCCCGCC
CCCCGATGGTACCNGGTTGNTNAGAGGTCCACAACGGNTCNNACGACNNNNTTAAGGCCTCANGCNTN
ACCCANNNCNGNNGNGTCCCGGCGCGGCGACCTTGAAGTCNGGGNNCAAGTATGGCCCCCCGGGAAGG
ACGCNGTAGTGCATNTGGAGTGCCGNTTCGTGGTCCAGAATGCCACCACGATGGTATGAGTNGCAAAG
GTACTTNGNNCACCACCCCGTTTTCTANTTCCAGANGCGGCCNTTCAGGAACGACGCGAATCGTAATC
TACCTGCCGCGTNCGCGGATCGTACANGCGTNTTCCAGCGTTCGAAATAGAGTNTGTCGTTCCGCTGA
CCNGGTTNCACGNNTACTNNGATAAGCATATTCTACTGGNGGACTGTAAAGGAATTGTATCCANNCNG
CTTCCTGTCAATTGGNCATGTGCATCTCTNGTTGTTCAGGNNACATTAATAACACACAGNATNAANTA
GATNAGTCTCCGATATAAGCGAACAGANCGTTGGTCAGAGACTATGGTTCAATTGTAGTGGTATAATG
TTTCCCAGNGNATAAGGAANTCAGGNACACTACTAGCATGGGCGAGGAGAGACCCTTGGGTGGCAANA
CGNAATAAGTGTTACCTAGTACATCAGCACGAGNNAGGGTTNGNCCATGTGTTTNTAGNNGACCCCGG
TCGCATTGAACCTGNCATTCNGAATGACGTGCTNTCAAGAATCAAGNTCCNCNCANNGAGGNTCATNN
GCTTCGCGGGTCAGCGTATGGNCATNNNNNT
>Kinh04
GTGTAGCCCAGTTANACACTNNNNAATCCNTNGTNACAGACATGCAGTCCCCTCGGCAGAGNCCCGCC
CCCCNATGGTACCGGGTTGTNNAGAGGTCCACAACGGNTCNCACGACNNNNNTAAGGCCTCANGCATN
ACCCAACNCGGGGGCGTCCCGGCGCGGNGACCTTGAAGTNNGGGGNCAAGTATGGCCCCCCGGGAAGG
ACGCCGTAGTGCANCTGGAGTGCCGNTTCGTGGTCNNGAATGNCACCANGATGGTATGAGTNGCAAAG
GTACTTNGGACACCACCCCGTTTTCTATTTCCAGANGCGGCCATTCAGGAACGACGCGAATCGTAATC
TACCTGCCGCGTNCGCGGATCGTACANGCGTNTTCCAGNNTTCGAAATAGAGTTNGTCGTTCCGCTGA
CCNGGTTNCACGGCTACTNNGATAAGNATATTCTACTGGNGGACTGTAAAGGAATTGTATCCACTCNG
CTTCCTGTCAATTGGCCATGTGCATCTCTNGTTGNTCAGGNNANATTAATAACACACAGNANNAAGTA
GATNAGTCTCCNNTATAAGCGAACAGANCGTTGGTNAGAGACTNTGGTTCAATTGTAGTGGTATAATG
TTTCCCAGTGCATAANNAANTCAGGGACACTACTAGCATGGNNGAGGAGAGACCCTTGGGTGGCANNA
CGNAATAAGTGTTNCCTAGTACATCAGCACNNGNNAGGGTTTGNCCATGTGTTTTTAGACGACCCCGG
TCGCATTGAACCTGGCATTCAGAATGACGTGCTNTCAANAATCAAGNTCCNNNCACTGAGGGNCANNN
NCTTCGCGGGTCNGCGTATNGTCATANNNAT
>Kinh05
GTGTAGCCCAGTTNNACACTTCTAAATNCNTNGTAACAGACATGCAGTNNNCNCNNCAGAGCNCCGCC
CCCCNATGGTACCGGGTTGNTNAGAAGTCCACAACGGNTCNNANGACNNNNTTAAGGCCTNANGCATG
ACCCANNNCNGNGGNGTCCCGGNGNNGCGACCTTNNANTCCGGGGNCAAGTATGGCCCCCCGNGAAGG
ACGCNGTAGTGCATNTGGANTGCCGATTCGTGGTCNAGAATGCCACCACGATGGTATGAGTGGCAAAG
GTACTNNGGACACNACCCNGTTNNCTANTTCCAGANGCGGCCNTTCAGGAACGACGCGAATCGTAATC
TACCTNCCGCGTNCGCGGATCGTACANGCGTNTTCCAGCGTTCGAAATAGAGTNTGTCGTTCCGCTGA
CCAGGTTNCACGGCTACTNNGATAAGCATATTCTACTGGNGGACTGTAAAGNAATTGTATCCANNCNG
CTTCCTGNCAATTGGNCATGTGCATCTCTNGTTGTTCAGGNNANATTAATAACNCACAGNAGNAAGTA
GNTNAGTCTCCGATATAAGCTAACNGANCGTTTGTNAGAGACTGTGGTTCAATTGTAGTGGTATAATG
TTTCCCAGNNCATAAGNNNNTCAGGGACACTACTAGCATGGGCGAGGAGNGACCCTTGGGTGGCAANA
CGNANNAAGTGTTNCCTNGTACACCAGCACNAGNNAGGNNTTGACCATGNNTTTTCAGACGACCCCGG
TCGCATTGAACCTGGCNTTCAGAATGNCGTGCTNTCAAGAATCAAGNTCCNNNNACTGAGGNNCANNT
GCTTCGCNNGTCAGCGTATNGTCACAAACNT
>Kinh06
GTGNAGCCCAGTTAGACACTTCNAAATCCNTNGTAACAGACATGCAGTCCCCTCNNCAGAGCCCCGCC
CCCCNATGGTACCGGGTTGNTNAGNGGTCCACAACGGGTCGNACGACNNNNTTAAGGCCTNANGCATN
ANNCNACNCNGNGGCGTCCCGGNGNNGCGACCTTGNANTCCGGGGACAAGTATGGCCCCCCGGGAAGG
ACGCCGTAGTGCATCTGGAGTGCCGATTNGTGGTCCAGAATGCCACCACGATGGTATGAGTGGCAAAG
GTACTTNNNNCACCACCCNGTTTTCTATTTCCAGACGCGGCCNTTCAGGAACGACGCGAATCGTAATC
TACCTGCCGCGTNCGCGGATNGTACATGCGTTTTCCAGCGTTCGANATAGAGTTTGTCGTTCCGCTGA
CCNGGTTNCACGGNTACTNNGATAAGCATATTCTACTGGGGGACTGTAAAGGAATTGTATCCACNCTG
CTTCCTGTCAATTGGNCATGTGCATCTCTGGTTGTTCAGGNNACATTAATAACACACAGCNTNAAGTA
GATAAGTCNCCGATATAAGCGAACAGANCGTTGGTCAGAGACTATGGTTCAATTGTAGTGGTATAATG
TTTCCCAGTNCATAAGGANCTCAGGNACACTACTAGCATGGNCGAGGAGAGACCCTTGGGTGGCAACA
CGCAATAAGTGTTACCTAGTACATCAGCACGAGNNAGGGTTTGNCCATGTGTTTNTAGACGACCCCGG

TCGCATTGAACCTGNCNTTCAGAATGACGTGCTNTCAAGAATCAAGNTCCNNNCACTGAGGGNCATCN
GCTTCGCGGNTCNGCGTATGGNCATANNNAT
>Kinh07
GTGNAGCCCAGTTNNNCACNNCTAAATNCNTGGTAACAGACATGCAGTCCCCTCNNCAGAGCCCCGNC
CCCCGATGGTACCGGGTTGNNNAGAGGTCCACAACGGNTCNNACGACNNNNTTAAGGCCNNANGCATG
ACCCANNNCNGGGGNGTCCCGGNGNGGNNACCTTNAANTCNGGGNNCAAGTATGGCCCCCCGNGAAGG
ACGCNGTAGTGCATCTGGAGTGCCGATTCGTGGTCNNGAATGCCACCACGATGGTATGAGTGGCAAAG
GTACTTNGGACACNACCCCGTTNNCTNTNTCCAGANGCGGCCNTTCAGGAACGACGCGAATNGTANTC
TACCTGCCGCGTACGCGGATNGTACNNGCGTNTTCCAGCGTTCGANATANAGTNTGTCGTTCCGCTGA
CCNGGTTNCACGGCTACTNNGATAAGCATATTCTACTGGGGGACTGTAAAGNAATTGTANCCACTCNG
CTTCCTGNCAATTGGNCATGTGCATCTCTGGTTGTTCAGGNNANATTAATAACACACAGCNTNAAGTA
GATNAGTCNCCGATATAAGCGAACAGANCGTTGGTNAGAGACTATGGTTCAATTGTAGTGGTATAATG
TTTCCCAGTNCNNNAGGANNTCAGGNACACTACTAGCANGGGCGAGGAGAGACCCTTGGGTGGCAANA
CGNGANAAGTGTTNNCTAGTACATCAGCACNAGNNAGGGTTNGACCATGTGTTTTTAGACGACCCCGG
TCGCATTGAACCTGGCNTNCAGAATGACGTGCTNTCANGAATNNAGNTCCTCNCACTGAGGGNCANNN
GNTTCGCGGNNCNGCGTATNGNCATNANCAT
>Kinh08
GNNTAGCCCAGNTNNACACTNNNNAANCCNTNGTAACAGACATGCAGTNCCCTCNNCAGAGCNCCGCN
CCCCGATGGTACCGGGTTGNTAAGAAGTCCACAACGGNTCNNACGACNNNNNTAAGGCCNCANGCATN
ANNCANNNCNGNNGNGTCCCGGNGNNGNGNCCNNGNANNNNGGGNNCAAGTATGGCCCNCCGNGAAGG
ACNCNGTAGTGCANNTGGNGTGCCGANNCNTGGTCCAGAATGCCACCACGATGGTATGAGTNGCAAAG
GTACTTNNNNCACNACCCNNTTNNCTNNNNCCAGANGCGGCCNTTCANGAACGACGCGAATCGTAATN
NNNCTGCCGCGTNCNCGGATNGTACNNGCGTNTTNCAGCGTTCGAAATAGNGTNTGTCGTTCCGCTGA
CCNGGTTNCACGGNTACTNCGNTANGCANNTNCTACTNGNGGACTGNAAANNNNTTGTANCCANNCNG
CNTCCTGNCAATTGGNCNTGTGCATCTCTNGNTGNTNAGGNNANATTNATNNCACANAGNNGNAAGTA
GGTNAGTCNCCGATNTAAGCTNACAGANCGTTTGTNAGAGACTGTGGNTCAATTGNAGTGGTATAANG
TTTCCCAGNGCATAANGANNTCAGGNACACNACTAGCANGGNCNAGNAGNGACCCTTGGGTGGCAANA
CGCANTAAGTGTTNNNTAGTACACCAGCACGAGNNAGGGTNNGNCCANGTGTTTNCAGNNGACCCCGG
TCGCATTGAACCTGNCATNCANNNNGNNNTGCTNTCAAGAATNNAGNTCCNNNCACNNNGGNNCANNN
NNNTCGCGGNTCNGCGTATNGTCACNANNAT
>Kinh10
GTGNAGCCCAGTTNGACACTNNNNAATNCNTNGTANCAGNCATGCAGTNCCCNCNNNAGAGNNCCGCC
CCCCGATGGTACCGGGNNGNTNAGAGGTCCACAACGGNNCNNANGACNNNNNTAAGGCCNNANGCATG
ACCCNNCNCNGGNGNGNCCCGGCGNNGNGACCTTNNNGNCNGGGGNCAAGTATNGCCCCNNGGGAAGG
ACGCCGTAGTGCATNTGGAGTGCCGNNNCNTGGTCNNGAATGCCACCACGATGGTNNGAGTGGCAANG
GTACTTNGGACACCACCCNGTTNNCTNNTTCCAGANGNNGCCNTTCAGGAACGACGCGAATCGNAATN
TANCTNCNGCGTNCGCGGATNGNACNNGCGTNTTCCAGCNTTCGANATAGANTNTGTCNNNCCGCTGA
CCNGGTTNCNNGGNNANTNNGATAANCATATTCTNCTNGNGGACNGTAAANNNNTTGTATCCACNCNG
NTNCCTGNCAATTNGNCATGTGCATCTCTGGTTGTTCANGNNNCANTAATAACACANAGNATNAANTA
GNTNAGTCTCCNNTNTAAGNNAANAGANCGTTGGTNANANACTATNGTTNNATTNTAGTGNNATAATG
TTTCCCAGNGCATAAGGAANTCAGGNACNCTACNAGNATGGNCGAGGAGAGACCCTTGGGTGGCAANA
CGNGANNAGTGTTNCCTNNTACATCAGCACNNGNNANGGTTTGNNCNTNNNTTNNNAGNNGNCCNNGG
TCGNATTGAANNTGNCNTTCNNAATGANGTNCTNTCANGAANNNANNTCCNCNCANNGAGGGNCANNN
NNTTCGCGGNNCNGCGTATNGNCATAAACAT
>Kinh11
GTGTAGCCCAGTTNGNCACNNNNAAATCCNTGGTAACAGACATGCAGTNCCNTCNNCAGANCCCCGCC
CCCCGATGGTACCGGGTTGNTNAGAGGTCCACCACGANTCNNANGACNNNNGCAAGGCCNNGNGCATN
ACCCANNNCNGGGGNGTCCCGGCNNGGCGACCTTGNAGTCCGGGNNCAAGTATGGCTCCCCGNGAAGG
ACGCNGTAGTGCATCTGGAGTGCCGANNCGTGGTCCNGAATGCCACCACGATGGTATGAGTNGCAAAG
GTACTNTNNNCACCACCCCGTTTTCTANTCCCAGANGCGGCCNTTCAGGAACGACGTGAATCGTAATC
TANCTNCCGCGTNCGCGGNTCGTACNNGCGTNTTCCAGCGTTCGANATANAGTNTGTCGTTCCGCTGA
CCNGGTTNCACGGNTACTGCGNTAAGCATACTCTACTGGGGGACTGNAAANNAATTGTATCCACTCTG
CTTCCTGTCAGTTGGCNATATGTATCTCTGGTTGTTCAGGGTANATTAATAACACACAGCNTNAAGTA
GATNAGTCTCCGATATAGGCGAACAGANCGTTGGTCAAAGACTGTGGTTCAACTGTAGTGGTATAATG

TTTCCCAGTNCATNAGNAANTCAGGNACACTACTAGCATGGNCGAGTANAGACCCTTGGGTGGCAANA
CGNAATAAGNGTTNNCTNGTACACCAACACGATNNANGGTTTGNCCATGTGTTTNTAGNNGACCCCGG
TCGCATTGAACCTGGCATTCATAATGACGTGCTNTCAAGAGTCAAGNTCCTNNCACTGNGGGTCANCN
GNTTCGCNGGTCATCGTATNGNCATNAACAT
>Kinh12
GTGTAGCCCAGTTAGACACTTCNAAATCCNTNGTAACAGACATGCAGTCCCCTCNNCAGAGCNCCGCC
CCCCGATGGTACCGGGTTGNTNAGAGGTCCACAACAGNTCNNACGACNNCATTAAGGCCTCATGCATG
ACCCANNNCNGGNGNGTCCCGGNGCGGCGACCTTGAANTCCGGGGNCAAGTATGGCCCCCCGGGAAGG
ACGCNGTAGTGCATCTGGAGTGCCGANNCNTGGTCNAGAATGCCACCACGATGGTATGAGTGGCAANG
GTACTTNNNNCACCACCCNGTTNNCTNTTTCCAGANGCGGCCNTTCAGGAACGACGCGAATCGTNATC
TACCTGCCGCGTACGCGGATCGTACNNGCGTTTTNCAGCGTTCGAAATAGAGTTTGTCGTTCCGCCGA
CCAGGTTNCACGGCTANTNCGATAAGCATATTCTACTGGGGGACTGTAAAGGAATTGTATCCACTCNA
CTTCCTGTCAATTGGNCATGTGCATCTCTNGTTGTTCAGGNNACATTAATAACACACAGCATTAAGTA
GATNAGTCTCCGATATAAGCGAACAGANCGTTGGTCAGAGACTGTGGTTCAATTGTAGTGGTATAATG
TTTCCCAGTGCATAAGGAACTTAGGNACATTACTAGCATGGNCGAGGAGAGACCCTTGGGTGGCAANA
CGCAATAAGTGTTANCTAGTACACCAGCACGAGNNANGGTTTGNCCATGTGTTTNTAGNNGACCCCGG
TCGCATTGAACCTGGCATTCAGAATGACGTGCTNTNAAGAATCNAGNTCCTCNCACTGAGGGTCATNN
GCTTCGCGGGTCNGCGTATNGTCANANACAT
>Kinh13
GTNTAGCCCAGTTNNACACTNNNNAATCCNTNGTAACAGACATGCAGTCCCCTCNNCAGAGNNCCGCC
CCCCGATGGTACCGGGTTGNTNAGAGGTCCACAACGGNTCNNACGACNNNNTTAAGGCCNCANGCATG
ACCCANCACNGGGGNGTCCCGGNGNGGCGACCTTGNAGTCCGGGGNCAAGTATGGCCCCCCGNGAAGG
ACGCCGTAGTGCATCTGGAGTGCCGNTTNGTGGTCCAGAATGCCACCACGATGGTATGAGTGGCAAAG
GTACTTNGGACACCACCCCGTTTTCTNTTNCCAGACGCGGCCNTTCAGGAACGACGCGAATCGTAATC
TACCTGCCGCGTNCGCGGATNGTACANGCGTNTTNCAGCGTTCGAAATAGAGTTTGTCGTTCCGCTGA
CCNGGTTNCACGGCTACTGNGATAAGCATATTCTACTGGGGGACTGTAAAGGAATTGTATCCACNCTG
CTTCCTGNCAATTGGNCATGTGCATCTCTNGTTGTTCAGGNNACATTAATAACACACAGNATNAAGTA
GATNAGTCTCCGATATAAGCGAACAGANCGTTGGTNAGAGACTATGGTTCAATTGTAGTGGTATAANG
TTTCCCAGTNCATAAGGAACTCAGGNACACTACTAGCANGGNCNAGGAGAGACCCTTGGGTGGCAACA
CGCGATAAGTGTTNCCTAGTACATCAGCACGAGNNAGGGTTTGNCCATGTGTTTTTAGACGNCCCCGG
TCGNATTGAACCTGGCATTCAGAATGACGTGCTNTCAAGAATCNAGGTCCNCNCACTGAGGGTCANNN
GNTTCGCGGGTCAGCGTATNGTCANAAACNT
>Kinh14
GTGTAGCCCAGTCAGACACTNNNNAATCCNTGGTAACAGACATGCAGTCCCCTCNNCAGAGCCCCGCC
CCCCGATGGTACCGGGTTGTTNAGAGGTCCACAACGGGTCNNACGACNNNNTTAAGGCCTNNNGCNTG
ACCCAANNCNGNGGNGTCCCGGCGCNGCGNCCTTGNANNCCGGGGNCAAGTATGGCCCCCCGGGAAGG
ACGCNGTAGTGCATCTGGAGTGCCGATTCGTGGTCCAGAATGCCACCACGATGGTATGAGTNGCAAAG
GTACTTNGGACACCACCCCGTTTTCTNTTTCCAGANGCGGCCNTTTAGGAACGACGCGAATCGTAATC
TACCTGCCGCGTNCGCGGATCGTACATGCGTTTTCCAGCGTTCGAAATAGAGTNTGTCGTTCCGCTGA
CCAGGTTNCACNGNTACTNCGATAAGCATATTCTACTGGGGGACTGTAAAGNAATTGTATCCATNCNG
CTTCCTGNCAATTGGNCATGTGCATCTCTGGTTGTTCAGGNNACATTAATANCACANAGCAGTAAGTA
GNTNAGTCTCCGATATAAGCGAACAGANCGTTTGTCAGAGACTGTGGTTCAANTGTAGTGGTATAATG
TTTCCCAGTGCATAAGGANNTCAGGGACACTACTAGCATGGGCGAGGAGAGACCCTTGGGTGGCAACA
CGCAATAAGTGTTNCCTNGTACACCAGCACGAGAGANGGTTTGACCATGTGTTTTTAGACGACCCCGG
TCGCATTGAACCTGGCNTTCANAATGACGTNCTNTCAAGAATCAAGGTCCNCNCACNGAGGNNCATNN
GCTTCGCGNGTCAGCGTATGGTCANAANNNT
>Kinh15
GTGTAGCCCAGTTNGACACTNCNAAATCCTTNGTAACAGACATGCAGTCCCCTCNNCAGAGCCCCGCC
CCCCGATGGTACCGGGTTGTTNAGAGGTCCACCACGGGTCNNACGACNNNNTTAAGGCCTNANGCATG
ACCCANNNCNGNGGNGTCCCGGCGNGGCGACCTTGNAGNCCGGGNNCAAGTATGGCCCCCCGGGAAGG
ACGCNGTAGTGCATCTGGAGTGCCGATTCGTGGTCCAGAATGCCACCACGATGGTATGAGTNGCAAAG
GTACTTTGGACACCACCCCGTTTTCTANTTCCAGANGCGGCCATTCAGGAACGACGCGAATCGTAATC
TACCTGCCGCGTACGCGGATCGTATATACGTTTTCCAGCGTTCGAAATAGAGTNTGTCGTTCCGCTGA
CCAGGTTNCACGGCTACTNNGATAAGCATNTTCTACTGGGGGACTGTAAAGNAATTGTATCCACTCNG

CTTCCTGTCAATTGGNCATGTGCATCTCTGGTTGTTCAGGNNACATNAATAACACACAGNNTNAAGTA
GNTNAGTCNCCGATATAAGCGAACAGANCGTTGGTCAGAGACTGTGGTTCAATTNTAGTGGTATAATG
TTTCCCAGTGCATAAGGAANTCAGGNACACTACTAGCATGGGCGAGGAGAGACCCTTGGGTGGCAACA
CGCAATAAGTGTTNCCTAGTACACCAGCACGAGNNAGGGTTTGACCANGTGTTTTAGACGACCCCGG
TCGCATTGAACCTGNCNTTCAGAATGACGTGCTNTCAAGAGTCAAGNTCCNCCCACTNAGNNTCANNN
NCTTCGCGNGTCAGCGTATNGNCATANACAT
>Kinh16
GTGTAGCCCAGTTANACACTNNNNAATNNNTNGTAACAGACATGCAGTCCCCTCNNCAGAGNCCCGCC
CCCCGATGGTACCGGGTTGNTAAGAGGTCCACAACGGNTCGNACGANNNNNTTAAGGCCNNNNGNNTG
ANNCNNNNCNGNNGNGTCCCGGNGNGGCGACCTNGNANNCNGGGGNCAAGTATGGCCCCCCGGGAAGG
ACGCNGTAGTGCATNTNGAGTGCCGATTCGTGGTCCNGAATGCCACCACGATGGTATGAGTGGCAAAG
GTACTNNGGNCACCACCCCGTTNNCTANTTCCAGANGCGGCCNTTCAGGAACGACGCGAATCGTAATC
TACCTGCCGCGTNCGCGGATCGTACANGCGTTTTNCAGCGTTCGAAATAGAGTNTGTCGTTCCGCTGA
CCNGGTTNCACGGNTANTNNGATANGCATNTTCTACTGGNGGACTGNAAAGNNATTGTATCCACNCNG
CTTCCTGTCAATTNGNNATGTGCATCTCTNGTTGTTCAGNNNANATTAATAACACACAGNNTNANGTA
GATNAGTCTCCGATNTAAGCGAACAGANCGTTGGTNAGAGACTATGGTTCAATTGTAGTGGTATAATG
TTTCCCAGNGCATAANNANNTCAGGNACACTACTAGCATGGNCGAGGAGNGACCCTTGGGTGGCAANA
CGNGANAAGTGTTNCCTAGTACATCAGCACGAGNNAGGGTTTGNCCANGNNTTTTAGNNGACCCCGG
TCGCATTGAACCTGNCNTTCANAATGACGTGCTNTCAAGAATCAAGNTCCNNNCACTGANGNTCANNN
GCTTCGCGGGTCAGCGTATNGTCANNNNNNT
>Kinh17
GNGNAGCCCAGTTANACACNNCTNAATCNNTNGTAACANACATGCAGTCCCCTCNNCAGAGNNCCGCN
CCCCGATGGTACCNGGTTGNTNAGAGGTCCACAACGGNTCNNACGACNNNNTTAAGGCCTNATGCATG
ANNCAACNCNGNGNNGTCCCGGNGCNGCGNCCTTGNANTNNGGGNNCAAGTATNGCCCCCCGNNAAGG
NCNCNGTAGTGCATCTGGAGTGCCGATTCGTGGTCNAGANTGCCACCACGATGGTATGAGTGGCAANG
GTACTTNNNNCACCACCCCGTTNNCTNNTNCCAGANGCGGCCNTTCAGGAACGACGCNAATCGTAATC
TANCTNCNGCGTNCGCGGATCGTACATGCNNTTTCCAGCGTTCGANATAGAGNNNGTCGTTCCGCTGA
CCAGGTTNNNCGGCTACTNNGATAAGNATNNTCTACTGGGGGNCTGNAAAGNAATTGTATCCANTNNN
CTTCCTGTCAATTGGNCATGTGCATCTCTGGNTGTTCAGGNNACATNANTAACNNACAGNATNANGTA
GNTNAGTCNCCGATATAAGCGAACAGAACGTTGGNNAGAGACTANGGTTNNATTGTAGTGGTATAATG
TTTCCCAGNNCNNNANGANNTCAGGNACACTACTAGCATGGNCGAGGNGNGACCCTTGGGTGGCAANN
NGNGNTAAGTGTTACCTAGTACATCAGCACGANNNNNGGTTTGACCATGTGTTNNTAGNNNNCCCCGG
TCGCATTGAACCTNGNNTTCAGAATGNCGTNCTNTCAAGAATCAAGNTCCNNNCACNGAGGNNCANNN
GCTTCGCGGNNNNGCGTATGGNCATAANNAT
>Kinh18
GTGNAGCCCAGTTAGACACTTNNAAATCCNTNGTAACAGACATGCAGTCCCCTCNNCNNAGCCCCGCC
CCCCGATGGTACCGGGTTGTNAAGNGGTCCACAACGGNTCGNACGACAACNTTAAGGCCTNATGCATG
ANNCANNNCNGGGGNGTCCCGGNGNGGCGACCTTGNAGTCCGGGNNCAAGTATNGCCCCCCGNGAAGG
ACGCNGTAGTGCATCTGGAGTGCCGANNCNTGNNCCANANTGNCACCACGATGGTATGAGTGGCAAAG
GTACTTNNNNCACCACCCCGTTTTNTNNTTCCAGACGCGGCCNTTCAGGAACGACGCGAATCGTAATC
TACCTGCCGCGTNCGCGGATNGTACNTGCNTNTTNCAGCGTTCGAAATAGAGTTTGTCGTTCCGCTGA
CCNGGTTNCACGGCNACTNCGATAAGCATATTCTACTGGGGGACTGTAAAGNAATTGTATCCACTCTG
CTTCCTGTCAATTGGNCATGTGCATCTCTNGTTGTTCAGGNNACATTAATAACACACAGCATNAAGTA
GATNAGTCNCCGATATAAGCGAACAGANCGTTGGTCNGAGACTATGGTTNNATTGTAGTGGTATNNTG
TTTCCCAGNNCATAAGGAACTCAGGGACACTACTANCANGGNCGAGGAGAGACCCTTGGGTGGCANNA
CGNGATAAGTGTTNCCTAGTACATCAGCACGAGNNANGGTTTGACCNNGNNTTNNTAGNNGNCCCCGG
TCGCATTGAACCTGGCATTCAGAATGACGTNCTNTNAANAATNNAGNTCCNNNCACTGAGGGTCANNN
NCTTCGCGNGNCNGCGTATGGNCATAAACAT
>Kinh19
GTGTAGCCCAGTCAGACACTTCTAAATCCTTGGTAACAGACATGCAGTCCCCTCGGCAGAGCCCCGCC
CCCCGATGGTACCGGGTTGTTNAGAGGTCCACAACGGGTCGCACGACAACATTAAGGCCTNATGCATG
ACCCAANNCGGGGGCGTCCCGGCGCGGCGACCTTGAAGTCCGGGGACAAGTATGGCCCCCCGGGAAGG
ACGCCGTAGTGCATCTGGAGTGCCGATTCNTGGTCNAGAATGNCACCACGATGGTATGAGTGGCAAAG
GTACTTNGGACACCACCCCGTTTTCTANTTCCAGACGCGGCCNTTTAGGAACGACGCGAATCGTAATC

TACCTGCCGCGTNCGCGGATCGTACANGCGTTTTCCAGCGTTCGAAATAGAGTTTGTCGTTCCGCTGA
CCAGGTTTCACGGCTACTGCGATAAGCATATTCTACTGGGGGACTGTAAAGGAATTGTATCCATTCNG
CTTCCTGTCAATTGGCCATGTGCATCTCTNGTTGTTCAGGNNACATTAATAACACACAGCAGTAAGTA
GGTAAGTCTCCGATATAAGCGAACAGANCGTTTGTCAGAGACTGTGGTTCAATTGTAGTGGTATAATG
TTTCCCAGTGCATAAGGAACTCAGGNACACTACTAGCATGGNCGAGGAGAGACCCTTGGGTGGCAANA
CGCAATAAGTGTTANCTAGTACACCAGCACGAGAGANGGTTTGNCCNTGTGTTTNTAGACGACCCCGG
TCGCATTGAACCTGGCATTCAGAATGACGTGCTCTCAAGAATCNAGGTCCNCCCACTGAGGGTCATCT
GCTTCGCGGGTCAGCGTATGGTCATAAACAT
>Kinh20
GNGNAGCCCAGTTANACACNTNNAAATCCNTNGTAACAGACATGCAGTNCCCTCNNCAGAGCNCCGCC
CCCCGATGGTACCNGGTTGNTNAGAAGTCCACAACGGNTCNNANGNCNNNNTTAAGGCCNNANGCNTN
ACCCANNNNNNNNGNGNCCCGGNNNNGNNNCCNNNNANNNNGGGGNCAAGTATGGCCCCCCGGGAAGG
ACNCNGTAGTGCATNNNGAGTGCCGATTCGTGGTCNAGAATGCCACCACGATGGTATNAGTNGCAAAG
GTACTNNNNNCACNACCCCGTTNNCTNNTNCCAGANGCGGCCATTCAGGNACGACGCGAATCGTAATC
TANCTGCCGCGTNCGCGGNTNGTANNTGCGTTTTCCAGCGTTCGANATAGANTNTGTCGTTCCGCTGA
CNNGGTTNCACGGCTANTNNGNTAANCANNNTCTACTGGNGNANTGNAAAGNAATTGTATCCANNCNG
CTNCCTGTCAATTGGNNNTGTGCATCTCTNGTTGTTCAGNNNANATNAATANCNCANAGNNGNANNTA
GNTNAGTCNCCGATNTAAGCTNACAGANCGTTTGTNANAGACTGTGGNTCAANTNTAGTGGTATAATG
TTTCCCAGNNCATAANGAANTNAGGNACACTACTAGCANGGGCGAGGAGNGACCCTTGGGTGGCAANA
NGNAANAAGTGTTNNNTNNTANACCAGCACNAGANAGGNTNNGNNCNTGTGTTNNCAGNNGACCCCGG
TCGCATTGAANCTGNCATTCANAATGANNTGCTNTCAAGAATNAANNTCCNNNNANNNAGGGTCNNNN
NNTTCGCNGGTCAGCGTATNGNCATNAACAT
>Kinh21
GTNTAGCCCAGTTNNACACTTCNAAATCCTTNGTAACAGACATGCAGTNCCCTCNNCAGAGCCCCGCC
CCCCGATGGTACCGGGTTGTTNAGAGGTCCACAACGGNTCNCANGACAANNTTAAGGCCTCANGCATG
ACCCANNACGGNNGNGTCCCGGNGNGGNGACCTTGNANTCNGGGNNCAAGTATGGCCCCCCGGGAAGG
ACNCNGTAGTGCATCTGGAGTGCCGNTTNGTGGTCNNGAATGCCACCACGATGGTATGAGTNGCAAAG
GTACTTNGGNCACCACCCCGTNNNCTNNTNCCAGANGCGGCCNTTCAGGAACGACGCGNANNGTAATC
TACCTNCCGCGTNCGCGGNTNGTANANGCGTTNNNCAGCGTTCGANATNNAGTNNGTCGTTCCGCTGA
CCNGGTTNCACGGCTANTNNGATAAGCATATTCTACTGGNGGACTGNAAAGGAATTGTATCCACTCTG
CTTCCTGTCAATTGGNCATGTGCATCTCTGGTTGTTCAGGNNANANTAATAACANACAGNNTNAANTA
GNTAAGTCNCCGATATAAGCGAACAGAACGTTGGTCAGANACTATGGTTCAATTGTAGTGGTATAATG
TTTCCCAGNNCATAAGGAACTCAGGGACACTACTANCATGGNCNAGGAGNNACCCTTGGGTGGCAANA
CGNGNTAAGTGTTNNNTNNTNCATCAGCACGAGNNANGGTTNGACCATGTGTTTTTAGNNGACCCCGG
TCGCATTGAACCTGGCATTCANAATGANNTGNTCTCAAGAATNNANGTCCNNNCACTGAGGNTCATNN
NCTTCGCGGGTNNGCGTATNGNCACAAACAT
>Kinh22
GNGTAGCCCAGTCAGNCACNNNNNAANCCNTNGTAACAGACATGCAGTCCCCTCNNCAGAGCNCNNCC
CCCCGATGGTACCNGGTTGNTAAGAGGTCCACAACGGNTCNNANGNNNNNNTTAAGGNCNNANGCATN
ACCCANNNCNGNNGNGTCCCGGNNNNGNGNCCTNGNANTCCGGGGNCAAGTATGGCCCCCCGNGAAGG
ACNCNGTAGTGCATNNNGAGNGCCGANNNGTGGTCNAGAATGCCACCACGATGGTATGAGTNGCAAAG
GTACTTNGGACACNACCCCGTTNNCTNNTTCCAGNNNCGGCCATTTANGAACGACGCGAATCGTANTC
TANCTGNNGCGTNCGCGGATCGTACANGCGTTTTCCAGCGTTCGANATAGAGTNTGTCGTTCCGCTGA
CCAGGTNNCACGGNTACTNNGANAAGCNTATTCTACTGGGGGACTGTAAAGNNNTTGTATCCATTCTG
CTTCCTGNCAATTNGNCATGNGCATCTCTNGTTGTTCAGGNNANATTAATNACNCANAGNAGNANNTA
NNTAAGTCTCCGATNTNAGCGAACAGANNGTTTGNNAGAGACTGTNNTTCAATNGTAGTGGTATAATG
TTTCCCAGTNCNTNAGGAACTCAGNNACACTACNAGCATGGNCGAGGANAGACCCTTGGGTGGCAANA
CGCANNAAGTGTTNCCTNGTNNACCAGCACNANANANGGTTNGACCATGTGNNNNTAGNNNNCCCCGG
TCGCNTTGAACCTGNCNTTCANAATNACGTGNTCTCAAGAATCNANNTCCNCNNACTNANNNNCANNN
NCTTCGCGNNNNNGCGTATGGTCANANNNAT
>Kinh23
GTGNAGCCCAGTTAGACACTTCTAAATCCNTGGTAACAGACATGCAGTCCCCTCNNCAGAGNCCCGCC
CCCCGATGGTACCGGGTTGTTNAGAAGTCCACAACGGGTCNCACGACAANATTAAGGCCTNANGCATG
ANNCAACACGGNGGNGTCCCGGNGNGGCGACCTTGAAGNCCGGGGNCAAGTATGGCCCCCCGNGAAGG

ACGCNGTAGTGCATCTGGAGTGCCGATTCGTGGTCCNGAATGCCACCACGATGGTATGAGTGGCAAAG
GTACTTNGGACACCACCCCGTTTTCTANTTCCAGANGCGGCCNTTCAGGAACGACGCGAATCGTAATC
TACCTGCCGCGTNCGCGGATCGTACANGCGTNTTCCAGCGTTCGAAATAGAGTTTGTCGTTCCGCTGA
CCAGGTTNCACGGCTACTNCGATAAGCATATTCTACTGGGGGACTGTAAAGGAATTGTATCCATTCNG
CTTCCTGTCAATTGGNCATGTGCATCTCTGGTTGTTCAGGNNACATTAATAACACACAGCAGNAAGTA
GGTNAGTCTCCGATATAAGCTAACAGANCGTTTGTCAGAGACTGTGGTTCAATTGTAGTGGTATAATG
TTTCCCAGTGCATAAGGAACTCAGGNACACTACTAGCATGGGCGAGGAGAGACCCTTGGGTGGCANCA
CGNAATAAGTGTTNCCTAGTACACCAGCACGAGANAGGGTTTGNCCATGNNTTTNCAGACGACCCCGG
TCGCATTGAACCTGGCATTCAGAATGACGTGCTCTNANGAATCAANNTCCNCCCACTGAGGGTCATNN
GCTTCGCGGGTCAGCGTATNGTCATAAACAT
>Kinh24
GTGNAGCTCAGTTNGACACNTCTAAATCCTTNGTAACAGACATGCAGTCCCCTCGGCAGAGCCCCGCC
CCCCNGTGGTACCGGGTTGNTAAGAGGTCCACCACGGNTCGNACGACAANNTCAAGGCCTNANGCATG
ANNCANNNCNGNGANGTACCGGNGCGGNGACCTTGAANNTNGGGGNCAAGTATNTCCCCCCGNGAAGG
ACGCNGTAGTGCATCTGGANTGCCGATTCNTGGTCNNGAATGNCACCACGATGGTATGAGTGGCAAAG
GTATTTTGGACNCCACCCCGTTNNCTANNTCCAGANGCGGCCNTTCAGGAACGACGCGAATCGTAATC
TACCTGCCGCGTNCGCGGATNGTACANGCGTTTTTCAGCGTTCGAAATAGAGTTTGTCGTTCCGCTGA
CCAGGTTNCACGGCTACTGCGATAAGCATATTCTACGGGGGGACTGTAAAGGAATTGTATCCACTCNG
CTCCCTGTCAATTGGNCATGTGCATCTCTNGTTGTTTAGGNNACATTAATAACACACAGCATTAGGTA
GATAAGTCNCCNATATAAGCGAACAGANCGTTGGTNAAAGACCGTGGTTCAATTGTAGTGGTATAACA
TTTCCCAGTGCATAAGGAACCCAGGNACACTACTANCATGGGCNAGGAGAGACCCTTGGGTGGCAACA
CGCANTAAGTGTTNNNCAGTACACCAGCACGATANANGGTTTGNCCANGNNTTTNTAGNNGNCCCCGG
TCGCATTGAACCTGGCATTCAGAATGACGTGCTCTCANGAGTCAAGNTCCNCNCACTGAGGGTCANNT
NCTTCGCGNNNTNGCGTATGGTCATAAACAT
>Kinh25
GTGNAGCCCAGTCAGACACNTCTAAATNCTTGGTAACAGACATGCAGTCCCCTCNNCAGAGCCCCGCC
CCCCNATGGTACCGGGTTGNTNAGAGGTCCACAACGGNTCNNACGACNNNNTTAAGGCCTNANGCATG
ANNCNNNACGGGGGNGTCCCGGNGNGGNGACCTTGAAGTCNGGGGACAAGTATGGCCCCCCGNGAAGG
ACGCNGTAGTGCATCTGGANTGCCGATTCGTNNNCCNGAATGNCACCACGATGGTATGAGTGGCAAAG
GTACTTNGGACACCACCCCGTTNNCTANTTCCAGANGCNGCCATTCAGGAACGACGCGAATCGTAATC
TACCTGCCGCGTNCGCGGATCGTACNNGCGTNTTCCAGCGTTCGAAATAGAGTTTGTCGTTCCGCTGA
CCNGGTTNCACGGCTACTNCGATAAGCATATTCTACTGGGGGACTGTAAAGGAATTGTATCCATTCNG
CTTCCTGTCAATTGGNCATGTGCATCTCTGGTTGTTCAGGNNACANTAATAACACACAGCAGNAAGTA
GGTNAGTCTCCGATATAAGCGAACAGANCGTTTGTNAGANACTGTGGTTCAATTGTAGTGGTATAATG
TTTCCCAGNGCATAAGGAACTCAGGNACACTACTAGCATGGGCGAGGAGAGACCCTTGGGTGGCAANA
CGCAATAAGTGTTANNTAGTACACCAGCACGAGANANGGTTTGNCCNTGTGTTTNTAGNNGACCCCGG
TCGCATTGAACCTGGCATTCAGAATGACGTGCTNTNANGAATCNAGGTCCNCNCACTGAGGGNCATCN
GCTTCGCGGGTCNGCGTATNGTCATAANNNT
>Kinh26
GTNNAGCCCAGTTNGACACTTCTAAATNCNTNGTAACAGACATGCAGTCCCCTCNNCAGAGNNCCGCC
CCCCGATGGTACCGGGTTGTTNAGAGGTCCACCACGAGTCNNACGACAANNGCAAGGCCTNGNGCATG
ACCCNNCACNGGGGCGTCCCGGCGNGGNGACCTTGAANTCNGGGANCAAGTATGGCTCCCCGNGAAGG
ACNCNGTAGTGCATCTGGANTGCCGANNNGTGGTCNAGAATGNCACCACGATGGTATGAGTNGCAAAG
GTACTTTGGACACCACCCCGTTTNCTANTCCCAGANGCGGCCNTTCAGGAACGACGTGAATCGTAATC
TACCTGCCGCGTACGCGGATCGTACANGCGTTTTCCAGCGTTCGAAATAGAGTTTGTCGTTCCGCTGA
CCAGGTTNCACGGCTACTNCGATAAGCATACTCTACTNGGGGACTGTAAAGGAATTGTATCCACTCNG
CTTCCTGTCAGTTGGNCATATGTATCTCTNGTTGTTCAGGNNANANTAATAACACACAGCATNAAGTA
GATNAGTCTCCGATATAGGCGNACAGANCGTTGGTCAAAGACTGTGGTTCAACTGTAGTGGTATAATG
TTTCCCAGTGCATAAGGAACTCAGGGACACTACTAGCANGGNCGAGTAGAGACCCTTGGGTGGCAANA
CGNAATAAGTGTTANNTAGTACACCAACACGATANAGGGTTTGACCNNGTGTTTNTAGNNGACCCCGG
TCGCATTGAACCTGGCATTCATAATGACGTGCTNTCAAGAGTCAAGGTCCNCCCACTGAGGGTCATNT
NCTTCGCGNGTCNTCGTATGGNCATAAACAT
>Kinh27
GTGTAGCCCAGTTNGACACTTCTAAATCCNTNGTAACAGACATGCAGTCCCCTCNNCAGAGCCCCGCC

CCCCGATGGTACCGGGTTGTTAAGAAGTCCACAACGGNTCGNACGACAACNTTAAGGCCTCATGCATG
ACCCANCNCNGGGGNGTCCCGGCGCGGCGACCTTGAANTCCGGGGNCAAGTATGGCCCCCCGGGAAGG
ACGCCGTAGTGCATCTGGANTGCCGATTNGTGGTCCANAATGCCACCACGATGGTATGAGTGGCAAAG
GTACTTNGGACACCACCCCGTTTTCTATTTCCAGACGCGGCCNTTCAGGAACGACGCGAATCGTAATC
TANCTGCCGCGTNCGCGGATCGTACANGCGTTTTCCAGCGTTCGAAATAGAGTTTGTCGTTCCGCTGA
CCAGGTTNCACGGCTACTNCGATAAGCATATTCTACTGGGGGACTGTAAAGGAATTGTATCCATTCNG
CTTCCTGTCAATTGGNCATGTGCATCTCTGGTTGTTCAGGNNACATTAATAACACACAGNAGTAAGTA
GGTAAGTCTCCGATATAAGCTAACAGANCGTTTGTNAGAGACTGTGGTTCAATTGTAGTGGTATAATG
TTTCCCAGTGCATAAGGAACTCAGGNACACTACTAGCANGGNCGAGGAGAGACCCTTGGGTGGCANNA
CGCAATAAGTGTTACCTAGTACACCAGCACGAGAGAGGGTTTGACCATGNNTTTTCAGACGACCCCGG
TCGCATTGAACCTGGCATTCAGAATGACGTGCTCTCAAGAATCAAGNTCCNCNCACTGAGGGTCATCN
NCTTCGCGGGTCNGCGTATGGNCATAAACAT
>Kinh28
GTGNAGCTCAGTTNNACACTTCTAAATCCNTNGTAACAGACATGCAGTCCCCTCNNCAGAGCNCCGCC
CCCCNGTGGTACCGGGTTGNNAAGNGGTCCACCACGGGTCGCACGACANNNTCAAGGCCTCANGCATG
ANNCAACNCGGGGACGTACCGGCGNGGCNACCTTGNAGGTCGGGGNCAAGTATGTCCCCCCGGGAAGG
ACGCCGTAGTGCATNTGGANTGCCGNTTCNTGGTCNAGANTGNCACCACGANGGTATGAGTGGCAANG
GTATTTNGGACACCACCCCGTTTTCTNTTTCCAGANGCGGCCNTTCAGGAACGACGCGANTCGTAATC
TANCTGCNGCGTNCGCGGATNGTACANGCGTTTTNCAGCGTTCGAAATAGAGTTTGTCGTTCCGCTGA
CCAGGTTNCACGGCTACTGCGATAAGCATATTCTACGGGGGGACTGTAAAGGAATTGTATCCACTCTG
CTCCCTNNCAATTGGCCATGTGCATCTCTNGTTGTTTAGGGTACATTAATAACACACAGNATNAGGTA
GATNAGTCTCCGNTATAAGCGAACAGANCGTTGGTNAAANACCGTGGTTCAATTGTAGTGGTATAACA
TTTCCCAGTGCATAAGGAACCCAGGNACACTACTANCANGGGCGAGGAGAGACCCTTGGGTGGCANNA
CGNAATAAGTGTTNCCCAGTACACCAGCACGATNNANGGTTTGACCNTGTNTTTTAGNNGACCCCGG
TCGCATTGAACCTGGCATTCANAATGACGTNCTNTCANNAGTCAANGTCCNCNCACTGAGGGNCANNN
GCTTCGCGNNNTAGCGTATNGTCATAAACAT
>Kinh29
GTGTAGCCCAGTTAGACACTTCTAAATCCTTGGTAACAGACATGCAGTCCCCTCGGCAGAGCCCCGCC
CCCCGATGGTACCGGGTTGTTAAGNAGTCCACAACGGGTCNNACGACAACATTAAGGCCTCATGCATG
ACNCNANNCGGGGGCGTCCCGGCGCGGCGACCTTGAANTCCGGGGNCAAGTATGGCCCCCCGGGAAGG
ACGCCGTAGTGCATCTGGAGTGCCGATTNNTGGTCNAGAATGCCACCACGATGGTATGAGTGGCAAAG
GTACTTNGGACACCACCCCGTTTTCTANTTCCAGACGCGGCCNTTCAGGAACGACGCGAATCGTAATC
TACCTGCCGCGTACGCGGATNGTACATGCGTNTTCCAGCGTTCGAAATAGAGTTTGTCGTTCCGCTGA
CCNGGTTNCACGGCTACTGCGATAAGCATATTCTACTGGGGGACTGTAAAGGAATTGTATCCATTCTG
CTTCCTGTCAATTGGNCATGTGCATCTCTNGTTGTTCAGGNNACATTAATAACACACAGCAGTAAGTA
GNTNAGTCTCCGATATAAGCTAACAGANCGTTTGTNAGAGACTGTGGTTCAATTGTAGTGGTATAATG
TTTCCCAGTGCATAAGGAACTCAGGGACACTACTAGCATGGGCGAGGAGAGACCCTTGGGTGGCAANA
CGNAANAAGTGTTNNNTAGTACACCAGCACGAGNNANGGTTTGNCCNNGTGTTTNCAGNNGACCCCGG
TCGCATTGAACCTGGCATTCAGAATGACGTGCTCTCAAGAATCNAGNTCCNNNCACTGAGGGTCANCN
NCTTCGCGGGTCNGCGTATGGTCATAAACAT
>Kinh30
GTGTAGCCCAGTCAGACACTNCTNAATCCNTNGTAACAGACATGCAGTCCCCTCNNCAGAGCCCCGCC
CCCCGATGGTACCGGGTTGTNAAGAGGTCCACAACGGNTCGCANGACNNNNNTAAGGCCTNANGCATG
ACCCAACACGGNGGNGTCCCGGNGCGGCGACCTTGAAGTCCGGGGNCAAGTATGGCCCCCCGGGAAGN
ACGCCGTAGTGCATCTGGAGTGCCGNTTCGTGGTCNAGAATGCCACCANGATGGTATGAGTNGCAAAG
GTACTTTGGACACCACCCCGTTTTCTANTTCCAGANGCGGCCNTTCAGGAACGACGCGAATCGTAATC
TACCTGCCGCGTACGCGGATCGTACATGCGTTTTCCAGCGTTCGANATAGAGTTTGTCGTTCCGCTGA
CCAGGTTTCACGGCTACTNCGATAAGCATATTCTACTGGGGGACTGTAAAGGAATTGTATCCATTCNG
CTTCCTGTCAATTGGCCATGTGCATCTCTGGTTGTTCAGGNNANATTAATAACACACAGNAGNAAGTA
GGTNAGTCTCCGATATAAGCGAACAGANCGTTTGTNAGAGACNGTGGTTCAATTGTAGTGGTATAATG
TTTCCCAGTGCATAAGGAACTCAGGGACACTACTAGCATGGNCGAGGAGAGACCCTTGGGTGGCAANA
CGCAATAAGTGTTNCCTAGTACACCAGCACGAGNNAGGGNTTGNCCATGTGTTTTTAGACGACCCCGG
TCGCATTGAACCTGGCATTCAGAATGACGTGCTCTCANNAATCAAGGTCCNCCCACTGAGGGTCANCN
NCTTCGCGGGTCNGCGTATNGTCATAAACNT

>Kinh31
GNNTAGCCCAGTTNGACACTNNNNAANCCNTNGTAACAGACATGCAGTCCCCTCNNCAGAGNCCCGCC
CCCCGATGGTACCGGGTTGNNNAGAAGTCCACAACGGNTCNNANGACNNNNNTAAGGCNNNANGCANG
ANNCNNNNCNGGGGNGTCCCGGNGNGGCGACCTTGAANTCNGGNGNCAAGTATGGCCCCCCGNNAAGN
NCNCNGTAGTGCATCTGGAGTGCCGANNNGTGGTCNAGAATGNCACCNNGATGGTATGAGTNGCAAAG
GTACTNTGGNCACCACCCCGTTTTCTNNNTCCAGANGCGGCCNTTCAGGAACGACGCGAATCGTAATC
TACCTGCCGCGTNCGCGGATCGTACNNGCGTNTTNCAGCGTTCGAAATAGAGTTTGTCGTTCCGCTGA
CCNGGTTNCACGNCTACNNNGATAAGNANANTCTACTNGGGGACTGNAAAGGNNTTGTATCNATNCNG
CNTCCTGNCAATTGGNCATGTGCATCTCTNGTTGTTCAGGNNANANTAATAACACACAGCANNANGTA
GGTNAGTCNCCGATANAAGCTAACAGANCGTTTGTNAGAGACTGTGGTTCAATTGTAGTGGTATAATG
TTNCCCAGNNCATAAGNAACTCAGGNACACTACTAGCANGGGNGAGGAGAGACCCTTGGGTGGCAANA
CGNANTAAGTGTTNNCTAGTACACCAGCACGAGNNNNGNTTTGNCCNNNNNNNTNCAGNNNACCCCGG
TCGCATTGAACCTGGCATTCAGAATGANGTGCTNTCANNAATCNAGNTCCNCCCANNGNGNNTCANNN
NCTTCGCGGGTCNGCGTATNGNCATAAACAT
>Kinh32
GTNTAGCTCAGTTANACACTNNNNAANCCNTGGTAACAGACATGCAGTCCCCTCGGCAGAGNNCCGCC
CCCCGGTGGTACCGGGTTGNNNAGAGGTCCACCACGGNTCNNACGACNNNATCAAGGCCTNANGCATG
ACCCANCGCNGNNANGTACCGGCGCGGCGACCTTGAANGTCGGGGNCAAGTATGTCCCCCCGNGAAGG
ACGCNGTAGTGCATNTGGAGTGCCGATTCGTGGNCCNGANTGCCANCANGATGGTATGAGTNGCAAAG
GTATTTNGGACACCACCCCGTTNNCTANTTCCAGANGCGGCCNTTCAGGAACGACGCGAATCGTAATC
TACCTGCCGCGTNCGCGGATCGTACANGCGTNTTTCAGCGTTCGAAATAGAGTTTGTCGTTCCGCTGA
CCAGGTTNCACGGCTACTNNGATAAGCATATTCTACGGGGGGACTGNAAAGNAATTGTATCCACTCNG
CTCCCTGTCAATTGGCCATGTGCATCTCTGGTTGTTTAGGNNACATTAATAACACACAGNATNAGGTA
GATAAGTCTCCGNTATAAGCGAACAGANCGTTGGTNAAAGACCGTGGTTCAATTGTAGTGGTATAACA
TTTCCCAGNGCATAAGGAANCCAGGGACACTACTAGCANGGNCGNGGAGAGACCCTTGGGTGGCAANA
CGNAATAAGTGTTANCCAGTACACCAGCACGATNNANGGTTTGNCCATNNNTTTNTAGNNGACCCCGG
TCGCATTGAACCTGGCANTCAGAATGACGTGCTNTCANGAGTCNAGNTCCTCCCACTGAGGGTCANCN
NCTTCGCGNNNTNGCGTATNGNCACAAACAT
>Kinh33
GTGNAGCCCAGTTNGACACTNCTNAATNCTTNGTAACAGACATGCAGTCCCCTCNNCAGAGCCCCGCC
CCCCNATGGTACCGGGTTGNTAAGAGGTCCACAACGGNTCNNANGACNNNNNTAAGGCCTNNNGCATG
ANNCNACNCNGNGGNGTCCCGGNGCGGCGACCTTGAAGTCNGGGGNCAAGTATGGCCCCCCGNGAAGG
ACGCCGTAGTGCATCTGGAGTGCCGATTCGTGGNCCAGAATGCCACCACGATGGTATGAGTNGCAAAG
GTACTTNNNNCACCACCCCGTTTNCTANTTCCAGANGCGGCCNTTCAGGAACGACGCGAATCGTAATC
TACCTGCCGCNTNCGCGGATCGTACATGCGTNTTCCAGCGTTCGAAATAGAGTTTGTCGTTCCGCTGA
CCAGGTTNCACGGCTACTNNGATAAGCATNTTCTACTGGGGGACTGTAAAGGAATTGTATCNANNCNG
CTTCCTNNCAATTGGNCATGTGCATCTCTGGTTGTTCAGGGTACATTAATAACACACAGNNTNAAGTA
GATNAGTCTCCGATATAAGCGAACAGANCGTTGGTNAGAGACTATGGTTCAATTGTAGTGGTATAATG
TTTCCCAGTNCATAAGGAANTCAGGNACACTACTAGCATGGNCGAGGAGAGACCCTTGGGTGGCAANA
CGNGATAAGTGTTNNCTNGTACATCAGCACNAGNNNNGGTTTGNCCATGTGTTTTTAGNNGACCCCGG
TCGCATTGAACCTGGCATTCANAATGACGTGCTNTCAAGAATCNAGNTCCNCNCACTGAGGGNCANCN
GCTTCGCGNGTCNGCGTATNGNCANNNNNNT
>Kinh34
GTGNAGCCCAGTTANACACTTCTNAATCCTTGGTAACAGACATGCAGTCCCCTCNNCAGAGNCCCGCC
CCCCNATGGTACCGGGTTGNTNAGAGGTCCACAACGGNTCGNACGACAANNTTAAGGCCTCATGCATG
ACCCNACNCGGNGGCGTCCCGGCGCGGNGACCTTGNAGTCNGGGGNCAAGTATGGCCCCCCGGGAAGG
ACGCCGTAGTGCATCTGGAGTGCCGATTCGTGGTCNAGANTGNCACCACGATGGTATGAGTNGCAAAG
GTACTTTGGNCACCACCCCGTTTTCTANTNCCAGANGCGGCCNTTCAGGAACGACGCGAATCGTAATC
TACCTGCCGCNTNCGCGGATNNTACATGCGTTTTCCAGCGTTCGAAATAGAGTTNGTCGTTCCGCTGA
CCNGGTTNCACGGCTACTGCGATAAGCATATTCTACTGGGGGACTGTAAAGGAATTGTATCCACTCNG
CTTCCTGTCAATTGGNCATGTGCATCTCTNGTTGTTCAGGNNACATTAATAACACACAGCATTAAGTA
GNTNAGTCNCCGATATAAGCGAACAGANCGTTGGTNNGANACTATGGTTCAATTGTAGTGGTATAATG
TTNCCCAGTGCATAAGGAACTCAGGGACACTACTAGCATGGGCGAGGAGAGACCCTTGGGTGGCAANA
CGNAATAAGTGTTANNTAGTACATCAGCACGAGNNANGGTTTGNCCATNTGTTTTTAGNNGACCCCGG

TCGCATTGAACCTGGCATTCAGAATGANGTGCTNTCANGAATCAAGNTCCNCCCACTGAGGGTCANCT
GCTTCGCGNGTCNGCGTATNGTCATAAACNT
>Kinh699
GTGNAGCCCAGTTAGACACTTCTAAATCCNTGGTAACAGACATGCAGTCCCCTCGGCAGAGNCCCGCC
CCCCNATGGTACCGGGTTGNTNAGAGGTCCACAACGGGTCGNACGACAACATTAAGGCCTNANGCATG
ACNCANCNCGGGNGNGTCCCGGNGNGGNGACCTTGNAGTCCGGGGNCAAGTATGGCCCCCCGGGAAGG
ACGCNGTAGTGCATCTGGAGTGCCGATTCNTGGTCCNGAATGCCACCACGATGGTATGAGTGGCAAAG
GTACTTTGNNCNCCACCCCGTTNNCTANTTCCAGACGCGGCCATTCAGGAACGACGCGAATCGTAATC
TANCTGCCGCGTNCGCGGATCGTACANGCGTTTTCCAGCGTTCGAAATAGAGTTTGTCGTTCCGCTGA
CCNGGTTNCACGGCTACTGCGATAAGCATATTCTACTGGGGGACTGTAAAGGAATTGTATCCACTCNG
CTTCCTGNCAATTGGCCATGTGCATCTCTGGTTGTTCAGGNNACATTAATAACACACAGCATTAAGTA
GATNAGTCTCCGATATAAGCGAACAGAACGTTGGTCAGAGACTATGGTTCAATTGTAGTGGTATAATG
TTTCCCAGTGCATAAGGAACTCAGGGACACTACTAGCANGGNCGAGGAGAGACCCTTGGGTGGCAANA
CGCGANAAGTGTTNNCTAGTACATCAGCACGAGANANGGTTTGNCCNTNNNTTTTAGACGNCCCCGG
TCGCATTGAACCTGGCATTCAGAATGACGTNCTCTNAAGAATCAAGNTCCNNNCACTGAGGGTCATNT
GCTTCGCGGGTCAGCGTATNGTCATAAACAT
>Kinh700
GTGNAGCCCAGTCAGACACTTCTAAATCCNTNGTAACAGACATGCAGTCCCCTCNNCAGAGCCCCGCC
CCCCNATGGTACCGGGTTGTTNAGAGGTCCACAACGGNTCNNACGACAACATTAAGGCCTNATGCATG
ACCCANNNCNGGGGCGTCCCGGCGCGGCGACCTTGAAGTCCGGGGNCAAGTATGGCCCCCCGGGAAGG
ACGCCGTAGTGCATCTGGAGTGCCGATTNNTGGTCCAGAATGCCACCACGATGGTATGAGTGGCAAAG
GTACTTNGGACACCACCCNGTTTTCTANTNCCAGACGCGGCCNTTCAGGAACGACGCGAATCGTAATC
TACCTGCCGCGTACGCGGATCGTACANGCGTTTTNCAGCGTTCGAAATAGAGTTTGTCGTTCCGCTGA
CCAGGTTNCACGGCTACTGCGATAAGCATATTCTACTGGGGGACTGTAAAGGAATTGTATCCATTCTG
CTTCCTGTCAATTGGCCATGTGCATCTCTGGTTGTTCAGGGTACATTAATAACACACAGCAGNAAGTA
GGTAAGTCTCCGATATAAGCGAACAGANCGTTTGTCAGAGACTGTGGTTCAATTGTAGTGGTATAATG
TTTCCCAGTGCATAAGGAACTCAGGNACACTACTAGCATGGGCGAGGAGAGACCCTTGGGTGGCAACA
CGNAATAAGTGTTNCCTAGTACACCAGCACGAGANANGGTTTGACCATGTGTTTNTAGNNGACCCCGG
TCGCATTGAACCTGGCATTCAGAATGACGTGCTCTCAAGAATCAAGGTCCTCNCACTGAGGGTCATNN
GCTTCGCGGGTCAGCGTATGGTCATAAACAT
>Kinh701
GTGTAGCCCAGTCNGACACTTCTAAATCCTTGGTAACAGACATGCAGTCCCCTCNNCAGAGCCCCGCC
CCCCGATGGTACCGGGTTGTTAAGAGGTCCACAACGGGTCGNACGACAACATTAAGGCCTCATGCATG
ACCCAACACGGNNGCGTCCCGGNGNGGCGACCTTGAAGTCCGGGGACAAGTATGGCCCCCCGGGAAGG
ACGCCGTAGTGCATCTGGAGTGCCGATTCGTGGTCCAGAATGCCACCACGATGGTATGAGTNGCAAAG
GTACTTTGGACACCACCCCGTTTTCTATTNCCAGACGCGGCCATTTAGGAACGACGCGAATCGTAATC
TACCTGCCGCGTACGCGGATCGTACANGCGTTTTNCAGCGTTCGAAATAGAGTTTGTCGTTCCGCTGA
CCAGGTTNCACGGCTACTGCGATAAGCATATTCTACTGGGGGACTGTAAAGGAATTGTATCCATTCTG
CTTCCTGTCAATTGGCCATGTGCATCTCTGGTTGTTCAGGGNACATTAATAACACACAGCAGNAAGTA
GGTAAGTCTCCGATATAAGCGAACAGAACGTTTGTCAGAGACTGTGGTTCAATTGTAGTGGTATAATG
TTTCCCAGTGCATAAGGAACTCAGGGACACTACTAGCANGGGCGAGGAGAGACCCTTGGGTGGCAANA
CGCAATAAGTGTTACCTAGTACACCAGCACGAGANAGGGTTTGACCATGNNTTTTAGNNGACCCCGG
TCGCATTGAACCTGGCATTCAGAATGACGTGCTCTCAAGAATCAANGTCCTCNCACTGAGGGTCATCN
GCTTCGCGGNNCNGCGTATNGTCATAANCAT
>Kinh702
GTGTAGCCCAGTTAGACACTTCTAAATCCNTNGTAACAGACATGCAGTCCCCTCGGCAGAGCCCCGCC
CCCCGATGGTACCGGGTTGTTAAGAGGTCCACAACGGNTCGCACGACAACATTAAGGCCTCATGCATG
ANNCANCACGGNGGCGTCCCGGNGCGGCGACCTTGAAGTCCGGGGNCAAGTATGGCCCCCCGGGAAGG
ACGCNGTAGTGCATCTGGAGTGCCGATTCGTGGTCCAGANTGCCACCACGATGGTATGAGTGGCAAAG
GTACTTTGGACACCACCCCGTTTTCTANTTCCAGACGCGGCCATTCAGGAACGACGCGAATCGTAATC
TACCTGCCGCGTACGCGGATCGTACATGCGTTTTCCAGCGTTCGAAATAGAGTTTGTCGTTCCGCTGA
CCAGGTTNCACGGCTACTNCGATAAGCATATTCTACTGGGGGACTGTAAAGGAATTGTATCCACTCTG
CTTCCTGTCAATTGGCCATGTGCATCTCTNGTTGTTCAGGGTACATTAATAACACACAGCATNAAGTA
GATNAGTCTCCGATATAAGCGAACAGAACGTTGGTCAGAGACTATGGTTCAATTGTAGTGGTATAATG

TTTCCCAGTGCATAAGGAACTCAGGGACACTACTAGCATGGGCGAGGAGAGACCCTTGGGTGGCAACA
CGCAATAAGTGTTACCTAGTACATCAGCACGAGANAGGGTTTGACCNTGTGTTTTTAGACGACCCCGG
TCGCATTGAACCTGGCATTCAGAATGACGTGCTCTCAAGAATCAAGNTCCTCCCACTGAGGGTCATCT
GCTTCGCGGNNCAGCGTATGGTCATAANNAT
>Kinh703
GTGTAGCCCAGTTAGACACTTCTAAATCCNTGGTAACAGACATGCAGTCCCCTCNNCAGAGCNCCGCC
CCCCNATGGTACCGGGTTGNTAAGAGGTCCACAACGGNTCGCACGACAACATTAAGGCCTCATGCATG
ACCCAACNCNGGGGCGTCCCGGCGCGGCGACCTTGAAGTCCGGGGNCAAGTATGGCCCCCCGNGAAGG
ACGCCGTAGTGCATCTGGAGTGCCGATTCGTGGTCCAGAATGCCACCACGATGGTATGAGTGGCAAAG
GTACTTTGGNCACCACCCCGTTTTCTANTTCCAGACGCGGCCATTCAGGAACGACGCGAATCGTAATC
TACCTGCCGCGTNCGCGGATCGTACATGCGTTTTCCAGCGTTCGAAATAGAGTTTGTCGTTCCGCTGA
CCAGGTTNCACGGCTACTNCGATAAGCATATTCTACTGGGGGACTGTAAAGGAATTGTATCCACTCTG
CTTCCTGTCAATTGGCCATGTGCATCTCTGGTTGTTCAGGNNACATTAATAACACACAGCATNAAGTA
GATAAGTCTCCGATATAAGCGAACAGANCGTTGGTCAGAGACTATGGTTCAATTGTAGTGGTATAATG
TTTCCCAGTGCATAAGGAACTCAGGGACACTACTAGCATGGGCGAGGAGAGACCCTTGGGTGGCAANA
CGNGATAAGTGTTNNCTAGTACATCAGCACGAGANAGGGTTTGACCANGTGTTTTTAGACGACCCCGG
TCGCATTGAACCTGGCATTCAGAATGACGTGCTCTCAAGAATCAAGNTCCTNNCACTGAGGGTCATCT
GCTTCGCGGGTCNGCGTATGGTCATAAACAT
>Kinh704
GTGTAGCCCAGTTAGACACTTCTAAATCCNTGGTAACAGACATGCAGTCCCCTCNNCAGAGCCCCGCC
CCCCGATGGTACCGGGTTGNTAAGAGGTCCACAACGGGTCGCACGACAACATTAAGGCCTCATGCATG
ACCCAACACGGGGGCGTCCCGGCGCGGCGACCTTGAANTCCGGGGACAAGTATGGCCCCCCGGGAAGG
ACGCCGTAGTGCATCTGGAGTGCCGATTCGTGGTCCAGAATGCCACCACGATGGTATGAGTGGCAAAG
GTACTTTGGACACCACCCCGTTTTCTATTTCCAGACGCGGCCATTCAGGAACGACGCGAATCGTAATC
TACCTGCCGCGTNCGCGGATCGTACATGCGTTTTCCAGCGTTCGAAATAGAGTTTGTCGTTCCGCTGA
CCAGGTTNCACGGCTACTGCGATAAGCATATTCTACTGGGGGACTGTAAAGGAATTGTATCCACTCNG
CTTCCTGTCAATTGGCCATGTGCATCTCTGGTTGTTCAGGGNACATTAATAACACACAGCATNAAGTA
GATAAGTCTCCGATATAAGCGAACAGAACGTTGGTCAGAGACTATGGTTCAATTGTAGTGGTATAATG
TTTCCCAGNGCATAAGGAACTCAGGGACACTACTAGCATGGGCGAGGAGAGACCCTTGGGTGGCAANA
CGCGATAAGTGTTACCTAGTACATCAGCACGAGAGAGGGTTTGACCATGNNTTTTTAGACGACCCCGG
TCGCATTGAACCTGGCATTCAGAATGACGTGCTCTCAAGAATCAAGGTCCTCCCACTGAGGGTCATCT
GCTTCGCGGGTCAGCGTATGGTCATAAACAT
>Kinh705
GTGTAGCCCAGTCAGACACTTCTAAATCCNTGGTAACAGACATGCAGTCCCCTCNNCAGAGCCCCGCC
CCCCGATGGTACCGGGTTGNTNAGAGGTCCACAACGGGTCGNACGACAANATTAAGGCCTNATGCATG
ACCCAANACNGGGGNGTCCCGGCGNGGCGACCTTGNAGTCCGGGGNCAAGTATGGCCCCCCGNGAAGG
ACGCCGTAGTGCATCTGGAGTGCCGNTTCGTGGTCCAGAATGCCACCACGATGGTATGAGTNGCAAAG
GTACTTTGGACACCACCCCGTTNNCTATTTCCAGANGCGGCCNTTCAGGAACGACGCGAATCGTAATC
TACCTGCCGCGTACGCGGATCGTACATGCGTTTTCCAGCGTTCGAAATAGAGTTTGTCGTTCCGCTGA
CCAGGTTNCACGGCTACTNCGATAAGCATATTCTACTGGGGGACTGTAAAGNAATTGTATCCATTCTG
CTTCCTGTCAATTGGNCATGTGCATCTCTNGTTGTTCAGGGTACATTAATAACACACAGNAGTAAGTA
GGTNAGTCTCCGATATAAGCGAACAGAACGTTTGTNAGAGACTGTGGTTCAATTGTAGTGGTATAATG
TTTCCCAGNGCATAAGGAANTCAGGGACACTACTAGCATGGGCGAGGAGAGACCCTTGGGTGGCAACA
CGCAATAAGTGTTACCTAGTACACCAGCACGAGNNAGGGTTTGNCCANGTGTTTTTAGNNGACCCCGG
TCGCATTGAACCTGGCATTCAGAATGACGTGCTCTCAAGAATCNAGGTCCTCCCACTGAGGGTCATCT
GCTTCGCGGGTCAGCGTATNGTCATNAACNT
>Kinh706
GTGTAGCCCAGTTAGACACTTCTAAATCCNTGGTAACAGACATGCAGTCCCCTCGGCAGAGNCCCGCC
CCCCNATGGTACCGGGTTGTTNAGAGGTCCACAACGGGTCNNACGACAACATTAAGGCCTCATGCATG
ACCCANCNCGGGGGNGTCCCGGCGCGGNGACCTTGAAGTCCGGGNNCAAGTATGGCCCCCCGGGAAGG
ACGCCGTAGTGCATCTGGANTGCCGATTNGTGGTCNAGAATGCCACCACGATGGTATGAGTGGCAAAG
GTACTTTGGACACCACCCCGTTNNCTANTNCCAGACGCGGCCNTTCAGGAACGACGCGAATCGTAATC
TACCTGCCGCGTACGCGGATCGTACANGCGTTTTCCAGCGTTCGAAATAGAGTTTGTCGTTCCGCTGA
CCAGGTTNCACGGCTACTNCGATAAGCATATTCTACTGGGGGACTGTAAAGNAATTGTATCCACTCNG

CTTCCTGTCAATTGGNCATGTGCATCTCTNGTTGTTCAGGGTACATTAATAACACACAGCATNAAGTA
GATAAGTCTCCGATATAAGCGAACAGAACGTTGGTCAGAGACTATGGTTCAATTGTAGTGGTATAATG
TTTCCCAGTGCATAAGGAACTCAGGGACACTACTAGCATGGGCGAGGAGAGACCCTTGGGTGGCAACA
CGCAATAAGTGTTACCTAGTACATCAGCACGAGNGAGGGTTTGACCATGTGTTTTTAGACGACCCCGG
TCGCATTGAACCTGGCATTCAGAATGACGTGCTCTNAAGAATCAAGGTCCTNCCACTGAGGGTCATCT
GCTTCGCGGGTCNGCGTATNGTCATAAANNT
>Kinh707
GTGTAGCCCAGTTAGACACTTCTAAATCCNTGGTAACAGACATGCAGTCCCCTCNNCAGAGCNCCGCC
CCCCNATGGTACCGGGTTGNTAAGAGGTCCACAACGGGTCNCACGACAANNTTAAGGCCTCATGCATG
ACCCANNACGGGGGCGTCCCGGCGNGGCGACCTTGAANTCCGGGGNCAAGTATGGCCCCCCGGGAAGG
ACGCCGTAGTGCATCTGGAGTGCCGANNCGTGGTCNAGAATGNCACCACGATGGTATGAGTNGCAAAG
GTACTTTGGACACCACCCCGTTTNCTANTTCCAGACGCGGCCATTCAGGAACGACGCGAATCGTAATC
TACCTGCCGCGTACGCGGATNGTACANGCGTNTTNCAGCGTTCGAAATAGAGTTTGTCGTTCCGCTGA
CCAGGTTNCACGGCTACTNCGATAAGCATATTCTACTGGGGGACTGTAAAGGAATTGTATCCACTCNG
CTTCCTGTCAATTGGNCATGTGCATCTCTNGTTGTTCAGGNNACATTAATAACACACAGCATTAAGTA
GATAAGTCTCCGATATAAGCGAACAGAACGTTGGTCAGAGACTATGGTTCAATTGTAGTGGTATAATG
TTTCCCAGTGCATAAGGAACTCAGGNACACTACTAGCATGGGCGAGGAGAGACCCTTGGGTGGCAACA
CGNGATAAGTGTTNCCTAGTACATCAGCACGAGNGANGGTTTGACCATGTGTTTTTAGANGACCCCGG
TCGCATTGAACCTGGCATTCAGAATGACGTGCTCTCAAGAATCNAGNTCCTCCCACTGAGGGTCATCT
GCTTCGCGGGTCNGCGTATGGNCATANNCAT
>Kinh708
GTGTAGCCCAGTTAGACACTTCTAAATCCTTGGTAACAGACATGCAGTCCCCTCGGCAGAGCCCCGCC
CCCCGATGGTACCGGGTTGNTNAGAGGTCCACAACGGNTCGNACGACAACATTAAGGCCTCATGCATG
ANNCAACACGGNGGCGTCCCGGCGCGGCGACCTTGAANTCCGGGGACAAGTATGGCCCCCCGGGAAGG
ACGCCGTAGTGCATCTGGAGTGCCGATTCGTGGTCCAGANTGCCACCACGATGGTATGAGTGGCAAAG
GTACTTTGGACACCACCCCGTTTTCTANTTCCAGACGCGGCCNTTCAGGAACGACGCGAATCGTAATC
TACCTGCCGCGTACGCGGATCGTACNTGCGTTTTNCAGCGTTCGAAATAGAGTNTGTCGTTCCGCTGA
CCAGGTTNCACGGCTACTGCGATAAGCATATTCTACTGGGGGACTGTAAAGGAATTGTATCCACTCNG
CTTCCTGTCAATTGGNCATGTGCATCTCTNGTTGTTCAGGNNACATTAATAACACACAGNATTAAGTA
GATAAGTCTCCGATATAAGCGAACAGAACGTTGGTNAGAGACTATGGTTCAATTGTAGTGGTATAATG
TTTCCCAGTGCATAAGGAACTCAGGGACACTACTAGCATGGGCGAGGAGAGACCCTTGGGTGGCAANA
CGCAATAAGTGTTNCCTAGTACATCAGCACGAGNGANGGTTTGNCCNTGTGTTTTTAGACGACCCCGG
TCGCATTGAACCTGGCATTCAGAATGACGTGCTCTCAAGAATCAAGGTCCTCCCACTGAGGGTCATCT
GCTTCGCGGGTCAGCGTATGGTCACAAACAT
>Kinh709
GTGTAGCCCAGTTAGACACTTCTAAATCCTTNGTAACAGACATGCAGTCCCCTCGGCAGAGCCCCGCC
CCCCGATGGTACCGGGTTGTTAAGAGGTCCACCACGGGTCGNACGACAANATTAAGGCCTCATGCATG
ACCCAACNCGGGGGCGTCCCGGCGCGGCGACCTTGNAGTCCGGGGACAAGTATGGCCCCCCGGGAAGG
ACGCCGTAGTGCATCTGGAGTGCCGATTCGTGGTCCAGAATGCCACCACGATGGTATGAGTGGCAAAG
GTACTTTGGACACCACCCCGTTTTCTANTTCCAGACGCGGCCATTCAGGAACGACGCGAATCGTAATC
TACCTGCCGCGTACGCGGATCGTATANACGTTTTCCAGCGTTCGAAATAGAGTTTGTCGTTCCGCTGA
CCAGGTTNCACGGCTACTNCGATAAGCATATTCTACTGGGGGACTGTAAAGGAATTGTATCCACTCTG
CTTCCTGTCAATTGGCCATGTGCATCTCTGGTTGTTCAGGNNACATTAATAACACACAGCATNAAGTA
GATNAGTCTCCGATATAAGCGAACAGAACGTTGGTCAGAGACTGTGGTTCAATTGTAGTGGTATAATG
TTTCCCAGTGCATAAGGAACTCAGGGACACTACTAGCATGGGCGAGGAGAGACCCTTGGGTGGCAANA
CGCAATAAGTGTTNCCTAGTACACCAGCACGAGANAGGGTTTGACCATGTGTTTTTAGACGACCCCGG
TCGCATTGAACCTGGCATTCAGAATGACGTGCTCTCAAGAGTCAANGTCCNCCCACTGAGGGTCATCT
GCTTCGCGGGTCNGCGTATGGNCATAAACAT
>Kinh710
GTGTAGCCCAGTTAGACACTTCTAAATCCNTGGTAACAGACATGCAGTCCCCTCGGCAGAGCCCCGCC
CCCCGATGGTACCGGGTTGTTAAGAGGTCCACAACGGNTCGNACGACAACNTTAAGGCCTCANGCATG
ACCCAACNCGGGGGCGTCCCGGCGNGGCGACCTTGAAGTCCGGGGNCAAGTATGGCCCCCCGGGAAGG
ACGCCGTAGTGCATCTGGAGTGCCGATTCGTGGTCCAGAATGCCACCACGATGGTATGAGTGGCAAAG
GTACTTTGGACACCACCCCGTTTTCTATTTCCAGACGCGGCCATTCAGGAACGACGCGAATCGTAATC

TACCTGCCGCGTACGCGGATCGTACANGCGTTTTNCAGCGTTCGAAATAGAGTTTGTCGTTCCGCTGA
CCAGGTTNCACGGCTACTGCGATAAGCATATTCTACTGGGGGACTGTAAAGNAATTGTATCCACTCNG
CTTCCTGTCAATTGGNCATGTGCATCTCTGGTTGTTCAGGGTACATTAATAACACACAGCATNAAGTA
GATAAGTCTCCGATATAAGCGAACAGAACGTTGGTCAGAGACTATGGTTCAATTGTAGTGGTATAATG
TTTCCCAGTGCATAAGGAACTCAGGGACACTACTAGCANGGGCGAGGAGAGACCCTTGGGTGGCAACA
CGCGATAAGTGTTACCTAGTACATCAGCACGAGANAGGGTTTGACCATGTGTTTTTAGACGACCCCGG
TCGCATTGAACCTGGCATTCAGAATGACGTGCTCTCAAGAATCAAGGTCCTCCCACTGAGGGTCATCT
GCTTCGCGGGTCAGCGTATNGTCACAANCAT
>Kinh711
GTGTAGCCCAGTTAGACACTTCTAAATCCTTNGTAACAGACATGCAGTCCCCTCGGCAGAGCCCCGCC
CCCCGATGGTACCGGGTTGTTAAGAGGTCCACAACGGGTCGNACGACAANATTAAGGCCTCATGCATG
ACCCNNCACGGGGGCGTCCCGGCGNGGCGACCTTGAAGTCCGGGGACAAGTATGGCCCCCCGGGAAGG
ACGCNGTAGTGCATCTGGAGTGCCGATTCNTGGTCNAGAATGNCACCACGATGGTATGAGTGGCAAAG
GTACTTTGGACACCACCCCGTTTTCTANTTCCAGACGCGGCCATTCAGGAACGACGCGAATCGTAATC
TACCTGCCGCGTNCGCGGATCGTACATGCGTTTTCCAGCGTTCGAAATAGAGTTTGTCGTTCCGCTGA
CCAGGTTNCACGGCTACTGCGATAAGCATATTCTACTGGGGGACTGTAAAGGAATTGTATCCACTCNG
CTTCCTGTCAATTGGNCATGTGCATCTCTGGTTGTTCAGGGTACATTAATAACACACAGCATNAAGTA
GATAAGTCTCCGATATAAGCGAACAGAACGTTGGTCAGAGACTATGGTTCAATTGTAGTGGTATAATG
TTTCCCAGTGCATAAGGAACTCAGGGACACTACTAGCATGGGCGAGGAGAGACCCTTGGGTGGCAACA
CGCGATAAGTGTTANCTAGTACATCAGCACGAGNGAGGGTTTGACCATGTGTTTTTAGNNGACCCCGG
TCGCATTGAACCTGGCATTCAGAATGACGTGCTCTCAAGAATCAAGNTCCNCNCACTGAGGGTCATCN
GCTTCGCGGGTCNGCGTATGGTCATAANNNT
>Kinh713
GTGTAGCCCAGTTAGACACTTCTAAATCCNTGGTAACAGACATGCAGTCCCCTCGGCAGAGCCCCGCC
CCCCGATGGTACCGGGTTGTTAAGAGGTCCACAACGGGTCNNACGACAACATTAAGGCCTCATGCATG
ACCCAACNCGGGGGCGTCCCGGCGNGGCGACCTTGNAGTCCGGGGNCAAGTATGGCCCCCCGGGAAGG
ACGCNGTAGTGCATCTGGAGTGCCGNTTCNTGGTCCAGAATGCCACCACGATGGTATGAGTNGCAAAG
GTACTTTGGACACCACCCCGTTTNCTANTTCCAGANGCGGCCNTTCAGGAACGACGCGAATCGTAATC
TACCTGCCGCGTNCGCGGATCGTACANGCGTTTTCCAGCGTTCGAAATAGAGTTTGTCGTTCCGCTGA
CCAGGTTNCACGGCTACTGCGATAAGCATATTCTACTGGGGGACTGTAAAGGAATTGTATCCACTCNG
CTTCCTGTCAATTGGNCATGTGCATCTCTGGTTGTTCAGGNNACATTAATAACACACAGCATTAAGTA
GATAAGTCTCCGATATAAGCGAACAGAACGTTGGTCAGAGACTATGGTTCAATTGTAGTGGTATAATG
TTTCCCAGTGCATAAGGAACTCAGGNACACTACTAGCATGGGCGAGGAGAGACCCTTGGGTGGCAACA
CGCAATAAGTGTTACCTAGTACATCAGCACGAGANAGGGTTTGACCATNNGTTTTTAGNNGACCCCGG
TCGCATTGAACCTGGCATTCAGAATGACGTGCTCTCAAGAATCAAGNTCCTCCCACTGAGGGTCATCT
GCTTCGCGGNNCAGCGTATNGTCATAAACAT
>Kinh714
GTGTAGCCCAGTTAGACACTTCTAAATCCTTGGTAACAGACATGCAGTCCCCTCGGCAGAGCCCCGCC
CCCCNATGGTACCGGGTTGTTAAGAGGTCCACAACAGNTCGCACGACAACNTTAAGGCCTCANGCATG
ACCCAACACGGGGGCGTCCCGGCGCGGCGACCTTGAAGTCCGGGNACAAGTATGGCCCCCCGGGAAGG
ACGCCGTAGTGCATCTGGANTGCCGATTCGTGGTCCAGAATGCCACCACGATGGTATGAGTGGCAAAG
GTACTTTGGACACCACCCCGTTNNCTANTTCCAGACGCGGCCNTTCAGGAACGACGCGAATCGTAATC
TACCTGCCGCGTACGCGGATCGTACANGCGTTTTCCAGCGTTCGAAATAGAGTTTGTCGTTCCGCCGA
CCAGGTTNCACGGCTACTGCGATAAGCATATTCTACTGGGGGACTGTAAAGGAATTGTATCCACTCNA
CTTCCTGTCAATTGGCCATGTGCATCTCTNGTTGTTCAGGGTACATTAATAACACACAGNATNAAGTA
GATAAGTCTCCGATATAAGCGAACAGANCGTTGGTNAGAGACTGTGGTTCAATTGTAGTGGTATAATG
TTTCCCAGTGCATAAGGAACTTAGGGACATTACTAGCANGGGCGAGGAGAGACCCTTGGGTGGCAANA
CGCAATAAGTGTTACCTAGTACACCAGCACGAGANAGGGTTTGACCATGTGTTTTTAGNNGACCCCGG
TCGCATTGAACCTGGCATTCAGAATGACGTGCTCTCAAGAATCAAGGTCCTCCCACTGAGGGTCATCN
GCTTCGCGGGTCNGCGTATGGTCATNCNNAT
>Kinh715
GTGTAGCCCAGTTAGACACTTCTAAATCCTTNGTAACAGACATGCAGTCCCCTCGGCAGAGCCCCGCC
CCCCNATGGTACCGGGTTGTTAAGAGGTCCACAACGGGTCGCACGACAACATTAAGGCCTCATGCATG
ACCCANCACGGGGGNGTCCCGGCGCGGCGACCTTGAAGTCCGGGGNCAAGTATGGCCCCCCGGGAAGG

ACGCNGTAGTGCATCTGGAGTGCCGATTCNTGGTCCAGAATGCCACCACGATGGTATGAGTNGCAAAG
GTACTTTGGACACCACCCCGTTTTCTANTTCCAGACGCGGCCNTTCAGGAACGACGCGAATCGTAATC
TACCTGCCGCGTNCGCGGATCGTACANGCGTTTTNCAGCGTTCGAAATAGAGTTTGTCGTTCCGCTGA
CCAGGTTNCACGGCTACTGCGATAAGCATATTCTACTGGGGGACTGTAAAGGAATTGTATCCACTCTG
CTTCCTGTCAATTGGCCATGTGCATCTCTGGTTGTTCAGGNNACATTAATAACACACAGCATNAAGTA
GATNAGTCTCCGATATAAGCGAACAGAACGTTGGTCAGAGACTGTGGTTCAATTGTAGTGGTATAATG
TTTCCCAGTGCATAAGGAACTCAGGGACACTACTAGCATGGGCGAGGAGAGACCCTTGGGTGGCAANA
CGCAATAAGTGTTACCTAGTACACCAGCACGAGNNAGGGTTTGACCATGTGTTTTTAGNNGACCCCGG
TCGCATTGAACCTGGCATTCAGAATGACGTGCTCTCAAGAATCAANNTCCTCNCACTGAGGGTCATCN
GCTTCGCGGGTCAGCGTATGGTCATAAACAT
>LaChi530
GTGTAGCCCAGTTAGACACNTCTAAATCCNTGGTAACAGACATGCAGTCCCCTCNNCAGAGCCCCGCC
CCCCGATGGTACCGGGTTGTTAAGAGGTCCACAACAGGTCGCACGACNANNTTAAGGCCTNANGCATG
ACCCNNCACNGNGGCGTCCCGGCGCGGNGACCTTGNANTCCGGGGNCAAGTATGGCCCCCCGNGAAGG
ACGCCGTAGTGCATCTGGAGTGCCGANNNGTGGTCNAGAATGCCACCACGATGGTATGAGTNGCAAAG
GTACTTTGGACACCACCCCGTTTTCTATTTCCAGANGCGGCCATTCAGGAACGACGCGAATCGTAATC
TACCTGCCGCGTACGCGGATCGTACATGCGTTTTCCAGCGTTCGAAATAGAGTTTGTCGTTCCGCCGA
CCNGGTTTCACGGCTACTNCGATAAGCATATTCTACTGGGGGACTGTAAAGGAATTGTATCCACTCTA
CTTCCTGTCAATTGGCCATGTGCATCTCTNGTTGTTCAGGNNACATTAATAACACACAGCATTAAGTA
GATAAGTCTCCGATATAAGCGAACAGANCGTTGGTNAGAGACTGTGGTTCAATTGTAGTGGTATAATG
TTTCCCAGTGCATAAGGAACTTAGGNACATTACTAGCATGGGCGAGGAGAGACCCTTGGGTGGCAACA
CGCAATAAGTGTTNCCTAGTACACCAGCACGAGNNANGGTTTGACCATGTGTTTTTAGACGACCCCGG
TCGCATTGAACCTGGCATTCAGAATGACGTGCTCTCAAGAATCAAGNTCCTCNCACTGAGGGTCATCN
GCTTCGCGGGTCNGCGTATGGTCATAANNAT
>LaChi531
GTGTAGCCCAGTTAGACACTTCTAAATCCTTGGTAACAGACATGCAGTCCCCTCNNCAGAGCCCCGCC
CCCCGATGGTACCGGGTTGNTAAGAGGTCCACAACGGGTCGCACGACNNCATTAAGGCCTCATGCATG
ACCCAACACNGGGGCGTCCCGGCGCGGCGACCTTGAAGTCCGGGGACAAGTATGGCCCCCCGGGAAGG
ACGCCGTAGTGCATCTGGAGTGCCGATTCGTGGTCCAGAATGCCACCACGATGGTATGAGTGGCAAAG
GTACTTTGGACACCACCCCGTTTTCTATTTCCAGACGCGGCCATTCAGGAACGACGCGAATCGTAATC
TACCTGCCGCGTACGCGGATCGTACATGCGTTTTNCAGCGTTCGAAATAGAGTTTGTCGTTCCGCTGA
CCAGGTTTCACGGCTACTNCGATAAGCATATTCTACTGGGGGACTGTAAAGGAATTGTATCCACTCNG
CTTCCTGTCAATTGGCCATGTGCATCTCTGGTTGTTCAGGGTACATTAATAACACACAGCATTAAGTA
GATNAGTCTCCGATATAAGCGAACAGAACGTTGGTCAGAGACTATGGTTCAATTGTAGTGGTATAATG
TTTCCCAGTGCATAAGGAACTCAGGGACACTACTAGCATGGGCGAGGAGAGACCCTTGGGTGGCAANA
CGNGATAAGTGTTACCTAGTACATCAGCACGAGNGAGGGTTTGACCATGTGTTTTTAGACGACCCCGG
TCGCATTGAACCTGGCATTCAGAATGACGTGCTCTCAAGAATCAAGNTCCTCCCACTGAGGGTCATCT
GCTTCGCGGGTCNGCGTATGGTCATAAACAT
>LaChi532
GTGTAGCCCAGTCAGACACTTCTAAATCCTTGGTAACAGACATGCAGTCCCCTCGGCAGAGCCCCGCC
CCCCGATGGTACCGGGTTGNTAAGAGGTCCACAACGGGTCGCACGACAACATTAAGGCCTCATGCATG
ACCCAACACGGGGGCGTCCCGGCGCGGCGACCTTGNAGTCCGGGGACAAGTATGGCCCCCCGGGAAGG
ACGCCGTAGTGCATCTGGAGTGCCGATTCGTGGTCCNGAATGNCACCACGATGGTATGAGTGGCAAAG
GTACTTTGGACACCACCCCGTTTTCTATTTCCAGANGCGGCCATTCAGGAACGACGCGAATCGTAATC
TACCTGCCGCGTACGCGGATCGTACATGCGTTTTNCAGCGTTCGAAATAGAGTTTGTCGTTCCGCTGA
CCAGGTTNCACGGCTACTGCGATAAGCATATTCTACTGGGGGACTGTAAAGGAATTGTATCCATTCTG
CTTCCTGTCAATTGGCCATGTGCATCTCTGGTTGTTCAGGNNACATTAATAACACACAGCAGTAAGTA
GGTNAGTCTCCGATATAAGCGAACAGAACGTTTGTCAGAGACTGTGGTTCAATTGTAGTGGTATAATG
TTTCCCAGTGCATAAGGAACTCAGGGACACTACTAGCATGGNCGAGGAGAGACCCTTGGGTGGCAACA
CGCAATAAGTGTTNCCTAGTACACCAGCACGAGAGANGGTTTGACCNTGTGTTTTTAGACGACCCCGG
TCGCATTGAACCTGGCATTCAGAATGACGTGCTCTCAAGAATCAAGGTCCTCCCACTGAGGGTCATCT
GCTTCGCGGGTCNGCGTATNGTCATAAACAT
>LaChi533
GTGTAGCCCAGTTAGACACTTCTAAATCCTTGGTAACAGACATGCAGTCCCCTCGGCAGAGCCCCGCC

CCCCGATGGTACCGGGTTGNTNAGAGGTCCACAACAGGTCGNACGACAANATTAAGGCCTCATGCATG
ANNCAACNCGGGGGCGTCCCGGNGCGGCGACCTTGAAGTCCGGGGACAAGTATGGCCCCCCGGGAAGG
ACGCCGTAGTGCATCTGGAGTGCCGATTCNTGGTCCAGANTGNCACCACGATGGTATGAGTGGCAAAG
GTACTTTGGACACCACCCCGTTNNCTATTTCCAGACGCGGCCNTTCAGGAACGACGCGAATCGTAATC
TACCTGCCGCGTACGCGGATCGTACATGCGTTTTCCAGCGTTCGAAATAGAGTTTGTCGTTCCGCCGA
CCAGGTTNCACGGCTACTGCGATAAGCATATTCTACTGGGGGACTGTAAAGGAATTGTATCCACTCNA
CTTCCTGTCAATTGGCCATGTGCATCTCTGGTTGTTCAGGNNACATTAATAACACACAGCATTAAGTA
GATAAGTCTCCGATATAAGCGAACAGAACGTTGGTNAGAGACTGTGGTTCAATTGTAGTGGTATAATG
TTTCCCAGTGCATAAGGAACTTAGGGACATTACTAGCATGGGCGAGGAGAGACCCTTGGGTGGCAACA
CGCAATAAGTGTTACCTAGTACACCAGCACGAGANANGGTTTGACCATGTGTTTTTAGACGACCCCGG
TCGCATTGAACCTGGCATTCAGAATGACGTGCTCTCAAGAATCAAGGTCCNCCCACTGAGGGTCANNN
GCTTCGCGGNTCAGCGTATGGTCATAAACAT
>LaChi534
GTGTAGCCCAGTCAGACACTNNNAAATNCNTGGTAACAGACATGCAGTCCCCTCGGCAGAGCNCCGCC
CCCCGATGGTACCGGGTTGNTNAGAGGTCCACAACGGGTCGNANGACNNNNTTAAGGCCTNANGCNTG
ACCCNANNCNGNNGCGTCCCGGNGCGGCNACCTTNNAGTCCGGGGNCAAGTATGGCCCCCCGGGAAGG
ACGCNGTAGTGCATCTGGAGTGCCGNTTCGTGGTCNAGAATGCCACCACGATGGTATGAGTNGCAAAG
GTACTTTGNNCACCACCCCGTTTTCTANTTCCAGANGCGGCCATTCAGGAACGACGCGAATCGTAATC
TACCTGCCGCGTNCGCGGATCGTACATGCGTNTTCCAGCGTTCGAAATAGAGTTTGTCGTTCCGCTGA
CCAGGTTNCACGGCTACTNCGATAAGCATATTCTACTGGGGGACTGTAAAGNAATTGTATCCATNCNG
CTTCCTGTCAATTGGNCATGTGCATCTCTGGTTGTTCAGGNNANATTAATAACACANAGNAGNAAGTA
GGTAAGTCTCCGATATAAGCGAACAGANCGTTTGNNAGAGACTGTGGTTCAATTGTAGTGGTATAATG
TTTCCCAGTGCATAANGAACTCAGGNACACTACTAGCATGGGCGAGGANAGACCCTTGGGTGGCAANA
CGCAATAAGTGTTNCCTAGTACACCAGCACGAGNNAGGGTTTGACCATGTGTTTTTAGNNGACCCCGG
TCGCATTGAACCTGGCATTCAGAATGACGTGCTNTCAAGAATCAAGGTCCNNNCACTGAGGGTCANNN
NCTTCGCNGGTCAGCGTATGGNCATNANNNT
>LaChi535
GTGTAGCCCAGTTAGACACNNCTAAATNCNTGGTAACAGACATGCAGTCCCCTCGGCAGAGCCCCGCC
CCCCGATGGTACCGGGTTGNTNAGAGGTCCACAACGGGTCNNACGACNNCATTAAGGCCTNANGCATG
ACCCAACNCGGGGGNGTCCCGGCGNGGCGACCTTGNAGTCCGGGNNCAAGTATNGCCCCCCGGGAAGG
ACGCNGTAGTGCATCTGGAGTGCCGNTTCGTGGTCNAGAATGCCACCACGATGGTATGAGTGGCAAAG
GTACTTTGGACACCACCCCGTTTTCTANTTCCAGACGCGGCCATTCAGGAACGACGCGAATCGTAATC
TACCTGCCGCGTACGCGGATCGTACATGCGTTTTCCAGCGTTCGAAATAGAGTTTGTCGTTCCGCTGA
CCNGGTTNCACGGCTACTNNGATAAGCATATTCTACTGGGGGACTGTAAAGNAATTGTATCCACTCTG
CTTCCTGTCAATTGGNCATGTGCATCTCTNGTTGTTCAGGNNACATTAATAACACACAGCATNAAGTA
GATNAGTCTCCGATATAAGCGAACAGAACGTTGGNNAGAGACTATGGTTCAATTGTAGTGGTATAATG
TTTCCCAGTNCATAAGGAANTCAGGGACACTACTAGCATGGGCGAGGAGAGACCCTTGGGTGGCAACA
CGCGATAAGTGTTACCTAGTACATCAGCACGAGAGAGGGTTTGNCCATGTNTTTTAGNNGACCCCGG
TCGCATTGAACCTGNCATTCAGAATGACGTGCTCTCANGAATCAANNTCCTCNCACTGAGGGNCATNN
GCTTCGCGGNNCNGCGTATGGTCANAANNNT
>LaChi536
GTGTAGCCCAGTTAGACACTTCTAAATCCTTGGTAACAGACATGCAGTCCCCTCGGCAGAGCCCCGCC
CCCCGATGGTACCGGGTTGNTAAGAGGTCCACAACGGGTCGCACGACAACATTAAGGCCTCATGCATG
ACCCAACACGGGGGCGTCCCGGCGNGGCGACCTTGNAGTCCGGGGACAAGTATGGCCCCCCGGGAAGG
ACGCCGTAGTGCATCTGGAGTGCCGATTNGTGGTCCAGAATGCCACCACGATGGTATGAGTGGCAAAG
GTACTTTGGACACCACCCCGTTTTCTANTTCCAGANGCGGCCNTTCAGGAACGACGCGAATCGTAATC
TACCTGCCGCGTACGCGGATCGTACATGCGTTTTCCAGCGTTCGAAATAGAGTTTGTCGTTCCGCTGA
CCAGGTTNCACGGCTACTNCGATAAGCATATTCTACTGGGGGACTGTAAAGGAATTGTATCCACTCTG
CTTCCTGTCAATTGGCCATGTGCATCTCTGGTTGTTCAGGGTACATTAATAACACACAGCATTAAGTA
GATAAGTCTCCGATATAAGCGAACAGANCGTTGGTCAGAGACTATGGTTCAATTGTAGTGGTATAATG
TTTCCCAGTGCATAAGGAACTCAGGGACACTACTAGCATGGGCGAGGAGAGACCCTTGGGTGGCAACA
CGCGATAAGTGTTNCCTAGTACATCAGCACGAGANANGGTTTGACCATGNNTTTTTAGACGACCCCGG
TCGCATTGAACCTGGCATTCAGAATGACGTNCTCTCAAGAATCAAGGTCCTCCCACTGAGGGTCATCT
GCTTCGCGGGTCNGCGTATNGTCATNANNAT

>LaChi537
GTGTAGCCCAGTTAGACACTTCTAAATCCTTNGTAACAGACATGCAGTCCCCTCNNCAGAGCCCCGCC
CCCCGATGGTACCGGGTTGTTAAGAGGTCCACAACGGGTCGCACGACAACATTAAGGCCTCATGCATG
ACCCAACACGGGGGCGTCCCGGCGCGGCGACCTTGNAGTCCGGGGNCAAGTATGGCCCCCCGGGAAGG
ACGCCGTAGTGCATCTGGAGTGCCGATTCGTGGTCCAGAATGCCACCACGATGGTATGAGTNGCAAAG
GTACTTTGGACACCACCCCGTTNNCTATTTCCAGACGCGGCCATTCAGGAACGACGCGAATCGTAATC
TACCTGCCGCGTACGCGGATCGTACANGCGTTTTCCAGCGTTCGAAATAGAGTTTGTCGTTCCGCTGA
CCNGGTTNCACGGCTACTGCGATAAGCATATTCTACTGGGGGACTGTAAAGGAATTGTATCNACTCTG
CTTCCTGTCAATTGGCCATGTGCATCTCTGGTTGTTCAGGNNACATTAATAACACACAGNATNAAGTA
GATNAGTCTCCGATATAAGCGAACAGANCGTTGGTNAGAGACTATGGTTCAATTGTAGTGGTATAATG
TTTCCCAGTGCATAAGGAACTCAGGGACACTACTAGCATGGGCGAGGAGAGACCCTTGGGTGGCAACA
CGNGATAAGTGTTACCTAGTACATCAGCACGAGNNAGGGTTTGACCATGTGTTTTTAGACGACCCCGG
TCGCATTGAACCTGGCATTCAGAATGACGTGCTNTCAAGAATCAAGNTCCTCCCACTGAGGGTCATCT
GCTTCGCGGGTCNGCGTATGGTCATAAACAT
>LaChi538
GTGTAGCCCAGTTAGACACTTCTAAATCCNTNGTAACAGACATGCAGTCCCCTCGGCAGAGCCCCGCC
CCCCNATGGTACCGGGTTGNTAAGAGGTCCACAACAGGTCNCACGACNNCATTAAGGCCTCATGCATG
ACCCNACACGGGGGCGTCCCGGCGCGGCGACCTTGNANTCCGGGGNCAAGTATGGCCCCCCGGGAAGG
ACNCCGTAGTGCATCTGGAGTGCCGATTCGTGGTCNAGAATGCCACCACGATGGTATGAGTNGCAAAG
GTACTTTGGACACCACCCCGTTTTCTANTTCCAGACGCGGCCNTTCAGGAACGACGCGAATCGTAATC
TANCTGCCGCGTACGCGGATCGTACATGCGTTTTCCAGCGTTCGAAATAGAGTTTGTCGTTCCGCCGA
CCAGGTTNCACGGCTACTNCGATAAGCATATTCTACTGGGGGACTGTAAAGGAATTGTATCCACTCTA
CTTCCTGTCAATTGGNCATGTGCATCTCTGGTTGTTCAGGNNACATTAATAACACACAGCATNAAGTA
GATNAGTCTCCGATATAAGCGAACAGAACGTTGGTCAGAGACTGTGGTTCAATTGTAGTGGTATAATG
TTTCCCAGNGCATAAGGAACTTAGGGACATTACTAGCATGGGCGAGGAGAGACCCTTGGGTGGCAACA
CGNAATAAGTGTTACCTAGTACACCAGCACGAGNGAGGGTTTGACCATGNNTTTTTAGACGACCCCGG
TCGCATTGAACCTGGCATTCAGAATGACGTGCTCTCAAGAATCAAGNTCCTCCCACTGAGGGTCATCT
GCTTCGCGGGTCNGCGTATGGTCATNANNNT
>LaChi539
GTGTAGCCCAGTTAGACACTTCTAAATCCNTGGTAACAGACATGCAGTCCCCTCGGCAGAGCCCCGCC
CCCCGATGGTACCGGGTTGTTAAGAGGTCCACAACGGGTCGNACGACNNNATTAAGGCCTNATGCATG
ACCCAANNCGGNGGNGTCCCGGNGCGGCGACCTTGNAGTCCGGGGNCAAGTATGGCCCCCCGGGAAGG
ACGCNGTAGTGCATCTGGAGTGCCGATTCGTGGTCCAGAATGNCACCACGATGGTATGAGTGGCAAAG
GTACTTTGGACACCACCCCGTTTTCTANTTCCAGACGCGGCCATTCAGGAACGACGCGAATCGTAATC
TACCTGCCGCGTACGCGGATCGTACANGCGTTTTCCAGCGTTCGAAATAGAGTTTGTCGTTCCGCTGA
CCAGGTTNCACGGCTACTNCGATAAGCATATTCTACTGGGGGACTGTAAAGNAATTGTATCCACTCTG
CTTCCTGTCAATTGGCCATGTGCATCTCTGGTTGTTCAGGGTACATTAATAACACACAGCATNAAGTA
GATNAGTCTCCGATATAAGCGAACAGAACGTTGGTCAGAGACTATGGTTCAATTGTAGTGGTATAATG
TTTCCCAGTGCATAAGGAACTCAGGGACACTACTAGCATGGGCGAGGAGAGACCCTTGGGTGGCAACA
CGCGATAAGTGTTNCCTAGTACATCAGCACGAGNNAGGGTTTGACCATGTGTTTNTAGACGACCCCGG
TCGCATTGAACCTGGCATTCAGAATGACGTGCTCTCAAGAATNAAGGTCCNCCCACTGAGGGNCATNN
GCTTCGCGGGNCAGCGTATGGTCATANNNAT
>LaChi540
GTGTAGCCCAGTCAGACACTTCTAAATCCTTGGTAACAGACATGCAGTCCCCTCGGCAGAGCCCCGCC
CCCCGATGGTACCGGGTTGTTAAGAGGTCCACAACGGNTCGCACGACAACATTAAGGCCTCATGCATG
ACCCANCNCGGNGGNGTCCCGGNGNGGNGACCTTGNANTCCGGGGACAAGTATGGCCCCCCGGGAAGG
ACGCCGTAGTGCATCTGGAGTGCCGATTCGTGGTCCAGAATGCCACCACGATGGTATGAGTGGCAAAG
GTACTTTGGACACCACCCCGTTTTCTANTTCCAGACGCGGCCATTCAGGAACGACGCGAATCGTAATC
TACCTGCCGCGTACGCGGATCGTACATGCGTTTTCCAGCGTTCGAAATAGAGTTTGTCGTTCCGCTGA
CCAGGTTTCACGGCTACTGCGATAAGCATATTCTACTGGGGGACTGTAAAGGAATTGTATCCATTCTG
CTTCCTGTCAATTGGNCATGTGCATCTCTGGTTGTTCAGGGNACATTAATAACACACAGCAGTAAGTA
GGTAAGTCTCCGATATAAGCGAACAGAACGTTTGTCAGAGACTGTGGTTCAATTGTAGTGGTATAATG
TTTCCCAGTGCATAAGGAACTCAGGNACACTACTAGCANGGGCGAGGAGAGACCCTTGGGTGGCAACA
CGCAATAAGTGTTACCTAGTACACCAGCACGAGAGANGGTTTGACCATGTGTTTTTAGACGACCCCGG

TCGCATTGAACCTGGCATTCAGAATGACGTGCTCTCAAGAATCAANGTCCTCCCACTGAGGGTCATCT
GCTTCGCGGGTCAGCGTATNGTCATAAACAT
>LaChi541
GTGTAGCCCAGTCAGACACTTCTAAATCCTTGGTAACAGACATGCAGTCCCCTCGGCAGAGCCCCGCC
CCCCGATGGTACCGGGTTGNTNAGAGGTCCACAACGGGTCNNACGACNNCATTAAGGCCTCATGCATG
ACCCAACACGGGGGCGTCCCGGCGCGGCGACCTTGAAGTCCGGGGACAAGTATGGCCCCCCGGGAAGG
ACGCCGTAGTGCATCTGGAGTGCCGATTCGTGGTCCAGAATGCCACCACGATGGTATGAGTGGCAAAG
GTACTTTGGACACCACCCCGTTTTCTATTTCCAGACGCGGCCATTCAGGAACGACGCGAATCGTAATC
TACCTGCCGCGTACGCGGATCGTACATGCGTTTTCCAGCGTTCGAAATAGAGTTTGTCGTTCCGCTGA
CCAGGTTNCACGGCTACTGCGATAAGCATATTCTACTGGGGGACTGTAAAGGAATTGTATCCATTCTG
CTTCCTGTCAATTGGCCATGTGCATCTCTGGTTGTTCAGGNNACATTAATAACACACAGCAGTAAGTA
GGTAAGTCTCCGATATAAGCGAACAGAACGTTTGTCAGAGACTGTGGTTCAATTGTAGTGGTATAATG
TTTCCCAGTGCATAAGGAACTCAGGGACACTACTAGCATGGGCGAGGAGAGACCCTTGGGTGGCAACA
CGCAATAAGTGTTACCTAGTACACCAGCACGAGNGAGGGTTTGACCATGTGTTTTTAGACGACCCCGG
TCGCATTGAACCTGGCATTCAGAATGACGTGCTCTCAAGAATCAAGGTCCTCCCACTGAGGGTCATCT
GCTTCGCGGGTCAGCGTATGGTCATNANCAT
>LaChi542
GTGTAGCCCAGTTAGACACTTCTAAATCCTTNGTAACAGACATGCAGTCCCCTCGGCAGAGCNCCGCC
CCCCGATGGTACCGGGTTGNNAAGAGGTCCACAACAGGTCGCACGACAACNTTAAGGCCTCATGCATG
ACCCNACACGGNGGNGTCCCGGCGNGGCGACCTTGNANTCCGGGGACAAGTATGGCCCCCCGGGAAGG
ACGCCGTAGTGCATCTGGANTGCCGATTCGTGGTCCAGAATGCCACCACGATGGTATGAGTGGCAAAG
GTACTTTGGACACCACCCCGTTTTCTATTTCCAGACGCGGCCATTCAGGAACGACGCGAATCGTAATC
TACCTGCCGCGTACGCGGATCGTACATGCGTTTTCCAGCGTTCGAAATAGAGTTTGTCGTTCCGCCGA
CCAGGTTNCACGGCTACTGCGATAAGCATATTCTACTGGGGGACTGTAAAGGAATTGTATCCACTCNA
CTTCCTGTCAATTGGCCATGTGCATCTCTGGTTGTTCAGGGNACATTAATAACACACAGNATTAAGTA
GATAAGTCTCCGATATAAGCGAACAGAACGTTGGTCAGAGACTGTGGTTCAATTGTAGTGGTATAATG
TTTCCCAGTGCATAAGGAACTTAGGGACATTACTAGCATGGGCGAGGAGAGACCCTTGGGTGGCAACA
CGCAATAAGTGTTACCTAGTACACCAGCACGAGNGANGGTTTGACCATGTGTTTTTAGNNGNCCCCGG
TCGCATTGAACCTGGCATTCAGAATGACGTGCTNTCANGAATCAAGGTCCTCCCACTGAGGGTCATCT
GCTTCGCGGGTCNGCGTATGGTCANNANNAT
>LaChi543
GTGTAGCCCAGTTAGACACTTCTAAATCCTTGGTAACAGACATGCAGTCCCCTCGGCAGAGCCCCGCC
CCCCNATGGTACCGGGTTGTTNAGAGGTCCACAACGGNTCGCACGACAACATTAAGGCCTCATGCATG
ACCCAANACGGNGGNGTCCCGGCGCGGCGACCTTGNAGTCCGGGGACAAGTATGGCCCCCCGGGAAGG
ACGCNGTAGTGCATCTGGAGTGCCGATTCGTGGTCCAGAATGCCACCACGATGGTATGAGTNGCAAAG
GTACTTNGGACACCACCCCGTTTTCTATTTCCAGACGCGGCCATTCAGGAACGACGCGAATCGTAATC
TACCTGCCGCGTACGCGGATCGTACATGCGTNTTCCAGCGTTCGAAATAGAGTTTGTCGTTCCGCTGA
CCAGGTTNCACGGCTACTNCGATAAGCATATTCTACTGGGGGACTGTAAAGGAATTGTATCCACTCNG
CTTCCTGTCAATTGGCCATGTGCATCTCTNGTTGTTCAGGNNACATTAATAACACACAGCATTAAGTA
GATAAGTCTCCGATATAAGCGAACAGAACGTTGGTCAGAGACTATGGTTCAATTGTAGTGGTATAATG
TTTCCCAGTGCATAAGGAACTCAGGGACACTACTAGCATGGGCGAGGAGAGACCCTTGGGTGGCAANA
CGNGATAAGTGTTACCTAGTACATCAGCACGAGNGANGGTTTGACCATGTGTTTTTAGACGACCCCGG
TCGCATTGAACCTGGCATTCAGAATGACGTGCTCTCAAGAATCAAGGTCCTCCCACTGAGGGTCATCT
GCTTCGCGGNTCNGCGTATGGTCATNANNNT
>LaChi544
GTGTAGCCCAGTTAGACACTTCTAAATCCTTNGTAACAGACATGCAGTCCCCTCGGCAGAGCCCCGCC
CCCCNATGGTACCGGGTTGTTAAGAGGTCCACAACAGGTCGCACGACNACNTTAAGGCCTNATGCATG
ACCCNACACGGNGGNGTCCCGGCGCGGCGACCTTGAAGTCCGGGGNCAAGTATGGCCCCCCGGGAAGG
ACGCCGTAGTGCATCTGGAGTGCCGATNCGTGGTCNAGAATGCCACCACGATGGTATGAGTNGCAAAG
GTACTTTGGACACCACCCCGTTTTCTATTTCCAGACGCGGCCATTCAGGAACGACGCGAATCGTAATC
TACCTGCCGCGTACGCGGATCGTACATGCGTTTTCCAGCGTTCGAAATAGAGTTTGTCGTTCCGCCGA
CCAGGTTNCACGGCTACTGCGATAAGCATATTCTACTGGGGGACTGTAAAGGAATTGTATCCACTCTA
CTTCCTGTCAATTGGCCATGTGCATCTCTGGTTGTTCAGGGTACATTAATAACACACAGNATTAAGTA
GATAAGTCTCCGATATAAGCGAACAGAACGTTGGTCAGAGACTGTGGTTCAATTGTAGTGGTATAATG

TTTCCCAGTGCATAAGGAACTTAGGGACATTACTAGCATGGGCGAGGAGAGACCCTTGGGTGGCAACA
CGCAATAAGTGTTACCTAGTACACCAGCACGAGNGANGGTTTGACCATGTGTTTTTAGACGACCCCGG
TCGCATTGAACCTGGCATTCAGAATGACGTGCTCTCAAGAATCAANGTCCTCCCACTGAGGGTCATCT
GCTTCGCGGGTCNGCGTATNGTCANNNNNNT
>LaChi545
GTGNAGCCCAGTTAGACACTTCTAAATCCTTGGTAACAGACATGCAGTCCCCTCNNCAGAGNCCCGCC
CCCCNATGGTACCGGGTTGTTNAGAGGTCCACAACAGGTCGCACGACAACATTAAGGCCTCANGCATG
ANNCAANNCNGGGGCGTCCCGGCGCGGCGACCTTGAAGTCCGGGGACAAGTATGGCCCCCCGGGAAGG
ACGCCGTAGTGCATCTGGAGTGCCGATTCGTGGTCCAGAATGCCACCACGATGGTATGAGTGGCAAAG
GTACTTTGGACACCACCCCGTTTTCTATTTCCAGANGCGGCCATTCAGGAACGACGCGAATCGTAATC
TACCTGCCGCGTACGCGGATCGTACANGCGTTTTCCAGCGTTCGAAATAGAGTTTGTCGTTCCGCCGA
CCAGGTTNCACGGCTACTNCGATAAGCATATTCTACTGGGGGACTGTAAAGGAATTGTATCCACTCTA
CTTCCTGTCAATTGGCCATGTGCATCTCTGGTTGTTCAGGNNACATTAATAACACACAGCATTAAGTA
GATAAGTCTCCGATATAAGCGAACAGAACGTTGGTCAGAGACTGTGGTTCAATTGTAGTGGTATAATG
TTTCCCAGTGCATAAGGAACTTAGGGACATTACTAGCATGGGCGAGGAGAGACCCTTGGGTGGCAANA
CGCAATAAGTGTTNCCTAGTACACCAGCACGAGAGANGGTTTGACCATGTGTTTTTAGACGACCCCGG
TCGCATTGAACCTGGCATTCAGAATGACGTGCTNTCAAGAATCAAGNTCCTCCCACTGAGGGTCATCN
GCTTCGCGGGTCAGCGTATNGTCATAAACAT
>LaChi546
GTGTAGCCCAGTTAGACACTTCTAAATCCTTGGTAACAGACATGCAGTCCCCTCGGCAGAGCCCCGCC
CCCCGATGGTACCGGGTTGTTNAGAGGTCCACAACAGGTCGCANGACNNCNTTAAGGCCTCATGCATG
ACCCAACACGGGGGCGTCCCGGCGCGGCNACCTTGNAGTCCGGGGACAAGTATGGCCCCCCGGGAAGG
ACGCCGTAGTGCATCTGGAGTGCCGNTTCGTGGTCCAGAATGCCACCACGATGGTATGAGTNGCAAAG
GTACTTTGGACACCACCCCGTTTTCTATTTCCAGANGCGGCCATTCAGGAACGACGCGAATCGTAATC
TACCTGCCGCGTACGCGGATCGTACATGCGTTTTCCAGCGTTCGAAATAGAGTTTGTCGTTCCGCCGA
CCAGGTTNCACGGCTACTGCGATAAGCATATTCTACTGGGGGACTGTAAAGGAATTGTATCNACTCTA
CTTCCTGTCAATTGGCCATGTGCATCTCTNGTTGTTCAGGNTACATTAATAACACACAGCATNAAGTA
GATAAGTCTCCGATATAAGCGAACAGAACGTTGGTCAGAGACTGTGGTTCAATTGTAGTGGTATAATG
TTTCCCAGTGCATAAGGAACTTAGGGACATTACTAGCATGGGCGAGGAGAGACCCTTGGGTGGCAACA
CGCAATAAGTGTTACCTAGTACACCAGCACGAGAGAGGGTTTGACCATGTGTTTTTAGACGACCCCGG
TCGCATTGAACCTGGCATTCAGAATGACGTGCTCTCAAGAATCAAGGTCCTCCCACTGAGGGTCATCT
GCTTCGCGGNNCAGCGTATGGTCATNAACNT
>LaChi547
GTGTAGCCCAGTCAGACACNTCTAAATCCTTGGTAACAGACATGCAGTCCCCTCGGCAGAGCCCCGCC
CCCCGATGGTACCGGGTTGNTAAGAGGTCCACAACGGGTCNNACGACNNCNTTAAGGCCTCANGCATG
ACCCAACACGGNGGCGTCCCGGNGNGGCGACCTTGAAGTCCGGGGNCAAGTATGGCCCCCCGNGAAGG
ACGCNGTAGTGCATCTGGAGTGCCGATTCGTGGTCCAGAATGCCACCACGATGGTATGAGTGGCAAAG
GTACTTNGGACACCACCCCGTTTTCTATTTCCAGACGCGGCCATTCAGGAACGACGCGAATCGTAATC
TACCTGCCGCGTACGCGGATCGTACATGCGTTTTCCAGCGTTCGAAATAGAGTTTGTCGTTCCGCTGA
CCAGGTTNCACGGCTACTGCGATAAGCATATTCTACTGGGGGACTGTAAAGGAATTGTATCCATTCNG
CTTCCTGTCAATTGGCCATGTGCATCTCTGGTTGTTCAGGNNACATTAATAACACACAGCAGTAAGTA
GGTAAGTCTCCGATATAAGCGAACAGAACGTTTGTCAGAGACTGTGGTTCAATTGTAGTGGTATAATG
TTTCCCAGTGCATAAGGAACTCAGGNACACTACTANCATGGGCGAGGAGAGACCCTTGGGTGGCAACA
CGCAATAAGTGTTACCTAGTACACCAGCACGAGNNANGGTTTGACCATGNNTTTTTAGACGACCCCGG
TCGCATTGAACCTGGCATTCAGAATGACGTGCTCTCAAGAATCAAGNTCCTNNCACTGAGGGTCATCT
GCTTCGCGGGTCAGCGTATGGTCATAANCAT
>LaChi548
GTGTAGCCCAGTTAGACACTTCTAAATCCTTGGTAACAGACATGCAGTCCCCTCGGCAGAGCCCCGCC
CCCCGATGGTACCGGGTTGTTAAGAGGTCCACAACGGGTCGCACGACAACATTAAGGCCTCATGCATG
ACCCAACACGGGGGCGTCCCGGCGCGGCGACCTTGAAGTCCGGGGACAAGTATGGCCCCCCGGGAAGG
ACGCCGTAGTGCATCTGGAGTGCCGATTCGTGGTCCAGAATGCCACCACGATGGTATGAGTNGCAAAG
GTACTTTGGACACCACCCCGTTTTCTATTTCCAGACGCGGCCATTCAGGAACGACGCGAATCGTAATC
TACCTGCCGCGTACGCGGATCGTACATGCGTTTTCCAGCGTTCGAAATAGAGTTTGTCGTTCCGCTGA
CCAGGTTNCACGGCTACTGCGATAAGCATATTCTACTGGGGGACTGTAAAGGAATTGTATCCACTCTG

CTTCCTGTCAATTGGCCATGTGCATCTCTGGTTGTTCAGGGTACATTAATAACACACAGCATTAAGTA
GATAAGTCTCCGATATAAGCGAACAGAACGTTGGTCAGAGACTATGGTTCAATTGTAGTGGTATAATG
TTTCCCAGTGCATAAGGAACTCAGGGACACTACTAGCATGGGCGAGGAGAGACCCTTGGGTGGCAACA
CGCGATAAGTGTTACCTAGTACATCAGCACGAGAGAGGGTTTGACCATGTGTTTTTAGACGACCCCGG
TCGCATTGAACCTGGCATTCAGAATGACGTGCTCTCAAGAATCAAGGTCCTCCCACTGAGGGTCATCT
GCTTCGCGGGTCNGCGTATGGTCANNAACNT
>LaChi549
GTGTAGCCCAGTTAGACACTTCTAAATCCNTGGTAACAGACATGCAGTCCCCTCGGCAGAGCCCCGCC
CCCCNATGGTACCGGGTTGNTAAGAGGTCCACAACAGNTCGNACGACAACATTAAGGCCTNANGCATG
ACCCAACACGGGGGNGTCCCGGNGCGGNGACCTTGAANTCCGGGGNCAAGTATGGCCCCCCGGGAAGG
ACGCNGTAGTGCATCTGGAGTGCCGATTCGTGGTCCAGAATGCCACCACGATGGTATGAGTNGCAAAG
GTACTTNGGACACCACCCCGTTTTCTATTTCCAGANGCGGCCNTTCAGGAACGACGCGAATCGTAATC
TACCTGCCGCGTACGCGGATCGTACATGCGTTTTCCAGCGTTCGAAATAGAGTTTGTCGTTCCGCCGA
CCAGGTTNCACGGCTACTGCGATAAGCATATTCTACTGGGGGACTGTAAAGNAATTGTATCCACTCNA
CTTCCTGTCAATTGGNCATGTGCATCTCTNGTTGTTCAGGGTACATTAATAACACACAGCATTAAGTA
GATAAGTCTCCGATATAAGCGAACAGANCGTTGGTNAGAGACTGTGGTTCAATTGTAGTGGTATAATG
TTTCCCAGNGCATAAGGAACTTAGGNACATTACTAGCATGGGCGAGGAGAGACCCTTGGGTGGCAANA
CGCAATAAGTGTTNNCTAGTACACCAGCACGAGNGANGGTTTGACCATGNNTTTTTAGNNGACCCCGG
TCGCATTGAACCTGGCATTCAGAATGACGTGCTCTCAAGAATNAAGNTCCTNNCACTGAGGGTCATCT
GCTTCGCGGGTCNGCGTATGGTCATNAACAT
>LaChi550
GTGTAGCCCAGTTAGACACTTCTAAATCCTTGGTAACAGACATGCAGTCCCCTCGGCAGAGCCCCGCC
CCCCGATGGTACCGGGTTGTTAAGAGGTCCACAACAGGTCGCACGACANNNTTAAGGCCTCANGCATG
ACCCAACACGGGGGCGTCCCGGCGNGGCGACCTTGNAGTCCGGGGNCAAGTATGGCCCCCCGGGAAGG
ACGCCGTAGTGCATCTGGAGTGCCGATTCGTGGTCCAGAATGCCACCACGATGGTATGAGTNGCAAAG
GTACTTTGGACACCACCCCGTTNNCTATTTCCAGACGCGGCCATTCAGGAACGACGCGAATCGTAATC
TANCTGCCGCGTACGCGGATCGTACATGCGTTTTCCAGCGTTCGAAATAGAGTTTGTCGTTCCGCCGA
CCAGGTTTCACGGCTACTGCGATAAGCATATTCTACTGGGGGACTGTAAAGGAATTGTATCCACTCTA
CTTCCTGTCAATTGGCCATGTGCATCTCTGGTTGTTCAGGGTACATTAATAACACACAGCATTAAGTA
GATAAGTCTCCGATATAAGCGAACAGAACGTTGGTCAGAGACTGTGGTTCAATTGTAGTGGTATAATG
TTTCCCAGTGCATAAGGAACTTAGGGACATTACTAGCATGGGCGAGGAGAGACCCTTGGGTGGCAACA
CGCAATAAGTGTTACCTAGTACACCAGCACGAGAGAGGGTTTGACCATGTGTTTTTAGACGACCCCGG
TCGCATTGAACCTGGCATTCAGAATGACGTGCTCTCAAGAATCAAGGTCCNCNCACTGAGGGTCATCT
GCTTCGCGGGTCNGCGTATGGTCATNNACAT
>LaChi551
GTGNAGCCCAGTTAGACACTTCTAAATCCTTGGTAACAGACATGCAGTCCCCTCNNCAGAGCCCCGCC
CCCCNATGGTACCGGGTTGTTAAGAGGTCCACAACAGGTCGNACGACANCNTTAAGGCCNCATGCATG
ACNCAACACGGNGGCGTCCCGGCGCGGCGACCTTGNAGTCCGGGGACAAGTATGGCCCCCCGGGAAGG
ACGCCGTAGTGCATCTGGANTGCCGATTCNTGGTCNNGAATGCCACCACGATGGTATGAGTGGCAAAG
GTACTTTGGACACCACCCCGTTTTCTATTTCCAGACGCGGCCATTCAGGAACGACGCGAATCGTAATC
TACCTGCCGCGTNCGCGGATCGTACATGCGTTTTCCAGCGTTCGAAATAGAGTTTGTCGTTCCGCCGA
CCAGGTTNCACGGCTACTNCGATAAGCATATTCTACTGGGGGACTGTAAAGGAATTGTATCCACTCTA
CTTCCTGTCAATTGGNCATGTGCATCTCTNGTTGTTCAGGGTACATTAATAACACACAGNATTAAGTA
GATAAGTCTCCGATATAAGCGAACAGAACGTTGGTCAGAGACTGTGGTTCAATTGTAGTGGTATAATG
TTTCCCAGTGCATAAGGAACTTAGGGACATTACTAGCATGGGCGAGGAGAGACCCTTGGGTGGCANCA
CGNAATAAGTGTTACCTAGTACACCAGCACGAGNNAGGGTTTGACCNTGNNTTTTTAGACGACCCCGG
TCGCATTGAACCTGGCATTCAGAATGACGTGCTNTCAAGAATNAANGTCCNCNCACTGAGGGTCATNN
GCTTCGCGGGTCNGCGTATNGTCATANNCAT
>LaChi552
GTGTAGCCCAGTTAGACACTTCTAAATCCTTGGTAACAGACATGCAGTCCCCTCGGCAGAGCCCCGCC
CCCCGATGGTACCGGGTTGTTAAGAGGTCCACAACAGGTCGNACGACAACNTTAAGGCCTCATGCATG
ACCCAACACGGGGGCGTCCCGGNGNGGCGACCTTGAAGTCCGGGGNCAAGTATGGCCCCCCGGGAAGG
ACGCCGTAGTGCATCTGGAGTGCCGATTCGTGGTCCAGAATGCCACCACGATGGTATGAGTNGCAAAG
GTACTTTGGACACCACCCCGTTTTCTATTTCCAGACGCGGCCATTCAGGAACGACGCGAATCGTAATC

TACCTGCCGCGTACGCGGATCGTACANGCGTTTTCCAGCGTTCGAAATAGAGTTTGTCGTTCCGCCGA
CCAGGTTTCACGGCTACTGCGATAAGCATATTCTACTGGGGGACTGTAAAGGAATTGTATCCACTCNA
CTTCCTGTCAATTGGCCATGTGCATCTCTGGTTGTTCAGGGTACATTAATAACACACAGCATTAAGTA
GATNAGTCTCCGATATAAGCGAACAGAACGTTGGTCAGAGACTGTGGTTCAATTGTAGTGGTATAATG
TTTCCCAGTGCATAAGGAACTTAGGNACATTACTAGCATGGGCGAGGAGAGACCCTTGGGTGGCAANA
CGCAATAAGTGTTNCCTAGTACACCAGCACGAGAGAGGGTTTGACCNTGTNTTTTAGACGACCCCGG
TCGCATTGAACCTGGCATTCAGAATGACGTGCTCTCAAGAATCAAGNTCCTCCCACTGAGGGTCATCT
GCTTCGCGGNTCNGCGTATGGTCATAAACNT
>LaChi553
GTGTAGCCCAGTTAGACACTTCTAAATCCNTGGTAACAGACATGCAGTCCCCTCNNCAGAGCNCCGCC
CCCCGATGGTACCGGGTTGTTNAGAGGTCCACAACAGGTCGCACGACANNNTTAAGGCCTNATGCATG
ANNCNNCACGGNGGCGTCCCGGNNNNGCGACCTTGAANTCCGGGGNCAAGTATGGCCCCCCGGGAAGG
ACGCCGTAGTGCATCTGGAGTGCCGNNNCGTGGTCNAGAATGCCACCACGATGGTATGAGTNGCAAAG
GTACTTNGGACACNACCCCGTTTTCTATTTCCAGANGCGGCCATTCAGGAACGACGCGAATCGTAATC
TACCTGCCGCGTACGCGGATCGTACATGCGTTTTCCAGCGTTCGAAATAGAGTTTGTCGTTCCGCCGA
CCAGGTTNCACGNCTACTNCGATAAGCATATTCTACTGGGGGACTGTAAAGGAATTGTATCCACTCNA
CTTCCTGTCAATTGGNCATGTGCATCTCTNGTTGTTCAGGNNACATTAATAACACACAGCATTAAGTA
GATNAGTCTCCGATATAAGCGAACAGAACGTTGGTCAGAGACTGTGGTTCAATTGTAGTGGTATAATG
TTTCCCAGTGCATAAGGAACTTAGGGACATTACTAGCATGGGCGAGGAGAGACCCTTGGGTGGCAACA
CGCAATAAGTGTTACCTAGTACACCAGCACGAGANANGGTTTGACCATNNNTTTTAGACNACCNCGG
TCGCATTGAACCTGGCATTCAGAATGACGTNCTNTCAAGAATNNAGGTCCNCCCACTGAGGNTCATCT
GCTTCGCGGNNCNGCGTATNGTCATNNNNAT
>LaChi554
GTGTAGCCCAGTTAGACACTTCTAAATCCNTGGTAACAGACATGCAGTCCCCTCNNCAGAGNCCCGCC
CCCCNATGGTACCGGGTTGNTAAGAGGTCCACAACGGGTCGCACGACAACNTTAAGGCCTCATGCATG
ACCCAANNCNGNGGCGTCCCGGCGNGGCGACCTTGAANTCCGGGGNCAAGTATNGCCCCCCGGGAAGG
ACGCCGTAGTGCATCTGGAGTGCCGATTNNTGGTCNAGAATGNCACCACGATGGTATGAGTGGCAAAG
GTACTTTGGACACCACCCCGTTTTCTANTTCCAGANGCGGCCNTTCAGGAACGACGCGAATCGTAATC
TACCTGCCGCGTACGCGGATCGTACATGCGTTTTCCAGCGTTCGAAATAGAGTTTGTCGTTCCGCTGA
CCAGGTTNCACGGCTACTNCGATAAGCATATTCTACTGGGGGACTGTAAAGGAATTGTATCNACTCTG
CTTCCTGTCAATTGGNCATGTGCATCTCTGGTTGTTCAGGGTACATTAATAACACACAGCATTAAGTA
GATAAGTCTCCGATATAAGCGAACAGANCGTTGGTCAGAGACTATGGTTCAATTGTAGTGGTATAATG
TTTCCCAGTGCATAAGGAACTCAGGGACACTACTAGCATGGNCGAGGAGAGACCCTTGGGTGGCAANA
CGCGNTAAGTGTTANCTAGTACATCAGCACGAGNNANGGTTTGACCATNNNNNTNTAGACGACCCCGG
TCGCATTGAACCTGGCATTCAGAATGACGTGCTCTCAAGAATCAAGGTCCTCCCACTGAGGGTCATCN
GCTTCGCNNNTCNGCGTATNGTCATNNNNAT
>LaChi555
GTGTAGCCCAGTTAGACACTTCTAAATCCTTNGTAACAGACATGCAGTCCCCTCGGCAGAGCCCCGCC
CCCCGATGGTACCGGGTTGNTAAGAGGTCCACAACGGGTCGCACGACAACATTAAGGCCTCANGCATG
ACCCAACACGGGGGCGTCCCGGCGCGGCGACCTTGAAGTCCGGGGACAAGTATGGCCCCCCGGGAAGG
ACGCCGTAGTGCATCTGGAGTGCCGATTCGTGGTCCAGANTGCCACCACGATGGTATGAGTNGCAAAG
GTACTTTGGACACCACCCCGTTTTCTATTTCCAGACGCGGCCATTCAGGAACGACGCGAATCGTAATC
TACCTGCCGCGTACGCGGATCGTACATGCGTTTTCCAGCGTTCGAAATAGAGTTTGTCGTTCCGCTGA
CCAGGTTTCACGGCTACTGCGATAAGCATATTCTACTGGGGGACTGTAAAGGAATTGTATCCACTCTG
CTTCCTGTCAATTGGCCATGTGCATCTCTGGTTGTTCAGGGTACATTAATAACACACAGCATTAAGTA
GATAAGTCTCCGATATAAGCGAACAGAACGTTGGTCAGAGACTATGGTTCAATTGTAGTGGTATAATG
TTTCCCAGTGCATAAGGAACTCAGGGACACTACTAGCATGGGCGAGGAGAGACCCTTGGGTGGCAACA
CGCAATAAGTGTTACCTAGTACATCAGCACGAGANAGGGTTTGACCATGTGTTTTTAGACGACCCCGG
TCGCATTGAACCTGGCATTCAGAATGACGTGCTCTCAAGAATCAAGNTCCTCCCACTGAGGGTCATCT
GCTTCGCGGGTCAGCGTATNGTCATNANCAT
>LaChi556
GTGTAGCCCAGTTAGACACTTCTAAATCCTTGGTAACAGACATGCAGTCCCCTCNNCAGAGCCCCGCC
CCCCGATGGTACCGGGTTGTTNAGAGGTCCACAACAGGTCGCACGACAACNTTAAGGCCTCANGCATG
ACCCAACACGGNGGNGTCCCGGCGCGGCGACCTTGNAGTCCGGGGACAAGTATGGCCCCCCGGGAAGG

ACGCCGTAGTGCATCTGGAGTGCCGATTCGTGGTCNAGAATGCCACCACGATGGTATGAGTNGCAAAG
GTACTTTGGACACCACCCCGTTTTCTATTNCCAGACGCGGCCATTCAGGAACGACGCGAATCGTAATC
TACCTGCCGCGTACGCGGATCGTACATGCGTTTTCCAGCGTTCGAAATAGAGTTTGTCGTTCCGCCGA
CCAGGTTTCACGGCTACTGCGATAAGCATATTCTACTGGGGGACTGTAAAGGAATTGTATCCACTCTA
CTTCCTGTCAATTGGCCATGTGCATCTCTGGTTGTTCAGGGTACATTAATAACACACAGCATTAAGTA
GATAAGTCTCCGATATAAGCGAACAGAACGTTGGTCAGAGACTGTGGTTCAATTGTAGTGGTATAATG
TTTCCCAGTGCATAAGGAACTTAGGGACATTACTAGCATGGGCGAGGAGAGACCCTTGGGTGGCAACA
CGCAATAAGTGTTACCTAGTACACCAGCACGAGAGAGGGTTTGACCATNTGTTTTTAGACGACCCCGG
TCGCATTGAACCTGGCATTCAGAATGACGTGCTNTCAAGAATCNAGGTCCNCCCACTGAGGGTCATCT
GCTTCGCGGGTCAGCGTATGGTCANNAACAT
>LaChi557
GTGTAGCCCAGTTAGACACTTCTAAATCCNTGGTAACAGACATGCAGTCCCCTCNNCAGAGCNCCGCC
CCCCNATGGTACCGGGTTGTTAAGAGGTCCACAACAGGTCGCACGACNNNATTAAGGCCTCANGCATG
ACCCAACACGGNGGCGTCCCGGNGCGGCGACCTTGNANTCCGGGGACAAGTATGGCCCCCCGGGAAGG
ACGCCGTAGTGCATCTGGAGTGCCGNTTCNTGGTCCAGAATGNCACCACGATGGTATGAGTGGCAAAG
GTACTTTGGACACCACCCCGTTTTCTATTNCCAGANGCGGCCATTCAGGAACGACGCGAATCGTAATC
TACCTNCCGCGTACGCGGATCGTACATGCGTTTTCCAGCGTTCGAAATAGAGTTTGTCGTTCCGCCGA
CCAGGTTNCACGGCTACTGCGATAAGCATATTCTACTGGGGGACTGTAAAGGAATTGTATCCACTCTA
CTTCCTGTCAATTGGCCATGTGCATCTCTGGTTGTTCAGGGTACATTAATAACACACAGCATNAAGTA
GATAAGTCTCCGATATAAGCGAACAGAACGTTGGTCAGANACTGTGGTTCAATTGTAGTGGTATAATG
TTTCCCAGTGCATAAGGAACTTAGGGACATTACTAGCATGGNCGAGGAGAGACCCTTGGGTGGCAACA
CGCAATAAGTGTTACCTAGTACACCAGCACGAGAGAGGGTTTGACCANGNNTTTTTAGACGNCCCCGG
TCGCATTGAACCTGGCATTCAGAATGACGTGCTCTCAAGAATCAAGGTCCTCCCACTGAGGGTCATCT
GCTTCGCGGGTCNGCGTATNGNCANAANNAT
>LaChi558
GTGTAGCCCAGTTAGACACTNNNAAANNCTTGGTAACAGACATGCAGTCCCCTCGGCAGAGNCCCGCC
CCCCGATGGTACCGGGTTGNTAAGAGGTCCACAACAGGTCNCACGACNNNNTTAAGGCCTNANGCATG
ACCCAACNCNGGGGCGTCCCGGCGNGGCGACCTTGNAGTCCGGGGACAAGTATGGCCCCCCGGGAAGG
ACGCNGTAGTGCATCTGGAGTGCCGATTCGTGGTCCAGAATGCCACCACGATGGTATGAGTNGCAAAG
GTACTTTGGACACCACCCCGTTTTCTATTTCCAGACGCGGCCNTTCAGGAACGACGCGAATCGTAATC
TACCTGCCGCGTACGCGGATCGTACATGCGTTTTCCAGCGTTCGAAATAGAGTTTGTCGTTCCGCCGA
CCAGGTTNCACNGNTACTNCGATAAGCATATTCTACTGGGGGACTGTAAAGNAATTGTATCCACTCNA
CTTCCTGTCAATTGGNCATGTGCATCTCTGGTTGTTCAGGNNACATTAATAACACACAGNATTAAGTA
GATNAGTCTCCGATATAAGCGAACAGANCGTTGGTNAGAGACTGTGGTTCAATTGTAGTGGTATAATG
TTTCCCAGTGCATAAGGAACTTAGGGACATTACTAGCATGGGCGAGGAGAGACCCTTGGGTGGCAANA
CGCAATAAGTGTTACCTAGTACACCAGCACGAGNNAGGGTTTGACCATGTGTTTTTAGNNGACCCCGG
TCGCATTGAACCTGGCATTCAGAATGACGTGCTNTCAAGAATCAAGNTCCNCCCACTGAGGNNCATCN
GCTTCGCGGGTCAGCGTATGGTCATAANNNT
>LaChi559
GTGTAGCCNAGTTAGACACTTCTAAATCCNTGGTAACAGACATGCAGTCCCCTCGGCAGAGCCCCGCC
CCCCGATGGTACCGGGTTGTTAAGAGGTCCACAACAGGTCGCACGACNNNNTTAAGGCCTNANGCATG
ACCCAACACGGGGGCGTCCCGGNGNNGCGACCTTGNANTCCGGGNACAAGTATGGCCCCCCGGGAAGG
ACGCCGTAGTGCATCTGGAGTGCCGATTCGTGGTCCAGAATGNCACCACGATGGTATGAGTNGCAAAG
GTACTTTGGACACCACCCCGTTTNCTATTNCCAGACGCGGCCATTCAGGAACGACGCGAATCGTAATC
TACCTGCCGCGTACGCGGATNGTACANGCGTTTTCCAGCGTTCGAAATAGAGTTTGTCGTTCCGCCGA
CCAGGTTTCACGGCTACTGCGATAAGCATATTCTACTGGGGGACTGTAAAGGAATTGTATCCACTCTA
CTTCCTGTCAATTGGNCATGTGCATCTCTNGTTGTTCAGGNNACATTAATAACACACAGNATTAAGTA
GNTAAGTCTCCGATATAAGCGAACAGANCGTTGGTCAGAGACTGTGGTTCAATTGTAGTGGTATAATG
TTTCCCAGTGCATAAGGAACTTAGGNACATTACTAGCATGGGCGAGGAGAGACCCTTGGGTGGCAACA
CGCAATAAGTGTTACCTAGTACACCAGCACGAGANAGGGTTTGACCNTNNNTTTTTAGACGACCCCGG
TCGCATTGAACCTGGCATTCAGAATGACGTGCTCTCAAGAATCAAGGTCCNCCCACTGAGGGTCATCT
GCTTCGCGGNNCAGCGTATNGTCANNAACAT
>LaChi560
GTGTAGCCCAGTTAGACACTTCTAAATCCTTGGTAACAGACATGCAGTCCCCTCNNCAGAGCCCCGCC

CCCCNATGGTACCGGGTTGTTNAGAGGTCCACAACGGNTCGCACGACNNNNTTAAGGCCTNANGCATG
ACCCAANACGGNGGNGTCCCGGNGNGGCGACCTTGNAGTCCGGGGACAAGTATGGCCCCCCGGGAAGG
ACGCCGTAGTGCATCTGGAGTGCCGATTNGTGGTCCAGAATGCCACCACGATGGTATGAGTNGCAAAG
GTACTTTGGACACCACCCCGTTTTCTATTTCCAGANGCGGCCATTCAGGAACGACGCGAATCGTAATC
TACCTGCCGCGTNCGCGGATNGTACATGCGTTTTCCAGCGTTCGAAATAGAGTTTGTCGTTCCGCTGA
CCAGGTTNCACGGNTACTGCGATAAGCATATTCTACTGGGGGACTGTAAAGGAATTGTATCCACTCTG
CTTCCTGTCAATTGGCCATGTGCATCTCTGGTTGTTCAGGGTACATTAATAACACACAGCATTAAGTA
GATAAGTCTCCGATATAAGCGAACAGANCGTTGGTCAGAGACTATGGTTCAATTGTAGTGGTATAATG
TTTCCCAGTGCATAAGGAACTCAGGGACACTACTAGCATGGGCGAGNAGAGACCCTTGGGTGGCAACA
CGNANTAAGTGTTACCTAGTACATCAGCACGAGNGANGGTTTGACCATGNNTTTTTAGACGNCCCCGG
TCGCATTGAACCTGGCATTCAGAATGACGTNNTCTCANGAATCAANNTCCTCNCACTGAGGGTCATNT
GCTTCGCGGGTCNGCGTATNGTCANNNACNT
>LaChi561
GTGTAGCCCAGTTAGACACTTCTAAATCCNTGGTAACAGACATGCAGTCCCCTCGGCAGAGCCCCGCC
CCCCGATGGTACCGGGTTGTTNAGNGGTCCACAACAGGTCGCACGACNNNNTTAAGGCCTCANGCATG
ACCCAACACGGGGGCGTCCCGGNGCGGCGACCTTGNAGTCCGGGGNCAAGTATGGCCCCCCGGGAAGG
ACGCCGTAGTGCATCTGGAGTGCCGANNCGTGGTCCAGAATGCCACCACGATGGTATGAGTNGCAAAG
GTACTTNGGACACCACCCCGTTTTCTATTTCCAGACGCGGCCATTCAGGAACGACGCGAATCGTAATC
TACCTGCCGCGTNCGCGGATCGTACANGCGTTTTCCAGCGTTCGANATAGAGTTTGTCGTTCCGCCGA
CCAGGTTNCACGGCTACTGCGATAAGCATATTCTACTGGGGGACTGTAAAGGAATTGTATCCACTCTA
CTTCCTGTCAATTGGNCATGTGCATCTCTGGTTGTTCAGGGTACATTAATAACACACAGCATTAAGTA
GATAAGTCTCCGATATAAGCGAACAGAACGTTGGTCAGAGACTGTGGTTCAATTGTAGTGGTATAATG
TTTCCCAGTGCATAAGGAACTTAGGGACATTACTAGCATGGGCGAGGAGAGACCCTTGGGTGGCAACA
CGCAATAAGTGTTACCTAGTACACCAGCACGAGAGAGGGTTTGACCATGNNTTTTTAGACGNCCCCGG
TCGCATTGAACCTGGCATTCAGAATGACGTNCTCTCAAGAATCAAGGTCCTCCCACTGAGGGTCATCT
GCTTCGCGGNNCAGCGTATGGTCATNNACAT
>LaChi562
GTGTAGCCCAGTCAGACACTTCTAAATCCNTGGTAACAGACATGCAGTCCCCTCGGCAGAGCCCCGCC
CCCCGATGGTACCGGGTTGNTNAGAGGTCCACAACGGNTCGCACGACAACNTTAAGGCCTCATGCATG
ACCCAACACGGNGGCGTCCCGGCGCGGCGACCTTGAAGTCCGGGGACAAGTATGGCCCCCCGGGAAGG
ACGCCGTAGTGCATCTGGAGTGCCGATTCGTGGTCCAGAATGCCACCACGATGGTATGAGTGGCAAAG
GTACTTTGGACACCACCCCGTTTTCTANTTCCAGACGCGGCCATTCAGGAACGACGCGAATCGTAATC
TACCTGCCGCGTACGCGGATCGTACATGCGTTTTCCAGCGTTCGAAATAGAGTTTGTCGTTCCGCTGA
CCAGGTTTCACGGCTACTNCGATAAGCATATTCTACTGGGGGACTGTAAAGGAATTGTATCCATTCTG
CTTCCTGTCAATTGGCCATGTGCATCTCTNGTTGTTCAGGGTACATTAATAACACACAGCAGTAAGTA
GGTNAGTCTCCGATATAAGCGAACAGAACGTTTGTCAGAGACTGTGGTTCAATTGTAGTGGTATAATG
TTTCCCAGTGCATAAGGAACTCAGGGACACTACTAGCATGGGCGAGGAGAGACCCTTGGGTGGCAANA
CGCAATAAGTGTTACCTAGTACACCAGCACGAGNGAGGGTTTGNCCATGTGTTTNTAGACGNCCCCGG
TCGCATTGAACCTGGCATTCAGAATGACGTGCTCTCAAGAATCAAGGTCCTCCCACTGAGGGTCATCT
GCTTCGCGGGTCAGCGTATNGTCANAAACAT
>LaChi563
GTGTAGCCCAGTCAGACACTTCTAAATCCTTGGTAACAGACATGCAGTCCCCTCNNCAGAGCCCCGCC
CCCCGATGGTACCGGGTTGTTNAGAGGTCCACAACGGGTCGCACGACAANATTAAGGCCTCANGCATG
ACCCAACACGGNGGNGTCCCGGNGNGGCGACCTTGNAGTCCGGGGACAAGTATGGCCCCCCGNGAAGG
ACGCCGTAGTGCATCTGGAGTGCCGATTCGTGGTCNAGAATGCCACCACGATGGTATGAGTGGCAAAG
GTACTTTGGACACCACCCCGTTTTCTATTTCCAGANGCGGCCATTCAGGAACGACGCGAATCGTAATC
TACCTGCCGCGTNCGCGGATCGTACATGCGTTTTCCAGCGTTCGAAATAGAGTNTGTCGTTCCGCTGA
CCAGGTTNCACGGCTACTGCGATAAGCATATTCTACTGGGGGACTGTAAAGGAATTGTATCCATTCNG
CTTCCTGTCAATTGGCCATGTGCATCTCTNGTTGTTCAGGGTACATTAATAACACACAGCAGTAAGTA
GGTAAGTCTCCGATATAAGCGAACAGAACGTTTGTCAGAGACTGTGGTTCAATTGTAGTGGTATAATG
TTTCCCAGTGCATAAGGAACTCAGGNACACTACTAGCATGGGCGAGGAGAGACCCTTGGGTGGCAACA
CGCAATAAGTGTTACCTAGTACACCAGCACGAGAGAGGGTTTGACCATGNNTTTTTAGACGACCCCGG
TCGCATTGAACCTGGCATTCAGAATGACGTGCTCTCAAGAATCAAGGTCCTCCCACTGAGGGTCATCT
GCTTCGCGGNNCAGCGTATGGTCANAANCAT

```
>LaChi564
GTGTAGCCCAGTCAGACACTTCTAAATCCTTGGTAACAGACATGCAGTCCCCTCNNCAGAGCNCCGCC
CCCCGATGGTACCGGGTTGTTNAGAGGTCCACAACGGGTCGCACGACNNCNTTAAGGCCTCANGCATG
ACCCAANACGGNGGCGTCCCGGCGCGGCGACCTTGAAGTCCGGGNACAAGTATGGCCCCCCGGGAAGG
ACGCCGTAGTGCATCTGGAGTGCCGATTCGTGGTCNAGANTGCCACCACGATGGTATGAGTGGCAAAG
GTACTTTGGACACCACCCCGTTTTCTATTTCCAGACGCGGCCNTTCAGGAACGACGCGAATCGTAATC
TACCTGCCGCGTNCGCGGATCGTACATGCGTTTTCCAGCGTTCGAAATAGAGTTTGTCGTTCCGCTGA
CCAGGTTNCACGGCTACTGCGATAAGCATATTCTACTGGGGGACTGTAAAGGAATTGTATCCATTCTG
CTTCCTGTCAATTGGNCATGTGCATCTCTGGTTGTTCAGGGNACATTAATAACACACAGCAGTAAGTA
GGTAAGTCTCCGATATAAGCGAACAGAACGTTTGTCAGAGACTGTGGTTCAATTGTAGTGGTATAATG
TTTCCCAGTGCATAAGGAACTCAGGNACACTACTAGCATGGGCGAGGAGAGACCCTTGGGTGGCAACA
CGCAATAAGTGTTACCTAGTACACCAGCACGAGAGAGGGTTTGACCATGTGTTTTTAGACGACCCCGG
TCGCATTGAACCTGGCATTCAGAATGACGTGCTCTCAAGAATCAAGGTCCTCCCACTGAGGGTCATCT
GCTTCGCGGNTCNGCGTATNGTCANNANNAT
>LaChi565
GTGTAGCCCAGTTAGACACTTCTAAATCCTTGGTAACAGACATGCAGTCCCCTCGGCAGAGCCCCGCC
CCCCNATGGTACCGGGTTGTTAAGAGGTCCACAACGGGTCGNACGACAACNTTAAGGCCTCATGCATG
ACCCAACACGGGGGCGTCCCGGCGCGGCGACCTTGNANTCCGGGGACAAGTATGGCCCCCCGGGAAGG
ACGCCGTAGTGCATCTGGAGTGCCGNTTCGTGGTCCAGAATGCCACCACGATGGTATGAGTNGCAAAG
GTACTTTGGACACCACCCCGTTTTCTANTTCCAGACGCGGCCATTCAGGAACGACGCGAATCGTAATC
TACCTGCCGCGTACGCGGATCGTACANGCGTTTTCCAGCGTTCGAAATAGAGTTTGTCGTTCCGCTGA
CCAGGTTNCACGGCTACTGCGATAAGCATATTCTACTGGGGGACTGTAAAGGAATTGTATCCACTCNG
CTTCCTGTCAATTGGNCATGTGCATCTCTGGTTGTTCAGGGTACATTAATAACACACAGNATTAAGTA
GATAAGTCTCCGATATAAGCGAACAGAACGTTGGTCAGAGACTATGGTTCAATTGTAGTGGTATAATG
TTTCCCAGTGCATAAGGAACTCAGGGACACTACTAGCATGGGCGAGGAGAGACCCTTGGGTGGCAACA
CGCAATAAGTGTTANCTAGTACATCAGCACGAGAGAGGGTTTGACCATGTGTTTTTAGACGACCCCGG
TCGCATTGAACCTGGCATTCAGAATGACGTGCTCTCAAGAATCAAGNTCCNCCCACTGAGGGTCATCT
GCTTCGCGGNTCAGCGTATNGTCATNAACAT
>LaHu342
GTGTAGCCCAGTCAGACACTTCTAAATCCNTNGTAACAGACATGCAGTCCCCTCNNCAGAGCCCCGCC
CCCCGATGGTACCGGGTTGTTAAGAGGTCCACAACGGGTCGNACGACAACATTAAGGCCTCANGCATG
ACCCANNNCGGNGGCGTCCCGGNGCGGNGACCTTGNAGTCCGGGGACAAGTATGGCCCCCCGGGAAGG
ACGCCGTAGTGCATCTGGAGTGCCGATTCNTGGTCCAGAATGCCACCACGATGGTATGAGTGGCAAAG
GTACTTTGGACACCACCCCGTTTTCTATTTCCAGACGCGGCCATTCAGGAACGACGCGAATCGTAATC
TACCTGCCGCGTACGCGGATCGTACATGCGTTTTCCAGCGTTCGAAATAGAGTTTGTCGTTCCGCTGA
CCAGGTTTCACGGCTACTNCGATAAGCATATTCTACTGGGGGACTGTAAAGGAATTGTATCCATTCTG
CTTCCTGTCAATTGGCCATGTGCATCTCTGGTTGTTCAGGGTACATTAATAACACACAGCAGTAAGTA
GGTNAGTCTCCGATATAAGCGAACAGAACGTTTGTCAGANACTGTGGTTCAATTGTAGTGGTATAATG
TTTCCCAGTGCATAAGGAACTCAGGGACACTACTAGCATGGGCGAGGAGAGACCCTTGGGTGGCAANA
CGCAATAAGTGTTACCTAGTACACCAGCACGAGNGAGGGTTTGACCATGTGTTTTTAGACGACCCCGG
TCGCATTGAACCTGGCATTCAGAATGACGTGCTNTCAAGAATCNANGTCCNCCCACTGAGGGTCATCT
GCTTCGCGGGTCNGCGTATGGTCATAAACNT
>LaHu343
GTGTAGCCCAGTCAGACACTTCTAAATCCTTGGTAACAGACATGCAGTCCCCTCGGCAGAGCCCCGCC
CCCCGATGGTACCGGGTTGTTAAGAGGTCCACAACGGGTCGNACGACAACATTAAGGCCTCATGCATG
ACCCAACNCGGGGGCGTCCCGGCGNGGCGACCTTGAANTCCGGGGNCAAGTATNGCCCCCCGNGAAGG
ACGCCGTAGTGCATCTGGAGTGCCGATTNGTGGTCCAGAATGCCACCACGATGGTATGAGTGGCAAAG
GTACTTTGGACACCACCCCGTTTTCTANTTCCAGANGCGGCCNTTCAGGAACGACGCGAATCGTAATC
TACCTGCCGCGTACGCGGATCGTACATGCGTTTTCCAGCGTTCGAAATAGAGTTTGTCGTTCCGCTGA
CCAGGTTTCACGGCTACTGCGATAAGCATATTCTACTGGGGGACTGTAAAGGAATTGTATCCATTCTG
CTTCCTGTCAATTGGCCATGTGCATCTCTNGTTGTTCAGGNNACATTAATAACACACAGCAGTAAGTA
GGTAAGTCTCCGATATAAGCGAACAGAACGTTTGTCAGAGACTGTGGTTCAATTGTAGTGGTATAATG
TTTCCCAGTGCATAAGGAACTCAGGGACACTACTAGCATGGNCGAGGAGAGACCCTTGGGTGGCAANA
CGCAATAAGTGTTACCTAGTACACCAGCACGAGNNAGGGTTTGACCATGTGTTTTTAGACGACCCCGG
```

TCGCATTGAACCTGGCATTCAGAATGACGTGCTCTCAAGAATCAAGNTCCNCCCACTGAGGGTCATCT
GCTTCGCGGGTCAGCGTATNGTCATAAACAT
>LaHu344
GTGTAGCCCAGTTAGACACTTCTAAATCCTTGGTAACAGACATGCAGTCCCCTCGGCAGAGNCCCGCC
CCCCGATGGTACCGGGTTGTTNAGAGGTCCACCACGGGTCGNACGACAACATTAAGGCCTCANGCATG
ACCCAANACGNNNGCGTCCCGGCGNGGCGACCTTGAAGTCCGGGNNCAAGTATGGCCCCCCGGGAAGG
ACGCCGTAGTGCATCTGGAGTGCCGATTCGTGGTCCAGAATGCCACCACGATGGTATGAGTGGCAAAG
GTACTTTGGACACCACCCCGTTTTCTATTTCCAGACGCGGCCNTTCAGGAACGACGCGAATCGTAATC
TACCTGCCGCGTNCGCGGATCGTATATACGTTTTCCAGCGTTCGAAATAGAGTTTGTCGTTCCGCTGA
CCAGGTTTCACGGCTACTGCGATAAGCATATTCTACTGGGGGACTGTAAAGGAATTGTATCCACTCNG
CTTCCTGTCAATTGGCCATGTGCATCTCTNGTTGTTCAGGNNACANTAATAACACACAGCATTAAGTA
GATAAGTCTCCGATATAAGCGAACAGANCGTTGGTCAGAGACTGTGGTTCAATTGTAGTGGTATAATG
TTTCCCAGTGCATAAGGAACTCAGGGACACTACTAGCATGGGCGAGGAGAGACCCTTGGGTGGCAANA
CGNAATAAGTGTTNCCTAGTACACCAGCACGAGAGAGGGTTTGACCATGNNTTTTTAGNNGACCCCGG
TCGCATTGAACCTGGCATTCAGAATGACGTGCTCTCAAGAGTNAAGGTCCNCCCACTGAGGGTCATCN
GCTTCGCGGGTCAGCGTATGGNCANAAACAT
>LaHu345
GTGTAGCCCAGTTAGACACTTCTAAATCCNTGGTAACAGACATGCAGTCCCCTCGGCAGAGCCCCGCC
CCCCNATGGTACCGGGTTGNTAAGAGGTCCACAACGGGTCGCACGACAANATTAAGGCCTCANGCATG
ACCCAANACGGGGGCGTCCCGGCGCGGCGACCTTGAAGTCCGGGGACAAGTATGGCCCCCCGGGAAGG
ACGCCGTAGTGCATCTGGAGTGCCGATTCGTGGTCNAGAATGNCACCACGATGGTATGAGTNGCAAAG
GTACTTTGGACACCACCCCGTTTTCTANTTCCAGACGCGGCCATTCAGGAACGACGCGAATCGTAATC
TACCTGCCGCGTACGCGGATCGTACATGCGTTTTCCAGCGTTCGAAATAGAGTTTGTCGTTCCGCTGA
CCAGGTTTCACGGCTACTGCGATAAGCATATTCTACTGGGGGACTGTAAAGGAATTGTATCCACTCTG
CTTCCTGTCAATTGGCCATGTGCATCTCTGGTTGTTCAGGGTACATTAATAACACACAGCATNAAGTA
GATAAGTCTCCGATATAAGCGAACAGAACGTTGGTCAGANACTATGGTTCAATTGTAGTGGTATAATG
TTTCCCAGTGCATAAGGAACTCAGGGACACTACTAGCATGGGCGAGGAGAGACCCTTGGGTGGCAACA
CGCGATAAGTGTTACCTAGTACATCAGCACGAGAGAGGGTTTGACCATGTGTTTTTAGACGACCCCGG
TCGCATTGAACCTGGCATTCAGAATGACGTGCTCTCAAGAATCAAGGTCCTCCCACTGAGGGTCATCN
GCTTCGCGGGTCAGCGTATGGTCATAAACAT
>LaHu346
GTGTAGCCCAGTTAGACACTTCTAAATCCNTGGTAACAGACATGCAGTCNCCTCGGCAGAGCNCCGCC
CCCCGGTGGTACCGGGTTGTTAAGAGGTCCACCACGGGTCGCACGACAACNTCAAGGCCTCANGCATG
ACCCAACACGGGGGCGTCCCGGCGNGGCGACCTTGNANGCCGGGGACAAGTATGGCCCCCCGGGAAGG
ACGCCGTAGTGCATCTGGAGTGCCGATTCGTGGNCCNGAATGNCACCACGATGGTATGAGTGGCAAAG
GTACTTTGGACACCACCCCGTTTTCTANTTCCAGACGCGGCCATTCAGGAACGACGCGAATCGTAATC
TACCTGCCGCGTACGCGGATCGTACATGCGTTTTNCAGCGTTCGAAATAGAGTTTGTCGTTCCGCTGA
CCAGGTTTCACGGCTACTGCGATAAGCATATTCTACTGGGGGACTGTAAAGGAATTGTATCCACTCTG
CTTCCTGTCAATTGGCCATGTGCATCTCTGGTTGTTCAGGGTACATTAATAACACACAGCATTAAGTA
GATAAGTCTCCGATATAAGCGAACAGAACGTTGGTCAAAGACTGTGGTTCAATTGTAGTGGTATAACG
TTTCCCAGTGCATAAGGAACTCAGGGACACTACTAGNATGGGCGAGGAGAGACCCTTGGGTGGCAACA
CGCAATAAGTGTTACCTAGTACACCAGCACGATAGANGGTTTGACCATNNNTTTTTAGACGACCCCGG
TCGCATTGAACCTGGCATTCAGAATGACGTGCTCTCAAGAGTCAAGGTCCNCCCACTGAGGGTCATCT
GCTTCGCGNGTCAGCGTATGGNCATANACAT
>LaHu347
GTGTAGCCCAGTTAGACACTTCTAAATCCTTGGTAACAGACATGCAGTCCCCTCGGCAGAGCCCCGCC
CCCCNATGGTACCGGGTTGTTAAGAGGTCCACAACGGGTCGCACGACAACATTAAGGCCTCATGCATG
ACCCAACACGGGGGCGTCCCGGCGCGGCGACCTTGAAGTCCGGGGACAAGTATGGCCCCCCGGGAAGG
ACGCCGTAGTGCATCTGGAGTGCCGATTCGTGGTCNAGAATGCCACCACGATGGTATGAGTGGCAAAG
GTACTTTGGACACCACCCCGTTTTCTATTTCCAGACGCGGCCATTCAGGAACGACGCGAATCGTAATC
TACCTGCCGCGTACGCGGATCGTACATGCGTTTTCCAGCGTTCGAAATAGAGTTTGTCGTTCCGCTGA
CCAGGTTNCACGGCTACTGCGATAAGCATATTCTACTGGGGGACTGTAAAGGAATTGTATCCACTCTG
CTTCCTGTCAATTGGCCATGTGCATCTCTGGTTGTTCAGGGTACATTAATAACACACAGCATTAAGTA
GATAAGTCTCCGATATAAGCGAACAGAACGTTGGTCAGAGACTATGGTTCAATTGTAGTGGTATAATG

TTTCCCAGTGCATAAGGAACTCAGGGACACTACTAGCATGGGCGAGGAGAGACCCTTGGGTGGCAACA
CGCGATAAGTGTTACCTAGTACATCAGCACGAGAGAGGGTTTGACCATGTGTTTTTAGACGACCCCGG
TCGCATTGAACCTGGCATTCAGAATGACGTGCTCTCAAGAATCAAGGTCCTCCCACTGAGGGTCATCT
GCTTCGCGGGTCAGCGTATGGTCATAAACAT
>LaHu348
GTGTAGCCCAGTTAGACACTTCTAAATCCTTGGTAACAGACATGCAGTCCCCTCNGCAGAGCCCCGCC
CCCCGGTGGTACCGGGTTGTTAAGAGGTCCACCACGGGTCGNACGACAACATCAAGGCCTCATGCATG
ACCCAACACGGGGGCGTCCCGGCGCGGCGACCTTGNAGGCCGGGGNCAAGTATGGCCCCCCGGGAAGG
ACGCCGTAGTGCATCTGGAGTGCCGATTCGTGGTCCAGAATGNCACCACGATGGTATGAGTGGCAAAG
GTACTTTGGACACCACCCCGTTTTCTANTTCCAGACGCGGCCATTCAGGAACGACGCGAATCGTAATC
TACCTGCCGCGTACGCGGATCGTACATGCGTTTTCCAGCGTTCGAAATAGAGTTTGTCGTTCCGCTGA
CCAGGTTTCACGGCTACTGCGATAAGCATATTCTACTGGGGGACTGTAAAGGAATTGTATCCACTCTG
CTTCCTGTCAATTGGCCATGTGCATCTCTGGTTGTTCAGGGTACATTAATAACACACAGCATTAAGTA
GNTAAGTCTCCGATATAAGCGAACAGAACGTTGGTCAAAGACTGTGGTTCAATTGTAGTGGTATAACG
TTTCCCAGTGCATAAGGAACTCAGGGACACTACTAGCATGGGCGAGGAGAGACCCTTGGGTGGCAACA
CGCAATAAGTGTTNCCTAGTACACCAGCACGATAGAGGGTTTGACCATGTGTTTTTAGACGACCCCGG
TCGCATTGAACCTGGCATTCAGAATGACGTGCTCTCAAGAGTCAAGGTCCTCCCACTGAGGGTCATCT
GCTTCGCGNGTCAGCGTATGGTCATAAACAT
>LaHu349
GTGTAGCCCAGTTAGACACTTCTAAATCCTTGGTAACAGACATGCAGTCCCCTCGGCAGAGCNCCGCC
CCCCNGTGGTACCGGGTTGTTNAGAGGTCCACCACGGGTCGCACGACAANNTCAAGGCCTNATGCATG
ACCCAACACGGNGGCGTCCCGGCGNGGCGACCTTGAAGGCCGGGGNCAAGTATGGCCCCCCGGGAAGG
ACGCCGTAGTGCATNTGGAGTGCCGNNNCGTGGTCCAGAATGCCACCACGATGGTATGAGTNGCAAAG
GTACTTTGGACACCACCCCGTTTTCTATTTCCAGANGCGGCCATTCAGGAACGACGCGAATCGTANTC
TACCTGCCGCGTACGCGGATCGTACATGCGTTTTCCAGCGTTCGAAATAGAGTTTGTCGTTCCGCTGA
CCAGGTTNCACGGCTACTGCGATAAGCATATTCTACTGGGGGACTGTAAAGGAATTGTATCCACTCNG
CTTCCTGTCAATTGGCCATGTGCATCTCTGGTTGTTCAGGNNACATTAATAACACACAGCATTAAGTA
GATAAGTCTCCGATATAAGCGAACAGAACGTTGGTCAAAGACTGTGGTTCAATTGTAGTGGTATAACG
TTTCCCAGTGCATAAGGAACTCAGGNACACTACTAGCATGGGCGAGGAGAGACCCTTGGGTGGCAACA
CGCANTAAGTGTTACCTAGTACACCAGCACGATAGAGGGTTTGACCNTGNNTTTTTAGACGNCCCCGG
TCGCATTGAACCTGGCATTCAGAATGACGTGCTNTCAAGAGTCNAGNTCCNCCCACTGAGGGTCATCT
GCTTCGCGNGTCNGCGTATNGTCATAANNAT
>LaHu350
GTGTAGCCCAGTCAGACACTTCTAAATCCTTNGTAACAGACATGCAGTCCCCTCGNCAGAGCCCCGCC
CCCCNATGGTACCGGGTTGTTAAGAGGTCCACAACGGGTCGNACGACAANATTAAGGCCTCATGCATG
ACCCAACACGGGGGCGTCCCGGCGCGGCGACCTTGAAGTCCGGGGACAAGTATGGCCCCCCGGGAAGG
ACGCCGTAGTGCATCTGGAGTGCCGATTCGTGGTCCAGAATGCCACCACGATGGTATGAGTGGCAAAG
GTACTTTGGACACCACCCCGTTTTCTATTTCCAGACGCGGCCATTCAGGAACGACGCGAATCGTAATC
TACCTGCCGCGTACGCGGATCGTACATGCGTTTTCCAGCGTTCGAAATAGAGTTTGTCGTTCCGCTGA
CCAGGTTNCACGGCTACTGCGATAAGCATATTCTACTGGGGGACTGTAAAGGAATTGTATCCATTCTG
CTTCCTGTCAATTGGCCATGTGCATCTCTGGTTGTTCAGGGTACATTAATAACACACAGCAGTAAGTA
GGTAAGTCTCCGATATAAGCGAACAGANCGTTTGTCAGAGACTGTGGTTCAATTGTAGTGGTATAATG
TTTCCCAGTGCATAAGGAACTCAGGGACACTACTAGCATGGGCGAGGAGAGACCCTTGGGTGGCAANA
CGCAATAAGTGTTACCTAGTACACCAGCACGAGNGAGGGTTTGACCATGTGTTTTTAGACGACCCCGG
TCGCATTGAACCTGGCATTCAGAATGACGTGCTCTCAAGAATCAAGNTCCTCCCACTGAGGGTCATCT
GCTTCGCGGGTCAGCGTATGGTCATAAACAT
>LaHu351
GTGTAGCCCAGTTAGACACTTCTAAATCCTTGGTAACAGACATGCAGTCCCCTCGGCAGAGCCCCGCC
CCCCNATGGTACCGGGTTGTTAAGAGGTCCACAACGGGTCGCACGACAANATTAAGGCCTCATGCATG
ACCCANCNCGGGGGCGTCCCGGCGNGGCGACCTTGNAGTCCGGGGACAAGTATGGCCCCCCGGGAAGG
ACGCCGTAGTGCATCTGGAGTGCCGATTCGTGGTCCAGAATGCCACCACGATGGTATGAGTGGCAAAG
GTACTTTGGNCACCACCCCGTTTTCTATTTCCAGANGCGGCCATTCAGGAACGACGCGAATCGTAATC
TACCTGCCGCGTACGCGGATCGTACATGCGTTTTCCAGCGTTCGAAATAGAGTTTGTCGTTCCGCTGA
CCAGGTTNCACGGCTACTGCGATAAGCATATTCTACTGGGGGACTGTAAAGGAATTGTATCCACTCTG

CTTCCTGTCAATTGGCCATGTGCATCTCTNGTTGTTCAGGGTACATTAATAACACACAGCATNAAGTA
GATAAGTCTCCGATATAAGCGAACAGAACGTTGGTCAGAGACTATGGTTCAATTGTAGTGGTATAATG
TTTCCCAGTGCATAAGGAACTCAGGGACACTACTAGCATGGGCGAGGAGAGACCCTTGGGTGGCAACA
CGCAATAAGTGTTACCTAGTACATCAGCACGAGANAGGGTTTGACCATGTGTTTTTAGACGACCCCGG
TCGCATTGAACCTGGCATTCAGAATGACGTGCTCTCAAGAATCAAGGTCCNCCCACTGAGGGTCATCT
GCTTCGCGGGTCAGCGTATGGTCACAAACAT
>LaHu352
GTGTAGCCCAGTTAGACACTTCTAAATCCNTGGTAACAGACATGCAGTCCCCTCNNCAGAGNCCCGCC
CCCCNGTGGTACCGGGTTGNTAAGAGGTCCACCACGGGTCGNACGACAANATCAAGGCCTNATGCATG
ANNCNNCNCGGGGGNGTCCCGGCGCGGCGACCTTGNANGCCGGGGACAAGTATGGCCCCCCGGGAAGG
ACGCCGTAGTGCATCTGGAGTGCCGATTCGTGGNCNAGAATGCCACCACGATGGTATGAGTNGCAAAG
GTACTTTGGACACCACCCCGTTTTCTANTTCCAGACGCGGCCNTTCAGGAACGACGCGAATCGTAATC
TACCTGCCGCGTNCGCGGATCGTACATGCGTTTTCCAGCGTTCGAAATAGAGTTTGTCGTTCCGCTGA
CCNGGTTTCACGGCTACTGCGATAAGCATATTCTACTGGGGGACTGTAAAGGAATTGTATCCACTCNG
CTTCCTGTCAATTGGCCATGTGCATCTCTNGTTGTTCAGGGTACATTAATAACACACAGNATTAAGTA
GATAAGTCTCCGATATAAGCGAACAGAACGTTGGTCAAAGACTGTGGTTCAATTGTAGTGGTATAACG
TTTCCCAGTGCATAAGGAACTCAGGGACACTACTAGCATGGGCGAGGAGAGACCCTTGGGTGGCAANA
CGCAATAAGTGTTANNTAGTACACCAGCACGATAGANGGTTTGACCATNTGTTTTTAGACGACCCCGG
TCGCATTGAACCTGGCATTCAGAATGACGTGCTCTCAAGAGTCNANNTCCTCNCACTGAGGGTCATCN
GCTTCGCGNGTCNGCGTATNGTCATAAACAT
>LaHu353
GTGTAGCCCAGTTANACACTTCTAAATCCNTGGTAACAGACATGCAGTCCCCTCGGCAGAGCCCCGCC
CCCCNATGGTACCGGGTTGTTAAGAGGTCCACCACGGGTCGCACGACAACATTAAGGCCTCATGCATG
ACCCAACACGNNNGCGTCCCGGCGCGGCGACCTTGAAGTCCGGGGNCAAGTATGGCCCCCCGGGAAGG
ACGCCGTAGTGCATCTGGANTGCCGATTCGTGGTCCAGANTGCCACCACGATGGTATGAGTGGCAAAG
GTACTTNGGACACCACCCCGTTTTCTANTTCCAGACGCGGCCATTCAGGAACGACGCGAATCGTAATC
TACCTGCCGCGTACGCGGATCGTATATACGTTTTCCAGCGTTCGAAATAGAGTTTGTCGTTCCGCTGA
CCAGGTTNCACGGCTACTGCGATAAGCATATTCTACTGGGGGACTGTAAAGGAATTGTATCCACTCTG
CTTCCTGTCAATTGGCCATGTGCATCTCTNGTTGTTCAGGGTACATTAATAACACACAGCATTAAGTA
GATAAGTCTCCGATATAAGCGAACAGAACGTTGGTCAGAGACTGTGGTTCAATTGTAGTGGTATAATG
TTTCCCAGTGCATAAGGAACTCAGGGACACTACTAGCATGGGCGAGGAGAGACCCTTGGGTGGCAANA
CGCAATAAGTGTTACCTAGTACACCAGCACGAGAGAGGGTTTGACCNTGTGTTTTTAGACGACCCCGG
TCGCATTGAACCTGGCATTCAGAATGACGTGCTCTCAAGAGTCAAGGTCCTCCCACTGAGGGTCATCT
GCTTCGCGGGTCNGCGTATNGNCACAAACAT
>LaHu354
GTGTAGCCCAGTCAGACACTTCTAAATCCNTGGTAACAGACATGCAGTCCCCTCGGCAGAGCCCCGCC
CCCCGATGGTACCGGGTTGTTAAGAGGTCCACAACGGNTCNNACGACAACNTTAAGGCCTCANGCATG
ANNCAACACGGNGGCGTCCCGGCGNGGCGACCTTGNANTCCGGGGNCAAGTATGGCCCCCCGGGAAGG
ACGCCGTAGTGCATCTGGAGTGCCGATTCNTGGTCNAGAATGNCACCACGATGGTATGAGTGGCAAAG
GTACTTTGGACACCACCCCGTTTTCTATTTCCAGANGCGGCCATTCAGGAACGACGCGAATCGTAATC
TACCTGCCGCGTACGCGGATCGTACANGCGTTTTCCAGCGTTCGAAATAGAGTTTGTCGTTCCGCTGA
CCAGGTTNCACGGCTACTGCGATAAGCATATTCTACTGGGGGACTGTAAAGGAATTGTATCCATTCTG
CTTCCTGTCAATTGGNCATGTGCATCTCTNGTTGTTCAGGNNACATTAATAACACACAGNAGTAAGTA
GGTAAGTCTCCGATATAAGCGAACAGANCGTTTGTCAGAGACTGTGGTTCAATTGTAGTGGTATAATG
TTTCCCAGTGCATAAGGAACTCAGGNACACTACTAGCATGGGCGAGGAGAGACCCTTGGGTGGCAACA
CGCAATAAGTGTTNCCTAGTACACCAGCACGAGNGAGGGTTTGACCATGNNTTTTTAGACGACCCCGG
TCGCATTGAACCTGGCATTCAGAATGACGTGCTCTCAAGAATCAAGNTCCTCCCACTGAGGGTCATCT
GCTTCGCGGGTCAGCGTATNGTCATAAACAT
>LaHu355
GTGNAGCCCAGTTAGACACTTCTAAATCCTTGGTAACAGACATGCAGTCCCCTCGGCAGAGCCCCGCC
CCCCNGTGGTACCGGGTTGNTNAGAGGTCCACCACGGGTCNCACGACAACNTCAAGGCCTCATGCATG
ACCCANCNCGGNGGCGTCCCGGNGCGGCGACCTTGAAGGCCGGGGNCAAGTATGGCCCCCCGNGAAGG
ACGCCGTAGTGCATCTGGAGTGCCGATTCGTGGTCNAGAATGNCACCACGATGGTATGAGTNGCAAAG
GTACTTTGGACACCACCCCGTTTNCTATTTCCAGACGCGGCCNTTCAGGAACGACGCGAATCGTAATC

TACCTGCCGCGTACGCGGATCGTACANGCGTNTTCCAGCGTTCGAAATAGAGTTTGTCGTTCCGCTGA
CCAGGTTNCACGGCTACTGCGATAAGCATATTCTACTGGGGGACTGTAAAGGAATTGTATCCACTCNG
CTTCCTGTCAATTGGCCATGTGCATCTCTGGTTGTTCAGGNNACATTAATAACACACAGCATNAAGTA
GATNAGTCTCCGATATAAGCGAACAGANCGTTGGTCAAAGACTGTGGTTCAATTGTAGTGGTATAACG
TTTCCCAGTGCATAAGGAACTCAGGGACACTACTAGCANGGGCGAGGAGAGACCCTTGGGTGGCAANA
CGNAANAAGTGTTANCTAGTACACCAGCACGATANANGGTTTGACCATGNNTTTTAGACGACCCCGG
TCGCATTGAACCTGGCATTCAGAATGACGTGCTCTCAAGAGTCAAGNTCCTCNCACTGAGGGTCATCT
GCTTCGCGNGTCNGCGTATNGTCATAANNAT
>LaHu356
GTGTAGCCCAGTTAGACACTTCTAAATCCTTGGTAACAGACATGCAGTCCCCTCGGCAGAGCCCCGCC
CCCCNATGGTACCGGGTTGTTAAGAGGTCCACAACGGGTCNCACGACNNCATTAAGGCCTCANGCATG
ACCCAACACGGGGGCGTCCCGGCGCGGCGACCTTGAANTCCGGGGACAAGTATGGCCCCCCGGGAAGG
ACGCCGTAGTGCATCTGGAGTGCCGANNCGTGGTCCAGAATGNCACCACGATGGTATGAGTGGCAAAG
GTACTTTGGACACCACCCCGTTNNCTANTTCCAGACGCGGCCATTCAGGAACGACGCGAATCGTAATC
TACCTGCCGCGTNCGCGGATCGTACATGCGTTTTCCAGCGTTCGAAATAGAGTTTGTCGTTCCGCTGA
CCAGGTTNCACGGCTACTGCGATAAGCATATTCTACTGGGGGACTGTAAAGGAATTGTATCCACTCTG
CTTCCTGTCAATTGGCCATGTGCATCTCTNGTTGTTCAGGGTACATTAATAACACACAGCATNAAGTA
GATAAGTCTCCGATATAAGCGAACAGAACGTTGGTCAGAGACTGTGGTTCAATTGTAGTGGTATAATG
TTTCCCAGTGCATAAGGAACTCAGGGACACTACTAGCATGGGCGAGGAGAGACCCTTGGGTGGCAANA
CGCAATAAGTGTTACCTAGTACACCAGCACGAGANAGGGTTTGACCATNNNTTTTAGACGACCCCGG
TCGCATTGAACCTGGCATTCAGAATGACGTGCTCTCAAGAATCAAGGTCCNCCCACTGAGGGTCANCT
GCTTCGCGGGTCNGCGTATNGTCACAAACAT
>LaHu357
GTGTAGCCCAGTTAGACACTTCTAAATCCNTGGTAACAGACATGCAGTCCCCTCGGCAGAGCCCCGCC
CCCCGGTGGTACCGGGTTGNTAAGAGGTCCACCACGGGTCGNACGACAACATCAAGGCCTCANGCATG
ACCCAACACGGNGGCGTCCCGGCGNGGCGACCTTGAAGGCCGGGGACAAGTATGGCCCCCCGGGAAGG
ACGCCGTAGTGCATCTGGAGTGCCGATTCGTGGTCNAGAATGCCACCACGATGGTATGAGTGGCAAAG
GTACTTTGGACACCACCCCGTTTTCTATTTCCAGACGCGGCCNTTCAGGAACGACGCGAATCGTAATC
TANCTGCCGCGTACGCGGATNGTACATGCGTTTTNCAGCGTTCGAAATAGAGTTTGTCGTTCCGCTGA
CCAGGTTNCACGGCTACTGCGATAAGCATATTCTACTGGGGGACTGTAAAGGAATTGTATCCACTCTG
CTTCCTGTCAATTGGCCATGTGCATCTCTNGTTGTTCAGGNNACATTAATAACACACAGCATNAAGTA
GATAAGTCTCCGATATAAGCGAACAGAACGTTGGTCAAAGACTGTGGTTCAATTGTAGTGGTATAACG
TTTCCCAGTGCATAAGGAACTCAGGGACACTACTAGCATGGGCGAGGAGAGACCCTTGGGTGGCAANA
CGCAATAAGTGTTACCTAGTACACCAGCACGATANAGGGTTTGACCATGTGTTTTTAGNNGACCCCGG
TCGCATTGAACCTGGCATTCAGAATGACGTGCTCTNAAGAGTCAAGGTCCTCCCACTGAGGGTCATCT
GCTTCGCGNGTCNGCGTATNGTCATAAACAT
>LaHu361
GTGTAGCCCAGTTAGACACTTCTAAATCCNTNGTAACAGACATGCAGTCCCCTCGGCAGAGCCCCGCC
CCCCGGTGGTACCGGGTTGTTNAGAGGTCCACCACGGGTCGCACGACAACATCAAGGCCTCANGCATG
ACCCAACACGGGGGCGTCCCGGCGCGGCGACCTTGAAGGCCGGGGACAAGTATGGCCCCCCGGGAAGG
ACGCCGTAGTGCATCTGGAGTGCCGATTCGTGGTCCAGAATGCCACCACGATGGTATGAGTNGCAAAG
GTACTTTGGACACCACCCCGTTNTCTATTTCCAGACGCGGCCATTCAGGAACGACGCGAATCGTAATC
TACCTGCCGCGTACGCGGATCGTACANGCGTTTTCCAGCGTTCGAAATAGAGTTTGTCGTTCCGCTGA
CCAGGTTTCACGGCTACTGCGATAAGCATATTCTACTGGGGGACTGTAAAGGAATTGTATCCACTCNG
CTTCCTGTCAATTGGNCATGTGCATCTCTGGTTGTTCAGGGTACATTAATAACACACAGCATNAAGTA
GATAAGTCTCCGATATAAGCGAACAGAACGTTGGTCAAAGACTGTGGTTCAATTGTAGTGGTATAACG
TTTCCCAGTGCATAAGGAACTCAGGGACACTACTAGCANGGGCGAGGAGAGACCCTTGGGTGGCAANA
CGCAATAAGTGTTANCTAGTACACCAGCACGATNGAGGGTTTGNCCATNNNTTTTAGACGACCCCGG
TCGCATTGAACCTGGCATTCAGAATGACGTGCTCTNAAGAGTCAAGGTCCTCCCACTGAGGGTCATCT
GCTTCGCGNGTCAGCGTATGGTCATAANCAT
>LaHu362
GTGTAGCCCAGTTAGACACTTCTAAATCCTTNGTAACAGACATGCAGTCCCCTCNNCAGAGCCCCGCC
CCCCGGTGGTACCGGGTTGNTAAGAGGTCCACCACGGGTCGCACGACAACATCAAGGCCTCATGCATG
ACCCAACACGGNGGNGTCCCGGCGNGGCGACCTTGNAGGCCGGGGACAAGTATGGCCCCCCGGGAAGG

ACGCCGTAGTGCATCTGGAGTGCCGATTCGTGGTCNAGAATGCCACCACGATGGTATGAGTGGCAAAG
GTACTTTGGACACCACCCCGTTTTCTATTTCCAGACGCGGCCNTTCAGGAACGACGCGAATCGTAATC
TACCTGCCGCGTNCGCGGATCGTACANGCGTTTTCCAGCGTTCGAAATAGAGTTTGTCGTTCCGCTGA
CCAGGTTNCACGGCTACTGCGATAAGCATATTCTACTGGGGGACTGTAAAGGAATTGTATCCACTCTG
CTTCCTGTCAATTGGNCATGTGCATCTCTGGTTGTTCAGGGTACATTAATAACACACAGCATNAAGTA
GATAAGTCTCCGATATAAGCGAACAGAACGTTGGTNAAAGACTGTGGTTCAATTGTAGTGGTATAACG
TTTCCCAGTGCATAAGGAACTCAGGGACACTACTAGCATGGGCGAGGAGAGACCCTTGGGTGGCAANA
CGCAATAAGTGTTNCCTAGTACACCAGCACGATANAGGGTTTGACCNTGTGTTTTTAGNNGACCCCGG
TCGCATTGAACCTGGCATTCAGAATGACGTGCTCTCAAGAGTCAANGTCCTCCCACTGAGGGTCATCT
GCTTCGCGNGTCNGCGTATNGTCATAAACAT
>LaHu363
GTGTAGCCCAGTTAGACACTTCTAAATCCTTGGTAACAGACATGCAGTCCCCTCGGCAGAGCCCCGCC
CCCCGATGGTACCGGGTTGNTAAGAGGTCCACAACGGGTCGCACGACAACATTAAGGCCTCATGCATG
ACCCNACNCGGGGGCGTCCCGGCGCGGCGACCTTGNAGTCCGGGGNCAAGTATGGCCCCCCGGGAAGG
ACGCCGTAGTGCATCTGGAGTGCCGATTCGTGGTCCAGAATGCCACCACGATGGTATGAGTGGCAAAG
GTACTTTGGACACCACCCCGTTNNCTATTTCCAGANGCGGCCATTCAGGAACGACGCGAATCGTAATC
TACCTGCCGCGTACGCGGATCGTACATGCGTTTTCCAGCGTTCGAAATAGAGTTTGTCGTTCCGCTGA
CCAGGTTNCACGGCTACTGCGATAAGCATATTCTACTGGGGGACTGTAAAGGAATTGTATCCACTCTG
CTTCCTGTCAATTGGNCATGTGCATCTCTGGTTGTTCAGGGTACATTAATAACACACAGNATTAAGTA
GATNAGTCTCCGATATAAGCGAACAGANCGTTGGTNAGAGACTGTGGTTCAATTGTAGTGGTATAATG
TTTCCCAGTGCATAAGGAACTCAGGGACACTACTAGCATGGNCGAGGAGAGACCCTTGGGTGGCAACA
CGCAATAAGTGTTACCTAGTACACCAGCACGAGNNAGGGTTTGACCATGTGTTTNTAGACGACCCCGG
TCGCATTGAACCTGGCATTCAGAATGACGTGCTCTCAAGAATCAAGGTCCTCCCACTGAGGGTCATCT
GCTTCGCGGGTCNGCGTATNGTCATAAACNT
>LaHu364
GTGTAGCCCAGTCAGACACTTCTAAATCCNTGGTAACAGACATGCAGTCCCCTCGGCAGAGCCCCGCC
CCCCNATGGTACCGGGTTGTTAAGAGGTCCACAACGGGTCGNACGACAACNTTAAGGCCTNATGCATG
ACCCAANACGGNGGCGTCCCGGCGNGGCGACCTTGAAGTCCGGGGNCAAGTATGGCCCCCCGNGAAGG
ACGCCGTAGTGCATCTGGAGTGCCGATTCGTGGTCNAGAATGCCACCACGATGGTATGAGTGGCAAAG
GTACTTTGGACACCACCCCGTTTTCTANTTCCAGACGCGGCCNTTCAGGAACGACGCGAATCGTAATC
TACCTGCCGCGTACGCGGATNGTACANGCGTTTTCCAGCGTTCGAAATAGAGTTTGTCGTTCCGCTGA
CCAGGTTNCACGGCTACTGCGATAAGCATATTCTACTGGGGGACTGTAAAGGAATTGTATCCATTCTG
CTTCCTGTCAATTGGCCATGTGCATCTCTGGTTGTTCAGGNNACATTAATAACACACAGCAGTAAGTA
GGTNAGTCTCCGATATAAGCGAACAGAACGTTTGTNAGAGACTGTGGTTCAATTGTAGTGGTATAATG
TTTCCCAGTGCATAAGGAACTCAGGGACACTACTAGCANGGGCGAGGAGAGACCCTTGGGTGGCANNA
CGCAATAAGTGTTACCTAGTACACCAGCACGAGANAGGGTTTGACCATGTGTTTTTAGACGACCCCGG
TCGCATTGAACCTGGCATTCAGAATGACGTGCTCTCAAGAATCAAGNTCCTCCCACTGAGGGTCATCT
GCTTCGCGGGTCNGCGTATGGTCATAAACAT
>LaHu365
GTGTAGCCCAGTTAGACACTTCTAAATCCTTGGTAACAGACATGCAGTCCCCTCGGCAGAGCCCCGCC
CCCCGATGGTACCGGGTTGTTAAGAGGTCCACCACGGGTCGCACGACAACATTAAGGCCTCATGCATG
ACCCAACACGGGGGCGTCCCGGCGCGGCGACCTTGAAGTCCGGGGACAAGTATGGCCCCCCGGGAAGG
ACGCCGTAGTGCATCTGGAGTGCCGATTCGTGGTCCAGAATGCCACCACGATGGTATGAGTGGCAAAG
GTACTTTGGACACCACCCCGTTTTCTATTTCCAGACGCGGCCATTCAGGAACGACGCGAATCGTAATC
TACCTGCCGCGTACGCGGATCGTATATACGTTTTCCAGCGTTCGAAATAGAGTTTGTCGTTCCGCTGA
CCAGGTTTCACGGCTACTGCGATAAGCATATTCTACTGGGGGACTGTAAAGGAATTGTATCCACTCTG
CTTCCTGTCAATTGGCCATGTGCATCTCTGGTTGTTCAGGGTACATTAATAACACACAGCATTAAGTA
GATAAGTCTCCGATATAAGCGAACAGAACGTTGGTCAGAGACTGTGGTTCAATTGTAGTGGTATAATG
TTTCCCAGTGCATAAGGAACTCAGGGACACTACTAGCATGGGCGAGGAGAGACCCTTGGGTGGCAACA
CGCAATAAGTGTTACCTAGTACACCAGCACGAGAGAGGGTTTGACCATGTGTTTTTAGACGACCCCGG
TCGCATTGAACCTGGCATTCAGAATGACGTGCTCTCAAGAGTCAAGGTCCTCCCACTGAGGGTCATCT
GCTTCGCGGGTCAGCGTATGGNCATAAACAT
>LaHu366
GTGTAGCCCAGTTAGACACTTCTAAATCCTTGGTAACAGACATGCAGTCCCCTCGGCAGAGCCCCGCC

CCCCNGTGGTACCGGGTTGTTAAGAGGTCCACCACGGGTCGCACGACAACATCAAGGCCTCATGCATG
ACCCANCNCGGGGGCGTCCCGGCGNGGCGACCTTGAAGGCCGGGGACAAGTATGGCCCCCCGGGAAGG
ACGCNGTAGTGCATCTGGAGTGCCGATTCGTGGTCCAGAATGCCACCACGATGGTATGAGTGGCAAAG
GTACTTTGGACACCACCCCGTTTTCTATTTCCAGACGCGGCCNTTCAGGAACGACGCGAATCGTAATC
TACCTGCCGCGTACGCGGATCGTACATGCGTTTTCCAGCGTTCGAAATAGAGTTTGTCGTTCCGCTGA
CCAGGTTTCACGGCTACTGCGATAAGCATATTCTACTGGGGGACTGTAAAGGAATTGTATCCACTCTG
CTTCCTGTCAATTGGCCATGTGCATCTCTGGTTGTTCAGGGTACATTAATAACACACAGCATTAAGTA
GATAAGTCTCCGATATAAGCGAACAGAACGTTGGTCAAAGACTGTGGTTCAATTGTAGTGGTATAACG
TTTCCCAGTGCATAAGGAACTCAGGGACACTACTAGCATGGGCGAGGAGAGACCCTTGGGTGGCAACA
CGCAATAAGTGTTACCTAGTACACCAGCACGATANAGGGTTTGACCATGTGTTTTTAGNNGACCCCGG
TCGCATTGAACCTGGCATTCAGAATGACGTGCTCTCAAGAGTCAAGNTCCTCCCACTGAGGGTCATCT
GCTTCGCGNGTCNGCGTATGGTCATAAACAT
>LaHu367
GTGTAGCCCAGTTAGACACTTCTAAATCCTTGGTAACAGACATGCAGTCCCCTCGGCAGAGCCCCGCC
CCCCGATGGTACCGGGTTGNTAAGAGGTCCACAACGGGTCGCACGACAANATTAAGGCCTCATGCATG
ACCCAACACGGGGGCGTCCCGGCGCGGCGACCTTGAAGTCCGGGGNCAAGTATGGCCCCCCGGGAAGG
ACGCCGTAGTGCATCTGGANTGCCGATTCGTGGTCNAGAATGCCACCACGATGGTATGAGTGGCAAAG
GTACTTTGGACACCACCCCGTTTTCTATTTCCAGACGCGGCCNTTCAGGAACGACGCGAATCGTAATC
TACCTGCCGCGTACGCGGATCGTACATGCGTTTTCCAGCGTTCGAAATAGAGTTTGTCGTTCCGCTGA
CCAGGTTNCACGGCTACTGCGATAAGCATATTCTACTGGGGGACTGTAAAGGAATTGTATCCACTCNG
CTTCCTGTCAATTGGCCATGTGCATCTCTGGTTGTTCAGGGTACATTAATAACACACAGCATTAAGTA
GATAAGTCTCCGATATAAGCGAACAGAACGTTGGTCAGAGACTATGGTTCAATTGTAGTGGTATAATG
TTTCCCAGTGCATAAGGAACTCAGGGACACTACTAGCATGGGCGAGGAGAGACCCTTGGGTGGCAACA
CGCGATAAGTGTTACCTAGTACATCAGCACGAGAGAGGGTTTGACCATGTGTTTTTAGACGACCCCGG
TCGCATTGAACCTGGCATTCAGAATGACGTGCTCTCAAGAATCAAGGTCCTCCCACTGAGGGTCATCT
GCTTCGCGGGTCAGCGTATGGTCANAAACAT
>LaHu368
GTGTAGCCCAGTTAGACACTTCTAAATCCNTGGTAACAGACATGCAGTCCCCTCNNCAGAGCCCCGCC
CCCCNATGGTACCGGGTTGTTAAGAGGTCCACAACGGGTCNCACGACANCATTAAGGCCTCATGCATG
ACCCAACACGGNGGNGTCCCGGCGCGGCGACCTTGAAGTCCGGGGACAAGTATGGCCCCCCGGGAAGG
ACGCCGTAGTGCATCTGGAGTGCCGNTTCGTGGTCNAGAATGNCACCACGATGGTATGAGTGGCAAAG
GTACTTTGGACACCACCCCGTTTTCTANTTCCAGACGCGGCCNTTCAGGAACGACGCGAATCGTAATC
TACCTGCCGCGTACGCGGATCGTACANGCGTTTTCCAGCGTTCGAAATAGAGTTTGTCGTTCCGCTGA
CCAGGTTNCACGGCTACTGCGATAAGCATATTCTACTGGGGGACTGTAAAGGAATTGTATCCACTCNG
CTTCCTGTCAATTGGCCATGTGCATCTCTGGTTGTTCAGGGTACATTAATAACACACAGCATTAAGTA
GATNAGTCTCCGATATAAGCGAACAGAACGTTGGTNAGAGACTATGGTTCAATTGTAGTGGTATAATG
TTTCCCAGTGCATAAGGAACTCAGGGACACTACTAGCATGGGCGAGGAGAGACCCTTGGGTGGCAANA
CGCGATAAGTGTTACCTAGTACATCAGCACGAGNGAGGGTTTGACCATGTGTTTTTAGACGACCCCGG
TCGCATTGAACCTGGCATTCAGAATGACGTGCTCTCAAGAATCAAGNTCCTCCCACTGAGGGTCATCT
GCTTCGCGGGTCNGCGTATNGTCATAAACNT
>LaHu369
GTGTAGCCCAGTTAGACACTTCTAAATCCNTGGTAACAGACATGCAGTCCCCTCGGCAGAGCCCCGCC
CCCCNATGGTACCGGGTTGTTAAGAGGTCCACAACGGGTCGCACGACAACATTAAGGCCTCATGCATG
ACCCAACACGGGGGNGTCCCGGNGCGGCGACCTTGAAGTCCGGGGACAAGTATGGCCCCCCGGGAAGG
ACGCCGTAGTGCATCTGGAGTGCCGATTCNTGGTCCAGAATGCCACCACGATGGTATGAGTNGCAAAG
GTACTTTGGACACCACCCCGTTTTCTANTTCCAGACGCGGCCATTCAGGAACGACGCGAATCGTAATC
TACCTGCCGCGTACGCGGATCGTACATGCGTTTTCCAGCGTTCGAAATAGAGTTTGTCGTTCCGCTGA
CCAGGTTTCACGGCTACTGCGATAAGCATATTCTACTGGGGGACTGTAAAGGAATTGTATCCACTCTG
CTTCCTGTCAATTGGCCATGTGCATCTCTNGTTGTTCAGGGTACATTAATAACACACAGCATTAAGTA
GATAAGTCTCCGATATAAGCGAACAGAACGTTGGTCAGAGACTATGGTTCAATTGTAGTGGTATAATG
TTTCCCAGTGCATAAGGAACTCAGGGACACTACTAGCATGGGCGAGGAGAGACCCTTGGGTGGCAACA
CGCAATAAGTGTTACCTAGTACATCAGCACGAGAGAGGGTTTGACCATGNNTTTTTAGACGACCCCGG
TCGCATTGAACCTGGCATTCAGAATGACGTGCTCTCAAGAATCAAGGTCCTCCCACTGAGGGTCATCT
GCTTCGCGGGTCAGCGTATGGTCATANACAT

>LaHu370
GTGTAGCCCAGTTAGACACTTCTAAATCCNTGGTAACAGACATGCAGTCCCCTCGGCAGAGCCCCGCC
CCCCNATGGTACCGGGTTGNTNAGAGGTCCACCACGGGTCGCACGACAANATTAAGGCCTNATGCATG
ANNCAACNCGNNNGCGTCCCGGCGCGGNGACCTTGAAGTCCGGGGNCAAGTATGGCCCCCCGGGAAGG
ACGCCGTAGTGCATCTGGAGTGCCGANNCNTGGTCCAGAATGCCACCACGATGGTATGAGTGGCAAAG
GTACTTTGGACACCACCCCGTTTTCTANTTCCAGANGCGGCCATTCAGGAACGACGCGAATCGTAATC
TACCTGCCGCGTACGCGGATCGTATANACGTTTTCCAGCGTTCGAAATAGAGTTTGTCGTTCCGCTGA
CCAGGTTNCACGGCTACTGCGATAAGCATATTCTACTGGGGGACTGTAAAGGAATTGTATCCACTCNG
CTTCCTGTCAATTGGCCATGTGCATCTCTGGTTGTTCAGGGTACATTAATAACACACAGCATNAAGTA
GATNAGTCTCCGATATAAGCGAACAGAACGTTGGTCAGAGACTGTGGTTCAATTGTAGTGGTATAATG
TTTCCCAGTGCATAAGGAACTCAGGGACACTACTAGCANGGGCGAGGAGAGACCCTTGGGTGGCAACA
CGNAATAAGTGTTANNTAGTACACCAGCACGAGANAGGGTTTGACCATGTGTTTTTAGACGACCCCGG
TCGCATTGAACCTGGCATTCAGAATGACGTGCTNTNANGAGTCAAGNTCCNCNCACTGAGGGTCATCT
GCTTCGCGGGTCAGCGTATNGNCATAAACAT
>LaHu371
GTGTAGCCCAGTTAGACACTTCTAAATCCNTNGTAACAGACATGCAGTCCCCTCNNCAGAGCCCCGCC
CCCCGATGGTACCGGGTTGTTNAGAGGTCCACCACGGGTCGNACGACAANATTAAGGCCTCATGCATG
ACCCAACACGGGGGCGTCCCGGCGCGGCGACCTTGAAGTCCGGGGACAAGTATGGCCCCCCGGGAAGG
ACGCCGTAGTGCATCTGGAGTGCCGATTCGTGGTCNAGAATGNCACCACGATGGTATGAGTGGCAAAG
GTACTTTGGACACCACCCCGTTTTCTANTTCCAGACGCGGCCATTCAGGAACGACGCGAATCGTAATC
TACCTGCCGCGTACGCGGATCGTATATACGTTTTCCAGCGTTCGAAATAGAGTTTGTCGTTCCGCTGA
CCAGGTTNCACGGCTACTGCGATAAGCATATTCTACTGGGGGACTGTAAAGGAATTGTATCCACTCNG
CTTCCTGTCAATTGGNCATGTGCATCTCTNGTTGTTCAGGGTACATTAATAACACACAGCATTAAGTA
GATAAGTCTCCGATATAAGCGAACAGAACGTTGGTCAGANACTGTGGTTCAATTGTAGTGGTATAATG
TTTCCCAGTGCATAAGGAACTCAGGGACACTACTAGCATGGGCGAGGAGAGACCCTTGGGTGGCAANA
CGCAATAAGTGTTANCTAGTACACCAGCACGAGNNANGGTTTGACCATGTGTTTTTAGACGACCCCGG
TCGCATTGAACCTGGCATTCAGAATGACGTGCTCTCAAGAGTCNAGNTCCNCCCACTGAGGGTCATCT
GCTTCGCGGGTCAGCGTATGGNCACAAANAT
>LaHu372
GTGTAGCCCAGTTAGACACTTCTAAATCCTTGGTAACAGACATGCAGTCCCCTCNNCAGAGCCCCGCC
CCCCGGTGGTACCGGGTTGTTAAGAGGTCCACCACGGGTCGNACGACAACATCAAGGCCTCATGCATG
ACCCAACACGGGGGCGTCCCGGCGNGGCGACCTTGNAGGCCGGGGNCAAGTATGGCCCCCCGGGAAGG
ACGCCGTAGTGCATCTGGAGTGCCGATTCGTGGTCCAGAATGCCACCACGATGGTATGAGTNGCAAAG
GTACTTTGGACACCACCCCGTTTTCTANTTCCAGACGCGGCCATTCAGGAACGACGCGAATCGTAATC
TACCTGCCGCGTACGCGGATCGTACATGCGTTTTCCAGCGTTCGAAATAGAGTTTGTCGTTCCGCTGA
CCAGGTTTCACGGCTACTGCGATAAGCATATTCTACTGGGGGACTGTAAAGGAATTGTATCCACTCTG
CTTCCTGTCAATTGGNCATGTGCATCTCTGGTTGTTCAGGGTACATTAATAACACACAGCATTAAGTA
GATAAGTCTCCGATATAAGCGAACAGAACGTTGGTCAAAGACTGTGGTTCAATTGTAGTGGTATAACG
TTTCCCAGTGCATAAGGAACTCAGGNACACTACTAGCATGGGCGAGGAGAGACCCTTGGGTGGCAACA
CGCAATAAGTGTTACCTAGTACACCAGCACGATAGAGGGTTTGACCATGNNTTTTTAGACGACCCCGG
TCGCATTGAACCTGGCATTCAGAATGACGTGCTCTCAAGAGTCAAGGTCCTCCCACTGAGGGTCATCT
GCTTCGCGNGTCNGCGTATGGTCACAAACAT
>LaHu373
GTGTAGCCCAGTTAGACACTTCTAAATCCTTGGTAACAGACATGCAGTCCCCTCGGCAGAGCCCCGCC
CCCCNATGGTACCGGGTTGTTAAGAGGTCCACCACGGGTCGCACGACAACATTAAGGCCTCATGCATG
ACCCAACACGNNNGCGTCCCGGCGCGGCGACCTTGNAGTCCGGGGACAAGTATGGCCCCCCGGGAAGG
ACGCCGTAGTGCATCTGGAGTGCCGATTCGTGGTCCAGAATGCCACCACGATGGTATGAGTGGCAAAG
GTACTTTGGACACCACCCCGTTTTCTATTTCCAGACGCGGCCATTCAGGAACGACGCGAATCGTAATC
TACCTGCCGCGTACGCGGATCGTATATACGTTTTCCAGCGTTCGAAATAGAGTTTGTCGTTCCGCTGA
CCAGGTTTCACGGCTACTGCGATAAGCATATTCTACTGGGGGACTGTAAAGGAATTGTATCCACTCTG
CTTCCTGTCAATTGGCCATGTGCATCTCTGGTTGTTCAGGGTACATTAATAACACACAGCATTAAGTA
GATAAGTCTCCGATATAAGCGAACAGAACGTTGGTCAGAGACTGTGGTTCAATTGTAGTGGTATAATG
TTTCCCAGTGCATAAGGAACTCAGGGACACTACTAGCATGGGCGAGGAGAGACCCTTGGGTGGCAACA
CGCAATAAGTGTTACCTAGTACACCAGCACGAGNGAGGGTTTGACCATGTGTTTTTAGACGACCCCGG

TCGCATTGAACCTGGCATTCAGAATGACGTGCTCTCAAGAGTCAAGGTCCTCCCACTGAGGGTCATCN
GCTTCGCGGGTCAGCGTATNGNCATAAACNT
>LaHu374
GTGTAGCCCAGTTAGACACTTCTAAATCCTTGGTAACAGACATGCAGTCCCCTCGGCAGAGCCCCGCC
CCCCNATGGTACCGGGTTGTTAAGAGGTCCACAACGGGTCGNACGACAACNTTAAGGCCTCATGCATG
ACCCNACNCGGGGGCGTCCCGGCGNGGCGACCTTGNAGTCCGGGGACAAGTATGGCCCCCCGGGAAGG
ACGCCGTAGTGCATCTGGAGTGCCGATTCGTGGTCCAGAATGCCACCACGATGGTATGAGTGGCAAAG
GTACTTTGGACACCACCCCGTTTTCTANTTCCAGANGCGGCCATTCAGGAACGACGCGAATCGTAATC
TACCTGCCGCGTNCGCGGATCGTACANGCGTTTTCCAGCGTTCGAAATAGAGTTTGTCGTTCCGCTGA
CCNGGTTNCACGGCTACTGCGATAAGCATATTCTACTGGGGGACTGTAAAGGAATTGTATCCACTCTG
CTTCCTGTCAATTGGCCATGTGCATCTCTNGTTGTTCAGGGTACATTAATAACACACAGCATTAAGTA
GATAAGTCTCCGATATAAGCGAACAGAACGTTGGTCAGAGACTATGGTTCAATTGTAGTGGTATAATG
TTTCCCAGTGCATAAGGAACTCAGGNACACTACTAGCATGGGCGAGGAGAGACCCTTGGGTGGCAACA
CGCAATAAGTGTTACCTAGTACATCAGCACGAGNGAGGGTTTGACCATGNNTTTTTAGACGACCCCGG
TCGCATTGAACCTGGCATTCAGAATGACGTGCTCTCAAGAATCNAGGTCCTCCCACTGAGGGTCATCN
GCTTCGCGGGTCAGCGTATNGTCACAAACAT
>LaHu375
GTGTAGCCCAGTTAGACACTTCTAAATCCNTGGTAACAGACATGCAGTCCCCTCGGCAGAGCNCCGCC
CCCCGATGGTACCGGGTTGTTNAGAGGTCCACCACGGGTCGCACGACAACATTAAGGCCTCATGCATG
ACCCAACACGNNNGCGTCCCGGNGCGGCGACCTTGAAGTCCGGGGACAAGTATGGCCCCCCGGGAAGG
ACGCCGTAGTGCATCTGGAGTGCCGNTTCGTGGTCNAGAATGCCACCACGATGGTATGAGTGGCAAAG
GTACTTTGGACACCACCCCGTTTTCTANTTCCAGACGCGGCCATTCAGGAACGACGCGAATCGTAATC
TACCTGCCGCGTNCGCGGATCGTATATACGTTTTCCAGCGTTCGAAATAGAGTTTGTCGTTCCGCTGA
CCAGGTTTCACGGCTACTGCGATAAGCATATTCTACTGGGGGACTGTAAAGGAATTGTATCCACTCTG
CTTCCTGTCAATTGGCCATGTGCATCTCTGGTTGTTCAGGGTACATTAATAACACACAGNATTAAGTA
GATNAGTCTCCGATATAAGCGAACAGAACGTTGGTCAGAGACTGTGGTTCAATTGTAGTGGTATAATG
TTTCCCAGTGCATAAGGAACTCAGGNACACTACTAGCATGGGCGAGGAGAGACCCTTGGGTGGCAANA
CGCAATAAGTGTTACCTAGTACACCAGCACGAGNNAGGGTTTGACCATGTGTTTTTAGACGACCCCGG
TCGCATTGAACCTGGCATTCAGAATGACGTGCTCTCAAGAGTCAAGNTCCTCCCACTGAGGGTCATCT
GCTTCGCGGGTCNGCGTATGGNCATAAACAT
>LoLo458
GTGTAGCCCAGTTAGACACTTCTAAATCCTTNGTAACAGACATGCAGTCCCCTCGGCAGAGCCCCGCC
CCCCGATGGTACCGGGTTGTTNAGAGGTCCACAACGGGTCGNACGACAACATTAAGGCCTCATGCATG
ACCCNNCNCGGGGGNGTCCCGGCGCGGCGACCTTGNAGTCCGGGNACAAGTATGGCCCCCCGGGAAGG
ACGCCGTAGTGCATCTGGAGTGCCGNNNCGTGGTCNAGAATGCCACCACGATGGTATGAGTNGCAAAG
GTACTTTGGACACCACCCCGTTTTCTATTTCCAGACGCGGCCNTTCAGGAACGACGCGAATCGTAATC
TACCTGCCGCGTACGCGGATCGTACATGCGTTTTCCAGCGTTCGAAATAGAGTTTGTCGTTCCGCTGA
CCAGGTTTCACGGCTACTGCGATAAGCATATTCTACTGGGGGACTGTAAAGGAATTGTATCCACTCNG
CTTCCTGTCAATTGGCCATGTGCATCTCTNGTTGTTCAGGNNACATTAATAACACACAGCATTAAGTA
GATNAGTCTCCGATATAAGCGAACAGANCGTTGGTNAGAGACTATGGTTCAATTGTAGTGGTATAATG
TTTCCCAGTGCATAAGGAACTCAGGGACACTACTAGCATGGNCGAGGAGAGACCCTTGGGTGGCAACA
CGCGATAAGTGTTNCCTAGTACATCAGCACGAGAGAGGGTTTGACCATGTGTTTTTAGACGACCCCGG
TCGCATTGAACCTGGCATTCAGAATGACGTGCTCTCAAGAATCAAGNTCCNCCCACTGAGGGTCATCT
GCTTCGCGGGTCNGCGTATGGTCANAAACAT
>LoLo459
GTGTAGCCCAGTTGGACACTTCTAAATCCTTGGTAACAGACATGCAGTCCCCTCGGCAGAGCCCCGCC
CCCCGGTGGTACCGGGTTGNTAAGAGGTCCACCACGGGTCGCACGACAACATCAAGGCCTCATGCATG
ACCCAACGCGGGGACGTACCGGCGCGGCGACCTTGAAGGTCGGGGACAAGTATGTCCCCCCGGGAAGG
ACGCCGTAGTGCATCTGGAGTGCCGANNCGTGGTCCAGAATGCCACCACGATGGTATGAGTGGCAAAG
GTATTTTGGACACCACCCCGTTTTCTATTTCCAGACGCGGCCATTCAGGAACGACGCGAATCGTAATC
TACCTGCCGCGTACGCGGATCGTACATGCGTTTTTCAGCGTTCGAAATAGAGTTTGTCGTTCCGCTGA
CCAGGTTNCACGGCTACTGCGATAAGCATATTCTACGGGGGGACTGTAAAGGAATTGTATCCACTCTG
CTCCCTGTCAATTGGCCATGTGCATCTCTGGTTGTTTAGGNNACATTAATAACACACAGCATGAGGTA
GATAAGTCTCCGATATAAGCGAACAGAACGTTGGTCAAAGACCGTGGTTCAATTGTAGTGGTATAACA

TTTCCCAGTGCATAAGGAACCCAGGGACACTATTAGCATGGGCGAGGAGAGACCCTTGGGTGGCAACA
CGCAATAAGTGTTACCCAGTACACCAGCACGATAGAGGGTTTGACCATGTNTTTTAGACGACCCCGG
TCGCATTGAACCTGGCATTCAGAATGACGTGCTCTCAAGAGTNAAGGTCCTCNCACTGAGGGTCATCT
GCTTCGCGNGTTAGCGTATGGTCATAAACAT
>LoLo460
GTGTAGCCCAGTTAGACACTTCTAAATCCTTNGTAACAGACATGCAGTCCCCTCGGCAGAGCCCCGCC
CCCCNATGGTACCGGGTTGNTAAGAGGTCCACAACGGGTCNNACGACAACNTTAAGGCCTCANGCATG
ACCCNACNCNGGGGCGTCCCGGCGCGGCGACCTTGNAGTCCGGGGACAAGTATGGCCCCCCGGGAAGG
ACGCNGTAGTGCATCTGGAGTGCCGATTCGTGGTCCAGAATGCCACCACGATGGTATGAGTNGCAAAG
GTACTTTGGACACCACCCCGTTTTCTANTTCCAGACGCGGCCATTCAGGAACGACGCGAATCGTAATC
TACCTGCCGCGTACGCGGATNGTACATGCGTNTTCCAGCGTTCGANATAGAGTTTGTCGTTCCGCTGA
CCAGGTTNCACGGCTACTNCGATAAGCATATTCTACTGGGGGACTGTAAAGGAATTGTATCCACTCNG
CTTCCTGTCAATTGGCCATGTGCATCTCTGGTTGTTCAGGGTACATTAATAACACACAGCATTAAGTA
GATNAGTCTCCGATATAAGCGAACAGAACGTTGGTNAGAGACTATGGTTCAATTGTAGTGGTATAATG
TTTCCCAGTGCATAAGGAACTCAGGGACACTACTAGCATGGGCGAGGAGAGACCCTTGGGTGGCAACA
CGCGATAAGTGTTANCTAGTACATCAGCACGAGNNAGGGTTTGACCATNNNTTTNTAGNNGACCCCGG
TCGCATTGAACCTGGCATTCAGAATGACGTGCTCTCAAGAATCAAGNTCCTCCCACTGAGGGTCATCN
GCTTCGCGGGTCAGCGTATGGNCATAAACAT
>LoLo461
GTGTAGCCCAGTTGGACACTTCTAAATCCTTGGTAACAGACATGCAGTCCCCTCGGCAGAGCCCCGCC
CCCCGGTGGTACCGGGTTGTTNAGAGGTCCACCACGGGTCGNACGACAACNTCAAGGCCTCANGCATG
ACCCAACGCGGGGACGTACCGGCGNGGCGACCTTGAAGGTCGGGNACAAGTATGTCCCCCCGGGAAGG
ACGCCGTAGTGCATCTGGAGTGCCGNTTCGTGGTCCAGAATGCCACCACGATGGTATGAGTGGCAAAG
GTATTTTGGACACCACCCCGTTTTCTATTTCCAGACGCGGCCATTCAGGAACGACGCGAATCGTAATC
TACCTGCCGCGTACGCGGATCGTACATGCGTTTTTCAGCGTTCGAAATAGAGTTTGTCGTTCCGCTGA
CCAGGTTNCACGGCTACTGCGATAAGCATATTCTACGGGGGGACTGTAAAGGAATTGTATCCACTCTG
CTCCCTGTCAATTGGCCATGTGCATCTCTGGTTGTTTAGGGTACATTAATAACACACAGCATGAGGTA
GATAAGTCTCCGATATAAGCGAACAGAACGTTGGTCAAAGACCGTGGTTCAATTGTAGTGGTATAACA
TTTCCCAGTGCATAAGGAACCCAGGGACACTATTAGCATGGGCGAGGAGAGACCCTTGGGTGGCAACA
CGCAATAAGTGTTACCCAGTACACCAGCACGATNGAGGGTTTGACCATGTGTTTTTAGNNGACCCCGG
TCGCATTGAACCTGGCATTCAGAATGACGTGCTCTCAAGAGTCAAGGTCCTCCCACTGAGGGTCATCN
GCTTCGCGNGTTNGCGTATGGTCATAAACAT
>LoLo462
GTGTAGCCCAGTTAGACACTTCTAAATCCNTGGTAACAGACATGCAGTCCCCTCGGCAGAGCCCCGCC
CCCCGATGGTACCGGGTTGTTAAGAGGTCCACAACGGGTCGCACGACAACATTAAGGCCTCATGCATG
ACCCAACACNGGGGNGTCCCGGCGCGGCGACCTTGNAGTCCGGGGACAAGTATGGCCCCCCGNGAAGG
ACGCNGTAGTGCATCTGGAGTGCCGATTCGTGGNCNAGAATGNCACCACGATGGTATGAGTGGCAAAG
GTACTTTGGACACCACCCCGTTTTCTATTTCCAGACGCGGCCNTTCAGGAACGACGCGAATCGTAATC
TACCTGCCGCGTACGCGGATCGTACANGCGTTTTCCAGCGTTCGAAATAGAGTTTGTCGTTCCGCTGA
CCAGGTTNCACGGCTACTGCGATAAGCATATTCTACTGGGGGACTGNAAAGGAATTGTATCCACTCTG
CTTCCTGTCAATTGGNCATGTGCATCTCTGGTTGTTCAGGGTACATTAATAACACACAGCATNAAGTA
GATAAGTCTCCGATATAAGCGAACAGAACGTTGGTCAGAGACTATGGTTCAATTGTAGTGGTATAATG
TTTCCCAGTGCATAAGGAACTCAGGGACACTACTAGCATGGGCGAGGAGAGACCCTTGGGTGGCAACA
CGCGATAAGTGTTNCCTAGTACATCAGCACGAGNGAGGGTTTGACCNTGNGTTTTTAGACGACCCCGG
TCGCATTGAACCTGGCATTCAGAATGACGTGCTCTCANGAATNAANGTCCTNNCACTGAGGGTCATCT
GCTTCGCGGGTCAGCGTATGGTCATAAACAT
>LoLo463
GTGTAGCCCAGTTAGACACTTCTAAATCCNTGGTAACAGACATGCAGTCCCCTCNNCAGAGCCCCGCC
CCCCNATGGTACCGGGTTGNTAAGAGGTCCACAACGGGTCGCACGACAACNTTAAGGCCTCATGCATG
ACCCAACNCGGGGGNGTCCCGGCGCGGCGACCTTGAAGTCCGGGGNCAAGTATGGCCCCCCGGGAAGG
ACGCCGTAGTGCATCTGGAGTGCCGATTCGTGGTCCAGAATGNCACCACGATGGTATGAGTGGCAAAG
GTACTTTGGACACCACCCCGTTTTCTANTTCCAGACGCGGCCNTTCAGGAACGACGCGAATCGTAATC
TANCTGCCGCGTACGCGGATCGTACATGCGTTTTCCAGCGTTCGAAATAGAGTTTGTCGTTCCGCTGA
CCAGGTTTCACGGCTACTGCGATAAGCATATTCTACTGGGGGACTGTAAAGGAATTGTATCCACTCTG

CTTCCTGTCAATTGGNCATGTGCATCTCTNGTTGTTCAGGGTACATTAATAACACACAGCATTAAGTA
GATAAGTCTCCGATATAAGCGAACAGAACGTTGGTCAGAGACTATGGTTCAATTGTAGTGGTATAATG
TTTCCCAGTGCATAAGGAANTCAGGNACACTACTAGCANGGNCGAGGAGAGACCCTTGGGTGGCAACA
CGCGATAAGTGTTACCTAGTACATCAGCACGAGNGANGGTTTGACCATGTGTTTTTAGACGACCCCGG
TCGCATTGAACCTGGCATTCAGAATGACGTGCTCTCAAGAATCNAGGTCCNCCCACTGAGGGTCATCT
GCTTCGCGGGTCAGCGTATGGNCANAAACAT
>LoLo464
GTGTAGCCCAGTTAGACACTTCTAAATCCNTGGTAACAGACATGCAGTCCCCTCGGCAGAGCCCCGCC
CCCCGATGGTACCGGGTTGTTAAGAGGTCCACAACGGGTCGCACGACAANATTAAGGCCTCATGCATG
ACCCAACACNGNGGNGTCCCGGCGCGGCGACCTTGAAGTCCGGGGNCAAGTATGGCCCCCCGGGAAGG
ACGCCGTAGTGCATCTGGAGTGCCGATTCGTGGTCCAGAATGCCACCACGATGGTATGAGTGGCAAAG
GTACTTTGGACACCACCCCGTTTTCTATTTCCAGACGCGGCCATTCAGGAACGACGCGAATCGTAATC
TACCTGCCGCGTACGCGGATCGTACATGCGTTTTCCAGCGTTCGAAATAGAGTTTGTCGTTCCGCTGA
CCAGGTTTCACGGCTACTNCGATAAGCATATTCTACTGGGGGACTGTAAAGGAATTGTATCCACTCTG
CTTCCTGTCAATTGGCCATGTGCATCTCTNGTTGTTCAGGGTACATTAATAACACACAGCATTAAGTA
GATAAGTCTCCGATATAAGCGAACAGAACGTTGGTCAGAGACTATGGTTCAATTGTAGTGGTATAATG
TTTCCCAGTGCATAAGGAACTCAGGGACACTACTAGCATGGGCGAGGAGAGACCCTTGGGTGGCAACA
CGCGATAAGTGTTACCTAGTACATCAGCACGAGANANGGTTTGACCATGTNTTTTAGACGACCCCGG
TCGCATTGAACCTGGCATTCAGAATGACGTGCTCTCAAGAATCAANGTCCNCCCACTGAGGGTCANCT
GCTTCGCGNGTCNGCGTATGGTCATANNNAT
>LoLo465
GTGTAGCCCAGTCAGACACTTCTAAATCCTTGGTAACAGACATGCAGTCCCCTCGGCAGAGCCCCGCC
CCCCGATGGTACCGGGTTGTTAAGAGGTCCACAACGGGTCGCACGACAACATTAAGGCCTCATGCATG
ACCCAACNCGGGGGNGTCCCGGCGCGGCGACCTTGAAGTCCGGGGNCAAGTATGGCCCCCCGGGAAGG
ACGCCGTAGTGCATCTGGAGTGCCGATTCGTGGTCCAGAATGNCACCACGATGGTATGAGTNGCAAAG
GTACTTNGGACACCACCCCGTTTTCTATTTCCAGACGCGGCCATTTAGGAACGACGCGAATCGTAATC
TACCTGCCGCGTACGCGGATCGTACATGCGTTTTCCAGCGTTCGAAATAGAGTTTGTCGTTCCGCTGA
CCAGGTTTCACGGCTACTGCGATAAGCATATTCTACTGGGGGACTGTAAAGGAATTGTATCCATTCNG
CTTCCTGTCAATTGGCCATGTGCATCTCTGGTTGTTCAGGGTACATTAATAACACACAGCAGTAAGTA
GGTAAGTCTCCGATATAAGCGAACAGAACGTTTGTCAGAGACTGTGGTTCAATTGTAGTGGTATAATG
TTTCCCAGTGCATAAGGAACTCAGGGACACTACTAGCATGGGCGAGGAGAGACCCTTGGGTGGCAANA
CGCAATAAGTGTTACCTAGTACACCAGCACGAGAGAGGGTTTGACCATGTGTTTTTAGACGACCCCGG
TCGCATTGAACCTGGCATTCAGAATGACGTGCTCTCAAGAATCAAGGTCCTCCCACTGAGGGTCATCT
GCTTCGCGGNTCNGCGTATGGTCATAAACNT
>LoLo466
GTGTAGCCCAGTTGGACACTTCTAAATCCTTGGTAACAGACATGCAGTCCCCTCGGCAGAGCCCCGCC
CCCCGGTGGTACCGGGTTGTTAAGAGGTCCACCACGGGTCGCACGACNACNTCAAGGCCTCATGCATG
ACCCNACGCGGGGACGTACCGGCGCGGCGACCTTGAANGTCGGGGACAAGTATGTCCCCCCGGGAAGG
ACGCCGTAGTGCATCTGGAGTGCCGATTCGTGGTCCNGAATGCCACCACGATGGTATGAGTGGCAAAG
GTATTTTGGACACCACCCCGTTTTCTATTTCCAGACGCGGCCATTCAGGAACGACGCGAATCGTAATC
TACCTGCCGCGTACGCGGATCGTACATGCGTTTTTCAGCGTTCGAAATAGAGTTTGTCGTTCCGCTGA
CCAGGTTTCACGGCTACTGCGATAAGCATATTCTACGGGGGGACTGTAAAGGAATTGTATCCACTCTG
CTCCCTGTCAATTGGCCATGTGCATCTCTGGTTGTTTAGGGTACATTAATAACACACAGCATGAGGTA
GATAAGTCTCCGATATAAGCGAACAGAACGTTGGTCAAAGACCGTGGTTCAATTGTAGTGGTATAACA
TTTCCCAGTGCATAAGGAACCCAGGGACACTATTAGCATGGGCGAGGAGAGACCCTTGGGTGGCAACA
CGCAATAAGTGTTACCCAGTACACCAGCACGATAGAGGGTTTGACCATGTGTTTTTAGACGACCCCGG
TCGCATTGAACCTGGCATTCAGAATGACGTGCTCTCAAGAGTCAAGGTCCTCCCACTGAGGGTCATCT
GCTTCGCGNGTTAGCGTATGGTCATAAACAT
>LoLo467
GTGTAGCCCAGTTAGACACTTCTAAATCCTTGGTAACAGACATGCAGTCCCCTCGGCAGAGCCCCGCC
CCCCNATGGTACCGGGTTGTTAAGAGGTCCACAACGGNTCGCACGACAACATTAAGGCCTCATGCATG
ACCCANCACGGGGGCGTCCCGGCGNGGCGACCTTGAANTCCGGGGNCAAGTATGGCCCCCCGGGAAGG
ACGCCGTAGTGCATCTGGAGTGCCGATTCGTGGTCCAGAATGCCACCACGATGGTATGAGTNGCAAAG
GTACTTTGGACACCACCCCGTTTTCTATTTCCAGACGCGGCCNTTCAGGAACGACGCGAATCGTAATC

TACCTGCCGCGTACGCGGATCGTACNNGCGTTTTCCAGCGTTCGAAATAGAGTTTGTCGTTCCGCTGA
CCAGGTTTCACGGCTACTGCGATAAGCATATTCTACTGGGGGACTGTAAAGGAATTGTATCCACTCTG
CTTCCTGTCAATTGGCCATGTGCATCTCTNGTTGTTCAGGGTACATTAATAACACACAGCATTAAGTA
GATAAGTCTCCGATATAAGCGAACAGAACGTTGGTCAGAGACTATGGTTCAATTGTAGTGGTATAATG
TTTCCCAGTGCATAAGGAACTCAGGGACACTACTAGCATGGGCGAGGAGAGACCCTTGGGTGGCAANA
CGCGATAAGTGTTACCTAGTACATCAGCACGAGNGAGGGTTTGACCATGTNTTTTAGACGACCCCGG
TCGCATTGAACCTGGCATTCAGAATGACGTGCTCTCAAGAATCAAGNTCCTCCCACTGAGGGTCATCT
GCTTCGCGGGTCAGCGTATNGTCACAAACAT
>LoLo468
GTGTAGCCCAGTTAGACACTTCTAAATCCTTNGTAACAGACATGCAGTCCCCTCGGCAGAGCCCCGCC
CCCCNATGGTACCGGGTTGTTAAGAGGTCCACAACGGGTCGCACGACAACATTAAGGCCTCATGCATG
ACCCAACACNGNGGCGTCCCGGCGCGGNGACCTTGAANTCCGGGGNCAAGTATGGCCCCCCGGGAAGG
ACGCCGTAGTGCATCTGGAGTGCCGANNCGTGGTCNAGAATGCCACCACGATGGTATGAGTGGCAAAG
GTACTTTGGACACCACCCCGTTTTCTANTTCCAGACGCGGCCATTCAGGAACGACGCGAATCGTAATC
TACCTGCCGCGTNCGCGGATCGTACATGCGTTTTNCAGCGTTCGAAATAGAGTTTGTCGTTCCGCTGA
CCAGGTTNCACGGCTACTGCGATAAGCATATTCTACTGGGGGACTGTAAAGGAATTGTATCCACTCNG
CTTCCTGTCAATTGGCCATGTGCATCTCTNGTTGTTCAGGGTACATTAATAACACACAGCATNAAGTA
GATAAGTCTCCGATATAAGCGAACAGAACGTTGGTCAGAGACTATGGTTCAATTGTAGTGGTATAATG
TTTCCCAGTGCATAAGGAACTCAGGGACACTACTAGCANGGGCGAGGAGAGACCCTTGGGTGGCAACA
CGCGATAAGTGTTACCTAGTACATCAGCACGAGNGANGGTTTGACCATGTGTTTTTAGACGACCCCGG
TCGCATTGAACCTGGCATTCAGAATGACGTGCTCTCAAGAATCAAGGTCCTCCCACTGAGGGTCATNT
GCTTCGCGGGTCAGCGTATNGTCATAAACAT
>LoLo469
GTGTAGCCCAGTTAGACACTTCTAAATCCTTGGTAACAGACATGCAGTNCCCTCNNCAGAGCCCCGCC
CCCCGATGGTACCGGGTTGNTAAGAGGTCCACAACGGGTCGCACGACAACATTAAGGCCTCANGCATG
ACCCAACACGGNGGNGTCCCGGCGCGGCGACCTTGAAGTCCGGGGNCAAGTATGGCCCCCCGNGAAGG
ACGCCGTAGTGCATCTGGAGTGCCGATTCGTGGTCCAGANTGNCACCACGATGGTATGAGTGGCAAAG
GTACTTNGGACACCACCCCGTTTTCTANTTCCAGACGCGGCCNTTCAGGAACGACGCGAATCGTAATC
TACCTGCCGCGTNCGCGGATCGTACANGCGTTTTCCAGCGTTCGAAATAGAGTTTGTCGTTCCGCTGA
CCAGGTTNCACGGCTACTGCGATAAGCATATTCTACTGGGGGACTGTAAAGGAATTGTATCCACTCTG
CTTCCTGTCAATTGGNCATGTGCATCTCTNGTTGTTCAGGNNACATTAATAACACACAGCATTAAGTA
GATAAGTCTCCGATATAAGCGAACAGAACGTTGGTCAGAGACTATGGTTCAATTGTAGTGGTATAATG
TTTCCCAGTGCATAAGGAACTCAGGNACACTACTAGCATGGGCGAGGAGAGACCCTTGGGTGGCAACA
CGCGATAAGTGTTACCTAGTACATCAGCACGAGAGAGGGTTTGACCATGNNTTTTTAGACGACCCCGG
TCGCATTGAACCTGGCATTCANAATGACGTGCTCTCAAGAATCAANGTCCTCNCACTGAGGGTCATCN
GCTTCGCGGGTCAGCGTATGGNCATAAANAT
>LoLo470
GTGTAGCCCAGTTAGACACTTCNAAATCCTTGGTAACAGACATGCAGTCCCCTCGGCAGAGCCCCGCC
CCCCGATGGTACCGGGTTGTTAAGAGGTCCACAACGGGTCGNACGACAACATTAAGGCCTCATGCATG
ACCCANCNCGGGGGNGTCCCGGNGNGGNGACCTTGNAGTCCGGGGACAAGTATGGCCCCCCGGGAAGG
ACGCNGTAGTGCATCTGGAGTGCCGATTCGTGGTCCAGAATGNCACCACGATGGTATGAGTGGCAAAG
GTACTTTGGACACCACCCCGTTTTCTANTTCCAGANGCGGCCNTTCAGGAACGACGCGAATCGTAATC
TACCTGCCGCGTACGCGGATNGTACANGCGTTTTCCAGCGTTCGAAATAGAGTTTGTCGTTCCGCTGA
CCAGGTTNCACGGCTACTGCGATAAGCATATTCTACTGGGGGACTGTAAAGGAATTGTATCCACTCNG
CTTCCTGTCAATTGGNCATGTGCATCTCTGGTTGTTCAGGNNACATTAATAACACACAGCATNAAGTA
GATAAGTCTCCGATATAAGCGAACAGAACGTTGGTNAGAGACTATGGTTCAATTGTAGTGGTATAATG
TTTCCCAGTGCATAAGGAACTCAGGNACACTACTAGCATGGGCGAGGAGAGACCCTTGGGTGGCAACA
CGCGATAAGTGTTACCTAGTACATCAGCACGAGNGAGGGTTTGNCCATGTGTTTTTAGACGACCCCGG
TCGCATTGAACCTGGCATTCAGAATGACGTGCTNTNAAGAATCAAGNTCCTCCCACTGAGGGTCATCT
GCTTCGCGGGTCAGCGTATNGNCATAAACAT
>LoLo471
GTGTAGCCCAGTTAGACACTTCTAAATCCNTGGTAACAGACATGCAGTCCCCTCNNCAGAGCCCCGCC
CCCCGATGGTACCGGGTTGTTAAGAGGTCCACAACGGGTCGNACGACAACATTAAGGCCTNATGCATG
ACCCANCACGGGGGNGTCCCGGNGNGGCGACCTTGAAGTCCGGGGNCAAGTATGGCCCCCCGGGAAGG

ACGCCGTAGTGCATCTGGAGTGCCGATTCGTGGTCCAGAATGNCACCACGATGGTATGAGTGGCAAAG
GTACTTTGGACACCACCCCGTTTTCTATTTCCAGACGCGGCCATTCAGGAACGACGCGAATCGTAATC
TACCTGCCGCGTACGCGGATCGTACATGCGTTTTCCAGCGTTCGAAATAGAGTTTGTCGTTCCGCTGA
CCAGGTTNCACGGCTACTGCGATAAGCATATTCTACTGGGGGACTGTAAAGGAATTGTATCCACTCTG
CTTCCTGTCAATTGGCCATGTGCATCTCTGGTTGTTCAGGNNACATTAATAACACACAGCATTAAGTA
GATAAGTCTCCGATATAAGCGAACAGANCGTTGGTCAGAGACTATGGTTCAATTGTAGTGGTATAATG
TTTCCCAGTGCATAAGGAACTCAGGGACACTACTAGCATGGGCGAGGAGAGACCCTTGGGTGGCAACA
CGCGATAAGTGTTACCTAGTACATCAGCACGAGAGANGGTTTGACCATGTGTTTTTAGACGACCCCGG
TCGCATTGAACCTGGCATTCAGAATGACGTGCTNTCAAGAATCAAGGTCCTCCCACTGAGGGTCATCT
GCTTCGCGGNTCNGCGTATGGNCATAAACAT
>LoLo472
GTGTAGCCCAGTTAGACACTTCTAAATCCTTGGTAACAGACATGCAGTCCCCTCGGCAGAGNCCCGCC
CCCCGATGGTACCGGGTTGTTAAGAGGTCCACAACGGGTCGNACGACAANATTAAGGCCTCATGCATG
ACCCANCACGGGGGCGTCCCGGNGCGGCGACCTTGAAGTCCGGGGNCAAGTATGGCCCCCCGGGAAGG
ACGCCGTAGTGCATCTGGAGTGCCGATNCGTGGTCCAGAATGCCACCACGATGGTATGAGTTGCAAAG
GTACTTTGGACACCACCCCGTTTTCTATTTCCAGACGCGGCCATTCAGGAACGACGCGAATCGTAATC
TACCTGCCGCGTNCGCGGATCGTACATGCGTTTTCCAGCGTTCGAAATAGAGTTTGTCGTTCCGCTGA
CCAGGTTTCACGGCTACTGCGATAAGCATATTCTACTGGGGGACTGTAAAGGAATTGTATCCACTCTG
CTTCCTGTCAATTGGCCATGTGCATCTCTNGTTGTTCAGGGTACATTAATAACACACAGCATTAAGTA
GATAAGTCTCCGATATAAGCGAACAGAACGTTGGTCAGAGACTATGGTTCAATTGTAGTGGTATAATG
TTTCCCAGTGCATAAGGAACTCAGGNACACTACTAGCATGGGCGAGGAGAGACCCTTGGGTGGCAACA
CGCGATAAGTGTTACCTAGTACATCAGCACGAGANAGGGTTTGACCATGTGTTTTTAGACGACCCCGG
TCGCATTGAACCTGGCATTCAGAATGACGTGCTCTCAAGAATCNAGNTCCTCCCACTGAGGGTCATCT
GCTTCGCGGGTCAGCGTATNGTCANAAACAT
>LoLo473
GTGTAGCCCAGTTAGACACTTCTAAATCCTTGGTAACAGACATGCAGTCCCCTCGGCAGAGCNCCGCC
CCCCGATGGTACCGGGTTGNTAAGAGGTCCACAACGGNTCGCACGACAACNTTAAGGCCTCATGCATG
ACCCNACNCNGNGGCGTCCCGGNGNGGCGACCTTGAAGTCCGGGGNCAAGTATGGCCCCCCGGGAAGG
ACGCCGTAGTGCATCTGGAGTGCCGATTCNTGGTCNAGAATGNCACCACGATGGTATGAGTGGCAAAG
GTACTTTGGACACCACCCCGTTTTCTANTTCCAGACGCGGCCATTCAGGAACGACGCGAATCGTAATC
TACCTGCCGCGTACGCGGATCGTACATGCGTTTTCCAGCGTTCGAAATAGAGTTTGTCGTTCCGCTGA
CCAGGTTTCACGGCTACTGCGATAAGCATATTCTACTGGGGGACTGTAAAGGAATTGTATCCACTCTG
CTTCCTGTCAATTGGCCATGTGCATCTCTGGTTGTTCAGGGTACATTAATAACACACAGCATTAAGTA
GATAAGTCTCCGATATAAGCGAACAGANCGTTGGTCAGAGACTATGGTTCAATTGTAGTGGTATAATG
TTTCCCAGTGCATAAGGAACTCAGGGACACTACTAGCATGGGCGAGGAGAGACCCTTGGGTGGCAACA
CGCGATAAGTGTTACCTAGTACATCAGCACGAGAGANGGTTTGACCNTGTGTTTTTAGNNGACCCCGG
TCGCATTGAACCTGGCATTCAGAATGACGTGCTCTCAAGAATCAAGNTCCNCCCACTGAGGGTCATCT
GCTTCGCGGGTCNGCGTATGGNCATAAACAT
>LoLo474
GTGTAGCCCAGTTAGACACTTCTAAATCCTTGGTAACAGACATGCAGTCCCCTCGGCAGAGCNCCGCC
CCCCGATGGTACCGGGTTGTTAAGAGGTCCACAACGGGTCGCACGACAANNTTAAGGCCTCATGCATG
ACCCAACACGGGGGCGTCCCGGCGNGGCGACCTTGAAGTCCGGGGNCAAGTATGGCCCCCCGGGAAGG
ACGCCGTAGTGCATCTGGAGTGCCGATTCGTGGTCCAGAATGCCACCACGATGGTATGAGTGGCAAAG
GTACTTTGGNCACCACCCCGTTTTCTATTTCCAGACGCGGCCATTCAGGAACGACGCGAATCGTAATC
TACCTGCCGCGTACGCGGATCGTACATGCGTTTTCCAGCGTTCGAAATAGAGTTTGTCGTTCCGCTGA
CCAGGTTNCACGGCTACTGCGATAAGCATATTCTACTGGGGGACTGTAAAGGAATTGTATCCACTCTG
CTTCCTGTCAATTGGCCATGTGCATCTCTGGTTGTTCAGGGTACATTAATAACACACAGCATNAAGTA
GATAAGTCTCCGATATAAGCGAACAGAACGTTGGTCAGAGACTATGGTTCAATTGTAGTGGTATAATG
TTTCCCAGTGCATAAGGAACTCAGGGACACTACTAGCATGGGCGAGGAGAGACCCTTGGGTGGCAACA
CGCGATAAGTGTTACCTAGTACATCAGCACGAGAGAGGGTTTGACCATGTGTTTNTAGACGACCCCGG
TCGCATTGAACCTGGCATTCAGAATGACGTGCTCTCAAGAATCAAGGTCCTCCCACTGAGGGTCATNN
GCTTCGCGGGTCAGCGTATGGTCATAAACNT
>LoLo475
GTGTAGCCCAGTTGGACACTTCTAAATCCNTGGTAACAGACATGCAGTCCCCTCNNCAGAGNCCCGCC

CCCCGGTGGTACCGGGTTGTTNAGAGGTCCACCACGGGTCGCACGACANNNTCAAGGCCTNATGCATG
ACCCAACGCGGNGACGTACCGGNGCGGCGACCTTGAAGGTCGGGGNCAAGTATGTCCCCCCGGGAAGG
ACGCCGTAGTGCATCTGGAGTGCCGATTNNTGGNCCAGAATGNCACCACGATGGTATGAGTGGCAAAG
GTATTTTGGACACCACCCCGTTNNCTATTTCCAGACGCGGCCATTCAGGAACGACGCGAATCGTAATC
TACCTGCCGCGTNCGCGGATCGTACANGCGTTTTTCAGCGTTCGAAATAGAGTTTGTCGTTCCGCTGA
CCAGGTTTCACGGCTACTGCGATAAGCATATTCTACGGGGGGACTGTAAAGGAATTGTATCCACTCNG
CTCCCTGTCAATTGGNCATGTGCATCTCTNGTTGTTTAGGGTACATTAATAACACACAGCATGAGGTA
GATAAGTCTCCGATATAAGCGAACAGANCGTTGGTCAAAGACCGTGGTTCAATTGTAGTGGTATAACA
TTTCCCAGTGCATAAGGAACCCAGGGACACTATTAGCATGGGCGAGGAGAGACCCTTGGGTGGCAACA
CGCAATAAGTGTTNCCCAGTACACCAGCACGATAGAGGGTTTGACCATNNNTTTTTAGACGACCCCGG
TCGCATTGAACCTGGCATTCAGAATGACGTGCTCTCANGAGTCAAGNTCCNCCCACTGAGGGTCATCT
GCTTCGCGNGTTAGCGTATNGTCATAAACAT
>LoLo476
GTGTAGCCCAGTTGGACACTTCTAAATCCTTGGTAACAGACATGCAGTCCCCTCNNCAGAGCCCCGCC
CCCCNGTGGTACCGGGTTGTTAAGAGGTCCACCACGGGTCGCACGACAACNTCAAGGCCTCANGCATG
ACCCAACGCGGGGACGTACCGGCGNGGCNACCTTGNAGGTCGGGGACAAGTATGTCCCCCCGGGAAGG
ACGCCGTAGTGCATCTGGAGTGCCGATTCGTGGTCNAGAATGNCACCACGATGGTATGAGTGGCAAAG
GTATTTTGGACACCACCCCGTTTTCTANTTCCAGACGCGGCCATTCAGGAACGACGCGAATCGTAATC
TACCTGCCGCGTNCGCGGATCGTACATGCGTTTTTCAGCGTTCGAAATAGAGTTTGTCGTTCCGCTGA
CCAGGTTNCACGGCTACTGCGATAAGCATATTCTACGGGGGGACTGTAAAGGAATTGTATCCACTCNG
CTCCCTGTCAATTGGCCATGTGCATCTCTGGTTGTTTAGGNNACATTAATAACACACAGCATGAGGTA
GATAAGTCTCCGATATAAGCGAACAGANCGTTGGTNAAAGACCGTGGTTCAATTGTAGTGGTATAACA
TTTCCCAGTGCATAAGGAACCCAGGGACACTATTAGCATGGGCGAGGAGAGACCCTTGGGTGGCAACA
CGNAATAAGTGTTACCCAGTACACCAGCACGATAGANGGTTTGACCANGTGTTTTTAGACGACCCCGG
TCGCATTGAACCTGGCATTCAGAATGACGTGCTCTNAAGAGTCAANGTCCNCCCACTGAGGGTCATCN
GCTTCGCGNGTTAGCGTATNGTCATANACAT
>LoLo477
GTGTAGCCCAGTTAGACACTTCTAAATCCTTGGTAACAGACATGCAGTCCCCTCGGCAGAGCCCCGCC
CCCCGATGGTACCGGGTTGTTAAGAGGTCCACAACGGGTCNCACGACAACATTAAGGCCTCATGCATG
ACCCAACACGGGGGCGTCCCGGCGCGGCGACCTTGNAGTCCGGGGACAAGTATGGCCCCCCGGGAAGG
ACGCCGTAGTGCATCTGGAGTGCCGATTCGTGGTCNAGAATGNCACCACGATGGTATGAGTGGCAAAG
GTACTTTGGACACCACCCCGTTTTCTANTTCCAGACGCGGCCNTTCAGGAACGACGCGAATCGTAATC
TACCTGCCGCGTACGCGGATNGTACANGCGTTTTCCAGCGTTCGAAATAGAGTTTGTCGTTCCGCTGA
CCAGGTTTCACGGCTACTGCGATAAGCATATTCTACTGGGGGACTGTAAAGGAATTGTATCCACTCNG
CTTCCTGTCAATTGGCCATGTGCATCTCTGGTTGTTCAGGGTACATTAATAACACACAGCATNAAGTA
GATAAGTCTCCGATATAAGCGAACAGAACGTTGGTCAGAGACTATGGTTCAATTGTAGTGGTATAATG
TTTCCCAGTGCATAAGGAACTCAGGGACACTACTAGCATGGGCGAGGAGAGACCCTTGGGTGGCAANA
CGCGATAAGTGTTACCTAGTACATCAGCACGAGAGAGGGTTTGACCATGNNTTTTTAGNNGACCCCGG
TCGCATTGAACCTGGCATTCAGAATGACGTGCTCTCAAGAATCAAGGTCCTCCCACTGAGGGTCATCT
GCTTCGCGGGTCNGCGTATNGNCATAAACAT
>LoLo478
GTGTAGCCCAGTTAGACACTTCTAAATCCTTGGTAACAGACATGCAGTCCCCTCGGCAGAGCCCCGCC
CCCCGATGGTACCGGGTTGTTAAGAGGTCCACAACGGGTCGCACGACAACATTAAGGCCTCATGCATG
ACCCAACACGGGGGNGTCCCGGNGNGGCGACCTTGNANTCCGGGGNCAAGTATGGCCCCCCGGGAAGG
ACGCNGTAGTGCATCTGGAGTGCCGATTCGTGGTCCAGAATGCCACCACGATGGTATGAGTGGCAAAG
GTACTTTGGACACCACCCCGTTTTCTANTTCCAGACGCGGCCATTCAGGAACGACGCGAATCGTAATC
TACCTGCCGCGTACGCGGATCGTACANGCGTTTTCCAGCGTTCGAAATAGAGTTTGTCGTTCCGCTGA
CCAGGTTNCACGGCTACTGCGATAAGCATATTCTACTGGGGGACTGTAAAGGAATTGTATCCACTCNG
CTTCCTGTCAATTGGCCATGTGCATCTCTGGTTGTTCAGGGTACATTAATAACACACAGCATTAAGTA
GATAAGTCTCCGATATAAGCGAACAGANCGTTGGTCAGAGANTATGGTTCAATTGTAGTGGTATAATG
TTTCCCAGTGCATAAGGAACTCAGGGACACTACTAGCATGGGCGAGGAGAGACCCTTGGGTGGCAACA
CGNGATAAGTGTTNCCTAGTACATCAGCACGAGAGAGGGTTTGACCATGTGTTTTTAGACGACCCCGG
TCGCATTGAACCTGGCATTCAGAATGACGTGCTCTCAAGAATCAAGNTCCTCCCACTGAGGGTCATCT
GCTTCGCGNGTCNGCGTATNGTCATAAANAT

>LoLo479
GTGTAGCCCAGTTAGACACTTCTAAATCCTTNGTAACAGACATGCAGTCCCCTCGGCAGAGCCCCGCC
CCCCGATGGTACCGGGTTGTTNAGAGGTCCACAACGGGTCGNACGACAANATTAAGGCCTCANGCATG
ACCCAACNCNGNGGCGTCCCGGCGCNGCGACCTTGAAGTCCGGGGNCAAGTATGGCCCCCCGNGAAGG
ACGCCGTAGTGCATCTGGAGTGCCGATTNGTGGTCCAGAATGCCACCACGATGGTATGAGTNGCAAAG
GTACTTTGGACACCACCCCGTTTTCTANTTCCAGACGCGGCCATTCAGGAACGACGCGAATCGTAATC
TACCTGCCGCGTACGCGGATCGTACATGCGTTTTCCAGCGTTCGAAATAGAGTTTGTCGTTCCGCTGA
CCAGGTTNCACGGCTACTGCGATAAGCATATTCTACTGGGGGACTGTAAAGGAATTGTATCCACTCNG
CTTCCTGTCAATTGGCCATGTGCATCTCTNGTTGTTCAGGGTACATTAATAACACACAGCATTAAGTA
GATNAGTCTCCGATATAAGCGAACAGAACGTTGGTNAGAGACTATGGTTCAATTGTAGTGGTATAATG
TTTCCCAGTGCATAAGGAACTCAGGGACACTACTAGCATGGGCGAGGAGAGACCCTTGGGTGGCAACA
CGCGNTAAGTGTTANNTAGTACATCAGCACGAGNNAGGGTTTGACCATGTGTTTTTAGACGACCCCGG
TCGCATTGAACCTGGCATTCAGAATGACGTGCTCTNAAGAATCAAGGTCCTCCCACTGAGGGNCATCT
GCTTCGCGGNTCAGCGTATGGTCATAAACAT
>LoLo480
GTGTAGCCCAGTTAGACACTTCTAAATCCNTNGTAACAGACATGCAGTCCCCTCNNCAGAGCCCCGCC
CCCCGATGGTACCGGGTTGTTAAGAGGTCCACAACGGGTCGNACGACAANNTTAAGGCCTCATGCATG
ACCCANCNCGGNGGCGTCCCGGCGCGGCNACCTTGAAGTCCGGGGNCAAGTATGGCCCCCCGGGAAGG
ACGCCGTAGTGCATCTGGAGTGCCGATTCNTGGTCNAGAATGCCACCACGATGGTATGAGTNGCAAAG
GTACTTTGGACACCACCCCGTTTTCTANTTCCAGANGCGGCCATTCAGGAACGACGCGAATCGTAATC
TACCTGCCGCGTNCGCGGATCGTACATGCGTTTTCCAGCGTTCGAAATAGAGTTTGTCGTTCCGCTGA
CCAGGTTNCACGGCTACTGCGATAAGCATATTCTACTGGGGGACTGTAAAGGAATTGTATCCACTCTG
CTTCCTGTCAATTGGCCATGTGCATCTCTNGTTGTTCAGGGTACATTAATAACACACAGCATTAAGTA
GATAAGTCTCCGATATAAGCGAACAGAACGTTGGTCAGAGACTATGGTTCAATTGTAGTGGTATAATG
TTTCCCAGTGCATAAGGAACTCAGGNACACTACTAGCATGGGCGAGGAGAGACCCTTGGGTGGCAACA
CGCGATAAGTGTTNCCTAGTACATCAGCACGAGANAGGGTTTGACCATGNNTTTTTAGACGACCCCGG
TCGCATTGAACCTGGCATTCAGAATGACGTGCTCTCAAGAATCAAGGTCCTCCCACTGAGGGTCATCN
NCTTCGCGGGTCNGCGTATGGTCANANNNAT
>LoLo481
GTGTAGCCCAGTTAGACACTTNTAAATCCNTGGTAACAGACATGCAGTCCCCTCNNCAGAGCCCCGCC
CCCCGATGGTACCGGGTTGTTNAGAGGTCCACAACGGGTCNCACGACAACNTTAAGGCCTCATGCATG
ACCCNACACGGGGGCGTCCCGGNGNGGCGACCTTGNANTCCGGGGNCAAGTATGGCCCCCCGGGAAGG
ACGCNGTAGTGCATCTGGAGTGCCGATTCGTGGTCCAGAATGNCACCACGATGGTATGAGTGGCAAAG
GTACTTTGGACACCACCCCGTTTTCTANTTCCAGACGCGGCCATTCAGGAACGACGCGAATCGTAATC
TACCTGCCGCGTACGCGGATCGTACATGCGTTTTCCAGCGTTCGAAATAGAGTTTGTCGTTCCGCTGA
CCAGGTTTCACGGCTACTGCGATAAGCATATTCTACTGGGGGACTGTAAAGGAATTGTATCCACTCNG
CTTCCTGTCAATTGGCCATGTGCATCTCTGGTTGTTCAGGGTACATTAATAACACACAGCATTAAGTA
GATAAGTCTCCGATATAAGCGAACAGAACGTTGGTCAGAGACTATGGTTCAATTGTAGTGGTATAATG
TTTCCCAGTGCATAAGGAACTCAGGGACACTACTAGCATGGGCGAGGAGAGACCCTTGGGTGGCAANA
CGCGATAAGTGTTNCCTAGTACATCAGCACGAGAGAGGGTTTGACCATGNNTTTTTAGACGNCCCCGG
TCGCATTGAACCTGGCATTCAGAATGACGTGCTCTCAAGAATCAAGNTCCTCCCACTGAGGGTCATCT
GCTTCGCGGGTCNGCGTATGGNCATAAACAT
>LoLo482
GTGTAGCCCAGTTAGACACTTCTAAATCCTTGGTAACAGACATGCAGTCCCCTCGGCAGAGCCCCGCC
CCCCGATGGTACCGGGTTGTTAAGAGGTCCACAACGGGTCGCACGACAACATTAAGGCCTCATGCATG
ACCCANCNCGGGGGNGTCCCGGCGCGGCGACCTTGAAGTCCGGGGACAAGTATGGCCCCCCGGGAAGG
ACGCCGTAGTGCATCTGGAGTGCCGATTCGTGGTCCAGAATGCCACCACGATGGTATGAGTGGCAAAG
GTACTTTGGACACCACCCCGTTTTCTATTTCCAGACGCGGCCATTCAGGAACGACGCGAATCGTAATC
TACCTGCCGCGTACGCGGATCGTACATGCGTTTTCCAGCGTTCGAAATAGAGTTTGTCGTTCCGCTGA
CCAGGTTNCACGGCTACTGCGATAAGCATATTCTACTGGGGGACTGTAAAGGAATTGTATCCACTCNG
CTTCCTGTCAATTGGCCATGTGCATCTCTGGTTGTTCAGGGTACATTAATAACACACAGCATNAAGTA
GATAAGTCTCCGATATAAGCGAACAGAACGTTGGTCAGAGACTATGGTTCAATTGTAGTGGTATAATG
TTTCCCAGTGCATAAGGAACTCAGGGACACTACTAGCATGGGCGAGGAGAGACCCTTGGGTGGCAANA
CGCGATAAGTGTTACCTAGTACATCAGCACGAGANAGGGTTTGACCATGTGTTTTTAGACGACCCCGG

TCGCATTGAACCTGGCATTCAGAATGACGTGCTCTCAAGAATCAAGNTCCTCCCACTGAGGGTCATCT
GCTTCGCGGGTCAGCGTATGGTCATAANNAT
>LoLo483
GTGTAGCCCAGTTAGACACTTCTAAATCCTTNGTAACAGACATGCAGTCCCCTCNNCAGAGCNCCGCC
CCCCGATGGTACCGGGTTGNTNAGAGGTCCACAACGGGTCGCACGACNANATTAAGGCCTCATGCATG
ACCCAACACGGNGGNGTCCCGGNGNGGCGACCTTGAAGTCCGGGNACAAGTATGGCCCCCCGGGAAGG
ACGCCGTAGTGCATCTGGAGTGCCGATTCGTGGTCCNGANTGCCACCACGATGGTATGAGTNGCAAAG
GTACTTTGGACACCACCCCGTTTTCTATTTCCAGACGCGGCCATTCAGGAACGACGCGAATCGTAATC
TANCTGCCGCGTNCGCGGATCGTACANGCGTTTTCCAGCGTTCGAAATAGAGTTTGTCGTTCCGCTGA
CCAGGTTNCACGGCTACTNCGATAAGCATATTCTACTGGGGGACTGTAAAGGAATTGTATCCACTCNG
CTTCCTGTCAATTGGCCATGTGCATCTCTGGTTGTTCAGGNNACATTAATAACACACAGNATTAAGTA
GATNAGTCTCCGATATAAGCGAACAGANCGTTGGTNAGAGACTATGGTTCAATTGTAGTGGTATAATG
TTTCCCAGTGCATAAGGAACTCAGGGACACTACTAGCANGGGCGAGGAGAGACCCTTGGGTGGCAACA
CGNGATAAGTGTTACCTAGTACATCAGCACGAGAGAGGGTTTGACCNTGTGTTTTTAGACGACCCCGG
TCGCATTGAACCTGGCATTCAGAATGACGTGCTCTCAAGAATCAAGGTCCNCCCACTGAGGGTCATCN
GCTTCGCGGGTCAGCGTATNGTCANAAACAT
>LoLo484
GTGTAGCCCAGTTAGACACTTCTAAATCCTTGGTAACAGACATGCAGTCCCCTCGGCAGAGCCCCGCC
CCCCGATGGTACCGGGTTGTTAAGAGGTCCACAACGGGTCGCACGACAACNTTAAGGCCTCATGCATG
ACCCAACNCGGGGGNGTCCCGGCGNGGCGACCTTGNAGTCCGGGGACAAGTATGGCCCCCCGGGAAGG
ACGCCGTAGTGCATCTGGAGTGCCGATTCGTGGTCCAGAATGNCACCACGATGGTATGAGTGGCAAAG
GTACTTTGGACACCACCCCGTTTTCTATTTCCAGACGCGGCCATTCAGGAACGACGCGAATCGTAATC
TACCTGCCGCGTACGCGGATCGTACANGCGTTTTCCAGCGTTCGAAATAGAGTTTGTCGTTCCGCTGA
CCAGGTTTCACGGCTACTNCGATAAGCATATTCTACTGGGGGACTGTAAAGGAATTGTATCCACTCNG
CTTCCTGTCAATTGGCCATGTGCATCTCTNGTTGTTCAGGNNACATTAATAACACACAGCATNAAGTA
GATAAGTCTCCGATATAAGCGAACAGANCGTTGGTNAGAGACTATGGTTCAATTGTAGTGGTATAATG
TTTCCCAGTGCATAAGGAACTCAGGNACACTACTAGCANGGGCGAGGAGAGACCCTTGGGTGGCAANA
CGCGATAAGTGTTANCTAGTACATCAGCACGAGAGANGGTTTGACCATGTGTTTTTAGNNGACCCCGG
TCGCATTGAACCTGGCATTCAGAATGACGTGCTCTCAAGAATCAAGGTCCTCCCACTGAGGGTCATNN
GCTTCGCGGGTCNGCGTATGGTCATNAACAT
>LoLo485
GTGTAGCCCAGTTAGACACTTCTAAATCCNTGGTAACAGACATGCAGTNCCCTCGGCAGAGCCCCGCC
CCCCNATGGTACCGGGTTGTTAAGAGGTCCACAACGGGTCNCACGACAACNTTAAGGCCTCANGCATG
ACCCANNACGGGGGCGTCCCGGCGCGGCGACCTTGAAGTCCGGGGNCAAGTATGGCCCCCCGGGAAGG
ACGCCGTAGTGCATCTGGAGTGCCGATTNGTGGTCCAGAATGCCACCACGATGGTATGAGTGGCAAAG
GTACTTTGGACACCACCCCGTTNNCTATTTCCAGACGCGGCCATTCAGGAACGACGCGAATCGTAATC
TACCTGCCGCGTNCGCGGATCGTACANGCGTTTTCCAGCGTTCGAAATAGAGTTTGTCGTTCCGCTGA
CCAGGTTNCACGGCTACTGCGATAAGCATATTCTACTGGGGGACTGTAAAGGAATTGTATCCACTCNG
CTTCCTGTCAATTGGCCATGTGCATCTCTGGTTGTTCAGGGTACATTAATAACACACAGCATTAAGTA
GATNAGTCTCCGATATAAGCGAACAGAACGTTGGTCAGAGACTATGGTTCAATTGTAGTGGTATAATG
TTTCCCAGTGCATAAGGAACTCAGGGACACTACTAGCATGGGCGAGGAGAGACCCTTGGGTGGCAACA
CGNGATAAGTGTTACCTAGTACATCAGCACGAGAGAGGGTTTGNCCATGNNTTTTTAGACGACCCCGG
TCGCATTGAACCTGGCATTCAGAATGACGTGCTCTCAAGAATCAAGNTCCTCCCACTGAGGGTCATNN
GCTTCGCGNGTCNGCGTATGGNCACAAACAT
>LoLo486
GTGTAGCCCAGTTAGACACTTCTAAATCCNTGGTAACAGACATGCAGTCCCCTCNGCAGAGCCCCGCC
CCCCGATGGTACCGGGTTGTTAAGAAGTCCACAACGGGTCGCACGACAANNTTAAGGCCTCATGCATG
ACCCNNCNCNGNGGNGTCCCGGCGCGGCGACCTTGAAGTCNGGGGNCAAGTATGGCCCCCCGGGAAGG
ACGCNGTAGTGCATCTGGAGTGCCGATTCGTGGTCCNGAATGCCACCACGATGGTATGAGTGGCAAAG
GTACTTNGGACACCACCCCGTTTTCTANTNCCAGACGCGGCCATTCAGGAACGACGCGAATCGTAATC
TACCTGCCGCGTACGCGGATCGTACATGCGTTTTCCAGCGTTCGAAATAGAGTTTGTCGTTCCGCTGA
CCAGGTTNCACGGCTACTNCGATAAGCATATTCTACTGGGGGACTGTAAAGGAATTGTATCCATTCTG
CTTCCTGTCAATTGGCCATGTGCATCTCTNGTTGTTCAGGNNACATTAATAACACACAGCAGTAAGTA
GGTAAGTCTCCGATATAAGCTAACAGANCGTTTGTCAGAGACTGTGGTTCAATTGTAGTGGTATAATG

TTTCCCAGTGCATAAGGAACTCAGGGACACTACTAGCATGGGCGAGGAGAGACCCTTGGGTGGCAANA
CGNAATAAGTGTTACCTAGTACACCAGCACGAGANANGGTTTGACCATGNGTTTTCAGACGACCCCGG
TCGCATTGAACCTGGCATTCAGAATGACGTGCTCTCAAGAATCAAGGTCCNCNCACTGAGGGTCATCN
GCTTCGCGGGTCAGCGTATGGTCATAAACAT
>LoLo487
GTGTAGCCCAGTTAGACACTTCTAAATCCTTNGTAACAGACATGCAGTCCCCTCGGCAGAGCCCCGCC
CCCCNATGGTACCGGGTTGTTAAGNGGTCCACAACGGGTCNCACGACAACATTAAGGCCTCATGCATG
ACCCAACNCGGGGGCGTCCCGGCGCGGNGACCTTGAANTCCGGGGACAAGTATGGCCCCCCGGGAAGG
ACGCCGTAGTGCATCTGGAGTGCCGATTNGTGGTCNAGAATGCCACCACGATGGTATGAGTGGCAAAG
GTACTTTGGACACCACCCCGTTNNCTATTTCCAGACGCGGCCATTCAGGAACGACGCGAATCGTAATC
TACCTGCCGCGTACGCGGATCGTACATGCGTTTTCCAGCGTTCGAAATAGAGTTTGTCGTTCCGCTGA
CCAGGTTTCACGGCTACTGCGATAAGCATATTCTACTGGGGGACTGTAAAGGAATTGTATCCACTCTG
CTTCCTGTCAATTGGCCATGTGCATCTCTNGTTGTTCAGGNTACATTAATAACACACAGCATTAAGTA
GATNAGTCNCCGATATAAGCGAACAGAACGTTGGTCAGAGACTATGGTTCAATTGTAGTGGTATAATG
TTTCCCAGTGCATAAGGAACTCAGGNACACTACTAGCATGGGCGAGGAGAGACCCTTGGGTGGCAACA
CGCGATAAGTGTTACCTAGTACATCAGCACGAGAGAGGGTTTGACCATGTGTTTTTAGACGACCCCGG
TCGCATTGAACCTGGCATTCAGAATGACGTGCTNTCAAGAATCAAGGTCCNCCCACTGAGGGTCATCT
GCTTCGCGGGTCAGCGTATNGTCATAANNNT
>LoLo488
GTGTAGCCCAGTCAGACACTTCTAAATCCNTNGTAACAGACATGCAGTCCCCTCGGCAGAGCNCCGCC
CCCCGATGGTACCGGGTTGTTNAGAGGTCCACAACGGGTCGCACGACAACNTTAAGGCCTCANGCATG
ACCCAACACNGGGGCGTCCCGGCGCGGCGACCTTGAAGTCCGGGGNCAAGTATGGCCCCCCGGGAAGG
ACGCCGTAGTGCATCTGGAGTGCCGNTTCNTGGTCCAGANTGCCACCACGATGGTATGAGTGGCAANG
GTACTTTGNNCACCACCCCGTTTTCTANTTCCAGACGCGGCCATTTAGGAACGACGCGAATCGTAATC
TACCTGCCGCGTNCGCGGATCGTACANGCGTTTTCCAGCGTTCGAAATAGAGTTTGTCGTTCCGCTGA
CCNGGTTNCACGGCTACTNCGATAAGCATATTCTACTGGGGGACTGTAAAGGAATTGTATCCATTCNG
CTTCCTGTCAATTGGCCATGTGCATCTCTGGTTGTTCAGGNNACATTAATAACACACAGCAGTAAGTA
GGTAAGTCTCCGATATAAGCGAACAGAACGTTTGTCAGAGACTGTGGTTCAATTGTAGTGGTATAATG
TTTCCCAGTGCATAAGGAACTCAGGNACACTACTAGCANGGGCGAGGAGAGACCCTTGGGTGGCAACA
CGCAATAAGTGTTACCTAGTACACCAGCACGAGAGANGGTTTGACCATGTNTTTNTAGACGACCCCGG
TCGCATTGAACCTGGCATTCAGAATGACGTGCTCTNAAGAATCAANNTCCTCCCACTGAGGGTCATNN
GCTTCGCGGGTCNGCGTATGGTCATAAACAT
>LoLo489
GTGTAGCCCAGTTGGACACTTCTAAATCCTTNGTAACAGACATGCAGTCCCCTCNNCAGAGCCCCGCC
CCCCNGTGGTACCGGGTTGTTAAGAGGTCCACCACGGGTCGNACGACNNCNTCAAGGCCTCATGCATG
ACCCAACGCGGNGACGTACCGGNGCGGCGACCTTGNAGGTCGGGGACAAGTATGTCCCCCCGGGAAGG
ACGCCGTAGTGCATCTGGAGTGCCGATTNGTGGTCNAGANTGCCACCACGATGGTATGAGTNGCAAAG
GTATTTTGGACACCACCCCGTTTTCTATTTCCAGANGCGGCCATTCAGGAACGACGCGAATCGTAATC
TACCTGCCGCGTACGCGGATCGTACATGCGTTTTTCAGCGTTCGAAATAGAGTTTGTCGTTCCGCTGA
CCAGGTTTCACGGCTACTGCGATAAGCATATTCTACGGGGGGACTGTAAAGGAATTGTATCCACTCTG
CTCCCTGTCAATTGGCCATGTGCATCTCTNGTTGTTTAGGGNACATTAATAACACACAGNATGAGGTA
GATAAGTCTCCGATATAAGCGAACAGANCGTTGGTCAAAGACCGTGGTTCAATTGTAGTGGTATAACA
TTTCCCAGTGCATAAGGAACCCAGGGACACTATTAGCATGGGCGAGGAGAGACCCTTGGGTGGCAACA
CGCAATAAGTGTTACCCAGTACACCAGCACGATAGAGGGTTTGACCATGTGTTTTTAGNNGACCCCGG
TCGCATTGAACCTGGCATTCAGAATGACGTGCTCTCAAGAGTCAAGNTCCNCCCACTGAGGGTCATCT
GCTTCGCGNGTTNGCGTATNGTCATANACNT
>LoLo490
GTGTAGCCCAGTTAGACACTTCTAAATCCTTGGTAACAGACATGCAGTCCCCTCGGCAGAGCCCCGCC
CCCCGGTGGTACCGGGTTGTTAAGAGGTCCACCACGGGTCGCACGACAACATCAAGGCCTCATGCATG
ACCCAACACGGGGGCGTCCCGGCGCGGCGACCTTGAAGGCCGGGGNCAAGTATGGCCCCCCGGGAAGG
ACGCCGTAGTGCATCTGGAGTGCCGATTCGTGGTCCAGAATGCCACCACGATGGTATGAGTGGCAAAG
GTACTTTGGACACCACCCCGTTTTCTATTTCCAGACGCGGCCATTCAGGAACGACGCGAATCGTAATC
TACCTGCCGCGTACGCGGATCGTACANGCGTTTTCCAGCGTTCGAAATAGAGTTTGTCGTTCCGCTGA
CCAGGTTNCACGGCTACTGCGATAAGCATATTCTACTGGGGGACTGTAAAGGAATTGTATCCACTCTG

CTTCCTGTCAATTGGCCATGTGCATCTCTNGTTGTTCAGGGTACATTAATAACACACAGCATTAAGTA
GATAAGTCTCCGATATAAGCGAACAGAACGTTGGTCAAAGACTGTGGTTCAATTGTAGTGGTATAACG
TTTCCCAGTGCATAAGGAACTCAGGGACACTACTAGCATGGGCGAGGAGAGACCCTTGGGTGGCAACA
CGCAATAAGTGTTACCTAGTACACCAGCACGATAGAGGGTTTGACCATGTGTTTTTAGACGACCCCGG
TCGCATTGAACCTGGCATTCAGAATGACGTGCTCTCAAGAGTCAAGGTCCTCCCACTGAGGGTCATCT
GCTTCGCGNGTCAGCGTATNGTCATAAACAT
>LoLo491
GTGTAGCCCAGTTAGACACTTCTAAATCCTTGGTAACAGACATGCAGTCCCCTCNNCAGAGCNCCGCC
CCCCGATGGTACCGGGTTGTTNAGAGGTCCACAACGGGTCGNACGACAANNTTAAGGCCTCANGCATG
ACCCAACACGGGGGCGTCCCGGNGNGGCGACCTTGNANTCCGGGGACAAGTATGGCCCCCCGGGAAGG
ACGCCGTAGTGCATCTGGAGTGCCGANNCNTGGTCCAGAATGCCACCACGATGGTATGAGTGGCAAAG
GTACTTTGGACACCACCCCGTTTTCTATTTCCAGANGCGGCCATTCAGGAACGACGCGAATCGTAATC
TACCTGCCGCGTACGCGGATCGTACATGCGTTTTCCAGCGTTCGAAATAGAGTTTGTCGTTCCGCTGA
CCAGGTTTCACGGCTACTGCGATAAGCATATTCTACTGGGGGACTGTAAAGGAATTGTATCCACTCTG
CTTCCTGTCAATTGGCCATGTGCATCTCTNGTTGTTCAGGGTACATTAATAACACACAGCATTAAGTA
GATAAGTCTCCGATATAAGCGAACAGANCGTTGGTCAGANACTATGGTTCAATTGTAGTGGTATAATG
TTTCCCAGTGCATAAGGAACTCAGGGACACTACTAGCATGGNCGAGGAGAGACCCTTGGGTGGCAACA
CGCGATAAGTGTTACCTAGTACATCAGCACGAGAGAGGGTTTGACCANNNNTTTTTAGACGACCCCGG
TCGCATTGAACCTGGCATTCAGAATGACGTGNTCTCAAGAATCAAGGTCCNCCCACTGAGGGTCATCT
GCTTCGCGGNNCAGCGTATNGTCANNAANNT
>LoLo492
GTGTAGCCCAGTTAGACACTTCTAAATCCTTGGTAACAGACATGCAGTCCCCTCGGCAGAGCCCCGCC
CCCCGATGGTACCGGGTTGTTAAGAGGTCCACCACGGGTCGNACGACAACNTTAAGGCCTCANGCATG
ANNCAANACGGGGGNGTCCCGGCGCGGCGACCTTGNAGTCCGGGNACAAGTATGGCCCCCCGGGAAGG
ACGCCGTAGTGCATCTGGAGTGCCGATTCGTGGTCNAGAATGCCACCACGATGGTATGAGTGGCAAAG
GTACTTTGGACACCACCCCGTTTTCTANTTCCAGACGCGGCCATTCAGGAACGACGCGAATCGTAATC
TACCTGCCGCGTACGCGGATCGTATATACGTTTTCCAGCGTTCGAAATAGAGTTTGTCGTTCCGCTGA
CCAGGTTNCACGGCTACTGCGATAAGCATATTCTACTGGGGGACTGTAAAGGAATTGTATCCACTCTG
CTTCCTGTCAATTGGCCATGTGCATCTCTGGTTGTTCAGGGTACATTAATAACACACAGCATTAAGTA
GATAAGTCTCCGATATAAGCGAACAGAACGTTGGTCAGAGACTGTGGTTCAATTGTAGTGGTATAATG
TTTCCCAGTGCATAAGGAACTCAGGGACACTACTAGCATGGGCGAGGAGAGACCCTTGGGTGGCAANA
CGCAATAAGTGTTACCTAGTACACCAGCACGAGAGAGGGTTTGACCATGTGTTTTTAGACGACCCCGG
TCGCATTGAACCTGGCATTCAGAATGACGTGCTCTCAAGAGTCAAGGTCCTCCCACTGAGGGTCATCT
GCTTCGCGGNNCNGCGTATNGNCATNAACAT
>LoLo493
GTGTAGCCCAGTTAGACACTTCTAAATCCNTGGTAACAGACATGCAGTCCCCTCGGCAGAGCCCCGCC
CCCCGATGGTACCGGGTTGNTNAGAGGTCCACAACGGNTCGNACGACNANNTTAAGGCCTCATGCATG
ACCCANCNCGGGGGCGTCCCGGCGCGGCGACCTTGAAGTCCGGGGACAAGTATGGCCCCCCGGGAAGG
ACGCCGTAGTGCATCTGGAGTGCCGATTCGTGGTCNAGAATGNCACCACGATGGTATGAGTGGCAAAG
GTACTTTGGACACCACCCCGTTNNCTATTTCCAGACGCGGCCATTCAGGAACGACGCGAATCGTAATC
TACCTGCCGCGTNCGCGGATCGTACANGCGTTTTCCAGCGTTCGAAATAGAGTTTGTCGTTCCGCTGA
CCAGGTTNCACGGCTACTGCGATAAGCATATTCTACTGGGGGACTGTAAAGGAATTGTATCCACTCNG
CTTCCTGTCAATTGGNCATGTGCATCTCTGGTTGTTCAGGGTACATTAATAACACACAGCATTAAGTA
GATAAGTCTCCGATATAAGCGAACAGAACGTTGGTCAGAGACTATGGTTCAATTGTAGTGGTATAATG
TTTCCCAGTGCATAAGGAACTCAGGNACACTACTAGCANGGGCGAGGAGAGACCCTTGGGTGGCAANA
CGCGATAAGTGTTACCTAGTACATCAGCACGAGAGANGGTTTGACCATNNNTTTTTAGACGACCCCGG
TCGCATTGAACCTGGCATTCAGAATGACGTNCTCTCAAGAATCAAGGTCCTCCCACTGAGGGTCATCT
GCTTCGCGNGTCAGCGTATGGTCACNNACNT
>Mang273
GTGTAGCCCAGTTAGACACTTCTAAATCCNTGGTAACAGACATGCAGTCCCCTCNNCAGAGCCCCGCC
CCCCNATGGTACCGGGTTGTTAAGAGGTCCACAACGGNTCGNACGACAACNTTAAGGCCTCATGCATG
ANCCAACACGGGGGCGTCCCGGNGNGGCGACCTTGAANTCCGGGGACAAGTATGGCCCCCCGGGAAGG
ACGCCGTAGTGCATCTGGAGTGCCGATTCGTGGTCNNGAATGCCACCACGATGGTATGAGTGGCAAAG
GTACTTTGGACACCACCCCGTTTTCTANTTCCAGACGCGGCCATTCAGGAACGACGCGAATCGTAATC

TACCTGCCGCGTACGCGGATCGTACATGCGTTTTCCAGCGTTCGAAATAGAGTTTGTCGTTCCGCTGA
CCAGGTTTCACGGCTACTNCGATAAGCATATTCTACTGGGGGACTGTAAAGGAATTGTATCCACTCTG
CTTCCTGTCAATTGGCCATGTGCATCTCTNGTTGTTCAGGNNACATTAATAACACACAGCATNAAGTA
GATAAGTCTCCGATATAAGCGAACAGAACGTTGGTCAGAGACTATGGTTCAATTGTAGTGGTATAATG
TTTCCCAGTGCATAAGGAACTCAGGGACACTACTAGCATGGGCGAGGAGAGACCCTTGGGTGGCAACA
CGCAATAAGTGTTNNCTAGTACATCAGCACGAGANAGGGTTTGACCATGTGTTTTTAGACGACCCCGG
TCGCATTGAACCTGGCATTCAGAATGACGTGCTCTNAAGAATCAAGNTCCNCCCACTGAGGGTCATCN
GCTTCGCGGGTCAGCGTATGGTCACANNNAT
>Mang274
GTGTAGCCCAGTTAGACACTTCTAAATCCTTGGTAACAGACATGCAGTCCCCTCGGCAGAGCCCCGCC
CCCCGATGGTACCGGGTTGTTAAGAGGTCCACAACGGGTCGCACGACAACATTAAGGCCTCATGCATG
ACCCAACACGGGGGCGTCCCGGCGCGGCGACCTTGAAGTCCGGGGACAAGTATGGCCCCCCGGGAAGG
ACGCCGTAGTGCATCTGGAGTGCCGATTCGTGGTCNAGAATGCCACCACGATGGTATGAGTNGCAAAG
GTACTTTGGACACCACCCCGTTTTCTATTTCCAGACGCGGCCATTCAGGAACGACGCGAATCGTAATC
TACCTGCCGCGTACGCGGATCGTACATGCGTTTTCCAGCGTTCGAAATAGAGTTTGTCGTTCCGCTGA
CCAGGTTTCACGGCTACTGCGATAAGCATATTCTACTGGGGGACTGTAAAGGAATTGTATCCACTCTG
CTTCCTGTCAATTGGCCATGTGCATCTCTNGTTGTTCAGGGTACATTAATAACACACAGCATTAAGTA
GATAAGTCTCCGATATAAGCGAACAGAACGTTGGTCAGAGACTATGGTTCAATTGTAGTGGTATAATG
TTTCCCAGTGCATAAGGAACTCAGGNACACTACTAGCATGGGCGAGGAGAGACCCTTGGGTGGCAACA
CGCAATAAGTGTTACCTAGTACATCAGCACGAGAGAGGGTTTGACCATGTGTTTTTAGACGACCCCGG
TCGCATTGAACCTGGCATTCAGAATGACGTGCTCTCAAGAATCAAGGTCCTCCCACTGAGGGTCATCT
GCTTCGCGGGTCAGCGTATGGTCATNAACAT
>Mang275
GTGTAGCCCAGTTAGACACTTCTAAATCCTTGGTAACAGACATGCAGTCCCCTCGGCAGAGCCCCGCC
CCCCGATGGTACCGGGTTGTTAAGAGGTCCACAACGGGTCGCACGACAACNTTAAGGCCTCATGCATG
ACCCAACACGGNGGCGTCCCGGCGCGGCGACCTTGNANTCCGGGGACAAGTATGGCCCCCCGGGAAGG
ACGCCGTAGTGCATCTGGAGTGCCGATTCNTGGTCCAGAATGCCACCACGATGGTATGAGTNGCAAAG
GTACTTTGGACACCACCCCGTTTTCTATTTCCAGACGCGGCCATTCAGGAACGACGCGAATCGTAATC
TACCTGCCGCGTACGCGGATCGTACATGCGTTTTCCAGCGTTCGAAATAGAGTTTGTCGTTCCGCTGA
CCAGGTTNCACGGCTACTGCGATAAGCATATTCTACTGGGGGACTGTAAAGGAATTGTATCCACTCTG
CTTCCTGTCAATTGGNCATGTGCATCTCTGGTTGTTCAGGNNACATTAATAACACACAGCATTAAGTA
GATAAGTCTCCGATATAAGCGAACAGAACGTTGGTNAGAGACTATGGTTCAATTGTAGTGGTATAATG
TTTCCCAGTGCATAAGGAACTCAGGNACACTACTAGCATGGGCGAGGAGAGACCCTTGGGTGGCAACA
CGCAATAAGTGTTACCTAGTACATCAGCACGAGAGANGGTTTGACCATGTGTTTTTAGACGACCCCGG
TCGCATTGAACCTGGCATTCAGAATGACGTGCTCTCAAGAATCAAGGTCCTCCCACTGAGGGTCATCT
GCTTCGCGGNTCAGCGTATGGTCATAAACAT
>Mang276
GTGTAGCCCAGTTAGACACTTCTAAATCCNTGGTAACAGACATGCAGTCCCCTCGGCAGAGCCCCGCC
CCCCNATGGTACCGGGTTGTTNAGAGGTCCACAACGGGTCGNACGACAACATTAAGGCCTCATGCATG
ACCCAACACGGGGGCGTCCCGGCGCGGCGACCTTGAANTCCGGGNNCAAGTATGGCCCCCCGGGAAGG
ACGCCGTAGTGCATCTGGAGTGCCGATTCGTGGTCCAGAATGCCACCACGATGGTATGAGTNGCAAAG
GTACTTTGGACACCACCCCGTTTTCTNTTTCCAGACGCGGCCATTCAGGAACGACGCGAATCGTAATC
TACCTGCCGCGTNCGCGGATCGTACATGCGTTTTCCAGCGTTCGAAATAGAGTTTGTCGTTCCGCTGA
CCAGGTTNCACGGCTACTNCGATAAGCATATTCTACTGGGGGACTGTAAAGGAATTGTATCCACTCTG
CTTCCTGTCAATTGGCCATGTGCATCTCTGGTTGTTCAGGNNACATTAATAACACACAGNATTAAGTA
GATAAGTCTCCGATATAAGCGAACAGANCGTTGGTCAGAGACTATGGTTCAATTGTAGTGGTATAATG
TTTCCCAGTGCATAAGGAACTCAGGGACACTACTAGCATGGGCGAGGAGAGACCCTTGGGTGGCANNA
CGCAATAAGTGTTACCTAGTACATCAGCACGAGANAGGGTTTGACCNTGTGTTTTTAGNNGACCCCGG
TCGCATTGAACCTGGCATTCAGAATGACGTGCTCTCAAGAATCAANGTCCNCCCACTGAGGGTCATCT
GCTTCGCGNGNCNGCGTATNGTCATAANNAT
>Mang277
GTGTAGCCCAGTTAGACACTTCTAAATCCNTGGTAACAGACATGCAGTCCCCTCGGCAGAGCCCCGCC
CCCCNATGGTACCGGGTTGTTNAGAGGTCCACAACGGGTCGNACGACAANATTAAGGCCTCANGCATG
ACCCAACNCGGNGGNGTCCCGGNGNGGCGACCTTGAANTCCGGGGNCAAGTATGGCCCCCCGGGAAGG

ACGCCGTAGTGCATCTGGAGTGCCGNTTCNTGGTCNAGAATGNCACCACGATGGTATGAGTGGCAAAG
GTACTTTGGACACCACCCCGTTTTCTANTTCCAGACGCGGCCATTCAGGAACGACGCGAATCGTAATC
TACCTGCCGCGTACGCGGATCGTACANGCGTTTTCCAGCGTTCGAAATAGAGTTTGTCGTTCCGCTGA
CCAGGTTNCACGGCTACTGCGATAAGCATATTCTACTGGGGGACTGTAAAGGAATTGTATCCACTCNG
CTTCCTGTCAATTGGCCATGTGCATCTCTGGTTGTTCAGGNNACATTAATAACACACAGNATTAAGTA
GATAAGTCTCCGATATAAGCGAACAGANCGTTGGTNAGANACTATGGTTCAATTGTAGTGGTATAATG
TTTCCCAGTGCATAAGGAACTCAGGGACACTACTAGCATGGGCGAGGAGAGACCCTTGGGTGGCAACA
CGCAATAAGTGTTNCCTAGTACATCAGCACGAGNGAGGGTTTGACCNTGTNTTTTAGACGACCCCGG
TCGCATTGAACCTGGCATTCAGAATGACGTGCTCTCAAGAATCAANNTCCNCCCACTGAGGGTCATCT
GCTTCGCGGGTCAGCGTATNGTCATAANNAT
>Mang278
GTGNAGCCCAGTTAGACACTTCTAAATCCNTGGTAACAGACATGCAGTCCCCTCNNCAGAGNNCCGCC
CCCCNATGGTACCGGGTTGTTNAGAGGTCCACAACGGGTCGCACGACAANATTAAGGCCTCATGCATG
ACCCAACACGGGGGNGTCCCGGCGNGGCGACCTTGAANTCCGGGGNCAAGTATGGCCCCCCGGGAAGG
ACGCCGTAGTGCATCTGGAGTGCCGATTCNTGGTCCNGAATGNCACCACGATGGTATGAGTNGCAAAG
GTACTTTGGACACCACCCCGTTTTCTATTTCCAGACGCGGCCNTTCAGGAACGACGCGAATCGTAATC
TACCTGCCGCGTACGCGGATCGTACATGCGTTTTCCAGCGTTCGAAATAGAGTTTGTCGTTCCGCTGA
CCAGGTTNCACGGCTACTGCGATAAGCATATTCTACTGGGGGACTGTAAAGGAATTGTATCCACTCTG
CTTCCTGTCAATTGGCCATGTGCATCTCTGGTTGTTCAGGGTACATTAATAACACACAGNATTAAGTA
GATAAGTCTCCGATATAAGCGAACAGANCGTTGGTCAGAGACTATGGTTCAATTGTAGTGGTATAATG
TTTCCCAGTGCATAAGGAACTCAGGGACACTACTAGCATGGGCGAGGAGAGACCCTTGGGTGGCAACA
CGNAATAAGTGTTACCTAGTACATCAGCACGAGANANGGTTTGACCATGTGTTTTTAGACGACCCCGG
TCGCATTGAACCTGGCATTCAGAATGACGTGCTCTCAAGAATCAANGTCCTCCCACTGAGGGTCATCT
GCTTCGCGGNNCNGCGTATGGTCATNAACAN
>Mang279
GTGTAGCCCAGTTAGACACTTCTAAATCCTTGGTAACAGACATGCAGTCCCCTCNNCAGAGCCCCGCC
CCCCGATGGTACCGGGTTGTTNAGAGGTCCACAACGGGTCGCACGACANNNTTAAGGCCTCATGCATG
ACCCAACACGGNGGNGTCCCGGCGCGGCGACCTTGAAGTCCGGGGNCAAGTATGGCCCCCCGGGAAGG
ACGCCGTAGTGCATCTGGAGTGCCGATTCGTGGTCNAGAATGNCACCACGATGGTATGAGTGGCAAAG
GTACTTTGGACACCACCCCGTTTTCTATTTCCAGANGCGGCCATTCAGGAACGACGCGAATCGTAATC
TACCTGCCGCGTACGCGGATCGTACATGCGTTTTCCAGCGTTCGAAATAGAGTTTGTCGTTCCGCTGA
CCAGGTTTCACGGCTACTGCGATAAGCATATTCTACTGGGGGACTGTAAAGGAATTGTATCCACTCTG
CTTCCTGTCAATTGGCCATGTGCATCTCTGGTTGTTCAGGGTACATTAATAACACACAGCATTAAGTA
GATAAGTCNCCGATATAAGCGAACAGAACGTTGGTCAGAGACTATGGTTCAATTGTAGTGGTATAATG
TTTCCCAGTGCATAAGGAACTCAGGGACACTACTAGCATGGGCGAGGAGAGACCCTTGGGTGGCAACA
CGCAATAAGTGTTANCTAGTACATCAGCACGAGANAGGGTTTGNCCATGTGTTTTTAGACGACCCCGG
TCGCATTGAACCTGGCATTCAGAATGACGTGCTCTNAAGAATCAAGGTCCTCCCACTGAGGGTCATCT
GCTTCGCGGGTCNGCGTATNGTCANNNNCAT
>Mang280
GTGTAGCCCAGTTAGACACTTCTAAATCCTTGGTAACAGACATGCAGTCCCCTCGGCAGAGCCCCGCC
CCCCNATGGTACCGGGTTGTTNAGAGGTCCACAACGGGTCGNACGACAANATTAAGGCCTCANGCATG
ACCCAACACGGGGGNGTCCCGGCGNNGNGACCTTGAAGTCCGGGGNCAAGTATGGCCCCCCGGGAAGG
ACGCCGTAGTGCATCTGGAGTGCCGATTCGTGGTCCAGAATGCCACCACGATGGTATGAGTGGCAAAG
GTACTTTGGACACCACCCCGTTTTCTANTTCCAGACGCGGCCATTCAGGAACGACGCGAATCGTAATC
TACCTGCCGCGTACGCGGATCGTACATGCGTTTTCCAGCGTTCGAAATAGAGTTTGTCGTTCCGCTGA
CCAGGTTTCACGGCTACTGCGATAAGCATATTCTACTGGGGGACTGTAAAGGAATTGTATCCACTCTG
CTTCCTGTCAATTGGCCATGTGCATCTCTGGTTGTTCAGGGTACATTAATAACACACAGCATTAAGTA
GATAAGTCTCCGATATAAGCGAACAGAACGTTGGTNAGAGACTATGGTTCAATTGTAGTGGTATAATG
TTTCCCAGTGCATAAGGAACTCAGGGACACTACTAGCATGGGCGAGGAGAGACCCTTGGGTGGCAACA
CGCAATAAGTGTTACCTAGTACATCAGCACGAGANAGGGTTTGACCATGNNTTTTTAGNNGACCCCGG
TCGCATTGAACCTGGCATTCAGAATGACGTGCTCTCAAGAATCAAGGTCCNCCCACTGAGGGTCATCT
GCTTCGCGGGTCNGCGTATGGTCACNAACNT
>Mang281
GTGTAGCCCAGTTAGACACTTCTAAATCCTTGGTAACAGACATGCAGTCCCCTCNNCAGAGCCCCGCC

CCCCGATGGTACCGGGTTGTTAAGAGGTCCACAACGGGTCGCACGACANNATTAAGGCCTCATGCATG
ACCCAACACGGGGGCGTCCCGGCGCGGCGACCTTGNAGTCCGGGGACAAGTATGGCCCCCCGGGAAGG
ACGCNGTAGTGCATCTGGAGTGCCGATTNNTGGTCCAGAATGCCACCACGATGGTATGAGTNGCAAAG
GTACTTTGGACACCACCCCGTTTTCTANTTCCAGANGCGGCCATTCAGGAACGACGCGAATCGTAATC
TACCTGCCGCGTACGCGGATCGTACATGCGTTTTCCAGCGTTCGAAATAGAGTTTGTCGTTCCGCTGA
CCAGGTTTCACGGCTACTGCGATAAGCATATTCTACTGGGGGACTGTAAAGGAATTGTATCCACTCNG
CTTCCTGTCAATTGGCCATGTGCATCTCTNGTTGTTCAGGGTACATTAATAACACACAGCATTAAGTA
GATAAGTCTCCGATATAAGCGAACAGAACGTTGGTCAGAGACTATGGTTCAATTGTAGTGGTATAATG
TTTCCCAGTGCATAAGGAACTCAGGNACACTACTAGCATGGGCGAGGAGAGACCCTTGGGTGGCAANA
CGCAATAAGTGTTACCTAGTACATCAGCACGAGAGANGGTTTGACCATGTGTTTTAGACGACCCCGG
TCGCATTGAACCTGGCATTCAGAATGACGTGCTCTCAAGAATCAAGNTCCTCCCACTGAGGGTCATCT
GCTTCGCGGGTCAGCGTATGGTCATAAACAT
>Mang283
GTGTAGCCCAGTTAGACACTTCTAAATCCTTGGTAACAGACATGCAGTCCCCTCGGCAGAGCCCCGCC
CCCCNATGGTACCGGGTTGTTNAGAGGTCCACAACGGGTCGCACGACAACATTAAGGCCTCATGCATG
ACCCAACACGGGGGCGTCCCGGCGCGGCGACCTTGAAGTCCGGGGACAAGTATGGCCCCCCGGGAAGG
ACGCCGTAGTGCATCTGGAGTGCCGATTCGTGGTCCAGAATGCCACCACGATGGTATGAGTGGCAAAG
GTACTTTGGACACCACCCCGTTTTCTATTTCCAGACGCGGCCATTCAGGAACGACGCGAATCGTAATC
TACCTGCCGCGTACGCGGATCGTACATGCGTTTTCCAGCGTTCGAAATAGAGTTTGTCGTTCCGCTGA
CCAGGTTTCACGGCTACTGCGATAAGCATATTCTACTGGGGGACTGTAAAGGAATTGTATCCACTCTG
CTTCCTGTCAATTGGCCATGTGCATCTCTGGTTGTTCAGGGTACATTAATAACACACAGCATTAAGTA
GATAAGTCTCCGATATAAGCGAACAGAACGTTGGTCAGAGACTATGGTTCAATTGTAGTGGTATAATG
TTTCCCAGTGCATAAGGAACTCAGGGACACTACTAGCANGGGCGAGGAGAGACCCTTGGGTGGCAACA
CGCAATAAGTGTTACCTAGTACATCAGCACGAGAGAGGGTTTGACCATGTGTTTTTAGACGACCCCGG
TCGCATTGAACCTGGCATTCAGAATGACGTGCTCTCAAGAATCAAGGTCCTCCCACTGAGGGTCATCT
GCTTCGCGGGTCAGCGTATGGTCATAAACAT
>Mang284
GTGTAGCCCAGTTAGACACTTCTAAATCCTTNGTAACAGACATGCAGTCCCCTCGGCAGAGCCCCGCC
CCCCNATGGTACCGGGTTGTTAAGAGGTCCACAACGGGTCNCACGACAACATTAAGGCCTCATGCATG
ACCCAACACGGGGGNGTCCCGGCGNGGCGACCTTGAAGTCCGGGGACAAGTATGGCCCCCCGGGAAGG
ACGCCGTAGTGCATCTGGAGTGCCGATTCGTGGTCCAGAATGCCACCACGATGGTATGAGTGGCAAAG
GTACTTTGGACACCACCCCGTTTTCTANTTCCAGACGCGGCCATTCAGGAACGACGCGAATCGTAATC
TANCTGCCGCGTACGCGGATCGTACATGCGTTTTCCAGCGTTCGAAATAGAGTTTGTCGTTCCGCTGA
CCAGGTTTCACGGCTACTGCGATAAGCATATTCTACTGGGGGACTGTAAAGGAATTGTATCCACTCNG
CTTCCTGTCAATTGGCCATGTGCATCTCTNGTTGTTCAGGGTACATTAATAACACACAGCATTAAGTA
GATAAGTCTCCGATATAAGCGAACAGAACGTTGGTCAGAGACTATGGTTCAATTGTAGTGGTATAATG
TTTCCCAGTGCATAAGGAACTCAGGNACACTACTAGCATGGGCGAGGAGAGACCCTTGGGTGGCAACA
CGCAATAAGTGTTACCTAGTACATCAGCACGAGAGANGGTTTGACCATGTGTTTNTAGACGACCCCGG
TCGCATTGAACCTGGCATTCAGAATGACGTGCTCTCAAGAATCAAGGTCCTCCCACTGAGGGTCANCT
GCTTCGCGGGTCNGCGTATGGTCATNAACAT
>Mang286
GTGTAGCCCAGTTAGACACTTCTAAATCCTTGGTAACAGACATGCAGTCCCCTCGGCAGAGCCCCGCC
CCCCGATGGTACCGGGTTGNTAAGAGGTCCACAACGGGTCGNACGACAACATTAAGGCCTCATGCATG
ACCCAACACGGGGGCGTCCCGGCGCGGCGACCTTGNAGTCCGGGGACAAGTATGGCCCCCCGNGAAGG
ACGCCGTAGTGCATCTGGAGTGCCGATTCGTGGTCCAGAATGCCACCACGATGGTATGAGTGGCAAAG
GTACTTTGGACACCACCCCGTTTTCTANTTCCAGACGCGGCCATTCAGGAACGACGCGAATCGTAATC
TACCTGCCGCGTNCGCGGATCGTACATGCGTTTTCCAGCGTTCGAAATAGAGTTTGTCGTTCCGCTGA
CCAGGTTNCACGGCTACTGCGATAAGCATATTCTACTGGGGGACTGTAAAGGAATTGTATCCACTCTG
CTTCCTGTCAATTGGCCATGTGCATCTCTNGTTGTTCAGGGTACATTAATAACACACAGCATTAAGTA
GATAAGTCTCCGATATAAGCGAACAGAACGTTGGTCAGAGACTATGGTTCAATTGTAGTGGTATAATG
TTTCCCAGTGCATAAGGAACTCAGGGACACTACTAGCANGGGCGAGGAGAGACCCTTGGGTGGCAACA
CGCAATAAGTGTTACCTAGTACATCAGCACGAGANANGGTTTGACCATGTGTTTTTAGACGACCCCGG
TCGCATTGAACCTGGCATTCAGAATGACGTGCTCTCAAGAATCAAGGTCCTCCCACTGAGGGTCATCT
GCTTCGCGGGTCAGCGTATGGTCATAAACAT

>Mang287
GTGTAGCCCAGTTAGACACTTCTAAATCCTTGGTAACAGACATGCAGTCCCCTCGGCAGAGCCCCGCC
CCCCGATGGTACCGGGTTGTTAAGAGGTCCACAACGGGTCGNACGACAACATTAAGGCCTCATGCATG
ACCCAACACGGGGGCGTCCCGGCGCGGCGACCTTGAAGTCCGGGGNCAAGTATGGCCCCCCGGGAAGG
ACGCNGTAGTGCATCTGGAGTGCCGATTCGTGGNCCAGAATGCCACCACGATGGTATGAGTNGCAAAG
GTACTTTGGACACCACCCCGTTTTCTATTTCCAGACGCGGCCNTTCAGGAACGACGCGAATCGTAATC
TACCTGCCGCGTACGCGGATNGTACANGCGTTTTCCAGCGTTCGAAATAGAGTTTGTCGTTCCGCTGA
CCAGGTTNCACGGCTACTGCGATAAGCATATTCTACTGGGGGACTGTAAAGGAATTGTATCCACTCNG
CTTCCTGTCAATTGGCCATGTGCATCTCTGGTTGTTCAGGGTACATTAATAACACACAGCATTAAGTA
GATAAGTCTCCGATATAAGCGAACAGANCGTTGGTCAGAGACTATGGTTCAATTGTAGTGGTATAATG
TTTCCCAGTGCATAAGGAACTCAGGGACACTACTAGCATGGGCGAGGAGAGACCCTTGGGTGGCAANA
CGCAATAAGTGTTACCTAGTACATCAGCACGAGAGAGGGTTTGACCATGNNTTTTAGACGACCCCGG
TCGCATTGAACCTGGCATTCAGAATGACGTGCTCTCAAGAANCAAGGTCCTCCCACTGAGGGTCATCT
GCTTCGCGGGTCAGCGTATGGTCANNANNAT
>Mang288
GTGTAGCCCAGTTAGACACTTCTAAATCCNTNGTAACAGACATGCAGTCCCCTCNNCAGAGCCCCGCC
CCCCGATGGTACCGGGNTGTTNAGAGGTCCACAACGGGTCGCACGACAACATTAAGGCCTCANGCATG
ACCCNACACGGGGGCGTCCCGGNGCGGNGACCTTGNAGTCCGGGGNCAAGTATGGCCCCCCGGGAAGG
ACGCCGTAGTGCATCTGGAGTGCCGATTCGTGGTCNAGAATGNCACCACGATGGTATGAGTNGCAAAG
GTACTTTGGACACCACCCCGTTTTCTANTTCCAGANGCGGCCATTCAGGAACGACGCGAATCGTAATC
TACCTGCCGCGTNCGCGGATCGTACATGCGTTTTCCAGCGTTCGAAATAGAGTTTGTCGTTCCGCTGA
CCAGGTTNCACGGCTACTGCGATAAGCATATTCTACTGGGGGACTGTAAAGNAATTGTATCNACTCNG
CTTCCTGTCAATTGGCCATGTGCATCTCTNGTTGTTCAGGGTACATTAATAACACACAGNATTAAGTA
GATAAGTCTCCGATATAAGCGAACAGAACGTTGGTCAGAGACTATGGTTCAATTGTAGTGGTATAATG
TTTCCCAGTGCATAAGGAACTCAGGNACACTACTAGCATGGGCGAGGAGAGACCCTTGGGTGGCAACA
CGCAATAAGTGTTNCCTAGTACATCAGCACGAGAGAGGGTTTGACCATNTNTTNNTAGACGACCCCGG
TCGCATTGAACCTGGCATTCAGAATGACGTGCTCTCAAGAATCAAGGTCCNCCCACTGAGGGTCATCT
GCTTCGCGGGTCAGCGTATNGNCACAAACAT
>Mang289
GTGTAGCCCAGTTAGACACTTCTAAATCCTTGGTAACAGACATGCAGTCCCCTCGGCAGAGCNCCGCC
CCCCGATGGTACCGGGTTGTTAAGAGGTCCACAACGGGTCGCACGACAACATTAAGGCCTCATGCATG
ACCCAANACGGGGGCGTCCCGGCGNGGCGACCTTGAANTCCGGGGACAAGTATGGCCCCCCGGGAAGG
ACGCCGTAGTGCATCTGGAGTGCCGATTCNTGGTCCAGAATGCCACCACGATGGTATGAGTGGCAAAG
GTACTTTGGACACCACCCCGTTTTCTATTTCCAGACGCGGCCATTCAGGAACGACGCGAATCGTAATC
TACCTGCCGCGTACGCGGATCGTACATGCGTTTTCCAGCGTTCGAAATAGAGTTTGTCGTTCCGCTGA
CCAGGTTNCACGGCTACTGCGATAAGCATATTCTACTGGGGGACTGTAAAGGAATTGTATCCACTCTG
CTTCCTGTCAATTGGCCATGTGCATCTCTGGTTGTTCAGGGTACATTAATAACACACAGCATTAAGTA
GATAAGTCTCCGATATAAGCGAACAGAACGTTGGTNAGAGACTATGGTTCAATTGTAGTGGTATAATG
TTTCCCAGTGCATAAGGAACTCAGGGACACTACTAGCATGGGCGAGGAGAGACCCTTGGGTGGCAACA
CGCAATAAGTGTTACCTAGTACATCAGCACGAGAGAGGGTTTGACCNTGNNTTTTAGACGACCCCGG
TCGCATTGAACCTGGCATTCAGAATGACGTGCTNTCAAGAATCAANGTCCTCCCACTGAGGGTCATCT
GCTTCGCGGGTCAGCGTATNGTCANAANCAT
>Mang290
GTGTAGCCCAGTTAGACACTTCTAAATCCTTGGTAACAGACATGCAGTCCCCTCGGCAGAGCCCCGCC
CCCCGATGGTACCGGGTTGTTAAGAGGTCCACAACGGGTCGCACGACAACATTAAGGCCTCATGCATG
ACCCAACACGGGGGCGTCCCGGCGCGGCGACCTTGAAGTCCGGGGACAAGTATGGCCCCCCGGGAAGG
ACGCCGTAGTGCATCTGGAGTGCCGATTCGTGGTCCAGAATGCCACCACGATGGTATGAGTNGCAAAG
GTACTTTGGACACCACCCCGTTTTCTANTTCCAGACGCGGCCATTCAGGAACGACGCGAATCGTAATC
TACCTGCCGCGTACGCGGATCGTACATGCGTTTTCCAGCGTTCGAAATAGAGTTTGTCGTTCCGCTGA
CCAGGTTTCACGGCTACTGCGATAAGCATATTCTACTGGGGGACTGTAAAGGAATTGTATCCACTCNG
CTTCCTGTCAATTGGCCATGTGCATCTCTGGTTGTTCAGGGTACATTAATAACACACAGCATTAAGTA
GATAAGTCTCCGATATAAGCGAACAGAACGTTGGTCAGAGACTATGGTTCAATTGTAGTGGTATAATG
TTTCCCAGTGCATAAGGAACTCAGGGACACTACTAGCATGGGCGAGGAGAGACCCTTGGGTGGCAACA
CGCAATAAGTGTTACCTAGTACATCAGCACGAGAGANGGTTTGACCATGTGTTTTTAGACGACCCCGG

TCGCATTGAACCTGGCATTCAGAATGACGTGCTCTCAAGAATCAAGNTCCTCCCACTGAGGGTCATCT
GCTTCGCGGGTCAGCGTATGGTCATNANNAT
>Mang291
GTGNAGCCCAGTTAGACACTTCTAAATCCTTGGTAACAGACATGCAGTCCCCTCNNCAGAGCCCCGCC
CCCCNATGGTACCGGGTTGNTNAGAGGTCCACAACGGGTCGCACGACAACATTAAGGCCTNATGCATG
ACCCNNCACNGGGGCGTCCCGGNGNGGCGACCTTGNAGTCCGGGGNCAAGTATGGCCCCCCGGGAAGG
ACGCCGTAGTGCATCTGGAGTGCCGATTCNTGGTCNAGAATGCCACCACGATGGTATGAGTGGCAANG
GTACTTTGGACACCACCCCGTTTTCTATTTCCAGACGCGGCCNTTCAGGAACGACGCGAATCGTAATC
TANCTGCCGCGTACGCGGATCGTACATGCGTTTTCCAGCGTTCGAAATAGAGTTTGTCGTTCCGCTGA
CCAGGTTNCACGGCTACTGCGATAAGCATATTCTACTGGGGGACTGTAAAGGAATTGTATCCACTCTG
CTTCCTGTCAATTGGCCATGTGCATCTCTNGTTGTTCAGGGTACATTAATAACACACAGCATTAAGTA
GATAAGTCTCCGATATAAGCGAACAGAACGTTGGTCAGAGACTATGGTTCAATTGTAGTGGTATAATG
TTTCCCAGTGCATAAGGAACTCAGGNACACTACTAGCATGGGCGAGGAGAGACCCTTGGGTGGCAANA
CGCAATAAGTGTTACCTAGTACATCAGCACGAGNGAGGGTTTGACCATGTGTTTTTAGACGACCCCGG
TCGCATTGAACCTGGCATTCAGAATGACGTGCTCTCAAGAATCAAGGTCCTCCCACTGAGGGTCATCT
GCTTCGCGGGTCNGCGTATGGTCATAANNAT
>Mang292
GTGTAGCCCAGTTAGACACTTCTAAATCCNTGGTAACAGACATGCAGTCCCCTCNNCAGAGCCCCGCC
CCCCGATGGTACCGGGTTGTTAAGAGGTCCACAACGGGTCGCACGACAACATTAAGGCCTCATGCATG
ACCCAACACGGGGGCGTCCCGGCGCGGCGACCTTGAAGTCCGGGGACAAGTATGGCCCCCCGGGAAGG
ACGCCGTAGTGCATCTGGAGTGCCGATTCGTGGTCCAGAATGCCACCACGATGGTATGAGTGGCAAAG
GTACTTTGGACACCACCCCGTTTTCTATTTCCAGACGCGGCCATTCAGGAACGACGCGAATCGTAATC
TACCTGCCGCGTACGCGGATCGTACATGCGTTTTCCAGCGTTCGAAATAGAGTTTGTCGTTCCGCTGA
CCAGGTTTCACGGCTACTGCGATAAGCATATTCTACTGGGGGACTGTAAAGGAATTGTATCCACTCTG
CTTCCTGTCAATTGGCCATGTGCATCTCTGGTTGTTCAGGGTACATTAATAACACACAGCATTAAGTA
GATAAGTCTCCGATATAAGCGAACAGAACGTTGGTCAGAGACTATGGTTCAATTGTAGTGGTATAATG
TTTCCCAGTGCATAAGGAACTCAGGGACACTACTAGCATGGGCGAGGAGAGACCCTTGGGTGGCAACA
CGCAATAAGTGTTACCTAGTACATCAGCACGAGAGAGGGTTTGACCATGTGTTTTTAGACGACCCCGG
TCGCATTGAACCTGGCATTCAGAATGACGTGCTCTCAAGAATCAAGGTCCTCCCACTGAGGGTCATCT
GCTTCGCGGGTCAGCGTATGGTCATAAACAT
>Mang293
GTGTAGCCCAGTTAGACACTTCTAAATCCTTGGTAACAGACATGCAGTCCCCTCGGCAGAGNCCCGCC
CCCCGATGGTACCGGGTTGTTAAGAGGTCCACAACGGGTCGCACGACANCNTTAAGGCCTCATGCATG
ACCCAACNCGGNGGNGTCCCGGCGCGGNGACCTTGAAGTCCGGGGACAAGTATGGCCCCCCGGGAAGG
ACGCCGTAGTGCATCTGGAGTGCCGATTCGTGGTCCAGAATGNCACCACGATGGTATGAGTNGCAAAG
GTACTTTGGACACCACCCCGTTTTCTATTTCCAGACGCGGCCATTCAGGAACGACGCGAATCGTAATC
TACCTGCCGCGTACGCGGATCGTACATGCGTTTTCCAGCGTTCGAAATAGAGTTTGTCGTTCCGCTGA
CCAGGTTTCACGGCTACTGCGATAAGCATATTCTACTGGGGGACTGTAAAGGAATTGTATCCACTCTG
CTTCCTGTCAATTGGCCATGTGCATCTCTGGTTGTTCAGGGTACATTAATAACACACAGNATTAAGTA
GATAAGTCTCCGATATAAGCGAACAGAACGTTGGTCAGAGACTATGGTTCAATTGTAGTGGTATAATG
TTTCCCAGTGCATAAGGAACTCAGGGACACTACTAGCATGGGCGAGGAGAGACCCTTGGGTGGCAACA
CGCAATAAGTGTTACCTAGTACATCAGCACGAGNGANGGTTTGACCATGTGTTTTTAGNNGACCCCGG
TCGCATTGAACCTGGCATTCAGAATGACGTGCTCTCAAGAATCAAGGTCCTCCCACTGAGGGTCATCT
GCTTCGCGGGTCAGCGTATGGTCATNNACAT
>Mang294
GTGTAGCCCAGTTANACACTTCTAAATCCTTGGTAACAGACATGCAGTCCCCTCGGCAGAGNCCCGCC
CCCCGATGGTACCGGGTTGTTAAGAGGTCCACAACGGGTCGCACGACAANNTTAAGGCCTCATGCATG
ACCCAACACGGGGGCGTCCCGGCGCGGCGACCTTGAANTCCGGGGNCAAGTATGGCCCCCCGGGAAGG
ACGCCGTAGTGCATCTGGAGTGCCGATTCGTGGTCNAGAATGCCACCACGATGGTATGAGTGGCAAAG
GTACTTTGGACACCACCCCGTTTTCTATTTCCAGANGCGGCCATTCAGGAACGACGCGAATCGTAATC
TACCTGCCGCGTNCGCGGATCGTACATGCGTTTTCCAGCGTTCGAAATAGAGTTTGTCGTTCCGCTGA
CCAGGTTNCACGGCTACTGCGATAAGCATATTCTACTGGGGGACTGTAAAGGAATTGTATCCACTCTG
CTTCCTGTCAATTGGCCATGTGCATCTCTGGTTGTTCAGGGTACATTAATAACACACAGCATTAAGTA
GATAAGTCTCCGATATAAGCGAACAGAACGTTGGTCAGAGACTATGGTTCAATTGTAGTGGTATAATG

TTTCCCAGTGCATAAGGAACTCAGGGACACTACTAGCATGGGCGAGGAGAGACCCTTGGGTGGCAACA
CGCAATAAGTGTTACCTAGTACATCAGCACGAGAGAGGGTTTGACCATGTGTTTTTAGNCGACCCCGG
TCGCATTGAACCTGGCATTCAGAATGACGTGCTCTCAAGAATCAAGGTCCTCCCACTGAGGGTCATCT
GCTTCGCGGGTCAGCGTATNGTCATAAAAAT
>Mang295
GTGTAGCCCAGTTAGACACNTCTAAATCCNTNGTAACAGACATGCAGTCCCCTCNNCAGAGNCCCGCC
CCCCGATGGTACCGGGNTGNTNAGAGGTCCACAACGGGTCGNACGACAACATTAAGGCCTCANGCATG
ANNCANNACNGGGGNGTCCCGGNGCGGCNACCTTGAANTCCGGGGNCAAGTATGGCCCCCCGGGAAGG
ACGCCGTAGTGCATCTGGAGTGCCGATTCNTGGTCCAGAATGCCACCACGATGGTATGAGTGGCAAAG
GTACTTNGGACACCACCCCGTTTTCTANTTCCAGACGCGGCCNTTCAGGAACGACGCGAATCGTAATC
TACCTGCCGCGTNCGCGGATNGTACANGCGTNTTNCAGCGTTCGAAATAGAGTTTGTCGTTCCGCTGA
CCAGGTTNCACGGCTACTNCGATAAGCATATTCTACTGGGGGACTGTAAAGGAATTGTATCCACTCNG
CTTCCTGTCAATTGGCCATGTGCATCTCTNGTTGTTCAGGGTACATTAATAACACACAGNATNAAGTA
GATNAGTCTCCNATATAAGCGAACAGANCGTTGGTNAGAGACTATGGTTCAATTGTAGTGGTATAATG
TTTCCCAGTGCATAAGGAACTCAGGNACACTACTAGCANGGGCGAGGAGAGACCCTTGGGTGGCAACA
CGNANTAAGTGTTANCTAGTACATCAGCACGAGANANGGTTTGACCNTGTGTTTTTAGNNGACCCCGG
TCGCATTGAACCTGGCATTCAGAATGACGTGCTCTCAAGAATCAAGNTCCNCCCACTGAGGGTCANCN
NCTTCGCGGNNCNGCGTATNGTCACANNNAT
>Mang296
GTGTAGCCCAGTTANACACTTCTNAATCCTTGGTAACAGACATGCAGTCCCCTCNNCAGAGCCCCGCC
CCCCGATGGTACCGGGTTGTTAAGAGGTCCACAACGGGTCGCACGACNNNATTAAGGCCTCATGCATG
ACCCNNCACGGGGGNGTCCCGGCGCGGCGACCTTGNAGTCCGGGGACAAGTATGGCCCCCCGGGAAGG
ACGCNGTAGTGCATCTGGAGTGCCGANNCGTGGTCCNGAATGCCACCACGATGGTATGAGTGGCAAAG
GTACTTTGGACACCACCCCGTTNTCTANTTCCAGANGCGGCCATTCAGGAACGACGCGAATCGTAATC
TACCTGCCGCGTNCGCGGATNGTACATGCGTTTTNCAGCGTTCGAAATAGAGTTTGTCGTTCCGCTGA
CCAGGTTNCACGGCTACTNCGATAAGCATATTCTACTGGGGGACTGTAAAGGAATTGTATCCACTCTG
CTTCCTGTCAATTGGNCATGTGCATCTCTGGTTGTTCAGGGTACATTAATAACACACAGCATTAAGTA
GATAAGTCNCCGATATAAGCGAACAGANCGTTGGTCAGANANTATGGTTCAATTGTAGTGGTATAATG
TTTCCCAGTGCATAAGGAACTCAGGGACACTACTAGCATGGGCGAGGAGAGACCCTTGGGTGGCAACA
CGCAATAAGTGTTACCTAGTACATCAGCACGAGNGAGGGTTTGACCNTNNNTTNNTAGACGACCCCGG
TCGNATTGAACCTGGCATTCAGAATGACGTNCTCTCAAGAATCAAGNTCCTCCCACTGAGGGTCANCT
GCTTCGCGGGTCNGCGTATNGTCATAAACAT
>Mang297
GTGTAGCCCAGTTAGACACTTCTAAATCCTTGGTAACAGACATGCAGTCCCCTCGGCAGAGCCCCGCC
CCCCGATGGTACCGGGTTGTTAAGAGGTCCACAACGGGTCGCACGACAACATTAAGGCCTCATGCATG
ACCCAACACGGGGGNGTCCCGGCGCGGCGACCTTGAAGTCCGGGGACAAGTATGGCCCCCCGGGAAGG
ACGCCGTAGTGCATCTGGAGTGCCGATTCGTGGTCCAGAATGCCACCACGATGGTATGAGTGGCAAAG
GTACTTTGGACACCACCCCGTTTTCTATTTCCAGACGCGGCCATTCAGGAACGACGCGAATCGTAATC
TACCTGCCGCGTACGCGGATCGTACATGCGTTTTCCAGCGTTCGAAATAGAGTTTGTCGTTCCGCTGA
CCAGGTTTCACGGCTACTGCGATAAGCATATTCTACTGGGGGACTGTAAAGGAATTGTATCCACTCTG
CTTCCTGTCAATTGGCCATGTGCATCTCTGGTTGTTCAGGGTACATTAATAACACACAGCATTAAGTA
GATAAGTCTCCGATATAAGCGAACAGAACGTTGGTCAGAGACTATGGTTCAATTGTAGTGGTATAATG
TTTCCCAGTGCATAAGGAACTCAGGGACACTACTAGCATGGGCGAGGAGAGACCCTTGGGTGGCAACA
CGCAATAAGTGTTACCTAGTACATCAGCACGAGAGAGGGTTTGACCATGTGTTTTTAGACGACCCCGG
TCGCATTGAACCTGGCATTCAGAATGACGTGCTCTCAAGAATCAAGGTCCTCCCACTGAGGGTCATCT
GCTTCGCGGGTCAGCGTATGGTCATAAACAT
>Mang298
GTGTAGCCCAGTTAGACACTTCTAAATCCTTGGTAACAGACATGCAGTCCCCTCNNCAGAGCCCCGCC
CCCCGATGGTACCGGGTTGTTAAGAGGTCCACAACGGGTCGCACGACAANATTAAGGCCTCATGCATG
ACCCAACNCGGGGGNGTCCCGGCGNGGCGACCTTGAAGTCCGGGGACAAGTATGGCCCCCCGGGAAGG
ACGCCGTAGTGCATCTGGAGTGCCGATTCGTGGTCCAGAATGNCACCACGATGGTATGAGTGGCAAAG
GTACTTTGGACACCACCCCGTTTTCTANTTCCAGACGCGGCCNTTCAGGAACGACGCGAATCGTAATC
TACCTGCCGCGTNCGCGGATCGTACATGCGTTTTNCAGCGTTCGAAATAGAGTTTGTCGTTCCGCTGA
CCAGGTTNCACGGCTACTGCGATAAGCATATTCTACTGGGGGACTGTAAAGGAATTGTATCCACTCTG

CTTCCTGTCAATTGGNCATGTGCATCTCTNGTTGTTCAGGGTACATTAATAACACACAGNATTAAGTA
GATNAGTCTCCGATATAAGCGAACAGAACGTTGGTCAGAGACTATGGTTCAATTGTAGTGGTATAATG
TTTCCCAGTGCATAAGGAACTCAGGGACACTACTAGCATGGGCGAGGAGAGACCCTTGGGTGGCAANA
CGCAATAAGTGTTACCTAGTACATCAGCACGAGANAGGGTTTGACCATGTGTTTTTAGACGACCCCGG
TCGCATTGAACCTGGCATTCAGAATGACGTGCTCTCAAGAATCAAGGTCCNCCCACTGAGGGTCATCT
GCTTCGCGNGTCNGCGTATGGTCATANACAT
>Mang300
GTGTAGCCCAGTTAGACACTTCTAAATCCTTGGTAACAGACATGCAGTCCCCTCGGCAGAGCCCCGCC
CCCCNATGGTACCGGGTTGNTAAGAGGTCCACAACGGGTCGNACGACAACNTTAAGGCCTCATGCATG
ACCCAACACGGNGGCGTCCCGGNGCGGCGACCTTGAAGTCCGGGGACAAGTATGGCCCCCCGGGAAGG
ACGCCGTAGTGCATCTGGAGTGCCGATTCGTGGTCCAGAATGCCACCACGATGGTATGAGTGGCAAAG
GTACTTTGGACACCACCCCGTTTTCTATTTCCAGACGCGGCCNTTCAGGAACGACGCGAATCGTAATC
TACCTGCCGCGTACGCGGATCGTACANGCGTTTTCCAGCGTTCGAAATAGAGTTTGTCGTTCCGCTGA
CCAGGTTTCACGGCTACTGCGATAAGCATATTCTACTGGGGGACTGTAAAGGAATTGTATCCACTCNG
CTTCCTGTCAATTGGCCATGTGCATCTCTGGTTGTTCAGGGTACATTAATAACACACAGCATNAAGTA
GATAAGTCTCCGATATAAGCGAACAGAACGTTGGTNAGAGACTATGGTTCAATTGTAGTGGTATAATG
TTTCCCAGTGCATAAGGAACTCAGGGACACTACTAGCANGGGCGAGGAGAGACCCTTGGGTGGCAACA
CGCAATAAGTGTTNCCTAGTACATCAGCACGAGANAGGGTTTGACCATGTGTTTTTAGACGACCCCGG
TCGCATTGAACCTGGCATTCAGAATGACGTGCTCTCANGAATCAAGNTCCTCCCACTGAGGGTCATCN
NCTTCGCGGGTCNGCGTATNGTCATAANNAT
>Mang301
GTGTAGCCCAGTTAGACACTTCTAAATCCTTGGTAACAGACATGCAGTCCCCTCGGCAGAGCCCCGCC
CCCCGATGGTACCGGGTTGTTAAGAGGTCCACAACGGGTCGCACGACAANNTTAAGGCCTCATGCATG
ACCCNANACGGGGGNGTCCCGGCGNGGCGACCTTGAANTCCGGGGACAAGTATGGCCCCCCGGGAAGG
ACGCNGTAGTGCATCTGGAGTGCCGATTNGTNNNCCAGAATGCCACCACGATGGTATGAGTGGCAAAG
GTACTTTGGACACCACCCCGTTTTCTANTTCCAGANGCGGCCATTCAGGAACGACGCGAATCGTAATC
TACCTGCCGCGTNCGCGGATCGTACATGCGTTTTCCAGCGTTCGAAATAGAGTTTGTCGTTCCGCTGA
CCAGGTTTCACGGCTACTGCGATAAGCATATTCTACTGGGGGACTGTAAAGGAATTGTATCCACTCTG
CTTCCTGTCAATTGGCCATGTGCATCTCTNGTTGTTCAGGNNACATTAATAACACACAGCATTAAGTA
GATAAGTCTCCGATATAAGCGAACAGANCGTTGGTCAGAGACTATGGTTCAATTGTAGTGGTATAATG
TTTCCCAGTGCATAAGGAACTCAGGGACACTACTAGCANGGGCGAGGAGAGACCCTTGGGTGGCAACA
CGCAATAAGTGTTACCTAGTACATCAGCACGAGNGANGGTTTGACCATGTGTTTTTAGACGACCCCGG
TCGCATTGAACCTGGCATTCAGAATGACGTGCTCTCAAGAATCAAGGTCCNCCCACTGAGGGTCATCT
GCTTCGCGGGTCAGCGTATGGTCATAAACAT
>Mang302
GTGTAGCCCAGTTAGACACTTCTAAATCCNTGGTAACAGACATGCAGTCCCCTCGGCAGAGCCCCGCC
CCCCGATGGTACCGGGTTGTTAAGAGGTCCACAACGGGTCGCACGACAANNTTAAGGCCTCATGCATG
ACCCAACACGGGGGNGTCCCGGNGCGGCGACCTTGAAGTCCGGGGNCAAGTATGGCCCCCCGGGAAGG
ACGCCGTAGTGCATCTGGAGTGCCGATTCNTGGNCCAGAATGNCACCACGATGGTATGAGTGGCAAAG
GTACTTTGGACACCACCCCGTTTTCTATTTCCAGACGCGGCCATTCAGGAACGACGCGAATCGTAATC
TACCTGCCGCGTACGCGGATCGTACATGCGTTTTCCAGCGTTCGAAATAGAGTTTGTCGTTCCGCTGA
CCAGGTTTCACGGCTACTGCGATAAGCATATTCTACTGGGGGACTGTAAAGGAATTGTATCCACTCTG
CTTCCTGTCAATTGGCCATGTGCATCTCTGGTTGTTCAGGGTACATTAATAACACACAGCATTAAGTA
GATAAGTCTCCGATATAAGCGAACAGAACGTTGGTCAGAGACTATGGTTCAATTGTAGTGGTATAATG
TTTCCCAGTGCATAAGGAACTCAGGGACACTACTAGCATGGGCGAGGAGAGACCCTTGGGTGGCAACA
CGCAATAAGTGTTACCTAGTACATCAGCACGAGAGAGGGTTTGACCATGTGTTTTTAGANGACCCCGG
TCGCATTGAACCTGGCATTCAGAATGACGTGCTCTCAAGAATCAAGNTCCTCCCACTGAGGGTCATCT
GCTTCGCGGGTCNGCGTATGGTCATAAACNT
>Mang303
GTGTAGCCCAGTTAGACACTTCTAAATCCTTGGTAACAGACATGCAGTCCCCTCGGCAGAGCCCCGCC
CCCCGATGGTACCGGGTTGTTNAGAGGTCCACAACGGGTCGCACGACAACATTAAGGCCTCATGCATG
ACCCNACACGGGGGCGTCCCGGCGCGGCGACCTTGAAGTCCGGGGACAAGTATGGCCCCCCGGGAAGG
ACGCCGTAGTGCATCTGGAGTGCCGATTCGTGGTCCAGAATGCCACCACGATGGTATGAGTGGCAAAG
GTACTTTGGACACCACCCCGTTTTCTATTTCCAGANGCGGCCATTCAGGAACGACGCGAATCGTAATC

TACCTGCCGCGTNCGCGGATCGTACATGCGTTTTCCAGCGTTCGAAATAGAGTTTGTCGTTCCGCTGA
CCAGGTTTCACGGCTACTGCGATAAGCATATTCTACTGGGGGACTGTAAAGGAATTGTATCCACTCTG
CTTCCTGTCAATTGGCCATGTGCATCTCTNGTTGTTCAGGGTACATTAATAACACACAGNATTAAGTA
GATAAGTCTCCGATATAAGCGAACAGAACGTTGGTCAGAGACTATGGTTCAATTGTAGTGGTATAATG
TTTCCCAGTGCATAAGGAACTCAGGGACACTACTAGCATGGGCGAGGAGAGACCCTTGGGTGGCAACA
CGCAATAAGTGTTACCTAGTACATCAGCACGAGAGAGGGTTTGACCATGTGTTTTTAGACGACCCCGG
TCGCATTGAACCTGGCATTCAGAATGACGTGCTCTCAAGAATCAAGGTCCTCCCACTGAGGGTCATCT
GCTTCGCGGGTCNGCGTATGGTCATAAACAT
>Mang305
GTGTAGCCCAGTTAGACACTTCTAAATCCTTGGTAACAGACATGCAGTCCCCTCNNCAGAGCCCCGCC
CCCCNATGGTACCGGGTTGTTNAGAGGTCCACAACGGGTCGNACGACAACNTTAAGGCCTCANGCATG
ACCCAACACGGNGGCGTCCCGGNGNGGCGACCTTGAANTCCGGGGACAAGTATGGCCCCCCGGGAAGG
ACGCCGTAGTGCATCTGGAGTGCCGATTCGTGGTCNAGAATGNCACCACGATGGTATGAGTGGCAAAG
GTACTTTGGACACCACCCCGTTTTCTATTTCCAGACGCGGCCNTTCAGGAACGACGCGAATCGTAATC
TACCTGCCGCGTNCGCGGATCGTACATGCGTTTTCCAGCGTTCGAAATAGAGTTTGTCGTTCCGCTGA
CCAGGTTNCACGGCTACTGCGATAAGCATATTCTACTGGGGGACTGTAAAGGAATTGTATCCACTCTG
CTTCCTGTCAATTGGNCATGTGCATCTCTGGTTGTTCAGGGTACATTAATAACACACAGCATTAAGTA
GATAAGTCTCCGATATAAGCGAACAGAACGTTGGTNAGAGACTATGGTTCAATTGTAGTGGTATAATG
TTTCCCAGTGCATAAGGAACTCAGGGACACTACTAGCATGGNCGAGGAGAGACCCTTGGGTGGCAANA
CGCAATAAGTGTTACCTAGTACATCAGCACGAGAGANGGTTTGACCATNNNTTTTTAGACGACCCCGG
TCGCATTGAACCTGGCATTCAGAATGACGTGCTCTCAAGAATCAANNTCCTCCCACTGAGGGTCATCN
GCTTCGCGNGTCAGCGTATGGTCANAAAAAT
>Mang306
GTGTAGCCCAGTTAGACACTTCTAAATCCTTGGTAACAGACATGCAGTCCCCTCGGCAGAGNNCCGCC
CCCCGATGGTACCGGGTTGTTAAGAGGTCCACAACGGGTCGCACGACAACATTAAGGCCTCATGCATG
ACCCAANACGGGGGCGTCCCGGCGCGGCGACCTTGAAGTCCGGGGNCAAGTATGGCCCCCCGGGAAGG
ACGCCGTAGTGCATCTGGAGTGCCGATTCGTGGTCCAGAATGNCACCACGATGGTATGAGTGGCAAAG
GTACTTTGGACACCACCCCGTTTTCTATTTCCAGACGCGGCCATTCAGGAACGACGCGAATCGTAATC
TACCTGCCGCGTACGCGGATCGTACATGCGTTTTCCAGCGTTCGAAATAGAGTTTGTCGTTCCGCTGA
CCAGGTTTCACGGCTACTGCGATAAGCATATTCTACTGGGGGACTGTAAAGGAATTGTATCCACTCTG
CTTCCTGTCAATTGGCCATGTGCATCTCTGGTTGTTCAGGGTACATTAATAACACACAGCATTAAGTA
GATAAGTCTCCGATATAAGCGAACAGAACGTTGGTCAGAGACTATGGTTCAATTGTAGTGGTATAATG
TTTCCCAGTGCATAAGGAACTCAGGGACACTACTAGCATGGGCGAGGAGAGACCCTTGGGTGGCAACA
CGCAATAAGTGTTACCTAGTACATCAGCACGAGNGAGGGTTTGACCATGTGTTTTTAGACGACCCCGG
TCGCATTGAACCTGGCATTCAGAATGACGTGCTCTCAAGAATNAAGGTCCTCCCACTGAGGGTCATCT
GCTTCGCGGGTCNGCGTATGGTCATAAACAT
>Mang307
GTGTAGCCCAGTTAGACACTTCTAAATCCTTGGTAACAGACATGCAGTCCCCTCGGCAGAGCCCCGCC
CCCCGATGGTACCGGGTTGTTAAGAGGTCCACAACGGGTCGNACGACAACATTAAGGCCTCATGCATG
ACCCAACACGGGGGCGTCCCGGCGCGGCGACCTTGAAGTCCGGGGACAAGTATGGCCCCCCGGGAAGG
ACGCCGTAGTGCATCTGGAGTGCCGATTCGTGGTCCAGAATGCCACCACGATGGTATGAGTNGCAAAG
GTACTTTGGACACCACCCCGTTTTCTATTTCCAGACGCGGCCATTCAGGAACGACGCGAATCGTAATC
TACCTGCCGCGTACGCGGATCGTACATGCGTTTTCCAGCGTTCGAAATAGAGTTTGTCGTTCCGCTGA
CCAGGTTTCACGGCTACTGCGATAAGCATATTCTACTGGGGGACTGTAAAGGAATTGTATCCACTCNG
CTTCCTGTCAATTGGCCATGTGCATCTCTGGTTGTTCAGGGTACATTAATAACACACAGCATTAAGTA
GATAAGTCTCCGATATAAGCGAACAGAACGTTGGTCAGAGACTATGGTTCAATTGTAGTGGTATAATG
TTTCCCAGTGCATAAGGAACTCAGGNACACTACTAGCATGGGCGAGGAGAGACCCTTGGGTGGCAACA
CGCAATAAGTGTTACCTAGTACATCAGCACGAGNGAGGGTTTGACCATGNNTTTTTAGACGACCCCGG
TCGCATTGAACCTGGCATTCAGAATGACGTGCTCTCAAGAATCAAGGTCCTCCCACTGAGGGTCATCT
GCTTCGCGGGTCAGCGTATGGTCATAANNAT
>Mang308
GTGTAGCCCAGTTAGACACTTCTAAATCCTTNGTAACAGACATGCAGTCCCCTCGGCAGAGCCCCGCC
CCCCGATGGTACCGGGTTGTTNAGAGGTCCACAACGGGTCGNACGACAACATTAAGGCCTCANGCATG
ACCCAACNCGGGGGNGTCCCGGCGCGGCGACCTTGAAGTCCGGGGNCAAGTATGGCCCCCCGNGAAGG

ACGCCGTAGTGCATCTGGAGTGCCGATTCGTGGTCCAGANTGCCACCACGATGGTATGAGTGGCAAAG
GTACTTTGGACACCACCCCGTTTNCTANTTCCAGACGCGGCCNTTCAGGAACGACGCGAATCGTAATC
TACCTGCCGCGTACGCGGATCGTACANGCGTTTTCCAGCGTTCGAAATAGAGTTTGTCGTTCCGCTGA
CCAGGTTTCACGGCTACTGCGATAAGCATATTCTACTGGGGGACTGTAAAGGAATTGTATCCACTCNG
CTTCCTGTCAATTGGCCATGTGCATCTCTGGTTGTTCAGGGTACATTAATAACACACAGCATTAAGTA
GATNAGTCTCCGATATAAGCGAACAGAACGTTGGTNAGAGACTATGGTTCAATTGTAGTGGTATAATG
TTTCCCAGTGCATAAGGAACTCAGGNACACTACTAGCATGGGCGAGGAGAGACCCTTGGGTGGCAANA
CGCAATAAGTGTTANCTAGTACATCAGCACGAGNNAGGGTTTGACCATGTGTTTTTAGACGACCCCGG
TCGCATTGAACCTGGCATTCAGAATGACGTGCTNTCAAGAATCAAGGTCCTCCCACTGAGGGTCANCN
GCTTCGCGGGTCAGCGTATGGTCATNAACAT
>Mang309
GTGTAGCCCAGTTAGACACTTCTAAATCCTTNGTAACAGACATGCAGTCCCCTCGGCAGAGCCCCGCC
CCCCGATGGTACCGGGTTGTTAAGAGGTCCACAACGGGTCGNACGACAACATTAAGGCCTCATGCATG
ACCCAACACGGGGGCGTCCCGGCGCGGCGACCTTGAANTCCGGGGACAAGTATGGCCCCCCGGGAAGG
ACGCCGTAGTGCATCTGGAGTGCCGATTCNTGGTCCAGAATGNCACCACGATGGTATGAGTGGCAAAG
GTACTTTGGACACCACCCCGTTTTCTATTTCCAGANGCGGCCATTCAGGAACGACGCGAATCGTAATC
TACCTGCCGCGTNCGCGGATCGTACATGCGTTTTCCAGCGTTCGAAATAGAGTTTGTCGTTCCGCTGA
CCAGGTTNCACGGCTACTGCGATAAGCATATTCTACTGGGGGACTGTAAAGGAATTGTATCCACTCTG
CTTCCTGTCAATTGGCCATGTGCATCTCTGGTTGTTCAGGGTACATTAATAACACACAGCATTAAGTA
GATAAGTCTCCGATATAAGCGAACAGAACGTTGGTCAGAGACTATGGTTCAATTGTAGTGGTATAATG
TTTCCCAGTGCATAAGGAACTCAGGGACACTACTAGCATGGGCGAGGAGAGACCCTTGGGTGGCAACA
CGCAATAAGTGTTACCTAGTACATCAGCACGAGAGAGGGTTTGACCATGNNTTTTTAGACGACCCCGG
TCGCATTGAACCTGGCATTCAGAATGACGTGCTCTCAAGAATCAAGGTCCTCCCACTGAGGGTCATCT
GCTTCGCGGGTCAGCGTATGGTCATAAACAT
>Mang310
GTGTAGCCCAGTTAGACACTTCTAAATCCNTGGTAACAGACATGCAGTCCCCTCGGCAGAGCCCCGCC
CCCCGATGGTACCGGGTTGNTAAGAGGTCCACAACGGGTCGCACGACAACATTAAGGCCTCANGCATG
ACCCAACNCGGNGGCGTCCCGGCGCGGCGACCTTGAANTCCGGGGACAAGTATGGCCCCCCGGGAAGG
ACGCCGTAGTGCATCTGGAGTGCCGATTCGTGGTCNAGAATGCCACCACGATGGTATGAGTGGCAAAG
GTACTTTGGNCACCACCCCGTTTTCTATTTCCAGACGCGGCCATTCAGGAACGACGCGAATCGTAATC
TACCTGCCGCGTACGCGGATCGTACANGCGTTTTCCAGCGTTCGAAATAGAGTTTGTCGTTCCGCTGA
CCAGGTTNCACGGCTACTGCGATAAGCATATTCTACTGGGGGACTGTAAAGGAATTGTATCCACTCNG
CTTCCTGTCAATTGGCCATGTGCATCTCTGGTTGTTCAGGGTACATTAATAACACACAGCATTAAGTA
GATAAGTCTCCGATATAAGCGAACAGAACGTTGGTCAGAGACTATGGTTCAATTGTAGTGGTATAATG
TTTCCCAGTGCATAAGGAACTCAGGNACACTACTAGCATGGGCGAGGAGAGACCCTTGGGTGGCAACA
CGCAATAAGTGTTACCTAGTACATCAGCACGAGAGAGGGTTTGACCATGNNTTTTTAGNNGACCCCGG
TCGCATTGAACCTGGCATTCAGAATGACGTGCTCTCAAGAATCAAGGTCCTCCCACTGAGGGTCATCT
GCTTCGCGGGTCAGCGTATGGTCATAAAAAT
>Mang311
GTGTAGCCCAGTTAGACACTTCTAAATCCTTGGTAACAGACATGCAGTCCCCTCGGCAGAGCCCCGCC
CCCCGATGGTACCGGGTTGTTAAGNGGTCCACAACGGGTCGCACGACAACATTAAGGCCTCATGCATG
ACCCAACACGGGGGCGTCCCGGCGCGGCGACCTTGAAGTCCGGGGACAAGTATGGCCCCCCGGGAAGG
ACGCCGTAGTGCATCTGGAGTGCCGATTNGTGGTCNAGAATGCCACCACGATGGTATGAGTNGCAAAG
GTACTTTGGACACCACCCCGTTTTCTATTTCCAGANGCGGCCATTCAGGAACGACGCGAATCGTAATC
TACCTGCCGCGTACGCGGATCGTACATGCGTTTTCCAGCGTTCGAAATAGAGTTTGTCGTTCCGCTGA
CCAGGTTNCACGGCTACTGCGATAAGCATATTCTACTGGGGGACTGTAAAGGAATTGTATCCACTCNG
CTTCCTGTCAATTGGCCATGTGCATCTCTGGTTGTTCAGGGTACATTAATAACACACAGCATTAAGTA
GATNAGTCTCCGATATAAGCGAACAGAACGTTGGTCAGAGACTATGGTTCAATTGTAGTGGTATAATG
TTTCCCAGTGCATAAGGAACTCAGGGACACTACTAGCATGGGCGAGGAGAGACCCTTGGGTGGCAACA
CGCAATAAGTGTTACCTAGTACATCAGCACGAGAGAGGGTTTGACCATGTGTTTTTAGACGACCCCGG
TCGCATTGAACCTGGCATTCAGAATGACGTGCTCTCAAGAATCAAGGTCCTCCCACTGAGGGTCATCT
GCTTCGCGGGTCAGCGTATNGTCATAAACAT
>Mang399
GTGTAGCCCAGTTAGACACTTCTAAATCCTTGGTAACAGACATGCAGTCCCCTCGGCAGAGNCCCGCC

CCCCNATGGTACCGGGTTGTTAAGAGGTCCACAACGGGTCGCACGACAACATTAAGGCCTCATGCATG
ACCCAACACGGGGGCGTCCCGGCGNGGCGACCTTGNAGTCCGGGGNCAAGTATGGCCCCCCGGGAAGG
ACGCCGTAGTGCATCTGGAGTGCCGATTCNTGGTCCAGAATGCCACCACGATGGTATGAGTNGCAAAG
GTACTTTGGACACCACCCCGTTTTCTATTTCCAGACGCGGCCATTCAGGAACGACGCGAATCGTAATC
TACCTGCCGCGTACGCGGATCGTACATGCGTTTTCCAGCGTTCGAAATAGAGTTTGTCGTTCCGCTGA
CCAGGTTNCACGGCTACTNCGATAAGCATATTCTACTGGGGGACTGTAAAGGAATTGTATCCACTCTG
CTTCCTGTCAATTGGCCATGTGCATCTCTGGTTGTTCAGGGTACATTAATAACACACAGNATTAAGTA
GATAAGTCTCCGATATAAGCGAACAGAACGTTGGTCAGAGACTATGGTTCAATTGTAGTGGTATAATG
TTTCCCAGTGCATAAGGAACTCAGGGACACTACTAGCATGGGCGAGGAGAGACCCTTGGGTGGCAACA
CGCAATAAGTGTTACCTAGTACATCAGCACGAGAGAGGGTTTGACCATGTGTTTTTAGACGACCCCGG
TCGCATTGAACCTGGCATTCAGAATGACGTGCTCTCAAGAATCAAGNTCCTCCCACTGAGGGTCATCT
GCTTCGCGGGTCAGCGTATGGTCANAAANNT
>Nung47
GTGNAGCCCAGTTAGACACNTCTAAATCCTTGGTAACAGACATGCAGTCCCCTCGGCAGAGCCCCGCC
CCCCNATGGTACCGGGTTGTTAAGAGGTCCACAACAGGTCNCACGACAACATTAAGGCCTCATGCATG
ACCCAANACGGNGGCGTCCCGGCGCGGCGACCTTGAAGTCCGGGGNCAAGTATGGCCCCCCGGGAAGG
ACGCCGTAGTGCATCTGGAGTGCCGATTCGTGGTCNAGANTGCCACCACGATGGTATGAGTNGCAAAG
GTACTTNGGACACCACCCCGTTTTCTATTTCCAGACGCGGCCATTCAGGAACGACGCGAATCGTAATC
TACCTGCCGCGTACGCGGATCGTACANGCGTTTTCCAGCGTTCGAAATAGAGTTTGTCGTTCCGCCGA
CCAGGTTTCACGGCTACTGCGATAAGCATATTCTACTGGGGGACTGTAAAGGAATTGTATCCACTCNA
CTTCCTGTCAATTGGCCATGTGCATCTCTGGTTGTTCAGGGTACATTAATAACACACAGCATNAAGTA
GATAAGTCTCCGATATAAGCGAACAGANCGTTGGTCAGAGAATGTGGTTCAATTGTAGTGGTATAATG
TTTCCCAGTGCATAAGGAACTTAGGGACATTACTAGCATGGGCGAGGAGAGACCCTTGGGTGGCAACA
CGCAATAAGTGTTACCTAGTACACCAGCACGAGAGAGGGTTTGACCATGTGTTTTTAGACGACCCCGG
TCGCATTGAACCTGGCATTCAGAATGACGTGCTCTCAAGAATCAAGNTCCTCACACTGAGGGTCATCT
GCTTCGCGGGTCAGCGTATGGTCATAAACAT
>Nung48
GTGTAGCTCAGTTAGACACTTNNAAATCCNTGGTAACAGACATGCAGTCCCCTCGGCAGAGCCCCGCC
CCCCGGTGGTACCGGGTTGNNAAGAGGTCCACCACGGGTCGNACGACAANNTCAAGGCCTNANGCATG
ANCCAACNCGGGGANGTACCGGCGNGGCGACCTTGAANGTCGGGGNCAAGTATGTCCCCCCGGGAAGG
ACGCCGTAGTGCATCTGGAGTGCCGATTCGTGGTCCAGAATGCCANCACGATGGTATGAGTNGCAAAG
GTATTTTNNNCACCACCCCGTTTTCTATTTCCAGACGCGGCCATTCAGGAACGACGCGAATCGTAATC
TACCTGCCGCGTNCGCGGATCGTACATGCGTNTTTCAGCGTTCGAAATAGAGTTTGTCGTTCCGCTGA
CCAGGTTNCACGGCTACTNCGATAAGCATATTCTACGGGGGGACTGTAAAGNAATTGTATCCACNCTG
CTCCCTGTCAATTGGNCATGTGCATCTCTGGTTGTTTAGGGTACATTAATAACACACAGNATNAGNTA
GATAAGTCTCCGATATAAGCGAACAGANCGTTGGTCAAAGACCGTGGTTCAATTGTAGTGGTATAACA
TTTCCCAGTGCATAAGGAACCCAGGGACACTACTAGCATGGGCGAGGAGAGACCCTTGGGTGGCAACA
CGCAATAAGTGTTACCCAGTACACCAGCACGATNNAGGGTTTGACCATGNNTTTTTAGACGACCCCGG
TCGCATTGAACCTGGCATTCAGAATGACGTGCTCTCAAGAGTCAAGNTCCNCCCACTGAGGGNCANCT
GCTTCGCGNNNTAGCGTATGGTCANANNNNT
>Nung49
GTGTAGCCCAGTCAGACACTTCTAAATCCTTGGTAACAGACATGCAGTCCCCTCGGCAGAGCCCCGCC
CCCCGATGGTACCGGGTTGTTNAGAGGTCCACAACGGGTCGCACGACAACATTAAGGCCTCANGCATG
ACCCAACACGGGGGNGTCCCGGCGNGGCGACCTTGAAGTCCGGGGNCAAGTATGGCCCCCCGGGAAGG
ACGCCGTAGTGCATCTGGAGTGCCGATTCGTGGTCNAGAATGCCACCACGATGGTATGAGTGGCAAAG
GTACTTTGGACACCACCCCGTTTTCTATTTCCAGACGCGGCCATTCAGGAACGACGCGAATCGTAATC
TACCTGCCGCGTACGCGGATCGTACATGCGTTTTCCAGCGTTCGAAATAGAGTTTGTCGTTCCGCTGA
CCAGGTTNCACGGCTACTGCGATAAGCATATTCTACTGGGGGACTGTAAAGGAATTGTATCCATTCTG
CTTCCTGTCAATTGGCCATGTGCATCTCTGGTTGTTCAGGGTACATTAATAACACACAGCAGTAAGTA
GGTNAGTCTCCGATATAAGCGAACAGAACGTTTGTCAGAGACTGTGGTTCAATTGTAGTGGTATAATG
TTTCCCAGTGCATAAGGAACTCAGGGACACTACTAGCATGGGCGAGGAGAGACCCTTGGGTGGCAACA
CGCAATAAGTGTTACCTAGTACACCAGCACGAGAGAGGGTTTGACCATGNGTTTTTAGACGACCCCGG
TCGCATTGAACCTGGCATTCAGAATGACGTGCTCTCAAGAATCAAGGTCCTCCCACTGAGGGTCATCT
GCTTCGCGGGTCAGCGTATNGTCATAAACAT

>Nung664
GTGTAGCCCAGTTAGACACTTCTAAATCCNTGGTAACAGACATGCAGTCCCCTCNNCAGAGCCCCGCC
CCCCGATGGTACCGGGTTGNTAAGAGGTCCACAACGGNTCNCACGACAANNTTAAGGCCTCATGCATG
ACNCANNACGGNGGNGTCCCGGCGNGGCGACCTTGAAGTCCGGGGNCAAGTATGGCCCCCCGGGAAGG
ACGCNGTAGTGCATCTGGAGTGCCGATTCGTGGTCCAGAATGCCACCACGATGGTATGAGTNGCAAAG
GTACTTNGGACACNACCCCGTTTTCTANTTCCAGANGCGGCCATTCAGGAACGACGCGAATCGTAATC
TACCTGCCGCGTACGCGGATCGTACANGCGTTTTCCAGCGTTCGAAATAGAGTTTGTCGTTCCGCTGA
CCAGGTTNCACGGCTACTNCGATAAGCATATTCTACTGGGGGACTGTAAAGGAATTGTATCCACTCTG
CTTCCTGTCAATTGGCCATGTGCATCTCTNGTTGTTCAGGGTACATTAATAACACACAGCATNAAGTA
GATAAGTCTCCGATATAAGCGAACAGAACGTTGGTCAGAGACTATGGTTCAATTGTAGTGGTATAATG
TTTCCCAGTGCATAAGGAACTCAGGGACACTACTAGCATGGNCGAGGAGAGACCCTTGGGTGGCAANA
CGCGATAAGTGTTACCTAGTACATCAGCACGAGNNAGGGTTTGACCATGTGTTTTTAGACGACCCCGG
TCGCATTGAACCTGGCATTCAGAATGACGTGCTCTCAAGAATCAAGNTCCTCCCACTGAGGGTCATCT
GCTTCGCGGGTCAGCGTATGGTCATAAACAT
>Nung665
GTGTAGCCCAGTTAGACACTTCTAAATCCNTGGTAACAGACATGCAGTCCCCTCGGCAGAGCCCCGCC
CCCCGATGGTACCGGGTTGTTAAGAGGTCCACAACGGGTCGCACGACAACNTTAAGGCCTCANGCATG
ACCCAACACGGGGGCGTCCCGGCGCGGCGACCTTGAAGTCCGGGGACAAGTATGGCCCCCCGGGAAGG
ACGCCGTAGTGCATCTGGAGTGCCGATTCGTGGTCCAGAATGCCACCACGATGGTATGAGTGGCAAAG
GTACTTTGGACACCACCCCGTTTTCTANTTCCAGACGCGGCCATTCAGGAACGACGCGAATCGTAATC
TACCTGCCGCGTNCGCGGATCGTACATGCGTTTTCCAGCGTTCGAAATAGAGTTTGTCGTTCCGCTGA
CCAGGTTNCACGGCTACTGCGATAAGCATATTCTACTGGGGGACTGTAAAGGAATTGTATCCACTCTG
CTTCCTGTCAATTGGCCATGTGCATCTCTGGTTGTTCAGGNNACATTAATAACACACAGCATTAAGTA
GATAAGTCTCCGATATAAGCGAACAGAACGTTGGTCAGAGACTATGGTTCAATTGTAGTGGTATAATG
TTTCCCAGTGCATAAGGAACTCAGGGACACTACTAGCANGGGCGAGGAGAGACCCTTGGGTGGCAACA
CGCAATAAGTGTTACCTAGTACATCAGCACGAGAGAGGGTTTGACCATGTGTTTNTAGACGACCCCGG
TCGCATTGAACCTGGCATTCAGAATGACGTGCTCTCAAGAATCAAGGTCCNCCCACTGAGGGTCATCT
GCTTCGCGNGTCNGCGTATGGTCANAANNAT
>Nung666
GTGNAGCCCAGTTAGACACTTCTAAATCCNTGGTAACAGACATGCAGTCCCCTCNNCAGAGCNCCGCC
CCCCGATGGTACCGGGTTGTTAAGAGGTCCACAACGGGTCGCACGACANCNTTAAGGCCTCANGCATG
ACCCANNACNGGGGCGTCCCGGNGCGGCGACCTTGAANTCCGGGGNCAAGTATGGCCCCCCGGGAAGG
ACGCCGTAGTGCATCTGGAGTGCCGATTNGTNGTCNAGANTGNCACCACGATGGTATGAGTGGCAAAG
GTACTTNGGACACCACCCCGTTTTCTATTTCCAGACGCGGCCATTCAGGAACGACGCGAATCGTAATC
TACCTGCCGCGTACGCGGATNGTACANGCGTTTTNCAGCGTTCGAAATAGAGTTTGTCGTTCCGCTGA
CCNGGTTNCACGGCTACTNCGATAAGCATATTCTACTGGGGGACTGTAAAGGAATTGTATCCACTCNG
CTTCCTGTCAATTGGCCATGTGCATCTCTGGTTGTTCAGGNNACATTAATAACACACAGCATNAAGTA
GATNAGTCNCCGATATAAGCGAACAGAACGTTGGTNAGAGACTATGGTTCAATTGTAGTGGTATAATG
TTTCCCAGTGCATAAGGAACTCAGGNACACTACTAGCANGGGCGAGGAGAGACCCTTGGGTGGCAACA
CGCGATAAGTGTTANCTAGTACATCAGCACGAGNNAGGGTTTGACCATGTGTTTNTAGACGACCCCGG
TCGCATTGAACCTGGCATTCAGAATGACGTGCTNTCAAGAATCAAGGTCCTCCCACTGAGGGTCATCN
GCTTCGCGGGTCAGCGTATNGTCATAANNAT
>Nung667
GTGTAGCCCAGTTAGACACTTCTAAATCCTTGGTAACAGACATGCAGTCCCCTCGGCAGAGCCCCGCC
CCCCGATGGTACCGGGTTGTTAAGAGGTCCACAACGGGTCGCACGACAACATTAAGGCCTCATGCATG
ACCCAACACGGGGGCGTCCCGGCGCGGCGACCTTGAAGTCCGGGGACAAGTATGGCCCCCCGGGAAGG
ACGCCGTAGTGCATCTGGAGTGCCGATTCGTGGTCCAGAATGCCACCACGATGGTATGAGTGGCAAAG
GTACTTTGGACACCACCCCGTTTTCTATTTCCAGACGCGGCCATTCAGGAACGACGCGAATCGTAATC
TACCTGCCGCGTNCGCGGATCGTACATGCGTTTTCCAGCGTTCGAAATAGAGTTTGTCGTTCCGCTGA
CCAGGTTTCACGGCTACTGCGATAAGCATATTCTACTGGGGGACTGTAAAGGAATTGTATCCACTCTG
CTTCCTGTCAATTGGCCATGTGCATCTCTGGTTGTTCAGGGTACATTAATAACACACAGCATTAAGTA
GATAAGTCTCCGATATAAGCGAACAGAACGTTGGTCAGAGACTATGGTTCAATTGTAGTGGTATAATG
TTTCCCAGTGCATAAGGAACTCAGGGACACTACTAGCATGGGCGAGGAGAGACCCTTGGGTGGCAACA
CGCGATAAGTGTTACCTAGTACATCAGCACGAGAGAGGGTTTGACCATGTGTTTTTAGACGACCCCGG

TCGCATTGAACCTGGCATTCAGAATGACGTGCTCTCAAGAATCAAGGTCCTCCCACTGAGGGTCATCT
GCTTCGCGGGTCAGCGTATGGTCATAAACAT
>Nung668
GTGTAGCCCAGTTAGACACTTCTAAATCCNTNGTAACAGACATGCAGTCCCCTCNNCAGAGCCCCGCC
CCCCNATGGTACCGGGTTGTTAAGAGGTCCACCACGGGTCNCACGACAACATTAAGGCCTCANGCATG
ACCCANCACGGGGGCGTCCCGGCGNGGCGACCTTGAAGTCCGGGGNCAAGTATGGCCCCCCGGGAAGG
ACGCCGTAGTGCATCTGGAGTGCCGATTCGTGGTCCAGAATGCCACCACGATGGTATGAGTGGCAAAG
GTACTTNGGACACCACCCCGTTTNCTANTTCCAGACGCGGCCATTCAGGAACGACGCGAATCGTAATC
TACCTGCCGCGTACGCGGATCGTATANACGTTTTCCAGCGTTCGAAATAGAGTTTGTCGTTCCGCTGA
CCNGGTTTCACGGCTACTGCGATAAGCATATTCTACTGGGGGACTGTAAAGGAATTGTATCCACTCTG
CTTCCTGTCAATTGGCCATGTGCATCTCTGGTTGTTCAGGNNACATTAATAACACACAGCATTAAGTA
GATAAGTCTCCGATATAAGCGAACAGANCGTTGGTCAGAGACTGTGGTTCAATTGTAGTGGTATAATG
TTTCCCAGTGCATAAGGAACTCAGGNACACTACTAGCATGGNCGAGGAGAGACCCTTGGGTGGCAACA
CGCAATAAGTGTTACCTAGTACACCAGCACGAGANANGGTTTGACCATGTGTTTTTAGACGACCCCGG
TCGCATTGAACCTGGCATTCAGAATGACGTGCTCTCAAGAGTCAAGGTCCTCCCACTGAGGGTCATCT
GCTTCGCGGGTCNGCGTATNGNCATAAACAT
>Nung669
GTGTAGCCCAGTTGGACACNTCTAAATCCTTGGTAACAGACATGCAGTCCCCTCNNCAGAGCNCCGCC
CCCCNGTGGTACCGGGNTGNTNAGAGGTCCACCACGGGTCGCACGACAACNTCAAGGCCTCATGCATG
ACCCAACNCGGGGANGTACCGGNGNGGCGACCTTGNAGGTCGGGGACAAGTATGTCCCCCCGGGAAGG
ACGCCGTAGTGCATCTGGAGTGCCGATTNGTGGTCCAGAATGCCACCACGATGGTATGAGTNGCAAAG
GTATTTTGGACACCACCCCGTTTTCTATTTCCAGACGCGGCCATTCAGGAACGACGCGAATCGTAATC
TACCTGCCGCGTNCGCGGATCGTACATGCGTTTTTCAGCGTTCGANATNGAGTTTGTCGTTCCGCTGA
CCAGGTTNCACGGCTACTGCGATAAGCATATTCTACGGGGGGACTGTAAAGGAATTGTATCCACTCTG
CTCCCTGTCAATTGGCCATGTGCATCTCTGGTTGTTTAGGGTACATTAATAACACACAGNATGAGGTA
GATAAGTCTCCGATATAAGCGAACAGAACGTTGGTCAAAGACCGTGGTTCAATTGTAGTGGTATAACA
TTTCCCAGTGCATAAGGAACCCAGGGACACTATTAGCATGGNCGAGGAGAGACCCTTGGGTGGCAACA
CGCAATAAGTGTTACCCAGTACACCAGCACGATAGANGGTTTGACCATGTGNNTNTAGACGACCCCGG
TCGCATTGAACCTGGCATTCAGAATGACGTGCTCTCAAGAGTCAANNTCCTCCCACTGAGGGTCATCN
GCTTCGCGNGTTAGCGTATNGTCATAAACAT
>Nung670
GTGTAGCCCAGTTAGACACTTCTAAATCCNTGGTAACAGACATGCAGTCCCCTCGGCAGAGNCCCGCC
CCCCNATGGTACCGGGTTGTTAAGAGGTCCACAACGGGTCGCACGACAACATTAAGGCCTCATGCATG
ACCCAACACNGGGGCGTCCCGGNGCGGCGACCTTGAAGTCCGGGGNCAAGTATGGCCCCCCGGGAAGG
ACGCCGTAGTGCATCTGGAGTGCCGATTCGTGGTCCAGAATGNCACCACGATGGTATGAGTGGCAAAG
GTACTTTGGACACCACCCCGTTTTCTATTTCCAGACGCGGCCATTCAGGAACGACGCGAATCGTAATC
TACCTGCCGCGTNCGCGGATCGTACANGCGTTTTCCAGCGTTCGAAATAGAGTTTGTCGTTCCGCTGA
CCAGGTTNCACGGCTACTGCGATAAGCATATTCTACTGGGGGACTGTAAAGGAATTGTATCCACTCNG
CTTCCTGTCAATTGGNCATGTGCATCTCTGGTTGTTCAGGGTACATTAATAACACACAGCATTAAGTA
GATNAGTCTCCGATATAAGCGAACAGAACGTTGGTCAGAGACTATGGTTCAATTGTAGTGGTATAATG
TTTCCCAGTGCATAAGGAACTCAGGNACACTACTAGCANGGGCGAGGAGAGACCCTTGGGTGGCAACA
CGCAATAAGTGTTACCTAGTACATCAGCACGAGNGAGGGTTTGACCATGNNTTTTTAGACGACCCCGG
TCGCATTGAACCTGGCATTCAGAATGACGTGCTCTCAAGAATCAAGGTCCTCCCACTGAGGGTCATCT
GCTTCGCGGGTCAGCGTATGGTCANNAACAT
>Nung671
GTGTAGCCCAGTTGGACACTTCTAAATCCTTNGTAACAGACATGCAGTCCCCTCNNCAGAGCCCCGCC
CCCCGGTGGTACCGGGTTGTTAAGAGGTCCACCACGGGTCGCACGACAACATCAAGGCCTCATGCATG
ACCCAACGCGGGGACGTACCGGCGCGGCGACCTTGAANGTCGGGGACAAGTATGTCCCCCCGGGAAGG
ACGCCGTAGTGCATCTGGAGTGCCGATTCGTGGTCCAGAATGCCACCACGATGGTATGAGTGGCAAAG
GTATTTTGGACACCACCCCGTTTTCTATTTCCAGACGCGGCCATTCAGGAACGACGCGAATCGTAATC
TACCTGCCGCGTACGCGGATCGTACATGCGTTTTTCAGCGTTCGAAATAGAGTTTGTCGTTCCGCTGA
CCAGGTTNCACGGCTACTNCGATAAGCATATTCTACGGGGGGACTGTAAAGGAATTGTATCCACTCTG
CTCCCTGTCAATTGGCCATGTGCATCTCTGGTTGTTTAGGGTACATTAATAACACACAGCATGAGGTA
GATAAGTCTCCGATATAAGCGAACAGAACGTTGGTNAAAGACCGTGGTTCAATTGTAGTGGTATAACA

TTTCCCAGTGCATAAGGAACCCAGGGACACTATTAGCATGGGCGAGGAGAGACCCTTGGGTGGCAACA
CGCAATAAGTGTTACCCAGTACACCAGCACGATNGAGGGTTTGACCATGTGTTTTTAGACGACCCCGG
TCGCATTGAACCTGGCATTCAGAATGACGTGCTCTCAAGAGTCAAGGTCCTCCCACTGAGGGTCATNT
GCTTCGCGNGTTNGCGTATNGTCATAANNAT
>Nung672
GTGTAGCCCAGTTAGACACTTCTAAATCCTTGGTAACAGACATGCAGTCCCCTCGGCAGAGCCCCGCC
CCCCGATGGTACCGGGTTGTTAAGAGGTCCACAACGGGTCGNACGACAACNTTAAGGCCTCATGCATG
ACCCANCACGGGGGCGTCCCGGNGCGGCGACCTTGAAGTCCGGGGNCAAGTATGGCCCCCCGGGAAGG
ACGCCGTAGTGCATCTGGAGTGCCGATTNGTGGTCCAGAATGNCACCACGATGGTATGAGTGGCAAAG
GTACTTTGGACACCACCCCGTTTTCTANTTCCAGACGCGGCCATTCAGGAACGACGCGAATCGTAATC
TACCTGCCGCGTACGCGGATCGTACATGCGTTTTCCAGCGTTCGAAATAGAGTTTGTCGTTCCGCTGA
CCAGGTTTCACGGCTACTGCGATAAGCATATTCTACTGGGGGACTGTAAAGGAATTGTATCCACTCNG
CTTCCTGTCAATTGGCCATGTGCATCTCTNGTTGTTCAGGGTACATTAATAACACACAGCATTAAGTA
GATAAGTCTCCGATATAAGCGAACAGAACGTTGGTCAGAGACTATGGTTCAATTGTAGTGGTATAATG
TTTCCCAGTGCATAAGGAACTCAGGGACACTACTAGCATGGGCGAGGAGAGACCCTTGGGTGGCAANA
CGCGATAAGTGTTACCTAGTACATCAGCACGAGAGANGGTTTGACCATGTGTTTTTAGACGACCCCGG
TCGCATTGAACCTGGCATTCAGAATGACGTGCTCTCAAGAATCAAGGTCCTCCCACTGAGGGTCATCT
GCTTCGCGGGTCAGCGTATGGTCATAAACAT
>Nung673
GTGTAGCCCAGTTAGACACTTCTAAATCCTTGGTAACAGACATGCAGTCCCCTCGGCAGAGCCCCGCC
CCCCGATGGTACCGGGTTGTTAAGAGGTCCACAACGGNTCGCACGACAACATTAAGGCCTCATGCATG
ACCCANNACGGGGGNGTCCCGGCGCGGCGACCTTGNAGTCCGGGGNCAAGTATGGCCCCCCGGGAAGG
ACGCCGTAGTGCATCTGGAGTGCCGATTCGTGGTCCAGAATGNCACCACGATGGTATGAGTGGCAAAG
GTACTTTGGACACCACCCCGTTTTCTANTTCCAGACGCGGCCNTTCAGGAACGACGCGAATCGTAATC
TACCTGCCGCGTNCGCGGATCGTACATGCGTTTTCCAGCGTTCGAAATAGAGTTTGTCGTTCCGCTGA
CCAGGTTNCACGGCTACTGCGATAAGCATATTCTACTGGGGGACTGTAAAGGAATTGTATCCACTCTG
CTTCCTGTCAATTGGCCATGTGCATCTCTNGTTGTTCAGGGNACATTAATAACACACAGCATNAAGTA
GATAAGTCTCCGATATAAGCGAACAGAACGTTGGTCAGAGACTATGGTTCAATTGTAGTGGTATAATG
TTTCCCAGTGCATAAGGAACTCAGGNACACTACTAGCANGGGCGAGGAGAGACCCTTGGGTGGCAACA
CGCAATAAGTGTTNCCTAGTACATCAGCACGAGNGAGGGTTTGACCATGNNTTTTAGNNGACCCCGG
TCGCATTGAACCTGGCATTCAGAATGACGTGCTCTCAAGAATCAAGGTCCTCCCACTGAGGGTCATCT
GCTTCGCGNGTCAGCGTATGGTCATAAACNT
>Nung674
GTGTAGCCCAGTTAGACACTTCTAAATCCTTGGTAACAGACATGCAGTCCCCTCNNCAGAGCCCCGCC
CCCCGATGGTACCGGGTTGTTNAGAGGTCCACAACGGGTCGCACGACAACATTAAGGCCTCATGCATG
ANNCANNACGGGGGCGTCCCGGCGCGGCGACCTTGAAGTCCGGGGACAAGTATGGCCCCCCGGGAAGG
ACGCCGTAGTGCATCTGGAGTGCCGATTCGTGGTCCAGAATGNCACCACGATGGTATGAGTNGCAAAG
GTACTTTGGACACCACCCCGTTTTCTANTTCCAGACGCGGCCNTTCAGGAACGACGCGAATCGTAATC
TACCTGCCGCGTNCGCGGATCGTACATGCGTTTTNCAGCGTTCGAAATAGAGTTTGTCGTTCCGCTGA
CCAGGTTNCACGGCTACTGCGATAAGCATATTCTACTGGGGGACTGTAAAGGAATTGTATCCACTCTG
CTTCCTGTCAATTGGCCATGTGCATCTCTGGTTGTTCAGGGTACATTAATAACACACAGCATTAAGTA
GATAAGTCTCCGATATAAGCGAACAGAACGTTGGTCAGANACTATGGTTCAATTGTAGTGGTATAATG
TTTCCCAGTGCATAAGGAACTCAGGNACACTACTAGCANGGGCGAGGAGAGACCCTTGGGTGGCAANA
CGCGATAAGTGTTACCTAGTACATCAGCACGAGNNANGGTTTGACCATGTGTTTTTAGNNGACCCCGG
TCGCATTGAACCTGGCATTCAGAATGACGTGCTCTCAAGAATCAAGGTCCNCCCACTGAGGGTCATCN
GCTTCGCGGGTCNGCGTATGGTCATAAACAT
>Nung675
GTGTAGCCCAGTTAGACACTTCTAAATCCTTGGTAACAGACATGCAGTCCCCTCGGCAGAGCCCCGCC
CCCCNATGGTACCGGGTTGTTAAGAGGTCCACAACGGGTCGCACGACAACATTAAGGCCTCATGCATG
ACCCAACACGGGGGCGTCCCGGCGCGGCGACCTTGAAGTCCGGGGACAAGTATGGCCCCCCGGGAAGG
ACGCCGTAGTGCATCTGGAGTGCCGATTCGTGGTCCAGAATGCCACCACGATGGTATGAGTGGCAAAG
GTACTTTGGACACCACCCCGTTTTCTATTTCCAGACGCGGCCATTCAGGAACGACGCGAATCGTAATC
TACCTGCCGCGTACGCGGATCGTACATGCGTTTTCCAGCGTTCGAAATAGAGTTTGTCGTTCCGCTGA
CCAGGTTTCACGGCTACTGCGATAAGCATATTCTACTGGGGGACTGTAAAGGAATTGTATCCACTCTG

CTTCCTGTCAATTGGCCATGTGCATCTCTGGTTGTTCAGGGTACATTAATAACACACAGCATTAAGTA
GATAAGTCTCCGATATAAGCGAACAGAACGTTGGTCAGAGACTATGGTTCAATTGTAGTGGTATAATG
TTTCCCAGTGCATAAGGAACTCAGGGACACTACTAGCATGGGCGAGGAGAGACCCTTGGGTGGCAACA
CGCAATAAGTGTTACCTAGTACATCAGCACGAGAGAGGGTTTGACCATGTGTTTTTAGACGACCCCGG
TCGCATTGAACCTGGCATTCAGAATGACGTGCTCTCAAGAATCAAGGTCCTCCCACTGAGGGTCATCT
GCTTCGCGGGTCAGCGTATGGTCATAAACAT
>Nung676
GTGTAGCCCAGTTAGACACTTCTAAATCCTTGGTAACAGACATGCAGTCCCCTCGGCAGAGCCCCGCC
CCCCGATGGTACCGGGTTGTTAAGAGGTCCACAACGGGTCGCACGACAACNTTAAGGCCTCATGCATG
ACCCAACACGGGGGCGTCCCGGNGNGGCGACCTTGAAGTCCGGGGACAAGTATGGCCCCCCGGGAAGG
ACGCCGTAGTGCATCTGGAGTGCCGATTCGTGGTCCAGAATGNCACCACGATGGTATGAGTNGCAAAG
GTACTTTGGACACCACCCCGTTTTCTATTTCCAGACGCGGCCATTCAGGAACGACGCGAATCGTAATC
TACCTGCCGCGTACGCGGATCGTACATGCGTTTTCCAGCGTTCGAAATAGAGTTTGTCGTTCCGCTGA
CCAGGTTTCACGGCTACTGCGATAAGCATATTCTACTGGGGGACTGTAAAGGAATTGTATCCACTCTG
CTTCCTGTCAATTGGCCATGTGCATCTCTGGTTGTTCAGGGTACATTAATAACACACAGCATTAAGTA
GATAAGTCTCCGATATAAGCGAACAGAACGTTGGTCAGAGACTATGGTTCAATTGTAGTGGTATAATG
TTTCCCAGTGCATAAGGAACTCAGGGACACTACTAGCATGGGCGAGGAGAGACCCTTGGGTGGCAACA
CGCGATAAGTGTTACCTAGTACATCAGCACGAGNNAGGGTTTGACCATGTGTTTTTAGACGACCCCGG
TCGCATTGAACCTGGCATTCAGAATGACGTGCTCTCAAGAATCAAGGTCCNCCCACTGAGGGTCATCT
GCTTCGCGGGTCAGCGTATGGTCATAAACNT
>Nung677
GTGTAGCCCAGTTGGACACTTCTAAATCCTTGGTAACAGACATGCAGTCCCCTCNNCAGAGCCCCGCC
CCCCNGTGGTACCGGGTTGNTAAGAGGTCCACCACGGGTCGCACGACNNNATCAAGGCCTCATGCATG
ACCCAACGCGGGGACGTACCGGCGNGGCGACCTTGNANGTCGGGGACAAGTATGTCCCCCCGGGAAGG
ACGCCGTAGTGCATCTGGAGTGCCGATTCGTGGTCNAGAATGNCACCACGATGGTATGAGTNGCAAAG
GTATTTTGGACACCACCCCGTTTTCTANTTCCAGACGCGGCCATTCAGGAACGACGCGAATCGTAATC
TACCTGCCGCGTNCGCGGATCGTACATGCGTTTTTCAGCGTTCGANATAGAGTTTGTCGTTCCGCTGA
CCAGGTTTCACGGCTACTGCGATAAGCATATTCTACGGGGGGACTGTAAAGGAATTGTATCCACTCTG
CTCCCTGTCAATTGGCCATGTGCATCTCTGGTTGTTTAGGGTACATTAATAACACACAGCATGAGGTA
GATAAGTCTCCGATATAAGCGAACAGANCGTTGGTCAAAGACCGTGGTTCAATTGTAGTGGTATAACA
TTTCCCAGTGCATAAGGAACCCAGGGACACTATTAGCATGGGCGAGGAGAGACCCTTGGGTGGCAACA
CGCAATAAGTGTTACCCAGTACACCAGCACGATAGANGGTTTGACCATGTGTTTTTAGACGACCCCGG
TCGCATTGAACCTGGCATTCAGAATGACGTNCTNTCAAGAGTCAAGGTCCTCCCACTGAGGGTCATCT
GCTTCGCGNGTTNGCGTATNGTCATAAACAT
>Nung678
GTGTAGCCCAGTTAGACACTTCTAAATCCTTGGTAACAGACATGCAGTCCCCTCGGCAGAGCNCCGCC
CCCCGATGGTACCGGGTTGTTAAGAGGTCCACAACGGGTCGNACGACAACATTAAGGCCTCATGCATG
ACCCAACACGGGGGCGTCCCGGCGCGGCGACCTTGAAGTCCGGGGNCAAGTATGGCCCCCCGGGAAGG
ACGCCGTAGTGCATCTGGAGTGCCGATTCGTGGNCCAGAATGNCACCACGATGGTATGAGTGGCAAAG
GTACTTTGGACACCACCCCGTTTTCTATTTCCAGACGCGGCCATTCAGGAACGACGCGAATCGTAATC
TACCTGCCGCGTACGCGGATCGTACATGCGTTTTCCAGCGTTCGAAATAGAGTTTGTCGTTCCGCTGA
CCAGGTTTCACGGCTACTGCGATAAGCATATTCTACTGGGGGACTGTAAAGGAATTGTATCCACTCNG
CTTCCTGTCAATTGGCCATGTGCATCTCTNGTTGTTCAGGGTACATTAATAACACACAGCATNAAGTA
GATAAGTCTCCGATATAAGCGAACAGANCGTTGGTNAGAGACTATGGTTCAATTGTAGTGGTATAATG
TTTCCCAGTGCATAAGGAACTCAGGGACACTACTAGCATGGGCGAGGAGAGACCCTTGGGTGGCAACA
CGCAATAAGTGTTACCTAGTACATCAGCACGAGANAGGGTTTGACCATGTGTTTTTAGACGACCCCGG
TCGCATTGAACCTGGCATTCAGAATGACGTGCTCTCAAGAATCAAGGTCCTCCCACTGAGGGTCATCT
GCTTCGCGGGTCNGCGTATGGTCANNANNAT
>Nung679
GTGTAGCCCAGTTAGACACTTCTAAATCCNTGGTAACAGACATGCAGTCCCCTCGGCAGAGCCCCGCC
CCCCGATGGTACCGGGTTGTTAAGAGGTCCACAACGGGTCGCACGACANCNTTAAGGCCTCATGCATG
ACCCAANNCGGGGGCGTCCCGGCGNGGCGACCTTGAAGTCCGGGGNCAAGTATGGCCCCCCGGGAAGG
ACGCCGTAGTGCATCTGGAGTGCCGATTCNTGGTCNAGAATGCCACCACGATGGTATGAGTGGCAAAG
GTACTTTGGACACCACCCCGTTTTCTANTTCCAGACGCGGCCATTCAGGAACGACGCGAATCGTAATC

TACCTGCCGCGTACGCGGATCGTACATGCGTTTTCCAGCGTTCGAAATAGAGTTTGTCGTTCCGCTGA
CCAGGTTTCACGGCTACTGCGATAAGCATATTCTACTGGGGGACTGTAAAGGAATTGTATCCACTCNG
CTTCCTGTCAATTGGCCATGTGCATCTCTGGTTGTTCAGGNNACATTAATAACACACAGCATTAAGTA
GATAAGTCTCCGATATAAGCGAACAGANCGTTGGTCAGAGACTATGGTTCAATTGTAGTGGTATAATG
TTTCCCAGTGCATAAGGAACTCAGGGACACTACTAGCATGGGCGAGGAGAGACCCTTGGGTGGCAACA
CGCGATAAGTGTTACCTAGTACATCAGCACGAGAGAGGGTTTGACCATGNNTTTTTAGACGACCCCGG
TCGCATTGAACCTGGCATTCAGAATGACGTGCTCTCAAGAATCAAGGTCCTCNCACTGAGGGTCATCT
GCTTCGCGNNNCAGCGTATGGNCATAAACAT
>Nung68
GTGTAGCCCAGTTAGACACTTCTAAATCCTTGGTAACAGACATGCAGTCCCCTCNNCAGAGCCCCGCC
CCCCGATGGTACCGGGTTGTTNAGAGGTCCACAACGGGTCGCACGACAACATTAAGGCCTCATGCATG
ACCCAACACNGGGGCGTCCCGGCGCGGCGACCTTGAANTCCGGGGACAAGTATGGCCCCCCGGGAAGG
ACGCCGTAGTGCATCTGGAGTGCCGATTCNTGGTCNAGAATGNCACCACGATGGTATGAGTNGCAAAG
GTACTTTGGACACCACCCCGTTTTCTANTTCCAGACGCGGCCATTCAGGAACGACGCGAATCGTAATC
TACCTGCCGCGTNCGCGGATCGTACANGCGTTTTCCAGCGTTCGAAATAGAGTTTGTCGTTCCGCTGA
CCAGGTTTCACGGCTACTGCGATAAGCATATTCTACTGGGGGACTGTAAAGGAATTGTATCCACTCTG
CTTCCTGTCAATTGGCCATGTGCATCTCTNGTTGTTCAGGGTACATTAATAACACACAGCATTAAGTA
GATAAGTCTCCGATATAAGCGAACAGAACGTTGGTCAGAGACTATGGTTCAATTGTAGTGGTATAATG
TTTCCCAGTGCATAAGGAACTCAGGGACACTACTAGCATGGGCGAGGAGAGACCCTTGGGTGGCAACA
CGCGATAAGTGTTACCTAGTACATCAGCACGAGNGANGGTTTGACCATGTGTTTTTAGACGACCCCGG
TCGCATTGAACCTGGCATTCAGAATGACGTGCTCTCAAGAATCAAGGTCCTCCCACTGAGGGTCATCT
GCTTCGCGGGTCNGCGTATGGTCATAANNNT
>Nung680
GTGTAGCCCAGTTAGACACTTCTAAATCCTTGGTAACAGACATGCAGTCCCCTCGGCAGAGCCCCGCC
CCCCGATGGTACCGGGTTGNTAAGAGGTCCACAACGGGTCGNACGACAACATTAAGGCCTCATGCATG
ACCCAACACGGGGGCGTCCCGGCGCGGCGACCTTGAAGTCCGGGGNCAAGTATGGCCCCCCGGGAAGG
ACGCCGTAGTGCATCTGGAGTGCCGATTCGTGGTCCAGAATGNCACCACGATGGTATGAGTGGCAAAG
GTACTTTGGACACCACCCCGTTTTCTATTTCCAGACGCGGCCATTCAGGAACGACGCGAATCGTAATC
TACCTGCCGCGTACGCGGATCGTACANGCGTTTTCCAGCGTTCGAAATAGAGTTTGTCGTTCCGCTGA
CCAGGTTTCACGGCTACTGCGATAAGCATATTCTACTGGGGGACTGTAAAGGAATTGTATCCACTCTG
CTTCCTGTCAATTGGCCATGTGCATCTCTGGTTGTTCAGGGTACATTAATAACACACAGCATNAAGTA
GATAAGTCTCCGATATAAGCGAACAGANCGTTGGTNAGAGACTATGGTTCAATTGTAGTGGTATAATG
TTTCCCAGTGCATAAGGAACTCAGGNACACTACTAGCATGGGCGAGGAGAGACCCTTGGGTGGCAACA
CGCGATAAGTGTTACCTAGTACATCAGCACGAGAGAGGGTTTGACCNTGTGTTTTTAGACGACCCCGG
TCGCATTGAACCTGGCATTCAGAATGACGTGCTCTCAAGAATCAAGGTCCTCCCACTGAGGGTCATCT
GCTTCGCGGGTCNGCGTATNGTCANAANCAT
>Nung683
GTGTAGCCCAGTCAGACACTTCTAAATCCTTNGTAACAGACATGCAGTCCCCTCGGCAGAGCCCCGCC
CCCCNATGGTACCGGGTTGTTNAGAGGTCCACAACGGGTCGNACGACAACATTAAGGCCTCANGCATG
ACCCAACACGGGGGCGTCCCGGCGCGGCGACCTTGNAGTCCGGGGACAAGTATGGCCCCCCGGGAAGG
ACGCCGTAGTGCATCTGGAGTGCCGATTCGTGGNCNAGAATGCCACCACGATGGTATGAGTGGCAAAG
GTACTTTGGACACCACCCCGTTTTCTATTTCCAGACGCGGCCATTTAGGAACGACGCGAATCGTAATC
TACCTGCCGCGTACGCGGATCGTACATGCGTTTTCCAGCGTTCGANATAGAGTTTGTCGTTCCGCTGA
CCAGGTTTCACGGCTACTGCGATAAGCATATTCTACTGGGGGACTGTAAAGGAATTGTATCCATTCTG
CTTCCTGTCAATTGGCCATGTGCATCTCTNGTTGTTCAGGNNACATTAATAACACACAGNAGTAAGTA
GGTAAGTCTCCGATATAAGCGAACAGANCGTTTGTCAGAGACTGTGGTTCAATTGTAGTGGTATAATG
TTTCCCAGTGCATAAGGAACTCAGGNACACTACTAGCANGGGCGAGGAGAGACCCTTGGGTGGCAACA
CGCAATAAGTGTTNCCTAGTACACCAGCACGAGAGANGGTTTGACCATGNNTTTTTAGACGACCCCGG
TCGCATTGAACCTGGCATTCAGAATGACGTGCTCTCAAGAATCNAGGTCCTCCCACTGAGGGTCATNN
GCTTCGCGGGTCNGCGTATGGTCATNANNAT
>Nung684
GTGTAGCCCAGTCAGACACTTCTAAATCCTTGGTAACAGACATGCAGTCCCCTCGGCAGAGCCCCGCC
CCCCGATGGTACCGGGTTGTTAAGAGGTCCACAACGGGTCGCACGACAACATTAAGGCCTCATGCATG
ACCCAACACGGGGGCGTCCCGGCGCGGCGACCTTGAAGTCCGGGGACAAGTATGGCCCCCCGGGAAGG

ACGCCGTAGTGCATCTGGAGTGCCGATTCGTGGTCCAGAATGCCACCACGATGGTATGAGTGGCAAAG
GTACTTTGGACACCACCCCGTTTTCTATTTCCAGACGCGGCCATTTAGGAACGACGCGAATCGTAATC
TACCTGCCGCGTACGCGGATCGTACATGCGTTTTCCAGCGTTCGAAATAGAGTTTGTCGTTCCGCTGA
CCAGGTTTCACGGCTACTGCGATAAGCATATTCTACTGGGGGACTGTAAAGGAATTGTATCCATTCTG
CTTCCTGTCAATTGGCCATGTGCATCTCTGGTTGTTCAGGGTACATTAATAACACACAGCAGTAAGTA
GGTAAGTCTCCGATATAAGCGAACAGAACGTTTGTCAGAGACTGTGGTTCAATTGTAGTGGTATAATG
TTTCCCAGTGCATAAGGAACTCAGGGACACTACTAGCATGGGCGAGGAGAGACCCTTGGGTGGCAACA
CGCAATAAGTGTTACCTAGTACACCAGCACGAGAGAGGGTTTGACCATGTGTTTTTAGACGACCCCGG
TCGCATTGAACCTGGCATTCAGAATGACGTGCTCTCAAGAATCAAGGTCCTCCCACTGAGGGTCATCT
GCTTCGCGGGTCAGCGTATGGTCATAAACAT
>Nung685
GTGTAGCCCAGTTAGACACTTCTAAATCCTTGGTAACAGACATGCAGTCCCCTCNNCAGAGCNCCGCC
CCCCGATGGTACCGGGTTGTTNAGAGGTCCACAACGGGTCGCACGACAACNTTAAGGCCTCATGCATG
ACCCAACACGGNGGCGTCCCGGCGNGGCGACCTTGNAGTCCGGGGACAAGTATGGCCCCCCGGGAAGG
ACGCCGTAGTGCATCTGGAGTGCCGATTCGTGGTCCAGAATGNCACCACGATGGTATGAGTNGCAAAG
GTACTTTGGACACCACCCCGTTTTCTANTTCCAGACGCGGCCATTCAGGAACGACGCGAATCGTAATC
TACCTGCCGCGTACGCGGATCGTACATGCGTTTTCCAGCGTTCGAAATAGAGTTTGTCGTTCCGCTGA
CCAGGTTTCACGGCTACTGCGATAAGCATATTCTACTGGGGGACTGTAAAGGAATTGTATCCACTCNG
CTTCCTGTCAATTGGCCATGTGCATCTCTGGTTGTTCAGGGNACATTAATAACACACAGCATTAAGTA
GATAAGTCTCCGATATAAGCGAACAGAACGTTGGTCAGAGACTATGGTTCAATTGTAGTGGTATAATG
TTTCCCAGTGCATAAGGAACTCAGGGACACTACTAGCATGGGCGAGGAGAGACCCTTGGGTGGCAANA
CGCGATAAGTGTTANCTAGTACATCAGCACGAGAGAGGGTTTGACCATNNNTTTTTAGACGACCCCGG
TCGCATTGAACCTGGCATTCAGAATGACGTGCTCTCAAGAATCAAGGTCCTCCCACTGAGGGTCATCT
GCTTCGCGGGTCAGCGTATGGTCATAAACAT
>Nung686
GTGTAGCCCAGTTAGACACTTCTAAATCCTTGGTAACAGACATGCAGTCCCCTCGGCAGAGCCCCGCC
CCCCNATGGTACCGGGTTGNTNAGAGGTCCACAACGGGTCGCACGACAANATTAAGGCCTCATGCATG
ACCCAACNCGGGGGCGTCCCGGNGNGGCGACCTTGNANTCCGGGGNCAAGTATGGCCCCCCGGGAAGG
ACGCCGTAGTGCATCTGGAGTGCCGNTTNNTGGTCNAGAATGCCACCACGATGGTATGAGTNGCAAAG
GTACTTTGGACACCACCCCGTTNNCTATTTCCAGACGCGGCCATTCAGGAACGACGCGAATCGTAATC
TACCTGCCGCGTACGCGGATCGTACANGCGTTTTCCAGCGTTCGAAATAGAGTTTGTCGTTCCGCTGA
CCNGGTTTCACGGCTACTGCGATAAGCATATTCTACTGGGGGACTGTAAAGGAATTGTATCCACTCTG
CTTCCTGTCAATTGGNCATGTGCATCTCTGGTTGTTCAGGNNACATTAATAACACACAGCATTAAGTA
GATAAGTCTCCGATATAAGCGAACAGANCGTTGGTCAGAGACTGTGGTTCAATTGTAGTGGTATAATG
TTTCCCAGTGCATAAGGAACTCAGGNACACTACTAGCATGGGCGAGGAGAGACCCTTGGGTGGCAACA
CGCAATAAGTGTTACCTAGTACACCAGCACGAGAGAGGGTTTGACCATGTGTTNNTAGACGACCCCGG
TCGCATTGAACCTGGCATTCAGAATGACGTNCTCTCAAGAATCAAGNTCCTCCCACTGAGGGTCATCT
GCTTCGCGGNTCAGCGTATNGTCANNANNAT
>Nung687
GTGTAGCCCAGTTAGACACTTCTAAATCCTTGGTAACAGACATGCAGTCCCCTCGGCAGAGCCCCGCC
CCCCNATGGTACCGGGTTGNTNAGAGGTCCACAACGGGTCGCACGACNACATTAAGGCCTCANGCATG
ACCCAACACGGGGGCGTCCCGGCGNGGCGACCTTGAAGTCCGGGGNCAAGTATGGCCCCCCGGGAAGG
ACGCCGTAGTGCATCTGGAGTGCCGATTNGTGGTCNAGAATGCCACCACGATGGTATGAGTGGCAAAG
GTACTTTGGACACCACCCCGTTTTCTATTTCCAGACGCGGCCATTCAGGAACGACGCGAATCGTAATC
TACCTGCCGCGTNCGCGGATCGTACATGCGTTTTCCAGCGTTCGAAATAGAGTTTGTCGTTCCGCTGA
CCAGGTTTCACGGCTACTGCGATAAGCATATTCTACTGGGGGACTGTAAAGGAATTGTATCCACTCNG
CTTCCTGTCAATTGGNCATGTGCATCTCTGGTTGTTCAGGGTACATTAATAACACACAGCATTAAGTA
GATAAGTCTCCGATATAAGCGAACAGAACGTTGGTCAGAGACTGTGGTTCAATTGTAGTGGTATAATG
TTTCCCAGTGCATAAGGAACTCAGGGACACTACTAGCATGGGCGAGGAGAGACCCTTGGGTGGCAACA
CGCAATAAGTGTTACCTAGTACACCAGCACGAGNGANGGTTTGACCATNTGTTTTTAGACGACCCCGG
TCGCATTGAACCTGGCATTCAGAATGACGTGCTCTCANGAATCAAGGTCCTCCCACTGAGGGTCATCT
GCTTCGCGGGTCNGCGTATGGNCATNAACNT
>Nung688
GTGTAGCCCAGTTAGACACTTCTAAATCCTTNGTAACAGACATGCAGTCCCCTCGGCAGAGCCCCGCC

CCCCGATGGTACCGGGTTGTTAAGAGGTCCACAACGGGTCGCACGACAACATTAAGGCCTCATGCATG
ACCCAACACGGGGGNGTCCCGGCGCGGCGACCTTGAAGTCCGGGGNCAAGTATGGCCCCCCGGGAAGG
ACGCCGTAGTGCATCTGGAGTGCCGATTCGTGGTCCAGAATGNCACCACGATGGTATGAGTGGCAAAG
GTACTTTGGNCACCACCCCGTTTTCTATTTCCAGACGCGGCCATTCAGGAACGACGCGAATCGTAATC
TACCTGCCGCGTACGCGGATCGTACATGCGTTTTNCAGCGTTCGAAATAGAGTTTGTCGTTCCGCTGA
CCAGGTTNCACGGCTACTGCGATAAGCATATTCTACTGGGGGACTGTAAAGGAATTGTATCCACTCTG
CTTCCTGTCAATTGGCCATGTGCATCTCTGGTTGTTCAGGGTACATTAATAACACACAGNATTAAGTA
GATAAGTCTCCGATATAAGCGAACAGAACGTTGGTCAGAGACTGTGGTTCAATTGTAGTGGTATAATG
TTTCCCAGTGCATAAGGAACTCAGGGACACTACTAGCATGGGCGAGGAGAGACCCTTGGGTGGCAACA
CGCAATAAGTGTTACCTAGTACACCAGCACGAGAGAGGGTTTGACCATNNNTTTNTAGNNGACCCCGG
TCGCATTGAACCTGGCATTCAGAATGACGTGCTNTCAAGAATCAAGGTCCTCCCACTGAGGGTCATCT
GCTTCGCGGGTCAGCGTATGGTCATAAACAT
>Nung689
GTGTAGCCCAGTCAGACACTTCTAAATCCTTGGTAACAGACATGCAGTCCCCTCGGCAGAGCCCCGCC
CCCCGATGGTACCGGGTTGTTAAGAGGTCCACAACGGGTCNCACGACAACATTAAGGCCTCATGCATG
ACCCAACNCGGGGGCGTCCCGGCGCGGCGACCTTGAAGTCCGGGGNCAAGTATGGCCCCCCGGGAAGG
ACGCCGTAGTGCATCTGGAGTGCCGATTCGTGGTCCAGAATGNCACCACGATGGTATGAGTGGCAAAG
GTACTTTGGACACCACCCCGTTTTCTANTTCCAGACGCGGCCATTTAGGAACGACGCGAATCGTAATC
TACCTGCCGCGTACGCGGATCGTACATGCGTTTTCCAGCGTTCGAAATAGAGTTTGTCGTTCCGCTGA
CCAGGTTNCACGGCTACTGCGATAAGCATATTCTACTGGGGGACTGTAAAGGAATTGTATCCATTCTG
CTTCCTGTCAATTGGCCATGTGCATCTCTGGTTGTTCAGGGTACATTAATAACACACAGCAGNAAGTA
GGTAAGTCTCCGATATAAGCGAACAGAACGTTTGTNAGAGACTGTGGTTCAATTGTAGTGGTATAATG
TTTCCCAGTGCATAAGGAACTCAGGGACACTACTAGCATGGGCGAGGAGAGACCCTTGGGTGGCAACA
CGCAATAAGTGTTACCTAGTACACCAGCACGAGAGAGGGTTTGACCATGTGTTTTTAGACGACCCCGG
TCGCATTGAACCTGGCATTCAGAATGACGTGCTCTCAAGAATCAAGGTCCTCCCACTGAGGGTCATCT
GCTTCGCGGGTCNGCGTATGGTCANAAACAT
>Nung690
GTGTAGCCCAGTTAGACACTTCTAAATCCNTGGTAACAGACATGCAGTCCCCTCGGCAGAGCCCCGCC
CCCCGATGGTACCGGGTTGTTAAGAGGTCCACAACGGGTCGNACGACAACATTAAGGCCTCATGCATG
ACCCAACACGGGGGCGTCCCGGCGNGGCGACCTTGAAGTCCGGGGACAAGTATGGCCCCCCGGGAAGG
ACGCCGTAGTGCATCTGGAGTGCCGATTCGTGGTCNAGAATGCCACCACGATGGTATGAGTNGCAAAG
GTACTTTGGACACCACCCCGTTTTCTANTTCCAGACGCGGCCATTCAGGAACGACGCGAATCGTAATC
TACCTGCCGCGTACGCGGATCGTACANGCGTTTTCCAGCGTTCGAAATAGAGTTTGTCGTTCCGCTGA
CCAGGTTNCACGGCTACTGCGATAAGCATATTCTACTGGGGGACTGTAAAGGAATTGTATCCACTCNG
CTTCCTGTCAATTGGCCATGTGCATCTCTGGTTGTTCAGGGTACATTAATAACACACAGCATTAAGTA
GATAAGTCTCCGATATAAGCGAACAGAACGTTGGTNAGAGACTATGGTTCAATTGTAGTGGTATAATG
TTTCCCAGTGCATAAGGAACTCAGGNACACTACTAGCANGGGCGAGGAGAGACCCTTGGGTGGCAANA
CGCAATAAGTGTTACCTAGTACATCAGCACGAGAGAGGGTTTGACCATGNNTTTTTAGACGACCCCGG
TCGCATTGAACCTGGCATTCAGAATGACGTGCTCTCAAGAATCAAGNTCCNCCCACTGAGGGTCATCN
GCTTCGCGGGTCAGCGTATGGTCATAAACAT
>Nung691
GTGTAGCCCAGTTAGACACTTCTAAATCCNTGGTAACAGACATGCAGTCCCCTCNNCAGAGCCCCGCC
CCCCNATGGTACCGGGTTGTTNAGAAGTCCACAACGGGTCGNACGACAACATTAAGGCCTCATGCATG
ACCCAACNCGGNGGCGTCCCGGCGCGGCGACCTTGAAGTCCGGGGNCAAGTATGGCCCCCCGGGAAGG
ACGCCGTAGTGCATCTGGAGTGCCGATTCGTGGTCCAGAATGCCACCACGATGGTATGAGTGGCAAAG
GTACTTTGGACACCACCCCGTTTTCTANTTCCAGANGCGGCCNTTCAGGAACGACGCGAATCGTAATC
TACCTGCCGCGTACGCGGATCGTACATGCGTTTTCCAGCGTTCGAAATAGAGTTTGTCGTTCCGCTGA
CCAGGTTTCACGGCTACTGCGATAAGCATATTCTACTGGGGGACTGTAAAGGAATTGTATCCATTCNG
CTTCCTGTCAATTGGCCATGTGCATCTCTNGTTGTTCAGGGTACATTAATAACACACAGCAGNAAGTA
GGTNAGTCTCCGATATAAGCTAACAGANCGTTTGTNAGAGACTGTGGTTCAATTGTAGTGGTATAATG
TTTCCCAGTGCATAAGGAACTCAGGNACACTACTAGCATGGGCGAGGAGAGACCCTTGGGTGGCAACA
CGCAATAAGTGTTACCTAGTACACCAGCACGAGAGAGGGTTTGACCATGTGTTTTCAGACGACCCCGG
TCGCATTGAACCTGGCATTCAGAATGACGTGCTCTCAAGAATCAAGNTCCTCCCACTGAGGGTCATCT
GCTTCGCGGGTCAGCGTATGGTCATAAACAT

>Nung692
GTGTAGCCCAGTTAGACACTTCTAAATCCTTGGTAACAGACATGCAGTCCCCTCGGCAGAGCCCCGCC
CCCCGATGGTACCGGGTTGTTAAGAGGTCCACAACGGGTCGCACGACAACATTAAGGCCTCATGCATG
ACCCAACACGGGGGCGTCCCGGCGCGGCGACCTTGAAGTCCGGGGACAAGTATGGCCCCCCGGGAAGG
ACGCCGTAGTGCATCTGGAGTGCCGATTCGTGGTCCAGAATGCCACCACGATGGTATGAGTGGCAAAG
GTACTTTGGACACCACCCCGTTTTCTATTTCCAGACGCGGCCATTCAGGAACGACGCGAATCGTAATC
TACCTGCCGCGTACGCGGATCGTACATGCGTTTTCCAGCGTTCGAAATAGAGTTTGTCGTTCCGCTGA
CCAGGTTNCACGGCTACTGCGATAAGCATATTCTACTGGGGGACTGTAAAGGAATTGTATCCACTCTG
CTTCCTGTCAATTGGCCATGTGCATCTCTGGTTGTTCAGGNNACATTAATAACACACAGCATTAAGTA
GATAAGTCTCCGATATAAGCGAACAGAACGTTGGTCAGAGACTGTGGTTCAATTGTAGTGGTATAATG
TTTCCCAGTGCATAAGGAACTCAGGGACACTACTAGCATGGGCGAGGAGAGACCCTTGGGTGGCAACA
CGCAATAAGTGTTACCTAGTACACCAGCACGAGAGAGGGTTTGACCATGTGTTTTTAGACGACCCCGG
TCGCATTGAACCTGGCATTCAGAATGACGTGCTCTCAAGAATCAAGGTCCTCCCACTGAGGGTCATCT
GCTTCGCGGGTCAGCGTATGGTCATAAACAT
>Nung693
GTGTAGCCCAGTTAGACACNTCTAAATCCNTNGTAACAGACATGCAGTNCCCTCNNCAGAGNCCCGCC
CCCCGATGGTACCGGGTTGTTAAGAGGTCCACAACGGGTCGNACGACANCNTTAAGGCCTCATGCATG
ACCCAACACGGNGGNGTCCCGGCGNGGCGACCTTGNANTCCGGGNNCAAGTATNGCCCCCCGGGAAGG
ACGCCGTAGTGCATCTGGAGTGCCGATTCGTGGTCCAGAATGCCACCACGATGGTATGAGTGGCAAAG
GTACTTTGGACACCACCCCGTTTTCTATTTCCAGACGCGGCCNTTCAGGAACGACGCGAATCGTAATC
TACCTGCCGCGTACGCGGATCGTACATGCGTTTTCCAGCGTTCGAAATAGAGTTTGTCGTTCCGCTGA
CCAGGTTNCACGGCTACTGCGATAAGCATATTCTACTGGGGGACTGTAAAGGAATTGTATCCACTCTG
CTTCCTGTCAATTGGNCATGTGCATCTCTNGTTGTTCAGGGTACATTAATAACACACAGCNTTAAGTA
GATAAGTCTCCGATATAAGCGAACAGAACGTTGGTNAGAGACTGTGGTTCAATTGTAGTGGTATAATG
TTTCCCAGTGCATAAGGAACTCAGGGACACTACTAGCATGGGCGAGGAGAGACCCTTGGGTGGCAACA
CGCAATAAGTGTTACCTAGTACACCAGCACGAGNNANGGTTTGACCATGTGTTTTTAGACGACCCCGG
TCGCATTGAACCTGGCATTCAGAATGACGTGCTCTCAAGAATCNANGTCCTCCCACTGAGGGTCATCN
GCTTCGCGGGTCAGCGTATNGTCATNAACAT
>Nung694
GTGTAGCCCAGTTAGACACNTCTAAATCCTTGGTAACAGACATGCAGTCCCCTCGGCAGAGCCCCGCC
CCCCNATGGTACCGGGTTGTTAAGAGGTCCACAACGGGTCGCACGACAANNTTAAGGCCTCATGCATG
ACCCAANACGGGGGCGTCCCGGCGNGGCGACCTTGNAGTCCGGGGACAAGTATGGCCCCCCGGGAAGG
ACGCCGTAGTGCATCTGGAGTGCCGATTCNTGGTCNAGANTGCCACCACGATGGTATGAGTGGCAAAG
GTACTTTGGACACCACCCCGTTTTCTATTTCCAGACGCGGCCATTCAGGAACGACGCGAATCGTAATC
TACCTGCCGCGTACGCGGATCGTACATGCGTTTTCCAGCGTTCGAAATAGAGTTTGTCGTTCCGCTGA
CCAGGTTTCACGGCTACTGCGATAAGCATATTCTACTGGGGGACTGTAAAGGAATTGTATCCACTCTG
CTTCCTGTCAATTGGCCATGTGCATCTCTGGTTGTTCAGGGTACATTAATAACACACAGCATTAAGTA
GATAAGTCTCCGATATAAGCGAACAGAACGTTGGTCAGAGACTGTGGTTCAATTGTAGTGGTATAATG
TTTCCCAGTGCATAAGGAACTCAGGNACACTACTAGCATGGGCGAGGAGAGACCCTTGGGTGGCAACA
CGCAATAAGTGTTACCTAGTACACCAGCACGAGAGAGGGTTTGACCATGTGTTTTTAGNNGACCCCGG
TCGCATTGAACCTGGCATTCAGAATGACGTGCTCTNAAGAATCAAGGTCCTCCCACTGAGGGTCATCT
GCTTCGCGGGTCAGCGTATGGNCATAAACAT
>Nung695
GTGTAGCCCAGTTAGACACNTCTAAATCCNTGGTAACAGACATGCAGTCCCCTCGGCAGAGCCCCGCC
CCCCNATGGTACCGGGNNNNTNAGAGGTCCACAACGGGTCGCANGACAACNTTAAGGCCTCATGCATG
ACCCANNACGGGGGCGTCCCGGCGCGGNGACCTTGNAGTCCGGGGNCAAGTATNGCCCCCCGGGAAGG
ACGCNGTAGTGCATCTGGAGTGCCGATTCGNGGTCNAGAATGNCACCACGATGGTATGAGTGGCAAAG
GTACTTTGGACACCACCCCGTTTTCTATTNCCAGANGCGGCCATTCAGGAACGACGCGAATCGTAATC
TACCTGCCGCGTACGCGGATCGTACANGCGTTTTCCAGCGTTCGAAATAGAGTTTGTCGTTCCGCTGA
CCAGGTTTCACGGCTACTGCGATAAGCATATTCTACTGGGGGACTGTAAAGNAATTGTATCNACTCTG
CTTCCTGTCAATTGGNCATGTGCATCTCTGGTTGTTCAGGGTACATTAATAACACACAGCNTTAAGTA
GATNAGTCTCCGATATAAGCGAACAGANCGTTGGTCAGAGACTATGGTTCAATTGTAGTGGTATAATG
TTTCCCAGTGCATAAGGAACTCAGGGACACTACTAGCATGGGCGAGGAGAGACCCTTGGGTGGCAACA
CGCGATAAGTGTTANNTAGTACATCAGCACGAGNNANGGTTTGNCCNTGTGTTTTTAGACGACCCCGG

TCGCATTGAACCTGGCATTCAGAATGACGTNCTCTCAAGAATCAAGGTCCTCCCACTGAGGGTCATCT
GCTTCGCGNNNCNGCGTATGGTCANAANNAT
>Nung696
GTGTAGCCCAGTTANACACTTCTAAATCCTTGGTAACAGACATGCAGTNCCCTCNNCAGAGCNCCGCC
CCCCNATGGTACCGGGTTGTTNAGNGGTCCACAACGGGTCGCACGACNNNATTAAGGCCTCANGCATG
ACCCAANACGGGGGCGTCCCGGNGNGGCGACCTTGNANTCCGGGGNCAAGTATGGCCCCCCGGGAAGG
ACGCCGTAGTGCATCTGGANTGCCGATTCGTGGTCNAGAATGNCACCACGATGGTATGAGTNGCAAAG
GTACTTTGGACACCACCCCGTTTTCTANTTCCAGACGCGGCCATTCAGGAACGACGCGAATCGTAATC
TACCTGCCGCGTNCGCGGATCGTACATGCGTTTTCCAGCGTTCGAAATAGAGTTTGTCGTTCCGCTGA
CCAGGTTTCACGGCTACTGCGATAAGCATATTCTACTGGGGGACTGTAAAGGAATTGTATCCACTCTG
CTTCCTGTCAATTGGCCATGTGCATCTCTGGTTGTTCAGGNNACATTAATAACACACAGNATTAAGTA
GNTAAGTCTCCGATATAAGCGAACAGANCGTTGGTCAGAGACTATGGTTCAATTGTAGTGGTATAATG
TTTCCCAGTGCATAAGGAACTCAGGGACACTACTAGCATGGGCGAGGAGAGACCCTTGGGTGGCAACA
CGCGNTAAGTGTTANCTAGTACATCAGCACGAGNNANGGTTTGNCCANGTGTTTTTAGACGACCCCGG
TCGCATTGAACCTGGCATTCAGAATGACGTGCTCTCAAGAATCAANGTCCTCCCACTGAGGGTCATCT
GCTTCGCGNGTCAGCGTATGGTCATAAACAT
>Nung697
GTGTAGCCCAGTTAGACACTTCTAAATCCTTGGTAACAGACATGCAGTCCCCTCGGCAGAGCCCCGCC
CCCCNATGGTACCGGGTTGNTNAGAGGTCCACAACGGGTCNCACGACAANATTAAGGCCTNATGCATG
ACCCANCACGGGGGNGTCCCGGCGCGGCGACCTTGAANTCCGGGGACAAGTATNGCCCCCCGGGAAGG
ACGCCGTAGTGCATCTGGAGTGCCGANNNNTGGTCCAGAATGCCACCACGATGGTATGAGTGGCAAAG
GTACTTTGGNCACCACCCCGTTTTCTANTTCCAGACGCGGCCATTCAGGAACGACGCGAATCGTAATC
TACCTGCCGCGTNCGCGGATCGTACANGCGTTTTCCAGCGTTCGAAATAGAGTNTGTCGNTCCGCTGA
CCAGGTTNCACGGCTACTGCGATAAGCATATTCTACTGGGGGACTGTAAAGGAATTGTATCCACTCTG
CTTCCTGTCAATTGGNCATGTGCATCTCTNGTTGTTCAGGGNACATTAATAACACACAGCATTAAGTA
GNTNAGTCTCCGATATAAGCGAACAGANCGTTGGTCAGAGACTATGGTTCAATTGTAGTGGTATAATG
TTTCCCAGNGCATAAGGAACTCAGGGACACTACTANCATGGGCGAGGAGAGACCCTTGGGTGGCAANA
CGNAATAAGTGTTACCTAGTACATCAGCACGAGNNAGGGTTTGACCATNNNTTTNTAGNNGACCCCGG
TCGCATTGAACCTGGCATTCAGAATGACGTNNTNTCAAGAATCNAGGTCCTCCCACTGAGGGTCATNT
GCTTCGCGGGNCNGCGTATGGNCATAAACAT
>Nung698
GTGTAGCCCAGTTAGACACTTCTAAATCCTTGGTAACAGACATGCAGTCCCCTCNGCAGAGCNCCGCC
CCCCNATGGTACCGGGTTGTTNAGAGGTCCACAACGGGTCGCACGACAACNTTAAGGCCTCANGCATG
ACCCNNCACGGNGGCGTCCCGGNGCGGCNACCTTGAAGTCCGGGGNCAAGTATGGCCCCCCGGGAAGG
ACGCCGTAGTGCATCTGGAGTGCCGATTCGTGGTCNAGANTGNCACCACGATGGTATGAGTGGCAAAG
GTACTTNGGACACCACCCCGTTTTCTANTTCCAGACGCGGCCATTCAGGAACGACGCGAATCGTAATC
TACCTGCCGCGTNCGCGGATNGTACATGCGTTTTCCAGCGTTCGAAATAGAGTTTGTCGTTCCGCTGA
CCAGGTTNCACGGCTACTGCGATAAGCATATTCTACTGGGGGACTGTAAAGGAATTGTATCCACTCNG
CTTCCTGTCAATTGGCCATGTGCATCTCTNGTTGTTCAGGGTACATTAATAACACACAGCATTAAGTA
GATAAGTCTCCGATATAAGCGAACAGANCGTTGGTCAGAGACTATGGTTCAATTGTAGTGGTATAATG
TTTCCCAGTGCATAANGAACTCAGGNACACTACTAGCANGGGCGAGGAGAGACCCTTGGGTGGCAACA
CGCANTAAGTGTTACCTAGTACATCAGCACGAGANANGGTTTGACCNTGTGTTTTTAGACGACCCCGG
TCGCATTGAACCTGGCATTCAGAATGACGTGCTCTCAAGAATCAAGNTCCTCCCACTGAGGGTCATCT
GCTTCGCGNGTCAGCGTATGGTCATAANCAT
>PaThen494
GTGTAGCCCAGTCAGACACNTCTAAATCCTTGGTAACAGACATGCAGTCCCCTCNNCAGAGCNCCGCC
CCCCNATGGTACCGGGTTGTTNAGAGGTCCACAACGGGTCGNACGACNNNNTTAAGGCCTCANGCATG
ACCCAANACGGNGGNGTCCCGGCGCGGCNACCTTGNAGTCCGGGGACAAGTATGGCCCCCCGGGAAGG
ACGCCGTAGTGCATCTGGAGTGCCGATTCGTGGTCNAGAATGNCACCACGATGGTATGAGTGGCAAAG
GTACTTTGGNCACCACCCCGTTTTCTATTTCCAGACGCGGCCATTTAGGAACGACGCGAATCGTAATC
TACCTGCCGCGTACGCGGATCGTACANGCGTTTTCCAGCGTTCGANATAGAGTTTGTCGTTCCGCTGA
CCAGGTTTCACGGCTACTGCGATAAGCATATTCTACTGGGGGACTGTAAAGGAATTGTATCCATTCTG
CTTCCTGTCAATTGGCCATGTGCATCTCTGGTTGTTCAGGGTACATTAATAACACACAGCAGTAAGTA
GGTAAGTCTCCGATATAAGCGAACAGAACGTTTGTCAGAGACTGTGGTTNNATTGTAGTGGTATAATG

TTTCCCAGTGCATAAGGAACTCAGGGACACTACTAGCATGGGCGAGNAGAGACCCTTGGGTGGCAACA
CGCAATAAGTGTTACCTAGTACACCAGCACGAGANAGGGTTTGACCATNNNTTTTTAGNNGACCCCGG
TCGCATTGAACCTGGCATTCAGAATGACGTGCTCTCAAGAATCNANNTCCTCCCACTGAGGGTCATNT
GCTTCGCGNGTCNGCGTATGGTCACAAACAT
>PaThen495
GTGTAGCCCAGTTAGACACNTCTNAATCCNTGGTAACAGACATGCAGTNNCCTCGGCAGAGNNCCGCC
CCCCNATGGTACCGGGNNNTTNAGAAGTCCACAACGGNTCGNACGACNNNNTTAAGGCCTCANGCATG
ACCCNANACNGNGGNGTCCCGGNGNGGCGACCTTGNANTCCGGGGACAAGTATNGCCCCCCGGGAAGG
ACGCCGTAGTGCATCTGGAGTGCCGATTNNTGNNCCAGANTNNCACCACGANGGTATGAGTGGCAAAG
GTACTTTGGACACCACCCCGTTNNCTATNTCCAGACGCNGCCATTCAGGAACGACGCGAATCGTAATC
TACCTGCCGCGTNCGCGGATCGTACATGCGTTTTCCAGCGTTCGAAATAGAGTNTGTCGTTCCGCTGA
CCAGGTTTCACGGCTNCTGCGATAAGCATATTCTACTGGGGGACTGTAAAGGAATTGTATCCATTCNG
CTTCCTGTCAATTGGCCATGTGCATCTCTNGTTGTTCAGGNNACATTAATAACACACAGNAGTAAGTA
GGTAAGTCTCCGATATAAGCTAACAGAACGTTTGTCAGANACTGTGGTTCAATTGTAGTGGTATAATG
TTTCCCAGTGCATAANGAACTCAGGGACACTACTAGCANGGGCGAGGAGAGACCCTTGGGTGGCAANA
CGCANTAAGTGTTNCCTAGTANACCAGCACGAGNNANGGTTTGACCANNNNTTTNCAGACGNCCCCGG
TCGCATTGAACCTGGCATTCAGAATGACGTGCTCTNAAGAANCAAGGTCCNNNCACTGAGGGTCATCT
GCTTCGCGNNNCNGCGTATGGTCATAAACAT
>PaThen496
GTGTAGCCCAGTCAGACACTTCTAAATCCTTGGTAACAGACATGCAGTCCCCTCGGCAGAGCCCCGCC
CCCCGATGGTACCGGGTTGTTAAGAGGTCCACAACGGGTCGCACGACAACATTAAGGCCTNANGCATG
ACCCNACACGGGGGNGTCCCGGCGCGGCGACCTTGAAGTCCGGGGACAAGTATGGCCCCCCGGGAAGG
ACGCCGTAGTGCATCTGGAGTGCCGATTNNTGGNCNAGAATGCCACCACGATGGTATGAGTGGCAAAG
GTACTTNGGACACCACCCCGTTTTCTATTTCCAGACGCGGCCATTTAGGAACGACGCGAATCGTAATC
TACCTGCCGCGTACGCGGATCGTACATGCGTTTTCCAGCGTTCGAAATAGAGTTTGTCGTTCCGCTGA
CCAGGTTTCACGGCTACTGCGATAAGCATATTCTACTGGGGGACTGTAAAGGAATTGTATCCATTCNG
CTTCCTGTCAATTGGCCATGTGCATCTCTNGTTGTTCAGGGTACATTAATAACACACAGCAGNAAGTA
GGTAAGTCNCCGATATAAGCGAACAGANCGTTTGTCAGAGACTGTGGTTCAATTGTAGTGGTATAATG
TTTCCCAGTGCATAAGGAACTCAGGGACACTACTAGCATGGGCGAGGAGAGACCCTTGGGTGGCAACA
CGCAATAAGTGTTACCTAGTACACCAGCACGAGANANGGTTTGACCATGNNTTTNTAGACGACCCCGG
TCGCATTGAACCTGGCATTCAGAATGACGTGCTCTCAAGAATCAAGNTCCTCCCACTGAGGGTCATCT
GCTTCGCGNGTCNGCGTATGGTCATAAACAT
>PaThen497
GTGTAGCCCAGTCAGACACTTCTAAATCCTTGGTAACAGACATGCAGTNNCCTCNNCAGAGCNCCGCC
CCCCNATGGTACCGGGTTGTTNAGNGGTCCACAACGGNTCGCACGACAANNTTAAGGCCTCANGCATG
ACCCNACACNGGGGNGTCCCGGCGCGGCGACCTTGNANTCCGGGGACAAGTATGGCCCCCCGNGAAGG
ACGCCGTAGTGCATCTGGAGTGCCGATTCGTNGNCCAGANTGNCACCACGATGGTATGAGTGGCAAAG
GTACTTTGGACACCACCCCGTTTTCTATTTCCAGANGCGGCCATTTAGGAACGACGCGAATCGTAATC
TACCTGNCGCGTACGCGGATCGTACANGCGTTTTCCAGCGTTCGANATAGAGTTTGTCGTTCCGCTGA
CCAGGTTTCACGGCTACTGCGATAAGCATATTCTACTGGGGGACTGTAAAGGAATTGTATCCATTCTG
CTTCCTGTCAATTGGNCATGTGCATCTCTGGTTGTTCAGGGTACATTAATAACACACAGCAGTAAGTA
GGTNAGTCNCCGATATAAGCGAACAGAACGTTTGTCAGAGACTGTGGTTCAATTGTAGTGGTATAATG
TTTCCCAGTGCATAAGGAACTCAGGNACACTACTAGCATGGGCGAGGAGAGACCCTTGGGTGGCAACA
CGCAATAAGTGTTACNTAGTACACCAGCACGAGNGAGGGTTTGACCATGNNTTTTTAGACGNCCCCGG
TCGCATTGAACCTGGCATTCAGAATGACGTGCTCTCAAGAATCAANGTCCTCCCACTGAGGGTCATCT
GCTTCGCGGNNCAGCGTATNGNCATAAACAT
>PaThen498
GTGTNGCCNAGTCAGACACNTCTAAATCCNTGGTAACAGACATGCAGTCCCCTCNNCAGAGNNCCGCC
CCCCNATGGTACCGGGNTGNTNAGNGGTCCACAACGGNTCGNACGACAANNTTAAGGCCTNANGCATG
ACCCNACACGGNGGNGTCCCGGNGNNGCGNCCTTGNANTCCGGGGNCAAGTATNGCCCCCCGNGAAGG
ACGCCGTAGTGCATCTGGAGTGCCGATTNNTGGTCNAGAATGNCACCACGATGGTATGAGTGGCAAAG
GTACTTTGGACACCACCCCGTTTTCTANTTCNAGACGCNGCCATTTAGGAACGACGCGAATCGTANTC
TACCTGCCGCGTACGCGGATCGTACATGCGTTTTCCAGCGTTCGAAATAGAGTTTGTCGTTCCGCTGA
CCAGGTTNCACGGNTACTGCGATANGCATATTCTACTGGGGGACTGTAAAGGAATTGTATCCATTCNG

CTTCCTGTCAATTNGCCATGTGCATCTCTGGTTGTTCAGGNNACATTAATAACACACAGCAGTAAGTA
GNTNAGTCNCCGATATAAGCGAACAGANCGTTTGTCAGAGANTGTGGTTCAATTGTAGTGGTATAATG
TTTCCCAGNNCATAAGGAACTCAGGNACACTACTAGCANGGGCGAGGAGAGACCCTTGGGTGGCAACA
CGCAATAAGTGTTACCTAGTACACCAGCACGAGAGAGGGTTTGACNATGTNTTTTAGACGACCCCGG
TCGCATTGAACCTGGCATTCAGAATGACGTNCTNTCAAGAATCNANGTCCNCCCACTGAGGGNCATCN
GCTTCGCGNNNCNGCGTATGGNCATNAACNT
>PaThen499
GTGTAGCTCAGTTAGACACTTCTAAATCCTTGGTAACAGACATGCAGTNCCCTCNNCAGAGCCCCGCC
CCCCGGTGGTACCGGGTTGTTAAGNGGTCCACCACGGGTCNNACGACAACATCAAGGCCTCANGCATG
ANNCNACNCGGGGACGTACCGGCGNGGCGACCTTGNAGGTCGGGGNCAAGTATGTCCCNCCGGGAAGG
ACGCCGTAGTGCATCTGGAGTGCCGANNCNTGGTCNAGAATGCCACCACGATGGTATGAGTGGCAAAG
GTATTTTGGACACCACCCCGTTTTCTATTTCCAGACGCGGCCATTCAGGAACGACGCGAATCGTAATC
TACCTGCCGCGTACGCGGATCGTACATGCGTTTTTCAGCGTTCGAAATAGAGTTTGTCGTTCCGCTGA
CCAGGTTTCACGGCTACTGCGATAAGCATATTCTACGGGGGGACTGTAAAGGAATTGTATCCACTCNG
CTCCCTGTCAATTGGCCATGTGCATCTCTNGTTGTTTAGGNNACATTAATAACACACAGNATTAGGTA
GATAAGTCTCCGATATAAGCGAACAGAACGTTGGTCAAAGACCGTGGTTCAATTGTAGTGGTATAACA
TTTCCCAGTGCATAAGGAACCCAGGGACACTACTAGCATGGGCGAGGAGAGACCCTTGGGTGGCAACA
CGCAATAAGTGTTNCCCAGTACACCAGCACGATAGANGGTTTGACCATGTGTTTTTAGACGACCCCGG
TCGCATTGAACCTGGCATTCAGAATGACGTNCTCTCAAGAGTCAAGGTCCTCCCACTGAGGGTCATCT
GCTTCGCGNNNTNGCGTATGGTCATNAACNT
>PaThen500
GTGTAGCCCAGTTAGACACNTCTAAATCCTTGGTAACAGACATGCAGTCCCCTCNNCAGAGCCCCGCC
CCCCGATGGTACCGGGTTGTTNAGAAGTCCACAACGGGTCGCACGACAACNTTAAGGCCTCANGCATG
ACCCAACACGGGGGCGTCCCGGNGNGGCGACCTTGNAGTCCGGGGACAAGTATGGCCCCCCGGGAAGG
ACGCCGTAGTGCATCTGGAGTGCCGATTNGTGGTCNAGAATGCCACCACGATGGTATGAGTGGCAAAG
GTACTTTGGACACCACCCCGTTTTCTATTTCCAGACGCGGCCATTCAGGAACGACGCGAATCGTAATC
TACCTGCCGCGTACGCGGATCGTACATGCGTTTTCCAGCGTTCGAAATAGAGTTTGTCGTTCCGCTGA
CCAGGTTNCACGGCTACTGCGATAAGCATATTCTACTGGGGGACTGTAAAGGAATTGTATCCATTCTG
CTTCCTGTCAATTGGCCATGTGCATCTCTGGTTGTTCAGGGTACATTAATAACACACAGCAGTAAGTA
GGTAAGTCTCCGATATAAGCTAACAGAACGTTTGTCAGAGACTGTGGTTCAATTGTAGTGGTATAATG
TTTCCCAGTGCATAAGGAACTCAGGNACACTACTAGCANGGGCGAGGAGAGACCCTTGGGTGGCAACA
CGCAATAAGTGTTACCTAGTACACCAGCACGAGAGAGGGTTTGACCATGTGTTTTCAGNNGACCCCGG
TCGCATTGAACCTGGCATTCAGAATGACGTGCTCTCAAGAATCAAGGTCCTCCCACTGAGGGTCATCN
GCTTCGCGNNNCAGCGTATGGTCATAAACNT
>PaThen501
GTGTAGCTCAGTTAGACACTTCTAAATCCTTNGTAACAGACATGCAGTCCNCTCNNCAGAGCCCCGCC
CCCCGGTGGTACCGGGTTGNTNAGAGGTCCACCACGGGTCNCACGACAACNTCAAGGCCTNANGCATG
ACCCNACGCGGNGACGTACCGGNGCGGCGACCTTGNANGTCGGGNACAAGTATGTCCCCCCGNGAAGG
ACGCCGTAGTGCATCTGGAGTGCCGANNCNTGGTCNAGAATGCCACCACGATGGTATGAGTNGCAAAG
GTATTTTGGACACCACCCCGTTNNCTANNTCCAGACGCGGCCATTCAGGAACGACGCGAATCGTAATC
TACCTGCCGCGTNCGCGGATCGTACANGCGTTTTTCAGCGTTCGANATAGAGTTTGTCGTTCCGCTGA
CCAGGTTTCACGGNTNCTGCGATAAGCATATTCTACGGGGGGACTGTAAAGNAATTGTATCCACTCTG
CTCCCTGTCAATTGGNCATGTGCATCTCTGGTTGTTTAGGNNACATTAATAACACACAGNATTAGGTA
GATAAGTCTCCGATATAAGCGAACAGANCGTTGGTNAAAGANCGTGGTTCAATTGTAGTGGTATAACA
TTTCCCAGNGCATAAGGAACCCAGGNACACTACTAGCANGGGCGAGGAGAGACCCTTGGGTGGCAANA
CGCAATAAGTGTTNCCCAGTACACCAGCACGATNNAGGGTTTGACNATNNNTTTTAGNNGNCCCCGG
TCGCATTGAACCTGGCATTCAGAATGACGTNCTCTNANGAGTCAAGNTCCNCCCACTGAGGGTCATCT
GCTTCGCGNNNTAGCGTATNGTCATNAACAT
>PaThen502
GTGTAGCTCAGTTAGACACNTCTAAATCCTTGGTAACAGACATGCAGTNNCCTCGGCAGAGNNCCGCC
CCCCGGTGGTACCGGGTTGTTNAGAGGTCCACCACGGGTCGCACGACANCNTCAAGGCCTCATGCATG
ACCCAACGCGGGGACGTACCGGCGCGGCGACCTTGAANGTCGGGGACAAGTATGTCCCCCCGNGAAGG
ACGCCGTAGTGCATCTGGAGTGCCGATTCNTGGNCNAGAATGCCACCACGATGGTATGAGTGGCAAAG
GTATTTTGGACACCACCCCGTTTTCTATTTCCAGACGCGGCCATTCAGGAACGACGCGAATCGTAATC

TACCTGCCGCGTACGCGGATCGTACATGCGTTTTTCAGCGTTCGAAATAGAGTTTGTCGTTCCGCTGA
CCAGGTTTCACGGCTACTGCGATAAGCATATTCTACGGGGGGACTGTAAAGGAATTGTATCCACTCTG
CTCCCTGTCAATTGGCCATGTGCATCTCTGGTTGTTTAGGGTACATTAATAACACACAGNATTAGGTA
GATAAGTCTCCGATATAAGCGAACAGAACGTTGGTCAAAGACCGTGGTTCAATTGTAGTGGTATAACA
TTTCCCAGTGCATAAGGAACCCAGGGACACTACTAGCATGGGCGAGGAGAGACCCTTGGGTGGCAANA
CGCAANAAGTGTTACCCAGTACACCAGCACGATAGANGGTTTGACCATGTGTTTTAGACGNCCCCGG
TCGCATTGAACCTGGCATTCAGAATGACGTGCTCTCAAGAGTCAANGTCCTCCCACTGAGGGTCATCT
GCTTCGCGNNNTAGCGTATGGTCACANACAT
>PaThen503
GTGTAGCCCAGTCAGACACNTCTAAATCCTTGGTAACAGACATGCAGTCCCCTCGGCAGAGCCCCGCC
CCCCNATGGTACCGGGTTGTTAAGAGGTCCACAACGGGTCGCACGACAACATTAAGGCCTNANGCATG
ACCCAANACGGGGGCGTCCCGGNGNGGCGACCTTGNANTCCGGGNACAAGTATGGCCCCCCGGGAAGG
ACGCCGTAGTGCATCTGGAGTGCCGATTNNTNGTCCAGANTGCCACCACGATGGTATGAGTGGCAAAG
GTACTTTGGACACCACCCCGTTTTCTANTTCCAGACGCGGCCATTTAGGAACGACGCGAATCGTAATC
TACCTGCCGCGTACGCGGATCGTACATGCGTTTTCCAGCGTTCGAAATAGAGTTNGTCGTTCCGCTGA
CCAGGTTTCACGGCTACTGCGATAAGCATATTCTACTGGGGGACTGTAAAGGAATTGTATCCATTCTG
CTTCCTGTCAATTGGCCATGTGCATCTCTGGTTGTTCAGGGTACATTAATAACACACAGCAGTAAGTA
GGTAAGTCTCCGATATAAGCGAACAGAACGTTTGTCAGAGACTGTGGTTCAATTGTAGTGGTATAATG
TTTCCCAGTGCATAAGGAACTCAGGGACACTACTAGCANGGGCGAGGAGAGACCCTTGGGTGGCAACA
CGCAATAAGTGTTACCTAGTACACCAGCACGAGNNANGGTTTGACCATGTGTTTTAGACGACCCCGG
TCGCATTGAACCTGGCATTCAGAATGACGTNCTCTCAAGAATCAAGGTCCTCCCACTGAGGGNCATCT
GCTTCGCNNNNNCAGCGTATGGTCACNAACAT
>PaThen504
GTGTAGCCCAGTTAGACACNTCTAAATCCNTGGTAACAGACATGCAGTCCCCTCGGCAGAGCNCCGCC
CCCCGATGGTACCGGGTTGTTAAGAGGTCCACAACGGGTCGCACGACAACATTAAGGCCTCATGCATG
ACCCAACACGGGGGCGTCCCGGNGCGGCGACCTTGNAGTCCGGGGACAAGTATGGCCCCCCGGGAAGG
ACGCCGTAGTGCATCTGGAGTGCCGATTNGTGGTCCAGAATGNCACCACGATGGTATGAGTGGCAAAG
GTACTTTGGACACCACCCCGTTTTCTATTTCCAGACGCGGCCATTCAGGAACGACGCGAATCGTAATC
TACCTGCCGCGTACGCGGATCGTACATGCGTTTTCCAGCGTTCGAAATAGAGTTTGTCGTTCCGCTGA
CCAGGTTNCACGGCTACTGCGATAAGCATATTCTACTGGGGGACTGTAAAGGAATTGTATCCACTCTG
CTTCCTGTCAATTGGCCATGTGCATCTCTGGTTGTTCAGGGTACATTAATAACACACAGNATTAAGTA
GATAAGTCTCCGATATAAGCGAACAGAACGTTGGTCAGANACTATGGTTCAATTGTAGTGGTATAATG
TTTCCCAGTGCATAAGGAACTCAGGGACACTACTAGCATGGGCGAGGAGAGACCCTTGGGTGGCAACA
CGCGATAAGTGTTNCCTAGTACATCAGCACGAGAGANGGTTTGACCATGTGTTTTAGACGACCCCGG
TCGCATTGAACCTGGCATTCAGAATGACGTGCTCTCAAGAATCAAGGTCCTCCCACTGAGGGTCATCT
GCTTCGCGNGTCAGCGTATNGTCATNAACAT
>PaThen505
GTGTAGCTCAGTTAGACACTTCTAAATCCTTGGTAACAGACATGCAGTCCCCTCGGCAGAGNNCCGCC
CCCCNGTGGTACCGGGTTGTTNAGAGGTCCACCACGGGTCGCACGACAACNTCAAGGCCTCANGCATG
ACCCANNGCNGGGANGTACCGGCGNGGCGACCTTGNAGGTCGGGGACAAGTATGTCCCCCCGGGAAGG
ACGCCGTAGTGCATCTGGAGTGCCGATTNGTGGTCCAGAATGNCACCACGATGGTATGAGTGGCAAAG
GTATTTNGGACACCACCCCGTTTTCTATTTCCAGACGCGGCCATTCAGGAACGACGCGAATCGTAATC
TACCTGCCGCGTACGCGGATCGTACATGCGTTTTTCAGCGTTCGANATAGAGTTTGTCGTTCCGCTGA
CCAGGTTTCACGGCTACTGCGATAAGCATATTCTACGGGGGGACTGTAAAGGAATTGTATCCACTCTG
CTCCCTGTCAATTGGCCATGTGCATCTCTNGTTGTTTAGGGTACATTAATAACACACAGCATTAGGTA
GATAAGTCTCCGATATAAGCGAACAGAACGTTGGTCAAAGACCGTGGTTCAATTGTAGTGGTATAACA
TTTCCCAGTGCATAAGGAACCCAGGNACACTACTAGCATGGGCGAGGAGAGACCCTTGGGTGGCAACA
CGCAATAAGTGTTACCCAGTACACCAGCACGATAGANGGTTTGACCATGTGTTTTAGACGACCCCGG
TCGCATTGAACCTGGCATTCAGAATGACGTGCTNTCAAGAGTCNAGNTCCTCCCACTGAGGGTCATCT
GCTTCGCNNNNNTAGCGTATGGTCANAAACAT
>PaThen506
GTGTAGCCCAGTCAGACACTTCTAAATCCTTGGTAACAGACATGCAGTCCCCTCGGCAGAGNCCCGCC
CCCCNATGGTACCGGGTTGNTNAGAGGTCCACAACGGGTCGNACGACANCATTAAGGCCTNATGCATG
ACCCAACNCGGGGGCGTCCCGGNGCGGCGACCTTGNAGTCCGGGGACAAGTATGGCCCCCCGGGAAGG

ACGCCGTAGTGCATCTGGAGTGCCGATTCGTGGTCCAGAATGNCACCACGATGGTATGAGTGGCAAAG
GTACTTTGGACACCACCCCGTTTTCTATTTCCAGACGCGGCCATTTAGGAACGACGCGAATCGTAATC
TACCTGCCGCGTNCGCGGATCGTACATGCGTTTTCCAGCGTTCGANATAGAGTTTGTCGTTCCGCTGA
CCAGGTTTCACGGCTACTGCGATAAGCATATTCTACTGGGGGACTGTAAAGGAATTGTATCCATTCTG
CTTCCTGTCAATTGGCCATGTGCATCTCTGGTTGTTCAGGGTACATTAATAACACACAGCAGTAAGTA
GGTAAGTCTCCGATATAAGCGAACAGAACGTTTGTCAGAGACTGTGGTTCAATTGTAGTGGTATAATG
TTTCCCAGTGCATAAGGAACTCAGGGACACTACTAGCATGGGCGAGGAGAGACCCTTGGGTGGCAACA
CGCAATAAGTGTTACCTAGTACACCAGCACGAGAGANGGTTTGACCATGTGTTTTTAGACGACCCCGG
TCGCATTGAACCTGGCATTCAGAATGACGTGCTCTCAAGAATCAAGGTCCTCCCACTGAGGGTCATCT
GCTTCGCGNGTCNGCGTATGGTCACAAACAT
>PaThen507
GTGTAGCCCAGTTAGACACTTCTAAATCCNTGGTAACAGACATGCAGTCCCCTCGGCAGAGCNCCGCC
CCCCGATGGTACCGGGTTGTTAAGNGGTCCACAACGGGTCNNACGACAANNTTAAGGCCTCANGCATG
ACCCAACACGGGGGCGTCCCGGNGCGGCGACCTTGAAGTCCGGGNACAAGTATNGCCCCCCGGGAAGG
ACGCCGTAGTGCATCTGGAGTGCCGATTCGTGGNCNAGANTGNCACCACGATGGTATGAGTGGCAAAG
GTACTTTGGACACCACCCCGTTTTCTATTTCCAGACGCGGCCATTCAGGAACGACGCGAATCGTAATC
TACCTGCCGCGTACGCGGATCGTACATGCGTTTTCCAGCGTTCGAAATAGAGTTTGTCGTTCCGCTGA
CCAGGTTNCACGGCTACTGCGATAAGCATATTCTACTGGGGGACTGTAAAGGAATTGTATCCACTCNG
CTTCCTGTCAATTGGCCATGTGCATCTCTNGNTGTTCAGGGTACATTAATAACACACAGNATTAAGTA
GATAAGTCTCCGATATAAGCGAACAGAACGTTGGTNAGAGACTATGGTTCAATTGTAGTGGTATAATG
TTTCCCAGTGCATAAGGAACTCAGGNACACTACTAGCATGGGCGAGGAGAGACCCTTGGGTGGCAACA
CGCGATAAGTGTTNCCTAGTACATCAGCACGAGAGANGGTTTGACCATGTGTTTTTAGACGACCCCGG
TCGCATTGANCCTGGCATTCAGAATGACGTGCTCTCAAGAATCNAGGTCCNCCCACTGAGGGTCATCN
GCTTCGCGNNTCNGCGTATGGTCATAAACAT
>PaThen508
GTGTAGCCCAGTTAGACACTTNNAAATCCTTGGTAACAGACATGCAGTCCCCTCGGCAGAGCNCCGCC
CCCCNATGGTACCGGGTTGTTAAGAGGTCCACAACAGGTCGCACGACNNNNTTAAGGCCTNATGCATG
ANNCNACNCGGNGGNGTCCCGGCGNGGCGACCTTGNAGTCCGGGGACAAGTATGGCCCCCCGGGAAGG
ACGCCGTAGTGCATCTGGAGTGCCGANTNGTGGTCNAGAATGNCACCACGATGGTATGAGTGGCAAAG
GTACTTNGGACACNACCCCGTTNNCTANTTCCAGACGCGGCCATTCAGGAACGACGCGAATCGTAATC
TACCTGCNGCGTACGCGGATCGTACATGCGTTTTCCAGCGTTCGANATAGAGTTTGTCGTTCCGCCGA
CCAGGTTNCACGGCTACTGCGATAAGCATATTCTACTGGGGGACTGTAAAGGAATTGTATCCACTCNA
CTTCCTGTCAATTGGNCATGTGCATCTCTGGNTGTTCAGGNNACATTAATAACACACAGCATTAAGTA
GNTAAGTCTCCGATATAAGCGAACAGAACGTTGGTCAGANACTGTGGTTCAATTGTAGTGGTATAATG
TTTCCCAGTGCATAAGGAANTTAGGNACATTACTAGNATGGGCGAGGAGAGACCCTTGGGTGGCAACA
CGCAATAAGTGTTACCTAGTACACCAGCACGAGAGANGGTTTGACCATGTNTTTTTAGACGACCCCGG
TCGCATTGAACCTGGCATTCAGAATGACGTGCTCTCAAGAATCNAGNTCCTCCCACTGAGGGTCATCT
GCTTCGCGNNNCNGCGTATGGTCANAAACAT
>PaThen509
GTGTAGCCCAGTCAGACACTTCNAAATCCTTGGTAACAGACATGCAGTCCCCTCGGCAGAGNNCCGCC
CCCCNATGGTACCGGGTTGTTAAGAGGTCCACAACGGGTCNCACGACAANNTTAAGGCCTCANGCATG
ANCCNACACGGGGGCGTCCCGGCGCGGNNACCTTGNAGTCCGGGGNCAAGTATGGCCCCCCGNGAAGG
ACGCCGTAGTGCATCTGGAGTGCCGANNCGTGGTCCAGAATGNCACCACGATGGTATGAGTGGCAAAG
GTACTTTGGACACCACCCCGTTNNCTATTTCCAGANGCGGCCATTTAGGAACGACGCGAATCGTAATC
TACCTGCCGCGTACGCGGATCGTACATGCGTTTTCCAGCGTTCGAAATAGAGTTTGTCGTTCCGCTGA
CCAGGTTTCACGGCTACTGCGATAAGCATATTCTACTGGGGGACTGTAAAGGAATTGTATCCATTCTG
CTTCCTGTCAATTGGCCATGTGCATCTCTNGTTGTTCAGGGTACATTAATAACACACAGNAGTAAGTA
GGTAAGTCTCCGATATAAGCGAACAGANCGTTTGTCAGAGACTGTGGTTCAATTGTAGTGGTATAATG
TTTCCCAGTGCATAAGGAACTCAGGGACACTACTAGCATGGGCGAGGAGAGACCCTTGGGTGGCAACA
CGCAATAAGTGTTNNNTAGTACACCAGCACGAGAGANGGTTTGACCATGNNTTTTTAGACGNCCCCGG
TCGCATTGAACCTGGCATTCAGAATGACGTNCTCTNANGAATCAANNTCCNCCCACTGAGGGTCATCT
GCTTCGCGNNNCNGCGTATGGNCANAAACAT
>PaThen510
GTGTAGCCCAGTTAGACANTTCTAAATCCNTGGTAACAGACATGCAGTCCCCTCGGCAGAGNCCCGCC

CCCCGATGGTACCGGGTTGTTNAGAAGTCCACAACGGGTCGNACGACANNATTAAGGCCTCATGCATG
ACCCNACACGGGGGNGTCCCGGCGCGGCGACCTTGAAGTCCGGGGACAAGTATGGCCCCCCGGGAAGG
ACGCCGTAGTGCATCTGGAGTGCCGATTNGTGGTCNAGAATGNCACCACGATGGTATGAGTGGCAAAG
GTACTTTGGACACCACCCCGTTTTCTATTTCCAGACGCGGCCATTCAGGAACGACGCGAATCGTAATC
TACCTGCCGCGTNCGCGGATCGTACANGCGTTTTCCAGCGTTCGAAATAGAGTTTGTCGTTCCGCTGA
CCAGGTTTCACGGCTACTNCGATAAGCATATTCTACTGGGGGACTGTAAAGGAATTGTATCCATTCTG
CTTCCTGTCAATTGGCCATGTGCATCTCTNGTTGTTCAGGGTACATTAATAACACACAGCAGTAAGTA
GNTAAGTCTCCGATATAAGCTAACAGAACGTTTGTCAGAGACTGTGGTTCAATTGTAGTGGTATAATG
TTTCCCAGTGCATAAGGAACTCAGGGACACTACTAGCATGGGCGAGGAGAGACCCTTGGGTGGCAACA
CGCAATAAGTGTTNCCTAGTACACCAGCACNAGAGAGGGTTTGACCATGTGNNNNCAGACGACCCCGG
TCGCATTGAACCTGGCATTCAGAATGACGTGCTCTCAAGAATCAAGGTCCNCCCACTGAGGGTCATCT
GCTTCGCGNGNCNGCGTATGGTCATAAACAT
>PaThen511
GTGNAGCCCAGTTGGACANNNCTNAATCCTTGGTAACAGACATGCAGTNNCCTCGGCAGAGCNCCGCC
CCCCNGTGGTACCGGGTTGTTNAGAGGTNCACCACGGNTCGNACGACAANATCAAGGCCTCANGCATN
ANNCAACNCGGNGACGTACCGGNGNGGNGACCTTGAANGTCGGGGACAAGTATGTCCCCCCGNGAAGG
ACGCCGTAGTGCATNTGGAGTGCCGATNCNNGGNCNAGANTGCCACCACGANGGTATGAGTNGCAAAG
GTATTTNGGACACCACCCCGTTTTCTATTTCCAGACGCGGCCATTCAGGAACGACGCGAATCGTAATC
TACCTGCCGCGTNCGCGGATCGTACANGCGTTTTTCAGNGTTCGANATAGAGTTNGTCGTTCCGCTGA
CCAGGTTNCACGGCTACTNCGATAAGCATATTCTACGGGGGGACTGTAAAGGAATTGTATCCACTCTG
CTCCCTGTCAATTGGNCATGTNCATCTCTGGTTGTTTAGGNNACATTAATAACACACAGCATGAGGTA
GNTNAGTCTCCGATATAANCGAACAGAACGTTGGTNAAAGANCGTGGTTCNATTGTAGTGGTATAACA
TTTCCCAGTNCATAAGGAACCCAGGNACACTATTAGCATGGGCGAGGAGAGACCCTTGGGTGGCAACA
CGCANTAAGTGTTNCCCNGTACACCAGCACGATANAGGGTTTGACCATNNNNNNTTAGACGACCCCGG
TCGCATTGAACCTGGCATTCAGAATGACGTNCTCTNANGAGTCAANGTCCTCCCACTGAGGGTCATNN
GCTTCGCGNGTTAGCGTATNGTCANAAACAT
>PaThen512
GTGTAGCCCAGTTAGACACTTCTAAATCCTTGGTAACAGACATGCAGTCCCCTCGGCAGAGCNCCGCC
CCCCNATGGTACCGGGTTGTTAAGAGGTCCACAACGGGTCGCACGACAANATTAAGGCCTCANGCATG
ANCCAACACGGGGGCGTCCCGGNGCGGCGACCTTGNANTCCGGGGACAAGTATGGCCCCCCGGGAAGG
ACGCCGTAGTGCATCTGGAGTGCCGATTCNTGGTCNAGAATGCCACCACGATGGTATGAGTGGCAAAG
GTACTTTGGACACCACCCCGTTTTCTATTTCCAGACGCGGCCATTCAGGAACGACGCGAATCGTAATC
TACCTGCCGCGTACGCGGATCGTACATGCGTTTTCCAGCGTTCGAAATAGAGTTTGTCGTTCCGCTGA
CCAGGTTTCACGGCTACTGCGATAAGCATATTCTACTGGGGGACTGTAAAGGAATTGTATCCACTCTG
CTTCCTGTCAATTGGCCATGTGCATCTCTGGTTGTTCAGGGTACATTAATAACACACAGCATTAAGTA
GATAAGTCTCCGATATAAGCGAACAGAACGTTGGTCAGAGACTATGGTTCAATTGTAGTGGTATAATG
TTTCCCAGTGCATAAGGAACTCAGGGACACTACTAGCATGGGCGAGGAGAGACCCTTGGGTGGCAACA
CGCAATAAGTGTTNCCTAGTACATCAGCACGAGANAGGGTTTGACCATGNGTTTTTAGACGACCCCGG
TCGCATTGAACCTGGCATTCAGAATGACGTGCTCTCAAGAATCAAGGTCCTCCCACTGAGGGTCATCT
GCTTCGCGGGTCAGCGTATGGTCATAAACAT
>PaThen513
GTGTAGCCCAGTTAGACACNTCTAAATCCTTGGTAACAGACATGCAGTNNNCNCNNCAGAGNCCCGCC
CCCCNATGGTACCGGGTTGNTNAGAAGTCCACAACGGGTCGCACGACNNNNTTAAGGCCTNANGCATG
ACCCAANACGGNGGNGTCCCGGCGCGGCGACCTTGAANTCCGGGGACAAGTATGGCCCCCCGGGAAGG
ACGCCGTAGTGCATCTGGAGTGCCGATTCNTGGTCNAGAATGNCACCACGATGGTATGAGTNGCAAAG
GTACTTTGGACACCACCCCGTTTTCTATTTCCAGACGCGGCCATTCAGGAACGACGCGAATCGTAATC
TACCTGCCGCGTNCGCGGATCGTACATGCGTTTTCCAGCGTTCGAAATAGAGTTTGTCGTTCCGCTGA
CCAGGTTTCACGGCTACTGCGATAAGCATATTCTACTGGGGGACTGTAAAGGAATTGTATCCATTCTG
CTTCCTGTCAATTGGCCATGTGCATCTCTNGTTGTTCAGGNNACATTAATAACACACAGNAGTAAGTA
GNTAAGTCTCCGATATAAGCTAACAGAACGTTTGTCAGAGACTGTGGTTCAATTGTAGTGGTATAATG
TTTCCCAGNGCATAAGGAACTCAGGGACACTACTAGCATGGGCGAGGAGAGACCCTTGGGTGGCAACA
CGCANTAAGTGTTNCCTAGTACACCAGCACGAGANANGGTTTGACCATGTNTTTTCAGACGACCCCGG
TCGCATTGAACCTGGCATTCAGAATGACGTGCTCTCAAGAATCAAGGTCCTCCCACTGAGGGTCATCN
GCTTCGCGNGTCAGCGTATNGTCACAANNNT

>PaThen514
GTGTAGCCCAGTCAGACACNTCTAAATNCTTGGTAACAGACATGCAGTCCCCTCNNCAGAGCNCCGCC
CCCCNATGGTACCGGGNTGNTAAGAGGTCCACAACGGGTCNNACGACNACNTTAAGGCCTCANGCATG
ANNCNACNCGGGGGCGTCCCGGNGNGGNNACCTTNNANTCCGGGGNCAAGTATNGCCCCCCGNGAAGG
ACGCNGTAGTGCATNTGGAGTGCCGNTTNGTGGTCCAGAATGCCACCACGATGGTATGAGTNGCAAAG
GTACTTNGGACNCCACCCCGTTNNCTANTNCCAGACGCGGCCATTTAGGAACGACGCGAATCGTAATC
TACCTGCNGCGTNCGCGGATCGTACATGCGTTTTCCAGCGTTCGANATAGAGTTTGTCGTTCCGCTGA
CCANGTTTCACGGCTANTNCGATAAGCATATTCTACTGGGGGACTGTAAAGGAATTGTATCCATTCTG
CTTCCTGTCAATTGGCCATGTGCATCTCTNGTTGTTCAGGGTACATTAATAACACACAGNAGTAAGTA
GGTAAGTCTCCGATATAAGCGAACAGAACGTTTGTCAGAGACTGTGGTTCAATTNTAGTGGTATAATG
TTTCCCAGTGCATAAGGAACTCAGGNACACTACTAGNATGGNCGAGGAGAGACCCTTGGGTGGCAACA
CGCAATAAGTGTTACCTNGTACACCAGCACGAGAGANGGTTTGACCATGTGTTTTTAGACGACCCCGG
TCGCATTGAACCTGGCATTCAGAATGACGTNCTCTCANGAATCAANGTCCNCCCACTGAGGGTCATCT
GCTTCGCGNNNCNGCGTATGGNCANNANNNT
>PaThen515
GTGTAGCCCAGTCAGACACTTCTAAATCCTTGGTAACAGACATGCAGTCCCCTCGGCAGAGCNCCGCC
CCCCGATGGTACCGGGTTGTTAAGAGGTCCACAACGGGTCGCACGACAANATTAAGGCCTCANGCATG
ACCCNACACGGGGGCGTCCCGGCGNGGCGACCTTGNAGTCCGGGGACAAGTATGGCCCCCCGNGAAGG
ACGCCGTAGTGCATCTGGAGTGCCGATTCNTGGTCCAGAATGCCACCACGATGGTATGAGTNGCAAAG
GTACTTTGGACACCACCCCGTTTTCTATTTCCAGACGCGGCCATTTAGGAACGACGCGAATCGTAATC
TACCTGCCGCGTNCGCGGATCGTACATGCGTTTTCCAGCGTTCGAAATAGAGTTTGTCGTTCCGCTGA
CCAGGTTTCACGGCTACTGCGATAAGCATATTCTACTGGGGGACTGTAAAGGAATTGTATCCATTCTG
CTTCCTGTCAATTGGCCATGTGCATCTCTNGTTGTTCAGGGTACATTAATAACACACAGCAGTAAGTA
GGTAAGTCTCCGATATAAGCGAACAGAACGTTTGTCAGAGACTGTGGTTCAATTGTAGTGGTATAATG
TTTCCCAGTGCATAAGGAACTCAGGGACACTACTAGCATGGGCGAGGAGAGACCCTTGGGTGGCAACA
CGCAATAAGTGTTACCTAGTACACCAGCACGAGAGAGGGTTTGACCATGTGTTTTTAGACGACCCCGG
TCGCATTGAACCTGGCATTCAGAATGACGTGCTCTCAAGAATCAAGGTCCTCCCACTGAGGGTCATCT
GCTTCGCGGGTCAGCGTATNGTCANNAACAT
>PaThen516
GTGTAGCCCAGTTAGACACTTCTAAATCCTTGGTAACAGACATGCAGTCCCCTCNGCAGAGCNCCGCC
CCCCNATGGTACCGGGTTGTTAAGAGGTCCACAACAGGTCGCACGACANNATTAAGGCCTNANGCATG
ACCCAACACGGGGGCGTCCCGGNGNGGCGACCTTGNAGTCCGGGGACAAGTATGGCCCCCCGGGAAGG
ACGCCGTAGTGCATCTGGAGTGCCGATTCGTGGTCCAGAATGNCACCACGATGGTATGAGTGGCAAAG
GTACTTTGGACACCACCCCGTTTTCTATTTCCAGACGCGGCCATTCAGGAACGACGCGAATCGTAATC
TACCTGCCGCGTACGCGGATCGTACATGCGTTTTCCAGCGTTCGAAATAGAGTTTGTCGTTCCGCCGA
CCAGGTTNCACGGCTACTGCGATAAGCATATTCTACTGGGGGACTGTAAAGGAATTGTATCCACTCTA
CTTCCTGTCAATTGGCCATGTGCATCTCTGGTTGTTCAGGGTACATTAATAACACACAGCATTAAGTA
GATAAGTCTCCGATATAAGCGAACAGAACGTTGGTCAGAGACTGTGGTTCAATTGTAGTGGTATAATG
TTTCCCAGTGCATAAGGAACTTAGGNACATTACTAGCATGGGCGAGGAGAGACCCTTGGGTGGCAACA
CGCAATAAGTGTTANCTAGTACACCAGCACGAGANANGGTTTGACCATGTGTTTTTAGACGACCCCGG
TCGCATTGAACCTGGCATTCAGAATGACGTGCTCTCAAGAATCAAGGTCCTCCCACTGAGGGTCATCT
GCTTCGCGGGTCAGCGTATGGNCATAAACAT
>PaThen517
GTGTNGCCCAGTCAGACACNTCTAAATCCTTGGTAACAGACATGCAGTCCCCTCGGCAGAGCCCCGCC
CCCCGATGGTACCGGGTTGTNAAGAGGTCCACAACGGGTCGCACGACAANNTTAAGGCCTCANGCATG
ACCCAANACGGGGGNGTCCCGGCGNGGCGACCTTGNANTCCGGGGNCAAGTATGGCCCCCCGGGAAGG
ACGCCGTAGTGCATCTGGAGTGCCGATTNGTGGTCNAGANTGCCACCACGATGGTATGAGTGGCAAAG
GTACTTTGGACNCNACCCCGTTNNCTATTTCCAGACGCGGCCATTCAGGAACGACGCGAATCGTAATC
TACCTGCCGCGTACGCGGATCGTACATGCGTTTTCCAGNGTTCGAAATAGAGTTTGTCGTTCCGCTGA
CCAGGTTNCACGGCTACTGCGATAAGCATATTCTACTGGGGGACTGTAAAGGAATTGTATCCATTCNG
CTTCCTGTCAATTGGCCATGTGCATCTCTNGTTGTTCAGGGTACATTAATAACACACAGCAGTAAGTA
GGTAAGTCTCCGATATAAGCGAACAGAACGTTTGTCAGANACTGTGGTTCAATTGTAGTGGTATAATG
TTTCCCAGTGCATAAGGANCTCAGGGACACTACTAGCATGGGCGAGGAGAGACCCTTGGGTGGCAANA
CGCAATAAGTGTTNNNTAGTACACCAGCACGAGAGAGGGTTTGACCATGTNTTTNTAGACGACCCCGG

TCGCATTGAACCTGGCATTCAGAATGACGTGCTNTCAAGAATCAAGGTCCTCCCACTGAGGGTCATCT
GCTTCGCGGGTCNGCGTATGGTCATAAACAT
>PaThen518
GTGTAGCTCAGTTAGACACTTCTAAATCCTTGGTAACAGACATGCAGTCCCCTCGGCAGAGCCCCGCC
CCCCNGTGGTACCGGGTTGTTNAGAGGTCCACCACGGGTCGCACGACANCATCAAGGCCTCANGCATG
ACCCAANGCGGGGANGTACCGGNGCGGCGACCTTGAANGTCGGGGACAAGTATGTCCCCCCGGGAAGG
ACGCCGTAGTGCATCTGGAGTGCCGATTNGTGGTCNAGAATGCCACCACGATGGTATGAGTTGCAAAG
GTATTTTGGACACCACCCCGTTTTCTATTTCCAGACGCGGCCATTCAGGAACGACGCGAATCGTAATC
TACCTGCCGCGTACGCGGATCGTACATGCGTTTTTCAGCGTTCGAAATAGAGTTTGTCGTTCCGCTGA
CCAGGTTTCACGGCTACTGCGATAAGCATATTCTACGGGGGGACTGTAAAGGAATTGTATCCACTCTG
CTCCCTGTCAATTGGCCATGTGCATCTCTNGTTGTTTAGGGNACATTAATAACACACAGCATTAGGTA
GATAAGTCNCCGATATAAGCGAACAGAACGTTGGTCAAAGACCGTGGTTCAATTGTAGTGGTATAACA
TTTCCCAGTGCATAAGGAACCCAGGGACACTACTAGCATGGGCGAGGAGAGACCCTTGGGTGGCAACA
CGCAATAAGTGTTACCCAGTACACCAGCACGATAGAGGGTTTGACCATGTGTTTTTAGACGACCCCGG
TCGCATTGAACCTGGCATTCAGAATGACGTGCTCTCAAGAGTCAAGGTCCNCCCACTGAGGGTCATCT
GCTTCGCGNNNTNGCGTATGGTCATAAACAT
>PaThen519
GTGTAGCCCAGTTAGACACTTCTAAATCCTTGGTAACAGACATGCAGTCCCCTCGGCAGAGNCCCGCC
CCCCGATGGTACCGGGTTGTTAAGAGGTCCACAACGGGTCGCACGACNNNATTAAGGCCTNATGCATG
ACCCANNACNGGGGCGTCCCGGCGCGGCGACCTTGAANTCCGGGGACAAGTATGGCCCCCCGGGAAGG
ACGCCGTAGTGCATCTGGAGTGCCGATTCGTGGTCNAGAATGNCACCACGATGGTATGAGTNGCAAAG
GTACTTTGGACACCACCCCGTTNNCTATTTCCAGACGCGGCCATTCAGGAACGACGCGAATCGTAATC
TACCTGCCGCGTACGCGGATCGTACATGCGTTTTCCAGCGTTCGAAATAGAGTTTGTCGTTCCGCTGA
CCAGGTTTCACGGCTACTGCGATAAGCATATTCTACTGGGGGACTGTAAAGGAATTGTATCCACTCTG
CTTCCTGTCAATTGGCCATGTGCATCTCTNGTTGTTCAGGGTACATTAATAACACACAGCATTAAGTA
GATAAGTCTCCGATATAAGCGAACAGAACGTTGGTNAGAGACTATGGTTCAATTGTAGTGGTATAATG
TTTCCCAGTGCATAAGGAACTCAGGGACACTACTAGCATGGGCGAGGAGAGACCCTTGGGTGGCAACA
CGCAATAAGTGTTACCTAGTACATCAGCACGAGANAGGGTTTGACCATGTGTTTTTAGACGACCCCGG
TCGCATTGAACCTGGCATTCAGAATGACGTGCTCTCAAGAATCAAGGTCCTCCCACTGAGGGTCATCT
GCTTCGCGNNTCNGCGTATGGTCACNAACNT
>PaThen520
GTGTAGCCCAGTCAGACACTTCTAAATCCTTGGTAACAGACATGCAGTCCCCTCNNCAGAGCCCCGCC
CCCCNATGGTACCGGGTTGNTAAGAGGTCCACAACGGGTCGCACGACAANATTAAGGCCTCATGCATG
ACCCAANNCGGNGGNGTCCCGGCGNGGCGACCTTGAAGTCCGGGGACAAGTATGGCCCCCCGGGAAGG
ACGCCGTAGTGCATCTGGAGTGCCGATTCNTGGTCCAGAATGNCACCACGATGGTATGAGTGGCAAAG
GTACTTTGGACACCACCCCGTTTTCTATTTCCAGACGCGGCCATTTAGGAACGACGCGAATCGTAATC
TACCTGCCGCGTACGCGGATCGTACATGCGTTTTCCAGCGTTCGAAATAGAGTTTGTCGTTCCGCTGA
CCAGGTTTCACGGCTACTGCGATAAGCATATTCTACTGGGGGACTGTAAAGGAATTGTATCCATTCTG
CTTCCTGTCAATTGGCCATGTGCATCTCTNGTTGTTCAGGGTACATTAATAACACACAGCAGTAAGTA
GGTAAGTCTCCGATATAAGCGAACAGAACGTTTGTCAGAGACTGTGGTTCAATTGTAGTGGTATAATG
TTTCCCAGTGCATAAGGAACTCAGGNACACTACTAGCATGGGCGAGGAGAGACCCTTGGGTGGCAACA
CGCAATAAGTGTTNCCTAGTACACCAGCACGAGANAGGGTTTGACCANGTGTTTTTAGACGACCCCGG
TCGCATTGAACCTGGCATTCAGAATGACGTGCTCTNAAGAATCNAGGTCCTCCCACTGAGGGTCATCT
GCTTCGCNGGTCAGCGTATGGTCATAAACAT
>PaThen521
GTGTAGCCCAGTCAGACACTTCTAAATCCTTGGTAACAGACATGCAGTCCCCTCGGCAGAGCCCCGCC
CCCCGATGGTACCGGGTTGTTAAGAGGTCCACAACGGGTCGCACGACANCNTTAAGGCCTCATGCATG
ACCCNACACGGGGGCGTCCCGGNGCGGCGACCTTGNANTCCGGGGACAAGTATGGCCCCCCGGGAAGG
ACGCCGTAGTGCATCTGGAGTGCCGATTNGTGGTCNAGANTGNCACCACGATGGTATGAGTNGCAAAG
GTACTTTGGACACCACCCCGTTTTCTATTTCCAGACGCGGCCATTTAGGAACGACGCGAATCGTAATC
TACCTGCCGCGTACGCGGATCGTACATGCGTTTTCCAGCGTTCGAAATAGAGTTTGTCGTTCCGCTGA
CCAGGTTTCACGGCTACTGCGATAAGCATATTCTACTGGGGGACTGTAAAGGAATTGTATCCATTCTG
CTTCCTGTCAATTGGCCATGTGCATCTCTGGTTGTTCAGGGTACATTAATAACACACAGCAGTAAGTA
GGTAAGTCTCCGATATAAGCGAACAGAACGTTTGTCAGAGACTGTGGTTCAATTGTAGTGGTATAATG

TTTCCCAGTGCATAAGGAACTCAGGGACACTACTAGCATGGGCGAGGAGAGACCCTTGGGTGGCAACA
CGCAATAAGTGTTACCTAGTACACCAGCACGAGAGAGGGTTTGACCATGTGTTTTAGACGACCCCGG
TCGCATTGAACCTGGCATTCAGAATGACGTGCTCTCAAGAATCAAGGTCCTCCCACTGAGGGTCATCT
GCTTCGCGGNNCAGCGTATGGNCATAAACAT
>PaThen522
GTGTAGCCCAGTCAGACACTTCTAAATCCNTNGTAACAGACATGCAGTCCCCTCNNCAGAGCNCCGCC
CCCCGATGGTACCGGGTTGTTNAGAGGTCCACAACGGGTCNNACGACNACATTAAGGCCTCANGCATG
ANNCANCACGGNGGCGTCCCGGNGNGGCGACCTTGAANTCCGGGNACAAGTATGGCCCNCCGGGAAGG
ACGCCGTAGTGCATCTGGAGTNNCGATTNGNGGTCNNGAATGCCACCACGNTGGTATGAGTGGCAAAG
GTACTTTGGACACCACCCCGTTTNCTANTTCCAGANGCGGCCATTTAGGAACGACGCGAATCGTAATC
TACCTGCCGCGTACGCGGATCGTACATGCGTTTTCCAGCGTTCGAAATAGAGTTTGTCNNNCCGCTGA
CCAGGTTTCACGGCTACTGCGATAAGCATATTCTACTGGGGGACTGTAAAGGAATTGTATCCATTCTG
CTTCCTGTCAATTGGCCATGTGCATCTCTNGTTGTTCAGGNNACATTAATAACACACAGNAGTAAGTA
GGTNAGTCTCCGATATAAGCGAACAGAACGTTTGTCAGAGACTGTGGTTCAATTGTAGTGGTATAATG
TTTCCCAGNGCATAAGGAACTCAGGNACACTACTAGCATGGGCGAGGAGAGACCCTTGGGTGGCAACA
CGCAATAAGTGTTNCNTAGTACACCAGCACGAGNGANGGTTTGACCNTGTGTTNNTAGACGACCCCGG
TCGCATTGAACCTGGCATTCAGAATGACGTGCTNTNAAGAATCAAGGTCCTCCCACTGAGGGTCATCT
GCTTCGCGNGTCNGCGTATNGTCATAAACAT
>PaThen523
GTGTAGCCCAGTCAGACACTTCTAAATCCTTGGTAACAGACATGCAGTCCCCTCGGCAGAGCNCCGCC
CCCCGATGGTACCGGGTTGTTAAGAGGTCCACAACGGGTCGCACGACAACNTTAAGGCCTCANGCATG
ACCCAACNCGGGGGCGTCCCGGCGNGGCGACCTTGNAGTCCGGGGACAAGTATGGCCCCCCGGGAAGG
ACGCCGTAGTGCATCTGGAGTGCCGATTCGTGNTCNAGAATGNCACCACGATGGTATGAGTNGCAAAG
GTACTTTGGACACCACCCCGTTTTCTATTTCCAGACGCGGCCATTTAGGAACGACGCGAATCGTAATC
TACCTGCCGCGTACGCGGATCGTACATGCGTTTTCCAGCGTTCGAAATAGAGTTTGTCGTTCCGCTGA
CCAGGTTTCACGGCTACTGCGATAAGCATATTCTACTGGGGGACTGTAAAGGAATTGTATCCATTCTG
CTTCCTGTCAATTGGCCATGTGCATCTCTGGTTGTTCAGGNNACATTAATAACACACAGCAGTAAGTA
GGTAAGTCTCCGATATAAGCGAACAGAACGTTTGTCAGAGACTGTGGTTCAATTGTAGTGGTATAATG
TTTCCCAGNGCATAAGGAACTCAGGGACACTACTAGCATGGGCGAGGAGAGACCCTTGGGTGGCAACA
CGCAATAAGTGTTACCTAGTACACCAGCACGAGANAGGGTTTGACCATGTGTTTNTAGACGACCCCGG
TCGCATTGAACCTGGCATTCAGAATGACGTGCTCTCAAGAATCAAGNTCCTCCCACTGAGGGTCATCT
GCTTCGCGGGTCAGCGTATGGTCATANACNT
>PaThen524
GTGTAGCCCAGTTAGACACNTCTAAATCCTTGGTAACAGACATGCAGTNNCCTCGGCAGAGCNCCGCC
CCCCGATGGTACCGGGTTGTTAAGAGGTCCACAACAGGTCGNACGACNNNNTTAAGGCCTCANGCATG
ACCCNACACNGGGGNGTCCCGGCGCGGCNACCTTGAANTCCGGGGNCAAGTATGGCCCCCCGGGAAGG
ACGCCGTAGTGCATCTGGAGTGCCGATTCNTGGTCCAGAATGCCACCACGATGGTATGAGTGGCAAAG
GTACTTTGGACACCACCCCGTTTTCTATTTCCAGACGCGGCCATTCAGGAACGACGCGAATCGTAATC
TANCTGCCGCGTACGCGGATCGTACATGCGTTTTCCAGCGTTCGAAATAGAGTTTGTCGTTCCGCCGA
CCAGGTTTCACGGCTACTGCGATAAGCATATTCTACTGGGGGACTGTAAAGGAATTGTATCCACTCTA
CTTCCTGTCAATTGGCCATGTGCATCTCTNGTTGTTCAGGNNACATTAATAACACACAGNATTAAGTA
GATAAGTCTCCGATATAAGCGAACAGAACGTTGGTNAGAGACTGTGGTTCAATTGTAGTGGTATAATG
TTTCCCAGTNCATAAGGAACTTAGGGACATTACTAGCATGGGCGAGGAGAGACCCTTGGGTGGCAACA
CGCAATAAGTGTTANCTAGTACACCAGCACGAGAGANGGTTTGACCANGTGTTTNTAGACGACCCCGG
TCGCATTGAACCTGGCATTCAGAATGACGTNCTCTCAAGAATCAAGGTCCTCCCACTGAGGGTCATCT
GCTTCGCGGGTCAGCGTATNGTCATNNACAT
>PaThen525
GTGTAGCTCAGTTAGACACTTCTAAATCCTTGGTAACAGACATGCAGTNCCCTCGGCAGAGCCCCGCC
CCCCNGTGGTACCGGGTTGTTAAGAGGTCCACCACGGGTCGCACGACAANNTCAAGGCCTCATGCATG
ACCCNACGCGGGGACGTACCGGCGNGGCGACCTTGAANGTCGGGGACAAGTATGTCCCCCCGGGAAGG
ACGCCGTAGTGCATCTGGAGTGCCGATTCGTGGTCCAGAATGCCACCACGATGGTATGAGTGGCAAAG
GTATTTTGGACACCACCCCGTTTTCTATTTCCAGACGCGGCCATTCAGGAACGACGCGAATCGTAATC
TACCTGCCGCGTACGCGGATCGTACATGCGTTTTTCAGCGTTCGAAATAGAGTTTGTCGTTCCGCTGA
CCAGGTTNCACGGCTACTGCGATAAGCATATTCTACGGGGGGACTGTAAAGGAATTGTATCCACTCTG

CTCCCTGTCAATTGGCCATGTGCATCTCTGGTTGTTTAGGGTACATTAATAACACACAGCATTAGGTA
GATAAGTCTCCGATATAAGCGAACAGAACGTTGGTCAAAGACCGTGGTTCAATTGTAGTGGTATAACA
TTTCCCAGTGCATAAGGAACCCAGGGACACTACTAGCATGGGCGAGGAGAGACCCTTGGGTGGCAACA
CGCAATAAGTGTTANNCAGTACACCAGCACGATAGAGGGTTTGACCATGTGTTTTTAGACGACCCCGG
TCGCATTGAACCTGGCATTCAGAATGACGTGCTCTCAAGAGTCAAGGTCCTCCCACTGAGGGTCATCT
GCTTCGCGNNNTAGCGTATGGTCATAAACNT
>PaThen526
GTGTAGCCCAGTCAGACACTTCTAAATCCTTGGTAACAGACATGCAGTCCCCTCGGCAGAGCNCCGCC
CCCCNATGGTACCGGGTTGTTNAGAGGTCCACAACGGGTCGCACGACANNNTTAAGGCCTNANGCATG
ACNCAANACGGGGGCGTCCCGGNGCGGCNACCTTGAAGTCCGGGGACAAGTATGGCCCCCCGGGAAGG
ACGCCGTAGTGCATCTGGAGTGCCGNTTNNTGGNCCAGAATGCCACCACGATGGTATGAGTGGCAAAG
GTACTTTGGACACCACCCCGTTTTCTANTTCCAGANGCGGCCATTTAGGAACGACGCGAATCGTAATC
TACCTGCCGCGTNCGCGGATCGTACATGCGTTTTCCAGCGTTCGAAATAGAGTTTGTCGTTCCGCTGA
CCAGGTTTCACGGCTACTNCGATAAGCATATTCTACTGGGGGACTGTAAAGGAATTGTATCCATTCTG
CTTCCTGTCAATTGGCCATGTGCATCTCTGGTTGTTCAGGNNACATTAATAACACACAGCAGTAAGTA
GGTAAGTCTCCGATATAAGCGAACAGAACGTTTGTCAGANACTGTGGTTCAATTGTAGTGGTATAATG
TTTCCCAGTGCATAAGGAACTCAGGGACACTACTAGCATGGGCGAGGAGAGACCCTTGGGTGGCAACA
CGCAATAAGTGTTACCTAGTACACCAGCACGAGAGAGGGTTTGACCATGTGTTTTTAGACGACCCCGG
TCGCATTGAACCTGGCATTCAGAATGACGTGCTCTCAAGAATCAAGGTCCTCCCACTGAGGGTCATCT
GCTTCGCGGGTCAGCGTATGGTCATAAACAT
>PaThen527
GTGTAGCCCAGTCAGACACTTCTAAATCCNTGGTAACAGACATGCAGTCCCCTCGGCAGAGCCCCGCC
CCCCNATGGTACCGGGTTGTTNAGAGGTCCACAACGGGTCGNACGACAANNTTAAGGCCTCATGCATG
ACCCAACACGGNGGCGTCCCGGCGCGGCGACCTTGNAGTCCGGGGNCAAGTATGGCCCCCCGGGAAGG
ACGCCGTAGTGCATCTGGAGTGCCGATTCGTGGTCNAGAATGNCACCACGATGGTATGAGTGGCAAAG
GTACTTTGGACACCACCCCGTTTTCTANTTCCAGACGCGGCCATTTAGGAACGACGCGAATCGTAATC
TACCTGCCGCGTACGCGGATCGTACATGCGTTTTCCAGCGTTCGAAATAGAGTTTGTCGTTCCGCTGA
CCAGGTTTCACGGCTACTNCGATAAGCATATTCTACTGGGGGACTGTAAAGGAATTGTATCCATTCTG
CTTCCTGTCAATTGGCCATGTGCATCTCTGGTTGTTCAGGGTACATTAATAACACACAGCAGTAAGTA
GGTNAGTCTCCGATATAAGCGAACAGAACGTTTGTCAGAGACTGTGGTTCAATTGTAGTGGTATAATG
TTTCCCAGTGCATAAGGAACTCAGGNACACTACTAGCANGGGCGAGGAGAGACCCTTGGGTGGCAACA
CGCAATAAGTGTTACCTAGTACACCAGCACGAGAGANGGTTTGACCATGTGTTTNTAGACGACCCCGG
TCGCATTGAACCTGGCATTCAGAATGACGTGCTNTCAAGAATCAAGGTCCTCCCACTGAGGGNCATCT
GCTTCGCGGNNCAGCGTATGGNCATAANCNT
>PaThen528
GTGTAGCCCAGTTAGACACTTCTAAATCCTTGGTAACAGACATGCAGTCCCCTCGGCAGAGNCCCGCC
CCCCGATGGTACCGGGTTGTTAAGAGGTCCACAACGGGTCGCACGACAACATTAAGGCCTCATGCATG
ACCCAACACGGGGGCGTCCCGGCGCGGCGACCTTGAAGTCCGGGGACAAGTATGGCCCCCCGGGAAGG
ACGCCGTAGTGCATCTGGAGTGCCGATTCGTGGTCNAGAATGCCACCACGATGGTATGAGTGGCAAAG
GTACTTTGGACACCACCCCGTTTTCTATTTCCAGACGCGGCCATTCAGGAACGACGCGAATCGTAATC
TACCTGCCGCGTACGCGGATCGTACATGCGTTTTCCAGCGTTCGAAATAGAGTTTGTCGTTCCGCTGA
CCAGGTTTCACGGCTACTGCGATAAGCATATTCTACTGGGGGACTGTAAAGGAATTGTATCCACTCTG
CTTCCTGTCAATTGGCCATGTGCATCTCTGGTTGTTCAGGGTACATTAATAACACACAGCATTAAGTA
GATAAGTCTCCGATATAAGCGAACAGAACGTTGGTCAGAGACTATGGTTCAATTGTAGTGGTATAATG
TTTCCCAGTGCATAAGGAACTCAGGGACACTACTAGCATGGGCGAGGAGAGACCCTTGGGTGGCAACA
CGCGATAAGTGTTACCTAGTACATCAGCACGAGAGAGGGTTTGACCATGTGTTTTTAGACGACCCCGG
TCGCATTGAACCTGGCATTCAGAATGACGTGCTCTCAAGAATCAAGGTCCTCCCACTGAGGGTCATCT
GCTTCGCGGGTCAGCGTATGGTCATAAACAT
>PaThen529
GTGTAGCCCAGTCAGACACTTCTAAATCCTTGGTAACAGACATGCAGTCCCCTCNNCAGAGNCCCGCC
CCCCNATGGTACCGGGTTGNTAAGAGGTCCACAACGGGTCGCACGACNNNNTTAAGGCCTCANGCATG
ACCCAACACGGGGGNGTCCCGGCGNGGNGACCTTGNANTCCGGGGACAAGTATGGCCCCCCGGGAAGG
ACGCCGTAGTGCATCTGGAGTGCCGATTNGTGGTCCAGAATGNCACCACGATGGTATGAGTGGCAAAG
GTACTTTGGACACCACCCCGTTTNCTATTTCCAGACGCGGCCATTTAGGAACGACGCGAATCGTAATC

TACCTGCCGCGTACGCGGATCGTACATGCGTTTTCCAGCGTTCGAAATAGAGTTTGTCGTTCCGCTGA
CCAGGTTTCACGGCTACTGCGATAAGCATATTCTACTGGGGGACTGTAAAGGAATTGTATCCATTCTG
CTTCCTGTCAATTGGCCATGTGCATCTCTNGTTGTTCAGGGTACATTAATAACACACAGNAGTAAGTA
GGTAAGTCTCCGATATAAGCGAACAGAACGTTTGTNAGAGACTGTGGTTCAATTGTAGTGGTATAATG
TTTCCCAGTGCATAAGGAACTCAGGGACACTACTAGCATGGGCGAGGAGAGACCCTTGGGTGGCAACA
CGCAATAAGTGTTACCTAGTACACCAGCACGAGAGAGGGTTTGACCATGTGTTTTTAGACGACCCCGG
TCGCATTGAACCTGGCATTCAGAATGACGTGCTNTCAAGAATCAAGGTCCTCCCACTGAGGGTCATCT
GCTTCGCGNGTCNGCGTATGGTCATAAACAT
>PhuLa141
GTGTAGCCCAGTTAGACACTTCTAAATCCNTNGTAACAGACATGCAGTCCCCNCNNCAGAGCNCCGCC
CCCCGATGGTACCGGGTTGNTAAGAGGTCCACAACGGGTCGCACGACNANNTTAAGGCCTCANGCATG
ACCCAACACGGGGGCGTCCCGGCGNGGCGACCTTGNAGTCCGGGGACAAGTATGGCCCCCCGGGAAGG
ACGCCGTAGTGCATCTGGAGTGCCGANNCGTGGTCNAGAATGNCACCACGATGGTATGAGTNGCAAAG
GTACTTTGGACACCACCCCGTTTTCTATTTCCAGANGCGGCCATTCAGGAACGACGCGAATCGTAATC
TACCTGCCGCGTACGCGGATCGTACATGCGTTTTCCAGNGTTCGAAATAGAGTTTGTCGTTCCGCTGA
CCAGGTTTCACGGCTACTGCGATAAGCATATTCTACTGGGGGACTGTAAAGGAATTGTATCCATTCTG
CTTCCTGTCAATTGGCCATGTGCATCTCTNGTTGTTCAGGGTACATTAATAACACACAGCANNAAGTA
GATAAGTCTCCGATATAAGCGAACAGAACGTTGGTNAGAGACTGTGGTTCAATTGTAGTGGTATAATG
TTTCCCAGTGCATAAGGAACTCAGGGACACTACTAGCATGGGCGAGGAGAGACCCTTGGGTGGCAACA
CGCAATAAGTGTTACCTAGTACACCAGCACGAGNNANGGTTTGACCATGTGTTTTTAGACGACCCCGG
TCGCATTGAACCTGGCATTCAGAATGACGTGCTCTCANGAATCNAGGTCCNCCCACTGAGGGTCATCN
NCTTCGCGNGNCNGCGTATNGTCATAANNAT
>PhuLa142
GTGTAGCCCAGTTNGACACTTCTAAATCCNTGGTAACAGACATGCAGTNNNCTCNNCAGAGCNCCGCC
CCCCNATGGTACCGGGTTGNTNAGAGGTCCACAACGGGTCGCACGACNANNTTAAGGCCTNATGCATG
ACCCNACACGGGGGCGTCCCGGCGCNGCGACCTTGNAGTCCGGGGACAAGTATGGCCCCCCGGGAAGG
ACGCCGTAGTGCATCTGGAGTGCCGANNNGTGGTCNAGAATGNCACCACGATGGTATGAGTGGCAAAG
GTACTTTGGACACCACCCCGTTTNCTATTTCCAGACGCGGCCATTCAGGAACGACGCGAATCGTAATC
TACCTGCCGCGTACGCGGATCGTACATGCGTTTTCCAGCGTTCGANATAGAGTTTGTCGTTCCGCTGA
CCAGGTTTCACGGCTACTGCGATAAGCATATTCTACTGGGGGACTGTAAAGGAATTGTATCCACTCNG
CTTCCTGTCAATTGGCCATGTGCATCTCTGGTTGTTCAGGGTACATTAATAACACACAGNATTAAGTA
GATNAGTCTCCGATATAAGCGAACAGAACGTTGGTNAGAGACTATGGTTCAATTGTAGTGGTATAATG
TTTCCCAGTGCATAAGGAACTCAGGNACACTACTAGCANGGGCGAGGAGAGACCCTTGGGTGGCANCA
CGCAATAAGTGTTNNNTAGTACATCAGCACGAGNNANGGTTTGACCATNNNTTTTTAGACGACCCCGG
TCGCATTGAACCTGGCATTCAGAATGACGTNCTCTCAAGAATCAAGNTCCNCCCACTGAGGGTCATNT
GCTTCGCGGNNCAGCGTATGGTCATNAACAT
>PhuLa143
GTGTAGCCCAGTCAGACACTTCTAAATCCTTGGTAACAGACATGCAGTNCCCTCNNCAGAGNCCCGCC
CCCCGATGGTACCGGGTTGTTNAGAGGTCCACAACGGGTCGCACGACAACNTTAAGGCCTCATGCATG
ACCCAACACGGGGGCGTCCCGGNGCGGCGACCTTGNANTCCGGGGACAAGTATGGCCCCCCGGGAAGG
ACGCCGTAGTGCATCTGGAGTGCCGATTCGTGGTCCAGAATGCCACCACGATGGTATGAGTGGCAAAG
GTACTTTGGACACCACCCCGTTTTCTATTTCCAGANGCGGCCATTTAGGAACGACGCGAATCGTAATC
TACCTGCCGCGTACGCGGATCGTACATGCGTTTTCCAGCGTTCGAAATAGAGTTTGTCGTTCCGCTGA
CCAGGTTTCACGGCTACTGCGATAAGCATATTCTACTGGGGGACTGTAAAGGAATTGTATCCATTCNG
CTTCCTGTCAATTGGCCATGTGCATCTCTGGTTGTTCAGGGTACATTAATAACACACAGCAGTAAGTA
GNTAAGTCTCCGATATAAGCGAACAGAACGTTTGTCAGAGANTGTGGTTCAATTGTAGTGGTATAATG
TTTCCCAGTNCATAAGGAACTCAGGGACACTACTAGCATGGGCGAGGAGAGACCCTTGGGTGGCAACA
CGCAATAAGTGTTACCTAGTACACCAGCACGAGANAGGGTTTGACCATNNNTTTTTAGACGACCCCGG
TCGCATTGAACCTGGCATTCAGAATGACGTGCTCTCAAGAATCAANGTCCTCCCACTGAGGGTCATCT
GCTTCGCGGNNCAGCGTATGGTCATAAACAT
>PhuLa144
GTGTAGCCCAGTTAGACACTTCTAAATCCNTNGTAACAGACATGCAGTCCCCTCNNCAGAGCNCCGCC
CCCCGGTGGTACCGGGTTGTTAAGAGGTCCACCACGGGTCGCACGACNNNNTCAAGGCCTCANGCATG
ACCCNACACGGGGGCGTCCCGGNGCGGNGACCTTGNANGCCGGGGACAAGTATGGCCCCCCGGGAAGG

ACGCCGTAGTGCATCTNGANTGCCGATTNNTGGTCCAGANTGNCACCACGATGGTATGAGTNGCAAAG
GTACTTTGGACACCACCCCGTTTTCTATTTCCAGANGCGGCCATTCAGGAACGACGCGAATCGTAATC
TACCTGCCGCGTACGCGGATCGTACATGCGTTTTCCAGNGTTCGAAATAGAGTTTGTCGTTCCGCTGA
CCAGGTTNCACGGCTACTNCGATAAGCATATTCTACTGGGGGACTGTAAAGGAATTGTATCCACTCTG
CTTCCTGTCAATTGGCCATGTGCATCTCTGGTTGTTCAGGNNACATTAATAACACACAGNATTAAGTA
GNTAAGTCTCCGNTATAAGCGAACAGAACGTTGGTCAAAGACTGTGGTTCAATTGTAGTGGTATAACG
TTTCCCAGTGCATAAGGAACTCAGGGACACTACTANCANGGGCGAGGAGAGACCCTTGGGTGGCAANA
CGCAATAAGTGTTACCTAGTACACCAGCACGATANAGGGTTTGACCATNNNTTTTAGACGACCCCGG
TCGCATTGAACCTGGCATTCAGAATGACGTGCTCTCAAGAGTCAAGGTCCNCCCACTGAGGGTCATCN
GCTTCGCGNGTCNGCGTATNGTCATAAACAT
>PhuLa145
GTGTAGCCCAGTTAGACACTTCTAAATCCTTGGTAACAGACATGCAGTCCCCTCNNCAGAGCCCCGCC
CCCCNGTGGTACCGGGTTGNTNAGAGGTCCACCACGGGTCGCACGACNANNTCAAGGCCTNANGCATG
ACCCAANACGGGGGCGTCCCGGCGCGGNGACCTTGAAGGCCGGGGACAAGTATGGCCCCCCGGGAAGG
ACGCCGTAGTGCATCTGGAGTGCCGATTNGTGGTCCAGANTGCCACCACGATGGTATGAGTNGCAAAG
GTACTTTGGACACCACCCCGTTTTCTATTTCCAGACGCGGCCATTCAGGAACGACGCGAATCGTAATC
TACCTGCCGCGTACGCGGATCGTACATGCGTTTTCCAGCGTTCGAAATAGAGTTTGTCGTTCCGCTGA
CCAGGTTNCACGGCTACTGCGATAAGCATATTCTACTGGGGGACTGTAAAGGAATTGTATCCACTCTG
CTTCCTGTCAATTGGCCATGTGCATCTCTGGTTGTTCAGGGTACATTAATAACACACAGCATTAAGTA
GATAAGTCTCCGATATAAGCGAACAGAACGTTGGTCAAAGACTGTGGTTCAATTGTAGTGGTATAACG
TTTCCCAGTGCATAAGGAACTCAGGNACACTACTAGCATGGGCGAGGAGAGACCCTTGGGTGGNAACA
CGCAATAAGTGTTACCTAGTACACCAGCACGATAGAGGGTTTGACCATGTNTTTTAGNNGACCCCGG
TCGCATTGAACCTGGCATTCAGAATGACGTGCTCTCAAGAGNCAAGGTCCTCCCACTGAGGGTCATCT
GCTTCGCGNGTCAGCGTATNGTCATAAACNT
>PhuLa146
GTGTAGCCCAGTTAGACACNTCTAAATCCTTGGTAACAGACATGCAGTCCCCTCGGCAGAGCCCCGCC
CCCCNGTGGTACCGGGTTGTTAAGAGGTCCACCACGGGTCGCACGACAACATCAAGGCCTCANGCATG
ACCCAANACGGGGGNGTCCCGGCGCGGCGACCTTGAAGGCCGGGGACAAGTATGGCCCCCCGGGAAGG
ACGCCGTAGTGCATCTGGAGTGCCGATTCGTGGTCNNGAATGNCACCACGATGGTATGAGTNGCAAAG
GTACTTTGGACACCACCCCGTTNTCTANTTCCAGACGCGGCCATTCAGGAACGACGCGAATCGTAATC
TACCTGCCGCGTACGCGGATCGTACATGCGTTTTCCAGCGTTCGAAATAGAGTTTGTCGTTCCGCTGA
CCAGGTTTCACGGCTACTGCGATAAGCATATTCTACTGGGGGACTGTAAAGGAATTGTATCCACTCTG
CTTCCTGTCAATTGGCCATGTGCATCTCTNGTTGTTCAGGGTACATTAATAACACACAGCATTAAGTA
GATAAGTCTCCGATATAAGCGAACAGAACGTTGGTCAAAGACTGTGGTTCAATTGTAGTGGTATAACG
TTTCCCAGNGCATAAGGAACTCAGGNACACTACTAGCATGGGCGAGGAGAGACCCTTGGGTGGCAACA
CGCAATAAGTGTTNNNTAGTACACCAGCACGATAGANGGTTTGACCATGTGTTTTTAGACGACCCCGG
TCGCATTGAACCTGGCATTCAGAATGACGTGCTCTCANGAGTCAAGGTCCTCCCACTGAGGGTCATCT
GCTTCGCGNGTCAGCGTATGGTCATNAACAT
>PhuLa147
GTGTAGCCCAGTTAGACACTTCTAAATCCTTGGTAACAGACATGCAGTCCCCTCNNCAGAGCCCCGCC
CCCCGGTGGTACCGGGTTGNTNAGAGGTCCACCACGGGTCGCACGACAACATCAAGGCCTCATGCATG
ANNCAACACGGNGGCGTCCCGGCGCGGCGACCTTGNAGGCCGGGGACAAGTATGGCCCCCCGGGAAGG
ACGCCGTAGTGCATCTGGAGTGCCGATTCNTGGTCCAGANTGNCACCACGATGGTATGAGTNGCAAAG
GTACTTTGGACACCACCCCGTTTTCTATTTCCAGACGCGGCCATTCAGGAACGACGCGAATCGTAATC
TACCTGCCGCGTACGCGGATCGTACATGCGTTTTCCAGCGTTCGAAATAGAGTTTGTCGTTCCGCTGA
CCAGGTTNCACGGCTACTGCGATAAGCATATTCTACTGGGGGACTGTAAAGGAATTGTATCCACTCTG
CTTCCTGTCAATTGGCCATGTGCATCTCTGGTTGTTCAGGGTACATTAATAACACACAGCATTAAGTA
GATAAGTCTCCGATATAAGCGAACAGAACGTTGGTCAAAGACTGTGGTTCAATTGTAGTGGTATAACG
TTTCCCAGTGCATAAGGAACTCAGGNACACTACTAGCATGGGCGAGGAGAGACCCTTGGGTGGCAACA
CGCAATAAGTGTTACCTAGTACACCAGCACGATANAGGGTTTGACCATGTGTTTTTAGACGACCCCGG
TCGCATTGAACCTGGCATTCAGAATGACGTNCTCTCAAGAGTCAAGGTCCTCCCACTGAGGGTCATCT
GCTTCGCGNGTCAGCGTATGGTCATAAACAT
>PhuLa148
GTGNAGCCCAGTTAGACACTTCTAAATCCTTGGTAACAGACATGCAGTCCCCTCNNCAGAGNCCCGCC

CCCCNATGGTACCGGGTTGTTAAGAGGTCCACAACGGGTCGCACGACNNCNTTAAGGCCTCATGCATG
ACCCAACACGGNGGNGTCCCGGCGNNGCGACCTTGAAGTCCGGGGACAAGTATGGCCCCCCGGGAAGG
ACGCCGTAGTGCATCTGGAGTGCCGATTCGTGGTCNAGAATGCCACCACGATGGTATGAGTGGCAAAG
GTACTTTGGACACCACCCCGTTTTCTATTTCCAGANGCGGCCATTCAGGAACGACGCGAATCGTAATC
TACCTGCCGCGTACGCGGATCGTACATGCGTTTTCCAGCGTTCGAAATAGAGTTTGTCGTTCCGCTGA
CCAGGTTTCACGGCTACTGCGATAAGCATATTCTACTGGGGGACTGTAAAGGAATTGTATCCACTCTG
CTTCCTGTCAATTGGNCATGTGCATCTCTGGTTGTTCAGGGTACATTAATAACACACAGCATTAAGTA
GATAAGTCTCCGATATAAGCGAACAGAACGTTGGTCAGAGACTATGGTTCAATTGTAGTGGTATAATG
TTTCCCAGTGCATAAGGAACTCAGGGACACTACTAGCATGGGCGAGGAGAGACCCTTGGGTGGCAACA
CGCAATAAGTGTTACNTAGTACATCAGCACGAGAGANGGTTTGACCATGTGTTTTAGACGACCCCGG
TCGCATTGAACCTGGCATTCAGAATGACGTGCTCTCAAGAATCAAGGTCCTCCCACTGAGGGTCATCT
GCTTCGCGGNNCAGCGTATNGTCATAANNAT
>PhuLa149
GTGTAGCCCAGTTAGACACTTCTAAATCCNTGGTAACAGACATGCAGTCCCCTCGGCAGAGCCCCGCC
CCCCNATGGTACCGGGTTGTTNAGAGGTCCACAACGGGTCGCACGACAANNTTAAGGCCTCATGCATG
ACCCAACACGGGGGCGTCCCGGCGCGGCGACCTTGAANTCCGGGGACAAGTATGGCCCCCCGGGAAGG
ACGCCGTAGTGCATCTGGAGTGCCGATTCGTGGNCCAGANTGNCACCACGNTGGTATGAGTGGCAAAG
GTACTTNGGACACCACCCCGTTTTCTATTTCCAGANGCGGCCATTCAGGAACGACGCGAATCGTAATC
TACCTGCCGCGTNCGCGGATNGTACATGCGTTTTCCAGCGTTCGAAATAGAGTTTGTCGTTCCGCTGA
CCAGGTTTCACGGCTACTGCGATAAGCATATTCTACTGGGGGACTGTAAAGGAATTGTATCCACTCNG
CTTCCTGTCAATTGGCCATGTGCATCTCTGGTTGTTCAGGGTACATTAATAACACACAGNATNAAGTA
GATAAGTCTCCGATATAAGCGAACAGANCGTTGGTCAGAGACTATGGTTCAATTGTAGTGGTATAATG
TTTCCCAGTGCATAAGGANCTCAGGGACACTACTAGCATGGNCGAGGAGAGACCCTTGGGTGGCAACA
CGCAATAAGTGTTACCTAGTACATCAGCACGAGAGAGGGTTTGACCANGTGTTTNTAGNCGACCCCGG
TCGCATTGAACCTGGCATTCAGAATGACGTGCTCTCAAGAATCAAGNTCCTCCCACTGAGGGTCATCN
GCTTCGCGGGTCAGCGTATNGTCATAAACAT
>PhuLa150
GTGTAGCCCAGTTAGACACTTCTAAATCCTTGGTAACAGACATGCAGTCCCCTCGNCAGAGCCCCGCC
CCCCNATGGTACCGGGTTGTTAAGAGGTCCACAACGGGTCGCACGACANNNTTAAGGCCTNANGCATG
ANNCAACNCNGGGGCGTCCCGGNGCGGCGACCTTGNANTCCGGGGACAAGTATGGCCCCCCGGGAAGG
ACGCCGTAGTGCATCTGGAGTGCCGATNNGTGGTCCAGAATGNCACCACGATGGTATGAGTNGCAAAG
GTACTTTGGACACCACCCCGTTTTCTANTNCCAGACGCGGCCATTCAGGAACGACGCGAATCGTAATC
TACCTGCCGCGTACGCGGATNGTACATGCGTTTTCCAGCGTTCGAAATAGAGTTTGTCNTNNCGCTGA
CCAGGTTTCACGGCTACTNCGATAAGCATATTCTACTGGGGGACTGTAAAGGAATTGTATCCACTCNG
CTTCCTGTCAATTGGCCATGTGCATCTCTGGTTGTTCAGGNNACATTAATAACACACAGNATTAAGTA
GATAAGTCNCCGATATAAGCGAACAGAACGTTGGTCAGAGACTATGGTTCAATTGTAGTGGTATAATG
TTTCCCAGTGCATAAGGAACTCAGGGACACTACTAGCANGGGCGAGGAGAGACCCTTGGGTGGCAACA
CGCANTAAGTGTTACCTAGTACATCAGCACGAGAGANGGTTTGACCATGNNTTTTTAGACGACCCCGG
TCGCATTGAACCTGGCATTCAGAATGACGTGCTCTCAAGAATCNANNTCCTCCCACTGAGGGTCATCN
GCTTCGCGNNNCAGCGTATNGTCATAAACAT
>PhuLa151
GTGTAGCCCAGTTAGACACTTCTAAATCCNTGGTAACAGACATGCAGTCCCCTCNNCAGAGCNCCGCC
CCCCNGTGGTACCGGGTTGNTNAGAGGTCCACCACGGGTCGCACGACAANATCAAGGCCNCATGCATG
ANNCANNNCGGGGGNGTCCCGGCGCGGCGACCTTGNAGGCCGGGGACAAGTATGGCCCCCCGNGAAGG
ACGCCGTAGTGCATCTGGAGTGCCGATTNGTGGTCCAGANTGNCACCACGATGGTATGAGTNGCAAAG
GTACTTTGGACACCACCCCGTTTTCTANTTCCAGACGCGGCCATTCAGGAACGACGCGAATCGTAATC
TACCTGCCGCGTACGCGGATCGTACATGCGTTTTCCAGCGTTCGAAATAGAGTTTGTCGTTCCGCTGA
CCAGGTTTCACGGCTACTGCGATAAGCATATTCTACTGGGGGACTGTAAAGGAATTGTATCCACTCTG
CTTCCTGTCAATTGGCCATGTGCATCTCTGGTTGTTCAGGGTACATTAATAACACACAGCATTAAGTA
GATNAGTCTCCGATATAAGCGAACAGAACGTTGGTNAAAGACTGTGGTTCAATTGTAGTGGTATAACG
TTTCCCAGTGCATAAGGAACTCAGGGACACTACTAGCATGGGCGAGGAGAGACCCTTGGGTGGCAACA
CGCAATAAGTGTTACCTAGTACACCAGCACGATAGANGGTTTGNCCATNNNTTTTTAGACGACCCCGG
TCGCATTGAACCTGGCATTCAGAATGACGTGCTCTCNAGAGNCAAGGTCCTCCCACTGAGGGTCATCT
GCTTCGCGNGTCAGCGTATGGTCACAAACAT

>PhuLa152
GTGTAGCCCAGTTAGACACTTCTAAATCCNTNGTAACAGACATGCAGTCCCCTCGGCAGAGCCCCGCC
CCCCGGTGGTACCGGGNNGTTAAGAGGTCCACCACGGGTCGCACGACAACATCAAGGCCTCATGCATG
ANCCNACACGGNGGNGTCCCGGCGCGGNGACCTTGAAGGCCGGGGNCAAGTATGGCCCCCCGGGAAGG
ACGCCGTAGTGCATCTGGAGTGCCGATTNGTGGTCNAGAATGNCACCACGATGGTATGAGTNGCAAAG
GTACTTTGGACACCACCCCGTTTTCTATTTCCAGACGCGGCCATTCAGGAACGACGCGAATCGTAATC
TACCTGCCGCGTACGCGGATCGTACANGCGTTTTCCAGCGTTCGAAATAGAGTTTGTCGTTCCGCTGA
CCAGGTTTCACGGCTACTNCGATAAGCATATTCTACTGGGGGACTGTAAAGGAATTGTATCCACTCTG
CTTCCTGTCAATTGGCCATGTGCATCTCTGGTTGTTCAGGGTACATTAATAACACACAGCATTAAGTA
GATAAGTCTCCGATATAAGCGAACAGAACGTTGGTCAAAGACTGTGGTTCAATTGTAGTGGTATAACG
TTTCCCAGTGCATAAGGAACTCAGGGACACTACTANCATGGNCGAGGAGAGACCCTTGGGTGGCAANA
CGCAATAAGTGTTNCCTAGTACACCAGCACGATNNANGGTTTGACCATGNNTTTTTAGACGACCCCGG
TCGCATTGAACCTGGCATTCAGAATGACGTGCTNTNAAGAGTCAAGGTCCTCCCACTGAGGGTCATCT
GCTTCGCGNGTCAGCGTATNGNCATAAACAT
>PhuLa153
GTGTAGCCCAGTTAGNCACTTCTAAATCCNTGGTAACAGACATGCAGTCCCCTCNNCAGAGCNCCGCC
CCCCGGTGGTACCGGGNTGNTNAGAGGTCCACCACGGGTCGCACGACNNCNTCAAGGCCTCANGCATG
ACCCAANACGGNGGCGTCCCGGCGNGGCGACCTTGNAGGCCGGGGACAAGTATGGCCCCCCGGGAAGG
ACGCCGTAGTGCATCTGGAGTGCCGATTCNTGGTCNAGANTGCCACCACGATGGTATGAGTNGCAAAG
GTACTTTGGACACCACCCCGTTTTCTATTTCCAGACGCGGCCATTCAGGAACGACGCGAATCGTAATC
TACCTGCCGCGTACGCGGATCGTACANGCGTTTTCCAGCGTTCGAAATAGAGTTTGTCGTTCCGCTGA
CCAGGTTNCACGGCTACTGCGATAAGCATATTCTACTGGGGGACTGTAAAGGAATTGTATCCACTCTG
CTTCCTGTCAATTGGCCATGTGCATCTCTNGTTGTTCAGGGTACATTAATAACACACAGCATNAAGTA
GATAAGTCTCCGATATAAGCGAACAGAACGTTGGTCAAAGACTGTGGTTCAATTGTAGTGGTATAACG
TTTCCCAGTGCATAAGGAACTCAGGNACACTACTAGCATGGGCGAGGAGAGACCCTTGGGTGGCAACA
CGCAATAAGTGTTACCTAGTACACCAGCACGATAGANGGTTTGACCATGTGTTTTTAGACGNCCCCGG
TCGCATTGAACCTGGCATTCAGAATGACGTNCTCTNAAGAGTCAAGGTCCTCCCACTGAGGGTCANCT
GCTTCGCGNGTCAGCGTATNGTCANANACAT
>PhuLa154
GTGTAGCCCAGTTAGACACTTCTAAATCCTTGGTAACAGACATGCAGTNCCCTCNGCAGAGCCCCGCC
CCCCNGTGGTACCGGGTTGTTAAGAGGTCCACCACGGGTCGCACGACANNATCAAGGCCTCANGCATG
ACCCAACNCGGGGGNGTCCCGGCGNGGNGACCTTGNANGCCGGGNACAAGTATGGCCCCCCGGGAAGG
ACGCCGTAGTGCATCTGGAGTGCCGATTCGTGGTCCAGAATGCCACCACGATGGTATGAGTGGCAAAG
GTACTTTGGACACCACCCCGTTTTCTANTTCCAGACGCGGCCATTCAGGAACGACGCGAATCGTAATC
TACCTGCCGCGTACGCGGATNGTACANGCGTTTTCCAGCGTTCGAAATAGAGTTTGTCGTTCCGCTGA
CCAGGTTTCACGGCTACTGCGATAAGCATATTCTACTGGGGGACTGTAAAGGAATTGTATCCACTCTG
CTTCCTGTCAATTGGNCATGTGCATCTCTNGTTGTTCAGGGTACATTAATAACACACAGCATTAAGTA
GATAAGTCTCCGATATAAGCGAACAGAACGTTGGTCAAANACTGTGGTTCAATTGTAGTGGTATAACG
TTTCCCAGTNCATAAGGANCTCAGGGACACTACTAGCATGGGCGAGGAGAGACCCTTGGGTGGCAACA
CGCAATAAGTGTTACCTAGTACACCAGCACGATNGAGGGTTTGNCCATGTGTTTTTAGACGACCCCGG
TCGCATTGAACCTGGCATTCAGAATGACGTGCTCTCANGAGTCAAGGTCCTCCCACTGAGGGTCATCT
GCTTCGCGNGTCNGCGTATGGNCATAAACAT
>PhuLa602
GTGTAGCCCAGTTAGACACTTCTAAATCCNTGGTAACAGACATGCAGTCCCCTCGGCAGAGCCCCGCC
CCCCGGTGGTACCGGGTTGNTNAGAGGTCCACCACGGGTCGCACGACAACNTCAAGGCCTCATGCATG
ACCCAACACGGGGGCGTCCCGGCGCGGCGACCTTGAAGGCCGGGGACAAGTATGGCCCCCCGGGAAGG
ACGCCGTAGTGCATCTGGAGTGCCGANNNGTGGTCNAGAATGNCACCACGATGGTATGAGTGGCAAAG
GTACTTTGGACACCACCCCGTTTTCTANTTCCAGACGCGGCCATTCAGGAACGACGCGAATCGTAATC
TACCTGCCGCGTACGCGGATCGTACATGCGTTTTCCAGCGTTCGAAATAGAGTTTGTCGTTCCGCTGA
CCAGGTTNCACGGCTACTGCGATAAGCATATTCTACTGGGGGACTGTAAAGGAATTGTATCCACTCTG
CTTCCTGTCAATTGGCCATGTGCATCTCTNGTTGTTCAGGGTACATTAATAACACACAGCATTAAGTA
GATAAGTCTCCGATATAAGCGAACAGAACGTTGGTCAAAGACTGTGGTTCAATTGTAGTGGTATAACG
TTTCCCAGTGCATAAGGAACTCAGGNACACTACTAGCATGGGCGAGGAGAGACCCTTGGGTGGCAACA
CGCAATAAGTGTTACCTAGTACACCAGCACGATNGANGGTTTGACCATGNNTTTTTAGNNGACCCCGG

TCGCATTGAACCTGGCATTCAGAATGACGTNCTCTCAAGAGTCAAGGTCCTCCCACTGAGGGTCATCT
GCTTCGCGNGTCAGCGTATGGTCATAAACAT
>PhuLa603
GTGTAGCCCAGTTAGACACTTCTAAATCCTTGGTAACAGACATGCAGTCCCCTCGGCAGAGCCCCGCC
CCCCNGTGGTACCGGGTTGTTAAGAGGTCCACCACGGGTCGCACGACAACATCAAGGCCTCATGCATG
ACCCAACACGGGGGCGTCCCGGCGCGGCGACCTTGAANGCCGGGGACAAGTATGGCCCCCCGGGAAGG
ACGCCGTAGTGCATCTGGAGTGCCGATTCGTGGTCCAGAATGNCACCACGATGGTATGAGTGGCAAAG
GTACTTTGGACACCACCCCGTTTTCTATTNCCAGACGCGGCCATTCAGGAACGACGCGAATCGTAATC
TACCTGCCGCGTACGCGGATCGTACATGCGTTTTCCAGCGTTCGAAATAGAGTTTGTCGTTCCGCTGA
CCAGGTTTCACGGCTACTGCGATAAGCATATTCTACTGGGGGACTGTAAAGGAATTGTATCCACTCTG
CTTCCTGTCAATTGGCCATGTGCATCTCTGGTTGTTCAGGGTACATTAATAACACACAGCATTAAGTA
GATAAGTCTCCGATATAAGCGAACAGAACGTTGGTCAAAGACTGTGGTTCAATTGTAGTGGTATAACG
TTTCCCAGTGCATAAGGAACTCAGGGACACTACTAGCATGGGCGAGGAGAGACCCTTGGGTGGCAANA
CGCAATAAGTGTTACCTAGTACACCAGCACGATANAGGGTTTGACCATGTGTTTTTAGACGACCCCGG
TCGCATTGAACCTGGCATTCAGAATGACGTGCTCTCAAGAGTCAAGNTCCTCCCACTGAGGGTCATCT
GCTTCGCGNGTCAGCGTATNGTCATAAACAT
>PhuLa604
GTGTAGCCCAGTTNGACACNTCTAAATCCTTGGTAACAGACATGCAGTCCCCTCGGCAGAGCNCCGCC
CCCCNGTGGTACCGGGTTGTTAAGAGGTCCACCACGGNTCGNACGACAACATCAAGGCCTCATGCATG
ACCCAACACGGGGGNGTCCCGGNGNGGCGACCTTGAANGCCGGGGACAAGTATGGCCCCCCGGGAAGG
ACGCCGTAGTGCATCTGGAGTGCCGATTNGTGGTCCAGAATGCCACCACGATGGTATGAGTGGCAAAG
GTACTTTGGACACCACCCCGTTTTCTATTTCCAGACGCGGCCATTCAGGAACGACGCGAATCGTAATC
TACCTGCCGCGTNCGCGGATCGTACATGCGTTTTCCAGCGTTCGAAATAGAGTTTGTCGTTCCGCTGA
CCAGGTTTCACGGCTACTGCGATAAGCATATTCTACTGGGGGACTGTAAAGGAATTGTATCCACTCNG
CTTCCTGTCAATTGGCCATGTGCATCTCTNGTTGTTCAGGGNACATTAATAACACACAGCATTAAGTA
GATAAGTCTCCGATATAAGCGAACAGAACGTTGGTCAAAGACTGTGGTTCAATTGTAGTGGTATAACG
TTTCCCAGTGCATAAGGAACTCAGGNACACTACTAGCATGGGCGAGGAGAGACCCTTGGGTGGCAACA
CGCAATAAGTGTTACCTAGTACACCAGCACGATANAGGGTTTGNCCATGTGTTTNTAGACGACCCCGG
TCGCATTGAACCTGGCATTCAGAATGACGTNCTCTCAAGAGTCAAGGTCCTCCCACTGAGGGTCATCT
GCTTCGCGNGTCNGCGTATNGTCATANACAT
>PhuLa605
GTGTAGCCCAGTTAGACACTTCTNAATCCTTGGTAACAGACATGCAGTCCCCTCNGCAGAGCCCCGCC
CCCCGGTGGTACCGGGTTGTTAAGAGGTCCACCACGGGTCGCACGACNNCNTCAAGGCCTCANGCATG
ACCCANCACGGNGGNGTCCCGGNGNGGNGACCTTGNANGCCGGGGACAAGTATGGCCCCCCGGGAAGG
ACGCCGTAGTGCATCTGGAGTGCCGATTCNTGGTCCAGANTGNCACCACGATGGTATGAGTNGCAAAG
GTACTTTGGACNCCACCCCGTTTTCTATTTCCAGACGCGGCCATTCAGGAACGACGCGAATCGTAATC
TACCTGCCGCGTACGCGGATNGTACATGCGTTTTCCAGCGTTCGAAATAGAGTTTGTCGTTCCGCTGA
CCAGGTTTCACGGCTACTGCGATAAGCATATTCTACTGGGGGACTGTAAAGGAATTGTATCCACTCTG
CTTCCTGTCAATTGGCCATGTGCATCTCTNGTTGTTCAGGGTACATTAATAACACACAGCATTAAGTA
GATAAGTCTCCGATATAAGCGAACAGAACGTTGGTCAAAGACTGTGGTTCAATTGTAGTGGTATAACG
TTTCCCAGTNCATAAGGAACTCAGGNACACTACTAGCATGGGCGAGGAGAGACCCTTGGGTGGCAACA
CGCAATAAGTGTTACNTAGTACACCAGCACGATNGANGGTTTGACCATGTGTTTTTAGACGACCCCGG
TCGCATTGAACCTGGCATTCAGAATGACGTGCTCTCAAGAGTCAANGTCCTNNCACTGAGGGTCATCT
GCTTCGCGNGTCAGCGTATGGTCACAAACAT
>PhuLa606
GTGTAGCCCAGTTAGACACNTCTAAATCCTTGGTAACAGACATGCAGTCCCCTCGGCAGAGCCCCGCC
CCCCNGTGGTACCGGGTTGTTNAGAGGTCCACCACGGGTCGCACGACAACATCAAGGCCTCATGCATG
ACCCAACACGGGGGCGTCCCGGCGCGGCGACCTTGNAGGCCGGGGACAAGTATGGCCCCCCGGGAAGG
ACGCCGTAGTGCATCTGGAGTGCCGATTCGTGGTCNAGAATGCCACCACGATGGTATGAGTGGCAAAG
GTACTTTGGACACCACCCCGTTTTCTATTTCCAGANGCGGCCATTCAGGAACGACGCGAATCGTAATC
TACCTGCCGCGTNCGCGGATCGTACANGCGTTTTCCAGCGTTCGAAATAGAGTTTGTCGTTCCGCTGA
CCAGGTTNCACGGCTACTGCGATAAGCATATTCTACTGGGGGACTGTAAAGGAATTGTATCCACTCTG
CTTCCTGTCAATTGGCCATGTGCATCTCTGGTTGTTCAGGGTACATTAATAACACACAGCATTAAGTA
GNTAAGTCTCCGATATAAGCGAACAGAACGTTGGTCAAAGACTGTGGTTCAATTGTAGTGGTATAACG

TTTCCCAGTGCATAAGGAACTCAGGGACACTACTAGCATGGGCGAGGAGAGACCCTTGGGTGGCAACA
CGCAATAAGTGTTACCTAGTACACCAGCACGATAGAGGGTTTGACCATGTGTTTTTAGACGACCCCGG
TCGCATTGAACCTGGCATTCAGAATGACGTGCTCTCAAGAGTCAAGGTCCTCCCACTGAGGGTCATCT
GCTTCGCGNGTCNGCGTATGGNCATAAACAT
>PhuLa607
GTGTAGCCCAGTTAGACACTTCTAAATCCTTGGTAACAGACATGCAGTCNCCTCNNCAGAGNCCCGCC
CCCCNGTGGTACCGGGTTGNTNAGNGGTCCACCACGGGTCNCACGACAACATCAAGGCCTCANGCATG
ACCCAACACGGGGGNGTCCCGGNGCNGCGACCTTGAANGCCGGGGNCAAGTATGGCCCCCCGGGAAGG
ACGCCGTAGTGCATCTGGAGTGCCGATTCGTGGTCCAGAATGCCACCACGATGGTATGAGTGGCAAAG
GTACTTTGGACACCACCCCGTTTTCTANTTCCAGACGCGGCCATTCAGGAACGACGCGAATCGTAATC
TACCTGCCGCGTNCGCGGATNGTACATGCGTTTTCCAGCGTTCGAAATAGAGTTTGTCGTTCCGCTGA
CCAGGTTTCACGGCTACTGCGATAAGCATATTCTACTGGGGGACTGTAAAGGAATTGTATCCACTCTG
CTTCCTGTCAATTGGCCATGTGCATCTCTNGTTGTTCAGGNNACATTAATAACACACAGCATTAAGTA
GATAAGTCTCCGATATAAGCGAACAGAACGTTGGTCAAAGACTGTGGTTCAATTGTAGTGGTATAACG
TTTCCCAGTGCATAAGGAACTCAGGNACACTACTAGCATGGGCGAGGAGAGACCCTTGGGTGGCAACA
CGCAATAAGTGTTACCTAGTACACCAGCACGATANANGGTTTGACCATGTGTTTTTAGACGACCCCGG
TCGCATTGAACCTGGCATTCAGAATGACGTNCTCTCAAGAGTCAANGTCCNCCCACTGAGGGTCATCT
GCTTCGCGNGTCAGCGTATNGTCATAACCAT
>PhuLa608
GTGTAGCCCAGTTAGACACTTCTAAATCCTTGGTAACAGACATGCAGTCCCCTCGGCAGAGCCCCGCC
CCCCGGTGGTACCGGGTTGTTNAGAGGTCCACCACGGGTCGCACGACAACATCAAGGCCTCATGCATG
ACCCAACACGGGGGCGTCCCGGCGCGGCGACCTTGNAGGCCGGGGACAAGTATGGCCCCCCGGGAAGG
ACGCCGTAGTGCATCTGGAGTGCCGATTCNTGGTCCAGANTGCCACCACGATGGTATGAGTGGCAAAG
GTACTTTGGACACCACCCCGTTTTCTATTTCCAGACGCGGCCATTCAGGAACGACGCGAATCGTAATC
TACCTGCCGCGTACGCGGATCGTACATGCGTTTTCCAGCGTTCGAAATAGAGTTTGTCGTTCCGCTGA
CCAGGTTTCACGGCTACTGCGATAAGCATATTCTACTGGGGGACTGTAAAGGAATTGTATCCACTCTG
CTTCCTGTCAATTGGCCATGTGCATCTCTGGTTGTTCAGGGTACATTAATAACACACAGCATTAAGTA
GATAAGTCTCCGATATAAGCGAACAGAACGTTGGTCAAAGACTGTGGTTCAATTGTAGTGGTATAACG
TTTCCCAGTGCATAAGGAACTCAGGGACACTACTAGCATGGGCGAGGAGAGACCCTTGGGTGGCAACA
CGCAATAAGTGTTACCTAGTACACCAGCACGATAGAGGGTTTGACCATGTGTTTTTAGACGACCCCGG
TCGCATTGAACCTGGCATTCAGAATGACGTGCTCTCAAGAGTCAAGGTCCNCCCACTGAGGGTCATCN
GCTTCGCGNGTCAGCGTATNGTCATAAACAT
>PhuLa609
GTGTAGCCCAGTTAGACACTTCTAAATCCTTGGTAACAGACATGCAGTCCCCTCGGCAGAGCCCCGCC
CCCCGGTGGTACCGGGTTGTTAAGAGGTCCACCACGGGTCGCACGACAACATCAAGGCCTCANGCATG
ACCCAACACGGNGGCGTCCCGGCGCGGCGACCTTGNAGGCCGGGGACAAGTATGGCCCCCCGGGAAGG
ACGCCGTAGTGCATCTGGAGTGCCGATTCGTGGTCCAGAATGCCACCACGATGGTATGAGTGGCAAAG
GTACTTTGGACACCACCCCGTTTTCTATTTCCAGACGCGGCCATTCAGGAACGACGCGAATCGTAATC
TACCTGCCGCGTACGCGGATCGTACANGCGTTTTCCAGCGTTCGAAATAGAGTTTGTCGTTCCGCTGA
CCAGGTTNCACGGCTACTGCGATAAGCATATTCTACTGGGGGACTGTAAAGGAATTGTATCCACTCTG
CTTCCTGTCAATTGGCCATGTGCATCTCTNGTTGTTCAGGNNACATTAATAACACACAGCATTAAGTA
GATAAGTCTCCGATATAAGCGAACAGAACGTTGGTCAAAGACTGTGGTTCAATTGTAGTGGTATAACG
TTTCCCAGTGCATAAGGAACTCAGGGACACTACTAGCANGGGCGAGGAGAGACCCTTGGGTGGCAACA
CGCAATAAGTGTTACCTAGTACACCAGCACGATNNAGGGTTTGACCATGNNTTTNTAGACGACCCCGG
TCGCATTGAACCTGGCATTCAGAATGACGTGCTCTCAAGAGTCAAGGTCCTCCCACTGAGGGTCATCT
GCTTCGCGNGTCAGCGTATGGTCATAAACAT
>PhuLa610
GTGTAGCCCAGTTAGACACTTCTAAATCCNTNGTAACAGACATGCAGTCCCCTCNNCAGAGCNCCGCC
CCCCGGTGGTACCGGGTTGNTAAGAGGTCCACCACGGGTCNCACGACAACNTCAAGGCCTNATGCATG
ACCCAACACGGGGGCGTCCCGGCGCGGCGACCTTGAANGCCGGGGACAAGTATGGCCCNCCGGGAAGG
ACGCCGTAGTGCATCTGGAGTGCCGATTCGTGGTCNAGAATGNCACCACGATGGTATGAGTNGCAAAG
GTACTTTGGACACCACCCCGTTNNCTATTNCCAGANGCGGCCATTCAGGAACGACGCGAATCGTAATC
TACCTGCCGCGTACGCGGATCGTACATGCGTTTTCCAGCGTTCGAAATAGAGTTTGTCGTTCCGCTGA
CCAGGTTNCACGGCTACTNCGATAAGCATATTCTACTGGGGGACTGTAAAGGAATTGTATCCACTCTG

CTTCCTGTCAATTGGCCATGTGCATCTCTGGTTGTTCAGGGTACATTAATAACACACAGCATTAAGTA
GATAAGTCTCCGATATAAGCGAACAGANCGTTGGTCAAAGACTGTGGTTCAATTGTAGTGGTATAACG
TTTCCCAGTGCATAAGGAACTCAGGGACACTACTANCATGGGCGAGGAGAGACCCTTGGGTGGCAACA
CGCAATAAGTGTTACCTAGTACACCAGCACGATNNAGGGTTTGACCATNNNTTTTAGACGACCCCGG
TCGCATTGAACCTGGCATTCAGAATGACGTNCTCTCAAGAGTCNAGGTCCNCCCACTGAGGGTCATCT
GCTTCGCGNGTCNGCGTATGGTCATAANNNT
>PhuLa611
GTGTAGCCCAGTTAGACACTTCTAAATCCTTGGTAACAGACATGCAGTCCCCTCGGCAGAGCCCCGCC
CCCCGGTGGTACCGGGTTGTTAAGAGGTCCACCACGGGTCGCACGACNNCATCAAGGCCTCATGCATG
ACCCAACACGGGGGCGTCCCGGCGCGGCGACCTTGAAGGCCGGGGACAAGTATGGCCCCCCGGGAAGG
ACGCCGTAGTGCATCTGGAGTGCCGATTCGTGGTCCNGAATGCCACCACGATGGTATGAGTGGCAAAG
GTACTTTGGACACCACCCCGTTTTCTATTTCCAGACGCGGCCATTCAGGAACGACGCGAATCGTAATC
TACCTGCCGCGTACGCGGATCGTACATGCGTTTTCCAGCGTTCGAAATAGAGTTTGTCGTTCCGCTGA
CCAGGTTTCACGGCTACTGCGATAAGCATATTCTACTGGGGGACTGTAAAGGAATTGTATCCACTCTG
CTTCCTGTCAATTGGCCATGTGCATCTCTNGTTGTTCAGGGNACATTAATAACACACAGCATTAAGTA
GATAAGTCTCCGATATAAGCGAACAGAACGTTGGTNAAAGACTGTGGTTCAATTGTAGTGGTATAACG
TTTCCCAGTGCATAAGGAACTCAGGGACACTACTAGCATGGGCGAGGAGAGACCCTTGGGTGGCAACA
CGCAATAAGTGTTNCCTAGTACACCAGCACGATAGANGGTTTGACCATGTGTTTTTAGACGACCCCGG
TCGCATTGAACCTGGCATTCAGAATGACGTGCTCTCAAGAGTCAAGGTCCNCCCACTGAGGGTCATCT
GCTTCGCGNGTCNGCGTATGGTCATAAACAT
>PhuLa612
GTGTAGCCCAGTCAGACACNTCTAAATCCTTNGTAACAGACATGCAGTCCCCTCGGCAGAGCCCCGCC
CCCCGATGGTACCGGGTTGTTNAGNGGTCCACAACGGGTCGCACGACAACATTAAGGCCTCATGCATG
ACCCAACACGGGGGCGTCCCGGCGCGGCGACCTTGNAGTCCGGGGACAAGTATGGCCCCCCGGGAAGG
ACGCCGTAGTGCATCTGGAGTGCCGATTCGTGGTCNAGAATGNCACCACGATGGTATGAGTGGCAAAG
GTACTTTGGACACCACCCCGTTTTCTATTTCCAGACGCGGCCATTCAGGAACGACGCGAATCGTAATC
TACCTGCCGCGTACGCGGATCGTACATGCGTTTTCCAGCGTTCGAAATAGAGTTTGTCGTTCCGCTGA
CCAGGTTTCACGGCTACTGCGATAAGCATATTCTACTGGGGGACTGTAAAGGAATTGTATCCATTCNG
CTTCCTGTCAATTGGCCATGTGCATCTCTGGTTGTTCAGGGNACATTAATAACACACAGCAGTAAGTA
GGTAAGTCTCCGATATAAGCGAACAGAACGTTTGTNAGAGACTGTGGTTCAATTGTAGTGGTATAATG
TTTCCCAGTGCATAAGGAACTCAGGNACACTACTAGCATGGGCGAGGAGAGACCCTTGGGTGGCAACA
CGCAATAAGTGTTACCTAGTACACCAGCACGAGANAGGGTTTGACCATGTGTTTTTAGNNGACCCCGG
TCGCATTGAACCTGGCATTCAGAATGACGTNCTCTCAAGAATCAAGGTCCTCCCACTGAGGGTCATCT
GCTTCGCGGGTCAGCGTATGGNCATNAACAT
>PhuLa613
GTGTAGCCCAGTCAGACACTTCTAAATCCTTNGTAACAGACATGCAGTCCCCTCNNCAGAGNNCCGCC
CCCCNATGGTACCGGGTTGTTAAGNGGTCCACAACGGGTCGCACGACAANATTAAGGCCTCATGCATG
ANNCAACNCGGGGGCGTCCCGGCGCGGCGACCTTGNAGTCCGGGGNCAAGTATGGCCCCCCGGGAAGG
ACGCCGTAGTGCATCTGGAGTGCCGATTCNTGGTCNAGAATGCCACCACGATGGTATGAGTGGCAANG
GTACTTTGGACACCACCCCGTTTTCTATTNCCAGANGCGGCCATTCAGGAACGACGCGAATCGTAATC
TACCTGCCGCGTACGCGGATCGTACATGCGTTTTCCAGCGTTCGAAATAGAGTTNGTCGTTCCGCTGA
CCAGGTTNCACGGCTACTGCGATAAGCATATTCTACTGGGGGACTGTAAAGGAATTGTATCCATTCNG
CTTCCTGTCAATTGGCCATGTGCATCTCTGGTTGTTCAGGNNACATTAATAACACACAGCAGNAAGTA
GGTNAGTCNCCGATATAAGCGAACAGAACGTTTGTCAGAGACTGTGGTTCAATTGTAGTGGTATAATG
TTTCCCAGTGCATAAGGAACTCAGGGACACTACTAGCATGGGCGAGGAGAGACCCTTGGGTGGCAACA
CGCAATAAGTGTTNCCTAGTACACCAGCACGAGANAGGGTTTGACCNTGTGTTTNTAGACGNCCCCGG
TCGCATTGAACCTGGCATTCAGAATGACGTNCTCTNAAGAATCAAGGTCCNCCCACTGAGGGTCATCN
GCTTCGCGGGTCAGCGTATGGTCATAAACAT
>PhuLa614
GTGTAGCCCAGTTAGACACTTCTAAATCCTTNGTAACAGACATGCAGTCCCCTCGGCAGAGCNCCGCC
CCCCNGTGGTACCGGGTTGNTNAGAGGTCCACCACGGGTCGCACGACAACNTCAAGGCCTCATGCATG
ACCCAACACGGNGGCGTCCCGGCGCGGCGACCTTGAAGGCCGGGGNCAAGTATGGCCCCCCGGGAAGG
ACGCCGTAGTGCATCTGGAGTGCCGATTCGTGGTCCAGANTGCCACCACGATGGTATGAGTNGCAAAG
GTACTTTGGACACCACCCCGTTTTCTATTNCCAGACGCGGCCATTCAGGAACGACGCGAATCGTAATC

TACCTGCCGCGTNCGCGGATCGTACATGCGTTTTCCAGCGTTCGAAATAGAGTTTGTCGTTCCGCTGA
CCAGGTTTCACGGCTACTGCGATAAGCATATTCTACTGGGGGACTGTAAAGNAATTGTATCCACTCTG
CTTCCTGTCAATTGGCCATGTGCATCTCTNGTTGTTCAGGGTACATTAATAACACACAGCATTAAGTA
GATNAGTCNCCGATATAAGCGAACAGAACGTTGGTCAAAGACTGTGGTTCAATTGTAGTGGTATAACG
TTTCCCAGTGCATAAGGAACTCAGGGACACTACTAGCANGGGCGAGGAGAGACCCTTGGGTGGCAACA
CGCAATAAGTGTTACCTAGTACACCAGCACGATNGAGGGTTTGACCNTGTGTTTNTAGACGACCCCGG
TCGCATTGAACCTGGCATTCAGAATGACGTGCTCTCAAGAGTCNAGGTCCTCCCACTGAGGGTCATCT
GCTTCGCGNGTCAGCGTATNGTCATAAACAT
>PhuLa615
GTGTAGCCCAGTTAGACACTTCTAAATCCTTGGTAACAGACATGCAGTNNCCTCGGCAGAGCCCCGCC
CCCCGGTGGTACCGGGTTGTTAAGAGGTCCACCACGGGTCGCACGACAANNTCAAGGCCTCANGCATG
ACCCAACACGGGGGCGTCCCGGCGCGGCGACCTTGAANGCCGGGGACAAGTATGGCCCCCCGGGAAGG
ACGCCGTAGTGCATCTGGAGTGCCGATTCGTGGTCCAGAATGCCACCACGATGGTATGAGTNGCAAAG
GTACTTTGGACACCACCCCGTTTTCTANTTCCAGACGCGGCCATTCAGGAACGACGCGAATCGTAATC
TACCTGCCGCGTACGCGGATCGTACATGCGTTTTCCAGCGTTCGAAATAGAGTTTGTCGTTCCGCTGA
CCAGGTTTCACGGCTACTGCGATAAGCATATTCTACTGGGGGACTGTAAAGGAATTGTATCCACTCTG
CTTCCTGTCAATTGGCCATGTGCATCTCTGGTTGTTCAGGGTACATTAATAACACACAGCATTAAGTA
GATAAGTCTCCGATATAAGCGAACAGAACGTTGGTCAAAGACTGTGGTTCAATTGTAGTGGTATAACG
TTTCCCAGTGCATAAGGANCTCAGGGACACTACTAGCATGGGCGAGGAGAGACCCTTGGGTGGCAACA
CGCAATAAGTGTTNCCTAGTACACCAGCACGATAGAGGGTTTGACCATGTGTTTTTAGACGNCCCCGG
TCGCATTGAACCTGGCATTCAGAATGACGTGCTCTCAAGAGTCAAGGTCCTCCCACTGAGGGTCATCT
GCTTCGCGNGTCAGCGTATNGTCATNNACAT
>PhuLa616
GTGTAGCCCAGTCAGACACTTCTAAATCCTTGGTAACAGACATGCAGTCCCCTCGGCAGAGCCCCGCC
CCCCGATGGTACCGGGTTGTTAAGAGGTCCACAACGGGTCGCACGACAACATTAAGGCCTNATGCATG
ACCCAACACGGGGGCGTCCCGGCGCGGCGACCTTGAAGTCCGGGGACAAGTATGGCCCCCCGGGAAGG
ACGCCGTAGTGCATCTGGAGTGCCGATTCGTGGTCCAGAATGCCACCACGATGGTATGAGTGGCAAAG
GTACTTTGGACACCACCCCGTTTTCTATTTCCAGACGCGGCCATTCAGGAACGACGCGAATCGTAATC
TACCTGCCGCGTACGCGGATCGTACATGCGTTTTCCAGCGTTCGAAATAGAGTTTGTCGTTCCGCTGA
CCAGGTTNCACGGCTACTGCGATAAGCATATTCTACTGGGGGACTGTAAAGGAATTGTATCCATTCTG
CTTCCTGTCAATTGGCCATGTGCATCTCTNGTTGTTCAGGGNACATTAATAACACACAGCAGTAAGTA
GGTAAGTCTCCGATATAAGCGAACAGAACGTTTGTCAGAGACTGTGGTTCAATTGTAGTGGTATAATG
TTTCCCAGTGCATAAGGAACTCAGGGACACTACTAGCATGGGCGAGGAGAGACCCTTGGGTGGCAACA
CGCAATAAGTGTTACCTAGTACACCAGCACGAGAGAGGGTTTGACCATGTGTTTTTAGACGACCCCGG
TCGCATTGAACCTGGCATTCAGAATGACGTGCTCTNAAGAATCAAGGTCCTCCCACTGAGGGTCATCT
GCTTCGCGGGTCAGCGTATGGTCATAAACAT
>PhuLa617
GTGTAGCCCAGTTAGACACTTCTAAATCCNTGGTAACAGACATGCAGTCCCCTCGGCAGAGCNCCGCC
CCCCGGTGGTACCGGGTTGTTAAGAGGTCCACCACGGGTCGNACGACAACATCAAGGCCTCANGCATG
ANNCANCACGGGGGCGTCCCGGNGCGGCGACCTTGNANGCCGGGGACAAGTATGGCCCCCCGGGAAGG
ACGCCGTAGTGCATCTGGAGTGCCGATTNGTGGTCNAGAATGNCACCACGATGGTATGAGTNGCAAAG
GTACTTTGGACACCACCCCGTTTTCTANTTCCAGACGCGGCCATTCAGGAACGACGCGAATCGTAATC
TACCTGCCGCGTACGCGGATCGTACATGCGTTTTCCAGCGTTCGAAATAGAGTTTGTCGTTCCGCTGA
CCAGGTTTCACGGCTACTGCGATAAGCATATTCTACTGGGGGACTGTAAAGGAATTGTATCNACTCNG
CTTCCTGTCAATTGGCCATGTGCATCTCTGGTTGTTCAGGNNACATTAATAACACACAGCATTAAGTA
GATAAGTCTCCGATATAAGCGAACAGAACGTTGGTCAAAGACTGTGGTTCAATTGTAGTGGTATAACG
TTTCCCAGTGCATAAGGAACTCAGGNACACTACTAGCATGGGCGAGGAGAGACCCTTGGGTGGCAACA
CGCAATAAGTGTTACCTAGTACACCAGCACGATANAGGGTTTGACCANGTGTTTTTAGNCGACCCCGG
TCGCATTGAACCTGGCATTCAGAATGACGTGCTNTCAAGAGTCAAGGTCCTCCCACTGAGGGTCATCN
GCTTCGCGNGTCAGCGTATGGTCATAAACNT
>PhuLa618
GTGTAGCCCAGTTAGACACTTCTAAATCCTTGGTAACAGACATGCAGTCCCCTCGGCAGAGCCCCGCC
CCCCGGTGGTACCGGGTTGTTAAGAGGTCCACCACGGGTCGCACGACAACATCAAGGCCTCATGCATG
ACCCAANNCGGGGGCGTCCCGGCGCGGCGACCTTGAAGGCCGGGGACAAGTATGGCCCCCCGGGAAGG

ACGCCGTAGTGCATCTGGAGTGCCGATTCGTGGTCCAGAATGCCACCACGATGGTATGAGTNGCAAAG
GTACTTTGGACACCACCCCGTTTTCTANTTCCAGACGCGGCCATTCAGGAACGACGCGAATCGTAATC
TACCTGCCGCGTNCGCGGATCGTACATGCGTTTTCCAGCGTTCGAAATAGAGTTTGTCGTTCCGCTGA
CCAGGTTTCACGGCTACTGCGATAAGCATATTCTACTGGGGGACTGTAAAGGAATTGTATCCACTCTG
CTTCCTGTCAATTGGCCATGTGCATCTCTGGTTGTTCAGGGTACATTAATAACACACAGCATTAAGTA
GATAAGTCTCCGATATAAGCGAACAGAACGTTGGTNAAAGACTGTGGTTCAATTGTAGTGGTATAACG
TTTCCCAGTGCATAAGGAACTCAGGGACACTACTAGCATGGGCGAGGAGAGACCCTTGGGTGGCAACA
CGCAATAAGTGTTACCTAGTACACCAGCACGATAGAGGGTTTGACCATGTGTTTTTAGACGACCCCGG
TCGCATTGAACCTGGCATTCAGAATGACGTGCTCTCAAGAGTCAAGGTCCTCCCACTGAGGGTCATCT
GCTTCGCGNGTCAGCGTATGGTCATAAACAT
>PhuLa619
GTGTAGCCCAGTTAGACACNTCTAAATCCTTGGTAACAGACATGCAGTNCCCTCNNCAGAGCNCCGCC
CCCCNGTGGTACCGGGTTGTTAAGAGGTCCACCACGGGTCGNACGACNNCATCAAGGCCTCANGCATG
ACCCAACACGGNGGCGTCCCGGNGCGGCGACCTTGNAGGCCGGGGACAAGTATGGCCCCCCGNGAAGG
ACGCCGTAGTGCATCTGGAGTGCCGATTCGTNGTCCAGAATGCCACCACGATGGTATGAGTGGCAAAG
GTACTTTGGACACCACCCCGTTTTCTATTTCCAGACGCGGCCATTCAGGAACGACGCGAATCGTAATC
TACCTGCCGCGTACGCGGATCGTACATGCGTTTTCCAGCGTTCGAAATAGAGTTTGTCGTTCCGCTGA
CCAGGTTTCACGGCTACTGCGATAAGCATATTCTACTGGGGGACTGTAAAGGAATTGTATCCACTCTG
CTTCCTGTCAATTGGCCATGTGCATCTCTGGTTGTTCAGGGTACATTAATAACACACAGCATTAAGTA
GATAAGTCTCCGATATAAGCGAACAGAACGTTGGTCAAAGACTGTGGTTCAATTGTAGTGGTATAACG
TTTCCCAGTGCATAAGGAACTCAGGGACACTACTAGCATGGGCGAGGAGAGACCCTTGGGTGGCAACA
CGCAATAAGTGTTACCTAGTACACCAGCACGATANANGGTTTGACCATGTGTTTTTAGACGACCCCGG
TCGCATTGAACCTGGCATTCAGAATGACGTGCTCTCAAGAGTCAAGGTCCNCCCACTGAGGGTCATCT
GCTTCGCGNGTCAGCGTATGGTCATAAACAT
>PhuLa620
GTGTAGCCCAGTTAGACACTTCTAAATCCTTGGTAACAGACATGCAGTCCCCTCNNCAGAGCCCCGCC
CCCCNGTGGTACCGGGTTGTTAAGAGGTCCACCACGGGTCGCACGACAACNTCAAGGCCTCATGCATG
ACCCAACACGGGGGCGTCCCGGCGCGGCNACCTTGAAGGCCGGGGACAAGTATGGCCCCCCGGGAAGG
ACGCCGTAGTGCATCTGGAGTGCCGATTNGTGGTCCAGAATGNCACCACGATGGTATGAGTNGCAAAG
GTACTTTGGACACCACCCCGTTNNCTATTTCCAGACGCGGCCATTCAGGAACGACGCGAATCGTAATC
TACCTGCCGCGTACGCGGATCGTACATGCGTTTTCCAGCGTTCGAAATAGAGTTTGTCGTTCCGCTGA
CCAGGTTTCACGGCTACTNCGATAAGCATATTCTACTGGGGGACTGTAAAGGAATTGTATCCACTCTG
CTTCCTGTCAATTGGCCATGTGCATCTCTGGTTGTTCAGGGTACATTAATAACACACAGCATTAAGTA
GATAAGTCTCCGATATAAGCGAACAGAACGTTGGTCAAAGACTGTGGTTCAATTGTAGTGGTATAACG
TTTCCCAGTGCATAAGGAACTCAGGGACACTACTAGCATGGGCGAGGAGAGACCCTTGGGTGGCAACA
CGCAATAAGTGTTANCTAGTACACCAGCACGATANAGGGTTTGACCATGTGTTTTTAGNNGACCCCGG
TCGCATTGAACCTGGCATTCAGAATGACGTGCTCTCAAGAGTCAAGGTCCTCCCACTGAGGGTCATCT
GCTTCGCGNGTCNGCGTATGGTCATAAACAT
>PhuLa621
GTGTAGCCCAGTTAGACACTTCTAAATCCTTGGTAACAGACATGCAGTCCCCTCNNCAGAGCCCCGCC
CCCCNGTGGTACCGGGTTGTTAAGAGGTCCACCACGGGTCGCACGACAANATCAAGGCCTCATGCATG
ACCCAACACGGGGGCGTCCCGGCGCGGCGACCTTGAAGGCCGGGGACAAGTATGGCCCCCCGGGAAGG
ACGCCGTAGTGCATCTGGAGTGCCGATTCGTGGTCCAGAATGCCACCACGATGGTATGAGTGGCAAAG
GTACTTTGGACACCACCCCGTTTTCTATTTCCAGACGCGGCCATTCAGGAACGACGCGAATCGTAATC
TACCTGCCGCGTACGCGGATCGTACATGCGTTTTCCAGCGTTCGAAATAGAGTTTGTCGTTCCGCTGA
CCAGGTTTCACGGCTACTGCGATAAGCATATTCTACTGGGGGACTGTAAAGGAATTGTATCCACTCTG
CTTCCTGTCAATTGGCCATGTGCATCTCTGGTTGTTCAGGGTACATTAATAACACACAGCATTAAGTA
GATAAGTCTCCGATATAAGCGAACAGAACGTTGGTCAAAGACTGTGGTTCAATTGTAGTGGTATAACG
TTTCCCAGTGCATAAGGAACTCAGGGACACTACTAGCATGGGCGAGGAGAGACCCTTGGGTGGCAACA
CGCAATAAGTGTTACCTAGTACACCAGCACGATNGAGGGTTTGACCATGTGTTTTTAGACGACCCCGG
TCGCATTGAACCTGGCATTCAGAATGACGTGNTCTCAAGAGTCAAGGTCCTCCCACTGAGGGTCATCT
GCTTCGCGNGTCAGCGTATGGTCATAAACAT
>PhuLa622
GTGTAGCCCAGTTAGACACTTCTAAATCCTTGGTAACAGACATGCAGTCCCCTCNNCAGAGCCCCGCC

CCCCGGTGGTACCGGGTTGTTAAGAGGTCCACCACGGGTCGCACGACAACNTCAAGGCCTCATGCATG
ACCCAACACGGGGGCGTCCCGGCGCGGCGACCTTGAAGGCCGGGGACAAGTATGGCCCCCCGGGAAGG
ACGCCGTAGTGCATCTGGAGTGCCGATTNGTGGTCNAGAATGNCACCACGATGGTATGAGTGGCAAAG
GTACTTTGGACACCACCCCGTTTTCTATTTCCAGANGCGGCCATTCAGGAACGACGCGAATCGTAATC
TACCTGCCGCGTACGCGGATCGTACATGCGTTTTCCAGCGTTCGAAATAGAGTTTGTCGTTCCGCTGA
CCAGGTTTCACGGCTACTGCGATAAGCATATTCTACTGGGGGACTGTAAAGGAATTGTATCCACTCTG
CTTCCTGTCAATTGGCCATGTGCATCTCTGGTTGTTCAGGGTACATTAATAACACACAGCATTAAGTA
GATAAGTCTCCGATATAAGCGAACAGAACGTTGGTCAAAGACTGTGGTTCAATTGTAGTGGTATAACG
TTTCCCAGTGCATAAGGAACTCAGGGACACTACTAGCATGGGCGAGGAGAGACCCTTGGGTGGCAACA
CGCAATAAGTGTTACCTAGTACACCAGCACGATAGAGGGTTTGACCATGTGTTTTTAGACGACCCCGG
TCGCATTGAACCTGGCATTCAGAATGACGTGCTCTCAAGAGTCAAGNTCCTCCCACTGAGGGTCATCT
GCTTCGCGNGTCAGCGTATGGTCATAAACAT
>SiLa427
GTGTAGCCCAGTTAGACACNTCTAAATCCTTGGTAACAGACATGCAGTCCCCTCGGCAGAGCCCCGCC
CCCCNATGGTACCGGGTTGNTAAGAGGTCCACAACGGGTCGCACGACNNCATTAAGGCCTNATGCATG
ACCCAACACGGGGGCGTCCCGGCGCGGCGACCTTGAANTCCGGGGNCAAGTATGGCCCCCCGGGAAGG
ACGCCGTAGTGCATCTGGAGTGCCGATTCGTGGTCCAGAATGCCACCACGATGGTATGAGTGGCAAAG
GTACTTNGGACACCACCCCGTTTTCTATTTCCAGACGCGGCCATTCAGGAACGACGCGAATCGTAATC
TACCTGCCGCGTACGCGGATCGTACATGCGTTTTCCAGCGTTCGAAATAGAGTTTGTCGTTCCGCTGA
CCAGGTTTCACGGCTACTGCGATAAGCATATTCTACTGGGGGACTGTAAAGGAATTGTATCCACTCTG
CTTCCTGTCAATTGGCCATGTGCATCTCTNGTTGTTCAGGGTACATTAATAACACACAGNATTAAGTA
GATAAGTCTCCGATATAAGCGAACAGAACGTTGGTCAGAGACTGTGGTTCAATTGTAGTGGTATAATG
TTTCCCAGTGCATAAGGAACTCAGGGACACTACTANCATGGGCGAGGAGAGACCCTTGGGTGGCAACA
CGCAATAAGTGTTACCTAGTACACCAGCACGAGAGAGGGTTTGACCATGTGTTTTTAGACGACCCCGG
TCGCATTGAACCTGGCATTCAGAATGACGTGCTCTCAAGAATCAAGGTCCTCCCACTGAGGGTCATCT
GCTTCGCGGGTCNGCGTATNGNCATNAACAT
>SiLa428
GTGTAGCTCAGTTAGACACTTCTAAATCCTTGGTAACAGACATGCAGTNCCCTCGGCAGAGCCCCGCC
CCCCGGTGGTACCGGGTTGTTAAGAGGTCCACCACGGGTCGCACGACAANATCAAGGCCTCATGCATG
ACCCAACGCGGGGACGTACCGGCGCGGCGACCTTGAAGGTCGGGGACAAGTATGTCCCCCCGGGAAGG
ACGCCGTAGTGCATCTGGAGTGCCGATTNGTGGTCCAGANTGNCACCACGATGGTATGAGTNGCAAAG
GTATTTTGGACACCACCCCGTTTTCTATTTCCAGACGCGGCCATTCAGGAACGACGCGAATCGTAATC
TACCTGCCGCGTACGCGGATCGTACATGCGTTTTTCAGCGTTCGAAATAGAGTTTGTCGTTCCGCTGA
CCAGGTTNCACGGCTACTGCGATAAGCATATTCTACGGGGGGACTGTAAAGGAATTGTATCCACTCTG
CTCCCTGTCAATTGGCCATGTGCATCTCTGGTTGTTTAGGGTACATTAATAACACACAGCATTAGGTA
GATAAGTCTCCGATATAAGCGAACAGAACGTTGGTCAAAGACCGTGGTTCAATTGTAGTGGTATAACA
TTTCCCAGTGCATAAGGAACCCAGGGACACTACTAGCATGGGCGAGGAGAGACCCTTGGGTGGCAACA
CGCAATAAGTGTTACCCAGTACACCAGCACGATAGAGGGTTTGACCATGTGTTTTTAGACGACCCCGG
TCGCATTGAACCTGGCATTCAGAATGACGTGCTCTCAAGAGTCAAGGTCCTCCCACTGAGGGTCATCT
GCTTCGCGNNNTAGCGTATGGTCATAAACAT
>SiLa429
GTGTAGCCCAGTTAGACACTTCTAAATCCTTNGTAACAGACATGCAGTCCCCTCGGCAGAGCCCCGCC
CCCCGATGGTACCGGGTTGNTNAGAGGTCCACAACGGGTCGNACGACAANNTTAAGGCCTNATGCATG
ACCCAACACGGGGGCGTCCCGGCGCGGCGACCTTGNANTCCGGGGNCAAGTATGGCCCCCCGGGAAGG
ACGCCGTAGTGCATCTGGANTGCCGATTCNTGGTCCAGANTGCCACCACGATGGTATGAGTNGCAAAG
GTACTTTGGACACCACCCCGTTTTCTATTTCCAGACGCGGCCATTCAGGAACGACGCGAATCGTAATC
TACCTGCCGCGTACGCGGATCGTACATGCGTTTTCCAGCGTTCGAAATAGAGTTTGTCGTTCCGCTGA
CCAGGTTTCACGGCTACTGCGATAAGCATATTCTACTGGGGGACTGTAAAGGAATTGTATCCACTCTG
CTTCCTGTCAATTGGCCATGTGCATCTCTGGTTGTTCAGGNNACATTAATAACACACAGCATTAAGTA
GATAAGTCTCCGATATAAGCGAACAGAACGTTGGTCAGAGACTGTGGTTCAATTGTAGTGGTATAATG
TTTCCCAGTGCATAAGGAACTCAGGGACACTACTAGCANGGGCGAGGAGAGACCCTTGGGTGGCAANA
CGCAATAAGTGTTACCTAGTACACCAGCACGAGANAGGGTTTGACCATGNNTTTTTAGACGACCCCGG
TCGCATTGAACCTGGCATTCAGAATGACGTGCTCTCAAGAATCAAGGTCCTCCCACTGAGGGTCATCT
GCTTCGCGGGTCAGCGTATGGTCACAAACAT

>SiLa430
GTGTAGCTCAGTTAGACACTTCTAAATCCTTGGTAACAGACATGCAGTCCCCTCGGCAGAGCCCCGCC
CCCCGGTGGTACCGGGTTGTTAAGAGGTCCACCACGGGTCGCACGACAACATCAAGGCCTCATGCATG
ACCCAACGCGGGGACGTACCGGCGCGGCGACCTTGAAGGTCGGGGACAAGTATGTCCCCCCGGGAAGG
ACGCCGTAGTGCATCTGGAGTGCCGATTNNTGGTCCAGAATGCCACCACGATGGTATGAGTGGCAAAG
GTATTTTGGACACCACCCCGTTTTCTATTTCCAGACGCGGCCATTCAGGAACGACGCGAATCGTAATC
TACCTGCCGCGTACGCGGATCGTACATGCGTTTTTCAGCGTTCGAAATAGAGTTTGTCGTTCCGCTGA
CCAGGTTTCACGGCTACTGCGATAAGCATATTCTACGGGGGGACTGTAAAGGAATTGTATCCACTCTG
CTCCCTGTCAATTGGCCATGTGCATCTCTGGTTGTTTAGGGTACATTAATAACACACAGCATTAGGTA
GATAAGTCTCCGATATAAGCGAACAGAACGTTGGTCAAAGACCGTGGTTCAATTGTAGTGGTATAACA
TTTCCCAGTGCATAAGGAACCCAGGGACACTACTAGCATGGGCGAGGAGAGACCCTTGGGTGGCAANA
CGCAATAAGTGTTACCCAGTACACCAGCACGATAGANGGTTTGACCATNNNTTTTAGACGACCCCGG
TCGCATTGAACCTGGCATTCAGAATGACGTGCTCTCAAGAGTCAAGGTCCTCCCACTGAGGGTCATCT
GCTTCGCGNNNTAGCGTATGGTCATAAANAT
>SiLa431
GTGTAGCCCAGTTAGACACTTCTAAATCCTTGGTAACAGACATGCAGTCCCCTCGGCAGAGCCCCGCC
CCCCGATGGTACCGGGTTGTTAAGAGGTCCACAACGGGTCNCACGACAACATTAAGGCCTNATGCATG
ACCCAACNCGGGGGNGTCCCGGCGCGGCGACCTTGAAGTCCGGGGACAAGTATGGCCCCCCGGGAAGG
ACGCCGTAGTGCATCTGGAGTGCCGATTCNTGGNCCAGAATGCCACCACGATGGTATGAGTGGCAAAG
GTACTTTGGACACCACCCCGTTTTCTATTTCCAGACGCGGCCATTCAGGAACGACGCGAATCGTAATC
TACCTGCCGCGTACGCGGATCGTACATGCGTTTTCCAGCGTTCGAAATAGAGTTTGTCGTTCCGCTGA
CCAGGTTTCACGGCTACTGCGATAAGCATATTCTACTGGGGGACTGTAAAGGAATTGTATCCACTCTG
CTTCCTGTCAATTGGCCATGTGCATCTCTNGTTGTTCAGGNNACATTAATAACACACAGCATTAAGTA
GATAAGTCTCCGATATAAGCGAACAGAACGTTGGTCAGAGACTGTGGTTCAATTGTAGTGGTATAATG
TTTCCCAGTGCATAAGGAACTCAGGNACACTACTAGCANGGGCGAGGAGAGACCCTTGGGTGGCAACA
CGCAATAAGTGTTACCTAGTACACCAGCACGAGAGANGGTTTGACCATGTGTTTTTAGACGACCCCGG
TCGCATTGAACCTGGCATTCAGAATGACGTGCTCTCAAGAATCAAGNTCCNCCCACTGAGGGTCATCT
GCTTCGCGGGTCAGCGTATGGTCATAAACAT
>SiLa432
GTGTAGCCCAGTTAGACACTTCTAAATCCTTGGTAACAGACATGCAGTCCCCTCGGCAGAGCCCCGCC
CCCCNATGGTACCGGGTTGTTNAGAGGTCCACAACGGGTCGCACGACAACATTAAGGCCTNATGCATG
ACCCAACACGGGGGCGTCCCGGCGCGGCGACCTTGAAGTCCGGGGACAAGTATGGCCCCCCGGGAAGG
ACGCCGTAGTGCATCTGGAGTGCCGATTNGTGGTCCAGAATGCCACCACGATGGTATGAGTGGCAAAG
GTACTTTGGACACCACCCCGTTTTCTATTTCCAGACGCGGCCATTCAGGAACGACGCGAATCGTAATC
TACCTGCCGCGTACGCGGATCGTACATGCGTTTTCCAGCGTTCGAAATAGAGTTTGTCGTTCCGCTGA
CCAGGTTTCACGGCTACTGCGATAAGCATATTCTACTGGGGGACTGTAAAGGAATTGTATCCACTCTG
CTTCCTGTCAATTGGCCATGTGCATCTCTNGTTGTTCAGGGTACATTAATAACACACAGCATTAAGTA
GATAAGTCTCCGATATAAGCGAACAGAACGTTGGTCAGAGACTGTGGTTCAATTGTAGTGGTATAATG
TTTCCCAGTGCATAAGGAACTCAGGGACACTACTAGCATGGGCGAGGAGAGACCCTTGGGTGGCAACA
CGCAATAAGTGTTACCTAGTACACCAGCACGAGAGAGGGTTTGACCATGTGTTTTTAGACGACCCCGG
TCGCATTGAACCTGGCATTCAGAATGACGTGCTCTCAAGAATCAAGGTCCTCCCACTGAGGGTCATCT
GCTTCGCGGGTCAGCGTATGGNCATAAACAT
>SiLa433
GTGTAGCCCAGTTAGACACTTCTAAATCCNTNGTAACAGACATGCAGTCCCCTCNNCAGAGCCCCGCC
CCCCGATGGTACCGGGTTGTTNAGAGGTCCACAACGGGTCGCANGACNNNNTTAAGGCCTCANGCATG
ANCCANCACGGGGGCGTCCCGGCGCGGCGACCTTGNAGTCCGGGGACAAGTATGGCCCCCCGGGAAGG
ACGCCGTAGTGCATCTGGAGTGCCGATTCGTGGTCCAGAATGCCACCACGATGGTATGAGTNGCAAAG
GTACTTTGGACACCACCCCGTTTTCTATTTCCAGANGCGGCCATTCAGGAACGACGCGAATCGTAATC
TACCTGCCGCGTNCGCGGATCGTACATGCGTTTTCCAGCGTTCGAAATAGAGTTTGTCGTTCCGCTGA
CCAGGTTNCACGGCTACTGCGATAAGCATATTCTACTGGGGGACTGTAAAGGAATTGTATCCACTCTG
CTTCCTGTCAATTGGCCATGTGCATCTCTGGTTGTTCAGGNNACATTAATAACACACAGCATTAAGTA
GATAAGTCTCCGATATAAGCGAACAGANCGTTGGTCAGAGACTGTGGTTCAATTGTAGTGGTATAATG
TTTCCCAGTGCATAAGGAACTCAGGNACACTACTAGCATGGGCGAGGAGAGACCCTTGGGTGGCAACA
CGCAATAAGTGTTACCTAGTACACCAGCACGAGANAGGGTTTGACCATGTGTTTTTAGACGNCCCCGG

TCGCATTGAACCTGGCATTCAGAATGACGTGCTCTCAAGAATCAAGGTCCTCCCACTGAGGGTCATCT
GCTTCGCGGGTCAGCGTATGGNCANAAANAT
>SiLa434
GTGTAGCCCAGTTAGACACTTCTAAATCCTTGGTAACAGACATGCAGTCCCCTCGGCAGAGCCCCGCC
CCCCNATGGTACCGGGTTGTTAAGAGGTCCACAACGGGTCNCACGACAANNTTAAGGCCTNATGCATG
ACNCAACACGGGGGNGTCCCGGNGCGGCGACCTTGNAGTCCGGGGACAAGTATGGCCCCCCGGGAAGG
ACGCCGTAGTGCATCTGGAGTGCCGATTCGTGGTCCAGAATGCCACCACGATGGTATGAGTGGCAAAG
GTACTTTGGACACCACCCCGTTTTCTATTTCCAGACGCGGCCATTCAGGAACGACGCGAATCGTAATC
TACCTGCCGCGTACGCGGATCGTACATGCGTTTTCCAGCGTTCGAAATAGAGTTTGTCGTTCCGCTGA
CCAGGTTTCACGGCTACTGCGATAAGCATATTCTACTGGGGGACTGTAAAGGAATTGTATCCACTCTG
CTTCCTGTCAATTGGCCATGTGCATCTCTGGTTGTTCAGGGTACATTAATAACACACAGCATTAAGTA
GATAAGTCTCCGATATAAGCGAACAGAACGTTGGTCAGANACTGTGGTTCAATTGTAGTGGTATAATG
TTTCCCAGTGCATAAGGAACTCAGGNACACTACTAGCATGGGCGAGGAGAGACCCTTGGGTGGCAACA
CGCAATAAGTGTTANCTAGTACACCAGCACGAGANAGGGTTTGACCATGTGTTTTTAGNNGACCCCGG
TCGCATTGAACCTGGCATTCAGAATGACGTGCTCTCAAGAATCAAGGTCCTCCCACTGAGGGTCATCT
GCTTCGCGGGTCNGCGTATGGNCATAAACAT
>SiLa435
GTGTAGCCCAGTTAGACACTTCTAAATCCTTGGTAACAGACATGCAGTCCCCTCNNCAGAGCNCCGCC
CCCCNATGGTACCGGGTTGTTAAGAGGTCCACAACGGGTCGNACGACNNCNTTAAGGCCTNANGCATG
ACCCAACACGGGGGCGTCCCGGNGCGGCGACCTTGAAGTCCGGGGACAAGTATGGCCCCCCGGGAAGG
ACGCCGTAGTGCATCTGGAGTGCCGATTCGTGGTCCAGAATGCCACCACGATGGTATGAGTGGCAAAG
GTACTTTGGACACCACCCCGTTTTCTATTTCCAGACGCGGCCATTCAGGAACGACGCGAATCGTAATC
TACCTGCCGCGTNCGCGGATCGTACATGCGTTTTCCAGCGTTCGAAATAGAGTTTGTCGTTCCGCTGA
CCAGGTTNCACGGCTACTGCGATAAGCATATTCTACTGGGGGACTGTAAAGGAATTGTATCCACTCTG
CTTCCTGTCAATTGGCCATGTGCATCTCTGGTTGTTCAGGGTACATTAATAACACACAGCATTAAGTA
GATAAGTCTCCGATATAAGCGAACAGAACGTTGGTCAGAGACTGTGGTTCAATTGTAGTGGTATAATG
TTTCCCAGTGCATAAGGAACTCAGGGACACTACTAGCATGGGCGAGGAGAGACCCTTGGGTGGCAACA
CGCAATAAGTGTTACCTAGTACACCAGCACGAGANANGGTTTGACCATGNNTTTNTAGNCGACCCCGG
TCGCATTGAACCTGGCATTCAGAATGACGTGCTCTCAAGAATCAAGGTCCTCCCACTGAGGGTCATCT
GCTTCGCGNGTCAGCGTATGGNCANAAACAT
>SiLa436
GTGTAGCTCAGTTAGACACTTCTAAATCCTTGGTAACAGACATGCAGTCCCCTCNNCAGAGCCCCGCC
CCCCNGTGGTACCGGGTTGTTNAGAGGTCCACCACGGGTCGNACGACAACATCAAGGCCTCATGCATG
ACCCAACGCGGGGACGTACCGGCGCGGCGACCTTGAAGGTCGGGGACAAGTATGTCCCCCCGGGAAGG
ACGCCGTAGTGCATCTGGAGTGCCGATTCGTGGTCCAGAATGCCACCACGATGGTATGAGTGGCAAAG
GTATTTTGGACACCACCCCGTTTTCTATTTCCAGACGCGGCCATTCAGGAACGACGCGAATCGTAATC
TACCTGCCGCGTACGCGGATCGTACATGCGTTTTTCAGCGTTCGAAATAGAGTTTGTCGTTCCGCTGA
CCAGGTTTCACGGCTACTNCGATAAGCATATTCTACGGGGGGACTGTAAAGGAATTGTATCCACTCTG
CTCCCTGTCAATTGGCCATGTGCATCTCTGGTTGTTTAGGGTACATTAATAACACACAGCATTAGGTA
GATAAGTCTCCGATATAAGCGAACAGAACGTTGGTCAAAGACCGTGGTTCAATTGTAGTGGTATAACA
TTTCCCAGTGCATAAGGAACCCAGGGACACTACTAGCATGGGCGAGGAGAGACCCTTGGGTGGCAACA
CGCAATAAGTGTTACCCAGTACACCAGCACGATAGAGGGTTTGACCATGTGTTTTTAGACGACCCCGG
TCGCATTGAACCTGGCATTCAGAATGACGTGCTCTCAAGAGTCAAGNTCCTCCCACTGAGGGTCATCT
GCTTCGCGNNNTAGCGTATGGTCATAAACAT
>SiLa437
GTGTAGCCCAGTTAGACACTTCTAAATCCTTGGTAACAGACATGCAGTCCCCTCGGCAGAGCCCCGCC
CCCCGATGGTACCGGGTTGTTNAGAGGTCCACAACGGGTCGCACGACAACATTAAGGCCTCATGCATG
ACCCANCACGGGGGCGTCCCGGCGCGGCGACCTTGAANTCCGGGGNCAAGTATGGCCCCCCGGGAAGG
ACGCCGTAGTGCATCTGGAGTGCCGATTCGTGGTCCAGANTGCCACCACGATGGTATGAGTGGCAAAG
GTACTTTGGACACCACCCCGTTTTCTATTTCCAGACGCGGCCATTCAGGAACGACGCGAATCGTAATC
TACCTGCCGCGTACGCGGATCGTACATGCGTTTTCCAGCGTTCGAAATAGAGTTTGTCGTTCCGCTGA
CCAGGTTTCACGGCTACTGCGATAAGCATATTCTACTGGGGGACTGTAAAGGAATTGTATCCACTCTG
CTTCCTGTCAATTGGCCATGTGCATCTCTNGTTGTTCAGGGTACATTAATAACACACAGCATTAAGTA
GATAAGTCTCCGATATAAGCGAACAGAACGTTGGTNAGAGACTGTGGTTCAATTGTAGTGGTATAATG

TTTCCCAGTGCATAAGGAACTCAGGGACACTACTAGCATGGGCGAGGAGAGACCCTTGGGTGGCAACA
CGCAATAAGTGTTACCTAGTACACCAGCACGAGNGANGGTTTGACCATGTGTTTTTAGACGACCCCGG
TCGCATTGAACCTGGCATTCAGAATGACGTGCTCTCAAGAATCAAGGTCCTCCCACTGAGGGTCATCT
GCTTCGCGGGTCAGCGTATGGNCATAAACAT
>SiLa438
GTGTAGCCCAGTTAGACACTTCTAAATCCTTGGTAACAGACATGCAGTCCCCTCGGCAGAGCCCCGCC
CCCCGATGGTACCGGGTTGTTNAGAGGTCCACAACGGGTCGCACGACAACATTAAGGCCTCATGCATG
ACCCAACACGGGGGCGTCCCGGCGCGGCGACCTTGNAGTCCGGGGACAAGTATGGCCCCCCGGGAAGG
ACGCCGTAGTGCATCTGGAGTGCCGATTCGTGGTCCAGAATGCCACCACGATGGTATGAGTGGCAAAG
GTACTTTGGACACCACCCCGTTTTCTANTTCCAGACGCGGCCATTCAGGAACGACGCGAATCGTAATC
TACCTGCCGCGTACGCGGATCGTACATGCGTTTTCCAGCGTTCGAAATAGAGTTTGTCGTTCCGCTGA
CCAGGTTTCACGGCTACTGCGATAAGCATATTCTACTGGGGGACTGTAAAGGAATTGTATCCACTCNG
CTTCCTGTCAATTGGCCATGTGCATCTCTGGTTGTTCAGGGTACATTAATAACACACAGCATTAAGTA
GATAAGTCTCCGATATAAGCGAACAGANCGTTGGTCAGAGACTGTGGTTCAATTGTAGTGGTATAATG
TTTCCCAGTGCATAAGGAACTCAGGGACACTACTAGCATGGGCGAGGAGAGACCCTTGGGTGGCAACA
CGCAATAAGTGTTACCTAGTACACCAGCACGAGAGAGGGTTTGACCATGTGTTTTTAGACGACCCCGG
TCGCATTGAACCTGGCATTCAGAATGACGTNCTCTCAAGAATCAAGNTCCTCCCACTGAGGGTCATCT
GCTTCGCGGGTCAGCGTATGGTCATNAACNT
>SiLa439
GTGTAGCCCAGTTAGACACTTCTAAATCCTTGGTAACAGACATGCAGTCCCCTCGGCAGAGNCCCGCC
CCCCGATGGTACCGGGTTGTTAAGAGGTCCACAACGGGTCGCACGACAANATTAAGGCCTNATGCATG
ACCCAACACGGGGGCGTCCCGGCGCGGCGACCTTGAAGTCCGGGGNCAAGTATGGCCCCCCGGGAAGG
ACGCCGTAGTGCATCTGGAGTGCCGATTCGTGGTCNAGAATGNCACCACGATGGTATGAGTGGCAAAG
GTACTTTGGACACCACCCCGTTTTCTATTTCCAGACGCGGCCATTCAGGAACGACGCGAATCGTAATC
TACCTGCCGCGTACGCGGATCGTACANGCGTTTTCCAGCGTTCGAAATAGAGTTTGTCGTTCCGCTGA
CCAGGTTTCACGGCTACTGCGATAAGCATATTCTACTGGGGGACTGTAAAGGAATTGTATCCACTCTG
CTTCCTGTCAATTGGCCATGTGCATCTCTGGTTGTTCAGGGTACATTAATAACACACAGCATTAAGTA
GNTAAGTCTCCGATATAAGCGAACAGAACGTTGGTCAGAGACTGTGGTTCAATTGTAGTGGTATAATG
TTTCCCAGTGCATAAGGAACTCAGGNACACTACTAGCATGGGCGAGGAGAGACCCTTGGGTGGCAACA
CGCAATAAGTGTTACCTAGTACACCAGCACGAGANANGGTTTGACCATGTGTTTTTAGACGACCCCGG
TCGCATTGAACCTGGCATTCAGAATGACGTGCTCTCAAGAATCAAGGTCCNCCCACTGAGGGTCATCT
GCTTCGCGNGTCAGCGTATGGTCATAAACAT
>SiLa440
GTGTAGCCCAGTTAGACACTTCTAAATCCTTGGTAACAGACATGCAGTCCCCTCNNCAGAGCCCCGCC
CCCCGATGGTACCGGGTTGTTAAGAGGTCCACAACGGGTCNCACGACAACNTTAAGGCCTCATGCATG
ACCCAACACGGNGGNGTCCCGGCGNGGCGACCTTGAAGTCCGGGGACAAGTATGGCCCCCCGGGAAGG
ACGCCGTAGTGCATCTGGAGTGCCGATTCGTGGTCCAGAATGCCACCACGATGGTATGAGTGGCAAAG
GTACTTTGGACACCACCCCGTTTNCTATTTCCAGANGCGGCCATTCAGGAACGACGCGAATCGTAATC
TACCTGCCGCGTNCGCGGATCGTACATGCGTTTTCCAGCGTTCGAAATAGAGTTTGTCGTTCCGCTGA
CCAGGTTTCACGGCTACTGCGATAAGCATATTCTACTGGGGGACTGTAAAGGAATTGTATCCACTCTG
CTTCCTGTCAATTGGCCATGTGCATCTCTGGTTGTTCAGGGTACATTAATAACACACAGNATNAAGTA
GATAAGTCTCCGATATAAGCGAACAGAACGTTGGTCAGANACTGTGGTTCAATTGTAGTGGTATAATG
TTTCCCAGTGCATAAGGAACTCAGGNACACTACTAGCATGGGCGAGGAGAGACCCTTGGGTGGCAACA
CGCAATAAGTGTTACCTAGTACACCAGCACGAGAGAGGGTTTGACCATGTGTTTTTAGACGACCCCGG
TCGCATTGAACCTGGCATTCAGAATGACGTGCTCTCAAGAATCNAGGTCCTCCCACTGAGGGTCATCT
GCTTCGCGGGTCAGCGTATGGNCATAAACAT
>SiLa441
GTGTAGCCCAGTTAGACACNTCTAAATCCTTGGTAACAGACATGCAGTCCCCTCNNCAGAGCCCCGCC
CCCCNATGGTACCGGGTTGTTAAGAGGTCCACAACGGGTCGCACGACAANNTTAAGGCCTCANGCATG
ACCCNACACGGNGGCGTCCCGGCGCGGCGACCTTGNANTCCGGGGACAAGTATGGCCCCCCGGGAAGG
ACGCCGTAGTGCATCTGGAGTGCCGATTNGTGGTCNAGANTGCCACCACGATGGTATGAGTGGCAAAG
GTACTTTGGACACCACCCCGTTTTCTATTTCCAGACGCGGCCATTCAGGAACGACGCGAATCGTAATC
TACCTGCCGCGTACGCGGATCGTACATGCGTTTTCCAGCGTTCGAAATAGAGTTTGTCGTTCCGCTGA
CCAGGTTNCACGGCTACTGCGATAAGCATATTCTACTGGGGGACTGTAAAGGAATTGTATCCACTCNG

CTTCCTGTCAATTGGCCATGTGCATCTCTNGTTGTTCAGGGNACATTAATAACACACAGCATTAAGTA
GATAAGTCTCCGATATAAGCGAACAGANCGTTGGTCAGANACTGTGGTTCAATTGTAGTGGTATAATG
TTTCCCAGTGCATAAGGAACTCAGGNACACTACTAGCATGGGCGAGGAGAGACCCTTGGGTGGCAACA
CGCAATAAGTGTTNCCTAGTACACCAGCACGAGAGAGGGTTTGACCATGTGTTTTTAGACGACCCCGG
TCGCATTGAACCTGGCATTCAGAATGACGTGCTCTCAAGAATCAAGGTCCTCCCACTGAGGGTCATCT
GCTTCGCGGGTCAGCGTATNGTCATAAACAT
>SiLa442
GTGTAGCCCAGTTAGACACTTCTAAATCCTTGGTAACAGACATGCAGTCCCCTCNNCAGAGCCCCGCC
CCCCGATGGTACCGGGTTGTTAAGAGGTCCACAACGGGTCGCACGACAACATTAAGGCCTCATGCATG
ACCCAACACGGGGGCGTCCCGGCGCGGCGACCTTGAAGTCCGGGGACAAGTATGGCCCCCCGGGAAGG
ACGCCGTAGTGCATCTGGAGTGCCGATTCGTGGTCCAGAATGCCACCACGATGGTATGAGTGGCAAAG
GTACTTTGGACACCACCCCGTTTTCTATTTCCAGACGCGGCCATTCAGGAACGACGCGAATCGTAATC
TACCTGCCGCGTACGCGGATCGTACATGCGTTTTCCAGCGTTCGAAATAGAGTTTGTCGTTCCGCTGA
CCAGGTTTCACGGCTACTGCGATAAGCATATTCTACTGGGGGACTGTAAAGGAATTGTATCCACTCTG
CTTCCTGTCAATTGGCCATGTGCATCTCTGGTTGTTCAGGGTACATTAATAACACACAGCATTAAGTA
GATAAGTCTCCGATATAAGCGAACAGAACGTTGGTCAGAGACTGTGGTTCAATTGTAGTGGTATAATG
TTTCCCAGTGCATAAGGAACTCAGGGACACTACTAGCATGGGCGAGGAGAGACCCTTGGGTGGCAACA
CGCAATAAGTGTTACCTAGTACACCAGCACGAGANAGGGTTTGACCATGTGTTTTTAGACGACCCCGG
TCGCATTGAACCTGGCATTCAGAATGACGTGCTCTCAAGAATCAAGGTCCTCCCACTGAGGGTCATCT
GCTTCGCGGGTCAGCGTATGGNCATAAACAT
>SiLa443
GTGTAGCCCAGTTAGACACTTCTAAATCCTTGGTAACAGACATGCAGTCCCCTCGGCAGAGNCCCGCC
CCCCGATGGTACCGGGTTGTTNAGAGGTCCACAACGGGTCGCACGACAANNTTAAGGCCTCATGCATG
ACCCANCACGGGGGCGTCCCGGCGCGGCGACCTTGNAGTCCGGGGACAAGTATGGCCCCCCGGGAAGG
ACGCCGTAGTGCATCTGGAGTGCCGATTCNTGGTCCAGANTGCCACCACGATGGTATGAGTGGCAAAG
GTACTTTGGACACCACCCCGTTTTCTATTTCCAGACGCGGCCATTCAGGAACGACGCGAATCGTAATC
TACCTGCCGCGTACGCGGATCGTACATGCGTTTTCCAGCGTTCGAAATAGAGTTTGTCGTTCCGCTGA
CCAGGTTTCACGGCTACTGCGATAAGCATATTCTACTGGGGGACTGTAAAGGAATTGTATCCACTCTG
CTTCCTGTCAATTGGCCATGTGCATCTCTNGTTGTTCAGGGTACATTAATAACACACAGCATTAAGTA
GATAAGTCTCCGATATAAGCGAACAGAACGTTGGTCAGAGACTGTGGTTCAATTGTAGTGGTATAATG
TTTCCCAGTGCATAAGGAACTCAGGGACACTACTAGCATGGGCGAGGAGAGACCCTTGGGTGGCAACA
CGCAATAAGTGTTACCTAGTACACCAGCACGAGNGAGGGTTTGACCATGTGTTTTTAGACGACCCCGG
TCGCATTGAACCTGGCATTCAGAATGACGTNCTCTCAAGAATCAAGGTCCNCCCACTGAGGGTCATCN
GCTTCGCGGGNCAGCGTATGGNCATAAANAT
>SiLa444
GTGTAGCCCAGTTAGACACNTCTAAATCCTTGGTAACAGACATGCAGTCCCCTCNNCAGAGCCCCGCC
CCCCNATGGTACCGGGTTGTTNAGAGGTCCACAACGGGTCGCACGACAACATTAAGGCCTCATGCATG
ACCCAACNCGGGGGCGTCCCGGNGCGGCGACCTTGNANTCCGGGNACAAGTATGGCCCCCCGGGAAGG
ACGCCGTAGTGCATCTGGAGTGCCGATTCGTGGNCNAGAATGNCACCACGATGGTATGAGTGGCAAAG
GTACTTTGGACACCACCCCGTTTTCTATTTCCAGACGCGGCCATTCAGGAACGACGCGAATCGTAATC
TACCTGCCGCGTACGCGGATCGTACATGCGTTTTCCAGCGTTCGAAATAGAGTTTGTCGTTCCGCTGA
CCAGGTTTCACGGCTACTGCGATAAGCATATTCTACTGGGGGACTGTAAAGGAATTGTATCCACTCTG
CTTCCTGTCAATTGGCCATGTGCATCTCTNGNTGTTCAGGNNACANTAATAACACACAGCATTAAGTA
GATAAGTCTCCGATATAAGCGAACAGAACGTTGGTCAGAGACTGTGGTTCAATTGTAGTGGTATAATG
TTTCCCAGTGCATAAGGAACTCAGGNACACTACTAGCATGGGCGAGGAGAGACCCTTGGGTGGCAACA
CGCAATAAGTGTTACCTAGTACACCAGCACGAGAGANGGTTTGACCATGTGTTTTTAGACGACCCCGG
TCGCATTGAACCTGGCATTCAGAATGACGTGCTCTNAAGAANCAANGTCCTCCCACTGAGGGTCATCT
GCTTCGCGNGTCAGCGTATGGNCANAANNAT
>SiLa445
GTGTAGCCCAGTTAGACACTTCTAAATCCTTNGTAACAGACATGCAGTCCCCTCGGCAGAGCNCCGCC
CCCCGATGGTACCGGGTTGTTAAGAGGTCCACAACGGGTCGCACGACAANNTTAAGGCCTCANGCATG
ACCCAACACGGGGGCGTCCCGGNGNGGCGACCTTGAAGTCCGGGGACAAGTATGGCCCCCCGGGAAGG
ACGCCGTAGTGCATCTGGAGTGCCGATNCGTGGTCNAGAATGCCACCACGATGGTATGAGTGGCAAAG
GTACTTTGGACACCACCCCGTTTTCTATTTCCAGACGCGGCCATTCAGGAACGACGCGAATCGTAATC

TACCTGCCGCGTACGCGGATCGTACATGCGTTTTCCAGCGTTCGAAATAGAGTTTGTCGTTCCGCTGA
CCAGGTTTCACGGCTACTGCGATAAGCATATTCTACTGGGGGACTGTAAAGGAATTGTATCCACTCTG
CTTCCTGTCAATTGGCCATGTGCATCTCTGGTTGTTCAGGGTACATTAATAACACACAGNATTAAGTA
GATAAGTCTNCGATATAAGCGAACAGAACGTTGGTCAGAGACTGTGGTTCAATTGTAGTGGTATAATG
TTTCCCAGTGCATAAGGAACTCAGGGACACTACTAGCANGGGCGAGGAGAGACCCTTGGGTGGCAACA
CGCAATAAGTGTTACCTAGTACACCAGCACGAGAGAGGGTTTGACCATGTGTTTTTAGACGACCCCGG
TCGCATTGAACCTGGCATTCAGAATGACGTGCTCTCAAGAATCAAGGTCCTCCCACTGAGGGTCATNT
GCTTCGCGGNNCAGCGTATGGTCANNNACAT
>SiLa446
GTGTAGCCCAGTTAGACACTTCTAAATCCTTGGTAACAGACATGCAGTCCCCTCNNCAGAGCCCCGCC
CCCCGATGGTACCGGGTTGTTNAGAGGTCCACAACGGGTCGCACGACNNNNTTAAGGCCTCATGCATG
ACCCNACACGGGGGCGTCCCGGCGCGGCGACCTTGAANTCCGGGGACAAGTATGGCCCCCCGGGAAGG
ACGCCGTAGTGCATCTGGAGTGCCGATTCGTGGTCCAGAATGNCACCACGATGGTATGAGTGGCAAAG
GTACTTTGGACACCACCCCGTTTTCTATTTCCAGACGCGGCCATTCAGGAACGACGCGAATCGTAATC
TACCTGCCGCGTNCGCGGATCGTACATGCGTTTTCCAGCGTTCGAAATAGAGTTTGTCGTTCCGCTGA
CCAGGTTTCACGGCTACTGCGATAAGCATATTCTACTGGGGGACTGTAAAGGAATTGTATCCACTCTG
CTTCCTGTCAATTGGCCATGTGCATCTCTGGTTGTTCAGGGTACATTAATAACACACAGNATTAAGTA
GATAAGTCTCCGATATAAGCGAACAGAACGTTGGTCAGAGACTGTGGTTCAATTGTAGTGGTATAATG
TTTCCCAGTGCATAAGGAACTCAGGGACACTACTAGCATGGGCGAGGAGAGACCCTTGGGTGGCAACA
CGCAATAAGTGTTACCTAGTACACCAGCACGAGNGAGGGTTTGACCATGTGTTTTTAGACGACCCCGG
TCGCATTGAACCTGGCATTCAGAATGACGTGCTCTCAAGAATCAAGGTCCTCCCACTGAGGGTCATCT
GCTTCGCGGGTCNGCGTATGGTCANAAACAT
>SiLa447
GTGTAGCCCAGTTAGACACTTCTAAATCCTTGGTAACAGACATGCAGTCCCCTCGGCAGAGCCCCGCC
CCCCGATGGTACCGGGTTGTTNAGAGGTCCACAACGGGTCGCACGACANCATTAAGGCCTCANGCATG
ACCCAACACGGGGGCGTCCCGGCGCGGCGACCTTGNANTCCGGGGACAAGTATGGCCCCCCGGGAAGG
ACGCCGTAGTGCATCTGGAGTGCCGATTCGTGGTCNAGAATGCCACCACGATGGTATGAGTGGCAAAG
GTACTTTGGACACCACCCCGTTTTCTATTTCCAGACGCGGCCATTCAGGAACGACGCGAATCGTAATC
TACCTGCCGCGTACGCGGATCGTACATGCGTTTTCCAGCGTTCGAAATAGAGTTTGTCGTTCCGCTGA
CCAGGTTTCACGGCTACTGCGATAAGCATATTCTACTGGGGGACTGTAAAGGAATTGTATCCACTCTG
CTTCCTGTCAATTGGCCATGTGCATCTCTGGTTGTTCAGGGTACATTAATAACACACAGCATTAAGTA
GATAAGTCTCCGATATAAGCGAACAGAACGTTGGTCAGAGACTGTGGTTCAATTGTAGTGGTATAATG
TTTCCCAGTGCATAAGGAACTCAGGGACACTACTAGCATGGGCGAGGAGAGACCCTTGGGTGGCAACA
CGCAATAAGTGTTACCTAGTACACCAGCACGAGAGAGGGTTTGACCATGTGTTTTTAGACGACCCCGG
TCGCATTGAACCTGGCATTCAGAATGACGTNCTCTCAAGAATCAAGGTCCTCCCACTGAGGGTCATCT
GCTTCGCGNGTCAGCGTATGGTCATAAACAT
>SiLa448
GTGTAGCCCAGTTAGACACTTCTAAATCCTTGGTAACAGACATGCAGTCCCCTCGGCAGAGCCCCGCC
CCCCGATGGTACCGGGTTGTTAAGAGGTCCACAACGGGTCGCACGACAANNTTAAGGCCTCATGCATG
ACCCAACACGGGGGCGTCCCGGNGCGGCGACCTTGAAGTCCGGGGACAAGTATGGCCCCCCGGGAAGG
ACGCCGTAGTGCATCTGGAGTGCCGATTCGTGGTCCAGAATGNCACCACGATGGTATGAGTNGCAAAG
GTACTTTGGACACCACCCCGTTTTCTATTTCCAGACGCGGCCATTCAGGAACGACGCGAATCGTAATC
TACCTGCCGCGTACGCGGATCGTACATGCGTTTTCCAGCGTTCGAAATAGAGTTTGTCGTTCCGCTGA
CCAGGTTTCACGGCTACTGCGATAAGCATATTCTACTGGGGGACTGTAAAGNAATTGTATCCACTCTG
CTTCCTGTCAATTGGCCATGTGCATCTCTGGTTGTTCAGGGTACATTAATAACACACAGCATTAAGTA
GATAAGTCTCCGATATAAGCGAACAGAACGTTGGTCAGAGACTGTGGTTCAATTGTAGTGGTATAATG
TTTCCCAGTGCATAAGGAACTCAGGGACACTACTAGCATGGGCGAGGAGAGACCCTTGGGTGGCAACA
CGCAATAAGTGTTACCTAGTACACCAGCACGAGAGAGGGTTTGACCATGNNTTTTTAGACGACCCCGG
TCGCATTGAACCTGGCATTCAGAATGACGTGCTCTCAAGAATCAANGTCCTCCCACTGAGGGTCATCT
GCTTCGCGNGTCNGCGTATGGTCATAAANNT
>SiLa449
GTGTAGCCCAGTTAGACACNTCTAAATCCTTGGTAACAGACATGCAGTCCCCTCNNCAGAGCCCCGCC
CCCCNATGGTACCGGGTTGTTNAGAGGTCCACAACGGGTCGCACGACAACATTAAGGCCTCANGCATG
ACCCAACACGGGGGCGTCCCGGCGCGGCGACCTTGAAGTCCGGGGACAAGTATGGCCCCCCGGGAAGG

ACGCCGTAGTGCATCTGGAGTGCCGATTCGTGGTCCAGAATGCCACCACGATGGTATGAGTGGCAAAG
GTACTTTGGACACCACCCCGTTTTCTATTTCCAGACGCGGCCATTCAGGAACGACGCGAATCGTAATC
TACCTGCCGCGTACGCGGATCGTACATGCGTTTTCCAGCGTTCGAAATAGAGTTTGTCGTTCCGCTGA
CCAGGTTTCACGGCTACTGCGATAAGCATATTCTACTGGGGGACTGTAAAGGAATTGTATCCACTCTG
CTTCCTGTCAATTGGCCATGTGCATCTCTGGTTGTTCAGGGTACATTAATAACACACAGCATTAAGTA
GATAAGTCTCCGATATAAGCGAACAGAACGTTGGTCAGANACTGTGGTTCAATTGTAGTGGTATAATG
TTTCCCAGTGCATAAGGAACTCAGGGACACTACTAGCANGGGCGAGGAGAGACCCTTGGGTGGCAACA
CGCAATAAGTGTTANNTAGTACACCAGCACGAGANANGGTTTGACCATGTGTTTTTAGACGACCCCGG
TCGCATTGAACCTGGCATTCAGAATGACGTGCTCTCAAGAATCAAGGTCCTCCCACTGAGGGTCATCT
GCTTCGCGGGTCAGCGTATGGNCANAAACAT
>SiLa450
GTGTAGCCCAGTTAGACACTTCTAAATCCTTGGTAACAGACATGCAGTCCCCTCGGCAGAGCCCCGCC
CCCCGATGGTACCGGGTTGTTAAGAGGTCCACAACAGGTCGCACGACAACATTAAGGCCTCATGCATG
ACCCANCACGGGGGCGTCCCGGCGCGGCGACCTTGNANTCCGGGGACAAGTATGGCCCCCCGGGAAGG
ACGCCGTAGTGCATCTGGAGTGCCGATTCGTGGTCNAGAATGNCACCACGATGGTATGAGTGGCAAAG
GTACTTTGGACACCACCCCGTTTTCTATTTCCAGACGCGGCCATTCAGGAACGACGCGAATCGTAATC
TACCTGCCGCGTACGCGGATCGTACATGCGTTTTCCAGCGTTCGAAATAGAGTTTGTCGTTCCGCCGA
CCAGGTTTCACGGCTACTGCGATAAGCATATTCTACTGGGGGACTGTAAAGGAATTGTATCCACTCNA
CTTCCTGTCAATTGGCCATGTGCATCTCTNGTTGTTCAGGGTACATTAATAACACACAGCATTAAGTA
GATAAGTCTCCGATATAAGCGAACAGAACGTTGGTCAGAGACTGTGGTTCAATTGTAGTGGTATAATG
TTTCCCAGTGCATAAGGAACTTAGGGACATTACTAGCATGGGCGAGGAGAGACCCTTGGGTGGCAACA
CGCAATAAGTGTTANCTAGTACACCAGCACGAGANANGGTTTGACCATGTGTTTTTAGACGACCCCGG
TCGCATTGAACCTGGCATTCAGAATGACGTGCTCTCAAGAATCAAGGTCCTCCCACTGAGGGTCATCT
GCTTCGCGGGTCAGCGTATGGTCATAANNAT
>SiLa451
GTGTAGCCCAGTTAGACACTTCTAAATCCTTGGTAACAGACATGCAGTCCCCTCGGCAGAGCNCCGCC
CCCCGATGGTACCGGGTTGTTAAGAGGTCCACCACGGGTCGCACGACANCATTAAGGCCTNANGCATG
ACCCAACACGGNGGCGTCCCGGCGCGGCGACCTTGAAGTCCGGGGNCAAGTATGGCCCCCCGGGAAGG
ACGCCGTAGTGCATCTGGAGTGCCGATTCGTGGTCCAGAATGCCACCACGATGGTATGAGTNGCAAAG
GTACTTTGGACACCACCCCGTTTNCTATTTCCAGACGCGGCCATTCAGGAACGACGCGAATCGTAATC
TACCTGCCGCGTACGCGGATCGTATATACGTTTTCCAGCGTTCGAAATAGAGTTTGTCGTTCCGCTGA
CCAGGTTTCACGGCTACTGCGATAAGCATATTCTACTGGGGGACTGTAAAGGAATTGTATCCACTCTG
CTTCCTGTCAATTGGCCATGTGCATCTCTGGTTGTTCAGGNNACATTAATAACACACAGCATTAAGTA
GATAAGTCTCCGATATAAGCGAACAGAACGTTGGTCAGAGACTGTGGTTCAATTGTAGTGGTATAATG
TTTCCCAGTGCATAAGGAACTCAGGGACACTACTAGCATGGGCGAGGAGAGACCCTTGGGTGGCAACA
CGCAATAAGTGTTACCTAGTACACCAGCACGAGNGAGGGTTTGACCATGTGTTTTTAGACGACCCCGG
TCGCATTGAACCTGGCATTCAGAATGACGTGCTCTCAAGAGTCAAGGTCCNCCCACTGAGGGTCATCT
GCTTCGCGGGTCAGCGTATNGNCATAAACAT
>SiLa452
GTGTAGCCCAGTTAGACACTTCTAAATCCTTGGTAACAGACATGCAGTCCCCTCNNCAGAGCNCCGCC
CCCCGATGGTACCGGGTTGTTAAGAGGTCCACAACGGGTCGNACGACAANATTAAGGCCTNATGCATG
ACCCAACACGGGGGCGTCCCGGCGCGGCGACCTTGNANTCCGGGGACAAGTATGGCCCCCCGGGAAGG
ACGCCGTAGTGCATCTGGAGTGCCGATTCGTGGTCCAGAATGNCACCACGATGGTATGAGTNGCAAAG
GTACTTTGGACACCACCCCGTTTTCTATTTCCAGACGCGGCCATTCAGGAACGACGCGAATCGTAATC
TACCTGCCGCGTACGCGGATCGTACATGCGTTTTCCAGCGTTCGAAATAGAGTTTGTCGTTCCGCTGA
CCAGGTTTCACGGCTACTGCGATAAGCATATTCTACTGGGGGACTGTAAAGGAATTGTATCCACTCTG
CTTCCTGTCAATTGGCCATGTGCATCTCTGGTTGTTCAGGNNACATTAATAACACACAGCATTAAGTA
GATAAGTCTCCGATATAAGCGAACAGANCGTTGGTCAGAGACTGTGGTTCAATTGTAGTGGTATAATG
TTTCCCAGTGCATAAGGAACTCAGGGACACTACTAGCATGGGCGAGGAGAGACCCTTGGGTGGCAACA
CGCAATAAGTGTTNCCTAGTACACCAGCACGAGNGANGGTTTGACCATGTGTTTTTAGACGACCCCGG
TCGCATTGAACCTGGCATTCAGAATGACGTGCTCTCAAGAATCAAGGTCCTCCCACTGAGGGTCATCT
GCTTCGCGGGTCAGCGTATGGNCATAANCAT
>SiLa455
GTGTAGCCCAGTTAGACACTTCTAAATCCTTGGTAACAGACATGCAGTCCCCTCGGCAGAGCCCCGCC

CCCCGATGGTACCGGGTTGTTAAGAGGTCCACAACGGGTCGCACGACAACATTAAGGCCTCATGCATG
ACCCAACACGGGGGCGTCCCGGCGCGGCGACCTTGAAGTCCGGGGACAAGTATGGCCCCCCGGGAAGG
ACGCCGTAGTGCATCTGGAGTGCCGATTCGTGGTCNAGAATGCCACCACGATGGTATGAGTNGCAAAG
GTACTTTGGACACCACCCCGTTTTCTANTTCCAGACGCGGCCATTCAGGAACGACGCGAATCGTAATC
TACCTGCCGCGTACGCGGATCGTACATGCGTTTTCCAGCGTTCGAAATAGAGTTTGTCGTTCCGCTGA
CCAGGTTTCACGGCTACTGCGATAAGCATATTCTACTGGGGGACTGTAAAGGAATTGTATCCACTCTG
CTTCCTGTCAATTGGCCATGTGCATCTCTGGTTGTTCAGGGTACATTAATAACACACAGCATTAAGTA
GATAAGTCTCCGATATAAGCGAACAGAACGTTGGTCAGAGACTGTGGTTCAATTGTAGTGGTATAATG
TTTCCCAGTGCATAAGGAACTCAGGNACACTACTAGCATGGGCGAGGAGAGACCCTTGGGTGGCAACA
CGCAATAAGTGTTACCTAGTACACCAGCACGAGAGAGGGTTTGACCATGTGTTTTTAGACGACCCCGG
TCGCATTGAACCTGGCATTCAGAATGACGTGCTCTCAAGAATCAAGGTCCNCCCACTGAGGGTCATCT
GCTTCGCGGGTCAGCGTATGGNCANNAACAT
>SiLa456
GTGTAGCCCAGTTAGACACTTCTAAATCCTTGGTAACAGACATGCAGTCCCCTCNNCAGAGCCCCGCC
CCCCGATGGTACCGGGTTGTTAAGAGGTCCACAACGGGTCGCACGACANCNTTAAGGCCTCATGCATG
ACCCAACACGGGGGNGTCCCGGCGCGGCGACCTTGAAGTCCGGGGACAAGTATGGCCCCCCGGGAAGG
ACGCCGTAGTGCATCTGGAGTGCCGATTCGTGGTCCAGAATGCCACCACGATGGTATGAGTNGCAAAG
GTACTTTGGACACCACCCCGTTTTCTATTTCCAGACGCGGCCATTCAGGAACGACGCGAATCGTAATC
TACCTGCCGCGTACGCGGATCGTACATGCGTTTTCCAGCGTTCGAAATAGAGTTTGTCGTTCCGCTGA
CCAGGTTTCACGGCTACTGCGATAAGCATATTCTACTGGGGGACTGTAAAGGAATTGTATCCACTCNG
CTTCCTGTCAATTGGCCATGTGCATCTCTGGTTGTTCAGGGTACATTAATAACACACAGCATTAAGTA
GATAAGTCTCCGATATAAGCGAACAGAACGTTGGTCAGAGACTGTGGTTCAATTGTAGTGGTATAATG
TTTCCCAGTGCATAAGGAACTCAGGNACACTACTAGCATGGGCGAGGAGAGACCCTTGGGTGGCAACA
CGCAATAAGTGTTACCTAGTACACCAGCACGAGNGAGGGTTTGACCATGTGTTTTTAGACGNCCCCGG
TCGCATTGAACCTGGCATTCAGAATGACGTGCTCTCAAGAATCAAGGTCCTCCCACTGAGGGTCATCT
GCTTCGCGGGTCAGCGTATGGTCATAAACNT
>SiLa457
GTGTAGCCCAGTTAGACACTTCTAAATCCTTGGTAACAGACATGCAGTCCCCTCGGCAGAGCCCCGCC
CCCCGATGGTACCGGGTTGTTAAGAGGTCCACAACGGGTCGCACGACAACNTTAAGGCCTCATGCATG
ANNCAACACGGGGGCGTCCCGGCGCGGCGACCTTGAANTCCGGGGACAAGTATGGCCCCCCGGGAAGG
ACGCCGTAGTGCATCTGGAGTGCCGATTNGTGGNCNAGAATGCCACCACGATGGTATGAGTGGCAAAG
GTACTTTGGACACCACCCCGTTTTCTATTTCCAGACGCGGCCATTCAGGAACGACGCGAATCGTAATC
TACCTGCCGCGTACGCGGATCGTACATGCGTTTTCCAGCGTTCGAAATAGAGTTTGTCGTTCCGCTGA
CCAGGTTTCACGGCTACTGCGATAAGCATATTCTACTGGGGGACTGTAAAGGAATTGTATCCACTCTG
CTTCCTGTCAATTGGCCATGTGCATCTCTGGTTGTTCAGGGTACATTAATAACACACAGCATTAAGTA
GATAAGTCTCCGATATAAGCGAACAGAACGTTGGTCAGAGACTGTGGTTCAATTGTAGTGGTATAATG
TTTCCCAGTGCATAAGGAACTCAGGGACACTACTAGCATGGGCGAGGAGAGACCCTTGGGTGGCAACA
CGCAATAAGTGTTACCTAGTACACCAGCACGAGAGAGGGTTTGACCATGTGTTTTTAGACGACCCCGG
TCGCATTGAACCTGGCATTCAGAATGACGTNCTCTCAAGAATCAAGGTCCTCCCACTGAGGGTCATCT
GCTTCGCGGGTCAGCGTATGGNCATAANNAT
>Tay103
GTGTAGCCCAGTTAGACACTTCTAAATCCTTGGTAACAGACATGCAGTCCCCTCGGCAGAGCCCCGCC
CCCCGATGGTACCGGGTTGTTAAGAGGTCCACAACGGGTCGCACGACNNCNTTAAGGCCTCATGCATG
ANNCAACACNGNGGCGTCCCGGCGCGGCGACCTTGAAGTCCGGGGACAAGTATGGCCCCCCGGGAAGG
ACGCCGTAGTGCATCTGGAGTGCCGATTCGTGGTCCAGAATGCCACCACGATGGTATGAGTGGCAAAG
GTACTTTGGACACCACCCCGTTTTCTATTTCCAGACGCGGCCATTCAGGAACGACGCGAATCGTAATC
TACCTGCCGCGTACGCGGATCGTACATGCGTTTTCCAGCGTTCGAAATAGAGTTTGTCGTTCCGCTGA
CCAGGTTTCACGGCTACTGCGATAAGCATATTCTACTGGGGGACTGTAAAGGAATTGTATCCACTCTG
CTTCCTGTCAATTGGCCATGTGCATCTCTGGTTGTTCAGGGTACATTAATAACACACAGCATTAAGTA
GATAAGTCTCCGATATAAGCGAACAGAACGTTGGTCAGAGACTATGGTTCAATTGTAGTGGTATAATG
TTTCCCAGTGCATAAGGAACTCAGGGACACTACTAGCATGGGCGAGGAGAGACCCTTGGGTGGCAACA
CGCAATAAGTGTTACCTAGTACATCAGCACGAGAGAGGGTTTGACCATGTGTTTTTAGACGACCCCGG
TCGCATTGAACCTGGCATTCAGAATGACGTGCTCTCAAGAATCAAGGTCCTCCCACTGAGGGTCATCT
GCTTCGCGGGTCAGCGTATGGTCATAAACAT

>Tay129
GTGTAGCCCAGTTAGACACTTCTAAATCCTTGGTAACAGACATGCAGTCCCCTCNNCAGAGCCCCGCC
CCCCGATGGTACCGGGTTGTTNAGAGGTCCACAACGGGTCGCACGACAACATTAAGGCCTNATGCATG
ACCCAACACGGGGGCGTCCCGGCGNGGCGACCTTGAAGTCCGGGGACAAGTATGGCCCCCCGGGAAGG
ACGCCGTAGTGCATCTGGAGTGCCGATTCGTGGTCCAGAATGNCACCACGATGGTATGAGTGGCAAAG
GTACTTTGGACACCACCCCGTTTTCTATTTCCAGACGCGGCCATTCAGGAACGACGCGAATCGTAATC
TACCTGCCGCGTACGCGGATCGTACATGCGTTTTCCAGCGTTCGAAATAGAGTTTGTCGTTCCGCTGA
CCAGGTTTCACGGCTACTGCGATAAGCATATTCTACTGGGGGACTGTAAAGGAATTGTATCCACTCTG
CTTCCTGTCAATTGGCCATGTGCATCTCTGGTTGTTCAGGGTACATTAATAACACACAGCATTAAGTA
GATAAGTCTCCGATATAAGCGAACAGAACGTTGGTCAGAGACTATGGTTCAATTGTAGTGGTATAATG
TTTCCCAGTGCATAAGGAACTCAGGGACACTACTAGCATGGGCGAGGAGAGACCCTTGGGTGGCAACA
CGCAATAAGTGTTACCTAGTACATCAGCACGAGAGAGGGTTTGACCATGTGTTTTTAGNCGACCCCGG
TCGCATTGAACCTGGCATTCAGAATGACGTNCTCTCAAGAATCAAGGTCCTCCCACTGAGGGTCATCT
GCTTCGCGGGTCAGCGTATGGTCATNAACAT
>Tay135
GTGTAGCCCAGTTAGACACTTCTAAATCCTTGGTAACAGACATGCAGTCCCCTCGGCAGAGCCCCGCC
CCCCGATGGTACCGGGTTGTTAAGAGGTCCACAACGGGTCGCACGACAACATTAAGGCCTCANGCATG
ACCCAACACGGGGGCGTCCCGGCGCGGCGACCTTGNAGTCCGGGGACAAGTATGGCCCCCCGGGAAGG
ACGCCGTAGTGCATCTGGAGTGCCGATTCGTGGTCCAGAATGCCACCACGATGGTATGAGTNGCAAAG
GTACTTTGGACACCACCCCGTTTTCTATTTCCAGACGCGGCCATTCAGGAACGACGCGAATCGTAATC
TACCTGCCGCGTNCGCGGATCGTACATGCGTTTTCCAGCGTTCGAAATAGAGTTTGTCGTTCCGCTGA
CCAGGTTTCACGGCTACTGCGATAAGCATATTCTACTGGGGGACTGTAAAGGAATTGTATCCACTCTG
CTTCCTGTCAATTGGCCATGTGCATCTCTGGTTGTTCAGGGTACATTAATAACACACAGCATTAAGTA
GATAAGTCTCCGATATAAGCGAACAGAACGTTGGTCAGAGACTGTGGTTCAATTGTAGTGGTATAATG
TTTCCCAGTGCATAAGGAACTCAGGGACACTACTAGCATGGGCGAGGAGAGACCCTTGGGTGGCAACA
CGCAATAAGTGTTACCTAGTACACCAGCACGAGAGAGGGTTTGACCATGTGTTTTTAGACGACCCCGG
TCGCATTGAACCTGGCATTCAGAATGACGTGCTCTCAAGAATCAAGGTCCTCCCACTGAGGGTCATCT
GCTTCGCGGGTCAGCGTATGGTCACNANNNT
>Tay138
GTGTAGCCCAGTTAGACACTTCTAAATCCTTGGTAACAGACATGCAGTCCCCTCGGCAGAGCCCCGCC
CCCCGATGGTACCGGGTTGTTAAGAGGTCCACAACGGGTCGCACGACNACATTAAGGCCTCANGCATG
ANNCAANACGGNGGNGTCCCGGCGNGGCGACCTTGNAGTCCGGGGACAAGTATGGCCCCCCGGGAAGG
ACGCCGTAGTGCATCTGGAGTGCCGATTNNTGGTCCAGANTGNCACCACGATGGTATGAGTGGCAAAG
GTACTTTGGACACCACCCCGTTTTCTANTTCCAGANGCGGCCATTCAGGAACGACGCGAATCGTAATC
TACCTGCCGCGTACGCGGATCGTACATGCGTTTTCCAGCGTTCGAAATAGAGTTTGTCGTTCCGCTGA
CCAGGTTNCACGGCTACTGCGATAAGCATATTCTACTGGGGGACTGTAAAGGAATTGTATCCACTCTG
CTTCCTGTCAATTGGCCATGTGCATCTCTNGTTGTTCAGGNNACATTAATAACACACAGNATTAAGTA
GATAAGTCTCCGATATAAGCGAACAGAACGTTGGTNAGAGACTATGGTTCAATTGTAGTGGTATAATG
TTTCCCAGTGCATAAGGAACTCAGGGACACTACTAGCATGGGCGAGGAGAGACCCTTGGGTGGCANCA
CGCAATAAGTGTTACCTAGTACATCAGCACGAGANANGGTTTGACCATNNNTTTTTAGACGACCCCGG
TCGCATTGAACCTGGCATTCAGAATGACGTGCTCTCAAGAATCAAGGTCCTCCCACTGAGGGTCATCT
GCTTCGCGGGTCAGCGTATNGTCATAAACAT
>Tay155
GTGTAGCCCAGTTAGACACTTCTAAATCCTTGGTAACAGACATGCAGTCCCCTCGGCAGAGCCCCGCC
CCCCGATGGTACCGGGTTGTTAAGAGGTCCACAACGGGTCGCACGACAANNTTAAGGCCTCANGCATG
ACCCAACACGGGGGCGTCCCGGCGNGGCGACCTTGNAGTCCGGGGNCAAGTATGGCCCCCCGGGAAGG
ACGCCGTAGTGCATCTGGAGTGCCGATTCNTGGTCNAGAATGCCACCACGATGGTATGAGTGGCAAAG
GTACTTTGGACACCACCCCGTTTTCTATTTCCAGACGCGGCCATTCAGGAACGACGCGAATCGTAATC
TACCTGCCGCGTNCGCGGATCGTACATGCGTTTTCCAGCGTTCGAAATAGAGTTTGTCGTTCCGCTGA
CCAGGTTTCACGGCTACTGCGATAAGCATATTCTACTGGGGGACTGTAAAGGAATTGTATCCACTCTG
CTTCCTGTCAATTGGCCATGTGCATCTCTGGTTGTTCAGGGTACATTAATAACACACAGCATTAAGTA
GATAAGTCTCCGATATAAGCGAACAGAACGTTGGTCAGAGACTATGGTTCAATTGTAGTGGTATAATG
TTTCCCAGTGCATAAGGAACTCAGGGACACTACTAGCATGGGCGAGGAGAGACCCTTGGGTGGCAACA
CGCAATAAGTGTTACCTAGTACATCAGCACGAGAGANGGTTTGACCATGTGTTTTTAGACGACCCCGG

TCGCATTGAACCTGGCATTCAGAATGACGTGCTCTCAAGAATCAAGGTCCTCCCACTGAGGGTCATCT
GCTTCGCGNGTCAGCGTATNGTCACAANNAT
>Tay156
GTGTAGCCCAGTTAGACACTTCTAAATCCTTGGTAACAGACATGCAGTCCCCTCGGCAGAGCCCCGCC
CCCCGATGGTACCGGGTTGTTNAGAGGTCCACAACGGGTCGCACGACAACATTAAGGCCTCATGCATG
ACCCAACACGGGGGCGTCCCGGCGCGGCGACCTTGAAGTCCGGGGACAAGTATGGCCCCCCGGGAAGG
ACGCCGTAGTGCATCTGGAGTGCCGATTCGTGGTCNAGAATGCCACCACGATGGTATGAGTNGCAAAG
GTACTTTGGACACCACCCCGTTTTCTATTTCCAGACGCGGCCATTCAGGAACGACGCGAATCGTAATC
TACCTGCCGCGTACGCGGATCGTACATGCGTTTTCCAGCGTTCGAAATAGAGTTTGTCGTTCCGCTGA
CCAGGTTTCACGGCTACTGCGATAAGCATATTCTACTGGGGGACTGTAAAGGAATTGTATCCACTCTG
CTTCCTGTCAATTGGCCATGTGCATCTCTGGTTGTTCAGGGTACATTAATAACACACAGCATTAAGTA
GATAAGTCTCCGATATAAGCGAACAGAACGTTGGTCAGAGACTATGGTTCAATTGTAGTGGTATAATG
TTTCCCAGTGCATAAGGAACTCAGGGACACTACTAGCATGGGCGAGGAGAGACCCTTGGGTGGCAACA
CGCGATAAGTGTTACCTAGTACATCAGCACGAGAGAGGGTTTGACCATGTGTTTTTAGACGACCCCGG
TCGCATTGAACCTGGCATTCAGAATGACGTGCTCTCAAGAATCAAGGTCCTCCCACTGAGGGTCATCT
GCTTCGCGGGTCAGCGTATGGTCANANACAT
>Tay157
GTGTAGCCCAGTTAGACACTTCTAAATCCTTGGTAACAGACATGCAGTCCCCTCGGCAGAGCCCCGCC
CCCCGATGGTACCGGGTTGTTAAGAGGTCCACAACGGGTCGCACGACAACATTAAGGCCTCATGCATG
ACCCAACACGGGGGCGTCCCGGCGCGGCGACCTTGAANTCCGGGGACAAGTATGGCCCCCCGGGAAGG
ACGCCGTAGTGCATCTGGAGTGCCGATTCGTGGTCCAGAATGCCACCACGATGGTATGAGTGGCAAAG
GTACTTTGGACACCACCCCGTTTTCTATTTCCAGACGCGGCCATTCAGGAACGACGCGAATCGTAATC
TACCTGCCGCGTACGCGGATCGTACATGCGTTTTCCAGCGTTCGAAATAGAGTTTGTCGTTCCGCTGA
CCAGGTTTCACGGCTACTGCGATAAGCATATTCTACTGGGGGACTGTAAAGGAATTGTATCCACTCTG
CTTCCTGTCAATTGGCCATGTGCATCTCTGGTTGTTCAGGGTACATTAATAACACACAGCATTAAGTA
GATAAGTCTCCGATATAAGCGAACAGAACGTTGGTCAGAGACTATGGTTCAATTGTAGTGGTATAATG
TTTCCCAGTGCATAAGGAACTCAGGGACACTACTAGCATGGGCGAGGAGAGACCCTTGGGTGGCAACA
CGCGATAAGTGTTACCTAGTACATCAGCACGAGAGAGGGTTTGACCATGTGTTTTTAGACGACCCCGG
TCGCATTGAACCTGGCATTCAGAATGACGTGCTCTCAAGAATCAAGGTCCTCCCACTGAGGGTCATCT
GCTTCGCGGGTCAGCGTATGGTCATNNNNNT
>Tay158
GTGTAGCCCAGTTAGACACTTCTAAATCCTTGGTAACAGACATGCAGTCCCCTCGGCAGAGCCCCGCC
CCCCNATGGTACCGGGTTGTTAAGAGGTCCACAACGGGTCGCACGACAACNTTAAGGCCTCATGCATG
ACCCAACACGGGGGCGTCCCGGCGCGGCGACCTTGNANTCCGGGGACAAGTATGGCCCCCCGGGAAGG
ACGCCGTAGTGCATCTGGAGTGCCGATTNNTGGTCCAGAATGCCACCACGATGGTATGAGTGGCAAAG
GTACTTTGGACACCACCCCGTTTTCTATTTCCAGACGCGGCCATTCAGGAACGACGCGAATCGTAATC
TACCTGCCGCGTACGCGGATCGTACATGCGTTTTCCAGCGTTCGAAATAGAGTTTGTCGTTCCGCTGA
CCAGGTTTCACGGCTACTGCGATAAGCATATTCTACTGGGGGACTGTAAAGGAATTGTATCCACTCTG
CTTCCTGTCAATTGGCCATGTGCATCTCTGGTTGTTCAGGGTACATTAATAACACACAGCATTAAGTA
GATAAGTCTCCGATATAAGCGAACAGAACGTTGGTCAGAGACTATGGTTCAATTGTAGTGGTATAATG
TTTCCCAGTGCATAAGGAACTCAGGGACACTACTAGCANGGGCGAGGAGAGACCCTTGGGTGGCAACA
CGCAATAAGTGTTACCTAGTACATCAGCACGAGAGAGGGTTTGACCATGTGTTTTTAGACGACCCCGG
TCGCATTGAACCTGGCATTCAGAATGACGTGCTCTCAAGAATCAAGGTCCTCCCACTGAGGGTCATCT
GCTTCGCGGGTCAGCGTATGGTCANAAACAT
>Tay159
GTGTAGCCCAGTTAGACACTTCTAAATCCTTGGTAACAGACATGCAGTCCCCTCGGCAGAGCCCCGCC
CCCCGATGGTACCGGGTTGTTAAGAGGTCCACAACGGGTCGCACGACAACATTAAGGCCTCATGCATG
ACCCAACACGGGGGCGTCCCGGCGNGGCGACCTTGAAGTCCGGGGACAAGTATGGCCCCCCGGGAAGG
ACGCCGTAGTGCATCTGGAGTGCCGATTNGTGGTCCAGAATGCCACCACGATGGTATGAGTGGCAAAG
GTACTTTGGACACCACCCCGTTTTCTATTTCCAGACGCGGCCATTCAGGAACGACGCGAATCGTAATC
TACCTGCCGCGTACGCGGATCGTACATGCGTTTTCCAGCGTTCGAAATAGAGTTTGTCGTTCCGCTGA
CCAGGTTTCACGGCTACTGCGATAAGCATATTCTACTGGGGGACTGTAAAGGAATTGTATCCACTCTG
CTTCCTGTCAATTGGCCATGTGCATCTCTGGTTGTTCAGGGTACATTAATAACACACAGCATTAAGTA
GATAAGTCTCCGATATAAGCGAACAGAACGTTGGTCAGAGACTATGGTTCAATTGTAGTGGTATAATG

TTTCCCAGTGCATAAGGAACTCAGGGACACTACTAGCATGGGCGAGGAGAGACCCTTGGGTGGCAACA
CGCAATAAGTGTTACCTAGTACATCAGCACGAGAGAGGGTTTGACCATGTGTTTTTAGACGACCCCGG
TCGCATTGAACCTGGCATTCAGAATGACGTGCTCTCAAGAATCAAGGTCCTCCCACTGAGGGTCATCT
GCTTCGCGNGTCAGCGTATGGTCATNNNNAT
>Tay160
GTGTAGCCCAGTTAGACACTTCTAAATCCTTGGTAACAGACATGCAGTCCCCTCGGCAGAGNCCCGCC
CCCCNGTGGTACCGGGTTGTTAAGAGGTCCACCACGGGTCGCACGACAANNTCAAGGCCTCATGCATG
ACCCAACACGGGGGCGTCCCGGNGCGGCGACCTTGNANGCCGGGGACAAGTATGGCCCCCCGGGAAGG
ACGCCGTAGTGCATCTGGAGTGCCGATTCGTGGTCCAGAATGCCACCACGATGGTATGAGTNGCAAAG
GTACTTTGGACACCACCCCGTTTTCTATTTCCAGACGCGGCCATTCAGGAACGACGCGAATCGTAATC
TACCTGCCGCGTACGCGGATCGTACATGCGTTTTCCAGCGTTCGAAATAGAGTTTGTCGTTCCGCTGA
CCAGGTTTCACGGCTACTGCGATAAGCATATTCTACTGGGGGACTGTAAAGGAATTGTATCCACTCTG
CTTCCTGTCAATTGGCCATGTGCATCTCTGGTTGTTCAGGGTACATTAATAACACACAGCATTAAGTA
GATAAGTCTCCGATATAAGCGAACAGAACGTTGGTCAAAGACTGTGGTTCAATTGTAGTGGTATAACG
TTTCCCAGTGCATAAGGAACTCAGGGACACTACTAGCATGGGCGAGGAGAGACCCTTGGGTGGCAACA
CGCAATAAGTGTTACCTAGTACACCAGCACGATAGANGGTTTGACCATGTGTTTTTAGACGACCCCGG
TCGCATTGAACCTGGCATTCAGAATGACGTGCTCTCAAGAGTCAAGGTCCTCCCACTGAGGGTCATCT
GCTTCGCGNGTCAGCGTATGGTCANNAACNT
>Tay161
GTGTAGCCCAGTTAGACACTTCTAAATCCTTGGTAACAGACATGCAGTCCCCTCGGCAGAGCCCCGCC
CCCCGATGGTACCGGGTTGTTAAGAGGTCCACAACGGGTCGCACGACAACATTAAGGCCTCANGCATG
ACCCAACACGGGGGCGTCCCGGNGCGGCGACCTTGAAGTCCGGGGACAAGTATGGCCCCCCGGGAAGG
ACGCCGTAGTGCATCTGGAGTGCCGATTCGTGGTCCAGAATGCCACCACGATGGTATGAGTGGCAAAG
GTACTTTGGACACCACCCCGTTTTCTATTTCCAGACGCGGCCATTCAGGAACGACGCGAATCGTAATC
TACCTGCCGCGTNCGCGGATCGTACATGCGTTTTCCAGCGTTCGAAATAGAGTTTGTCGTTCCGCTGA
CCAGGTTTCACGGCTACTGCGATAAGCATATTCTACTGGGGGACTGTAAAGGAATTGTATCCACTCTG
CTTCCTGTCAATTGGCCATGTGCATCTCTGGTTGTTCAGGGTACATTAATAACACACAGCATTAAGTA
GATAAGTCTCCGATATAAGCGAACAGAACGTTGGTCAGAGACTATGGTTCAATTGTAGTGGTATAATG
TTTCCCAGTGCATAAGGAACTCAGGGACACTACTAGCATGGGCGAGGAGAGACCCTTGGGTGGCAACA
CGCAATAAGTGTTACCTAGTACATCAGCACGAGAGAGGGTTTGACCATGTGTTTTTAGACGACCCCGG
TCGCATTGAACCTGGCATTCAGAATGACGTGCTCTCAAGAATCAAGGTCCTCCCACTGAGGGTCATCT
GCTTCGCGGGTCAGCGTATGGTCATNANNNT
>Tay162
GTGTAGCCCAGTTAGACACTTCTAAATCCTTGGTAACAGACATGCAGTCCCCTCGGCAGAGCCCCGCC
CCCCGATGGTACCGGGTTGTTNAGAGGTCCACAACGGGTCGCACGACAACNTTAAGGCCTCATGCATG
ACCCANCNCGGGGGCGTCCCGGNGCGGCGACCTTGAAGTCCGGGGACAAGTATGGCCCCCCGGGAAGG
ACGCCGTAGTGCATCTGGAGTGCCGATTCGTGGTCCAGAATGNCACCACGATGGTATGAGTGGCAAAG
GTACTTTGGACACCACCCCGTTTTCTATTTCCAGACGCGGCCATTCAGGAACGACGCGAATCGTAATC
TACCTGCCGCGTACGCGGATCGTACATGCGTTTTCCAGCGTTCGAAATAGAGTTTGTCGTTCCGCTGA
CCAGGTTTCACGGCTACTGCGATAAGCATATTCTACTGGGGGACTGTAAAGGAATTGTATCCACTCTG
CTTCCTGTCAATTGGCCATGTGCATCTCTGGTTGTTCAGGNNACATTAATAACACACAGCATTAAGTA
GATAAGTCTCCGATATAAGCGAACAGAACGTTGGTCAGAGACTATGGTTCAATTGTAGTGGTATAATG
TTTCCCAGTGCATAAGGAACTCAGGGACACTACTAGCATGGGCGAGGAGAGACCCTTGGGTGGCAACA
CGCAATAAGTGTTACCTAGTACATCAGCACGAGAGAGGGTTTGACCATGTGTTTTTAGACGACCCCGG
TCGCATTGAACCTGGCATTCAGAATGACGTGCTCTCAAGAATCAAGGTCCTCCCACTGAGGGTCATCT
GCTTCGCGGGTCAGCGTATNGTCATAANNNT
>Tay163
GTGTAGCCCAGTTAGACACTTCTAAATCCTTGGTAACAGACATGCAGTCCCCTCGGCAGAGCCCCGCC
CCCCGATGGTACCGGGTTGTTAAGAGGTCCACAACGGGTCGCACGACAACATTAAGGCCTCATGCATG
ACCCAACACGGGGGCGTCCCGGCGCGGCGACCTTGAAGTCCGGGGACAAGTATGGCCCCCCGGGAAGG
ACGCCGTAGTGCATCTGGAGTGCCGATTCGTGGTCCAGAATGCCACCACGATGGTATGAGTNGCAAAG
GTACTTTGGACACCACCCCGTTTTCTATTTCCAGACGCGGCCATTCAGGAACGACGCGAATCGTAATC
TACCTGCCGCGTACGCGGATCGTACATGCGTTTTCCAGCGTTCGAAATAGAGTTTGTCGTTCCGCTGA
CCAGGTTTCACGGCTACTGCGATAAGCATATTCTACTGGGGGACTGTAAAGGAATTGTATCCACTCTG

CTTCCTGTCAATTGGCCATGTGCATCTCTGGTTGTTCAGGGTACATTAATAACACACAGCATTAAGTA
GATAAGTCTCCGATATAAGCGAACAGAACGTTGGTCAGAGACTATGGTTCAATTGTAGTGGTATAATG
TTTCCCAGTGCATAAGGAACTCAGGGACACTACTAGCATGGGCGAGGAGAGACCCTTGGGTGGCAACA
CGCAATAAGTGTTACCTAGTACATCAGCACGAGAGAGGGTTTGACCATGTGTTTTTAGACGACCCCGG
TCGCATTGAACCTGGCATTCAGAATGACGTGCTCTCAAGAATCAAGGTCCTCCCACTGAGGGTCATCT
GCTTCGCGNGTCAGCGTATGGTCANAAANAT
>Tay164
GTGTAGCCCAGTTAGACACTTCTAAATCCTTGGTAACAGACATGCAGTCCCCTCNNCAGAGNCCCGCC
CCCCGATGGTACCGGGTTGTTAAGAGGTCCACAACGGGTCGCACGACAACATTAAGGCCTNATGCATG
ACCCAACACGGGGGCGTCCCGGNGCGGCGACCTTGAAGTCCGGGGACAAGTATGGCCCCCCGGGAAGG
ACGCCGTAGTGCATCTGGAGTGCCGATTCGTGGTCCAGANTGNCACCACGATGGTATGAGTGGCAAAG
GTACTTTGGACACCACCCCGTTTTCTATTTCCAGACGCGGCCATTCAGGAACGACGCGAATCGTAATC
TACCTGCCGCGTACGCGGATCGTACATGCGTTTTCCAGCGTTCGAAATAGAGTTTGTCGTTCCGCTGA
CCAGGTTTCACGGCTACTGCGATAAGCATATTCTACTGGGGGACTGTAAAGGAATTGTATCCACTCTG
CTTCCTGTCAATTGGCCATGTGCATCTCTNGTTGTTCAGGGTACATTAATAACACACAGCATTAAGTA
GATAAGTCTCCGATATAAGCGAACAGAACGTTGGTCAGAGACTATGGTTCAATTGTAGTGGTATAATG
TTTCCCAGTGCATAAGGAACTCAGGGACACTACTAGCATGGGCGAGGAGAGACCCTTGGGTGGCAACA
CGCAATAAGTGTTACCTAGTACATCAGCACGAGAGANGGTTTGACCATGTGTTTTTAGACGACCCCGG
TCGCATTGAACCTGGCATTCAGAATGACGTGCTCTCAAGAATCNAGGTCCTCCCACTGAGGGTCATCT
GCTTCGCGGGTCAGCGTATGGTCATAAACAT
>Tay165
GTGTAGCCCAGTTAGACACTTCTAAATCCTTGGTAACAGACATGCAGTCCCCTCGGCAGAGCCCCGCC
CCCCGATGGTACCGGGTTGTTAAGAGGTCCACAACGGGTCGNACGACAACNTTAAGGCCTCATGCATG
ACCCAACACGGGGGCGTCCCGGCGCGGCGACCTTGAAGTCCGGGGACAAGTATGGCCCCCCGGGAAGG
ACGCCGTAGTGCATCTGGAGTGCCGATTNGTGGTCCAGAATGCCACCACGATGGTATGAGTGGCAAAG
GTACTTTGGACACCACCCCGTTTNCTATTTCCAGACGCGGCCATTCAGGAACGACGCGAATCGTAATC
TACCTGCCGCGTACGCGGATCGTACATGCGTTTTCCAGCGTTCGAAATAGAGTTTGTCGTTCCGCTGA
CCAGGTTTCACGGCTACTGCGATAAGCATATTCTACTGGGGGACTGTAAAGGAATTGTATCCACTCTG
CTTCCTGTCAATTGGCCATGTGCATCTCTNGTTGTTCAGGGTACATTAATAACACACAGCATTAAGTA
GATAAGTCTCCGATATAAGCGAACAGAACGTTGGTCAGAGACTATGGTTCAATTGTAGTGGTATAATG
TTTCCCAGTGCATAAGGAACTCAGGNACACTACTAGCATGGGCGAGGAGAGACCCTTGGGTGGCAACA
CGCAATAAGTGTTACCTAGTACATCAGCACGAGAGAGGGTTTGACCATGTGTTTTTAGACGACCCCGG
TCGCATTGAACCTGGCATTCAGAATGACGTNCTCTCAAGAATCAAGGTCCTCCCACTGAGGGTCATCT
GCTTCGCGGGTCAGCGTATGGTCATANACAT
>Tay166
GTGTAGCCCAGTTAGACACTTCTAAATCCTTGGTAACAGACATGCAGTCCCCTCGGCAGAGCCCCGCC
CCCCGATGGTACCGGGTTGTTAAGAGGTCCACAACGGGTCGCACGACAACNTTAAGGCCTCANGCATG
ACCCAACACGGGGGCGTCCCGGCGCGGCGACCTTGAAGTCCGGGGACAAGTATGGCCCCCCGGGAAGG
ACGCCGTAGTGCATCTGGAGTGCCGATTNGTGGTCCAGAATGCCACCACGATGGTATGAGTNGCAAAG
GTACTTTGGACACCACCCCGTTTTCTATTTCCAGACGCGGCCATTCAGGAACGACGCGAATCGTAATC
TACCTGCCGCGTNCGCGGATCGTACATGCGTTTTCCAGCGTTCGAAATAGAGTTTGTCGTTCCGCTGA
CCAGGTTTCACGGCTACTGCGATAAGCATATTCTACTGGGGGACTGTAAAGGAATTGTATCCACTCTG
CTTCCTGTCAATTGGCCATGTGCATCTCTGGTTGTTCAGGGTACATTAATAACACACAGCATTAAGTA
GATAAGTCTCCGATATAAGCGAACAGAACGTTGGTCAGAGACTATGGTTCAATTGTAGTGGTATAATG
TTTCCCAGTGCATAAGGAACTCAGGGACACTACTAGCATGGGCGAGGAGAGACCCTTGGGTGGCAACA
CGCAATAAGTGTTACCTAGTACATCAGCACGAGAGAGGGTTTGACCATGTGTTTTTAGACGACCCCGG
TCGCATTGAACCTGGCATTCAGAATGACGTGCTCTCAAGAATCAAGGTCCTCCCACTGAGGGTCATCT
GCTTCGCGGGTCAGCGTATGGTCANAANNNT
>Tay167
GTGTAGCCCAGTCAGACACTTCTAAATCCTTGGTAACAGACATGCAGTCCCCTCNNCAGAGCCCCGCC
CCCCGATGGTACCGGGTTGTTAAGAGGTCCACAACGGGTCGCACGACAANNTTAAGGCCTCATGCATG
ACCCAACACGGGGGCGTCCCGGCGCGGCGACCTTGAANTCCGGGGACAAGTATGGCCCCCCGGGAAGG
ACGCCGTAGTGCATCTGGAGTGCCGATTCGTGGTCCAGAATGCCACCACGATGGTATGAGTGGCAAAG
GTACTTTGGACACCACCCCGTTTTCTANTTCCAGACGCGGCCATTCAGGAACGACGCGAATCGTAATC

TACCTGCCGCGTACGCGGATCGTACATGCGTTTTCCAGCGTTCGAAATAGAGTTTGTCGTTCCGCTGA
CCAGGTTTCACGGCTACTGCGATAAGCATATTCTACTGGGGGACTGTAAAGGAATTGTATCCATTCTG
CTTCCTGTCAATTGGCCATGTGCATCTCTGGTTGTTCAGGGTACATTAATAACACACAGNAGTAAGTA
GGTAAGTCTCCGATATAAGCGAACAGAACGTTTGTCAGAGACTGTGGTTCAATTGTAGTGGTATAATG
TTTCCCAGTGCATAAGGAACTCAGGNACACTACTAGCANGGGCGAGGAGAGACCCTTGGGTGGCAACA
CGCAATAAGTGTTACCTAGTACACCAGCACGAGNNAGGGTTTGACCATGNNTTTTAGACGACCCCGG
TCGCATTGAACCTGGCATTCAGAATGACGTGCTCTCAAGAATCAAGNTCCTCCCACTGAGGGTCATCT
GCTTCGCGGGTCAGCGTATNGTCANNNNNNT
>Tay168
GTGTAGCCCAGTTAGACACTTCTAAATCCTTGGTAACAGACATGCAGTCCCCTCGGCAGAGNCCCGCC
CCCCGGTGGTACCGGGTTGTTAAGAGGTCCACCACGGGTCGCACGACNACNTCAAGGCCTNATGCATG
ACCCAACACGGGGGCGTCCCGGCGCGGCGACCTTGAAGGCCGGGGACAAGTATGGCCCCCCGGGAAGG
ACGCCGTAGTGCATCTGGAGTGCCGATTCGTGGTCNAGAATGCCACCACGATGGTATGAGTGGCAAAG
GTACTTTGGACACCACCCCGTTTTCTATTTCCAGACGCGGCCATTCAGGAACGACGCGAATCGTAATC
TACCTGCCGCGTACGCGGATCGTACATGCGTTTTCCAGCGTTCGAAATAGAGTTTGTCGTTCCGCTGA
CCAGGTTTCACGGCTACTGCGATAAGCATATTCTACTGGGGGACTGTAAAGGAATTGTATCCACTCTG
CTTCCTGTCAATTGGCCATGTGCATCTCTGGTTGTTCAGGGTACATTAATAACACACAGCATTAAGTA
GATAAGTCTCCGATATAAGCGAACAGAACGTTGGTCAAAGACTGTGGTTCAATTGTAGTGGTATAACG
TTTCCCAGTGCATAAGGAACTCAGGGACACTACTAGCATGGGCGAGGAGAGACCCTTGGGTGGCAACA
CGCAATAAGTGTTACCTAGTACACCAGCACGATAGAGGGTTTGACCATGTGTTTTTAGACGACCCCGG
TCGCATTGAACCTGGCATTCAGAATGACGTGCTCTCAAGAGTCNAGGTCCTCCCACTGAGGGTCATCT
GCTTCGCGNGTCAGCGTATGGTCATNAACAT
>Tay169
GTGTAGCCCAGTCAGACACTTCTAAATCCTTGGTAACAGACATGCAGTCCCCTCNNCAGAGNCCCGCC
CCCCGATGGTACCGGGTTGTTAAGAGGTCCACAACGGGTCGCACGACAACATTAAGGCCTCANGCATG
ACCCANCACGGGGGNGTCCCGGCGCGGCGACCTTGAANTCCGGGGACAAGTATGGCCCCCCGGGAAGG
ACGCCGTAGTGCATCTGGAGTGCCGATTNGTGGTCCAGANTGCCACCACGATGGTATGAGCGGCAAAG
GTACTTTGGACACCACCCCGTTTTCTATTTCCAGACGCGGCCATTTAGGAACGACGCGAATCGTAATC
TACCTGCCGCGTACGCGGATCGTACATGCGTTTTCCAGCGTTCGAAATAGAGTTTGTCGTTCCGCTGA
CCAGGTTTCACGGCTACTGCGATAAGCATATTCTACTGGGGGACTGTAAAGGAATTGTATCCATTCTG
CTTCCTGTCAATTGGCCATGTGCATCTCTNGTTGTTCAGGNTACATTAATAACACACAGCAGTAAGTA
GGTAAGTCTCCGATATAAGCGAACAGAACGTTTGTCAGAGACTGTGGTTCAATTGTAGTGGTATAATG
TTTCCCAGTGCATAAGGAACTCAGGGACACTACTAGCATGGGCGAGGAGAGACCCTTGGGTGGCAACA
CGCAATAAGTGTTACCTAGTACACCAGCACGAGAGAGGGTTTGACCATGTGTTTTTAGNNGACCCCGG
TCGCATTGAACCTGGCATTCAGAATGACGTGCTCTCAAGAATCAAGGTCCTCCCACTGAGGGTCATCT
GCTTCGCGGGTCAGCGTATGGTCATNAACAT
>Tay170
GTGTAGCCCAGTCAGACACTTCTAAATCCTTGGTAACAGACATGCAGTCCCCTCGGCAGAGCCCCGCC
CCCCGATGGTACCGGGTTGTTAAGAGGTCCACAACGGGTCGCACGACAANNTTAAGGCCTCATGCATG
ANNCAACACGGGGGCGTCCCGGCGCGGCGACCTTGAAGTCCGGGGACAAGTATGGCCCCCCGGGAAGG
ACGCCGTAGTGCATCTGGAGTGCCGATTCGTGGNCCAGAATGCCACCACGATGGTATGAGTGGCAAAG
GTACTTTGGACACCACCCCGTTTTCTATTTCCAGACGCGGCCATTCAGGAACGACGCGAATCGTAATC
TACCTGCCGCGTACGCGGATCGTACATGCGTTTTCCAGCGTTCGAAATAGAGTTTGTCGTTCCGCTGA
CCAGGTTTCACGGCTACTGCGATAAGCATATTCTACTGGGGGACTGTAAAGGAATTGTATCCATTCTG
CTTCCTGTCAATTGGCCATGTGCATCTCTGGTTGTTCAGGGTACATTAATAACACACAGCAGTAAGTA
GGTAAGTCTCCGATATAAGCGAACAGAACGTTTGTCAGAGACTGTGGTTCAATTGTAGTGGTATAATG
TTTCCCAGTGCATAAGGAACTCAGGNACACTACTAGCATGGGCGAGGAGAGACCCTTGGGTGGCAACA
CGCAATAAGTGTTACCTAGTACACCAGCACGAGAGANGGTTTGACCATGTGTTTTTAGACGACCCCGG
TCGCATTGAACCTGGCATTCAGAATGACGTGCTCTCAAGAATCAAGGTCCTCCCACTGAGGGTCATCT
GCTTCGCGGGTCAGCGTATGGTCANAAACAT
>Tay171
GTGTAGCCCAGTCAGACACNTCTAAATCCTTGGTAACAGACATGCAGTCCCCTCGGCAGAGCCCCGCC
CCCCGATGGTACCGGGTTGTTAAGAGGTCCACAACGGGTCGNACGACAACNTTAAGGCCTCATGCATG
ACCCAACACGGGGGCGTCCCGGNGCGGCGACCTTGAAGTCCGGGGACAAGTATGGCCCCCCGGGAAGG

ACGCCGTAGTGCATCTGGAGTGCCGATTCGTGGTCCAGAATGCCACCACGATGGTATGAGTGGCAAAG
GTACTTTGGACACCACCCCGTTTTCTATTTCCAGACGCGGCCATTCAGGAACGACGCGAATCGTAATC
TACCTGCCGCGTACGCGGATCGTACATGCGTTTTCCAGCGTTCGAAATAGAGTTTGTCGTTCCGCTGA
CCAGGTTTCACGGCTACTGCGATAAGCATATTCTACTGGGGGACTGTAAAGGAATTGTATCCATTCNG
CTTCCTGTCAATTGGCCATGTGCATCTCTNGTTGTTCAGGGTACATTAATAACACACAGNAGTAAGTA
GGTAAGTCTCCGATATAAGCGAACAGAACGTTTGTCAGAGACTGTGGTTCAATTGTAGTGGTATAATG
TTTCCCAGTGCATAAGGAACTCAGGGACACTACTAGCATGGGCGAGGAGAGACCCTTGGGTGGCAACA
CGCAATAAGTGTTACCTAGTACACCAGCACGAGAGAGGGTTTGACCATGTGTTTTTAGACGACCCCGG
TCGCATTGAACCTGGCATTCAGAATGACGTGCTCTCAAGAATCNANGTCCTCCCACTGAGGGTCATCT
GCTTCGCGGGTCNGCGTATGGTCANAAACAT
>Tay172
GTGTAGCCCAGTTAGACACTTCTAAATCCTTGGTAACAGACATGCAGTCCCCTCGGCAGAGCCCCGCC
CCCCNATGGTACCGGGTTGTTAAGAGGTCCACAACAGGTCGCACGACAACATTAAGGCCTCANGCATG
ACCCNACACGGGGGCGTCCCGGCGCGGCGACCTTGNAGTCCGGGGACAAGTATGGCCCCCCGGGAAGG
ACGCCGTAGTGCATCTGGAGTGCCGATTCGTGGTCCAGAATGNCACCACGATGGTATGAGTTGCAAAG
GTACTTTGGACACCACCCCGTTTTCTATTTCCAGACGCGGCCATTCAGGAACGACGCGAATCGTAATC
TACCTGCCGCGTNCGCGGATCGTACATGCGTTTTCCAGCGTTCGAAATAGAGTTTGTCGTTCCGCCGA
CCAGGTTTCACGGCTACTGCGATAAGCATATTCTACTGGGGGACTGTAAAGGAATTGTATCCACTCTA
CTTCCTGTCAATTGGCCATGTGCATCTCTNGTTGTTCAGGGTACATTAATAACACACAGCATTAAGTA
GATAAGTCTCCGATATAAGCGAACAGAACGTTGGTCAGAGACTGTGGTTCAATTGTAGTGGTATAATG
TTTCCCAGTGCATAAGGAACTTAGGGACATTACTAGCATGGGCGAGGAGAGACCCTTGGGTGGCAACA
CGCAATAAGTGTTACCTAGTACACCAGCACGAGAGAGGGTTTGACCATGTGTTTTTAGACGACCCCGG
TCGCATTGAACCTGGCATTCAGAATGACGTGCTCTCAAGAATCAANGTCCTCCCACTGAGGGTCATCT
GCTTCGCGGGTCAGCGTATGGTCANNNNNAT
>Tay173
GTGTAGCCCAGTTAGACACTTCTAAATCCTTGGTAACAGACATGCAGTCCCCTCGGCAGAGCCCCGCC
CCCCGATGGTACCGGGTTGTTAAGAGGTCCACAACAGGTCGCACGACAACATTAAGGCCTCATGCATG
ACCCAACACGGGGGCGTCCCGGCGCGGCGACCTTGAANTCCGGGGACAAGTATGGCCCCCCGGGAAGG
ACGCCGTAGTGCATCTGGAGTGCCGATTCGTGGTCCAGAATGNCACCACGATGGTATGAGTGGCAAAG
GTACTTTGGACACCACCCCGTTTTCTATTTCCAGACGCGGCCATTCAGGAACGACGCGAATCGTAATC
TACCTGCCGCGTACGCGGATCGTACATGCGTTTTCCAGCGTTCGAAATAGAGTTTGTCGTTCCGCCGA
CCAGGTTTCACGGCTACTGCGATAAGCATATTCTACTGGGGGACTGTAAAGGAATTGTATCCACTCNA
CTTCCTGTCAATTGGCCATGTGCATCTCTGGTTGTTCAGGNNACATTAATAACACACAGCATTAAGTA
GATAAGTCTCCGATATAAGCGAACAGAACGTTGGTCAGAGACTGTGGTTCAATTGTAGTGGTATAATG
TTTCCCAGTGCATAAGGAACTTAGGGACATTACTAGCATGGGCGAGGAGAGACCCTTGGGTGGCAACA
CGCAATAAGTGTTACCTAGTACACCAGCACGAGAGAGGGTTTGACCATGTGTTTTTAGACGACCCCGG
TCGCATTGAACCTGGCATTCAGAATGACGTGCTCTCAAGAATCAAGGTCCTCCCACTGAGGGTCATCT
GCTTCGCGGGTCAGCGTATGGTCANNAAAAT
>Tay174
GTGTAGCCCAGTCAGNCACTTCTAAATCCTTGGTAACAGACATGCAGTCCCCTCGGCAGAGCCCCGCC
CCCCGATGGTACCGGGTTGTTAAGAGGTCCACAACGGGTCGCACGACAACATTAAGGCCTCATGCATG
ACCCAACACGGGGGCGTCCCGGCGCGGCGACCTTGNANTCCGGGGNCAAGTATGGCCCCCCGGGAAGG
ACGCCGTAGTGCATCTGGAGTGCCGANTNGTGGTCNAGAATGCCACCACGATGGTATGAGTGGCAAAG
GTACTTTGGACACCACCCCGTTTTCTATTTCCAGACGCGGCCATTCAGGAACGACGCGAATCGTAATC
TACCTGCCGCGTACGCGGATCGTACATGCGTTTTCCAGCGTTCGAAATAGAGTTTGTCGTTCCGCTGA
CCAGGTTTCACGGCTACTGCGATAAGCATATTCTACTGGGGGACTGTAAAGGAATTGTATCCATTCTG
CTTCCTGTCAATTGGCCATGTGCATCTCTGGTTGTTCAGGGTACATTAATAACACACAGCAGTAAGTA
GGTAAGTCTCCGATATAAGCGAACAGAACGTTTGTCAGAGACTGTGGTTCAATTGTAGTGGTATAATG
TTTCCCAGTGCATAAGGAACTCAGGGACACTACTAGCATGGGCGAGGAGAGACCCTTGGGTGGCAACA
CGCAATAAGTGTTACCTAGTACACCAGCACGAGNGANGGTTTGACCATGTNTTTTTAGACGNCCCCGG
TCGCATTGAACCTGGCATTCAGAATGACGTGCTCTCANGAATCAAGGTCCTCCCACTGAGGGTCATCT
GCTTCGCGGGTCAGCGTATGGTCANNAACNT
>Tay175
GTGTAGCCCAGTCAGACACTTCTAAATCCTTGGTAACAGACATGCAGTCCCCTCGGCAGAGNCCCGCC

CCCCGATGGTACCGGGTTGTTAAGAGGTCCACAACGGGTCGCACGACAACATTAAGGCCTCANGCATG
ACCCAACACGGGGGCGTCCCGGCGCGGCGACCTTGAAGTCCGGGGACAAGTATGGCCCCCCGGGAAGG
ACGCCGTAGTGCATCTGGAGTGCCGATTCNTGGNCCAGAATGNCACCACGATGGTATGAGCGGCAAAG
GTACTTTGGACACCACCCCGTTTTCTATTTCCAGACGCGGCCATTTAGGAACGACGCGAATCGTAATC
TACCTGCCGCGTACGCGGATCGTACATGCGTTTTCCAGCGTTCGAAATAGAGTTTGTCGTTCCGCTGA
CCAGGTTTCACGGCTACTGCGATAAGCATATTCTACTGGGGGACTGTAAAGGAATTGTATCCATTCTG
CTTCCTGTCAATTGGCCATGTGCATCTCTNGTTGTTCAGGGTACATTAATAACACACAGCAGTAAGTA
GGTAAGTCTCCGATATAAGCGAACAGAACGTTTGTCAGAGACTGTGGTTCAATTGTAGTGGTATAATG
TTTCCCAGTGCATAAGGAACTCAGGGACACTACTAGCATGGGCGAGGAGAGACCCTTGGGTGGCAACA
CGCAATAAGTGTTACCTAGTACACCAGCACGAGAGAGGGTTTGACCATGTGTTTTTAGACGACCCCGG
TCGCATTGAACCTGGCATTCAGAATGACGTGCTCTCAAGAATCAAGGTCCTCCCACTGAGGGTCATCT
GCTTCGCGGGNCAGCGTATGGTCATNNNNNT
>Tay176
GTGTAGCCCAGTCAGACACTTCTAAATCCTTGGTAACAGACATGCAGTCCCCTCNGCAGAGCCCCGCC
CCCCGATGGTACCGGGTTGTTNAGAGGTCCACAACGGGTCGCACGACAACATTAAGGCCTCATGCATG
ACCCAACACGGGGGCGTCCCGGCGCGGCGACCTTGAAGTCCGGGGACAAGTATGGCCCCCCGGGAAGG
ACGCCGTAGTGCATCTGGANTGCCGATTCGTGGTCCAGAATGCCACCACGATGGTATGAGTNGCAAAG
GTACTTTGGACACCACCCCGTTTTCTATTTCCAGACGCGGCCATTCAGGAACGACGCGAATCGTAATC
TACCTGCCGCGTNCGCGGATCGTACATGCGTTTTCCAGCGTTCGAAATAGAGTTTGTCGTTCCGCTGA
CCAGGTTTCACGGCTACTGCGATAAGCATATTCTACTGGGGGACTGTAAAGGAATTGTATCCATTCTG
CTTCCTGTCAATTGGCCATGTGCATCTCTGGTTGTTCAGGGTACATTAATAACACACAGCAGTAAGTA
GGTAAGTCTCCGATATAAGCGAACAGAACGTTTGTCAGAGACTGTGGTTCAATTGTAGTGGTATAATG
TTTCCCAGTGCATAAGGAACTCAGGGACACTACTAGCATGGGCGAGGAGAGACCCTTGGGTGGCAACA
CGCAATAAGTGTTACCTAGTACACCAGCACGAGNNAGGGTTTGACCATGTGTTTTTAGACGACCCCGG
TCGCATTGAACCTGGCATTCAGAATGACGTNCTCTCAAGAATCAAGGTCCTCCCACTGAGGGTCATCT
GCTTCGCGGGTCAGCGTATGGTCATAAACAT
>Tay177
GTGTAGCCCAGTCAGACACNTCTAAATCCTTGGTAACAGACATGCAGTCCCCTCGGCAGAGCCCCGCC
CCCCGATGGTACCGGGTTGTTAAGAGGTCCACAACGGGTCGCACGACNNCNTTAAGGCCTCANGCATG
ACCCNACACGGNGGCGTCCCGGCGCGGCGACCTTGNAGTCCGGGGACAAGTATGGCCCCCCGGGAAGG
ACGCCGTAGTGCATCTGGAGTGCCGATTCGTGGTCNAGAATGNCACCACGATGGTATGAGTNGCAAAG
GTACTTTGGACACCACCCCGTTTTCTATTTCCAGACGCGGCCATTCAGGAACGACGCGAATCGTAATC
TACCTGCCGCGTACGCGGATCGTACATGCGTTTTCCAGCGTTCGAAATAGAGTTTGTCGTTCCGCTGA
CCAGGTTTCACGGCTACTGCGATAAGCATATTCTACTGGGGGACTGTAAAGGAATTGTATCCATTCNG
CTTCCTGTCAATTGGCCATGTGCATCTCTGGTTGTTCAGGNNACATTAATAACACACAGCAGTAAGTA
GGTAAGTCTCCGATATAAGCGAACAGAACGTTTGTCAGAGACTGTGGTTCAATTGTAGTGGTATAATG
TTTCCCAGTGCATAAGGAACTCAGGNACACTACTAGCATGGGCGAGGAGAGACCCTTGGGTGGCAACA
CGCAATAAGTGTTACCTAGTACACCAGCACGAGAGANGGTTTGACCATGTGTTTTTAGACGNCCCCGG
TCGCATTGAACCTGGCATTCAGAATGACGTGCTCTCAAGAATCNAGGTCCTCCCACTGAGGGTCATCT
GCTTCGCGNGTCAGCGTATGGTCATNNNNNT
>Tay178
GTGTAGCCCAGTTAGACACTTCTAAATCCTTGGTAACAGACATGCAGTCCCCTCNNCAGAGCCCCGCC
CCCCGGTGGTACCGGGTTGTTAAGAGGTCCACCACGGGTCGCACGACNNCATCAAGGCCTCANGCATG
ACCCAACACGGGGGNGTCCCGGCGCGGCGACCTTGNANGCCGGGGACAAGTATGGCCCCCCGGGAAGG
ACGCCGTAGTGCATCTGGAGTGCCGATTCGTGGTCCAGAATGCCACCACGATGGTATGAGTNGCAAAG
GTACTTTGGACACCACCCCGTTTTCTATTTCCAGACGCGGCCATTCAGGAACGACGCGAATCGTAATC
TACCTGCCGCGTNCGCGGATCGTACATGCGTTTTCCAGCGTTCGAAATAGAGTTTGTCGTTCCGCTGA
CCAGGTTTCACGGCTACTGCGATAAGCATATTCTACTGGGGGACTGTAAAGGAATTGTATCCACTCTG
CTTCCTGTCAATTGGCCATGTGCATCTCTGGTTGTTCAGGGTACATTAATAACACACAGCATTAAGTA
GATAAGTCTCCGATATAAGCGAACAGAACGTTGGTCAAANACTGTGGTTCAATTGTAGTGGTATAACG
TTTCCCAGTGCATAAGGAACTCAGGGACACTACTAGCANGGGCGAGGAGAGACCCTTGGGTGGCAACA
CGCAATAAGTGTTACCTAGTACACCAGCACGATAGAGGGTTTGACCATGTGTTTTTAGACGACCCCGG
TCGCATTGAACCTGGCATTCAGAATGACGTGCTCTCAAGAGTCAAGGTCCTCCCACTGAGGGTCATCT
GCTTCGCGNGTCAGCGTATGGTCANAAACAT

>Tay378
GTGTAGCCCAGTTAGACACTTCTAAATCCTTGGTAACAGACATGCAGTCCCCTCNNCAGAGCCCCGCC
CCCCNATGGTACCGGGTTGTTAAGAGGTCCACAACGGGTCGCACGACNNCATTAAGGCCTCATGCATG
ACCCAACACGGGGGCGTCCCGGNGNGGCGACCTTGAANTCCGGGGNCAAGTATGGCCCCCCGGGAAGG
ACGCCGTAGTGCATCTGGAGTGCCGATTCGTGGTCCAGAATGCCACCACGATGGTATGAGTNGCAAAG
GTACTTTGGACACCACCCCGTTTTCTATTTCCAGACGCGGCCATTCAGGAACGACGCGAATCGTAATC
TACCTGCCGCGTNCGCGGATCGTACATGCGTTTTCCAGCGTTCGAAATAGAGTTTGTCGTTCCGCTGA
CCAGGTTTCACGGCTACTGCGATAAGCATATTCTACTGGGGGACTGTAAAGGAATTGTATCCACTCNG
CTTCCTGTCAATTGGCCATGTGCATCTCTGGTTGTTCAGGGTACATTAATAACACACAGCATTAAGTA
GATAAGTCTCCGATATAAGCGAACAGAACGTTGGTCAGAGACTATGGTTCAATTGTAGTGGTATAATG
TTTCCCAGTGCATAAGGAACTCAGGGACACTACTAGCATGGGCGAGGAGAGACCCTTGGGTGGCAACA
CGCAATAAGTGTTANCTAGTACATCAGCACGAGAGAGGGTTTGACCATGTGTTTTTAGACGACCCCGG
TCGCATTGAACCTGGCATTCAGAATGACGTGCTCTCAAGAATCAAGGTCCTCCCACTGAGGGTCATCT
GCTTCGCGGNTCAGCGTATGGTCACANNNAT
>Tay379
GTGTAGCCCAGTTAGACACTTCTAAATCCTTGGTAACAGACATGCAGTCCCCTCGGCAGAGCNCCGCC
CCCCNATGGTACCGGGTTGTTAAGAGGTCCACAACGGGTCGCACGACAACATTAAGGCCTNATGCATG
ANCCNNCACGGGGGCGTCCCGGCGCGGCGACCTTGAAGTCCGGGGACAAGTATGGCCCCCCGGGAAGG
ACGCCGTAGTGCATCTGGAGTGCCGATTCGTGGTCCAGAATGNCACCACGATGGTATGAGTGGCAAAG
GTACTTTGGACACCACCCCGTTTTCTATTTCCAGANGCGGCCATTCAGGAACGACGCGAATCGTAATC
TACCTGCCGCGTACGCGGATCGTACATGCGTTTTCCAGCGTTCGAAATAGAGTTTGTCGTTCCGCTGA
CCAGGTTTCACGGCTACTGCGATAAGCATATTCTACTGGGGGACTGTAAAGGAATTGTATCCACTCTG
CTTCCTGTCAATTGGCCATGTGCATCTCTGGTTGTTCAGGGTACATTAATAACACACAGCATTAAGTA
GATAAGTCTCCGATATAAGCGAACAGANCGTTGGTCAGAGACTATGGTTCAATTGTAGTGGTATAATG
TTTCCCAGTGCATAAGGAACTCAGGGACACTACTAGCATGGGCGAGGAGAGACCCTTGGGTGGCAACA
CGCAATAAGTGTTACCTAGTACATCAGCACGAGAGANGGTTTGACCATGTGTTTTTAGACGACCCCGG
TCGCATTGAACCTGGCATTCAGAATGACGTGCTCTCAAGAATCAAGGTCCTCCCACTGAGGGTCATCT
GCTTCGCGGGTCAGCGTATGGTCATAAACAT
>Tay380
GTGTAGCCCAGTTAGACACTTCTAAATCCTTGGTAACAGACATGCAGTCCCCTCGGCAGAGCCCCGCC
CCCCGATGGTACCGGGTTGTTAAGAGGTCCACAACGGGTCGNACGACANNATTAAGGCCTCATGCATG
ACCCAACNCGGGGGCGTCCCGGCGCGGCGACCTTGNAGTCCGGGGACAAGTATGGCCCCCCGGGAAGG
ACGCCGTAGTGCATCTGGAGTGCCGATTCGTGGTCCAGANTGNCACCACGATGGTATGAGTGGCAAAG
GTACTTTGGACACCACCCCGTTTTCTATTTCCAGACGCGGCCATTCAGGAACGACGCGAATCGTAATC
TACCTGCCGCGTACGCGGATCGTACATGCGTTTTCCAGCGTTCGAAATAGAGTTTGTCGTTCCGCTGA
CCAGGTTTCACGGCTACTGCGATAAGCATATTCTACTGGGGGACTGTAAAGGAATTGTATCCACTCTG
CTTCCTGTCAATTGGCCATGTGCATCTCTNGTTGTTCAGGGTACATTAATAACACACAGCATTAAGTA
GATAAGTCTCCGATATAAGCGAACAGAACGTTGGTCAGAGACTATGGTTCAATTGTAGTGGTATAATG
TTTCCCAGTGCATAAGGAACTCAGGGACACTACTAGCATGGGCGAGGAGAGACCCTTGGGTGGCAACA
CGCAATAAGTGTTACCTAGTACATCAGCACGAGAGAGGGTTTGACCATGTGTTTTTAGACGACCCCGG
TCGCATTGAACCTGGCATTCAGAATGACGTGCTCTCAAGAATCAAGGTCCTCCCACTGAGGGTCATCT
GCTTCGCGGGTCAGCGTATGGTCANNANNAT
>Tay387
GTGTAGCCCAGTTAGACACTTCTAAATCCTTGGTAACAGACATGCAGTCCCCTCGGCAGAGNCCCGCC
CCCCNATGGTACCGGGTTGTTAAGAGGTCCACAACAGGTCGCACGACAACNTTAAGGCCTCATGCATG
ACCCAACACGGGGGCGTCCCGGCGCGGCGACCTTGAAGTCCGGGGACAAGTATGGCCCCCCGGGAAGG
ACGCCGTAGTGCATCTGGAGTGCCGATTNNTGGTCCAGAATGCCACCACGATGGTATGAGTNGCAAAG
GTACTTTGGACACCACCCCGTTTTCTATTTCCAGACGCGGCCATTCAGGAACGACGCGAATCGTAATC
TACCTGCCGCGTACGCGGATCGTACATGCGTTTTCCAGCGTTCGAAATAGAGTTTGTCGTTCCGCCGA
CCAGGTTTCACGGCTACTGCGATAAGCATATTCTACTGGGGGACTGTAAAGGAATTGTATCCACTCTA
CTTCCTGTCAATTGGCCATGTGCATCTCTGGTTGTTCAGGGTACATTAATAACACACAGCATTAAGTA
GATAAGTCTCCGATATAAGCGAACAGAACGTTGGTNAGAGACTGTGGTTCAATTGTAGTGGTATAATG
TTTCCCAGTGCATAAGGAACTTAGGGACATTACTAGCATGGGCGAGGAGAGACCCTTGGGTGGCAACA
CGCAATAAGTGTTACCTAGTACACCAGCACGAGAGAGGGTTTGACCATGTGTTTTTAGACGACCCCGG

TCGCATTGAACCTGGCATTCAGAATGACGTGCTCTCAAGAATCAAGGTCCTCCCACTGAGGGTCATCT
GCTTCGCGGGTCAGCGTATNGTCATAAACNT
>Tay388
GTGTAGCCCAGTTAGACACTTCTAAATCCTTGGTAACAGACATGCAGTCCCCTCGGCAGAGCCCCGCC
CCCCNATGGTACCGGGTTGTTAAGAGGTCCACAACGGGTCGCACGACAACATTAAGGCCTCATGCATG
ACCCAACACGGNGGCGTCCCGGCGNGGCGACCTTGNANTCCGGGGACAAGTATGGCCCCCCGGGAAGG
ACGCCGTAGTGCATCTGGAGTGCCGATTCGTGGTCCAGAATGCCACCACGATGGTATGAGTGGCAAAG
GTACTTTGGACACCACCCCGTTTTCTATTTCCAGACGCGGCCATTCAGGAACGACGCGAATCGTAATC
TACCTGCCGCGTACGCGGATCGTACATGCGTTTTCCAGCGTTCGAAATAGAGTTTGTCGTTCCGCTGA
CCAGGTTTCACGGCTACTGCGATAAGCATATTCTACTGGGGGACTGTAAAGGAATTGTATCCACTCTG
CTTCCTGTCAATTGGCCATGTGCATCTCTGGTTGTTCAGGGTACATTAATAACACACAGCATTAAGTA
GATAAGTCTCCGATATAAGCGAACAGAACGTTGGTCAGAGACTATGGTTCAATTGTAGTGGTATAATG
TTTCCCAGTGCATAAGGAACTCAGGGACACTACTAGCATGGGCGAGGAGAGACCCTTGGGTGGCAACA
CGCGATAAGTGTTACCTAGTACATCAGCACGAGAGAGGGTTTGACCNTGTGTTTTTAGACGACCCCGG
TCGCATTGAACCTGGCATTCAGAATGACGTGCTCTCAAGAATCAAGGTCCTCCCACTGAGGGTCATCT
GCTTCGCGGGTCAGCGTATGGTCATAAACAT
>Tay390
GTGTAGCCCAGTTAGACACTTCTAAATCCTTGGTAACAGACATGCAGTCCCCTCNNCAGAGCCCCGCC
CCCCNATGGTACCGGGTTGTTAAGAGGTCCACAACGGGTCGCACGACAACATTAAGGCCTNANGCATG
ACCCNACACGGGGGCGTCCCGGCGCGGCGACCTTGAANTCCGGGGACAAGTATGGCCCCCCGGGAAGG
ACGCCGTAGTGCATCTGGAGTGCCGATTCGTGGNCCNGAATGCCACCACGATGGTATGAGTGGCAAAG
GTACTTTGGACACCACCCCGTTTTCTATTTCCAGANGCGGCCATTCAGGAACGACGCGAATCGTAATC
TACCTGCCGCGTACGCGGATCGTACATGCGTTTTCCAGCGTTCGAAATAGAGTTTGTCGTTCCGCTGA
CCAGGTTTCACGGCTACTGCGATAAGCATATTCTACTGGGGGACTGTAAAGGAATTGTATCCACTCTG
CTTCCTGTCAATTGGCCATGTGCATCTCTNGTTGTTCAGGGTACATTAATAACACACAGCATTAAGTA
GATAAGTCTCCGATATAAGCGAACAGAACGTTGGTCAGANACTATGGTTCAATTGTAGTGGTATAATG
TTTCCCAGTGCATAAGGAACTCAGGGACACTACTANCATGGGCGAGGAGAGACCCTTGGGTGGCAACA
CGCAATAAGTGTTANCTAGTACATCAGCACGAGANAGGGTTTGACCNTGTGTTTTTAGACGACCCCGG
TCGCATTGAACCTGGCATTCAGAATGACGTGCTCTCAAGAATCAAGGTCCTCCCACTGAGGGTCATCT
GCTTCGCGGATCAGCGTATGGTCACAAACAT
>Tay391
GTGTAGCCCAGTTAGACACNTCTAAATCCTTGGTAACAGACATGCAGTCCCCTCGGCAGAGCCCCGCC
CCCCNATGGTACCGGGTTGTTAAGAGGTCCACCACGGGTCGNACGACAACATTAAGGCCTCANGCATG
ACCCAACACGGGGGCGTCCCGGCGCGGCGACCTTGAAGTCCGGGGACAAGTATGGCCCCCCGNGAAGG
ACGCNGTAGTGCATCTGGAGTGCCGATTNGTGGTCCAGANTGCCACCACGATGGTATGAGTGGCAAAG
GTACTTTGGACACCACCCCGTTTTCTATTTCCAGACGCGGCCATTCAGGAACGACGCGAATCGTAATC
TACCTGCCGCGTACGCGGATCGTATATACGTTTTCCAGCGTTCGAAATAGAGTTTGTCGTTCCGCTGA
CCAGGTTTCACGGCTACTGCGATAAGCATATTCTACTGGGGGACTGTAAAGGAATTGTATCCACTCNG
CTTCCTGTCAATTGGCCATGTGCATCTCTGGTTGTTCAGGNNACATTAATAACACACAGCATTAAGTA
GATAAGTCTCCGATATAAGCGAACAGAACGTTGGTCAGAGACTGTGGTTCAATTGTAGTGGTATAATG
TTTCCCAGTGCATAAGGAACTCAGGGACACTACTAGCANGGGCGAGGAGAGACCCTTGGGTGGCAACA
CGCAATAAGTGTTACCTAGTACACCAGCACGAGAGAGGGTTTGACCATGTGTTTTTAGACGACCCCGG
TCGCATTGAACCTGGCATTCAGAATGACGTGCTCTCAAGAGTCAAGGTCCTCCCACTGAGGGTCATCT
GCTTCGCGGGTCAGCGTATGGNCATNAACAT
>Tay392
GTGTAGCCCAGTTAGACACTTCTAAATCCTTGGTAACAGACATGCAGTCCCCTCGGCAGAGCCCCGCC
CCCCGATGGTACCGGGTTGTTAAGAGGTCCACAACGGGTCGCACGACAACNTTAAGGCCTCATGCATG
ACCCANCACGGGGGCGTCCCGGCGNGGCGACCTTGAAGTCCGGGGACAAGTATGGCCCCCCGGGAAGG
ACGCCGTAGTGCATCTGGAGTGCCGATTCGTGGTCNAGAATGNCACCACGATGGTATGAGTGGCAAAG
GTACTTTGGACACCACCCCGTTTTCTATTTCCAGACGCGGCCATTCAGGAACGACGCGAATCGTAATC
TACCTGCCGCGTNCGCGGATCGTACATGCGTTTTCCAGCGTTCGAAATAGAGTTTGTCGTTCCGCTGA
CCAGGTTTCACGGCTACTGCGATAAGCATATTCTACTGGGGGACTGTAAAGGAATTGTATCCACTCTG
CTTCCTGTCAATTGGCCATGTGCATCTCTGGTTGTTCAGGGTACATTAATAACACACAGCATTAAGTA
GATAAGTCTCCGATATAAGCGAACAGAACGTTGGTCAGAGACTATGGTTCAATTGTAGTGGTATAATG

TTTCCCAGTGCATAAGGAACTCAGGGACACTACTAGCATGGGCGAGGAGAGACCCTTGGGTGGCAACA
CGCAATAAGTGTTACCTAGTACATCAGCACGAGAGAGGGTTTGACCATGNNTTTTTAGACGACCCCGG
TCGCATTGAACCTGGCATTCAGAATGACGTNCTCTCAAGAATCAAGGTCCTCCCACTGAGGGTCATCT
GCTTCGCGGGTCAGCGTATGGTCATAAACAT
>Tay394
GTGTAGCCCAGTTAGACACTTCTAAATCCTTGGTAACAGACATGCAGTCCCCTCGGCAGAGCCCCGCC
CCCCGATGGTACCGGGTTGTTNAGAGGTCCACAACGGGTCNCACGACNANNTTAAGGCCTCATGCATG
ACCCAACACGGGGGCGTCCCGGCGCGGCGACCTTGAANTCCGGGGACAAGTATGGCCCCCCGGGAAGG
ACGCCGTAGTGCATCTGGAGTGCCGATTCGTGGTCNAGAATGCCACCACGATGGTATGAGTTGCAAAG
GTACTTTGGACACCACCCCGTTTTCTATTTCCAGACGCGGCCATTCAGGAACGACGCGAATCGTAATC
TACCTGCCGCGTACGCGGATCGTACATGCGTTTTCCAGCGTTCGAAATAGAGTTTGTCGTTCCGCTGA
CCAGGTTTCACGGCTACTGCGATAAGCATATTCTACTGGGGGACTGTAAAGGAATTGTATCCACTCNG
CTTCCTGTCAATTGGCCATGTGCATCTCTGGTTGTTCAGGGTACATTAATAACACACAGCATTAAGTA
GATAAGTCTCCGATATAAGCGAACAGAACGTTGGTCAGAGACTATGGTTCAATTGTAGTGGTATAATG
TTTCCCAGTGCATAAGGAANTCAGGGACACTACTAGCATGGGCGAGGAGAGACCCTTGGGTGGCAACA
CGCAATAAGTGTTACCTAGTACATCAGCACGAGNGAGGGTTTGACCATGTGTTTTTAGACGACCCCGG
TCGCATTGAACCTGGCATTCAGAATGACGTGCTCTCAAGAATCAAGGTCCTCCCACTGAGGGTCATCT
GCTTCGCGGGTCAGCGTATGGTCACAAANAT
>Tay395
GTGTAGCCCAGTTAGACACNTCTAAATCCTTGGTAACAGACATGCAGTCCCCTCNNCNNAGCCCCGCC
CCCCNATGGTACCGGGTTGTTNAGAGGTCCACAACGGGTCNNACGACAACNTTAAGGCCTNANGCATG
ANNCANNACGGNGGCGTCCCGGCNCGGCGACCTTGAANTCCGGGGNCAAGTATGGCCCCCCGNGAAGG
ACGCNGTAGTGCATCTGGANTGCCGATTNGTGGNCCAGANTGNCACNACGNTGGTATGAGTNGCAAAG
GTACTTTGGACACCACCCNGTTTTCTATTTCCAGACGCGGCCATTCAGGAACGACGCGAATCGTAATC
TACCTGCCGCGTNCGCGGATCGTACATGCGTTTTCCAGCGTTCGAAATAGAGTTTGTCGTTCCGCTGA
CCAGGTTNCACGGCTACTGCGATAAGCATNTTCTACTGGGGGACTGTAAAGGAATTGTATCCACTCNG
CTTCCTGTCAATTGGCCATGTGCATCTCTGGTTGTTCAGGGTACATTAATAACACACAGNATTAAGTA
GATAAGTCTCCGATATAAGCGAACAGAACGTTGGTNAGANACTATGGTTCAATTGTAGTGGTATAATG
TTTCCCAGTGCATAAGGAACTCAGGNACACTACTANCATGGGCGAGGAGAGACCCTTGGGTGGCAACA
CGCGATAAGTGTTACCTNGTACATCAGCACGAGANAGGGTTTGACCNTGTGTTTTTAGACGACCCCGG
TCGCATTGAACCTGGCATTCAGAATGACGTGCTCTCAAGAATCAAGGTCCNCCCACTGAGGGTCATCT
GCTTCGCGGGTCAGCGTATGGNCANACACNT
>Tay396
GTGTAGCCCAGTTAGACACTTCTAAATCCTTGGTAACAGACATGCAGTCCCCTCGGCAGAGNCCCGCC
CCCCGATGGTACCGGGTTGTTAAGAGGTCCACAACGGGTCGCACGACAANATTAAGGCCTCATGCATG
ACCCAACACGGGGGCGTCCCGGCGCGGCGACCTTGAAGTCCGGGGACAAGTATGGCCCCCCGGGAAGG
ACGCCGTAGTGCATCTGGAGTGCCGATTCGTGGTCCAGAATGCCACCACGATGGTATGAGTNGCAAAG
GTACTTTGGACACCACCCCGTTTTCTATTTCCAGACGCGGCCATTCAGGAACGACGCGAATCGTAATC
TACCTGCCGCGTACGCGGATCGTACATGCGTTTTCCAGCGTTCGAAATAGAGTTTGTCGTTCCGCTGA
CCAGGTTTCACGGCTACTGCGATAAGCATATTCTACTGGGGGACTGTAAAGGAATTGTATCCACTCTG
CTTCCTGTCAATTGGCCATGTGCATCTCTGGTTGTTCAGGGTACATTAATAACACACAGCATTAAGTA
GATAAGTCTCCGATATAAGCGAACAGANCGTTGGTCAGAGACTATGGTTCAATTGTAGTGGTATAATG
TTTCCCAGTGCATAAGGAACTCAGGGACACTACTAGCATGGGCGAGGAGAGACCCTTGGGTGGCAACA
CGCAATAAGTGTTACCTAGTACATCAGCACGAGANAGGGTTTGACCATGTGTTTTTAGACGACCCCGG
TCGCATTGAACCTGGCATTCAGAATGACGTGCTCTCAAGAATCAAGGTCCTCCCACTGAGGGTCATCT
GCTTCGCGGGTCAGCGTATGGTCATANNCAT
>Tay397
GTGTAGCCCAGTTAGACACNTCTAAATCCTTGGTAACAGACATGCAGTCCCCTCGGCAGAGCCCCGCC
CCCCGATGGTACCGGGTTGTTAAGAGGTCCACAACGGGTCGCACGACNACNTTAAGGCCTCATGCATG
ACCCAACACGGGGGCGTCCCGGCGCGGCGACCTTGNAGTCCGGGGACAAGTATGGCCCCCCGGGAAGG
ACGCCGTAGTGCATCTGGAGTGCCGATTNGTGGTCCAGAATGNCACCACGATGGTATGAGTGGCAAAG
GTACTTTGGACACCACCCCGTTTTCTATTTCCAGANGCGGCCATTCAGGAACGACGCGAATCGTAATC
TACCTGCCGCGTACGCGGATCGTACATGCGTTTTCCAGCGTTCGAAATAGAGTTTGTCGTTCCGCTGA
CCAGGTTTCACGGCTACTGCGATAAGCATATTCTACTGGGGGACTGTAAAGGAATTGTATCCACTCNG

CTTCCTGTCAATTGGCCATGTGCATCTCTGGTTGTTCAGGGTACATTAATAACACACAGCATTAAGTA
GATAAGTCTCCGATATAAGCGAACAGAACGTTGGTNAGAGACTATGGTTCAATTGTAGTGGTATAATG
TTTCCCAGTGCATAAGGAACTCAGGGACACTACTAGCANGGGCGAGGAGAGACCCTTGGGTGGCAACA
CGCAATAAGTGTTACCTAGTACATCAGCACGAGAGANGGTTTGACCATGNNTTTTAGACGACCCCGG
TCGCATTGAACCTGGCATTCAGAATGACGTGCTCTCAAGAATCNAGGTCCTCCCACTGAGGGTCATCT
GCTTCGCGGNTCAGCGTATGGTCATAAACAT
>Tay401
GTGTAGCCCAGTCAGACACNTCTAAATCCTTGGTAACAGACATGCAGTCCCCTCGGCAGAGCCCCGCC
CCCCGATGGTACCGGGTTGTTNAGAGGTCCACAACGGGTCGCACGACAACATTAAGGCCTCATGCATG
ACCCAANACGGGGGCGTCCCGGCGCGGCGACCTTGAAGTCCGGGGACAAGTATGGCCCCCCGGGAAGG
ACGCCGTAGTGCATCTGGAGTGCCGATTCGTGGTCCAGAATGCCACCACGATGGTATGAGTGGCAAAG
GTACTTTGGACACCACCCCGTTTTCTATTTCCAGACGCGGCCATTCAGGAACGACGCGAATCGTAATC
TACCTGCCGCGTACGCGGATCGTACATGCGTTTTCCAGCGTTCGAAATAGAGTTTGTCGTTCCGCTGA
CCAGGTTTCACGGCTACTGCGATAAGCATATTCTACTGGGGGACTGTAAAGGAATTGTATCCATTCTG
CTTCCTGTCAATTGGCCATGTGCATCTCTGGTTGTTCAGGGTACATTAATAACACACAGCAGTAAGTA
GGTAAGTCTCCGATATAAGCGAACAGAACGTTTGTCAGAGACTGTGGTTCAATTGTAGTGGTATAATG
TTTCCCAGTGCATAAGGAACTCAGGGACACTACTAGCATGGGCGAGGAGAGACCCTTGGGTGGCAACA
CGCAATAAGTGTTACCTAGTACACCAGCACGAGAGAGGGTTTGACCATGTGTTTTTAGACGACCCCGG
TCGCATTGAACCTGGCATTCAGAATGACGTGCTCTCAAGAATCAAGGTCCTCCCACTGAGGGTCATCT
GCTTCGCGGGTCAGCGTATGGTCANNAACNT
>Tay406
GTGTAGCCCAGTTAGACACTTCTAAATCCTTGGTAACAGACATGCAGTCCCCTCGGCAGAGCCCCGCC
CCCCGATGGTACCGGGTTGTTAAGAGGTCCACAACGGGTCGCACGACAANATTAAGGCCTCANGCATG
ACCCAACACNGGGGCGTCCCGGCGCGGCGACCTTGAAGTCCGGGGACAAGTATGGCCCCCCGGGAAGG
ACGCCGTAGTGCATCTGGAGTGCCGATTNGTGGTCCAGAATGCCACCACGATGGTATGAGTGGCAAAG
GTACTTTGGACACCACCCCGTTTTCTANTTCCAGANGCGGCCATTCAGGAACGACGCGAATCGTAATC
TACCTGCCGCGTACGCGGATCGTACATGCGTTTTCCAGCGTTCGAAATAGAGTTTGTCGTTCCGCTGA
CCAGGTTTCACGGCTACTGCGATAAGCATATTCTACTGGGGGACTGTAAAGGAATTGTATCCACTCTG
CTTCCTGTCAATTGGCCATGTGCATCTCTGGTTGTTCAGGGTACATTAATAACACACAGCATTAAGTA
GATAAGTCTCCGATATAAGCGAACAGAACGTTGGTNAGAGACTATGGTTCAATTGTAGTGGTATAATG
TTTCCCAGTGCATAAGGAACTCAGGNACACTACTAGCANGGGCGAGGAGAGACCCTTGGGTGGCAACA
CGCAATAAGTGTTACCTAGTACATCAGCACGAGAGAGGGTTTGACCATGTGTTTTTAGACGACCCCGG
TCGCATTGAACCTGGCATTCAGAATGACGTGCTCTCAAGAATCAAGGTCCTCCCACTGAGGGTCATCT
GCTTCGCGGGTCAGCGTATNGTCATAAACAT
>Tay72
GTGTAGCCCAGTTAGACACTTCTAAATCCTTGGTAACAGACATGCAGTNNNCTCNNCAGAGCCCCGCC
CCCCNATGGTACCGGGTTGTTNAGAGGTCCACAACGGGTCGNACGACANCNTTAAGGCCTCANGCATG
ACCCAACACGGGGGCGTCCCGGNGCGGCGACCTTGAANTCCGGGGACAAGTATGGCCCCCCGGGAAGG
ACGCCGTAGTGCATCTGGAGTGCCGATTNGTGGTCCAGAATGCCACCACGATGGTATGAGTGGCAAAG
GTACTTTGGACACCACCCCGTTTTCTATTTCCAGACGCGGCCATTCAGGAACGACGCGAATCGTAATC
TACCTGCCGCGTNCGCGGATCGTACATGCGTTTTCCAGCGTTCGAAATAGAGTTTGTCGTTCCGCTGA
CCAGGTTTCACGGCTACTGCGATAAGCATATTCTACTGGGGGACTGTAAAGGAATTGTATCCACTCTG
CTTCCTGTCAATTGGCCATGTGCATCTCTGGTTGTTCAGGGTACATTAATAACACACAGCATTAAGTA
GATAAGTCTCCGATATAAGCGAACAGAACGTTGGTCAGAGACTATGGTTCAATTGTAGTGGTATAATG
TTTCCCAGTGCATAAGGAACTCAGGNACACTACTAGCANGGGCGAGGAGAGACCCTTGGGTGGCAACA
CGCAATAAGTGTTACCTAGTACATCAGCACGAGANANGGTTTGACCATGTGTTTTTAGNNGACCCCGG
TCGCATTGAACCTGGCATTCAGAATGACGTGCTCTCAAGAATCAAGGTCCTCCCACTGAGGGTCATCT
GCTTCGCGGGTCAGCGTATGGNCATAAACAT
>Tay78
GTGTAGCCCAGTTAGACACNTCTAAATCCTTGGTAACAGACATGCAGTCCCCTCGGCAGAGCCCCGCC
CCCCGATGGTACCGGGTTGTTAAGAGGTCCACAACAGGTCGNACGACAACATTAAGGCCTNANGCATG
ACCCAACACGGNGGCGTCCCGGCGNGGCGACCTTGNAGTCCGGGGACAAGTATGGCCCCCCGGGAAGG
ACGCCGTAGTGCATCTGGAGTGCCGATTCGTGGTCCAGAATGCCACCACGATGGTATGAGTNGCAAAG
GTACTTTGGACACCACCCCGTTTTCTATTTCCAGACGCGGCCATTCAGGAACGACGCGAATCGTAATC

TACCTGCCGCGTACGCGGATCGTACATGCGTTTTCCAGCGTTCGAAATAGAGTTTGTCGTTCCGCCGA
CCAGGTTTCACGGCTACTGCGATAAGCATATTCTACTGGGGGACTGTAAAGGAATTGTATCCACTCTA
CTTCCTGTCAATTGGCCATGTGCATCTCTGGTTGTTCAGGGTACATTAATAACACACAGCATTAAGTA
GATAAGTCTCCGATATAAGCGAACAGAACGTTGGTCAGAGACTGTGGTTCAATTGTAGTGGTATAATG
TTTCCCAGTGCATAAGGAACTTAGGNACATTACTAGCATGGGCGAGGAGAGACCCTTGGGTGGCAACA
CGCAATAAGTGTTANNTAGTACACCAGCACGAGNGAGGGTTTGACCATGTGTTTTTAGACGACCCCGG
TCGCATTGAACCTGGCATTCAGAATGACGTGCTCTCAAGAATCAAGGTCCTCCCACTGAGGGTCATCT
GCTTCGCGGGTCAGCGTATGGTCANAANNAT
>Tay82
GTGTAGCCCAGTTAGACACTTCTAAATCCTTGGTAACAGACATGCAGTCCCCTCNGCAGAGCCCCGCC
CCCCGATGGTACCGGGTTGTTNAGAGGTCCACAACGGGTCGCACGACAACATTAAGGCCTCATGCATG
ACCCAACACGGGGGCGTCCCGGNGCGGCGACCTTGNANTCCGGGGACAAGTATGGCCCCCCGGGAAGG
ACGCCGTAGTGCATCTGGAGTGCCGATTCGTGGTCCAGAATGCCACCACGATGGTATGAGTGGCAAAG
GTACTTTGGACACCACCCCGTTTTCTATTTCCAGANGCGGCCATTCAGGAACGACGCGAATCGTAATC
TACCTGCCGCGTACGCGGATCGTACATGCGTTTTCCAGCGTTCGAAATAGAGTTTGTCGTTCCGCTGA
CCAGGTTTCACGGCTACTGCGATAAGCATATTCTACTGGGGGACTGTAAAGGAATTGTATCCACTCNG
CTTCCTGTCAATTGGCCATGTGCATCTCTGGTTGTTCAGGNNACATTAATAACACACAGCATTAAGTA
GATAAGTCTCCGATATAAGCGAACAGAACGTTGGTCAGAGACTATGGTTCAATTGTAGTGGTATAATG
TTTCCCAGNGCATAAGGAACTCAGGNACACTACTAGCANGGGCGAGGAGAGACCCTTGGGTGGCAACA
CGCGATAAGTGTTANNTAGTACATCAGCACGAGANANGGTTTGACCATGTGTTTTTAGACGACCCCGG
TCGCATTGAACCTGGCATTCAGAATGACGTGCTCTCAAGAATCAAGGTCCTCCCACTGAGGGTCATCT
GCTTCGCGNGTCAGCGTATGGTCANNNNNAT
>Tay84
GTGTAGCCCAGTCAGACACTTCTAAATCCTTGGTAACAGACATGCAGTCCCCTCGGCAGAGNCCCGCC
CCCCGATGGTACCGGGTTGTTNAGAGGTCCACAACGGGTCGCACGACAACNTTAAGGCCTCANGCATG
ANNCAACACGGGGGCGTCCCGGCGCGGCGACCTTGAAGTCCGGGGACAAGTATGGCCCCCCGGGAAGG
ACGCCGTAGTGCATCTGGAGTGCCGATTCGTGGTCNAGAATGCCACCACGATGGTATGAGTGGCAAAG
GTACTTTGGACACCACCCCGTTTTCTATTTCCAGACGCGGCCATTCAGGAACGACGCGAATCGTAATC
TACCTGCCGCGTNCGCGGATCGTACATGCGTTTTCCAGCGTTCGAAATAGAGTTTGTCGTTCCGCTGA
CCAGGTTTCACGGCTACTGCGATAAGCATATTCTACTGGGGGACTGTAAAGGAATTGTATCCATTCTG
CTTCCTGTCAATTGGCCATGTGCATCTCTGGTTGTTCAGGGTACATTAATAACACACAGNAGTAAGTA
GGTAAGTCTCCGATATAAGCGAACAGAACGTTTGTCAGAGACTGTGGTTCAATTGTAGTGGTATAATG
TTTCCCAGTGCATAAGGAACTCAGGNACACTACTAGCANGGGCGAGGAGAGACCCTTGGGTGGCAACA
CGCAATAAGTGTTACCTAGTACACCAGCACGAGAGANGGTTTGACCATGTGTTTTTAGACGACCCCGG
TCGCATTGAACCTGGCATTCAGAATGACGTGCTCTCAAGAATCAAGGTCCTCCCACTGAGGGTCATCT
GCTTCGCGNGTCAGCGTATGGTCATNNNNNT
>Tay87
GTGTAGCCCAGTCAGACACTTCTAAATCCTTGGTAACAGACATGCAGTCCCCTCNNCAGAGCCCCGCC
CCCCGATGGTACCGGGTTGTTAAGAGGTCCACAACGGGTCGNACGACAANATTAAGGCCTCANGCATG
ACCCAACACGGGGGCGTCCCGGNGNGGCGACCTTGAAGTCCGGGGACAAGTATGGCCCCCCGGGAAGG
ACGCCGTAGTGCATCTGGAGTGCCGATTCGTGGTCNAGANTGNCACCACGATGGTATGAGTNGCAAAG
GTACTTTGGACACCACCCCGTTTTCTANTTCCAGACGCGGCCATTTAGGAACGACGCGAATCGTAATC
TACCTGCCGCGTACGCGGATCGTACATGCGTTTTCCAGCGTTCGAAATAGAGTTTGTCGTTCCGCTGA
CCAGGTTTCACGGCTACTGCGATAAGCATATTCTACTGGGGGACTGTAAAGGAATTGTATCCATTCNG
CTTCCTGTCAATTGGCCATGTGCATCTCTGGTTGTTCAGGGTACATTAATAACACACAGCAGTAAGTA
GGTAAGTCTCCGATATAAGCGAACAGAACGTTTGTCAGAGACTGTGGTTCAATTGTAGTGGTATAATG
TTTCCCAGTGCATAAGGAACTCAGGGACACTACTAGCATGGGCGAGGAGAGACCCTTGGGTGGCAACA
CGCAATAAGTGTTACCTAGTACACCAGCACGAGAGAGGGTTTGACCNTGTGTTTTTAGACGACCCCGG
TCGCATTGAACCTGGCATTCAGAATGACGTGCTCTCAAGAATCAAGGTCCTCCCACTGAGGGTCATCT
GCTTCGCGGGTCAGCGTATGGTCATNNNCNT
>Thai130
GTGTAGCCCAGTTAGACACTTCTAAATCCTTGGTAACAGACATGCAGTCCCCTCNNCAGAGCCCCGCC
CCCCGATGGTACCGGGTTGTTAAGAGGTCCACAACGGGTCGNACGACAANNTTAAGGCCTCANGCATG
ACCCAACACGGNGGNGTCCCGGNGCGGCGACCTTGAAGTCCGGGGACAAGTATGGCCCCCCGGGAAGG

ACGCCGTAGTGCATCTGGAGTGCCGATTCGTGGTCCAGANTGNCACCACGATGGTATGAGTGGCAAAG
GTACTTTGGACACCACCCCGTTTTCTATTTCCAGACGCGGCCATTCAGGAACGACGCGAATCGTAATC
TACCTGCCGCGTACGCGGATCGTACATGCGTTTTCCAGCGTTCGAAATAGAGTTTGTCGTTCCGCTGA
CCAGGTTTCACGGCTACTGCGATAAGCATATTCTACTGGGGGACTGTAAAGGAATTGTATCCACTCTG
CTTCCTGTCAATTGGCCATGTGCATCTCTNGTTGTTCAGGNNACATTAATAACACACAGCATTAAGTA
GATAAGTCTCCGATATAAGCGAACAGAACGTTGGTCAGAGACTATGGTTCAATTGTAGTGGTATAATG
TTTCCCAGTGCATAAGGAACTCAGGNACACTACTAGCATGGGCGAGGAGAGACCCTTGGGTGGCAACA
CGCGATAAGTGTTACCTAGTACATCAGCACGAGAGAGGGTTTGACCATGTGTTTTTAGACGACCCCGG
TCGCATTGAACCTGGCATTCAGAATGACGTGCTCTCAAGAATCAAGGTCCTCCCACTGAGGGTCATCT
GCTTCGCGGGTCAGCGTATNGTCATANNCAT
>Thai136
GTGTAGCCCAGTTANNCACNNNNNAANNCNTNGTNACAGACATGCAGTNNNCTCNNCAGAGNNCCGCC
CCCCNATGGTACCGGGNNGTTNAGAGGTCCACAACGGGTCGNANGANNNNNTTNAGGCCNCANGCATN
ANNCNNNNCNGNGGNGTCCCGGNGNNGNNACCTTGNAGTCNGGGNNCAAGTATNGCCCCCCGNGAAGG
ACGCNGTAGTGCATCTGNANTGCCGNTTNNTGGNCNNNANTNNCACNANGNTGGTATGAGTGGCAAAG
GTACTTNGGACACCACCCCGTTNNCTANTTCCAGACGCGGCCATTCANGAANGACGCGAATCGTAATC
TACCTGNCGCGTNCGCGGATCGTACANGCGTTTTCCNGNNTTCGANATANAGTNNNTCGTTCCGCTGA
CCAGGTTNCACGGCTACTNCGATAAGCATATTCTACTGGNGGACTGTAAAGNAATTGTATCCACTCTG
CTTCCTGTCANTTGGNCATGTGCATCTCTNNTTGTTCAGGNNACATTAANAACACACAGCATNAAGTA
GATAAGTCNCCGNTATAAGNGAACAGANCNTTGGTNAGAGNCTATGGTTNNATTGTAGTGGTATAATG
TTTCCCAGTNCATAAGNAANTCAGGNANACTACTANCANGGGCGAGNAGNGACCCTTGGGTGNNNACA
CGNANNAAGTGTTNNNTAGTANATCAGNACGAGNNAGGGTTNGNCCNTGTGTTNNTAGACNNCCCCGG
TCGCATTGAACCTGNCATTCAGAATGACGTNCTNNNNNNAATCNAGNTCCNNNNACTGAGGGTCATNN
GCTTCNCNNNNCNGCGTATNGNCATAAACAT
>Thai248
GTGTAGCCCAGTTAGACACTTCTAAATCCTTGGTAACAGACATGCAGTCCCCTCGGCAGAGCCCCGCC
CCCCNATGGTACCGGGTTGTTNAGAGGTCCACAACGGGTCGCACGACAANNTTAAGGCCTNANGCATG
ACCCAACACGGNGGCGTCCCGGCGCGGCGACCTTGAAGTCCGGGGACAAGTATGGCCCCCCGGGAAGG
ACGCCGTAGTGCATCTGGAGTGCCGATTCGTGGTCNAGANTGCCACCACGATGGTATGAGTGGCAAAG
GTACTTTGGACACCACCCCGTTTTCTANTTCCAGACGCGGCCATTCAGGAACGACGCGAATCGTAATC
TACCTGCCGCGTACGCGGATCGTACATGCGTTTTCCAGCGTTCGAAATAGAGTTTGTCGTTCCGCTGA
CCAGGTTTCACGGCTACTGCGATAAGCATATTCTACTGGGGGACTGTAAAGGAATTGTATCCACTCTG
CTTCCTGTCAATTGGNCATGTGCATCTCTGGTTGTTCAGGGTACATTAATAACACACAGCATTAAGTA
GATAAGTCTCCGATATAAGCGAACAGAACGTTGGTCAGAGACTATGGTTCAATTGTAGTGGTATAATG
TTTCCCAGTGCATAAGGAACTCAGGGACACTACTAGCATGGGCGAGGAGAGACCCTTGGGTGGCAACA
CGCAATAAGTGTTACCTAGTACATCAGCACGAGAGANGGTTTGACCATGTGTTTTTAGACGACCCCGG
TCGCATTGAACCTGGCATTCAGAATGACGTNCTCTCAAGAATCAAGGTCCTCCCACTGAGGGTCATCT
GCTTCGCGGGTCNGCGTATGGTCANANNNNN
>Thai249
GTGTAGCCCAGTCAGACACTTCTAAATCCTTGGTAACAGACATGCAGTCCCCTCGGCAGAGNNCCGCC
CCCCGATGGTACCGGGTTGTTNAGAGGTCCACAACGGGTCGCACGACAACNTTAAGGCCTCANGCATG
ACCCNACACGGGGGCGTCCCGGCGCGGCGACCTTGNAGTCCGGGGACAAGTATGGCCCCCCGGGAAGG
ACGCCGTAGTGCATCTGGAGTGCCGATTNGTGGTCCAGAATGNCACCACGATGGTATGAGTGGCAAAG
GTACTTTGGACACCACCCCGTTTTCTATTTCCAGACGCGGCCATTTAGGAACGACGCGAATCGTAATC
TACCTGCCGCGTACGCGGATCGTACATGCGTTTTCCAGCGTTCGAAATAGAGTTTGTCGTTCCGCTGA
CCAGGTTTCACGGCTACTGCGATAAGCATATTCTACTGGGGGACTGTAAAGGAATTGTATCCATTCTG
CTTCCTGTCAATTGGCCATGTGCATCTCTGGTTGTTCAGGGTACATTAATAACACACAGCAGTAAGTA
GGTAAGTCTCCGATATAAGCGAACAGAACGTTTGTCAGAGACTGTGGTTCAATTGTAGTGGTATAATG
TTTCCCAGTGCATAAGGAACTCAGGGACACTACTAGCATGGGCGAGGAGAGACCCTTGGGTGGCAACA
CGCAATAAGTGTTACCTAGTACACCAGCACGAGAGANGGTTTGACCATGTGTTTTTAGACGNCCCCGG
TCGCATTGAACCTGGCATTCAGAATGACGTGCTCTCAAGAATCNAGGTCCTCCCACTGAGGGTCATCT
GCTTCGCGGGTCAGCGTATGGTCATNNACNT
>Thai250
GTGTAGCCCAGTTAGACACTTCTAAATCCTTNGTAACAGACATGCAGTCCCCTCGGCAGAGNCCCGCC

CCCCGATGGTACCGGGTTGTTAAGAGGTCCACAACGGGTCNCACGACAANNTTAAGGCCTCATGCATG
ACCCAANACGGGGGCGTCCCGGCGCGGCGACCTTGAAGTCCGGGGACAAGTATGGCCCCCCGGGAAGG
ACGCCGTAGTGCATCTGGAGTGCCGATTCGTGGTCCAGANTGCCACCACGATGGTATGAGTGGCAAAG
GTACTTTGGACACCACCCCGTTTTCTATTTCCAGACGCGGCCATTCAGGAACGACGCGAATCGTAATC
TACCTGCCGCGTACGCGGATCGTACATGCGTTTTCCAGCGTTCGAAATAGAGTTTGTCGTTCCGCTGA
CCAGGTTTCACGGCTACTGCGATAAGCATATTCTACTGGGGGACTGTAAAGGAATTGTATCCACTCTG
CTTCCTGTCAATTGGCCATGTGCATCTCTNGTTGTTCAGGGTACATTAATAACACACAGCATTAAGTA
GATAAGTCTCCGATATAAGCGAACAGAACGTTGGTCAGAGACTATGGTTCAATTGTAGTGGTATAATG
TTTCCCAGTGCATAAGGAACTCAGGNACACTACTAGCANGGGCGAGGAGAGACCCTTGGGTGGCAACA
CGCAATAAGTGTTACCTAGTACATCAGCACGAGAGAGGGTTTGACCATGTNTTTTAGNNGACCCCGG
TCGCATTGAACCTGGCATTCAGAATGACGTGCTCTCAAGAATCAAGGTCCTCCCACTGAGGGTCATCT
GCTTCGCGGGTCAGCGTATGGTCACNANCAT
>Thai251
GTGTAGCCCAGTTNGACACTNNNNAANNCNTNGTAACAGACATGCAGTCCCCTCNNCAGAGNNCCGCC
CCCCGATGGTACCGGGNNGNNNAGAGGTCCACAACGGGTCGNACGACNNNNNTAAGGCCTNANGCNNG
ACCCANCNCGGNGGNGTCCCGGNGNNGCNACCTTGNANTCCGGGNNCAAGTATGGCCCCCCGGGAAGG
ACGCCGTAGTGCATCTGGAGTGCCGNTTCGTGGTCNAGAATGCCACCANGATGGTATGAGTNGCAAAG
GTACTTNNNNCACCACCCCGTTTTCTATTTCCAGANGCGGCCATTCAGGAACGACGCGAATCGTAATC
TACCTNCCGCGTACGCGGATCGTACATGCGTTTNCCAGCGTTCGAAATAGAGTNNGTCGTTCCGCTGA
CCAGGTTTCANGGNTACTNCNATAAGNATANTCTACTGGGGGACNGNAAAGNAATTGTATCCACTCNG
NTTCCTGTCAATTGGNCATGTGCATCTCTGGTTGTTCAGGNNACATTAATAACNNACAGNATTAAGNA
GATNAGTCTCCGATANAAGCGAANAGANCGTTGGNCAGAGACTATGGTTCAATTGTAGTGGTATAATG
TTTCCCAGTNCATAAGGAACTCAGGGACACTACTAGCATGGGCGAGGAGAGACCCTTGGGTGGCAACA
CGCANTAAGTGTTACCTAGTACATCAGCACNAGNNAGGGTNNGACCATGNNTTTTAGNNGACCCCGG
TCGCATTGAACCTGGCATTCANAATGACGTGCTNTCAANAATNNAGNTCCNCCCACTGAGGGNCANNT
GCTTCNCGNNNCNGCGTATNGTCATNNNNNN
>Thai252
GTGTAGCCCAGTTAGACACNTCTAAATCCTTGGTAACAGACATGCAGTCCCCTCGGCAGAGCCCCGCC
CCCCGGTGGTACCGGGTTGTTAAGAGGTCCACCACGGGTCGCANGACNNNNTCAAGGCCTNANGCATG
ACCCAACACGGGGGCGTCCCGGCGNGGCGACCTTGAANGCCGGGGACAAGTATGGCCCCCCGNGAAGG
ACGCCGTAGTGCATCTGGAGTGCCGATTCGTGGTCCAGAATGCCACCACGATGGTATGAGTNGCAAAG
GTACTTTGGACACCACCCCGTTTTCTATTTCCAGACGCGGCCATTCAGGAACGACGCGAATCGTAATC
TACCTGCCGCGTACGCGGATCGTACATGCGTTTTCCAGCGTTCGANATAGAGTTTGTCGTTCCGCTGA
CCAGGTTTCACGGCTACTGCGATAAGCATATTCTACTGGGGGACTGTAAAGGAATTGTATCCACTCTG
CTTCCTGTCAATTGGCCATGTGCATCTCTGGTTGTTCAGGGTACATTAATAACACACAGCATTAAGTA
GATAAGTCTCCGATATAAGCGAACAGAACGTTGGTCAAAGACTGTGGTTCAATTGTAGTGGTATAACG
TTTCCCAGTGCATAAGGAACTCAGGGACACTACTAGCATGGGCGAGGAGAGACCCTTGGGTGGCAACA
CGCAATAAGTGTTACCTAGTACACCAGCACGATAGAGGGTTTGACCATGTGTTTTTAGACGACCCCGG
TCGCATTGAACCTGGCATTCAGAATGACGTGNTCTCAAGAGTCAAGGTCCTCCCACTGAGGGTCATNN
GCTTCGCGNGTCAGCGTATGGTCANNNNNAT
>Thai253
GTGTAGCCCAGTTAGACACNTCTAAATCCTTGGTAACAGACATGCAGTCCCCTCGGCAGAGCNCCGCC
CCCCGATGGTACCGGGTTGTTAAGAGGTCCACAACGGGTCGNACGACNNCNTTAAGGCCTCANGCATG
ACCCAACACGGGGGNGTCCCGGCGCGGCGACCTTGAAGTCCGGGGACAAGTATGGCCCCCCGGGAAGG
ACGCCGTAGTGCATCTGGAGTGCCGATTNNTGGTCCAGAATGNCACCACGATGGTATGAGTGGCAAAG
GTACTTTGGACACCACCCCGTTTTCTATTTCCAGACGCGGCCATTCAGGAACGACGCGAATCGTAATC
TACCTGCCGCGTACGCGGATCGTACATGCGTTTTCCAGCGTTCGAAATAGAGTTTGTCGTTCCGCTGA
CCAGGTTTCACGGCTACTGCGATAAGCATATTCTACTGGGGGACTGTAAAGGAATTGTATCCACTCTG
CTTCCTGTCAATTGGCCATGTGCATCTCTNGTTGTTCAGGNTACATTAATAACACACAGCATTAAGTA
GATAAGTCTCCGATATAAGCGAACAGAACGTTGGTCAGAGACTATGGTTCAATTGTAGTGGTATAATG
TTTCCCAGTGCATAAGGAACTCAGGGACACTACTAGCATGGGCGAGGAGAGACCCTTGGGTGGCAACA
CGCAATAAGTGTTACCTAGTACATCAGCACGAGAGAGGGTTTGACCATGTGTTTTTAGACGNCCCCGG
TCGCATTGAACCTGGCATTCAGAATGACGTNCTCTCAAGAATCAAGGTCCTCCCACTGAGGGTCATCT
GCTTCGCGGGTCAGCGTATGGTCATNANNAT

>Thai254
GTGTAGCCCAGTCAGACACTTCTAAATCCTTGGTAACAGACATGCAGTCCCCTCGGCAGAGCNCCGCC
CCCCGATGGTACCGGGTTGTTNAGAGGTCCACAACGGGTCGCACGACNNCATTAAGGCCTCANGCATG
ACCCAACACGGGGGCGTCCCGGCGCGGCGACCTTGAAGTCCGGGGACAAGTATGGCCCCCCGGGAAGG
ACGCCGTAGTGCATCTGGAGTGCCGATTCNTGGTCCAGANTGNCACCACGATGGTATGAGTGGCAAAG
GTACTTTGGACACCACCCCGTTTTCTATTTCCAGACGCGGCCATTTAGGAACGACGCGAATCGTAATC
TACCTGCCGCGTACGCGGATCGTACATGCGTTTTCCAGCGTTCGAAATAGAGTTTGTCGTTCCGCTGA
CCAGGTTTCACGGCTACTGCGATAAGCATATTCTACTGGGGGACTGTAAAGGAATTGTATCCATTCTG
CTTCCTGTCAATTGGCCATGTGCATCTCTGGTTGTTCAGGGTACATTAATAACACACAGCAGTAAGTA
GGTAAGTCTCCGATATAAGCGAACAGAACGTTTGTCAGAGACTGTGGTTCAATTGTAGTGGTATAATG
TTTCCCAGTGCATAAGGAACTCAGGGACACTACTAGCATGGGCGAGGAGAGACCCTTGGGTGGCAACA
CGCAATAAGTGTTACCTAGTACACCAGCACGAGAGAGGGTTTGACCATGTGTTTTTAGACGACCCCGG
TCGCATTGAACCTGGCATTCAGAATGACGTGCTCTCAAGAATCAAGGTCCTCCCACTGAGGGTCATCT
GCTTCGCGGNNCAGCGTATGGTCANNNNNAT
>Thai255
GTGTAGCCCAGTCAGACACTTCTAAATCCTTGGTAACAGACATGCAGTCCCCTCGGCAGAGCCCCGCC
CCCCGATGGTACCGGGTTGTTAAGAGGTCCACAACGGGTCGCACGACAANNTTAAGGCCTCATGCATG
ACCCNACACGGNGGCGTCCCGGCGCGGCGACCTTGAANTCCGGGGACAAGTATGGCCCCCCGGGAAGG
ACGCCGTAGTGCATCTGGAGTGCCGATTCGTGGTCCAGAATGNCACCACGATGGTATGAGTGGCAAAG
GTACTTTGGACACCACCCCGTTTTCTATTTCCAGACGCGGCCATTCAGGAACGACGCGAATCGTAATC
TACCTGCCGCGTNCGCGGATCGTACATGCGTTTTCCAGCGTTCGAAATAGAGTTTGTCGTTCCGCTGA
CCAGGTTTCACGGCTACTGCGATAAGCATATTCTACTGGGGGACTGTAAAGGAATTGTATCCATTCTG
CTTCCTGTCAATTGGCCATGTGCATCTCTNGTTGTTCAGGGTACATTAATAACACACAGNAGTAAGTA
GGTAAGTCTCCGATATAAGCGAACAGAACGTTTGTNAGAGACTGTGGTTCAATTGTAGTGGTATAATG
TTTCCCAGTGCATAAGGAACTCAGGGACACTACTAGCATGGGCGAGGAGAGACCCTTGGGTGGCAACA
CGCAATAAGTGTTACCTAGTACACCAGCACGAGAGAGGGTTTGACCATGTGTTTTTAGACGNCCCCGG
TCGCATTGAACCTGGCATTCAGAATGACGTGCTCTCAAGAATCAAGGTCCTCCCACTGAGGGTCATCT
GCTTCGCGGGNCAGCGTATGGTCATAAANNT
>Thai257
GTGTAGCCCAGTTAGACACNTCTAAATCCTTGGTAACAGACATGCAGTCCCCTCGGCAGAGNNCCGCC
CCCCGATGGTACCGGGTTGTTNAGAGGTCCACAACGGGTCGCACGACNACATTAAGGCCTCATGCATG
ACCCAACACGGGGGCGTCCCGGNGCGGCGACCTTGAANTCCGGGGACAAGTATGGCCCCCCGGGAAGG
ACGCCGTAGTGCATCTGGAGTGCCGATTNGTGGTCNAGAATGCCACCACGATGGTATGAGTGGCAAAG
GTACTTTGGACACCACCCCGTTTTCTATTTCCAGACGCGGCCATTCAGGAACGACGCGAATCGTAATC
TACCTGCCGCGTACGCGGATCGTACATGCGTTTTCCAGCGTTCGAAATAGAGTTTGTCGTTCCGCTGA
CCAGGTTTCACGGCTACTGCGATAAGCATATTCTACTGGGGGACTGTAAAGGAATTGTATCCACTCTG
CTTCCTGTCAATTGGCCATGTGCATCTCTGGTTGTTCAGGGTACATTAATAACACACAGCATTAAGTA
GATAAGTCTCCGATATAAGCGAACAGAACGTTGGTCAGAGACTATGGTTCAATTGTAGTGGTATAATG
TTTCCCAGTGCATAAGGAACTCAGGGACACTACTAGCANGGGCGAGGAGAGACCCTTGGGTGGCAACA
CGCAATAAGTGTTACCTAGTACATCAGCACGAGAGAGGGTTTGACCATGTGTTTTTAGACGACCCCGG
TCGCATTGAACCTGGCATTCAGAATGACGTGCTCTCAAGAATCAAGGTCCTCCCACTGAGGGTCATCT
GCTTCGCGGNNCAGCGTATGGTCANNAACAT
>Thai258
GTGTAGCCCAGTTAGACACTTCTAAATCCTTGGTAACAGACATGCAGTCCCCTCGGCAGAGCCCCGCC
CCCCGATGGTACCGGGTTGTTAAGAGGTCCACAACGGGTCGCACGACAACNTTAAGGCCTNATGCATG
ACCCAACACGGGGGCGTCCCGGCGCGGCGACCTTGAAGTCCGGGGACAAGTATGGCCCCCCGGGAAGG
ACGCCGTAGTGCATCTGGAGTGCCGATTCNTGGTCNAGAATGCCACCACGATGGTATGAGTGGCAAAG
GTACTTTGGACACCACCCCGTTTTCTATTTCCAGACGCGGCCATTCAGGAACGACGCGAATCGTAATC
TACCTGCCGCGTACGCGGATCGTACATGCGTTTTCCAGCGTTCGAAATAGAGTTTGTCGTTCCGCTGA
CCAGGTTTCACGGCTACTGCGATAAGCATATTCTACTGGGGGACTGTAAAGGAATTGTATCCACTCTG
CTTCCTGTCAATTGGCCATGTGCATCTCTGGTTGTTCAGGGTACATTAATAACACACAGCATTAAGTA
GATAAGTCTCCGATATAAGCGAACAGAACGTTGGTCAGAGACTATGGTTCAATTGTAGTGGTATAATG
TTTCCCAGTGCATAAGGAACTCAGGGACACTACTAGCATGGGCGAGGAGAGACCCTTGGGTGGCAACA
CGCAATAAGTGTTANCTAGTACATCAGCACGAGAGAGGGTTTGACCATGTGTTTTTAGACGACCCCGG

TCGCATTGAACCTGGCATTCAGAATGACGTGCTCTCAAGAATCAAGGTCCTCCCACTGAGGGTCATCT
GCTTCGCGNGTCAGCGTATGGTCATAAACNN
>Thai262
GTGTAGCCCAGTCAGACACTTCTAAATCCTTGGTAACAGACATGCAGTCCCCTCGGCAGAGNCCCGCC
CCCCGATGGTACCGGGTTGTTAAGAGGTCCACAACGGGTCGCACGACAANATTAAGGCCTCANGCATG
ACCCAACACGGGGGCGTCCCGGCGCGGCGACCTTGNANTCCGGGGACAAGTATGGCCCCCCGNGAAGG
ACGCCGTAGTGCATCTGGAGTGCCGATTCGTGGTCCAGAATGNCACCACGATGGTATGAGTGGCAAAG
GTACTTTGGACACCACCCCGTTTTCTATTTCCAGACGCGGCCATTTAGGAACGACGCGAATCGTAATC
TACCTGCCGCGTACGCGGATCGTACATGCGTTTTCCAGCGTTCGAAATAGAGTTTGTCGTTCCGCTGA
CCAGGTTTCACGGCTACTGCGATAAGCATATTCTACTGGGGGACTGTAAAGGAATTGTATCCATTCNG
CTTCCTGTCAATTGGCCATGTGCATCTCTNGTTGTTCAGGGNACATTAATAACACACAGCAGTAAGTA
GGTAAGTCTCCGATATAAGCGAACAGAACGTTTGTCAGAGACTGTGGTTCAATTGTAGTGGTATAATG
TTTCCCAGTGCATAAGGAACTCAGGGACACTACTAGCATGGGCGAGGAGAGACCCTTGGGTGGCAACA
CGCAATAAGTGTTACCTAGTACACCAGCACGAGAGAGGGTTTGACCATGTGTTTTTAGACGACCCCGG
TCGCATTGAACCTGGCATTCAGAATGACGTGCTCTCAAGAATCAAGGTCCNCCCACTGAGGGTCATCT
GCTTCGCGGGTCAGCGTATGGTCATAAACAT
>Thai263
GTGTAGCCCAGTTAGACACTTCTAAATCCTTGGTAACAGACATGCAGTCCCCTCGGCAGAGCCCCGCC
CCCCNATGGTACCGGGTTGTTAAGAGGTCCACAACGGGTCGCACGACANCATTAAGGCCTNATGCATG
ACCCAACACGGGGGNGTCCCGGCGCGGCGACCTTGAAGTCCGGGGACAAGTATGGCCCCCCGGGAAGG
ACGCCGTAGTGCATCTGGAGTGCCGATTCNTGGTCCAGAATGCCACCACGATAGTATGAGTGGCAAAG
GTACTTTGGACACCACCCCGTTTTCTATTTCCAGACGCGGCCATTCAGGAACGACGCGAATCGTAATC
TACCTGCCGCGTACGCGGATCGTACATGCGTTTTCCAGCGTTCGAAATAGAGTTTGTCGTTCCGCTGA
CCAGGTTTCACGGCTACTGCGATAAGCATATTCTACTGGGGGACTGTAAAGGAATTGTATCCATTCNG
CTTCCTGTCAATTGGCCATGTGCATCTCTNGTTGTTCAGGNNACATTAATAACACACAGNATTAAGTA
GATAAGTCTCCGATATAAGCGAACAGAACGTTGGTCAGAGACTGTGGTTCAATTGTAGTGGTATAATG
TTTCCCAGTGCATAAGGAACTCAGGNACACTACTAGCATGGGCGAGGAGAGACCCTTGGGTGGCAACA
CGCAATAAGTGTTACCTAGTACACCAGCACGAGAGAGGGTTTGACCATGTGTTTTTAGACGACCCCGG
TCGCATTGAACCTGGCATTCAGAATGACGTGCTCTCAAGAATCAAGGTCCTCCCACTGAGGGTCATCT
GCTTCGCGGGTCAGCGTATGGTCATAAACAT
>Thai268
GTGTAGCCCAGTTAGACACTTCTAAATCCTTGGTAACAGACATGCAGTCCCCTCGGCAGAGCNCCGCC
CCCCGATGGTACCGGGTTGTTAAGAGGTCCACAACGGGTCGCACGACAACATTAAGGCCTCANGCATG
ANNCAACACGGNGGCGTCCCGGCGCGGCGACCTTGAAGTCCGGGGACAAGTATGGCCCCCCGGGAAGG
ACGCCGTAGTGCATCTGGAGTGCCGATTNGTGGTCCAGAATGNCACCACGATGGTATGAGTGGCAAAG
GTACTTTGGACACCACCCCGTTTTCTATTTCCAGACGCGGCCATTCAGGAACGACGCGAATCGTAATC
TACCTGCCGCGTACGCGGATCGTACATGCGTTTTCCAGCGTTCGAAATAGAGTTTGTCGTTCCGCTGA
CCAGGTTTCACGGCTACTGCGATAAGCATATTCTACTGGGGGACTGTAAAGGAATTGTATCCACTCTG
CTTCCTGTCAATTGGCCATGTGCATCTCTGGTTGTTCAGGGTACATTAATAACACACAGNATTAAGTA
GATAAGTCTCCGATATAAGCGAACAGAACGTTGGTCAGAGACTATGGTTCAATTGTAGTGGTATAATG
TTTCCCAGTGCATAAGGAACTCAGGGACACTACTAGCATGGGCGAGGAGAGACCCTTGGGTGGCAACA
CGCAATAAGTGTTACCTAGTACATCAGCACGAGAGAGGGTTTGACCATGTGTTTTTAGACGACCCCGG
TCGCATTGAACCTGGCATTCAGAATGACGTGCTCTCAAGAATCNAGGTCCTCCCACTGAGGGTCATCT
GCTTCGCGNGTCAGCGTATGGTCACAAACAT
>Thai269
GTGTAGCCNAGTTAGACACTTCTAAATCCTTGGTAACAGACATGCAGTCCCCTCGGCAGAGNCCCGCC
CCCCGATGGTACCGGGTTGTTNAGAGGTCCACAACGGGTCGCACGACNNNNTTAAGGCCTCANGCATG
ACCCNACACGGGGGCGTCCCGGNGNGGCGACCTTGAANTCCGGGGNCAAGTATGGCCCCCCGGGAAGG
ACGCCGTAGTGCATCTGGAGTGCCGATTCGTGGTCNAGAATGNCACCACGATGGTATGAGTNGCAAAG
GTACTTTGGACNCCACCCCGTTTTCTATTTCCAGACGCGGCCATTCAGGAACGACGCGAATCGTAATC
TACCTGCCGCGTNCGCGGATCGTACATGCGTTTTCCAGCGTTCGANATAGAGTTTGTCGTTCCGCTGA
CCAGGTTTCACGGCTACTGCGATAAGCATATTCTACTGGGGGACTGTAAAGGAATTGTATCCACTCNG
CTTCCTGTCAATTGGCCATGTGCATCTCTGGTTGTTCAGGGTACATTAATAACACACAGNATTAAGTA
GATAAGTCTCCGATATAAGCGAACAGAACGTTGGTCAGAGACTATGGTTCAATTGTAGTGGTATAATG

TTTCCCAGTGCATAAGGAACTCAGGGACACTACTANCATGGGCGAGGAGAGACCCTTGGGTGGCAACA
CGCANTAAGTGTTACCTAGTACATCAGCACGAGAGAGGGTTTGACCATGTGTTTTTAGACGNCCCCGG
TCGCATTGAACCTGGCATTCAGAATGACGTNNTCTCAAGAATCAAGGTCCTCCCACTGAGGGTCATCT
GCTTCGCGGGTCAGCGTATGGTCANAAACAN
>Thai270
GTGTAGCCCAGTTAGACACNTCTAAATCCTTGGTAACAGACATGCAGTCCCCTCGGCAGAGCCCCGCC
CCCCNATGGTACCGGGTTGTTNAGAGGTCCACAACGGGTCGCACGACAACATTAAGGCCTNANGCATG
ACCCAACACGGNGGNGTCCCGGNGNGGCGACCTTGAAGTCCGGGGACAAGTATGGCCCCCCGGGAAGG
ACGCCGTAGTGCATCTGGAGTGCCGATTNGTGGTCCAGAATGCCACCACGATGGTATGAGTGGCAAAG
GTACTTTGGACACCACCCCGTTTTCTATTTCCAGACGCGGCCATTCAGGAACGACGCGAATCGTAATC
TACCTGCCGCGTNCGCGGATCGTACATGCGTTTTCCAGCGTTCGAAATAGAGTTTGTCGTTCCGCTGA
CCAGGTTTCACGGCTACTGCGATAAGCATATTCTACTGGGGGACTGTAAAGGAATTGTATCCACTCTG
CTTCCTGTCAATTGGCCATGTGCATCTCTNGTTGTTCAGGGTACATTAATAACACACAGNATTAAGTA
GATAAGTCTCCGATATAAGCGAACAGAACGTTGGTCAGAGACTATGGTTCAATTGTAGTGGTATAATG
TTTCCCAGTGCATAAGGAACTCAGGNACACTACTAGCATGGGCGAGGAGAGACCCTTGGGTGGCAACA
CGCAATAAGTGTTANCTAGTACATCAGCACGAGAGAGGGTTTGACCATGTGTTTTTAGACGACCCCGG
TCGCATTGAACCTGGCATTCAGAATGACGTGCTCTCAAGAATCAAGGTCCTCCCACTGAGGGTCATCT
GCTTCGCGGGTCAGCGTATGGTCACAAACAT
>Thai271
GTGTAGCCCAGTTAGACACTTCTAAATCCTTGGTAACAGACATGCAGTCCCCTCGGCAGAGNCCCGCC
CCCCNATGGTACCGGGTTGTTAAGAGGTCCACAACGGGTCGCACGACAACATTAAGGCCTCANGCATG
ANNCANNNCGGGGGCGTCCCGGNGCGGNGACCTTGNAGTCCGGGGACAAGTATGGCCCCCCGGGAAGG
ACGCCGTAGTGCATCTGGAGTGCCGATTCNTGGTCCNGAATGCCACCACGATGGTATGAGTGGCAAAG
GTACTTTGGACACCACCCCGTTTTCTATTTCCAGACGCGGCCATTCAGGAACGACGCGAATCGTAATC
TACCTGCCGCGTACGCGGATCGTACATGCGTTTTCCAGCGTTCGAAATAGAGTTTGTCGTTCCGCTGA
CCAGGTTTCACGGCTACTGCGATAAGCATATTCTACTGGGGGACTGTAAAGGAATTGTATCCACTCTG
CTTCCTGTCAATTGGCCATGTGCATCTCTNGTTGTTCAGGNNACATTAATAACACACAGCATTAAGTA
GATAAGTCTCCGATATAAGCGAACAGAACGTTGGTCAGAGACTATGGTTCAATTGTAGTGGTATAATG
TTTCCCAGTGCATAAGGAACTCAGGGACACTACTAGCATGGGCGAGGAGAGACCCTTGGGTGGCAACA
CGCAATAAGTGTTACCTAGTACATCAGCACGAGAGAGGGTTTGACCATGTGTTTTTAGACGACCCCGG
TCGCATTGAACCTGGCATTCAGAATGACGTGCTCTCAAGAATCAAGGTCCTCCCACTGAGGGTCATCT
GCTTCGCGGGTCAGCGTATGGTCACAAACAT
>Thai272
GTGTAGCCCAGTTAGACACNTCTAAATCCTTGGTAACAGACATGCAGTCCCCTCGNCAGAGNCCCGCC
CCCCNATGGTACCGGGTTGTTAAGAGGTCCACAACGGGTCGCACGACAANATTAAGGCCTCATGCATG
ACNCAANACGGNGGCGTCCCGGCGNGGCGACCTTGNANTCCGGGGACAAGTATGGCCCCCCGGGAAGG
ACGCCGTAGTGCATCTGGAGTGCCGATTNGTGNNCNAGANTGCCACCACGATAGTATGAGTGGCAAAG
GTACTTTGGACACCACCCCGTTTTCTATTTCCAGACGCGGCCATTCAGGAACGACGCGAATCGTAATC
TACCTGCCGCGTACGCGGATCGTACATGCGTTTTCCAGCGTTCGAAATAGAGTTTGTCGTTCCGCTGA
CCAGGTTTCACGGCTACTGCGATAAGCATATTCTACTGGGGGACTGTAAAGGAATTGTATCCATTCTG
CTTCCTGTCAATTGGCCATGTGCATCTCTGGTTGTTCAGGGTACATTAATAACACACAGNATTAAGTA
GATAAGTCTCCGATATAAGCGAACAGAACGTTGGTCAGAGACTGTGGTTCAATTGTAGTGGTATAATG
TTTCCCAGTGCATAAGGAACTCAGGGACACTACTAGCATGGGCGAGGAGAGACCCTTGGGTGGCAACA
CGCAATAAGTGTTACCTAGTACACCAGCACGAGAGAGGGTTTGACCATGTGTTTTTAGACGACCCCGG
TCGCATTGAACCTGGCATTCAGAATGACGTGCTCTCAAGAATCAANGTCCTCCCACTGAGGGTCATCT
GCTTCGCGGGTCAGCGTATGGTCATNANCAT
>Thai393
GTGTNGCCCAGTTANACANNTCNNAATNCTTNGTAACAGACATGCAGTCCNCNNNNCAGAGNNCCGCC
CCCCNATGGTACCGGGNNNNTNAGNGGTNCACNACGGGTCGNANGACNNNNTTAAGGCCNNANGNATG
ANNCNNNNCNGNNGNGTCCCGNNGNNGCNACCTTGAANTCNGGGNNCAAGTATNGCCCNNCGGGAAGG
ACNCCGTAGTGCATCTGGAGNGNCNNNNCNNGGNCNAGANTGNCACCACGANGGTATGAGTNGCAAAG
GTACTNTGGACNCCACCCNGTTTNCTANTTCNAGANGCNGCCNTTCAGGAACGACGNGNATCGTAATN
TACCTGCNGCGTNCGCGGATCGTACANGCGTTTTCCAGNNTTCGANATAGAGTTNGTCGTTNNNNTGA
NNAGGTTNCACGGNTNCTGNGATAAGCNTNTTCTACTNGNGGACTGTAAAGNAATTGTATCNACTCNG

CTTCCTGTCAATTGGCCATGTGCATCTCTNNNTGTTCAGGNNACATTAATANCACACAGNATNAAGTA
GATNAGTCTNCNATATAAGCGAACNGANCGTTGNTNAGNNANTATGGTNCAATTNNAGTGGTATAATG
TTTCCCAGNNCATNAGGANNTCAGGNACACTACNANCANGGGCGAGNAGAGACCCTTGGGTGGCANCA
CGNANNAAGTGTTANNTAGTANATCAGCACGAGANNNNGTTNGNCCNNNNNNNNNNAGACGNCCCCGG
TCGCATTGAACCTGGCATTCAGAATNACGTNCTNTCANGAANNNANNTCCNNNCACTGAGGNNCANNN
NCTTCNNGNNNCAGCGTATNGTCANNANNNT
>Thai55
GTGTAGCCCAGTTAGACACTTCTAAATCCTTGGTAACAGACATGCAGTCCCCTCGGCAGAGCCCCGCC
CCCCNATGGTACCGGGTTGTTAAGAGGTCCACAACGGGTCGCACGACAACATTAAGGCCTCANGCATG
ACNCAACACGGGGGNGTCCCGGCGNGGCGACCTTGNAGTCCGGGGACAAGTATGGCCCCCCGGGAAGG
ACGCCGTAGTGCATCTGGAGTGCCGATTCGTGGTCNAGAATGCCACCACGATGGTATGAGTNGCAAAG
GTACTTTGGACACCACCCCGTTTTCTATTTCCAGACGCGGCCATTCAGGAACGACGCGAATCGTAATC
TACCTGCCGCGTACGCGGATCGTACATGCGTTTTCCAGCGTTCGAAATAGAGTTTGTCGTTCCGCTGA
CCAGGTTTCACGGCTACTGCGATAAGCATATTCTACTGGGGGACTGTAAAGGAATTGTATCCACTCTG
CTTCCTGTCAATTGGCCATGTGCATCTCTGGTTGTTCAGGGTACATTAATAACACACAGCATTAAGTA
GATAAGTCTCCGATATAAGCGAACAGAACGTTGGTCAGAGACTATGGTTCAATTGTAGTGGTATAATG
TTTCCCAGTGCATAAGGAACTCAGGGACACTACTAGCATGGGCGAGGAGAGACCCTTGGGTGGCAACA
CGCAATAAGTGTTACCTAGTACATCAGCACGAGAGANGGTTTGACCATGTGTTTTTAGACGACCCCGG
TCGCATTGAACCTGGCATTCAGAATGACGTGCTCTCAAGAATCAAGGTCCTCCCACTGAGGGTCATCT
GCTTCGCGGGTCAGCGTATGGTCANANNNAT
>Thai56
GTGTAGCCCAGTCAGACACTTCTAAATCCTTGGTAACAGACATGCAGTCCCCTCNNCAGAGNCCCGCC
CCCCNATGGTACCGGGTTGTTAAGAGGTCCACAACGGGTCGNACGACAANATTAAGGCCTNANGCATG
ACCCAANACGGGGGNGTCCCGGCGNGGCGACCTTGAANTCCGGGGACAAGTATGGCCCCCCGGGAAGG
ACGCCGTAGTGCATCTGGAGTGCCGATTCGTGGTCNAGAATGNCACCACGATGGTATGAGTGGCAAAG
GTACTTTGGACACCACCCCGTTTTCTATTTCCAGACGCGGCCATTCAGGAACGACGCGAATCGTAATC
TACCTGCCGCGTACGCGGATCGTACATGCGTTTTCCAGCGTTCGAAATAGAGTTTGTCGTTCCGCTGA
CCAGGTTTCACGGCTACTGCGATAAGCATATTCTACTGGGGGACTGTAAAGGAATTGTATCCATTCTG
CTTCCTGTCAATTGGCCATGTGCATCTCTNGTTGTTCAGGGTACATTAATAACACACAGCAGTAAGTA
GGTAAGTCTCCGATATAAGCGAACAGAACGTTTGTCAGAGACTGTGGTTCAATTGTAGTGGTATAATG
TTTCCCAGTGCATAAGGAACTCAGGGACACTACTAGCATGGGCGAGGAGAGACCCTTGGGTGGCAACA
CGCAATAAGTGTTANCTAGTACACCAGCACGAGAGAGGGTTTGACCATGTGTTTTTAGACGACCCCGG
TCGCATTGAACCTGGCATTCAGAATGACGTNCTCTCAAGAATCAAGGTCCTCCCACTGAGGGTCATCN
GCTTCGCGNGTCAGCGTATGGTCACNAACNT
>Thai57
GTGTAGCCCAGTTAGACACTTCTAAATCCTTGGTAACAGACATGCAGTCCCCTCGGCAGAGNCCCGCC
CCCCGATGGTACCGGGTNGTTNAGAGGTCCACAACGGGTCGNACGACNNNATTAAGGCCTCATGCATG
ACCCAACACGGNGGCGTCCCGGCGCGGCGACCTTGAAGTCCGGGNACAAGTATGGCCCCCCGGGAAGG
ACGCCGTAGTGCATCTGGAGTGCCGATTCNTGGTCNAGAATGNCACCACGATGGTATGAGTNGCAAAG
GTACTTTGGACACCACCCCGTTTTCTATTTCCAGACGCGGCCATTCAGGAACGACGCGAATCGTAATC
TACCTGCCGCGTNCGCGGATCGTACATGCGTTTTCCAGCGTTCGAAATAGAGTTTGTCGTTCCGCTGA
CCAGGTTTCACGGCTACTGCGATAAGCATATTCTACTGGGGGACTGTAAAGGAATTGTATCCACTCTG
CTTCCTGTCAATTGGCCATGTGCATCTCTGGTTGTTCAGGGTACATTAATAACACACAGCATTAAGTA
GATAAGTCTCCGATATAAGCGAACAGAACGTTGGTCAGAGACTATGGTTCAATTGTAGTGGTATAATG
TTTCCCAGTGCATAAGGAACTCAGGGACACTACTAGCATGGGCGAGGAGAGACCCTTGGGTGGCAACA
CGCAATAAGTGTTACCTAGTACATCAGCACGAGAGAGGGTTTGACCATGTGTTTTTAGACGNCCCCGG
TCGCATTGAACCTGGCATTCAGAATGACGTGCTCTCAAGAATCAAGGTCCTCCCACTGAGGGTCATCT
GCTTCGCGGNNCAGCGTATGGTCATNAANNT
>Thai99
GTGTAGCCCAGTTAGACACTTCTAAATCCTTGGTAACAGACATGCAGTCCCCTCGGCAGAGCCCCGCC
CCCCNATGGTACCGGGTTGTTAAGAGGTCCACAACGGGTCGCACGACAACNTTAAGGCCTCATGCATG
ACCCAACACGGGGGCGTCCCGGNGCGGCGACCTTGNAGTCCGGGGACAAGTATGGCCCCCCGGGAAGG
ACGCCGTAGTGCATCTGGAGTGCCGATTCGTGGTCCAGAATGCCACCACGATGGTATGAGTGGCAAAG
GTACTTTGGACACCACCCCGTTTTCTATTTCCAGACGCGGCCATTCAGGAACGACGCGAATCGTAATC

TACCTGCCGCGTACGCGGATCGTACATGCGTTTTCCAGCGTTCGAAATAGAGTTTGTCGTTCCGCTGA
CCAGGTTTCACGGCTACTGCGATAAGCATATTCTACTGGGGGACTGTAAAGGAATTGTATCCACTCTG
CTTCCTGTCAATTGGCCATGTGCATCTCTNGTTGTTCAGGGTACATTAATAACACACAGCATTAAGTA
GATAAGTCTCCGATATAAGCGAACAGAACGTTGGTCAGAGACTATGGTTCAATTGTAGTGGTATAATG
TTTCCCAGTGCATAAGGAACTCAGGGACACTACTAGCATGGGCGAGGAGAGACCCTTGGGTGGCAACA
CGCAATAAGTGTTACCTAGTACATCAGCACGAGAGANGGTTTGACCATGTGTTTTTAGACGACCCCGG
TCGCATTGAACCTGGCATTCAGAATGACGTGCTCTCAAGAATCAAGGTCCTCCCACTGAGGGTCATCT
GCTTCGCGGNNCAGCGTATGGTCANAAACAT
